# International Collaboration Patterns and Effecting Factors of Emerging Technologies

**DOI:** 10.1371/journal.pone.0167772

**Published:** 2016-12-02

**Authors:** Xu Bai, Yun Liu

**Affiliations:** School of Management and Economics, Beijing Institute of Technology, Beijing, China; West Virginia University, UNITED STATES

## Abstract

With the globalization of the world economy, international innovation collaboration has taken place all over the world. This study selects three emerging technologies (3D printing, big data and carbon nanotubes and graphene technology) among 20 countries as the research objects, using three patent-based indicators and network relationship analysis to reflect international collaboration patterns. Then we integrate empirical analyses to show effecting factors of international collaboration degrees by using panel data. The results indicate that while 3D printing technology is associated with a “balanced collaboration” mode, big data technology is more accurately described by a radial pattern, centered on the United States, and carbon nanotubes and graphene technology exhibits “small-world” characteristics in this respect. It also shows that the factors GDP per capita (GPC), R&D expenditure (RDE) and the export of global trade value (ETV) negatively affect the level of international collaboration. It could be useful for China and other developing countries to make international scientific and technological collaboration strategies and policies in the future.

## Introduction

With the globalization of the world economy and the internationalization of innovation resources, international collaboration has taken place all over the world [1]. Both professional progress and knowledge extension could be provided by scientific collaboration [2]. Vessuri [3] concluded that international innovation collaboration has a positive impact on increasing technology professionalization. There is a growing consensus that emerging technologies enormously improve productivity, create more employment opportunities, and significantly stimulate economic growth [4], which appears to be the future trend and may be the important factor of one developing country (region) realizing its economic catch-up strategy [5]. As one form of technology professionalization, emerging technologies need international innovation collaboration to fully develop.

The latest technological achievements could be reflected by patents, which include meaningful information of international innovation collaboration [6, 7]. Many scholars have characterized the growth and trends in emerging technologies using patents data mining and patents content visualization tools [8]. The patent citation is helpful to understand the comparative development of technological innovation [9]. Also, co-invented patents could be used for analyzing international collaboration characteristics [10].

Because emerging technologies are an important way for different countries to engage in technological competitiveness [11], major countries put many human and financial resources into emerging technology research and participate in international collaboration [12], while the degree of international collaboration seems to vary considerably among countries [13]. So there is great significance in studying the international collaboration pattern characteristics of emerging technologies for countries, mainly for China and other developing countries, to identify international scientific and technological collaboration strategies and policies, because collaboration patterns influence collaboration level to some extent [10].

To study the collaboration patterns of emerging technologies, international co-inventions (patent-based) data are used to do indicator analysis and network relationship analysis. In addition, determinants of international collaboration level via empirical analysis are explored. To be specific, this paper aims to discuss the following two questions:

1. What is the international collaboration pattern characteristic of emerging technologies?
2. What are the effecting factors of emerging technologies’ international collaboration levels?

Despite the proliferation of studies on various dimensions of international collaboration, there is still a dearth of empirical analysis on international collaboration of emerging technologies [14]. The existing studies either focus on analysis at the enterprise level [15, 16] or present research at the industry level [17, 18] or apply case studies at the country level [19, 20]. Developed countries are the main research objects [21, 22]. Only a few studies consider developing counties [23] because data from developing countries are not easy to look up [24]. The number of studies that display international collaboration patterns of emerging technologies is great [25, 26]. However, only a few studies explicitly investigate the relationship between international collaboration patterns and the collaboration levels of emerging technologies by empirical analysis [27]. To fill this gap, this study analyzes the characteristics of international collaboration patterns and determinants of collaboration levels by empirical analysis via panel data among 20 countries which include not only developed countries but also developing countries.

The rest of this paper is organized as follows: Section 2 introduces the data and methods. Section 3 analyzes international collaboration patterns. Section 4 discusses effecting factors of international collaboration levels by using empirical analysis. Section 5 concludes with the findings and suggestions for making technology strategies and policies.

## Data and Methods

### Data source

Three representative emerging technologies are selected for this study: 3D printing, big data, and carbon nanotubes and graphene technology. 3D printing technology takes the digital model as a template to create solid objects through addition processes [28]. Recently, the conceptions of “Smart City”, Internet of Things (IoT) and Cloud computing [29, 30] are proposed so that information data has been in a massive growth and big data technology have emerged to meet the present-day requirements. It is soon becoming a tool to forecast possibilities of an event. [31, 32]. Due to the advantageous structural and functional properties of carbon nanotubes and graphene, it has broad range of applications in diverse fields, such as medical, sensors and computers [33]. These technologies were chosen as representative because first, all of them are disruptive and emerging technologies, that are going to dramatically change the way human beings live and the products human being use. [34] Second, these technologies represent different industries. Last but not least, the three emerging technologies have different collaboration patterns.

Sixteen member countries of the Organization for Economic Co-operation and Development (OECD)—the Unite States, Germany, Japan, South Korea, the United Kingdom, Canada, Israel, the Netherlands, Switzerland, France, Australia, Italy, Sweden, Belgium, Spain and Denmark and four non-OECD member countries—China, India, Russia and Brazil are investigated, as all of them owned selected emerging technology patents and nearly all countries participate in international collaboration.

Our data source is the European Patent Office (EPO) Worldwide Patent Statistical Database (PATSTAT), November 2015 version (https://worldwide.espacenet.com/advancedSearch?locale=en_EP). In this database, the patent applications are submitted to about 90 Patent Offices worldwide. Therefore, patents related to emerging technologies in the PATSTAT database are representative, comprehensive, and international [35]. The time period covers from January 1st, 2004 to December 31st, 2015.

A key word query approach was used to identify emerging technology patents from the PATSTAT database. The key words are based on the definition of emerging technologies. Therefore, the retrieved strategy for each emerging technology were compiled and listed in Text H in S1 File. Then we use Vantage Point [36] software to clean the patents. For example, in carbon nanotubes and graphene technology field, we use the key-word “nano tube*”. However, we need to refine the sample of patents whose topic descriptor contains the word “tube*”, but whose content has nothing to do with the nanotubes (for example tuberculosis). This refinement of the samples necessities two types of actions. The first dictates that patents whose keywords include “tube” (for example turbine) should be removed. The second requires that patents be removed whose keywords only contain a word with the root “tube,*” but no other keywords related to the carbon nanotubes. After cleaning up, there are 7385 patents in the 3D printing technology field, 6524 patents in the big data technology field, and 11324 patents in the carbon nanotube and graphene technology field.

Based on the cleaning data, we mainly use items “applicants” and “inventors” which both include the nationalities to do indicator analysis and regression analysis. Then we defined patents whose inventors’ nationalities are equal to or greater than 2 as “collaboration patents”. We then extracted those patents and established a new patents database, with a patent amount for 3D printing technology of 6277, big data of 5154, and carbon nanotube and graphene of 9965.

### Methods

#### Indicator analysis

Indicator analysis [37] is usually applied in the quantitative studies of science and technology. Following the work of Guellee [10], three indicators were calculated to reflect international collaboration patterns, which mirror each other. We introduce them in detail in the following paragraphs.

SHII is the share for a given country of patents with a foreign resident as a co-inventor in the population of patents with a domestic inventor. The computation formula is as follows:

$$SHII_{i}=PC_{i}/PI_{i}$$ (1)

where PC_*i*_ is the total number of patents invented by the residents of country *i* in collaboration with foreign researchers and PI_*i*_ is the total number of patents invented by the residents of country *i*.

SHIA is the share for a given country of patents with a domestic inventor and a foreign applicant in the country’s total domestic inventions. It reflects the extent to which foreign firms control (own) domestic inventions. The computation formula is as follows:

$$SHIA_{i}=PFA_{i}/PI_{i}$$ (2)

where PFA_*i*_ is the number of patents invented by the residents of country *i* and at least partly owned by the residents of country *i* and PI_*i*_ is the total number of patents invented by the residents of country *i*. PFA_*i*_ indicates the total fractional number of patents invented by the residents of country *i* and controlled by foreign residents.

SHAI is the share for a given country of patents with a foreign inventor and a domestic applicant in the country’s total domestic applications. It reflects the extent to which domestic firms control (own) foreign inventions. The computation formula is as follows:

$$SHAI_{i}=PFI_{i}/PA_{i}$$ (3)

where PFI_*i*_ is the total number of patents controlled by the residents of country *i* and invented by foreign residents and PA_*i*_ is the total number of patents owned by the residents of country *i*.

#### Network relationship analysis

Drawing maps is a relatively common method of displaying network relationships [38]; the maps use visualization and can efficiently reflect and reveal the complex relationships of international collaboration [39]. Maps describe the overall characteristics of emerging technology cooperation networks, while centrality analysis is used to measure the importance of nodes in the network structure. In social network analysis, degree centrality, betweenness centrality, and closeness centrality are common indicators [40]. Degree centrality is often used as a first step, while betweenness centrality elaborates the ability of a given node to control interactions between pairs of other nodes and closeness centrality describes degree of a node that is not subject to any other node’s control [41].

#### Regression analysis

Regression analysis is a statistical process for estimating the relationships among variables and helps one understand how the typical value of the dependent variable changes when any one of the independent variables is varied, while the other independent variables are held fixed [42]. We integrate Ordinary Least-Squares regression analysis of panel data to explore the effecting factors of international collaboration level.

## Analysis of Collaboration Patterns

In this section, we focus on international collaboration characteristic of three emerging technologies among 20 countries.

### Indicator analysis

From the perspective of collaboration indicators:

1. **Indicator SHII:** Except for South Korea and Japan, there is a certain degree of international collaboration among other countries in 3D printing technology. For big data technology, there is a polarization trend, namely, the collaboration proportion is extremely high or low. There is no cooperation among South Korea, Russia, and Denmark, but the patents in Italy and Brazil are all products of international collaboration. In the field of carbon nanotubes and graphene, the frequency of collaboration is higher than in the other two fields.
2. **Indicators SHIA and SHAI:** The level of SHAI in developed countries is higher than the level of SHIA, which indicates that developed countries mainly depend on independent innovation in emerging technology fields. However, developing countries have the opposite situation, which confirms that developing countries weakly control the ownership of co-patents and are in the position of being workers for developed countries.

From the perspective of countries, as reported in Table 1, there is a striking heterogeneity among the 20 countries studied. First, the SHII indicators of the United States and Germany are at the intermediate position, indicating that they have moderate levels of cooperation, while their levels of SHAI are obviously higher than that of SHIA, showing that these countries’ emerging technology innovation capability is significantly higher than that of other countries. On the other hand, very few inventions of Japan and South Korea are controlled by foreign firms or invented in co-operation with foreign researchers. It has long been recognized that Japan has little cooperation with other countries in emerging technology fields. So does South Korea. Amongst the developed countries, the Netherlands and Australia are characterized by a relatively high degree of international collaboration, with ratios of nearly 50%. Other developed countries (the United Kingdom, Switzerland) are less highly internationalized.

**Table 1:** Indicator analysis of 20 countries.

	3D printing			Big data			Carbon nanotubes and graphene		
	SHII	SHIA	SHAI	SHII	SHIA	SHAI	SHII	SHIA	SHAI
US	8.69%	12.97%	18.89%	1.30%	2.26%	15.96%	13.94%	2.64%	14.62%
Germany	8.88%	10.29%	14.23%	12.50%	7.50%	21.95%	31.48%	8.14%	39.41%
Japan	0.79%	4.16%	2.87%	1.64%	3.02%	3.48%	4.36%	3.41%	4.14%
South Korea	1.63%	12.23%	20.29%	0.00%	0.00%	0.00%	5.31%	1.98%	5.41%
UK	27.71%	42.17%	19.30%	32.00%	26.00%	30.43%	25.53%	14.04%	26.19%
Canada	23.81%	42.86%	18.75%	17.39%	10.43%	15.00%	15.00%	35.00%	13.33%
Israel	5.56%	11.11%	0.00%	25.00%	25.00%	18.18%	0.18%	0.00%	2.00%
Netherlands	37.93%	17.24%	46.88%	9.09%	26.36%	22.22%	8.00%	7.02%	20.00%
Switzerland	22.45%	40.82%	48.15%	40.00%	20.00%	40.00%	25.00%	50.00%	25.00%
France	21.69%	31.33%	21.05%	0.59%	0.00%	12.50%	15.52%	13.79%	15.52%
Australia	50.00%	33.33%	60.00%	10.00%	20.00%	0.00%	44.44%	24.32%	44.44%
Italy	16.00%	20.00%	4.76%	100.00%	30.00%	35.58%	60.00%	20.00%	33.33%
Sweden	17.78%	24.44%	19.05%	25.00%	0.00%	40.00%	50.00%	50.00%	50.00%
Belgium	23.53%	35.29%	26.67%	0.21%	0.00%	0.00%	42.86%	28.57%	42.86%
Spain	18.52%	18.52%	15.38%	0.12%	0.00%	0.00%	60.00%	20.00%	66.67%
Denmark	22.22%	30.00%	40.00%	0.00%	0.00%	0.00%	20.00%	10.00%	30.00%
China	11.70%	13.43%	2.23%	10.30%	12.41%	2.21%	14.65%	18.25%	3.59%
India	25.00%	25.00%	0.00%	81.82%	90.91%	11.82%	60.00%	50.00%	9.41%
Russia	5.71%	40.00%	4.55%	0.00%	0.00%	0.00%	42.86%	38.10%	7.23%
Brazil	40.00%	30.00%	2.05%	100.00%	100.00%	2.25%	0.25%	0.00%	0.00%

Data compiled by authors for this study.

Developing countries (China, India, Russia and Brazil) have strong SHII and SHIA and weak SHAI, which shows that the control ability of patent ownership in developing countries is rather weak; the inventors in these countries are in a migrant worker position and a large proportion of patent ownership is controlled by developed countries. The reason is as follows: It is very common that researchers from a developing country have fewer intelligent local colleagues in emerging technology fields and need to look abroad for collaboration [43]. Furthermore, internationalization of research and development (R&D) activities of multinational companies is very active in the domestic regions of the developing countries. Once they start international cooperation, however, developed countries will hold patent ownership in the end, since they provide a great amount of human and financial resources. On the other hand, developing countries set up fewer R&D institutions abroad, as they lack capital and qualifications.

### Network relationship analysis

**Fig 1:**
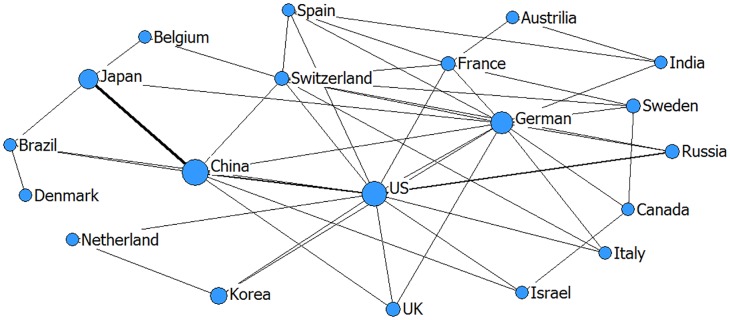
The network relationship of 3D printing technology (drawn by netdraw software).

**Fig 2:**
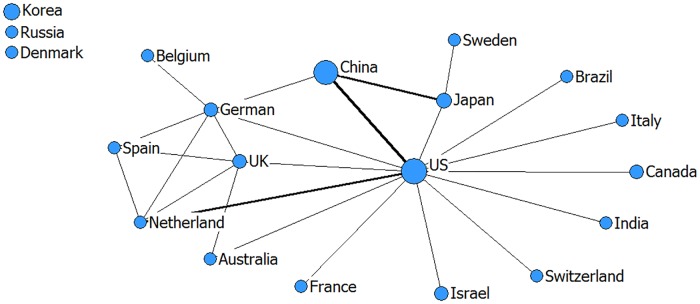
The network relationship of big data technology (drawn by netdraw software).

**Fig 3:**
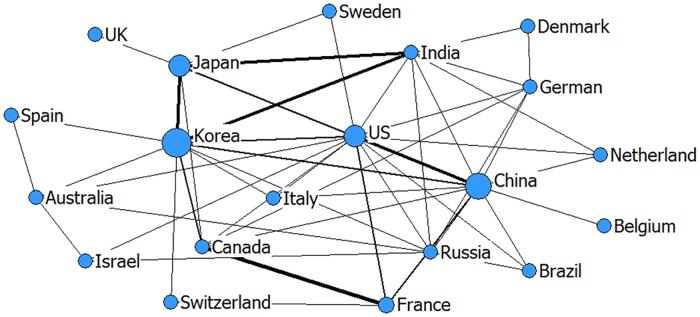
The network relationship of carbon nanotubes and graphene technology (drawn by netdraw software).

Network relationship can reflect the international collaboration patterns of emerging technologies on the other side [44]. In Figs 1–3, the larger the node (one node = one country), the greater is the number of co-patents associated with that country. Thicker lines between two nodes demonstrate that the two countries have closer cooperation. In addition, in order to better understand Figs 1–3, we calculated the centrality indicators as an auxiliary interpretation which is listed in Tables A-C in S1 File.

In the field of 3D printing (Fig 1), combining the analysis of centrality indicators (Table A in S1 File), we find that the United States and Germany have an obvious advantage: the two countries lie in the centricity, which indicates that they have a larger influence on the international cooperation network. It is important to note, though, that the US ranks first in the degree centrality indicator, and the betweenness centrality of Germany is the biggest. Japan and China are the other two main collaboration countries in 3D printing technology. The relationships between China and the other countries are tighter than those of Japan and the other countries. China is characterized by high degree centrality and low closeness centrality. The network illustrates the characteristics of “balanced collaboration,” which means that most countries exhibit cooperation though not a close cooperation.

Fig 2 presents that, in the field of big data, the United States is the core of the radial network, which shows that nearly every country has technology cooperation with the United States. South Korea, Russia, and Denmark are the isolated points, which means these three countries have no collaboration with other countries. Combining the analysis of centrality indicators (Table B in S1 File), the United Kingdom has strong betweenness centricity in the international big data cooperative network. Though the United Kingdom was not the core country, it maintained a strong mediation centricity role, which embodies the United Kingdom acting as the intermediary and in a bridge role in the international cooperative network. The degree centrality of China and Japan are strong in big data, but the betweenness centrality of each shows weakly.

In the carbon nanotubes and graphene technology field, the network relationship shows characteristics of a “small world”, which refers to having higher clustering coefficients and shorter average path length [45] (Fig 3). A small-world network is widely considered to stimulate creativity and improve overall global performance [46]. There is high interaction between India, South Korea and Japan in this field and they form a significant and strong leading group in terms of their domestic volume and international collaboration. Canada has a mutually collaborative relationship with France. As one of the Brazil, Russia, India and China (BRICs) economies, the most obvious upward trend is seen in China, which plays a more and more important role in the international collaboration network of emerging technologies. Combining the analysis of centrality indicators (Table C in S1 File), the degree centrality of China is the second biggest in this field. The other obvious upward trend is seen in India. And from the Table C in S1 File, it also shows the betweenness centrality of India is quite strong, indicating that India acts as an intermediary in the carbon nanotubes and graphene technology cooperative network.

All in all, the capacity of collaboration depends not only on the capability and quality of the national research base but also on the existing volume of activity. If there is little activity, thus leading to a limited output, then the opportunity for collaboration is naturally constrained. China is a good example: it holds a large number of patents, so its international collaboration level is higher than that of other developing countries. So the developing countries should improve both the volume of activity and the quality of their national research. Furthermore, it is advisable that China, India, Russia and Brazil increase their betweenness centrality and improve their control ability. Only in this way can they boost their collaboration ability and control ability.

## Results and Discussion

### Hypotheses

By studying the collaboration patterns of 20 countries, we found that the international collaboration patterns of different countries are rather different from each other in the three emerging technologies of 3D printing, big data, and carbon nanotubes and grapheme technology. In this section we discuss the effecting factors of international collaboration levels among 20 countries. There is a wide range of rationales for why countries and their scientific communities enter into cross-country science, technology and innovation (STI) cooperation [47]. A few studies on the factors have essentially focused on country size and stage of R&D development [48]. Based on discussion thus far in this paper, we propose the following hypotheses:

Generally speaking, there is an inverse relationship between the scale of the scientific community in a given country and the amount of international collaboration for innovation associated with that country [49]. And researchers in smaller countries are more likely to turn to international collaboration than those in larger ones [21].

**Hypothesis 1**. **Country size negatively affects the country’s level of international collaboration in emerging technologies**.

Researchers or scientists from countries with high technical expertise do not necessarily have appropriate incentives to collaborate with foreign scientists or researchers because their own knowledge reserves are already large enough [50]. So, the stage of R&D development has an effect on the international collaboration level to some extent.

**Hypothesis 2**. **The stage of a country’s R&D level negatively affects this country’s level of international collaboration in emerging technologies**.

The larger the R&D full-time equivalent (FTE) of one country, the more inventors participate in research activities [48]. Countries with more FTE do not need much cooperation with foreign researchers since their own scientific research strength is enough.

**Hypothesis 3**. **A country’s FTE negatively affects the level of international collaboration in emerging technologies**.

The geographical concentration discussed in a previous study [51] indicated that the choice of a preferred country for collaboration may depend on its geographical position. Indeed, increasing spatial distance could reduce the knowledge transmission and increase the coordination costs [52]. So, geographical proximity may affect international collaboration in emerging technology fields.

**Hypothesis 4**. **Geographical proximity negatively affects the level of international collaboration in emerging technologies**.

An important strategy for acquiring advanced technology by developing countries is to encourage FDI from developed countries [53]. International collaboration happens in science and technology research during the process of transferring technology.

**Hypothesis 5**. **Out-flows of FDI positively affect the level of international collaboration in emerging technologies**.

When an existing industry of one country improves in terms of quality metrics (e.g., higher quality raw materials or intermediate products), technology-oriented international collaboration may concomitantly happen [54].

**Hypothesis 6**. **International trade positively affects the level of international collaboration in emerging technologies**.

### Measures

#### Dependent variable

The indicators of SHII (there is a SHII indicator for each country for each year) of 20 countries as the measurement of international collaboration level in three emerging technologies are used. Since the early data are not complete, the choosing period is from 2008 to 2015.

#### Independent variables

1. Gross Domestic Product (GDP) per capita (GPC). GDP represents a country’s advanced degree, namely, country size. The variable we want to focus on and compare over time and across countries is GDP per capita rather than GDP itself, since we are unable to define the GDP of each emerging technology field. We get the data from the Organization for Economic Co-operation and Development (OECD) statistic database [55].
2. **R&D Expenditure (RDE)**. R&D expenditure per capita is selected to represent a country’s R&D level; we get this data from the Main Science and Technology Indicator (MSTI) of the OECD database (http://stats.oecd.org/viewhtml.aspx?datasetcode=MSTI_PUB&lang=en.), as it provides more than 100 countries’ science and technology input and output index data since 1981.
3. **Full-time equivalent (FTE)**. We get R&D FTE data from the MSTI database.
4. **Geographic proximity (GEO)**. We calculate the average space distance between one country (capital) and other countries (capitals) inspired by the work of Knack [56]. The algorithm is as follows:
  - We use the latitude and longitude of one country to calculate the distance between two countries:
    $$D_{AB}=R*arccos\;\left[cos\;A_{w}*cos\;B_{w}*cos\;\left(A_{j}-B_{j}\right)+sin\;A_{w}*sin\;B_{w}\right]$$ (4)
    where,
    D_AB_ is the distance between country A and country B,
    *R* indicates the radius of the earth,
    *A*_*w*_ and *B*_*w*_ are the latitude of country A and country B,
    *A*_*j*_ and *B*_*j*_ are the longitude of country A and country B.
  - We seek the latitude and longitude of each country (capital) and put them into the formula above, then the calculation process is realized by C Language programming.
  - We calculate the average distance between each country and the others.
5. **Flows of foreign direct investment (FDI)**. We consider outflows of FDI indicator to explain this variable since the outflows could better reflect the international collaboration capability of a country. We get the data from the OECD statistic database.
6. **The export of global trade value (ETV)**. The international trade situation is proxied by the export of global trade value. We get the data from the OECD statistic database.

#### Control variables

The control variables are as follows:

1. Degree centrality (DEG).
2. Betweenness centrality (BET).
3. Closeness centrality (CLO).

The three centrality indicators of each country from the years 2008–2015 in one emerging technology field can be calculated by Ucinet software [40].

For the sake of getting linear effects, all the independent variables and control variables are taken in logarithmic form. Table 2 shows the description of variables.

**Table 2:** Variables description.

Variables		Source
Acronym	Full name	
SHII	International collaboration level	SHII_*i*_ = PC_*i*_/PI_*i*_
GPC	GDP per capita	OECD Statistic Database
RDE	R&D Expenditure	MSTI Database
FTE	Full-time equivalent	MSTI Database
GEO	The average space distance between one country and other countries	C language programming
FDI	Flows of foreign direct investment	OECD Statistic Database
ETV	The export of global trade value	OECD Statistic Database
DEG	Degree centrality	Compiled by Unicet software
BET	Betweenness centrality	Compiled by Unicet software
CLO	Closeness centrality	Compiled by Unicet software

### Statistical model

In general, since the panel data include both cross-sectional data and time-series data, parameter estimation may be simultaneously affected by the inter-group effect (which here refers to differences between countries) and the inner effect group (in this case the same differences between countries in different years). Because eliminating differences between country panel data may cause a heteroscedasticity problem in the regression model, we used White’s heteroscedasticity [14] correction method to deal with this problem and the Ordinary Least Squares-fixed-effect models to estimate, because the random effect specification could be rejected by the Hausman test (*p* = 0.0012).

To methodically investigate the effecting factors of the international collaboration levels among 20 countries, eight models were estimated in each emerging technology field. We refer to the existing models [57] and our estimation process as follows: First, the baseline model, which only includes control variables, was estimated. Second, six independent variables were separately introduced to the baseline model step by step. Finally, all the control and independent variables were introduced simultaneously into an integral model.

### Results

The baseline model only considers the influence of control variables, and nearly all the control variables passed the significance test in the three emerging technology fields. We can draw the conclusion that degree centrality and betweenness centrality are positively correlated with SHII, and closeness centrality is negatively related to SHII by the estimating coefficients. Compared with the baseline model, model 2–7 (see Tables 3–5) which added explanatory variables, has better performance according to the likelihood value. Meanwhile, in order to check the robustness of our estimations, we used the sys-GMM regression model (due to the limited number of observations) with the same explanatory and control variables. The GMM model could perfectly solve the endogeneity problems. The results are very similar to those obtained under the OLS model in three emerging technology fields, indicating that the OLS model is robust. The results of robustness analysis are shown in Tables D-F in S1 File

**Table 3:** Analysis result in 3D printing technology field, 2008–2015^a^.

	Baseline Model	Model 1	Model 2	Model 3	Model 4	Model 5	Model 6	Model 7
Log(DEG)	0.1849***	0.2027***	0.2296***	0.1678***	0.1412***	0.1870***	0.2129***	0.2093***
Log(BET)	0.0359***	0.0082	0.0194*	0.0131	0.0542*	0.0135*	0.0071	0.0193**
Log(CLO)	-0.1839***	-0.0444*	-0.0531	-0.0219	-0.0593*	-0.0344	-0.0452*	-0.0525*
Log(GPC)		-0.6741*						0.4379*
Log(RDE)			-0.3730***					0.5532*
Log(FTE)				0.0153				0.0048***
Log(GEO)					0.0102			0.0458
Log(FDI)						0.0078		0.0146
Log(ETV)							-0.2402**	0.0736
Constant	0.9146	-2.5987	-0.4647	0.3364	0.2256	0.5322	-0.2677	0.8588
Log Likelihood	192.8580	222.2928	198.6446	232.5478	200.7458	216.8651	223.8207	232.96
No. Observations	160	160	160	160	160	160	160	160

^a^ OLS-fixed-effect model estimates compiled by stata 12.0 software.

*the parameters that are significantly different from zero at a 10% probability threshold,

**the parameters that are significantly different from zero at a 5% probability threshold,

***the parameters that are significantly different from zero at a 1% probability threshold.

**Table 4:** Analysis result in big data technology field, 2008–2015^a^.

	Baseline Model	Model 8	Model 9	Model 10	Model 11	Model 12	Model 13	Model 14
Log(DEG)	0.3521***	0.4459***	0.4428***	0.4353***	0.2671***	0.4854***	0.4017***	0.4838***
Log(BET)	0.0563*	0.0241*	-0.0371	0.0358*	0.0677**	0.0349*	0.0287*	0.01791*
Log(CLO)	-0.0432*	0.0119	0.0577	-0.1575*	-0.0569	-0.2101	-0.0052	0.06778
Log(GPC)		-1.0824***						-1.7619*
Log(RDE)			-1.0568**					-0.2584*
Log(FTE)				0.0015				-0.0085
Log(GEO)					0.0064			0.0072
Log(FDI)						0.0744		0.0967
Log(ETV)							-1.0167**	-0.0712*
Constant	0.4855	5.3836	1.6651	0.3998	0.9586	0.8269	2.5580	7.2827
Log Likelihood	196.2679	182.2023	197.8779	197.5234	198.2254	180.1938	188.5064	207.1326
No. Observations	160	160	160	160	160	160	160	160

^a^ OLS-fixed-effect model estimates compiled by stata 12.0 software.

*the parameters that are significantly different from zero at a 10% probability threshold,

**the parameters that are significantly different from zero at a 5% probability threshold,

***the parameters that are significantly different from zero at a 1% probability threshold.

**Table 5:** Analysis result in carbon nanotubes and graphene technology field, 2008–2015^a^.

	Baseline Model	Model 15	Model 16	Model 17	Model 18	Model 19	Model 20	Model 21
Log(DEG)	0.0313***	0.0833***	0.0756***	0.0194	0.0103**	0.0328*	0.0453**	0.0728**
Log(BET)	0.0374***	0.0212*	0.0214	0.0258*	0.0536	0.0165	0.0207	0.0217*
Log(CLO)	-0.0711*	-0.0529	-0.0782*	-0.0507	-0.059	-0.0319	-0.0696	-0.0595*
Log(GPC)		-0.9420*						-0.9865**
Log(RDE)			-0.6590**					-0.4622*
Log(FTE)				0.0148				0.0087
Log(GEO)					0.0078			0.0029
Log(FDI0						0.0088		0.0306
Log(ETV)							-0.0758	0.4012**
Constant	0.3628	0.7073	1.0601	0.2141	1.0635	0.1339	0.2012	5.6734
Log Likelihood	242.1368	263.13	257.0432	223.3211	210.3352	214.0577	248.0877	266.2321
No. Observations	160	160	160	160	160	160	160	160

^a^ OLS-fixed-effect model estimates compiled by stata 12.0 software.

*the parameters that are significantly different from zero at a 10% probability threshold,

**the parameters that are significantly different from zero at a 5% probability threshold,

***the parameters that are significantly different from zero at a 1% probability threshold.

From Tables 3, 4 and 5, we can draw the conclusion that despite the independent variables having different levels of impact on SHII in the three technologies, the overall results are nearly consistent. In order to illustrate intuitively, we summarize the analysis results of three emerging technologies, as shown in Table 6:

1. **GPC:** The coefficient is negative indicating that smaller countries are more tend to have international innovation cooperation than larger ones.
2. **RDE:** The three fields have consistent test results, which is consistent with the traditional result [10] (RDE has a negative correlation with SHII), showing that the higher R&D level of a country, the fewer are the researchers who engage in international innovation collaboration.
3. **FTE:** This variable did not pass the test, which indicates that the number of patent inventors has no correlation with the international collaboration level in emerging technologies.
4. **GEO:** This variable did not pass the test in three fields, either.
5. **FDI:** This variable did not pass the test in three fields, either.
6. **ETV:** This variable did not pass the test in carbon nanotubes and graphene field, but in the other two fields it passes the test and the coefficients are negative. The result shows that international trade value is inversely proportional to the degree of international patent cooperation.

**Table 6:** Effecting factors of SHII.

	3D printing (SHII)	Big data (SHII)	Carbon nanotubes and graphene technology (SHII)
DEG	+++	+++	+++
BET	++	+	+
CLO	-	-	-
GPC	-	---	-
RDE	---	--	--
FTE	none	none	none
GEO	none	none	none
FDI	none	none	none
ETV	--	--	none

+: small influence, ++: general influence, +++: great influence

-: small negative influence, --: general negative influence, ---: great negative influence

With regard to the results, the related interpretation is as follows:

1. **GDP:** As the reflection of one country’s comprehensive economic strength, the higher the country’s GDP per capita, the stronger the overall level of scientific research. So, larger countries tend to use their own research facilities to do sophisticated technical research, and the level of international collaboration is lower.
2. **RDE:** Low R&D level countries depend more on research collaboration with foreign innovative countries because their innovation capacities are rather weak. Thus, they can learn emerging and frontier technologies from international scientific collaboration [59].
3. **FTE:** Developed countries pay more attention to innovation and especially to the independent R&D of emerging technologies. So, more research workforce does not matter with the level of international collaboration.
4. **GEO:** Geographic proximity cannot be a determinant of international collaboration in emerging technologies since core technical communication is more important than the cost on geography.
5. **FDI:** Developed countries put emphasis on the protection of intellectual property rights. Although they make foreign investments, they transfer less core technology and make fewer patent collaborations with host countries.
6. **ETV:** A country’s high volume of international trade indicates that the country has a strong core technology in a certain industry [58]. In order to gain more profit, they need to keep possession of the unique technology. So they are reluctant to cooperate with other countries. A good example is Apple in the United States.

In summary, our empirical results support Hypothesis 1 and Hypothesis 2, and we fail to identify the relationships between SHII and FTE, GEO, and FDI. In addition, ETV is negatively correlated with SHII.

## Conclusions

This paper selects patents of three emerging technologies as the research objects, analyzes the international collaboration patterns through three indicators (SHII, SHIA, and SHAI) and network relationship analysis, then finds effecting factors of international collaboration levels by constructing regression modes. The conclusions are as follows.

First, international innovation collaboration patterns across 20 countries exist striking heterogeneities: developed countries, like the US and Germany, tend to have more international cooperation with other countries. However, Japan and South Korea have little cooperation with other countries. These differences are related to national technology development strategies. Take Japan, for example, it is known that Japan has implemented a technology statehood policy since the mid-1980s. Moreover, they have the self-contained technology system [59]. So in order to take advantage of the international innovation collaboration benefits, developing countries should clearly know what positions they locate in the international innovation collaboration network. Moreover, they should know what other countries’ core emerging technology advantages and international technology collaboration strategies are.

Second, the collaboration patterns of three emerging technologies exhibit as follows: the balanced collaboration mode (3D printing), the radial mode (big data) and the “small-world” mode (carbon nanotubes and graphene). Different patterns are primarily related to the characteristics of the technology itself. Specifically, the United States masters the main core technology of big data, so other countries need to cooperate with them to cooperatively invent patents. Therefore, the international collaboration pattern of big data technology forms a radial mode, centered on the United States.

Last but not the least, the effecting factors of international collaboration levels among different emerging technologies are similar. From the results of empirical analysis, the main factors, GPC, RDE, and ETV negatively associated with international collaboration levels, indicating that the existing international cooperation mode is not conducive for developing countries to cooperate on emerging technology patent inventions. Developing countries need to make efforts on strategic transformation, namely, from imitation to innovation and implement R&D investment strategies to adapt to the new economic and social situation. Then they should strengthen the rational allocation of resources and management of R&D to accelerate the transformation of economic development. Doing so will have theoretical and practical significance for enhancing the international competitiveness of developing countries.

## Supporting Information

S1 File This contains Tables A-G and Text H. Table A. The centrality indicators of 3D printing technology. Table B. The centrality indicators of big data technology. Table C. The centrality indicators of carbon nanotubes and graphene technology. Table D. Robust analysis results of 3D printing technology. Table E. Robust analysis results of big data technology. Table F. Robust analysis results of carbon nanotubes and graphene technology. Table G. Raw data of three emerging technologies. Text H. The retrieved strategy for three emerging technologies.
(DOCX)
