# Supplementary material for: International Collaboration Patterns and Effecting Factors of Emerging Technologies
Source: PLoS One. 2016 Dec 2;11(12):e0167772. doi: 10.1371/journal.pone.0167772 (PMC5135128; doi:10.1371/journal.pone.0167772)
Supplement: S1 File — Table A. The centrality indicators of 3D printing technology. Table B. The centrality indicators of big data technology. Table C. The centrality indicators of carbon nanotubes and graphene technology. Table D. Robust analysis results of 3D printing technology. Table E. Robust analysis results of big data technology. Table F. Robust analysis results of carbon nanotubes and graphene technology. Table G. Raw data of three emerging technologies. Text H. The retrieved strategy for three emerging technologies. (DOCX) [file pone.0167772.s001.docx]

# Table A. The centrality indicators of 3D printing technology.

| Country | Degree centrality | Betweenness centrality | Closeness centrality |
| --- | --- | --- | --- |
| US | 3.424 | 23.342 | 449.000 |
| Germany | 2.778 | 23.681 | 447.000 |
| Japan | 2.584 | 0.511 | 472.000 |
| South Korea | 0.258 | 4.111 | 467.000 |
| UK | 1.550 | 6.432 | 459.000 |
| Canada | 0.388 | 0.879 | 473.000 |
| Israel | 0.038 | 0.000 | 522.000 |
| Netherlands | 0.711 | 0.609 | 463.000 |
| Switzerland | 0.904 | 2.688 | 469.000 |
| France | 1.550 | 10.847 | 454.000 |
| Australia | 0.258 | 0.000 | 476.000 |
| Italy | 0.258 | 3.691 | 488.000 |
| Sweden | 0.581 | 0.000 | 467.000 |
| Belgium | 0.258 | 1.255 | 475.000 |
| Spain | 0.664 | 0.000 | 467.000 |
| Denmark | 0.194 | 0.000 | 475.000 |
| China | 3.411 | 5.169 | 459.000 |
| India | 0.065 | 0.000 | 482.000 |
| Russia | 0.129 | 0.000 | 480.000 |
| Brazil | 0.194 | 0.083 | 475.000 |

Data processed by Ucinet 6.0 software.

# Table B. The centrality indicators of big data technology.

| Country | Degree centrality | Betweenness centrality | Closeness centrality |
| --- | --- | --- | --- |
| US | 11.936 | 18.935 | 324.000 |
| Germany | 1.857 | 2.984 | 334.000 |
| Japan | 3.979 | 1.478 | 536.000 |
| South Korea | 2.122 | 1.478 | 841.000 |
| UK | 2.387 | 7.262 | 327.000 |
| Canada | 0.796 | 0.000 | 342.000 |
| Israel | 0.265 | 0.000 | 342.000 |
| Netherlands | 2.918 | 0.708 | 337.000 |
| Switzerland | 0.265 | 0.000 | 870.000 |
| France | 0.265 | 0.000 | 870.000 |
| Australia | 1.592 | 0.000 | 340.000 |
| Italy | 0.531 | 0.000 | 342.000 |
| Sweden | 0.265 | 0.000 | 870.000 |
| Belgium | 0.265 | 0.000 | 870.000 |
| Spain | 0.796 | 0.000 | 349.000 |
| Denmark | 0.265 | 0.000 | 870.000 |
| China | 10.631 | 1.693 | 335.000 |
| India | 0.531 | 0.000 | 342.000 |
| Russia | 0.265 | 0.000 | 870.000 |
| Brazil | 0.265 | 0.000 | 870.000 |

Data processed by Ucinet 6.0 software.

# Table C. The centrality indicators of carbon nanotubes and graphene technology.

| Country | Degree centrality | Betweenness centrality | Closeness centrality |
| --- | --- | --- | --- |
| US | 2.133 | 11.274 | 352.000 |
| Germany | 0.213 | 1.675 | 362.000 |
| Japan | 1.733 | 2.675 | 362.000 |
| South Korea | 3.904 | 5.359 | 356.000 |
| UK | 0.071 | 0.142 | 359.000 |
| Canada | 0.373 | 0.243 | 361.000 |
| Israel | 0.089 | 0.000 | 366.000 |
| Netherlands | 0.089 | 0.000 | 365.000 |
| Switzerland | 0.089 | 0.144 | 371.000 |
| France | 0.515 | 0.781 | 365.000 |
| Australia | 0.196 | 1.010 | 363.000 |
| Italy | 0.071 | 0.000 | 364.000 |
| Sweden | 0.036 | 0.000 | 368.000 |
| Belgium | 0.053 | 0.000 | 371.000 |
| Spain | 0.213 | 0.000 | 371.000 |
| Denmark | 0.005 | 0.000 | 374.000 |
| China | 3.492 | 5.340 | 354.000 |
| India | 0.302 | 3.866 | 359.000 |
| Russia | 0.231 | 2.105 | 374.000 |
| Brazil | 0.160 | 0.000 | 366.000 |

Data processed by Ucinet 6.0 software.

# Table D. Robust analysis result of 3D printing technology field, 2008-2015^b^.

|  | **Baseline**  **Model** | **Model 1** | **Model 2** | **Model 3** | **Model 4** | **Model 5** | **Model 6** | **Model 7** |
| --- | --- | --- | --- | --- | --- | --- | --- | --- |
| Log(DEG) | 0.2846*** | 0.2855*** | 0.2813*** | 0.2786*** | 0.1341*** | 0.2842*** | 0.2925*** | 0.2847*** |
| Log(BET) | 0.0715*** | 0.0697**** | 0.0639*** | 0.0685*** | 0.1532* | 0.0723*** | 0.05648** | 0.0451** |
| Log(CLO) | -0.0193** | -0.2029 | -0.0329** | -0.1888* | -0.0685* | -0.0201 | -0.0206* | -0.0333* |
| Log(GPC) |  | -0.3297* |  |  |  |  |  | -0.0642* |
| Log(RDE) |  |  | -0.1337*** |  |  |  |  | -0.1089** |
| Log(FTE) |  |  |  | 0.0153 |  |  |  | 0.0322 |
| Log(GEO) |  |  |  |  | 0.1021 |  |  | 0.0069 |
| Log(FDI) |  |  |  |  |  | 0.0286 |  | 0.0478* |
| Log(ETV) |  |  |  |  |  |  | -0.1387** | -0.1352*** |
| Constant | 2.7915 | 4.2597 | -0.1948 | 1.9485 | 1.3586 | 1.9349 | 2.7007 | 3.4990 |
| Sargan test (P) | 0.17 | 0.13 | 0.22 | 0.12 | 0.18 | 0.15 | 0.29 | 0.33 |
| No. Observations | 160 | 160 | 160 | 160 | 160 | 160 | 160 | 160 |

b GMM model estimates compiled by stata 12.0 software.

*the parameters that are significantly different from zero at a 10% probability threshold,

**the parameters that are significantly different from zero at a 5% probability threshold,

***the parameters that are significantly different from zero at a 1% probability threshold.

# Table E. Robust analysis result of big data technology field, 2008-2015b.

|  | **Baseline**  **Model** | **Model 8** | **Model 9** | **Model 10** | **Model 11** | **Model 12** | **Model 13** | **Model 14** |
| --- | --- | --- | --- | --- | --- | --- | --- | --- |
| Log(DEG) | 1.4567*** | 1.4599*** | 1.4315*** | 1.4469*** | 1.2372** | 1.4766*** | 1.4409*** | 1.4515*** |
| Log(BET) | 0.2545* | 0.2346** | 0.2573*** | 0.2508*** | 0.16273** | 0.2738*** | 0.2513** | 0.2609*** |
| Log(CLO) | -0.0145** | -0.0127 | -0.0208* | -0.0095* | -0.0451 | -0.0089* | -0.0194* | -0.0075** |
| Log(GPC) |  | -0.2752** |  |  |  |  |  | -0.2782** |
| Log(RDE) |  |  | -0.1512* |  |  |  |  | -0.1671** |
| Log(FTE) |  |  |  | -0.0326 |  |  |  | -0.0008 |
| Log(GEO) |  |  |  |  | 0.0012 |  |  | 0.0012 |
| Log(FDI) |  |  |  |  |  | 0.1058* |  | 0.1209 |
| Log(ETV) |  |  |  |  |  |  | -0.0369* | -0.0303** |
| Constant | 0.6438 | 7.5664 | 0.6422 | 0.9431 | 1.4856 | 0.0876 | 0.4935 | 0.9441 |
| Sargan test (P) | 0.21 | 0.32 | 0.27 | 0.25 | 0.15 | 0.11 | 0.15 | 0.37 |
| No. Observations | 160 | 160 | 160 | 160 | 160 | 160 | 160 | 160 |

b GMM model estimates compiled by stata 12.0 software.

*the parameters that are significantly different from zero at a 10% probability threshold,

**the parameters that are significantly different from zero at a 5% probability threshold,

***the parameters that are significantly different from zero at a 1% probability threshold.

# Table F. Robust analysis result of carbon nanotubes and graphene technology field, 2008-2015b.

|  | **Baseline**  **Model** | **Model 15** | **Model 16** | **Model 17** | **Model 18** | **Model 19** | **Model 20** | **Model 21** |
| --- | --- | --- | --- | --- | --- | --- | --- | --- |
| Log(DEG) | 0.6479*** | 0.0583*** | 0.1248*** | 0.0551* | 0.0196** | 0.0502* | 0.0196** | 0.0939** |
| Log(BET) | 0.0181*** | 0.0183* | 0.0478* | 0.0182* | 0.0133 | 0.0063 | 0.0372** | 0.0268*** |
| Log(CLO) | -0.4667* | -0.0025 | -0.0234 | -0.0565 | -0.403 | -0.1861 | -0.0112 | -0.1321* |
| Log(GPC) |  | -0.7275* |  |  |  |  |  | -0.1413** |
| Log(RDE) |  |  | -0.2086** |  |  |  |  | -0.2900*** |
| Log(FTE) |  |  |  | 0.0023 |  |  |  | 0.0039 |
| Log(GEO) |  |  |  |  | 0.0001 |  |  | 0.0011 |
| Log(FDI) |  |  |  |  |  | 0.0133 |  | 0.0221 |
| Log(ETV) |  |  |  |  |  |  | -0.1601** | 0.1596** |
| Constant | 0.3431 | 0.6321 | 1.1809 | 0.3578 | 0.01325 | 0.4581 | 0.6014 | 0.7099 |
| Sargan test (P) | 20 | 23 | 22 | 23 | 12 | 15 | 24 | 27 |
| No. Observations | 160 | 160 | 160 | 160 | 160 | 160 | 160 | 160 |

b GMM model estimates compiled by stata 12.0 software .

*the parameters that are significantly different from zero at a 10% probability threshold,

**the parameters that are significantly different from zero at a 5% probability threshold,

***the parameters that are significantly different from zero at a 1% probability threshold.

# Table G. Raw data of three emerging technologies.

| **Number** | **Category** | **Title** | **Publication number** | **Inventor(s)** | **Applicant(s)** | **International classification** |
| --- | --- | --- | --- | --- | --- | --- |
| 1 | 3D printing | METHOD AND DEVICE FOR MANUFACTURING TITANIUM OBJECTS | [AU2012100393A4](https://worldwide.espacenet.com/publicationDetails/biblio?adjacent=true&locale=en_EP&FT=D&date=20161020&CC=US&NR=2016306266A1&KC=A1) | GULDBERG SIGRID (NO) | NORSK TITANIUM COMPONENTS AS (NO) | B23K9/04 |
| 2 | 3D printing | REFLECTIVE ROADSTUD AND MANUFACTURE OF THE SAME\ | [AU2012200151A1](https://worldwide.espacenet.com/publicationDetails/biblio?adjacent=true&locale=en_EP&FT=D&date=20161020&CC=US&NR=2016303779A1&KC=A1) | LEE HUNG-CHEN (TW) | TECHEYE OPTICS TECHNOLOGIES CO LTD (TW) | B29C45/00\ E01F9/06 |
| 3 | 3D printing | CLOSURE DEVICE FOR TUBULAR PACKAGING FOR E.G. ROLLED UP CARPET USES ADHESIVE TAPE TO SEAL HEAD FORMED AT OPEN END OF PACKAGING\[NL] INRICHTING EN WERKWIJZE VOOR HET AFSLUITEN VAN EEN BUISVORMIGE VERPAKKING. | [BE1017152A6](https://worldwide.espacenet.com/publicationDetails/biblio?adjacent=true&locale=en_EP&FT=D&date=20161020&CC=US&NR=2016303805A1&KC=A1) | HANDSAEME OLIVIER (BE) | HANDSAEME MACHINERY BVBA (BE) | Empty Field |
| 4 | 3D printing | FINISHING DEVICE FOR SECURING TOGETHER CORNERS OF CARPETS AFTER EDGING INCLUDES MEANS FOR AUTOMATICALLY CUTTING CONNECTING THREAD AND SECURING THREAD ENDS TO CARPETS\[NL] INRICHTING VOOR HET AFWERKEN VAN DE HOEKEN VAN EEN TAPIJT EN WERKWIJZE DAARBIJ TOEGEPAST. | [BE1017433A3](https://worldwide.espacenet.com/publicationDetails/biblio?adjacent=true&locale=en_EP&FT=D&date=20161020&CC=US&NR=2016303795A1&KC=A1) | Empty Field US | EVILO NV (BE) | Empty Field |
| 5 | 3D printing | METHOD OF ULTRASONICALLY WELDING PLASTIC SHEETS TO A NON-PLASTIC COVER\[FR] METHODE DE SOUDAGE AUX ULTRA-SONS DE FEUILLES EN PLASTIQUE A UNE POCHETTE NON CONSTITUEE DE PLASTIQUE | [CA2246977A1](https://worldwide.espacenet.com/publicationDetails/biblio?adjacent=true&locale=en_EP&FT=D&date=20161020&CC=US&NR=2016302469A1&KC=A1) | DESMARAIS RICHARD (CA) | ALBUMS DF LTEE (CA) | B29C59/00\ B29C65/08\ B29C53/06\ B42D1/08 |
| 6 | 3D printing | METHOD AND APPARATUS FOR REPAIRING SYNTHETIC TEXTILES\[FR] METHODE ET APPAREIL DE REPARATION DE TEXTILES SYNTHETIQUES | [CA2442522A1](https://worldwide.espacenet.com/publicationDetails/biblio?adjacent=true&locale=en_EP&FT=D&date=20161020&CC=US&NR=2016303807A1&KC=A1) | CAMMACK DAWN E (CA) | CAMMACK DAWN E (CA) | D06M10/02\ D21F1/00\ B29C73/10 |
| 7 | 3D printing | A METHOD FOR ULTRASONIC WELDING AND ULTRASONIC WELDING FASTENER AND JOINING SYSTEMS\[FR] UNE METHODE POUR LE SOUDAGE PAR ULTRASONS LIANT DE SOUDAGE PAR ULTRASONS ET SYSTEMES D'ASSEMBLAGE | [CA2590812A1](https://worldwide.espacenet.com/publicationDetails/biblio?adjacent=true&locale=en_EP&FT=D&date=20161020&CC=US&NR=2016303803A1&KC=A1) | BOURBEAU ALAIN (CA)\ BELANGER GERMAIN (CA) | 9163 9934 QUEBEC INC (CA) | B29C65/08 |
| 8 | 3D printing | WINDOW FOR CLOSING BOX OF E.G. WRIST-WATCH HAS PERIPHERAL RIM INCLUDING AREA IN WHICH WINDOW IS ULTRASONICALLY WELDED TO BOX OF PORTABLE OBJECT WHERE PERIPHERAL RIM EXTENDS ALONG PART OF PERIMETER OF WINDOW\[FR] GLACE SERVANT à FERMER PAR LE HAUT LA BO?TE D'UN OBJET PORTABLE. | [CH703966A2](https://worldwide.espacenet.com/publicationDetails/biblio?adjacent=true&locale=en_EP&FT=D&date=20161020&CC=US&NR=2016303616A1&KC=A1) | POLI GIAN-CARLO (CH) | SWATCH GROUP RES & DEV LTD (CH) | B29C65/08\ G04B39/02 |
| 9 | 3D printing | METHOD FOR BONDING NANOMETER MATERIAL ON METAL ELECTRODE | [CN100336192C](https://worldwide.espacenet.com/publicationDetails/biblio?adjacent=true&locale=en_EP&FT=D&date=20161020&CC=US&NR=2016303796A1&KC=A1) | ZHANG YAFEI CHEN (CN) | UNIV SHANGHAI JIAOTONG (CN) | H01L21/28\ H01L21/768\ H01L21/607 |
| 10 | 3D printing | WELDING APPARATUS WITH MICROPICTURE AUTOMATIC-COMPUTING POSITIONING SYSTEM AND OPERATION MODE\ | [CN100351721C](https://worldwide.espacenet.com/publicationDetails/biblio?adjacent=true&locale=en_EP&FT=D&date=20161020&CC=US&NR=2016307083A1&KC=A1) | GUANG LI (CN) | GUANG LI (CN) | G05B19/4097\ G05B19/409\ G05B19/19\ G05B15/02 |
| 11 | 3D printing | FACE-DOWN BONDING STRUCTURE WITH LOW-MELTING POINT CONVEX POINT AND MAKING METHOD\ | [CN100359679C](https://worldwide.espacenet.com/publicationDetails/biblio?adjacent=true&locale=en_EP&FT=D&date=20161020&CC=WO&NR=2016168421A1&KC=A1) | PINGJUAN NIU (CN)\ HAIQIANG JIA (CN)\ HONG CHEN (CN)\ HONGWEI LIU (CN)\ GUANGHUA YANG (CN)\ TIECHENG GAO (CN)\ HUIYING LUO (CN) | UNIV TIANJIN POLYTECHNIC (CN) | H01L21/60\ H01L23/48\ H01L21/48 |
| 12 | 3D printing | METHOD AND APPARATUS FOR MANUFACTURING TUBULAR FILTER FILM TUBE\ | [CN100428983C](https://worldwide.espacenet.com/publicationDetails/biblio?adjacent=true&locale=en_EP&FT=D&date=20161020&CC=WO&NR=2016168822A1&KC=A1) | FUZHAO HUANG (CN)\ AOQUAN WU (CN) | HUANG LIYAO (CN) | B01D69/04 |
| 13 | 3D printing | A WELDING PROCESS OF BRIDGING FIBRIL OF ELECTRIC DETONATOR FIRING DEVICES AND A JOINT STRUCTURE THEREOF\ | [CN100465574C](https://worldwide.espacenet.com/publicationDetails/biblio?adjacent=true&locale=en_EP&FT=D&date=20161020&CC=WO&NR=2016167239A1&KC=A1) | FAN SHI (CN)\ XIAO GU (CN)\ RONG SHI (CN)\ YIN GU (CN) | SHI FAN GU XIAO SHI RONG GU YI (CN) | F42C19/12 |
| 14 | 3D printing | MATERIAL FILLING TYPE SUPERSONIC WELDING PLASTIC UPTAKE CLAD PLATE\ | [CN100467933C](https://worldwide.espacenet.com/publicationDetails/biblio?adjacent=true&locale=en_EP&FT=D&date=20161020&CC=WO&NR=2016168718A1&KC=A1) | LUHAO LENG (CN) | LUHAO LENG (CN) | B32B27/10\ B32B3/12\ B32B7/08\ B32B3/04\ B29C65/08\ F16S1/10 |
| 15 | 3D printing | METHOD FOR MANUFACTURING PLATE TYPE HEAT PIPE WITH ULTRASONIC WELDING\ | [CN100488694C](https://worldwide.espacenet.com/publicationDetails/biblio?adjacent=true&locale=en_EP&FT=D&date=20161020&CC=WO&NR=2016165043A1&KC=A1) | XIUWEI YANG (CN)\ PEIPEI CHEN (CN)\ WENHUA YU (CN)\ ZHAOXIANG ZHENG (CN) | ASIA VITAL COMPONENTS CO LTD (CN) | B23K20/10\ B23P15/26\ B23K20/26\ B23K101/14 |
| 16 | 3D printing | ALUMINUM ALUMINUM ALLOY AND COMPOSITE MATERIAL ULTRASONIC VIBRATION LIQUID PHASE WELDING EQUIPMENT\ | [CN100500357C](https://worldwide.espacenet.com/publicationDetails/biblio?adjacent=true&locale=en_EP&FT=D&date=20161013&CC=US&NR=2016297935A1&KC=A1) | ZHIWU XU (CN)\ YANG ZHANG (CN)\ WEIWEI ZHAO (CN)\ JIUCHUN YAN (CN)\ DACHENG LI (CN)\ HANCHEN YU (CN)\ SHIQIN YANG (CN) | HARBIN INST OF TECHNOLOGY (CN) | B23K20/10 |
| 17 | 3D printing | AN AUTOMATIC OPERATION SYSTEM OF INNER LEADING BONDING DEVICE OF SEMICONDUCTOR DEVICE\ | [CN100505198C](https://worldwide.espacenet.com/publicationDetails/biblio?adjacent=true&locale=en_EP&FT=D&date=20161013&CC=US&NR=2016297149A1&KC=A1) | YUEHONG GAO (CN)\ ZHI SONG (CN)\ FUZHI ZHENG (CN)\ LI WEI (CN) | CHANGCHUN OPTICS FINE MECH (CN) | H01L21/607 |
| 18 | 3D printing | POLY ETHER ETHER KETONE PLASTIC HIGH COMPACTNESS JOINT PREHEATING ULTRASONIC WELDING DEVICE AND METHOD\ | [CN100519153C](https://worldwide.espacenet.com/publicationDetails/biblio?adjacent=true&locale=en_EP&FT=D&date=20161013&CC=US&NR=2016297006A1&KC=A1) | JIUCHUN YAN (CN)\ SHIQIN YANG (CN)\ XIAOLIN WANG (CN)\ RUIQI LI (CN) | UNIV HARBIN ENG (CN) | B29C65/08 |
| 19 | 3D printing | CYLINDRICAL LITHIUM ION CELL CAP AND ASSEMBLY METHOD\ | [CN100561771C](https://worldwide.espacenet.com/publicationDetails/biblio?adjacent=true&locale=en_EP&FT=D&date=20161013&CC=US&NR=2016298833A1&KC=A1) | JIANGFENG WU (CN)\ PENGKUN GAO (CN)\ YAO CHEN (CN) | SHANGHAI DLG BATTERY CO LTD (CN) | H01M2/30\ H01M2/10\ H01M2/34\ H01M2/00\ H01M2/04\ H01M2/06\ H01M2/08\ H01M2/12\ H01M10/40 |
| 20 | 3D printing | MULTIPLE PROBE POWER SYSTEMS AND METHODS FOR ULTRASONIC WELDING\ | [CN101011775A](https://worldwide.espacenet.com/publicationDetails/biblio?adjacent=true&locale=en_EP&FT=D&date=20161013&CC=US&NR=2016298213A1&KC=A1) | JOHANSEN DAVID K (US) | DUKANE CORP (US) | B23K20/10\ B29C65/08 |
| 21 | 3D printing | PC/ABS ALLOY AND ITS PRODUCTION\ | [CN101029168A](https://worldwide.espacenet.com/publicationDetails/biblio?adjacent=true&locale=en_EP&FT=D&date=20161013&CC=US&NR=2016297142A1&KC=A1) | LI XIAOBO XU (CN) | KEJU NEW MATERIAL CO LTD SHENZ (CN) | C08L69/00\ C08L67/00\ C08K5/524\ C08L55/02 |
| 22 | 3D printing | FLOW-CONTROLLED ELECTROMAGNETIC VALVE WITH NEW STRUCTURE AND CONNECTION STYLE\ | [CN101074622A](https://worldwide.espacenet.com/publicationDetails/biblio?adjacent=true&locale=en_EP&FT=D&date=20161013&CC=US&NR=2016297143A1&KC=A1) | WEN WEIGUANG (CN) | WEN WEIGUANG (CN) | F01N3/30\ F02M23/06 |
| 23 | 3D printing | PROCESS FOR PRODUCING A PLASTIC LENS\ | [CN101096117A](https://worldwide.espacenet.com/publicationDetails/biblio?adjacent=true&locale=en_EP&FT=D&date=20161013&CC=US&NR=2016297104A1&KC=A1) | SHINICHIRO KADOWAKI (JP) | HOYA CORP (JP) | B29C39/02\ B29L11/00\ B29C65/08\ B29C39/22 |
| 24 | 3D printing | PLASTIC PLATE RIVET WELDING THERMAL SYNTHESIS KIT AND ITS WORKING METHOD\ | [CN101105193A](https://worldwide.espacenet.com/publicationDetails/biblio?adjacent=true&locale=en_EP&FT=D&date=20161013&CC=US&NR=2016298215A1&KC=A1) | MINGZHI LIAO (CN) | MINGFENG TECHNOLOGY TIANJIN CO (CN) | B29C65/08\ F16B5/08 |
| 25 | 3D printing | METHOD FOR MANUFACTURING DRAINAGE BOTTLE\ | [CN101172394A](https://worldwide.espacenet.com/publicationDetails/biblio?adjacent=true&locale=en_EP&FT=D&date=20160919&CC=KR&NR=20160107980A&KC=A) | HAITAO MA (CN)\ RONGPING YAO (CN)\ JIANHUA TANG (CN) | RONGPING YAO (CN) | B29C45/00\ B29C69/00\ B29C65/08\ A61M1/00\ A61M27/00 |
| 26 | 3D printing | ULTRASONIC SOLDERING DEVICE AND METHOD FOR USING THE DEVICE TO WELD PRODUCT\ | CN101190475A | YISONG FU (CN) | BYD CO LTD (CN) | B23K20/10\ B29C65/08 |
| 27 | 3D printing | MECHANICAL TYPE PRESSURE REGULATOR\ | CN101202176A | ZHONGYI HUANG (CN) | ZHONGYI HUANG (CN) | H01H35/34\ H01H35/26 |
| 28 | 3D printing | PLASTIC SHELL ZINC-MANGANESE DRY CELL AND FABRICATION TECHNOLOGY AS WELL AS SPECIAL-PURPOSE DIE\ | CN101207206A | SHISONG CAI (CN) | JIAXING HUARONG BATTERY CO LTD (CN) | H01M2/02\ H01M6/00 |
| 29 | 3D printing | METHOD AND APPARATUS FOR AUTOMATIC CUTTING GRIDDING RING\ | CN101255651A | YIHONG WU (CN) | JIANGYIN HUAFANG ADVANCED TECH (CN) | D06H7/02\ D06C25/00 |
| 30 | 3D printing | TUBE WINDING MACHINE AND PIPE MEMBRANE PRODUCTION EQUIPMENT COMPOSED OF THE SAME\ | CN101269300B | ENHUA LIU (CN)\ QIYUN DU (CN) | TIANJIN MOTIAN MEMBRANE ENGINEERING & TECHNOLOGY CO LTD US | B01D69/04 |
| 31 | 3D printing | VACUUM FORMING AND INJECTION MOLDING COMPOSITE BOARD OF SUPERSONIC WELDING\ | CN101275707A | LUHAO LENG (CN) | LUHAO LENG (CN) | B29C69/00\ F16S1/10 |
| 32 | 3D printing | FLEXURAL VIBRATIONS TYPE ULTRASONIC BAR\ | CN101279317A | JINXUAN YAN (CN)\ ZHIZHONG YANG (CN)\ ZHUOLI YAN (CN)\ ZHUOSHENG YAN (CN) | GUANGZHOU NEW POWER ULTRASONIC (CN) | B06B1/02\ B06B1/00\ B06B3/02 |
| 33 | 3D printing | METHOD FOR BONDING DOUBLE-LAYER SLITTINGS OF UNDERWEAR BY ULTRASONIC\ | CN101301132A | FUKAI HUANG (CN) | DONGGUAN HEXUN GARMENT DESIGN (CN) | D06H5/00\ A41H43/04 |
| 34 | 3D printing | PLASTIC CAP BONDING METHOD FOR ZINC-MANGANESE VINYL COVER DRY CELL\ | CN101318381A | ZHECHEN LEI (CN) | ZHECHEN LEI (CN) | H01M2/02\ B29C65/08 |
| 35 | 3D printing | METHOD FOR ULTRASONIC WELDING PLASTIC PRODUCTS\ | CN101337430A | QIHONG YAN (CN) | SHENZHEN XWODA ELECTRONICS CO (CN) | B29C65/08 |
| 36 | 3D printing | MANUFACTURING METHOD FOR MINIATURE MICROPHONE\ | CN101360356A | ZHENGMIN PAN (CN) | AAC ACOUSTIC TECHNOLOGY CHANGZ (CN) | H04R31/00 |
| 37 | 3D printing | PRECISE ULTRASONIC ASSISTANT BRAZING DEVICE OF MAGNESIUM ALLOY AND COMPOSITE MATERIAL THEREOF AND DEVICE THEREOF\ | CN101362240A | HONG LI (CN)\ ZHUOXIN LI (CN)\ SHUYING BAN (CN)\ CHEN GAO (CN) | UNIV BEIJING TECHNOLOGY (CN) | B23K1/06\ B23K1/19\ B23K1/20\ B23K3/00 |
| 38 | 3D printing | OVERALL-PLASTIC WATER FEED APPARATUS\ | CN101367559A | XINSHI WEI (CN) | XINSHI WEI (CN) | C02F1/00 |
| 39 | 3D printing | IMPROVED FLOTATION TOOL CONSTRUCTION\ | CN101386168A | ZHIQING XIE (CN) | ZHIQING XIE (CN) | B25G1/00\ B25G1/10\ B25G3/00 |
| 40 | 3D printing | PIEZO-ELECTRIC ACOUSTIC GENERATOR MANUFACTURING METHOD\ | CN101389160A | HONGHUA XUN (CN) | AAC ACOUSTIC TECHNOLOGY CHANGZ (CN) | H04R31/00 |
| 41 | 3D printing | EYE-MASK ULTRASONIC-WAVE FORMING DEVICE AND EYE-MASK PROCESSING METHOD\ | CN101390786A | JIAN XUE (CN) | JIAN XUE (CN) | A61F9/04 |
| 42 | 3D printing | QUICK SWITCHING DEVICE OF CLAMP OF ULTRASONIC WELDING MACHINE\ | CN101391343A | HONGQI LI (CN)\ ZHENGXING ZHOU (CN) | YAGUANG NYPRO PREC MOLDING TIA (CN) | B23K20/10\ B23K37/04\ B23K20/26 |
| 43 | 3D printing | ASSEMBLY CONSTRUCTION AND METHOD FOR FUEL CELL\ | CN101393993A | ZHIJUN GU (CN)\ KE JIN (CN) | HORIZON FUEL CELL TECHNOLOGIES (CN) | H01M8/10\ H01M8/24 |
| 44 | 3D printing | ABSORPTION ULTRASONIC TOOL HEAD APPLIED FOR CONNECTING THE POLYMERS MICRO-DEVICES\ | CN101396866A | XIAODONG WANG (CN)\ YIBO SUN (CN)\ YI LUO (CN)\ PENG QIAO (CN)\ LIDING WANG (CN) | DALIAN UNIVERSITY OF SCIENCE A (CN) | B29C65/08 |
| 45 | 3D printing | AUTOMOBILE CONTROL LEVER DUST-PROOF COVER WELDING DEVICE THEREOF AND MANUFACTURING METHOD THEREOF\ | CN101462490A | ZHIMING XU (CN)\ CHUNLEI SUN (CN) | ZHIMING XU (CN) | B60K20/02 |
| 46 | 3D printing | MOBILE PHONE COMPOUND LENS AND ITS MANUFACTURING PROCESS\ | CN101464532A | LINA CHEN (CN)\ BAODI CHEN (CN)\ QIANG GAO (CN)\ GUOSHENG LI (CN)\ GAOFENG HU (CN) | TIANJIN ZHONGHUAN HI TECH CO L (CN) | G02B3/00 |
| 47 | 3D printing | TECHNIQUE FOR PRODUCING FLAT-PLATE TV SET CASE WITH HIGH-GLOSS MATERIAL\ | CN101503003A | GUOQING LIU (CN) | SHENZHEN KEXIN ULTRASONIC BOND (CN) | B29C69/00\ B29C65/08\ H04N5/64 |
| 48 | 3D printing | ULTRASONIC WELDING USING AMPLITUDE PROFILING\ | CN101512786A | Empty Field US | BRANSON ULTRASONICS CORP (US) | H01L41/04 |
| 49 | 3D printing | OPTICAL FIBER ADAPTER AND MANUFACTURING METHOD THEREOF\ | CN101526649A | LIBO SHAO US | CHANGZHOU WEIPU ELECTRONIC TEC US | G02B6/38 |
| 50 | 3D printing | ULTRASONIC LEAD COMPRESSION JOINTING MACHINE AND COMPRESSION JOINTING METHOD\ | CN101557065A | FULIANG LI (CN) | NINGBO ZHONGWANG ELECTRONIC SC (CN) | B23K20/10\ H01R43/02 |
| 51 | 3D printing | PARALLELISM ADJUSTING METHOD OF WELDING HEAD AND SAMPLE APPLICABLE TO ULTRASONIC NANO WELDING\ | CN101559547A | DONG XU (CN)\ YAFEI ZHANG (CN)\ BO ZHAO (CN)\ QIMING MAO (CN)\ XIANG CHEN (CN)\ DANNONG HE (CN) | UNIV SHANGHAI JIAOTONG (CN) | B23K20/10\ B23K37/00 |
| 52 | 3D printing | METHOD FOR PREVENTING CERAMIC LINER OF OPTICAL FIBER ADAPTER FROM BEING BROKEN DURING ULTRASONIC WELDING\ | CN101566708A | ZHONGMING MAO (CN)\ JIAN LEI (CN) | ZHONGMING MAO (CN) | G02B6/38\ B29L31/34\ B29C65/08 |
| 53 | 3D printing | CONTROL SYSTEM OF ULTRASONIC WELDING MACHINE\ | CN101574757B | CHEN JIALE US | TAIZHOU JULONG ULTRASONIC EQUIPMENT CO LTD US\ ZHEJIANG GREAT DRAGON AUTOMATION EQUIPMENT CO LTD US | B23K20/10\ B29C65/08\ H02M1/14\ H02M7/42 |
| 54 | 3D printing | NON-ENERGY-ORIENTED RIDGE POLYMER ULTRASONIC BONDING METHOD BASED ON TEMPERATURE COMPENSATION\ | CN101607688A | XIAODONG WANG (CN)\ YI LUO (CN)\ LIDING WANG (CN)\ ZONGBO ZHANG (CN)\ YINGSONG ZHENG (CN)\ YANGUO ZHANG (CN) | UNIV DALIAN TECH (CN) | B81C3/00 |
| 55 | 3D printing | WELDING METHOD OF LARGE-SIZE TARGET ASSEMBLY\ | CN101648305A | LIJUN YAO US\ JIE PAN US\ XUEZE WANG US\ LIN OUYANG US\ QING LIU US | NINGBO KONFOONG MATERIALS INTE US | B23K1/06 |
| 56 | 3D printing | SEMIACTIVE SUSPENDING DEVICE WITH ELECTROMAGNETIC VALVE\ | CN101670776A | XIAOCHENG DUAN US | NINGBO TUOPU VIBRATION CONTROL US | F16F13/10\ B60K5/12 |
| 57 | 3D printing | ULTRASONIC WELDING MACHINE AND CONTROL METHOD THEREOF\ | CN101683645A | KAIXING MA US | SHANGHAI FURONG INDUSTRY HEATI US | B23K20/10\ B29C65/08\ D06H5/00\ B06B1/02\ B06B1/06\ A41H43/04 |
| 58 | 3D printing | FULL-AUTOMATIC PIPETTE ULTRASONIC WELDING DEVICE\ | CN101698353A | JIANHUA YUAN US\ YE YUAN US\ CHANGPING GUO US | JIANHUA YUAN US\ YE YUAN US\ CHANGPING GUO US | B29C65/08\ B29C65/78\ B29C65/82 |
| 59 | 3D printing | PORTAL FRAME MOVABLE TYPE HORIZONTAL COORDINATE NUMERICAL CONTROL ULTRASONIC WELDING MACHINE\ | CN101700602A | HENGGUO LI US | NINGBO KAIBO NC MACHINERY CO L US | B23K20/10\ B29C65/08\ B23K20/26 |
| 60 | 3D printing | SEMI-AUTOMATIC ULTRASONIC WELDING MACHINE\ | CN101704161A | XINGRONG CHEN US\ HU JUN US | SHENZHEN OCEANSUN BATTERY CO L US | B23K20/10\ B23K20/26 |
| 61 | 3D printing | CLEAVER STRUCTURE WITH ELECTROSTATIC ADSORPTION ULTRASONIC WELDING EQUIPMENT AND BONDING METHOD\ | CN101712097B | YONGFEI SONG US | SHENZHEN CHUANGWEIXING AUTOMATION EQUIPMENT CO LTD US | B23K20/10\ B23K20/26 |
| 62 | 3D printing | METHOD FOR FIXING VELCRO CONNECTING PIECE ON AUTOMOTIVE PEDAL PAD\ | CN101733931A | JINGJING HAN US\ LIHUI SHEN US\ YUFENG WAN US\ MINGHUA YAO US\ YUAN YAO US | SHANGHAI CAR CARPET PLANT US | B29C65/08 |
| 63 | 3D printing | METHOD FOR FIXING VELCRO CONNECTING PIECE TO AUTOMOTIVE PEDAL PAD\ | CN101734186A | JINGJING HAN US\ LIHUI SHEN US\ MINGHUA YAO US\ YUAN YAO US\ DUDI GONG US\ MUGEN JIN US | SHANGHAI CAR CARPET PLANT US | B60N3/06 |
| 64 | 3D printing | MANUFACTURING METHOD OF CONNECTING WIRE WITH HIGH-DEFINITION MULTIMEDIA INTERFACE\ | CN101752769A | HAO JIN US | KAIXINTONG ELECTRONIC NANJING US | H01R43/20\ H01R43/00\ H01R43/28 |
| 65 | 3D printing | ASSEMBLING METHOD OF ELECTRIC CONNECTOR ASSEMBLY\ | CN101764336A | SHIMIN QU US | AAC ACOUSTIC TECHNOLOGIES SHEN US\ AAC ACOUSTIC TECHNOLOGIES CHAN (CN) | H01R43/20\ H01R43/02 |
| 66 | 3D printing | ULTRASONIC WELDING METHOD AND PRODUCT OF HIGH-MELTING METAL\ | CN101767249A | GUOLIANG SHI US | SHI GUOQING US | B23K20/10\ B23K20/24\ F28F1/12 |
| 67 | 3D printing | FULL AUTOMATIC ULTRASONIC WELDING MACHINE FOR PLASTIC PEN\ | CN101767456B | JINHUA LI US | WENZHOU DONGFENG JINGGONG ELECTRONIC INSTR FACTORY US | B29C65/08\ B29C65/78 |
| 68 | 3D printing | NEGATIVE PRESSURE WORKPIECE STABILIZING DOUBLE-SURFACE RAPID AND SYNCHRONOUS ULTRASONIC WAVE WELDING DEVICE\ | CN101774076A | MINGLIANG OU US\ RUKUN YANG US\ LINGZHONG ZENG US\ XINGTAO ZHONG US\ JINXUAN YAN (CN)\ ZHUOLI YAN (CN)\ ZHUOSHENG YAN (CN) | JINXUAN YAN (CN)\ ZHUOLI YAN (CN)\ ZHUOSHENG YAN (CN) | B23K20/10 |
| 69 | 3D printing | METHOD FOR WELDING POLYFLUORTETRAETHYLENE MEMBRANE BY ULTRASONIC WAVE ON MEDICAL APPLIANCE\ | CN101774278A | SHUANGJUN REN US | SHIJIAZHUANG NUOLIDA MEDICAL I US | B29C65/08 |
| 70 | 3D printing | HIGH-MAGNIFICATION CYLINDRICAL EXPLOSION-PROOF COMPOSITE CAP FOR LITHIUM ION BATTERY\ | CN101777634A | WEIBING ZHOU US | WEIBING ZHOU US | H01M2/04\ H01M2/08\ H01M2/12\ H01M2/22 |
| 71 | 3D printing | CFAST MEMORY CARD\ | CN101782973A | CHONGMING MA US\ ZHIQIANG QI US\ ZHANGMING XIE US | ITT IND CHINA INVEST CO LTD US | G06K19/077 |
| 72 | 3D printing | POLYPHENYL ETHER RESIN MATERIAL USED FOR PROCESSING CHARGER CASE\ | CN101792590A | WEN YUAN US\ HAITAO LI US\ JIAN LI US\ DALIAN MAO US\ YIWEI SUI US\ LIANHE WANG US | NANTONG XINGCHEN SYNTHETIC MATERIAL CO LTD US\ NANTONG ZHONGLAN ENGINEERING PLASTICS CO LTD US | C08L83/04\ C08L25/06\ C08L23/08\ C08L53/02\ C08L23/06\ C08L71/12\ C08K5/134\ C08K5/526 |
| 73 | 3D printing | MAHJONG AND METHOD FOR PRODUCING SAME\ | CN101804253A | YONGJIAN GUAN US | YONGJIAN GUAN US | A63F9/20 |
| 74 | 3D printing | LITHIUM ION CYLINDER BATTERY WELDING CLAMP AND USE METHOD THEREOF\ | CN101814593A | FENG CHEN US\ HUAHONG CHEN US\ JIAN HU US\ GUOFENG KAN US\ LIBIN SUN US | HUNAN GELIN NEW ENERGY CO LTD US | B23K20/10\ H01M2/26\ B23K20/26 |
| 75 | 3D printing | WELDING METHOD OF PLASTIC PARTS\ | CN101817232A | JIUYAN CUI US\ HONGXU FU US\ HAIREN JIANG US\ YU LI US\ ZHAOQI SHI US\ NIANYOU WANG US\ MING YI US\ YONG YU US\ DEXING ZHANG US | HARBIN DONGAN ENGINE GROUP CO US | B29C65/08 |
| 76 | 3D printing | FALCATE/AXE-SHAPED MASK SHELL FABRIC ULTRASONIC MAKING MACHINE\ | CN101828779A | QIONG WU US\ SHENGRONG WU US | SHANGHAI DASHENG SANITARY ARTICLE MFG CO LTD US | B32B37/00\ A41D13/11\ D06H7/00 |
| 77 | 3D printing | INTEGRAL FIGURE EIGHT-SHAPED SAND BLOCK MESH ASSEMBLING MACHINE\ | CN101838982B | XIE AILIANG US | XIE AILIANG US | E02D3/00 |
| 78 | 3D printing | HEMOPERFUSION CARTRIDGE\ | CN101843932A | ZHANGKAI LUO US\ JING TIAN US\ ZHANREN WANG US\ WEN ZHANG US | CHONGQING XI ERKANG BLOOD PURIFICATION EQUIPMENT RES & DEV CO LTD US | A61M1/36\ A61M1/00 |
| 79 | 3D printing | ULTRASONIC WELDING METHOD OF AL/TI DISSIMILAR METAL TIG (TUNGSTEN INERT GAS) ELECTRICAL ARC MICRO-MELTING BRAZING AND FOLLOWING WELDING\ | CN101850462A | JINLI CHENG US\ YONGXIAN HUANG US\ XIAOJUN JING US\ SHIXIONG LU US\ JIE QU US\ JINGWEI SHI US\ TAO YANG (US) | HARBIN INST OF TECHNOLOGY (CN) | B23K9/167\ B23K9/235\ B23K9/23\ B23K9/02\ B23K9/32 |
| 80 | 3D printing | WEAR-RESISTING ULTRASONIC WELDING MOULD AND MANUFACTURING METHOD THEREOF\ | CN101850469A | MIAOZHU LUO US\ JINXUAN YAN (CN)\ ZHUOLI YAN (CN)\ ZHUOSHENG YAN (CN) | JINXUAN YAN (CN)\ ZHUOLI YAN (CN)\ ZHUOSHENG YAN (CN)\ MIAOZHU LUO US | B23K20/10\ B23K20/26 |
| 81 | 3D printing | HAND BAG AND MOULD SEPARATING DEVICE\ | CN101850614A | ZHANGYIN CHAI US\ WEI CHEN (CN) | UNIV XIAMEN US\ XIAMEN CHUANGLING PACKAGING EQUIPMENT CO LTD US | B29C65/08 |
| 82 | 3D printing | LIGHT-EMITTING SEMICONDUCTOR CONNECTION STRUCTURE AND METHOD THEREOF\ | CN101859819A | FENGREN JIAN US\ XIMING PAN US | SHANDONG CANYUAN OPTO ELECTRONIC TECHNOLOGY CO LTD US | H01L33/00 |
| 83 | 3D printing | METHOD FOR BONDING COPPER WIRE OF POWER DEVICE\ | CN101862897B | LI XIPING US\ LIU WEIGUANG US\ YAN WEN US | SHENZHEN GUIHONGDA ELECTRONIC CO LTD US | H01L21/607 |
| 84 | 3D printing | METHOD FOR PREPARING ALUMINUM BASED INTELLIGENT COMPOSITE MATERIAL BY ULTRASONIC WELDING\ | CN101879656A | ZHENGQIANG ZHU US\ YIFU ZHANG US | UNIV NANCHANG US | B23K20/10\ B23K20/24 |
| 85 | 3D printing | SPECIAL TABULAR CONTACT ULTRASONIC WELDING EQUIPMENT FOR LOW-VOLTAGE ELECTRICAL APPLIANCE AND ULTRASONIC WELDING METHOD THEREOF\ | CN101905376A | LIQIANG LIU US\ WEI WENG US\ XIAOPING BAI US\ SHIHUA MU US | FUDA ALLOY MATERIALS CO LTD US | B23K20/10\ B23K20/26\ B23K20/24 |
| 86 | 3D printing | AUTOMATIC WELDING MACHINE OF ALUMINUM-NICKEL COMPOUND PIECE OF LITHIUM ION BATTERY COVER PLATE\ | CN101913029B | ZHOU JUN US | ZHOU JUN US | B23K28/02 |
| 87 | 3D printing | REUSABLE BAG AND MANUFACTURING METHOD THEREOF\ | CN101913454B | XIKUO LIU US | XIKUO LIU US | B65D30/10\ B31B29/00\ B65D33/08 |
| 88 | 3D printing | ALUMINUM COMPOSITE MATERIAL REINFORCED WITH CARBON NANO TUBES BY ULTRASONIC WELDING\ | CN101914735B | ZHENGQIANG ZHU US\ ZHILIN XIONG US | UNIV NANCHANG US | B23K20/10\ B23K20/24\ C22C47/02\ C22C49/06\ C22C101/10 |
| 89 | 3D printing | ULTRASONIC ROLL WELDING STAND\ | CN101920394A | MINGHONG HUANG US\ SHUINIAN DU US\ JUNJIE HUANG US\ YUCHENG ZHENG US | CONTREL TECHNOLOGY CO LTD (TW) | B23K20/10\ B23K20/26 |
| 90 | 3D printing | METHOD FOR SEALING PLAIN FILTER MEMBRANE COMPONENT\ | CN101940881A | GUOMING LIANG US\ FULE LOU US\ GUOYING TONG US | SHANGHAI SINAP MEMBRANE SCIENCE AND TECHNOLOGY CO LTD US | B01D69/06 |
| 91 | 3D printing | MULTIFUNCTIONAL AUTOMATIC ROTARY TABLE MACHINE\ | CN101947857A | QIFENG TAN US | QIFENG TAN US | B29C65/08 |
| 92 | 3D printing | PRODUCTION METHOD FOR WELDING HEAD COVER ASSEMBLY OF WASHING MACHINE BY UTILIZING ULTRASONIC WAVES\ | CN101954741B | JIANCHANG SHAO US | WUXI XINCHANG PLASTIC MACHINERY FACTORY US | B29C65/08 |
| 93 | 3D printing | BAGGED SPRING MACHINE CAPABLE OF CONTINUOUSLY ROLLING\ | CN101955150A | MINGLIANG OU US\ LINGZHONG ZENG US\ YANMIAO HE US\ ZHUOSHENG YAN (CN) | ZHUOSHENG YAN (CN)\ YAN ZHUOLI (CN)\ YAN JINXUAN (CN) | B68G9/00 |
| 94 | 3D printing | HIGH-STRENGTH COMPOSITE GRID\ | CN101956350A | WENPING LI US\ WEIFENG JIANG US | YIXING HUATAI EARTHWORK MATERIAL CO LTD US | E02D17/20\ E01C3/00\ E01C11/16 |
| 95 | 3D printing | MANUFACTURE METHOD FOR INTEGRAL SPLAYED SAND BLOCKING NET\ | CN101961918A | AILIANG XIE US | AILIANG XIE US | B29L28/00\ B29C65/08 |
| 96 | 3D printing | WATERPROOF SOCKET\ | CN101976784A | LIANG JIN US | LIANG JIN US | H01R13/52 |
| 97 | 3D printing | EMBEDDING RIVETING BRACKET FOR CONNECTING AUTOMOBILE INTERIOR INSTRUMENT BOARD WITH SAFE AIR BAG CABIN\ | CN101979263A | YI BAO US | NINGBO MINGFEI MOULD PLASTICS CO LTD US | B60R21/205 |
| 98 | 3D printing | FURNITURE FASTENER\ | CN101979885A | ZHIYONG WU US | ZHIYONG WU US | F16B12/10 |
| 99 | 3D printing | PRODUCTIVE TECHNOLOGY OF COMPOSITE EPOXY RESIN COMPOUND TYPE CABLE BRIDGE\ | CN101985991A | HUAISHU XU US | HUAISHU XU US | B29C45/76\ F16L3/00 |
| 100 | 3D printing | REINFORCED CONCRETE INTERNAL MOLD WITH MULTI-DIRECTION FIXED SUPPORTS\ | CN101988309A | RONG LIN US | RONG LIN US | E04B5/36\ E04C1/00\ E04G15/06 |
| 101 | 3D printing | POLYPROPYLENE OVERALL FOAMING FLAT-PLATE SOLAR COLLECTOR\ | CN102022842A | CAILIANG WEI US | ZHEJIANG MEIDIYA NEW ENERGY TECHNOLOGY CO LTD US | F24J2/46\ F24J2/20 |
| 102 | 3D printing | POWER SUPPLY CHANGEOVER BOX OF VEHICLE-MOUNTED WIRING HARNESS\ | CN102036528A | YUANTAO HE US\ FUTAO CUI US | DONGGUAN SHIJIEZHIYE ELECTRONICS CO LTD US | H05K5/06 |
| 103 | 3D printing | POLYETHYLENE TEREPHTHALATE COMPOSITE MATERIAL AND METHOD FOR PREPARING SAME\ | CN102040809A | YONG XU US\ HANG SUN US\ DONG XU (CN) | POLYMER SCI SHENZHEN NEW MAT US | C08K3/26\ C08K5/098\ C08K3/34\ C08L51/04\ B29C47/92\ C08L51/06\ B29B9/06\ C08L67/02\ C08K9/06\ C08K13/06 |
| 104 | 3D printing | METHOD FOR CONNECTING POLE LUG OF LITHIUM ION BATTERY WITH POLE POST AND BATTERY\ | CN102054956A | WANLI FANG US\ KANG WU US\ FUJIE LIAO US | SHENZHEN BAK BATTERY CO LTD (CN) | H01M2/26\ H01M10/0585\ H01M10/0525 |
| 105 | 3D printing | ASSEMBLY METHOD OF ELECTRONIC DEVICE\ | CN102056473A | XIANGSEN ZHANG US\ LIYING CHEN US | HUANXU ELECTRONICS CO LTD US\ HUANHONG SCIENCE & TECHNOLOGY CO LTD US | H05K5/02\ H01R4/02\ H05K13/04 |
| 106 | 3D printing | PURIFICATION WELDING MACHINE FOR POSITIVE PLATE OF LITHIUM CELL\ | CN102059447B | ZHOU JUNXIONG US\ ZHOU JUNHAO US\ ZHOU JUNJIE US | ZHOU JUNXIONG US | B23K20/10\ B23K20/26 |
| 107 | 3D printing | LED LAMP PANEL AND MANUFACTURING METHOD THEREOF\ | CN102062367A | MINHANG ZHANG US | XIANGSHAN ZHONGXIANG ELECTRONIC TECHNOLOGY CO LTD US | F21Y101/02\ B23K20/10\ F21V19/00 |
| 108 | 3D printing | MOULDING METHOD OF SINGLE-PIECE MATERIAL PACKAGE BOX OF FLUID FOOD AND BEVERAGE FLEXIBLE PACKAGE BOX\ | CN102085729A | BAOLUO LIU US\ WENKANG ZHANG US\ JIANZHEN LI US\ JINGDONG YU US | QINGDAO RENMIN PRINTING CO LTD US | B31B17/14\ B31B17/26\ B31B17/60 |
| 109 | 3D printing | LINEAR ACTUATOR ASSEMBLING MECHANISM WITH SELF-LOCKING FUNCTION\ | CN102088222B | XINAN JIANG US\ DONGXING CHEN US\ TAIHONG LIN US\ SHIYUAN ZENG US\ GUOEN ZENG US | HIWIN MIKROSYSTEM CORP US | H02K7/06\ F16H25/20 |
| 110 | 3D printing | HIGH-POWER MODULE WITH NOVEL PACKAGING STRUCTURE\ | CN102097416A | ZHEN LV US\ XIAOHANG JIN US | JIAXING SIDA ELECTRONICS CO LTD US | H01L23/48\ H01L25/00\ H01L23/04\ H01L23/488 |
| 111 | 3D printing | HIGH-PERFORMANCE MOTORCYCLE BATTERY AND MANUFACTURING METHOD THEREOF\ | CN102097625A | XIAOMENG PENG US\ FANG YUAN US\ TIANBAO LIANG US\ BO YIN US | JIANGSU LEOCH BATTERY CO LTD US | H01M2/30\ H01M10/12\ H01M4/68\ B62M6/90\ H01M10/14 |
| 112 | 3D printing | FULL-AUTOMATIC OVERSLEEVE MANUFACTURING MACHINE\ | CN102100418A | DAOQI BIE US | XIANTAO DAOQI PLASTIC CO LTD US | A41H43/04\ A41D27/10 |
| 113 | 3D printing | METHOD FOR CONNECTING SOLAR BATTERY SHEETS\ | CN102122681A | KENGO NAKANO US | LDK SOLAR CO LTD US | B23K20/10\ H01L31/0224\ H01L31/18\ B23K20/24\ H01L31/05 |
| 114 | 3D printing | FULL-AUTOMATIC DOUBLE-HEAD ULTRASONIC PLASTIC BOTTLE CAP WELDING DEVICE\ | CN102126287A | JIUPING LIU US\ MINGLIANG ZHANG US\ JUNGANG HUO US\ CHUNLONG ZHANG US | SHANDONG LIPENG CO LTD US | B29C65/08\ B29L31/56 |
| 115 | 3D printing | TUBE-TYPE SLAB LATTICE PASTE EXTRUSION PRODUCTION LINE\ | CN102136571B | WANG FUQUAN US\ WANG SHOUBAO US | ZIBO XINXU POWER SUPPLY TECHNOLOGY CO LTD US | H01M4/20 |
| 116 | 3D printing | METHOD FOR MANUFACTURING LITHIUM-IRON DISULFIDE BATTERY ANODE STRUCTURE\ | CN102142529A | QINGSHUN ZHANG US\ JIANXING LIN US\ HAITAO CHANG US\ SHENG SU US\ JINTIAN CHEN US | FUJIAN NANPING NANFU BATTERY US | B23K20/10\ H01M2/26 |
| 117 | 3D printing | ULTRASONIC WELDING DEVICE WITH QUALITY CONTROL FUNCTION\ | CN102151978A | XIANGGUO LIU US | XIANGGUO LIU US | B23K20/10 |
| 118 | 3D printing | FIBER REINFORCED INORGANIC-SUBSTANCE DOPED PERFLUORINATED PROTON EXCHANGE MEMBRANE\ | CN102153827A | SHUFANG YU US | SHUFANG YU US | C08K3/00\ C08J5/22\ H01M2/16\ H01M8/10\ H01M8/02\ C08L27/18\ C08K7/02 |
| 119 | 3D printing | SPECIAL ULTRAHIGH-POWER RECTIFICATION POWER ELECTRONIC DEVICE MODULE FOR ULTRASONIC WELDING MACHINE\ | CN102163928A | XUHAI YANG US\ LEI YANG US\ SHANYI YANG US | CHANGZHOU XIZHENG ELECTRONIC TECHNOLOGY CO LTD US | H01L23/14\ H01L23/373\ H01L25/07\ H02M7/06 |
| 120 | 3D printing | SPEED-REDUCING MOTOR AND METHOD FOR REGULATING MOTOR SHAFT EXTRUSION OF SPEED-REDUCING MOTOR\ | CN102170197B | GAO DONG US\ HAN GANG US\ LIU SHUN US\ XIE DEZHI US\ LU ZHIJUN US | BYD CO LTD (CN) | H02K15/00 |
| 121 | 3D printing | MANUFACTURING METHOD AND EQUIPMENT OF UNEQUAL-SECTION BIMETAL TRANSMISSION PART\ | CN102179637A | JIAN LUO US | JIAN LUO US | B23K37/00\ B23K31/00 |
| 122 | 3D printing | FULL-AUTOMATIC ULTRASONIC WELDING MACHINE FOR LITHIUM ION BATTERY CELL MULTI-LAYER LUG\ | CN102189328A | AIGUO ZHANG US\ LIELONG WEN US\ DENGHONG LUO US\ KAIGUI CAI US | AMPEREX TECHNOLOGY LTD US\ NINGDE NEW ENERGY TECHNOLOGY CO LTD US | B23K20/10\ B23K20/26 |
| 123 | 3D printing | STAINLESS STEEL NOZZLE OF HANGING-IRONING CLEANER\ | CN102191679B | HUANG JIAN US\ ZENG XIANGHE US\ LIU HUAYONG US\ LIANG CHAOHUA US | MIDEA GROUP CO LTD US | B05B1/14\ D06F75/06\ B05B1/12\ D06F75/22 |
| 124 | 3D printing | FILTER ELEMENT FOR ENVIRONMENT-FRIENDLY OIL FILTER AND MANUFACTURING PROCESS THEREOF\ | CN102198343B | WU JIESHU US | BENGBU PHOENIX FILTER CO LTD US | B01D29/13\ B32B5/06 |
| 125 | 3D printing | CURRENT GUIDER OF HIGH-POWER AND LARGE-CAPACITY CAPACITANCE BATTERY\ | CN102201561A | JIANG CHEN US\ LIANKUAN XU US\ XIAN MA US\ LI ZHANG US\ YI XIE US | ZHONGDA IND GROUP CO LTD US\ GUANGZHOU JIELI NEW ENERGY TECHNOLOGY CO LTD US | H01M2/26 |
| 126 | 3D printing | PREPARATION TECHNOLOGY OF HIGH STRENGTH NET FALSE ROOF FOR UNDERGROUND COAL MINING\ | CN102206378A | YING LIU US\ QINGSHAN TIAN US | HENGSHUI BAOLI ENGINEERING PLASTIC CO LTD US | B29C69/00\ C08L23/12\ B29C47/92\ B29C65/08\ E21D19/00 |
| 127 | 3D printing | METHOD FOR PROCESSING LUGS OF POLYMER SOFT-PACKAGING LITHIUM BATTERY\ | CN102208687A | BIN YANG US\ MING PAN US\ CONGMEI ZHANG US | JIANGSU CEL BATTERY CO LTD US | H01M2/26\ H01M10/058 |
| 128 | 3D printing | PAPER FILTER ELEMENT PAPER-JOINTING PROCESS\ | CN102225633A | YU ZHANG US | ZHEJIANG WEITAI AUTO PARTS CO LTD US | B31F5/06 |
| 129 | 3D printing | ULTRASONIC BONDING METHOD AND SPECIAL ULTRASONIC WELDING MACHINE USED FOR OPTOELECTRONIC DEVICE PACKAGING\ | CN102225839B | ZHANG JIANHUA US\ LI MAOYU US\ LAI YUNENG US\ HUANG YUANHAO US\ CHEN ZUNMIAO US | UNIV SHANGHAI US | C03B23/24\ H01L51/56 |
| 130 | 3D printing | ENHANCED FAUCET AND PREPARATION METHOD THEREOF\ | CN102226479A | SHUI YU US\ JINSHU ZHANG US\ LING CHEN US\ XIANMING HUANG US\ HUAMEI LIN US\ MINGREN LI US | XIAMEN RUNNER INDUSTRY CO LTD US | F16K27/00 |
| 131 | 3D printing | METHOD AND EQUIPMENT FOR MANUFACTURING GRADIENT MATERIAL MOULD\ | CN102240860B | WANG YING US\ LUO JIAN US | LUO JIAN US | B23K20/10\ B23K28/02\ B23K9/095\ B23K9/32\ B23K9/08\ B23K9/16 |
| 132 | 3D printing | METHOD AND EQUIPMENT FOR MANUFACTURING GRADIENT FUNCTIONAL STRUCTURE\ | CN102240861A | JIAN LUO US\ YING WANG US\ KELIANG XUE US\ JUNFENG XIANG US\ FEI LI US\ XIAOMING WANG US\ DEJIA LIU US | UNIV CHONGQING US | B23K20/10\ B23K28/02\ B23K9/32\ B23K9/08\ B23K9/16 |
| 133 | 3D printing | METHOD FOR ULTRASONICALLY WELDING BLOCK AMORPHOUS ALLOY MATERIAL\ | CN102248275A | ZHENGQIANG ZHU US\ YONG LIU US\ XIAOLONG WANG US | UNIV NANCHANG US | B23K20/10 |
| 134 | 3D printing | No Title Available\ | CN102284215A | Empty Field US | FORD GLOBAL TECH INC (US) | B01D46/02 |
| 135 | 3D printing | DOUBLE-ZERO ALUMINUM FOIL CONNECTOR WELDING METHOD\ | CN102284799B | JIA JIANXIN US\ LIU WENZHONG US | DARE TECHNOLOGY GROUP US | B23K31/02\ B23K35/362 |
| 136 | 3D printing | ULTRASONIC INNER SHEET MANUFACTURING MACHINE\ | CN102303409A | YAMIN SHI US | WUXI LINGSHAN JOSS STICKS ART CRAFT PRODUCT CO LTD US | B29C65/08\ B42F5/02 |
| 137 | 3D printing | KEY\ | CN102306579A | JINZHANG HUANG US | HUIZHOU CITY DESAY XIWEI AUTOMOTIVE ELECTRONICS CO LTD US | H01H13/14 |
| 138 | 3D printing | PRODUCTION TECHNIQUE OF HIGH-BARRIER ULTRASONIC WELD FOR DISPOSABLE MEDICAL PROTECTIVE CLOTHING\ | CN102309081A | MIN TANG US\ HUANLONG ZHANG US\ CHAOQUN YANG US\ HAISU FANG US | SHIYUAN TECHNOLOGY JIAXING MEDICAL ELECTRONICS CO LTD US | A41H43/04 |
| 139 | 3D printing | TECHNOLOGY OF IMPLANTING RFID CHIP INTO SOLAR MODULE\ | CN102315322A | XIE LI US\ JUAN GAO US\ QICONG CHEN US\ ZHAOTING LI US | DONGXU GROUP CO LTD US\ CHENGDU TAIYISI SOLAR TECH CO US | H01L31/18\ H01L31/20 |
| 140 | 3D printing | UPPER-LOWER SHELL WELDING STRUCTURE APPLIED TO ELECTRONIC PRODUCT\ | CN102316689A | YUHUA ZHANG US | TAIDEXING PREC ELECTRONIC KUNSHAN CO LTD US | H05K5/00\ H05K5/02 |
| 141 | 3D printing | NON-LEAD SEMICONDUCTOR LEAD FRAME ALUMINUM FOIL WELDING METHOD\ | CN102324391A | WUXIA XI US\ ZHENJIE XU US\ SHAOYONG TAO US\ SHENGQI CHEN US\ JIANGWEI LI US\ ZHOU CAO US | GREAT TEAM BACKEND FOUNDRY DONG GUAN LTD US | H01L21/48 |
| 142 | 3D printing | FUSE AND LITHIUM ION SECONDARY BATTERY WITH SAME\ | CN102332373A | TING TAN US\ PINGHUA DENG US\ YANHUA LU US\ QUANKUN LI US | AMPEREX TECHNOLOGY LTD US\ DONGGUAN AMPEREX TECH LTD US | H01M2/34\ H01H85/22\ H01H85/20 |
| 143 | 3D printing | CHIP ATTACHING METHOD\ | CN102332506A | JINMING LI US | DONGGUAN WANFENG NANOMETER MATERIALS CO LTD US | H01L33/00 |
| 144 | 3D printing | CHIP ATTACHING METHOD\ | CN102332507A | JINMING LI US | DONGGUAN WANFENG NANOMETER MATERIALS CO LTD US | H01L33/00 |
| 145 | 3D printing | ULTRASONIC WELDING METHOD FOR PREPARING TITANIUM ALLOY STRUCTURE\ | CN102350583A | ZHENGQIANG ZHU US\ ZONGHUI WU US\ QIAN WANG US | UNIV NANCHANG US | B23K20/10\ B23K20/24\ B23K103/14 |
| 146 | 3D printing | AUTOMATION DEVICE FOR PRODUCING MEDICAL PRODUCTS\ | CN102358040A | FAFU GUO US\ HONGMING YU US | SUZHOU MEIER AUTOMATION EQUIPMENT CO LTD US | B29C65/08\ B29C65/78 |
| 147 | 3D printing | CRYSTAL AND NON-CRYSTAL ULTRASONIC WELDING METHOD\ | CN102407402A | ZHENGQIANG ZHU US\ YONG LIU US\ CHENYANG XIE US | UNIV NANCHANG US | B23K20/10 |
| 148 | 3D printing | SOLAR TERMINAL BOX\ | CN102420266A | ZEPENG CHEN US | DONGGUAN ZERUN ELECTRONIC TECHNOLOGY CO LTD US | H02S40/34 |
| 149 | 3D printing | INFRARED PHOTOELECTRIC SWITCH SHELL\ | CN102426947B | DAI BAITE US\ ZHENG XIAOFENG US\ ZHANG NANNAN US\ CHEN WEI (CN) | ESPROS CHANGSHU SENSOR CO LTD US | H01H9/02 |
| 150 | 3D printing | CLOTHES HANGER AND MANUFACTURING METHOD THEREOF\ | CN102440656A | WEIJUN GUO US | ZHONGSHAN WEI JIA PLASTIC PRODUCTS CO LTD US | B29C65/08\ A47G25/28 |
| 151 | 3D printing | VACUUM GLASS SEALING METHOD AND PRODUCT THEREOF\ | CN102452801A | YANBING LI US\ ZHANGSHENG WANG US | LUOYANG LANDGLASS TECH CO LTD US | C03C27/08 |
| 152 | 3D printing | LEAD FOR ULTRASONIC WELDING AND PREPARATION METHOD THEREOF\ | CN102456432B | XIE LIBO US\ FU XIAODAN US | JIAXING TITION ELECTRONIC WIRE CO LTD US | H01B7/02\ H01B3/44\ H01B13/14 |
| 153 | 3D printing | ABS (ACRYLONITRILE BUTADIENE STYRENE) MATERIAL WITH ULTRASONIC WELDING CAPABILITY\ | CN102464858A | WEIQIN ZHANG US\ BAOHAI MA US\ JIANRONG ZHANG US | WEIQIN ZHANG US | C08K13/04\ C08K3/22\ C08K7/06\ C08L55/02 |
| 154 | 3D printing | JUMPING-BEAN TOY\ | CN102475970A | ZHONGMING LIANG US | DONGGUAN LC TECHNOLOGY CO LTD US | A63H11/06 |
| 155 | 3D printing | ROTARY DISPENSING DEVICE FOR PRODUCING MEDICAL DROPPING BOTTLE\ | CN102489430A | YANHAI XIANG US | SUZHOU HUARI JINLING MACHINERY CO LTD US | B05C11/10\ B05C13/02\ B05C5/02 |
| 156 | 3D printing | MICROPART POWDER COMPRESSION MOLDING METHOD\ | CN102490301A | XIAOYU WU US\ TAIJIANG PENG US\ XIONG LIANG US\ RONG CHENG US\ FENG LUO US\ GUIJIAN HUANG US | UNIV SHENZHEN US | B29C43/36\ B29C43/52\ B29C43/02\ B22F3/03 |
| 157 | 3D printing | POLE PIECE AND POLE TAB WELDING METHOD AND DEVICE\ | CN102500925A | RUKUN YANG US\ LINGZHONG ZENG US | SHENZHEN GEESUN AUTOMATION TECHNOLOGY CO LTD US | B23P17/00\ B23K26/21\ B23K26/70 |
| 158 | 3D printing | METHOD FOR MANUFACTURING SHAPE MEMORY ALLOY-ALUMINUM METAL MATRIX COMPOSITE MATERIAL THROUGH ULTRASONIC WELDING\ | CN102501006A | YIQING WANG US\ WANHUA ZHAO US\ YUGANG DUAN US\ YAXIONG LIU US\ BINGHENG LU US | UNIV XI AN JIAOTONG (CN) | B23K20/10\ B23P15/00\ C23C28/00\ B23K20/24\ C22F1/04\ C23C14/18\ C23C22/64 |
| 159 | 3D printing | PRODUCTION METHOD FOR PAPER PACKAGING CONTAINER USED FOR FOOD BY ULTRASONIC WELDING\ | CN102501403A | CHUNHUA CAI US | SHANGHAI JINYE PACKAGING MATERIAL CO LTD US | B31B1/60 |
| 160 | 3D printing | ULTRASONIC WELDING HEAD ULTRASONIC WELDING EQUIPMENT PROVIDED WITH SAME AND METHOD FOR WELDING BATTERY POLES BY UTILIZING ULTRASONIC WELDING EQUIPMENT\ | CN102513686A | RENZHI HUANG US\ YU ZENG US | SUZHOU GOLDEN CROWN NEW ENERGY US\ GOLDEN CROWN NEW ENERGY HONGKONG CO LTD US | B23K20/10 |
| 161 | 3D printing | ULTRASONIC WELDING PROCESS OF MULTI-BENDING PRODUCT\ | CN102554452A | ZHIQIANG CAO US | WUXI NICLE ULTRASONIC EQUIPMENT LTD COMPANY US | B23K20/10 |
| 162 | 3D printing | ULTRASONIC WELDING EQUIPMENT FOR POROUS SOUND ATTENUATION AIR INLET TUBE OF NON-WOVEN FABRIC HELICAL AIR FILTER\ | CN102555212A | JINSONG XIAO US\ BINGRONG LIU US\ YI ZHANG US | JIANGSU XINGCHENXING AUTO ACCESSORIES CO LTD US | B29C65/08 |
| 163 | 3D printing | MANUFACTURING METHOD FOR PLASTIC TAP FAUCET BODY AND PRODUCT\ | CN102555219A | ZHAODA YANG US | SHENZHEN GLOBE UNION IND CORP US | B29L31/00\ B29C69/00 |
| 164 | 3D printing | HIGH-RELIABILITY HIGH-POWER INSULATED GATE BIPOLAR TRANSISTOR (IGBT) MODULE\ | CN102569271A | ZHEN LV US | JIAXING STARR MICROELECTRONICS CO LTD US | H01L25/00\ H01L23/00 |
| 165 | 3D printing | AUTOMATIC CHEF CAP PRODUCTION LINE\ | CN102578738A | XIAOYIN ZHU US | SHANGHAI XUEYA INDUSTRY AND TRADE CO LTD US | A42C1/00\ A42C1/04\ A42C1/06 |
| 166 | 3D printing | WELD LINE PRESSURE PLATE\ | CN102581532B | SUN DEBO US\ XU XIA US\ LI NINGBO US | GOERTEK INC US | B23K20/10\ B23K37/04 |
| 167 | 3D printing | ULTRASONIC WELDING DEVICE USED FOR SOLAR ENERGY COLLECTING PANEL AND PROVIDED WITH WELDING MARK COATING MECHANISM\ | CN102601513A | GUOGONG GAN US\ GUANG LIU US\ SHENGYANG XU US\ DEWU CHEN US | GUOGONG GAN US | B05C11/10\ B23K20/10\ B05C5/00\ B23K20/26 |
| 168 | 3D printing | DEVICE FOR REALIZING TRANSMITTING AND CUTTING FOR HANDLE TAPE AND POSITIONING FOR BAG BODY OF NON-WOVEN HANDBAG\ | CN102602050A | YAZHOU CHEN US\ YINTAI GUI US\ TIANYI ZHENG US\ PI JUN US\ CAN WEI US\ JIANHUA SUN US\ SHUMIN XING US\ XIAOMING ZHANG US\ QUANMING ZHANG US | XIAMEN CYR INDUSTRY & TRADE CO LTD US | B31B1/86 |
| 169 | 3D printing | ULTRASONIC BONDING METHOD FOR J-SHAPED RULER\ | CN102615825A | XIUYING YANG US\ HUA CHEN US\ MINGYUAN ZHU US | NANCHANG NAVIATION GROUP CULTURAL AND SPORTS SUPPLIES CO LTD US | B29C65/08 |
| 170 | 3D printing | MANUFACTURING METHOD OF TAP\ | CN102644783A | ZHONGWEI SHU US | ZHONGWEI SHU US | B29C45/14\ B29C45/76\ B29K69/00\ F16K27/00 |
| 171 | 3D printing | BATTERY CORE LAMINATING MACHINE OF LITHIUM BATTERY AND MEMBRANE UNREELING DEVICE\ | CN102646849A | WEIXI WANG US\ HONGYAN WENREN US | NINGBO ZIYU AUTOMATION EQUIPMENT DEV CO LTD US | H01M10/058 |
| 172 | 3D printing | BATTERY CORE LAMINATION MACHINE OF LITHIUM BATTERY\ | CN102646851B | WANG WEIXI US\ WENREN HONGYAN US | NINGBO ZIYU AUTOMATION EQUIPMENT DEV CO LTD US | H01M6/14\ H01M10/058 |
| 173 | 3D printing | HIGH JUNCTION TEMPERATURE SEMICONDUCTOR CONTROLLABLE SILICON\ | CN102651391A | LIN WU US\ JIANYING PAN US\ YABING ZUO US | YIXING HUANZHOU MICRO ELECTRONICS CO LTD US | H01L23/31\ H01L23/495\ H01L29/74 |
| 174 | 3D printing | CONTAINING DEVICE AND MANUFACTURING METHOD THEREOF\ | CN102652641A | CHENGRUI MA US | CHENGRUI MA US | A47G29/00\ A45C7/00\ A47G25/00\ A47G25/54 |
| 175 | 3D printing | METHOD FOR FORMING SINGLE-INTERMETALLIC COMPOUND WELDING SPOTS OF MULTILAYER STACKED CHIPS IN LOW-TEMPERATURE ULTRASONIC BONDING MODE\ | CN102672296A | WEI LIU US\ YANHONG TIAN US\ LINGCHAO KONG US\ CHUNQING WANG US | HARBIN INST OF TECHNOLOGY (CN) | H01L21/60\ H01L21/607\ B23K1/06\ B23K1/20 |
| 176 | 3D printing | WELDING PROCESS OF MULTILAYER FOIL TYPE COILS OF RESONANCE-ELIMINATING COMPENSATION TRANSFORMATION DEVICE\ | CN102672338A | YUELIN WANG US\ LIPING ZHOU US | JIANGSU UONONE ELECTRIC AUTOMATION CO LTD US | B23K20/10 |
| 177 | 3D printing | LONGITUDINAL ULTRASONIC WELDING DEVICE FOR POWER BATTERY ELECTRODES AND WORKING METHOD OF LONGITUDINAL ULTRASONIC WELDING DEVICE\ | CN102672339A | GUANGWEI LEI US | GUANGWEI LEI US | B23K20/10 |
| 178 | 3D printing | GOLD WIRE BALL ULTRASONIC WELDING PROCESS DIRECTLY PERFORMED ON ALUMINUM PRODUCT\ | CN102672340A | GUANG LI (CN) | GUANG LI (CN) | B23K20/10\ B23K20/24 |
| 179 | 3D printing | METHOD FOR WELDING COPPER WIRE AND ALUMINUM WIRE\ | CN102684035A | KENG XIAO US\ ZHENGGUO ZHOU US\ HAIFENG ZENG US\ YIN HUANG US | SHENZHEN CLICK TECHNOLOGY LTD US\ XINFENG CLICK TECHNOLOGY CO LTD US\ HUIZHOU CLICK TECHNOLOGY CO LTD US | H01R43/02 |
| 180 | 3D printing | No Title Available\ | CN102699518B | Empty Field US | FEDERAL MOGUL POWERTRAIN INC US | B23K20/10\ H01M2/26 |
| 181 | 3D printing | SEMI-AUTOMATIC ULTRASONIC SPOT WELDING MACHINE\ | CN102700134A | WEI SU US | SUZHOU KEBER PREC MACHINERY CO US | B29C65/08 |
| 182 | 3D printing | ULTRASONIC WELDING DEVICE FOR POLYMER MICRO-COMPONENT AND WELDING METHOD OF DEVICE\ | CN102700137A | YI LUO (CN)\ XIAODONG WANG (CN)\ YIBO SUN (CN) | UNIV DALIAN JIAOTONG US | B29C65/08 |
| 183 | 3D printing | HOUSING AND METHOD FOR MANUFACTURING THE SAME\ | CN102740618A | ZHIRONG CHEN US | INVENTEC CORP US | B29C45/00\ H05K5/00\ B29C65/08 |
| 184 | 3D printing | MULTI-UNIT ULTRASONIC WELDING MACHINE\ | CN102744881A | WEI SU US | SUZHOU KEBER PREC MACHINERY CO US | B23K20/10\ B29C65/08 |
| 185 | 3D printing | ULTRASONIC WELDING METHOD SUITABLE FOR ELECTRIC ENERGY METER CALIBRATION SEAL\ | CN102744882A | XINRONG XU US | HUZHOU XINRONG ELECTROMECHANICAL EQUIPMENT MFG CO LTD US | B29C65/08 |
| 186 | 3D printing | FULL-AUTOMATIC WELDING MACHINE FOR PRODUCING LIGHTER\ | CN102773602A | JIABAO BAI US\ XINYA LIANG US\ ZENGGUO WANG US | LANGXI DONGHAI PHOTOELECTRIC TECHNOLOGY CO LTD US | B23K20/10\ B23K20/26 |
| 187 | 3D printing | ULTRASONIC WELDING DEVICE SUITABLE FOR MAGNETIC LATCHING RELAY\ | CN102785021A | XIAODONG WAN US\ ZIYOU ZHANG US | YUEQING LIHUI ELECTRICAL APPLIANCE CO LTD US | B23K20/10\ B23K20/26 |
| 188 | 3D printing | GRABBING MECHANISM OF ULTRASONIC WELDING DEVICE OF MAGNETIC LATCHING RELAY\ | CN102785022A | XIAODONG WAN US\ ZIYOU ZHANG US | YUEQING LIHUI ELECTRICAL APPLIANCE CO LTD US | B23K20/10\ B23K20/26 |
| 189 | 3D printing | ULTRASONIC BATTEN MACHINE\ | CN102785360A | LIPING XIA US | BENGBU HUAHONG ELECTRONIC TECHNOLOGY CO LTD US\ ANHUI BENGBU DEMO FILTRATION TECHNOLOGY CO LTD US | B29C65/08 |
| 190 | 3D printing | ULTRASONIC WELDING DEVICE AND PRODUCTION METHOD THEREOF FOR WELDING AUTOMOBILE PLAQUE\ | CN102794906A | HAO ZHANG US\ WEIQING WANG US\ LIMIN QIN US\ GUOJIAN TANG US | CHANGSHU AUTOMOTIVE TRIM CO LTD US | B29C65/08 |
| 191 | 3D printing | MANUFACTURE METHOD OF ENVIRONMENTAL-FRIENDLY PACKAGING BODY\ | CN102795388A | WEIBING DING US | ZHONGSHAN JIULI PACKAGING MATERIAL CO LTD US | B65D30/02 |
| 192 | 3D printing | SIGNAL LAMP\ | CN102798011A | GUOLIANG WANG US\ JIANYU ZHAO US\ WENLI JIANG US | SHANGHAI UPUN INDUSTRY CONTROL SYSTEM CO LTD US | F21S2/00\ F21V17/12\ F21V23/06 |
| 193 | 3D printing | LIGHT-EMITTING DIODE WITH INVERTED CHIP AND MANUFACTURING METHOD OF LIGHT-EMITTING DIODE\ | CN102800778A | WEIYUN WANG US\ YONGDE LI US | DONGGUAN FORTUNE ELECTRONIC MATERIAL CO LTD US | H01L33/62\ H01L33/00\ H01L33/42\ H01L33/48 |
| 194 | 3D printing | SAND FILLED DUMBBELL PIECE\ | CN102814023A | GUAN YONGZHONG US | NANTONG IRONMAN SPORTING IND CO LTD US | A63B21/075 |
| 195 | 3D printing | THREE-STATION ULTRASONIC WELDING MACHINE FOR FRONT BUMPER\ | CN102814984A | SU WEI US | SUZHOU KEBER PREC MACHINERY CO US | B29C65/08 |
| 196 | 3D printing | METHOD FOR FIXING BUS TAPE (STRIP) ON BZO (BORON-DOPED ZINC OXIDE) THIN FILM OF THIN FILM SOLAR MODULE\ | CN102825379A | NI JIANXIONG US\ HUANG YUELONG US\ WANG YALI US\ MA YUNXIANG US\ WANG XIAOLI US\ LIU XING US | BAODING TIANWEI SOLARFILMS CO LTD US | B23K20/10\ H01L31/18 |
| 197 | 3D printing | WELDING-FREE OPTICAL FIBER ADAPTER\ | CN102830468A | ZHANG CHUANLING US | ZHANG ZUANXING US | G02B6/38 |
| 198 | 3D printing | NOVEL PLASTIC GRID PRODUCTION DEVICE\ | CN102848591A | LIU BAOYU US\ LIU MING US\ ZHANG HUIDI US | YIXING CITY MINGHUI PLASTIC PRODUCTS CO LTD US | B29D28/00 |
| 199 | 3D printing | PACKAGING METHOD OF VERTICAL SENSOR\ | CN102849674A | DING LIGUO US\ WANG PING US | HANGZHOU SILAN INTEGRATED CIRCUIT CO LTD US | B81C1/00 |
| 200 | 3D printing | NEW PLASTIC GRILLE\ | CN102852062A | LIU BAOYU US\ LIU MING US\ ZHANG HUIDI US | YIXING CITY MINGHUI PLASTIC PRODUCTS CO LTD US | E01C3/04 |
| 201 | 3D printing | FOUR-HEAD ULTRASONIC WELDING EQUIPMENT FOR POSITIVE AND NEGATIVE POLES OF POWER BATTERY\ | CN102861984A | LEI GUANGWEI US | LEI GUANGWEI US | B23K20/10\ B23K20/26 |
| 202 | 3D printing | ULTRASONIC WELDING MACHINE WITH ROBOT\ | CN102862292A | SU WEI US | SUZHOU KEBER PREC MACHINERY CO US | B29C65/08 |
| 203 | 3D printing | THREE-AXIS LINKAGE ULTRASONIC WELDER\ | CN102862293A | SU WEI US | SUZHOU KEBER PREC MACHINERY CO US | B29C65/08 |
| 204 | 3D printing | FLAT PLATE COLLECTOR WITH CLOSED SNAKELIKE HEAT PIPE\ | CN102878696A | WANG CHAOSHENG US\ RUAN GUOCHENG US | ANHUI HAITAIKE NEW ENERGY TECHNOLOGY CO LTD US | F24J2/46\ F24J2/32\ F24J2/26 |
| 205 | 3D printing | FLAT PLATE COLLECTOR WITH HEAT PIPES\ | CN102878697A | WANG CHAOSHENG US\ RUAN GUOCHENG US | ANHUI HAITAIKE NEW ENERGY TECHNOLOGY CO LTD US | F24J2/46\ F24J2/32\ F24J2/26 |
| 206 | 3D printing | FLAT PLATE COLLECTOR WITH WORM PIPES\ | CN102878701A | WANG CHAOSHENG US\ RUAN GUOCHENG US | ANHUI HAITAIKE NEW ENERGY TECHNOLOGY CO LTD US | F24J2/20\ F24J2/32 |
| 207 | 3D printing | POSITIVE POLAR TAB AND NEGATIVE POLAR TAB WELDING INTEGRATED MACHINE FOR LITHIUM ION LAMINATED ELECTRIC CORE\ | CN102881857A | LIU LIN US\ LIU YONG US\ YANG SHIJUN US\ ZHANG YURU US\ SHEN CHENGLONG US | HUBEI CAMEL BATTERY RES INST CO LTD US | H01M2/26\ B23K37/04 |
| 208 | 3D printing | No Title Available\ | CN102896772B | Empty Field US | Empty Field US | B29C69/00\ B29C65/08 |
| 209 | 3D printing | NON-WOVEN FABRIC COVERING MACHINE FOR WATER DISCHARGING PLATE OR WATER DISCHARGING BELT\ | CN102897610A | WU FAZHOU US | WU FAZHOU US | B65H81/06 |
| 210 | 3D printing | FAST FORMER BASED ON LASER CUTTING OF SINGLE PIECE OF PAPER\ | CN102910481A | ZHANG HAIYAN US\ CHENG CHUANXIN US | UNIV XI AN TECHNOLOGY US | B65H35/10 |
| 211 | 3D printing | ULTRASONIC WELDING JIG OF T25 CELL CULTURE BOTTLE\ | CN102941673A | XU DONGLIANG US | VANGUARD TECHNOLOGY WUXI CO US | B29C65/08 |
| 212 | 3D printing | COMPLETE MULTICHANNEL WHITE BODY WELDING SPOT DETECTION SYSTEM\ | CN102944609A | CHENG AIGUO US\ HE ZHICHENG US\ HU CHAOHUI US\ SONG KAI US | HUNAN UNIVERSITY AISHENG AUTO TECHNOLOGY DEV CO LTD US | G01N29/04\ G01N29/44 |
| 213 | 3D printing | LITHIUM ION POWER BATTERY AND PROCESSING METHOD THEREOF\ | CN102945940A | YANG XUANKUN US | SHANDONG FOREVER NEW ENERGY CO LTD US | H01M2/26\ H01M10/058\ H01M10/0525 |
| 214 | 3D printing | DEVICE AND METHOD FOR ADDING SOLDER WIRES ON PLAIN FILMS OF METAL HONEYCOMB CARRIER\ | CN102950356A | CHEN XUEHONG US\ QIU XIAOZHEN US\ CHEN QUNHUI US | TAIZHOU OUXIN ENVIRONMENTAL PROT PURIFIER CO LTD US | B23K20/10\ B23K3/06 |
| 215 | 3D printing | MANUFACTURING METHOD FOR ELECTRODES OF CRYSTALLINE SILICON SOLAR CELL\ | CN102969406A | XU JIASHU US\ JIA YUNTAO US\ SHANGGUAN QUANYUAN US\ DING XINGLONG US | CHANGZHOU BITAI TECHNOLOGY CO LTD US | H01L31/0224\ H01L31/18\ B23K1/06 |
| 216 | 3D printing | LITHIUM ION BATTERY AND TAB AND POLE CONNECTING PIECES THEREOF\ | CN102969477B | SUN JING US\ YU YU US\ ZHANG YONGJIE US\ ZHANG GUOJUN US\ DU PEIPEI US | CHINA AVIAT LITHIUM BATTERY LUOYANG CO LTD US | Empty Field |
| 217 | 3D printing | CONNECTING POINT PLATE WELDING DEVICE\ | CN102975364A | DUAN HENG US | SUZHOU LING OU AUTOMATION EQUIPMENT CO LTD US | B29C65/08 |
| 218 | 3D printing | LIGHT-SHADING METHOD FOR CONTROLLING CHARACTER LIGHT-EMITTING AREA OF LIGHT GUIDE PANEL MADE OF TRANSPARENT ORGANIC MATERIAL\ | CN103000098A | ZENG WEIMING US\ XU JUN US\ XU HUACHANG US\ LI XIANZAO US | SHANGHAI AVIAT ELECTRIC CO LTD US | F21V8/00\ G09F13/04 |
| 219 | 3D printing | ULTRASONIC CLOTH SEWING METHOD\ | CN103005764A | SHEN JIANXIN US | JIANGSU EBUN CLEANING SUPPLIES CO LTD US | A41H43/04\ D06B1/02 |
| 220 | 3D printing | ELECTRONIC TAG MANUFACTURING METHOD\ | CN103020696A | LU HONGMEI US | SHANGHAI ZHENXIAN ELECTRONIC TECHNOLOGY CO LTD US | G06K19/07 |
| 221 | 3D printing | ROTARY SEALING MECHANISM OF BATTERY COMPARTMENT\ | CN103022397A | HAN ANMENG US\ YANG ZHONG US\ LIU ZHIQIANG US | SHENZHEN STAR INSTR CO LTD US | H01M2/10 |
| 222 | 3D printing | No Title Available\ | CN103028805B | Empty Field US | Empty Field US | B23K20/10\ H01Q1/22\ H01R43/02\ B23K20/00\ B23K1/06 |
| 223 | 3D printing | ULTRASONIC WELDING MACHINE\ | CN103029295A | SU WEI US | SUZHOU KEBER PREC MACHINERY CO US | B23K20/10\ B29C65/08 |
| 224 | 3D printing | WELDING METHOD OF CURRENT COLLATORS FOR HIGH-CAPACITY LITHIUM ION POWER BATTERIES\ | CN103042313A | LI LINGJUN US\ YIN DONGXING US | LISHEN MILES POWER BATTERY SYS US | B23K26/346 |
| 225 | 3D printing | VEHICLE NON-CONTACT ELECTRONIC SPEAKER ADOPTING ULTRASONIC WELDING TECHNOLOGY\ | CN103065617A | ZHAO HONGWEI US | HARBIN GOODTIME ELECTRONICS CO US | G10K9/12 |
| 226 | 3D printing | TRANSFORMER\ | CN103065767A | QU WENJUN US | NINGBO ZHONGCE E T ELECTRONICS CO LTD US | B29C65/08\ H01F27/02 |
| 227 | 3D printing | HIGH CAPACITY AND HIGH RATE TYPE SOFT-PACKAGE LITHIUM IRON PHOSPHATE BATTERY\ | CN103066321A | LIU YONGHUA US\ CAI HUISHENG US\ LI BING US\ HU XIAOZHUAN US\ FENG XIANGMING US\ ZHAO GUANGJIN US\ WANG GANG US | HUANYU NEW ENERGY TECHNOLOGY CO LTD US\ HENAN ELECTRIC POWER CORP ELECTRIC POWER RES INST US\ STATE GRID CORP CHINA US | H01M4/62\ H01M10/058\ H01M10/0525 |
| 228 | 3D printing | SEDAN QUARTER WINDOW ULTRASONIC HOT RIVETING AND WELDING INTEGRATED MACHINE\ | CN103071936A | PAN BAOPING US | WUHU XINBAO ULTRASONIC DEVICE CO LTD US | B23K20/10\ B23K28/02 |
| 229 | 3D printing | METHOD OF FLEXIBLE PUNCH HEAD ULTRASONIC MICRO DEEP DRAWING STEP-BY-STEP MOLDING AND DEVICE THEREOF\ | CN103084466A | LUO FENG US\ WU XIAOYU US\ ZHONG JINMING US\ ZHANG GUANJIN US\ LI KAIHUI US\ GU XIAOMENG US | UNIV SHENZHEN US | B21D33/00 |
| 230 | 3D printing | THERMAL RADIATION WELDING MACHINE OF AUTOMOBILE INSTRUMENT\ | CN103085271A | PAN BAOPING US | WUHU XINBAO ULTRASONIC EQUIPMENT CO LTD US | B29C65/02\ B29C65/08 |
| 231 | 3D printing | AUTOMATIC PROCESSING SYSTEM FOR ROLLED CLOTH\ | CN103086186A | YU CHUNMING US\ PAN XIUDONG US | MAXCLEAN WUXI TECHNOLOGY CO LTD US | B65H37/00\ B65H23/032\ B65H23/04 |
| 232 | 3D printing | ANTI-REFLUX STRUCTURE OF BLOOD TAKING NEEDLE\ | CN103126688A | XIONG YANFANG US | XIONG YANFANG US | A61B5/154 |
| 233 | 3D printing | PREPARATION METHOD AND STRUCTURE OF REFLECTIVE SIGN\ | CN103132472A | LI HONGZHEN US | TECHEYE OPTICS TECHOLOGIES CO LTD US | C08K3/22\ B29C69/00\ C08K3/36\ C08L69/00\ C08K7/14\ C08L55/02\ E01F9/06\ C08K7/10 |
| 234 | 3D printing | ULTRASONIC WELDING MACHINE\ | CN103143832A | PAN BAOPING US | WUHU XINBAO ULTRASONIC EQUIPMENT CO LTD US | B23K20/10\ B23K37/02\ B23K20/26 |
| 235 | 3D printing | MANUAL ULTRASONIC WELDING TOOL\ | CN103143833A | PAN BAOPING US | WUHU XINBAO ULTRASONIC EQUIPMENT CO LTD US | B23K20/10\ B23K20/26 |
| 236 | 3D printing | LIGHT DISTRIBUTION LENS FOR LED (LIGHT EMITTING DIODE) LAMP\ | CN103148444A | LIU GUOXUE US\ WANG BO US | CHANGZHOU TONGBAO LIGHTING CO LTD US | F21Y101/02\ F21V5/04\ F21V13/02\ F21W101/02 |
| 237 | 3D printing | LIQUID CRYSTAL TELEVISION SET HOUSING PROCESSING PROCESS\ | CN103158261A | ZHANG XIAOQIN US | WUXI DAYAN TECHNOLOGY CO LTD US | B29C69/02\ B29C65/08 |
| 238 | 3D printing | MOTORCYCLE LICENSE-PLATE LAMP\ | CN103171660A | XIAO HUIYU US | XIAO HUIYU US | B62J6/00 |
| 239 | 3D printing | TRIPLE-CORE-WIRE AUTOMATIC ASSEMBLING EQUIPMENT\ | CN103178424A | LIU LIANQIAO US\ WANG SHOUMING US\ XIN RUI US\ SUN XIANG US\ WANG CHI US | KUNSHAN KSTOPD AUTOMATION EQUIPMENT TECHNOLOGY CO LTD US | H01R43/20\ H01R43/00\ H01R43/052\ H01R43/05 |
| 240 | 3D printing | ULTRASONIC WELDING MACHINE\ | CN103192176A | KAN YANPING US\ SU YUMING US\ LIU JIA US | TIANJIN TAISHIKANG PHARMACEUTICALS SCIENCE TECHNIC CO LTD US | B23K20/10\ B29C65/08 |
| 241 | 3D printing | BACKWARD-INCLINING TYPE CENTRIFUGAL FAN BLADE\ | CN103195751A | GAO WENMING US\ HAN XIAOHONG US\ SHI JIANLI US | NINGBO LANGDI IMPELLER MACH CO US | F04D29/30 |
| 242 | 3D printing | LICENSE PLATE LAMP WITH LIGHT-EMITTING DIODE (LED) AUTOMATIC DIAGNOSTIC FUNCTION\ | CN103196088A | SU CHAOPIN US | JIANGSU NEW TIANYANG MACHINERY MFG CO LTD US | F21S8/10\ F21Y101/02\ F21W101/02\ F21V23/02 |
| 243 | 3D printing | SEX TOY\ | CN103202760A | ZHANG YIN US | SUZHOU GANTAI PREC MACHINERY CO LTD US | A61H23/02\ A61H19/00 |
| 244 | 3D printing | ULTRASONIC WELDING CHOPPER WITH MULTIPLE BONDING WIRE GROOVES\ | CN103219255A | LI SHANGZHE US | LI SHANGZHE US | H01L21/607 |
| 245 | 3D printing | EQUIPMENT FOR LAYING WELDING AND CUTTING ALUMINUM FOILS ON SURFACE OF CYLINDRICAL CORE DIE\ | CN103240889A | WANG LIMING US\ LI WEIPING US\ LIU MINGHONG US\ SUN BINGJUN US\ WU LEI US | RES INST PHYSICAL & CHEMICAL ENG NUCLEAR INDUSTRY US | B29C70/54\ B29C70/32 |
| 246 | 3D printing | DEVICE FOR FORMING TIN ELECTRODE WIRE ON SOLAR CELL SHEET\ | CN103258890A | LIU YONG US\ SHANGGUAN QUANYUAN US\ LI WEI (CN) | CHANGZHOU BITAI TECHNOLOGY CO LTD US | B23K1/06\ H01L31/05 |
| 247 | 3D printing | FLEXIBLE COMPOSITE TUBE CONNECTOR AND CONNECTION METHOD THEREOF\ | CN103267192A | GAO RUIMIN US\ ZHANG YONGQIANG US\ WANG KE US\ LI JIANDONG US\ LIU JIE US\ LI HUI US\ HE FEI US\ SI XIAOMING US\ NAN BEIBEI US | SHAANXI YANCHANG PETROLEUM GROUP RES INST US | F16L13/02\ F16L33/28 |
| 248 | 3D printing | BATTERY ASSEMBLY JIG OF ENERGY STORAGE POWER STATION MODULE\ | CN103273241A | FAN HAOJIE US\ TIAN YINGCHUN US | SHANDONG REALFORCE ENTPR CO US | B23K37/04\ H01M10/058 |
| 249 | 3D printing | ULTRASONIC WELDING STRUCTURE OF PLASTIC PLANETARY GEARBOX\ | CN103277489A | DENG SHUPING US | WUXI LONGSHENG TECHNOLOGY CO US | B29C65/08\ F16H57/02 |
| 250 | 3D printing | CROSS-FLOW FAN WELDING MACHINE\ | CN103286946A | ZHANG ZHI US | CHUZHOU CHENGYE MACHINERY MFG CO LTD US | B29C65/08 |
| 251 | 3D printing | WELDING UNIT USED FOR FOUR-AXIS ULTRASONIC WELDING MACHINE\ | CN103302394A | SU WEI US | SUZHOU KEBER PREC MACHINERY CO US | B23K20/10\ B29C65/08 |
| 252 | 3D printing | AUTOMATIC DIE CHANGING MECHANISM FOR FOUR-AXIS ULTRASONIC WELDING MACHINE\ | CN103302397A | SU WEI US | SUZHOU KEBER PREC MACHINERY CO US | B23K37/02\ B23K20/26 |
| 253 | 3D printing | AUTOMATIC SELECTION WORKBENCH FOR FOUR-SHAFT ULTRASONIC WELDING MACHINE\ | CN103302853A | SU WEI US | SUZHOU KEBER PREC MACHINERY CO US | B23K20/10\ B29C65/08 |
| 254 | 3D printing | ULTRASONIC WELDING HEAD ASSEMBLY AND ULTRASONIC WELDING MACHINE\ | CN103317223A | ZHANG XIUCHONG US\ YANG RUKUN (CN) | SHENZHEN GEESUN AUTOMATION TECHNOLOGY CO LTD US | B23K20/10\ B23K37/02\ B23K20/26 |
| 255 | 3D printing | FOUR-AXIS ULTRASONIC WELDING MACHINE\ | CN103317224A | SU WEI US | SUZHOU KEBER PREC MACHINERY CO US | B23K20/10\ B29C65/08 |
| 256 | 3D printing | AUTOMOBILE REAR BUMPER ULTRASONIC WELDING MACHINE\ | CN103317720A | SU WEI US | SUZHOU KEBER PREC MACHINERY CO US | B29C65/08 |
| 257 | 3D printing | AUTOMOBILE FRONT BUMPER ULTRASONIC WELDING MACHINE\ | CN103317721A | SU WEI US | SUZHOU KEBER PREC MACHINERY CO US | B29C65/08\ B26F1/00 |
| 258 | 3D printing | ULTRASONIC BIOLOGICAL BOX WELDING MACHINE\ | CN103317722A | SU WEI US | SUZHOU KEBER PREC MACHINERY CO US | B23K20/10\ B29C65/08 |
| 259 | 3D printing | ULTRASONIC WELDING PROCESS FOR ACOUSTIC WOOL OF ACOUSTIC PART ON AUTOMOBILE\ | CN103341970A | WANG HUA US | WUXI JIXING AUTO PARTS CO LTD US | B29C65/08\ B60R13/08 |
| 260 | 3D printing | VALVE CAPABLE OF CONTROLLING TWO WATER OUTLETS SIMULTANEOUSLY AND MANUFACTURING METHOD AND ASSEMBLY METHOD THEREOF\ | CN103343824A | SU KE US\ YE XIAODONG US\ ZHOU ZHENMING US | NINGBO DAYE GARDEN INDUSTRY CO LTD US | F16K11/08 |
| 261 | 3D printing | TEMPERATURE SENSOR IN ULTRASONIC WELDING MODE\ | CN103344353A | TAN LIGUO US\ SHI GUIYANG US | QUFU TEMB AUTO PARTS MFG CO US | G01K7/22 |
| 262 | 3D printing | ULTRASONIC WAVE WELDING STRUCTURE AND ULTRASONIC WAVE WELDING METHOD\ | CN103358015A | WANG BAISHENG US\ XU JUNXIANG US\ XIE QINGFENG US | ASKEY ELECTRONIC TECHNOLOGY JIANGSU CO LTD US\ ASKEY COMP CO LTD US | B23K20/10\ B23K33/00 |
| 263 | 3D printing | ULTRASONIC WELDING METHOD FOR WOOD-PLASTIC COMPOSITE\ | CN103358543A | ZHAO HUI US\ WANG QINGWEN US\ ZHU HONGYUAN US\ WANG WEIHONG US\ ZHOU YILONG US\ WANG HAIGANG US | UNIV NORTHEAST FORESTRY US | B29C65/08 |
| 264 | 3D printing | LED LAMP AND MANUFACTURING METHOD THEREOF\ | CN103363320A | ZHOU MINGJIE US\ LI XIULI US | OCEANS KING DONGGUAN LIGHTING TECH CO LTD US\ OCEANS KING LIGHTING SCIENCE US | F21Y101/02\ F21V5/04\ F21S2/00\ F21V23/00\ F21V19/00\ F21V31/00\ F21V17/10 |
| 265 | 3D printing | MANUFACTURING METHOD OF MEDICAL SYRINGE NEEDLE FILTER AND MEDICAL INJECTION FILTER\ | CN103372247A | HU HEBIN US | AMERICAN MEMBRANE NANTONG CO LTD US | A61M5/165 |
| 266 | 3D printing | SPLIT-METAL GRAVITY HEAT PIPE WITH MICRO-ARC-WELDED RIB PLATE\ | CN103376006A | GUAN SHAOQIN US\ YANG YAONING US\ GUAN FANG US\ YANG YINGDAN US\ QI ZHAOJU US | GUAN SHAOQIN US | B23K9/00\ F28D15/02 |
| 267 | 3D printing | FIXING METHOD FOR WIRING OF FLEXIBLE ELECTRIC HOT LINE ON SURFACE OF IRREGULAR-SHAPE OBJECT\ | CN103385568A | CHEN WEI (CN) | GUANGZHOU WITECH CO LTD US | A41H43/04 |
| 268 | 3D printing | FLEXIBLE CIRCULAR MICRO-FLUIDIC CHIP\ | CN103386335A | YE JIAMING US | SUZHOU YOUNGCHIP SCIENCE & TECHNOLOGY CO LTD US | B01L3/00 |
| 269 | 3D printing | INTELLIGENT ANNULAR TRACK COIN-SHAPED TICKET CARD AND PROCESSING TECHNOLOGY THEREOF\ | CN103390189A | SHI WENBIN US | BEIJING YICHENG XINTONG SMART CARD CO LTD US | G06K19/077 |
| 270 | 3D printing | NOVEL BRUSH TYPE SEAL MANUFACTURING METHOD\ | CN103406739A | TIAN BO US | TIAN BO US | B23K20/10\ B23P17/06 |
| 271 | 3D printing | SUBMARINE POWER CABLE FLOATING ELEMENT\ | CN103413602A | XIA HANG US | XIA HANG US | H01B7/12\ H01B7/14\ H01B9/00 |
| 272 | 3D printing | ELECTRONIC CIGARETTE ULTRASONIC WELDING STRUCTURE ULTRASONIC WELDING DIE AND ULTRASONIC WELDING METHOD\ | CN103416853A | LIN GUANGRONG US | LIN GUANGRONG US | A24F47/00 |
| 273 | 3D printing | FULL-AUTOMATIC INTERMEDIATE-BULK-CONTAINER POST-PROCESSING DEVICE AND PROCESSING METHOD THEREOF\ | CN103425114A | CHEN LVYI US | YUSHU HUAWANG INVEST CO LTD US | G05B19/418\ G01D21/02 |
| 274 | 3D printing | LEAD-ACID BATTERY GRID SEAL PRODUCTION LINE\ | CN103441244A | WANG QUNHUA US\ YIN HUIYANG US | JIANGSU SANHUAN HOLDINGS INC US | H01M4/20 |
| 275 | 3D printing | NOVEL AUTOMATIC FREQUENCY TRACKING CONTROL CIRCUIT\ | CN103441752A | ZHANG CHAOHAI US\ LU YAN US | NANJING LAOLUNSI ELECTRONIC SCIENCE & TECHNOLOGY CO LTD US | H03K17/567 |
| 276 | 3D printing | AUTOMATIC WELDING DEVICE FOR CYLINDRICAL BATTERY CAP\ | CN103447684A | MENG XIANGHUI US\ ZHAO CHANGFENG US | SHANDONG AOYU POWER SUPPLY CO LTD US | B23K20/10\ B23K20/26 |
| 277 | 3D printing | METHOD FOR PROCESSING LED (LIGHT-EMITTING DIODE) VARIABLE MESSAGE SIGN\ | CN103456246A | JIAO WEIQI US\ ZHOU SHUANGJUN US\ ZHOU XU US\ WANG ZHANMING US | YAHAM OPTOELECTRONICS CO LTD US | F21V5/04\ F21V31/00\ G09F9/33 |
| 278 | 3D printing | HORN SEAL STRUCTURE AND PROCESSING METHOD THEREOF\ | CN103458326A | YU WENFEI US | SHANGHAI CHUANGGONG COMM TECHNOLOGY CO LTD US | H04R31/00\ H04R1/02 |
| 279 | 3D printing | PRODUCTION METHOD OF SPONGE WIPE WITH GRID MESH\ | CN103462569A | LU YAXIANG US | LU YAXIANG US | A47L13/16 |
| 280 | 3D printing | TITANIUM ALLOY WELDING METHOD BASED ON ARC ULTRASONIC PROCESS\ | CN1267230C | WU MINSHENG LI (CN) | UNIV TSINGHUA (CN) | B23K20/10\ B23K9/06\ B23K9/067 |
| 281 | 3D printing | ULTRASONIC VIBRATION ASSISTING RESISTANCE IMPLANTATION WELDING METHOD FOR THERMOPLASTIC RESIN BASE COMPOSITE MATERIAL\ | CN1285460C | YAN JIUCHUN WANG (CN) | HARBIN INST OF TECHNOLOGY (CN) | B29C65/34\ B29C65/08 |
| 282 | 3D printing | REFRIGERATOR DOOR BODY AND MANUFACTURING METHOD THEREOF\ | CN1288407C | LI HENGGUO LI (CN) | HENAN FRESTECH ELECTRIC APPLIA (CN) | F25D23/02 |
| 283 | 3D printing | TECHNICAL METHOD FOR RAPID SHAPING SILICON CARBIDE CERAMIC PART UTILIZING LAMINATED SOLID BODY\ | CN1303038C | LI DICHEN CUI (CN) | UNIV XI AN JIAOTONG (CN) | C04B35/622\ C04B35/565 |
| 284 | 3D printing | BICYCLE FRAME MADE FROM CARBON FIBER COMPOSITE\ | CN1321856C | SHA JIANGANG (CN) | SHA JIANGANG (CN) | B62K19/16 |
| 285 | 3D printing | STRUCTURAL IMPROVED INJECTION MAGNIFIER AND INJECTING METHOD THEREOF\ | CN1482471A | CHEN GUOQING (CN) | DONGGUAN CHANGCHENG OPTICAL PL (CN) | G02B3/12\ G02B25/00 |
| 286 | 3D printing | APPARATUS FOR MOUNTING SEMICONDUCTOR CHIP\ | CN1542936A | KENJI KOBAYASHI HIROSHI YAMAKA (JP) | FUJITSU LTD (JP) | B23K20/10\ G11B5/00\ H01L21/60\ H05K3/34\ H01L21/607\ G11B5/48\ B23K31/00\ H01L21/58 |
| 287 | 3D printing | AIR-BLOWING FAN AND ANTIINSECTS NET ASSEMBLING STRUCTURE FOR AIR BLOWING SYSTEM\ | CN1548836A | CHENG SHIQING (CN)\ LI DONGHE (CN) | LG ELECTRONICS TIANJIN (CN) | F24F13/00 |
| 288 | 3D printing | BEARING FIXTURE CONJUNCTION STRUCTURE OF DYNAMO\ | CN1601861A | LI HAOZAI (CN) | LG ELECTRONICS TIANJIN (CN) | H02K5/16\ H02K5/167 |
| 289 | 3D printing | ANTICORROSIVE HEAT SHRINK SLEEVE FOR MOUTH-REPAIRING FOR WELDING ENGINEERING PIPELINE\ | CN1664431A | ZUO JUN (CN)\ GUO PENGFEI (CN)\ SU QINGSHAN (CN)\ LIANG XIAOZHUANG (CN)\ CHEN DAIJIU (CN) | SICHUAN JIUYUAN SCIENCE AND TE (CN) | F16L47/02\ F16L58/00 |
| 290 | 3D printing | ULTRASONIC WAVE WIRE SOLDERING METHOD AND WIRE SOLDERING APPARATUS\ | CN1702847A | CHEN LUKUN (CN) | ESSENCE MACHINERY SHENZHEN CO (CN) | H01L21/607 |
| 291 | 3D printing | HEAT PIPE VACUUM SEALING METHOD AND APPARATUS THEREOF\ | CN1737484A | LIN XIANGWU (CN) | LIN XIANGWU (CN) | B23K20/10\ B23K20/12\ B23K101/14\ F28D15/02 |
| 292 | 3D printing | SUPERSONIC WAVE WELDING AND SEALING CONNECTION METHOD FOR MEDICINE MOUTHPIECE AND ASSEMBLED LID\ | CN1799942A | TANG YUE ZENG (CN) | CHANGSHA CHUTIAN SCI & TECH CO (CN) | B65B51/22 |
| 293 | 3D printing | FLAT-TYPE HEAT PIPE MANUFACTURING METHOD USING ULTRASONIC WELDING\ | CN1845321A | CHEN PEIPEI YANG (CN) | ASIA VITAL COMPONENTS CO LTD (CN) | F28D15/02\ H01L23/427 |
| 294 | 3D printing | WATER CUTOFF STRUCTURE OF COVERED WIRE\ | CN1848528A | TETSURO ASAKURA NOBUYUKI IDE (JP) | YAZAKI CORP (JP) | H01R4/70\ H02G15/08\ H01R13/52 |
| 295 | 3D printing | HOT-PIPE VACUUM SEALING METHOD AND ITS SEALER\ | CN1854670A | LIN XIANGWU (CN) | LIN XIANGWU (CN) | F28D15/02 |
| 296 | 3D printing | PLASTIC CASING FOR FIBER OPTIC ADAPTOR\ | CN1900752A | ZHANG CHANGBO XU (CN) | PUTIAN COMM CO LTD NANJING (CN) | G02B6/36 |
| 297 | 3D printing | CLAMPING CARTRIDGE POLYMER POLE EAR WELDING METHOD\ | CN1967907A | HE ANXUAN HUANG (CN) | ZENG JIANYI (CN) | B23K20/10\ B29C65/08\ H01M2/26 |
| 298 | 3D printing | AUTOMATIC WELDING CONTROL METHOD AND DEVICE\ | CN1981974A | YAN XUEXIN (CN) | TONGLI HIGH TECHNOLOGY CO LTD (CN) | B23K20/10\ B23K37/00\ B23K20/26\ B23K20/24\ B23K37/047 |
| 299 | 3D printing | COLOR BAND ULTRASONIC WELDING METHOD AND WELDING APPARATUS THEREOF\ | CN1990215A | HUANG NAN WEI (CN) | ZHUHAI TIANWEI TECH DEV CO LTD (CN) | B29C65/08 |
| 300 | 3D printing | ADULT INCONTINENCE TROUSERS CONVEYING CLAMPING WELDING AND CUTTING DEVICE\ | CN200954178Y | WANG GUODONG XU (CN) | KERUITE MECHANICAL MFG CO LTD (CN) | A61F13/49\ A61F13/15 |
| 301 | 3D printing | GEARSHIFT OPERATING HANDLE OF AUTOMOBILE\ | CN200957769Y | DONG YIFENG JIANG (CN) | CHANG AN MOTOR CO LTD CHONGQIN (CN) | B60K20/02 |
| 302 | 3D printing | SOLDERING DEVICE\ | CN200977584Y | YAO LIJUN PAN (CN) | NINGBO HUIFENG ELECTRONIC MATE (CN) | B23K1/06\ B23K3/04\ B23K3/08 |
| 303 | 3D printing | SAFETY WARNING GARMENT CAPABLE OF ACTIVE ILLUMINATING\ | CN200990872Y | WANJUN JIANG (HK)\ ZHONGSHENG CUI (HK) | CHAOEN INTERNAT TRADE CO LTD (HK) | A41D13/01 |
| 304 | 3D printing | FORMING EQUIPMENT OF PAPER CELLULAR LUNCH BOX\ | CN200995498Y | DIANJI FENG (CN) | DIANJI FENG (CN) | B31B7/46\ B31B1/12\ B31B1/46\ B31B1/64\ B31B7/60\ B31B43/00 |
| 305 | 3D printing | FORMING EQUIPMENT OF PAPER CELLULAR LUNCH BOX\ | CN200995499Y | DIANJI FENG (CN) | DIANJI FENG (CN) | B31B7/46\ B31B1/12\ B31B1/46\ B31B1/64\ B31B7/60\ B31B43/00 |
| 306 | 3D printing | FORMING EQUIPMENT OF PAPER CELLULAR LUNCH BOX\ | CN200995500Y | DIANJI FENG (CN) | DIANJI FENG (CN) | B31B7/46\ B31B1/12\ B31B1/46\ B31B1/64\ B31B7/60\ B31B43/00 |
| 307 | 3D printing | WATERPROOF WATCH\ | CN200997051Y | LI CHEN (CN) | LI CHEN (CN) | G04B37/00\ G04B37/11 |
| 308 | 3D printing | PLASTICS AIR INLET MANIFOLD BRANCH USED FOR CAR ENGINE\ | CN200999680Y | DEXIN LIU (CN) | TIANJIN HUIDE VEHICLE AIR INTA (CN) | F02M35/104 |
| 309 | 3D printing | LED NEON LIGHT\ | CN200999998Y | YONG LI (CN) | YONG LI (CN) | F21Y101/02\ F21W121/00\ F21S4/00\ F21V27/00\ F21V31/00 |
| 310 | 3D printing | PET SAFETY WAIST CAPABLE OF LIGHTING ACTIVELY\ | CN201008300Y | WANJUN JIANG (HK)\ ZHONGSHENG CUI (HK) | JOEN INTERNAT TRADE CO LTD (HK) | A01K13/00 |
| 311 | 3D printing | PLASTIC INDUCTION MANIFOLD FOR AUTOMOBILE ENGINE\ | CN201013496Y | DEXIN LIU (CN) | TIANJIN HUIDE AUTOMOBILE INTAK (CN) | B29C65/08\ B29C65/06\ F02M35/104 |
| 312 | 3D printing | EYE SHIELD FOR NEONATE\ | CN201019913Y | ZHANGSHENG YU (CN) | ZHANGSHENG YU (CN) | A61F9/04 |
| 313 | 3D printing | BENDING VIBRATION TYPE ULTRASONIC STICK\ | CN201020440Y | JINXUAN YAN (CN)\ ZHIZHONG YANG (CN)\ ZHUOLI YAN (CN)\ ZHUOSHENG YAN (CN) | GUANGZHOU NEW DONGLI ULTRASONI (CN) | B06B1/02\ B06B1/00\ B06B3/02 |
| 314 | 3D printing | ELECTRIC CAP PRIMING DEVICE BRIDGING FIBRIL WELDING ENDS STRUCTURE\ | CN201034508Y | FAN SHI (CN)\ XIAO GU (CN)\ RONG SHI (CN)\ YIN GU (CN) | SHI FAN GU XIAO SHI RONG GU YI (CN) | F42C19/08 |
| 315 | 3D printing | T-TYPE BENCH USED FOR ULTRASONIC WELDING\ | CN201064851Y | HUAJIAN JIANG (CN)\ WANGBAO REN (CN)\ ZHIWEI CHEN (CN) | SHENZHEN B & K TECHNOLOGY CO L (CN) | B23K20/10\ H01M2/00\ B23K37/04\ B23K20/26 |
| 316 | 3D printing | ULTRASONIC LINE STRUCTURE FOR WELDING PLASTIC PIECE\ | CN201064946Y | YU WANG (CN) | BYD CO LTD (CN) | B29C45/14\ B29C65/08 |
| 317 | 3D printing | COMPOSITE LAYER STRUCTURE FOR GAUZE MASK\ | CN201067099Y | ZHIJUN TONG (CN) | ZHIJUN TONG (CN) | A41D13/11 |
| 318 | 3D printing | AUTOMATIC ULTRASONIC WELDING MACHINE\ | CN201075394Y | YI LI (CN)\ BIN ZHANG (CN) | YI LI (CN) | B23K20/10\ H01L31/18\ B23K20/24 |
| 319 | 3D printing | WELDING HEAD FOR SUPERSONIC WAVE SEAMLESS SEAMING MACHINE CORE\ | CN201080009Y | YUANPING CHEN (CN) | YUANPING CHEN (CN) | B29C65/08 |
| 320 | 3D printing | BATTERY SHELL AND BATTERY INCLUDING SAME\ | CN201084760Y | JIAN DU (CN)\ YANFEI ZHOU (CN) | BYD CO LTD (CN) | H01M2/02 |
| 321 | 3D printing | ULTRASOUND WAVE ULTRASONIC WELDING MACHINE\ | CN201086400Y | JIANQIANG CAI (CN)\ YINLIANG FENG (CN)\ QIUWEI ZHANG (CN) | JIASHAN YIJIA MACHINERY CO LTD (CN) | B29C65/08 |
| 322 | 3D printing | FORMER BLOCK DEVICE OF ULTRASONIC WELDING MACHINE\ | CN201086401Y | JIANQIANG CAI (CN)\ YINLIANG FENG (CN)\ QIUWEI ZHANG (CN) | JIASHAN YIJIA MACHINERY CO LTD (CN) | B29C37/00\ B29C65/08 |
| 323 | 3D printing | PISTON TYPE LIFTING AND LOWERING MECHANISM OF ULTRASONIC WELDING MACHINE\ | CN201086402Y | JIANQIANG CAI (CN)\ YINLIANG FENG (CN)\ QIUWEI ZHANG (CN) | JIASHAN YIJIA MACHINERY CO LTD (CN) | B29C65/08 |
| 324 | 3D printing | WATER-PROOF PACKAGE BATTERY CASING\ | CN201112433Y | FANGXIAO XIAO (CN) | HUIZHOU DESAY BATTERY CO LTD (CN) | H01M2/02 |
| 325 | 3D printing | FLEXIBLE PACKING MULTI-LAMINATION STRUCTURE LITHIUM ION POWER CELL\ | CN201117740Y | LUNFENG HUANG (CN)\ XIANG BAO (CN)\ JIANHUA CAO (CN)\ JUNFANG LAN (CN) | SHENZHEN B & K TECHNOLOGY CO L (CN) | H01M10/40\ H01M2/20 |
| 326 | 3D printing | REFRIGERATION COMPRESSOR WITH NOVEL ELECTROMOTOR INSULATION BUSHING STRUCTURE\ | CN201118301Y | BINGHONG LI (CN) | BINGHONG LI (CN) | H01B17/58\ H02K3/00 |
| 327 | 3D printing | METALLIC BUILT-IN SPRAY GUN\ | CN201120352Y | BAIMING ZHANG (CN) | BAIMING ZHANG (CN) | B05B15/00 |
| 328 | 3D printing | ULTRASONIC WELDING CLAMP FOR LITHIUM IONIC BATTERY COVER PLATE\ | CN201124294Y | HONGMEI MA (CN)\ RONGGUANG HUANG (CN) | SHENZHEN BAK BATTERY CO LTD (CN) | B23K20/10\ B23K20/26\ B23K37/053 |
| 329 | 3D printing | HOLLOW BOARD SEPARATED BOX\ | CN201124970Y | HAN ZHOU (CN)\ TING ZHAO (CN) | SUZHOU KESIJIA PLASTICATION TE (CN) | B65D6/02\ B65D25/04\ B65D25/30\ B65D43/14 |
| 330 | 3D printing | CRAMPING APPARATUS FOR SUPERSONIC WELDING WITH POSITION SHIFTER\ | CN201128047Y | YU WANG (CN)\ LIMING SONG (CN) | CHONGQING ZONGSHEN TECH RES (CN) | B23K20/10\ B29C65/08\ B23K20/26 |
| 331 | 3D printing | SUPERSONIC WELDING APPARATUS\ | CN201136080Y | XIAOHANG FENG (CN) | XIAOHANG FENG (CN) | B23K20/10\ G01B7/26 |
| 332 | 3D printing | ULTRASONIC BRAZING UNIT WITH ADJUSTABLE OSCILLATING PRESSURE\ | CN201136081Y | XIAOHANG FENG (CN) | XIAOHANG FENG (CN) | B23K20/10 |
| 333 | 3D printing | HOLE-DISC TYPE ULTRASONIC WELDING HEAD DEVICE\ | CN201136082Y | RONGZAI ZHOU (CN) | RINCO STRONG ULTRASONIC MACHIN (CN) | B23K20/10\ B29C65/08 |
| 334 | 3D printing | SWITCH AND SOCKET FOR PURE PLANAR WALL\ | CN201146334Y | ZHONGFANG BAI (CN) | ZHONGFANG BAI (CN) | H01H9/02\ H02B1/40\ H01R13/46 |
| 335 | 3D printing | HIGH PURE GRAPHITE ELECTROLYTIC WATER HEATER\ | CN201149367Y | YONGQIAN LI (CN)\ JIHAO ZOU (CN) | YONGQIAN LI (CN) | H05B6/00\ F24H1/22\ F24H9/00\ F24H9/18\ F24H9/20 |
| 336 | 3D printing | MACHINE FOR MAKING DISC PACKING FILM BAG\ | CN201151213Y | YUEWEN CHEN (CN) | YUEWEN CHEN (CN) | B31B39/02\ B31B39/14\ B31B39/60 |
| 337 | 3D printing | COMPLEX TYPE WATER FILTERING CORE\ | CN201171927Y | YUNBIAO XIA (CN)\ RUIGUANG HUANG (CN)\ HONGCHANG ZHANG (CN) | YUNBIAO XIA (CN) | B01D24/02\ B01D15/00\ B01D29/33\ C02F1/00 |
| 338 | 3D printing | LIGHTER GAS CHARGING VALVE\ | CN201173322Y | FENGJING HUANG (CN) | FENGJING HUANG (CN) | F16K15/20 |
| 339 | 3D printing | SEAMLESS SWIMMING CLOTHES\ | CN201182216Y | YAMING LIU (CN) | YAMING LIU (CN) | A41D7/00 |
| 340 | 3D printing | CUTTING KNIFE STRUCTURE FOR ULTRASONICWELDING UNIT\ | CN201189594Y | SHUNRU ZHANG (CN) | SHENZHEN CHUANGWEIXING AUTOMAT (CN) | B23K20/26\ B23D27/00 |
| 341 | 3D printing | ULTRASONIC SPOT WELDING MACHINE FOR BACK COVER LOCK\ | CN201192755Y | YAQUN ZHU (CN)\ JINGWEN CAI (CN)\ YANGYONG ZHU (CN) | VAST KUNSHAN SECURE SYSTEMS LT (CN) | B23K20/10 |
| 342 | 3D printing | ACCUMULATOR SEALED BY ULTRASONIC SOLDERING MACHINE\ | CN201196963Y | YUNXIANG ZHOU (CN) | YUNXIANG ZHOU (CN) | H01M2/02\ H01M10/04\ H01M2/04\ H01M2/08 |
| 343 | 3D printing | ULTRASONIC BUTT FUSION SEWING MACHINE\ | CN201199972Y | ZHINAN JIANG (HK) | ZHINAN JIANG (HK) | D06H5/00\ A41H43/04 |
| 344 | 3D printing | COMBINED ZINC HOLLOW DRY BATTERY\ | CN201204229Y | XIANGUO CHEN (CN)\ YIJUN WANG (CN) | JINGZHOU LANYU NAVIGATION MARK (CN) | H01M6/06\ H01M12/06 |
| 345 | 3D printing | ULTRASONIC BONDING CLEAVER FOR INCREASING GOLD-REMAINING ANGLE\ | CN201226353Y | WEILIN BU (CN) | KEWEI ZHAOQING SEMICONDUCTOR C (CN) | H01L21/60\ H01R43/02 |
| 346 | 3D printing | PACKAGING STRUCTURE FOR ELECTRONIC MEMORY DEVICE\ | CN201226355Y | YUEMING DONG (CN)\ JIAMING YANG (CN)\ XIUNI CAI (CN)\ YUANWEI LIU (CN)\ JINGYING YAN (CN) | ORIENT SEMICONDUCTOR ELECT LTD (CN) | H01L25/16\ H01L23/31\ G06K19/077\ H01L25/00\ H01L23/04\ H01L23/488\ G11C5/00\ G11C5/04\ G11C5/06 |
| 347 | 3D printing | ONE-ROTATION MULTI-CHIP MULTIPLE WELDING HIGH-SPEED HIGH-INTENSITY ULTRASONIC WELDING DEVICE\ | CN201226829Y | SHIJIN LI (CN) | HANGZHOU CREATOR MACHINERY MAN (CN) | B23K20/10\ B29C65/08\ A41H43/04 |
| 348 | 3D printing | CYLINDRICAL LITHIUM IONIC CELL CAP\ | CN201233920Y | JIANGFENG WU (CN)\ PENGKUN GAO (CN)\ YAO CHEN (CN) | SHANGHAI DLG BATTERY CO LTD (CN) | H01M2/10\ H01M2/34\ H01M2/00\ H01M2/04\ H01M2/06\ H01M2/08\ H01M2/12\ H01M10/40 |
| 349 | 3D printing | POWER LITHIUM IONIC CELL MODULE\ | CN201233924Y | ZHENGYAO LIU (CN)\ LU QI (CN)\ XUE SUN (CN)\ LIANG ZHOU (CN)\ JIE LU (CN) | CITIC GUOAN MENGGULI NEW ENERG (CN) | H01M2/10\ H01M10/42\ H01M2/20\ H01M10/613\ H01M10/6563 |
| 350 | 3D printing | ENVIRONMENT PROTECTION BAG MAKING COMPLETE EQUIPMENT\ | CN201244931Y | CHENG ZHANG (CN) | CHENG ZHANG (CN) | B31B23/02\ B31B23/14\ B31B23/60 |
| 351 | 3D printing | IMPEDANCE CONVERSION TYPE ELECTRO-ACOUSTIC TRANSDUCER FOR AN ULTRASONIC WELDING MACHINE\ | CN201249168Y | MINGXI LU (CN) | MINGXI LU (CN) | B06B1/06 |
| 352 | 3D printing | MECHANICAL PRESSURE REGULATOR\ | CN201259866Y | ZHONGYI HUANG (CN) | ZHONGYI HUANG (CN) | H01H35/34 |
| 353 | 3D printing | WING SHAPED HEATING VENTILATION RADIATOR FOR NON-METAL HEADER PIPE\ | CN201266027Y | SHIWEI PEI (CN) | SHIWEI PEI (CN) | F28D1/053 |
| 354 | 3D printing | ZIPPER BAG MAKING MACHINE\ | CN201268117Y | MING GUO (CN) | MING GUO (CN) | B31B1/90 |
| 355 | 3D printing | WIND WHEEL ULTRASONIC WELDING MACHINE OF AXIAL-FLOW FAN\ | CN201271780Y | JIANZHONG GUO (CN) | GUANGDONG SHUNWEI PREC PLASTIC (CN) | B23K20/10 |
| 356 | 3D printing | SUPERSONIC WELDING SEALING ARRANGEMENT FOR NONWOVEN CLOTH PACKAGE BAG\ | CN201272053Y | HONGBING HOU (CN) | GANSU QIZHENG TIBETAN MEDICINE (CN) | B31B1/64 |
| 357 | 3D printing | WATER WINDOW SEALING STRUCTURE OF ELECTRIC HEATING KETTLE\ | CN201274991Y | ZAIJIE TANG (CN)\ ZHENGNAN JIANG (CN) | GUANGDONG GALANZ GROUP CO LTD (CN) | A47J27/21 |
| 358 | 3D printing | BOTTLE CAP WITH WIRELESS RADIO FREQUENCY RECOGNIZABLE ANTIFAKE LABEL\ | CN201276257Y | LIANGHENG XU (CN)\ DEQIU CHEN (CN)\ LE LIU (CN)\ XUEWEN XU (CN) | SHANGHAI FUDAN TECHSUN NEW TEC (CN) | B65D55/06 |
| 359 | 3D printing | QUICK SWITCHING DEVICE FOR CLAMPER OF ULTRASONIC WELDING MACHINE\ | CN201283476Y | HONGQI LI (CN)\ ZHENGXING ZHOU (CN) | YAGUANG NYPRO PREC MOLDING TIA (CN) | B23K20/10\ B23K37/04\ B23K20/26 |
| 360 | 3D printing | NEGATIVE-PRESSURE TYPE PLASTIC-CASE BALL-VALVE LITHIUM ION BATTERY\ | CN201289872Y | HEQING HUANG (CN)\ JIMING LONG (CN) | JIMING LONG (CN) | H01M4/02\ H01M2/30\ H01M2/06\ H01M2/12\ H01M10/40\ H01M4/48\ H01M4/58 |
| 361 | 3D printing | WATER KETTLE WITH A CUP COVER\ | CN201299234Y | YANGLIN ZHU (CN) | YANGLIN ZHU (CN) | A45F3/16 |
| 362 | 3D printing | BOUNCING-COVER WATER KETTLE WITH A SUCTION PIPE\ | CN201299363Y | YANGLIN ZHU (CN) | YANGLIN ZHU (CN) | A47G19/12 |
| 363 | 3D printing | DRINKING CUP WITH A SQUARE SEAT\ | CN201299371Y | YANGLIN ZHU (CN) | YANGLIN ZHU (CN) | A47G19/22 |
| 364 | 3D printing | SAFETY DRIVING CUP BEING OPERATED BY A SINGLE HAND\ | CN201299372Y | YANGLIN ZHU (CN) | YANGLIN ZHU (CN) | A47G19/22 |
| 365 | 3D printing | KEYSTROKE CUP OPERATED BY A SINGLE HAND\ | CN201299373Y | YANGLIN ZHU (CN) | YANGLIN ZHU (CN) | A47G19/22 |
| 366 | 3D printing | NOVEL ULTRASONIC WELDING MACHINE POWER SUPPLY\ | CN201304544Y | XINHONG JIANG (CN) | DONGGUAN CHANGJIANG ULTRASONIC (CN) | B23K20/10 |
| 367 | 3D printing | FULL AUTOMATIC ROTATING DISC TYPE ULTRASONIC WELDING MACHINE\ | CN201304546Y | XINHONG JIANG (CN) | DONGGUAN CHANGJIANG ULTRASONIC (CN) | B23K20/10 |
| 368 | 3D printing | POWER CONTROLLER CIRCUIT STRUCTURE OF ULTRASONIC WELDING MACHINE\ | CN201304719Y | XINHONG JIANG (CN) | DONGGUAN CHANGJIANG ULTRASONIC (CN) | B29C65/08 |
| 369 | 3D printing | MOBILE PHONE LENS\ | CN201311495Y | KECONG ZANG (CN) | BYD CO LTD (CN) | G02B3/00 |
| 370 | 3D printing | LENS DECORATION RING\ | CN201311512Y | ZHICONG LI (CN)\ ZHONGGUO HOU (CN) | SICHUAN CHANGHONG ELECTRIC CO (CN) | G02B7/02 |
| 371 | 3D printing | LIQUID CRYSTAL DISPLAY SCREEN CLEANING EQUIPMENT CAPABLE OF AUTOMATICALLY CHANGING BELTS\ | CN201316721Y | QIUFENG SHANG (CN)\ CHUNFENG LU (CN) | ZHUANG TIANCAI (CN) | B08B1/00 |
| 372 | 3D printing | ULTRASONIC WELDING MANIPULATOR\ | CN201320660Y | HUIQING LU US | BIELOMATIK MACHINERY DONGGUAN (CN) | B23K20/10\ B23K20/26\ B25J18/00\ B25J19/00 |
| 373 | 3D printing | CUTTING ULTRASONIC WELDING MACHINE\ | CN201340881Y | HAITAO DING (CN) | TIANJIN LISHEN BATTERY (CN) | H01M10/38\ H01M2/20 |
| 374 | 3D printing | WATERPROOF FLASH DISK\ | CN201345228Y | HUA ZHENG (CN) | HUA ZHENG (CN) | H05K5/00\ G11C7/10 |
| 375 | 3D printing | ULTRASONIC WELDING DEVICE OF THICK MEMBRANE INTEGRATED CIRCUIT\ | CN201348992Y | YONGQING ZHOU (CN) | YIXING DINGKE ELECTRONIC & ELE (CN) | B23K20/10\ H01L21/60 |
| 376 | 3D printing | WATERPROOF FLASH DISK\ | CN201352458Y | HUA ZHENG (CN) | HUA ZHENG (CN) | H05K5/00\ G11C7/10 |
| 377 | 3D printing | MEMORY CARD CONNECTOR\ | CN201352645Y | XIANJUN LIU (CN) | KUNSHAN SHANGZHENG ELECTRONICS (CN) | H01R13/629\ G06K7/06\ H01R13/633 |
| 378 | 3D printing | MEDIUM KNOT WITH IMPROVED MEDIAN PLATE OF CROSS FLOW FAN\ | CN201354759Y | JIAN XU (CN)\ SHIJUN LONG (CN)\ XIN YANG (CN) | GUANGDONG SUNWILL GROUP (CN) | F04D29/28\ F04D17/04\ F04D29/62 |
| 379 | 3D printing | CENTRIFUGAL WIND WHEEL WITH IMPROVED BLADE\ | CN201354760Y | JIAN XU (CN) | GUANGDONG SUNWILL GROUP (CN) | F04D29/30\ F04D29/28\ F04D29/60 |
| 380 | 3D printing | ADAPTER SUITABLE FOR SC OPTICAL FIBER CONNECTING LINE INTERFACE\ | CN201364401Y | ZHONGBING ZHAO (CN)\ YUMING LUO (CN)\ SHUGANG ZHANG (CN)\ WEIDONG TAN (CN) | SUNSTAR COMM TECHNOLOGY CO LTD (CN) | G02B6/38 |
| 381 | 3D printing | ELECTRO-ACOUSTIC ENERGY SOUND GENERATOR\ | CN201369817Y | WENTAO JIANG (CN) | AAC ACOUSTIC TECHNOLOGIES CHAN (CN) | H04R9/02\ H04R9/06 |
| 382 | 3D printing | HIGHLIGHT MATERIAL FLAT-PLATE TELEVISION CASING BASE WELDING MACHINE\ | CN201371578Y | GUOQING LIU (CN) | SHENZHEN KEXIN ULTRASONIC BONDERS CO LTD US | B29C65/02\ B29C65/08\ B29L12/00 |
| 383 | 3D printing | HIGHLIGHT MATERIAL FLAT-PLATE TELEVISION CASING WELDING MACHINE\ | CN201371579Y | GUOQING LIU (CN) | SHENZHEN KEXIN ULTRASONIC BONDERS CO LTD US | B29C65/02\ B29C65/08\ B29L12/00 |
| 384 | 3D printing | HIGHLIGHT MATERIAL FLAT-PLATE TELEVISION CASING WELDING MACHINE\ | CN201371581Y | GUOQING LIU (CN) | SHENZHEN KEXIN ULTRASONIC BONDERS CO LTD US | B29C65/08\ B29L12/00 |
| 385 | 3D printing | OPTICAL FIBER ADAPTER\ | CN201383016Y | LIBO SHAO US | CHANGZHOU WAIPU ELECTRONICS TECHNOLOGY CO LTD US | G02B6/38 |
| 386 | 3D printing | LIFT TYPE ULTRASONIC WELDING PLATFORM\ | CN201385193Y | XIAOAN LIU US\ SIPING YI US\ NIJIA MAO US | TIANJIN LISHEN BATTERY (CN) | B23K20/10\ B23K37/04 |
| 387 | 3D printing | FRONT PILLAR INNER PANEL OF AUTOMOBILE\ | CN201390212Y | LI XIONG US\ ZHIMING LUO US\ JINGHONG LIU US | WUHAN MECA MACHINERY PLASTIC CO LTD US | B60R13/02 |
| 388 | 3D printing | CAR CENTER POST INNER PROTECTING PLATE\ | CN201390288Y | LI XIONG US\ ZHIMING LUO US\ JINGHONG LIU US | WUHAN MECA MACHINERY PLASTIC CO LTD US | B62D25/04 |
| 389 | 3D printing | FULL-PLASTIC VALVE BODY OF COLD AND HOT WATER MIXING VALVE\ | CN201391640Y | XUEFEN CHEN US | NINGBO FENGYUAN SANITARY WARE CO LTD US | F16K27/06\ F16K11/08 |
| 390 | 3D printing | CLAMP TYPE HDMI PLUG\ | CN201392941Y | XIFAN ZHANG US\ SHENGRONG GAO US | JACK COMM TECHNOLOGY DONGGUAN CO LTD G US | H01R13/502\ H01R13/506 |
| 391 | 3D printing | AUTOMATIC ULTRASONIC WELDING ALUMINONICKEL COMPOUND BELT DEVICE\ | CN201402843Y | WUFU BAO US | SHENZHEN ZKSHINE ENERGY TECHNOLOGY CO LTD US | H01M10/38 |
| 392 | 3D printing | FLOATING BALL OF FUEL SENSOR\ | CN201408053Y | HUAIDE XU US\ AIMEI HE US\ QINGLIAN FANG US\ XUEWEN XU (CN) | HUANGSHAN TIANXIANG VALVE CO LTD US | G01F23/76 |
| 393 | 3D printing | PRESSURE-TRIGGERED ULTRASONIC WELDING MACHINE\ | CN201410603Y | TONGFEI SHENG US | TONGFEI SHENG US | B23K20/10 |
| 394 | 3D printing | ULTRASONIC WELDING SYSTEM USED FOR CONTINUOUS HEAT SEAL OF COLD DRINK PAPER CONTAINER\ | CN201411075Y | YANCHEN FU US\ QIWEI ZHANG US\ YAXING CHEN US | HONGBAOLAI GROUP CORP US | B65B51/22\ B65B43/52 |
| 395 | 3D printing | NET EDGE FOLDING WELDER\ | CN201415313Y | SHUJIN LI US | SHANGHAI HUGONG ELECTRIC WELDING MACHINE MFG CO LTD US | B23K20/10 |
| 396 | 3D printing | MANUAL ADJUSTMENT MECHANISM FOR BONDING WIRE WIDTH OF ULTRASONIC LEAD PRESSING MACHINE\ | CN201419302Y | FULIANG LI (CN) | NINGBO ZHONGWANG ELECTRONIC SCIENT TECHNOLOGY CO LTD US | B23K20/10\ B23K20/26 |
| 397 | 3D printing | NATURAL CONVECTION TYPE AND THERMAL RADIATION TYPE HEAT PIPE RADIATOR\ | CN201421285Y | GUANSHENG CHEN US\ JINGLIAN HUANG US | JINGLIAN HUANG US | F28F13/18\ F28D15/02\ F28D15/04\ C09K5/04\ F28F1/20 |
| 398 | 3D printing | ULTRASONIC METAL SPOT-WELDING MACHINE\ | CN201423497Y | FULIANG LI (CN) | NINGBO ZHONGWANG ELECTRONIC SCIENT TECHNOLOGY CO LTD US | B23K20/10 |
| 399 | 3D printing | DETECTING DEVICE OF ULTRASONIC WELDING STRENGTH OF PLASTIC PARTS\ | CN201425579Y | JIBIN ZHANG US\ JIKUN HOU US | UNIVERSAL WUHU IND CO LTD US | G01N19/04 |
| 400 | 3D printing | ULTRASONIC CONDUCTOR COMPRESSION JOINTING MACHINE\ | CN201455540U | FULIANG LI (CN) | NINGBO ZHONGWANG ELECTRONIC SCIENT TECHNOLOGY CO LTD US | B23K20/10\ H01L21/607 |
| 401 | 3D printing | CROSS-FLOW FAN BLADE MIDDLE JOINT PART FOR IMPROVING DISTRIBUTION OF FAN BLADES\ | CN201461528U | JIAN XU (CN)\ XIN YANG (CN) | GUANGDONG SUNWILL PREC PLASTIC CO LTD US | F04D29/26 |
| 402 | 3D printing | DIGITAL HIGH POWER MULTIPURPOSE ULTRASONIC POWER SYSTEM\ | CN201464854U | PENG DU US\ SHUIYUAN DU US\ QIAN LI US\ SONGFAN XU US\ LICHENG YU US | WUHAN JIAPENG ELECTRONICS CO LTD US | G05B19/042\ H02M1/00 |
| 403 | 3D printing | PHOTOVOLTAIC COMPONENT TERMINAL BOX\ | CN201466598U | XIAOJUN TAI US | SUZHOU XUNTONG SOLAR TECHNOLOGY CO LTD US | H02G3/08\ H02G3/18\ H02S40/34 |
| 404 | 3D printing | SMD CRYSTAL RESONATOR\ | CN201479091U | XIAOWEI WANG US\ CHENGXIU WU US\ YAHUA WU US | ANHUI TONGFENG ELECTRONICS CO US | H03H9/13\ H03H9/05 |
| 405 | 3D printing | LIFTING DEVICE FOR ULTRASONIC WELDING MACHINE\ | CN201483770U | XINHONG JIANG (CN) | DONGGUAN CHANGO ULTRASONIC CO LTD US | B23K20/10\ B29C65/08\ B23K20/26 |
| 406 | 3D printing | REINFORCED CONCRETE INTERNAL MOLD WITH MULTIDIRECTIONAL FIXED BRACKETS\ | CN201486139U | RONG LIN US | RONG LIN US | E04G15/06 |
| 407 | 3D printing | SC FIBER ADAPTER\ | CN201489138U | SHENGEN DING US\ QIXING SONG US\ BO WANG (CN) | SHENZHEN SUNSEA TELECOMM CO US | G02B6/38 |
| 408 | 3D printing | NOZZLE BODY OF DUST COLLECTOR\ | CN201510238U | ZHENGMING CHEN US | ZHENGMING CHEN US | A47L9/00 |
| 409 | 3D printing | WELDING-PACKAGED ELECTRONIC THROTTLE SENSOR\ | CN201514188U | LIANG CHEN US\ NIANQUAN HE US\ MEILONG HUANG US | RUILI GROUP CO LTD US | G01B7/00\ F02D41/00 |
| 410 | 3D printing | STORAGE BATTERY UPPER COVER WITH ULTRASONIC WELDING\ | CN201514963U | PENGFEI CUI US\ WENLIN LIU US\ JIHUI NIU US\ LIZHAI WANG US | FENGFAN CO LTD US | H01M2/04 |
| 411 | 3D printing | COMPOSITE NONWOVEN FABRIC\ | CN201516710U | YONGLAN ZHONG US | YONGLAN ZHONG US | B32B27/02\ B32B27/12 |
| 412 | 3D printing | POST FORMING CENTRIFUGAL WIND WHEEL\ | CN201521480U | JIAN XU (CN) | GUANGDONG SUNWILL PREC PLASTIC CO LTD US | F04D29/30 |
| 413 | 3D printing | COSMETIC BOTTLE BODY WITH INNER CONTAINER ASSEMBLED AT LOWER PORTION\ | CN201525529U | RUOSHAN WU US | RUOSHAN WU US | B65D8/04\ B65D8/06\ B65D23/00\ B65D41/02 |
| 414 | 3D printing | OIL FILLED PRESSURE GAUGE\ | CN201540186U | JIGUO TONG US | JIGUO TONG US | G01L19/14 |
| 415 | 3D printing | ULTRASONIC WELDED PRODUCT OF HIGH MELTING METAL\ | CN201543965U | GUOLIANG SHI US | XU LIMING US | B23K20/10\ B32B15/01 |
| 416 | 3D printing | ULTRASONIC WELDING TOOL FOR AUTOMOBILE DOOR GUARD PLATE\ | CN201543967U | GAN ZHA US\ WENKANG SHI US\ YUANBIN WANG US\ YOUWEI WU US | JOHNSON CONTROLS WUHU AUTOMOTIVE INTERIORS CO LTD US | B29C65/08\ B23K20/26\ B23K37/053 |
| 417 | 3D printing | ANTI-ELECTRICITY-THEFT ELECTRIC ENERGY METER\ | CN201548591U | HUIYUN CHEN US\ NING HU US\ LIUDONG SHAO US\ ZHONGXIANG ZHOU US | NINGBO SANXING ELECTRIC CO LTD US | G01R11/04\ G01R11/24 |
| 418 | 3D printing | ANTI-ELECTRICITY-THEFT ELECTRIC ENERGY METER\ | CN201548595U | HUIYUN CHEN US\ NING HU US\ LIUDONG SHAO US\ ZHONGXIANG ZHOU US | NINGBO SANXING ELECTRIC CO LTD US | G01R11/04 |
| 419 | 3D printing | HIGH-EFFICIENCY ULTRASONIC WELDING MACHINE\ | CN201559027U | LIN LUO US\ WENXUAN TANG US | SHENZHEN WEIHENG AUTOMATION EQUIPMENTS CO LTD US | B23K20/10 |
| 420 | 3D printing | GANTRY-TRAVELLING TYPE PLANE COORDINATE NUMERICAL CONTROL ULTRASONIC WELDING MACHINE TOOL\ | CN201559028U | HENGGUO LI US | NINGBO KAIBO NC MACHINERY CO LTD US | B23K20/10\ B29C65/08\ B23K20/26 |
| 421 | 3D printing | RADIATING AND SOUND-INSULATING COVER LINKED WITH ULTRASONIC WELDING HEAD\ | CN201565718U | CHANGZHENG CHEN US\ CHAOYANG FEI US\ YANLING GU US\ YANBIN LI US\ ZIQIANG SUN US | UNIV SHENYANG TECHNOLOGY US | B23K20/10\ B29C65/08\ B23K20/26\ G10K11/16 |
| 422 | 3D printing | WELDING HEAD OF WELDING MACHINE FOR MULTI-DIRECTION WELDING BUS BAR\ | CN201565722U | WANXUE LIU US\ JING YU US\ BING ZHANG US\ JIZE ZHANG US\ FUJIAN ZHONG US\ TENG ZUO US\ HAITAO LIU (CN) | JILIN QINGDA NEW ENERGY ELECTRIC POWER CO LTD US | B23K20/10\ B23K20/26 |
| 423 | 3D printing | CASING OF NOVEL WATCH\ | CN201569857U | RENXU LI US | SHISHI XINJIA ELECTRONICS CO LTD US | G04B37/00\ G04B39/00 |
| 424 | 3D printing | DEVICE FOR ULTRASONIC WELDING OF PIPETTE\ | CN201573338U | JIANHUA YUAN US\ YE YUAN US\ CHANGPING GUO US | JIANHUA YUAN US\ YE YUAN US\ CHANGPING GUO US | B29C65/08\ B29C65/78 |
| 425 | 3D printing | FULL-AUTOMATIC ULTRASONIC PLASTIC PEN WELDING MACHINE\ | CN201579986U | JINHUA LI US | WENZHOU DONGFENG JINGGONG ELECTRONIC INSTR FACTORY US | B29C65/08\ B29C65/78 |
| 426 | 3D printing | SEALING MECHANISM OF ICE CREAM PAPER CUP MACHINE\ | CN201580020U | FUMING SONG US\ FUWU SONG US | RUIAN LUZHOU MACHINERY CO LTD US | A47G19/22\ B31B1/64 |
| 427 | 3D printing | WATERPROOF SOCKET\ | CN201584572U | YUANPING QI US | YUANPING QI US | H01R13/52 |
| 428 | 3D printing | ULTRASONIC WELDING MACHINE\ | CN201587120U | JICHANG JIANG US | JICHANG JIANG US | B29C65/08 |
| 429 | 3D printing | DEVICE FOR PRODUCING CDS/CDTE SOLAR BATTERY BY EMPLOYING MAGNETRON SPUTTERING METHOD\ | CN201590428U | RENYI HOU US | CHENGDU COE APOLLO SOLAR CO LTD US | H01L31/18 |
| 430 | 3D printing | COMPOSITE BOARD FOR PACKAGING ELECTRIC APPLIANCES\ | CN201592858U | SHAOSHANG LIAN US\ RONGKUI WU US | XIAMEN SANDEXIN ELECTRON TECHNOLOGY CO LTD US | B65D65/40\ B65D85/68\ B65D77/26\ B65D81/127\ B65D85/30 |
| 431 | 3D printing | SOLAR PV JUNCTION BOX\ | CN201623417U | WEIHUI WANG US | WEIHUI WANG US | H02G3/08 |
| 432 | 3D printing | INTEGRATED SPLAYED SAND-BLOCKING NET ASSEMBLING MACHINE\ | CN201626121U | AILIANG XIE US | AILIANG XIE US | B29C65/08\ B29C65/78 |
| 433 | 3D printing | SERVO MOTOR ULTRASONIC WELDING MACHINE\ | CN201626122U | JIANFENG PAN US | SHANGHAI CHEN FENG INDUSTRY DEV CO LTD US | B29C65/08 |
| 434 | 3D printing | PLASTIC COMPOSITE GEOGRID\ | CN201627128U | HONGZHU ZHANG US | HONGZHU ZHANG US | E01C11/16 |
| 435 | 3D printing | EXPLOSION PROOF COMBINED CAP OF HIGH-RATE CYLINDRICAL LITHIUM-ION BATTERY\ | CN201629358U | WEIBING ZHOU US | WEIBING ZHOU US | H01M2/04\ H01M2/12\ H01M2/22 |
| 436 | 3D printing | ULTRASONIC WELDING MACHINE OF CURRENT COLLECTING BELT OF POSITIVE PLATE\ | CN201629367U | ZHONG ZHANG US\ SHAOPING XU US\ YONGGANG YANG US\ ZHONGLIANG CHANG US\ YIJUN MA US\ XIAOLONG ZHAO US\ HENGZHI FENG US\ JUNLIN LIU US\ ZHUOPENG LIU US | INNER MONGOLIA RARE EARTH OVONIC HIGH POWER MH NI BATTERY CO LTD US | B23K20/10\ H01M2/26 |
| 437 | 3D printing | AUTOMATIC DUST REMOVAL DEVICE OF LITHIUM-ION BATTERY SOLDERING STATION\ | CN201632760U | XIAOAN LIU US\ SIPING YI US\ NIJIA MAO US\ BIN SU US | TIANJIN LISHEN BATTERY (CN) | B23K20/10\ B23K20/26 |
| 438 | 3D printing | FOLLOW-UP PRESSING MECHANISM FOR ULTRASONIC WELDING MACHINE\ | CN201632761U | QIGAO HUANG US\ WEIDONG LU US\ LIN SHI US\ JIANHUA NI US | BRANSON ULTRASONICS SHANGHAI CORP US | B23K20/10\ B23K20/26 |
| 439 | 3D printing | CFAST MEMORY CARD\ | CN201638254U | CHONGMING MA US\ ZHIQIANG QI US\ CHANGMING XIE US | ITT CANNON ELECTRONICS SHENZHEN CO LTD US | G06K19/077 |
| 440 | 3D printing | ULTRASONIC WELDING ELECTRIC MOSQUITO SWATTER\ | CN201640256U | JIANWEI DENG US | JIANWEI DENG US | A01M3/02 |
| 441 | 3D printing | FULLY AUTOMATIC ULTRASONIC WELDING EQUIPMENT FOR PIPETTE\ | CN201645830U | JIANHUA YUAN US\ YE YUAN US\ CHANGPING GUO US | JIANHUA YUAN US\ YE YUAN US\ CHANGPING GUO US | B29C65/08\ B29C65/78\ B29C65/82 |
| 442 | 3D printing | SOLAR TRACK SPIKE\ | CN201648989U | JIANGUO CHEN US | JIANGUO CHEN US | F21Y101/02\ F21V23/00\ F21V17/00\ E01F9/06\ F21S9/03\ F21V13/00\ F21W111/02 |
| 443 | 3D printing | BINDER-REINFORCED ULTRASONIC-WELDED COPPER TUBE AND ALUMINUM FIN PLATE ABSORBER\ | CN201652949U | XIA CHEN US | XIA CHEN US | C09J9/00\ F24J2/48 |
| 444 | 3D printing | PORTAL TRAVELING CRANE FAULT DETECTION MACHINE\ | CN201653987U | ZHONGKUI LIU US | ANSHAN CHANGFENG NDT EQUIPMENT CO LTD US | G01N29/04\ G01N29/26 |
| 445 | 3D printing | FILTER ELEMENT NON-WOVEN FABRIC ULTRASONIC WELDING STRUCTURE IN AUTOMOTIVE AIR CONDITIONER FILTER\ | CN201658897U | WENYOU YANG US | SHANGHAI YONGHONG AUTOMOTIVE PARTS CO LTD US | B01D46/00\ B60H3/00\ B60H3/06\ F24F3/16 |
| 446 | 3D printing | CURRENT CONDUCTING BODY FOR HIGH-CAPACITY HIGH-POWER BATTERY CAPACITOR\ | CN201663187U | JIANG CHEN US\ LIANKUAN XU US\ XIAN MA US\ LI ZHANG US\ YI XIE US | ZHONGDA IND GROUP CO LTD US\ GUANGZHOU JIELI NEW ENERGY TECHNOLOGY CO LTD US | H01M2/26\ H01G9/004 |
| 447 | 3D printing | ULTRASONIC MAKING MACHINE FOR FALCATE OR AXE-SHAPED MASK FABRICS\ | CN201671000U | QIONG WU US\ SHENGRONG WU US | SHANGHAI DASHENG MASK CO LTD US | A41D13/11\ D06H7/00\ D06B23/02 |
| 448 | 3D printing | HEAT-INSULATING CONNECTING STRUCTURE OF ELECTRIC KETTLE\ | CN201675737U | YINGWU LU US | DONGGUAN BUBUGAO HOME APPLIANCES CO LTD US | A47J27/21\ A47J36/00 |
| 449 | 3D printing | OXYGEN HUMIDIFICATION BOTTLE\ | CN201676376U | JINGHAI LIU US\ RUJU LV US\ YUNYONG HU US\ QING LIN US\ YONGJIAN CUI US\ HONGMIN LV US | SHANDONG WEGO NEW LIFE MEDICAL DEVICES CO LTD US | A61M16/16 |
| 450 | 3D printing | WELDING MECHANISM OF AUTOMATIC ASSEMBLY SYSTEM OF FOUNTAIN PEN\ | CN201677511U | HANPING ZHANG US | HANPING ZHANG US | B29C65/08 |
| 451 | 3D printing | ANTI-EXPLOSION AND ANTI-CORROSION OPERATING SWITCH\ | CN201681732U | SHENGYU ZHANG US\ NAIYUE ZHANG US | HOP LUNG EXPLOSION PROOF ELECTRIC CO LTD US | H01H9/02 |
| 452 | 3D printing | MULTI-STATION ROTATION SELF-LEVELING ULTRASONIC WELDING AND CUTTING EQUIPMENT\ | CN201683070U | WEI ZHANG US | DONGGUAN SOUTH NEKON MACHINERY CO LTD US | A41D13/11 |
| 453 | 3D printing | BLOOD PERFUSION DEVICE PRESSING COVER\ | CN201684227U | ZHIGANG WANG US\ ZHANGKAI LUO US\ ZHANREN WANG US\ WEN ZHANG US | CHONGQING XI ERKANG BLOOD PURIFICATION EQUIPMENT RES & DEV CO LTD US | A61M1/36\ A61M1/00 |
| 454 | 3D printing | DISPOSABLE RESIN ABSORBING COLUMN\ | CN201684229U | ZHANGKAI LUO US\ JING TIAN US\ ZHANREN WANG US\ WEN ZHANG US | CHONGQING XI ERKANG BLOOD PURIFICATION EQUIPMENT RES & DEV CO LTD US | B01D15/08\ A61M1/38 |
| 455 | 3D printing | WEAR-RESISTANT ULTRASONIC WELDING DIE\ | CN201685028U | MIAOZHU LUO US\ JINXUAN YAN (CN)\ ZHUOLI YAN (CN)\ ZHUOSHENG YAN (CN) | JINXUAN YAN (CN)\ ZHUOLI YAN (CN)\ ZHUOSHENG YAN (CN)\ MIAOZHU LUO US | B23K20/10\ B23K20/26 |
| 456 | 3D printing | DISPOSABLE ANION RESIN BLOOD PLASMA ADSORPTION COLUMN\ | CN201701553U | ZHANGKAI LUO US\ JING TIAN US\ ZHANREN WANG US\ WEN ZHANG US | CHONGQING XIERKANG BLOOD PURIFYING EQUIPMENT RES AND DEV CO LTD US | A61M1/38 |
| 457 | 3D printing | PROBE SPACER SLEEVE USED FOR PRODUCTS WITH DETECTION FUNCTION\ | CN201710348U | WEIJING XIA US | WEIJING XIA US | A61B5/01 |
| 458 | 3D printing | DOUBLE-END SYNCHRONOUS ULTRASONIC METAL WELDING DEVICE\ | CN201711667U | RUKUN YANG US\ XINGTAO ZHONG US\ XIAOBIN LU US\ WEISHU HUANG US\ JINXUAN YAN (CN)\ ZHUOLI YAN (CN)\ ZHUOSHENG YAN (CN) | JINXUAN YAN (CN)\ ZHUOLI YAN (CN)\ ZHUOSHENG YAN (CN) | B23K20/10 |
| 459 | 3D printing | ULTRASONIC WELDING MACHINE\ | CN201711668U | JIANFENG PAN US | SHANGHAI CHENFENG INDUSTRY DEV CO LTD US | B23K20/10\ B23K20/26 |
| 460 | 3D printing | SOLAR PANEL CORE\ | CN201715739U | XIONGFENG ZHOU US | ZHENENEGHE SHANGHAI SOLAR ENERGY EQUIPMENT CO LTD US | F24J2/46\ F24J2/48 |
| 461 | 3D printing | DOUBLE-SIDED FAST SYNCHRONOUS ULTRASONIC WELDING DEVICE FOR NEGATIVE PRESSURE FIRMWARE\ | CN201744784U | MINGLIANG OU US\ RUKUN YANG US\ LINGZHONG ZENG US\ XINGTAO ZHONG US\ JINXUAN YAN (CN)\ ZHUOLI YAN (CN)\ ZHUOSHENG YAN (CN) | JINXUAN YAN (CN)\ ZHUOLI YAN (CN)\ ZHUOSHENG YAN (CN) | B23K20/10 |
| 462 | 3D printing | NOVEL ULTRASONIC METAL SPOT-WELDING MACHINE\ | CN201744785U | MIAOZHU LUO US\ JINXUAN YAN (CN)\ ZHUOLI YAN (CN)\ ZHUOSHENG YAN (CN) | JINXUAN YAN (CN)\ ZHUOLI YAN (CN)\ ZHUOSHENG YAN (CN)\ MIAOZHU LUO US | B23K20/10 |
| 463 | 3D printing | ULTRASONIC WELDING MOULD\ | CN201744786U | DONGPING QIAN US\ ZHENGQIU XU US | CHANGSHU INTIER AUTOMOTIVE INTERIORS CO LTD US | B23K20/10\ B23K20/26 |
| 464 | 3D printing | TEXTILE COMPOSITE FABRICS\ | CN201752943U | QIHUI LIANG US | QIHUI LIANG US | B32B5/16\ B32B7/04 |
| 465 | 3D printing | PVC FILM OR PVC FOLDER MESH WELDER\ | CN201761084U | HONGWANG ZHANG US\ WANNENG HUANG US\ DERONG LAI US\ SHISHENG JIANG US\ QIANG DENG US | FUJIAN SIJIA IND MATERIAL CO LTD US | B29L7/00\ B29C65/10\ B29C65/78\ B29K27/06 |
| 466 | 3D printing | HEMOPERFUSION APPARATUS\ | CN201768206U | ZHANGKAI LUO US\ JING TIAN US\ ZHANREN WANG US\ WEN ZHANG US | CHONGQING XIERKANG BLOOD PURIFICATION EQUIPMENT R & D CO LTD US | A61M1/38 |
| 467 | 3D printing | FULL-AUTOMATIC MATERIAL FEEDING AND RECEIVING ULTRASONIC WELDING MACHINE\ | CN201776561U | YIWU ZHU US | JIANGMEN CITY XINQIAO OPTOELECTRONIC TECHNOLOGY CO LTD US | B23K20/10\ B23K20/26 |
| 468 | 3D printing | CONTINUOUSLY-ROLLING POCKET SPRING MACHINE\ | CN201777876U | MINGLIANG OU US\ LINGZHONG ZENG US\ YANMIAO HE US\ ZHUOSHENG YAN (CN) | ZHUOSHENG YAN (CN)\ LUO MIAOZHU US\ YAN ZHUOLI (CN)\ YAN JINXUAN (CN) | B68G9/00 |
| 469 | 3D printing | NOVEL ANTI-COUNTERFEIT BOTTLE COVER\ | CN201784887U | XIAOLONG DENG US\ SHIXIONG CAI US | ZHUHAI SPECIAL ECONOMIC ZONE DRAGON & LION CAP CO LTD US | B65D55/06\ B65D51/28 |
| 470 | 3D printing | SEALING STRUCTURE OF DIAPHRAGM AIR PUMP\ | CN201786631U | HANJI LIN US | XIAMEN YANGMING INDUSTRY AND TRADING CO LTD US | F04B45/047\ F04B53/00 |
| 471 | 3D printing | DEVICE FOR OPTIMIZING RADIAN IN GOLD WIRE BALL WELDER AND WELDING MACHINE\ | CN201792111U | HUICAI LUO US\ XIAOYU CHEN US\ XIAJUN HU US\ GANGQIANG YI US\ JUNMAN CHEN US | SHENZHEN INWORK TECHNOLOGY CO LTD US | B23K37/00 |
| 472 | 3D printing | BUCKLE TYPE BUTTON SWITCH\ | CN201796772U | JIANZHONG ZHENG US\ XUEHUI TIAN US | ZHEJIANG ZHONGXUN ELECTRONICS CO LTD US | H01H13/04\ H01H13/10 |
| 473 | 3D printing | FULLY AUTOMATIC SLEEVE MAKING MACHINE\ | CN201798065U | DAOQI BIE US | XIANTAO DAOQI PLASTIC CO LTD US | A41D27/10\ A41D13/08 |
| 474 | 3D printing | VEHICULAR WATER PUMP POWER SUPPLY\ | CN201805360U | XIACHEN TENG US\ CHANGSEN HENG US\ WEIXING PAN US | DEFA TECHNOLOGY WUXI COMPANY LTD US | H05K7/20\ H02M7/21\ H02M3/335 |
| 475 | 3D printing | WELDED TYPE DECORATIVE BLOCK FOR AUTOMOBILE REAR COMBINED LAMP\ | CN201808507U | ZONGLIANG LIU US | TAIZHOU XUANJIN AUTOMOTIVE LAMP MFG CO LTD US | B60Q1/30 |
| 476 | 3D printing | DISPOSABLE SEALING LOCK\ | CN201812301U | ZHANGLU TAN US\ BOQIANG SHI US | CHINA MINE QINENG TECHNOLOGY BEIJING CO LTD US | G06K17/00\ G06K19/077 |
| 477 | 3D printing | PROCESSING DEVICE FOR CUTTING AND WELDING UNDERWEAR BACK BUTTONS\ | CN201813903U | JINGLIAN WANG US | JINGLIAN WANG US | A41F1/00 |
| 478 | 3D printing | ALUMINUM STRIP TRANSMISSION DEVICE FOR THIN-FILM SOLAR CELL ELECTRODE WELDING MACHINE\ | CN201824056U | HUI WANG US\ CEN CAI US\ MINGXIAN XU US\ YULAN LI US\ FENGTAO CHEN US | HENAN XINNENG PHOTOVOLTAIC CO LTD US | B23K20/10\ B23K20/26 |
| 479 | 3D printing | FLOUR SIEVE\ | CN201832781U | JIEJUN BAO US | WINGS PRODUCT WORKSHOP LTD US | B07B1/18 |
| 480 | 3D printing | RIVET-EMBEDDED BRACKET ARRANGED ON AUTOMOBILE INTERIOR TRIM INSTRUMENT PANEL AND USED FOR CONNECTING SAFETY AIR BAG BIN\ | CN201833978U | YI BAO US | NINGBO MINGFEI MOULD PLASTICS CO LTD US | B60R21/205 |
| 481 | 3D printing | AUTO SOUND BUTTON\ | CN201837912U | YAJIE HUANG US\ WEIXIN LIU US | HUIZHOU DESAY SV AUTOMOTIVE CO LTD US | G05G1/02 |
| 482 | 3D printing | ELASTIC MEMBRANE PRESSING FILTER\ | CN201840727U | JIANQIANG MA US\ WEIDONG SU US\ SHENGUI LI US\ FANGMING XU US\ JIAYI CHAI US\ LEIFENG ZHU US\ YUQIN SHEN US | ZHEJIANG FERT MEDICAL DEVICE CO LTD US | A61M5/165 |
| 483 | 3D printing | MOBILE NUMERICAL CONTROL ULTRASONIC WELDING GUN\ | CN201841335U | QINZHONG CHEN US\ KE ZHANG US | FUZHOU ARSHICHUANG ELECTRONIC MATERIAL CO LTD US | B23K1/06\ B23K3/00 |
| 484 | 3D printing | ULTRASONIC WELDING MACHINE FOR MULTISTATION CELL CULTURE FLASK\ | CN201841672U | JIANHUA YUAN US\ YE YUAN US | JIANHUA YUAN US\ YE YUAN US | B29C65/08\ B29C65/82\ B29C65/80 |
| 485 | 3D printing | WATERPROOF SOCKET\ | CN201853889U | LIANG JIN US | LIANG JIN US | H01R13/52 |
| 486 | 3D printing | WEAR-RESISTING WELDING HEAD\ | CN201862917U | BAOPING PAN US | WUHU XINBAO ULTRASOUND EQUIPMENT CO LTD US | B23K20/10 |
| 487 | 3D printing | ULTRASONIC HOT RIVETING WELDER\ | CN201863392U | CHENGHUA LI US | SUZHOU KEB PREC CO LTD US | B29C65/72 |
| 488 | 3D printing | DOUBLE FACED ADHESIVE TAPE CUTTING DEVICE FOR THIN-FILM SOLAR CELL WELDING MACHINE\ | CN201868461U | CEN CAI US\ MINGXIAN XU US\ YULAN LI US\ FENGTAO CHEN US | HENAN XINNENG PHOTOVOLTAIC CO LTD US | H01L31/18\ B29C65/08\ B29C65/74 |
| 489 | 3D printing | DUST PROTECTING DEVICE OF FULL-AUTOMATIC WINDING MACHINE\ | CN201877535U | XIAOAN LIU US\ SIPING YI US\ PENG ZHANG US\ YANGFENG PAN US | TIANJIN LISHEN BATTERY (CN) | B23K20/10\ B23K20/26\ H01M10/0587 |
| 490 | 3D printing | MANUAL CLOTH BAG WELDING MACHINE\ | CN201883329U | LIFEI GU US\ JUNJIE YANG US\ WEIDONG WU (CN) | ZHEJIANG FEILI HARDWARE SPRING CO LTD US | D06H5/00 |
| 491 | 3D printing | ULTRASONIC WELDING POWER SUPPLY SYSTEM\ | CN201886318U | JIHUA LIAO US | SUZHOU KAIERBO PREC MACHINERY CO LTD US | G05B19/042\ G01H13/00\ G01N29/12 |
| 492 | 3D printing | POWER SWITCHING BOX OF VEHICULAR WIRE HARNESS\ | CN201888058U | YUANTAO HE US\ FUTAO CUI US | DONGGUAN SHIJIEZHIYE ELECTRONICS CO LTD US | H05K5/06 |
| 493 | 3D printing | PORTAL-FRAME TYPE ULTRASONIC WELDING DEVICE OF COMMERCIAL CLIP PRODUCTION LINE\ | CN201889582U | HANGUANG XU US\ XIHUA ZHENG US | SHANTOU DONGHE MACHINERY CO LTD US | B23K20/10\ B23K20/26 |
| 494 | 3D printing | TWO-STAGE SHIFTING MECHANISM FOR WELDING HEAD OF ULTRASONIC WELDING MACHINE\ | CN201900723U | XINHONG JIANG (CN) | DONGGUAN CHANGO ULTRASONIC CO LTD US | B29C65/08 |
| 495 | 3D printing | BUFFER FOR ROTATING COMPONENT\ | CN201902490U | JIANGANG GUO US\ ZHENGJUN QU US\ XIAOFENG DENG US | XINBAO ELEC EQUIPMENTS CO LTD US | F16F9/12 |
| 496 | 3D printing | LIQUID FLOW SENSOR\ | CN201903372U | ZIXING LIU US | SHENZHEN BOTELANG TECHNOLOGY CO LTD US | G01F1/58 |
| 497 | 3D printing | FANCY YARNS MOPPING CLOTH\ | CN201905841U | ZHENGLONG CHEN US | HANGZHOU LIN AN TIANFU NON WOVEN PRODUCTS FACTORY US | D02G3/04\ A47L13/24\ D04H11/04 |
| 498 | 3D printing | PLATE BACK TYPE EXPLOSION-PROOF CORROSION-PROOF CONTROL BUTTON\ | CN201910347U | SHENGYU ZHANG US\ NAIYUE ZHANG US | HELON EXPLOSION PROOF ELECTRIC CO LTD US | H01H13/04\ H01H13/06 |
| 499 | 3D printing | STABLE UNIVERSAL MOVING MECHANISM OF ULTRASONIC WELDING HEAD\ | CN201913809U | XINHONG JIANG (CN) | DONGGUAN CHANGO ULTRASONIC CO LTD US | B29C65/08 |
| 500 | 3D printing | HOT RIVETING WELDING SPOT\ | CN201922561U | BAOPING PAN US | WUHU XINBAO ULTRASONIC EQUIPMENT CO LTD US | B29C65/08 |
| 501 | 3D printing | BOARD BACK TYPE EXPLOSION-PROOF AND CORROSION-RESISTANT SIGNAL LAMP\ | CN201925785U | SHENGYU ZHANG US\ NAIYUE ZHANG US | HELON EXPLOSION PROOF ELECTRIC CO LTD US | F21S2/00\ F21V31/00 |
| 502 | 3D printing | FLAT SOLAR HEAT ABSORPTION FIN\ | CN201926174U | XINJIAN LUO US | SHANDONG JULI SOLAR ENERGY CO LTD US | F24J2/46 |
| 503 | 3D printing | FLAP-COVERED CLAMPING PLATE OF DUST FILTRATION BAG\ | CN201930663U | SHUXUE YANG US\ JIANMING YOU US\ SIXIONG LI US\ WANG JUN (CN) | TONGXIANG JIANMIN FILTER MATERIALS CO LTD US | B01D46/42\ B01D46/02 |
| 504 | 3D printing | ULTRASONIC SEALING AND CONTINUOUSLY CHARGING MACHINE\ | CN201931621U | GUOHUI JI US\ WENJUN CAO US | BEIJING JINGMEI CHEMICAL INDUSTRY CO LTD US | B29C65/08\ B31B1/60 |
| 505 | 3D printing | TUBULAR GRID PASTE-EXTRUSION PRODUCTION LINE\ | CN201936943U | FUQUAN WANG US\ SHOUBAO WANG US\ CUICUI LI US | ZIBO XINXU MECHANICAL AND ELECTRICAL EQUIPMENT CO LTD US | H01M4/20 |
| 506 | 3D printing | HIGH-POWER BATTERY\ | CN201936945U | KE BAI US\ XINGFU ZHOU US | KE BAI US\ XINGFU ZHOU US | H01M2/02\ H01M2/30\ H01M4/70 |
| 507 | 3D printing | LITHIUM ION BATTERY WITH NOVEL COVER PLATE DEVICE\ | CN201936959U | MAOCONG ZHANG US\ XUEFENG YAN US\ NENGTONG CHEN US\ HENIAN WU US\ FENG LIANG US | SHENZHEN DONG FANG HUA LIAN TECHNOLOGY CO LTD US | H01M2/26\ H01M2/04\ H01M10/0525 |
| 508 | 3D printing | SHELL FOR ACCOMMODATING SOUNDING COMPONENT\ | CN201937791U | XIONG ZHANG US | AAC PHOTOELECTRIC TECHNOLOGY CHANGZHOU CO LTD US\ AAC ACOUSTIC TECH SHENZHEN CO US | H04R1/02 |
| 509 | 3D printing | DECORATIVE LIGHTING WALL-HUNG SHOWER HEAD\ | CN201949946U | XIAOFA LIN US | QUANZHOU JOMOO SANITARY WARE CO LTD US | B05B15/00\ F21Y101/02\ F21V33/00\ F21V7/00\ B05B1/18 |
| 510 | 3D printing | ULTRASONIC WELDING MACHINE\ | CN201950366U | HUAMIN LIU US\ CHUNYIN LIU US\ BIN LI US\ CHEN WANG US | TIANJIN JINRUNTIAN SOLAR ENERGY TECHNOLOGY CO LTD US | B23K20/10 |
| 511 | 3D printing | POLYMER LITHIUM ION BUILT-IN BATTERY PACKAGE STRUCTURE\ | CN201956397U | WEIPING ZHANG US\ YIPING GUO US\ SHUQING GAO US\ ZHOUDONG WANG US | HUIZHOU TAIKELI GROUP CO LTD US\ TCL CORP US | H01M2/02 |
| 512 | 3D printing | NUT CAP FOR HINTING POLARITY OF BATTERY\ | CN201956414U | JIAYONG WANG US | ZHONGYU LI ION BATTERY CO LTD US | H01M2/30\ H01M2/34 |
| 513 | 3D printing | PRECISE LIQUID MEDICINE FILTER\ | CN201959341U | JINGYUAN ZHU US\ YONGCUN LV US\ MAOCHUN GUO US | SHANGHAI ZHENPU MEDICAL APPARATUS CO LTD US | A61M5/165\ A61M5/31 |
| 514 | 3D printing | AIR FILTER\ | CN201959682U | BIN LI US\ YONGCUN LV US\ GONGFEN XIN US | SHANGHAI ZHENPU MEDICAL APPARATUS CO LTD US | B01D46/42\ A61M1/14\ B01D46/54\ A61B5/15 |
| 515 | 3D printing | PLATE BACK TYPE ANTI-EXPLOSION CORROSION-RESISTANT POTENTIOMETER\ | CN201966010U | SHENGYU ZHANG US\ NAIYUE ZHANG US | HELON EXPLOSION PROOF ELECTRIC CO LTD US | H01C1/024 |
| 516 | 3D printing | REFRIGERATOR DOOR\ | CN201973994U | ZHICHENG YANG US\ YONGQING WANG US | GUANGDONG HOMA APPLIANCES CO US | F25D23/02 |
| 517 | 3D printing | MAGNETIC SEAL STRIP\ | CN201977315U | LIDING ZHANG US | LIDING ZHANG US | A47C29/00\ A47H23/01\ E04H15/58 |
| 518 | 3D printing | CAR COVER\ | CN201980061U | LIDING ZHANG US | LIDING ZHANG US | B60J11/04 |
| 519 | 3D printing | FULL-AUTOMATIC DOUBLE-HEADED ULTRASONIC WELDING DEVICE FOR PLASTIC BOTTLE CAPS\ | CN201989320U | JIUPING LIU US\ MINGLIANG ZHANG US\ JUNGANG HUO US\ CHUNLONG ZHANG US | SHANDONG LIPENG CO LTD US | B29C65/08 |
| 520 | 3D printing | PIPE JOINT CONNECTED BY UTILIZING ULTRASONIC WELDING\ | CN201992251U | JIANXIANG ZHOU US | JIANXIANG ZHOU US | F16L47/02 |
| 521 | 3D printing | POWDER-CLEARING WELDING MACHINE FOR POSITIVE PLATE OF LITHIUM BATTERY\ | CN201997844U | JUNXIONG ZHOU US\ JUNHAO ZHOU US\ JUNJIE ZHOU US | JUNXIONG ZHOU US | B23K20/10\ B23K20/26 |
| 522 | 3D printing | BOARD BACK CABLE TYPE EXPLOSION-PROOF AND CORROSION-RESISTANT SIGNAL LAMP\ | CN202001897U | SHENGYU ZHANG US\ NAIYUE ZHANG US | CHINA HELON EXPLOSION PROOF ELECTRIC CO LTD US | F21S2/00\ F21V31/00 |
| 523 | 3D printing | BATTERY\ | CN202004053U | JIANFENG CHEN US\ WEISHENG AO US\ BAO YANG US\ PENGFEI LEI US\ ZHIPING CHEN (CN) | HYTERA COMM CO LTD US | H01M2/02\ H01M10/42 |
| 524 | 3D printing | CONNECTION STRUCTURE OF CONDUCTIVE TERMINALS OF LITHIUM ION BATTERY\ | CN202004074U | JIANGTAO TIAN US | QINHUANGDAO XINCHI OPTOELECTRONICS TECHNOLOGY CO LTD US | H01M2/30\ H01M2/26 |
| 525 | 3D printing | HORN SEAL CHAMBER BODY\ | CN202009476U | FEN XIAO US\ SHIYING CHEN US\ BEIJIN WEI US | SHENZHEN FENDA TECHNOLOGY CO LTD US | H04R1/02 |
| 526 | 3D printing | MATCHING STRUCTURE FOR UPPER PIECE AND MIDDLE COVER OF STORAGE BATTERY\ | CN202019009U | JIANCHENG LIU US | B B BATTERY CO LTD US | H01M2/04 |
| 527 | 3D printing | CAPACITIVE SENSOR\ | CN202024771U | RUIYU HE US | TRULYIN INSTR SHANWEI CO LTD US | B23K20/10\ G01D5/24\ G01D11/24 |
| 528 | 3D printing | MINIATURE FASTENER FOR MOP\ | CN202027534U | BROWN DAN US | QINGDAO AIXING CLEANING APPLIANCE CO LTD US | A47L13/256 |
| 529 | 3D printing | ADAPTER\ | CN202034600U | ZHIYAO CHEN US\ JUXIANG LONG US | WELL SHIN ELECTRONIC KUNSHAN CO LTD (CN)\ WELL SHIN TECHNOLOGY CO LTD (TW) | H01R31/06\ H01R13/502\ H01R13/42 |
| 530 | 3D printing | ACCOMMODATING DEVICE AND MANUFACTURING METHOD THEREOF\ | CN202035952U | CHENGRUI MA US\ SHIZHE CHEN US | CHENGRUI MA US\ SHIZHE CHEN US | A47G29/00\ A45C7/00\ A47G25/00\ A47G25/54 |
| 531 | 3D printing | AVEOLATE SHEET SPLICING APPARATUS\ | CN202038749U | CHUN HUANG US\ YUEWEN CHEN (CN) | CHUN HUANG US\ YUEWEN CHEN (CN) | B65H21/00 |
| 532 | 3D printing | SPLICING DEVICE FOR AVEOLATE SHEETS\ | CN202038750U | CHUN HUANG US\ YUEWEN CHEN (CN) | CHUN HUANG US\ YUEWEN CHEN (CN) | B65H21/00 |
| 533 | 3D printing | PLASTIC CASING OF FIREPROOFING PROOF BOX\ | CN202039719U | HANDONG LING US\ XIAODONG FEI US\ RUIHONG CAI US | WUJIANG TIANWU SECURITY MACHINERY CO LTD US | E05G1/02\ E05G1/024 |
| 534 | 3D printing | CONNECTOR OF SOLAR PHOTOVOLTAIC JUNCTION BOX\ | CN202050075U | SHENGXIN JIANG US\ HUI SHEN US\ LIXIA PAN US\ QINCHENG CAI US | JIANGSU LIYANG ELECTRONICS CO LTD US | H01R4/18\ H01R4/02\ H01R13/52\ H01R24/00\ H01R13/502\ H01R13/02 |
| 535 | 3D printing | CAPSULE THERMAL INSULATION QUILT\ | CN202055349U | FENGSHAN XU US | FENGSHAN XU US | E04B1/78\ A01G9/14 |
| 536 | 3D printing | ULTRASONIC WELDED COMPOUND METALLIC HEAT CONDUCTIVE HEATING FLOOR\ | CN202055461U | XUEQIN QIU US | XUEQIN QIU US | E04F15/02 |
| 537 | 3D printing | STRUCTURE FOR WELDING UPPER SHELL AND LOWER SHELL APPLIED IN ELECTRONIC PRODUCT\ | CN202059701U | YUHUA ZHANG US | TAITECH PREC ELECTRONIC KUNSHAN CO LTD US | H05K5/00 |
| 538 | 3D printing | LED LIGHT STRIP BACKLIGHT SOURCE AND LIQUID CRYSTAL PANEL\ | CN202065761U | CHANGJIA FU US\ RUI HAN US | BOE TECHNOLOGY GROUP CO LTD US | F21Y101/02\ G02F1/13357\ F21V29/00\ F21V19/00\ F21S4/00\ F21S8/00 |
| 539 | 3D printing | HIGH-SPEED ROLLING AND BEATING ACCUMULATED FORMING APPARATUS FOR LEADING SCREW\ | CN202070705U | MINGSHUN YANG US\ QILONG YUAN US\ JIANMING ZHENG US\ MING WANG US\ ZHIHUA YIN US\ YAN LI (CN) | UNIV XIAN TECHNOLOGY US | B21H3/04 |
| 540 | 3D printing | CLAMP FOR ULTRASONIC WELDING OF BATTERY CELL\ | CN202070876U | HAIJUN ZHANG US\ LEI GUO US\ CHENGWANG ZHAO US | CHERY AUTOMOBILE CO LTD US | B23K20/10\ B23K37/04 |
| 541 | 3D printing | MULTIFUNCTIONAL FRUIT BEVERAGE DEVICE\ | CN202072123U | JUNZHI ZHAI US | JUNZHI ZHAI US | B65D51/24\ B65D81/32\ B65D85/72 |
| 542 | 3D printing | OPTICAL FIBER ADAPTER\ | CN202075464U | QIYUE WANG US\ GUO YANG US | SUNSEA TELECOMM CO LTD (CN) | G02B6/38 |
| 543 | 3D printing | CLAMP FOR ULTRASONIC WELDED PLASTIC SILK RIBBON BUCKLE\ | CN202079784U | ZHIHAO CHEN US | TIANJIN HUIYUAN PRINTING CO LTD US | B29C65/08 |
| 544 | 3D printing | PULSE PORT MULTIPLEXING CIRCUIT\ | CN202102102U | HAIFENG CHEN US\ BIN WU US\ XUEJUN WANG US | SHENZHEN WISMEI TECHNOLOGY CO LTD US | G01R35/04 |
| 545 | 3D printing | SEALING STRUCTURE OF AUTOMOBILE SPEED MILEAGE SENSOR\ | CN202119446U | YONGHUA XU US\ WEI XIANG US | HEFEI CHANGHUI AUTO ELECTRONIC CO LTD US | G01D11/24\ G01C23/00 |
| 546 | 3D printing | ALUMINUM FOIL POSITIVE LUG LITHIUM ION BATTERY\ | CN202121016U | YU LI US\ XIAOYING ZHAO US\ YUMEI ZHANG US | ZHEJIANG GOFEEL COMM EQUIPMENT CO LTD US | H01M2/26\ H01M10/0525 |
| 547 | 3D printing | STRUCTURE FOR CONNECTING POWER TYPE LITHIUM ION BATTERY COVER WITH CURRENT COLLECTING BODIES OF POLE GROUP\ | CN202127051U | YANG LI US\ HUOCHENG ZHANG US\ NA ZHANG US\ YONGWU WANG US | LISHEN MILES POWER BATTERY SYS US | H01M2/26\ H01M2/04 |
| 548 | 3D printing | ULTRASONIC WELDING DEVICE FOR AUTOMOBILE SEALING STRIPS AND SIMILAR ARTICLES THEREOF\ | CN202156034U | CHAO YANG US\ DECAI YANG US | BEIJING WANYUAN HENNIGES SEALING PRODUCTS CO LTD US | B29C65/08 |
| 549 | 3D printing | LIGHT EMITTING DIODE WITH FLIP CHIP\ | CN202159699U | WEIYUN WANG US\ YONGDE LI US | DONGGUAN FORTUNE ELECTRONIC MATERIAL CO LTD US | H01L33/64\ H01L33/62\ H01L33/42\ H01L33/48 |
| 550 | 3D printing | AUTOMATIC ULTRASONIC WELDING APPARATUS\ | CN202162499U | TAOTAO LIN US\ WEI GUO US\ RUI WANG US\ ZHIHONG ZHAN US\ HAIJUN JIANG US\ XIAOHUA LI US | JTR SHANGHAI AUTOMOTIVE ELECTRONICS CO LTD US | B23K20/10\ B23K20/26 |
| 551 | 3D printing | AUTOMOBILE CENTRAL CONTROL LOCK\ | CN202164896U | YI YU US\ DAWEI YAN US | SHENZHEN CREATIVE & BRIGHT PREC PLASTIC MOULD CO LTD US | E05B45/06\ E05B47/00\ E05B77/48 |
| 552 | 3D printing | WELDING AND SHEARING INTEGRATED ULTRASONIC WELDING HEAD\ | CN202169437U | BAOPING PAN US | WUHU XINBAO ULTRASONIC EQUIPMENT CO LTD US\ BAOPING PAN US | B23K20/10 |
| 553 | 3D printing | ULTRASONIC WELDING CONNECTION WELDING HEAD SUITABLE FOR NARROW SPACE\ | CN202169440U | BAOPING PAN US | WUHU XINBAO ULTRASONIC EQUIPMENT CO LTD US\ BAOPING PAN US | B23K20/10\ B23K20/26 |
| 554 | 3D printing | SEPARATING TYPE DIE QUICKLY-CHANGING MECHANISM\ | CN202169702U | CHANGYIN CHAI US\ SHAOJUN LI US | CHANGYIN CHAI US | B29C65/08 |
| 555 | 3D printing | ULTRASONIC FILM WELDING DEVICE\ | CN202169703U | HANYANG YANG US\ MINGCONG CHEN US | JINJIANG LIANXING REFLECTIVE MATERIAL CO LTD US | B29C65/08 |
| 556 | 3D printing | GANTRY NUMERICAL CONTROL ULTRASONIC WELDER WITHOUT BEHIND BEAM\ | CN202174339U | XIANJIE YANG US | XIANJIE YANG US | B23K20/10\ B23K20/26 |
| 557 | 3D printing | PLANER-TYPE NUMERICAL CONTROL ULTRASONIC WELDING MACHINE WITH UNDERLYING CROSSBEAM\ | CN202174340U | XIANJIE YANG US | XIANJIE YANG US | B23K20/10\ B23K37/00 |
| 558 | 3D printing | MULTIFUNCTIONAL ULTRASONIC WELDING MACHINE\ | CN202180292U | JIASHENG HE US | JIASHENG HE US | B23K20/10 |
| 559 | 3D printing | ULTRASONIC WELDING HEAD FOR OPEN TYPE MOTORCYCLE BATTERY\ | CN202180293U | XIAOMENG PENG US\ FANG YUAN US\ FULIN YUAN US | JIANGSU LEOCH BATTERY CO LTD US | B23K20/10 |
| 560 | 3D printing | CANTILEVER TYPE NUMERICAL CONTROL ULTRASONIC WELDING MACHINE WITHOUT BOTTOM CROSS BEAM\ | CN202180294U | XIANJIE YANG US | XIANJIE YANG US | B23K20/10 |
| 561 | 3D printing | CANTILEVER TYPE NUMERICAL CONTROL ULTRASONIC WELDING MACHINE WITH LOWER CROSSBEAM\ | CN202180295U | XIANJIE YANG US | XIANJIE YANG US | B23K20/10\ B23K20/26 |
| 562 | 3D printing | AUTOMATIC MATERIAL FEEDING AND TRIMMING DEVICE USING LITHIUM BATTERY COVER AND NICRAL STRIP\ | CN202180296U | DEJIN ZHU US\ LEIHUA ZHANG US\ DEZE SUN US\ GANG TIAN US\ XUEHUA ZHANG US\ ZIJING LIU US | SHANDONG RONGDA NEW ENERGY CO LTD US | B23K20/10\ B23K20/26\ H01M2/04 |
| 563 | 3D printing | FILM WELDING DEVICE\ | CN202180594U | HANYANG YANG US\ MINGCONG CHEN US | JINJIANG LIANXING REFLECTIVE MATERIAL CO LTD US | B29C65/08 |
| 564 | 3D printing | WATERPROOF LED LIGHTING MODULE\ | CN202188471U | HUAFENG WANG US | RIGHTLEDS CO LTD US | F21Y101/02\ F21V31/00 |
| 565 | 3D printing | NOVEL TV ADAPTER\ | CN202189982U | QINGSONG XIANG US | QINGSONG XIANG US | H01R13/66\ H01R31/06 |
| 566 | 3D printing | AUTOMATIC OIL INJECTION SEALING MACHINE OF SLIDE RAIL DAMPER\ | CN202194987U | SUZI WANG US | DONGGUAN SHILONG WANTONG IND CO LTD US | F16F9/14 |
| 567 | 3D printing | JUNCTION BOX FOR SOLAR CURTAIN WALL\ | CN202196794U | YUEJING SUN US | YUEJING SUN US | H05K7/20\ H02S40/34 |
| 568 | 3D printing | INTEGRATED MACHINE FOR WELDING POSITIVE AND NEGATIVE ELECTRODE LUGS OF LITHIUM ION LAMINATED BATTERY CORE\ | CN202199940U | LIN LIU US\ YONG LIU US\ SHIJUN YANG US\ YURU ZHANG US\ CHENGLONG SHEN US | HUBEI LUOTUO BATTERY RES INST CO LTD US | B23K31/02\ B23K37/00\ H01M2/26\ B23K37/04 |
| 569 | 3D printing | POSITIONING CLAMPING APPARATUS FOR ULTRASONIC WELDING OF TAB AND GUIDING PLATE\ | CN202199980U | ZHE MA US\ XIAOKUN JI US | LISHEN MILES POWER BATTERY SYS US | B23K20/10\ B23K37/04\ B23K20/26 |
| 570 | 3D printing | NOVEL PRODUCTION DEVICE OF PLASTIC GRILLE\ | CN202200557U | BAOYU LIU US\ MING LIU US\ HUIDI ZHANG US | YIXING MINGHUI PLASTIC PRODUCTS CO LTD US | B29D28/00 |
| 571 | 3D printing | ULTRASONIC RADIAL WELDING AND CUTTING MACHINE\ | CN202202188U | BILIE WEI US | MING JIUN ULTRASONIC MACHINERY CO LTD US | D06H5/00\ D06H7/22 |
| 572 | 3D printing | INFRARED PHOTOELECTRIC SWITCH SHELL\ | CN202205607U | BAITE DAI US\ XIAOFENG ZHENG US\ NANNAN ZHANG US\ WEI CHEN (CN) | ESPROS CHANGSHU SENSOR CO LTD US | H01H9/02 |
| 573 | 3D printing | EXTERNAL OXYGEN BLOCKING FILTER FOR OXYGEN HUMIDIFYING FILTER\ | CN202207375U | SHUANGJUN REN US | SHUANGJUN REN US | A61M16/00 |
| 574 | 3D printing | ULTRASONIC WELDING MACHINE FOR ALUMINUM RESISTORS\ | CN202207860U | BENXIN ZHANG US\ ZHIGUANG SI US\ YONGJUN CAO US | YICHANG XINFENG MECHANICAL AND ELECTRICAL EQUIPMENT MFG CO LTD US | B23K28/02 |
| 575 | 3D printing | LIGHT-EMITTING DIODE (LED) ENERGY-SAVING WATERPROOF LAMP\ | CN202215966U | PEIZAI LIU US | BEIJING TRUWIN OPTOELECTRONIC CO LTD US | F21Y101/02\ F21V5/04\ F21S2/00\ F21V19/00\ F21V31/00 |
| 576 | 3D printing | MOTORCYCLE ELECTRIC FUEL PUMP ASSEMBLY\ | CN202220693U | HAIRANG TIAN US\ WEIZHONG KONG US | SHANGHAI YESON ELECTRIC CO LTD US | F02M37/08 |
| 577 | 3D printing | CYLINDRICAL LITHIUM ION BATTERY POLE PIECE\ | CN202221793U | YAOXIN GUAN US\ WEILIANG SU US\ TAO YUAN US | SHENZHEN BOFUNENG BATTERY CO LTD US | H01M2/26\ H01M4/13 |
| 578 | 3D printing | ULTRASONIC WELDING MOULD\ | CN202224838U | WENJIA TANG US | CHANGZHOU RONGJIA VEHICLE ENTPR CO LTD US | B23K20/10\ B23K20/26 |
| 579 | 3D printing | DEVICE FOR AUTOMATICALLY WELDING NASAL CLIP OF MASK\ | CN202224839U | RENJIE WU US | DONGGUAN HONGXIANG MACHINERY EQUIPMENT CO LTD US | B23K20/10\ B23K20/26 |
| 580 | 3D printing | ULTRASONIC WELDING FIXTURE FOR BATTERIES\ | CN202224842U | ZHONGBO ZHENG US\ HUAJUN SHI US\ YIN ZHANG US | JIANGSU FREY NEW ENERGY CO LTD US | B23K20/26 |
| 581 | 3D printing | AUTOMATIC MANUFACTURE MACHINE FOR FOLDING MASK\ | CN202233130U | SHENGRONG WU US\ XIAOMING WANG US\ YAGEN CAI US\ ZHIWEI YAO US\ FENG ZHU US\ QISHENG CHEN US\ SANG WU US | SHANGHAI DASHENG HEALTH PRODUCTS MFG CO LTD US | A41D13/11 |
| 582 | 3D printing | ULTRASONIC WELDING HEAD CONTROLLING WIELDING BY UTILIZING SHARP CHANGE OF ENERGY\ | CN202240146U | BAOPING PAN US | WUHU XINBAO ULTRASOUND EQUIPMENT CO LTD US\ BAOPING PAN US | B23K20/10 |
| 583 | 3D printing | WELDING ROD OF ULTRASONIC WELDING MACHINE\ | CN202240147U | WEIDONG ZHANG US\ LIANBING HAN US\ PEIGANG YUAN US\ SUGUO LIU US | WEIDONG NEW ENERY CO LTD US | B23K20/10 |
| 584 | 3D printing | ULTRASONIC INSIDE PAGE MANUFACTURING MACHINE\ | CN202241993U | YAMIN SHI US | WUXI LINGSHAN INCENSE & CANDLE HANDICRAFT CO LTD US | B29C65/08\ B42F5/02 |
| 585 | 3D printing | ELECTRONIC LOCK\ | CN202249209U | XINWEI MIAO US | LEADTECH METAL MFG DONGGUAN CO LTD US | E05B17/22\ E05B15/00 |
| 586 | 3D printing | INTEGRAL DUAL LC OPTICAL FIBER ADAPTER WITH COVER\ | CN202256766U | XINMEI WU US | SHENZHEN FIBERCAN OPTICAL CO LTD US | G02B6/36\ G02B6/38 |
| 587 | 3D printing | INTEGRAL OUTER FRAME OF INFRARED TOUCH SCREEN\ | CN202257519U | ZUOHENG YIN US | ZUOHENG YIN US | G06F3/042 |
| 588 | 3D printing | ULTRASONIC WELDING POWER SOURCE SYSTEM\ | CN202275295U | ZHENGZHONG LI US\ GUANGPING ZHOU US\ ZHAOFENG LIANG US | SHENZHEN POLYTECHNIC US | B23K20/10\ G05B19/042 |
| 589 | 3D printing | BRIEFS USING ULTRASONIC WELDING SEAM\ | CN202286312U | HUI XU US | HUI XU US | A41B9/00 |
| 590 | 3D printing | PRECISE FILTER SYRINGE\ | CN202289080U | CHAO YANG US\ ZIHANG HUANG US\ HEJUN DONG US | CHENGDU XINJIN SHIFENG MED APP US | A61M5/31\ A61M5/178 |
| 591 | 3D printing | ULTRASONIC WELDING HEAD\ | CN202293351U | LEI YANG US\ XIAOJUN SU US\ FULI CHEN US\ JUNMING NIU US | PELZER AUTOMOTIVE INTERIOR SYSTEMS TAICANG CO LTD US | B29C65/08 |
| 592 | 3D printing | GLASS TRANSFER FRAME GUIDE ROD\ | CN202296391U | XIANG LUO US | XIAMEN JIEYOU FLAT DISPLAY TECHNOLOGY CO LTD US | B65G49/06 |
| 593 | 3D printing | SOLAR HEAT COLLECTOR PANEL\ | CN202304031U | WEI ZHANG US\ MINFENG LI US\ CHENGXIN WANG US | HANGZHOU UNITED NEW MATERIAL TECHNOLOGY CO LTD US | F24J2/46\ F24J2/48 |
| 594 | 3D printing | HIGH-RATE LITHIUM ION BATTERY WITH NOVEL POLE LUGS\ | CN202308190U | YU ZHANG US\ CHENGSHI LIU US\ JINBAO SONG US | HEFEI GUOXUAN HIGH TECH POWER ENERGY CO LTD US | H01M2/26\ H01M4/70\ H01M10/0525 |
| 595 | 3D printing | ULTRASONIC WELDING LEAD DISTRIBUTION DEVICE\ | CN202317433U | JIAN FANG US | WUHU TIANHAI ELECTRIC FITTING US | B23K20/10\ B23K20/26 |
| 596 | 3D printing | DEVICE FOR WELDING POLE PIECE WITH POLE LUG\ | CN202317439U | RUKUN YANG US\ LINGZHONG ZENG US | SHENZHEN GEESUN AUTOMATION TECHNOLOGY CO LTD US | B23K26/21\ B23K26/70 |
| 597 | 3D printing | AUTOMOBILE DOOR LOCK LOCKING DEVICE WITH CENTRALIZED CONTROL SWITCH\ | CN202324898U | YIHU DAI US | WENZHOU DONGFENG GENERAL MACHANICAL AND ELECTRICAL FACTORY US | E05B47/00\ E05B77/34\ E05B79/02\ E05B85/02 |
| 598 | 3D printing | SOLAR CONNECTOR\ | CN202340001U | ZEPENG CHEN US | DONGGUAN ZERUN ELECTRONICS TECHNOLOGY CO LTD US | H01R13/02\ H01R13/40\ H01R13/639 |
| 599 | 3D printing | IMPROVED STRUCTURE OF ULTRASONIC WELDING MACHINE\ | CN202344845U | WENDA YANG US | WENDA YANG US\ WANG ZHENGHUI US | B29C65/08 |
| 600 | 3D printing | COMPOSITE FILTERING MASK\ | CN202354420U | ZHUANGXIN ZHOU US | CHIEFDON XIAMEN TECHNOLOGY CO LTD US | A41D13/11 |
| 601 | 3D printing | END SEALING MEMBER AND DEVELOPER BOX\ | CN202360757U | JIANQIANG SU US\ JIZHONG WANG US | PRINT RITE UNICORN IMAGE PROD (CN) | G03G15/08\ F16J15/16 |
| 602 | 3D printing | RECTIFIER ASSEMBLY MACHINE\ | CN202363914U | YANGWEI TONG US | HUIZHOU AODE ELECTRONIC EQUIPMENT CO LTD US | H02J7/00\ B29C65/08 |
| 603 | 3D printing | WATER SEPARATOR FOR TAP\ | CN202371216U | XIAOFA LIN US\ XIAOSHAN LIN US | FUJIAN XIHE SANITARY WARE TECH US | F16K11/074 |
| 604 | 3D printing | TRIPLE BATHTUB FAUCET\ | CN202371251U | SENWEN ZHANG US | TAIZHOU ZHUOTAI INDUSTRY AND TRADE CO LTD US | F16K27/00\ F16K11/00 |
| 605 | 3D printing | LED LAMPSHADE\ | CN202371629U | FEIHONG LONG US | BYD CO LTD (CN) | F21Y101/02\ F21S2/00\ F21V3/04\ F21V17/10\ F21V3/02 |
| 606 | 3D printing | SECONDARY PRESSURE ULTRASONIC PLASTIC WELDING MACHINE\ | CN202378293U | WEICHENG ZHENG US | WUXI BOPUDA TECHNOLOGY CO LTD US | B29C65/08 |
| 607 | 3D printing | BRUSHLESS ELECTRIC CIRCULATING WATER PUMP\ | CN202381401U | YANGGUAN KE US\ BINGJIN KE US\ LIXIAN SUN US | BINGJIN KE US | F04D29/22\ F04D29/42\ H02P6/08\ H02P6/16 |
| 608 | 3D printing | REAR TAIL LAMP FOR AUTOMOBILE\ | CN202392651U | HUI SUN US | LANCE VEHICLE TECHNOLOGY CO LTD US | F21S8/10\ F21W101/14\ F21Y101/02\ F21V31/00\ F21V17/10 |
| 609 | 3D printing | SOLAR ABSORBER PLATE\ | CN202393043U | WEI ZHANG US\ MINFENG LI US\ CHENGXIN WANG US | HANGZHOU UNITED NEW MATERIAL TECHNOLOGY CO LTD US | F24J2/46\ F24J2/48 |
| 610 | 3D printing | OPTICAL FIBER ADAPTER\ | CN202393946U | QIYUE WANG US\ GUO YANG US | SUNSEA TELECOMM CO LTD (CN) | G02B6/38 |
| 611 | 3D printing | ULTRASONIC COVER PLATE FOR STORAGE BATTERY\ | CN202395057U | KAIHONG ZHANG US\ CHANGGUI SUN US\ ZIQIANG SHI US | TIANNENG BATTERY WUHU CO LTD US | H01M2/04 |
| 612 | 3D printing | ULTRASONIC WELDING SEAT FOR MULTI-LAYERED METAL\ | CN202398937U | AIGUO ZHANG US\ DENGHONG LUO US\ KAIGUI CAI US\ FANG LI US | AMPEREX TECHNOLOGY LTD US\ NINGDE AMPEREX TECHNOLOGY LTD US | B23K20/10 |
| 613 | 3D printing | CASE STRUCTURE OF CYLINDRICAL LITHIUM-ION BATTERY\ | CN202405330U | FENG ZHANG US\ BO WANG (CN) | QINGDAO HIPOWER ENERGY GROUP CO LTD US | H01M2/02\ H01M2/04\ H01M10/0525 |
| 614 | 3D printing | ULTRASONIC WELDING EQUIPMENT FOR HELICAL POROUS SILENCING AIR INLET PIPES MADE OF NON-WOVEN FABRICS FOR AIR FILTERS\ | CN202412702U | JINSONG XIAO US\ BINGRONG LIU US\ YI ZHANG US | JIANGSU XINGCHENXING AUTO ACCESSORIES CO LTD US | B29C65/08 |
| 615 | 3D printing | AUXILIARY DEVICE FOR WELDING AND POSITIONING LITHIUM ION BATTERY LUG\ | CN202428141U | ZHENGTAO ZHANG US\ WUSHENG YANG US | TIANJIN LISHEN BATTERY (CN) | B23K20/10\ H01M2/26\ B23K37/04 |
| 616 | 3D printing | AUTOMATIC STIGMATIZING AND ULTRASONIC WELDING-MISSING DETECTING MACHINE\ | CN202428853U | ZUCHUN ZHANG US | DONGGUAN YI LI SHA METAL PRODUCT CO LTD US | G01N29/04\ B41K3/04 |
| 617 | 3D printing | BRAND HEAD REGULATION LIFTING DEVICE FOR AUTOMATIC BRAND ULTRASONIC WELDING MISSING CHECKING MACHINE\ | CN202428854U | ZUCHUN ZHANG US | DONGGUAN YILISHA HARDWARE & PLASTIC PRODUCTS CO LTD US | B41K3/04\ B41K3/62 |
| 618 | 3D printing | ULTRASONIC WELDING DEVICE FOR CYLINDRICAL FILM PACKAGES\ | CN202429407U | BIN WANG US | BEIJING YANGHANG TECHNOLOGY TRADE CO LTD US | B65B61/06\ B65B51/22 |
| 619 | 3D printing | HIGH-RELIABILITY HIGH-POWER INSULATED GATE BIPOLAR TRANSISTOR MODULE\ | CN202434507U | ZHEN LV US | STARPOWER MICROELECTRONICS CO LTD US | H01L25/00\ H01L23/00 |
| 620 | 3D printing | NOVEL SOLAR TERMINAL BOX\ | CN202434544U | ZEPENG CHEN US | DONGGUAN ZERUN ELECTRONICS TECHNOLOGY CO LTD US | H05K7/20\ H02S40/34 |
| 621 | 3D printing | TOOL HEAD FOR ULTRASONIC COPPER PIPE CUTTING SEALING MACHINE\ | CN202438838U | WEICHENG ZHENG US | WUXI BOPUDA TECHNOLOGY CO LTD US | B23P23/04 |
| 622 | 3D printing | CONNECTING STRUCTURE FOR INTERNAL DECORATIVE PLATE MAIN BODY OF FRONT DOOR AND INTERIOR LINING PLATE OF AUTOMOBILE\ | CN202439640U | JINGMING LI US\ WENXING LI US\ LONGMEI SONG US\ CHUANXIAO YANG US | HARBIN HAQING PLASTIC CO LTD US | B60R13/02 |
| 623 | 3D printing | FIXING STRUCTURE OF HARDWARE DECORATION SHEET AND SHEET MATERIAL\ | CN202444520U | YUANQING ZENG US | GUANGDONG OPPO MOBILE TELECOMM US | H04M1/02 |
| 624 | 3D printing | AUTOMATIC THREE-WELDING-HEAD ULTRASONIC WELDING MACHINE\ | CN202447808U | LANLAN ZHANG US\ WENSONG XU US\ MIN LV US\ JINFENG ZHAO US | SHANDONG ZHONGCAI PACKAGING CO LTD US | B23K20/10\ B29C65/08 |
| 625 | 3D printing | NOVEL EASY-TO-PROCESS LITHIUM BATTERY\ | CN202454648U | ZHIJIAN LI US\ YAOPING DENG US\ ZHIAN SHE US\ XIAOLIN WANG (CN) | FOSHAN SHUNDE DISTR ELITE BATTERY TECHNOLOGY CO LTD US | H01M2/26 |
| 626 | 3D printing | ELECTRIC CONNECTOR REALIZING SEALING THROUGH ULTRASONIC WELDING\ | CN202454808U | WENBO KANG US | CHINA AVIAT OPTICAL ELEC TECH US | B23K20/10\ H01R13/516\ H01R43/18 |
| 627 | 3D printing | ULTRASONIC WELDING GUN HEAD\ | CN202461801U | XIANGFENG LEI US\ PUWEN LIU US\ XIUJUN LI US\ ANHUI HE US\ CHUNGUANG ZHANG US\ BAITUN SHEN US | CHINA RAILWAY FIRST GROUP CO US | B23K20/10 |
| 628 | 3D printing | NOVEL ULTRASONIC WELDING HEAD SUPPORT FRAME\ | CN202461803U | ZHIQIANG CAO US | WUXI NICLE ULTRASONIC EQUIPMENT CO LTD US | B23K20/10\ B23K20/26 |
| 629 | 3D printing | NOVEL ULTRASONIC WELDING MACHINE TOOL\ | CN202461804U | ZHIQIANG CAO US | WUXI NICLE ULTRASONIC EQUIPMENT CO LTD US | B23K20/10\ B23K20/26 |
| 630 | 3D printing | UPPER TRIM PANEL STRUCTURE FOR REAR DOOR PANEL OF AUTOMOBILE\ | CN202463728U | FUQING LIN US\ JIANGHONG ZHU US\ ZHENGPING ZHU US | NINGBO ANTOLIN HUAXIANG AUTOMOBILE PRODUCTS CO LTD US | B60R13/02 |
| 631 | 3D printing | WATERPROOF STRUCTURE OF POWER SUPPLY ADAPTER\ | CN202475996U | ZHIXIONG OU US | FOSHAN SHUNDE GUANYUDA POWER SUPPLY CO LTD US | H05K5/06 |
| 632 | 3D printing | LITHIUM BATTERY ULTRASONIC WELDING POSITIONING DEVICE\ | CN202479691U | JIAN LI US\ MINGFEI XU US\ DANDAN FAN US\ ZHIXIONG ZENG US | ZHUHAI GREE NEW ENERGY TECHNOLOGY CO LTD US | B23K20/10\ B23K20/26 |
| 633 | 3D printing | COLD STORAGE DEVICE OF ICE CREAM MAKER\ | CN202489148U | HOULIN ZHU US | HOULIN ZHU US | A23G9/22 |
| 634 | 3D printing | TOILET BOWL COVER STRUCTURE\ | CN202489873U | YONGHUA LI US\ XIAODONG ZHOU US | ZHOU YUJIA US | A47K13/12 |
| 635 | 3D printing | PACKAGING BAG\ | CN202492004U | CHANGSAN MIAO US | CHANGSAN MIAO US | B65D30/10\ B65D33/18 |
| 636 | 3D printing | ULTRASONIC WELDING HEAD AND ULTRASONIC WELDING MACHINE\ | CN202498308U | XIAOMENG PENG US\ JIANPING MA US | ANHUI LEOCH BATTERY TECH CO US | B23K20/10 |
| 637 | 3D printing | OPTICAL FIBRE ADAPTER\ | CN202502274U | YONG LIU US\ PENG ZHANG US\ QIANG WANG US | ANHUI TELECOMM ENGINEERING CO LTD US | G02B6/38 |
| 638 | 3D printing | AUTOMATIC LIQUID STOPPING PRECISE FILTRATION DEVICE FOR INFUSION SET WITH DRIPPING FUNNEL\ | CN202505894U | YICHEN ZHOU US | YICHEN ZHOU US | A61M5/165\ A61M5/38 |
| 639 | 3D printing | SOLAR ENERGY HEAT COLLECTING PLATE ULTRASONIC WELDING DEVICE WITH WELD MARK COATING MECHANISM\ | CN202517187U | GUOGONG GAN US\ GUANG LIU US\ SHENGYANG XU US\ DEWU CHEN US | GUOGONG GAN US | B05C11/10\ B23K20/10\ B05C5/00\ B23K20/26 |
| 640 | 3D printing | ULTRASONIC WELDING DEVICE FOR WELDING SOLAR ENERGY HEAT COLLECTING PLATE CORE WITH HEAT EXCHANGE FINS\ | CN202517188U | GUOGONG GAN US\ GUANG LIU US\ SHENGYANG XU US\ DEWU CHEN US | GUOGONG GAN US | B23K20/10\ B23K20/26 |
| 641 | 3D printing | MOBILE TYPE WELDING STORAGE DEVICE FOR ULTRASONIC WELDING MACHINE\ | CN202517723U | XINHONG JIANG (CN) | DONGGUAN CHANGO ULTRASONIC CO LTD US | B29C65/08 |
| 642 | 3D printing | HIGH-STRENGTH GEOTEXTILE SPECIALLY USED FOR BALLASTLESS TRACK\ | CN202519541U | LINGJIN MENG US\ ZHANMING CUI US | SHANDONG HONGXIANG CHEMICAL FIBER GROUP CO LTD US | E02D17/20\ E01B1/00 |
| 643 | 3D printing | ENVIRONMENT-FRIENDLY MEMORY TYPE OIL FILLING GAUGE WITH PLASTIC SHELL\ | CN202522363U | JIGUO TONG US\ XIAOJUN ZHOU US | JIGUO TONG US | G01L19/00\ G01L19/08 |
| 644 | 3D printing | CONTROL SYSTEM OF ULTRASONIC WELDING MACHINE\ | CN202527847U | JIJIAN HUANG US | JIJIAN HUANG US | B23K20/10 |
| 645 | 3D printing | TURNTABLE TYPE ULTRASONIC WELDING MACHINE\ | CN202527848U | JIJIAN HUANG US | JIJIAN HUANG US | B23K20/10\ B23K20/26 |
| 646 | 3D printing | ULTRASONIC SPRAY HEAD WELDING MACHINE\ | CN202527859U | JIJIAN HUANG US | JIJIAN HUANG US | B23K20/10\ B23K28/02\ B23K20/26 |
| 647 | 3D printing | FULL-AUTOMATIC CUP TYPE MASK MACHINE\ | CN202528471U | DEZHI WEN US | DONGGUAN LIHAN MACHINERY CO LTD US | B29C69/00\ B29C31/08\ B29C65/08\ B26D7/18\ B26F1/38 |
| 648 | 3D printing | HEATING PAPER BOX USED FOR MICROWAVE OVENS\ | CN202529227U | FAZHANG ZHOU US\ JIUXIU WANG US\ HENGSEN LEI US\ LILI TANG US | BEIJING JINTIANMAI INTERNAT FOOD CO LTD US | B65D5/20 |
| 649 | 3D printing | ANNULAR-RAIL COIN-SHAPED INTELLIGENT TICKET CARD\ | CN202533985U | WENBIN SHI US | BEIJING YICHENG XINTONG SMART CARD CO LTD US | G06K19/077 |
| 650 | 3D printing | WATERPROOF BABY ANTITHEFT LABEL\ | CN202534102U | YIPING GE US\ JIALIN QIU US\ GUOHAO YAO US\ JIE QIU US\ QI CAO US | HANGZHOU YINJIANG WISDOM MEDICAL GROUP CO LTD US | G08B21/02 |
| 651 | 3D printing | HAND-HELD UMBRELLA EDGE BONDER\ | CN202536325U | MUSHOU TU US | XIAMEN RENNENG ENVIRONMENTAL PROT TECHNOLOGY CO LTD US | A45B25/04 |
| 652 | 3D printing | CENTRAL VENOUS CATHETER\ | CN202538119U | BENGANG LIU US\ ZUOWEN LV US\ SHULONG HU US\ WEIDONG GAO US\ GUOWEI LI US\ HE XING US\ ZHUANG LIU US\ AIWEN ZHANG US | SHANDONG KANGLI MEDICAL EQUIPMENT TECHNOLOGY CO LTD US | A61M25/00 |
| 653 | 3D printing | CUTTER FOR ULTRASONIC WELDING WIRE\ | CN202539800U | YUANWEI HUANG US | GREAT TEAM BACKEND FOUNDRY LTD US | B23K20/10 |
| 654 | 3D printing | ULTRASONIC TERMINAL WELDING MACHINE\ | CN202539802U | KUI YANG US\ YAO ZHOU US\ QUN CHEN US\ TING WANG US | JING JIN ELECTRIC SHANGHAI CO LTD US | B23K20/10\ B23K20/26 |
| 655 | 3D printing | IMPROVED ULTRASONIC MACHINING MACHINE STRUCTURE\ | CN202540746U | RUITIAN LIN US | RUITIAN LIN US | B29C65/08 |
| 656 | 3D printing | DEVICE FOR REALIZING TRANSMITTING AND CUTTING FOR HANDLE TAPE AND POSITIONING FOR BAG BODY OF NON-WOVEN HANDBAG\ | CN202540818U | YAZHOU CHEN US\ YINTAI GUI US\ TIANYI ZHENG US\ PI JUN US\ CAN WEI US\ JIANHUA SUN US\ SHUMIN XING US\ XIAOMING ZHANG US\ QUANMING ZHANG US | XIAMEN CYRINDUSTRIAL & TRADE CO LTD US | B31B1/86 |
| 657 | 3D printing | MEDICAL IN-VIVO IMPLANTED DOSER\ | CN202554731U | XIAOYUN ZHAO US | BEIJING ZHAOSHI MEDICAL DEVICE CO LTD US | A61M31/00 |
| 658 | 3D printing | ULTRASONIC WELDING MECHANISM FOR WELDING UPPER COVER AND LOWER COVER OF CELL\ | CN202555965U | XIUHUI LAN US | SUZHOU LINGOU AUTOMATION EQUIPMENT CO LTD US | B23K20/10\ B23K37/04 |
| 659 | 3D printing | PELVIC CAVITY MEMBRANE FIXING CLAMP\ | CN202569213U | LI YUNDONG US\ GUO RAN US\ XIA XIUJIE US | WANG HONGLUN US | A61N5/00 |
| 660 | 3D printing | ROTARY DISPENSING DEVICE FOR PRODUCING MEDICAL DROPPING BOTTLE\ | CN202570520U | XIANG YANHAI US | SUZHOU RIJIN MACHINERY CO LTD US | B05C11/00\ B05C11/10\ B05C5/02 |
| 661 | 3D printing | LOWER MOULD OF ULTRASONIC WELDING MACHINE\ | CN202571587U | HE PING US\ PENG YEJUN US | AMPEREX TECHNOLOGY LTD US | B23K20/10 |
| 662 | 3D printing | ULTRASONIC WELDING DEVICE OF CUP BODY AND CUP BOTTOM OF SEPARATING CUP\ | CN202571588U | LI FEI US\ JIANG LIN US\ YANG YONG US | SICHUAN NIGALE BIOMEDICAL INC US | B23K20/10\ B23K20/26 |
| 663 | 3D printing | ULTRASONIC WELDING MACHINE\ | CN202571589U | HUANG JIJIAN US | HUANG JIJIAN US | B23K20/10\ B29C65/08 |
| 664 | 3D printing | ULTRASONIC WELDING HEAD FOR ASSEMBLING GAS EXHAUST MEMBRANE\ | CN202572989U | CHEN WEN US\ CAI KUN US\ CUI JING US\ WANG DEQING US\ SONG LITAO US\ ZHANG MIN US\ GONG JIAFU US | SHANDONG ZHONGBAOKANG MEDICAL IMPLEMENTS CO LTD US | B29C65/08 |
| 665 | 3D printing | NOVEL ULTRASONIC WELDING HEAD\ | CN202572990U | CHEN WEN US\ WANG DEQING US\ SONG LITAO US\ YU YANG US\ XU AIHUA US\ LU ZHIHAO US | SHANDONG ZHONGBAOKANG MEDICAL IMPLEMENTS CO LTD US | B29C65/08\ B29L31/14 |
| 666 | 3D printing | NOVEL COMPOSITE NON-WOVEN FABRIC\ | CN202573190U | LIN ZHAOXIONG US | LONGZHIBU FUJIAN LIGHT TEXTILE PRODUCTS CO LTD US | B32B27/02\ B32B27/12 |
| 667 | 3D printing | ADJUSTABLE NOVEL VIBRATION-REDUCING QUICKLY-INSTALLED GUY CABLE CONNECTOR\ | CN202579607U | XIAO JIAXIN US\ QIU GUANGHONG US | SHANGHAI DIYOU CONTROL SYSTEM CO LTD US | F16C1/14 |
| 668 | 3D printing | LIGHTING COLOR-MATCHING MECHANISM OF CAR BACKUP LIGHT\ | CN202581139U | XU PENG US | CHONGQING QINCHUAN IND GROUP CO LTD US | F21W101/14\ F21V9/10 |
| 669 | 3D printing | ENVIRONMENT-FRIENDLY ENGINE OIL FILTER CORE PROVIDED WITH NO GLUE\ | CN202590454U | GAO YANG US | DONGGUAN HAISHA FILTER CO LTD US | B01D27/06 |
| 670 | 3D printing | MOVABLE WELDING AND FIXED WELDING INTEGRATION ULTRASONIC WELDING MACHINE\ | CN202591828U | JIANG XINHONG US | DONGGUAN CHANGO ULTRASONIC CO LTD US | B23K20/10\ B23K20/26 |
| 671 | 3D printing | NOVEL HIGH-FREQUENCY TRANSFORMER\ | CN202601380U | YU HAI US | YU HAI US | H01F27/32\ H01F27/30\ H01F27/24 |
| 672 | 3D printing | DUST BAG CLAMPING PLATE STRUCTURE\ | CN202604722U | LI SIXIONG US\ GE WEIWEI US\ SHEN JIANCHANG US\ LIU CHAOJUN US\ YOU JIANMING US\ FANG MING US | ZHEJIANG ZHAOHUI FILTER TECHNOLOGY CO LTD US | A47L9/14 |
| 673 | 3D printing | SHOWER NOZZLE\ | CN202606318U | ZHONG WENJUN US | ZHONG WENJUN US | B05B1/18\ B05B15/06 |
| 674 | 3D printing | SHOWER HEAD\ | CN202621336U | ZHONG WENJUN US | ZHONG WENJUN US | B05B1/18\ B05B15/06 |
| 675 | 3D printing | SEMI-AUTOMATIC ULTRASONIC SPOT WELDING MACHINE\ | CN202623301U | SU WEI US | SUZHOU KEBER PREC MACHINERY CO US | B29C65/08 |
| 676 | 3D printing | NOVEL HIGH-RELIABILITY POWER MODULE\ | CN202633270U | YAO LIJUN US | JIAXING STARPOWER MICROELECTRONICS CO LTD US | H01L23/48\ H01L23/31\ H01L23/488 |
| 677 | 3D printing | ULTRASONIC WELDING MACHINE\ | CN202639643U | DU ZHONGWEI US | SUZHOU SURUN MACHINERY TECHNOLOGY CO LTD US | B23K20/10\ B29C65/08 |
| 678 | 3D printing | WELDING EQUIPMENT OF HOLLOW FIBER MEMBRANE SUPPORTING TUBE\ | CN202640769U | CHEN YILI US\ QI YULIANG US\ WEN JIANPING US\ PENG WENJUAN US\ HE SHUIPING US | BEIJING ORIGIN WATER TECHNOLOGY CO US | B29C65/08 |
| 679 | 3D printing | WINE GLASS COVER TEARING AND PULLING CLOTH ULTRASONIC WELDING TOOL\ | CN202640770U | YE LIFANG US | SHAOXING YUXING PLASTIC PACKING CO LTD US | B29C65/08 |
| 680 | 3D printing | RECYCLED NON-WOVEN FABRIC\ | CN202640900U | ZHENG GUANGFENG US | WENZHOU XIEDECHANG NONWOVEN CO LTD US | B32B27/02\ B32B27/12 |
| 681 | 3D printing | HIGH-POWER LITHIUM ION BATTERY COVER STRUCTURE\ | CN202651223U | JIN QUANLIANG US\ LIU FENGLONG US\ WANG YONGWU US | TIANJIN LISHEN BATTERY (CN) | H01M2/04 |
| 682 | 3D printing | IDLING MOTOR\ | CN202651962U | XU NA US | XU NA US | H02K5/16 |
| 683 | 3D printing | AUTOMATIC CONVEYING MECHANISM FOR LOOSE-LEAF BINDER MANUFACTURING MACHINE\ | CN202656346U | YU QINGPENG US\ ZHANG XUNJIA US | GAIN HOW PRINTING CO LTD US\ WEIPENG TECHNOLOGY CO LTD US | B29C65/08\ B29C53/04\ B29C31/00 |
| 684 | 3D printing | ULTRASONIC LONGITUDINAL WELDING DEVICE FOR POWER BATTERY ELECTRODES\ | CN202667914U | LEI GUANGWEI US | LEI GUANGWEI US | B23K20/10 |
| 685 | 3D printing | AUTOMATIC ASSEMBLY PRODUCTION LINE FOR MOBILE PHONES\ | CN202668050U | CAI JIANXUN US | PELOS GUANGZHOU ELECTRONICS COMPONENTS CO LTD US | B23P21/00 |
| 686 | 3D printing | ULTRASONICALLY FUSION-WELDED SEAL APPLICABLE TO KILOWATT-HOUR METER CALIBRATION\ | CN202677722U | XU XINRONG US | HUZHOU XINRONG ELECTROMECHANICAL EQUIPMENT MFG CO LTD US | G09F3/03\ G01R11/24 |
| 687 | 3D printing | ULTRASONIC WELDING SEAL OF ELECTRIC ENERGY METER PROGRAMMING SWITCH\ | CN202677724U | XU XINRONG US | HUZHOU XINRONG ELECTROMECHANICAL EQUIPMENT MFG CO LTD US | G09F3/03\ G01R11/24 |
| 688 | 3D printing | DISPOSABLE HIGH-FREQUENCY OPERATION ELECTRODE WITH DOUBLE-LAYER ANTI-HIGH-FREQUENCY ANTI-HIGH-VOLTAGE WATERPROOF STRUCTURE\ | CN202682047U | LI HAOBIN US | RONGJIA HUIZHOU MEDICAL EQUIPMENT MFG CO LTD US | A61B18/14 |
| 689 | 3D printing | OCCLUSION TYPE COMMODITY ANTI-THEFT SIGNBOARD\ | CN202694501U | ZHAO BING US | SHENZHEN RONGHUA ELECTRONIC CO LTD US | G08B13/24 |
| 690 | 3D printing | ULTRASONIC WELDING MACHINE FOR PLASTIC WELDING\ | CN202702624U | DOU HUALI US | TIANJIN LISHUN PLASTIC PRODUCTS CO LTD US | B29C65/08 |
| 691 | 3D printing | ULTRASONIC WAVE WELDING LINE MOLD\ | CN202702625U | KANG LIANGHUA US | XIAMEN YJC POLYMER TECH CO LTD US | B29C65/08 |
| 692 | 3D printing | DISPLACEMENT SENSOR ON MOTOR-DRIVEN EGR VALVE\ | CN202710019U | ZHANG HUA US\ NI MING US\ CHEN KAI US\ XIAO LIN US\ SHAO XINGLONG US | WUXI LONGSHENG TECHNOLOGY CO US | G01B21/02 |
| 693 | 3D printing | SMALL OUTDOOR WATERPROOF ANTENNA\ | CN202721265U | HUANG JINKANG US\ YANG YONGHUA US\ HAN SHAOFEI US | SHENZHEN HOWKING TECHNOLOGY CO LTD US | H01Q1/42 |
| 694 | 3D printing | ULTRASONIC METAL WELDING MACHINE FOR LITHIUM ION POWER BATTERY PACK\ | CN202726310U | LUO MIAOZHU US\ YAN ZHUOSHENG (CN)\ YAN ZHUOLI (CN)\ YAN JINXUAN (CN) | GUANGZHOU NEW POWER ULTRASONIC ELECTRONIC EQUIPMENT CO LTD US | B23K20/10\ B23K20/26 |
| 695 | 3D printing | DIE OF ULTRASONIC WELDING DEVICE OF MAGNETIC LATCHING RELAY\ | CN202726311U | WAN XIAODONG US\ ZHANG ZIYOU US | YUEQING LIHUI ELECTRICAL APPLIANCE CO LTD US | B23K20/10 |
| 696 | 3D printing | ANTI-EXPLOSIVE IMPACT PLASTIC ULTRASONIC WELDING SPLICING FACE STRUCTURE\ | CN202727348U | CHEN XIUMING US | NINGHAI XINCHENG AUTOMOBILE FITTINGS CO LTD US | B29C65/08 |
| 697 | 3D printing | WELDING UNIT OF ULTRASONIC BATTEN MACHINE\ | CN202727414U | XIA LIPING US | BENGBU HUAHONG ELECTRONIC TECHNOLOGY CO LTD US\ BENGBU DEMO FILTRATION TECHNOLOGY CO LTD US | B31F5/00 |
| 698 | 3D printing | REFLEX REFLECTOR ASSEMBLY\ | CN202728083U | LI YOUZHI US\ GUO KAIPING US\ ZUO MINGYANG US | SHIYAN GUANDA AUTOMOBILE COMPONENTS AND PARTS CO LTD US | B60Q9/00 |
| 699 | 3D printing | ULTRASONIC WELDING AND CUTTING EQUIPMENT COMBINATION\ | CN202738896U | HUANG FUKAI US\ HUANG JIANQI US\ YANG ZHUOXIONG US\ YIN HAIQING US\ ZHANG YANPING US | P H GRAMENT DONGGUAN MFG CO LTD US | A41H43/02 |
| 700 | 3D printing | MAGNETIC-THERAPY HEALTHCARE BED MATTRESS WITH GABLE-SHAPED STRIP FOR ULTRASONIC WELDING\ | CN202739384U | LI YINXIANG US | QINHUANGDAO KANGZIBAIDE ADVANCED TECHNOLOGY CO LTD US | A47C27/00\ B68G7/05 |
| 701 | 3D printing | FULL AUTOMATION WELDING MACHINE IN LIGHTER PRODUCTION\ | CN202741922U | BAI JIABAO US\ LIANG XINYA US\ WANG ZENGGUO US | LANGXI DONGHAI PHOTOELECTRIC TECHNOLOGY CO LTD US | B23K20/10\ B23K20/26 |
| 702 | 3D printing | ULTRASONIC WELDING DEVICE FOR NON-WOVEN FABRIC FILTER\ | CN202742676U | DU HAO US\ KANG DESHENG US | LAERDAL MEDICAL SUZHOU CO LTD US | B29C65/08\ B29C65/78 |
| 703 | 3D printing | WELDING MECHANISM OF LIGHTER BASEBOARD AND LIGHTER SHELL\ | CN202742677U | BAI JIABAO US\ LIANG XINYA US\ WANG ZENGGUO US | LANGXI DONGHAI PHOTOELECTRIC TECHNOLOGY CO LTD US | B29C65/08\ B29C65/78 |
| 704 | 3D printing | ULTRASONIC WELDING DIE HEAD DEVICE\ | CN202744329U | LI YINXIANG US | QINHUANGDAO KANGZIBAIDE ADVANCED TECHNOLOGY CO LTD US | B68G7/00\ B68G15/00 |
| 705 | 3D printing | ANTI-BLOCKING ULTRASONIC WELDING CYLINDRICAL LITHIUM BATTERY ANTI-EXPLOSION COMBINED CAP\ | CN202749425U | YANG XUEXIN US | CHANGZHOU WUJIN ZHONGRUI ELECTRONIC CO LTD US | H01M2/04\ H01M2/12 |
| 706 | 3D printing | ULTRASONIC SEMI-AUTOMATIC WELDING DEVICE USED FOR CLOCK SPRING\ | CN202752743U | CUI HONGHONG US\ REN XIAOLI US\ GUO CHAO US | SHANXI RONGCHANG AUTO PARTS CO LTD US | B23K20/10 |
| 707 | 3D printing | DRINK BOTTLE CAP\ | CN202754294U | ZHAO BAOMIN US | JIANGSU CHINA PALARICH FOOD CO LTD US | B65D81/32\ B65D51/28\ B65D85/72 |
| 708 | 3D printing | FILAMENT-PLATE FLY KILLER\ | CN202760045U | SU XIAOLEI US | SU XIAOLEI US | A01M3/02 |
| 709 | 3D printing | POSITION-VARIABLE ULTRASONIC WELDING DEVICE\ | CN202764216U | SU WEI US | SUZHOU KEBER PREC MACHINERY CO US | B23K20/10\ B29C65/08 |
| 710 | 3D printing | MULTI-UNIT ULTRASONIC WELDING MACHINE\ | CN202764217U | SU WEI US | SUZHOU KEBER PREC MACHINERY CO US | B29C65/08 |
| 711 | 3D printing | SIGNAL LAMP\ | CN202769346U | WANG GUOLIANG US\ ZHAO JIANYU US\ JIANG WENLI US | SHANGHAI YOUBANG INDUSTRY CONTROL SYSTEM CO LTD US | F21S2/00\ F21V19/00\ F21V17/10 |
| 712 | 3D printing | SPEAKER DEVICE\ | CN202773071U | GUO XIAODONG US | GOERTEK INC US | H04R9/02\ H04R9/06 |
| 713 | 3D printing | CERAMIC FILTER DEVICE\ | CN202778067U | SU KAIFENG US | SU KAIFENG US | B01D35/04\ B01D36/02 |
| 714 | 3D printing | PAINT POLLUTION RESISTANT ULTRASONIC WELDING POINT BAFFLE PLATE\ | CN202778899U | WANG XINZHI US | CHUZHOU HONGYUAN SPRAYING CO LTD US | B05D1/32 |
| 715 | 3D printing | SPLIT TYPE ULTRASONIC WELDING DEVICE\ | CN202780223U | LV HUIQING US | BIELOMATIK MACHINERY DONGGUAN CO LTD US | B23K20/10 |
| 716 | 3D printing | MATERIAL HOLDING MECHANISM OF ULTRASONIC WELDING DEVICE OF MAGNETIC LATCHING RELAY\ | CN202780227U | WAN XIAODONG US\ ZHANG ZIYOU US | YUEQING LIHUI ELECTRIC CO LTD US | B23K20/10\ B23K20/26 |
| 717 | 3D printing | THREE-POSITION FRONT GUARD ULTRASONIC WELDING MACHINE\ | CN202781831U | SU WEI US | SUZHOU KEBER PREC MACHINERY CO US | B29C65/08 |
| 718 | 3D printing | COOLING STRUCTURE OF ULTRASONIC WELDING HEAD\ | CN202781832U | JIANG XINHONG US | DONGGUAN CHANGO ULTRASONIC CO LTD US | B29C65/08 |
| 719 | 3D printing | ULTRASONIC WELDING MACHINE WITH ROBOT\ | CN202781834U | SU WEI US | SUZHOU KEBER PREC MACHINERY CO US | B29C65/08 |
| 720 | 3D printing | THREE-AXIS LINKAGE ULTRASONIC WELDING MACHINE\ | CN202781835U | SU WEI US | SUZHOU KEBER PREC MACHINERY CO US | B29C65/08 |
| 721 | 3D printing | ULTRASONIC WELDING EQUIPMENT WITH MECHANICAL ARMS\ | CN202781836U | LV HUIQING US | BIELOMATIK MACHINERY DONGGUAN CO LTD US | B29C65/08 |
| 722 | 3D printing | CRANKSHAFT POSITION SENSOR\ | CN202793412U | JIA DONGHUI US\ ZHOU XIAOGANG US\ WANG QIANG (CN) | HARBIN DONG AN ZHIYANG AUTOMOBILE ELECTRIC CO LTD US | G01D5/12 |
| 723 | 3D printing | PHASE SENSOR\ | CN202793413U | ZHOU XIAOGANG US\ WANG QIANG (CN) | HARBIN DONG AN ZHIYANG AUTOMOBILE ELECTRIC CO LTD US | G01D5/12\ G01B7/30 |
| 724 | 3D printing | POWER MODULE ELECTRODE TERMINAL\ | CN202796921U | ZHANG MIN US\ ZHENG JUN US\ MA CHANGSHENG US\ YAO YUSHUANG US\ WANG XIAOBAO US | JIANGSU MACMIC SCIENCE & TECHNOLOGY CO LTD US | H01L23/488 |
| 725 | 3D printing | ULTRASONIC CONTINUOUS ROLLING WELDER FOR ELECTRODES OF POWER BATTERIES\ | CN202804473U | LEI GUANGWEI US\ PAN JINGUANG US\ ZHENG WEILIANG US\ ZHU WENTING US | LEI GUANGWEI US | B23K20/10\ H01M2/26 |
| 726 | 3D printing | ULTRASONIC WELDING MACHINE ACOUSTIC ENCLOSURE\ | CN202804475U | LIN JIAXIANG US\ CAI WEIQIANG US\ WU YAOTING US\ TANG JIANWU US\ ZHANG QINGSHAN US\ FANG YANMEI US | XIAMEN JIADA ENVIRONMENTAL PROT CONSTRUCTION ENGINEERING CO LTD US | B23K20/10\ B23K20/26 |
| 727 | 3D printing | ULTRASONIC-BASED PACKAGING FILM HEAT-SEALING DEVICE\ | CN202807177U | LI ZHIFENG US | CHANGSHA QIQING MACHINERY EQUIPMENT CO LTD US | B65B51/22 |
| 728 | 3D printing | NOVEL TAB-WELDED MODE POLYMER FLEXIBLY-PACKAGED BATTERY\ | CN202817075U | WANG YOUPING US\ WANG XIUFENG (CN) | SHENZHEN JUHEYUAN TECHNOLOGY CO LTD US | H01M2/26 |
| 729 | 3D printing | SPRINKLER\ | CN202823679U | WANG DAOYU US | WUHU RAIN FLOWER BATH CO LTD US | B05B1/18 |
| 730 | 3D printing | CLEANING MECHANISM OF ULTRASONIC WAVE MACHINE\ | CN202824989U | TONG YANGWEI US | HUIZHOU AODE ELECTRONICS CO LTD US | B23K20/10\ B23K20/26 |
| 731 | 3D printing | WELDING DEVICE OF SOFT FILM IN AUTOMOBILE THROTTLE VALVE COVER PLATE COMPONENT\ | CN202826404U | SHEN QIAN US | CIXI XINYUE ELECTRIC APPLIANCE CO LTD US | B29C65/08\ B29C65/78 |
| 732 | 3D printing | ULTRASONIC WELDING STRUCTURE\ | CN202826405U | HAN FENG US\ PANG MINGDUO US\ YANG SHUYONG US | TIANNENG BATTERY WUHU CO LTD US | B29C65/08 |
| 733 | 3D printing | ULTRASONIC WELDING MOULD\ | CN202826544U | GUO CONG US\ ZHANG YUNHUA US | SHANGHAI HAWK FILTER CO LTD US | B31F5/00 |
| 734 | 3D printing | CENTRIFUGAL FAN BLADES\ | CN202833294U | GAO WENMING US\ HAN XIAOHONG US\ CHAI SHUIHUA US | NINGBO LANGDI IMPELLER MACH CO US | F04D29/28 |
| 735 | 3D printing | OBLIQUE FLOW CENTRIFUGE BLADE\ | CN202833296U | GAO WENMING US\ HAN XIAOHONG US\ CHAI SHUIHUA US | NINGBO LANGDI IMPELLER MACH CO US | F04D29/28 |
| 736 | 3D printing | COMBINED SOFT PACKAGE SUPER CAPACITOR\ | CN202839315U | GUAN DAOAN US\ FAN JING US\ YUAN BIN US\ GUO LI US\ DING GANGQIANG US | HUBEI GREATSEA NEWPOWER TECHNOLOGY CO LTD US | H01G9/008\ H01G9/08\ H01G9/26 |
| 737 | 3D printing | LOUDSPEAKER MODULE\ | CN202841502U | QU HONGJU US\ SHI HUA US | GOERTEK INC US | H04R9/02 |
| 738 | 3D printing | DUMBBELL PIECE WITH SAND FILLED INSIDE\ | CN202844457U | GUAN YONGZHONG US | NANTONG IRONMASTER SPORTING IND CO LTD US | A63B21/075 |
| 739 | 3D printing | ULTRASONIC WELDING PLASTIC MOLD\ | CN202846892U | LI XINXING US\ FANG XIN US\ LI SHIWU US | QINGDAO HIWITS METER CO LTD US | B29C65/08 |
| 740 | 3D printing | CATALYTIC VALVE ASSEMBLING DEVICE OF LEAD-ACID STORAGE BATTERY\ | CN202846895U | LIU TAOSONG US\ GUO ZHIQIU US\ DANG ZHIMIN US\ HUANG DEJIN US\ ZHANG YING US\ WANG YU US | ZHEJIANG NARADA POWER SOURCE US\ HANGZHOU NARADA BATTERY COMPANY US\ HANGZHOU NARADA ENERGY TECHNOLOGY CO LTD US | B29C65/40 |
| 741 | 3D printing | WELDING-FREE TYPE FIBER ADAPTER\ | CN202854370U | ZHANG CHUANLING US | ZHANG ZUANXING US | G02B6/38 |
| 742 | 3D printing | DOUBLE-HEAD CONNECTOR SPECIAL FOR TRANSFUSION BAG\ | CN202859664U | ZHAO JINGDONG US\ DONG GUOHUI US\ LI JICHENG US | JIANGSU YANGZI PACKING TECHNOLOGY CO LTD US | A61J1/10\ A61J1/14 |
| 743 | 3D printing | ANTI-FREEZING HEAT PIPE FLAT PLATE COLLECTOR\ | CN202869036U | WANG CHAOSHENG US\ RUAN GUOCHENG US | ANHUI HAITAIKE NEW ENEYGY TECHNOLOGY CO LTD US | F24J2/46\ F24J2/32\ F24J2/26 |
| 744 | 3D printing | HOT PIPE FLAT PLATE HEAT COLLECTOR\ | CN202869037U | WANG CHAOSHENG US\ RUAN GUOCHENG US | ANHUI HAITAIKE NEW ENEYGY TECHNOLOGY CO LTD US | F24J2/46\ F24J2/32\ F24J2/26 |
| 745 | 3D printing | SPIRAL TUBE TYPE HEAT TUBE FLAT PLATE COLLECTOR\ | CN202869038U | WANG CHAOSHENG US\ RUAN GUOCHENG US | ANHUI HAITAIKE NEW ENEYGY TECHNOLOGY CO LTD US | F24J2/20\ F24J2/32 |
| 746 | 3D printing | LITHIUM ION POWER BATTERY\ | CN202871898U | YANG XUANKUN US | SHANDONG FOREVER NEW ENERGY CO LTD US | H01M2/26\ H01M10/0525 |
| 747 | 3D printing | POSITIVE TAB WELDING NICKEL SHEET LITHIUM ION POLYMER BATTERY\ | CN202871919U | WANG PENG US\ LIU SUGUO US | WIDO NEW ENERGY CO LTD US | H01M4/66\ H01M2/26\ H01M10/0525 |
| 748 | 3D printing | ULTRASONIC DEGRADED MATERIAL FUSION CUTTING DEVICE\ | CN202878617U | CHEN SHICHANG US\ LU XIAOLI US | DONGGUAN JUSHENG PLASTIC & ELECTRONIC PRODUCTS CO LTD US | B29C45/38 |
| 749 | 3D printing | SEAMLESS MOBILE PHONE\ | CN202889434U | LIN TIANNENG US\ WU MING US | AMGOO TELECOM SHENZHEN CO LTD US | H04M1/02 |
| 750 | 3D printing | ULTRASONIC WELDING MACHINE FOR WHEEL CASING SAFE EDGE\ | CN202894588U | XU WEI US\ ZHANG CHUN US\ GUO YIPING US\ MA JI US | CHANGCHUN ENGLEY MOLD MFG CO LTD US | B23K20/10\ B23K20/26 |
| 751 | 3D printing | COMPENSATION TYPE EMITTER INSTALLATION PLACE ROTATION TYPE AUTOMATIC ASSEMBLING MACHINE\ | CN202895117U | ZHANG LIANG US | LAIWU SPRING RAIN DRIP IRRIGATION TECHNOLOGY CO LTD US | B25B27/00 |
| 752 | 3D printing | DEVICE FOR ULTRASONIC WELDING OF HIGH-DENSITY POLYETHYLENE (HDPE) WATERPROOF COILED MATERIAL\ | CN202895694U | HE HAICHAO US\ JIANG JUN US | SHANGHAI JWELL EXTRUSION MACHINERY CO LTD US\ SHANGHAI JWELL PIPE EQUIPMENT CO LTD US\ SHANGHAI JWELL PLATE & SHEET EQUIPMENT CO LTD US\ SHANGHAI JWELL CHEMICAL FIBER MACHINERY CO LTD US | B29C65/08 |
| 753 | 3D printing | NON-WOVEN FABRIC COVERING MACHINE FOR DRAIN BOARD OR DRAIN STRIP\ | CN202897693U | WU FAZHOU US | WU FAZHOU US | B65H81/06 |
| 754 | 3D printing | STARTING TYPE SUPER CAPACITOR MODULE\ | CN202905479U | ZHANG HUA US\ SU YINGDONG US\ XIONG TINGYU US\ YANG CONGLI US\ XU QIAN US | SHOTO GROUP CO LTD US | H01G11/76\ H01G11/10\ H01G11/82 |
| 755 | 3D printing | CLAMPING-BUCKLE-TYPE MOBILE CHARGING POWER SUPPLY SHELL\ | CN202907375U | WANG YONG US\ LIU RUNTIAN US | DONGGUAN VIAFINE ELECTRONIC & TECHNOLOGY LTD US | H05K5/06 |
| 756 | 3D printing | POWER BATTERY POSITIVE-NEGATIVE ELECTRODE TERMINAL FOUR-HEAD ULTRASONIC WELDING DEVICE\ | CN202910454U | LEI GUANGWEI US | LEI GUANGWEI US | B23K20/10\ B23K20/26 |
| 757 | 3D printing | ULTRASONIC PLASTIC TUBE FORMING MACHINE\ | CN202911150U | WANG WEI US | SHANTOU INNOVATION TECHNOLOGY ELECTRONICS CO LTD US | B29C69/00\ B29C65/08 |
| 758 | 3D printing | MELTING DEVICE FOR SPRUE OF INJECTION MOLDING PRODUCT\ | CN202911152U | CUI WENHAO US\ LI YONG US\ YE QINZHONG US\ CHEN BINQING US | SHANGHAI NO 1 FINE MOLDING CO LTD US | B29C65/08 |
| 759 | 3D printing | THREADED CONNECTION TYPE ULTRASONIC WELDING HEAD WITH LONG SERVICE LIFE\ | CN202911153U | CUI WENHAO US\ LI YONG US\ YE QINZHONG US\ CHEN BINQING US | SHANGHAI NO 1 FINE MOLDING CO LTD US | B23K20/10\ B29C65/08 |
| 760 | 3D printing | ULTRASONIC WELDING MACHINE\ | CN202911154U | LIU XINHUAI US\ YANG TINGGUI US\ WANG WANHONG US | DONGGUAN LANGDI GELINTE ELECTRIC APPLIANCES CO LTD US | B29C65/08 |
| 761 | 3D printing | STRONG-OFF TYPE CONNECTING STRUCTURE INSIDE CAR LIGHT\ | CN202911619U | GUAN LING US | CHANGCHUN HELLA AUTOMOTIVE LIGHTING LTD US | B60Q1/00 |
| 762 | 3D printing | MOTOR AND ASSEMBLY OF THREE-PHASE LEAD-OUT WIRE AND WIRING TERMINAL OF MOTOR\ | CN202918107U | YU PING US\ YANG KUI US\ YU ZHIMING US\ CAI WEI US | JINGJIN BAISITE ELECTRIC SHANGHAI CO LTD US | H01R4/02\ H02K5/22 |
| 763 | 3D printing | ULTRASONIC WELDING JIG FOR FILTER SCREEN\ | CN202922976U | WU ZHIYONG US | SHENZHEN JINSANWEI MOLD CO LTD US | B29C65/08\ B29L31/14 |
| 764 | 3D printing | ULTRASONIC WELDING HEAD FOR AUTOMOBILE INTERIOR AND EXTERIOR TRIMMING PRODUCTS\ | CN202922977U | ZHOU WEI US\ SONG ZHIBING US\ ZHANG HUILI US\ LI HONGPING US\ WANG SHOUPENG US\ JIN SHAOFENG US\ HU YU US | DONGFENG VISTEON SHIYAN AUTOMOTIVE TRIM SYSTEMS CO LTD US | B29C65/08 |
| 765 | 3D printing | TRANSFORMER\ | CN202940096U | QU WENJUN US | NINGBO ZHONGCE E T ELECTRONICS CO LTD US | B29C65/08\ H01F27/02 |
| 766 | 3D printing | POWER SUPPLY TOTAL SWITCH\ | CN202948869U | ZHANG RONGHUA US\ LU JIANGLI US\ ZHANG YANWEI US\ WANG TINGPING US\ MENG GUOLONG US\ SHI YUNTING US\ ZHANG LINGHUA US\ HAN QINGFU US\ HAN DAWEI US\ HAN HUI US\ TAN ZHENG US\ ZHAO JIE US | SINO TRUK JINAN POWER CO LTD US | H01H27/06 |
| 767 | 3D printing | NOVEL BRASSIE CAP SLEEVE SIGNBOARD\ | CN202956990U | JIN HAIYING US | JIN HAIYING US | G09F7/00 |
| 768 | 3D printing | FOLDABLE NAIL FILE\ | CN202959245U | LI YIQIANG US | LI YIQIANG US | A45D29/04 |
| 769 | 3D printing | ULTRASONIC SKELETON REPAIRING AND CUTTING WELDING DEVICE\ | CN202960716U | WU LIYE US\ HU LONGYUN US\ YAO HAIBIN US | WU LIYE US\ HU LONGYUN US\ YAO HAIBIN US | A61B17/58 |
| 770 | 3D printing | STABLE HIGH-EFFICIENCY WELDING WIRE MACHINE\ | CN202963780U | ZOU ZHIFENG US | ZOU ZHIFENG US | B23K20/10\ B23K20/26 |
| 771 | 3D printing | WELDING DEVICE FOR CONTACT PLATE\ | CN202965203U | DUAN HENG US | SUZHOU LINGOU AUTOMATION EQUIPMENT CO LTD US | B29C65/08 |
| 772 | 3D printing | ULTRASONIC BANDING DEVICE\ | CN202967664U | XUE YULONG US | KUNSHAN YONG AN NON WOVEN EX FACTORY US | B65H21/00 |
| 773 | 3D printing | FASTENER AND ELECTRIC DOOR LOCK USING FASTENER\ | CN202970165U | DAI CHUANG US | ZHAOQING ZHONGJING IND CO LTD US | E05B47/00\ E05B15/00 |
| 774 | 3D printing | VEHICLE-USED CONTACTLESS ELECTRONIC LOUDSPEAKER ADOPTING ULTRASONIC WELDING TECHNOLOGY\ | CN202976823U | ZHAO HONGWEI US | HARBIN GUTAI ELECTRONIC CO LTD US | G10K9/12 |
| 775 | 3D printing | AUTOMOTIVE ALARMING HORN\ | CN202976824U | LIU XUENAN US | LIU XUENAN US | G10K9/20 |
| 776 | 3D printing | DEVICE FOR CUTTING OFF CARBON NANO TUBES\ | CN202985804U | LI ZHENQI US\ LIU LIJUN US\ YAN JUN US | LI ZHENQI US\ LIU LIJUN US | B28D1/22 |
| 777 | 3D printing | ULTRASONIC WELDING MACHINE FOR SOUND BOX SHELL\ | CN202986103U | LONG FU US | JIANGXI RIZENG SCIENCE AND TECHNOLOGY CO LTD US | B29C65/08 |
| 778 | 3D printing | FLEXIBLE PACKAGE BATTERY SERIES-PARALLEL CONNECTING STRUCTURE\ | CN202996950U | GU YEMING US\ ZHANG YINGTAO US\ XIAO NINGQIANG US | BEIQI FOTON MOTOR CO LTD US | H01M2/26 |
| 779 | 3D printing | AIR FILTER\ | CN203002132U | ZHANG HONG US | PHILIPS CHINA INVEST CO LTD US | B01D46/10\ B01D53/02 |
| 780 | 3D printing | DEVICE FOR REMOVING METAL DEBRIS PRODUCED BY WELDING LITHIUM ION BATTERY POLE\ | CN203003334U | GU MINGHAI US\ HAN CHENGXIANG US\ SHI YANG US | HEFEI GUOXUAN HIGH TECH POWER ENERGY CO LTD US | B23K20/10\ B23K20/26\ B08B15/04 |
| 781 | 3D printing | INNER PLATE OF AUTOMOBILE PILLAR A\ | CN203005306U | THIERRY PIERRE CHARLES MANNI US | MECAPLAST CAR COMPONENTS SHANGHAI CO LTD US | B60R13/02\ B60R13/08 |
| 782 | 3D printing | HIGH-INTENSITY QUICK-POSITIONING B-SHAPED TRIM PILLAR OF CAR\ | CN203005545U | ZHU GUOHUA US\ ZHU XIAOMING US | SHANGHAI HUASHI MECHANICAL ELECTRICAL CO LTD US | B62D25/04 |
| 783 | 3D printing | WATERPROOF TELEPHONE WITH TOUCH DIALING\ | CN203014873U | LIN KUANNONG US | LIN KUANNONG US | H04M1/02 |
| 784 | 3D printing | SOUND BOX ULTRASONIC WELDING STRUCTURE\ | CN203015060U | XIAO GANG US | GUOGUANG ELECTRIC CO LTD US\ GUANGZHOU YANGCHENG PREC ELECTRONIC CO LTD US | H04R31/00\ H04R1/02\ B29C65/08 |
| 785 | 3D printing | VOICE HORN FOR VEHICLE\ | CN203015111U | LIU XUENAN US | LIU XUENAN US | H04R9/02\ H04R9/06 |
| 786 | 3D printing | ULTRASONIC TOOL HEAD\ | CN203018298U | CHEN YUANPING US\ HUA DACHENG US\ CHEN CHUAN US\ XU PAN US | HANGZHOU SUCCESS ULTRASONIC EQUIPMENT CO LTD US | B06B3/00\ B06B1/02 |
| 787 | 3D printing | CURRENT COLLECTOR WELDING STRUCTURE OF HIGH-POWER LITHIUM ION POWER BATTERY\ | CN203026587U | LIU YANG US\ CHU BAIQING US | QINHUANGDAO XINCHI PHOTOELECTRICITY TECHNOLOGY CO LTD US | H01M4/70\ H01M4/64 |
| 788 | 3D printing | ULTRASONIC WELDING JOINT FOR AUTOMOBILE PLASTIC PART\ | CN203031956U | PAN BAOPING US | WUHU XINBAO ULTRASONIC EQUIPMENT CO LTD US | B29C65/08 |
| 789 | 3D printing | END PORTION PHASE-TO-PHASE INSULATION PART\ | CN203039456U | LI WEN (US) | ZHUHAI HAOTIAN ELECTRICAL TECHNOLOGY CO LTD US | H02K3/38\ H02K3/34 |
| 790 | 3D printing | BELT MOUNTING DEVICE OF GAUZE MASK PIECE\ | CN203040760U | LIU JING US | LIU JING US | A41D13/11 |
| 791 | 3D printing | UPPER-INCOMING LOWER OUTGOING TYPE ANTI-THEFT AMMETER\ | CN203054036U | HU DONGFANG US\ HU DONGJUN US\ LIU GUANG US | ZHEJIANG RISESUN SCIENCE AND TECHNOLOGY CO LTD US | G01R11/04\ G01R11/24 |
| 792 | 3D printing | INFANT ANTI-THEFT FOOT RING\ | CN203055096U | ZHANG BANGQUN US\ ZHANG JIE US\ WU GUANGZE US | XIAMEN ANMENG NETWORK INFORMATION CO LTD US | G08B21/02 |
| 793 | 3D printing | FUSE STRUCTURE WITH HIGH VOLTAGE LEVEL\ | CN203071028U | HU XINGHUA US | HOLLY CHINA ELECTRONIC TECHNOLOGY CO LTD US | H01H85/055\ H01H85/042 |
| 794 | 3D printing | AUTOMOBILE INSTRUMENT PANEL HEAT RADIATION WELDING MACHINE\ | CN203077619U | PAN BAOPING US | WUHU XINBAO ULTRASONIC EQUIPMENT CO LTD US | B29C65/02\ B29C65/08 |
| 795 | 3D printing | WATER-PROOF ELECTRONIC TAG\ | CN203084754U | ZHANG JIE US\ SI HAITAO US\ WU JUN US\ GAO BO US | NINGBO LIXIN RADIO FREQUENCY CO LTD US | G06K19/02 |
| 796 | 3D printing | ANTIHALATION STRUCTURE OF TRANSPARENT ORGANIC MATERIAL LIGHT GUIDE PANEL\ | CN203085103U | ZENG WEIMING US\ XU JUN US\ XU HUACHANG US\ LI XIANZAO US | SHANGHAI AVIAT ELECTRIC CO LTD US | F21V8/00\ G09F13/04 |
| 797 | 3D printing | ELECTROACOUSTIC TRANSDUCER\ | CN203086725U | SUN LIGUO US\ ZHANG CHENGFEI US | GOERTEK INC US | H04R9/02\ H04R9/06 |
| 798 | 3D printing | BLOWING NOZZLE OF CCD (CHARGE COUPLED DEVICE) COLOR SELECTOR\ | CN203091300U | HU XIANWEN US\ YAN TIANXIN US\ XU DAHONG US | HEFEI TAIHE OPTOELECTRONIC TECHNOLOGY CO LTD US | B07C5/342 |
| 799 | 3D printing | FUEL DISTRIBUTION PIPE OF AUTOMOBILE ENGINE\ | CN203098113U | LIU TAO US | WUHAN MEIJIA MACHINERY PLASTIC CO LTD US | F02M55/02 |
| 800 | 3D printing | WATERPROOF TYPE INTERPHONE BATTERY BOX\ | CN203103372U | HONG YUEZHI US | QUANZHOU FEIJIE ELECTRONIC CO LTD US | H04M1/02\ H01M2/10 |
| 801 | 3D printing | ULTRASONIC WELDING EQUIPMENT SAFETY PROTECTION DEVICE\ | CN203103901U | WU ZHONGKAI US\ WANG LIANGCHENG US | SHANGHANG XIANGHUI ELECTRONIC CO LTD US | H02H3/00 |
| 802 | 3D printing | ULTRASONIC WELDING MACHINE\ | CN203109436U | SU WEI US | SUZHOU KEBER PREC MACHINERY CO US | B23K20/10\ B29C65/08 |
| 803 | 3D printing | CAR QUARTER WINDOW ULTRASONIC HOT RIVETING WELDING ALL-IN-ONE MACHINE\ | CN203109456U | PAN BAOPING US | WUHU XINBAO ULTRASONIC EQUIPMENT CO LTD US | B23K20/10\ B23K28/02 |
| 804 | 3D printing | SPRING CUSHION\ | CN203121739U | HUANG JINBO US | SHENZHEN BLOSUN HARDWARE PRODUCTS CO LTD US | A47C27/04 |
| 805 | 3D printing | ULTRASONIC WELDING JIG DEVICE OF NOVEL LITHIUM-ION POWER BATTERY\ | CN203125024U | TIAN LE US\ DENG SHI US\ FENG QUANSHAN US | LISHEN MILES POWER BATTERY SYS US | B23K20/10\ B23K37/04 |
| 806 | 3D printing | TEMPERATURE SENSOR\ | CN203132712U | FU YU US | FU YU US | G01K1/08 |
| 807 | 3D printing | TENSILE STRENGTH EXPERIMENT CLAMP OF ULTRASONIC WELDING POINT\ | CN203132916U | KU YUAN US\ REN ZHEN AN US\ CAO LEI US\ MENG FANRUI US\ WANG YANHONG US | CHANGCHUN FAWAY JOHNSON CONTROLS AUTOMOTIVE SYSTEMS CO LTD US | G01N3/04 |
| 808 | 3D printing | HORIZONTAL ULTRASONIC WELDING DIE\ | CN203141847U | CHEN BO US | SHENZHEN KUNXING TECHNOLOGY CO LTD US | B29C65/08 |
| 809 | 3D printing | THREE-CORE-WIRE AUTOMATIC ASSEMBLING EQUIPMENT\ | CN203150871U | LIU LIANQIAO US\ WANG SHOUMING US\ XIN RUI US\ SUN XIANG US\ WANG CHI US | TOPA AUTOMATION EQUIPMENT & TECHNOLOGY CO LTD US | H01R43/20\ H01R43/00\ H01R43/052\ H01R43/05 |
| 810 | 3D printing | ENERGY-SAVING AND ENVIRONMENT-FRIENDLY FLOATING TOY\ | CN203154816U | ZHENG XIAOPENG US | ZHENG XIAOPENG US | A63H23/10 |
| 811 | 3D printing | ULTRASONIC WELDING WORKBENCH\ | CN203156227U | ZHANG MI US\ MING XIN US\ HU CHUNQUAN US | TIANJIN EMPIRETEST CORP US | B23K20/10\ B29C65/08 |
| 812 | 3D printing | LICENSE PLATE LAMP WITH FUNCTION OF LED AUTOMATICALLY DIAGNOSING\ | CN203162797U | SU CHAOPIN US | JIANGSU XINTIANYANG MACHINERY MFG CO LTD US | F21S8/10\ F21Y101/02\ F21W101/02\ F21V23/02 |
| 813 | 3D printing | PACKAGING DEVICE FOR PLASTIC HOUSING OF LITHIUM-ION BATTERY SINGLE CELL\ | CN203166021U | CHEN YANZHONG US | DONGGUAN RUNGUANG BATTERY TECHNOLOGY CO LTD US | H01M10/058 |
| 814 | 3D printing | MANUFACTURING DEVICE\ | CN203169431U | MATSUMOTO YOSHIHIKO US | UNI CHARM CORP US | A61F13/15\ D06H5/00 |
| 815 | 3D printing | FACE CARE DEVICE\ | CN203169553U | LU KEYAN US | LU KEYAN US | A61H23/00 |
| 816 | 3D printing | BACKWARD-STYLE CENTRIFUGAL FAN BLADE\ | CN203175975U | GAO WENMING US\ HAN XIAOHONG US\ SHI JIANLI US | NINGBO LANGDI IMPELLER MACH CO US | F04D29/30\ F04D29/28 |
| 817 | 3D printing | SC TYPE OPTICAL FIBER ADAPTER\ | CN203178523U | ZHANG CHANGBO US | NANJING SEEME OPTOELECTRONIC EQUIPMENTS CO LTD US | G02B6/38 |
| 818 | 3D printing | PRECISION SPOT WELDING MACHINE\ | CN203185649U | ZENG XUEZHEN US | ZENG XUEZHEN US | B29C65/08 |
| 819 | 3D printing | RED WINE STOPPER\ | CN203186760U | HUANG SHAODONG US | HUANG SHAODONG US | B65D39/00\ B65D39/16 |
| 820 | 3D printing | WIND COLLECTION PIECE OF THREE-DIMENSIONAL FLOW FAN AND THREE-DIMENSIONAL FLOW FAN\ | CN203189307U | DENG XIANGTAO US | SUZHOU IND PARK SIP CINDERSON MOTOR CO LTD US | F04D29/28\ F04D29/00\ F04D25/08\ H02K7/14 |
| 821 | 3D printing | STORAGE BATTERY MIDDLE COVER WITH COVER PLATE EASY TO MOUNT\ | CN203192887U | LI JUN US\ FANG MINGXUE US | JIANGSU NEW ENERGY CO LTD ZHEJIANG TIANNENG BATTERY US | H01M2/10 |
| 822 | 3D printing | CUP COVER WITH INNER PLUG\ | CN203195274U | YANG GUIQIU US | SHANGHAI SUIXIN TRADE CO LTD US | A47G19/22 |
| 823 | 3D printing | ULTRASONIC WELDING HEAD\ | CN203197454U | CHEN YUANPING US\ HUA DACHENG US\ CHEN CHUAN US\ WANG JIANFENG US | HANGZHOU SUCCESS ULTRASONIC EQUIPMENT CO LTD US | B23K20/10\ B29C65/08\ A41H43/04 |
| 824 | 3D printing | LIGHT DISTRIBUTION LENS FOR LED LAMP\ | CN203202906U | LIU GUOXUE US\ WANG BO US | CHANGZHOU TONGBAO LIGHTING CO LTD US | F21Y101/02\ F21V5/04\ F21W101/02\ B60Q1/56 |
| 825 | 3D printing | LED (LIGHT-EMITTING DIODE) LAMP PANEL\ | CN203215587U | YAN YANYAN US | YAN YANYAN US | F21Y101/02\ F21V19/00 |
| 826 | 3D printing | ANTI-REFLUX STRUCTURE OF BLOOD TAKING NEEDLE\ | CN203220372U | XIONG YANFANG US | XIONG YANFANG US | A61B5/155 |
| 827 | 3D printing | BACKFLOW-PREVENTION CONNECTING DEVICE USED FOR FLUSHING ASPIRATOR\ | CN203220628U | ZHENG XIQING US | ZHEJIANG GEYI MEDICAL INSTR CO LTD US | A61M1/00\ A61M3/02 |
| 828 | 3D printing | SAVE-TYPE PHASE SEGREGATED PAPER\ | CN203243141U | ZHANG YAODONG US\ LI ZHUOQI US\ QIAN LIFENG US\ ZHANG YI US\ SHEN GANG US\ ZHU XINYU US | NANTONG YANGTZE RIVER ELECTRIC IND CO LTD US | H02K3/34 |
| 829 | 3D printing | DISPOSABLE WATERPROOF ANTI-COUNTERFEITING BUCKLE\ | CN203243823U | QU LE US | SUZHOU JINHETONG SOFTWARE CO LTD US | A01K61/00 |
| 830 | 3D printing | HYDRAULIC RELEASE BEARING UTILIZING WELDING PLUGS\ | CN203248916U | SHI HAOFENG US\ ZHANG DINGLONG US\ ZHANG SHUFENG US | NINGBO HONGXIE CLUTCH CO LTD US | F16L55/11 |
| 831 | 3D printing | DISPOSABLE WATERPROOF BINDING BOARD\ | CN203250476U | QU LE US | SUZHOU JINHETONG SOFTWARE CO LTD US | G09F3/14 |
| 832 | 3D printing | REAR COVER STRUCTURE OF LOUDSPEAKER\ | CN203251403U | YAN JUN US\ SHI SONGFU US | NINGBO TONWEL AUDIO CO LTD US | H04R1/02 |
| 833 | 3D printing | OVEN FOOD PACKAGING BAG CONVENIENT TO USE\ | CN203255521U | GAO HU US\ ZOU YANHONG US | HUIZHOU ALCAN PACKAGING PROPACK CO LTD US | B65D33/00\ B65D81/34 |
| 834 | 3D printing | THROTTLE KNOB DEVICE\ | CN203257546U | QIAN JIANDONG US | CHANGZHOU DINGXING ELECTRONIC CO LTD US | F02D11/02 |
| 835 | 3D printing | LOCK CATCHES FOR MAKING EYES AND NOSE OF TOY\ | CN203264264U | LI YONGBIN US | ANHUI TANYU TOYS CO LTD US | A63H3/36 |
| 836 | 3D printing | WELDING UNIT FOR FOUR-AXIS ULTRASONIC WELDING MACHINE\ | CN203265879U | SU WEI US | SUZHOU KEBER PREC MACHINERY CO US | B23K20/10\ B29C65/08 |
| 837 | 3D printing | AUTOMATIC SELECTION WORKBENCH FOR FOUR-AXIS ULTRASONIC WELDING MACHINE\ | CN203265882U | SU WEI US | SUZHOU KEBER PREC MACHINERY CO US | B23K20/10\ B23K20/26 |
| 838 | 3D printing | AUTOMATIC DIE CHANGING MECHANISM FOR FOUR-AXIS ULTRASONIC WELDING MACHINE\ | CN203265883U | SU WEI US | SUZHOU KEBER PREC MACHINERY CO US | B23K37/02\ B23K20/26 |
| 839 | 3D printing | ULTRASONIC WELDING MACHINE FOR VEHICLE TAIL BUMPERS\ | CN203267213U | SU WEI US | SUZHOU KEBER PREC MACHINERY CO US | B29C65/08 |
| 840 | 3D printing | MULTILAYER THERMAL INSULATION SEALING COVER\ | CN203268578U | HU GANG US | HU GANG US | A47J41/02\ B65D41/02 |
| 841 | 3D printing | ULTRASONIC WELDING MACHINE MATERIAL ROLLING DEVICE\ | CN203269180U | SONG GUOFANG US | SONG GUOFANG US | B65H35/02\ B65H18/20 |
| 842 | 3D printing | PET NECKLACE POWER CONTROLLER\ | CN203273774U | YE YANGCHUN US | YE YANGCHUN US | F21V23/00\ F21V31/00\ H05B37/02 |
| 843 | 3D printing | FIXING MECHANISM OF DIAPHRAGM AND PARALLEL PLATE OF GAS METER\ | CN203274833U | LIN YING US | ANHUI HONGLING INTELLIGENT METER TECHNOLOGY CO LTD US | G01F15/16 |
| 844 | 3D printing | ELECTRIC LEAKAGE PROTECTION PLUG\ | CN203277803U | QIAN JIACAN US | YUYAO CITY JIARONG ELECTRONIC ELECTRICAL APPLIANCE CO LTD US | H01R13/52 |
| 845 | 3D printing | NOVEL SWITCH POWER SUPPLY ADAPTER\ | CN203278610U | ZHANG WEIMING US | SOMETHING HIGH ELECTRIC XIAMEN CO LTD US | H02M1/00 |
| 846 | 3D printing | ULTRASONIC WELDING MACHINE\ | CN203282041U | KAN YANPING US\ SU YUMING US\ LIU JIA US | TIANJIN TAISHIKANG PHARMACEUTICALS SCIENCE TECHNIC CO LTD US | B23K20/10\ B29C65/08 |
| 847 | 3D printing | LC ADAPTER HOUSING AND LC ADAPTER\ | CN203287564U | GAO LIQING US\ YAO YUAN US | SUZHOU KELANGUANG COMM CO LTD US | G02B6/38 |
| 848 | 3D printing | AUTOMOBILE WIRE HARNESS\ | CN203288805U | ZHENG JUN US\ REN WEIXING US\ ZHANG KAIJUN US\ JIN TAO US\ YE FEIHONG US | NINGBO HENGFU AUTO PARTS DEV CO LTD US | H01R4/02\ H01R13/52\ H01R31/06\ H01R13/02 |
| 849 | 3D printing | AUTOMATIC TRANSFERRING DEVICE OF POLYMER BATTERY ALUMINUM LEAD AND NICKEL ALUMINUM COMPOSITE BELT\ | CN203292694U | ZHANG WANLIANG US\ YU SHENJUN US | HANGZHOU PEOPLE POWER BATTERY CO LTD US | B23K20/10\ B23K20/26 |
| 850 | 3D printing | ULTRASONIC WELDING HEAD ASSEMBLY AND ULTRASONIC WELDING MACHINE\ | CN203292695U | ZHANG XIUCHONG US\ YANG RUKUN (CN) | SHENZHEN GEESUN AUTOMATION TECHNOLOGY CO LTD US | B23K20/10\ B23K37/02\ B23K20/26 |
| 851 | 3D printing | THIN USB PLUG\ | CN203300859U | TU ZHENGHUI US | TU ZHENGHUI US | H01R13/504\ H01R13/03\ H01R13/40 |
| 852 | 3D printing | WELDING DEVICE OF BATTERY ELECTRODE TAB\ | CN203304777U | JIA YULONG US | TIANJIN LISHEN BATTERY (CN) | B23K20/10\ B23K20/26\ B23D31/00 |
| 853 | 3D printing | TORSION TYPE ULTRASONIC WELDING DEVICE\ | CN203304778U | LIN SHUYU US\ JIANG XIAOMING US\ YAN ZHUOSHENG (CN)\ YAN ZHUOLI (CN)\ HUANG WEISHU (CN) | GUANGZHOU NEWPOWER ULTRASONIC ELECTRONIC EQUIPMENT CO LTD (CN) | B23K20/10\ B06B1/02 |
| 854 | 3D printing | ULTRASONIC WELDING STRUCTURE FOR PLASTIC PLANETARY GEARBOX\ | CN203309073U | DENG SHUPING US | WUXI LONGSHENG TECHNOLOGY CO US | B29C65/08\ F16H57/02 |
| 855 | 3D printing | FRONT COVERED PROTECTING AND REAR FOLDED SUPPORTING MID HOUSING\ | CN203311311U | HUANG ZHENG US | HUANG ZHENG US | G06F1/16 |
| 856 | 3D printing | ASSEMBLY STRUCTURE OF PEDESTAL AND YOKE IRON OF RELAY WITH ADJUSTABLE OVER TRAVEL\ | CN203312172U | HE LIANHUI US | HELLA XIAMEN AUTOMOTIVE ELECTRONICS CO LTD US | H01H50/02 |
| 857 | 3D printing | PROTECTIVE CAP FOR INFUSION JOINT\ | CN203315443U | CHEN MIN US\ XIAO WEITAO US\ BAI XICHUAN US | SHENGGUANG MEDICAL INSTR CO LTD US | A61M5/14 |
| 858 | 3D printing | HAND-HELD TYPE ANGLE GRINDER\ | CN203317187U | WU HAODONG US | ZHEJIANG BODA IND CO LTD US | B24B23/02 |
| 859 | 3D printing | VACUUM ONE-WAY VALVE\ | CN203318395U | ZHU XIANQIAO US\ JIANG DAPING US\ YANG JIANLI US\ SANG LANFEN US\ SHI YAQIONG US | NINGBO QIAOSHI RUBBER & PLASTICS CO LTD US | F16K27/10\ B60T13/57 |
| 860 | 3D printing | WATERPROOF ELECTRIC LEAKAGE PROTECTION PLUG\ | CN203326256U | ZHANG ZUDE US | CHANGSHU LIDE ELECTRICAL APPLIANCES CO LTD US | H01R13/52 |
| 861 | 3D printing | GLOVE BOX ASSEMBLY\ | CN203331969U | XIA BINGMEI US\ RONG JIAN US\ LV LICHENG US | TIANJIN HUATAO AUTO PLASTIC DECORATION PARTS CO LTD US | B60R7/06 |
| 862 | 3D printing | AUTOMATIC PACKAGING MACHINE FOR ULTRASONIC WELDING FILMS OF ALUMINUM ALLOY SECTIONS\ | CN203332472U | LI GUANHUA US | LI GUANHUA US | B65B51/22 |
| 863 | 3D printing | FOUR-SHAFT ULTRASONIC WELDING MACHINE\ | CN203343610U | SU WEI US | SUZHOU KEBER PREC MACHINERY CO US | B23K20/10\ B29C65/08 |
| 864 | 3D printing | ULTRASONIC WELDING MACHINE FOR CAR FRONT BUMPER\ | CN203344318U | SU WEI US | SUZHOU KEBER PREC MACHINERY CO US | B29C65/08\ B26F1/00 |
| 865 | 3D printing | FLOOR MOP REDUCING PRODUCTION COST\ | CN203354489U | DAN BROWN US | QINGDAO AIXING CLEANING PRODUCTS CO LTD US | B32B5/24\ A47L13/16 |
| 866 | 3D printing | ULTRASONIC WAVE MASK WIRE WELDING EQUIPMENT\ | CN203356843U | WU RENJIE US | BROADFAIR NON WOVEN CONVERTING MACHINERY CO LTD US | B23K20/10 |
| 867 | 3D printing | WELDING DEVICE OF ULTRASONIC MASK EAR BELT\ | CN203356844U | WU RENJIE US | BROADFAIR NON WOVEN CONVERTING MACHINERY CO LTD US | B23K20/10\ B23K20/26 |
| 868 | 3D printing | ULTRASONIC WELDING MACHINE\ | CN203356845U | CHEN XIAOMING US | JIANGSU MARITIME INST US | B23K20/10\ B23K37/02\ B23K37/047 |
| 869 | 3D printing | ULTRASONIC WELDING DEVICE FOR RESPIRATOR BELTS\ | CN203357891U | WU RENJIE US | BROADFAIR NON WOVEN CONVERTING MACHINERY CO LTD US | B29C65/08 |
| 870 | 3D printing | ULTRASONIC RESPIRATOR EAR BAND WELDING MACHINE\ | CN203357892U | WU RENJIE US | BROADFAIR NON WOVEN CONVERTING MACHINERY CO LTD US | B29C65/08\ B29C65/78\ A41D13/11 |
| 871 | 3D printing | ULTRASONIC BIOLOGICAL BOX WELDING MACHINE\ | CN203357893U | SU WEI US | SUZHOU KEBER PREC MACHINERY CO US | B23K20/10\ B29C65/08 |
| 872 | 3D printing | MULTIFUNCTIONAL BAG MAKING MACHINE\ | CN203357952U | HUANG SUFEI US\ LIN XINGSHENG US | HUANG SUFEI US | B31B1/64\ B31B1/00\ B31B1/20\ B31B1/74 |
| 873 | 3D printing | ROTARY FULL-AUTOMATIC DETONATING TUBE MACHINE\ | CN203359667U | LIU YI US\ DUAN XIAOXU US\ LIU ZHENYU US\ WANG KUAN US\ GAO HONGWEI US | CHANGCHUN HUIWEI SCIENCE & TECHNOLOGY DEV CO LTD US | B65H54/10\ B65B27/06 |
| 874 | 3D printing | FLANGE PLATE OPTICAL FIBER ADAPTER AUTOMATIC ASSEMBLING AND WELDING MACHINE\ | CN203365721U | FU XINYONG US | CIXI BOSHI AUTOMATION EQUIPMENT FACTORY US | G02B6/255 |
| 875 | 3D printing | LOUDSPEAKER MODULE\ | CN203368684U | WU DI US\ SHE XIAODONG US | GOERTEK INC US | H04R1/02 |
| 876 | 3D printing | LOUDSPEAKER MODULE\ | CN203368694U | LIU YITAO US\ MIAO QING US\ SHAO SHUAI US\ SONG XUEPING US | GOERTEK INC US | H04R1/04 |
| 877 | 3D printing | TRIANGLE WARNING SIGN\ | CN2600893Y | WANG WENCAI (CN) | TIANJIN SHENGUANG NEW TECHNOLO (CN) | G09F13/16\ G09F13/20 |
| 878 | 3D printing | PLASTIC FILM PACKAGING BAG MAKING MACHINE\ | CN2604309Y | CHEN YUEWEN (CN) | CHEN YUEWEN (CN) | B29C65/08 |
| 879 | 3D printing | GAS VALVE ASSEMBLY FOR LIGHTER\ | CN2605485Y | HUANG ZIQIANG (CN) | FULIU SCIENCE TECHNOLOGY CO LT (CN) | F23Q2/173 |
| 880 | 3D printing | PAPER-FIREWORK AIR-STORAGE DEVICE\ | CN2642380Y | QI WEIWEI DING (CN) | TIANTAI COUNTY DAZHIXI HANDICR (CN) | A63H33/18\ A63H37/00 |
| 881 | 3D printing | BUFFER COMPENSATION TYPE DROPLET HEAD\ | CN2643645Y | SUN ZHENHAI (CN) | SUN ZHENHAI (CN) | A01G25/02 |
| 882 | 3D printing | SEALING STRUCTURE OF CIGARETTE LIGHTERS\ | CN2658594Y | DAI GUANG (CN) | DAI GUANG (CN) | F23Q2/16\ F23Q2/34 |
| 883 | 3D printing | ASSEMBLIED CASE DEVICE OF CONNECTOR\ | CN2678179Y | LIN SHU (CN)\ PENG XIAOBING (CN) | LIANZHAN SCIENCE & TECHNOLOGY (CN) | H01R13/46 |
| 884 | 3D printing | STEPPED BACK-STACK-SEALED HAGGED SPRING CONVEYING DRUM DEVICE\ | CN2687098Y | CHEN CHAOGUANG (CN)\ HUANG YUYING (CN) | CHEN CHAOGUANG (CN) | B68G9/00 |
| 885 | 3D printing | ALUMINIUM PIPE FORMING DIE FOR ALUMINIUM PLASTIC COMPOSITE PIPE PRODUCED BY ULTRASONIC WELDING\ | CN2715874Y | SHI JINGDONG (CN)\ CHEN ZHONGZHI (CN)\ SHI ZHONGBEN (CN) | SHI JINGDONG (CN) | B23K20/10\ B29C47/02\ B21C37/08 |
| 886 | 3D printing | EARTHWORK GRILLE ULTRASONIC WELDING MACHINE\ | CN2753569Y | HOU SHULIN (CN)\ WANG AIMIN (CN)\ ZHAO YONGSHOU (CN)\ CAI JUN (CN) | HOU SHULIN (CN) | B29C65/08 |
| 887 | 3D printing | HEAT TRANSFER CORE STRIP OF SOLAR ENERGY COLLECTOR\ | CN2757035Y | PAN GE (CN) | PAN GE (CN) | F24J2/46 |
| 888 | 3D printing | ULTRASONIC CASTING-CONNECTING MACHINE FOR ROPE-THROUGH BUCKLE OF PLASTIC TENT CLOTH\ | CN2763026Y | ZHENG DAOZHEN (CN) | JIAOHEJIGONG PLASTIC CO LTD QI (CN) | B29C65/08 |
| 889 | 3D printing | ULTRASONIC PRESS WELDING MACHINE HAVING PRESSURE ADAPTIVE FUNCTION\ | CN2776616Y | LUO LIN (CN)\ TANG WENXUAN (CN)\ PANG XIAODONG (CN) | WEIXUN AUTOMATION APPARATUS CO (CN) | B23K20/10 |
| 890 | 3D printing | SUPPORT DEVICE FOR ULTRASONIC WELDING APPARATUS\ | CN2778455Y | ZHOU BISHAN (CN) | ZHOU BISHAN (CN) | B23K20/10\ B23K37/02 |
| 891 | 3D printing | WINE BOTTLE CAP WITH ALUMINIUM-PLASTIC MEMBRANE\ | CN2786006Y | XU RONGGUI (CN) | XU RONGGUI (CN) | B65D41/34 |
| 892 | 3D printing | DOUBLE-LAYER INSULATION DISMOUNTABLE CUP BODY\ | CN2788683Y | YE FANGYING (CN) | BEIQIN BABY S ARTICLES SHANGHA (CN) | A47G19/22\ A47J41/00\ A61J9/00 |
| 893 | 3D printing | AUTOMATIC ASSEMBLING SYSTEM OF HARD DISK HEAD PILE OF COMPUTER\ | CN2793876Y | PAN LIMING (CN)\ SHI JIEFU (CN)\ YU HUARONG (CN)\ WU YUZHEN (CN)\ HE LIU (CN)\ LI ZHENHUA (CN)\ YE WEI (CN)\ XIONG YONGFANG (CN)\ ZHOU YONG (CN)\ WANG DONGMIN (CN)\ CHENG JINZHENG (CN)\ LING SHENGLING (CN)\ LIU GUOAN (CN) | SHENZHEN DEV SCIENCE AND TECHN (CN) | G11B21/00\ G11B33/00 |
| 894 | 3D printing | ELECTRIC ENERGY METER\ | CN2804879Y | HU SHENG (CN)\ LU HANXI (CN)\ SHAO QUANYU (CN) | LINYANG ELECTRONIC CO LTD JIAN (CN) | G01R11/04 |
| 895 | 3D printing | TRANSLATION WELDING TYPE ULTRASONIC WELDING UNIT\ | CN2806034Y | CHEN YUEWEN (CN) | CHEN YUEWEN (CN) | B23K20/10\ B29C65/08 |
| 896 | 3D printing | ULTRASONIC WELDING PIN\ | CN2818049Y | LING SHENGLING (CN)\ JIANG PING (CN)\ WU CHUANGHUA (CN) | SHENZHEN DEV SCIENCE AND TECHN (CN) | B23K20/10 |
| 897 | 3D printing | CUP WITH POURING SUBSTANCE\ | CN2819976Y | CHEN QIZHE (CN) | CHEN QIZHE (CN) | A47G19/16\ A47J31/06 |
| 898 | 3D printing | LITHIUM CELL\ | CN2821882Y | ZHU DEHONG (CN) | AIBOER NEW ENERGY SOURE CO LTD (CN) | H01M6/14\ H01M2/02\ H01M6/42 |
| 899 | 3D printing | ELECTRIC ISOLATION SAFETY WATER PIPE JOINT\ | CN2823909Y | WANG BOHONG (CN) | HUNAN WUJIANG QINGHUA GROUP CO (CN) | F24H9/00 |
| 900 | 3D printing | MACHINE FOR INTEGRAL SEALING PLASTIC CAP ON BOTTLE\ | CN2828001Y | WANG YANJUN (CN) | WANG YANJUN (CN) | B67B3/00 |
| 901 | 3D printing | PAPERWEIGHT\ | CN2843899Y | SONG PING (CN) | SONG PING (CN) | B43M9/00 |
| 902 | 3D printing | ULTRASONIC WELDING LINE APPTS.\ | CN2882878Y | SHEN YUNDE (CN) | ZHOU XUZHENG (CN) | B23K20/10\ B23K37/02\ H01L21/607\ B23K37/04\ B23Q7/00 |
| 903 | 3D printing | ULTRASONIC WAVE WELDING APPARATUS FOR PAPER CUP BOWL MAKING MACHINERY\ | CN2892352Y | CHEN ZIPING PAN (CN) | CHEN ZIPING (CN) | B23K20/10\ B29C65/08 |
| 904 | 3D printing | TRANSLATIONAL WELDING TYPE ULTRASONIC WELDED TEMPLATE\ | CN2894917Y | CHEN YUEWEN (CN) | CHEN YUEWEN (CN) | B23K20/10\ B29C65/08 |
| 905 | 3D printing | HOLLOW SURFACE STICKING MACHINE FOR PAPER CUP AND PAPER BOWL\ | CN2900155Y | CHEN ZHIPING PAN (CN) | CHEN ZHIPING (CN) | B31C1/04\ B31C1/08 |
| 906 | 3D printing | COLUMN LITHIUM ION CELL\ | CN2904320Y | FU LIPING (CN) | FU LIPING (CN) | H01M2/16\ H01M4/66\ H01M10/38\ H01M10/40 |
| 907 | 3D printing | ASSEMBLING PRODUCTION LINE FOR ELECTRONIC PRODUCTS\ | CN2904602Y | DU RUIZHAO (CN) | HUANLONG ELECTRICAL CO LTD (CN) | H05K13/08\ H05K13/04 |
| 908 | 3D printing | STRENGTHENING STEEL-PLASTIC MESH FOR SINGLE-SIDE BACK-FOLD WELDING ENVELOPED EDGE AND ULTRASONIC SELVAGE FOLD WELDING MACHINE\ | CN2913614Y | CHEN SHULIN (CN) | CHEN SHULIN (CN) | E21F15/04\ B29C35/10\ B29C53/04\ E21D19/06 |
| 909 | 3D printing | DISPOSABLE STERILE STRAIGHT TYPE NEEDLE-FREE TRANSFUSION JOINT\ | CN2917663Y | JING WENPU CHEN (CN) | MA LIXUN (CN) | A61M5/00 |
| 910 | 3D printing | ANALOGUE ADAPTIVE CARD STRUCTURE\ | CN2924649Y | SHI WENSHUAN (CN) | QUANXIN CO LTD (CN) | G06K17/00\ H01R12/16\ H01R27/00\ H01R31/06 |
| 911 | 3D printing | ELECTRONIC CANDLE\ | CN2926762Y | BIN (NL) | BIN (NL) | F21Y101/02\ F21V23/00\ F21W121/00\ F21L4/08\ H02J7/02\ H05B37/02 |
| 912 | 3D printing | AUTOMATIC LITHIUM-ION BATTERY PELLETER\ | CN2927328Y | SUN KECHENG ZHANG (CN) | HUAGUAN ELECTRONIC SCIENCE & T (CN) | H01M4/04\ H01M10/38\ H01M10/40 |
| 913 | 3D printing | FEED GEAR OF ULTRASONIC HEAT SEALING MACHINE\ | CN2933836Y | CHEN YINGSHENG (CN) | HURG IND CO LTD (CN) | B29C65/08\ B29C65/78 |
| 914 | 3D printing | ULTRASONIC WELDING DEVICE FOR WELDING LITHIUM ION CELL POLE EAR AND LID MODULE\ | CN2936531Y | LI WEILING (CN) | BIKE BATTERY CO LTD SHENZHEN C (CN) | B23K20/10\ B23K37/04\ B23K20/26 |
| 915 | 3D printing | WELDER FOR LITHIUM ION CELL POSITIVE AND NEGATIVE POLE\ | CN2936536Y | FAN WANYONG DING (CN) | LISHENG BATTERY CO LTD TIANJIN (CN) | B23K11/00\ B23K20/10\ B23K28/02 |
| 916 | 3D printing | VEHICLE PANEL CONNECTING STRUCTURE\ | CN2937594Y | LI XIREN (CN) | YANFENG WEISHITONG VEHICLE ORN (CN) | F16B5/02 |
| 917 | 3D printing | OPTICAL FIBRE ADAPTIVE\ | CN2938150Y | HONG JIAN ZHANG (CN) | HONG JIAN (CN) | G02B6/36 |
| 918 | 3D printing | FIXTURE FOR GRIPPING PLASTIC PARTS DURING VIBRATION WELDING PARTICULARLY DURING ULTRASONIC WELDING\ | CZ14043U1 | CECH JAN ING (CZ)\ HERMA ALES ING (CZ) | ALFA PLASTIK (CZ) | B29C65/08 |
| 919 | 3D printing | FIXTURE FOR GRIPPING PLASTIC PARTS DURING VIBRATION WELDING PARTICULARLY DURING ULTRASONIC WELDING\ | CZ14091U1 | CECH JAN ING (CZ)\ HERMA ALES ING (CZ) | ALFA PLASTIK (CZ) | B29C65/00\ B29C65/08 |
| 920 | 3D printing | WELDING FIXTURE FOR ULTRASONIC WELDING OF THERMOPLASTIC TEST SPECIMENS\ | CZ22897U1 | HUSEK DANIEL (CZ) | TECHNICKA UNIVERZITA V LIBERCI (CZ) | B29C65/08 |
| 921 | 3D printing | FIXTURE FOR FIXING PLASTIC BODIES FOR THEIR LONGITUDINAL ULTRASONIC WELDING\ | CZ23810U1 | KOPAC DALIBOR (CZ)\ KOTERA ONDREJ (CZ) | TECHNICKA UNIVERZITA V LIBERCI (CZ) | B29C65/00\ B29C65/08 |
| 922 | 3D printing | ABDECKUNG\[EN] COVER FOR A MOTOR VEHICLE DASHBOARD DISPLAY COMPRISES A FLEXIBLE TRANSPARENT PLASTIC COVER THAT IS ULTRASONICALLY WELDED TO THE SURFACE DISPLAY AFTER INITIAL POSITIONING USING MOUNTING ELEMENTS IN A ROBUST MANNER\ | DE10058527A1 | SCHACH HARALD (DE) | BOSCH GMBH ROBERT (DE) | B60K37/02\ B60K37/04 |
| 923 | 3D printing | FLACHES KABEL UND MODULARER DREHAMBO? ZUR HERSTELLUNG DAVON\ | DE10058754A1 | SCHILSON TOM (US)\ KAMPS STEVE (US) | METHODE ELECTRONICS INC (US) | B29C65/00\ B29C65/02\ H01B7/00\ B29C65/08\ B60R16/02\ H01B7/08 |
| 924 | 3D printing | NON-AQUEOUS ELECTROLYTE SECONDARY BATTERY FORMS CONCAVE PORTION ON PROTRUDING SURFACE OF ANODE AND CATHODE PLATES IN WHICH ELECTROCONDUCTIVE CONNECTION TABS ARE FIXED BY ULTRASONIC WELDING AFTER WINDING ANODE PLATE\[DE] SEKUND?RBATTERIE MIT NICHTW?SSRIGEM ELECTROLYTEN UND VERFAHREN ZUM VERBINDEN EINES ELEKTRODENANSATZES\ | DE10066064B4 | SUZUKI HIROMI (JP)\ KITAMI TSUYOSHI (JP)\ INOI TAKAYUKI (JP)\ SAITO SUSUMU (JP) | NEC TOKIN CORP (JP) | H01M4/70 |
| 925 | 3D printing | KUPPELEINRICHTUNG MIT EINER KUPPELSCHALE UND EINEM MIT DER KUPPELSCHALE ZU VERBINDENDEN VERBINDUNGSPROFIL DAS VERBINDUNGSPROFIL ALS SOLCHES UND EIN VERFAHREN ZUM VERBINDEN DER KUPPELSCHALE MIT EINEM DERARTIGEN VERBINDUNGSPROFIL\[EN] DOME USED AS SKYLIGHT COUPLED TO CONNECTOR PROFILE WITH WELDING TRACK USED TO FORM AN ULTRASONICALLY WELDED JOINT\ | DE10100056A1 | GLORIEUX RIK (BE) | PLASTICS N V AG (BE) | B29C65/08\ E04D13/03 |
| 926 | 3D printing | SELBSTSCHMIERMODUL EINER LINEARFüHRUNG\ | DE102004008780B4 | CHI-MENG LIAO (TW)\ CHEN SCOTTE (TW) | HIWIN TECH CORP (TW) | F16C33/66\ F16C29/04\ F16C29/08\ F16N7/02 |
| 927 | 3D printing | VERFAHREN ZUR HERSTELLUNG VON VERBINDUNGEN VON FOLIENLEITERN DURCH ULTRASCHALLSCHWEI?EN\[EN] MAKING CONNECTIONS BETWEEN FILM CONDUCTORS E.G. FOR VEHICLES INVOLVES ULTRASONIC WELDING OF LAMINATED OR EXTRUDED FILM CONDUCTORS WITH SIMULTANEOUS PROCESS CONTROL; FFC CONDUCTORS AND FPC CIRCUIT PARTS ARE COMBINED INTO F-HYBRID CIRCUIT\ | DE102004012099B3 | KOENIG KARL-HEINZ (DE)\ MUELLER DIETER (DE) | VOLKSWAGEN BORDNETZE GMBH (DE) | B23K20/10\ B29C65/02\ H05K3/36\ H05K3/32\ B29C65/08\ H01R43/02\ H01R12/61 |
| 928 | 3D printing | ULTRASCHALLSCHWEI?VORRICHTUNG\[EN] ULTRASONIC WELDING TOOL FOR WELDING PLASTICS FILM MATERIALS HAS AIR OUTFLOW OPENINGS AT THE ANVIL LAYING SURFACE TO INDICATE WHEN A WORKPIECE IS INCORRECTLY POSITIONED\ | DE102004013048B4 | MOEGLICH HARTMUT (DE) | HERRMANN ULTRASCHALLTECHNIK (DE) | B23K20/10\ B29C65/08\ B29C65/78 |
| 929 | 3D printing | ULTRASCHALLSCHWEI?VORRICHTUNG\[EN] ULTRASONIC WELDING APPARATUS TO WELD AND SEAL PLASTICS FILM BAGS HAS A CUTTING EDGE AT THE SONOTRODE SURFACE TO SEPARATE THE SEALED BAG FROM A CONTINUOUS TUBULAR FILM\ | DE102004013049A1 | MOEGLICH HARTMUT (DE) | HERRMANN ULTRASCHALLTECHNIK (DE) | B23K20/10\ B29C65/08 |
| 930 | 3D printing | ULTRASCHALLSCHWEI?VORRICHTUNG\[EN] ULTRASONIC WELDING APPARATUS FOR CLOSING PLASTIC PACKAGES HAS CUTTING EDGE SPACED FROM WELDING SURFACE AND FULL BLADE HEIGHT EXTENDS ABOVE ANVIL BASE SURFACE\ | DE102004013050A1 | MOEGLICH HARTMUT (DE) | HERRMANN ULTRASCHALLTECHNIK (DE) | B23K20/10\ B29C65/00\ B29C65/08\ B29C65/74 |
| 931 | 3D printing | VERFAHREN ZUM VERSCHWEI?EN VON GURTBAND MIT EINER DECKFOLIE UND ANORDNUNG ZUR DURCHFüHRUNG DES VERFAHRENS\[EN] METHOD FOR CONTINUOUS CONTACT WELDING OF A PERIODICALLY MOVING MATERIAL TO BE WELDED(MWM) E.G. SYNTHETIC PLASTIC MATERIAL GENERALLY USEFUL IN WELDING SYNTHETIC PLASTIC MATERIALS\ | DE102004017297A1 | NEUBERT HOLGER (DE)\ GRETZSCHEL HANS-JOACHIM (DE) | UNIV DRESDEN TECH (DE) | B29C65/18\ B29C65/22\ B29C65/24\ B29C65/32\ B65B9/04\ B65B15/04\ B65B51/16\ B65B51/26 |
| 932 | 3D printing | VERFAHREN ZUR HERSTELLUNG EINER SCHWEI?VERBINDUNG SOWIE SCHWEI?VERBINDUNG\[EN] METHOD FOR WELDING ULTRASONIC LITZ WIRE INVOLVING A PREFERABLY CUBOID CARRIER INSERTED IN A COMPRESSION SPACE WHICH ON WELDING IS DECREASED IN DIAMETER USEFUL FOR USEFUL FOR ULTRASONIC AND RESISTANCE WELDING OF JOINTS\ | DE102004044480A1 | STEINER ERNST (DE)\ STROH DIETER (DE)\ THOMS ENNO (DE) | SCHUNK ULTRASCHALLTECHNIK GMBH (DE) | B23K20/10\ B23K26/02\ H01R43/02 |
| 933 | 3D printing | VORRICHTUNG UND VERFAHREN ZUM DURCHTRENNENDEN UND DIE ENDEN DER ENTSTEHENDEN EINZELSTüCKE VERSCHLIE?ENDEN ULTRASCHALLVERSCHWEI?EN VON L?NGLICHEN HOHLK?RPERNINSBESONDERE ROHREN\ | DE102005004771B4 | DOBERNECKER KLAUS ECKHART (DE) | FORSCH & ENTWICKLUNG KLAUS DOB (DE) | B23K20/10\ B23P15/26 |
| 934 | 3D printing | ULTRASCHALLSCHWEI?VORRICHTUNG\[EN] ULTRASONIC WELDER FOR PLASTICS AND/OR METALS HAS ITS FUNCTIONAL PARTS (E.G. SONOTRODE AND BOOSTER) CONNECTED BY A TAPERED THREAD\ | DE102005005480B3 | STEINER ERNST (DE)\ DIETERLE HORST (DE) | SCHUNK ULTRASCHALLTECHNIK GMBH (DE) | B23K20/10\ B29C65/08 |
| 935 | 3D printing | SCHIRM\[EN] PARASOL USED ESPECIALLY AS AN UMBRELLA COMPRISES A TWO-PART CROWN CONSISTING OF A BASE PART COUPLED TO A HEAD PART WHICH FORM WITH FACING SIDES A SOCKET CONNECTED TO THE OUTER PERIPHERY OF THE CROWN BY RADIAL SLOTS\ | DE102005013747A1 | GOEBEL EBERHARD (DE) | EBERHARD GOEBEL GMBH & CO (DE) | A45B25/22 |
| 936 | 3D printing | VERFAHREN ZUM VERBINDEN VON TEILEN\[EN] PLASTICS WITH A CROSS LINKED FOAM STRUCTURE CAN BE CONNECTED USING AN ULTRASONIC WELDING UNIT AND A SONOTRODE AN OPPOSING ELECTRODE\ | DE102005020718A1 | GAEBLER JOERG (DE) | STAPLA ULTRASCHALLTECHNIK GMBH (DE) | B23K20/10\ B29C65/08\ B06B3/00 |
| 937 | 3D printing | JALOUSIENREINIGUNGSGER?T\[EN] SHUTTER CLEANING DEVICE HAS CLEANING FINGERS CARRYING RESPECTIVE CLEANING FLEECES THAT ARE MELTED AT RESPECTIVE FINGER BY ULTRASONIC WELDING WHERE FLEECES ARE CONNECTED WITH FINGERS AND ARE IMPLEMENTED AS MICRO NON-WOVEN FABRIC\ | DE102005027351A1 | GROSS CHRISTIAN (DE)\ FISCHER KLAUS JUERGEN (DE) | LEIFHEIT AG (DE) | B29C65/08\ A47L4/02\ A47L13/38 |
| 938 | 3D printing | VERFAHREN ZUM HERSTELLEN EINES FLACHROHR- W?RMETAUSCHERS DURCH ULTRASCHALLSCHWEI?EN\[EN] METHOD FOR MANUFACTURING FLAT TUBE HEAT EXCHANGER BY ULTRASONIC WELDING ENTAILS MOVING WELDING HEAD MOVED ALONG LINE TO INTERCONNECT FIRST COMPONENT AND SECOND COMPONENT AND THEN MOVED TRANSVERSELY TO AFORESAID LINE\ | DE102005028348A1 | DING PEI-PEI (TW)\ YANG HSIU-WEI (TW)\ LIN JAO-CHING (TW)\ YU WEN-HWA (TW)\ CHEN YEN-WEN (TW) | ASIA VITAL COMPONENTS CO LTD (TW) | B23K20/10 |
| 939 | 3D printing | VERFAHREN UND VORRICHTUNG ZUM AUTOMATISCHEN POSITIVWUCHTEN EINES LAUFRADS\[EN] FLYWHEEL POSITIVE BALANCING METHOD FOR USE IN COOLING FAN OF MOTOR VEHICLE INVOLVES POSITIONING BALANCING UNITS WITH PRESET MASS AT PREDETERMINED POSITION IN FLYWHEEL AND WELDING BALANCING UNITS AT PRESET POSITION IN FLYWHEEL\ | DE102005057750A1 | HESSDOERFER ROBERT (DE)\ HUSSY THOMAS (DE) | SIEMENS AG (DE) | G01M1/32\ F16F15/32 |
| 940 | 3D printing | BRENNSTOFFZELLE VERFAHREN ZUR HERSTELLUNG DERSELBEN UND VORRICHTUNG ZUM AUSFüHREN DES HERSTELLVERFAHRENS EINER BRENNSTOFFZELLE\[EN] FUEL CELL AND ROLL-TO-ROLL PROCESS TO MANUFACTURE FUEL CELL ASSEMBLY WITH ELECTRICAL MEMBRANE UNITS ULTRASONICALLY WELDED TO SURROUNDING FRAME\ | DE102005058370A1 | MAJOR FRITZ (DE)\ CLAUSNITZER PETER (DE)\ ROSER JOCHEN (DE) | HOEFLIGER HARRO VERPACKUNG (DE) | B23K20/10\ H01M8/02\ B29C65/08 |
| 941 | 3D printing | STURZ- UND K?RPERSCHUTZPROTEKTOR SOWIE VERFAHREN ZUR HERSTELLUNG EINES STURZ- UND K?RPERPROTEKTORS\[EN] BODY PROTECTOR COMPRISES AN ANATOMICALLY SHAPED LAMINATE COMPRISING AN OUTER LAYER OF BREATHABLE FOAM OR A FOAM- OR FLUID-FILLED CUSHION MATERIAL AND AN INNER LAYER OF AIR-PERMEABLE AND BREATHABLE SPACER FABRIC\ | DE102005060624A1 | HEIDE GEB SCHUETT (DE) | TITV GREIZ (DE) | A41D31/02\ A44B18/00\ A41D31/00\ A41D13/015\ A41D13/05\ D04B21/14 |
| 942 | 3D printing | ULTRASCHALL-SCHWEI?VORRICHTUNG MIT DYNAMISCH AKTIVEM WERKSTüCKHALTER\[EN] ULTRASONIC WELDING DEVICE COMPRISES A WORKPIECE HOLDER THAT ACTS AS A MECHANICAL VIBRATORY SYSTEM TO SUPPORT BOND FORMATION DURING WELDING\ | DE102006006344A1 | KOENIG KARL-HEINZ (DE)\ HEDEMANN STEFAN (DE)\ KLOHR MICHAEL (DE) | SUMITOMO ELECTRIC BORDNETZE GM (DE) | B23K20/10\ B29C65/08 |
| 943 | 3D printing | GAS- UND FLüSSIGKEITSDICHT VERSCHLOSSENER BEH?LTER SOWIE VERFAHREN ZU DESSEN HERSTELLUNG\[EN] MASS E.G. FLUID RETAINING CYLINDRICAL CONTAINER FOR USE IN AUTOMOBILE INDUSTRY HAS COVER WITH ONE FIXING SECTION JOINED WITH OTHER FIXING SECTION OF CONTAINER WALL BY ULTRASONIC WELDING BOTH FIXING SECTIONS DO NOT PROTRUDE OUTWARD\ | DE102006007133A1 | FOERDERER GUIDO (DE)\ HOLIK BERND (DE) | HENKEL KGAA (DE) | B65D83/76\ B05C17/005\ B65D35/30\ E04F21/165 |
| 944 | 3D printing | ULTRASCHALL-SCHWEI?STATION SOWIE VERFAHREN ZUM BETREIBEN EINER ULTRASCHALL-SCHWEI?STATION\ | DE102006021651B4 | FINK DANIEL (DE) | ULTRASONICS STECKMANN GMBH (DE) | B23K20/10\ B29C65/08 |
| 945 | 3D printing | VERFAHREN ZUM VERSCHLIE?EN EINES GER?TEGEH?USES GER?TEGEH?USE UND SET VON GER?TEGEH?USEN\[EN] SEALING METHOD FOR EQUIPMENT BOXES E.G. FOR CAR PARTS COMPRISES FITTING PLASTIC SHEET ON TOP OF BOX SO THAT ITS EDGE FITS EXACTLY ON ITS RIM AND LASER-WELDING SHEET IN PLACE\ | DE102006035197A1 | BAUR RICHARD (DE) | BAYERISCHE MOTOREN WERKE AG (DE) | B29C65/16\ H05K5/03 |
| 946 | 3D printing | SENSORVORRICHTUNG MIT STRUKTURIERTER DURCHFLUSSZELLE\[EN] SENSOR DEVICE USEFUL IN CLINICAL DIAGNOSTICS FOR THE CONTINUOUS AND SEMI-CONTINUOUS MEASUREMENT OF SUBSTANCES IN BLOOD OR INTERSTITIAL LIQUID COMPRISES POLYCARBONATE FOIL WITH FLOW CELL ARRANGED PARALLELLY TO PLANAR STRUCTURED SENSOR\ | DE102006038271A1 | GRUENDIG BERND (DE)\ HELLMICH ROBERT (DE)\ WEDIG HEIKO (DE) | SENSLAB GES ZUR ENTWICKLUNG UN (DE) | B01L3/00\ G01N27/403\ G01N35/08\ G01N21/05\ G01N27/413\ G01N33/483 |
| 947 | 3D printing | VERFAHREN ZUM HERSTELLEN VON FLACHROHR-W?RMETAUSCHER DURCH ULTRASCHALLSCHWEI?EN\[EN] - PROCESS FOR PRODUCTION OF A FLAT TUBE HEAT EXCHANGER BY ULTRASONIC SPOT WELDING USEFUL IN HEAT EXCHANGER PRODUCTION IS QUICKER THAN PREVIOUS PROCESSES AND GIVES INCREASED PLATE BONDING STRENGTH\ | DE102006039605A1 | DING PEI-PEI (TW)\ YANG HSIU-WEI (TW)\ YU WEN-HWA (TW)\ CHENG TSAO-HSIANG (TW) | ASIA VITAL COMPONENTS CO LTD (TW) | B23K20/10\ B21D53/08 |
| 948 | 3D printing | ELEKTRISCHE VERBINDUNG ZWISCHEN RUND- UND FLACHLEITERN\ | DE102006046350B4 | SCHLOMS MARTIN (DE) | AUTO KABEL MAN GMBH (DE) | H01R4/02\ H01R12/63 |
| 949 | 3D printing | REIBUNGSSCHWEI?VERFAHREN UND REIBUNGSSCHWEI?ANLAGE\ | DE102006051691B4 | JUNKES ROLAND (DE) | FAURECIA INNENRAUM SYS GMBH (DE) | B23K20/12\ B29C65/08\ B29C65/06 |
| 950 | 3D printing | REINIGUNGSKARTE\[EN] CLEANING CARD FOR E.G. DATA CARRIER CARD READER`S READING HEAD HAS SECTION FLOCK ATTACHED TO CARRIER UNIT BY ULTRASONIC-WELDING WHERE FLOCK LAYER OF FLOCK INCLUDES CLEANING FIBERS THAT ARE DIMENSIONED IN LENGTH REFINEMENT AND PROFILE\ | DE102007016949A1 | SCHOENHEIT WALTER (DE)\ DIETSCH STEFFEN (DE)\ KRIENBUEHL WALTER (CH) | OSTTHUERINGISCHE MATERIALPRUEF (DE) | G11B5/41 |
| 951 | 3D printing | SONNENSCHUTZFOLIE SOWIE VERFAHREN UND EINRICHTUNG ZUM AUSRüSTEN EINER DERARTIGEN FOLIE\[EN] MULTILAYERED SUN PROTECTION- AND/OR SOLAR ABSORBING COMPOSITE FILM FOR SELECTIVE FILTRATION OF SUN RADIATION FROM THERMOPLASTIC COMPRISES REINFORCEMENT MATERIAL CONNECTED TO AREA TO BE CHARGED OR PROCESSED FOR PARTIAL STABILIZATION\ | DE102007021651A1 | LANG KLAUS (DE) | LANG INDUSTRIESERVICE GMBH (DE) | B32B7/00\ B32B27/08\ B32B27/12\ B29C65/08\ B32B3/08\ E04F10/02\ E06B9/24 |
| 952 | 3D printing | VERFAHREN ZUM ELEKTRISCH LEITENDEN VERBINDEN VON LITZEN SOWIE ULTRASCHALLSCHWEI?VORRICHTUNG\ | DE102007026707B8 | STROH DIETER (DE)\ STROH HEIKO (DE) | SCHUNK ULTRASCHALLTECHNIK GMBH (DE) | B23K20/10 |
| 953 | 3D printing | VORRICHTUNG ZUR ABSTANDSMESSUNG UND ULTRASCHALL-SCHWEI?VERFAHREN ZUR FIXIERUNG DER VORRICHTUNG\[EN] MOTOR VEHICLE'S DISTANCE MONITORING DEVICE HAS FASTENER FIXABLE IN BODY REGION OF MOTOR VEHICLE AND CONTACT SURFACE AND/OR SURFACE OF BODY REGION PROVIDED WITH PINS WHERE SURFACE OF BODY REGION IS TURNED TOWARDS CONTACT SURFACE\ | DE102007034412A1 | BILLEWICZ STEFAN (DE)\ PIANTADOSI NICOLA (DE)\ MERTENS THOMAS (DE)\ SCHARNBACH MICHAEL (DE) | VOLKSWAGEN AG (DE) | B60R19/48\ G01B17/02 |
| 954 | 3D printing | VERFAHREN ZUR INTERNEN KONTAKTIERUNG EINES LEISTUNGSHALBLEITERMODULS\ | DE102007044046B4 | SIEPE DIRK DR (DE) | INFINEON TECHNOLOGIES AG (DE) | H01L21/60\ H01L23/498\ H01L25/07\ H01L23/492 |
| 955 | 3D printing | VERFAHREN ZUR VERBINDUNG EINES ERSTEN VARISTORBLOCKES MIT EINEM ZWEITEN VARISTORBLOCK SOWIE üBERSPANNUNGSABLEITER\[EN] VARISTOR BLOCKS CONNECTING METHOD INVOLVES CONNECTING VARISTOR BLOCKS WITH ELECTRICALLY CONDUCTIVE INTERMEDIATE LAYER BY FRICTION WELDING I.E. ULTRASONIC WELDING AT EDGE REGION OF CONTACT SURFACE\ | DE102007049988A1 | KRUSKA BERND (DE) | SIEMENS AG (DE) | H01C7/12\ H01C17/00 |
| 956 | 3D printing | RINGMAGNET FüR EINE DREHMOMENT- ODER DREHWINKELSENSORANORDNUNG\[EN] RING MAGNET FOR USE IN ROTATION ANGLE OR TORQUE SENSOR ARRANGEMENT AT STEERING SHAFT IN MOTOR VEHICLE HAS MAGNET PARTICLES LOCATED ON CARRIER AND GEOMETRY EMBEDDED WITH RECESS INTO CASING WHICH ENCLOSES AREAS IN DIRECTION OF SURFACE\ | DE102007050258A1 | RACHUI DIRK (DE)\ THOM JENS (DE)\ FROEHLICH EKKEHART (DE) | VALEO SCHALTER & SENSOREN GMBH (DE) | G01B7/00\ H01F7/02\ G01P3/487 |
| 957 | 3D printing | PAPIERMASCHINENBESPANNUNG UND VERFAHREN ZU DEREN HERSTELLUNG\[EN] BELT FOR USE AS E.G. VOLTAGE PRESSING BELT IN PAPER-MAKING MACHINE HAS ADDITIONAL CONNECTION PROVIDED BY ULTRASONIC WELDING IN PART OF SEAM OR SEAM REGION AND LONGITUDINAL THREAD AND/OR TRANSVERSE THREADS IN CONTACT WITH ONE ANOTHER\ | DE102008000066A1 | KOECKRITZ UWE (DE) | VOITH PATENT GMBH (DE) | D03D1/00\ D21F1/00\ D21F7/08\ D21F7/10\ D21F1/10 |
| 958 | 3D printing | VERFAHREN UND VORRICHTUNG ZUR HERSTELLUNG VON FILAMENTVERBINDUNGEN AN EINEM LAUFENDEN MULTIFILEN FADEN\ | DE102008014530A1 | STUENDL MATHIAS (DE)\ KAULITZKI MARCO (DE) | OERLIKON TEXTILE GMBH & CO KG (DE) | B65H69/08 |
| 959 | 3D printing | FAHRZEUGLENKRAD\[EN] STEERING WHEEL FOR MOTOR VEHICLE I.E. CAR HAS FLEXIBLE COVER WITH CUT WHOSE SECTIONS EXTEND INTO GROOVE FORMED IN RIM AND ULTRASONICALLY WELDED WITH SIDE WALLS OF GROOVE FOR CONNECTING GROOVE WITH RIM\ | DE102008027777B3 | DOURSOUX HUGUES (FR)\ BOULLE PIERRE (FR)\ BONNIN JONATHAN (FR) | AUTOLIV DEV (SE) | B62D1/06 |
| 960 | 3D printing | SONOTRODENWERKZEUG MIT KüHLEINRICHTUNG\[EN] SONOTRODE TOOL FOR ULTRASONIC WELDING HAS INTERNAL COOLING SYSTEM IN SONOTRODE BODY TO ACCELERATE COOLING AND REDUCE RESIDENCE TIME OF SONOTRODE ON WORKPIECE\ | DE102008029769A1 | NAGEL DIETER (DE)\ LICHTENBERGER KLAUS (DE) | SONOTRONIC NAGEL GMBH (DE) | B23K20/10\ B29C65/08\ B06B3/02 |
| 961 | 3D printing | MONTAGEVERFAHREN FüR EIN LAGER AN EINER WELLE\[EN] BEARING E.G. CONNECTING ROD BEARING MOUNTING METHOD FOR RECIPROCATING PISTON ENGINE OF MOTOR VEHICLE INVOLVES FIXEDLY CONNECTING BEARING HOUSING PARTS BY WELDING PROCESS WHERE WELDING OF PARTS IS PERFORMED IN DEFINED EXTERNAL REGION\ | DE102008030897B3 | WILL FRANK (AU) | FORD GLOBAL TECH LLC (US) | B23P13/00\ B23B31/00\ B23P19/04\ B26F3/00\ F16C9/04 |
| 962 | 3D printing | HERSTELLUNGSVERFAHREN FüR EINWEG-Fü?LINGE\ | DE102008030941B4 | ROSEMANN STEFAN (DE) | ROSEMANN STEFAN (DE) | D04H1/42\ A41H43/04\ A41H43/00\ A41B11/00 |
| 963 | 3D printing | VORRICHTUNG ZUM SELBSTST?NDIGEN AUSWUCHTEN\[EN] AUTOMATIC BALANCING DEVICE I.E. BALANCING ELEMENT FOR ROTATING MACHINE PART I.E. GRINDING DISK OF ANGULAR GRINDER HAS IMBALANCE COMPENSATING DISK COMPRISING TWO SEMI SHELLS MADE OF FIBER-REINFORCED PLASTIC\ | DE102008031105A1 | BAUER JOERG (DE)\ NUISL CHRISTIAN (DE)\ STOELZLE JUERGEN (DE) | SCHAEFFLER KG (DE) | G01M1/36 |
| 964 | 3D printing | VERFAHREN UND VORRICHTUNG ZUM ULTRASCHALLVERSCHWEI?EN EINER VERSCHLUSSVORRICHTUNG AUF DEM AUSGUSSSTUTZEN EINES BEH?LTERS\ | DE102008031379B4 | SCHMITT WOLFGANG (DE)\ SCHMITT ROBERT (DE) | F W SCHMITT GMBH (DE) | B29C65/00\ B29C65/08 |
| 965 | 3D printing | SELBSTKLEBEND AUSGERüSTETER STREIFEN FüR DEN TRANSPORTSCHUTZ\[EN] SELF-ADHESIVE MOUNTING STRIP USEFUL AS TRANSPORTATION PROTECTOR OF ANY KIND SUCH AS EDGE- AND/OR DOOR EDGE PROTECTOR OF AUTOMOBILES COMPRISES A SHOCK ABSORBING CARRIER PROVIDED ON ONE SIDE WITH AN ADHESIVE COATING AND A GROOVE\ | DE102008038473A1 | HOPF MARTIN (DE)\ ERDMANN BJOERN (DE)\ GRODD GERD (DE) | TESA SE (DE) | C09J7/02 |
| 966 | 3D printing | URFORMKERNE ZUR HERSTELLUNG UMFANGREICH KONTURIERTER HINTERSCHNITTENER HOHLR?UME IN URFORMTEILEN DAMIT HERGESTELLTE URFORMTEILE SOWIE VERFAHREN ZUR HERSTELLUNG ANWENDUNG UND ENTFERNUNG DER KERNE\[EN] CORE FOR USE IN PROTOTYPING ESPECIALLY FOR PRESSURE CASTING OR POWDER METALLURGY IS OBTAINED USING METAL SHEATH MOLDING MATERIAL MECHANICAL SHAPING DEVICE AND HIGH PRESSURE COMPRESSION TUBE\ | DE102008039208A1 | Empty Field US | HEPPES FRANK (DE) | B22C9/06\ B22C9/10 |
| 967 | 3D printing | VERFAHREN ZUR BEWERTUNG EINER SCHWEI?NAHT\[EN] METHOD FOR EVALUATING WELDING SEAM DURING JOINING SURFACES OF WORKPIECES BY ULTRASONIC WELDING PROCESS INVOLVES COMPARING DETERMINED CHARACTERISTIC VALUE OF REFLECTION WITH CALIBRATION CURVE\ | DE102008062503A1 | HOFFMANN THOMAS (DE)\ LINDENAU DIRK (DE)\ PAASCH RUDOLF (DE) | DAIMLER AG (DE) | G01N29/07\ G01N23/02\ G01N23/18 |
| 968 | 3D printing | HüLSE FüR ELEKTRISCHE ODER ELEKTRONISCHE GER?TE\[EN] SLEEVE I.E. ROTATION SYMMETRIC AND/OR CYLINDRICAL SLEEVE MANUFACTURING METHOD FOR E.G. APPROXIMATION SENSOR DEVICE INVOLVES PROVIDING HALF SHELLS OF SLEEVE IN LONGITUDINAL DIRECTION BY DEEP DRAWING AND CONNECTING HALF SHELLS TO SLEEVE\ | DE102008063691A1 | MAY THOMAS (DE)\ HORNSTEIN ANDREAS (DE)\ KATHAN BENNO (DE) | IFM ELECTRONIC GMBH (DE) | G01D11/24\ G12B9/02 |
| 969 | 3D printing | VERFAHREN ZUR SCHICHTAPPLIKATION FOLIENF?RMIGER LOTWERKSTOFFE MITTELS ULTRASCHALLSCHWEI?EN\[EN] METHOD FOR LAYER APPLICATION OF FILM-SHAPED SOLDERING MATERIALS BY ULTRASONIC WELDING ON SAME OR DIFFERENT SURFACES OF COMPONENT COMPRISES COUPLING THE ULTRASONICS ON COMPONENT SURFACE TO ITS ACTIVATION AND WETTABILITY\ | DE102009000892A1 | HERRMANN JOERG (DE)\ BRUNS CLAAS (DE) | SLV HALLE GMBH (DE) | H01L21/60\ H01R43/02\ B23K1/06\ B23K1/20\ H01L23/488 |
| 970 | 3D printing | VERFAHREN ZUM HERSTELLEN EINER DICHTUNGSANORDNUNG ZUM ELEKTRISCH ISOLIERENDEN ABDICHTEN ZWISCHEN BAUTEILEN EINES BRENNSTOFFZELLENSTAPELS UND BAUGRUPPE MIT EINER SOLCHEN DICHTUNGSANORDNUNG\[EN] METHOD FOR MANUFACTURING SEAL ARRANGEMENT UTILIZED FOR ELECTRICALLY ISOLATINGLY SEALING OF HOUSING LOWER PART AND INTERMEDIATE ELEMENT OF FUEL CELL STACK INVOLVES CONNECTING COMPONENTS WITH EACH OTHER BY DUCTILE MATERIAL\ | DE102009008986A1 | KIEFER THOMAS (DE)\ MAIER UWE (DE)\ LANGJAHR AXEL (DE)\ GRUENLING HERMANN (DE) | ELRINGKLINGER AG (DE) | H01M8/02 |
| 971 | 3D printing | ULTRASCHALL-SCHWEI?WERKZEUG\[EN] ULTRASONIC-WELDING TOOL E.G. ANVIL FOR PRODUCTION OF THIN WELDING SEAM FOR CONNECTING TWO PLASTIC FOILS HAS MULTIPLE ELEVATIONS ARRANGED NEXT TO EACH OTHER AND SEPARATED FROM EACH OTHER BY GAPS\ | DE102009024258A1 | NAGEL INGO (DE) | SONOTRONIC NAGEL GMBH (DE) | B29C65/08 |
| 972 | 3D printing | VERFAHREN ZUM EINSCHWEI?EN EINES SPOUTS IN EINEN BEUTEL UND ULTRASCHALLSCHWEI?WERKZEUG ZUM DURCHFüHREN DIESES VERFAHRENS\[EN] ULTRASONIC WELDING OF TWO POUCH FILMS WITH A SPOUT COMPRISES WELDING THE POUCH FILMS WITH ONE ANOTHER AT THEIR SIDE EDGES JOINTLY TOGETHER WITH THE SPOUT WHERE THREE OF THE OTHER SIDE EDGES OF THE POUCH FILMS ARE WELDED WITH ONE ANOTHER\ | DE102009043143A1 | GOETTERT BENJAMIN (DE) | WIDMANN MASCHINEN E K (DE) | B23K20/10\ B29C65/00\ B31B19/60\ B65B51/22\ B31B19/90\ B31B1/90\ B31B19/66 |
| 973 | 3D printing | SONOTRODENANORDNUNG FüR KUNSTSTOFFBE- UND -VERARBEITUNGSWERKZEUGE\[EN] SONOTRODE ARRANGEMENT FOR E.G. PLASTIC MACHINING AND PROCESSING TOOLS USED IN ULTRASONIC WELDING OF WORKPIECE HAS FITTING GROOVE ARRANGED IN END REGION THAT LIES OPPOSITE TO CLAMPING POSITION OF SLOT \ | DE102009048696B3 | KAZMIRZAK WOLFGANG (DE)\ BLOS PETER (DE)\ ZWICKER THOMAS (DE) | KUNSTSTOFF ZENTRUM LEIPZIG (DE) | B29C65/08\ B06B3/00\ B06B3/02 |
| 974 | 3D printing | GETR?NKEDOSE DIE WEITGEHEND AUS ZELLSTOFFHALTIGEM MATERIAL BESTEHT UND EINE LEBENSMITTELVERTR?GLICHE BARRIERESCHICHT AUFWEIST FüR UNTER üBERDRUCK STEHENDE GETR?NKE\[EN] BEVERAGE CAN FOR CONTAINING BEVERAGES WITH CARBONIC ACID UNDER HIGH PRESSURE COMPRISES A CASING CLOSED BY BOTTOM- AND COVER-ELEMENTS WHERE THE CASING CONSISTS OF A PARALLELY WRAPPED LAMINATE FROM CELLULOSE CONTAINING MATERIAL\ | DE102010000562A1 | KEIENBURG GUENTER (DE) | KEIENBURG GUENTER (DE) | B65D65/40\ B32B7/00\ B65D3/04\ B65D17/00\ B65D65/42 |
| 975 | 3D printing | SONOTRODE FüR EINE ULTRASCHALL-SCHWEI?ANLAGE\[EN] SONOTRODE FOR ULTRASONIC WELDING MACHINE COMPRISES ULTRASONIC GENERATOR WHICH GUIDES HIGH-FREQUENCY ELECTRICAL ENERGY TO CONVERTER CONVERTS ELECTRICAL ENERGY INTO MECHANICAL VIBRATIONS AND GUIDES TO THE SONOTRODE VIA BOOSTER\ | DE102010000968A1 | MARQUARDT OTTO (DE)\ WEILANDT WERNER (DE)\ SIMON JOERG (DE) | FRIMO GROUP GMBH (DE) | B23K20/10\ B29C65/08\ B06B3/02 |
| 976 | 3D printing | VORMISCHGEBL?SE\[EN] PREMIXING COMPRESSOR FOR SUPPLYING AIR AND GAS MIXTURE INTO COMBUSTION CHAMBER OF GAS BURNER TO HEAT WATER IN E.G. COMMERCIAL APPLICATION HAS ELECTRIC MOTOR WHOSE OUTPUT SHAFT IS COUPLED TO IMPELLER THAT IS FASTENED TO OUTPUT SHAFT\ | DE102010008988A1 | KWOK LO CHING (HK)\ LIU DONG XIA (HK)\ YU WEI (HK) | JOHNSON ELECTRIC SA (CH) | F04D29/00 |
| 977 | 3D printing | VERFAHREN ZUR HERSTELLUNG EINER GETR?NKEDOSE\[EN] METHOD FOR MANUFACTURING BEVERAGE CAN WITH CARBONIC ACID PRESSURIZED BEVERAGES SUCH AS BEER SOFT DRINK OR MINERAL WATER INVOLVES PROVIDING CYLINDRICAL MULTI-LAYER SHELL WITH LIQUID-TIGHT BARRIER AND LAYER OF CARDBOARD OR PAPER\ | DE102010009117A1 | TSCHECH FLORIAN DR (DE) | NUCLEUS GMBH (DE) | B29C65/08\ B31C1/00\ B29D23/00 |
| 978 | 3D printing | GETR?NKEDOSE DIE WEITGEHEND AUS ZELLSTOFFHALTIGEM MATERIAL BESTEHT UND EINE LEBENSMITTELVERTR?GLICHE BARRIERESCHICHT AUFWEIST FüR UNTER üBERDRUCK STEHENDE GETR?NKE\[EN] BEVERAGE CAN FOR CONTAINING BEVERAGES WITH CARBONIC ACID UNDER HIGH PRESSURE COMPRISES A CASING CLOSED BY BOTTOM- AND COVER-ELEMENTS WHERE THE CASING CONSISTS OF A PARALLELY WRAPPED LAMINATE FROM CELLULOSE CONTAINING MATERIAL\ | DE102010037965A1 | Empty Field US | KEIENBURG GUENTER (DE) | B65D65/40\ B65D3/22\ B65D17/28 |
| 979 | 3D printing | SCHWEI?VORRICHTUNG\[EN] WELDING DEVICE FOR MULTI-LAYER PLASTIC FILMS HAS ULTRASONIC WELDING SONOTRODE AND COUNTER TOOL WHERE WELDED METAL SHEET IS ARRANGED BETWEEN ULTRASONIC WELDING SONOTRODE AND COUNTER TOOL\ | DE102010039089A1 | GOETTERT BENJAMIN (DE)\ GRAS WERNER (DE)\ KACHEL GUENTER (DE) | WIDMANN MASCHINEN E K (DE) | B29C65/08\ B31B1/66 |
| 980 | 3D printing | KURBELGEH?USEENTLüFTUNGSVORRICHTUNG FüR EINE BRENNKRAFTMASCHINE\[EN] CRANKCASE VENTILATION DEVICE FOR REMOVAL OF OIL-AIR MIXTURE FROM CRANKCASE OF INTERNAL COMBUSTION ENGINE HAS NON-RETURN VALVES ARRANGED IN RESPECTIVE DIVERTING APERTURES AND CLAMPED BETWEEN SEPARATOR PLATE AND UPPER HOUSING PORTION\ | DE102010054347A1 | WEBER ANDREAS (DE)\ SCHLEIDEN THOMAS (DE) | MANN & HUMMEL GMBH (DE) | F01M13/04 |
| 981 | 3D printing | KOTFLüGELANORDNUNG FüR EIN FAHRZEUG\[EN] FENDER ARRANGEMENT FOR COMMERCIAL MOTOR VEHICLE HAS SUPPORT MEMBER CONNECTED WITH FENDER PARTS BY CONNECTING FLANGE THROUGH CORRESPONDING RETAINERS WHERE FENDER PARTS ARE CONNECTED TO RESPECTIVE CONNECTION REGIONS\ | DE102010056216A1 | VINCON FLORIAN DIPL ING (DE) | DAIMLER AG (DE) | B62D25/16 |
| 982 | 3D printing | VERFAHREN ZUR HERSTELLUNG GESCHWEISSTER FORMK?RPER AUS POLYAMID-6.10\[EN] PRODUCING WELDED MOLDED BODY USEFUL E.G. AS FENDER COMPRISES ASSEMBLING AND WELDING PREFABRICATED MOLDED PARTS COMPRISING POLYAMIDE-6.10 POLYETHYLENEIMINE HOMO- OR COPOLYMER FILLER COPPER-CONTAINING STABILIZER AND PHENOL AND ADDITIVE\ | DE102010062538A1 | URANO YUICHI (DE)\ SCHEIBITZ MATTHIAS (DE) | BASF SE (DE) | C08L77/00\ B29C65/02\ B29C65/06 |
| 983 | 3D printing | VERSTELLANTRIEB\[EN] ADJUSTING DRIVE PARTICULARLY ADJUSTING DEVICE FOR STEERING COLUMN ADJUSTMENT OR VEHICLE SEAT OF MOTOR VEHICLE HAS HOUSING AND OUTPUT SHAFT THAT IS ROTATABLY SUPPORTED IN HOUSING\ | DE102011000972A1 | STRZODKA MANFRED (DE)\ ARNDT THOMAS (DE) | HAMMERSTEIN GMBH C ROB (DE) | F16H57/12 |
| 984 | 3D printing | VERSTELLANTRIEB SOWIE VERFAHREN ZUR HERSTELLUNG EINES VERSTELLANTRIEBS\[EN] DRIVE FOR ADJUSTING E.G. STEERING COLUMN OF MOTOR VEHICLE HAS WEDGE-SHAPED COMPENSATING ELEMENT CONNECTED WITH HOUSING BY ULTRASONIC WELDING FOR SECURING AXIAL PLAY-FREE ARRANGEMENT OF OUTPUT SHAFT IN OPERATING POSITION\ | DE102011001971A1 | STRZODKA MANFRED (DE)\ ARNDT THOMAS (DE)\ PHILIP TONY (DE)\ POCZIK TAMAS (DE) | HAMMERSTEIN GMBH C ROB (DE) | F16H57/12\ B62D1/181\ F16H57/023 |
| 985 | 3D printing | ANSCHLUSSDOSE FüR EIN SOLARMODUL UND VERFAHREN ZUM ANSCHLIE?EN EINER VERBINDUNGSLEITUNG AN EIN SOLARMODUL\[EN] CONNECTION BOX USED FOR CONNECTING ELECTRICAL CONNECTION LINE TO THIN LAYER SOLAR MODULE HAS LID THAT IS FIRMLY SECURED AT CONTACT SPACE BY WELDED PORTION OBTAINED BY PERFORMING ULTRASONIC WELDING PROCESS\ | DE102011005064A1 | BRANDT HOLGER (DE)\ SCHALICH THORSTEN (DE)\ NOWAK AXEL (DE) | INVENTUX TECHNOLOGIES AG (DE) | H02N6/00\ H02G3/16 |
| 986 | 3D printing | SCHWEI?VORRICHTUNG UND SCHWEI?BLECH\[EN] WELDING APPARATUS FOR MULTI-LAYER PLASTIC FILMS HAS WELDING PLATE HAVING RAISED STRUCTURE WITH CUTTING EDGE WHICH IS ARRANGED BETWEEN WELDING SONOTRODE AND COUNTER TOOL\ | DE102011006930A1 | GOETTERT BENJAMIN (DE)\ GRAS WERNER (DE)\ KACHEL GUENTHER (DE) | WIDMANN MASCHINEN E K (DE) | B29C65/08\ B31B1/66 |
| 987 | 3D printing | VORRICHTUNG ZUM SCHWEI?EN UND/ODER SCHNEIDEN VON WERKSTüCKEN\ | DE102011006932B4 | G?TTERT BENJAMIN (DE)\ GRASS WERNER (DE)\ KACHEL GüNTER (DE) | WIDMANN MASCHINEN GMBH & CO KG (DE) | B23K20/10 |
| 988 | 3D printing | HITZEAKTIVIERBARES FASERVERST?RKTES STRUKTURELLES KLEBEMITTEL\[EN] HEAT-ACTIVABLE FIBER-REINFORCED STRUCTURAL ADHESIVE USEFUL AS A SPLICING BAND FOR ABRASIVE BELTS COMPRISES A CARRIER FILM A FIXING ADHESIVE LAYER APPLIED WITH REINFORCING FIBER AND A HEAT-ACTIVATABLE ADHESIVE LAYER\ | DE102011008430A1 | KUEHL OLIVER (DE)\ SIEBERT JOACHIM (DE) | LOHMANN GMBH & CO KG (DE) | C09J7/02\ C09J5/06\ B24D11/06 |
| 989 | 3D printing | LAUFRAD EINER KREISELPUMPE\[EN] RUNNING WHEEL FOR USE IN CENTRIFUGAL PUMP HAS BLADES ARRANGED BETWEEN FRONT-SIDE SUPPORT PLATE AND REAR COVER PLATE WHICH IS WELDED AS INDIVIDUAL PART AT TUBULAR SHAFT COAXIAL SEAT BY ULTRASONIC WELDING PROCESS\ | DE102011012074A1 | ADLER ANDREAS (DE) | WILO SE (DE) | F04D29/22 |
| 990 | 3D printing | VERFAHREN ZUM VERSCHWEI?EN VON LEITERN INSBESONDERE ZUR HERSTELLUNG EINES KABELBAUMS EINES FAHRZEUGBORDNETZES\[EN] WELDING OF CONDUCTORS USEFUL FOR PRODUCING CABLE HARNESS OF VEHICLE ELECTRICAL SYSTEM COMPRISES WELDING OF CONDUCTOR FOR THE FORMATION OF NODE BY COMPRESSION CHAMBER HAVING WELDING DEVICE WELDING OF MULTIPLE CONDUCTORS TO FIRST NODE\ | DE102011014801A1 | FINK HANS-PETER (DE)\ HUMMEL HELMUT DIPL ING (DE)\ LUTZENBERGER MARKUS (DE)\ SPIES ANTON (DE) | AUDI AG (DE) | B23K20/10\ B23K31/02\ H01B13/012 |
| 991 | 3D printing | VERFAHREN ZUR OPTIMIERUNG VON SCHWEI?N?HTEN\[EN] OPTIMIZING WELD SEAMS IN PLASTIC COMPONENT INCLUDING E.G. FUEL FILTER AND/OR FUEL FILTER HOUSING BY DETERMINING WELD SEAM GEOMETRY AND DETERMINING LOADING CAPACITY/STRENGTH OF WELD SEAM DEFINING PARAMETERS SUCH AS BURSTING PRESSURE\ | DE102011076637A1 | RUDOLPH THOMAS (DE) | MAHLE INT GMBH (DE) | B29C65/02\ B29C65/82 |
| 992 | 3D printing | VERFAHREN ZUM VERSCHLIE?EN EINER BOHRUNG EINES WERKSTüCKES UND BOHRUNGSVERSCHLUSSVORRICHTUNG\[EN] CLOSING METHOD FOR CLOSING BORE OF WORKPIECE USING BORE CLOSING DEVICE INVOLVES PLACING BORE IN WORKPIECE WHERE BORE IN AREA OF WORKPIECE NEAR TO SURFACE HAS BIGGER DIAMETER THAN IN AREA OF WORKPIECE AWAY FROM SURFACE\ | DE102011086791A1 | DILALAN UEMIT (DE) | CONTINENTAL AUTOMOTIVE GMBH (DE) | B23P23/04 |
| 993 | 3D printing | DURCHFüHRUNGEN INSBESONDERE FüR BATTERIEN MITTELS ULTRASCHALLSCHWEI?EN UND VERFAHREN ZUM EINBRINGEN DER DURCHFüHRUNG IN EIN GEH?USE\[EN] FEED-THROUGH USEFUL IN A COMPONENT OF A BATTERY CELL HOUSING OF AN ACCUMULATOR E.G. A LITHIUM ION BATTERY WHERE THE FEED-THROUGH PASSES THROUGH THE HOUSING COMPONENT COMPRISING AN OPENING THROUGH WHICH A PIN-SHAPED CONDUCTOR IS GUIDED\ | DE102011106873A1 | ESEMANN HAUKE DR (DE)\ ROTERS ANDREAS (DE)\ HARTL HELMUT (AT)\ KROLL FRANK (DE) | SCHOTT AG (DE) | B23K20/10\ H01M2/06 |
| 994 | 3D printing | ELEKTRISCHE KONTAKTEINRICHTUNG\[EN] ELECTRICAL CONTACT DEVICE FOR USE IN MOTOR VEHICLE HAS ELECTRICAL CONDUCTOR MADE FROM ALUMINUM AND COMPRISING END PIECE AND CONTACT PART CONNECTED WITH VEHICLE-SPECIFICALLY DESIGNED CONTACT PART DESIGNED AS PUNCHING OR CASTING PART\ | DE102011112936A1 | Empty Field US | KROMBERG & SCHUBERT KG (DE) | H01R4/00\ H01R43/16 |
| 995 | 3D printing | KüHLVORRICHTUNG UND VERFAHREN ZU DEREN HERSTELLUNG\[EN] COOLING DEVICE FOR ELECTRONIC COMPONENTS E.G. CPU HAS COOLING STRUCTURE THAT IS CONNECTED WITH CERAMIC STRUCTURE BY BRAZE WELDING ULTRASONIC WELDING SOLDERING OR DIFFUSION BONDING\ | DE102011114770A1 | YANG HSIU-WEI (TW) | ASIA VITAL COMPONENTS CO LTD (TW) | H05K7/20\ H01L23/34\ H01L23/12\ H01L21/58 |
| 996 | 3D printing | VAPOR-CHAMBER-KüHLER UND VERFAHREN ZU DEREN HERSTELLUNG\[EN] VAPOR CHAMBER RADIATOR FOR USE IN ELECTRONIC PRODUCT HAS CAPILLARY AND SUPPORTING STRUCTURES PROVIDED IN CAVITY WHERE CAPILLARY STRUCTURE IS FORMED AT WALLS OF CAVITY AND SUPPORTING STRUCTURE IS CONNECTED WITH METAL AND CERAMIC PLATES\ | DE102011115784A1 | YANG HSIU-WEI (TW) | ASIA VITAL COMPONENTS CO LTD (TW) | H05K7/20\ H01L23/467\ F28F21/04 |
| 997 | 3D printing | VERFAHREN ZUM MONTIEREN EINES BEPLANKUNGSBAUTEILS FüR EINEN KRAFTWAGEN\[EN] MOUNTING A COVERING COMPONENT FOR A FOLDING ROOF OF A MOTOR VEHICLE BY APPLYING A FOAM-SHAPED ADHESIVE BEAD TO A RECEIVING AREA OF A CARRIER COMPONENT AND PLACING A DIAPHRAGM COMPONENT ON THE CARRIER COMPONENT PROVIDED WITH THE BEAD\ | DE102011120341A1 | BRUNSTEIN HEINRICH (DE)\ GUCKEL MARTIN (DE)\ HASSELGRUBER ANDREAS (DE)\ SCHENK BERNHARD (DE) | DAIMLER AG (DE)\ PEGUFORM GMBH (DE) | F16B11/00\ C09J5/00\ B60J7/12 |
| 998 | 3D printing | ELEKTRISCHER STECKVERBINDER ZUR ELEKTRISCHEN VERBINDUNG MITTELS ULTRASCHALLSCHWEI?EN\ | DE102012002910B4 | HOLLEY DANICA (DE)\ SCHULZ STEFAN (DE)\ LANGHOFF WOLFGANG (DE)\ BEDNARCZYK PETER (DE) | AMPHENOL TUCHEL ELECT (DE) | H01R4/02 |
| 999 | 3D printing | MULTIFUNKTIONELLE MEHRLAGENLEITERPLATTE MIT EINER ELEKTRISCH LEITF?HIGEN HOCHSTROM-LEITSTRUKTUR\[EN] MULTI-FUNCTIONAL MULTILAYER PRINTED CIRCUIT BOARD FOR HANDBOOK HAS WIRE ELEMENTS ELECTRIC CONDUCTIVELY INTERCONNECTED WITH ELECTRICALLY CONDUCTIVE STRUCTURE OF BOARD BY WELDING PROCESS E.G. ULTRASONIC WELDING PROCESS\ | DE102012002945A1 | JANESCH RUDOLF (AT)\ JARISCH CHRISTOPH (AT) | HAEUSERMANN GMBH (AT) | H05K3/10\ H05K1/02 |
| 1000 | 3D printing | STAUBBEUTEL MIT EINER SAUGLOCH?FFNUNG UND EINER VERSCHLUSSVORRICHTUNG\[EN] VACUUM CLEANER BAG FOR COLLECTING E.G. DUST HAS APERTURE FOR RETAINING PARTICLES INSIDE BAG AND COMPRISING WALLS THAT ARE ASSEMBLED FROM NON-WOVEN MATERIAL LAYERS AND NARROW SEAMS ARRANGED AT PREDETERMINED DISTANCE FROM APERTURE EDGE\ | DE102012008225A1 | WULBRANDT HERBERT (DE) | ELKU BAUTEILE GMBH (DE) | A47L9/14 |
| 1001 | 3D printing | VERFAHRENSKOMBINATION ZUM WIDERSTANDSSCHWEI?EN\[EN] WELDING DEVICE USEFUL FOR CONNECTING SIMILAR AND/OR DISSIMILAR METALS COMPRISES A FIRST MAIN ELECTRODE A SECOND MAIN ELECTRODE OR PARTIAL ELECTRODES A CURRENT SOURCE ULTRASONIC WELDING SOURCE AND A THIRD WELDING HEAD\ | DE102012015241A1 | KARAKAS ERDOGAN (DE)\ KARAKAS TIMUR (DE) | KARAKAS ERDOGAN (DE)\ KARAKAS TIMUR (DE) | B23K28/02 |
| 1002 | 3D printing | VERFAHREN ZUR HERSTELLUNG EINES FOLIENSTAPELS\[EN] PREPARING FILM STACK BY FORMING FILM STACK FORM A NUMBER OF FIRMLY BONDED METAL FOILS CONNECTED TO EACH OTHER WHERE THE FOILS ARE ARRANGED ONE ABOVE THE OTHER IN A PROCESS STEP AND COMPACTED AND PREFIXED BY A HIGH FREQUENCY ULTRASOUND\ | DE102012018912A1 | LINDENAU DIRK (DE)\ BRANDNER MICHAEL (DE)\ LEITZ ANDREAS (DE)\ HOEFER TILLMANN (DE)\ STEINMETZ HEIKO (DE) | DAIMLER AG (DE) | B23K20/10\ B23K26/32\ B23K37/04\ H01M10/0585 |
| 1003 | 3D printing | GEH?USE INSBESONDERE FüR EINEN ELEKTRONISCHEN SCHLüSSEL\[EN] HOUSING FOR ELECTRONIC KEY OF LOCKING SYSTEM OF DOOR USED IN MOTOR VEHICLE HAS APERTURE AND THIN SECTION FOR RECEPTACLE THAT ARE PROVIDED IN LOWER HOUSING\ | DE102012025062A1 | DUFNER MICHAEL (DE)\ BRUSS BRUNO (DE)\ LANISNIK-BENSCH FRANZ (DE) | MARQUARDT GMBH (DE) | H05K5/02 |
| 1004 | 3D printing | SPULE MIT MEDIENDICHTER UMMANTELUNG\[EN] COIL FOR ELECTROMAGNET UTILIZED IN E.G. ELECTROMAGNETIC ACTUATOR HAS COVER COMPRISING TWO PARTS THAT ARE FIRMLY CONNECTED WITH EACH OTHER AT IMPACT EDGE VIA PROVISION OF ENERGY WHERE ONE OF PARTS COMPRISE MECHANICAL STRUCTURE\ | DE102012205738A1 | BURKART HARALD (DE)\ STROBEL CHRISTIAN (DE)\ KNOEBEL WOLFGANG (DE)\ HEINGL RALF (DE) | KENDRION VILLINGEN GMBH (DE) | H01F5/06\ H01F7/16\ H01F7/128\ H01H50/44 |
| 1005 | 3D printing | WISCHBLATTVORRICHTUNG\[EN] WIPER BLADE DEVICE FOR WIPER BLADE USED FOR WIPING WINDOWS E.G. MOTOR CAR WINDOWS HAS WIPER BLADE ADAPTER WHICH IS CONNECTED WITH SPRING RAIL BY ULTRASONIC WELDING METHOD AND IS PROVIDED FOR CONTROLLED GUIDANCE OF MELT\ | DE102012205955A1 | BONROY JAN (BE)\ VANDERHEYDEN GERT (BE) | BOSCH GMBH ROBERT (DE) | B60S1/38\ B60S1/40 |
| 1006 | 3D printing | SCHLEIFTELLERFEDERPLATTE\ | DE102012213749A1 | EGLIN THOMAS (CH) | BOSCH GMBH ROBERT (DE) | B24D9/00\ B24B23/00 |
| 1007 | 3D printing | PASSIV GED?MPFTES VIBRATIONSSCHWEISSSYSTEM UND -VERFAHREN\[EN] VIBRATION WELDING SYSTEM I.E. ULTRASONIC WELDING SYSTEM FOR FORMING WELDED JOINT USING ULTRASONIC VIBRATION HAS PASSIVE DAMPING MECHANISM COMPRISING CALIBRATED SET OF DAMPING PROPERTIES THAT DAMPS VIBRATION IN UNDESIRABLE DIRECTION\ | DE102012217436A1 | TAN CHIN-AN (US)\ KANG BONGSU (US)\ CAI WAYNE W (US)\ WU TAO (US) | GM GLOBAL TECH OPERATIONS INC (US) | B23K20/10\ B29C65/08 |
| 1008 | 3D printing | ABSORBER UND VERFAHREN ZU SEINER HERSTELLUNG\ | DE10232387B4 | HINDENBURG CARSTEN (DE)\ RENNER GERD (DE)\ KALLWELLIS VOLKER (DE)\ DOTZLER WOLFGANG (DE) | FRAUNHOFER GES FORSCHUNG (DE)\ GRAMMER SOLAR GMBH (DE) | B23K20/10\ B23K26/22\ F24J2/04\ F24J2/20 |
| 1009 | 3D printing | VERFAHREN ZUM HERSTELLEN VON ORGANISATIONSMITTELN UND ORGANISATIONSMITTEL\ | DE10241331B4 | WEIHE GEORG (DE)\ WEIHE VOLKER (DE) | SIEGERLAENDER PLASTIK GMBH (DE) | B42F13/40 |
| 1010 | 3D printing | VERFAHREN ZUM BEFESTIGEN EINES FL?CHENF?RMIGEN MATERIALS UND ANORDNUNG MIT FL?CHENF?RMIGEM MATERIAL\[EN] METHOD FOR ATTACHING FLEXIBLE NET TO VEHICLE SEAT BACK COMPRISES FASTENING IT AT POINTS AROUND ITS EDGE BY ULTRASONIC WELDING GLUING SEALING OR HOT-RIVETING\ | DE10247585A1 | JUERGEN FRANK (DE) | SEEBER AG & CO KG (DE) | B60R7/00 |
| 1011 | 3D printing | KAROSSERIEELEMENT FüR EIN FAHRZEUG INSBESONDERE DACHMODUL\ | DE10249412B4 | BRAUN ROBERT (DE)\ DICHTL MATTHIAS (DE)\ ELBS NORBERT (DE)\ GARRABOU LAURENT (FR)\ KOELBL MICHAEL (DE)\ LEROY ALAIN (DE)\ OEZKOEK KERIM (DE)\ WEYL PETER (DE) | WEBASTO AG (DE) | B62D65/00\ B29C65/08\ B62D25/06\ B62D27/02 |
| 1012 | 3D printing | BEHEIZTES SCHWINGWERKZEUG ZUM EINSATZ IN SCHWINGENDEN SYSTEMEN\ | DE10250741B4 | KAZMIRZAK WOLFGANG (DE)\ RICHTER BERNHARD (DE) | KUNSTSTOFF ZENTRUM LEIPZIG (DE) | B29C65/08\ B06B3/02 |
| 1013 | 3D printing | VERFAHREN ZUM VERBINDEN ELEKTRISCHER LEITER SOWIE ULTRASCHALLSCHWEI?VORRICHTUNG\[EN] CONNECTING ELECTRICAL CONDUCTOR FOR MOTOR VEHICLES INVOLVES REMOVING EXTERNAL INSULATION OF ONE OR MORE FLAT CABLES E.G. BY RADIATION AND/OR MELTING BY ULTRASONICALLY OSCILLATING SONOTRODE ULTRASONIC WELDING\ | DE10260897A1 | STROH DIETER (DE)\ STROH HEIKO (DE) | SCHUNK ULTRASCHALLTECHNIK GMBH (DE) | H01R43/02 |
| 1014 | 3D printing | ULTRASCHALLSCHWEI?ANLAGE UND VERFAHREN ZUR POSITIONIERUNG VON SONOTRODEN UND BAUTEILAUFNAHMEN\[EN] ULTRASONIC WELDING ASSEMBLY ESPECIALLY FOR PLASTICS HOUSING FOR ELECTRICAL/ELECTRONIC SENSORS HAS A SONOTRODE PROJECTING FROM A STACK WITH A GUIDE PIN TO WORK WITH A GUIDE DRILLING AT THE COMPONENT HOLDER\ | DE10301423A1 | WEIS CHRISTOF (DE)\ MERDES MARTIN (DE)\ ADRIAN JOERG (DE) | FRAUNHOFER GES FORSCHUNG (DE) | B23K20/10\ B29C65/00\ B29C65/08 |
| 1015 | 3D printing | ULTRASCHALLSCHWEI?ANLAGE UND VERFAHREN ZUR ERFASSUNG DER POSITION VON SONOTRODEN ZU BAUTEILAUFNAHMEN\ | DE10301433B4 | WEIS CHRISTOF (DE)\ MERDES MARTIN (DE)\ ADRIAN JOERG (DE) | FRAUNHOFER GES FORSCHUNG (DE) | B23K20/10\ B29C65/00\ B29C65/08\ B06B1/02 |
| 1016 | 3D printing | VERFAHREN ZUR VERKAPSELUNG VON FUNKTIONELLEN KOMPONENTEN EINES OPTOELEKTRONISCHEN BAUELEMENTS\ | DE10309607B4 | HEUSER KARSTEN (DE)\ PAETZOLD RALPH (DE)\ HENSELER DEBORA (DE)\ WITTMANN GEORG (DE) | OSRAM OPTO SEMICONDUCTORS GMBH (DE) | H05B33/04\ H01L51/40\ H01L21/50\ H01L51/52\ H01L51/10 |
| 1017 | 3D printing | VERFAHREN UND VORRICHTUNG ZUR HERSTELLUNG EINES VERKLEIDUNGSELEMENTS\[EN] VEHICLE CLADDING COMPONENT E.G. FOR DOORS AND INSTRUMENT PANELS HAS NONWOVEN INSERT IN INJECTION MOLDED FRAME BONDED BY ULTRASONIC WELDING AT SIDE OF FABRIC AWAY FROM FRAME\ | DE10339435A1 | WITTIG CHRISTIAN (DE) | VOLKSWAGEN AG (DE) | B29C65/08\ B60R13/02 |
| 1018 | 3D printing | VORRICHTUNG ZUM VERDICHTEN UND/ODER VERSCHWEI?EN VON ELEKTRISCHEN LEITERN\ | DE10342534B4 | STEINER ERNST (DE)\ STEINER CHRISTIAN (DE) | SCHUNK ULTRASCHALLTECHNIK GMBH (DE) | B23K11/00\ B23K20/10\ H01R43/02 |
| 1019 | 3D printing | ULTRASCHALLSCHWEI?ANORDNUNG SOWIE VERFAHREN ZUM BETREIBEN EINER SOLCHEN\[EN] ULTRASONIC WELDING EQUIPMENT FOR JOINING METALS OR PLASTICS HAS A WELDING MODULE WITH INTEGRAL DATA STORAGE UNIT FOR STORING MODULE RELATED PARAMETERS WHICH ARE READ BY THE PROCESS CONTROL UNIT\ | DE10349725A1 | EBERBACH JOST (DE) | SCHUNK ULTRASCHALLTECHNIK GMBH (DE) | B23K20/10\ B29C65/00\ B29C65/08 |
| 1020 | 3D printing | VERFAHREN ZUM VERSCHWEISSEN VON LEITERN\[EN] ULTRASONIC WELDING OF ELECTRICAL CONDUCTORS E.G. STRANDED WIRES APPLIES PRESSURE DURING WELDING AND FURTHER ULTRASONIC ACTION WHEN RELIEVED AND MEASURING GAP BETWEEN SONOTRODE AND COUNTER ELECTRODE\ | DE10350809B3 | EBERBACH JOST (DE) | SCHUNK ULTRASCHALLTECHNIK GMBH (DE) | B23K20/10\ H01R4/02\ H01R43/02 |
| 1021 | 3D printing | VERFAHREN ZUM VERSCHWEI?EN EINES BECHERARTIGEN BEH?LTERS UND WERKZEUG ZUR DURCHFüHRUNG DES VERFAHRENS\ | DE10359036B4 | MOEGLICH HARTMUT (DE) | HERRMANN ULTRASCHALLTECHNIK (DE) | B29C59/00\ B29C65/08\ B29C65/74\ B65B61/18\ B65B51/22\ B65B7/28\ B65B7/16\ B65B51/14\ B65B51/30 |
| 1022 | 3D printing | ULTRASONIC WELDING OF ELECTRICAL CONDUCTORS E.G. STRANDED WIRES APPLIES PRESSURE DURING WELDING AND FURTHER ULTRASONIC ACTION WHEN RELIEVED AND MEASURING GAP BETWEEN SONOTRODE AND COUNTER ELECTRODE\[DE] VERFAHREN ZUM VERSCHWEI?EN VON LEITERN\ | DE10359368A1 | EBERBACH JOST (DE) | SCHUNK ULTRASCHALLTECHNIK GMBH (DE) | B23K20/10\ H01R4/02\ H01R43/02 |
| 1023 | 3D printing | SCHALTGABEL MIT GLEITSCHUHEN AUS KUNSTSTOFF\[EN] GEAR SELECTOR FORK FOR MOTOR VEHICLE GEARBOX COMPRISES SLIDE SHOES WITH TWO PLASTIC SLIDE ELEMENTS WHICH ARE JOINED TO ONE ANOTHER BY ULTRASONIC WELDING VIA MATING MUTUAL POSITIONING ELEMENTS\ | DE19901674A1 | BECK BRUNO (DE)\ KRAEMER KLAUS (DE)\ TRISLER ARNOLD (DE) | SCHAEFFLER WAELZLAGER OHG (DE) | B60K20/00\ F16H63/32 |
| 1024 | 3D printing | VORRICHTUNG ZUM FüGEN VON MATERIALLAGEN DURCH ULTRASCHALLSCHWEI?EN\[EN] ULTRASONIC WELDING DEVICE FOR JOINING LAYERS OF MATERIAL WITH TWO ULTRASOUND UNITS SUPPLYING ULTRASONIC ENERGY TO OPPOSITE SIDES SIMULTANEOUSLY\ | DE19906873A1 | LANG GEORG (DE) | BRANSON ULTRASCHALL (DE) | B23K20/10\ B29C65/00\ B29C65/08 |
| 1025 | 3D printing | DURCH ULTRASCHALLSCHWEI?EN VERBINDBARE BAUGRUPPE\ | DE19950512B4 | BLAUDSCHUN DIETMAR (DE)\ LUTTERS ANDREAS (DE)\ LINDNER ANDREAS (DE) | STIEBEL ELTRON GMBH & CO KG (DE) | B29C65/00\ B29C65/08 |
| 1026 | 3D printing | WARNWESTE\[EN] SAFETY VEST ENSURES VISIBILITY OF WEARER AND HAS OUTER NONWOVEN LAYER MADE OF CONTINUOUS POLYMER FILAMENTS WHICH COMPRISE FLUORESCENT COLOR PIGMENTS\ | DE202004003109U1 | Empty Field US | BBA NONWOVENS BERLIN GMBH (DE) | A41D13/01 |
| 1027 | 3D printing | SONOTRODENWERKZEUG FüR EINE SONOTRODE\[EN] SONOTRODE TOOL ASSEMBLY USEFUL IN ULTRASONIC WELDING INCLUDES VIBRATING SYSTEM SONOTRODE TOOL BOOSTERS AND SUPPORT\ | DE202004003917U1 | NOVIKOV V M US\ SHKULIN A N US | SONOTRONIC NAGEL GMBH (DE) | B23K20/10 |
| 1028 | 3D printing | SILIZIUMHALTIGER STAHL FüR EINE FAHRRADGENERATORNABE\[EN] SILICON-CONTAINING STEEL ELEMENT FOR BICYCLE GENERATOR HUB IS WOUND INTO ROLL IN ONE PIECE FROM THIN METAL PLATE ARRANGED ABOUT WHEEL AXLE AND INSERTED INTO STATOR WITH SINGLE CYLINDRICAL FIXING BORE\ | DE202004014206U1 | TAKEDA KATSUHIRO US\ INUTSUKA TAKAMITSU US | TSAI MING TA (TW) | H02K1/24\ H02K1/28\ H02K1/30 |
| 1029 | 3D printing | SONOTRODE FüR DIE ULTRASCHALL-VERSCHWEI?UNG VON THERMOPLASTISCHEN WERKSTüCKEN\[EN] SONIC HORN FOR ULTRASONIC WELDING OF THIN WALL THERMOPLASTIC COMPONENTS HAS TAPERED STUDS FACING THE WORKPIECE\ | DE202005003067U1 | Empty Field US | KLN ULTRASCHALL GMBH (DE) | B29C65/00\ B29C65/02\ B29C65/08 |
| 1030 | 3D printing | KLOSETTABDECKUNG\[EN] DECORATIVE SEAT AND COVER FOR A WC ARE EACH MADE OF UPPER AND LOWER MOULDINGS OF TRANSPARENT PLASTIC WITH INTERNAL COMPARTMENTS FILLED WITH COLOURED LIQUIDS AND OTHER INCLUSIONS\ | DE202005003525U1 | SINCLAIR IAIN (GB) | TSAI JUI AN (TW) | A47K13/02 |
| 1031 | 3D printing | STAUBFILTERBEUTEL\[EN] DUST FILTRATION BAG E.G. FOR VACUUM CLEANER INCLUDES A VERY OPEN UPSTREAM LAYER ONLY LOOSELY ATTACHED TO FINE FILTRATION DOWNSTREAM LAYERS\ | DE202005018003U1 | Empty Field US | FIBERMARK GESSNER GMBH & CO (DE) | B01D39/16\ A47L9/14 |
| 1032 | 3D printing | BEH?LTER ZUM AUFTRAGEN EINES MEDIUMS\[EN] CONTAINER DELIVERING E.G. VISCOUS OR FOAMING SUBSTANCE INCLUDES FLEXIBLE TUBING POUCH SEALED TO TOP OF CONTAINER AT ONE END WITH E.G. RING CLOSURE AT LOWER END\ | DE202006010434U1 | FURLONG MICHAEL THOMAS (GB)\ HOPKINSON NEIL (GB)\ MUMTAZ KAMRAN AAMIR (GB) | LINDAL VENTIL GMBH (DE) | B65D83/38\ B65D83/14\ B65D83/62 |
| 1033 | 3D printing | FALT- UND üBERGABEVORRICHTUNG FüR EINE N?HMASCHINE\ | DE202006020176U1 | LEE BO IL (KR) | RSG ROMBOLD SYSTEM GMBH & CO K (DE) | D05B35/08 |
| 1034 | 3D printing | BüGELEISEN MIT EINEM GRIFF DER EIN VERFORMBARES KISSEN AUFWEIST\ | DE202009007301U1 | LEE NAM KI (KR) | SEB SA (FR) | D06F75/34 |
| 1035 | 3D printing | ULTRASCHALLVERSCHWEI?TES OPTIKGEH?USE\ | DE202013007706U1 | TUNG YUEH-MING (TW) | VALEO VISION (FR) | B29C65/08 |
| 1036 | 3D printing | FILTEREINSATZ ZUM EINBAU IN EINEN STR?MUNGSKANAL\[EN] FILTER INSERT FOR INSTALLATION IN ROOM VENTILATION DUCT HAS COMPRESSIBLE SEAL WITH SMOOTH SURFACE AT EDGES\ | DE20316367U1 | Empty Field US | B & S INDUSTRIESERVICE GMBH (DE) | B01D46/10\ B01D46/00\ B01D46/52 |
| 1037 | 3D printing | VORRICHTUNG ZUM SCHWEISSEN MIT ULTRASCHALL\[EN] APPARATUS FOR ULTRASONIC WELDING\[FR] DISPOSITIF POUR SOUDAGE PAR ULTRASONS\ | EP0941774A2 | DOBMANN GERD (DE)\ PAUL MICHAEL (DE)\ EIFLER DIETMAR (DE)\ FASSBENDER SILIVIA (DE) | FRAUNHOFER GES FORSCHUNG (DE) | B23K20/10 |
| 1038 | 3D printing | SYSTEM ZUM VERSCHWEISSEN BLISTERARTIGER VERPACKUNGEN UND DERGLEICHEN\[EN] SYSTEM FOR WELDING BLISTER TYPE CONTAINERS AND SIMILAR\[FR] SYSTèME POUR LE SOUDAGE D'EMBALAGES DU TYPE BLISTER ET SIMILAIRES\ | EP1110701A2 | MIRET GAYET XAVIER (ES) | MIRET GAYET XAVIER (ES) | B29C65/00\ B29C65/08\ B65B51/22 |
| 1039 | 3D printing | ANSAUGSYSTEM FüR BRENNKRAFTMASCHINEN\[EN] INTAKE SYSTEM FOR COMBUSTION ENGINES\[FR] SYSTèME D'ADMISSION POUR MOTEURS à COMBUSTION\ | EP1162362A2 | BAUMANN CHRISTOPH (DE)\ PRICKEN FRANC (DE) | MANN & HUMMEL FILTER (DE) | F02M35/14 |
| 1040 | 3D printing | VERFAHREN UND VORRICHTUNG ZUM HERSTELLEN MEHRLAGIGER PRODUKTE UND ZUGEH?RIGES PRODUKT\[EN] PROCESS AND APPARATUS FOR MAKING MULTILAYERED PRODUCTS AND PRODUCTS OBTAINED THEREBY\[FR] PROCéDé ET DISPOSITIF POUR LA FABRICATION DE PRODUITS MULTI-COUCHES ET PRODUITS AINSI OBTENUS\ | EP1163998A3 | POMMERANZ WILFRIED (DE)\ RUETTERMANN ROLF (DE)\ UEFFING HEINZ (DE) | EUL & GUENTHER GMBH (DE) | B29C59/00\ B29C65/00\ B29C65/08\ B29C65/74 |
| 1041 | 3D printing | ELEKTRISCHE VERBINDUNG MITTELS ULTRASCHALL-L?TEN\[EN] ELECTRICAL CONNECTION BY ULTRASONIC WELDING\[FR] CONNEXION éLECTRIQUE PAR SOUDAGE ULTRASONIQUE\ | EP1236563A1 | KREUTER MARKUS (AT)\ REISS THOMAS (AT)\ FEDER ROLAND (AT) | HIRSCHMANN AUSTRIA GMBH (AT) | B23K20/10\ H01R4/02\ B29C65/08\ H01R13/504\ H01R43/02 |
| 1042 | 3D printing | METHOD FOR JOINING ARTICLES AND CORRESPONDING TOOL\[DE] VORRICHTUNG ZUM VERBINDEN VON GEGENST?NDEN SOWIE WERKZEUG\[FR] DISPOSITIF POUR LE RACCORDEMENT D'OBJETS ET OUTIL CORRESPONDANT\ | EP1339521A1 | STEINER ERNST (DE)\ STROH DIETER (DE)\ DIETERLE HORST (DE) | SCHUNK ULTRASCHALLTECHNIK GMBH (DE) | B23K20/10\ H01R43/02\ B23K20/00 |
| 1043 | 3D printing | VERFAHREN ZUR HERSTELLUNG EINES ROHRFILTERS DURCH WICKELN SOWIE ROHRFILTER\[EN] PROCESS FOR MAUFACTURING OF A TUBULAR FILTER BY WINDING AND TUBULAR FILTER\[FR] PROCéDé DE FABRICATION D'UN FILTRE TUBULAIRE PAR ENROULEMENT ET FILTRE TUBULAIRE\ | EP1371405A1 | PFEIL BERTHOLD (DE) | INTENSIV FILTER GMBH (DE) | B01D39/16\ B29C65/00\ B01D46/00\ B29C65/08\ B01D46/24\ B01D46/36\ B01D63/06\ B29C53/60 |
| 1044 | 3D printing | VERFAHREN ZUM ULTRASCHALLSCHWEISSEN VON METALLISCHEN BAUTEILEN\[EN] METHOD OF ULTRASONIC WELDING METAL PARTS\[FR] PROCéDé DE SOUDAGE PAR ULTRASONS DES PIECES MéTALLIQUES\ | EP1371441A1 | WEBER PETER (DE) | BUDERUS HEIZTECHNIK GMBH (DE) | B23K20/10 |
| 1045 | 3D printing | SAUGF?HIGER ARTIKEL MIT GüRTEL UND VERFAHREN ZUR HERSTELLUNG EINES VERBUNDMATERIALS ZUR VERWENDUNG ALS GüRTELMATERIAL\[EN] BELTED ABSORBENT ARTICLE AND A METHOD OF PRODUCING A LAMINATE FOR USE AS BELT MATERIAL\[FR] ARTICLE ABSORBANT A CEINTURE ET PROCEDE DE PRODUCTION D'UN LAMINE UTILISE COMME MATERIAU DE CEINTURE\ | EP1423077A1 | ALMBERG CHRISTIAN (SE)\ KUSIBOJOSKA LILJANA (SE) | SCA HYGIENE PROD AB (SE) | A61F13/49\ A61F13/15\ B32B3/28\ B32B5/26\ A61F13/56\ A61F13/64\ B32B37/14 |
| 1046 | 3D printing | DAS SCHWEISSEN EINES KUPPLUNGSELEMENTES MITTELS ULTRASCHALL AUF EINE INTEGRIERTE OBERE POLYMERSCHAUMSCHICHT EINER K?RPERSEITIGEN SCHEIBE FüR EIN ZWEITEILIGES OSTOMIESYSTEM\[EN] THE ULTRASONIC WELDING OF A COUPLING MEMBER ONTO AN INTEGRATED TOP LAYER OF POLYMERIC FOAM OF A STOMA BODY SIDE WAFER FOR A TWO-PIECE STOMA SYSTEM\[FR] LE SOUDAGE PAR ULTRASONS D'UN éLéMENT D'ACCOUPLEMENT SUR UNE COUCHE SUPéRIEURE EN MOUSSE POLYMéRIQUE INTéGRéE DANS UNE PLAQUETTE COTé CORP POUR UN SYSTèME D'OSTOMIE EN DEUX PARTIES\ | EP1428647A1 | GIJSBERT VAN DER LEDEN ARIE (BE) | EUROTEC BEHEER B V (NL) | B29C65/08\ A61F5/448 |
| 1047 | 3D printing | ABSORBENT ARTICLE\[FR] ARTICLE ABSORBANT\[DE] SAUGF?HIGER ARTIKEL\ | EP1441680A1 | ALMBERG CHRISTIAN (SE) | SCA HYGIENE PROD AB (SE) | A61F13/49\ A61F13/15\ A61F5/44\ A61F13/56\ A61F13/64 |
| 1048 | 3D printing | ROTIERENDE FüLLMASCHINE MIT VERSCHLIESSVORRICHTUNG\[EN] ROTARY FILLING MACHINE WITH CLOSURE DEVICE\[FR] MACHINE ROTATIF DE REMPLISSAGE AVEC DISPOSITIF DE FERMETURE\ | EP1466829A1 | VOLLENKEMPER WILLI (DE) | HAVER & BOECKER (DE) | B29C65/08\ B65B51/22\ B65B1/18 |
| 1049 | 3D printing | PILE WEATHERSTRIPPING AND METHODS OF MAKING SAME\[DE] FENSTERDICHTBAND MIT FLOR UND VERFAHREN ZU DESSEN HERSTELLUNG\[FR] BOURRELET DE POIL ET PROCEDES DE REALISATION\ | EP1507909A1 | LOUGHNEY DAVID M (US) | ULTRAFAB INC (US) | B29C65/08\ E06B7/22\ E06B7/23\ F16J15/16 |
| 1050 | 3D printing | ULTRASCHALLSCHWEISSVORRICHTUNG UND VERFAHREN ZUM ULTRASCHALLVERBINDEN VON GEGENST?NDEN WIE ELEKTRISCHEN LEITERN INSBESONDERE VON LITZEN\[EN] DEVICE AND METHOD OF ULTRASONIC WELDING OF WORKPIECES LIKE ELECTRICAL WIRES IN PARTICULAR STRANDS\[FR] DISPOSITIF ET MéTHODE DE SOUDAGE PAR ULTRASONS DE PIèCES COMME DES FILS éLECTRIQUES EN PARTICULIER DES TORONS\ | EP1516689A1 | KAMMERER MICHAEL (CH)\ BUETTIKER ALBERT (CH) | TELSONIC HOLDING AG (CH) | B23K20/10\ H01R43/02\ B06B3/02 |
| 1051 | 3D printing | VORRICHTUNG ZUM VERSCHWEISSEN VON MEHRLAGIGEN LAMINATEN\[EN] APPARATUS FOR WELDING MULTILAYERED SHEETS\[FR] DISPOSITIF POUR LE SOUDAGE DE FEUILLES MULTICOUCHES\ | EP1535723A2 | MOEGLICH HARTMUT (DE) | HERRMANN ULTRASCHALLTECHNIK (DE) | B29C65/00\ B29C65/08\ B65D75/00 |
| 1052 | 3D printing | VORRICHTUNG ZUM FLUIDDICHTEN ABDICHTEN EINES ROHRABSCHNITTS\[EN] DEVICE FOR FLUID TIGHT SEALING OF TUBULAR SECTIONS\[FR] DISPOSITIF POUR RENDRE éTANCHE AUX FLUIDES UNE SECTION TUBULAIR à L'AIDE D'ULTRASONS\ | EP1543911A1 | NEIS DAN (DE) | STAPLA ULTRASCHALLTECHNIK GMBH (DE) | B23K20/10\ B29C65/08\ B29C65/74\ B23K101/06\ F25B45/00 |
| 1053 | 3D printing | VORDERE MOTORRAUMVERKLEIDUNG EINES KRAFTFAHRZEUGES MIT EINER FUSSG?NGERAUFPRALLSCHUTZFUNKTION\[EN] FRONT LINING FOR VEHICLE ENGINE COMPARTMENT WITH PEDESTRIAN IMPACT PROTECTION\[FR] REVêTEMENT POUR COMPARTIMENT MOTEUR D'UN VéHICULE AVEC PROTECTION DES PIETONS CONTRE LES CHOCS\ | EP1564088A1 | HAEGELE KLAUS (DE)\ KLEINEBERG WOLFGANG (DE)\ WOGH ERWIN (DE) | DAIMLER CHRYSLER AG (DE) | B60R21/34 |
| 1054 | 3D printing | REASSEMBLED TONER CARTRIDGE AND METHOD OF MANUFACTURE\[DE] NEUZUSAMMENGEBAUTE TONERKASSETTE UND HERSTELLUNGSVERFAHREN\[FR] CARTOUCHE DE TONER REMONTEE ET SON PROCEDE DE FABRICATION\ | EP1570317A1 | WAZANA YOEL (US)\ PAULUS JODA (US) | WAZANA BROTHERS INTERNATIONAL (US)\ WAZANA YOEL (US)\ PAULUS JODA (US) | G03G15/08\ B29C65/00\ G03G21/18 |
| 1055 | 3D printing | PROTHESENIMPLANTAT ZUM VERSCHLIESSEN.\[EN] PROSTHETIC IMPLANT FOR OBSTRUCTING.\[FR] IMPLANT PROTHETIQUE OBTURATEUR\ | EP1625831A1 | HOUARD WILLIAM (FR) | TEXTILE HI TEC (FR) | A61F2/00 |
| 1056 | 3D printing | ELEKTRISCHE VORRICHTUNG\[EN] ELECTRIC DEVICE\[FR] DISPOSITIF éLECTRIQUE\ | EP1655999A1 | KORTWIG CARSTEN (DE)\ STIER MARTIN (DE)\ RUNGE OLAF (DE)\ BOEHLAND HEIKO (DE) | DELPHI TECH INC (US) | H05B3/84 |
| 1057 | 3D printing | STRUKTUR ZUR BEFESTIGUNG EINER FASERIGEN MATTE\[EN] FIBROUS MAT FIXING STRUCTURE\[FR] STRUCTURE DE FIXATION DE TAPIS FIBREUX\ | EP1674242A1 | SASAKI KEI (JP) | KASAI KOGYO KK (JP) | B29C65/08\ B60R13/02\ F02B77/13 |
| 1058 | 3D printing | VERFAHREN ZUM TEMPOR?REN VERBINDEN VON VIEHMARKENTEILEN VIEHMARKENTEIL UND STREIFEN VON VIEHMARKEN\[EN] A METHOD OF PROVISIONALLY JOINING LIVESTOCK TAG PARTS TOGETHER A LIVESTOCK TAG PART AND STRIP OF LIVESTOCK TAGS\[FR] PROCéDé DE JONCTION PROVISOIRE D'éLéMENTS D'éTIQUETTE POUR BéTAIL éLéMENT D'éTIQUETTE POUR BéTAIL ET BANDE D'éTIQUETTES POUR BéTAIL\ | EP1690451A1 | WIKAN EGIL (NO) | OS HUSDYRMERKEFABRIKK AS (NO) | A01K11/00\ A01K |
| 1059 | 3D printing | VERFAHREN ZUM ABQUETSCHEN UND ABDICHTEN EINES ROHRES\[EN] METHOD FOR CONSOLIDATING AND SEALING A TUBE\[FR] PROCEDE POUR COMPRIMER ET BOUCHER UN TUBE\ | EP1708844A1 | NEIS DAN (DE)\ FRANK HARALD (DE) | STAPLA ULTRASCHALLTECHNIK GMBH (DE) | B23K20/10\ B29C65/00\ B29C65/08\ B29C65/74\ B29C57/10 |
| 1060 | 3D printing | ULTRASONIC WELDING STRUCTURE AND ULTRASONIC WELDING METHOD\[DE] ULTRASCHALLSCHWEISSKONSTRUKTION UND ULTRASCHALLSCHWEISSVERFAHREN\[FR] STRUCTURE DE SOUDAGE PAR ULTRASONS ET PROCEDE DE SOUDAGE PAR ULTRASONS\ | EP1712342A4 | TOMINAGA JUN (JP)\ SHIGEMATSU RYOHEI (JP)\ FURUKAWA KAZUO (JP) | NHK SPRING CO LTD (JP) | B29C65/56\ B29C65/08\ B29C65/60 |
| 1061 | 3D printing | EINBAUGER?T FüR EIN KRAFTFAHRZEUG\[EN] BUILT-IN APPARATUS FOR A VEHICLE\[FR] APPAREIL à ENCASTRER POUR VéHICULE\ | EP1732076B1 | SLAWIK RALF (DE)\ SCHIRMER THORSTEN (DE) | DELPHI TECH INC (US) | G11B17/04 |
| 1062 | 3D printing | DüSE FüR EINEN DRUCKBEH?LTER\[EN] AEROSOL NOZZLE\[FR] BUSE POUR RéCIPIENT AéROSOL\ | EP1754540A2 | LAGERWERF MARKUS LEENDERT (NL) | PLASTICUM GROUP B V (NL) | B05B1/34\ B65D83/16 |
| 1063 | 3D printing | DISPOSITIF DE SOUDAGE PAR ULTRASONS\[DE] ULTRASCHALLSCHWEISSVORRICHTUNG\[EN] AN ULTRASONIC WELDING DEVICE\ | EP1758728A1 | BOLDRINI FULVIO (IT)\ GHIOTTI ROBERTO (IT)\ CAVALLARI STEFANO (IT) | AZIONARIA COSTRUZIONI ACMA SPA (IT) | B29C65/00\ B29C65/08\ B31B1/64\ B31B3/00 |
| 1064 | 3D printing | VORRICHTUNG ZUM ULTRASCHALLVERBINDEN VON ELEKTRISCHEN LEITERN MIT EINER SICH üBER DIE ARBEITSFL?CHE DER SONOTRODE HINAUS ERSTRECKENDEN FüHRUNG\[EN] DEVICE FOR ULTRASONIC WELDING OF ELECTRICAL CONDUCTORS WITH ANVIL GUIDING MEANS EXTENDING ABOVE THE SONOTRODE WORKING SURFACE\[FR] DISPOSITIF DE SOUDAGE PAR ULTRASONS DE CONDUCTEURS éLECTRIQUES AVEC DES MOYENS DE GUIDAGE DE L'ENCLUME S'éTENDANT AU-DELà DE LA SURFACE DE TRAVAIL DE LA SONOTRODE\ | EP1772223A1 | KAMMERER MICHAEL (CH)\ MUELLER RALF (CH) | TELSONIC HOLDING AG (CH) | B23K20/10\ H01R43/02 |
| 1065 | 3D printing | VERFAHREN UND VORRICHTUNG ZUM VERSCHLIESSEN VON KUNSTSTOFFBEUTELN\[EN] METHOD AND APPARATUS FOR CLOSING PLASTIC BAGS\[FR] MéTHODE ET APPAREIL POUR FERMER DES SACS PLASTIQUES\ | EP1772381A1 | HOENIGS MICHAEL (DE)\ KEPPEL JUERGEN (DE) | H E T ANKE FILTERTECHNIK GMBH (DE)\ EM SYSTEME GMBH (DE) | B29C65/08\ B65B51/22\ B65B9/20 |
| 1066 | 3D printing | WISCHERARM SOWIE VERFAHREN ZUR HERSTELLUNG EINES WISCHERARMS\[EN] WIPER ARM AND METHOD FOR PRODUCING A WIPER ARM\[FR] BRAS D'ESSUIE-GLACE ET PROCEDES DE PRODUCTION D'UN BRAS D'ESSUIE-GLACE\ | EP1799518A1 | WEILER MICHAEL (KR)\ ZIMMER JOACHIM (DE) | BOSCH GMBH ROBERT (DE) | B60S1/32\ B60S1/34 |
| 1067 | 3D printing | VERFAHREN UND VORRICHTUNG ZUM VERSCHWEISSEN VON WERKSTüCKEN\[EN] PROCESS AND DEVICE FOR WELDING WORKPIECES\[FR] PROCéDé ET DISPOSITIF POUR SOUDER DES PIèCES\ | EP1800837A3 | VOLLENKEMPER WILLI (DE) | HAVER & BOECKER OHG (DE) | B29C65/08\ B65B7/02 |
| 1068 | 3D printing | VORRICHTUNG ZUR DICHTVERSIEGELUNG VON VERBUNDFOLIENBEUTELN\[EN] DEVICE FOR TIGHTLY SEALING BAGS MADE OF LAMINATED FILMS\[FR] DISPOSITIF POUR SCELLER HERMéTIQUEMENT DES SACS FABRIQUéS à PARTIR DE FILMS STRATIFIéS\ | EP1808375A1 | ODENTHAL HEINZ F (DE) | HILCONA AG (LI) | B65B51/14 |
| 1069 | 3D printing | VERBESSERUNGEN VON GIESSTüLLENVORRICHTUNGEN ODER DIESE BETREFFEND\[EN] IMPROVEMENTS IN OR RELATING TO POUR SPOUT DEVICES\[FR] AMELIORATIONS APPORTEES OU LIEES A DES DISPOSITIFS DE BEC VERSEUR\ | EP1812228A2 | ADLER NILS PETER (NO)\ KIESER WOLFGANG (DE) | ELOPAK SYSTEMS (CH) | B31B1/84\ B65D5/74 |
| 1070 | 3D printing | BAHNENWARE ZUM SCHUTZ VON PFLANZENKULTUREN\[EN] SHEET MATERIAL FOR PROTECTING CROPS\[FR] BANDE POUR LA PROTECTION DES CULTURES VéGéTALES\ | EP1817954A3 | GEISER DIETMAR (DE)\ GOETTEL MATTHIAS (DE)\ MAYR PETER DR (DE) | FREUDENBERG CARL KG (DE) | A01G13/02 |
| 1071 | 3D printing | GAP ADJUSTMENT FOR AN ULTRASONIC WELDING SYSTEM\[DE] SPALTEINSTELLUNG FüR EIN ULTRASCHALLSCHWEISSSYSTEM\[FR] REGLAGE D'INTERVALLE POUR UN SYSTEME DE SOUDURE ULTRASONORE\ | EP1833658A1 | NAYAR SATINDER K (US)\ POCHARDT DONALD L (US)\ OBLAK DONALD S (US)\ MLINAR JOHN R (US)\ FETTIG PAUL M (US) | 3M INNOVATIVE PROPERTIES CO (US) | B29C65/08 |
| 1072 | 3D printing | ULTRASCHALL-SCHWEI?BARE THERMOPLASTISCHE POLYOLEFINFOLIE\[EN] ULTRASONICALLY WELDABLE THERMOPLASTIC POLYOLEFIN FILMS\[FR] FEUILLE DE POLYOLéFINE THERMOPLASTIQUE POUVANT êTRE BLANCHIE PAR ULTRASONS\ | EP1837162B1 | PFEIFFER MARKUS (DE) | RKW AG (DE) | C08J5/18\ B29C65/08\ B65B51/22 |
| 1073 | 3D printing | VORRICHTUNG UND VERFAHREN ZUM APPLIZIEREN VON AUSGIESSELEMENTEN AUF KARTON/KUNSTSTOFF-VERBUNDPACKUNGEN UND/ODER ZUM VERSCHLIESSEN SOLCHER PACKUNGEN MITTELS ULTRASCHALL-SCHWEISSWERKZEUGEN\[EN] DEVICE AND METHOD FOR APPLYING POURING ELEMENTS TO CARDBOARD/PLASTIC COMPOSITE PACKAGINGS AND/OR CLOSING SAID PACKAGINGS BY ULTRASOUND WELDING TOOLS\[FR] DISPOSITIF ET PROCEDE POUR APPLIQUER DES ELEMENTS VERSEURS SUR DES EMBALLAGES COMPOSITES CARTON/PLASTIQUE ET/OU FERMER CES DERNIERS AU MOYEN D'OUTILS DE SOUDAGE PAR ULTRASONS\ | EP1846299A1 | BALTES KLAUS (DE) | SIG TECHNOLOGY AG (CH) | B29C65/08\ B29C65/78\ B65B61/18\ B65B51/22\ B31B1/84 |
| 1074 | 3D printing | ULTRASCHALL-SCHWEISSVORRICHTUNG ZUR BILDUNG VON SCHWEISSN?HTEN AN TEXTILIEN\[EN] ULTRASONIC WELDING DEVICE FOR FORMING WELDED SEAMS ON TEXTILES\[FR] DISPOSITIF DE SOUDAGE PAR ULTRASONS DESTINE A FORMER DES CORDONS DE SOUDURE SUR DES TEXTILES\ | EP1851037A1 | HEIL DIETMAR (DE) | JENTSCHMANN AG ZUERICH (CH) | B29C65/08 |
| 1075 | 3D printing | FILTEREINRICHTUNG UND VERFAHREN ZUR HERSTELLUNG EINER FILTEREINRICHTUNG\[EN] FILTER DEVICE AND METHOD FOR ITS MANUFACTURE\[FR] DISPOSITIF DE FILTRE ET PROCéDé DE FABRICATION D'UN DISPOSITIF DE FILTRE\ | EP1867377B1 | FRIEDRICH MATTHIAS (DE)\ SCHOEN MIRCO (DE)\ KEIBEL THORSTEN (DE) | MANN & HUMMEL INNENRAUMFILTER GMBH & CO KG (DE) | B01D46/10\ B01D46/52 |
| 1076 | 3D printing | DISTANZSCHWEISSEN\[EN] DISTANCE WELDING\[FR] SOUDAGE EN DISTANCE\ | EP1867462B1 | ALBERT BRUNO (DE) | FAURECIA EXTERIORS GMBH (DE) | B29C65/08 |
| 1077 | 3D printing | ULTRASCHALLGEBER MIT KüHLFLüSSIGKEIT ULTRASCHALL-SCHWEISSANORDNUNG UND VERFAHREN ZUM BETREIBEN EINES ULTRASCHALLGEBERS\[EN] ULTRASONIC GENERATOR WITH COOLING-FLUID A WELDING SYSTEM AND A METHOD FOR OPERATING AN ULTRASONIC GENERATOR\[FR] GéNéRATEUR D'ULTRASONS AVEC LIQUIDE DE REFROIDISSEMENT SYSTèME DE SOUDURE ET PROCéDé D'OPéRATION D'UN GéNéRATEUR D'ULTRASONS\ | EP1868182A1 | SOLENTHALER PETER (CH) | TELSONIC HOLDING AG (CH) | G10K11/00 |
| 1078 | 3D printing | HERSTELLUNGSVERFAHREN FüR EINEN VERDAMPFER\[EN] PRODUCTION METHOD OF EVAPORATOR\[FR] PROCéDé DE PRODUCTION D'éVAPORATEUR\ | EP1906113A1 | YILMAZ UMUT (TR)\ TURAN HALIL (TR) | VESTEL BEYAZ ESYA SANAYI VE TI (TR) | F25B39/02\ F28F1/22 |
| 1079 | 3D printing | ULTRASONIC WELDING DEVICE\[FR] DISPOSITIF DE SOUDAGE PAR ULTRASONS\[DE] ULTRASCHALLSCHWEISSVORRICHTUNG\ | EP1923201A1 | HEIL DIETMAR (DE) | JENTSCHMANN AG ZUERICH (CH) | B29C65/08 |
| 1080 | 3D printing | VERFAHREN ZUM BILDEN EINER BEH?LTERANORDNUNG AUS KUNSTSTOFF UND LAMINATMATERIALEN\[EN] METHOD FOR ASSEMBLYING OF CONTAINERS MADE OF PLASTICS AND LAMINATE MATERIAL COMPONENTS\[FR] PROCéDé POUR L'ASSEMBLAGE DE ENSEMBLE-CONTENEURS EN MATIèRE PLASTIQUE ET MATéRIAUX STRATIFIéS\ | EP1925559A8 | MCDOWELL CRAIG (GB)\ VON SPRECKELSEN HENNING (GB)\ FOWELL NEIL (GB) | BAPCO CLOSURES RES LTD (GB) | B29C45/14\ B65D8/04 |
| 1081 | 3D printing | BESCHLEUNIGUNGSSENSOR\[EN] ACCELERATION SENSOR\[FR] CAPTEUR D'ACCELERATION\ | EP1949041A1 | SCHEDLER CHRISTIAN (DE)\ BRANDT TOBBY (DE) | BOSCH GMBH ROBERT (DE) | G01P15/00\ G01P1/02\ B60R19/48 |
| 1082 | 3D printing | STüTZWENDEL\[EN] HELICAL SUPPORT\[FR] HéLICE D'APPUI\ | EP1975494A3 | ROEDEL SILVIO (DE) | REHAU AG & CO (DE) | B29C65/08\ B29C65/76\ H02G15/18\ B29C53/78\ B29C61/06\ F16L11/16\ F16L11/20 |
| 1083 | 3D printing | SCHWEISSVERFAHREN UND GEOSYNTHETISCHE PRODUKTE DARAUS\[EN] WELDING PROCESS AND GEOSYNTHETIC PRODUCTS THEREOF\[FR] PROCESSUS DE SOUDAGE ET PRODUITS GéOSYNTHéTIQUES ASSOCIéS\ | EP1981697A1 | HALAHMI IZHAR (IL)\ EREZ ODED (IL)\ EREZ ADI (IL) | P R S MEDITERRANEAN LTD (IL) | B29C65/08\ E02D17/00 |
| 1084 | 3D printing | VERFAHREN UND VORRICHTUNG ZUM VERSCHWEI?EN EINER SAMMELSCHIENE MIT KABELN DURCH VIBRATIONEN\[EN] METHOD AND DEVICE FOR WELDING A BUSBAR AND CABLES BY VIBRATIONS\[FR] PROCéDé ET DISPOSITIF POUR SOUDER UNE BARRE BUS ET DES CABLES PAR VIBRATIONS\ | EP1997580B1 | MOLY JOSE (FR) | LEONI WIRING SYSTEMS FRANCE (FR) | B23K20/10\ B29C65/00\ H01R4/62\ B29C65/08\ H01R43/02\ B23K101/32\ B23K103/18\ H01R11/11 |
| 1085 | 3D printing | MONTAGESYSTEM EINER BLENDE AUF EINEM SCHEINWERFERGEH?USE FüR KRAFTFAHRZEUGE\[EN] SYSTEM FOR INSTALLING A MASK ON A HEADLIGHT CASING FOR AN AUTOMOBILE\[FR] SYSTèME D'ASSEMBLAGE D'UN MASQUE SUR UN BO?TIER DE PROJECTEUR POUR VéHICULE AUTOMOBILE\ | EP2017527A1 | PERON RODOLPHE (FR) | PEUGEOT CITROEN AUTOMOBILES SA (FR) | F21W101/14\ F21V17/00\ F21W101/10 |
| 1086 | 3D printing | BAUTEILANORDNUNG\[EN] COMPONENT ASSEMBLY\[FR] AGENCEMENT DE COMPOSANT\ | EP2020285A2 | WAIBEL THORSTEN (DE) | PORSCHE AG (DE) | B29C65/08\ B29C65/60 |
| 1087 | 3D printing | POWDER METAL FRICTION STIR WELDING TOOL AND METHOD OF MANUFACTURE THEREOF\[DE] PULVERMETALL-REIBRüHRSCHWEISSWERKZEUG UND HERSTELLUNGSVERFAHREN DAFüR\[FR] OUTIL DE SOUDAGE PAR AGITATION ET FRICTION EN MéTAL EN POUDRE ET PROCéDé DE MANUFACTURE ASSOCIé\ | EP2021140A2 | CHRISTOPHERSON DENIS JR (US) | FEDERAL MOGUL CORP (US) | B22F3/00\ B23K20/12 |
| 1088 | 3D printing | IMPROVED BATTERY PACK\[DE] VERBESSERTES BATTERIEPACK\[FR] ENSEMBLE BATTERIE AMéLIORé\ | EP2025018A1 | KIM TAE IL (KR)\ JUNG SOONKWANG (KR) | LG CHEMICAL LTD (KR) | H01M2/02\ H01M2/10 |
| 1089 | 3D printing | SCHWEISSPISTOLENANORDNUNG\[EN] WELDING GUN ASSEMBLY\[FR] ENSEMBLE D'UN PISTOLET DE SOUDURE\ | EP2032342B1 | VIRELIZIER FRANCOIS (FR)\ BRIZIN JEROME (FR) | PLASTIC OMNIUM CIE (FR) | B29C65/08 |
| 1090 | 3D printing | FUTTERKORB\[EN] SWIMFEEDER\[FR] DISPOSITIF D'AMORCAGE POUR LA PêCHE\ | EP2047745A1 | DRENNAN PETER JOHN (GB) | DRENNAN INT LTD (GB) | A01K97/02 |
| 1091 | 3D printing | PULLEY WITH ROLLING BEARING\[DE] W?LZGELAGERTE ROLLE\[FR] POULIE AVEC ROULEMENT\ | EP2058535B1 | DUNGS JENS (DE) | SCHAEFFLER TECHNOLOGIES GMBH (DE) | B29C45/14\ F16C13/00\ F16H55/48 |
| 1092 | 3D printing | SCHALTUNGSTR?GER VERFAHREN ZUR HERSTELLUNG EINES SCHALTUNGSTR?GERS SOWIE BONDVERFAHREN\[EN] CIRCUIT CARRIER METHOD FOR MANUFACTURING A CIRCUIT CARRIER AND BONDING METHOD\[FR] PORTE-CIRCUIT PROCéDé DE FABRICATION D'UN PORTE-CIRCUIT ET PROCéDé DE LIAISON\ | EP2073259A3 | BERNHARDT ANDREAS (DE)\ SCHINGALE ANGELIKA (DE) | CONTINENTAL AUTOMOTIVE GMBH (DE) | H01L21/60 |
| 1093 | 3D printing | SCHUH INSBESONDERE SPORTSCHUH UND/ODER FREIZEITSCHUH\[EN] SHOE IN PARTICULAR SPORT SHOE AND/OR LEISURE SHOE\[FR] CHAUSSURE NOTAMMENT CHAUSSURE DE SPORT ET/OU DE LOISIR\ | EP2084981B1 | FISCHER HEINZ-JOERG (DE) | INNOVATEC MICROFIBRE TECHNOLOGY GMBH & CO KG (DE) | B32B27/40\ B32B27/12\ A43B9/12\ A43B13/04\ A43B19/00\ A43B23/02 |
| 1094 | 3D printing | DREHSONOTRODE FüR DAS ULTRASCHALLSCHWEISSEN\[EN] ROTARY SONOTRODE FOR ULTRASONIC WELDING\[FR] SONOTRODE ROTATIVE POUR SOUDAGE ULTRASONIQUE\ | EP2089214A1 | MONTI MASSIMILIANO (IT)\ SALA WALTER (IT) | STARTEC S R L (IT) | B29C65/08 |
| 1095 | 3D printing | ULTRASCHALLPRESSE MIT SERVOMOTOR UND VERZ?GERTER BEWEGUNGSTECHNIK\[EN] ULTRASONIC PRESS USING SERVO MOTOR AND DELAYED MOTION TECHNIQUE\[FR] PRESSE ULTRASONIQUE UTILISANT UN SERVOMOTEUR ET TECHNIQUE DE MOUVEMENT DIFFéRéE\ | EP2106899A3 | KLINSTEIN LEO (US)\ JURKOWSKI WILLIAM E (US)\ GOLKO PAUL (US) | DUKANE CORP (US) | B29C65/08 |
| 1096 | 3D printing | METHOD FOR SECURING A GETTER FOR A VACUUM SWITCHING TUBE AND VACUUM SWITCHING TUBE\[DE] VERFAHREN ZUR BEFESTIGUNG EINES GETTERS FüR EINE VAKUUMSCHALTR?HRE SOWIE VAKUUMSCHALTR?HRE\[FR] PROCéDé DE FIXATION D'UN GETTER POUR UN INTERRUPTEUR à VIDE ET INTERRUPTEUR à VIDE\ | EP2140468A1 | SCHUEMANN ULF (DE)\ SPOERER HENRYK (DE) | SIEMENS AG (DE) | H01H33/66 |
| 1097 | 3D printing | DREHHORN ZUM ULTRASCHALLSCHWEISSEN\[EN] ROTARY HORN FOR ULTRASONIC WELDING\[FR] PAVILLON ROTATIF POUR SOUDAGE PAR ULTRASONS\ | EP2227371B1 | MONTI MASSIMILIANO (IT)\ SALA WALTER (IT) | STARTEC S R L (IT) | B29C65/08 |
| 1098 | 3D printing | ANBAUTEIL ZUR VERBINDUNG MIT EINEM BAUTEIL DURCH TORSIONALES ULTRASCHALLSCHWEI?EN\[EN] EXTENSION COMPONENT FOR CONNECTION WITH A COMPONENT BY MEANS OF TORSIONAL ULTRASOUND WELDING\[FR] PIèCE RAPPORTéE à CONNECTER à UN COMPOSANT à L'AIDE D'UNE SOUDURE PAR ULTRASONS TORSIONNELLE\ | EP2246178B1 | SAUTNER ANTON (DE)\ HELFENBERGER ANDREAS (DE)\ BUECKER DIRK (DE)\ FENZEL ROBERT (DE)\ GOERSE HERGEN (DE) | FAURECIA EXTERIORS GMBH (DE) | B29C65/08\ B29C65/06 |
| 1099 | 3D printing | GEP?CKNETZ MIT RAHMEN INSBESONDERE FAHRZEUG-GEP?CKNETZ SOWIE VERFAHREN UND VORRICHTUNG ZU DESSEN HERSTELLUNG\[EN] LUGGAGE NET WITH FRAME IN PARTICULAR VEHICLE LUGGAGE NET AND METHOD AND DEVICE FOR PRODUCING SAME\[FR] FILET à BAGAGES DOTé D'UN CADRE NOTAMMENT FILET à BAGAGES DE VéHICULE AINSI QUE PROCéDé ET DISPOSITIF DESTINéS à SA FABRICATION\ | EP2246218A3 | NACHBAUR JOERG (AT) | NATEX SPITZEN GMBH & CO (AT) | B60R7/00 |
| 1100 | 3D printing | VERFAHREN ZUR HERSTELLUNG VON VIEHOHRMARKEN\[EN] METHOD FOR MANUFACTURING EAR TAGS FOR CATTLE\[FR] PROCéDé DE FABRICATION DE MARQUES AURICULAIRES POUR BéTAIL\ | EP2272330A2 | FIERET GILLES FRANCOIS (NL) | PLASTIFRAN B V (NL) | A01K11/00 |
| 1101 | 3D printing | NACHBEARBEITUNGSSYSTEM FüR SFF-TEILE (SFF - SOLID FREEFORM FABRICATION)\[EN] POST-PROCESSING SYSTEM FOR SOLID FREEFORM FABRICATION PARTS\[FR] SYSTèME DE POST-TRAITEMENT POUR PIèCES DE FABRICATION DE SOLIDES à FORME LIBRE\ | EP2293925A2 | REICHENTAL ABRAHAM N (US)\ MOUSSA KHALIL (US)\ SPERRY CHARLES R (US)\ MCNAMARA DENNIS F (US)\ SCOTT SUZANNE M (US)\ MUSKUS KRZYSZTOF J (US)\ GREGORY RICHARD O (US)\ BAEHL DONALD E (US) | 3D SYSTEMS INC (US) | B29C67/00\ B29C71/04\ B08B3/04\ B08B7/00 |
| 1102 | 3D printing | ANZEIGEEINRICHTUNG\[EN] A DISPLAY DEVICE\[FR] DISPOSITIF D AFFICHAGE\ | EP2318750A1 | GURSES AYLIN (TR)\ AVCI SERDAL KORKUT (TR)\ GULER CEMAL (TR)\ CELIKKOL ULKU (TR)\ FERADOGLU ALEV (TR) | ARCELIK AS (TR) | B29C65/08\ F16M11/00 |
| 1103 | 3D printing | VERFAHREN ZUR HERSTELLUNG EINES HEIZK?RPERS UNTER VERWENDUNG EINES ULTRASCHALLSCHWEI?ENS; DANACH HERGESTELLTER HEIZK?RPER\[EN] METHOD FOR PRODUCING A HEATER BY MEANS OF ULTRASOUND WELDING; HEATER PRODUCED ACCORDING TO THE METHOD\[FR] PROCéDé DE FABRICATION D'UN CORPS DE CHAUFFAGE EN UTILISANT UN SOUDAGE à ULTRASONS; RADITEUR DE CHAUFFAGE FABRIQUé PAR CETTE MéTHODE\ | EP2338631A1 | GRAULS ROGER (BE)\ WOJCIK GUY (BE)\ BENZLER TOBIAS DR (DE)\ MEYER ROLF HEINRICH (DE)\ PRITZLAFF REINHARD (DE)\ WEHNER TORSTEN (DE) | CARADON STELRAD B V (BE)\ HYDRO ALUMINIUM ROLLED PROD (DE) | B23K20/10\ F28F3/02\ F24H9/00\ F24D19/00\ F28D1/03 |
| 1104 | 3D printing | ELASTISCHES VERBUNDMATERIAL\[EN] COMPOSITE ELASTIC MATERIAL\[FR] MATéRIAU éLASTIQUE COMPOSITE\ | EP2352471B1 | COTTON STEPHEN (GB) | BRIGHTWAKE LTD (GB) | A61F13/00\ B32B5/18\ B32B27/40\ B32B27/36\ D04H1/54\ D04H1/559\ A61L15/16\ D04H1/593\ A61L15/22\ A61L15/42\ D04H1/52\ D04H1/555 |
| 1105 | 3D printing | VERFAHREN ZUR HERSTELLUNG EINER GETR?NKEDOSE\[EN] METHOD FOR PRODUCING A BEVERAGE CAN\[FR] PROCéDé DE FABRICATION D'UNE CANETTE DE BOISSON\ | EP2360007A2 | TSCHECH FLORIAN DR (DE) | NUCLEUS GMBH (DE) | B29L9/00\ B29L23/00\ B29C65/08\ B31C1/00\ B29C53/50\ B29K711/00 |
| 1106 | 3D printing | GETR?NKEDOSE DIE WEITGEHEND AUS ZELLSTOFFHALTIGEM MATERIAL BESTEHT UND EINE LEBENSMITTELVERTR?GLICHE BARRIERESCHICHT AUFWEIST FüR UNTER üBERDRUCK STEHENDE GETR?NKE\[EN] DRINKS CAN MAINLY COMPRISED OF MATERIAL CONTAINING CELLULOSE AND A FOOD-SAFE BARRIER COATING FOR PRESSURISED DRINKS\[FR] BRIQUE DE BOISSON PRINCIPALEMENT CONSTITUéE DE MATéRIAUX CONTENANT DE LA MATIèRE CELLULAIRE ET UNE COUCHE DE BARRIèRE TOLéRANT LES ALIMENTS POUR DES BOISSONS SOUS SURPRESSION\ | EP2384886A1 | KEIENBURG GUENTER (DE) | KEIENBURG GUENTER (DE) | B32B27/30\ B32B27/10\ B65D3/04\ B65D3/14\ B65D3/22 |
| 1107 | 3D printing | VORRICHTUNG ZUM SCHWEI?EN UND TRENNEN VON PACKSTOFFEN\[EN] DEVICE FOR WELDING AND SEPARATING PACKAGING MATERIALS\[FR] DISPOSITIF DE SOUDAGE ET DE SéPARATION DE MATéRIAUX D'EMBALLAGE\ | EP2387495A1 | WIEDUWILT ULRICH (DE)\ LOECHT HEINRICH (DE) | BOSCH GMBH ROBERT (DE) | B29C65/08\ B29C65/74\ B65B51/22\ B65B51/30 |
| 1108 | 3D printing | W?RMEüBERGANGSLEITER\[EN] HEAT TRANSFER CONDUCTOR\[FR] CONDUCTEUR DE TRANSFERT DE CHALEUR\ | EP2396611A2 | ENGERT TOBIAS (DE)\ KOJOUHAROV IVAN (DE)\ GERL JUERGEN (DE) | GSI HELMHOLTZZENTR SCHWERIONEN (DE) | H01L31/024\ F25D19/00\ F17C3/08\ G01J5/06 |
| 1109 | 3D printing | VERFAHREN ZUR BEFESTIGUNG EINES BAUTEILS AN EIN KUNSTSTOFFTEIL EINES KRAFTFAHRZEUGS MIT DEM TORSIONALEN ULTRASCHALLSCHWEISSVERFAHREN\[EN] METHOD FOR FASTENING A COMPONENT TO A PLASTIC PART OF A MOTOR VEHICLE USING THE TORSIONAL ULTRASONIC WELDING METHOD\[FR] PROCéDé POUR LA FIXATION D'UN COMPOSANT à UNE PIèCE EN PLASTIQUE D'UN VéHICULE AUTOMOBILE AVEC UN PROCéDé DE SOUDAGE AUX ULTRASONS PAR TORSION\ | EP2403704A1 | LANG GEORG (DE)\ SAUTNER ANTON (DE)\ BUECKER DIRK (DE)\ GOERSE HERGEN (DE)\ REGENBERG CLAUS (DE) | PLASTAL GMBH (DE)\ TELSONIC GMBH (DE) | B23K20/10\ B29C65/08\ B60G17/015 |
| 1110 | 3D printing | SCHALTER FüR ELEKTRISCHE INSTALLATIONEN\[EN] SWITCH FOR ELECTRICAL INSTALLATIONS\[FR] COMMUTATEUR POUR INSTALLATIONS éLECTRIQUES\ | EP2423937A3 | ARIZ ARNEDO FCO JAVIER (ES)\ GONZALEZ IRIBAS AITOR (ES)\ GONI ITURMENDI JULIO JOSE (ES)\ REBOLE OLLETA JOSE ANTONIO (ES) | SCHNEIDER ELECTRIC ESPANA SA (ES) | H01H23/02\ H01H23/04\ H01H23/24\ H01H23/20\ H01H23/14 |
| 1111 | 3D printing | ORIGINALIT?TSSICHERUNG BEI EINEM KUNSTSTOFFBEH?LTER ZUR AUFNAHME VON LEBENSMITTELN\[EN] TAMPER-PROOF CLOSURE FOR A PLASTIC CONTAINER FOR ACCOMMODATING FOOD\[FR] FERMETURE INVIOLABLE POUR UN RECIPIENT EN MATIERE PLASTIQUE DESTINE A CONTENIR DES DENREES ALIMENTAIRES\ | EP2437982A1 | HOFMANN GEORG (DE) | FUERST GMBH (DE) | B65B51/22\ B65B7/28\ B65D43/02 |
| 1112 | 3D printing | BEFESTIGUNG VON MONTIERBAREN ELEMENTEN AUF KABELMANTEL\[EN] FIXING FOR ELEMENTS THAT CAN BE FITTED ONTO CABLE SHEATH\[FR] FIXATION D'éLéMENTS POUVANT êTRE MONTéS SUR UNE GAINE\ | EP2439109A1 | JANC THOMAS (AT)\ KOPF HUBERT (AT) | HIRSCHMANN AUTOMOTIVE GMBH (AT) | B60R16/02\ H02G3/08\ F02M25/07\ G01D5/14 |
| 1113 | 3D printing | VERPACKUNGSMASCHINE MIT EINER ULTRASCHALL-SCHWEISSVORRICHTUNG\[EN] PACKAGING MACHINE WITH ULTRASONIC WELDING DEVICE\[FR] MACHINE D' EMBALLAGE MUNIE D' UN APPAREIL DE SOUDAGE à ULTRASONS.\ | EP2445795A2 | RUMERY GLENN (US)\ POUBLON KENNETH ARCHIE (US) | ELOPAK SYSTEMS (CH) | B65B61/18\ B31B1/84\ B23Q1/56 |
| 1114 | 3D printing | KAPSEL FüR INFUSIONSPRODUKTE\[EN] CAPSULE FOR INFUSION PRODUCTS\[FR] CAPSULE POUR PRODUITS à INFUSER\ | EP2465793B1 | RONDELLI RAFFAELE (IT) | MACCHIAVELLI S R L (IT) | B65D85/804 |
| 1115 | 3D printing | VERFAHREN ZUM HERSTELLEN EINES DREIDIMENSIONALEN OBJEKTES IM WEGE EINES GENERATIVEN HERSTELLUNGSPROZESSES NACH DEM LOM-VERFAHREN\[EN] METHOD FOR PRODUCING A THREE-DIMENSIONAL OBJECT IN THE COURSE OF A GENERATIVE PRODUCTION PROCESS BASED ON THE LOM METHOD\[FR] PROCéDé DE PRODUCTION D'UN OBJET TRI-DIMENSIONNEL AU COURS D'UN PROCéDé DE PRODUCTION GéNéRATIF SELON LE PROCéDé DE FABRICATION STRATIFIéE D'OBJET - LOM -\ | EP2473335A1 | GRZESIAK ANDRZEJ (DE)\ BECKER RALF (DE)\ REFLE OLIVER (DE) | FRAUNHOFER GES FORSCHUNG (DE) | B29C67/00\ B29C70/38 |
| 1116 | 3D printing | AMBOSS FüR ULTRASCHALLSCHWEI?VORRICHTUNG DIE MIT MINDESTENS EINER SONOTRODE AUSGESTATTET IST\[EN] ANVIL FOR AN ULTRASOUND WELDING DEVICE INCLUDING AT LEAST ONE SONOTRODE\[FR] ENCLUME POUR DISPOSITIF DE SOUDAGE PAR ULTRASONS COMPRENANT AU MOINS UNE SONOTRODE\ | EP2474407A1 | DE CAMPREDON JEAN-MARIE (FR)\ MAGNAN SEBASTIEN (FR) | MV 2 (FR) | B29C65/08 |
| 1117 | 3D printing | ULTRASONIC WELDING DEVICE AND METHOD\[DE] ULTRASCHALL-SCHWEI?VORRICHTUNG UND ULTRASCHALL-SCHWEI?VERFAHREN\[FR] DISPOSITIF ET PROCéDé DE SOUDURE PAR ULTRASONS\ | EP2484511A1 | DE CAMPREDON JEAN-MARIE (FR) | MV 2 (FR) | B29C65/08 |
| 1118 | 3D printing | HALTER FüR ANHALTEKELLE\[EN] HOLDER FOR STOP SIGNALLING DISC\[FR] SUPPORT POUR PALETTE D'ARRêT\ | EP2490191A3 | SCHULZE DIRK (DE) | KAMEI AUTOMOTIVE GMBH (DE) | G08B5/00 |
| 1119 | 3D printing | BUCHSE UND VERFAHREN ZUM FERTIGEN VON BUCHSEN\[EN] SOCKET AND METHOD FOR MANUFACTURING SOCKETS\[FR] DOUILLE ET PROCéDé DE FABRICATION DE DOUILLES\ | EP2522878B1 | WERNER PHILIPP DR (DE)\ SELL HENDRIK DR (DE) | TRELLEBORGVIBRACOUSTIC GMBH (DE) | F16F13/14 |
| 1120 | 3D printing | ULTRASCHALLSCHWEISSGER?T IM BESONDEREN ZUM SCHWEISSEN EINES STAPELS AUS ZELLLAGEN\[EN] ULTRASONIC WELDING DEVICE IN PARTICULAR FOR WELDING A STACK OF CELLULAR SHEETS\[FR] DISPOSITIF DE SOUDAGE PAR ULTRASONS NOTAMMENT POUR LE SOUDAGE D'UN EMPILEMENT DE FEUILLES ALVéOLéES\ | EP2531343A1 | BEZAULT YVES (FR)\ MARTIN CLAUDE (FR) | HAMON THERMAL EUROP FRANCE (FR) | B29C65/08\ F28F21/06 |
| 1121 | 3D printing | ANPASSUNG UND INDIVIDUALISIERUNG DER OBERFL?CHENKONTUR ZU VERBINDENDER K?RPER SPEZIELL BEIM TORSIONALEN ULTRASCHALLSCHWEISSEN\[EN] ADAPTATION AND CUSTOMIZATION OF THE SURFACE CONTOUR OF BODIES TO BE CONNECTED IN PARTICULAR DURING TORSIONAL ULTRASONIC WELDING\[FR] ADAPTATION ET INDIVIDUALISATION DU CONTOUR DE SURFACE DE CORPS à RELIER NOTAMMENT LORS DE SOUDURES TORSIONNELLES PAR ULTRASONS\ | EP2536550A1 | BUECKER DIRK (DE)\ GOERSE HERGEN (DE) | FAURECIA EXTERIORS GMBH (DE) | B23K33/00\ B29C65/48\ B29C65/08\ B60R19/48 |
| 1122 | 3D printing | KUNSTSTOFFVERSCHLUSSVORRICHTUNG FüR SCHLAUCHBEUTEL\[EN] PLASTIC CLOSURE DEVICE FOR TUBULAR BAGS\[FR] DISPOSITIF DE FERMETURE EN PLASTIQUE POUR SACHET TUBULAIRE\ | EP2547593A1 | DOCHERTY TOM (CH)\ BARRON DAN (CH) | BOSCH GMBH ROBERT (DE) | B29C65/00\ B29C65/08\ B29C65/78\ B65D5/74\ B65D41/34\ B65D75/58\ B65D77/06 |
| 1123 | 3D printing | TURBINE FüR PUMPE MIT HOHEM LUFTSTROM\[EN] TURBINE FOR HIGH AIR FLOW PUMP\[FR] TURBINE DE POMPE à éCOULEMENT D'AIR éLEVé\ | EP2581606A1 | SOLER ALVAREZ MARINA (ES) | DESARROLLOS EMPRESARIALES VALAR S L (ES) | F04D29/02\ F04D29/22 |
| 1124 | 3D printing | TELESKOPIERBARE HUBS?ULE EINES M?BELTEILS\[EN] TELESCOPIC LIFTING COLUMN OF A PIECE OF FURNITURE\[FR] COLONNE éLéVATRICE TéLESCOPIQUE D'UNE PARTIE DE MEUBLE\ | EP2594157A3 | SCHAEL OLIVER (DE)\ SOBOLEWSKI UWE (DE)\ ANDSCHUS STEFAN (DE)\ PRIOR THOMAS (DE)\ GORGES ALEXANDER (DE) | HETTICH PAUL GMBH & CO KG (DE) | A47B9/04 |
| 1125 | 3D printing | VERFAHREN ZUM VERBINDEN EINES SENSORTEILS MIT EINEM WELLENTEIL EINES KRAFTFAHRZEUGS UND ANORDNUNG MIT EINEM WELLENTEIL UND EINEM SENSORTEIL\[EN] METHOD FOR CONNECTING A SENSOR PART WITH A SHAFT SECTION OF A MOTOR VEHICLE AND ASSEMBLY WITH A SHAFT SECTION AND A SENSOR PART\[FR] PROCéDé DE LIAISON D'UNE PIèCE DE CAPTEUR AVEC UNE PIèCE D'ARBRE D'UN VéHICULE AUTOMOBILE ET AGENCEMENT COMPORTANT UNE PIèCE D'ARBRE ET UNE PIèCE DE CAPTEUR\ | EP2594915A2 | FROEHLICH EKKEHART (DE)\ SCHWADERER JUERGEN (DE) | VALEO SCHALTER & SENSOREN GMBH (DE) | G01L3/10\ G01D5/14\ G01L5/22 |
| 1126 | 3D printing | ULTRASONIC WELDING DEVICE AND METHOD\[DE] ULTRASCHALLSCHWEISSVORRICHTUNG UND VERFAHREN\[FR] DISPOSITIF DE SOUDAGE PAR ULTRASONS ET PROCéDé ASSOCIé\ | EP2763837A1 | BULATOW MICHAEL (DE)\ RADTKE CHRISTOPH (DE) | CONTINENTAL AUTOMOTIVE GMBH (DE) | B23K20/10\ B29C65/08\ B29C65/78 |
| 1127 | 3D printing | A METHOD FOR MANUFACTURING A PASSIVE DIRECT METHANOL FUEL CELL AND A PASSIVE DIRECT METHANOL FUEL CELL\[DE] VERFAHREN ZUR HERSTELLUNG EINER PASSIVEN DIREKT-METHANOL-BRENNSTOFFZELLE UND PASSIVE DIREKT-METHANOL-BRENNSTOFFZELLE\[FR] PILE à COMBUSTIBLE PASSIVE AU MéTHANOL DIRECT ET SON PROCéDé DE FABRICATION\ | EP2774200A1 | TUOMINEN AULIS (FI)\ SUOMINEN ARHO (FI)\ JOKINEN RAIMO (FI)\ FONSELL MARJO (FI)\ LAGERCRANTZ HENRIK (FI)\ M?KIL? JANNE (FI)\ ZHENG WUKUI (CN) | TURUN YLIOPISTO (FI) | H01M8/10\ H01M8/02 |
| 1128 | 3D printing | HOLLOW FIBER MEMBRANE MODULE\[DE] HOHLFASERMEMBRANENMODUL\[FR] MODULE DE MEMBRANES à FIBRES CREUSES\ | EP2832423A1 | HASHIMOTO KAZUYUKI (JP)\ INUZUKA HAYATO (JP)\ NAKANO YOSHINORI (JP)\ NAKAMATSU OSAMU (JP) | TORAY INDUSTRIES (JP) | B01D63/00\ A61M1/18\ B01D63/02 |
| 1129 | 3D printing | INDUSTRIAL FABRIC INCLUDING SPIRALLY WOUND MATERIAL STRIPS WITH REINFORCEMENT\[DE] TECHNISCHES GEWEBE MIT SPIRALF?RMIG GEWICKELTEN MATERIALBAHNEN MIT VERST?RKUNG\[FR] TISSU INDUSTRIEL COMPORTANT DES BANDES DE MATéRIAU ENROULéES EN SPIRALE AVEC RENFORT\ | EP2847379A1 | EAGLES DANA (US)\ KARLSSON JONAS (SE)\ HANSEN ROBERT (US) | ALBANY INT CORP (US) | D21F1/00\ D21F7/08\ D21F3/02 |
| 1130 | 3D printing | INDUSTRIAL FABRIC INCLUDING SPIRALLY WOUND MATERIAL STRIPS WITH REINFORCEMENT\[DE] TECHNISCHES GEWEBE MIT SPIRALF?RMIG GEWICKELTEN MATERIALBAHNEN MIT VERST?RKUNG\[FR] TISSU INDUSTRIEL COMPORTANT DES BANDES DE MATéRIAU ENROULéES EN SPIRALE AVEC RENFORT\ | EP2847380A1 | EAGLES DANA (US)\ KARLSSON JONAS (SE)\ HANSEN ROBERT (US) | ALBANY INT CORP (US) | D21F1/00\ D21F7/08\ D21F3/02 |
| 1131 | 3D printing | DISPOSITIF DE SOUDAGE PAR ULTRASONS PROCEDE ET EMBALLAGE ASSOCIES\[EN] ULTRASONIC WELDING DEVICE FOR MAKING FOOD PACKAGING ETC. BY WELDING FLEXIBLE PLASTIC SHEETS HAS PASSAGE BETWEEN ULTRASONIC SOURCE AND ANVIL WITH PROJECTING RIDGE PARALLEL TO SHEET MOVEMENT\ | FR2807392A1 | BLANCHARD DANIEL (FR)\ FAUL PATRICK (FR) | UNISABI SA (FR) | B29C65/00\ B29C65/74 |
| 1132 | 3D printing | COMPOSITION PYROTECHNIQUE FAIBLEMENT SENSIBLE A L'ELECTRICITE STATIQUE ET AUX ULTRASONS.\ | FR2861389B1 | MOUSSIER PATRICK US | DAVEY BICKFORD (FR) | B60R21/26\ C06B33/06\ C06C9/00\ F42B3/11\ F42B3/18 |
| 1133 | 3D printing | METHODE DE FABRICATION DE TUBE A CHALEUR PLAN AU MOYEN DE SOUDURE A ULTRASONS\ | FR2891172B3 | CHEN YEN WEN US\ DING PEI PEI (TW)\ YANG HSIU WEI (TW)\ LIN JAO CHING (TW)\ YU WEN HWA (TW) | ASIA VITAL COMPONENTS CO LTD (TW) | B23K20/10\ B23P15/26\ F28D15/04\ G06F1/20 |
| 1134 | 3D printing | MEMORY CARD FOR STORING E.G. DATA HAS CIRCUIT CARD RECEIVED IN RECEPTION SPACES BY RIBS WHEN INSULATION SHELLS ARE ASSEMBLED WHERE CARD HAS FRONT AND REAR EDGES FORMED BY UNIVERSAL SERIAL BUS AND MEMORY CARD CONNECTORS RESPECTIVELY\[FR] CARTE MEMOIRE AVEC CONNECTEUR USB\ | FR2903516A3 | HSUEH SUNG CHENG (TW) | HSUEH SUNG CHENG (TW) | G06K19/077\ G06F13/00 |
| 1135 | 3D printing | ADHESIVE BAND FOR BINDING DECORATIVE PRODUCTS SUCH AS PROTECTION/DECORATION STICKERS COMPRISES STRIPPABLE PROTECTIVE FILM HAVING SHEETS SUPERIMPOSED AND LINKED WITH EACH OTHER BY POINTS LINES/ZONES WHICH ARE DISTRIBUTED LONGITUDINALLY\[FR] BANDE ADHESIVE AVEC PELLICULE DE PROTECTION PELABLE ET PRODUIT COMPORTANT UNE TELLE BANDE ADHESIVE\ | FR2912755A3 | CORDIER PHILIPPE US | RENAULT SAS (FR) | C09J7/02\ B32B7/06\ C09J5/00 |
| 1136 | 3D printing | CABLE ELECTRIQUE\ | FR2920588B1 | DEMANGEON MICHEL (FR) | PEUGEOT CITROEN AUTOMOBILES SA (FR) | H01B7/285 |
| 1137 | 3D printing | FLOATING HAND TOOL FOR MAINTAINING E.G. HYDRAULIC PUMP HAS FUNCTIONAL ELEMENTS ARRANGED IN RECEIVING SPACES THAT ARE FORMED IN TWO ENDS OF HANDLE WHERE SPACE FORMED IN HANDLE IS ISOLATED FORM RECEIVING SPACES\[FR] OUTIL A MAIN FLOTTANT.\ | FR2922135A3 | HSIEH CHIH CHING (TW) | HSIEH CHIH CHING (TW) | B25G1/10 |
| 1138 | 3D printing | MATERIAU D'INSONORISATION\ | FR2942830A1 | RAWAS CLEMENCE (FR)\ RUOL DANIEL (FR) | DUFLOT IND (FR) | E04F15/18\ E04F15/20 |
| 1139 | 3D printing | OVERPACKAGE I.E. POUCH PACKAGE MANUFACTURING DEVICE HAS CYLINDER ARRANGED OPPOSITE TO SONOTRODE AND FILM STRIPS WHERE ROTATION OF CYLINDER IS CONTROLLED BY CONTROL UNITS BETWEEN TWO ANGULAR VELOCITIES DURING EACH REVOLUTION OF CYLINDER\[FR] DISPOSITIF DE FABRICATION D'EMPAQUETAGES DE CONDITIONNEMENT PAR ULTRASON\ | FR2953200A1 | BOILLOT DOMINIQUE (FR) | MV 2 (FR) | B65B9/04 |
| 1140 | 3D printing | PROCEDE DE FABRICATION D'UN FILTRE\ | FR2973258B1 | PERON BENOIT (FR)\ LEBRUN MICHEL (FR)\ PINEAU PHILIPPE (FR)\ FRADET ERIC (FR) | MECAPLAST SA (MC) | B01D46/52\ F02M35/024\ B01D27/06 |
| 1141 | 3D printing | PROCEDE DE REMPLISSAGE D'UN RECIPIENT SOUPLE\ | FR2978426B1 | CAPITAINE FRANCOIS (FR) | TECHNOFLEX (FR) | B65B7/02\ B65B3/00\ B65B55/02 |
| 1142 | 3D printing | FLAT CABLE AND MODULAR ROTARY ANVIL TO MAKE SAME\ | GB0221403D0 | GIM YEONG SU (KR) | METHODE ELECTRONICS INC (US) | B29C65/00\ B29C65/02\ H01B7/00\ B29C65/08\ H01B7/08 |
| 1143 | 3D printing | HEAT RECOVERY SYSTEM\ | GB0408470D0 | SERGENT FABIEN (FR)\ CUNHA ANDRE (BR) | TRINCAS NICHOLAS J US\ BOWKETT TREVOR G US | F24J2/20\ F24D3/08\ F24D11/00\ F24J2/23\ F24J2/24\ F28F21/06 |
| 1144 | 3D printing | METHOD OF MANUFACTURE OF A ROTOR\ | GB0602997D0 | CHOI WON HO (KR) | ATKINSON DESIGN ASS LTD (GB) | F04D29/24\ B65G33/26\ F03D3/02 |
| 1145 | 3D printing | A METHOD APPARATUS COMPUTER READABLE STORAGE MEDIUM AND COMPUTER PROGRAM FOR FORMING AN OBJECT\ | GB0914621D0 | TRINCAS NICHOLAS JOHN (GB) | UNIV LOUGHBOROUGH (GB) | Empty Field |
| 1146 | 3D printing | MEN'S AND BOY'S MOULDED UNDERGARMENTS SWIMWEAR AND THERMAL GARMENTS\ | GB0919577D0 | WANG WEI US | WOODHALL DAVID J US\ FRANCIS LEE B US\ KWANSUNGNOENTAN JANTARARAT (TH) | Empty Field |
| 1147 | 3D printing | FOLDING POCKET KNIFE\ | GB201014473D0 | NEUDAKHIN ALEKSANDR VALENTINOV (RU)\ ZOTOV NIKOLAJ ALEKSANDROVICH (RU) | SINCLAIR IAIN (GB) | Empty Field |
| 1148 | 3D printing | THIN FILM ULTRASONIC WELDING\ | GB201103139D0 | WANG AIMIN US\ ZHANG CHUNQING US | WEISSFELD CONSULTING LTD US | Empty Field |
| 1149 | 3D printing | MANUFACTURE OF A HEAT PIPE USING ULTRASONIC WELDING\ | GB2427582A | DING PEI-PEI (TW)\ YANG HSIU-WEI (TW)\ LIN JAO-CHING (TW)\ YU WEN-HWA (TW)\ CHEN YEN-WEN (TW) | ASIA VITAL COMPONENTS CO LTD (TW) | B23K20/10\ B23P15/26\ B23K101/14\ F28D15/02\ B21D53/02 |
| 1150 | 3D printing | WATERPROOF BREATHABLE STRETCHABLE GARMENT\ | GB2435631A | MCFARLANE JAMES BROWN (GB) | ENDURA LTD (GB) | B32B37/12\ B32B37/06\ B29C65/50\ A41D31/00\ B32B25/10\ A41D1/08\ A41D3/04 |
| 1151 | 3D printing | FLUID CARRYING ARRANGEMENT AND ITS MANUFACTURE USING A SOLID FREEFORM FABRICATION PROCESS\ | GB2440546A | NICHOLSON RICHARD (GB) | ROLLS ROYCE PLC (GB) | B22F3/105\ B23K26/34\ B22F5/10\ B23K15/00\ B23K101/06\ B23K103/14\ F16L9/19 |
| 1152 | 3D printing | A FLUID CARRYING ARRANGEMENT\ | GB2440547A | NICHOLSON RICHARD (GB) | ROLLS ROYCE PLC (GB) | B22F3/105\ B23K26/34\ B22F5/10\ B23K15/00\ B23K101/06\ B23K103/14\ F16L59/02 |
| 1153 | 3D printing | DRAUGHT EXCLUDER FOR A PET FLAP\ | GB2462580A | KIRK CHRISTOPHER PETER (GB) | PET MATE LTD (GB) | E06B7/26\ E06B7/32 |
| 1154 | 3D printing | METHOD OF WELDING SHEET MATERIAL\ | GB2475886A | BEER DAVID (GB) | BEER DAVID (GB) | B29C65/08 |
| 1155 | 3D printing | CONNECTING CONTACT LEADS TO LITHIUM-BASED ELECTRODES\ | GB2495581A | SZCZERBA MAREK JOZEF (GB)\ KOLOSNITSYN VLADIMIR (GB)\ IVANOV GLEB (GB)\ LILLEY SCOTT JOSEPH (GB) | OXIS ENERGY LTD (GB) | H01M2/20 |
| 1156 | 3D printing | METHODS OF JOINING SUPERCONDUCTING WIRES\ | GB2498961A | BLUMENTHAL MARK DAVID (GB)\ LAKRIMI M HAMED (GB) | SIEMENS PLC (GB) | H01R4/68\ H01R43/02 |
| 1157 | 3D printing | A REFLECTIVE ROAD STUD\ | GB2499188A | HUNG-CHEN LEE (CN) | TECHEYE OPTICS TECHNOLOGIES CO LTD (TW) | E01F9/06 |
| 1158 | 3D printing | BATTERY PACK\ | JP11007926A | YAMANOBE TERUJI US\ TANAKA TAKABUMI US | TOSHIBA BATTERY US\ TOSHIBA AVE KK US | B29L22/00\ B29C65/08\ H01M2/10 |
| 1159 | 3D printing | REEL FOR MAGNETIC TAPE CARTRIDGE\ | JP11053862A | MORITA KIYOO US\ OMORI SHOZO US\ NAITO HIROYUKI US | FUJI PHOTO FILM CO LTD (US) | B29L31/00\ B29C45/26\ G11B23/107\ B29C65/08\ G11B23/087 |
| 1160 | 3D printing | THIN BATTERY\ | JP2000058014A | SHIBUYA MASHIO US\ TOMITA TAKASHI US | SONY CORP (JP) | H01M2/08\ H01M10/05\ H01M6/18\ H01M10/052\ H01M10/0565\ H01M10/0585 |
| 1161 | 3D printing | TERMINAL PART OF FLAT CABLE\ | JP2000077116A | WADA KEITARO US\ UENO SEIICHI US | FURUKAWA ELECTRIC CO LTD (JP) | Empty Field |
| 1162 | 3D printing | BATTERY\ | JP2000082486A | INOUE TAKEFUMI US | JAPAN STORAGE BATTERY CO LTD (JP) | H01M10/04\ H01M2/26\ H01M10/0587\ H01M10/05\ H01M4/64 |
| 1163 | 3D printing | BATTERY\ | JP2000082487A | INOUE TAKEFUMI US | JAPAN STORAGE BATTERY CO LTD (JP) | H01M10/04\ H01M2/26\ H01M10/0587\ H01M10/05\ H01M4/64 |
| 1164 | 3D printing | ULTRASONIC WELDING METHOD\ | JP2000117834A | MIURA MORIMICHI US | TOYOTA MOTOR CORP US | B29C65/08 |
| 1165 | 3D printing | SEALED BATTERY AND MANUFACTURE THEREOF\ | JP2000123822A | KATO MASAHIRO US\ HONMA KATSUHISA US\ OGUCHI YUJI US\ HAGIWARA OSAMU US\ YAMANAKA MASAHARU US | TOSHIBA CORP US\ AT BATTERY KK US | H01M2/02\ H01M2/04\ H01M2/36 |
| 1166 | 3D printing | DEVICE FOR FIXING FACING MATERIAL ON PANEL BODY\ | JP2000127247A | FUKUHARA ATSUSHI US | ITOKI CREBIO CORP US | B29L9/00\ E04C2/24\ B29L31/10\ B29C65/08 |
| 1167 | 3D printing | ULTRASONIC BONDING METHOD\ | JP2000127248A | MIURA MORIMICHI US | TOYOTA MOTOR CORP US | B23K20/10\ B29C65/56\ B29C65/08 |
| 1168 | 3D printing | RESIN-MADE FUEL DELIVERY PIPE\ | JP2000161179A | YAMAMOTO TAKUMI US\ OYA SHINICHI US | AISIN SEIKI (JP) | F16L21/00\ F02M55/02 |
| 1169 | 3D printing | WELDING SECTION AND ULTRASONIC WELDING DEVICE PROVIDED THEREWITH\ | JP2000219215A | KARATA KAZUHIRO US | FUJI IMPULSE KK US | B29C65/00\ B29C65/02\ B29C65/08\ B29C65/74\ B65B51/10\ B65B51/22 |
| 1170 | 3D printing | ULTRASONIC WELDING-CUTTING DEVICE\ | JP2000246803A | ISHIWATARI SHOICHI US\ NODA HARUNORI US | CHO ONPA KOGYO CO US\ YAMAZAKI BAKING CO US | B29L22/00\ B29C65/08\ B29C65/78\ B29C65/74\ B65B51/10\ B65B51/24 |
| 1171 | 3D printing | WIRE CONNECTING METHOD ITS STRUCTURE AND COIL PARTS\ | JP2000263249A | KOBAYASHI KAZUMI US\ SASAKI HITOSHI US\ HASHIMOTO KIYOMI US | TDK CORP (JP) | B23K20/10\ B23K103/12\ B23K20/00\ B23K101/36\ B23K103/08 |
| 1172 | 3D printing | NONAQUEOUS ELECTROLYTE BATTERY\ | JP2001006746A | MORISANE YUICHIRO US | NEC MOBILE ENERGY KK US | H01M2/30\ H01M10/0566\ H01M10/058\ H01M10/05 |
| 1173 | 3D printing | SEALING DEVICE FOR ROLLING BEARING\ | JP2001065582A | ARAI YAMATO US | KOYO SEIKO CO US | F16C33/78\ F16C33/76 |
| 1174 | 3D printing | POLYMER ELECTROLYTIC CELL\ | JP2001126709A | YAMAMOTO HIROSHI US | HITACHI MAXELL (JP) | H01M2/30\ H01M2/26\ H01M10/058\ H01M10/05\ H01M10/0565\ H01M2/32 |
| 1175 | 3D printing | SEMICONDUCTOR CHIP ELEMENT SEMICONDUCTOR CHIP ELEMENT MOUNTING DEVICE AND MOUNTING METHOD THEREFOR\ | JP2001189339A | KAINUMA NORIO (JP)\ BABA SHUNJI (JP)\ KIRA HIDEHIKO (JP)\ OKADA TORU (JP) | FUJITSU LTD (JP) | H01L21/60 |
| 1176 | 3D printing | MOLDED ARTICLE HAVING SPONGY MEMBER AND METHOD OF MANUFACTURING THE MOLDED ARTICLE\ | JP2001239588A | UCHIYAMA YASUSHI US\ SUGANO TADASHI US | SHINOHARA KK US | B29C65/08\ A45D33/34\ A45D34/04 |
| 1177 | 3D printing | TOOTHBRUSH AND PRODUCING PROCESS OF TOOTHBRUSH HANDLE\ | JP2001275751A | KINOSHITA ATSUSHI US | SUNSTAR INC US | B29C65/08\ A46B5/00\ A46B5/02 |
| 1178 | 3D printing | HAIR IMPLANT EQUIPMENT FOR MACHINING AND DECORATING TOOTHBRUSH\ | JP2001275756A | KINOSHITA ATSUSHI US | SUNSTAR INC US | A46D3/04\ B29C65/08\ A46B5/00 |
| 1179 | 3D printing | NON-AQUEOUS ELECTROLYTE SECONDARY BATTERY AND ITS SAFETY VALVE\ | JP2001307706A | KOJIMA TETSUYA US | SONY CORP (JP) | H01M2/30\ H01M2/04\ H01M2/12\ H01M10/0566\ H01M10/058\ H01M10/05 |
| 1180 | 3D printing | BAG ARTICLE METHOD FOR PRODUCING THE SAME AND APPARATUS THEREFOR\ | JP2002038318A | YAMAZAKI TATSUO US | TOKIWA SANGYO KK US | B29C65/08\ A41D19/00\ A41D19/04 |
| 1181 | 3D printing | BATTERY FOR HIGH POWER OUTPUT APPLICATION\ | JP2002050338A | OGUMA MIKIO US\ TORITSUKA TAKAHISA US | SHIN KOBE ELECTRIC MACHINERY US | H01M2/26\ H01M10/0587\ H01M10/05\ H01M2/22 |
| 1182 | 3D printing | ELECTRIC JUNCTION BOX\ | JP2002058136A | NAKAMURA MASAYOSHI US | SUMITOMO WIRING SYSTEMS (JP) | B60R16/02\ H01R9/03\ H01R12/28\ H02G3/16 |
| 1183 | 3D printing | LIQUID SUPPLYING DEVICE INK JET RECORDING HEAD AND METHOD FOR MANUFACTURING THEM\ | JP2002067342A | TAKADA MASAYUKI US | BROTHER IND LTD (JP) | B41J2/16\ B41J2/045\ B41J2/055 |
| 1184 | 3D printing | WELDING DEVICE AND WELDING METHOD FOR COLLECTOR TAB AND COLLECTOR IN BATTERY\ | JP2002075336A | AOKI YASUNORI US\ SUZUKI HIROMI (JP) | MISHIMA DAIJI US\ NEC CORP (JP) | B23K20/10\ H01M4/04\ H01M2/26\ H01M10/0587\ H01M4/36 |
| 1185 | 3D printing | ULTRASONIC VIBRATION WELDING APPARATUS\ | JP2002190500A | AMANO MASAMI US\ HERAI MASAYASU US | MISHIMA DAIJI US\ SONY CORP (JP) | B23K20/10\ H01L21/60\ B23K101/42\ H01L21/607\ B06B1/02 |
| 1186 | 3D printing | BATTERY\ | JP2002231214A | MATSUBARA TAKEHITO US\ SHIMOZONO TAKESHI US\ KOJIMA TETSUZO US\ MUNENAGA TAKEYOSHI US | JAPAN STORAGE BATTERY CO LTD (JP) | H01M2/10\ H01M2/26\ H01M10/0587\ H01M10/05\ H01M2/22 |
| 1187 | 3D printing | PRINTED CIRCUIT BOARD\ | JP2002231853A | WATANABE KOSUKE US | MITSUI MINING & SMELTING CO (JP) | H05K1/09\ H01L23/12\ H05K3/24 |
| 1188 | 3D printing | ULTRASONIC WELDING/FUSING APPARATUS\ | JP2002273792A | INOUE MASATO (JP) | INOUE SHOTEN KK US | B29L9/00\ B29L7/00\ B29C65/08\ B29C65/78 |
| 1189 | 3D printing | INKJET RECORDING HEAD AND INKJET RECORDER\ | JP2003011384A | KANETANI MUNEHIDE US\ KOIZUMI YOSHIHIRO US | SEIKO EPSON CORP (JP) | B41J2/175 |
| 1190 | 3D printing | ULTRASONIC HORN AND ULTRASONIC WELDING OPERATION ASSEMBLY HAVING THE SAME\ | JP2003062908A | ABE TOSHIHIRO US\ MIDORIKAWA KOJI US\ TETSU YASUHIRO US\ SEYA KIYOMI US | KUREHA CHEMICAL IND CO LTD (JP) | B29C65/08\ B65B51/10\ B65B51/22\ B06B1/02\ B31B1/90\ B65D75/62\ B06B3/04 |
| 1191 | 3D printing | CYLINDRICAL TONER CONTAINER AND ITS MANUFACTURING METHOD\ | JP2003066702A | OMA MASANORI US\ NAKAGAWA KEN US\ OTA YUKIO US | SHIIZU KK US | G03G15/08\ B29L22/00\ B29C65/08\ B65D83/06 |
| 1192 | 3D printing | CURRENT CUTOFF ELEMENT AND BATTERY PACK BUILT-IN WITH THE SAME\ | JP2003132860A | WATABE KOJI US | SANYO ELECTRIC CO (JP) | B29C39/10\ B29L31/34\ B29C65/08\ H01M2/10\ H01M2/34\ H02H5/04 |
| 1193 | 3D printing | BATTERY\ | JP2003197174A | IWATA MIKIO US\ MATSUBARA TAKEHITO US | JAPAN STORAGE BATTERY CO LTD (JP) | H01M2/26\ H01M10/058\ H01M10/05 |
| 1194 | 3D printing | TERMINAL CONNECTING STRUCTURE FOR POLYMER SECONDARY BATTERY AND ITS TERMINAL CONNECTING METHOD\ | JP2003257409A | WATARAI YUSUKE US\ MIZUGUCHI AKIO US\ HIGAMI AKIHIRO US\ SUGIHARA TADASHI US | MITSUBISHI MATERIALS CORP US | H01M2/26\ H01M10/058\ H01M10/05\ H01M10/0565 |
| 1195 | 3D printing | ULTRASONIC WELDING DEVICE\ | JP2003320584A | INOUE MASATO (JP) | INOUE SHOTEN KK US | B29C65/00\ B29L7/00\ B29C65/08 |
| 1196 | 3D printing | IC CARD ADAPTER\ | JP2003331222A | KATAYAMA YOSUKE US\ TAKAHASHI TAKEHIRO US\ TERAI KATSUMI US | MAXELL SEIKI KK US\ HIKARI KOGYO SEISAKUSHO KK US | G06K17/00\ B42D15/10 |
| 1197 | 3D printing | ULTRASONIC WELDING DEVICE\ | JP2003334670A | TAKADA HAJIME (JP) | YAZAKI CORP (JP) | B23K20/10\ H01R43/02\ B23K101/38 |
| 1198 | 3D printing | DRIPPER\ | JP2004000711A | SUZUKI KYOSUKE US\ OSADA SHINICHI US\ KYOTANI HITOSHI US | DAINIPPON PRINTING CO LTD (JP) | A47J31/06\ A47J31/02 |
| 1199 | 3D printing | DISCHARGE PART FOR POT\ | JP2004016485A | OSUGA TAKESHI US | ZOJIRUSHI CORP US | A47J27/21 |
| 1200 | 3D printing | TEXTILE PRODUCT SET AND METHOD FOR PRODUCING THE TEXTILE PRODUCT\ | JP2004027412A | NAGAO MITSUO US\ MORI YOSHINORI US | NAGAO TSUSHO KK US | A41D13/00\ G09B21/00\ A41B15/00\ A41D23/00\ A45C13/08\ D06Q1/10 |
| 1201 | 3D printing | TAPE REEL AND TAPE CARTRIDGE PROVIDED WITH IT\ | JP2004030752A | SAKURAI MITSUE US\ KIKUCHI SHUICHI US\ SASAKI KAZUO US\ CHIBA HITOMI US\ SANPEI TAKAAKI US | SONY CORP (JP) | G11B23/107 |
| 1202 | 3D printing | METHOD FOR ASSEMBLING NON-CONTACT TYPE LIQUID LEVEL SENSOR\ | JP2004037195A | FUKUHARA TOSHIAKI US\ TAKAHASHI YUKIO US | YAZAKI CORP (JP) | G01F23/38 |
| 1203 | 3D printing | METHOD AND STRUCTURE FOR CONNECTING TERMINALS AND ELECTRIC JUNCTION BOX COMPRISING IT\ | JP2004040888A | HARA YASUHIRO US | YAZAKI CORP (JP) | H01R4/02\ H01R9/03\ H02G3/16 |
| 1204 | 3D printing | SOLID STRUCTURE COMPOSED OF MIXED MOLDING OF SYNTHETIC RESIN AND WOOD FLOUR AND ITS MANUFACTURING METHOD\ | JP2004043740A | MINAMI KOJI US\ USUI HIROAKI US\ OKAMOTO KIYOSHI US\ TSUKAMOTO MASASUKE US | MATSUSHITA ELECTRIC WORKS LTD (JP) | C08L101/00\ B29K105/16\ C08J5/12\ B29C65/08\ B29K311/14\ C08L23/26\ C08L97/02 |
| 1205 | 3D printing | ULTRASONIC WELDING METHOD AND CABLE SUITABLE FOR ULTRASONIC WELDING\ | JP2004050204A | KAMIYAMA HIDEKI US | FURUKAWA ELECTRIC CO LTD (JP) | B23K20/10\ H01R43/00\ H01B7/28\ B23K101/38\ H01B7/282 |
| 1206 | 3D printing | CAR INTERIOR PARTS\ | JP2004050862A | ENDO TOYOKAZU US | KASAI KOGYO KK (JP) | B60R13/02 |
| 1207 | 3D printing | RICE SCOOP\ | JP2004057227A | MIYAGUCHI JUNICHI US | TOSHIBA HOME TECH CORP US | A47J43/28\ A47J36/00 |
| 1208 | 3D printing | MANUFACTURING METHOD INORGANIC OXIDE SEMICONDUCTOR ELECTRODE FOR PHOTOELECTRIC CONVERSION\ | JP2004071252A | ANDOU MUNENORI US\ KURATA RYUICHIRO US | TOYO INK MFG CO US | H01L31/04\ H01M14/00 |
| 1209 | 3D printing | INCONTINENCE PAD AND INCONTINENCE PANTY WITH THE PAD ATTACHED THERETO\ | JP2004073555A | KUDO SHOICHI US | KATAKURA IND CO LTD US | A61F5/44\ B68G7/05 |
| 1210 | 3D printing | ULTRASONIC WELDING HORN FOR ELECTROLYTIC CAPACITOR AND METHOD OF MANUFACTURING ELECTROLYTIC CAPACITOR\ | JP2004079563A | KUBONAI TATSURO US\ KUBOTA TAKESHI US | NIPPON CHEMICON US | H01G9/008\ H01G9/00\ H01G9/10 |
| 1211 | 3D printing | ULTRASONIC WELDING HORN FOR ELECTROLYTIC CAPACITOR AND METHOD OF MANUFACTURING ELECTROLYTIC CAPACITOR\ | JP2004079564A | KUBONAI TATSURO US\ KUBOTA TAKESHI US | NIPPON CHEMICON US | H01G9/008\ H01G9/00\ H01G9/10 |
| 1212 | 3D printing | BATTERY LAMINATED AGGREGATE AND BATTERY USED FOR IT\ | JP2004087337A | TONO TATSUYA US | NISSAN MOTOR (JP) | H01M2/26 |
| 1213 | 3D printing | POWER MODULE\ | JP2004096135A | YAGI YUJI US\ TSUKADA ATSUSHI US\ NAGASE HIROSHI US | TOYOTA CENTRAL RES & DEV US | H01L25/07\ H01L25/18 |
| 1214 | 3D printing | ALUMINUM-NICKEL CLAD MEMBER MANUFACTURING METHOD THEREOF AND EXTERNAL TERMINAL FOR BATTERY\ | JP2004106059A | FUNAMOTO KENICHI (JP)\ SHIOMI KAZUHIRO (JP)\ ISHIO MASAAKI (JP) | SUMITOMO SPEC METALS (JP) | B32B15/01\ B23K20/00\ H01M2/30\ B23K103/18 |
| 1215 | 3D printing | ULTRASONIC WELDING METHOD AND DEVICE\ | JP2004106242A | ARAI KAZUYA US | RICOH KK (JP) | B29L22/00\ B29C65/08\ B65B51/10\ B65B51/22\ B65B7/28 |
| 1216 | 3D printing | ULTRASONIC WELDING EQUIPMENT\ | JP2004114136A | MIURA YUTAKA US | SONY CORP (JP) | B23K20/10 |
| 1217 | 3D printing | FRAME FOR MANUFACTURING BOX-TYPE CASE BOX-TYPE CASE MANUFACTURING DEVICE AND METHOD USING THE FRAME\ | JP2004122592A | SAKUMA KAZUHIKO US\ ANZAI CHIAKI US | SONY CORP (JP) | B29L22/00\ H01M2/02\ B29C65/08\ B65D77/20 |
| 1218 | 3D printing | JOINT STRUCTURE DEVELOPMENT DEVICE AND PROCESS CARTRIDGE\ | JP2004126182A | UENO TAKAHITO US\ NUMAGAMI ATSUSHI US\ FUJITA AKIRA US | CANON KK (JP) | G03G15/08\ B23K20/10 |
| 1219 | 3D printing | NONAQUEOUS ELECTROLYTE SECONDARY BATTERY\ | JP2004127599A | ISHIZAWA MASATSUGU US\ HATTORI HIROSHI US | HITACHI MAXELL (JP) | H01M4/62\ H01M2/26\ H01M10/05\ H01M4/13\ H01M4/139 |
| 1220 | 3D printing | DRAIN STRUCTURE OF DOORPHONE SLAVE UNIT\ | JP2004129113A | MORIKAWA MASAYUKI US\ YAMADA MANABU US | AIPHONE CO LTD US | H04M1/02\ H04M1/03\ H04M1/18 |
| 1221 | 3D printing | METHOD OF SOLDERING ALUMINUM MATERIAL\ | JP2004134452A | YAMADA TSUYOSHI US | FUJIKURA LTD US | H05K3/34\ B23K101/42\ B23K103/10\ B23K3/04\ B23K1/19 |
| 1222 | 3D printing | ELECTRONIC COMPONENT AND METHOD AND APPARATUS FOR MANUFACTURING THE SAME\ | JP2004134518A | SHIBUYA AKIHIRO US\ OKADA YASUHIRO US | NISSAN MOTOR (JP) | H01L21/60\ H01L21/607 |
| 1223 | 3D printing | ULTRASONIC WELDING METHOD FOR RESIN PACKAGE\ | JP2004142136A | SAKAI RYOSUKE US\ KITANI AKIHIRO US | MURATA MANUFACTURING CO (JP) | B29C65/08\ B29C65/78\ H01L23/02\ H01L23/08 |
| 1224 | 3D printing | HOME ELECTRIC APPLIANCE\ | JP2004147858A | OKADA NAOKI US\ SHINODA JUN US\ YAMADA SHINYA US\ MATSUDA YOSHIHISA US\ YOSHIDA JUNJI US | INAX CORP US | A47K13/30\ A62C3/16\ E03D9/08 |
| 1225 | 3D printing | ULTRASONIC WELDING APPARATUS FOR METAL FOIL\ | JP2004148373A | ARITA SATOSHI US | MITSUI MINING & SMELTING CO (JP) | B23K20/10 |
| 1226 | 3D printing | INSTRUMENT PANEL FOR AUTOMOBILE\ | JP2004168112A | HASEGAWA YOSHITOSHI US | HITACHI CHEMICAL CO LTD US | B29L9/00\ B29C65/08\ B29C65/06\ B60K37/00\ B29L31/58\ B60R21/20 |
| 1227 | 3D printing | ELECTRODE CONNECTING METHOD FOR SECONDARY SHEET BATTERY\ | JP2004178860A | TANJO YUJI US\ HORIE HIDEAKI US\ SHIMAMURA OSAMU US\ ITO TAKANORI US\ WATANABE KYOICHI US\ OZAWA HIRONORI US\ TAKASAKI TAKAO US\ OZAWA KAZUNORI US | ENAX INC US\ NISSAN MOTOR (JP) | H01M2/30\ H01M2/20\ B60L11/18 |
| 1228 | 3D printing | ROTATING CONNECTOR\ | JP2004178907A | SASAKI KUNIHIKO US\ RI HOOCHURU US | ALPS ELECTRIC CO LTD (JP) | H01R4/02\ B60R16/02\ B60R16/027\ H01R35/04\ B62D1/04\ H02G11/02 |
| 1229 | 3D printing | METHOD FOR ULTRASONIC WELDING OF CASE MADE OF RESIN\ | JP2004181654A | MIYAGAWA YASUO US | ALPS ELECTRIC CO LTD (JP) | B29C65/08 |
| 1230 | 3D printing | ULTRASONIC WELDING HORN ULTRASONIC WELDING DEVICE AND METHOD FOR MANUFACTURING TAPE REEL\ | JP2004202828A | KIKUCHI SHUICHI US\ ABE YASUYUKI US | SONY CORP (JP) | B29C65/08\ B29C65/78\ B29C65/60\ G11B23/087 |
| 1231 | 3D printing | OPTICAL UNIT\ | JP2004205774A | YAMAMOTO SHOGO US\ SHIN YUICHI US | KONICA MINOLTA HOLDINGS INC (JP) | G02B7/02\ G02B13/00\ G11B7/135 |
| 1232 | 3D printing | ULTRASONIC WELDING APPARATUS\ | JP2004209659A | ARAI KAZUYA US | RICOH KK (JP) | B29C65/08 |
| 1233 | 3D printing | WATER STOP STRUCTURE OF SHEATHED WIRE\ | JP2004215403A | IDE TETSUO US | YAZAKI CORP (JP) | H01B7/282\ H02G1/14 |
| 1234 | 3D printing | VISCOUS LIQUID-FILLED DAMPER AND ITS MANUFACTURING METHOD\ | JP2004218831A | OGURA HIROSHI US\ TANII SHIHORI US | HOKUSHIN IND US | F16F15/023\ F16F13/08\ F16F9/10 |
| 1235 | 3D printing | ULTRASONIC WELDING DEVICE AND ULTRASONIC WELDING METHOD FOR ELECTRIC WIRE\ | JP2004220933A | ICHIKAWA YOJI US | YAZAKI CORP (JP) | B23K20/10\ H01R43/02\ B06B1/02\ B23K101/38 |
| 1236 | 3D printing | ULTRASONIC WELDING METHOD FOR ELECTRONIC CIRCUIT UNIT\ | JP2004223982A | SHIBATA HIRONORI US | ALPS ELECTRIC CO LTD (JP) | H05K5/00\ B29C65/08 |
| 1237 | 3D printing | CONTAINER\ | JP2004224348A | OKURA YOSHIMASA US | KYOCERA MITA CORP US | G03G15/00\ G03G15/08\ B65D83/06\ B65D77/20 |
| 1238 | 3D printing | RESIN COMPOSITION WITH IMPROVED ULTRASONIC WELDING PROCESSABILITY\ | JP2004231898A | KURIBAYASHI RYOTARO US\ HOSHIBA TAKAO US | KURARAY CO (JP) | C08L67/00\ C08J5/12\ B29K67/00\ C08L87/00\ B29C65/08 |
| 1239 | 3D printing | CHIP TYPE THERMAL FUSE\ | JP2004235047A | YOSHIKAWA TOKIHIRO US | NEC SCHOTT COMPONENTS CORP US | H01H37/76 |
| 1240 | 3D printing | METAL FOIL JOINING METHOD AND METAL FOIL CONNECTING DEVICE\ | JP2004243402A | KOJIMA KAZUNORI US\ TSUSHIMA TAKAAKI US\ MATSUDA TOSHINORI US | MITSUI CHEMICALS INC US | B23K20/10\ B23K20/16 |
| 1241 | 3D printing | HUMOR TREATING APPARATUS\ | JP2004255044A | ABE MICHIO US | KAWASUMI LAB INC US | C08J5/12\ A61M1/18\ A61M1/02\ B01D63/02 |
| 1242 | 3D printing | ULTRASONIC WELDING HORN\ | JP2004255580A | FUJII TAKAMASA US\ KITAJIMA HIROSHI US | TOPPAN PRINTING CO LTD US\ SHIKOKU KAKOKI CO LTD US | B29C65/00\ B29L22/00\ B29C65/08\ B65B51/22\ B31B1/84\ B31B1/64 |
| 1243 | 3D printing | MANUFACTURING METHOD OF INORGANIC FORMED PRODUCT\ | JP2004260130A | MASUKO HIDEAKI US\ KUDO KAZUO US\ YOSHIDA MASAYUKI US\ SUDO JUNICHI US\ AOKI SHUNJI US | JSR CORP (JP) | G03F7/20\ H05K3/20 |
| 1244 | 3D printing | METHOD FOR ULTRASONICALLY JOINING RESIN COMPONENTS\ | JP2004262041A | SUGANO MASAHIKO US\ SHINOHARA TORU US | KASAI KOGYO KK (JP) | B29C65/56 |
| 1245 | 3D printing | THERMISTOR\ | JP2004266058A | MARUHASHI TSUTOMU US\ FUKAYA TAKESHI US\ ISHITANI SHOZO US | RINNAI KK US | H01C7/04 |
| 1246 | 3D printing | ULTRASONIC WELDING DEVICE REPAIR MEMBER AND ULTRASONIC WELDING METHOD FOR REPAIR\ | JP2004268520A | KOIZUMI TAKESHI US | JAPAN PALLET RENTAL CORP US | B29C65/08\ B29C73/10\ B29C73/34 |
| 1247 | 3D printing | MANUFACTURING METHOD AND MANUFACTURING DEVICE OF LEADER BUCKLE\ | JP2004273018A | SODA HIROSHI US | FUJI PHOTO FILM CO LTD (US) | G11B23/26\ B29C65/08 |
| 1248 | 3D printing | ULTRASONIC WELDING DEVICE\ | JP2004276255A | SODA HIROSHI US | FUJI PHOTO FILM CO LTD (US) | G11B23/26\ B29C65/08 |
| 1249 | 3D printing | SWITCHING VALVE\ | JP2004278631A | NOMURA KENICHI US\ ITO MASAHARU US | FUJI KOKI KK US | F16K27/06\ F16K11/087 |
| 1250 | 3D printing | AUTOMATIC PACKAGING METHOD OF CARD\ | JP2004280778A | WANG JIUE-HUNG (TW) | YUANCISAN SCIENCE & TECHNOL CO US | G06K19/00\ H05K3/30\ B42D15/10\ G06K19/077 |
| 1251 | 3D printing | FLAT CABLE AND ITS MANUFACTURING METHOD\ | JP2004281108A | YAMANO YOSHIAKI (JP)\ FUKUMOTO KOJI (JP) | SUMITOMO WIRING SYSTEMS (JP) | H01B13/00\ H01B7/08 |
| 1252 | 3D printing | MOUNTING STRUCTURE OF BELTLINE INNER WEATHER STRIP\ | JP2004284420A | NAITO MAKOTO US\ HAMANAKA HITOSHI US | NISHIKAWA RUBBER CO LTD US | B60J10/04 |
| 1253 | 3D printing | SQUARE-SHAPED CLOSED-TYPE BATTERY AND ITS MANUFACTURING METHOD\ | JP2004288656A | IZUMI YOSHIAKI US\ SOMATOMO YOSHIKI US\ WATANABE OSAMU (JP) | HITACHI MAXELL (JP) | H01M2/22 |
| 1254 | 3D printing | MANUFACTURING METHOD OF FLAT CABLE\ | JP2004296402A | YAMANO YOSHIAKI (JP)\ FUKUMOTO KOJI (JP) | SUMITOMO WIRING SYSTEMS (JP) | H01B13/00 |
| 1255 | 3D printing | ELECTROLYTIC CAPACITOR AND ITS MANUFACTURING METHOD\ | JP2004303814A | CHINO KAORU US | NIPPON CHEMICON US | H01G9/008\ H01G9/00 |
| 1256 | 3D printing | ELECTROLYTIC CAPACITOR AND METHOD AND APPARATUS FOR MANUFACTURING SAME\ | JP2004304059A | KUBONAI TATSURO US\ NAKAMURA TOSHIO US | NIPPON CHEMICON US | H01G9/008\ H01G9/00\ H01G13/00 |
| 1257 | 3D printing | ULTRASONIC WELDING STRUCTURE FOR PLASTIC PRODUCT\ | JP2004306366A | FUJIMOTO FUTOSHI US\ YAMAUCHI TAKAHIRO US | NAGOYA NAKANO BUSSAN KK US | B29K23/00\ B29C65/08\ B29C65/50\ B65B51/10 |
| 1258 | 3D printing | UPHOLSTERY FOR VEHICLE\ | JP2004306741A | TOMONO HIROTA US\ WATABE AKINORI US | KASAI KOGYO KK (JP) | B29L31/30\ B29C65/08\ B29C65/50\ B60R16/02\ B60R13/02 |
| 1259 | 3D printing | LIGHT GUIDE STRUCTURE FOR STATUS INDICATOR\ | JP2004309629A | SHIROYAMA YUSUKE US\ MIURA SEIICHI US | KOKUSAI DENKI ENGINEERING KK US | G09F9/00\ G09F13/20\ G09F13/18 |
| 1260 | 3D printing | REINFORCING MEMBER\ | JP2004323037A | OKAZAKI YOSHIKAZU US\ MAKINO MINORU US\ MIYAGI KENICHI US\ GOSHI ISATO US | TOTO LTD US | B65D5/44\ B65D5/462 |
| 1261 | 3D printing | WRAPPED SANDWICH AND WRAPPING METHOD OF THE SAME\ | JP2004323084A | SUZUKI MAKOTO US\ SUZUKI EIICHI US | SUZUKI MAKOTO US\ SUZUKI EIICHI US | B65D85/50\ A23L1/48\ B65B11/48\ B65D65/10\ B65D65/34\ B65D75/20 |
| 1262 | 3D printing | HOLLOW FIBER MODULE\ | JP2004351319A | YAMAMOTO MASARU US\ KAWAMURA FUSAO US | NIKKISO CO LTD US | B29C65/08\ B01D63/00\ A61M1/18\ B01D63/02 |
| 1263 | 3D printing | ULTRASONIC WELDING DEVICE\ | JP2004351428A | TAKADA HAJIME (JP) | YAZAKI CORP (JP) | B23K20/10\ H01R43/02\ B06B3/04\ H01R43/00\ B23K101/38\ B23K101/34 |
| 1264 | 3D printing | BATTERY AND BATTERY PACK\ | JP2004355915A | MATSUSHITA SHOHEI US | TOYOTA MOTOR CORP US | H01M2/02 |
| 1265 | 3D printing | METHOD FOR PRODUCING HOT AIR DUCT FOR HEATER\ | JP2005000017A | INOUE MASATO (JP) | INOUE SHOTEN KK US | A01G9/24 |
| 1266 | 3D printing | METHOD FOR FITTING RESIN BRACKET BY ULTRASONIC WELDING AND ULTRASONIC HORN USED FOR THE METHOD\ | JP2005001295A | TSUZUKIYAMA KENJI US | TAKANICHI KK US | B29C65/08 |
| 1267 | 3D printing | FIRE SENSOR\ | JP2005011136A | YAMAZAKI RYUICHI US | NITTAN CO LTD US | G08B17/10 |
| 1268 | 3D printing | ELECTRIC DOUBLE LAYER CAPACITOR AND SECONDARY BATTERY\ | JP2005019658A | OMURA SEIJI US\ KAWADA YOHEI US\ KIMOTO MAMORU US | SANYO ELECTRIC CO (JP) | H01M2/04\ H01M10/28\ H01M10/05\ H01M10/0585\ H01G9/10\ H01G11/00\ H01G11/74\ H01G11/78\ H01G11/84 |
| 1269 | 3D printing | SHIELDING TREATMENT METHOD FOR SHIELDING ELECTRIC WIRE AND TOOL FOR SHIELDING TREATMENT TO SHIELDING ELECTRIC WIRE USED FOR THE SHIELDING TREATMENT METHOD\ | JP2005028431A | IDE TETSUO US\ MITA AKIRA US | YAZAKI CORP (JP) | B23K20/10\ H01R43/02\ B23K101/38\ H01R4/64\ H02G1/14\ H01B7/17\ H02G15/188 |
| 1270 | 3D printing | FRAME OF PROCESS CARTRIDGE\ | JP2005031487A | SUZUKI TATSUYA US\ HOSHI NOBUHARU US\ TSUZUKI MASATOMO US | CANON KK (JP) | B29C65/08\ G03G21/18 |
| 1271 | 3D printing | MANUFACTURING METHOD OF WIRE HARNESS AND ITS MANUFACTURING DEVICE\ | JP2005032459A | MIYAGAWA TAKUYA US\ TAKADA HAJIME (JP) | YAZAKI CORP (JP) | H01B13/00\ H01B13/012\ H01R43/02\ H01R43/00 |
| 1272 | 3D printing | DEFLECTION SCANNER\ | JP2005033892A | KONUMA TAKU US | CANON KK (JP) | B41J2/44\ G02B26/10\ H02K5/16\ H02K7/08\ G02B26/12\ H04N1/113 |
| 1273 | 3D printing | DOUBLE GLAZING WITH GLAZING CHANNEL\ | JP2005042385A | TSUJI MASAAKI US | CENTRAL GLASS CO LTD US | E06B3/66\ E06B3/62 |
| 1274 | 3D printing | DOUBLE GLAZING WITH GLAZING CHANNEL\ | JP2005042386A | TSUJI MASAAKI US | CENTRAL GLASS CO LTD US | E06B3/66\ E06B3/62\ E06B3/96 |
| 1275 | 3D printing | PROCESS CARTRIDGE AND DRUM SUPPORT MEMBER\ | JP2005049762A | KIKUCHI TAKESHI US | CANON KK (JP) | G03G21/18\ F16C13/04 |
| 1276 | 3D printing | ULTRASONIC WELDING MACHINE AND SUPPORT STRUCTURE THEREOF\ | JP2005053063A | TERADA MASAAKI US | SUZUKI MOTOR CO US | B29C65/08\ H04R1/30 |
| 1277 | 3D printing | SURFACE-MOUNTED THIN CAPACITOR AND MANUFACTURING METHOD THEREFOR\ | JP2005072106A | KAWAI HARUHIRO US\ NAGASAWA TOSHIHISA US\ MARUKO YUICHI US | NEC TOKIN TOYAMA LTD US\ NEC TOKIN CORP (JP) | H01G9/00\ H01G9/14\ H01G9/012 |
| 1278 | 3D printing | JOINING METHOD OF MICROCHIP SUBSTRATES AND MICROCHIP\ | JP2005077239A | ARAI SUSUMU US | SUMITOMO BAKELITE CO US | B29C65/08\ G01N37/00\ G01N31/20 |
| 1279 | 3D printing | TAPE CARTRIDGE\ | JP2005078719A | AMANO KOSUKE US | HITACHI MAXELL (JP) | G11B23/107\ G11B23/087 |
| 1280 | 3D printing | TAPE CARTRIDGE\ | JP2005078784A | AMANO KOSUKE US | HITACHI MAXELL (JP) | G11B23/107\ G11B23/087 |
| 1281 | 3D printing | FLEXIBLE FLAT CABLE COVERED WITH SHIELDING MATERIAL AND ITS MANUFACTURING METHOD\ | JP2005093367A | YAMANOBE HIROSHI US\ WASHIMI TORU US\ ICHIKAWA TAKAO US\ ITO MASATO US\ KOBAYASHI HIDENORI US | HITACHI CABLE (JP) | H01B13/00\ H01B7/08\ H02G1/14 |
| 1282 | 3D printing | ELECTRIC DOUBLE-LAYERED CAPACITOR\ | JP2005093859A | OTA HIDETOSHI US | NICHICON CORP US | H01G11/74\ H01G11/84\ H01G9/14\ H01G11/66\ H01G11/22\ H01G11/24\ H01G11/42\ H01G11/70\ H01G11/86 |
| 1283 | 3D printing | BATTERY PACK\ | JP2005100689A | TSUTSUMI TAKUYA US\ MATSUDA TAKUYA US\ INOUE YASUSHI US | SANYO ELECTRIC CO (JP) | H01M2/10 |
| 1284 | 3D printing | FLYBACK TRANSFORMER\ | JP2005101570A | YOSHINARI HIROSHI US\ NOMURA TAKESHI US\ NAGAI TAKAYA US\ YAMASHITA HISAO US | MATSUSHITA ELECTRIC IND CO LTD (JP) | H01F38/42 |
| 1285 | 3D printing | PULSING DIAPHRAGM FUEL PUMP\ | JP2005106031A | KOBAYASHI YASUSHI US\ KONAGAI GENSAKU US | NIKKI CO LTD US | F02M37/06\ F04B43/02\ F04B43/06 |
| 1286 | 3D printing | ULTRASONIC WELDING MACHINE\ | JP2005111547A | SHIMIZU AKIRA US\ MINAGAWA HAJIME US\ OGAMI ETSUO US | NISSAN MOTOR (JP) | B23K20/10 |
| 1287 | 3D printing | WELDING METHOD OF BATTERY ELECTRODE AND BATTERY PACK\ | JP2005116434A | TONO TATSUYA US | NISSAN MOTOR (JP) | H01M2/20 |
| 1288 | 3D printing | POLYESTER NONWOVEN FABRIC\ | JP2005120542A | TANAKA SHIGEKI US | TOYO BOSEKI (JP) | D04H1/74\ B32B27/36\ B32B5/08\ D04H1/4374\ D04H1/435 |
| 1289 | 3D printing | PLATE TYPE HEAT PIPE AND METHOD FOR PRODUCING IT\ | JP2005121345A | KOYATA KEN US | FURUKAWA ELECTRIC CO LTD (JP) | B23K20/10\ B23K20/12\ F28F3/04\ B23K101/14\ F28D15/02\ B21D53/04\ B23K9/20\ B23K101/08 |
| 1290 | 3D printing | TURBOFAN AND MANUFACTURING METHOD FOR TURBOFAN\ | JP2005127176A | KINOSHITA KIYOSHI US\ OKUYA TAKASHI US\ IWASHITA TETSUO US\ YOSHIMATSU KENJI US\ SAKAI KOICHI US | MATSUSHITA ELECTRIC IND CO LTD (JP) | B29C45/14\ B29C45/00\ B29C49/42\ B29L31/08\ F04D29/30 |
| 1291 | 3D printing | ULTRASONIC WELDING DEVICE\ | JP2005132000A | KOYAMA TAKESHI US\ HARADA TOSHIHIKO US\ KOSUGI HIROTERU US | YAZAKI CORP (JP) | B29C65/08 |
| 1292 | 3D printing | ULTRASONIC WELDING DEVICE AND ULTRASONIC WELDING METHOD\ | JP2005135803A | KOYAMA TAKESHI US\ HARADA TOSHIHIKO US\ KOSUGI HIROTERU US | YAZAKI CORP (JP) | H01R43/02 |
| 1293 | 3D printing | PACK CELL AND ITS MANUFACTURING METHOD\ | JP2005135851A | TANAKA AKIHITO US\ AMEZUTSUMI TORU US\ MORITA SEIJI US\ URUSHIBARA KANJI US\ SAKAGUCHI MAKOTO US\ KURIMURA MASAAKI US | SANYO ENERGY TOTTORI KK US\ SANYO ELECTRIC CO (JP) | H01M2/10 |
| 1294 | 3D printing | ANTENNA MODULE FOR IC CARD AND ITS MANUFACTURING METHOD AND THE IC CARD AND ITS MANUFACTURING METHOD\ | JP2005136901A | OKABE KOJI US | SONY CORP (JP) | H01P11/00\ B42D15/10\ G06K19/07\ G06K19/077\ H01Q1/38\ H01Q7/00 |
| 1295 | 3D printing | ULTRASONIC WELDING APPARATUS AND METHOD\ | JP2005138172A | MURAKAMI KAZUHIRO (JP)\ HANAZAKI KENICHI (JP) | YAZAKI CORP (JP) | B23K20/10\ B06B3/00 |
| 1296 | 3D printing | ULTRASONIC WELDING EQUIPMENT AND ULTRASONIC WELDING METHOD\ | JP2005144532A | WATANABE KUNIHIKO US\ HIRAMITSU HIROMI US | AUTO NETWORK GIJUTSU KENKYUSHO US\ SUMITOMO ELECTRIC INDUSTRIES (JP)\ SUMITOMO WIRING SYSTEMS (JP) | B23K20/10 |
| 1297 | 3D printing | ULTRASONIC WELDING DEVICE\ | JP2005144930A | MIYAMOTO TOSHIAKI US\ KOSAKA HIDEO US\ MAKINO MASANOBU US\ SHIBUYA NOBUNAGA US | HONDA ELECTRONIC US | B29C65/08 |
| 1298 | 3D printing | FILM EXTERIOR PACKAGED BATTERY AND MANUFACTURING METHOD THEREFOR\ | JP2005149938A | KIKUCHI HIROSHI US\ OIKAWA KIYOKAZU US | NEC LAMILION ENERGY LTD US | H01M2/34\ H01M2/26\ H01M2/22 |
| 1299 | 3D printing | ELECTRIC DOUBLE LAYER CAPACITOR ELECTROLYTIC BATTERY AND THEIR MANUFACTURING METHODS\ | JP2005166974A | OMURA SEIJI US\ ITO KIYOTAKA US | SANYO ELECTRIC CO (JP) | H01M2/02\ H01M10/04\ H01G9/10\ H01G13/00\ H01G11/00\ H01G11/78\ H01G11/84\ H01G11/42\ H01G11/40\ H01G11/80 |
| 1300 | 3D printing | TERMINAL JOINTING STRUCTURE AND PACKED BATTERY\ | JP2005183070A | TANAKA HIROYUKI (JP) | TOYOTA MOTOR CORP US | H01M2/22\ H01M2/20 |
| 1301 | 3D printing | KEY-SHEET-INTEGRATED CHASSIS PANEL\ | JP2005190743A | NISHIMURA TAKESHI US | POLYMATECH CO LTD US | H01H13/70\ G06F3/02\ H01H13/04\ H01H13/14 |
| 1302 | 3D printing | METHOD AND DEVICE FOR DETERMINING WELDING QUALITY\ | JP2005205421A | KOMAI TADASHI US | NISSAN MOTOR (JP) | B23K20/10\ G01B21/18\ G01B21/20 |
| 1303 | 3D printing | ULTRASONIC WELDING METHOD AND APPARATUS THEREFOR\ | JP2005219064A | KURINAGA KAORU US | DENSO CORP (JP) | B23K20/10 |
| 1304 | 3D printing | FINE SILICA PARTICLE-DISPERSED HYDROPHILIC POLYURETHANE RESIN COMPOSITION AND METHOD FOR PRODUCING THE SAME\ | JP2005220322A | HANADA KAZUYUKI US | DAINICHISEIKA COLOR CHEM US\ UKIMA CHEM & COLOUR MFG US | C08L75/04\ B41J2/01\ C08K3/36\ C08G18/65 |
| 1305 | 3D printing | MANUFACTURING METHOD OF ELECTRIC WIRE WITH TERMINAL\ | JP2005222849A | OGIWARA SHIGERU US | AUTO NETWORK GIJUTSU KENKYUSHO US\ SUMITOMO ELECTRIC INDUSTRIES (JP)\ SUMITOMO WIRING SYSTEMS (JP) | B23K20/10\ H01R4/02\ H01R43/02\ B23K101/38 |
| 1306 | 3D printing | ULTRASONIC WELDING EQUIPMENT\ | JP2005224821A | SOEJIMA JUNICHIRO US\ YAMAMOTO AKIO US\ MAEDA TAKASHI (JP) | KAIJO KK US | B23K20/10\ H01L21/60\ B23K20/26 |
| 1307 | 3D printing | RESIN PRODUCT WELDING METHOD\ | JP2005224966A | NOGUCHI YASUO US\ SHIBATA MASARU US\ SAKAGUCHI YUKIHIKO US | SUMITOMO BAKELITE CO US | B29C65/40\ B29C65/08 |
| 1308 | 3D printing | CLOTHING AND METHOD FOR PRODUCING THE SAME\ | JP2005226173A | TAJIMA KAZUHIKO US\ KIMURA TADASHI US | PHOENIX KK US | A41D27/24\ A41D27/00\ A41D27/08 |
| 1309 | 3D printing | INNER BAG FOR TRANSPORTATION TANK AND MANUFACTURING METHOD FOR THE SAME\ | JP2005239263A | SHIGEIZUMI YOSHITSUGU US | FUJI PHOTO FILM CO LTD (US) | B65D88/22 |
| 1310 | 3D printing | INNER BAG FOR TRANSPORTATION TANK AND MANUFACTURING METHOD FOR THE SAME\ | JP2005239264A | SHIGEIZUMI YOSHITSUGU US | FUJI PHOTO FILM CO LTD (US) | B65D88/22 |
| 1311 | 3D printing | LEADER TAPE LEADER TAPE CUTTER METHOD OF CUTTING BOTH END PARTS OF ROLL FOR LEADER TAPE AND MAGNETIC TAPE CARTRIDGE\ | JP2005243085A | OMORI SHOZO US | FUJI PHOTO FILM CO LTD (US) | G11B23/107 |
| 1312 | 3D printing | ULTRASONIC WELDING TIP AND WELDING METHOD\ | JP2005254323A | SASABE SEIJI US\ IWASE SATORU US | KOBE STEEL LTD US | B23K20/10\ B23K103/10\ B23K103/04 |
| 1313 | 3D printing | ULTRASONIC WELDING METHOD OF ALUMINUM MATERIAL AND STEEL MATERIAL\ | JP2005254324A | SASABE SEIJI US\ IWASE SATORU US | KOBE STEEL LTD US | B23K20/10\ B23K103/20 |
| 1314 | 3D printing | LEADER TAPE TIP REINFORCING MEMBER AND MAGNETIC TAPE CARTRIDGE EQUIPPED WITH THE SAME\ | JP2005259288A | OMORI SHOZO US | FUJI PHOTO FILM CO LTD (US) | G11B23/107 |
| 1315 | 3D printing | CONTAINER COVER\ | JP2005263307A | IMOTANI MITSURU US | IMOTANI KK US | B65D51/24 |
| 1316 | 3D printing | MAGNETIC TAPE CARTRIDGE\ | JP2005267819A | YAMAMOTO HIDETOSHI US | FUJI PHOTO FILM CO LTD (US) | G11B23/107\ G11B23/087 |
| 1317 | 3D printing | ULTRASONIC WELDING EQUIPMENT\ | JP2005271028A | KUME HIROSHI US\ SUZUKI KAZUO US | ALPS ELECTRIC CO LTD (JP) | B23K20/10\ B29C65/08\ B06B1/02 |
| 1318 | 3D printing | ULTRASONIC WELDING EQUIPMENT\ | JP2005271029A | KUME HIROSHI US\ SUZUKI KAZUO US | ALPS ELECTRIC CO LTD (JP) | B23K20/10\ B29C65/08\ B29C65/82 |
| 1319 | 3D printing | SIMPLE BODY FREE GUARD BRUSH AND A PRODUCTION METHOD THEREOF\ | JP2005296555A | TAKEUCHI TOSHIBUMI US | NARUTOYA KK US\ TAKEUCHI TOSHIBUMI US | A46D3/05 |
| 1320 | 3D printing | ULTRASONIC WELDING DEVICE\ | JP2005296954A | AOKI YASUNORI US\ ARAI MASAYOSHI (JP) | MISHIMA DAIJI US | B23K20/10\ B29C65/08 |
| 1321 | 3D printing | DEVICE FOR STORING WELDING TREATMENT INFORMATION IN ULTRASONIC WELDING MACHINE AND METHOD FOR ADMINISTRATING THE INFORMATION\ | JP2005297309A | SATO HIROKAZU US\ HAMAGUCHI CHUKICHI US | MISHIMA DAIJI US | G06K17/00\ B29C65/08 |
| 1322 | 3D printing | ULTRASONIC WELDING EQUIPMENT\ | JP2005319483A | SHIGETA YASUNOBU US\ ASAMI KAZUHIKO US | FURUKAWA AUTOMOTIVE PARTS INC US\ FURUKAWA ELECTRIC CO LTD (JP) | B23K20/10\ H01R43/02\ B23K101/38 |
| 1323 | 3D printing | MANUFACTURING METHOD OF WIRE WITH TERMINAL\ | JP2005319497A | OGIWARA SHIGERU US | AUTO NETWORK GIJUTSU KENKYUSHO US\ SUMITOMO ELECTRIC INDUSTRIES (JP)\ SUMITOMO WIRING SYSTEMS (JP) | B23K20/10\ H01R4/02\ H01R43/02 |
| 1324 | 3D printing | CONNECTION OF INSULATED ELECTRIC WIRES AND METHOD FOR CONNECTING INSULATED ELECTRIC WIRES\ | JP2005322544A | SHUKUTANI TAKASHI US | FURUKAWA AUTOMOTIVE PARTS INC US\ FURUKAWA ELECTRIC CO LTD (JP) | H01R4/02\ H01R43/02\ H02G1/14 |
| 1325 | 3D printing | ULTRASONIC WELDING PIPE JOINT\ | JP2005325945A | HARADA TAKESHI US | BRIDGESTONE CORP (JP) | F16L47/02\ F16L21/08 |
| 1326 | 3D printing | JOINTING METHOD OF ELECTRODE AND ELECTRODE JUNCTION\ | JP2005327677A | MATSUZAKA YOSHIHIKO US\ SHIMODA MAMORU US\ FUJIKI KOJI US\ AWANO NAOMI US | DENSO CORP (JP) | H01M2/22 |
| 1327 | 3D printing | HORN OF ULTRASONIC WELDING MACHINE ULTRASONIC WELDING MACHINE AND JOINING METHOD OF THERMOPLASTIC RESIN SHEET USING IT\ | JP2005335105A | KURIHARA MASAMI US | OLYMPUS CORP (JP) | B29C65/08\ G09F7/16 |
| 1328 | 3D printing | INTAKE AIR QUANTITY CONTROL DEVICE\ | JP2005337041A | YAMAZAKI SHIGERU US | MIKUNI KOGYO KK US | F02D9/10\ F02D9/00\ F02D35/00 |
| 1329 | 3D printing | ULTRASONIC WELDING METHOD CONNECTION STRUCTURE OF TERMINAL FITTING TO WIRE\ | JP2005340106A | HAYASHI HIROYUKI US | AUTO NETWORK GIJUTSU KENKYUSHO US\ SUMITOMO ELECTRIC INDUSTRIES (JP)\ SUMITOMO WIRING SYSTEMS (JP) | H01R4/02\ H01R43/02 |
| 1330 | 3D printing | TERMINAL FITTING\ | JP2005340107A | HAYASHI HIROYUKI US | AUTO NETWORK GIJUTSU KENKYUSHO US\ SUMITOMO ELECTRIC INDUSTRIES (JP)\ SUMITOMO WIRING SYSTEMS (JP) | B23K20/10\ H01R4/02 |
| 1331 | 3D printing | ULTRASONIC WELDING APPARATUS PACKAGE MANUFACTURING APPARATUS AND PACKAGE MANUFACTURING METHOD\ | JP2005343496A | MIDORIKAWA KOJI US | KUREHA CHEMICAL IND CO LTD (JP) | B29C65/00\ B29C65/08\ B65B51/10\ B65B9/12 |
| 1332 | 3D printing | ULTRASONIC JOINING METHOD AND ULTRASONIC JOINING DEVICE\ | JP2005349448A | WATANABE SHINICHI US\ NAKUI SHIN US\ CHIN MIN US | SEIDENSHA ELECTRONICS US\ NISSAN MOTOR (JP) | B23K20/10\ H01M2/30\ H01M2/20 |
| 1333 | 3D printing | ULTRASONIC JOINING METHOD AND ULTRASONIC JOINING DEVICE\ | JP2005349449A | WATANABE SHINICHI US | NISSAN MOTOR (JP) | B23K20/10\ H01M2/20 |
| 1334 | 3D printing | ULTRASONIC JOINING METHOD AND ULTRASONIC JOINING DEVICE\ | JP2005349450A | WATANABE SHINICHI US | NISSAN MOTOR (JP) | B23K20/10\ B29C65/08 |
| 1335 | 3D printing | MAGNETIC TAPE CARTRIDGE\ | JP2006004565A | FUKUDA TAIZO US | SONY CORP (JP) | G11B23/107 |
| 1336 | 3D printing | METHOD OF CONNECTING LEADER TAPE TO LEADING PIECE IN SINGLE REEL MAGNETIC TAPE CARTRIDGE\ | JP2006012230A | INUKAI YASUO US\ KATAYAMA TAKUYA (JP) | HITACHI MAXELL (JP) | G11B23/26\ G11B23/107\ G11B23/113 |
| 1337 | 3D printing | STRUCTURE OF SECONDARY BATTERY AND SECONDARY BATTERY PACK\ | JP2006012657A | ENDO TOSHIAKI US | SONY CORP (JP) | H01M2/10 |
| 1338 | 3D printing | ULTRASONIC WELDING APPARATUS CORRECTING MEMBER FOR ULTRASONIC WELDING AND ULTRASONIC WELDING METHOD\ | JP2006015354A | HIGUCHI MASARU US\ SAKA MASAMI US | SUMITOMO WIRING SYSTEMS (JP) | B23K20/10\ H01R43/02\ B23K101/38 |
| 1339 | 3D printing | TURBOFAN AND MANUFACTURING METHOD OF TURBOFAN\ | JP2006029319A | KINOSHITA KIYOSHI US\ OKUYA TAKASHI US | MATSUSHITA ELECTRIC IND CO LTD (JP) | F04D29/28\ F04D29/58 |
| 1340 | 3D printing | NON-AQUEOUS ELECTROLYTE BATTERY\ | JP2006040813A | MURAI TETSUYA US\ OKUDA MORIHIKO US | SANYO GS SOFT ENERGY CO LTD US\ SANYO ELECTRIC CO (JP) | H01M4/02\ H01M10/058\ H01M10/05\ H01M4/13\ H01M10/052\ H01M4/48\ H01M4/485\ H01M4/58\ H01M10/0567 |
| 1341 | 3D printing | METHOD FOR MANUFACTURING DISPOSABLE PANTS AND DISPOSABLE PANTS\ | JP2006061188A | FUJIOKA MASARU US | LIVEDO CORP US | A61F13/49\ A61F13/15\ A61F13/496\ A61F13/58 |
| 1342 | 3D printing | ULTRASONIC WELDING APPARATUS ULTRASONIC WELDING METHOD AND CASE MEMBER\ | JP2006075994A | ARAI KAZUYA US | RICOH KK (JP) | B29C65/08 |
| 1343 | 3D printing | PIPE JOINT\ | JP2006083999A | NAKABAYASHI KUNIAKI US\ NAGASE AKIHIKO US | BRIDGESTONE FLOWTEC US | F16L37/12\ F02M37/00 |
| 1344 | 3D printing | MANUFACTURING METHOD OF WIRE WITH TERMINAL\ | JP2006107882A | OGIWARA SHIGERU US | AUTO NETWORK GIJUTSU KENKYUSHO US\ SUMITOMO ELECTRIC INDUSTRIES (JP)\ SUMITOMO WIRING SYSTEMS (JP) | B23K20/10\ H01R4/02\ H01R43/02\ B23K101/38 |
| 1345 | 3D printing | LIGHT EMITTING SEMICONDUCTOR CONNECTION STRUCTURE AND METHOD THEREOF\ | JP2006108471A | HAN SHAKUMEI US\ KAN HONIN US | SANEN KODEN KOFUN YUGENKOSHI US | H01L33/32\ H01L33/62 |
| 1346 | 3D printing | METHOD FOR MANUFACTURING ELECTRIC CABLE WITH TERMINAL\ | JP2006114343A | OGIWARA SHIGERU US | AUTO NETWORK GIJUTSU KENKYUSHO US\ SUMITOMO ELECTRIC INDUSTRIES (JP)\ SUMITOMO WIRING SYSTEMS (JP) | H01R43/02 |
| 1347 | 3D printing | MICROMINIATURE POWER CONVERTER\ | JP2006114792A | KAWASHIMA TETSUYA US\ AMANO AKIRA US | FUJI ELEC DEVICE TECH CO LTD US | H01F17/00\ H01L21/60\ H01L25/00\ H01F27/28\ H01F10/20\ H01F37/00\ H01G4/40\ H02M3/00 |
| 1348 | 3D printing | CONNECTED ELECTRIC WIRE AND METHOD FOR JOINING ELECTRIC WIRE\ | JP2006116559A | HANAZAKI KENICHI (JP) | YAZAKI CORP (JP) | B23K20/10\ H01R43/02\ B23K101/38 |
| 1349 | 3D printing | PIPE JOINT\ | JP2006132747A | NAKABAYASHI KUNIAKI US\ NAGASE AKIHIKO US | BRIDGESTONE FLOWTEC US | F16L37/06\ F16L37/40 |
| 1350 | 3D printing | DISPOSABLE ABSORBENT PRODUCT AND ITS PRODUCING METHOD\ | JP2006136623A | NAKAOKA KENJI US | LIVEDO CORP US | A61F13/49\ A61F13/15\ A61F5/44 |
| 1351 | 3D printing | METHOD FOR JOINING FLUOROCARBON RESIN MOLDING AND FLUOROCARBON RESIN JOINED BODY\ | JP2006137069A | OMA MASANORI US\ OTA YUKIO US | SHIIZU KK US | H01M8/04\ B29L23/00\ B29L31/34\ F16L47/02\ B29C65/08\ B29K27/12 |
| 1352 | 3D printing | MICROCHEMICAL DEVICE AND ITS PRODUCTION METHOD\ | JP2006142198A | KUROKAWA MASAYA US\ OIKO MASATO US | STARLITE IND US | B81B1/00\ B01J19/00\ G01N37/00 |
| 1353 | 3D printing | DEHUMIDIFICATION DEVICE AND OPTICAL APPLIANCE\ | JP2006159091A | TSUSHIMA HIROYUKI US\ HISAMORI YOICHI US | MITSUBISHI ELECTRIC CORP (JP) | B01D53/26\ G03B15/00\ G03B17/02\ G03B17/08 |
| 1354 | 3D printing | ULTRASONIC WELDING METHOD ULTRASONIC WELDING EQUIPMENT AND CONDUCTOR UNIT\ | JP2006159277A | MURAKAMI KAZUHIRO (JP) | YAZAKI CORP (JP) | B23K20/10\ B23K103/10\ H01R43/02\ B23K101/38 |
| 1355 | 3D printing | BATTERY\ | JP2006173079A | MORITA HIROYUKI US | SONY CORP (JP) | H01M4/06\ H01M6/16\ H01M2/02\ H01M2/30 |
| 1356 | 3D printing | LIGHT WAVELENGTH DETECTOR MANUFACTURING METHOD THEREOF AND WAVELENGTH STABILIZING LIGHT SOURCE\ | JP2006173341A | OSHIMA SHIGERU US | TOSHIBA CORP US | H01S5/0687\ H01S3/137 |
| 1357 | 3D printing | INTERIOR PARTS FOR AUTOMOBILE\ | JP2006176010A | OGURA TOSHIHIDE US\ KUBO JUNICHI US | KASAI KOGYO KK (JP) | B60R13/02\ B60J5/00 |
| 1358 | 3D printing | BATTERY PACK\ | JP2006179204A | KOMIYA SADAHIRO US | NEC TOKIN TOCHIGI LTD US\ NEC TOKIN CORP (JP) | H01M2/10 |
| 1359 | 3D printing | LEAD MATERIAL FOR WIRING CONNECTION AND ITS MANUFACTURING METHOD\ | JP2006179228A | ODA YOSHIMITSU US\ ISHIO MASAAKI (JP) | NEOMAX MATERIAL KK US | H01M2/30 |
| 1360 | 3D printing | CONNECTING STRUCTURE OF MEDICAL APPLIANCE ITS CONNECTING METHOD AND TUBE WELDING TOOL FOR ULTRASONIC WELDING MACHINE USED THEREFOR\ | JP2006181222A | UKAI TADASHI US\ KOGASHIWA SHIGERU US | SEIDENSHA ELECTRONICS US\ NIPRO CORP US | A61M39/02 |
| 1361 | 3D printing | WELDING STRUCTURE FOR POSITIONING OF RESIN COMPONENT\ | JP2006181999A | ISHIGAKI MASATOSHI US\ YAMADA TOMOJI US\ YAMADA SHUJI US\ SAGISAKA RYUJI US\ HIKASA TAKASHI US\ UEZONO KOICHIRO US | NIHON PLAST CO LTD US | B29C65/08\ B60K37/00\ B29L31/58 |
| 1362 | 3D printing | WELDING STRUCTURE\ | JP2006182395A | OSE KAZUMA US\ OMURA TAKASHI US | PENTEL KK US | B29C65/08\ B65D53/00 |
| 1363 | 3D printing | JOINING STRUCTURE AND PIPE JOINT\ | JP2006183782A | NAKABAYASHI KUNIAKI US | BRIDGESTONE FLOWTEC US | B29C65/08\ F16L37/08\ F16L37/40 |
| 1364 | 3D printing | FLAME-RETARDANT POLYCARBONATE RESIN COMPOSITION HAVING EXCELLENT ULTRASONIC WELDABILITY AND BATTERY CASE PRODUCED BY MOLDING THE SAME\ | JP2006193669A | OKADA KOJI US\ SAKAKI YOICHIRO US | SUMITOMO DOW LTD US | C08L101/00\ C08K3/22\ C08K5/521\ C08L27/18\ C08L69/00\ H01M2/02 |
| 1365 | 3D printing | WATER REPELLENT FILTER ATTACHING STRUCTURE AND ELECTRONIC APPARATUS PROVIDED WITH WATER REPELLENT FILTER\ | JP2006196596A | IWAMIYA HIROKI US\ YOSHIMURA SATOSHI US | DENSO CORP (JP) | H05K5/06\ F16B5/08\ F16B11/00 |
| 1366 | 3D printing | CERAMIC CIRCUIT BOARD\ | JP2006196916A | FURUKUWA TAKESHI US | KYOCERA CORP (JP) | H05K3/32\ H01L23/12\ H01L23/13\ H05K1/11 |
| 1367 | 3D printing | HYDRODYNAMIC BEARING DEVICE\ | JP2006200624A | SATOJI FUMITADA US\ ITO KENJI (JP) | NTN TOYO BEARING CO LTD (JP) | F16C17/10\ F16C35/02\ H02K7/08\ H02K21/22\ F16C17/02\ F16C33/74\ G11B19/20\ F16C33/08 |
| 1368 | 3D printing | APPARATUS FOR ULTRASONIC WELDING\ | JP2006212687A | MORIYA TAKASHI US\ TAKAHASHI SUSUMU US\ HAYASHI TATSURO US | NISSAN MOTOR (JP) | B23K20/10\ B23K103/18 |
| 1369 | 3D printing | HOUSING METHOD OF MANUFACTURING THIS HOUSING AND PORTABLE TERMINAL DEVICE HAVING THIS HOUSING\ | JP2006222112A | MASUDA TAKESHI US\ KUROKAWA SUSUMU US | SONY ERICSSON MOBILE COMM JP US | H04M1/02\ H05K5/02 |
| 1370 | 3D printing | TAPE REEL AND TAPE CARTRIDGE EQUIPPED WITH THE SAME\ | JP2006228296A | KIKUCHI SHUICHI US\ SANPEI TAKAAKI US\ ABE YASUYUKI US | SONY CORP (JP) | G11B23/107\ G11B23/087 |
| 1371 | 3D printing | ULTRASONIC WELDING DEVICE\ | JP2006231698A | FUJITA KAZUHIKO US | KYOTO SEISAKUSHO US | B29C65/08 |
| 1372 | 3D printing | SWITCHING DEVICE\ | JP2006236825A | MORITA KIMIYUKI US\ HASEGAWA SHIGEKI US | FUJISOKU ELECTRIC US | H01H9/02\ H01H11/00\ H01H19/04 |
| 1373 | 3D printing | METHOD FOR MEASURING AMPLITUDE OF BONDING TOOL\ | JP2006237408A | OZAKI YUJI US | SONY CORP (JP) | H01L21/60 |
| 1374 | 3D printing | LIQUID CONTAINER\ | JP2006240646A | SAKURADA MASATAKA US\ SHIBATA TOMIHIRO US\ MATSUMOTO TAKIHIRO US | TOPPAN PRINTING CO LTD US\ ASIA STENCIL PAPER US | B65D47/36 |
| 1375 | 3D printing | LIQUID-FILLED TYPE VIBRATION ISOLATOR\ | JP2006250304A | YAMAMOTO KENTARO US | TOYO TIRE & RUBBER CO US | F16F13/18\ F16F13/06 |
| 1376 | 3D printing | BATTERY PACK AND MANUFACTURING METHOD OF BATTERY PACK\ | JP2006253153A | TANABE MASAO US\ OKANO RENJIROU US\ WATANABE GOTARO US | SONY CORP (JP) | H01M2/10\ B65B7/28 |
| 1377 | 3D printing | LIQUID-SEALED VIBRATION CONTROL DEVICE\ | JP2006258152A | YAMAMOTO KENTARO US | TOYO TIRE & RUBBER CO US | F16F13/18\ F16F13/06 |
| 1378 | 3D printing | LIQUID-SEALED VIBRATION CONTROL DEVICE\ | JP2006258153A | YAMAMOTO KENTARO US | TOYO TIRE & RUBBER CO US | F16F13/18\ F16F13/06\ F16F13/10 |
| 1379 | 3D printing | ULTRASONIC WELDING DEVICE MANUFACTURING METHOD OF WELDED POWER TRANSMISSION CABLE AND MANUFACTURING METHOD OF MOTOR\ | JP2006260921A | MATSUMOTO KATSUNARI US | TOYOTA MOTOR CORP US | B23K20/10\ H01R43/02\ B23K101/38\ H02K15/04 |
| 1380 | 3D printing | ULTRASONIC WELDING METHOD AND APPARATUS USING THE SAME\ | JP2006263816A | UENISHI TAKASHI US\ OKADA SHIRO US\ IMAI KOICHI US\ NISHIMURA KOJI US | TORAY ENG CO LTD US | B23K20/10\ B23K101/42 |
| 1381 | 3D printing | ULTRASONIC WELDING STRUCTURE\ | JP2006264699A | FUJIMOTO FUTOSHI US | NAGOYA NAKANO BUSSAN KK US | B29C65/08\ B65D6/32 |
| 1382 | 3D printing | ULTRASONIC WELDING METHOD AND EQUIPMENT AND ULTRASONICALLY WELDED TUBE\ | JP2006272444A | AOKI YUICHI US\ SHIMIZU AKINORI US\ UENO MASAKATSU US | DENSO CORP (JP) | B23K20/10\ B23K101/04 |
| 1383 | 3D printing | CEILING MATERIAL FOR VEHICLE AND ITS MANUFACTURING METHOD\ | JP2006272709A | SHODA HIROSHI US\ TSUCHIYA TADASHI US | SHIGERU CO LTD US | B32B27/40\ B32B5/24\ B29C65/08\ B60R13/02 |
| 1384 | 3D printing | DEVELOPER CARTRIDGE AND RECYCLING METHOD THEREOF\ | JP2006276690A | NISHIMURA SOICHIRO US\ SATO FUMIKAZU US | BROTHER IND LTD (JP) | G03G15/08 |
| 1385 | 3D printing | MANUFACTURING DEVICE OF FLAT CABLE\ | JP2006278350A | YAMANO YOSHIAKI (JP)\ FUKUMOTO KOJI (JP) | SUMITOMO WIRING SYSTEMS (JP) | H01B13/00 |
| 1386 | 3D printing | ULTRASONIC WELDING HORN AND MANUFACTURING METHOD OF ELECTROLYTIC CAPACITOR USING THE SAME\ | JP2006278849A | YASUSAKA TAKESHI US\ KUROKI NOBUO US | NICHICON CORP US | B23K20/10\ H01G9/008\ H01G13/00 |
| 1387 | 3D printing | SEMICONDUCTOR DEVICE AND ITS MANUFACTURING METHOD\ | JP2006278951A | SHIMOYAMA MIKITO US\ YANAGIDA YUICHI US\ KOGURE SATOSHI US | MITSUBA CORP US | H01L23/40 |
| 1388 | 3D printing | ULTRASONIC WELDING METHOD\ | JP2006297407A | SHIMIZU AKIRA US\ MATSUYAMA HIDENOBU US\ MARUKUNI HITOSHI US | NISSAN MOTOR (JP) | B23K20/10\ B23K103/18\ B23K101/36 |
| 1389 | 3D printing | HANDLE AND SWITCH INCLUDING IT\ | JP2006302583A | KONDO TAKESHI US\ KAWAMOTO TAKASHI (JP)\ KAWAMOTO TETSUYASU (JP)\ YANO MANABU (JP) | MATSUSHITA ELECTRIC WORKS LTD (JP) | H01H3/12 |
| 1390 | 3D printing | MANUFACTURING METHOD OF FLAT CABLE\ | JP2006313757A | YAMANO YOSHIAKI (JP)\ FUKUMOTO KOJI (JP) | SUMITOMO WIRING SYSTEMS (JP) | H01B13/00 |
| 1391 | 3D printing | PREMIUM CARD FOR GAME\ | JP2006325641A | HOTTA TAKASHI US | HITACHI MAXELL (JP) | A63F7/02 |
| 1392 | 3D printing | INCOMING CALL INDICATION EMITTING SECTION OF SMALL-SIZED RADIO AND PORTABLE TELEPHONE AND ITS MOUNTING METHOD\ | JP2006345391A | KOMAKI EISUKE US | NEC SAITAMA LTD US | H04M1/02\ H05K7/12 |
| 1393 | 3D printing | WELDING HORN IN ULTRASONIC WELDING DEVICE AND METHOD OF USING THE SAME\ | JP2006346951A | MARUZOE TOMIO US\ SHINODA TAKAHIRO US\ ANZAI MICHIO US\ AZUMA TETSUSHI US | ITO YOSHIHIKO US\ PIOLAX INC (JP) | B29C65/08 |
| 1394 | 3D printing | METHOD OF MANUFACTURING FLAT PLATE TYPE THERMOTUBE\ | JP2007003102A | LIN JAOCHING US\ CHEN PEI-PEI (MY)\ YANG HSIU-WEI (TW)\ YU WEN-HWA (TW)\ CHEN YEN-WEN (TW) | KIKO KAGI KOFUN YUGENKOSHI US | B23K20/10\ F28D15/02\ H01L23/427 |
| 1395 | 3D printing | POROUS UNIT AND ITS PRODUCTION METHOD\ | JP2007007971A | ITO MASA US\ KITAGAWA AKIICHI US | CKD CORP US | B29C65/08\ B23Q3/08\ B29L31/14\ B65G49/06\ B65G49/07\ H01L21/677 |
| 1396 | 3D printing | ANTICORROSION PROTECTIVE LAYER FORMING METHOD AND ANTICORROSION COATING STRUCTURE\ | JP2007009563A | HASHIGUCHI YOSHIYO US\ YOSHIDA MICHIO US | SUMIKO ECO ENGINEERING CO LTD US | E02D31/06\ C23F11/00\ E02B1/00 |
| 1397 | 3D printing | CONNECTION TERMINAL ALUMINUM CABLE HAVING THE SAME ULTRASONIC WELDING METHOD AND ULTRASONIC CONNECTING APPARATUS\ | JP2007012329A | WASHIMI TORU US\ HORIKOSHI TOSHIYUKI US\ SHIMIZU MICHIAKI US | HITACHI CABLE (JP) | H01R4/62\ H01R4/02\ H01R13/03 |
| 1398 | 3D printing | MOUNTING METHOD OF IC (INTEGRATED CIRCUIT) MANUFACTURING METHOD OF OSCILLATOR AND THE OSCILLATOR FORMED THEREBY\ | JP2007027629A | MURATA KAZUO US | CITIZEN MIYOTA CO LTD US | H01L21/60\ H01L25/00\ H01L21/607\ H01L23/02 |
| 1399 | 3D printing | ULTRASONIC WELDING STRUCTURE OF SEALED CONTAINER\ | JP2007030332A | OMURA TAKASHI US | PENTEL KK US | B29L22/00\ B29C65/08\ B65D53/00 |
| 1400 | 3D printing | ULTRASONIC WELDING METHOD FOR RESIN COMPONENT\ | JP2007038422A | SHIOTANI TSUNAMASA US | KASAI KOGYO KK (JP) | B29C65/08 |
| 1401 | 3D printing | CAGE FOR ROLLING BEARING\ | JP2007040421A | AOKI MAMORU US | NSK LTD US | F16C33/66\ F16C33/38\ F16C33/44 |
| 1402 | 3D printing | SEAL CUTTING APPARATUS FOR POCKET COIL ROW\ | JP2007045518A | ETO HIROYUKI US | KYUSHU MATSUSHITA KOGYO KK US | B65B61/06\ B65B51/10 |
| 1403 | 3D printing | REFLECTOR AND ITS MANUFACTURING METHOD\ | JP2007057934A | SAITO JUNICHI US | THREE M INNOVATIVE PROPERTIES US | G02B5/124\ E01F9/00 |
| 1404 | 3D printing | INK CARTRIDGE AND CASE FORMED OF RESIN\ | JP2007090729A | HATTORI SHINGO US\ SASAKI TOYOKI US\ KANBE TOSHIHIRO US | BROTHER IND LTD (JP) | B29C33/42\ B41J2/175\ B29C45/37\ B29L31/34\ B29C65/08 |
| 1405 | 3D printing | STRUCTURE BY ULTRASONIC WELDING\ | JP2007090824A | KATAYAMA SHINKICHI US | PENTEL KK US | B23K20/10\ B29C65/08\ B43K3/00 |
| 1406 | 3D printing | LITHIUM ION BATTERY PACK\ | JP2007103284A | KOMIYA SADAHIRO US | NEC TOKIN CORP (JP) | H01M2/10\ H01M10/052 |
| 1407 | 3D printing | ULTRASONIC WELDING METHOD FOR RESIN COMPONENT AND ULTRASONIC HORN USED IN THIS METHOD\ | JP2007112044A | SHIOTANI TSUNAMASA US | KASAI KOGYO KK (JP) | B29C65/08 |
| 1408 | 3D printing | APPARATUS OF MANUFACTURING FLAT CABLE\ | JP2007128911A | YAMANO YOSHIAKI (JP)\ FUKUMOTO KOJI (JP) | SUMITOMO WIRING SYSTEMS (JP) | H01B13/00 |
| 1409 | 3D printing | LIQUID JETTING HEAD AND LIQUID JETTING APPARATUS\ | JP2007136737A | OKUBO KATSUHIRO US | SEIKO EPSON CORP (JP) | B41J2/175 |
| 1410 | 3D printing | WELDING STRUCTURE OF JOINING MEMBER ELECTRONIC INSTRUMENT AND METHOD FOR PRODUCING ELECTRONIC INSTRUMENT\ | JP2007136834A | KUROKAWA SUSUMU US\ SASAMORI KEIICHI US\ HAYAZAKI YOSUKE US | SONY ERICSSON MOBILE COMM JP US\ SONY CORP (JP) | B29C65/08 |
| 1411 | 3D printing | ULTRASONIC WELDING DEVICE AND CONTROL METHOD THEREFOR\ | JP2007160336A | WATANABE SHINICHI US | NISSAN MOTOR (JP) | B23K20/10\ B23K101/36\ B23K101/10 |
| 1412 | 3D printing | ULTRASONIC WELDING DEVICE AND CONTROL METHOD THEREFOR\ | JP2007160337A | WATANABE SHINICHI US | NISSAN MOTOR (JP) | B23K20/10\ B23K103/10\ B06B1/02\ B23K101/36 |
| 1413 | 3D printing | PIEZOELECTRIC ELEMENT TYPE INKJET RECORDING HEAD AND METHOD FOR MANUFACTURING THE SAME\ | JP2007160869A | ASAI NAOTO US\ TAKIMOTO MASAFUMI US | CANON KK (JP) | B41J2/16\ B41J2/045\ B41J2/055 |
| 1414 | 3D printing | BATTERY MODULE\ | JP2007172943A | TANJO YUJI US | NISSAN MOTOR (JP) | H01M2/02\ H01M2/26\ H01M2/20 |
| 1415 | 3D printing | ULTRASONIC WELDING DEVICE\ | JP2007175876A | YAMAMOTO KAZUHISA US\ KUBOTA KAZUMASA US\ TOYODA MASASUKE US\ MURAMATSU KIWA US | HONDA ELECTRONIC US | A61J3/00\ B29C65/08 |
| 1416 | 3D printing | ULTRASONIC WELDING DEVICE\ | JP2007175877A | YAMAMOTO KAZUHISA US\ KUBOTA KAZUMASA US\ TOYODA MASASUKE US\ MURAMATSU KIWA US | HONDA ELECTRONIC US | B29C65/08\ B65B51/10\ B06B1/02 |
| 1417 | 3D printing | ULTRASONIC WELDING DEVICE\ | JP2007175878A | YAMAMOTO KAZUHISA US\ KUBOTA KAZUMASA US\ TOYODA MASASUKE US\ MURAMATSU KIWA US | HONDA ELECTRONIC US | B29C65/08 |
| 1418 | 3D printing | ULTRASONIC WELDING DEVICE\ | JP2007175879A | YAMAMOTO KAZUHISA US\ KUBOTA KAZUMASA US\ TOYODA MASASUKE US\ MURAMATSU KIWA US | HONDA ELECTRONIC US | B29C65/08 |
| 1419 | 3D printing | SPOUT FOR PAPER PACK\ | JP2007230608A | WATABE ATSUSHI US\ SASO TAKAYUKI US | CROWN CORK JAPAN (JP) | B65D5/74 |
| 1420 | 3D printing | METHOD FOR MANUFACTURING LIQUID JET HEAD LIQUID JET HEAD AND LIQUID JET DEVICE\ | JP2007245464A | KOIKE YASUNORI US\ FUNASAKA KENJI US | SEIKO EPSON CORP (JP) | B41J2/175\ B41J2/16\ B41J2/045\ B41J2/055 |
| 1421 | 3D printing | FILM-ARMORED ELECTRIC DEVICE AND METHOD OF MANUFACTURING SAME\ | JP2007250310A | OIKAWA KIYOKAZU US\ NODA TOSHIHARU US\ YAGATA HIROSHI US | NEC LAMILION ENERGY LTD US | H01M2/26\ H01M2/06\ H01G9/08 |
| 1422 | 3D printing | CHLORINE ION SELECTIVE ELECTRODE\ | JP2007263798A | URANO HIKARI US\ TAKAHASHI SHUICHI US\ NIRASAWA MITSUHARU US | FUJIFILM CORP (JP) | G01N27/28\ G01N27/333\ G01N27/416 |
| 1423 | 3D printing | MOTOR WITH REDUCTION GEAR MECHANISM\ | JP2007274767A | OHASHI YASUO US\ ISHIKAWA MASARU US\ ONO HIDEYUKI US | MITSUBA CORP US | H02K5/22\ H02K7/116\ H02K5/24\ H02K11/00 |
| 1424 | 3D printing | DUST COLLECTION FILTER FOR VACUUM CLEANER AND VACUUM CLEANER USING THE SAME\ | JP2007275397A | MIYAHARA TOSHIFUMI US\ KUROKI YOSHITAKA US\ NAGAOKA HIROKAZU US\ KOGA MICHIMOTO US | MATSUSHITA ELECTRIC IND CO LTD (JP) | A47L9/14 |
| 1425 | 3D printing | RESIN LAMINATED BODY AND ITS MANUFACTURING METHOD\ | JP2007283677A | OTA YUKIO US\ ITO OSAMU US | SHIIZU KK US | H01M8/10\ B29C65/08\ H01M8/24 |
| 1426 | 3D printing | WELDING METHOD OF ELECTRIC WIRE HAVING TERMINAL\ | JP2007294154A | TORIMOTO YUKIHIRO (JP) | FURUKAWA AUTOMOTIVE PARTS INC US\ FURUKAWA ELECTRIC CO LTD (JP) | B23K20/10\ H01R4/02\ H01R43/02\ B23K101/38 |
| 1427 | 3D printing | SEMICONDUCTOR DEVICE PACKAGE LEAD FRAME FORMED WITH A PLURALITY OF METAL LAYERS\ | JP2007300088A | HARNDEN JAMES (US)\ CHIA ANTHONY (SG)\ WONG LIMING (CN)\ YANG HONGBO (CN) | GEM SERVICES INC (US) | H01L23/50 |
| 1428 | 3D printing | CABLE WITH TERMINAL MANUFACTURING METHOD THEREFOR ULTRASONIC WELDING METHOD FOR JOINING TERMINAL AND CABLE AND ULTRASONIC WELDER\ | JP2007305314A | WASHIMI TORU US\ HORIKOSHI TOSHIYUKI US\ SHIMIZU MICHIAKI US | HITACHI CABLE (JP) | H01R4/62\ H01R43/02 |
| 1429 | 3D printing | BATTERY AND ITS MANUFACTURING METHOD\ | JP2007305322A | TANAKA HIROYUKI (JP) | TOYOTA MOTOR CORP US | H01M4/04\ H01M2/30\ H01M2/26\ H01M10/0587\ H01M10/05\ H01M4/13\ H01M4/64\ H01M4/139 |
| 1430 | 3D printing | TERMINAL AND ALUMINUM ELECTRIC WIRE THEREWITH AND ITS MANUFACTURING METHOD\ | JP2007305355A | ASHIDA TETSUYA US\ TERASAWA KAZUHIKO US | MITSUBISHI CABLE IND LTD US | H01R4/02\ H01R43/02\ H01R11/12 |
| 1431 | 3D printing | VEHICULAR SUN VISOR\ | JP2007320375A | KANASHIKI AKIRA US\ SAKURAZAWA KIYOSHI US | KASAI KOGYO KK (JP) | B60J3/02 |
| 1432 | 3D printing | BATTERY PACK\ | JP2007323952A | OKADA WATARU US\ YOKOYA KAZUNOBU US\ YANO JUNYA US | SANYO ELECTRIC CO (JP) | H01M2/10\ H01M2/20 |
| 1433 | 3D printing | ULTRASONIC WELDING METHOD ULTRASONIC WELDING APPARATUS MANUFACTURING METHOD OF LIQUID DISCHARGE HEAD LIQUID DISCHARGE HEAD AND LIQUID DISCHARGE DEVICE\ | JP2008000899A | BIZEN RYOICHI US\ ONO KENICHI US\ ANAMI MAKOTO US | SEIKO EPSON CORP (JP) | B41J2/16\ B29C65/08\ B29C65/06 |
| 1434 | 3D printing | LIQUID OBJECT DELIVERING HEAD METHOD FOR MANUFACTURING LIQUID OBJECT DELIVERING HEAD AND LIQUID OBJECT DELIVERING APPARATUS\ | JP2008000900A | BIZEN RYOICHI US\ ONO KENICHI US | SEIKO EPSON CORP (JP) | B41J2/175\ B41J2/16 |
| 1435 | 3D printing | CROSS FLOW FAN\ | JP2008002401A | KAWAI TAKASHI US\ KATO TAKAHISA US | NIPPON KOBUNSHI KK US | F04D17/04 |
| 1436 | 3D printing | SKIN FOR FOAM BODY METHOD OF MANUFACTURING SKIN FOR FOAM BODY AND PRESSER ROLLER FOR ULTRASONIC WELDING\ | JP2008006074A | AOKI NORICHIKA US | INOUE MTP KK US | B29K105/04\ B29C65/08\ B68G7/05\ B29L31/58 |
| 1437 | 3D printing | AUXILIARY KNOB MOUNTING METHOD AND MOUNTING DEVICE IN VEHICULAR SUN VISOR\ | JP2008006874A | TAGUCHI KIYOTAKA US | KASAI KOGYO KK (JP) | B60J3/02 |
| 1438 | 3D printing | LIQUID FEEDING PATH AND ITS MANUFACTURING METHOD\ | JP2008018625A | SAITO TAKEMITSU US\ MARUYAMA KEN US | SEIKO EPSON CORP (JP) | B41J2/175\ B41J2/16 |
| 1439 | 3D printing | METHOD FOR TREATING PERIPHERAL EDGE OF PILE FABRIC AND THE PILE FABRIC USING THE METHOD\ | JP2008031582A | SAKAGUCHI EIJI US\ TAKEDA JUNICHI US | SUMINOE TEXTILE US | A47G27/02\ D06C25/00\ D06C27/00 |
| 1440 | 3D printing | HOLLOW STRUCTURE HEAD UNIT AND INKJET APPARATUS\ | JP2008044298A | OBA SHINICHIRO US\ SAITO TOSHIHIRO US | SEIKO EPSON CORP (JP) | B41J2/175 |
| 1441 | 3D printing | METHOD OF MANUFACTURING PLATE-LIKE HEAT PIPE\ | JP2008045820A | JENG JAU-SHIANG US\ CHEN PEI-PEI (MY)\ YANG HSIU-WEI (TW)\ YU WEN-HWA (TW) | KIKO KAGI KOFUN YUGENKOSHI US | F28D15/02\ H01L23/427 |
| 1442 | 3D printing | BRUSH\ | JP2008054779A | ISHIGURO SHINJI US\ MIZUTANI FUJIO US | KOWA CO US | A46B3/06\ B24D13/10 |
| 1443 | 3D printing | STORAGE ELEMENT\ | JP2008060407A | MIWA TOSHIYUKI US\ KATSUBE YASUYUKI US\ UEKI SHINICHI US\ OGAWA TAKUSHI US | FDK CORP US | H01M2/30\ H01M2/26\ H01G9/008\ H01G11/74\ H01G11/84\ H01G11/66\ H01G11/86\ H01G4/232\ H01G11/68\ H01G11/76 |
| 1444 | 3D printing | ULTRASONIC WELDING METHOD\ | JP2008068261A | TANAKA KOJI US\ SUZUKI MINORU US\ FUJISAWA YASUNARI US\ NOMURA KOJI US\ OIKAWA HIDENORI US | TOYOTA MOTOR CORP US\ SUMITOMO LIGHT METAL IND US | B23K20/10\ B23K103/20 |
| 1445 | 3D printing | CARBON BRUSH JOINED BODY AND ITS MANUFACTURING METHOD\ | JP2008071497A | YOSHIDA KEN US | AUPACK CO LTD US | H01R4/02\ H01R43/02\ H01R39/36\ H01R43/12\ H01R43/28\ H02K13/00 |
| 1446 | 3D printing | TURBO FAN AND AIR CONDITIONER PROVIDED THEREWITH\ | JP2008075626A | TSUJIKAWA YUEI US\ MORI NOBUYUKI US\ HORIUCHI YOSHIYASU US | FUJITSU GENERAL LTD US | B29C65/08\ B29L31/08\ F24F1/00\ F04D29/30\ F04D17/16 |
| 1447 | 3D printing | ULTRASONIC WELDING JOINING METHOD FOR SPEAKER CABINET\ | JP2008078967A | RYU TOKUKO US | FOSTER ELECTRIC CO HONG KONG L US | H04R31/00\ H04R1/02\ B29L22/00\ B29C65/08 |
| 1448 | 3D printing | OIL STRAINER\ | JP2008082314A | ENOKIDA TOMOSHI US\ KANEYASU TOSHIYA US | DAIKYONISHIKAWA KK US | F01M11/03 |
| 1449 | 3D printing | LOW-PROFILE BATTERY\ | JP2008091268A | YAMAMURA AKIRA US | NISSAN MOTOR (JP) | H01M2/26\ H01M10/05\ H01M10/0585 |
| 1450 | 3D printing | HEMODIAFILTRATION MODULE\ | JP2008093518A | UMEDA MARIKO US\ TOYODA KYOKO US | ASAHI KASEI KURARAY MEDICAL CO US | A61M1/18\ B01D63/02\ A61M1/34\ B01D61/28 |
| 1451 | 3D printing | ULTRASONIC WELDING DEVICE\ | JP2008119837A | HAZAMA YOSHIHIKO US\ NISHI NOBUYUKI US | KIWA KINZOKU KK US | B29C65/08\ B29C65/74\ B26D7/08\ B26D7/10 |
| 1452 | 3D printing | MANUFACTURING METHOD OF FLAT CABLE\ | JP2008140599A | SHIODA RYOSUKE US\ SATO OSAMU (JP) | AUTO NETWORK GIJUTSU KENKYUSHO US\ SUMITOMO ELECTRIC INDUSTRIES (JP)\ SUMITOMO WIRING SYSTEMS (JP) | H01B13/00 |
| 1453 | 3D printing | ULTRASONIC WELDING APPARATUS AND ITS CONTROL METHOD\ | JP2008142738A | KISHIMOTO HIROAKI (JP) | NISSAN MOTOR (JP) | B23K20/10\ B23K103/18\ B23K101/36 |
| 1454 | 3D printing | ULTRASONIC WELDER AND METHOD FOR CONTROLLING THE SAME AND WELDING INSPECTION APPARATUS FOR ULTRASONIC WELDING AND WELDING INSPECTION METHOD THEREFOR\ | JP2008142739A | WATANABE SHINICHI US\ ENOKIDA TAKESHI US | NISSAN MOTOR (JP) | B23K20/10\ G01N27/04 |
| 1455 | 3D printing | APPARATUS AND METHOD FOR INSPECTING ULTRASONIC WELDING AND APPARATUS AND METHOD FOR ULTRASONIC WELDING\ | JP2008145252A | TAKAYAMA HAJIME US | NISSAN MOTOR (JP) | B23K20/10\ B29C65/08\ B23K101/36\ G01N25/72 |
| 1456 | 3D printing | CONNECTION TAB FOR BATTERY\ | JP2008159555A | ABE HIROSHI US | ALFACT CO LTD US | H01M2/26 |
| 1457 | 3D printing | BOTTLE TO BE OPENED SINGLE WAY\ | JP2008179390A | OHASHI SHOZO US\ FUKUMOTO SHINJI US | TIGER VACUUM BOTTLE CO LTD US | B65D47/08\ A47J41/02\ B65D47/06 |
| 1458 | 3D printing | FUEL CARTRIDGE FOR METHANOL FUEL CELL AND PORTABLE ELECTRONIC DEVICE USING SAME\ | JP2008181764A | NOZUE MITSURU US | KURITA WATER IND LTD US | H01M8/10\ H01M8/04\ H01M8/00 |
| 1459 | 3D printing | ULTRASONIC WELDING DEVICE\ | JP2008183622A | TAKADA HAJIME (JP) | YAZAKI CORP (JP) | B23K20/10\ B29C65/08\ H01R43/02 |
| 1460 | 3D printing | SOLID ELECTROLYTIC CAPACITOR ELEMENT AND ITS MANUFACTURING METHOD\ | JP2008187115A | SATO RIE US | HITACHI AIC INC US | H01G9/00\ H01G9/012\ H01G9/04 |
| 1461 | 3D printing | METHOD FOR ULTRASONICALLY WELDING DEVELOPING CARTRIDGE CASE\ | JP2008209593A | SEKIOKA TADAYASU US\ KAWAI NOBUYUKI US\ IMAIZUMI SHOJI US | KONICA MINOLTA BUSINESS TECH US | G03G15/08\ B29C65/08\ B65D83/06 |
| 1462 | 3D printing | RADIO EQUIPMENT FOR WATER METER\ | JP2008215959A | NISHIURA MASAHITO US | TAKAHATA SEIKO KK US | G01F1/00\ H05K7/00 |
| 1463 | 3D printing | SUPPORT DEVICE FOR ULTRASONIC WELDING\ | JP2008229948A | FUJIMOTO RYOICHI US | KONICA MINOLTA BUSINESS TECH US | G03G15/08\ B29C65/08\ B29C65/78 |
| 1464 | 3D printing | ULTRASONIC WELDING METHOD\ | JP2008230033A | MUKAI HIROAKI US | TOYOTA BOSHOKU CORP US | B29C65/08\ F16B5/08 |
| 1465 | 3D printing | METHOD FOR MANUFACTURING CONTAINER\ | JP2008238437A | OKUDA HIROYUKI US\ SOTOMOTO KOSUKE US | TORAY INDUSTRIES (JP) | B29C65/08\ A61M1/14 |
| 1466 | 3D printing | COMPACT SELF-BALLASTED FLUORESCENT LAMP AND LIGHTING SYSTEM\ | JP2008270174A | IKADA KUNIHIKO US\ MATSUNAGA HIROYUKI US\ SANPEI TOMOHIRO US | TOSHIBA LIGHTING & TECHNOLOGY US | F21S2/00 |
| 1467 | 3D printing | REPAIR METHOD OF PALLET\ | JP2008279692A | HAGIWARA TOSHIO US | TOYO UNSO KK US | B29C65/08\ B29C73/10\ B65D19/38 |
| 1468 | 3D printing | ULTRASONIC WELDING METHOD HAVING INTERMEDIATE INSERTION LAYER\ | JP2008284862A | KIYU TAKETERU US\ GO YUHA US | KIYU TAKETERU US | B23K20/10\ B29C65/08 |
| 1469 | 3D printing | MANUFACTURING PROCESS OF FOAMED RESIN PRODUCT\ | JP2008296513A | NAKAJIMA YOSHIYUKI US | TOYOTA MOTOR CORP US | B29C45/17\ B29C45/00\ B29K105/04\ B60K37/00\ B29L31/58\ B29C45/44 |
| 1470 | 3D printing | BATTERY\ | JP2008305756A | MATSUBARA TAKEHITO US\ HIRATA MINORU US\ MUKAI HIROSHI US | GS YUASA CORP (JP) | H01M10/04\ H01M2/26 |
| 1471 | 3D printing | SOLENOID CONTROLLED VALVE\ | JP2008309237A | ASAI MIKIO US | TOYOOKI KOGYO KK US | F16K31/06\ F16K37/00\ H01F7/16 |
| 1472 | 3D printing | SEAM BELT\ | JP2009002980A | NAKADA SHIGEKI US\ TAKEUCHI YUICHI US\ OGAWA HIDEYUKI US\ NIIDATE TORU US\ KOBAYASHI TAKESHI US\ TAKASHIMA NAGAMITSU US | SEIKO EPSON CORP (JP) | G03G15/16 |
| 1473 | 3D printing | COVER MEMBER AND ELECTRONIC EQUIPMENT USING THE SAME\ | JP2009005113A | MIYAGAWA TATSUYA US | CASIO HITACHI MOBILE COMM CO US | H04M1/02\ H05K5/03 |
| 1474 | 3D printing | TERMINAL CONNECTION STRUCTURE TO ALUMINUM WIRE\ | JP2009009736A | HAGA KOKICHI US | AUTO NETWORK GIJUTSU KENKYUSHO US\ SUMITOMO ELECTRIC INDUSTRIES (JP)\ SUMITOMO WIRING SYSTEMS (JP) | H01R4/62\ H01R4/18\ H01R4/70 |
| 1475 | 3D printing | CYLINDRICAL SHRINK LABEL CONTAINER HAVING CYLINDRICAL SHRINK LABEL AND METHOD FOR MANUFACTURING THEM\ | JP2009012779A | MATSUSHITA TAEKO US\ ITO KATSUNOBU US\ MASUDA KENICHI US | DAINIPPON PRINTING CO LTD (JP) | B29K25/00\ B29K67/00\ B29C65/08\ B65D23/08\ B65D65/04\ G09F3/04 |
| 1476 | 3D printing | ULTRASONIC WELDING APPARATUS AND MANUFACTURING METHOD OF LITHIUM ION SECONDARY BATTERY USING THE SAME\ | JP2009022977A | SUZUKI YASUYUKI US\ NISHIMURA TAKASHI US | NEC TOKIN CORP (JP) | B23K20/10\ H01M2/26 |
| 1477 | 3D printing | LITHIUM ION BATTERY PACK\ | JP2009026631A | KOMIYA SADAHIRO US | NEC TOKIN CORP (JP) | H01M2/10 |
| 1478 | 3D printing | ULTRASONIC SEALING TIP\ | JP2009034699A | YONETANI YUKIHIRO US\ SAKAGUCHI TADAHISA US | YAZAKI CORP (JP) | B23K20/10\ H01R43/02\ H01R4/64\ H02G1/14 |
| 1479 | 3D printing | PHOTOELECTRIC CONVERTER AND MANUFACTURING METHOD THEREOF\ | JP2009043904A | MUROZONO MIKIO US\ HIBINO TAKESHI US | CLEAN VENTURE 21 CORP US | H01L31/04 |
| 1480 | 3D printing | MANUFACTURING METHOD OF RELIEF VALVE\ | JP2009074607A | HAYAKAWA YOSHIHIRO US\ HATTORI MITSURU US\ YAMADA KIYOHIRO US\ MURAKAMI TOMOHIRO US\ HIBINO SOKICHI US\ KATO KIMIO US | TOYOTA IND CORP US\ TOKYU CO LTD US | F16K17/04 |
| 1481 | 3D printing | NONAQUEOUS ELECTROLYTE SECONDARY BATTERY\ | JP2009076301A | MATSUYAMA TAKESHI US\ NIIDA YOSHIHIRO US\ KOJIMA YASUHISA US | NEC TOKIN CORP (JP) | H01M2/26 |
| 1482 | 3D printing | DEVICE OF MANUFACTURING PLASTIC LENS\ | JP2009083248A | SUNAGAWA YUKIAKI US\ GOMI YOSHIMITSU US\ KADOWAKI SHINICHIRO (JP) | HOYA CORP (JP) | B29C39/02\ B29L11/00\ B29C39/24 |
| 1483 | 3D printing | SYNTHETIC RESIN HOUSING FOR VALVE APPARATUS\ | JP2009085301A | ITO MASAHARU US | FUJI KOKI KK US | F16K27/06\ F16K11/087 |
| 1484 | 3D printing | TERMINAL CONNECTION METHOD AND TERMINAL CONNECTION DEVICE OF FLAT CABLE\ | JP2009087831A | YOSHIMURA KENJI US\ ISHIYAMA HIROMI US\ OMOTEYA TAKESHI US | FURUKAWA ELECTRIC CO LTD (JP) | H01B13/00\ H01R43/02\ H01R43/00 |
| 1485 | 3D printing | HEAT DISSIPATING SUBSTRATE AND METHOD OF MANUFACTURING THE SAME\ | JP2009088205A | SATO HIROYUKI US\ NUMATA MAKOTO US\ SAITO HIROSHI US\ TAKEUCHI GORO US\ TANAKA RYUICHI US | TDK CORP (JP) | H01L23/12 |
| 1486 | 3D printing | BUOYANCY TOOL\ | JP2009090432A | SHA CHIKEI US | SHA CHIKEI US | B25G1/00\ B25G1/10\ B25B13/02 |
| 1487 | 3D printing | FINE PASSAGE STRUCTURE AND ITS MANUFACTURING METHOD\ | JP2009095800A | OTA YUKIO US\ ITO OSAMU US\ FUKUMURA RYUICHI US | SATO LIGHT KOGYO KK US | B81B1/00\ C12M1/00\ B81C3/00\ B29C65/08\ B01J19/00\ G01N37/00 |
| 1488 | 3D printing | POLYAMIDE RESIN COMPOSITION\ | JP2009097011A | YOSHIDA TETSUYU US\ YAMADA MOTONOBU US\ SUGATA KOJI US | TORAY INDUSTRIES (JP) | C08K3/32\ C08L77/00\ C08K7/14\ B29C65/06\ C08K5/524 |
| 1489 | 3D printing | ULTRASONIC WELDING DEFECT FLAW DETECTION APPARATUS AND METHOD\ | JP2009097972A | HIRASAWA TAIJI US\ KAWAHARADA YOSHIYUKI US\ IWAI OSAMU US | TOSHIBA CORP US | G01N29/04 |
| 1490 | 3D printing | FUEL FILTER\ | JP2009106878A | NONOYAMA HAYASHI US\ OYA YOSHIHIKO US\ TAKAMIZAWA OSAMU US | DENSO CORP (JP)\ KYOSAN DENKI KK (JP) | B01D39/16\ B01D39/18\ D04H1/593\ F02M37/22 |
| 1491 | 3D printing | ULTRASONIC WELDING STRUCTURE AND ITS METHOD\ | JP2009107140A | KIDA HIROHISA US | KIDA HIROHISA US | B29C65/08\ A41H43/04 |
| 1492 | 3D printing | SEALED BATTERY\ | JP2009110808A | YAMASHITA SHUICHI US\ OGI MASANORI US\ MATSUURA SHUICHI US | SANYO ELECTRIC CO (JP) | H01M2/34\ H01M2/12 |
| 1493 | 3D printing | PACKAGING BAG BODY AND ITS MANUFACTURING METHOD\ | JP2009120240A | OZAKI IKUKO US\ TAKAGI TAKEO US | MYUUPAKKU OZAKI KK US | B65D33/16\ B31B1/90\ B31B1/64\ B65D33/25\ B31B1/14\ B31B1/22\ B65D30/22\ B65D33/14\ B65D33/18 |
| 1494 | 3D printing | METHOD FOR JOINING RESIN MOLDINGS AND RESIN JOINED BODY\ | JP2009126085A | OTA YUKIO US\ ITO OSAMU US\ FUKUMURA RYUICHI US | SATO LIGHT KOGYO KK US | B29C65/08 |
| 1495 | 3D printing | METHOD OF ULTRASONIC WELDING BETWEEN POSITIVE ELECTRODE COLLECTOR FOR STORAGE BATTERY AND TAB AND LITHIUM ION BATTERY\ | JP2009134971A | YOSHIDA HIROYUKI US\ YAMAMOTO MASAHIRO US\ HIRUTA TERUSHIGE US | FURUKAWA BATTERY CO LTD US | H01M2/26 |
| 1496 | 3D printing | RESIN MOLDING OPTICAL LENS\ | JP2009139190A | KONDO JUN (JP)\ TANAKA MOTOKI (JP)\ HIRAO TOSHINORI (JP)\ KIMURA SHINGO (JP) | NIPPON CERAMIC KK (JP) | G01J1/02\ G02B1/04\ G01V8/12\ G01J1/04 |
| 1497 | 3D printing | GAME MACHINE\ | JP2009160026A | ARAI MASASATO US | SOPHIA CO LTD US | A63F7/02 |
| 1498 | 3D printing | VALVE BODY STRUCTURE FOR VALVE DEVICE AND ITS MANUFACTURING METHOD\ | JP2009174690A | MOCHIZUKI KENICHI US | FUJI KOKI KK US | F16K1/42\ F16K1/32\ F16K31/04 |
| 1499 | 3D printing | ULTRASONIC WELDING APPARATUS\ | JP2009178981A | FUKUOKA TAKASUKE US | YAMATAKE CORP US | B29C65/08 |
| 1500 | 3D printing | METHOD OF WELDING SHEET-LIKE ELECTRODE IN LAMINATED STATE TO TAB WITH ULTRASONIC WAVE\ | JP2009187674A | OGAWA TAKUSHI US | FDK CORP US | B23K20/10\ H01R43/02 |
| 1501 | 3D printing | SOLID ELECTROLYTIC CAPACITOR AND MANUFACTURING METHOD THEREFOR\ | JP2009194258A | ANDO SUSUMU US\ ICHIHARA HIROKAZU US\ KAWAMURA ATSUSHI US | NIPPON CHEMICON US | H01G9/00\ H01G9/012\ H01G9/04 |
| 1502 | 3D printing | METHOD OF BONDING MULTILAYERED METALLIC FOIL USING ULTRASONIC WELDING MACHINE\ | JP2009195979A | TAMURA TERUO US | SONOMAC JAPAN KK US | B23K20/10\ B23K103/10 |
| 1503 | 3D printing | METHOD FOR FABRICATING FILTER ASSEMBLY AND INKJET HEAD\ | JP2009196292A | BIZEN RYOICHI US | SEIKO EPSON CORP (JP) | B41J2/175 |
| 1504 | 3D printing | BATTERY PACK\ | JP2009199911A | MARUOKA KAZUNORI US | NEC TOKIN CORP (JP) | H01M2/10\ H01M2/12 |
| 1505 | 3D printing | ALCOHOL-RESISTANT MOLDING MATERIAL AND MOLDED ARTICLE\ | JP2009215523A | ICHIHASHI TAKAO US\ SUGIYAMA FUMITAKA US | TECHNO POLYMER CO LTD US | H01M8/10\ H01M8/04\ C08L23/00\ B65D1/00\ C08L51/06\ C08L23/26\ C08K9/04 |
| 1506 | 3D printing | HEMODIAFILTRATION MODULE\ | JP2009240499A | UMEDA MARIKO US\ WASAI KYOKO US | ASAHI KASEI KURARAY MEDICAL CO US | A61M1/18 |
| 1507 | 3D printing | SHUNT RESISTOR AND TERMINAL FITTING METHOD FOR SHUNT RESISTOR\ | JP2009244065A | DOI SHOGO US | FURUKAWA ELECTRIC CO LTD (JP) | G01R15/14 |
| 1508 | 3D printing | METHOD FOR MANUFACTURING ELECTRIC WIRE WITH TERMINAL METAL FITTING AND ELECTRIC WIRE WITH TERMINAL METAL FITTING\ | JP2009259702A | SUETANI MASAHARU US\ MIYAZAKI TADASHI US | SUMITOMO ELECTRIC INDUSTRIES (JP)\ AUTONETWORKS TECHNOLOGIES LTD (JP)\ SUMITOMO WIRING SYSTEMS (JP) | H01R4/02\ H01R43/02 |
| 1509 | 3D printing | METHOD FOR ULTRASONIC WELDING BETWEEN ELECTRIC WIRE AND TERMINAL\ | JP2009277445A | YAMAGUCHI MAKOTO US | YAZAKI CORP (JP) | B23K20/10\ H01R43/02\ B23K20/24 |
| 1510 | 3D printing | BATTERY PACK AND METHOD FOR MANUFACTURING THE SAME\ | JP2009277673A | TANJO YUJI US\ WATANABE MASAJI US\ ENOKIDA TAKESHI US\ AMETANI RYUICHI US\ HAYAMI MUNEHITO US\ OTA MASAYASU (JP)\ KISHIMOTO HIROAKI (JP)\ SEGAWA TERUO (JP) | NISSAN MOTOR (JP) | H01M2/02\ H01M2/30\ H01M2/10\ H01M2/20 |
| 1511 | 3D printing | COMPOSITE CONTAINER\ | JP2009280270A | UMEYAMA HIROSHI US\ IIJIMA SHUNJI US | TOPPAN PRINTING CO LTD US | B65D3/22\ B65D3/28\ B65D77/00 |
| 1512 | 3D printing | METHOD FOR MANUFACTURING HEAT ROLLER HEAT ROLLER AND IMAGE FORMING APPARATUS\ | JP2009288331A | SOJO YOSHIHIRO US\ OYA KAZUYUKI US | MURATA MACHINERY LTD US | G03G15/20 |
| 1513 | 3D printing | AUTOMOBILE INTERIOR COMPONENT\ | JP2009292438A | SATO YOKO US | KASAI KOGYO KK (JP) | B60R11/02\ B60J5/00\ B60J5/04 |
| 1514 | 3D printing | ULTRASONIC WELDER\ | JP2009297938A | KURIHASHI KATSUNORI US | NEC TOKIN CORP (JP) | B23K20/10\ B29C65/08 |
| 1515 | 3D printing | COMPACT ELECTRONIC DEVICE AND METHOD OF MANUFACTURING COMPACT ELECTRONIC DEVICE\ | JP2009300395A | MORITA KATSUMI US\ TOMIOKA MASAKI US\ ONISHI YOSHIHIDE US | OMRON HEALTHCARE CO LTD US | G01K7/00\ G06M3/00 |
| 1516 | 3D printing | METHOD OF MANUFACTURING FLEXIBLE PIPE AND GOOSE NECK TYPE MICROPHONE SYSTEM\ | JP2009302905A | AKINO YUTAKA US | AUDIO TECHNICA KK US | F16M11/00\ H04R1/00\ B21F45/06\ B21F99/00\ F16M11/40 |
| 1517 | 3D printing | COTTON SWAB\ | JP2010000410A | HORAGUCHI KATSUNORI US | TOWA KASEI KK US | A47L25/00\ B08B1/00 |
| 1518 | 3D printing | AIR CONDITIONER\ | JP2010014317A | KOYATA ATSUSHI US | MITSUBISHI ELECTRIC CORP (JP) | F24F13/14 |
| 1519 | 3D printing | CONDENSER MICROPHONE UNIT AND METHOD OF MANUFACTURING THE SAME\ | JP2010016679A | AKINO YUTAKA US | AUDIO TECHNICA KK US | H04R31/00\ H04R19/04 |
| 1520 | 3D printing | ATTACHMENT METHOD AND APPARATUS OF ASSEMBLY\ | JP2010036513A | KAWADA SATOSHI US | SHIGERU CO LTD US | B29C65/08 |
| 1521 | 3D printing | WIRE HARNESS AND METHOD OF MANUFACTURING THE SAME AND METHOD OF CONNECTING INSULATION WIRE\ | JP2010044887A | SATO OSAMU (JP) | SUMITOMO ELECTRIC INDUSTRIES (JP)\ AUTONETWORKS TECHNOLOGIES LTD (JP)\ SUMITOMO WIRING SYSTEMS (JP) | H01B7/00\ H01B13/012\ H01R4/02\ H01R43/02\ H01R4/20 |
| 1522 | 3D printing | CAPACITOR MICROPHONE UNIT\ | JP2010050868A | AKINO YUTAKA US | AUDIO TECHNICA KK US | H04R19/04 |
| 1523 | 3D printing | METHOD FOR SEWING DUST-PROOF GARMENT\ | JP2010059590A | NAGARA YOSHITAKA US | UNITIKA LTD US | D06H5/00\ A41D27/24\ D06C25/00\ A41D13/02 |
| 1524 | 3D printing | METHOD OF MANUFACTURING HEAD CASE FOR MICROPHONE\ | JP2010068287A | AKINO YUTAKA US | AUDIO TECHNICA KK US | H04R1/02 |
| 1525 | 3D printing | RESIN COMPONENT PROCESS CARTRIDGE FORMED OF RESIN COMPONENT AND IMAGE FORMING APPARATUS PROVIDED WITH PROCESS CARTRIDGE FORMED OF RESIN COMPONENT\ | JP2010072331A | TAKASE MAKOTO US\ YOSHIKAWA KIYOYUKI US\ TSUKAMOTO HIDEAKI US | KONICA MINOLTA BUSINESS TECH US | G03G15/08\ G03G21/18\ B65D83/06 |
| 1526 | 3D printing | FILTER APPARATUS AND METHOD OF MANUFACTURING FILTER BODY USED FOR THE SAME\ | JP2010075881A | KIKUCHI TAKESHI US\ ISHII JUNKO US | KYOSAN DENKI KK (JP) | B01D39/00\ B01D39/16 |
| 1527 | 3D printing | JOINING STRUCTURE AND ITS MANUFACTURING METHOD\ | JP2010082668A | IKEDA UKYO US\ NAKAMURA MASATO US\ YAMASHITA SHIRO US | HITACHI AUTOMOTIVE SYSTEMS LTD US | B23K20/10\ B23K101/36 |
| 1528 | 3D printing | CAPACITOR MODULE AND METHOD OF MANUFACTURING THE SAME\ | JP2010087318A | OKADA NOBORU US\ HONDA IKUFUMI US | NIPPON CHEMICON US | H01G11/00\ H01G11/74\ H01G11/78\ H01G11/84\ H01G9/26\ H01G2/04 |
| 1529 | 3D printing | CAPACITOR MODULE AND METHOD OF MANUFACTURING THE SAME\ | JP2010087319A | OKADA NOBORU US\ ICHIKURA OSAMU US\ YOKOYAMA SHINICHI US\ HAYAKAWA KAZUHIDE US | NIPPON CHEMICON US | H01G11/74\ H01G11/78\ H01G11/66\ H01G9/26\ H01G2/04\ H01G11/10 |
| 1530 | 3D printing | METHOD FOR PRODUCING FELT FOR PAPERMAKING AND FELT FOR PAPERMAKING\ | JP2010100961A | AOKI SHIGETO US | NIPPON FELT CO LTD US | D21F7/10 |
| 1531 | 3D printing | ELECTRIC DOUBLE LAYER CAPACITOR AND METHOD OF MANUFACTURING THE SAME\ | JP2010114365A | OYA MASAKO US\ HADO YUKINORI US | NEC TOKIN CORP (JP) | H01G11/00\ H01G11/78\ H01G11/10\ H01G11/12\ H01G11/82 |
| 1532 | 3D printing | RESIN ENCAPSULATED SEMICONDUCTOR DEVICE AND METHOD OF MANUFACTURING THE SAME\ | JP2010118577A | SHINKAI JIRO US | SUMITOMO ELECTRIC INDUSTRIES (JP) | H01L21/60\ H01L21/56\ H01L23/50 |
| 1533 | 3D printing | ULTRASONIC WELDING MACHINE\ | JP2010131847A | MOTODA YOSHISUKE US | SAN AI IND INC (JP) | B29C65/08\ B65B51/10\ B65C7/00 |
| 1534 | 3D printing | SHIELD PROCESSING METHOD FOR SHIELDED CABLE\ | JP2010140741A | YONETANI YUKIHIRO US | YAZAKI CORP (JP) | B23K20/10\ B29C65/08\ H01R43/02\ H02G1/14\ H01B7/17\ H02G15/188 |
| 1535 | 3D printing | ULTRASONIC WELDING PROCESS\ | JP2010143156A | FUJITA TAKAHIDE US | FUJITA KIKAI KK US | B29C65/08 |
| 1536 | 3D printing | RELIEF VALVE\ | JP2010144921A | MURAKAMI TOMOHIRO US\ KATO KIMIO US\ MURAO KAZUE US\ MIYACHI HIDENAO US | TOYOTA IND CORP US\ TOKYU CO LTD US | F16K17/04\ F16K27/00\ F16K27/02 |
| 1537 | 3D printing | RELIEF VALVE\ | JP2010144922A | MURAKAMI TOMOHIRO US\ KATO KIMIO US\ MURAO KAZUE US\ MIYACHI HIDENAO US | TOYOTA IND CORP US\ TOKYU CO LTD US | F16K17/04\ F16K27/00 |
| 1538 | 3D printing | PRODUCTION PROCESS OF AUTOMOBILE INTERIOR COMPONENT AND MOLDING DIE USED FOR THE PROCESS\ | JP2010149410A | ONO TATSUFUMI US\ AIZAWA HIROYUKI US | KASAI KOGYO KK (JP) | B29C43/18\ B60R13/02 |
| 1539 | 3D printing | CONNECTION METHOD OF ELECTRIC WIRE\ | JP2010153074A | TAKAYASHIKI YOSUKE US\ TAKADA HAJIME (JP) | YAZAKI CORP (JP) | H01R43/02 |
| 1540 | 3D printing | END CAP STRUCTURE OF VEHICLE DOOR SASH MOLDING\ | JP2010195064A | MAKITA JUN US\ OKADA TAKAYUKI US | SHIROKI CORP US | B60R13/04 |
| 1541 | 3D printing | FLAT SECONDARY BATTERY\ | JP2010205701A | TAKAI TOKU US | HITACHI MAXELL (JP) | H01M2/02\ H01M2/30\ H01M2/26 |
| 1542 | 3D printing | WELDING METHOD WELDER SHEET-SHAPED MEMBER WITH WELDED RESIN MOLDING\ | JP2010208031A | KOGASHIWA SHIGERU US | SEIDENSHA ELECTRONICS US | B29C65/08\ B01D63/00 |
| 1543 | 3D printing | FLAT SECONDARY BATTERY\ | JP2010212206A | TAKAI TOKU US | HITACHI MAXELL (JP) | H01M4/66\ H01M2/02\ H01M2/26\ H01M2/04\ H01M10/0566\ H01M10/0585 |
| 1544 | 3D printing | METHOD AND DEVICE FOR BINDING PAPER SHEET\ | JP2010253870A | OTA SETSUZO US | SANKYO CO US | B42B4/00 |
| 1545 | 3D printing | ELECTRIC BLOWER AND VACUUM CLEANER USING THE SAME\ | JP2010255505A | NISHIMURA TAKESHI US\ TOKUDA TAKESHI US | PANASONIC CORP (JP) | F04D29/28\ F04D25/08 |
| 1546 | 3D printing | ULTRASONIC TYPE WELDING APPARATUS FOR THERMOPLASTIC BAG MATERIAL STOCK ROLL AND METHOD FOR THE SAME\ | JP2010269548A | OOKA YOSHIO US | MURATA KK US\ ST SANGYO KK US | B29C65/08 |
| 1547 | 3D printing | METHOD AND DEVICE FOR MANUFACTURING SPRING\ | JP2010270806A | SAKATA MOTONORI US | NHK SPRING CO LTD (JP) | B29C65/08\ B29C63/04\ F16F1/02 |
| 1548 | 3D printing | ULTRASONIC WELDING APPARATUS\ | JP2010274296A | TANAKA TOSHIHARU US | NISSAN MOTOR (JP) | B23K20/10 |
| 1549 | 3D printing | ULTRASONIC WELDING METHOD ULTRASONIC WELDING MACHINE AND ELECTRONICS WITH RESIN MOLDING\ | JP2010274516A | MATSUGISHI NORIAKI US\ KUROKI YOSHIHITO US | SEIDENSHA ELECTRONICS US | B29C65/08 |
| 1550 | 3D printing | SECONDARY BATTERY AND METHOD OF MANUFACTURING THE SAME\ | JP2010282846A | MATSUURA TOMOHIRO US\ SOKI TAKAHIRO US | TOYOTA MOTOR CORP US | H01M2/26 |
| 1551 | 3D printing | SUBSTRATE FIXING TABLE AND ULTRASONIC WELDING APPARATUS\ | JP2011005500A | YOSHIDA AKIO US\ OGURA MASAHISA US | TOSHIBA MITSUBISHI ELEC INC US | B23K20/10\ H01L21/607 |
| 1552 | 3D printing | ULTRASONIC WELDING METHOD ULTRASONIC WELDING DEVICE AND TERMINAL METAL FITTING\ | JP2011018597A | KASHIYAMA MASAHISA US\ NAGASAWA MASANORI US | YAZAKI CORP (JP) | H01R4/02 |
| 1553 | 3D printing | ULTRASONIC WELDING MACHINE\ | JP2011031516A | MORIMURA TAKESHI US | SEIDENSHA ELECTRONICS US | B29C65/08 |
| 1554 | 3D printing | CLOTH AND BAG\ | JP2011036463A | ONO KOICHIRO US | ASAKURA KK US | A45C5/02 |
| 1555 | 3D printing | METHOD FOR PRODUCING FOOTWEAR\ | JP2011036669A | CHEN MING TE (TW) | CHEN MING TE (TW) | A43D86/00\ A43D25/06 |
| 1556 | 3D printing | PNEUMATIC TIRE AND METHOD FOR MANUFACTURING SAME\ | JP2011037399A | TANNO ATSUSHI US | YOKOHAMA RUBBER CO LTD US | B60C19/12\ B29D30/30\ B60C5/14 |
| 1557 | 3D printing | STRUCTURE FOR FIXING FELT MATERIAL TO INTERIOR COMPONENT FOR AUTOMOBILE\ | JP2011042321A | HARADA TASUKU US | KASAI KOGYO KK (JP) | B60R13/02 |
| 1558 | 3D printing | GASKET STRUCTURE AND METHOD FOR MANUFACTURING THE SAME\ | JP2011047488A | WATABE SHIGERU US | NOK CORP US | F16J15/10\ F16J15/00 |
| 1559 | 3D printing | APPARATUS AND METHOD FOR INSPECTING ULTRASONIC WELDING\ | JP2011056557A | WATANABE SHINICHI US | NISSAN MOTOR (JP) | B23K20/10\ B23K31/00 |
| 1560 | 3D printing | CONNECTION TERMINAL AND ALUMINUM CABLE WITH THE SAME\ | JP2011060778A | WASHIMI TORU US\ HORIKOSHI TOSHIYUKI US\ SHIMIZU MICHIAKI US | HITACHI CABLE (JP) | H01B7/00\ H01R4/62\ H01R4/02\ H01R4/58\ H01R4/72 |
| 1561 | 3D printing | ULTRASONIC WELDING METHOD ULTRASONIC WELDING MACHINE AND PACKAGING MACHINE\ | JP2011063002A | KIYOTA YUSUKE US\ UEKAKIUCHI AKIRA US | ISHIDA SEISAKUSHO US | B29C65/08\ B65B51/10\ B65B9/10 |
| 1562 | 3D printing | LEAD ACID BATTERY\ | JP2011070905A | OSAKI MAKOTO US\ UMETANI HIROBUMI US\ HOJO EIJI US\ NAKAYAMA TAKAHIDE US | GS YUASA CORP (JP) | H01M2/04\ H01M2/12 |
| 1563 | 3D printing | INTERIOR COMPONENT FOR VEHICLE\ | JP2011073607A | CHIGAI YOICHI US | KASAI KOGYO KK (JP) | B60R13/02 |
| 1564 | 3D printing | BATTERY HOUSING CASE AND ITS JOINING METHOD\ | JP2011076968A | SUZUKI KAZUYUKI US\ IMAI TADASHI US | PANASONIC CORP (JP) | H01M2/02 |
| 1565 | 3D printing | WATER STOP STRUCTURE OF ELECTRIC WIRE AND METHOD OF FORMING THE WATER STOP STRUCTURE\ | JP2011081918A | SAWAMURA NAOTO US | SUMITOMO WIRING SYSTEMS (JP) | H01R4/18\ H01R4/70 |
| 1566 | 3D printing | CUSHION PAD AND METHOD OF MANUFACTURING CUSHION PAD\ | JP2011083321A | OKABE NORIYUKI US | TOYO TIRE & RUBBER CO US | B68G7/05 |
| 1567 | 3D printing | TOOL HORN AND ULTRASONIC WELDING APPARATUS USING THE SAME\ | JP2011110839A | KUROKI YOSHIHITO US\ SHIBATA YU US | SEIDENSHA ELECTRONICS US | B23K20/10\ B29C65/08\ B06B1/02\ B06B1/06 |
| 1568 | 3D printing | POWER STORAGE ELEMENT HAVING LAMINATE FILM OUTER PACKAGE\ | JP2011113929A | YAMAMOTO TAKAHIRO US\ SATO KATSUYA US\ TSUBATA TOSHIO US | ASAHI KASEI CORP US | H01M2/02\ H01M2/30\ H01M2/26\ H01M10/0566\ H01M10/0585\ H01G11/00\ H01G11/74\ H01G11/78\ H01G11/66\ H01G11/80\ H01G11/82 |
| 1569 | 3D printing | ULTRASONIC WELDING METHOD OF METAL THIN PLATE\ | JP2011115814A | HORI HIROFUMI US | HITACHI VEHICLE ENERGY LTD (JP) | B23K20/10\ B23K103/10\ B23K103/12\ B23K101/36 |
| 1570 | 3D printing | CABLE CONNECTION CHECK SYSTEM IDENTIFICATION TAG MANUFACTURING METHOD AND RELATED DEVICE\ | JP2011130652A | MORI SHIGERU US | HITACHINAKA TECHNO CT KK US | H02G1/00 |
| 1571 | 3D printing | ELECTRIC WIRE WITH TERMINAL FITTING AND MANUFACTURING METHOD THEREOF\ | JP2011134515A | SUETANI MASAHARU US | SUMITOMO ELECTRIC INDUSTRIES (JP)\ AUTONETWORKS TECHNOLOGIES LTD (JP)\ SUMITOMO WIRING SYSTEMS (JP) | H01B13/00\ H01B7/00\ H01R4/18\ H01R4/02 |
| 1572 | 3D printing | METHOD OF MANUFACTURING WIRE HARNESS\ | JP2011138641A | SATO OSAMU (JP)\ HIRANO NOBUYUKI (JP)\ MURATA ATSUSHI (JP) | SUMITOMO ELECTRIC INDUSTRIES (JP)\ AUTONETWORKS TECHNOLOGIES LTD (JP)\ SUMITOMO WIRING SYSTEMS (JP) | H01B13/012\ B60R16/02 |
| 1573 | 3D printing | FILTER DEVICE\ | JP2011140905A | UEKI KOJI US | NIFCO INC US | B01D29/11\ B01D35/02\ B01D29/13\ F02M37/22\ F02M37/10 |
| 1574 | 3D printing | JOINING METHOD AND METHOD FOR MANUFACTURING MICROCHANNEL DEVICE\ | JP2011161578A | KATO KOTA US | FUJIFILM CORP (JP) | B81C3/00\ G01N35/08\ B01J19/00\ G01N37/00 |
| 1575 | 3D printing | WIRING DEVICE ULTRASONIC BONDING APPARATUS AND ULTRASONIC WELDING DEVICE\ | JP2011171429A | NAKAJIMA TOMOHIKO US | SEIDENSHA ELECTRONICS US | B23K20/10\ H01L21/60\ B29C65/08\ H01L21/607 |
| 1576 | 3D printing | APPARATUS AND METHOD FOR ULTRASONIC WELDING\ | JP2011173158A | NAKAMURA KENTARO US | TOKYO INST TECH US | B23K20/10\ B29C65/08\ B06B1/06\ B06B3/04 |
| 1577 | 3D printing | ULTRASONIC WELDING METHOD ULTRASONIC WELDING MACHINE USED FOR THE SAME AND HORN FOR ULTRASONIC WELDING MACHINE\ | JP2011183675A | YAMADA TSUNEJI US | BRIGHT CORP US | B29C65/08 |
| 1578 | 3D printing | LITHIUM ION SECONDARY BATTERY\ | JP2011192476A | ARIGA TOSHIYUKI US\ NISHIHARA SHOJI US\ KASHIWANO HIROSHI US\ ARISHIMA YASUO US\ SHINAMI MASAFUMI US | HITACHI VEHICLE ENERGY LTD (JP) | H01M2/02\ H01M2/30\ H01M2/34\ H01M2/26\ H01M2/04\ H01M10/0587\ H01M4/13\ H01M10/0525 |
| 1579 | 3D printing | ULTRASONIC WELDING MACHINE AND ULTRASONIC WELDING SYSTEM\ | JP2011194454A | KOGASHIWA SHIGERU US\ MATSUGISHI NORIAKI US\ ONOZUKA YOSHIFUMI US\ SERA MANABU US\ MAKI TATSUYA US | SEIDENSHA ELECTRONICS US | B23K20/10\ B29C65/08\ B06B1/02 |
| 1580 | 3D printing | METHOD OF MANUFACTURING METAL-RESIN COMPOSITE\ | JP2011224974A | PARK EUN KYOU (KR) | SUMITOMO CHEMICAL CO (JP) | B23K20/10\ B29C65/08\ B23K20/24 |
| 1581 | 3D printing | SECONDARY BATTERY\ | JP2011233257A | Empty Field US | HITACHI VEHICLE ENERGY LTD (JP) | H01M2/02\ H01M10/04\ H01M2/26 |
| 1582 | 3D printing | JOINT REINFORCEMENT METHOD OF MODULE HEADER AND REINFORCED MODULE\ | JP2011255380A | CHEN BIN US | ASAHI KASEI KURARAY MEDICAL CO US | B29C65/08\ B01D63/00 |
| 1583 | 3D printing | PUMP\ | JP2012002134A | KIM GIL HO US | PANASONIC ELEC WORKS CO LTD (JP) | F04D29/22\ F04D29/044\ F04D13/06\ F04D29/04 |
| 1584 | 3D printing | AIRTIGHT PRESSURE VESSEL AND MANUFACTURING METHOD OF THE SAME\ | JP2012002342A | LEE KYUNG MIN (KR)\ SHIN YOUNG JOON (KR) | SATO LIGHT KOGYO KK US | B29C65/08\ B65B7/28\ B65D43/06\ F16J12/00\ B65D35/44\ B65D43/08 |
| 1585 | 3D printing | ULTRASONIC WELDING DEVICE\ | JP2012024790A | WOODHALL DAVID JOHN (TH) | YAZAKI CORP (JP) | B23K20/10\ B29C65/08\ B06B1/02 |
| 1586 | 3D printing | LAMINATED FILM SHEATHED LAMINATED BATTERY AND METHOD OF MANUFACTURING THE SAME\ | JP2012033449A | IM SEONG TEAC (KR) | NEC ENERGY DEVICES LTD US | H01M2/02\ H01M2/06 |
| 1587 | 3D printing | CHIP BONDER\ | JP2012054583A | AIRI NAKAKAWA US | AKIM KK US | H01L21/60\ H01L21/607\ H05K13/04 |
| 1588 | 3D printing | ELECTRIC ELEMENT MANUFACTURING METHOD THEREFOR AND ULTRASONIC PROCESSING DEVICE\ | JP2012069480A | Empty Field US | BROTHER IND LTD (JP) | G09F9/00\ H01L51/50\ H05B33/04\ H05B33/10\ G09F9/30\ B29C65/08\ H05B33/02 |
| 1589 | 3D printing | MOTOR AND MANUFACTURING METHOD OF MOTOR\ | JP2012110204A | IM SEONG TEAC (KR) | NIHON DENSAN KK US | H02K3/46 |
| 1590 | 3D printing | CIRCUIT BOARD CASE AND GAME MACHINE\ | JP2012110516A | Empty Field US | HEIWA CORP US | A63F7/02 |
| 1591 | 3D printing | ULTRASONIC SEAM WELDING EQUIPMENT AND ULTRASONIC SEAM WELDING METHOD\ | JP2012115842A | ROH KWANG CHUL (KR) | TOSHIBA MITSUBISHI ELEC INC US | B23K20/10 |
| 1592 | 3D printing | VOLTAGE DETECTION TERMINAL PLATE WITH TERMINAL AND BATTERY MODULE\ | JP2012134016A | Empty Field US | AUTO NETWORK GIJUTSU KENKYUSHO US\ SUMITOMO ELECTRIC INDUSTRIES (JP)\ SUMITOMO WIRING SYSTEMS (JP)\ NISSAN MOTOR (JP) | H01M2/10\ H01M2/20 |
| 1593 | 3D printing | PACKAGING CONTAINER WITH LID\ | JP2012136282A | RYU NAE GEOL (KR) | TOYO ALUMINIUM EKCO PROD KK US | B65D1/26\ B65D55/02\ B65D43/16 |
| 1594 | 3D printing | VOLTAGE DETECTION TERMINAL PLATE WITH TERMINAL AND BATTERY MODULE\ | JP2012138268A | Empty Field US | AUTO NETWORK GIJUTSU KENKYUSHO US\ SUMITOMO ELECTRIC INDUSTRIES (JP)\ SUMITOMO WIRING SYSTEMS (JP)\ NISSAN MOTOR (JP) | H01M2/10 |
| 1595 | 3D printing | ELECTRIC WIRE CONDUCTOR AND INSULATED ELECTRIC WIRE\ | JP2012146431A | Empty Field US | AUTO NETWORK GIJUTSU KENKYUSHO US\ SUMITOMO ELECTRIC INDUSTRIES (JP)\ SUMITOMO WIRING SYSTEMS (JP) | H01B7/00\ C22C9/02\ H01B1/02\ H01B5/08\ C22C9/00\ C22C9/06 |
| 1596 | 3D printing | CUTLERY WITH ADVERTISEMENT USING GRIP AS ADVERTISING SPACE\ | JP2012159813A | LEE KYUNG MIN (KR) | SAIKYU SEIKI KK US | G09F23/00\ A47G21/00 |
| 1597 | 3D printing | RECTANGULAR SECONDARY BATTERY AND METHOD OF MANUFACTURING THE SAME\ | JP2012169064A | Empty Field US | HITACHI VEHICLE ENERGY LTD (JP) | H01M10/04\ H01M2/26\ H01M2/04 |
| 1598 | 3D printing | TEMPORARILY FIXING STRUCTURE OF AUTOMOBILE EXTERIOR EQUIPMENT\ | JP2012176710A | SEO GI WON (KR) | TOYODA GOSEI KK US | B62D25/10\ B29C65/56\ B29C65/08\ B29C65/72\ B60J5/00\ B60J5/04 |
| 1599 | 3D printing | ULTRASONIC WELDING METHOD\ | JP2012187597A | Empty Field US | HITACHI CABLE (JP) | B23K20/10\ H01R43/02 |
| 1600 | 3D printing | BATTERY BACK AND JOINING METHOD OF SEPARATION CASES\ | JP2012209174A | KAMPS STEVE (US) | PANASONIC CORP (JP) | H01M2/10 |
| 1601 | 3D printing | BATTERY\ | JP2012209260A | PAUTET FRANCK (FR)\ LOPRETE STEPHANE (FR) | TOSHIBA CORP US | H01M2/26 |
| 1602 | 3D printing | BATTERY\ | JP2012209261A | PARK JI WON (KR) | TOSHIBA CORP US | H01M10/04\ H01M2/26 |
| 1603 | 3D printing | METHOD AND APPARATUS FOR ULTRASONIC-WELDING OF METAL\ | JP2012223780A | GUANGWEI LEI US\ JINGUANG PAN US\ WEILIANG ZHENG US\ WENTING ZHU US | MITSUBISHI ELECTRIC CORP (JP) | B23K20/10 |
| 1604 | 3D printing | CONTAINER FOR INK REFILLING\ | JP2012232492A | KWON JE HYEONG (KR) | PILOT INK CO LTD US | B43K5/14\ B43K7/02 |
| 1605 | 3D printing | WARNING STREAMER AND METHOD FOR MANUFACTURING THE SAME\ | JP2012242608A | JUNCAI LIU US | OZU SANGYO KK US\ KOJINSHA KK US | F21V7/22\ G02B5/128\ F21V7/00\ F21V7/10\ G02B5/13 |
| 1606 | 3D printing | CONTAINER ARRANGED WITH CONTENTS\ | JP2013000921A | KIM SOOK HWAN (KR)\ KIM SUNG WOOK (KR) | PENTEL KK US | B43K8/02\ B43K8/03\ B65D51/26 |
| 1607 | 3D printing | MANUFACTURING METHOD OF WIRE WITH TERMINAL\ | JP2013004406A | Empty Field US | HITACHI CABLE (JP) | H01R4/02\ H01R43/02 |
| 1608 | 3D printing | AC ADAPTER\ | JP2013016288A | JUNG JEAN MAN (KR) | PANASONIC CORP (JP) | H01R13/52\ H01R31/06 |
| 1609 | 3D printing | SEALANT FILM LAMINATE USING THE SEALANT FILM PACKAGING CONTAINER USING THE LAMINATE AND METHOD FOR PRODUCING THE SEALANT FILM\ | JP2013028732A | Empty Field US | KYODO PRINTING CO LTD US | C08J5/18\ B65D65/40\ C08L23/08\ B32B27/28\ C08L23/26 |
| 1610 | 3D printing | ULTRASONIC WELDING APPARATUS AND METHOD FOR PROCESSING KNURLING SURFACE OF ULTRASONIC JOINT TOOL USED IN ULTRASONIC WELDING APPARATUS\ | JP2013031869A | JANG JUN HWAN (KR) | NISSAN MOTOR (JP) | B23K20/10\ B24B9/00 |
| 1611 | 3D printing | KNURLING SURFACE REPROCESSING METHOD OF ULTRASONIC WELDING TOOL\ | JP2013031903A | JANG JUN HWAN (KR) | NISSAN MOTOR (JP) | B23K20/10\ B24B9/00\ B24B53/00\ B24C1/02 |
| 1612 | 3D printing | CASE AND METHOD FOR WELDING CASE MEMBER FILTER UNIT WITH THE CASE LIQUID EJECTION HEAD AND IMAGE FORMING APPARATUS\ | JP2013063520A | LAWS JONATHAN RODGER (GB) | RICOH CO LTD (JP) | B29C65/08 |
| 1613 | 3D printing | ULTRASONIC WELDING DEVICE ULTRASONIC WELDING METHOD WIRING DEVICE\ | JP2013063521A | LEE SEUNG WOOK (KR) | SEIDENSHA ELECTRONICS US | B23K20/10\ B29C65/08 |
| 1614 | 3D printing | INK REPLENISHING TOOL\ | JP2013086340A | KIM YONG KEON (KR) | PILOT CORP US | B43K11/00 |
| 1615 | 3D printing | OPERATION PANEL\ | JP2013086561A | ZENIN VIKTOR VASIL EVICH (RU) | YUSHIN SEIKI KOGYO KK US\ EMBLEM CO LTD US | B60H1/00\ H01H9/18 |
| 1616 | 3D printing | SEALED CONTAINER\ | JP2013086823A | RYU JAE HYUNG (KR) | TOSOH CORP US | C08K3/08\ C08K3/28\ C08K3/22\ C08K3/34\ C08K3/38\ B65D1/00\ B65D53/00\ C08L81/02 |
| 1617 | 3D printing | METHOD FOR MANUFACTURING INK CARTRIDGE FOR WRITING UTENSIL\ | JP2013095017A | KIM SUN DALL (KR) | PILOT CORP US | B43K5/14\ B43K11/00 |
| 1618 | 3D printing | RESIN-MADE CELL CULTURE VESSEL AND METHOD FOR MANUFACTURING THE SAME\ | JP2013099279A | Empty Field US | DAINIPPON PRINTING CO LTD (JP) | C12M3/00\ C12M1/00 |
| 1619 | 3D printing | METHOD FOR MANUFACTURING CELL CULTURE CONTAINER\ | JP2013099884A | Empty Field US | DAINIPPON PRINTING CO LTD (JP) | B29C45/14\ C12M3/00\ B29C65/08 |
| 1620 | 3D printing | VACUUM LAMINATION DEVICE AND METHOD OF FORMING INSULATION LAYER USING THE SAME\ | JP2013110143A | PARK HWAN KI (KR) | TOPPAN PRINTING CO LTD US | H05K3/46\ H05K3/00 |
| 1621 | 3D printing | CONNECTION METHOD OF WIRE\ | JP2013118196A | WOO IN YOUNG US | YAZAKI CORP (JP) | H01R43/02 |
| 1622 | 3D printing | ２重構造容器\ | JP5616202B2 | Empty Field US | Empty Field US | B65D81/38\ A47G19/22 |
| 1623 | 3D printing | TUBE WELD MOLDING AND TUBE END HOLDER\ | JP7052904A | KOGANEZAWA AKIRA US\ FUJITA MAMORU US | YOSHINO KOGYOSHO CO LTD (JP) | B29C65/00\ B29C65/08\ B65B7/14 |
| 1624 | 3D printing | SECONDARY BATTERY\ | JP7326336A | FUJIWARA NOBUHIRO US\ SUZUKI KOJI US | SONY CORP (JP) | H01M2/26\ H01M10/40 |
| 1625 | 3D printing | BATTERY EQUIPPED WITH EXPLOSION-PROOF SAFETY DEVICE AND ITS MANUFACTURE\ | JP8007866A | OGAWA TAKUSHI US\ HARADA YOSHIRO US\ KITAKATA MASAKAZU US\ YAMAMOTO KOHEI US | FUJI ELECTROCHEMICAL CO LTD US | H01M2/34\ H01M2/12 |
| 1626 | 3D printing | POSITION SIGNAL GENERATION SWITCH\ | JP8036946A | IDA TOKUHIRO US | MATSUSHITA ELECTRIC WORKS LTD (JP) | H01H11/00\ H01H21/18\ H01H19/58 |
| 1627 | 3D printing | SEALED TYPE BATTERY\ | JP8045488A | FUKUNAGA TAKAO US | JAPAN STORAGE BATTERY CO LTD (JP) | H01M2/04\ H01M2/36 |
| 1628 | 3D printing | EQUIPMENT AND METHOD FOR UNITING ELECTRIC CONDUCTION AND HEAT CONDUCTION\ | JP8046099A | RORUFU MAIAA US\ GERUHARUTO PUFURIYUUGAA US\ ARUNO ARUTOPEETAA US\ IERUKU SHIYUTORERAA US\ RUUTOBUITSUHI BORUTSU US\ MANFUREETO RESURAA US\ RIHIYARUTO SHIYUPITSUTSU US\ HERUBERUTO GEEBERU US\ BUESUNA BIARASU US\ HENINGU SHIYUTEIRUKE US\ GODEHARUTO SHIYUMITSUTSU US\ RAINAA BURATSUHIERUTO US\ TOOMASU RIHIYARUTO US\ JIIKUFURIITO SHIYUURAA US\ HORUGAA HAUSUMAN US | BOSCH GMBH ROBERT (DE) | H01L21/48\ H01L21/607\ H01L23/40\ H02K11/04 |
| 1629 | 3D printing | NET FOR ULTRASONIC WELDING AND ULTRASONIC WELDING METHOD\ | JP8150669A | TASAKA TAKAYUKI US\ MAKIHATA KAZUMASA US | SHIN KOBE ELECTRIC MACHINERY US | B29L9/00\ B29C65/40\ B29D28/00\ B29C65/08\ B29C65/50 |
| 1630 | 3D printing | INSULATING PAPER AT JOINT OF LEAD WIRE OF MOTOR AND INSULATING METHOD\ | JP9084288A | YAMANOI MASAAKI US\ MURATA EIICHI US\ KURAMOTO EIJI US | SANYO ELECTRIC CO (JP) | H02K3/38\ H02K3/34\ H02K3/50\ H02K5/22 |
| 1631 | 3D printing | BOARD DEVICE AND MANUFACTURE THEREOF\ | JP9130066A | SAITO MASAYASU US\ SHIMIZU YOSHIMASA US\ IIDA TSUKASA US\ IDA MASAHIRO US\ MURAMATSU YUJI US | AISIN SEIKI (JP) | B23K20/10\ H05K5/00\ H01L21/60\ H01R12/04\ H05K7/14 |
| 1632 | 3D printing | METHOD AND APPARATUS FOR ULTRASONIC WELDING\ | JP9234795A | NAGASHIMA FUMIHIDE US | TOSHIBA CORP US | B29L23/00\ B29C65/08\ B29C65/78 |
| 1633 | 3D printing | BAG-MAKING DEVICE\ | JP9323708A | HOUSHIYOU AKIRA US\ SUNADA MASAHIRO US\ SHIKADA MOTOO US\ MATSUI HIROYUKI US | FAB TOYAMA KK US\ FUJIMORI KOGYO CO US | B29C65/00\ B29C65/02\ B29C65/08\ B65B51/10\ B29C53/48\ B65B9/06\ B65B9/073 |
| 1634 | 3D printing | BATTERY WITH LEAD PLATE\ | JPH09330696A | TAMAOKI AKIYOSHI US\ MORISHITA TAKUMA US | SANYO ELECTRIC CO (JP) | H01M2/30 |
| 1635 | 3D printing | INTAKE MANIFOLD FOR INTERNAL COMBUSTION ENGINE\ | JPH10122070A | FUJIMORI MAKOTO US\ AKASAKA YASUYUKI US | AISAN IND US | F02M35/104 |
| 1636 | 3D printing | ULTRASONIC WELDING METHOD AND ITS APPARATUS\ | JPH10202751A | SUEOKA YOSHIHISA US\ KAWASAKI HIROSHI US\ KITANO EIICHI US | DAIKIN IND LTD (JP) | B29C65/00\ B29C65/02\ B29C65/08\ B29L31/08 |
| 1637 | 3D printing | CASE FOR PACKAGE AND ULTRASONIC WELDING METHOD THEREOF\ | JPH10236438A | TANABE MASAO US\ OKANO RENJIROU US\ WATANABE GOTARO US | SONY CORP (JP) | B29L22/00\ B29C65/08\ B65B51/10 |
| 1638 | 3D printing | AIR DIRECTION VARYING DEVICE FOR AIR-CONDITIONER AND MANUFACTURE THEREOF\ | JPH11108433A | SUDA SHOICHI US\ TAMURA YOSHIHISA US\ MAKINO MASAZUMI US\ HIGANO KAZUHIRO US\ OKAWA KAZUNOBU US | SANYO ELECTRIC CO (JP) | F24F1/00\ F24F13/14\ F24F13/20 |
| 1639 | 3D printing | CONNECTING METHOD FOR BATTERY ELECTRODE\ | JPH11233096A | YOSHIHARA YASUO US\ FUKUIRI TAKASHI US\ OGAWA MASAHIKO US\ EDA NOBUO US | MATSUSHITA ELECTRIC IND CO LTD (JP) | H01M2/26\ H01M2/22 |
| 1640 | 3D printing | FITTING DEVICE FOR SPOUT PLUG\ | JPH11235766A | NOMURA TOSHIAKI US\ MIYOSHI MASAKI US | TOPPAN PRINTING CO LTD US | B29C65/08\ B29C65/78\ B31B1/84 |
| 1641 | 3D printing | ELECTRICAL EQUIPMENT CASE\ | JPH11284359A | KAJIYA SHIYUUJIRO US\ UEHARA YUJI US | SANYO ELECTRIC CO (JP) | B29C65/00\ H05K5/02\ B29C65/08\ H01M2/10 |
| 1642 | 3D printing | ULTRASONIC WELDING APPARATUS\ | JPH11348131A | TANAKA YASUHIRO (JP) | MURATA MANUFACTURING CO (JP) | B29C65/00\ B29C65/08 |
| 1643 | 3D printing | ULTRASONIC BONDING MACHINE FOR BATTERY CASE\ | KR100508411B1 | Empty Field US | Empty Field US | B29C65/08 |
| 1644 | 3D printing | METHOD TO MANUFACTURE FLEXIBLE TUBE SHEET FOR ULTRASONIC WELDING\ | KR100575366B1 | Empty Field US | Empty Field US | B32B27/08 |
| 1645 | 3D printing | FINISHING MATERIAL AND METHOD FOR CONSTRUCTION WORK TILE USING FINISHING MATERIAL THEREOF\ | KR100679243B1 | Empty Field US | Empty Field US | E04F13/08 |
| 1646 | 3D printing | CONCURRENT AUTOMATIC ADHESION METHOD FOR ASSORTED KINDS OF JEWELS\ | KR100689113B1 | LIM TAE SIK (KR) | LIM TAE SIK (KR) | A44C27/00 |
| 1647 | 3D printing | ULTRASONIC WELDING APPARATUS\ | KR100697101B1 | Empty Field US | GUANGWEI LEI US | B29C65/08\ B29C65/04 |
| 1648 | 3D printing | THE CLICK UNIT OF ULTRASONIC WELDING MACHINE\ | KR100715714B1 | AHN YONG WOO (KR) | AHN YONG WOO (KR) | B29C65/08 |
| 1649 | 3D printing | HORN FOR ULTRASONIC WELDING WITH PLURAL TIP\ | KR100724065B1 | KIM SANG KON (KR) | MECHS CO LTD G (KR) | B23K20/10\ B23K1/06 |
| 1650 | 3D printing | EXTENDABLE CARD PACKAGE OF PERSONAL COMPUTER ETC. AND METHOD OF FABRICATION OF THAT\ | KR100790403B1 | LEE JONG WOO (KR)\ PARK BEOM JOON (KR)\ OH SE UK (KR)\ LEE CHEON YEOL (KR)\ SEO YEOL GU (KR)\ HAN JI SOUCK (KR) | WOOJEON TECH CO LTD (KR) | G06F1/16\ G06F1/00 |
| 1651 | 3D printing | HORN OF THE ULTRASONIC WAVE WELDER MAKING METHOD\ | KR100804255B1 | SEO OK SOON (KR) | SEO OK SOON (KR) | B23P15/00\ B23P17/02 |
| 1652 | 3D printing | METAL SEPARATOR ASSEMBLY FOR FUEL CELL THAT SEALED AND ASSEMBLED BY ULTRASONIC-WELDING\ | KR100817858B1 | JEONG YEON SOO (KR)\ JEON YOO TAEK (KR) | HYUNDAI HYSCO (KR) | H01M8/02 |
| 1653 | 3D printing | THE DRUM WASHING MACHINE DOOR MANUFACTURE METHOD\ | KR100836030B1 | Empty Field US | Empty Field US | B29C67/24\ B29C45/00\ D06F37/28 |
| 1654 | 3D printing | RADIO FREQUENCY IDENTIFICATION TAG AND MANUFACTURING METHOD THEREOF\ | KR100848173B1 | Empty Field US | Empty Field US | G06K19/07\ G06K19/077 |
| 1655 | 3D printing | No Title Available\ | KR100875108B1 | Empty Field US | Empty Field US | H01M2/22 |
| 1656 | 3D printing | APPARATUS FOR INSERTING INSULATING FILM INTO STATOR CORE\ | KR100900763B1 | CHOI WON HO (KR) | TAE HWA ENTPR CO LTD (KR) | H02K15/10\ H02K1/04\ H02K3/32 |
| 1657 | 3D printing | PACKING METHOD FOR FOOD CONTAINER ADOPTTING THE ULTRSONIC-WAVE WELDING\ | KR100918823B1 | O PIL JAE (KR)\ PARK YONG UK (KR) | O PIL JAE (KR)\ PARK YONG UK (KR) | B65B51/22\ B65B51/02 |
| 1658 | 3D printing | PROCESS FOR PREPARATION OF SECONDARY BATTERY OF IMPROVED SAFETY\ | KR100919389B1 | Empty Field US | Empty Field US | H01M2/02\ H01M2/34 |
| 1659 | 3D printing | PLASTIC ADAPTER USING ULTRA SONIC WELDING\ | KR100939851B1 | Empty Field US | Empty Field US | F16L47/02\ F16L47/16 |
| 1660 | 3D printing | ULTRASONIC WAVES METAL WELDER HORN\ | KR100939975B1 | SEO JUNG SEOK (KR) | SEO JUNG SEOK (KR) | B23K20/10 |
| 1661 | 3D printing | ULTRASONIC WELDING MACHINE FOR MANUFACTURING FILM OF TONER FEEDER AND PRODUCING METHOD USING THE SAME\ | KR100942282B1 | Empty Field US | Empty Field US | G03G15/06\ G03G21/00 |
| 1662 | 3D printing | ULTRASONIC WELDING MACHINE FOR MANUFACTURING FILM OF TONER FEEDER\ | KR100942283B1 | Empty Field US | Empty Field US | G03G15/06\ G03G21/00 |
| 1663 | 3D printing | METHOD FOR WELDING SOCKET PIN AND STRANDING WIRES\ | KR100950156B1 | Empty Field US | Empty Field US | H01R4/02 |
| 1664 | 3D printing | MANUFACTURING METHOD OF GLASS COMPONENTS AND GLASS COMPONENTS\ | KR100950609B1 | Empty Field US | Empty Field US | G02C5/00\ G02C13/00\ B29D12/02 |
| 1665 | 3D printing | ELECTRICAL CONNECTION METHOD OF CONDUCTING MEMBERS\ | KR101029020B1 | Empty Field US | Empty Field US | H01R4/02 |
| 1666 | 3D printing | NON OVERLAPPING SEWING METHOD AND THE CLOTH OF THE SAME\ | KR101033396B1 | Empty Field US | Empty Field US | D05B35/02\ D05B35/00 |
| 1667 | 3D printing | No Title Available\ | KR101048690B1 | Empty Field US | Empty Field US | H01M2/02\ H01M10/38 |
| 1668 | 3D printing | SONOTRODE AND MANUFACTURING METHOD THEREOF AND ULTRASONIC WAVE WELDING METHOD USING THE SAME\ | KR101088472B1 | Empty Field US | Empty Field US | C21D1/00\ B23K35/00\ C21D9/00 |
| 1669 | 3D printing | A THERMAL TRANSFER PROCESS METHOD OF PATTERN FILM\ | KR101162349B1 | KIM GUN CHUL (KR) | DAEWOO METAL INDUSTRY CO LTD (KR) | B41M5/26\ B41M1/28 |
| 1670 | 3D printing | LITHIUM ION CAPACITOR AND METHOD FOR BONDING TERMINAL THEREOF\ | KR101173078B1 | LEE CHANG HYUP (KR)\ HAN SANG JIN (KR)\ WOO DAE JOONG (KR)\ LEE DONG YEOUL (KR) | VINA TECHNOLOY CO LTD (KR) | B23K20/10\ H01G9/016 |
| 1671 | 3D printing | THERMAL FUSE\ | KR101197036B1 | Empty Field US | GIM YEONG SU (KR) | H01H37/76\ H01M2/10\ H01H85/02 |
| 1672 | 3D printing | A PACKING MACHINE HAVE ULTRASONIC JOINING DEVICE\ | KR101253662B1 | Empty Field US | Empty Field US | B65B9/08\ B65B51/16\ B65B61/08 |
| 1673 | 3D printing | HIERARCHICAL MODULATION AND DEMODULATION SYSTEM TO ASSURE ENVIRONMENT AND ADAPTIVE RECEIVE OF TERRESTRIAL 3D HDTV BASED SFN\ | KR101279948B1 | Empty Field US | Empty Field US | H04N13/04\ H04L27/00\ H04N5/44 |
| 1674 | 3D printing | ULTRASONIC WAVE WELDING APPARATUS FOR WELDING METAL SEPARATOR OF FUEL CELL\ | KR101294039B1 | Empty Field US | Empty Field US | B23K20/10\ H01M8/02 |
| 1675 | 3D printing | METHODE AND DEVICE FOR INSPECTION OF INTENSITY OF ULTRASONIC WELDING\ | KR101305255B1 | Empty Field US | Empty Field US | B23K31/12\ G01B11/28\ G01N21/88 |
| 1676 | 3D printing | METHOD FOR FORMING A STOP-END FOR A SLIDER IN A ZIPPER BY MEANS OF ULTRA SONIC WELDING\ | KR101327993B1 | Empty Field US | Empty Field US | A44B19/36\ A44B19/60 |
| 1677 | 3D printing | WATERPROOF TEXTILE TRANSPORT SYSTEM OF ULTRASONIC WELDING MACHINE HAVING PRESSURE AND ANGLE ADJUSTING STRUCTURE\ | KR101331335B1 | Empty Field US | Empty Field US | D06H5/00 |
| 1678 | 3D printing | LUMINESCENT ROAD SIGN\ | KR101338101B1 | Empty Field US | Empty Field US | G09F13/22\ E01F9/00 |
| 1679 | 3D printing | ULTRASONIC WAVES WELDING APPARATUS\ | KR101354142B1 | Empty Field US | Empty Field US | B23K20/10 |
| 1680 | 3D printing | ULTRASONIC WAVES WELDING APPARATUS\ | KR101433196B1 | Empty Field US | Empty Field US | B23K20/10\ H01M2/30\ H01M10/04\ H01M2/26 |
| 1681 | 3D printing | APPARATUS AND PROCESS FOR INSERTING INSULATING FILM INTO STATOR CORE\ | KR101455873B1 | Empty Field US | Empty Field US | H02K15/02\ H02K15/10\ H02K1/04 |
| 1682 | 3D printing | MANUFACTURING METHOD OF SUPERCAPACITOR ELECTRODE\ | KR101484926B1 | Empty Field US | Empty Field US | H01G11/22\ H01G9/042 |
| 1683 | 3D printing | ULTRASONIC WELDING MACHINE\ | KR200376565Y1 | Empty Field US | GILLETTE CO US | B29C65/08 |
| 1684 | 3D printing | ULTRASONIC BONDING DEVICE FOR COVER PAPER FOR CARD\ | KR20040003104A | KIL YONG HO US\ KIM JANG GON US\ LEE GANG HYEON US\ LEE JONG SEOK US\ MUN BYEONG GI US\ SONG YEONG HO US\ YOON JAE HWAN US | KDNSMARTEC CO LTD US\ S J ENGINEERING CO LTD US | B29C65/08 |
| 1685 | 3D printing | METHOD FOR MANUFACTURING HEAT SINK BY BONDING FINS THROUGH ULTRASONIC WELDING\ | KR20040026943A | JUNG GU BONG US\ LEE JONG HYEON US\ LEE JONG MAHN US\ PARK SE JEONG US | LEE JONG MAHN US | B23K20/10 |
| 1686 | 3D printing | No Title Available\ | KR200454915Y1 | Empty Field US | DAVEY BICKFORD (FR)\ DAVEY BICKFORD ROUEN (FR) | H01L23/12\ G06K19/07\ G11C7/00 |
| 1687 | 3D printing | STRUCTURE FOR ASSEMBLING OPERATION STATE DISPLAY WINDOW TO COVER PANEL OF INDOOR UNIT FOR AIR CONDITIONER\ | KR20050007067A | KANG JIN IL US | LG ELECTRONICS INC (KR) | F24F1/00 |
| 1688 | 3D printing | ROLLER FOR INLINE ROLLER SKATE CAPABLE OF PREVENTING A PAIR OF INJECTION MOLDS VERTICALLY SEPARATED FROM FRAME FROM BEING TWISTED BY INCORPORATING INTO ONE BODY USING ULTRASONIC WELDING\ | KR20050015667A | KWON MAN HYUN US | KWON MAN HYUN US | A63C17/22 |
| 1689 | 3D printing | SECONDARY BATTERY WITH COPPER NEGATIVE TAP AND METHOD FOR ATTACHMENT OF COPPER NEGATIVE TAP TO NEGATIVE PLATE USING ULTRASONIC WAVE\ | KR20050018268A | UM JAE CHUL US | SAMSUNG SDI CO LTD (KR) | H01M2/30 |
| 1690 | 3D printing | METHOD TO BUILD THIN METAL FILM WITHOUT ADHESIVE BY USING ION SPUTTERING AND ULTRASONIC WELDING\ | KR20050029656A | YOU JEONG WON (KR) | YOU JEONG WON (KR) | C23C14/22 |
| 1691 | 3D printing | DEVICE TO ATTACH BACK SHEET TO RETROREFLECTIVE SHEET\ | KR20060116084A | KIM IK JUNG (KR) | SUE HEUNG REF CO LTD (KR)\ KIM IK JUNG (KR) | B32B37/00\ B32B27/08\ B32B7/02 |
| 1692 | 3D printing | MANUFACTURE METHOD OF FLAT PLATE HEAT PIPE USING ULTRASOUND WELDING\ | KR20060135370A | DING PEI PEI (TW)\ YANG HSIU WEI (TW)\ LIN JAO CHING (TW)\ YU WEN HWA (TW)\ CHEN YEIN WEN (TW) | ASIA VITAL COMPONENTS CO LTD (TW) | B23K20/10 |
| 1693 | 3D printing | MANUFACTURING METHOD OF PLANAR HEAT PIPE BY USING ULTRASONIC WELDING\ | KR20080010020A | DING PEI PEI (TW)\ YANG HSIU WEI (TW)\ YU WEN HWA (TW)\ CHENG TSAO HSIANG (TW) | ASIA VITAL COMPONENTS CO LTD (TW) | B23K20/10 |
| 1694 | 3D printing | SPACER TAPE FOR SEMICONDUCTOR MOUNTED CIRCUIT TAPES AND METHOD FOR MANUFACTURING THE SAME\ | KR20080037520A | GOTO KEIICHI (JP)\ UCHIYAMA MASAO (JP) | KAWAMURA SANGYO CO LTD (JP) | H01L21/60 |
| 1695 | 3D printing | DEPOSITION DATA DISPLAY DEVICE IN DEPOSITION APPARATUS AND COMPUTER PROGRAM\ | KR20080047133A | HAMAGUCHI TADAYOSHI (JP)\ KUDO HAJIME (JP)\ SATO HIROICHI (JP)\ ARAI MASAYOSHI (JP) | ITOH HITOHIKO (JP) | G06F17/50\ G06Q50/04 |
| 1696 | 3D printing | PLATE OF SOLAR COLLECTOR STRUCTURE FOR VACUUM-TUBE TYPE CONCENTRATING SOLAR COLLECTOR\ | KR20090002404A | SONG KYU SOP (KR)\ KIM KUANG SOO (KR) | APACK INC (KR) | F24J2/24\ F24J2/32 |
| 1697 | 3D printing | A SEAM JOINING TOGETHER AT LEAST TWO WEB MATERIALS\ | KR20090028702A | BACK LUCAS (SE) | SCA HYGIENE PROD AB (SE) | B29C65/02\ B29C65/08 |
| 1698 | 3D printing | CAN SYSTEM OF VEHICLES\ | KR20090067703A | PARK YOUNG JOO (KR) | RENAULT SAMSUNG MOTORS CO LTD (KR) | B60R16/02\ H04L12/40 |
| 1699 | 3D printing | SLIDE MODULE FOR MOBILE DEVICE\ | KR20100008660A | LEE SOO JIN (KR) | LEE SOO JIN (KR) | H04B1/38 |
| 1700 | 3D printing | WEIGHTING ASSEMBLY FOR WINDOW BLIND\ | KR20100009834A | KIM SUNG JIN (KR) | LEAD SENSE CO LTD (KR) | E06B9/42\ E06B9/40 |
| 1701 | 3D printing | ULTRASONIC WELDING METHOD\ | KR20100071510A | KIM SOOK HWAN (KR)\ LEE MOK YOUNG (KR) | RES INST IND SCIENCE & TECH (KR)\ POSCO (KR) | B23K20/10\ B23K20/22\ B23K20/227 |
| 1702 | 3D printing | MULTI CHANNEL ULTRASONIC WELDING INSPECTION SYSTEM AND CONTROL METHOD\ | KR20100076636A | KWON OH KUN (KR) | KWON OH KUN (KR) | G01N29/04\ G01N29/26 |
| 1703 | 3D printing | A THERMAL FUSE AND THE MANUFACTURING METHOD THEREOF\ | KR20100084367A | PARK CHUN HWAN (KR)\ KIM YOUNG SUN (KR) | PARK CHUN HWAN (KR)\ KIM YOUNG SUN (KR) | H01H85/22\ H01H85/00 |
| 1704 | 3D printing | A ULTRASONIC WELDER\ | KR20110089621A | HONG SUNG CHEOL (KR) | HONG SUNG CHEOL (KR) | B29C65/08 |
| 1705 | 3D printing | AIR POCKET HOT WATER FLOORING PANEL\ | KR20120023167A | WOO KYUNG SIK (KR) | WOO KYUNG SIK (KR) | E04B1/78\ E04F15/00\ F24D3/12 |
| 1706 | 3D printing | ULTRASONIC WELDING DEVICE\ | KR20120038270A | KIM DO HUNG (KR) | KIM DO HUNG (KR) | B23K20/10\ B29C65/08 |
| 1707 | 3D printing | SECONDARY BATTERY INCLUDING FUSE-INSERTED ELECTRODE LEAD\ | KR20120048404A | PARK SEOK JUNG (KR)\ KIM JONG HEE (KR)\ LIM HYUNG KYU (KR)\ KIM IN JUNG (KR)\ YANG IN SEOK (KR) | LG CHEMICAL LTD (KR) | H01M2/02\ H01M2/30 |
| 1708 | 3D printing | BATTERY HAVING CELL TAB CONNECTING STRUCTURE USING ULTRASONIC WELDING\ | KR20120080391A | LEE WON JUN (KR) | SK INNOVATION CO LTD (KR) | H01M2/26\ H01M2/20 |
| 1709 | 3D printing | ULTRASONIC WAVES WELDING APPARATUS\ | KR20120133021A | Empty Field US | Empty Field US | B23K20/10\ H01M2/30\ H01M2/26 |
| 1710 | 3D printing | METHOD OF LIVE INSPECTING WELDING STATE ON ULTRASONIC WELDING\ | KR20130036104A | HWANG DONG SOO (KR) | MONITECH CO LTD (KR) | B23K20/10\ B23K31/12 |
| 1711 | 3D printing | SECONDARY BATTERY FOR PREVENTING DISCONNECTION BETWEEN LEAD AND TAB\ | KR20130050654A | KIM SOO YOUNG (KR)\ SHIN YOUNG JOON (KR)\ PARK HYO SEOK (KR) | LG CHEMICAL LTD (KR) | H01M2/30\ H01M2/10\ H01M2/26 |
| 1712 | 3D printing | BATTERY CELL INCLUDING FRAME AND MANUFACTURING METHOD OF THE SAME\ | KR20130051035A | KIM SANG BUM (KR)\ KIM KWAN YONG (KR)\ JI YOUNG SU (KR)\ SHIN BYUNG HEON (KR)\ BAE DONG GWAN (KR) | SK INNOVATION CO LTD (KR) | H01M2/02\ H01M2/10 |
| 1713 | 3D printing | WATERPROOF CONNECTOR\ | KR20130066453A | PARK JONG WOOK (KR)\ JOO HONG SUCK (KR) | YURA CORP CO LTD (KR) | H01R13/52 |
| 1714 | 3D printing | WELDING INSPECTION SYSTEM OF CELL MODULE ASSEMBLY FOR ELECTRIC VEHICLE AND THE INSPECTION METHOD\ | KR20130068981A | KIM TAE KUN (KR) | V ENS (KR) | B23K20/10\ B23K37/00\ G01H1/00 |
| 1715 | 3D printing | STORAGE BOX OF PARTICLE FOAM PLASTIC MATERIAL\ | KR20130104119A | KIM YOUNG TAEK (KR)\ LEE KYUNG CHUL (KR) | GPS KOREA CO LTD (KR) | B65D81/38\ B65D43/16\ B65D6/18 |
| 1716 | 3D printing | JIG ASSEMBLY FOR ULTRASONIC WELDING MACHINE AND ULTRASONIC WELDING METHOD USING THE SAME\ | KR20130104292A | LEE TAE JAE (KR)\ LEE SEOK JAE (KR)\ PARK TAE JUNG (KR)\ LEE KYOUNG G (KR)\ KIM BYEONG IL (KR) | KOREA ADVANCED INST SCI & TECH (KR) | B23K20/10\ B29C65/08\ B23K20/26 |
| 1717 | 3D printing | METHOD FOR POWER CONTROL IN THE PROCESS OF ULTRASONIC WELDING\ | PL214478B1 | BRYLSKI MACIEJ (PL) | INST TELE I RADIOTECH (PL) | B29C65/08\ B06B1/02 |
| 1718 | 3D printing | METHOD FOR CONTINUOUS ULTRASONIC WELDING AND SONOTRODE FOR CONTINUOUS ULTRASONIC WELDING\ | PL214616B1 | CHMIELEWSKI EDWARD (PL)\ MLYNARSKI BOHDAN (PL)\ KRZEMINSKI LUKASZ (PL) | INST TELE I RADIOTECH (PL) | B29C65/08 |
| 1719 | 3D printing | METHOD FOR CONTROLLING ULTRASONIC WELDING PROCESS\ | PL215708B1 | BRYLSKI MACIEJ (PL)\ BARMUTA PAWEL (PL) | INST TELE I RADIOTECH (PL) | B23K20/10\ B29C65/08 |
| 1720 | 3D printing | METHOD FOR THE MANUFACTURE OF CELLULAR GEO MESH AS WELL AS EQUIPMENT FOR THE MANUFACTURE OF GEO MESH\ | PL380523A1 | KRUK ZBIGNIEW (PL)\ KRUK-CAPIK DOBROCHNA (PL)\ BENECKI PIOTR (PL) | GEO GLOBE POLSKA SPO & LSTROK (PL) | B29C65/08\ E02D3/00 |
| 1721 | 3D printing | ULTRASONIC WELDING AND RIVETING PROCESS\ | RO122957B1 | PAUSAN DORU VIRGIL (RO) | PAUSAN DORU VIRGIL (RO) | B23K20/10\ B23K1/06 |
| 1722 | 3D printing | No Title Available\ | RU2002122774A | Empty Field US | ELENTEC CO LTD US | B23K20/10\ B29C65/14 |
| 1723 | 3D printing | No Title Available\ | RU2004100792A | Empty Field US | DELPHI TECH INC (US) | B23K9/235 |
| 1724 | 3D printing | No Title Available\ | RU2004111308A | Empty Field US | DELONG INFORMATION TECHNOLOGY SUZHOU CO LTD US | B23K20/10\ B23K103/18 |
| 1725 | 3D printing | METHOD OF ULTRASONIC WELDING OF THERMOPLASTICS\ | RU2229382C1 | LJASHKO F E (RU)\ SOKOLOVA O F (RU) | MILLIKEN & CO (US) | B29C65/08 |
| 1726 | 3D printing | METHOD FOR PRODUCING CONTACT PAD OF THIN-FILM INTEGRATED CIRCUIT\ | RU2231237C2 | LUGIN A N (RU)\ VLASOV G S (RU)\ LUGINA V V (RU) | OPTOFORM SARL PROCEDES DE PROT (FR) | H05K1/02\ H01C17/24 |
| 1727 | 3D printing | METHOD AND DEVICE FOR APPLYING VIBRATING RAREFACTION MASSAGE DOUCHE\ | RU2231352C1 | STEPANOV V N (RU) | SPAICHINGEN GMBH MASCHF (DE) | A61H9/00 |
| 1728 | 3D printing | METHOD FOR ENCAPSULATING ELECTRONIC DEVICE CASE\ | RU2233568C1 | BEREZINA I E (RU)\ KONDRATENKOV S V (RU)\ LEGKIJ N M (RU)\ KUMANAEV V V (RU) | AUTONICS CORP (KR) | H05K5/06 |
| 1729 | 3D printing | METHOD OF ULTRASONIC WELDING OF PLASTICS\ | RU2240917C2 | LJASHKO F E (RU)\ SOKOLOVA O F (RU) | MARS INC (US)\ MASTERFOODS (FR) | B29C65/08 |
| 1730 | 3D printing | APPARATUS FOR MAKING CORD WITH FILLER\ | RU2245237C1 | KAZAKOV JU V (RU)\ PARSHIN S G (RU)\ GORDEEV K P (RU)\ MIKHEEV M E (RU) | LEE BO IL (KR) | B23K35/40 |
| 1731 | 3D printing | ULTRASONIC WELDING PRESS\ | RU2261177C1 | NOVIK A A (RU) | RFCAMP LTD (KR) | B29C65/08 |
| 1732 | 3D printing | METAL-POLYMER TUBE ULTRASONIC SEAM WELDING METHOD\ | RU2262426C1 | BUKHAREV E JU (RU)\ MIRZOJAN A M (RU)\ RYZHOV N N (RU) | CRYDOM TECHNOLOGIES US | B23K20/10\ B21C37/08 |
| 1733 | 3D printing | METAL-POLYMER TUBE ULTRASONIC SEAM WELDING METHOD\ | RU2262427C1 | BUKHAREV E JU (RU)\ MIRZOJAN A M (RU)\ RYZHOV N N (RU) | COLLINS DAVID C (US)\ HEWLETT PACKARD DEVELOPMENT CO (US) | B23K20/10\ B21C37/08 |
| 1734 | 3D printing | MODE OF DETECTION AND ANALYSIS OF RADIO SIGNALS\ | RU2296349C9 | DROGALIN VALERIJ VASIL EVICH (RU)\ MERKULOV VLADIMIR IVANOVICH (RU)\ FRANTSEV VLADIMIR VASIL EVICH (RU)\ SURKOV IGOR KONSTANTINOVICH (RU)\ CHELEJ GALINA SERGEEVNA (RU) | KORPORATSIJA FAZOTRON NII RADI (RU) | G01S13/72 |
| 1735 | 3D printing | APPARATUS FOR ULTRASONIC WELDING\ | RU2301731C1 | NOVIK ALEKSANDR ALEKSEEVICH (RU) | OOO UL TRAZVUKOVAJA TEKHN INLA (RU) | B23K20/10 |
| 1736 | 3D printing | CELLULAR GROUND SURFACE CONSOLIDATION GRID PRODUCTION METHOD\ | RU2322551C1 | KHMELEV VLADIMIR NIKOLAEVICH (RU)\ TSYGANOK SERGEJ NIKOLAEVICH (RU)\ SLIVIN ALEKSEJ NIKOLAEVICH (RU)\ BARSUKOV ROMAN VLADISLAVOVICH (RU)\ LEVIN SERGEJ VIKTOROVICH (RU)\ ABRAMOV ALEKSEJ DMITRIEVICH (RU) | G OBRAZOVATEL NOE UCHREZHDENIE (RU) | B29C65/02\ E02D17/20\ B06B1/06\ B06B3/02\ B29C65/18 |
| 1737 | 3D printing | METHOD OF PACKING OF ITEMS AND PACKING FOR ITEM\ | RU2329928C1 | SVETLOV SERGEJ ALEKSEEVICH (RU)\ GOLOMIDOV MIKHAIL ALEKSANDROVICH (RU) | G OBRAZOVATEL NOE UCHREZHDENIE (RU) | B32B27/30\ B32B15/08\ B65B9/02\ B65D75/28\ A61M15/00 |
| 1738 | 3D printing | METOD OCH ANORDNING VID F?RSEGLING AV F?RPACKNINGS?MNE\[EN] ULTRASONIC WELDING METHOD FOR SEALING PACKAGING CONTAINING E.G. FOODSTUFFS INVOLVES PRESSING PACKAGING MATERIAL BETWEEN ULTRASONIC TOOLS INTRODUCED INTO PASSAGE DEFINED BY PAIR OF MOVABLE CLAMPS\ | SE529661C2 | NILSSON GOERAN US\ NEDSTEDT ULF US | TETRA LAVAL HOLDINGS & FINANCE (CH) | B29C65/08\ B29C65/78\ B65B51/22\ B29C57/10\ B65B51/30 |
| 1739 | 3D printing | FLOW PATTERN SWITCHING STRUCTURE FOR HORTICULTURAL SPRAY NOZZLE\ | TW200403107A | WANG JIN-ZHONG (TW) | WANG JIN-ZHONG (TW) | B05B1/02 |
| 1740 | 3D printing | METHOD OF AND APPARATUS FOR TESTING A WIRE BOND CONNECTION\ | TW200540407A | FRANK WALTHER (DE) | HESSE & KNIPPS GMBH (DE) | Empty Field |
| 1741 | 3D printing | THIN-CHIP ELECTROLYTIC CAPACITOR STRUCTURE\ | TW200540894A | LIN JIE-FU (TW) | LIN JIE-FU (TW) | H01G9/15 |
| 1742 | 3D printing | ELECTRICAL CONNECTOR AND COMBINATION METHOD THEREOF\ | TW200917597A | WANG CHIEN-CHUN (TW)\ WANG WEN-LUN (TW) | P TWO IND INC (TW) | H01R43/26 |
| 1743 | 3D printing | LIGHT MODULE STRUCTURE AND LIGHT TUBE FRAME THEREOF\ | TW200928191A | CHEN TANG-BO (TW)\ LI CHI-YUNG (TW)\ SHIAU DENG-KUEN (TW)\ HUANG SHUN-CHIEH (TW) | AU OPTRONICS CORP (TW) | G02F1/1333\ F21V19/00 |
| 1744 | 3D printing | ULTRASONIC WELDING MACHINE AND ULTRASONIC WELDING METHOD\ | TW200946323A | JIANG GUO-CHANG (TW) | JIANG GUO-CHANG (TW) | B29C65/08\ D05B17/00 |
| 1745 | 3D printing | COMBINED DEVICE OF ULTRASONIC WELDING AND SPIN WELDING\ | TW200948520A | XU KUN-MING (TW) | UNIV NAT FORMOSA (TW) | B23K20/10\ B23K20/12\ B29C65/08\ B29C65/06 |
| 1746 | 3D printing | METHOD TO JOIN THE LIGHT-EMITTING DIODE TO HEAT-DISSIPATION BASE MATERIAL\ | TW201010123A | ZHANG MIN-GU (TW) | CHI LONG MACHINERY CO LTD (TW) | H01L23/34\ B23K20/10\ H01L33/00 |
| 1747 | 3D printing | ULTRASONIC ROLL WELDING MACHINE\ | TW201043368A | HUANG MING-HONG (TW)\ DU SHUI-NIAN (TW)\ HUANG JUN-JIE (TW)\ ZHENG YU-CHENG (TW) | CONTREL TECHNOLOGY CO LTD (TW) | B23K20/10 |
| 1748 | 3D printing | HIGH AIR-TIGHTNESS FLIP-CHIP PACKAGE COMPONENTS AND ITS METHOD FOR FORMING THE SAME.\ | TW201123316A | FAN JIN-DA (TW)\ ZHOU YU-JIE (TW)\ WEI JIAN-CHENG (TW)\ JIANG DA-XIANG (TW) | TONG HSING ELECTRIC IND LTD (TW) | H01L21/56\ H01L23/28 |
| 1749 | 3D printing | METHOD FOR FIXING LENS SET\ | TW201208342A | YANG CHIA-PIN (TW)\ WU TSU-JEN (TW) | ASIA TECH IMAGE INC (TW) | H04N1/028 |
| 1750 | 3D printing | PREPARATION METHOD OF REFLECTIVE MARKERS AND STRUCTURE THEREOF\ | TW201321579A | LI HONG-ZHEN (TW) | TECHEYE OPTICS TECHNOLOGIES CO LTD (TW) | B29K105/08\ B29C45/00\ B29K55/02\ E01F9/04\ B29K105/30\ B29K709/08 |
| 1751 | 3D printing | BATTERY AND ULTRASONIC WELDING METHOD AND DEVICE FOR THE BATTERY\ | TW201322534A | LIN DIAN-YONG (TW)\ WU LUNG-TIEN (TW)\ HUANG SHIH-TSUNG (TW) | METAL IND RES & DEV CT (TW) | H01M2/20 |
| 1752 | 3D printing | TWINS LIGHT GUIDING PLATE AND MANUFACTURING METHOD THEREOF AND BACKLIGHT MODULE WITH TWINS LIGHT GUIDING PLATE\ | TW201416731A | CHEN CHUN-YUAN (TW)\ CHEN CHIA-HUNG (TW) | AU OPTRONICS CORP (TW) | G02F1/13357\ G02B6/00 |
| 1753 | 3D printing | BONDING STRUCTURE OF LIGHT-EMITTING SEMICONDUCTOR AND ITS METHOD\ | TWI239109B | PAN SHYI-MING (TW)\ CHIEN FEN-REN (TW) | FORMOSA EPITAXY INC (TW) | H01L33/00 |
| 1754 | 3D printing | TWO-STAGED DOUBLE LAYER ULTRASONIC WELDING APPARATUS USED FOR AN UPPER COVER AND A BOTTLE PART\ | TWI264406B | LIU YU-JU (TW) | LIU YU-JU (TW) | B65D8/22\ A45D33/00 |
| 1755 | 3D printing | PROCESS OF A FLAT THIN-PLATE HEAT PIPE THROUGH ULTRASONIC WELDING\ | TWI291540B | CHEN PEI-PEI (MY)\ YANG SHIOU-WEI (TW)\ LIN JAU-CHING (US)\ YU WEN-HUA (TW)\ CHEN YAN-WEN (TW) | ASIA VITAL COMPONENTS CO LTD (TW) | B23K1/06\ F28D15/02 |
| 1756 | 3D printing | METHOD OF FABRICATING SUBSTRATE WHERE PATTERNS ARE FORMED\ | TWI387003B | KWON SUNG-HOON (KR)\ YOON EUI-JOON (KR) | SNU R&DB FOUNDATION (KR) | H01L21/31 |
| 1757 | 3D printing | DIRECT DRIVE FEEDING STRUCTURE FOR ULTRASONIC WELDING MACHINE\ | TWM308811U | CHEN YING-SHENG (TW) | HURG CO LTD (TW) | B23K20/10 |
| 1758 | 3D printing | ULTRASONIC WELDING ROLLER\ | TWM314677U | LI CHING-FEN (TW) | KING ULTRASONIC CO LTD (TW) | B29C65/08 |
| 1759 | 3D printing | A DISK-TYPED ULTRASONIC WELDING HEAD WITH APERTURES\ | TWM333977U | ZHOU RONG-ZAI (TW) | STRONG ULTRASONIC MACHINERY CO LTD (TW) | B23K20/10 |
| 1760 | 3D printing | IMPROVED STRUCTURE OF AN ULTRASONIC WELDING MACHINE\ | TWM336154U | GUO JI-CHUN (TW) | GUO JI-CHUN (TW) | B29C65/08 |
| 1761 | 3D printing | IMPROVED STRUCTURE OF ULTRASONIC WELDING DEVICE\ | TWM409912U | LI QING-FEN (TW) | KING ULTRASONIC CO LTD (TW) | B23K20/10 |
| 1762 | 3D printing | TESTING MACHINE FOR ADAPTER PLUG\ | TWM419108U | CHEN JING-JIANG (CN) | WELL SHIN TECHNOLOGY CO LTD (TW)\ WELL SHIN ELECTRONIC KUNSHAN CO LTD (CN) | B23K20/10\ G01N3/00\ G01R19/00 |
| 1763 | 3D printing | PLASTIC PART WELDED CONSTRUCTION USING ULTRASONIC WELDING\ | TWM423011U | HUANG CHIEN-FA (TW) | CELESTICA INT INC (CA) | B23K20/10 |
| 1764 | 3D printing | GEO-GRID FOR SOIL SURFACE FIXATION\ | UA18496U | Empty Field US | FORD GLOBAL TECH LLC (US) | E02D17/20 |
| 1765 | 3D printing | RESPIRATOR\ | UA83565U | KOCHKIN ANDRII ARKADIIOVYCH (UA) | RESPPHARM LTD LIABILITY COMPANY (UA) | A62B7/00\ A62B7/10 |
| 1766 | 3D printing | PROCESS FOR SEALING A FILLED SACK MADE OF THERMOPLASTIC PLASTIC AND PROVIDED WITH GUSSETS\ | US2002011048A1 | KOEHN UWE (DE) | LG CHEMICAL LTD (KR) | B29C65/00\ B29C65/08\ B65B51/14 |
| 1767 | 3D printing | COMPOSITE BOX WRENCH WITH REINFORCING AND RETAINING STRUCTURE\ | US2002011136A1 | EGGERT DANIEL M (US)\ DEVECCHIS MARCO E (US)\ THOMPSON MICHAEL Q (US)\ SPRACKLIN STACY T (US) | SNAP ON TECH INC (US) | B25B13/04\ B25B13/46 |
| 1768 | 3D printing | METHOD OF CONNECTING ELECTRIC WIRES\ | US2002017550A1 | MURAKAMI KAZUHIRO (JP)\ WATANABE YOSHIHIKO (JP)\ HANAZAKI KENICHI (JP)\ MURAKOSHI HIROYUKI (JP) | YAZAKI CORP (JP) | B23K20/10\ H01R43/02\ B23K101/38\ H02G1/14 |
| 1769 | 3D printing | TEXTILE LAMINATE AND ARTICLES MADE USING THE LAMINATE\ | US2002056502A1 | BORDES YVES A (FR) | TEXTINOV LTD (US) | B29C65/00\ B32B37/12\ A41D31/02\ B29C65/08\ B29C65/74\ A41D27/24\ B32B5/04\ B32B37/20 |
| 1770 | 3D printing | APPARATUS FOR ULTRASONIC WELDING\ | US2002088525A1 | JAHN RAY (US)\ POPOOLA OLUDELE OLUSEGUN (US)\ REATHERFORD LARRY VAN (US)\ COOPER RONALD PAUL (US) | INTEL CORP (US) | B29C65/08\ B29C65/06 |
| 1771 | 3D printing | METHOD AND APPARATUS FOR ULTRASONIC PROCESSING OF WORKPIECES\ | US2002100534A1 | DISTEL ARMIN (DE) | DANA CORP (US) | B23K20/10\ B29C65/00\ B29C65/08\ B29C57/00\ B29C65/04\ B29C65/60 |
| 1772 | 3D printing | PRINTED-THERMOPLASTIC TAMPER-RESISTANT PACKAGE\ | US2002162764A1 | SHERLINE DREW (US)\ HACKER MARK G (US) | SIEMENS AG (DE) | B65D75/32 |
| 1773 | 3D printing | AUTOMATIC DETERMINATION AND SELECTION OF BUILD PARAMETERS FOR SOLID FREEFORM FABRICATION TECHNIQUES BASED ON AUTOMATIC PART FEATURE RECOGNITION\ | US2002188369A1 | MANNERS CHRIS R (US)\ GUERTIN MICHELLE D (US) | ORIENT SEMICONDUCTOR ELECT LTD (TW) | B29C67/00\ G05B19/4099 |
| 1774 | 3D printing | HEAD ASSEMBLY OF A DISK APPARATUS HAVING A HEAD IC CHIP MOUNTED ON A SUSPENSION BY ULTRASONIC BONDING\ | US2002195476A1 | BABA SHUNJI (JP)\ KIRA HIDEHIKO (JP)\ KAINUMA NORIO (JP)\ OKADA TORU (JP) | FUJITSU LTD (JP) | H01L21/60\ B23K1/00\ H01L21/607\ G11B21/00\ G11B5/48\ G11B33/12 |
| 1775 | 3D printing | DAMPED FLEXIBLE PROTECTIVE SLEEVING\ | US2003029627A1 | FATATO FRANCIS B (US) | HEWLETT PACKARD DEVELOPMENT CO (US) | B60R16/02\ F16L3/26\ F16L57/00\ H02G3/04 |
| 1776 | 3D printing | METHOD OF PRODUCING FLUID UNIT\ | US2003062092A1 | NAGAI SHIGEKAZU (JP)\ MATSUSHIMA HIROSHI (JP) | SMC KK (JP) | B29C65/08\ F15B11/00\ F04F5/44\ F15B13/00\ F15B13/08\ F16B11/00\ F16J15/00\ F16K27/00 |
| 1777 | 3D printing | SONOTRODE FOR ULTRASONIC WELDING APPARATUS\ | US2003066863A1 | JAHN RAY (US)\ POPOOLA OLUDELE OLUSEGUN (US)\ REATHERFORD LARRY VAN (US)\ SKOGSMO JAN BIRGER (US)\ KIRIDENA VIJITHA SENAKA (US) | JIANGSU FILTER MATERIAL CO LTD US | B23K20/10 |
| 1778 | 3D printing | PACKAGE WITH ZIPPER CLOSURE\ | US2003074860A1 | MIKOL MARK CHARLES (US)\ DUTRA HERBERTO (US) | KRAFT FOODS HOLDINGS INC (US) | B65B61/18\ B65B51/22\ B31B19/90\ B65B9/08 |
| 1779 | 3D printing | CONCEALED TYPE SLIDE FASTENER WITH SEPARABLE BOTTOM END STOP\ | US2003140465A1 | YUKI KENJI (JP)\ IGARASHI OSAMU (JP) | YKK CORP (JP) | B29C65/08\ A44B19/60\ A44B19/34\ A44B19/38 |
| 1780 | 3D printing | ULTRASONIC SEALING DEVICE RELATED METHOD AND PACKAGE\ | US2003168149A1 | BLANCHARD DANIEL (FR)\ FAUL PATRICK (FR) | BRIDGESTONE CORP (JP) | B29C65/00\ B29C65/08\ B29C65/74\ B65B51/10 |
| 1781 | 3D printing | PROCESS FOR BONDING SEVERAL LAYERS OF PLASTIC FILM\ | US2003173016A1 | SITZMANN STEFAN (DE) | HUHTAMAKI FORCHHEIM (DE) | B29L22/00\ B29C65/02\ B65D30/10\ B29C65/08\ B29C65/78\ B29C65/74\ B65B61/00\ B65D33/00\ B31B1/64 |
| 1782 | 3D printing | PASTE FILLED WITH METAL POWDER AND METAL PRODUCTS OBTAINED WITH SAME\ | US2003175621A1 | HINCZEWSKI CATHERINE (US) | HIGH TECHNO CELL CO LTD (KR) | B29C67/00\ G03F7/00\ B22F3/105\ B22F3/16\ G03F7/004\ C08F2/46\ B22F1/00\ B22F3/02\ B22F3/10\ B22F3/22\ B22F1/02\ H05K1/09 |
| 1783 | 3D printing | METHOD AND APPARATUS FOR ULTRASONICALLY STOMPING SLIDER END STOPS ON ZIPPER\ | US2003183315A1 | SHARE LAWRENCE (US)\ MCMAHON MICHAEL J (US)\ POLLACK MICHAEL A (US)\ PIOTROWSKI STANLEY (US)\ CREVIER DONALD L (US)\ KNIGHT NIGEL D (US) | SHANTOU INNOVATION TECHNOLOGY ELECTRONICS CO LTD US | B29C65/00\ B29C65/08\ B31B19/90\ B65D33/25\ B29C65/18 |
| 1784 | 3D printing | THERMOPLASTIC POLYMER FILLED PASTES\ | US2003190472A1 | MOUSSA KHALIL M (US) | 3D SYSTEMS INC (US) | C08F2/50\ C08J3/12\ C08L101/00\ B29K77/00\ C08L5/00\ B29K69/00\ B29B9/12\ C08G81/00\ B29B9/00\ B29K81/00\ C08F290/00\ C08G64/42\ C08G85/00 |
| 1785 | 3D printing | METHOD AND APPARATUS FOR TYING ROLLS OF FABRIC\ | US2003209307A1 | GARTLAND ROBERT JOHN (US)\ KOT KENNETH MICHAEL (US) | GOODYEAR TIRE & RUBBER (US) | B29C65/02\ B29C65/08\ B65H19/18\ B65H19/28\ B65H19/29 |
| 1786 | 3D printing | RECORDING MEDIUM CARTRIDGE\ | US2004013081A1 | ODAGIRI HIROKAZU (JP) | SAMSUNG ELECTRONICS INC (KR) | G11B23/02\ G11B23/03\ G11B23/38\ G11B23/40 |
| 1787 | 3D printing | ELECTRICAL CONNECTOR INCORPORATING TERMINALS HAVING ULTRASONICALLY WELDED WIRES\ | US2004018777A1 | VANDEN WYMELENBERG MARK J (US)\ HSIEH SHAO C (US)\ BUNGO EDWARD M (US) | SUED CHEMIE INC (US) | H01R43/20\ H01R43/02\ H01R43/28\ H01R43/052\ H01R43/055 |
| 1788 | 3D printing | FLEXIBLE FLAT CABLE CONNECTING METHOD AND A HORN CONSTRUCTION OF AN ULTRASONIC WELDING MACHINE\ | US2004020580A1 | OISHI AKINORI (JP)\ KIHARA TAKESHI (JP) | SUMITOMO WIRING SYSTEMS (JP) | B23K20/10\ H01R43/02\ H01R43/00 |
| 1789 | 3D printing | ENGINEERED THERMAL MANAGEMENT DEVICES AND METHODS OF THE SAME\ | US2004024482A1 | WHITE DAWN (US)\ CARMEIN DAVID (US) | TSUBAKIMOTO CHAIN CO (JP) | G06F19/00\ H01L21/48\ H01L23/427\ F28D15/00\ H05B6/10 |
| 1790 | 3D printing | FILTER ELEMENT PARTICULARLY FOR SEPARATING A LIQUID FROM A GAS STREAM\ | US2004031253A1 | STAUDENMAYER BERNHARD (DE)\ BRANDT DETLEF (DE)\ HEIKAMP WOLFGANG (DE) | MANN & HUMMEL GMBH (DE) | B01D39/20\ F01M13/04\ B01D46/02 |
| 1791 | 3D printing | DEVICE AND METHOD FOR WELDING THIN-WALLED WORK PIECES USING ULTRASOUND\ | US2004031554A1 | MOGLICH HARTMUT (DE) | HERRMANN ULTRASCHALLTECHNIK (DE) | B29C65/00\ B29C65/08\ B29C65/78 |
| 1792 | 3D printing | METHOD OF FORMING A DOUBLE CONTAINER AND RELATED ASSEMBLY\ | US2004031803A1 | MATSUI YUTAKA (US)\ MURASE JUN (US) | PROUDFIT ERIC (US)\ BELL AUTOMOTIVE PRODUCTS INC (US) | B65D21/02\ B65D81/32 |
| 1793 | 3D printing | METHOD AND DEVICE FOR CONNECTING CONDUCTORS\ | US2004038598A1 | STEINER ERNST (DE)\ STROH DIETER (DE)\ DIETERLE HORST (DE) | SONY CORP (JP) | B23K20/10\ H05K3/36\ H05K3/32\ H01R4/02\ H01R43/02\ H05K3/40 |
| 1794 | 3D printing | BRUSH FILAMENT BUNDLES AND PREPARATION THEREOF\ | US2004048063A1 | DEPUYDT JOSEPH A (US)\ PORTMAN DANIEL C (US)\ ZIMMET HELGE (US)\ BROWN WILLIAM R (US) | D N TECH CO LTD (KR)\ KIM YONG RAE (KR) | A46D1/08 |
| 1795 | 3D printing | ULTRASONIC-WELDING APPARATUS OPTICAL SENSOR AND ROTATION SENSOR FOR THE ULTRASONIC-WELDING APPARATUS\ | US2004051032A1 | KITAMURA KOUTA (JP)\ TAKAHARA TADASHI (JP)\ ISHIKAWA KENJI (JP)\ TAKAHASHI NOVUO (JP) | FUJI PHOTO FILM CO LTD (US) | G01V8/12 |
| 1796 | 3D printing | FUSE\ | US2004057225A1 | IWATA MASASHI (JP)\ MASUDA TOSHIKO (JP)\ OHASHI NORIHIRO (JP) | YAZAKI CORP (US) | H01H85/02\ H01H85/041 |
| 1797 | 3D printing | PILE WEATHERSTRIPPING AND METHODS OF MAKING SAME\ | US2004074719A1 | LOUGHNEY DAVID M (US) | MORGAN CRUCIBLE CO (GB) | E06B7/22 |
| 1798 | 3D printing | STRUCTURE OF ANTENNA\ | US2004085252A1 | JU U-LIN (TW) | BENQ CORP (US) | H01Q1/24\ H01Q1/36\ H01Q9/30\ H01Q11/08 |
| 1799 | 3D printing | IMPELLER AND METHOD USING SOLID FREE FORM FABRICATION\ | US2004086388A1 | GIGAS BERND (US)\ HALE STEVE (US) | SPX CORP (US) | F04D29/02\ F04D29/38 |
| 1800 | 3D printing | TENSIONER\ | US2004092350A1 | HASHIMOTO HIROSHI (JP)\ YOSHIDA OSAMU (JP)\ KATO MASAKI (JP) | HEWLETT PACKARD DEVELOPMENT CO (US) | B29C65/08\ F16B5/08\ F16H7/08 |
| 1801 | 3D printing | ULTRASONIC WELDED HINGE DAMPER\ | US2004103746A1 | ANTON JAMISON J (US)\ BIVENS STEVEN L (US)\ LEVEY KENNETH R (US)\ DOORNBOS DAVID A (US)\ BELLA JOSEPH J (US) | 3D SYSTEMS INC (US) | B23K20/10\ B29C65/00\ B29C65/08\ F16F9/34\ F16F9/14\ F16F9/32\ F16F9/516 |
| 1802 | 3D printing | AUTOMATED RAPID PROTOTYPING COMBINING ADDITIVE AND SUBTRACTIVE PROCESSES\ | US2004107019A1 | WHITE DAWN (US)\ KESHAVMURTHY SHYAM (US)\ CARMEIN JUDY ANN (US) | WINDMOELLER & HOELSCHER (DE) | G06F19/00\ G05B19/4097 |
| 1803 | 3D printing | SPOOL FOR WINDING OPTICAL FIBER\ | US2004108407A1 | JEONG CHANG-WOONG (KR)\ LEE YEONG-SEOP (KR)\ JUNG KI-TAE (KR) | KRAMER LAURA (US) | G02B6/00\ B29C65/00\ B29C65/08\ B29C65/58\ B65H75/14\ B65H75/06\ B65H75/28\ B65H75/50\ C03B37/03 |
| 1804 | 3D printing | PRODUCTION METHOD FOR PHOTOSENSITIVE DRUM-USE SUBSTRATE AND PHOTOSENSITIVE DRUM-USE SUBSTRATE\ | US2004115548A1 | IIZUKA MUNENORI (JP)\ SUZUKI TAKAHIRO (JP)\ MACHIDA KUNIO (JP) | ILLNOIS TOOL WORKS INC (US) | B29C65/08\ G03G5/10 |
| 1805 | 3D printing | FLOW FIELD PLATES AND A METHOD FOR FORMING A SEAL BETWEEN THEM\ | US2004151972A1 | TURPIN MARK CHRISTOPHER (GB)\ BILTON BRENDAN MICHAEL (GB)\ BOFF JAMES CHARLES (GB) | STAPLA ULTRASCHALLTECHNIK GMBH (DE) | H01M8/10\ H01M8/02\ C25B9/00\ C25B9/04\ H01M8/24 |
| 1806 | 3D printing | WATER CUTOFF STRUCTURE OF COVERED WIRE\ | US2004163729A1 | IDE TETSURO (JP)\ TAKAYAMA TSUTOMU (JP) | YAZAKI CORP (US) | H01B7/28 |
| 1807 | 3D printing | METHODS AND SYSTEMS FOR PRODUCING AN OBJECT THROUGH SOLID FREEFORM FABRICATION USING IMMISCIBLE FLUIDS\ | US2004169699A1 | HUNTER SHAWN D (US)\ KASPERCHIK VLADEK P (US) | ILLINOIS TOOL WORKS (US) | B29C67/00 |
| 1808 | 3D printing | LEAD FRAME AND ELECTRONIC COMPONENT USING SAME\ | US2004183168A1 | MIYAMOTO TADASHI (JP)\ UEKAWA YOSHIHIRO (JP)\ ASADA KENICHI (JP)\ SUGAWARA SHIGEAKI (JP) | HIMEJI TOSHIBA E P CORP (JP) | H01L23/48\ H01L21/48\ H01L23/495 |
| 1809 | 3D printing | COMPOSITION AND METHOD FOR ENHANCING THE WELD STRENGTH OF POLYPHENYLENE ETHER COMPOSITIONS\ | US2004214960A1 | KANNAN GANESH (IN)\ MHETAR VIJAY (US) | GEN ELECTRIC (US) | B29C65/00\ B29C65/08\ B29C65/06 |
| 1810 | 3D printing | SYSTEMS AND METHODS FOR WELDING OF PARTS\ | US2004216829A1 | GORDON KEVIN (US) | STAPLA ULTRASONICS CORP INC (US) | B23K20/10\ B29C65/00\ B29C65/08 |
| 1811 | 3D printing | ACCESSORIES FOR VEHICLES AND METHOD OF MANUFACTURING IMPROVED ACCESSORIES FOR VEHICLES\ | US2004226400A1 | PROUDFIT ERIC (US) | SEJIN ENTPR CO LTD (KR) | B32B27/30\ B62D1/06 |
| 1812 | 3D printing | HIGH SURFACE AREA POROUS ALUMINUM ELECTRODE IN ELECTROLYTIC CAPACITORS USING SOLID FREEFORM FABRICATION\ | US2004246659A1 | KESER MILAN (US) | LEE SEUNG WOOK (KR) | H01G9/04\ H01G9/048 |
| 1813 | 3D printing | MICRO- AND NANO-FABRICATION USING FOCUSED PLASMA ASSISTED VAPOR DEPOSITION\ | US2004251581A1 | JANG BOR Z (US)\ SONG LULU (CN) | JUNG JEAN MAN (KR) | B29C67/00\ B22F3/00 |
| 1814 | 3D printing | MACRO-POROUS HYDROXYAPATITE SCAFFOLD COMPOSITIONS AND FREEFORM FABRICATION METHOD THEREOF\ | US2004254668A1 | JANG BOR Z (US)\ YANG LAIXIA (US) | KEY TRANDING CO LTD US | A61F2/00\ A61F2/02\ A61F2/28\ A61F2/30\ A61L27/12\ G06F19/00\ A61F2/46 |
| 1815 | 3D printing | CALCIUM ALUMINATE CEMENT COMPOSITIONS FOR SOLID FREEFORM FABRICATION\ | US2004261664A1 | LAMBRIGHT TERRY M (US)\ ORIAKHI CHRIST PHER (US) | LG CHEMICAL LTD (KR) | B28B1/00\ C04B24/26\ C04B22/04\ C04B22/08\ C04B22/10\ C04B24/32\ C04B28/06\ C04B41/50 |
| 1816 | 3D printing | FOLDABLE AUTOMOBILE WINDOW SHADE WITH ULTRASONICALLY FORMED FOLD\ | US2005000664A1 | HUANG SUNNY E L (US) | HYUNDAI MOTOR CO LTD (KR) | B60J1/20 |
| 1817 | 3D printing | AIR BAG AND METHOD FOR MAKING AN AIR BAG\ | US2005006882A1 | WANG YUNZHANG (US) | TAE HWA ENTPR CO LTD (KR) | B60R21/16\ B60R21/232\ B60R21/233 |
| 1818 | 3D printing | SYSTEMS AND METHODS FOR USING MULTI-PART CURABLE MATERIALS\ | US2005012247A1 | LAMBRIGHT TERRY M (US)\ KASPERCHIK VLADEK P (US)\ KRAMER LAURA (US) | LG CHEMICAL LTD (KR) | B29C67/00\ B29K63/00\ B29C35/04\ G06F19/00\ B29K75/00\ B29K45/00 |
| 1819 | 3D printing | SEPARATE SOLIDIFICATION OF BUILD MATERIAL AND SUPPORT MATERIAL IN SOLID FREEFORM FABRICATION SYSTEM\ | US2005023719A1 | HUNTER SHAWN D (US)\ COLLINS DAVID C (US)\ NIELSEN JEFFREY ALLEN (US) | IAC IN NAT UNIV CHUNGNAM (KR) | B29C35/08\ B29C67/00\ B41J2/01\ B29C71/02\ B29C41/02\ B41J2/04 |
| 1820 | 3D printing | PAVEMENT MARKER WITH ENHANCED DAYTIME SIGNAL\ | US2005025570A1 | SNAGEL PAUL (US) | AVERY DENNISON (US) | E01F9/06 |
| 1821 | 3D printing | CHEMICAL-RESISTANT PROTECTIVE CLOTHING\ | US2005037683A1 | KONISHI TAKESHI (JP)\ KAWASAKI YASUTOSHI (JP)\ MAKI MOTOCHIKA (JP) | KURARAY CO (JP)\ MEDITEC JAPAN CO LTD (JP) | B32B27/06\ A41D13/00\ B32B27/12\ A41D31/02\ A41D31/00\ A62B17/00\ B32B27/28 |
| 1822 | 3D printing | SYSTEMS AND METHODS FOR USING NORBORNENE BASED CURABLE MATERIALS\ | US2005040564A1 | FARR ISAAC (US)\ LAMBRIGHT TERRY M (US)\ OLIVER JONES (US) | HEWLETT PACKARD DEVELOPMENT CO (US) | B29C35/08\ B29C67/00 |
| 1823 | 3D printing | BONDING METHOD FOR A PLURALITY OF COMPONENTS BONDING METHOD FOR CONTAINER AND LID MEMBER AND ULTRASONIC WELDING APPARATUS\ | US2005045266A1 | TSUCHIHASHI KAZUO (JP) | CANON KK (JP) | B23K20/10\ B29C65/00\ B29L22/00\ B29C65/08\ B29C65/78\ B65B7/28\ B23K37/04 |
| 1824 | 3D printing | INORGANIC PHOSPHATE CEMENT COMPOSITIONS FOR SOLID FREEFORM FABRICATION\ | US2005046067A1 | FARR ISAAC (US)\ LAMBRIGHT TERRY M (US)\ ORIAKHI CHRISTOPHER (US) | HEWLETT PACKARD DEVELOPMENT CO (US) | B28B1/00\ C04B28/34 |
| 1825 | 3D printing | SECONDARY CELL\ | US2005058908A1 | IMACHI NAOKI (JP)\ YOSHIMURA SEIJI (JP) | SANYO ELECTRIC CO (JP) | H01M2/02\ H01M2/30\ H01M10/04\ H01M2/26\ H01M10/0587\ H01M10/05\ H01M4/64 |
| 1826 | 3D printing | USES OF SUPPORT MATERIAL IN SOLID FREEFORM FABRICATION SYSTEMS\ | US2005072113A1 | FARR ISAAC (US)\ ORIAKHI CHRISTOPHER (US)\ COLLINS DAVID C (US)\ NIELSEN JEFFREY ALLEN (US) | HEWLETT PACKARD DEVELOPMENT CO (US) | B29C67/00 |
| 1827 | 3D printing | METHOD AND SYSTEM FOR USING POROUS STRUCTURES IN SOLID FREEFORM FABRICATION\ | US2005074596A1 | NIELSEN JEFFREY A (US)\ COLLINS DAVID C (US)\ CRUZ-URIBE TONY S (US) | RES INST IND SCIENCE & TECH (KR)\ G & C TECHNOLOGY CO LTD (KR) | B29C35/08\ B29C67/00 |
| 1828 | 3D printing | POLYMER SYSTEMS WITH REACTIVE AND FUSIBLE PROPERTIES FOR SOLID FREEFORM FABRICATION\ | US2005080191A1 | LAMBRIGHT TERRY M (US)\ BOYD MELISSA D (US)\ KASPERCHIK VLADEK P (US)\ KRAMER LAURA (US) | LG CHEMICAL LTD (KR) | C08F8/00 |
| 1829 | 3D printing | ELECTRO-PYROTECHNIC INITIATOR\ | US2005081731A1 | PHELEP STEPHANE (FR)\ PINET PHILIPPE (FR) | SAMSUNG SDI CO LTD US | B29C65/08\ F42B3/12\ F42B3/195 |
| 1830 | 3D printing | HYBRID ORGANIC-INORGANIC COMPOSITION FOR SOLID FREEFORM FABRICATION\ | US2005082710A1 | FARR ISAAC (US)\ LAMBRIGHT TERRY M (US)\ ORIAKHI CHRISTOPHER (US)\ COLLINS DAVID C (US)\ KASPERCHIK VLADEK P (US) | HEWLETT PACKARD DEVELOPMENT CO (US) | B29C67/00\ A61C13/00\ C04B35/622\ A61K6/083\ A61K6/06\ C08K3/40\ C04B28/28\ C08L101/08 |
| 1831 | 3D printing | ALUMINUM HEAT SINK FOR A SOLID STATE RELAY HAVING ULTRASONICALLY WELDED COPPER FOIL\ | US2005086797A1 | POPESCU EUGEN (US) | SCHUNK ULTRASCHALLTECHNIK GMBH (DE) | B23K20/10\ H01L\ H01H11/00\ H01L23/02\ H05K7/02 |
| 1832 | 3D printing | SYSTEMS AND METHODS FOR REDUCING WASTE IN SOLID FREEFORM FABRICATION\ | US2005087897A1 | NIELSEN JEFFREY A (US)\ COLLINS DAVID C (US) | PUREECHEM CO LTD (KR)\ KOREA INST CERAMIC ENG & TECH (KR) | B29C67/00 |
| 1833 | 3D printing | ALGINATE-BASED MATERIALS METHODS OF APPLICATION THEREOF AND SYSTEMS FOR USING THE ALGINATE-BASED MATERIALS\ | US2005087902A1 | FARR ISAAC (US)\ LAMBRIGHT TERRY M (US)\ HOVAGIMIAN HOWARD (US) | HEWLETT PACKARD DEVELOPMENT CO (US) | B29C67/00\ B28B1/00\ C04B26/28 |
| 1834 | 3D printing | HOUSING OR HOUSING PART AND METHOD FOR PRODUCING A HOUSING OR HOUSING PART AND TOOL FOR CARRYING OUT SAID METHOD\ | US2005089159A1 | SCHRACK CORNELIA (DE) | SEOUL ENGINEERING CO LTD (KR) | H04M1/02\ B29C65/08 |
| 1835 | 3D printing | RESIN-MODIFIED INORGANIC PHOSPHATE CEMENT FOR SOLID FREEFORM FABRICATION\ | US2005089636A1 | FARR ISAAC (US)\ LAMBRIGHT TERRY M (US)\ ORIAKHI CHRISTOPHER (US) | HEWLETT PACKARD DEVELOPMENT CO (US) | B29C67/00\ B28B1/00\ C04B24/26\ C04B28/34\ C04B28/28 |
| 1836 | 3D printing | CLOCKSPRING WITH FLAT CABLE\ | US2005090135A1 | SCHILSON TOM (US)\ KAMPS STEVE (US) | SEO GI WON (KR) | H01B13/00\ B29C65/00\ H05K3/28\ B29C65/02\ H01B7/00\ B29C65/08\ H01B7/08\ H05K1/00\ H01R39/00 |
| 1837 | 3D printing | METHOD AND A SYSTEM FOR SOLID FREEFORM FABRICATION OF A THREE-DIMENSIONAL OBJECT\ | US2005093208A1 | BOYD MELISSA D (US)\ KASPERCHIK VLADEK (US) | BTM AND P CO LTD (KR) | B29C67/00 |
| 1838 | 3D printing | DEVICE FOR THE ULTRASONIC SEALING AND SEPARATION OF A PIPE SECTION\ | US2005167054A1 | WAGENBACH UDO (DE)\ MORSCHNER ROLF (DE) | SUNMOON INTERNAT CO LTD (KR) | B23K20/10\ B29C65/08\ B29C65/74\ B23K37/00 |
| 1839 | 3D printing | ULTRASONIC BONDING APPARATUS AND METHOD\ | US2005194423A1 | OKITA TAKANORI (JP) | RENESAS TECH CORP (JP) | H01L21/60\ H01L21/607\ B23K31/00\ B23K1/06 |
| 1840 | 3D printing | FUSED SUTURE KNOT\ | US2005209639A1 | GIDWANI SURESH (US)\ CHIN YEM (US) | BOSTON SCIENT SCIMED INC (US) | A61B17/32\ A61B17/04\ A61B17/06 |
| 1841 | 3D printing | METHOD AND DEVICE FOR IMPROVING ADHESION OF THE INDIVIDUAL LAYERS OF A COMPOSITE MATERIAL\ | US2005224168A1 | MAINZ HANS-WILLI (DE) | MAINZ HANS-WILLI (DE) | B65D65/40\ B32B27/08\ B65B61/18\ B32B15/20\ B65D85/80 |
| 1842 | 3D printing | IDENTIFIABLE STRUCTURES AND SYSTEMS AND METHODS FOR FORMING THE SAME IN A SOLID FREEFORM FABRICATION SYSTEM\ | US2005225004A1 | MAY GREGORY J (US) | MAY GREGORY J (US) | B29C67/00\ A61F2/28\ G01N29/00\ G01N23/00\ E05B49/00 |
| 1843 | 3D printing | WATERPROOFING MATERIAL\ | US2005227044A1 | FLYNN BRYAN N (GB)\ ROSTAMI SHAMSEDEAU (GB) | FLYNN BRYAN N (GB)\ ROSTAMI SHAMSEDEAU (GB) | B29C59/02\ B29C65/02\ B32B3/24\ B29C65/08\ E04B1/66\ B32B13/12\ E02D31/00 |
| 1844 | 3D printing | STOP SECTION FOR SLIDE FASTENER AND SLIDE FASTENER HAVING SUCH STOP SECTION\ | US2005257350A1 | TAKAMATSU SATOSHI (JP)\ FUDA MASAAKI (JP)\ TAGA YUKIO (JP) | YKK CORP (JP) | A44B19/12\ A44B19/36\ A44B19/60 |
| 1845 | 3D printing | WELDING DEVICE\ | US2005257895A1 | MOTODA SHUHO (JP) | MOTODA SHUHO (JP) | B29C65/00\ B29C65/08\ B25F5/02 |
| 1846 | 3D printing | METHOD FOR BONDING A BODY SIDE WAFER OF A STOMA SYSTEM AND A FURTHER COMPONENT OF SAID STOMA SYSTEM WITH EACH OTHER\ | US2005277901A1 | GIJSBERT VAN DER LEDEN ARIE (BE) | GIJSBERT VAN DER LEDEN ARIE (BE) | A61F5/44\ B29C65/08\ A61F5/445 |
| 1847 | 3D printing | MULTIPLE PROBE POWER SYSTEMS AND METHODS FOR ULTRASONIC WELDING\ | US2006011707A1 | JOHANSEN DAVID K (US) | DUKANE CORP (US) | B23K20/10\ B29C65/08\ B23K1/06 |
| 1848 | 3D printing | POLYMERIC MANIFOLD ASSEMBLY AND METHOD\ | US2006016416A1 | STULL RANDY (US)\ KIM DUKHYUNG (KR)\ KEIGHRON TIMOTHY (US)\ RHEE JAE M (US) | R & B INC (US) | F02M35/10 |
| 1849 | 3D printing | ASSEMBLY STRUCTURE\ | US2006053623A1 | I YA-TUNG (TW) | LITE ON TECHNOLOGY CORP (TW) | H05K13/04 |
| 1850 | 3D printing | ALUMINUM END CAPS ULTRASONICALLY WELDED TO END OF ALUMINUM TUBE\ | US2006071054A1 | BOLSER DAVID R (US)\ GRAFF KARL F (US)\ TRAPP TIMOTHY J (US) | BOLSER DAVID R (US)\ GRAFF KARL F (US)\ TRAPP TIMOTHY J (US) | B23K31/02 |
| 1851 | 3D printing | RETRIEVING INFORMATION ON MATERIAL USED IN SOLID FREEFORM FABRICATION\ | US2006091199A1 | LOUGHRAN STEPHEN A (GB) | LOUGHRAN STEPHEN A (US) | G06F7/00 |
| 1852 | 3D printing | RECEIVING SOLID FREEFORM FABRICATION (SFF) JOB\ | US2006095152A1 | LOUGHRAN STEPHEN A (GB) | LOUGHRAN STEPHEN A (US) | G06F19/00 |
| 1853 | 3D printing | LASER PENETRATION WELD\ | US2006096958A1 | ZHAO HAILIANG (US)\ LUND JEFFREY S (US) | ZHAO HAILIANG (US)\ LUND JEFFREY S (US) | B23K26/22 |
| 1854 | 3D printing | WINDOW PANE AND A METHOD OF BONDING A CONNECTOR TO THE WINDOW PANE\ | US2006105589A1 | ACKERMAN MARK S (US)\ HOEPFNER TIMOTHY P (US) | AGC AUTOMOTIVE AMERICAS R & D (US) | H01R33/00 |
| 1855 | 3D printing | SECURE PHYSICAL CONNECTIONS FORMED BY A KINETIC SPRAY PROCESS\ | US2006113359A1 | TEETS RICHARD E (US)\ VAN STEENKISTE THOMAS H (US)\ KRUGER DUANE D (US)\ BEER ROBERT C (US) | TEETS RICHARD E (US)\ VAN STEENKISTE THOMAS H (US)\ KRUGER DUANE D (US)\ BEER ROBERT C (US) | B23K31/02 |
| 1856 | 3D printing | DEVICE FOR ULTRASONIC WELDING\ | US2006124690A1 | DOBERNECKER KLAUS-ECKHART (DE) | DOBERNECKER KLAUS-ECKHART (DE) | B23K20/10\ B29C65/00\ B29C65/08\ H01R43/02\ B23K1/06 |
| 1857 | 3D printing | FIBROUS MAT FIXING STRUCTURE\ | US2006141221A1 | SASAKI KEI (JP) | SASAKI KEI (JP) | B32B3/00 |
| 1858 | 3D printing | SEPARATOR MATERIAL FOR FORMING A SEPARATOR FOR AN ACID ACCUMULATOR\ | US2006141350A1 | DREYER DANIEL (FR)\ CAUBLOT JEAN-MARTIAL (FR) | DREYER DANIEL (FR)\ CAUBLOT JEAN-MARTIAL (FR) | B32B3/30\ H01M2/16\ H01M\ H01M2/14\ H01M2/18\ H01M10/06 |
| 1859 | 3D printing | ULTRASONIC LAMINATION\ | US2006141890A1 | LINK EBERHARD (US)\ MASON CHARLES R (US)\ FRASCH JAMES (US)\ TOSTI AMELIA (US)\ KARNIK ASHUTOSH (US) | LINK EBERHARD (US)\ MASON CHARLES R (US)\ FRASCH JAMES (US)\ TOSTI AMELIA (US)\ KARNIK ASHUTOSH (US) | D04H1/00\ D04H1/46\ B32B5/26 |
| 1860 | 3D printing | ULTRASONIC WELDER WITH HIGH-Q TOOL\ | US2006144906A1 | SHEEHAN JAMES F (US)\ GRISWOLD ROBERT S (US) | SHEEHAN JAMES F (US)\ GRISWOLD ROBERT S (US) | B23K20/10 |
| 1861 | 3D printing | TEXTURED GEOCELL\ | US2006147275A1 | LIN CHIN-TAI (TW)\ CHEN FERDIE (TW) | LIN CHIN-TAI (TW)\ CHEN FERDIE (TW) | E02D17/20 |
| 1862 | 3D printing | TEXTURED GEOCELL\ | US2006147276A1 | LIN CHIN-TAI (TW)\ CHEN FERDIE (TW) | LIN CHIN-TAI (TW)\ CHEN FERDIE (TW) | E02D3/00 |
| 1863 | 3D printing | APPARATUS METHOD AND ARTICLE FOR DIRECT SLICING OF STEP BASED NURBS MODELS FOR SOLID FREEFORM FABRICATION\ | US2006155418A1 | GAYLO CHRISTOPHER M (US)\ LAU WING K (US)\ SUN WEI (US)\ BRADBURY THOMAS J (US)\ STARLY BINIL (US)\ LAU ALAN C (US)\ YOUSSEF ADOLPHE H (US) | THERICS INC (US) | G06F19/00 |
| 1864 | 3D printing | REACTIVE MATERIALS SYSTEMS AND METHODS FOR SOLID FREEFORM FABRICATION OF THREE-DIMENSIONAL OBJECTS\ | US2006159869A1 | FARR ISAAC (US)\ LAMBRIGHT TERRY M (US)\ ORIAKHI CHRISTOPHER (US)\ KRAMER LAURA (US) | ORIAKHI CHRISTOPHER (US)\ FARR ISAAC (US)\ LAMBRIGHT TERRY M (US)\ KRAMER LAURA (US) | B44F7/00 |
| 1865 | 3D printing | ANCHOR SHEET\ | US2006162269A1 | PACIONE JOSEPH R (CA)\ PACIONE JOHN A (CA) | PACIONE JOSEPH R (CA)\ PACIONE JOHN A (CA) | B32B27/32\ A47G27/04\ E04F11/16\ E04F15/22 |
| 1866 | 3D printing | ELASTIC LAMINATE MATERIAL AND METHOD OF MAKING\ | US2006169387A1 | NAYAR SATINDER K (US)\ POCHARDT DONALD L (US)\ NIELSEN SHARON K (US) | NAYAR SATINDER K (US)\ POCHARDT DONALD L (US)\ NIELSEN SHARON K (US) | B32B37/00\ B32B27/12\ B32B5/26 |
| 1867 | 3D printing | CORE-SHELL SOLID FREEFORM FABRICATION\ | US2006192315A1 | FARR ISAAC (US)\ LAMBRIGHT TERRY M (US) | FARR ISAAC (US)\ LAMBRIGHT TERRY M (US) | B29C41/02 |
| 1868 | 3D printing | COVER FOR A TOOTHBRUSH HEAD\ | US2006195998A1 | CALATAYUD AUGUSTIN O (ES) | CALATAYUD AUGUSTIN O (ES) | A46B17/04\ A46B7/02 |
| 1869 | 3D printing | MANUFACTURING METHOD FOR EYELET AND STRUCTURE THEREOF\ | US2006200954A1 | CHUNG DO-JIN (KR) | QINGDAO GYOHA EN TECH CO LTD (CN) | A44B99/00\ A43C5/00 |
| 1870 | 3D printing | MULTIPLE PROBE POWER SYSTEMS AND METHODS FOR ULTRASONIC WELDING\ | US2006213952A1 | JOHANSEN DAVID K (US) | DUKANE CORP (US) | B23K1/06 |
| 1871 | 3D printing | METHOD FOR MANUFACTURING A SEAM CONNECTION BETWEEN TWO TEXTILE SHEET FORMATIONS AND ULTRASONIC WELDING DEVICE\ | US2006216454A1 | HEIL DIETMAR (DE) | HEIL DIETMAR (DE) | B32B37/00 |
| 1872 | 3D printing | FILTER SYSTEM\ | US2006236664A1 | STAHL ULRICH (DE)\ FELBER UWE (DE) | FREUDENBERG CARL KG (DE) | B01D47/00 |
| 1873 | 3D printing | BATTERY PACK HAVING VERY THIN THICKNESS\ | US2006244416A1 | YONG SEOKJU (KR)\ LEE DONGCHEOL (KR)\ BAEK JU-HWAN (KR) | YONG SEOKJU (KR)\ LEE DONGCHEOL (KR)\ BAEK JU-HWAN (KR) | H02J7/00 |
| 1874 | 3D printing | CORRUGATED POLYMERIC VOID BOARD\ | US2006283142A1 | PECK LAWRENCE R (US)\ VARMA TILAK R (US)\ SHARE LAWRENCE (US) | ILLINOIS TOOL WORKS (US) | E04C2/54 |
| 1875 | 3D printing | MIRROR HEATER ASSEMBLY\ | US2006289423A1 | MARTINEZ JOSE (US)\ MORETH CARY P (US)\ NEENAN MICHAEL N (US)\ RODAWOLD JOSEPH L JR (US) | MARTINEZ JOSE (US)\ NEENAN MICHAEL N (US)\ MORETH CARY P (US)\ RODAWOLD JOSEPH L JR (US) | H05B1/00 |
| 1876 | 3D printing | POROUS FILM CARTRIDGE\ | US2007017914A1 | SHIGESADA KEIJI (JP)\ FUJIWARA MORIO (JP) | SHIGESADA KEIJI (JP)\ FUJIWARA MORIO (JP) | B01L3/00\ B01D61/14\ B01D63/08\ B01D29/01\ B01D61/18\ B01D63/00\ B01D65/00\ B01D69/06\ B65D6/00 |
| 1877 | 3D printing | MULTICOMPONENT FUSED SUTURE LOOP AND APPARATUS FOR MAKING SAME\ | US2007021780A1 | WESTHAVER PAUL (US)\ FENTON PAUL (US)\ HARRINGTON FRANCIS (US)\ SCHMITT PETER (US)\ GRAY JOHN R JR (US) | GRAY JOHN R JR (US)\ HARRINGTON FRANCIS (US)\ FENTON PAUL (US)\ WESTHAVER PAUL (US)\ SCHMITT PETER (US) | A61L17/00 |
| 1878 | 3D printing | SYSTEMS AND METHODS OF SOLID FREEFORM FABRICATION WITH IMPROVED POWDER SUPPLY BINS\ | US2007026102A1 | FARR ISAAC (US)\ LAMBRIGHT TERRY M (US)\ NIELSEN JEFFREY A (US)\ COLLINS DAVID C (US)\ DEVOS JOHN A (US)\ CRUZ-URIBE TONY S (US) | NIELSEN JEFFREY A (US)\ DEVOS JOHN A (US)\ CRUZ-URIBE TONY S (US)\ COLLINS DAVID C (US)\ FARR ISAAC (US)\ LAMBRIGHT TERRY M (US) | B28B21/42 |
| 1879 | 3D printing | DEFORMING TOOL AND PROCESS FOR MANUFACTURE\ | US2007062245A1 | FULLER KARL-HEINZ (DE)\ KNUPPEL MICHAEL (DE) | DAIMLER CHRYSLER AG (DE) | B21D37/02 |
| 1880 | 3D printing | SYSTEMS AND METHODS OF SOLID FREEFORM FABRICATION WITH INTERCHANGEABLE POWDER BINS\ | US2007063372A1 | NIELSEN JEFFREY A (US)\ HUNTER SHAWN D (US)\ BOYD MELISSA D (US)\ COLLINS DAVID C (US) | HUNTER SHAWN D (US)\ NIELSEN JEFFREY A (US)\ BOYD MELISSA D (US)\ COLLINS DAVID C (US) | B29C67/00 |
| 1881 | 3D printing | ULTRASONIC WELDING SYSTEM\ | US2007068991A1 | HANDEL JEFFREY M (US)\ CLARK MATTHEW A (US) | HANDEL JEFFREY M (US)\ CLARK MATTHEW A (US) | B23K37/00\ B23K5/20 |
| 1882 | 3D printing | ULTRASONIC OR HEAT STAKING ATTACHMENT OR PLANT TAG\ | US2007079536A1 | HALL WALTER J (US) | HALL WALTER J (US) | G09F3/10 |
| 1883 | 3D printing | CLAMPED QUANTIZED FEED SYSTEM FOR SOLID FREEFORM FABRICATION\ | US2007090568A1 | TEAL BRENT (US)\ THEAL RYAN J (US)\ PETCH DAVE B (US)\ O'REGAN THOMAS (US)\ CUNNINGHAM STEPHEN M (US) | 3D SYSTEMS INC (US) | B29C41/02 |
| 1884 | 3D printing | METHODS AND SYSTEMS FOR THE ANNOTATION OF BIOMOLECULE PATTERNS IN CHROMATOGRAPHY/MASS-SPECTROMETRY ANALYSIS\ | US2007095757A1 | BJORKESTEN LENNART (SE)\ KAPLAN ANDERS (SE) | KAPLAN ANDERS (SE)\ BJORKESTEN LENNART (SE) | G01N30/72\ G01N30/86\ G01N33/68\ B01D15/08 |
| 1885 | 3D printing | ULTRASONIC WELDING HEAD\ | US2007102489A1 | GOEBL CHRISTIAN (DE)\ AUGUSTIN KARLHEINZ (DE) | SEMIKRON ELEKTRONIK GMBH (DE) | A47J36/02 |
| 1886 | 3D printing | NON-WOVEN FABRIC LAMINATE/ELASTOMER/NON-WOVEN FABRIC\ | US2007105472A1 | MARCHE THIERRY (FR) | MARCHE THIERRY (US) | B32B3/00\ B32B3/28\ B32B27/12\ B32B25/10 |
| 1887 | 3D printing | CONCURRENT WELDING AND SEVERING BELT FORMING PROCESS\ | US2007107829A1 | ROETKER MICHAEL S (US)\ MARTIN DAVID W (US)\ DARCY JOHN J III (US) | XEROX CORP (US) | B32B37/00\ B32B38/04\ B32B37/06\ B31C1/00 |
| 1888 | 3D printing | HIGH STRENGTH CURABLE COMPOSITIONS FOR THE SOLID FREEFORM FABRICATION OF HEARING AIDS\ | US2007116311A1 | KADZIELA VIC (US)\ LITKE ALAN E (US) | HENKEL CORP (US) | H04R25/00 |
| 1889 | 3D printing | SEMICONDUCTOR DEVICE PACKAGE LEADFRAME FORMED FROM MULTIPLE METAL LAYERS\ | US2007130759A1 | HARNDEN JAMES (US)\ CHIA ANTHONY (SG)\ WONG LIMING (CN)\ YANG HONGBO (CN) | GEM SERVICES INC (US) | H01L21/00 |
| 1890 | 3D printing | ULTRASONIC WELDING STRUCTURE AND ULTRASONIC WELDING METHOD\ | US2007158011A1 | TOMINAGA JUN (JP)\ SHIGEMATSU RYOHEI (JP)\ FURUKAWA KAZUO (JP) | TOMINAGA JUN (JP)\ SHIGEMATSU RYOHEI (JP)\ FURUKAWA KAZUO (JP) | B32B37/00 |
| 1891 | 3D printing | ULTRASOUND WELDING DEVICE\ | US2007158012A1 | HEIL DIETMAR (DE)\ BARTHOLDI PIERRE (CH) | JENTSCHMANN AG ZURICH (CH) | B32B37/00\ B29C65/00\ B29C65/08\ B29C65/50 |
| 1892 | 3D printing | PLASTIC RELIEF VALVE HOUSING WITH ANTI-DRAIN BACK VALVE\ | US2007163931A1 | CLINE L S (US) | ARVIN TECHNOLOGIES INC US | B01D35/14 |
| 1893 | 3D printing | PROCESS FOR MANUFACTURING INSULATED SIDING\ | US2007212970A1 | GRAHAM W D (US)\ ROCKWELL ANTHONY L (US)\ VERMILION DONN R (US)\ CHAKRABARTY KAUSHIK (US) | GRAHAM W D (US)\ ROCKWELL ANTHONY L (US)\ VERMILION DONN R (US)\ CHAKRABARTY KAUSHIK (US) | D04H1/00\ B32B27/12 |
| 1894 | 3D printing | ROLLER HOLDER\ | US2007214589A1 | JEWELL GARLAND M (US) | EVERCARE CO (US) | A47L25/00 |
| 1895 | 3D printing | ULTRASONIC JOINING METHOD ULTRASONIC JOINING DEVICE AND UNIT JOINED BY ULTRASONIC JOINING\ | US2007228107A1 | AOKI YUUICHI (JP)\ MORIKAWA HIROYASU (JP)\ SHIMIZU ARINORI (JP) | DENSO CORP (JP) | B23K20/10 |
| 1896 | 3D printing | BIOCOMPATIBLE MEMBRANE AND PROCESS FOR PRODUCING THE SAME\ | US2007231364A1 | NISHIMOTO KUNIO (JP)\ KATO HIROMU (JP)\ KATSURAGI YASUHIRO (JP)\ KAZUYOSHI KITA (JP) | KAZUYOSHI KITA (JP)\ NISHIMOTO KUNIO (JP)\ KATSURAGI YASUHIRO (JP)\ KATO HIROMU (JP) | A61L27/00\ A61F2/00\ A61K9/00\ A61K6/00\ A61L31/00 |
| 1897 | 3D printing | TAMPER-EVIDENT CLOSURE\ | US2007267381A1 | STALIONS STEPHEN E (US)\ BRANDON DAVID A (US)\ LAVEAULT RICHARD A (US)\ SCHMIDT WAYNE L (US) | SCHMIDT WAYNE L (US)\ LAVEAULT RICHARD A (US)\ BRANDON DAVID A (US)\ STALIONS STEPHEN E (US) | B65D51/00 |
| 1898 | 3D printing | METHOD FOR CONTROLLING A COMPENSATION CYLINDER UNIT IN PARTICULAR FOR A WELDING DEVICE AND ASSOCIATED COMPENSATION CYLINDER UNIT\ | US2007295697A1 | BRAUN FLORIAN (DE)\ LAUBACHER THOMAS (DE)\ SCHNUR FRANK (DE)\ SCHNEIDER-KONIG HELMUT (DE) | SCHNEIDER-KONIG HELMUT (DE)\ BRAUN FLORIAN (DE)\ LAUBACHER THOMAS (DE)\ SCHNUR FRANK (DE) | B23K11/31 |
| 1899 | 3D printing | METHOD FOR ATTACHING A SPOUT TO A FLEXIBLE FILM POUCH\ | US2007297700A1 | BERMAN RONALD H (US) | SADDLESPRINGS BEVERAGE COMPANY (US) | B65D33/16 |
| 1900 | 3D printing | PROCESS FOR PRODUCING A PLASTIC LENS\ | US2008001319A1 | KADOWAKI SHINICHIRO (JP) | KADOWAKI SHINICHIRO (JP) | B29D11/00 |
| 1901 | 3D printing | REFRIGERATING UNIT COMPRISING AN ULTRASOUND-WELDED SUCTION TUBE AND A THROTTLING TUBE\ | US2008016904A1 | KRANZ THOMAS (DE)\ SCHMID WERNER (DE)\ EBERLE JURGEN (DE) | BSH BAOSCH AND SIEMENS HAUSGER (DE) | B23P15/26\ F25B39/02\ F25B41/06\ F25B40/00 |
| 1902 | 3D printing | SOLID FREEFORM FABRICATION METHODS AND SYSTEMS\ | US2008018018A1 | FARR ISAAC (US)\ LAMBRIGHT TERRY M (US)\ NIELSEN JEFFREY A (US)\ DEVOS JOHN A (US)\ CRUZ-URIBE TONY S (US) | NIELSEN JEFFREY A (US)\ DEVOS JOHN A (US)\ CRUZ-URIBE TONY S (US)\ FARR ISAAC (US)\ LAMBRIGHT TERRY M (US) | B29C41/02 |
| 1903 | 3D printing | HIGH STRENGTH INFLATABLE MATTRESS\ | US2008022460A1 | YANG CHOU-LIAN (TW) | YANG CHOU-LIAN (TW) | A47C27/08 |
| 1904 | 3D printing | ULTRASONIC WELDING APPARATUS\ | US2008023529A1 | JAHN RAY (US)\ REATHERFORD LARRY VAN (US)\ WILKOSZ DANIEL EDWARD (US)\ SKOGSMO JAN (SE)\ HETRICK ELIZABETH THERESE (US) | REATHERFORD LARRY VAN (US)\ SKOGSMO JAN (SE)\ WILKOSZ DANIEL EDWARD (US)\ HETRICK ELIZABETH THERESE (US)\ JAHN RAY (US) | B23K20/10 |
| 1905 | 3D printing | GAS CATALYTIC COMBUSTION ELEMENT AND A GAS POWERED HEATING DEVICE\ | US2008041360A1 | PETER ALFRED (IE)\ OGLESBY JOHN P (IE) | OGLESBY & BUTLER RES & DEV LTD (IE) | B23K3/02 |
| 1906 | 3D printing | HYBRID RESISTANCE/ULTRASONIC WELDING SYSTEM AND METHOD\ | US2008041922A1 | FORREST MARIANA G (US)\ LU FENG (US)\ LOGAN STEPHEN D (US) | FORREST MARIANA G (US)\ LU FENG (US)\ LOGAN STEPHEN D (US) | B21D35/00 |
| 1907 | 3D printing | PALLET-TYPE MEMBRANE ELECTRODE ASSEMBLY LAYER STRUCTURE\ | US2008044689A1 | SHU HSI-MING (TW)\ CHANG TSANG-MING (TW)\ HUANG WEI-LI (TW) | SHU HSI-MING (TW)\ CHANG TSANG-MING (KY)\ HUANG WEI-LI (KY) | H01M8/00 |
| 1908 | 3D printing | LIGHT GUIDE TEST SENSOR FOR USE IN DETERMINING AN ANALYTE IN A FLUID SAMPLE AND METHODS FOR MANUFACTURING THE SAME\ | US2008064939A1 | JUNG SUNG-KWON (US)\ GEORGE SUNY J (US)\ CHARLTON STEVEN C (US)\ REYNOLDS JEFFREY S (US) | REYNOLDS JEFFREY S (US)\ CHARLTON STEVEN C (US)\ JUNG SUNG-KWON (US)\ GEORGE SUNY J (US) | B29C65/08\ A61B5/1455 |
| 1909 | 3D printing | SELF SUPPORTED FLUID FILTER\ | US2008066437A1 | SOLBERG CHARLES H JR (US)\ ANDERSON ADAM (US) | SOLBERG MFG INC (US) | B32B37/00\ B32B37/12\ B29C65/08 |
| 1910 | 3D printing | METHOD FOR MANUFACTURING SEALED BATTERY\ | US2008078815A1 | TANIGUCHI YASUTOMO (JP)\ YAMAUCHI YASUHIRO (JP)\ NAKANISHI NAOYA (JP)\ NOHMA TOSHIYUKI (JP) | SANYO ELECTRIC CO (JP) | H01M10/04\ H01M2/26\ H01M10/0566\ H01M10/058\ H01M10/0587\ B23K1/06 |
| 1911 | 3D printing | FUEL CELL STRUCTURE\ | US2008096084A1 | SHU HSI-MING (TW)\ CHANG TSANG-MING (TW)\ HUANG WEI-LI (TW) | SHU HSI-MING (TW)\ CHANG TSANG-MING (KY)\ HUANG WEI-LI (KY) | H01M8/02 |
| 1912 | 3D printing | ANVIL FOR ULTRASONIC WELDING AND DEVICE FOR ULTRASONIC WELDING\ | US2008105385A1 | WILD HANS-PETER (DE)\ KRAFT EBERHARD (DE)\ LECHERT FRANK (DE) | WILD HANS-PETER (DE)\ KRAFT EBERHARD (DE)\ LECHERT FRANK (DE) | B29C65/08 |
| 1913 | 3D printing | SEALING AND CUTTING DEVICE FOR POCKET COIL ROW\ | US2008110574A1 | ETOH HIROYUKI (JP) | ETOH HIROYUKI (JP) | B29C65/08 |
| 1914 | 3D printing | PROCESS FOR MAKING THREE DIMENSIONAL OBJECTS FROM DISPERSIONS OF POLYMER COLLOIDAL PARTICLES\ | US2008111282A1 | XIE BAOJUN (CN)\ SMAY JIM (US)\ WARREN WILLIAM (US)\ PARKHILL ROBERT (US) | XIE BAOJUN (CN)\ SMAY JIM (US)\ WARREN WILLIAM (US)\ PARKHILL ROBERT (US) | B29C35/08 |
| 1915 | 3D printing | BATTERY HOUSING STRUCTURES\ | US2008118823A1 | YANG CHI-SHAN (TW)\ CHANG SU NING (TW)\ HO HSIEN PENG (TW)\ HSIEH CHIH YUEN (TW)\ KUO SHENG JUNG (TW) | DYNAPACK INTERNAT TECHNOLOGY C (TW) | H01M2/10 |
| 1916 | 3D printing | ULTRASONIC WELDING DEVICE WITH OPPOSITE WELDING AND LATERAL SURFACES THAT DELIMIT A COMPRESSION SPACE AND COMPRESSION SPACE\ | US2008128471A1 | STROH DIETER (DE)\ DIETERLE HORST (DE)\ EBERBACH JOST (DE)\ STROH HEIKO (DE) | SCHUNK ULTRASCHALLTECHNIK GMBH (DE) | B23K20/10 |
| 1917 | 3D printing | AIR PACKING MACHINE USING ULTRASONIC SEALING AND METHODS AND PRODUCTS RELATING TO SAME\ | US2008141620A1 | SZABO BELA (US) | SZABO BELA (US) | B65D81/18 |
| 1918 | 3D printing | RUBBER SHOT PACKING\ | US2008141892A1 | KEMPF CHRISTIAN (CH) | SALTECH AG (CH) | F42B30/00 |
| 1919 | 3D printing | PRODUCTION PROCESS FOR PARTS OF A PERFLUOROPOLYMER COMPOSITION\ | US2008152924A1 | DEAKYNE CLIFFORD K (US)\ BOOZE J DAVID (US)\ GANNETT THOMAS P (US) | BOOZE J DAVID (US)\ DEAKYNE CLIFFORD K (US)\ GANNETT THOMAS P (US) | B32B27/06\ B29C65/06 |
| 1920 | 3D printing | RESOLVER MANUFACTURING METHOD THEREOF AND MOTOR USING THE RESOLVER\ | US2008169714A1 | KATAOKA NAKABA (JP)\ HATTORI TAKAHIRO (JP)\ NAKANISHI KEITA (JP) | NIDEC CORP (JP) | H02K24/00\ H02K15/00 |
| 1921 | 3D printing | ULTRASONIC WELDING SYSTEM\ | US2008173694A1 | WILD HANS-PETER (DE)\ KRAFT EBERHARD (DE)\ LECHERT FRANK (DE) | WILD HANS-PETER (DE)\ KRAFT EBERHARD (DE)\ LECHERT FRANK (DE) | B23K1/06 |
| 1922 | 3D printing | ATHLETIC PROTECTIVE UNDERGARMENT\ | US2008184467A1 | BRASSILL PATRICK (US) | BRASSILL PATRICK (US) | A41D1/00\ A41D13/00\ A41D27/00\ A41D1/06\ A41D27/26 |
| 1923 | 3D printing | WELDING PROCESS AND GEOSYNTHETIC PRODUCTS THEREOF\ | US2008210359A1 | HALAHMI IZHAR (IL)\ EREZ ODED (IL)\ EREZ ADI (IL) | PRS MEDITERRANEAN LTD (IL) | B32B37/00 |
| 1924 | 3D printing | BATTERY PACK\ | US2008286642A1 | NAITO MASAKAZU (JP)\ SUZUKI HIDEYUKI (JP) | SONY CORP (JP) | H01M2/10 |
| 1925 | 3D printing | PACKAGING-TYPE VACUUM-FORMING COMPOSITE BOARD WELDED BY ULTRASONIC WELDING\ | US2008295454A1 | LENG LUHAO (CN) | LENG LUHAO (CN) | B65B31/02 |
| 1926 | 3D printing | METHOD FOR ULTRASONIC WELDING AND ULTRASONIC WELDING FASTENER AND JOINING SYSTEMS\ | US2008295947A1 | BOURBEAU ALAIN (CA)\ BELANGER GERMAIN (CA) | BOURBEAU ALAIN (CA)\ BELANGER GERMAIN (CA) | B29C65/08 |
| 1927 | 3D printing | METHOD OF ATTACHING AN RFID TAG TO A COMPONENT A COMPONENT COMPRISING AN RFID TAG AND RFID TAG\ | US2008309497A1 | BRYANT KEITH C (GB) | BRYANT KEITH C (GB) | G06K19/04\ G06K19/077 |
| 1928 | 3D printing | FIBER OPTIC ADAPTER WITH INTEGRATED SHUTTER\ | US2009028507A1 | JONES ASHLEY WESLEY (US)\ LEYVA JR DANIEL (US)\ MUDD RONALD LEE (US)\ PARKMAN III LOUIS EDWARD (US)\ TRAVIS PETER TIMOTHY (US) | JONES ASHLEY WESLEY (US)\ LEYVA JR DANIEL (US)\ MUDD RONALD LEE (US)\ PARKMAN III LOUIS EDWARD (US)\ TRAVIS PETER TIMOTHY (US) | G02B6/38 |
| 1929 | 3D printing | METHOD OF MAKING RFID DEVICES\ | US2009038735A1 | KIAN KOUROCHE (US) | KIAN KOUROCHE (US) | B29C65/08 |
| 1930 | 3D printing | SUPPORT ARCHITECTURE\ | US2009039570A1 | CLARK DANIEL (GB) | ROLLS ROYCE PLC (GB) | C04B35/653 |
| 1931 | 3D printing | AUTOMATED EVACUATION AND SEALING PROCESS\ | US2009057376A1 | HOOVER WILLIAM S (US)\ FRIBLEY JOSH M (US)\ MORAN MICHAEL E (US) | HONEYWELL INT INC (US) | B23K20/10 |
| 1932 | 3D printing | ADHESIVE TAPE COMPRISING A SPUNBONDED BACKING ONE-SIDEDLY COATED AT LEAST PARTIALLY WITH A PRESSURE-SENSITIVE ADHESIVE\ | US2009075052A1 | HOPF MARTIN (DE) | TESA AG (DE) | B32B5/02\ C09J7/00\ B32B37/04 |
| 1933 | 3D printing | FILTER ELEMENT AND ARRANGEMENT\ | US2009078117A1 | SCHROTH THOMAS (DE)\ RIVERA SAMUEL (US)\ JULCH KARL (DE)\ DRIESEN ECKHART (DE) | FREUDENBERG CARL KG (DE) | B01D53/04\ B01D46/06 |
| 1934 | 3D printing | MANUFACTURING METHOD OF OPTICAL SHEETS FOR DISPLAY\ | US2009078366A1 | ENDO KEISUKE (JP)\ TAKEDA AKIHIKO (JP) | FUJIFILM CORP (JP) | B32B38/04\ B32B37/06 |
| 1935 | 3D printing | ATTACHMENT STRUCTURES LIQUID EJECTION HEAD UNIT AND METHOD FOR MANUFACTURING A LIQUID EJECTION HEAD UNIT\ | US2009085958A1 | IMAI KAZUYOSHI (JP) | SEIKO EPSON CORP (JP) | B41J2/015\ B23P17/00 |
| 1936 | 3D printing | END STOPS FOR ZIPPERS FOR RECLOSABLE PACKAGES\ | US2009094803A1 | BENTSEN PER (US) | ILLINOIS TOOL WORKS (US) | A44B19/00 |
| 1937 | 3D printing | MEDICATION PORT FOR MEDICAL FLUID CONTAINER\ | US2009105684A1 | BALTEAU PATRICK (BE)\ PESCI GIAMPIERO (IT)\ DEWEZ JEAN LUC (BE) | BAXTER INT (US)\ BAXTER HEALTHCARE SA (CH) | A61B19/00 |
| 1938 | 3D printing | RELIEF VALVE IN A TURBOMACHINE\ | US2009121172A1 | MAJEUX CLAUDE MARCEL ROBERT (FR)\ COMIN FRANCOIS JEAN (FR) | SNECMA (FR) | F16K5/00 |
| 1939 | 3D printing | PRINTED CIRCUIT BOARD ASSEMBLY AND MANUFACTURING METHOD FOR THE SAME\ | US2009139758A1 | SHIN HYO YOUNG (KR)\ JUNG SEUNG BOO (KR)\ MOON YOUNG JUN (KR)\ HONG SOON MIN (KR)\ LEE CHANG YONG (KR)\ KOO JA MYEONG (KR)\ KIM HYUN TAE (KR)\ LEE JONG BUM (KR)\ HAN HYUN JOO (KR) | SAMSUNG ELECTRONICS CO LTD (KR) | H05K3/36\ H01R12/04 |
| 1940 | 3D printing | MEDICAL SYNTHESIS SYSTEM AND METHOD OF CONNECTING A MULTIPART MEDICAL SYNTHESIS SYSTEM\ | US2009143780A1 | GABELE LORENZ (DE)\ REINAUER FRANK (DE) | GABELE LORENZ (DE)\ REINAUER FRANK (DE) | A61B17/00 |
| 1941 | 3D printing | PLASTIC COMPOSITE BOARD WITH ULTRASONIC WELDED STRUCTURE\ | US2009148668A1 | LENG LUHAO (CN) | LENG LUHAO (CN) | B32B3/30 |
| 1942 | 3D printing | METHOD FOR CONSOLIDATING AND SEALING A TUBE\ | US2009152325A1 | NEIS DAN (DE)\ FRANK HARALD (DE) | FRANK HARALD (DE)\ NEIS DAN (DE) | B23K20/10\ B29C65/00\ B29C65/08\ B29C65/74\ B29C57/10\ B23K31/12 |
| 1943 | 3D printing | ULTRASONIC WELDING-BASED MICROFLUIDIC DEVICE AND METHOD OF MANUFACTURING THE SAME\ | US2009152326A1 | SHIN DONG HO (KR)\ KIM YOUNG JUN (KR)\ JEONG MIN SUK (KR)\ KIM SANG HEE (KR)\ KIM HYE YOON (KR)\ JUNG MOON YOUN (KR)\ PARK SEON HEE (KR) | KOREA ELECTRONICS TELECOMM (KR) | B23K20/10\ B23K1/06 |
| 1944 | 3D printing | PUMP USING UNIMORPH DIAPHRAGM\ | US2009162224A1 | WAKABAYASHI MASANORI (JP)\ OKI YASUMASA (JP) | WAKABAYASHI MASANORI (JP)\ OKI YASUMASA (JP) | F04B17/00 |
| 1945 | 3D printing | RE-WORKABLE PRESSURE VESSELS FOR SUPERCONDUCTING MAGNET ARRANGEMENTS\ | US2009162584A1 | RAYNER EDGAR CHARLES MALCOLM (GB)\ HOBBS MATTHEW (GB)\ LEFEUVRE MARK JAMES (GB) | SIEMENS MAGNET TECHNOLOGY LTD (GB) | B32B1/02\ B32B38/10\ B02C23/00 |
| 1946 | 3D printing | MICROFLUIDIC DEVICE AND METHOD OF FABRICATING THE SAME\ | US2009170189A1 | NAMKOONG KAK (KR)\ PARK CHIN-SUNG (KR)\ LEE YOUNG-SUN (KR) | SAMSUNG ELECTRONICS CO LTD (KR) | C12M1/34\ C12M1/00\ B32B37/06 |
| 1947 | 3D printing | POWDER METAL ULTRASONIC WELDING TOOL AND METHOD OF MANUFACTURE THEREOF\ | US2009212089A1 | CHRISTOPHERSON JR DENIS (US) | CHRISTOPHERSON JR DENIS (US) | B22F7/02\ B22F1/00\ B23K20/10\ B22F3/12 |
| 1948 | 3D printing | MULTICOMPONENT FUSED SUTURE LOOP AND APPARATUS FOR MAKING SAME\ | US2009216269A1 | GRAY JOHN R (US)\ WESTHAVER PAUL (US)\ FENTON PAUL (US)\ HARRINGTON FRANCIS (US)\ SCHMITT PETER (US) | AXYA MEDICAL INC (US) | B32B37/00\ A61B17/04 |
| 1949 | 3D printing | HEATED MOTORCYCLE GAS TANK COVER\ | US2009218334A1 | WALLACE KYIE (US) | WALLACE KYIE (US) | H05B3/34 |
| 1950 | 3D printing | HEATED MOTORCYCLE GAS TANK COVER\ | US2009218335A1 | WALLACE KYIE (US) | WALLACE KYIE (US) | H05B3/34 |
| 1951 | 3D printing | KEY ASSEMBLY\ | US2009241621A1 | SHI ZHENG (CN)\ CHANG CHIH-CHIANG (TW) | FIH HONG KONG LTD (HK)\ SHENZHEN FUTAIHONG PREC IND CO (CN) | E05B19/00 |
| 1952 | 3D printing | METHOD OF MAKING HEAT SINK\ | US2009249624A1 | GUO QING-LEI (CN)\ ZHU SHOU-LI (CN) | FU ZHUN PRECISION IND SHENZHEN (CN)\ FOXCONN TECH CO LTD (TW) | B21D53/02 |
| 1953 | 3D printing | ULTRASONIC WELDING TOOL WITH FLUID DRIVE\ | US2009250170A1 | AUST VOLKER (DE) | HERMANN ULTRASCHALLTECHNIK GMB (DE) | B32B37/00 |
| 1954 | 3D printing | PROCESS AND DEVICE FOR AUTOMATICALLY DETERMINING THE BEGINNING OF A WELDING PROCESS FOR MAKING TUBULAR LABELS\ | US2009266469A1 | ZACCHE VANNI (IT)\ MARASTONI DANIELE (IT)\ PHUCHAKR PRINGPUANGKEO (IT) | SIDEL SPA (IT) | B32B37/00 |
| 1955 | 3D printing | SELECTIVE DEPOSITION MODELING USING CW UV LED CURING\ | US2009267269A1 | CLAY JOHN D (US)\ MOUSSA KHALIL (US)\ LIM JIN HONG (US)\ ANGULO HERNANDO VINCENTE (US) | ANGULO HERNANDO VINCENTE (US)\ LIM JIN HONG (US)\ CLAY JOHN D (US)\ MOUSSA KHALIL (US) | B29C35/08 |
| 1956 | 3D printing | FENCE SUCH AS A HIGH VISIBILITY ANIMAL FENCE AND METHOD OF MAKING\ | US2009278104A1 | MARRECAU WILLY LOUIS (US)\ ASPINWALL DAVID MICHAEL (US)\ FERRINGER MICHAEL (US) | MARRECAU WILLY LOUIS (US)\ ASPINWALL DAVID MICHAEL (US)\ FERRINGER MICHAEL (US) | B29C65/08\ B29C65/50\ E04H17/14 |
| 1957 | 3D printing | METHODS FOR CLEANING AND CURING SOLID FREEFORM FABRICATION PARTS\ | US2009283109A1 | REICHENTAL ABRAHAM N (US)\ MOUSSA KHALIL (US)\ SPERRY CHARLES R (US)\ MCNAMARA DENNIS F (US)\ SCOTT SUZANNE M (US)\ MUSKUS KRZYSZTOF J (US) | SPERRY CHARLES R (US)\ REICHENTAL ABRAHAM N (US)\ MOUSSA KHALIL (US)\ MCNAMARA DENNIS F (US)\ SCOTT SUZANNE M (US)\ MUSKUS KRZYSZTOF J (US) | B08B3/04\ B08B3/10 |
| 1958 | 3D printing | POST-PROCESSING SYSTEM FOR SOLID FREEFORM FABRICATION PARTS\ | US2009283119A1 | REICHENTAL ABRAHAM N (US)\ MOUSSA KHALIL (US)\ MCNAMARA DENNIS F (US)\ SCOTT SUZANNE M (US)\ MUSKUS KRZYSZTOF J (US)\ PERRY CHARLES R (US) | PERRY CHARLES R (US)\ REICHENTAL ABRAHAM N (US)\ MOUSSA KHALIL (US)\ MCNAMARA DENNIS F (US)\ SCOTT SUZANNE M (US)\ MUSKUS KRZYSZTOF J (US) | B08B7/04\ B08B3/12 |
| 1959 | 3D printing | SEAM JOINING TOGETHER AT LEAST TWO WEB MATERIALS\ | US2009292266A1 | BACK LUCAS (SE) | SCA HYGIENCE PRODUCTS AB (SE) | B29C65/02\ B29C65/08\ A61L15/58 |
| 1960 | 3D printing | ULTRASONIC WELDING MEMBER FOR SMOOTHING FLEXIBLE BELT SEAMS AND METHOD FOR USING\ | US2009294513A1 | ROETKER MICHAEL S (US)\ MARTIN DAVID W (US)\ DEHOLLANDER DAVID A (US)\ HERKO JONATHAN H (US)\ GRIFFIN SCOTT J (US) | XEROX CORP (US) | B23K20/10 |
| 1961 | 3D printing | FOIL BASED SEMICONDUCTOR PACKAGE\ | US2009305076A1 | WONG WILL (US)\ TU NGHIA THUC (US)\ BAYAN JAIME (US)\ CHIN DAVID (US) | NAT SEMICONDUCTOR CORP (US) | B32B37/00\ B32B15/01 |
| 1962 | 3D printing | MICROCHIP MANUFACTURING METHOD\ | US2010012255A1 | SEKIHARA KANJI (JP)\ UEHIRA MASAYOSHI (JP) | SEKIHARA KANJI (JP)\ UEHIRA MASAYOSHI (JP) | B32B38/10\ B29C65/08 |
| 1963 | 3D printing | METHOD AND APPARATUS FOR MANUFACTURING THIN FILM AND METHOD FOR MANUFACTURING ELECTRONIC DEVICE\ | US2010040771A1 | KADONO KOJI (JP) | SONY CORP (JP) | B05D5/12\ B05C9/12 |
| 1964 | 3D printing | ANISOTROPICALLY COMPLIANT HORNS FOR ULTRASONIC VIBRATORY SOLID-STATE BONDING\ | US2010040903A1 | KALT ANDREW S (US)\ KESHNER MARVIN (US)\ RICE DONALD WINSTON (US)\ STAWITCKE FREDERICK A (US)\ SANCHEZ JESSIE RAMIREZ (US) | OPTISOLAR INC A DELAWARE CORP (US) | B23K20/10\ B32B15/01 |
| 1965 | 3D printing | SINGLE-REEL TYPE TAPE CARTRIDGE\ | US2010050413A1 | INUGAI YASUO (JP)\ KATAYAMA TAKUYA (JP) | INUGAI YASUO (JP)\ KATAYAMA TAKUYA (JP) | G11B23/26\ G11B23/107\ B29C65/00\ B29C65/02\ B29C65/08\ B29C53/42\ G11B15/67\ B23P11/00 |
| 1966 | 3D printing | WELDING PROCESS AND GEOSYNTHETIC PRODUCTS THEREOF\ | US2010055443A1 | HALAHMI IZHAR (IL)\ EREZ ODED (IL)\ EREZ ADI (IL) | PRS MEDITERRANEAN LTD (IL) | B32B37/00\ B32B27/00 |
| 1967 | 3D printing | METHOD AND MEANS FOR THE HIGH-SPEED MANUFACTURING OF INDIVIDUAL PACKAGING FOR A LIQUID OR SOLID PRODUCT\ | US2010065187A1 | VAILLANT RENAUD (FR)\ PETEUIL PIERRE (FR) | CRYOLOG S A (FR) | B29C65/08 |
| 1968 | 3D printing | FLUID-CONTAINING POUCHES WITH REDUCED GAS EXCHANGE AND METHODS FOR MAKING SAME\ | US2010068097A1 | ADE RHONDA (CA)\ POIRIER PHILIPPE (CA)\ DICKE BILL (CA)\ BREEZE STEVEN R (CA) | I STAT CORP (US) | B01L3/00\ B32B37/00\ B32B38/00\ B01J19/00\ F16J15/02 |
| 1969 | 3D printing | ULTRASONIC SYSTEM AND METHOD FOR AFFIXING A SCREEN SUB-ASSEMBLY TO A PLATE\ | US2010071194A1 | DERRICK ROBERT G (US)\ SCHILLER RONALD J (US) | DERRICK ROBERT G (US)\ SCHILLER RONALD J (US) | H05K13/00 |
| 1970 | 3D printing | USE OF A DEVICE FOR TORSIONAL ULTRASONIC WELDING\ | US2010078115A1 | LANG GEORG (DE) | TELSONIC HOLDING AG (CH) | B29C65/08\ B28B1/087 |
| 1971 | 3D printing | THIN FOIL FOR USE IN PACKAGING INTEGRATED CIRCUITS\ | US2010084748A1 | TU NGHIA THUC (US)\ BAYAN JAIME A (US)\ WONG WILL K (US)\ PHAM KEN (US)\ PODDAR ANINDYA (US) | NAT SEMICONDUCTOR CORP (US) | B32B3/00\ B32B15/04\ H01L23/48\ H01L21/50\ B23K1/06 |
| 1972 | 3D printing | WIRING HARNESS AND A METHOD FOR PRODUCING THE SAME AND A METHOD FOR CONNECTING INSULATED WIRES\ | US2010096185A1 | OTSUKA YASUYUKI (JP)\ HOSOKAWA TAKEHIRO (JP)\ INOUE MASATO (JP)\ IMASATO FUMITOSHI (JP) | SUMITOMO ELECTRIC INDUSTRIES (JP)\ SUMITOMO WIRING SYSTEMS (JP)\ AUTONETWORKS TECHNOLOGIES LTD (JP) | H01R4/02\ H01R43/00 |
| 1973 | 3D printing | SYSTEMS AND METHODS FOR MANUFACTURING REINFORCED WEATHERSTRIP\ | US2010136317A1 | HUNTRESS JOHN E (US)\ MERTINOOKE PETER (US) | AMESBURY GROUP (US) | B32B37/00\ B05D5/00\ B32B3/26\ B32B38/00\ B28B19/00 |
| 1974 | 3D printing | COMPOSITIONS FOR 3D PRINTING\ | US2010140850A1 | NAPADENSKY EDUARDO (IL)\ RAVICH DIANA (IL) | OBJET GEOMETRIES LTD (IL) | B29C35/08\ C08F2/46 |
| 1975 | 3D printing | VEHICLE SEAT STRUCTURE\ | US2010141009A1 | KIRCH ECKHARD (DE)\ UTSCH HEIKO (DE) | KIRCH ECKHARD (DE)\ UTSCH HEIKO (DE) | B23K20/10\ B60N2/68 |
| 1976 | 3D printing | POLARIZED GLASS AND METHOD FOR MANUFACTURING THE POLARIZED GLASS\ | US2010167904A1 | ICHIMURA MASAHIRO (JP)\ MORIMOTO SHOZO (JP)\ KANAYA YOHSUKE (JP) | POLATECHNO CO LTD (JP) | C03C14/00\ C03C15/00 |
| 1977 | 3D printing | METHOD OF REMOVING SUPPORT STRUCTURE FROM 3-D OBJECTS MADE BY SOLID FREEFORM FABRICATION\ | US2010170540A1 | KRITCHMAN ELIAHU M (IL)\ COHEN AVI (IL)\ KLEINHENDLER CHAIM (IL)\ TARAN DAVID (IL)\ NAROVLYANSKY VLADIMIR (IL)\ SOFFER BEVERLEY (IL) | COHEN AVI (IL)\ KRITCHMAN ELIAHU M (IL)\ KLEINHENDLER CHAIM (IL)\ TARAN DAVID (IL)\ NAROVLYANSKY VLADIMIR (IL)\ SOFFER BEVERLEY (IL) | B08B3/00 |
| 1978 | 3D printing | METHOD AND APPARATUS FOR MONITORING ELECTRO-MAGNETIC RADIATION POWER IN SOLID FREEFORM FABRICATION SYSTEMS\ | US2010173096A1 | KRITCHMAN ELIAHU M (IL)\ MENCHIK GUY (IL)\ LEVI MOSHE (IL)\ LIBINSON ALEXANDER (IL) | LIBINSON ALEXANDER (IL)\ KRITCHMAN ELIAHU M (IL)\ LEVI MOSHE (IL)\ MENCHIK GUY (IL) | B05D3/06\ B05C11/02 |
| 1979 | 3D printing | ULTRASONIC WELDING USING AMPLITUDE PROFILING\ | US2010176184A1 | GREWELL DAVID A (US)\ SHEEHAN JAMES F (US) | BRANSON ULTRASONICS CORP (US) | B23K20/10 |
| 1980 | 3D printing | FLUID BEARING DEVICE\ | US2010177998A1 | SATOJI FUMINORI (JP)\ ITOU KENJI (JP) | NTN TOYO BEARING CO LTD (JP) | B29C45/00\ B29C65/08\ F16C17/10\ F16C33/10\ F16C35/02\ H02K7/08\ F16C17/02\ F16C33/20\ F16C33/74\ G11B19/20\ F16C32/06 |
| 1981 | 3D printing | GOLF CLUB ASSEMBLY USING ULTRASONIC WELDING\ | US2010181368A1 | GALLOWAY J ANDREW (US) | CALLAWAY GOLF CO (US) | B23K20/10 |
| 1982 | 3D printing | METHOD FOR PRODUCING A COMPOSITE PLASTIC SHEET THE COMPOSITE PLASTIC SHEET AND THE SEAT BACK OF A CHAIR MADE OF THE COMPOSITE PLASTIC SHEET\ | US2010187892A1 | LENG LUHAO (CN) | LENG LUHAO (CN) | B29D99/00\ B32B7/08\ B32B1/00\ A47C7/16 |
| 1983 | 3D printing | RADIATION CURABLE COMPOSITIONS USEFUL IN SOLID FREEFORM FABRICATION SYSTEMS\ | US2010190881A1 | STEINMANN BETTINA (CH) | 3D SYSTEMS INC (US) | C08F291/10\ C08F4/42\ C08F299/04 |
| 1984 | 3D printing | SOLID FREEFORM FABRICATION USING A PLURALITY OF MODELING MATERIALS\ | US2010191360A1 | NAPADENSKY EDUARDO (IL)\ KLEINHENDLER CHAIM (IL)\ SHTILERMAN SLAVA (IL) | OBJECT GEOMETRIES LTD (IL) | G06F17/50 |
| 1985 | 3D printing | DISPENSING DEVICE\ | US2010206911A1 | BERNHARD JASPER (DE) | SEAQUIST PERFECT DISPENSING (DE) | B65D83/62 |
| 1986 | 3D printing | BIODEGRADABLE RESIN COMPOSITION UTILIZED IN THE MANUFACTURE OF BIODEGRADABLE CONTAINERS BIODEGRADABLE CONTAINERS AND METHOD OF MANUFACTURE\ | US2010230405A1 | STRAIT III JAMES LEE (US) | NUVISION BIOPLASTICS LLC (US) | B29C65/06\ B65D6/28\ C08L1/00 |
| 1987 | 3D printing | COMPONENT JOINING STRUCTURE IC CARD AND CONNECTOR\ | US2010232118A1 | TOCHI AKIHIRO (JP)\ TSUJIMOTO MASAKI (JP) | J S T MFG CO LTD (JP) | H05K5/00\ G06K19/077\ H01R13/648\ H01R13/40 |
| 1988 | 3D printing | INDUSTRIAL FABRIC INCLUDING SPIRALLY WOUND MATERIAL STRIPS\ | US2010236034A1 | BOTELHO JOSEPH (US)\ EAGLES DANA (US)\ KARLSSON JONAS (SE)\ STOWE BRUCE (US)\ MOURAD SABRI (FR)\ O'CONNOR JERRY (US)\ MONTCRIEFF JON (US)\ HANSEN ROBERT (US) | BOTELHO JOSEPH (US)\ EAGLES DANA (US)\ KARLSSON JONAS (SE)\ STOWE BRUCE (US)\ MOURAD SABRI (FR)\ O'CONNOR JERRY (US)\ MONTCRIEFF JON (US)\ HANSEN ROBERT (US) | D06M10/00\ B65H81/08 |
| 1989 | 3D printing | METHOD OF FASTENING A CIRCUIT ASSEMBLY TO A LIGHT GUIDE IN A MANUFACTURING PROCESS OF A LCD-DISPLAY\ | US2010245702A1 | ROBERTS TOM (NL)\ VERWEG FRANS (NL)\ AMORY RALPH (BE) | TPO DISPLAYS CORP (TW) | G02F1/1333\ H01J9/20 |
| 1990 | 3D printing | METHOD AND APPARATUS FOR THE MANUFACTURE OF POUCHES\ | US2010262273A1 | DOSE THOMAS (DE)\ STORCKS ANDREAS (DE)\ GESKE HAGEN (DE) | FOCKE & CO (DE) | G06F19/00 |
| 1991 | 3D printing | SEAM JOINING TOGETHER AT LEAST TWO WEB MATERIALS\ | US2010268183A1 | EEN HANS (SE)\ LEHTO MARCUS (SE)\ CARLSON ULRIKA (SE) | SCA HYGIENE PROD AB (SE) | B32B3/10\ A61F13/49 |
| 1992 | 3D printing | CANTILEVERED BAR GAP ADJUSTMENT FOR AN ULTRASONIC WELDING SYSTEM\ | US2010276061A1 | NAYAR SATINDER K (US)\ POCHARDT DONALD L (US)\ OBLAK DONALD S (US)\ MLINAR JOHN R (US)\ FETTIG PAUL M (US) | 3M INNOVATIVE PROPERTIES CO (US) | B32B37/06 |
| 1993 | 3D printing | METHOD OF PRODUCING A DEVICE COMPRISING AT LEAST TWO DISTINCT COMPONENTS THAT ARE INTERCONNECTED BY INTERCONNECTING WIRES AND A DEVICE THEREBY OBTAINED\ | US2010276497A1 | SEBAN FREDERICK (FR)\ FIDALGO JEAN-CHRISTOPHE (FR) | GEMALTO SA (FR) | G06K19/06\ H05K3/36\ G06K19/077\ H05K1/18 |
| 1994 | 3D printing | TWO PART SLIP BOBBER AND METHOD OF ASSEMBLY\ | US2010281757A1 | BENNIS GARY (US) | BENNIS GARY (US) | A01K93/00 |
| 1995 | 3D printing | ERGONOMIC HORN\ | US2010282821A1 | COTO GUILLERMO (US)\ HANSEN RICHARD R (US) | COTO GUILLERMO (US)\ HANSEN RICHARD R (US) | B23K1/06 |
| 1996 | 3D printing | METHOD AND DEVICE FOR CONNECTING A CABLE TO AN ELECTRICAL CONNECTION ELEMENTS\ | US2010288819A1 | LANG GEORG (DE)\ HUENIG THOMAS (DE)\ SPENGER PETER (CH) | TELSONIC HOLDING AG (CH) | B23K20/10 |
| 1997 | 3D printing | BACK WING FOR BRASSIERE\ | US2010297911A1 | LIU ZHEN QIANG (HK) | LIU ZHEN QIANG (HK) | A41C3/00 |
| 1998 | 3D printing | DEVICE AND METHOD FOR ULTRASONIC TREATMENT\ | US2010307660A1 | MOEGLICH HARTMUT (DE) | TELSONIC HOLDING AG (CH) | B29C65/08 |
| 1999 | 3D printing | SINGLE-LAYER PLASTIC COMPOSITE PANEL\ | US2010310811A1 | LENG LUHAO (CN) | LENG LUHAO (CN) | B32B3/10\ B32B3/02\ B32B3/00\ B32B3/30\ B32B1/04 |
| 2000 | 3D printing | DEVICE USE OF THE DEVICE AND METHOD FOR TORSIONAL ULTRASONIC WELDING\ | US2010326586A1 | MOEGLICH HARTMUT (DE) | TELSONIC HOLDING AG (CH) | B29C65/08 |
| 2001 | 3D printing | METHOD AND ARRANGEMENT FOR MANUFACTURING A LAMINATED STRUCTURE\ | US2011004178A1 | FERNKVIST MARIA (SE)\ TONDKAR MARYAM (SE) | SCA HYGIENE PROD AB (SE) | B32B3/26\ B32B7/04\ B29C65/08\ A61F13/51 |
| 2002 | 3D printing | METHOD TO INTEGRATE AN RFID COMPONENT IN A PROSTHESIS OR IMPLANT SUBSTITUTE OR IN A SURGICAL INSTRUMENT IN POLYMER OR PLASTIC\ | US2011009962A1 | GRIMARD JEAN-CHRISTOPHE (FR) | SFERIC STELLITE (FR) | A61F2/02\ A61B17/00\ B32B37/04 |
| 2003 | 3D printing | THERMOPLASTIC FENCING CONSTRUCTION\ | US2011012078A1 | FATTORI JAMES (US) | LMT MERCER GROUP INC (US) | E04H17/14 |
| 2004 | 3D printing | ULTRASONIC WELDING APPARATUS AND ULTRASONIC WELDING METHOD\ | US2011042444A1 | OHNUMA KENTARO (JP) | YAZAKI CORP (JP) | B23K20/10 |
| 2005 | 3D printing | APPARATUS AND METHOD FOR ELECTRICAL CONNECTION CLAMPING\ | US2011059660A1 | KONKLE NICHOLAS RYAN (US)\ MORITZ MICHAEL RICHARD (US)\ CORCORAN THOMAS MICHAEL (US) | GEN ELECTRIC (US) | H01R43/00\ H01R4/44\ H01R4/28 |
| 2006 | 3D printing | INTEGRAL DOUBLE BAG FOR VACUUM BAGGING A COMPOSITE PART AND METHOD OF USING THE SAME\ | US2011067359A1 | KULESHA RICHARD L (US) | BOEING CO (US) | B32B38/04\ B29C65/48\ B65D30/08\ B65B31/00 |
| 2007 | 3D printing | ELECTROWETTING MICROARRAY PRINTING SYSTEM AND METHODS FOR BIOACTIVE TISSUE CONSTRUCT MANUFACTURING\ | US2011076734A1 | ZHOU GONGYAO (US)\ LELKES PETER I (US)\ FAIR ROBERT B (US) | UNIV DREXEL (US)\ UNIV DUKE (US) | C12M3/00\ C12N13/00 |
| 2008 | 3D printing | WIRE HARNESS AND METHOD OF MANUFACTURING THE SAME\ | US2011104943A1 | SUZUKI SACHIO (JP)\ TAKEHARA HIDEAKI (JP)\ FUKUDA KUNIHIRO (JP)\ KATAOKA YUTA (JP) | HITACHI CABLE (JP) | B29C65/08\ H01R13/52 |
| 2009 | 3D printing | CONNECTION OF AERONAUTICAL STRUCTURAL ELEMENTS WITH OTHER THERMOPLASTIC ELEMENTS\ | US2011120621A1 | SANCHEZ BLAZQUEZ ANGELES (ES) | AIRBUS OPERATIONS SL (ES) | B32B9/04\ B32B27/02\ B29C65/08 |
| 2010 | 3D printing | APPARATUS AND METHOD FOR WELDING A WORKPIECE\ | US2011120622A1 | MOEGLICH HARTMUT (DE) | TELSONIC HOLDING AG (CH) | B29C65/00\ B29C65/06 |
| 2011 | 3D printing | RETAINING FRAME COMPRISING AT LEAST ONE OPTICAL ELEMENT\ | US2011122617A1 | FREY PETER (DE)\ VOLLMER RALF (DE) | OSRAM GMBH (DE) | G02B7/02\ B23P17/00\ F21V7/00 |
| 2012 | 3D printing | IMPROVEMENTS IN OR RELATING TO JOINTS AND/OR IMPLANTS\ | US2011125284A1 | GABBRIELLI RUGGERO (GB)\ TURNER IRENE GLADYS (GB)\ BOWEN CHRISTOPHER RHYS (GB)\ MAGALINI EMANUELE (IT) | UNIV BATH (GB) | A61F2/02\ A61F2/30\ B23P17/00 |
| 2013 | 3D printing | FIBERS AND METHODS FOR USE IN SOLID FREEFORM FABRICATION\ | US2011143108A1 | FRUTH CARL JOHANNES (DE)\ FEULNER ROBERT (DE)\ SCHMACHTENBERG ERNST (DE)\ RIETZEL DOMINIK (DE) | FIT FRUTH INNOVATIVE TECHNOLOGIEN GMBH (DE) | B32B5/02\ B29C70/28\ B32B7/14 |
| 2014 | 3D printing | COULOMB DAMPING FEATURES USING ULTRASONIC WELDING\ | US2011143161A1 | SCHROTH JAMES G (US)\ PERRY THOMAS A (US) | GM GLOBAL TECH OPERATIONS INC (US) | B23K20/10\ B32B15/01\ B23K31/02 |
| 2015 | 3D printing | CHAMBER\ | US2011155615A1 | MARKHAM LEE (GB)\ TICCI ANDREA (IT) | PETROTECHNIK LTD (GB) | B65D21/00\ B23P17/04 |
| 2016 | 3D printing | PROSTHESIS COMPRISING KNITTED MATERIAL LAYERS AND METHOD OF MANUFACTURING BY ULTRASONIC WELDING\ | US2011166494A1 | CALVEZ XAVIER (FR)\ EVRARD FREDERIC (FR) | SOFRADIM PRODUCTION (FR) | A61M5/00\ B32B37/10\ B32B5/26\ B32B7/04\ B32B37/14\ B32B37/02 |
| 2017 | 3D printing | METHOD OF ATTACHING TIES TO A MEDICAL GOWN AND A MEDICAL GOWN HAVING TIES SECURED IN ACCORDANCE WITH THE METHOD\ | US2011167534A1 | WONG NANCY (CA)\ LEEUW CHRISTOPHER (CA)\ MARUSYK RAYMOND (CA)\ ETHIER LUCAS (CA) | PRIMED MEDICAL PRODUCTS INC (CA) | A41D13/12 |
| 2018 | 3D printing | METHOD FOR THE PRODUCTION OF SELF-VENTING PACKAGING\ | US2011179754A1 | UTZ HELMAR (CH) | AMCOR FLEXIBLES TRANSPAC NV (BE) | B65B5/00 |
| 2019 | 3D printing | WIREBONDING PROCESS\ | US2011192885A1 | HOCHSTENBACH HENDRIK PIETER (NL)\ VAN DRIEL WILLEM DIRK (NL)\ OOMS ERIC (NL) | NXP BV (NL) | B23K31/02\ B23K1/20 |
| 2020 | 3D printing | JOINT DESIGN FOR WELDING PLASTIC ASSEMBLIES\ | US2011206898A1 | HAYSLIP JASON M (US) | GM GLOBAL TECH OPERATIONS INC (US) | B32B3/10\ B29C65/08 |
| 2021 | 3D printing | CONNECTION OF ELECTRICAL CABLES BY ULTRASONIC WELDING\ | US2011220385A1 | MARTENS SEBASTIAN (DE)\ LIETZ FRANZ-JOSEF (DE) | AUTO KABEL MAN GMBH (DE) | H01R43/02\ H02G3/06 |
| 2022 | 3D printing | ULTRASONIC WELD PAD FOR WELDING BATTERY CELL TABS\ | US2011248069A1 | KHAKHALEV ALEXANDER D (US)\ SPACHER PAUL F (US)\ BRACEY JENNIFER (US) | GM GLOBAL TECH OPERATIONS INC (US) | B23K20/10 |
| 2023 | 3D printing | BUOYANT MEMBER\ | US2011269357A1 | CARROLL JOHN (GB) | SUNDRIDGE HOLDINGS LTD (GB) | B32B5/18\ B32B3/26\ B63C9/08 |
| 2024 | 3D printing | MEMBRANE KEYBOARD WITH PARTS BEING FIXEDLY SECURED TOGETHER\ | US2011290628A1 | TSAI HUO-LU (TW) | TSAI HUO-LU (TW)\ SUNREX TECHNOLOGY CORP (TW) | H01H13/76 |
| 2025 | 3D printing | METHOD OF CONNECTING ELECTRIC WIRES\ | US2011296683A1 | WATANABE YOSHIHIKO (JP)\ MATSUSHITA KOUICHIROU (JP)\ TAKAYASHIKI YOUSUKE (JP)\ TAKADA HAJIME (JP) | YAZAKI CORP (JP)\ TAKAYASHIKI YOUSUKE (JP)\ TAKADA HAJIME (JP)\ WATANABE YOSHIHIKO (JP)\ MATSUSHITA KOUICHIROU (JP) | H01R43/00 |
| 2026 | 3D printing | FERROELECTRET TWO-LAYER AND MULTILAYER COMPOSITE AND METHOD FOR PRODUCTION THEREOF\ | US2011309716A1 | JENNINGER WERNER (DE)\ WAGNER JOACHIM (DE)\ SCHAPELER DIRK (DE)\ PUDLEINER HEINZ (DE)\ STOLLWERCK GUNTHER (DE)\ GERHARD REIMUND (DE)\ WIRGES WERNER (DE)\ ALTAFIM RUY ALBERTO (BR)\ WALZE GUNTER (TW) | JENNINGER WERNER (DE)\ WAGNER JOACHIM (DE)\ WALZE GUNTER (TW)\ SCHAPELER DIRK (DE)\ PUDLEINER HEINZ (DE)\ STOLLWERCK GUNTHER (DE)\ GERHARD REIMUND (DE)\ WIRGES WERNER (DE)\ ALTAFIM RUY ALBERTO (BR) | B32B38/06\ B32B7/08\ H02N1/00\ B44C3/08 |
| 2027 | 3D printing | METHOD FOR PRODUCING A SHROUDED IMPELLER FROM TWO OR MORE COMPONENTS\ | US2011318183A1 | NORONHA MARIAN B (US)\ NORONHA TIMOTHY P (US) | TURBOCAM INC (US)\ NORONHA MARIAN B (US)\ NORONHA TIMOTHY P (US) | B23P15/04\ F04D29/38\ F04D29/32 |
| 2028 | 3D printing | ELECTROMAGNETIC LINEAR VALVE\ | US2012001108A1 | ZHANG KUNLIN (CN) | XIAMEN KOGE MICRO TECH CO LTD (CN)\ ZHANG KUNLIN (CN) | F16K31/02 |
| 2029 | 3D printing | POLYMER BATTERY PACK AND METHOD FOR MANUFACTURING THE SAME\ | US2012015217A1 | YOON HEUI SANG (KR)\ LEE HYUNG BOK (KR) | LEE HYUNG BOK (KR)\ YOON HEUI SANG (KR) | H01M2/02\ H01M2/10\ H01M2/34\ H01M10/42 |
| 2030 | 3D printing | COMPOSITE ABSORBENT MATERIALS AND METHODS FOR THEIR PRODUCTION\ | US2012034432A1 | COTTON STEPHEN (GB) | BRIGHTWAKE LTD (GB)\ COTTON STEPHEN (GB) | B32B3/00\ B32B37/06\ B29C65/08 |
| 2031 | 3D printing | SYSTEM FOR ENHANCING SONOTRODE PERFORMANCE IN ULTRASONIC ADDITIVE MANUFACTURING APPLICATIONS\ | US2012061027A1 | SHORT MATTHEW A (US) | EDISON WELDING INST INC (US)\ SHORT MATTHEW A (US) | B29C65/08 |
| 2032 | 3D printing | APPARATUS FOR WELDING AND SEPARATING PACKAGING MATERIALS\ | US2012090283A1 | WIEDUWILT ULRICH (DE)\ IPPERS JUERGEN (DE)\ BROOS CLAUDIA (DE) | BOSCH GMBH ROBERT (DE)\ WIEDUWILT ULRICH (DE)\ IPPERS JUERGEN (DE)\ BROOS CLAUDIA (DE) | B29C65/74\ B65B51/22\ B65B51/30\ B65B9/207 |
| 2033 | 3D printing | SONOTRODE AND ANVIL ENERGY DIRECTOR GRIDS FOR NARROW/COMPLEX ULTRASONIC WELDS OF IMPROVED DURABILITY\ | US2012097339A1 | HULL GORDON (US) | HULL GORDON (US) | B32B37/06 |
| 2034 | 3D printing | FLUID TREATMENT FILTER CARDRIDGE WITH HIGH-PRESSURE RESISTANT WOVEN COVER\ | US2012097592A1 | RUBIO CHARLES ARTHUR (US) | RUBIO CHARLES ARTHUR (US) | B01D27/00\ B01D35/30 |
| 2035 | 3D printing | COULOMB DAMPING AND/OR VISCOUS DAMPING INSERT USING ULTRASONIC WELDING\ | US2012107546A1 | SCHROTH JAMES G (US)\ PERRY THOMAS A (US)\ RIEFE MARK T (US) | GM GLOBAL TECH OPERATIONS INC (US)\ SCHROTH JAMES G (US)\ PERRY THOMAS A (US)\ RIEFE MARK T (US) | B23K20/10\ B32B15/00\ F16F7/00 |
| 2036 | 3D printing | ATTRITION RESISTANT HARDENED ZEOLITE MATERIALS FOR AIR FILTRATION MEDIA\ | US2012118160A1 | HEFFES RUTH G (US)\ SINCLAIR FITZGERALD A (US)\ WITHIAM MICHAEL C (US)\ NASSIVERA TERRY W (US) | HEFFES RUTH G (US)\ SINCLAIR FITZGERALD A (US)\ WITHIAM MICHAEL C (US)\ NASSIVERA TERRY W (US) | B29C47/00\ B01D53/02 |
| 2037 | 3D printing | PROCESS FOR REDUCING ALUMINIUM PICK-UP AND ULTRASONIC WELDING DEVICE\ | US2012125976A1 | STEINER ERNST (DE) | SCHUNK SONOSYSTEMS GMBH (DE)\ STEINER ERNST (DE) | B23K20/10 |
| 2038 | 3D printing | METHOD AND APARATUS FOR RADIAL ULTRASONIC WELDING INTERCONNECTED COAXIAL CONNECTOR\ | US2012129384A1 | VAN SWEARINGEN KENDRICK (US) | ANDREW LLC (US)\ VAN SWEARINGEN KENDRICK (US) | H01R9/05\ H01R43/16\ H01R13/58 |
| 2039 | 3D printing | ULTRASONIC WELD COAXIAL CONNECTOR AND INTERCONNECTION METHOD\ | US2012129390A1 | VAN SWEARINGEN KENDRICK (US)\ ISLAM NAHID (US) | ANDREW LLC (US)\ VAN SWEARINGEN KENDRICK (US)\ ISLAM NAHID (US) | B23K20/10\ B23K31/02\ H01R9/05 |
| 2040 | 3D printing | DISPOSABLE HYGIENE ARTICLE SURGICAL COVERING ITEM OR SURGICAL GARMENT\ | US2012143164A1 | FRANK BERND (DE)\ GROENER RUDOLF (DE)\ KOCH CHRISTIAN (DE)\ KESSELMEIER RUEDIGER (DE) | FRANK BERND (DE)\ KESSELMEIER RUEDIGER (DE)\ KOCH CHRISTIAN (DE)\ GROENER RUDOLF (DE) | B29C65/08\ A61F13/45 |
| 2041 | 3D printing | ULTRASONIC WELDING SYSTEM AND METHOD FOR FORMING A WELD JOINT\ | US2012153006A1 | KOETTING WILLIAM (US)\ NIELSON MICHAEL (US)\ KHAKHALEV ALEXANDER (US) | LG CHEMICAL LTD (KR)\ KOETTING WILLIAM (US)\ KHAKHALEV ALEXANDER (US)\ NIELSON MICHAEL (US) | B23K20/10\ B23K1/06 |
| 2042 | 3D printing | ADD-ON PART FOR CONNECTING TO A COMPONENT BY TORSIONAL ULTRASONIC WELDING\ | US2012155954A1 | SAUTNER ANTON (DE)\ BUECKER DIRK (DE)\ GOERSE HERGEN (DE)\ ORTNER FLORIAN (DE) | BUECKER DIRK (DE)\ GOERSE HERGEN (DE)\ SAUTNER ANTON (DE)\ ORTNER FLORIAN (DE) | B23K20/10\ B29C65/08\ F16B11/00 |
| 2043 | 3D printing | COIL CAPTURE APPARATUS AND PILOT OPERATED WATER VALVE INCORPORATING SAME\ | US2012168657A1 | HENTSCHEL MARK A (US)\ KLOPP JEROME C (US)\ WARDEH JESSE (US) | HENTSCHEL MARK A (US)\ KLOPP JEROME C (US)\ WARDEH JESSE (US)\ ROBERTSHAW CONTROLS CO (US) | F16K31/02 |
| 2044 | 3D printing | DEVICE AND METHOD FOR WELDING AND SEPARATING PACKAGING MATERIALS FOR A PACKAGE\ | US2012178604A1 | WIEDUWILT ULRICH (DE)\ IPPERS JUERGEN (DE) | BOSCH GMBH ROBERT (DE)\ WIEDUWILT ULRICH (DE)\ IPPERS JUERGEN (DE) | B31B1/14 |
| 2045 | 3D printing | ULTRASONIC WELDING SYSTEM AND METHOD FOR FORMING A WELD JOINT UTILIZING THE ULTRASONIC WELDING SYSTEM\ | US2012180929A1 | KOETTING WILLIAM (US)\ NIELSON MICHAEL (US)\ KHAKHALEV ALEXANDER (US) | LG CHEMICAL LTD (KR)\ KOETTING WILLIAM (US)\ KHAKHALEV ALEXANDER (US)\ NIELSON MICHAEL (US) | B23K20/10\ B32B41/00\ B32B37/02 |
| 2046 | 3D printing | METHOD OF ATTACHING GRIP TABS TO THE CARRIER LAYER OF A FILM DRESSING\ | US2012184891A1 | JOHANNISON ULF (SE) | MOELNLYCKE HEALTH CARE AB (SE)\ JOHANNISON ULF (SE) | B32B37/06\ A61F13/02 |
| 2047 | 3D printing | METHOD FOR PERFORMING AN ULTRASONIC WELDING PROCESS ON AT LEAST ONE WEB\ | US2012186719A1 | VAN DEN AKER MARTINUS CORNELUS ADRIANUS (NL) | BEILER BEHEER BV (NL)\ VAN DEN AKER MARTINUS CORNELUS ADRIANUS (NL) | B32B37/10\ B32B41/00\ B32B38/18 |
| 2048 | 3D printing | METHOD AND DEVICE FOR MANUFACTURING TITANIUM OBJECTS\ | US2012193335A1 | GULDBERG SIGRID (NO) | GULDBERG SIGRID (NO) | B23K9/00 |
| 2049 | 3D printing | METHOD OF FASTENING ARRANGEMENT FOR THE POSITIONALLY CORRECT FASTENING OF A DEVICE TO A STRUCTURAL PART OF A MOTOR VEHICLE\ | US2012213574A1 | GRAF HUBERT (DE)\ KRAUS TOBIAS (DE)\ GRIEBENOW FRANK (DE)\ MUELLER MICHAEL (DE)\ SEILER THORSTEN (DE)\ THUMBACH HANS-PETER (DE)\ WILLMANN JAKOB (DE)\ SCHMEIDER HANSJOERG (DE) | PROGRESS WERK OBERKIRCH AG (DE)\ GRAF HUBERT (DE)\ KRAUS TOBIAS (DE)\ GRIEBENOW FRANK (DE)\ MUELLER MICHAEL (DE)\ SCHMEIDER HANSJOERG (DE)\ SEILER THORSTEN (DE)\ THUMBACH HANS-PETER (DE)\ WILLMANN JAKOB (DE) | B32B37/12\ F16B11/00\ B32B37/02 |
| 2050 | 3D printing | HOLDING PLATE FOR A VACUUM CLEANER FILTER BAG\ | US2012216494A1 | SAUER RALF (BE)\ SCHULTINK JAN (BE) | SCHULTINK JAN (BE)\ SAUER RALF (BE) | B29C45/16\ B32B37/06\ A47L9/14 |
| 2051 | 3D printing | ORGANIZATION DEVICE AND MANUFACTURING METHOD OF THE SAME\ | US2012217248A1 | MA CHEN-JUI (TW) | MA CHEN-JUI (TW) | B32B38/10\ B65D25/04\ B32B37/02\ B65D33/14\ B32B37/16\ B65D85/18 |
| 2052 | 3D printing | HEARING AID AND METHOD OF FABRICATING THE SAME\ | US2012224734A1 | COHEN AVI (IL) | COHEN AVI (IL)\ OBJET LTD (IL) | H04R25/00\ B05D5/00\ H05K13/00 |
| 2053 | 3D printing | COPPER FOIL AND METHOD FOR PRODUCING COPPER FOIL\ | US2012258281A1 | SHINOZAKI KENSAKU (JP)\ SUZUKI AKITOSHI (JP)\ TSURUTA TAKAHIRO (JP) | FURUKAWA ELECTRIC CO LTD (JP)\ SHINOZAKI KENSAKU (JP)\ SUZUKI AKITOSHI (JP)\ TSURUTA TAKAHIRO (JP) | B32B5/00\ B32B15/04\ B05D5/00\ B32B15/20 |
| 2054 | 3D printing | PREPARATION OF BUILDING MATERIAL FOR SOLID FREEFORM FABRICATION\ | US2012276233A1 | NAPADENSKY EDUARDO (IL) | NAPADENSKY EDUARDO (IL)\ OBJET LTD (IL) | B29C49/78 |
| 2055 | 3D printing | COMPACT FILTER METHOD FOR PRODUCING A COMPACT FILTER AND FILTER MEDIUM\ | US2012279186A1 | HEIM MICHAEL (DE)\ SIEBER THOMAS (DE)\ ROHRMEIER JOSEF (DE)\ GEHWOLF KLAUS (DE)\ KRIEGER JOACHIM-PAUL (DE)\ EPLI SVEN (DE)\ MAJER TANJA (DE)\ LINARES ANDRES MORENO (DE)\ EISENGRAEBER-PABST JOBST (DE) | MANN & HUMMEL GMBH (DE)\ HEIM MICHAEL (DE)\ SIEBER THOMAS (DE)\ ROHRMEIER JOSEF (DE)\ GEHWOLF KLAUS (DE)\ KRIEGER JOACHIM-PAUL (DE)\ EPLI SVEN (DE)\ MAJER TANJA (DE)\ LINARES ANDRES MORENO (DE)\ EISENGRAEBER-PABST JOBST (DE) | B01D39/14\ B01D39/18\ B21D53/00 |
| 2056 | 3D printing | FLOW PASSAGE ASSEMBLY AND METHOD FOR MANUFACTURING FLOW PASSAGE ASSEMBLY INKJET RECORDING HEAD AND RECORDING APPARATUS\ | US2012293588A1 | SHIMAMURA RYO (JP)\ SHIBATA TAKESHI (JP)\ YAMAMOTO AKIRA (JP)\ HIROSAWA TOSHIAKI (JP)\ TAJIMA HIROKI (JP) | CANON KK (JP)\ SHIMAMURA RYO (JP)\ SHIBATA TAKESHI (JP)\ YAMAMOTO AKIRA (JP)\ HIROSAWA TOSHIAKI (JP)\ TAJIMA HIROKI (JP) | B41J2/17\ B29C65/08 |
| 2057 | 3D printing | METHOD AND ASSEMBLY FOR THE ELECTRICALLY CONDUCTIVE CONNECTION OF WIRES\ | US2012298645A1 | STROH DIETER (DE)\ WAGNER PETER (DE)\ WAGENBACH UDO (DE)\ MOOS ANDREAS (DE)\ ZIESLER ALEXANDER (DE)\ KLEESPIESS BJOERN (DE) | SCHUNK SONOSYSTEMS GMBH (DE)\ STROH DIETER (DE)\ WAGENBACH UDO (DE)\ WAGNER PETER (DE)\ KLEESPIESS BJOERN (DE)\ MOOS ANDREAS (DE)\ ZIESLER ALEXANDER (DE) | B23K20/10\ B23K33/00\ B23K31/02 |
| 2058 | 3D printing | ULTRASONIC WELDING METHOD AND WELDING SECTION\ | US2013000949A1 | TORIMOTO YUKIHIRO (JP)\ YAMAGUCHI SHIZUKA (JP) | FURUKAWA ELECTRIC CO LTD (JP)\ FURUKAWA AUTOMOTIVE SYS INC (JP)\ TORIMOTO YUKIHIRO (JP)\ YAMAGUCHI SHIZUKA (JP) | B23K20/10\ H01B13/00\ H01B7/00 |
| 2059 | 3D printing | BATTERY AND ULTRASONIC WELDING SYSTEM USED IN PRODUCING THEREOF\ | US2013011717A1 | YOTSUMOTO HIROAKI (JP) | MITSUBISHI HEAVY IND LTD (JP)\ YOTSUMOTO HIROAKI (JP) | B23K20/10\ H01M10/02\ H01M2/22\ B23K31/12 |
| 2060 | 3D printing | BATTERY MODULES HAVING INTERCONNECT MEMBERS WITH VIBRATION DAMPENING PORTIONS\ | US2013029204A1 | KHAKHALEV ALEX (US)\ ISAYEV IGOR (US) | LG CHEMICAL LTD (KR)\ KHAKHALEV ALEX (US)\ ISAYEV IGOR (US) | H01M2/10\ H01M2/22 |
| 2061 | 3D printing | SOLID FREEFORM FABRICATION OF SHELLED OBJECTS\ | US2013040091A1 | NAPADENSKY EDUARDO (IL)\ DIKOVSKY DANIEL (IL)\ LEVY AVRAHAM (IL) | NAPADENSKY EDUARDO (IL)\ OBJET LTD (IL)\ DIKOVSKY DANIEL (IL)\ LEVY AVRAHAM (IL) | B28B17/00\ B29C35/02\ B32B3/02 |
| 2062 | 3D printing | ULTRASONIC WELDING MACHINE AND METHOD OF ASSEMBLING THE ULTRASONIC WELDING MACHINE\ | US2013048698A1 | KHAKHALEV ALEX (US) | LG CHEMICAL LTD (KR)\ KHAKHALEV ALEX (US) | B23K20/10\ B23P11/00 |
| 2063 | 3D printing | PASSIVELY DAMPED VIBRATION WELDING SYSTEM AND METHOD\ | US2013081753A1 | TAN CHIN-AN (US)\ KANG BONGSU (US)\ CAI WAYNE W (US)\ WU TAO (US) | GM GLOBAL TECH OPERATIONS INC (US)\ CAI WAYNE W (US)\ TAN CHIN-AN (US)\ KANG BONGSU (US)\ WU TAO (US) | B29C65/04 |
| 2064 | 3D printing | INDUSTRIAL FABRIC INCLUDING SPIRALLY WOUND MATERIAL STRIPS WITH REINFORCEMENT\ | US2013081772A1 | EAGLES DANA (US)\ KARLSSON JONAS (SE)\ HANSEN ROBERT (US) | ALBANY INT CORP (US)\ EAGLES DANA (US)\ KARLSSON JONAS (SE)\ HANSEN ROBERT (US) | D21F7/08\ D21F11/00 |
| 2065 | 3D printing | ULTRASONIC WELD INTERCONNECTION COAXIAL CONNECTOR AND INTERCONNECTION WITH COAXIAL CABLE\ | US2013095695A1 | VAN SWEARINGEN KENDRICK (US) | ANDREW LLC (US) | H01R9/05 |
| 2066 | 3D printing | MOULDING FOR VEHICLE AND METHOD FOR ATTACHING MOULDING END CAP\ | US2013097962A1 | YOSHIDA KOJI (JP)\ OKAZAKI HIROSHI (JP)\ NAGATA KANEHIRO (JP)\ HANANO SHINGO (JP) | YOSHIDA KOJI (JP)\ AISIN SEIKI (JP)\ OKAZAKI HIROSHI (JP)\ NAGATA KANEHIRO (JP)\ HANANO SHINGO (JP) | B29C65/08\ B60R13/04 |
| 2067 | 3D printing | WATER HAMMER ARRESTER\ | US2013098492A1 | COSLEY JAMES (US)\ CHASE JACOB (US) | COSLEY JAMES (US)\ CHASE JACOB (US)\ KING BROTHERS IND INC (US) | B32B37/04\ F16L55/045 |
| 2068 | 3D printing | ULTRASONICALLY-WELDED JUNCTION BOX\ | US2013104982A1 | GONZALEZ PEDRO (US)\ BECK MARKUS (US) | FIRST SOLAR INC (US) | H01L31/02\ H01L31/18 |
| 2069 | 3D printing | QUALITY STATUS DISPLAY FOR A VIBRATION WELDING PROCESS\ | US2013105557A1 | BRACEY JENNIFER (US)\ WANG HUI (US)\ ABELL JEFFREY A (US)\ SPICER JOHN PATRICK (US)\ WINCEK MICHAEL ANTHONY (US)\ CHAKRABORTY DEBEJYO (US)\ UTZ SHAWN (US)\ TAVORA PETER W (US)\ DAVIS JEFFREY S (US)\ HUTCHINSON DANIEL C (US)\ REARDON RONALD L (US) | GM GLOBAL TECH OPERATIONS INC (US) | B23K20/10 |
| 2070 | 3D printing | CONNECTOR APPARATUS FOR BIDET WAND\ | US2013106098A1 | XIE BAIAN (CN)\ KUANG QIFENG (CN) | SHANGHAI KOHLER ELECTRONICS (CN) | F16L39/00 |
| 2071 | 3D printing | METHOD FOR ASSEMBLING AT LEAST TWO PLASTIC COMPONENTS TO EACH OTHER\ | US2013112336A1 | ROBIN STEPHANE (FR)\ JUAN PASCAL (FR)\ MOREL MERRY (FR) | FAURECIA INTERIEUR IND (FR)\ ROBIN STEPHANE (FR)\ JUAN PASCAL (FR)\ MOREL MERRY (FR) | B29C65/08 |
| 2072 | 3D printing | COMPOSITE FILM AND PACKAGING PRODUCED THEREFROM\ | US2013118128A1 | LUECKE ANDREAS (DE)\ EBBERS ANDRE (DE)\ LETTMANN CHRISTIAN (DE) | LUECKE ANDREAS (DE)\ EBBERS ANDRE (DE)\ LETTMANN CHRISTIAN (DE) | B65D65/40\ B32B15/08\ B65B51/22\ B65B3/16 |
| 2073 | 3D printing | METHOD OF ASSEMBLY OF THERMOPLASTIC FENCING\ | US2013126068A1 | FATTORI JAMES (US) | LMT MERCER GROUP INC (US) | B29C65/08 |
| 2074 | 3D printing | METHOD FOR JOINING WIRE ENDS\ | US2013139924A1 | NABETA YASUNORI (JP)\ ITO NAOKI (JP) | YAZAKI CORP (JP)\ NABETA YASUNORI (JP)\ ITO NAOKI (JP) | H01R43/02 |
| 2075 | 3D printing | PORTABLE BEVERAGE CONTAINER WITH ULTRASONIC WELDED JOINT AND METHOD OF MAKING THE SAME\ | US2013140316A1 | FLOWERS SEAN T (US)\ SHEELY JAMES M (US)\ VOYLES WILLIAM D (US) | TERVIS TUMBLER COMPANY (US) | A47G19/22\ B29C65/08 |
| 2076 | 3D printing | CRYSTAL USED FOR CLOSING THE TOP OF THE CASE OF A PORTABLE OBJECT AND METHOD OF WELDING A CRYSTAL OF THIS TYPE\ | US2013153289A1 | POLI GIAN-CARLO (CH) | SWATCH GROUP RES & DEV LTD (CH)\ POLI GIAN-CARLO (CH) | B29C65/56\ H05K1/03\ H02G3/14 |
| 2077 | 3D printing | ELECTRODE ASSEMBLY WITH HYBRID WELD\ | US2013164582A1 | HUMPHRYS SETH M (US)\ BUENDORF DAVID J (US)\ ZHAO HAILING (US) | HUMPHRYS SETH M (US)\ BUENDORF DAVID J (US)\ ZHAO HAILING (US) | B23K20/10\ B23K26/22\ H01M10/0587\ H01M10/36 |
| 2078 | 3D printing | REFLECTIVE ROADSTUD AND MANUFACTURE OF THE SAME\ | US2013170906A1 | LEE HUNG-CHEN (TW) | LEE HUNG-CHEN (TW) | B29C65/08\ E01F9/06 |
| 2079 | 3D printing | SOLID ELECTROLYTIC CAPACITOR AND METHOD FOR PRODUCING THE SAME\ | US2013182374A1 | SAKATA KOJI (JP)\ MIZUKOSHI TAKASHI (JP) | NEC TOKIN CORP (JP) | H01G9/00\ H01G9/048 |
| 2080 | 3D printing | FORMING OF CONSOLIDATION REGIONS IN A WEB AND A WEB COMPRISING SUCH REGIONS\ | US2013183476A1 | SCHMITZ CHRISTOPH (DE) | CONCEPTS FOR SUCCESS C4S (DE)\ SCHMITZ CHRISTOPH (DE) | B29C35/02\ D04H1/544\ D04H1/559\ D04H1/555 |
| 2081 | 3D printing | CONNECTION STRUCTURE OF HOUSING OF ELECTRONIC APPARATUS AND CONNECTION METHOD OF FORMING HOUSING\ | US2013189024A1 | KOBAYASHI MINORU (JP) | OMRON TATEISI ELECTRONICS CO (JP) | H05K5/02\ B29C65/08 |
| 2082 | 3D printing | METHOD FOR DISASSEMBLY OF TWO ELEMENTS WELDED BY ULTRASOUND AND ELEMENT ABLE TO BE WELDED BY ULTRASOUND TO ANOTHER ELEMENT TO THE IMPLEMENTATION OF SAID METHOD\ | US2013192433A1 | CHARLES JULIEN (FR)\ GALLANT GUILLAUME (FR)\ SOULA DENIS (FR) | AIRBUS OPERATIONS SAS (FR) | B32B38/00\ B26D1/547 |
| 2083 | 3D printing | FRAME\ | US2013195543A1 | MAHAT MOHD KAMAL (MY)\ KWAK LIP KEONG (MY)\ HASSAN MUHD SHUHAIL (MY)\ NASIR M NOORASHIDI M (MY) | LIEBHERR HAUSGERATE LIENZ GMBH (AT) | F16B12/06 |
| 2084 | 3D printing | MANUFACTURING METHOD FOR MICROVALVES\ | US2013206319A1 | STADELBAUER BIRGIT (DE)\ SCHEUFLER SVEN (DE) | STADELBAUER BIRGIT (DE)\ SCHEUFLER SVEN (DE) | B29C69/00 |
| 2085 | 3D printing | DROPLET ACTUATOR ASSEMBLIES AND METHODS OF MAKING SAME\ | US2013206597A1 | WANG TIH-HONG (US)\ BRACKETT GEORGE (US)\ CLEVENGER DAVID (US)\ BORT DONOVAN E (US) | ADVANCED LIQUID LOGIC INC (US)\ WANG TIH-HONG (US)\ BRACKETT GEORGE (US)\ CLEVENGER DAVID (US)\ BORT DONOVAN E (US) | C07K1/26 |
| 2086 | 3D printing | GLOSS-CONTROLLABLE RADIATION-CURABLE INKJET INK\ | US2013222499A1 | CONG LIANHUI (US)\ GLOSTER DANIEL FRANCIS (US)\ EDWARDS PAUL ANDREW (US) | CONG LIANHUI (US)\ GLOSTER DANIEL FRANCIS (US)\ EDWARDS PAUL ANDREW (US) | C09D11/10\ B41J2/01 |
| 2087 | 3D printing | APPARATUS FOR CUTTING A PACKAGING MATERIAL FOR A PACKAGE\ | US2013228288A1 | WIEDUWILT ULRICH (DE)\ IPPERS JUERGEN (DE) | BOSCH GMBH ROBERT (DE)\ WIEDUWILT ULRICH (DE)\ IPPERS JUERGEN (DE) | B65B61/06\ B65B51/22 |
| 2088 | 3D printing | METHOD FOR MOUNTING A COMPONENT IN OR ON A CIRCUIT BOARD AND CIRCUIT BOARD\ | US2013233603A1 | WEICHSLBERGER GUENTHER (AT)\ DROFENIK DIETMAR (AT) | AUSTRIA TECH & SYSTEM TECH (AT)\ WEICHSLBERGER GUENTHER (AT)\ DROFENIK DIETMAR (AT) | H05K3/32\ H05K1/18 |
| 2089 | 3D printing | HOLLOW TIP WELDING TOOL\ | US2013240152A1 | CHANG CHIH-CHI (TW)\ LEE KUO-HUNG (TW)\ REGAN PATRICK CONALL (TW) | REGAN PATRICK CONALL (TW)\ LEE KUO-HUNG (TW)\ CHANG CHIH-CHI (TW)\ NIKE INC (US) | B23K20/10\ B32B37/06 |
| 2090 | 3D printing | TOOL FOR ULTRASONIC WELDING DEVICE\ | US2013240606A1 | LANG GEORG (DE)\ ROSIWAL STEFAN (DE)\ LODES MATTHIAS (DE)\ NEUERER KATHARINA (DE) | LANG GEORG (DE)\ ROSIWAL STEFAN (DE)\ LODES MATTHIAS (DE)\ NEUERER KATHARINA (DE) | B23K20/10 |
| 2091 | 3D printing | ADJUSTING DRIVE OF A MOTOR VEHICLE ADJUSTING ELEMENT\ | US2013242484A1 | SCHWEICHART ANDREAS (DE)\ KLINGLER PETER (DE) | BROSE FAHRZEUGTEILE (DE)\ WUERZBURG BROSE FAHRZEUGTEILE GMBH & CO KG (US) | H05K5/06\ H05K5/03 |
| 2092 | 3D printing | ULTRASONIC WELD COAXIAL CONNECTOR\ | US2013244487A1 | VAN SWEARINGEN KENDRICK (US)\ ISLAM NAHID (US) | ANDREW LLC (US) | H01R4/02 |
| 2093 | 3D printing | ULTRASONIC WELDING STRUCTURE AND ULTRASONIC WELDING METHOD\ | US2013263998A1 | WANG PO-SHENG (TW)\ HSU CHUN-HSIANG (TW)\ HSIEH CHING-FENG (TW) | WANG PO-SHENG (TW)\ HSU CHUN-HSIANG (TW)\ HSIEH CHING-FENG (TW)\ ASKEY COMPUTER CORP (TW)\ ASKEY TECHNOLOGY JIANGSU LTD (CN) | B23K20/10\ B32B37/06\ B29C65/08 |
| 2094 | 3D printing | ULTRASONIC WELDING DEVICE AND METHOD OF OPERATING SAID DEVICE\ | US2013263999A1 | WEILAND FRANK (DE)\ GUBERNATIS STEFAN (DE)\ BEIER UWE (DE)\ WEIMER CHRISTIAN (DE) | EUROCOPTER DEUTSCHLAND (DE) | B29C65/08 |
| 2095 | 3D printing | ELECTRICAL COMPONENT\ | US2013269987A1 | KOEPSELL MARTIN (DE) | JOHNSON ELECTRIC SA (CH) | H05K3/00\ H05K1/11 |
| 2096 | 3D printing | WATCH COMPRISING A ZEBRA CONNECTOR FOR ELECTRICAL CONNECTION BETWEEN A DIGITAL DISPLAY DEVICE AND A PRINTED CIRCUIT BOARD AND METHOD OF ASSEMBLING SUCH A WATCH\ | US2013279303A1 | KALTENRIEDER ANDRE (CH)\ JUFER MARTIN (CH)\ RACINE CHRISTIAN (CH)\ BOVET JEAN-LUC (CH)\ GROSJEAN JEROME (CH) | ETA SA MFT HORLOGERE SUISSE (CH)\ KALTENRIEDER ANDRE (CH)\ JUFER MARTIN (CH)\ RACINE CHRISTIAN (CH)\ BOVET JEAN-LUC (CH)\ GROSJEAN JEROME (CH) | B29C65/08\ G04G17/04 |
| 2097 | 3D printing | WELDED-IN SPOUT\ | US2013284769A1 | SCHICK WERNER (DE) | POEPPELMANN HOLDING GMBH & CO (US) | B65D47/06 |
| 2098 | 3D printing | STRAPS PRODUCED FROM RENEWABLE RAW MATERIALS\ | US2013291741A1 | EBERLE ULRICH (DE) | MOSCA G MASCHF (DE)\ EBERLE ULRICH (DE) | C08G63/02\ B65B13/02 |
| 2099 | 3D printing | METHOD FOR WELDING RENEWABLE RAW MATERIALS\ | US2013294821A1 | EBERLE ULRICH (DE) | MOSCA G MASCHF (DE)\ EBERLE ULRICH (DE) | B32B37/04 |
| 2100 | 3D printing | WATCH INCLUDING A BEZEL AND METHOD OF ATTACHING SUCH BEZEL\ | US2013329535A1 | KALTENRIEDER ANDRE (CH)\ JUFER MARTIN (CH)\ RACINE CHRISTIAN (CH)\ BOVET JEAN-LUC (CH)\ GROSJEAN JEROME (CH) | ETA SA MFT HORLOGERE SUISSE (CH)\ KALTENRIEDER ANDRE (CH)\ JUFER MARTIN (CH)\ RACINE CHRISTIAN (CH)\ BOVET JEAN-LUC (CH)\ GROSJEAN JEROME (CH) | G04B39/02 |
| 2101 | 3D printing | FEED-THROUGH\ | US2013330599A1 | ROTERS ANDREAS (DE)\ HARTL HELMUT (AT)\ KROLL FRANK (DE)\ ESEMANN HAUKE (DE)\ PICHLER-WILHELM SABINE (DE)\ LANDENDINGER MARTIN (DE)\ DAHLMANN ULF (DE)\ GOEDEKE DIETER (DE)\ BACKNAES LINDA JOHANNA (DE) | SCHOTT AG (DE) | H01M2/06 |
| 2102 | 3D printing | STEERING WHEEL\ | US2014013898A1 | DOURSOUX HUGUES (FR)\ BERTRAND OLIVIER (FR)\ GUERIN PATRICK (FR)\ BALOGE EMMANUELLE (FR)\ GROLEAU LAURENT (FR) | AUTOLIV DEV (SE)\ DOURSOUX HUGUES (FR)\ BERTRAND OLIVIER (FR)\ GUERIN PATRICK (FR)\ BALOGE EMMANUELLE (FR)\ GROLEAU LAURENT (FR) | B62D1/06 |
| 2103 | 3D printing | DEPOSITION OF MATERIALS FOR EDIBLE SOLID FREEFORM FABRICATION\ | US2014013962A1 | LIPTON JEFFREY I (US)\ LIPSON HOD (US) | LIPTON JEFFREY I (US)\ LIPSON HOD (US) | A47J43/04 |
| 2104 | 3D printing | METHOD OF PREPARING A FUEL CELL UNITIZED ELECTRODE ASSEMBLY BY ULTRASONIC WELDING\ | US2014014254A1 | SKIBA TOMMY (US)\ PAIK CHI-HUM (US)\ JARVI THOMAS D (US) | SKIBA TOMMY (US)\ PAIK CHI-HUM (US)\ JARVI THOMAS D (US) | B29C65/72 |
| 2105 | 3D printing | ULTRASONIC WELDING ASSEMBLY AND METHOD OF ATTACHING AN ANVIL TO A BRACKET OF THE ASSEMBLY\ | US2014026386A1 | JOHNSTON BRUCE (US)\ TOM KWOK (US) | LG CHEMICAL LTD (KR) | B23K20/10 |
| 2106 | 3D printing | WIRE HARNESS MANUFACTURING METHOD\ | US2014033524A1 | SATO OSAMU (JP)\ HIRANO NOBUYUKI (JP)\ MURATA ATSUSHI (JP) | SUMITOMO ELECTRIC INDUSTRIES (JP)\ SUMITOMO WIRING SYSTEMS (JP)\ AUTONETWORKS TECHNOLOGIES LTD (JP)\ SATO OSAMU (JP)\ HIRANO NOBUYUKI (JP)\ MURATA ATSUSHI (JP) | H01B13/012 |
| 2107 | 3D printing | METHOD AND ARRANGEMENT FOR BUILDING METALLIC OBJECTS BY SOLID FREEFORM FABRICATION\ | US2014061165A1 | STEMPFER FERDINAND (AT) | NORSK TITANIUM COMPONENTS AS (NO)\ STEMPFER FERDINAND (AT) | B22F3/105\ B23K9/04\ C23C16/52\ C23C16/513 |
| 2108 | 3D printing | ULTRASONIC WELDING DEVICE WITH ROTARY COUPLER\ | US2014083623A1 | JURZITZA DIETER (DE) | JURZITZA DIETER (DE)\ HERMANN ULTRASCHALLTECHNIK GMBH & CO KG (DE) | B23K20/10\ B32B37/06 |
| 2109 | 3D printing | ULTRASONIC WELDING APPARATUS AND SECONDARY BATTERY WITH ENHANCED ELECTRODE STRUCTURE\ | US2014087245A1 | LEE KYUNG-MIN (KR)\ SHIN YOUNG-JOON (KR)\ PARK HYUN-WOO (KR)\ LEE JAE-PIL (KR)\ PARK JIN-YOUNG (KR)\ PARK JUN-KYU (KR) | LG CHEMICAL LTD (KR) | B23K20/10\ H01M2/30\ H01M10/04\ H01M2/06 |
| 2110 | 3D printing | LED MODULE AND METHOD OF BONDING THEREOF\ | US2014112009A1 | YEH WEI-YU (TW) | TSMC SOLID STATE LIGHTING LTD (TW) | F21V29/00 |
| 2111 | 3D printing | SPUNLACED NONWOVENS AS HOOK AND LOOP FASTENER COMPONENT\ | US2014115838A1 | KIRSCH ANDREAS (DE)\ B?RMANN LUDWIG (DE)\ SCHREINER GüNTER (DE) | RKW SE (DE)\ KIRSCH ANDREAS (DE)\ B?RMANN LUDWIG (DE)\ SCHREINER GüNTER (DE) | D04H3/16\ A44B18/00\ D04H1/498 |
| 2112 | 3D printing | METHOD FOR PRODUCING DRIP IRRIGATION TUBES\ | US2014166187A1 | KERTSCHER EBERHARD (CH)\ LAMBERT CEDRIC (CH)\ BERSIER ROMAIN (CH) | MACH YVONAND SA (CH)\ KERTSCHER EBERHARD (CH)\ LAMBERT CEDRIC (CH)\ BERSIER ROMAIN (CH) | A01G25/02 |
| 2113 | 3D printing | ULTRASONIC WELDING OF FABRICS FOR SLEEP APNEA TREATMENT\ | US2014190486A1 | DUNN JESSICA LEA (AU)\ OVZINSKY GRANT MILTON (AU)\ BARBARA ANTHONY PAUL (AU)\ HASKARD KIRRILY MICHELE (AU)\ FORMICA JUSTIN JOHN (AU)\ LAGUZZA MICHAEL CHARLES (AU) | RESMED LTD (AU)\ DUNN JESSICA LEA (AU)\ OVZINSKY GRANT MILTON (AU)\ BARBARA ANTHONY PAUL (AU)\ HASKARD KIRRILY MICHELE (AU)\ FORMICA JUSTIN JOHN (AU)\ LAGUZZA MICHAEL CHARLES (AU) | A61M16/06 |
| 2114 | 3D printing | UNITARY HOUSING FOR ELECTRONIC DEVICE\ | US2014218850A1 | PREST CHRISTOPHER D (US)\ ZADESKY STEPHEN P (US)\ WEBER TRENT (US)\ BROWNING LUCY E (US) | APPLE INC (US) | H05K5/00\ H05K13/00 |
| 2115 | 3D printing | PLASTIC CLOSURE DEVICE FOR A TUBULAR BAG\ | US2014233871A1 | BARRON DAN (CH)\ EKENHORST DIRKü (DE) | BOSCH GMBH ROBERT (DE) | B65D33/16 |
| 2116 | 3D printing | BINARY CLASSIFICATION OF ITEMS OF INTEREST IN A REPEATABLE PROCESS\ | US2014236874A1 | WANG HUI (US)\ ABELL JEFFREY A (US)\ SPICER JOHN PATRICK (US)\ WINCEK MICHAEL ANTHONY (US)\ CHAKRABORTY DEBEJYO (US) | GM GLOBAL TECH OPERATIONS INC (US) | G06N99/00 |
| 2117 | 3D printing | LAYERED MANUFACTURING UTILIZING FOAM AS A SUPPORT AND MULTIFUNCTIONAL MATERIAL FOR THE CREATION OF PARTS AND FOR TISSUE ENGINEERING\ | US2014335141A1 | SUN WEI (US)\ NAM JAE HYUN (US) | UNIV DREXEL (US) | A61L31/14 |
| 2118 | 3D printing | DRINKS CONTAINER WHICH CONSISTS LARGELY OF CELLULOSE-CONTAINING MATERIAL AND HAS A FOOD-COMPATIBLE BARRIER LAYER FOR PRESSURIZED DRINKS\ | US2014339230A1 | KEIENBURG GUENTER (DE) | KEIENBURG GUENTER (DE) | B65D1/40 |
| 2119 | 3D printing | ELECTRICAL PLUG CONNECTOR FOR ELECTRICAL CONNECTION BY MEANS OF ULTRASONIC WELDING\ | US2014378010A1 | HOLLEY DANICA (DE)\ SCHULZ STEFAN (DE)\ LANGHOFF WOLFGANG (DE)\ BEDNARCZYK PETER (DE)\ BECK TILL (DE) | AMPHENOL TUCHEL ELECT (DE) | H01R4/02\ H01R43/02 |
| 2120 | 3D printing | METHOD OF FILLING AND SEALING AN AEROSOL DISPENSER\ | US2015033675A1 | SMITH SCOTT EDWARD (US) | PROCTER & GAMBLE (US) | B65B31/02 |
| 2121 | 3D printing | INTERNAL TRIM PART COMPRISING A DECORATIVE SKIN HAVING A DECORATIVE ELEMENT THAT IS ARRANGED IN A DEPRESSION IN THE DECORATIVE SKIN AND IS CONNECTED TO THE DECORATIVE SKIN BY MEANS OF ULTRASONIC WELDING AND METHOD FOR PRODUCING SAID INTERNAL TRIM PART WITH DECORATIVE ELEMENT\ | US2015048639A1 | MUSY MAXIME (DE)\ MEYER ANDREAS (DE)\ SCHULZ MARTIN (DE) | FAURECIA INNENRAUM SYS GMBH (DE) | B29C65/00\ B29C65/08\ B60R13/02 |
| 2122 | 3D printing | BACKPLANE OPTICAL FIBER CONNECTOR FOR ENGAGING BOARDS OF DIFFERENT THICKNESSES AND METHOD OF USE\ | US5530783A | BELOPOLSKY YAKOV (US)\ STONER STUART C (US) | BERG TECH INC (US) | G02B6/38 |
| 2123 | 3D printing | ELECTROLUMINESCENT MODULAR LAMP UNIT\ | US5565733A | MCGUIGAN RALPH (US)\ KRAFCIK ROBERT J (US)\ WHEELER DOUGLAS T (US)\ PACIOREK WALTER J (US)\ ECKERSLEY RODNEY T (US)\ MUNSON HAROLD T (US)\ JOHNSON DEE L (US) | DUREL CORP (US) | B29C51/00\ H05B33/10\ B29C43/18\ B29C51/12\ G09F9/30\ H05K3/32\ H05B33/00\ H01H9/02\ B29C43/10\ B29C43/20\ G09F13/00\ G09F13/04\ G09F13/12\ G09F13/22\ H04M1/22\ H05B33/02\ H05B33/06\ H05B33/12 |
| 2124 | 3D printing | APPARATUS AND METHOD FOR RELEASABLY FUSING LENS MOLD PIECES\ | US5593620A | GALAS STEPHEN (US) | JOHNSON & JOHNSON VISION PROD (US) | B29C43/00\ B29C39/02\ B29C33/40\ B29C33/00\ B29L11/00\ B29D11/00\ B29C65/08\ B29C65/76\ B29C65/78\ G02C7/04 |
| 2125 | 3D printing | PHOTOVOLTAIC DEVICE AND MANUFACTURING METHOD OF THE SAME\ | US5667596A | TSUZUKI KOJI (JP)\ INOUE KATSUHIKO (JP)\ TAKADA TAKESHI (JP)\ TAKEYAMA YOSHIFUMI (JP) | CANON KK (JP) | H01L31/04\ H01L31/02\ H01L31/05\ H01L31/0392\ H01L31/048 |
| 2126 | 3D printing | SUPPORT CORE RIBBON FOR COLD-SHRINK TUBE\ | US5670223A | SADLO JAMES L (US)\ MELANCON EUGENE J (US) | MINNESOTA MINING & MFG (US) | B29C65/08\ B29C65/76\ H02G15/18\ B29C53/78\ B29C61/06\ B29C65/58\ B29C65/72 |
| 2127 | 3D printing | DISPLACEMENT DETECTING SWITCH DEVICE\ | US5693925A | YAMADA KENICHI (JP)\ UTSUNOMIYA HIROTOSHI (JP)\ NAKAZAWA TAKASHI (JP) | FUJI HEAVY IND LTD (JP)\ NILES PARTS CO LTD (JP) | F16H59/10\ H01H9/02\ H01H11/00\ H01H21/02 |
| 2128 | 3D printing | METHOD OF DETERMINING THE COLLAPSE OF PLASTIC PARTS\ | US5788791A | GREWELL DAVID A (US) | BRANSON ULTRASONICS CORP (US) | B23K20/10\ B29C65/00\ B29C65/08\ B29C65/06 |
| 2129 | 3D printing | METHOD OF BONDING AN ELASTIC MATERIAL TO A SUBSTRATE AND ARTICLE MANUFACTURED BY SAID METHOD\ | US5797895A | WIDLUND URBAN (SE)\ KLING ROBERT (SE) | MOELNLYCKE AB (SE) | B29C65/00\ B29C65/48\ A61F13/49\ B29C65/02\ A61F13/15\ A61F5/44\ B29C65/08\ B29C65/06\ B29K21/00\ B29L31/48 |
| 2130 | 3D printing | ELECTRONIC PART COMPRISING A CASING WITH A NARROW GROOVE FORMED IN THE CASING\ | US5824951A | TANAKA YASUHIRO (JP) | MURATA MANUFACTURING CO (JP) | H03H9/02\ H01L23/10\ H03H9/10\ H03H9/13\ H03H9/17 |
| 2131 | 3D printing | METHOD OF ULTRASONIC WELDING FOR A RESIN CASE\ | US5830300A | SUZUKI KAZUSHI (JP)\ SONE TAKAHIRO (JP)\ FUJINAMI HIROSHI (JP) | STAR MFG CO (JP) | B29C65/08\ B29C65/06\ G10K9/13\ G10K9/22\ H04R13/00 |
| 2132 | 3D printing | SIMULTANEOUS AMPLITUDE AND FORCE PROFILING DURING ULTRASONIC WELDING OF THERMOPLASTIC WORKPIECES\ | US5855706A | GREWELL DAVID A (US) | BRANSON ULTRASONICS CORP (US) | B29C65/00\ B29C65/08\ B29C65/60\ B06B1/02\ C08F214/18\ G10K11/02 |
| 2133 | 3D printing | PROCESS AND A DEVICE FOR CLOSING FILLED FOLDED-CARTON PACKAGES\ | US5924267A | MAINZ HANS-WILLI (DE)\ BOEMER HANS (DE) | SIG COMBIBLOC GMBH (DE) | B29C65/00\ B29C65/08\ B65B51/10\ B65B51/22 |
| 2134 | 3D printing | ULTRASONIC WELDING OF COPPER FOIL\ | US5942314A | FISHER BRIAN K (US)\ FISHER ALBERT R (US) | OAK MITSUI (JP) | B23K20/10\ H05K1/09\ B32B15/01\ B23K101/18\ B23K103/10\ B23K103/12\ B23K103/22\ H05K3/02 |
| 2135 | 3D printing | ULTRASONIC WELDING APPARATUS\ | US5947364A | TAMURA NAOKI (JP)\ SUZUKI TOSHIAKI (JP) | SUMITOMO ELECTRIC INDUSTRIES (JP)\ SUMITOMO WIRING SYSTEMS (JP)\ HARNESS SYST TECH RES LTD (JP) | B23K20/10\ H01R43/02\ B06B1/02\ B21F15/00\ B23K101/38 |
| 2136 | 3D printing | OSMOTIC SYSTEM FOR DELIVERY OF FLUID-SENSITIVE SOMATOTROPINS TO BOVINE ANIMALS\ | US5980509A | MAGRUDER JUDY A (US)\ CORTESE RICHARD (US)\ WRIGHT JEREMY C (US)\ PEERY JOHN R (US)\ PIKE JAMES B (US)\ ROBINSON URANO A (US)\ SMITH JONATHAN P (US)\ ZIEMANN LYLE E (US)\ ECKENHOFF JAMES B (US) | ALZA CORP (US) | A61K9/00\ A61D7/00\ A61K9/22\ A61K9/52\ A61M31/00 |
| 2137 | 3D printing | NONAQUEOUS ELECTROLYTE SECONDARY BATTERY\ | US6040086A | YOSHIDA HIROAKI (JP)\ HAGIWARA ZENZO (JP)\ TERASAKI MASANAO (JP) | JAPAN STORAGE BATTERY CO LTD (JP) | H01M10/04\ H01M2/26\ H01M10/0587\ H01M4/70\ H01M10/05 |
| 2138 | 3D printing | MESA FORMING WELD DEPTH LIMITATION FEATURE FOR USE WITH ENERGY DIRECTOR IN ULTRASONIC WELDING\ | US6066216A | RUPPEL JR EDWARD F (US) | BIOMETRIC IMAGING INC (US) | B01L3/00\ B29C65/00\ B29C65/08\ B06B1/02 |
| 2139 | 3D printing | ROTARY CONNECTOR\ | US6109942A | BANNAI HIROYUKI (JP) | ALPS ELECTRIC CO LTD (JP) | B60R16/02\ H01R43/00\ B60R16/027\ H01R35/02\ H01R35/04 |
| 2140 | 3D printing | SIMPLE ENCLOSURE FOR ELECTRONIC COMPONENTS\ | US6111760A | NIXON PATRICK EDWARD (US) | ERICSSON INC (US) | H04M1/02\ H05K5/00\ H05K5/02\ B29C65/08\ B29C65/58\ H04B1/38\ H04M1/725\ H05K7/14 |
| 2141 | 3D printing | METHOD OF MANUFACTURING HOLLOW BLADE AND HOLLOW BLADE MANUFACTURED BY SAME\ | US6132826A | KAWASAKI HIRAKU (JP)\ SIMOMURA TAKEO (JP) | DAIKIN IND LTD (JP) | F04D29/02\ B29C65/02\ B29C65/08\ F04D29/38 |
| 2142 | 3D printing | TEXTILE LENGTH PROCESS FOR PRODUCING ONE SUCH TEXTILE LENGTH AND A DEVICE FOR EXECUTING THIS PROCESS\ | US6162518A | KORFER STEFAN (DE) | HEIMBACH GMBH THOMAS JOSEF (DE) | B29C65/00\ B29C65/08\ B29C65/50\ D03D3/04\ D06H5/00\ D21F1/00\ D21F7/08 |
| 2143 | 3D printing | SEALED LEAD-ACID STORAGE BATTERY\ | US6218045B1 | IDE MASAYUKI (JP)\ SASAKI TAKEHIRO (JP)\ NAKAYAMA TAKUROU (JP)\ INOUE TOSHIHIRO (JP) | MATSUSHITA ELECTRIC IND CO LTD (US) | H01M2/02\ H01M2/04\ H01M2/12 |
| 2144 | 3D printing | OIL SUMP WITH INTEGRAL FILTER\ | US6290843B1 | LEE BRIAN THOMAS (US)\ COVINGTON EDWARD ALLEN (US) | LG CHEMICAL LTD (KR) | F01M11/03\ B01D35/027\ F01M11/00 |
| 2145 | 3D printing | ULTRASONIC WELDING STRUCTURE\ | US6313407B1 | OHASHI NORIHIRO (JP)\ SHINCHI AKIRA (JP) | YAZAKI CORP (US) | B29C65/08\ H01R13/504\ H01R43/00 |
| 2146 | 3D printing | RAPID FABRIC FORMING FOR PENETRATION RESISTANT FABRIC\ | US6323145B1 | YNGVE PAUL WESLEY (US)\ WALKER WILLIAM CHARLES (US)\ POPPER PETER (US)\ TAM ALBERT S (US) | DU PONT (US) | D04H13/00\ D04H3/05\ D04H3/07\ D04H3/14\ B32B5/08\ D04H3/04\ A41D31/00\ B63H9/06\ F41H5/04 |
| 2147 | 3D printing | METHOD OF PACKAGING SPRINGS\ | US6357209B1 | WELLS THOMAS J (US)\ MOSSBECK NIELS S (US) | L & P PROPERTY MANAGEMENT CO (US) | B65B63/04\ B65B9/02\ B65B63/02 |
| 2148 | 3D printing | SUCTION MUFFLER AND HERMETIC COMPRESSOR\ | US6361290B1 | IDE TERUMASA (JP) | MATSUSHITA REFRIGERATION (JP) | F04B39/00\ F16B5/08 |
| 2149 | 3D printing | ANTICHAFING STRIP FOR SHAVING EQUIPMENT HEAD AND SHAVING EQUIPMENT HEAD COMPRISING SAME\ | US6405438B1 | DUEZ JOSE (FR)\ REBAUDIERES JEAN (FR) | BIC SOC (FR) | C08J5/00\ B26B21/44 |
| 2150 | 3D printing | FORCE SENSING APPARATUS\ | US6425514B1 | OU GANG (SG)\ WIDDOWSON GARY PETER (HK)\ KWAN KA SHING KENNY (SG) | ASM TECH SINGAPORE PTE LTD (US) | B23K20/10\ B23K20/00 |
| 2151 | 3D printing | ELECTRODE TAB FOR A NONAQUEOUS ELECTROLYTE SECONDARY BATTERY AND METHOD OF FORMING THE SAME\ | US6432574B1 | SUZUKI HIROMI (JP)\ KITAMI TSUYOSHI (JP)\ INOI TAKAYUKI (JP)\ SAITO SUSUMU (JP) | NEC CORP (JP) | H01M2/26\ H01M10/0566\ H01M10/0587\ H01M4/70\ H01M10/05\ H01M2/22\ H01M4/13 |
| 2152 | 3D printing | ROTARY CONNECTOR AND CONNECTING STRUCTURE OF FLEXIBLE CABLE AND LEAD BLOCK USED THEREIN\ | US6435885B2 | ARAKI SHUNJI (JP)\ SHIRAKO ICHIRO (JP) | ALPS ELECTRIC CO LTD (JP) | H01R43/02\ B60R16/027\ H01R35/02\ H01R35/04\ B62D1/04 |
| 2153 | 3D printing | METHOD AND DEVICE FOR FIXING PIECES OF MATERIAL ON A CONTINUOUS WEB\ | US6471804B1 | TENNBY ANDERS (SE)\ THORNSTROEM BO (SE)\ HENRIKSSON KENNETH (SE)\ HANSSON ROY (SE)\ AABERG THOMAS (SE) | SCA HYGIENE PROD AB (SE) | B29C69/00\ B29C65/00\ A61F13/15\ B29C65/08\ B32B37/22 |
| 2154 | 3D printing | APPARATUS FOR MANUFACTURING A FLEXIBLE CURTAIN\ | US6523596B1 | MULLET WILLIS JAY (US)\ KYLE DONALD BRUCE (US)\ GREEN KELLY RAY (US)\ ASBURY HARRY EDWARD (US) | WAYNE DALTON CORP (US) | E06B9/266 |
| 2155 | 3D printing | ULTRASONIC WELDING APPARATUS\ | US6523732B1 | POPOOLA OLUDELE OLUSEGUN (US)\ VAN REATHERFORD LARRY (US)\ DE VRIES EDGAR (US)\ WILKOSZ DANIEL EDWARD (US)\ SKOGSMO JAN BIRGER (US)\ WEXLER ARNON (US) | FORD GLOBAL TECH INC (US) | B23K20/10\ B29C65/00\ B29C65/08 |
| 2156 | 3D printing | METHOD FOR JOINING WIRING BOARDS AND MANUFACTURING DATA CARRIER AND DEVICE FOR MOUNTING ELECTRONIC COMPONENT MODULES\ | US6523734B1 | KAWAI WAKAHIRO (JP)\ OKADA MASANOBU (JP) | OMRON TATEISI ELECTRONICS CO (JP) | B23K20/10\ H01L21/60\ H05K3/36\ H05K3/32\ H01R43/02\ G06K19/077 |
| 2157 | 3D printing | SECONDARY BATTERY AND METHOD OF MANUFACTURING SAME\ | US6537693B1 | MORITA SHIGETOMI (JP)\ SUZUKI HIROMI (JP) | NEC TOKIN CORP (US) | H01M2/34\ H01M2/00\ H01M10/02\ H01M10/04\ H01M10/38 |
| 2158 | 3D printing | ULTRASONIC WELDING APPARATUS\ | US6554931B1 | BLANCHARD DANIEL (FR)\ FAUL PATRICK (FR) | MASTERFOODS SCS (FR) | B23K20/10\ B29C65/00\ B29C65/08\ B29C65/74\ B65B51/10 |
| 2159 | 3D printing | REASSEMBLED TONER CARTRIDGE AND METHOD OF MANUFACTURE\ | US6577830B1 | WAZANA YOEL (US)\ PAULUS JODA (US) | WAZANA BROTHERS INTERNATIONAL (US) | G03G15/08 |
| 2160 | 3D printing | FLAT CABLE AND A MANUFACTURING METHOD THEREOF\ | US6585836B2 | INAGAKI TOMONORI (JP)\ YAMANO YOSHIAKI (JP)\ FUKUMOTO KOJI (JP) | SUMITOMO WIRING SYSTEMS (JP) | H05K3/28\ H05K3/38\ H01B7/08\ H05K1/00 |
| 2161 | 3D printing | GUARANTEE SEAL FOR FOOD PRODUCTS AND A MACHINE FOR ITS AUTOMATIC APPLICATION TO THE PRODUCT\ | US6595550B1 | MEAZZI ALBERTO (CH) | RENIO COM E CONSULTADORIA LDA (US) | B65D33/26\ B65B51/06\ B65C3/26\ B65D33/16 |
| 2162 | 3D printing | APPARATUS AND METHOD FOR JOINING LAYERS OF MATERIALS\ | US6612479B2 | POPOOLA OLUDELE OLUSEGUN (US)\ REATHERFORD LARRY VAN (US)\ WILKOSZ DANIEL EDWARD (US)\ SKOGSMO JAN BIRGER (US)\ KOEHL ROBERT (US)\ COOPER RONALD P (US)\ WEXLER ARNON (US) | FORD GLOBAL TECH LLC (US) | B23K20/10 |
| 2163 | 3D printing | PROCESS FOR JOINING METALS\ | US6644534B2 | HANAZAKI KENICHI (JP) | YAZAKI CORP (JP) | B23K20/10\ H01R4/02\ H01R43/02\ B23K20/24\ B23K101/38 |
| 2164 | 3D printing | REASSEMBLED PROCESS CARTRIDGE AND METHOD OF MANUFACTURE\ | US6684039B1 | WAZANA YOEL (US)\ PAULUS JODA (US) | WAZANA BROTHERS INTERNATIONAL (US) | G03G21/18 |
| 2165 | 3D printing | DEVELOPING DEVICE PROCESS CARTRIDGE ELECTROPHOTOGRAPHIC IMAGE FORMING APPARATUS AND DEVELOPER CONTAINER AND METHOD OF ASSEMBLING THE DEVELOPER CONTAINER\ | US6684040B2 | OGUMA TORU (JP)\ YOKOI AKIYOSHI (JP)\ WATANABE KAZUSHI (JP) | CANON KK (JP) | G03G15/08\ G03G21/18\ B65D83/06 |
| 2166 | 3D printing | COVER FOR AIR BAG DEVICE\ | US6692016B2 | YOKOTA KEISHI (JP)\ IGAWA TADAHIRO (JP)\ KOBAYASHI ICHIZO (JP) | TAKATA CORP (JP) | B60R21/2165\ B29C45/14\ B60R13/00\ B62D1/04\ B60K37/00\ B60R21/215\ B62D1/11 |
| 2167 | 3D printing | OPTICAL MODULE AND PRODUCTION METHOD THEREFOR\ | US6695492B2 | IDE TSUGIO (JP)\ KITAMURA SHOJIRO (JP)\ HARADA ATSUSHI (JP) | SEIKO EPSON CORP (JP) | G02B6/42\ H01L31/0232\ H01S5/022\ H01S5/183 |
| 2168 | 3D printing | COMPONENT FOR MULTILAYER PRINTED CIRCUIT BOARD METHOD OF PRODUCTION THEREOF AND ASSOCIATED MULTILAYER PRINTED CIRCUIT BOARD\ | US6709769B1 | PEDRETTI GIUSEPPE (IT) | ZINCOCELERE SPA (US) | B23K20/10\ B32B15/08\ B32B15/01\ H05K3/02 |
| 2169 | 3D printing | INFILTRATION OF THREE-DIMENSIONAL OBJECTS FORMED BY SOLID FREEFORM FABRICATION\ | US6713125B1 | XU PINGYONG (US)\ SHERWOOD MICHAEL THOMAS (US) | 3D SYSTEMS INC (US) | B29C67/00\ B29C71/00 |
| 2170 | 3D printing | ELECTRON TUBE AND METHOD OF MANUFACTURING THE SAME\ | US6717350B2 | YONEZAWA YOSHIHISA (JP)\ OGAWA YUKIO (JP)\ NOHARA YASUHIRO (JP)\ KAWASAKI HIROAKI (JP) | FUTABA DENSHI KOGYO KK (JP) | H01J1/18\ H01J1/88\ H01J9/04\ H01J9/18\ H01J29/04\ H01J29/82\ H01J31/12\ H01J31/15 |
| 2171 | 3D printing | APPARATUS FOR FORMING AND/OR SEALING A PACKAGING UNIT\ | US6745543B2 | TILLACK BERND (DE)\ DIETRICH NORBERT (DE)\ DREIER HANS BERND (DE) | GREIF VELOX MASCHF GMBH (DE) | B29C65/08\ B29C65/74\ B65B51/22\ B65B51/30 |
| 2172 | 3D printing | ULTRASONIC WELDING AND CUTTING DEVICE FOR USE IN THE MANUFACTURE OF A FLAT CABLE\ | US6749102B2 | YAMANO YOSHIAKI (JP)\ FUKUMOTO KOJI (JP) | SUMITOMO WIRING SYSTEMS (JP) | H01B13/00\ H01B7/08 |
| 2173 | 3D printing | ULTRASONIC WELDING METHOD AND APPARATUS FOR WELDING REINFORCING TAPE PIECE TO FASTENER TAPE\ | US6749703B2 | IWASHITA KEISUKE (JP)\ MAEDA HIDEJI (JP)\ MATSUMURA SATOSHI (JP) | YKK CORP (JP) | A44B19/42\ B29C65/00\ B29C65/08 |
| 2174 | 3D printing | SOLID FREEFORM FABRICATION OF LIGHTWEIGHT LITHOGRAPHY STAGE\ | US6764619B2 | BERNAS JAMES J (US)\ BOWDEN BRADLEY F (US)\ HRDINA KENNETH E (US)\ WIGHT JR JOHN F (US) | CORNING INC (US) | G03F7/20\ G03B27/48 |
| 2175 | 3D printing | SONOTRODE FOR ULTRASOUND WELDING OF PLASTICS\ | US6776323B2 | SCHMIDT GERHARD (DE) | DYNAMIT NOBEL KUNSTSTOFF GMBH (DE) | B29C65/02\ B29C65/08 |
| 2176 | 3D printing | METHOD OF PRODUCING A BRUSH HAVING A BRISTLE PLATE\ | US6779851B2 | BOUCHIERE BART GERARD (BE) | BOUCHERIE NV G B (BE) | A46D3/04 |
| 2177 | 3D printing | SEALER WITH CONTINUOUS MOTION ULTRASONIC ZIPPER WELDING\ | US6780263B2 | DELISLE VICTOR (US) | ILLINOIS TOOL WORKS (US) | B29C65/00\ B29C65/08\ B31B19/90\ B29C65/18 |
| 2178 | 3D printing | ULTRASONIC HORN MOUNT\ | US6786384B1 | HAREGOPPA GOPAL B (US) | 3M INNOVATIVE PROPERTIES CO (US) | B23K20/10\ B29C65/08\ B06B3/00 |
| 2179 | 3D printing | ENERGY MANAGEMENT SYSTEM AND WELDING PROCESS THEREFOR\ | US6797089B2 | GIFFORD ROBERT (US)\ BRAHM JOERG (US) | BRANSON ULTRASONICS CORP (US) | B29C65/00\ F16F7/00\ B29C65/08\ B29C65/06\ B60R13/02\ B60R21/04 |
| 2180 | 3D printing | SEALING STRUCTURE FOR AN OIL PIPE FEMALE COUPLER\ | US6799747B1 | LAI LARRY (TW) | PLUM IND CO LTD (US) | F16L37/127\ F16L37/33\ F16L37/35 |
| 2181 | 3D printing | MULTI-SLIDE ASSEMBLY INCLUDING SLIDE FRAME AND STRIP CAP AND METHODS THEREOF\ | US6803239B2 | BUNN KENNETH E (US)\ KHAN ABDUL WAHID (US)\ NANDA SUSAN K W (US)\ SULLIVAN BARBARA MYSZKIEWICZ (US)\ WHITTLINGER KEITH O (US)\ WILLIAMSON KATHLEEN R (US)\ MATTHAI HOYT E (US) | NALGE NUNC INT CORP (US) | B01L3/00\ B01L3/14\ G01N1/28 |
| 2182 | 3D printing | MAGNETIC DISC CARTRIDGE METHOD OF MANUFACTURING THE SAME AND METHOD OF CLEANING LINERS OF THE SAME\ | US6805761B1 | KATO SHINICHI (JP)\ MIZUTA AKIRA (JP)\ HAYASHI YOICHI (JP) | FUJI PHOTO FILM CO LTD (JP) | B29L22/00\ B29C65/08\ G11B23/03\ G11B23/033\ G11B23/50 |
| 2183 | 3D printing | CONDUCTIVE THIN FILM SHEET SHIELD HARNESS AND METHOD OF MANUFACTURING THE SAME\ | US6806417B2 | MURAKAMI KAZUHIRO (JP)\ WATANABE YOSHIHIKO (JP)\ BABA AKIRA (JP)\ IKEDA TOMOHIRO (JP)\ SAITO SATOSHI (JP) | YAZAKI CORP (JP) | H01R43/20\ H01B7/00\ H01B13/012\ H01R43/02\ B60R16/02\ H01R9/03\ H01B11/10\ H01R4/64\ H01B9/02\ H01B13/26\ H01R13/648\ H01R13/658\ H02G3/38 |
| 2184 | 3D printing | OBJECT CONSOLIDATION THROUGH SEQUENTIAL MATERIAL DEPOSITION\ | US6814823B1 | WHITE DAWN (US) | SOLIDICA INC (US) | B29C67/00\ G05B19/4099\ B23K11/00\ B23K20/10\ B23K20/12\ B29C73/00 |
| 2185 | 3D printing | ULTRASONIC TOOL AND METHOD FOR SECURING A COVERING TO A FRAME\ | US6821367B1 | MACDONALD DOUGLAS B (US)\ CONSTANTINO DANIEL V (US)\ HUBBARD DEAN K (US)\ RUTHVEN ROBERT T (US)\ URSUL DANIEL M (US)\ HANIFY LARRY B (US) | STEELCASE DEV CORP (US) | B29C65/08\ B29C65/74\ B29C63/04 |
| 2186 | 3D printing | PROCESS FOR SPLICING A CONTINUOUS STRIP OF PACKETS\ | US6829876B1 | YOUNG ROBERT W (US)\ KIMURA TATESHI (US)\ DICK STEFAN O (US) | YIYUAN IND CO (US) | B65B61/00\ B65B61/06\ B65D75/42 |
| 2187 | 3D printing | ULTRASONIC WELD PATTERN FOR ADSORBENT CONTAINING PACKAGE\ | US6835257B2 | PERRINE GLENN D (US) | FLOW DRY TECHNOLOGY LTD (US) | B29C65/02\ B29C65/08\ B65D75/20\ B65D75/56 |
| 2188 | 3D printing | CABLE-ENROLLING CONDUCTIVE THIN-FILM SHEET AND MANUFACTURING METHOD THEREOF\ | US6858804B2 | MURAKAMI KAZUHIRO (JP)\ WATANABE YOSHIHIKO (JP)\ BABA AKIRA (JP) | YAZAKI CORP (JP) | H05K3/28\ H05K3/32\ H01R4/02\ H01R43/02\ H01B11/10 |
| 2189 | 3D printing | FUEL FILL SYSTEM\ | US6880593B1 | SWANE JEFFREY A (US) | DAYCO PRODUCTS LLC (US) | B60K15/04\ B65B1/04 |
| 2190 | 3D printing | HOUSING ASSEMBLY\ | US6884941B2 | BIRT DAVID ALAN (GB) | ARVINMERITOR LIGHT VEHICLE SYS (GB) | F16C1/26\ E05B53/00\ E05B65/12 |
| 2191 | 3D printing | SELF-ADJUSTING DYNAMIC FLOATING FIXTURE\ | US6892652B2 | JALBERT ROBERT (US)\ ANDREWS WILLIAM J (US) | BRANSON ULTRASONICS CORP (US) | B29C65/00\ B29C65/08\ B29C65/78 |
| 2192 | 3D printing | GAS IMPERMEABLE TUBE JOINT AND METHOD OF FORMING SAME\ | US6902208B1 | MOBLEY JOHN E (US)\ THURSTON MICHAEL O (US)\ SMITH CHRISTOPHER W (US) | DAYCO PRODUCT LLC (US) | B29C65/00\ B29C65/02\ F16L47/02\ B29C65/08\ B29C65/06\ B29C65/20\ F16L13/02\ F16L13/14 |
| 2193 | 3D printing | QUANTIZED FEED SYSTEM FOR SOLID FREEFORM FABRICATION\ | US6902246B2 | FONG JON JODY (US)\ VARNON DAVID MONTGOMERY (US)\ BISHOP RAYMOND J (US) | 3D SYSTEMS INC (US) | B29C67/00\ B05D3/00 |
| 2194 | 3D printing | SUPPORT VOLUME CALCULATION FOR A CAD MODEL\ | US6907307B2 | KULKARNI RAJEEV B (US)\ CHEN YONG (US) | 3D SYSTEMS INC (US) | G05B19/4099\ G06F19/00 |
| 2195 | 3D printing | METHOD OF MANUFACTURING A FOOD PACKAGING ARTICLE\ | US6913185B2 | HILLEBRAND CORY (US) | HIGH TECHNO CELL CO LTD (KR) | B65D75/42\ B65D75/58\ B65D85/36 |
| 2196 | 3D printing | POST PROCESSING THREE-DIMENSIONAL OBJECTS FORMED BY SOLID FREEFORM FABRICATION\ | US6916441B2 | SCHMIDT KRIS ALAN (US)\ NEWELL KENNETH J (US)\ SHERWOOD MICHAEL THOMAS (US) | 3D SYSTEMS INC (US) | B29C67/00 |
| 2197 | 3D printing | FUSED FILTER SCREEN FOR USE IN INK JET CARTRIDGE AND METHOD OF ASSEMBLING SAME\ | US6923530B2 | ANDERSON STEPHEN A (US)\ CARTER PATRICK D (US)\ JONES BRUCE S (US) | NU KOTE INT INC (US) | B41J2/175 |
| 2198 | 3D printing | LACE-LIKE STRUCTURE OF A HIVE-SHAPED VENETIAN BLIND\ | US6941995B2 | HSU PEY-SON (TW) | CHING FENG BLINDS IND CO LTD (TW) | E06B9/262 |
| 2199 | 3D printing | METHOD AND COMPONENTS FOR FLIP-CHIP BONDING\ | US6946745B2 | HESSE HANS (DE) | HESSE & KNIPPS GMBH (DE) | H01L21/60\ H01L21/56 |
| 2200 | 3D printing | METHOD FOR PRODUCING AN AIR-PERMEABLE LAMINATE FILM WITH A TEXTILE SURFACE WHICH HAS ELASTIC AND NON-ELASTIC REGIONS\ | US6974514B2 | HAMULSKI MARKUS (DE)\ SCHOENBECK MARCUS (DE)\ BALDAUF GEORG (DE) | NORDENIA DEUTSCHLAND GRONAU (DE) | D04H13/00\ B32B5/26\ B32B7/04\ B29C65/08\ B32B5/04 |
| 2201 | 3D printing | METHOD FOR CONTOURING BONE RECONSTRUCTION PLATES\ | US6978188B1 | CHRISTENSEN ANDREW M (US) | MEDICAL MODELING LLC (US) | G06F19/00 |
| 2202 | 3D printing | METHOD FOR MAKING A BIO-COMPATIBLE SCAFFOLD\ | US6993406B1 | CESARANO III JOSEPH (US)\ STUECKER JOHN N (US)\ DELLINGER JENNIFER G (US)\ JAMISON RUSSELL D (US) | SANDIA CORP (US) | A61F2/00\ A61F2/28 |
| 2203 | 3D printing | DETOXIFICATION OF SOLID FREEFORM FABRICATION MATERIALS\ | US6996245B2 | HANNA STEPHEN D (US) | 3D SYSTEMS INC (US) | B29C67/00\ B29K63/00\ A61L27/00\ B29K33/00\ H04R25/00 |
| 2204 | 3D printing | CONVEYOR BELT MODULES WITH EMBEDDED ROLLERS RETAINED IN THE MODULES AND ASSOCIATED METHOD\ | US6997306B2 | SOFRANEC RICHARD J (US)\ RAU BRIEN G (US) | LAITRAM LLC (US) | B65G17/08\ B65G17/32\ B65G39/20\ B65G17/00 |
| 2205 | 3D printing | ULTRASONIC WELDING METHOD FOR THE MANUFACTURE OF A POLISHING PAD COMPRISING AN OPTICALLY TRANSMISSIVE REGION\ | US6997777B2 | NEWELL KELLY J (US) | CABOT MICROELECTRONICS CORP (US) | B24D18/00\ B24D7/12\ B24B49/00 |
| 2206 | 3D printing | MAGNETIC TAPE CARTRIDGE\ | US7000861B2 | FUJII AKIHIKO (JP)\ TSUYUKI SEIJI (JP) | FUJI PHOTO FILM CO LTD (JP) | G11B23/107 |
| 2207 | 3D printing | COOLING TECHNIQUES IN SOLID FREEFORM FABRICATION\ | US7011783B2 | FONG JON JODY (US) | 3D SYSTEMS INC (US) | B29C67/00\ B29C41/36\ B29C41/46\ B28B1/30\ B29C35/16 |
| 2208 | 3D printing | ULTRASONIC APPARATUS WITH NON-ROTATING HORN AND ROTATING ANVIL FOR WELDING PLASTIC PARTS\ | US7025841B2 | OWEN KEVIN (US) | ILLINOIS TOOL WORKS (US) | B29C65/00\ B29C65/08\ B31B19/90\ B29C65/18 |
| 2209 | 3D printing | ULTRASOUND WELDING OF PLASTICS COMPONENTS\ | US7025842B2 | MONSHEIMER SYLVIA (DE)\ GOERING RAINER (DE)\ BRUDNY GUENTER (DE)\ RIES HANS (DE) | DEGUSSA (DE) | B32B37/00\ B23K20/10\ B29L23/00\ F16L47/02\ B29C65/08\ B29C65/64 |
| 2210 | 3D printing | WATER CUTOFF STRUCTURE OF COVERED WIRE\ | US7030320B2 | IDE TETSURO (JP)\ ASAKURA NOBUYUKI (JP) | YAZAKI CORP (JP) | H01R4/70\ H02G15/013\ H02G15/08\ H02G15/113\ H01R4/00 |
| 2211 | 3D printing | HIGH STRENGTH ALUMINUM ALLOY AND METHOD OF PRODUCING SAME\ | US7032644B2 | BRICE CRAIG A (US) | LOCKHEED CORP (US) | C22C21/00\ C22C21/06\ B22D23/06\ C22C1/02\ B22D11/00 |
| 2212 | 3D printing | POWER SEMICONDUCTOR MODULE AND METHOD FOR PRODUCING IT\ | US7042074B2 | GOEBL CHRISTIAN (DE)\ HEILBRONNER HEINRICH (DE) | SEMIKRON ELEKTRONIK GMBH (DE) | H01L23/34\ H01L21/60\ H01L25/00\ H01L25/07\ H01L21/607\ H01L23/538\ H01L25/18\ H01L23/02 |
| 2213 | 3D printing | SOLID FREEFORM FABRICATION SYSTEM AND METHOD\ | US7073561B1 | HENN DAVID S (US) | HENN DAVID S (US) | B22D23/00 |
| 2214 | 3D printing | VARIABLE FOCUS OPTICAL APPARATUS\ | US7085065B2 | SILVER JOSHUA D (GB) | SILVER JOSHUA D (GB) | G02B1/00\ G02B3/14\ G02B3/12\ G02B1/06 |
| 2215 | 3D printing | ADJUSTABLE KEYBOARD TRAY\ | US7086634B1 | KIRCHHOFF KENNETH J (US) | 3M INNOVATIVE PROPERTIES CO (US) | G06F3/02\ A47B21/03\ E04G3/00 |
| 2216 | 3D printing | FUSED LOOP OF FILAMENTOUS MATERIAL AND APPARATUS FOR MAKING SAME\ | US7090111B2 | EGAN THOMAS D (US)\ FENTON JR PAUL V (US) | AXYA MEDICAL INC (US) | A61B17/32\ A61L17/00\ A61B17/04\ A61B17/00\ A61B17/06\ B23K1/06\ A61B17/08 |
| 2217 | 3D printing | PRESSURE DIFFERENTIAL REGULATING VALVE UNIT A LIQUID CARTRIDGE AND A METHOD FOR ASSEMBLING A LIQUID CARTRIDGE\ | US7097293B2 | ICHIHASHI AKIRA (JP)\ ITO KENJI (JP)\ SHINADA SATOSHI (JP)\ SAKAI YASUTO (JP)\ ISHIZAWA TAKU (JP) | SEIKO EPSON CORP (JP) | B41J2/175 |
| 2218 | 3D printing | DEVELOPER CARTRIDGE AND IMAGE FORMING APPARATUS\ | US7106982B2 | ISHII MAKOTO (JP) | BROTHER IND LTD (JP) | G03G15/08 |
| 2219 | 3D printing | METHOD AND A SYSTEM FOR SOLID FREEFORM FABRICATION USING NON-REACTIVE POWDER\ | US7120512B2 | LAMBRIGHT TERRY M (US)\ BOYD MELISSA D (US)\ KASPERCHIK VLADEK P (US)\ KRAMER LAURA (US) | HEWLETT PACKARD DEVELOPMENT CO (US) | B29C67/00\ G06F19/00 |
| 2220 | 3D printing | ULTRASONIC WELDING APPARATUS\ | US7121447B2 | MOSCA TIMO (DE)\ LUEDTKE PETER (DE) | MOSCA G MASCHF (DE) | B32B37/00\ B29C65/00\ B29C65/08\ B65B13/32\ B23K37/00\ B23K5/20 |
| 2221 | 3D printing | ULTRASOUND HORN\ | US7122097B2 | RABE MAGNUS (SE) | TETRA LAVAL HOLDINGS & FINANCE (CH) | B32B37/00\ B29C65/08\ B06B3/00 |
| 2222 | 3D printing | METHOD OF AND APPARATUS FOR TESTING A WIRE BOND CONNECTION\ | US7134591B2 | WALTHER FRANK (DE) | HESSE & KNIPPS GMBH (DE) | B23K20/10\ H01L21/60\ B23K31/02\ B23K20/00\ G01N3/00\ B23K31/12\ G01N3/08 |
| 2223 | 3D printing | MAGNETIC SOURCE AND FERROMAGNETIC DEVICE FOR ULTRASONIC WELDING\ | US7152775B2 | JACKSON EUGENE A (US)\ GABRIEL MARK F (US) | BOEING CO (US) | B23K20/10\ B29C65/00\ B29C65/08\ B29C53/42\ B23K1/06 |
| 2224 | 3D printing | SOLID FREEFORM FABRICATION APPARATUS AND METHODS\ | US7168935B1 | TAMINGER KAREN M (US)\ WATSON J KEVIN (US)\ HAFLEY ROBERT A (US)\ PETERSEN DANIEL D (US) | NASA (US) | B29C35/04\ B28B17/00\ B23K15/00 |
| 2225 | 3D printing | THERMISTOR HAVING IMPROVED LEAD STRUCTURE AND SECONDARY BATTERY HAVING THE THERMISTOR\ | US7173512B2 | KO CHANG-MO (KR)\ CHOI SU-AN (KR)\ HAN JUN-KU (KR)\ LEE AN-NA (KR)\ LEE JONG-HWAN (KR)\ LEE JONG-HO (KR)\ YOON JONG-SEO (KR)\ KIM JU-DAM (KR) | LG CABLE LTD (KR) | H01M2/10\ H01M2/34\ H01C7/02\ H01C1/144\ H01C7/00\ H01C7/10\ H01C7/13\ H01M10/48 |
| 2226 | 3D printing | BACK LIGHT UNIT FOR LIQUID CRYSTAL DISPLAY DEVICE AND METHOD FOR MANUFACTURING THE UNIT\ | US7173677B2 | KITAMURA TAKESHI (JP)\ KONDO YASUKAZU (JP) | ROHM CO LTD (JP) | G02F1/1335\ F21V8/00\ G02F1/1333 |
| 2227 | 3D printing | TOOTHBRUSH AND PROCESS FOR PRODUCING SUCH A TOOTHBRUSH\ | US7174596B2 | FISCHER FRANZ (CH)\ STRAEHLER RETO (CH) | TRISA HOLDING AG (CH) | A61C17/00\ A46B9/04\ A46B7/06\ A46D3/00\ A46B9/06\ A46B15/00 |
| 2228 | 3D printing | ULTRASONICALLY WELDED STAKED OR SWAGED COMPONENTS IN AN IMPLANTABLE MEDICAL DEVICE\ | US7187974B2 | HAEG DANIEL C (US)\ KELLEY JAMES F (US)\ WIKLUND CRAIG L (US) | MEDTRONIC INC (US) | A61N1/375 |
| 2229 | 3D printing | THERMOPLASTIC FENCING CONSTRUCTION AND METHOD OF ASSEMBLY THEREOF\ | US7204898B2 | FATTORI JAMES (US) | LMT MERCER GROUP INC (US) | B32B37/00 |
| 2230 | 3D printing | SYSTEM AND METHOD FOR FABRICATING A THREE-DIMENSIONAL METAL OBJECT USING SOLID FREE-FORM FABRICATION\ | US7220380B2 | FARR ISAAC (US)\ LAMBRIGHT TERRY M (US)\ KEARL DANIEL A (US) | HEWLETT PACKARD DEVELOPMENT CO (US) | B29C67/00\ B22F3/00\ B22F3/16\ B29C35/02\ B22F7/00\ B22F1/00\ B22F3/02\ B29K33/04 |
| 2231 | 3D printing | MULTIPLE PROBE POWER SYSTEMS AND METHODS FOR ULTRASONIC WELDING\ | US7225965B2 | JOHANSEN DAVID K (US) | DUKANE CORP (US) | B23K20/10\ B29C65/08\ B23K1/06\ B23K5/20 |
| 2232 | 3D printing | TREATMENT FOR ULTRASONIC WELDING\ | US7229523B2 | MARTIN DAVID W (US)\ SANTANA FELIX J (US)\ GRIFFIN SCOTT (US)\ DARCY III JOHN JOSEPH (US)\ ROETKER MICHAEL STEPHEN (US) | XEROX CORP (US) | B32B37/00 |
| 2233 | 3D printing | ELECTROCHEMICAL SENSOR\ | US7241369B2 | VAN HOORN HENDRIK (NL) | METTLER TOLEDO AG (CH) | G01N27/414\ G01N27/333\ H01L23/58 |
| 2234 | 3D printing | SYSTEMS AND METHODS OF SOLID FREEFORM FABRICATION WITH TRANSLATING POWDER BINS\ | US7296990B2 | LAMBRIGHT TERRY M (US)\ NIELSEN JEFFREY A (US)\ COLLINS DAVID C (US)\ DEVOS JOHN A (US)\ CRUZ-URIBE TONY S (US) | HEWLETT PACKARD DEVELOPMENT CO (US) | B28B1/16 |
| 2235 | 3D printing | METHODS AND SYSTEMS FOR CONTROLLING PRINTHEAD TEMPERATURE IN SOLID FREEFORM FABRICATION\ | US7306758B2 | NIELSEN JEFFREY A (US)\ CASTLE STEVEN T (US)\ CUMMINGS DARREL H (US)\ LOUGHRAN STEPHEN A (GB) | HEWLETT PACKARD DEVELOPMENT CO (US) | B29C67/00\ B28B1/00\ B28B1/16\ B41J2/175\ B28B1/32\ B28B11/08\ B28B23/00 |
| 2236 | 3D printing | COMPOSITIONS FOR SOLID FREEFORM FABRICATION\ | US7326759B2 | FARR ISAAC (US)\ LAMBRIGHT TERRY M (US)\ ORIAKHI CHRISTOPHER (US)\ KASPERCHIK VLADEK (US)\ AKINMADE ADE (DE)\ NEEL DAVID A (US)\ ENGELBRECHT JURGEN (DE) | HEWLETT PACKARD DEVELOPMENT CO (US) | G03F7/00\ C08L33/08\ C08K13/02\ C08K7/02\ C08K7/14\ C08F24/00\ C08K3/20 |
| 2237 | 3D printing | ULTRASONIC BONDING HONE AND ULTRASONIC WELDING METHOD\ | US7337938B2 | NORO TAKASHI (JP) | FUJIFILM CORP (JP) | B32B37/00\ B23K1/06 |
| 2238 | 3D printing | ABRASIVE ARTICLE AND METHODS OF MAKING AND USING THE SAME\ | US7338355B2 | NAYAR SATINDER K (US)\ ROWELL TIMOTHY J (US)\ SANDERS JR RUFUS C (US) | 3M INNOVATIVE PROPERTIES CO (US) | B24D11/00 |
| 2239 | 3D printing | SANDWICH HOUSING FOR AN ANTENNA AMPLIFIER\ | US7358438B2 | ZOLLER ORTWIN (DE) | HIRSCHMANN ELECTRONICS GMBH (DE) | H05K5/00\ B60R16/02\ H05K7/14\ B60R16/023\ H02G3/08 |
| 2240 | 3D printing | ULTRASONIC WELDING HORN FOR WELDING A SEAM IN AN IMAGING RECEPTOR BELT\ | US7377416B2 | ROETKER MICHAEL S (US)\ MARTIN DAVID W (US)\ YU ROBERT C U (US) | XEROX CORP (US) | B29C65/08\ B23K1/06 |
| 2241 | 3D printing | ULTRASONIC WELDING MACHINE AS WELL AS TOOL FOR AN ULTRASONIC WELDING MACHINE\ | US7392923B2 | STROH DIETER (DE)\ DIETERLE HORST (DE)\ WAGENBACH UDO (DE) | SCHUNK ULTRASCHALLTECHNIK GMBH (DE) | B23K20/10\ H01R43/20\ H01R43/00 |
| 2242 | 3D printing | ULTRASONIC WELDED INITIATOR AND CONNECTOR SOCKET\ | US7401810B2 | BRISIGHELLA JR DARIO G (US)\ SMITH BRADLEY W (US)\ ELLIS KELLY (US)\ RASMUSSEN KIRK (US)\ COX MATT (US) | AUTOLIV ASP INC (US) | B60R21/26\ B60R21/264 |
| 2243 | 3D printing | FLASH CURING IN SELECTIVE DEPOSITION MODELING\ | US7402273B2 | SHERWOOD MICHAEL THOMAS (US) | 3D SYSTEMS INC (US) | B29C35/08\ B29C67/00\ G03F7/00\ B29C41/02 |
| 2244 | 3D printing | METHOD FOR FORMING A BUTTONHOLE IN A FABRIC\ | US7404368B2 | LAMPE DOMINIQUE (BE) | BVBA SHADOW BELGIUM (BE) | D05B17/00 |
| 2245 | 3D printing | METHODS AND SYSTEMS FOR PRODUCING AN OBJECT THROUGH SOLID FREEFORM FABRICATION\ | US7435072B2 | HUNTER SHAWN D (US)\ COLLINS DAVID C (US)\ NIELSEN JEFFREY ALLEN (US) | HEWLETT PACKARD DEVELOPMENT CO (US) | B29C67/00\ B29C47/00 |
| 2246 | 3D printing | SOLID FREEFORM COMPOSITIONS METHODS OF APPLICATION THEREOF AND SYSTEMS FOR USE THEREOF\ | US7435763B2 | FARR ISAAC (US)\ LAMBRIGHT TERRY M (US)\ ORIAKHI CHRISTOPHER (US)\ KASPERCHIK VLADEK P (US)\ NEEL DAVID A (US) | HEWLETT PACKARD DEVELOPMENT CO (US) | B29C67/00\ G03F7/00\ G03F7/004\ B29C35/04\ G03C5/00\ C08F20/00\ G03C1/725 |
| 2247 | 3D printing | METHOD AND APPARATUS FOR SWITCHING ON AN ULTRASOUND OSCILLATION SYSTEM\ | US7439815B2 | SCHIEF DIETER (DE) | WALTER MARTIN ULTRASCHALLTECH (DE) | H02N2/00\ H03L7/00 |
| 2248 | 3D printing | PRODUCTION METHOD OF ULTRASONIC WELD ASSEMBLY\ | US7445140B2 | AOKI YUUICHI (JP)\ MATSUOKA SHIN-ICHI (JP) | DENSO CORP (JP) | B23K20/10\ B23K37/00\ B23K1/06\ B23K5/20 |
| 2249 | 3D printing | WEB WELDING SYSTEM\ | US7449084B2 | NAKAKADO MASAKI (JP) | ZUIKO CORP (JP) | B32B37/00\ B29C65/08\ B65H23/188 |
| 2250 | 3D printing | SYSTEMS FOR PROVIDING CONTROLLED POWER TO ULTRASONIC WELDING PROBES\ | US7475801B2 | JOHANSEN DAVID K (US)\ LEONARD CHARLES L (US)\ KLINSTEIN LEO (US)\ JURKOWSKI WILLIAM E (US)\ RE JOSEPH (US) | DUKANE CORP (US) | B29C65/08\ B23K1/06 |
| 2251 | 3D printing | BATTERY PACK CASING WITH LOCK TYPE CONNECTOR\ | US7479758B2 | MOON KI EOB (KR) | LG CHEMICAL LTD (KR) | H02J7/00\ H01M2/02\ H01M2/00\ H01M2/04\ H01M2/08 |
| 2252 | 3D printing | BELT AND METHOD OF MAKING SAME\ | US7491281B2 | ROETKER MICHAEL S (US)\ MARTIN DAVID W (US)\ DARCY III JOHN J (US) | XEROX CORP (US) | B32B37/00 |
| 2253 | 3D printing | METHODS AND SYSTEMS FOR PRODUCING AN OBJECT THROUGH SOLID FREEFORM FABRICATION BY VARYING A CONCENTRATION OF EJECTED MATERIAL APPLIED TO AN OBJECT LAYER\ | US7497977B2 | NIELSEN JEFFREY A (US)\ COLLINS DAVID C (US)\ CASTLE STEVEN T (US)\ HUNTER SHAWN (US) | HEWLETT PACKARD DEVELOPMENT CO (US) | B29C67/00\ B29C41/02 |
| 2254 | 3D printing | WATER-STOP STRUCTURE OF SHEATHED WIRE\ | US7504584B2 | IDE TETSURO (JP) | YAZAKI CORP (JP) | H02G15/113\ H01B17/58\ H01B7/285 |
| 2255 | 3D printing | SOLID FREEFORM FABRICATION OF STRUCTURALLY ENGINEERED MULTIFUNCTIONAL DEVICES\ | US7509240B2 | DAS SUMAN (US)\ HOLLISTER SCOTT J (US)\ KREBSBACH PAUL H (US)\ SANTOSA JAMES K (US) | UNIV MICHIGAN (US) | B29C67/00\ G06F17/50\ G06F19/00 |
| 2256 | 3D printing | PIPING CONNECTOR AND METHOD OF FABRICATING THE SAME\ | US7516989B2 | YOSHIDA NAOKI (JP) | PIOLAX INC (JP) | F16L37/08\ F16L37/14\ F16L37/088 |
| 2257 | 3D printing | TWO-PIECE CONNECTOR FOR FLAT BLADE WINDSHIELD WIPER\ | US7523522B2 | KOPPEN SCOTT A (US)\ LYTLE JAMES L (US)\ HERRING LEGAL REPRESENTATIVE DEBORAH (US) | FEDERAL MOGUL WORLD WIDE INC (US) | B60S1/38\ B60S1/40 |
| 2258 | 3D printing | ULTRASONIC WELDING METHOD ULTRASONIC WELDING DEVICE AND PIPE JOINED BY THE SAME\ | US7523849B2 | AOKI YUUICHI (JP)\ MATSUSAKA YOSHIHIKO (JP)\ MORIKAWA HIROYASU (JP)\ SHIMIZU ARINORI (JP)\ HASHIMOTO HIROKAZU (JP) | DENSO CORP (JP) | B29C65/00\ B23K1/06 |
| 2259 | 3D printing | ARRANGEMENT FOR WELDING WORKPIECES BY MEANS OF AN ULTRASONIC DEVICE\ | US7533791B2 | STEINER ERNST (DE)\ STROH DIETER (DE)\ DIETERLE HORST (DE)\ WAGNER PETER (DE)\ STROH HEIKO (DE) | SCHUNK ULTRASCHALLTECHNIK GMBH (DE) | B23K20/10\ B29C65/08\ H01R43/02\ B23K37/00\ B23K1/06 |
| 2260 | 3D printing | APPARATUS AND METHOD FOR WELDING A POUR SPOUT FITMENT TO A CONTAINER\ | US7568607B2 | ADLER NILS PETER (NO) | ELOPAK SYSTEMS (CH) | B23K20/10\ B29C65/00\ B29C65/08\ B65B61/18\ B65B51/22\ B31B1/84 |
| 2261 | 3D printing | VEHICLE LIGHTING DEVICE\ | US7568824B2 | WATANABE SHIGEYUKI (JP)\ INABA TETSUAKI (JP)\ YAMAMICHI TATSUHIKO (JP) | KOITO MFG CO LTD (JP) | B60Q1/04 |
| 2262 | 3D printing | WELDING DEVICE AND METHOD FOR WELDING WORKPIECES\ | US7600664B2 | DIETERLE HORST (DE)\ WAGNER PETER (DE) | SCHUNK ULTRASCHALLTECHNIK GMBH (DE) | B23K20/10\ H01R43/02\ B23K37/00\ B23K1/06\ B23K5/20 |
| 2263 | 3D printing | REWELDED CARTRIDGE AND METHOD OF MANUFACTURE\ | US7618507B2 | WAZANA YOEL (US)\ PEREZ JESUS GONZALEZ (US) | WAZANA BROTHERS INTERNATIONAL (US) | B32B37/00 |
| 2264 | 3D printing | DIRECT-MANUFACTURED DUCT INTERCONNECTS\ | US7623940B2 | BAGWILL TRACY L (US)\ HUSKAMP CHRIS (US) | BOEING CO (US) | G06F17/50\ G06F19/00\ F16L9/18\ F16L21/02\ F16L23/00 |
| 2265 | 3D printing | METHOD AND A SYSTEM FOR PRODUCING AN OBJECT USING SOLID FREEFORM FABRICATION\ | US7625512B2 | COLLINS DAVID C (US)\ NIELSEN JEFFREY ALLEN (US)\ CRUZ-URIBE ANTONIO S (US) | HEWLETT PACKARD DEVELOPMENT CO (US) | B29C67/00\ B29C41/02 |
| 2266 | 3D printing | BRASSIERE CONSTRUCTION\ | US7641536B2 | FILDAN GERHARD (AT)\ WANZENBOECK KARL (AT) | FILDAN ACCESSORIES CORP (US) | A41C3/00 |
| 2267 | 3D printing | METHOD OF IMPROVING COLOR QUALITY IN AN OBJECT PRODUCED BY SOLID FREEFORM FABRICATION\ | US7645403B2 | COLLINS DAVID C (US)\ NIELSEN JEFFREY ALLEN (US)\ CASTLE STEVEN T (US) | HEWLETT PACKARD DEVELOPMENT CO (US) | B29C67/00\ G06F17/50\ B29C35/00\ B29C41/02\ B41J2/04\ B29C41/32 |
| 2268 | 3D printing | METHOD FOR MEASURING AND/OR REGULATING THE OSCILLATION AMPLITUDE OF AN ULTRASONIC TRANSMITTER AND AN ULTRASONIC WELDING DEVICE\ | US7647828B2 | GASSERT FRANK (DE) | SCHUNK ULTRASCHALLTECHNIK GMBH (DE) | G01N29/04\ B23K20/10\ B29C65/00\ B29C65/08\ B06B1/00\ G01H5/00 |
| 2269 | 3D printing | CARBON FIBER TOW TERMINATION AND METHOD FOR MAKING\ | US7662002B2 | JOHNSTON JAMES J (US) | METHODE ELECTRONICS INC (US) | H02G15/02 |
| 2270 | 3D printing | METHODS OF CONSTRUCTING A BETATRON VACUUM CHAMBER AND INJECTOR\ | US7675252B2 | CHEN FELIX K (US)\ CORRIS GARY W (US)\ WONG JOYCE (US)\ ZHOU ZILU (US)\ BALKUNAS STEPHEN (US)\ HAUG JAMES G (US) | SCHLUMBERGER TECHNOLOGY CORP (US) | H05H7/00 |
| 2271 | 3D printing | ROLLED-PRINT-MEDIUM HOLDER DEVICE\ | US7677823B2 | HOSHINO TERUMASA (JP)\ NAKAMURA YASUNORI (JP)\ KATO TSUTOMU (JP)\ MATSUMOTO HARUKI (JP)\ NONOMURA YOSHIHITO (JP) | BROTHER IND LTD (JP) | B23K20/10\ G11B23/107\ B29C65/08\ B41J15/02 |
| 2272 | 3D printing | METHOD AND APPARATUS FOR FILLING BAGS\ | US7681378B2 | BRORMANN HUBERT (DE)\ NEUMANN ROBERT (DE) | HAVER & BOECKER OHG (DE) | B29C65/02\ B65B3/00 |
| 2273 | 3D printing | APPARATUS FOR ADJUSTING THE POSITION OF AN ULTRASONIC WELDING HORN\ | US7690548B2 | OBLAK DONALD S (US)\ MLINAR JOHN R (US)\ FETTIG PAUL M (US) | 3M INNOVATIVE PROPERTIES CO (US) | B29C65/00\ B29C65/08\ B23K1/06 |
| 2274 | 3D printing | METHODS FOR PRODUCING AN OBJECT THROUGH SOLID FREEFORM FABRICATION\ | US7700020B2 | NIELSEN JEFFREY A (US)\ KASPERCHIK VLADEK (US) | HEWLETT PACKARD DEVELOPMENT CO (US) | B29C67/00\ B29C41/02 |
| 2275 | 3D printing | TOOTHBRUSH WITH ENHANCED CLEANING EFFECTS\ | US7703163B2 | JIMENEZ EDUARDO (US)\ GATZEMEYER JOHN J (US)\ MINTEL THOMAS (US)\ SORRENTINO ALAN V (US) | COLGATE PALMOLIVE CO (US) | A46B9/04\ A46B15/00\ A46B13/02\ A61C17/22\ A46B9/02 |
| 2276 | 3D printing | INK SUPPLY NEEDLE FOR A LIQUID EJECTING HEAD AND LIQUID EJECTING APPARATUS\ | US7726788B2 | AKAHANE FUJIO (JP) | SEIKO EPSON CORP (JP) | B41J2/175 |
| 2277 | 3D printing | ULTRASONIC WELDING APPARATUS\ | US7748590B2 | REATHERFORD LARRY (US)\ HETRICK ELIZABETH T (US)\ WILKOSZ DANIEL E (US)\ WALSH JOSEPH (US)\ SKOGSMO JAN (SE) | FORD GLOBAL TECH LLC (US)\ JAGUAR CARS (GB) | B23K37/00\ B23K1/06 |
| 2278 | 3D printing | BATTERY PACK\ | US7768231B2 | KIM TAE IL (KR)\ JUNG SOONKWANG (KR) | LG CHEMICAL LTD (KR) | H02J7/00 |
| 2279 | 3D printing | ADAPTIVE CONTINUOUS ACOUSTIC WELDING SYSTEM FOR INCOMPATIBLE MATERIALS\ | US7771551B2 | SCHROEDER TERRENCE K (US) | SWCE (US) | B32B37/00 |
| 2280 | 3D printing | CANTILEVERED BAR GAP ADJUSTMENT FOR AN ULTRASONIC WELDING SYSTEM\ | US7775413B2 | NAYAR SATINDER K (US)\ POCHARDT DONALD L (US)\ OBLAK DONALD S (US)\ MLINAR JOHN R (US)\ FETTIG PAUL M (US) | 3M INNOVATIVE PROPERTIES CO (US) | B23K20/10\ B29C65/00\ B29C65/08\ B23K1/06 |
| 2281 | 3D printing | FORMATION OF A FABRIC SEAM BY ULTRASONIC GAP WELDING OF A FLAT WOVEN FABRIC\ | US7794555B2 | LAFOND JOHN J (US)\ BOWDEN JENNIFER L (US)\ KROLL LYNN F (US)\ BOTELHO JOSEPH (US) | ALBANY INT CORP (US) | B32B37/00 |
| 2282 | 3D printing | METHODS TO PRODUCE AN OBJECT THROUGH SOLID FREEFORM FABRICATION\ | US7794636B2 | COLLINS DAVID C (US)\ KRAMER LAURA (US) | HEWLETT PACKARD DEVELOPMENT CO (US) | B29C67/00\ B29C41/12\ B29C41/36\ B29C35/04\ B27N5/00\ B29C41/02 |
| 2283 | 3D printing | DAMPER DEVICE AND MANUFACTURING METHOD FOR DAMPER DEVICE\ | US7798296B2 | IWASHITA HIROYUKI (JP) | NIDEC SANKYO CORP (JP) | F16D57/00 |
| 2284 | 3D printing | ULTRASONIC PRESS USING SERVO MOTOR WITH INTEGRATED LINEAR ACTUATOR\ | US7819158B2 | KLINSTEIN LEO (US)\ JURKOWSKI WILLIAM E (US)\ GOLKO PAUL (US) | DUKANE CORP (US) | B32B41/00 |
| 2285 | 3D printing | TRAILER WALL COMPOSITE LINER WITH INTEGRAL SCUFF PANEL\ | US7829165B2 | GRANDOMINICO GARY ALAN (US)\ MCDONALD JR RAYMOND A (US)\ GRANDOMINICO DOMINIC PLAUCHE (US) | RIDGE CORP (US) | B32B3/00\ B62D53/06\ E04B5/00 |
| 2286 | 3D printing | ULTRASONIC WELDING OF RESILIENT LAYERS TO PLATES\ | US7879168B2 | PACIONE JOSEPH ROCCO (CA)\ PACIONE JOHN ANTHONY (CA)\ HOHENBICHLER JURGEN (AT)\ RITTMANNSBERGER FRANZ (CA) | TAC FAST GEORGIA L L C (US) | B32B37/00\ B29C65/16\ B32B27/32\ B29C65/02\ B32B37/06\ B29C65/08\ B32B37/04 |
| 2287 | 3D printing | AUTOMOTIVE LAMP PROVIDED WITH A BULB LOCKED TO THE CAP OF THE LAMP\ | US7887224B2 | PELSMA JELLE (NL)\ DOREE VANESSA (FR)\ RIOU PHILIPPE (FR)\ THEELEN ANTONIUS NICOLAAS (NL) | KONINKL PHILIPS ELECTRONICS NV (NL) | B60Q1/00 |
| 2288 | 3D printing | ROTARY ULTRASONIC SEALING\ | US7892375B2 | BLANCHARD DANIEL (FR)\ WICKS DAVID (GB)\ MONTOYA MICHEL (FR) | MARS INC (US) | B32B37/00 |
| 2289 | 3D printing | METHOD AND APPARATUS FOR ULTRASONIC WELDING OF TERMINALS\ | US7896219B2 | SCHEUERMAN ROBERT J (US)\ ROURKE RICK F (US) | GM GLOBAL TECH OPERATIONS INC (US) | B23K20/10 |
| 2290 | 3D printing | MEDICAL CAPSULE HOUSING FORMED BY THERMAL WELDING\ | US7931584B2 | AKAGI TOSHIMASA (JP)\ SEGAWA HIDETAKE (JP) | OLYMPUS CORP (JP) | A61B1/00\ A61B1/05 |
| 2291 | 3D printing | STRUCTURE OF A KEY\ | US7952010B2 | MINAKAWA MAKOTO (JP)\ UNO SHIRO (JP)\ TAKATA YUKIHIDE (JP) | ROLAND CORP (JP) | G10D13/02 |
| 2292 | 3D printing | BRASSIERE WITH REINFORCED EDGES\ | US7963822B2 | SIMPSON ELISABETH (CN) | TRIUMPH INTERTRADE AG (CH) | A41C3/00 |
| 2293 | 3D printing | ULTRASONIC DEVICE WITH LONGITUDINAL AND TORSIONAL SONOTRODES\ | US7984839B2 | BUETTIKER ALBERT (CH) | TELSONIC HOLDING AG (CH) | B29C65/00\ B23K1/06 |
| 2294 | 3D printing | HYDRODYNAMIC BEARING DEVICE\ | US7985025B2 | ITO KENJI (JP)\ SATOJI FUMINORI (JP) | NTN TOYO BEARING CO LTD (JP) | F16C17/08\ F16C17/10\ F16C33/10\ F16C35/02\ H02K5/16\ H02K7/08\ H02K21/22\ F16C32/06 |
| 2295 | 3D printing | ULTRASONIC WELDING APPARATUS AND METHOD FOR PRODUCING WELDED PRODUCT\ | US7985312B2 | SHIMIZU YOICHIRO (JP)\ TANIGUCHI AKIHIKO (JP)\ HARA KOICHIRO (JP)\ NAKAMURA HIROTAKE (JP) | BROTHER IND LTD (JP) | B32B37/00 |
| 2296 | 3D printing | POWER SEMICONDUCTOR MODULE AND METHOD FOR PRODUCING THE SAME\ | US7986034B2 | KEMPER ALFRED (DE)\ STROTMANN GUIDO (DE) | INFINEON TECHNOLOGIES AG (DE) | H01L21/00\ H01L23/498\ H01L23/49 |
| 2297 | 3D printing | ELECTROCHEMICALLY ACTIVATED ANOLYTE AND CATHOLYTE LIQUID\ | US8007654B2 | FIELD BRUCE F (US)\ GRONLUND PATRICK J (US) | TENNANT CO (US) | C02F1/467\ C02F1/461 |
| 2298 | 3D printing | STACKED SOLID ELECTROLYTIC CAPACITOR\ | US8014129B2 | SAITO TAKESHI (JP)\ KASUGA TAKEO (JP)\ SAKATA KOJI (JP) | NEC TOKIN CORP (JP) | H01G9/04\ H01G4/228 |
| 2299 | 3D printing | REGION-BASED SUPPORTS FOR PARTS PRODUCED BY SOLID FREEFORM FABRICATION\ | US8046097B2 | HULL CHARLES W (US)\ KULKARNI RAJEEV (US)\ WANG HONGQING V (US)\ WEST JOHN CORBIN (US)\ MOJDEH MEDHI (US) | 3D SYSTEMS INC (US) | B29C67/00\ B29C35/04\ G06F19/00\ G09G5/00\ B28B1/14\ D04H1/16\ H05B6/00\ G05B19/04\ G06F17/15 |
| 2300 | 3D printing | METHOD FOR PRODUCING A WELDED JOINT BETWEEN ELECTRICAL CONDUCTORS BY MEANS OF AN ULTRASONIC WELDING METHOD\ | US8047420B2 | STROH DIETER (DE) | ULTRASCHALLTECHNIK GMBH (DE) | B23K20/10 |
| 2301 | 3D printing | ULTRASONIC PRESS USING SERVO MOTOR WITH DELAYED MOTION\ | US8052816B2 | KLINSTEIN LEO (US)\ JURKOWSKI WILLIAM E (US)\ GOLKO PAUL (US) | DUKANE CORP (US) | B32B41/00 |
| 2302 | 3D printing | CASE CLAMP\ | US8070897B2 | TOENNIGS BODO (DE) | LIGMATECH AUTOMATIONSSYSTEME (DE)\ TOENNIGS BODO (DE) | B32B37/00 |
| 2303 | 3D printing | ULTRASONIC WELDING DEVICE\ | US8113258B2 | PATRIKIOS MIKE (US)\ RUSHTON ANDREW (US) | PATRIKIOS MIKE (US)\ RUSHTON ANDREW (US)\ SONICS & MATERIALS INC (US) | B32B37/00 |
| 2304 | 3D printing | BRASSIERES\ | US8133091B2 | LIU ZHEN QIANG (HK) | REGINA MIRACLE INT LTD (HK)\ LIU ZHEN QIANG (HK) | A41C3/00 |
| 2305 | 3D printing | ULTRASONIC WELDING DEVICE AND PACKAGING MACHINE WITH AN ULTRASONIC WELDING DEVICE\ | US8156984B2 | WIEDUWILT ULRICH (DE)\ LOECHT HEINRICH (DE) | BOSCH GMBH ROBERT (DE)\ WIEDUWILT ULRICH (DE)\ LOECHT HEINRICH (DE) | B32B37/00\ B65B9/20 |
| 2306 | 3D printing | PART SENSING HORN\ | US8215359B2 | JALBERT ROBERT D (US)\ ABLAMSKY JR JOHN J (US) | BRANSON ULTRASONICS CORP (US)\ JALBERT ROBERT D (US)\ ABLAMSKY JR JOHN J (US) | B32B37/00 |
| 2307 | 3D printing | CONTAINER AND PACKAGING METHOD FOR VEGETATION TRIMMER LINE\ | US8230995B2 | ANDREWS JEFF (US)\ PHILLIPS ROBERT (US)\ CHRISTMANN ALLEN W (US)\ THOMPSON TERRY J (US) | DESERT EXTRUSION CORP (US)\ ANDREWS JEFF (US)\ PHILLIPS ROBERT (US)\ CHRISTMANN ALLEN W (US)\ THOMPSON TERRY J (US) | B65D85/671 |
| 2308 | 3D printing | BACK LIGHTED MEMBRANE KEYBOARD WITH COMPONENTS BEING SECURED TOGETHER BY SUBJECTING TO ULTRASONIC WELDING\ | US8247714B2 | TSAI HUO-LU (TW) | TSAI HUO-LU (TW)\ SUNREX TECHNOLOGY CORP (TW) | H01H13/70 |
| 2309 | 3D printing | METHOD AND APPARATUS FOR ULTRASONIC WELDING OR BONDING\ | US8317078B1 | STROTMANN GUIDO (DE)\ CORDES ACHIM (DE) | INFINEON TECHNOLOGIES AG (DE)\ STROTMANN GUIDO (DE)\ CORDES ACHIM (DE) | B23K1/06 |
| 2310 | 3D printing | METHOD AND APPARATUS FOR ULTRASONIC WELDING\ | US8360300B1 | STROTMANN GUIDO (DE)\ KOECHLING PETER (DE)\ STEININGER CHRISTIAN (DE) | INFINEON TECHNOLOGIES AG (DE)\ STROTMANN GUIDO (DE)\ KOECHLING PETER (DE)\ STEININGER CHRISTIAN (DE) | B23K1/06 |
| 2311 | 3D printing | ULTRASONIC WELDING ASSEMBLY AND METHOD OF ATTACHING AN ANVIL TO A BRACKET OF THE ASSEMBLY\ | US8403019B2 | KHAKHALEV ALEX (US) | LG CHEMICAL LTD (KR)\ KHAKHALEV ALEX (US) | B32B37/00 |
| 2312 | 3D printing | ELECTRONIC HOUSING ASSEMBLIES THEREFOR AND METHODS OF MAKING SAME\ | US8406001B2 | CHAO MICHAEL CHY (US) | CHAO MICHAEL CHY (US)\ DUALSONIC INC (US) | H05K5/00 |
| 2313 | 3D printing | SYNCHRONOUS ULTRASONIC METAL WELDING DEVICE WITH DOUBLE-HEADS\ | US8413874B2 | YAN ZHUOSHENG (CN)\ YAN ZHUOLI (CN)\ YANG RUKUN (CN)\ LU XIAOBIN (CN)\ YAN JINXUAN (CN)\ ZHONG XINGTAO (CN)\ HUANG WEISHU (CN) | YAN ZHUOSHENG (CN)\ YAN ZHUOLI (CN)\ YAN JINXUAN (CN)\ YANG RUKUN (CN)\ LU XIAOBIN (CN)\ ZHONG XINGTAO (CN)\ HUANG WEISHU (CN)\ GUANGZHOU NEWPOWER ULTRASONIC ELECTRONIC EQUIPMENT CO LTD (CN) | B23K37/00\ B23K1/06 |
| 2314 | 3D printing | SYSTEM AND METHOD FOR DETERMINING WHETHER AN ULTRASONIC HORN IS ALIGNED WITH AN ANVIL\ | US8433433B2 | KHAKHALEV ALEX (US) | LG CHEMICAL LTD (KR)\ KHAKHALEV ALEX (US) | G06F17/00\ B29C65/08 |
| 2315 | 3D printing | DEVICE FOR ULTRASONIC WELDING OF AT LEAST TWO PLASTIC FOIL LAYERS\ | US8435368B2 | ROMIJN BAREND BASTIAAN (NL)\ JANSSEN IVAN JACOBUS (NL)\ KOEHLER HANNES (DE) | ROBERT BOSCH PACKAGING TECHNOLOGY B V (NL)\ ROMIJN BAREND BASTIAAN (NL)\ JANSSEN IVAN JACOBUS (NL)\ KOEHLER HANNES (DE) | B32B37/00 |
| 2316 | 3D printing | ULTRASONIC WELDING SYSTEM WITH DYNAMIC PRESSURE CONTROL\ | US8439247B1 | ABELL JEFFREY A (US)\ SPICER JOHN PATRICK (US)\ CHRISTIAN BENJAMIN R (US) | GM GLOBAL TECH OPERATIONS INC (US)\ SPICER JOHN PATRICK (US)\ CHRISTIAN BENJAMIN R (US)\ ABELL JEFFREY A (US) | B23K20/10\ B23K20/00 |
| 2317 | 3D printing | TORSIONAL ULTRASONIC WELDING\ | US8448836B2 | SCHLOMS MARTIN (DE)\ MARTENS SEBASTIAN (DE)\ LIETZ FRANZ-JOSEF (DE) | AUTO KABEL MAN GMBH (DE)\ LIETZ FRANZ-JOSEF (DE)\ SCHLOMS MARTIN (DE)\ MARTENS SEBASTIAN (DE) | B23K20/10 |
| 2318 | 3D printing | MULTI-MODE ULTRASONIC WELDING CONTROL AND OPTIMIZATION\ | US8450644B2 | CAI WAYNE W (US)\ TANG JASON C H (US) | GM GLOBAL TECH OPERATIONS INC (US)\ TANG JASON C H (US)\ CAI WAYNE W (US) | B23K15/00\ B23K31/02\ B23K9/12\ B23K1/06\ B23K11/10\ B23K20/08 |
| 2319 | 3D printing | USE OF ADHESIVE TAPE COMPRISING A SPUNBONDED BACKING ONE-SIDEDLY COATED AT LEAST PARTIALLY WITH A PRESSURE-SENSITIVE ADHESVIE\ | US8460492B2 | HOPF MARTIN (DE) | TESA SE (DE)\ HOPF MARTIN (DE) | B29C65/08\ B29C65/50\ B29C65/04 |
| 2320 | 3D printing | DISPOSABLE CHAIR COVERS\ | US8500199B2 | PAULIN VICTORIA (US) | PAULIN VICTORIA (US) | A47C31/00\ A47B13/08\ A47C31/11 |
| 2321 | 3D printing | MOTOR\ | US8525379B2 | KOKUBU HIROSHI (JP) | ASMO CO LTD (JP)\ KOKUBU HIROSHI (JP) | H02K11/00 |
| 2322 | 3D printing | METHOD OF FORMING A TURBINE ENGINE COMPONENT\ | US8533947B2 | MORELAND THOMAS R (US) | MORELAND THOMAS R (US)\ PCC AIRFOILS INC (US) | B21K25/00 |
| 2323 | 3D printing | DEVICE FOR CONNECTING PRODUCTS MUTUALLY AND AN ULTRASONIC WELDING DEVICE\ | US8534340B2 | STOLZE MARTIN (NL) | MARTIN STOLZE B V (NL)\ STOLZE MARTIN (NL) | B32B37/00 |
| 2324 | 3D printing | RETROFIT OF A FORM-FILL-SEAL MACHINE HEAT STATION WITH AN ADVANCED ULTRASONIC WELDING KIT\ | US8591679B1 | HULL GORDON (US) | HULL GORDON (US)\ RINCO ULTRASONICS USA INC US | B65B9/20 |
| 2325 | 3D printing | APPARATUS AND METHOD FOR SOLID FREEFORM FABRICATION\ | US8609204B2 | KRITCHMAN ELIAHU M (IL) | STRATASYS LTD (IL)\ KRITCHMAN ELIAHU M (IL) | B29C35/08\ B29C35/04\ H05B6/00\ B05D5/00\ B05D1/36\ C23C14/28 |
| 2326 | 3D printing | MANUFACTURING METHOD AND MANUFACTURING APPARATUS OF INSULATION CAP\ | US8632645B2 | SANO YUSUKE (JP)\ DAITOU KOUJI (JP)\ OISHI YASUSADA (JP) | YAZAKI CORP (JP)\ DAITOU KOUJI (JP)\ OISHI YASUSADA (JP)\ SANO YUSUKE (JP) | B32B37/00 |
| 2327 | 3D printing | ULTRASONIC WELDING MACHINE AND METHOD OF ALIGNING AN ULTRASONIC WELDING HORN RELATIVE TO AN ANVIL\ | US8640760B2 | KHAKHALEV ALEX (US)\ COLE LEE NELSON (US)\ HICKS ERIC (US) | LG CHEMICAL LTD (KR)\ KHAKHALEV ALEX (US)\ COLE LEE NELSON (US)\ HICKS ERIC (US) | B32B37/06 |
| 2328 | 3D printing | METHOD OF MANUFACTURING A FIBRE REINFORCED METAL MATRIX COMPOSITE ARTICLE\ | US8647453B2 | DOORBAR PHILIP J (GB) | ROLLS ROYCE PLC (GB)\ DOORBAR PHILIP J (GB) | B23K20/10\ B23K15/00\ B23K20/00\ B23K20/24 |
| 2329 | 3D printing | TORSION SONOTRODE ULTRASONIC WELDING DEVICE AND METHOD FOR PRODUCING A WELDED CONNECTION BY MEANS OF ULTRASONIC SOUND\ | US8657182B2 | BUETTIKER ALBERT (CH) | TELSONIC HOLDING AG (CH)\ BUETTIKER ALBERT (CH) | B23K1/06 |
| 2330 | 3D printing | SECONDARY CELL\ | US8673470B2 | AOTA KINYA (JP)\ FUJITA TOSHIRO (JP)\ KAJIWARA KOUICHI (JP)\ IWASA MASAAKI (JP) | HITACHI VEHICLE ENERGY LTD (JP)\ AOTA KINYA (JP)\ FUJITA TOSHIRO (JP)\ KAJIWARA KOUICHI (JP)\ IWASA MASAAKI (JP) | H01M6/10 |
| 2331 | 3D printing | WELDED DIAPHRAGM VALVE\ | US8689817B2 | LEYS JOHN A (US)\ SCHLEICHER MIKE (US) | ENTEGRIS INC (US)\ LEYS JOHN A (US)\ SCHLEICHER MIKE (US) | F16K51/00 |
| 2332 | 3D printing | PEDESTAL MOUNTED ULTRASONIC WELDING DEVICE\ | US8689850B2 | HULL GORDON (US) | HULL GORDON (US)\ RINCO ULTRASONICS INC US | B32B37/00 |
| 2333 | 3D printing | POLYMER BATTERY PACK AND MANUFACTURING METHOD OF THE SAME\ | US8691425B2 | YOON HEUISANG (KR) | SAMSUNG SDI CO LTD (KR)\ YOON HEUISANG (KR) | H01M2/02\ H01M2/10 |
| 2334 | 3D printing | PRESS FOR ULTRASONIC WELDING DEVICE\ | US8714081B2 | PATRIKIOS MIKE (US)\ RUSHTON ANDREW (US) | PATRIKIOS MIKE (US)\ RUSHTON ANDREW (US)\ SONICS & MATERIALS INC (US) | F16P3/00 |
| 2335 | 3D printing | INDUSTRIAL FABRIC INCLUDING SPIRALLY WOUND MATERIAL STRIPS WITH REINFORCEMENT\ | US8728280B2 | EAGLES DANA (US)\ KARLSSON JONAS (SE)\ HANSEN ROBERT (US) | ALBANY INT CORP (US)\ EAGLES DANA (US)\ KARLSSON JONAS (SE)\ HANSEN ROBERT (US) | B29C53/58\ D21F1/44\ B31F1/16 |
| 2336 | 3D printing | BATTERY PACK\ | US8741471B2 | PARK SANG-HUN (KR)\ MOON DEA-YON (KR) | SAMSUNG SDI CO LTD (KR)\ PARK SANG-HUN (KR)\ MOON DEA-YON (KR) | H01M2/26 |
| 2337 | 3D printing | CIRCULAR STAPLER AND STAPLE LINE REINFORCEMENT MATERIAL\ | US8789737B2 | HODGKINSON GERALD N (US)\ STEVENSON RICHARD (US)\ WHIFFEN JENNIFER (US) | HODGKINSON GERALD N (US)\ STEVENSON RICHARD (US)\ WHIFFEN JENNIFER (US)\ COVIDIEN LP (US) | A61B17/04\ A61B17/10 |
| 2338 | 3D printing | TORSION SONOTRODE AND ULTRASONIC WELDING PROCESS USING A TORSION SONOTRODE\ | US8801882B2 | LANG GEORG (DE)\ REGENBERG CLAUS (DE) | TELSONIC HOLDING AG (CH)\ LANG GEORG (DE)\ REGENBERG CLAUS (DE) | B32B37/00\ B29C65/00\ B29C65/08\ B06B3/00 |
| 2339 | 3D printing | DOSAGE FORMS AND METHODS OF USE THEREOF\ | US8828411B2 | WEST THOMAS G (US)\ YOO JAE D (US)\ SURPRENANT HENRY L (US)\ MONKHOUSE DONALD C (US) | YOO JAE D (US)\ SURPRENANT HENRY L (US)\ MONKHOUSE DONALD C (US)\ WEST THOMAS G (US)\ APRECIA PHARMACEUTICALS COMPANY (US) | A61K9/00\ A61K9/20 |
| 2340 | 3D printing | METHOD FOR PRODUCING A TOOTHBRUSH AND TOOTHBRUSH\ | US8840192B2 | TREVISAN OSKAR (CH)\ LOTSCHER JOST (CH) | TRISA HOLDING AG (CH)\ LOTSCHER JOST (CH)\ TREVISAN OSKAR (CH) | A46D3/04\ A46B3/04 |
| 2341 | 3D printing | SEMICONDUCTOR DEVICE AND METHOD FOR MANUFACTURING THE SAME\ | US8860220B2 | MOMOSE FUMIHIKO (JP)\ KIDO KAZUMASA (JP)\ NISHIMURA YOSHITAKA (JP)\ SHIGETA FUMIO (JP) | FUJI ELECTRIC CO LTD (JP) | H01L23/52\ H01L23/48 |
| 2342 | 3D printing | SOLID FREEFORM FABRICATION OF EASILY REMOVEABLE SUPPORT CONSTRUCTIONS\ | US8865047B2 | SELLA NADAV (IL) | STRATASYS LTD (IL)\ SELLA NADAV (IL) | B29C67/00\ B29C41/02\ B29C31/10 |
| 2343 | 3D printing | METHOD AND ARRANGEMENT FOR WELDING ELECTRICAL CONDUCTORS\ | US8870052B2 | STROH DIETER (DE)\ WAGNER PETER (DE)\ STROBEL HEIKO (DE)\ KOC SINAN (DE)\ RUHL SEBASTIAN (DE) | SCHUNK SONOSYSTEMS GMBH (DE)\ STROH DIETER (DE)\ WAGNER PETER (DE)\ RUHL SEBASTIAN (DE)\ STROBEL HEIKO (DE)\ KOC SINAN (DE) | B23K20/10\ H01R43/02\ B23K20/26\ B23K1/06 |
| 2344 | 3D printing | IN-LINE CLEANING METHOD FOR ULTRASONIC WELDING TOOLS\ | US8876983B2 | WIDHALM HUMI (US) | FORD GLOBAL TECH LLC (US)\ WIDHALM HUMI (US) | B08B3/08\ B23K20/10\ B08B3/00\ B29C65/08\ B23K20/26\ B23K20/24\ B08B1/00 |
| 2345 | 3D printing | ULTRASONIC VIBRATION UNIT\ | US8887784B2 | THAERIGEN JAN (CH) | BOSCH GMBH ROBERT (DE)\ THAERIGEN JAN (CH) | B32B37/00\ B29C65/00\ B29C65/08\ B06B3/00 |
| 2346 | 3D printing | SYNTHETIC RESIN SPOUT STOPPER\ | US8899442B2 | MURAMOTO KATSUHIRO (JP)\ NAGASE MASAKI (JP)\ ANBE NAOYUKI (JP)\ HASHIMOTO KATSUMI (JP) | CROWN CORK JAPAN (JP)\ MURAMOTO KATSUHIRO (JP)\ NAGASE MASAKI (JP)\ ANBE NAOYUKI (JP)\ HASHIMOTO KATSUMI (JP) | B65D5/74\ B65D47/36\ B65D30/00 |
| 2347 | 3D printing | INCONTINENCE ARTICLE IN PANTS FORM\ | US8905990B2 | MALOWANIEC KRZYSZTOF D (DE) | HARTMANN PAUL AG (DE)\ MALOWANIEC KRZYSZTOF D (DE) | A61F13/49\ A61F13/496 |
| 2348 | 3D printing | METHOD OF FABRICATING SUBSTRATE WHERE PATTERNS ARE FORMED\ | US8936828B2 | YOON EUIJOON (KR)\ KWON SUNG-HOON (KR) | SNU R&DB FOUNDATION (KR)\ YOON EUIJOON (KR)\ KWON SUNG-HOON (KR) | C30B33/00\ B05D1/12\ H01L33/22\ B05D1/24 |
| 2349 | 3D printing | METHOD FOR ATTACHING RFID TAG OF MEMORY CASSETTE FOR TISSUE SPECIMEN AND MEMORY CASSETTE FOR TISSUE SPECIMEN HAVING RFID TAG\ | US8945483B2 | PARK BYUNG GYU (KR)\ KIM YONG PILL (KR)\ CHOI KWANG IL (KR) | PARK BYUNG GYU (KR)\ KIM YONG PILL (KR)\ CHOI KWANG IL (KR)\ TIME SYSTEM CO LTD (KR) | A61B19/00\ A61B10/00 |
| 2350 | 3D printing | METHOD OF MAKING A WEB FROM WHICH ELASTIC DIAPER CLOSURES CAN BE STAMPED\ | US8956477B2 | SCHOENBECK MARCUS (DE) | MONDI CONSUMER PACKAGING TECHNOLOGIES GMBH (DE)\ SCHOENBECK MARCUS (DE) | B32B5/02\ B32B37/00\ B29C65/00\ B29C65/48\ B29C65/02\ A61F13/15\ B29C65/08\ B29L31/48\ B29C65/50\ B32B5/04\ A61F13/62 |
| 2351 | 3D printing | PLUG ASSEMBLY\ | USD501447S1 | GREENWOOD MARK (US)\ HEISLER ALLAN (US) | PYROMATION INC (US) | H01R13/504\ H01R13/66 |
| 2352 | 3D printing | END STOP FOR RECLOSABLE FASTENER AND METHOD OF INSTALLATION THEREON\[FR] BUTEE D'EXTREMITE POUR FERMETURE REFERMABLE ET SON PROCEDE D'INSTALLATION\ | WO0208066A3 | SAVICKI ALAN F (US) | GLAD PRODUCTS CO (US) | A44B19/26\ A44B19/38\ B65D33/25 |
| 2353 | 3D printing | MOUNTING STRUCTURE AND MOUNTING METHOD FOR SEMICONDUCTOR CHIP\[FR] STRUCTURE DE MONTAGE ET PROCEDE DE MONTAGE D'UNE PUCE DE SEMI-CONDUCTEUR\ | WO02093638A1 | KIRA HIDEHIKO (JP)\ KAINUMA NORIO (JP)\ KOBAE KENJI (JP)\ KOBAYASHI HIROSHI (JP) | FUJITSU LTD (JP)\ KIRA HIDEHIKO (JP)\ KOBAE KENJI (JP)\ KAINUMA NORIO (JP)\ KOBAYASHI HIROSHI (JP) | H01L21/60 |
| 2354 | 3D printing | METHOD FOR CONTACTING THIN-FILM ELECTRODES\[FR] PROCEDE POUR LA MISE EN CONTACT D'ELECTRODES A FILM MINCE\ | WO03012883A2 | KARG FRANZ (DE)\ VOGT HELMUT (DE)\ UFERT KLAUS DIETER (DE) | SHELL SOLAR GMBH (DE)\ KARG FRANZ (DE)\ UFERT KLAUS DIETER (DE)\ VOGT HELMUT (DE) | B23K20/10\ H01R43/02\ H01L31/05 |
| 2355 | 3D printing | ULTRASONIC-SEALED LAMINATE AND METHOD OF PRODUCING THE SAME AND ULTRASONIC WELDING MACHINE USING THE SAME\[FR] STRATIFIE SCELLE AUX ULTRASONS PROCEDE DE PRODUCTION CORRESPONDANT ET MACHINE DE SOUDAGE AUX ULTRASONS CORRESPONDANTE\ | WO03076272A1 | ISHIWATARI KIYOKAZU (JP)\ SAITO KATSUNORI (JP) | SHOWA DENKO PLASTIC PROD CO (JP)\ SAITO KATSUNORI (JP)\ ISHIWATARI KIYOKAZU (JP) | B29C65/00\ B29L22/00\ B29C65/08\ B29C65/74\ B65B51/10\ B65B51/22\ B65B61/04 |
| 2356 | 3D printing | VERFAHREN ZUR HERSTELLUNG EINES ROHRFILTERS DURCH WICKELN SOWIE ROHRFILTER\[EN] METHOD FOR PRODUCING A TUBULAR FILTER BY WINDING AND TUBULAR FILTER\[FR] PROCEDE DE FABRICATION D'UN FILTRE TUBULAIRE PAR ENROULEMENT ET FILTRE TUBULAIRE\ | WO03103798A1 | PFEIL BERTHOLD (DE) | INTENSIV FILTER GMBH (DE)\ PFEIL BERTHOLD (DE) | B01D39/16\ B01D46/00\ B01D46/24\ B01D63/06\ B01D29/11 |
| 2357 | 3D printing | ALUMINUM/NICKEL CLAD MATERIAL AND METHOD FOR MANUFACTURE THEREOF AND EXTERIOR TERMINAL FOR ELECTRIC CELL\[FR] MATIERE A REVETEMENT EN ALUMINIUM/NICKEL ET SON PROCEDE DE FABRICATION ET BORNE EXTERNE POUR PILE ELECTRIQUE\ | WO2004020191A1 | SHIOMI KAZUHIRO (JP)\ ISHIO MASAAKI (JP)\ FUNAMOTO KENICHI (JP) | SUMITOMO SPECIAL METALS C0 LTD (JP)\ SHIOMI KAZUHIRO (JP)\ ISHIO MASAAKI (JP)\ FUNAMOTO KENICHI (JP) | B23K20/02\ B23K35/00\ H01M2/30 |
| 2358 | 3D printing | ULTRASONIC WELDING PRODUCT\[FR] PRODUIT DE SOUDAGE PAR ULTRASONS\ | WO2005009722A1 | KIKUCHI TATSUYA (JP) | ITOCHU CORP (JP)\ KIKUCHI TATSUYA (JP) | B29C65/00\ B29C65/02\ B29C65/08\ B29C65/50\ A41D27/24 |
| 2359 | 3D printing | METHODS AND SYSTEMS FOR CHROMATOGRAPHY/MASS-SPECTROMETRY ANALYSIS\[FR] PROCEDES ET SYSTEMES POUR UNE ANALYSE DE CHROMATOGRAPHIE /SPECTROMETRIE DE MASSE\ | WO2005015199A1 | BJORKESTEN LENNART (SE)\ KAPLAN ANDERS (SE) | AMERSHAM BIOSCIENCES AB (SE)\ KAPLAN ANDERS (SE)\ BJORKESTEN LENNART (SE) | G01N30/72\ G01N30/86 |
| 2360 | 3D printing | A METHOD OF MAKING HONEYCOMB STRUCTURE\[FR] PROCEDE POUR FABRIQUER UNE STRUCTURE A NIDS D'ABEILLE\ | WO2005028194A1 | PARK KIHO (KR) | PARK KIHO (KR) | B29D99/00 |
| 2361 | 3D printing | NURSING PAD\[FR] COUSSINET D'ALLAITEMENT\ | WO2005034824A1 | LEWIS REBECCA (GB) | DAILYS LTD (GB)\ LEWIS REBECCA (GB) | A61F13/15\ A61F13/14 |
| 2362 | 3D printing | METHOD OF RETREADING TIRES\[FR] PROCEDE DE RECHAPAGE DE PNEUS\ | WO2005039865A1 | WIMMER RALPH EDWARD (US) | MICHELIN RECH TECH (CH)\ MICHELIN SOC TECH (FR)\ WIMMER RALPH EDWARD (US) | B29C65/08\ B29D30/56 |
| 2363 | 3D printing | ASSEMBLED BATTERY AND MANUFACTURING METHOD THEREOF\[FR] BATTERIE ASSEMBLéE ET MéTHODE DE FABRICATION\ | WO2005117163A1 | KISHIMOTO HIROAKI (JP)\ TANJOU YUUJI (JP)\ WATANABE MASASHI (JP)\ SEGAWA TERUO (JP)\ AMAGAI RYUICHI (JP)\ OTA MASAYASU (JP)\ ENOKIDA TSUYOSHI (JP)\ HAYAMI MUNEO (JP) | NISSAN MOTOR (JP)\ WATANABE MASASHI (JP)\ TANJOU YUUJI (JP)\ OTA MASAYASU (JP)\ ENOKIDA TSUYOSHI (JP)\ KISHIMOTO HIROAKI (JP)\ AMAGAI RYUICHI (JP)\ SEGAWA TERUO (JP)\ HAYAMI MUNEO (JP) | H01M2/02\ H01M2/30\ H01M2/10\ H01M2/26\ H01M6/46 |
| 2364 | 3D printing | REFLECTIVE SHEET FOR ROAD MARKER\[FR] FEUILLE REFLECHISSANTE DESTINEE A UN PANNEAU D'IDENTIFICATION DES ROUTES\ | WO2006010293A1 | LEE HUNGCHEN (CN) | LEE HUNGCHEN (CN) | Empty Field |
| 2365 | 3D printing | KONTAKTIERUNG THERMOELEKTRISCHER MATERIALIEN DURCH ULTRASCHALLSCHWEI?EN\[EN] CONTACTING THERMOELECTRIC MATERIALS BY MEANS OF ULTRASONIC WELDING\[FR] MISE EN CONTACT DE MATERIAUX THERMOELECTRIQUES PAR SOUDAGE AUX ULTRASONS\ | WO2006037498A2 | STERZEL HANS-JOSEF (DE)\ KUEHLING KLAUS (DE) | BASF AG (DE)\ STERZEL HANS-JOSEF (DE)\ KUEHLING KLAUS (DE) | B23K20/10\ H01L35/08 |
| 2366 | 3D printing | A PROCESS FOR MAKING WATERPROOF CLOTHING FROM CHEMICAL FIBRE TEXTILE\[FR] PROCEDE POUR FABRIQUER UN VETEMENT IMPERMEABLE A L'EAU A PARTIR DE TEXTILES EN FIBRES CHIMIQUES\ | WO2006045218A1 | CHEN NGSANG (CN) | CHEN NGSANG (CN) | Empty Field |
| 2367 | 3D printing | AN AUTOMATIC WELDING MACHINE\[FR] MACHINE DE SOUDAGE AUTOMATIQUE\ | WO2006128334A1 | IAU YING FAI (CN) | IAU YING FAI (CN) | B32B37/00\ B29C65/08\ A43D43/00 |
| 2368 | 3D printing | PRESSURE SENSOR AND PRODUCTION METHOD THEREOF\[FR] CAPTEUR DE PRESSION ET PROCéDé DE PRODUCTION CORRESPONDANT\ | WO2007004400A1 | SUZUKI KAZUSHIGE (JP)\ WATANABE HAJIME (JP) | SAGINOMIYA SEISAKUSHO INC (JP)\ SUZUKI KAZUSHIGE (JP)\ WATANABE HAJIME (JP) | G01L19/14 |
| 2369 | 3D printing | COSMETICS CONTAINER WITH A PART FOR ACCOMMODATING OIL PAPERS\[FR] CONTENANT POUR PRODUIT COSMéTIQUE POURVU D'UN éLéMENT CON?U POUR RECEVOIR DES PAPIERS HUILéS\ | WO2007094566A1 | CHUNG IN-YONG (US) | CTK CO LTD (KR)\ CHUNG IN-YONG (US) | A45D33/26 |
| 2370 | 3D printing | MANUFACTURE AND APPARATUS FOR SAFETY GARMENTS USING ULTRASOUND FUSION\[FR] FABRICATION ET APPAREIL POUR VêTEMENTS DE SéCURITé AU MOYEN DE LA FUSION ULTRASONORE\ | WO2007126287A1 | AN JAE-SANG (KR)\ LEE DAE-HOON (KR)\ CHUNG GI-SOO (KR) | KOREA IND TECH INST (KR)\ AN JAE-SANG (KR)\ LEE DAE-HOON (KR)\ CHUNG GI-SOO (KR) | A41H43/04 |
| 2371 | 3D printing | TWO-PIECE CONNECTOR FOR FLAT BLADE WINDSHIELD WIPER\[FR] CONNECTEUR EN DEUX PARTIES POUR UN ESSUIE-GLACE à BALAI PLAT\ | WO2008036894A3 | HERRING RICHARD A (US)\ KOPPEN SCOTT ALLEN (US)\ LYTLE JAMESE LYNN (US) | FEDERAL MOGUL CORP (US)\ KOPPEN SCOTT ALLEN (US)\ LYTLE JAMESE LYNN (US) | B60S1/38\ B60S1/40 |
| 2372 | 3D printing | METHOD FOR PRODUCING A PLURALITY OF INTERIOR MEMBERS AND SERIES OF INTERIOR MEMBERS FOR AN AUTOMOBILE\[FR] PROCEDE DE FABRICATION DE PLUSIEURS ELEMENTS INTERIEURS ET SERIE D'ELEMENTS INTERIEURS D'AUTOMOBILE\ | WO2008053094A1 | GORGES ANTOINE (FR) | FAURECIA INTERIEUR IND (FR)\ GORGES ANTOINE (FR) | B60R13/02\ B62D65/06 |
| 2373 | 3D printing | LIGHT ASSEMBLY\[FR] ENSEMBLE D'éCLAIRAGE\ | WO2008129321A1 | GRAY RICHARD (GB) | BRITAX P M G LTD (GB)\ GRAY RICHARD (GB) | B60Q3/02\ H03K17/955\ H03K17/96 |
| 2374 | 3D printing | IMPROVEMENTS IN COALESCING FILTERS\[FR] FILTRES COALESCEURS AMéLIORéS\ | WO2008146045A1 | SPENCE DEBORAH MICHELE (GB) | PSI GLOBAL LTD (GB)\ SPENCE DEBORAH MICHELE (GB) | B01D46/00\ B01D46/24 |
| 2375 | 3D printing | ULTRASCHALL-SCHWEISSSTATION\[EN] ULTRASONIC WELDING STATION\[FR] STATION DE SOUDAGE à ULTRASONS\ | WO2008155013A1 | STECKMANN HELGE (DE) | ULTRASONICS STECKMANN GMBH (DE)\ STECKMANN HELGE (DE) | B23K20/10\ B23K20/24 |
| 2376 | 3D printing | A METHOD FOR LABELING A CONTAINER\[FR] PROCéDé D'éTIQUETAGE D'UN CONTENANT\ | WO2008155443A1 | MITCHELL NOEL (DE) | UPM RAFLATAC OY (FI)\ MITCHELL NOEL (DE) | B65C3/08 |
| 2377 | 3D printing | DEVICE FOR ULTRASONIC WELDING IN PARTICULAR OF LAYERED ELEMENTS\[FR] DISPOSITIF POUR SOUDAGE ULTRASONIQUE EN PARTICULIER D'éLéMENTS EN COUCHE\ | WO2009019723A9 | MAZZINI SAMUELE (IT) | S M R E ENGINEERING DI MAZZINI (IT)\ MAZZINI SAMUELE (IT) | B29C65/08 |
| 2378 | 3D printing | DEVICE FOR MULTIPLE ULTRASONIC WELDING IN PARTICULAR OF LAYERED ELEMENTS\[FR] DISPOSITIF POUR LE SOUDAGE MULTIPLE PAR ULTRASONS NOTAMMENT D'éLéMENTS MULTICOUCHES\ | WO2009019724A9 | MAZZINI SAMUELE (IT) | S M R E ENGINEERING DI MAZZINI (IT)\ MAZZINI SAMUELE (IT) | B29C65/08 |
| 2379 | 3D printing | METHOD FOR PRODUCING A DECORATIVE ARTICLE\[FR] PROCéDé DE FABRICATION D'UN ARTICLE DéCORATIF\ | WO2009120113A1 | MAKAREVICH IGOR OLEGOVICH (RU) | MAKAREVICH IGOR OLEGOVICH (RU) | B44C5/00 |
| 2380 | 3D printing | IMPROVEMENTS IN OR RELATING TO JOINING COPPER AND NICKEL CONTAINING METALS\[FR] AMéLIORATIONS APPORTéES à LA LIAISON DE MéTAUX CONTENANT DU CUIVRE ET DU NICKEL\ | WO2009121614A1 | SHELDON SIMON (GB) | AMBERJAC PROJECTS LTD (GB)\ SHELDON SIMON (GB) | B23K20/10\ H01M2/10\ H01M2/20\ B23K103/18\ B23K101/36\ H01M2/24 |
| 2381 | 3D printing | VERFAHREN ZUR HERSTELLUNG UND AUFBAU EINES LEISTUNGSMODULS\[EN] METHOD FOR PRODUCING AND ASSEMBLY OF A POWER MODULE\[FR] PROCéDé DE FABRICATION ET STRUCTURE D'UN MODULE DE PUISSANCE\ | WO2009127179A1 | GRAF WERNER (DE)\ FELBER BERND (DE)\ HORNSTEIN CHRISTOPH (DE)\ GREUL ROLAND (DE) | CONTI TEMIC MICROELECTONIC GMB (DE)\ GRAF WERNER (DE)\ FELBER BERND (DE)\ HORNSTEIN CHRISTOPH (DE)\ GREUL ROLAND (DE) | H01L25/07 |
| 2382 | 3D printing | DISPOSABLE PATIENT TRANSFER ASSEMBLY\[FR] ENSEMBLE TRANSFERT DE PATIENT JETABLE\ | WO2010020818A2 | NASIR MUHAMMED ASLAM (GB) | NASIR MUHAMMED ASLAM (GB) | A61G7/10\ A61G1/01 |
| 2383 | 3D printing | VEHICLE TYRES WITH INTEGRAL STUDS\[FR] PNEUS POUR VéHICULES à ERGOTS INTéGRéS\ | WO2010029319A1 | ROLFE NICHOLAS (GB) | ROLFE NICHOLAS (GB)\ GOLF INNOVATIONS LTD (GB) | B60B15/24\ B60C11/16 |
| 2384 | 3D printing | EXTRACTION BAG FILLING/PACKING MACHINE\[FR] MACHINE DE REMPLISSAGE/EMBALLAGE DE SAC D'EXTRACTION\ | WO2010055591A1 | TSUJI YOSHIYUKI (JP)\ TAKEMOTO TOMOHIRO (JP) | TSUBAKIMOTO KOGYO CO LTD (JP)\ TOYAMA FAB CORP (JP)\ TSUJI YOSHIYUKI (JP)\ TAKEMOTO TOMOHIRO (JP) | B65B51/10\ B65B9/10\ B65B29/02 |
| 2385 | 3D printing | DIAPHRAGM FOR MICROSPEAKER AND MANUFACTURING METHOD THEREOF\[FR] DIAPHRAGME POUR HAUT-PARLEURS MINIATURES ET SON PROCéDé DE FABRICATION\ | WO2010104250A1 | LEE HAN-RYANG (KR)\ KIM CHANG-WON (KR)\ DOH SUNG-HWAN (KR)\ LEE SANG-HO (KR)\ KWON CHEOL-YOUNG (KR) | BSE CO LTD (KR)\ LEE HAN-RYANG (KR)\ KIM CHANG-WON (KR)\ DOH SUNG-HWAN (KR)\ LEE SANG-HO (KR)\ KWON CHEOL-YOUNG (KR) | H04R7/02 |
| 2386 | 3D printing | ULTRASONIC MULTI-WELDING SYSTEM AND METHOD FOR GEOCELL SHEETS\[FR] SYSTèME ET PROCéDé DE SOUDAGE MULTIFONCTIONNEL PAR ULTRASONS DE FEUILLES DE GéOTEXTILE CELLULAIRE\ | WO2010137886A2 | BYEON YOUNG SIK (KR) | BYEON YOUNG SIK (KR) | B29C65/08 |
| 2387 | 3D printing | ULTRASCHALL-SCHWEISSWERKZEUG\[EN] ULTRASONIC WELDING TOOL\[FR] OUTIL DE SOUDAGE PAR ULTRASONS\ | WO2010139464A1 | NAGEL INGO (DE) | SONOTRONIC NAGEL GMBH (DE)\ NAGEL INGO (DE) | B29C65/08\ B65B51/22 |
| 2388 | 3D printing | VORRICHTUNG UND VERFAHREN ZUR HERSTELLUNG EINER EINZELZELLE FüR EINE BATTERIE\[EN] DEVICE AND METHOD FOR PRODUCING A SINGLE CELL FOR A BATTERY\[FR] PROCéDé ET DISPOSITIF DE FABRICATION D'UNE CELLULE UNITAIRE POUR UNE BATTERIE\ | WO2011012207A1 | SCHROETER DIRK (DE)\ WARTHMANN WOLFGANG (DE)\ KAUFMANN RAINER (DE)\ MEINTSCHEL JENS (DE)\ REINHARDT RUDOLF (DE)\ HOHENTHANNER CLAUS-RUPERT (DE)\ BRASSE CLAUDIA (DE)\ EICHINGER GUENTER (DE)\ KAISER JOERG (DE)\ TRANTOW SOEREN (DE)\ GNANN MICHAEL (DE) | DAIMLER AG (DE)\ SCHROETER DIRK (DE)\ WARTHMANN WOLFGANG (DE)\ KAUFMANN RAINER (DE)\ MEINTSCHEL JENS (DE)\ REINHARDT RUDOLF (DE)\ HOHENTHANNER CLAUS-RUPERT (DE)\ BRASSE CLAUDIA (DE)\ EICHINGER GUENTER (DE)\ KAISER JOERG (DE)\ TRANTOW SOEREN (DE)\ GNANN MICHAEL (DE) | H01M2/02\ B29C65/08\ H01M10/04\ H01M2/26 |
| 2389 | 3D printing | DüBEL UND VERFAHREN ZUR MONTAGE EINES DüBELS IN EINE LEICHTBAUPLATTE\[EN] ANCHOR AND METHOD FOR MOUNTING AN ANCHOR IN A LIGHTWEIGHT BUILDING BOARD\[FR] CHEVILLE ET PROCéDé DE MONTAGE D'UNE CHEVILLE DANS UN PANNEAU SANDWICH\ | WO2011042326A1 | SCHAEL OLIVER (DE)\ SOBOLEWSKI UWE (DE) | HETTICH HEINZE GMBH & CO KG (DE)\ SOBOLEWSKI UWE (DE)\ SCHAEL OLIVER (DE) | F16B5/01 |
| 2390 | 3D printing | CIRCUIT DEVICE AND PRODUCTION METHOD THEREFOR\[FR] DISPOSITIF DE CIRCUIT ET PROCéDé DE FABRICATION DE CELUI-CI\ | WO2011046036A1 | KADOWAKI TAKAHIRO US\ KANAMORI AKITAKA US\ ISHIBASHI HIROKI US | KOITO MFG CO LTD (JP)\ KADOWAKI TAKAHIRO US\ KANAMORI AKITAKA US\ ISHIBASHI HIROKI US | H05K3/12\ H05K3/32\ H01L23/50 |
| 2391 | 3D printing | VERFAHREN ZUM VERSCHLIE?EN EINES SACKK?RPERS\[EN] METHOD FOR CLOSING A BAG BODY\[FR] PROCéDé DE FERMETURE D'UN CORPS DE SAC\ | WO2011050380A1 | WORFF HERWIG (AT)\ SCHOBESBERGER ERNESTO (AT) | WORFF HERWIG (AT)\ SCHOBESBERGER ERNESTO (AT) | B29C65/08\ B65B51/22\ B65B51/14\ B65D75/20\ B31B23/00\ B65B7/08 |
| 2392 | 3D printing | METHOD FOR THE ULTRASONIC WELDING OF TWO PLASTIC COMPONENTS\[DE] VERFAHREN ZUM ULTRASCHALLSCHWEISSEN ZWEIER KUNSTSTOFF-BAUTEILE\[FR] PROCéDé DE SOUDAGE AUX ULTRASONS DE DEUX COMPOSANTS EN MATIèRE PLASTIQUE\ | WO2011060971A1 | FISCHER WOLFGANG (DE)\ HUG DANIEL (DE)\ FAERBER CHRISTINE (DE)\ SCHOENFELD STEFFEN (DE) | FISCHER WOLFGANG (DE)\ BOSCH GMBH ROBERT (DE)\ HUG DANIEL (DE)\ FAERBER CHRISTINE (DE)\ SCHOENFELD STEFFEN (DE) | B29C65/08\ B29C65/06\ F16K37/00 |
| 2393 | 3D printing | A SAFE AND IMPROVED OSTOMY DEVICE ADAPTABLE TO PATIENTS HAVING COLOSTOMY OR ILEOSTOMY\[FR] DISPOSITIF DE STOMIE S?R ET AMéLIORé POUVANT S'ADAPTER à DES PATIENTS AYANT SUBI UNE COLOSTOMIE OU UNE ILéOSTOMIE\ | WO2011067779A1 | HITENDRA DINESHBHAI PUROHIT (IN)\ SARVESH PRADIPKUMAR CHINAGI (IN)\ CHARUDATTA CHANDRAKANT ARADHYE (IN) | JOHNSON & JOHNSON LTD (IN)\ HITENDRA DINESHBHAI PUROHIT (IN)\ SARVESH PRADIPKUMAR CHINAGI (IN)\ CHARUDATTA CHANDRAKANT ARADHYE (IN) | A61F5/44\ A61F5/445\ A61F5/441 |
| 2394 | 3D printing | WINDABLE PHOTOVOLTAIC CURTAIN\[FR] RIDEAU PHOTOVOLTA?QUE POUVANT êTRE ENROULé\ | WO2011095304A1 | ROMANI MASSIMO (IT) | KF SRL (IT)\ ROMANI MASSIMO (IT) | H01L31/048\ E04F10/06\ A47H23/10\ H01L31/045 |
| 2395 | 3D printing | ELECTRIC WIRE WITH TERMINAL FITTING AND PRODUCTION METHOD FOR SAME\[FR] FIL éLECTRIQUE AVEC RACCORD MéTALLIQUE D'EXTRéMITé FEMELLE ET PROCéDé DE FABRICATION ASSOCIé\ | WO2011118416A1 | MORIKAWA SATOSHI (JP)\ KOBAYASHI KAZUMASA (JP)\ OTSUKA TAKUJI (JP)\ SHIMODA HIROKI (JP) | SUMITOMO ELECTRIC INDUSTRIES (JP)\ SUMITOMO WIRING SYSTEMS (JP)\ AUTONETWORKS TECHNOLOGIES LTD (JP)\ MORIKAWA SATOSHI (JP)\ KOBAYASHI KAZUMASA (JP)\ OTSUKA TAKUJI (JP)\ SHIMODA HIROKI (JP) | H01R4/62\ H01R4/18\ H01R43/02\ H01R43/00 |
| 2396 | 3D printing | OMEGA-ULTRASCHALLSCHWEISSMASCHINE\[EN] OMEGA ULTRASONIC WELDING MACHINE\[FR] MACHINE DE SOUDAGE PAR ULTRASONS DE TYPE OMEGA\ | WO2011128118A1 | STECKMANN HELGE (DE)\ KARPEL IRINA (DE) | ULTRASONICS STECKMANN GMBH (DE)\ STECKMANN HELGE (DE)\ KARPEL IRINA (DE) | B23K20/10 |
| 2397 | 3D printing | BIMETALLIC TARGET\[FR] CIBLE BIMéTALLIQUE\ | WO2011162813A1 | IVANOV EUGENE Y (US)\ DEL-RIO EDUARDO (US) | TOSOH SMD INC (US)\ IVANOV EUGENE Y (US)\ DEL-RIO EDUARDO (US) | C23C14/34\ H01J37/34 |
| 2398 | 3D printing | CHIP BONDER\[FR] SYSTèME DE REPORT DE PUCE\ | WO2012002211A1 | MOMOSE KAZUHISA (JP)\ NAKAHARA ISAO (JP) | AKIM CORP (JP)\ MOMOSE KAZUHISA (JP)\ NAKAHARA ISAO (JP) | H01L21/60\ H01L21/607\ H05K13/04 |
| 2399 | 3D printing | MOBILE PHONE DEVICE\[FR] DISPOSITIF DE TéLéPHONE MOBILE\ | WO2012065486A1 | TANG MINGBING (CN) | ZTE CORP (CN)\ TANG MINGBING (CN) | H04R1/02\ H04M1/02 |
| 2400 | 3D printing | VERFAHREN UND VORRICHTUNG ZUR HERSTELLUNG EINES FILTERELEMENTES UND FILTERELEMENT\[EN] METHOD AND DEVICE FOR PRODUCING A FILTER ELEMENT AND FILTER ELEMENT\[FR] PROCéDé ET DISPOSITIF DE FABRICATION D'UN éLéMENT FILTRANT ET éLéMENT FILTRANT\ | WO2012100960A1 | BRENNEIS DANIEL (DE)\ VEIT JOCHEN (DE) | BOSCH GMBH ROBERT (DE)\ BRENNEIS DANIEL (DE)\ VEIT JOCHEN (DE) | B29C65/08\ B01D29/11\ B29L31/14 |
| 2401 | 3D printing | LITHIUM ION CELL\[FR] PILE AU LITHIUM-ION\ | WO2012114586A1 | KAGA YUSUKE (JP) | HITACHI LTD (JP)\ KAGA YUSUKE (JP) | H01M2/26\ H01M10/0587\ H01M10/0525 |
| 2402 | 3D printing | VERFAHREN ZUM ANBRINGEN EINER ?FFNUNGS- UND SCHLIESSVORRICHTUNG AUF EINEN SCHLAUCHBEUTEL MITTELS ULTRASCHALLSCHWEISSUNG\[EN] METHOD FOR ATTACHING AN OPENING AND CLOSING DEVICE TO A TUBULAR BAG BY MEANS OF ULTRASONIC WELDING\[FR] PROCéDé POUR MONTER UN DISPOSITIF D'OUVERTURE ET DE FERMETURE SUR UN SACHET TUBULAIRE AU MOYEN D'UN SOUDAGE PAR ULTRASONS\ | WO2012126732A1 | BARRON DAN (CH) | BOSCH GMBH ROBERT (DE)\ BARRON DAN (CH) | B29C65/08\ B65D75/58 |
| 2403 | 3D printing | NANO MICROPOROUS DIAPHRAGM OF POST-CROSSLINKED RUBBER AND POLYOLEFIN COMPOSITE AND MANUFACTURING METHOD THEREOF\[FR] DIAPHRAGME MICROPOREUX DE DIMENSION NANOMéTRIQUE CONSTITUé DE CAOUTCHOUC POST-RéTICULé ET DE COMPOSITE DE POLYOLéFINE ET SON PROCéDé DE FABRICATION\ | WO2012142802A1 | LI XIN (CN)\ LI JIANHUA (CN)\ CHEN WEI (CN)\ JIAO YONGJUN (CN)\ LI LONG (CN)\ DENG XINJIAN (CN) | TIANJIN DG MEMBRANE CO LTD (CN)\ LI XIN (CN)\ LI JIANHUA (CN)\ CHEN WEI (CN)\ JIAO YONGJUN (CN)\ LI LONG (CN)\ DENG XINJIAN (CN) | H01M2/16\ B32B27/18\ B32B27/32\ C08L23/16\ C08L23/06\ C08L51/06\ B32B3/24\ C08J9/28 |
| 2404 | 3D printing | SHAFT-TYPE CROSS-FLOW FAN AND METHOD FOR MANUFACTURING SAME\[FR] VENTILATEUR TANGENTIEL DE TYPE à ARBRE ET SON PROCéDé DE FABRICATION\ | WO2012147182A1 | OHNO HIROYUKI (JP) | FANTEC CO LTD (JP)\ OHNO HIROYUKI (JP) | F04D17/04\ F04D29/60 |
| 2405 | 3D printing | A RAPID VACUUM POWDER MATERIAL FEEDER THAT FEEDS FROM THE BOTTOM\[FR] DISPOSITIF D'ALIMENTATION DE MATéRIAU EN POUDRE à VIDE RAPIDE QUI EFFECTUE UNE ALIMENTATION à PARTIR DU BAS\ | WO2012155530A1 | Empty Field US | BEIJING SAIDELI TECHNOLOGY INC COMPANY LTD (CN) | B65G53/24\ B65G53/46\ B65G53/50 |
| 2406 | 3D printing | DRUCKSPEICHEREINRICHTUNG DRUCKSENSOREINRICHTUNG FüR EINE DRUCKSPEICHEREINRICHTUNG UND VERFAHREN ZUR HERSTELLUNG EINER DRUCKSPEICHEREINRICHTUNG\[EN] PRESSURE RESERVOIR PRESSURE SENSOR FOR A PRESSURE RESERVOIR AND METHOD FOR PRODUCING A PRESSURE RESERVOIR\[FR] DISPOSITIF D'ACCUMULATION DE PRESSION DISPOSITIF DE DéTECTION DE PRESSION POUR UN DISPOSITIF D'ACCUMULATION DE PRESSION ET PROCéDé DE FABRICATION D'UN DISPOSITIF D'ACCUMULATION DE PRESSION\ | WO2012163454A1 | STICHLMEIR MAXIMILIAN (DE)\ TROESTER SVEN (DE)\ BECKER JUERGEN (DE) | AUDI AG (DE)\ STICHLMEIR MAXIMILIAN (DE)\ TROESTER SVEN (DE)\ BECKER JUERGEN (DE) | B29C65/06\ F02M55/02\ G01L19/00\ G01L19/14\ F02M69/46 |
| 2407 | 3D printing | VERFAHREN ZUR HERSTELLUNG EINER ELEKTROCHEMISCHEN ZELLE EINE ELEKTROCHEMISCHE ZELLE UND EINE ENERGIESPEICHERVORRICHTUNG MIT WENIGSTENS ZWEI ELEKTROCHEMISCHEN ZELLEN\[EN] METHOD FOR PRODUCING AN ELECTROCHEMICAL CELL ELECTROCHEMICAL CELL AND ENERGY STORAGE DEVICE HAVING AT LEAST TWO ELECTROCHEMICAL CELLS\[FR] PROCéDé DE PRODUCTION D'UNE CELLULE éLECTROCHIMIQUE CELLULE éLECTROCHIMIQUE ET DISPOSITIF ACCUMULATEUR D'éNERGIE DOTé D'AU MOINS DEUX CELLULES éLECTROCHIMIQUES\ | WO2013023760A2 | MEINTSCHEL JENS (DE)\ HOHENTHANNER CLAUS-RUPERT (DE)\ HOERNIG ALEXANDER (DE)\ REICHE HARALD (DE) | MEINTSCHEL JENS (DE)\ HOHENTHANNER CLAUS-RUPERT (DE)\ LI TEC BATTERY GMBH (DE)\ HOERNIG ALEXANDER (DE)\ REICHE HARALD (DE) | H01M2/30\ H01M10/04\ H01M2/26 |
| 2408 | 3D printing | ENERGIESPEICHERVORRICHTUNG KONTAKTELEMENT FüR EINE SOLCHE ENERGIESPEICHERVORRICHTUNG UND VERFAHREN ZUR HERSTELLUNG EINER SOLCHEN ENERGIESPEICHERVORRICHTUNG\[EN] ENERGY ACCUMULATOR DEVICE CONTACT ELEMENT FOR SAID ENERGY ACCUMULATOR DEVICE AND METHOD FOR PRODUCING SAID ENERGY ACCUMULATOR DEVICE\[FR] DISPOSITIF ACCUMULATEUR D'éNERGIE éLéMENT DE CONTACT POUR UN TEL DISPOSITIF ACCUMULATEUR D'éNERGIE ET PROCéDé DE PRODUCTION D'UN TEL DISPOSITIF ACCUMULATEUR D'éNERGIE\ | WO2013023768A1 | MEINTSCHEL JENS (DE)\ HOHENTHANNER CLAUS-RUPERT (DE)\ ZSCHECH DANILO (DE)\ HOERNIG ALEXANDER (DE)\ REICHE HARALD (DE) | MEINTSCHEL JENS (DE)\ HOHENTHANNER CLAUS-RUPERT (DE)\ LI TEC BATTERY GMBH (DE)\ HOERNIG ALEXANDER (DE)\ REICHE HARALD (DE)\ ZSCHECH DANILO (DE) | H01M2/30\ H01M2/26 |
| 2409 | 3D printing | SCHWEI?ELEMENT ZUM VERSCHWEI?EN EINES KONTAKTELEMENTES MIT EINEM ANSCHLUSSELEMENT\[EN] WELDING ELEMENT FOR WELDING A CONTACT ELEMENT TO A CONNECTING ELEMENT\[FR] éLéMENT DE SOUDAGE POUR SOUDER UN éLéMENT DE CONTACT à UN éLéMENT DE CONNEXION\ | WO2013053553A1 | KERSTEN EIKE-CHRISTIAN (DE)\ ELSER ARMIN (DE)\ JURETKO MARTIN (DE) | BOSCH GMBH ROBERT (DE)\ KERSTEN EIKE-CHRISTIAN (DE)\ ELSER ARMIN (DE)\ JURETKO MARTIN (DE) | B23K20/10\ H01R39/00\ H02K13/04 |
| 2410 | 3D printing | END-SEALING COMPONENT DEVELOPER CARTRIDGE AND MANUFACTURING METHOD THEREFOR\[FR] éLéMENT D'éTANCHéITé D'EXTRéMITé CARTOUCHE DE DéVELOPPEUR ET PROCéDé DE FABRICATION POUR CEUX-CI\ | WO2013075610A1 | SO KIN KEUNG (CN)\ WANG JIZHONG (CN) | PRINT RITE UNICORN IMAGE PROD (CN) | G03G15/08 |
| 2411 | 3D printing | ASSEMBLING APPARATUS OF MOTOR FOR ASSEMBLING STATOR ASSEMBLY TO PRINTED CIRCUIT BOARD\[FR] APPAREIL D'ASSEMBLAGE DE MOTEUR POUR ASSEMBLER UN ENSEMBLE STATOR SUR UNE CARTE DE CIRCUIT IMPRIMé\ | WO2013103191A1 | LEE DEOK KYU (KR) | NEW MOTECH CO LTD (KR)\ LEE DEOK KYU (KR) | B23K20/10\ H02K15/00 |
| 2412 | 3D printing | OPTICAL FIBRE ADAPTER AND ASSEMBLY METHOD THEREFOR\[FR] ADAPTATEUR DE FIBRE OPTIQUE ET PROCéDé D'ASSEMBLAGE ASSOCIé\ | WO2013104137A1 | YANG GUO (CN)\ WANG QIYUE (CN) | SUNSEA TELECOMM CO LTD (CN)\ YANG GUO (CN)\ WANG QIYUE (CN) | G02B6/36\ G02B6/38 |
| 2413 | 3D printing | ULTRASONIC WELDING TIP ULTRASONIC WELDING MACHINE AND METHOD FOR PRODUCING BATTERY\[FR] BEC DE SOUDAGE PAR ULTRASONS MACHINE DE SOUDAGE PAR ULTRASONS ET PROCéDé DE PRODUCTION DE BATTERIE\ | WO2013105361A1 | UENO TOMOHIRO US | HITACHI MAXELL (JP) | B23K20/10\ H01M4/66\ B29C65/08\ H01L21/607\ H01M2/26 |
| 2414 | 3D printing | METHOD FOR PRODUCING BATTERY\[FR] PROCéDé PERMETTANT DE PRODUIRE UNE BATTERIE\ | WO2013105362A1 | UENO TOMOHIRO US | HITACHI MAXELL (JP) | H01M2/26\ H01M4/64 |
| 2415 | 3D printing | COLOUR 3-DIMENSIONAL PRINTING\[FR] IMPRESSION TRIDIMENSIONNELLE EN COULEURS\ | WO2013174526A1 | TRAVERS JAMES (IE)\ KEENAN PHILIP (GB)\ MACCORMACK FINTAN (IE)\ MACCORMACK CONOR (IE) | MCOR TECHNOLOGIES LTD (IE) | B29C67/00\ B41J3/60 |
| 2416 | 3D printing | APPAREIL POUR LA SOUDURE ET/OU LA COUPE PAR ULTRASONS D'UN ARTICLE THERMOFUSIBLE\[EN] APPARATUS FOR ULTRASONIC WELDING AND/OR CUTTING OF A THERMOFUSIBLE ARTICLE\ | WO2013175109A1 | MAGNERON FREDERIC (FR)\ PFISTER FREDERIC (FR) | SPOOLEX (FR) | B29C65/08\ B29C65/74 |
| 2417 | 3D printing | IMPROVEMENT TO PACKAGING SHEET FOR EDIBLE PRODUCTS AND METHOD FOR MANUFACTURING THE SAME\[FR] AMéLIORATION APPORTéE à UN EMBALLAGE LAMELLAIRE POUR PRODUITS COMESTIBLES ET PROCéDé POUR SA FABRICATION\[PT] APERFEI?OAMENTO EM EMBALAGEM LAMINAR PARA PRODUTOS COMESTíVEIS E PROCESSO PARA SUA FABRICA??O\ | WO2013177634A1 | GAMBARINI HECTOR ERNESTO (BR) | ARCOR DO BRASIL LTDA (BR)\ GAMBARINI HECTOR ERNESTO (BR) | B65B61/18\ B65D75/10\ B65B11/14\ B65B11/34\ B65D65/14 |
| 2418 | 3D printing | MELTFUSION BONDED ABSORBENT STRUCTURE COMPRISING FIBRES AND SUPERABSORBENT PARTICLES AND METHOD FOR MANUFACTURING SUCH STRUCTURE\[FR] STRUCTURE ABSORBANTE COLLéE PAR FUSION à CHAUD COMPRENANT DES FIBRES ET DES PARTICULES SUPERABSORBANTES ET PROCéDé DE FABRICATION D'UNE TELLE STRUCTURE\ | WO2014001487A1 | SCHMITZ CHRISTOPH (DE) | SCHMITZ CHRISTOPH (DE)\ CONCEPTS FOR SUCCESS C4S E K (DE) | D04H1/54\ A61F13/533 |
| 2419 | 3D printing | COLOUR 3-DIMENSIONAL PRINTING WITH 3D GAMUT MAPPING\[FR] IMPRESSION TRIDIMENSIONNELLE (3D) EN COULEURS à MAPPAGE DE GAMMES 3D\ | WO2014015994A1 | TRAVERS JAMES (IE)\ KEENAN PHILIP (GB)\ MACCORMACK FINTAN (IE)\ MACCORMACK CONOR (IE) | MCOR TECHNOLOGIES LTD (IE) | B29C67/00 |
| 2420 | 3D printing | TACK AND BOND ADHESIVE SYSTEM AND METHOD FOR LAYERED OBJECT MANUFACTURE\[FR] SYSTèME ET PROCéDé D'APPLICATION D'ADHéSIFS POUR LIAISON ET ADHéSION INSTANTANéE DESTINéS à LA FABRICATION D'OBJETS EN COUCHES\ | WO2014131848A3 | KEENAN PHILIP (GB)\ MACCORMACK FINTAN (IE)\ MACCORMACK CONOR (IE) | MCOR TECHNOLOGIES LTD (IE) | B29C67/00 |
| 2421 | 3D printing | INTEGRALLY COMBINED SPRING PAD PRODUCER\[FR] MOYEN DE PRODUCTION DE COUSSINS à RESSORTS COMBINéS D'UN SEUL TENANT\ | WO2014176866A1 | TAN ZHIMING (CN) | GUANGZHOU LIANROU MACHINERY & EQUIPMENT CO LTD (CN) | B68G9/00\ A47C23/043 |
| 2422 | 3D printing | METHOD AND APPARATUS FOR LAYER MANUFACTURING OF ARTEFACTS\[FR] PROCéDé ET APPAREIL DE FABRICATION EN COUCHES D'ARTéFACTS\ | WO2011064725A1 | POTGIETER PAUL (ZA) | AEROSUD INNOVATION & TRAINING CT PTY LTD (ZA)\ POTGIETER PAUL (ZA) | B29C67/00\ B22F3/105 |
| 2423 | 3D printing | SUBLIMATION INKS FOR THERMAL INKJET PRINTERS USING THERMALLY STABLE DYE PARTICLES\ | US2012162332A1 | MCKEAN DENNIS (HK)\ LEE FRANCIS CHEE-SHUEN (HK) | HK APPLIED SCIENCE & TECH RES (HK)\ MCKEAN DENNIS (HK)\ LEE FRANCIS CHEE-SHUEN (HK) | B41J2/01\ C09D11/02 |
| 2424 | 3D printing | PHASE CHANGE SUPPORT MATERIAL COMPOSITION\ | US7176253B2 | SCHMIDT KRIS ALAN (US)\ XU PINGYONG (US)\ RUATTA STEPHEN A (US)\ DOAN VU A (US) | 3D SYSTEMS INC (US) | B29C67/00\ C09D175/16\ C09D11/10\ C09D11/00\ B41J2/01\ B41M5/00\ C08L93/04\ C08K5/05 |
| 2425 | 3D printing | PHASE CHANGE SUPPORT MATERIAL COMPOSITION\ | US7399796B2 | SCHMIDT KRIS ALAN (US)\ XU PINGYONG (US)\ RUATTA STEPHEN A (US)\ DOAN VU A (US) | 3D SYSTEMS INC (US) | B29C67/00\ C09D175/16\ B05D3/06\ C09D11/10\ C09D11/00\ B41J2/01\ B41M5/00 |
| 2426 | 3D printing | METHODS AND APPARATUS FOR FABRICATING POROUS 3-DIMENSIONAL CELL CULTURE CONSTRUCT FOR CELL CULTURE AND OTHER BIOMEDICAL APPLICATIONS\ | US8463418B2 | LIU QING (US)\ LAU WING K (US) | 3D BIOTEK LLC (US)\ LIU QING (US)\ LAU WING K (US) | G06F19/00 |
| 2427 | 3D printing | TARGET-LOCKING ACQUISITION WITH REAL-TIME CONFOCAL (TARC) MICROSCOPY\ | US2010195868A1 | LU PETER J (US) | LU PETER J (US) | G06T7/00 |
| 2428 | 3D printing | PRINT FUSER AND PROCESS WITH MULTIPLE CLEANING BLADES\ | US7184696B2 | COKELEY JEFFREY H (US) | EASTMAN KODAK CO (US) | G03G15/20 |
| 2429 | 3D printing | PRINT FUSER AND PROCESS WITH A REVERSE CLEANING BLADE\ | US2006045581A1 | ECK EDWARD M (US)\ MILLS BORDEN H III (US) | ECK EDWARD M (US)\ MILLS BORDEN H III (US) | G03G15/20 |
| 2430 | 3D printing | FUSIBLE INK-JET RECORDING MATERIALS CONTAINING HOLLOW BEADS AND ULTRAFINE POLYMER PARTICLES\ | US2006045999A1 | CHEN TIENTEH (US)\ SEN RADHA (US) | CHEN TIENTEH (US)\ SEN RADHA (US) | B41M5/00 |
| 2431 | 3D printing | METHOD AND DEVICE FOR PRODUCING THREE-DIMENSIONAL OBJECTS\ | US2010163405A1 | ACKELID ULF (SE) | ARCAM AB (SE) | B29C35/08\ H01J37/305\ C23C14/34 |
| 2432 | 3D printing | DIGITAL THERMOGRAPHIC METHODS SYSTEMS AND PRODUCTS\ | US2013280500A1 | KIY MICHAEL (CH) | VISTAPRINT TECHNOLOGIES LTD (BM) | B41M5/00 |
| 2433 | 3D printing | METHOD AND DEVICE FOR PRINTING COLOR IMAGES ON CHOCOLATE PRODUCTS\ | US2009081341A1 | MANDELBOUM ISAR (IL) | MANDELBOUM ISAR (IL) | A23P1/08\ A23G1/22 |
| 2434 | 3D printing | FILM LAMINATE\ | US2008188154A1 | LEIS TERRY (US)\ FORSTER JAMES (US)\ HOPKINS DAVID (US) | JEN COAT INC (US) | B32B5/16\ B32B7/12\ B32B27/12 |
| 2435 | 3D printing | METHOD AND APPARATUS FOR REPAIRING TURBINE COMPONENTS\ | US8140179B2 | DRECHSLER DAVID E (US)\ COBB JAMES N (US)\ HUMBER J MICHAEL (US) | DRECHSLER DAVID E (US)\ COBB JAMES N (US)\ HUMBER J MICHAEL (US)\ HUFFMAN S E CORP (US) | G06F19/00\ B23K9/12 |
| 2436 | 3D printing | ION FUSION FORMATION\ | US6680456B2 | ADAMS ROBBIE (US) | HONEYWELL INT INC (US) | B23K10/02 |
| 2437 | 3D printing | FUSIBLE INKJET RECORDING MATERIALS CONTAINING HOLLOW BEADS SYSTEM USING THE RECORDING MATERIALS AND METHODS OF USING THE RECORDING MATERIALS\ | US2005287311A1 | CHEN TIENTEH (US)\ SEN RADHA (US)\ WEBB STEVEN L (US)\ KELLY-ROWLEY ANNE M (US)\ PAGE LORETTA ANN G (US) | CHEN TIENTEH (US)\ SEN RADHA (US)\ WEBB STEVEN L (US)\ KELLY-ROWLEY ANNE M (US)\ PAGE LORETTA ANN G (US) | B41M5/00\ B41M5/50\ B41M5/52\ B41M7/00\ B41J11/00 |
| 2438 | 3D printing | FUSIBLE INKJET RECORDING MATERIALS CONTAINING HOLLOW BEADS SYSTEM USING THE RECORDING MATERIALS AND METHODS OF USING THE RECORDING MATERIALS\ | US7651216B2 | CHEN TIENTEH (US)\ SEN RADHA (US)\ WEBB STEVEN L (US)\ KELLY-ROWLEY ANNE M (US)\ PAGE LORETTA ANN GREZZO (US) | HEWLETT PACKARD DEVELOPMENT CO (US) | B41J2/01\ B41M5/52\ B41M7/00 |
| 2439 | 3D printing | POLYESTER POWDER COMPOSITIONS METHODS AND ARTICLES\ | US2014221566A1 | MARTINONI RAFFAELE (CH) | VALSPAR SOURCING INC (US) | B29C67/00 |
| 2440 | 3D printing | SINGLE SIDE BI-DIRECTIONAL FEED FOR LASER SINTERING\ | US2005263933A1 | HANNA CHRISTOPHER R (US)\ WELCH ROBERT H IV (US)\ CHUNG TAE M (US) | 3D SYSTEMS INC (US) | B29C35/08\ B29C67/00\ B29C41/36\ B29C35/02 |
| 2441 | 3D printing | PREPARATION OF MICROPOROUS FILMS FROM IMMISCIBLE BLENDS VIA MELT PROCESSING AND STRETCHING\ | US6824680B2 | CHANDAVASU CHAIYA (TH)\ XANTHOS MARINO (US)\ SIRKAR KAMALESH K (US)\ GOGOS COSTAS (US) | NEW JERSEY TECH INST (US) | B01D69/14\ B01D39/16\ B29C47/00\ B01D67/00\ B01D71/26\ B29C55/00 |
| 2442 | 3D printing | BENDING RECEIVER USING HEAT-SHRINKABLE FILM\ | US8227165B2 | TYAGI DINESH (US) | EASTMAN KODAK CO (US)\ TYAGI DINESH (US) | G03G5/00 |
| 2443 | 3D printing | ANISOTROPIC CONDUCTIVE MATERIAL\ | US7785494B2 | YEH SYH-TAU (TW) | TEAMCHEM COMPANY (TW) | H01B1/00\ B32B5/16\ H01B1/02\ G03C1/76\ H01B1/12 |
| 2444 | 3D printing | ION FUSION FORMATION PROCESS FOR LARGE SCALE THREE-DIMENSIONAL FABRICATION\ | US2009026175A1 | ADAMS ROBBIE J (US) | HONEYWELL INT INC (US) | B23K9/04\ B23K10/00\ B05D1/08 |
| 2445 | 3D printing | METAL POWDER LAYERED APPARATUS FOR DOWNHOLE USE\ | US2008075618A1 | MARTIN GARY A (US)\ BELLIVEAU DAVID J (US)\ THERIAULT DANIEL B (US) | SCHLUMBERGER TECHNOLOGY CORP (US) | G06F19/00\ B22F7/02\ G06G7/48\ E21B1/00 |
| 2446 | 3D printing | POLYMERIC ADDITIVES TO IMPROVE PRINT QUALITY AND PERMANENCE ATTRIBUTES IN INK-JET INKS\ | US2002156153A1 | TSANG JOSEPH W (US)\ MOFFATT JOHN R (US) | CONCEPT LASER GMBH (DE) | C09D11/00\ B41J2/01\ B41M5/00\ B41J2/21\ B41J11/00 |
| 2447 | 3D printing | NON-CONTACT PRINTED COMESTIBLE PRODUCTS AND APPARATUS AND METHOD FOR PRODUCING SAME\ | US2007231425A1 | GLEFFE WILLIAM R (US)\ REAM RONALD L (US)\ VALDEZ ENRIQUE (US) | WRIGLEY W M JUN CO (US) | A23G4/00 |
| 2448 | 3D printing | COLORLESS TONER FORMULATED TO IMPROVE LIGHT FASTNESS OF INK JET INK PRINTS\ | US2002120040A1 | LIN AN-CHUNG ROBERT (US)\ LEE MICHAEL H (US) | Empty Field US | B41J2/01\ B41M5/00\ G03G9/087\ G03G9/08\ G03G9/09\ G03G9/097\ G03G8/00 |
| 2449 | 3D printing | METHOD OF BUILDING THREE-DIMENSIONAL OBJECT WITH MODIFIED ABS MATERIALS\ | US2009295032A1 | HOPKINS PAUL E (US) | STRATASYS INC (US) | B28B1/14 |
| 2450 | 3D printing | APPARATUS FOR CONTROLLED FREEZING OF MELTED SOLID INK IN A SOLID INK PRINTER\ | US2011211010A1 | SHRADER ERIC J (US)\ PASCHKEWITZ JOHN S (US) | PALO ALTO RES CT INC (US) | B41J2/175\ B41J29/377 |
| 2451 | 3D printing | PROCESS FOR PRODUCING AND APPLYING A LASER HEAT TRANSFER CAPABLE OF PRINTING ON FLAT CYLINDRICAL CURVED AND IRREGULARLY SHAPED OBJECTS\ | US7495162B1 | POKALLUS KEVIN (US) | POKALLUS KEVIN (US) | G10D13/02 |
| 2452 | 3D printing | PARYLENE COATED MICROFLUIDIC COMPONENTS AND METHODS FOR FABRICATION THEREOF\ | US2006204699A1 | MALTEZOS GEORGE (US)\ TANG HONGXING (US)\ ROUKES MICHAEL (US)\ SCHERER AXEL (US) | MALTEZOS GEORGE (US)\ TANG HONGXING (US)\ ROUKES MICHAEL (US)\ SCHERER AXEL (US) | B29C33/76\ B29C33/40\ B28B7/38\ F16L11/04 |
| 2453 | 3D printing | NANOTECHNOLOGICAL PROCESSING OF CATALYTIC SURFACES\ | US2006115389A1 | INDECH ROBERT (US) | INDECH ROBERT (US) | B01D53/34 |
| 2454 | 3D printing | POROUS FUSIBLE INKJET MEDIA WITH FUSIBLE CORE-SHELL COLORANT-RECEIVING LAYER\ | US7086732B2 | KASPERCHIK VLADEK P (US)\ UNGEFUG GARY ALLAN (US) | HEWLETT PACKARD DEVELOPMENT CO (US) | B41J2/01\ B41M5/00\ B41M5/50\ B41M5/52\ B41M7/00 |
| 2455 | 3D printing | METHOD OF FORMING HEAT RESISTANT RAISED PRINT FROM RADIATION CURABLE SOLID PARTICULATE COMPOSITIONS\ | US2005013932A1 | DULANY MARGARET A (US)\ WALDO ROSALYN M (US)\ DENNIS DEREK J (US) | Empty Field US | B05D3/02\ B05D3/06\ C09D11/10\ B05D5/06\ B05D7/24\ B41M3/16\ B41M7/00\ C08F299/02\ C09D133/10 |
| 2456 | 3D printing | ULTRATOUGH CVD SINGLE CRYSTAL DIAMOND AND THREE DIMENSIONAL GROWTH THEREOF\ | US7594968B2 | YAN CHIH-SHIUE (US)\ MAO HO-KWANG (US)\ HEMLEY RUSSELL J (US) | CARNEGIE INST OF WASHINGTON (US) | C30B29/04 |
| 2457 | 3D printing | PROCESS FOR PRODUCING A FILTER IN PARTICULAR FOR A ROTARY SEPARATOR\ | US8292982B2 | GENNARO PAOLO (IT)\ ROMEO MAURIZIO (IT)\ BENE GIUSEPPE (IT)\ TORTAROLO FRANCO (IT)\ ZANON GIOVANNI PAOLO (IT) | AVIO SPA (IT)\ GENNARO PAOLO (IT)\ ROMEO MAURIZIO (IT)\ BENE GIUSEPPE (IT)\ TORTAROLO FRANCO (IT)\ ZANON GIOVANNI PAOLO (IT) | B01D46/18 |
| 2458 | 3D printing | CONTROLLED DENSIFICATION OF FUSIBLE POWDERS IN LASER SINTERING\ | US2006119012A1 | RUATTA STEPHEN A (US)\ MOUSSA KHALIL M (US) | 3D SYSTEMS INC (US) | B29C35/08\ B29C67/00\ B29C41/02\ C23C24/10\ C23C26/02 |
| 2459 | 3D printing | MANUFACTURING PROCESS SUCH AS THREE DIMENSIONAL PRINTING INCLUDING BINDING OF WATER-SOLUBLE MATERIAL FOLLOWED BY SOFTENING AND FLOWING AND FORMING FILMS OF ORGANIC-SOLVENT-SOLUBLE MATERIAL\ | US2007009606A1 | SACHS EMANUEL M (US)\ SERDY JAMES G (US)\ MATERNA PETER A (US)\ WEST THOMAS G (US)\ SAINI SUNIL (US)\ CAI JIE (US)\ CARUSO ANDREA B (US)\ SHAROBIEM JOHN (US) | SHAROBIEM JOHN (US)\ SERDY JAMES G (US)\ SACHS EMANUEL M (US)\ WEST THOMAS G (US)\ SAINI SUNIL (US)\ CAI JIE (US)\ CARUSO ANDREA B (US)\ MATERNA PETER A (US) | A61F2/00\ A61F2/02\ A61F2/28\ A61F2/30\ G03C1/00\ A61K9/50 |
| 2460 | 3D printing | MELTING DEVICE FOR INCREASED PRODUCTION OF MELTED INK IN A SOLID INK PRINTER\ | US8052264B2 | HAYS ANDREW WAYNE (US)\ LEO MICHAEL F (US)\ LEIGHTON ROGER G (US) | XEROX CORP (US) | B41J2/175 |
| 2461 | 3D printing | METHOD FOR PREVENTING NOZZLE CONTAMINATION DURING WARM-UP\ | US7762656B2 | SNYDER TREVOR JAMES (US) | XEROX CORP (US) | B41J2/165\ B41J2/175 |
| 2462 | 3D printing | APPARATUS FOR THREE DIMENSIONAL PRINTING USING IMAGED LAYERS\ | US8119053B1 | BEDAL BRYAN (US)\ SCHELL STEVEN E (US)\ BEERS ROSS D (US) | 3D SYSTEMS INC (US)\ BEDAL BRYAN (US)\ SCHELL STEVEN E (US)\ BEERS ROSS D (US) | B29C35/08\ B28B1/16 |
| 2463 | 3D printing | APPARATUS FOR THREE DIMENSIONAL PRINTING USING IMAGED LAYERS\ | US2010244333A1 | BEDAL BRYAN (US)\ SCHELL STEVEN E (US)\ BEERS ROSS D (US) | BEDAL BRYAN (US)\ SCHELL STEVEN E (US)\ BEERS ROSS D (US) | B29C35/08\ B29C67/00\ B29C47/92 |
| 2464 | 3D printing | APPARATUS FOR MAKING ENGINEERED TISSUE USING SELF-ASSEMBLING CELL AGGREGATES\ | US2015004273A1 | FORGACS GABOR (US)\ JAKAB KAROLY (US)\ NEAGU ADRIAN (US)\ MIRONOV VLADIMIR (US) | MUSC FOUND FOR RES DEV (US)\ UNIV MISSOURI (US) | B29C67/00\ C12N5/071 |
| 2465 | 3D printing | MICROFABRICATED CELLULAR TRAPS BASED ON THREE-DIMENSIONAL MICRO-SCALE FLOW GEOMETRIES\ | US2007178582A1 | KOSER HUR (US) | KOSER HUR (US) | C12M1/34 |
| 2466 | 3D printing | PROCESS FOR LAMINATING AN INK JET PRINT\ | US6454896B1 | WEXLER ALLAN (US) | EASTMAN KODAK CO (US) | B41J2/01\ B41M7/00\ G03C11/08\ G03G8/00 |
| 2467 | 3D printing | DEICER SALT SHAPED FORMS\ | US2010308255A1 | ROBERTS MARK L (US)\ ENGLISH ROBERT S (US) | ROBERTS MARK L (US)\ ENGLISH ROBERT S (US) | C09K3/18 |
| 2468 | 3D printing | CONTROL OF METALLIC APPEARANCE IN AUTOMOTIVE CAST PAINT FILMS\ | US5653927A | FLYNN MICHAEL D (US)\ TRUOG KEITH L (US) | AVERY DENNISON CORP (US) | B29C37/00\ B05D5/06\ B05D7/24\ B05D7/14\ B32B15/08 |
| 2469 | 3D printing | FAST RESPONSE ELECTROCHEMICAL ORGANOPHOSPHATE SENSOR\ | US2010294662A1 | ZHENG ZHI (CN)\ ZHAO LINAN (CN)\ WANG MARILYN (CN) | HONEYWELL INT INC (US) | H01B13/00\ C25B11/04\ B05D5/12 |
| 2470 | 3D printing | OFFSET PRINTING METHOD FOR INK JET PRINTER AND INK JET PRINTER\ | JP2005096468A | BURR RONALD F US\ SEGERSTOM ERIC C US\ TITTERINGTON DONALD R US | FUJI XEROX CO LTD US | C09D11/00\ B41J2/01\ B41M5/00 |
| 2471 | 3D printing | METHOD AND APPARATUS FOR REDUCING DROP PLACEMENT ERROR IN PRINTERS\ | US6367909B1 | LEAN MENG H (US) | XEROX CORP (US) | B41J2/015\ B41J2/06\ B41J2/07 |
| 2472 | 3D printing | PHOTOVOLTAIC DEVICES FABRICATED FROM NANOSTRUCTURED TEMPLATE\ | US2005098204A1 | PICHLER KARL (US)\ ROSCHEISEN MARTIN R (US)\ SAGER BRIAN M (US) | NANOSOLAR INC (US) | H01L31/0256\ B81B1/00\ C09D5/24\ H01L31/042\ B82B3/00\ C07C61/00\ C07C63/42\ C08G61/00\ H01L31/00\ H01L31/0352\ H01L31/06\ H01L51/00\ H01L51/30 |
| 2473 | 3D printing | THREE-DIMENSIONAL FABRICS AND FILMS AND THE PROCESS FOR MAKING THE SAME\ | US2004222552A1 | HOUSLER JOHN (US)\ CARTER NICK (US)\ DALE ROBERT (US)\ SCHAEFFER JAMES (US)\ MOODY RALPH (US) | Empty Field US | D04H3/03\ B29C43/22\ B29C47/00\ B29C47/88\ B29C51/22\ B29C59/06\ D04H3/05\ D04H3/08 |
| 2474 | 3D printing | UNITARY CONDUIT FOR TRANSPORTING FLUIDS AND METHOD OF MANUFACTURING\[FR] CONDUIT UNITAIRE POUR LE TRANSPORT DE FLUIDES ET PROCéDé DE FABRICATION\ | WO2009148680A3 | MCMASTERS MARIE ANN (US)\ BENJAMIN MICHAEL A (US)\ MANCINI ALFRED (US) | GEN ELECTRIC (US)\ MCMASTERS MARIE ANN (US)\ BENJAMIN MICHAEL A (US)\ MANCINI ALFRED (US) | F02C7/22\ F23R3/14\ F23R3/28\ F23R3/34 |
| 2475 | 3D printing | VENTURI AND METHOD OF MANUFACTURING\[FR] VENTURI ET PROCéDé DE FABRICATION\ | WO2009126404A3 | MCMASTERS MARIE ANN (US)\ BENJAMIN MICHAEL A (US)\ MANCINI ALFRED (US) | GEN ELECTRIC (US)\ MCMASTERS MARIE ANN (US)\ BENJAMIN MICHAEL A (US)\ MANCINI ALFRED (US) | F23R3/14\ F23R3/28 |
| 2476 | 3D printing | SWIRLERS AND METHOD OF MANUFACTURING\[FR] COUPELLES ROTATIVES ET PROCéDé DE FABRICATION\ | WO2009126403A3 | MCMASTERS MARIE ANN (US)\ BENJAMIN MICHAEL A (US)\ MANCINI ALFRED (US) | GEN ELECTRIC (US)\ MCMASTERS MARIE ANN (US)\ BENJAMIN MICHAEL A (US)\ MANCINI ALFRED (US) | F02C7/22\ F23R3/14\ F23C7/00\ F23R3/28\ F23R3/34 |
| 2477 | 3D printing | REPAIR OF FUEL NOZZLE COMPONENT\ | US2009255102A1 | MCMASTERS MARIE ANN (US)\ BENJAMIN MICHAEL A (US)\ MANCINI ALFRED (US) | MCMASTERS MARIE ANN (US)\ BENJAMIN MICHAEL A (US)\ MANCINI ALFRED (US) | B23P6/00 |
| 2478 | 3D printing | HIGH RESOLUTION MULTICOLOR INK JET PRINTER\ | US7690779B2 | HOISINGTON PAUL A (US)\ SPEHRLEY JR CHARLES W (US) | FUJIFILM DIMATIX INC (US) | B41J2/01\ G01D11/00\ B41J2/175\ B41J2/21\ B41J2/195\ B41J11/00\ B41J2/205\ B41J13/22\ B41J19/16 |
| 2479 | 3D printing | LASER-PRODUCED POROUS SURFACE\ | US2004191106A1 | SUTCLIFFE CHRISTOPHER J (GB)\ JONES ERIC (IE)\ O'NEILL WILLIAM (GB) | HOWMEDICA OSTEONICS CORP (US) | A61F2/00\ A61F2/30\ B22F3/105\ B22F7/00\ C23C24/10\ A61F2/38\ C23C26/02\ C23C4/12\ A61F2/36\ C23C24/04\ C23C4/18\ A61F2/34\ B23K26/38\ C23C4/02 |
| 2480 | 3D printing | SYSTEM AND METHOD FOR MELTING SOLID INK STICKS IN A PHASE CHANGE INK PRINTER\ | US7581827B2 | JONES BRENT RODNEY (US)\ PLATT DAVID PAUL (US) | XEROX CORP (US) | G01D11/00\ B41J2/175 |
| 2481 | 3D printing | METAL POWDER COMPOSITION FOR LASER SINTERING\ | US2004226405A1 | SCHMIDT KRIS ALAN (US)\ NEWELL KENNETH J (US)\ GEVING BRAD (US) | 3D SYSTEMS INC (US) | C22C33/02 |
| 2482 | 3D printing | METHODS FOR FABRICATING THREE-DIMENSIONAL ALL ORGANIC INTERCONNECT STRUCTURES\ | US2004000425A1 | WHITE GEORGE E (US)\ DALMIA SIDHARTH (US)\ SUNDARAM VENKATESH (US)\ SWAMINATHAN MADHAVAN (US) | Empty Field US | H01F17/00\ H05K3/46\ H01F41/04\ H01L21/48\ H01L25/065\ H01L27/08\ H05K1/16 |
| 2483 | 3D printing | INTEGRATABLE ASSISTED COOLING SYSTEM FOR PRECISION EXTRUSION DEPOSITION IN THE FABRICATION OF 3D SCAFFOLDS\ | US2012080814A1 | SUN WEI (US)\ HAMID QUDUS (US) | SUN WEI (US)\ HAMID QUDUS (US)\ UNIV DREXEL (US) | B29C47/78\ F28F9/00 |
| 2484 | 3D printing | ADDITIVE MANUFACTURING METHOD FOR BUILDING THREE-DIMENSIONAL OBJECTS WITH CORE-SHELL ARRANGEMENTS AND THREE-DIMENSIONAL OBJECTS THEREOF\ | US2013224423A1 | MIKULAK JAMES K (US)\ DECKARD CARL R (US)\ ZINNIEL ROBERT L (US) | STRATASYS INC (US) | B29C67/00\ B32B3/26 |
| 2485 | 3D printing | COMPOSITE POLYMERIC ARTICLES FORMED FROM EXTRUDED SHEETS CONTAINING A LIQUID CRYSTAL POLYMER\ | US2012021212A1 | HOFMANN ACHIM (DE)\ YUNG PAUL C (US) | TICONA GMBH (DE)\ HOFMANN ACHIM (DE)\ YUNG PAUL C (US) | B29C51/02\ B32B5/00\ B29C47/78 |
| 2486 | 3D printing | NON-FIBROUS ADHESIVE MATRIX AND LAMINATE FORMED THEREFROM\ | US7803459B2 | KEUCHEL HERBERT WILLIE (US)\ KEUCHEL KENNETH (US) | KEUCHEL HERBERT WILLIE (US)\ KEUCHEL KENNETH (US) | B32B27/06\ B32B3/26\ B32B7/12\ B32B37/10\ A41D31/02\ B32B37/08\ C09J5/08 |
| 2487 | 3D printing | THREE-DIMENSIONAL NON-WOVEN FILTER\ | US2003080050A1 | ROLCHIGO PHILIP M (US)\ STIFTER TRAVIS G (US)\ KOPP CLINTON V (US)\ AUNE THOMAS M (US)\ MADSEN MICHAEL J (US) | OSMONICS INC (US) | B01D39/16\ B29C47/30\ D04H3/07 |
| 2488 | 3D printing | METHODS FOR FABRICATING COMPONENTS\ | US2007163114A1 | JOHNSON MICHAEL R (US) | GEN ELECTRIC (US) | B23P15/04 |
| 2489 | 3D printing | RASTER METHODOLOGY APPARATUS AND SYSTEM FOR ELECTRON BEAM LAYER MANUFACTURING USING CLOSED LOOP CONTROL\ | US2014158667A1 | WOLLENHAUPT PHILLIP E (US)\ STECKER SCOTT (US) | SCIAKY INC (US) | B23K15/00\ B23K15/06\ B23K15/02 |
| 2490 | 3D printing | PROCESS FOR PRODUCING A 3-DIMENSIONAL COMPONENT BY SELECTIVE LASER MELTING (SLM)\ | US2012018115A1 | STANKOWSKI ALEXANDER (CH)\ RICKENBACHER LUKAS (CH)\ HOEVEL SIMONE (CH) | HOEVEL SIMONE (CH)\ STANKOWSKI ALEXANDER (CH)\ RICKENBACHER LUKAS (CH) | B22D25/02\ B22D23/06\ B22D27/02 |
| 2491 | 3D printing | METHOD FOR SIMULATION OF LASER MATERIAL DEPOSITION\ | US6813533B1 | SEMAK VLADIMIR V (US) | VANTICO GMBH US | G06F17/50\ B23K26/34\ G06F19/00 |
| 2492 | 3D printing | BINDER REMOVAL IN SELECTIVE LASER SINTERING\ | US2004081573A1 | NEWELL KENNETH J (US) | 3D SYSTEMS INC (US) | B22F1/00\ B22F3/26\ B22F3/11 |
| 2493 | 3D printing | METHOD OF APPLYING MULTIPLE MATERIALS WITH SELECTIVE LASER MELTING ON A 3D ARTICLE\ | US2011106290A1 | STANKOWSKI ALEXANDER (CH)\ RICKENBACHER LUKAS (CH)\ HOEVEL SIMONE (CH) | HOEVEL SIMONE (CH)\ STANKOWSKI ALEXANDER (CH)\ RICKENBACHER LUKAS (CH) | G06F17/00 |
| 2494 | 3D printing | METHOD AND APPARATUS FOR CREATING A FREE-FORM THREE-DIMENSIONAL METAL PART USING HIGH-TEMPERATURE DIRECT LASER MELTING\ | US6621039B2 | WANG XINHUA (SG)\ WANG YUNGAN (SG)\ LUO JIHUAN (SG)\ YU YUEFENG (SG)\ ZHANG XIANGLIN (SG)\ TANG YAXIN (SG)\ LU LI (SG)\ WANG XUAN (SG)\ FUH YING HSI JERRY (SG)\ LIM KUAK CHOI LEALIC (SG)\ WONG YOKE SAN (SG)\ CHENG HONGBIN (SG) | KINERGY PTE LTD (SG)\ UNIV SINGAPORE (SG) | B22F3/00\ B22F3/105 |
| 2495 | 3D printing | HIGH DENSITY PERFORMANCE PROCESS\ | US2007085241A1 | HUSMANN CHRISTOPHER H (US)\ STEIN GREGORY N (US) | NORTHROP GRUMMAN CORP (US) | H05B6/00 |
| 2496 | 3D printing | METHOD ENABLING A STANDARD CMOS FAB TO PRODUCE AN IC TO SENSE THREE-DIMENSIONAL INFORMATION USING AUGMENTED RULES CREATING MASK PATTERNS NOT OTHERWISE EXPRESSIBLE WITH EXISTING FAB RULES\ | US7464351B2 | BAMJI CYRUS (US)\ LIU XINQIAO (US) | CANESTA INC (US) | G06F17/50\ A61B6/00\ G01B11/26\ A61N5/00\ G01C3/00\ G01N23/00\ G01S7/491\ G01S7/493\ G01S17/08\ G01S17/36\ G01S17/89\ G21G5/00\ G21K1/12\ H01L\ H01L27/146\ H01L31/02\ H04N3/14\ H05G1/60 |
| 2497 | 3D printing | FLUORESCENT SOLID INK MADE WITH FLUORESCENT NANOPARTICLES\ | US2010086683A1 | IFTIME GABRIEL (CA)\ VANBESIEN DARYL W (CA)\ WAGNER CHRISTOPHER A (CA)\ ALLEN C GEOFFREY (CA)\ BIRAU MARIA M (CA)\ WOSNICK JORDAN H (CA) | XEROX CORP (US) | B05D5/06\ C09K11/02 |
| 2498 | 3D printing | PHASE CHANGE INKS\ | US2010080922A1 | TUREK CAROLINE M (CA)\ BRETON MARCEL P (CA) | XEROX CORP (US) | C09D175/04\ C09D11/02\ B05D5/00\ B05D1/04 |
| 2499 | 3D printing | RAPID PROTOTYPING AND MANUFACTURING SYSTEM AND METHOD\ | US7690909B2 | WAHLSTROM BEN (US) | 3D SYSTEMS INC (US) | B28B1/16\ B05C11/02\ B29B13/08 |
| 2500 | 3D printing | WATER-BASED MATERIAL SYSTEMS AND METHODS FOR 3D PRINTING\ | US2004038009A1 | LEYDEN RICHARD NOEL (US)\ CLEARY TIMOTHY MARVIN (US)\ LI JOHN XIAOSAN (US)\ OBUCHOWICZ JACEK (GB)\ PEACE RICHARD J (GB) | Empty Field US | B29C67/00 |
| 2501 | 3D printing | OSTEOIMPLANT AND METHOD OF MAKING SAME\[FR] IMPLANT OSSEUX ET SON PROCEDE DE FABRICATION\[DE] OSTEOIMPLANTAT UND VERFAHREN ZU SEINER HERSTELLUNG\ | EP1301222A2 | SCARBOROUGH NELSON L (US)\ MANRIQUE ALBERT (US)\ EDWARDS JEAN T (US)\ BODEN SCOTT D (US) | OSTEOTECH INC (US) | A61F2/00\ A61F2/28\ A61F2/30\ A61B17/86\ A61L31/00\ A61L27/36\ A61F2/44\ A61F2/46 |
| 2502 | 3D printing | DIRECT SELECTIVE LASER SINTERING OF METALS\ | US6676892B2 | BEAMAN JOSEPH J (US)\ DAS SUMAN (US) | REGENTS UNIVERSITY TEXAS SYSTE (US) | B22F3/105\ B23K26/34\ C23C26/02\ G05B19/4062 |
| 2503 | 3D printing | METHODS AND APPARATUS FOR INCREASING IMAGE GLOSS\ | US6679600B1 | REGIMBAL LAURENT A (US) | HEWLETT PACKARD DEVELOPMENT CO (US) | G03G15/20 |
| 2504 | 3D printing | THERMOCHROMIC BUILD MATERIALS\ | US2013292881A1 | STEINER ROBERT J (US)\ VON SICE CORRIE M (US) | STEINER ROBERT J (US)\ VON SICE CORRIE M (US) | B41M5/34 |
| 2505 | 3D printing | ADDITITVE MANUFACTURING OF COMPONENTS FOR DOWNHOLE WIRELINE TUBING AND DRILL PIPE CONVEYED TOOLS\ | US2013310961A1 | INTRIAGO VELEZ JUAN CARLOS (US) | CORP SCHLUMBERGER TECHNOLOGY (US)\ SCHLUMBERGER TECHNOLOGY CORP (US) | G06F17/50 |
| 2506 | 3D printing | FILM TRANSFER METHOD AND APPEARANCE MEMBER MANUFACTURED BY USING THE SAME\ | US2013264727A1 | LEE JIH-HOUNG (TW)\ PENG MING-CHUNG (TW) | COMPAL ELECTRONICS INC (TW) | B29C45/02\ B29D11/00\ B29C45/40 |
| 2507 | 3D printing | METHODS AND APPARATUS FOR ACTUATED FABRICATOR\ | US2013292039A1 | PETERS BENJAMIN (US)\ OXMAN NERI (US) | MASSACHUSETTS INST TECHNOLOGY (US) | B32B37/12\ B32B38/10 |
| 2508 | 3D printing | LASER THERMAL PRINTING ON MICROPOROUS PLASTIC SUBSTRATES\ | US2013202828A1 | JACOBY PHILIP (US) | JACOBY PHILIP (US)\ MAYZO CORP (US) | B32B33/00\ B32B3/26\ B29C59/16 |
| 2509 | 3D printing | LASER WELDED BONDING PADS FOR PIEZOELECTRIC PRINT HEADS\ | US8857021B2 | GERNER BRADLEY JAMES (US)\ NYSTROM PETER J (US)\ DOLAN BRYAN R (US) | XEROX CORP (US)\ GERNER BRADLEY JAMES (US)\ NYSTROM PETER J (US)\ DOLAN BRYAN R (US) | H05K3/10\ B41J2/16\ B41J2/14\ B23K26/22 |
| 2510 | 3D printing | METHOD AND APPARATUS FOR INCREASING THE RESOLUTION IN ADDITIVELY MANUFACTURED THREE-DIMENSIONAL ARTICLES\ | US2014370323A1 | ACKELID ULF (SE) | ARCAM AB (SE) | B22F3/105\ B22F3/24\ B22F3/10 |
| 2511 | 3D printing | METHOD AND APPARATUS FOR DETECTING DEFECTS IN FREEFORM FABRICATION\ | US2014308153A1 | LJUNGBLAD ULRIC (SE) | ARCAM AB (SE) | B22F3/105 |
| 2512 | 3D printing | MELT-EXTRUDED SUBSTRATE FOR USE IN THERMOFORMED ARTICLES\ | US8852730B2 | NAIR KAMLESH P (US)\ GRAY STEVEN D (US)\ CANO CAMILO (US) | NAIR KAMLESH P (US)\ GRAY STEVEN D (US)\ CANO CAMILO (US)\ TICONA LLC (US) | C08K5/20\ B29C43/24\ C09K19/30\ C09K19/38\ C09K19/04\ C09K19/22\ C09K19/32\ C09K19/34\ C09K19/48 |
| 2513 | 3D printing | HOT MELT CALENDERED OR EXTRUDED WEAR LAYER FOR EMBOSSED SUBSTRATES AND METHOD OF MANUFACTURE\ | US2004048044A1 | CHRISTIE PETER A (US)\ MCQUATE WILLIAM M (US)\ SCHNEIDER DONALD E (US) | ARMSTRONG WORLD IND INC (US) | B32B3/00\ B32B38/06\ B29C43/24\ B32B37/15\ D06N7/00 |
| 2514 | 3D printing | NICHTFASERIGE KLEBSTOFFMATRIX UND DARAUS HERGESTELLTES LAMINAT\[EN] NON-FIBROUS ADHESIVE MATRIX AND LAMINATE FORMED THEREFROM\[FR] MATRICE ADHESIVE NON-FIBREUSE ET LAMINE FORME A PARTIR DE CETTE MATRICE\ | EP1729955A4 | KEUCHEL HERBERT WILLIE (US)\ KEUCHEL KENNETH (US) | SPUNFAB LTD (US) | B32B27/06\ B32B5/18\ B32B3/26 |
| 2515 | 3D printing | METHOD OF FABRICATING BATH-MODELS FOR NICKEL SHELLS\ | US2010102478A1 | MASCHERA FLAVIO (IT)\ BARROTE PEDRO (PT) | MASCHERA FLAVIO (IT)\ BARROTE PEDRO (PT) | B29C33/38 |
| 2516 | 3D printing | RETICULATED MESH ARRAYS AND DISSIMILAR ARRAY MONOLITHS BY ADDITIVE LAYERED MANUFACTURING USING ELECTRON AND LASER BEAM MELTING\ | US2010291401A1 | WICKER RYAN (US)\ MEDINA FRANK (US)\ MURR LAWRENCE (US)\ GAYTAN SARA (MX) | UNIV TEXAS (US) | B23K26/36\ B32B3/12\ B32B15/02 |
| 2517 | 3D printing | OBJECT CONSOLIDATION THROUGH SEQUENTIAL MATERIAL DEPOSITION\ | US6814823B1 | WHITE DAWN (US) | SOLIDICA INC (US) | B29C67/00\ G05B19/4099\ B23K11/00\ B23K20/10\ B23K20/12\ B29C73/00 |
| 2518 | 3D printing | IMAGING MODULE FOR HOT MELT WAX INK JET PRINTER\ | US2010208017A1 | FLORENCE RICHARD N (US)\ MACQUEEN JR RAYMOND J (US)\ PULLEN ELAINE A (US)\ WALTER GRAHAM D (US)\ WIITA ROBERT L (US)\ WRIGHT WILLIAM PARKER ALEXANDER (US) | BLACK DOT TECHNOLOGY INC (US) | B41J2/175 |
| 2519 | 3D printing | INK CONSUMPTION DETERMINATION\ | US7425061B2 | KORN SCOTT J (US)\ BUEHLER JAMES D (US)\ KNIERIM DAVID L (US)\ YUSEM GUSTAVO J (US) | XEROX CORP (US) | G01D11/00\ B41J2/175 |
| 2520 | 3D printing | POLYESTER POWDER COMPOSITIONS METHODS AND ARTICLES\[DE] POLYESTERPULVERZUSAMMENSETZUNGEN VERFAHREN UND ARTIKEL\[FR] COMPOSITIONS DE POUDRE DE POLYESTER PROCéDéS ET ARTICLES AFFéRENTS\ | EP2379311A2 | MARTINONI RAFFAELE (CH) | VALSPAR SOURCING INC (US) | B29C67/00\ B29C70/58 |
| 2521 | 3D printing | VASCULARIZED TISSUE REGENERATION MATRICES FORMED BY SOLID FREE FORM FABRICATION TECHNIQUES\ | US6176874B1 | VACANTI JOSEPH P (US)\ CIMA MICHAEL J (US)\ CIMA LINDA G (US) | CHILDRENS MEDICAL CENTER (US)\ MASSCHUSETTS INST OF TECHNOLOG (US) | B29C67/00\ A61L27/00\ A61F2/00\ A61F2/02\ A61F2/06\ A61F2/10\ A61F2/28\ A61F2/30\ A61K9/00\ A61K9/16\ A61L27/38\ A61L27/40\ A61L27/56\ A61L27/60\ B82B1/00 |
| 2522 | 3D printing | THREE-DIMENSIONAL INTEGRATED HETEROGENEOUS SEMICONDUCTOR STRUCTURE\ | US7741645B2 | ZHU HUILONG (US) | IBM (US) | H01L29/76\ H01L31/036\ H01L31/112 |
| 2523 | 3D printing | POST PROCESSOR FOR THREE-DIMENSIONAL OBJECTS\ | US7114943B1 | FONG JON JODY (US)\ SOLIZ RAYMOND M (US) | 3D SYSTEMS INC (US) | B28B17/00\ B29C35/00 |
| 2524 | 3D printing | PROCESSLESS DIGITALLY IMAGED PRINTING PLATE USING MICROSPHERES\ | US2003180636A1 | ROSEN DANIEL (US)\ KANGA RUSTOM SAM (US) | Empty Field US | G03F7/027\ B41C1/05\ B41N1/12 |
| 2525 | 3D printing | METHOD OF MANUFACTURING COMBUSTOR COMPONENTS\ | US2009255256A1 | MCMASTERS MARIE ANN (US)\ LOHMUELLER STEVEN JOSEPH (US)\ BOEHM RANDALL CHARLES (US) | GEN ELECTRIC (US) | B22F7/00\ F02C7/22 |
| 2526 | 3D printing | COMBUSTOR COMPONENT AND METHOD OF MANUFACTURE\[FR] COMPOSANT DE CHAMBRE DE COMBUSTION ET SON PROCéDé DE FABRICATION\ | WO2009126534A1 | MCMASTERS MARIE ANN (US)\ LOHMUELLER STEVEN JOSEPH (US)\ BOEHM RANDALL CHARLES (US) | GEN ELECTRIC (US)\ MCMASTERS MARIE ANN (US)\ LOHMUELLER STEVEN JOSEPH (US)\ BOEHM RANDALL CHARLES (US) | B23K26/34 |
| 2527 | 3D printing | RAPID OBJECT CONSTRUCTION SYSTEM WITH GRADIENT COMPOSITIONS PROGRAMMABLE UNDER HIGH TEMPERATURE\ | TW201208869A | LIN KUN-YI (TW)\ CHANG HUI-CHU (TW)\ LIN PAI-CHI (TW) | LIN KUN-YI (TW)\ CHANG HUI-CHU (TW)\ LIN PAI-CHI (TW) | B29C67/02 |
| 2528 | 3D printing | HOUSING OF ELECTRONIC DEVICE AND METHOD FOR MAKING THE HOUSING\ | TW201110457A | WANG QIANG (CN) | FIH HONG KONG LTD (HK) | H01Q1/22 |
| 2529 | 3D printing | MOLDING TECHNIQUE OF ELEVATED PLASTIC FLOOR TILE AND THE STRUCTURE THEREOF\ | TW201026940A | YOU WEN-BANG (TW) | TAD CHIN IND CORP (TW) | E04F15/10 |
| 2530 | 3D printing | SHELL AND METHOD OF MANUFACTURING THE SAME\ | TW200950651A | LEE CHIEN-HUNG (TW) | LEE CHIEN-HUNG (TW) | H05K5/06 |
| 2531 | 3D printing | METHOD FOR MANUFACTURING POLYCRYSTALLINE TILES\ | TW200846531A | LIN RONG-DE (TW) | CHAMPION BUILDING MATERIALS CO LTD (TW) | E04C2/04\ C04B18/02 |
| 2532 | 3D printing | No Title Available\ | TWI325814B | Empty Field US | Empty Field US | B44C5/00\ B32B17/10\ C03C27/12 |
| 2533 | 3D printing | METHOD AND SYSTEM FOR LEADING MACROMOLECULE SUBSTANCES INTO LIVING TARGET CELLS\ | US2005085711A1 | TIEN DER-YANG (TW) | Empty Field US | A61M37/00\ A61B19/00\ A61K48/00\ A61M5/14\ A61B17/22\ A61K9/10\ A61K45/00\ A61K49/00 |
| 2534 | 3D printing | A MANUFACTURE METHOD AND PRODUCT OF AN ARTIFICIAL BOARD WITH DUAL-COLOR AND THREE-DIMENSIONAL CONVEX PATTERNS\ | TW585959B | TSAI JE-AN (TW) | TSAI JE-AN (TW) | E04C2/00 |
| 2535 | 3D printing | BUBBLE PRINT HEAD WITH INK FEED CHAMBER MADE BY INJECTION MOLDING\ | TW200409702A | SHI WANG-PING (TW)\ WEI REN-JIE (TW)\ FANG DUN-YING (TW)\ XIE SHU-PIN (TW)\ YE FENG-CHUN (TW) | MICRO BASE TECHNOLOGY CORP (TW) | Empty Field |
| 2536 | 3D printing | METHOD FOR MANUFACTURING RIM OF THERMOPLASTIC COMPOSITE MATERIAL\ | TW201313456A | ZHOU WEN-SHOU (TW) | ZHOU WEN-SHOU (TW) | B29C70/10\ B29C70/40\ B29L31/32 |
| 2537 | 3D printing | METHOD OF LASER CASTING COPPER-BASED COMPOSITES\ | US6723278B1 | LU LI (SG)\ WONG YOKE SAN (SG)\ FUH JERRY YING (SG)\ CHEN ZHENDA (SG)\ LIM GNIAN CHER (SG) | UNIV SINGAPORE (US)\ SINGAPORE INST OF MFG TECHNOLO (US) | B22F3/105 |
| 2538 | 3D printing | METHOD FOR PRODUCTION OF A THREE-DIMENSIONAL BODY\ | US2013300035A1 | SNIS ANDERS (SE) | ARCAM AB (SE)\ SNIS ANDERS (SE) | B29C67/00 |
| 2539 | 3D printing | METHOD AND APPARATUS FOR PRODUCING THREE-DIMENSIONAL OBJECTS\ | US2010270708A1 | SNIS ANDERS (SE)\ LARSSON MORGAN (SE)\ ACKELID ULF (SE)\ JONASSON DANIEL (SE) | SNIS ANDERS (SE)\ JONASSON DANIEL (SE)\ LARSSON MORGAN (SE)\ ACKELID ULF (SE) | B29C35/08 |
| 2540 | 3D printing | METHOD AND DEVICE FOR PRODUCING THREE-DIMENSIONAL OBJECTS\ | US8187521B2 | SNIS ANDERS (SE)\ LARSSON MORGAN (SE) | ARCAM AB (SE)\ SNIS ANDERS (SE)\ LARSSON MORGAN (SE) | H01J37/30 |
| 2541 | 3D printing | METHOD OF PRODUCING OBJECTS CONTAINING NANO METAL OR COMPOSITE METAL\ | US8333922B2 | LANGLET ABRAHAM (SE)\ SKOGLUND PETER (SE) | LANGLET ABRAHAM (SE)\ EXMET AB (SE)\ SKOGLUND PETER (SE) | B22F3/105\ C22C45/00 |
| 2542 | 3D printing | METHOD OF PRODUCING PRODUCTS OF AMORPHOUS METAL\ | US8052923B2 | LANGLET ABRAHAM (SE) | LANGLET ABRAHAM (SE) | B22F3/105\ C22C45/00 |
| 2543 | 3D printing | ANORDNING F?R DR?NERING\ | SE0501521L | MASSET GILLES US | EPSCEMENT INTERNAT AB (SE) | Empty Field |
| 2544 | 3D printing | PULVER F?R SNABBTILLVERKNING AV VOLYMKROPPAR SAMT METOD ATT FRAMST?LLA PULVRET OCH KROPPAR TILLVERKADE AV PULVRET\ | SE0300422D0 | LARSON RALF US | SPEEDPART RP AB (SE) | B22F3/105\ B22F9/08\ B22F |
| 2545 | 3D printing | ARRANGEMENT AND METHOD FOR PRODUCING A THREE-DIMENSIONAL PRODUCT\ | US7713454B2 | LARSSON MORGAN (SE) | ARCAM AB (SE) | B29C67/00\ B22F3/105\ B29C35/02 |
| 2546 | 3D printing | ARRANGEMENT AND METHOD FOR PRODUCTION OF A THREE DIMENSIONAL OBJECT\ | US7454262B2 | SNIS ANDERS (SE)\ LARSSON MORGAN (SE) | ARCAM AB (SE) | B29C67/00\ G06F19/00 |
| 2547 | 3D printing | ARRANGEMENT AND METHOD FOR PRODUCING A THREE-DIMENSIONAL PRODUCT\ | US7833465B2 | LARSSON MORGAN (SE) | ARCAM AB (SE) | B29C35/08\ B29C67/00\ B29C41/52\ B29C41/02 |
| 2548 | 3D printing | ARRANGEMENT AND METHOD FOR PRODUCING A THREE-DIMENSIONAL PRODUCT\ | US7635825B2 | LARSSON MORGAN (SE) | ARCAM AB (SE) | B29C67/00\ B23K26/08\ H05B6/00 |
| 2549 | 3D printing | ARRANGEMENT FOR THE PRODUCTION OF A THREE-DIMENSIONAL PRODUCT\ | US2006141089A1 | LARSSON MORGAN (SE)\ LARSSON MIKAEL (SE) | ARCAM AB (SE) | B29C67/00\ B29C35/00 |
| 2550 | 3D printing | DEVICE AND ARRANGEMENT FOR PRODUCING A THREE-DIMENSIONAL OBJECT\ | US2004026807A1 | ANDERSSON LARS-ERIK (SE)\ LARSSON MORGAN (SE) | CHEN WEN-ZHENG (TW) | B29C35/08\ B29C67/00\ B22F3/105\ B29C35/02 |
| 2551 | 3D printing | METHOD FOR PRODUCING THREE-DIMENSIONAL ARTICLES FROM POWDERS AND APPARATUS FOR CARRYING OUT SAID METHOD\[FR] PROCéDé DE FABRICATION D'ARTICLES VOLUMIQUES à PARTIR DE POUDRES ET DISPOSITIF DE SA MISE EN OEUVRE\ | WO2013080030A1 | CHIVEL YURI (BY) | CHIVEL YURI (BY) | B22F3/105 |
| 2552 | 3D printing | No Title Available\ | RU2010135856A | Empty Field US | Empty Field US | B64G1/66 |
| 2553 | 3D printing | METHOD OF COATING ARTICLE BY LASER LAYER-BY-LAYER SYNTHESIS\ | RU2443506C2 | KHARANZHEVSKIJ EVGENIJ VIKTOROVICH (RU)\ IPATOV ALEKSEJ GENNAD EVICH (RU)\ GALENKO PETR KONSTANTINOVICH (RU)\ KRIVILEV MIKHAIL DMITRIEVICH (RU)\ DANILOV DENIS ANATOL EVICH (RU) | G OBRAZOVATEL NOE UCHREZHDENIE VYSSHEGO PROFESSIONAL NOGO OBRAZOVANIJA UDMURTSKIJ GU UDGU (RU) | B22F3/105 |
| 2554 | 3D printing | No Title Available\ | RU2009117927A | Empty Field US | Empty Field US | B22F1/00 |
| 2555 | 3D printing | VERFAHREN ZUR HERSTELLUNG EINES WERKSTüCKS DURCH SCHMELZEN ODER SELEKTIVES SINTERN MIT HILFE EINES LASERS VON PULVERN AUS UNTERSCHIEDLICHEN MATERIALIEN\[EN] METHOD FOR MANUFACTURING A PART BY SELECTIVE LASER FUSION OR SINTERING OF POWDERS OF DIFFERENT MATERIALS\[FR] PROCéDé DE RéALISATION D'UNE PIèCE PAR FUSION OU FRITTAGE SéLECTIF PAR LASER DE POUDRES DE MATéRIAUX DIFFéRENTS\ | EP2156942B1 | CHIVEL YURI ALEXANDROVICH (BY)\ SMUROV IGOR (FR)\ LAGET BERNARD (FR)\ YADROITSEV IGOR (FR) | ECOLE NATIONALE D INGENIEURS DE SAINT ETIENNE (FR)\ INST PHYSIQUE B I STEPANOV DE L ACADEMIE NATIONALE DES SCIENCES DE BIELORUSSIE (BY) | B29C67/00 |
| 2556 | 3D printing | METHOD OF MANUFACTURING OF BEARING COMBINED REINFORCED IN SITU PILE\ | RU2334048C1 | SAURIN ANATOLIJ NIKIFOROVICH (RU)\ IL ICHEV VJACHESLAV ALEKSANDRO (RU)\ ZHADANOVSKIJ BORIS VASIL EVICH (RU)\ SAURIN JAROSLAV ANATOL EVICH (RU)\ RED KINA JULIJA VJACHESLAVOVNA (RU)\ MILJUTIN DENIS NIKOLAEVICH (RU) | SAURIN ANATOLIJ NIKIFOROVICH (RU) | E02D5/34 |
| 2557 | 3D printing | PLASMATRON\ | RU2225084C1 | SUSLOV V I (RU) | ZHUZHOU SMELTER GROUP CO LTD US\ UNIV CENTRAL SOUTH US\ HUNAN NONFERROUS METALS HOLDING GROUP CO LTD US | H05H1/42\ H05B7/20\ B05B7/22 |
| 2558 | 3D printing | POLYCRYSTALLINE MAGNETOSTRICTIVE MATERIAL OF A TB-FE-B-TYPE AND PROCESS FOR PREPARING THE SAME\ | RO123154B1 | LUPU NICOLETA (RO)\ V&ABREVELEANU MIHAELA CRISTINA (RO)\ CHIRIAC HORIA (RO) | INST NA&TCEDILIONAL DE CERCETARE DEZVOLTARE PENTRU FIZIC&ABREVE TEHNIC&ABREVE IFT IA&SCEDILI (RO) | H01F1/053\ C22C33/04 |
| 2559 | 3D printing | PRECIOUS METAL LAYER CERAMIC MOLDINGS AND METHOD OF MANUFACTURE\ | KR101315344B1 | SHIN HONG DAI (KR) | SHIN HONG DAI (KR) | B44C5/00\ C04B41/88 |
| 2560 | 3D printing | METHOD FOR MANUFACTURING ARTIFICIAL FINGERPRINT USING QR CODE AND QR CODE MANUFACTURED BY THE SAME\ | KR101332682B1 | KIM BYUNG JUN (KR)\ KIM JUNG HYUN (KR)\ LEE SEUNG YUN (KR) | NAT UNIV HANBAT INDUSTRY (KR) | G06K7/10\ G06K9/46\ G06K9/18\ G06K9/20 |
| 2561 | 3D printing | METHOD FOR MANUFACTURING BIODEGRADABLE BOARD AND BIODEGRADABLE BOARD MANUFACTURED THEREBY\[FR] PROCéDé DE FABRICATION DE PLANCHE BIODéGRADABLE ET PLANCHE BIODéGRADABLE FABRIQUéE ASSOCIéE\ | WO2013118930A1 | YOON SANG-JUN (KR)\ CHEY WON-JUN (KR)\ KIM TAE-KYUN (KR)\ PARK HYUNG-JOON (KR) | DOWNNARA CO LTD (KR)\ YOON SANG-JUN (KR)\ CHEY WON-JUN (KR)\ KIM TAE-KYUN (KR)\ PARK HYUNG-JOON (KR) | B27N3/00\ C08L67/04\ B27N3/08 |
| 2562 | 3D printing | THE METHOD OF TREATING AUTO'S PADDALE SUFACE TO PREVENT SLIP WIST MEATAL COMPCSI TION\ | KR101190513B1 | LEE BO HYUN (KR)\ LEE CHANG YAP (KR) | LEE CHANG YAP (KR)\ LEE BO HYUN (KR) | B22F3/00\ C23C28/00\ B60K26/00 |
| 2563 | 3D printing | THREE-DIMENSIONAL NET-LIKE STRUCTURE\ | US2014370769A1 | OSAKI HIROKO (JP) | C ENG CO LTD (JP) | A47C27/12\ A47C31/00 |
| 2564 | 3D printing | PHOTO-BOOK LINKED TO WEB-SITE AND MANUFACTURING METHOD THEREOF\ | KR20130082544A | SONG CHANG HOON (KR) | ABYZ CO LTD (KR) | G06K9/18\ G06Q50/10 |
| 2565 | 3D printing | THE GILT PRINTING METHOD AND THE TEXTILE\ | KR20130063329A | LEE YOUNG JAE (KR)\ CHOI JAE YOUNG (KR) | LEE YOUNG JAE (KR)\ CHOI JAE YOUNG (KR) | D06P5/10\ D06P1/52\ D06Q1/04 |
| 2566 | 3D printing | SYSTEM AND ITS MAKING METHOD OF COLLECTION NOTE USING QR CODE\ | KR101283310B1 | Empty Field US | INFO VISION OPTOELECTRONICS KUNSHAN CO LTD US | G06F3/12\ B41J3/36\ G06K15/02 |
| 2567 | 3D printing | PHOSPHORESCENT PAINT COMPOSITION AND ITS PREPARATION METHOD\ | KR20120067139A | SEO SAM SANG (KR) | CHANGDAE HOTMELTSHEET CO LTD (KR) | B32B27/20\ B32B37/00\ C08J5/18\ B32B27/08 |
| 2568 | 3D printing | METHOD FOR MANUFACTURING REDUCER OF COAL PULVERIZER\ | KR101057668B1 | SONG JONG SEOP (KR) | SECO LTD (KR) | B02C15/16\ B02C4/42\ B02C15/04 |
| 2569 | 3D printing | NYLON FILM HAVING IMPROVED PRINTING ADHESIVE STRENGTH\ | KR20110085651A | CHO HYUN (KR)\ SONG GI SANG (KR)\ KIM SI MIN (KR) | KOLON INC (KR) | C08J5/18\ B29D7/01 |
| 2570 | 3D printing | A MANUFACTURING METHOD FOR NON-FLAMABLE SHEET\ | KR101238799B1 | Empty Field US | KB KHIMAVTOMATIKI AOOT (RU) | B29C43/24\ B29C43/28 |
| 2571 | 3D printing | PHOTOCATALYTIC THERMO-RESPONSIVE 3-DIMENSIONAL COPOLYMER AND FABRICATING METHOD OF THE SAME\ | KR101157527B1 | Empty Field US | Empty Field US | C02F1/72\ B01J31/08\ B01J37/02\ C02F1/32 |
| 2572 | 3D printing | FILM ENVELOP HAND STRAP MADE OF POLYPROPYLENE AND MANUFACTURING METHOD THEREOF\ | KR20110059496A | CHOI OK BOM (KR)\ HA JAE YOUNG (KR) | CHOI OK BOM (KR)\ HA JAE YOUNG (KR) | B29D7/00\ B29C47/02\ B65D33/06 |
| 2573 | 3D printing | WHITE POROUS POLYESTER FILM AND PREPARATION METHOD THEREOF\ | US2011046254A1 | SHIN HEON JUNG (KR)\ JU JAE SEOG (KR)\ KIM MIN JU (KR) | SKC CO LTD (KR) | C08J9/00 |
| 2574 | 3D printing | PRINT CIRCUIT BOARD HAVING EXCELLENT HEAT DISSIPATING AND LIGHT EMITTING DIODE MODULE USING THEREOF\ | KR100919975B1 | SIM SANG YONG (KR) | LI TECH CO LTD (KR) | H01L33/64 |
| 2575 | 3D printing | MULTIPLE PURPOSE MATRIX OF 3D WEB STRUCTRED\ | KR101020878B1 | Empty Field US | KIM MOON OK (KR) | B29C47/00\ B29D28/00 |
| 2576 | 3D printing | ADHISIVE RESINS FOR COATING OF STEEL PLATE\ | KR20100082077A | HONG CHANG IL (KR)\ NAM SOON HEE (KR) | HONG CHANG IL (KR) | C09J7/02\ C09J133/04 |
| 2577 | 3D printing | ANISOTROPIC CONDUCTIVE MATERIAL\ | KR20100028901A | YEH SYH TAU (TW) | TEAMCHEM COMPANY (TW) | H01B1/22\ H01B1/02 |
| 2578 | 3D printing | METAL JET UNIT\ | KR100975627B1 | Empty Field US | KOREA INST SCI & TECH (KR) | B22D23/00 |
| 2579 | 3D printing | LOW MELTING POINT FRIT PASTE COMPOSITION AND SEALING METHOD FOR ELECTRIC ELEMENT USING THE SAME\ | US7923393B2 | SON JUNG-HYUN (KR)\ LEE SANG-KYU (KR)\ JOO HAN-BOK (KR) | DONGJIN SEMICHEM CO LTD (KR) | C03C8/24\ C03C8/08\ B29C65/16\ C03B23/00\ C03C3/21\ C03C8/14\ C03C8/16\ H01J9/26 |
| 2580 | 3D printing | ENVIRONMENT-FRIENDLY MULTILEVEL PLASTIC ENVELOPE AND THAT PRODUCTION METHODS\ | KR100831377B1 | BAK YEONG GI (KR)\ KANG JOO HO (KR)\ KIM SUN WOOK (KR) | HAESUNG INDUSTRY CO LTD (KR)\ BAK YEONG GI (KR)\ KANG JOO HO (KR)\ KIM SUN WOOK (KR) | B65D30/14\ B65D30/10 |
| 2581 | 3D printing | VIA USING ZN OR ZN ALLOYS AND ITS MAKING METHOD 3D CHIP STACK PACKAGES USING THEREOF\ | US2010240174A1 | YU JIN (KR)\ JEE YOUNG-KUN (KR) | YU JIN (KR)\ JEE YOUNG-KUN (KR) | H01L21/768\ H01L21/50 |
| 2582 | 3D printing | MANUFACTURING METHOD OF FRINTING FILM FOR INSERT MOLDING\ | KR100877184B1 | KIM KWANG WON (KR)\ LEE HONG SOO (KR)\ KWACK SE HOON (KR) | SANGBO CORP (KR) | C08J5/18\ B29D7/01 |
| 2583 | 3D printing | FRINTING FILM FOR INSERT MOLDING\ | KR100877183B1 | KIM KWANG WON (KR)\ LEE HONG SOO (KR)\ KWACK SE HOON (KR) | SANGBO CORP (KR) | C08J5/18\ B29D7/01 |
| 2584 | 3D printing | PACKCING SHEET MANUFACTURING MATHOD AND PACKING SHEET THEREBY\ | KR20080107832A | KIM DO EON (KR) | KIM DO EON (KR) | B31B19/60\ B31B21/60 |
| 2585 | 3D printing | CLAY FOR RUBBER BOARD\ | KR20080104627A | KIM HYUN MOOK (KR) | KIM HYUN MOOK (KR) | B44D3/18 |
| 2586 | 3D printing | CERAMIC FOAM WHICH HAS SUPERIOR INSULATING EFFECT AND SOUNDPROOFING EFFECT AND THE METHOD FOR MANUFACTURING THE SAME\[FR] MOUSSE CéRAMIQUE à EFFET ISOLANT ET INSONORISANT SUPéRIEUR ET PROCéDé DE FABRICATION\ | WO2008123671A2 | PARK MIN-HWA (KR) | PARK MIN-HWA (KR) | C04B38/00\ C04B38/10 |
| 2587 | 3D printing | No Title Available\ | JP2010518958A | Empty Field US | Empty Field US | B44C5/00\ A44C17/00\ A44C13/00 |
| 2588 | 3D printing | PACK FOR PREVENTING FALSIFICATION\ | KR100853559B1 | Empty Field US | KOREA MACH & MATERIALS INST (KR) | B65D33/22\ B65D33/20 |
| 2589 | 3D printing | HOLLOW THREE-DIMENSIONAL MANUFACTURING METHOD OF A CASTING PATTERN MODEL AND THE APPARATUS THEREOF\ | KR100837109B1 | LEE IN GU (KR)\ LEE YOON JU (KR)\ I YEONG CHEOL (KR) | FUTURECAST CO LTD (KR)\ LEE IN GU (KR)\ LEE YOON JU (KR)\ I YEONG CHEOL (KR) | B29C45/76\ B29C45/16 |
| 2590 | 3D printing | MANUFACTURE METHOD OF FOAM CERAMICS\ | KR100760040B1 | PARK MIN HWA (KR) | PARK MIN HWA (KR) | C04B38/00\ C04B14/10\ C04B35/16\ C04B38/02 |
| 2591 | 3D printing | A MANUFACTURING METHOD OF FILM FOR INSERT INMOLDING AND FILM FOR INSERT INMOLDING\ | KR20080066889A | JUNG HAE OK (KR)\ KIM WOO HWEAN (KR) | JUNG HAE OK (KR)\ KIM WOO HWEAN (KR) | B29D7/01 |
| 2592 | 3D printing | MANUFACTURE METHOD OF WOOD FOSSILS\ | KR100804471B1 | PARK MIN HWA (KR) | POZOPIA (KR) | B27K3/00\ B27K3/16\ B27K3/34 |
| 2593 | 3D printing | A METHOD WHICH PRINT A LOGO ON SILICON\ | KR100813479B1 | LEE BYONG SUP (KR) | LEE BYONG SUP (KR) | B41M1/30\ B41M1/32 |
| 2594 | 3D printing | METAL-JET UNIT\ | KR100735115B1 | LEE TAIK MIN (KR)\ KIM DONG SOO (KR)\ KIM KWANG YOUNG (KR)\ KANG TAE GOO (KR)\ YANG JEONG SOON (KR)\ CHOI BYUNG OH (KR)\ JO JEONG DAI (KR)\ YUN SO NAM (KR)\ HAM YOUNG BOG (KR)\ KIM CHUNG HWAN (KR) | KOREA MACH & MATERIALS INST (KR) | B22D23/00 |
| 2595 | 3D printing | EMBLEM OF AUTOMOBILE PART AND METHOD OF MANUFACTURING THE SAME\ | US7998383B2 | LEE CHANGWOO (KR)\ SHIM SANGWOO (KR)\ LEE HOJIN (KR)\ PARK SEUNGMOK (KR) | AUTOLIV DEV (SE) | B29C59/00 |
| 2596 | 3D printing | INK JET PRINTER AND PRINT HEAD DEVICE THEREOF\ | US7654639B2 | PARK JEONG-YEON (KR)\ PARK SANG-CHEOL (KR) | SAMSUNG ELECTRONICS CO LTD (KR) | B41J2/16\ B41J2/14 |
| 2597 | 3D printing | CUSHIONING SHEET FOR FRUIT PACKAGING BOX HAVING EXCELLENT CUSHIONING EFFECT AND BEAUTIFUL APPEARANCE AND MANUFACTURING METHOD THEREOF\ | KR20040082245A | KIM YONG KWAN US | KIM YONG KWAN US | B65D85/34 |
| 2598 | 3D printing | A BRAZING METHOD OF A THIN METAL PLATE FOR COOLING AN ELECTRONIC DEVICE\ | KR100524048B1 | HELSA AUTOMOTIVE GMBH & CO KG (DE) | MACDERMID GRAPHIC ARTS INC (US) | B23K1/008 |
| 2599 | 3D printing | METHOD FOR MANUFACTURING AROMATIC POROUS SINTERED METAL COMPACT\ | KR20040013342A | CHOI SUNG JO US | CHOI SUNG JO US | B22F3/11 |
| 2600 | 3D printing | DISPLAY PANEL FOR WASHING MACHINE\ | KR20040006269A | CHOI DONG HA US\ PARK HYE YONG US | LG ELECTRONICS INC (KR) | D06F39/00 |
| 2601 | 3D printing | METHODS FOR MAKING A PASTE OF MATERIALS TO FORM BUMPS FOR SCREEN PRINT PROCESSES\ | US6783801B2 | YI SANG-DON (KR) | SAMSUNG ELECTRONICS CO LTD (US) | H01L21/60\ H01L23/485 |
| 2602 | 3D printing | CASTING DIES FOR CYLINDER HEAD\ | KR20030031774A | KWAK HYEONG SIK (KR) | HYUNDAI MOTOR CO LTD (KR) | B22D15/00 |
| 2603 | 3D printing | TREATING LIQUID FOR FORMING LUBRICATING FILM\ | JP9169990A | UCHIDA YUKIO US\ TAKETSU HIROBUMI US\ FURUKAWA SHINYA US\ FUKUOKA TAKAYUKI US | NISSHIN STEEL CO LTD US\ NIPPON PAINT CO LTD US | B05D5/08\ B21D22/20\ C10M169/04\ C10M173/02\ C10N20/00\ C10N30/00\ C10N30/06\ C10N30/08\ C10N40/20\ C10N40/24\ C10N50/02 |
| 2604 | 3D printing | WATERPROOF TOOL FOR FITTING\ | JP9155896A | MATSUNO TAKEMI US | NAKATA COATING KK US | B29C41/46\ B29C41/20\ B29L31/10\ B29K105/12\ E06B1/62\ B29C41/18\ B29C41/38 |
| 2605 | 3D printing | MANUFACTURE OF DECORATIVE SHEET\ | JP8224796A | SUZUKI YUKIO US\ NISHIMURA IKUMASA US\ AIZAWA TETSUO US | TOPPAN PRINTING CO LTD US | B29C59/00\ B29L9/00\ B29C69/00\ B32B27/00\ B05D3/04\ B32B33/00\ B32B27/32\ B29C47/88\ B29D7/01\ B29K23/00\ B29L7/00\ B29C59/04\ B41M1/24\ B29C47/14\ B41M1/30 |
| 2606 | 3D printing | INK-JET PRINTER HEAD AND MANUFACTURE THEREOF\ | JP8174851A | HAMABUCHI KAZUFUMI US | MATSUSHITA ELECTRIC IND CO LTD (JP) | B41J2/16\ B41J2/05 |
| 2607 | 3D printing | POROUS METAL BODY PROCESS FOR PRODUCING THE SAME AND BATTERY PLATE FORMED THEREFROM\ | US5597665A | HARADA KEIZO (JP)\ ISHII MASAYUKI (JP)\ YAMANAKA SHOSAKU (JP) | SUMITOMO ELECTRIC INDUSTRIES (JP) | B22F3/11\ B22F5/00\ C22C1/08\ B01D39/20\ H01M4/80\ C23C14/20\ H01M4/02\ H01M4/66\ H01M6/16 |
| 2608 | 3D printing | NONWOVEN FABRIC OF POLYLACTATE-BASED FILAMENT AND ITS PRODUCTION\ | JPH0995850A | NAGAOKA KOICHI US\ MATSUOKA FUMIO US\ ICHINOSE NAOJI US | UNITIKA LTD US | D01F6/84\ D04H3/00\ D04H5/00 |
| 2609 | 3D printing | MELT-ADHESIVE COMPOSITE FIBERS PROCESS FOR PRODUCING THE SAME AND FUSED FABRIC OR SURFACE MATERIAL OBTAINED THEREFROM\[DE] SCHMETZKLEBENDE VERBUNDFASERN VERFAHREN ZU IHRER HERSTELLUNG UND DARAUS HERGESTELLTER SCHMELZGEBUNDENER STOFF ODER OBERFL?CHENMATERIAL\[FR] FIBRES COMPOSéES THERMOADHéSIVES PROCéDé POUR LEUR PRODUCTION ET éTOFFE FONDUE OU MATéRIAU DE SURFACE à PARTIR DE CELLES-CI\ | EP0696655A1 | SEI ISHIZAWA (JP)\ MASAYASU SUZUKI (JP)\ HIROKAZU TERADA (JP) | CHISSO CORP (JP) | A61F13/511\ A61F13/49\ D01F8/06\ D02G1/20\ D04H1/541\ D04H1/544 |
| 2610 | 3D printing | CHEMICAL VAPOR DEPOSITION RAW MATERIAL COMPRISING ORGANIC NICKEL COMPOUND AND CHEMICAL VAPOR DEPOSITION METHOD USING SAID CHEMICAL VAPOR DEPOSITION RAW MATERIAL\[FR] MATIèRE PREMIèRE DE DéP?T CHIMIQUE EN PHASE VAPEUR COMPRENANT UN COMPOSé ORGANIQUE DU NICKEL ET PROCéDé DE DéP?T CHIMIQUE EN PHASE VAPEUR UTILISANT LADITE MATIèRE PREMIèRE DE DéP?T CHIMIQUE EN PHASE VAPEUR\ | WO2014188629A1 | SUZUKI KAZUHARU (JP)\ SAITO MASAYUKI (JP)\ HARADA RYOSUKE (JP)\ NABEYA SHUNICHI (JP)\ MIYAZAKI SATOSHI (JP) | TANAKA PRECIOUS METAL IND (JP) | C07F15/04\ C23C16/18 |
| 2611 | 3D printing | THREE-DIMENSIONAL NETWORK STRUCTURE METHOD FOR PRODUCING THREE-DIMENSIONAL NETWORK STRUCTURE AND APPARATUS FOR PRODUCING THREE-DIMENSIONAL NETWORK STRUCTURE\ | JP2013108211A | SUMITOMO CHEMICAL CO US | SHIIENJI KK US | D04H1/736\ D04H3/14 |
| 2612 | 3D printing | METHOD FOR MANUFACTURING THREE-DIMENSIONAL SHAPED OBJECT\ | US2015017055A1 | HIGASHI YOSHIKAZU (JP)\ YOSHIDA NORIO (JP)\ ABE SATOSHI (JP) | PANASONIC CORP (JP) | B29C67/00\ B22F3/00\ B22F3/105\ B23K26/34 |
| 2613 | 3D printing | EXTERIOR CASE FOR ELECTRONIC APPARATUS\ | JP2012138594A | ALPS ELECTRIC CO LTD US | ALPS ELECTRIC CO LTD (JP) | B29C45/14\ H04M1/02\ H05K5/02 |
| 2614 | 3D printing | METHOD OF MANUFACTURING OA EQUIPMENT EXTERIOR COMPONENT\ | JP2012076462A | YUPO CORP US | TEIJIN LTD US\ TEIJIN CHEMICALS LTD US | B29C45/00\ C08J5/00\ C08L101/16\ C08K3/34\ C08L67/04\ B29K67/00 |
| 2615 | 3D printing | DECORATIVE SHEET FOR INJECTION MOLDING DECORATIVE MOLDED ARTICLE AND METHOD FOR SIMULTANEOUSLY CARRYING OUT INJECTION MOLDING AND DECORATING WORK\ | JP2012045938A | DAINIPPON PRINTING CO LTD US | DAINIPPON PRINTING CO LTD (JP) | B29C45/14\ B29C51/10\ B44C3/02\ B32B27/00\ B32B33/00 |
| 2616 | 3D printing | METHOD FOR MANUFACTURING INTEGRAL MOLDING AND INTEGRAL MOLDING\ | JP2013018226A | DAICEL PACK SYSTEMS LTD US\ POLYPLASTICS CO US\ DAIPLA SYSTEM TECHNOLOGY KK US | DAICEL PACK SYSTEMS LTD US\ POLYPLASTICS CO US\ DAIPLA SYSTEM TECHNOLOGY KK US | B29C45/14\ B29C51/10 |
| 2617 | 3D printing | METHOD FOR MANUFACTURING OF LIGHT GUIDE PLATE WITH PROTECTIVE FILM\ | JP2012252906A | TEIJIN CHEMICALS LTD US\ TEIJIN LTD US | SUMITOMO CHEMICAL CO (JP) | F21Y101/02\ G02B6/00\ F21S2/00 |
| 2618 | 3D printing | METHOD AND APPARATUS FOR MANUFACTURING THREE-DIMENSIONAL OBJECT\ | JP2012246541A | PARTHY KAI (DE) | PANASONIC CORP (JP) | B29C67/00\ B22F3/105\ B22F3/16 |
| 2619 | 3D printing | METHOD FOR PRODUCING THREE-DIMENSIONAL SHAPED OBJECT\ | US2014147328A1 | HIGASHI YOSHIKAZU (JP)\ TAKENAMI MASATAKA (JP)\ FUWA ISAO (JP)\ YOSHIDA NORIO (JP)\ ABE SATOSHI (JP)\ MATSUMOTO ISAMU (JP)\ UCHINONO YOSHIYUKI (JP) | PANASONIC CORP (JP)\ FUWA ISAO (JP)\ ABE SATOSHI (JP)\ TAKENAMI MASATAKA (JP)\ HIGASHI YOSHIKAZU (JP)\ YOSHIDA NORIO (JP)\ UCHINONO YOSHIYUKI (JP)\ MATSUMOTO ISAMU (JP) | B22F3/105\ B22F3/24 |
| 2620 | 3D printing | METHOD FOR MANUFACTURING ARTIFICIAL ORGAN AND ARTIFICIAL ORGAN\ | JP2012137514A | NICHIA CORP US | JMC KK US | G09B23/34 |
| 2621 | 3D printing | THERMOPLASTIC RESIN MOLDING MOLD FOR INJECTION-MOLDING THE MOLDING AND INJECTION MOLDING METHOD\ | JP2012086453A | PARTHY KAI (DE) | ONO SANGYO KK US | B29C45/37\ B29C45/72 |
| 2622 | 3D printing | VAPOR DEPOSITION MASK MANUFACTURING METHOD OF THE SAME AND VAPOR DEPOSITION METHOD\ | JP2012077328A | ONO SANGYO KK US | MITSUBISHI PLASTICS INC US\ LASER GIJUTSU SOGO KENKYUSHO US | B23K26/00\ B23K26/38\ C23C14/04\ B23K26/32 |
| 2623 | 3D printing | 癒光装置\ | JP5619533B2 | JMC KK US | PARK KYOUNG HOON (KR) | C03C8/08\ C03C8/14\ H01L33/56\ C03C8/04\ C03C8/10\ C03C8/20 |
| 2624 | 3D printing | LIGHT EMITTING DEVICE\ | JP2010268013A | OGAWA SATORU US | NICHIA CORP US | H01L33/56 |
| 2625 | 3D printing | WATER VAPOR PERMEATION PREVENTION POROUS FILM VENTILATION STRUCTURE AND CASE\ | JP2012030184A | PANASONIC CORP US | NIPPON SHEET GLASS CO LTD US | B01D71/26\ B01D53/22 |
| 2626 | 3D printing | INJECTION MOLDING COMPOSITE CONTAINER AND METHOD OF MANUFACTURING THE SAME\ | JP2012012019A | ZHUZHOU SMELTER GROUP CO LTD US\ UNIV CENTRAL SOUTH US\ HUNAN NONFERROUS METALS HOLDING GROUP CO LTD US | YUPO CORP US\ SANYO KAGAKU KOGYO KK US | B29C45/14\ B65D25/20 |
| 2627 | 3D printing | METHOD FOR MANUFACTURING THREE-DIMENSIONALLY SHAPED OBJECT THREE-DIMENSIONALLY SHAPED OBJECT OBTAINED THEREBY AND METHOD FOR MANUFACTURING MOLDED ARTICLE\ | US2013069282A1 | FUWA ISAO (JP)\ YOSHIDA NORIO (JP)\ ABE SATOSHI (JP)\ UCHINONO YOSHIYUKI (JP)\ MORIMOTO KAZUHO (JP) | PANASONIC CORP (JP)\ FUWA ISAO (JP)\ OPM LAB CO LTD (JP)\ ABE SATOSHI (JP)\ YOSHIDA NORIO (JP)\ UCHINONO YOSHIYUKI (JP)\ MORIMOTO KAZUHO (JP) | B29C33/38\ B29C45/00 |
| 2628 | 3D printing | METHOD FOR MANUFACTURING THREE-DIMENSIONALLY SHAPED OBJECT AND THREE-DIMENSIONALLY SHAPED OBJECT\ | US2013075575A1 | FUWA ISAO (JP)\ YOSHIDA NORIO (JP)\ ABE SATOSHI (JP)\ UCHINONO YOSHIYUKI (JP)\ MORIMOTO KAZUHO (JP) | PANASONIC CORP (JP)\ FUWA ISAO (JP)\ OPM LAB CO LTD (JP)\ ABE SATOSHI (JP)\ YOSHIDA NORIO (JP)\ UCHINONO YOSHIYUKI (JP)\ MORIMOTO KAZUHO (JP) | B29C33/02 |
| 2629 | 3D printing | DRAWING METHOD OF SOLID FOOD\ | JP2011239765A | NIPPON SHEET GLASS CO LTD US | KUWABARA YUJI US\ KUWAHARA TAKU US | A23L1/00 |
| 2630 | 3D printing | METHOD FOR MANUFACTURING THREE-DIMENSIONAL SHAPED OBJECT AND THREE-DIMENSIONAL SHAPED OBJECT\ | US2012308781A1 | HIGASHI YOSHIKAZU (JP)\ TAKENAMI MASATAKA (JP)\ FUWA ISAO (JP)\ YOSHIDA NORIO (JP)\ ABE SATOSHI (JP)\ MATUMOTO ISAMU (JP) | PANASONIC CORP (JP)\ FUWA ISAO (JP)\ ABE SATOSHI (JP)\ TAKENAMI MASATAKA (JP)\ HIGASHI YOSHIKAZU (JP)\ YOSHIDA NORIO (JP)\ MATUMOTO ISAMU (JP) | B22F3/105\ B32B7/04 |
| 2631 | 3D printing | 複合体およびその裊造方法\ | JP5640190B2 | INFO VISION OPTOELECTRONICS KUNSHAN CO LTD US | NEGOPACK CO LTD (KR)\ LEE BO YOUNG (KR) | D04H3/16\ B32B3/24 |
| 2632 | 3D printing | SPHERICAL HYDROTALCITE COMPOUND AND RESIN COMPOSITION FOR SEALING ELECTRONIC COMPONENT\ | US2012298912A1 | ONO YASUHARU (JP) | ONO YASUHARU (JP) | C08K3/26 |
| 2633 | 3D printing | METHOD OF MANUFACTURING LENS SHEET LENS SHEET ILLUMINATOR AND DISPLAY\ | JP2011145515A | NAKAGOME TOMOHIRO US\ SAKAKI YUICHI US\ SAKAI NATSUKA US\ KIMURA KAZUTERU US | TOPPAN PRINTING CO LTD US | G02F1/1335\ G02B3/00\ F21S2/00\ G02B5/02\ F21V5/00\ F21Y103/00 |
| 2634 | 3D printing | HIGH HEAT-RESISTANCE POWDER FOR FORMING INKJET POWDER LAMINATION MOLD\ | JP2011051010A | TOBA ATSUYA US\ INANO HIROYUKI US\ HIRANO SHIGEKI US | HOKKAIDO RES ORGANIZATION US | B22C1/10\ B22C9/02\ B22C1/18\ B22C1/00 |
| 2635 | 3D printing | METHOD OF MANUFACTURING POWER RECEIVING SYSTEM HOUSING IN WIRELESS CHARGING SYSTEM\ | JP2011109546A | TAKEUCHI SHINYA US | NISSHA PRINTING US | H04M1/02\ H02J7/00 |
| 2636 | 3D printing | RESIN FOAMED SHEET\[FR] FEUILLE DE MOUSSE DE RéSINE\ | WO2011062141A1 | IWATA MASAYOSHI (JP)\ KOGI TOSHINOBU (JP) | SEKISUI PLASTICS (JP)\ IWATA MASAYOSHI (JP)\ KOGI TOSHINOBU (JP) | B32B5/18\ B32B27/32\ C08J9/06\ C08L23/10 |
| 2637 | 3D printing | PROCESS FOR PRODUCING THREE-DIMENSIONALLY SHAPED OBJECT AND DEVICE FOR PRODUCING SAME\ | US2012251378A1 | HIGASHI YOSHIKAZU (JP)\ FUWA ISAO (JP)\ YOSHIDA NORIO (JP)\ ABE SATOSHI (JP) | PANASONIC CORP (JP)\ FUWA ISAO (JP)\ ABE SATOSHI (JP)\ HIGASHI YOSHIKAZU (JP)\ YOSHIDA NORIO (JP) | B29C35/08\ B22F3/105\ B29C67/24 |
| 2638 | 3D printing | METHOD AND APPARATUS FOR MANUFACTURING THREE-DIMENSIONAL PATTERN SHEET AND FILM USING ULTRASONIC GENERATOR\ | JP2011062972A | HOSOYA SHUHEI US | TOPPAN PRINTING CO LTD US | B29C47/88\ B29L7/00\ B29L11/00\ B29C59/04 |
| 2639 | 3D printing | ELECTROCONDUCTIVE SHEET\ | JP2011056668A | TERUI TOMOKO US\ TERAMAE KIMITO US | LONSEAL KOGYO KK US | B29L9/00\ B32B27/18\ H01B1/20\ B29K101/12\ B29K105/16\ B29C43/24\ B29C43/18\ B29L7/00\ B29C43/34\ C08K7/02\ C08L101/12\ B29K105/12 |
| 2640 | 3D printing | METHOD FOR PRODUCING IMPLANT MADE OF TITANIUM ALLOY\ | JP2011052289A | KURAMOTO KOICHI US\ DOI KENJI US\ ISHIZAKA HARUHIKO US\ SUGINO ATSUSHI US\ FUKUDA EIJI US | NAKASHIMA MEDICAL CO LTD US | B22F3/105\ B22F3/16\ B22F1/00\ C22C14/00 |
| 2641 | 3D printing | MO COMPACT HAVING THREE-DIMENSIONAL SHAPE AND METHOD FOR MANUFACTURING THE SAME\ | JP2011068983A | YANAGIYA AKIHIKO US\ SAWADA TOSHIYUKI US\ KISHIDA ATSUSHI US | SANYO SPECIAL STEEL CO LTD US | B22F3/15\ C22C1/04\ C22C27/04 |
| 2642 | 3D printing | POWDER MATERIAL FOR LAMINATE MOLDING AND POWDER LAMINATE MOLDING METHOD\ | JP2011021218A | KYOGOKU HIDEKI US\ YOSHIDA HIRONOBU US\ HAGIWARA TADASHI US | ASPECT INC US\ UNIV KINKI US | B22F3/105\ B22F3/16\ B22F1/00 |
| 2643 | 3D printing | METHOD FOR MANUFACTURING THREE-DIMENSIONAL SHAPED OBJECT AND THREE-DIMENSIONAL SHAPED OBJECT OBTAINED BY THE SAME\ | US2012093674A1 | HIGASHI YOSHIKAZU (JP)\ TAKENAMI MASATAKA (JP)\ FUWA ISAO (JP)\ ABE SATOSHI (JP) | FUWA ISAO (JP)\ PANASONIC ELEC WORKS CO LTD (JP)\ ABE SATOSHI (JP)\ TAKENAMI MASATAKA (JP)\ HIGASHI YOSHIKAZU (JP) | B22F3/105\ B22C9/00\ B29C33/38\ B22F7/00\ H05B6/00 |
| 2644 | 3D printing | METHOD FOR FORMING SHAPE WITH ELECTRON BEAM\ | JP2010261072A | TATSUMI RYUJI US | HTL KK US | B22F3/105\ B22F3/16\ H01J37/305 |
| 2645 | 3D printing | MATTRESS FOR PREVENTING DEW CONDENSATION AND METHOD OF MANUFACTURING THE MATTRESS\ | JP2010279687A | TAKAOKA NOBUYUKI (JP) | SHIIENJI KK US | A47C27/12 |
| 2646 | 3D printing | INJECTION MOLDING IN-MOLD DECORATION METHOD\ | JP2009143247A | NAGI YOSHIYUKI US\ ATAKE HIROYUKI (JP)\ SUGA KAZUHIRO (JP) | DAINIPPON PRINTING CO LTD (JP) | B29C45/14\ B29L9/00\ B32B27/00\ B32B33/00 |
| 2647 | 3D printing | DECORATIVE SHEET DECORATIVE MOLDED PRODUCT AND INJECTION MOLDING IN-MOLD DECORATION METHOD\ | JP2009143246A | NAGI YOSHIYUKI US\ ATAKE HIROYUKI (JP)\ SUGA KAZUHIRO (JP) | DAINIPPON PRINTING CO LTD (JP) | B32B27/00\ B29C69/02 |
| 2648 | 3D printing | NATURAL MARBLE-LIKE CRYSTALLIZED GLASS NATURAL MARBLE-LIKE CRYSTALLIZED GLASS ARTICLE AND METHOD FOR MANUFACTURING THE SAME\ | JP2010248056A | YAMADA AKIHITO US | NIPPON ELECTRIC GLASS CO US | C03C10/04\ C03B31/00\ C03B32/02 |
| 2649 | 3D printing | METHOD OF PRODUCING THREE-DIMENSIONAL SHAPED ARTICLE AND THREE-DIMENSIONAL SHAPED ARTICLE OBTAINED FROM THE SAME\ | JP2010215971A | YOSHIDA TOKUO US\ FUWA ISAO (JP)\ ABE SATOSHI (JP)\ TAKENAMI MASATAKA (JP) | PANASONIC ELEC WORKS CO LTD (JP) | B22F3/105\ B22F3/16\ B22F3/24\ C22C38/00 |
| 2650 | 3D printing | METHOD FOR MANUFACTURING THREE-DIMENSIONAL SHAPED OBJECT AND THREE-DIMENSIONAL SHAPED OBJECT OBTAINED BY THE SAME\ | US2012041586A1 | HIGASHI YOSHIKAZU (JP)\ TAKENAMI MASATAKA (JP)\ FUWA ISAO (JP)\ YOSHIDA NORIO (JP)\ ABE SATOSHI (JP) | FUWA ISAO (JP)\ PANASONIC ELEC WORKS CO LTD (JP)\ ABE SATOSHI (JP)\ TAKENAMI MASATAKA (JP)\ HIGASHI YOSHIKAZU (JP)\ YOSHIDA NORIO (JP) | G06F19/00 |
| 2651 | 3D printing | RESIN PARTICLE COMPOSITION\ | JP2010174068A | KAWASHIMA NAOYUKI US\ MUTSUKA YASUAKI US\ MIYAKI NOBUYUKI US | JSR CORP (JP) | C08L33/00\ C08L65/00\ C08L45/00 |
| 2652 | 3D printing | LAMINATION FORMING DEVICE AND METHOD FOR FORMING THREE-DIMENSIONAL MOLDING\ | JP2010132960A | YOSHIDA TOKUO US\ AZUMA YOSHIKAZU US\ FUWA ISAO (JP)\ ABE SATOSHI (JP)\ TAKENAMI MASATAKA (JP) | PANASONIC ELEC WORKS CO LTD (JP) | B22F3/16\ B22F3/11 |
| 2653 | 3D printing | THREE-DIMENSIONAL SHAPED ARTICLE AND METHOD FOR PRODUCING THE SAME\ | JP2010121187A | YOSHIDA TOKUO US\ SUGITA TOSHIO US\ ABE SATOSHI (JP) | PANASONIC ELEC WORKS CO LTD (JP) | B29C67/00\ B22F3/105\ B22F7/06\ B22F5/10\ B29C33/40 |
| 2654 | 3D printing | METHOD AND APPARATUS FOR PRODUCING A THREE-DIMENSIONALLY SHAPED OBJECT\ | US8282377B2 | HIGASHI YOSHIKAZU (JP)\ TAKENAMI MASATAKA (JP)\ YOSHIDA NORIO (JP)\ ABE SATOSHI (JP) | PANASONIC CORP (JP)\ ABE SATOSHI (JP)\ TAKENAMI MASATAKA (JP)\ HIGASHI YOSHIKAZU (JP)\ YOSHIDA NORIO (JP) | B29C35/08 |
| 2655 | 3D printing | APPARATUS FOR PRODUCING A LAMINATED OBJECT\ | US2010031882A1 | HIGASHI YOSHIKAZU (JP)\ TAKENAMI MASATAKA (JP)\ FUWA ISAO (JP)\ YOSHIDA NORIO (JP)\ ABE SATOSHI (JP) | PANASONIC ELEC WORKS CO LTD (JP) | B05C9/12\ B05C19/00 |
| 2656 | 3D printing | VEGETABLE-ORIGINATED COMPONENT-CONTAINING ADHESIVE SHEET\ | JP2009280676A | KUNUKATANA MASAHIRO US\ YAMAKAWA YASUKO US | KUREHA SENI KK US | C09J7/04\ C09J177/00 |
| 2657 | 3D printing | MANUFACTURING APPARATUS AND METHOD OF MOLDING\ | JP2008201137A | ITO HIROSHI US\ YAKIMOTO KAZUTOSHI US\ SHIROGANEYA TSUKASA US\ NAITO AKIHIRO US | JAPAN STEEL WORKS LTD US | B29C43/02\ B29C43/34 |
| 2658 | 3D printing | METHOD OF PRODUCING TONER\ | JP2009175632A | IGA TAKASHI US\ KANEDA TAKUYA US | NIPPON ZEON CO US | G03G9/087\ G03G9/08 |
| 2659 | 3D printing | METHOD OF INJECTION MOLDING AND INJECTION MOLDING MACHINE\ | JP2009119700A | MATSUSHITA YOSHIYUKI US | ASMO CO LTD (JP) | B29C45/23 |
| 2660 | 3D printing | REINFORCED POLYAMIDE RESIN COMPOSITION FOR BLOW MOLDING AND BLOW MOLDED PRODUCT USING IT\ | JP2009132908A | FUJII YASUHITO US\ SHIGA KENJI US\ SAKAI SATOSHI US | TOYO BOSEKI (JP) | C08L63/00\ B29L31/30\ C08L77/00\ B29K77/00\ B29C49/00\ B29K105/06\ C08K7/02 |
| 2661 | 3D printing | METHOD FOR MODIFYING SURFACE OF PLASTIC MOLDED BODY METHOD FOR FORMING METAL FILM INCLUDING THE SAME AND PLASTIC COMPONENT THEREOF\ | JP2009113266A | ANO TETSUYA US\ YUSA ATSUSHI US\ NOMURA YOSHIYUKI US | HITACHI MAXELL (JP) | B29C45/00\ B29L31/34\ C23C18/22 |
| 2662 | 3D printing | METHOD FOR SPINNING POLYESTER MONOFILAMENT HAVING SMALL FINENESS\ | JP2009084712A | YAMAMOTO HIROSHI US\ OGUCHI TOMOHIRO US\ KUROKAWA HIROYUKI US | TORAY INDUSTRIES (JP) | D01D5/088\ D01F6/62 |
| 2663 | 3D printing | BIAXIALLY ORIENTED LAMINATED POLYESTER FILM FOR MOLDING AND SIMULTANEOUS DECORATION\ | JP2009029073A | OKUYAMA SHUNSUKE US\ KIMURA MANABU US | TEIJIN DUPONT FILMS JAPAN LTD US | B29C45/14\ B29L9/00\ B29L7/00\ B32B27/36\ B29C55/12\ B29K67/00 |
| 2664 | 3D printing | OPTICAL MOLDED LAMINATE\ | JP2008276150A | NAKAKOSHI YASUNOBU US | NAKAKOSHI YASUNOBU US | B29C45/14\ B29L9/00\ B29C69/00\ B29L11/00\ G02B5/30\ G02C7/12\ B29D11/00 |
| 2665 | 3D printing | LAMINATION SHAPING APPARATUS\ | US2010006228A1 | HIGASHI YOSHIKAZU (JP)\ FUWA ISAO (JP)\ YOSHIDA NORIO (JP)\ ABE SATOSHI (JP) | ABE SATOSHI (JP)\ HIGASHI YOSHIKAZU (JP)\ YOSHIDA NORIO (JP)\ FUWA ISAO (JP) | B32B37/00 |
| 2666 | 3D printing | METHOD FOR MANUFACTURING THREE-DIMENSIONALLY SHAPED STRUCTURE\ | JP2008280582A | AZUMA YOSHIKAZU US\ KUSANO NOBORU US\ FUWA ISAO (JP)\ ABE SATOSHI (JP)\ TAKENAMI MASATAKA (JP)\ SHIMIZU TAKASHI (JP) | MATSUSHITA ELECTRIC WORKS LTD (JP) | B22F3/105\ B22F3/16 |
| 2667 | 3D printing | METHOD FOR MANUFACTURING THREE-DIMENSIONALLY SHAPED STRUCTURE\ | JP2008280581A | FUWA ISAO (JP)\ ABE SATOSHI (JP) | MATSUSHITA ELECTRIC WORKS LTD (JP) | B29C67/00\ B22F3/16 |
| 2668 | 3D printing | DIRECT MOLDING METHOD AND DIRECT MOLDING DEVICE OF POLYMER MATERIAL\ | JP2008194968A | NISHIMURA OSAMU US\ IMOTO TOSHIYUKI US\ HOJO MASAKI US\ ADACHI TAIJI US\ NISHINO TAKASHI US | IMOTO SEISAKUSHO KK US\ UNIV KYOTO US\ UNIV KOBE US | B29C67/00\ A61L27/00 |
| 2669 | 3D printing | MANUFACTURING METHOD OF OPERATION PANEL\ | JP2008183848A | HAYASHI SEIJI US\ TAKAHASHI TATSUMI US\ ITO YOSHIHIRO US | SANWA SCREEN MEIBAN KK US | B29C45/14\ B29C69/02\ D06F39/00 |
| 2670 | 3D printing | METHOD FOR PRODUCING THREE-DIMENSIONAL MOLDING AND MATERIAL\ | JP2008184623A | FUWA ISAO (JP) | MATSUSHITA ELECTRIC WORKS LTD (JP) | B22F3/105\ B22F3/16\ B22F3/24\ B22F3/02\ C22C38/00 |
| 2671 | 3D printing | METHOD FOR PRODUCING THREE-DIMENSIONAL MOLDING\ | JP2008184622A | FUWA ISAO (JP) | MATSUSHITA ELECTRIC WORKS LTD (JP) | B22F3/105\ B22F1/00 |
| 2672 | 3D printing | THERMOPLASTIC TRANSPARENT RESIN POWDER METHOD FOR PRODUCING THE SAME METHOD FOR APPLYING THE SAME AND METHOD FOR CREATING PRESSURE-BONDED PRINT USING THE SAME\ | JP2008181027A | TAMAGAWA YOICHI US\ KANEMURA TOSHIAKI US\ YORIFUJI TAKAO US\ MAEDA KATSUMI US\ MITSUI GOSHI US\ IEGAKI YUICHIRO US\ NIINUMA HIDEYOSHI US\ TAKAHASHI HIDEKI US | CASIO COMPUTER CO LTD US\ CASIO ELECTRONICS CO LTD US | G03G9/087\ G03G9/08\ G03G9/097\ G03G15/20\ G03G8/00\ B42D15/02 |
| 2673 | 3D printing | INK COMPOSITION RECORDING METHOD AND RECORD AND METHOD FOR JUDGING AUTHENTICITY\ | JP2007177248A | NAKAMURA HIROTO US | SEIKO EPSON CORP (JP) | C09D11/00\ B41J2/01\ B41M5/00 |
| 2674 | 3D printing | QUARTZ-BASED OPTICAL WAVEGUIDE AND MANUFACTURING METHOD FOR THE SAME\ | JP2008165014A | TANAKA KOTARO US | HITACHI CABLE (JP) | G02B6/12\ G02B6/13\ G02F1/01 |
| 2675 | 3D printing | THERMOPLASTIC RESIN CASING AND METHOD FOR FABRICATING THE SAME\ | US2008145578A1 | YUBA TAKASHI (JP)\ AKIEDA SHINICHIRO (JP)\ ARITA TAKASHI (JP) | FUJITSU COMPONENT LTD (JP) | B29C45/00\ B32B1/04 |
| 2676 | 3D printing | METHOD OF FORMING THREE-DIMENSIONAL PRINTING IMAGE\[FR] PROCéDé DE FORMATION D'UNE IMAGE D'IMPRESSION EN TROIS DIMENSIONS\ | WO2008075450A1 | OZAWA CHIZUO (JP) | MASTERMIND CO LTD (JP)\ OZAWA CHIZUO (JP) | B41J2/01\ B41M5/00 |
| 2677 | 3D printing | METHOD OF MANUFACTURING FILM INSERTED MOLDED ARTICLE\ | JP2008168613A | HAYASHI SEIJI US\ MATSUOKA KIYOMI US\ GOTO YOSHIHIDE US\ GOTO EIICHIRO US | SANWA SCREEN MEIBAN KK US | B29C45/14\ B29L9/00\ B29C59/02\ B29C69/00 |
| 2678 | 3D printing | METHOD AND APPARATUS FOR PRODUCING CELLULOSE RESIN FILM AND CELLULOSE RESIN FILM AND FUNCTIONAL FILM\ | US2008075922A1 | UEDA TADASHI (JP) | FUJIFILM CORP (JP) | C08J5/18 |
| 2679 | 3D printing | APPARATUS FOR PRODUCING TONER PRECURSOR AND METHOD FOR THE SAME FIBROUS TONER PRECURSOR APPARATUS FOR PRODUCING TONER AND ELECTROPHOTOGRAPHIC TONER\ | JP2008096974A | KINOSHITA NAOTOSHI US\ TANAKA TETSUYA US\ KAWAMOTO MASAHIRO US | RICOH KK (JP) | G03G9/087\ G03G9/08 |
| 2680 | 3D printing | IMAGE FORMING APPARATUS\ | JP2008065156A | TAKAMATSU MASAHIRO US\ NOGAMI YUTAKA US | FUJI XEROX CO LTD US | G03G15/01 |
| 2681 | 3D printing | METHOD AND APPARATUS FOR PRODUCING GRANULAR CRYSTAL\ | US2008029020A1 | SUGAWARA SHIN (JP)\ TAKAHASHI EIGO (JP)\ ARIMUNE HISAO (JP) | KYOCERA CORP (JP) | C30B30/06 |
| 2682 | 3D printing | HOLLOW BODY MOLDING DEVICE\ | JP2007296700A | YUSHIMO MASASHI US\ TANIGAKI KENJI US\ NISHIDA SHOZO US\ KAMIMURA TAKASHI US | JAPAN STEEL WORKS LTD US\ AISIN SEIKI (JP) | B29C45/17\ B29C69/02\ B29L22/00\ B29C65/14 |
| 2683 | 3D printing | ELECTRONIC PICTURE IMAGE-RECEIVING MATERIAL\ | JP2007249126A | YAMAZAKI CHIKAMASA US | KONICA MINOLTA HOLDINGS INC (JP) | G03G7/00 |
| 2684 | 3D printing | OA EQUIPMENT EXTERIOR PART\ | JP2007191549A | KONDO FUMITAKA US\ HIRONAKA KATSUHIKO US\ INO KEIICHIRO US\ MATSUNO YUICHI US\ TOYOHARA KIYOTSUNA US\ SUZUKI HIROTAKA US\ NONOKAWA RYUJI US\ MATSUDA TAKAAKI US | TEIJIN LTD US\ TEIJIN CHEMICALS LTD US | B29C45/00\ C08J5/00\ C08L101/16\ C08K3/34\ C08L67/04 |
| 2685 | 3D printing | ELECTRONIC EQUIPMENT EXTERIOR PART\ | JP2007191548A | KONDO FUMITAKA US\ HIRONAKA KATSUHIKO US\ INO KEIICHIRO US\ MATSUNO YUICHI US\ TOYOHARA KIYOTSUNA US\ SUZUKI HIROTAKA US\ NONOKAWA RYUJI US\ MATSUDA TAKAAKI US | TEIJIN LTD US\ TEIJIN CHEMICALS LTD US | C08J5/00\ C08L101/16\ C08K3/34\ C08L67/04 |
| 2686 | 3D printing | ELECTRIC/ELECTRONIC COMPONENT\ | JP2007191547A | KONDO FUMITAKA US\ HIRONAKA KATSUHIKO US\ INO KEIICHIRO US\ MATSUNO YUICHI US\ TOYOHARA KIYOTSUNA US\ SUZUKI HIROTAKA US\ NONOKAWA RYUJI US\ MATSUDA TAKAAKI US | TEIJIN LTD US\ TEIJIN CHEMICALS LTD US | B29C45/00\ C08J5/00\ C08L101/16\ C08K3/34\ C08L67/04\ B29L31/34\ B29K67/00 |
| 2687 | 3D printing | AUTOMOTIVE COMPONENT\ | JP2007191546A | KONDO FUMITAKA US\ HIRONAKA KATSUHIKO US\ INO KEIICHIRO US\ MATSUNO YUICHI US\ TOYOHARA KIYOTSUNA US\ SUZUKI HIROTAKA US\ NONOKAWA RYUJI US\ MATSUDA TAKAAKI US | TEIJIN LTD US\ TEIJIN CHEMICALS LTD US | B29C45/73\ C08J5/00\ C08L101/16\ C08K3/34\ C08L67/04\ B29K67/00 |
| 2688 | 3D printing | ELECTROPHOTOGRAPHIC IMAGE-RECEIVING MATERIAL\[FR] MATéRIAU DE RéCEPTION D'IMAGE éLECTROPHOTOGRAPHIQUE\ | WO2007083467A1 | YAMAZAKI KATSUMASA (JP) | KONICA MINOLTA HOLDINGS INC (JP)\ YAMAZAKI KATSUMASA (JP) | B32B27/10\ B32B5/18\ D21H27/00\ D21H27/30\ B32B7/02\ G03G7/00\ D21H19/70\ D21H21/56 |
| 2689 | 3D printing | MANUFACTURING METHOD OF RESIN COMPOSITE FORMED PRODUCT\ | JP2008100491A | HASEGAWA KATSUYUKI US | ALMIGHTY KK US | C08L101/00\ C08K3/22\ B29K105/16\ B29C43/02\ C08L101/16 |
| 2690 | 3D printing | METHOD FOR MANUFACTURING RESIN COMPOSITE FORMED PRODUCT\[DE] HERSTELLUNGSVERFAHREN FüR EIN HARZVERBUND-FORMPRODUKT\[FR] PROCéDé DE FABRICATION D'UN PRODUIT FORMé DE COMPOSITE RéSINE\ | EP1795319A2 | HASEGAWA KATUYUKI (JP) | HASEGAWA KATUYUKI (JP) | B27N5/00\ B27N3/00 |
| 2691 | 3D printing | METHOD FOR MANUFACTURING RESIN COMPOSITE FORMED PRODUCT\ | US2007132133A1 | HASEGAWA KATUYUKI (JP) | HASEGAWA KATUYUKI (JP) | D04H1/00 |
| 2692 | 3D printing | METHOD FOR MANUFACTURING RESIN COMPOSITE FORMED PRODUCT\ | CN1978165A | KATUYUKI HASEGAWA (JP) | KATUYUKI HASEGAWA (JP) | B27N5/00\ B27N3/02 |
| 2693 | 3D printing | THERMAL TRANSFER ACCEPTANCE SHEET\ | JP2007152697A | SHINOHARA HIDEAKI US\ TANAKA YOSHIMASA US | OJI PAPER CO US | B41M5/50\ B41M5/52\ B41M5/382 |
| 2694 | 3D printing | THERMALLY INSULATED CONTAINER MANUFACTURING METHOD THEREFOR AND LAMINATED LABEL ATTACHED TO THE CONTAINER\ | JP2007153359A | YAMADA MINORU US\ YAMAZAKI DAISUKE US\ KOBAYASHI SATOSHI US\ MASHITA ATSUSHI US\ MURAWAKI YASUHIRO US | KISHIMOTO SANGYO CO US | G09F3/02\ B65D81/38\ B65D1/28\ B65D25/20\ B65D25/36 |
| 2695 | 3D printing | METHOD AND APPARATUS FOR PRODUCING THREE-DIMENSIONAL STRUCTURE\ | US2009014916A1 | NAGAI HISAO (JP)\ YOSHIDA HIDEHIRO (JP)\ FURUKAWA TAKAYUKI (JP)\ INOUE TAKASHI (JP) | MATSUSHITA ELECTRIC IND CO LTD (JP) | B81C99/00\ B29C41/08\ B05B13/02 |
| 2696 | 3D printing | METHOD FOR MANUFACTURING RING-INTEGRATED DISPLAY BOARD\ | JP2007125832A | KOJIMA TAKUO US\ YOSHIZAKA KIMIYASU US | SAKAIYA KK US | B29C45/14\ B29L31/00\ B29C33/12 |
| 2697 | 3D printing | INK JET RECORDING METHOD AND RECORDED MATTER\ | JP2007076228A | NOJIMA TAKAHIKO US\ ITO HIROAKI US | KONICA MINOLTA PHOTO IMAGING US | C09D11/00\ B41J2/01\ B41M5/00\ B41M5/50\ B41M5/52 |
| 2698 | 3D printing | SYSTEM\ | JP2007017922A | YOSHINO KAZUTORA US | YOSHINO KAZUTORA US | G09G1/00 |
| 2699 | 3D printing | ORIENTED FILM AND POLARIZING PLATE USING THE SAME\ | JP2007031537A | ONO MITSUMASA US | TEIJIN DUPONT FILMS JAPAN LTD US | G02F1/1335\ C08J5/18\ B29C55/02\ B29K33/04\ B29K79/00\ C08F220/18\ C08F222/40\ G02B5/30 |
| 2700 | 3D printing | UNIFORM MELT SIMULATION METHOD OF EXTRUSION SCREW COMPUTER PROGRAM FOR EXECUTING IT AND UNIFORM MELT SIMULATION APPARATUS OF EXTRUSION SCREW\ | JP2007007951A | ENDO KUNIAKI US\ HIROSE TAKASHI US\ NOGI TAKAYUKI US\ YOSHIKAWA HIDEO US | TOSHIBA MACHINE CO LTD US | B29C47/38 |
| 2701 | 3D printing | HIGH-STRENGTH COMPLEX SOFT MAGNETIC MATERIAL HAVING HIGH STRENGTH HIGH MAGNETIC FLUX DENSITY HIGH RESISTANCE AND LESS IRON LOSS AND MANUFACTURING METHOD THEREOF\ | JP2006332525A | IGARASHI KAZUNORI US\ WATANABE MUNEAKI US\ NAKAYAMA RYOJI US\ MORIMOTO KOICHIRO US | MITSUBISHI MATERIALS PMG CORP US | B22F3/00\ B22F3/24\ B22F1/02\ H01F1/33\ H01F1/24 |
| 2702 | 3D printing | HIGH-STRENGTH COMPLEX SOFT MAGNETIC MATERIAL HAVING HIGH STRENGTH HIGH MAGNETIC FLUX DENSITY HIGH RESISTANCE AND LESS IRON LOSS AND MANUFACTURING METHOD THEREOF\ | JP2006332524A | IGARASHI KAZUNORI US\ WATANABE MUNEAKI US\ NAKAYAMA RYOJI US\ MORIMOTO KOICHIRO US | MITSUBISHI MATERIALS PMG CORP US | B22F3/00\ B22F1/02\ H01F1/33\ H01F1/24 |
| 2703 | 3D printing | INK JET PRINT HEAD SUBSTRATE INK JET PRINT HEAD INK JET PRINTING APPARATUS AND METHOD OF MANUFACTURING INK JET PRINT HEAD SUBSTRATE\ | US2006256160A1 | OZAKI TERUO (JP)\ SAITO ICHIRO (JP)\ YOKOYAMA SAKAI (JP)\ ONO KENJI (JP)\ IBE SATOSHI (JP)\ SHIBATA KAZUAKI (JP)\ SAKAI TOSHIYASU (JP) | CANON KK (JP) | B41J2/04 |
| 2704 | 3D printing | RE-REMOVABLE PAPER LABEL AND CONTAINER WITH RE-REMOVABLE PAPER LABEL\ | JP2006267990A | ARITA YOICHI US\ TASHIRO CHIYO US | KOTOBUKI SEIHAN PRINTING CO US | C09J201/00\ B65D65/40\ C09J7/02\ B65D25/20\ G09F3/10 |
| 2705 | 3D printing | METHOD FOR PRODUCING DEPOSITED DIAMOND FILM\ | JP2006290687A | ISSHIKI HIDEO US | UNIV ELECTRO COMMUNICATIONS US\ CAMPUS CREATE CO LTD US | H04R31/00\ C30B29/04\ H04R7/02 |
| 2706 | 3D printing | METHOD FOR PRODUCING POLYACETAL RESIN COMPOSITION REDUCED IN FORMALDEHYDE CONTENT\ | JP2006291001A | SUNAGA DAISUKE US\ NAGAI SATOSHI US\ OKAMURA AKIRA US | MITSUBISHI GAS CHEMICAL CO US | C08G2/28 |
| 2707 | 3D printing | POWDERY MATERIAL TO BE SINTERED BY LASER MANUFACTURING METHOD THEREFOR THREE-DIMENSIONAL STRUCTURE AND MANUFACTURING METHOD THEREFOR\ | JP2006257463A | SUDO HAJIME US\ NAGUMO TSUTOMU US\ KAWASHIMA YOSHINARI US\ MORIMOTO JUNJI US\ TAKAHASHI HIDEKAZU US\ KATORI KENJI US\ MOTOMURA ISATO US\ MATSUKI HIROSHI US | SONY CORP (JP) | B22F3/105\ B22F3/16\ C22C38/00\ B22F1/02 |
| 2708 | 3D printing | INK DEAERATOR FOR INK-JET PRINTER\ | JP2006231809A | KOSEKI OSAMU US | SII PRINTEK INC US | B41J2/175 |
| 2709 | 3D printing | FOAMING TOOL FOR POWDER SOAP\ | JP2006204390A | OTA TANTO US\ SAITO TATSUKI US\ NAKANISHI YUMIKO US | INOUE MTP KK US | A47K5/14 |
| 2710 | 3D printing | MOLTEN RESIN EXTRUDING LAMINATING AND SHAPING METHOD AND APPARATUS THEREFOR\ | JP2006192710A | ISHITANI SHIGEKI US | SEKISUI CHEMICAL CO LTD US | B29C67/00 |
| 2711 | 3D printing | MOLTEN RESIN EXTRUDING LAMINATING AND SHAPING METHOD AND APPARATUS THEREFOR\ | JP2006192616A | ISHITANI SHIGEKI US\ IWASAKI JUNYA US | SEKISUI CHEMICAL CO LTD US | B29C67/00 |
| 2712 | 3D printing | BATTERY\ | JP2006164638A | MIYAZAWA SHOTA US | MIYAZAWA SHOTA US | H01M4/02\ H01M6/04\ H01M4/04 |
| 2713 | 3D printing | AU-SN ALLOY LAMINATE SOLDER AND ITS MANUFACTURING METHOD\ | JP2006110626A | OBATA MOTOKI US\ MIZUMA ISAO US\ HORIKANE YOSHIHIKO US\ TSUCHIYA JUNJI US | CITIZEN FINE TECH CO LTD US | B23K35/30\ C23C14/14\ H05K3/34\ B23K35/22\ B23K35/14\ B23K35/40\ C22C5/02 |
| 2714 | 3D printing | PRINTING SHEET\ | JP2006104460A | OKAWACHI ICHIRO US\ IWAI AKIRA US\ KATO MINORU US | YUPO CORP US | B29K105/04\ C08J9/00\ G09F3/02\ B29L7/00\ B32B27/36\ B29K67/00\ B29C55/04 |
| 2715 | 3D printing | THREE-DIMENSIONAL NETWORK STRUCTURE AND METHOD FOR PRODUCING THREE-DIMENSIONAL NETWORK STRUCTURE\ | JP2006097223A | SHIRAKI MOTOYUKI US | JATECX KK US | A47C27/12\ D04H3/07 |
| 2716 | 3D printing | PHOTOSENSITIVE MATERIAL PACKAGING MATERIAL PHOTOSENSITIVE MATERIAL PACKAGE AND MANUFACTURING METHOD THEREOF\ | JP2006053365A | GOI KATSUNORI US\ SHIMA TETSUO US\ TSUJI NOBUAKI US\ GOTO SHIGETO US\ KIMURA AKIZUMI US | KONICA MINOLTA MED & GRAPHIC US | G03C3/00 |
| 2717 | 3D printing | LAYERING SHAPING METHOD\ | JP2006045584A | TOKUNAGA TAKESHI US | MEDIA PLUS INC US\ TOKUNAGA TAKESHI US | B29C67/00\ B22F3/105\ B22F3/16\ B23K26/00\ H01S3/00 |
| 2718 | 3D printing | INSULATING FILM FOR ELECTRIC WIRE AND ELECTRIC WIRE USING IT\ | JP2006024473A | MATSUNAMI TAIZO US\ IMOTO HARUJI US\ MATSUNAMI TAKAAKI US | NIPPON SHEET GLASS CO LTD US | C08L23/00\ H01B17/56\ C08J9/26\ H01B7/02 |
| 2719 | 3D printing | MELTING HEAT TRANSFER RIBBON FOR METALLIC PRINT\ | JP2005335319A | SOGABE ATSUSHI US\ FUJIMOTO KOTARO US | FUJI KAGAKU SHIKOGYO US | B41M5/30\ B41M5/42\ B41J31/00\ B41J31/05\ B41M5/382\ B41M5/385\ B41M5/39\ B41M5/40\ B41M5/41 |
| 2720 | 3D printing | METHOD FOR MANUFACTURING THREE-DIMENSIONAL SHAPE\ | JP2005335203A | AZUMA YOSHIKAZU US\ TOUGEYAMA HIROHIKO US\ FUWA ISAO (JP)\ ABE SATOSHI (JP)\ TAKENAMI MASATAKA (JP)\ SHIMIZU TAKASHI (JP) | MATSUSHITA ELECTRIC WORKS LTD (JP) | B29C67/00\ B22F3/105\ B22F3/16\ B22F3/24\ B22F3/11 |
| 2721 | 3D printing | METHOD FOR PRODUCTION OF ELECTRONIC CIRCUIT BOARD\ | US8038051B2 | SAKAI TAKEKAZU (JP)\ SHOJI TAKASHI (JP) | SHOWA DENKO KK (JP) | H05K3/34\ B23K31/02 |
| 2722 | 3D printing | MANUFACTURING METHOD OF CERAMIC COMPOSITE MATERIAL\ | JP2005298310A | KOMIYAMA TSUNEO US\ SUZUKI IWAO US | NGK ADREC CO LTD US\ NGK INSULATORS LTD (JP) | C04B35/573\ C04B41/88\ C04B41/91 |
| 2723 | 3D printing | PRESS FIT CONNECTOR ATTACHMENT METHOD\ | JP2005276705A | WANDA YOSHIHIRO US | DENSO CORP (JP) | H01R43/20\ H05K3/32 |
| 2724 | 3D printing | APPARATUS FOR PRODUCING FOOD AND METHOD FOR PRODUCING FOOD\ | JP2005261286A | MIZUNO MICHIMASA US\ TERAMOTO TOSHIO (JP) | CHUBU NIPPON KOGYO KK US\ JSR CORP (JP) | A23P1/10\ A23P1/08 |
| 2725 | 3D printing | SOUND INSULATING MATERIAL METHOD FOR MANUFACTURING SOUND INSULATING MATERIAL AND FILLER FOR SOUND INSULATING MATERIAL\ | JP2005241888A | KONDO YASUHIRO US\ TAKAOKA NOBUYUKI (JP) | SHIIENJI KK US | C04B38/00\ E04B1/86\ C04B28/02\ C04B38/10\ G10K11/162 |
| 2726 | 3D printing | THREE-DIMENSIONAL STRUCTURE MANUFACTURING METHOD\ | JP2005238488A | SHINNO TOSHIKI US\ HAYANO SEIJI US | SHINNO TOSHIKI US\ ASPECT KK US | B29K77/00\ B29C39/02\ B29K25/00\ B29C39/26 |
| 2727 | 3D printing | ALCOHOL VOLATILIZER PACKAGE\ | JP2005231717A | TAKADA MASAO US\ TAKADA SHINYA US | DORENCY KK US | B32B5/02\ B65D65/40\ B65D30/02\ A23L3/3409\ B65D75/26 |
| 2728 | 3D printing | TEMPERATURE-SENSING MATERIAL AND TEMPERATURE-SENSING METHOD\ | JP2005214930A | OKUDA KATSUMI US\ ONO SHINICHI US\ TAKEUCHI HIDENAO US\ MORIYA KOJI US\ AMAMIYA MASAHIRO US\ KOJIMA SEIJI US\ MACHIDA KENICHI US | OSAKA SEALING LABEL PRINT US\ MACHIDA KENICHI US | G01K11/06 |
| 2729 | 3D printing | CIRCUIT BOARD MANUFACTURING METHOD CIRCUIT BOARD THIN FILM TRANSISTOR ELECTRO-OPTICAL DEVICE AND ELECTRONIC EQUIPMENT\ | JP2005217359A | KAWASE TAKEO US\ MORIYA SOICHI US\ HARADA MITSUAKI US | SEIKO EPSON CORP (JP) | G09F9/00\ H01L23/52\ H05K3/10\ H01L21/288\ H01L51/00\ G02F1/167\ G09F9/30\ H01L21/28\ H01L21/3205\ H01L29/423\ H01L29/49\ H01L29/786\ H01L51/05\ H01L51/40 |
| 2730 | 3D printing | METHOD FOR MANUFACTURING THREE-DIMENSIONAL INJECTION MOLDING CIRCUIT COMPONENT\ | JP2005217279A | NISHIKAWA SHINYA US\ HAYAMI HIROSHI US\ SAKAMOTO YOSHITO US\ KITAMURA TAKAHIKO US\ HOSOE AKIHISA US | SUMITOMO ELECTRIC INDUSTRIES (JP) | B29C45/14\ B29K77/00\ C25D5/56\ C25D5/02\ H01L23/14\ H05K3/00\ H05K3/18\ B29L31/34\ C23C18/28\ C25D7/00\ H05K1/03 |
| 2731 | 3D printing | OPTICAL DEVICE MANUFACTURING METHOD AND OPTICAL DEVICE MANUFACTURING MOLD\ | JP2005212154A | YOSHIDA HIROYUKI US | MATSUSHITA ELECTRIC WORKS LTD (JP) | B29C45/26\ B29L11/00\ B29C45/02 |
| 2732 | 3D printing | METAL BOND GRINDSTONE AND ITS MANUFACTURING METHOD\ | JP2005161510A | TANIGAWA YOSHIHIRO US\ NAKAMURA NORIKAZU US | TANIGAWA YOSHIHIRO US\ NAKAMURA NORIKAZU US | B24D3/00\ B24D3/06 |
| 2733 | 3D printing | METHOD AND APPARATUS FOR SHAPING THREE-DIMENSIONAL OBJECT\ | JP2005169878A | MIYAWAKI KUNIO US\ KAWACHI JOSUKE US\ KUNISHIO KAZUYOSHI US\ SUNAYAMA KAZUYUKI US\ SAITO YUKIO US\ MATSUMOTO YUJI US | HITACHI SHIPBUILDING ENG CO US | B29C67/00 |
| 2734 | 3D printing | SHOCK ABSORBING APPARATUS\ | JP2005172130A | NISHIBORI SADAO (JP)\ NAKAMURA YUICHIRO (JP) | AIN KK SOGO KENKYUSHO US | F16F7/00 |
| 2735 | 3D printing | POLYESTER RESIN FOR TONER TONER COMPOSITION\ | CN100504628C | YASUHIRO ONO (JP)\ SHOWA IWATA (JP)\ TAKASHI AKUTAGAWA (JP)\ TSUYOSHI IZUMI (JP)\ TADAO TAKIGAWA (JP)\ SHUHEI YAHIRO (JP) | SANYO CHEMICAL IND LTD (JP) | G03G9/087 |
| 2736 | 3D printing | RELAXATION SLEEP-INDUCING BEDDING\ | JP2005143522A | TAKAKURA JAKUKO US\ MURATA TOSHIHIRO US | KYOTO NISHIKAWA & CO US\ ONKYOORIBU KK US | A61H23/02\ A61F7/08\ A61M21/02\ B06B1/04 |
| 2737 | 3D printing | DECORATIVE SHEET\ | JP2005119021A | TAKASAKI YUTAKA US | TOPPAN PRINTING CO LTD US | B32B27/20\ B29L9/00\ B32B27/00\ B29L7/00\ B29C59/04 |
| 2738 | 3D printing | DECORATING SHEET DECORATING MOLDED ARTICLE AND IN-MOLD DECORATING INJECTION MOLDING\ | US2007026197A1 | SUGA KAZUHIRO (JP)\ MEIKI YOSHIYUKI (JP)\ ATAKE HIROYUKI (JP) | DAINIPPON PRINTING CO LTD (JP) | B29C45/14\ B29C51/02\ B29L9/00\ B32B3/00\ B29C51/10\ B29C45/26\ B44C3/02\ B32B27/06\ B32B27/00\ B32B3/28\ B29C33/18\ B29K105/20 |
| 2739 | 3D printing | PULP MOULD PAPER CONTAINER\ | JP2005096790A | UEDA YOSHIKI US | TOPPAN PRINTING CO LTD US | B32B1/02\ B65D25/34\ B32B29/00\ B65D1/26\ B65D1/40\ B65D25/14\ B65D81/38 |
| 2740 | 3D printing | METHOD FOR PRODUCING BULKY POLYESTER FIBER\ | JP2005097768A | YAMADA TOSHIHIRO US\ TSUKAMOTO RYOJI US | TEIJIN FIBERS LTD US | D01F6/62 |
| 2741 | 3D printing | RECURSIVE REFLECTION PRINT SHEET OF ELECTRONIC IMAGE AND PRINTING METHOD THEREFOR\ | JP2005049383A | YAMAMOTO KAORU US | KOOWATEKUNOA KK US | D06P5/00\ G09F3/02\ G02B5/128\ G09F13/16 |
| 2742 | 3D printing | HOLOGRAM FILM FOR INSERT MOLDING\ | JP2005003853A | SHIRAISHI KAZUO US\ OKAMOTO KOJI US\ OSATO MASAHIKO US | TOPPAN PRINTING CO LTD US | B29C45/14\ G03H1/18\ B32B7/02 |
| 2743 | 3D printing | MANUFACTURING METHOD FOR INFORMATION-COMMUNICATION MEDIUM\ | JP2004306575A | KIMURA YOSHIKAZU US\ TSUCHIYA MASAHITO US | KYOTO DAIICHI KAGAKU KK US | B42D15/02 |
| 2744 | 3D printing | METHOD FOR PROVIDING GLOSSY PAPER AND GLOSSY PAPER\ | JP2004308034A | YOSHIDA MASAHIKO US\ NAKAMURA MISAO US\ UCHIDA TAIZO US | OSAKA SEALING LABEL PRINT US | B41M5/26\ B41M5/28\ B05D7/00\ D21H27/00\ B32B29/00\ B41M5/30\ B41M5/337\ B41M5/42\ D21H19/36\ D21H27/30 |
| 2745 | 3D printing | THREE-DIMENSIONAL LAMINATING MOLDING DEVICE\ | US7104773B2 | MAEKAWA TSUTOMU (JP)\ OUCHI AKEMI (JP)\ FUJII HIDETOSHI (JP)\ TAMAHASHI KUNIHIRO (JP)\ SHOJI YUTAKA (JP)\ TAKAHAGI HIROSHI (JP) | RICOH PRINTING SYS LTD (JP) | B29C35/08\ B29C67/00\ B29C41/36\ B28B17/00\ B41J2/01\ B29C41/46\ B29C41/52\ B41M5/00\ B29C41/02 |
| 2746 | 3D printing | METAL POWDER COMPOSITION FOR USE IN SELECTIVE LASER SINTERING\ | US7258720B2 | HIGASHI YOSHIKAZU (JP)\ IWANAGA TOSHIHARU (JP)\ TAKENAMI MASATAKA (JP)\ TOGEYAMA HIROHIKO (JP)\ FUWA ISAO (JP)\ YOSHIDA NORIO (JP)\ UENAGA SHUSHI (JP)\ ABE SATOSHI (JP)\ SHIMIZU TAKASHI (JP) | MATSUSHITA ELECTRIC WORKS LTD (JP) | B22F1/00 |
| 2747 | 3D printing | METAL POWDER FOR METAL LIGHT MOLD MOLDING METHOD OF PRODUCING THE SAME METHOD OF PRODUCING MOLDING WITH THREE-DIMENSIONAL SHAPE BY METAL LIGHT MOLDING AND METAL LIGHT MOLDING\ | JP2004277877A | YOSHIDA TOKUO US\ KAMINAGA SHUJI US\ AZUMA YOSHIKAZU US\ TOUGEYAMA HIROHIKO US\ FUWA ISAO (JP)\ IWANAGA TOSHIHARU (JP)\ ABE SATOSHI (JP)\ TAKENAMI MASATAKA (JP)\ SHIMIZU TAKASHI (JP) | MATSUSHITA ELECTRIC WORKS LTD (JP) | B22F3/105\ B22F1/00 |
| 2748 | 3D printing | LIQUID EJECTOR AND PROCESS FOR FORMING SMALL PROTRUSION BY LIQUID EJECTION\ | JP2004249685A | TOGASHI TAKAYUKI US | SEIKO EPSON CORP (JP) | C09D11/00\ B41J2/01\ C09D11/02\ B41J3/32\ B41J2/015 |
| 2749 | 3D printing | LAYERING SHAPING METHOD\ | JP2004232043A | TOKUNAGA TAKESHI US\ YOSHIZAWA TORU US | MEDIA PLUS INC US\ TOKUNAGA TAKESHI US | B22F3/105\ B22F3/16 |
| 2750 | 3D printing | FALSE PORCELAIN TILE AND MANUFACTURING METHOD THEREFOR\ | JP2004225379A | TSUKAMOTO KAZUO US\ YAMAMOTO TADAHIKO US | MITSUBOSHI CO LTD US | E04F15/10 |
| 2751 | 3D printing | METHOD FOR PRODUCING DIE FOR PRESS\ | JP2004211162A | HIRAI KAZUO US | JKB KK US | B22F3/105\ B22F3/16\ B22F7/04\ B22F3/02\ B22F5/00\ B21D37/20 |
| 2752 | 3D printing | MANUFACTURING METHOD OF THREE DIMENSIONAL SINTERED BODY AND THREE DIMENSIONAL SINTERED BODY\ | JP2004168610A | KATSUKI KENJI US\ KAKIUCHI EISAKU US | TOYOTA MOTOR CORP US | B22F3/105\ C04B35/64\ B22F3/26\ B22F3/11\ C04B41/88 |
| 2753 | 3D printing | EXHAUST EMISSION CONTROL STRUCTURE FOR MOTORCYCLE ENGINE\ | JP2004132345A | SHIRASAGI SADAO US\ SATO TATSUO US | OVER RACING PROJECTS KK US | F01N3/20\ B01D53/86\ F01N3/10\ F01N3/24\ F01N3/28 |
| 2754 | 3D printing | INK JET HEAD WITH BUBBLE COLLECTOR AND RECORDER EMPLOYING THE INK JET HEAD\ | JP2004142330A | NOZU TOMOHITO US\ NOJIRI HIDEAKI US\ AKAIKE MASATAKE US\ KAWASAKI TAKEHIKO US\ KURASHIMA TAMAYOSHI US\ TOYAMA KOZO US\ WADA TAKATSUGI US | CANON KK (JP) | B41J2/045\ B41J2/055\ B41J2/015 |
| 2755 | 3D printing | INK JET RECORDER USING HOT MELT INK AND ITS INK EJECTION RECOVERING METHOD\ | JP2004142329A | NOZU TOMOHITO US\ NOJIRI HIDEAKI US\ AKAIKE MASATAKE US\ KAWASAKI TAKEHIKO US\ KURASHIMA TAMAYOSHI US\ TOYAMA KOZO US\ WADA TAKATSUGI US | CANON KK (JP) | B41J2/18\ B41J2/185 |
| 2756 | 3D printing | INK JET RECORDER USING HOT MELT INK\ | JP2004142328A | NOZU TOMOHITO US\ NOJIRI HIDEAKI US\ AKAIKE MASATAKE US\ KAWASAKI TAKEHIKO US\ KURASHIMA TAMAYOSHI US\ TOYAMA KOZO US\ WADA TAKATSUGI US | CANON KK (JP) | B41J2/01\ B41J2/045\ B41J2/055\ B41J2/015 |
| 2757 | 3D printing | NONWOVEN FABRIC\ | JP2004122693A | HIROTSU TOSHIAKI US | HONEY STEEL KK US | B32B5/02\ B32B27/00\ D04H1/46\ B32B27/12\ B32B7/14\ D04H1/4374 |
| 2758 | 3D printing | POLYAMIDE RESIN SOLUBLE IN ORGANIC ACID METHOD FOR PRODUCING THE SAME AND METHOD FOR PRODUCING THREE-DIMENSIONAL INJECTION MOLDED CIRCUIT PART\ | JP2004059829A | NISHIKAWA SHINYA US\ HAYAMI HIROSHI US\ SAKAMOTO YOSHITO US\ KITAMURA TAKAHIKO US\ FUJIWARA YASUHIRO US\ UEDA KOJI US\ UEDA HIDEHARU US | CHIKUNO SHOKUHIN KOGYO KK US\ SUMITOMO ELECTRIC INDUSTRIES (JP) | G03F7/037\ C08G69/48\ H05K3/00\ H05K3/18\ H05K3/38 |
| 2759 | 3D printing | SHEET FOR MOLDING DECORATION AND METHOD FOR SIMULTANEOUS DECORATION WITH INJECTION MOLDING\ | JP2004042351A | HIRANO TAMAMI US\ KAMIYAMA HIRONORI US\ ATAKE HIROYUKI (JP) | DAINIPPON PRINTING CO LTD (JP) | B29C45/14\ B32B27/30\ B29L9/00\ B32B33/00\ B29K105/20\ B29K627/12 |
| 2760 | 3D printing | INK FILM FOR INSERT MOLDING AND INSERT MOLDED ARTICLE\ | JP2004002629A | SEKINE YASUYOSHI US | TEIKOKU PRINTING INK MFG US | B29C45/14\ C09D11/02\ B29K25/00\ B29K69/00\ B29K105/20 |
| 2761 | 3D printing | MOLDING METHOD OF A RESIN MOLDED ARTICLE BY A MOLD APPARATUS THE MOLD APPARATUS THE RESIN MOLDED ARTICLE AND A MOLDING MACHINE HAVING THE MOLD APPARATUS\ | US2003099794A1 | SASAKI TSUYOSHI (JP)\ WATANABE OSAMU (JP)\ TAKIGAWA NAOKI (JP) | Empty Field US | B29C45/14\ B29C45/26\ B29C33/00\ B29C45/70\ B29C45/56 |
| 2762 | 3D printing | THREE-DIMENSIONAL DECORATIVE OBJECT AND METHOD FOR MANUFACTURING THE SAME\ | JP2003127225A | OSHIMA NORIHIRO US\ UEDA YUJI US\ KATAOKA MASATOSHI US | MEIWA GRAVURE CO LTD US | B29C59/00\ B29L9/00\ B29C69/00\ B44C1/17\ B29C65/02\ E04F15/10\ B29C39/20\ B29C47/04\ B29L31/10 |
| 2763 | 3D printing | INJECTION FOAMING METHOD USING THERMOPLASTIC ELASTOMER COMPOSITION FOR CORE BACK TYPE INJECTION FOAMING\ | JP2008013780A | IEDA KATSUMASA US\ MORIKAWA AKIHIKO (JP)\ KANAE KENTAROU (JP)\ ABE TOMOKAZU (JP) | JSR CORP (JP)\ HONDA MOTOR CO LTD (JP) | C08J9/34 |
| 2764 | 3D printing | THERMOPLASTIC ELASTOMER COMPOSITION FOR CORE-BACK SYSTEM FOAM INJECTION MOLDING AND FOAM INJECTION MOLDING METHOD USING THE SAME\ | US7867416B2 | MORIKAWA AKIHIKO (JP)\ KANAE KENTAROU (JP)\ IEDA KATUMASA (JP)\ ABE TOMOKAZU (JP) | JSR CORP (JP)\ HONDA MOTOR CO LTD (JP) | B32B37/00\ B29C44/04 |
| 2765 | 3D printing | FILM FOR PRINT LAMINATE\ | JP2003062957A | YAMASHITA TAKASHI US | JAPAN POLYCHEM CORP US | B32B27/32\ C08L23/04\ B29C47/06\ B29K23/00\ B29L7/00 |
| 2766 | 3D printing | INK COMPOSITION RECORDING METHOD AND RECORD AND METHOD FOR JUDGING AUTHENTICITY\ | JP2003026971A | NAKAMURA HIROTO US | SEIKO EPSON CORP (JP) | C09D11/00\ B41J2/01\ B41M5/00 |
| 2767 | 3D printing | MOUNTING PARTS PEEL SUPPRESSING SOLDERING METHOD ELECTRONIC CIRCUIT BASEBOARD AND ELECTRONIC INSTRUMENT\ | US2002170947A1 | IGARASHI MINORU (JP)\ MUKAI KATSUHIKO (JP)\ ISODA MASASHI (JP)\ KOBAYASHI TAKAYUKI (JP) | Empty Field US | H05K3/30\ B23K1/00\ H05K3/34\ B23K1/08\ B23K31/02\ B23K101/42\ H05K3/28 |
| 2768 | 3D printing | METHOD FOR MANUFACTURING MOLDING OF THREE-DIMENSIONAL PRODUCT\ | JP2002249805A | KAMINAGA SHUJI US\ ABE RON US\ MACHIDA SEIZO US\ FUWA ISAO (JP)\ TAKENAMI MASATAKA (JP) | MATSUSHITA ELECTRIC WORKS LTD (JP) | B29C67/00\ B22F3/105\ B29C33/38\ B23K26/00\ B22F3/26 |
| 2769 | 3D printing | FORGERY-PREVENTING FILM\ | JP2012000991A | YUPO CORP US\ SANYO KAGAKU KOGYO KK US | YUPO CORP US | B32B27/00\ B32B27/32\ B42D15/10 |
| 2770 | 3D printing | INK-JET PRINTING METHOD AND PRINTED GOODS\ | US2002025379A1 | NAKAMURA SHUNGAKU (JP)\ YAMAZAKI AYUMI (JP) | SEIREN CO LTD (US) | D06P1/642\ D06P1/647\ D06P3/54\ D06P3/66\ D06P3/82\ D06P3/872\ D06P5/30 |
| 2771 | 3D printing | THERMOPLASTIC ELASTOMER COMPOSITION FOAM MADE FROM THE SAME AND PROCESS FOR PRODUCING FOAM\ | US6841582B2 | MORIKAWA AKIHIKO (JP)\ KANAE KENTAROU (JP)\ NAKANISHI HIDEO (JP)\ MAEDA MINORU (JP) | JSR CORP (JP) | C08L53/00\ C08J9/00\ C08L23/04\ C08L23/08\ C08L23/16\ C08L53/02 |
| 2772 | 3D printing | NONWOVEN FABRIC FOR THERMOFORMING\ | JPH1037056A | YAMAMOTO NOBUYUKI US\ YAMADA IWAHIRO US | TEIJIN LTD US | D01D5/34\ D01F8/14\ D01F1/10\ D04H3/011\ D04H3/147\ D04H3/153 |
| 2773 | 3D printing | COMPOSITE MATERIAL AND SEMICONDUCTOR DEVICE USING THE SAME\ | US6737168B1 | FUKUI AKIRA (JP)\ YAMAGATA SHINICHI (JP)\ KAMITAKE KAZUYA (JP)\ ABE YUGAKU (JP) | SUMITOMO ELECTRIC INDUSTRIES (JP) | C04B41/50\ C04B35/573\ C04B41/51\ C04B41/85\ C04B41/88\ H01L23/14\ H01L23/15\ H01L23/373 |
| 2774 | 3D printing | POLYPROPYLENE NONWOVEN FABRIC\ | JPH10292256A | HIKASA KATSUJI US\ YAMAOKA YASUSHI US | ASAHI CHEMICAL IND US | D04H3/16\ D04H3/007 |
| 2775 | 3D printing | PROCESS FOR THE PRODUCTION OF A BRAKING BAND OF A BRAKE DISK WITH VENTILATION DUCTS AND A BRAKING BAND PRODUCED BY THIS PROCESS\ | US7134476B2 | PACCHIANA GIOVANNI PAOLO (IT)\ GOLLER RALF SIEGFRIED (IT) | FRENI BREMBO SPA (IT) | B22D19/00\ B22C9/04\ B22C9/10\ B29C43/02\ B29C33/00\ B22D21/00\ F16D65/12\ B29C33/52\ C04B33/34 |
| 2776 | 3D printing | MAGNETIC AND ELECTROSTATIC NUCLEAR FUSION REACTOR\ | US2011200153A1 | FERREIRA JR MOACIR L (BR) | FERREIRA JR MOACIR L (BR) | H05H1/11\ G21B1/00 |
| 2777 | 3D printing | METHOD TO CREATE REMOVABLE DENTAL PROSTHESIS AND THE DENTAL PROSTHESIS MAKING THEREOF\ | US2012261848A1 | HARASZATI GYOERGY (HU) | HARASZATI GYOERGY (HU) | A61C13/20 |
| 2778 | 3D printing | PRINTING PROCESS\ | US6902268B1 | BRENTNALL ROGER LESLIE (GB)\ KING JEFFREY RONALD (GB)\ LEGGETT SIMON RICHARD JOHN (CH)\ TAYLOR KAREN (GB)\ SCHUETTEL STEFAN (CH) | ILFORD IMAGING UK LTD (CH) | B41M5/00\ B41M5/52\ B41M7/00 |
| 2779 | 3D printing | ADDITIVE LAYER MANUFACTURING METHOD\ | US2014367367A1 | TODD IAIN (GB)\ WOOD SCOTT DAVID (GB)\ BLACKMORE MICHAEL LEWIS (GB) | ROLLS ROYCE PLC (GB) | B23K15/00 |
| 2780 | 3D printing | ADDED LAYER MANUFACTURE\ | GB2491472A | COLEGROVE PAUL (GB)\ WILLIAMS STEWART (GB) | UNIV CRANFIELD (GB) | B23K5/18 |
| 2781 | 3D printing | PROCEDURE AND APPARATUS FOR IN-SITU MONITORING AND FEEDBACK CONTROL OF SELECTIVE LASER POWDER PROCESSING\ | US2009206065A1 | KRUTH JEAN-PIERRE (BE)\ MERCELIS PETER (BE) | KRUTH JEAN-PIERRE (BE)\ MERCELIS PETER (BE) | B23K26/00\ B23K26/02 |
| 2782 | 3D printing | VERFAHREN ZUR HERSTELLUNG VON EINER KAUTSCHUK-/GEWEBE-VERBUNDPRODUKTE\[EN] METHODS FOR MAKING RUBBER/FABRIC COMPOSITE PRODUCTS\[FR] PROCEDES POUR LA FABRICATION DE PRODUITS COMPOSITES A BASE DE CAOUTCHOUC/TISSU\ | EP1778464A2 | WALTERS IAN (GB)\ HOUGH PHILIP (GB) | HAYDALE LTD (GB) | B29D99/00\ B29C65/48\ B29C70/74 |
| 2783 | 3D printing | POWDER-COATED GLASS PRODUCTS\ | US8287988B2 | LEACH ROGER JOHN (GB) | THORSTONE BUSINESS MAN LTD (GB)\ LEACH ROGER JOHN (GB) | C08F2/46\ C09D11/10\ B05D1/12\ C09D5/03\ B32B17/06\ C03C17/00\ C03C17/32\ C03C17/38 |
| 2784 | 3D printing | METHOD OF MANUFACTURING THREE-DIMENSIONAL PRODUCTS THROUGH DEPOSITS OF SUCCESSIVE LAYERS OF A WIRE MELTED BY ELECTRON BEAM\[FR] PROCEDE DE FABRICATION D'ARTICLES TRIDIMENSIONNELS PAR DEPOT DE COUCHES SUCCESSIVES DE FIL FONDU PAR FAISCEAU D'ELECTRONS\ | WO2005077586A1 | PLUMB PAUL NIGEL (GB)\ DUMONT CONRAD FREDERICK (GB) | AQUASIUM TECHNOLOGY LTD (GB)\ PLUMB PAUL NIGEL (GB)\ DUMONT CONRAD FREDERICK (GB) | B23K15/00\ B23K15/06 |
| 2785 | 3D printing | METHOD OF INK-JET PRINTING\ | US2008284836A1 | BAKER JULIE (GB)\ HIGGINS JOHN M (GB) | HIGGINS JOHN M (GB)\ BAKER JULIE (GB) | B41J3/407\ B41M5/52\ B41M7/00\ B41J11/00 |
| 2786 | 3D printing | METHOD OF MANUFACTURING NONWOVEN ABRASIVE ARTICLES USING DRY PARTICULATE MATERIAL\[DE] VERFAHREN ZUR HERSTELLUNG VON VLIESSTOFFSCHLEIFARTIKELN MIT TROCKENEM K?RNIGEM MATERIAL\[FR] PROCEDE PERMETTANT DE PRODUIRE DES ARTICLES ABRASIFS EN NON-TISSE AU MOYEN D'UN MATERIAU EN PARTICULES SEC\ | EP1617973A1 | POLLAUD GUY M (FR)\ MALJEAN SANDRINE (FR)\ MARTIN RIVERA CARMEN (ES)\ CABRERO GOMEZ ESTRELLA (ES) | 3M INNOVATIVE PROPERTIES CO (US) | B24D11/00 |
| 2787 | 3D printing | SELECTIVE LASER SINTERING WITH INTERLEAVED FILL SCAN\ | US6694207B2 | DARRAH JAMES F (US)\ ZU XIAOSHU (US) | 3D SYSTEMS INC (US) | B29C67/00\ G05B19/4099 |
| 2788 | 3D printing | SELECTIVE LASER SINTERING WITH OPTIMIZED RASTER SCAN DIRECTION\ | US6677554B2 | DARRAH JAMES F (US)\ ZU XIAOSHU (US) | 3D SYSTEMS INC (US) | B29C67/00\ B22F3/105\ G05B19/4099 |
| 2789 | 3D printing | METHOD AND APPARATUS FOR PRODUCING THREE-DIMENSIONAL OBJECTS WITH IMPROVED PROPERTIES\ | US2015064048A1 | BESSAC CHRISTOPHE (FR)\ WALRAND GILLES (FR)\ BROCHET STEPHANIE (FR)\ PIALOT FREDERIC (FR) | MICHELIN RECH TECH (CH)\ MICHELIN & CIE (FR) | B22F3/105\ B22F3/24\ B22F5/00\ C21D1/00 |
| 2790 | 3D printing | METHOD AND APPARATUS FOR PRODUCING THREE-DIMENSIONAL OBJECTS\ | US2014348692A1 | BESSAC CHRISTOPHE (FR)\ PIALOT FRéDéRIC (FR)\ WALRAND GILLES (FR) | MICHELIN RECH TECH (CH)\ MICHELIN & CIE (FR) | B29C67/00\ B22F3/105 |
| 2791 | 3D printing | METHOD AND APPARATUS FOR PRODUCING THREE-DIMENSIONAL OBJECTS\[FR] PROCEDE ET APPAREIL POUR REALISER DES OBJETS TRIDIMENSIONNELS\ | WO2013092994A1 | BESSAC CHRISTOPHE (FR)\ WALRAND GILLES (FR)\ BROCHET STEPHANIE (FR)\ PIALOT FREDERIC (FR) | MICHELIN RECH TECH (CH)\ MICHELIN & CIE (FR) | B29C67/00\ B22F3/00\ B22F3/105 |
| 2792 | 3D printing | MANUFACTURING BONE PROSTHESIS FOR FILLING GAPS OF ALL/PART OF BONE OF PATIENT COMPRISES DIGITALIZING PATIENT USING THREE-DIMENSIONAL MEDICAL ACQUISITION DEVICE BY MAGNETIC RESONANCE DEFINING PROSTHESIS AND MANUFACTURING THE PROSTHESIS\[FR] PROCEDE DE FABRICATION D'UNE PROTHESE PAR PROTOTYPAGE RAPIDE\ | FR2979817A1 | BERTIN JEAN-MICHEL (FR) | BERTIN JEAN-MICHEL (FR) | B29C67/00\ A61F2/28\ B22F3/105 |
| 2793 | 3D printing | FILM FOR PACKAGING PRODUCT ESPECIALLY AN ENVELOPE\ | US2012193402A1 | MARTINEZ DAVID (FR)\ CHAPALAIN FLORIAN (FR) | GASCOGNE LAMINATES (FR)\ CHAPALAIN FLORIAN (FR)\ MARTINEZ DAVID (FR) | B32B3/00\ B32B38/14\ B65D27/00 |
| 2794 | 3D printing | OUTIL DE SERRAGE D'UN ORGANE DE FIXATION\ | FR2970200B1 | CHATENET LUC HENRI (FR)\ BABOEUF SEBASTIEN PIERRE LOUIS (FR)\ LE QUELLEC JOHN (FR) | SNECMA (FR) | B25B23/15 |
| 2795 | 3D printing | THERMOPLASTIC POWDER COMPOSITION AND THREE-DIMENSIONAL OBJECTS MANUFACTURED BY SINTERING SUCH A COMPOSITION\ | US2013052453A1 | FILOU GREGORY (FR)\ LEMAITRE ARNAUD (FR)\ MALET FREDERIC (FR)\ MATHIEU CYRILLE (FR) | ARKEMA FRANCE (FR)\ FILOU GREGORY (FR)\ MALET FREDERIC (FR)\ LEMAITRE ARNAUD (FR)\ MATHIEU CYRILLE (FR) | B32B5/30\ B32B5/16\ B29C45/72\ B02C19/18 |
| 2796 | 3D printing | ELECTROMAGNETIC RADIATION DETECTOR AND METHOD FOR MANUFACTURING SUCH A DETECTOR\ | US7999227B2 | VILAIN MICHEL (FR) | ULIS (FR) | G01J5/02 |
| 2797 | 3D printing | DEMOULAGE PAR CIRE PERDUE D'UNE BAUDRUCHE DE RESTRUCTURATION\ | FR2911079B1 | GAUTREAU NICOLAS US\ CHARRON YVES US | INST FRANCAIS DU PETROLE (FR) | B22C9/04\ B29C33/52\ B05D7/22\ B29C39/36 |
| 2798 | 3D printing | CORE-SHELL POLYAMIDE POWDER\ | US2010098880A1 | SENFF HOLGER (FR) | ARKEMA FRANCE (FR) | C08G69/00\ B05D3/00 |
| 2799 | 3D printing | TEXTILE COMPLEX INTENDED TO BE USED AS REINFORCING LAYER FOR THE MANUFACTURE OF COMPOSITE PARTS AND PROCESS FOR MANUFACTURING SUCH A COMPLEX\ | US2008305701A1 | GUIGNER DELPHINE (FR)\ BOUTIER SEBASTIEN (FR) | CHOMARAT COMPOSITES (FR) | B29C35/02\ B32B27/04\ B32B27/12 |
| 2800 | 3D printing | PHOTON-EMISSION SCANNING TUNNELING MICROSCOPY\ | US8006315B2 | CHARRA FABRICE (FR)\ SILLY MATTHIEU (FR)\ SOUKIASSIAN PATRICK (FR) | COMMISSARIAT ENERGIE ATOMIQUE (FR)\ UNIV PARIS SUD PARIS II (FR) | G01Q10/00\ G01Q30/00\ G01Q60/10\ G01Q60/12\ G01N13/00\ G12B1/00 |
| 2801 | 3D printing | PLANE STRUCTURE COMPOSED OF A COHESIVE ASSEMBLY OF CONTIGUOUS CELLULAR CELLS\ | US2008193711A1 | DEHENNAU CLAUDE (BE)\ GRANDJEAN DOMINIQUE (BE)\ LENG PHILIPPE-JACQUES (BE) | SOLVAY (BE) | B29D99/00\ B29C39/02\ B32B3/12 |
| 2802 | 3D printing | METHOD AND MACHINE FOR PRODUCING THREE-DIMENSIONAL OBJECTS BY MEANS OF SUCCESSIVE LAYER DEPOSITION\ | US7597835B2 | MARSAC NICOLAS (FR) | MARSAC NICOLAS (FR) | B29C35/08\ B29C67/00\ B28B1/14\ B28B1/02 |
| 2803 | 3D printing | METHOD FOR COATING THE SURFACE OF A METALLIC MATERIAL DEVICE FOR CARRYING OUT SAID METHOD\ | US2006096674A1 | CHOQUET PATRICK (FR)\ CHALEIX DANIEL (FR) | CHOQUET PATRICK (FR)\ CHALEIX DANIEL (FR) | C23C28/00\ C23C14/14\ C23C2/26\ C23C28/02\ C21D1/09 |
| 2804 | 3D printing | POWDER DISTRIBUTION IN ADDITIVE MANUFACTURING\ | US2015054191A1 | LJUNGBLAD ULRIC (SE) | ARCAM AB (SE)\ LJUNGBLAD ULRIC (SE) | B29C67/00\ B01J6/00 |
| 2805 | 3D printing | SAFETY PROTECTION METHOD AND APPARATUS FOR ADDITIVE MANUFACTURING DEVICE\ | US2013278920A1 | LOEWGREN LARS (SE) | ARCAM AB (SE) | G01B11/14 |
| 2806 | 3D printing | DREIDIMENSIONALES BAUTEIL UND VERFAHREN ZUR HERSTELLUNG DESSELBEN\[EN] THREE-DIMENSIONAL COMPONENT AND METHOD FOR PRODUCING THE SAME\[FR] COMPOSANT TRIDIMENSIONNEL ET PROCéDé POUR SA FABRICATION\ | EP2051839A1 | SCHLARB ANDREAS (DE) | SCHREINER GROUP GMBH & CO KG (DE) | B29C45/14\ B32B7/12 |
| 2807 | 3D printing | USE OF MOULDING COMPOSITIONS\ | US2012193839A1 | MARGRAF GUENTER (DE)\ JOACHIMI DETLEV (DE) | LANXESS DEUTSCHLAND GMBH (DE)\ MARGRAF GUENTER (DE)\ JOACHIMI DETLEV (DE) | B29C45/00\ B29C49/00\ B29C47/00\ B29C49/04 |
| 2808 | 3D printing | METHOD FOR PRODUCING COATING MATERIAL\ | US2013014882A1 | SCHMID JOHANNES (DE) | HOMAG HOLZBEARBEITUNGSSYSTEME (DE)\ SCHMID JOHANNES (DE) | B05D5/10\ B32B37/12 |
| 2809 | 3D printing | PA 12 POWDER FOR USE IN A GENERATIVE LAYER-WISE MANUFACTURING METHOD\ | US2014329953A1 | PATERNOSTER STEFAN (DE) | EOS ELECTRO OPTICAL SYST (DE) | B29C67/00\ C08L77/06 |
| 2810 | 3D printing | VORRICHTUNG ZUR HERSTELLUNG VON WERKSTüCKEN DURCH BEAUFSCHLAGEN VON PULVERSCHICHTEN MIT ELEKTROMAGNETISCHER STRAHLUNG ODER TEILCHENSTRAHLUNG\[EN] DEVICE FOR MANUFACTURING WORKPIECES BY APPLYING POWDER LAYERS WITH ELECTROMAGNETIC RADIATION OR PARTICLE RADIATION\[FR] DISPOSITIF DE FABRICATION DE PIèCES USINéES EN INJECTANT DES COUCHES DE POUDRE à L'AIDE D'UN RAYONNEMENT éLECTROMAGNéTIQUE OU D'UN RAYONNEMENT DE PARTICULES\ | EP2386404A1 | WIESNER ANDREAS (DE)\ SCHOENEBORN HENNER DIPL-ING (DE) | SLM SOLUTIONS GMBH (DE) | B29C67/00\ B22F3/105 |
| 2811 | 3D printing | LASER- UND THERMISCH BESCHREIBBARE OBERFL?CHENBESCHICHTUNG FüR MATERIALIEN\[EN] LASER AND THERMALLY WRITABLE SURFACE COATING FOR MATERIALS\[FR] REVêTEMENT DE SURFACE POUR MATéRIAUX POUVANT RECEVOIR UNE INSCRIPTION PAR LASER OU PAR VOIE THERMIQUE\ | EP2429826A1 | PALKOVITS ROLAND (AT)\ SCHALKHAMMER THOMAS (AT)\ KORNHERR ANDREAS (AT) | MONDI UNCOATED FINE & KRAFT PAPER GMBH (AT) | C09D7/12\ B41M5/26\ B41M5/28\ C04B41/80\ C09D5/26\ D21H17/66\ D21H17/67\ D21H19/38\ D21H19/80\ D21H21/28\ D21H23/22 |
| 2812 | 3D printing | METHOD OF MAKING A THERMOPLASTIC FILM WITH A THREE-DIMENSIONALLY STRUCTURED SURFACE\ | US2011189437A1 | BADER HERBERT (DE)\ HAGEMANN ANDREAS (DE)\ MARCEL GRUENDKEN (DE) | MARCEL GRUENDKEN (DE)\ BADER HERBERT (DE)\ HAGEMANN ANDREAS (DE) | B32B3/00\ B29C55/18 |
| 2813 | 3D printing | VERFAHREN ZUR HERSTELLUNG EINES FILTERS INBESONDERE FüR EINEN DREHABSCHEIDER UND DADURCH HERGESTELLTER FILTER\[EN] PROCESS FOR PRODUCING A FILTER IN PARTICULAR FOR A ROTARY SEPARATOR AND FILTER OBTAINED THEREBY\[FR] PROCéDé DE PRODUCTION D'UN FILTRE EN PARTICULIER POUR UN SéPARATEUR ROTATIF ET FILTRE AINSI OBTENU\ | EP2156941B1 | GENNARO PAOLO (IT)\ ROMEO MAURIZIO (IT)\ BENE GIUSEPPE (IT)\ TORTAROLO FRANCO (IT)\ ZANON GIOVANNI PAOLO (IT) | AVIO SPA (IT) | B29C67/00 |
| 2814 | 3D printing | FE&MDASH;SI&MDASH;LA ALLOY HAVING EXCELLENT MAGNETO-CALORIC PROPERTIES\ | US8808468B2 | WAECKERLE THIERRY (FR)\ FRAISSE HERVE (FR)\ BALLI MOHAMED (FR)\ DE RANGO PATRICIA (FR)\ FRUCHART DANIEL (FR)\ GIGNOUX DAMIEN (FR)\ MIRAGLIA SALVATORE (FR)\ ROSCA MARIANA (FR)\ ARTIGAS ALAVA MIGUEL JOSE (ES) | WAECKERLE THIERRY (FR)\ FRAISSE HERVE (FR)\ BALLI MOHAMED (FR)\ DE RANGO PATRICIA (FR)\ FRUCHART DANIEL (FR)\ GIGNOUX DAMIEN (FR)\ MIRAGLIA SALVATORE (FR)\ ROSCA MARIANA (FR)\ ARTIGAS ALAVA MIGUEL JOSE (ES)\ APERAM ALLOYS IMPHY (FR) | B22F9/08\ B22F9/04\ H01F1/055\ H01F1/20 |
| 2815 | 3D printing | METHOD OF FORMING A PHASE CHANGE INK IMAGE ON A SELF-LAMINATING RECORDING MEDIUM\ | US2008049090A1 | WILLEMS GUIDO G (NL)\ ROOIJAKKERS JOHANNES P J C (NL)\ STURME RUDOLF A H M (NL) | OCE TECH BV (NL) | B41J2/01 |
| 2816 | 3D printing | BILDERZEUGUNGSVERFAHREN DAS EINE PHASENAUSTAUSCHTINTE AUF EINEM SELBSTLAMINIERENDEN AUFZEICHNUNGSMEDIUM VERWENDET\[EN] METHOD OF FORMING A PHASE CHANGE INK IMAGE ON A SELF-LAMINATING RECORDING MEDIUM\[FR] PROCéDé DE FORMATION D'IMAGE UTILISANT UNE ENCRE à CHANGEMENT DE PHASE SUR UN MATéRIAU AUTO-LAMINANT\ | EP1892115A1 | WILLEMS GUIDO G (NL)\ ROOIJAKKERS JOHANNES P J C (NL)\ STURME RUDOLF A H M (NL) | OCE TECH BV (NL) | B41M7/00 |
| 2817 | 3D printing | VERWENDUNG VON POLYAMID-FORMMASSEN ZUR HERSTELLUNG VON FORMTEILEN MIT REDUZIERTER OBERFL?CHENVERKOHLUNG\[EN] USE OF POLYAMIDE MOULDING MATERIALS FOR THE FABRICATION OF MOULDED ARTICLES WITH REDUCED SUPERFICIAL CHARRING\[FR] UTILISATION DE COMPOSITIONS à MOULER DE POLYAMIDE POUR LA FABRICATION D'ARTICLES MOULéS à CARBONISATION SUPERFICIELLE RéDUITE\ | EP1780241A1 | STOEPPELMANN GEORG DR RER NAT (CH)\ KETTL RALPH DIPL-ING (CH)\ HOFFMANN BOTHO DR RER NAT (CH)\ EBERT MARTINA DR RER NAT DIPL (CH)\ HEWEL MANFRED DR RER NAT DIPL (CH) | EMS CHEMIE AG (CH) | C08L77/00\ C08K3/34 |
| 2818 | 3D printing | METHOD OF TREATING IMAGE RECEIVING SHEETS AND A HOT MELT INK JET PRINTER EMPLOYING THIS METHOD\ | US2006114302A1 | HOLTMAN LODEWIJK T (NL) | OCE TECH BV (NL) | B41J2/175 |
| 2819 | 3D printing | VERFAHREN ZUM BEHANDELN VON BILDAUFZEICHNUNGSBL?TTERN UND DIESES VERFAHREN ANWENDENDER TINTENSTRAHLDRUCKER FüR W?RMESCHMELZBARE TINTE\[EN] METHOD OF TREATING IMAGE RECEIVING SHEETS AND HOT MELT INK JET PRINTER EMPLOYING THIS METHOD\[FR] PROCéDé POUR TRAITER DES FEUILLES DESTINéES à RECEVOIR DES IMAGES ET IMPRIMANTE à JET D'ENCRE POUR ENCRE THERMOFUSIBLE UTILISANT CE PROCéDé\ | EP1661723A3 | HOLTMAN LODEWIJK T (NL) | OCE TECH BV (NL) | B41J2/175\ B41J11/00 |
| 2820 | 3D printing | PROCESS FOR MANUFACTURING A SHAPED ARTICLE IN PARTICULAR POWDER STEREOLITHOGRAPHIC OR SINTERING PROCESS\ | US2004099996A1 | HERZOG FRANK (DE) | Empty Field US | B29C67/00\ B22F3/105\ B22F3/16\ B23K26/34 |
| 2821 | 3D printing | VERFAHREN ZUR HERSTELLUNG EINES METALLISCHEN K?RPERS\[EN] METAL OBJECT IN PARTICULAR INJECTION MOLDING TOOL MANUFACTURE INVOLVES REPEATED ELECTROLYTIC DEPOSITION OF METAL ONTO A MASKED FLAT SURFACE TO BUILD UP A THREE-DIMENSIONAL STRUCTURE\ | DE10361119A1 | KURENBACH TORSTEN (DE) | SOLIDTOOL GMBH (DE) | C25D1/10\ C25D5/02\ C25D5/10 |
| 2822 | 3D printing | METHOD FOR THE DIRECT AND CONTINUOUS PRODUCTION OF HOLLOW ARTICLES FROM A POLYMER MELT\ | US7547406B2 | OTTO BRIGITTA (DE)\ SCHAEFER ROLAND (DE)\ BACHMANN HOLGER (DE)\ HOELTING LUDWIG (DE)\ DEISS STEFAN (DE) | LURGI ZIMMER GMBH (DE) | C08G63/78\ B29C45/00\ C08J5/00\ B29B13/00\ B29C31/04\ B29D22/00\ B29B7/32\ B29B7/88\ B29C45/18\ B29C49/00\ C08G63/08\ C08G63/88\ C08J3/205 |
| 2823 | 3D printing | DRUCKKOPF UND DESSEN VERWENDUNG\ | DE10353112B4 | SCHWESINGER NORBERT (DE)\ SCHAEFER EUGEN (DE) | METEC INGENIEUR AG (DE) | B41J2/175\ B41M3/16\ B41J2/14\ B41J3/32\ B41J2/38\ B41J2/475 |
| 2824 | 3D printing | VERFAHREN ZUR FREIFORMENDEN HERSTELLUNG VON DREIDIMENSIONALEN HOHLR?UMEN ODER HINTERSCHNEIDUNGEN AUFWEISENDEN MIKROBAUTEILEN\[EN] MICRO COMPONENTS MANUFACTURING METHOD INVOLVES MANUFACTURING THREE-DIMENSIONAL MASTER RAW COMPONENTS OF VARIOUS SHAPES AND CHEMICALLY PHYSICALLY OR MECHANICALLY SEPARATING MASTER COMPONENTS AND METAL LAYER FROM MICRO COMPONENT\ | DE10353060A1 | KOCH KAI UWE (DE)\ FETZER HANS-JOCHEN (DE)\ GEMMLER ARMIN (DE)\ GOTTWALD BERNHARD (DE) | FRAUNHOFER GES FORSCHUNG (DE) | C25D1/10\ B81C1/00\ C25D1/02 |
| 2825 | 3D printing | FILTERMATERIAL\[EN] HEAVY DUTY FILTRATION MATERIAL REMOVING DUST FROM INDUSTRIAL GASES INCLUDES BASE FELT AND COVER FELT WITH THINNER FIBERS CONSOLIDATED BY FLUID-JET NEEDLING\ | DE20321162U1 | BWF TEC GMBH & CO KG (DE) | BWF TEC GMBH & CO KG (DE) | B01D46/42\ B01D39/16\ D04H3/02\ B01D46/10 |
| 2826 | 3D printing | POROUS CERAMIC BODY AND METHOD FOR PRODUCTION THEREOF\ | US2006236668A1 | STOBBE PER (DK)\ HAOK UDO (DE) | HAOK UDO (DE)\ STOBBE PER (DK) | C04B38/00\ B01D71/02\ B01D46/00 |
| 2827 | 3D printing | PROCESS AND DEVICE FOR PRODUCING SOLID BODIES BY SEQUENTIAL LAYER BUILDUP\ | US7285234B2 | PFEIFER ROLF (DE)\ SHEN JIALIN (DE)\ ZEPPELIN DIDIER VON (DE) | DAIMLER CHRYSLER AG (DE) | B29C67/00\ B22F3/00\ B32B37/00\ D04H1/16\ B32B38/04\ B27J5/00 |
| 2828 | 3D printing | METHOD OF MANUFACTURING METAL- OR CERAMIC MICROPARTS\ | US2005140070A1 | FINNAH GUIDO (DE)\ JOHANN THOMAS (DE)\ PIOTTER VOLKER (DE)\ RUPRECHT ROBERT (DE)\ SCHANZ GERHARD (DE)\ HAUSSELT JURGEN (DE)\ HOLSTEIN NIIS (DE) | YEOM JANG YUL (KR) | C25D1/10\ B29C45/00\ B29C33/40\ B29C45/16\ C25D1/08\ C25D1/14 |
| 2829 | 3D printing | VERFAHREN ZUR HERSTELLUNG VON METALL- ODER KERAMIK-MIKROBAUTEILEN\[EN] METHOD FOR PRODUCING METAL OR CERAMIC MICROCOMPONENTS\[FR] PROCEDE POUR LA PRODUCTION DE MICROCOMPOSANTS METALLIQUES OU CERAMIQUES\ | EP1527214A2 | HAUSSELT JUERGEN (DE)\ FINNAH GUIDO (DE)\ HOLSTEIN NILS (DE)\ JOHANN THOMAS (DE)\ PIOTTER VOLKER (DE)\ RUPRECHT ROBERT (DE)\ SCHANZ GERHARD (DE) | KARLSRUHE FORSCHZENT (DE) | B81C1/00\ B29C45/00\ B29C45/16 |
| 2830 | 3D printing | METHOD AND DEVICE FOR THE PRODUCTION OF A THREE-DIMENSIONAL OBJECT BY MEANS OF SINTERING\ | US7419632B2 | KELLER PETER (DE) | EOS ELECTRO OPTICAL SYST (DE) | B29C35/08\ B29C67/00\ B29C41/52\ B23K26/00\ B29C67/04\ B29C35/00\ B29C41/02\ B22F3/10\ B28B11/24 |
| 2831 | 3D printing | MECHANISCH STABILER POR?SER AKTIVKOHLEFORMK?RPER\[EN] MECHANICALLY STABLE POROUS SHAPED ACTIVATED CARBON ARTICLE COMPRISES FIRST THREE-DIMENSIONAL FRAMEWORK STRUCTURE BASED ON CARBONIZED RESIN AND SECOND THREE-DIMENSIONAL INORGANIC FRAMEWORK STRUCTURE\ | DE20221547U1 | MITSUBISHI PLASTICS INC US\ LASER GIJUTSU SOGO KENKYUSHO US | HELSA AUTOMOTIVE GMBH & CO KG (DE) | B01J20/20\ C01B31/08\ C04B35/524 |
| 2832 | 3D printing | POLYMER POWDER WITH MODIFIED MELTING BEHAVIOUR\ | US2013274435A1 | GREBE MAIK (DE)\ BAUMANN FRANZ-ERICH (DE)\ DIEKMANN WOLFGANG (DE) | GREBE MAIK (DE)\ DIEKMANN WOLFGANG (DE)\ BAUMANN FRANZ-ERICH (DE) | B29C67/00\ C08G69/26\ C08G69/28 |
| 2833 | 3D printing | PROCESS FOR MELTING/SINTERING POWDER PARTICLES FOR THE LAYER-BY-LAYER PRODUCTION OF THREE-DIMENSIONAL OBJECTS\ | US2013216836A1 | GREBE MAIK (DE)\ DIEKMANN WOLFGANG (DE)\ HESSEL-GELDMANN SIGRID (DE)\ KREUTZ JUERGEN (DE) | GREBE MAIK (DE)\ DIEKMANN WOLFGANG (DE)\ HESSEL-GELDMANN SIGRID (DE)\ KREUTZ JUERGEN (DE) | B29C35/08\ B32B5/16 |
| 2834 | 3D printing | VERFAHREN ZUR BEFüLLUNG EINER DOSIERKAMMER SOWIE VORRICHTUNG HIERFüR\[EN] DEVICE FOR PRODUCING THREE-DIMENSIONAL OBJECTS BY E.G. CONSECUTIVELY SOLIDIFYING LAYERS OF A POWDER COMPRISES SUPPORT DEVICE COATING DEVICE IRRADIATION DEVICE FOR IRRADIATING BUILDING MATERIAL LAYERS DOSING CHAMBER AND REFILL DEVICE\ | DE102012008664A1 | HERZOG FRANK (DE)\ SCHOEDEL FRANK (DE)\ FLORIAN BECHMANN (DE) | CL SCHUTZRECHTSVERWALTUNGS GMBH (DE) | B22F3/105 |
| 2835 | 3D printing | ADSORPTIVE FILTER MEDIUM\ | US2015053080A1 | BOEHRINGER BERTRAM (DE)\ GONZALEZ OMAR GUERRA (DE)\ ECKLE ISEBILL (DE) | BLüCHER GMBH (DE) | B01D53/04 |
| 2836 | 3D printing | MODELING MATERIAL METHOD AND APPARATUS FOR MANU-FACTURING A THREE-DIMENSIONAL OBJECT BY MELT LAYERING\ | US2013209739A1 | FRUTH CARL (DE) | FIT FRUTH INNOVATIVE TECHNOLOGIEN GMBH (DE)\ GMBH FIT FRUTH INNOVATIVE TECHNOLOGIEN (US) | B05D3/00 |
| 2837 | 3D printing | 3D-DRUCKKOPF MIT TEMPERATUR-GESTEUERTER AUSTRITTS?FFNUNG FüR VERKüRZTE HEIZ- UND ABKüHLZEITEN SOWIE OPTIMIERTES AUSSEHEN DER DRUCKSTüCKE\[EN] THREE-DIMENSIONAL PRINTHEAD FOR MELTING AND DEPOSITING LAYERS OF WIRE-SHAPED MATERIALS HAS MELTING CHAMBER INCLUDING OUTLET DUCT LEADING INTO COOLING ZONES SUCH THAT FLOW VELOCITY OF MOLTEN MATERIAL IS REDUCED TO ZERO\ | DE102012000873A1 | PARTHY KAI (DE) | PARTHY KAI (DE) | B41J3/407\ B41J3/32 |
| 2838 | 3D printing | VERFAHREN ZUR HERSTELLUNG EINES HEI?GASSPIEGELS ZUM ERW?RMEN VON KUNSTSTOFFEN\[EN] HOT GAS MIRROR MANUFACTURING METHOD FOR INJECTION MOLDING TOOL DURING MANUFACTURING E.G. PLASTIC COMPONENTS FOR PRODUCTION OF DOOR OF PASSENGER CAR INVOLVES SEPARATING MOLD AND COMPONENT INCLUDING INLETS AND CHANNELS AND HEATING COMPONENT\ | DE102011120014A1 | FUETTERER MICHAEL (DE) | DAIMLER AG (DE) | B29C67/00\ B29C45/00\ B29C65/10 |
| 2839 | 3D printing | 3D-DRUCKKOPF MIT KüHLBAREM BESCHICKUNGSKANAL AUS HOCHW?RMELEITF?HIGEM MATERIAL\[EN] THREE DIMENSIONAL PRINTING HEAD USEFUL FOR MELTING AND DEPOSITING LAYERS OF WIRE-SHAPED MATERIALS COMPRISES FEED CHANNEL CONSISTING OF ARRAY OF HIGHLY HEAT-CONDUCTIVE MATERIALS E.G. ALUMINUM AND LOW HEAT-CONDUCTIVE MATERIALS E.G. PTFE\ | DE102011110804A1 | PARTHY KAI (DE) | PARTHY KAI (DE) | B29C67/00\ B22F3/115 |
| 2840 | 3D printing | VORRICHTUNG ZUR HERSTELLUNG VON DREIDIMENSIONALEN OBJEKTEN\[EN] DEVICE USEFUL FOR PRODUCING THREE-DIMENSIONAL OBJECTS COMPRISES A CARRYING DEVICE FOR CARRYING THE OBJECT A COATING DEVICE FOR APPLYING LAYERS OF BUILDING MATERIAL ON THE CARRYING DEVICE AND AN IRRADIATION SOURCE FOR IRRADIATING LAYER\ | DE102011106380A1 | HERZOG FRANK (DE) | CL SCHUTZRECHTSVERWALTUNGS GMBH (DE) | B22F3/105 |
| 2841 | 3D printing | NEBENKREISLAUF FüR EINE VORRICHTUNG ZUR HERSTELLUNG DREIDIMENSIONALER OBJEKTE\[EN] SECONDARY CIRCUIT FOR DEVICE FOR PRODUCING THREE-DIMENSIONAL METAL OBJECT USED IN BEAM FUSION PLANT HAS RETURN LINE THAT IS PROVIDED FOR RECYCLING OF POWDER FROM OVERFLOW CONTAINER IN MAIN CIRCUIT OF DEVICE\ | DE102011088158A1 | MEIXLSPERGER MAXIMILIAN (DE) | BAYERISCHE MOTOREN WERKE AG (DE) | B29C67/00\ B22F3/105\ B23K26/34 |
| 2842 | 3D printing | APPARATUS FOR AVOIDING DEPOSITS ON OPTICAL COMPONENTS IN THE LASER SINTERING PROCESS\ | US2013101803A1 | GREBE MAIK (DE)\ DIEKMANN WOLFGANG (DE)\ HESSEL-GELDMANN SIGRID (DE) | GREBE MAIK (DE)\ DIEKMANN WOLFGANG (DE)\ HESSEL-GELDMANN SIGRID (DE) | B29C35/08\ B29C67/24\ B32B3/26 |
| 2843 | 3D printing | COMPONENT PROPERTIES THROUGH BEAM SHAPING IN THE LASER SINTERING PROCESS\ | US2013183493A1 | MONSHEIMER SYLVIA (DE)\ GREBE MAIK (DE)\ DIEKMANN WOLFGANG (DE)\ KREUTZ JUERGEN (DE) | EVONIK DEGUSSA GMBH (DE)\ GREBE MAIK (DE)\ DIEKMANN WOLFGANG (DE)\ MONSHEIMER SYLVIA (DE)\ KREUTZ JUERGEN (DE) | B29C35/08\ B32B27/16 |
| 2844 | 3D printing | COMPONENT PROPERTIES THROUGH OPTIMIZED PROCESS MANAGEMENT IN LASER SINTERING\ | US2013183494A1 | MONSHEIMER SYLVIA (DE)\ GREBE MAIK (DE)\ DIEKMANN WOLFGANG (DE)\ KREUTZ JUERGEN (DE) | EVONIK DEGUSSA GMBH (DE)\ GREBE MAIK (DE)\ DIEKMANN WOLFGANG (DE)\ MONSHEIMER SYLVIA (DE)\ KREUTZ JUERGEN (DE) | B29C35/08\ B32B27/16 |
| 2845 | 3D printing | MULTICOLOURED FUSED DEPOSITION MODELLING PRINT\ | US2014088751A1 | POPPE DIRK (DE)\ KOHLSTRUK STEPHAN (DE)\ HAMMANN BENJAMIN (DE)\ CREMER SONJA (DE)\ BEKS KRIS (BE)\ DEWAELHEYNS LUDO (BE)\ PRIDOEHL MARKUS (DE)\ SCHMITT GUENTER (DE) | EVONIK ROEHM GMBH (DE)\ PRIDOEHL MARKUS (DE)\ SCHMITT GUENTER (DE)\ POPPE DIRK (DE)\ KOHLSTRUK STEPHAN (DE)\ HAMMANN BENJAMIN (DE)\ CREMER SONJA (DE)\ BEKS KRIS (BE)\ DEWAELHEYNS LUDO (BE) | G05B19/042 |
| 2846 | 3D printing | UNIVERSELLER 3D-DRUCKKOPF MIT MEHREREN ARTEN DER DRAHTZUFüHRUNG UND DRAHT MIT ZUG-SEELE\[EN] THREE DIMENSIONAL PRINTING HEAD FOR MELTING AND DEPOSITING LAYERS OF WIRE-SHAPED MATERIALS HAS VARIOUS TYPES OF WIRE FEEDERS AND FEED CHANNELS FOR WIRES WHERE SOFT WIRE IS MADE OF THERMOPLASTIC ELASTOMERS\ | DE102011017310A1 | ROSSIJSKAJA KORPORATSIJA RAKETNO KOSM PRIBOROSTROENIJA I INF SISTEM OAO ROSSIJSKIE KOSM SISTEMY AOOT (RU) | PARTHY KAI (DE) | B05C5/04 |
| 2847 | 3D printing | PRESSE ZUR HERSTELLUNG VON DR?HTEN FüR 3D-DRUCKER\[EN] THERMAL MELTING WIRE FOR THREE-DIMENSIONAL DRUCKER IS OBTAINED BY MOLDING PLASTIC MATERIAL OR MELTING THERMOPLASTIC MATERIAL IN HEATED MOLD COOLING MULTI-COMPONENT CASTING RESIN INTO MOLD AND CURING\ | DE102011016270A1 | SHIIENJI KK US | PARTHY KAI (DE) | B29C39/02 |
| 2848 | 3D printing | WASSERSTOFFSPEICHERMATERIALIEN\[EN] PRODUCT COMPRISES CARBON MICROSTRUCTURE WITH OPEN-CELL BACKBONE WHICH IS DEFINED BY CARBON SEGMENT AND METAL HYDRIDE MATERIAL OR CARBON MICROSTRUCTURE WITH OPEN-CELL BACKBONE WHICH IS DEFINED BY CARBON SEGMENT WHICH PENETRATE AT NODES\ | DE102011008387A1 | LI WEN (US)\ GROSS ADAM F (US)\ JACOBSEN ALAN J (US)\ VAJO JOHN C (US) | GM GLOBAL TECH OPERATIONS INC (US) | B01J20/28\ B01J20/20\ B01J20/02 |
| 2849 | 3D printing | DREIDIMENSIONAL KONTURIERTE SANDWICHSTRUKTUR\[EN] PRODUCING A THREE DIMENSIONAL CONTOURED SANDWICH STRUCTURE INCLUDING FIRST THERMOPLASTIC COVERING LAYER SECOND THERMOPLASTIC COVERING LAYER AND CORE MATERIAL COMPRISES E.G. MACHINING OR CARRYING OUT SEPARATE PROCESSING OF CORE MATERIAL\ | DE102011006819A1 | PAESLER ERIK (DE)\ KROLL LOTHAR PROF DR (DE)\ KAUFMANN JOERG (DE)\ MEYER MATTHIAS (DE)\ MUELLER MARCO (DE) | UNIV CHEMNITZ TECH (DE) | B29C45/14\ B32B3/12\ B32B27/08\ B32B37/10\ B32B38/10 |
| 2850 | 3D printing | BAUTEIL MIT EINEM GEFüLLTEN HOHLRAUM VERWENDUNG DIESES BAUTEILS UND VERFAHREN ZU DESSEN HERSTELLUNG\[EN] COMPONENT HAVING A FILLED CAVITY USE OF SAID COMPONENT AND METHOD FOR PRODUCING SAME\[FR] éLéMENT COMPRENANT UNE CHAMBRE CREUSE REMPLIE UTILISATION DE CET éLéMENT ET SON PROCéDé DE FABRICATION\ | WO2012084688A1 | SCHAEFER MARTIN (DE)\ AVDOVIC PAJAZIT (SE)\ GRAICHEN ANDREAS (SE)\ REHME OLAF (DE) | SIEMENS AG (DE)\ AVDOVIC PAJAZIT (SE)\ GRAICHEN ANDREAS (SE)\ REHME OLAF (DE)\ SCHAEFER MARTIN (DE) | B22F3/105\ B23K26/34\ B22F5/10\ B23B27/00\ B23B27/10\ B23B29/02\ B23B29/12\ B23C5/00\ B23Q11/00\ F16F15/023\ F16F15/173 |
| 2851 | 3D printing | VERFAHREN ZUM HERSTELLEN EINER DREHSTABFEDER ODER EINES WANKSTABILISATOR\[EN] MANUFACTURING TORSION BAR SPRING OR ROLLING STABILIZER FOR MOTOR VEHICLE WITH TUBULAR CROSS-SECTION COMPRISES PRODUCING COMPONENT BY BRAIDING AND/OR WRAPPING CORE WITH FIBER BUNDLES AND THERMAL MELTING OF MATRIX MATERIAL\ | DE102011011577A1 | BUSCHBECK FRANK (DE)\ NAGEL GUENTER DIPL ING (DE)\ REESE ECKHARD DIPL ING (DE)\ RUPPRECHT JOCHEN DIPL ING (DE)\ STEINERT HANS-RUDOLF DIPL ING (DE) | DAIMLER AG (DE) | B29C70/50\ B29C33/76\ B29C53/02\ B29C70/00\ B60G21/055\ C08J5/04\ F16F1/14 |
| 2852 | 3D printing | VERFAHREN ZUR HERSTELLUNG VON ELEKTRONIKMODULEN IN 3D-TECHNOLOGIE\[EN] MANUFACTURING METHOD OF ELECTRONIC MODULE BASED ON THREE-DIMENSIONAL TECHNOLOGY INVOLVES APPLYING SOLDER PASTE ON SOLDER SURFACES OF INDIVIDUAL PRINTED CIRCUIT BOARDS (PCBS) AND HEATING PCB IN REFLOW OVEN FOR MELTING THE SOLDER PASTE\ | DE102011079278A1 | SCHNEIDER BERNHARD (DE) | IFM ELECTRONIC GMBH (DE) | H05K3/36\ H01L25/16 |
| 2853 | 3D printing | VERFAHREN ZUR HERSTELLUNG EINER SCHRAUBGEWINDEAUFNAHME\ | DE102011009682A1 | ABMAYR UTE (DE) | ABMAYR UTE (DE) | B29C45/14\ F16B37/00\ B29C65/56\ F16B33/02\ F16B37/12 |
| 2854 | 3D printing | PLATTE MIT DREIDIMENSIONAL STRUKTURIERTER OBERFL?CHE AUF DER BASIS VON HOLZWERKSTOFFEN UND VERFAHREN ZUR HERSTELLUNG EINER SOLCHEN PLATTE\[EN] PLATE USEFUL FOR PRODUCING DOORS AND FURNITURE PIECES COMPRISES A THREE-DIMENSIONAL TEXTURED SURFACE PRODUCED VIA COMPRESSION CARRIER PLATE MADE UP OF WOODEN MATERIAL AND A DECORATIVE LAYER COATED ON CARRIER PLATE\ | DE102010005350A1 | BECKERS WILHELM DIPL ING (DE) | WESTAG & GETALIT AG (DE) | B27N7/00\ B32B21/02\ B32B27/10\ B32B33/00\ B32B27/04\ B32B21/06 |
| 2855 | 3D printing | METHOD FOR PRODUCING CONTINUOUS-FIBER-REINFORCED MOLDED PARTS FROM THERMOPLASTIC PLASTIC AND MOTOR VEHICLE MOLDED PART\ | US2012269999A1 | MICHEL PETER (DE)\ KIND FRANZ-GEORG (DE) | REHAU AG & CO (DE)\ KIND FRANZ-GEORG (DE)\ MICHEL PETER (DE) | B29C35/08\ B32B5/02\ B29C37/00\ B29C45/00\ B29C69/00\ B32B9/00\ B32B1/00\ B32B17/02\ B62D25/00 |
| 2856 | 3D printing | HERSTELLUNG VON HOHLK?RPERN ODER SCHICHTEN MIT HOHLR?UMEN\[EN] PRODUCING HOLLOW BODY WITH CAVITY COMPRISES PRODUCING MOLDED BODY WITH OUTER MOLD PARTIALLY HAVING SHAPE OF HOLLOW CAVITY WHERE MOLDED BODY IS DISPLACEABLE BY SOLVENT/MELT IN FLOWABLE STATE AND COATING MOLDED BODY WITH COATING MATERIAL\ | DE102009037893A1 | HERTTER MANUEL (DE)\ JAKIMOV ANDREAS (DE)\ WACHTER WOLFGANG (DE) | MTU AERO ENGINES GMBH (DE) | B29C45/00\ C04B35/638\ B05D1/00 |
| 2857 | 3D printing | VERFAHREN UND VORRICHTUNG ZUM FARBIGEN BEDRUCKEN VON PERSONALISIERUNGSDOKUMENTEN\[EN] METHOD FOR COLOR PRINTING OF PERSONALIZATION DOCUMENT INVOLVES TIGHTENING PERSONALIZATION DOCUMENT UNDER PRINT HEAD AND CONTROLLING UV LEDS IN SECTIONS BY ELECTRONIC CONTROL SO THAT UV LEDS ARE ILLUMINATED\ | DE102009021634A1 | HOTZEL MARIO (DE)\ BARTEL MARKO (DE)\ SCHMIDT REINHARD (DE) | RUHLAMAT GMBH (DE) | B41M3/14\ B41J2/21\ B41J3/407\ B41F11/02\ B41J2/525 |
| 2858 | 3D printing | VERFAHREN ZUR GENERATIVEN HERSTELLUNG DREIDIMENSIONALER FORMTEILE\[EN] METHOD FOR GENERATIVE PRODUCTION OF THREE-DIMENSIONAL SHAPED PLASTIC PART I.E. MOLDED BODY INVOLVES OBTAINING CONNECTION OF APPLIED POWDER LAYER WITH UNDERLYING LAYER AND/OR SUBSTRATE BY MELTING LAYER AND/OR SUBSTRATE\ | DE102009016128A1 | HOFMANN ROBERT (DE)\ MISCHKE PETER (DE) | MODELLBAU ROBERT HOFMANN GMBH (DE) | B29C67/00 |
| 2859 | 3D printing | HOCHMOLEKULARE POLYAMIDE\[EN] THERMOPLASTIC MOLDING COMPOSITION USEFUL TO PRODUCE FIBERS FILMS AND MOLDINGS INCLUDING E.G. INTAKE MANIFOLDS INTERCOOLER AND CIRCUIT BOARD COMPONENTS COMPRISES THERMOPLASTIC POLYAMIDE POLYETHYLENEIMINE HOMO OR COPOLYMER AND ADDITIVES\ | DE102008058246A1 | JAIN SACHIN (DE)\ GABRIEL CLAUS (DE)\ STAAL BASTIAAN BRAM PIETER (DE)\ LIEW CHEE CHIN (DE) | BASF SE (DE) | C08L77/00\ C08L79/00 |
| 2860 | 3D printing | METHOD FOR IDENTIFYING LASER SINTERING POWDERS\ | US2010140550A1 | KELLER PETER (DE)\ MATTES THOMAS (DE)\ GERSCH MANDY (DE)\ OBERHOFER JOHANN (DE) | EOS ELECTRO OPTICAL SYST (DE) | C09K11/78\ C09K11/84\ C09K11/86 |
| 2861 | 3D printing | METHOD FOR INJECTION MOLDING OF THERMOPLASTIC POLYMER MATERIAL WITH CONTINUOUS PROPERTY TRANSITIONS\ | US2010112267A1 | STENZEL HOLGER (DE)\ RIDDER HELMUT (DE)\ SCHNIEDERS JOACHIM (DE) | KURARAY EUROPE GMBH (DE) | B29C45/16\ B32B5/14 |
| 2862 | 3D printing | VERFAHREN ZUR HERSTELLUNG EINES W?RMEüBERTRAGERS UND W?RMEüBERTRAGER HERSTELLBAR NACH DEM VERFAHREN\[EN] PRODUCING A PART OF A HEAT EXCHANGER COMPRISING ALUMINUM AND/OR ALUMINUM ALLOY AND HAVING A CORROSION PROTECTED SURFACE COMPRISES APPLYING ZINC OR ZINC-CONTAINING LAYER TO THE SURFACE OR PART OF THE SURFACE\ | DE102008033222A1 | GHIANI FRANCO (DE) | BEHR GMBH & CO KG (DE) | C23C24/06\ B05D7/14\ B23P15/26\ F28F19/06\ F28F21/08 |
| 2863 | 3D printing | VERFAHREN UND VORRICHTUNG ZUR HERSTELLUNG EINES FILAMENTE AUFWEISENDEN VLIESSTOFFES\[EN] METHOD AND DEVICE FOR PRODUCING A NON-WOVEN FABRIC COMPRISING FILAMENTS\[FR] PROCéDé ET DISPOSITIF DESTINé à FABRIQUER UN NON-TISSé PRéSENTANT DES FILAMENTS\ | WO2009152791A1 | MUENSTERMANN ULLRICH (DE) | FLEISSNER GMBH (DE)\ MUENSTERMANN ULLRICH (DE) | D04H3/11\ B26F1/26 |
| 2864 | 3D printing | VERFAHREN ZUM HERSTELLEN EINES FASERVERBUNDBAUTEILS\ | DE102008026161B4 | ILZHOEFER KARL-HEINZ DIPL ING (DE)\ SALKIC ASMIR DIPL ING (DE) | DAIMLER AG (DE) | B29C70/34\ B29C70/50\ C08J5/04\ B29B15/12\ B62D29/04 |
| 2865 | 3D printing | SELEKTIVES SINTERN VON STRUKTURELL MODIFIZIERTEN POLYMEREN\[EN] PREPARING A THREE-DIMENSIONAL OBJECT FROM A POWDER COMPRISING POLYMER OR COPOLYMER CONTAINING AN AROMATIC GROUP THAT NON-LINEARLY LINKS TO THE MAIN CHAIN COMPRISES SELECTIVE SINTERING OF THE POWDER BY ELECTROMAGNETIC RADIATION\ | DE102008024288A1 | MUELLER FRANK (DE)\ PFISTER ANDREAS (DE)\ LEUTERER MARTIN (DE) | EOS ELECTRO OPTICAL SYST (DE) | C08J3/28\ B29C67/04 |
| 2866 | 3D printing | SELEKTIVES SINTERN VON STRUKTURELL MODIFIZIERTEN POLYMEREN\[EN] PRODUCING A THREE-DIMENSIONAL OBJECT BY SELECTIVELY SINTERING A POLYMER POWDER COMPRISES USING A POLYMER THAT HAS A BRANCHING GROUP IN THE MAIN CHAIN HAS A MODIFIED TERMINAL GROUP AND/OR HAS A BULKY GROUP IN THE MAIN CHAIN\ | DE102008024281A1 | MUELLER FRANK (DE)\ PFISTER ANDREAS (DE)\ LEUTERER MARTIN (DE) | EOS ELECTRO OPTICAL SYST (DE) | C08J3/28\ B29C67/04 |
| 2867 | 3D printing | KRAFTSCHLüSSIG MITEINANDER VERBINDBARE BAUTEILE\ | DE102008017029B4 | BRODA MAIK (DE)\ VERPOORT CLEMENS (DE)\ MUESCHEN ANDREAS (DE) | FORD GLOBAL TECH LLC (US) | C23C4/12\ C23C4/06\ C23C4/10\ F16H55/36 |
| 2868 | 3D printing | VERFAHREN ZUR HERSTELLUNG EINES HYBRIDFORMTEILS\[EN] METHOD FOR THE PRODUCTION OF HYBRID MOLDED PART COMPRISES PLACING A CONTOUR MASK ON A BASE BODY COATING THE SURFACE OF THE MASK AND THE BODY WITH A POWDERY MATERIAL AND EXPOSING THE MATERIAL TO BE APPLIED IN AREA WISE MANNER\ | DE102008012063A1 | HERZOG FRANK (DE) | CL SCHUTZRECHTSVERWALTUNGS GMB (DE) | B22F3/105\ B23K26/34\ B05D3/06\ B05D1/38\ B29C70/74\ B05D1/32 |
| 2869 | 3D printing | VERFAHREN ZUR HERSTELLUNG VON PHARMAZEUTISCHEN ZUBEREITUNGEN DURCH AUFBAU MITTELS APPLIKATION VON DüNNEN SCHICHTEN\[EN] PRODUCING PHARMACEUTICAL PREPARATIONS BY BUILDUP BY MEANS OF APPLICATION OF THIN LAYERS USEFUL AS MEDICAMENTS OR DIETARY SUPPLEMENTS FOR HUMANS AND ANIMALS\ | DE102008003999A1 | ECKER FELIX (DE) | ECKER FELIX (DE) | A61K9/00\ A61L15/44 |
| 2870 | 3D printing | COPOLYAMIDE POWDER AND ITS PREPARATION USE OF COPOLYAMIDE POWDER IN A SHAPING PROCESS AND MOULDINGS PRODUCED FROM THIS COPOLYAMIDE POWDER\ | US8591797B2 | MONSHEIMER SYLVIA (DE)\ GREBE MAIK (DE)\ BAUMANN FRANZ-ERICH (DE)\ ALTKEMPER STEFAN (DE)\ SIMON ULRICH (DE)\ HESSEL-GELDMANN SIGRID (DE)\ WARNKE KRISTIANE (DE)\ STEMMER HEIKE (DE)\ MUEGGE JOACHIM (DE)\ TUERKE MICHAEL (DE) | EVONIK DEGUSSA GMBH (DE)\ GREBE MAIK (DE)\ ALTKEMPER STEFAN (DE)\ MONSHEIMER SYLVIA (DE)\ BAUMANN FRANZ-ERICH (DE)\ HESSEL-GELDMANN SIGRID (DE)\ WARNKE KRISTIANE (DE)\ STEMMER HEIKE (DE)\ MUEGGE JOACHIM (DE)\ TUERKE MICHAEL (DE)\ SIMON ULRICH (DE) | B29C35/08 |
| 2871 | 3D printing | VERFAHREN UND EINRICHTUNG ZUR HOCHLEISTUNGS-MIKROBEARBEITUNG EINES K?RPERS ODER EINER PULVERSCHICHT MIT EINEM LASER HOHER BRILLANZ\ | DE102007057129B4 | EBERT ROBBY (DE)\ DRECHSEL JAN (DE)\ SCHILLE JOERG (DE)\ STREEK ANDRE (DE)\ HARTWIG LARS (DE)\ KLOETZER SASCHA (DE)\ LOESCHNER UDO (DE)\ EXNER HORST (DE)\ SUES TINO (DE) | HOCHSCHULE MITTWEIDA FH (DE) | B29C67/00\ B22F3/105\ B23K26/34\ B23K26/10\ B23K26/08\ B23K26/36 |
| 2872 | 3D printing | BLASKOPF FüR EINE BLASFOLIENEXTRUSIONSANLAGE\ | DE102007050694B4 | BUETTEL ULRICH (DE)\ SCHNEIDER JUERGEN DIPL ING (DE) | BUETTEL ULRICH (DE) | B29C47/92\ B29D7/01\ B29C47/20 |
| 2873 | 3D printing | PAEK POWDER IN PARTICULAR FOR THE USE IN A METHOD FOR A LAYER-WISE MANUFACTURING OF A THREE-DIMENSIONAL OBJECT AS WELL AS METHOD FOR PRODUCING IT\ | US2008258330A1 | PFISTER ANDREAS (DE)\ LEUTERER MARTIN (DE)\ MULLER FRANK (DE) | EOS ELECTRO OPTICAL SYST (DE) | B29C41/02\ C08G8/02 |
| 2874 | 3D printing | VERWENDUNG EINER EXTRUSIONSFOLIE ZUM EINRICHTEN EINER DRUCKVORRICHTUNG\[EN] EXTRUSION FILM FOR INSTALLATION OF PRINTING DEVICE IN LABEL PRINTING MACHINE HAS DRIVING DEVICE PROVIDED FOR MATERIAL WEB AND PRINTING UNIT WHERE FILM IS FORMED IN SINGLE LAYER OR MULTILAYER AND MADE OF POLYOLEFIN\ | DE102007009110A1 | NIEPELT RALF (DE) | NORDENIA DEUTSCHLAND GRONAU (DE) | B41M5/52\ B31D1/02\ G09F3/00 |
| 2875 | 3D printing | VERFAHREN ZUM STRAHLUNGSGESTüTZTEN URFORMEN UMFORMEN FüGEN UND TRENNEN MITTELS AUFGESETZTER KRAFTüBERTRAGENDER KONTAKTOPTIK\[EN] PROCEDURE FOR RADIATION SUPPORT FORMING REMODELING JOINING AND SEPARATION OF WORKPIECES BY MOUNTED POWER TRANSMISSION CONTACT OPTICS COMPRISES SUBJECTING RADIATION ENERGY INTO THE WORKPIECE OVER THE CONTACT OPTICS\ | DE102006051333A1 | WULFSBERG JENS PETER (DE)\ TERZI MAHDI (DE)\ KUHN ANDRE (DE)\ BRUHNS FRANZ-LUDWIG (DE) | WULFSBERG JENS PETER (DE) | B23K26/42 |
| 2876 | 3D printing | GRO?TECHNISCHES VERFAHREN UND VORRICHTUNG ZUR PLASTISCHEN VERFORMUNG VON POLYMEREN DURCH ELEKTROMAGNETISCHE STRAHLUNG\[EN] PLASTICIZATION OF POLYMER SHOWING STRONG INTERMOLECULAR INTERACTIONS E.G. CELLULOSE INVOLVES APPLICATION OF ELECTROMAGNETIC RADIATION IN PLASTICIZING UNIT WITH RADIATION-PERMEABLE COMPONENT\ | DE102006050831A1 | SCHROETER JOHANNES (DE)\ FELIX FLORIAN (DE) | SCHROETER JOHANNES (DE)\ FELIX FLORIAN (DE) | B29C47/82\ B29C45/74\ B29C47/84 |
| 2877 | 3D printing | DEVICE AND METHOD FOR A LAYERWISE MANUFACTURING OF A THREE-DIMENSIONAL OBJECT FROM A BUILDING MATERIAL IN POWDER FORM\ | US8658078B2 | WEIDINGER JOCHEN (DE)\ PFEFFERKORN FLORIAN (DE)\ MULLER FRANK (DE) | EOS ELECTRO OPTICAL SYST (DE)\ WEIDINGER JOCHEN (DE)\ MULLER FRANK (DE)\ PFEFFERKORN FLORIAN (DE) | B29C35/08 |
| 2878 | 3D printing | VERFAHREN ZUR ABFORMUNG VON DREIDIMENSIONALEN FREIFORMFL?CHEN MIT MIKROSTRUKTURIERTEN OBERFL?CHEN\[EN] METHOD FOR CASTING 3-D FREELY FORMABLE SHAPES WITH MICROSTRUCTURED SURFACES\[FR] PROCéDé DE PRISE D'EMPREINTE DE SURFACES DE FORME LIBRE EN TROIS DIMENSIONS DOTéES DE SURFACES MICROSTRUCTURéES\ | EP1854568B1 | SAUERESSIG KILIAN (DE) | K1 GMBH (DE) | B22C9/00 |
| 2879 | 3D printing | POLYMER POWDER PROCESS FOR PRODUCTION OF AND USE OF THIS POWDER AND RESULTANT SHAPED ARTICLES\ | US2007232753A1 | MONSHEIMER SYLVIA (DE)\ KOMADA HAJIME (JP)\ MATSUI HIDEKI (JP)\ NAKAIE YOSHIKI (JP) | DEGUSSA (DE) | C08L77/00\ C08L61/00 |
| 2880 | 3D printing | FLAMEPROOFED IMPACT-STRENGTH-MODIFIED POLY(ESTER)CARBONATE COMPOSITIONS\ | US7833448B2 | BUCHHOLZ VERA (DE)\ WENZ ECKHARD (DE)\ ECKEL THOMAS (DE)\ THUERMER BURKHARD (DE)\ WITTMANN DIETER (DE) | BAYER MATERIALSCIENCE AG (DE) | B28B3/20 |
| 2881 | 3D printing | SCHMELZ-AGGREGAT EINTHALTEND EINEN INJEKTOR BLASLANZE ODER BRENNER\[EN] SMELTING UNIT COMPRISING AN INJECTOR A LANCE OR A BURNER\[FR] DISPOSITIF DE FUSION AVEC UN INJECTEUR UNE LANCE OU UN BR?LEUR\ | EP1835039B1 | MEYN MATTHIAS (DE) | SMS SIEMAG AG (DE) | C21C5/52\ F27B3/20\ C21C5/56\ F27B3/22 |
| 2882 | 3D printing | USE OF POLYMER POWDER PRODUCED FROM A DISPERSION IN A SHAPING PROCESS AND MOLDINGS PRODUCED FROM THIS POLYMER POWDER\ | US2007182070A1 | MONSHEIMER SYLVIA (DE)\ GREBE MAIK (DE)\ KOMADA HAJIME (JP)\ MATSUI HIDEKI (JP) | DEGUSSA (DE) | B29C35/08\ B29C41/02 |
| 2883 | 3D printing | USE OF POLYESTER POWDER IN A SHAPING PROCESS AND MOLDINGS PRODUCED FROM THIS POLYESTER POWDER\ | US8834777B2 | MONSHEIMER SYLVIA (DE)\ GREBE MAIK (DE)\ SIMON ULRICH (DE)\ TEMME HEINRICH (DE) | EVONIK DEGUSSA GMBH (DE)\ GREBE MAIK (DE)\ MONSHEIMER SYLVIA (DE)\ SIMON ULRICH (DE)\ TEMME HEINRICH (DE) | B29C35/08\ B29C67/00\ B29C41/02\ B29C71/04\ B29K67/00 |
| 2884 | 3D printing | PROCESS FOR PRODUCING SHEATH-CORE STAPLE FIBERS WITH A THREE-DIMENSIONAL CRIMP AND A CORRESPONDING SHEATH-CORE STAPLE FIBER\ | US2009124155A1 | TIEMEIER HENDRIK (DE)\ LABITZKE EKKEHARD (DE) | OERLIKON TEXTILE GMBH & CO KG (DE) | B32B5/02\ D04H1/00\ B29C47/08 |
| 2885 | 3D printing | W?SSRIGE MAGENTAFARBENE FARBSTOFFE IHRE HERSTELLUNG UND VERWENDUNG\[EN] NEW HETEROCYCLIC DYE COMPOUNDS USEFUL E.G. FOR COLORING AND PRINTING NATURAL AND SYNTHETIC FIBER MATERIALS FOR COLORING PAPER OR CELLULOSES IN THE MASS AND IN THE MANUFACTURE OF MAGENTA COLOR RECORDING FLUIDS\ | DE102005033584A1 | BORCHERT TILL (DE)\ KLEINERT MARKUS (DE)\ SCHNEIDER STEFFEN (DE)\ GEISENBERGER JOSEF (DE) | CLARIANT PRODUKTE DEUTSCHLAND (DE) | C09D11/02\ C09B56/20\ C09B67/36\ D06P1/41 |
| 2886 | 3D printing | THREE-DIMENSIONAL LAYER-BY-LAYER PRODUCTION PROCESS WITH POWDERS BASED ON CYCLIC OLIGOMERS\ | US7988906B2 | MONSHEIMER SYLVIA (DE)\ RICHTER ALEXANDER (DE) | EVONIK DEGUSSA GMBH (DE) | B29C35/08\ H05B6/00\ B27N3/00 |
| 2887 | 3D printing | METHOD AND DEVICE FOR PRODUCING A 3D OBJECT BY MEANS OF A GENERATIVE 3D-METHOD\ | US8414281B2 | SCHLEISS PETER (CH)\ LEVY GIDEON (CH) | EOS ELECTRO OPTICAL SYST (DE)\ SCHLEISS PETER (CH)\ LEVY GIDEON (CH) | B29C35/08\ B29C67/00 |
| 2888 | 3D printing | THERMOFORMBARES VERPACKUNGSMATERIAL MIT INNENLIEGENDEM DRUCKBILD\[EN] THERMOFORMABLE PACKAGING MATERIAL WITH AN INTERNAL PRINTED IMAGE\[FR] MATERIAU D'EMBALLAGE THERMOFORMABLE COMPRENANT UNE IMAGE IMPRIMéE à L'INTéRIEUR\ | EP1724105A1 | TOIVOLA TIMO (FI)\ LEHTO KEIJO (FI)\ JUNNILA JARMO (FI)\ SILANDER HARRI (FI)\ SAARISTO ESKO (FI)\ KASCHEL GREGOR DR (DE)\ BRANDT RAINER DR (DE) | WIPAK WALSRODE GMBH & CO KG (DE) | B32B27/00 |
| 2889 | 3D printing | METHOD FOR PRODUCING DUROPLASTIC FINE-FIBER NON-WOVENS HAVING A HIGH FLAME-RETARDANT THERMAL PROTECTIVE AND SOUND INSULATING EFFECT\ | US2008203602A1 | RIEDEL BERND (DE)\ BUCKA HARTMUT (AT) | AMI AGROLINZ MELAMINE INT GMBH (AT) | D04H3/03\ D04H3/02\ D04C1/00 |
| 2890 | 3D printing | POLYMER POWDER WITH BLOCK POLYETHERAMIDE USE IN A SHAPING PROCESS AND MOLDINGS PRODUCED FROM THIS POLYMER POWDER\ | US2006189784A1 | MONSHEIMER SYLVIA (DE)\ GREBE MAIK (DE)\ BAUMANN FRANZ-ERICH (DE)\ SIMON ULRICH (DE)\ HESSEL SIGRID (DE) | DEGUSSA (DE) | C08G69/14 |
| 2891 | 3D printing | HERSTELLUNGSVERFAHREN FüR EINEN KERAMISCHEN FORMK?RPER UND ENTSPRECHENDER KERAMISCHER FORMK?RPER SOWIE DESSEN VERWENDUNG\[EN] PRODUCTION OF CERAMIC MOLD USEFUL E.G. AS CAST FOR CERAMIC SLIP CASTING OR AS CARRIER FOR CULTURING MICROORGANISMS COMPRISES E.G. MIXING BIOMORPHOUS CARBON FIBERS BINDING MATERIAL AND METAL-CONTAINING MATERIAL AND CROSSLINKING\ | DE102005058034A1 | FEY TOBIAS (DE)\ HOEFNER THOMAS (DE)\ GREIL PETER (DE) | UNIV FRIEDRICH ALEXANDER ER (DE) | C04B35/565\ B28B7/34\ C04B35/571\ C03B19/02\ C12M1/16 |
| 2892 | 3D printing | POLYMER POWDER WITH POLYAMIDE USE IN A SHAPING PROCESS AND MOLDINGS PRODUCED FROM THIS POLYMER POWDER\ | US8840829B2 | MONSHEIMER SYLVIA (DE)\ BAUMANN FRANZ-ERICH (DE) | EVONIK DEGUSSA GMBH (DE)\ MONSHEIMER SYLVIA (DE)\ BAUMANN FRANZ-ERICH (DE) | H05B6/00\ B27N3/00\ B29B9/00\ D04H1/20 |
| 2893 | 3D printing | USE OF POLYARYLENE ETHER KETONE POWDER IN A THREE-DIMENSIONAL POWDER-BASED MOLDLESS PRODUCTION PROCESS AND MOLDINGS PRODUCED THEREFROM\ | US2006134419A1 | MONSHEIMER SYLVIA (DE)\ GREBE MAIK (DE)\ KREIDLER PATRICK (DE)\ RICHTER ALEXANDER (DE) | DEGUSSA (DE) | C08G8/02\ B32B15/02 |
| 2894 | 3D printing | KRAFTSTOFFINJEKTOR MIT KüHLEINRICHTUNG\ | DE102004049703B4 | LEWENTZ GUENTER (DE) | CONTINENTAL AUTOMOTIVE GMBH (DE) | F02M53/00 |
| 2895 | 3D printing | POWDER WITH IMPROVED RECYCLING PROPERTIES PROCESS FOR ITS PRODUCTION AND USE OF THE POWDER IN A PROCESS FOR PRODUCING THREE-DIMENSIONAL OBJECTS\ | US8173258B2 | MONSHEIMER SYLVIA (DE)\ GREBE MAIK (DE)\ BAUMANN FRANZ-ERICH (DE)\ ALTKEMPER STEFAN (DE)\ CHRISTOPH WOLFGANG (DE)\ SIMON ULRICH (DE) | EVONIK DEGUSSA GMBH (DE)\ GREBE MAIK (DE)\ ALTKEMPER STEFAN (DE)\ MONSHEIMER SYLVIA (DE)\ BAUMANN FRANZ-ERICH (DE)\ SIMON ULRICH (DE)\ CHRISTOPH WOLFGANG (DE) | C08J3/14\ C08G69/26\ C08L\ C08F6/00 |
| 2896 | 3D printing | VERFAHREN ZUR HERSTELLUNG VON KUNSTSTOFFSPRITZGIE?TEILEN UND VORRICHTUNG ZUR DURCHFüHRUNG DES VERFAHRENS\ | DE102004043253B4 | MANZ BRUNO (DE) | SCHUSTER KUNSTSTOFFTECHNIK GMB (DE) | B29C45/14\ B29C51/00\ B29C45/00\ B29C69/00 |
| 2897 | 3D printing | METALLIZED OR CERAMIC-COATED POLYESTER FILMS COMPRISING POLY(M-XYLENEADIPAMIDE)\ | US2005287378A1 | KLEIN OLIVER (DE)\ PEIFFER HERBERT (DE)\ DAVIS RICHARD L (DE) | KLEIN OLIVER (DE)\ PEIFFER HERBERT (DE)\ DAVIS RICHARD L (DE) | C08K3/00\ C08J7/04\ C08L77/06\ B32B15/08\ B32B27/36\ C08L67/02 |
| 2898 | 3D printing | METHOD AND DEVICE FOR PRODUCTION OF THREE-DIMENSIONAL OBJECTS BY MEANS OF ELECTROMAGNETIC RADIATION OF ELECTROMAGNETIC RADIATION AND APPLICATION OF AN ABSORBER BY MEANS OF AN INK-JET METHOD\ | US2007238056A1 | MONSHEIMER SYLVIA (DE)\ GREBE MAIK (DE)\ BAUMANN FRANZ-ERICH (DE) | DEGUSSA (DE) | B29C35/08\ B29C67/00\ B22F3/105 |
| 2899 | 3D printing | METHOD AND DEVICE FOR PRODUCING THREE-DIMENSIONAL OBJECTS USING LASER TECHNOLOGY AND FOR APPLYING AN ABSORBER USING AN INK JET METHOD\ | US2007183918A1 | MONSHEIMER SYLVIA (DE)\ BAUMANN FRANZ-ERICH (DE)\ GORING RAINER (DE)\ GREBE MARK (DE) | DEGUSSA (DE) | B29C35/08\ B29C67/00\ B22F3/105\ B22F3/10\ C22C1/04\ C22C33/02 |
| 2900 | 3D printing | VERKLEBEN VON KUNSTSTOFFEXTRUDATEN MIT WERKSTOFFOBERFL?CHEN\ | DE10144524B4 | ERB ARMIN (DE)\ SCHROEDEL JUERGEN (DE)\ TERFLOTH CHRISTIAN (DE) | JOWAT AG (DE) | C08L75/04\ B29C69/00\ B29C65/48\ B32B37/15\ B29C69/02\ B29C43/28\ B27D5/00\ C09J5/06\ B32B7/12\ B29C47/02\ B29C63/00\ B29C63/48\ B29C65/02\ C09J175/04\ B05C5/02\ B27D1/00\ C09J5/10\ C09J177/00 |
| 2901 | 3D printing | PROCESS AND APPARATUS FOR PRODUCING PRECISION CASTINGS\ | US6725902B2 | BLUM MATTHIAS (DE)\ JARCZYK GEORG (DE)\ FELLMANN HANS-GUENTHER (DE)\ BUSSE PETER (DE) | ALD VACUUM TECHN AG (DE) | B22C9/04\ B22D13/00\ B22D13/06\ B22D13/10\ B22C9/02\ B22D21/00\ B22D21/06 |
| 2902 | 3D printing | METHOD FOR PRODUCING THREE-DIMENSIONAL SINTERED WORK PIECES\ | US2005142024A1 | HERZOG FRANK (DE) | Empty Field US | B29C67/00\ B22F3/105\ B22F3/16 |
| 2903 | 3D printing | NANOCRYSTALLINE COMPOSITE MATERIAL BASED ON AL2O3 - ZRO2 - SIO2 AND ITS PRODUCTION METHOD\[FR] MATéRIAU COMPOSITE NANOCRISTALLIN à BASE DE AL&LT;SB&GT;2&LT;/SB&GT;O&LT;SB&GT;3&LT;/SB&GT; - ZRO&LT;SB&GT;2&LT;/SB&GT; - SIO&LT;SB&GT;2 &LT;/SB&GT;ET SON PROCéDé DE PRODUCTION\ | WO2009033435A1 | NEUFUSS KAREL (CZ)\ CHRASKA TOMAS (CZ) | INST OF PLASMA PHYSICS AS CR V (CZ)\ INST OF INORGANIC CHEMISTRY AS (CZ)\ EUTIT LTD (CZ)\ CHRASKA TOMAS (CZ)\ NEUFUSS KAREL (CZ) | C04B35/622\ C04B35/10\ C04B35/05\ C04B35/109 |
| 2904 | 3D printing | EXTRUSION DEVICE OF 3D (THREE DIMENSIONAL) PRINTER APPLIED TO FUSE WIRE ACCUMULATION TECHNOLOGY\ | CN203282708U | YUAN ZHENGUO US | PANOWIN TECHNOLOGIES CO LTD US | B29C67/00 |
| 2905 | 3D printing | 3D (THREE-DIMENSIONAL) PRINTING RAPID FORMING DEVICE BASED ON MAGNETORHEOLOGICAL MATERIAL\ | CN203344319U | CHEN XI US\ HE GUOTIAN US\ GU MINGXIN US\ WANG ZHONGXUN US\ ZHU XIAOQIANG US\ LIN YUANCHANG US\ XU ZEYU US\ ZHAO JIAN US\ LIU YONGFU US | CHONGQING INST OF GREEN AND INTELLIGENT TECHNOLOGY CHINESE ACADEMY OF SCIENCES US | B29C67/00 |
| 2906 | 3D printing | FIRST MIRROR SURFACE IMPURITY ANALYSIS DEVICE BASED ON REFLECTION TYPE TERAHERTZ SPECTRUM\ | CN203037573U | HAI RAN US\ ZHANG CHENFEI US\ XIN YU US\ DING HONGBIN US | UNIV DALIAN TECH (CN) | G01B11/06\ G01N21/31 |
| 2907 | 3D printing | COMPRESSION RESISTING AND WATER DIVERSION THREE-DIMENSIONAL WATER FILTERING AND DRAINING DEVICE\ | CN203034439U | YANG JINGLIN US | TAI AN YONGJIA CIVIL ENGINEERING MATERIALS CO LTD US | E02D3/10 |
| 2908 | 3D printing | STEREOSCOPIC ANTI-FAKE PACKING BOX WITH HIGH BAR CODE LEVEL\ | CN203064295U | ZHOU LIQUAN US\ ZOU YAOBANG US\ JIANG ZONGZHENG US\ YANG PEIXIANG US\ LIU KUNHONG US\ LI HONGYE US\ WANG MINGJUN US\ LIU GUANGTIAN US | SICHUAN YIBIN PUSH GROUP 3D CO LTD US | G09F3/02\ B65D5/00 |
| 2909 | 3D printing | CALCULUS 3D (THREE DIMENSIONAL) CONSTRUCTING METHOD AND DEVICE\ | CN103465475A | YANG WEIMIN US\ YANG HUAGUANG US\ QIN LIU US\ JIAO ZHIWEI US\ DING YUMEI US | UNIV BEIJING CHEMICAL US | B29C67/00 |
| 2910 | 3D printing | GLASS HIGH TEMPERATURE MELTING MOLDING SPRAYER BASED ON 3D (THREE DIMENSIONAL) PRINTING TECHNOLOGY\ | CN103395973A | PENG SHOU US\ ZHANG JIALIN US | BENGBU GLASS IND DESIGN INST US\ CHINA TRIUMPH INT ENG CO LTD US | C03B19/00 |
| 2911 | 3D printing | METHOD USING 3D PRINTING TECHNOLOGY TO PREPARE DROPPING PILLS\ | CN103393543A | WEI XUEWEN US | WEI XUEWEN US | A61J3/06\ A61K9/20 |
| 2912 | 3D printing | METHOD FOR ACHIEVING HIGH-MELTING-POINT MATERIAL 3D PRINTING THROUGH NANOMETER INK TOGETHER WITH LASER MELTING\ | CN103407296A | ZHANG SHANGFENG US\ ZHANG YANGMING US\ ZHANG SHIZHU US\ SHI XINCHAO US\ LIU CHUANG US\ LIU YUBO US\ LIU YAN US\ ZHANG LIANBIN US | NANJING DINGKE NANOTECHNOLOGY INST CO LTD US | B41J5/00 |
| 2913 | 3D printing | MANUFACTURING METHOD OF ENVIRONMENT-FRIENDLY CONTAINER WITH 3D COLOR PRINTING PICTURE AND ENVIRONMENT-FRIENDLY CONTAINER\ | CN103332352A | YANG RONGSHI US | DONGGUAN MORNING LIGHT PRINTING COMPANY LTD US | B65D3/02\ B65D3/30\ B65D25/34 |
| 2914 | 3D printing | TEXTURED YARN LONG-FIBER REINFORCED THERMOPLASTIC PLASTIC GRANULES AND PRODUCTION METHOD THEREOF\ | CN103407169A | LU ZHIHONG US\ ZENG TIANQUAN US | JURONG BASTEP COMPOSITE MATERIALS CO LTD US | B29B9/06\ B29C70/16\ B29C70/52 |
| 2915 | 3D printing | LASER 3D (THREE-DIMENSIONAL) PRINTING METHOD OF CERAMIC FUNCTIONAL GRADIENT COMPONENT\ | CN103317590A | WU DONGJIANG US\ NIU FANGYONG US\ MA GUANGYI US\ GUO DONGMING US | UNIV DALIAN TECH (CN) | B28B1/24 |
| 2916 | 3D printing | 3D PRINTING METHOD FOR MACHINING WORKPIECE WITH INNER HOLLOW STRUCTURE\ | CN103341629A | LI QIU US | LI QIU US | B29C67/00\ B22F5/10 |
| 2917 | 3D printing | 3D (THREE-DIMENSIONAL) STRUCTURE RELEASE FILM USED FOR FORMING BICYCLE\ | CN103264501A | LIU LIEXIN US\ WU ZHENYU US\ FU GUOZHENG US | XIAMEN XINWANG NEW MATERIAL SCIENCE & TECHNOLOGY CO LTD US | B29L9/00\ B29K77/00\ B29K25/00\ B29K23/00\ B29L7/00\ B29C55/28 |
| 2918 | 3D printing | No Title Available\ | CN103266235B | KB KHIMAVTOMATIKI AOOT (RU) | ROSSIJSKAJA KORPORATSIJA RAKETNO KOSM PRIBOROSTROENIJA I INF SISTEM OAO ROSSIJSKIE KOSM SISTEMY AOOT (RU) | C22C1/05 |
| 2919 | 3D printing | PRODUCTION PROCESS OF COLORFULLY-PAINTED AND BACK-ENGRAVED CRYSTAL GLASS\ | CN103253057A | JI QINGKAI US | ARIAMOTION DALIAN CO LTD US | B44C1/22\ C03B19/00\ C03C3/089 |
| 2920 | 3D printing | MULTISTAGE-TEMPERATURE-CONTROL-BASED FUSED DEPOSITION MODELING (FDM) TYPE 3D PRINTING SPRAYER AND TEMPERATURE CONTROL METHOD\ | CN103240883A | PENG YONGGANG US\ WEI WEI US | UNIV ZHEJIANG US | B29C67/00 |
| 2921 | 3D printing | DIRECT LASER FORMING METHOD FOR UNEQUAL-WIDTH MEMBER\ | CN103231056A | ZHU GANGXIAN US\ WANG LIFANG US\ SHI SHIHONG (CN)\ FU GEYAN (CN) | UNIV SOOCHOW (CN) | B22F3/105\ B22F7/02 |
| 2922 | 3D printing | 3D (THREE DIMENSIONAL) PRINTER EXTRUSION APPARATUS APPLIED IN FUSE STACKING TECHNOLOGY\ | CN103231517A | YUAN ZHENGUO US | WU JIE US | B29C67/00 |
| 2923 | 3D printing | DOUBLE-ALLOY NDFEB RARE EARTH PERMANENT MAGNETIC MATERIAL AND MANUFACTURING METHOD THEREOF\ | US2014328713A1 | SUN HAOTIAN (CN) | CHINA NORTH MAGNETIC & ELECTRONIC TECHNOLOGY CO LTD (CN) | B22F3/24\ B22F9/08\ H01F1/053 |
| 2924 | 3D printing | ARTIFICIAL BONE MATERIAL AND PREPARATION METHOD THEREOF\ | CN103285428A | LAN HAI US\ SHI SHENGTAO US\ REN FANGPING US\ LI GUOKANG US | CHINA INTERNAT TOURISM & TRADE CO LTD US | A61L27/48\ A61L27/46 |
| 2925 | 3D printing | PREPARATION METHOD OF MULLITE/SILICON CARBIDE COMPLEX-PHASE FOAMED CERAMIC\ | CN103253981A | YAN HONG US\ AI FANRONG US\ CHEN WEI (CN) | UNIV NANCHANG US | C04B35/622\ C04B35/565\ C04B38/06\ C04B35/185 |
| 2926 | 3D printing | NOVEL 3D (THREE DIMENSIONAL) PRINTED BODY SURFACE TREATMENT PROCESS\ | CN103192524A | PENG ZHENYUN US\ XU QIMING US | WOWMAN INFORMATION SERVICES CO LTD SUZHOU US | B29C59/00 |
| 2927 | 3D printing | THREE-DIMENSIONAL PRINTING PEN\ | CN103350507A | ZHOU SHUANG US\ WU JIE US | WU JIE US\ ZHOU SHUANG US | B29C67/00 |
| 2928 | 3D printing | METHOD AND EQUIPMENT FOR PREPARING STRIP STRUCTURES ON CONJUGATED POLYMER SURFACES THROUGH PULSE LASERS\ | CN103193999A | LIU JIAN US\ LI XIAOHONG US\ LIU ZIYI US\ XU DANDAN US\ LI YUANYUAN US\ ZHU MIN US | UNIV SW SCI & TECH SWUST US | C08J7/00\ C08J5/18\ C08L65/00\ C08L79/02\ C08L79/04 |
| 2929 | 3D printing | MAGNETORHEOLOGICAL-MATERIAL-BASED 3D (THREE-DIMENSIONAL) PRINTING TYPE RAPID PROTOTYPING DEVICE AND METHOD\ | CN103213281A | CHEN XI US\ HE GUOTIAN US\ GU MINGXIN US\ WANG ZHONGXUN US\ ZHU XIAOQIANG US\ LIN YUANCHANG US\ XU ZEYU US\ ZHAO JIAN US\ LIU YONGFU US | CHONGQING INST OF GREEN AND INTELLIGENT TECHNOLOGY US | B29C67/00 |
| 2930 | 3D printing | RAPID PROTOTYPING SHAPE MEMORY HIGH POLYMER MATERIAL AND PREPARATION METHOD AND APPLICATION THEREOF\ | CN103160948A | LUO HONGSHENG US\ LUO XIAOFAN US | SUZHOU JUFU POLYMER MATERIAL CO LTD US | B29C67/00\ C08G18/32\ C08G18/42\ C08G18/10\ C08G18/48\ C08G18/66\ D01F6/78 |
| 2931 | 3D printing | SIDE BY SIDE COMPOSITE FIBER SPINNERET PLATE METHOD OF PREPARING THREE-DIMENSIONAL CRIMP ANTIBACTERIAL FIBER WITH SPINNERET PLATE AND APPLICATION OF THREE-DIMENSIONAL CRIMP ANTIBACTERIAL FIBER\ | CN103225118A | YAN ZHIYONG US\ HU YING US\ YE HONGFU US\ ZHAO GANGHUA US\ WANG LIGANG US\ SHEN XIAOJUN US\ WANG SHANGDONG US | UNIV JIAXING US | D01D5/22\ D01F8/14\ D01F1/10\ D01D5/253\ D01D5/32\ D01F8/12 |
| 2932 | 3D printing | THREE-DIMENSIONAL (3D) PRINTING METHOD\ | CN103129140A | CHEN XUGUO US | CHEN XUGUO US | B41J2/01\ B41J3/00 |
| 2933 | 3D printing | METHOD FOR IMPROVING METAL 3D (THREE-DIMENSIONAL) PRINTING COMPACTNESS BY UTILIZING SYNCHRONOUS DOUBLE-BEAM LASER\ | CN103160825A | ZHANG CHONGHAO US\ LIU ANMIN US\ SHAO GUOFENG US\ YANG JIAN US | ZHANG CHONGHAO US | B22F3/105\ B22F7/04\ C23C24/10 |
| 2934 | 3D printing | METHOD FOR IMPROVING COMPACTNESS OF 3D (THREE DIMENSIONAL) PRINTING METAL PART BY ADOPTING SECOND LASER BEAM\ | CN103173760A | ZHANG CHONGHAO US\ LIU ANMIN US\ SHAO GUOFENG US\ YANG JIAN US | ZHANG CHONGHAO US | B22F3/105\ B22F7/04\ C23C24/10 |
| 2935 | 3D printing | 3D (THREE DIMENSIONAL) PRINTING MANUFACTURING METHOD OF SEAL\ | CN103143706A | WU XINHUA US\ MEI JUNFA US\ WANG ANQI US\ SUN MEI US\ YANG ZHIYI US\ YOU LINHUA US | WUXI ANDI LIJIE TRADE CO LTD US | B29C67/00\ B22F3/105\ C04B35/622 |
| 2936 | 3D printing | 3D LASER PRINTING DEVICE TAKING LOW-MELTING-POINT GLASS POWDER AS RAW MATERIAL\ | CN103073174A | ZHANG QIAN US | SUZHOU BAINASI OPTICAL TECHNOLOGY CO LTD US | C03B19/06 |
| 2937 | 3D printing | PLASTIC SOLE WITH THREE-DIMENSIONAL PATTERNS\ | CN202950080U | CHEN XIAOLIN US | JIEYANG JIAYI INDUSTRY AND TRADE CO LTD US | A43B13/12 |
| 2938 | 3D printing | TOUCH SCREEN FUNCTIONAL FILM\ | CN202952611U | CHEN JIANHUI US | ZHEJIANG ZHAOYI TECHNOLOGY CO LTD US | G06F3/041\ B32B27/06\ B32B27/36 |
| 2939 | 3D printing | A THREE-DIMENSIONAL PRINTER EXTRUSION HEAD CAPABLE OF FEEDING IN A SWITCHING MODE\ | CN203157146U | LI YAOTANG US\ YE SHIDONG US\ ZHANG HAIQIANG US\ LIU YUPENG US | GUANGZHOU ELECTRONIC TECHNOLOGY CO LTD CHINESE ACADEMY OF SCIENCES US | B29C67/00 |
| 2940 | 3D printing | THREE-DIMENSIONAL COMPOSITE PLASTIC WATER DISCHARGING BODY\ | CN202787227U | XUAN GEN US\ ZHANG JIANHUA US\ HE XUEJUN US\ ZHAO XUEFENG US\ BIAN FEIJING US\ XU HAIYONG US\ YU JINGJING US\ ZHANG PEIGUANG US\ XU QUAN US | ZHEJIANG ZHUAN CONSTRUCTION GROUP CO LTD US\ ZHEJIANG HUAHUI MECHANICAL AND ELECTRICAL EQUIPMENT INSTALLATION CO LTD US\ ZHEJIANG GUANGCHUAN CONSTRUCTION CO LTD US | E02D3/10 |
| 2941 | 3D printing | THREE-DIMENSIONAL HOLLOW POLYESTER STAPLE FIBER\ | CN202849604U | ZHENG QUAN US\ YAN DONGNING US\ CUI HUI US | JIANGSU DESAI CHEMICAL FIBER CO LTD US | D01D5/24\ D01D5/253 |
| 2942 | 3D printing | THREE-DIMENSIONAL (3D) GLASSES\ | CN202548438U | MING ZHAO US | BOE TECHNOLOGY GROUP CO LTD US | G02B27/22\ G09G3/34\ G02B26/02 |
| 2943 | 3D printing | THREE-DIMENSIONAL RAPID PROTOTYPING METHOD AND DEVICE BASED ON PULSE SMALL HOLE DROPLET INJECTION\ | CN103056367A | DONG WEI US\ TAN YI US\ LU DONG US | UNIV DALIAN TECH (CN) | B22F3/115 |
| 2944 | 3D printing | LOOSE-LEAF INSERTED PIN FOR ENABLING GREENING CONTAINERS CONNECTED LEFT AND RIGHT TO ROTATE RANDOMLY\ | CN203067518U | PANG QIFU US | PANG QIFU US | F16C11/00 |
| 2945 | 3D printing | PLASTIC PIECE SURFACE AND METAL ORNAMENT COMPOSITE SEAMLESS PRIMARY INJECTION MOLDING AND FORMATION PROCESS\ | CN102962945A | LI QI US\ ZENG XIAODONG US\ WANG HUI (US) | TIANJIN ZHONGHUAN HI TECH CO US | B29C45/14\ B29B11/00 |
| 2946 | 3D printing | CORROSION AND SCALE RESISTING COATING\ | CN102936446A | CHEN HONGGEN US\ ZHANG JUSHENG US\ QU XINGWANG US\ FAN YUZHEN US | CHEN HONGGEN US | C09D5/00\ C09D163/00\ C09D5/08 |
| 2947 | 3D printing | THREE-DIMENSIONAL PRINTER EXTRUSION HEAD CAPABLE OF FEEDING IN SWITCHING MODE\ | CN102922745A | LI YAOTANG US\ YE SHIDONG US\ ZHANG HAIQIANG US\ LIU YUPENG US | GUANGZHOU ELECTRONIC TECHNOLOGY CO LTD CHINESE ACADEMY OF SCIENCES US | B29C67/00 |
| 2948 | 3D printing | ELECTROMAGNETIC FLEXIBLE COMPOSITE DEPOSITION DIRECT PREPARATION FORMING METHOD OF MULTIFUNCTIONAL GRADIENT COMPONENT\ | CN102962451A | LEI LEI US\ BAI XINGWANG US\ WANG XIANGPING US\ ZHANG HAIOU (CN)\ WANG GUILAN (CN) | UNIV HUAZHONG SCIENCE TECH (CN) | B22F3/105\ B22F7/02\ C23C24/10\ B22D27/02 |
| 2949 | 3D printing | PREPARATION METHOD OF NANO MODIFIED ULTRA-HIGH MOLECULAR WEIGHT POLYETHYLENE (UHMWPE) ANTISTATIC COMPOSITE MATERIAL\ | CN102888039A | ZHANG WEI US\ LI ZHI US\ HONG WEI US\ XU ZHENHUA US\ ZHAO CHUNBAO US\ LI JIANLONG US | SHANGHAI RES INST CHEMICAL IND US | C08K3/08\ C08K3/04\ B29C47/92\ C08L23/06\ C08K7/06\ B29B9/06\ C08K9/10 |
| 2950 | 3D printing | PREPARATION METHOD OF DETACHABLE PLANT THREE-DIMENSIONAL SPECIMEN\ | CN102835390A | WANG YING US\ CAI BAIYAN US\ LI YAN US | UNIV EAST HEILONGJIANG US | A01N3/00 |
| 2951 | 3D printing | METHOD FOR PRODUCING MICROCRYSTAL GLASS CERAMICS COMPOSITE BOARD\ | CN102775074A | DONGSHENG XIE US\ GUIPING DU US\ WEIHUA LV US\ WENFENG DENG US | GUANGDONG GOLD MEDAL CERAMICS CO LTD US | C03C17/02 |
| 2952 | 3D printing | PRODUCTION METHOD OF ACTIVE CARBON THREE-COMPONENT MELT-BLOWN NON-WOVEN FABRIC\ | CN102808288A | QU YAOHUA US | SHENZHEN CHINA TEXTILE FILTER MATERIAL NON WOVEN FABRICS CO LTD US | D04H5/06\ D04H5/08 |
| 2953 | 3D printing | SINGLE-SPINNERET-PLATE BINARY SKIN-CORE-TYPE COMPOSITE-SPINNING MULTIPLE-DIFFERENT-YARN PRODUCTION PROCESS\ | CN103060979A | ZHAN HAIHUA US\ SHEN YUNGEN US | UNIV SHAOXING US\ SHAOXING YUNXIANG CHEMICAL FIBER CO LTD US | D01D5/34\ D01D10/02\ D01D1/04\ D02G3/04\ D01D1/06\ D01D7/00\ D02G1/02 |
| 2954 | 3D printing | PREPARATION METHOD OF DESIGNABLE POLYMER-BASED CONDUCTIVE COMPOSITE MATERIAL\ | CN102837430A | GUO SHAOYUN US\ SHEN JIABIN US\ LI JIANG US\ ZHU JIAMING US\ YANG ZHI US | UNIV SICHUAN (CN) | C08L77/02\ C08K3/04\ B29C70/28\ C08L23/12\ C08L23/06\ C08L51/06 |
| 2955 | 3D printing | WIND TURBINE BLADE MOULD\ | CN102785315A | XINGDONG CAO US | CHANGZHOU SHENHUI ELECTRIC MATERIAL CO LTD US | B29C33/38 |
| 2956 | 3D printing | NEAR-NET-SHAPE FORMING METHOD OF REFRACTORY METAL PART\ | CN102773479A | JINHUI LIU US\ HUANAN LIU US\ SHENGBING XIAO US\ TONG WU US\ WENJUAN XIE US | HEILONGJIANG INST SCI & TECH US | B22F3/00\ B22F3/105 |
| 2957 | 3D printing | PRODUCING PROCESS FOR ANTIBACTERIAL FLAME-RETARDANT SINGLE AND MULTI-PORE THREE-DIMENSIONAL CRIMPED POLYESTER STAPLE FIBER\ | CN102704031A | QINRONG TONG US\ JIANQIU XU US\ ZUAN SUN US\ GUOZHI CAI US\ WENQIN WU US | SHANGHAI XINDILUN IND CO LTD US | C08G63/78\ D01F6/92\ D01F8/14\ D01D1/00\ D01F1/10\ D01D5/08\ D01D5/12\ D01F1/07\ D02J13/00\ C08G63/692 |
| 2958 | 3D printing | THERMAL TRANSFER IN-MOULD DECORATION AND PREPARATION METHOD THEREOF\ | CN102673293B | FU JINGUO US\ XIANG WEIWEI US | TEC HI PRINTING TECHNOLOGY CO LTD US | B44C3/02\ B32B27/00\ B44C5/00\ B44C1/16 |
| 2959 | 3D printing | METHOD FOR PRODUCING PTT (POLYTRIMETHYLENE TEREPHTHALATE) SPIRAL THREE-DIMENSIONAL HOLLOW FIBERS\ | CN102618947A | AIGUO MIAO US\ YUNCHANG LIN US\ AIZHEN LIN US\ YUZHONG HUO US\ AIMIN YANG US\ HUANMING XU US | AIGUO MIAO US | D01D5/24\ D02G1/00\ D01D1/04\ D01D5/096\ D01D5/092\ D02J1/00\ D02J13/00 |
| 2960 | 3D printing | POLYMERIC ULTRAVIOLET (UV) DECORATIVE BOARD AND PROCESSING TECHNOLOGY THEREOF\ | CN102616076A | GUOXIANG LI US | GUOXIANG LI US | B32B27/06\ B44C5/04 |
| 2961 | 3D printing | GRAPHENE/ STANNIC OXIDE NANOMETER COMPOUNDING RESISTANCE TYPE FILM GAS SENSOR AND MANUFACTURING METHOD THEREOF\ | CN102636522A | YANG LI US\ MUJIE YANG US\ QIANQIAN LIN US | UNIV ZHEJIANG US | G01N27/04 |
| 2962 | 3D printing | 3D (THREE DIMENSIONAL) LASER PRINTER\ | CN102608890A | ZITENG ZHU US | ZITENG ZHU US | G03G15/00\ G03G15/08 |
| 2963 | 3D printing | NANO-POROUS PTSI MATERIAL AND PREPARATION METHOD THEREOF\ | CN102593476A | JINSHAN YU US\ CHANGRUI ZHANG (CN)\ XINGUI ZHOU (CN) | NAT UNIV DEFENSE TECHNOLOGY (CN) | H01M4/92\ B01J23/42\ B01J35/10\ C25D11/02 |
| 2964 | 3D printing | METHOD FOR DIRECTLY MANUFACTURING POROUS PART OF COMPLICATED CAVITY BY USING LASER\ | CN102554474A | JICHANG LIU US | JICHANG LIU US | B23K26/34 |
| 2965 | 3D printing | PREPARATION METHOD OF HIGH-STRENGTH BIOLOGICAL GLASS BONE BRACKET WITH REGULAR-HOLE DISTRIBUTION\ | CN102526797A | WENCHAN HUANG US\ LINNAN LU US\ DEPING WANG US\ NAI ZHOU US\ XU CUI US\ LE LI US | UNIV TONGJI (CN) | A61L27/56\ A61L27/10\ A61L27/50 |
| 2966 | 3D printing | METHOD FOR PREPARING THREE-DIMENSIONAL NETWORK NANOPOROUS COPPER\ | CN102628112A | QI ZHOU US\ HAITAO WU US\ XINMIN ZHANG US\ QUAN ZHOU US | UNIV LANZHOU TECH US | C22C1/08\ B82Y40/00\ B22D11/06\ C22C9/05 |
| 2967 | 3D printing | STEREO PROJECTION SYSTEM\ | CN202306131U | WENJIN LIAO US\ JINLIANG HUANG US\ JIANPING HE US | SHENZHEN SAIYE EXHIBITION DISPLAY CO LTD US | H04N13/00\ G03B35/18\ G03B21/60\ G06F3/14 |
| 2968 | 3D printing | EMBEDDED TYPE MOULD\ | CN202292992U | ZHIYU HU US\ CHUANG JIANG US\ WENYONG CHENG US | SHANGHAI TONGCHUANG CERAMICS CO LTD US | B28B7/00 |
| 2969 | 3D printing | DEVICE FOR MANUFACTURING ALLOY-METAL-BASED PARTS BY SPRAY FORMING\ | CN202317021U | XIANJIE ZHANG US\ JUNBIAO WANG US\ CHAORUN SI US\ YUJUN LI US\ XIAOBAO CAO US | UNIV NORTHWESTERN POLYTECHNIC US | B22F3/115 |
| 2970 | 3D printing | ELECTRIC MULTIDIRECTIONAL METAL POWDER INJECTION FORMING MACHINE\[FR] MACHINE éLECTRIQUE MULTIDIRECTIONNELLE DE MOULAGE PAR INJECTION D'UNE POUDRE MéTALLIQUE\ | WO2013020496A1 | LIU JUN (CN) | LIU JUN (CN) | B22F3/115 |
| 2971 | 3D printing | OMNIBEARING ELECTRIC NUMERICAL CONTROL INJECTION MOLDING MACHINE\ | CN202213120U | MENTIONED THE INVENTOR HAS WAIVED THE RIGHT TO BE US | LIU JUN (CN) | B29C45/17\ B29C45/76\ B29C45/70\ B29C45/07\ B29C45/66 |
| 2972 | 3D printing | PRINT-TYPE FLEXIBLE DISPLAY SCREEN\ | CN202189208U | QIAN JUN US\ CHAOQUN TANG US\ JICHENG HU US\ YIHUA ZHOU US\ XINZHI SHI US\ CHANG QI US\ NA CHEN US\ LISHENG MA US | BEIJING 359 INVEST CO LTD US | G02F1/1335\ G02F1/1343\ G02F1/1333\ G02F1/1334 |
| 2973 | 3D printing | SELECTIVE LASER MELTING RAPID MOLDING EQUIPMENT FOR DIRECTLY FABRICATING LARGE-SIZED PARTS\ | CN202291409U | MING GAO US\ XIAOYAN ZENG (CN)\ ZEMIN WANG (CN)\ KAI GUAN (CN)\ QIANWU HU (CN)\ XIANGYOU LI (CN) | UNIV HUAZHONG SCIENCE TECH (CN) | B22F3/105\ B29C67/04 |
| 2974 | 3D printing | LASER-INDUCTION HYBRID MELTING DIRECT-FORMING DEVICE\ | CN202052935U | DENGZHI WANG US\ MINGMING MA US\ XIAOYAN ZENG (CN)\ ZEMIN WANG (CN)\ QIANWU HU (CN) | UNIV HUAZHONG SCIENCE TECH (CN) | B22F3/105 |
| 2975 | 3D printing | SINGLE-NOZZLE MELT-EXTRUSION-TYPE THREE-DIMENSIONAL PRINTER\ | CN103182781A | WANG YUNGAN (SG)\ WANG XUAN (SG) | SHANGHAI FOCHIF MECHATRONICS TECHNOLOGY CO LTD US | B29C47/92\ B29C69/02\ B29C47/08\ B29C47/12\ B29C47/34\ B29C53/56 |
| 2976 | 3D printing | HOLLOW FIBROUS MEMBRANE AND PREPARATION METHOD THEREOF\ | CN102527250B | ZHU WENBING US\ ZHANG TING US | HEBEI NORN WATER PURIFICATION EQUIPMENT CO LTD US | B01D69/08\ B01D67/00\ B01D71/34 |
| 2977 | 3D printing | METHOD FOR PREPARING CERAMIC PARTICLE REINFORCED STEEL-BASED MESH MATERIAL\ | CN102513522A | YEHUA JIANG US\ YUDONG SUI US\ ZULAI LI US\ RONG ZHOU US\ QIHONG CEN US\ QUAN SHAN US | UNIV KUNMING SCIENCE & TECH US | B22D19/14 |
| 2978 | 3D printing | FORMING METHOD FOR 3D (THREE-DIMENSIONAL) SHOE BODY OF SKATING SHOE\ | CN103182766A | YANG XINZHI US | SAKURAI SPORTS MFG CO LTD US | B29C45/14\ B29C45/26\ B29B11/00\ B29C33/12\ B29L31/50 |
| 2979 | 3D printing | PRODUCTION TECHNIQUE OF THREE-DIMENSIONAL CURLY HOLLOW POLYESTER STAPLE FIBERS\ | CN102517675A | YANQIONG WEI US\ JUNJIE YU US\ JIANNONG SHAO US\ YIPING DING US\ HONGBO XU US\ MINGGANG LI US\ ZHICHAO SUN US\ LIJUN WANG US | HANGZHOU BEST CHEMICAL FIBER CO LTD US | D01D5/088\ D01D5/22\ D01D5/24\ D01F6/84\ D01D1/00\ D01D10/02\ D01D5/08\ D01D5/12\ D01D1/04\ D01D5/096\ D01F11/08\ D06M13/46\ D06M15/643 |
| 2980 | 3D printing | HIGH CARBON AND HIGH TUNGSTEN SELF-MELTING ALLOY POWDER\ | CN102433497A | CHUNCHANG WANG US\ JIAJIA ZHAO US | SHAANXI TIAN YUAN MATERIALS PROT TECHNOLOGY CO LTD US | B22F1/00\ C22C38/12\ C23C4/06\ B22F9/04 |
| 2981 | 3D printing | No Title Available\ | CN102495509B | KUWABARA YUJI US\ KUWAHARA TAKU US | SPEC CO LTD US | H04N13/00\ G02B27/22\ G02F1/29\ G02F1/03 |
| 2982 | 3D printing | WEAR-RESISTANT CROSSLINKED PE (POLYETHYLENE) COMPOSITE PIPE AND MANUFACTURING METHOD THEREOF\ | CN102417650B | ZHANG WEIJIAO US\ GAO LI US\ JIANG JIANPING US | ZHEJIANG WEIXING NEW BUILDING MATERIALS CO LTD US | C08J3/24\ C08K5/098\ C08K13/04\ C08L27/18\ C08K3/04\ C08L23/08\ C08L23/16\ C08L23/06\ C08K3/16\ C08K7/06\ F16L9/12 |
| 2983 | 3D printing | DRY POWDER FIRE EXTINGUISHING AGENT\ | CN102512779B | DI GUOQING US\ BIAN SHUSONG US | UNIV SOOCHOW (CN) | A62D1/00 |
| 2984 | 3D printing | SPRAY FORMING SYSTEM FOR PREPARING ALLOY AND METAL-BASED COMPOSITE PARTS\ | CN102319898A | XIANJIE ZHANG US\ JUNBIAO WANG US\ CHAORUN SI US\ YUJUN LI US\ XIAOBAO CAO US | UNIV NORTHWESTERN POLYTECHNIC US | B22F3/115 |
| 2985 | 3D printing | METHOD FOR MANUFACTURING RAPID SHUTTER SPECTACLES USED IN STEREOSCOPIC DISPLAY\ | CN102323673A | JIHONG ZHENG US\ ZENGJUN ZHOU US\ LIJIA SUN US\ PINGYU TANG US\ SONGLIN ZHUANG US | UNIV SHANGHAI SCIENCE & TECH US | G02B27/22\ G02F1/1333 |
| 2986 | 3D printing | METHOD FOR RAPIDLY FORMING THREE-DIMENSIONAL METAL PARTS BY HIGH-POWER LASERS\ | CN102328081A | MINGMING MA US\ XIAOYAN ZENG (CN)\ ZEMIN WANG (CN)\ QIANWU HU (CN)\ XIANGYOU LI (CN) | UNIV HUAZHONG SCIENCE TECH (CN) | B22F3/105\ C23C24/10 |
| 2987 | 3D printing | LIQUID CRYSTAL COMPOSITION SUITABLE FOR 3D GLASSES\ | CN102433131A | HAIGUANG CHEN US\ DEYU HANG US\ TIANMENG JIANG US\ CHUNYAN YANG US\ GUANGTAO WANG US\ HUIQIANG TIAN US\ SHIHONG CHU US | BEIJING BAYI SPACE LCD MATERIALS TECHNOLOGY CO LTD US | G02B27/22\ C09K19/30\ C09K19/12\ C09K19/16\ C09K19/18\ C09K19/20\ C09K19/58 |
| 2988 | 3D printing | WATER CHANNEL PRE-FILLING METHOD USED DURING LOST FOAM CASTING OF ENGINE CYLINDER BODY\ | CN102343417A | ZHENGSONG MA US\ YUSHENG ZHANG US\ QIAO XU US | CHUZHOU JINNUO IND CO LTD US | B22C9/04 |
| 2989 | 3D printing | PRODUCTION TECHNOLOGY OF THREE-DIMENSIONAL HOLLOW FIBER\ | CN102443863A | HAIBIN CAI US | QUANZHOU SHENGXIN FIBER PRODUCT CO LTD US | D01D5/088\ D01D5/24\ D01D1/00\ D01D1/10\ D01D10/02\ D01D5/08\ D01D5/12 |
| 2990 | 3D printing | METHOD FOR PREPARING SURFACE MICROSTRUCTURE OF SUPER-HYDROPHOBIC AMORPHOUS ALLOY\ | CN102328902B | LI NING US\ XIA TING US\ XU WEI US\ LIU LIN US | UNIV HUAZHONG SCIENCE TECH (CN) | B81C1/00\ C22C45/00\ C22C45/10 |
| 2991 | 3D printing | ELECTRIC MULTI-AZIMUTH METAL POWDER INJECTION MOLDING MACHINE\ | CN102921949A | THE INVENTOR HAS WAIVED THE RIGHT TO BE MENTIONED US | LIU YA US | B22F3/115 |
| 2992 | 3D printing | ELECTRICAL ALL-DIMENSIONAL NUMERICAL CONTROL INJECTION MOLDING MACHINE\ | US2014141112A1 | LIU JUN (CN) | LIU JUN (CN) | B29C45/72\ B29C45/80 |
| 2993 | 3D printing | THREE-DIMENSIONAL DISPLAY APPARATUS AND METHOD FOR MANUFACTURING THE SAME\ | US2014118643A1 | HUANG HONG-JI (CN) | HUANG HONG-JI (CN)\ SHENZHEN CHINA STAR OPTOELECT (CN) | G02B27/22\ G02F1/1335 |
| 2994 | 3D printing | DEFECTIVE TOTAL STERNUM REPLACEMENT PART FOR TOTAL STERNUM DEFECT OPERATION TREATMENT AND METHOD FOR MANUFACTURING THE SAME\ | CN102389343A | HUA TANG US\ ZHIFEI XU US\ BIN WU US\ MINGMING JI US | HUA TANG US\ ZHIFEI XU US\ BIN WU US\ MINGMING JI US | A61F2/28\ A61L27/18\ A61L27/12\ A61B17/56 |
| 2995 | 3D printing | THREE-DIMENSIONAL VISUALIZATION METHOD FOR ELECTROMAGNETIC ENVIRONMENT BODY DATA\ | CN102254347A | LINGDA WU US\ CHAO YANG US\ RONGHUAN YU US | EQUIPMENT COMMAND AND TECHNOLOGY COLLEGE OF PLA US | G06T17/00 |
| 2996 | 3D printing | CATALYST COMPONENT FOR HIGH TEMPERATURE OLEFIN POLYMERIZATION AND PREPARATION METHOD THEREOF\ | CN102838697B | XU XIUDONG US\ TAN ZHONG US\ ZHOU QILONG US\ YAN LIAN US\ YU JINHUA US\ LI FENGKUI US\ YIN SHANSHAN US\ SONG WEIWEI US | CHINA PETROLEUM & CHEMICAL US\ BEIJING CHEM INST CN PETROLEUM US | C08F10/00\ C08F4/646\ C08F4/649\ C08F4/651\ C08F4/654\ C08F10/06 |
| 2997 | 3D printing | METHOD FOR REMANUFACTURING DIE WITH GRADIENT FUNCTION BY REPAIRING MICRO ZONE THROUGH LASER\ | CN102248164A | JIAN SUN US\ ZHENGYANG LI US\ DEYING GENG US\ GANG DING US\ JIAWEI DING US\ JILONG YAO US\ HUAPING TANG US | DANYANG HUIDA MOLD MATERIAL TECHNOLOGY CO LTD US | B22F3/105\ B22F7/08 |
| 2998 | 3D printing | LASER-INDUCTION HYBRID MELTING DIRECT FORMING METHOD AND DEVICE\ | CN102179517A | DENGZHI WANG US\ MINGMING MA US\ XIAOYAN ZENG (CN)\ ZEMIN WANG (CN)\ QIANWU HU (CN) | UNIV HUAZHONG SCIENCE TECH (CN) | B22F3/105 |
| 2999 | 3D printing | RAPID PROTOTYPING METHOD AND DEVICE BASED ON SUPERSATURATION SALINE SOLUTION CRYSTALLIZATION\ | CN102649020A | YUEGUANG DENG US\ JING LIU US | CHINESE ACAD TECH INST PHYSICS US | B01D9/00 |
| 3000 | 3D printing | MEDICAL C-ARM THREE-DIMENSIONAL IMAGING SYNCHRONOUS CONTROL DEVICE\ | CN102934999A | LI JINSHENG US\ GAO SONG US | NANJING PERLOVE RADIAL VIDEO EQUIPMENT CO LTD US | A61B6/00 |
| 3001 | 3D printing | RAPID MOLDING METHOD BASED ON ELECTRORHEOLOGICAL FLUID AND APPARATUS THEREOF\ | CN102615830A | YUEGUANG DENG US\ JING LIU US | CHINESE ACAD TECH INST PHYSICS US | B29C67/00 |
| 3002 | 3D printing | THREE-DIMENSIONAL (3D) DISPLAY PANEL 3D DISPLAY SYSTEM AND 3D DISPLAY\ | CN202019419U | YUANYUAN LI US | KONKA GROUP CO LTD US | H04N13/00\ G09G3/32 |
| 3003 | 3D printing | LOCOMOTIVE CRANKSHAFT LASER CLADDING SYSTEM WITH YAG (YTTRIUM ALUMINUM GARNET) LASER DEVICE AS LIGHT SOURCE\ | CN201915147U | BING AN US | GANSU JINGCHENG PHOTOELECTRIC TECHNOLOGY CO LTD US | C23C24/10 |
| 3004 | 3D printing | ADSORPTION ROLLING BEARING\ | CN201827235U | ZHEN HUANG US | ZHEN HUANG US | F16C33/32\ F16C33/58\ F16C33/66\ F16C19/00\ F16C19/06\ F16C19/26\ F16C33/34 |
| 3005 | 3D printing | HIGH-POLYMER ELASTIC AND VENTILATING PILLOW INNER\ | CN201641314U | XIAOLIANG ZHUANG US | XIAOLIANG ZHUANG US | A47G9/10 |
| 3006 | 3D printing | METHOD FOR MANUFACTURING IMPELLER MOULD FOR GAS COMPRESSOR OF TURBOCHARGER\ | CN102125998A | GUOWEN CHEN US\ JIANFEI YAN US\ FUXING DONG US\ AIGUO ZHU US\ YUNHU FENG US\ GANG CHEN (CN) | DATONG NORTH TIANLI TURBOCHARGING TECHNOLOGY CO LTD US | B22C9/06\ B22C9/22 |
| 3007 | 3D printing | FLAME-RETARDING POLYPHENYL ETHER COMPOSITE AND PREPARATION METHOD AND USE THEREOF\ | CN102101941A | HENGMEI HUANG US\ LIN WANG US\ JIANZHONG LIU US\ SHAOHUA GUO US\ JINFENG FU US\ ZHIHUA HU US\ NANBIAO YE US | KINGFA SCI & TECH CO LTD US\ SHANGHAI KINGFA SCI & TECH CO US\ CHANG XIN MIANYANG NEW MATERIALS LTD US | C08K5/20\ C08L9/00\ C08L23/00\ C08L23/08\ C08L23/16\ C08L53/02\ C08K5/523\ B29C47/92\ C08L71/12\ C08K5/00\ C08K5/3492\ C08L9/02\ C08L25/12\ H01B7/295\ H01B7/36 |
| 3008 | 3D printing | NEW PROCESS FOR SPRAY-COATING POWDER ON SURFACE OF METAL\ | CN101992183A | ZHENJIANG SUN US | TIANJIN CITY YIJIANG ALUMINUM MATERIAL CO LTD US | B05D3/02\ B05D7/24\ B05D7/14\ B05D5/00\ B05D3/00 |
| 3009 | 3D printing | METHOD FOR PREPARING MELT SPUN CHLORINE-RESISTANCE HIGH RESILIENT POLYURETHANE FIBER\ | CN102011211A | MINCHAO LI US\ LIMING CHENG US\ HAIFENG CHEN US\ DONGZHI ZHANG US\ WENMING WANG US\ GAOQIANG LI US | ZHANGJIAGANG WOSITING RES INST CO LTD US | C08G18/42\ B29C47/92\ D01D5/088\ D01F6/94\ C08G18/48\ C08G18/66\ D01D1/00\ D01D1/10\ D01D10/02\ D01F1/10 |
| 3010 | 3D printing | SPECIAL POLYCARBONATE FILM FOR 3D AUTOMOBILE INSTRUMENT PANEL AND PREPARATION METHOD THEREOF\ | CN102019854A | WEI LUO US\ YUEZHANG REN US | SUZHOU AOMEI MATERIALS TECHNOLOGY CO LTD US | B60K35/00\ B29C47/92\ B29L7/00\ B29K69/00 |
| 3011 | 3D printing | TECHNOLOGY FOR APPLYING INLAID METAL FINISHING COMPONENT TO IN-MOLD LABELLING\ | CN102029683A | JINGYAN LIU US\ XIAODONG ZENG US\ HUI WANG US\ QI LI US | TIANJIN ZHONGHUAN HI TECH CO US | B29C45/14 |
| 3012 | 3D printing | PRODUCTION METHOD FOR MANUFACTURING ALUMINUM ALLOY EYEWEAR ACCESSORIES\ | CN101912955A | XIUJUAN YANG US | XIUJUAN YANG US | B22D17/10\ G02C5/00 |
| 3013 | 3D printing | SANDALWOOD TYPE SOLID WOOD GRAIN SPRAYING PROCESS\ | CN101961706A | HAOHONG XU US | HAOHONG XU US | B05D5/00\ B05D1/38\ B05D7/06 |
| 3014 | 3D printing | LIQUID MOLDING CORE-MELTING FORMING PROCESS FOR COMPLICATED RESIN-BASED COMPOSITE MATERIAL STRUCTURAL MEMBER\ | CN101913251A | GUIZHE ZHAO US\ YANFEI ZHANG US\ RUIKUI DU US\ YAQING LIU US | UNIV NORTH CHINA US | B29C70/54\ B29C70/36 |
| 3015 | 3D printing | MANUFACTURING METHOD OF SUPER THIN BIDIRECTIONAL STRETCHED POLYPROPYLENE COARSE THIN FILM FOR CAPACITOR\ | CN101863120B | KE XIBIN US\ LI GAOCONG US\ SHEN SHILIN US\ SU LIYONG US\ XIE JIN US | SICHUAN EM TECHNOLOGY CO LTD US | B29C47/92\ B29C47/88\ B29C47/06\ B29K23/00\ B29C55/14\ B29L7/00 |
| 3016 | 3D printing | METHOD FOR PRODUCING MULTICOLOR CHOCOLATE\ | CN101912023A | GUOXIONG LI US | GUOXIONG LI US | A23G1/04 |
| 3017 | 3D printing | PRODUCTION METHOD OF DOUBLE METAL NETWORK-INTERPENETRATED MULTIPHASE SECTION\ | CN101825218B | WANG YONG US\ CUI MIN US\ MA RU US\ WANG SHOUREN US | UNIV JINAN US | F16S5/00 |
| 3018 | 3D printing | METHOD FOR MANUFACTURING METAL PARTS AND MOLDS AND MICRO-ROLLER USED THEREFOR\ | US2013197683A1 | ZHANG HAIOU (CN)\ WANG GUILAN (CN) | UNIV HUAZHONG SCIENCE TECH (CN) | B23K26/34\ B23K9/04\ B23K10/00 |
| 3019 | 3D printing | ADSORPTION ROLLING BEARING\ | CN102213262A | ZHEN HUANG US | ZHEN HUANG US | F16C33/32\ F16C33/58\ F16C33/66 |
| 3020 | 3D printing | ASIA1 TYPE MULTI-EPITOPE RECOMBINANT VACCINE OF BOVINE FOOT-AND-MOUTH DISEASE VIRUSES AND PREPARATION METHOD THEREOF\ | CN101775399A | HUIYUN CHANG US\ GUOZHENG CONG US\ JUNZHENG DU US\ SHANDIAN GAO US\ TONG LIN US\ JUNJUN SHAO US\ JINGFENG WANG US | LANZHOU VETERINARY RES INST CH US | C12N15/62\ A61K48/00\ C12N15/42\ A61K39/135\ A61K47/48\ A61P31/14\ C07K14/09 |
| 3021 | 3D printing | MANUFACTURING METHOD AND DEVICE OF INORGANIC PLATES AND INORGANIC PLATES\ | CN102190424A | CHANGLIN WANG US\ JIANBIN QI US\ SENBEN GENG US\ CHANGXING WANG US | JINGNIU GLASS CERAMICS GROUP US | B28B1/00\ C03B13/00\ C03B15/02\ C03B17/06\ C03B25/00\ C04B35/14 |
| 3022 | 3D printing | HIGH MOLECULAR ELASTIC VENTILATED PILLOW INNER AND PREPARATION METHOD THEREOF\ | CN102133017A | XIAOLIANG ZHUANG US | XIAOLIANG ZHUANG US | A47G9/10 |
| 3023 | 3D printing | METHOD FOR FORMING REFRACTORY METAL PARTS BY USING LASER\ | CN101780544A | JINHUI LIU US\ CAN ZHAO US\ JIBING CHEN US\ RUIDI LI US\ DANQING ZHANG US | HEILONGJIANG INST OF SCIENCE A US | B22F3/105\ B23K26/34\ C23C24/10 |
| 3024 | 3D printing | LOW TEMPERATURE THERMOPLASTIC MATERIAL AND PREPARING METHOD THEREOF\ | US8859691B2 | ZHANG ZHONGQUN (CN) | KLARITY MEDICAL & EQUIPMENT GZ CO LTD (CN)\ ZHANG ZHONGQUN (CN) | B29C35/08\ C08L75/04\ C08F283/00\ C08L67/04 |
| 3025 | 3D printing | DEVICE FOR PREPARING SEMI-SOLID ALLOY MELT\ | CN201512567U | JIANBO TAN US\ ZENGMIN LI US\ LIXIN LI US | UNIV HEBEI SCIENCE & TECH US | B22D17/08\ C22C1/00 |
| 3026 | 3D printing | THREE-DIMENSIONAL PICTURE LUMINOUS CUP\ | CN201436933U | YINGCHAO TANG US | SHENZHEN XINCHANG PLASTIC PRODUCT CO LTD US | B44D2/00\ A47G19/22\ B44F1/06\ H05B33/00 |
| 3027 | 3D printing | THREE-DIMENSIONAL THREE-U ENERGY-SAVING LAMP TUBE WITH ONE GAP BRIDGE\ | CN201383484Y | WENYUAN QIAN US | WENYUAN QIAN US | H01J61/00\ H01J61/28\ H01J61/30\ H01J61/36 |
| 3028 | 3D printing | NEAR-NET-SHAPE METHOD FOR POROUS METAL PART\ | CN101722306A | RUIDI LI US\ ZHIGANG WANG US\ YUSHENG SHI (CN)\ QINGSONG WEI (CN) | UNIV HUAZHONG SCIENCE TECH (CN) | B22F3/11 |
| 3029 | 3D printing | SHEEP APHTHOVIRUS ASIAL TYPE MULTI-EPITOPE RECOMBINANT VACCINE AND PREPARATION METHOD THEREOF\ | CN101864434A | HUIYUN CHANG US\ GUOZHENG CONG US\ JUNZHENG DU US\ SHANDIAN GAO US\ TONG LIN US\ JUNJUN SHAO US\ JINGFENG WANG US | LANZHOU VETERINARY RES INST CHINESE ACADEMY OF AGRICULTURAL SCIENCES US | C12N15/62\ A61K48/00\ C12N15/42\ A61K39/135\ A61P31/14\ C07K14/09 |
| 3030 | 3D printing | CERAMIC/METALLIC DOUBLE CONTINUOUS PHASE COMPOSITE MATERIAL BRAKE PAD AND PREPARATION METHOD THEREOF\ | CN102102720B | ZHANG JINSONG US\ CAO XIAOMING US\ ZHANG YUNAN US\ TIAN CHONG US\ YANG ZHENMING US | INST METAL RES CHINESE ACAD SC (CN) | F16D69/02 |
| 3031 | 3D printing | DIGITAL PRE-LAMINATING FILM\ | CN101705060A | ZHAOZHAO ZHANG US | HU MINSHENG US | C09J123/08\ C09J7/02\ C09J157/02 |
| 3032 | 3D printing | MANUFACTURING METHOD FOR MAKING ANTIQUE ARTISTIC FLOWERPOT\ | CN101755632A | XIANLIAN LIU (CN) | XIANLIAN LIU (CN) | A01G9/02\ B44D5/10 |
| 3033 | 3D printing | THREE-DIMENSIONAL BRONZING METHOD\ | CN101670719A | RUIJUN WU US | SHENZHEN BAIXINGLONG CREATIVE US | G03C1/73\ B41M5/26\ B41M1/22\ B41F19/06 |
| 3034 | 3D printing | TRANSPARENT GLASS CERAMIC WITH TUNABLE LIGHT EMITTING COLORS AND PREPARATION TECHNIQUE THEREOF\ | CN102092952A | PING HUANG US\ DAQIN CHEN US\ YUANSHENG WANG US | FUJIAN INST RES STR MATTER CAS US | C03B32/02\ C03C10/16 |
| 3035 | 3D printing | MOULD STRUCTURE WITH CONFORMAL COOLING PIPE\ | CN101670627A | JINHUI LIU US\ CAN ZHAO US\ JIBING CHEN US\ RUIDI LI US | UNIV ZHEJIANG SCIENCES US | B29C33/38\ B29C33/04\ B29C45/73\ B22F3/22\ B29C45/26 |
| 3036 | 3D printing | MOULD MAKING METHOD WITH CONFORMAL COOLING PIPE\ | CN101670437A | JINHUI LIU US\ CAN ZHAO US\ JIBING CHEN US\ RUIDI LI US | UNIV ZHEJIANG SCIENCES US | B22F3/22 |
| 3037 | 3D printing | CLOTHING PROTECTION STRUCTURE AND PRODUCTION METHOD THEREOF\ | CN101647605A | ZHAO JUN US | HANGZHOU FUXING INDUSTRY & TRA US | A41D13/00 |
| 3038 | 3D printing | PREPARATION METHOD OF DYEABLE POLYESTER COMPOSITE FIBER OF CATIONIC DYE\ | CN101613894B | XIUHUA WANG US\ YUYUAN YAO US\ JICHENG MENG US | UNIV ZHEJIANG SCIENCE & TECH US | D01D5/34\ D01F8/14 |
| 3039 | 3D printing | INJECTION MOULD PRODUCT IN-MOULD PRINTING MOLDING METHOD\ | CN101474849A | WEIXIA ZHU (CN) | WEIXIA ZHU (CN) | B29C45/17 |
| 3040 | 3D printing | INJECTION MOULD PRODUCT IN-MOULD PRINTING MOLDING EQUIPMENT\ | CN101474848A | WEIXIA ZHU (CN) | WEIXIA ZHU (CN) | B29C45/17 |
| 3041 | 3D printing | DEVICE FOR PREPARING SEMI-SOLID ALLOY MELT AND PROCESS FOR PREPARING ALSI9MG SEMI-SOLID ALLOY\ | CN101660063A | JIANBO TAN US\ ZENGMIN LI US\ LIXIN LI US | UNIV HEBEI SCIENCE & TECH US | C22C1/00 |
| 3042 | 3D printing | NUMERICALLY-CONTROLLED PLASTIC THIN-LAYER SEDIMENT MOULDING MACHINE\ | CN101693407A | YUHUI LING US | YUHUI LING US | B29C67/00 |
| 3043 | 3D printing | METHOD FOR PREPARING THREE-DIMENSIONAL PHOTOELASTICITY ANALYSIS MODEL BY APPLYING FAST-FORMING PLASTER MOLD TECHNIQUE\ | CN101623907A | GUANGHUI CHEN US\ GUOQI CUI US | TIANJIN INTERNAL COMB ENGINE R US | B29C39/42 |
| 3044 | 3D printing | METHOD FOR PACKAGING DYE-SENSITIZED SOLAR BATTERY\ | CN101582335A | RUIXING GAO (CN)\ ZHIJUN YANG (CN)\ KE YU (CN) | IRICO GROUP CORP (CN) | H01G9/20\ H01L51/48\ H01M14/00 |
| 3045 | 3D printing | INTEGRAL QUICK FORMING METHOD FOR CERAMIC PARTS BASED ON PARAFFIN\ | CN101550007A | XIUFENG WANG (CN)\ WENJIE XU (CN)\ HONGTAO JIANG (CN)\ CHENGLONG YU (CN) | UNIV SHAANXI SCIENCE & TECH (CN) | C04B35/622\ B28B7/34\ C04B35/632\ B28B1/54 |
| 3046 | 3D printing | ENVIRONMENT-PROTECTION STEREO AUTOMOBILE FOOT PAD\ | CN201254118Y | YUNBO GUO (CN) | DONGGUAN HONG EN PLASTIC PRODU (CN) | B60N3/06 |
| 3047 | 3D printing | APPARATUS FOR PRODUCING FINE THREE-DIMENSIONAL FIGURED PLATE GLASS WITH FLOATING METHOD\ | CN201236175Y | XIAOXIAO YANG (CN)\ DENING YANG (CN) | DENING YANG (CN) | C03B18/12\ C03B18/14 |
| 3048 | 3D printing | METAL MASTER MOLD FOR ELECTROFORMING AND USE THEREOF\ | CN101403125A | ZHONGYAO YU (CN)\ GUANNAN LI (CN)\ JING CHENG (CN) | BOAO BIOTECH CO LTD (CN) | C25D1/10 |
| 3049 | 3D printing | PRODUCTION METHOD OF FLAME-RETARDANT SINGLE-HOLE HOLLOW OR MULTI-HOLE THREE-DIMENSIONAL CRIMPED POLYESTER STAPLE FIBER\ | CN101676451A | JIANJUN ZHANG US\ QINRONG TONG US\ JIANQIU XU US\ GUANGZE WU US\ ZUAN SUN US\ HAITIAN XIA US\ GUOZHI CAI US\ HENG ZHANG US\ WENQIN WU US | SHANGHAI XINDILUN IND CO LTD US\ TOPRET IND DESIGN SHANGHAI CO US | D01D5/24\ D01F6/84\ D02G1/00 |
| 3050 | 3D printing | METHOD FOR QUICKLY PRODUCING REMOVALBE PARTIAL DENTURE BRACKET\ | CN101401746B | XIAOYAN ZENG (CN)\ ZEMIN WANG (CN)\ KAI GUAN (CN)\ QIANWU HU (CN)\ GUANGXIA CHEN (CN) | UNIV HUAZHONG SCIENCE TECH (CN) | A61C13/12 |
| 3051 | 3D printing | ENTIRETY QUICK MANUFACTURE METHOD OF HOT ISOSTATIC PRESSING METAL WRAPPS\ | CN101391302A | YUSHENG SHI (CN)\ QINGSONG WEI (CN)\ BINGBING QU (CN)\ YANYING DU (CN)\ JIE LIU (CN) | UNIV HUAZHONG SCIENCE TECH (CN) | B22F3/105 |
| 3052 | 3D printing | MANUFACTURING AND REPAIRING METHOD FOR CONDUCTIVE CIRCUIT OF THREE DIMENSIONAL MOLD INTERCONNECTING DEVICE\ | CN101394710A | XIAOYAN ZENG (CN)\ YU CAO (CN)\ XIANGYOU LI (CN)\ JIANGUO LIU (CN) | UNIV HUAZHONG SCIENCE TECH (CN) | H05K3/10\ H05K3/20 |
| 3053 | 3D printing | NO-MOLD FUSION STACKING MANUFACTURE METHOD OF PARTS OR MOLD\ | CN101362272A | HAIOU ZHANG (CN)\ GUILAN WANG (CN)\ ZHONGCHAO WEI (CN) | UNIV HUAZHONG SCIENCE TECH (CN) | B23K26/34\ B23K37/06\ B23K9/04 |
| 3054 | 3D printing | METHOD FOR MODELING THREE-DIMENSIONAL PATTERN\ | CN101722784A | JIANGANG HE US | JIANGANG HE US | B44C1/20 |
| 3055 | 3D printing | TECHNIQUE FOR MANUFACTURING AIR PRESSURE TYPE NON-CUTTING QUICK-MOLDING LOW MELTING-TEMPERATURE ALLOY MOLD\ | CN101433952A | KEFEI XIE (CN) | KEFEI XIE (CN) | B22D18/04\ B22C9/02 |
| 3056 | 3D printing | ELECTRON BEAM MELTING PREPARATION OF PERSONALIZED TITANIUM MADE MATERIAL CRANIOMAXILLARY BONE REPAIRING BODY\ | CN101249028A | XIAOFENG LI (CN)\ CAN LIU (CN)\ QIAN JIANG (CN)\ XING WU (CN) | BEIJING GIMMAFEI TECHNOLOGY DE (CN) | G06F17/50\ G06T17/00\ A61F2/28\ A61B17/56\ G06T15/00\ A61B6/03\ C22C14/00\ A61L27/06\ C22C1/02 |
| 3057 | 3D printing | SILICON WITH THREE-DIMENSIONAL DEPRESSION STRUCTURE AND METHOD FOR PREPARING SAME\ | CN101311350A | ZHONGHE XI (CN)\ YUE WU (CN)\ GENGMIN ZHANG (CN)\ HONGYU CUI (CN)\ DENGZHU GUO (CN) | UNIV BEIJING (CN) | C30B29/60\ C30B29/06 |
| 3058 | 3D printing | SILICON WITH THREE-DIMENSIONAL DEPRESSION STRUCTURE AND METHOD FOR PREPARING SAME\ | CN101311347A | ZHONGHE XI (CN)\ YUE WU (CN)\ GENGMIN ZHANG (CN)\ HONGYU CUI (CN)\ DENGZHU GUO (CN) | UNIV BEIJING (CN) | C30B29/60\ C30B29/06 |
| 3059 | 3D printing | PRECISION-INVESTMENT CASTING METHOD\ | CN101406932A | YUANHUA DENG (CN) | QUANZHOU TENGDA FINE CASTING C (CN) | B22C9/04\ B22D15/00\ B22D31/00 |
| 3060 | 3D printing | HAFNIUM IRON MANGANESE THREE-DOPED LITHIUM NIOBATE CRYSTAL AND METHOD OF PRODUCING THE SAME\ | CN101319392A | XIAOCHUN LI (CN)\ XUEZHUAN YI (CN)\ XIONGYING XIA (CN)\ ZHE WANG (CN)\ JINWEN ZHOU (CN)\ FENG SHI (CN)\ LONGSHENG WANG (CN) | UNIV TAIYUAN TECHNOLOGY (CN) | C30B29/30\ C30B15/00 |
| 3061 | 3D printing | THREE-DIMENSIONAL STEPHANOPORATE ORGANIZATION ENGINEERING BRACKET MATERIAL FIBRE CEMENTING METHOD PREPARING SAME AND APPLICATIONS THEREOF\ | CN101249277A | QING YANG (CN)\ ZHIQING TAN (CN)\ XINYUAN SHEN (CN)\ LANLAN PENG (CN)\ SISHI CHEN (CN) | UNIV DONGHUA (CN) | A61F2/28\ A61L27/56\ A61L27/18 |
| 3062 | 3D printing | METHOD FOR PREPARING HIGH TEMPERATURE FLUENT METAL LOOP BY QUARTZ AND SICF/SIC COMPOSITE MATERIAL\ | CN101320600A | XINGUI ZHOU (CN)\ YICAN WU (CN)\ WANG JUN (CN)\ CHANGRUI ZHANG (CN)\ QUNYING HUANG (CN)\ YINGBIN CAO (CN)\ RONGJUN LIU (CN)\ HONGLEI WANG (CN)\ HAIJIAO YU (CN)\ SHUANG ZHAO (CN)\ ZHIYI WANG (CN)\ ZELAN HUANG (CN) | NAT UNIV DEFENSE TECHNOLOGY (CN) | C04B41/50\ G21B1/11\ C04B35/66 |
| 3063 | 3D printing | ENVIRONMENT-PROTECTIVE STEREO AUTOMOBILE FOOT PAD AND MANUFACTURING METHOD THEREOF\ | CN101376351A | YUNBO GUO (CN) | DONGGUAN HONGEN RUBBER AND PLA (CN) | B32B3/30\ B32B27/06\ B32B5/18\ B60N3/06\ B29C43/18\ B29C43/28\ B29K23/00\ B29K96/00\ B32B7/08 |
| 3064 | 3D printing | SELECTIVE LASER FUSION RAPID MOLDING METHOD AND DEVICE OF MEDICAL IMPLANTATION MATERIAL WAX MOULD\ | CN101310964B | YONGQIANG YANG (CN)\ SHUFAN WANG (CN)\ WEIHUI WU (CN) | UNIV SOUTH CHINA TECH (CN) | B29C51/00\ B29C73/00\ A61B6/03\ B29K91/00\ B29L22/00 |
| 3065 | 3D printing | AN EQUIPMENT FOR PRODUCING GLASS WITH REFINED THREE-DIMENSIONAL PATTERN BY FLOAT PROCESS AND THE METHOD FOR PRODUCING THE GLASS\[FR] MATéRIEL PERMETTANT DE PRODUIRE PAR PROCESSUS FLOAT DU VERRE AVEC UN MOTIF EN TROIS DIMENSIONS RAFFINé ET PROCéDé DE PRODUCTION DE VERRE\ | WO2009124465A1 | YANG DENING (CN)\ YANG XIAOXIAO (CN) | YANG DENING (CN)\ YANG XIAOXIAO (CN) | C03B18/12\ C03B18/14\ C03B5/173 |
| 3066 | 3D printing | COMPOSITE ELECTRODE FOR FINE ELECTRIC SPARK PROCESSING\ | CN101259548A | YUANGANG WANG (CN)\ FULING ZHAO (CN)\ JIN WANG (CN) | UNIV DALIAN TECH (CN) | B23H1/04 |
| 3067 | 3D printing | SILICON CARBIDE FOAMED CERAMIC CORRUGATED PLATE AND PREPARATION METHOD THEREOF\ | CN101555138B | ZHANG JINSONG US\ CAO XIAOMING US\ TIAN CHONG US\ YANG ZHENMING US\ LIU QIANG US | INST METAL RES CHINESE ACAD SC (CN) | C04B35/565\ B28B3/12\ C04B38/00\ C04B41/85\ B28B1/29 |
| 3068 | 3D printing | METHOD OF MANUFACTURING 300M ULTRAHIGH STRENGTH STEEL AIRCRAFT LANDING GEAR\ | CN101229586A | YUNGE WANG (CN)\ XIANGMING WANG (CN)\ HUAMING WANG (CN) | YUNGE WANG (CN) | B22F3/105 |
| 3069 | 3D printing | METHOD OF MANUFACTURING AF1410 ULTRAHIGH STRENGTH STEEL AIRPLANE HORIZONTAL TAIL AXIS\ | CN101229585A | YUNGE WANG (CN)\ XIANGMING WANG (CN)\ HUAMING WANG (CN) | YUNGE WANG (CN) | B22F3/105 |
| 3070 | 3D printing | MIXED POWDER ALLOY WITH HIGH MELTING POINT PARTICLES OR FIBERS AND LOW MELTING POINT PARTICLES AS WELL AS METHOD FOR PREPARING SAME\ | CN101519737A | GUANGWU WANG US | GUANGWU WANG US | C22C1/08\ C22C47/12\ C22C49/00 |
| 3071 | 3D printing | PRINTING MEMBRANE\ | CN201075278Y | WENJIAN LUO (CN) | LUOTIE MACHINERY CO LTD (CN) | G09F23/00 |
| 3072 | 3D printing | HAMMER HEAD STRUCTURE OF ORE CRUSHER\ | CN201098622Y | MAOXUN ZHANG (CN)\ YUQUN LIN (CN) | FUZHOU HONGLIAN ENGINEERING MA (CN) | B02C13/28 |
| 3073 | 3D printing | SPUMY CERAMIC FILTER CAPABLE OF HIGH SLAG RESISTANCE AND THERMAL STABILITY AND FABRICATING METHOD THEREOF\ | CN101224361A | DONGQING HE (CN)\ HAIJUN SUN (CN)\ HE LI (CN)\ QI HE (CN)\ LI XU (CN) | ELITE CERAMICS IND BEIJING CO (CN) | C04B35/565\ C04B38/06\ B01D39/20 |
| 3074 | 3D printing | LIQUID STATE MICRO-FORMING METHOD FOR THREE-DIMENSIONAL COMPLEX MICRO-COMPONENT\ | CN100515609C | BANGSHENG LI (CN)\ MINGXING REN (CN)\ CHUANG YANG (CN) | HARBIN INST OF TECHNOLOGY (CN) | B22D17/08 |
| 3075 | 3D printing | METHOD FOR IMPROVING INGOT SURFACE QUALITY\ | CN100457322C | KUN XU (CN)\ DENGQUAN CHEN (CN)\ XIMIN LUO (CN)\ ZEGUANG LIU (CN) | SINO PLATINUM METALS CO LTD (CN) | B22C3/02 |
| 3076 | 3D printing | WAVEGUIDE OPTICAL SWITCH AND MAKING METHOD THEREOF\ | CN100445783C | SHULIN E (CN)\ GUANGMING KONG (CN)\ HUDAN ZHAO (CN)\ WENYUAN DENG (CN) | CHANGCHUN OPTICS FINE MECH (CN) | G02B6/12\ G02B6/13\ G02B6/35 |
| 3077 | 3D printing | MICROSPRING LASER PROCESSING METHOD\ | CN100455841C | HONGBO SUN (CN) | JINLIN UNIV (CN) | F16F1/06 |
| 3078 | 3D printing | THREE-DIMENSION POROUS TISSUE ENGINEERING CARRIER MATERIAL AND PREPARATION AND APPLICATION\ | CN101147812A | QING YANG (CN)\ XIJIAN WANG (CN)\ ZHIQING TAN (CN)\ XINYUAN SHEN (CN)\ LANLAN PENG (CN)\ SISHI CHEN (CN) | UNIV DONGHUA (CN) | A61L27/56\ A61L27/26 |
| 3079 | 3D printing | HAMMERHEAD STRUCTURE OF THE ORE CRACKING MECHANISM AND PROCESSING METHOD THEREOF\ | CN101116836A | MAOXUN ZHANG (CN)\ YUQUN LIN (CN) | FUZHOU HONGLIAN ENGINEERING MA (CN) | B02C13/28\ B22D19/16 |
| 3080 | 3D printing | SECTION BAR SURFACE PATTERN MOLDING DEVICE\ | CN200981338Y | JIANMIN HAN (CN) | JIANMIN HAN (CN) | B29C47/08\ B29C59/04 |
| 3081 | 3D printing | PAPER PLASTIC GLUE-FREE COMPOSITE BIDIRECTIONAL STRETCHING POLYPROPYLENE FILM AND ITS PREPARATION METHOD\ | CN101058247A | XU WENSHU ZHU (CN) | GUANGDONG DECRO PACKAGE FILMS (CN) | B29C71/00\ B29C71/02\ B32B27/06\ B32B27/18\ B32B27/10\ B32B27/32\ B29C47/06\ B29C69/02\ B29D7/01 |
| 3082 | 3D printing | PROCESS FOR MANUFACTURING WAREF WITH DOUBLE-WALL BODY\ | CN101152752A | KUNCHE LI (CN) | KUNCHE LI (CN) | B29C45/14\ B29B11/00\ B65D1/00 |
| 3083 | 3D printing | MANUFACTURING METHOD OF FINE PERSONALIZED SKULL MODEL CAPABLE OF DESCRIBING TEETH OCCLUDING RELATION\ | CN100569195C | XIAOFENG LI (CN)\ HUIZHI CAO (CN)\ CAN LIU (CN)\ QIAN JIANG (CN) | XIAOFENG LI (CN) | G09B23/30\ G06F17/00\ A61C19/00\ A61B6/00\ G06Q50/00 |
| 3084 | 3D printing | 3D ELASTIC FILLER\ | CN1861531A | LIU DUANHUA (CN) | LIU DUANHUA (CN) | C02F3/10 |
| 3085 | 3D printing | FAST METHOD FOR PREPARING HIGH TEMPERATURE COMPONENT PARTS IN COMPOSITE MATERIAL OF MOLYBDENUM AND COPPER\ | CN1958196A | BAI PEIKANG LIU (CN) | UNIV ZHONGBEI (CN) | B22F3/105\ B22F3/26 |
| 3086 | 3D printing | EQUIPMENT FOR PRODUCING PLASTIC CONTAINER WITH STEREO ANAGLYPH PATTERN ON SURFACE AND WITHOUT SEAM LINE\ | CN100453298C | XIANLIAN LIU (CN) | XIANLIAN LIU (CN) | B29C49/30\ B29C49/02\ B29C49/42\ B29C49/54\ B29C49/58\ B29C49/64 |
| 3087 | 3D printing | LABEL WHEEL ROTARY PRINTING MACHINE\ | CN100595061C | HELIANG QI US\ ZE YANG US | SHANGHAI TAIYO KIKAI CO LTD US | B41F17/00\ B41F13/02\ B32B39/00\ B41F13/54\ B41F19/00\ B41F23/00\ B65H5/06\ G09F3/02 |
| 3088 | 3D printing | PRODUCTION METHOD FOR TWO-MONOLAYER PAPER SUPPORTING TO PRINT AND PROCESS INTO STICKERS\ | CN101054010A | QI HELIANG YANG (CN) | SHANGHAI SUIXUAN ENTPR DEV CO (CN) | B41F17/00\ B41F13/008\ B41F13/02\ B32B39/00\ B41F13/54\ B41F19/00\ B41F23/00\ B65H5/06\ G09F3/02 |
| 3089 | 3D printing | METHOD FOR RAPIDLY MANUFACTURING INJECTION-MOULD INSERT WITH FOLLOW-COOLING PASSAGEWAY\ | CN1850396A | SHI YUSHENG HUANG (CN) | UNIV HUAZHONG SCIENCE TECH (CN) | B22F3/105\ B22F3/24\ B29C33/38 |
| 3090 | 3D printing | WATER SOLUBLE FLEXIBLE PRINT PLATE AND ITS PRODUCING METHOD\ | CN1900822A | ZHAO XIAOJIE (CN) | ZHONGYUAN PRINTING SCIENCE AND (CN) | G03F7/09\ B41N1/24 |
| 3091 | 3D printing | APPTS. FOR PRODUCING WATER WAVE GLASS MOSAIC\ | CN2856043Y | SHEN RONGFANG XU (CN) | SHEN RONGFANG (CN) | C03B19/02\ C03B11/00 |
| 3092 | 3D printing | BRA CUP WITH GOOD SHAPE KEEPING FUNCTION\ | CN2829399Y | ZHENG BIHAO (CN) | ANLIFANG CHINA GARMENT CO LTD (CN) | A41C3/12\ A41C3/00 |
| 3093 | 3D printing | HIGH-PRECISION THREE-DIMENSIONAL MICRO-ASSEMBLING METHOD AND ASSEMBLY PARTS BASED ON MEMS\ | CN1803577A | CHEN JING YANG (CN) | UNIV BEIJING (CN) | B81C3/00\ G01C19/00\ G01P15/00 |
| 3094 | 3D printing | REGENERATION TECHNOLOGY ADAPTED FOR INK POX WITH SPONGE WITHOUT PRINTING HEAD\ | CN100357108C | LANGMING WANG (CN) | LANGMING WANG (CN) | B41J2/175 |
| 3095 | 3D printing | FAST SHAPING METHOD OF CORAMIC COMPONENT PORT\ | CN100351062C | XIUFENG WANG (CN)\ HONGTAO JIANG (CN)\ CHENGLONG YU (CN)\ LIANJUAN SHAN (CN)\ YONG SUN (CN) | SHAANXI UNIV OF SCIENCE AND TE (CN) | B28B1/00\ B44B1/00 |
| 3096 | 3D printing | SPECIAL MOULD FOR MAKING CLOTHES PATTERN AND METHOD FOR MAKING PATTERN\ | CN1857919A | CAI JINGXIANG (CN) | CAI JINGXIANG (CN) | B44C1/16\ B41F16/02 |
| 3097 | 3D printing | BALL MOUNTING METHOD AND APPARATUS FOR BALL GRID ARRAY PACKAGING SUBSTRATE\ | CN100382266C | FUJIANG SUN (CN) | HUAWEI TECH CO LTD (CN) | H01L21/60 |
| 3098 | 3D printing | DRAINAGE GEOTECHNICAL GRILLAGE\ | CN2752326Y | BAO SHAOZHENG (CN) | BAO SHAOZHENG (CN) | B29C69/00\ E02D19/20 |
| 3099 | 3D printing | BUILT-IN LASER SMELTING SPRAY NOZZLE\ | CN2705236Y | HU QIANWU (CN)\ ZENG XIAOYAN (CN) | CENTRAL CHINA SCIENCE & TECH (CN) | B23K26/34\ B05B7/16\ C23C4/00 |
| 3100 | 3D printing | CONTINUOUS SCREWED MULTI-CELLULAR PLASTIC SHAPING STRUCTURE\ | CN2714295Y | ZHANG BINGRONG (CN) | ZHANG BINGRONG (CN) | B29C53/58\ B29C53/74 |
| 3101 | 3D printing | METHOD AND ITS SYSTEM FOR LEADING LARGE MOLECULAR MATTER INTO TARGET CELL\ | CN100435851C | DEYANG TIAN (CN) | DEYANG TIAN (CN) | A61B8/00\ A61B6/00\ A61K48/00\ A61K49/06\ A61M5/00\ C12N15/87\ C12N15/89 |
| 3102 | 3D printing | HOT MELT ADHESIVES BONDING FIBER\ | US2005170143A1 | YAU PAK C (CN) | BISQUIT DEV LTD (HK) | B32B3/30\ B32B1/00\ B32B5/24\ B32B7/12\ B32B27/02\ D06N7/04 |
| 3103 | 3D printing | POLISHING BRICK\ | CN2671826Y | LIU YIJUN (CN) | MENGNAISHA CERAMICS CO LTD GUA (CN) | B44C3/00\ B44C1/00\ B44F7/00\ B44C5/00 |
| 3104 | 3D printing | MANUFACTURING METHOD OF COLOUR STEREO MOULD CANDY AND ITS PRODUCT\ | CN1319461C | HUANG ZHENGLONG HUANG (CN) | YONGXIN TRADE CO LTD (CN) | A23G3/54\ A23G1/54\ A23G3/00 |
| 3105 | 3D printing | MAGNETIC RAIL COLOURED PATTERN CARPET CAPABLE OF ASSEMBLING AT WILL\ | CN2604128Y | WANG MANZHU (CN) | WANG MANZHU (CN) | A47G27/02\ A63H18/10 |
| 3106 | 3D printing | METHOD AND EQUIPMENT FOR DIRECTLY AND QUICKLY MANUFACTURING MOULD AND PARTS\ | CN1298780A | ZHANG HAIOU (CN)\ WANG GUILAN (CN) | UNIV HUAZHONG SCIENCE TECH (CN) | B23K28/02\ B23P23/04 |
| 3107 | 3D printing | INK JET TRANSFER SYSTEMS PROCESS FOR PRODUCING THE SAME AND THEIR USE IN A PRINTING PROCESS\ | US6638604B1 | BAMBERG ULF (DE)\ KUMMER PETER (CH)\ STIBUREK ILONA (CH) | ARKWRIGHT INC (US) | D06P5/20\ B41J2/01\ B41M5/00\ D06P5/00\ B41M5/50\ B41M5/52\ D06P5/24 |
| 3108 | 3D printing | PROCESS FOR THE PRODUCTION OF ARTICLES MADE OF A GAMMA-PRIME PRECIPITATION-STRENGTHENED NICKEL-BASE SUPERALLOY BY SELECTIVE LASER MELTING (SLM)\ | US2013228302A1 | RICKENBACHER LUKAS EMANUEL (CH)\ SPIERINGS ADRIAAN BERNARDUS (CH) | ALSTOM TECHNOLOGY LTD (CH) | B22F3/00 |
| 3109 | 3D printing | VERFAHREN ZUR HERSTELLUNG VON SCHMUCKSTüCKEN.\[EN] PRODUCING THREE-DIMENSIONAL JEWELRY PIECES MADE OF METAL COMPRISES REPEATEDLY APPLYING A METAL POWDER ON A SUBSTRATE AND/OR HIGHLY-ADJUSTABLE PLATFORM IN A LAYER-WISE MANNER AND PARTIALLY MELTING THE POWDER LAYER AT A PREDETERMINED POINT\ | CH700273A1 | OTT HANSPETER (CH) | OTT HANSPETER (CH) | B22F3/11\ A44C27/00 |
| 3110 | 3D printing | COMPOSITION AND METHODS FOR THE PRODUCTION OF BIOPOLYMERIC MATERIALS THAT BIODEGRADE RAPIDLY AND ARE FLEXIBLE AND RIGID WITH USE OF THE XANTHAN BIOPLASTIC AS A COMPONENT OF THE BIOPOLYMETRIC MATRIX AND OPTIONALLY FILLERS AND/OR NANOFILLERS AND OTHER CONSTITUENTS THE RESULTING PRODUCTS AND THE USES THEREOF\[FR] COMPOSITION ET PROCéDéS DE PRODUCTION DE MATIèRES BIOPOLYMèRES à BIODéGRADATION RAPIDE SOUPLES ET RIGIDES AVEC UTILISATION DU BIOPLASTIQUE XANTHANE COMPOSANT LA MATRICE BIOPOLYMèRE ET éVENTUELLEMENT DES CHARGES ET/OU DES NANOCHARGES ET D'AUTRES CONSTITUANTS PRODUITS OBTENUS ET LEURS UTILISATIONS\[PT] COMPOSI??O E MéTODOS DE PRODU??O DE MATERIAIS BIOPOLIMéRICOS DE RáPIDA BIODEGRADA??O FLEXíVEIS E RíGIDOS COM USO DO BIOPLáSTICO XANTANA COMPONDO A MATRIZ BIOPOLIMéRICA E OPCIONALMENTE CARGAS E/OU NANOCARGAS ê OUTROS CONSTITUINTES; PRODUTOS OBTIDOS E SEUS USOS\ | WO2013181731A1 | TONDO VENDRUSCOLO CLAIRE (BR)\ DA SILVA MOREIRA ANGELITA (BR)\ DE ANDRADE SALLES CLEIA (BR)\ CALCAGNO CARMEN IARA WALTER (BR) | IFSUL INST FED DE EDUCACAO CIENCIA E TECNOLOGIA SUL RIO GRANDENSE (BR)\ UFPEL UNIVERSIDADE FED DE PELOTAS (BR) | C08K3/32\ A61K8/73\ A61Q11/00\ C08K3/34\ C12P19/04\ A23L1/0522\ A23L1/054\ A61K31/685\ A61K31/722\ A61P19/02\ A61P19/04\ C07H3/00\ C07K14/195\ C08B37/00\ C08L3/02\ C09K8/02\ C09K8/08\ C12N1/20\ C12N9/10\ C12P19/06\ C12R1/64 |
| 3111 | 3D printing | ION FUSION FORMATION\ | US6680456B2 | ADAMS ROBBIE (US) | HONEYWELL INT INC (US) | B23K10/02 |
| 3112 | 3D printing | ION FUSION FORMATION PROCESS FOR LARGE SCALE THREE-DIMENSIONAL FABRICATION\ | US2009026175A1 | ADAMS ROBBIE J (US) | HONEYWELL INT INC (US) | B23K9/04\ B23K10/00\ B05D1/08 |
| 3113 | 3D printing | METHOD FOR PRODUCING PTT (POLYTRIMETHYLENE TEREPHTHALATE) SPIRAL THREE-DIMENSIONAL HOLLOW FIBERS\ | CN102618947A | AIGUO MIAO US\ YUNCHANG LIN US\ AIZHEN LIN US\ YUZHONG HUO US\ AIMIN YANG US\ HUANMING XU US | AIGUO MIAO US | D01D5/24\ D02G1/00\ D01D1/04\ D01D5/096\ D01D5/092\ D02J1/00\ D02J13/00 |
| 3114 | 3D printing | SUPPORT MATERIAL TO STABILIZE EARTH FOR FOUNDATIONS AND PREVENT EROSION IS COMPOSED OF A WOVEN FABRIC OF SYNTHETIC FILAMENTS BONDED TO A NONWOVEN THREE-DIMENSIONAL SYNTHETIC FIBER BASE\[FR] NATTE DE RETENTION ET DE FILTRATION POUR LE SOUTENNEMENT DES TERRES ET SUPPORTS\ | FR2850675A1 | ANDRE HENRI (FR) | ANDRE HENRI (FR) | D04H13/00\ D04H1/00\ D03D15/00\ B32B5/26\ B32B5/08\ D03D1/00\ E02B3/12\ E02D17/20\ E02D29/02 |
| 3115 | 3D printing | BRASSIERE\[FR] SOUTIEN-GORGE\ | WO2012077232A1 | ANNAKA SHIGEO (JP) | YAGI & CO LTD (JP)\ ANNAKA SHIGEO (JP) | A41C3/12 |
| 3116 | 3D printing | METHOD FOR MODIFYING SURFACE OF PLASTIC MOLDED BODY METHOD FOR FORMING METAL FILM INCLUDING THE SAME AND PLASTIC COMPONENT THEREOF\ | JP2009113266A | ANO TETSUYA US\ YUSA ATSUSHI US\ NOMURA YOSHIYUKI US | HITACHI MAXELL (JP) | B29C45/00\ B29L31/34\ C23C18/22 |
| 3117 | 3D printing | THREE-DIMENSIONAL NON-WOVEN MEDIA FILTER AND PROCESS\ | CN100582343C | AUNE THOMAS M (US)\ KOPP CLINTON V (US)\ MADSEN MICHAEL J (US)\ ROLCHIGO PHILIP M (US)\ STIFTER TRAVIS G (US) | OSMONICS INC (US) | D04H1/56 |
| 3118 | 3D printing | METHOD OF MAKING A THERMOPLASTIC FILM WITH A THREE-DIMENSIONALLY STRUCTURED SURFACE\ | US2011189437A1 | BADER HERBERT (DE)\ HAGEMANN ANDREAS (DE)\ MARCEL GRUENDKEN (DE) | MARCEL GRUENDKEN (DE)\ BADER HERBERT (DE)\ HAGEMANN ANDREAS (DE) | B32B3/00\ B29C55/18 |
| 3119 | 3D printing | ENVIRONMENT-FRIENDLY MULTILEVEL PLASTIC ENVELOPE AND THAT PRODUCTION METHODS\ | KR100831377B1 | BAK YEONG GI (KR)\ KANG JOO HO (KR)\ KIM SUN WOOK (KR) | HAESUNG INDUSTRY CO LTD (KR)\ BAK YEONG GI (KR)\ KANG JOO HO (KR)\ KIM SUN WOOK (KR) | B65D30/14\ B65D30/10 |
| 3120 | 3D printing | METHOD OF INK-JET PRINTING\ | US2008284836A1 | BAKER JULIE (GB)\ HIGGINS JOHN M (GB) | HIGGINS JOHN M (GB)\ BAKER JULIE (GB) | B41J3/407\ B41M5/52\ B41M7/00\ B41J11/00 |
| 3121 | 3D printing | INK JET TRANSFER SYSTEMS PROCESS FOR PRODUCING THE SAME AND THEIR USE IN A PRINTING PROCESS\ | US6638604B1 | BAMBERG ULF (DE)\ KUMMER PETER (CH)\ STIBUREK ILONA (CH) | ARKWRIGHT INC (US) | D06P5/20\ B41J2/01\ B41M5/00\ D06P5/00\ B41M5/50\ B41M5/52\ D06P5/24 |
| 3122 | 3D printing | METHOD ENABLING A STANDARD CMOS FAB TO PRODUCE AN IC TO SENSE THREE-DIMENSIONAL INFORMATION USING AUGMENTED RULES CREATING MASK PATTERNS NOT OTHERWISE EXPRESSIBLE WITH EXISTING FAB RULES\ | US7464351B2 | BAMJI CYRUS (US)\ LIU XINQIAO (US) | CANESTA INC (US) | G06F17/50\ A61B6/00\ G01B11/26\ A61N5/00\ G01C3/00\ G01N23/00\ G01S7/491\ G01S7/493\ G01S17/08\ G01S17/36\ G01S17/89\ G21G5/00\ G21K1/12\ H01L\ H01L27/146\ H01L31/02\ H04N3/14\ H05G1/60 |
| 3123 | 3D printing | LIQUID STATE MICRO-FORMING METHOD FOR THREE-DIMENSIONAL COMPLEX MICRO-COMPONENT\ | CN100515609C | BANGSHENG LI (CN)\ MINGXING REN (CN)\ CHUANG YANG (CN) | HARBIN INST OF TECHNOLOGY (CN) | B22D17/08 |
| 3124 | 3D printing | DRAINAGE GEOTECHNICAL GRILLAGE\ | CN2752326Y | BAO SHAOZHENG (CN) | BAO SHAOZHENG (CN) | B29C69/00\ E02D19/20 |
| 3125 | 3D printing | DIRECT SELECTIVE LASER SINTERING OF METALS\ | US6676892B2 | BEAMAN JOSEPH J (US)\ DAS SUMAN (US) | REGENTS UNIVERSITY TEXAS SYSTE (US) | B22F3/105\ B23K26/34\ C23C26/02\ G05B19/4062 |
| 3126 | 3D printing | PLATTE MIT DREIDIMENSIONAL STRUKTURIERTER OBERFL?CHE AUF DER BASIS VON HOLZWERKSTOFFEN UND VERFAHREN ZUR HERSTELLUNG EINER SOLCHEN PLATTE\[EN] PLATE USEFUL FOR PRODUCING DOORS AND FURNITURE PIECES COMPRISES A THREE-DIMENSIONAL TEXTURED SURFACE PRODUCED VIA COMPRESSION CARRIER PLATE MADE UP OF WOODEN MATERIAL AND A DECORATIVE LAYER COATED ON CARRIER PLATE\ | DE102010005350A1 | BECKERS WILHELM DIPL ING (DE) | WESTAG & GETALIT AG (DE) | B27N7/00\ B32B21/02\ B32B27/10\ B32B33/00\ B32B27/04\ B32B21/06 |
| 3127 | 3D printing | APPARATUS FOR THREE DIMENSIONAL PRINTING USING IMAGED LAYERS\ | US8119053B1 | BEDAL BRYAN (US)\ SCHELL STEVEN E (US)\ BEERS ROSS D (US) | 3D SYSTEMS INC (US)\ BEDAL BRYAN (US)\ SCHELL STEVEN E (US)\ BEERS ROSS D (US) | B29C35/08\ B28B1/16 |
| 3128 | 3D printing | APPARATUS FOR THREE DIMENSIONAL PRINTING USING IMAGED LAYERS\ | US2010244333A1 | BEDAL BRYAN (US)\ SCHELL STEVEN E (US)\ BEERS ROSS D (US) | BEDAL BRYAN (US)\ SCHELL STEVEN E (US)\ BEERS ROSS D (US) | B29C35/08\ B29C67/00\ B29C47/92 |
| 3129 | 3D printing | MANUFACTURING BONE PROSTHESIS FOR FILLING GAPS OF ALL/PART OF BONE OF PATIENT COMPRISES DIGITALIZING PATIENT USING THREE-DIMENSIONAL MEDICAL ACQUISITION DEVICE BY MAGNETIC RESONANCE DEFINING PROSTHESIS AND MANUFACTURING THE PROSTHESIS\[FR] PROCEDE DE FABRICATION D'UNE PROTHESE PAR PROTOTYPAGE RAPIDE\ | FR2979817A1 | BERTIN JEAN-MICHEL (FR) | BERTIN JEAN-MICHEL (FR) | B29C67/00\ A61F2/28\ B22F3/105 |
| 3130 | 3D printing | METHOD AND APPARATUS FOR PRODUCING THREE-DIMENSIONAL OBJECTS\ | US2014348692A1 | BESSAC CHRISTOPHE (FR)\ PIALOT FRéDéRIC (FR)\ WALRAND GILLES (FR) | MICHELIN RECH TECH (CH)\ MICHELIN & CIE (FR) | B29C67/00\ B22F3/105 |
| 3131 | 3D printing | METHOD AND APPARATUS FOR PRODUCING THREE-DIMENSIONAL OBJECTS WITH IMPROVED PROPERTIES\ | US2015064048A1 | BESSAC CHRISTOPHE (FR)\ WALRAND GILLES (FR)\ BROCHET STEPHANIE (FR)\ PIALOT FREDERIC (FR) | MICHELIN RECH TECH (CH)\ MICHELIN & CIE (FR) | B22F3/105\ B22F3/24\ B22F5/00\ C21D1/00 |
| 3132 | 3D printing | METHOD AND APPARATUS FOR PRODUCING THREE-DIMENSIONAL OBJECTS\[FR] PROCEDE ET APPAREIL POUR REALISER DES OBJETS TRIDIMENSIONNELS\ | WO2013092994A1 | BESSAC CHRISTOPHE (FR)\ WALRAND GILLES (FR)\ BROCHET STEPHANIE (FR)\ PIALOT FREDERIC (FR) | MICHELIN RECH TECH (CH)\ MICHELIN & CIE (FR) | B29C67/00\ B22F3/00\ B22F3/105 |
| 3133 | 3D printing | PROCESS AND APPARATUS FOR PRODUCING PRECISION CASTINGS\ | US6725902B2 | BLUM MATTHIAS (DE)\ JARCZYK GEORG (DE)\ FELLMANN HANS-GUENTHER (DE)\ BUSSE PETER (DE) | ALD VACUUM TECHN AG (DE) | B22C9/04\ B22D13/00\ B22D13/06\ B22D13/10\ B22C9/02\ B22D21/00\ B22D21/06 |
| 3134 | 3D printing | METHOD FOR MANUFACTURING PERFORATED NONWOVEN FABRICS\ | US2010291343A1 | BOCCHIO RINALDO (IT) | UNION IND S P A (IT) | B29C67/00\ B32B3/10\ D04H1/4291 |
| 3135 | 3D printing | ADSORPTIVE FILTER MEDIUM\ | US2015053080A1 | BOEHRINGER BERTRAM (DE)\ GONZALEZ OMAR GUERRA (DE)\ ECKLE ISEBILL (DE) | BLüCHER GMBH (DE) | B01D53/04 |
| 3136 | 3D printing | W?SSRIGE MAGENTAFARBENE FARBSTOFFE IHRE HERSTELLUNG UND VERWENDUNG\[EN] NEW HETEROCYCLIC DYE COMPOUNDS USEFUL E.G. FOR COLORING AND PRINTING NATURAL AND SYNTHETIC FIBER MATERIALS FOR COLORING PAPER OR CELLULOSES IN THE MASS AND IN THE MANUFACTURE OF MAGENTA COLOR RECORDING FLUIDS\ | DE102005033584A1 | BORCHERT TILL (DE)\ KLEINERT MARKUS (DE)\ SCHNEIDER STEFFEN (DE)\ GEISENBERGER JOSEF (DE) | CLARIANT PRODUKTE DEUTSCHLAND (DE) | C09D11/02\ C09B56/20\ C09B67/36\ D06P1/41 |
| 3137 | 3D printing | MONOCOMPONENT MONOLAYER MELTBLOWN WEB AND MELTBLOWING APPARATUS\ | US7902096B2 | BRANDNER JOHN M (US)\ KOPECKY WILLIAM J (US)\ ANGADJIVAND SEYED A (US)\ SPRINGETT JAMES E (US)\ LINDQUIST TIMOTHY J (US) | 3M INNOVATIVE PROPERTIES CO (US) | D04H1/56\ D04H3/16\ D04H3/00 |
| 3138 | 3D printing | PRINTING PROCESS\ | US6902268B1 | BRENTNALL ROGER LESLIE (GB)\ KING JEFFREY RONALD (GB)\ LEGGETT SIMON RICHARD JOHN (CH)\ TAYLOR KAREN (GB)\ SCHUETTEL STEFAN (CH) | ILFORD IMAGING UK LTD (CH) | B41M5/00\ B41M5/52\ B41M7/00 |
| 3139 | 3D printing | KRAFTSCHLüSSIG MITEINANDER VERBINDBARE BAUTEILE\ | DE102008017029B4 | BRODA MAIK (DE)\ VERPOORT CLEMENS (DE)\ MUESCHEN ANDREAS (DE) | FORD GLOBAL TECH LLC (US) | C23C4/12\ C23C4/06\ C23C4/10\ F16H55/36 |
| 3140 | 3D printing | FLAMEPROOFED IMPACT-STRENGTH-MODIFIED POLY(ESTER)CARBONATE COMPOSITIONS\ | US7833448B2 | BUCHHOLZ VERA (DE)\ WENZ ECKHARD (DE)\ ECKEL THOMAS (DE)\ THUERMER BURKHARD (DE)\ WITTMANN DIETER (DE) | BAYER MATERIALSCIENCE AG (DE) | B28B3/20 |
| 3141 | 3D printing | BLASKOPF FüR EINE BLASFOLIENEXTRUSIONSANLAGE\ | DE102007050694B4 | BUETTEL ULRICH (DE)\ SCHNEIDER JUERGEN DIPL ING (DE) | BUETTEL ULRICH (DE) | B29C47/92\ B29D7/01\ B29C47/20 |
| 3142 | 3D printing | OFFSET PRINTING METHOD FOR INK JET PRINTER AND INK JET PRINTER\ | JP2005096468A | BURR RONALD F US\ SEGERSTOM ERIC C US\ TITTERINGTON DONALD R US | FUJI XEROX CO LTD US | C09D11/00\ B41J2/01\ B41M5/00 |
| 3143 | 3D printing | VERFAHREN ZUM HERSTELLEN EINER DREHSTABFEDER ODER EINES WANKSTABILISATOR\[EN] MANUFACTURING TORSION BAR SPRING OR ROLLING STABILIZER FOR MOTOR VEHICLE WITH TUBULAR CROSS-SECTION COMPRISES PRODUCING COMPONENT BY BRAIDING AND/OR WRAPPING CORE WITH FIBER BUNDLES AND THERMAL MELTING OF MATRIX MATERIAL\ | DE102011011577A1 | BUSCHBECK FRANK (DE)\ NAGEL GUENTER DIPL ING (DE)\ REESE ECKHARD DIPL ING (DE)\ RUPPRECHT JOCHEN DIPL ING (DE)\ STEINERT HANS-RUDOLF DIPL ING (DE) | DAIMLER AG (DE) | B29C70/50\ B29C33/76\ B29C53/02\ B29C70/00\ B60G21/055\ C08J5/04\ F16F1/14 |
| 3144 | 3D printing | SPECIAL MOULD FOR MAKING CLOTHES PATTERN AND METHOD FOR MAKING PATTERN\ | CN1857919A | CAI JINGXIANG (CN) | CAI JINGXIANG (CN) | B44C1/16\ B41F16/02 |
| 3145 | 3D printing | COLORED POLYESTER TRANSITION SILK COLORFUL CARPET HAND-KNITTED YARN AND MANUFACTURING METHOD THEREOF\ | CN102888688A | CAO XINYANG US\ QIAN ZHANGBAO US\ ZHOU QUANZHONG US\ ZHAO JIANGFENG US\ DUAN YAFENG US\ HE JIA US\ GAN FENGMEI US\ ZHENG DI US\ YU HAIXIANG US | ZHEJIANG HUAXIN ADVANCED MATERIALS CO LTD US | D02G3/40\ D02G3/04\ D02G3/38 |
| 3146 | 3D printing | PREPARATION OF MICROPOROUS FILMS FROM IMMISCIBLE BLENDS VIA MELT PROCESSING AND STRETCHING\ | US6824680B2 | CHANDAVASU CHAIYA (TH)\ XANTHOS MARINO (US)\ SIRKAR KAMALESH K (US)\ GOGOS COSTAS (US) | NEW JERSEY TECH INST (US) | B01D69/14\ B01D39/16\ B29C47/00\ B01D67/00\ B01D71/26\ B29C55/00 |
| 3147 | 3D printing | MANUFACTURING METHOD AND DEVICE OF INORGANIC PLATES AND INORGANIC PLATES\ | CN102190424A | CHANGLIN WANG US\ JIANBIN QI US\ SENBEN GENG US\ CHANGXING WANG US | JINGNIU GLASS CERAMICS GROUP US | B28B1/00\ C03B13/00\ C03B15/02\ C03B17/06\ C03B25/00\ C04B35/14 |
| 3148 | 3D printing | PHOTON-EMISSION SCANNING TUNNELING MICROSCOPY\ | US8006315B2 | CHARRA FABRICE (FR)\ SILLY MATTHIEU (FR)\ SOUKIASSIAN PATRICK (FR) | COMMISSARIAT ENERGIE ATOMIQUE (FR)\ UNIV PARIS SUD PARIS II (FR) | G01Q10/00\ G01Q30/00\ G01Q60/10\ G01Q60/12\ G01N13/00\ G12B1/00 |
| 3149 | 3D printing | OUTIL DE SERRAGE D'UN ORGANE DE FIXATION\ | FR2970200B1 | CHATENET LUC HENRI (FR)\ BABOEUF SEBASTIEN PIERRE LOUIS (FR)\ LE QUELLEC JOHN (FR) | SNECMA (FR) | B25B23/15 |
| 3150 | 3D printing | TOUCH SCREEN FUNCTIONAL FILM\ | CN202952611U | CHEN JIANHUI US | ZHEJIANG ZHAOYI TECHNOLOGY CO LTD US | G06F3/041\ B32B27/06\ B32B27/36 |
| 3151 | 3D printing | HIGH-PRECISION THREE-DIMENSIONAL MICRO-ASSEMBLING METHOD AND ASSEMBLY PARTS BASED ON MEMS\ | CN1803577A | CHEN JING YANG (CN) | UNIV BEIJING (CN) | B81C3/00\ G01C19/00\ G01P15/00 |
| 3152 | 3D printing | FUSIBLE INK-JET RECORDING MATERIALS CONTAINING HOLLOW BEADS AND ULTRAFINE POLYMER PARTICLES\ | US2006045999A1 | CHEN TIENTEH (US)\ SEN RADHA (US) | CHEN TIENTEH (US)\ SEN RADHA (US) | B41M5/00 |
| 3153 | 3D printing | FUSIBLE INKJET RECORDING MATERIALS CONTAINING HOLLOW BEADS SYSTEM USING THE RECORDING MATERIALS AND METHODS OF USING THE RECORDING MATERIALS\ | US2005287311A1 | CHEN TIENTEH (US)\ SEN RADHA (US)\ WEBB STEVEN L (US)\ KELLY-ROWLEY ANNE M (US)\ PAGE LORETTA ANN G (US) | CHEN TIENTEH (US)\ SEN RADHA (US)\ WEBB STEVEN L (US)\ KELLY-ROWLEY ANNE M (US)\ PAGE LORETTA ANN G (US) | B41M5/00\ B41M5/50\ B41M5/52\ B41M7/00\ B41J11/00 |
| 3154 | 3D printing | FUSIBLE INKJET RECORDING MATERIALS CONTAINING HOLLOW BEADS SYSTEM USING THE RECORDING MATERIALS AND METHODS OF USING THE RECORDING MATERIALS\ | US7651216B2 | CHEN TIENTEH (US)\ SEN RADHA (US)\ WEBB STEVEN L (US)\ KELLY-ROWLEY ANNE M (US)\ PAGE LORETTA ANN GREZZO (US) | HEWLETT PACKARD DEVELOPMENT CO (US) | B41J2/01\ B41M5/52\ B41M7/00 |
| 3155 | 3D printing | MAGNETORHEOLOGICAL-MATERIAL-BASED 3D (THREE-DIMENSIONAL) PRINTING TYPE RAPID PROTOTYPING DEVICE AND METHOD\ | CN103213281A | CHEN XI US\ HE GUOTIAN US\ GU MINGXIN US\ WANG ZHONGXUN US\ ZHU XIAOQIANG US\ LIN YUANCHANG US\ XU ZEYU US\ ZHAO JIAN US\ LIU YONGFU US | CHONGQING INST OF GREEN AND INTELLIGENT TECHNOLOGY US | B29C67/00 |
| 3156 | 3D printing | 3D (THREE-DIMENSIONAL) PRINTING RAPID FORMING DEVICE BASED ON MAGNETORHEOLOGICAL MATERIAL\ | CN203344319U | CHEN XI US\ HE GUOTIAN US\ GU MINGXIN US\ WANG ZHONGXUN US\ ZHU XIAOQIANG US\ LIN YUANCHANG US\ XU ZEYU US\ ZHAO JIAN US\ LIU YONGFU US | CHONGQING INST OF GREEN AND INTELLIGENT TECHNOLOGY CHINESE ACADEMY OF SCIENCES US | B29C67/00 |
| 3157 | 3D printing | PLASTIC SOLE WITH THREE-DIMENSIONAL PATTERNS\ | CN202950080U | CHEN XIAOLIN US | JIEYANG JIAYI INDUSTRY AND TRADE CO LTD US | A43B13/12 |
| 3158 | 3D printing | PRINTING FORMING GOODS USING PET\ | KR20040071807A | CHO HAN YONG US | CHO HAN YONG US | B41M1/30 |
| 3159 | 3D printing | NYLON FILM HAVING IMPROVED PRINTING ADHESIVE STRENGTH\ | KR20110085651A | CHO HYUN (KR)\ SONG GI SANG (KR)\ KIM SI MIN (KR) | KOLON INC (KR) | C08J5/18\ B29D7/01 |
| 3160 | 3D printing | DISPLAY PANEL FOR WASHING MACHINE\ | KR20040006269A | CHOI DONG HA US\ PARK HYE YONG US | LG ELECTRONICS INC (KR) | D06F39/00 |
| 3161 | 3D printing | FILM ENVELOP HAND STRAP MADE OF POLYPROPYLENE AND MANUFACTURING METHOD THEREOF\ | KR20110059496A | CHOI OK BOM (KR)\ HA JAE YOUNG (KR) | CHOI OK BOM (KR)\ HA JAE YOUNG (KR) | B29D7/00\ B29C47/02\ B65D33/06 |
| 3162 | 3D printing | METHOD FOR MANUFACTURING AROMATIC POROUS SINTERED METAL COMPACT\ | KR20040013342A | CHOI SUNG JO US | CHOI SUNG JO US | B22F3/11 |
| 3163 | 3D printing | METHOD FOR COATING THE SURFACE OF A METALLIC MATERIAL DEVICE FOR CARRYING OUT SAID METHOD\ | US2006096674A1 | CHOQUET PATRICK (FR)\ CHALEIX DANIEL (FR) | CHOQUET PATRICK (FR)\ CHALEIX DANIEL (FR) | C23C28/00\ C23C14/14\ C23C2/26\ C23C28/02\ C21D1/09 |
| 3164 | 3D printing | HOT MELT CALENDERED OR EXTRUDED WEAR LAYER FOR EMBOSSED SUBSTRATES AND METHOD OF MANUFACTURE\ | US2004048044A1 | CHRISTIE PETER A (US)\ MCQUATE WILLIAM M (US)\ SCHNEIDER DONALD E (US) | ARMSTRONG WORLD IND INC (US) | B32B3/00\ B32B38/06\ B29C43/24\ B32B37/15\ D06N7/00 |
| 3165 | 3D printing | PRINT FUSER AND PROCESS WITH MULTIPLE CLEANING BLADES\ | US7184696B2 | COKELEY JEFFREY H (US) | EASTMAN KODAK CO (US) | G03G15/20 |
| 3166 | 3D printing | ADDED LAYER MANUFACTURE\ | GB2491472A | COLEGROVE PAUL (GB)\ WILLIAMS STEWART (GB) | UNIV CRANFIELD (GB) | B23K5/18 |
| 3167 | 3D printing | PLANE STRUCTURE COMPOSED OF A COHESIVE ASSEMBLY OF CONTIGUOUS CELLULAR CELLS\ | US2008193711A1 | DEHENNAU CLAUDE (BE)\ GRANDJEAN DOMINIQUE (BE)\ LENG PHILIPPE-JACQUES (BE) | SOLVAY (BE) | B29D99/00\ B29C39/02\ B32B3/12 |
| 3168 | 3D printing | LASER-INDUCTION HYBRID MELTING DIRECT FORMING METHOD AND DEVICE\ | CN102179517A | DENGZHI WANG US\ MINGMING MA US\ XIAOYAN ZENG (CN)\ ZEMIN WANG (CN)\ QIANWU HU (CN) | UNIV HUAZHONG SCIENCE TECH (CN) | B22F3/105 |
| 3169 | 3D printing | METHOD AND ITS SYSTEM FOR LEADING LARGE MOLECULAR MATTER INTO TARGET CELL\ | CN100435851C | DEYANG TIAN (CN) | DEYANG TIAN (CN) | A61B8/00\ A61B6/00\ A61K48/00\ A61K49/06\ A61M5/00\ C12N15/87\ C12N15/89 |
| 3170 | 3D printing | THREE-DIMENSIONAL RAPID PROTOTYPING METHOD AND DEVICE BASED ON PULSE SMALL HOLE DROPLET INJECTION\ | CN103056367A | DONG WEI US\ TAN YI US\ LU DONG US | UNIV DALIAN TECH (CN) | B22F3/115 |
| 3171 | 3D printing | METHOD FOR PRODUCING MICROCRYSTAL GLASS CERAMICS COMPOSITE BOARD\ | CN102775074A | DONGSHENG XIE US\ GUIPING DU US\ WEIHUA LV US\ WENFENG DENG US | GUANGDONG GOLD MEDAL CERAMICS CO LTD US | C03C17/02 |
| 3172 | 3D printing | PRINT FUSER AND PROCESS WITH A REVERSE CLEANING BLADE\ | US2006045581A1 | ECK EDWARD M (US)\ MILLS BORDEN H III (US) | ECK EDWARD M (US)\ MILLS BORDEN H III (US) | G03G15/20 |
| 3173 | 3D printing | VERFAHREN ZUR HERSTELLUNG EINER SCHRAUBGEWINDEAUFNAHME\ | DE102011009682A1 | CHAOPING CHEN US\ CAIJIAN ZHANG US | ABMAYR UTE (DE) | B29C45/14\ F16B37/00\ B29C65/56\ F16B33/02\ F16B37/12 |
| 3174 | 3D printing | EXTERIOR CASE FOR ELECTRONIC APPARATUS\ | JP2012138594A | CHEN WEN-ZHENG (TW) | ALPS ELECTRIC CO LTD (JP) | B29C45/14\ H04M1/02\ H05K5/02 |
| 3175 | 3D printing | FILTERMATERIAL\[EN] HEAVY DUTY FILTRATION MATERIAL REMOVING DUST FROM INDUSTRIAL GASES INCLUDES BASE FELT AND COVER FELT WITH THINNER FIBERS CONSOLIDATED BY FLUID-JET NEEDLING\ | DE20321162U1 | Empty Field US | BWF TEC GMBH & CO KG (DE) | B01D46/42\ B01D39/16\ D04H3/02\ B01D46/10 |
| 3176 | 3D printing | METHOD FOR MANUFACTURING INTEGRAL MOLDING AND INTEGRAL MOLDING\ | JP2013018226A | Empty Field US | DAICEL PACK SYSTEMS LTD US\ POLYPLASTICS CO US\ DAIPLA SYSTEM TECHNOLOGY KK US | B29C45/14\ B29C51/10 |
| 3177 | 3D printing | DECORATIVE SHEET FOR INJECTION MOLDING DECORATIVE MOLDED ARTICLE AND METHOD FOR SIMULTANEOUSLY CARRYING OUT INJECTION MOLDING AND DECORATING WORK\ | JP2012045938A | Empty Field US | DAINIPPON PRINTING CO LTD (JP) | B29C45/14\ B29C51/10\ B44C3/02\ B32B27/00\ B32B33/00 |
| 3178 | 3D printing | PHOTOCATALYTIC THERMO-RESPONSIVE 3-DIMENSIONAL COPOLYMER AND FABRICATING METHOD OF THE SAME\ | KR101157527B1 | Empty Field US | HEWLETT PACKARD CO (US) | C02F1/72\ B01J31/08\ B01J37/02\ C02F1/32 |
| 3179 | 3D printing | No Title Available\ | JP2010518958A | Empty Field US | HEWLETT PACKARD CO (US) | B44C5/00\ A44C17/00\ A44C13/00 |
| 3180 | 3D printing | No Title Available\ | TWI325814B | Empty Field US | HOUSLER JOHN US | B44C5/00\ B32B17/10\ C03C27/12 |
| 3181 | 3D printing | SYSTEM AND ITS MAKING METHOD OF COLLECTION NOTE USING QR CODE\ | KR101283310B1 | Empty Field US | INFO VISION OPTOELECTRONICS KUNSHAN CO LTD US | G06F3/12\ B41J3/36\ G06K15/02 |
| 3182 | 3D printing | A MANUFACTURING METHOD FOR NON-FLAMABLE SHEET\ | KR101238799B1 | Empty Field US | KIM MOON OK (KR) | B29C43/24\ B29C43/28 |
| 3183 | 3D printing | MULTIPLE PURPOSE MATRIX OF 3D WEB STRUCTRED\ | KR101020878B1 | Empty Field US | KOREA INST SCI & TECH (KR) | B29C47/00\ B29D28/00 |
| 3184 | 3D printing | METAL JET UNIT\ | KR100975627B1 | Empty Field US | KOREA MACH & MATERIALS INST (KR) | B22D23/00 |
| 3185 | 3D printing | PACK FOR PREVENTING FALSIFICATION\ | KR100853559B1 | Empty Field US | MACDERMID GRAPHIC ARTS INC (US) | B65D33/22\ B65D33/20 |
| 3186 | 3D printing | A BRAZING METHOD OF A THIN METAL PLATE FOR COOLING AN ELECTRONIC DEVICE\ | KR100524048B1 | Empty Field US | MORIMURA KOSAN KK US | B23K1/008 |
| 3187 | 3D printing | 複合体およびその裊造方法\ | JP5640190B2 | Empty Field US | NEGOPACK CO LTD (KR)\ LEE BO YOUNG (KR) | D04H3/16\ B32B3/24 |
| 3188 | 3D printing | No Title Available\ | CN102691117B | Empty Field US | PARK KYOUNG HOON (KR) | D01D5/24\ D01D5/23 |
| 3189 | 3D printing | No Title Available\ | CN102495509B | Empty Field US | RICOH KK (JP) | H04N13/00\ G02B27/22\ G02F1/29\ G02F1/03 |
| 3190 | 3D printing | MECHANISCH STABILER POR?SER AKTIVKOHLEFORMK?RPER\[EN] MECHANICALLY STABLE POROUS SHAPED ACTIVATED CARBON ARTICLE COMPRISES FIRST THREE-DIMENSIONAL FRAMEWORK STRUCTURE BASED ON CARBONIZED RESIN AND SECOND THREE-DIMENSIONAL INORGANIC FRAMEWORK STRUCTURE\ | DE20221547U1 | Empty Field US | HELSA AUTOMOTIVE GMBH & CO KG (DE) | B01J20/20\ C01B31/08\ C04B35/524 |
| 3191 | 3D printing | METHOD FOR MANUFACTURING ARTIFICIAL ORGAN AND ARTIFICIAL ORGAN\ | JP2012137514A | Empty Field US | JMC KK US | G09B23/34 |
| 3192 | 3D printing | VAPOR DEPOSITION MASK MANUFACTURING METHOD OF THE SAME AND VAPOR DEPOSITION METHOD\ | JP2012077328A | Empty Field US | MITSUBISHI PLASTICS INC US\ LASER GIJUTSU SOGO KENKYUSHO US | B23K26/00\ B23K26/38\ C23C14/04\ B23K26/32 |
| 3193 | 3D printing | THERMOPLASTIC RESIN MOLDING MOLD FOR INJECTION-MOLDING THE MOLDING AND INJECTION MOLDING METHOD\ | JP2012086453A | Empty Field US | ONO SANGYO KK US | B29C45/37\ B29C45/72 |
| 3194 | 3D printing | 3D-DRUCKKOPF MIT TEMPERATUR-GESTEUERTER AUSTRITTS?FFNUNG FüR VERKüRZTE HEIZ- UND ABKüHLZEITEN SOWIE OPTIMIERTES AUSSEHEN DER DRUCKSTüCKE\[EN] THREE-DIMENSIONAL PRINTHEAD FOR MELTING AND DEPOSITING LAYERS OF WIRE-SHAPED MATERIALS HAS MELTING CHAMBER INCLUDING OUTLET DUCT LEADING INTO COOLING ZONES SUCH THAT FLOW VELOCITY OF MOLTEN MATERIAL IS REDUCED TO ZERO\ | DE102012000873A1 | Empty Field US | PARTHY KAI (DE) | B41J3/407\ B41J3/32 |
| 3195 | 3D printing | 3D-DRUCKKOPF MIT KüHLBAREM BESCHICKUNGSKANAL AUS HOCHW?RMELEITF?HIGEM MATERIAL\[EN] THREE DIMENSIONAL PRINTING HEAD USEFUL FOR MELTING AND DEPOSITING LAYERS OF WIRE-SHAPED MATERIALS COMPRISES FEED CHANNEL CONSISTING OF ARRAY OF HIGHLY HEAT-CONDUCTIVE MATERIALS E.G. ALUMINUM AND LOW HEAT-CONDUCTIVE MATERIALS E.G. PTFE\ | DE102011110804A1 | HAMAMATSU TOYOHIRO (JP) | PARTHY KAI (DE) | B29C67/00\ B22F3/115 |
| 3196 | 3D printing | UNIVERSELLER 3D-DRUCKKOPF MIT MEHREREN ARTEN DER DRAHTZUFüHRUNG UND DRAHT MIT ZUG-SEELE\[EN] THREE DIMENSIONAL PRINTING HEAD FOR MELTING AND DEPOSITING LAYERS OF WIRE-SHAPED MATERIALS HAS VARIOUS TYPES OF WIRE FEEDERS AND FEED CHANNELS FOR WIRES WHERE SOFT WIRE IS MADE OF THERMOPLASTIC ELASTOMERS\ | DE102011017310A1 | KIM MOON OK (KR) | PARTHY KAI (DE) | B05C5/04 |
| 3197 | 3D printing | THREE-DIMENSIONAL NETWORK STRUCTURE METHOD FOR PRODUCING THREE-DIMENSIONAL NETWORK STRUCTURE AND APPARATUS FOR PRODUCING THREE-DIMENSIONAL NETWORK STRUCTURE\ | JP2013108211A | LEE BO YOUNG (KR) | SHIIENJI KK US | D04H1/736\ D04H3/14 |
| 3198 | 3D printing | THREE-DIMENSIONAL NETWORK STRUCTURE METHOD FOR PRODUCING THREE-DIMENSIONAL NETWORK STRUCTURE AND APPARATUS FOR PRODUCING THREE-DIMENSIONAL NETWORK STRUCTURE\ | JP2012082568A | LEE SANG HUI US | SHIIENJI KK US | D04H3/16\ D04H3/033 |
| 3199 | 3D printing | METHOD FOR MANUFACTURING OF LIGHT GUIDE PLATE WITH PROTECTIVE FILM\ | JP2012252906A | LEE SANG HYUP (KR) | SUMITOMO CHEMICAL CO (JP) | F21Y101/02\ G02B6/00\ F21S2/00 |
| 3200 | 3D printing | METHOD OF MANUFACTURING OA EQUIPMENT EXTERIOR COMPONENT\ | JP2012076462A | LEE TAIK MIN (KR) | TEIJIN LTD US\ TEIJIN CHEMICALS LTD US | B29C45/00\ C08J5/00\ C08L101/16\ C08K3/34\ C08L67/04\ B29K67/00 |
| 3201 | 3D printing | NET-LIKE STRUCTURE\ | JP2013091862A | MORIMURA NORIKI US | TOYO BOSEKI (JP) | D01F6/92\ D04H3/07\ D04H3/011\ D04H3/14\ D01F6/62\ D04H3/018 |
| 3202 | 3D printing | CUSHION\ | JP2013090662A | PARK KYOUNG HOON (KR) | TOYO BOSEKI (JP) | A47C27/12\ D04H3/011\ D04H3/14\ B68G11/03 |
| 3203 | 3D printing | FORGERY-PREVENTING FILM\ | JP2012000991A | XIUZHI ZHAO US | YUPO CORP US | B32B27/00\ B32B27/32\ B42D15/10 |
| 3204 | 3D printing | INJECTION MOLDING COMPOSITE CONTAINER AND METHOD OF MANUFACTURING THE SAME\ | JP2012012019A | YEOM JANG YUL (KR) | YUPO CORP US\ SANYO KAGAKU KOGYO KK US | B29C45/14\ B65D25/20 |
| 3205 | 3D printing | UNIFORM MELT SIMULATION METHOD OF EXTRUSION SCREW COMPUTER PROGRAM FOR EXECUTING IT AND UNIFORM MELT SIMULATION APPARATUS OF EXTRUSION SCREW\ | JP2007007951A | ENDO KUNIAKI US\ HIROSE TAKASHI US\ NOGI TAKAYUKI US\ YOSHIKAWA HIDEO US | TOSHIBA MACHINE CO LTD US | B29C47/38 |
| 3206 | 3D printing | VERKLEBEN VON KUNSTSTOFFEXTRUDATEN MIT WERKSTOFFOBERFL?CHEN\ | DE10144524B4 | ERB ARMIN (DE)\ SCHROEDEL JUERGEN (DE)\ TERFLOTH CHRISTIAN (DE) | JOWAT AG (DE) | C08L75/04\ B29C69/00\ B29C65/48\ B32B37/15\ B29C69/02\ B29C43/28\ B27D5/00\ C09J5/06\ B32B7/12\ B29C47/02\ B29C63/00\ B29C63/48\ B29C65/02\ C09J175/04\ B05C5/02\ B27D1/00\ C09J5/10\ C09J177/00 |
| 3207 | 3D printing | NONWOVEN FABRIC FOR JOINING\ | JP9158025A | EZAKI KOJI US\ MINETA YOSHIHIKO US | UNITIKA LTD US | D04H13/00\ D04H3/00\ D04H3/14\ A44B18/00 |
| 3208 | 3D printing | ANTIBACTERIAL 3D WARP-KNITTED SPACER FABRIC PREPARED FROM ANTIBACTERIAL POLYESTER FILAMENTS\ | CN102031585A | FEI WANG US | FEI WANG US | C08K3/32\ C08K13/02\ D01F6/92\ C08G63/183\ C08K3/24\ D04B21/16\ D04B21/20 |
| 3209 | 3D printing | MAGNETIC AND ELECTROSTATIC NUCLEAR FUSION REACTOR\ | US2011200153A1 | FERREIRA JR MOACIR L (BR) | FERREIRA JR MOACIR L (BR) | H05H1/11\ G21B1/00 |
| 3210 | 3D printing | METHOD OF MANUFACTURING METAL- OR CERAMIC MICROPARTS\ | US2005140070A1 | FINNAH GUIDO (DE)\ JOHANN THOMAS (DE)\ PIOTTER VOLKER (DE)\ RUPRECHT ROBERT (DE)\ SCHANZ GERHARD (DE)\ HAUSSELT JURGEN (DE)\ HOLSTEIN NIIS (DE) | SUMITOMO HEAVY INDUSTRIES (JP) | C25D1/10\ B29C45/00\ B29C33/40\ B29C45/16\ C25D1/08\ C25D1/14 |
| 3211 | 3D printing | TOOTHBRUSH AND PROCESS FOR PRODUCING THE SAME\ | US2014075696A1 | FISCHER FRANZ (CH)\ PFENNIGER PHILIPP (CH) | TRISA HOLDING AG (CH) | A46B3/06\ A46B9/04\ A46D3/04 |
| 3212 | 3D printing | IMAGING MODULE FOR HOT MELT WAX INK JET PRINTER\ | US2010208017A1 | FLORENCE RICHARD N (US)\ MACQUEEN JR RAYMOND J (US)\ PULLEN ELAINE A (US)\ WALTER GRAHAM D (US)\ WIITA ROBERT L (US)\ WRIGHT WILLIAM PARKER ALEXANDER (US) | BLACK DOT TECHNOLOGY INC (US) | B41J2/175 |
| 3213 | 3D printing | CONTROL OF METALLIC APPEARANCE IN AUTOMOTIVE CAST PAINT FILMS\ | US5653927A | FLYNN MICHAEL D (US)\ TRUOG KEITH L (US) | AVERY DENNISON CORP (US) | B29C37/00\ B05D5/06\ B05D7/24\ B05D7/14\ B32B15/08 |
| 3214 | 3D printing | POST PROCESSOR FOR THREE-DIMENSIONAL OBJECTS\ | US7114943B1 | FONG JON JODY (US)\ SOLIZ RAYMOND M (US) | 3D SYSTEMS INC (US) | B28B17/00\ B29C35/00 |
| 3215 | 3D printing | APPARATUS FOR MAKING ENGINEERED TISSUE USING SELF-ASSEMBLING CELL AGGREGATES\ | US2015004273A1 | FORGACS GABOR (US)\ JAKAB KAROLY (US)\ NEAGU ADRIAN (US)\ MIRONOV VLADIMIR (US) | MUSC FOUND FOR RES DEV (US)\ UNIV MISSOURI (US) | B29C67/00\ C12N5/071 |
| 3216 | 3D printing | MODELING MATERIAL METHOD AND APPARATUS FOR MANU-FACTURING A THREE-DIMENSIONAL OBJECT BY MELT LAYERING\ | US2013209739A1 | FRUTH CARL (DE) | FIT FRUTH INNOVATIVE TECHNOLOGIEN GMBH (DE)\ GMBH FIT FRUTH INNOVATIVE TECHNOLOGIEN (US) | B05D3/00 |
| 3217 | 3D printing | METHOD FOR FEEDING WIRE IN LASER AND CLADDING AND IN-LASER WIRE FEEDER\[FR] PROCéDé POUR DISTRIBUER UN FIL DANS UN LASER ET REVêTEMENT ET DISPOSITIF DE DISTRIBUTION DE FIL DANS UN LASER\ | WO2009039753A1 | FU GEYAN (CN)\ SHI SHIHONG (CN)\ HU JIN (CN)\ YANG GUOJIA (CN)\ HAN XUELEI (CN) | UNIV SUZHOU (CN)\ FU GEYAN (CN)\ SHI SHIHONG (CN)\ HU JIN (CN)\ YANG GUOJIA (CN)\ HAN XUELEI (CN) | B23K26/34\ B23K26/06 |
| 3218 | 3D printing | THERMAL TRANSFER IN-MOULD DECORATION AND PREPARATION METHOD THEREOF\ | CN102673293B | FU JINGUO US\ XIANG WEIWEI US | TEC HI PRINTING TECHNOLOGY CO LTD US | B44C3/02\ B32B27/00\ B44C5/00\ B44C1/16 |
| 3219 | 3D printing | VERFAHREN ZUR HERSTELLUNG EINES HEI?GASSPIEGELS ZUM ERW?RMEN VON KUNSTSTOFFEN\[EN] HOT GAS MIRROR MANUFACTURING METHOD FOR INJECTION MOLDING TOOL DURING MANUFACTURING E.G. PLASTIC COMPONENTS FOR PRODUCTION OF DOOR OF PASSENGER CAR INVOLVES SEPARATING MOLD AND COMPONENT INCLUDING INLETS AND CHANNELS AND HEATING COMPONENT\ | DE102011120014A1 | FUETTERER MICHAEL (DE) | DAIMLER AG (DE) | B29C67/00\ B29C45/00\ B29C65/10 |
| 3220 | 3D printing | BALL MOUNTING METHOD AND APPARATUS FOR BALL GRID ARRAY PACKAGING SUBSTRATE\ | CN100382266C | FUJIANG SUN (CN) | HUAWEI TECH CO LTD (CN) | H01L21/60 |
| 3221 | 3D printing | REINFORCED POLYAMIDE RESIN COMPOSITION FOR BLOW MOLDING AND BLOW MOLDED PRODUCT USING IT\ | JP2009132908A | FUJII YASUHITO US\ SHIGA KENJI US\ SAKAI SATOSHI US | TOYO BOSEKI (JP) | C08L63/00\ B29L31/30\ C08L77/00\ B29K77/00\ B29C49/00\ B29K105/06\ C08K7/02 |
| 3222 | 3D printing | COMPOSITE MATERIAL AND SEMICONDUCTOR DEVICE USING THE SAME\ | US6737168B1 | FUKUI AKIRA (JP)\ YAMAGATA SHINICHI (JP)\ KAMITAKE KAZUYA (JP)\ ABE YUGAKU (JP) | SUMITOMO ELECTRIC INDUSTRIES (JP) | C04B41/50\ C04B35/573\ C04B41/51\ C04B41/85\ C04B41/88\ H01L23/14\ H01L23/15\ H01L23/373 |
| 3223 | 3D printing | DEMOULAGE PAR CIRE PERDUE D'UNE BAUDRUCHE DE RESTRUCTURATION\ | FR2911079B1 | GAUTREAU NICOLAS US\ CHARRON YVES US | INST FRANCAIS DU PETROLE (FR) | B22C9/04\ B29C33/52\ B05D7/22\ B29C39/36 |
| 3224 | 3D printing | LASER WELDED BONDING PADS FOR PIEZOELECTRIC PRINT HEADS\ | US8857021B2 | GERNER BRADLEY JAMES (US)\ NYSTROM PETER J (US)\ DOLAN BRYAN R (US) | XEROX CORP (US)\ GERNER BRADLEY JAMES (US)\ NYSTROM PETER J (US)\ DOLAN BRYAN R (US) | H05K3/10\ B41J2/16\ B41J2/14\ B23K26/22 |
| 3225 | 3D printing | NON-CONTACT PRINTED COMESTIBLE PRODUCTS AND APPARATUS AND METHOD FOR PRODUCING SAME\ | US2007231425A1 | GLEFFE WILLIAM R (US)\ REAM RONALD L (US)\ VALDEZ ENRIQUE (US) | WRIGLEY W M JUN CO (US) | A23G4/00 |
| 3226 | 3D printing | PHOTOSENSITIVE MATERIAL PACKAGING MATERIAL PHOTOSENSITIVE MATERIAL PACKAGE AND MANUFACTURING METHOD THEREOF\ | JP2006053365A | GOI KATSUNORI US\ SHIMA TETSUO US\ TSUJI NOBUAKI US\ GOTO SHIGETO US\ KIMURA AKIZUMI US | KONICA MINOLTA MED & GRAPHIC US | G03C3/00 |
| 3227 | 3D printing | APPARATUS FOR AVOIDING DEPOSITS ON OPTICAL COMPONENTS IN THE LASER SINTERING PROCESS\ | US2013101803A1 | GREBE MAIK (DE)\ DIEKMANN WOLFGANG (DE)\ HESSEL-GELDMANN SIGRID (DE) | GREBE MAIK (DE)\ DIEKMANN WOLFGANG (DE)\ HESSEL-GELDMANN SIGRID (DE) | B29C35/08\ B29C67/24\ B32B3/26 |
| 3228 | 3D printing | THREE-DIMENSIONALLY STRUCTURED FIBROUS WEB AND A METHOD FOR ITS MANUFACTURE\ | US6723416B1 | GROITZSCH DIETER (DE)\ SCHAUT GERHARD (DE)\ GRIMM HANS-JOERG (DE) | FREUDENBERG CARL FA (US) | D04H13/00\ D04H1/46\ B32B5/26\ D04H1/54 |
| 3229 | 3D printing | METHOD FOR PREPARING THREE-DIMENSIONAL PHOTOELASTICITY ANALYSIS MODEL BY APPLYING FAST-FORMING PLASTER MOLD TECHNIQUE\ | CN101623907A | GUANGHUI CHEN US\ GUOQI CUI US | TIANJIN INTERNAL COMB ENGINE R US | B29C39/42 |
| 3230 | 3D printing | TEXTILE COMPLEX INTENDED TO BE USED AS REINFORCING LAYER FOR THE MANUFACTURE OF COMPOSITE PARTS AND PROCESS FOR MANUFACTURING SUCH A COMPLEX\ | US2008305701A1 | GUIGNER DELPHINE (FR)\ BOUTIER SEBASTIEN (FR) | CHOMARAT COMPOSITES (FR) | B29C35/02\ B32B27/04\ B32B27/12 |
| 3231 | 3D printing | LIQUID MOLDING CORE-MELTING FORMING PROCESS FOR COMPLICATED RESIN-BASED COMPOSITE MATERIAL STRUCTURAL MEMBER\ | CN101913251A | GUIZHE ZHAO US\ YANFEI ZHANG US\ RUIKUI DU US\ YAQING LIU US | UNIV NORTH CHINA US | B29C70/54\ B29C70/36 |
| 3232 | 3D printing | PREPARATION METHOD OF DESIGNABLE POLYMER-BASED CONDUCTIVE COMPOSITE MATERIAL\ | CN102837430A | GUO SHAOYUN US\ SHEN JIABIN US\ LI JIANG US\ ZHU JIAMING US\ YANG ZHI US | UNIV SICHUAN (CN) | C08L77/02\ C08K3/04\ B29C70/28\ C08L23/12\ C08L23/06\ C08L51/06 |
| 3233 | 3D printing | METHOD FOR MANUFACTURING IMPELLER MOULD FOR GAS COMPRESSOR OF TURBOCHARGER\ | CN102125998A | GUOWEN CHEN US\ JIANFEI YAN US\ FUXING DONG US\ AIGUO ZHU US\ YUNHU FENG US\ GANG CHEN (CN) | DATONG NORTH TIANLI TURBOCHARGING TECHNOLOGY CO LTD US | B22C9/06\ B22C9/22 |
| 3234 | 3D printing | POLYMERIC ULTRAVIOLET (UV) DECORATIVE BOARD AND PROCESSING TECHNOLOGY THEREOF\ | CN102616076A | GUOXIANG LI US | GUOXIANG LI US | B32B27/06\ B44C5/04 |
| 3235 | 3D printing | METHOD FOR PRODUCING MULTICOLOR CHOCOLATE\ | CN101912023A | GUOXIONG LI US | GUOXIONG LI US | A23G1/04 |
| 3236 | 3D printing | FIRST MIRROR SURFACE IMPURITY ANALYSIS DEVICE BASED ON REFLECTION TYPE TERAHERTZ SPECTRUM\ | CN203037573U | HAI RAN US\ ZHANG CHENFEI US\ XIN YU US\ DING HONGBIN US | UNIV DALIAN TECH (CN) | G01B11/06\ G01N21/31 |
| 3237 | 3D printing | PRODUCTION TECHNOLOGY OF THREE-DIMENSIONAL HOLLOW FIBER\ | CN102443863A | HAIBIN CAI US | QUANZHOU SHENGXIN FIBER PRODUCT CO LTD US | D01D5/088\ D01D5/24\ D01D1/00\ D01D1/10\ D01D10/02\ D01D5/08\ D01D5/12 |
| 3238 | 3D printing | NO-MOLD FUSION STACKING MANUFACTURE METHOD OF PARTS OR MOLD\ | CN101362272A | HAIOU ZHANG (CN)\ GUILAN WANG (CN)\ ZHONGCHAO WEI (CN) | UNIV HUAZHONG SCIENCE TECH (CN) | B23K26/34\ B23K37/06\ B23K9/04 |
| 3239 | 3D printing | INK-JET PRINTER HEAD AND MANUFACTURE THEREOF\ | JP8174851A | HAMABUCHI KAZUFUMI US | MATSUSHITA ELECTRIC IND CO LTD (JP) | B41J2/16\ B41J2/05 |
| 3240 | 3D printing | SINGLE SIDE BI-DIRECTIONAL FEED FOR LASER SINTERING\ | US2005263933A1 | HANNA CHRISTOPHER R (US)\ WELCH ROBERT H IV (US)\ CHUNG TAE M (US) | 3D SYSTEMS INC (US) | B29C35/08\ B29C67/00\ B29C41/36\ B29C35/02 |
| 3241 | 3D printing | POROUS METAL BODY PROCESS FOR PRODUCING THE SAME AND BATTERY PLATE FORMED THEREFROM\ | US5597665A | HARADA KEIZO (JP)\ ISHII MASAYUKI (JP)\ YAMANAKA SHOSAKU (JP) | SUMITOMO ELECTRIC INDUSTRIES (JP) | B22F3/11\ B22F5/00\ C22C1/08\ B01D39/20\ H01M4/80\ C23C14/20\ H01M4/02\ H01M4/66\ H01M6/16 |
| 3242 | 3D printing | METHOD TO CREATE REMOVABLE DENTAL PROSTHESIS AND THE DENTAL PROSTHESIS MAKING THEREOF\ | US2012261848A1 | HARASZATI GYOERGY (HU) | HARASZATI GYOERGY (HU) | A61C13/20 |
| 3243 | 3D printing | THREE-DIMENSIONAL COFORM NONWOVEN WEB\ | US2003211802A1 | HARRIS CHARLENE BENDU (US)\ FRAZIER NINA (US)\ KECK LAURA ELIZABETH (US) | KIMBERLY CLARK CO (US) | D04H11/00\ A47L13/16 |
| 3244 | 3D printing | VERFAHREN ZUR HERSTELLUNG VON METALL- ODER KERAMIK-MIKROBAUTEILEN\[EN] METHOD FOR PRODUCING METAL OR CERAMIC MICROCOMPONENTS\[FR] PROCEDE POUR LA PRODUCTION DE MICROCOMPOSANTS METALLIQUES OU CERAMIQUES\ | EP1527214A2 | HAUSSELT JUERGEN (DE)\ FINNAH GUIDO (DE)\ HOLSTEIN NILS (DE)\ JOHANN THOMAS (DE)\ PIOTTER VOLKER (DE)\ RUPRECHT ROBERT (DE)\ SCHANZ GERHARD (DE) | KARLSRUHE FORSCHZENT (DE) | B81C1/00\ B29C45/00\ B29C45/16 |
| 3245 | 3D printing | METHOD OF MANUFACTURING FILM INSERTED MOLDED ARTICLE\ | JP2008168613A | HAYASHI SEIJI US\ MATSUOKA KIYOMI US\ GOTO YOSHIHIDE US\ GOTO EIICHIRO US | SANWA SCREEN MEIBAN KK US | B29C45/14\ B29L9/00\ B29C59/02\ B29C69/00 |
| 3246 | 3D printing | MANUFACTURING METHOD OF OPERATION PANEL\ | JP2008183848A | HAYASHI SEIJI US\ TAKAHASHI TATSUMI US\ ITO YOSHIHIRO US | SANWA SCREEN MEIBAN KK US | B29C45/14\ B29C69/02\ D06F39/00 |
| 3247 | 3D printing | MELTING DEVICE FOR INCREASED PRODUCTION OF MELTED INK IN A SOLID INK PRINTER\ | US8052264B2 | HAYS ANDREW WAYNE (US)\ LEO MICHAEL F (US)\ LEIGHTON ROGER G (US) | XEROX CORP (US) | B41J2/175 |
| 3248 | 3D printing | LABEL WHEEL ROTARY PRINTING MACHINE\ | CN100595061C | HELIANG QI US\ ZE YANG US | SHANGHAI TAIYO KIKAI CO LTD US | B41F17/00\ B41F13/02\ B32B39/00\ B41F13/54\ B41F19/00\ B41F23/00\ B65H5/06\ G09F3/02 |
| 3249 | 3D printing | FLAME-RETARDING POLYPHENYL ETHER COMPOSITE AND PREPARATION METHOD AND USE THEREOF\ | CN102101941A | HENGMEI HUANG US\ LIN WANG US\ JIANZHONG LIU US\ SHAOHUA GUO US\ JINFENG FU US\ ZHIHUA HU US\ NANBIAO YE US | KINGFA SCI & TECH CO LTD US\ SHANGHAI KINGFA SCI & TECH CO US\ CHANG XIN MIANYANG NEW MATERIALS LTD US | C08K5/20\ C08L9/00\ C08L23/00\ C08L23/08\ C08L23/16\ C08L53/02\ C08K5/523\ B29C47/92\ C08L71/12\ C08K5/00\ C08K5/3492\ C08L9/02\ C08L25/12\ H01B7/295\ H01B7/36 |
| 3250 | 3D printing | HERSTELLUNG VON HOHLK?RPERN ODER SCHICHTEN MIT HOHLR?UMEN\[EN] PRODUCING HOLLOW BODY WITH CAVITY COMPRISES PRODUCING MOLDED BODY WITH OUTER MOLD PARTIALLY HAVING SHAPE OF HOLLOW CAVITY WHERE MOLDED BODY IS DISPLACEABLE BY SOLVENT/MELT IN FLOWABLE STATE AND COATING MOLDED BODY WITH COATING MATERIAL\ | DE102009037893A1 | HERTTER MANUEL (DE)\ JAKIMOV ANDREAS (DE)\ WACHTER WOLFGANG (DE) | MTU AERO ENGINES GMBH (DE) | B29C45/00\ C04B35/638\ B05D1/00 |
| 3251 | 3D printing | VORRICHTUNG ZUR HERSTELLUNG VON DREIDIMENSIONALEN OBJEKTEN\[EN] DEVICE USEFUL FOR PRODUCING THREE-DIMENSIONAL OBJECTS COMPRISES A CARRYING DEVICE FOR CARRYING THE OBJECT A COATING DEVICE FOR APPLYING LAYERS OF BUILDING MATERIAL ON THE CARRYING DEVICE AND AN IRRADIATION SOURCE FOR IRRADIATING LAYER\ | DE102011106380A1 | HERZOG FRANK (DE) | CL SCHUTZRECHTSVERWALTUNGS GMBH (DE) | B22F3/105 |
| 3252 | 3D printing | POLYPROPYLENE NONWOVEN FABRIC\ | JPH10292256A | HIKASA KATSUJI US\ YAMAOKA YASUSHI US | ASAHI CHEMICAL IND US | D04H3/16\ D04H3/007 |
| 3253 | 3D printing | ELASTIC BODY SUPPORTING APPARATUS\ | JP2009050344A | HIRAI YOSHIHISA US\ WATASHIBA HIDEMASA US | KAWASHIMA SELKON TEXTILES CO US | A47C27/12 |
| 3254 | 3D printing | SHEET FOR MOLDING DECORATION AND METHOD FOR SIMULTANEOUS DECORATION WITH INJECTION MOLDING\ | JP2004042351A | HIRANO TAMAMI US\ KAMIYAMA HIRONORI US\ ATAKE HIROYUKI (JP) | DAINIPPON PRINTING CO LTD (JP) | B29C45/14\ B32B27/30\ B29L9/00\ B32B33/00\ B29K105/20\ B29K627/12 |
| 3255 | 3D printing | COMPOSITE POLYMERIC ARTICLES FORMED FROM EXTRUDED SHEETS CONTAINING A LIQUID CRYSTAL POLYMER\ | US2012021212A1 | HOFMANN ACHIM (DE)\ YUNG PAUL C (US) | TICONA GMBH (DE)\ HOFMANN ACHIM (DE)\ YUNG PAUL C (US) | B29C51/02\ B32B5/00\ B29C47/78 |
| 3256 | 3D printing | HIGH RESOLUTION MULTICOLOR INK JET PRINTER\ | US7690779B2 | HOISINGTON PAUL A (US)\ SPEHRLEY JR CHARLES W (US) | FUJIFILM DIMATIX INC (US) | B41J2/01\ G01D11/00\ B41J2/175\ B41J2/21\ B41J2/195\ B41J11/00\ B41J2/205\ B41J13/22\ B41J19/16 |
| 3257 | 3D printing | METHOD OF TREATING IMAGE RECEIVING SHEETS AND A HOT MELT INK JET PRINTER EMPLOYING THIS METHOD\ | US2006114302A1 | HOLTMAN LODEWIJK T (NL) | OCE TECH BV (NL) | B41J2/175 |
| 3258 | 3D printing | VERFAHREN ZUM BEHANDELN VON BILDAUFZEICHNUNGSBL?TTERN UND DIESES VERFAHREN ANWENDENDER TINTENSTRAHLDRUCKER FüR W?RMESCHMELZBARE TINTE\[EN] METHOD OF TREATING IMAGE RECEIVING SHEETS AND HOT MELT INK JET PRINTER EMPLOYING THIS METHOD\[FR] PROCéDé POUR TRAITER DES FEUILLES DESTINéES à RECEVOIR DES IMAGES ET IMPRIMANTE à JET D'ENCRE POUR ENCRE THERMOFUSIBLE UTILISANT CE PROCéDé\ | EP1661723A3 | HOLTMAN LODEWIJK T (NL) | OCE TECH BV (NL) | B41J2/175\ B41J11/00 |
| 3259 | 3D printing | ADHISIVE RESINS FOR COATING OF STEEL PLATE\ | KR20100082077A | HONG CHANG IL (KR)\ NAM SOON HEE (KR) | HONG CHANG IL (KR) | C09J7/02\ C09J133/04 |
| 3260 | 3D printing | MICROSPRING LASER PROCESSING METHOD\ | CN100455841C | HONGBO SUN (CN) | JINLIN UNIV (CN) | F16F1/06 |
| 3261 | 3D printing | METHOD OF BUILDING THREE-DIMENSIONAL OBJECT WITH MODIFIED ABS MATERIALS\ | US2009295032A1 | HOPKINS PAUL E (US) | STRATASYS INC (US) | B28B1/14 |
| 3262 | 3D printing | METHOD AND APPARATUS FOR MANUFACTURING THREE-DIMENSIONAL PATTERN SHEET AND FILM USING ULTRASONIC GENERATOR\ | JP2011062972A | HOSOYA SHUHEI US | TOPPAN PRINTING CO LTD US | B29C47/88\ B29L7/00\ B29L11/00\ B29C59/04 |
| 3263 | 3D printing | VERFAHREN UND VORRICHTUNG ZUM FARBIGEN BEDRUCKEN VON PERSONALISIERUNGSDOKUMENTEN\[EN] METHOD FOR COLOR PRINTING OF PERSONALIZATION DOCUMENT INVOLVES TIGHTENING PERSONALIZATION DOCUMENT UNDER PRINT HEAD AND CONTROLLING UV LEDS IN SECTIONS BY ELECTRONIC CONTROL SO THAT UV LEDS ARE ILLUMINATED\ | DE102009021634A1 | HOTZEL MARIO (DE)\ BARTEL MARKO (DE)\ SCHMIDT REINHARD (DE) | RUHLAMAT GMBH (DE) | B41M3/14\ B41J2/21\ B41J3/407\ B41F11/02\ B41J2/525 |
| 3264 | 3D printing | THREE-DIMENSIONAL FABRICS AND FILMS AND THE PROCESS FOR MAKING THE SAME\ | US2004222552A1 | HOUSLER JOHN (US)\ CARTER NICK (US)\ DALE ROBERT (US)\ SCHAEFFER JAMES (US)\ MOODY RALPH (US) | Empty Field US | D04H3/03\ B29C43/22\ B29C47/00\ B29C47/88\ B29C51/22\ B29C59/06\ D04H3/05\ D04H3/08 |
| 3265 | 3D printing | BUILT-IN LASER SMELTING SPRAY NOZZLE\ | CN2705236Y | HU QIANWU (CN)\ ZENG XIAOYAN (CN) | CENTRAL CHINA SCIENCE & TECH (CN) | B23K26/34\ B05B7/16\ C23C4/00 |
| 3266 | 3D printing | DEFECTIVE TOTAL STERNUM REPLACEMENT PART FOR TOTAL STERNUM DEFECT OPERATION TREATMENT AND METHOD FOR MANUFACTURING THE SAME\ | CN102389343A | HUA TANG US\ ZHIFEI XU US\ BIN WU US\ MINGMING JI US | HUA TANG US\ ZHIFEI XU US\ BIN WU US\ MINGMING JI US | A61F2/28\ A61L27/18\ A61L27/12\ A61B17/56 |
| 3267 | 3D printing | THREE-DIMENSIONAL DISPLAY APPARATUS AND METHOD FOR MANUFACTURING THE SAME\ | US2014118643A1 | HUANG HONG-JI (CN) | HUANG HONG-JI (CN)\ SHENZHEN CHINA STAR OPTOELECT (CN) | G02B27/22\ G02F1/1335 |
| 3268 | 3D printing | MANUFACTURING METHOD OF COLOUR STEREO MOULD CANDY AND ITS PRODUCT\ | CN1319461C | HUANG ZHENGLONG HUANG (CN) | YONGXIN TRADE CO LTD (CN) | A23G3/54\ A23G1/54\ A23G3/00 |
| 3269 | 3D printing | ASIA1 TYPE MULTI-EPITOPE RECOMBINANT VACCINE OF BOVINE FOOT-AND-MOUTH DISEASE VIRUSES AND PREPARATION METHOD THEREOF\ | CN101775399A | HUIYUN CHANG US\ GUOZHENG CONG US\ JUNZHENG DU US\ SHANDIAN GAO US\ TONG LIN US\ JUNJUN SHAO US\ JINGFENG WANG US | LANZHOU VETERINARY RES INST CH US | C12N15/62\ A61K48/00\ C12N15/42\ A61K39/135\ A61K47/48\ A61P31/14\ C07K14/09 |
| 3270 | 3D printing | SHEEP APHTHOVIRUS ASIAL TYPE MULTI-EPITOPE RECOMBINANT VACCINE AND PREPARATION METHOD THEREOF\ | CN101864434A | HUIYUN CHANG US\ GUOZHENG CONG US\ JUNZHENG DU US\ SHANDIAN GAO US\ TONG LIN US\ JUNJUN SHAO US\ JINGFENG WANG US | LANZHOU VETERINARY RES INST CHINESE ACADEMY OF AGRICULTURAL SCIENCES US | C12N15/62\ A61K48/00\ C12N15/42\ A61K39/135\ A61P31/14\ C07K14/09 |
| 3271 | 3D printing | INJECTION FOAMING METHOD USING THERMOPLASTIC ELASTOMER COMPOSITION FOR CORE BACK TYPE INJECTION FOAMING\ | JP2008013780A | IEDA KATSUMASA US\ MORIKAWA AKIHIKO (JP)\ KANAE KENTAROU (JP)\ ABE TOMOKAZU (JP) | JSR CORP (JP)\ HONDA MOTOR CO LTD (JP) | C08J9/34 |
| 3272 | 3D printing | PROCESS FOR PRODUCING AN ELECTRONIC CARD FOR PRODUCT QUALITY PROTECTION\ | RO122027B1 | IERCESCU CONSTANTIN (RO)\ MANDULA GHEORGHE CONSTANTIN (DE) | IERCESCU CONSTANTIN (RO)\ MANDULA GHEORGHE CONSTANTIN (DE) | B42D15/10\ G06K19/07 |
| 3273 | 3D printing | FLUORESCENT SOLID INK MADE WITH FLUORESCENT NANOPARTICLES\ | US2010086683A1 | IFTIME GABRIEL (CA)\ VANBESIEN DARYL W (CA)\ WAGNER CHRISTOPHER A (CA)\ ALLEN C GEOFFREY (CA)\ BIRAU MARIA M (CA)\ WOSNICK JORDAN H (CA) | XEROX CORP (US) | B05D5/06\ C09K11/02 |
| 3274 | 3D printing | METHOD OF PRODUCING TONER\ | JP2009175632A | IGA TAKASHI US\ KANEDA TAKUYA US | NIPPON ZEON CO US | G03G9/087\ G03G9/08 |
| 3275 | 3D printing | HIGH-STRENGTH COMPLEX SOFT MAGNETIC MATERIAL HAVING HIGH STRENGTH HIGH MAGNETIC FLUX DENSITY HIGH RESISTANCE AND LESS IRON LOSS AND MANUFACTURING METHOD THEREOF\ | JP2006332525A | IGARASHI KAZUNORI US\ WATANABE MUNEAKI US\ NAKAYAMA RYOJI US\ MORIMOTO KOICHIRO US | MITSUBISHI MATERIALS PMG CORP US | B22F3/00\ B22F3/24\ B22F1/02\ H01F1/33\ H01F1/24 |
| 3276 | 3D printing | HIGH-STRENGTH COMPLEX SOFT MAGNETIC MATERIAL HAVING HIGH STRENGTH HIGH MAGNETIC FLUX DENSITY HIGH RESISTANCE AND LESS IRON LOSS AND MANUFACTURING METHOD THEREOF\ | JP2006332524A | IGARASHI KAZUNORI US\ WATANABE MUNEAKI US\ NAKAYAMA RYOJI US\ MORIMOTO KOICHIRO US | MITSUBISHI MATERIALS PMG CORP US | B22F3/00\ B22F1/02\ H01F1/33\ H01F1/24 |
| 3277 | 3D printing | MOUNTING PARTS PEEL SUPPRESSING SOLDERING METHOD ELECTRONIC CIRCUIT BASEBOARD AND ELECTRONIC INSTRUMENT\ | US2002170947A1 | IGARASHI MINORU (JP)\ MUKAI KATSUHIKO (JP)\ ISODA MASASHI (JP)\ KOBAYASHI TAKAYUKI (JP) | GEORGIA TECH RES INST (US) | H05K3/30\ B23K1/00\ H05K3/34\ B23K1/08\ B23K31/02\ B23K101/42\ H05K3/28 |
| 3278 | 3D printing | VERFAHREN ZUM HERSTELLEN EINES FASERVERBUNDBAUTEILS\ | DE102008026161B4 | ILZHOEFER KARL-HEINZ DIPL ING (DE)\ SALKIC ASMIR DIPL ING (DE) | DAIMLER AG (DE) | B29C70/34\ B29C70/50\ C08J5/04\ B29B15/12\ B62D29/04 |
| 3279 | 3D printing | NANOTECHNOLOGICAL PROCESSING OF CATALYTIC SURFACES\ | US2006115389A1 | INDECH ROBERT (US) | INDECH ROBERT (US) | B01D53/34 |
| 3280 | 3D printing | ADDITITVE MANUFACTURING OF COMPONENTS FOR DOWNHOLE WIRELINE TUBING AND DRILL PIPE CONVEYED TOOLS\ | US2013310961A1 | INTRIAGO VELEZ JUAN CARLOS (US) | CORP SCHLUMBERGER TECHNOLOGY (US)\ SCHLUMBERGER TECHNOLOGY CORP (US) | G06F17/50 |
| 3281 | 3D printing | MOLTEN RESIN EXTRUDING LAMINATING AND SHAPING METHOD AND APPARATUS THEREFOR\ | JP2006192710A | ISHITANI SHIGEKI US | SEKISUI CHEMICAL CO LTD US | B29C67/00 |
| 3282 | 3D printing | MOLTEN RESIN EXTRUDING LAMINATING AND SHAPING METHOD AND APPARATUS THEREFOR\ | JP2006192616A | ISHITANI SHIGEKI US\ IWASAKI JUNYA US | SEKISUI CHEMICAL CO LTD US | B29C67/00 |
| 3283 | 3D printing | METHOD FOR PRODUCING DEPOSITED DIAMOND FILM\ | JP2006290687A | ISSHIKI HIDEO US | UNIV ELECTRO COMMUNICATIONS US\ CAMPUS CREATE CO LTD US | H04R31/00\ C30B29/04\ H04R7/02 |
| 3284 | 3D printing | MANUFACTURING APPARATUS AND METHOD OF MOLDING\ | JP2008201137A | ITO HIROSHI US\ YAKIMOTO KAZUTOSHI US\ SHIROGANEYA TSUKASA US\ NAITO AKIHIRO US | JAPAN STEEL WORKS LTD US | B29C43/02\ B29C43/34 |
| 3285 | 3D printing | RESIN FOAMED SHEET\[FR] FEUILLE DE MOUSSE DE RéSINE\ | WO2011062141A1 | IWATA MASAYOSHI (JP)\ KOGI TOSHINOBU (JP) | SEKISUI PLASTICS (JP)\ IWATA MASAYOSHI (JP)\ KOGI TOSHINOBU (JP) | B32B5/18\ B32B27/32\ C08J9/06\ C08L23/10 |
| 3286 | 3D printing | LASER THERMAL PRINTING ON MICROPOROUS PLASTIC SUBSTRATES\ | US2013202828A1 | JACOBY PHILIP (US) | JACOBY PHILIP (US)\ MAYZO CORP (US) | B32B33/00\ B32B3/26\ B29C59/16 |
| 3287 | 3D printing | HOCHMOLEKULARE POLYAMIDE\[EN] THERMOPLASTIC MOLDING COMPOSITION USEFUL TO PRODUCE FIBERS FILMS AND MOLDINGS INCLUDING E.G. INTAKE MANIFOLDS INTERCOOLER AND CIRCUIT BOARD COMPONENTS COMPRISES THERMOPLASTIC POLYAMIDE POLYETHYLENEIMINE HOMO OR COPOLYMER AND ADDITIVES\ | DE102008058246A1 | JAIN SACHIN (DE)\ GABRIEL CLAUS (DE)\ STAAL BASTIAAN BRAM PIETER (DE)\ LIEW CHEE CHIN (DE) | BASF SE (DE) | C08L77/00\ C08L79/00 |
| 3288 | 3D printing | COVER MOUNTING OF 3D MAT AND SADDLE STITCHING METHOD\ | KR101337739B1 | JEONG MI SU (KR) | JEONG MI SU (KR) | D05B11/00 |
| 3289 | 3D printing | PRODUCTION PROCESS OF COLORFULLY-PAINTED AND BACK-ENGRAVED CRYSTAL GLASS\ | CN103253057A | JI QINGKAI US | ARIAMOTION DALIAN CO LTD US | B44C1/22\ C03B19/00\ C03C3/089 |
| 3290 | 3D printing | DEVICE FOR PREPARING SEMI-SOLID ALLOY MELT AND PROCESS FOR PREPARING ALSI9MG SEMI-SOLID ALLOY\ | CN101660063A | JIANBO TAN US\ ZENGMIN LI US\ LIXIN LI US | UNIV HEBEI SCIENCE & TECH US | C22C1/00 |
| 3291 | 3D printing | DEVICE FOR PREPARING SEMI-SOLID ALLOY MELT\ | CN201512567U | JIANBO TAN US\ ZENGMIN LI US\ LIXIN LI US | UNIV HEBEI SCIENCE & TECH US | B22D17/08\ C22C1/00 |
| 3292 | 3D printing | METHOD FOR MODELING THREE-DIMENSIONAL PATTERN\ | CN101722784A | JIANGANG HE US | JIANGANG HE US | B44C1/20 |
| 3293 | 3D printing | PRODUCTION METHOD OF FLAME-RETARDANT SINGLE-HOLE HOLLOW OR MULTI-HOLE THREE-DIMENSIONAL CRIMPED POLYESTER STAPLE FIBER\ | CN101676451A | JIANJUN ZHANG US\ QINRONG TONG US\ JIANQIU XU US\ GUANGZE WU US\ ZUAN SUN US\ HAITIAN XIA US\ GUOZHI CAI US\ HENG ZHANG US\ WENQIN WU US | SHANGHAI XINDILUN IND CO LTD US\ TOPRET IND DESIGN SHANGHAI CO US | D01D5/24\ D01F6/84\ D02G1/00 |
| 3294 | 3D printing | SECTION BAR SURFACE PATTERN MOLDING DEVICE\ | CN200981338Y | JIANMIN HAN (CN) | JIANMIN HAN (CN) | B29C47/08\ B29C59/04 |
| 3295 | 3D printing | PET/PET (POLYETHYLENE GLYCOL TEREPHTHALATE) HIGH-PRECISION FILTER MATERIAL WITH SEA-ISLAND FIBER LAYER\ | CN202654812U | JIN SHUANGLIN US | FUSHUN HENGYI TECHNOLOGY FILTER CO LTD US | B01D39/16 |
| 3296 | 3D printing | TECHNOLOGY FOR APPLYING INLAID METAL FINISHING COMPONENT TO IN-MOLD LABELLING\ | CN102029683A | JINGYAN LIU US\ XIAODONG ZENG US\ HUI WANG US\ QI LI US | TIANJIN ZHONGHUAN HI TECH CO US | B29C45/14 |
| 3297 | 3D printing | MOULD MAKING METHOD WITH CONFORMAL COOLING PIPE\ | CN101670437A | JINHUI LIU US\ CAN ZHAO US\ JIBING CHEN US\ RUIDI LI US | UNIV ZHEJIANG SCIENCES US | B22F3/22 |
| 3298 | 3D printing | MOULD STRUCTURE WITH CONFORMAL COOLING PIPE\ | CN101670627A | JINHUI LIU US\ CAN ZHAO US\ JIBING CHEN US\ RUIDI LI US | UNIV ZHEJIANG SCIENCES US | B29C33/38\ B29C33/04\ B29C45/73\ B22F3/22\ B29C45/26 |
| 3299 | 3D printing | METHOD FOR FORMING REFRACTORY METAL PARTS BY USING LASER\ | CN101780544A | JINHUI LIU US\ CAN ZHAO US\ JIBING CHEN US\ RUIDI LI US\ DANQING ZHANG US | HEILONGJIANG INST OF SCIENCE A US | B22F3/105\ B23K26/34\ C23C24/10 |
| 3300 | 3D printing | SYSTEM AND METHOD FOR MELTING SOLID INK STICKS IN A PHASE CHANGE INK PRINTER\ | US7581827B2 | JONES BRENT RODNEY (US)\ PLATT DAVID PAUL (US) | XEROX CORP (US) | G01D11/00\ B41J2/175 |
| 3301 | 3D printing | A MANUFACTURING METHOD OF FILM FOR INSERT INMOLDING AND FILM FOR INSERT INMOLDING\ | KR20080066889A | JUNG HAE OK (KR)\ KIM WOO HWEAN (KR) | JUNG HAE OK (KR)\ KIM WOO HWEAN (KR) | B29D7/01 |
| 3302 | 3D printing | POROUS FUSIBLE INKJET MEDIA WITH FUSIBLE CORE-SHELL COLORANT-RECEIVING LAYER\ | US7086732B2 | KASPERCHIK VLADEK P (US)\ UNGEFUG GARY ALLAN (US) | HEWLETT PACKARD DEVELOPMENT CO (US) | B41J2/01\ B41M5/00\ B41M5/50\ B41M5/52\ B41M7/00 |
| 3303 | 3D printing | CIRCUIT BOARD MANUFACTURING METHOD CIRCUIT BOARD THIN FILM TRANSISTOR ELECTRO-OPTICAL DEVICE AND ELECTRONIC EQUIPMENT\ | JP2005217359A | KAWASE TAKEO US\ MORIYA SOICHI US\ HARADA MITSUAKI US | SEIKO EPSON CORP (JP) | G09F9/00\ H01L23/52\ H05K3/10\ H01L21/288\ H01L51/00\ G02F1/167\ G09F9/30\ H01L21/28\ H01L21/3205\ H01L29/423\ H01L29/49\ H01L29/786\ H01L51/05\ H01L51/40 |
| 3304 | 3D printing | MANUFACTURING METHOD OF SUPER THIN BIDIRECTIONAL STRETCHED POLYPROPYLENE COARSE THIN FILM FOR CAPACITOR\ | CN101863120B | KE XIBIN US\ LI GAOCONG US\ SHEN SHILIN US\ SU LIYONG US\ XIE JIN US | SICHUAN EM TECHNOLOGY CO LTD US | B29C47/92\ B29C47/88\ B29C47/06\ B29K23/00\ B29C55/14\ B29L7/00 |
| 3305 | 3D printing | TECHNIQUE FOR MANUFACTURING AIR PRESSURE TYPE NON-CUTTING QUICK-MOLDING LOW MELTING-TEMPERATURE ALLOY MOLD\ | CN101433952A | KEFEI XIE (CN) | KEFEI XIE (CN) | B22D18/04\ B22C9/02 |
| 3306 | 3D printing | NON-FIBROUS ADHESIVE MATRIX AND LAMINATE FORMED THEREFROM\ | US7803459B2 | KEUCHEL HERBERT WILLIE (US)\ KEUCHEL KENNETH (US) | KEUCHEL HERBERT WILLIE (US)\ KEUCHEL KENNETH (US) | B32B27/06\ B32B3/26\ B32B7/12\ B32B37/10\ A41D31/02\ B32B37/08\ C09J5/08 |
| 3307 | 3D printing | NICHTFASERIGE KLEBSTOFFMATRIX UND DARAUS HERGESTELLTES LAMINAT\[EN] NON-FIBROUS ADHESIVE MATRIX AND LAMINATE FORMED THEREFROM\[FR] MATRICE ADHESIVE NON-FIBREUSE ET LAMINE FORME A PARTIR DE CETTE MATRICE\ | EP1729955A4 | KEUCHEL HERBERT WILLIE (US)\ KEUCHEL KENNETH (US) | SPUNFAB LTD (US) | B32B27/06\ B32B5/18\ B32B3/26 |
| 3308 | 3D printing | METHOD FOR MANUFACTURING ARTIFICIAL FINGERPRINT USING QR CODE AND QR CODE MANUFACTURED BY THE SAME\ | KR101332682B1 | KIM BYUNG JUN (KR)\ KIM JUNG HYUN (KR)\ LEE SEUNG YUN (KR) | NAT UNIV HANBAT INDUSTRY (KR) | G06K7/10\ G06K9/46\ G06K9/18\ G06K9/20 |
| 3309 | 3D printing | PACKCING SHEET MANUFACTURING MATHOD AND PACKING SHEET THEREBY\ | KR20080107832A | KIM DO EON (KR) | KIM DO EON (KR) | B31B19/60\ B31B21/60 |
| 3310 | 3D printing | CLAY FOR RUBBER BOARD\ | KR20080104627A | KIM HYUN MOOK (KR) | KIM HYUN MOOK (KR) | B44D3/18 |
| 3311 | 3D printing | WIRELESS TELEPHONE KEYPAD AND THE METHOD\ | KR20060110083A | KIM JONG WAN (KR) | MOGEM CO LTD (KR) | H01H13/70 |
| 3312 | 3D printing | MANUFACTURING METHOD OF FRINTING FILM FOR INSERT MOLDING\ | KR100877184B1 | KIM KWANG WON (KR)\ LEE HONG SOO (KR)\ KWACK SE HOON (KR) | SANGBO CORP (KR) | C08J5/18\ B29D7/01 |
| 3313 | 3D printing | FRINTING FILM FOR INSERT MOLDING\ | KR100877183B1 | KIM KWANG WON (KR)\ LEE HONG SOO (KR)\ KWACK SE HOON (KR) | SANGBO CORP (KR) | C08J5/18\ B29D7/01 |
| 3314 | 3D printing | CUSHIONING SHEET FOR FRUIT PACKAGING BOX HAVING EXCELLENT CUSHIONING EFFECT AND BEAUTIFUL APPEARANCE AND MANUFACTURING METHOD THEREOF\ | KR20040082245A | KIM YONG KWAN US | KIM YONG KWAN US | B65D85/34 |
| 3315 | 3D printing | MANUFACTURING METHOD FOR INFORMATION-COMMUNICATION MEDIUM\ | JP2004306575A | KIMURA YOSHIKAZU US\ TSUCHIYA MASAHITO US | KYOTO DAIICHI KAGAKU KK US | B42D15/02 |
| 3316 | 3D printing | APPARATUS FOR PRODUCING TONER PRECURSOR AND METHOD FOR THE SAME FIBROUS TONER PRECURSOR APPARATUS FOR PRODUCING TONER AND ELECTROPHOTOGRAPHIC TONER\ | JP2008096974A | KINOSHITA NAOTOSHI US\ TANAKA TETSUYA US\ KAWAMOTO MASAHIRO US | RICOH KK (JP) | G03G9/087\ G03G9/08 |
| 3317 | 3D printing | METALLIZED OR CERAMIC-COATED POLYESTER FILMS COMPRISING POLY(M-XYLENEADIPAMIDE)\ | US2005287378A1 | KLEIN OLIVER (DE)\ PEIFFER HERBERT (DE)\ DAVIS RICHARD L (DE) | KLEIN OLIVER (DE)\ PEIFFER HERBERT (DE)\ DAVIS RICHARD L (DE) | C08K3/00\ C08J7/04\ C08L77/06\ B32B15/08\ B32B27/36\ C08L67/02 |
| 3318 | 3D printing | VERFAHREN ZUR FREIFORMENDEN HERSTELLUNG VON DREIDIMENSIONALEN HOHLR?UMEN ODER HINTERSCHNEIDUNGEN AUFWEISENDEN MIKROBAUTEILEN\[EN] MICRO COMPONENTS MANUFACTURING METHOD INVOLVES MANUFACTURING THREE-DIMENSIONAL MASTER RAW COMPONENTS OF VARIOUS SHAPES AND CHEMICALLY PHYSICALLY OR MECHANICALLY SEPARATING MASTER COMPONENTS AND METAL LAYER FROM MICRO COMPONENT\ | DE10353060A1 | KOCH KAI UWE (DE)\ FETZER HANS-JOCHEN (DE)\ GEMMLER ARMIN (DE)\ GOTTWALD BERNHARD (DE) | FRAUNHOFER GES FORSCHUNG (DE) | C25D1/10\ B81C1/00\ C25D1/02 |
| 3319 | 3D printing | STRETCHABLE NONWOVEN FABRIC AND ARTICLE GIVEN BY USING THE SAME\ | JP2007100274A | KOJIMA MITSURU US | CHISSO POLYPRO SENI KK US\ CHISSO CORP (JP) | D01F8/06\ D01F8/14\ D04H3/011\ D04H3/16\ D04H3/007\ D04H3/147\ D04H3/153\ D01F8/08 |
| 3320 | 3D printing | METHOD FOR MANUFACTURING RING-INTEGRATED DISPLAY BOARD\ | JP2007125832A | KOJIMA TAKUO US\ YOSHIZAKA KIMIYASU US | SAKAIYA KK US | B29C45/14\ B29L31/00\ B29C33/12 |
| 3321 | 3D printing | GREENING MAT STRUCTURE\ | JP2007151449A | KOKUBU TOKIO US\ HIGUCHI YASUO US\ YOKOYAMA TAMIO US | KUREHA SENI KK US | B32B27/32\ B32B5/26\ A01G1/00 |
| 3322 | 3D printing | CLEANING STRUCTURAL ARTICLE\ | JP2004313323A | KOKUBU TOKIO US\ MORII SATORU US\ INATOMI SHINICHIRO US | TOYO BOSEKI (JP) | D04H3/07\ D04H3/16\ A47L13/16\ A47L13/20 |
| 3323 | 3D printing | MANUFACTURING METHOD OF CERAMIC COMPOSITE MATERIAL\ | JP2005298310A | KOMIYAMA TSUNEO US\ SUZUKI IWAO US | NGK ADREC CO LTD US\ NGK INSULATORS LTD (JP) | C04B35/573\ C04B41/88\ C04B41/91 |
| 3324 | 3D printing | OA EQUIPMENT EXTERIOR PART\ | JP2007191549A | KONDO FUMITAKA US\ HIRONAKA KATSUHIKO US\ INO KEIICHIRO US\ MATSUNO YUICHI US\ TOYOHARA KIYOTSUNA US\ SUZUKI HIROTAKA US\ NONOKAWA RYUJI US\ MATSUDA TAKAAKI US | TEIJIN LTD US\ TEIJIN CHEMICALS LTD US | B29C45/00\ C08J5/00\ C08L101/16\ C08K3/34\ C08L67/04 |
| 3325 | 3D printing | ELECTRONIC EQUIPMENT EXTERIOR PART\ | JP2007191548A | KONDO FUMITAKA US\ HIRONAKA KATSUHIKO US\ INO KEIICHIRO US\ MATSUNO YUICHI US\ TOYOHARA KIYOTSUNA US\ SUZUKI HIROTAKA US\ NONOKAWA RYUJI US\ MATSUDA TAKAAKI US | TEIJIN LTD US\ TEIJIN CHEMICALS LTD US | C08J5/00\ C08L101/16\ C08K3/34\ C08L67/04 |
| 3326 | 3D printing | ELECTRIC/ELECTRONIC COMPONENT\ | JP2007191547A | KONDO FUMITAKA US\ HIRONAKA KATSUHIKO US\ INO KEIICHIRO US\ MATSUNO YUICHI US\ TOYOHARA KIYOTSUNA US\ SUZUKI HIROTAKA US\ NONOKAWA RYUJI US\ MATSUDA TAKAAKI US | TEIJIN LTD US\ TEIJIN CHEMICALS LTD US | B29C45/00\ C08J5/00\ C08L101/16\ C08K3/34\ C08L67/04\ B29L31/34\ B29K67/00 |
| 3327 | 3D printing | AUTOMOTIVE COMPONENT\ | JP2007191546A | KONDO FUMITAKA US\ HIRONAKA KATSUHIKO US\ INO KEIICHIRO US\ MATSUNO YUICHI US\ TOYOHARA KIYOTSUNA US\ SUZUKI HIROTAKA US\ NONOKAWA RYUJI US\ MATSUDA TAKAAKI US | TEIJIN LTD US\ TEIJIN CHEMICALS LTD US | B29C45/73\ C08J5/00\ C08L101/16\ C08K3/34\ C08L67/04\ B29K67/00 |
| 3328 | 3D printing | SOUND INSULATING MATERIAL METHOD FOR MANUFACTURING SOUND INSULATING MATERIAL AND FILLER FOR SOUND INSULATING MATERIAL\ | JP2005241888A | KONDO YASUHIRO US\ TAKAOKA NOBUYUKI (JP) | SHIIENJI KK US | C04B38/00\ E04B1/86\ C04B28/02\ C04B38/10\ G10K11/162 |
| 3329 | 3D printing | INK CONSUMPTION DETERMINATION\ | US7425061B2 | KORN SCOTT J (US)\ BUEHLER JAMES D (US)\ KNIERIM DAVID L (US)\ YUSEM GUSTAVO J (US) | XEROX CORP (US) | G01D11/00\ B41J2/175 |
| 3330 | 3D printing | INK DEAERATOR FOR INK-JET PRINTER\ | JP2006231809A | KOSEKI OSAMU US | SII PRINTEK INC US | B41J2/175 |
| 3331 | 3D printing | MICROFABRICATED CELLULAR TRAPS BASED ON THREE-DIMENSIONAL MICRO-SCALE FLOW GEOMETRIES\ | US2007178582A1 | KOSER HUR (US) | KOSER HUR (US) | C12M1/34 |
| 3332 | 3D printing | PROCESS OF PRODUCING A COMPOSITE COMPONENT AND INTERMEDIATE PRODUCT THEREOF\ | US7837914B2 | KOSTAR TIMOTHY DANIEL (US)\ CARPER DOUGLAS MELTON (US)\ SUBRAMANIAN SURESH (US)\ STEIBEL JAMES DALE (US) | GEN ELECTRIC (US) | C04B35/00\ B29C44/04 |
| 3333 | 3D printing | METHOD FOR IMPROVING INGOT SURFACE QUALITY\ | CN100457322C | KUN XU (CN)\ DENGQUAN CHEN (CN)\ XIMIN LUO (CN)\ ZEGUANG LIU (CN) | SINO PLATINUM METALS CO LTD (CN) | B22C3/02 |
| 3334 | 3D printing | PROCESS FOR MANUFACTURING WAREF WITH DOUBLE-WALL BODY\ | CN101152752A | KUNCHE LI (CN) | KUNCHE LI (CN) | B29C45/14\ B29B11/00\ B65D1/00 |
| 3335 | 3D printing | VEGETABLE-ORIGINATED COMPONENT-CONTAINING ADHESIVE SHEET\ | JP2009280676A | KUNUKATANA MASAHIRO US\ YAMAKAWA YASUKO US | KUREHA SENI KK US | C09J7/04\ C09J177/00 |
| 3336 | 3D printing | VERFAHREN ZUR HERSTELLUNG EINES METALLISCHEN K?RPERS\[EN] METAL OBJECT IN PARTICULAR INJECTION MOLDING TOOL MANUFACTURE INVOLVES REPEATED ELECTROLYTIC DEPOSITION OF METAL ONTO A MASKED FLAT SURFACE TO BUILD UP A THREE-DIMENSIONAL STRUCTURE\ | DE10361119A1 | KURENBACH TORSTEN (DE) | SOLIDTOOL GMBH (DE) | C25D1/10\ C25D5/02\ C25D5/10 |
| 3337 | 3D printing | CASTING DIES FOR CYLINDER HEAD\ | KR20030031774A | KWAK HYEONG SIK (KR) | HYUNDAI MOTOR CO LTD (KR) | B22D15/00 |
| 3338 | 3D printing | ARTIFICIAL BONE MATERIAL AND PREPARATION METHOD THEREOF\ | CN103285428A | LAN HAI US\ SHI SHENGTAO US\ REN FANGPING US\ LI GUOKANG US | CHINA INTERNAT TOURISM & TRADE CO LTD US | A61L27/48\ A61L27/46 |
| 3339 | 3D printing | REGENERATION TECHNOLOGY ADAPTED FOR INK POX WITH SPONGE WITHOUT PRINTING HEAD\ | CN100357108C | LANGMING WANG (CN) | LANGMING WANG (CN) | B41J2/175 |
| 3340 | 3D printing | METHOD AND APPARATUS FOR REDUCING DROP PLACEMENT ERROR IN PRINTERS\ | US6367909B1 | LEAN MENG H (US) | XEROX CORP (US) | B41J2/015\ B41J2/06\ B41J2/07 |
| 3341 | 3D printing | A METHOD WHICH PRINT A LOGO ON SILICON\ | KR100813479B1 | LEE BYONG SUP (KR) | LEE BYONG SUP (KR) | B41M1/30\ B41M1/32 |
| 3342 | 3D printing | EMBLEM OF AUTOMOBILE PART AND METHOD OF MANUFACTURING THE SAME\ | US7998383B2 | LEE CHANGWOO (KR)\ SHIM SANGWOO (KR)\ LEE HOJIN (KR)\ PARK SEUNGMOK (KR) | AUTOLIV DEV (SE) | B29C59/00 |
| 3343 | 3D printing | SHELL AND METHOD OF MANUFACTURING THE SAME\ | TW200950651A | LEE CHIEN-HUNG (TW) | LEE CHIEN-HUNG (TW) | H05K5/06 |
| 3344 | 3D printing | HOLLOW THREE-DIMENSIONAL MANUFACTURING METHOD OF A CASTING PATTERN MODEL AND THE APPARATUS THEREOF\ | KR100837109B1 | LEE IN GU (KR)\ LEE YOON JU (KR)\ I YEONG CHEOL (KR) | FUTURECAST CO LTD (KR)\ LEE IN GU (KR)\ LEE YOON JU (KR)\ I YEONG CHEOL (KR) | B29C45/76\ B29C45/16 |
| 3345 | 3D printing | FILM TRANSFER METHOD AND APPEARANCE MEMBER MANUFACTURED BY USING THE SAME\ | US2013264727A1 | LEE JIH-HOUNG (TW)\ PENG MING-CHUNG (TW) | COMPAL ELECTRONICS INC (TW) | B29C45/02\ B29D11/00\ B29C45/40 |
| 3346 | 3D printing | METAL MASK AND METHOD OF MANUFACTURING THE SAME WITHOUT GENERATION OF AIR POCKET AND METHOD OF MANUFACTURING MULTI-LAYERED PRINTED CIRCUIT BOARD USING THE SAME\ | KR20040110565A | LEE SEONG HEON US | DAP CORP US | H05K3/46 |
| 3347 | 3D printing | METAL-JET UNIT\ | KR100735115B1 | LEE TAIK MIN (KR)\ KIM DONG SOO (KR)\ KIM KWANG YOUNG (KR)\ KANG TAE GOO (KR)\ YANG JEONG SOON (KR)\ CHOI BYUNG OH (KR)\ JO JEONG DAI (KR)\ YUN SO NAM (KR)\ HAM YOUNG BOG (KR)\ KIM CHUNG HWAN (KR) | KOREA MACH & MATERIALS INST (KR) | B22D23/00 |
| 3348 | 3D printing | ELECTROMAGNETIC FLEXIBLE COMPOSITE DEPOSITION DIRECT PREPARATION FORMING METHOD OF MULTIFUNCTIONAL GRADIENT COMPONENT\ | CN102962451A | LEI LEI US\ BAI XINGWANG US\ WANG XIANGPING US\ ZHANG HAIOU (CN)\ WANG GUILAN (CN) | UNIV HUAZHONG SCIENCE TECH (CN) | B22F3/105\ B22F7/02\ C23C24/10\ B22D27/02 |
| 3349 | 3D printing | FILM LAMINATE\ | US2008188154A1 | LEIS TERRY (US)\ FORSTER JAMES (US)\ HOPKINS DAVID (US) | JEN COAT INC (US) | B32B5/16\ B32B7/12\ B32B27/12 |
| 3350 | 3D printing | KRAFTSTOFFINJEKTOR MIT KüHLEINRICHTUNG\ | DE102004049703B4 | LEWENTZ GUENTER (DE) | CONTINENTAL AUTOMOTIVE GMBH (DE) | F02M53/00 |
| 3351 | 3D printing | MEDICAL C-ARM THREE-DIMENSIONAL IMAGING SYNCHRONOUS CONTROL DEVICE\ | CN102934999A | LI JINSHENG US\ GAO SONG US | NANJING PERLOVE RADIAL VIDEO EQUIPMENT CO LTD US | A61B6/00 |
| 3352 | 3D printing | METHOD FOR PREPARING SURFACE MICROSTRUCTURE OF SUPER-HYDROPHOBIC AMORPHOUS ALLOY\ | CN102328902B | LI NING US\ XIA TING US\ XU WEI US\ LIU LIN US | UNIV HUAZHONG SCIENCE TECH (CN) | B81C1/00\ C22C45/00\ C22C45/10 |
| 3353 | 3D printing | PLASTIC PIECE SURFACE AND METAL ORNAMENT COMPOSITE SEAMLESS PRIMARY INJECTION MOLDING AND FORMATION PROCESS\ | CN102962945A | LI QI US\ ZENG XIAODONG US\ WANG HUI (US) | TIANJIN ZHONGHUAN HI TECH CO US | B29C45/14\ B29B11/00 |
| 3354 | 3D printing | SPECIAL-SHAPED FILAMENT WITH RANDOM THERMAL-CONGLUTINATION\ | US2013101844A1 | LI SHUIRONG (CN)\ LING RONGGEN (CN)\ LU ZHANHUA (CN)\ YUON WENCHONG (CN)\ DUAN SHUJUN (CN)\ WU WEIGUANG (CN)\ XU GUANGHUA (CN)\ WU JINLIANG (CN)\ ZHOU XIANHE (CN) | LI SHUIRONG (CN)\ LING RONGGEN (CN)\ LU ZHANHUA (CN)\ YUON WENCHONG (CN)\ DUAN SHUJUN (CN)\ WU WEIGUANG (CN)\ XU GUANGHUA (CN)\ WU JINLIANG (CN)\ ZHOU XIANHE (CN)\ RONGSHENG PETROCHEMICAL CO LTD (CN) | D01D1/09 |
| 3355 | 3D printing | A THREE-DIMENSIONAL PRINTER EXTRUSION HEAD CAPABLE OF FEEDING IN A SWITCHING MODE\ | CN203157146U | LI YAOTANG US\ YE SHIDONG US\ ZHANG HAIQIANG US\ LIU YUPENG US | GUANGZHOU ELECTRONIC TECHNOLOGY CO LTD CHINESE ACADEMY OF SCIENCES US | B29C67/00 |
| 3356 | 3D printing | THREE-DIMENSIONAL PRINTER EXTRUSION HEAD CAPABLE OF FEEDING IN SWITCHING MODE\ | CN102922745A | LI YAOTANG US\ YE SHIDONG US\ ZHANG HAIQIANG US\ LIU YUPENG US | GUANGZHOU ELECTRONIC TECHNOLOGY CO LTD CHINESE ACADEMY OF SCIENCES US | B29C67/00 |
| 3357 | 3D printing | COLORLESS TONER FORMULATED TO IMPROVE LIGHT FASTNESS OF INK JET INK PRINTS\ | US2002120040A1 | LIN AN-CHUNG ROBERT (US)\ LEE MICHAEL H (US) | DEN TOKUYO US | B41J2/01\ B41M5/00\ G03G9/087\ G03G9/08\ G03G9/09\ G03G9/097\ G03G8/00 |
| 3358 | 3D printing | RAPID OBJECT CONSTRUCTION SYSTEM WITH GRADIENT COMPOSITIONS PROGRAMMABLE UNDER HIGH TEMPERATURE\ | TW201208869A | LIN KUN-YI (TW)\ CHANG HUI-CHU (TW)\ LIN PAI-CHI (TW) | LIN KUN-YI (TW)\ CHANG HUI-CHU (TW)\ LIN PAI-CHI (TW) | B29C67/02 |
| 3359 | 3D printing | THREE-DIMENSIONAL VISUALIZATION METHOD FOR ELECTROMAGNETIC ENVIRONMENT BODY DATA\ | CN102254347A | LINGDA WU US\ CHAO YANG US\ RONGHUAN YU US | EQUIPMENT COMMAND AND TECHNOLOGY COLLEGE OF PLA US | G06T17/00 |
| 3360 | 3D printing | 3D ELASTIC FILLER\ | CN1861531A | LIU DUANHUA (CN) | LIU DUANHUA (CN) | C02F3/10 |
| 3361 | 3D printing | METHOD AND EQUIPMENT FOR PREPARING STRIP STRUCTURES ON CONJUGATED POLYMER SURFACES THROUGH PULSE LASERS\ | CN103193999A | LIU JIAN US\ LI XIAOHONG US\ LIU ZIYI US\ XU DANDAN US\ LI YUANYUAN US\ ZHU MIN US | UNIV SW SCI & TECH SWUST US | C08J7/00\ C08J5/18\ C08L65/00\ C08L79/02\ C08L79/04 |
| 3362 | 3D printing | ELECTRICAL ALL-DIMENSIONAL NUMERICAL CONTROL INJECTION MOLDING MACHINE\ | US2014141112A1 | LIU JUN (CN) | LIU JUN (CN) | B29C45/72\ B29C45/80 |
| 3363 | 3D printing | ELECTRIC MULTIDIRECTIONAL METAL POWDER INJECTION FORMING MACHINE\[FR] MACHINE éLECTRIQUE MULTIDIRECTIONNELLE DE MOULAGE PAR INJECTION D'UNE POUDRE MéTALLIQUE\ | WO2013020496A1 | LIU JUN (CN) | LIU JUN (CN) | B22F3/115 |
| 3364 | 3D printing | 3D (THREE-DIMENSIONAL) STRUCTURE RELEASE FILM USED FOR FORMING BICYCLE\ | CN103264501A | LIU LIEXIN US\ WU ZHENYU US\ FU GUOZHENG US | XIAMEN XINWANG NEW MATERIAL SCIENCE & TECHNOLOGY CO LTD US | B29L9/00\ B29K77/00\ B29K25/00\ B29K23/00\ B29L7/00\ B29C55/28 |
| 3365 | 3D printing | METHODS AND APPARATUS FOR FABRICATING POROUS 3-DIMENSIONAL CELL CULTURE CONSTRUCT FOR CELL CULTURE AND OTHER BIOMEDICAL APPLICATIONS\ | US8463418B2 | LIU QING (US)\ LAU WING K (US) | 3D BIOTEK LLC (US)\ LIU QING (US)\ LAU WING K (US) | G06F19/00 |
| 3366 | 3D printing | POWDER DISTRIBUTION IN ADDITIVE MANUFACTURING\ | US2015054191A1 | LJUNGBLAD ULRIC (SE) | ARCAM AB (SE)\ LJUNGBLAD ULRIC (SE) | B29C67/00\ B01J6/00 |
| 3367 | 3D printing | TARGET-LOCKING ACQUISITION WITH REAL-TIME CONFOCAL (TARC) MICROSCOPY\ | US2010195868A1 | LU PETER J (US) | LU PETER J (US) | G06T7/00 |
| 3368 | 3D printing | TEXTURED YARN LONG-FIBER REINFORCED THERMOPLASTIC PLASTIC GRANULES AND PRODUCTION METHOD THEREOF\ | CN103407169A | LU ZHIHONG US\ ZENG TIANQUAN US | JURONG BASTEP COMPOSITE MATERIALS CO LTD US | B29B9/06\ B29C70/16\ B29C70/52 |
| 3369 | 3D printing | NOVEL DOUBLE-LAYER COATED JACQUARD ROPEFOR JACQUARD LOOM\ | CN202202067U | LUNGUAN NI US | LUNGUAN NI US | D03C3/40 |
| 3370 | 3D printing | RAPID PROTOTYPING SHAPE MEMORY HIGH POLYMER MATERIAL AND PREPARATION METHOD AND APPLICATION THEREOF\ | CN103160948A | LUO HONGSHENG US\ LUO XIAOFAN US | SUZHOU JUFU POLYMER MATERIAL CO LTD US | B29C67/00\ C08G18/32\ C08G18/42\ C08G18/10\ C08G18/48\ C08G18/66\ D01F6/78 |
| 3371 | 3D printing | POLYCRYSTALLINE MAGNETOSTRICTIVE MATERIAL OF A TB-FE-B-TYPE AND PROCESS FOR PREPARING THE SAME\ | RO123154B1 | LUPU NICOLETA (RO)\ V&ABREVELEANU MIHAELA CRISTINA (RO)\ CHIRIAC HORIA (RO) | INST NA&TCEDILIONAL DE CERCETARE DEZVOLTARE PENTRU FIZIC&ABREVE TEHNIC&ABREVE IFT IA&SCEDILI (RO) | H01F1/053\ C22C33/04 |
| 3372 | 3D printing | THREE-DIMENSIONAL LAMINATING MOLDING DEVICE\ | US7104773B2 | MAEKAWA TSUTOMU (JP)\ OUCHI AKEMI (JP)\ FUJII HIDETOSHI (JP)\ TAMAHASHI KUNIHIRO (JP)\ SHOJI YUTAKA (JP)\ TAKAHAGI HIROSHI (JP) | RICOH PRINTING SYS LTD (JP) | B29C35/08\ B29C67/00\ B29C41/36\ B28B17/00\ B41J2/01\ B29C41/46\ B29C41/52\ B41M5/00\ B29C41/02 |
| 3373 | 3D printing | PARYLENE COATED MICROFLUIDIC COMPONENTS AND METHODS FOR FABRICATION THEREOF\ | US2006204699A1 | MALTEZOS GEORGE (US)\ TANG HONGXING (US)\ ROUKES MICHAEL (US)\ SCHERER AXEL (US) | MALTEZOS GEORGE (US)\ TANG HONGXING (US)\ ROUKES MICHAEL (US)\ SCHERER AXEL (US) | B29C33/76\ B29C33/40\ B28B7/38\ F16L11/04 |
| 3374 | 3D printing | METHOD AND DEVICE FOR PRINTING COLOR IMAGES ON CHOCOLATE PRODUCTS\ | US2009081341A1 | MANDELBOUM ISAR (IL) | MANDELBOUM ISAR (IL) | A23P1/08\ A23G1/22 |
| 3375 | 3D printing | VERFAHREN ZUR HERSTELLUNG VON KUNSTSTOFFSPRITZGIE?TEILEN UND VORRICHTUNG ZUR DURCHFüHRUNG DES VERFAHRENS\ | DE102004043253B4 | MANZ BRUNO (DE) | SCHUSTER KUNSTSTOFFTECHNIK GMB (DE) | B29C45/14\ B29C51/00\ B29C45/00\ B29C69/00 |
| 3376 | 3D printing | HAMMER HEAD STRUCTURE OF ORE CRUSHER\ | CN201098622Y | MAOXUN ZHANG (CN)\ YUQUN LIN (CN) | FUZHOU HONGLIAN ENGINEERING MA (CN) | B02C13/28 |
| 3377 | 3D printing | HAMMERHEAD STRUCTURE OF THE ORE CRACKING MECHANISM AND PROCESSING METHOD THEREOF\ | CN101116836A | MAOXUN ZHANG (CN)\ YUQUN LIN (CN) | FUZHOU HONGLIAN ENGINEERING MA (CN) | B02C13/28\ B22D19/16 |
| 3378 | 3D printing | USE OF MOULDING COMPOSITIONS\ | US2012193839A1 | MARGRAF GUENTER (DE)\ JOACHIMI DETLEV (DE) | LANXESS DEUTSCHLAND GMBH (DE)\ MARGRAF GUENTER (DE)\ JOACHIMI DETLEV (DE) | B29C45/00\ B29C49/00\ B29C47/00\ B29C49/04 |
| 3379 | 3D printing | METHOD AND MACHINE FOR PRODUCING THREE-DIMENSIONAL OBJECTS BY MEANS OF SUCCESSIVE LAYER DEPOSITION\ | US7597835B2 | MARSAC NICOLAS (FR) | MARSAC NICOLAS (FR) | B29C35/08\ B29C67/00\ B28B1/14\ B28B1/02 |
| 3380 | 3D printing | FILM FOR PACKAGING PRODUCT ESPECIALLY AN ENVELOPE\ | US2012193402A1 | MARTINEZ DAVID (FR)\ CHAPALAIN FLORIAN (FR) | GASCOGNE LAMINATES (FR)\ CHAPALAIN FLORIAN (FR)\ MARTINEZ DAVID (FR) | B32B3/00\ B32B38/14\ B65D27/00 |
| 3381 | 3D printing | METHOD OF FABRICATING BATH-MODELS FOR NICKEL SHELLS\ | US2010102478A1 | MASCHERA FLAVIO (IT)\ BARROTE PEDRO (PT) | MASCHERA FLAVIO (IT)\ BARROTE PEDRO (PT) | B29C33/38 |
| 3382 | 3D printing | POLYLACTIC ACID-BASED NONWOVEN FABRIC\ | JP2006057197A | MATSUNAGA ATSUSHI US\ EZAKI KOJI US | UNITIKA LTD US | D04H3/011\ B32B27/36\ B32B5/26\ D04H3/105 |
| 3383 | 3D printing | METHOD OF INJECTION MOLDING AND INJECTION MOLDING MACHINE\ | JP2009119700A | MATSUSHITA YOSHIYUKI US | ASMO CO LTD (JP) | B29C45/23 |
| 3384 | 3D printing | OMNIBEARING ELECTRIC NUMERICAL CONTROL INJECTION MOLDING MACHINE\ | CN202213120U | MENTIONED THE INVENTOR HAS WAIVED THE RIGHT TO BE US | LIU JUN (CN) | B29C45/17\ B29C45/76\ B29C45/70\ B29C45/07\ B29C45/66 |
| 3385 | 3D printing | SCHMELZ-AGGREGAT EINTHALTEND EINEN INJEKTOR BLASLANZE ODER BRENNER\[EN] SMELTING UNIT COMPRISING AN INJECTOR A LANCE OR A BURNER\[FR] DISPOSITIF DE FUSION AVEC UN INJECTEUR UNE LANCE OU UN BR?LEUR\ | EP1835039B1 | MEYN MATTHIAS (DE) | SMS SIEMAG AG (DE) | C21C5/52\ F27B3/20\ C21C5/56\ F27B3/22 |
| 3386 | 3D printing | METHOD FOR PRODUCING CONTINUOUS-FIBER-REINFORCED MOLDED PARTS FROM THERMOPLASTIC PLASTIC AND MOTOR VEHICLE MOLDED PART\ | US2012269999A1 | MICHEL PETER (DE)\ KIND FRANZ-GEORG (DE) | REHAU AG & CO (DE)\ KIND FRANZ-GEORG (DE)\ MICHEL PETER (DE) | B29C35/08\ B32B5/02\ B29C37/00\ B29C45/00\ B29C69/00\ B32B9/00\ B32B1/00\ B32B17/02\ B62D25/00 |
| 3387 | 3D printing | ADDITIVE MANUFACTURING METHOD FOR BUILDING THREE-DIMENSIONAL OBJECTS WITH CORE-SHELL ARRANGEMENTS AND THREE-DIMENSIONAL OBJECTS THEREOF\ | US2013224423A1 | MIKULAK JAMES K (US)\ DECKARD CARL R (US)\ ZINNIEL ROBERT L (US) | STRATASYS INC (US) | B29C67/00\ B32B3/26 |
| 3388 | 3D printing | METHOD FOR PREPARING MELT SPUN CHLORINE-RESISTANCE HIGH RESILIENT POLYURETHANE FIBER\ | CN102011211A | MINCHAO LI US\ LIMING CHENG US\ HAIFENG CHEN US\ DONGZHI ZHANG US\ WENMING WANG US\ GAOQIANG LI US | ZHANGJIAGANG WOSITING RES INST CO LTD US | C08G18/42\ B29C47/92\ D01D5/088\ D01F6/94\ C08G18/48\ C08G18/66\ D01D1/00\ D01D1/10\ D01D10/02\ D01F1/10 |
| 3389 | 3D printing | THREE-DIMENSIONAL (3D) GLASSES\ | CN202548438U | MING ZHAO US | BOE TECHNOLOGY GROUP CO LTD US | G02B27/22\ G09G3/34\ G02B26/02 |
| 3390 | 3D printing | METHOD FOR RAPIDLY FORMING THREE-DIMENSIONAL METAL PARTS BY HIGH-POWER LASERS\ | CN102328081A | MINGMING MA US\ XIAOYAN ZENG (CN)\ ZEMIN WANG (CN)\ QIANWU HU (CN)\ XIANGYOU LI (CN) | UNIV HUAZHONG SCIENCE TECH (CN) | B22F3/105\ C23C24/10 |
| 3391 | 3D printing | METHOD AND APPARATUS FOR SHAPING THREE-DIMENSIONAL OBJECT\ | JP2005169878A | MIYAWAKI KUNIO US\ KAWACHI JOSUKE US\ KUNISHIO KAZUYOSHI US\ SUNAYAMA KAZUYUKI US\ SAITO YUKIO US\ MATSUMOTO YUJI US | HITACHI SHIPBUILDING ENG CO US | B29C67/00 |
| 3392 | 3D printing | APPARATUS FOR PRODUCING FOOD AND METHOD FOR PRODUCING FOOD\ | JP2005261286A | MIZUNO MICHIMASA US\ TERAMOTO TOSHIO (JP) | CHUBU NIPPON KOGYO KK US\ JSR CORP (JP) | A23P1/10\ A23P1/08 |
| 3393 | 3D printing | METHOD AND DEVICE FOR PRODUCING THREE-DIMENSIONAL OBJECTS USING LASER TECHNOLOGY AND FOR APPLYING AN ABSORBER USING AN INK JET METHOD\ | US2007183918A1 | MONSHEIMER SYLVIA (DE)\ BAUMANN FRANZ-ERICH (DE)\ GORING RAINER (DE)\ GREBE MARK (DE) | DEGUSSA (DE) | B29C35/08\ B29C67/00\ B22F3/105\ B22F3/10\ C22C1/04\ C22C33/02 |
| 3394 | 3D printing | METHOD AND DEVICE FOR PRODUCTION OF THREE-DIMENSIONAL OBJECTS BY MEANS OF ELECTROMAGNETIC RADIATION OF ELECTROMAGNETIC RADIATION AND APPLICATION OF AN ABSORBER BY MEANS OF AN INK-JET METHOD\ | US2007238056A1 | MONSHEIMER SYLVIA (DE)\ GREBE MAIK (DE)\ BAUMANN FRANZ-ERICH (DE) | DEGUSSA (DE) | B29C35/08\ B29C67/00\ B22F3/105 |
| 3395 | 3D printing | COPOLYAMIDE POWDER AND ITS PREPARATION USE OF COPOLYAMIDE POWDER IN A SHAPING PROCESS AND MOULDINGS PRODUCED FROM THIS COPOLYAMIDE POWDER\ | US8591797B2 | MONSHEIMER SYLVIA (DE)\ GREBE MAIK (DE)\ BAUMANN FRANZ-ERICH (DE)\ ALTKEMPER STEFAN (DE)\ SIMON ULRICH (DE)\ HESSEL-GELDMANN SIGRID (DE)\ WARNKE KRISTIANE (DE)\ STEMMER HEIKE (DE)\ MUEGGE JOACHIM (DE)\ TUERKE MICHAEL (DE) | EVONIK DEGUSSA GMBH (DE)\ GREBE MAIK (DE)\ ALTKEMPER STEFAN (DE)\ MONSHEIMER SYLVIA (DE)\ BAUMANN FRANZ-ERICH (DE)\ HESSEL-GELDMANN SIGRID (DE)\ WARNKE KRISTIANE (DE)\ STEMMER HEIKE (DE)\ MUEGGE JOACHIM (DE)\ TUERKE MICHAEL (DE)\ SIMON ULRICH (DE) | B29C35/08 |
| 3396 | 3D printing | COMPONENT PROPERTIES THROUGH BEAM SHAPING IN THE LASER SINTERING PROCESS\ | US2013183493A1 | MONSHEIMER SYLVIA (DE)\ GREBE MAIK (DE)\ DIEKMANN WOLFGANG (DE)\ KREUTZ JUERGEN (DE) | EVONIK DEGUSSA GMBH (DE)\ GREBE MAIK (DE)\ DIEKMANN WOLFGANG (DE)\ MONSHEIMER SYLVIA (DE)\ KREUTZ JUERGEN (DE) | B29C35/08\ B32B27/16 |
| 3397 | 3D printing | USE OF POLYMER POWDER PRODUCED FROM A DISPERSION IN A SHAPING PROCESS AND MOLDINGS PRODUCED FROM THIS POLYMER POWDER\ | US2007182070A1 | MONSHEIMER SYLVIA (DE)\ GREBE MAIK (DE)\ KOMADA HAJIME (JP)\ MATSUI HIDEKI (JP) | DEGUSSA (DE) | B29C35/08\ B29C41/02 |
| 3398 | 3D printing | USE OF POLYARYLENE ETHER KETONE POWDER IN A THREE-DIMENSIONAL POWDER-BASED MOLDLESS PRODUCTION PROCESS AND MOLDINGS PRODUCED THEREFROM\ | US2006134419A1 | MONSHEIMER SYLVIA (DE)\ GREBE MAIK (DE)\ KREIDLER PATRICK (DE)\ RICHTER ALEXANDER (DE) | DEGUSSA (DE) | C08G8/02\ B32B15/02 |
| 3399 | 3D printing | USE OF POLYESTER POWDER IN A SHAPING PROCESS AND MOLDINGS PRODUCED FROM THIS POLYESTER POWDER\ | US8834777B2 | MONSHEIMER SYLVIA (DE)\ GREBE MAIK (DE)\ SIMON ULRICH (DE)\ TEMME HEINRICH (DE) | EVONIK DEGUSSA GMBH (DE)\ GREBE MAIK (DE)\ MONSHEIMER SYLVIA (DE)\ SIMON ULRICH (DE)\ TEMME HEINRICH (DE) | B29C35/08\ B29C67/00\ B29C41/02\ B29C71/04\ B29K67/00 |
| 3400 | 3D printing | POLYMER POWDER PROCESS FOR PRODUCTION OF AND USE OF THIS POWDER AND RESULTANT SHAPED ARTICLES\ | US2007232753A1 | MONSHEIMER SYLVIA (DE)\ KOMADA HAJIME (JP)\ MATSUI HIDEKI (JP)\ NAKAIE YOSHIKI (JP) | DEGUSSA (DE) | C08L77/00\ C08L61/00 |
| 3401 | 3D printing | THERMOPLASTIC ELASTOMER COMPOSITION FOR CORE-BACK SYSTEM FOAM INJECTION MOLDING AND FOAM INJECTION MOLDING METHOD USING THE SAME\ | US7867416B2 | MORIKAWA AKIHIKO (JP)\ KANAE KENTAROU (JP)\ IEDA KATUMASA (JP)\ ABE TOMOKAZU (JP) | JSR CORP (JP)\ HONDA MOTOR CO LTD (JP) | B32B37/00\ B29C44/04 |
| 3402 | 3D printing | THERMOPLASTIC ELASTOMER COMPOSITION FOAM MADE FROM THE SAME AND PROCESS FOR PRODUCING FOAM\ | US6841582B2 | MORIKAWA AKIHIKO (JP)\ KANAE KENTAROU (JP)\ NAKANISHI HIDEO (JP)\ MAEDA MINORU (JP) | JSR CORP (JP) | C08L53/00\ C08J9/00\ C08L23/04\ C08L23/08\ C08L23/16\ C08L53/02 |
| 3403 | 3D printing | VERFAHREN UND VORRICHTUNG ZUR HERSTELLUNG EINES FILAMENTE AUFWEISENDEN VLIESSTOFFES\[EN] METHOD AND DEVICE FOR PRODUCING A NON-WOVEN FABRIC COMPRISING FILAMENTS\[FR] PROCéDé ET DISPOSITIF DESTINé à FABRIQUER UN NON-TISSé PRéSENTANT DES FILAMENTS\ | WO2009152791A1 | MUENSTERMANN ULLRICH (DE) | FLEISSNER GMBH (DE)\ MUENSTERMANN ULLRICH (DE) | D04H3/11\ B26F1/26 |
| 3404 | 3D printing | ANTI-FLAMING EXTRA-WHITE REGENERATED THREE-DIMENSIONAL CRIMP DACRON STAPLE FIBER AND MANUFACTURING METHOD THEREOF\ | CN102605457A | MUSHENG ZHANG US | HANGZHOU HANBANG CHEMICAL FIBER CO LTD US | D01F6/92\ D01F1/10\ D01F1/07 |
| 3405 | 3D printing | METHOD AND APPARATUS FOR PRODUCING THREE-DIMENSIONAL STRUCTURE\ | US2009014916A1 | NAGAI HISAO (JP)\ YOSHIDA HIDEHIRO (JP)\ FURUKAWA TAKAYUKI (JP)\ INOUE TAKASHI (JP) | MATSUSHITA ELECTRIC IND CO LTD (JP) | B81C99/00\ B29C41/08\ B05B13/02 |
| 3406 | 3D printing | NONWOVEN FABRIC OF POLYLACTATE-BASED FILAMENT AND ITS PRODUCTION\ | JPH0995850A | NAGAOKA KOICHI US\ MATSUOKA FUMIO US\ ICHINOSE NAOJI US | UNITIKA LTD US | D01F6/84\ D04H3/00\ D04H5/00 |
| 3407 | 3D printing | INJECTION MOLDING IN-MOLD DECORATION METHOD\ | JP2009143247A | NAGI YOSHIYUKI US\ ATAKE HIROYUKI (JP)\ SUGA KAZUHIRO (JP) | DAINIPPON PRINTING CO LTD (JP) | B29C45/14\ B29L9/00\ B32B27/00\ B32B33/00 |
| 3408 | 3D printing | DECORATIVE SHEET DECORATIVE MOLDED PRODUCT AND INJECTION MOLDING IN-MOLD DECORATION METHOD\ | JP2009143246A | NAGI YOSHIYUKI US\ ATAKE HIROYUKI (JP)\ SUGA KAZUHIRO (JP) | DAINIPPON PRINTING CO LTD (JP) | B32B27/00\ B29C69/02 |
| 3409 | 3D printing | MELT-EXTRUDED SUBSTRATE FOR USE IN THERMOFORMED ARTICLES\ | US8852730B2 | NAIR KAMLESH P (US)\ GRAY STEVEN D (US)\ CANO CAMILO (US) | NAIR KAMLESH P (US)\ GRAY STEVEN D (US)\ CANO CAMILO (US)\ TICONA LLC (US) | C08K5/20\ B29C43/24\ C09K19/30\ C09K19/38\ C09K19/04\ C09K19/22\ C09K19/32\ C09K19/34\ C09K19/48 |
| 3410 | 3D printing | METHOD OF MANUFACTURING LENS SHEET LENS SHEET ILLUMINATOR AND DISPLAY\ | JP2011145515A | NAKAGOME TOMOHIRO US\ SAKAKI YUICHI US\ SAKAI NATSUKA US\ KIMURA KAZUTERU US | TOPPAN PRINTING CO LTD US | G02F1/1335\ G02B3/00\ F21S2/00\ G02B5/02\ F21V5/00\ F21Y103/00 |
| 3411 | 3D printing | OPTICAL MOLDED LAMINATE\ | JP2008276150A | NAKAKOSHI YASUNOBU US | NAKAKOSHI YASUNOBU US | B29C45/14\ B29L9/00\ B29C69/00\ B29L11/00\ G02B5/30\ G02C7/12\ B29D11/00 |
| 3412 | 3D printing | INK COMPOSITION RECORDING METHOD AND RECORD AND METHOD FOR JUDGING AUTHENTICITY\ | JP2003026971A | NAKAMURA HIROTO US | SEIKO EPSON CORP (JP) | C09D11/00\ B41J2/01\ B41M5/00 |
| 3413 | 3D printing | INK COMPOSITION RECORDING METHOD AND RECORD AND METHOD FOR JUDGING AUTHENTICITY\ | JP2007177248A | NAKAMURA HIROTO US | SEIKO EPSON CORP (JP) | C09D11/00\ B41J2/01\ B41M5/00 |
| 3414 | 3D printing | INK-JET PRINTING METHOD AND PRINTED GOODS\ | US2002025379A1 | NAKAMURA SHUNGAKU (JP)\ YAMAZAKI AYUMI (JP) | SEIREN CO LTD (US) | D06P1/642\ D06P1/647\ D06P3/54\ D06P3/66\ D06P3/82\ D06P3/872\ D06P5/30 |
| 3415 | 3D printing | SPRING STRUCTURAL RESIN MOLDED PRODUCT AND METHOD AND DEVICE FOR FORMING SURFACE LAYER ON THE SPRING STRUCTURE RESIN MOLDED PRODUCT\ | US2006116045A1 | NAKAMURA YUICHIRO (JP)\ KOUNO IWAO (JP)\ NISHIBORI SADAO (JP) | NISHIBORI SADAO (JP)\ KOUNO IWAO (JP)\ NAKAMURA YUICHIRO (JP) | D04H13/00\ D04H1/00\ D04H3/03\ D04H3/16\ B32B27/02\ B29C63/00 |
| 3416 | 3D printing | BINDER REMOVAL IN SELECTIVE LASER SINTERING\ | US2004081573A1 | NEWELL KENNETH J (US) | 3D SYSTEMS INC (US) | B22F1/00\ B22F3/26\ B22F3/11 |
| 3417 | 3D printing | VERWENDUNG EINER EXTRUSIONSFOLIE ZUM EINRICHTEN EINER DRUCKVORRICHTUNG\[EN] EXTRUSION FILM FOR INSTALLATION OF PRINTING DEVICE IN LABEL PRINTING MACHINE HAS DRIVING DEVICE PROVIDED FOR MATERIAL WEB AND PRINTING UNIT WHERE FILM IS FORMED IN SINGLE LAYER OR MULTILAYER AND MADE OF POLYOLEFIN\ | DE102007009110A1 | NIEPELT RALF (DE) | NORDENIA DEUTSCHLAND GRONAU (DE) | B41M5/52\ B31D1/02\ G09F3/00 |
| 3418 | 3D printing | SHOCK ABSORBING APPARATUS\ | JP2005172130A | NISHIBORI SADAO (JP)\ NAKAMURA YUICHIRO (JP) | AIN KK SOGO KENKYUSHO US | F16F7/00 |
| 3419 | 3D printing | METHOD OF MANUFACTURING SOLAR CELL SEALING MATERIAL\ | JP2006186237A | NISHIJIMA KOICHI US\ KAWAMOTO MANABU US\ SATO RIE US | MITSUI DU PONT POLYCHEMICAL US\ MITSUI CHEMICAL FABRO INC US | H01L31/04 |
| 3420 | 3D printing | POLYAMIDE RESIN SOLUBLE IN ORGANIC ACID METHOD FOR PRODUCING THE SAME AND METHOD FOR PRODUCING THREE-DIMENSIONAL INJECTION MOLDED CIRCUIT PART\ | JP2004059829A | NISHIKAWA SHINYA US\ HAYAMI HIROSHI US\ SAKAMOTO YOSHITO US\ KITAMURA TAKAHIKO US\ FUJIWARA YASUHIRO US\ UEDA KOJI US\ UEDA HIDEHARU US | CHIKUNO SHOKUHIN KOGYO KK US\ SUMITOMO ELECTRIC INDUSTRIES (JP) | G03F7/037\ C08G69/48\ H05K3/00\ H05K3/18\ H05K3/38 |
| 3421 | 3D printing | METHOD FOR MANUFACTURING THREE-DIMENSIONAL INJECTION MOLDING CIRCUIT COMPONENT\ | JP2005217279A | NISHIKAWA SHINYA US\ HAYAMI HIROSHI US\ SAKAMOTO YOSHITO US\ KITAMURA TAKAHIKO US\ HOSOE AKIHISA US | SUMITOMO ELECTRIC INDUSTRIES (JP) | B29C45/14\ B29K77/00\ C25D5/56\ C25D5/02\ H01L23/14\ H05K3/00\ H05K3/18\ B29L31/34\ C23C18/28\ C25D7/00\ H05K1/03 |
| 3422 | 3D printing | DIRECT MOLDING METHOD AND DIRECT MOLDING DEVICE OF POLYMER MATERIAL\ | JP2008194968A | NISHIMURA OSAMU US\ IMOTO TOSHIYUKI US\ HOJO MASAKI US\ ADACHI TAIJI US\ NISHINO TAKASHI US | IMOTO SEISAKUSHO KK US\ UNIV KYOTO US\ UNIV KOBE US | B29C67/00\ A61L27/00 |
| 3423 | 3D printing | INK JET RECORDING METHOD AND RECORDED MATTER\ | JP2007076228A | NOJIMA TAKAHIKO US\ ITO HIROAKI US | KONICA MINOLTA PHOTO IMAGING US | C09D11/00\ B41J2/01\ B41M5/00\ B41M5/50\ B41M5/52 |
| 3424 | 3D printing | NONWOVEN FABRIC HAVING BELTLIKE SPLIT-FIBER AREA AND ITS PRODUCTION\ | JP9268462A | NOZU TAKASHI US | UNITIKA LTD US | D04H3/02\ D04H3/14\ A47L13/16 |
| 3425 | 3D printing | INK JET HEAD WITH BUBBLE COLLECTOR AND RECORDER EMPLOYING THE INK JET HEAD\ | JP2004142330A | NOZU TOMOHITO US\ NOJIRI HIDEAKI US\ AKAIKE MASATAKE US\ KAWASAKI TAKEHIKO US\ KURASHIMA TAMAYOSHI US\ TOYAMA KOZO US\ WADA TAKATSUGI US | CANON KK (JP) | B41J2/045\ B41J2/055\ B41J2/015 |
| 3426 | 3D printing | INK JET RECORDER USING HOT MELT INK AND ITS INK EJECTION RECOVERING METHOD\ | JP2004142329A | NOZU TOMOHITO US\ NOJIRI HIDEAKI US\ AKAIKE MASATAKE US\ KAWASAKI TAKEHIKO US\ KURASHIMA TAMAYOSHI US\ TOYAMA KOZO US\ WADA TAKATSUGI US | CANON KK (JP) | B41J2/18\ B41J2/185 |
| 3427 | 3D printing | INK JET RECORDER USING HOT MELT INK\ | JP2004142328A | NOZU TOMOHITO US\ NOJIRI HIDEAKI US\ AKAIKE MASATAKE US\ KAWASAKI TAKEHIKO US\ KURASHIMA TAMAYOSHI US\ TOYAMA KOZO US\ WADA TAKATSUGI US | CANON KK (JP) | B41J2/01\ B41J2/045\ B41J2/055\ B41J2/015 |
| 3428 | 3D printing | AU-SN ALLOY LAMINATE SOLDER AND ITS MANUFACTURING METHOD\ | JP2006110626A | OBATA MOTOKI US\ MIZUMA ISAO US\ HORIKANE YOSHIHIKO US\ TSUCHIYA JUNJI US | CITIZEN FINE TECH CO LTD US | B23K35/30\ C23C14/14\ H05K3/34\ B23K35/22\ B23K35/14\ B23K35/40\ C22C5/02 |
| 3429 | 3D printing | BIAXIALLY ORIENTED LAMINATED POLYESTER FILM FOR MOLDING AND SIMULTANEOUS DECORATION\ | JP2009029073A | OKUYAMA SHUNSUKE US\ KIMURA MANABU US | TEIJIN DUPONT FILMS JAPAN LTD US | B29C45/14\ B29L9/00\ B29L7/00\ B32B27/36\ B29C55/12\ B29K67/00 |
| 3430 | 3D printing | ORIENTED FILM AND POLARIZING PLATE USING THE SAME\ | JP2007031537A | ONO MITSUMASA US | TEIJIN DUPONT FILMS JAPAN LTD US | G02F1/1335\ C08J5/18\ B29C55/02\ B29K33/04\ B29K79/00\ C08F220/18\ C08F222/40\ G02B5/30 |
| 3431 | 3D printing | THREE-DIMENSIONAL NET-LIKE STRUCTURE\ | US2014370769A1 | OSAKI HIROKO (JP) | C ENG CO LTD (JP) | A47C27/12\ A47C31/00 |
| 3432 | 3D printing | THREE-DIMENSIONAL DECORATIVE OBJECT AND METHOD FOR MANUFACTURING THE SAME\ | JP2003127225A | OSHIMA NORIHIRO US\ UEDA YUJI US\ KATAOKA MASATOSHI US | MEIWA GRAVURE CO LTD US | B29C59/00\ B29L9/00\ B29C69/00\ B44C1/17\ B29C65/02\ E04F15/10\ B29C39/20\ B29C47/04\ B29L31/10 |
| 3433 | 3D printing | METHOD FOR THE DIRECT AND CONTINUOUS PRODUCTION OF HOLLOW ARTICLES FROM A POLYMER MELT\ | US7547406B2 | OTTO BRIGITTA (DE)\ SCHAEFER ROLAND (DE)\ BACHMANN HOLGER (DE)\ HOELTING LUDWIG (DE)\ DEISS STEFAN (DE) | LURGI ZIMMER GMBH (DE) | C08G63/78\ B29C45/00\ C08J5/00\ B29B13/00\ B29C31/04\ B29D22/00\ B29B7/32\ B29B7/88\ B29C45/18\ B29C49/00\ C08G63/08\ C08G63/88\ C08J3/205 |
| 3434 | 3D printing | INK JET PRINT HEAD SUBSTRATE INK JET PRINT HEAD INK JET PRINTING APPARATUS AND METHOD OF MANUFACTURING INK JET PRINT HEAD SUBSTRATE\ | US2006256160A1 | OZAKI TERUO (JP)\ SAITO ICHIRO (JP)\ YOKOYAMA SAKAI (JP)\ ONO KENJI (JP)\ IBE SATOSHI (JP)\ SHIBATA KAZUAKI (JP)\ SAKAI TOSHIYASU (JP) | CANON KK (JP) | B41J2/04 |
| 3435 | 3D printing | METHOD OF FORMING THREE-DIMENSIONAL PRINTING IMAGE\[FR] PROCéDé DE FORMATION D'UNE IMAGE D'IMPRESSION EN TROIS DIMENSIONS\ | WO2008075450A1 | OZAWA CHIZUO (JP) | MASTERMIND CO LTD (JP)\ OZAWA CHIZUO (JP) | B41J2/01\ B41M5/00 |
| 3436 | 3D printing | PROCESS FOR THE PRODUCTION OF A BRAKING BAND OF A BRAKE DISK WITH VENTILATION DUCTS AND A BRAKING BAND PRODUCED BY THIS PROCESS\ | US7134476B2 | PACCHIANA GIOVANNI PAOLO (IT)\ GOLLER RALF SIEGFRIED (IT) | FRENI BREMBO SPA (IT) | B22D19/00\ B22C9/04\ B22C9/10\ B29C43/02\ B29C33/00\ B22D21/00\ F16D65/12\ B29C33/52\ C04B33/34 |
| 3437 | 3D printing | DREIDIMENSIONAL KONTURIERTE SANDWICHSTRUKTUR\[EN] PRODUCING A THREE DIMENSIONAL CONTOURED SANDWICH STRUCTURE INCLUDING FIRST THERMOPLASTIC COVERING LAYER SECOND THERMOPLASTIC COVERING LAYER AND CORE MATERIAL COMPRISES E.G. MACHINING OR CARRYING OUT SEPARATE PROCESSING OF CORE MATERIAL\ | DE102011006819A1 | PAESLER ERIK (DE)\ KROLL LOTHAR PROF DR (DE)\ KAUFMANN JOERG (DE)\ MEYER MATTHIAS (DE)\ MUELLER MARCO (DE) | UNIV CHEMNITZ TECH (DE) | B29C45/14\ B32B3/12\ B32B27/08\ B32B37/10\ B32B38/10 |
| 3438 | 3D printing | LASER- UND THERMISCH BESCHREIBBARE OBERFL?CHENBESCHICHTUNG FüR MATERIALIEN\[EN] LASER AND THERMALLY WRITABLE SURFACE COATING FOR MATERIALS\[FR] REVêTEMENT DE SURFACE POUR MATéRIAUX POUVANT RECEVOIR UNE INSCRIPTION PAR LASER OU PAR VOIE THERMIQUE\ | EP2429826A1 | PALKOVITS ROLAND (AT)\ SCHALKHAMMER THOMAS (AT)\ KORNHERR ANDREAS (AT) | MONDI UNCOATED FINE & KRAFT PAPER GMBH (AT) | C09D7/12\ B41M5/26\ B41M5/28\ C04B41/80\ C09D5/26\ D21H17/66\ D21H17/67\ D21H19/38\ D21H19/80\ D21H21/28\ D21H23/22 |
| 3439 | 3D printing | LOOSE-LEAF INSERTED PIN FOR ENABLING GREENING CONTAINERS CONNECTED LEFT AND RIGHT TO ROTATE RANDOMLY\ | CN203067518U | PANG QIFU US | PANG QIFU US | F16C11/00 |
| 3440 | 3D printing | INK JET PRINTER AND PRINT HEAD DEVICE THEREOF\ | US7654639B2 | PARK JEONG-YEON (KR)\ PARK SANG-CHEOL (KR) | SAMSUNG ELECTRONICS CO LTD (KR) | B41J2/16\ B41J2/14 |
| 3441 | 3D printing | MANUFACTURE METHOD OF WOOD FOSSILS\ | KR100804471B1 | PARK MIN HWA (KR) | POZOPIA (KR) | B27K3/00\ B27K3/16\ B27K3/34 |
| 3442 | 3D printing | GLASS HIGH TEMPERATURE MELTING MOLDING SPRAYER BASED ON 3D (THREE DIMENSIONAL) PRINTING TECHNOLOGY\ | CN103395973A | PENG SHOU US\ ZHANG JIALIN US | BENGBU GLASS IND DESIGN INST US\ CHINA TRIUMPH INT ENG CO LTD US | C03B19/00 |
| 3443 | 3D printing | MULTISTAGE-TEMPERATURE-CONTROL-BASED FUSED DEPOSITION MODELING (FDM) TYPE 3D PRINTING SPRAYER AND TEMPERATURE CONTROL METHOD\ | CN103240883A | PENG YONGGANG US\ WEI WEI US | UNIV ZHEJIANG US | B29C67/00 |
| 3444 | 3D printing | NOVEL 3D (THREE DIMENSIONAL) PRINTED BODY SURFACE TREATMENT PROCESS\ | CN103192524A | PENG ZHENYUN US\ XU QIMING US | WOWMAN INFORMATION SERVICES CO LTD SUZHOU US | B29C59/00 |
| 3445 | 3D printing | METHODS AND APPARATUS FOR ACTUATED FABRICATOR\ | US2013292039A1 | PETERS BENJAMIN (US)\ OXMAN NERI (US) | MASSACHUSETTS INST TECHNOLOGY (US) | B32B37/12\ B32B38/10 |
| 3446 | 3D printing | PROCESS AND DEVICE FOR PRODUCING SOLID BODIES BY SEQUENTIAL LAYER BUILDUP\ | US7285234B2 | PFEIFER ROLF (DE)\ SHEN JIALIN (DE)\ ZEPPELIN DIDIER VON (DE) | DAIMLER CHRYSLER AG (DE) | B29C67/00\ B22F3/00\ B32B37/00\ D04H1/16\ B32B38/04\ B27J5/00 |
| 3447 | 3D printing | PHOTOVOLTAIC DEVICES FABRICATED FROM NANOSTRUCTURED TEMPLATE\ | US2005098204A1 | PICHLER KARL (US)\ ROSCHEISEN MARTIN R (US)\ SAGER BRIAN M (US) | NANOSOLAR INC (US) | H01L31/0256\ B81B1/00\ C09D5/24\ H01L31/042\ B82B3/00\ C07C61/00\ C07C63/42\ C08G61/00\ H01L31/00\ H01L31/0352\ H01L31/06\ H01L51/00\ H01L51/30 |
| 3448 | 3D printing | METHOD OF MANUFACTURING THREE-DIMENSIONAL PRODUCTS THROUGH DEPOSITS OF SUCCESSIVE LAYERS OF A WIRE MELTED BY ELECTRON BEAM\[FR] PROCEDE DE FABRICATION D'ARTICLES TRIDIMENSIONNELS PAR DEPOT DE COUCHES SUCCESSIVES DE FIL FONDU PAR FAISCEAU D'ELECTRONS\ | WO2005077586A1 | PLUMB PAUL NIGEL (GB)\ DUMONT CONRAD FREDERICK (GB) | AQUASIUM TECHNOLOGY LTD (GB)\ PLUMB PAUL NIGEL (GB)\ DUMONT CONRAD FREDERICK (GB) | B23K15/00\ B23K15/06 |
| 3449 | 3D printing | METHOD OF MANUFACTURING NONWOVEN ABRASIVE ARTICLES USING DRY PARTICULATE MATERIAL\[DE] VERFAHREN ZUR HERSTELLUNG VON VLIESSTOFFSCHLEIFARTIKELN MIT TROCKENEM K?RNIGEM MATERIAL\[FR] PROCEDE PERMETTANT DE PRODUIRE DES ARTICLES ABRASIFS EN NON-TISSE AU MOYEN D'UN MATERIAU EN PARTICULES SEC\ | EP1617973A1 | POLLAUD GUY M (FR)\ MALJEAN SANDRINE (FR)\ MARTIN RIVERA CARMEN (ES)\ CABRERO GOMEZ ESTRELLA (ES) | 3M INNOVATIVE PROPERTIES CO (US) | B24D11/00 |
| 3450 | 3D printing | MULTICOLOURED FUSED DEPOSITION MODELLING PRINT\ | US2014088751A1 | POPPE DIRK (DE)\ KOHLSTRUK STEPHAN (DE)\ HAMMANN BENJAMIN (DE)\ CREMER SONJA (DE)\ BEKS KRIS (BE)\ DEWAELHEYNS LUDO (BE)\ PRIDOEHL MARKUS (DE)\ SCHMITT GUENTER (DE) | EVONIK ROEHM GMBH (DE)\ PRIDOEHL MARKUS (DE)\ SCHMITT GUENTER (DE)\ POPPE DIRK (DE)\ KOHLSTRUK STEPHAN (DE)\ HAMMANN BENJAMIN (DE)\ CREMER SONJA (DE)\ BEKS KRIS (BE)\ DEWAELHEYNS LUDO (BE) | G05B19/042 |
| 3451 | 3D printing | METHOD AND APPARATUS FOR LAYER MANUFACTURING OF ARTEFACTS\[FR] PROCéDé ET APPAREIL DE FABRICATION EN COUCHES D'ARTéFACTS\ | WO2011064725A1 | POTGIETER PAUL (ZA) | AEROSUD INNOVATION & TRAINING CT PTY LTD (ZA)\ POTGIETER PAUL (ZA) | B29C67/00\ B22F3/105 |
| 3452 | 3D printing | CONSTRUCTION MATERIALS AND METHODS FOR PARAFOILS AND PARACHUTES\ | US2005077430A1 | PRESTON DANIEL (US) | ATAIR AEROSPACE INC (US) | B29C70/08\ B32B27/12\ B64D17/02\ B29C70/30 |
| 3453 | 3D printing | PRODUCTION METHOD FOR TWO-MONOLAYER PAPER SUPPORTING TO PRINT AND PROCESS INTO STICKERS\ | CN101054010A | QI HELIANG YANG (CN) | SHANGHAI SUIXUAN ENTPR DEV CO (CN) | B41F17/00\ B41F13/008\ B41F13/02\ B32B39/00\ B41F13/54\ B41F19/00\ B41F23/00\ B65H5/06\ G09F3/02 |
| 3454 | 3D printing | PRINT-TYPE FLEXIBLE DISPLAY SCREEN\ | CN202189208U | QIAN JUN US\ CHAOQUN TANG US\ JICHENG HU US\ YIHUA ZHOU US\ XINZHI SHI US\ CHANG QI US\ NA CHEN US\ LISHENG MA US | BEIJING 359 INVEST CO LTD US | G02F1/1335\ G02F1/1343\ G02F1/1333\ G02F1/1334 |
| 3455 | 3D printing | CHEMICAL TREATING METHOD FOR MELT POLYCONDENSATION HIGH POLYMER OLD MATERIAL REGENERATION\ | CN101805445A | QIANG CHENG US | QIANG CHENG US | C08G63/78\ C08G69/00\ C08G64/20\ C08J11/10 |
| 3456 | 3D printing | COMPOSITE EIDERDOWN SILK\ | CN201062201Y | QIGUANG LIAO (CN)\ MAOPENG WU (CN) | GUANGZHOU QIJUN TEXTILE CO LTD (CN) | B68G3/00\ A41G9/00 |
| 3457 | 3D printing | THREE-DIMENSION POROUS TISSUE ENGINEERING CARRIER MATERIAL AND PREPARATION AND APPLICATION\ | CN101147812A | QING YANG (CN)\ XIJIAN WANG (CN)\ ZHIQING TAN (CN)\ XINYUAN SHEN (CN)\ LANLAN PENG (CN)\ SISHI CHEN (CN) | UNIV DONGHUA (CN) | A61L27/56\ A61L27/26 |
| 3458 | 3D printing | THREE-DIMENSIONAL STEPHANOPORATE ORGANIZATION ENGINEERING BRACKET MATERIAL FIBRE CEMENTING METHOD PREPARING SAME AND APPLICATIONS THEREOF\ | CN101249277A | QING YANG (CN)\ ZHIQING TAN (CN)\ XINYUAN SHEN (CN)\ LANLAN PENG (CN)\ SISHI CHEN (CN) | UNIV DONGHUA (CN) | A61F2/28\ A61L27/56\ A61L27/18 |
| 3459 | 3D printing | PRODUCING PROCESS FOR ANTIBACTERIAL FLAME-RETARDANT SINGLE AND MULTI-PORE THREE-DIMENSIONAL CRIMPED POLYESTER STAPLE FIBER\ | CN102704031A | QINRONG TONG US\ JIANQIU XU US\ ZUAN SUN US\ GUOZHI CAI US\ WENQIN WU US | SHANGHAI XINDILUN IND CO LTD US | C08G63/78\ D01F6/92\ D01F8/14\ D01D1/00\ D01F1/10\ D01D5/08\ D01D5/12\ D01F1/07\ D02J13/00\ C08G63/692 |
| 3460 | 3D printing | METHODS AND APPARATUS FOR INCREASING IMAGE GLOSS\ | US6679600B1 | REGIMBAL LAURENT A (US) | HEWLETT PACKARD DEVELOPMENT CO (US) | G03G15/20 |
| 3461 | 3D printing | METHOD FOR PRODUCING DUROPLASTIC FINE-FIBER NON-WOVENS HAVING A HIGH FLAME-RETARDANT THERMAL PROTECTIVE AND SOUND INSULATING EFFECT\ | US2008203602A1 | RIEDEL BERND (DE)\ BUCKA HARTMUT (AT) | AMI AGROLINZ MELAMINE INT GMBH (AT) | D04H3/03\ D04H3/02\ D04C1/00 |
| 3462 | 3D printing | THREE-DIMENSIONAL NON-WOVEN FILTER\ | US2003080050A1 | ROLCHIGO PHILIP M (US)\ STIFTER TRAVIS G (US)\ KOPP CLINTON V (US)\ AUNE THOMAS M (US)\ MADSEN MICHAEL J (US) | OSMONICS INC (US) | B01D39/16\ B29C47/30\ D04H3/07 |
| 3463 | 3D printing | PROCESSLESS DIGITALLY IMAGED PRINTING PLATE USING MICROSPHERES\ | US2003180636A1 | ROSEN DANIEL (US)\ KANGA RUSTOM SAM (US) | Empty Field US | G03F7/027\ B41C1/05\ B41N1/12 |
| 3464 | 3D printing | METHOD FOR PACKAGING DYE-SENSITIZED SOLAR BATTERY\ | CN101582335A | RUIXING GAO (CN)\ ZHIJUN YANG (CN)\ KE YU (CN) | IRICO GROUP CORP (CN) | H01G9/20\ H01L51/48\ H01M14/00 |
| 3465 | 3D printing | MOLDING METHOD OF A RESIN MOLDED ARTICLE BY A MOLD APPARATUS THE MOLD APPARATUS THE RESIN MOLDED ARTICLE AND A MOLDING MACHINE HAVING THE MOLD APPARATUS\ | US2003099794A1 | SASAKI TSUYOSHI (JP)\ WATANABE OSAMU (JP)\ TAKIGAWA NAOKI (JP) | CHEN WEN-ZHENG (TW) | B29C45/14\ B29C45/26\ B29C33/00\ B29C45/70\ B29C45/56 |
| 3466 | 3D printing | VERFAHREN ZUR ABFORMUNG VON DREIDIMENSIONALEN FREIFORMFL?CHEN MIT MIKROSTRUKTURIERTEN OBERFL?CHEN\[EN] METHOD FOR CASTING 3-D FREELY FORMABLE SHAPES WITH MICROSTRUCTURED SURFACES\[FR] PROCéDé DE PRISE D'EMPREINTE DE SURFACES DE FORME LIBRE EN TROIS DIMENSIONS DOTéES DE SURFACES MICROSTRUCTURéES\ | EP1854568B1 | SAUERESSIG KILIAN (DE) | K1 GMBH (DE) | B22C9/00 |
| 3467 | 3D printing | METHOD OF MANUFACTURING OF BEARING COMBINED REINFORCED IN SITU PILE\ | RU2334048C1 | SAURIN ANATOLIJ NIKIFOROVICH (RU)\ IL ICHEV VJACHESLAV ALEKSANDRO (RU)\ ZHADANOVSKIJ BORIS VASIL EVICH (RU)\ SAURIN JAROSLAV ANATOL EVICH (RU)\ RED KINA JULIJA VJACHESLAVOVNA (RU)\ MILJUTIN DENIS NIKOLAEVICH (RU) | SAURIN ANATOLIJ NIKIFOROVICH (RU) | E02D5/34 |
| 3468 | 3D printing | OSTEOIMPLANT AND METHOD OF MAKING SAME\[FR] IMPLANT OSSEUX ET SON PROCEDE DE FABRICATION\[DE] OSTEOIMPLANTAT UND VERFAHREN ZU SEINER HERSTELLUNG\ | EP1301222A2 | SCARBOROUGH NELSON L (US)\ MANRIQUE ALBERT (US)\ EDWARDS JEAN T (US)\ BODEN SCOTT D (US) | OSTEOTECH INC (US) | A61F2/00\ A61F2/28\ A61F2/30\ A61B17/86\ A61L31/00\ A61L27/36\ A61F2/44\ A61F2/46 |
| 3469 | 3D printing | DREIDIMENSIONALES BAUTEIL UND VERFAHREN ZUR HERSTELLUNG DESSELBEN\[EN] THREE-DIMENSIONAL COMPONENT AND METHOD FOR PRODUCING THE SAME\[FR] COMPOSANT TRIDIMENSIONNEL ET PROCéDé POUR SA FABRICATION\ | EP2051839A1 | SCHLARB ANDREAS (DE) | SCHREINER GROUP GMBH & CO KG (DE) | B29C45/14\ B32B7/12 |
| 3470 | 3D printing | METHOD AND DEVICE FOR PRODUCING A 3D OBJECT BY MEANS OF A GENERATIVE 3D-METHOD\ | US8414281B2 | SCHLEISS PETER (CH)\ LEVY GIDEON (CH) | EOS ELECTRO OPTICAL SYST (DE)\ SCHLEISS PETER (CH)\ LEVY GIDEON (CH) | B29C35/08\ B29C67/00 |
| 3471 | 3D printing | METHOD FOR PRODUCING COATING MATERIAL\ | US2013014882A1 | SCHMID JOHANNES (DE) | HOMAG HOLZBEARBEITUNGSSYSTEME (DE)\ SCHMID JOHANNES (DE) | B05D5/10\ B32B37/12 |
| 3472 | 3D printing | PHASE CHANGE SUPPORT MATERIAL COMPOSITION\ | US7176253B2 | SCHMIDT KRIS ALAN (US)\ XU PINGYONG (US)\ RUATTA STEPHEN A (US)\ DOAN VU A (US) | 3D SYSTEMS INC (US) | B29C67/00\ C09D175/16\ C09D11/10\ C09D11/00\ B41J2/01\ B41M5/00\ C08L93/04\ C08K5/05 |
| 3473 | 3D printing | PHASE CHANGE SUPPORT MATERIAL COMPOSITION\ | US7399796B2 | SCHMIDT KRIS ALAN (US)\ XU PINGYONG (US)\ RUATTA STEPHEN A (US)\ DOAN VU A (US) | 3D SYSTEMS INC (US) | B29C67/00\ C09D175/16\ B05D3/06\ C09D11/10\ C09D11/00\ B41J2/01\ B41M5/00 |
| 3474 | 3D printing | VERFAHREN ZUR HERSTELLUNG VON ELEKTRONIKMODULEN IN 3D-TECHNOLOGIE\[EN] MANUFACTURING METHOD OF ELECTRONIC MODULE BASED ON THREE-DIMENSIONAL TECHNOLOGY INVOLVES APPLYING SOLDER PASTE ON SOLDER SURFACES OF INDIVIDUAL PRINTED CIRCUIT BOARDS (PCBS) AND HEATING PCB IN REFLOW OVEN FOR MELTING THE SOLDER PASTE\ | DE102011079278A1 | SCHNEIDER BERNHARD (DE) | IFM ELECTRONIC GMBH (DE) | H05K3/36\ H01L25/16 |
| 3475 | 3D printing | GRO?TECHNISCHES VERFAHREN UND VORRICHTUNG ZUR PLASTISCHEN VERFORMUNG VON POLYMEREN DURCH ELEKTROMAGNETISCHE STRAHLUNG\[EN] PLASTICIZATION OF POLYMER SHOWING STRONG INTERMOLECULAR INTERACTIONS E.G. CELLULOSE INVOLVES APPLICATION OF ELECTROMAGNETIC RADIATION IN PLASTICIZING UNIT WITH RADIATION-PERMEABLE COMPONENT\ | DE102006050831A1 | SCHROETER JOHANNES (DE)\ FELIX FLORIAN (DE) | SCHROETER JOHANNES (DE)\ FELIX FLORIAN (DE) | B29C47/82\ B29C45/74\ B29C47/84 |
| 3476 | 3D printing | DRUCKKOPF UND DESSEN VERWENDUNG\ | DE10353112B4 | SCHWESINGER NORBERT (DE)\ SCHAEFER EUGEN (DE) | METEC INGENIEUR AG (DE) | B41J2/175\ B41M3/16\ B41J2/14\ B41J3/32\ B41J2/38\ B41J2/475 |
| 3477 | 3D printing | MELT-ADHESIVE COMPOSITE FIBERS PROCESS FOR PRODUCING THE SAME AND FUSED FABRIC OR SURFACE MATERIAL OBTAINED THEREFROM\[DE] SCHMETZKLEBENDE VERBUNDFASERN VERFAHREN ZU IHRER HERSTELLUNG UND DARAUS HERGESTELLTER SCHMELZGEBUNDENER STOFF ODER OBERFL?CHENMATERIAL\[FR] FIBRES COMPOSéES THERMOADHéSIVES PROCéDé POUR LEUR PRODUCTION ET éTOFFE FONDUE OU MATéRIAU DE SURFACE à PARTIR DE CELLES-CI\ | EP0696655A1 | SEI ISHIZAWA (JP)\ MASAYASU SUZUKI (JP)\ HIROKAZU TERADA (JP) | CHISSO CORP (JP) | A61F13/511\ A61F13/49\ D01F8/06\ D02G1/20\ D04H1/541\ D04H1/544 |
| 3478 | 3D printing | INK FILM FOR INSERT MOLDING AND INSERT MOLDED ARTICLE\ | JP2004002629A | SEKINE YASUYOSHI US | TEIKOKU PRINTING INK MFG US | B29C45/14\ C09D11/02\ B29K25/00\ B29K69/00\ B29K105/20 |
| 3479 | 3D printing | METHOD FOR SIMULATION OF LASER MATERIAL DEPOSITION\ | US6813533B1 | SEMAK VLADIMIR V (US) | SPEC CO LTD US | G06F17/50\ B23K26/34\ G06F19/00 |
| 3480 | 3D printing | CORE-SHELL POLYAMIDE POWDER\ | US2010098880A1 | SENFF HOLGER (FR) | ARKEMA FRANCE (FR) | C08G69/00\ B05D3/00 |
| 3481 | 3D printing | APPTS. FOR PRODUCING WATER WAVE GLASS MOSAIC\ | CN2856043Y | SHEN RONGFANG XU (CN) | SHEN RONGFANG (CN) | C03B19/02\ C03B11/00 |
| 3482 | 3D printing | BUBBLE PRINT HEAD WITH INK FEED CHAMBER MADE BY INJECTION MOLDING\ | TW200409702A | SHI WANG-PING (TW)\ WEI REN-JIE (TW)\ FANG DUN-YING (TW)\ XIE SHU-PIN (TW)\ YE FENG-CHUN (TW) | MICRO BASE TECHNOLOGY CORP (TW) | Empty Field |
| 3483 | 3D printing | METHOD FOR RAPIDLY MANUFACTURING INJECTION-MOULD INSERT WITH FOLLOW-COOLING PASSAGEWAY\ | CN1850396A | SHI YUSHENG HUANG (CN) | UNIV HUAZHONG SCIENCE TECH (CN) | B22F3/105\ B22F3/24\ B29C33/38 |
| 3484 | 3D printing | COSMETIC COMPOSITION FOR CARING AND MAKING UP THE SKIN AND LIPS E.G. LIP MAKEUP AND CARE COMPOSITION COMPRISES ESTER OF DIMERDILINOLEIC ACID AND OF POLYOL\[FR] COMPOSITION COSMETIQUE COMPRENANT UN ESTER D'ACIDE DIMERDILINOLEIQUE ET DE POLYOL(S) ET UN POLYMERE SEMI-CRISTALLIN\ | FR2894472A1 | SHIMIZU MOMOKO (JP)\ TOKUNAGA EMIKO (JP) | OREAL (FR) | A61K8/37\ A61Q19/00\ A61K8/895\ A61Q1/04 |
| 3485 | 3D printing | WHITE POROUS POLYESTER FILM AND PREPARATION METHOD THEREOF\ | US2011046254A1 | SHIN HEON JUNG (KR)\ JU JAE SEOG (KR)\ KIM MIN JU (KR) | SKC CO LTD (KR) | C08J9/00 |
| 3486 | 3D printing | PRECIOUS METAL LAYER CERAMIC MOLDINGS AND METHOD OF MANUFACTURE\ | KR101315344B1 | SHIN HONG DAI (KR) | SHIN HONG DAI (KR) | B44C5/00\ C04B41/88 |
| 3487 | 3D printing | THERMAL TRANSFER ACCEPTANCE SHEET\ | JP2007152697A | SHINOHARA HIDEAKI US\ TANAKA YOSHIMASA US | OJI PAPER CO US | B41M5/50\ B41M5/52\ B41M5/382 |
| 3488 | 3D printing | HOLOGRAM FILM FOR INSERT MOLDING\ | JP2005003853A | SHIRAISHI KAZUO US\ OKAMOTO KOJI US\ OSATO MASAHIKO US | TOPPAN PRINTING CO LTD US | B29C45/14\ G03H1/18\ B32B7/02 |
| 3489 | 3D printing | THREE-DIMENSIONAL NETWORK STRUCTURE AND METHOD FOR PRODUCING THREE-DIMENSIONAL NETWORK STRUCTURE\ | JP2006097223A | SHIRAKI MOTOYUKI US | JATECX KK US | A47C27/12\ D04H3/07 |
| 3490 | 3D printing | EXHAUST EMISSION CONTROL STRUCTURE FOR MOTORCYCLE ENGINE\ | JP2004132345A | SHIRASAGI SADAO US\ SATO TATSUO US | OVER RACING PROJECTS KK US | F01N3/20\ B01D53/86\ F01N3/10\ F01N3/24\ F01N3/28 |
| 3491 | 3D printing | APPARATUS FOR CONTROLLED FREEZING OF MELTED SOLID INK IN A SOLID INK PRINTER\ | US2011211010A1 | SHRADER ERIC J (US)\ PASCHKEWITZ JOHN S (US) | PALO ALTO RES CT INC (US) | B41J2/175\ B41J29/377 |
| 3492 | 3D printing | WAVEGUIDE OPTICAL SWITCH AND MAKING METHOD THEREOF\ | CN100445783C | SHULIN E (CN)\ GUANGMING KONG (CN)\ HUDAN ZHAO (CN)\ WENYUAN DENG (CN) | CHANGCHUN OPTICS FINE MECH (CN) | G02B6/12\ G02B6/13\ G02B6/35 |
| 3493 | 3D printing | METHOD FOR PRODUCTION OF A THREE-DIMENSIONAL BODY\ | US2013300035A1 | SNIS ANDERS (SE) | ARCAM AB (SE)\ SNIS ANDERS (SE) | B29C67/00 |
| 3494 | 3D printing | METHOD FOR PREVENTING NOZZLE CONTAMINATION DURING WARM-UP\ | US7762656B2 | SNYDER TREVOR JAMES (US) | XEROX CORP (US) | B41J2/165\ B41J2/175 |
| 3495 | 3D printing | MELTING HEAT TRANSFER RIBBON FOR METALLIC PRINT\ | JP2005335319A | SOGABE ATSUSHI US\ FUJIMOTO KOTARO US | FUJI KAGAKU SHIKOGYO US | B41M5/30\ B41M5/42\ B41J31/00\ B41J31/05\ B41M5/382\ B41M5/385\ B41M5/39\ B41M5/40\ B41M5/41 |
| 3496 | 3D printing | PHOTO-BOOK LINKED TO WEB-SITE AND MANUFACTURING METHOD THEREOF\ | KR20130082544A | SONG CHANG HOON (KR) | ABYZ CO LTD (KR) | G06K9/18\ G06Q50/10 |
| 3497 | 3D printing | METHOD FOR MANUFACTURING REDUCER OF COAL PULVERIZER\ | KR101057668B1 | SONG JONG SEOP (KR) | SECO LTD (KR) | B02C15/16\ B02C4/42\ B02C15/04 |
| 3498 | 3D printing | VERFAHREN UND VORRICHTUNG ZUR HERSTELLUNG DREIDIMENSIONAL STRUKTURIERTER VLIESSTOFFE\[EN] MASS PRODUCTION OF THREE-DIMENSIONALLY-TEXTURED FABRIC PLACES FLEECE AGAINST MOVING TEXTURED SUPPORT PRE-COMPACTS AND APPLIES STEAM HEATING ON OUTER SURFACE\ | DE102007043523A1 | STEINBACH ULLRICH (DE)\ KREBS STEFAN (DE)\ GHORBEL LOTFI (DE) | FLEISSNER GMBH (DE) | D04H13/00\ D04H1/541\ D04H3/11\ D04H1/54\ D04H3/14\ D04H1/48\ D04H1/49\ D04H1/495\ D04H1/55 |
| 3499 | 3D printing | THERMOCHROMIC BUILD MATERIALS\ | US2013292881A1 | STEINER ROBERT J (US)\ VON SICE CORRIE M (US) | STEINER ROBERT J (US)\ VON SICE CORRIE M (US) | B41M5/34 |
| 3500 | 3D printing | METHOD FOR INJECTION MOLDING OF THERMOPLASTIC POLYMER MATERIAL WITH CONTINUOUS PROPERTY TRANSITIONS\ | US2010112267A1 | STENZEL HOLGER (DE)\ RIDDER HELMUT (DE)\ SCHNIEDERS JOACHIM (DE) | KURARAY EUROPE GMBH (DE) | B29C45/16\ B32B5/14 |
| 3501 | 3D printing | POROUS CERAMIC BODY AND METHOD FOR PRODUCTION THEREOF\ | US2006236668A1 | STOBBE PER (DK)\ HAOK UDO (DE) | HAOK UDO (DE)\ STOBBE PER (DK) | C04B38/00\ B01D71/02\ B01D46/00 |
| 3502 | 3D printing | VERWENDUNG VON POLYAMID-FORMMASSEN ZUR HERSTELLUNG VON FORMTEILEN MIT REDUZIERTER OBERFL?CHENVERKOHLUNG\[EN] USE OF POLYAMIDE MOULDING MATERIALS FOR THE FABRICATION OF MOULDED ARTICLES WITH REDUCED SUPERFICIAL CHARRING\[FR] UTILISATION DE COMPOSITIONS à MOULER DE POLYAMIDE POUR LA FABRICATION D'ARTICLES MOULéS à CARBONISATION SUPERFICIELLE RéDUITE\ | EP1780241A1 | STOEPPELMANN GEORG DR RER NAT (CH)\ KETTL RALPH DIPL-ING (CH)\ HOFFMANN BOTHO DR RER NAT (CH)\ EBERT MARTINA DR RER NAT DIPL (CH)\ HEWEL MANFRED DR RER NAT DIPL (CH) | EMS CHEMIE AG (CH) | C08L77/00\ C08K3/34 |
| 3503 | 3D printing | DECORATING SHEET DECORATING MOLDED ARTICLE AND IN-MOLD DECORATING INJECTION MOLDING\ | US2007026197A1 | SUGA KAZUHIRO (JP)\ MEIKI YOSHIYUKI (JP)\ ATAKE HIROYUKI (JP) | DAINIPPON PRINTING CO LTD (JP) | B29C45/14\ B29C51/02\ B29L9/00\ B32B3/00\ B29C51/10\ B29C45/26\ B44C3/02\ B32B27/06\ B32B27/00\ B32B3/28\ B29C33/18\ B29K105/20 |
| 3504 | 3D printing | METHOD AND APPARATUS FOR PRODUCING GRANULAR CRYSTAL\ | US2008029020A1 | SUGAWARA SHIN (JP)\ TAKAHASHI EIGO (JP)\ ARIMUNE HISAO (JP) | KYOCERA CORP (JP) | C30B30/06 |
| 3505 | 3D printing | INTEGRATABLE ASSISTED COOLING SYSTEM FOR PRECISION EXTRUSION DEPOSITION IN THE FABRICATION OF 3D SCAFFOLDS\ | US2012080814A1 | SUN WEI (US)\ HAMID QUDUS (US) | SUN WEI (US)\ HAMID QUDUS (US)\ UNIV DREXEL (US) | B29C47/78\ F28F9/00 |
| 3506 | 3D printing | METHOD FOR PRODUCING POLYACETAL RESIN COMPOSITION REDUCED IN FORMALDEHYDE CONTENT\ | JP2006291001A | SUNAGA DAISUKE US\ NAGAI SATOSHI US\ OKAMURA AKIRA US | MITSUBISHI GAS CHEMICAL CO US | C08G2/28 |
| 3507 | 3D printing | PLASMATRON\ | RU2225084C1 | SUSLOV V I (RU) | YEOM JANG YUL (KR) | H05H1/42\ H05B7/20\ B05B7/22 |
| 3508 | 3D printing | LASER-PRODUCED POROUS SURFACE\ | US2004191106A1 | SUTCLIFFE CHRISTOPHER J (GB)\ JONES ERIC (IE)\ O'NEILL WILLIAM (GB) | HOWMEDICA OSTEONICS CORP (US) | A61F2/00\ A61F2/30\ B22F3/105\ B22F7/00\ C23C24/10\ A61F2/38\ C23C26/02\ C23C4/12\ A61F2/36\ C23C24/04\ C23C4/18\ A61F2/34\ B23K26/38\ C23C4/02 |
| 3509 | 3D printing | CHEMICAL VAPOR DEPOSITION RAW MATERIAL COMPRISING ORGANIC NICKEL COMPOUND AND CHEMICAL VAPOR DEPOSITION METHOD USING SAID CHEMICAL VAPOR DEPOSITION RAW MATERIAL\[FR] MATIèRE PREMIèRE DE DéP?T CHIMIQUE EN PHASE VAPEUR COMPRENANT UN COMPOSé ORGANIQUE DU NICKEL ET PROCéDé DE DéP?T CHIMIQUE EN PHASE VAPEUR UTILISANT LADITE MATIèRE PREMIèRE DE DéP?T CHIMIQUE EN PHASE VAPEUR\ | WO2014188629A1 | SUZUKI KAZUHARU (JP)\ SAITO MASAYUKI (JP)\ HARADA RYOSUKE (JP)\ NABEYA SHUNICHI (JP)\ MIYAZAKI SATOSHI (JP) | TANAKA PRECIOUS METAL IND (JP) | C07F15/04\ C23C16/18 |
| 3510 | 3D printing | MANUFACTURE OF DECORATIVE SHEET\ | JP8224796A | SUZUKI YUKIO US\ NISHIMURA IKUMASA US\ AIZAWA TETSUO US | TOPPAN PRINTING CO LTD US | B29C59/00\ B29L9/00\ B29C69/00\ B32B27/00\ B05D3/04\ B32B33/00\ B32B27/32\ B29C47/88\ B29D7/01\ B29K23/00\ B29L7/00\ B29C59/04\ B41M1/24\ B29C47/14\ B41M1/30 |
| 3511 | 3D printing | RELAXATION SLEEP-INDUCING BEDDING\ | JP2005143522A | TAKAKURA JAKUKO US\ MURATA TOSHIHIRO US | KYOTO NISHIKAWA & CO US\ ONKYOORIBU KK US | A61H23/02\ A61F7/08\ A61M21/02\ B06B1/04 |
| 3512 | 3D printing | THREE-DIMENSIONAL NET-LIKE STRUCTURE AND METHOD AND DEVICE FOR PRODUCING THREE DIMENSIONAL NET-LIKE STRUCTURE\ | US2003092335A1 | TAKAOKA NOBUYUKI (JP) | Empty Field US | D04H13/00\ B29C43/02\ D04H3/03\ B29C43/22\ B29C47/30\ D04H3/07\ B29C47/12\ D04H3/16\ B29C43/34 |
| 3513 | 3D printing | MATTRESS FOR PREVENTING DEW CONDENSATION AND METHOD OF MANUFACTURING THE MATTRESS\ | JP2010279687A | TAKAOKA NOBUYUKI (JP) | SHIIENJI KK US | A47C27/12 |
| 3514 | 3D printing | DECORATIVE SHEET\ | JP2005119021A | TAKASAKI YUTAKA US | TOPPAN PRINTING CO LTD US | B32B27/20\ B29L9/00\ B32B27/00\ B29L7/00\ B29C59/04 |
| 3515 | 3D printing | METHOD OF MANUFACTURING POWER RECEIVING SYSTEM HOUSING IN WIRELESS CHARGING SYSTEM\ | JP2011109546A | TAKEUCHI SHINYA US | NISSHA PRINTING US | H04M1/02\ H02J7/00 |
| 3516 | 3D printing | QUARTZ-BASED OPTICAL WAVEGUIDE AND MANUFACTURING METHOD FOR THE SAME\ | JP2008165014A | TANAKA KOTARO US | HITACHI CABLE (JP) | G02B6/12\ G02B6/13\ G02F1/01 |
| 3517 | 3D printing | METHOD FOR COMPREHENSIVELY IMPROVING FUNCTIONS OF AN ELECTROGRAPHIC IMAGING DEVICE\[FR] PROCéDé PERMETTANT D'AMéLIORER DE FA?ON COMPLèTE LES FONCTIONS D'UN DISPOSITIF D'IMAGERIE éLECTROPHOTOGRAPHIQUE\ | WO2013078938A1 | TANG FUGEN (CN)\ ZHOU HONGHUI (CN) | PRINT RITE UNICORN IMAGE PROD (CN) | G03G21/16 |
| 3518 | 3D printing | METAL BOND GRINDSTONE AND ITS MANUFACTURING METHOD\ | JP2005161510A | TANIGAWA YOSHIHIRO US\ NAKAMURA NORIKAZU US | TANIGAWA YOSHIHIRO US\ NAKAMURA NORIKAZU US | B24D3/00\ B24D3/06 |
| 3519 | 3D printing | SUPPORT BODY\[ES] CUERPO DE DESCANSO\[FR] ARTICLE DE REPOS\ | WO2010018270A1 | TEROL CONTRERAS NICOLAS (ES) | MANTEROL S A (ES)\ TEROL CONTRERAS NICOLAS (ES) | A47C27/12\ A47G9/10 |
| 3520 | 3D printing | ELECTROCONDUCTIVE SHEET\ | JP2011056668A | TERUI TOMOKO US\ TERAMAE KIMITO US | LONSEAL KOGYO KK US | B29L9/00\ B32B27/18\ H01B1/20\ B29K101/12\ B29K105/16\ B29C43/24\ B29C43/18\ B29L7/00\ B29C43/34\ C08K7/02\ C08L101/12\ B29K105/12 |
| 3521 | 3D printing | ELECTRIC MULTI-AZIMUTH METAL POWDER INJECTION MOLDING MACHINE\ | CN102921949A | THE INVENTOR HAS WAIVED THE RIGHT TO BE MENTIONED US | LIU YA US | B22F3/115 |
| 3522 | 3D printing | PROCESS FOR PRODUCING SHEATH-CORE STAPLE FIBERS WITH A THREE-DIMENSIONAL CRIMP AND A CORRESPONDING SHEATH-CORE STAPLE FIBER\ | US2009124155A1 | TIEMEIER HENDRIK (DE)\ LABITZKE EKKEHARD (DE) | OERLIKON TEXTILE GMBH & CO KG (DE) | B32B5/02\ D04H1/00\ B29C47/08 |
| 3523 | 3D printing | METHOD AND SYSTEM FOR LEADING MACROMOLECULE SUBSTANCES INTO LIVING TARGET CELLS\ | US2005085711A1 | TIEN DER-YANG (TW) | FUJIAN JUNLUN FIBER SHAREHOLDING CO LTD US | A61M37/00\ A61B19/00\ A61K48/00\ A61M5/14\ A61B17/22\ A61K9/10\ A61K45/00\ A61K49/00 |
| 3524 | 3D printing | THREE-DIMENSIONAL GEOMETRIC BIO-COMPATIBLE POROUS ENGINEERED STRUCTURE FOR USE AS A BONE MASS REPLACEMENT OR FUSION AUGMENTATION DEVICE\ | US6206924B1 | TIMM JENS PETER (US) | INTERPORE CROSS INTERNAT (US) | A61F2/00\ A61F2/28\ A61F2/30\ A61F2/44 |
| 3525 | 3D printing | LIQUID EJECTOR AND PROCESS FOR FORMING SMALL PROTRUSION BY LIQUID EJECTION\ | JP2004249685A | TOGASHI TAKAYUKI US | SEIKO EPSON CORP (JP) | C09D11/00\ B41J2/01\ C09D11/02\ B41J3/32\ B41J2/015 |
| 3526 | 3D printing | THERMOFORMBARES VERPACKUNGSMATERIAL MIT INNENLIEGENDEM DRUCKBILD\[EN] THERMOFORMABLE PACKAGING MATERIAL WITH AN INTERNAL PRINTED IMAGE\[FR] MATERIAU D'EMBALLAGE THERMOFORMABLE COMPRENANT UNE IMAGE IMPRIMéE à L'INTéRIEUR\ | EP1724105A1 | TOIVOLA TIMO (FI)\ LEHTO KEIJO (FI)\ JUNNILA JARMO (FI)\ SILANDER HARRI (FI)\ SAARISTO ESKO (FI)\ KASCHEL GREGOR DR (DE)\ BRANDT RAINER DR (DE) | WIPAK WALSRODE GMBH & CO KG (DE) | B32B27/00 |
| 3527 | 3D printing | COMPOSITION AND METHODS FOR THE PRODUCTION OF BIOPOLYMERIC MATERIALS THAT BIODEGRADE RAPIDLY AND ARE FLEXIBLE AND RIGID WITH USE OF THE XANTHAN BIOPLASTIC AS A COMPONENT OF THE BIOPOLYMETRIC MATRIX AND OPTIONALLY FILLERS AND/OR NANOFILLERS AND OTHER CONSTITUENTS THE RESULTING PRODUCTS AND THE USES THEREOF\[FR] COMPOSITION ET PROCéDéS DE PRODUCTION DE MATIèRES BIOPOLYMèRES à BIODéGRADATION RAPIDE SOUPLES ET RIGIDES AVEC UTILISATION DU BIOPLASTIQUE XANTHANE COMPOSANT LA MATRICE BIOPOLYMèRE ET éVENTUELLEMENT DES CHARGES ET/OU DES NANOCHARGES ET D'AUTRES CONSTITUANTS PRODUITS OBTENUS ET LEURS UTILISATIONS\[PT] COMPOSI??O E MéTODOS DE PRODU??O DE MATERIAIS BIOPOLIMéRICOS DE RáPIDA BIODEGRADA??O FLEXíVEIS E RíGIDOS COM USO DO BIOPLáSTICO XANTANA COMPONDO A MATRIZ BIOPOLIMéRICA E OPCIONALMENTE CARGAS E/OU NANOCARGAS ê OUTROS CONSTITUINTES; PRODUTOS OBTIDOS E SEUS USOS\ | WO2013181731A1 | TONDO VENDRUSCOLO CLAIRE (BR)\ DA SILVA MOREIRA ANGELITA (BR)\ DE ANDRADE SALLES CLEIA (BR)\ CALCAGNO CARMEN IARA WALTER (BR) | IFSUL INST FED DE EDUCACAO CIENCIA E TECNOLOGIA SUL RIO GRANDENSE (BR)\ UFPEL UNIVERSIDADE FED DE PELOTAS (BR) | C08K3/32\ A61K8/73\ A61Q11/00\ C08K3/34\ C12P19/04\ A23L1/0522\ A23L1/054\ A61K31/685\ A61K31/722\ A61P19/02\ A61P19/04\ C07H3/00\ C07K14/195\ C08B37/00\ C08L3/02\ C09K8/02\ C09K8/08\ C12N1/20\ C12N9/10\ C12P19/06\ C12R1/64 |
| 3528 | 3D printing | A MANUFACTURE METHOD AND PRODUCT OF AN ARTIFICIAL BOARD WITH DUAL-COLOR AND THREE-DIMENSIONAL CONVEX PATTERNS\ | TW585959B | TSAI JE-AN (TW) | TSAI JE-AN (TW) | E04C2/00 |
| 3529 | 3D printing | POLYMERIC ADDITIVES TO IMPROVE PRINT QUALITY AND PERMANENCE ATTRIBUTES IN INK-JET INKS\ | US2002156153A1 | TSANG JOSEPH W (US)\ MOFFATT JOHN R (US) | C ENG CO LTD (JP)\ TAKAOKA NOBUYUKI (JP) | C09D11/00\ B41J2/01\ B41M5/00\ B41J2/21\ B41J11/00 |
| 3530 | 3D printing | FALSE PORCELAIN TILE AND MANUFACTURING METHOD THEREFOR\ | JP2004225379A | TSUKAMOTO KAZUO US\ YAMAMOTO TADAHIKO US | MITSUBOSHI CO LTD US | E04F15/10 |
| 3531 | 3D printing | PHASE CHANGE INKS\ | US2010080922A1 | TUREK CAROLINE M (CA)\ BRETON MARCEL P (CA) | XEROX CORP (US) | C09D175/04\ C09D11/02\ B05D5/00\ B05D1/04 |
| 3532 | 3D printing | BENDING RECEIVER USING HEAT-SHRINKABLE FILM\ | US8227165B2 | TYAGI DINESH (US) | EASTMAN KODAK CO (US)\ TYAGI DINESH (US) | G03G5/00 |
| 3533 | 3D printing | METHOD AND APPARATUS FOR PRODUCING CELLULOSE RESIN FILM AND CELLULOSE RESIN FILM AND FUNCTIONAL FILM\ | US2008075922A1 | UEDA TADASHI (JP) | FUJIFILM CORP (JP) | C08J5/18 |
| 3534 | 3D printing | PULP MOULD PAPER CONTAINER\ | JP2005096790A | UEDA YOSHIKI US | TOPPAN PRINTING CO LTD US | B32B1/02\ B65D25/34\ B32B29/00\ B65D1/26\ B65D1/40\ B65D25/14\ B65D81/38 |
| 3535 | 3D printing | VASCULARIZED TISSUE REGENERATION MATRICES FORMED BY SOLID FREE FORM FABRICATION TECHNIQUES\ | US6176874B1 | VACANTI JOSEPH P (US)\ CIMA MICHAEL J (US)\ CIMA LINDA G (US) | CHILDRENS MEDICAL CENTER (US)\ MASSCHUSETTS INST OF TECHNOLOG (US) | B29C67/00\ A61L27/00\ A61F2/00\ A61F2/02\ A61F2/06\ A61F2/10\ A61F2/28\ A61F2/30\ A61K9/00\ A61K9/16\ A61L27/38\ A61L27/40\ A61L27/56\ A61L27/60\ B82B1/00 |
| 3536 | 3D printing | ELECTROMAGNETIC RADIATION DETECTOR AND METHOD FOR MANUFACTURING SUCH A DETECTOR\ | US7999227B2 | VILAIN MICHEL (FR) | ULIS (FR) | G01J5/02 |
| 3537 | 3D printing | RAPID PROTOTYPING AND MANUFACTURING SYSTEM AND METHOD\ | US7690909B2 | WAHLSTROM BEN (US) | 3D SYSTEMS INC (US) | B28B1/16\ B05C11/02\ B29B13/08 |
| 3538 | 3D printing | VERFAHREN ZUR HERSTELLUNG VON EINER KAUTSCHUK-/GEWEBE-VERBUNDPRODUKTE\[EN] METHODS FOR MAKING RUBBER/FABRIC COMPOSITE PRODUCTS\[FR] PROCEDES POUR LA FABRICATION DE PRODUITS COMPOSITES A BASE DE CAOUTCHOUC/TISSU\ | EP1778464A2 | WALTERS IAN (GB)\ HOUGH PHILIP (GB) | HAYDALE LTD (GB) | B29D99/00\ B29C65/48\ B29C70/74 |
| 3539 | 3D printing | PRESS FIT CONNECTOR ATTACHMENT METHOD\ | JP2005276705A | WANDA YOSHIHIRO US | DENSO CORP (JP) | H01R43/20\ H05K3/32 |
| 3540 | 3D printing | HOUSING OF ELECTRONIC DEVICE AND METHOD FOR MAKING THE HOUSING\ | TW201110457A | WANG QIANG (CN) | FIH HONG KONG LTD (HK) | H01Q1/22 |
| 3541 | 3D printing | THREE-DIMENSIONAL PRINTER PRINTHEAD CAPABLE OF RAPIDLY CROSSING FILAMENTS\ | CN103358554A | WANG RUIGANG US\ YU HUA US | SUZHOU JIANGNAN JIAJIE ELECTROMECHANICAL TECHNOLOGY RES INST CO LTD US | B29C67/00 |
| 3542 | 3D printing | METHOD AND APPARATUS FOR CREATING A FREE-FORM THREE-DIMENSIONAL METAL PART USING HIGH-TEMPERATURE DIRECT LASER MELTING\ | US6621039B2 | WANG XINHUA (SG)\ WANG YUNGAN (SG)\ LUO JIHUAN (SG)\ YU YUEFENG (SG)\ ZHANG XIANGLIN (SG)\ TANG YAXIN (SG)\ LU LI (SG)\ WANG XUAN (SG)\ FUH YING HSI JERRY (SG)\ LIM KUAK CHOI LEALIC (SG)\ WONG YOKE SAN (SG)\ CHENG HONGBIN (SG) | KINERGY PTE LTD (SG)\ UNIV SINGAPORE (SG) | B22F3/00\ B22F3/105 |
| 3543 | 3D printing | PREPARATION METHOD OF DETACHABLE PLANT THREE-DIMENSIONAL SPECIMEN\ | CN102835390A | WANG YING US\ CAI BAIYAN US\ LI YAN US | UNIV EAST HEILONGJIANG US | A01N3/00 |
| 3544 | 3D printing | PRODUCTION METHOD OF DOUBLE METAL NETWORK-INTERPENETRATED MULTIPHASE SECTION\ | CN101825218B | WANG YONG US\ CUI MIN US\ MA RU US\ WANG SHOUREN US | UNIV JINAN US | F16S5/00 |
| 3545 | 3D printing | SINGLE-NOZZLE MELT-EXTRUSION-TYPE THREE-DIMENSIONAL PRINTER\ | CN103182781A | WANG YUNGAN (SG)\ WANG XUAN (SG) | SHANGHAI FOCHIF MECHATRONICS TECHNOLOGY CO LTD US | B29C47/92\ B29C69/02\ B29C47/08\ B29C47/12\ B29C47/34\ B29C53/56 |
| 3546 | 3D printing | SPECIAL POLYCARBONATE FILM FOR 3D AUTOMOBILE INSTRUMENT PANEL AND PREPARATION METHOD THEREOF\ | CN102019854A | WEI LUO US\ YUEZHANG REN US | SUZHOU AOMEI MATERIALS TECHNOLOGY CO LTD US | B60K35/00\ B29C47/92\ B29L7/00\ B29K69/00 |
| 3547 | 3D printing | INJECTION MOULD PRODUCT IN-MOULD PRINTING MOLDING METHOD\ | CN101474849A | WEIXIA ZHU (CN) | WEIXIA ZHU (CN) | B29C45/17 |
| 3548 | 3D printing | INJECTION MOULD PRODUCT IN-MOULD PRINTING MOLDING EQUIPMENT\ | CN101474848A | WEIXIA ZHU (CN) | WEIXIA ZHU (CN) | B29C45/17 |
| 3549 | 3D printing | PRINTING MEMBRANE\ | CN201075278Y | WENJIAN LUO (CN) | LUOTIE MACHINERY CO LTD (CN) | G09F23/00 |
| 3550 | 3D printing | STEREO PROJECTION SYSTEM\ | CN202306131U | WENJIN LIAO US\ JINLIANG HUANG US\ JIANPING HE US | SHENZHEN SAIYE EXHIBITION DISPLAY CO LTD US | H04N13/00\ G03B35/18\ G03B21/60\ G06F3/14 |
| 3551 | 3D printing | PROCESS FOR LAMINATING AN INK JET PRINT\ | US6454896B1 | WEXLER ALLAN (US) | EASTMAN KODAK CO (US) | B41J2/01\ B41M7/00\ G03C11/08\ G03G8/00 |
| 3552 | 3D printing | OBJECT CONSOLIDATION THROUGH SEQUENTIAL MATERIAL DEPOSITION\ | US6814823B1 | WHITE DAWN (US) | SOLIDICA INC (US) | B29C67/00\ G05B19/4099\ B23K11/00\ B23K20/10\ B23K20/12\ B29C73/00 |
| 3553 | 3D printing | METHODS FOR FABRICATING THREE-DIMENSIONAL ALL ORGANIC INTERCONNECT STRUCTURES\ | US2004000425A1 | WHITE GEORGE E (US)\ DALMIA SIDHARTH (US)\ SUNDARAM VENKATESH (US)\ SWAMINATHAN MADHAVAN (US) | Empty Field US | H01F17/00\ H05K3/46\ H01F41/04\ H01L21/48\ H01L25/065\ H01L27/08\ H05K1/16 |
| 3554 | 3D printing | METHOD OF FORMING A PHASE CHANGE INK IMAGE ON A SELF-LAMINATING RECORDING MEDIUM\ | US2008049090A1 | WILLEMS GUIDO G (NL)\ ROOIJAKKERS JOHANNES P J C (NL)\ STURME RUDOLF A H M (NL) | OCE TECH BV (NL) | B41J2/01 |
| 3555 | 3D printing | BILDERZEUGUNGSVERFAHREN DAS EINE PHASENAUSTAUSCHTINTE AUF EINEM SELBSTLAMINIERENDEN AUFZEICHNUNGSMEDIUM VERWENDET\[EN] METHOD OF FORMING A PHASE CHANGE INK IMAGE ON A SELF-LAMINATING RECORDING MEDIUM\[FR] PROCéDé DE FORMATION D'IMAGE UTILISANT UNE ENCRE à CHANGEMENT DE PHASE SUR UN MATéRIAU AUTO-LAMINANT\ | EP1892115A1 | WILLEMS GUIDO G (NL)\ ROOIJAKKERS JOHANNES P J C (NL)\ STURME RUDOLF A H M (NL) | OCE TECH BV (NL) | B41M7/00 |
| 3556 | 3D printing | RASTER METHODOLOGY APPARATUS AND SYSTEM FOR ELECTRON BEAM LAYER MANUFACTURING USING CLOSED LOOP CONTROL\ | US2014158667A1 | WOLLENHAUPT PHILLIP E (US)\ STECKER SCOTT (US) | SCIAKY INC (US) | B23K15/00\ B23K15/06\ B23K15/02 |
| 3557 | 3D printing | LASER 3D (THREE-DIMENSIONAL) PRINTING METHOD OF CERAMIC FUNCTIONAL GRADIENT COMPONENT\ | CN103317590A | WU DONGJIANG US\ NIU FANGYONG US\ MA GUANGYI US\ GUO DONGMING US | UNIV DALIAN TECH (CN) | B28B1/24 |
| 3558 | 3D printing | FOLDING GRID CLOTH\ | CN201915275U | XI ZHANG US | SHANGHAI EXH INTERNAT TRADE CO LTD US | D06J1/12 |
| 3559 | 3D printing | SPRAY FORMING SYSTEM FOR PREPARING ALLOY AND METAL-BASED COMPOSITE PARTS\ | CN102319898A | XIANJIE ZHANG US\ JUNBIAO WANG US\ CHAORUN SI US\ YUJUN LI US\ XIAOBAO CAO US | UNIV NORTHWESTERN POLYTECHNIC US | B22F3/115 |
| 3560 | 3D printing | DEVICE FOR MANUFACTURING ALLOY-METAL-BASED PARTS BY SPRAY FORMING\ | CN202317021U | XIANJIE ZHANG US\ JUNBIAO WANG US\ CHAORUN SI US\ YUJUN LI US\ XIAOBAO CAO US | UNIV NORTHWESTERN POLYTECHNIC US | B22F3/115 |
| 3561 | 3D printing | MANUFACTURING METHOD FOR MAKING ANTIQUE ARTISTIC FLOWERPOT\ | CN101755632A | XIANLIAN LIU (CN) | XIANLIAN LIU (CN) | A01G9/02\ B44D5/10 |
| 3562 | 3D printing | EQUIPMENT FOR PRODUCING PLASTIC CONTAINER WITH STEREO ANAGLYPH PATTERN ON SURFACE AND WITHOUT SEAM LINE\ | CN100453298C | XIANLIAN LIU (CN) | XIANLIAN LIU (CN) | B29C49/30\ B29C49/02\ B29C49/42\ B29C49/54\ B29C49/58\ B29C49/64 |
| 3563 | 3D printing | MANUFACTURING METHOD OF FINE PERSONALIZED SKULL MODEL CAPABLE OF DESCRIBING TEETH OCCLUDING RELATION\ | CN100569195C | XIAOFENG LI (CN)\ HUIZHI CAO (CN)\ CAN LIU (CN)\ QIAN JIANG (CN) | XIAOFENG LI (CN) | G09B23/30\ G06F17/00\ A61C19/00\ A61B6/00\ G06Q50/00 |
| 3564 | 3D printing | HIGH-POLYMER ELASTIC AND VENTILATING PILLOW INNER\ | CN201641314U | XIAOLIANG ZHUANG US | XIAOLIANG ZHUANG US | A47G9/10 |
| 3565 | 3D printing | HIGH MOLECULAR ELASTIC VENTILATED PILLOW INNER AND PREPARATION METHOD THEREOF\ | CN102133017A | XIAOLIANG ZHUANG US | XIAOLIANG ZHUANG US | A47G9/10 |
| 3566 | 3D printing | APPARATUS FOR PRODUCING FINE THREE-DIMENSIONAL FIGURED PLATE GLASS WITH FLOATING METHOD\ | CN201236175Y | XIAOXIAO YANG (CN)\ DENING YANG (CN) | DENING YANG (CN) | C03B18/12\ C03B18/14 |
| 3567 | 3D printing | MANUFACTURING AND REPAIRING METHOD FOR CONDUCTIVE CIRCUIT OF THREE DIMENSIONAL MOLD INTERCONNECTING DEVICE\ | CN101394710A | XIAOYAN ZENG (CN)\ YU CAO (CN)\ XIANGYOU LI (CN)\ JIANGUO LIU (CN) | UNIV HUAZHONG SCIENCE TECH (CN) | H05K3/10\ H05K3/20 |
| 3568 | 3D printing | WIND TURBINE BLADE MOULD\ | CN102785315A | XINGDONG CAO US | CHANGZHOU SHENHUI ELECTRIC MATERIAL CO LTD US | B29C33/38 |
| 3569 | 3D printing | METHOD FOR PREPARING HIGH TEMPERATURE FLUENT METAL LOOP BY QUARTZ AND SICF/SIC COMPOSITE MATERIAL\ | CN101320600A | XINGUI ZHOU (CN)\ YICAN WU (CN)\ WANG JUN (CN)\ CHANGRUI ZHANG (CN)\ QUNYING HUANG (CN)\ YINGBIN CAO (CN)\ RONGJUN LIU (CN)\ HONGLEI WANG (CN)\ HAIJIAO YU (CN)\ SHUANG ZHAO (CN)\ ZHIYI WANG (CN)\ ZELAN HUANG (CN) | NAT UNIV DEFENSE TECHNOLOGY (CN) | C04B41/50\ G21B1/11\ C04B35/66 |
| 3570 | 3D printing | INTEGRAL QUICK FORMING METHOD FOR CERAMIC PARTS BASED ON PARAFFIN\ | CN101550007A | XIUFENG WANG (CN)\ WENJIE XU (CN)\ HONGTAO JIANG (CN)\ CHENGLONG YU (CN) | UNIV SHAANXI SCIENCE & TECH (CN) | C04B35/622\ B28B7/34\ C04B35/632\ B28B1/54 |
| 3571 | 3D printing | PREPARATION METHOD OF DYEABLE POLYESTER COMPOSITE FIBER OF CATIONIC DYE\ | CN101613894B | XIUHUA WANG US\ YUYUAN YAO US\ JICHENG MENG US | UNIV ZHEJIANG SCIENCE & TECH US | D01D5/34\ D01F8/14 |
| 3572 | 3D printing | PRODUCTION METHOD FOR MANUFACTURING ALUMINUM ALLOY EYEWEAR ACCESSORIES\ | CN101912955A | XIUJUAN YANG US | XIUJUAN YANG US | B22D17/10\ G02C5/00 |
| 3573 | 3D printing | PAPER PLASTIC GLUE-FREE COMPOSITE BIDIRECTIONAL STRETCHING POLYPROPYLENE FILM AND ITS PREPARATION METHOD\ | CN101058247A | XU WENSHU ZHU (CN) | GUANGDONG DECRO PACKAGE FILMS (CN) | B29C71/00\ B29C71/02\ B32B27/06\ B32B27/18\ B32B27/10\ B32B27/32\ B29C47/06\ B29C69/02\ B29D7/01 |
| 3574 | 3D printing | THREE-DIMENSIONAL COMPOSITE PLASTIC WATER DISCHARGING BODY\ | CN202787227U | XUAN GEN US\ ZHANG JIANHUA US\ HE XUEJUN US\ ZHAO XUEFENG US\ BIAN FEIJING US\ XU HAIYONG US\ YU JINGJING US\ ZHANG PEIGUANG US\ XU QUAN US | ZHEJIANG ZHUAN CONSTRUCTION GROUP CO LTD US\ ZHEJIANG HUAHUI MECHANICAL AND ELECTRICAL EQUIPMENT INSTALLATION CO LTD US\ ZHEJIANG GUANGCHUAN CONSTRUCTION CO LTD US | E02D3/10 |
| 3575 | 3D printing | METHOD FOR ETCHING CHINESE SILK\ | CN100556713C | YALONG ZHENG (CN) | YALONG ZHENG (CN) | B41M3/00\ B44C1/22 |
| 3576 | 3D printing | NATURAL MARBLE-LIKE CRYSTALLIZED GLASS NATURAL MARBLE-LIKE CRYSTALLIZED GLASS ARTICLE AND METHOD FOR MANUFACTURING THE SAME\ | JP2010248056A | YAMADA AKIHITO US | NIPPON ELECTRIC GLASS CO US | C03C10/04\ C03B31/00\ C03B32/02 |
| 3577 | 3D printing | THERMALLY INSULATED CONTAINER MANUFACTURING METHOD THEREFOR AND LAMINATED LABEL ATTACHED TO THE CONTAINER\ | JP2007153359A | YAMADA MINORU US\ YAMAZAKI DAISUKE US\ KOBAYASHI SATOSHI US\ MASHITA ATSUSHI US\ MURAWAKI YASUHIRO US | KISHIMOTO SANGYO CO US | G09F3/02\ B65D81/38\ B65D1/28\ B65D25/20\ B65D25/36 |
| 3578 | 3D printing | METHOD FOR PRODUCING BULKY POLYESTER FIBER\ | JP2005097768A | YAMADA TOSHIHIRO US\ TSUKAMOTO RYOJI US | TEIJIN FIBERS LTD US | D01F6/62 |
| 3579 | 3D printing | METHOD FOR SPINNING POLYESTER MONOFILAMENT HAVING SMALL FINENESS\ | JP2009084712A | YAMAMOTO HIROSHI US\ OGUCHI TOMOHIRO US\ KUROKAWA HIROYUKI US | TORAY INDUSTRIES (JP) | D01D5/088\ D01F6/62 |
| 3580 | 3D printing | RECURSIVE REFLECTION PRINT SHEET OF ELECTRONIC IMAGE AND PRINTING METHOD THEREFOR\ | JP2005049383A | YAMAMOTO KAORU US | KOOWATEKUNOA KK US | D06P5/00\ G09F3/02\ G02B5/128\ G09F13/16 |
| 3581 | 3D printing | NONWOVEN FABRIC FOR THERMOFORMING\ | JPH1037056A | YAMAMOTO NOBUYUKI US\ YAMADA IWAHIRO US | TEIJIN LTD US | D01D5/34\ D01F8/14\ D01F1/10\ D04H3/011\ D04H3/147\ D04H3/153 |
| 3582 | 3D printing | ELASTIC NET-LIKE STRUCTURE HAVING SOFT RESILIENCE\ | JP2006200118A | YAMANAKA MASAKI (JP)\ MATSUI YOSHIHIRO (JP) | TOYO BOSEKI (JP) | A47C27/12\ D04H3/07\ D04H3/007\ B68G7/06 |
| 3583 | 3D printing | ELASTIC NET-LIKE STRUCTURE HAVING EXCELLENT CUSHION CHARACTERISTICS AT LOW TEMPERATURE\ | JP2006200120A | YAMANAKA MASAKI (JP)\ MATSUI YOSHIHIRO (JP) | TOYO BOSEKI (JP) | A47C27/12\ D04H3/07\ D04H3/007 |
| 3584 | 3D printing | LIGHTWEIGHT ELASTIC NET-LIKE STRUCTURE HAVING EXCELLENT CHEMICAL RESISTANCE\ | JP2006200119A | YAMANAKA MASAKI (JP)\ MATSUI YOSHIHIRO (JP) | TOYO BOSEKI (JP) | D04H3/16\ D04H3/007 |
| 3585 | 3D printing | ELASTIC NET-LIKE STRUCTURE HAVING EXCELLENT LIGHT RESISTANCE\ | JP2006200117A | YAMANAKA MASAKI (JP)\ MATSUI YOSHIHIRO (JP) | TOYO BOSEKI (JP) | A47C27/12\ D04H3/07\ D04H3/007\ B68G5/00 |
| 3586 | 3D printing | FILM FOR PRINT LAMINATE\ | JP2003062957A | YAMASHITA TAKASHI US | JAPAN POLYCHEM CORP US | B32B27/32\ C08L23/04\ B29C47/06\ B29K23/00\ B29L7/00 |
| 3587 | 3D printing | ELECTRONIC PICTURE IMAGE-RECEIVING MATERIAL\ | JP2007249126A | YAMAZAKI CHIKAMASA US | KONICA MINOLTA HOLDINGS INC (JP) | G03G7/00 |
| 3588 | 3D printing | ELECTROPHOTOGRAPHIC IMAGE-RECEIVING MATERIAL\[FR] MATéRIAU DE RéCEPTION D'IMAGE éLECTROPHOTOGRAPHIQUE\ | WO2007083467A1 | YAMAZAKI KATSUMASA (JP) | KONICA MINOLTA HOLDINGS INC (JP)\ YAMAZAKI KATSUMASA (JP) | B32B27/10\ B32B5/18\ D21H27/00\ D21H27/30\ B32B7/02\ G03G7/00\ D21H19/70\ D21H21/56 |
| 3589 | 3D printing | ULTRATOUGH CVD SINGLE CRYSTAL DIAMOND AND THREE DIMENSIONAL GROWTH THEREOF\ | US7594968B2 | YAN CHIH-SHIUE (US)\ MAO HO-KWANG (US)\ HEMLEY RUSSELL J (US) | CARNEGIE INST OF WASHINGTON (US) | C30B29/04 |
| 3590 | 3D printing | SIDE BY SIDE COMPOSITE FIBER SPINNERET PLATE METHOD OF PREPARING THREE-DIMENSIONAL CRIMP ANTIBACTERIAL FIBER WITH SPINNERET PLATE AND APPLICATION OF THREE-DIMENSIONAL CRIMP ANTIBACTERIAL FIBER\ | CN103225118A | YAN ZHIYONG US\ HU YING US\ YE HONGFU US\ ZHAO GANGHUA US\ WANG LIGANG US\ SHEN XIAOJUN US\ WANG SHANGDONG US | UNIV JIAXING US | D01D5/22\ D01F8/14\ D01F1/10\ D01D5/253\ D01D5/32\ D01F8/12 |
| 3591 | 3D printing | AN EQUIPMENT FOR PRODUCING GLASS WITH REFINED THREE-DIMENSIONAL PATTERN BY FLOAT PROCESS AND THE METHOD FOR PRODUCING THE GLASS\[FR] MATéRIEL PERMETTANT DE PRODUIRE PAR PROCESSUS FLOAT DU VERRE AVEC UN MOTIF EN TROIS DIMENSIONS RAFFINé ET PROCéDé DE PRODUCTION DE VERRE\ | WO2009124465A1 | YANG DENING (CN)\ YANG XIAOXIAO (CN) | YANG DENING (CN)\ YANG XIAOXIAO (CN) | C03B18/12\ C03B18/14\ C03B5/173 |
| 3592 | 3D printing | COMPRESSION RESISTING AND WATER DIVERSION THREE-DIMENSIONAL WATER FILTERING AND DRAINING DEVICE\ | CN203034439U | YANG JINGLIN US | TAI AN YONGJIA CIVIL ENGINEERING MATERIALS CO LTD US | E02D3/10 |
| 3593 | 3D printing | GRAPHENE/ STANNIC OXIDE NANOMETER COMPOUNDING RESISTANCE TYPE FILM GAS SENSOR AND MANUFACTURING METHOD THEREOF\ | CN102636522A | YANG LI US\ MUJIE YANG US\ QIANQIAN LIN US | UNIV ZHEJIANG US | G01N27/04 |
| 3594 | 3D printing | MANUFACTURING METHOD OF ENVIRONMENT-FRIENDLY CONTAINER WITH 3D COLOR PRINTING PICTURE AND ENVIRONMENT-FRIENDLY CONTAINER\ | CN103332352A | YANG RONGSHI US | DONGGUAN MORNING LIGHT PRINTING COMPANY LTD US | B65D3/02\ B65D3/30\ B65D25/34 |
| 3595 | 3D printing | CALCULUS 3D (THREE DIMENSIONAL) CONSTRUCTING METHOD AND DEVICE\ | CN103465475A | YANG WEIMIN US\ YANG HUAGUANG US\ QIN LIU US\ JIAO ZHIWEI US\ DING YUMEI US | UNIV BEIJING CHEMICAL US | B29C67/00 |
| 3596 | 3D printing | FORMING METHOD FOR 3D (THREE-DIMENSIONAL) SHOE BODY OF SKATING SHOE\ | CN103182766A | YANG XINZHI US | SAKURAI SPORTS MFG CO LTD US | B29C45/14\ B29C45/26\ B29B11/00\ B29C33/12\ B29L31/50 |
| 3597 | 3D printing | PRODUCTION TECHNIQUE OF THREE-DIMENSIONAL CURLY HOLLOW POLYESTER STAPLE FIBERS\ | CN102517675A | YANQIONG WEI US\ JUNJIE YU US\ JIANNONG SHAO US\ YIPING DING US\ HONGBO XU US\ MINGGANG LI US\ ZHICHAO SUN US\ LIJUN WANG US | HANGZHOU BEST CHEMICAL FIBER CO LTD US | D01D5/088\ D01D5/22\ D01D5/24\ D01F6/84\ D01D1/00\ D01D10/02\ D01D5/08\ D01D5/12\ D01D1/04\ D01D5/096\ D01F11/08\ D06M13/46\ D06M15/643 |
| 3598 | 3D printing | HOT MELT ADHESIVES BONDING FIBER\ | US2005170143A1 | YAU PAK C (CN) | BISQUIT DEV LTD (HK) | B32B3/30\ B32B1/00\ B32B5/24\ B32B7/12\ B32B27/02\ D06N7/04 |
| 3599 | 3D printing | SELF ASSEMBLING PROTEINS\ | US6756039B1 | YEATES TODD O (US)\ PADILLA JENNIFER (US)\ COLOVOS CHRIS (US) | UNIV CALIFORNIA (US) | C07K14/11\ C12N9/18 |
| 3600 | 3D printing | ANISOTROPIC CONDUCTIVE MATERIAL\ | KR20100028901A | YEH SYH TAU (TW) | TEAMCHEM COMPANY (TW) | H01B1/22\ H01B1/02 |
| 3601 | 3D printing | ANISOTROPIC CONDUCTIVE MATERIAL\ | US7785494B2 | YEH SYH-TAU (TW) | TEAMCHEM COMPANY (TW) | H01B1/00\ B32B5/16\ H01B1/02\ G03C1/76\ H01B1/12 |
| 3602 | 3D printing | SELECTIVE LASER FUSION RAPID MOLDING METHOD AND DEVICE OF MEDICAL IMPLANTATION MATERIAL WAX MOULD\ | CN101310964B | YONGQIANG YANG (CN)\ SHUFAN WANG (CN)\ WEIHUI WU (CN) | UNIV SOUTH CHINA TECH (CN) | B29C51/00\ B29C73/00\ A61B6/03\ B29K91/00\ B29L22/00 |
| 3603 | 3D printing | METHOD FOR MANUFACTURING BIODEGRADABLE BOARD AND BIODEGRADABLE BOARD MANUFACTURED THEREBY\[FR] PROCéDé DE FABRICATION DE PLANCHE BIODéGRADABLE ET PLANCHE BIODéGRADABLE FABRIQUéE ASSOCIéE\ | WO2013118930A1 | YOON SANG-JUN (KR)\ CHEY WON-JUN (KR)\ KIM TAE-KYUN (KR)\ PARK HYUNG-JOON (KR) | DOWNNARA CO LTD (KR)\ YOON SANG-JUN (KR)\ CHEY WON-JUN (KR)\ KIM TAE-KYUN (KR)\ PARK HYUNG-JOON (KR) | B27N3/00\ C08L67/04\ B27N3/08 |
| 3604 | 3D printing | OPTICAL DEVICE MANUFACTURING METHOD AND OPTICAL DEVICE MANUFACTURING MOLD\ | JP2005212154A | YOSHIDA HIROYUKI US | MATSUSHITA ELECTRIC WORKS LTD (JP) | B29C45/26\ B29L11/00\ B29C45/02 |
| 3605 | 3D printing | METHOD FOR PROVIDING GLOSSY PAPER AND GLOSSY PAPER\ | JP2004308034A | YOSHIDA MASAHIKO US\ NAKAMURA MISAO US\ UCHIDA TAIZO US | OSAKA SEALING LABEL PRINT US | B41M5/26\ B41M5/28\ B05D7/00\ D21H27/00\ B32B29/00\ B41M5/30\ B41M5/337\ B41M5/42\ D21H19/36\ D21H27/30 |
| 3606 | 3D printing | SYSTEM\ | JP2007017922A | YOSHINO KAZUTORA US | YOSHINO KAZUTORA US | G09G1/00 |
| 3607 | 3D printing | MOLDING TECHNIQUE OF ELEVATED PLASTIC FLOOR TILE AND THE STRUCTURE THEREOF\ | TW201026940A | YOU WEN-BANG (TW) | TAD CHIN IND CORP (TW) | E04F15/10 |
| 3608 | 3D printing | VIA USING ZN OR ZN ALLOYS AND ITS MAKING METHOD 3D CHIP STACK PACKAGES USING THEREOF\ | US2010240174A1 | YU JIN (KR)\ JEE YOUNG-KUN (KR) | YU JIN (KR)\ JEE YOUNG-KUN (KR) | H01L21/768\ H01L21/50 |
| 3609 | 3D printing | THREE-DIMENSIONAL FOAM CUTTING MACHINE\ | CN1919554A | YUAN GUOQING YUAN (CN) | YUAN GUOQING (CN) | B26F3/12\ B26D5/20\ B26D7/22\ B26D7/26 |
| 3610 | 3D printing | EXTRUSION DEVICE OF 3D (THREE DIMENSIONAL) PRINTER APPLIED TO FUSE WIRE ACCUMULATION TECHNOLOGY\ | CN203282708U | YUAN ZHENGUO US | PANOWIN TECHNOLOGIES CO LTD US | B29C67/00 |
| 3611 | 3D printing | 3D (THREE DIMENSIONAL) PRINTER EXTRUSION APPARATUS APPLIED IN FUSE STACKING TECHNOLOGY\ | CN103231517A | YUAN ZHENGUO US | WU JIE US | B29C67/00 |
| 3612 | 3D printing | COMPOSITE ELECTRODE FOR FINE ELECTRIC SPARK PROCESSING\ | CN101259548A | YUANGANG WANG (CN)\ FULING ZHAO (CN)\ JIN WANG (CN) | UNIV DALIAN TECH (CN) | B23H1/04 |
| 3613 | 3D printing | PRECISION-INVESTMENT CASTING METHOD\ | CN101406932A | YUANHUA DENG (CN) | QUANZHOU TENGDA FINE CASTING C (CN) | B22C9/04\ B22D15/00\ B22D31/00 |
| 3614 | 3D printing | THREE-DIMENSIONAL (3D) DISPLAY PANEL 3D DISPLAY SYSTEM AND 3D DISPLAY\ | CN202019419U | YUANYUAN LI US | KONKA GROUP CO LTD US | H04N13/00\ G09G3/32 |
| 3615 | 3D printing | THERMOPLASTIC RESIN CASING AND METHOD FOR FABRICATING THE SAME\ | US2008145578A1 | YUBA TAKASHI (JP)\ AKIEDA SHINICHIRO (JP)\ ARITA TAKASHI (JP) | FUJITSU COMPONENT LTD (JP) | B29C45/00\ B32B1/04 |
| 3616 | 3D printing | RAPID PROTOTYPING METHOD AND DEVICE BASED ON SUPERSATURATION SALINE SOLUTION CRYSTALLIZATION\ | CN102649020A | YUEGUANG DENG US\ JING LIU US | CHINESE ACAD TECH INST PHYSICS US | B01D9/00 |
| 3617 | 3D printing | RAPID MOLDING METHOD BASED ON ELECTRORHEOLOGICAL FLUID AND APPARATUS THEREOF\ | CN102615830A | YUEGUANG DENG US\ JING LIU US | CHINESE ACAD TECH INST PHYSICS US | B29C67/00 |
| 3618 | 3D printing | NUMERICALLY-CONTROLLED PLASTIC THIN-LAYER SEDIMENT MOULDING MACHINE\ | CN101693407A | YUHUI LING US | YUHUI LING US | B29C67/00 |
| 3619 | 3D printing | CRISSCROSS ECDP/PET COMPOUND FLAME-RETARDANT FIBER\ | CN201713619U | YULI HOU US\ HAIBIN GUO US\ XINGMEI GUO US\ SHENGCHUN LI US | SHENGCHUN LI US | D01F8/14\ D01D5/253 |
| 3620 | 3D printing | ENVIRONMENT-PROTECTION STEREO AUTOMOBILE FOOT PAD\ | CN201254118Y | YUNBO GUO (CN) | DONGGUAN HONG EN PLASTIC PRODU (CN) | B60N3/06 |
| 3621 | 3D printing | ENVIRONMENT-PROTECTIVE STEREO AUTOMOBILE FOOT PAD AND MANUFACTURING METHOD THEREOF\ | CN101376351A | YUNBO GUO (CN) | DONGGUAN HONGEN RUBBER AND PLA (CN) | B32B3/30\ B32B27/06\ B32B5/18\ B60N3/06\ B29C43/18\ B29C43/28\ B29K23/00\ B29K96/00\ B32B7/08 |
| 3622 | 3D printing | METHOD OF MANUFACTURING 300M ULTRAHIGH STRENGTH STEEL AIRCRAFT LANDING GEAR\ | CN101229586A | YUNGE WANG (CN)\ XIANGMING WANG (CN)\ HUAMING WANG (CN) | YUNGE WANG (CN) | B22F3/105 |
| 3623 | 3D printing | METHOD OF MANUFACTURING AF1410 ULTRAHIGH STRENGTH STEEL AIRPLANE HORIZONTAL TAIL AXIS\ | CN101229585A | YUNGE WANG (CN)\ XIANGMING WANG (CN)\ HUAMING WANG (CN) | YUNGE WANG (CN) | B22F3/105 |
| 3624 | 3D printing | METHOD FOR MANUFACTURING THREE-DIMENSIONAL ARTISTIC GLAZED BRICK\ | CN102040399A | YUNSHU ZHANG US | YUNSHU ZHANG US | C04B41/89 |
| 3625 | 3D printing | HOLLOW BODY MOLDING DEVICE\ | JP2007296700A | YUSHIMO MASASHI US\ TANIGAKI KENJI US\ NISHIDA SHOZO US\ KAMIMURA TAKASHI US | JAPAN STEEL WORKS LTD US\ AISIN SEIKI (JP) | B29C45/17\ B29C69/02\ B29L22/00\ B29C65/14 |
| 3626 | 3D printing | SINGLE-SPINNERET-PLATE BINARY SKIN-CORE-TYPE COMPOSITE-SPINNING MULTIPLE-DIFFERENT-YARN PRODUCTION PROCESS\ | CN103060979A | ZHAN HAIHUA US\ SHEN YUNGEN US | UNIV SHAOXING US\ SHAOXING YUNXIANG CHEMICAL FIBER CO LTD US | D01D5/34\ D01D10/02\ D01D1/04\ D02G3/04\ D01D1/06\ D01D7/00\ D02G1/02 |
| 3627 | 3D printing | CONTINUOUS SCREWED MULTI-CELLULAR PLASTIC SHAPING STRUCTURE\ | CN2714295Y | ZHANG BINGRONG (CN) | ZHANG BINGRONG (CN) | B29C53/58\ B29C53/74 |
| 3628 | 3D printing | METHOD FOR MANUFACTURING METAL PARTS AND MOLDS AND MICRO-ROLLER USED THEREFOR\ | US2013197683A1 | ZHANG HAIOU (CN)\ WANG GUILAN (CN) | UNIV HUAZHONG SCIENCE TECH (CN) | B23K26/34\ B23K9/04\ B23K10/00 |
| 3629 | 3D printing | METHOD AND EQUIPMENT FOR DIRECTLY AND QUICKLY MANUFACTURING MOULD AND PARTS\ | CN1298780A | ZHANG HAIOU (CN)\ WANG GUILAN (CN) | UNIV HUAZHONG SCIENCE TECH (CN) | B23K28/02\ B23P23/04 |
| 3630 | 3D printing | CERAMIC/METALLIC DOUBLE CONTINUOUS PHASE COMPOSITE MATERIAL BRAKE PAD AND PREPARATION METHOD THEREOF\ | CN102102720B | ZHANG JINSONG US\ CAO XIAOMING US\ ZHANG YUNAN US\ TIAN CHONG US\ YANG ZHENMING US | INST METAL RES CHINESE ACAD SC (CN) | F16D69/02 |
| 3631 | 3D printing | METHOD FOR ACHIEVING HIGH-MELTING-POINT MATERIAL 3D PRINTING THROUGH NANOMETER INK TOGETHER WITH LASER MELTING\ | CN103407296A | ZHANG SHANGFENG US\ ZHANG YANGMING US\ ZHANG SHIZHU US\ SHI XINCHAO US\ LIU CHUANG US\ LIU YUBO US\ LIU YAN US\ ZHANG LIANBIN US | NANJING DINGKE NANOTECHNOLOGY INST CO LTD US | B41J5/00 |
| 3632 | 3D printing | PREPARATION METHOD OF NANO MODIFIED ULTRA-HIGH MOLECULAR WEIGHT POLYETHYLENE (UHMWPE) ANTISTATIC COMPOSITE MATERIAL\ | CN102888039A | ZHANG WEI US\ LI ZHI US\ HONG WEI US\ XU ZHENHUA US\ ZHAO CHUNBAO US\ LI JIANLONG US | SHANGHAI RES INST CHEMICAL IND US | C08K3/08\ C08K3/04\ B29C47/92\ C08L23/06\ C08K7/06\ B29B9/06\ C08K9/10 |
| 3633 | 3D printing | WEAR-RESISTANT CROSSLINKED PE (POLYETHYLENE) COMPOSITE PIPE AND MANUFACTURING METHOD THEREOF\ | CN102417650B | ZHANG WEIJIAO US\ GAO LI US\ JIANG JIANPING US | ZHEJIANG WEIXING NEW BUILDING MATERIALS CO LTD US | C08J3/24\ C08K5/098\ C08K13/04\ C08L27/18\ C08K3/04\ C08L23/08\ C08L23/16\ C08L23/06\ C08K3/16\ C08K7/06\ F16L9/12 |
| 3634 | 3D printing | LOW TEMPERATURE THERMOPLASTIC MATERIAL AND PREPARING METHOD THEREOF\ | US8859691B2 | ZHANG ZHONGQUN (CN) | KLARITY MEDICAL & EQUIPMENT GZ CO LTD (CN)\ ZHANG ZHONGQUN (CN) | B29C35/08\ C08L75/04\ C08F283/00\ C08L67/04 |
| 3635 | 3D printing | CLOTHING PROTECTION STRUCTURE AND PRODUCTION METHOD THEREOF\ | CN101647605A | ZHAO JUN US | HANGZHOU FUXING INDUSTRY & TRA US | A41D13/00 |
| 3636 | 3D printing | WATER SOLUBLE FLEXIBLE PRINT PLATE AND ITS PRODUCING METHOD\ | CN1900822A | ZHAO XIAOJIE (CN) | ZHONGYUAN PRINTING SCIENCE AND (CN) | G03F7/09\ B41N1/24 |
| 3637 | 3D printing | DIGITAL PRE-LAMINATING FILM\ | CN101705060A | ZHAOZHAO ZHANG US | HU MINSHENG US | C09J123/08\ C09J7/02\ C09J157/02 |
| 3638 | 3D printing | ADSORPTION ROLLING BEARING\ | CN201827235U | ZHEN HUANG US | ZHEN HUANG US | F16C33/32\ F16C33/58\ F16C33/66\ F16C19/00\ F16C19/06\ F16C19/26\ F16C33/34 |
| 3639 | 3D printing | ADSORPTION ROLLING BEARING\ | CN102213262A | ZHEN HUANG US | ZHEN HUANG US | F16C33/32\ F16C33/58\ F16C33/66 |
| 3640 | 3D printing | BRA CUP WITH GOOD SHAPE KEEPING FUNCTION\ | CN2829399Y | ZHENG BIHAO (CN) | ANLIFANG CHINA GARMENT CO LTD (CN) | A41C3/12\ A41C3/00 |
| 3641 | 3D printing | THREE-DIMENSIONAL HOLLOW POLYESTER STAPLE FIBER\ | CN202849604U | ZHENG QUAN US\ YAN DONGNING US\ CUI HUI US | JIANGSU DESAI CHEMICAL FIBER CO LTD US | D01D5/24\ D01D5/253 |
| 3642 | 3D printing | FAST RESPONSE ELECTROCHEMICAL ORGANOPHOSPHATE SENSOR\ | US2010294662A1 | ZHENG ZHI (CN)\ ZHAO LINAN (CN)\ WANG MARILYN (CN) | HONEYWELL INT INC (US) | H01B13/00\ C25B11/04\ B05D5/12 |
| 3643 | 3D printing | WATER CHANNEL PRE-FILLING METHOD USED DURING LOST FOAM CASTING OF ENGINE CYLINDER BODY\ | CN102343417A | ZHENGSONG MA US\ YUSHENG ZHANG US\ QIAO XU US | CHUZHOU JINNUO IND CO LTD US | B22C9/04 |
| 3644 | 3D printing | NEW PROCESS FOR SPRAY-COATING POWDER ON SURFACE OF METAL\ | CN101992183A | ZHENJIANG SUN US | TIANJIN CITY YIJIANG ALUMINUM MATERIAL CO LTD US | B05D3/02\ B05D7/24\ B05D7/14\ B05D5/00\ B05D3/00 |
| 3645 | 3D printing | EMBEDDED TYPE MOULD\ | CN202292992U | ZHIYU HU US\ CHUANG JIANG US\ WENYONG CHENG US | SHANGHAI TONGCHUANG CERAMICS CO LTD US | B28B7/00 |
| 3646 | 3D printing | SILICON WITH THREE-DIMENSIONAL DEPRESSION STRUCTURE AND METHOD FOR PREPARING SAME\ | CN101311350A | ZHONGHE XI (CN)\ YUE WU (CN)\ GENGMIN ZHANG (CN)\ HONGYU CUI (CN)\ DENGZHU GUO (CN) | UNIV BEIJING (CN) | C30B29/60\ C30B29/06 |
| 3647 | 3D printing | SILICON WITH THREE-DIMENSIONAL DEPRESSION STRUCTURE AND METHOD FOR PREPARING SAME\ | CN101311347A | ZHONGHE XI (CN)\ YUE WU (CN)\ GENGMIN ZHANG (CN)\ HONGYU CUI (CN)\ DENGZHU GUO (CN) | UNIV BEIJING (CN) | C30B29/60\ C30B29/06 |
| 3648 | 3D printing | METAL MASTER MOLD FOR ELECTROFORMING AND USE THEREOF\ | CN101403125A | ZHONGYAO YU (CN)\ GUANNAN LI (CN)\ JING CHENG (CN) | BOAO BIOTECH CO LTD (CN) | C25D1/10 |
| 3649 | 3D printing | STEREOSCOPIC ANTI-FAKE PACKING BOX WITH HIGH BAR CODE LEVEL\ | CN203064295U | ZHOU LIQUAN US\ ZOU YAOBANG US\ JIANG ZONGZHENG US\ YANG PEIXIANG US\ LIU KUNHONG US\ LI HONGYE US\ WANG MINGJUN US\ LIU GUANGTIAN US | SICHUAN YIBIN PUSH GROUP 3D CO LTD US | G09F3/02\ B65D5/00 |
| 3650 | 3D printing | THREE-DIMENSIONAL PRINTING PEN\ | CN103350507A | ZHOU SHUANG US\ WU JIE US | WU JIE US\ ZHOU SHUANG US | B29C67/00 |
| 3651 | 3D printing | THREE-DIMENSIONAL INTEGRATED HETEROGENEOUS SEMICONDUCTOR STRUCTURE\ | US7741645B2 | ZHU HUILONG (US) | IBM (US) | H01L29/76\ H01L31/036\ H01L31/112 |
| 3652 | 3D printing | HOLLOW FIBROUS MEMBRANE AND PREPARATION METHOD THEREOF\ | CN102527250B | ZHU WENBING US\ ZHANG TING US | HEBEI NORN WATER PURIFICATION EQUIPMENT CO LTD US | B01D69/08\ B01D67/00\ B01D71/34 |
| 3653 | 3D printing | ION FUSION FORMATION\ | US6680456B2 | ADAMS ROBBIE (US) | HONEYWELL INT INC (US) | B23K10/02 |
| 3654 | 3D printing | ION FUSION FORMATION PROCESS FOR LARGE SCALE THREE-DIMENSIONAL FABRICATION\ | US2009026175A1 | ADAMS ROBBIE J (US) | HONEYWELL INT INC (US) | B23K9/04\ B23K10/00\ B05D1/08 |
| 3655 | 3D printing | METHOD FOR PRODUCING PTT (POLYTRIMETHYLENE TEREPHTHALATE) SPIRAL THREE-DIMENSIONAL HOLLOW FIBERS\ | CN102618947A | AIGUO MIAO US\ YUNCHANG LIN US\ AIZHEN LIN US\ YUZHONG HUO US\ AIMIN YANG US\ HUANMING XU US | AIGUO MIAO US | D01D5/24\ D02G1/00\ D01D1/04\ D01D5/096\ D01D5/092\ D02J1/00\ D02J13/00 |
| 3656 | 3D printing | METHOD FOR MODIFYING SURFACE OF PLASTIC MOLDED BODY METHOD FOR FORMING METAL FILM INCLUDING THE SAME AND PLASTIC COMPONENT THEREOF\ | JP2009113266A | ANO TETSUYA US\ YUSA ATSUSHI US\ NOMURA YOSHIYUKI US | HITACHI MAXELL (JP) | B29C45/00\ B29L31/34\ C23C18/22 |
| 3657 | 3D printing | METHOD OF MAKING A THERMOPLASTIC FILM WITH A THREE-DIMENSIONALLY STRUCTURED SURFACE\ | US2011189437A1 | BADER HERBERT (DE)\ HAGEMANN ANDREAS (DE)\ MARCEL GRUENDKEN (DE) | MARCEL GRUENDKEN (DE)\ BADER HERBERT (DE)\ HAGEMANN ANDREAS (DE) | B32B3/00\ B29C55/18 |
| 3658 | 3D printing | ENVIRONMENT-FRIENDLY MULTILEVEL PLASTIC ENVELOPE AND THAT PRODUCTION METHODS\ | KR100831377B1 | BAK YEONG GI (KR)\ KANG JOO HO (KR)\ KIM SUN WOOK (KR) | HAESUNG INDUSTRY CO LTD (KR)\ BAK YEONG GI (KR)\ KANG JOO HO (KR)\ KIM SUN WOOK (KR) | B65D30/14\ B65D30/10 |
| 3659 | 3D printing | METHOD OF INK-JET PRINTING\ | US2008284836A1 | BAKER JULIE (GB)\ HIGGINS JOHN M (GB) | HIGGINS JOHN M (GB)\ BAKER JULIE (GB) | B41J3/407\ B41M5/52\ B41M7/00\ B41J11/00 |
| 3660 | 3D printing | INK JET TRANSFER SYSTEMS PROCESS FOR PRODUCING THE SAME AND THEIR USE IN A PRINTING PROCESS\ | US6638604B1 | BAMBERG ULF (DE)\ KUMMER PETER (CH)\ STIBUREK ILONA (CH) | ARKWRIGHT INC (US) | D06P5/20\ B41J2/01\ B41M5/00\ D06P5/00\ B41M5/50\ B41M5/52\ D06P5/24 |
| 3661 | 3D printing | METHOD ENABLING A STANDARD CMOS FAB TO PRODUCE AN IC TO SENSE THREE-DIMENSIONAL INFORMATION USING AUGMENTED RULES CREATING MASK PATTERNS NOT OTHERWISE EXPRESSIBLE WITH EXISTING FAB RULES\ | US7464351B2 | BAMJI CYRUS (US)\ LIU XINQIAO (US) | CANESTA INC (US) | G06F17/50\ A61B6/00\ G01B11/26\ A61N5/00\ G01C3/00\ G01N23/00\ G01S7/491\ G01S7/493\ G01S17/08\ G01S17/36\ G01S17/89\ G21G5/00\ G21K1/12\ H01L\ H01L27/146\ H01L31/02\ H04N3/14\ H05G1/60 |
| 3662 | 3D printing | LIQUID STATE MICRO-FORMING METHOD FOR THREE-DIMENSIONAL COMPLEX MICRO-COMPONENT\ | CN100515609C | BANGSHENG LI (CN)\ MINGXING REN (CN)\ CHUANG YANG (CN) | HARBIN INST OF TECHNOLOGY (CN) | B22D17/08 |
| 3663 | 3D printing | DRAINAGE GEOTECHNICAL GRILLAGE\ | CN2752326Y | BAO SHAOZHENG (CN) | BAO SHAOZHENG (CN) | B29C69/00\ E02D19/20 |
| 3664 | 3D printing | DIRECT SELECTIVE LASER SINTERING OF METALS\ | US6676892B2 | BEAMAN JOSEPH J (US)\ DAS SUMAN (US) | REGENTS UNIVERSITY TEXAS SYSTE (US) | B22F3/105\ B23K26/34\ C23C26/02\ G05B19/4062 |
| 3665 | 3D printing | PLATTE MIT DREIDIMENSIONAL STRUKTURIERTER OBERFL?CHE AUF DER BASIS VON HOLZWERKSTOFFEN UND VERFAHREN ZUR HERSTELLUNG EINER SOLCHEN PLATTE\[EN] PLATE USEFUL FOR PRODUCING DOORS AND FURNITURE PIECES COMPRISES A THREE-DIMENSIONAL TEXTURED SURFACE PRODUCED VIA COMPRESSION CARRIER PLATE MADE UP OF WOODEN MATERIAL AND A DECORATIVE LAYER COATED ON CARRIER PLATE\ | DE102010005350A1 | BECKERS WILHELM DIPL ING (DE) | WESTAG & GETALIT AG (DE) | B27N7/00\ B32B21/02\ B32B27/10\ B32B33/00\ B32B27/04\ B32B21/06 |
| 3666 | 3D printing | APPARATUS FOR THREE DIMENSIONAL PRINTING USING IMAGED LAYERS\ | US8119053B1 | BEDAL BRYAN (US)\ SCHELL STEVEN E (US)\ BEERS ROSS D (US) | 3D SYSTEMS INC (US)\ BEDAL BRYAN (US)\ SCHELL STEVEN E (US)\ BEERS ROSS D (US) | B29C35/08\ B28B1/16 |
| 3667 | 3D printing | APPARATUS FOR THREE DIMENSIONAL PRINTING USING IMAGED LAYERS\ | US2010244333A1 | BEDAL BRYAN (US)\ SCHELL STEVEN E (US)\ BEERS ROSS D (US) | BEDAL BRYAN (US)\ SCHELL STEVEN E (US)\ BEERS ROSS D (US) | B29C35/08\ B29C67/00\ B29C47/92 |
| 3668 | 3D printing | MANUFACTURING BONE PROSTHESIS FOR FILLING GAPS OF ALL/PART OF BONE OF PATIENT COMPRISES DIGITALIZING PATIENT USING THREE-DIMENSIONAL MEDICAL ACQUISITION DEVICE BY MAGNETIC RESONANCE DEFINING PROSTHESIS AND MANUFACTURING THE PROSTHESIS\[FR] PROCEDE DE FABRICATION D'UNE PROTHESE PAR PROTOTYPAGE RAPIDE\ | FR2979817A1 | BERTIN JEAN-MICHEL (FR) | BERTIN JEAN-MICHEL (FR) | B29C67/00\ A61F2/28\ B22F3/105 |
| 3669 | 3D printing | METHOD AND APPARATUS FOR PRODUCING THREE-DIMENSIONAL OBJECTS\ | US2014348692A1 | BESSAC CHRISTOPHE (FR)\ PIALOT FRéDéRIC (FR)\ WALRAND GILLES (FR) | MICHELIN RECH TECH (CH)\ MICHELIN & CIE (FR) | B29C67/00\ B22F3/105 |
| 3670 | 3D printing | METHOD AND APPARATUS FOR PRODUCING THREE-DIMENSIONAL OBJECTS WITH IMPROVED PROPERTIES\ | US2015064048A1 | BESSAC CHRISTOPHE (FR)\ WALRAND GILLES (FR)\ BROCHET STEPHANIE (FR)\ PIALOT FREDERIC (FR) | MICHELIN RECH TECH (CH)\ MICHELIN & CIE (FR) | B22F3/105\ B22F3/24\ B22F5/00\ C21D1/00 |
| 3671 | 3D printing | METHOD AND APPARATUS FOR PRODUCING THREE-DIMENSIONAL OBJECTS\[FR] PROCEDE ET APPAREIL POUR REALISER DES OBJETS TRIDIMENSIONNELS\ | WO2013092994A1 | BESSAC CHRISTOPHE (FR)\ WALRAND GILLES (FR)\ BROCHET STEPHANIE (FR)\ PIALOT FREDERIC (FR) | MICHELIN RECH TECH (CH)\ MICHELIN & CIE (FR) | B29C67/00\ B22F3/00\ B22F3/105 |
| 3672 | 3D printing | BLAST FURNACE BURDEN FACE IMAGING SYSTEM BASED ON MULTISOURCE HETEROGENEOUS DATA FUSION\ | CN102409123A | BIN CHU US\ XIANZHONG CHEN US\ HONGJIN DU US\ YANG XIAO US | BAOSHAN IRON & STEEL US\ UNIV BEIJING SCIENCE & TECH (CN) | C21B7/24 |
| 3673 | 3D printing | PROCESS AND APPARATUS FOR PRODUCING PRECISION CASTINGS\ | US6725902B2 | BLUM MATTHIAS (DE)\ JARCZYK GEORG (DE)\ FELLMANN HANS-GUENTHER (DE)\ BUSSE PETER (DE) | ALD VACUUM TECHN AG (DE) | B22C9/04\ B22D13/00\ B22D13/06\ B22D13/10\ B22C9/02\ B22D21/00\ B22D21/06 |
| 3674 | 3D printing | ADSORPTIVE FILTER MEDIUM\ | US2015053080A1 | BOEHRINGER BERTRAM (DE)\ GONZALEZ OMAR GUERRA (DE)\ ECKLE ISEBILL (DE) | BLüCHER GMBH (DE) | B01D53/04 |
| 3675 | 3D printing | W?SSRIGE MAGENTAFARBENE FARBSTOFFE IHRE HERSTELLUNG UND VERWENDUNG\[EN] NEW HETEROCYCLIC DYE COMPOUNDS USEFUL E.G. FOR COLORING AND PRINTING NATURAL AND SYNTHETIC FIBER MATERIALS FOR COLORING PAPER OR CELLULOSES IN THE MASS AND IN THE MANUFACTURE OF MAGENTA COLOR RECORDING FLUIDS\ | DE102005033584A1 | BORCHERT TILL (DE)\ KLEINERT MARKUS (DE)\ SCHNEIDER STEFFEN (DE)\ GEISENBERGER JOSEF (DE) | CLARIANT PRODUKTE DEUTSCHLAND (DE) | C09D11/02\ C09B56/20\ C09B67/36\ D06P1/41 |
| 3676 | 3D printing | 3-D CONSTRUCTION MODULES\ | US7124547B2 | BRAVINSKI LEONID G (CA) | BRAVINSKI LEONID G (CA) | E04B1/16\ E04B2/86\ E02D27/02\ E04B1/61\ E04B2/56\ E04C5/16\ E04B2/00 |
| 3677 | 3D printing | PRINTING PROCESS\ | US6902268B1 | BRENTNALL ROGER LESLIE (GB)\ KING JEFFREY RONALD (GB)\ LEGGETT SIMON RICHARD JOHN (CH)\ TAYLOR KAREN (GB)\ SCHUETTEL STEFAN (CH) | ILFORD IMAGING UK LTD (CH) | B41M5/00\ B41M5/52\ B41M7/00 |
| 3678 | 3D printing | KRAFTSCHLüSSIG MITEINANDER VERBINDBARE BAUTEILE\ | DE102008017029B4 | BRODA MAIK (DE)\ VERPOORT CLEMENS (DE)\ MUESCHEN ANDREAS (DE) | FORD GLOBAL TECH LLC (US) | C23C4/12\ C23C4/06\ C23C4/10\ F16H55/36 |
| 3679 | 3D printing | FLAMEPROOFED IMPACT-STRENGTH-MODIFIED POLY(ESTER)CARBONATE COMPOSITIONS\ | US7833448B2 | BUCHHOLZ VERA (DE)\ WENZ ECKHARD (DE)\ ECKEL THOMAS (DE)\ THUERMER BURKHARD (DE)\ WITTMANN DIETER (DE) | BAYER MATERIALSCIENCE AG (DE) | B28B3/20 |
| 3680 | 3D printing | BLASKOPF FüR EINE BLASFOLIENEXTRUSIONSANLAGE\ | DE102007050694B4 | BUETTEL ULRICH (DE)\ SCHNEIDER JUERGEN DIPL ING (DE) | BUETTEL ULRICH (DE) | B29C47/92\ B29D7/01\ B29C47/20 |
| 3681 | 3D printing | OFFSET PRINTING METHOD FOR INK JET PRINTER AND INK JET PRINTER\ | JP2005096468A | BURR RONALD F US\ SEGERSTOM ERIC C US\ TITTERINGTON DONALD R US | FUJI XEROX CO LTD US | C09D11/00\ B41J2/01\ B41M5/00 |
| 3682 | 3D printing | VERFAHREN ZUM HERSTELLEN EINER DREHSTABFEDER ODER EINES WANKSTABILISATOR\[EN] MANUFACTURING TORSION BAR SPRING OR ROLLING STABILIZER FOR MOTOR VEHICLE WITH TUBULAR CROSS-SECTION COMPRISES PRODUCING COMPONENT BY BRAIDING AND/OR WRAPPING CORE WITH FIBER BUNDLES AND THERMAL MELTING OF MATRIX MATERIAL\ | DE102011011577A1 | BUSCHBECK FRANK (DE)\ NAGEL GUENTER DIPL ING (DE)\ REESE ECKHARD DIPL ING (DE)\ RUPPRECHT JOCHEN DIPL ING (DE)\ STEINERT HANS-RUDOLF DIPL ING (DE) | DAIMLER AG (DE) | B29C70/50\ B29C33/76\ B29C53/02\ B29C70/00\ B60G21/055\ C08J5/04\ F16F1/14 |
| 3683 | 3D printing | SPECIAL MOULD FOR MAKING CLOTHES PATTERN AND METHOD FOR MAKING PATTERN\ | CN1857919A | CAI JINGXIANG (CN) | CAI JINGXIANG (CN) | B44C1/16\ B41F16/02 |
| 3684 | 3D printing | DEVICE FOR FUSING TONER ON PRINT MEDIUM\ | US2005286939A1 | CHAE YOUNG-MIN (KR)\ HAN SANG-YONG (KR)\ KWON JOONG-GI (KR)\ KIM HWAN-GUEM (KR)\ CHO DURK-HYUN (KR) | SAMSUNG ELECTRONICS CO LTD (KR) | G03G15/20\ H05B6/14 |
| 3685 | 3D printing | PREPARATION OF MICROPOROUS FILMS FROM IMMISCIBLE BLENDS VIA MELT PROCESSING AND STRETCHING\ | US6824680B2 | CHANDAVASU CHAIYA (TH)\ XANTHOS MARINO (US)\ SIRKAR KAMALESH K (US)\ GOGOS COSTAS (US) | NEW JERSEY TECH INST (US) | B01D69/14\ B01D39/16\ B29C47/00\ B01D67/00\ B01D71/26\ B29C55/00 |
| 3686 | 3D printing | MANUFACTURING METHOD AND DEVICE OF INORGANIC PLATES AND INORGANIC PLATES\ | CN102190424A | CHANGLIN WANG US\ JIANBIN QI US\ SENBEN GENG US\ CHANGXING WANG US | JINGNIU GLASS CERAMICS GROUP US | B28B1/00\ C03B13/00\ C03B15/02\ C03B17/06\ C03B25/00\ C04B35/14 |
| 3687 | 3D printing | PHOTON-EMISSION SCANNING TUNNELING MICROSCOPY\ | US8006315B2 | CHARRA FABRICE (FR)\ SILLY MATTHIEU (FR)\ SOUKIASSIAN PATRICK (FR) | COMMISSARIAT ENERGIE ATOMIQUE (FR)\ UNIV PARIS SUD PARIS II (FR) | G01Q10/00\ G01Q30/00\ G01Q60/10\ G01Q60/12\ G01N13/00\ G12B1/00 |
| 3688 | 3D printing | OUTIL DE SERRAGE D'UN ORGANE DE FIXATION\ | FR2970200B1 | CHATENET LUC HENRI (FR)\ BABOEUF SEBASTIEN PIERRE LOUIS (FR)\ LE QUELLEC JOHN (FR) | SNECMA (FR) | B25B23/15 |
| 3689 | 3D printing | TOUCH SCREEN FUNCTIONAL FILM\ | CN202952611U | CHEN JIANHUI US | ZHEJIANG ZHAOYI TECHNOLOGY CO LTD US | G06F3/041\ B32B27/06\ B32B27/36 |
| 3690 | 3D printing | HIGH-PRECISION THREE-DIMENSIONAL MICRO-ASSEMBLING METHOD AND ASSEMBLY PARTS BASED ON MEMS\ | CN1803577A | CHEN JING YANG (CN) | UNIV BEIJING (CN) | B81C3/00\ G01C19/00\ G01P15/00 |
| 3691 | 3D printing | FUSIBLE INK-JET RECORDING MATERIALS CONTAINING HOLLOW BEADS AND ULTRAFINE POLYMER PARTICLES\ | US2006045999A1 | CHEN TIENTEH (US)\ SEN RADHA (US) | CHEN TIENTEH (US)\ SEN RADHA (US) | B41M5/00 |
| 3692 | 3D printing | FUSIBLE INKJET RECORDING MATERIALS CONTAINING HOLLOW BEADS SYSTEM USING THE RECORDING MATERIALS AND METHODS OF USING THE RECORDING MATERIALS\ | US2005287311A1 | CHEN TIENTEH (US)\ SEN RADHA (US)\ WEBB STEVEN L (US)\ KELLY-ROWLEY ANNE M (US)\ PAGE LORETTA ANN G (US) | CHEN TIENTEH (US)\ SEN RADHA (US)\ WEBB STEVEN L (US)\ KELLY-ROWLEY ANNE M (US)\ PAGE LORETTA ANN G (US) | B41M5/00\ B41M5/50\ B41M5/52\ B41M7/00\ B41J11/00 |
| 3693 | 3D printing | FUSIBLE INKJET RECORDING MATERIALS CONTAINING HOLLOW BEADS SYSTEM USING THE RECORDING MATERIALS AND METHODS OF USING THE RECORDING MATERIALS\ | US7651216B2 | CHEN TIENTEH (US)\ SEN RADHA (US)\ WEBB STEVEN L (US)\ KELLY-ROWLEY ANNE M (US)\ PAGE LORETTA ANN GREZZO (US) | HEWLETT PACKARD DEVELOPMENT CO (US) | B41J2/01\ B41M5/52\ B41M7/00 |
| 3694 | 3D printing | MAGNETORHEOLOGICAL-MATERIAL-BASED 3D (THREE-DIMENSIONAL) PRINTING TYPE RAPID PROTOTYPING DEVICE AND METHOD\ | CN103213281A | CHEN XI US\ HE GUOTIAN US\ GU MINGXIN US\ WANG ZHONGXUN US\ ZHU XIAOQIANG US\ LIN YUANCHANG US\ XU ZEYU US\ ZHAO JIAN US\ LIU YONGFU US | CHONGQING INST OF GREEN AND INTELLIGENT TECHNOLOGY US | B29C67/00 |
| 3695 | 3D printing | 3D (THREE-DIMENSIONAL) PRINTING RAPID FORMING DEVICE BASED ON MAGNETORHEOLOGICAL MATERIAL\ | CN203344319U | CHEN XI US\ HE GUOTIAN US\ GU MINGXIN US\ WANG ZHONGXUN US\ ZHU XIAOQIANG US\ LIN YUANCHANG US\ XU ZEYU US\ ZHAO JIAN US\ LIU YONGFU US | CHONGQING INST OF GREEN AND INTELLIGENT TECHNOLOGY CHINESE ACADEMY OF SCIENCES US | B29C67/00 |
| 3696 | 3D printing | PLASTIC SOLE WITH THREE-DIMENSIONAL PATTERNS\ | CN202950080U | CHEN XIAOLIN US | JIEYANG JIAYI INDUSTRY AND TRADE CO LTD US | A43B13/12 |
| 3697 | 3D printing | MOLDING METHOD AND APPARATUS OF THREE-DIMENSIONAL CAVITY MODEL FOR TRUE THREE-DIMENSIONAL MODEL TEST\ | CN102508316B | CHEN XUGUANG US\ WANG YUAN US\ LI SHUCAI US\ NI XIAODONG US\ ZHANG QIANGYONG US | UNIV HOHAI US | G01V9/00 |
| 3698 | 3D printing | NYLON FILM HAVING IMPROVED PRINTING ADHESIVE STRENGTH\ | KR20110085651A | CHO HYUN (KR)\ SONG GI SANG (KR)\ KIM SI MIN (KR) | KOLON INC (KR) | C08J5/18\ B29D7/01 |
| 3699 | 3D printing | DISPLAY PANEL FOR WASHING MACHINE\ | KR20040006269A | CHOI DONG HA US\ PARK HYE YONG US | LG ELECTRONICS INC (KR) | D06F39/00 |
| 3700 | 3D printing | FILM ENVELOP HAND STRAP MADE OF POLYPROPYLENE AND MANUFACTURING METHOD THEREOF\ | KR20110059496A | CHOI OK BOM (KR)\ HA JAE YOUNG (KR) | CHOI OK BOM (KR)\ HA JAE YOUNG (KR) | B29D7/00\ B29C47/02\ B65D33/06 |
| 3701 | 3D printing | METHOD FOR MANUFACTURING AROMATIC POROUS SINTERED METAL COMPACT\ | KR20040013342A | CHOI SUNG JO US | CHOI SUNG JO US | B22F3/11 |
| 3702 | 3D printing | METHOD FOR COATING THE SURFACE OF A METALLIC MATERIAL DEVICE FOR CARRYING OUT SAID METHOD\ | US2006096674A1 | CHOQUET PATRICK (FR)\ CHALEIX DANIEL (FR) | CHOQUET PATRICK (FR)\ CHALEIX DANIEL (FR) | C23C28/00\ C23C14/14\ C23C2/26\ C23C28/02\ C21D1/09 |
| 3703 | 3D printing | HOT MELT CALENDERED OR EXTRUDED WEAR LAYER FOR EMBOSSED SUBSTRATES AND METHOD OF MANUFACTURE\ | US2004048044A1 | CHRISTIE PETER A (US)\ MCQUATE WILLIAM M (US)\ SCHNEIDER DONALD E (US) | ARMSTRONG WORLD IND INC (US) | B32B3/00\ B32B38/06\ B29C43/24\ B32B37/15\ D06N7/00 |
| 3704 | 3D printing | PRINT FUSER AND PROCESS WITH MULTIPLE CLEANING BLADES\ | US7184696B2 | COKELEY JEFFREY H (US) | EASTMAN KODAK CO (US) | G03G15/20 |
| 3705 | 3D printing | ADDED LAYER MANUFACTURE\ | GB2491472A | COLEGROVE PAUL (GB)\ WILLIAMS STEWART (GB) | UNIV CRANFIELD (GB) | B23K5/18 |
| 3706 | 3D printing | NOVEL DESK CALENDAR\ | CN201998597U | CUIMING WANG US | CUIMING WANG US | B42D5/04 |
| 3707 | 3D printing | THREE-LAYERED LIGHTWEIGHT METALLIC MIRROR AND METHOD FOR MANUFACTURING THE SAME\ | RU1840410C | DANCHENKO JURIJ VALENTINOVICH (RU)\ ANTSIFEROV VLADIMIR NIKITOVICH (RU)\ VOLEGOV ANDREJ NIKOLAEVICH (RU)\ KOVALEV JURIJ GENNAD EVICH (RU)\ FILIPPOV ANDREJ IGOREVICH (RU)\ EGOROV VALENTIN NIKITICH (RU) | YEOM JANG YUL (KR) | G02B5/08 |
| 3708 | 3D printing | PLANE STRUCTURE COMPOSED OF A COHESIVE ASSEMBLY OF CONTIGUOUS CELLULAR CELLS\ | US2008193711A1 | DEHENNAU CLAUDE (BE)\ GRANDJEAN DOMINIQUE (BE)\ LENG PHILIPPE-JACQUES (BE) | SOLVAY (BE) | B29D99/00\ B29C39/02\ B32B3/12 |
| 3709 | 3D printing | LASER-INDUCTION HYBRID MELTING DIRECT FORMING METHOD AND DEVICE\ | CN102179517A | DENGZHI WANG US\ MINGMING MA US\ XIAOYAN ZENG (CN)\ ZEMIN WANG (CN)\ QIANWU HU (CN) | UNIV HUAZHONG SCIENCE TECH (CN) | B22F3/105 |
| 3710 | 3D printing | METHOD AND ITS SYSTEM FOR LEADING LARGE MOLECULAR MATTER INTO TARGET CELL\ | CN100435851C | DEYANG TIAN (CN) | DEYANG TIAN (CN) | A61B8/00\ A61B6/00\ A61K48/00\ A61K49/06\ A61M5/00\ C12N15/87\ C12N15/89 |
| 3711 | 3D printing | THREE-DIMENSIONAL RAPID PROTOTYPING METHOD AND DEVICE BASED ON PULSE SMALL HOLE DROPLET INJECTION\ | CN103056367A | DONG WEI US\ TAN YI US\ LU DONG US | UNIV DALIAN TECH (CN) | B22F3/115 |
| 3712 | 3D printing | METHOD FOR PRODUCING MICROCRYSTAL GLASS CERAMICS COMPOSITE BOARD\ | CN102775074A | DONGSHENG XIE US\ GUIPING DU US\ WEIHUA LV US\ WENFENG DENG US | GUANGDONG GOLD MEDAL CERAMICS CO LTD US | C03C17/02 |
| 3713 | 3D printing | PRINT FUSER AND PROCESS WITH A REVERSE CLEANING BLADE\ | US2006045581A1 | ECK EDWARD M (US)\ MILLS BORDEN H III (US) | ECK EDWARD M (US)\ MILLS BORDEN H III (US) | G03G15/20 |
| 3714 | 3D printing | VERFAHREN ZUR HERSTELLUNG EINER SCHRAUBGEWINDEAUFNAHME\ | DE102011009682A1 | CHEN CHAOPING US\ ZHANG CAIJIAN US | ABMAYR UTE (DE) | B29C45/14\ F16B37/00\ B29C65/56\ F16B33/02\ F16B37/12 |
| 3715 | 3D printing | EXTERIOR CASE FOR ELECTRONIC APPARATUS\ | JP2012138594A | CHEN WEN-ZHENG (TW) | ALPS ELECTRIC CO LTD (JP) | B29C45/14\ H04M1/02\ H05K5/02 |
| 3716 | 3D printing | TIG WELDING APPARATUS\ | JP2012200740A | Empty Field US | BAB HITACHI IND CO US | B23K9/167\ B23K9/028\ B23K9/00\ B23K37/02 |
| 3717 | 3D printing | FILTERMATERIAL\[EN] HEAVY DUTY FILTRATION MATERIAL REMOVING DUST FROM INDUSTRIAL GASES INCLUDES BASE FELT AND COVER FELT WITH THINNER FIBERS CONSOLIDATED BY FLUID-JET NEEDLING\ | DE20321162U1 | Empty Field US | BWF TEC GMBH & CO KG (DE) | B01D46/42\ B01D39/16\ D04H3/02\ B01D46/10 |
| 3718 | 3D printing | METHOD FOR MANUFACTURING INTEGRAL MOLDING AND INTEGRAL MOLDING\ | JP2013018226A | Empty Field US | DAICEL PACK SYSTEMS LTD US\ POLYPLASTICS CO US\ DAIPLA SYSTEM TECHNOLOGY KK US | B29C45/14\ B29C51/10 |
| 3719 | 3D printing | DECORATIVE SHEET FOR INJECTION MOLDING DECORATIVE MOLDED ARTICLE AND METHOD FOR SIMULTANEOUSLY CARRYING OUT INJECTION MOLDING AND DECORATING WORK\ | JP2012045938A | Empty Field US | DAINIPPON PRINTING CO LTD (JP) | B29C45/14\ B29C51/10\ B44C3/02\ B32B27/00\ B32B33/00 |
| 3720 | 3D printing | PHOTOCATALYTIC THERMO-RESPONSIVE 3-DIMENSIONAL COPOLYMER AND FABRICATING METHOD OF THE SAME\ | KR101157527B1 | Empty Field US | HEWLETT PACKARD DEVELOPMENT CO (US) | C02F1/72\ B01J31/08\ B01J37/02\ C02F1/32 |
| 3721 | 3D printing | No Title Available\ | JP2010518958A | Empty Field US | INFO VISION OPTOELECTRONICS KUNSHAN CO LTD US | B44C5/00\ A44C17/00\ A44C13/00 |
| 3722 | 3D printing | No Title Available\ | TWI325814B | Empty Field US | KIM MOON OK (KR) | B44C5/00\ B32B17/10\ C03C27/12 |
| 3723 | 3D printing | No Title Available\ | RU2010135856A | Empty Field US | KOREA INST SCI & TECH (KR) | B64G1/66 |
| 3724 | 3D printing | SYSTEM AND ITS MAKING METHOD OF COLLECTION NOTE USING QR CODE\ | KR101283310B1 | Empty Field US | KOREA MACH & MATERIALS INST (KR) | G06F3/12\ B41J3/36\ G06K15/02 |
| 3725 | 3D printing | A MANUFACTURING METHOD FOR NON-FLAMABLE SHEET\ | KR101238799B1 | Empty Field US | MACDERMID GRAPHIC ARTS INC (US) | B29C43/24\ B29C43/28 |
| 3726 | 3D printing | ENERGY HARVESTER FOR THREE-DIMENSIONAL COMPLEX\ | KR101199466B1 | Empty Field US | MORIMURA KOSAN KK US | H02N2/00\ H02J7/35 |
| 3727 | 3D printing | MULTIPLE PURPOSE MATRIX OF 3D WEB STRUCTRED\ | KR101020878B1 | Empty Field US | NEGOPACK CO LTD (KR)\ LEE BO YOUNG (KR) | B29C47/00\ B29D28/00 |
| 3728 | 3D printing | METAL JET UNIT\ | KR100975627B1 | Empty Field US | PARK KYOUNG HOON (KR) | B22D23/00 |
| 3729 | 3D printing | PACK FOR PREVENTING FALSIFICATION\ | KR100853559B1 | Empty Field US | POLYMER GROUP INC (US) | B65D33/22\ B65D33/20 |
| 3730 | 3D printing | A BRAZING METHOD OF A THIN METAL PLATE FOR COOLING AN ELECTRONIC DEVICE\ | KR100524048B1 | Empty Field US | REES ANNE DU (SE) | B23K1/008 |
| 3731 | 3D printing | 複合体およびその裊造方法\ | JP5640190B2 | Empty Field US | RICOH KK (JP) | D04H3/16\ B32B3/24 |
| 3732 | 3D printing | No Title Available\ | CN102495509B | Empty Field US | ROSSIJSKAJA KORPORATSIJA RAKETNO KOSM PRIBOROSTROENIJA I INF SISTEM OAO ROSSIJSKIE KOSM SISTEMY AOOT (RU) | H04N13/00\ G02B27/22\ G02F1/29\ G02F1/03 |
| 3733 | 3D printing | SNOW MELTING METHOD FOR ROOF AND SNOW MELTING APPARATUS\ | JP2012229602A | Empty Field US | HARADA SHINPEI US | E04D13/10 |
| 3734 | 3D printing | MECHANISCH STABILER POR?SER AKTIVKOHLEFORMK?RPER\[EN] MECHANICALLY STABLE POROUS SHAPED ACTIVATED CARBON ARTICLE COMPRISES FIRST THREE-DIMENSIONAL FRAMEWORK STRUCTURE BASED ON CARBONIZED RESIN AND SECOND THREE-DIMENSIONAL INORGANIC FRAMEWORK STRUCTURE\ | DE20221547U1 | Empty Field US | HELSA AUTOMOTIVE GMBH & CO KG (DE) | B01J20/20\ C01B31/08\ C04B35/524 |
| 3735 | 3D printing | METHOD FOR MANUFACTURING ARTIFICIAL ORGAN AND ARTIFICIAL ORGAN\ | JP2012137514A | Empty Field US | JMC KK US | G09B23/34 |
| 3736 | 3D printing | VAPOR DEPOSITION MASK MANUFACTURING METHOD OF THE SAME AND VAPOR DEPOSITION METHOD\ | JP2012077328A | KIM MOON OK (KR) | MITSUBISHI PLASTICS INC US\ LASER GIJUTSU SOGO KENKYUSHO US | B23K26/00\ B23K26/38\ C23C14/04\ B23K26/32 |
| 3737 | 3D printing | THERMOPLASTIC RESIN MOLDING MOLD FOR INJECTION-MOLDING THE MOLDING AND INJECTION MOLDING METHOD\ | JP2012086453A | KIM SANG WOO (KR)\ LEE GEUN YOUNG (KR) | ONO SANGYO KK US | B29C45/37\ B29C45/72 |
| 3738 | 3D printing | 3D-DRUCKKOPF MIT TEMPERATUR-GESTEUERTER AUSTRITTS?FFNUNG FüR VERKüRZTE HEIZ- UND ABKüHLZEITEN SOWIE OPTIMIERTES AUSSEHEN DER DRUCKSTüCKE\[EN] THREE-DIMENSIONAL PRINTHEAD FOR MELTING AND DEPOSITING LAYERS OF WIRE-SHAPED MATERIALS HAS MELTING CHAMBER INCLUDING OUTLET DUCT LEADING INTO COOLING ZONES SUCH THAT FLOW VELOCITY OF MOLTEN MATERIAL IS REDUCED TO ZERO\ | DE102012000873A1 | LEE BO YOUNG (KR) | PARTHY KAI (DE) | B41J3/407\ B41J3/32 |
| 3739 | 3D printing | 3D-DRUCKKOPF MIT KüHLBAREM BESCHICKUNGSKANAL AUS HOCHW?RMELEITF?HIGEM MATERIAL\[EN] THREE DIMENSIONAL PRINTING HEAD USEFUL FOR MELTING AND DEPOSITING LAYERS OF WIRE-SHAPED MATERIALS COMPRISES FEED CHANNEL CONSISTING OF ARRAY OF HIGHLY HEAT-CONDUCTIVE MATERIALS E.G. ALUMINUM AND LOW HEAT-CONDUCTIVE MATERIALS E.G. PTFE\ | DE102011110804A1 | LEE SANG HUI US | PARTHY KAI (DE) | B29C67/00\ B22F3/115 |
| 3740 | 3D printing | UNIVERSELLER 3D-DRUCKKOPF MIT MEHREREN ARTEN DER DRAHTZUFüHRUNG UND DRAHT MIT ZUG-SEELE\[EN] THREE DIMENSIONAL PRINTING HEAD FOR MELTING AND DEPOSITING LAYERS OF WIRE-SHAPED MATERIALS HAS VARIOUS TYPES OF WIRE FEEDERS AND FEED CHANNELS FOR WIRES WHERE SOFT WIRE IS MADE OF THERMOPLASTIC ELASTOMERS\ | DE102011017310A1 | LEE SANG HYUP (KR) | PARTHY KAI (DE) | B05C5/04 |
| 3741 | 3D printing | PRESSE ZUR HERSTELLUNG VON DR?HTEN FüR 3D-DRUCKER\[EN] THERMAL MELTING WIRE FOR THREE-DIMENSIONAL DRUCKER IS OBTAINED BY MOLDING PLASTIC MATERIAL OR MELTING THERMOPLASTIC MATERIAL IN HEATED MOLD COOLING MULTI-COMPONENT CASTING RESIN INTO MOLD AND CURING\ | DE102011016270A1 | LEE TAIK MIN (KR) | PARTHY KAI (DE) | B29C39/02 |
| 3742 | 3D printing | DRAHT MIT STRUKTURIERTER OBERFL?CHE FüR 3D-DRUCKER\[EN] THERMALLY MELTING WIRE PARTICULARLY WELDING WIRES FOR THREE-DIMENSIONAL PRINTER IN FREQUENCY DIVISION MULTIPLEXING PROCESS HAS SURFACE WITH SPECIFIC IRREGULARITIES OR SHAPE DEVIATION IN SCORING GROOVES RAILS NUBS OR CRACKS\ | DE102011013961A1 | MORIMURA NORIKI US\ KOHAMA ATSUSHI US | PARTHY KAI (DE) | B41M99/00 |
| 3743 | 3D printing | THREE-DIMENSIONAL NETWORK STRUCTURE METHOD FOR PRODUCING THREE-DIMENSIONAL NETWORK STRUCTURE AND APPARATUS FOR PRODUCING THREE-DIMENSIONAL NETWORK STRUCTURE\ | JP2013108211A | PARK KYOUNG HOON (KR) | SHIIENJI KK US | D04H1/736\ D04H3/14 |
| 3744 | 3D printing | METHOD FOR MANUFACTURING OF LIGHT GUIDE PLATE WITH PROTECTIVE FILM\ | JP2012252906A | TOYOHIRO HAMAMATSU US | SUMITOMO CHEMICAL CO (JP) | F21Y101/02\ G02B6/00\ F21S2/00 |
| 3745 | 3D printing | METHOD OF MANUFACTURING OA EQUIPMENT EXTERIOR COMPONENT\ | JP2012076462A | URLICHICH JURIJ MATEHVICH (RU) | TEIJIN LTD US\ TEIJIN CHEMICALS LTD US | B29C45/00\ C08J5/00\ C08L101/16\ C08K3/34\ C08L67/04\ B29K67/00 |
| 3746 | 3D printing | FORGERY-PREVENTING FILM\ | JP2012000991A | YEOM JANG YUL (KR) | YUPO CORP US | B32B27/00\ B32B27/32\ B42D15/10 |
| 3747 | 3D printing | INJECTION MOLDING COMPOSITE CONTAINER AND METHOD OF MANUFACTURING THE SAME\ | JP2012012019A | YUKI IKEDA US | YUPO CORP US\ SANYO KAGAKU KOGYO KK US | B29C45/14\ B65D25/20 |
| 3748 | 3D printing | UNIFORM MELT SIMULATION METHOD OF EXTRUSION SCREW COMPUTER PROGRAM FOR EXECUTING IT AND UNIFORM MELT SIMULATION APPARATUS OF EXTRUSION SCREW\ | JP2007007951A | ENDO KUNIAKI US\ HIROSE TAKASHI US\ NOGI TAKAYUKI US\ YOSHIKAWA HIDEO US | TOSHIBA MACHINE CO LTD US | B29C47/38 |
| 3749 | 3D printing | VERKLEBEN VON KUNSTSTOFFEXTRUDATEN MIT WERKSTOFFOBERFL?CHEN\ | DE10144524B4 | ERB ARMIN (DE)\ SCHROEDEL JUERGEN (DE)\ TERFLOTH CHRISTIAN (DE) | JOWAT AG (DE) | C08L75/04\ B29C69/00\ B29C65/48\ B32B37/15\ B29C69/02\ B29C43/28\ B27D5/00\ C09J5/06\ B32B7/12\ B29C47/02\ B29C63/00\ B29C63/48\ B29C65/02\ C09J175/04\ B05C5/02\ B27D1/00\ C09J5/10\ C09J177/00 |
| 3750 | 3D printing | MAGNETIC AND ELECTROSTATIC NUCLEAR FUSION REACTOR\ | US2011200153A1 | FERREIRA JR MOACIR L (BR) | FERREIRA JR MOACIR L (BR) | H05H1/11\ G21B1/00 |
| 3751 | 3D printing | METHOD OF MANUFACTURING METAL- OR CERAMIC MICROPARTS\ | US2005140070A1 | FINNAH GUIDO (DE)\ JOHANN THOMAS (DE)\ PIOTTER VOLKER (DE)\ RUPRECHT ROBERT (DE)\ SCHANZ GERHARD (DE)\ HAUSSELT JURGEN (DE)\ HOLSTEIN NIIS (DE) | SUMITOMO HEAVY INDUSTRIES (JP) | C25D1/10\ B29C45/00\ B29C33/40\ B29C45/16\ C25D1/08\ C25D1/14 |
| 3752 | 3D printing | IMAGING MODULE FOR HOT MELT WAX INK JET PRINTER\ | US2010208017A1 | FLORENCE RICHARD N (US)\ MACQUEEN JR RAYMOND J (US)\ PULLEN ELAINE A (US)\ WALTER GRAHAM D (US)\ WIITA ROBERT L (US)\ WRIGHT WILLIAM PARKER ALEXANDER (US) | BLACK DOT TECHNOLOGY INC (US) | B41J2/175 |
| 3753 | 3D printing | CONTROL OF METALLIC APPEARANCE IN AUTOMOTIVE CAST PAINT FILMS\ | US5653927A | FLYNN MICHAEL D (US)\ TRUOG KEITH L (US) | AVERY DENNISON CORP (US) | B29C37/00\ B05D5/06\ B05D7/24\ B05D7/14\ B32B15/08 |
| 3754 | 3D printing | POST PROCESSOR FOR THREE-DIMENSIONAL OBJECTS\ | US7114943B1 | FONG JON JODY (US)\ SOLIZ RAYMOND M (US) | 3D SYSTEMS INC (US) | B28B17/00\ B29C35/00 |
| 3755 | 3D printing | APPARATUS FOR MAKING ENGINEERED TISSUE USING SELF-ASSEMBLING CELL AGGREGATES\ | US2015004273A1 | FORGACS GABOR (US)\ JAKAB KAROLY (US)\ NEAGU ADRIAN (US)\ MIRONOV VLADIMIR (US) | MUSC FOUND FOR RES DEV (US)\ UNIV MISSOURI (US) | B29C67/00\ C12N5/071 |
| 3756 | 3D printing | MODELING MATERIAL METHOD AND APPARATUS FOR MANU-FACTURING A THREE-DIMENSIONAL OBJECT BY MELT LAYERING\ | US2013209739A1 | FRUTH CARL (DE) | FIT FRUTH INNOVATIVE TECHNOLOGIEN GMBH (DE)\ GMBH FIT FRUTH INNOVATIVE TECHNOLOGIEN (US) | B05D3/00 |
| 3757 | 3D printing | METHOD FOR FEEDING WIRE IN LASER AND CLADDING AND IN-LASER WIRE FEEDER\[FR] PROCéDé POUR DISTRIBUER UN FIL DANS UN LASER ET REVêTEMENT ET DISPOSITIF DE DISTRIBUTION DE FIL DANS UN LASER\ | WO2009039753A1 | FU GEYAN (CN)\ SHI SHIHONG (CN)\ HU JIN (CN)\ YANG GUOJIA (CN)\ HAN XUELEI (CN) | UNIV SUZHOU (CN)\ FU GEYAN (CN)\ SHI SHIHONG (CN)\ HU JIN (CN)\ YANG GUOJIA (CN)\ HAN XUELEI (CN) | B23K26/34\ B23K26/06 |
| 3758 | 3D printing | THERMAL TRANSFER IN-MOULD DECORATION AND PREPARATION METHOD THEREOF\ | CN102673293B | FU JINGUO US\ XIANG WEIWEI US | TEC HI PRINTING TECHNOLOGY CO LTD US | B44C3/02\ B32B27/00\ B44C5/00\ B44C1/16 |
| 3759 | 3D printing | VERFAHREN ZUR HERSTELLUNG EINES HEI?GASSPIEGELS ZUM ERW?RMEN VON KUNSTSTOFFEN\[EN] HOT GAS MIRROR MANUFACTURING METHOD FOR INJECTION MOLDING TOOL DURING MANUFACTURING E.G. PLASTIC COMPONENTS FOR PRODUCTION OF DOOR OF PASSENGER CAR INVOLVES SEPARATING MOLD AND COMPONENT INCLUDING INLETS AND CHANNELS AND HEATING COMPONENT\ | DE102011120014A1 | FUETTERER MICHAEL (DE) | DAIMLER AG (DE) | B29C67/00\ B29C45/00\ B29C65/10 |
| 3760 | 3D printing | BALL MOUNTING METHOD AND APPARATUS FOR BALL GRID ARRAY PACKAGING SUBSTRATE\ | CN100382266C | FUJIANG SUN (CN) | HUAWEI TECH CO LTD (CN) | H01L21/60 |
| 3761 | 3D printing | REINFORCED POLYAMIDE RESIN COMPOSITION FOR BLOW MOLDING AND BLOW MOLDED PRODUCT USING IT\ | JP2009132908A | FUJII YASUHITO US\ SHIGA KENJI US\ SAKAI SATOSHI US | TOYO BOSEKI (JP) | C08L63/00\ B29L31/30\ C08L77/00\ B29K77/00\ B29C49/00\ B29K105/06\ C08K7/02 |
| 3762 | 3D printing | COMPOSITE MATERIAL AND SEMICONDUCTOR DEVICE USING THE SAME\ | US6737168B1 | FUKUI AKIRA (JP)\ YAMAGATA SHINICHI (JP)\ KAMITAKE KAZUYA (JP)\ ABE YUGAKU (JP) | SUMITOMO ELECTRIC INDUSTRIES (JP) | C04B41/50\ C04B35/573\ C04B41/51\ C04B41/85\ C04B41/88\ H01L23/14\ H01L23/15\ H01L23/373 |
| 3763 | 3D printing | METHOD FOR PRODUCING THREE-DIMENSIONAL STRUCTURE\ | JP2008133497A | FUWA ISAO (JP) | MATSUSHITA ELECTRIC WORKS LTD (JP) | B22F3/105 |
| 3764 | 3D printing | DEMOULAGE PAR CIRE PERDUE D'UNE BAUDRUCHE DE RESTRUCTURATION\ | FR2911079B1 | GAUTREAU NICOLAS US\ CHARRON YVES US | INST FRANCAIS DU PETROLE (FR) | B22C9/04\ B29C33/52\ B05D7/22\ B29C39/36 |
| 3765 | 3D printing | LASER WELDED BONDING PADS FOR PIEZOELECTRIC PRINT HEADS\ | US8857021B2 | GERNER BRADLEY JAMES (US)\ NYSTROM PETER J (US)\ DOLAN BRYAN R (US) | XEROX CORP (US)\ GERNER BRADLEY JAMES (US)\ NYSTROM PETER J (US)\ DOLAN BRYAN R (US) | H05K3/10\ B41J2/16\ B41J2/14\ B23K26/22 |
| 3766 | 3D printing | NON-CONTACT PRINTED COMESTIBLE PRODUCTS AND APPARATUS AND METHOD FOR PRODUCING SAME\ | US2007231425A1 | GLEFFE WILLIAM R (US)\ REAM RONALD L (US)\ VALDEZ ENRIQUE (US) | WRIGLEY W M JUN CO (US) | A23G4/00 |
| 3767 | 3D printing | PHOTOSENSITIVE MATERIAL PACKAGING MATERIAL PHOTOSENSITIVE MATERIAL PACKAGE AND MANUFACTURING METHOD THEREOF\ | JP2006053365A | GOI KATSUNORI US\ SHIMA TETSUO US\ TSUJI NOBUAKI US\ GOTO SHIGETO US\ KIMURA AKIZUMI US | KONICA MINOLTA MED & GRAPHIC US | G03C3/00 |
| 3768 | 3D printing | METHOD FOR PREPARING PBT/PET 3-D CRIMPED FIBER AND USE THEREOF\ | CN1962968A | GONG JINGHUA (CN) | UNIV DONGHUA (CN) | D01F8/14\ D01D5/32\ D02G1/18 |
| 3769 | 3D printing | APPARATUS FOR AVOIDING DEPOSITS ON OPTICAL COMPONENTS IN THE LASER SINTERING PROCESS\ | US2013101803A1 | GREBE MAIK (DE)\ DIEKMANN WOLFGANG (DE)\ HESSEL-GELDMANN SIGRID (DE) | GREBE MAIK (DE)\ DIEKMANN WOLFGANG (DE)\ HESSEL-GELDMANN SIGRID (DE) | B29C35/08\ B29C67/24\ B32B3/26 |
| 3770 | 3D printing | METHOD FOR PREPARING THREE-DIMENSIONAL PHOTOELASTICITY ANALYSIS MODEL BY APPLYING FAST-FORMING PLASTER MOLD TECHNIQUE\ | CN101623907A | GUANGHUI CHEN US\ GUOQI CUI US | TIANJIN INTERNAL COMB ENGINE R US | B29C39/42 |
| 3771 | 3D printing | ILLUMINATED ORNAMENT WITH MUSICAL CANDLE\ | US2008176176A1 | GUE JIMMY J (US)\ ZHANG PATTY H (US) | ZHANG PATTY H (US)\ GUE JIMMY J (US) | F23D3/18 |
| 3772 | 3D printing | TEXTILE COMPLEX INTENDED TO BE USED AS REINFORCING LAYER FOR THE MANUFACTURE OF COMPOSITE PARTS AND PROCESS FOR MANUFACTURING SUCH A COMPLEX\ | US2008305701A1 | GUIGNER DELPHINE (FR)\ BOUTIER SEBASTIEN (FR) | CHOMARAT COMPOSITES (FR) | B29C35/02\ B32B27/04\ B32B27/12 |
| 3773 | 3D printing | LIQUID MOLDING CORE-MELTING FORMING PROCESS FOR COMPLICATED RESIN-BASED COMPOSITE MATERIAL STRUCTURAL MEMBER\ | CN101913251A | GUIZHE ZHAO US\ YANFEI ZHANG US\ RUIKUI DU US\ YAQING LIU US | UNIV NORTH CHINA US | B29C70/54\ B29C70/36 |
| 3774 | 3D printing | PREPARATION METHOD OF DESIGNABLE POLYMER-BASED CONDUCTIVE COMPOSITE MATERIAL\ | CN102837430A | GUO SHAOYUN US\ SHEN JIABIN US\ LI JIANG US\ ZHU JIAMING US\ YANG ZHI US | UNIV SICHUAN (CN) | C08L77/02\ C08K3/04\ B29C70/28\ C08L23/12\ C08L23/06\ C08L51/06 |
| 3775 | 3D printing | METHOD FOR MANUFACTURING IMPELLER MOULD FOR GAS COMPRESSOR OF TURBOCHARGER\ | CN102125998A | GUOWEN CHEN US\ JIANFEI YAN US\ FUXING DONG US\ AIGUO ZHU US\ YUNHU FENG US\ GANG CHEN (CN) | DATONG NORTH TIANLI TURBOCHARGING TECHNOLOGY CO LTD US | B22C9/06\ B22C9/22 |
| 3776 | 3D printing | POLYMERIC ULTRAVIOLET (UV) DECORATIVE BOARD AND PROCESSING TECHNOLOGY THEREOF\ | CN102616076A | GUOXIANG LI US | GUOXIANG LI US | B32B27/06\ B44C5/04 |
| 3777 | 3D printing | METHOD FOR PRODUCING MULTICOLOR CHOCOLATE\ | CN101912023A | GUOXIONG LI US | GUOXIONG LI US | A23G1/04 |
| 3778 | 3D printing | FIRST MIRROR SURFACE IMPURITY ANALYSIS DEVICE BASED ON REFLECTION TYPE TERAHERTZ SPECTRUM\ | CN203037573U | HAI RAN US\ ZHANG CHENFEI US\ XIN YU US\ DING HONGBIN US | UNIV DALIAN TECH (CN) | G01B11/06\ G01N21/31 |
| 3779 | 3D printing | PRODUCTION TECHNOLOGY OF THREE-DIMENSIONAL HOLLOW FIBER\ | CN102443863A | HAIBIN CAI US | QUANZHOU SHENGXIN FIBER PRODUCT CO LTD US | D01D5/088\ D01D5/24\ D01D1/00\ D01D1/10\ D01D10/02\ D01D5/08\ D01D5/12 |
| 3780 | 3D printing | LIQUID CRYSTAL COMPOSITION SUITABLE FOR 3D GLASSES\ | CN102433131A | HAIGUANG CHEN US\ DEYU HANG US\ TIANMENG JIANG US\ CHUNYAN YANG US\ GUANGTAO WANG US\ HUIQIANG TIAN US\ SHIHONG CHU US | BEIJING BAYI SPACE LCD MATERIALS TECHNOLOGY CO LTD US | G02B27/22\ C09K19/30\ C09K19/12\ C09K19/16\ C09K19/18\ C09K19/20\ C09K19/58 |
| 3781 | 3D printing | ANTERIOR THREE-DIMENSIONAL REDUCTION INSTRUMENT FOR CERVICAL VERTEBRA BILATERAL FACET JOINT DISLOCATION\ | CN202397574U | HAILONG HE US\ XIAOJIAN YE US\ WEN YUAN US\ JUNMING TAN US\ ZHENGHUA HONG US\ JIASHUN LI US | UNIV PLA 2ND MILITARY MEDICAL US | A61B17/56 |
| 3782 | 3D printing | NO-MOLD FUSION STACKING MANUFACTURE METHOD OF PARTS OR MOLD\ | CN101362272A | HAIOU ZHANG (CN)\ GUILAN WANG (CN)\ ZHONGCHAO WEI (CN) | UNIV HUAZHONG SCIENCE TECH (CN) | B23K26/34\ B23K37/06\ B23K9/04 |
| 3783 | 3D printing | INK-JET PRINTER HEAD AND MANUFACTURE THEREOF\ | JP8174851A | HAMABUCHI KAZUFUMI US | MATSUSHITA ELECTRIC IND CO LTD (JP) | B41J2/16\ B41J2/05 |
| 3784 | 3D printing | SINGLE SIDE BI-DIRECTIONAL FEED FOR LASER SINTERING\ | US2005263933A1 | HANNA CHRISTOPHER R (US)\ WELCH ROBERT H IV (US)\ CHUNG TAE M (US) | 3D SYSTEMS INC (US) | B29C35/08\ B29C67/00\ B29C41/36\ B29C35/02 |
| 3785 | 3D printing | POROUS METAL BODY PROCESS FOR PRODUCING THE SAME AND BATTERY PLATE FORMED THEREFROM\ | US5597665A | HARADA KEIZO (JP)\ ISHII MASAYUKI (JP)\ YAMANAKA SHOSAKU (JP) | SUMITOMO ELECTRIC INDUSTRIES (JP) | B22F3/11\ B22F5/00\ C22C1/08\ B01D39/20\ H01M4/80\ C23C14/20\ H01M4/02\ H01M4/66\ H01M6/16 |
| 3786 | 3D printing | METHOD TO CREATE REMOVABLE DENTAL PROSTHESIS AND THE DENTAL PROSTHESIS MAKING THEREOF\ | US2012261848A1 | HARASZATI GYOERGY (HU) | HARASZATI GYOERGY (HU) | A61C13/20 |
| 3787 | 3D printing | VERFAHREN ZUR HERSTELLUNG VON METALL- ODER KERAMIK-MIKROBAUTEILEN\[EN] METHOD FOR PRODUCING METAL OR CERAMIC MICROCOMPONENTS\[FR] PROCEDE POUR LA PRODUCTION DE MICROCOMPOSANTS METALLIQUES OU CERAMIQUES\ | EP1527214A2 | HAUSSELT JUERGEN (DE)\ FINNAH GUIDO (DE)\ HOLSTEIN NILS (DE)\ JOHANN THOMAS (DE)\ PIOTTER VOLKER (DE)\ RUPRECHT ROBERT (DE)\ SCHANZ GERHARD (DE) | KARLSRUHE FORSCHZENT (DE) | B81C1/00\ B29C45/00\ B29C45/16 |
| 3788 | 3D printing | METHOD OF MANUFACTURING FILM INSERTED MOLDED ARTICLE\ | JP2008168613A | HAYASHI SEIJI US\ MATSUOKA KIYOMI US\ GOTO YOSHIHIDE US\ GOTO EIICHIRO US | SANWA SCREEN MEIBAN KK US | B29C45/14\ B29L9/00\ B29C59/02\ B29C69/00 |
| 3789 | 3D printing | MANUFACTURING METHOD OF OPERATION PANEL\ | JP2008183848A | HAYASHI SEIJI US\ TAKAHASHI TATSUMI US\ ITO YOSHIHIRO US | SANWA SCREEN MEIBAN KK US | B29C45/14\ B29C69/02\ D06F39/00 |
| 3790 | 3D printing | MELTING DEVICE FOR INCREASED PRODUCTION OF MELTED INK IN A SOLID INK PRINTER\ | US8052264B2 | HAYS ANDREW WAYNE (US)\ LEO MICHAEL F (US)\ LEIGHTON ROGER G (US) | XEROX CORP (US) | B41J2/175 |
| 3791 | 3D printing | LABEL WHEEL ROTARY PRINTING MACHINE\ | CN100595061C | HELIANG QI US\ ZE YANG US | SHANGHAI TAIYO KIKAI CO LTD US | B41F17/00\ B41F13/02\ B32B39/00\ B41F13/54\ B41F19/00\ B41F23/00\ B65H5/06\ G09F3/02 |
| 3792 | 3D printing | FLAME-RETARDING POLYPHENYL ETHER COMPOSITE AND PREPARATION METHOD AND USE THEREOF\ | CN102101941A | HENGMEI HUANG US\ LIN WANG US\ JIANZHONG LIU US\ SHAOHUA GUO US\ JINFENG FU US\ ZHIHUA HU US\ NANBIAO YE US | KINGFA SCI & TECH CO LTD US\ SHANGHAI KINGFA SCI & TECH CO US\ CHANG XIN MIANYANG NEW MATERIALS LTD US | C08K5/20\ C08L9/00\ C08L23/00\ C08L23/08\ C08L23/16\ C08L53/02\ C08K5/523\ B29C47/92\ C08L71/12\ C08K5/00\ C08K5/3492\ C08L9/02\ C08L25/12\ H01B7/295\ H01B7/36 |
| 3793 | 3D printing | HERSTELLUNG VON HOHLK?RPERN ODER SCHICHTEN MIT HOHLR?UMEN\[EN] PRODUCING HOLLOW BODY WITH CAVITY COMPRISES PRODUCING MOLDED BODY WITH OUTER MOLD PARTIALLY HAVING SHAPE OF HOLLOW CAVITY WHERE MOLDED BODY IS DISPLACEABLE BY SOLVENT/MELT IN FLOWABLE STATE AND COATING MOLDED BODY WITH COATING MATERIAL\ | DE102009037893A1 | HERTTER MANUEL (DE)\ JAKIMOV ANDREAS (DE)\ WACHTER WOLFGANG (DE) | MTU AERO ENGINES GMBH (DE) | B29C45/00\ C04B35/638\ B05D1/00 |
| 3794 | 3D printing | VORRICHTUNG ZUR HERSTELLUNG VON DREIDIMENSIONALEN OBJEKTEN\[EN] DEVICE USEFUL FOR PRODUCING THREE-DIMENSIONAL OBJECTS COMPRISES A CARRYING DEVICE FOR CARRYING THE OBJECT A COATING DEVICE FOR APPLYING LAYERS OF BUILDING MATERIAL ON THE CARRYING DEVICE AND AN IRRADIATION SOURCE FOR IRRADIATING LAYER\ | DE102011106380A1 | HERZOG FRANK (DE) | CL SCHUTZRECHTSVERWALTUNGS GMBH (DE) | B22F3/105 |
| 3795 | 3D printing | POLYPROPYLENE NONWOVEN FABRIC\ | JPH10292256A | HIKASA KATSUJI US\ YAMAOKA YASUSHI US | ASAHI CHEMICAL IND US | D04H3/16\ D04H3/007 |
| 3796 | 3D printing | SHEET FOR MOLDING DECORATION AND METHOD FOR SIMULTANEOUS DECORATION WITH INJECTION MOLDING\ | JP2004042351A | HIRANO TAMAMI US\ KAMIYAMA HIRONORI US\ ATAKE HIROYUKI (JP) | DAINIPPON PRINTING CO LTD (JP) | B29C45/14\ B32B27/30\ B29L9/00\ B32B33/00\ B29K105/20\ B29K627/12 |
| 3797 | 3D printing | COMPOSITE POLYMERIC ARTICLES FORMED FROM EXTRUDED SHEETS CONTAINING A LIQUID CRYSTAL POLYMER\ | US2012021212A1 | HOFMANN ACHIM (DE)\ YUNG PAUL C (US) | TICONA GMBH (DE)\ HOFMANN ACHIM (DE)\ YUNG PAUL C (US) | B29C51/02\ B32B5/00\ B29C47/78 |
| 3798 | 3D printing | HIGH RESOLUTION MULTICOLOR INK JET PRINTER\ | US7690779B2 | HOISINGTON PAUL A (US)\ SPEHRLEY JR CHARLES W (US) | FUJIFILM DIMATIX INC (US) | B41J2/01\ G01D11/00\ B41J2/175\ B41J2/21\ B41J2/195\ B41J11/00\ B41J2/205\ B41J13/22\ B41J19/16 |
| 3799 | 3D printing | METHOD OF TREATING IMAGE RECEIVING SHEETS AND A HOT MELT INK JET PRINTER EMPLOYING THIS METHOD\ | US2006114302A1 | HOLTMAN LODEWIJK T (NL) | OCE TECH BV (NL) | B41J2/175 |
| 3800 | 3D printing | VERFAHREN ZUM BEHANDELN VON BILDAUFZEICHNUNGSBL?TTERN UND DIESES VERFAHREN ANWENDENDER TINTENSTRAHLDRUCKER FüR W?RMESCHMELZBARE TINTE\[EN] METHOD OF TREATING IMAGE RECEIVING SHEETS AND HOT MELT INK JET PRINTER EMPLOYING THIS METHOD\[FR] PROCéDé POUR TRAITER DES FEUILLES DESTINéES à RECEVOIR DES IMAGES ET IMPRIMANTE à JET D'ENCRE POUR ENCRE THERMOFUSIBLE UTILISANT CE PROCéDé\ | EP1661723A3 | HOLTMAN LODEWIJK T (NL) | OCE TECH BV (NL) | B41J2/175\ B41J11/00 |
| 3801 | 3D printing | ADHISIVE RESINS FOR COATING OF STEEL PLATE\ | KR20100082077A | HONG CHANG IL (KR)\ NAM SOON HEE (KR) | HONG CHANG IL (KR) | C09J7/02\ C09J133/04 |
| 3802 | 3D printing | MICROSPRING LASER PROCESSING METHOD\ | CN100455841C | HONGBO SUN (CN) | JINLIN UNIV (CN) | F16F1/06 |
| 3803 | 3D printing | METHOD OF BUILDING THREE-DIMENSIONAL OBJECT WITH MODIFIED ABS MATERIALS\ | US2009295032A1 | HOPKINS PAUL E (US) | STRATASYS INC (US) | B28B1/14 |
| 3804 | 3D printing | METHOD AND APPARATUS FOR MANUFACTURING THREE-DIMENSIONAL PATTERN SHEET AND FILM USING ULTRASONIC GENERATOR\ | JP2011062972A | HOSOYA SHUHEI US | TOPPAN PRINTING CO LTD US | B29C47/88\ B29L7/00\ B29L11/00\ B29C59/04 |
| 3805 | 3D printing | VERFAHREN UND VORRICHTUNG ZUM FARBIGEN BEDRUCKEN VON PERSONALISIERUNGSDOKUMENTEN\[EN] METHOD FOR COLOR PRINTING OF PERSONALIZATION DOCUMENT INVOLVES TIGHTENING PERSONALIZATION DOCUMENT UNDER PRINT HEAD AND CONTROLLING UV LEDS IN SECTIONS BY ELECTRONIC CONTROL SO THAT UV LEDS ARE ILLUMINATED\ | DE102009021634A1 | HOTZEL MARIO (DE)\ BARTEL MARKO (DE)\ SCHMIDT REINHARD (DE) | RUHLAMAT GMBH (DE) | B41M3/14\ B41J2/21\ B41J3/407\ B41F11/02\ B41J2/525 |
| 3806 | 3D printing | THREE-DIMENSIONAL FABRICS AND FILMS AND THE PROCESS FOR MAKING THE SAME\ | US2004222552A1 | HOUSLER JOHN (US)\ CARTER NICK (US)\ DALE ROBERT (US)\ SCHAEFFER JAMES (US)\ MOODY RALPH (US) | Empty Field US | D04H3/03\ B29C43/22\ B29C47/00\ B29C47/88\ B29C51/22\ B29C59/06\ D04H3/05\ D04H3/08 |
| 3807 | 3D printing | BUILT-IN LASER SMELTING SPRAY NOZZLE\ | CN2705236Y | HU QIANWU (CN)\ ZENG XIAOYAN (CN) | CENTRAL CHINA SCIENCE & TECH (CN) | B23K26/34\ B05B7/16\ C23C4/00 |
| 3808 | 3D printing | DEFECTIVE TOTAL STERNUM REPLACEMENT PART FOR TOTAL STERNUM DEFECT OPERATION TREATMENT AND METHOD FOR MANUFACTURING THE SAME\ | CN102389343A | HUA TANG US\ ZHIFEI XU US\ BIN WU US\ MINGMING JI US | HUA TANG US\ ZHIFEI XU US\ BIN WU US\ MINGMING JI US | A61F2/28\ A61L27/18\ A61L27/12\ A61B17/56 |
| 3809 | 3D printing | DEVICE FOR SLIDING FIXATION OF POSTERIOR SPINAL PART\ | CN202113154U | HUAN WANG US\ SHAOQIAN CUI US | HUAN WANG US\ SHAOQIAN CUI US | A61B17/70 |
| 3810 | 3D printing | THREE-DIMENSIONAL DISPLAY APPARATUS AND METHOD FOR MANUFACTURING THE SAME\ | US2014118643A1 | HUANG HONG-JI (CN) | HUANG HONG-JI (CN)\ SHENZHEN CHINA STAR OPTOELECT (CN) | G02B27/22\ G02F1/1335 |
| 3811 | 3D printing | MANUFACTURING METHOD OF COLOUR STEREO MOULD CANDY AND ITS PRODUCT\ | CN1319461C | HUANG ZHENGLONG HUANG (CN) | YONGXIN TRADE CO LTD (CN) | A23G3/54\ A23G1/54\ A23G3/00 |
| 3812 | 3D printing | ASIA1 TYPE MULTI-EPITOPE RECOMBINANT VACCINE OF BOVINE FOOT-AND-MOUTH DISEASE VIRUSES AND PREPARATION METHOD THEREOF\ | CN101775399A | HUIYUN CHANG US\ GUOZHENG CONG US\ JUNZHENG DU US\ SHANDIAN GAO US\ TONG LIN US\ JUNJUN SHAO US\ JINGFENG WANG US | LANZHOU VETERINARY RES INST CH US | C12N15/62\ A61K48/00\ C12N15/42\ A61K39/135\ A61K47/48\ A61P31/14\ C07K14/09 |
| 3813 | 3D printing | SHEEP APHTHOVIRUS ASIAL TYPE MULTI-EPITOPE RECOMBINANT VACCINE AND PREPARATION METHOD THEREOF\ | CN101864434A | HUIYUN CHANG US\ GUOZHENG CONG US\ JUNZHENG DU US\ SHANDIAN GAO US\ TONG LIN US\ JUNJUN SHAO US\ JINGFENG WANG US | LANZHOU VETERINARY RES INST CHINESE ACADEMY OF AGRICULTURAL SCIENCES US | C12N15/62\ A61K48/00\ C12N15/42\ A61K39/135\ A61P31/14\ C07K14/09 |
| 3814 | 3D printing | INJECTION FOAMING METHOD USING THERMOPLASTIC ELASTOMER COMPOSITION FOR CORE BACK TYPE INJECTION FOAMING\ | JP2008013780A | IEDA KATSUMASA US\ MORIKAWA AKIHIKO (JP)\ KANAE KENTAROU (JP)\ ABE TOMOKAZU (JP) | JSR CORP (JP)\ HONDA MOTOR CO LTD (JP) | C08J9/34 |
| 3815 | 3D printing | FLUORESCENT SOLID INK MADE WITH FLUORESCENT NANOPARTICLES\ | US2010086683A1 | IFTIME GABRIEL (CA)\ VANBESIEN DARYL W (CA)\ WAGNER CHRISTOPHER A (CA)\ ALLEN C GEOFFREY (CA)\ BIRAU MARIA M (CA)\ WOSNICK JORDAN H (CA) | XEROX CORP (US) | B05D5/06\ C09K11/02 |
| 3816 | 3D printing | METHOD OF PRODUCING TONER\ | JP2009175632A | IGA TAKASHI US\ KANEDA TAKUYA US | NIPPON ZEON CO US | G03G9/087\ G03G9/08 |
| 3817 | 3D printing | HIGH-STRENGTH COMPLEX SOFT MAGNETIC MATERIAL HAVING HIGH STRENGTH HIGH MAGNETIC FLUX DENSITY HIGH RESISTANCE AND LESS IRON LOSS AND MANUFACTURING METHOD THEREOF\ | JP2006332525A | IGARASHI KAZUNORI US\ WATANABE MUNEAKI US\ NAKAYAMA RYOJI US\ MORIMOTO KOICHIRO US | MITSUBISHI MATERIALS PMG CORP US | B22F3/00\ B22F3/24\ B22F1/02\ H01F1/33\ H01F1/24 |
| 3818 | 3D printing | HIGH-STRENGTH COMPLEX SOFT MAGNETIC MATERIAL HAVING HIGH STRENGTH HIGH MAGNETIC FLUX DENSITY HIGH RESISTANCE AND LESS IRON LOSS AND MANUFACTURING METHOD THEREOF\ | JP2006332524A | IGARASHI KAZUNORI US\ WATANABE MUNEAKI US\ NAKAYAMA RYOJI US\ MORIMOTO KOICHIRO US | MITSUBISHI MATERIALS PMG CORP US | B22F3/00\ B22F1/02\ H01F1/33\ H01F1/24 |
| 3819 | 3D printing | MOUNTING PARTS PEEL SUPPRESSING SOLDERING METHOD ELECTRONIC CIRCUIT BASEBOARD AND ELECTRONIC INSTRUMENT\ | US2002170947A1 | IGARASHI MINORU (JP)\ MUKAI KATSUHIKO (JP)\ ISODA MASASHI (JP)\ KOBAYASHI TAKAYUKI (JP) | HEWLETT PACKARD CO (US) | H05K3/30\ B23K1/00\ H05K3/34\ B23K1/08\ B23K31/02\ B23K101/42\ H05K3/28 |
| 3820 | 3D printing | SENSOR AND FLOW SENSOR\ | JP2009085973A | IKE SHINICHI US | YAMATAKE CORP US | G01F1/692 |
| 3821 | 3D printing | VERFAHREN ZUM HERSTELLEN EINES FASERVERBUNDBAUTEILS\ | DE102008026161B4 | ILZHOEFER KARL-HEINZ DIPL ING (DE)\ SALKIC ASMIR DIPL ING (DE) | DAIMLER AG (DE) | B29C70/34\ B29C70/50\ C08J5/04\ B29B15/12\ B62D29/04 |
| 3822 | 3D printing | NANOTECHNOLOGICAL PROCESSING OF CATALYTIC SURFACES\ | US2006115389A1 | INDECH ROBERT (US) | INDECH ROBERT (US) | B01D53/34 |
| 3823 | 3D printing | ADDITITVE MANUFACTURING OF COMPONENTS FOR DOWNHOLE WIRELINE TUBING AND DRILL PIPE CONVEYED TOOLS\ | US2013310961A1 | INTRIAGO VELEZ JUAN CARLOS (US) | CORP SCHLUMBERGER TECHNOLOGY (US)\ SCHLUMBERGER TECHNOLOGY CORP (US) | G06F17/50 |
| 3824 | 3D printing | MOLTEN RESIN EXTRUDING LAMINATING AND SHAPING METHOD AND APPARATUS THEREFOR\ | JP2006192710A | ISHITANI SHIGEKI US | SEKISUI CHEMICAL CO LTD US | B29C67/00 |
| 3825 | 3D printing | MOLTEN RESIN EXTRUDING LAMINATING AND SHAPING METHOD AND APPARATUS THEREFOR\ | JP2006192616A | ISHITANI SHIGEKI US\ IWASAKI JUNYA US | SEKISUI CHEMICAL CO LTD US | B29C67/00 |
| 3826 | 3D printing | FLAME RETARDANT SEAT AND ITS MANUFACTURE\ | JP8033782A | ISODA HIDEO US\ YAMADA YASUSHI US | TOYO BOSEKI (JP) | D04H3/07\ D04H3/16\ A47C7/00\ B32B25/08\ B68G7/00 |
| 3827 | 3D printing | METHOD FOR PRODUCING DEPOSITED DIAMOND FILM\ | JP2006290687A | ISSHIKI HIDEO US | UNIV ELECTRO COMMUNICATIONS US\ CAMPUS CREATE CO LTD US | H04R31/00\ C30B29/04\ H04R7/02 |
| 3828 | 3D printing | MANUFACTURING APPARATUS AND METHOD OF MOLDING\ | JP2008201137A | ITO HIROSHI US\ YAKIMOTO KAZUTOSHI US\ SHIROGANEYA TSUKASA US\ NAITO AKIHIRO US | JAPAN STEEL WORKS LTD US | B29C43/02\ B29C43/34 |
| 3829 | 3D printing | RESIN FOAMED SHEET\[FR] FEUILLE DE MOUSSE DE RéSINE\ | WO2011062141A1 | IWATA MASAYOSHI (JP)\ KOGI TOSHINOBU (JP) | SEKISUI PLASTICS (JP)\ IWATA MASAYOSHI (JP)\ KOGI TOSHINOBU (JP) | B32B5/18\ B32B27/32\ C08J9/06\ C08L23/10 |
| 3830 | 3D printing | LASER THERMAL PRINTING ON MICROPOROUS PLASTIC SUBSTRATES\ | US2013202828A1 | JACOBY PHILIP (US) | JACOBY PHILIP (US)\ MAYZO CORP (US) | B32B33/00\ B32B3/26\ B29C59/16 |
| 3831 | 3D printing | HOCHMOLEKULARE POLYAMIDE\[EN] THERMOPLASTIC MOLDING COMPOSITION USEFUL TO PRODUCE FIBERS FILMS AND MOLDINGS INCLUDING E.G. INTAKE MANIFOLDS INTERCOOLER AND CIRCUIT BOARD COMPONENTS COMPRISES THERMOPLASTIC POLYAMIDE POLYETHYLENEIMINE HOMO OR COPOLYMER AND ADDITIVES\ | DE102008058246A1 | JAIN SACHIN (DE)\ GABRIEL CLAUS (DE)\ STAAL BASTIAAN BRAM PIETER (DE)\ LIEW CHEE CHIN (DE) | BASF SE (DE) | C08L77/00\ C08L79/00 |
| 3832 | 3D printing | POLYPEPTIDE MATERIAL WITH ADJUSTABLE PORE PROPERTIES\[FR] MATéRIAU PEPTIDIQUE à PROPRIéTéS DE PORES AJUSTABLES\ | WO2011046520A1 | JERALA ROMAN (SI)\ FEKONJA OTA (SI)\ POHAR JELKA (SI)\ GRADISAR HELENA (SI)\ BENCINA MOJCA (SI)\ HAFNER BRATKOVIC IVA (SI)\ BREMSAK ROBERT (SI)\ MIKLAVIC SPELA (SI)\ JELERCIC URSKA (SI)\ LUKAN ANJA (SI)\ DOLES TIBOR (SI)\ BOZIC SABINA (SI)\ VERCE MARKO (SI)\ DEBELJAK NIKA (SI) | KEMIJSKI INST (SI)\ JERALA ROMAN (SI)\ FEKONJA OTA (SI)\ POHAR JELKA (SI)\ GRADISAR HELENA (SI)\ BENCINA MOJCA (SI)\ HAFNER BRATKOVIC IVA (SI)\ BREMSAK ROBERT (SI)\ MIKLAVIC SPELA (SI)\ JELERCIC URSKA (SI)\ LUKAN ANJA (SI)\ DOLES TIBOR (SI)\ BOZIC SABINA (SI)\ VERCE MARKO (SI)\ DEBELJAK NIKA (SI) | C07K14/00\ C12N15/62 |
| 3833 | 3D printing | PRODUCTION PROCESS OF COLORFULLY-PAINTED AND BACK-ENGRAVED CRYSTAL GLASS\ | CN103253057A | JI QINGKAI US | ARIAMOTION DALIAN CO LTD US | B44C1/22\ C03B19/00\ C03C3/089 |
| 3834 | 3D printing | DEVICE FOR PREPARING SEMI-SOLID ALLOY MELT AND PROCESS FOR PREPARING ALSI9MG SEMI-SOLID ALLOY\ | CN101660063A | JIANBO TAN US\ ZENGMIN LI US\ LIXIN LI US | UNIV HEBEI SCIENCE & TECH US | C22C1/00 |
| 3835 | 3D printing | DEVICE FOR PREPARING SEMI-SOLID ALLOY MELT\ | CN201512567U | JIANBO TAN US\ ZENGMIN LI US\ LIXIN LI US | UNIV HEBEI SCIENCE & TECH US | B22D17/08\ C22C1/00 |
| 3836 | 3D printing | METHOD FOR MODELING THREE-DIMENSIONAL PATTERN\ | CN101722784A | JIANGANG HE US | JIANGANG HE US | B44C1/20 |
| 3837 | 3D printing | PRODUCTION METHOD OF FLAME-RETARDANT SINGLE-HOLE HOLLOW OR MULTI-HOLE THREE-DIMENSIONAL CRIMPED POLYESTER STAPLE FIBER\ | CN101676451A | JIANJUN ZHANG US\ QINRONG TONG US\ JIANQIU XU US\ GUANGZE WU US\ ZUAN SUN US\ HAITIAN XIA US\ GUOZHI CAI US\ HENG ZHANG US\ WENQIN WU US | SHANGHAI XINDILUN IND CO LTD US\ TOPRET IND DESIGN SHANGHAI CO US | D01D5/24\ D01F6/84\ D02G1/00 |
| 3838 | 3D printing | SECTION BAR SURFACE PATTERN MOLDING DEVICE\ | CN200981338Y | JIANMIN HAN (CN) | JIANMIN HAN (CN) | B29C47/08\ B29C59/04 |
| 3839 | 3D printing | METHOD FOR MANUFACTURING RAPID SHUTTER SPECTACLES USED IN STEREOSCOPIC DISPLAY\ | CN102323673A | JIHONG ZHENG US\ ZENGJUN ZHOU US\ LIJIA SUN US\ PINGYU TANG US\ SONGLIN ZHUANG US | UNIV SHANGHAI SCIENCE & TECH US | G02B27/22\ G02F1/1333 |
| 3840 | 3D printing | TECHNOLOGY FOR APPLYING INLAID METAL FINISHING COMPONENT TO IN-MOLD LABELLING\ | CN102029683A | JINGYAN LIU US\ XIAODONG ZENG US\ HUI WANG US\ QI LI US | TIANJIN ZHONGHUAN HI TECH CO US | B29C45/14 |
| 3841 | 3D printing | MOULD MAKING METHOD WITH CONFORMAL COOLING PIPE\ | CN101670437A | JINHUI LIU US\ CAN ZHAO US\ JIBING CHEN US\ RUIDI LI US | UNIV ZHEJIANG SCIENCES US | B22F3/22 |
| 3842 | 3D printing | MOULD STRUCTURE WITH CONFORMAL COOLING PIPE\ | CN101670627A | JINHUI LIU US\ CAN ZHAO US\ JIBING CHEN US\ RUIDI LI US | UNIV ZHEJIANG SCIENCES US | B29C33/38\ B29C33/04\ B29C45/73\ B22F3/22\ B29C45/26 |
| 3843 | 3D printing | METHOD FOR FORMING REFRACTORY METAL PARTS BY USING LASER\ | CN101780544A | JINHUI LIU US\ CAN ZHAO US\ JIBING CHEN US\ RUIDI LI US\ DANQING ZHANG US | HEILONGJIANG INST OF SCIENCE A US | B22F3/105\ B23K26/34\ C23C24/10 |
| 3844 | 3D printing | SYSTEM AND METHOD FOR MELTING SOLID INK STICKS IN A PHASE CHANGE INK PRINTER\ | US7581827B2 | JONES BRENT RODNEY (US)\ PLATT DAVID PAUL (US) | XEROX CORP (US) | G01D11/00\ B41J2/175 |
| 3845 | 3D printing | A MANUFACTURING METHOD OF FILM FOR INSERT INMOLDING AND FILM FOR INSERT INMOLDING\ | KR20080066889A | JUNG HAE OK (KR)\ KIM WOO HWEAN (KR) | JUNG HAE OK (KR)\ KIM WOO HWEAN (KR) | B29D7/01 |
| 3846 | 3D printing | POROUS FUSIBLE INKJET MEDIA WITH FUSIBLE CORE-SHELL COLORANT-RECEIVING LAYER\ | US7086732B2 | KASPERCHIK VLADEK P (US)\ UNGEFUG GARY ALLAN (US) | HEWLETT PACKARD DEVELOPMENT CO (US) | B41J2/01\ B41M5/00\ B41M5/50\ B41M5/52\ B41M7/00 |
| 3847 | 3D printing | CIRCUIT BOARD MANUFACTURING METHOD CIRCUIT BOARD THIN FILM TRANSISTOR ELECTRO-OPTICAL DEVICE AND ELECTRONIC EQUIPMENT\ | JP2005217359A | KAWASE TAKEO US\ MORIYA SOICHI US\ HARADA MITSUAKI US | SEIKO EPSON CORP (JP) | G09F9/00\ H01L23/52\ H05K3/10\ H01L21/288\ H01L51/00\ G02F1/167\ G09F9/30\ H01L21/28\ H01L21/3205\ H01L29/423\ H01L29/49\ H01L29/786\ H01L51/05\ H01L51/40 |
| 3848 | 3D printing | MANUFACTURING METHOD OF SUPER THIN BIDIRECTIONAL STRETCHED POLYPROPYLENE COARSE THIN FILM FOR CAPACITOR\ | CN101863120B | KE XIBIN US\ LI GAOCONG US\ SHEN SHILIN US\ SU LIYONG US\ XIE JIN US | SICHUAN EM TECHNOLOGY CO LTD US | B29C47/92\ B29C47/88\ B29C47/06\ B29K23/00\ B29C55/14\ B29L7/00 |
| 3849 | 3D printing | TECHNIQUE FOR MANUFACTURING AIR PRESSURE TYPE NON-CUTTING QUICK-MOLDING LOW MELTING-TEMPERATURE ALLOY MOLD\ | CN101433952A | KEFEI XIE (CN) | KEFEI XIE (CN) | B22D18/04\ B22C9/02 |
| 3850 | 3D printing | NON-FIBROUS ADHESIVE MATRIX AND LAMINATE FORMED THEREFROM\ | US7803459B2 | KEUCHEL HERBERT WILLIE (US)\ KEUCHEL KENNETH (US) | KEUCHEL HERBERT WILLIE (US)\ KEUCHEL KENNETH (US) | B32B27/06\ B32B3/26\ B32B7/12\ B32B37/10\ A41D31/02\ B32B37/08\ C09J5/08 |
| 3851 | 3D printing | NICHTFASERIGE KLEBSTOFFMATRIX UND DARAUS HERGESTELLTES LAMINAT\[EN] NON-FIBROUS ADHESIVE MATRIX AND LAMINATE FORMED THEREFROM\[FR] MATRICE ADHESIVE NON-FIBREUSE ET LAMINE FORME A PARTIR DE CETTE MATRICE\ | EP1729955A4 | KEUCHEL HERBERT WILLIE (US)\ KEUCHEL KENNETH (US) | SPUNFAB LTD (US) | B32B27/06\ B32B5/18\ B32B3/26 |
| 3852 | 3D printing | BLADE DRIVING APPARATUS FOR CAMERAS\ | JP2007264617A | KIHARA TOMOKAZU US | NIDEC COPAL CORP US | G03B9/10\ G03B9/18 |
| 3853 | 3D printing | METHOD FOR MANUFACTURING ARTIFICIAL FINGERPRINT USING QR CODE AND QR CODE MANUFACTURED BY THE SAME\ | KR101332682B1 | KIM BYUNG JUN (KR)\ KIM JUNG HYUN (KR)\ LEE SEUNG YUN (KR) | NAT UNIV HANBAT INDUSTRY (KR) | G06K7/10\ G06K9/46\ G06K9/18\ G06K9/20 |
| 3854 | 3D printing | THE METHOD WHICH FORMS A THREE-DIMENSIONAL DECORATION IN THE RE-APPLICATION PLASTICS FORMATION WATER AND THAT PRODUCT\ | KR100752034B1 | KIM CHA GON (KR) | DEAHAN IND CO LTD (KR) | B29C59/00\ B29C59/08 |
| 3855 | 3D printing | PACKCING SHEET MANUFACTURING MATHOD AND PACKING SHEET THEREBY\ | KR20080107832A | KIM DO EON (KR) | KIM DO EON (KR) | B31B19/60\ B31B21/60 |
| 3856 | 3D printing | CLAY FOR RUBBER BOARD\ | KR20080104627A | KIM HYUN MOOK (KR) | KIM HYUN MOOK (KR) | B44D3/18 |
| 3857 | 3D printing | MANUFACTURING METHOD OF FRINTING FILM FOR INSERT MOLDING\ | KR100877184B1 | KIM KWANG WON (KR)\ LEE HONG SOO (KR)\ KWACK SE HOON (KR) | SANGBO CORP (KR) | C08J5/18\ B29D7/01 |
| 3858 | 3D printing | FRINTING FILM FOR INSERT MOLDING\ | KR100877183B1 | KIM KWANG WON (KR)\ LEE HONG SOO (KR)\ KWACK SE HOON (KR) | SANGBO CORP (KR) | C08J5/18\ B29D7/01 |
| 3859 | 3D printing | CUSHIONING SHEET FOR FRUIT PACKAGING BOX HAVING EXCELLENT CUSHIONING EFFECT AND BEAUTIFUL APPEARANCE AND MANUFACTURING METHOD THEREOF\ | KR20040082245A | KIM YONG KWAN US | KIM YONG KWAN US | B65D85/34 |
| 3860 | 3D printing | MANUFACTURING METHOD FOR INFORMATION-COMMUNICATION MEDIUM\ | JP2004306575A | KIMURA YOSHIKAZU US\ TSUCHIYA MASAHITO US | KYOTO DAIICHI KAGAKU KK US | B42D15/02 |
| 3861 | 3D printing | APPARATUS FOR PRODUCING TONER PRECURSOR AND METHOD FOR THE SAME FIBROUS TONER PRECURSOR APPARATUS FOR PRODUCING TONER AND ELECTROPHOTOGRAPHIC TONER\ | JP2008096974A | KINOSHITA NAOTOSHI US\ TANAKA TETSUYA US\ KAWAMOTO MASAHIRO US | RICOH KK (JP) | G03G9/087\ G03G9/08 |
| 3862 | 3D printing | THREE-DIMENSIONAL FREE SHAPING METHOD FREE COATING METHOD AND APPARATUS THEREFOR\ | JP2005054197A | KIRIHARA SATOHIDE US\ MIYAMOTO YOSHIO US\ MATSUURA KIYOTAKA US\ WADA KAZUO US | MIYAMOTO YOSHIO US\ KIRIHARA SATOHIDE US\ MATSUURA KIYOTAKA US\ SERUBAKKU KK US | C23C28/00\ B23K9/04 |
| 3863 | 3D printing | METALLIZED OR CERAMIC-COATED POLYESTER FILMS COMPRISING POLY(M-XYLENEADIPAMIDE)\ | US2005287378A1 | KLEIN OLIVER (DE)\ PEIFFER HERBERT (DE)\ DAVIS RICHARD L (DE) | KLEIN OLIVER (DE)\ PEIFFER HERBERT (DE)\ DAVIS RICHARD L (DE) | C08K3/00\ C08J7/04\ C08L77/06\ B32B15/08\ B32B27/36\ C08L67/02 |
| 3864 | 3D printing | VERFAHREN ZUR FREIFORMENDEN HERSTELLUNG VON DREIDIMENSIONALEN HOHLR?UMEN ODER HINTERSCHNEIDUNGEN AUFWEISENDEN MIKROBAUTEILEN\[EN] MICRO COMPONENTS MANUFACTURING METHOD INVOLVES MANUFACTURING THREE-DIMENSIONAL MASTER RAW COMPONENTS OF VARIOUS SHAPES AND CHEMICALLY PHYSICALLY OR MECHANICALLY SEPARATING MASTER COMPONENTS AND METAL LAYER FROM MICRO COMPONENT\ | DE10353060A1 | KOCH KAI UWE (DE)\ FETZER HANS-JOCHEN (DE)\ GEMMLER ARMIN (DE)\ GOTTWALD BERNHARD (DE) | FRAUNHOFER GES FORSCHUNG (DE) | C25D1/10\ B81C1/00\ C25D1/02 |
| 3865 | 3D printing | METHOD FOR MANUFACTURING RING-INTEGRATED DISPLAY BOARD\ | JP2007125832A | KOJIMA TAKUO US\ YOSHIZAKA KIMIYASU US | SAKAIYA KK US | B29C45/14\ B29L31/00\ B29C33/12 |
| 3866 | 3D printing | MANUFACTURING METHOD OF CERAMIC COMPOSITE MATERIAL\ | JP2005298310A | KOMIYAMA TSUNEO US\ SUZUKI IWAO US | NGK ADREC CO LTD US\ NGK INSULATORS LTD (JP) | C04B35/573\ C04B41/88\ C04B41/91 |
| 3867 | 3D printing | OA EQUIPMENT EXTERIOR PART\ | JP2007191549A | KONDO FUMITAKA US\ HIRONAKA KATSUHIKO US\ INO KEIICHIRO US\ MATSUNO YUICHI US\ TOYOHARA KIYOTSUNA US\ SUZUKI HIROTAKA US\ NONOKAWA RYUJI US\ MATSUDA TAKAAKI US | TEIJIN LTD US\ TEIJIN CHEMICALS LTD US | B29C45/00\ C08J5/00\ C08L101/16\ C08K3/34\ C08L67/04 |
| 3868 | 3D printing | ELECTRONIC EQUIPMENT EXTERIOR PART\ | JP2007191548A | KONDO FUMITAKA US\ HIRONAKA KATSUHIKO US\ INO KEIICHIRO US\ MATSUNO YUICHI US\ TOYOHARA KIYOTSUNA US\ SUZUKI HIROTAKA US\ NONOKAWA RYUJI US\ MATSUDA TAKAAKI US | TEIJIN LTD US\ TEIJIN CHEMICALS LTD US | C08J5/00\ C08L101/16\ C08K3/34\ C08L67/04 |
| 3869 | 3D printing | ELECTRIC/ELECTRONIC COMPONENT\ | JP2007191547A | KONDO FUMITAKA US\ HIRONAKA KATSUHIKO US\ INO KEIICHIRO US\ MATSUNO YUICHI US\ TOYOHARA KIYOTSUNA US\ SUZUKI HIROTAKA US\ NONOKAWA RYUJI US\ MATSUDA TAKAAKI US | TEIJIN LTD US\ TEIJIN CHEMICALS LTD US | B29C45/00\ C08J5/00\ C08L101/16\ C08K3/34\ C08L67/04\ B29L31/34\ B29K67/00 |
| 3870 | 3D printing | AUTOMOTIVE COMPONENT\ | JP2007191546A | KONDO FUMITAKA US\ HIRONAKA KATSUHIKO US\ INO KEIICHIRO US\ MATSUNO YUICHI US\ TOYOHARA KIYOTSUNA US\ SUZUKI HIROTAKA US\ NONOKAWA RYUJI US\ MATSUDA TAKAAKI US | TEIJIN LTD US\ TEIJIN CHEMICALS LTD US | B29C45/73\ C08J5/00\ C08L101/16\ C08K3/34\ C08L67/04\ B29K67/00 |
| 3871 | 3D printing | SOLID INK JET PRINTER\ | JPH10129003A | KONDO NORIMASA US | HITACHI KOKI KK US | B41J2/175\ B41J2/015 |
| 3872 | 3D printing | SOUND INSULATING MATERIAL METHOD FOR MANUFACTURING SOUND INSULATING MATERIAL AND FILLER FOR SOUND INSULATING MATERIAL\ | JP2005241888A | KONDO YASUHIRO US\ TAKAOKA NOBUYUKI (JP) | SHIIENJI KK US | C04B38/00\ E04B1/86\ C04B28/02\ C04B38/10\ G10K11/162 |
| 3873 | 3D printing | INK CONSUMPTION DETERMINATION\ | US7425061B2 | KORN SCOTT J (US)\ BUEHLER JAMES D (US)\ KNIERIM DAVID L (US)\ YUSEM GUSTAVO J (US) | XEROX CORP (US) | G01D11/00\ B41J2/175 |
| 3874 | 3D printing | INK DEAERATOR FOR INK-JET PRINTER\ | JP2006231809A | KOSEKI OSAMU US | SII PRINTEK INC US | B41J2/175 |
| 3875 | 3D printing | MICROFABRICATED CELLULAR TRAPS BASED ON THREE-DIMENSIONAL MICRO-SCALE FLOW GEOMETRIES\ | US2007178582A1 | KOSER HUR (US) | KOSER HUR (US) | C12M1/34 |
| 3876 | 3D printing | WIRE FEEDING DEVICE OF LASER FUSION-COATING SYSTEM\ | CN201390782Y | KU SI US | UNIV DONGGUAN TECHNOLOGY US | B23K26/34\ C23C24/10 |
| 3877 | 3D printing | METHOD FOR IMPROVING INGOT SURFACE QUALITY\ | CN100457322C | KUN XU (CN)\ DENGQUAN CHEN (CN)\ XIMIN LUO (CN)\ ZEGUANG LIU (CN) | SINO PLATINUM METALS CO LTD (CN) | B22C3/02 |
| 3878 | 3D printing | PROCESS FOR MANUFACTURING WAREF WITH DOUBLE-WALL BODY\ | CN101152752A | KUNCHE LI (CN) | KUNCHE LI (CN) | B29C45/14\ B29B11/00\ B65D1/00 |
| 3879 | 3D printing | VEGETABLE-ORIGINATED COMPONENT-CONTAINING ADHESIVE SHEET\ | JP2009280676A | KUNUKATANA MASAHIRO US\ YAMAKAWA YASUKO US | KUREHA SENI KK US | C09J7/04\ C09J177/00 |
| 3880 | 3D printing | VERFAHREN ZUR HERSTELLUNG EINES METALLISCHEN K?RPERS\[EN] METAL OBJECT IN PARTICULAR INJECTION MOLDING TOOL MANUFACTURE INVOLVES REPEATED ELECTROLYTIC DEPOSITION OF METAL ONTO A MASKED FLAT SURFACE TO BUILD UP A THREE-DIMENSIONAL STRUCTURE\ | DE10361119A1 | KURENBACH TORSTEN (DE) | SOLIDTOOL GMBH (DE) | C25D1/10\ C25D5/02\ C25D5/10 |
| 3881 | 3D printing | CASTING DIES FOR CYLINDER HEAD\ | KR20030031774A | KWAK HYEONG SIK (KR) | HYUNDAI MOTOR CO LTD (KR) | B22D15/00 |
| 3882 | 3D printing | ARTIFICIAL BONE MATERIAL AND PREPARATION METHOD THEREOF\ | CN103285428A | LAN HAI US\ SHI SHENGTAO US\ REN FANGPING US\ LI GUOKANG US | CHINA INTERNAT TOURISM & TRADE CO LTD US | A61L27/48\ A61L27/46 |
| 3883 | 3D printing | VORRICHTUNG ZUR HERSTELLUNG VON KRISTALLEN AUS ELEKTRISCH LEITENDEN SCHMELZEN\[EN] DEVICE FOR PRODUCING CRYSTALS FROM ELECTROCONDUCTIVE MELTS\[FR] DISPOSITIF DE PRODUCTION DE CRISTAUX à PARTIR DE BAINS FONDUS éLECTRIQUEMENT CONDUCTEURS\ | EP2162570A2 | LANGE RALPH-PETER (DE)\ JOCKEL DIETMAR (DE)\ ZIEM MARIO (DE)\ RUDOLPH PETER (DE)\ KIESSLING FRANK (DE)\ FRANK-ROTSCH CHRISTIANE (DE)\ CZUPALLA MATTHIAS (DE)\ NACKE BERNARD (DE)\ KASJANOW HELENE (DE) | FORSCHUNGSVERBUND BERLIN EV (DE) | C30B15/14\ C30B11/00 |
| 3884 | 3D printing | REGENERATION TECHNOLOGY ADAPTED FOR INK POX WITH SPONGE WITHOUT PRINTING HEAD\ | CN100357108C | LANGMING WANG (CN) | LANGMING WANG (CN) | B41J2/175 |
| 3885 | 3D printing | METHOD AND APPARATUS FOR REDUCING DROP PLACEMENT ERROR IN PRINTERS\ | US6367909B1 | LEAN MENG H (US) | XEROX CORP (US) | B41J2/015\ B41J2/06\ B41J2/07 |
| 3886 | 3D printing | CONSUMABLE WELDING PRODUCT E.G. WIRE CONDITIONING ASSEMBLY FOR E.G. TRUCK HAS CONTAINER CONTAINING CONSUMABLE WELDING PRODUCT AND SURMOUNTED BY CAP WHICH IS MADE OF DEFORMABLE MATERIAL WHICH DEFORMS UNDER EFFECT OF FORCE APPLIED ON CAP\[FR] ENSEMBLE DE CONDITIONNEMENT DE PRODUIT DE SOUDAGE A COIFFE POINTUE\ | FR2910445A1 | LEDUEY BRUNO (FR)\ CANNET GILLES (FR) | AIR LIQUIDE (FR)\ AIR LIQUIDE WELDING FRANCE (FR) | B65D71/06\ B65D19/22\ B65D85/20\ B65D85/26 |
| 3887 | 3D printing | A METHOD WHICH PRINT A LOGO ON SILICON\ | KR100813479B1 | LEE BYONG SUP (KR) | LEE BYONG SUP (KR) | B41M1/30\ B41M1/32 |
| 3888 | 3D printing | EMBLEM OF AUTOMOBILE PART AND METHOD OF MANUFACTURING THE SAME\ | US7998383B2 | LEE CHANGWOO (KR)\ SHIM SANGWOO (KR)\ LEE HOJIN (KR)\ PARK SEUNGMOK (KR) | AUTOLIV DEV (SE) | B29C59/00 |
| 3889 | 3D printing | SHELL AND METHOD OF MANUFACTURING THE SAME\ | TW200950651A | LEE CHIEN-HUNG (TW) | LEE CHIEN-HUNG (TW) | H05K5/06 |
| 3890 | 3D printing | HOLLOW THREE-DIMENSIONAL MANUFACTURING METHOD OF A CASTING PATTERN MODEL AND THE APPARATUS THEREOF\ | KR100837109B1 | LEE IN GU (KR)\ LEE YOON JU (KR)\ I YEONG CHEOL (KR) | FUTURECAST CO LTD (KR)\ LEE IN GU (KR)\ LEE YOON JU (KR)\ I YEONG CHEOL (KR) | B29C45/76\ B29C45/16 |
| 3891 | 3D printing | FILM TRANSFER METHOD AND APPEARANCE MEMBER MANUFACTURED BY USING THE SAME\ | US2013264727A1 | LEE JIH-HOUNG (TW)\ PENG MING-CHUNG (TW) | COMPAL ELECTRONICS INC (TW) | B29C45/02\ B29D11/00\ B29C45/40 |
| 3892 | 3D printing | METAL-JET UNIT\ | KR100735115B1 | LEE TAIK MIN (KR)\ KIM DONG SOO (KR)\ KIM KWANG YOUNG (KR)\ KANG TAE GOO (KR)\ YANG JEONG SOON (KR)\ CHOI BYUNG OH (KR)\ JO JEONG DAI (KR)\ YUN SO NAM (KR)\ HAM YOUNG BOG (KR)\ KIM CHUNG HWAN (KR) | KOREA MACH & MATERIALS INST (KR) | B22D23/00 |
| 3893 | 3D printing | ELECTROMAGNETIC FLEXIBLE COMPOSITE DEPOSITION DIRECT PREPARATION FORMING METHOD OF MULTIFUNCTIONAL GRADIENT COMPONENT\ | CN102962451A | LEI LEI US\ BAI XINGWANG US\ WANG XIANGPING US\ ZHANG HAIOU (CN)\ WANG GUILAN (CN) | UNIV HUAZHONG SCIENCE TECH (CN) | B22F3/105\ B22F7/02\ C23C24/10\ B22D27/02 |
| 3894 | 3D printing | FILM LAMINATE\ | US2008188154A1 | LEIS TERRY (US)\ FORSTER JAMES (US)\ HOPKINS DAVID (US) | JEN COAT INC (US) | B32B5/16\ B32B7/12\ B32B27/12 |
| 3895 | 3D printing | KRAFTSTOFFINJEKTOR MIT KüHLEINRICHTUNG\ | DE102004049703B4 | LEWENTZ GUENTER (DE) | CONTINENTAL AUTOMOTIVE GMBH (DE) | F02M53/00 |
| 3896 | 3D printing | MEDICAL C-ARM THREE-DIMENSIONAL IMAGING SYNCHRONOUS CONTROL DEVICE\ | CN102934999A | LI JINSHENG US\ GAO SONG US | NANJING PERLOVE RADIAL VIDEO EQUIPMENT CO LTD US | A61B6/00 |
| 3897 | 3D printing | METHOD FOR PREPARING SURFACE MICROSTRUCTURE OF SUPER-HYDROPHOBIC AMORPHOUS ALLOY\ | CN102328902B | LI NING US\ XIA TING US\ XU WEI US\ LIU LIN US | UNIV HUAZHONG SCIENCE TECH (CN) | B81C1/00\ C22C45/00\ C22C45/10 |
| 3898 | 3D printing | PLASTIC PIECE SURFACE AND METAL ORNAMENT COMPOSITE SEAMLESS PRIMARY INJECTION MOLDING AND FORMATION PROCESS\ | CN102962945A | LI QI US\ ZENG XIAODONG US\ WANG HUI (US) | TIANJIN ZHONGHUAN HI TECH CO US | B29C45/14\ B29B11/00 |
| 3899 | 3D printing | SWING SYSTEM FOR HIGH FREQUENCY MELTING FURNACE\ | CN201680716U | LI WEI (CN) | FENG ZHONGDA US\ LI WEI (CN) | F27B14/08 |
| 3900 | 3D printing | A THREE-DIMENSIONAL PRINTER EXTRUSION HEAD CAPABLE OF FEEDING IN A SWITCHING MODE\ | CN203157146U | LI YAOTANG US\ YE SHIDONG US\ ZHANG HAIQIANG US\ LIU YUPENG US | GUANGZHOU ELECTRONIC TECHNOLOGY CO LTD CHINESE ACADEMY OF SCIENCES US | B29C67/00 |
| 3901 | 3D printing | THREE-DIMENSIONAL PRINTER EXTRUSION HEAD CAPABLE OF FEEDING IN SWITCHING MODE\ | CN102922745A | LI YAOTANG US\ YE SHIDONG US\ ZHANG HAIQIANG US\ LIU YUPENG US | GUANGZHOU ELECTRONIC TECHNOLOGY CO LTD CHINESE ACADEMY OF SCIENCES US | B29C67/00 |
| 3902 | 3D printing | MULTI-HOLE SOLID CERAMIC FILTER SCREEN\ | CN202751888U | LIAO LIANG US\ LI XUEYAN US\ GUO HONGZHAO US\ WANG DONGMING US\ LI LANZHEN US\ SUN YONGRUI US\ WAN YANGWEI US\ LIU JIUPENG US | HENAN HONGDA FURNACE IND CO US | B01D39/20\ B22C9/08 |
| 3903 | 3D printing | COLORLESS TONER FORMULATED TO IMPROVE LIGHT FASTNESS OF INK JET INK PRINTS\ | US2002120040A1 | LIN AN-CHUNG ROBERT (US)\ LEE MICHAEL H (US) | Empty Field US | B41J2/01\ B41M5/00\ G03G9/087\ G03G9/08\ G03G9/09\ G03G9/097\ G03G8/00 |
| 3904 | 3D printing | RAPID OBJECT CONSTRUCTION SYSTEM WITH GRADIENT COMPOSITIONS PROGRAMMABLE UNDER HIGH TEMPERATURE\ | TW201208869A | LIN KUN-YI (TW)\ CHANG HUI-CHU (TW)\ LIN PAI-CHI (TW) | LIN KUN-YI (TW)\ CHANG HUI-CHU (TW)\ LIN PAI-CHI (TW) | B29C67/02 |
| 3905 | 3D printing | L D S (LASER DIRECT STRUCTURING) LASER ROTARY FIXTURE\ | CN202764220U | LING LEI US\ ZHANG XINSHENG US\ WANG ZHOUYAN US\ LIU WENGANG US | CHENGDU HOMIN SHUANGXIN TECHNOLOGY CO LTD US | B29C65/16 |
| 3906 | 3D printing | THREE-DIMENSIONAL VISUALIZATION METHOD FOR ELECTROMAGNETIC ENVIRONMENT BODY DATA\ | CN102254347A | LINGDA WU US\ CHAO YANG US\ RONGHUAN YU US | EQUIPMENT COMMAND AND TECHNOLOGY COLLEGE OF PLA US | G06T17/00 |
| 3907 | 3D printing | 3D ELASTIC FILLER\ | CN1861531A | LIU DUANHUA (CN) | LIU DUANHUA (CN) | C02F3/10 |
| 3908 | 3D printing | METHOD AND EQUIPMENT FOR PREPARING STRIP STRUCTURES ON CONJUGATED POLYMER SURFACES THROUGH PULSE LASERS\ | CN103193999A | LIU JIAN US\ LI XIAOHONG US\ LIU ZIYI US\ XU DANDAN US\ LI YUANYUAN US\ ZHU MIN US | UNIV SW SCI & TECH SWUST US | C08J7/00\ C08J5/18\ C08L65/00\ C08L79/02\ C08L79/04 |
| 3909 | 3D printing | ELECTRICAL ALL-DIMENSIONAL NUMERICAL CONTROL INJECTION MOLDING MACHINE\ | US2014141112A1 | LIU JUN (CN) | LIU JUN (CN) | B29C45/72\ B29C45/80 |
| 3910 | 3D printing | ELECTRIC MULTIDIRECTIONAL METAL POWDER INJECTION FORMING MACHINE\[FR] MACHINE éLECTRIQUE MULTIDIRECTIONNELLE DE MOULAGE PAR INJECTION D'UNE POUDRE MéTALLIQUE\ | WO2013020496A1 | LIU JUN (CN) | LIU JUN (CN) | B22F3/115 |
| 3911 | 3D printing | 3D (THREE-DIMENSIONAL) STRUCTURE RELEASE FILM USED FOR FORMING BICYCLE\ | CN103264501A | LIU LIEXIN US\ WU ZHENYU US\ FU GUOZHENG US | XIAMEN XINWANG NEW MATERIAL SCIENCE & TECHNOLOGY CO LTD US | B29L9/00\ B29K77/00\ B29K25/00\ B29K23/00\ B29L7/00\ B29C55/28 |
| 3912 | 3D printing | METHODS AND APPARATUS FOR FABRICATING POROUS 3-DIMENSIONAL CELL CULTURE CONSTRUCT FOR CELL CULTURE AND OTHER BIOMEDICAL APPLICATIONS\ | US8463418B2 | LIU QING (US)\ LAU WING K (US) | 3D BIOTEK LLC (US)\ LIU QING (US)\ LAU WING K (US) | G06F19/00 |
| 3913 | 3D printing | POWDER DISTRIBUTION IN ADDITIVE MANUFACTURING\ | US2015054191A1 | LJUNGBLAD ULRIC (SE) | ARCAM AB (SE)\ LJUNGBLAD ULRIC (SE) | B29C67/00\ B01J6/00 |
| 3914 | 3D printing | TARGET-LOCKING ACQUISITION WITH REAL-TIME CONFOCAL (TARC) MICROSCOPY\ | US2010195868A1 | LU PETER J (US) | LU PETER J (US) | G06T7/00 |
| 3915 | 3D printing | TEXTURED YARN LONG-FIBER REINFORCED THERMOPLASTIC PLASTIC GRANULES AND PRODUCTION METHOD THEREOF\ | CN103407169A | LU ZHIHONG US\ ZENG TIANQUAN US | JURONG BASTEP COMPOSITE MATERIALS CO LTD US | B29B9/06\ B29C70/16\ B29C70/52 |
| 3916 | 3D printing | RAPID PROTOTYPING SHAPE MEMORY HIGH POLYMER MATERIAL AND PREPARATION METHOD AND APPLICATION THEREOF\ | CN103160948A | LUO HONGSHENG US\ LUO XIAOFAN US | SUZHOU JUFU POLYMER MATERIAL CO LTD US | B29C67/00\ C08G18/32\ C08G18/42\ C08G18/10\ C08G18/48\ C08G18/66\ D01F6/78 |
| 3917 | 3D printing | POLYCRYSTALLINE MAGNETOSTRICTIVE MATERIAL OF A TB-FE-B-TYPE AND PROCESS FOR PREPARING THE SAME\ | RO123154B1 | LUPU NICOLETA (RO)\ V&ABREVELEANU MIHAELA CRISTINA (RO)\ CHIRIAC HORIA (RO) | INST NA&TCEDILIONAL DE CERCETARE DEZVOLTARE PENTRU FIZIC&ABREVE TEHNIC&ABREVE IFT IA&SCEDILI (RO) | H01F1/053\ C22C33/04 |
| 3918 | 3D printing | PASSIVE COMPONENTS FOR MILLIMETER SUBMILLIMETER AND TERAHERTZ ELECTROMAGNETIC WAVES MADE BY PILING UP SUCCESSIVE LAYERS OF MATERIAL\[FR] COMPOSANTS PASSIFS POUR ONDES éLECTROMAGNéTIQUES DE L'ORDRE DU MILLIMèTRE DU SOUS-MILLIMèTRE OU TéRAHERTZIENNES OBTENUS PAR EMPILEMENT DE COUCHES SUCCESSIVES DE MATéRIAU\ | WO2012076994A1 | MACOR ALESSANDRO (CH)\ ANSERMET JEAN-PHILIPPE (CH)\ DE RIJK EMILE (CH) | ECOLE POLYTECH (CH)\ MACOR ALESSANDRO (CH)\ ANSERMET JEAN-PHILIPPE (CH)\ DE RIJK EMILE (CH) | H01P3/123\ H01P11/00\ H01Q13/02 |
| 3919 | 3D printing | THREE-DIMENSIONAL LAMINATING MOLDING DEVICE\ | US7104773B2 | MAEKAWA TSUTOMU (JP)\ OUCHI AKEMI (JP)\ FUJII HIDETOSHI (JP)\ TAMAHASHI KUNIHIRO (JP)\ SHOJI YUTAKA (JP)\ TAKAHAGI HIROSHI (JP) | RICOH PRINTING SYS LTD (JP) | B29C35/08\ B29C67/00\ B29C41/36\ B28B17/00\ B41J2/01\ B29C41/46\ B29C41/52\ B41M5/00\ B29C41/02 |
| 3920 | 3D printing | PARYLENE COATED MICROFLUIDIC COMPONENTS AND METHODS FOR FABRICATION THEREOF\ | US2006204699A1 | MALTEZOS GEORGE (US)\ TANG HONGXING (US)\ ROUKES MICHAEL (US)\ SCHERER AXEL (US) | MALTEZOS GEORGE (US)\ TANG HONGXING (US)\ ROUKES MICHAEL (US)\ SCHERER AXEL (US) | B29C33/76\ B29C33/40\ B28B7/38\ F16L11/04 |
| 3921 | 3D printing | METHOD AND DEVICE FOR PRINTING COLOR IMAGES ON CHOCOLATE PRODUCTS\ | US2009081341A1 | MANDELBOUM ISAR (IL) | MANDELBOUM ISAR (IL) | A23P1/08\ A23G1/22 |
| 3922 | 3D printing | VERFAHREN ZUR HERSTELLUNG VON KUNSTSTOFFSPRITZGIE?TEILEN UND VORRICHTUNG ZUR DURCHFüHRUNG DES VERFAHRENS\ | DE102004043253B4 | MANZ BRUNO (DE) | SCHUSTER KUNSTSTOFFTECHNIK GMB (DE) | B29C45/14\ B29C51/00\ B29C45/00\ B29C69/00 |
| 3923 | 3D printing | HAMMER HEAD STRUCTURE OF ORE CRUSHER\ | CN201098622Y | MAOXUN ZHANG (CN)\ YUQUN LIN (CN) | FUZHOU HONGLIAN ENGINEERING MA (CN) | B02C13/28 |
| 3924 | 3D printing | HAMMERHEAD STRUCTURE OF THE ORE CRACKING MECHANISM AND PROCESSING METHOD THEREOF\ | CN101116836A | MAOXUN ZHANG (CN)\ YUQUN LIN (CN) | FUZHOU HONGLIAN ENGINEERING MA (CN) | B02C13/28\ B22D19/16 |
| 3925 | 3D printing | USE OF MOULDING COMPOSITIONS\ | US2012193839A1 | MARGRAF GUENTER (DE)\ JOACHIMI DETLEV (DE) | LANXESS DEUTSCHLAND GMBH (DE)\ MARGRAF GUENTER (DE)\ JOACHIMI DETLEV (DE) | B29C45/00\ B29C49/00\ B29C47/00\ B29C49/04 |
| 3926 | 3D printing | METHOD AND MACHINE FOR PRODUCING THREE-DIMENSIONAL OBJECTS BY MEANS OF SUCCESSIVE LAYER DEPOSITION\ | US7597835B2 | MARSAC NICOLAS (FR) | MARSAC NICOLAS (FR) | B29C35/08\ B29C67/00\ B28B1/14\ B28B1/02 |
| 3927 | 3D printing | FILM FOR PACKAGING PRODUCT ESPECIALLY AN ENVELOPE\ | US2012193402A1 | MARTINEZ DAVID (FR)\ CHAPALAIN FLORIAN (FR) | GASCOGNE LAMINATES (FR)\ CHAPALAIN FLORIAN (FR)\ MARTINEZ DAVID (FR) | B32B3/00\ B32B38/14\ B65D27/00 |
| 3928 | 3D printing | METHOD OF FABRICATING BATH-MODELS FOR NICKEL SHELLS\ | US2010102478A1 | MASCHERA FLAVIO (IT)\ BARROTE PEDRO (PT) | MASCHERA FLAVIO (IT)\ BARROTE PEDRO (PT) | B29C33/38 |
| 3929 | 3D printing | INSULATING FILM FOR ELECTRIC WIRE AND ELECTRIC WIRE USING IT\ | JP2006024473A | MATSUNAMI TAIZO US\ IMOTO HARUJI US\ MATSUNAMI TAKAAKI US | NIPPON SHEET GLASS CO LTD US | C08L23/00\ H01B17/56\ C08J9/26\ H01B7/02 |
| 3930 | 3D printing | METHOD OF INJECTION MOLDING AND INJECTION MOLDING MACHINE\ | JP2009119700A | MATSUSHITA YOSHIYUKI US | ASMO CO LTD (JP) | B29C45/23 |
| 3931 | 3D printing | SUBLIMATION INKS FOR THERMAL INKJET PRINTERS USING THERMALLY STABLE DYE PARTICLES\ | US2012162332A1 | MCKEAN DENNIS (HK)\ LEE FRANCIS CHEE-SHUEN (HK) | HK APPLIED SCIENCE & TECH RES (HK)\ MCKEAN DENNIS (HK)\ LEE FRANCIS CHEE-SHUEN (HK) | B41J2/01\ C09D11/02 |
| 3932 | 3D printing | OMNIBEARING ELECTRIC NUMERICAL CONTROL INJECTION MOLDING MACHINE\ | CN202213120U | MENTIONED THE INVENTOR HAS WAIVED THE RIGHT TO BE US | LIU JUN (CN) | B29C45/17\ B29C45/76\ B29C45/70\ B29C45/07\ B29C45/66 |
| 3933 | 3D printing | SCHMELZ-AGGREGAT EINTHALTEND EINEN INJEKTOR BLASLANZE ODER BRENNER\[EN] SMELTING UNIT COMPRISING AN INJECTOR A LANCE OR A BURNER\[FR] DISPOSITIF DE FUSION AVEC UN INJECTEUR UNE LANCE OU UN BR?LEUR\ | EP1835039B1 | MEYN MATTHIAS (DE) | SMS SIEMAG AG (DE) | C21C5/52\ F27B3/20\ C21C5/56\ F27B3/22 |
| 3934 | 3D printing | METHOD FOR PRODUCING CONTINUOUS-FIBER-REINFORCED MOLDED PARTS FROM THERMOPLASTIC PLASTIC AND MOTOR VEHICLE MOLDED PART\ | US2012269999A1 | MICHEL PETER (DE)\ KIND FRANZ-GEORG (DE) | REHAU AG & CO (DE)\ KIND FRANZ-GEORG (DE)\ MICHEL PETER (DE) | B29C35/08\ B32B5/02\ B29C37/00\ B29C45/00\ B29C69/00\ B32B9/00\ B32B1/00\ B32B17/02\ B62D25/00 |
| 3935 | 3D printing | ADDITIVE MANUFACTURING METHOD FOR BUILDING THREE-DIMENSIONAL OBJECTS WITH CORE-SHELL ARRANGEMENTS AND THREE-DIMENSIONAL OBJECTS THEREOF\ | US2013224423A1 | MIKULAK JAMES K (US)\ DECKARD CARL R (US)\ ZINNIEL ROBERT L (US) | STRATASYS INC (US) | B29C67/00\ B32B3/26 |
| 3936 | 3D printing | METHOD FOR PREPARING MELT SPUN CHLORINE-RESISTANCE HIGH RESILIENT POLYURETHANE FIBER\ | CN102011211A | MINCHAO LI US\ LIMING CHENG US\ HAIFENG CHEN US\ DONGZHI ZHANG US\ WENMING WANG US\ GAOQIANG LI US | ZHANGJIAGANG WOSITING RES INST CO LTD US | C08G18/42\ B29C47/92\ D01D5/088\ D01F6/94\ C08G18/48\ C08G18/66\ D01D1/00\ D01D1/10\ D01D10/02\ D01F1/10 |
| 3937 | 3D printing | THREE-DIMENSIONAL (3D) GLASSES\ | CN202548438U | MING ZHAO US | BOE TECHNOLOGY GROUP CO LTD US | G02B27/22\ G09G3/34\ G02B26/02 |
| 3938 | 3D printing | METHOD FOR RAPIDLY FORMING THREE-DIMENSIONAL METAL PARTS BY HIGH-POWER LASERS\ | CN102328081A | MINGMING MA US\ XIAOYAN ZENG (CN)\ ZEMIN WANG (CN)\ QIANWU HU (CN)\ XIANGYOU LI (CN) | UNIV HUAZHONG SCIENCE TECH (CN) | B22F3/105\ C23C24/10 |
| 3939 | 3D printing | METHOD AND APPARATUS FOR SHAPING THREE-DIMENSIONAL OBJECT\ | JP2005169878A | MIYAWAKI KUNIO US\ KAWACHI JOSUKE US\ KUNISHIO KAZUYOSHI US\ SUNAYAMA KAZUYUKI US\ SAITO YUKIO US\ MATSUMOTO YUJI US | HITACHI SHIPBUILDING ENG CO US | B29C67/00 |
| 3940 | 3D printing | APPARATUS FOR PRODUCING FOOD AND METHOD FOR PRODUCING FOOD\ | JP2005261286A | MIZUNO MICHIMASA US\ TERAMOTO TOSHIO (JP) | CHUBU NIPPON KOGYO KK US\ JSR CORP (JP) | A23P1/10\ A23P1/08 |
| 3941 | 3D printing | METHOD AND DEVICE FOR PRODUCING THREE-DIMENSIONAL OBJECTS USING LASER TECHNOLOGY AND FOR APPLYING AN ABSORBER USING AN INK JET METHOD\ | US2007183918A1 | MONSHEIMER SYLVIA (DE)\ BAUMANN FRANZ-ERICH (DE)\ GORING RAINER (DE)\ GREBE MARK (DE) | DEGUSSA (DE) | B29C35/08\ B29C67/00\ B22F3/105\ B22F3/10\ C22C1/04\ C22C33/02 |
| 3942 | 3D printing | METHOD AND DEVICE FOR PRODUCTION OF THREE-DIMENSIONAL OBJECTS BY MEANS OF ELECTROMAGNETIC RADIATION OF ELECTROMAGNETIC RADIATION AND APPLICATION OF AN ABSORBER BY MEANS OF AN INK-JET METHOD\ | US2007238056A1 | MONSHEIMER SYLVIA (DE)\ GREBE MAIK (DE)\ BAUMANN FRANZ-ERICH (DE) | DEGUSSA (DE) | B29C35/08\ B29C67/00\ B22F3/105 |
| 3943 | 3D printing | COPOLYAMIDE POWDER AND ITS PREPARATION USE OF COPOLYAMIDE POWDER IN A SHAPING PROCESS AND MOULDINGS PRODUCED FROM THIS COPOLYAMIDE POWDER\ | US8591797B2 | MONSHEIMER SYLVIA (DE)\ GREBE MAIK (DE)\ BAUMANN FRANZ-ERICH (DE)\ ALTKEMPER STEFAN (DE)\ SIMON ULRICH (DE)\ HESSEL-GELDMANN SIGRID (DE)\ WARNKE KRISTIANE (DE)\ STEMMER HEIKE (DE)\ MUEGGE JOACHIM (DE)\ TUERKE MICHAEL (DE) | EVONIK DEGUSSA GMBH (DE)\ GREBE MAIK (DE)\ ALTKEMPER STEFAN (DE)\ MONSHEIMER SYLVIA (DE)\ BAUMANN FRANZ-ERICH (DE)\ HESSEL-GELDMANN SIGRID (DE)\ WARNKE KRISTIANE (DE)\ STEMMER HEIKE (DE)\ MUEGGE JOACHIM (DE)\ TUERKE MICHAEL (DE)\ SIMON ULRICH (DE) | B29C35/08 |
| 3944 | 3D printing | COMPONENT PROPERTIES THROUGH BEAM SHAPING IN THE LASER SINTERING PROCESS\ | US2013183493A1 | MONSHEIMER SYLVIA (DE)\ GREBE MAIK (DE)\ DIEKMANN WOLFGANG (DE)\ KREUTZ JUERGEN (DE) | EVONIK DEGUSSA GMBH (DE)\ GREBE MAIK (DE)\ DIEKMANN WOLFGANG (DE)\ MONSHEIMER SYLVIA (DE)\ KREUTZ JUERGEN (DE) | B29C35/08\ B32B27/16 |
| 3945 | 3D printing | USE OF POLYMER POWDER PRODUCED FROM A DISPERSION IN A SHAPING PROCESS AND MOLDINGS PRODUCED FROM THIS POLYMER POWDER\ | US2007182070A1 | MONSHEIMER SYLVIA (DE)\ GREBE MAIK (DE)\ KOMADA HAJIME (JP)\ MATSUI HIDEKI (JP) | DEGUSSA (DE) | B29C35/08\ B29C41/02 |
| 3946 | 3D printing | USE OF POLYARYLENE ETHER KETONE POWDER IN A THREE-DIMENSIONAL POWDER-BASED MOLDLESS PRODUCTION PROCESS AND MOLDINGS PRODUCED THEREFROM\ | US2006134419A1 | MONSHEIMER SYLVIA (DE)\ GREBE MAIK (DE)\ KREIDLER PATRICK (DE)\ RICHTER ALEXANDER (DE) | DEGUSSA (DE) | C08G8/02\ B32B15/02 |
| 3947 | 3D printing | USE OF POLYESTER POWDER IN A SHAPING PROCESS AND MOLDINGS PRODUCED FROM THIS POLYESTER POWDER\ | US8834777B2 | MONSHEIMER SYLVIA (DE)\ GREBE MAIK (DE)\ SIMON ULRICH (DE)\ TEMME HEINRICH (DE) | EVONIK DEGUSSA GMBH (DE)\ GREBE MAIK (DE)\ MONSHEIMER SYLVIA (DE)\ SIMON ULRICH (DE)\ TEMME HEINRICH (DE) | B29C35/08\ B29C67/00\ B29C41/02\ B29C71/04\ B29K67/00 |
| 3948 | 3D printing | POLYMER POWDER PROCESS FOR PRODUCTION OF AND USE OF THIS POWDER AND RESULTANT SHAPED ARTICLES\ | US2007232753A1 | MONSHEIMER SYLVIA (DE)\ KOMADA HAJIME (JP)\ MATSUI HIDEKI (JP)\ NAKAIE YOSHIKI (JP) | DEGUSSA (DE) | C08L77/00\ C08L61/00 |
| 3949 | 3D printing | THERMOPLASTIC ELASTOMER COMPOSITION FOR CORE-BACK SYSTEM FOAM INJECTION MOLDING AND FOAM INJECTION MOLDING METHOD USING THE SAME\ | US7867416B2 | MORIKAWA AKIHIKO (JP)\ KANAE KENTAROU (JP)\ IEDA KATUMASA (JP)\ ABE TOMOKAZU (JP) | JSR CORP (JP)\ HONDA MOTOR CO LTD (JP) | B32B37/00\ B29C44/04 |
| 3950 | 3D printing | THERMOPLASTIC ELASTOMER COMPOSITION FOAM MADE FROM THE SAME AND PROCESS FOR PRODUCING FOAM\ | US6841582B2 | MORIKAWA AKIHIKO (JP)\ KANAE KENTAROU (JP)\ NAKANISHI HIDEO (JP)\ MAEDA MINORU (JP) | JSR CORP (JP) | C08L53/00\ C08J9/00\ C08L23/04\ C08L23/08\ C08L23/16\ C08L53/02 |
| 3951 | 3D printing | INTEGRATED 3D DIGITAL FILM PROJECTION APPARATUS\ | CN202995249U | MOU DUNXIAO US | MOU DUNXIAO US | G02B27/26\ G03B35/26\ G03B21/32\ G11B31/00 |
| 3952 | 3D printing | VERFAHREN UND VORRICHTUNG ZUR HERSTELLUNG EINES FILAMENTE AUFWEISENDEN VLIESSTOFFES\[EN] METHOD AND DEVICE FOR PRODUCING A NON-WOVEN FABRIC COMPRISING FILAMENTS\[FR] PROCéDé ET DISPOSITIF DESTINé à FABRIQUER UN NON-TISSé PRéSENTANT DES FILAMENTS\ | WO2009152791A1 | MUENSTERMANN ULLRICH (DE) | FLEISSNER GMBH (DE)\ MUENSTERMANN ULLRICH (DE) | D04H3/11\ B26F1/26 |
| 3953 | 3D printing | ANTI-FLAMING EXTRA-WHITE REGENERATED THREE-DIMENSIONAL CRIMP DACRON STAPLE FIBER AND MANUFACTURING METHOD THEREOF\ | CN102605457A | MUSHENG ZHANG US | HANGZHOU HANBANG CHEMICAL FIBER CO LTD US | D01F6/92\ D01F1/10\ D01F1/07 |
| 3954 | 3D printing | METHOD AND APPARATUS FOR PRODUCING THREE-DIMENSIONAL STRUCTURE\ | US2009014916A1 | NAGAI HISAO (JP)\ YOSHIDA HIDEHIRO (JP)\ FURUKAWA TAKAYUKI (JP)\ INOUE TAKASHI (JP) | MATSUSHITA ELECTRIC IND CO LTD (JP) | B81C99/00\ B29C41/08\ B05B13/02 |
| 3955 | 3D printing | NONWOVEN FABRIC OF POLYLACTATE-BASED FILAMENT AND ITS PRODUCTION\ | JPH0995850A | NAGAOKA KOICHI US\ MATSUOKA FUMIO US\ ICHINOSE NAOJI US | UNITIKA LTD US | D01F6/84\ D04H3/00\ D04H5/00 |
| 3956 | 3D printing | INJECTION MOLDING IN-MOLD DECORATION METHOD\ | JP2009143247A | NAGI YOSHIYUKI US\ ATAKE HIROYUKI (JP)\ SUGA KAZUHIRO (JP) | DAINIPPON PRINTING CO LTD (JP) | B29C45/14\ B29L9/00\ B32B27/00\ B32B33/00 |
| 3957 | 3D printing | DECORATIVE SHEET DECORATIVE MOLDED PRODUCT AND INJECTION MOLDING IN-MOLD DECORATION METHOD\ | JP2009143246A | NAGI YOSHIYUKI US\ ATAKE HIROYUKI (JP)\ SUGA KAZUHIRO (JP) | DAINIPPON PRINTING CO LTD (JP) | B32B27/00\ B29C69/02 |
| 3958 | 3D printing | MELT-EXTRUDED SUBSTRATE FOR USE IN THERMOFORMED ARTICLES\ | US8852730B2 | NAIR KAMLESH P (US)\ GRAY STEVEN D (US)\ CANO CAMILO (US) | NAIR KAMLESH P (US)\ GRAY STEVEN D (US)\ CANO CAMILO (US)\ TICONA LLC (US) | C08K5/20\ B29C43/24\ C09K19/30\ C09K19/38\ C09K19/04\ C09K19/22\ C09K19/32\ C09K19/34\ C09K19/48 |
| 3959 | 3D printing | METHOD OF MANUFACTURING LENS SHEET LENS SHEET ILLUMINATOR AND DISPLAY\ | JP2011145515A | NAKAGOME TOMOHIRO US\ SAKAKI YUICHI US\ SAKAI NATSUKA US\ KIMURA KAZUTERU US | TOPPAN PRINTING CO LTD US | G02F1/1335\ G02B3/00\ F21S2/00\ G02B5/02\ F21V5/00\ F21Y103/00 |
| 3960 | 3D printing | OPTICAL MOLDED LAMINATE\ | JP2008276150A | NAKAKOSHI YASUNOBU US | NAKAKOSHI YASUNOBU US | B29C45/14\ B29L9/00\ B29C69/00\ B29L11/00\ G02B5/30\ G02C7/12\ B29D11/00 |
| 3961 | 3D printing | INK COMPOSITION RECORDING METHOD AND RECORD AND METHOD FOR JUDGING AUTHENTICITY\ | JP2003026971A | NAKAMURA HIROTO US | SEIKO EPSON CORP (JP) | C09D11/00\ B41J2/01\ B41M5/00 |
| 3962 | 3D printing | INK COMPOSITION RECORDING METHOD AND RECORD AND METHOD FOR JUDGING AUTHENTICITY\ | JP2007177248A | NAKAMURA HIROTO US | SEIKO EPSON CORP (JP) | C09D11/00\ B41J2/01\ B41M5/00 |
| 3963 | 3D printing | INK-JET PRINTING METHOD AND PRINTED GOODS\ | US2002025379A1 | NAKAMURA SHUNGAKU (JP)\ YAMAZAKI AYUMI (JP) | SEIREN CO LTD (US) | D06P1/642\ D06P1/647\ D06P3/54\ D06P3/66\ D06P3/82\ D06P3/872\ D06P5/30 |
| 3964 | 3D printing | BINDER REMOVAL IN SELECTIVE LASER SINTERING\ | US2004081573A1 | NEWELL KENNETH J (US) | 3D SYSTEMS INC (US) | B22F1/00\ B22F3/26\ B22F3/11 |
| 3965 | 3D printing | VERWENDUNG EINER EXTRUSIONSFOLIE ZUM EINRICHTEN EINER DRUCKVORRICHTUNG\[EN] EXTRUSION FILM FOR INSTALLATION OF PRINTING DEVICE IN LABEL PRINTING MACHINE HAS DRIVING DEVICE PROVIDED FOR MATERIAL WEB AND PRINTING UNIT WHERE FILM IS FORMED IN SINGLE LAYER OR MULTILAYER AND MADE OF POLYOLEFIN\ | DE102007009110A1 | NIEPELT RALF (DE) | NORDENIA DEUTSCHLAND GRONAU (DE) | B41M5/52\ B31D1/02\ G09F3/00 |
| 3966 | 3D printing | MOSS-FIXING BASE\ | JP2004097202A | NISHIBORI SADAO (JP) | AIN KK SOGO KENKYUSHO US | A01G1/00\ E04D1/30 |
| 3967 | 3D printing | SHOCK ABSORBING APPARATUS\ | JP2005172130A | NISHIBORI SADAO (JP)\ NAKAMURA YUICHIRO (JP) | AIN KK SOGO KENKYUSHO US | F16F7/00 |
| 3968 | 3D printing | METHOD OF MANUFACTURING SOLAR CELL SEALING MATERIAL\ | JP2006186237A | NISHIJIMA KOICHI US\ KAWAMOTO MANABU US\ SATO RIE US | MITSUI DU PONT POLYCHEMICAL US\ MITSUI CHEMICAL FABRO INC US | H01L31/04 |
| 3969 | 3D printing | POLYAMIDE RESIN SOLUBLE IN ORGANIC ACID METHOD FOR PRODUCING THE SAME AND METHOD FOR PRODUCING THREE-DIMENSIONAL INJECTION MOLDED CIRCUIT PART\ | JP2004059829A | NISHIKAWA SHINYA US\ HAYAMI HIROSHI US\ SAKAMOTO YOSHITO US\ KITAMURA TAKAHIKO US\ FUJIWARA YASUHIRO US\ UEDA KOJI US\ UEDA HIDEHARU US | CHIKUNO SHOKUHIN KOGYO KK US\ SUMITOMO ELECTRIC INDUSTRIES (JP) | G03F7/037\ C08G69/48\ H05K3/00\ H05K3/18\ H05K3/38 |
| 3970 | 3D printing | METHOD FOR MANUFACTURING THREE-DIMENSIONAL INJECTION MOLDING CIRCUIT COMPONENT\ | JP2005217279A | NISHIKAWA SHINYA US\ HAYAMI HIROSHI US\ SAKAMOTO YOSHITO US\ KITAMURA TAKAHIKO US\ HOSOE AKIHISA US | SUMITOMO ELECTRIC INDUSTRIES (JP) | B29C45/14\ B29K77/00\ C25D5/56\ C25D5/02\ H01L23/14\ H05K3/00\ H05K3/18\ B29L31/34\ C23C18/28\ C25D7/00\ H05K1/03 |
| 3971 | 3D printing | DIRECT MOLDING METHOD AND DIRECT MOLDING DEVICE OF POLYMER MATERIAL\ | JP2008194968A | NISHIMURA OSAMU US\ IMOTO TOSHIYUKI US\ HOJO MASAKI US\ ADACHI TAIJI US\ NISHINO TAKASHI US | IMOTO SEISAKUSHO KK US\ UNIV KYOTO US\ UNIV KOBE US | B29C67/00\ A61L27/00 |
| 3972 | 3D printing | MANUFACTURING METHOD OF SILICON NANO WIRE\ | JP2004296750A | NODA TETSUJI US\ KO MASATOSHI US\ SE TAKA US\ SUZUKI YUTAKA US\ ARAKI HIROSHI US | NAT INST FOR MATERIALS SCIENCE US | H01L29/06\ B82B3/00\ C01B33/02 |
| 3973 | 3D printing | INK JET RECORDING METHOD AND RECORDED MATTER\ | JP2007076228A | NOJIMA TAKAHIKO US\ ITO HIROAKI US | KONICA MINOLTA PHOTO IMAGING US | C09D11/00\ B41J2/01\ B41M5/00\ B41M5/50\ B41M5/52 |
| 3974 | 3D printing | INK JET HEAD WITH BUBBLE COLLECTOR AND RECORDER EMPLOYING THE INK JET HEAD\ | JP2004142330A | NOZU TOMOHITO US\ NOJIRI HIDEAKI US\ AKAIKE MASATAKE US\ KAWASAKI TAKEHIKO US\ KURASHIMA TAMAYOSHI US\ TOYAMA KOZO US\ WADA TAKATSUGI US | CANON KK (JP) | B41J2/045\ B41J2/055\ B41J2/015 |
| 3975 | 3D printing | INK JET RECORDER USING HOT MELT INK AND ITS INK EJECTION RECOVERING METHOD\ | JP2004142329A | NOZU TOMOHITO US\ NOJIRI HIDEAKI US\ AKAIKE MASATAKE US\ KAWASAKI TAKEHIKO US\ KURASHIMA TAMAYOSHI US\ TOYAMA KOZO US\ WADA TAKATSUGI US | CANON KK (JP) | B41J2/18\ B41J2/185 |
| 3976 | 3D printing | INK JET RECORDER USING HOT MELT INK\ | JP2004142328A | NOZU TOMOHITO US\ NOJIRI HIDEAKI US\ AKAIKE MASATAKE US\ KAWASAKI TAKEHIKO US\ KURASHIMA TAMAYOSHI US\ TOYAMA KOZO US\ WADA TAKATSUGI US | CANON KK (JP) | B41J2/01\ B41J2/045\ B41J2/055\ B41J2/015 |
| 3977 | 3D printing | AU-SN ALLOY LAMINATE SOLDER AND ITS MANUFACTURING METHOD\ | JP2006110626A | OBATA MOTOKI US\ MIZUMA ISAO US\ HORIKANE YOSHIHIKO US\ TSUCHIYA JUNJI US | CITIZEN FINE TECH CO LTD US | B23K35/30\ C23C14/14\ H05K3/34\ B23K35/22\ B23K35/14\ B23K35/40\ C22C5/02 |
| 3978 | 3D printing | CONNECTION STRUCTURE BETWEEN ALUMINUM WIRE AND COPPER TERMINAL AND ALUMINUM WIRE WITH COPPER TERMINAL HAVING THE STRUCTURE\ | JP2009277674A | OGIWARA SHIGERU US | SUMITOMO ELECTRIC INDUSTRIES (JP)\ AUTONETWORKS TECHNOLOGIES LTD (JP)\ SUMITOMO WIRING SYSTEMS (JP) | H01R4/62\ H01R4/18 |
| 3979 | 3D printing | BIAXIALLY ORIENTED LAMINATED POLYESTER FILM FOR MOLDING AND SIMULTANEOUS DECORATION\ | JP2009029073A | OKUYAMA SHUNSUKE US\ KIMURA MANABU US | TEIJIN DUPONT FILMS JAPAN LTD US | B29C45/14\ B29L9/00\ B29L7/00\ B32B27/36\ B29C55/12\ B29K67/00 |
| 3980 | 3D printing | ORIENTED FILM AND POLARIZING PLATE USING THE SAME\ | JP2007031537A | ONO MITSUMASA US | TEIJIN DUPONT FILMS JAPAN LTD US | G02F1/1335\ C08J5/18\ B29C55/02\ B29K33/04\ B29K79/00\ C08F220/18\ C08F222/40\ G02B5/30 |
| 3981 | 3D printing | THREE-DIMENSIONAL NET-LIKE STRUCTURE\ | US2014370769A1 | OSAKI HIROKO (JP) | C ENG CO LTD (JP) | A47C27/12\ A47C31/00 |
| 3982 | 3D printing | THREE-DIMENSIONAL DECORATIVE OBJECT AND METHOD FOR MANUFACTURING THE SAME\ | JP2003127225A | OSHIMA NORIHIRO US\ UEDA YUJI US\ KATAOKA MASATOSHI US | MEIWA GRAVURE CO LTD US | B29C59/00\ B29L9/00\ B29C69/00\ B44C1/17\ B29C65/02\ E04F15/10\ B29C39/20\ B29C47/04\ B29L31/10 |
| 3983 | 3D printing | METHOD OF FORMING PAPER-MAKING NET\ | JP2002266298A | OTANI KENICHI US\ KOBAYASHI HIROAKI US\ TSUURA TOKUO US\ KUMAMOTO YOSHIAKI US | KAO CORP US | B29C51/08\ D21J3/00 |
| 3984 | 3D printing | METHOD FOR THE DIRECT AND CONTINUOUS PRODUCTION OF HOLLOW ARTICLES FROM A POLYMER MELT\ | US7547406B2 | OTTO BRIGITTA (DE)\ SCHAEFER ROLAND (DE)\ BACHMANN HOLGER (DE)\ HOELTING LUDWIG (DE)\ DEISS STEFAN (DE) | LURGI ZIMMER GMBH (DE) | C08G63/78\ B29C45/00\ C08J5/00\ B29B13/00\ B29C31/04\ B29D22/00\ B29B7/32\ B29B7/88\ B29C45/18\ B29C49/00\ C08G63/08\ C08G63/88\ C08J3/205 |
| 3985 | 3D printing | INK JET PRINT HEAD SUBSTRATE INK JET PRINT HEAD INK JET PRINTING APPARATUS AND METHOD OF MANUFACTURING INK JET PRINT HEAD SUBSTRATE\ | US2006256160A1 | OZAKI TERUO (JP)\ SAITO ICHIRO (JP)\ YOKOYAMA SAKAI (JP)\ ONO KENJI (JP)\ IBE SATOSHI (JP)\ SHIBATA KAZUAKI (JP)\ SAKAI TOSHIYASU (JP) | CANON KK (JP) | B41J2/04 |
| 3986 | 3D printing | METHOD OF FORMING THREE-DIMENSIONAL PRINTING IMAGE\[FR] PROCéDé DE FORMATION D'UNE IMAGE D'IMPRESSION EN TROIS DIMENSIONS\ | WO2008075450A1 | OZAWA CHIZUO (JP) | MASTERMIND CO LTD (JP)\ OZAWA CHIZUO (JP) | B41J2/01\ B41M5/00 |
| 3987 | 3D printing | PROCESS FOR THE PRODUCTION OF A BRAKING BAND OF A BRAKE DISK WITH VENTILATION DUCTS AND A BRAKING BAND PRODUCED BY THIS PROCESS\ | US7134476B2 | PACCHIANA GIOVANNI PAOLO (IT)\ GOLLER RALF SIEGFRIED (IT) | FRENI BREMBO SPA (IT) | B22D19/00\ B22C9/04\ B22C9/10\ B29C43/02\ B29C33/00\ B22D21/00\ F16D65/12\ B29C33/52\ C04B33/34 |
| 3988 | 3D printing | DREIDIMENSIONAL KONTURIERTE SANDWICHSTRUKTUR\[EN] PRODUCING A THREE DIMENSIONAL CONTOURED SANDWICH STRUCTURE INCLUDING FIRST THERMOPLASTIC COVERING LAYER SECOND THERMOPLASTIC COVERING LAYER AND CORE MATERIAL COMPRISES E.G. MACHINING OR CARRYING OUT SEPARATE PROCESSING OF CORE MATERIAL\ | DE102011006819A1 | PAESLER ERIK (DE)\ KROLL LOTHAR PROF DR (DE)\ KAUFMANN JOERG (DE)\ MEYER MATTHIAS (DE)\ MUELLER MARCO (DE) | UNIV CHEMNITZ TECH (DE) | B29C45/14\ B32B3/12\ B32B27/08\ B32B37/10\ B32B38/10 |
| 3989 | 3D printing | LASER- UND THERMISCH BESCHREIBBARE OBERFL?CHENBESCHICHTUNG FüR MATERIALIEN\[EN] LASER AND THERMALLY WRITABLE SURFACE COATING FOR MATERIALS\[FR] REVêTEMENT DE SURFACE POUR MATéRIAUX POUVANT RECEVOIR UNE INSCRIPTION PAR LASER OU PAR VOIE THERMIQUE\ | EP2429826A1 | PALKOVITS ROLAND (AT)\ SCHALKHAMMER THOMAS (AT)\ KORNHERR ANDREAS (AT) | MONDI UNCOATED FINE & KRAFT PAPER GMBH (AT) | C09D7/12\ B41M5/26\ B41M5/28\ C04B41/80\ C09D5/26\ D21H17/66\ D21H17/67\ D21H19/38\ D21H19/80\ D21H21/28\ D21H23/22 |
| 3990 | 3D printing | LOOSE-LEAF INSERTED PIN FOR ENABLING GREENING CONTAINERS CONNECTED LEFT AND RIGHT TO ROTATE RANDOMLY\ | CN203067518U | PANG QIFU US | PANG QIFU US | F16C11/00 |
| 3991 | 3D printing | INK JET PRINTER AND PRINT HEAD DEVICE THEREOF\ | US7654639B2 | PARK JEONG-YEON (KR)\ PARK SANG-CHEOL (KR) | SAMSUNG ELECTRONICS CO LTD (KR) | B41J2/16\ B41J2/14 |
| 3992 | 3D printing | MANUFACTURE METHOD OF WOOD FOSSILS\ | KR100804471B1 | PARK MIN HWA (KR) | POZOPIA (KR) | B27K3/00\ B27K3/16\ B27K3/34 |
| 3993 | 3D printing | GLASS HIGH TEMPERATURE MELTING MOLDING SPRAYER BASED ON 3D (THREE DIMENSIONAL) PRINTING TECHNOLOGY\ | CN103395973A | PENG SHOU US\ ZHANG JIALIN US | BENGBU GLASS IND DESIGN INST US\ CHINA TRIUMPH INT ENG CO LTD US | C03B19/00 |
| 3994 | 3D printing | MULTISTAGE-TEMPERATURE-CONTROL-BASED FUSED DEPOSITION MODELING (FDM) TYPE 3D PRINTING SPRAYER AND TEMPERATURE CONTROL METHOD\ | CN103240883A | PENG YONGGANG US\ WEI WEI US | UNIV ZHEJIANG US | B29C67/00 |
| 3995 | 3D printing | NOVEL 3D (THREE DIMENSIONAL) PRINTED BODY SURFACE TREATMENT PROCESS\ | CN103192524A | PENG ZHENYUN US\ XU QIMING US | WOWMAN INFORMATION SERVICES CO LTD SUZHOU US | B29C59/00 |
| 3996 | 3D printing | METHODS AND APPARATUS FOR ACTUATED FABRICATOR\ | US2013292039A1 | PETERS BENJAMIN (US)\ OXMAN NERI (US) | MASSACHUSETTS INST TECHNOLOGY (US) | B32B37/12\ B32B38/10 |
| 3997 | 3D printing | PROCESS AND DEVICE FOR PRODUCING SOLID BODIES BY SEQUENTIAL LAYER BUILDUP\ | US7285234B2 | PFEIFER ROLF (DE)\ SHEN JIALIN (DE)\ ZEPPELIN DIDIER VON (DE) | DAIMLER CHRYSLER AG (DE) | B29C67/00\ B22F3/00\ B32B37/00\ D04H1/16\ B32B38/04\ B27J5/00 |
| 3998 | 3D printing | PHOTOVOLTAIC DEVICES FABRICATED FROM NANOSTRUCTURED TEMPLATE\ | US2005098204A1 | PICHLER KARL (US)\ ROSCHEISEN MARTIN R (US)\ SAGER BRIAN M (US) | NANOSOLAR INC (US) | H01L31/0256\ B81B1/00\ C09D5/24\ H01L31/042\ B82B3/00\ C07C61/00\ C07C63/42\ C08G61/00\ H01L31/00\ H01L31/0352\ H01L31/06\ H01L51/00\ H01L51/30 |
| 3999 | 3D printing | METHOD OF MANUFACTURING THREE-DIMENSIONAL PRODUCTS THROUGH DEPOSITS OF SUCCESSIVE LAYERS OF A WIRE MELTED BY ELECTRON BEAM\[FR] PROCEDE DE FABRICATION D'ARTICLES TRIDIMENSIONNELS PAR DEPOT DE COUCHES SUCCESSIVES DE FIL FONDU PAR FAISCEAU D'ELECTRONS\ | WO2005077586A1 | PLUMB PAUL NIGEL (GB)\ DUMONT CONRAD FREDERICK (GB) | AQUASIUM TECHNOLOGY LTD (GB)\ PLUMB PAUL NIGEL (GB)\ DUMONT CONRAD FREDERICK (GB) | B23K15/00\ B23K15/06 |
| 4000 | 3D printing | METHOD AND DEVICE FOR A STREAM VORTEX TRANSFORMATION\ | US2009214339A1 | POLEACOV MIHAIL (MD)\ POLEACOVA NINA (MD) | POLEACOV MIHAIL (MD)\ POLEACOVA NINA (MD) | F01D5/14 |
| 4001 | 3D printing | METHOD OF MANUFACTURING NONWOVEN ABRASIVE ARTICLES USING DRY PARTICULATE MATERIAL\[DE] VERFAHREN ZUR HERSTELLUNG VON VLIESSTOFFSCHLEIFARTIKELN MIT TROCKENEM K?RNIGEM MATERIAL\[FR] PROCEDE PERMETTANT DE PRODUIRE DES ARTICLES ABRASIFS EN NON-TISSE AU MOYEN D'UN MATERIAU EN PARTICULES SEC\ | EP1617973A1 | POLLAUD GUY M (FR)\ MALJEAN SANDRINE (FR)\ MARTIN RIVERA CARMEN (ES)\ CABRERO GOMEZ ESTRELLA (ES) | 3M INNOVATIVE PROPERTIES CO (US) | B24D11/00 |
| 4002 | 3D printing | MULTICOLOURED FUSED DEPOSITION MODELLING PRINT\ | US2014088751A1 | POPPE DIRK (DE)\ KOHLSTRUK STEPHAN (DE)\ HAMMANN BENJAMIN (DE)\ CREMER SONJA (DE)\ BEKS KRIS (BE)\ DEWAELHEYNS LUDO (BE)\ PRIDOEHL MARKUS (DE)\ SCHMITT GUENTER (DE) | EVONIK ROEHM GMBH (DE)\ PRIDOEHL MARKUS (DE)\ SCHMITT GUENTER (DE)\ POPPE DIRK (DE)\ KOHLSTRUK STEPHAN (DE)\ HAMMANN BENJAMIN (DE)\ CREMER SONJA (DE)\ BEKS KRIS (BE)\ DEWAELHEYNS LUDO (BE) | G05B19/042 |
| 4003 | 3D printing | METHOD AND APPARATUS FOR LAYER MANUFACTURING OF ARTEFACTS\[FR] PROCéDé ET APPAREIL DE FABRICATION EN COUCHES D'ARTéFACTS\ | WO2011064725A1 | POTGIETER PAUL (ZA) | AEROSUD INNOVATION & TRAINING CT PTY LTD (ZA)\ POTGIETER PAUL (ZA) | B29C67/00\ B22F3/105 |
| 4004 | 3D printing | VORRICHTUNG UND VERFAHREN ZUM AUFTRAGEN VON FLUID AUF EIN BEWEGBARES SUBSTRAT\[EN] FLUID E.G. SEALING MATERIAL APPLICATION DEVICE HAS APPLICATION HEAD COUPLED WITH DRIVE DEVICE E.G. HYDRAULIC ACTUATOR FOR ACHIEVING PERIODIC TRANSLATION MOVEMENT WHERE APPLICATION HEAD IS MOUNTED ON LINEAR GUIDE IN FORM OF LINEAR RAIL\ | DE102007006600A1 | PUFFE WOLFGANG (DE)\ ZAEHLE FRANK (DE) | NORDSON CORP (US) | B05C5/02 |
| 4005 | 3D printing | PRODUCTION METHOD FOR TWO-MONOLAYER PAPER SUPPORTING TO PRINT AND PROCESS INTO STICKERS\ | CN101054010A | QI HELIANG YANG (CN) | SHANGHAI SUIXUAN ENTPR DEV CO (CN) | B41F17/00\ B41F13/008\ B41F13/02\ B32B39/00\ B41F13/54\ B41F19/00\ B41F23/00\ B65H5/06\ G09F3/02 |
| 4006 | 3D printing | PRINT-TYPE FLEXIBLE DISPLAY SCREEN\ | CN202189208U | QIAN JUN US\ CHAOQUN TANG US\ JICHENG HU US\ YIHUA ZHOU US\ XINZHI SHI US\ CHANG QI US\ NA CHEN US\ LISHENG MA US | BEIJING 359 INVEST CO LTD US | G02F1/1335\ G02F1/1343\ G02F1/1333\ G02F1/1334 |
| 4007 | 3D printing | THREE-DIMENSION POROUS TISSUE ENGINEERING CARRIER MATERIAL AND PREPARATION AND APPLICATION\ | CN101147812A | QING YANG (CN)\ XIJIAN WANG (CN)\ ZHIQING TAN (CN)\ XINYUAN SHEN (CN)\ LANLAN PENG (CN)\ SISHI CHEN (CN) | UNIV DONGHUA (CN) | A61L27/56\ A61L27/26 |
| 4008 | 3D printing | THREE-DIMENSIONAL STEPHANOPORATE ORGANIZATION ENGINEERING BRACKET MATERIAL FIBRE CEMENTING METHOD PREPARING SAME AND APPLICATIONS THEREOF\ | CN101249277A | QING YANG (CN)\ ZHIQING TAN (CN)\ XINYUAN SHEN (CN)\ LANLAN PENG (CN)\ SISHI CHEN (CN) | UNIV DONGHUA (CN) | A61F2/28\ A61L27/56\ A61L27/18 |
| 4009 | 3D printing | PRODUCING PROCESS FOR ANTIBACTERIAL FLAME-RETARDANT SINGLE AND MULTI-PORE THREE-DIMENSIONAL CRIMPED POLYESTER STAPLE FIBER\ | CN102704031A | QINRONG TONG US\ JIANQIU XU US\ ZUAN SUN US\ GUOZHI CAI US\ WENQIN WU US | SHANGHAI XINDILUN IND CO LTD US | C08G63/78\ D01F6/92\ D01F8/14\ D01D1/00\ D01F1/10\ D01D5/08\ D01D5/12\ D01F1/07\ D02J13/00\ C08G63/692 |
| 4010 | 3D printing | TEETH-CLEANSING MEANS\ | US6250313B1 | REES ANNE DU (SE) | DEN TOKUYO US | A61C15/04 |
| 4011 | 3D printing | METHODS AND APPARATUS FOR INCREASING IMAGE GLOSS\ | US6679600B1 | REGIMBAL LAURENT A (US) | HEWLETT PACKARD DEVELOPMENT CO (US) | G03G15/20 |
| 4012 | 3D printing | METHOD FOR PRODUCING DUROPLASTIC FINE-FIBER NON-WOVENS HAVING A HIGH FLAME-RETARDANT THERMAL PROTECTIVE AND SOUND INSULATING EFFECT\ | US2008203602A1 | RIEDEL BERND (DE)\ BUCKA HARTMUT (AT) | AMI AGROLINZ MELAMINE INT GMBH (AT) | D04H3/03\ D04H3/02\ D04C1/00 |
| 4013 | 3D printing | THREE-DIMENSIONAL NON-WOVEN FILTER\ | US2003080050A1 | ROLCHIGO PHILIP M (US)\ STIFTER TRAVIS G (US)\ KOPP CLINTON V (US)\ AUNE THOMAS M (US)\ MADSEN MICHAEL J (US) | OSMONICS INC (US) | B01D39/16\ B29C47/30\ D04H3/07 |
| 4014 | 3D printing | PROCESSLESS DIGITALLY IMAGED PRINTING PLATE USING MICROSPHERES\ | US2003180636A1 | ROSEN DANIEL (US)\ KANGA RUSTOM SAM (US) | Empty Field US | G03F7/027\ B41C1/05\ B41N1/12 |
| 4015 | 3D printing | MOLDING METHOD OF A RESIN MOLDED ARTICLE BY A MOLD APPARATUS THE MOLD APPARATUS THE RESIN MOLDED ARTICLE AND A MOLDING MACHINE HAVING THE MOLD APPARATUS\ | US2003099794A1 | SASAKI TSUYOSHI (JP)\ WATANABE OSAMU (JP)\ TAKIGAWA NAOKI (JP) | Empty Field US | B29C45/14\ B29C45/26\ B29C33/00\ B29C45/70\ B29C45/56 |
| 4016 | 3D printing | VERFAHREN ZUR ABFORMUNG VON DREIDIMENSIONALEN FREIFORMFL?CHEN MIT MIKROSTRUKTURIERTEN OBERFL?CHEN\[EN] METHOD FOR CASTING 3-D FREELY FORMABLE SHAPES WITH MICROSTRUCTURED SURFACES\[FR] PROCéDé DE PRISE D'EMPREINTE DE SURFACES DE FORME LIBRE EN TROIS DIMENSIONS DOTéES DE SURFACES MICROSTRUCTURéES\ | EP1854568B1 | SAUERESSIG KILIAN (DE) | K1 GMBH (DE) | B22C9/00 |
| 4017 | 3D printing | METHOD OF MANUFACTURING OF BEARING COMBINED REINFORCED IN SITU PILE\ | RU2334048C1 | SAURIN ANATOLIJ NIKIFOROVICH (RU)\ IL ICHEV VJACHESLAV ALEKSANDRO (RU)\ ZHADANOVSKIJ BORIS VASIL EVICH (RU)\ SAURIN JAROSLAV ANATOL EVICH (RU)\ RED KINA JULIJA VJACHESLAVOVNA (RU)\ MILJUTIN DENIS NIKOLAEVICH (RU) | SAURIN ANATOLIJ NIKIFOROVICH (RU) | E02D5/34 |
| 4018 | 3D printing | OSTEOIMPLANT AND METHOD OF MAKING SAME\[FR] IMPLANT OSSEUX ET SON PROCEDE DE FABRICATION\[DE] OSTEOIMPLANTAT UND VERFAHREN ZU SEINER HERSTELLUNG\ | EP1301222A2 | SCARBOROUGH NELSON L (US)\ MANRIQUE ALBERT (US)\ EDWARDS JEAN T (US)\ BODEN SCOTT D (US) | OSTEOTECH INC (US) | A61F2/00\ A61F2/28\ A61F2/30\ A61B17/86\ A61L31/00\ A61L27/36\ A61F2/44\ A61F2/46 |
| 4019 | 3D printing | DREIDIMENSIONALES BAUTEIL UND VERFAHREN ZUR HERSTELLUNG DESSELBEN\[EN] THREE-DIMENSIONAL COMPONENT AND METHOD FOR PRODUCING THE SAME\[FR] COMPOSANT TRIDIMENSIONNEL ET PROCéDé POUR SA FABRICATION\ | EP2051839A1 | SCHLARB ANDREAS (DE) | SCHREINER GROUP GMBH & CO KG (DE) | B29C45/14\ B32B7/12 |
| 4020 | 3D printing | METHOD AND DEVICE FOR PRODUCING A 3D OBJECT BY MEANS OF A GENERATIVE 3D-METHOD\ | US8414281B2 | SCHLEISS PETER (CH)\ LEVY GIDEON (CH) | EOS ELECTRO OPTICAL SYST (DE)\ SCHLEISS PETER (CH)\ LEVY GIDEON (CH) | B29C35/08\ B29C67/00 |
| 4021 | 3D printing | METHOD FOR PRODUCING COATING MATERIAL\ | US2013014882A1 | SCHMID JOHANNES (DE) | HOMAG HOLZBEARBEITUNGSSYSTEME (DE)\ SCHMID JOHANNES (DE) | B05D5/10\ B32B37/12 |
| 4022 | 3D printing | FIBER MATERIAL WEB\ | US2008249492A1 | SCHMIDT ANDREAS (DE) | MCAIRLAID S VLIESSTOFFE GMBH & (DE) | A61F13/15\ D21H27/00 |
| 4023 | 3D printing | PHASE CHANGE SUPPORT MATERIAL COMPOSITION\ | US7176253B2 | SCHMIDT KRIS ALAN (US)\ XU PINGYONG (US)\ RUATTA STEPHEN A (US)\ DOAN VU A (US) | 3D SYSTEMS INC (US) | B29C67/00\ C09D175/16\ C09D11/10\ C09D11/00\ B41J2/01\ B41M5/00\ C08L93/04\ C08K5/05 |
| 4024 | 3D printing | PHASE CHANGE SUPPORT MATERIAL COMPOSITION\ | US7399796B2 | SCHMIDT KRIS ALAN (US)\ XU PINGYONG (US)\ RUATTA STEPHEN A (US)\ DOAN VU A (US) | 3D SYSTEMS INC (US) | B29C67/00\ C09D175/16\ B05D3/06\ C09D11/10\ C09D11/00\ B41J2/01\ B41M5/00 |
| 4025 | 3D printing | VERFAHREN ZUR HERSTELLUNG VON ELEKTRONIKMODULEN IN 3D-TECHNOLOGIE\[EN] MANUFACTURING METHOD OF ELECTRONIC MODULE BASED ON THREE-DIMENSIONAL TECHNOLOGY INVOLVES APPLYING SOLDER PASTE ON SOLDER SURFACES OF INDIVIDUAL PRINTED CIRCUIT BOARDS (PCBS) AND HEATING PCB IN REFLOW OVEN FOR MELTING THE SOLDER PASTE\ | DE102011079278A1 | SCHNEIDER BERNHARD (DE) | IFM ELECTRONIC GMBH (DE) | H05K3/36\ H01L25/16 |
| 4026 | 3D printing | GRO?TECHNISCHES VERFAHREN UND VORRICHTUNG ZUR PLASTISCHEN VERFORMUNG VON POLYMEREN DURCH ELEKTROMAGNETISCHE STRAHLUNG\[EN] PLASTICIZATION OF POLYMER SHOWING STRONG INTERMOLECULAR INTERACTIONS E.G. CELLULOSE INVOLVES APPLICATION OF ELECTROMAGNETIC RADIATION IN PLASTICIZING UNIT WITH RADIATION-PERMEABLE COMPONENT\ | DE102006050831A1 | SCHROETER JOHANNES (DE)\ FELIX FLORIAN (DE) | SCHROETER JOHANNES (DE)\ FELIX FLORIAN (DE) | B29C47/82\ B29C45/74\ B29C47/84 |
| 4027 | 3D printing | DRUCKKOPF UND DESSEN VERWENDUNG\ | DE10353112B4 | SCHWESINGER NORBERT (DE)\ SCHAEFER EUGEN (DE) | METEC INGENIEUR AG (DE) | B41J2/175\ B41M3/16\ B41J2/14\ B41J3/32\ B41J2/38\ B41J2/475 |
| 4028 | 3D printing | MELT-ADHESIVE COMPOSITE FIBERS PROCESS FOR PRODUCING THE SAME AND FUSED FABRIC OR SURFACE MATERIAL OBTAINED THEREFROM\[DE] SCHMETZKLEBENDE VERBUNDFASERN VERFAHREN ZU IHRER HERSTELLUNG UND DARAUS HERGESTELLTER SCHMELZGEBUNDENER STOFF ODER OBERFL?CHENMATERIAL\[FR] FIBRES COMPOSéES THERMOADHéSIVES PROCéDé POUR LEUR PRODUCTION ET éTOFFE FONDUE OU MATéRIAU DE SURFACE à PARTIR DE CELLES-CI\ | EP0696655A1 | SEI ISHIZAWA (JP)\ MASAYASU SUZUKI (JP)\ HIROKAZU TERADA (JP) | CHISSO CORP (JP) | A61F13/511\ A61F13/49\ D01F8/06\ D02G1/20\ D04H1/541\ D04H1/544 |
| 4029 | 3D printing | INK FILM FOR INSERT MOLDING AND INSERT MOLDED ARTICLE\ | JP2004002629A | SEKINE YASUYOSHI US | TEIKOKU PRINTING INK MFG US | B29C45/14\ C09D11/02\ B29K25/00\ B29K69/00\ B29K105/20 |
| 4030 | 3D printing | METHOD FOR SIMULATION OF LASER MATERIAL DEPOSITION\ | US6813533B1 | SEMAK VLADIMIR V (US) | SPEC CO LTD US | G06F17/50\ B23K26/34\ G06F19/00 |
| 4031 | 3D printing | CORE-SHELL POLYAMIDE POWDER\ | US2010098880A1 | SENFF HOLGER (FR) | ARKEMA FRANCE (FR) | C08G69/00\ B05D3/00 |
| 4032 | 3D printing | APPTS. FOR PRODUCING WATER WAVE GLASS MOSAIC\ | CN2856043Y | SHEN RONGFANG XU (CN) | SHEN RONGFANG (CN) | C03B19/02\ C03B11/00 |
| 4033 | 3D printing | BUBBLE PRINT HEAD WITH INK FEED CHAMBER MADE BY INJECTION MOLDING\ | TW200409702A | SHI WANG-PING (TW)\ WEI REN-JIE (TW)\ FANG DUN-YING (TW)\ XIE SHU-PIN (TW)\ YE FENG-CHUN (TW) | MICRO BASE TECHNOLOGY CORP (TW) | Empty Field |
| 4034 | 3D printing | METHOD FOR RAPIDLY MANUFACTURING INJECTION-MOULD INSERT WITH FOLLOW-COOLING PASSAGEWAY\ | CN1850396A | SHI YUSHENG HUANG (CN) | UNIV HUAZHONG SCIENCE TECH (CN) | B22F3/105\ B22F3/24\ B29C33/38 |
| 4035 | 3D printing | WHITE POROUS POLYESTER FILM AND PREPARATION METHOD THEREOF\ | US2011046254A1 | SHIN HEON JUNG (KR)\ JU JAE SEOG (KR)\ KIM MIN JU (KR) | SKC CO LTD (KR) | C08J9/00 |
| 4036 | 3D printing | PRECIOUS METAL LAYER CERAMIC MOLDINGS AND METHOD OF MANUFACTURE\ | KR101315344B1 | SHIN HONG DAI (KR) | SHIN HONG DAI (KR) | B44C5/00\ C04B41/88 |
| 4037 | 3D printing | THERMAL TRANSFER ACCEPTANCE SHEET\ | JP2007152697A | SHINOHARA HIDEAKI US\ TANAKA YOSHIMASA US | OJI PAPER CO US | B41M5/50\ B41M5/52\ B41M5/382 |
| 4038 | 3D printing | HOLOGRAM FILM FOR INSERT MOLDING\ | JP2005003853A | SHIRAISHI KAZUO US\ OKAMOTO KOJI US\ OSATO MASAHIKO US | TOPPAN PRINTING CO LTD US | B29C45/14\ G03H1/18\ B32B7/02 |
| 4039 | 3D printing | THREE-DIMENSIONAL NETWORK STRUCTURE AND METHOD FOR PRODUCING THREE-DIMENSIONAL NETWORK STRUCTURE\ | JP2006097223A | SHIRAKI MOTOYUKI US | JATECX KK US | A47C27/12\ D04H3/07 |
| 4040 | 3D printing | EXHAUST EMISSION CONTROL STRUCTURE FOR MOTORCYCLE ENGINE\ | JP2004132345A | SHIRASAGI SADAO US\ SATO TATSUO US | OVER RACING PROJECTS KK US | F01N3/20\ B01D53/86\ F01N3/10\ F01N3/24\ F01N3/28 |
| 4041 | 3D printing | APPARATUS FOR CONTROLLED FREEZING OF MELTED SOLID INK IN A SOLID INK PRINTER\ | US2011211010A1 | SHRADER ERIC J (US)\ PASCHKEWITZ JOHN S (US) | PALO ALTO RES CT INC (US) | B41J2/175\ B41J29/377 |
| 4042 | 3D printing | WAVEGUIDE OPTICAL SWITCH AND MAKING METHOD THEREOF\ | CN100445783C | SHULIN E (CN)\ GUANGMING KONG (CN)\ HUDAN ZHAO (CN)\ WENYUAN DENG (CN) | CHANGCHUN OPTICS FINE MECH (CN) | G02B6/12\ G02B6/13\ G02B6/35 |
| 4043 | 3D printing | METHOD FOR PRODUCTION OF A THREE-DIMENSIONAL BODY\ | US2013300035A1 | SNIS ANDERS (SE) | ARCAM AB (SE)\ SNIS ANDERS (SE) | B29C67/00 |
| 4044 | 3D printing | METHOD FOR PREVENTING NOZZLE CONTAMINATION DURING WARM-UP\ | US7762656B2 | SNYDER TREVOR JAMES (US) | XEROX CORP (US) | B41J2/165\ B41J2/175 |
| 4045 | 3D printing | MELTING HEAT TRANSFER RIBBON FOR METALLIC PRINT\ | JP2005335319A | SOGABE ATSUSHI US\ FUJIMOTO KOTARO US | FUJI KAGAKU SHIKOGYO US | B41M5/30\ B41M5/42\ B41J31/00\ B41J31/05\ B41M5/382\ B41M5/385\ B41M5/39\ B41M5/40\ B41M5/41 |
| 4046 | 3D printing | PHOTO-BOOK LINKED TO WEB-SITE AND MANUFACTURING METHOD THEREOF\ | KR20130082544A | SONG CHANG HOON (KR) | ABYZ CO LTD (KR) | G06K9/18\ G06Q50/10 |
| 4047 | 3D printing | METHOD FOR MANUFACTURING REDUCER OF COAL PULVERIZER\ | KR101057668B1 | SONG JONG SEOP (KR) | SECO LTD (KR) | B02C15/16\ B02C4/42\ B02C15/04 |
| 4048 | 3D printing | THERMOCHROMIC BUILD MATERIALS\ | US2013292881A1 | STEINER ROBERT J (US)\ VON SICE CORRIE M (US) | STEINER ROBERT J (US)\ VON SICE CORRIE M (US) | B41M5/34 |
| 4049 | 3D printing | METHOD FOR INJECTION MOLDING OF THERMOPLASTIC POLYMER MATERIAL WITH CONTINUOUS PROPERTY TRANSITIONS\ | US2010112267A1 | STENZEL HOLGER (DE)\ RIDDER HELMUT (DE)\ SCHNIEDERS JOACHIM (DE) | KURARAY EUROPE GMBH (DE) | B29C45/16\ B32B5/14 |
| 4050 | 3D printing | POROUS CERAMIC BODY AND METHOD FOR PRODUCTION THEREOF\ | US2006236668A1 | STOBBE PER (DK)\ HAOK UDO (DE) | HAOK UDO (DE)\ STOBBE PER (DK) | C04B38/00\ B01D71/02\ B01D46/00 |
| 4051 | 3D printing | VERWENDUNG VON POLYAMID-FORMMASSEN ZUR HERSTELLUNG VON FORMTEILEN MIT REDUZIERTER OBERFL?CHENVERKOHLUNG\[EN] USE OF POLYAMIDE MOULDING MATERIALS FOR THE FABRICATION OF MOULDED ARTICLES WITH REDUCED SUPERFICIAL CHARRING\[FR] UTILISATION DE COMPOSITIONS à MOULER DE POLYAMIDE POUR LA FABRICATION D'ARTICLES MOULéS à CARBONISATION SUPERFICIELLE RéDUITE\ | EP1780241A1 | STOEPPELMANN GEORG DR RER NAT (CH)\ KETTL RALPH DIPL-ING (CH)\ HOFFMANN BOTHO DR RER NAT (CH)\ EBERT MARTINA DR RER NAT DIPL (CH)\ HEWEL MANFRED DR RER NAT DIPL (CH) | EMS CHEMIE AG (CH) | C08L77/00\ C08K3/34 |
| 4052 | 3D printing | W?RMEBEHANDLUNGSOFEN MIT INFRAROTSTRAHLERN\[EN] HEAT-TREATMENT FURNACE WITH INFRARED EMITTERS\[FR] FOUR DE TRAITEMENT THERMIQUE AVEC éMETTEURS DE RAYONS INFRAROUGES\ | WO2007134346A1 | SUESS HARALD (AT)\ HEMEDINGER GERALD (AT)\ SCHMIDT THOMAS (AT) | TIGERWERK LACK UND FARBENFABRI (AT)\ SUESS HARALD (AT)\ HEMEDINGER GERALD (AT)\ SCHMIDT THOMAS (AT) | B05D3/02\ F27B9/06\ F26B3/30\ F27D11/12 |
| 4053 | 3D printing | DECORATING SHEET DECORATING MOLDED ARTICLE AND IN-MOLD DECORATING INJECTION MOLDING\ | US2007026197A1 | SUGA KAZUHIRO (JP)\ MEIKI YOSHIYUKI (JP)\ ATAKE HIROYUKI (JP) | DAINIPPON PRINTING CO LTD (JP) | B29C45/14\ B29C51/02\ B29L9/00\ B32B3/00\ B29C51/10\ B29C45/26\ B44C3/02\ B32B27/06\ B32B27/00\ B32B3/28\ B29C33/18\ B29K105/20 |
| 4054 | 3D printing | ROTATIONAL SPEED DETECTOR\ | JP2004108968A | SUGANUMA TAKESHI US | TAMAGAWA SEIKI CO LTD US | G01P3/488\ G01P1/02 |
| 4055 | 3D printing | METHOD AND APPARATUS FOR PRODUCING GRANULAR CRYSTAL\ | US2008029020A1 | SUGAWARA SHIN (JP)\ TAKAHASHI EIGO (JP)\ ARIMUNE HISAO (JP) | KYOCERA CORP (JP) | C30B30/06 |
| 4056 | 3D printing | INTEGRATABLE ASSISTED COOLING SYSTEM FOR PRECISION EXTRUSION DEPOSITION IN THE FABRICATION OF 3D SCAFFOLDS\ | US2012080814A1 | SUN WEI (US)\ HAMID QUDUS (US) | SUN WEI (US)\ HAMID QUDUS (US)\ UNIV DREXEL (US) | B29C47/78\ F28F9/00 |
| 4057 | 3D printing | METHOD FOR PRODUCING POLYACETAL RESIN COMPOSITION REDUCED IN FORMALDEHYDE CONTENT\ | JP2006291001A | SUNAGA DAISUKE US\ NAGAI SATOSHI US\ OKAMURA AKIRA US | MITSUBISHI GAS CHEMICAL CO US | C08G2/28 |
| 4058 | 3D printing | PLASMATRON\ | RU2225084C1 | SUSLOV V I (RU) | UNIV SUNGKYUNKWAN FOUND (KR) | H05H1/42\ H05B7/20\ B05B7/22 |
| 4059 | 3D printing | LASER-PRODUCED POROUS SURFACE\ | US2004191106A1 | SUTCLIFFE CHRISTOPHER J (GB)\ JONES ERIC (IE)\ O'NEILL WILLIAM (GB) | HOWMEDICA OSTEONICS CORP (US) | A61F2/00\ A61F2/30\ B22F3/105\ B22F7/00\ C23C24/10\ A61F2/38\ C23C26/02\ C23C4/12\ A61F2/36\ C23C24/04\ C23C4/18\ A61F2/34\ B23K26/38\ C23C4/02 |
| 4060 | 3D printing | CHEMICAL VAPOR DEPOSITION RAW MATERIAL COMPRISING ORGANIC NICKEL COMPOUND AND CHEMICAL VAPOR DEPOSITION METHOD USING SAID CHEMICAL VAPOR DEPOSITION RAW MATERIAL\[FR] MATIèRE PREMIèRE DE DéP?T CHIMIQUE EN PHASE VAPEUR COMPRENANT UN COMPOSé ORGANIQUE DU NICKEL ET PROCéDé DE DéP?T CHIMIQUE EN PHASE VAPEUR UTILISANT LADITE MATIèRE PREMIèRE DE DéP?T CHIMIQUE EN PHASE VAPEUR\ | WO2014188629A1 | SUZUKI KAZUHARU (JP)\ SAITO MASAYUKI (JP)\ HARADA RYOSUKE (JP)\ NABEYA SHUNICHI (JP)\ MIYAZAKI SATOSHI (JP) | TANAKA PRECIOUS METAL IND (JP) | C07F15/04\ C23C16/18 |
| 4061 | 3D printing | PREFORM FOR OPTICAL FIBER AND ITS PRODUCTION\ | JP2000264662A | SUZUKI SHINJI US\ SHIMADA TADAKATSU US\ HIRASAWA HIDEO US | SHINETSU CHEMICAL CO US | G02B6/00\ C03B37/012 |
| 4062 | 3D printing | MANUFACTURE OF DECORATIVE SHEET\ | JP8224796A | SUZUKI YUKIO US\ NISHIMURA IKUMASA US\ AIZAWA TETSUO US | TOPPAN PRINTING CO LTD US | B29C59/00\ B29L9/00\ B29C69/00\ B32B27/00\ B05D3/04\ B32B33/00\ B32B27/32\ B29C47/88\ B29D7/01\ B29K23/00\ B29L7/00\ B29C59/04\ B41M1/24\ B29C47/14\ B41M1/30 |
| 4063 | 3D printing | PD-COATED COPPER BALL BONDING WIRE\[FR] FIL DE SOUDAGE PAR BOULE EN CUIVRE REVêTU DE PALLADIUM\ | WO2013094482A1 | TAKADA MITSUO (JP)\ YAMASHITA TSUTOMU (JP)\ SHIGYOU HIROYUKI (JP)\ KUWAHARA TAKASHI (JP)\ OKAZAKI JUNICHI (JP)\ SAITOU SHIGERU (JP) | TANAKA ELECTRONICS IND (JP) | H01L21/60 |
| 4064 | 3D printing | RELAXATION SLEEP-INDUCING BEDDING\ | JP2005143522A | TAKAKURA JAKUKO US\ MURATA TOSHIHIRO US | KYOTO NISHIKAWA & CO US\ ONKYOORIBU KK US | A61H23/02\ A61F7/08\ A61M21/02\ B06B1/04 |
| 4065 | 3D printing | MATTRESS FOR PREVENTING DEW CONDENSATION AND METHOD OF MANUFACTURING THE MATTRESS\ | JP2010279687A | TAKAOKA NOBUYUKI (JP) | SHIIENJI KK US | A47C27/12 |
| 4066 | 3D printing | DECORATIVE SHEET\ | JP2005119021A | TAKASAKI YUTAKA US | TOPPAN PRINTING CO LTD US | B32B27/20\ B29L9/00\ B32B27/00\ B29L7/00\ B29C59/04 |
| 4067 | 3D printing | AUTOMOBILE WIRE HARNESS AND METHOD FOR MANUFACTURING WIRE HARNESS FOR AUTOMOBILE\ | JP2009104889A | TAKASE SHINICHI US\ NAKAGAKI KAZUYUKI US | SUMITOMO ELECTRIC INDUSTRIES (JP)\ AUTONETWORKS TECHNOLOGIES LTD (JP)\ SUMITOMO WIRING SYSTEMS (JP) | H01B7/00\ H01B13/012 |
| 4068 | 3D printing | METHOD OF MANUFACTURING POWER RECEIVING SYSTEM HOUSING IN WIRELESS CHARGING SYSTEM\ | JP2011109546A | TAKEUCHI SHINYA US | NISSHA PRINTING US | H04M1/02\ H02J7/00 |
| 4069 | 3D printing | QUARTZ-BASED OPTICAL WAVEGUIDE AND MANUFACTURING METHOD FOR THE SAME\ | JP2008165014A | TANAKA KOTARO US | HITACHI CABLE (JP) | G02B6/12\ G02B6/13\ G02F1/01 |
| 4070 | 3D printing | HAIR ORNAMENT\ | JP2007215697A | TANAKA TAMAEI US | TANAKA TAMAEI US | A45D8/00 |
| 4071 | 3D printing | METAL BOND GRINDSTONE AND ITS MANUFACTURING METHOD\ | JP2005161510A | TANIGAWA YOSHIHIRO US\ NAKAMURA NORIKAZU US | TANIGAWA YOSHIHIRO US\ NAKAMURA NORIKAZU US | B24D3/00\ B24D3/06 |
| 4072 | 3D printing | ELECTROCONDUCTIVE SHEET\ | JP2011056668A | TERUI TOMOKO US\ TERAMAE KIMITO US | LONSEAL KOGYO KK US | B29L9/00\ B32B27/18\ H01B1/20\ B29K101/12\ B29K105/16\ B29C43/24\ B29C43/18\ B29L7/00\ B29C43/34\ C08K7/02\ C08L101/12\ B29K105/12 |
| 4073 | 3D printing | ELECTRIC MULTI-AZIMUTH METAL POWDER INJECTION MOLDING MACHINE\ | CN102921949A | THE INVENTOR HAS WAIVED THE RIGHT TO BE MENTIONED US | LIU YA US | B22F3/115 |
| 4074 | 3D printing | METHOD OF FORMING A COATING OR OF THREE-DIMENSIONAL STRUCTURAL ELEMENTS ON SUBSTRATE SURFACES WHICH IS/ARE FORMED BY TIAL BY LASER BUILD-UP WELDING\ | US2015041442A1 | THIEME SEBASTIAN (DE)\ LEYENS CHRISTOPH (DE)\ NOWOTNY STEFFEN (DE) | FRAUNHOFER GES FORSCHUNG (DE) | B23K26/34\ B23K35/02\ B23K35/28\ B23K35/32 |
| 4075 | 3D printing | PROCESS FOR PRODUCING SHEATH-CORE STAPLE FIBERS WITH A THREE-DIMENSIONAL CRIMP AND A CORRESPONDING SHEATH-CORE STAPLE FIBER\ | US2009124155A1 | TIEMEIER HENDRIK (DE)\ LABITZKE EKKEHARD (DE) | OERLIKON TEXTILE GMBH & CO KG (DE) | B32B5/02\ D04H1/00\ B29C47/08 |
| 4076 | 3D printing | METHOD AND SYSTEM FOR LEADING MACROMOLECULE SUBSTANCES INTO LIVING TARGET CELLS\ | US2005085711A1 | TIEN DER-YANG (TW) | GEORGIA TECH RES INST (US) | A61M37/00\ A61B19/00\ A61K48/00\ A61M5/14\ A61B17/22\ A61K9/10\ A61K45/00\ A61K49/00 |
| 4077 | 3D printing | LIQUID EJECTOR AND PROCESS FOR FORMING SMALL PROTRUSION BY LIQUID EJECTION\ | JP2004249685A | TOGASHI TAKAYUKI US | SEIKO EPSON CORP (JP) | C09D11/00\ B41J2/01\ C09D11/02\ B41J3/32\ B41J2/015 |
| 4078 | 3D printing | THERMOFORMBARES VERPACKUNGSMATERIAL MIT INNENLIEGENDEM DRUCKBILD\[EN] THERMOFORMABLE PACKAGING MATERIAL WITH AN INTERNAL PRINTED IMAGE\[FR] MATERIAU D'EMBALLAGE THERMOFORMABLE COMPRENANT UNE IMAGE IMPRIMéE à L'INTéRIEUR\ | EP1724105A1 | TOIVOLA TIMO (FI)\ LEHTO KEIJO (FI)\ JUNNILA JARMO (FI)\ SILANDER HARRI (FI)\ SAARISTO ESKO (FI)\ KASCHEL GREGOR DR (DE)\ BRANDT RAINER DR (DE) | WIPAK WALSRODE GMBH & CO KG (DE) | B32B27/00 |
| 4079 | 3D printing | COMPOSITION AND METHODS FOR THE PRODUCTION OF BIOPOLYMERIC MATERIALS THAT BIODEGRADE RAPIDLY AND ARE FLEXIBLE AND RIGID WITH USE OF THE XANTHAN BIOPLASTIC AS A COMPONENT OF THE BIOPOLYMETRIC MATRIX AND OPTIONALLY FILLERS AND/OR NANOFILLERS AND OTHER CONSTITUENTS THE RESULTING PRODUCTS AND THE USES THEREOF\[FR] COMPOSITION ET PROCéDéS DE PRODUCTION DE MATIèRES BIOPOLYMèRES à BIODéGRADATION RAPIDE SOUPLES ET RIGIDES AVEC UTILISATION DU BIOPLASTIQUE XANTHANE COMPOSANT LA MATRICE BIOPOLYMèRE ET éVENTUELLEMENT DES CHARGES ET/OU DES NANOCHARGES ET D'AUTRES CONSTITUANTS PRODUITS OBTENUS ET LEURS UTILISATIONS\[PT] COMPOSI??O E MéTODOS DE PRODU??O DE MATERIAIS BIOPOLIMéRICOS DE RáPIDA BIODEGRADA??O FLEXíVEIS E RíGIDOS COM USO DO BIOPLáSTICO XANTANA COMPONDO A MATRIZ BIOPOLIMéRICA E OPCIONALMENTE CARGAS E/OU NANOCARGAS ê OUTROS CONSTITUINTES; PRODUTOS OBTIDOS E SEUS USOS\ | WO2013181731A1 | TONDO VENDRUSCOLO CLAIRE (BR)\ DA SILVA MOREIRA ANGELITA (BR)\ DE ANDRADE SALLES CLEIA (BR)\ CALCAGNO CARMEN IARA WALTER (BR) | IFSUL INST FED DE EDUCACAO CIENCIA E TECNOLOGIA SUL RIO GRANDENSE (BR)\ UFPEL UNIVERSIDADE FED DE PELOTAS (BR) | C08K3/32\ A61K8/73\ A61Q11/00\ C08K3/34\ C12P19/04\ A23L1/0522\ A23L1/054\ A61K31/685\ A61K31/722\ A61P19/02\ A61P19/04\ C07H3/00\ C07K14/195\ C08B37/00\ C08L3/02\ C09K8/02\ C09K8/08\ C12N1/20\ C12N9/10\ C12P19/06\ C12R1/64 |
| 4080 | 3D printing | A MANUFACTURE METHOD AND PRODUCT OF AN ARTIFICIAL BOARD WITH DUAL-COLOR AND THREE-DIMENSIONAL CONVEX PATTERNS\ | TW585959B | TSAI JE-AN (TW) | TSAI JE-AN (TW) | E04C2/00 |
| 4081 | 3D printing | POLYMERIC ADDITIVES TO IMPROVE PRINT QUALITY AND PERMANENCE ATTRIBUTES IN INK-JET INKS\ | US2002156153A1 | TSANG JOSEPH W (US)\ MOFFATT JOHN R (US) | CHEN WEN-ZHENG (TW) | C09D11/00\ B41J2/01\ B41M5/00\ B41J2/21\ B41J11/00 |
| 4082 | 3D printing | FALSE PORCELAIN TILE AND MANUFACTURING METHOD THEREFOR\ | JP2004225379A | TSUKAMOTO KAZUO US\ YAMAMOTO TADAHIKO US | MITSUBOSHI CO LTD US | E04F15/10 |
| 4083 | 3D printing | PHASE CHANGE INKS\ | US2010080922A1 | TUREK CAROLINE M (CA)\ BRETON MARCEL P (CA) | XEROX CORP (US) | C09D175/04\ C09D11/02\ B05D5/00\ B05D1/04 |
| 4084 | 3D printing | BENDING RECEIVER USING HEAT-SHRINKABLE FILM\ | US8227165B2 | TYAGI DINESH (US) | EASTMAN KODAK CO (US)\ TYAGI DINESH (US) | G03G5/00 |
| 4085 | 3D printing | METHOD AND APPARATUS FOR PRODUCING CELLULOSE RESIN FILM AND CELLULOSE RESIN FILM AND FUNCTIONAL FILM\ | US2008075922A1 | UEDA TADASHI (JP) | FUJIFILM CORP (JP) | C08J5/18 |
| 4086 | 3D printing | PULP MOULD PAPER CONTAINER\ | JP2005096790A | UEDA YOSHIKI US | TOPPAN PRINTING CO LTD US | B32B1/02\ B65D25/34\ B32B29/00\ B65D1/26\ B65D1/40\ B65D25/14\ B65D81/38 |
| 4087 | 3D printing | PRODUCTION PROCESS OF A NONWOVEN FABRIC SHEET\ | US2014174628A1 | UEMATSU KATSUHIRO (JP)\ OBA TORU (JP)\ MIZUTANI SATOSHI (JP) | UNICHARM CORP (JP) | D04H13/00\ A61F13/511\ A61F13/49\ A61F13/15\ A61F13/53\ D04H1/559\ D04H1/70 |
| 4088 | 3D printing | SPINNERET PACK FOR MELT SPINNING AND METHOD FOR PRODUCING THERMOPLASTIC RESIN FIBER\ | JP2004292988A | UTSUNOMIYA KUNIHIRO US | TORAY INDUSTRIES (JP) | D01D4/08 |
| 4089 | 3D printing | VASCULARIZED TISSUE REGENERATION MATRICES FORMED BY SOLID FREE FORM FABRICATION TECHNIQUES\ | US6176874B1 | VACANTI JOSEPH P (US)\ CIMA MICHAEL J (US)\ CIMA LINDA G (US) | CHILDRENS MEDICAL CENTER (US)\ MASSCHUSETTS INST OF TECHNOLOG (US) | B29C67/00\ A61L27/00\ A61F2/00\ A61F2/02\ A61F2/06\ A61F2/10\ A61F2/28\ A61F2/30\ A61K9/00\ A61K9/16\ A61L27/38\ A61L27/40\ A61L27/56\ A61L27/60\ B82B1/00 |
| 4090 | 3D printing | ELECTROMAGNETIC RADIATION DETECTOR AND METHOD FOR MANUFACTURING SUCH A DETECTOR\ | US7999227B2 | VILAIN MICHEL (FR) | ULIS (FR) | G01J5/02 |
| 4091 | 3D printing | RAPID PROTOTYPING AND MANUFACTURING SYSTEM AND METHOD\ | US7690909B2 | WAHLSTROM BEN (US) | 3D SYSTEMS INC (US) | B28B1/16\ B05C11/02\ B29B13/08 |
| 4092 | 3D printing | VERFAHREN ZUR HERSTELLUNG VON EINER KAUTSCHUK-/GEWEBE-VERBUNDPRODUKTE\[EN] METHODS FOR MAKING RUBBER/FABRIC COMPOSITE PRODUCTS\[FR] PROCEDES POUR LA FABRICATION DE PRODUITS COMPOSITES A BASE DE CAOUTCHOUC/TISSU\ | EP1778464A2 | WALTERS IAN (GB)\ HOUGH PHILIP (GB) | HAYDALE LTD (GB) | B29D99/00\ B29C65/48\ B29C70/74 |
| 4093 | 3D printing | PRESS FIT CONNECTOR ATTACHMENT METHOD\ | JP2005276705A | WANDA YOSHIHIRO US | DENSO CORP (JP) | H01R43/20\ H05K3/32 |
| 4094 | 3D printing | HOUSING OF ELECTRONIC DEVICE AND METHOD FOR MAKING THE HOUSING\ | TW201110457A | WANG QIANG (CN) | FIH HONG KONG LTD (HK) | H01Q1/22 |
| 4095 | 3D printing | METHOD FOR DRIVING PERIODIC FLOW OF METAL MELT ON BASIS OF SPIRAL MAGNETIC FIELD OF ELECTRIFIED COIL\ | CN102980415A | WANG XIAODONG US | GUCAS US | F27D27/00 |
| 4096 | 3D printing | METHOD AND APPARATUS FOR CREATING A FREE-FORM THREE-DIMENSIONAL METAL PART USING HIGH-TEMPERATURE DIRECT LASER MELTING\ | US6621039B2 | WANG XINHUA (SG)\ WANG YUNGAN (SG)\ LUO JIHUAN (SG)\ YU YUEFENG (SG)\ ZHANG XIANGLIN (SG)\ TANG YAXIN (SG)\ LU LI (SG)\ WANG XUAN (SG)\ FUH YING HSI JERRY (SG)\ LIM KUAK CHOI LEALIC (SG)\ WONG YOKE SAN (SG)\ CHENG HONGBIN (SG) | KINERGY PTE LTD (SG)\ UNIV SINGAPORE (SG) | B22F3/00\ B22F3/105 |
| 4097 | 3D printing | LIGHT TURNING DEVICE\ | CN2842463Y | WANG YANBIN (CN) | LASER FUSION RES CT CHINA ENGI (CN) | G02B17/04 |
| 4098 | 3D printing | PREPARATION METHOD OF DETACHABLE PLANT THREE-DIMENSIONAL SPECIMEN\ | CN102835390A | WANG YING US\ CAI BAIYAN US\ LI YAN US | UNIV EAST HEILONGJIANG US | A01N3/00 |
| 4099 | 3D printing | PRODUCTION METHOD OF DOUBLE METAL NETWORK-INTERPENETRATED MULTIPHASE SECTION\ | CN101825218B | WANG YONG US\ CUI MIN US\ MA RU US\ WANG SHOUREN US | UNIV JINAN US | F16S5/00 |
| 4100 | 3D printing | SINGLE-NOZZLE MELT-EXTRUSION-TYPE THREE-DIMENSIONAL PRINTER\ | CN103182781A | WANG YUNGAN (SG)\ WANG XUAN (SG) | SHANGHAI FOCHIF MECHATRONICS TECHNOLOGY CO LTD US | B29C47/92\ B29C69/02\ B29C47/08\ B29C47/12\ B29C47/34\ B29C53/56 |
| 4101 | 3D printing | SPECIAL POLYCARBONATE FILM FOR 3D AUTOMOBILE INSTRUMENT PANEL AND PREPARATION METHOD THEREOF\ | CN102019854A | WEI LUO US\ YUEZHANG REN US | SUZHOU AOMEI MATERIALS TECHNOLOGY CO LTD US | B60K35/00\ B29C47/92\ B29L7/00\ B29K69/00 |
| 4102 | 3D printing | 2.5-TIMES THREE-DIMENSIONAL SEISMIC EXPLORATION METHOD OF COMPLEX GEOLOGICAL TARGET\ | CN102707316A | WEI ZHANG US\ XIANZHENG ZHAO US\ XUMING BAI US\ CHUANZHANG TANG US\ ZHIWEN DENG US\ FENG YAN US\ QIUYAN YE US\ WANG LIU US\ FENGMING JIN US\ ZHANZU LIU US\ YI QIU US | PETROCHINA CO LTD US | G01V1/28\ G01V1/30 |
| 4103 | 3D printing | INJECTION MOULD PRODUCT IN-MOULD PRINTING MOLDING METHOD\ | CN101474849A | WEIXIA ZHU (CN) | WEIXIA ZHU (CN) | B29C45/17 |
| 4104 | 3D printing | INJECTION MOULD PRODUCT IN-MOULD PRINTING MOLDING EQUIPMENT\ | CN101474848A | WEIXIA ZHU (CN) | WEIXIA ZHU (CN) | B29C45/17 |
| 4105 | 3D printing | PRINTING MEMBRANE\ | CN201075278Y | WENJIAN LUO (CN) | LUOTIE MACHINERY CO LTD (CN) | G09F23/00 |
| 4106 | 3D printing | STEREO PROJECTION SYSTEM\ | CN202306131U | WENJIN LIAO US\ JINLIANG HUANG US\ JIANPING HE US | SHENZHEN SAIYE EXHIBITION DISPLAY CO LTD US | H04N13/00\ G03B35/18\ G03B21/60\ G06F3/14 |
| 4107 | 3D printing | THREE-DIMENSIONAL THREE-U ENERGY-SAVING LAMP TUBE WITH ONE GAP BRIDGE\ | CN201383484Y | WENYUAN QIAN US | WENYUAN QIAN US | H01J61/00\ H01J61/28\ H01J61/30\ H01J61/36 |
| 4108 | 3D printing | PROCESS FOR LAMINATING AN INK JET PRINT\ | US6454896B1 | WEXLER ALLAN (US) | EASTMAN KODAK CO (US) | B41J2/01\ B41M7/00\ G03C11/08\ G03G8/00 |
| 4109 | 3D printing | OBJECT CONSOLIDATION THROUGH SEQUENTIAL MATERIAL DEPOSITION\ | US6814823B1 | WHITE DAWN (US) | SOLIDICA INC (US) | B29C67/00\ G05B19/4099\ B23K11/00\ B23K20/10\ B23K20/12\ B29C73/00 |
| 4110 | 3D printing | METHODS FOR FABRICATING THREE-DIMENSIONAL ALL ORGANIC INTERCONNECT STRUCTURES\ | US2004000425A1 | WHITE GEORGE E (US)\ DALMIA SIDHARTH (US)\ SUNDARAM VENKATESH (US)\ SWAMINATHAN MADHAVAN (US) | Empty Field US | H01F17/00\ H05K3/46\ H01F41/04\ H01L21/48\ H01L25/065\ H01L27/08\ H05K1/16 |
| 4111 | 3D printing | METHOD OF FORMING A PHASE CHANGE INK IMAGE ON A SELF-LAMINATING RECORDING MEDIUM\ | US2008049090A1 | WILLEMS GUIDO G (NL)\ ROOIJAKKERS JOHANNES P J C (NL)\ STURME RUDOLF A H M (NL) | OCE TECH BV (NL) | B41J2/01 |
| 4112 | 3D printing | BILDERZEUGUNGSVERFAHREN DAS EINE PHASENAUSTAUSCHTINTE AUF EINEM SELBSTLAMINIERENDEN AUFZEICHNUNGSMEDIUM VERWENDET\[EN] METHOD OF FORMING A PHASE CHANGE INK IMAGE ON A SELF-LAMINATING RECORDING MEDIUM\[FR] PROCéDé DE FORMATION D'IMAGE UTILISANT UNE ENCRE à CHANGEMENT DE PHASE SUR UN MATéRIAU AUTO-LAMINANT\ | EP1892115A1 | WILLEMS GUIDO G (NL)\ ROOIJAKKERS JOHANNES P J C (NL)\ STURME RUDOLF A H M (NL) | OCE TECH BV (NL) | B41M7/00 |
| 4113 | 3D printing | CS PADLOCK SEAL\ | SG178641A1 | WILLY KOH KEE JOO (SG) | RACER TECHNOLOGY PTE LTD (SG) | Empty Field |
| 4114 | 3D printing | RASTER METHODOLOGY APPARATUS AND SYSTEM FOR ELECTRON BEAM LAYER MANUFACTURING USING CLOSED LOOP CONTROL\ | US2014158667A1 | WOLLENHAUPT PHILLIP E (US)\ STECKER SCOTT (US) | SCIAKY INC (US) | B23K15/00\ B23K15/06\ B23K15/02 |
| 4115 | 3D printing | FUSE\ | US2014218159A1 | WOSGIEN JOACHIM (DE)\ LANGE RALF (DE) | WOSGIEN JOACHIM (DE)\ LANGE RALF (DE) | H01H85/36\ H01H85/143\ H01H85/30 |
| 4116 | 3D printing | LASER 3D (THREE-DIMENSIONAL) PRINTING METHOD OF CERAMIC FUNCTIONAL GRADIENT COMPONENT\ | CN103317590A | WU DONGJIANG US\ NIU FANGYONG US\ MA GUANGYI US\ GUO DONGMING US | UNIV DALIAN TECH (CN) | B28B1/24 |
| 4117 | 3D printing | MANUFACTURING METHOD OF SILICON NITRIDE HEATING PLATE\ | CN102329134A | XIANGLING DENG US\ BIN MA US | XIANGLING DENG US | C04B35/622\ C04B35/584 |
| 4118 | 3D printing | SPRAY FORMING SYSTEM FOR PREPARING ALLOY AND METAL-BASED COMPOSITE PARTS\ | CN102319898A | XIANJIE ZHANG US\ JUNBIAO WANG US\ CHAORUN SI US\ YUJUN LI US\ XIAOBAO CAO US | UNIV NORTHWESTERN POLYTECHNIC US | B22F3/115 |
| 4119 | 3D printing | DEVICE FOR MANUFACTURING ALLOY-METAL-BASED PARTS BY SPRAY FORMING\ | CN202317021U | XIANJIE ZHANG US\ JUNBIAO WANG US\ CHAORUN SI US\ YUJUN LI US\ XIAOBAO CAO US | UNIV NORTHWESTERN POLYTECHNIC US | B22F3/115 |
| 4120 | 3D printing | MANUFACTURING METHOD FOR MAKING ANTIQUE ARTISTIC FLOWERPOT\ | CN101755632A | XIANLIAN LIU (CN) | XIANLIAN LIU (CN) | A01G9/02\ B44D5/10 |
| 4121 | 3D printing | EQUIPMENT FOR PRODUCING PLASTIC CONTAINER WITH STEREO ANAGLYPH PATTERN ON SURFACE AND WITHOUT SEAM LINE\ | CN100453298C | XIANLIAN LIU (CN) | XIANLIAN LIU (CN) | B29C49/30\ B29C49/02\ B29C49/42\ B29C49/54\ B29C49/58\ B29C49/64 |
| 4122 | 3D printing | MANUFACTURING METHOD OF FINE PERSONALIZED SKULL MODEL CAPABLE OF DESCRIBING TEETH OCCLUDING RELATION\ | CN100569195C | XIAOFENG LI (CN)\ HUIZHI CAO (CN)\ CAN LIU (CN)\ QIAN JIANG (CN) | XIAOFENG LI (CN) | G09B23/30\ G06F17/00\ A61C19/00\ A61B6/00\ G06Q50/00 |
| 4123 | 3D printing | HIGH-POLYMER ELASTIC AND VENTILATING PILLOW INNER\ | CN201641314U | XIAOLIANG ZHUANG US | XIAOLIANG ZHUANG US | A47G9/10 |
| 4124 | 3D printing | HIGH MOLECULAR ELASTIC VENTILATED PILLOW INNER AND PREPARATION METHOD THEREOF\ | CN102133017A | XIAOLIANG ZHUANG US | XIAOLIANG ZHUANG US | A47G9/10 |
| 4125 | 3D printing | APPARATUS FOR PRODUCING FINE THREE-DIMENSIONAL FIGURED PLATE GLASS WITH FLOATING METHOD\ | CN201236175Y | XIAOXIAO YANG (CN)\ DENING YANG (CN) | DENING YANG (CN) | C03B18/12\ C03B18/14 |
| 4126 | 3D printing | MANUFACTURING AND REPAIRING METHOD FOR CONDUCTIVE CIRCUIT OF THREE DIMENSIONAL MOLD INTERCONNECTING DEVICE\ | CN101394710A | XIAOYAN ZENG (CN)\ YU CAO (CN)\ XIANGYOU LI (CN)\ JIANGUO LIU (CN) | UNIV HUAZHONG SCIENCE TECH (CN) | H05K3/10\ H05K3/20 |
| 4127 | 3D printing | WIND TURBINE BLADE MOULD\ | CN102785315A | XINGDONG CAO US | CHANGZHOU SHENHUI ELECTRIC MATERIAL CO LTD US | B29C33/38 |
| 4128 | 3D printing | METHOD FOR PREPARING HIGH TEMPERATURE FLUENT METAL LOOP BY QUARTZ AND SICF/SIC COMPOSITE MATERIAL\ | CN101320600A | XINGUI ZHOU (CN)\ YICAN WU (CN)\ WANG JUN (CN)\ CHANGRUI ZHANG (CN)\ QUNYING HUANG (CN)\ YINGBIN CAO (CN)\ RONGJUN LIU (CN)\ HONGLEI WANG (CN)\ HAIJIAO YU (CN)\ SHUANG ZHAO (CN)\ ZHIYI WANG (CN)\ ZELAN HUANG (CN) | NAT UNIV DEFENSE TECHNOLOGY (CN) | C04B41/50\ G21B1/11\ C04B35/66 |
| 4129 | 3D printing | INTEGRAL QUICK FORMING METHOD FOR CERAMIC PARTS BASED ON PARAFFIN\ | CN101550007A | XIUFENG WANG (CN)\ WENJIE XU (CN)\ HONGTAO JIANG (CN)\ CHENGLONG YU (CN) | UNIV SHAANXI SCIENCE & TECH (CN) | C04B35/622\ B28B7/34\ C04B35/632\ B28B1/54 |
| 4130 | 3D printing | PREPARATION METHOD OF DYEABLE POLYESTER COMPOSITE FIBER OF CATIONIC DYE\ | CN101613894B | XIUHUA WANG US\ YUYUAN YAO US\ JICHENG MENG US | UNIV ZHEJIANG SCIENCE & TECH US | D01D5/34\ D01F8/14 |
| 4131 | 3D printing | PRODUCTION METHOD FOR MANUFACTURING ALUMINUM ALLOY EYEWEAR ACCESSORIES\ | CN101912955A | XIUJUAN YANG US | XIUJUAN YANG US | B22D17/10\ G02C5/00 |
| 4132 | 3D printing | THERMAL PRINT HEAD AND METHOD FOR MANUFACTURING THE SAME\ | CN101412323A | XIWEI WANG (CN)\ GUOJIAN ZHAO (CN)\ ZHE HE (CN) | SHANGDONG HUALING ELECTRONICS (CN) | B41J2/335 |
| 4133 | 3D printing | PAPER PLASTIC GLUE-FREE COMPOSITE BIDIRECTIONAL STRETCHING POLYPROPYLENE FILM AND ITS PREPARATION METHOD\ | CN101058247A | XU WENSHU ZHU (CN) | GUANGDONG DECRO PACKAGE FILMS (CN) | B29C71/00\ B29C71/02\ B32B27/06\ B32B27/18\ B32B27/10\ B32B27/32\ B29C47/06\ B29C69/02\ B29D7/01 |
| 4134 | 3D printing | THREE-DIMENSIONAL COMPOSITE PLASTIC WATER DISCHARGING BODY\ | CN202787227U | XUAN GEN US\ ZHANG JIANHUA US\ HE XUEJUN US\ ZHAO XUEFENG US\ BIAN FEIJING US\ XU HAIYONG US\ YU JINGJING US\ ZHANG PEIGUANG US\ XU QUAN US | ZHEJIANG ZHUAN CONSTRUCTION GROUP CO LTD US\ ZHEJIANG HUAHUI MECHANICAL AND ELECTRICAL EQUIPMENT INSTALLATION CO LTD US\ ZHEJIANG GUANGCHUAN CONSTRUCTION CO LTD US | E02D3/10 |
| 4135 | 3D printing | NATURAL MARBLE-LIKE CRYSTALLIZED GLASS NATURAL MARBLE-LIKE CRYSTALLIZED GLASS ARTICLE AND METHOD FOR MANUFACTURING THE SAME\ | JP2010248056A | YAMADA AKIHITO US | NIPPON ELECTRIC GLASS CO US | C03C10/04\ C03B31/00\ C03B32/02 |
| 4136 | 3D printing | THERMALLY INSULATED CONTAINER MANUFACTURING METHOD THEREFOR AND LAMINATED LABEL ATTACHED TO THE CONTAINER\ | JP2007153359A | YAMADA MINORU US\ YAMAZAKI DAISUKE US\ KOBAYASHI SATOSHI US\ MASHITA ATSUSHI US\ MURAWAKI YASUHIRO US | KISHIMOTO SANGYO CO US | G09F3/02\ B65D81/38\ B65D1/28\ B65D25/20\ B65D25/36 |
| 4137 | 3D printing | METHOD FOR PRODUCING BULKY POLYESTER FIBER\ | JP2005097768A | YAMADA TOSHIHIRO US\ TSUKAMOTO RYOJI US | TEIJIN FIBERS LTD US | D01F6/62 |
| 4138 | 3D printing | METHOD FOR SPINNING POLYESTER MONOFILAMENT HAVING SMALL FINENESS\ | JP2009084712A | YAMAMOTO HIROSHI US\ OGUCHI TOMOHIRO US\ KUROKAWA HIROYUKI US | TORAY INDUSTRIES (JP) | D01D5/088\ D01F6/62 |
| 4139 | 3D printing | RECURSIVE REFLECTION PRINT SHEET OF ELECTRONIC IMAGE AND PRINTING METHOD THEREFOR\ | JP2005049383A | YAMAMOTO KAORU US | KOOWATEKUNOA KK US | D06P5/00\ G09F3/02\ G02B5/128\ G09F13/16 |
| 4140 | 3D printing | NONWOVEN FABRIC FOR THERMOFORMING\ | JPH1037056A | YAMAMOTO NOBUYUKI US\ YAMADA IWAHIRO US | TEIJIN LTD US | D01D5/34\ D01F8/14\ D01F1/10\ D04H3/011\ D04H3/147\ D04H3/153 |
| 4141 | 3D printing | ELASTIC NETWORK STRUCTURE\ | US7892991B2 | YAMANAKA MASAKI (JP)\ MATSUI YOSHIHIRO (JP) | TOYO BOSEKI (JP) | D04H3/03\ D04H3/05\ D04H3/16 |
| 4142 | 3D printing | FILM FOR PRINT LAMINATE\ | JP2003062957A | YAMASHITA TAKASHI US | JAPAN POLYCHEM CORP US | B32B27/32\ C08L23/04\ B29C47/06\ B29K23/00\ B29L7/00 |
| 4143 | 3D printing | ELECTRONIC PICTURE IMAGE-RECEIVING MATERIAL\ | JP2007249126A | YAMAZAKI CHIKAMASA US | KONICA MINOLTA HOLDINGS INC (JP) | G03G7/00 |
| 4144 | 3D printing | ELECTROPHOTOGRAPHIC IMAGE-RECEIVING MATERIAL\[FR] MATéRIAU DE RéCEPTION D'IMAGE éLECTROPHOTOGRAPHIQUE\ | WO2007083467A1 | YAMAZAKI KATSUMASA (JP) | KONICA MINOLTA HOLDINGS INC (JP)\ YAMAZAKI KATSUMASA (JP) | B32B27/10\ B32B5/18\ D21H27/00\ D21H27/30\ B32B7/02\ G03G7/00\ D21H19/70\ D21H21/56 |
| 4145 | 3D printing | ULTRATOUGH CVD SINGLE CRYSTAL DIAMOND AND THREE DIMENSIONAL GROWTH THEREOF\ | US7594968B2 | YAN CHIH-SHIUE (US)\ MAO HO-KWANG (US)\ HEMLEY RUSSELL J (US) | CARNEGIE INST OF WASHINGTON (US) | C30B29/04 |
| 4146 | 3D printing | NON-BLENDING SPINAL SIDE BENDING CORRECTOR\ | CN100360092C | YAN WANG (CN)\ XUESONG ZHANG (CN)\ ZHENG WANG (CN) | YAN WANG (CN) | A61B17/58\ A61B17/70 |
| 4147 | 3D printing | SIDE BY SIDE COMPOSITE FIBER SPINNERET PLATE METHOD OF PREPARING THREE-DIMENSIONAL CRIMP ANTIBACTERIAL FIBER WITH SPINNERET PLATE AND APPLICATION OF THREE-DIMENSIONAL CRIMP ANTIBACTERIAL FIBER\ | CN103225118A | YAN ZHIYONG US\ HU YING US\ YE HONGFU US\ ZHAO GANGHUA US\ WANG LIGANG US\ SHEN XIAOJUN US\ WANG SHANGDONG US | UNIV JIAXING US | D01D5/22\ D01F8/14\ D01F1/10\ D01D5/253\ D01D5/32\ D01F8/12 |
| 4148 | 3D printing | MICROSPHERE THREE-DIMENSIONAL ROLLING COATING DEVICE\ | CN201030355Y | YANBIN WANG (CN)\ YONGJIAN TANG (CN)\ LIN ZHANG (CN)\ WEIDONG WU (CN)\ HAILE LEI (CN) | LASER FUSION RES CT CHINA ACAD (CN) | B05C13/02 |
| 4149 | 3D printing | AN EQUIPMENT FOR PRODUCING GLASS WITH REFINED THREE-DIMENSIONAL PATTERN BY FLOAT PROCESS AND THE METHOD FOR PRODUCING THE GLASS\[FR] MATéRIEL PERMETTANT DE PRODUIRE PAR PROCESSUS FLOAT DU VERRE AVEC UN MOTIF EN TROIS DIMENSIONS RAFFINé ET PROCéDé DE PRODUCTION DE VERRE\ | WO2009124465A1 | YANG DENING (CN)\ YANG XIAOXIAO (CN) | YANG DENING (CN)\ YANG XIAOXIAO (CN) | C03B18/12\ C03B18/14\ C03B5/173 |
| 4150 | 3D printing | COMPRESSION RESISTING AND WATER DIVERSION THREE-DIMENSIONAL WATER FILTERING AND DRAINING DEVICE\ | CN203034439U | YANG JINGLIN US | TAI AN YONGJIA CIVIL ENGINEERING MATERIALS CO LTD US | E02D3/10 |
| 4151 | 3D printing | GRAPHENE/ STANNIC OXIDE NANOMETER COMPOUNDING RESISTANCE TYPE FILM GAS SENSOR AND MANUFACTURING METHOD THEREOF\ | CN102636522A | YANG LI US\ MUJIE YANG US\ QIANQIAN LIN US | UNIV ZHEJIANG US | G01N27/04 |
| 4152 | 3D printing | MANUFACTURING METHOD OF ENVIRONMENT-FRIENDLY CONTAINER WITH 3D COLOR PRINTING PICTURE AND ENVIRONMENT-FRIENDLY CONTAINER\ | CN103332352A | YANG RONGSHI US | DONGGUAN MORNING LIGHT PRINTING COMPANY LTD US | B65D3/02\ B65D3/30\ B65D25/34 |
| 4153 | 3D printing | CALCULUS 3D (THREE DIMENSIONAL) CONSTRUCTING METHOD AND DEVICE\ | CN103465475A | YANG WEIMIN US\ YANG HUAGUANG US\ QIN LIU US\ JIAO ZHIWEI US\ DING YUMEI US | UNIV BEIJING CHEMICAL US | B29C67/00 |
| 4154 | 3D printing | FORMING METHOD FOR 3D (THREE-DIMENSIONAL) SHOE BODY OF SKATING SHOE\ | CN103182766A | YANG XINZHI US | SAKURAI SPORTS MFG CO LTD US | B29C45/14\ B29C45/26\ B29B11/00\ B29C33/12\ B29L31/50 |
| 4155 | 3D printing | PRODUCTION TECHNIQUE OF THREE-DIMENSIONAL CURLY HOLLOW POLYESTER STAPLE FIBERS\ | CN102517675A | YANQIONG WEI US\ JUNJIE YU US\ JIANNONG SHAO US\ YIPING DING US\ HONGBO XU US\ MINGGANG LI US\ ZHICHAO SUN US\ LIJUN WANG US | HANGZHOU BEST CHEMICAL FIBER CO LTD US | D01D5/088\ D01D5/22\ D01D5/24\ D01F6/84\ D01D1/00\ D01D10/02\ D01D5/08\ D01D5/12\ D01D1/04\ D01D5/096\ D01F11/08\ D06M13/46\ D06M15/643 |
| 4156 | 3D printing | HOT MELT ADHESIVES BONDING FIBER\ | US2005170143A1 | YAU PAK C (CN) | BISQUIT DEV LTD (HK) | B32B3/30\ B32B1/00\ B32B5/24\ B32B7/12\ B32B27/02\ D06N7/04 |
| 4157 | 3D printing | COPPER COATED PLATE WITH HIGH GLASS-TRANSITION TEMPERATURE STRUCTURE\ | CN202271583U | YONGJUN GUO US\ YUESONG GONG US\ YANGJIE ZHU US\ XIAOLONG QI US | GUANGZHOU LIANMAO ELECTRONIC TECHNOLOGY CO LTD US | B32B15/04\ B32B27/04\ B32B3/22\ B32B9/04 |
| 4158 | 3D printing | SELECTIVE LASER FUSION RAPID MOLDING METHOD AND DEVICE OF MEDICAL IMPLANTATION MATERIAL WAX MOULD\ | CN101310964B | YONGQIANG YANG (CN)\ SHUFAN WANG (CN)\ WEIHUI WU (CN) | UNIV SOUTH CHINA TECH (CN) | B29C51/00\ B29C73/00\ A61B6/03\ B29K91/00\ B29L22/00 |
| 4159 | 3D printing | METHOD FOR MANUFACTURING BIODEGRADABLE BOARD AND BIODEGRADABLE BOARD MANUFACTURED THEREBY\[FR] PROCéDé DE FABRICATION DE PLANCHE BIODéGRADABLE ET PLANCHE BIODéGRADABLE FABRIQUéE ASSOCIéE\ | WO2013118930A1 | YOON SANG-JUN (KR)\ CHEY WON-JUN (KR)\ KIM TAE-KYUN (KR)\ PARK HYUNG-JOON (KR) | DOWNNARA CO LTD (KR)\ YOON SANG-JUN (KR)\ CHEY WON-JUN (KR)\ KIM TAE-KYUN (KR)\ PARK HYUNG-JOON (KR) | B27N3/00\ C08L67/04\ B27N3/08 |
| 4160 | 3D printing | OPTICAL DEVICE MANUFACTURING METHOD AND OPTICAL DEVICE MANUFACTURING MOLD\ | JP2005212154A | YOSHIDA HIROYUKI US | MATSUSHITA ELECTRIC WORKS LTD (JP) | B29C45/26\ B29L11/00\ B29C45/02 |
| 4161 | 3D printing | METHOD FOR PROVIDING GLOSSY PAPER AND GLOSSY PAPER\ | JP2004308034A | YOSHIDA MASAHIKO US\ NAKAMURA MISAO US\ UCHIDA TAIZO US | OSAKA SEALING LABEL PRINT US | B41M5/26\ B41M5/28\ B05D7/00\ D21H27/00\ B32B29/00\ B41M5/30\ B41M5/337\ B41M5/42\ D21H19/36\ D21H27/30 |
| 4162 | 3D printing | METAL FOAMED BODY AND ITS MANUFACTURING METHOD\ | JP2007061865A | YOSHIKAWA KATSUYUKI US\ FURUTA SEISHI US\ NISHI SEIJI US\ IMAI RYOTA US\ YOSHIMURA YUKIHIKO US\ MIYOSHI TETSUJI US | KOBE STEEL LTD US\ SHINKO WIRE CO LTD US | B22D25/02\ B22D21/04\ C22C1/08 |
| 4163 | 3D printing | SYSTEM\ | JP2007017922A | YOSHINO KAZUTORA US | YOSHINO KAZUTORA US | G09G1/00 |
| 4164 | 3D printing | MOLDING TECHNIQUE OF ELEVATED PLASTIC FLOOR TILE AND THE STRUCTURE THEREOF\ | TW201026940A | YOU WEN-BANG (TW) | TAD CHIN IND CORP (TW) | E04F15/10 |
| 4165 | 3D printing | ACRYLIC RESIN CULTURE MEDIUM FOR PLANTING PLANT AND PREPARATION METHOD THEREOF\ | CN102283091B | YOUXIANG CHEN US\ ZHIFENG ZU US | CHONGQING TIANKAI LANDSCAPE ENGINEERING CO LTD US | A01G31/00 |
| 4166 | 3D printing | VIA USING ZN OR ZN ALLOYS AND ITS MAKING METHOD 3D CHIP STACK PACKAGES USING THEREOF\ | US2010240174A1 | YU JIN (KR)\ JEE YOUNG-KUN (KR) | YU JIN (KR)\ JEE YOUNG-KUN (KR) | H01L21/768\ H01L21/50 |
| 4167 | 3D printing | EXTRUSION DEVICE OF 3D (THREE DIMENSIONAL) PRINTER APPLIED TO FUSE WIRE ACCUMULATION TECHNOLOGY\ | CN203282708U | YUAN ZHENGUO US | PANOWIN TECHNOLOGIES CO LTD US | B29C67/00 |
| 4168 | 3D printing | 3D (THREE DIMENSIONAL) PRINTER EXTRUSION APPARATUS APPLIED IN FUSE STACKING TECHNOLOGY\ | CN103231517A | YUAN ZHENGUO US | WU JIE US | B29C67/00 |
| 4169 | 3D printing | COMPOSITE ELECTRODE FOR FINE ELECTRIC SPARK PROCESSING\ | CN101259548A | YUANGANG WANG (CN)\ FULING ZHAO (CN)\ JIN WANG (CN) | UNIV DALIAN TECH (CN) | B23H1/04 |
| 4170 | 3D printing | PRECISION-INVESTMENT CASTING METHOD\ | CN101406932A | YUANHUA DENG (CN) | QUANZHOU TENGDA FINE CASTING C (CN) | B22C9/04\ B22D15/00\ B22D31/00 |
| 4171 | 3D printing | THREE-DIMENSIONAL (3D) DISPLAY PANEL 3D DISPLAY SYSTEM AND 3D DISPLAY\ | CN202019419U | YUANYUAN LI US | KONKA GROUP CO LTD US | H04N13/00\ G09G3/32 |
| 4172 | 3D printing | THERMOPLASTIC RESIN CASING AND METHOD FOR FABRICATING THE SAME\ | US2008145578A1 | YUBA TAKASHI (JP)\ AKIEDA SHINICHIRO (JP)\ ARITA TAKASHI (JP) | FUJITSU COMPONENT LTD (JP) | B29C45/00\ B32B1/04 |
| 4173 | 3D printing | RAPID PROTOTYPING METHOD AND DEVICE BASED ON SUPERSATURATION SALINE SOLUTION CRYSTALLIZATION\ | CN102649020A | YUEGUANG DENG US\ JING LIU US | CHINESE ACAD TECH INST PHYSICS US | B01D9/00 |
| 4174 | 3D printing | RAPID MOLDING METHOD BASED ON ELECTRORHEOLOGICAL FLUID AND APPARATUS THEREOF\ | CN102615830A | YUEGUANG DENG US\ JING LIU US | CHINESE ACAD TECH INST PHYSICS US | B29C67/00 |
| 4175 | 3D printing | NUMERICALLY-CONTROLLED PLASTIC THIN-LAYER SEDIMENT MOULDING MACHINE\ | CN101693407A | YUHUI LING US | YUHUI LING US | B29C67/00 |
| 4176 | 3D printing | ENVIRONMENT-PROTECTION STEREO AUTOMOBILE FOOT PAD\ | CN201254118Y | YUNBO GUO (CN) | DONGGUAN HONG EN PLASTIC PRODU (CN) | B60N3/06 |
| 4177 | 3D printing | ENVIRONMENT-PROTECTIVE STEREO AUTOMOBILE FOOT PAD AND MANUFACTURING METHOD THEREOF\ | CN101376351A | YUNBO GUO (CN) | DONGGUAN HONGEN RUBBER AND PLA (CN) | B32B3/30\ B32B27/06\ B32B5/18\ B60N3/06\ B29C43/18\ B29C43/28\ B29K23/00\ B29K96/00\ B32B7/08 |
| 4178 | 3D printing | METHOD OF MANUFACTURING 300M ULTRAHIGH STRENGTH STEEL AIRCRAFT LANDING GEAR\ | CN101229586A | YUNGE WANG (CN)\ XIANGMING WANG (CN)\ HUAMING WANG (CN) | YUNGE WANG (CN) | B22F3/105 |
| 4179 | 3D printing | METHOD OF MANUFACTURING AF1410 ULTRAHIGH STRENGTH STEEL AIRPLANE HORIZONTAL TAIL AXIS\ | CN101229585A | YUNGE WANG (CN)\ XIANGMING WANG (CN)\ HUAMING WANG (CN) | YUNGE WANG (CN) | B22F3/105 |
| 4180 | 3D printing | HOLLOW BODY MOLDING DEVICE\ | JP2007296700A | YUSHIMO MASASHI US\ TANIGAKI KENJI US\ NISHIDA SHOZO US\ KAMIMURA TAKASHI US | JAPAN STEEL WORKS LTD US\ AISIN SEIKI (JP) | B29C45/17\ B29C69/02\ B29L22/00\ B29C65/14 |
| 4181 | 3D printing | SINGLE-SPINNERET-PLATE BINARY SKIN-CORE-TYPE COMPOSITE-SPINNING MULTIPLE-DIFFERENT-YARN PRODUCTION PROCESS\ | CN103060979A | ZHAN HAIHUA US\ SHEN YUNGEN US | UNIV SHAOXING US\ SHAOXING YUNXIANG CHEMICAL FIBER CO LTD US | D01D5/34\ D01D10/02\ D01D1/04\ D02G3/04\ D01D1/06\ D01D7/00\ D02G1/02 |
| 4182 | 3D printing | CONTINUOUS SCREWED MULTI-CELLULAR PLASTIC SHAPING STRUCTURE\ | CN2714295Y | ZHANG BINGRONG (CN) | ZHANG BINGRONG (CN) | B29C53/58\ B29C53/74 |
| 4183 | 3D printing | METHOD FOR MANUFACTURING METAL PARTS AND MOLDS AND MICRO-ROLLER USED THEREFOR\ | US2013197683A1 | ZHANG HAIOU (CN)\ WANG GUILAN (CN) | UNIV HUAZHONG SCIENCE TECH (CN) | B23K26/34\ B23K9/04\ B23K10/00 |
| 4184 | 3D printing | METHOD AND EQUIPMENT FOR DIRECTLY AND QUICKLY MANUFACTURING MOULD AND PARTS\ | CN1298780A | ZHANG HAIOU (CN)\ WANG GUILAN (CN) | UNIV HUAZHONG SCIENCE TECH (CN) | B23K28/02\ B23P23/04 |
| 4185 | 3D printing | CERAMIC/METALLIC DOUBLE CONTINUOUS PHASE COMPOSITE MATERIAL BRAKE PAD AND PREPARATION METHOD THEREOF\ | CN102102720B | ZHANG JINSONG US\ CAO XIAOMING US\ ZHANG YUNAN US\ TIAN CHONG US\ YANG ZHENMING US | INST METAL RES CHINESE ACAD SC (CN) | F16D69/02 |
| 4186 | 3D printing | METHOD FOR ACHIEVING HIGH-MELTING-POINT MATERIAL 3D PRINTING THROUGH NANOMETER INK TOGETHER WITH LASER MELTING\ | CN103407296A | ZHANG SHANGFENG US\ ZHANG YANGMING US\ ZHANG SHIZHU US\ SHI XINCHAO US\ LIU CHUANG US\ LIU YUBO US\ LIU YAN US\ ZHANG LIANBIN US | NANJING DINGKE NANOTECHNOLOGY INST CO LTD US | B41J5/00 |
| 4187 | 3D printing | PREPARATION METHOD OF NANO MODIFIED ULTRA-HIGH MOLECULAR WEIGHT POLYETHYLENE (UHMWPE) ANTISTATIC COMPOSITE MATERIAL\ | CN102888039A | ZHANG WEI US\ LI ZHI US\ HONG WEI US\ XU ZHENHUA US\ ZHAO CHUNBAO US\ LI JIANLONG US | SHANGHAI RES INST CHEMICAL IND US | C08K3/08\ C08K3/04\ B29C47/92\ C08L23/06\ C08K7/06\ B29B9/06\ C08K9/10 |
| 4188 | 3D printing | WEAR-RESISTANT CROSSLINKED PE (POLYETHYLENE) COMPOSITE PIPE AND MANUFACTURING METHOD THEREOF\ | CN102417650B | ZHANG WEIJIAO US\ GAO LI US\ JIANG JIANPING US | ZHEJIANG WEIXING NEW BUILDING MATERIALS CO LTD US | C08J3/24\ C08K5/098\ C08K13/04\ C08L27/18\ C08K3/04\ C08L23/08\ C08L23/16\ C08L23/06\ C08K3/16\ C08K7/06\ F16L9/12 |
| 4189 | 3D printing | LOW TEMPERATURE THERMOPLASTIC MATERIAL AND PREPARING METHOD THEREOF\ | US8859691B2 | ZHANG ZHONGQUN (CN) | KLARITY MEDICAL & EQUIPMENT GZ CO LTD (CN)\ ZHANG ZHONGQUN (CN) | B29C35/08\ C08L75/04\ C08F283/00\ C08L67/04 |
| 4190 | 3D printing | CLOTHING PROTECTION STRUCTURE AND PRODUCTION METHOD THEREOF\ | CN101647605A | ZHAO JUN US | HANGZHOU FUXING INDUSTRY & TRA US | A41D13/00 |
| 4191 | 3D printing | WATER SOLUBLE FLEXIBLE PRINT PLATE AND ITS PRODUCING METHOD\ | CN1900822A | ZHAO XIAOJIE (CN) | ZHONGYUAN PRINTING SCIENCE AND (CN) | G03F7/09\ B41N1/24 |
| 4192 | 3D printing | DIGITAL PRE-LAMINATING FILM\ | CN101705060A | ZHAOZHAO ZHANG US | HU MINSHENG US | C09J123/08\ C09J7/02\ C09J157/02 |
| 4193 | 3D printing | ADSORPTION ROLLING BEARING\ | CN201827235U | ZHEN HUANG US | ZHEN HUANG US | F16C33/32\ F16C33/58\ F16C33/66\ F16C19/00\ F16C19/06\ F16C19/26\ F16C33/34 |
| 4194 | 3D printing | ADSORPTION ROLLING BEARING\ | CN102213262A | ZHEN HUANG US | ZHEN HUANG US | F16C33/32\ F16C33/58\ F16C33/66 |
| 4195 | 3D printing | BRA CUP WITH GOOD SHAPE KEEPING FUNCTION\ | CN2829399Y | ZHENG BIHAO (CN) | ANLIFANG CHINA GARMENT CO LTD (CN) | A41C3/12\ A41C3/00 |
| 4196 | 3D printing | THREE-DIMENSIONAL HOLLOW POLYESTER STAPLE FIBER\ | CN202849604U | ZHENG QUAN US\ YAN DONGNING US\ CUI HUI US | JIANGSU DESAI CHEMICAL FIBER CO LTD US | D01D5/24\ D01D5/253 |
| 4197 | 3D printing | FAST RESPONSE ELECTROCHEMICAL ORGANOPHOSPHATE SENSOR\ | US2010294662A1 | ZHENG ZHI (CN)\ ZHAO LINAN (CN)\ WANG MARILYN (CN) | HONEYWELL INT INC (US) | H01B13/00\ C25B11/04\ B05D5/12 |
| 4198 | 3D printing | WATER CHANNEL PRE-FILLING METHOD USED DURING LOST FOAM CASTING OF ENGINE CYLINDER BODY\ | CN102343417A | ZHENGSONG MA US\ YUSHENG ZHANG US\ QIAO XU US | CHUZHOU JINNUO IND CO LTD US | B22C9/04 |
| 4199 | 3D printing | NEW PROCESS FOR SPRAY-COATING POWDER ON SURFACE OF METAL\ | CN101992183A | ZHENJIANG SUN US | TIANJIN CITY YIJIANG ALUMINUM MATERIAL CO LTD US | B05D3/02\ B05D7/24\ B05D7/14\ B05D5/00\ B05D3/00 |
| 4200 | 3D printing | EMBEDDED TYPE MOULD\ | CN202292992U | ZHIYU HU US\ CHUANG JIANG US\ WENYONG CHENG US | SHANGHAI TONGCHUANG CERAMICS CO LTD US | B28B7/00 |
| 4201 | 3D printing | SILICON WITH THREE-DIMENSIONAL DEPRESSION STRUCTURE AND METHOD FOR PREPARING SAME\ | CN101311350A | ZHONGHE XI (CN)\ YUE WU (CN)\ GENGMIN ZHANG (CN)\ HONGYU CUI (CN)\ DENGZHU GUO (CN) | UNIV BEIJING (CN) | C30B29/60\ C30B29/06 |
| 4202 | 3D printing | SILICON WITH THREE-DIMENSIONAL DEPRESSION STRUCTURE AND METHOD FOR PREPARING SAME\ | CN101311347A | ZHONGHE XI (CN)\ YUE WU (CN)\ GENGMIN ZHANG (CN)\ HONGYU CUI (CN)\ DENGZHU GUO (CN) | UNIV BEIJING (CN) | C30B29/60\ C30B29/06 |
| 4203 | 3D printing | METAL MASTER MOLD FOR ELECTROFORMING AND USE THEREOF\ | CN101403125A | ZHONGYAO YU (CN)\ GUANNAN LI (CN)\ JING CHENG (CN) | BOAO BIOTECH CO LTD (CN) | C25D1/10 |
| 4204 | 3D printing | STEREOSCOPIC ANTI-FAKE PACKING BOX WITH HIGH BAR CODE LEVEL\ | CN203064295U | ZHOU LIQUAN US\ ZOU YAOBANG US\ JIANG ZONGZHENG US\ YANG PEIXIANG US\ LIU KUNHONG US\ LI HONGYE US\ WANG MINGJUN US\ LIU GUANGTIAN US | SICHUAN YIBIN PUSH GROUP 3D CO LTD US | G09F3/02\ B65D5/00 |
| 4205 | 3D printing | THREE-DIMENSIONAL PRINTING PEN\ | CN103350507A | ZHOU SHUANG US\ WU JIE US | WU JIE US\ ZHOU SHUANG US | B29C67/00 |
| 4206 | 3D printing | THREE-DIMENSIONAL INTEGRATED HETEROGENEOUS SEMICONDUCTOR STRUCTURE\ | US7741645B2 | ZHU HUILONG (US) | IBM (US) | H01L29/76\ H01L31/036\ H01L31/112 |
| 4207 | 3D printing | HOLLOW FIBROUS MEMBRANE AND PREPARATION METHOD THEREOF\ | CN102527250B | ZHU WENBING US\ ZHANG TING US | HEBEI NORN WATER PURIFICATION EQUIPMENT CO LTD US | B01D69/08\ B01D67/00\ B01D71/34 |
| 4208 | 3D printing | INDUCTIVE COIL INSULATING TREATMENT OF VACUUM FURNACE\ | CN101030475A | ZHU XINGFA (CN) | ZHU XINGFA (CN) | H01F41/12\ H01F5/06\ H01F27/32 |
| 4209 | 3D printing | METHOD AND APPARATUS TO DECODE A STREAMING FILE DIRECTLY TO DISPLAY DRIVERS\ | US2005195205A1 | ABRAMS THOMAS A JR (US) | MICROSOFT CORP (US) | G09G5/36 |
| 4210 | 3D printing | MULTIWELL PLATE DEVICE\ | US8470590B2 | ADRIEN CHRIS (US)\ BART JOHN C (US)\ CLEMENTS JAMES (US)\ MOORE DAVID (US)\ NELSON BRYCE P (US) | ERIE SCIENT CO (US)\ ADRIEN CHRIS (US)\ BART JOHN C (US)\ CLEMENTS JAMES (US)\ MOORE DAVID (US)\ NELSON BRYCE P (US) | C12M1/22 |
| 4211 | 3D printing | GENERATING THREE-DIMENSIONAL VIRTUAL TOURS FROM TWO-DIMENSIONAL IMAGES\ | US2012099804A1 | AGUILERA JAIME (ES)\ ALONSO FERNANDO (ES)\ GOMEZ JUAN BAUTISTA (ES) | 3DITIZE SL (ES)\ AGUILERA JAIME (ES)\ ALONSO FERNANDO (ES)\ GOMEZ JUAN BAUTISTA (ES) | G06K9/36 |
| 4212 | 3D printing | AUDIO DEVICE\ | JP2006217210A | AKIYASU MASAICHI US\ OWAKI TATSUO US\ TAKASHIMA NORIYUKI (JP) | ALPINE ELECTRONICS INC (JP) | H04S5/02\ H04S7/00 |
| 4213 | 3D printing | METHOD AND APPARATUS FOR PRODUCTION OF HIGH RESOLUTION THREE-DIMENSIONAL OBJECTS BY STEREOLITHOGRAPHY\ | US5870307A | ALBERT DAVID J (US)\ HARLOW RICHARD A (US)\ TARNOFF HARRY L (US)\ NGUYEN HOP D (US)\ VORGITCH TOM J (US)\ REMBA DAVID Z (US)\ SMALLEY DENNIS R (US)\ HULL CHARLES W (US)\ STINEBAUGH PHIL (US)\ SPENCE STUART T (US)\ LEWIS CHARLES W (US) | 3D SYSTEMS INC (US) | B29C67/00\ G01J1/42\ G03F7/00\ G03F7/20\ G06T17/00\ G06T17/10\ G06T17/20 |
| 4214 | 3D printing | METHODS AND SYSTEMS OF THERMAL SIMULATION FOR FIRE ANALYSIS OF OBJECTS\ | US2008126038A1 | ALVAREZ MARTA BERMEJO (ES)\ BERMEJO VICTOR PEREZ (ES)\ ESPINOSA PATRICIA PINEDA (ES) | AIRBUS ESPANA SL (ES) | G06N7/00 |
| 4215 | 3D printing | CUSTOM ORTHODONTIC APPLIANCE FORMING METHOD AND APPARATUS\ | US6846179B2 | ANDREIKO CRAIG A (US)\ PAYNE MARK A (US)\ CHAPOULAUD ERIC (US) | ORMCO CORP (US) | G06F17/50\ A61C13/00\ A61C7/00\ A61C7/14\ A61C19/04 |
| 4216 | 3D printing | MIKROSTRUKTURAPPARAT MIT OPTISCHER OBERFL?CHENGüTE SOWIE VERFAHREN ZUR HERSTELLUNG DESSELBEN\[EN] MICROSTRUCTURED APPARATUS WITH OPTICAL SURFACE QUALITY AND METHOD FOR THE PRODUCTION THEREOF\[FR] APPAREIL à MICROSTRUCTURE DOTé D'UNE QUALITé DE SURFACE OPTIQUE ET SON PROCéDé DE FABRICATION\ | EP2653222A3 | ANURJEW EUGEN (DE)\ BRANDNER JUERGEN (DE)\ HANSJOSTEN EDGAR (DE)\ LI YONGLI (FR)\ NEWPORT DAVID (IE) | KARLSRUHER INST TECHNOLOGIE (DE) | G03F7/00\ B01L3/00 |
| 4217 | 3D printing | STEREOLITHOGRAPHY APPARATUS AND METHOD\ | JP2008201135A | ARAI MASATO US\ SHINNO TOSHIKI US\ NAKAGAWA TAKEO US | RIKAGAKU KENKYUSHO US\ NABTESCO CORP (JP) | B29C67/00 |
| 4218 | 3D printing | DIRECT-MANUFACTURED DUCT INTERCONNECTS\ | US7623940B2 | BAGWILL TRACY L (US)\ HUSKAMP CHRIS (US) | BOEING CO (US) | G06F17/50\ G06F19/00\ F16L9/18\ F16L21/02\ F16L23/00 |
| 4219 | 3D printing | METHOD FOR PRODUCING HUMAN BODY MODEL FROM BONE ASH\ | CN1987931A | BAI SHUANSUO ZHU (CN) | BAI SHUANSUO (CN) | B29C67/00\ G06T17/00\ G05B19/4099 |
| 4220 | 3D printing | APPARATUS FOR ELECTRONICALLY CONTROLLABLE TRANSMISSION\ | US2010294067A1 | BAK JEONG-HO (KR)\ KIM BYUNG-KI (KR) | SL CORP (KR) | F16H59/04 |
| 4221 | 3D printing | DOCUMENT CREATION SYSTEM AND RELATED METHODS\ | US2006026504A1 | BALINSKY HELEN (GB) | BALINSKY HELEN (GB) | G06F17/21\ G06F17/24 |
| 4222 | 3D printing | DOCUMENT CREATION SYSTEM AND RELATED METHODS\ | US7747947B2 | BALINSKY HELEN (GB) | HEWLETT PACKARD DEVELOPMENT CO (US) | G06F17/21\ G06F7/00\ G06F7/14 |
| 4223 | 3D printing | DOCUMENT CREATION\ | GB2422703A | BALINSKY HELEN (GB) | HEWLETT PACKARD DEVELOPMENT CO (US) | G06F3/12 |
| 4224 | 3D printing | BAUTEIL MIT MINDESTENS EINER SOLLBRUCHSTELLE UND VERFAHREN ZU DESSEN HERSTELLUNG\[EN] STRUCTURAL MEMBER E.G. VEHICLE AIRBAG COVER HAS DEVELOPED LAYERS FORMED WITH BREAK SECTION FOR RELIEVING OR ATTENUATING FORCES ACTED ON MEMBER\ | DE102005015094A1 | BAREIS MARC (DE) | DAIMLER CHRYSLER AG (DE) | B60R21/2165 |
| 4225 | 3D printing | PRINTED PUBLICATION WITH 3-D OBJECT\ | US2005227208A1 | BEASCOA SANTIAGO J (ES)\ LOO PENG C (US) | BUNAMIR S L (ES) | A63H33/38\ G09B5/00\ G09B27/08 |
| 4226 | 3D printing | ORTHODONTIC APPLIANCE A SYSTEM AND A METHOD FOR AUTOMATICALLY STRAIGHTENING TEETH\ | US8870566B2 | BERGERSEN EARL O (US) | ORTHO TAIN INC (US)\ BERGERSEN EARL OLAF (US) | A61C13/00\ A61C7/00\ A61C7/08\ A61C3/00 |
| 4227 | 3D printing | STEREO LITHOGRAPHY RAPID PROTOTYPING PHOTOSENSITIVE RESIN AND PREPARATION METHOD THEREOF\ | CN102436145A | BIWU HUANG US\ XINPING CHEN US | UNIV NANCHANG US | G03F7/027 |
| 4228 | 3D printing | PRODUCTION OF INDIVIDUAL DENTAL PROSTHESES VIA CAD/CAM AND RAPID MANUFACTURING / RAPID PROTOTYPING BASED ON DATA OF THE SITUATION IN THE MOUTH OBTAINED BY DIGITAL MEANS\ | US2013326878A1 | BOEHM UWE (DE)\ RUPPERT KLAUS (DE)\ BEYER MARIO (DE) | HERAEUS KULZER GMBH (DE) | A61C13/00 |
| 4229 | 3D printing | BEARING COMPONENT IN PARTICULAR ROLLER BEARING CAGE AND METHOD FOR THE PRODUCTION THEREOF\ | US2013216174A1 | BRAUN WOLFGANG (DE) | SCHAEFFLER TECHNOLOGIES AG (DE)\ BRAUN WOLFGANG (DE) | F16C33/46 |
| 4230 | 3D printing | COMPUTER ASSISTED METHOD FOR THE ADVANCED DESIGN OF BENT PARTS OF COMPOSITE MATERIAL\ | US2010042243A1 | BURGOS GALLEGO RAUL (ES)\ DE JULIAN AGUADO ANTONIO (ES) | AIRBUS ESPANA SL (ES) | G06F17/50 |
| 4231 | 3D printing | METHOD FOR DESIGNING A COMPOSITE MATERIAL PART WITH A CURVED SURFACE\ | US2008208540A1 | BURGOS GALLEGO RAUL (ES)\ VILLARRUBIA CORTES JOSE (ES) | AIRBUS ESPANA SL (ES) | G06F17/50 |
| 4232 | 3D printing | VERFAHREN ZUM HERSTELLEN EINER DREHSTABFEDER ODER EINES WANKSTABILISATOR\[EN] MANUFACTURING TORSION BAR SPRING OR ROLLING STABILIZER FOR MOTOR VEHICLE WITH TUBULAR CROSS-SECTION COMPRISES PRODUCING COMPONENT BY BRAIDING AND/OR WRAPPING CORE WITH FIBER BUNDLES AND THERMAL MELTING OF MATRIX MATERIAL\ | DE102011011577A1 | BUSCHBECK FRANK (DE)\ NAGEL GUENTER DIPL ING (DE)\ REESE ECKHARD DIPL ING (DE)\ RUPPRECHT JOCHEN DIPL ING (DE)\ STEINERT HANS-RUDOLF DIPL ING (DE) | DAIMLER AG (DE) | B29C70/50\ B29C33/76\ B29C53/02\ B29C70/00\ B60G21/055\ C08J5/04\ F16F1/14 |
| 4233 | 3D printing | A PATTERNED RETARDER TYPE 3D DISPLAY DEVICE HAVING BLACK STRIPS AND MANUFACTURING METHOD THEREFOR\ | KR20130028350A | CHAE HEE YOUNG (KR) | LG DISPLAY CO LTD (KR) | H04N13/04\ G02F1/13\ G02B27/26 |
| 4234 | 3D printing | PATTERNED RETARDER WITH BLACK STRIPS\ | GB2501780A | CHAE HEEYOUNG (KR) | LG DISPLAY CO LTD (KR) | G02F1/1335\ G02F1/1362 |
| 4235 | 3D printing | THREE-DIMENSIONAL (3D) PRINTING METHOD\ | CN103129140A | CHEN XUGUO US | CHEN XUGUO US | B41J2/01\ B41J3/00 |
| 4236 | 3D printing | MANUFACTURING METHOD FOR 3D STRUCTURE OF BIOMATERIALS USING STEREOLITHOGRAPHY TECHNOLOGY AND PRODUCTS BY THE SAME\ | US8512622B2 | CHO DONG-WOO (KR)\ KANG HYUN-WOOK (KR) | POSTECH ACAD IND FOUND (KR)\ CHO DONG-WOO (KR)\ KANG HYUN-WOOK (KR) | B29C35/04 |
| 4237 | 3D printing | 3-D NONVOLATILE MEMORY DEVICE AND METHOD OF MANUFACTURING THE SAME\ | US2013099306A1 | CHOI EUN SEOK (KR)\ YOO HYUN SEUNG (KR) | SK HYNIX INC (KR)\ CHOI EUN SEOK (KR)\ YOO HYUN SEUNG (KR) | H01L21/336\ H01L29/78 |
| 4238 | 3D printing | PROCESSING METHOD FOR THREE DIMENSIONAL IMAGE AND PROJECTION SYSTEM\ | TW201316749A | CHUNG YAU-WING (US)\ LU YI-CHUN (TW) | OPTOMA CORP (TW) | H04N13/00\ G02B27/22 |
| 4239 | 3D printing | DEVICE FOR NONDESTRUCTIVE MATERIAL TESTING OF A TEST SUBJECT USING ULTRASONIC WAVES\ | US2011000301A1 | CLOSSEN-VON LANKEN SCHULZ MICHAEL (DE)\ OBERMAYR STEFAN (DE)\ OPHEYS MICHAEL (DE) | CLOSSEN-VON LANKEN SCHULZ MICHAEL (DE)\ OBERMAYR STEFAN (DE)\ OPHEYS MICHAEL (DE) | B29C35/08\ G01N29/04\ G01D21/00 |
| 4240 | 3D printing | METHOD FOR REDUCING DIFFERENTIAL SHRINKAGE IN STEREOLITHOGRAPHY\ | US8845949B2 | COECK SAM (BE) | MATERIALISE NV (BE)\ COECK SAM (BE) | B29C35/08\ B29C67/00\ B29C41/02\ B29C71/04 |
| 4241 | 3D printing | REVISED DESIGN INNOVATION OF TRANSPORTABLE SLAB THAT WILL ELIMINATE THE NEED TO USE CONCRETE DISKS(DONOUGHNUTS) ON SITE REDUCE SLAB WEIGHT INCREASE SLAB INSTALLATION SPEED ELIMINATE THE NEED TO SWAP JACKS DURING THE PROCESS OF INSTALLING SLAB ELIMINATE THE RISK OF CORING THROUGH RING BEAM AND REDUCING SLAB STRUCTURAL INTEGRITY. REVISED DESIGN INNOVATION WILL REDUCE MANUAL HANDLING OF CONCRETE DISKS ON SITE.\ | AU2012101774A4 | COLLINS WILLIAM US | COLLINS WILLIAM US | E04B5/04 |
| 4242 | 3D printing | STEREOLITHOGRAPHY SYSTEMS AND METHODS USING INTERNAL LASER MODULATION\[DE] STEREOLITHOGRAFISCHE SYSTEME UND VERFAHREN MIT INTERNER LASERMODULATION\[FR] SYSTèMES ET PROCéDéS DE STéRéOLITHOGRAPHIE UTILISANT UNE MODULATION DE LASER INTERNE\ | EP2646223A1 | COOPER GUTHRIE (US) | 3D SYSTEMS INC (US) | B29C67/00 |
| 4243 | 3D printing | STEREOLITHOGRAPHY SYSTEMS AND METHODS USING INTERNAL LASER MODULATION\ | US2013154160A1 | COOPER GUTHRIE (US) | 3D SYSTEMS INC (US)\ COOPER GUTHRIE (US) | B29C35/08 |
| 4244 | 3D printing | STEREOLITHOGRAPHY MACHINE FOR PRODUCING A THREE-DIMENSIONAL OBJECT AND STEREOLITHOGRAPHY METHOD APPLICABLE TO SAID MACHINE\ | US8894400B2 | COSTABEBER ETTORE MAURIZIO (IT) | DWS SRL (IT)\ COSTABEBER ETTORE MAURIZIO (IT) | B29C67/00 |
| 4245 | 3D printing | IDENTIFICATION OF UNITS IN CUSTOMIZED PRODUCTION\ | US7611058B2 | CULP JAMES C (US)\ HO KWAN (US)\ SAMBU SHIVA PRASAD (US)\ KAZA SRINIVAS (US)\ FARREN CRAIG E (US)\ KASS SAMUEL J (US)\ NIKOLSKY SERGEY (RU) | ALIGN TECHNOLOGY INC (US) | G06K7/10\ G06K19/06 |
| 4246 | 3D printing | NETWORK DEVICE\ | US2012182900A1 | DAVARI SHAHRAM (US) | DAVARI SHAHRAM (US) | H04L12/28 |
| 4247 | 3D printing | METHODS AND SYSTEMS FOR IMPROVING MESHES USED IN COMPUTATIONAL FLUID SIMULATIONS\ | US2009112527A1 | DE PABLO FOUCE VALENTIN (ES)\ ESCUDERO GUIJARRO JAIRO (ES)\ MORA ORDONEZ CORAL AIDA (ES) | AIRBUS ESPANA SL (ES) | G06F17/50 |
| 4248 | 3D printing | FABRICATION OF A POLYMERIC PROSTHETIC IMPLANT\ | US2002171178A1 | DEAN DAVID (US)\ COOKE MALCOLM (US) | UNIV CASE WESTERN RESERVE US | B29C67/00\ A61F2/00\ A61F2/02\ A61F2/28\ A61F2/30\ A61L27/56\ A61B17/86\ A61L27/18\ A61L27/48\ G05B19/4099 |
| 4249 | 3D printing | PROJECTION DEVICE FOR PROJECTING HOLOGRAPHIC IMAGE E.G. TEXT IMAGE AT REAR PART OF VEHICLE I.E. CAR HAS POSITIONING UNIT FOR POSITIONING LIGHT SOURCE AND PLATES RELATIVE TO SUPPORT UNIT TO GENERATE IMAGE IN SELECTED PLACE\[FR] DISPOSITIF DE PROJECTION D'IMAGE(S) HOLOGRAPHIQUE(S) A REGLAGES MULTIPLES\ | FR2987909A1 | DERANLOT CHRISTOPHE (FR)\ AUTEGARDEN RONAN (FR) | PEUGEOT CITROEN AUTOMOBILES SA (FR) | G02B27/22\ G03H1/22\ B60K35/00\ F16M11/04 |
| 4250 | 3D printing | RAPID INTERSECTION METHOD FOR STL (STEREO LITHOGRAPHY) MODELS OF PRODUCTS\ | CN102508973A | DIANZHU SUN US\ YONGWEI SUN US\ YANRUI LI US\ YANG SONG US | UNIV SHANDONG TECHNOLOGY (CN) | G06F17/50 |
| 4251 | 3D printing | TWO-WAY PHOTODYNAMIC THERAPY STEREO COLPOSCOPE FOR DIAGNOSING AND TREATING DISEASES OF THE FEMALE GENITAL TRACT.\[ES] COLPOESTEREOSCOPIO DE FOTOTERAPIA DINAMICA POR DOS VIAS PARA DIAGNOSTICO Y TRATAMIENTO DE LAS ENFERMEDADES DEL TRACTO GENITAL FEMENINO.\ | MX2013010351A | DIAZ SANCHEZ JOEL GERARDO (MX)\ ZERTUCHE ZUANI JOSE GERARDO (MX) | DIAZ SANCHEZ JOEL GERARDO (MX) | A61B1/303\ A61B1/06\ A61B1/07\ G01N21/64 |
| 4252 | 3D printing | TWO-WAY PHOTODYNAMIC THERAPY STEREO COLPOSCOPE FOR DIAGNOSING AND TREATING DISEASES OF THE FEMALE GENITAL TRACT\ | US2014031702A1 | DIAZ SANCHEZ JOEL GERARDO (MX)\ ZERTUCHE ZUANI JOSE GERARDO (MX) | DIAZ SANCHEZ JOEL GERARDO (MX)\ ZERTUCHE ZUANI JOSE GERARDO (MX) | A61B1/303\ A61B1/06\ A61B1/07\ A61B1/00\ A61B1/05\ A61B5/00\ A61N1/00\ A61N5/06 |
| 4253 | 3D printing | PRODUCTION METHOD OF HOLLOW TURBINE VANE WITH SHAPED AIR FILM HOLE\ | CN101306465A | DICHEN LI (CN)\ HAIHUA WU (CN)\ NANNAN GUO (CN)\ FENGLU CUI (CN)\ BO SUN (CN)\ DONGYANG XU (CN) | UNIV XI AN JIAOTONG (CN) | B22D25/02\ B22C9/00 |
| 4254 | 3D printing | PROCESS FOR THE PRODUCTION OF A DENTAL MODEL A DENTAL MODEL WITH A CERAMIC LAYER DEPOSITED THEREON AND A DENTAL MOULDING DENTAL MODELAND USE OF 3D PRINTER AND A KIT\ | US2006131770A1 | DIERKES STEPHAN (DE)\ WIEST THOMAS (DE) | HERBST BREMER GOLDSCHLAEGEREI (DE) | B29C35/08\ A61C13/00\ B29C41/02 |
| 4255 | 3D printing | STEREO SPEAKER SYSTEM\ | JP2010199802A | EBATO AKIHIKO US\ HIRUMA TAKAHIRO US | TOSHIBA CORP US | H04S1/00\ H04R5/02\ H04R1/32 |
| 4256 | 3D printing | METHOD AND APPARATUS FOR AUTOMATIC SUPPORT GENERATION FOR AN OBJECT MADE BY MEANS OF A RAPID PROTOTYPE PRODUCTION METHOD\ | US8903533B2 | EGGERS GERALD (BE)\ RENAP KURT (BE) | MATERIALISE NV (BE)\ EGGERS GERALD (BE)\ RENAP KURT (BE) | B29C67/00\ G06F17/50\ G06T17/00\ G06T17/10\ G06T19/00 |
| 4257 | 3D printing | 3-DIMENSIONAL NON-VOLATILE MEMORY DEVICE AND METHOD OF FABRICATING THE SAME\ | KR101297088B1 | CHEN CHANGYING US | SNU R&DB FOUNDATION (KR) | H01L21/8247\ H01L27/115 |
| 4258 | 3D printing | APPARATUS AND METHOD FOR RECORDING ACCIDENT IMAGE\ | KR101284169B1 | Empty Field US | SL CORP (KR) | B62D41/00\ G01D9/00 |
| 4259 | 3D printing | APPARATUS AND METHOD FOR DISPLAYING ARROUND IMAGE OF VEHICLE\ | KR101278654B1 | Empty Field US | SL CORP (KR) | B60R1/00\ H04N5/225\ H04N5/262 |
| 4260 | 3D printing | PROCESSING METHOD OF LARGE AREA STRUCTURE IN LOW COST TYPE STEREOLITHOGRAPHY SYSTEM USING SMALL DMD AND UV-LED\ | KR101199496B1 | Empty Field US | CA TECH CO LTD (KR) | B29C67/00 |
| 4261 | 3D printing | METHOD FOR PRODUCING A DENTAL PROSTHESIS USING A MEDICAL IMAGE INSTRUMENT\ | KR101170056B1 | HWANG CHEOL SEONG (KR)\ SEOK JUN YEONG (KR) | SHIN MI CHONG (KR)\ CHOI SL GI (KR) | A61C13/34\ A61C8/00\ A61B6/14\ A61C19/05 |
| 4262 | 3D printing | AUDITORY OSSICLES SHAPE STRUCTURE OF CERAMIC MATERIAL AND MANUFACTURING METHOD OF THE SAME\ | KR101137940B1 | KANG HYUN WOOK (KR)\ CHO DONG WOO (KR) | POSTECH ACAD IND FOUND (KR) | A61F2/28\ A61L27/12\ A61L27/02 |
| 4263 | 3D printing | 3-DIMENSION CERAMIC POROUS SCAFFOLD AND MANUFACTURING METHOD OF THE SAME\ | KR101132747B1 | KEATING MICHAEL (GB) | POSTECH ACAD IND FOUND (KR) | A61F2/28\ A61L27/12\ A61L27/02 |
| 4264 | 3D printing | A DEVICE FOR PREPARATION OF MICROSTRUCTURE WITH MULTI COMPLEX RESIN USING MICROSTEREOLITHOGRAPHY\ | KR101109288B1 | LEE JONG WOOK (KR)\ LEE SUNG HYUN (KR) | PUSAN NAT UNIV IND COOP FOUND (KR) | B29C67/00 |
| 4265 | 3D printing | MANUFACTURING METHOD FOR 3D STRUCTURE OF BIOMATERIALS USING STEREO LITHOGRAPHY TECHNOLOGY AND PRODUCTS BY THE SAME\ | KR101016384B1 | LEE SEOK HEE (KR) | POSTECH ACAD IND FOUND (KR) | B29C35/08\ B29C67/00 |
| 4266 | 3D printing | 複合部材、複合部材を具備した光造形システム\ | JP5610114B2 | LEE SUNG HYUN (KR) | RICOH KK US | G02F1/361\ G02F1/35 |
| 4267 | 3D printing | No Title Available\ | CN102263978A | PARK IN BAEK (KR)\ HA YOUNG MYUNG (KR) | UNIV JINAN US | H04N13/00\ H04N5/74 |
| 4268 | 3D printing | STEREOSCOPIC VIDEO DISPLAY DEVICE\ | JP2012230142A | SEOL YOUNG JOON (KR) | JAPAN BROADCASTING CORP US | G02B27/22\ H04N13/04\ G03B35/18\ G02B3/00\ G03B35/00\ G09F9/00 |
| 4269 | 3D printing | METHOD FOR MANUFACTURING ARTIFICIAL ORGAN AND ARTIFICIAL ORGAN\ | JP2012137514A | SEOL YOUNG JOON (KR) | JMC KK US | G09B23/34 |
| 4270 | 3D printing | IMPROVEMENTS IN AND RELATING TO GRAVURE PRINTING AND SECURITY PRINTING\ | GB0525187D0 | SHIN MI CHONG (KR)\ CHOI SL GI (KR) | KEATING GRAVURE SYSTEMS UK LTD (GB) | B41M1/10\ B41M3/14 |
| 4271 | 3D printing | IMAGE DISPLAY DEVICE IMAGE BRIGHTNESS CORRECTION METHOD OF IMAGE DISPLAY DEVICE SHUTTER GLASSES DEVICE AND IMAGE BRIGHTNESS CORRECTION METHOD OF SHUTTER GLASSES DEVICE\ | JP2011239164A | TOMURA TATSUYA US | SONY CORP (JP) | G02B27/22\ H04N13/04\ G02F1/13\ G02F1/133 |
| 4272 | 3D printing | VERFAHREN ZUR CHEMISCHEN BEHANDLUNG VON POR?SEN SILICIUMOBERFL?CHEN\[EN] METHOD OF CHEMICAL TREATMENT OF POROUS SILICON SURFACES\[FR] PROCéDé DE TRAITEMENT CHIMIQUE DE SURFACES DE SILICIUM POREUSES\ | EP2185480A4 | ERVIN JOHN LAWRENCE (US)\ TIGLI HUS (US)\ KENNEDY MARK (US) | TREX ENTPR CORP (US) | G01N33/543\ C03C17/30\ C30B33/00 |
| 4273 | 3D printing | PRINTER\ | CN103158369A | FAN ZHIGUO US | LENOVO BEIJING LTD US | B41J2/435 |
| 4274 | 3D printing | THREE-DIMENSIONAL MICROFABRICATED BIOREACTORS WITH EMBEDDED CAPILLARY NETWORK\ | US2011033887A1 | FANG NICHOLAS X (US)\ XIA CHUNGUANG (US)\ COX ANDREW (US) | FANG NICHOLAS X (US)\ XIA CHUNGUANG (US)\ COX ANDREW (US) | G03F7/20\ C12M3/00\ C12P1/00\ G01D18/00 |
| 4275 | 3D printing | 3D (3-DIMENSIONAL) HIGH DEFINITION (HD) VIDEO CONVERTER\ | CN102638666A | FENG HUANG US | SHENZHEN KALIF ELECTRONICS CO LTD US | H04N13/00\ H04N5/765\ H04N5/775\ H04N7/01 |
| 4276 | 3D printing | LOW VISCOSITY ALLOPHANATES CONTAINING ACTINICALLY CURABLE GROUPS\ | US2007191570A1 | FISCHER WOLFGANG (DE)\ WEIKARD JAN (DE)\ RICHTER FRANK (DE)\ MUNDSTOCK HOLGER (DE)\ GURTLER CHRISTOPH (DE)\ SCHMITZ JORG (DE) | WEIKARD JAN (DE)\ RICHTER FRANK (DE)\ GURTLER CHRISTOPH (DE)\ SCHMITZ JORG (DE)\ MUNDSTOCK HOLGER (DE)\ FISCHER WOLFGANG (DE) | C08G18/22 |
| 4277 | 3D printing | THREE-DIMENSIONAL MATRICES OF STRUCTURED POROUS MONETITE FOR TISSUE ENGINEERING AND BONE REGENERATION AND METHOD OF THE PREPARATION THEREOF\ | US2011158963A1 | FONT PEREZ JULIO (ES)\ DEL OLMO BASTERRECHEA MAITE (ES)\ GARCIA VAZQUEZ MARIA DOLORES (ES)\ RUBIO RETAMA JORGE (ES)\ LOPEZ CABARCOS ENRIQUE (ES)\ RUEDA RODRIGUEZ CARMEN (ES)\ CASTROFEO MARIA BEGONA (ES)\ TAMINI MARINO FALEH (ES)\ ALKHRAISAT MOHAMMAD HAMDAN ALI (ES) | HISTOCELL SL (ES) | A61K9/00\ A61K35/12\ A61P19/00\ B29B11/06\ B29C39/00\ B32B3/20 |
| 4278 | 3D printing | MAGENTA DYES AND INKS FOR USE IN INK-JET PRINTING\ | US7270700B2 | FOSTER CLIVE EDWIN (GB) | FUJIFILM IMAGING COLORANTS LTD (GB) | C09D11/00\ B41J2/01\ C07C323/22\ C07C323/66\ C09D11/02\ C07C50/36 |
| 4279 | 3D printing | STEREOLITHOGRAPHY METHOD AND STEREOLITHOGRAPHY APPARATUS\[FR] PROCéDé ET APPAREIL DE STéRéOLITHOGRAPHIE\ | WO2012111655A1 | FURUKAWA HIDEMITSU (JP)\ HIDEMA RURI (JP)\ KAMATA YOSHITAKA (JP)\ MUROI HISATO (JP) | SUNARROW LTD (JP)\ FURUKAWA HIDEMITSU (JP)\ HIDEMA RURI (JP)\ KAMATA YOSHITAKA (JP)\ MUROI HISATO (JP) | B29C67/00 |
| 4280 | 3D printing | METAL POWDER FOR METAL STEREO LITHOGRAPHY\ | JP2008050671A | FUWA ISAO (JP) | MATSUSHITA ELECTRIC WORKS LTD (JP) | B22F3/105\ B22F3/16\ B22F1/00 |
| 4281 | 3D printing | ROTATION FITTING FOR THE EMPENNAGE OF AN AIRCRAFT\ | US2007147953A1 | GARCIA SACRISTAN ANGEL (ES) | AIRBUS ESPANA SL (ES) | F16B9/00 |
| 4282 | 3D printing | TRUE THREE-DIMENSIONAL IMAGE DISPLAY SYSTEM\ | CN203217233U | GENG ZHENG (CN) | GENG ZHENG (CN) | G02B27/22\ H04N13/04\ G03B35/18 |
| 4283 | 3D printing | TRUE THREE-DIMENSIONAL IMAGE DISPLAY SYSTEM\ | CN203217232U | GENG ZHENG (CN) | GENG ZHENG (CN) | G02B27/22\ H04N13/04\ G03B35/18 |
| 4284 | 3D printing | TRUE 3D IMAGE DISPLAY SYSTEM\ | CN202975584U | GENG ZHENG (CN) | GENG ZHENG (CN) | G02B27/22\ H04N13/04\ G03B35/18 |
| 4285 | 3D printing | VERFAHREN ZUM HERSTELLEN KERAMISCHER BAUTEILE INSBESONDERE VON IMPLANTATEN\[EN] RAPID PROTOTYPING PROCESS TO FABRICATE CERAMIC SURGICAL IMPLANT WITH CERAMIC POWDER AND BINDING AGENT SUSPENSION DEVOID OF SOLVENT\ | DE102005058116A1 | GIA KHANH PHAM (DE)\ SCHWARZ MARTINA (DE)\ SCHAEFER MARTIN (DE)\ WESSLER BERIT (DE) | SIEMENS AG (DE) | C04B35/622\ A61C8/00\ A61L27/10 |
| 4286 | 3D printing | SYSTEM AND METHOD FOR SPEAKER RECOGNITION ON MOBILE DEVICES\ | US2013225128A1 | GOMAR MARTA GARCIA (ES) | AGNITIO SL (US)\ AGNITIO SL (ES) | H04W12/06 |
| 4287 | 3D printing | OPTICAL SHAPING APPARATUS USING VISIBLE LIGHT\ | JP2005014581A | GOTO HIKARI US | CHUBU NIPPON KOGYO KK US\ GRAN WERK KK US | B29C67/00 |
| 4288 | 3D printing | STEREO SIGNAL GENERATING APPARATUS AND STEREO SIGNAL GENERATING METHOD\ | US8019087B2 | GOTO MICHIYO (JP)\ TEO CHUN WOEI (SG)\ NEO SUA HONG (SG)\ YOSHIDA KOJI (JP) | PANASONIC CORP (JP) | G10L19/00\ H04R5/00\ G10L13/00 |
| 4289 | 3D printing | STEREO AUDIO ENCODING APPARATUS STEREO AUDIO DECODING APPARATUS AND METHOD THEREOF\ | US2009276210A1 | GOTO MICHIYO (JP)\ YOSHIDA KOJI (JP) | PANASONIC CORP (JP) | G10L19/00\ G10L19/008\ G10L19/14 |
| 4290 | 3D printing | INK JET INK AND RECORDING PROCESS\ | GB2401872A | GOULD JAYNE MAURA (GB)\ ROSE PETER EDWARD (GB)\ WALKER NICHOLAS ALEXANDER (GB) | ILFORD IMAGING UK LTD (GB) | C09D11/00 |
| 4291 | 3D printing | THREE-DIMENSIONAL OPTICAL AND MATERIAL STRUCTURES\[FR] STRUCTURES MATéRIELLES ET OPTIQUES TRIDIMENSIONNELLES\ | WO2011098609A1 | GREEN ROGER (GB)\ COVINGTON JAMES (GB)\ HUTCHINS DAVID (GB)\ BILLSON DUNCAN (GB)\ LEIGH SIMON (GB) | UNIV WARWICK (GB)\ GREEN ROGER (GB)\ COVINGTON JAMES (GB)\ HUTCHINS DAVID (GB)\ BILLSON DUNCAN (GB)\ LEIGH SIMON (GB) | B29C67/00 |
| 4292 | 3D printing | VORRICHTUNG UND VERFAHREN ZUM HERSTELLEN EINES DREIDIMENSIONALEN GEGENSTANDES MITTELS STEREOLITHOGRAPHIE\[EN] THREE-DIMENSIONAL COMPONENT IS FORMED USING A STEREOLITHOGRAPHY UNIT WHICH COMPRISES A CONTAINER FOR MATERIAL TO BE SOLIDIFIED AND AN IRRADIATION UNIT\ | DE102005003218B4 | GREINER RALPH (DE) | GREINER RALPH (DE) | B29C67/00\ G03F7/20 |
| 4293 | 3D printing | IMPROVEMENTS FOR RAPID PROTOTYPING APPARATUS\ | US8573958B2 | GRELIN JEROME (FR)\ POUGEOISE EMILIE (FR)\ HANGAARD OLE (DK)\ LARSEN NIELS HOLM (CH) | 3D SYSTEMS INC (US)\ HANGAARD OLE (DK)\ LARSEN NIELS HOLM (CH)\ POUGEOISE EMILIE (FR)\ GRELIN JEROME (FR) | B29C35/08 |
| 4294 | 3D printing | METHOD FOR MANUFACTURING PRODUCTS BY MEANS OF LASER STEREO-LITHOGRAPHY AND DEVICE FOR REALIZATION OF SAID METHOD\ | RU2269416C2 | GRIGOR JANTS ALEKSANDR GRIGOR (RU)\ SHIGANOV IGOR NIKOLAEVICH (RU)\ MALOV IL JA EVGEN EVICH (RU)\ SHEPELEV GENNADIJ VASIL EVICH (RU)\ EVSTROPOV GEORGIJ MIKHAJLOVICH (RU)\ ZAPADINSKIJ BORIS ISAAKOVICH (RU)\ LJUBIMOV ALEKSANDR VLADIMIROVI (RU) | Empty Field US | C08F2/46\ B29C41/02 |
| 4295 | 3D printing | MILLING HEAD IN TRICEPTS WITH ELECTRO-MECHANICAL DEPTH CONTROL\ | US2009088886A1 | GRILLE SACALUGA BENITO (ES)\ ASTORGA RAMIREZ JUAN RAMON (ES) | GRILLE SACALUGA BENITO (ES)\ ASTORGA RAMIREZ JUAN RAMON (ES) | G06F19/00 |
| 4296 | 3D printing | VERFAHREN ZUR DREIDIMENSIONALEN REKONSTRUKTION VON OBJEKTEN UNTER VERWENDUNG VON STREIFENPROJEKTIONSMUSTERN\[EN] METHOD FOR RECONSTRUCTING THREE-DIMENSIONAL OBJECTS USED IN QUALITY CONTROL APPLICATION INVOLVES GENERATING GRAY VALUE PROFILES FROM SUBSET OF PATTERN IMAGE SEQUENCE AND SUBSET OF SURFACE OF OBJECT SHIFTED FRINGE PATTERNS\ | DE102011010265A1 | GROSE MARCUS (DE)\ KOWARSCHIK RICHARD PROF DR (DE)\ SCHAFFER MARTIN (DE) | UNIV SCHILLER JENA (DE) | G01B11/25\ G06K9/46 |
| 4297 | 3D printing | STEREOLITHOGRAPHY RAPID PROTOTYPING PHOTOSENSITIVE RESIN PREPARATION METHOD AND APPLICATION THEREOF\ | CN101592859A | HAITAO LIU (CN)\ HANRONG ZHONG (CN)\ JIDONG XUE (CN) | GUANGZHOU MECHANICAL ENGINEERI (CN) | G03F7/004 |
| 4298 | 3D printing | ULTRASOUND TRANSDUCER MANUFACTURING USING RAPID-PROTOTYPING METHOD\ | US2013289593A1 | HALL TIMOTHY L (US)\ MAXWELL ADAM (US)\ CAIN CHARLES A (US)\ KIM YOHAN (US)\ XU ZHEN (US) | HALL TIMOTHY L (US)\ MAXWELL ADAM (US)\ CAIN CHARLES A (US)\ KIM YOHAN (US)\ XU ZHEN (US) | A61B17/32\ A61B17/225 |
| 4299 | 3D printing | DETOXIFICATION OF SOLID FREEFORM FABRICATION MATERIALS\ | US6996245B2 | HANNA STEPHEN D (US) | 3D SYSTEMS INC (US) | B29C67/00\ B29K63/00\ A61L27/00\ B29K33/00\ H04R25/00 |
| 4300 | 3D printing | IMAGE PROCESSING\ | US2006045329A1 | HANSARD MILES (GB)\ JONES GRAHAM R (GB) | JONES GRAHAM R (GB)\ HANSARD MILES (GB) | H04N13/00\ G06K9/00\ G06T15/20 |
| 4301 | 3D printing | IMAGE FORMING APPARATUS AND PRINT HEAD\ | JP2005059300A | HASEGAWA TOMOAKI US | FUJI XEROX CO LTD US | B41J2/44\ B41J2/45\ B41J2/455\ G03G15/04\ H01L33/08\ H01L33/58\ H04N1/036 |
| 4302 | 3D printing | STEREO LITHOGRAPHIC DEVICE\ | JP2000158545A | HATAYAMA SHOZO US | TEIJIN SEIKI CO LTD (JP) | B29C35/08\ B29C67/00\ B29K105/24 |
| 4303 | 3D printing | APPARATUS AND METHOD FOR PRODUCING PACKS OF FLEXIBLE FLAT OBJECTS\ | US8733752B2 | HEINIGER RETO (CH) | FERAG AG (CH)\ HEINIGER RETO (CH) | B65H33/00\ B65H33/12\ B65H33/16 |
| 4304 | 3D printing | BESCHICHTEREINRICHTUNG FüR EINE BAUVORRICHTUNG ZUR HERSTELLUNG VON FORMTEILEN AUS PULVERMATERIAL\[EN] COATING DEVICE FOR AN APPARATUS FOR PRODUCING ARTICLES MADE OF PULVERULENT MATERIAL\[FR] APPLICATEUR POUR UN APPAREIL DE FABRICATION DE PIèCES PRODUITES à PARTIR D'UN MATéRIAU PULVéRULENT\ | EP1439050A1 | HERZOG FRANK (DE) | CONCEPT LASER GMBH (DE) | B29C67/00\ B22F3/00\ B29C41/12\ B29C41/36\ B29C37/02 |
| 4305 | 3D printing | METHOD FOR PRODUCING THREE-DIMENSIONAL WORK PIECES IN A LASER MATERIAL MACHINING UNIT OR A STEREOLITHOGRAPHY UNIT AND UNIT FOR PERFORMING THE METHOD\ | US2004217095A1 | HERZOG FRANK (DE) | CONCEPT LASER GMBH (US) | B29C67/00\ B22F3/105\ B22F3/16\ B22F3/24 |
| 4306 | 3D printing | PROCESS FOR MANUFACTURING A SHAPED ARTICLE IN PARTICULAR POWDER STEREOLITHOGRAPHIC OR SINTERING PROCESS\ | US2004099996A1 | HERZOG FRANK (DE) | CONCEPT LASER GMBH US | B29C67/00\ B22F3/105\ B22F3/16\ B23K26/34 |
| 4307 | 3D printing | METHOD FOR PRODUCING THREE-DIMENSIONAL SINTERED WORK PIECES\ | US2005142024A1 | HERZOG FRANK (DE) | CL SCHUTZRECHTSVERWALTUNGS GMB (DE) | B29C67/00\ B22F3/105\ B22F3/16 |
| 4308 | 3D printing | CUSTOM-MOULDED EAR-PLUG AND PROCESS FOR PRODUCING A CUSTOM-MOULDED EAR-PLUG DEVICE\ | US6766878B2 | HESSEL HANS (CH)\ WEIDMANN MARKUS (CH)\ WIDMER CHRISTOPH (CH) | PHONAK AG (CH) | H04R25/00\ A61F11/00\ A61F11/08 |
| 4309 | 3D printing | DEVICE AND METHOD FOR THE PRODUCTION OF THREE-DIMENSIONAL OBJECTS BY MEANS OF GENERATIVE PRODUCTION METHOD\ | US7665979B2 | HEUGEL MARTIN (DE) | EOS ELECTRO OPTICAL SYST (DE) | B29C35/08\ B29C67/00 |
| 4310 | 3D printing | INK COMPOSITIONS CONTAINING ETHYLENE GLYCOL MONOBUTYL ETHER\ | US7427320B2 | HOLBROOK MARK (GB)\ YUSUF SHAKILA (GB)\ RIDING VICKY (GB) | FUJIFILM IMAGING COLORANTS LTD (GB) | C09D11/00\ C09D11/02 |
| 4311 | 3D printing | THREE DIMENSIONAL RECONSTRUCTION METROLOGY\ | US6714892B2 | HOUGE ERIK CHO (US)\ MCINTOSH JOHN MARTIN (US)\ PLEW LARRY E (US) | AGERE SYSTEMS INC (US) | G03F7/20\ G01Q30/02\ G01Q30/04\ G01Q80/00 |
| 4312 | 3D printing | STEREOLITHOGRAPHIC APPARATUS\ | US7785093B2 | HULL CHARLES W (US)\ HOLMBOE SCOTT B (US)\ REYNOLDS GARY L (US)\ STONESMITH MATTHEW K (US)\ REICHENTAL ABRAHAM N (US) | 3D SYSTEMS INC (US) | B28B17/00\ B29B13/08\ B28B1/14 |
| 4313 | 3D printing | RAPID PROTOTYPING AND MANUFACTURING SYSTEM AND METHOD\ | US7621733B2 | HUNTER DON FREDERICK (US)\ REYNOLDS GARY LEE (US) | 3D SYSTEMS INC (US) | B29C35/08\ B29C41/52\ B29C41/02 |
| 4314 | 3D printing | METHOD FOR JOINING PARTS FABRICATED VIA SELECTIVE LASER SINTERING WHILE MAINTAINING PROPER ALIGNMENT\ | US2007236018A1 | HUSMANN CHRISTOPHER H (US)\ STEIN GREGORY N (US) | NORTHROP GRUMMAN CORP (US) | F16L21/00 |
| 4315 | 3D printing | BUILT-IN ATTACHMENT DEVICE USING SELECTIVE LASER SINTERING\ | US7514649B2 | HUSMANN CHRISTOPHER H (US)\ STEIN GREGORY N (US) | NORTHROP GRUMMAN CORP (US) | B29C35/08\ B23K26/00 |
| 4316 | 3D printing | LIGHT DIFFUSER FOR LIQUID CRYSTAL DISPLAY USING STEREOLITHOGRAPHY AND METHOD FOR FABRICATING THE SAME\ | KR20040032514A | HWANG YUN IL US\ MUN JE MIN US | LG CHEMICAL LTD (KR) | G02F1/13357 |
| 4317 | 3D printing | THREE-DIMENSIONAL POLYMER-METAL COMPLEX MICROSTRUCTURE AND METHOD FOR PRODUCING THE SAME\ | US2013136924A1 | IKUTA KOJI (JP)\ IKEUCHI MASASHI (JP) | JAPAN SCIENCE & TECH AGENCY (JP)\ IKUTA KOJI (JP)\ IKEUCHI MASASHI (JP) | B29C67/00 |
| 4318 | 3D printing | CYTOCOMPATIBLE THREE-DIMENSIONAL STRUCTURES FABRICATED BY MICROSTEREOLITHOGRAPHY\ | US2011268948A1 | IKUTA KOJI (JP)\ INOUE YOSHINORI (JP) | JAPAN SCIENCE & TECH AGENCY (JP) | B29C35/08\ B32B3/00\ B32B5/00 |
| 4319 | 3D printing | ADDITITVE MANUFACTURING OF COMPONENTS FOR DOWNHOLE WIRELINE TUBING AND DRILL PIPE CONVEYED TOOLS\ | US2013310961A1 | INTRIAGO VELEZ JUAN CARLOS (US) | CORP SCHLUMBERGER TECHNOLOGY (US)\ SCHLUMBERGER TECHNOLOGY CORP (US) | G06F17/50 |
| 4320 | 3D printing | APPARATUS AND METHOD FOR EXECUTING STEREO LITHOGRAPHY\ | JP2002144437A | INUBUSHI TATSUO US | TEIJIN SEIKI CO LTD (JP) | B29C67/00 |
| 4321 | 3D printing | RESIN COMPOSITION FOR STEREOLITHOGRAPHY\ | US2010152314A1 | ITO TAKASHI (JP)\ HAGIWARA TSUNEO (JP) | CMET INC (JP) | C08F2/46 |
| 4322 | 3D printing | THREE-DIMENSIONAL OBJECT HAVING SMOOTHED FABRICATED EDGE SURFACE\ | US2010104804A1 | ITO TAKASHI (JP)\ HAGIWARA TSUNEO (JP) | CMET INC (JP) | B29C35/08\ C08L63/00\ B32B3/10\ B32B3/02\ C08L33/00 |
| 4323 | 3D printing | RESIN COMPOSITION FOR STEREOLITHOGRAPHY\ | US2009209674A1 | ITO TAKASHI (JP)\ HAGIWARA TSUNEO (JP)\ HIROTSU KENJI (JP)\ MURAKAMI TADASHI (JP) | CMET INC (JP)\ UBE INDUSTRIES (JP) | C08F2/46 |
| 4324 | 3D printing | MICROPHONE AND SOUND IMAGE PROCESSING SYSTEM\ | US2006222187A1 | JARRETT SCOTT (US)\ DAY THOMAS W (US) | JARRETT SCOTT (US)\ DAY THOMAS W (US) | H04R3/00 |
| 4325 | 3D printing | QUICK MANUFACTURING METHOD OF TITANIUM-ALUMINUM ALLOY COMPOSITE PART\ | CN102941343A | JIA WENPENG US\ YANG GUANGYU US\ ZHAO PEI US\ HE WEIWEI US\ HUANG YU US\ JIA LIANG US | NW INST NON FERROUS METAL RES US | B22F3/105 |
| 4326 | 3D printing | NUMERICAL SIMULATION TECHNOLOGY FOR HOLLOW SECTION PORTHOLE DIE EXTRUSION WELDING PROCESS\ | CN101604350A | JIANXIN XIE (CN)\ DONGNAN HUANG (CN)\ JINGYUAN LI (CN)\ ZHIHAO ZHANG (CN) | UNIV BEIJING SCIENCE & TECH (CN) | G06F17/50\ B21C25/02 |
| 4327 | 3D printing | METHOD FOR PREPARING TITANIUM PRODUCT\ | CN102120261A | JINXIN LIN US | JINXIN LIN US | B22F3/105\ C22F1/02\ C22F1/18 |
| 4328 | 3D printing | SYSTEME DE POSITIONNEMENT DE FIBRES OPTIQUES ET PROCEDES DE FABRICATION\ | FR2890455B1 | JOFFRE PASCAL YVON US\ SANCHOU FREDERIC DIDIER US | MICRO MODULE SARL (FR) | G02B6/40\ F21V8/00 |
| 4329 | 3D printing | ACOUSTIC SYSTEM\ | JP2010004129A | KADOWAKI NOBUSATO US | PANASONIC ELEC WORKS CO LTD (JP) | H04S5/02\ H04R1/02 |
| 4330 | 3D printing | METHOD AND APPARATUS FOR IMAGE DATA PROCESSING\ | US2003235343A1 | KAGAYA MAKOTO (JP) | FUJI PHOTO FILM CO LTD (US) | G06T5/20\ G06T5/00\ G06T5/10\ H04N1/407\ H04N1/41\ H04N5/20\ H04N5/21 |
| 4331 | 3D printing | NETWORK SLA CONTROL SYSTEM\ | JP2002325100A | KATO KEI US | OKI ELECTRIC IND CO LTD US | H04L12/56 |
| 4332 | 3D printing | ENDLAGENSCHALTUNG FüR STEREOMIKROSKOPE MIT MOTORISIERTEM FOKUSSIERTRIEB\[EN] LIMIT STOP SWITCH FOR STEREO-MICROSCOPES WITH MOTORIZED FOCUSING\[FR] INTERRUPTEUR DE BOUT DE COURSE POUR STéRéO-MICROSCOPE AVEC FOCALISATION MOTORISéE\ | EP1643287B1 | KAUFHOLD TOBIAS (DE)\ HEIN DETLEF (DE)\ FECHTI JUERGEN (DE) | ZEISS CARL MICROIMAGING GMBH (DE) | G02B21/24 |
| 4333 | 3D printing | HUMAN BODY AFFECTED PART ENTITY MODEL AND MANUFACTURING METHOD THEREOF\ | JP2006078604A | KAWASHIMA NORIMICHI US\ IIDA YUKIYASU US\ SATO TOSHIO US\ TSUJI KIICHI US\ TAKEUCHI MASAAKI US\ IMAMURA EISAKU US | TOIN GAKUEN US | B29C67/00\ G09B23/30 |
| 4334 | 3D printing | MANUFACTURING METHOD FOR HUMAN BODY AFFECTED PART ENTITY MODEL\ | JP2006119435A | KAWASHIMA NORIMICHI US\ IIDA YUKIYASU US\ SATO TOSHIO US\ TSUJI KIICHI US\ TAKEUCHI MASAAKI US\ NIIKURA YOSHIHIRO US | TOIN GAKUEN US | G09B23/30 |
| 4335 | 3D printing | FORMING STRUCTURES FROM CAD SOLID MODELS\ | US6811744B2 | KEICHER DAVID M (US)\ LOVE JAMES W (US)\ DULLEA KEVIN J (US)\ BULLEN JAMES L (US)\ GORMAN PIERRETTE H (US)\ SMITH MARK E (US) | OPTOMEC DESIGN (US) | B29C67/00\ B22F3/105\ B23K26/34\ B29C33/38\ B05B7/14\ B29C33/02\ B29C33/04\ B29C41/20\ B29C45/73\ F28F7/02 |
| 4336 | 3D printing | UTILISATION D'UN ALLIAGE METALLIQUE COMPLEXE A BASE D'ALUMINIUM POUR LA STEREOLITHOGRAPHIE\[EN] USE OF A COMPLEX METAL ALLOY CONTAINING ALUMINUM FOR STEREOLITHOGRAPHY\ | WO2013167448A1 | KENZARI SAMUEL (FR)\ SAKLY ADNENE (TN)\ BONINA DAVID (FR)\ CORBEL SERGE (FR)\ FOURNEE VINCENT (FR) | UNIV LORRAINE (FR)\ CENTRE NAT RECH SCIENT (FR) | B29C67/00\ B22F3/105 |
| 4337 | 3D printing | STEREOLITHOGRAPHY APPARATUS\ | US2009196946A1 | KIHARA NOBUHIRO (JP)\ KUZUSAKO JUNICHI (JP)\ HONDA KATSUHISA (JP) | SONY CORP (JP) | B29C35/08 |
| 4338 | 3D printing | METHOD FOR INSERTING AND EXTRACTING WATERMARK OF THREE-DIMENSIONAL SHAPE\ | KR20040003185A | KIM SUN HYUNG US | KIM SUN HYUNG US | G06T1/00 |
| 4339 | 3D printing | THREE-DIMENSIONAL VIDEO SCANNER\ | US7756323B2 | KIMMEL RON (IL) | TECHNION RES & DEV FOUNDATION (IL) | G01B11/25\ G06K9/00\ G01N21/86\ G02B5/32\ H01J3/14 |
| 4340 | 3D printing | THREE DIMENSIONAL PERIODIC STRUCTURE AND METHOD OF PRODUCING THE SAME\ | US2005221100A1 | KIRIHARA SOSHU (JP)\ MIYAMOTO YOSHINARI (JP)\ NAKAGAWA TAKUJI (JP)\ TANAKA KATSUHIKO (JP) | MURATA MANUFACTURING CO (JP) | B29C67/00\ G02B6/12\ C30B5/00\ G02B1/02\ G02B5/18\ G02B6/13 |
| 4341 | 3D printing | CURABLE RESIN COMPOSITION AND SHAPED PRODUCT USING THE SAME\ | JP2006282714A | KITANO TAKAHIRO US\ KUBO KEIJI US\ KAWASAKI MASAHIRO US | KURARAY CO (JP) | B29C67/00\ G03F7/027\ G03F7/004\ C08F2/44\ C08F287/00\ G03F7/033 |
| 4342 | 3D printing | LOW-VISCOUS RADIATION CURABLE FORMULATION PARTICULARLY FOR THE STEREO-LITHOGRAPHICAL PRODUCTION OF EARPIECE\ | US2006264526A1 | KLARE MARTIN (DE)\ ALTMANN REINER (DE)\ MEISSNER GEORGIA (DE)\ VEIT THOMAS (DE)\ KUTSHCINSKI MICHAEL (DE) | KUTSHCINSKI MICHAEL (DE)\ KLARE MARTIN (DE)\ ALTMANN REINER (DE)\ MEISSNER GEORGIA (DE)\ VEIT THOMAS (DE) | B29C67/00\ G03F7/00\ G03F7/027\ C08F290/14 |
| 4343 | 3D printing | METHOD AND SYSTEM FOR PRODUCTION OF FIBROUS COMPOSITE PROTOTYPES USING ACOUSTIC MANIPULATION IN STEREOLITHOGRAPHY\ | US2002185782A1 | KOCH ROBERT M (US)\ KUKLINSKI ROBERT (US) | US NAVY (US) | B29C67/00\ B29C70/54\ B29C70/62 |
| 4344 | 3D printing | NIEDERDRUCK-SPRITZGUSSVERFAHREN\[EN] INJECTION MOLD FOR LOW PRESSURE MOLDING OF THERMOPLASTIC MATERIALS IS PRODUCED BY A RAPID TOOLING PROCESS FOR COST REDUCTION\ | DE10238597A1 | KOLLER STEFAN (DE)\ HABERSTROH STEFAN (DE)\ HOFMANN ROBERT (DE)\ COINTEREAU JEAN-FRANCOIS (FR) | VALEO KLIMASYSTEME GMBH (DE)\ HOFMANN ROBERT (DE) | B29C67/00\ B29C33/38\ B29C33/56\ B29C44/58\ B29C45/17 |
| 4345 | 3D printing | VERFAHREN ZUR DREIDIMENSIONALEN REKONSTRUKTION VON OBJEKTEN\[EN] METHOD FOR THREE-DIMENSIONAL RECONSTRUCTION OF OBJECTS FOR PROJECTING PATTERN SEQUENCES ON RECONSTRUCTING OBJECT INVOLVES DETECTING PATTERN SEQUENCES IN FORM OF PATTERN VIEWS WHICH ARE COMPARED WITH EACH OTHER IN DIFFERENT IMAGE VIEWS\ | DE102009040981A1 | KOWARSCHIK RICHARD (DE)\ GROSE MARCUS (DE) | UNIV SCHILLER JENA (DE) | G01B11/25 |
| 4346 | 3D printing | PHOTO-FABRICATION APPARATUS\ | US7083405B2 | KOYAGI YASUYUKI (JP)\ SHIMOZUMA HIROKO (JP) | DAINIPPON SCREEN MFG (JP) | B29C67/00\ B28B17/00 |
| 4347 | 3D printing | THREE-DIMENSION MEASURING SYSTEM FOR DYNAMIC OBJECT\ | CN101825445A | KUI HUANG US\ ZHONGWEI LI US\ CONGJUN WANG US\ CHENGCHENG ZHAN US\ WEI ZHANG US\ KAI ZHONG US\ GANG ZHOU US\ XIAOPENG ZHU US\ YUSHENG SHI (CN) | UNIV HUAZHONG SCIENCE TECH (CN) | G01B11/25\ G01B11/00 |
| 4348 | 3D printing | PLANAR STEREO LITHOGRAPHY APPARATUS AND METHOD\ | CN103203875A | LE GUANG US\ WANG ZHANQIN US\ TONG ZHIQIANG US\ WANG LIJUN US\ CHENG YONGLI US\ WANG YONGXIN US | SHAANXI HENGTONG INTELLIGENT MACHINE CO LTD US | B29C67/00 |
| 4349 | 3D printing | MULTI-SIZE STEREOLITHOGRAPHY\ | KR100930788B1 | LEE BYUNG KEUK (KR) | CARIMA CO LTD (KR) | B29C35/08\ G03F7/004\ H01L21/027\ B29C35/00 |
| 4350 | 3D printing | PLANARIZATION APPARATUS FOR STEREO LITHOGRAPHY\ | KR20060034848A | LEE JIN WOO (KR)\ CHO DONG WOO (KR)\ LEE IN HWAN (KR) | POSTECH FOUNDATION (KR) | B29C59/00\ B29C59/02 |
| 4351 | 3D printing | LIQUID RADIATION-CURABLE COMPOSITION ESPECIALLY FOR STEREOLITHOGRAPHY\ | US2001046642A1 | LEYDEN RICHARD (US)\ PATEL RANJANA C (GB)\ JOHNSON DAVID L (US) | VANTICO AG US | B29C67/00\ G03F7/00\ G03F7/038\ G03F7/027\ B29K33/00\ C08G59/00\ C08G65/00\ G03F7/004 |
| 4352 | 3D printing | PHOTOSENSITIVE RESIN FOR STEREO LITHOGRAPHY FORMING AND PREPARATION METHOD THEREOF\ | CN103246164A | LI HOUMIN US | SUZHOU TAISULEI ELECTRONIC TECHNOLOGY CO LTD US | G03F7/027\ G03F7/004 |
| 4353 | 3D printing | TRAJECTORY GENERATION SYSTEM FOR COMPLEX CURVED SURFACE PROCESSING\ | CN203164681U | LI RUNWEI US\ LI LIN US\ ZOU YANBIAO US | UNIV SOUTH CHINA TECH (CN) | G05B19/4097 |
| 4354 | 3D printing | 3D STEREO LITHOGRAPHY WATCH\ | TWM458578U | LIN YU-SHENG (TW)\ LI MENG-KUN (TW)\ XU MING-FENG (TW)\ HUANG HONG-ZHANG (TW)\ SONG JIA-CHEN (TW)\ ZHANG SHENG-XIONG (TW) | UNIV FAR EAST (TW) | G04B19/00 |
| 4355 | 3D printing | VERFAHREN ZUM LASERSCHNEIDEN EINES NICHTMETALLISCHEN WERKSTüCKS\[EN] METHOD FOR LASER CUTTING OF A NONMETAL WORKPIECE\[FR] PROCéDé DE DéCOUPE AU LASER D'UNE PIèCE NON MéTALLIQUE\ | WO2009097987A1 | LISSOTSCHENKO VITALIJ (DE) | LIMO PATENTVERWALTUNG GMBH (DE)\ LISSOTSCHENKO VITALIJ (DE) | B23K26/40\ B28D5/00 |
| 4356 | 3D printing | PHOTOPOLYMERIZABLE COMPOSITION FOR VISIBLE-REGION LASER STEREOLITHOGRAPHY\ | RU2244335C2 | LJUBIMOV A V (RU)\ ZAPADINSKIJ B I (RU)\ SHIGANOV I N (RU)\ MALOV I E (RU)\ GRIGORJANTS A G (RU) | Empty Field US | G03F7/004\ C08F291/06 |
| 4357 | 3D printing | SYSTEM ZUR VIRTUELLEN SIMULATION VON R?UMEN SZENERIEN UND DERGLEICHEN\[EN] SYSTEM OF VIRTUAL SIMULATION OF SPACES SCENERIES AND SIMILAR\[FR] SYSTèME DE SIMULATION VIRTUELLE DES ESPACES DES DéCORS ET SIMILAIRE\ | EP1912195A1 | LLORENS MIRAVET SALVADOR (ES) | INSCA INT SL (ES) | G09B25/06\ G03B21/60 |
| 4358 | 3D printing | PREPARATION METHOD OF METAL TOUGHENED CERAMIC-BASED COMPOSITE MATERIAL TURBINE BLADE\ | CN103113112B | LU ZHONGLIANG US\ LI DICHEN US\ LIU TAO US\ JING HUI US\ LU BINGHENG US | UNIV XI AN JIAOTONG (CN) | C04B35/622\ C04B35/565\ C04B35/74\ F01D5/12 |
| 4359 | 3D printing | PROCESS FOR THE PREPARATION OF PARTICULARLY REACTIVE AND LOW-VISCOSITY ALLOPHANATES HAVING ACTINIC-CURABLE GROUPS AND THE USE THEREOF FOR THE PREPARATION OF PARTICULARLY SCRATCH-RESISTANT COATINGS\ | US2010204434A1 | LUDEWIG MICHAEL (DE)\ FISCHER WOLFGANG (DE)\ KUCZEWSKI HELMUT (DE) | BAYER MATERIALSCIENCE AG (DE) | C08G69/00\ C07C269/02 |
| 4360 | 3D printing | PROCESS FOR PREPARING LOW-VISCOSITY ALLOPHANATES CONTAINING ACTINICALLY CURABLE GROUPS\ | US2009062500A1 | LUDEWIG MICHAEL (DE)\ FISCHER WOLFGANG (DE)\ KUCZEWSKI HELMUT (DE) | FISCHER WOLFGANG (DE)\ LUDEWIG MICHAEL (DE)\ KUCZEWSKI HELMUT (DE) | C08G18/10 |
| 4361 | 3D printing | MEMORY ARCHITECTURE OF 3D ARRAY WITH DIODE IN MEMORY STRING\ | US2012327714A1 | LUE HANG-TING (TW) | MACRONIX INT CO LTD (TW)\ LUE HANG-TING (TW) | G11C16/04 |
| 4362 | 3D printing | PASSIVE COMPONENTS FOR MILLIMETER SUBMILLIMETER AND TERAHERTZ ELECTROMAGNETIC WAVES MADE BY PILING UP SUCCESSIVE LAYERS OF MATERIAL\[FR] COMPOSANTS PASSIFS POUR ONDES éLECTROMAGNéTIQUES DE L'ORDRE DU MILLIMèTRE DU SOUS-MILLIMèTRE OU TéRAHERTZIENNES OBTENUS PAR EMPILEMENT DE COUCHES SUCCESSIVES DE MATéRIAU\ | WO2012076994A1 | MACOR ALESSANDRO (CH)\ ANSERMET JEAN-PHILIPPE (CH)\ DE RIJK EMILE (CH) | ECOLE POLYTECH (CH)\ MACOR ALESSANDRO (CH)\ ANSERMET JEAN-PHILIPPE (CH)\ DE RIJK EMILE (CH) | H01P3/123\ H01P11/00\ H01Q13/02 |
| 4363 | 3D printing | NON-VOLATILE SEMICONDUCTOR STORAGE DEVICE\ | US2011063916A1 | MAEDA TAKASHI (JP) | TOSHIBA KK (JP) | G11C16/02 |
| 4364 | 3D printing | THREE DIMENSIONAL STACKED NONVOLATILE SEMICONDUCTOR MEMORY\ | US8952426B2 | MAEJIMA HIROSHI (JP) | TOSHIBA KK (JP)\ MAEJIMA HIROSHI (JP) | H01L27/115\ H01L29/76 |
| 4365 | 3D printing | METHOD AND APPARATUS ASSOCIATED WITH ANISOTROPIC SHRINK IN SINTERED CERAMIC ITEMS\ | US2014284848A9 | MAGUIRE MICHAEL (US)\ WITHEY PAUL (GB)\ BALDWIN MICHAEL D (US)\ BERGAN FRENCH NINA (US)\ SCHLIENGER M ERIC (US) | ROLLS ROYCE CORP (US) | B28B1/00 |
| 4366 | 3D printing | MICRO-SLICING CONTOUR SMOOTHING TECHNIQUE\ | US6678571B1 | MANNERS CHRIS R (US)\ KULKARNI RAJEEV B (US) | 3D SYSTEMS INC (US) | B29C67/00\ G06F17/50 |
| 4367 | 3D printing | VERFAHREN UND VORRICHTUNG ZUR DATENVER?NDERUNG UND SYSTEMSTEUERUNG BEI EINER MODELLIERVORRICHTUNG DURCH SELEKTIVE MATERIALABLAGERUNG\[EN] METHOD AND APPARATUS FOR DATA MANIPULATION AND SYSTEM CONTROL IN A SELECTIVE DEPOSITION MODELING SYSTEM\[FR] PROCéDé ET DISPOSITIF POUR LA MANIPULATION DE DONNéES ET UNITé DE COMMANDE DE SYSTèME POUR UN APPAREIL DE MODELAGE PAR DéPOSITION SéLECTIVE\ | EP1013407A2 | MANNERS CHRIS R (US)\ THAYER JEFFREY S (US)\ EARL JOCELYN M (US)\ KEREKES THOMAS A (US)\ MARYGOLD PAUL H (US) | 3D SYSTEMS INC (US) | B29C35/08\ B29C67/00\ G06T17/00\ B22F3/00\ B29C41/12\ B29C41/36\ B29K63/00 |
| 4368 | 3D printing | OBJECTS FABRICATED WITH INTEGRAL AND CONTOURED REAR PROJECTION SUBSTRATES\ | US2014340744A1 | MARTIN GILLES J (US) | MARTIN GILLES J (US)\ DISNEY ENTPR INC (US) | G03B21/62 |
| 4369 | 3D printing | FABRICATION OF A SOFT-SILICONE COVER FOR A HEARING INSTRUMENT SHELL\ | US7875223B2 | MASTERS MARTIN W (US) | SIEMENS HEARING INSTR INC (US) | B29C45/14 |
| 4370 | 3D printing | MULTIBEAM STEREOLITHOGRAPHY AND DEVICE FOR MICROSTRUCTURE USING DIFFERENT WAVELENGTH LASER BEAM\ | JP2005219400A | MASUHARA HIROSHI (JP)\ ITO SHOJI (JP) | D MEC KK US\ JAPAN SCIENCE & TECH AGENCY (JP) | B29C67/00\ B81C99/00 |
| 4371 | 3D printing | MANUFACTURING METHOD OF THREE-DIMENSIONAL STRUCTURE\ | JP2007290248A | MATSUMOTO NAOKI US\ NAKAGAWA TAKUJI (JP) | MURATA MANUFACTURING CO (JP) | B29C67/00\ B29C33/38\ B28B7/34 |
| 4372 | 3D printing | FEATURE PROTECTION FOR STEREO LITHOGRAPHIC MANUFACTURING PROCESSES\ | US2008175424A1 | MCBAGONLURI FRED (US)\ SALTYKOV OLEG (US)\ PARSI SALMAN (US) | SIEMENS HEARING INSTR INC (US) | H04R25/00 |
| 4373 | 3D printing | METHODS AND SYSTEMS FOR INTEGRATING FLUID DISPENSING TECHNOLOGY WITH STEREOLITHOGRAPHY\ | US7658603B2 | MEDINA FRANCISCO (US)\ WICKER RYAN (US)\ PALMER JEREMY A (US)\ DAVIS DON W (US)\ CHAVEZ BART D (US)\ GALLEGOS PHILLIP L (US) | UNIV TEXAS (US)\ SANDIA CORP OPERATOR OF SANDIA (US) | B28B1/16 |
| 4374 | 3D printing | CURABLE COMPOSITION\ | US2010104832A1 | MESSE LOIC (FR)\ CHAPELAT CAROLE (FR) | MESSE LOIC (FR)\ CHAPELAT CAROLE (FR) | C08F8/14\ B32B3/00\ C08J7/04 |
| 4375 | 3D printing | EVACUATED RECORD PLAYER PLATTER\ | GB2471369A | MILNES CRAIG (GB) | MILNES CRAIG (GB) | G11B3/61 |
| 4376 | 3D printing | APPARATUS AND METHOD FOR OPTICAL SHAPING\ | JP2001252986A | MIYOSHI TAKASHI US\ HAYASHI TERUTAKE US | JAPAN SCIENCE & TECH CORP US | B29C67/00\ G02F1/13\ G03F7/24 |
| 4377 | 3D printing | METHOD FOR PROCESSING STEREO LITHOGRAPHIC SHAPE DATA METHOD AND APPARATUS FOR STEREO LITHOGRAPHY\ | JP2004001531A | MORIYA MASUMI US\ OZAWA MASAHIKO US\ OKUBO MASAKATSU US\ ENDO TOSHIRO US\ GOTO NORIO (JP) | HITACHI LTD (JP) | B29C67/00 |
| 4378 | 3D printing | APPARATUS AND METHOD FOR PHOTOSETTING STEREO LITHOGRAPHY AND PHOTOSETTING STEREO LITHOGRAPHIC SYSTEM\ | JP2004009574A | MOROHOSHI KIMITAKA (JP) | JSR CORP (JP) | B29C67/00 |
| 4379 | 3D printing | METHOD AND APPARATUS FOR PHOTOSETTING STEREO LITHOGRAPHY\ | JP2004009573A | MOROHOSHI KIMITAKA (JP) | JSR CORP (JP) | B29C67/00 |
| 4380 | 3D printing | FACE IMAGE DISPLAY DEVICE AND FACE IMAGE COMMUNICATION SYSTEM\ | JP9233442A | MURATA YOSHIYUKI US | CASIO COMPUTER CO LTD US | H04N7/14\ G10L11/02\ G10L15/04\ G10L15/22\ G10L15/24\ H04M11/06 |
| 4381 | 3D printing | METHOD FOR MANUFACTURING THREE-DIMENSIONAL PHOTONIC STRUCTURE\ | US7682551B2 | NAKAGAWA TAKUJI (JP)\ WAKINO KIKUO (JP) | MURATA MANUFACTURING CO (JP) | B29C35/08\ B29C67/00\ G02B1/02\ H01L29/15\ H01L31/0256\ B29C41/02\ B29C41/20 |
| 4382 | 3D printing | FORTSCHRITTLICHE BAUTECHNIKEN IN STEREOLITHOGRAFIE\[EN] ENHANCED BUILDING TECHNIQUES IN STEREOLITHOGRAPHY\[FR] TECHNIQUES DE CONSTRUCTION AMELIOREES PAR STEREOLITHOGRAPHIE\ | EP1120227A2 | NGUYEN HOP D (US)\ GIGL JOHN J (US)\ PANG THOMAS H (US)\ SMALLEY DENNIS R (US)\ ALLEN KERRY J (US)\ VORGITCH THOMAS J (US)\ MANNERS CHRIS R (US)\ BEDAL BRYAN J L (US)\ JACOBS PAUL F (US)\ LEYDEN RICHARD N (US)\ HULL CHARLES W (US)\ VANDORIN STACIE L (US)\ EARL JOCELYN M (US) | 3D SYSTEMS INC (US) | B29C35/08\ B29C67/00\ G06F17/50\ G06T17/00 |
| 4383 | 3D printing | ON-VEHICLE STEREO SPEAKER DEVICE\ | JP2005022546A | NISHIKAWA AKIRA US\ HIDAKA NOBORU US | FUJITSU TEN LTD US | B60R11/02 |
| 4384 | 3D printing | SIGNAL PROCESSING APPARATUS AND METHOD\ | US2007098181A1 | NOGUCHI MASAYOSHI (JP)\ ICHIMURA GEN (JP) | SONY CORP (JP) | H04R5/00\ H04R1/40 |
| 4385 | 3D printing | THREE-DIMENSIONAL SHAPE MEASUREMENT APPARATUS AND THREE-DIMENSIONAL SHAPE MEASUREMENT METHOD\ | US2012218562A1 | OGINO HIROTAKA (JP)\ HONMA YUKI (JP)\ WADA HIROTAKA (JP) | OMRON TATEISI ELECTRONICS CO (JP)\ OGINO HIROTAKA (JP)\ HONMA YUKI (JP)\ WADA HIROTAKA (JP) | G01B11/25 |
| 4386 | 3D printing | 3-D NONVOLATILE MEMORY DEVICES AND METHODS OF MANUFACTURING THE SAME\ | US2013107602A1 | OH SANG HYUN (KR)\ LEE SANG BUM (KR)\ ARITOME SEIICHI (KR) | SK HYNIX INC (KR)\ OH SANG HYUN (KR)\ ARITOME SEIICHI (KR)\ LEE SANG BUM (KR) | G11C5/02\ H01L21/336\ H01L27/088 |
| 4387 | 3D printing | AMPLIFIER\ | JP2003087064A | OSHITA AKIHIRO US | SHARP KK (JP) | H03F3/68\ H03F1/30\ H03F3/181 |
| 4388 | 3D printing | STEREOLITHOGRAPHY APPARATUS\ | JP2010094938A | OZAWA MASAHIKO US\ NONAKA TAKAHIDE US\ HAMAOKA AKIO US\ MIYAZAKI AKIHIDE US\ KAWAKAMI KATSUYUKI US | HITACHI LTD (JP) | B29C67/00 |
| 4389 | 3D printing | LOCAL ENFORCEMENT OF ACCURACY IN FABRICATED MODELS\ | US7698014B2 | PALEY ERIC B (US)\ ROSENBLOOM MICAH J (US)\ DUNNE PATRICK COLM (US)\ GIRARD MICHAEL PATRICK (CA) | 3M INNOVATIVE PROPERTIES CO (US) | G06F19/00\ A61C8/00\ A61C3/02 |
| 4390 | 3D printing | LIQUID RADIATION-CURABLE COMPOSITION ESPECIALLY FOR PRODUCING CURED ARTICLES BY STEREOLITHOGRAPHY HAVING HEAT DEFLECTION TEMPERATURES\ | US6413696B1 | PANG THOMAS H (US)\ FONG JOHN W (US)\ MELISARIS ANASTASIOS P (US)\ RENYI WANG (US) | VANTICO INC (US) | B29C67/00\ G03F7/00\ C08G59/14\ C08G59/20\ C08G59/68\ G03F7/038 |
| 4391 | 3D printing | LIQUID RADIATION-CURABLE COMPOSITION ESPECIALLY FOR PRODUCING FLEXIBLE CURED ARTICLES BY STEREOLITHOGRAPHY\ | US6413697B1 | PANG THOMAS H (US)\ MELISARIS ANASTASIOS P (US)\ RENYI WANG (US) | VANTICO AT & T US INC (US) | B29C67/00\ G03F7/00\ C08G59/20\ G03F7/038\ G03F7/027 |
| 4392 | 3D printing | SELECTIVE DEPOSITION MODELING METHOD AND APPARATUS FOR FORMING THREE-DIMENSIONAL OBJECTS AND SUPPORTS\ | US2004075196A1 | PANG THOMAS H (US)\ SMALLEY DENNIS R (US)\ EARL JOCELYN M (GB)\ BEDAL BRYAN J L (US)\ LEYDEN RICHARD N (US)\ HULL CHARLES W (US)\ THAYER JEFFREY S (US)\ ALMQUIST THOMAS A (US)\ KEREKES THOMAS A (US)\ MEROT CHRISTIAN M (US)\ FEDCHENKO RICHARD P (US)\ LOCKARD MICHAEL S (US)\ THAT DINH TON (US) | 3D SYSTEMS INC (US) | B29C67/00\ B29C41/12\ B29C41/36 |
| 4393 | 3D printing | LAMP FOR VEHICLE\ | KR20110040070A | PARK HYO SANG (KR)\ AHN HONG GEUL (KR) | SL CORP (KR) | F21S8/10\ F21V8/00\ F21V5/04 |
| 4394 | 3D printing | PHOTOCURABLE COMPOSITIONS FOR PREPARING ABS-LIKE ARTICLES\ | US2010119835A1 | PATEL RANJANA (GB)\ MESSE LOIC (FR)\ CHAPELAT CAROLE (FR)\ MESSE LAURENCE (FR) | HUNTSMAN INT LLC (US) | G03F7/20\ G03F7/004\ C08L63/00\ B32B27/38 |
| 4395 | 3D printing | VERFAHREN ZUM INDIVIDUELLEN ANFERTIGEN UND/ODER RESTAURIEREN VON KNOCHENSTRUKTUREN SOWIE ZUM BEREITSTELLEN VON INDIVIDUELLEN KNOCHENIMPLANTATEN\[EN] METHOD FOR INDIVIDUAL MAKING AND RESTORING OF DAMAGED OR TO RESTORE OR TO REINFORCE BONE STRUCTURES AND FOR PREPARING INDIVIDUAL BONE IMPLANT FOR SURGEON INVOLVES COLLECTING DAMAGED ZONE OF PART TO BE OBTAINED\ | DE102007013282A1 | PEPPEL WERNER (DE) | PEPPEL WERNER (DE) | A61F2/28\ G06F19/00\ A61C8/00\ A61L27/10\ A61B17/56 |
| 4396 | 3D printing | POWER SAVING PROCESS APPLIED TO PRESSURIZED (AIR WATER) FACILITIES.\[ES] PROCEDIMIENTO ECONOMIZADOR DE ENERGIA APLICABLE A INSTALACIONES PRESURIZADOS. (AIRE AGUA).\ | MX2008004485A | PEREZ JUAN ALBERTO DE JESUS OROZCO (MX) | PEREZ JUAN ALBERTO DE JESUS OR (MX) | H01H33/94\ G05D16/00 |
| 4397 | 3D printing | DEVICE AND METHOD FOR THE MANUFACTURE OF ELEMENTS IN COMPOSITE MATERIAL\ | US2009320995A1 | PEREZ PASTOR AUGUSTO (ES)\ MENENDEZ MARTIN JOSE MANUEL (ES)\ MIGUES CHARINES YOLANDA (ES) | AIRBUS ESPANA SL (ES) | B29D99/00\ B28B3/12\ B28B5/00\ B29C53/54 |
| 4398 | 3D printing | LIGHTING UNIT HAVING LIGHT GUIDE(S) INSERTED BEWTEEN A REFLECTOR AND A SCREEN\ | US2012250345A1 | PERON RODOLPHE (FR)\ MOYNIER GILLES (FR) | PEUGEOT CITROEN AUTOMOBILES SA (FR)\ PERON RODOLPHE (FR)\ MOYNIER GILLES (FR) | G02B6/00 |
| 4399 | 3D printing | CONTINUOUS HIGH-ACCURACY LOCATING METHOD AND APPARATUS\ | US2011273324A1 | PETILLON JEAN-PAUL (FR) | EUROCOPTER FRANCE (FR) | G01S13/08\ G01C3/08\ G01S3/80 |
| 4400 | 3D printing | METHOD OF CALIBRATING AN APPARATUS FOR PRODUCING A THREE-DIMENSIONAL OBJECT CALIBRATION APPARATUS AND METHOD AND APPARATUS FOR PRODUCING A THREE-DIMENSIONAL OBJECT\ | US6483596B1 | PHILIPPI JOCHEN (DE)\ LOHNER ANDREAS (DE) | EOS ELECTRO OPTICAL SYST (US) | B29C67/00\ B23K26/00\ B23K26/10\ G05B19/401 |
| 4401 | 3D printing | DEVICE FOR ADJUSTING THE TEMPERATURE OF A PHYSIOLOGICAL FLUID\ | US7841189B2 | POCH JAIME ARBOS (ES) | CAIR ESPANA S L LAB (ES) | F25B21/02 |
| 4402 | 3D printing | CHANNEL FOR A SEMICONDUCTOR DIE AND METHODS OF FORMATION\ | US2010052157A1 | PRATT DAVID S (US) | MICRON TECHNOLOGY INC (US) | B29C35/00\ H01L23/34\ H01L21/00\ H01L23/52 |
| 4403 | 3D printing | HONEYCOMB CORES FOR AEROSPACE APPLICATIONS\ | US2004048027A1 | RICE CHRISTIAN V (US)\ POLUS JEFFREY E (US)\ HAYES MICHAEL W (US)\ DEGRANGE JEFFREY E (US) | BOEING CO US | B29C67/00 |
| 4404 | 3D printing | PHOTODETEKTOR-ANORDNUNG UND VERFAHREN ZUR ST?RLICHTKOMPENSATION\[EN] PHOTODETECTOR STRUCTURE FOR E.G. 3D TELEMETRIC IMAGE CAMERAS FOR MOTOR VEHICLES HAS A PHOTODETECTOR UNIT TO RECORD AND DETERMINE A PHOTOELECTRIC CURRENT AND AN INTEGRATED UNIT TO COMPENSATE FOR UNWANTED SIGNAL PORTIONS\ | DE10301598A1 | RIEDEL HELMUT (DE)\ LANG CHRISTIAN (DE)\ DAHL ANDREAS VON (DE)\ ZYWITZA FRIEDRICH (DE) | CONTI TEMIC MICROELECTRONIC (DE) | G01J1/42\ G01J1/10\ G01J1/46\ H04N5/363\ H04N5/3745 |
| 4405 | 3D printing | SILICONE RECEPTACLE WITH THREE-DIMENSIONAL REINFORCEMENT AND METHOD FOR THE PRODUCTION THEREOF\[ES] RECIPIENTE DE SILICONA CON REFUERZO TRIDIMENSIONAL Y PROCEDIMIENTO DE FABRICACIóN DEL MISMO\[FR] RéCIPIENT EN SILICONE à RENFORCEMENT TRIDIMENSIONNEL ET SON PROCéDé DE FABRICATION\ | WO2013140000A1 | RODRIGUEZ ESTEBAN MANUEL (ES)\ OLMOS AQUILUE ALEJANDRO (ES)\ PURROY LASHERAS RAUL (ES) | ARAVEN SL (ES)\ RODRIGUEZ ESTEBAN MANUEL (ES)\ OLMOS AQUILUE ALEJANDRO (ES)\ PURROY LASHERAS RAUL (ES) | B65D25/00\ B65D57/00 |
| 4406 | 3D printing | VERFAHREN ZUR HERSTELLUNG VON STRAHLUNGSH?RTBAREN (METH)ACRYLATEN\[EN] RADIATION CURABLE MIXTURE OBTAINED BY REACTING (METH)ACRYLIC ACID WITH PROPOXYLATED GLYCEROL REMOVING EXCESS (METH)ACRYLIC ACID FROM MIXTURE BY AQUEOUS EXTRACTION AND OPTIONALLY ADDING PRIMARY OR SECONDARY AMINE USEFUL E.G. IN COATINGS\ | DE102010044206A1 | ROESCH CHRISTINE (DE)\ GRUBER NICK (DE)\ MENZEL KLAUS (DE)\ NEUMANN SUSANNE (DE) | BASF SE (DE) | C08G63/78\ C08G63/91 |
| 4407 | 3D printing | VERFAHREN ZUR HERSTELLUNG EINES OHREINSATZES OHREINSATZANORDNUNG UND VERWENDUNG EINER OHREINSATZANORDNUNG\[EN] EAR INSERT E.G. HEARING AID OR MINIATURE LOUDSPEAKER ADAPTED TO INDIVIDUAL OUTER EAR CONTOURS IS OBTAINED USING DIGITALIZED GEOMETRY OF HARDENED MOLDING OF OUTER EAR REGION IN IMPRESSION MATERIAL\ | DE102006007032A1 | ROESCH SEBASTIAN (DE)\ KAMRAN RAVI (DE) | ACCUSTA GMBH (DE) | B29C67/00\ A61F11/08\ H04R1/10\ H04R25/02 |
| 4408 | 3D printing | SELF-CROSSLINKING INTERPENETRATING NETWORK THERMOPLASTIC ELASTOMER: SL-TPEX\ | CN101643535A | RUIHUA GONG US | SOKN TECHNOLOGY SHANGHAI CO LT US | C08F291/00 |
| 4409 | 3D printing | ACOUSTIC REPRODUCTION SYSTEM\ | JP2007274069A | SAITO IKUO US | VICTOR COMPANY OF JAPAN US | H04S5/02 |
| 4410 | 3D printing | ELASTOMER-DICHTUNGEN UND VERFAHREN ZU DEREN HERSTELLUNG\[EN] PREPARATION OF ELASTOMER SEAL USEFUL E.G. IN ENGINE COMPRISES MOLDING STEREOLITHOGRAPHY PART/MODEL ELEMENT OPENING THE MOLD POURING ELASTOMER SEALING POLYMERIZING CAST MATERIAL AND REMOVING SILICON RUBBER AND/OR POLYURETHANE\ | DE102005002970A1 | SALAMEH RALF (DE) | FEDERAL MOGUL SEALING SYS SPA (DE) | B29C67/00\ F16J15/10\ B29C35/02\ B29C67/24\ C08J5/02\ C09K3/12 |
| 4411 | 3D printing | REMOTE PROGRAM CONTROL DEVICE FOR COPIERS AND PRINTERS\[ES] DISPOSITIVO DE CONTROL EN REMOTO POR PROGRAMA DE COPIADORAS E IMPRESORAS\[FR] DISPOSITIF DE CONTR?LE à DISTANCE PAR PROGRAMME DE COPIEURS ET D'IMPRIMANTES\ | WO2013160488A1 | SANCHEZ ANTONIO (ES) | NUBE PRINT SL (ES)\ SANCHEZ ANTONIO (ES) | G06F3/12\ G03G15/00\ G06F11/00 |
| 4412 | 3D printing | CLEAR AND COLORLESS THREE-DIMENSIONAL ARTICLES MADE VIA STEREOLITHOGRAPHY AND METHOD OF MAKING SAID ARTICLES\ | US2009004579A1 | SARMAH SATYENDRA KUMAR (US)\ ZARAS KEVIN ANDREW (US) | DSM IP ASSETS BV (NL) | G03C5/00\ G03C1/04 |
| 4413 | 3D printing | METHOD FOR PRODUCTION OF A THREE-DIMENSIONAL STRUCTURED SURFACE WITH HAPTICALLY AND/OR OPTICALLY DETECTABLE MICROSTRUCTURES\ | US7727431B2 | SAUERESSIG KILIAN (DE) | K1 GMBH (DE) | B29C51/00\ B21D22/00\ B29C51/10 |
| 4414 | 3D printing | OBJECT DATA INPUT APPARATUS AND OBJECT RECONSTRUCTION APPARATUS\ | US2005274913A1 | SAWADA YASUHIRO (JP) | CANON KK (JP) | G01B11/25\ G01B11/24\ G01J3/36\ G01N21/31\ G01N21/86\ G01V8/00 |
| 4415 | 3D printing | SYSTEM METHOD AND APPARATUS FOR CLEANING A CERAMIC COMPONENT\ | US8864914B2 | SCHLIENGER MAX ERIC (US) | ROLLS ROYCE CORP (US)\ SCHLIENGER MAX ERIC (US) | B28B1/00\ B08B3/08\ B08B3/04\ B08B5/04 |
| 4416 | 3D printing | SYSTEM APPARATUS AND METHOD FOR RESIN LEVEL MAINTENANCE IN A STEREO-LITHOGRAPHY DEVICE\ | US2011049767A1 | SCHLIENGER MAX ERIC (US)\ WITHEY PAUL ANTHONY (GB) | SCHLIENGER MAX ERIC (US)\ WITHEY PAUL ANTHONY (GB) | B29C35/08\ B29C59/10 |
| 4417 | 3D printing | PULVERDOSIERUNG - AUTOMATISCHE PULVERZUSTELLUNG\ | DE10360094B4 | SCHOEDEL FRANK (DE) | CL SCHUTZRECHTSVERWALTUNGS GMB (DE) | B29C67/00\ B22F3/105\ B23K26/34\ B22C7/00 |
| 4418 | 3D printing | STEREOSCOPIC VIDEO SIGNAL PROCESSOR WITH ENHANCED 3D EFFECT\ | US2013033570A1 | SHAND MARK (FR)\ ETORRE GUILLAUME (FR) | ZORAN FRANCE (FR)\ SHAND MARK (FR)\ ETORRE GUILLAUME (FR) | H04N13/00 |
| 4419 | 3D printing | FABRICATION AIDING APPARATUS\ | US2007203600A1 | SHIBATA TSUTOMU (JP)\ MATSUDA YOSHINORI (JP) | SHIBATA TSUTOMU (JP)\ MATSUDA YOSHINORI (JP) | G06F17/50\ A61C13/00\ A61C5/10\ G06F19/00 |
| 4420 | 3D printing | THREE-DIMENSIONAL CAD SYSTEM AND RECORDING MEDIUM FOR THREE-DIMENSIONAL CAD SYSTEM\ | US6720963B2 | SHIIBA EIJI (JP)\ YOSHIDA YASUHIKO (JP) | CITEC CORP (JP)\ YOSHIDA YASUHIKO (JP) | G06F17/50\ G06T17/30 |
| 4421 | 3D printing | METHOD OF MAKING AN OPTICAL MANIFOLD\ | US6655848B2 | SIMMONS RICHARD L (US)\ SHOEMAKER CURTIS L (US)\ KORNELE KENNETH L (US) | 3M INNOVATIVE PROPERTIES CO (US) | G02B6/36\ G02B6/38\ G02B6/44 |
| 4422 | 3D printing | METHOD OF MAKING A THREE DIMENSIONAL OPTICAL CIRCUIT\ | US6549710B2 | SIMMONS RICHARD L (US)\ SHOEMAKER CURTIS L (US)\ KORNELE KENNETH L (US)\ LODER HARRY A (US)\ LURIE EDWARD B (US) | 3M INNOVATIVE PROPERTIES CO (US) | G02B6/36\ G02B6/38\ G02B6/44\ G02B6/04\ G02B6/40\ G02B6/43 |
| 4423 | 3D printing | METHOD OF USING MINIMAL SURFACES AND MINIMAL SKELETONS TO MAKE HEAT EXCHANGER COMPONENTS\ | US7866377B2 | SLAUGHTER VICTOR BLAKEMORE (US) | BOEING CO (US) | F28D19/00 |
| 4424 | 3D printing | METHOD OF MAKING A HEAT EXCHANGER CORE COMPONENT\ | US7866372B2 | SLAUGHTER VICTOR BLAKEMORE (US) | BOEING CO (US) | H05K7/20 |
| 4425 | 3D printing | APPARATUS AND METHOD FOR CONTROLLING EXPOSURE OF A SOLIDIFIABLE MEDIUM USING A PULSED RADIATION SOURCE IN BUILDING A THREE-DIMENSIONAL OBJECT USING STEREOLITHOGRAPHY\ | US6215095B1 | SMALLEY DENNIS R (US)\ PARTANEN JOUNI P (US) | 3D SYSTEMS INC (US) | B29C35/08\ B29C67/00 |
| 4426 | 3D printing | BOOLEAN LAYER COMPARISON SLICE\ | US6333741B1 | SMALLEY DENNIS R (US)\ VORGITCH THOMAS J (US)\ SNEAD DAVID E (US)\ COHEN ADAM L (US)\ ALLISON JOSEPH W (US)\ CHEN THOMAS P (US) | 3D SYSTEMS INC (US) | B29C35/08\ B29C67/00\ B29K105/24\ G01J1/42\ G03C9/08\ G03F7/00\ G03F7/20\ G06F17/50\ G06T17/00\ G06T17/10\ G06T17/20 |
| 4427 | 3D printing | NANOPARTICLE-FILLED STEREOLITHOGRAPHIC RESINS\ | US2005040562A1 | STEINMANN BETTINA (CH)\ STEINMANN ALFRED (CH) | 3D SYSTEMS INC (US) | G03F7/00\ G03F7/038\ G03F7/004\ C08G59/18\ B81C99/00 |
| 4428 | 3D printing | METHOD OF COATING AN SLA PART\ | US2005074543A1 | STEVENS RANDAL A (US)\ INSIXIENGMAY SID C (US) | IN TECH IND INC (US) | B05D3/02\ B05D3/06\ B05D7/02 |
| 4429 | 3D printing | METHOD OF DYEING AN SLA PART\ | US2006174425A1 | STEVENS RANDAL A (US)\ INSIXIENGMAY SID C (US) | STEVENS RANDAL A (US)\ INSIXIENGMAY SID C (US) | D06P1/00 |
| 4430 | 3D printing | METHODS OF MAKING A NEGATIVE HEARING AID MOLD\ | US2005035498A1 | STEVENS RANDAL ALAN (US) | Empty Field US | B29C67/00\ H04R25/00\ B29C33/38 |
| 4431 | 3D printing | RAPID SLICING METHOD OF STL (STEREO LITHOGRAPHY) MODEL BASED ON SORTING BY TRIANGLE ADJACENCY IN LAYERS IN RAPID PROTOTYPING MANUFACTURING\ | CN102446368A | SU WANG US\ HENG LIU US | SU WANG US\ HENG LIU US | G06T19/00 |
| 4432 | 3D printing | METHOD AND DEVICE FOR GENERATING DATA FOR OPTICAL STEREO LITHOGRAPHY FROM RESULT OF STRUCTURAL ANALYSIS\ | JP2004160858A | SUZUKI ATSUSHI US\ TAKAYAMA HARUYOSHI US\ NUMATA MASANORI US | HITACHI ENG CO LTD US | B29C67/00\ G06F17/50 |
| 4433 | 3D printing | PHOTOCURABLE COMPOSITION FOR STEREO LITHOGRAGHY CARBONIZED SHAPED ARTICLE AND METHOD FOR PRODUCING THE SAME\ | JP2009138017A | TAKASE KATSUYUKI (JP)\ KUROSAWA TAKAHIKO (JP) | JSR CORP (JP) | B29C67/00\ C08G59/68 |
| 4434 | 3D printing | PHOTOCURABLE RESIN COMPOSITION FOR OPTICAL STEREOLITHOGRAPHY AND THREE-DIMENSIONAL ARTICLE\ | JP2008266551A | TAKASE KATSUYUKI (JP)\ UEDA MASAHIRO (JP)\ KUROSAWA TAKAHIKO (JP) | JSR CORP (JP)\ JAPAN FINE COATINGS CO LTD (JP) | B29C67/00\ C08G59/68\ C08G59/18 |
| 4435 | 3D printing | SURROUND GENERATION APPARATUS\ | US2009279706A1 | TAKASHIMA NORIYUKI (JP) | ALPINE ELECTRONICS (JP) | H04R5/00\ G10L21/00 |
| 4436 | 3D printing | AUDIO DEVICE\ | JP2008219246A | TAKASHIMA NORIYUKI (JP) | ALPINE ELECTRONICS INC (JP) | H04S5/02 |
| 4437 | 3D printing | SURROUND PRODUCING APPARATUS\ | JP2007336118A | TAKASHIMA NORIYUKI (JP) | ALPINE ELECTRONICS INC (JP) | H04S5/02 |
| 4438 | 3D printing | AUDIO APPARATUS\ | US2008226085A1 | TAKASHIMA NORIYUKI (JP) | TAKASHIMA NORIYUKI (JP) | H04R5/00 |
| 4439 | 3D printing | AUDIO DEVICE AND METHOD FOR GENERATING SURROUND SOUND HAVING FIRST AND SECOND SURROUND SIGNAL GENERATION UNITS\ | US7920711B2 | TAKASHIMA NORIYUKI (JP)\ AKIHO MASAICHI (JP)\ HAMADA HAREO (JP) | ALPINE ELECTRONICS INC (JP)\ DIMAGIC CO LTD (JP) | H04R5/02 |
| 4440 | 3D printing | FLOCKMATERIAL UND ANWENDUNGSVERFAHREN\ | DE102005016875A1 | TALAVERA BARCELO JOSE (ES) | TALPA IND SL (ES) | B32B5/02\ B05D1/16\ B29C45/14\ D04H11/00 |
| 4441 | 3D printing | ADAPTIVE BIT ALLOCATION FOR MULTI-CHANNEL AUDIO ENCODING\ | US2008262850A1 | TALEB ANISSE (SE)\ ANDERSSON STEFAN (SE) | TALEB ANISSE (SE)\ ANDERSSON STEFAN (SE) | G10L19/00 |
| 4442 | 3D printing | PHOTOCURABLE LIQUID COMPOSITION THREE-DIMENSIONAL ARTICLE AND METHOD FOR PRODUCING THEM\ | JP2007291393A | TATARA AKITSUGU US\ YASHIRO TAKAO US\ TANABE TAKAYOSHI US\ UKAJI TAKASHI US | JSR CORP (JP)\ JAPAN FINE COATINGS CO LTD (JP) | B29C67/00\ C08F2/44\ B28B1/30\ C08F20/20 |
| 4443 | 3D printing | METHOD AND APPARATUS FOR REDUCING FRAME REPETITION IN STEREOSCOPIC 3D IMAGING\ | US2013038693A1 | TCHOUKALEYSKY EMIL (CA) | THOMSON LICENSING (FR)\ TCHOUKALEYSKY EMIL (CA) | H04N13/02\ G06T15/00 |
| 4444 | 3D printing | STEREOLITHOGRAPHY METHOD\ | US2009133800A1 | TERAMOTO TOSHIO (JP)\ MOROHOSHI KIMITAKA (JP) | JSR CORP (JP) | G03F7/00 |
| 4445 | 3D printing | STEREOLITHOGRAPHY APPARATUS AND STEREOLITHOGRAPHY METHOD\ | US7736577B2 | TERAMOTO TOSHIO (JP)\ MOROHOSHI KIMITAKA (JP) | JSR CORP (JP) | B29C35/04 |
| 4446 | 3D printing | VERFAHREN ZUR HERSTELLUNG VON GROSSEN BAUTEILEN AUS TEILSEGMENTEN UND ZUM ZUSAMMENFüGEN DIESEN TEILSEGMENTEN\[EN] METHOD FOR PRODUCING LARGE PARTS FROM INTERMEDIATE PARTS AND FOR ASSEMBLING SAID INTERMEDIATE PARTS\[FR] PROCéDé DE FABRICATION DE GRANDES PIèCES à PARTIR DE PIèCES INTERMéDIAIRES ET D'ASSEMBLAGE DE CES PIèCES INTERMéDIAIRES\ | EP1614526B1 | THIELE FRANK (DE)\ LEHNER KLAUS-DIETER (DE) | PORSCHE AG (DE) | B29C67/00 |
| 4447 | 3D printing | VORRICHTUNG ZUM REINIGEN VON KAN?LEN\[EN] APPARATUS FOR CLEANING CHANNELS IN MODELS ESPECIALLY STEREO-LITHOGRAPHY MODELS E.G. FOR WIND TUNNEL TESTS COMPRISING SOURCES OF COMPRESSED AIR AND CLEANING AGENT CONNECTABLE TO CHANNEL INLETS\ | DE102004053719B3 | THIELE FRANK (DE)\ LEHNER KLAUS-DIETER (DE) | PORSCHE AG (DE) | B29C67/00\ B08B5/02 |
| 4448 | 3D printing | SYSTEM AND METHOD FOR FORMING A CUSTOM MEDICAL MASK FROM A THREE-DIMENSIONAL ELECTRONIC MODEL\ | US2015045926A1 | THORNTON W KEITH (US) | THORNTON W KEITH (US)\ AIRWAY TECHNOLOGIES LLC (US) | B29C67/00\ G06F17/50 |
| 4449 | 3D printing | PHOTOSENSITIZED COMPOSITE MATERIAL THREE-DIMENSIONAL MEMORY MATERIAL AND RECORDING MEDIUM OPTICAL POWER LIMITING MATERIAL AND ELEMENT AND PHOTOCURING MATERIAL AND STEREOLITHOGRAPHY SYSTEM AND FLUORESCENT MATERIAL FOR MULTIPHOTON FLUORESCENCE MICROSCOPE AND MULTIPHOTON FLUORESCENCE MICROSCOPE\ | US2009323508A1 | TOMURA TATSUYA (JP)\ SATO TSUTOMU (JP)\ MIKI TAKESHI (JP)\ TAKADA MIKIKO (JP)\ SASAKI MASAOMI (JP) | TOMURA TATSUYA (JP)\ SATO TSUTOMU (JP)\ MIKI TAKESHI (JP)\ TAKADA MIKIKO (JP)\ SASAKI MASAOMI (JP) | G11B7/26\ C07D207/32\ C07D213/89\ C07F1/12 |
| 4450 | 3D printing | RECEIVER RECEPTION CONTROL METHOD RECEPTION CONTROL PROGRAM AND RECORDING MEDIUM\ | JP2009164932A | TSUBOI YOSHIMI US | PIONEER ELECTRONIC CORP US | H04B1/10\ H04H40/72 |
| 4451 | 3D printing | VIDEO AUDIO REPRODUCING DEVICE\ | JP9322300A | TSUBOOKA TAKEO US\ IIDA TOSHIYUKI US | VICTOR COMPANY OF JAPAN US | H04S1/00\ H04S5/02\ H04R3/12\ H04N5/93 |
| 4452 | 3D printing | OPTICAL STEREO LITHOGRAPHIC METHOD OPTICAL STEREO LITHOGRAPHIC APPARATUS AND COMPUTER PROGRAM FOR OPTICAL STEREO LITHOGRAPHY APPLIED TO OPTICAL STEREO LITHOGRAPHIC APPARATUS\ | JP2009298023A | UCHIYAMA SHIGEO US\ KINOSHITA MOTOKUNI US | ROLAND DG CORP US | B29C67/00 |
| 4453 | 3D printing | AUDIO SIGNAL PROCESSING DEVICE\ | US2007073427A1 | UDAGAWA KAZUHIKO (JP) | FUNAI ELECTRIC CO (JP) | G06F17/00 |
| 4454 | 3D printing | STEREOLITHOGRAPHY RESIN COMPOSITIONS AND THREE-DIMENSIONAL OBJECTS MADE THEREFROM\[FR] COMPOSITIONS DE RéSINE STéRéOLITHOGRAPHIQUE ET OBJETS TRIDIMENTIONNELS PRODUITS à PARTIR DE CES DERNIèRES\ | WO2008115057A1 | UEDA MASAHIRO (JP)\ TAKASE KATSUYUKI (JP)\ KUROSAWA TAKAHIKO (JP) | JSR CORP (JP)\ DSM IP ASSETS BV (NL)\ JAPAN FINE COATINGS (JP)\ UEDA MASAHIRO (JP)\ TAKASE KATSUYUKI (JP)\ KUROSAWA TAKAHIKO (JP) | G03F7/00\ G03F7/038 |
| 4455 | 3D printing | STEREOLITHOGRAPHY RESIN COMPOSITIONS AND THREE-DIMENSIONAL OBJECTS MADE THEREFROM\ | US2013316154A1 | UEDA MASAHIRO (JP)\ TAKASE KATSUYUKI (JP)\ KUROSAWA TAKAHIKO (JP) | JSR CORP (JP)\ DSM IP ASSETS BV (NL)\ JAPAN FINE COATINGS CO LTD (JP) | G03F7/00 |
| 4456 | 3D printing | STEREOLITHOGRAPHIC APPARATUS AND METHOD FOR MANUFACTURING THREE-DIMENSIONAL OBJECT WITH PHOTOHARDENABLE RESIN\ | US7090484B2 | UENO TAKAKUNI (JP) | TEIJIN SEIKI CO LTD (JP) | B29C67/00\ G03C9/08\ B28B17/00 |
| 4457 | 3D printing | STEREOLITHOGRAPHIC APPARATUS AND METHOD FOR MANUFACTURING THREE-DIMENSIONAL OBJECT\ | US7318718B2 | UENO TAKAKUNI (JP) | TEIJIN SEIKI CO LTD (JP) | B29C35/08\ B29C67/00\ G03F7/20 |
| 4458 | 3D printing | METHOD AND APPARATUS FOR STEREO LITHOGRAPHY\ | JP9267398A | UENO TAKAKUNI (JP) | TEIJIN SEIKI CO LTD (JP) | B29C35/08\ B29C67/00\ B29K105/24 |
| 4459 | 3D printing | PARTICLE MEASURING DEVICE AND METHOD\ | JP2007298327A | UESUGI TOMOHIRO US\ KAWAHASHI MASAAKI US\ HIRAHARA HIROYUKI US\ ZAMA YOSHIO US | UNIV SAITAMA US | G01B11/00\ G01B11/08\ G01P3/36 |
| 4460 | 3D printing | VASCULARIZED TISSUE REGENERATION MATRICES FORMED BY SOLID FREE FORM FABRICATION TECHNIQUES\ | US6176874B1 | VACANTI JOSEPH P (US)\ CIMA MICHAEL J (US)\ CIMA LINDA G (US) | CHILDRENS MEDICAL CENTER (US)\ MASSCHUSETTS INST OF TECHNOLOG (US) | B29C67/00\ A61L27/00\ A61F2/00\ A61F2/02\ A61F2/06\ A61F2/10\ A61F2/28\ A61F2/30\ A61K9/00\ A61K9/16\ A61L27/38\ A61L27/40\ A61L27/56\ A61L27/60\ B82B1/00 |
| 4461 | 3D printing | POROSITY REFERENCE STANDARD UTILIZING A MESH\ | US7752882B2 | VACCARO CHRISTOPHER M (US)\ BEISIEGEL APRIL L (US)\ DEFONCE RON (US) | BOEING CO (US) | G01N29/30 |
| 4462 | 3D printing | PSEUDO POROSITY REFERENCE STANDARD FOR CORED COMPOSITE LAMINATES\ | US2008134749A1 | VACCARO CHRISTOPHER M (US)\ BEISIEGEL APRIL L (US)\ ENGELBART ROGER W (US) | BOEING CO (US) | G01N29/30\ G01N29/04 |
| 4463 | 3D printing | PSEUDO POROSITY REFERENCE STANDARD FOR METALLIC INTERLEAVED COMPOSITE LAMINATES\ | US7770457B2 | VACCARO CHRISTOPHER M (US)\ BEISIEGEL APRIL L (US)\ ENGELBART ROGER W (US) | BOEING CO (US) | G01N29/04 |
| 4464 | 3D printing | POROSITY REFERENCE STANDARD UTILIZING ONE OR MORE HOLLOW NON-CYLINDRICAL SHAFTS\ | US2008196475A1 | VACCARO CHRISTOPHER M (US)\ BEISIEGEL APRIL L (US)\ ENGELBART ROGER W (US) | BOEING CO (US) | G01N29/30\ G01N29/04 |
| 4465 | 3D printing | ULTRASONIC INSPECTION REFERENCE STANDARD FOR POROUS COMPOSITE MATERIALS\ | US7424818B2 | VACCARO CHRISTOPHER M (US)\ LILIENTHAL DAVID (US) | BOEING CO (US) | G01M1/14 |
| 4466 | 3D printing | ULTRASONIC INSPECTION REFERENCE STANDARD FOR COMPOSITE MATERIALS\ | US7216544B2 | VACCARO CHRISTOPHER M (US)\ LILIENTHAL DAVID A (US) | BOEING CO (US) | G01N29/30\ G01N33/44 |
| 4467 | 3D printing | POROSITY REFERENCE STANDARD UTILIZING ONE OR MORE DISCRETE WIRES\ | US2008121039A1 | VACCARO CHRISTOPHER M (US)\ LILIENTHAL DAVID A (US)\ BEISIEGEL APRIL L (US) | BOEING CO (US) | G01N29/11 |
| 4468 | 3D printing | TAPERED ULTRASONIC REFERENCE STANDARD\ | US7762120B2 | VACCARO CHRISTOPHER M (US)\ LILIENTHAL DAVID A (US)\ BEISIEGEL APRIL L (US) | BOEING CO (US) | G01N29/30 |
| 4469 | 3D printing | PROCEDES DE FABRICATION D'UN MOULE FEMELLE POUR PROTHESE AUDITIVE\[DE] VERFAHREN ZUR HERSTELLUNG EINER NEGATIVFORM FüR H?RGER?TE\[EN] METHOD OF MAKING A NEGATIVE HEARING AID MOLD\ | EP1668957A1 | VASSSERMAN REMIR M (US)\ STEVENS RANDAL ALAN (US) | INTERTON USA (US)\ IN TECH IND INC (US) | H04R25/00 |
| 4470 | 3D printing | STEREOLITHOGRAPHIE-SYSTEM\[EN] STEREOLITHOGRAPHY SYSTEM\[FR] SYSTèME DE STéRéOLITHOGRAPHIE\ | WO2013182913A3 | VEHSE MARK (DE)\ SEITZ HERMANN (DE)\ HENNIG JOERG (DE) | UNIV ROSTOCK (DE) | B29C67/00\ B22F3/105 |
| 4471 | 3D printing | DUAL-CONTRAST MR IMAGING USING FLUID-ATTENUATION INVERSION RECOVERY (FLAIR)\ | US2012046539A1 | VISSER FREDERIK (NL) | KONINKL PHILIPS ELECTRONICS NV (NL)\ VISSER FREDERIK (NL) | A61B5/055 |
| 4472 | 3D printing | KOMPOSITHARZ-ZUSAMMENSETZUNG UND VERFAHREN ZUR HERSTELLUNG DENTALER BAUTEILE MITTELS STEREOLITHOGRAPHIE\[EN] COMPOSITE RESIN COMPOSITION AND METHOD FOR PRODUCING DENTAL COMPONENTS BY MEANS OF STEREO-LITHOGRAPHY\[FR] COMPOSITION DE RéSINE COMPOSITE ET PROCéDé DE FABRICATION DE COMPOSANTS DENTAIRES PAR STéRéOLITHOGRAPHIE\ | EP2751618A2 | WACHTER WOLFGANG (LI)\ EBERT J?RG (CH)\ VOSER DIETER (LI)\ MOSZNER NORBERT (LI)\ RHEINBERGER VOLKER (LI)\ STAMPFL JüRGEN (AT) | IVOCLAR VIVADENT AG (LI)\ TECH UNIVERSIT?T WIEN (AT) | G03F7/00\ G03F7/027\ A61C13/00\ A61K6/083\ G03F7/029 |
| 4473 | 3D printing | APPARATUS A SYSTEM AND A METHOD FOR ENABLING AN IMPEDANCE MEASUREMENT\ | US2008218180A1 | WAFFENSCHMIDT EBERHARD (DE)\ IGNEY CLAUDIA (DE)\ BRAUERS ANDREAS (DE) | KONINKL PHILIPS ELECTRONICS NV (NL) | A61B5/053\ G01R27/04 |
| 4474 | 3D printing | RAPID PROTOTYPING AND MANUFACTURING SYSTEM AND METHOD\ | US7690909B2 | WAHLSTROM BEN (US) | 3D SYSTEMS INC (US) | B28B1/16\ B05C11/02\ B29B13/08 |
| 4475 | 3D printing | RAPID PROTOTYPING AND MANUFACTURING SYSTEM AND METHOD\ | US2007075458A1 | WAHLSTROM BEN (US)\ HUNTER DON F (US) | 3D SYSTEMS INC (US) | B29C35/04 |
| 4476 | 3D printing | RAPID PROTOTYPING AND MANUFACTURING SYSTEM AND METHOD\ | US7520740B2 | WAHLSTROM BEN (US)\ HUNTER DON FREDERICK (US)\ STONESMITH MATTHEW (US) | 3D SYSTEMS INC (US) | B28B17/00 |
| 4477 | 3D printing | RAPID PROTOTYPING AND MANUFACTURING SYSTEM AND METHOD\ | US2007075461A1 | WAHLSTROM BEN (US)\ REYNOLDS GARY L (US)\ HUNTER DON F (US) | 3D SYSTEMS INC (US) | B29C35/08\ B29C35/04\ B29C35/02 |
| 4478 | 3D printing | RAPID PROTOTYPING AND MANUFACTURING SYSTEM AND METHOD\ | US2007077323A1 | WAHLSTROM BEN (US)\ REYNOLDS GARY L (US)\ STONESMITH MATTHEW (US)\ HUNTER DON F (US)\ STOUT LANSING V (US) | 3D SYSTEMS INC (US) | B29C35/08 |
| 4479 | 3D printing | LIGHT-CURING THREE-DIMENSIONAL PRINTER BASED ON DIGITAL LIGHT PROCESSING (DLP) PROJECTION\ | CN103302860A | WANG JIANXI US\ GU TIANBO US | WANG JIANXI US | B29C67/00 |
| 4480 | 3D printing | MILLING CUTTER ABRASION ON-MACHINE THREE-DIMENSIONAL MEASUREMENT DEVICE USING DIGITAL LIGHT PROCESSING (DLP)\ | CN202607422U | WANG ZHONGREN US\ LIU ZHOU US\ LI XIANGYI US\ ZHANG MING (CN) | UNIV XIANGFAN US | B23Q17/09\ B23Q17/24 |
| 4481 | 3D printing | SLA (STEREO LITHOGRAPHY) TECHNOLOGY OF LIGHT CURING STEREO HOMOCENTRIC SPHERE\ | CN102029715A | WENXUE ZHANG US\ JIANSHENG LU US\ XINQIAO WU US\ FENG XUE US\ WEI LIU US\ MINGMIN ZHANG US\ XIANG LIU US\ FENG JUN US\ JIANXIN YUAN US\ WEI WANG US\ LI CHEN (CN) | JIANGSU WENGUANG VEHICLE ACCESSORIES CO LTD US | B29C67/00 |
| 4482 | 3D printing | DISPLAY APPARATUS HAVING AUTOSTEREOSCOPIC 3D OR 2D/3D SWITCHABLE PIXEL ARRANGEMENT\ | US2013194521A1 | WHANGBO SANG-WOO (KR)\ KIM MYUNG-HWAN (KR) | SAMSUNG DISPLAY CO LTD (KR)\ WHANGBO SANG-WOO (KR)\ KIM MYUNG-HWAN (KR) | G02B27/22 |
| 4483 | 3D printing | OBJECT CONSOLIDATION THROUGH SEQUENTIAL MATERIAL DEPOSITION\ | US6814823B1 | WHITE DAWN (US) | SOLIDICA INC (US) | B29C67/00\ G05B19/4099\ B23K11/00\ B23K20/10\ B23K20/12\ B29C73/00 |
| 4484 | 3D printing | RADIATION CURABLE RESIN COMPOSITION AND RAPID PROTOTYPING PROCESS USING THE SAME\ | US7183040B2 | WINMILL DAVID L (US)\ XU JIGENG (US)\ YOU XIAORONG (US)\ THIES JENS C (NL)\ DIAS AYLVIN J A A (NL)\ LAWTON JOHN A (US) | DSM IP ASSETS BV (NL) | G03F7/00\ G03F7/038\ G03F7/027\ C08G65/18\ B29C35/04\ C08F283/02\ C08G59/17\ G03C1/73 |
| 4485 | 3D printing | STEREOPHONIC SOUND SYSTEM\ | US6122381A | WINTERER MARTIN (DE) | MICRONAS INTERUETALL GMBH (DE) | H04S5/02\ H04R3/00\ H04S3/00\ G10K15/12\ H04R3/12 |
| 4486 | 3D printing | METHOD AND DEVICE FOR ESTIMATING DEVELOPMENT PARAMETERS OF PLANTS\[FR] PROCéDé ET DISPOSITIF D'ESTIMATION DE PARAMèTRES DE DéVELOPPEMENT DE PLANTES\ | WO2013083146A1 | WOERGOETTER FLORENTIN (DE)\ ABRAMOV ALEXEY (DE)\ AKSOY EREN ERDAL (DE)\ DELLEN BABETTE (ES) | GEORG AUGUST UNI GOETTINGEN STIFTUNG OEFFENTLICHEN RECHTS (DE)\ UNIV CATALUNYA POLITECNICA (ES)\ WOERGOETTER FLORENTIN (DE)\ ABRAMOV ALEXEY (DE)\ AKSOY EREN ERDAL (DE)\ DELLEN BABETTE (ES) | G06T7/00 |
| 4487 | 3D printing | LIGHT EMITTING DEVICE AND LED PRINT HEAD USING THE SAME\ | TW201210849A | WU MING-CHE (TW) | UNIVERSAL SCIENT IND SHANGHAI (CN)\ UNIVERSAL GLOBAL SCIENT IND CO LTD (TW) | B41J2/45 |
| 4488 | 3D printing | LAYER-BY-LAYER AUTOMATIC FORMING METHOD AND DEVICE FOR MICRO PHOTO-CURING THICK FILM\ | CN101344723A | XIANG WANG (CN)\ GANG ZHAO (CN)\ JIARU CHU (CN) | USTC UNIV SCIENCE TECH CN (CN) | G03F7/00\ G03F7/16 |
| 4489 | 3D printing | LIGHT PATH ADJUSTABLE OPTICAL DISPLAY REAR PROJECTION TV SET\ | CN201001162Y | XIAOJUAN LIU (CN) | SHENZHEN SKYWORTH RGB ELECT CO (CN) | H04N5/74\ H04N9/31 |
| 4490 | 3D printing | LIGHT-CURED COMPONENT FOR LASER STEREO LITHOGRAPHY\ | CN101776846B | XIAOSAN LI US\ KUNPING YU US | BEIJING GUANGCHUANG WUCHENG MATERIAL TECHNOLOGY CO LTD US | G03F7/00\ G03F7/028\ G03F7/031 |
| 4491 | 3D printing | DEVELOPMENT APPARATUS AND IMAGE FORMING APPARATUS\ | US7885563B2 | YAMAMURA AKIHIRO (JP) | OKI DATA KK (JP) | G03G15/06 |
| 4492 | 3D printing | METHOD FOR MANUFACTURING ELECTRICAL DISCHARGE MACHINING COPPER-BASED TOOL ELECTRODE\ | CN101693311A | YANLU LIU US\ JINGANG CHEN US\ JIANG LIU US | YANLU LIU US\ JINGANG CHEN US\ JIANG LIU US | B29C67/00\ B23H1/04\ C25D1/10 |
| 4493 | 3D printing | STEREO-LITHOGRAPHY DEVICE WITH DETACHABLE GROOVE\ | CN101850617A | YAQING DONG US\ GUOYUAN XI US | MICROJET TECHNOLOGY CO LTD (TW) | B29C67/00\ B05C11/00\ B05C11/10\ B05C11/11\ B05C15/00\ B05C19/04\ B05C21/00\ B27N5/00\ B28B1/00\ B28B17/00 |
| 4494 | 3D printing | STEREOSCOPIC IMAGE DISPLAY DEVICE\ | US2013314629A1 | YASUI HIRONOBU (JP)\ HANAI MASAAKI (JP)\ YOSHII HIDEKI (JP) | MITSUBISHI ELECTRIC CORP (JP) | G02B27/22 |
| 4495 | 3D printing | THREE-DIMENSIONAL STEREOLITHOGRAPHY APPARATUS THREE-DIMENSIONAL STEREOLITHOGRAPHY METHOD AND THREE-DIMENSIONAL OBJECT\ | US8540501B2 | YASUKOCHI HIROYUKI (JP) | SONY CORP (JP)\ YASUKOCHI HIROYUKI (JP) | B28B7/36 |
| 4496 | 3D printing | ENCODER AND DECODER\ | US2010250244A1 | YOSHIDA KOJI (JP)\ ZHONG HAISHAN (SG)\ LIU ZONGXIAN (SG)\ CHONG KOK SENG (SG) | PANASONIC CORP (JP) | G10L19/00\ G10L19/008\ G10L19/02\ G10L19/04\ G10L19/14\ G10L19/24 |
| 4497 | 3D printing | 3-DIMENSIONAL DIGITAL MULTIMEDIA BROADCASTING SYSTEM\[DE] DREIDIMENSIONALES DIGITALES MULTIMEDIA-AUSSTRAHLUNGSSYSTEM\[FR] SYSTEME DE DIFFUSION MULTIMEDIA NUMERIQUE TRIDIMENSIONNEL\ | EP1776832A1 | YUN KUG-JIN (KR)\ CHO SUK-HEE (KR)\ SEO JEONG-IL (KR)\ PARK GI-YOON (KR)\ UM GI-MUN (KR)\ CHANG EUN-YOUNG (KR)\ KANG KYEONGOK (KR)\ AHN CHUNG-HYUN (KR)\ LEE SOO-IN (KR) | KOREA ELECTRONICS TELECOMM (KR) | H04N13/00\ H04N7/24\ H04N7/26 |
| 4498 | 3D printing | MODELLING PLATE FOR A STEREOLITHOGRAPHY MACHINE STEREOLITHOGRAPHY MACHINE USING SAID MODELLING PLATE AND TOOL FOR CLEANING SAID MODELLING PLATE\ | US2015054198A1 | ZENERE SERGIO (IT) | DWS SRL (IT) | B29C67/00 |
| 4499 | 3D printing | METHOD FOR PRODUCING A THREE-DIMENSIONAL OBJECT BY MEANS OF A STEREOLITHOGRAPHY PROCESS INCLUDING A COMPUTER GRAPHIC DESIGN OF SAID OBJECT\ | US2014316549A1 | ZENERE SERGIO (IT) | DWS SRL (IT) | G06F17/50 |
| 4500 | 3D printing | PERFECTED METHOD FOR MANUFACTURING THREE-DIMENSIONAL OBJECTS IN LAYERS AND PERFECTED STEREOLITHOGRAPHY MACHINE USING SAID METHOD\[FR] PROCéDé PERFECTIONNé POUR LA FABRICATION D'OBJETS TRIDIMENSIONNELS EN COUCHES ET MACHINE DE STéRéOLITHOGRAPHIE PERFECTIONNéE UTILISANT CE PROCéDé\ | WO2012150497A1 | ZENERE SERGIO (IT) | DWS SRL (IT)\ ZENERE SERGIO (IT) | B29C67/00 |
| 4501 | 3D printing | METHOD FOR PRODUCING A THREE-DIMENSIONAL OBJECT AND STEREOLITHOGRAPHY MACHINE EMPLOYING SAID METHOD\ | US2013249146A1 | ZENERE SERGIO (IT)\ FORTUNATO ROBERTO (IT) | DWS SRL (IT)\ ZENERE SERGIO (IT)\ FORTUNATO ROBERTO (IT) | B29C67/00 |
| 4502 | 3D printing | METHOD AND DEVICE FOR NON-CONTACT COLLECTION OF THREE-DIMENSIONAL PALM PRINT AND HAND SHAPE\ | CN102831414A | ZHANG ZONGHUA US\ WANG ZHAOHUI US\ HUANG SHUJUN US | UNIV HEBEI TECHNOLOGY US | G06K9/00 |
| 4503 | 3D printing | RIB STRUCTURE FOR TORSION BOXES OF A WING OR HORIZONTAL STABILISER OF AN AIRCRAFT\ | US2008283675A1 | ZUNIGA SAGREDO JUAN (ES) | ZUNIGA SAGREDO JUAN (ES) | B64C3/18\ B64C3/00 |
| 4504 | 3D printing | MIKROSTRUKTURAPPARAT MIT OPTISCHER OBERFL?CHENGüTE SOWIE VERFAHREN ZUR HERSTELLUNG DESSELBEN\[EN] MICROSTRUCTURED APPARATUS WITH OPTICAL SURFACE QUALITY AND METHOD FOR THE PRODUCTION THEREOF\[FR] APPAREIL à MICROSTRUCTURE DOTé D'UNE QUALITé DE SURFACE OPTIQUE ET SON PROCéDé DE FABRICATION\ | EP2653222A3 | ANURJEW EUGEN (DE)\ BRANDNER JUERGEN (DE)\ HANSJOSTEN EDGAR (DE)\ LI YONGLI (FR)\ NEWPORT DAVID (IE) | KARLSRUHER INST TECHNOLOGIE (DE) | G03F7/00\ B01L3/00 |
| 4505 | 3D printing | ENDOPROSTHESIS WITH THREE-DIMENSIONAL DISINTEGRATION CONTROL\ | US8052743B2 | ATANASOSKA LILIANA (US)\ WEBER JAN (NL) | BOSTON SCIENT SCIMED INC (US) | A61F2/82 |
| 4506 | 3D printing | DIRECT-MANUFACTURED DUCT INTERCONNECTS\ | US7623940B2 | BAGWILL TRACY L (US)\ HUSKAMP CHRIS (US) | BOEING CO (US) | G06F17/50\ G06F19/00\ F16L9/18\ F16L21/02\ F16L23/00 |
| 4507 | 3D printing | DIRECT SELECTIVE LASER SINTERING OF METALS\ | US6676892B2 | BEAMAN JOSEPH J (US)\ DAS SUMAN (US) | REGENTS UNIVERSITY TEXAS SYSTE (US) | B22F3/105\ B23K26/34\ C23C26/02\ G05B19/4062 |
| 4508 | 3D printing | ELEKTRONIKGEH?USE MIT PARTIELLEM KüHLK?RPER\[EN] ELECTRONICS HOUSING WITH PARTIAL COOLING BODY\[FR] BO?TIER éLECTRONIQUE DOTé D'UN CORPS DE REFROIDISSEMENT PARTIEL\ | EP2242344B1 | BEST DIETER (DE)\ SAUER THOMAS (DE)\ LIPP HELMUT (DE)\ FUTTERLIEB GERD (DE)\ ZIERLEIN RAINER (DE)\ EHMANN TOBIAS (DE) | EBM PAPST MULFINGEN GMBH & CO (DE) | H05K7/20\ H02K5/18 |
| 4509 | 3D printing | LOCOMOTIVE CRANKSHAFT LASER CLADDING SYSTEM WITH YAG (YTTRIUM ALUMINUM GARNET) LASER DEVICE AS LIGHT SOURCE\ | CN201915147U | BING AN US | GANSU JINGCHENG PHOTOELECTRIC TECHNOLOGY CO LTD US | C23C24/10 |
| 4510 | 3D printing | HOLOGRAPHIC PRINTER\ | US2010067076A1 | BROTHERTON-RATCLIFFE DAVID (GB) | BROTHERTON-RATCLIFFE DAVID (GB) | G03H1/20 |
| 4511 | 3D printing | HOLOGRAPHIC IMAGE DISPLAY SYSTEMS\ | US2011157667A1 | BUCKLEY EDWARD (GB)\ CABLE ADRIAN JAMES (GB)\ GIL-LEYVA DIEGO (GB)\ STINDT DOMINIK (GB)\ LACOSTE LILIAN (GB) | STINDT DOMINIK (GB)\ LACOSTE LILIAN (GB)\ BUCKLEY EDWARD (GB)\ CABLE ADRIAN JAMES (GB)\ GIL-LEYVA DIEGO (GB) | G03H1/22\ G03H1/08 |
| 4512 | 3D printing | IMAGING SYSTEMS WITH PIXELATED SPATIAL LIGHT MODULATORS\[FR] SYSTEMES D'IMAGERIE A MODULATEURS DE LUMIERE SPATIALE PIXELISES\ | WO2006102201A8 | CATHEY WADE THOMAS JR (US)\ DOWSKI EDWARD RAYMOND JR (US)\ COGSWELL CAROL JEAN (US) | CDM OPTICS INC (US)\ CATHEY WADE THOMAS JR (US)\ DOWSKI EDWARD RAYMOND JR (US)\ COGSWELL CAROL JEAN (US) | G02B27/46\ G02B27/50 |
| 4513 | 3D printing | MULTI-FUNCTION COAXIAL NOZZLE FOR LASER MATERIAL PROCESSING\ | TWI252789B | CHEN CHUN (TW)\ WU HSIEN-CHENG (TW) | CHEN CHUN (TW)\ WU HSIEN-CHENG (TW) | B23K26/14 |
| 4514 | 3D printing | SYSTEM AND METHOD FOR MANUFACTURING THREE DIMENSIONAL INTEGRATED CIRCUITS\ | US2012026478A1 | CHEN JANG FUNG (US)\ LAIDIG THOMAS (US) | CHEN JANG FUNG (US)\ LAIDIG THOMAS (US)\ PINEBROOK IMAGING SYSTEMS CORP (US) | G03B27/42 |
| 4515 | 3D printing | SYSTEM AND METHOD FOR MANUFACTURING THREE DIMENSIONAL INTEGRATED CIRCUIT\ | TW201214515A | CHEN JANG FUNG (US)\ LAIDIG THOMAS (US) | PINEBROOK IMAGING SYSTEMS CORP (US) | G03F7/20\ H01L21/027 |
| 4516 | 3D printing | THREE-DIMENSIONAL (3D) PRINTING METHOD\ | CN103129140A | CHEN XUGUO US | CHEN XUGUO US | B41J2/01\ B41J3/00 |
| 4517 | 3D printing | METHOD FOR PRODUCING THREE-DIMENSIONAL ARTICLES FROM POWDERS AND APPARATUS FOR CARRYING OUT SAID METHOD\[FR] PROCéDé DE FABRICATION D'ARTICLES VOLUMIQUES à PARTIR DE POUDRES ET DISPOSITIF DE SA MISE EN OEUVRE\ | WO2013080030A1 | CHIVEL YURI (BY) | CHIVEL YURI (BY) | B22F3/105 |
| 4518 | 3D printing | THREE DIMENSIONAL IMAGE DISPLAY APPARATUS USING DIGITAL HOLOGRAM\ | KR101292370B1 | CHOI JAE WU (KR) | UNIV KYUNG HEE UNIV IND COOP (KR) | H04N13/04\ G03H1/04 |
| 4519 | 3D printing | SINTERING USING THERMAL IMAGE FEEDBACK\ | US6815636B2 | CHUNG MARK (US)\ ALLANIC ANDRE-LUC (US) | 3D SYSTEMS INC (US) | B29C67/00\ B22F3/105\ B22F3/16\ C04B35/64\ B29C35/02\ G05D23/27 |
| 4520 | 3D printing | SELECTIVE LASER SINTERING POWDER RECYCLE SYSTEM\ | US7887316B2 | COX BRIAN DAVID (US) | 3D SYSTEMS INC (US) | B28B17/00\ B29B17/00\ B29B7/66 |
| 4521 | 3D printing | SELECTIVE LASER SINTERING WITH INTERLEAVED FILL SCAN\ | US6694207B2 | DARRAH JAMES F (US)\ ZU XIAOSHU (US) | 3D SYSTEMS INC (US) | B29C67/00\ G05B19/4099 |
| 4522 | 3D printing | SELECTIVE LASER SINTERING WITH OPTIMIZED RASTER SCAN DIRECTION\ | US6677554B2 | DARRAH JAMES F (US)\ ZU XIAOSHU (US) | 3D SYSTEMS INC (US) | B29C67/00\ B22F3/105\ G05B19/4099 |
| 4523 | 3D printing | METHOD FOR PROCESSING PAEK AND ARTICLES MANUFACTURED FROM THE SAME\ | US2013217838A1 | DEFELICE SCOTT F (US)\ DECARMINE ANTHONY (US) | DEFELICE SCOTT F (US)\ DECARMINE ANTHONY (US) | B29C67/00 |
| 4524 | 3D printing | SINTERABLE SEMI-CRYSTALLINE POWDER AND NEAR-FULLY DENSE ARTICLE FORMED THEREWITH\ | US5990268A | DICKENS JR ELMER DOUGLAS (US)\ LEE BIING LIN (US)\ TAYLOR GLENN ALFRED (US)\ MAGISTRO ANGELO JOSEPH (US)\ NG HENDRA (US) | DTM CORP (US) | B29C35/08\ B29C67/00\ B29C41/00\ B29C67/04\ C08J3/12\ C08J9/24\ C08G69/14 |
| 4525 | 3D printing | GRID FOR SELECTIVE TRANSMISSION OF ELECTROMAGNETIC RADIATION WITH STRUCTURAL ELEMENT BUILT BY SELECTIVE LASER SINTERING\ | US2010061520A1 | DORSCHEID RALF (NL)\ VOGTMEIER GEREON (DE) | KONINKL PHILIPS ELECTRONICS NV (NL) | B22F7/04\ G21K1/10 |
| 4526 | 3D printing | VERBESSERUNGEN FüR VORRICHTUNG ZUR SCHNELLEN ERSTELLUNG VON PROTOTYPEN UND VERFAHREN\[EN] IMPROVEMENTS FOR RAPID PROTOTYPING APPARATUS AND METHOD\[FR] AMéLIORATIONS POUR APPAREIL DE PROTOTYPAGE RAPIDE ET PROCéDé\ | EP2537665A1 | Empty Field US | 3D SYSTEMS INC (US) | B29C67/00\ G03F7/00 |
| 4527 | 3D printing | ３Ｄコンテンツ生成システム、その方法、及び通信方法\ | JP2014209247A | Empty Field US | CONCEPT LASER GMBH (DE)\ HERZOG FRANK (DE) | G03H1/22\ G03H1/08\ G03H1/04 |
| 4528 | 3D printing | No Title Available\ | RU2004130984A | Empty Field US | LG ELECTRONICS INC (KR) | B22F3/105 |
| 4529 | 3D printing | CCD MODULE STRUCTURE AND HOLOGRAM STORAGE CONSISTING OF THEREOF\ | KR100706316B1 | Empty Field US | QINETIQ LTD (GB) | G11B7/0065 |
| 4530 | 3D printing | THREE-DIMENSIONAL COAXIAL LASER POWDER-TRANSMITTING HEAD\ | CN201236210Y | FEI XING (CN)\ YIFEI ZHANG (CN)\ MINGHUI GONG (CN)\ PENG YANG (CN) | SHENYANG SIASUN ROBOT & AUTOMA (CN) | C23C24/10 |
| 4531 | 3D printing | AIR CURTAIN TYPE THREE-DIMENSIONAL COAXIAL LASER FEEDING HEAD\ | CN101643900A | FEI XING (CN)\ YIFEI ZHANG (CN)\ MINGHUI GONG (CN)\ PENG YANG (CN) | SHENYANG SIASUN ROBOT & AUTOMA (CN) | C23C24/10 |
| 4532 | 3D printing | METHOD FOR CONTROLLING DEPTH-OF-FOCUS IN 3D IMAGE RECONSTRUCTIONS IN PARTICULAR FOR SYNTHESIZING THREE-DIMENSIONAL DYNAMIC SCENE FOR THREE-DIMENSIONAL HOLOGRAPHY DISPLAY AND HOLOGRAPHIC APPARATUS UTILIZING SUCH A METHOD\ | US2012162733A1 | FERRARO PIETRO (IT)\ PATURZO MELANIA (IT)\ MEMMOLO PASQUALE (IT)\ FINIZIO ANDREA (IT) | CONSIGLIO NAZIONALE RICERCHE (IT)\ FERRARO PIETRO (IT)\ PATURZO MELANIA (IT)\ MEMMOLO PASQUALE (IT)\ FINIZIO ANDREA (IT) | G03H1/08 |
| 4533 | 3D printing | DEVICE AND METHOD FOR MANUFACTURING MOULDED PARTS FROM FIBROUS MATERIAL\[DE] VORRICHTUNG UND VERFAHREN ZUM HERSTELLEN VON FORMTEILEN AUS FASERMATERIAL\[FR] DISPOSITIF ET PROCéDé DE FABRICATION DE PIèCES DE FORMAGE à PARTIR DE MATéRIAUX EN FIBRES\ | EP2305869B1 | FOERSTER EGON (DE)\ HEINL MANFRED (DE) | BUERKLE GMBH ROBERT (DE) | D04H1/732\ D04H1/736\ D04H1/74\ D04H1/00 |
| 4534 | 3D printing | SYSTEM AND RESIN FOR RAPID PROTOTYPING\[FR] SYSTèME ET RéSINE POUR PROTOTYPAGE RAPIDE\[DE] SYSTEM UND HARZ ZUR SCHNELLEN PROTOTYPISIERUNG\ | EP2436510A1 | FRANTZ RICHARD (FR)\ BELKACEM JEMMEL (FR)\ CHERKAOUI ZOUBAIR (CH)\ DA COSTA RICARDO (CH) | 3D SYSTEMS INC (US) | B29C67/00\ G03F7/00\ G03F7/004 |
| 4535 | 3D printing | METHOD FOR FEEDING WIRE IN LASER AND CLADDING AND IN-LASER WIRE FEEDER\[FR] PROCéDé POUR DISTRIBUER UN FIL DANS UN LASER ET REVêTEMENT ET DISPOSITIF DE DISTRIBUTION DE FIL DANS UN LASER\ | WO2009039753A1 | FU GEYAN (CN)\ SHI SHIHONG (CN)\ HU JIN (CN)\ YANG GUOJIA (CN)\ HAN XUELEI (CN) | UNIV SUZHOU (CN)\ FU GEYAN (CN)\ SHI SHIHONG (CN)\ HU JIN (CN)\ YANG GUOJIA (CN)\ HAN XUELEI (CN) | B23K26/34\ B23K26/06 |
| 4536 | 3D printing | METAL POWDER FOR SELECTIVE LASER SINTERING METHOD FOR MANUFACTURING THREE-DIMENSIONAL SHAPED OBJECT BY USING THE SAME AND THREE-DIMENSIONAL SHAPED OBJECT OBTAINED THEREFROM\ | US2013065073A1 | FUWA ISAO (JP) | PANASONIC CORP (JP)\ FUWA ISAO (JP) | B22F3/105\ B22F1/00\ B32B5/30 |
| 4537 | 3D printing | REAL 3D HIGH DEFINITION DISPLAY METHOD AND DEVICE\ | CN1971340A | GENG ZHENG (CN) | GENG ZHENG (CN) | H04N13/00\ G02B27/22 |
| 4538 | 3D printing | METHOD FOR GENERATING COMPUTER-GENERATED VIDEO HOLOGRAMS IN REAL TIME BY MEANS OF PROPAGATION\ | US2010033780A1 | GITTER KURT (DE) | SEE REAL TECHNOLOGIES S A (LU) | G03H1/08 |
| 4539 | 3D printing | VERFAHREN ZUM GENERIEREN COMPUTER-GENERIERTER VIDEOHOLOGRAMME IN ECHTZEIT MITTELS PROPAGATION\ | DE102006042323B4 | GITTER KURT DIPL ING (DE) | SEEREAL TECHNOLOGIES SA (LU) | G03H1/08 |
| 4540 | 3D printing | PROCESS FOR LAYER-BY-LAYER PRODUCTION OF THREE-DIMENTIONAL OBJECTS\ | US2013316145A1 | GREBE MAIK (DE)\ DIEKMANN WOLFGANG (DE) | GREBE MAIK (DE)\ DIEKMANN WOLFGANG (DE) | B29C67/00 |
| 4541 | 3D printing | PROCESS FOR THE PRODUCTION OF A THREE DIMENSIONAL SYSTEM\ | US2012202012A1 | GREBE MAIK (DE)\ DIEKMANN WOLFGANG (DE)\ ALTKEMPER STEFAN (DE) | EVONIK DEGUSSA GMBH (DE)\ GREBE MAIK (DE)\ DIEKMANN WOLFGANG (DE)\ ALTKEMPER STEFAN (DE) | B29C41/34\ B29C41/52\ B29C41/22\ B32B3/30 |
| 4542 | 3D printing | RADIANT HEATING APPARATUS FOR PROVIDING UNIFORM SURFACE TEMPERATURE USEFUL IN SELECTIVE LASER SINTERING\ | US5155321A | GRUBE KRIS W (US)\ BEAMAN JOSEPH J (US) | DTM CORP (US) | B29C67/00\ B22F3/105\ B23K26/34\ G05B19/4099\ C04B35/64\ B29C35/02\ B23K26/00\ H05B3/00\ B22F3/10\ C23C24/10 |
| 4543 | 3D printing | PREPARATION METHOD OF NANO SILICON CARBIDE REINFORCED ALUMINUM-BASED COMPOSITE MATERIAL\ | CN103045914A | GU DONGDONG US\ WANG HONGQIAO US\ DAI DONGHUA US\ ZHANG GUOQUAN US | UNIV NANJING AERONAUTICS US | B22F3/105\ C22C21/00\ C22C32/00\ C22C1/05 |
| 4544 | 3D printing | VERFAHREN ZUR HERSTELLUNG EINER SCHABLONE\[EN] VEHICLE COMPONENT I.E. PROPELLER SHAFT TUNNEL MOLD MANUFACTURING METHOD INVOLVES PRODUCING MOLD BASED ON THREE-DIMENSIONAL DATASET THAT IS PROVIDED VIA STANDARD INTERFACE OF COMPUTER-AIDED CONSTRUCTION PROGRAM\ | DE102009005080A1 | HABERMEIER HOLGER (DE)\ LOEFFLER THOMAS (DE)\ STOEFFLER ROLF (DE) | DAIMLER AG (DE) | G06F17/50 |
| 4545 | 3D printing | VERFAHREN ZUM ELIMINIEREN EINER IN HOMOGENEN HELLIGKEITSWAHRNEHMUNG BEI EINER HOLOGRAPHISCHEN REKONSTRUKTION VON SZENEN\[EN] INHOMOGENEOUS BRIGHTNESS PERCEPTION COMPENSATION METHOD FOR THREE-DIMENSIONAL SCENE INVOLVES ESTIMATING LOCAL ERRORS OF RECONSTRUCTED THREE-DIMENSIONAL SCENE CAUSED BY AFFECTED ILLUMINATION REGIONS BASED ON PREDETERMINED PARAMETER\ | DE102006031942A1 | HAEUSLER RALF (DE) | SEEREAL TECHNOLOGIES SA (LU) | G03H1/22\ G03H1/08 |
| 4546 | 3D printing | HOLOGRAM PRODUCTION METHOD AND HOLOGRAM PRODUCTION SYSTEM EMPLOYING SILVER HALIDE PHOTOSENSITIVE MATERIAL\ | US2007134559A1 | HARA TAKEFUMI (JP) | FUJIFILM CORP (JP) | G03H1/04 |
| 4547 | 3D printing | DIRECTIONAL DISPLAY APPARATUS\ | US7796318B2 | HARROLD JONATHAN (GB)\ WOODGATE GRAHAM JOHN (GB) | AU OPTRONICS CORP (TW) | H04N13/00\ G02B27/22\ H04N13/04\ H04N15/00\ G02B3/00\ G02F1/00\ G09G3/00 |
| 4548 | 3D printing | METHOD FOR THE COMPENSATION OF AN INHOMOGENEOUS BRIGHTNESS PERCEPTION IN HOLOGRAPHICALLY RECONSTRUCTED SCENES\ | US2009180165A1 | HAUSSLER RALF (DE) | SEE REAL TECHNOLOGIES S A (LU) | G03H1/08 |
| 4549 | 3D printing | APPARATUS AND METHOD FOR SELECTIVE LASER SINTERING\ | JP2008037024A | HAYANO SEIJI US\ SASA MASAYOSHI US\ HORIBA YOSHINORI US | ASPECT INC US | B29C67/00 |
| 4550 | 3D printing | METHOD FOR PRODUCING THREE-DIMENSIONAL SINTERED WORK PIECES\ | US2005142024A1 | HERZOG FRANK (DE) | Empty Field US | B29C67/00\ B22F3/105\ B22F3/16 |
| 4551 | 3D printing | FILTEREINRICHTUNG ZUM ANSCHLUSS AN EINE LASERSINTER- ODER LASERSCHMELZANLAGE\[EN] FILTER DEVICE FOR USE WITH E.G. LASER SINTER SYSTEM TO MANUFACTURE THREE-DIMENSIONAL OBJECT FROM METALLIC POWDERS HAS INLET AND OUTLET PROVIDED AT FILTER MODULE TO MOISTEN FILTER BEFORE OPENING MODULE AND FILTER LOADED WITH RESIDUES\ | DE102012004587A1 | HERZOG FRANK (DE)\ SCHOEDEL FRANK (DE)\ BECHMANN FLORIAN (DE) | CL SCHUTZRECHTSVERWALTUNGS GMBH (DE) | B01D46/42 |
| 4552 | 3D printing | DEVICE AND METHOD FOR THE PRODUCTION OF THREE-DIMENSIONAL OBJECTS BY MEANS OF GENERATIVE PRODUCTION METHOD\ | US7665979B2 | HEUGEL MARTIN (DE) | EOS ELECTRO OPTICAL SYST (DE) | B29C35/08\ B29C67/00 |
| 4553 | 3D printing | METAL POWDER COMPOSITION FOR USE IN SELECTIVE LASER SINTERING\ | US7258720B2 | HIGASHI YOSHIKAZU (JP)\ IWANAGA TOSHIHARU (JP)\ TAKENAMI MASATAKA (JP)\ TOGEYAMA HIROHIKO (JP)\ FUWA ISAO (JP)\ YOSHIDA NORIO (JP)\ UENAGA SHUSHI (JP)\ ABE SATOSHI (JP)\ SHIMIZU TAKASHI (JP) | MATSUSHITA ELECTRIC WORKS LTD (JP) | B22F1/00 |
| 4554 | 3D printing | METHOD FOR THE PRECISE FABRICATION OF DENTAL COMPONENTS USING A SINTERING OR SELECTIVE LASER MELTING PROCESS\ | US2010143868A1 | HINTERSEHR JOSEF (DE) | HINTERSEHR JOSEF (DE) | G06F17/50\ A61C13/08\ A61C13/003 |
| 4555 | 3D printing | BUILT-IN LASER SMELTING SPRAY NOZZLE\ | CN2705236Y | HU QIANWU (CN)\ ZENG XIAOYAN (CN) | CENTRAL CHINA SCIENCE & TECH (CN) | B23K26/34\ B05B7/16\ C23C4/00 |
| 4556 | 3D printing | MANUFACTURE METHOD AND DEVICE OF THREE-DIMENSIONAL WORKPIECE\ | CN101422963A | HUAXING TANG (CN) | OUKESI INTERNAT CO LTD (CN) | B29C67/00\ B22F3/105\ C04B35/64\ B05C5/00\ B05C11/04 |
| 4557 | 3D printing | METHOD FOR JOINING PARTS FABRICATED VIA SELECTIVE LASER SINTERING WHILE MAINTAINING PROPER ALIGNMENT\ | US2007236018A1 | HUSMANN CHRISTOPHER H (US)\ STEIN GREGORY N (US) | NORTHROP GRUMMAN CORP (US) | F16L21/00 |
| 4558 | 3D printing | BUILT-IN ATTACHMENT DEVICE USING SELECTIVE LASER SINTERING\ | US7514649B2 | HUSMANN CHRISTOPHER H (US)\ STEIN GREGORY N (US) | NORTHROP GRUMMAN CORP (US) | B29C35/08\ B23K26/00 |
| 4559 | 3D printing | ADDITITVE MANUFACTURING OF COMPONENTS FOR DOWNHOLE WIRELINE TUBING AND DRILL PIPE CONVEYED TOOLS\ | US2013310961A1 | INTRIAGO VELEZ JUAN CARLOS (US) | CORP SCHLUMBERGER TECHNOLOGY (US)\ SCHLUMBERGER TECHNOLOGY CORP (US) | G06F17/50 |
| 4560 | 3D printing | EXPOSURE APPARATUS AND METHODS\ | US2011292363A1 | IVEY PETER ANTHONY (GB)\ MCWILLIAM RICHARD PETER (GB)\ PURVIS ALAN (GB)\ WILLIAMS GAVIN LEWIS (GB)\ CURRY RICHARD IAN (GB)\ TORIZ-GARCIA JOSE JUAN DE JESUS (GB)\ SEED NICHOLAS LUKE (GB) | IVEY PETER ANTHONY (GB)\ MCWILLIAM RICHARD PETER (GB)\ PURVIS ALAN (GB)\ WILLIAMS GAVIN LEWIS (GB)\ SEED NICHOLAS LUKE (GB)\ CURRY RICHARD IAN (GB)\ TORIZ-GARCIA JOSE JUAN DE JESUS (GB) | G03B27/52 |
| 4561 | 3D printing | ADJUSTING MECHANISM OF INSIDE-LASER POWDER FEEDING DEVICE FOR LASER CLADDING FORMING\ | CN201713576U | JIA LIU US\ CHUNSHENG LI US\ QIFEI CAI US\ GEYAN FU (CN)\ SHIHONG SHI (CN) | UNIV SUZHOU (CN) | C23C24/10 |
| 4562 | 3D printing | MOULD STRUCTURE WITH CONFORMAL COOLING PIPE\ | CN101670627A | JINHUI LIU US\ CAN ZHAO US\ JIBING CHEN US\ RUIDI LI US | UNIV ZHEJIANG SCIENCES US | B29C33/38\ B29C33/04\ B29C45/73\ B22F3/22\ B29C45/26 |
| 4563 | 3D printing | METHOD FOR FORMING REFRACTORY METAL PARTS BY USING LASER\ | CN101780544A | JINHUI LIU US\ CAN ZHAO US\ JIBING CHEN US\ RUIDI LI US\ DANQING ZHANG US | HEILONGJIANG INST OF SCIENCE A US | B22F3/105\ B23K26/34\ C23C24/10 |
| 4564 | 3D printing | NEAR-NET-SHAPE FORMING METHOD OF REFRACTORY METAL PART\ | CN102773479A | JINHUI LIU US\ HUANAN LIU US\ SHENGBING XIAO US\ TONG WU US\ WENJUAN XIE US | HEILONGJIANG INST SCI & TECH US | B22F3/00\ B22F3/105 |
| 4565 | 3D printing | DEVICE AND METHOD FOR PRODUCING A THREE-DIMENSIONAL OBJECT BY MEANS OF MASK EXPOSURE\ | US7783371B2 | JOHN HENDRIK (DE)\ SCHILLEN VOLKER (DE)\ EL-SIBLANI ALI (US) | ENVISIONTEC GMBH (DE) | G06F19/00 |
| 4566 | 3D printing | ASYMMETRIC CHARACTER MAXILLOFACIAL PROSTHESIS PRODUCING METHOD BASED ON THREE-DIMENSIONAL VISUAL SENSATION MEASUREMENT\ | CN101292915A | JUNTONG XI (CN)\ JIN SUN (CN)\ YAOYANG XIONG (CN)\ XIAOBO CHEN (CN)\ FUQIANG ZHANG (CN) | UNIV SHANGHAI JIAOTONG (CN) | G06F17/50\ A61F2/28\ A61B19/00 |
| 4567 | 3D printing | SYMMETRICAL CHARACTER MAXILLOFACIAL PROSTHESIS PRODUCING METHOD BASED ON THREE-DIMENSIONAL VISUAL SENSATION MEASUREMENT\ | CN101292914A | JUNTONG XI (CN)\ JIN SUN (CN)\ YAOYANG XIONG (CN)\ XIAOBO CHEN (CN)\ FUQIANG ZHANG (CN) | UNIV SHANGHAI JIAOTONG (CN) | G06F17/50\ A61F2/28\ A61B19/00 |
| 4568 | 3D printing | LASER MICROSCOPE\ | US2013250391A1 | KATO JUN (JP)\ SASAKI HIROSHI (JP)\ ARAI YUJIN (JP) | OLYMPUS CORP (JP) | G02B21/06\ G02B21/36 |
| 4569 | 3D printing | NETWORK STRUCTURE\ | JP2010004965A | KAWAI KUNIAKI US | EIHEIJI SIZING KK US | A47C27/12 |
| 4570 | 3D printing | METHOD FOR IDENTIFYING LASER SINTERING POWDERS\ | US2010140550A1 | KELLER PETER (DE)\ MATTES THOMAS (DE)\ GERSCH MANDY (DE)\ OBERHOFER JOHANN (DE) | EOS ELECTRO OPTICAL SYST (DE) | C09K11/78\ C09K11/84\ C09K11/86 |
| 4571 | 3D printing | METHOD OF COATING ARTICLE BY LASER LAYER-BY-LAYER SYNTHESIS\ | RU2443506C2 | KHARANZHEVSKIJ EVGENIJ VIKTOROVICH (RU)\ IPATOV ALEKSEJ GENNAD EVICH (RU)\ GALENKO PETR KONSTANTINOVICH (RU)\ KRIVILEV MIKHAIL DMITRIEVICH (RU)\ DANILOV DENIS ANATOL EVICH (RU) | G OBRAZOVATEL NOE UCHREZHDENIE VYSSHEGO PROFESSIONAL NOGO OBRAZOVANIJA UDMURTSKIJ GU UDGU (RU) | B22F3/105 |
| 4572 | 3D printing | HOLOGRAPHIC STEREOGRAM CREATION DEVICE AND ITS METHOD\ | US2007019266A1 | KIHARA NOBUHIRO (JP)\ SHIRAKURA AKIRA (JP)\ BABA SHIGEYUKI (JP) | SONY CORP (JP) | G03H1/26 |
| 4573 | 3D printing | NORMALIZATION OF RETINAL NERVE FIBER LAYER THICKNESS MEASUREMENTS MADE BY TIME DOMAIN-OPTICAL COHERENCE TOMOGRAPHY\ | US8911089B2 | KIM JONG S (US)\ ISHIKAWA HIROSHI (US)\ SCHUMAN JOEL S (US)\ WOLLSTEIN GADI (US) | KIM JONG S (US)\ ISHIKAWA HIROSHI (US)\ SCHUMAN JOEL S (US)\ WOLLSTEIN GADI (US)\ UNIV PITTSBURGH&mdashOF THE COMMONWEALTH SYSTEM OF HIGHER EDUCATION (US) | G01B11/06\ A61B3/10\ A61B3/14\ A61B3/00\ G01B9/02 |
| 4574 | 3D printing | LASER CLADDING FORMING DEVICE AND LASER CLADDING FORMING METHOD OF METAL PART\ | CN101885063A | KU SI US | UNIV DONGGUAN TECHNOLOGY US | B22F3/105 |
| 4575 | 3D printing | LASER CLADDING AND SHAPING EQUIPMENT\ | CN201817550U | KU SI US | UNIV DONGGUAN TECHNOLOGY US | C23C24/08\ G05B19/414 |
| 4576 | 3D printing | METHODS AND SYSTEMS TO COMPENSATE FOR A STITCHING DISTURBANCE OF A PRINTED PATTERN IN A MASKLESS LITHOGRAPHY SYSTEM UTILIZING OVERLAP OF EXPOSURE ZONES WITH ATTENUATION OF THE AERIAL IMAGE IN THE OVERLAP REGION\ | US7133121B2 | LATYPOV AZAT (US)\ BLEEKER ARNO (NL)\ CEBUHAR WENCESLAO A (US)\ KREUZER JUSTIN (US)\ VLADIMIRSKY YULI (US) | ASML HOLDING NV (NL) | G03F7/20\ G06F17/50\ G03C5/00\ H01L21/027\ G02F1/01\ G03B27/54\ G03B27/32\ G02B26/08\ G03B27/00\ G03B21/18\ G03B27/68\ G03F9/00 |
| 4577 | 3D printing | METHODS SYSTEMS AND COMPUTER PROGRAM PRODUCTS FOR PRINTING PATTERNS ON PHOTOSENSITIVE SURFACES\ | US2008079923A1 | LATYPOV AZAT (US)\ MAST KAREL V D (NL) | ASML HOLDING NV (NL) | G03F7/20\ H01L21/027\ G03B27/54\ G03B27/72 |
| 4578 | 3D printing | METHOD AND SYSTEM FOR A MASKLESS LITHOGRAPHY RASTERIZATION TECHNIQUE BASED ON GLOBAL OPTIMIZATION\ | US7469058B2 | LATYPOV AZAT (US)\ POULTNEY SHERMAN (US)\ CEBUHAR WENCESLAO (US) | ASML HOLDING NV (NL) | G06K9/00\ G02F1/1335\ G03B27/54\ G02F1/135\ G21K5/00\ G03H1/12\ G02B27/42\ G03C5/20 |
| 4579 | 3D printing | METHOD AND SYSTEM FOR MASKLESS LITHOGRAPHY REAL-TIME PATTERN RASTERIZATION AND USING COMPUTATIONALLY COUPLED MIRRORS TO ACHIEVE OPTIMUM FEATURE REPRESENTATION\ | US7209275B2 | LATYPOV AZAT M (US)\ BASELMANS JOHANNES JACOBUS MAT (NL)\ TROOST KARS Z (NL) | ASML HOLDING NV (NL) | G02B26/00 |
| 4580 | 3D printing | MULTI-SHAFT NUMERICAL CONTROL DOUBLE-WORKBENCH LASER PROCESSING SYSTEM\ | CN101342637A | LEI HONG (CN)\ WEIJIAN MI (CN)\ GANG WU (CN) | UNIV SHANGHAI MARITIME (CN) | B23K26/00\ B23K26/067\ B23K26/08\ B23Q1/25 |
| 4581 | 3D printing | MULTI-SHAFT NUMERICAL CONTROL AND DOUBLE WORKTABLE LASER MACHINING APPARATUS\ | CN201186370Y | LEI HONG (CN)\ WEIJIAN MI (CN)\ GANG WU (CN) | UNIV SHANGHAI MARITIME (CN) | B23K26/00\ B23K26/067\ B23K26/08\ B23Q1/25\ B23K26/06 |
| 4582 | 3D printing | METHOD AND DEVICE FOR HOLOGRAPHICALLY RECONSTRUCTING A SCENE\ | US8441703B2 | LEISTER NORBERT (DE) | SEEREAL TECHNOLOGIES SA (LU)\ LEISTER NORBERT (DE) | G03H1/08 |
| 4583 | 3D printing | VERFAHREN ZUR DATENKOMPRESSION COMPUTERGENERIERTER VIDEOHOLOGRAMME\[EN] METHOD FOR DATA COMPRESSION OF COMPUTER-GENERATED VIDEO HOLOGRAMMES\[FR] PROCéDéS DE COMPRESSION DE DONNéES D'HOLOGRAMMES VIDéO PRODUITS PAR ORDINATEUR\ | WO2007085233A2 | LEISTER NORBERT (DE) | SEEREAL TECHNOLOGIES SA (LU)\ LEISTER NORBERT (DE) | H04N7/26\ G03H1/08 |
| 4584 | 3D printing | THREE-DIMENSIONAL LIGHT MODULATION ARRANGEMENT FOR MODULATING A WAVE FIELD HAVING COMPLEX INFORMATION\ | US2012092735A1 | LEISTER NORBERT (DE)\ LAZAREV GRIGORY (DE)\ HAUSSLER RALF (DE)\ FUTTERER GERALD (DE) | SEEREAL TECHNOLOGIES SA (LU)\ FUTTERER GERALD (DE)\ LEISTER NORBERT (DE)\ HAUSSLER RALF (DE)\ LAZAREV GRIGORY (DE) | G03H1/12 |
| 4585 | 3D printing | LIGHTING UNIT FOR A HOLOGRAPHIC RECONSTRUCTION SYSTEM\ | US2010296143A1 | LEISTER NORBERT (DE)\ REICHELT STEPHAN (DE)\ SAHM HAGEN (DE) | SEEREAL TECHNOLOGIES SA (LU) | G03H1/22 |
| 4586 | 3D printing | METHOD FOR THE MULTIMODAL REPRESENTATION OF IMAGE CONTENTS ON A DISPLAY UNIT FOR VIDEO HOLOGRAMS AND MULTIMODAL DISPLAY UNIT\ | US8208012B2 | LEISTER NORBERT (DE)\ RENAUD-GOUD PHILIPPE (FR)\ HAUSSLER RALF (DE) | SEEREAL TECHNOLOGIES SA (LU)\ LEISTER NORBERT (DE)\ HAUSSLER RALF (DE)\ RENAUD-GOUD PHILIPPE (FR) | H04N13/04 |
| 4587 | 3D printing | DEVICE AND METHOD FOR REALIZING THREE-DIMENSIONAL DYNAMIC DISPLAY OF COMPUTER GENERATED HOLOGRAM\ | CN101707724A | LIHUI GUO US\ XIANZHONG JIAN US\ HAISHENG WANG US | UNIV SHANGHAI SCIENCE & TECH US | H04N13/00\ G03H1/22 |
| 4588 | 3D printing | SYSTEM FOR TREATING LASER COATING\ | CN102925938A | LIN XUECHUN US\ YANG YINGYING US\ ZHAO SHUSEN US\ YU HAIJUAN US | INST SEMICONDUCTORS CAS US | C23C24/10\ C25D5/00\ B23K26/352\ C23C18/31\ C25D5/18 |
| 4589 | 3D printing | LOW-COST HIGH-PRECISION HOLOGRAPHIC 3D TELEVISION TECHNOLOGY IMPLEMENTED USING CHROMINANCE CLAMPING METHOD\[FR] TECHNOLOGIE DE TéLéVISION EN 3D HOLOGRAPHIQUE DE HAUTE PRéCISION à FAIBLE CO?T MISE EN OEUVRE à L'AIDE D'UN PROCéDé DE CALAGE DE CHROMINANCE\ | WO2013143083A1 | LIU TRAVIS (AU)\ LIU SHICHANG (CN)\ LIU YUN (CA) | LIU TRAVIS (AU)\ LIU SHICHANG (CN)\ LIU YUN (CA) | H04N15/00 |
| 4590 | 3D printing | PASSIVE COMPONENTS FOR MILLIMETER SUBMILLIMETER AND TERAHERTZ ELECTROMAGNETIC WAVES MADE BY PILING UP SUCCESSIVE LAYERS OF MATERIAL\[FR] COMPOSANTS PASSIFS POUR ONDES éLECTROMAGNéTIQUES DE L'ORDRE DU MILLIMèTRE DU SOUS-MILLIMèTRE OU TéRAHERTZIENNES OBTENUS PAR EMPILEMENT DE COUCHES SUCCESSIVES DE MATéRIAU\ | WO2012076994A1 | MACOR ALESSANDRO (CH)\ ANSERMET JEAN-PHILIPPE (CH)\ DE RIJK EMILE (CH) | ECOLE POLYTECH (CH)\ MACOR ALESSANDRO (CH)\ ANSERMET JEAN-PHILIPPE (CH)\ DE RIJK EMILE (CH) | H01P3/123\ H01P11/00\ H01Q13/02 |
| 4591 | 3D printing | POWDER COMPOSITIONS AND METHODS OF MANUFACTURING ARTICLES THEREFROM\ | US2011052927A1 | MARTINONI RAFFAELE (CH)\ BOEHLER PAUL (CH)\ STENSON PAUL (CH)\ SPINDLER JURGEN (CH)\ KINKELIN EBERHARD (CH)\ POSSNECKER GERHARD (CH) | VALSPAR SOURCING INC (US) | C08G69/00\ C08L77/00\ C08G69/26\ B32B27/34\ B05D1/12\ C08G69/08\ C08G69/14 |
| 4592 | 3D printing | METHOD FOR HIGH PRECISION PRINTING OF PATTERNS\ | US7755657B2 | MARTINSSON HANS (SE)\ SANDSTROM TORBJORN (SE) | MICRONIC LASER SYSTEMS AB (SE) | G03F7/20\ B41J2/47\ G21K5/10 |
| 4593 | 3D printing | DEVICE AND METHOD FOR PRODUCING A THREE-DIMENSIONAL OBJECT BY MEANS OF A GENERATIVE PRODUCTION METHOD\ | US2006108712A1 | MATTES THOMAS (DE) | EOS ELECTRO OPTICAL SYST (DE) | B29C67/00\ B22F3/105\ B23K26/34\ C04B35/622\ B28B1/14 |
| 4594 | 3D printing | METHOD OF PROVIDING AN IDENTIFIABLE POWDER AMOUNT AND METHOD OF MANUFACTURING AN OBJECT\ | US2010161102A1 | MATTES THOMAS (DE)\ HUEMMELER LUDGER (DE)\ FROHNMAIER MARKUS (DE) | EOS ELECTRO OPTICAL SYST (DE) | G06F17/00 |
| 4595 | 3D printing | FLEXIBLER MEDIZINTECHNISCHER FORMK?RPER SOWIE VERFAHREN ZU DESSEN HERSTELLUNG\[EN] FLEXIBLE MEDICINAL FORM BODY AND METHOD FOR ITS MANUFACTURE\[FR] CORPS DE MOULAGE MéDICAL FLEXIBLE ET SON PROCéDé DE FABRICATION\ | EP2030762A1 | MESARIC EMANUEL (DE) | DELTAMED GMBH (DE) | B29C67/00\ H04R25/00 |
| 4596 | 3D printing | SELECTIVE LASER MELTING RAPID MOLDING EQUIPMENT FOR DIRECTLY FABRICATING LARGE-SIZED PARTS\ | CN202291409U | MING GAO US\ XIAOYAN ZENG (CN)\ ZEMIN WANG (CN)\ KAI GUAN (CN)\ QIANWU HU (CN)\ XIANGYOU LI (CN) | UNIV HUAZHONG SCIENCE TECH (CN) | B22F3/105\ B29C67/04 |
| 4597 | 3D printing | LASER CLADDING QUICK PRECISION MANUFACTURING METHOD AND DEVICE BASED ON LASER MILLING\ | CN102304712A | MINGDI WANG US\ CHENGFA SONG US\ XIUBO LIU US\ KAIBO GUO US\ GEYAN FU (CN)\ SHIHONG SHI (CN) | UNIV SOOCHOW (CN) | C23C24/10 |
| 4598 | 3D printing | POWDERY COMPOSITION OF A POLYMER AND A FLAMEPROOFING AGENT CONTAINING AMMONIUM POLYPHOSPHATE METHOD FOR THE PRODUCTION THEREOF AND MOULDED BODY PRODUCED FROM SAID POWDER\ | US7795339B2 | MONSHEIMER SYLVIA (DE)\ GREBE MAIK (DE)\ BAUMANN FRANZ-ERICH (DE) | DEGUSSA (DE) | C08K3/32 |
| 4599 | 3D printing | LASER SINTER POWDER WITH A METAL SALT AND A FATTY ACID DERIVATIVE PROCESS FOR ITS PRODUCTION AND MOLDINGS PRODUCED FROM THIS LASER SINTER POWDER\ | US2005027050A1 | MONSHEIMER SYLVIA (DE)\ GREBE MAIK (DE)\ BAUMANN FRANZ-ERICH (DE) | DEGUSSA (DE) | B29C67/04\ C08J3/20\ C08K5/101\ C08F22/36\ C08G73/02\ C08J3/00\ C08J3/12\ C08J3/14\ C08J5/00\ C08K3/08\ C08K3/26\ C08K5/04\ C08K5/09\ C08K5/098\ C08K5/10\ C08K5/138\ C08K5/20\ C08L77/00 |
| 4600 | 3D printing | VERFAHREN UND VORRICHTUNG ZUR HERSTELLUNG VON DREIDIMENSIONALEN OBJEKTEN MITTELS MIKROWELLENSTRAHLUNG SOWIE DADURCH HERGESTELLTER FORMK?RPER\[EN] METHOD AND APPARATUS FOR MANUFACTURING THREE DIMENSIONAL OBJECTS USING MICROWAVE RADIATION AND SHAPED BODY PRODUCED ACCORDING TO THIS METHOD\[FR] PROCéDé ET DISPOSITIF POUR LA FABRICATION DES OBJETS TRIDIMENSIONNELS AU MOYEN DE RADIATION à MICRO-ONDES ET PIèCE MOULéE AINSI OBTENUE\ | EP1459871A2 | MONSHEIMER SYLVIA (DE)\ GREBE MAIK (DE)\ BAUMANN FRANZ-ERICH (DE) | DEGUSSA (DE) | B29C35/08\ B29C67/00\ H05B6/74\ H05B6/80 |
| 4601 | 3D printing | VERFAHREN ZUR HERSTELLUNG VON DREIDIMENSIONALEN OBJEKTEN MITTELS MIKROWELLENSTRAHLUNG\ | DE10356193A1 | MONSHEIMER SYLVIA (DE)\ GREBE MAIK (DE)\ BAUMANN FRANZ-ERICH (DE) | DEGUSSA (DE) | B29C67/00 |
| 4602 | 3D printing | PROCESS FOR PRODUCING THREE-DIMENSIONAL OBJECTS BY MEANS OF MICROWAVE RADIATION\ | US7708929B2 | MONSHEIMER SYLVIA (DE)\ GREBE MAIK (DE)\ BAUMANN FRANZ-ERICH (DE) | EVONIK DEGUSSA GMBH (DE) | B29C35/08\ B29C67/00\ B29C41/02\ H05B6/64 |
| 4603 | 3D printing | POLYMER POWDERS FOR SIB PROCESSES\ | US2004137228A1 | MONSHEIMER SYLVIA (DE)\ GREBE MAIK (DE)\ BAUMANN FRANZ-ERICH (DE)\ GERTH CHRISTIAN (DE) | DEGUSSA (DE) | B29C67/00\ B29C67/04\ C08J7/04\ B29B13/08\ B29C65/34 |
| 4604 | 3D printing | POLYMERPULVER FüR SIV-VERFAHREN\ | DE10311446A1 | MONSHEIMER SYLVIA (DE)\ GREBE MAIK (DE)\ BAUMANN FRANZ-ERICH (DE)\ GERTH CHRISTIAN (DE) | DEGUSSA (DE) | B29C67/00\ C08L77/00 |
| 4605 | 3D printing | HOLOGRAPHIC 3D PRINTING APPARATUS AND METHOD OF DRIVING THE SAME\ | US2013050789A1 | MOROZOV ALEXANDER (RU)\ PUTILIN ANDREW (RU)\ PYUN KYOUNG-SEOK (KR)\ SUNG GEE-YOUNG (KR) | SAMSUNG ELECTRONICS CO LTD (KR)\ PYUN KYOUNG-SEOK (KR)\ PUTILIN ANDREW (RU)\ MOROZOV ALEXANDER (RU)\ SUNG GEE-YOUNG (KR) | G02B5/32\ G03H1/04 |
| 4606 | 3D printing | GRADUALLY LAYERED CLOCK\ | CN202649687U | MOU SHANKE US | CT CLOCK IND CO LTD US | G04B19/10\ G04B19/14\ G04B45/00 |
| 4607 | 3D printing | METHOD AND APPARATUS FOR FAST GENERATION HOLOGRAM\ | US2013100510A1 | NAM DONG KYUNG (KR)\ LEE SEOK (KR)\ WEY HO CHEON (KR) | SAMSUNG ELECTRONICS CO LTD (KR)\ NAM DONG KYUNG (KR)\ LEE SEOK (KR)\ WEY HO CHEON (KR) | G03H1/08 |
| 4608 | 3D printing | BINDER REMOVAL IN SELECTIVE LASER SINTERING\ | US2004081573A1 | NEWELL KENNETH J (US) | 3D SYSTEMS INC (US) | B22F1/00\ B22F3/26\ B22F3/11 |
| 4609 | 3D printing | INFILTRATED ALUMINUM PREFORMS\ | US2005161189A1 | NEWELL KENNETH J (US)\ SERCOMBE TIMOTHY B (AU)\ SCHMIDT KRIS A (US)\ SCHAFFER GRAHAM B (AU) | UNIV QUEENSLAND (AU) | B22F3/105\ B22F3/26\ C22C1/04 |
| 4610 | 3D printing | WETTING AGENT FOR INFILTRATED ALUMINUM PREFORMS\ | US6848494B2 | NEWELL KENNETH J (US)\ ZENUK CHARLES H (US) | 3D SYSTEMS INC (US) | B22F1/00\ B22F3/26\ B22D19/14 |
| 4611 | 3D printing | OPTICAL THREE-DIMENSIONAL SHAPING METHOD AND APPARATUS THEREFOR\ | JP2006043953A | ONO TOMOYA US | NABTESCO CORP (JP) | B29C67/00 |
| 4612 | 3D printing | METHOD FOR MANUFACTURING THREE-DIMENSIONAL SHAPED ARTICLE\ | JP2005007572A | OSAWA SADAO US\ KASAI KIYOSUKE US | FUJI PHOTO FILM CO LTD (US) | B29C67/00 |
| 4613 | 3D printing | CONTINUOUS CALIBRATION OF A NON-CONTACT THERMAL SENSOR FOR LASER SINTERING\ | US6930278B1 | PARTANEN JOUNI P (US)\ CHUNG TAE MARK (US) | 3D SYSTEMS INC (US) | B29C35/08\ B29C67/00\ B23K26/34\ G01J5/52 |
| 4614 | 3D printing | SYSTEM AND RESIN FOR RAPID PROTOTYPING\ | US2011195237A1 | PATEL RANJANA C (GB)\ CHAPELAT CAROLE (FR)\ DOBLER BEAT (CH)\ FRANTZ RICHARD (FR)\ LAGREF JEAN-JACQUES (CH)\ RHODES MICHAEL (CH)\ CHERKAOUI ZOUBAI M (CH) | HUNTSMAN INT LLC (US) | G03F7/20\ G03F7/004\ B32B3/10\ G03B27/54 |
| 4615 | 3D printing | REPLAY OPTICS FOR HOLOGRAPHIC DISPLAYS\ | US2004246547A1 | PAYNE DOUGLAS (GB) | BOEING CO (US) | G03H1/22\ G02B5/32 |
| 4616 | 3D printing | GAS TURBINE ENGINE BLADE WITH INNER MESHED STRUCTURE\ | CN103470312A | PENG HUI US\ GUO HONGBO US\ GONG SHENGKAI US\ XU HUIBIN US | UNIV BEIHANG US | F01D5/18 |
| 4617 | 3D printing | OPTICAL SYSTEM AND METHOD FOR INCREASING IMAGE RESOLUTION AND/OR DITHERING IN PRINTING APPLICATIONS\ | US2006176362A1 | PENN STEVEN M (US)\ MARSHALL STEPHEN W (US)\ PORADISH FRANK J (US)\ BOMMERSBACH WILLIAM M (US)\ WHITNEY DONALD C (US)\ WEDEMEIER FREDERICK C (US)\ CARVER ROGER S (US)\ POWELL DONALD A (US) | PENN STEVEN M (US)\ MARSHALL STEPHEN W (US)\ PORADISH FRANK J (US)\ BOMMERSBACH WILLIAM M (US)\ WHITNEY DONALD C (US)\ WEDEMEIER FREDERICK C (US)\ CARVER ROGER S (US)\ POWELL DONALD A (US) | B41J2/47 |
| 4618 | 3D printing | METHOD OF PRINTING HOLOGRAPHIC 3D IMAGE\ | US2013050790A1 | PYUN KYOUNG-SEOK (KR)\ SUNG GEE-YOUNG (KR) | SAMSUNG ELECTRONICS CO LTD (KR)\ PYUN KYOUNG-SEOK (KR)\ SUNG GEE-YOUNG (KR) | G03H1/26 |
| 4619 | 3D printing | MULTIDIMENSIONAL LASER PRINTING DEVICE AND METHOD CAPABLE OF MANUFACTURING LARGE-ANGLE CANTILEVER STRUCTURE WORKPIECE\ | CN103394693A | QI HAIBO US\ QI FANGJUAN US\ ZHENG ZHEWEN US\ ZHENG MENGMENG US\ ZHANG YANG US | UNIV SHIJIAZHUANG TIEDAO US | B22F3/105 |
| 4620 | 3D printing | DIGITAL/OPTICAL HYBRID PRINTER UTILIZING REFLECTIVE SPATIAL LIGHT MODULATION TECHNOLOGY\ | US2004201864A1 | RAMANUJAN SUJATHA (US) | EASTMAN KODAK CO (US) | G02F1/13\ B41J2/445\ G03B27/32\ G03B27/52\ H04N1/23 |
| 4621 | 3D printing | HONEYCOMB CORES FOR AEROSPACE APPLICATIONS\ | US2004048027A1 | RICE CHRISTIAN V (US)\ POLUS JEFFREY E (US)\ HAYES MICHAEL W (US)\ DEGRANGE JEFFREY E (US) | G NAUCHNOE UCHREZHDENIE I PORO (BY)\ G NAUCHNOE UCHREZHDENIE I FIZ (BY) | B29C67/00 |
| 4622 | 3D printing | PROCESS FOR THE PRODUCTION OF ARTICLES MADE OF A GAMMA-PRIME PRECIPITATION-STRENGTHENED NICKEL-BASE SUPERALLOY BY SELECTIVE LASER MELTING (SLM)\ | US2013228302A1 | RICKENBACHER LUKAS EMANUEL (CH)\ SPIERINGS ADRIAAN BERNARDUS (CH) | ALSTOM TECHNOLOGY LTD (CH) | B22F3/00 |
| 4623 | 3D printing | MULTIPLEXED OPTICAL SCANNER TECHNOLOGY\ | US6687036B2 | RIZA NABEEL AGHA (US) | NUONICS INC (US) | G02B26/10 |
| 4624 | 3D printing | AUTOSTEREOSCOPIC DISPLAY SYSTEM\ | US8587641B2 | ROTH ALEXANDER (US) | ROTH ALEXANDER (US) | H04N13/04\ H04N9/47 |
| 4625 | 3D printing | NEAR-NET-SHAPE METHOD FOR POROUS METAL PART\ | CN101722306A | RUIDI LI US\ ZHIGANG WANG US\ YUSHENG SHI (CN)\ QINGSONG WEI (CN) | UNIV HUAZHONG SCIENCE TECH (CN) | B22F3/11 |
| 4626 | 3D printing | METHOD OF PART SINTERING BY LASER LAYER-BY-LAYER SYNTHESIS\ | RU2450891C1 | RYTSEV SERGEJ BORISOVICH (RU)\ FILIPPOV EVGENIJ IVANOVICH (RU)\ TIMOFEEV ANATOLIJ IVANOVICH (RU)\ LEVIN ARKADIJ MORDUKHOVICH (RU) | ROSSIJSKAJA FEDERATSIJA OT IM KOTOROJ VYSTUPAET MIN PROMY I TORGOVLI RF MINPROMTORG ROSSII (RU) | B22F3/105\ B23K26/00 |
| 4627 | 3D printing | POLYSTYRELEN POWDER USABLE FOR THREE-DIMENSIONAL ARTICLE PRODUCED BY SELECTIVE LASER SINTERING\ | JP2002265619A | SAKAMOTO KATSUHIKO US\ TAKAYAMA HIROKAZU US | EBARA CORP US | B29C67/00\ B29C67/04\ C08J3/12\ B22C7/02\ B29K25/00 |
| 4628 | 3D printing | GRIP FIXTURE SEMIFINISHED PART AND METHOD FOR PROVIDING DENTAL PROSTHESES\[FR] MONTURE DE PRéHENSION PIèCE SEMI-FINIE ET PROCéDé DE FOURNITURE DE PROTHèSES DENTAIRES\ | WO2011124474A1 | SANDI ANDREA (IT) | 3D FAST S R L (IT)\ SANDI ANDREA (IT) | A61C13/00\ A61C13/12 |
| 4629 | 3D printing | METHOD FOR HIGH PRECISION PRINTING OF PATTERNS\ | US2004251430A1 | SANDSTROM TORBJORN (SE) | MICRONIC LASER SYSTEMS AB (SE) | G03F7/20 |
| 4630 | 3D printing | METHOD AND DEVICE FOR PRODUCING A 3D OBJECT BY MEANS OF A GENERATIVE 3D-METHOD\ | US8414281B2 | SCHLEISS PETER (CH)\ LEVY GIDEON (CH) | EOS ELECTRO OPTICAL SYST (DE)\ SCHLEISS PETER (CH)\ LEVY GIDEON (CH) | B29C35/08\ B29C67/00 |
| 4631 | 3D printing | METHOD AND DEVICE FOR RENDERING AND GENERATING COMPUTER-GENERATED VIDEO HOLOGRAMS\ | US2011096145A1 | SCHWERDTNER ALEXANDER (DE) | SEE REAL TECHNOLOGIES S A (LU) | G06T15/00\ H04N5/89 |
| 4632 | 3D printing | METHOD FOR GENERATING VIDEO HOLOGRAMS IN REAL-TIME FOR ENHANCING A 3D-RENDERING GRAPHIC PIPELINE\ | US2010149609A1 | SCHWERDTNER ALEXANDER (DE) | SEEREAL TECHNOLOGIES SA (LU) | G03H1/08 |
| 4633 | 3D printing | ANALYTICAL METHOD FOR COMPUTING VIDEO HOLOGRAMS IN REAL TIME\ | US2010149312A1 | SCHWERDTNER ALEXANDER (DE) | SEEREAL TECHNOLOGIES SYRDALL (LU) | H04N13/00\ G03H1/10 |
| 4634 | 3D printing | A HOLOGRAPHIC DISPLAY COMMUNICATIONS SYSTEM\ | GB2482076A | SCHWERDTNER ALEXANDER (DE)\ KROLL BO (GB)\ MISSBACH ROBERT (DE) | SEEREAL TECHNOLOGIES SA (LU) | G03H1/22\ G09G3/20\ G02B26/08\ H04M7/00 |
| 4635 | 3D printing | VERFAHREN ZUM GENERIEREN COMPUTER-GENERIERTER VIDEOHOLOGRAMME IN ECHTZEIT MITTELS TEILHOLOGRAMMEN\ | DE102006042324B4 | SCHWERDTNER ALEXANDER (DE)\ ZSCHAU ENRICO (DE)\ KROELL BO (GB)\ SCHWERDTNER ARMIN DR (DE) | SEEREAL TECHNOLOGIES SA (LU) | G03H1/08 |
| 4636 | 3D printing | METHOD AND DEVICE FOR COMPUTING COMPUTER-GENERATED VIDEO HOLOGRAMS\ | US7636184B2 | SCHWERDTNER ARMIN (DE) | SEEREAL TECHNOLOGIES GMBH (DE) | G03H1/08\ G03H1/16 |
| 4637 | 3D printing | HOLOGRAPHIC RECONSTRUCTION SYSTEM WITH AN ARRANGEMENT OF CONTROLLABLE MICROCELLS\ | US2010194854A1 | SCHWERDTNER ARMIN (DE)\ KROLL BO (GB)\ FUTTERER GERALD (DE) | SEEREAL TECHNOLOGIES SA (LU) | G03H1/22\ H04N5/89 |
| 4638 | 3D printing | HOLOGRAPHIC RECONSTRUCTION SYSTEM WITH A TRACKING DEVICE FOR THE RECONSTRUCTION\ | US2010149313A1 | SCHWERDTNER ARMIN (DE)\ KROLL BO (GB)\ OLAYA JEAN-CHRISTOPHE (DE) | KROLL BO (GB)\ SCHWERDTNER ARMIN (DE)\ OLAYA JEAN-CHRISTOPHE (DE) | H04N13/00\ H04N7/18 |
| 4639 | 3D printing | METHOD FOR GENERATING VIDEO HOLOGRAMS IN REAL TIME BY MEANS OF SUBHOLOGRAMS\ | US2010073744A1 | SCHWERDTNER ARMIN (DE)\ KROLL BO (GB)\ ZSCHAU ENRICO (DE) | SEEREAL TECHNOLOGIES SA (LU) | G03H1/08 |
| 4640 | 3D printing | DEVICE FOR HOLOGRAPHIC RECONSTRUCTION OF THREE-DIMENSIONAL SCENES\ | US8526088B2 | SCHWERDTNER ARMIN (DE)\ LEISTER NORBERT (DE)\ HAUSSLER RALF (DE) | SEEREAL TECHNOLOGIES GMBH (DE)\ LEISTER NORBERT (DE)\ HAUSSLER RALF (DE)\ SCHWERDTNER ARMIN (DE) | G02B5/32\ G03H1/08 |
| 4641 | 3D printing | METHOD FOR GENERATING VIDEO HOLOGRAMS FOR A HOLOGRAPHIC DISPLAY DEVICE WITH RANDOM ADDRESSING\ | US2010149610A1 | SCHWERDTNER ARMIN (DE)\ SCHWERDTNER ALEXANDER (DE) | SEEREAL TECHNOLOGIES SA (LU) | G03H1/22 |
| 4642 | 3D printing | METHOD FOR RENDERING AND GENERATING COLOR VIDEO HOLOGRAMS IN REAL TIME\ | US2010149314A1 | SCHWERDTNER ARMIN (DE)\ SCHWERDTNER ALEXANDER (DE) | SEEREAL TECHNOLOGIES SA (LU) | H04N15/00 |
| 4643 | 3D printing | METHOD FOR SIMULATION OF LASER MATERIAL DEPOSITION\ | US6813533B1 | SEMAK VLADIMIR V (US) | SEEREAL TECHNOLOGIES SA (LU) | G06F17/50\ B23K26/34\ G06F19/00 |
| 4644 | 3D printing | INFILTRATED ALUMINUM PREFORMS\ | US6823928B2 | SERCOMBE TIMOTHY BARRY (AU)\ SCHAFFER GRAHAM BARRY (AU) | UNIV QUEENSLAND (AU) | B22F3/105\ B22F3/24\ B22F3/02\ B22F3/10\ B22D19/00\ B22D21/04\ B22F3/26\ C22C1/04\ C22C9/01\ C22C21/00\ C22C21/02\ C22C21/06\ C22C21/12 |
| 4645 | 3D printing | INFILTRATED ALUMINUM PREFORMS\ | US7036550B2 | SERCOMBE TIMOTHY BARRY (AU)\ SCHAFFER GRAHAM BARRY (AU)\ SCHMIDT KRIS ALAN (US)\ NEWELL KENNETH J (US) | UNIV QUEENSLAND (AU) | B22F3/105\ B22F3/24\ B22F3/02\ B22F3/10\ B22D19/00\ B22D21/04\ B22F3/14\ B22F3/15\ B22F3/26\ C22C1/04\ C22C1/10\ C22C9/01\ C22C21/00\ C22C21/02\ C22C21/06\ C22C21/12\ C22C32/00 |
| 4646 | 3D printing | LASER RAPID-FORMING METHOD BASED ON CONTOUR SCANNING OF COATED POWDER MATERIALS\ | CN100386173C | SHAN YAO (CN)\ FENG ZENG (CN)\ CHANGKE YE (CN)\ BAOQING CHEN (CN) | UNIV DALIAN TECH (CN) | G06F17/50\ B22F3/105\ B23K26/00\ B29C67/02\ G05B15/00 |
| 4647 | 3D printing | RESIN MIXED LIQUOR COATING BASED ON THREE-DIMENSIONAL PRINTING RESIN MOULD PRINTING TECHNOLOGY\ | CN103342942A | SHANG XIAOFENG US\ SUN YONGHE US\ SHANG JIN US\ YANG SHENBO US\ LIU GUANGKE US | JILIN XUFENG LASER TECHNOLOGY CO LTD US | C09D163/00 |
| 4648 | 3D printing | METHOD OF PREPARING ORGANIZATION BRACKET PERFORMING SELECTIVITY LASER SINTERING BY USING MACROMOLECULE MICROSPHERE\ | CN101138651A | SHENGMIN ZHANG (CN)\ JIAOJIAO ZHOU (CN)\ ZHIYE QIU (CN) | UNIV HUAZHONG SCIENCE TECH (CN) | A61F2/28\ A61L27/40\ A61L27/18\ A61L27/12 |
| 4649 | 3D printing | METHOD FOR NEAR-PURIFICATION FORMATION OF PARTS\ | CN1861296A | SHI YUSHENG HUANG (CN) | HUAZHONG SCIENCE & TECH UNIV (CN) | B22F3/105\ B22F3/16 |
| 4650 | 3D printing | METHOD FOR RAPIDLY MANUFACTURING INJECTION-MOULD INSERT WITH FOLLOW-COOLING PASSAGEWAY\ | CN1850396A | SHI YUSHENG HUANG (CN) | UNIV HUAZHONG SCIENCE TECH (CN) | B22F3/105\ B22F3/24\ B29C33/38 |
| 4651 | 3D printing | REMANUFACTURING METHOD FOR AUTOMATIC LASER CLADDING OF GRAY CAST IRON CYLINDER COVER\ | CN102615431A | SHIYUN DONG US\ BINSHI XU US\ SHIXING YAN US\ YUJIANG WANG US\ JINXIANG FANG US | ARMORED FORCES ENGINEERING ACADEMY OF PLA US | C23C24/10\ B23K33/00\ B23K35/30 |
| 4652 | 3D printing | PREPARATION METHOD OF BIONIC ARTIFICIAL BONE WITH MULTISTAGE [MICROMETER/NANOMETER] PORE STRUCTURE\ | CN103462729A | SHUAI CIJUN US\ PENG SHUPING US\ GAO CHENGDE US\ HU HUANLONG US | UNIV CENTRAL SOUTH US | A61F2/28\ A61L27/56\ A61L27/18 |
| 4653 | 3D printing | SELECTIVE LASER SINTERING INDIRECT MANUFACTURING METHOD OF INDIVIDUATION TONGUE SIDE ORTHODONTIC BRACKET\ | CN101284302A | SHUFAN WANG (CN)\ YONGQIANG YANG (CN)\ DAWEI CHENG (CN) | UNIV SOUTH CHINA TECH (CN) | A61C7/14\ G05B19/4097\ A61B6/14\ B22C9/04 |
| 4654 | 3D printing | METHOD OF MAKING AN OPTICAL MANIFOLD\ | US6655848B2 | SIMMONS RICHARD L (US)\ SHOEMAKER CURTIS L (US)\ KORNELE KENNETH L (US) | 3M INNOVATIVE PROPERTIES CO (US) | G02B6/36\ G02B6/38\ G02B6/44 |
| 4655 | 3D printing | METHOD OF MAKING A THREE DIMENSIONAL OPTICAL CIRCUIT\ | US6549710B2 | SIMMONS RICHARD L (US)\ SHOEMAKER CURTIS L (US)\ KORNELE KENNETH L (US)\ LODER HARRY A (US)\ LURIE EDWARD B (US) | 3M INNOVATIVE PROPERTIES CO (US) | G02B6/36\ G02B6/38\ G02B6/44\ G02B6/04\ G02B6/40\ G02B6/43 |
| 4656 | 3D printing | CROSS BLADE FLEXURE PIVOT AND METHODS OF USE THEREOF\ | US2013308997A1 | SMITH WAYNE A (US) | FLEX BEARING CO INC C (US) | F16C11/12 |
| 4657 | 3D printing | PROCESS FOR PRODUCING A 3-DIMENSIONAL COMPONENT BY SELECTIVE LASER MELTING (SLM)\ | US2012018115A1 | STANKOWSKI ALEXANDER (CH)\ RICKENBACHER LUKAS (CH)\ HOEVEL SIMONE (CH) | HOEVEL SIMONE (CH)\ STANKOWSKI ALEXANDER (CH)\ RICKENBACHER LUKAS (CH) | B22D25/02\ B22D23/06\ B22D27/02 |
| 4658 | 3D printing | METHOD OF APPLYING MULTIPLE MATERIALS WITH SELECTIVE LASER MELTING ON A 3D ARTICLE\ | US2011106290A1 | STANKOWSKI ALEXANDER (CH)\ RICKENBACHER LUKAS (CH)\ HOEVEL SIMONE (CH) | HOEVEL SIMONE (CH)\ STANKOWSKI ALEXANDER (CH)\ RICKENBACHER LUKAS (CH) | G06F17/00 |
| 4659 | 3D printing | MAKING METHOD OF RAPID FORMING WAX PATTERN OF DIFFUSER\ | CN102019346B | SUCHUN BAI US\ TIEJUN WANG US\ XIAOXUAN WANG US\ MINZHENG ZHANG US\ JING FEI US | SHENYANG LIMING AERO ENGINE US | B22C7/02 |
| 4660 | 3D printing | APPARATUS AND METHOD FOR DISPLAYING HOLOGRAPHIC IMAGE USING COLLIMATED DIRECTIONAL BLACKLIGHT UNIT\ | US2012105929A1 | SUNG GEE YOUNG (KR)\ KIM YUN TAE (KR)\ NAM DONG KYUNG (KR)\ LEE SEUNG KYU (KR) | SAMSUNG ELECTRONICS CO LTD (KR)\ SUNG GEE YOUNG (KR)\ KIM YUN TAE (KR)\ NAM DONG KYUNG (KR)\ LEE SEUNG KYU (KR) | G03H1/08 |
| 4661 | 3D printing | LASER-PRODUCED IMPLANTS\ | US2008004709A1 | SUTCLIFFE CHRISTOPHER J (GB)\ JONES ERIC (IE)\ O'NEILL WILLIAM (GB) | HOWMEDICA OSTEONICS CORP (US) | A61F2/38 |
| 4662 | 3D printing | MULTICHANNEL INTELLIGENT MICRO-SPRAYING POWDER FEED LASER CLADDING APPARATUS\ | CN101407913A | TAO WANG (CN)\ JIANQUAN YAO (CN)\ XIAOWEN LIU (CN)\ FUZENG LI (CN)\ KAIKAI YANG (CN)\ ZHONGWEI JIN (CN)\ CHAONAN ZHU (CN)\ JING LU (CN) | WUXI HAOBO OPTOELECTRONIC CO L (CN) | C23C24/10 |
| 4663 | 3D printing | MULTICHANNEL INTELLIGENT MICRO JET POWDER-FEEDING CLADDING DEVICE\ | CN201132854Y | TAO WANG (CN)\ JIANQUAN YAO (CN)\ XIAOWEN LIU (CN)\ FUZENG LI (CN)\ KAIKAI YANG (CN)\ ZHONGWEI JIN (CN)\ CHAONAN ZHU (CN)\ JING LU (CN) | WUXI HOPE ELECTRONIC CO LTD (CN) | C23C24/10 |
| 4664 | 3D printing | MEASUREMENT STANDARD SPECIMEN OF NANOMETER SCALE AND METHOD OF CALIBRATING SCANNING TYPE MICROSCOPE USING THE MEASUREMENT STANDARD SPECIMEN OF NANO METER SCALE\ | JP2010078582A | TOKUDA NORIO US\ YAMAZAKI SATOSHI US\ KURIHARA KENICHI US\ GONTA SATOSHI US\ OTA TOSHITAKA US | NAT INST OF ADVANCED IND SCIEN US | G01Q40/02\ G01B1/00\ G01B3/30\ G01B15/00 |
| 4665 | 3D printing | PROJECTION DISPLAY SYSTEM AND METHOD WITH MULTIPLE CONVERTIBLE DISPLAY MODES\ | TW201344243A | TSAO CHE-CHIH (TW) | TSAO CHE-CHIH (TW) | H04N13/00\ G02B27/22\ G02B27/18 |
| 4666 | 3D printing | PATTERN PROJECTION TECHNIQUES FOR VOLUMETRIC 3D DISPLAYS AND 2D DISPLAYS\ | US6961045B2 | TSAO CHE-CHIH (US) | TSAO CHE-CHIH (TW) | H04N5/74\ G02B27/22\ H04N15/00\ H04N9/31\ G03B35/18\ G02F1/13\ G02F1/13357\ G02B27/18\ G03B21/00\ H04N17/00 |
| 4667 | 3D printing | DATA WRITING METHODS FOR VOLUMETRIC 3D DISPLAYS\ | US7714803B2 | TSAO CHE-CHIH (US) | TSAO CHE-CHIH (TW) | G09G5/00 |
| 4668 | 3D printing | SECTION WRITING METHOD FOR VOLUMETRIC 3D DISPLAYS\ | US2007165024A1 | TSAO CHE-CHIH (US) | TSAO CHE-CHIH (TW) | G06T17/00 |
| 4669 | 3D printing | PROJECTION DISPLAY SYSTEM AND METHOD WITH MULTIPLE CONVERTIBLE DISPLAY MODES\ | US2012092337A1 | TSAO CHE-CHIH (US) | TSAO CHE-CHIH (US) | G02B27/22\ G06T15/00\ G03B21/14\ G06F3/041 |
| 4670 | 3D printing | SECTION WRITING METHOD FOR VOLUMETRIC 3D DISPLAY\ | JP2009015271A | TSUAO CHE-CHII US | ACT RES CORP US | G02B27/22 |
| 4671 | 3D printing | DATA WRITING METHOD OF VOLUME 3D DISPLAY\ | JP2007188028A | TSUAO CHE-CHII US | ACT RES CORP US | G09G3/20\ G09G3/34\ G09G3/36 |
| 4672 | 3D printing | KARL MAYER CLOTH\[FR] TOILE KARL MAYER\ | WO2006100749A1 | TSUJIKAWA KEIZO (JP)\ TSUJIKAWA TOSHIZO (JP) | TSUJIKAWA SENKO CO LTD (JP)\ TSUJIKAWA KEIZO (JP)\ TSUJIKAWA TOSHIZO (JP) | A47G9/06\ D04B1/02\ D04B21/02 |
| 4673 | 3D printing | VASCULARIZED TISSUE REGENERATION MATRICES FORMED BY SOLID FREE FORM FABRICATION TECHNIQUES\ | US6176874B1 | VACANTI JOSEPH P (US)\ CIMA MICHAEL J (US)\ CIMA LINDA G (US) | CHILDRENS MEDICAL CENTER (US)\ MASSCHUSETTS INST OF TECHNOLOG (US) | B29C67/00\ A61L27/00\ A61F2/00\ A61F2/02\ A61F2/06\ A61F2/10\ A61F2/28\ A61F2/30\ A61K9/00\ A61K9/16\ A61L27/38\ A61L27/40\ A61L27/56\ A61L27/60\ B82B1/00 |
| 4674 | 3D printing | VORRICHTUNG ZUR HERSTELLUNG VON WERKSTüCKEN DURCH BEAUFSCHLAGEN VON PULVERSCHICHTEN MIT ELEKTROMAGNETISCHER STRAHLUNG ODER TEILCHENSTRAHLUNG\[EN] DEVICE FOR MANUFACTURING WORKPIECES BY APPLYING POWDER LAYERS WITH ELECTROMAGNETIC RADIATION OR PARTICLE RADIATION\[FR] DISPOSITIF DE FABRICATION DE PIèCES USINéES EN INJECTANT DES COUCHES DE POUDRE à L'AIDE D'UN RAYONNEMENT éLECTROMAGNéTIQUE OU D'UN RAYONNEMENT DE PARTICULES\ | EP2386404A1 | WIESNER ANDREAS (DE)\ SCHOENEBORN HENNER DIPL-ING (DE) | SLM SOLUTIONS GMBH (DE) | B29C67/00\ B22F3/105 |
| 4675 | 3D printing | OPTICAL IRRADIATION DEVICE FOR A SYSTEM FOR PRODUCING THREE-DIMENSIONAL WORK PIECES BY IRRADIATING POWDER LAYERS OF A POWDERED RAW MATERIAL USING LASER RADIATION\ | US2013134637A1 | WIESNER ANDREAS (DE)\ SCHWARZE DIETER (DE)\ SCHOENEBORN HENNER (DE) | SLM SOLUTIONS GMBH (DE) | B29C67/00 |
| 4676 | 3D printing | HEATING UNIT\ | US2006249499A1 | WINKLER WOLFGANG A (DE) | WINKLER WOLFGANG A (DE) | B60L1/02 |
| 4677 | 3D printing | HIGH-RESOLUTION MICROSCOPE AND METHOD FOR DETERMINING THE TWO- OR THREE-DIMENSIONAL POSITIONS OF OBJECTS\ | US2013010098A1 | WOLLESCHENSKY RALF (DE)\ KALKBRENNER THOMAS (DE) | KALKBRENNER THOMAS (DE)\ WOLLESCHENSKY RALF (DE) | H04N7/18 |
| 4678 | 3D printing | METHOD FOR LASER ENGINEERED NET SHAPING OF AL2O3-ZRO2 EUTECTIC CERAMIC STRUCTURE\ | CN103193486A | WU DONGJIANG US\ ZHANG YAOLEI US\ SUN BEI US\ NIU FANGYONG US\ MA GUANGYI US\ GUO DONGMING US | UNIV DALIAN TECH (CN) | C04B35/622\ C04B35/10 |
| 4679 | 3D printing | THREE-DIMENSIONAL CIRCUIT MANUFACTURING PROCESS AND COMPOSITE COMPONENTS OF LASER PLASTIC MATERIAL AND MANUFACTURING METHOD\ | CN101859613A | XIAOSHU XU US\ HONGWEI ZHOU US | HUNAN MEINA TECHNOLOGY CO LTD US | B29C45/00\ C08K3/26\ H01B13/00\ C08K5/41\ C08K7/20\ C08K13/02\ C08K13/04\ C08L101/00\ H01B1/00\ H01B17/64 |
| 4680 | 3D printing | MULTI-CHANNEL HOLOGRAPHIC RECORDING METHOD FOR DIGITAL INFORMATION STEREO DISPLAY\ | CN101661265A | XIAOYAN YU US\ XIANWEI RONG US\ BIAO ZHANG US\ CHENGXIANG GUAN US\ WENJUN SUN US\ QIANGHUA LI US\ XUHONG WANG US | UNIV HARBIN NORMAL US | G03H1/12 |
| 4681 | 3D printing | LASER CLADDING MACHINING HEAD\ | CN202643843U | XIE XIA US\ WANG RUIZE US\ FAN WEIGUO US\ SUN XIAOYU US | SHENYANG SIASUN ROBOT & AUTOMA (CN) | C23C24/10 |
| 4682 | 3D printing | METHOD FOR PREPARING TEMPERATURE COMPENSATION CONTROLLABLE NON-HOMOGENEOUS MATERIAL PART BASED ON SLS (SELECTIVE LASER SINTERING) FORMING\ | CN102120260A | XINGTAO DONG US\ CHUNBO WANG US\ WENJUN LI US\ LEI SUN US | UNIV ZHEJIANG TECHNOLOGY US | B22F3/105 |
| 4683 | 3D printing | PREPARATION METHOD FOR PART MADE OF CONTROLLABLE HETEROGENEOUS MATERIALS BASED ON SLS (SELECTIVE LASER SINTERING) FORMING PROCESS\ | CN102000821A | XINGTAO DONG US\ CHUNBO WANG US\ WENJUN LI US\ LEI SUN US | UNIV ZHEJIANG TECHNOLOGY US | B22F3/105 |
| 4684 | 3D printing | PREHEATING TEMPERATURE CONTROLLABLE METHOD FOR PREPARING HETEROGENEOUS MATERIAL COMPONENTS BASED ON SLS PROTOTYPING\ | CN101985176A | XINGTAO DONG US\ CHUNBO WANG US\ WENJUN LI US\ LEI SUN US | UNIV ZHEJIANG TECHNOLOGY US | B22F3/105 |
| 4685 | 3D printing | LASER ENERGY CONTROLLABLE METHOD FOR PREPARING HETEROGENEOUS MATERIAL COMPONENTS BASED ON SLS PROTOTYPING\ | CN101985175A | XINGTAO DONG US\ CHUNBO WANG US\ WENJUN LI US\ LEI SUN US | UNIV ZHEJIANG TECHNOLOGY US | B22F3/105 |
| 4686 | 3D printing | METHOD FOR MAKING FOUR TYPES OF BONE MODELS BY SLS (SELECTIVE LASER SINTERING)\ | CN102407331A | XINGTAO DONG US\ HAISONG PAN US\ JIA YOU US | HANGZHOU LIUWEI DENTAL MEDICAL TECHNOLOGY CO LTD US | B22F3/105 |
| 4687 | 3D printing | ONCE-FORMING DIRECT MAKING METHOD OF ASSEMBLY-FREE MECHANICAL MECHANISM AND APPLICATION THEREOF\ | CN101941070A | XUBIN SU US\ DI WANG US\ YONGQIANG YANG (CN) | UNIV SOUTH CHINA TECH (CN) | B22F5/00\ B22F3/115 |
| 4688 | 3D printing | SYSTEM FOR DIAGNOSING BLOODFLOW CHARACTERISTICS METHOD THEREOF AND COMPUTER SOFTWARE PROGRAM\ | US2015032435A1 | YAGI TAKANOBU (JP)\ PARK YOUNG-KWANG (JP) | EBM CORP (JP)\ YAGI TAKANOBU (JP)\ PARK YOUNG-KWANG (JP) | G06T17/00\ G06F19/00\ A61B5/00\ A61B5/026 |
| 4689 | 3D printing | DIRECT MANUFACTURING METHOD OF SELECTIVE LASER MELTING OF CUSTOMIZED TONGUE-SIDE ORTHODONTIC SUPPORT GROOVES\ | US2010324715A1 | YANG YONGQIANG (CN)\ WANG SHUFAN (CN) | YANG YONGQIANG (CN)\ WANG SHUFAN (CN) | G06F17/50\ G06F19/00 |
| 4690 | 3D printing | METHOD FOR PREPARING ELECTRIC SPARK COPPER BASE TOOL ELECTRODE BY SELECTIVE LASER SINTERING AND MOULDING TECHNOLOGY\ | CN101695779A | YANLU LIU US\ JINGANG CHEN US\ JIANG LIU US | YANLU LIU US\ JINGANG CHEN US\ JIANG LIU US | B22F3/105\ B23H1/04 |
| 4691 | 3D printing | METHOD FOR PREPARING POROUS TANTALUM MEDICAL IMPLANT MATERIAL THROUGH SELECTIVE LASER SINTERING FORMING\[FR] PROCéDé DE PRéPARATION D'UN MATéRIAU D'IMPLANT MéDICAL EN TANTALE POREUX AU MOYEN D'UNE FORMATION PAR FRITTAGE LASER SéLECTIF\ | WO2013113249A1 | YE LEI (CN) | CHONGQING RUNZE PHARMACEUTICAL COMPANY LTD (CN) | A61L27/56\ B22F3/11\ C22C1/08\ A61L27/04\ C22C27/02 |
| 4692 | 3D printing | FULLY AUTOMATIC STEPPING DIGITAL LITHOGRAPHY DEVICE\ | CN102163006A | YIQING GAO US\ PINGQI WANG US\ NINGNING LUO US\ YONGQING GONG US\ GANG LEI US\ YUFANG FU US\ MENGCHAO XIAO US\ WENDONG ZOU US | UNIV NANCHANG HANGKONG US | G03F7/20 |
| 4693 | 3D printing | OPTIMIZATION METHOD OF LIGHT DISTRIBUTION FOR UNDERTAKING COLOR THREE-DIMENSIONAL HOLOGRPHIC IMAGE BY USING UNIFORM SCATTERING MEDIA AND DISPLAY SYSTEM OF COLOR THREE-DIMENSIONAL HOLOGRPHIC IMAGE\ | CN102566390A | YONGTIAN WANG US\ JIA JIA US\ JUAN LIU US\ YIJIE PAN US\ XIN LI US\ JINGHUI XIE US | BEIJING INST TECHNOLOGY US | G03H1/22\ G02B27/10\ G03H1/12\ G03H1/04 |
| 4694 | 3D printing | DUAL HOLOGRAPHY 3D DISPLAY DEVICE\ | US2013120525A1 | YOON MINSUNG (KR)\ KIM SUNWOO (KR)\ SHIN MINYOUNG (KR) | LG DISPLAY CO LTD (KR) | G03H1/26 |
| 4695 | 3D printing | METAL POWDER FOR METAL LIGHT MOLD MOLDING METHOD OF PRODUCING THE SAME METHOD OF PRODUCING MOLDING WITH THREE-DIMENSIONAL SHAPE BY METAL LIGHT MOLDING AND METAL LIGHT MOLDING\ | JP2004277877A | YOSHIDA TOKUO US\ KAMINAGA SHUJI US\ AZUMA YOSHIKAZU US\ TOUGEYAMA HIROHIKO US\ FUWA ISAO (JP)\ IWANAGA TOSHIHARU (JP)\ ABE SATOSHI (JP)\ TAKENAMI MASATAKA (JP)\ SHIMIZU TAKASHI (JP) | MATSUSHITA ELECTRIC WORKS LTD (JP) | B22F3/105\ B22F1/00 |
| 4696 | 3D printing | THREE DIMENSIONAL DISPLAYS\[FR] ECRANS TRIDIMENSIONNELS\ | WO2005120084A2 | YOUNG RUPERT CHARLES DAVID (GB)\ CHATWIN CHRISTOPHER REGINALD (GB) | UNIV SUSSEX (GB)\ CHATWIN CHRISTOPHER REGINALD (GB)\ YOUNG RUPERT CHARLES DAVID (GB) | H04N13/00\ G03H1/22\ G03H1/08\ G03H1/32 |
| 4697 | 3D printing | LASER OPTICAL PATH GUIDE SYSTEM FOR LASER SINTER MOLDING DEVICE\ | CN203091969U | YU ZHENXIN US | YU ZHENXIN US | B22F3/105\ B23K26/064\ B23K26/0622 |
| 4698 | 3D printing | SELECTIVE LASER SINTERING FORMING METHOD\ | CN103192080A | YU ZHENXIN US | YU ZHENXIN US | B22F3/105 |
| 4699 | 3D printing | LASER OPTICAL PATH GUIDING SYSTEM OF LASER SINTER MOLDING EQUIPMENT\ | CN103111756A | YU ZHENXIN US | YU ZHENXIN US | B22F3/105\ B23K26/064\ B23K26/082 |
| 4700 | 3D printing | MANUFACTURING METHOD OF AUTOMOBILE SIDE DOOR ANTI-COLLISION BEAM OF SKELETON-SIMULATED MICROSTRUCTURE\ | CN103353915A | YUAN YOUWEI US\ ZENG XIAOHONG US\ WANG YONG US | UNIV HANGZHOU DIANZI US | G06F17/50\ B60R19/02\ G01M7/08\ G01M17/007 |
| 4701 | 3D printing | ENTIRETY QUICK MANUFACTURE METHOD OF HOT ISOSTATIC PRESSING METAL WRAPPS\ | CN101391302A | YUSHENG SHI (CN)\ QINGSONG WEI (CN)\ BINGBING QU (CN)\ YANYING DU (CN)\ JIE LIU (CN) | UNIV HUAZHONG SCIENCE TECH (CN) | B22F3/105 |
| 4702 | 3D printing | MOVABLE LASER CLADDING AND REPAIRING SYSTEM\ | CN103074625A | ZHANG KE US\ LI ZHUGUO US\ ZHANG YUE US\ HUANG JIAN US | UNIV SHANGHAI JIAOTONG (CN) | C23C24/10 |
| 4703 | 3D printing | GRADIENT LAMINATED POROUS SCAFFOLD BASED ON MICROSPHERE SELECTIVE LASER SINTERING AND PREPARATION METHOD THEREOF\ | CN102935019A | ZHANG SHENGMIN US\ DU YINGYING US\ LIU HAOMING US | UNIV HUAZHONG SCIENCE TECH (CN) | A61F2/28\ A61L27/56\ A61L27/18\ A61L27/12\ A61L27/20\ A61L27/24 |
| 4704 | 3D printing | VERFAHREN UND VORRICHTUNG ZUR HERSTELLUNG EINER DREIDIMENSIONALEN STRUKTUR AUF EINER GEKRüMMTEN BASISEBENE\[EN] METHOD AND DEVICE FOR CREATING A THREE-DIMENSIONAL STRUCTURE ON A CURVED BASE LEVEL\[FR] PROCéDé ET DISPOSITIF DE FABRICATION D'UNE STRUCTURE TRIDIMENSIONNELLE SUR UNE SURFACE DE BASE INCURVéE\ | EP2399695A1 | ZILIAN CERSTEN (DE)\ HAACK BODO (DE)\ SCHOENEBORN DIPL-ING HENNER (DE) | SLM SOLUTIONS GMBH (DE) | B29C67/00\ B22F3/105\ B29D30/06 |
| 4705 | 3D printing | MATERIAL FOR THREE-DIMENSIONAL MODELING PROCESS FOR PRODUCING THREE-DIMENSIONAL MODEL AND THREE-DIMENSIONAL MODEL\ | US2010247938A1 | ABE JUNYA (JP)\ KAKINO RYUKI (JP) | FUJIFILM CORP (JP) | B32B27/30\ B32B37/24 |
| 4706 | 3D printing | CONDUCTIVE FOAMED MATERIAL\ | JP2004063481A | AIDA TOMOHIKO US\ ITO SHINICHIRO US\ TAKEDA SHUNJI US | SEKISUI CHEMICAL CO LTD US | H05K9/00\ C08J9/36\ H01B1/20 |
| 4707 | 3D printing | METHOD OF IMPROVING THE TASTE AND TEXTURE OF SOYBEAN PROTEIN-GLUCOMANNAN COAGULATION AND ENABLING LONG-TIME STORAGE THEREOF\[FR] PROCéDé POUR AMéLIORER LE GO?T ET LA TEXTURE D'UNE COAGULATION DE PROTéINES DE SOJA-GLUCOMANNAN ET PERMETTRE UN STOCKAGE LONGUE DURéE DE CELLE-CI\ | WO2007110907A1 | AKITA MICHIHIDE (JP) | STC SYSTEM JAPAN CO LTD (JP)\ AKITA MICHIHIDE (JP) | A23L1/20\ A23J3/00 |
| 4708 | 3D printing | VERFAHREN ZUR HERSTELLUNG EINES GEB?UDES\[EN] METHOD FOR MANUFACTURING OF BUILDINGS INVOLVES LAYERING FORMABLE AND HARDENABLE MASS WITH HORIZONTAL AND VERTICAL POSITIONING SUPPLY SYSTEM MEASURING DOSAGE OF HARD MATERIAL AND BUILDING WITH WIDE SLIT NOZZLE\ | DE102004060096A1 | ASCHAUER LOTHAR (DE) | ASCHAUER LOTHAR (DE) | E04G21/00\ E04G21/02 |
| 4709 | 3D printing | VERAHREN ZUR HERSTELLUNG VON OXIDISCHER ZAHNKERAMIK\[EN] METHOD FOR THE PRODUCTION OF OXIDE DENTAL CERAMICS\[FR] PROCéDé POUR PRODUIRE UNE CéRAMIQUE DENTAIRE OXYDéE\ | EP2309947A1 | BAUER WERNER (DE)\ KNITTER REGINA (DE)\ RITZHAUPT-KLEISSL HANS-JOACHIM (DE)\ HAUSSELT JUERGEN (DE)\ BINDER JOACHIM R (DE)\ SCHLECHTRIEMEN NADJA (AT) | KARLSRUHER INST TECHNOLOGIE (DE) | A61C13/00\ A61C13/20 |
| 4710 | 3D printing | METHOD AND BINDER FOR POROUS ARTICLES\ | US2011129640A1 | BEALL GEORGE HALSEY (US)\ GALLAGHER MICHAEL THOMAS (US)\ HOUSE KEITH LEONARD (US)\ IYER SRIRAM RANGARAJAN (US)\ SHUSTACK PAUL JOHN (US) | BEALL GEORGE HALSEY (US)\ GALLAGHER MICHAEL THOMAS (US)\ HOUSE KEITH LEONARD (US)\ IYER SRIRAM RANGARAJAN (US)\ SHUSTACK PAUL JOHN (US) | B32B18/00\ B32B3/12\ B29C44/24\ C03C6/00\ C04B35/195 |
| 4711 | 3D printing | SYSTEM AND METHOD FOR PRODUCING A THREE-DIMENSIONAL BODY COMPRISING BONE OR TISSUE-COMPATIBLE MATERIAL\ | US2005154483A1 | BERGGREN CARINA (SE) | NOBEL BIOCARE AB (SE) | A61C13/00\ C23C4/12\ C23C4/18 |
| 4712 | 3D printing | A PHOTOELECTRIC DEVELOPING APPARATUS SCRAPER\ | TW200537262A | BI JIAN-ZHONG (TW)\ YANG CHEN-JEN (TW) | SINONAR CORP (TW) | G03G21/00 |
| 4713 | 3D printing | MOULDING PASTE\ | CN103146261A | BI JING US | DONGGUAN GAOBU DAWEI ART & CRAFT ORNAMENT MATERIALS FACTORY US | C09D17/00 |
| 4714 | 3D printing | ADVANCED DOUBLE-ROW SLIP CASTING DUCTULE DEVICE\ | CN201071726Y | BO WANG (CN)\ HENG KONG (CN)\ XIANXUE ZHU (CN)\ LEI DING (CN) | BEIJING MUNICIPAL BUILDING GRO (CN) | E21D11/10 |
| 4715 | 3D printing | PREPARATION METHOD OF CONDUCTING FIBER\ | CN101845679A | BOWEN CHENG US\ PENG XI US\ LEI XIA US | UNIV TIANJIN POLYTECHNIC (CN) | D01F6/46\ D01D5/11\ D01F1/09\ D01F6/48\ D01F6/92 |
| 4716 | 3D printing | POWDER REMOVAL SYSTEM FOR THREE-DIMENSIONAL OBJECT FABRICATOR\ | US2004084814A1 | BOYD MELISSA D (US)\ NIELSEN JEFFREY (US) | ZHOU TAO US\ YUNNAN YINFENG NEW MATERIALS CO LTD US | B29C67/00 |
| 4717 | 3D printing | IN-SERVICE INSULATED TANK CERTIFICATION\ | US2006009929A1 | BOYETTE ROGER L JR (US)\ HEBERT CASTILLE A (US)\ NEDELEA MIHAI (US)\ WOODELL THOMAS W (US)\ ZAMBRANO ALIRIO J (US) | BOYETTE ROGER L JR (US)\ HEBERT CASTILLE A (US)\ NEDELEA MIHAI (US)\ WOODELL THOMAS W (US)\ ZAMBRANO ALIRIO J (US) | G01F17/00 |
| 4718 | 3D printing | THERMOPLASTIC POWDER MATERIAL SYSTEM FOR APPEARANCE MODELS FROM 3D PRINITNG SYSTEMS\ | US2005003189A1 | BREDT JAMES F (US)\ WILLIAMS DEREK X (US)\ DICOLOGERO MATTHEW J (US)\ CLARK SARAH L (US) | CHEIL IND INC (KR) | B29C67/00\ B29C\ C08L101/00\ B32B5/16 |
| 4719 | 3D printing | ABSORBENT FILLERS FOR THREE-DIMENSIONAL PRINTING\ | US2005059757A1 | BREDT JAMES F (US)\ WILLIAMS DEREK X (US)\ DICOLOGERO MATTHEW J (US)\ CLARK SARAH L (US)\ SHAMBLEY WILLIAM B (US)\ TENNENHOUSE LAURA (US) | CORP Z (US) | B29C67/00\ C08K3/00\ C04B14/00 |
| 4720 | 3D printing | MASS PRODUCTION OF DENTAL RESTORATIONS BY SOLID FREE-FORM FABRICATION METHODS\ | US6322728B1 | BRODKIN DMITRI (US)\ PANZERA CARLINO (US)\ PANZERA PAUL (US) | JENERIC PENTRON INC (US) | A61C13/00\ A61K6/083\ A61K6/06\ A61K6/087 |
| 4721 | 3D printing | SECURE METHOD FOR PROVIDING VERIFYING AND CLEARING NEGOTIABLE DISCOUNT COUPONS UTILIZING A DISTRIBUTED PROCESSING NETWORK\ | US2006053437A1 | BRUNER DAVID W (US) | BRUNER DAVID W (US) | G07G1/14\ G06Q30/00\ G07F17/26\ G07F17/42\ G07G1/00\ H04N7/173 |
| 4722 | 3D printing | SYNTHETIC BONE MODEL AND METHOD FOR PROVIDING SAME\ | US2013085590A1 | BRYAN JASON A (US)\ KLATTE RYAN S (US)\ O'NEILL PETER D (US) | BRYAN JASON A (US)\ KLATTE RYAN S (US)\ O'NEILL PETER D (US) | G06F17/50 |
| 4723 | 3D printing | METHOD AND SYSTEM FOR GENERATING MECHANICAL WAVES\ | US2013216400A1 | BUCHER IZHAK (IL)\ SETTER EYAL (IL) | TECHNION RES & DEV FOUNDATION (IL)\ LTD TECHNION RES & DEV FOUNDATION (US) | F04B43/12 |
| 4724 | 3D printing | VERFAHREN UND VORRICHTUNG ZUR HERSTELLUNG EINES BETONFERTIGTEILES\[EN] METHOD AND APPARATUS FOR PRODUCING A PRECAST CONCRETE PART\[FR] PROCéDé ET DISPOSITIF POUR LA RéALISATION D'UN éLéMENT PRéFABRIQUé EN BéTON\ | WO2012054948A1 | BUERGLER WOLFGANG (AT) | BETONWERK RIEDER GES M B H (AT)\ BUERGLER WOLFGANG (AT) | B28B13/02 |
| 4725 | 3D printing | ABRASIVE PRODUCTS AND METHODS FOR FINISHING SURFACES\ | US2013298471A1 | CAI YING (US)\ MANNING JAMES J (US)\ WANG JIANNA (US) | CAI YING (US)\ MANNING JAMES J (US)\ WANG JIANNA (US) | B24D3/28 |
| 4726 | 3D printing | GRANULAT FüR 3-D-BINDERDRUCK\[EN] AGGLOMERATE FOR 3D-BINDER PRINTING\[FR] GRANULE POUR L'IMPRESSION à TROIS DIMENSIONS\ | EP1770176A1 | CARRENO-MORELLI EFRAIN (CH)\ MARTINERIE SEBASTIEN (CH)\ BIDAUX JACQUES-ERIC (CH) | HAUTE ECOLE VALAISANNE (CH) | B22F3/00\ B22F1/00\ C22C1/04 |
| 4727 | 3D printing | ARTWARE FINISHED WITH GOLD AND SILVER MICA PARTICLES\ | CN101758722A | CHAO XU US | TIANJIN SHIJI DONGFANG LANDSCA US | B44C1/00\ B05D5/06\ B05D7/24\ B44D5/00 |
| 4728 | 3D printing | MULTI-FUNCTION COAXIAL NOZZLE FOR LASER MATERIAL PROCESSING\ | TWI252789B | CHEN CHUN (TW)\ WU HSIEN-CHENG (TW) | CHEN CHUN (TW)\ WU HSIEN-CHENG (TW) | B23K26/14 |
| 4729 | 3D printing | METHOD FOR DETERMINING CONTINUOUS CASTING CRYSTALLIZER INNER CAVITY TAPER\ | CN103433442A | CHEN DENGFU US\ ZHANG LEILEI US\ LONG MUJUN US\ XIE XIN US\ MA YOUGUANG US\ ZHAO YAN US\ SHEN JIALONG US\ ZHANG XIANGUANG US\ ZHANG XING US | UNIV CHONGQING US | G06F17/50\ G06F19/00\ B22D11/057 |
| 4730 | 3D printing | LASER FAST SHAPING SYSTEM WITH HEXA-AXLE FOR POWDERED MATERIAL\ | CN2780382Y | CHEN JIMIN (CN)\ WANG YINGNA (CN)\ ZUO TIEXUN (CN)\ YANG JIANHUA (CN) | UNIV BEIJING TECHNOLOGY (CN) | B22F3/105 |
| 4731 | 3D printing | MULTIFUNCTIONAL COAXIAL LASER NOZZLE\ | CN100512977C | CHEN JUN (CN)\ XIANZHENG WU (CN) | CHEN JUN WU XIANZHENG (CN) | B05B7/02 |
| 4732 | 3D printing | STEREO PROTOTYPING MECHANISM\ | CN102189686B | CHEN ZHIZHONG US\ HUANG KEMING US\ DONG YAQING US\ XI GUOYUAN US | MICROJET TECHNOLOGY CO LTD (TW) | B29C67/00 |
| 4733 | 3D printing | COLORED THREE-DIMENSIONAL QUICK SAND TABLE SPRAY PRINTING MACHINE\ | CN201960834U | CHENG WEN US\ QING MU US\ BAO LI US\ YUBO MIAO US\ ZHEYUN HUANG US\ XUAN WANG US\ YUNGAN WANG (SG) | JIANGSU RABILY CO LTD US\ FOCHIF MECHATRONICS TECHNOLOGY CO LTD US | B41J2/01\ B41J3/00 |
| 4734 | 3D printing | QUICK JET PRINTING MACHINE FOR COLORIZED THREE-DIMENSIONAL SAND TABLE\ | CN102363393A | CHENG WEN US\ QING MU US\ BAO LI US\ YUBO MIAO US\ ZHEYUN HUANG US\ XUAN WANG US\ YUNGAN WANG (SG) | JIANGSU RABILY CO LTD US\ FOCHIF MECHATRONICS TECHNOLOGY CO LTD US | B41J2/01\ B41J3/00 |
| 4735 | 3D printing | SYNTHESIS OF ANTIMONY TELLURIDE (SB-TE) PASTE AND P-TYPE THERMOELECTRIC MATERIAL FORMING METHOD USING PASTE\ | KR20130116689A | CHO BYUNG JIN (KR)\ WE JU HYUNG (KR)\ KIM SUN JIN (KR)\ KIM GYUNG SOO (KR) | KOREA ADVANCED INST SCI & TECH (KR) | H01L35/14\ H01L35/16\ H01L35/18 |
| 4736 | 3D printing | METHOD AND SYSTEM OF GRAPHICAL SIMULATION OF THIN SHELL OBJECTS\ | US2009228256A1 | CHOI MIN GYU (KR)\ WOO SEUNG YONG (KR)\ KO HYEONG-SEOK (KR) | CHOI MIN GYU (KR)\ WOO SEUNG YONG (KR)\ KO HYEONG-SEOK (KR) | G06G7/48\ G06T13/00 |
| 4737 | 3D printing | METHOD FOR MANUFACTURING AROMATIC POROUS SINTERED METAL COMPACT\ | KR20040013342A | CHOI SUNG JO US | CHOI SUNG JO US | B22F3/11 |
| 4738 | 3D printing | PREPARATION METHOD OF THREE-DIMENSIONAL ELECTRODE MATERIAL SPECIAL FOR SEWAGE TREATMENT BY ELECTROCHEMICAL OXIDATION METHOD AND ELECTROCHEMICAL OXIDATION SYSTEM\ | CN101955246A | CHUNHUI ZHANG US\ QIANG QIN US\ JIANBING WANG US\ CHUNRONG WANG US\ LIYUAN LIU US\ XUWEN HE US | UNIV CHINA MINING US | C02F1/46\ C02F1/72 |
| 4739 | 3D printing | PROCEDE DE REALISATION D'UN SUPPORT COMPORTANT DES PARTICULES EN TROIS DIMENSIONS\ | FR2922155B1 | CYMES JACQUES ANTOINE (FR) | PRESTI DIAM GRAPHIC (FR) | B41M3/00\ B44C1/26 |
| 4740 | 3D printing | METHOD FOR PRODUCING POROUS SINTERED METAL MATERIAL\ | JP2008291365A | DATE KENJI US | HITACHI METALS LTD US\ HITACHI METALS PRECISION LTD US | B22F3/11\ C22C1/08\ C22C38/00\ C22C38/18 |
| 4741 | 3D printing | MAGNETIC INORGANIC NANO PARTICLE/LARGE-APERTURE ORDERED MESOPORE OXIDE NUCLEAR SHELL MICROSPHERES AND PREPARATION METHOD THEREOF\ | CN102989398A | DENG YONGHUI US\ WANG MINGHONG US | UNIV FUDAN US | B01J13/02\ H01F1/01 |
| 4742 | 3D printing | VERFAHREN ZUM STRANGGIESSEN EINES METALLSTRANGS\[EN] METHOD FOR THE CONTINUOUS CASTING OF A METAL STRAND\[FR] PROCéDé DE COULéE CONTINUE D'UNE BARRE MéTALLIQUE\ | EP2279053A1 | DITTENBERGER KURT (AT) | SIEMENS VAI METALS TECH GMBH (AT) | B22D11/22 |
| 4743 | 3D printing | SPUMY CERAMIC FILTER CAPABLE OF HIGH SLAG RESISTANCE AND THERMAL STABILITY AND FABRICATING METHOD THEREOF\ | CN101224361A | DONGQING HE (CN)\ HAIJUN SUN (CN)\ HE LI (CN)\ QI HE (CN)\ LI XU (CN) | ELITE CERAMICS IND BEIJING CO (CN) | C04B35/565\ C04B38/06\ B01D39/20 |
| 4744 | 3D printing | KOSMETISCHE ZUSAMMENSETZUNGEN ENTHALTEND KONKAVE PARTIKEL\[EN] COSMETIC COMPOSITIONS COMPRISING CONCAVE PARTICLES.\[FR] COMPOSITIONS COSMéTIQUES COMPRENANT DES PARTICULES CONCAVES\ | EP1579849A1 | DUMOUSSEAUX CHRISTOPHE (JP) | OREAL (FR) | A61K8/18\ A61Q1/08\ A61K8/00\ A61K8/02\ A61K8/11\ A61K8/19\ A61K8/23\ A61K8/24\ A61K8/25\ A61K8/26\ A61K8/29\ A61K8/31\ A61K8/34\ A61K8/36\ A61K8/37\ A61K8/44\ A61K8/58\ A61K8/64\ A61K8/73\ A61K8/89\ A61K8/891\ A61K8/92\ A61K8/97\ A61K8/98\ A61Q1/00\ A61Q1/02\ A61Q1/10\ A61Q1/12\ A61Q17/04\ A61Q19/08\ C08L83/04 |
| 4745 | 3D printing | PATIENTENINDIVIDUELLES KNOCHENAUFBAUMITTEL UND VERFAHREN ZU SEINER HERSTELLUNG\ | DE102006026000B4 | DüRR HOLGER PROF DR ING (DE)\ H?NEL T DIPL ING (DE)\ PETERS FABIAN DR (DE) | CURASAN AG (DE) | A61L27/00\ A61L27/56\ A61L27/12\ A61L27/20\ A61L27/54\ C07C55/06\ C07C59/245 |
| 4746 | 3D printing | VORRICHTUNG ZUM PUDERN VON BOGEN IN EINER DRUCKMASCHINE\[EN] PAPER SPREADING POWDER PRINTED SHEET PRINTING DEVICE HAS NOZZLE HEAD PROVIDED WITH TWO AIR FLOW NOZZLES WHERE ONE OF NOZZLES PASSES ALONG CONVEYING DIRECTION AND PRINTING SHEET ARRANGED BETWEEN NOZZLES TOWARD CONVEYING DIRECTION\ | DE102011108279A1 | EBEL BJOERN (DE)\ FRICKE ANDREAS DR (DE) | HEIDELBERGER DRUCKMASCH AG (DE) | B41F23/06 |
| 4747 | 3D printing | MAGNETIC TONER\ | JP2012002986A | AHN SUNG HOON (KR)\ CAHU DOO MAN (KR)\ KIM CHUNG SOO (KR) | CANON KK (JP) | G03G9/087\ G03G9/08\ G03G9/083 |
| 4748 | 3D printing | MANUFACTURING METHOD OF THREE DIMENSIONAL OBJECTS\ | KR101282888B1 | Empty Field US | SAMSUNG SDI CO LTD (KR) | B22F3/24\ B22F5/12\ B22F3/12 |
| 4749 | 3D printing | EXPANDABLE COATING COMPOSITION FOR PRINTING AND CONTAINER USING THEREOF\ | KR101236734B1 | Empty Field US | SNU R&AMP DB FOUNDATION (KR) | B32B1/02\ B32B5/18\ B32B27/40 |
| 4750 | 3D printing | A POROUS 3-DIMENSIONAL BIPOLAR ELECTRODE AN ELECTROLYZER HAVING THE POROUS 3-DIMENSIONAL BIPOLAR ELECTRODE AND WATER TREATMENT METHOD USING THE ELECTROLYZER HAVING THE POROUS 3-DIMENSIONAL BIPOLAR ELECTRODE\ | KR101220891B1 | Empty Field US | SUMMERFIELD MARTIN RAYMOND (GB) | C02F1/467\ C02F103/08\ C25B11/02\ C25B11/04 |
| 4751 | 3D printing | ULTRA PRECISION MACHINING APPARATUS USING NANO-SCALE THREE DIMENSIONAL PRINTING AND METHOD USING THE SAME\ | KR101172859B1 | Empty Field US | TRUMPF WERKZEUGMASCHINEN GMBH (DE) | H01L21/027\ H01L21/3065 |
| 4752 | 3D printing | COMPOSITION OF PASTE FOR FABRICATING THE ELECTRODE\ | KR100923741B1 | Empty Field US | UNIV SOUTHERN CALIFORNIA (US) | H01B1/22\ H01B3/08 |
| 4753 | 3D printing | No Title Available\ | CN1743578B | KIM YONG HYUN (KR)\ PARK SANG HEE (KR)\ CHOI DEOK YOUNG (KR) | UNIV STUTTGART INST FUER PHYSI (DE) | E04B5/21 |
| 4754 | 3D printing | No Title Available\ | CN102280207A | MOON SANG BONG (KR)\ LEE TAE LIM (KR) | VANTICO GMBH US | H01B13/00\ H01B1/22\ H01L31/0224 |
| 4755 | 3D printing | SCHMELZIMPR?GNIEREND VEREDELTE FASER-PLATTENWERKSTOFFE\[EN] PLATE SHAPED MATERIAL E.G. FIBER BOARD MANUFACTURING METHOD INVOLVES EXPOSING SURFACE OF PLATE WITH COMPONENTS TO TEMPERATURE AND/OR TEMPERATURE AND PRESSURE AND PROVIDING UPPER LAYER FOR BINDING COMPONENTS ARRANGED AT PLATE SURFACE\ | DE102005044648A1 | PARK DAE WON (KR) | HELD KURT (DE) | B27N3/06 |
| 4756 | 3D printing | PRESSE ZUR HERSTELLUNG VON DR?HTEN FüR 3D-DRUCKER\[EN] THERMAL MELTING WIRE FOR THREE-DIMENSIONAL DRUCKER IS OBTAINED BY MOLDING PLASTIC MATERIAL OR MELTING THERMOPLASTIC MATERIAL IN HEATED MOLD COOLING MULTI-COMPONENT CASTING RESIN INTO MOLD AND CURING\ | DE102011016270A1 | QIU ZEYOU (CN) | PARTHY KAI (DE) | B29C39/02 |
| 4757 | 3D printing | PRINTER\ | JP2012218339A | YANG YOUNG IL (KR)\ GU JA EUN (KR)\ KOO JA JUNG (KR) | SEIKO EPSON CORP (JP) | B41J2/01\ B41J11/26 |
| 4758 | 3D printing | SEAL AND METHOD FOR MANUFACTURING THE SAME\ | JP2012201108A | ZHOU TAO US\ ZHAO SHUCHUN US | TOHOKUDO INSATSU KK US | B41K1/02 |
| 4759 | 3D printing | METHOD FOR PRODUCING PHOTONIC CRYSTAL AND PHOTONIC CRYSTAL\ | US2004255841A1 | ENOKIDO YASUSHI (JP)\ TSUCHIYA HIROYASU (JP) | MASSACHUSETTS INST TECHNOLOGY (US) | C30B29/60\ C30B7/00\ C30B29/68 |
| 4760 | 3D printing | JETTING DEVICE FOR PRODUCING ZINC POWDER IN SIPHON SPRAYING FORM\ | CN202317027U | ERGU GE US | CHANGSHA ENG & RES INST NONFERROUS METALLURGY CO LTD US | B22F9/08 |
| 4761 | 3D printing | METHOD AND DEVICE FOR FORMING A DEPOSIT OF ONE OR MORE FRAGILE MATERIALS ON A SUBSTRATE BY SPRAYING A POWDER\ | US2014291885A1 | ETCHEGOYEN GREGORY (FR)\ DURAND OLIVIER (FR)\ ROSSIGNOL FABRICE (FR) | CT DE TRANSFERT DE TECHNOLOGIES CERAMIQUES C T T C (FR) | B29C67/00 |
| 4762 | 3D printing | LAGERKARTUSCHE INSBESONDERE FüR DEN GELENKARM EINER DREIDIMENSIONALEN KOORDINATENMESSVORRICHTUNG\[EN] BEARING CARTRIDGE IN PARTICULAR FOR THE ARTICULATED ARM OF A THREE-DIMENSIONAL COORDINATE MEASURING DEVICE\[FR] PALIER CARTOUCHE EN PARTICULIER POUR LE BRAS ARTICULé D?UN DISPOSITIF DE MESURE DE COORDONNéES TRIDIMENSIONNEL\ | WO2011003819A1 | FACKELMANN ROBERT (DE)\ HOECHERL NORBERT (DE)\ BOHR ANDREAS-JOHANN (DE)\ GRUNAU ARBOGAST (DE) | SCHAEFFLER TECHNOLOGIES GMBH (DE)\ BOHR ANDREAS-JOHANN (DE)\ FACKELMANN ROBERT (DE)\ HOECHERL NORBERT (DE)\ GRUNAU ARBOGAST (DE) | F16C19/38\ F16C33/36\ F16C35/063\ F16C35/067\ F16C43/06 |
| 4763 | 3D printing | SILVER OR GOLD AND SILVER PEONY PREPARED BY FRESH FLOWER AND PREPARATION PROCESS THEREOF\ | CN102862440A | FAN SHEQIANG US | FAN SHEQIANG US | B44C5/06\ B44C1/20\ C25D5/56 |
| 4764 | 3D printing | CORE-SHELL SOLID FREEFORM FABRICATION\ | US2006192315A1 | FARR ISAAC (US)\ LAMBRIGHT TERRY M (US) | FARR ISAAC (US)\ LAMBRIGHT TERRY M (US) | B29C41/02 |
| 4765 | 3D printing | ALGINATE-BASED MATERIALS METHODS OF APPLICATION THEREOF AND SYSTEMS FOR USING THE ALGINATE-BASED MATERIALS\ | US2005087902A1 | FARR ISAAC (US)\ LAMBRIGHT TERRY M (US)\ HOVAGIMIAN HOWARD (US) | HEWLETT PACKARD DEVELOPMENT CO (US) | B29C67/00\ B28B1/00\ C04B26/28 |
| 4766 | 3D printing | SYSTEMS AND METHODS OF SOLID FREEFORM FABRICATION WITH IMPROVED POWDER SUPPLY BINS\ | US2007026102A1 | FARR ISAAC (US)\ LAMBRIGHT TERRY M (US)\ NIELSEN JEFFREY A (US)\ COLLINS DAVID C (US)\ DEVOS JOHN A (US)\ CRUZ-URIBE TONY S (US) | NIELSEN JEFFREY A (US)\ DEVOS JOHN A (US)\ CRUZ-URIBE TONY S (US)\ COLLINS DAVID C (US)\ FARR ISAAC (US)\ LAMBRIGHT TERRY M (US) | B28B21/42 |
| 4767 | 3D printing | HERSTELLUNGSVERFAHREN FüR EINEN KERAMISCHEN FORMK?RPER UND ENTSPRECHENDER KERAMISCHER FORMK?RPER SOWIE DESSEN VERWENDUNG\[EN] PRODUCTION OF CERAMIC MOLD USEFUL E.G. AS CAST FOR CERAMIC SLIP CASTING OR AS CARRIER FOR CULTURING MICROORGANISMS COMPRISES E.G. MIXING BIOMORPHOUS CARBON FIBERS BINDING MATERIAL AND METAL-CONTAINING MATERIAL AND CROSSLINKING\ | DE102005058034A1 | FEY TOBIAS (DE)\ HOEFNER THOMAS (DE)\ GREIL PETER (DE) | UNIV FRIEDRICH ALEXANDER ER (DE) | C04B35/565\ B28B7/34\ C04B35/571\ C03B19/02\ C12M1/16 |
| 4768 | 3D printing | BOOK WITH EDIBLE PAGES E.G. OF CHOCOLATE HAS EDGES OF PAGES PIERCED TO RECEIVE BINDING RINGS OR RIBBON\[FR] LIVRE COMESTIBLE.\ | FR2890830A1 | FOURNET FAYARD ANNE CECILE (FR) | COM EDIT ENTPR UNIPERSONNELLE (FR) | A23P1/00\ B42D1/00 |
| 4769 | 3D printing | PRE-CAST PLASTIC RIB CEMENT-POLYPHENYL HEAT-INSULATION WALL PLATE\ | CN201190372Y | FUCUN ZHU (CN) | FUCUN ZHU (CN) | E04C2/30\ E04B1/80 |
| 4770 | 3D printing | THREE-DIMENSIONAL SHAPING DEVICE AND THREE-DIMENSIONAL SHAPING METHOD\ | JP2009107244A | FUJISAWA KAZUTOSHI (JP) | SEIKO EPSON CORP (JP) | B29C67/00 |
| 4771 | 3D printing | THREE-DIMENSIONAL SHAPING DEVICE AND THREE-DIMENSIONAL SHAPING METHOD\ | JP2009101651A | FUJISAWA KAZUTOSHI (JP) | SEIKO EPSON CORP (JP) | B29C67/00 |
| 4772 | 3D printing | THREE-DIMENSIONAL MOLDING APPARATUS AND THREE-DIMENSIONAL MOLDING METHOD\ | US2009004381A1 | FUJISAWA KAZUTOSHI (JP)\ KUMAGAI TOSHIO (JP) | SEIKO EPSON CORP (JP) | B05D1/38 |
| 4773 | 3D printing | THREE-DIMENSIONAL OBJECT FORMING APPARATUS AND METHOD FOR FORMING THREE DIMENSIONAL OBJECT\ | US7862320B2 | FUJISAWA KAZUTOSHI (JP)\ KUMAGAI TOSHIO (JP)\ ITO AKIO (JP) | SEIKO EPSON CORP (JP) | B29C35/08\ B29C67/00\ G06F19/00\ B29C31/04 |
| 4774 | 3D printing | CONDUCTIVE PASTE AND PRINTING METHOD AS WELL AS MANUFACTURING METHOD OF CERAMIC MULTILAYER CIRCUIT BOARD\ | JP2004047856A | FUKAYA MASASHI US\ NAKAMURA SADAHIRO US\ ONOZUMI SHIGEKAZU US | SUMITOMO METAL ELECTRONICS DEV US\ TANAKA PRECIOUS METAL IND (JP) | H01B1/22\ H05K3/46\ H05K1/09 |
| 4775 | 3D printing | COATED ABRASIVE GRAINS METHOD AND FOR THE PRODUCTION THEREOF AS WELL AS THE USE THEREOF FOR PRODUCING ABRASIVES\ | US2009100766A1 | GEBHARDT KNUTH (AT) | GEBHARDT KNUTH (AT) | C09G1/02\ B24D18/00 |
| 4776 | 3D printing | VERFAHREN ZUR HERSTELLUNG EINES W?RMEüBERTRAGERS UND W?RMEüBERTRAGER HERSTELLBAR NACH DEM VERFAHREN\[EN] PRODUCING A PART OF A HEAT EXCHANGER COMPRISING ALUMINUM AND/OR ALUMINUM ALLOY AND HAVING A CORROSION PROTECTED SURFACE COMPRISES APPLYING ZINC OR ZINC-CONTAINING LAYER TO THE SURFACE OR PART OF THE SURFACE\ | DE102008033222A1 | GHIANI FRANCO (DE) | BEHR GMBH & CO KG (DE) | C23C24/06\ B05D7/14\ B23P15/26\ F28F19/06\ F28F21/08 |
| 4777 | 3D printing | VERFAHREN ZUM HERSTELLEN KERAMISCHER BAUTEILE INSBESONDERE VON IMPLANTATEN\[EN] RAPID PROTOTYPING PROCESS TO FABRICATE CERAMIC SURGICAL IMPLANT WITH CERAMIC POWDER AND BINDING AGENT SUSPENSION DEVOID OF SOLVENT\ | DE102005058116A1 | GIA KHANH PHAM (DE)\ SCHWARZ MARTINA (DE)\ SCHAEFER MARTIN (DE)\ WESSLER BERIT (DE) | SIEMENS AG (DE) | C04B35/622\ A61C8/00\ A61L27/10 |
| 4778 | 3D printing | PROCESS FOR THE PRODUCTION OF A THREE DIMENSIONAL SYSTEM\ | US2012202012A1 | GREBE MAIK (DE)\ DIEKMANN WOLFGANG (DE)\ ALTKEMPER STEFAN (DE) | EVONIK DEGUSSA GMBH (DE)\ GREBE MAIK (DE)\ DIEKMANN WOLFGANG (DE)\ ALTKEMPER STEFAN (DE) | B29C41/34\ B29C41/52\ B29C41/22\ B32B3/30 |
| 4779 | 3D printing | METHOD FOR MANUFACTURING GYPSUM SILICON RUBBER PRECISION CAST TURBOCHARGER DIFFUSER\ | CN102101155A | GUOWEN CHEN US\ GUOQIANG HAN US\ JIANFEI YAN US\ XIAOJIE GAO US\ BINGWEN ZHANG US\ GANG CHEN (CN) | DATONG NORTH TIANLI TURBO CHARGING TECHNOLOGY CO LTD US | B22C9/04\ B22D18/04 |
| 4780 | 3D printing | VORRICHTUNG UND VERFAHREN ZUM BEST?UBEN VON BEDRUCKTEN BOGEN ODER EINER ENDLOSBAHN MIT PUDER\[EN] POWDER SPRAYING DEVICE FOR USE IN PRINTING MACHINE HAS SPRAY NOZZLES WHERE POWDER DENSITY IS INCREASED IN BOUNDARY REGION OF ELBOW OR WEB AS NOZZLES ARE INCLINED IN BOUNDARY REGION\ | DE102005039078B3 | HAAS RAINER (DE)\ GOETZ REINER (DE) | WEITMANN & KONRAD FA (DE) | B41F23/06 |
| 4781 | 3D printing | METHOD OF MANUFACTURING SLEEVE FOR FLUID-DYNAMIC BEARING AND SLEEVE MANUFACTURED BY THE METHOD\ | US2008206497A1 | HACHIGA SHOUJI (JP)\ SHINA YOSHIHIRO (JP) | JUKI KK (JP) | C04B35/64\ B28B1/30\ B29D22/00 |
| 4782 | 3D printing | FIRE EXTINGUISHING SPRAY NOZZLE USED FOR SUSPENSION TYPE DRY POWDER EXTINGUISHING EQUIPMENT\ | CN202154967U | HAIRONG WANG US\ RUIQING LI US | YANG LAE US | A62C31/05 |
| 4783 | 3D printing | BAHNF?RMIGE BESCHICHTUNGSFOLIE\[EN] TRACK FORMS USEFUL FOR APPLYING ON A SUBSTRATE OBTAINED BY A DECORATIVE AND ROLLED- OR ROLLABLE CONDITION EXISTING COATING FOIL WITH A DECORATIVELY PRESSED SUBSTRATE LAYER OF PAPER AND/OR PLASTICS\ | DE102005001773A1 | HALLER HEINZ (DE) | KUNZ HOLDING GMBH & CO KG (DE) | C08J3/24\ C09D4/02\ C09J7/04\ B32B27/10 |
| 4784 | 3D printing | STERILIZED MICRO NANO-SILVER FILM HAVING NONPOISONOUS AND THE PROCESS THEREOF\ | US2008008875A1 | HAN SANG OH (KR) | HAN SANG OH (KR) | C08K3/00\ C08J5/18\ B43K19/00 |
| 4785 | 3D printing | GEFORMTES FLUORHARZ\[EN] MOLDED FLUORORESIN\[FR] RESINE FLUOREE MOULEE\ | EP1950229A4 | HANDA KOICHI (JP)\ SUBIANTORO (JP)\ TSUKADA TAKAYUKI (JP)\ OKUBO TSUYOSHI (JP)\ SHAN JIAYI (JP)\ YAMAUCHI AKIRA (JP)\ NAGASHIMA MANABU (JP) | MITSUI BUSSAN (JP) | C08F14/26\ C01B31/00\ C08K7/04\ C08L27/18 |
| 4786 | 3D printing | MANUFACTURING METHOD OF RESIN COMPOSITE FORMED PRODUCT\ | JP2008100491A | HASEGAWA KATSUYUKI US | ALMIGHTY KK US | C08L101/00\ C08K3/22\ B29K105/16\ B29C43/02\ C08L101/16 |
| 4787 | 3D printing | METHOD FOR MANUFACTURING RESIN COMPOSITE FORMED PRODUCT\[DE] HERSTELLUNGSVERFAHREN FüR EIN HARZVERBUND-FORMPRODUKT\[FR] PROCéDé DE FABRICATION D'UN PRODUIT FORMé DE COMPOSITE RéSINE\ | EP1795319A2 | HASEGAWA KATUYUKI (JP) | HASEGAWA KATUYUKI (JP) | B27N5/00\ B27N3/00 |
| 4788 | 3D printing | METHOD FOR MANUFACTURING RESIN COMPOSITE FORMED PRODUCT\ | US2007132133A1 | HASEGAWA KATUYUKI (JP) | HASEGAWA KATUYUKI (JP) | D04H1/00 |
| 4789 | 3D printing | TONER CARTRIDGE REGENERATION METHOD FOR LASER PRINTER\ | CN103324060A | HE YONGGANG (CN) | PRINT RITE UNICOM IMAGE PRODUCTS CO LTD ZHUHAI US | G03G15/08 |
| 4790 | 3D printing | HERSTELLUNG VON HOHLK?RPERN ODER SCHICHTEN MIT HOHLR?UMEN\[EN] PRODUCING HOLLOW BODY WITH CAVITY COMPRISES PRODUCING MOLDED BODY WITH OUTER MOLD PARTIALLY HAVING SHAPE OF HOLLOW CAVITY WHERE MOLDED BODY IS DISPLACEABLE BY SOLVENT/MELT IN FLOWABLE STATE AND COATING MOLDED BODY WITH COATING MATERIAL\ | DE102009037893A1 | HERTTER MANUEL (DE)\ JAKIMOV ANDREAS (DE)\ WACHTER WOLFGANG (DE) | MTU AERO ENGINES GMBH (DE) | B29C45/00\ C04B35/638\ B05D1/00 |
| 4791 | 3D printing | VORRICHTUNG ZUR HERSTELLUNG VON DREIDIMENSIONALEN BAUTEILEN\[EN] DEVICE USED TO MAKE THREE-DIMENSIONAL HYBRID COMPONENT COMPRISES SUPPORTING UNIT APPLYING UNIT AND RADIATION UNIT WHERE PLATE-LIKE BODY IS DISPOSED ON UPPER SIDE OF SUPPORT IS FORMED AS CONTOURED PLUG HOLDER AND COMPRISES RECESSES\ | DE102012011217A1 | HERZOG FRANK (DE) | CL SCHUTZRECHTSVERWALTUNGS GMBH (DE) | B22F3/105\ B29C67/04 |
| 4792 | 3D printing | WAX-MODIFIED COATING COMPOSITIONS WITH IMPROVED ABRASION RESISTANCE\ | US2004096665A1 | HOEHNE JOERG (DE)\ WATERKAP PAUL-LUDWIG (DE)\ CRHISTOPH WOLFGANG (DE) | DEGUSSA (DE) | C09D175/04\ C09D5/00\ C09D7/12\ C08L77/00\ C09D191/06\ C08G18/80\ C08L91/00\ C09D175/06\ C09D177/00\ C09D177/02\ C09D201/06 |
| 4793 | 3D printing | METHOD FOR METALLIZATION OF CERAMIC PLATES\ | UA51817U | HOFMAN BORYS HERSHEVYCH (UA)\ NEMESH VIKTOR HEORHIIOVYCH (UA)\ OSECHKIN SERHII IVANOVYCH (UA) | HOFMAN BORYS HERSHEVYCH (UA)\ NEMESH VIKTOR HEORHIIOVYCH (UA)\ OSECHKIN SERHII IVANOVYCH (UA) | H05K3/10 |
| 4794 | 3D printing | LIGHT-DUTY STEEL FRAME-PLATE SHELL STRUCTURE\ | CN201437611U | HONGCHAO CUI US\ YUEMING YAO US\ XIUZHEN XU US | CHINA MAJESTY STRUCTURAL ENGINEERING DESIGN & CONSULTANT INC US\ SHANGHAI ZHENGCHENG MACHINERY AND ELECTRONICS CO LTD US | E04B1/24\ E04B1/18 |
| 4795 | 3D printing | PIEZOELECTRIC THREE-DIMENSIONAL PRINTING SHAPING SYSTEM\ | CN202448496U | HONGHAI ZHANG US\ SHU CAO US\ TIANZHU ZHU US\ YULI XU US\ XIAYUN SHU US\ ZHIMIN ZHAN US\ YAN HU US\ BO SUN (CN) | UNIV HUAZHONG SCIENCE TECH (CN) | B41J2/01\ B41J3/00 |
| 4796 | 3D printing | BUILT-IN LASER SMELTING SPRAY NOZZLE\ | CN2705236Y | HU QIANWU (CN)\ ZENG XIAOYAN (CN) | CENTRAL CHINA SCIENCE & TECH (CN) | B23K26/34\ B05B7/16\ C23C4/00 |
| 4797 | 3D printing | STEREO CASTING COPPER MOULD\ | CN2705247Y | HUO YANQUAN (CN) | HUO YANQUAN (CN) | B22D7/06 |
| 4798 | 3D printing | MANUFACTURE METHOD OF INORGANIC FOAM USING GEOPOLYMER\ | KR20120053411A | I DONG HUI (KR)\ JU CHEL WAN (KR) | I DONG HUI (KR)\ JU CHEL WAN (KR) | C04B38/00\ C04B14/04\ C04B28/24\ C04B18/08 |
| 4799 | 3D printing | MAGNETIC RECORDING MEDIUM\ | US2005079389A1 | ICHIKAWA TOMOHIRO (JP)\ MURAO NAOTO (JP) | FUJI PHOTO FILM CO LTD (US) | G11B5/70\ G11B5/708\ G11B5/73\ G11B5/738\ G11B5/84 |
| 4800 | 3D printing | MAGNETIC RECORDING MEDIUM\ | JP2005243063A | ICHIKAWA TOMOHIRO (JP)\ MURAO NAOTO (JP) | FUJI PHOTO FILM CO LTD (US) | G11B5/70\ G11B5/71 |
| 4801 | 3D printing | SCREEN-PRINT PASTE FOR MULTILAYER CERAMIC ELECTRONIC COMPONENT\ | JP2007281306A | II DAIZO US\ TAKAHASHI HIDEYUKI US | SEKISUI CHEMICAL CO LTD US | C09D11/10\ H01G4/12\ H01G4/30\ H01B1/22 |
| 4802 | 3D printing | VERFAHREN ZUM HERSTELLEN EINES FASERVERBUNDBAUTEILS\ | DE102008026161B4 | ILZHOEFER KARL-HEINZ DIPL ING (DE)\ SALKIC ASMIR DIPL ING (DE) | DAIMLER AG (DE) | B29C70/34\ B29C70/50\ C08J5/04\ B29B15/12\ B62D29/04 |
| 4803 | 3D printing | METHOD OF FABRICATING OPTICAL CERAMICS CONTAINING COMPOSITIONALLY TAILORED REGIONS IN THREE DIMENSION\ | US2012225767A1 | IMHOLT JIYUN C (US)\ GENTILMAN RICHARD L (US)\ HARTNETT THOMAS M (US) | IMHOLT JIYUN C (US)\ GENTILMAN RICHARD L (US)\ HARTNETT THOMAS M (US) | C04B35/00\ G02B1/00 |
| 4804 | 3D printing | APPARATUS SYSTEMS AND METHODS FOR USE IN THREE-DIMENSIONAL PRINTING\ | US6986654B2 | IMIOLEK IRENEUSZ J (US)\ GAYLO CHRISTOPHER M (US) | THERICS INC (US) | B29C35/08\ B29C67/00\ B29C41/36\ B29C41/34\ B29C41/46\ B29C41/52\ B29C35/02\ B29C41/02\ H05B6/00\ B27N3/02\ B29C31/04\ B41F1/34\ B41J3/407\ B41L5/16 |
| 4805 | 3D printing | SAND CORE AND METHOD FOR MANUFACTURING SAND CORE\ | JP2005334906A | INOUE KATSUNORI US | TOKYO LIGHT ALLOY CO LTD US | B22C9/10 |
| 4806 | 3D printing | CYAN TONER AND METHOD FOR FORMING AN IMAGE\ | US7138213B2 | ITAKURA TAKAYUKI (JP)\ BABA YOSHINOBU (JP)\ SATO YUKO (JP)\ SUGAHARA NOBUYOSHI (JP)\ TERAUCHI KAZUO (JP)\ KOTAKI TAKAAKI (JP)\ TANIKAWA HIROHIDE (JP) | CANON KK (JP) | G03G9/087\ G03G9/08\ G03G9/09\ G03G9/097\ G03G15/01 |
| 4807 | 3D printing | PRODUCTION METHOD OF STRUCTURES PRODUCTION METHOD OF MAGNETIC SUBSTANCE AND MANUFACTURING APPARATUS OF STRUCTURES\ | JP2004083999A | IWATA YUJI US | SEIKO EPSON CORP (JP) | B22F3/00\ B22F3/02\ H01F1/053\ H01F1/08\ H01F41/02 |
| 4808 | 3D printing | THREE-DIMENSIONAL PRINTING TECHNOLOGY BASED METHOD FOR FORMING MEDICAL POROUS PURE TITANIUM IMPLANT\ | CN101927346A | JIAN SUN US\ PING CHEN US\ YAOYANG XIONG (CN)\ FUQIANG ZHANG (CN) | JIAOTONG 9TH PEOPLES HOSPITAL US | B22F3/00 |
| 4809 | 3D printing | METHOD FOR REMANUFACTURING DIE WITH GRADIENT FUNCTION BY REPAIRING MICRO ZONE THROUGH LASER\ | CN102248164A | JIAN SUN US\ ZHENGYANG LI US\ DEYING GENG US\ GANG DING US\ JIAWEI DING US\ JILONG YAO US\ HUAPING TANG US | DANYANG HUIDA MOLD MATERIAL TECHNOLOGY CO LTD US | B22F3/105\ B22F7/08 |
| 4810 | 3D printing | METHOD FOR CASTING THIN-SHELL NODULAR-CAST-IRON TURBOCHARGER\ | CN101716668A | JIANBIN LU US\ ZEMAO QU US\ XIANGYANG WANG US\ WEIKE XIE US\ GUOQIANG XU US\ QINGLING YOU US\ XIAOWEI YU US\ XIAOLEI YUAN US\ LIPING LIU (CN) | NINGXIA CHANGCHENG SUZAKI MACH US\ NINGXIA KOCEL GROUP CO LTD US | B22D15/02\ B22D1/00 |
| 4811 | 3D printing | FASTENER\ | CN202233379U | JIANDONG GU US | JIASHAN YIHUI GARMENT ACCESSORY CO LTD US | A44B1/02 |
| 4812 | 3D printing | METHOD FOR MANUFACTURING MULTI-ROD JUNCTION LAMELLA TYPE SPACE NET SHELL CAST STEEL NODE\ | CN101402165A | JIANGPING MEI (CN)\ TIAN HUANG (CN)\ YABIN DING (CN)\ YUAN GONG (CN)\ JIANDA GONG (CN) | UNIV TIANJIN (CN) | G06F17/50\ B23P15/00\ B23P9/04\ E04B1/342\ E04B1/58 |
| 4813 | 3D printing | METHOD FOR PREPARING CARBON POWDER BY UTILIZING DISPERSION LIQUID\ | CN102063028A | JIANQIANG SU US | ZHUHAI PRINT RITE CO LTD US | G03G9/08 |
| 4814 | 3D printing | CARBON POWDER BOTTLE EASY TO ADD POWDER\ | CN201707549U | JIANQIANG SU US\ YUXIA ZHENG US\ YONGGANG HE US | ZHUHAI PRINT RITE CO LTD US | G03G15/08 |
| 4815 | 3D printing | PVC-BASED PICTURES WITH METAL COATING COATING COMPOSITIONS AND METHODS FOR MAKING SAME\[FR] IMAGES à BASE DE PVC AYANT UN REVêTEMENT DE MéTAL COMPOSITIONS DE REVêTEMENT ET PROCéDéS SERVANT à FABRIQUER CELLES-CI\ | WO2007051368A1 | JIN AILAN (CN)\ FU XIAOPING (CN)\ LIN DAOGUANG (CN) | JIN AILAN (CN)\ FU XIAOPING (CN)\ LIN DAOGUANG (CN) | B44C1/00\ B05D5/06\ C09D5/08\ B44F1/08 |
| 4816 | 3D printing | EPOXY CONDUCTIVE RESIN APPLICABLE TO BINDING OF LED (LIGHT-EMITTING DIODE) CHIPS AND PREPARATION METHOD THEREOF\ | CN102634313A | JINBO JIANG US\ WENHAO CHEN US\ GUANQI ZHANG US\ HENGCHAO HUANG US | GUANGZHOU BAIYUN CHEMICAL INDUSTRY CO LTD US | C09J163/00\ C09J9/02\ C09J163/02 |
| 4817 | 3D printing | MOULD MAKING METHOD WITH CONFORMAL COOLING PIPE\ | CN101670437A | JINHUI LIU US\ CAN ZHAO US\ JIBING CHEN US\ RUIDI LI US | UNIV ZHEJIANG SCIENCES US | B22F3/22 |
| 4818 | 3D printing | MOULD STRUCTURE WITH CONFORMAL COOLING PIPE\ | CN101670627A | JINHUI LIU US\ CAN ZHAO US\ JIBING CHEN US\ RUIDI LI US | UNIV ZHEJIANG SCIENCES US | B29C33/38\ B29C33/04\ B29C45/73\ B22F3/22\ B29C45/26 |
| 4819 | 3D printing | COMPOUND (E)-4-HYDROXY-35-DIMETHOXYSTILBENE AND PREPARATION THEREOF\ | CN101225027A | JINXIAN WANG (CN)\ WENJUN YAN (CN) | UNIV NORTHWEST NORMAL (CN) | C07C43/23\ C07C41/30 |
| 4820 | 3D printing | FIBER REINFORCED FILTER FOR MOLTEN METAL FILTRATION\ | US7621408B2 | JUMA KASSIM (GB) | SUD CHEMIE HITECH INC (US) | C04B38/06\ B01D19/04\ B01D39/20\ B01D39/00\ B01D39/16\ B01F17/50\ C04B35/80\ C04B38/00\ B01D29/00 |
| 4821 | 3D printing | THREE-DIMENSIONAL SHAPE FORMING METHOD OF METAL AND CERAMIC BY POWDER METALLURGY\ | KR20040060634A | JUN MAN SU US\ KWON YEONG SAM US | CETATECH INC US | B22F3/00 |
| 4822 | 3D printing | MANUFACTURE METHOD OF ZIRCONIA CERAMICS NOZZLE USED FOR SPINNING SPRAY PRINTING\ | CN101564935A | KAIFENG ZHANG (CN)\ ZHEN LU (CN)\ ZHANGRUI WANG (CN) | HARBIN INST OF TECHNOLOGY (CN) | C04B35/622\ B41J2/16\ B28C1/12\ B28C3/00\ B28C7/02\ C04B35/48 |
| 4823 | 3D printing | MATERIAL FOR THREE-DIMENSIONAL MODELING METHOD OF PRODUCING THREE-DIMENSIONAL MODEL AND THREE-DIMENSIONAL MODEL\[FR] MATéRIAU DE MODELAGE TRIDIMENSIONNEL PROCéDé DE PRODUCTION D'UN MODèLE TRIDIMENSIONNEL ET MODèLE TRIDIMENSIONNEL\ | WO2009139393A1 | KAKINO RYUKI (JP) | FUJIFILM CORP (JP)\ KAKINO RYUKI (JP) | B29C67/00\ C08F2/44 |
| 4824 | 3D printing | MATERIAL FOR THREE-DIMENSIONAL MODELING PRODUCTION METHOD FOR THREE-DIMENSIONAL MODEL AND THREE-DIMENSIONAL MODEL\ | JP2010229170A | KAKINO TATSUTERU US | FUJIFILM CORP (JP) | B29C67/00\ C08F291/00\ C08F2/44\ C08L57/00 |
| 4825 | 3D printing | METHOD OF MANUFACTURING THREE-DIMENSIONAL SHAPED ARTICLE\ | JP2010228316A | KAKINO TATSUTERU US | FUJIFILM CORP (JP) | B29C67/00 |
| 4826 | 3D printing | MATERIAL FOR THREE-DIMENSIONAL MOLDING METHOD OF MANUFACTURING THREE-DIMENSIONAL MOLDING AND THREE-DIMENSIONAL MOLDING\ | JP2009274259A | KAKINO TATSUTERU US | FUJIFILM CORP (JP) | B29C67/00 |
| 4827 | 3D printing | THREE-DIMENSIONAL SHAPING MATERIAL AND METHOD FOR MANUFACTURING THE SAME\ | JP2009255479A | KAKINO TATSUTERU US | FUJIFILM CORP (JP) | B29C67/00 |
| 4828 | 3D printing | MATERIAL FOR THREE-DIMENSIONAL SHAPING AND METHOD OF MANUFACTURING THREE-DIMENSIONAL SHAPED OBJECT USING THE SAME\ | JP2009102527A | KAKINO TATSUTERU US | FUJIFILM CORP (JP) | B29C67/00\ C08F292/00\ C08F265/06 |
| 4829 | 3D printing | MAGNETIC RECORDING MEDIUM MAGNETIC SIGNAL REPRODUCTION METHOD AND MAGNETIC SIGNAL REPRODUCTION SYSTEM\ | US7995309B2 | KAKUISHI YUTAKA (JP)\ SUZUKI RYOTA (JP)\ USUI MASAYUKI (JP) | FUJIFILM CORP (JP) | G11B5/735 |
| 4830 | 3D printing | CONDUCTIVE PASTE FOR INTAGLIO OFFSET PRINTING AND METHOD OF MANUFACTURING ELECTRODE SUBSTRATE USING THE SAME\ | JP2008293904A | KAMATA TOSHIO US | SUMITOMO RUBBER IND US | B41M1/10\ H01B13/00\ B41M3/00\ H01B1/22\ H05K3/12\ B41M1/34 |
| 4831 | 3D printing | METHOD AND APPARATUS FOR MANUFACTURING THREE-DIMENSIONAL MOLDING\ | JP2005144870A | KANI AKIRA US\ NISHIWAKI SUMIKIMI US\ ITO YUSUKE US | NORITAKE CO LTD US | B29C67/00 |
| 4832 | 3D printing | PROCESS FOR PRODUCING SINTERED SIALON CERAMIC\[DE] VERFAHREN ZUR HERSTELLUNG VON GESINTERTER SIALONKERAMIK\[FR] PROCéDé DE FABRICATION DE CéRAMIQUE FRITTéE DE SIALON\ | EP2394974A4 | KANKAWA YOSHIMITSU (JP)\ NAKAGAWA KATSUNORI (JP) | IHI CORP (JP) | B28B1/24\ C04B35/634\ C04B35/638\ C04B35/645\ C04B35/599\ C04B35/632 |
| 4833 | 3D printing | MOLDING DIE AND MANUFACTURING METHOD OF SEPARATOR FOR FUEL CELL USING THE SAME\ | JP2005093162A | KARIYA TOSHIHIKO US\ TODA NAOKI US\ HASHIZUME KATSUHIRO US | MITSUBISHI HEAVY IND LTD (JP) | B29L31/00\ H01M8/10\ H01M8/02\ B29C45/37\ B29K101/12\ B29K105/16\ B29K507/04 |
| 4834 | 3D printing | METHOD FOR MANUFACTURING RESIN COMPOSITE FORMED PRODUCT\ | CN1978165A | KATUYUKI HASEGAWA (JP) | KATUYUKI HASEGAWA (JP) | B27N5/00\ B27N3/02 |
| 4835 | 3D printing | METHOD OF MANUFACTURING FERRITE POROUS BODY\ | JP2004115333A | KAWAKAMI HIDEKI US\ MATSUO YOSHIO US | FDK CORP US | C04B38/00 |
| 4836 | 3D printing | THREE-PHASE MULTI-MATERIAL THREE-DIMENSIONAL PRESSURIZED CLASH COAL GASIFICATION DEVICE AND PROCESS THEREOF\ | CN101760243A | KEHUI JIA US\ FALIN LI US\ RONGLIANG LI US\ YUCHENG LI US\ HUANFA LIU US\ DESHENG PAN US\ GEYONG REN US\ HOUCHANG SU US\ FENGGANG SUN US\ ZHENFENG YANG US\ HUAXIN ZHANG US\ KEFENG ZHANG US\ XINJIANG ZHAO US\ GUANGSHAN ZHUANG US\ YU ZOU US\ YONG ZHANG (CN) | SHANDONG HUALU HENGSHENG CHEMI US | C10J3/54\ C10J3/56 |
| 4837 | 3D printing | METHOD FOR IDENTIFYING LASER SINTERING POWDERS\ | US2010140550A1 | KELLER PETER (DE)\ MATTES THOMAS (DE)\ GERSCH MANDY (DE)\ OBERHOFER JOHANN (DE) | EOS ELECTRO OPTICAL SYST (DE) | C09K11/78\ C09K11/84\ C09K11/86 |
| 4838 | 3D printing | PROTON-CONDUCTING CERAMIC/POLYMER COMPOSITE MEMBRANE FOR THE TEMPERATURE RANGE UP TO 300°C\ | US2002093008A1 | KERRES JOCHEN (DE)\ NICOLOSO NORBERT (DE)\ SCHAFER GUNTHER (DE) | ELCHEM TECH CO LTD (KR) | C08J5/22\ B01D69/14\ B01J31/06\ B01J35/06\ C08K9/02\ H01M2/16\ H01M8/10 |
| 4839 | 3D printing | METALLIC PARTS FABRICATION USING SELECTIVE INHIBITION OF SINTERING (SIS)\ | US2004018107A1 | KHOSHNEVIS BEHROKH (US) | CORP Z (US) | B22F3/00\ B22F3/105\ B22F3/16\ B23K26/00\ B22F3/02\ B22F7/08 |
| 4840 | 3D printing | DIFFUSION ELECTRODE FOR FUEL CELL\ | US2005069754A1 | KIM JI-RAE (KR)\ CHOI KYOUNG-HWAN (KR) | HEWLETT PACKARD DEVELOPMENT CO (US) | H01M8/10\ H01M4/86\ H01M4/96\ H01M4/88\ H01M4/92\ H01M8/02 |
| 4841 | 3D printing | METHOD FOR MANUFACTURING LARGE-SCALE THREE-DIMENSIONAL WALL PAPER\ | KR100420861B1 | KIM JONG YEOUL US | KIM JONG YEOUL US | B41M3/18 |
| 4842 | 3D printing | SILVER PASTE COMPOSITION AND SOLAR CELL USING THE SAME\ | US2012325308A1 | KIM MIN-SEO (KR)\ HEO SOO-YEON (KR) | LG CHEMICAL LTD (KR)\ KIM MIN-SEO (KR)\ HEO SOO-YEON (KR) | H01B1/22\ H01L31/0224 |
| 4843 | 3D printing | NATURAL BINDER FOR PREPARING INK\ | KR100782049B1 | KIM SE CHANG (KR) | SE CHANG CHEMICAL CO LTD (KR) | C09J103/02\ C09J103/00 |
| 4844 | 3D printing | PROCESS FOR MAKING REUSABLE TOOLING\ | US2008105996A1 | KLOEB JAMES TODD (US)\ ELLIS LAWRENCE E (US)\ SEARS HAROLD (US) | FORD MOTOR CO (US) | B29C33/38\ B29C33/56 |
| 4845 | 3D printing | DOUBLE-SIDE PRINTING DEVICE\ | JP2006130752A | KOBAYASHI KAZUYOSHI US | TOHOKU RIKO KK US | B41L13/04\ B41F35/06 |
| 4846 | 3D printing | MEMBRANE-ELECTRODE ASSEMBLY AND MANUFACTURING METHOD THEREFOR AND POLYMER ELECTROLYTE FUEL CELL\ | JP2008078051A | KONNO YOSHIHARU US\ KOMA SATOSHI US\ KANO MASASHI US\ MIYAMA TOSHIHITO US | SEKISUI CHEMICAL CO LTD US | H01M8/10\ H01M8/02 |
| 4847 | 3D printing | MIXING DEVICE\[FR] DISPOSITIF DE MéLANGEAGE\ | WO2012015335A1 | KOVALEV SERGEY IVANOVICH (RU) | KOVALEV SERGEY IVANOVICH (RU) | B01F7/04\ B01F7/08\ B01F13/00\ B29B7/14 |
| 4848 | 3D printing | MATERIAL ZUR REVERSIBLEN SAUERSTOFF-SPEICHERUNG SOWIE DESSEN VERWENDUNG\[EN] CERAMIC MATERIAL FOR REVERSIBLE OXYGEN STORAGE COMPRISES A MIXED OXIDE OF CALCIUM AND MANGANESE\ | DE102005034071A1 | KRIEGEL RALF (DE)\ MUELLER FRANK (DE)\ REUNER WOLFGANG (DE)\ SOMMER ELMAR (DE) | UNIV WEIMAR BAUHAUS (DE) | C04B35/01\ B01D53/96\ B32B18/00 |
| 4849 | 3D printing | THREE-DIMENSIONAL MOLDING APPARATUS AND THREE-DIMENSIONAL MOLDING METHOD\ | US2002105114A1 | KUBO NAOKI (JP)\ NORITA TOSHIO (JP)\ MIYAZAKI MAKOTO (JP)\ WADA AKIRA (JP) | MINOLTA CO LTD (US) | B29C35/08\ B29C67/00 |
| 4850 | 3D printing | APPARATUS FOR FORMING A THREE-DIMENSIONAL PRODUCT\ | US6799959B1 | KUBO NAOKI (JP)\ TOCHIMOTO SHIGEAKI (JP) | MINOLTA CO LTD (JP) | B29C67/00\ B29C41/12\ B29C37/00\ B29C31/08 |
| 4851 | 3D printing | PASTE COMPOSITION ELECTRONIC PARTS AND CERAMIC GREEN SHEET AND METHOD OF MANUFACTURING MULTILAYER CERAMIC BOARD\ | JP2001155543A | KUBOTA MASAHIRO US | MURATA MANUFACTURING CO (JP) | C08K3/00\ C04B35/00\ C08K5/20\ H01B1/22\ H05K3/46\ C04B35/495\ C08L101/02 |
| 4852 | 3D printing | NANOPARTICLE SUSPENSIONS FOR USE IN THE THREE-DIMENSIONAL PRINTING PROCESS\[FR] SUSPENSIONS DE NANOPARTICULES UTILISéES DANS L'IMPRESSION TRIDIMENSIONNELLE\ | WO2009017648A1 | KUHN HOWARD A (US)\ BAI JOHN G (US)\ CREEHAN KEVIN D (US) | KUHN HOWARD A (US)\ EX ONE COMPANY LLC (US)\ BAI JOHN G (US)\ CREEHAN KEVIN D (US) | C09D11/00 |
| 4853 | 3D printing | METHOD FOR COATING A HEAT EXCHANGER STRUCTURE COATED HEAT EXCHANGER STRUCTURE AND USE THEREOF\[DE] VERFAHREN ZUR BESCHICHTUNG EINER W?RMETAUSCHERSTRUKTUR BESCHICHTETE W?RMETAUSCHERSTRUKTUR UND DEREN VERWENDUNG\[FR] PROCéDé POUR APPLIQUER UN REVêTEMENT SUR UNE STRUCTURE D'éCHANGEUR DE CHALEUR STRUCTURE D'éCHANGEUR DE CHALEUR REVêTUE ET UTILISATION DE CETTE DERNIèRE\ | EP2675856A1 | KUMMER HARRY (DE)\ HENNINGER STEFAN (DE) | FRAUNHOFER GES FORSCHUNG (DE) | C09D183/04\ B01J20/28\ B01J20/32\ F28F13/18 |
| 4854 | 3D printing | MICRO MAGNETIC SHELL INTEGRALLY MAGNETIZING APPARATUS\ | CN201804655U | KUNXI CHANG US\ XIANGHAO HUANG US | NINGBO HANHUA MAGNETOELECTRICITY CO LTD US | H01F13/00 |
| 4855 | 3D printing | MAGNETIC RECORDING MEDIUM\ | US2005106420A1 | KUROSE SHIGEO (JP)\ TANAKA HIROYUKI (JP)\ SHIRASUNA KENJI (JP) | TDK CORP (JP) | G11B5/70\ G11B5/738\ G11B5/702\ G11B5/00\ G11B5/17\ G11B5/74 |
| 4856 | 3D printing | MAGNETIC RECORDING MEDIUM\ | US2005106421A1 | KUROSE SHIGEO (JP)\ TANAKA HIROYUKI (JP)\ SHIRASUNA KENJI (JP) | TDK CORP (JP) | G11B5/70\ G11B5/702\ G11B5/714\ G11B5/706 |
| 4857 | 3D printing | MANUFACTURING METHOD OF ELECTRONIC COMPONENT\ | JP2007096013A | KUSANO KAZUTAKA US\ IWANAGA KEIJI US | TORAY INDUSTRIES (JP) | H01L21/288\ H01L21/60 |
| 4858 | 3D printing | THREE-DIMENSIONAL MODELING APPARATUS AND THREE-DIMENSIONAL OBJECT\ | US2010228381A1 | KUZUSAKO JUNICHI (JP)\ YASUKOCHI HIROYUKI (JP)\ MATSUI TAKESHI (JP) | SONY CORP (JP) | G06F17/00 |
| 4859 | 3D printing | HEAT-ABSORBING STRUCTURAL MATERIAL\ | US2012061065A1 | LACOMBE ROSS M (US) | LACOMBE ROSS M (US) | B32B3/00\ F28F7/00\ B32B15/08\ E04B1/76 |
| 4860 | 3D printing | RAPID PROTOTYPING MATERIAL SYSTEMS\ | US2004094058A1 | LAMBRIGHT TERRY M (US)\ KASPERCHIK VLADEK (US) | DAIMLER CHRYSLER AG (DE) | C04B35/624 |
| 4861 | 3D printing | SYSTEMS AND METHODS OF SOLID FREEFORM FABRICATION WITH TRANSLATING POWDER BINS\ | US7296990B2 | LAMBRIGHT TERRY M (US)\ NIELSEN JEFFREY A (US)\ COLLINS DAVID C (US)\ DEVOS JOHN A (US)\ CRUZ-URIBE TONY S (US) | HEWLETT PACKARD DEVELOPMENT CO (US) | B28B1/16 |
| 4862 | 3D printing | CALCIUM ALUMINATE CEMENT COMPOSITIONS FOR SOLID FREEFORM FABRICATION\ | US2004261664A1 | LAMBRIGHT TERRY M (US)\ ORIAKHI CHRIST PHER (US) | HEWLETT PACKARD DEVELOPMENT CO (US) | B28B1/00\ C04B24/26\ C04B22/04\ C04B22/08\ C04B22/10\ C04B24/32\ C04B28/06\ C04B41/50 |
| 4863 | 3D printing | METHOD FOR THE PRODUCTION OF A COATING MATERIAL\ | US2010092686A1 | LARYEA NORA (DE)\ THURN CAROLIN (DE)\ SEPEUR STEFAN (DE) | LARYEA NORA (DE)\ THURN CAROLIN (DE)\ SEPEUR STEFAN (DE) | B05D3/06\ C09D183/04\ B05D1/04\ C09D189/00\ C23C16/44 |
| 4864 | 3D printing | THE METHOD OF TREATING AUTO'S PADDALE SUFACE TO PREVENT SLIP WIST MEATAL COMPCSI TION\ | KR101190513B1 | LEE BO HYUN (KR)\ LEE CHANG YAP (KR) | LEE CHANG YAP (KR)\ LEE BO HYUN (KR) | B22F3/00\ C23C28/00\ B60K26/00 |
| 4865 | 3D printing | METHOD OF CASTING A COMPONENT HAVING INTERIOR PASSAGEWAYS\ | US2011293434A1 | LEE CHING-PANG (US)\ MERRILL GARY B (US)\ BURNS ANDREW J (US) | LEE CHING-PANG (US)\ MERRILL GARY B (US)\ BURNS ANDREW J (US) | B22D25/02\ B32B3/10\ B22C9/10\ B22C9/02\ F01D5/14\ B32B15/00 |
| 4866 | 3D printing | SURFACE COATING APPARATUS OF PRO-FILE COATING METHOD THE SAME\ | KR100801433B1 | LEE SUK HO (KR)\ JUNG EUN CHUN (KR) | LG CHEMICAL LTD (KR) | B05B7/14\ B05C1/10 |
| 4867 | 3D printing | METAL-JET UNIT\ | KR100735115B1 | LEE TAIK MIN (KR)\ KIM DONG SOO (KR)\ KIM KWANG YOUNG (KR)\ KANG TAE GOO (KR)\ YANG JEONG SOON (KR)\ CHOI BYUNG OH (KR)\ JO JEONG DAI (KR)\ YUN SO NAM (KR)\ HAM YOUNG BOG (KR)\ KIM CHUNG HWAN (KR) | KOREA MACH & MATERIALS INST (KR) | B22D23/00 |
| 4868 | 3D printing | THE GILT PRINTING METHOD AND THE TEXTILE\ | KR20130063329A | LEE YOUNG JAE (KR)\ CHOI JAE YOUNG (KR) | LEE YOUNG JAE (KR)\ CHOI JAE YOUNG (KR) | D06P5/10\ D06P1/52\ D06Q1/04 |
| 4869 | 3D printing | HAFTENDES MITTEL ZUR APPLIKATION AUF EINEM SANIT?RGEGENSTAND\[EN] AGENT USEFUL E.G. AS COLD ADHESIVE IN SANITARY APPLICATIONS COMPRISES FILLERS COMPRISING A SURFACTANT AND A BONDING AGENT COMPRISING E.G. POLYALKYLENE DERIVATIVE POLYALKYLENE CHAINS AND POLYGLYCERIN POLYETHER ALKYL CARBONIC ACID\ | DE102008051173A1 | LEIPOLD JOACHIM (DE)\ JAESCHKE EDGAR (DE)\ FRITZ MATTHIAS (DE) | BUCK CHEMIE GMBH (DE) | C11D17/00\ C09J9/00\ C09J11/08 |
| 4870 | 3D printing | HAFTENDES MITTEL ZUR APPLIKATION AUF EINEM SANIT?RGEGENSTAND\[EN] AGENT USEFUL E.G. AS COLD ADHESIVE IN SANITARY APPLICATIONS COMPRISES FILLERS COMPRISING A SURFACTANT AND A BONDING AGENT COMPRISING E.G. POLYALKYLENE DERIVATIVE POLYALKYLENE CHAINS AND POLYGLYCERIN POLYETHER ALKYL CARBONIC ACID\ | DE102008012092A1 | LEIPOLD JOACHIM (DE)\ JAESCHKE EDGAR (DE)\ FRITZ MATTHIAS (DE) | BUCK CHEMIE GMBH (DE) | C11D3/37\ C09J9/00 |
| 4871 | 3D printing | ADHESIVE AGENT FOR APPLICATION ON A SANITARY OBJECT\ | US8461093B2 | LEIPOLD JOACHIM (DE)\ JAESCHKE EDGAR (DE)\ FRITZ MATTHIAS (DE) | BUCK CHEMIE GMBH (DE)\ LEIPOLD JOACHIM (DE)\ JAESCHKE EDGAR (DE)\ FRITZ MATTHIAS (DE) | C08L33/00\ C11D3/37\ C11D17/00\ C08L39/00\ B32B27/32 |
| 4872 | 3D printing | N ALPHA-(MENTHANECARBONYL)AMINO ACID AMIDES AND USE THEREOF AS PHYSIOLOGICAL COOLING ACTIVE INGREDIENTS\ | US2008096969A1 | LEY JAKOB (DE) | SYMRISE GMBH & CO KG (DE) | A61K8/42\ A61K31/16\ A61P43/00\ A61Q19/00\ C07C233/57 |
| 4873 | 3D printing | WATER-BASED MATERIAL SYSTEMS AND METHODS FOR 3D PRINTING\ | US2004038009A1 | LEYDEN RICHARD NOEL (US)\ CLEARY TIMOTHY MARVIN (US)\ LI JOHN XIAOSAN (US)\ OBUCHOWICZ JACEK (GB)\ PEACE RICHARD J (GB) | PENTRON LAB TECHNOLOGIES LLC (US)\ JENERIC PENTRON INC (US) | B29C67/00 |
| 4874 | 3D printing | ARTIFICIAL TOOTH AND PREPARATION METHOD THEREOF\ | CN103143711A | LI SHANGKUI US\ HUANG ZHENGHUA US | LI SHANGKUI US\ HUANG ZHENGHUA US | B22F3/16\ A61C13/08\ B22F3/22 |
| 4875 | 3D printing | POLYPEPTIDE PROTEIN CLASS-MEDICAMENT UNDER-TONGUE INSTANT NANOMETER EMULSION AND 3D PRINTING PREPARING METHOD THEREOF\ | CN100579575C | LIMIN ZHU (CN)\ DENGGUANG YU (CN)\ XIAXIA SHEN (CN)\ JIE HAN (CN) | UNIV DONGHUA (CN) | A61J3/00\ A61K9/70\ A61K38/16\ A61P3/10 |
| 4876 | 3D printing | METHOD FOR MEASURING PRINTING PERFORMANCE OF LIQUID SYSTEM THREE-DIMENSIONAL PRINTER SYSTEM\ | CN101439584B | LIMIN ZHU (CN)\ DENGGUANG YU (CN)\ XIAXIA SHEN (CN)\ XUELIAN LI (CN) | UNIV DONGHUA (CN) | B29C67/00\ B41J2/01\ A61J3/00 |
| 4877 | 3D printing | MOBILE LASER REPAIRING EQUIPMENT AND REPAIRING METHOD THEREOF\ | CN101214582A | LIN YANG (CN)\ BO LIU (CN)\ JIANLIN LI (CN)\ JUNCHUAN LIU (CN) | BEIJING ZISEGUANG LASER TECHNO (CN) | B23K26/34\ C23C24/08 |
| 4878 | 3D printing | APPARATUS AND PROCESS FOR PRODUCING A THREE-DIMENSIONAL SHAPED BODY\ | US2005116391A1 | LINDEMANN MARKUS (DE)\ HUTFLESS JOACHIM (DE)\ HAMMANN GERHARD (DE)\ RENZ BERND H (DE) | Empty Field US | B29C67/00\ B22F3/105\ B22F3/16\ C04B35/64 |
| 4879 | 3D printing | MULTI-LAYER REPRESSED WOOD ART DOOR STRUCTURE\ | CN201059144Y | MAOHUA WANG (CN) | MAOHUA WANG (CN) | E06B3/74\ B32B21/13 |
| 4880 | 3D printing | MAGNETIC INK AND PRINT COATED THEREWITH\ | JP9012954A | MARUYAMA TORU US | TOPPAN MOORE KK US | C09D11/10\ C09D11/00\ G11B5/73\ G11B5/702\ G07F7/08 |
| 4881 | 3D printing | METHOD OF FABRICATING BATH-MODELS FOR NICKEL SHELLS\ | US2010102478A1 | MASCHERA FLAVIO (IT)\ BARROTE PEDRO (PT) | MASCHERA FLAVIO (IT)\ BARROTE PEDRO (PT) | B29C33/38 |
| 4882 | 3D printing | ANORDNING F?R DR?NERING\ | SE0501521L | MASSET GILLES US | EPSCEMENT INTERNAT AB (SE) | Empty Field |
| 4883 | 3D printing | INTELLIGENT MODELING METHOD AND SYSTEM FOR EARMOLD SHELL AND HEARING AID DESIGN\ | US7605812B2 | MCBAGONLURI FRED (US)\ BINDNER JOERG (DE) | SIEMENS AUDIOLOGISCHE TECHNIK (DE) | G06T15/00 |
| 4884 | 3D printing | METHOD OF MANUFACTURING COMBUSTOR COMPONENTS\ | US2009255256A1 | MCMASTERS MARIE ANN (US)\ LOHMUELLER STEVEN JOSEPH (US)\ BOEHM RANDALL CHARLES (US) | GEN ELECTRIC (US) | B22F7/00\ F02C7/22 |
| 4885 | 3D printing | ANATOMICALLY REALISTIC THREE DIMENSIONAL PHANTOMS FOR MEDICAL IMAGING\ | US2010202001A1 | MILLER MICHAEL A (US)\ HUTCHINS GARY D (US) | MILLER MICHAEL A (US)\ HUTCHINS GARY D (US) | G06K9/00\ G06F15/00 |
| 4886 | 3D printing | METHOD MANUFACTURING OF ABSORBER FOR NFC TAG ANTENNA\ | KR101308595B1 | MIN SONG KI (KR) | MIN SONG KI (KR)\ TSP CO LTD (KR) | H05K9/00\ H01Q17/00 |
| 4887 | 3D printing | LASER CLADDING QUICK PRECISION MANUFACTURING METHOD AND DEVICE BASED ON LASER MILLING\ | CN102304712A | MINGDI WANG US\ CHENGFA SONG US\ XIUBO LIU US\ KAIBO GUO US\ GEYAN FU (CN)\ SHIHONG SHI (CN) | UNIV SOOCHOW (CN) | C23C24/10 |
| 4888 | 3D printing | LIQUID EJECTION HEAD AND ITS MANUFACTURING METHOD\ | JP2005280349A | MITA TAKESHI US\ NIHEI YASUKAZU (JP) | FUJI PHOTO FILM CO LTD (US) | B41J2/16\ B41J2/045\ B41J2/055 |
| 4889 | 3D printing | PRINT PASTE FOR ABSORBING ELECTRODE STEP DIFFERENCE AND MANUFACTURING METHOD OF STACKED CERAMIC ELECTRONIC COMPONENT\ | JP2008091514A | MIURA SHUICHI US\ MARUNO TETSUJI US\ ODA KAZUHIKO (JP) | TDK CORP (JP) | C04B35/00\ H01G4/12\ H01G4/30 |
| 4890 | 3D printing | INKJET RECORDING BODY\ | JP2011042062A | MIYAZAKI SAKURA US\ TAKANO TAKASHI US\ SATO KATAHIKO US\ MINAMI TAKEHIRO US | OJI PAPER CO US | B41M5/00\ B41M5/50\ B41M5/52 |
| 4891 | 3D printing | SHUTTERING METHOD FOR FORMING CONCRETE FOUNDATION USES THREE DIMENSIONAL PROFILED SHUTTERING SECTIONS WITH INTERCONNECTED CAVITIES\[NL] WERKWIJZE VOOR HET VERVAARDIGEN VAN EEN FUNDERING WAARBIJ IN HOGE MATE ARBEID EN MATERIAAL BESPAARD WORDT.\ | NL1028400C1 | MOONEN SERVATIUS PETRUS GERTRU (NL)\ KLEINE JANTIENUS DE (NL)\ BOUWHUIJS MAARTEN BONIFATIUS L (NL) | KOOLEN & LICHTENBERG DEV INTER (NL) | E04G13/00\ E02D27/01 |
| 4892 | 3D printing | ELECTROSTATIC CHUCK HEATER\ | US8008602B2 | MORI YUTAKA (JP)\ HATTORI AKIYOSHI (JP)\ TORIGOE TAKERU (JP) | NGK INSULATORS LTD (JP) | H05B3/68\ C23C16/00\ H05B3/18 |
| 4893 | 3D printing | ELECTROPHOTOGRAPHIC PHOTORECEPTOR AND METHOD FOR MANUFACTURING SAME\ | JP2008009001A | MORITA MAKOTO US\ NIIGAE RYUICHI US\ YASUI TATSUYA US | MATSUSHITA ELECTRIC IND CO LTD (JP) | G03G5/00\ G03G5/14 |
| 4894 | 3D printing | IMAGE FORMING METHOD\ | JP2010152234A | MORIYAMA SHINJI US\ KATAYAMA KOHEI US | KAO CORP US | G03G9/08\ G03G15/20 |
| 4895 | 3D printing | IMAGE FORMING METHOD\ | JP2008116666A | MORIYAMA SHINJI US\ KATAYAMA KOHEI US | KAO CORP US | G03G9/087\ G03G9/08\ G03G15/20 |
| 4896 | 3D printing | CASTING METHOD FOR GUARANTEEING WALL THICKNESS PRECISION OF BLAST FURNACE HOT-BLAST STOVE PILLAR\ | CN102836965B | MU CHENGSHUANG US | ANGANG HEAVY MACHINERY CO LTD US | B22C9/10\ B22C9/24 |
| 4897 | 3D printing | METHODS FOR PREPARATION OF THREE-DIMENSIONAL BODIES\ | US2004238999A1 | MULLIGAN ANTHONY C (US)\ RIGALI MARK J (US)\ SUTARIA MANISH P (US)\ ARTZ GREGORY J (US)\ GAFNER FELIX H (US)\ VAIDYANATHAN K RANJI (US) | ADVANCED CERAMICS RES INC (US) | B22F3/00\ B28B17/00\ B29C43/00\ C04B35/80\ B28B3/20\ B29C47/54\ C04B35/634\ C04B35/638\ C04B35/645 |
| 4898 | 3D printing | METHOD FOR MANUFACTURING THREE-DIMENSIONAL STRUCTURE AND SINTERED CERAMIC COMPACT USING THE SAME\ | JP2004315340A | MURAMOTO YASUTO US\ FUKUII AKIO US | KYOCERA CORP (JP) | C04B35/622\ B22F3/02\ C04B35/63 |
| 4899 | 3D printing | METHOD OF MANUFACTURING THREE DIMENSIONAL STRUCTURE CERAMIC SINTERED COMPACT USING THE SAME AND MEMBER FOR SEMICONDUCTOR/LIQUID CRYSTAL MANUFACTURING APPARATUS USING THE SAME\ | JP2005097078A | MURAMOTO YASUTO US\ FUKUII AKIO US\ MISHIMA HIROMITSU US | KYOCERA CORP (JP) | C04B35/64\ B28B11/00 |
| 4900 | 3D printing | METHOD OF PREPARING PRINTED OR COATED IMAGE AND PRINTED OR COATED IMAGE OBJECT BY THIS METHOD\ | JP2006069167A | MURAOKA KOJI US | SHUHO KK US | B05D3/02\ B41M7/02 |
| 4901 | 3D printing | METHOD OF MAKING A COMPOSITE MATERIAL BY THREE-DIMENSIONAL INK-JET PRINTING\ | US8932511B2 | NAPADENSKY EDUARDO (IL) | NAPADENSKY EDUARDO (IL)\ STRATASYS LTD (IL) | B29C35/08\ B29C67/00\ G03C9/08\ B41J2/01\ B29C41/52\ B29C35/02\ B29C37/00\ B29C\ B29C41/02\ B29C41/48\ B41M3/00\ B41M3/16 |
| 4902 | 3D printing | BINDER REMOVAL IN SELECTIVE LASER SINTERING\ | US2004081573A1 | NEWELL KENNETH J (US) | 3D SYSTEMS INC (US) | B22F1/00\ B22F3/26\ B22F3/11 |
| 4903 | 3D printing | SYSTEMS AND METHODS OF SOLID FREEFORM FABRICATION WITH INTERCHANGEABLE POWDER BINS\ | US2007063372A1 | NIELSEN JEFFREY A (US)\ HUNTER SHAWN D (US)\ BOYD MELISSA D (US)\ COLLINS DAVID C (US) | HUNTER SHAWN D (US)\ NIELSEN JEFFREY A (US)\ BOYD MELISSA D (US)\ COLLINS DAVID C (US) | B29C67/00 |
| 4904 | 3D printing | FABRICATING A THREE-DIMENSIONAL OBJECT\ | US2006071367A1 | NIELSEN JEFFREY A (US)\ HUNTER SHAWN D (US)\ COLLINS DAVID C (US)\ KASPERCHIK VLADEK P (US)\ CRUZ-URIBE TONY (US) | HUNTER SHAWN D (US)\ NIELSEN JEFFREY A (US)\ KASPERCHIK VLADEK P (US)\ COLLINS DAVID C (US)\ CRUZ-URIBE TONY (US) | B28B1/14 |
| 4905 | 3D printing | LIQUID DISCHARGE HEAD AND MANUFACTURING METHOD THEREOF\ | US7245017B2 | NIHEI YASUKAZU (JP)\ MITA TSUYOSHI (JP) | FUJIFILM CORP (JP) | H01L23/52\ B41J2/16\ B41J2/045\ B41J2/055\ H01L21/4763\ H01L23/48\ H01L29/40 |
| 4906 | 3D printing | METHOD AND APPARATUS FOR MANUFACTURING ORNAMENT FROM TITANIUM POWDER\ | JP2010240369A | NISHIHARA AKIO US\ NISHIKAWA HISAHIDE US | NISHIHARA AKIO US\ TOSHIN CO LTD US | A44C27/00 |
| 4907 | 3D printing | MANUFACTURING METHOD OF THREE-DIMENSIONAL PAPER OBJECT\ | JP2004262159A | NOMURA TAKANORI US | GIFU PREFECTURE US | B31D5/00\ D21B1/10 |
| 4908 | 3D printing | METHOD TO MANUFACTURE CONDUCTIVE PASTE FOR INTAGLIO OFFSET PRINTING AND CONDUCTIVE PATTERN\ | JP2007250892A | OCHI ATSUSHI US | SUMITOMO RUBBER IND US | B41M1/10\ C09D11/02\ H05K1/09\ H05K3/12 |
| 4909 | 3D printing | LEADER TAPE MAGNETIC TAPE CARTRIDGE AND MAGNETIC RECORDING AND REPRODUCTION METHOD\ | US2005214594A1 | OHNO MIKIO (JP) | FUJI PHOTO FILM CO LTD (US) | B32B5/16\ G11B5/627\ G11B23/26\ G11B15/18\ G11B23/107 |
| 4910 | 3D printing | POWDER BLAST TOOL POWDER RESERVOIR INSERT FOR POWDER RESERVOIR AND METHOD OF DENTAL TREATMENT\ | US7980923B2 | OLMO OLIVIER (CH)\ DONNET MARCEL (FR) | FERTON HOLDING SA (CH) | B24C9/00 |
| 4911 | 3D printing | BUILDING ENVELOPE DETERMINATION\ | US2013197868A1 | OLSSON THOMAS (US)\ MITCHELL JOHN (US) | OLSSON THOMAS (US)\ MITCHELL JOHN (US)\ AUTODESK INC (US) | G06F17/50 |
| 4912 | 3D printing | CEMENT SYSTEM INCLUDING A BINDER FOR USE IN FREEFORM FABRICATION\ | US6930144B2 | ORIAKHI CHRISTOPHER (US) | HEWLETT PACKARD DEVELOPMENT CO (US) | B29C67/00\ B28B1/00\ B28B7/46\ C04B14/04\ C04B14/10\ C04B14/30\ C04B14/36\ C04B22/16\ C04B24/00\ C04B24/04\ C04B24/26\ C04B26/02\ C04B28/34\ G03G9/087 |
| 4913 | 3D printing | TONER\ | JP2004109716A | OTA GENICHI US | NIPPON ZEON CO US | G03G9/08\ G03G9/097 |
| 4914 | 3D printing | VERFAHREN ZUR HERSTELLUNG VON SCHMUCKSTüCKEN.\[EN] PRODUCING THREE-DIMENSIONAL JEWELRY PIECES MADE OF METAL COMPRISES REPEATEDLY APPLYING A METAL POWDER ON A SUBSTRATE AND/OR HIGHLY-ADJUSTABLE PLATFORM IN A LAYER-WISE MANNER AND PARTIALLY MELTING THE POWDER LAYER AT A PREDETERMINED POINT\ | CH700273A1 | OTT HANSPETER (CH) | OTT HANSPETER (CH) | B22F3/11\ A44C27/00 |
| 4915 | 3D printing | TONERPARTIKEL SOWIE EIN VERFAHREN UND EINE ANLAGE ZU DEREN HERSTELLUNG\[EN] TONER POWDER E.G. BLACK TONER POWDER MANUFACTURING METHOD FOR E.G. LASER PRINTER INVOLVES DISSOLVING OR DISPERSING POLYMER MATERIAL AND COLORANT IN LIQUID MEDIUM AND HARDENING COMPONENT IN SOLUTION OR DISPERSION BY EVAPORATING MEDIUM\ | DE102005034407A1 | PAFFHAUSEN HANS (CH) | PELIKAN HARDCOPY PRODUCTION AG (CH) | G03G9/08 |
| 4916 | 3D printing | LASER- UND THERMISCH BESCHREIBBARE OBERFL?CHENBESCHICHTUNG FüR MATERIALIEN\[EN] LASER AND THERMALLY WRITABLE SURFACE COATING FOR MATERIALS\[FR] REVêTEMENT DE SURFACE POUR MATéRIAUX POUVANT RECEVOIR UNE INSCRIPTION PAR LASER OU PAR VOIE THERMIQUE\ | EP2429826A1 | PALKOVITS ROLAND (AT)\ SCHALKHAMMER THOMAS (AT)\ KORNHERR ANDREAS (AT) | MONDI UNCOATED FINE & KRAFT PAPER GMBH (AT) | C09D7/12\ B41M5/26\ B41M5/28\ C04B41/80\ C09D5/26\ D21H17/66\ D21H17/67\ D21H19/38\ D21H19/80\ D21H21/28\ D21H23/22 |
| 4917 | 3D printing | SOLID FREE-FORM FABRICATION METHODS FOR THE PRODUCTION OF DENTAL RESTORATIONS\ | US2002125592A1 | PANZERA CARLINO (US)\ SCHULMAN MARTIN L (US) | K F TECH CO LTD (KR) | A61C13/00\ A61K6/083\ A61K6/06\ A61K6/087 |
| 4918 | 3D printing | MASS PRODUCTION OF SHELLS AND MODELS FOR DENTAL RESTORATIONS PRODUCED BY SOLID FREE-FORM FABRICATION METHODS\ | US2002064745A1 | PANZERA CARLINO (US)\ SCHULMAN MARTIN L (US) | QIU ZEYOU (CN) | A61C13/00\ A61K6/083\ A61K6/06\ A61K6/087 |
| 4919 | 3D printing | SIMULTANEOUS NEGATIVE CAST AND SHELL FABRICATION FOR CUSTOM HEARING AIDS\ | US7875232B2 | PARSI SALMAN (US)\ BARRY SCOTT (US) | SIEMENS HEARING INSTR INC (US) | B29C35/04 |
| 4920 | 3D printing | GYPSUM BASE MATERIAL SYSTEM FOR 3-D SPRAYING BINDING AND PREPARING METHOD THEREFOR\ | CN100352643C | PEIKANG BAI (CN)\ JIANHONG WANG (CN)\ BIN LIU (CN)\ CHENG JUN (CN) | UNIV ZHONGBEI (CN) | C04B26/02\ B32B27/04\ C04B11/00\ C04B22/14\ C04B24/02\ C04B24/24 |
| 4921 | 3D printing | DIGITALLY INJECTED DESIGNS IN POWDER SURFACES\ | US2011250404A1 | PERVAN DARKO (SE)\ JACOBSSON JAN (SE)\ RYBERG MELKER (SE)\ H KANSSON NICLAS (SE)\ ZIEGLER GORAN (SE) | CERALOC INNOVATION BELGIUM (BE) | B05D3/12\ B32B3/30\ B05D1/36\ B32B7/00 |
| 4922 | 3D printing | DIGITALLY INJECTED DESIGNS IN POWDER SURFACES\[DE] DIGITAL INJIZIERTES DESIGN IN PULVEROBERFL?CHEN\[FR] MOTIFS RéALISéS PAR INJECTION NUMéRIQUE DANS DES COUCHES DE POUDRE\ | EP2558306A4 | PERVAN DARKO (SE)\ JACOBSSON JAN (SE)\ RYBERG MELKER (SE)\ H?KANSSON NICLAS (SE)\ ZIEGLER G?RAN (SE) | V?LINGE INNOVATION AB (SE) | B44C5/04\ B27N7/00\ B32B21/02\ B32B21/08\ B32B37/24\ B32B38/04\ B32B38/14\ B44F9/02\ E04C2/24\ E04F15/02 |
| 4923 | 3D printing | COATED POWDER PARTICLES FOR PRODUCING THREE-DIMENSIONAL BODIES BY MEANS OF A LAYER CONSTITUTING METHOD\ | US7722802B2 | PFEIFER ROLF (DE)\ SHEN JIALIN (DE) | DAIMLER AG (DE) | B29C67/00\ B29C41/00\ B29C67/24\ B22F7/00\ B22F1/02 |
| 4924 | 3D printing | PROCESS FOR COATING PARTICLES FOR GENERATIVE RAPID PROTOTYPING\ | US7611756B2 | PFEIFER ROLF (DE)\ SHEN JIALIN (DE) | DAIMLER AG (DE) | B29C67/00\ B22F3/00\ B22F1/00\ B01J2/00\ B01J2/16\ H05H1/00 |
| 4925 | 3D printing | PROCESS AND DEVICE FOR PRODUCING SOLID BODIES BY SEQUENTIAL LAYER BUILDUP\ | US7285234B2 | PFEIFER ROLF (DE)\ SHEN JIALIN (DE)\ ZEPPELIN DIDIER VON (DE) | DAIMLER CHRYSLER AG (DE) | B29C67/00\ B22F3/00\ B32B37/00\ D04H1/16\ B32B38/04\ B27J5/00 |
| 4926 | 3D printing | PROCESS FOR QUALITY CONTROL FOR A POWDER BASED LAYER BUILDING UP PROCESS\ | US2004173946A1 | PFEIFER ROLF (DE)\ SHEN JIALIN (DE)\ ZEPPELIN DIDIER VON (DE) | PARK DAE WON (KR) | B29C67/00 |
| 4927 | 3D printing | METHOD OF MANUFACTURING NONWOVEN ABRASIVE ARTICLES USING DRY PARTICULATE MATERIAL\[DE] VERFAHREN ZUR HERSTELLUNG VON VLIESSTOFFSCHLEIFARTIKELN MIT TROCKENEM K?RNIGEM MATERIAL\[FR] PROCEDE PERMETTANT DE PRODUIRE DES ARTICLES ABRASIFS EN NON-TISSE AU MOYEN D'UN MATERIAU EN PARTICULES SEC\ | EP1617973A1 | POLLAUD GUY M (FR)\ MALJEAN SANDRINE (FR)\ MARTIN RIVERA CARMEN (ES)\ CABRERO GOMEZ ESTRELLA (ES) | 3M INNOVATIVE PROPERTIES CO (US) | B24D11/00 |
| 4928 | 3D printing | WASTE DUST COLLECTION BAG\[FR] SAC DE COLLECTE DE POUSSIèRES DE DéCHETS\ | WO2013044727A1 | PU HONGLI (CN) | PRINT RITE UNICORN IMAGE PRODUCTS CO LTD ZHUHAI (CN)\ PU HONGLI (CN) | G03G21/10 |
| 4929 | 3D printing | SURFACE LAYER FOR PREVENTING OBJECT SURFACE FROM BEING PASTED AND SCRAWLED AND MANUFACTURING METHOD OF SURFACE LAYER\ | CN102828586A | QI JIANZONG US | QI JIANZONG US | E04F13/00 |
| 4930 | 3D printing | DOUBLE-GROUP SPRAY HEAD THREE-DIMENSIONAL POWDER FEEDING DEVICE\ | CN203245985U | QIAN BO US\ ZHENG YUTING US\ LIU JINHUI US\ XIAO SHENGBING US\ WU TONG US | HARBIN DEYU JIANXING TECHNOLOGY CO LTD US | B28B13/02 |
| 4931 | 3D printing | COMPOSITE ANTIBACTERIAL FAR INFRARED MEDICAL STONE POROUS CERAMIC BALL AND PREPARATION METHOD THEREOF\ | CN102153365A | QIANG LV US\ HAIQUAN HU US | ZHEJIANG HEARS VACUUM UTENSILS CO LTD US | C04B35/622\ C04B38/06\ C04B35/16\ C04B35/636 |
| 4932 | 3D printing | MECHANOCHEMICAL METHOD FOR PREPARING HIGH-PERFORMANCE REGENERATED RUBBER\ | CN101747528A | QISHENG WU US\ YONGJU YAO US | YANCHENG YUQIU RUBBER PRODUCTS US | C08J11/28\ C08L17/00\ C08L21/00 |
| 4933 | 3D printing | STEEL REINFORCED CONCRETE STEREO FORCE BEARING STRUCTURE FLOOR SLAB\ | CN100350111C | QIU ZEYOU (CN) | QIU ZEYOU (CN) | E04B5/21\ E04B5/18 |
| 4934 | 3D printing | STEREO BEARING SHUTTERING FOR STEEL REINFORCED CONCRETE\ | CN100346041C | QIU ZEYOU (CN) | QIU ZEYOU (CN) | E04B5/36\ E04B5/17\ E04C1/00\ E04G15/06 |
| 4935 | 3D printing | STEEL REINFORCED CONCRETE THREE-DIMENSIONAL FORCE-BEARING STRUCTURE FLOORSLAB\ | CN1743584A | QIU ZEYOU (CN) | QIU ZEYOU (CN) | E04B5/21 |
| 4936 | 3D printing | STEEL REINFORCED CONCRETE THREE-DIMENSIONAL FORCE-BEARING STRUCTURE FLOORSLAB\ | CN1743582A | QIU ZEYOU (CN) | QIU ZEYOU (CN) | E04B5/21 |
| 4937 | 3D printing | THREE-DIMENSIONAL BEARING BUILDING ROOF OF STEEL-REINFORCED CONCRETE\ | CN101029516A | QIU ZEYOU (CN) | QIU ZEYOU (CN) | E04B5/21 |
| 4938 | 3D printing | THREE-DIMENSIONAL BEARING BUILDING ROOF OF STEEL-REINFORCED CONCRETE\ | CN101029515A | QIU ZEYOU (CN) | QIU ZEYOU (CN) | E04B5/21 |
| 4939 | 3D printing | THREE-DIMENSIONAL BEARING BUILDING ROOF OF STEEL-REINFORCED CONCRETE\ | CN101029514A | QIU ZEYOU (CN) | QIU ZEYOU (CN) | E04B5/21 |
| 4940 | 3D printing | SOLID LOAD CARRYING STRUCTURED STORIED BUILDING CONSTRUCTED BY CONCRETE OF REINFORCING STEEL BAR\ | CN1873147A | QIU ZEYOU (CN) | QIU ZEYOU (CN) | E04B5/21 |
| 4941 | 3D printing | SOLID LOAD CARRYING STRUCTURED STORIED BUILDING CONSTRUCTED BY CONCRETE OF REINFORCING STEEL BAR\ | CN1873146A | QIU ZEYOU (CN) | QIU ZEYOU (CN) | E04B5/21 |
| 4942 | 3D printing | REINFORCED CONCRETE ROOFING WITH 3-D BEARING STRUCTURE\ | CN1834361A | QIU ZEYOU (CN) | QIU ZEYOU (CN) | E04B5/36\ E04B5/21\ E04B5/18 |
| 4943 | 3D printing | MECHANICAL ARM\ | CN102189275A | QIUHUA MENG US | QIUHUA MENG US | B23B15/00\ B25J9/02 |
| 4944 | 3D printing | CELLULATED WIREMESH CARRIER WITH ALUMINA COAT AND PREPARATION METHOD\ | CN101049577A | QUAN XIE SUN (CN) | UNIV DALIAN TECH (CN) | B01J32/00\ B01J35/04 |
| 4945 | 3D printing | CO-BURNING METHOD FOR OXYGEN SENSOR ELECTROLYTE AND PALATINUM ELECTRODE\ | CN100405052C | QUN WEI (CN)\ TAO JIANG (CN)\ YAN LI (CN)\ LIJUN JIANG (CN) | GCI SCIENCE & TECH CO LTD (CN) | H01L49/00\ G01N27/409\ G01N27/28 |
| 4946 | 3D printing | PRINTED GELATIN CAPSULE AND METHOD OF MANUFACTURE\ | US2013186293A1 | RATKO MICHAEL J (CA)\ MAMIZADEH NADER (CA)\ DANTA JEFFREY MARTIN (US) | RATKO MICHAEL J (CA)\ MAMIZADEH NADER (CA)\ DANTA JEFFREY MARTIN (US)\ GI SPORTZ INC (CA) | B41J2/01\ B41M5/00\ B41F17/00\ B41F13/008\ B41F13/02\ B41F17/36\ F42B12/40 |
| 4947 | 3D printing | METHOD AND DEVICE FOR PREPARING DIAMOND-LIKE CARBON COATING BY CLADDING FIBER LASER\ | CN102409339B | REN XUDONG US\ HUANGFU YONGZHUO US\ RUAN LIANG US\ ZHANG YONGKANG US\ ZHAN QIUBO US\ YANG HUIMIN US\ ZHOU JIANZHONG US\ DAI FENGZE US\ SUN GUIFANG US | UNIV JIANGSU US | C23C24/10\ B82Y30/00\ B82Y40/00 |
| 4948 | 3D printing | METAL-BEARING DENTAL RESTORATIONS BY THREE-DIMENSIONAL PRINTING\[FR] RESTAURATION DENTAIRE CONTENANT UN MéTAL ET OBTENUE PAR IMPRESSION EN TROIS DIMENSIONS\ | WO2007097747A1 | RHOADES LAWRENCE J (US)\ KUHN HOWARD A (US) | EX ONE CORP (US)\ RHOADES LAWRENCE J (US)\ KUHN HOWARD A (US) | B22F7/00 |
| 4949 | 3D printing | COMPOSITION OF LEAD-FREE SILVER ELECTROCONDUTION SLURRY USED FOR FORMING CRYSTAL SILICON SOLAR CELL BACK ELECTRODE AS WELL AS PREPARATION METHOD THEREOF\ | CN102768871A | RONGCHUN YANG US\ RUICHANG YANG US\ SHENGHU XIONG US\ HUA YANG US | HANGZHOU RIGHTSILVER ELECTRONIC MATERIAL CO LTD US | H01B13/00\ H01B1/22\ H01L31/0224\ C03C12/00 |
| 4950 | 3D printing | THREE-DIMENSIONAL PRINTER\ | US2009011066A1 | RUSSELL DAVID B (US)\ DAVIDSON THOMAS (US)\ PHILLIPS ROBERT A (US)\ HERNANDEZ ANDRES T (US)\ ROCHE KEVIN (US)\ ZENGERLE III WALTER H (US)\ BERLIN ANDREW (US)\ KINSLEY JOSHUA P (US)\ SWEET-BLOCK BENJAMIN DANIEL (US)\ KISAI DARUL (MY) | CORP Z (US) | B29C67/00\ B29C41/12\ B29C41/36\ B41J2/01\ B41J2/155\ B41J2/165\ B41J2/175 |
| 4951 | 3D printing | MANUFACTURING PROCESS SUCH AS THREE DIMENSIONAL PRINTING INCLUDING BINDING OF WATER-SOLUBLE MATERIAL FOLLOWED BY SOFTENING AND FLOWING AND FORMING FILMS OF ORGANIC-SOLVENT-SOLUBLE MATERIAL\ | US2007009606A1 | SACHS EMANUEL M (US)\ SERDY JAMES G (US)\ MATERNA PETER A (US)\ WEST THOMAS G (US)\ SAINI SUNIL (US)\ CAI JIE (US)\ CARUSO ANDREA B (US)\ SHAROBIEM JOHN (US) | SHAROBIEM JOHN (US)\ SERDY JAMES G (US)\ SACHS EMANUEL M (US)\ WEST THOMAS G (US)\ SAINI SUNIL (US)\ CAI JIE (US)\ CARUSO ANDREA B (US)\ MATERNA PETER A (US) | A61F2/00\ A61F2/02\ A61F2/28\ A61F2/30\ G03C1/00\ A61K9/50 |
| 4952 | 3D printing | PRECISION CASTING METHOD ITS CAST PRODUCT AND ROTARY MACHINE\ | JP2004090046A | SAKAMOTO KATSUHIKO US\ TAKAYAMA HIROKAZU US\ KUDO SATOSHI US\ OGAWA TOSHIYUKI US | EBARA CORP US | B22C9/04\ B22C9/10\ F04D29/02\ F04D29/22\ F04D29/42 |
| 4953 | 3D printing | METHOD FOR MANUFACTURING THREE-DIMENSIONAL SHAPED ARTICLE\ | JP2005254583A | SANO MASAJIRO US | FUJI PHOTO FILM CO LTD (US) | B29C67/00 |
| 4954 | 3D printing | METHOD FOR MANUFACTURING THREE-DIMENSIONAL SHAPED ARTICLE\ | JP2005254534A | SANO MASAJIRO US | FUJI PHOTO FILM CO LTD (US) | B29C67/00 |
| 4955 | 3D printing | METHOD FOR MANUFACTURING THREE-DIMENSIONAL SHAPED ARTICLE\ | JP2005254523A | SANO MASAJIRO US | FUJI PHOTO FILM CO LTD (US) | B29C67/00 |
| 4956 | 3D printing | THREE-DIMENSIONAL SHAPED ARTICLE AND ITS MANUFACTURING METHOD\ | JP2005254521A | SANO MASAJIRO US | FUJI PHOTO FILM CO LTD (US) | B29C67/00 |
| 4957 | 3D printing | MANUFACTURING METHOD OF THREE-DIMENSIONAL SHAPED ARTICLE\ | JP2005088429A | SANO MASAJIRO US | FUJI PHOTO FILM CO LTD (US) | B29C67/00 |
| 4958 | 3D printing | MANUFACTURING METHOD OF THREE-DIMENSIONAL SHAPED ARTICLE\ | JP2005088392A | SANO MASAJIRO US | FUJI PHOTO FILM CO LTD (US) | B29C67/00 |
| 4959 | 3D printing | MANUFACTURING METHOD OF THREE-DIMENSIONAL SHAPED ARTICLE\ | JP2005074904A | SANO MASAJIRO US | FUJI PHOTO FILM CO LTD (US) | B29C67/00 |
| 4960 | 3D printing | MANUFACTURING METHOD OF THREE-DIMENSIONAL SHAPED ARTICLE\ | JP2005074824A | SANO MASAJIRO US | FUJI PHOTO FILM CO LTD (US) | B29C67/00 |
| 4961 | 3D printing | MANUFACTURING METHOD OF THREE-DIMENSIONAL SHAPED ARTICLE\ | JP2005074665A | SANO MASAJIRO US | FUJI PHOTO FILM CO LTD (US) | B29C67/00\ B29K35/00 |
| 4962 | 3D printing | METHOD FOR MANUFACTURING THREE-DIMENSIONAL SHAPED ARTICLE\ | JP2004330743A | SANO MASAJIRO US | FUJI PHOTO FILM CO LTD (US) | B29C67/00 |
| 4963 | 3D printing | METHOD FOR MANUFACTURING THREE-DIMENSIONAL SHAPED ARTICLE\ | JP2004330730A | SANO MASAJIRO US | FUJI PHOTO FILM CO LTD (US) | B29C67/00 |
| 4964 | 3D printing | METHOD AND APPARATUS FOR MANUFACTURING THREE-DIMENSIONAL SHAPED ARTICLE\ | JP2004330702A | SANO MASAJIRO US | FUJI PHOTO FILM CO LTD (US) | B29C67/00 |
| 4965 | 3D printing | METHOD FOR MANUFACTURING THREE-DIMENSIONAL SHAPED ARTICLE\ | JP2004330694A | SANO MASAJIRO US | FUJI PHOTO FILM CO LTD (US) | B29C67/00 |
| 4966 | 3D printing | METHOD FOR MANUFACTURING THREE-DIMENSIONAL SHAPED ARTICLE\ | JP2004330679A | SANO MASAJIRO US | FUJI PHOTO FILM CO LTD (US) | B29C67/00 |
| 4967 | 3D printing | MANUFACTURING METHOD OF THREE-DIMENSIONAL SHAPED ARTICLE AND MANUFACTURING APPARATUS USED THEREIN\ | JP2005096199A | SANO MASAJIRO US\ SERA HIDEFUMI (JP) | FUJI PHOTO FILM CO LTD (US) | B29C67/00\ B29K63/00\ B29K75/00 |
| 4968 | 3D printing | MANUFACTURING METHOD OF THREE-DIMENSIONAL SHAPED ARTICLE AND MANUFACTURING APPARATUS USED THEREIN\ | JP2005088432A | SANO MASAJIRO US\ SERA HIDEFUMI (JP) | FUJI PHOTO FILM CO LTD (US) | B29C67/00 |
| 4969 | 3D printing | PROCESS FOR PRODUCING THREE-DIMENSIONAL MODEL\ | US7674423B2 | SANO SHOJIRO (JP) | FUJIFILM CORP (JP) | B29C35/08\ B29C67/00\ B29C41/02 |
| 4970 | 3D printing | PROCESS FOR PRODUCING THREE-DIMENSIONAL SHAPED ARTICLE\ | US2006290032A1 | SANO SHOJIRO (JP) | SANO SHOJIRO (JP) | B29C35/08\ B29C67/00 |
| 4971 | 3D printing | METHOD OF PRODUCTING THREE-DIMENSIONAL MODEL\ | US2007007698A1 | SANO SHOJIRO (JP) | SANO SHOJIRO (JP) | B29C35/08\ B29C67/00 |
| 4972 | 3D printing | PROCESS FOR PRODUCING THREE-DIMENSIONAL MODEL AND THREE-DIMENSIONAL MODEL\ | US7300613B2 | SANO SHOJIRO (JP)\ SERA HIDEFUMI (JP) | FUJIFILM CORP (JP) | B29C67/00\ B29C31/04\ G03G9/00 |
| 4973 | 3D printing | COLOR IMAGE FORMING METHOD\ | JP2010107540A | SATO AKIO US\ YOSHIDA HIDEKAZU US\ HIRAMOTO RYUICHI US | KONICA MINOLTA BUSINESS TECH US | G03G15/00\ G03G9/08\ G03G9/09\ G03G15/01 |
| 4974 | 3D printing | MULTILAYERT CERAMIC DEVICE AND THE PRODUCTION METHOD THEREOF\ | US2006158826A1 | SATOU SHIGEKI (JP)\ TOKUOKA YASUMICHI (JP)\ SATOU TATSUMORI (JP) | TDK CORP (JP) | H01G4/12\ H01G4/30\ H01G4/06 |
| 4975 | 3D printing | VERFAHREN ZUR ABFORMUNG VON DREIDIMENSIONALEN FREIFORMFL?CHEN MIT MIKROSTRUKTURIERTEN OBERFL?CHEN\[EN] METHOD FOR CASTING 3-D FREELY FORMABLE SHAPES WITH MICROSTRUCTURED SURFACES\[FR] PROCéDé DE PRISE D'EMPREINTE DE SURFACES DE FORME LIBRE EN TROIS DIMENSIONS DOTéES DE SURFACES MICROSTRUCTURéES\ | EP1854568B1 | SAUERESSIG KILIAN (DE) | K1 GMBH (DE) | B22C9/00 |
| 4976 | 3D printing | METAL POWDER COMPOSITION FOR LASER SINTERING\ | US2004226405A1 | SCHMIDT KRIS ALAN (US)\ NEWELL KENNETH J (US)\ GEVING BRAD (US) | 3D SYSTEMS INC (US) | C22C33/02 |
| 4977 | 3D printing | FIRE-EXTINGUISHING GAS-DISPERSED COMPOSITION A FIRE-FIGHTING METHOD AND A DEVICE FOR CARRYING OUT SAID METHOD\[FR] COMPOSITION D'EXTINCTION D'INCENDIES à GAZ DISPERSéS PROCéDé D'EXTINCTION D'INCENDIES ET DISPOSITIF DE RéALISATION CORRESPONDANT\ | WO2007117168A1 | SELIVERSTOV VLADIMIR IVANOVICH (RU)\ STENKOVOY VLADIMIR ILYICH (RU)\ VERETINSKY PAVEL GENNADIEVICH (RU)\ IVASHKOV VLADIMIR PETROVICH (RU)\ KUSKOV NIKOLAY ARSENTIEVICH (RU)\ RZHAVSKY LEV VLADISLAVOVICH (RU)\ TRUBNIKOVA GALINA VLADIMIROVNA (RU) | SELIVERSTOV VLADIMIR IVANOVICH (RU)\ STENKOVOY VLADIMIR ILYICH (RU)\ VERETINSKY PAVEL GENNADIEVICH (RU)\ IVASHKOV VLADIMIR PETROVICH (RU)\ KUSKOV NIKOLAY ARSENTIEVICH (RU)\ RZHAVSKY LEV VLADISLAVOVICH (RU)\ TRUBNIKOVA GALINA VLADIMIROVNA (RU) | A62D1/06\ A62C2/00\ A62C13/66 |
| 4978 | 3D printing | FLüSSIGHARZ UND VERFAHREN ZUR HERSTELLUNG EINES POLYMERK?RPERS UNTER VERWENDUNG DES FLüSSIGHARZES\[EN] LOW-VISCOSITY LIQUID RESIN USEFUL AS BINDERS IN THE PREPARATION OF MASSIVE/POROUS POLYMER BODY AND FIBER COMPOSITE COMPRISES STYRENE HYDROXYETHYL METHACRYLATE MALEIC ACID ANHYDRIDE AND A BIFUNCTIONAL ACRYLATE OR METHACRYLATE\ | DE102007061445A1 | SHEN JIALIN (DE)\ AHLERS JAN (DE)\ CHEN FENG (DE)\ LORENZ REINHARD (DE) | DAIMLER AG (DE) | B29C67/00\ C08F220/20\ B29C70/42\ C08J5/24 |
| 4979 | 3D printing | SELBSTAUSH?RTENDES MATERIAL UND VERFAHREN ZUM SCHICHTWEISEN AUFBAU DREIDIMENSIONALER BAUTEILE\[EN] SELF-HARDENING MATERIAL USEFUL TO BUILD THREE-DIMENSIONAL LAYER COMPONENT COMPRISES PARTICLE MATERIAL COMPRISING POLYMETHYLMETHACRYLATE AND CROSS-LINKING INITIATOR; AND BINDER COMPRISING CATALYST AND COMPONENT CONTAINING E.G. STYRENE\ | DE102007025656A1 | SHEN JIALIN (DE)\ BALKAN OSMAN (DE)\ EISELT WOLFGANG (DE)\ GUENTHER JOHANNES F G (DE)\ KASHANI KAVEH (DE) | DAIMLER AG (DE)\ GDF GES DENTALE FORSCH (DE)\ VOXELJET TECHNOLOGY GMBH (DE) | B29C67/00\ C08L33/12\ C08L25/04 |
| 4980 | 3D printing | UV-CURABLE RESIN FOR POWDER COATING\ | TW576858B | SHEN WAN-FA (TW)\ LI GANG (TW) | CHAN SIEH ENTPR CO LTD (TW) | C09D5/03\ C08L67/06\ C09D131/06 |
| 4981 | 3D printing | THREE-DIMENSIONAL OBJECT FORMING APPARATUS\ | US8342833B2 | SHI KWO YUAN (TW)\ TUNG YA CHING (TW) | MICROJET TECHNOLOGY CO LTD (TW)\ SHI KWO YUAN (TW)\ TUNG YA CHING (TW) | B28B17/00 |
| 4982 | 3D printing | PRINTING MODULE USED FOR A THREE DIMENSIONAL PROTOTYPING APPARATUS\ | TWI430892B | SHI KWO YUAN (TW)\ TUNG YA CHING (TW)\ LIN CHING SUNG (TW)\ TAI HSIEN CHUNG (TW)\ HUANG CHI FENG (TW) | MICROJET TECHNOLOGY CO LTD (TW) | B41J2/135\ B41J2/005 |
| 4983 | 3D printing | STAINLESS STEEL ZIPPER MANUFACTURED BY METAL INJECTION MOLDING AND PREPARATION METHOD THEREOF\ | CN101579160A | SHIHUA LIU (CN) | SHIHUA LIU (CN) | B29C45/00\ B22F3/22\ A44B19/26\ A44B19/42\ B29K103/06 |
| 4984 | 3D printing | BIOMATERIAL FOR ARTIFICIAL CARTILAGE\ | JP2008264562A | SHIKINAMI YASUO US | TAKIRON CO US | A61L27/00\ A61F2/30\ A61L27/56\ B29C43/00\ A61L27/44\ A61L27/46\ A61L27/58 |
| 4985 | 3D printing | MAKING METHOD OF PANEL AND PANEL MADE THEREBY\ | KR20100033798A | SHIM BEOUM SOO (KR) | BEACHE C0 LTD (KR) | B32B21/04\ B32B19/02\ B32B19/04 |
| 4986 | 3D printing | MANUFACTURE OF SINTERED SILICON ALLOY\ | US2011052440A1 | SHIMIZU KOUKI (JP)\ MATSUSHITA MASAFUMI (JP)\ WATANABE TOSHIYUKI (JP)\ MATSUZONO OSAMU (JP)\ YOSHIDA TAKASHI (JP)\ YANAGINO FUTOSHI (JP)\ SHITARA TAKUMI (JP)\ SAITO KUNIO (JP)\ MATSUSHITA AKIKO (JP)\ KUBOTA YUMIKO (JP)\ SHINDO AYUMI (JP)\ SHINDO SETSUKO (JP)\ MISAKI TAKAYOSHI (JP)\ MATSUSHITA YOKO (JP)\ SATO KAZUYA (JP)\ SAKURAI TOSHITAKA (JP)\ MIZUSHIMA TAKASHI (JP) | ISMAN J CORP (JP) | B22F1/00\ B22F3/12 |
| 4987 | 3D printing | METHOD OF MANUFACTURING CARBON MATERIAL AND METHOD OF PURIFYING THE SAME\ | JP2006232565A | SHINRYU KAZU US | TOSHIBA CERAMICS CO US | C04B35/52\ C01B31/04 |
| 4988 | 3D printing | MAGNETIC RECORDING MEDIUM\ | JP2005149622A | SHIRASAGO KENJI US\ KUROSE SHIGEO (JP)\ TANAKA HIROYUKI (JP) | TDK CORP (JP) | G11B5/70\ G11B5/702\ G11B5/714 |
| 4989 | 3D printing | MAGNETIC RECORDING MEDIUM\ | JP2005149621A | SHIRASAGO KENJI US\ KUROSE SHIGEO (JP)\ TANAKA HIROYUKI (JP) | TDK CORP (JP) | G11B5/70\ G11B5/702\ G11B5/714 |
| 4990 | 3D printing | UNDER-SAMPLED 3D MRI USING A SHELLS K-SPACE SAMPLING TRAJECTORY\ | US7245124B2 | SHU YUNHONG (US)\ BERNSTEIN MATTHEW A (US) | MAYO FOUNDATION (US) | G01V3/00 |
| 4991 | 3D printing | METHOD AND DEVICE FOR PRODUCING PRECISION INVESTMENT-CAST NE METAL ALLOY MEMBERS AND NE METAL ALLOYS FOR CARRYING OUT SAID METHOD\ | US2005279481A1 | SMARSLY WILFRIED (DE)\ RENKEL MANFRED (DE) | MTU AERO ENGINES GMBH (DE) | B22C9/04\ F01D5/28\ B22D13/04\ B22D13/06\ B22D13/10\ C22C14/00\ F02C7/00 |
| 4992 | 3D printing | METHOD FOR PRODUCING PLANAR METALLISED TEXTILE STRUCTURES PLANAR METALLISED TEXTILE STRUCTURE AND USE OF THE THUS PRODUCED PLANAR METALLISED TEXTILE STRUCTURE\ | US2009011674A1 | SOBOTKA BETTINA (DE)\ DURIS TIBOR (DE)\ LOCHTMAN RENE (DE)\ WAGNER NORBERT (DE)\ SCHNEIDER NORBERT (DE)\ PFISTER JURGEN (DE)\ ADDAMO ANTONINO RAFFAELE (DE)\ NORENBERG RALF (DE) | BASF SE (DE) | C08K3/08\ D03D15/00\ B32B15/14\ B05D5/12 |
| 4993 | 3D printing | LOW MELTING POINT FRIT PASTE COMPOSITION AND SEALING METHOD FOR ELECTRIC ELEMENT USING THE SAME\ | US7923393B2 | SON JUNG-HYUN (KR)\ LEE SANG-KYU (KR)\ JOO HAN-BOK (KR) | DONGJIN SEMICHEM CO LTD (KR) | C03C8/24\ C03C8/08\ B29C65/16\ C03B23/00\ C03C3/21\ C03C8/14\ C03C8/16\ H01J9/26 |
| 4994 | 3D printing | MODULARES SOL-GEL-SYSTEM UND VERFAHREN ZUM EINSTELLEN DER EIGENSCHAFTEN DES SOL-GEL-SYSTEMS\[EN] MODULAR SOLVENT-FREE WATER-BASED SOL-GEL SYSTEM WITH PREDETERMINABLE PROPERTIES FOR USE E.G. AS ANTI-CORROSION PRIMER CONTAINS AT LEAST 4 MATCHED COMPOUNDS AT LEAST ONE BEING A BASE COMPONENT\ | DE102007058712A1 | STANKE BURKHARD (DE)\ JENKNER PETER (DE)\ BORUP BJOERN (DE)\ MONKIEWICZ JAROSLAW (DE)\ WASMER CHRISTIAN (DE) | EVONIK DEGUSSA GMBH (DE) | C09D5/00\ C09D183/04\ B01J13/00\ C08G77/10\ C08J3/075\ C08L101/14\ C09D5/12\ C09D7/14\ C09D201/10\ C09K3/18 |
| 4995 | 3D printing | USE OF ADSORBENTS TO FACILITATE FILM ADHESION\ | US6750285B1 | STEWART JOHN H (US) | HEWLETT PACKARD DEVELOPMENT CO (US) | C08K3/36\ C09D133/06 |
| 4996 | 3D printing | CONDUCTIVE PASTE FOR SOLAR CELL AND MANUFACTURING METHOD THEREFOR\ | JP2010199034A | SUENAGA WATARU US\ SUGANO TSUTOMU US | DAINIPPON INK & CHEMICALS US | H01B13/00\ H01B1/00\ H01B1/22\ H01L31/04 |
| 4997 | 3D printing | ADSORPTION HEAT EXCHANGER AND METHOD OF MANUFACTURING THE SAME\ | JP2010270973A | SUEOKA YOSHIHISA US\ MATSUSHITA HIROHIKO US\ KO ARASHI US\ IKEGAMI SHUJI (JP) | DAIKIN IND LTD (JP) | F25B35/04\ B01D53/26\ B01J20/26\ B01J20/34\ F25B17/08 |
| 4998 | 3D printing | METALLIC POROUS BODY AND ITS PRODUCTION\ | JP7118706A | SUGIKAWA HIROFUMI US | KATAYAMA TOKUSHU KOGYO KK US | B22F1/00\ B22F3/11\ B22F5/00\ H01M4/80 |
| 4999 | 3D printing | CERAMIC POWDER TRANSFER PROCESS\ | US2004094260A1 | SUMMERFIELD MARTIN RAYMOND (GB) | JENERIC PENTRON INC (US) | B41M3/12\ B41M5/025\ B44C1/175 |
| 5000 | 3D printing | MAGNETIC TAPE\ | US2012196156A1 | SUZUKI RYOTA (JP) | FUJIFILM CORP (JP)\ SUZUKI RYOTA (JP) | G11B5/708 |
| 5001 | 3D printing | METHOD FOR FORMING ARTIFICIAL BONE\ | JP2004202126A | SUZUKI SHIGEKI US\ NISHIMURA KAZUTOSHI US | NEXT KK US\ SINTOBRATOR LTD US | B29C67/00\ A61L27/00\ A61F2/28\ B28B1/32 |
| 5002 | 3D printing | LIGHT SHIELDING BARRIER WRAPPING MATERIAL\ | JP2005001753A | TAKAHARA TAKESHI US | TOPPAN PRINTING CO LTD US | B65D65/16\ B32B9/00\ B65D65/40 |
| 5003 | 3D printing | METHOD OF MANUFACTURING OPTICAL RECORDING MEDIUM\ | JP2007199125A | TAKANO HIROAKI US\ YAMASHITA NORIKO US | FUJIFILM CORP (JP) | G11B7/26\ G03H1/02 |
| 5004 | 3D printing | DIE-ATTACH PASTE AND SEMICONDUCTOR DEVICE\ | JP2003347322A | TANAKA NOBUKI US\ SAITO KEIICHIRO US | SUMITOMO BAKELITE CO US | C08L101/00\ C08L83/04\ C09J183/04\ C09J201/00\ H01L21/52 |
| 5005 | 3D printing | VERFAHREN UND VORRICHTUNG ZUR HERSTELLUNG VON DREIDIMENSIONALEN TEILEN\[EN] METHOD AND APPARATUS FOR MAKING THREE-DIMENSIONAL PARTS\[FR] PROCéDé ET APPAREIL DE FABRICATION DE PIèCES TRIDIMENSIONNELLES\ | EP2263861A1 | TANG HWA-HSING (TW) | TANG LEE HUEY RU (TW) | B29C67/00 |
| 5006 | 3D printing | METHOD FOR PRODUCING A THREE DIMENSIONAL GREEN ARTICLE\ | US2013307175A1 | TANG HWA-HSING (TW) | UNIV NAT TAIPEI TECHNOLOGY (TW)\ TANG HWA-HSING (TW) | C04B35/64 |
| 5007 | 3D printing | PROCESS AND APPARATUS FOR MAKING THREE DIMENSIONAL PARTS\ | TW200936365A | TANG HWA-HSING (TW) | ZONACUS INTERNAT CO LTD (TW) | B29C67/04\ B29C70/02\ B29C70/28 |
| 5008 | 3D printing | METAL BOND GRINDSTONE AND ITS MANUFACTURING METHOD\ | JP2005161510A | TANIGAWA YOSHIHIRO US\ NAKAMURA NORIKAZU US | TANIGAWA YOSHIHIRO US\ NAKAMURA NORIKAZU US | B24D3/00\ B24D3/06 |
| 5009 | 3D printing | FAST SOFT FOUNDATION DYKE BUILDING METHOD AND TECHNOLOGY\ | CN1773022A | TAO SONGLEI TAO (CN) | TAO JUNBING (CN) | E02B3/10\ E02D3/02 |
| 5010 | 3D printing | PHOTOCURABLE LIQUID COMPOSITION THREE-DIMENSIONAL ARTICLE AND METHOD FOR PRODUCING THEM\ | JP2007291393A | TATARA AKITSUGU US\ YASHIRO TAKAO US\ TANABE TAKAYOSHI US\ UKAJI TAKASHI US | JSR CORP (JP)\ JAPAN FINE COATINGS CO LTD (JP) | B29C67/00\ C08F2/44\ B28B1/30\ C08F20/20 |
| 5011 | 3D printing | PHOTOCURABLE LIQUID COMPOSITION THREE-DIMENSIONAL FORM AND MANUFACTURING METHOD THEREOF\ | JP2004143247A | TATARA AKITSUGU US\ YASHIRO TAKAO US\ TANABE TAKAYOSHI US\ UKAJI TAKASHI US | JSR CORP (JP)\ JAPAN FINE COATINGS CO LTD (JP) | B29C67/00\ C08F291/00\ C08F2/44 |
| 5012 | 3D printing | SUPERCONDUCTING CONNECTION BETWEEN MGB2 SUPERCONDUCTING WIRES VIA A COMPRESSED ELEMENT MADE FROM HTS POWDER\ | US2010216645A1 | TENBRINK FELICITAS (DE)\ AUBELE ANDRE (DE)\ GLUECKLICH VOLKER (DE)\ SAILER BERND (DE)\ SCHLENGA KLAUS (DE) | BRUKER HTS GMBH (DE) | H01B12/04\ H01L39/24 |
| 5013 | 3D printing | METHOD FOR LAMINATING MULTIPLE CORE ASSEMBLIES OF SHELL CASTING CHAPLET AT INTERVALS\ | CN102380581A | TIANPING LIU US\ AILI WANG US | TIANPING LIU US | B22C9/10 |
| 5014 | 3D printing | PREPARATION METHOD FOR THREE-DIMENSIONAL WOVEN FIBER REINFORCED CERAMIC MATRIX COMPOSITE\ | CN101880172A | TIANQI GAO US\ GUOHONG ZHOU US\ YIWEN ZHENG US\ YANG HAN US\ KUN ZHANG US\ SHIWEI WANG US\ YIPING QIU US | UNIV DONGHUA (CN) | C04B35/622\ C04B35/80 |
| 5015 | 3D printing | HIGH HEAT-RESISTANCE POWDER FOR FORMING INKJET POWDER LAMINATION MOLD\ | JP2011051010A | TOBA ATSUYA US\ INANO HIROYUKI US\ HIRANO SHIGEKI US | HOKKAIDO RES ORGANIZATION US | B22C1/10\ B22C9/02\ B22C1/18\ B22C1/00 |
| 5016 | 3D printing | DISCHARGE EXAMINATION DEVICE FLUID DISCHARGE DEVICE HAVING THE SAME AND DISCHARGE EXAMINATION METHOD\ | JP2010076289A | UCHIYAMA MASAKI US | SEIKO EPSON CORP (JP) | B41J2/01\ B41J2/165 |
| 5017 | 3D printing | METHOD FOR PRINTING POWDER OF ORE METHOD FOR PRODUCING HEAT TRANSFER SHEET WITH PRINTED POWDER OF ORE METHOD FOR HEAT TRANSFER TO CLOTH BY USING HEAT TRANSFER SHEET CLOTH AND HEAT TRANSFER SHEET WITH PRINTED POWDER OF ORE\ | JP2004036036A | UEMURA ISOO US | UEMURA KK US | D06P5/20\ B44C1/17\ A61F13/00\ D06M11/00\ D06M11/79\ D06M15/564\ D06P1/44\ D06P1/673\ D06P5/00\ D06Q1/14 |
| 5018 | 3D printing | METHOD FOR MANUFACTURING MOLD\ | US7281566B2 | UKAI HIDEMI (JP)\ OGAWA KOSHICHI (JP)\ CHIKUGO KAZUYOSHI (JP)\ MURAKAMI KOICHI (JP) | ISHIKAWAJIMA HARIMA HEAVEY IND (JP) | A61L27/00\ A61F2/28\ B22D25/02\ A61C13/20\ B22C9/04\ B22C7/02\ C04B38/06 |
| 5019 | 3D printing | FLOOR PLATE FOR USE IN SINTERING OF POROUS BODY AND METHOD FOR MANUFACTURING POROUS SINTERED BODY\ | JP2008106299A | WADA MASAHIRO US\ OMORI SHINICHI US\ SHIBUYA TAKUMI US\ TSUJIMOTO TETSUSHI US\ UENO HIROCHIKA US | MITSUBISHI MATERIALS CORP US | B22F3/10\ B22F3/11\ C22C1/08\ C04B41/87 |
| 5020 | 3D printing | THREE-DIMENSIONAL POWDER MOLDING\ | US2012193841A1 | WANG HSIN-PANG (US)\ PERRY ERIN MARIE (US) | WANG HSIN-PANG (US)\ PERRY ERIN MARIE (US) | C04B35/64\ B29C45/72 |
| 5021 | 3D printing | TWO-DIMENSIAONL ROTARY SPRAY NOZZLE DEVICE\ | TW586971B | WANG JIUE-KUAN (TW) | WANG JIUE-KUAN (TW) | B05B7/00 |
| 5022 | 3D printing | CONCRETE MANHOLE COVER AND MANUFACTURING METHOD THEREOF\ | CN102852163A | WANG ZENGCHUN US\ SHEN CHENGXI US\ YE JUN US | UNIV SHANGHAI JIAOTONG (CN) | E02D29/14\ B28B23/02 |
| 5023 | 3D printing | BESCHICHTUNGSMATERIAL BESCHICHTETES TR?GERMATERIAL UND VERFAHREN ZUR HERSTELLUNG\[EN] MATERIAL FOR COATING SUBSTRATES E.G. SANITARY ARTICLES AND BUILDING SLABS COMPRISES A METAL POWDER WHICH CONSISTS OF METALS EARTH METALS OR METAL ALLOYS AND A BINDING AGENT WHICH CONTAINS A SOLVENT\ | DE102007047065A1 | WEDI STEPHAN (DE) | WEDI GMBH (DE) | C09D5/10\ A47K1/04\ A47K3/02\ A47K3/40 |
| 5024 | 3D printing | METHOD AND STRUCTURE FOR CONSTRUCTING LARGE-SCALE LIQUID STORAGE TANK ENERGY-DISSIPATING SHOCK ABSORPTION AND SHOCK INSULATION FOUNDATION\ | CN101349075A | WEI CHEN (CN) | WEI CHEN (CN) | E04B1/98\ E02D27/44 |
| 5025 | 3D printing | METHOD FOR CALCULATING CONTINUOUS CASTING FLOW TEMPERATURE AND SOLID PHASE RATE DISTRIBUTION\ | CN102110188A | WEI LIU US\ YONG TIAN US\ XIANGCHUN ZHANG US\ SHILONG WU US\ PENG WANG US\ LONG JIAN US\ LIANQI AN US | ANGANG STEEL CO LTD US | G06F19/00\ B22D11/16 |
| 5026 | 3D printing | VERFAHREN ZUR HERSTELLUNG VON PULVERF?RMIGEN WASSERL?SLICHEN UND/ODER WASSERQUELLBAREN POLYMERISATEN UND IHRE VERWENDUNG ALS VERDICKUNGSMITTEL\[EN] PREPARATION OF POWDERED WATER-SOLUBLE AND/OR WATER-SWELLABLE POLYMERS USEFUL AS THICKENERS FOR E.G. PAPER COATING MASSES COMPRISES DRYING AQUEOUS DISPERSIONS OF ETHYLENICALLY UNSATURATED ANIONIC POLYMERS IN STANDARD METHODS\ | DE102004051540A1 | WEIDL CHRISTIAN HUBERT (DE)\ MUKHERJEE PULAKESH (DE)\ BRAIG VOLKER (DE)\ BAYER ROBERT (DE) | BASF AG (DE) | C08F6/14\ A61K8/81\ A61K47/32\ C08F20/00\ C09D5/04\ C09D133/08\ C14C3/18\ D21H19/56 |
| 5027 | 3D printing | METHOD FOR LASER ENGINEERED NET SHAPING OF AL2O3-ZRO2 EUTECTIC CERAMIC STRUCTURE\ | CN103193486A | WU DONGJIANG US\ ZHANG YAOLEI US\ SUN BEI US\ NIU FANGYONG US\ MA GUANGYI US\ GUO DONGMING US | UNIV DALIAN TECH (CN) | C04B35/622\ C04B35/10 |
| 5028 | 3D printing | POWDER SPRAYER FOR PRINTING MACHINES\ | CN201784242U | XIANGGUI CHEN US\ CHANGHE YU US | XIANGGUI CHEN US\ YU ZHANGHE US | B41F23/06 |
| 5029 | 3D printing | DECORATIVE MATERIAL OF ENCHASING WATERMARK PROCESS AND MANUFACTURING PROCESS THEREOF\ | CN102173144A | XIANGMIN HUANG US | SHENZHEN DWPH ELECTRONIC TECHNOLOGY CO LTD US | B32B27/06\ B32B27/18\ B44C3/06\ B44F7/00 |
| 5030 | 3D printing | COMPOSITE HIGH-CALORIFIC-VALUE CLEAN FUEL BLOCK\ | CN102358859A | XIAOLING ZHENG US | XIAOLING ZHENG US | C10L5/04\ C10L5/36 |
| 5031 | 3D printing | METHOD FOR REPLICATING METAL RELIC WITH HIGH FIDELITY\ | CN101554647A | XIAOSAN GONG (CN)\ YEQI HU (CN)\ XIAOHONG GE (CN) | XIAMEN UNIVERSITY OF TECHNOLOG (CN) | B22C9/04 |
| 5032 | 3D printing | NEGATIVE ACTIVE SUBSTANCE AND ELECTROLYTE OF COLLOID LEAD-CARBON BATTERY\ | CN103337623A | XU DONGMING US\ WANG JINGCHUAN US\ LU YASHAN US\ YANG BAOFENG US\ YANG SHANJI US | SHUANGDENG GROUP CO LTD US | H01M4/57\ H01M4/62\ H01M10/10 |
| 5033 | 3D printing | DECORATIVE SHEET AND DECORATIVE MATERIAL\ | JP2003266610A | YAGISHITA NOBUNAO US\ TAKAHASHI MASARU US\ NAKAMURA TAKESHI US | TOPPAN PRINTING CO LTD US | C09D11/02\ B32B27/20\ E04F13/00\ E04F13/07\ B32B27/00\ C09D201/00 |
| 5034 | 3D printing | ELECTRODE LEVEL DIFFERENCE ABSORBING PRINT PASTE AND METHOD OF PRODUCING ELECTRONIC DEVICE\ | US7641727B2 | YAMAGUCHI AKIRA (JP)\ SATOU SHIGEKI (JP)\ TOKUOKA YASUMICHI (JP) | TDK CORP (JP) | C04B35/634\ C04B35/468\ H01G4/12\ H01G4/30\ C04B14/48 |
| 5035 | 3D printing | PRECISION CASTING METHOD FOR GRAPHITE MOULD-CERAMIC CORE TITANIUM ALLOY\ | CN101733383A | YANCHUN LOU US\ YUFU QU US\ KUN SHI US\ ZHIHUA WANG US\ HUASHENG XIE US\ CHUNHUI ZHANG US\ ZHAO JUN US | SHENYANG RES INST FOUNDRY US | B22D25/02\ B22D13/00\ B22C1/10\ B22C1/12\ B22C9/10 |
| 5036 | 3D printing | ULTRALOW-VOLTAGE PLASMA SPRAYING EQUIPMENT\ | CN101956154A | YANG GAO US | UNIV DALIAN MARITIME US | C23C4/00\ H05H1/42 |
| 5037 | 3D printing | SENSOR FOR ONLINE DETECTING COMBINATION PROPERTY OF MOLTEN METAL\ | CN200947090Y | YANG SHANLIN LING (CN) | SHUANGFA INFORMATION SYSTEM TE (CN) | G01N25/06\ G01N25/20 |
| 5038 | 3D printing | METHOD FOR PREPARING POROUS TANTALUM MEDICAL IMPLANT MATERIAL\[FR] PROCéDé DE PRéPARATION D'UN MATéRIAU D'IMPLANT MéDICAL EN TANTALE POREUX\ | WO2013113251A1 | YE LEI (CN) | CHONGQING RUNZE PHARMACEUTICAL COMPANY LTD (CN) | A61L27/56\ C22C1/08\ A61L27/04\ C22C27/02 |
| 5039 | 3D printing | METHOD FOR PREPARING BIOLOGICAL MEDICAL POROUS IMPLANT MATERIAL\[FR] PROCéDé DE PRéPARATION D'UN MATéRIAU D'IMPLANT MéDICAL BIOLOGIQUE POREUX\ | WO2013113250A1 | YE LEI (CN) | CHONGQING RUNZE PHARMACEUTICAL COMPANY LTD (CN) | A61L27/56\ C22C1/08\ A61L27/04\ C22C27/02 |
| 5040 | 3D printing | POROUS NIOBIUM USED AS METAL MEDICAL IMPLANT MATERIAL AND PREPARATION METHOD THEREOF\[FR] NIOBIUM POREUX UTILISé COMME MATéRIAU POUR IMPLANTS MéDICAUX ET SON PROCéDé DE PRéPARATION\ | WO2011147139A1 | YE LEI (CN)\ RUAN JIANMING (CN)\ XIE JIANQUAN (CN)\ JIE YUNFENG (CN)\ WANG ZHIQIANG (CN)\ FENG HUA (CN)\ YOU CHAO (CN)\ PANG QI (CN)\ ZHOU JIAN (CN)\ LIU BIN (CN)\ ZHANG YAZHUO (CN) | CHONGQING RUNZE MEDICAL EQUIPMENT & SUPPLIES CO LTD (CN)\ RUAN JIANMING (CN)\ XIE JIANQUAN (CN)\ JIE YUNFENG (CN)\ WANG ZHIQIANG (CN)\ FENG HUA (CN)\ YOU CHAO (CN)\ PANG QI (CN)\ ZHOU JIAN (CN)\ YE LEI (CN)\ LIU BIN (CN)\ ZHANG YAZHUO (CN) | A61L27/56\ A61L27/04 |
| 5041 | 3D printing | POROUS TANTALUM USED FOR MEDICAL IMPLANTATION AND METHOD FOR PREPARING THE SAME\ | US2013011691A1 | YE LEI (CN)\ RUAN JIANMING (CN)\ XIE JIANQUAN (CN)\ JIE YUNFENG (CN)\ WANG ZHIQIANG (CN)\ FENG HUA (CN)\ YOU CHAO (CN)\ PANG QI (CN)\ ZHOU JIAN (CN)\ LIU BIN (CN)\ ZHANG YAZHUO (CN) | RUAN JIANMING (CN)\ XIE JIANQUAN (CN)\ JIE YUNFENG (CN)\ WANG ZHIQIANG (CN)\ FENG HUA (CN)\ YOU CHAO (CN)\ PANG QI (CN)\ ZHOU JIAN (CN)\ YE LEI (CN)\ LIU BIN (CN)\ ZHANG YAZHUO (CN) | B22F3/11\ B32B3/26 |
| 5042 | 3D printing | REFLEX LED LAMP\ | CN201636605U | YINGHUA WANG US\ LIANG GUO US\ YE YE US | SHANGHAI SANSI ELECTRONIC ENG US\ SHANGHAI SANSI TECHNOLOGY CO US\ JIASHAN JINGHUI OPTOELECTRONIC TECHNOLOGY CO LTD US | F21Y101/02\ F21S2/00\ F21V7/22\ F21V29/00\ F21V3/04\ F21V7/04 |
| 5043 | 3D printing | REFLECTING LED LAMP\ | CN102192412A | YINGHUA WANG US\ LIANG GUO US\ YE YE US | SHANGHAI SANSI ELECTRONIC ENG US\ SHANGHAI SANSI TECHNOLOGY CO US\ JIASHAN JINGHUI OPTOELECTRONIC TECHNOLOGY CO LTD US | F21Y101/02\ F21S2/00\ F21V7/22\ F21V15/02\ F21V29/00 |
| 5044 | 3D printing | PRECISION CASTING METHOD FOR LARGE CASTINGS\ | CN101966558A | YINGLIU DU US | ANHUI YINGLIU GROUP HUOSHAN FOUNDRY CO LTD US | B22C9/04\ B22C7/02 |
| 5045 | 3D printing | FORMING DIE FOR CASTING DIE OF MOTOR SHELL AND METHOD USING FORMING DIE TO FORM CASTING DIE\ | CN102658353A | YIPING ZHAO US | YIPING ZHAO US | B22C7/00\ B22C9/24 |
| 5046 | 3D printing | MANUFACTURING METHOD OF LOST FOAM CASTING FOAMING MOLD\ | CN101554645A | YONG ZHANG (CN) | UNIV SHANDONG TECHNOLOGY (CN) | B22C9/04 |
| 5047 | 3D printing | DRYING DEVICE OF FLEXIBLE PLATE PRINTING ELEMENT\ | CN201214304Y | YONGQI DANG (CN)\ HONG WU (CN) | SHAANXI BEIREN PRINTING MACHIN (CN) | B41F23/04 |
| 5048 | 3D printing | MAKING PROCESS OF 3D COLOR OBJECT\ | CN100344440C | YUE DONG YANG (CN) | UNIV NANJING (CN) | B29C67/00\ B28B1/00\ B44C3/00\ B44C3/02\ B44C3/04 |
| 5049 | 3D printing | ELECTROSTATIC CHUCK HEATER\ | CN101110382A | YUTAKA MORI (JP)\ AKIYOSHI HATTORI (JP)\ TAKERU TORIGOE (JP) | NGK INSULATORS LTD (JP) | H01L21/683 |
| 5050 | 3D printing | BUILDING CONSTRUCTION SYSTEM WITH THE USE OF STRUCTURAL ELEMENTS OF NON-DETACHABLE FORMWORK.\ | GR1006023B | ZAVLIARIS KONSTANTINOS DIMITRIOU US | ZAVLIARIS KONSTANTINOS DIMITRIOU US | E04B1/16\ E04B2/86\ E04C3/34 |
| 5051 | 3D printing | THREE-DIMENSIONAL FORCE-BEARING SHUTTERING FOR STEEL REINFORCED CONCRETE\ | CN1743589B | ZEYOU QIU (CN) | HUNAN QIU ZEYOU PATENT STRATEG US | E04B5/36 |
| 5052 | 3D printing | SOLID LOAD CARRYING STRUCTURED STORIED BUILDING CONSTRUCTED BY CONCRETE OF REINFORCING STEEL BAR\ | CN1873149B | ZEYOU QIU (CN) | HUNAN QIUZEYOU PATENT STRATEGY CO LTD US | E04B5/21 |
| 5053 | 3D printing | THREE-DIMENSIONAL FORCE-BEARING SHUTTERING FOR STEEL REINFORCED CONCRETE\ | CN100357542C | ZEYOU QIU (CN) | ZEYOU QIU (CN) | E04B5/36 |
| 5054 | 3D printing | STEREO BEARING SHUTTERING FOR REINFORCED CONCRETE\ | CN100400760C | ZEYOU QIU (CN) | ZEYOU QIU (CN) | E04B5/36\ E04B5/17\ E04B5/16 |
| 5055 | 3D printing | REINFORCED CONCRETE ROOFING WITH 3-D BEARING STRUCTURE\ | CN100420803C | ZEYOU QIU (CN) | ZEYOU QIU (CN) | E04B5/36\ E04B5/21\ E04B5/18 |
| 5056 | 3D printing | HOLLOW CHAMBER MOLD SHELL MEMBER\ | CN100467753C | ZEYOU QIU (CN) | ZEYOU QIU (CN) | E04B5/36\ E04B5/18\ E04B5/16\ E04C1/40 |
| 5057 | 3D printing | THREE-DIMENSIONAL BEARING BUILDING ROOF OF STEEL-REINFORCED CONCRETE\ | CN101029513B | ZEYOU QIU (CN) | ZEYOU QIU (CN) | E04B5/21 |
| 5058 | 3D printing | MULTI-NOZZLE LASER 3D PRINTING DEVICE\ | CN203109234U | ZHANG CHONGHAO US\ LIU ANMIN US\ SHAO GUOFENG US\ YANG JIAN US\ LU YIN US | ZHANG CHONGHAO US | B22F3/105 |
| 5059 | 3D printing | POSITIVE ELECTRODE FOAM-TYPE LITHIUM-MANGANESE BATTERY AND MANUFACTURING METHOD THEREOF\ | CN102881922A | ZHANG HONGBING US\ DENG JIANGNAN US | ZHANG HONGBING US | H01M6/14\ H01M4/06\ H01M4/08 |
| 5060 | 3D printing | PIEZOELECTRIC-TYPE THREE-DIMENSIONAL PRINTING FORMING SYSTEM AND FORMING METHOD THEREOF\ | CN102602146B | ZHANG HONGHAI US\ CAO SHU US\ ZHU TIANZHU US\ XU YULI US\ SHU XIAYUN US\ ZHAN ZHIMIN US\ HU YAN US\ SUN BO (TW) | UNIV HUAZHONG SCIENCE TECH (CN) | B41J2/01\ B41J3/00 |
| 5061 | 3D printing | JET MILL WITH NOZZLE GROUP DISTRIBUTED IN THREE-DIMENSIONAL SPACE\ | CN203124062U | ZHANG JINGJIE US\ JIA WENHAO US\ ZHANG JINGBAO US\ LIU WENWEI US | WEIFANG EL FACTION POWDER TECHNOLOGY EQUIPMENT CO LTD US | B02C19/06 |
| 5062 | 3D printing | FEATHER DOWN PINNULE WIRE\ | CN2599029Y | ZHANG WENLI (CN) | ZHANG WENLI (CN) | D02G3/40 |
| 5063 | 3D printing | NOVEL CASING POWDER PRILLING MACHINE FOR EMBEDDED DIAMOND PRODUCT\ | CN2794723Y | ZHANG XINGLIANG (CN) | ZHANG XINGLIANG (CN) | B23P5/00 |
| 5064 | 3D printing | MOLDING MOLD FOR MOTOR SHELL CASTING MOLD\ | CN202639233U | ZHAO YIPING US | ZHAO YIPING US | B22C9/24 |
| 5065 | 3D printing | TONER BOTTLE\[FR] BOUTEILLE DE TONER\ | WO2011147278A1 | ZHENG YUXIA (CN)\ SO KINKEUNG (CN)\ HE YONGGANG (CN) | PRINT RITE UNICORN IMAGE PRODUCTS CO LTD ZHUHAI (CN)\ ZHENG YUXIA (CN)\ SO KINKEUNG (CN)\ HE YONGGANG (CN) | G03G15/08 |
| 5066 | 3D printing | ELECTRONIC DEVICE SHELL SURFACE COVERING STRUCTURE\ | CN202503831U | ZHENGJIAN XU US | SHANGHAI HAISEI ELECTRONIC CO LTD US | B44C5/04\ H05K5/00\ B32B15/04 |
| 5067 | 3D printing | BORON-CONTAINING METALLIC BOND SPECIAL FOR CUBIC BORON NITRIDE PRODUCT AND PREPARING METHOD THEREOF\ | CN102642023A | ZHIYING WU US | HENAN CUT STONE SCIENCE AND TECHNOLOGY CO LTD US | B22F1/00\ C22C1/05\ C04B28/00\ C22C29/16 |
| 5068 | 3D printing | RAPID COMPOSITE PRECISION MANUFACTURING METHOD FOR A METAL PART\ | CN102451882A | ZHONGDE SHAN US\ FENG LIU US\ XIANYI XU US\ ZHAOXIAN GU US | ADVANCED MFT TECH CT CN CAMST US | B22C9/04 |
| 5069 | 3D printing | COATING REINFORCING AGENT PREPARATION METHOD THEREOF AND PHOSPHATE-METAL ALUMINUM POWDER COATING USING REINFORCING AGENT\ | CN102492324A | ZHONGGUANG ZHOU US\ ZHIWEI LU US\ YONG HE US | ZHEJIANG MOTORBACS AUTOPARTS CO LTD US\ NO 52 INST CN NORTH IND GROUP US | C09D7/12\ C09D1/00\ C09D5/10 |
| 5070 | 3D printing | METHOD FOR PREPARING ZINC CATHODE SHEET OF ALKALINE ZINC BASE SECONDARY BATTERY\ | CN102208630A | ZHONGKUI LI US\ XUNYE WANG US\ GUOJI LU US\ CHONGSI ZENG US | GUOJI LU US\ ZHOU YAPING US | H01M4/26 |
| 5071 | 3D printing | MANUFACTURING METHOD OF NEODYMIUM IRON BORON BASED ON THREE-DIMENSIONAL (3D) PRINTING TECHNOLOGY\ | CN103170628A | ZHOU GAOFENG US | JIN KE MAGNETICS CO LTD US | B22F7/02\ B22F3/115 |
| 5072 | 3D printing | METHOD AND DEVICE FOR SPRAYING DIGITAL THREE-DIMENSIONAL CERAMICS\ | CN102101315A | ZHOU TONG US\ JING ZHAO US | SHANGHAI TECKWIN DEV CO LTD US | B28B1/32 |
| 5073 | 3D printing | MAGNETIC POWDER WARM CHAMBER PELLETING DEVICE\ | CN202762396U | ZHOU YONG (CN) | CHENGDU TO NAN ELECTRONICS CO LTD US | B01J2/10 |
| 5074 | 3D printing | PRECISION CASTING BLADE GUIDE WHEEL STRUCTURE\ | CN202883459U | ZHUANG CHAORONG US\ DONG SHUDONG US | TIANJIN LUTAI PREC CASTING CO LTD US | F04D29/18 |
| 5075 | 3D printing | FACE-CHANGED (DEFORMED) DISPLAY SHELL AND PREPARATION METHOD THEREOF\ | CN101975532A | ZICHUN KANG US | ZICHUN KANG US | F42B4/04\ F42B4/30 |
| 5076 | 3D printing | VORRICHTUNG UND SCHICHTBAUVERFAHREN ZUM HERSTELLEN VON DREIDIMENSIONALEN FORMTEILEN\[EN] DEVICE FOR MANUFACTURING THREE-DIMENSIONAL MOLD PARTS FOR E.G. PROTOTYPING HAS INSTALLATION SPACE WHERE LAYER OF POWDER MATERIAL IS SOLIDIFYABLE BY ADDING BINDER TO LAYERS OF MOLD PART AND LOW PRESSURE IS GENERATED IN INSTALLATION SPACE\ | DE102011082873A1 | ZIRNGIBL MARTIN (DE) | BAYERISCHE MOTOREN WERKE AG (DE) | B29C67/00 |
| 5077 | 3D printing | LASER QUICK FORMING SYSTEM AND FORMING METHOD WITH SIX SHAFT MACHINE HAND FOR POWDER MATERIAL\ | CN1586769A | ZUO TIECHUAN (CN)\ CHEN JIMIN (CN)\ WANG YINGNA (CN) | UNIV BEIJING POLYTECHNIC (CN) | B22F3/105 |
| 5078 | 3D printing | SYSTEMS FOR MANUFACTURING ORAL-BASED HEARING AID APPLIANCES\ | US2008070181A1 | ABOLFATHI AMIR (US)\ PHAN LOC X (US) | SONITUS MEDICAL INC (US) | A61C5/11\ A61C5/08 |
| 5079 | 3D printing | ASSAYS AND METHODS USING BIOMARKERS\ | US2008182277A1 | ASHKENAZI AVI J (US)\ WAGNER KLAUS W (US) | GENENTECH INC (US) | G01N33/574 |
| 5080 | 3D printing | RECOVERY OF DIMETHYLFORMAMIDE AND OTHER SOLVENTS FROM PROCESS STREAMS OF MANUFACTURE OF TRICHLOROGALACTOSUCROSE\ | US2009264640A1 | AURORA SUNDEEP (IN)\ RATNAM RAKESH (IN)\ SUBRAMANIYAN N A (IN) | VB MEDICARE PVT LTD (IN) | C07H3/04\ C07C231/24 |
| 5081 | 3D printing | DRILL BIT WITH DIAMOND IMPREGNATED CUTTER ELEMENT\ | US7469757B2 | AZAR MICHAEL G (US)\ BEATON TIMOTHY (US) | SMITH INTERNATIONAL (US) | E21B10/46\ E21B10/54\ E21B10/55\ E21B10/56\ E21B10/573 |
| 5082 | 3D printing | MIXTURE AND METHOD FOR REDUCING CHOLESTEROL USING HYDROPHOBIC MICROPARTICLES\ | US2010048508A1 | BEN-SHALOM NOACH (IL) | BEN BAR TECHNOLOGY 2006 LTD (US) | A61K31/722\ A61P3/06 |
| 5083 | 3D printing | WATER-SWELLABLE HOT-MELT-TYPE ADHESIVE\ | US6803400B1 | BUTTERBACH RUEDIGER (DE)\ MAASSEN ULRICKE (DE)\ KOPANNIA SIEGFRIED (DE) | HENKEL KGAA (DE) | C09K3/10\ C08L33/08\ G02B6/46\ C09J9/00\ C09J123/08\ C09J11/08\ C09J201/00\ C08L23/08\ A61F13/49\ C08L71/02\ C09J7/02\ A61F13/53\ H01B7/282\ C09J133/08\ D04H1/58\ C08F2/16\ C08L33/10\ C09J131/04\ C09J157/00\ D04H1/587\ H02G1/06\ H02G9/06 |
| 5084 | 3D printing | DEER RATTLING DEVICE\[FR] APPEAU DE CHASSE A CLIQUETIS POUR CHEVREUILS\ | CA2665632A1 | CASTNER MICHAEL (US) | CASTNER MICHAEL (US) | A01M31/00 |
| 5085 | 3D printing | SYSTEM AND METHOD OF MONITORING AND QUANTIFYING PERFORMANCE OF AN AUTOMATED MANUFACTURING FACILITY\ | US7379782B1 | COCCO DENNIS P (CA) | ACTIVPLANT CORP (CA) | G06F19/00 |
| 5086 | 3D printing | FRONT ELECTRODE FOR USE IN PHOTOVOLTAIC DEVICE AND METHOD OF MAKING SAME\ | US2009084438A1 | DEN BOER WILLEM (US)\ LU YIWEI (US)\ BROADWAY DAVID (US)\ CORSNER BRYCE (US) | GUARDIAN INDUSTRIES (US) | H01L31/00 |
| 5087 | 3D printing | RING SPRING BINDER CAPABLE OF TRANSMITTING SIGNAL\ | CN1526571A | DU ZHENYUAN (CN)\ DU ZHEN AN (CN)\ ZHENG HONGRU (CN) | INTERNAT STATIONERY MANUFACTUR (CN) | B42F3/04\ B42F13/16 |
| 5088 | 3D printing | DUSCHEINRICHTUNG MIT W?RMESTRAHLUNGSQUELLE\[EN] SHOWER DEVICE WITH THERMAL RADIANT SOURCE\[FR] DISPOSITIF DE DOUCHE AVEC SOURCE THERMIQUE RADIANTE\ | EP2353478A3 | EBNER OLIVER (AT)\ NACHTMANN MARKUS (AT)\ TSCHUDNIG HERBERT (AT)\ WEBERNDORFER BIRGIT (AT) | MANUFACTUR FUER GLAS UND SPIEGEL GMBH (AT) | A61N5/06\ A47K3/28\ A61H33/06 |
| 5089 | 3D printing | EINRICHTUNG MIT W?RMESTRAHLUNGSQUELLE\[EN] DEVICE HAVING A THERMAL RADIATION SOURCE\[FR] DISPOSITIF éQUIPé D'UNE SOURCE DE RAYONNEMENT THERMIQUE\ | EP2642907A1 | EBNER OLIVER (AT)\ NACHTMANN MARKUS (AT)\ TSCHUDNIG HERBERT (AT)\ WEBERNDORFER BIRGIT (AT) | MANUFACTUR FUER GLAS UND SPIEGEL GMBH (AT) | A61N5/06\ A47K3/28\ A61H33/06 |
| 5090 | 3D printing | KIMCHI MANUFACTUR ING METHOD USING MACKEREL\ | KR101287792B1 | Empty Field US | SONG YOUNG SEOG (KR) | A23L1/212\ A23B7/10\ A23L1/221\ A23L1/326 |
| 5091 | 3D printing | No Title Available\ | JP3128160U | Empty Field US | WAVIN BV (NL) | G11B7/26 |
| 5092 | 3D printing | SELF-ADHESIVE SECURITY SEAL STICKER WTIH AN INCORPORATED GRAPHITE OR GRANULATED LAYER FOR FINGERPRINT AND DNA COLLECTIONPROCEDURE OR LAMINATION TO MANUFACTUR\ | GB0625183D0 | Empty Field US | SALVA CALCAGNO EDUARDO L US | Empty Field |
| 5093 | 3D printing | LASER INTERFEROMETRIC ACCELEROMETER\[FR] ACCELEROMETRE D'INTERFEROMETRIE A LASER\ | CA2641704A1 | ERDMANN ERICH (CA)\ ERDMANN ADOLF (CA) | ERDMANN ERICH (CA)\ ERDMANN ADOLF (CA) | G01P15/00\ G01C19/58 |
| 5094 | 3D printing | USE OF 2345-TETRAHYDRO-1H-3-BENZAZEPINES FOR THE MANUFACTURE OF A PHARMACEUTICAL COMPOSITION FOR THE TREATMENT OF SLEEP DISORDERS\ | US6211173B1 | FINK-JENSEN ANDERS (DK)\ FOGED CHRISTIAN (DK) | CENES LTD (US) | C07D223/16\ A61K31/55\ A61P25/20\ C07D405/04 |
| 5095 | 3D printing | STENT HAVING A MULTIPLICITY OF UNDULATING LONGITUDINALS\ | US8157856B2 | FISCHELL ROBERT E (US)\ FISCHELL DAVID R (US)\ FISCHELL TIM A (US) | FISCHELL ROBERT (US)\ FISCHELL DAVID R (US)\ FISCHELL TIM A (US) | A61F2/00\ A61F2/82\ A61F2/90 |
| 5096 | 3D printing | SYSTEM FOR THE MANUFACTUR OF CHOPPED STRANDS MADE OF THERMOPLASTIC MATERIAL\ | US2011008482A1 | FONT DOMINIQUE (FR)\ BASTARD MICHEL (FR) | FONT DOMINIQUE (FR)\ BASTARD MICHEL (FR) | B28B11/14 |
| 5097 | 3D printing | SERVICE CABINET FOR USE WITH A UTILITY POLE\ | US7851702B2 | FOURNIER PAUL W (CA)\ LEGAULT JEAN-JACQUES (CA)\ GEOFFROY GUY (CA) | FOURNIER PAUL W (CA)\ GEOFFROY GUY (CA)\ LEGAULT JEAN-JACQUES (CA) | H02B1/30\ H02G7/20 |
| 5098 | 3D printing | FLAT TV WALL HANGING DEVICE\ | CN200944640Y | GAO YUGEN (CN) | SUZHOU VICTORY PREC MANUFACTUR (CN) | H04N5/64 |
| 5099 | 3D printing | HUMANIZED ANTIBODY\ | US2005226883A1 | GEMMELL JACK (CA)\ AVERBACK PAUL (CA) | AVERBACK PAUL (CA)\ GEMMELL JACK (CA) | A61K47/48\ A61K51/10\ A61K39/395\ C07K16/30\ C07K16/44\ G01N33/574 |
| 5100 | 3D printing | APPARATUS TO SHAPE THE INTERIOR SURFACE OF A PIPE MADE OF THERMOPLASTIC MATERIAL\ | US2007259065A1 | HABERLE ADALBERT (DE)\ HETZNER CLAUS (DE)\ KELLNER MICHAEL (DE) | DROSSBACH GMBH & CO KG (DE) | A21C3/00 |
| 5101 | 3D printing | ACTIVE PROGRAMMABLE ELECTRONIC DEVICES FOR MOLECULAR BIOLOGICAL ANALYSIS AND DIAGNOSTICS\ | US5605662A | HELLER MICHAEL J (US)\ TU EUGENE (US) | NANOGEN INC (US) | B82B1/00\ B01L3/00\ G01N21/64\ H01L21/336\ H01L29/78\ B82B3/00\ H01L51/30\ H01L51/05\ H01L51/40\ H05K1/02\ B01J19/00\ C12Q1/68\ C07B61/00\ C07H21/00\ C07K1/04\ C12N15/09\ C40B40/06\ C40B40/10\ C40B40/12\ C40B60/14\ G01N27/447\ G01N33/566\ G11C13/02\ G11C19/00\ H01L21/98 |
| 5102 | 3D printing | WIDTH WIRE SELF-FEEDING HOPPER DEVICE OF WIRE NET MANUFACTUR EQUIPMENT\ | KR101056629B1 | HONG HYEON GYU (KR) | HEUNG CHANG STEEL CO LTD (KR) | B21F23/00\ B21F27/00 |
| 5103 | 3D printing | FERMENTED MILK FOR SKIN IMPROVEMENT AND/OR TREATMENT AND PROCESS FOR PRODUCING THE SAME\ | US2010015110A1 | ITO HIROYUKI (JP)\ ISAWA KAKUHEI (JP)\ IKEGAMI SHUJI (JP)\ NOMA TERUYUKI (JP)\ KANO HIROSHI (JP) | MEIJI DAIRIES CORP (JP) | A61K35/74\ A61K35/20\ A61P17/00 |
| 5104 | 3D printing | RADIATION GENERATING DEVICE LITHOGRAPHIC APPARATUS DEVICE MANUFACTURING METHOD AND DEVICE MANUFACTURED THEREBY\ | US7335900B2 | IVANOV VLADIMIR VITALEVITCH (RU)\ KOROB EVGENII DMITREEVITCH (RU)\ ZUKAVISHVILI GIVI GEORGIEVITCH (RU)\ GAYAZOV ROBERT RAFILEVITCH (RU)\ KRIVTSUN VLADIMIR MIHAILOVITCH (RU)\ KOSHELEV KONSTANTIN NIKOLAEVIT (RU) | ASML NETHERLANDS BV (NL) | H05G2/00\ H01J65/04 |
| 5105 | 3D printing | LIGNOCELLULOSE FIBER FILLER FOR THERMOPLASTIC COMPOSITE COMPOSITIONS\ | US2002005602A1 | JACOBSEN WILLIAM W (US) | POLYMUN SCIENT IMMUNBIO FORSCH US | C08J5/04\ C08H8/00 |
| 5106 | 3D printing | MANUFACTURE METHOD OF WET-TISSUE WITH ANTIMICROBIAL AND ANTI-FUNGUS FUNCTION\ | US2010180413A1 | JEONG GU-WAN (KR) | NANOPOLY CO LTD (KR)\ PAUL MEDI CORP (KR) | D04H3/00 |
| 5107 | 3D printing | LEFTOVER RESOURCE PULPER\ | CN201720087U | JINCHANG CAO US\ JUNFANG GAO US\ SIJIN ZHOU US\ ZHAOLONG WANG US\ JIANRONG FU US\ JUNWEI MA US | SHANGHAI YUHUI MACHINE MANUFACTUR CO LTD US | B02C13/02\ B02C13/26\ B02C13/286 |
| 5108 | 3D printing | READY-TO-EAT DRY FRUIT PRODUCTS AND PROCESS\[FR] PRODUITS PRETS-A-MANGER A BASE DE FRUITS SECS ET METHODE DE FABRICATION CONNEXE\ | CA2559380C | JORDAN J KIRK (US) | GRAINNOVATION INC (US) | A23L1/212\ A23L3/40\ A23N12/08\ A23P1/12 |
| 5109 | 3D printing | TUMOR AND SENESCENCE MARKER\ | US2004086963A1 | KATINGER HERMANN (AT)\ GRABHERR REINGARD (AT)\ GILLARI JOHANNES (AT) | JACOBSEN WILLIAM W (US) | C07K14/47 |
| 5110 | 3D printing | HEARING AID ADAPTED FOR EMBEDDED ELECTRONICS\ | US2010034410A1 | LINK DOUGLAS F (US)\ PRCHAL DAVID (US)\ HIGGINS SIDNEY A (US) | STARKEY LAB INC (US) | H04R25/00 |
| 5111 | 3D printing | FLOATING STANDOFF ASSEMBLY\ | US2014044491A1 | MEYERS FRANK (US)\ BROWN JOHN A (US) | KEPNER PLASTICS FABRICAT INC (US) | E02B3/26 |
| 5112 | 3D printing | SYSTEM AND APPARATUS FOR RAPIDLY INSTALLED BREAKWATER\ | US6767162B2 | MEYERS FRANK (US)\ BROWN JOHN A (US) | KEPNER PLASTICS FABRICAT INC (US) | E02B3/06\ E02B15/04 |
| 5113 | 3D printing | FUNCTIONAL LAMINATE\ | US2009291271A1 | MICHALK MANFRED (DE)\ HOFMANN FRANZISKA (DE)\ GRIESBACH ANDREAS (DE) | HID GLOBAL GMBH (DE) | B32B37/00\ B32B3/10 |
| 5114 | 3D printing | HEAT RESISTANT POLYPROPYLENE FILM\ | US8440319B2 | MIER RODOLFO (US)\ MILLER MARK (US) | FINA TECHNOLOGY (US)\ MIER RODOLFO (US)\ MILLER MARK (US) | C08G64/00\ C08G63/02 |
| 5115 | 3D printing | BULK HYDROPROCESSING CATALYST COMPRISING A GROUP VIII METAL AND MOLYBDENUM PREPARATION AND USE THEREOF\ | US2009139904A1 | MISEO SABATO (US)\ LOUWEN JACOBUS NICOLAAS (NL)\ SOLED STUART LEON (US)\ EIJSBOUTS-SPICKOVA SONA (NL)\ PLANTENGA FRANS L (NL)\ LELIVELD ROBERTUS GERARDUS (NL) | ALBEMARLE NETHERLANDS BV (NL) | B01J23/883\ C10G45/04\ B01J23/882 |
| 5116 | 3D printing | FOUNTAIN THAT FLOWS WITH FLUIDIC MATERIAL\[FR] FONTAINE DANS LAQUELLE CIRCULE UNE MATIERE FLUIDIQUE\[DE] MIT FLUIDMATERIAL FLIESSENDER BRUNNEN\ | EP1677616A1 | MUIR RICHARD B (US)\ LIANG WU C (TW)\ POLLEY WILLIAM F (US)\ MUIR DEVAN B (US) | SEPHRA LLC (US) | A23G1/20\ A47J37/12\ A47J43/046\ B05B17/08\ B67D3/00\ E03B9/20 |
| 5117 | 3D printing | METHOD FOR THE MANUFACTURE OF L-THREONINE-0-(11-DIMETHYLETHYL)-11-DIMETHYLETHYL ESTER\ | US2006194983A1 | MUKARRAM MOHAMMED SIDDIQUI J (IN)\ BHARGAV KRISHNAJL U (IN)\ HEDGE DEEP V (IN) | MUKARRAM MOHAMMED SIDDIQUI J (IN)\ BHARGAV KRISHNAJL U (IN)\ HEDGE DEEP V (IN) | C07C227/18\ C07C229/22\ C07C227/14 |
| 5118 | 3D printing | TONER SUPPLY ROLL AND ITS MANUFACTURE\ | JP11038749A | NOZAWA AKITOSHI US\ SHIRAKI KEITA US | TOKAI RUBBER IND LTD US | C08L75/04\ G03G15/08\ B32B5/18\ C08G18/48\ B32B1/08\ F16C13/00 |
| 5119 | 3D printing | METHOD AND APPARATUS FOR ROLLING METAL PLATE\ | JP2011025289A | OGAWA SHIGERU US\ ISHII ATSUSHI US\ KASAI DAISUKE US | NIPPON STEEL CORP US | B21B37/30\ B21B37/00\ B21B37/58 |
| 5120 | 3D printing | MANUFACTUR METHOD OF SEMI-TOPPING FOR SINTERED ROLLED GEAR\ | KR20130039602A | PARK JONG KWAN (KR)\ KIM JIN HYUN (KR) | KOREA SINTERED METAL CO LTD (KR) | B22F3/12\ B21H5/00\ B23P15/14 |
| 5121 | 3D printing | BRACKET OF MANUFACTUR METHOD FOR HYDRAULIC BREAKER AND THE BRACKET\ | KR20120095027A | PARK YEON HWA (KR) | PARK YEON HWA (KR) | B21D53/00\ B21D5/01\ E21B1/26 |
| 5122 | 3D printing | METHOD OF MANUFACTURING SECURITY ELEMENTS FOR ELECTRONIC ARTICLE SURVEILLANCE AND SECURITY ELEMENT\ | US6585857B2 | RUEHRIG MANFRED (DE) | METO INTERNATIONAL GMBH (DE) | G08B13/24 |
| 5123 | 3D printing | ZIRCITE TEMPERATURE SENSOR\ | CN101281065B | SENAN ZHOU (CN)\ AIJUN WU (CN)\ JINWU GUO (CN)\ KEMING GENG (CN)\ YUQING ZHANG (CN) | LUOYANG SIGMA INSTR MANUFACTURE CO LTD US | G01K7/02 |
| 5124 | 3D printing | ALPHAMETHYLSTYRENE-BASED COPOLYMER HAVING EXCELLENT HEAT RESISTANCE AND METHOD FOR PREPARING THE SAME\ | KR20100062418A | SEO JAE BUM (KR)\ YI MIN JU (KR)\ HWANG YOUNG YOUNG (KR) | LG CHEMICAL LTD (KR) | C08F212/10\ C08F2/02\ C08F222/08\ C08F222/30 |
| 5125 | 3D printing | PHOTOCELL MATRIX TYPE MATERIAL CHOOSING RACK\ | CN201010286Y | SHAOSONG WANG (CN) | WUXI MAIRII MACHINE MANUFACTUR (CN) | B65G1/137 |
| 5126 | 3D printing | ACOUSTIC ABSORPTION SOUNDPROOF AND MANUFACTUR METHOD THEREOF\ | KR20130102320A | SHIN KYUNG HAK (KR) | WOOJIN TECH CO LTD (KR) | B60R13/00\ B60R13/08 |
| 5127 | 3D printing | THIN DOUBLE-SIDED PACKAGE SUBSTRATE AND MANUFACTURE METHOD THEREOF\ | US2008315239A1 | SUN BO (TW)\ LIN CHI CHIH (TW)\ WANG HUNG JEN (TW)\ TSENG JEN FENG (TW) | TAIWAN SOLUTIONS SYSTEM CORP (TW) | H01L23/48\ H01L33/48\ H01L33/60\ H05K3/42 |
| 5128 | 3D printing | HIGH AND LOW VOLTAGE ALUMINUM TRANSFORMER STRIP ASSEMBLY AND MACHINING TECHNIQUE THEREOF\ | CN101476064A | TAO WANG (CN)\ LIHUA WANG (CN)\ XIAOMING XIONG (CN)\ MIN LI (CN)\ ZUOWEN GAO (CN)\ BAOPING HOU (CN)\ XINTAO LI (CN)\ XIN SHI (CN)\ ZHIPING CHEN (CN) | CHALCO HENAN ALUMINUM FABRICAT (CN) | C22C21/00 |
| 5129 | 3D printing | OPTICAL MEMBER WITH HIGH AND LOW REFRACTIVE INDEX LAYERS\ | US8148672B2 | TODA ATSUSHI (JP) | SONY CORP (JP)\ TODA ATSUSHI (JP) | G02B5/18\ H01J3/14\ G02B3/08\ H04N5/369\ H01L27/14\ H04N5/335\ H04N5/361\ H04N5/3728\ H04N5/374\ G01D5/36\ G02B27/44 |
| 5130 | 3D printing | DERIVATIVES OF DI-CARBONYL AROMACHEMICALS\ | US2009162495A1 | TURIN LUCA (GB) | FLEXITRAL INC (US) | A23L1/22\ A23L1/237\ B65D85/00 |
| 5131 | 3D printing | METHODS AND DEVICES FOR THE PRODUCTION OF A PIPE FROM BIAXIALY ORIENTED THERMOPLASTIC MATERIAL HAVING AN INTEGRATED SOCKET\ | US2004016100A1 | VAN LENTHE ARJAN DIRK (NL)\ PRENGER JAN HENDRIK (NL)\ VISSCHER JAN (NL)\ WORMALD DUNCAN HAMILTON (GB) | AUTOMATION DESIGN & MANUFACTUR (TW) | F16L21/00\ B29L23/00\ B29C57/02\ B29C57/04\ B29C57/08 |
| 5132 | 3D printing | OXYGEN-SCAVENGING COMPOSITIONS AND ARTICLES\ | US2002086929A1 | VENKATESHWARAN LAKSHMI N (US)\ TSAI BOH C (US)\ CHEN STEPHEN Y (US)\ CHIANG WEILONG L (US) | CIBA SC HOLDING AG (US) | C08K3/00\ C08K3/32\ C08L101/00\ C08K5/00\ B01D53/04\ B65D81/24\ A23L3/3436\ B01D53/46\ C08J3/22\ C08K3/30 |
| 5133 | 3D printing | PROTECTIVE DEVICE AGAINST BITING OF ARTHROPODS\ | US2006010589A1 | VOLGYESI GEORGE A (CA) | VOLGYESI GEORGE A (CA) | A41D13/00\ A01K13/00\ A41D13/11\ A41D13/12\ A01K55/00 |
| 5134 | 3D printing | THREAT-RESISTANT AIRCRAFT TRAY\ | US7004430B2 | WEEKLY ROY L (US) | WEEKLY ROY L (US) | B64C1/00\ B60N3/00\ B64D11/06\ B64D45/00\ F41H5/08 |
| 5135 | 3D printing | CORIOLIS GYRO\ | US2010116050A1 | WOLFRAM GEIGER (DE) | LITEF GMBH (DE) | G01C19/56 |
| 5136 | 3D printing | METAL FRAME FOR LIQUID CRYSTAL TELEVISION\ | CN201260207Y | XIAOJUN TAO (CN) | SUZHOU VICTORY PREC MANUFACTUR (CN) | H04N5/64 |
| 5137 | 3D printing | ALUMINUM ALLOY STRIP ASSEMBLY FOR UMBRELLA FRAMEWORK AND MACHINING TECHNIQUE THEREOF\ | CN101476067A | XINTAO LI (CN)\ HAIXU ZHANG (CN)\ YUZHU CHEN (CN)\ JIANSHENG CHAI (CN)\ JIANFENG ZHAO (CN)\ ZUOQI CHEN (CN) | CHALCO HENAN ALUMINUM FABRICAT (CN) | C22C21/06 |
| 5138 | 3D printing | METAL PLATE FAST REPAIR DRAWING COMBINED TOOL\ | CN201283563Y | YANXIA LI (CN)\ JIFU SUN (CN)\ YIGUO WANG (CN) | DALIAN NEWSTAR TOOL MANUFACTUR (CN) | B25B33/00\ B21D1/06 |
| 5139 | 3D printing | HIGH VOLTAGE POWER CABLE TERMINATION\ | US2009071684A1 | ZHANG MING (CN) | ZHANG MING (CN) | H02G15/064 |
| 5140 | 3D printing | INDUCTION COOKING VESSEL\ | JP2007330391A | AIKAWA TAKAYUKI US\ FUJITA HAGINO US | TOYO SEIKAN KAISHA LTD US | B65D81/34\ A47J27/00\ A47J27/04 |
| 5141 | 3D printing | BIOLOGISCH WIRKSAMER NAHRUNGSMITTELZUSATZSTOFF UND BIOLOGISCH WIRKSAMER FUTTERZUSATZSTOFF ZUR VERHINDERUNG VON IODMANGEL SOWIE NAHRUNGS- UND FUTTERMITTEL DIE DIESE ZUSATZSTOFFE ENTHALTEN\[EN] BIOLOGICALLY ACTIVE FOOD ADDITIVE AND BIOLOGICALLY ACTIVE FORAGE ADDITIVE FOR PREVENTING IODINE DEFICIENCY AND OPTIMISING IODINE METABOLISM FOOD AND FORAGE PRODUCTS CONTAINING SAID ADDITIVES\[FR] ADDITIF ALIMENTAIRE BIOLOGIQUEMENT ACTIF ET ADDITIF FOURRAGER BIOLOGIQUEMENT ACTIF SERVANT A PREVENIR LA CARENCE EN IODE ET A OPTIMISER LE METABOLISME DE L'IODE PRODUITS ALIMENTAIRES ET FOURRAGERS CONTENANT CES ADDITIFS\ | EP1393636A1 | ANDREICHUK VASILY PETROVICH (RU)\ ANDREICHUK DMITRY VASILIEVICH (RU)\ TIGRANYAN RUBEN AMBARTSUMOVICH (RU)\ ANDREICHUK ELENA VIKTOROVNA (RU) | ANDREICHUK VASILY PETROVICH (RU) | A23G3/00\ A23K1/16\ A23L1/314\ A23K1/18\ A21D2/02\ A21D2/26\ A23C9/15\ A23G3/36\ A23G3/44\ A23K1/175\ A23L1/304\ A23L1/317 |
| 5142 | 3D printing | BELT-DRIVING ROLLER FOR DRIVING WIRE MESH BELT AND FOOD MANUFACTURING APPARATUS USING THE SAME\ | US2012292160A1 | ARAI SEIICHI (JP) | ARAI SEIICHI (JP) | B65G23/06 |
| 5143 | 3D printing | MACHINE AND SYSTEM FOR APPLYING CONTAINER CARRIERS TO CONTAINERS\ | US2013263557A1 | BATES STEPHEN PAUL (GB) | BRITISH POLYTHENE LTD (GB)\ BATES STEPHEN PAUL (GB) | B65B17/02 |
| 5144 | 3D printing | STEREO LITHOGRAPHY RAPID PROTOTYPING PHOTOSENSITIVE RESIN AND PREPARATION METHOD THEREOF\ | CN102436145A | BIWU HUANG US\ XINPING CHEN US | UNIV NANCHANG US | G03F7/027 |
| 5145 | 3D printing | HOT DOG CUTTING DEVICE\ | US2008201956A1 | BOUTON TODD R (US)\ BOUTON ANGELA K (US) | BOUTON TODD R (US)\ BOUTON ANGELA K (US) | B26B3/04 |
| 5146 | 3D printing | NAHRUNGSMITTELPRODUKTSPENDER\[EN] FOOD PRODUCT DISPENSER\[FR] DISTRIBUTEUR DE PRODUITS ALIMENTAIRES\ | EP1850714A1 | CARRARA OSVALDO (IT)\ PERSONENI SILVANO (IT) | N&W GLOBAL VENDING SPA (IT) | A47J31/40 |
| 5147 | 3D printing | PROJECTOR SYSTEMS AND METHODS FOR PRODUCING DIGITALLY AUGMENTED INTERACTIVE CAKES AND OTHER FOOD PRODUCTS\ | US2010315491A1 | CARTER CHARITA Y (US)\ MINE MARK RAYMOND (US)\ ADELEYE MODUPE (US)\ RUNCO CHRISTOPHER RAYNARD (US)\ LADUKE THOMAS FRAISER (US)\ YANG BEI (US) | DISNEY ENTPR INC (US) | H04N13/04\ H04N7/18\ H04N5/64 |
| 5148 | 3D printing | CASTING PROCESS FOR HIGH-EXPLOSIVE PRIMER\ | CN102788534A | CHAOSHENG YANG US\ XUEJIN WANG US\ BEIFENG WANG US\ ZHANLI LIU US\ CAN WANG US\ SONGPO SONG US\ SONGXI ZHAO US\ NIANGUO WANG US\ SULI QU US\ JUANJUAN WANG US | QIANJIN CIVILIAN BLASTING MATERIAL CO LTD US | F42B3/195 |
| 5149 | 3D printing | PICKLED VEGETABLE PREPARED THROUGH SOAKING FRESH VEGETABLES IN RECYCLED SALTING WATER\ | CN103349254B | CHEN GONG US\ LI JIEZHI US\ ZHANG QISHENG US | SICHUAN ACADEMY OF FOOD AND FERMENTATION IND US | Empty Field |
| 5150 | 3D printing | PAPER-MADE FOOD STORAGE CASE WITH 3D ENHANCEMENT STRUCTURE\ | TW595599U | CHEN GUO-JEN (TW) | CHEN GUO-JEN (TW) | B65D5/20 |
| 5151 | 3D printing | METHOD FOR MAKING COLLOIDAL NANOCRYSTALS\ | US7846216B2 | CHEN WEI (CN)\ LI YA-DONG (CN)\ BAI FENG (CN)\ WANG DING-SHENG (CN)\ HUO ZI-YANG (CN)\ LIU LI-PING (CN) | UNIV TSINGHUA (CN)\ HON HAI PREC IND CO LTD (TW) | C01B15/013 |
| 5152 | 3D printing | THREE-DIMENSIONAL CHITOSAN HYDROGEL AND PREPARATION METHOD THEREOF\ | CN102604149A | CHENGDONG JI US | SHANGHAI HUAYI BIO LAB CO LTD US | A61L27/60\ C08J3/24\ A61L27/20\ C08J3/075\ C08J9/36\ A61K47/36\ A61L27/52\ C08L5/08 |
| 5153 | 3D printing | DEPOSITING APPARATUS FOR FORMING THIN FILM\ | US2012260855A1 | CHO WHANG SIN (KR)\ SONG KI CHUL (KR)\ JUNG SEUNG CHUL (KR)\ AHN WOO JUNG (KR) | CHO WHANG SIN (KR)\ SONG KI CHUL (KR)\ JUNG SEUNG CHUL (KR)\ AHN WOO JUNG (KR) | C23C16/52\ C23C16/448 |
| 5154 | 3D printing | METHOD FOR PRODUCING ANTITYPE-4-ALKYLTHIO CINNAMIC ACID\ | CN101219981A | CHUNXIANG KUANG (CN)\ WENSHENG ZHANG (CN)\ YUBO JIANG (CN)\ TINGHUA HU (CN) | UNIV TONGJI (CN) | C07C323/62\ C07C319/14 |
| 5155 | 3D printing | PRESSURE BASED LIQUID FEED SYSTEM FOR SUSPENSION PLASMA SPRAY COATINGS\ | US2013270355A1 | COTLER ELLIOT M (US)\ MOLZ RONALD (US) | SULZER METCO US INC (US)\ COTLER ELLIOT M (US)\ MOLZ RONALD (US) | B05C11/00\ B05B15/02 |
| 5156 | 3D printing | COMPOSITE HEALTHY MATTRESS\ | CN2688160Y | DING ZHENMIN (CN)\ LIU ZHIGUO (CN)\ LI SIYUAN (CN) | JIANLONG HEALTH FOOD CO LTD TI (CN) | A61N5/06\ A47C27/12\ A47C27/00\ A61N2/08 |
| 5157 | 3D printing | ANLAGE ZUM BEFüLLEN VON NAHRUNGSMITTELBECHERN MEHRFACHDüSE UND DESSERTPRODUKT\[EN] FOOD CONTAINER FILLING DEVICE ESPECIALLY FOR DESERT PRODUCTS USES VALVE FREE PRESSURE REDUCING SYSTEM TO PREVENT FOOD DRIPPING FROM OPEN DISPENSING NOZZLES\ | DE202004020481U1 | Empty Field US | CAMPINA GMBH & CO KG (DE) | B65B3/32\ B65B3/14\ B65B39/00\ B65B43/62\ B65B3/12 |
| 5158 | 3D printing | No Title Available\ | RU2009133322A | Empty Field US | Empty Field US | A01N1/02 |
| 5159 | 3D printing | No Title Available\ | RU2009132281A | Empty Field US | Empty Field US | C07C4/00 |
| 5160 | 3D printing | VACUUM FORMING APPARATUS AND METHOD OF THE SAME\ | KR101364715B1 | Empty Field US | Empty Field US | B29C45/18\ B29C45/23\ B29D7/01 |
| 5161 | 3D printing | SYSTEM FOR MANAGING PRODUCTS IN REFRIGERATOR\ | KR101156733B1 | KIM YOUNG HUN (KR)\ MIN HWI KIM (NZ) | Empty Field US | F25D29/00\ H04B1/40 |
| 5162 | 3D printing | 通電加熱装置\ | JP5608946B2 | LI GUOZHONG US | Empty Field US | H05B3/00 |
| 5163 | 3D printing | No Title Available\ | CN103351718B | LUNITSYN VASILIJ GERASIMOVICH (RU) | Empty Field US | B41M5/42\ C09D11/106\ C09D11/03 |
| 5164 | 3D printing | METHOD FOR AUTOMATICALLY DILUTING CHLORINE DIOXIDE AQUEOUS SOLUTION WITH WATER TO CONCENTRATION FITTED TO INTENDED USE IN EACH USE\ | JP2012200589A | PALMAS PAOLO (US)\ MAJERS DEHNIEL NOL (US)\ MITCHELL TODD FILIP (US)\ AL TOFF DZHEJMS UEHJN (US) | SEIKEN CO US | A61L2/18\ B01F1/00\ B01F3/08\ B01F5/04\ C01B11/02 |
| 5165 | 3D printing | DIESEL FUEL MANUFACTURING REACTOR AND DIESEL FUEL MANUFACTURING SYSTEM\ | JP2012219141A | WON YUN JAE (KR) | TAKUMA KK US | C10G3/00\ C10G11/10 |
| 5166 | 3D printing | VORRICHTUNG SOWIE VERFAHREN ZUR DREIDIMENSIONALEN ABBILDUNG EINES RELATIV ZUR SENSORANORDNUNG BEWEGTEN OBJEKTS\[EN] METHOD FOR THREE-DIMENSIONAL IMAGING E.G. WEAPON HIDDEN UNDER CLOTH OF PEOPLE INVOLVES PRODUCING THREE-DIMENSIONAL RADAR IMAGE OF OBJECT FROM RADAR DATA FOR IMAGE REPRESENTATION IN E.G. CORRECT POSITION ON THREE-DIMENSIONAL SURFACE MODEL\ | DE102010051207A1 | ENDER JOACHIM DR (DE)\ HAEGELEN MANFRED (DE)\ HANTSCHER SEBASTIAN DR (DE)\ LANG STEFAN (DE) | FRAUNHOFER GES FORSCHUNG (DE) | G06T17/00\ G01S17/89\ G01S13/89\ G01S7/04\ G01S13/86\ G01S13/90\ G06T15/04 |
| 5167 | 3D printing | METHOD FOR PRODUCING A DHA-CONTAINING FATTY ACID COMPOSITION\ | US2008175975A1 | FABRITIUS DIRK (DE) | NUTRINOVA GMBH (DE) | A23D7/00\ C11B1/04 |
| 5168 | 3D printing | METHOD FOR SUSPENDING LIQUID PLASMA SPRAYING PREPARATION OF HYDROXYAPATITE BIOACTIVITY COATINGS\ | CN101250681A | FANG WU (CN)\ YI HUANG (CN)\ XIAOGUANG LIU (CN)\ YAO WU (CN)\ YANFENG XIAO (CN)\ LEI SONG (CN)\ JIYONG CHEN (CN) | UNIV SICHUAN (CN) | A61L27/04\ C23C4/04 |
| 5169 | 3D printing | DEVICE FOR FISH FILLET CUTTING\ | RU2453120C1 | FATYKHOV JURIJ ADGAMOVICH (RU)\ AGEEV OLEG VJACHESLAVOVICH (RU)\ MATSONKO ALEKSANDR ZENONOVICH (RU)\ EVTROPKOV VLADIMIR MIKHAJLOVICH (RU) | FEDERAL NOE G OBRAZOVATEL NOE UCHREZHDENIE VYSSHEGO PROFESSIONAL NOGO OBRAZOVANIJA KALININGR GTU (RU) | A22C25/18 |
| 5170 | 3D printing | ELECTRIC OVEN WITH ADJUSTABLE HEATING ELEMENT\ | US8330080B2 | FERNANDEZ JUAN (US)\ LONDONO RESTREPO JUAN DAVID (ES) | FERNANDEZ JUAN (US)\ LONDONO RESTREPO JUAN DAVID (ES)\ FOTOCRISTAL 3D S L (ES) | F27D5/00 |
| 5171 | 3D printing | HARDLY SOLUBLE SUSPENSION LIQUID INJECT METHOD AND APPARATUS FOR PREPARING COMPOSITE MATERIAL SAMPLE ROOM\ | CN1603011A | GAO CHEN (CN)\ CHEN LEI (CN)\ HUANG SUNXIANG (CN) | USTC UNIV SCIENCE TECH CN (CN) | G05B15/00\ B05C5/00\ B05D1/12\ B06B1/06\ B05B12/00\ B05C9/06 |
| 5172 | 3D printing | TETRAHEDRAL SHAPE PACKAGING CONTAINER AND A TETRAHEDRAL SHAPE PACKAGING CONTAINER METHOD\ | US8939643B2 | GOTO NORIO (JP) | GOTO NORIO (JP) | B65D33/00\ B65D33/25\ B65D75/58\ B65D75/50\ B65D65/26 |
| 5173 | 3D printing | METHOD FOR PRODUCING SMOKED PRODUCT AND SMOKING DEVICE\ | JP2004313080A | GOTO TAKASHI US\ YAMAMOTO TOSHIMASA US | THERMOS KK US | A23B4/044\ A23B4/052\ A47J27/00\ A47J39/02 |
| 5174 | 3D printing | COMPREHENSIVE CARBONIZING DEVICE\ | CN201981162U | GUANDE LI US | GUANDE LI US | C10B53/02\ C10B57/00\ C10B57/10\ C10C5/00\ C10K1/00 |
| 5175 | 3D printing | VERFAHREN ZUM BETRIEB EINER ANLAGE ZUR ROHEISEN- UND/ODER STAHLERZEUGUNG\[EN] OPERATING SYSTEM FOR MAKING IRON AND/OR STEEL BY RECOVERING CRUDE IRON AND/OR STEEL FROM IRON ORE CONTAINING RAW MATERIALS AND GENERATING OXYGEN BY REACTION IN WHICH OXYGEN FROM OXYGEN CONTAINING GAS IS BOUND TO CARRIER MATERIAL\ | DE102011077092A1 | GUENSTER WERNER (DE)\ HUBER NORBERT (DE)\ ZIMMERMANN GERHARD (DE)\ MILLNER ROBERT (AT) | SIEMENS AG (DE) | F27D17/00\ C01B13/08 |
| 5176 | 3D printing | CHEMICAL SENSORS BASED ON CUBIC NANOPARTICLES CAPPED WITH AN ORGANIC COATING\ | US2010273665A1 | HAICK HOSSAM (IL)\ DOVGOLEVSKY EKATERINA (IL) | TECHNION RES & DEV FOUNDATION (IL) | H01B1/02\ C40B30/02\ C40B60/10 |
| 5177 | 3D printing | METHOD OF MANUFACTURING A FOOD CONTAINER\ | US2006293158A1 | HAMANO SHINKICHI (JP) | ISAMU SATO (JP) | B31B1/62 |
| 5178 | 3D printing | METHOD TO CREATE REMOVABLE DENTAL PROSTHESIS AND THE DENTAL PROSTHESIS MAKING THEREOF\ | US2012261848A1 | HARASZATI GYOERGY (HU) | HARASZATI GYOERGY (HU) | A61C13/20 |
| 5179 | 3D printing | METHOD FOR USING BUDDING BROWN RICE TO PRODUCE NATURAL EATABLE FIBER GABA IP6 AND USEFUL BACTERIA IN INTESTINES\ | TW200300658A | HONG JIAN-LONG (TW)\ CHEN CANG-HAI (TW) | HONG JIAN-LONG (TW) | A23L1/30\ A23L1/308 |
| 5180 | 3D printing | PREPARATION METHOD OF THREE-DIMENSIONAL ORDERED MACROPOROUS LA1-XSRXCRO3 WITH MESOPOROUS PORE WALL\ | CN102060534A | HONGXING DAI US\ KEMENG JI US\ YUXI LIU US\ LEI ZHANG US\ HUICHAO XU US\ LINGLONG SHAN US\ RUOLIN CHEN US | UNIV BEIJING TECHNOLOGY (CN) | C04B35/622\ C04B38/00\ C04B35/50 |
| 5181 | 3D printing | SCARIFICATEUR DE PATONS ADAPTE POUR DIMINUER LA PENIBILITE DE LA SCARIFICATION\ | FR2884389B1 | HOREL JEAN CHRISTIAN (FR)\ FONTAINE JEROME (FR) | HOREL JEAN CHRISTIAN (FR)\ FONTAINE JEROME (FR) | A21C11/12 |
| 5182 | 3D printing | FLOTATION APPARATUS\ | US8181790B2 | ISHIDA MINORU (JP)\ KASHIBO MASAMI (JP)\ SONODA MUNETAKA (JP)\ KOYANAGI TOMOAKI (JP)\ HOSAKA TATSUMI (JP)\ AIKAWA YOSHIHIKO (JP) | JUJO PAPER CO LTD (JP)\ AIKAWA TEKKO (JP)\ ISHIDA MINORU (JP)\ KASHIBO MASAMI (JP)\ SONODA MUNETAKA (JP)\ KOYANAGI TOMOAKI (JP)\ HOSAKA TATSUMI (JP)\ AIKAWA YOSHIHIKO (JP) | B03D1/14 |
| 5183 | 3D printing | BUILDING FOR HOUSING POULTRY INCORPORATES SEVERAL FLOORS ONE ABOVE OTHER TOGETHER WITH ACCESS ARRANGEMENTS FOR AT LEAST SOME BIRDS TO FLOORS\[NL] INRICHTING VOOR HET HOUDEN VAN PLUIMVEE GEBOUW VOORZIEN VAN EEN DERGELIJKE INRICHTING EN WERKWIJZE.\ | NL1024938C2 | JANSEN ALBRECHT HENDRIK (NL) | A H JANSEN HOLDING B V (NL) | A01K31/22 |
| 5184 | 3D printing | PRODUCT E.G. BEVERAGE DISPLAY CASE FOR E.G. EXHIBITION HAS COMPARTMENTS STORING PRODUCTS AND FORMED OF STRUCTURES COMPRISING PLATES WHICH ARE FORMED OF THERMALLY INSULATING MATERIAL AND OPENINGS PROVIDED ON SIDES OF COMPARTMENTS\[FR] PRESENTOIR ISOTHERME DE PRODUITS.\ | FR2884810A1 | JARMASSON JEROME (FR)\ JARMASSON LIONEL (FR) | JARMASSON JEROME (FR)\ JARMASSON LIONEL (FR) | B65D81/38\ A47F3/04\ A47F10/02\ B65D88/74 |
| 5185 | 3D printing | REFRIGERATION BOARD CONTIUNANCE PRODUCTION METHOD AND DEVICE\ | KR100826098B1 | JEONG YONG CHAE (KR) | DUT KOREA CO LTD (KR) | B29D7/00 |
| 5186 | 3D printing | BARREL AUTOMATIC CLAMPING AND TILTING DEVICE\ | CN201647974U | JIAGUI XU US\ ZHANQI AN US\ GUOLIN TAO US\ JI JUN US\ SHEQING YU US | BAOYING RENHENG INDUSTRY CO LTD US | B67C9/00 |
| 5187 | 3D printing | DENDROBE CHROMATOGRAM FINGER PRINT MEASURING METHOD\ | CN101726547A | JINLE CHENG US\ XIANXIA LI US\ YIZENG LIANG US\ JIYIN XU US\ LUNCHAO YI US | ZHONGSHAN ZEUS MEDICINE GROUP US | G01N30/02\ G01N30/06 |
| 5188 | 3D printing | FOOD PACKAGING BOX\ | CN201592806U | KAIMING HUANG US | KAIMING HUANG US | B65D8/00\ B65D85/60 |
| 5189 | 3D printing | DEVICE FOR INSPECTING 3-D SHAPE OF FOOT AND METHOD FOR THE SAME\ | KR20100122419A | KANG CHEOL GWON (KR) | KANG CHEOL GWON (KR) | G01B11/24\ G01B11/26\ G01L19/00 |
| 5190 | 3D printing | LIGHT FLEXIBLE LIQUID STAB-PROOF MATERIAL AND PREPARATION METHOD THEREOF\ | CN102692161A | KEJING YU US\ KUN QIAN US\ HAIJIAN CAO US | UNIV JIANGNAN US | F41H1/02 |
| 5191 | 3D printing | METHOD FOR REMOVING ANISOTROPIC CONDUCTIVE FILM FROM PCB\ | KR20130104616A | KIM DO WHAN (KR) | TRON CO LTD L (KR) | G02F1/13\ G02F1/1345 |
| 5192 | 3D printing | SOLID-LIQUID SEPARATOR FOR FOOD GARBAGE\ | KR20100135102A | KIM YOUNG AE (KR) | KIM YOUNG AE (KR) | B09B3/00\ B04B1/20\ C02F11/12 |
| 5193 | 3D printing | 3D HAMMERTONE EFFECT\[FR] EFFET MARTELé 3D\ | WO2010128842A1 | KIONG KEK KEN (MY) | KIONG KEK KEN (MY) | C09D5/28 |
| 5194 | 3D printing | FOOD STORAGE CONTAINER\ | JP2005145483A | KOBAYASHI SHIGENOBU US | HARIMA SHOKUHIN KK US | B65D1/26\ B65D85/50\ B65D81/32\ B65D77/08 |
| 5195 | 3D printing | A PREPARING PROCESS OF LIQUID CATALYST CONTAINING TITANIUM FOR POLYESTER OR COPOLYESTER\[FR] PROCéDé DE PRéPARATION DE CATALYSEUR LIQUIDE CONTENANT DU TITANE POUR LE POLYESTER OU LE COPOLYESTER\ | WO2008113230A1 | KONG FANTAO (CN)\ MA XINSHENG (CN)\ WU QIUFANG (CN)\ LI FUQING (CN)\ YANG JINGHUI (CN)\ CHEN GANG (CN)\ MA RUI (CN)\ DONG QIHUA (CN) | SHANGHAI HUAMING HI TECH GROUP (CN)\ KONG FANTAO (CN)\ MA XINSHENG (CN)\ WU QIUFANG (CN)\ LI FUQING (CN)\ YANG JINGHUI (CN)\ CHEN GANG (CN)\ MA RUI (CN)\ DONG QIHUA (CN) | C08G63/85\ C08G63/82\ C08G63/83\ C08G63/84\ C08G63/87 |
| 5196 | 3D printing | DEVICE FOR BATCH DISPENSING LIQUID DETERGENT\ | UA56570U | KRASILNIKOV ANDRIEI ALIEKSIEIEVICH (BY)\ KLIMOVICH DIENIS ANATOLIEVICH (BY) | BELASEPTIKA CLOSED JOINT STOCK COMPANY (BY) | B05B11/02 |
| 5197 | 3D printing | PREPARATION FOR NATURAL COLORED COTTON FAR INFRARED YARN\ | CN101275320A | KUN WANG (CN)\ BAOSHENG LI (CN)\ RUIZHOU LI (CN)\ DAIPING LI (CN)\ XIANGHONG LI (CN)\ JIANHUA YIN (CN)\ LIMIN AO (CN)\ JUNQIAO CUI (CN)\ FANG MA (CN) | HEBEI HONGRUN NOVEL MATERIAL C (CN) | D06M11/00\ D02G3/02\ D06M101/06 |
| 5198 | 3D printing | HEAT INSULATION VERTICAL SILICON INGOT MOLD\ | CN2672032Y | LAI HUAJUN (CN)\ WANG YINGJIANG (CN) | WANG YINGJIANG (CN) | C01B33/02\ C01B33/021 |
| 5199 | 3D printing | MANUFACTURING METHOD OF THREE-DIMENSIONAL FOOD BY RAPID PROTOTYPING\ | US2008260918A1 | LAI WEI-HSIANG (TW)\ CHENG CHUN-I (TW) | LAI WEI-HSIANG (TW)\ CHENG CHUN-I (TW) | A23P1/00\ A23P1/08 |
| 5200 | 3D printing | A MANUFACTURING METHOD FOR 3D FOOD BY RAPID PROTOTYPING TECHNOLOGY\ | TWI279193B | LAI WEI-HSIANG (TW)\ CHENG CHUN-I (TW) | LAI WEI-HSIANG (TW)\ CHENG CHUN-I (TW) | A23P1/00 |
| 5201 | 3D printing | GUINEA PIG RAISING HOUSE\ | CN203194266U | LIN JUN US | FUJIAN YUTANG AGRICULTURE STOCK RAISING DEV CO LTD US | A01K1/02\ A01K1/00 |
| 5202 | 3D printing | METHOD TO FEED BIOMASS TABLETS AND LOGS INTO BURNERS\ | US2006272557A1 | LIU HENRY (US)\ LIN YUYI (US) | FREIGHT PIPELINE COMPANY (US) | F23K3/00 |
| 5203 | 3D printing | LOW-MELTING-POINT ALLOY CASTING POSITIONING TECHNOLOGY IN TURBINE BLADE MACHINING\ | CN103350213A | LIU MING US\ LIU XIAOZHONG US | CHANGZHOU 3D TECHNOLOGY COMPLETE SET EQUIPMENT CO LTD US | B22D19/00 |
| 5204 | 3D printing | SPUN LACED FABRIC AND PROCESSING METHOD THEREOF\ | CN101235579A | LIWEN ZHANG (CN) | LIWEN ZHANG (CN) | D04H1/46\ D01C3/00 |
| 5205 | 3D printing | SYNTHESIS OF TYPE OF THERMOSENSITIVE FUNCTIONALIZED ION LIQUID\ | CN101774964A | LU BAI US\ SUOJIANG ZHANG US\ XIANGPING ZHANG US\ YANSONG ZHAO US | INST PROCESS ENG CAS US | C07D211/82 |
| 5206 | 3D printing | ANGELICA KEISKEI WINE BREWING METHOD\ | CN103468454A | LU JUAN US | ZHENJIANG GAOGUAN FOOD CO LTD US | C12G1/022 |
| 5207 | 3D printing | COATED PAPER AND METHOD FOR PRODUCING THE SAME\ | JP2007186812A | MATSUI TAKASHI US\ YAMANAKA SHIGEO US | JSR CORP (JP) | D21H19/78\ D21H19/82 |
| 5208 | 3D printing | MASTICATOR\ | JP2007089701A | MATSUMURA YOSHIHIRO US\ IKEDA JUNJI US | MATSUSHITA ELECTRIC WORKS LTD (JP) | A61C19/04\ A61B5/11 |
| 5209 | 3D printing | STORAGE WRAP MATERIAL\ | US2007178262A1 | MCGUIRE KENNETH S (US) | PROCTER & GAMBLE (US) | B32B27/32 |
| 5210 | 3D printing | MICROWAVE HEATING APPARATUS\ | JP2010080185A | MIHARA MAKOTO US\ NOBUE TOMOTAKA US\ YASUI KENJI US\ OMORI YOSHIHARU US | PANASONIC CORP (JP) | H05B6/64\ H05B6/68 |
| 5211 | 3D printing | LIQUID EJECTION HEAD AND ITS MANUFACTURING METHOD\ | JP2005280349A | MITA TAKESHI US\ NIHEI YASUKAZU (JP) | FUJI PHOTO FILM CO LTD (US) | B41J2/16\ B41J2/045\ B41J2/055 |
| 5212 | 3D printing | POLYMER POWDER PROCESS FOR PRODUCTION OF AND USE OF THIS POWDER AND RESULTANT SHAPED ARTICLES\ | US2007232753A1 | MONSHEIMER SYLVIA (DE)\ KOMADA HAJIME (JP)\ MATSUI HIDEKI (JP)\ NAKAIE YOSHIKI (JP) | DEGUSSA (DE) | C08L77/00\ C08L61/00 |
| 5213 | 3D printing | CONTINUOUS MIXER FOR RUBBER COMPOUNDING\ | KR100874585B1 | MOON DONG SEOK (KR) | KUMHO TIRE CO INC (KR) | B29B7/18\ B29D30/08 |
| 5214 | 3D printing | ROTATING MOLD FOR E.G. AUTOMOBILE INDUSTRY HAS PLASTIC MATERIALS INJECTED SIMULTANEOUSLY BY PLASTIC MATERIAL INJECTING UNITS RESPECTIVELY WHEN INJECTION MOLDING MACHINE IS IN CLOSED POSITION AND PARTS MOUNTED ON RESPECTIVE PLATES\[FR] MOULE ROTATIF POUR L'INJECTION SIMULTANEE DE PIECES SURMOULEES EN MATIERES PLASTIQUES\ | FR2888152A1 | MOUSNIER MICHEL US | SM 3D SARL (FR) | B29C45/16 |
| 5215 | 3D printing | CHEMICALS RECYCLING METHOD OF RECOVERED FIRE EXTINGUISHERS\ | JP2006035118A | MUKUHASHI MASAKI US | HATSUTA SEISAKUSHO US | B09B5/00 |
| 5216 | 3D printing | FOOD\ | JP2010088329A | NAKAMURA TOSHIHIRO US | NAKAMURA TOSHIHIRO US | A23G1/00\ A23L1/00\ A23G1/30 |
| 5217 | 3D printing | NEUTRAL NEWSPRINT PAPER FOR OFFSET PRINTING\ | JP2007092203A | NAKANO TOMOYUKI US\ OCHI TAKASHI US | JUJO PAPER CO LTD (JP) | D21H17/67\ D21H27/00\ D21H17/29\ D21H17/45 |
| 5218 | 3D printing | VERFAHREN ZUM POLIEREN EINES GLASSGEGENSTANDS\[EN] METHOD FOR POLISHING A GLASS ARTICLE\[FR] PROCEDE DE POLISSAGE D'UN ARTICLE EN VERRE\ | EP0964772A1 | NGUYEN DAVID D (US)\ VISSER ROBERT G (US)\ CHRISTIANSON TODD J (US) | MINNESOTA MINING & MFG (US) | B24D3/00\ B24D11/00\ B24D3/28\ B24B13/01 |
| 5219 | 3D printing | HEAT INSULATING SHEET AND ITS MANUFACTURING METHOD AND COVERING MATERIAL ITS MANUFACTURING METHOD AND ITS INSTALLATION METHOD\ | JP2009101635A | OGURA YASUHIRO US\ ISHIDA SHUNPEI US\ NAKANO MINORU US\ TSUCHIZAWA KENICHI US\ IWAZAWA TOSHIO US\ YAMAMOTO OSAMU US | ASUKA KK US\ NITTO BOSEKI CO LTD US | B29K105/08\ B29C43/02\ B32B5/24\ B32B7/02\ B29K21/00\ F16L59/02 |
| 5220 | 3D printing | SYSTEM FOR AUTOMATICALLY PREPARING LIQUID FOR PROCESSING PRINT SURFACE\ | JP2006241345A | ONISHI HIROTETSU US | FUJI PHOTO FILM CO LTD (US) | B05D5/06\ C09D7/14\ B41M7/02\ B05D3/00\ B41F23/08 |
| 5221 | 3D printing | METHOD FOR THE DIRECT AND CONTINUOUS PRODUCTION OF HOLLOW ARTICLES FROM A POLYMER MELT\ | US7547406B2 | OTTO BRIGITTA (DE)\ SCHAEFER ROLAND (DE)\ BACHMANN HOLGER (DE)\ HOELTING LUDWIG (DE)\ DEISS STEFAN (DE) | LURGI ZIMMER GMBH (DE) | C08G63/78\ B29C45/00\ C08J5/00\ B29B13/00\ B29C31/04\ B29D22/00\ B29B7/32\ B29B7/88\ B29C45/18\ B29C49/00\ C08G63/08\ C08G63/88\ C08J3/205 |
| 5222 | 3D printing | THREE-DIMENSIONAL POROUS PRESSURE-RESISTANT AND EXPANSION-LIMITING TYPE BENTONITE ADSORBING MATERIAL AND PREPARATION METHOD THEREOF\ | CN102824898A | PENG LONGGUI US\ ZHANG ZAIYONG US | UNIV XI AN SCI & TECHNOLOGY US | B01J20/26\ B01J20/24\ B01J20/30 |
| 5223 | 3D printing | MULTIFUNCTIONAL FOOD PRINTER\ | CN203136994U | PENG YUHUI US\ ZENG JINRONG US | ZENGCHENG LEGO FOODS CO LTD US | A23P1/08\ A21C15/00 |
| 5224 | 3D printing | HIGH-ADHESION BENZENE-FREE ENVIRONMENTALLY-FRIENDLY UNIVERSAL JET INK AND PREPARATION METHOD THEREOF\ | CN101698764A | PIHUI PI US\ JIANHUA NIE US\ JIANLI LUO US\ DAFENG ZHENG US\ XIUFANG WEN US\ JIANG CHENG US\ ZHUORU YANG US | UNIV SOUTH CHINA TECH (CN) | C09D11/30 |
| 5225 | 3D printing | PROCESS OF CASTING GEAR PUMP REAR COVER\ | CN101885050A | QIAOSHENG PU US | WUJIANG HYDRAULIC COMPONENTS FOUNDRY CO LTD US | B22C9/00\ B22D18/02\ F04C2/18\ F04C15/00 |
| 5226 | 3D printing | LIQUID O-PHTHALIC ANHYDRIDE TANK CAR\ | CN202038217U | QINGFU YANG US | ZHONGSHAN UNICIZERS IND CO LTD US | B60P3/22 |
| 5227 | 3D printing | ENCODER-SIGNAL TIMING UNCERTAINTY ADJUSTED TO IMPROVE ESTHETIC QUALITY IN BIDIRECTIONAL INKJET PRINTING\ | US5519415A | RASKIN GREGORY D (US) | HEWLETT PACKARD CO (US) | B41J2/21\ B41J2/51\ B41J19/14 |
| 5228 | 3D printing | DIRECTION-INDEPENDENT ENCODER READING; POSITION LEADING AND DELAY AND UNCERTAINTY TO IMPROVE BIDIRECTIONAL PRINTING\ | US5426457A | RASKIN GREGORY D (US) | HEWLETT PACKARD CO (US) | B41J2/05\ B41J2/51\ B41J19/14\ B41J19/18 |
| 5229 | 3D printing | ANLAGE ZUM BEFüLLEN VON NAHRUNGSMITTELBECHERN\[EN] APPARATUS FOR FILLING FOOD PRODUCT CUPS\[FR] APPAREIL POUR REMPLIR DES GOBELETS POUR PRODUITS ALIMENTAIRES\ | EP1602579B1 | REUSS NORBERT (DE) | CAMPINA GMBH (DE) | B65B3/32\ B65B3/14\ B65B39/00\ B65B43/62\ B65D77/08 |
| 5230 | 3D printing | BRIDGE MANDRELS FOR ANILOX AND PRINT ROLLER APPLICATIONS AND TECHNIQUES FOR MAKING THEM\ | US2009165662A1 | RITCHIE JOHN E (US) | NIM COR INC (US) | B21D53/00\ B41F13/10 |
| 5231 | 3D printing | MULTI-FUNCTIONAL CARDBOARD BOX\[FR] BO?TE EN CARTON MULTIFONCTION\ | WO2012156766A2 | ROZSA BALAZS (HU)\ SZABO ZOLTAN (HU)\ BERTA HUBERT (HU) | ROZSA BALAZS (HU)\ SZABO ZOLTAN (HU)\ BERTA HUBERT (HU) | Empty Field |
| 5232 | 3D printing | HOLLOW TIO2 MICROSPHERE SYNTHESIZING METHOD\ | CN102701276A | RULIN DONG US\ CAIXIA WANG US\ SHUYUN LIU US\ ZHIDONG CHEN US\ CHANGCHUN JIN US\ WENCHANG WANG US | UNIV CHANGZHOU US | C01G23/053 |
| 5233 | 3D printing | DRUM AMMONIFICATION GRANULATOR\ | CN202284186U | SANZHOU TANG US\ SHAOHUA WANG US | HUBEI NEWYANGFENG FERTILIZER STOCK CO LTD US | C05G5/00 |
| 5234 | 3D printing | PREPARATION METHOD AND APPLICATION OF COMPOUND POROUS SUPERMOLECULE MIXED POLYMER\ | CN102311737A | SHANQUAN YAN US | SHANQUAN YAN US | C09K17/50\ C09K17/42\ C09K101/00\ C09K109/00 |
| 5235 | 3D printing | METHOD FOR PREPARING ALUMINUM SILICON CARBIDE COMPOSITE MATERIAL\ | CN102500748A | SHENGMING JIN US\ KUIXIN CUI US\ XIPENG LI US\ XINGHUA CHANG US | UNIV CENTRAL SOUTH US | B22D19/00\ C04B38/02 |
| 5236 | 3D printing | AUTOMATIC FEEDING DEVICE FOR DOG AND CAT\ | JP2004065220A | SHINADA AKINOBU US | SHINADA AKINOBU US | A01K5/02 |
| 5237 | 3D printing | BRIDGE TYPE PALLETIZER\ | CN201144092Y | SHUILONG XU (CN)\ YIQING SHEN (CN) | WUJIANG FOOD PROC MACHINERY CO (CN) | B65G15/22\ B65G1/04\ B65G57/00 |
| 5238 | 3D printing | PROCESSING METHOD OF CRUCIAN CARP\ | CN102934677A | SONG QIXIANG US | CHONGQING CITY HECHUAN DISTR HESHUANG TECHNOLOGY CO LTD US | A23B4/023\ A23B4/03 |
| 5239 | 3D printing | COMPOUNDS AND METHODS FOR THERAPEUTIC INTERVENTION IN PREVENTING DIABETIC COMPLICATIONS AND PROCEDURES FOR ASSESSING A DIABETIC'S RISK OF DEVELOPING COMPLICATIONS AND DETERMINING THE EFFICACY OF THERAPEUTIC INTERVENTION\ | US6004958A | SU BANGYING (US)\ LAL SUNDEEP (US)\ SZWERGOLD BENJAMIN (US)\ BROWN TRUMAN R (US)\ KAPPLER FRANCIS (US) | FOX CHASE CANCER CENTER (US) | A61P3/10\ A61P25/02\ G01N33/50\ A61K31/13\ A61K31/197\ A61K31/198\ A61K31/275\ A61K31/5375\ A61K31/7028\ A61P27/02\ C07C215/14\ C07C229/26\ C07C255/26\ C07H15/04\ C07H19/20 |
| 5240 | 3D printing | APPARATUS AND METHOD FOR PRODUCING NANOFIBERS\ | JP2009209485A | SUMITA HIROTO US\ KUROKAWA TAKAHIRO US\ ISHIKAWA KAZUNOBU US\ TOMINAGA YOSHIAKI US\ TAKEZAWA MIKIO US\ TAKAHASHI MITSUHIRO US | PANASONIC CORP (JP) | D01F6/00\ D01D5/04\ D04H1/728 |
| 5241 | 3D printing | MILK METER\ | JP2010038737A | TAKIZAWA HIROMI US\ HIRATA AKIRA US\ GOTO YUTAKA US | ORION MACHINERY CO LTD US\ NAT AGRICULTURE & FOOD RES ORG US | G01F1/00\ G01F11/28 |
| 5242 | 3D printing | METHOD FOR COMPUTING FOOD VOLUME IN A METHOD FOR ANALYZING FOOD\ | US8345930B2 | TAMRAKAR AMIR (US)\ SAWHNEY HARPREET SINGH (US)\ YU QIAN (US)\ DIVAKARAN AJAY (US) | STANFORD RES INST INT (US)\ TAMRAKAR AMIR (US)\ SAWHNEY HARPREET SINGH (US)\ YU QIAN (US)\ DIVAKARAN AJAY (US) | G06K9/00 |
| 5243 | 3D printing | PROPELLANT FILLING METHOD FOR DOUBLE AEROSOL DEVICE\ | JP2004131187A | TANAKA MASAZUMI US | DAIZO KK US | B65D83/38\ B65B31/00\ B65D83/46 |
| 5244 | 3D printing | NON-INTERPENETRATING CHIRAL MOF STATIONARY PHASE ITS PREPARATION METHOD AND APPLICATION IN ENANTIOMER SEPARATION IN HPLC\ | CN103331151A | TANG BO US\ MA YU US\ KUANG XUAN US\ SU HAO US | UNIV SHANDONG NORMAL US | B01J20/30\ B01J20/29\ C07B57/00\ C07C29/74\ C07C33/22\ C07C45/79\ C07C49/83\ C07C51/47\ C07C57/30\ C07C209/88\ C07C211/27\ C07F3/06 |
| 5245 | 3D printing | MACHINE IMAGING APPARATUS AND METHOD FOR DETECTING FOREIGN MATERIALS\ | US2009080706A1 | TAO YANG (US)\ CHEN XIN (US)\ JING HANSONG (US)\ ZHU BIN (US)\ JIN FENGHUA (US) | INDUSTRY VISION AUTOMATION COR (US) | G06K9/00 |
| 5246 | 3D printing | THERMOFORMED FOOD STUFF PACKAGE FOR E.G. AGRO-FOOD INDUSTRY HAS COMPARTMENTS COMPRISING RESPECTIVE BODIES AT OPENING AND COVERING SIDES AND INCLUDING COVERS CONSTITUTING TOTALITY OF OUTER PERIPHERAL SURFACE OF PACKAGE\[FR] EMBALLAGE THERMOFORME A COMPARTIMENTS DISPOSES EN FAISCEAU\ | FR2882989A1 | THIOT VIRGINIE US | CGL PACK SERVICE SOC PAR ACTIO (FR) | B65D6/18\ B65D6/34 |
| 5247 | 3D printing | GRAPHENE OXIDE MICROSPHERE AND GRAPHENE MICROSPHERE AND PREPARATION METHODS THEREOF\ | CN103121672A | TIAN YAYANG US\ ZHOU LISHA US\ CHEN WEI (CN) | SUZHOU INST NANO TECH & NANO B US | C01B31/04 |
| 5248 | 3D printing | METHOD FOR PRODUCTION OF FERMENTATIVE HYDROLYSATE OF FRESH WHITE CABBAGE AND NUTRITIVE MEDIUM FOR CULTIVATION LACTOBACTERIA BASED THEREON\ | RU2415923C1 | TIMCHENKO LJUDMILA DMITRIEVNA (RU)\ PEN KOVA NADEZHDA IVANOVNA (RU)\ KATUNINA LJUDMILA SEMENOVNA (RU)\ VAKULIN VALERIJ NIKOLAEVICH (RU)\ TARAN TAT JANA VIKTOROVNA (RU) | G OBSHCHEOBRAZOVATEL NOE UCHREZHDENIE VYSSHEGO PROFESSIONAL NOGO OBRAZOVANIJA STAVROPOL SKIJ GU (RU) | C12N1/20\ C12R1/225 |
| 5249 | 3D printing | PATTERNED BOILED EGG AND PROCESS FOR PRODUCING THE SAME\ | US6773742B1 | UMEHARA HATSUYO (JP) | Empty Field US | A23L1/32 |
| 5250 | 3D printing | 3D-FOOD PRODUCT FORMING APPARATUS AND PROCESS\[DE] VORRICHTUNG UND VERFAHREN ZUR HERSTELLUNG EINES DREIDIMENSIONALEN NAHRUNGSMITTELS\[FR] APPAREIL ET PROCéDé DE FORMATION DE PRODUIT ALIMENTAIRE EN 3D\ | EP2380442A3 | VAN GERWEN HENDRIKUS PETRUS GERARDUS (NL) | CFS BAKEL BV (NL) | A22C7/00 |
| 5251 | 3D printing | 3D-FOOD PRODUCT FORMING APPARATUS AND PROCESS\[DE] VORRICHTUNG UND VERFAHREN ZUR HERSTELLUNG EINES DREIDIMENSIONALEN NAHRUNGSMITTELS\[FR] PRODUIT ALIMENTAIRE EN 3D ET APPAREIL ET PROCéDé DE FORMATION\ | EP2560495A2 | VAN GERWEN HENDRIKUS PETRUS GERARDUS (NL)\ BOOGERS GEORGE LAMBERTUS JOSEPHUS MARIA (NL)\ RIGHOLT HENDRIK JAN (NL) | CFS BAKEL BV (NL) | A22C7/00 |
| 5252 | 3D printing | 3D-FOOD PRODUCT FORMING APPARATUS AND PROCESS\ | US2013045294A1 | VAN GERWEN HENDRIKUS PETRUS GERARDUS (NL)\ BOOGERS GEORGE LAMBERTUS JOSEPHUS MARIA (NL)\ RIGHOLT HENDRIK JAN (NL) | CFS BAKEL BV (NL)\ VAN GERWEN HENDRIKUS PETRUS GERARDUS (NL)\ BOOGERS GEORGE LAMBERTUS JOSEPHUS MARIA (NL)\ RIGHOLT HENDRIK JAN (NL) | A22C17/00\ A21C11/00 |
| 5253 | 3D printing | EXTRACTS OF LEAVES BARKS AND FLOWERS OF VARIOUS ENDEMIC GREEK VARIETIES OF THE PLANT OLEA EUROPEA IN SERIES OF POLAR AND NON POLAR SOLVENTS WITH IMMUNE REINFORCING ANTI-COAGULATING ANTI-PLATELETSDERIVED ANTI-LIPIDEMIC ANTI-AGEING ANTI-CANCEROGENIC NEURO-PROTECTIVE ANTI-CANCER X-RAY PROTECTIVE ANTI-RHEUMATIC ANTI-INFLAMMATORY SKIN-PROTECTIVE CEREBRAL-PROTECTIVE AND ANTI-OXIDISING PROPERTIES AS WELL AS PROTECTION FROM THE OXIDATION OF LIPOPROTEINS\ | GR1006199B | VERGINADIS IOANNIS US | ETAIREIA EREVNON VIOTECHNOLOGI US | A61P29/00\ A61K36/63\ A61K31/05 |
| 5254 | 3D printing | PREPARATION TECHNOLOGY OF SOYBEAN PASTE\ | CN102366085B | WANG SHENGLI US | DONGGUAN BAIWEIJIA FOOD CO LTD US | A23L1/24 |
| 5255 | 3D printing | 3D TOUCH CONTROL PRODUCT INQUIRING AND ORDERING INTEGRATED MACHINE WITH GREETING FUNCTION\ | CN203260126U | WANG ZIXING US | WANG ZIXING US | G07F17/40 |
| 5256 | 3D printing | NOVEL COLUMN-SHAPED THREE-DIMENSIONAL CULTIVATION METHOD AND DEVICE FOR MEDICAL DENDROBIUM\ | CN102172205A | WANGLI YANG US\ YUJIN YANG US | WANGLI YANG US | A01G31/00\ A01G1/00\ A01G31/02 |
| 5257 | 3D printing | MULTISPEKTRALSENSOR\[EN] MULTI-SPECTRAL SENSOR\[FR] CAPTEUR MULTISPECTRAL\ | EP2208033A2 | WEBER NORBERT (DE)\ JUNGER STEPHAN (DE)\ TSCHEKALINSKIJ WLADIMIR (DE) | FRAUNHOFER GES FORSCHUNG (DE) | H01L27/146\ G01J3/28\ G01J3/51 |
| 5258 | 3D printing | PRODUCTION METHOD OF THREE-DIMENSIONAL SKELETON COMPOSITE BOARD FOR BUILDING\ | CN103086658A | WEI FENG US\ ZHANG JUN US | SHANXI VK SCIENCE AND TECHNOLOGY DEV CO LTD US | C04B28/00\ C04B14/18 |
| 5259 | 3D printing | CALCIUM PHOSPHATE-BASED COMPOSITE MICROSPHERE SUPPORT AND PREPARATION METHOD THEREOF\ | CN103394120A | WEI KUN US\ XU WEIKANG US | UNIV SOUTH CHINA TECH (CN) | A61L27/56\ A61L27/18\ A61L27/12 |
| 5260 | 3D printing | MACHINE FOR AUTOMATICALLY CLEANING SILTS FROM GRANULAR FOODS\ | CN202162063U | WENGE GAO US | TIANJIN CHUANQI FOOD CO LTD US | B03B5/32 |
| 5261 | 3D printing | PYROLYZATION AND DEBROMINATION METHOD OF WASTE PRINTED CIRCUIT BOARD\ | CN101829666A | WENSHENG DONG US\ CHUNLING LIU US\ XIN LIU US\ ERTAO YANG US\ YAN WANG (CN) | UNIV SHANXI NORMAL US | C08J11/10\ B09B3/00\ C07C37/00 |
| 5262 | 3D printing | VORRICHTUNG ZUR NATURNAHEN KONTROLLE VON INSEKTEN\ | DE102010032785A1 | WINKLER CONSTANZE (DE) | WINKLER CONSTANZE (DE) | A01M1/14 |
| 5263 | 3D printing | INSECT CONTROLLING DEVICES AND METHODS\ | US2012055075A1 | WINKLER CONSTANZE (DE)\ FRIEND HERMAN L (US)\ BIRON ANTOINE (FR)\ ZHENG JIE SHAN (CN) | WINKLER CONSTANZE (DE)\ FRIEND HERMAN L (US)\ BIRON ANTOINE (FR)\ ZHENG JIE SHAN (CN) | A01M1/14\ A01M1/02\ A01M1/20\ A01M99/00 |
| 5264 | 3D printing | REAL-TIME INTERACTIVE 3D ENTERTAINMENT DEVICE AND 3D REPLICATION\ | TW201238326A | WU TUNG-FA (TW) | WU TUNG-FA (TW) | H04N13/00\ G02B27/22\ G03B35/00 |
| 5265 | 3D printing | EQUILIBRATED NUTRITION MEAT VEGETABLES EATING LIVESTOCK AND BIRD PRODUCT PROCESSING METHOD\ | CN101138421B | XINBIAO ZHANG (CN) | XINBIAO ZHANG (CN) | A23L1/31\ A23L1/29\ A23L1/311\ A23L1/314\ A23L1/315 |
| 5266 | 3D printing | SOFT FOOD 3D (THREE-DIMENSION) PRINTING SYSTEM\ | CN103315371A | XU DAN US\ WANG DONGHAI US\ WU HANGZONG US\ XU JINHONG US | KUNSHAN XIANGHUAJING BIOTECHNOLOGY CO LTD US | A23P1/10 |
| 5267 | 3D printing | HIGH-REDUCTION MODIFIED ALIPHATIC WATER REDUCING AGENT AND PREPARATION METHOD THEREOF\ | CN102898057A | XU YOUJUAN US | XU YOUJUAN US | C04B24/16\ C04B103/30 |
| 5268 | 3D printing | EDIBLE AND BIOCOMPATIBLE METAL-ORGANIC FRAMEWORKS\ | US2010286022A1 | YAGHI OMAR M (US)\ LI QIAOWEI (US)\ FANG QIANRONG (US)\ EL-KADRI OUSSAMA M (CA) | UNIV CALIFORNIA (US) | C07K14/00\ C07H21/00\ A61K38/00\ A61K31/7088\ A61K47/22\ C07C69/76\ C07F5/00 |
| 5269 | 3D printing | METHOD AND DEVICE FOR MONITORING PRODUCTION OF FLUID FILM\ | US2012235313A1 | YAN TAK CHUNG (CN)\ YAN TAK KIN ANDREW (CN) | YAN TAK KIN ANDREW (CN)\ YAN TAK CHUNG (CN) | B29C41/52 |
| 5270 | 3D printing | 3D (THREE-DIMENSIONAL) IMAGE CHOCOLATE\ | CN202873718U | YANG PING US | YANG PING US | A23G1/54 |
| 5271 | 3D printing | CROSSLINKED POLYCATION OIL DISPLACEMENT AGENT AND PREPARATION METHOD THEREOF\ | CN101805599A | YING TANG US\ JIAYE XU US\ GANG CHEN (CN) | UNIV XI AN SHIYOU US | C09K8/588 |
| 5272 | 3D printing | POLYOLEFIN THIN FILM HOT MELT ISOLATING AGENT AND PREPARATION METHOD AND USING METHOD THEREOF\ | CN102504306B | YOU JUN US | YOU JUN US | C08J7/04\ C08L23/00 |
| 5273 | 3D printing | METHOD FOR POND THREE-DIMENSIONAL (3D) CULTURE OF CHINESE PRAWNS\ | CN102177868A | YUMAN ZHANG US\ JIQIAO WANG US\ XIANGLI LIANG US\ DELING WANG US | MEIRI AGRICULTURE GROUP CO LTD US | A01K63/00\ A23K1/18 |
| 5274 | 3D printing | BOX WITH 3D DYNAMIC GRAPHIC PATTERN USED FOR FRESH-KEEPING OR FOOD OR MICROWAVE OVEN OR STORAGE\ | CN201086885Y | YUNGUO ZHANG (CN) | YUNGUO ZHANG (CN) | B65D25/34\ B65D43/02\ B65D45/04 |
| 5275 | 3D printing | PRODUCTION TECHNOLOGY OF BATIK CALLIGRAPHY WORKS\ | CN103072419A | ZHANG YIQUAN US | ZHANG YIQUAN US | B44C5/02\ D06B11/00 |
| 5276 | 3D printing | GAS METER WITH DOUBLE-DISPLAY MODE\ | CN201548263U | ZHEN WANG (--)\ JUNJUN XU (--) | ZHEJIANG WEIXING INTELLIGENT METER STOCK CO LTD (--) | G01F1/075 |
| 5277 | 3D printing | FLAME RETARDANT STEREO LITHOGRAPHY APPARATUS PHOTOSENSITIVE RESIN USED FOR THREE-DIMENSIONAL PRINTING, COMPRISES PHOSPHORUS-CONTAINING ACRYLATE PREPOLYMER, ACRYLATE, FREE-RADICAL INITIATOR, ADDITIVE AND REACTIVE DILUENT | CN105068378A | WELCH DAVID F (US)\ NAGARAJAN RADHAKRISHNAN L (US)\ CHIANG TING-KUANG (US)\ DOMINIC VINCENT G (US)\ KISH JR FRED A (US) |  | C08F-230/02\ C08F-283/00\ C08F-283/01\ C08F-283/10\ G03F-007/004\ G03F-007/027 |
| 5278 | 3D printing | BOLT INSTALLATION STRUCTURE, HAS SLS TECHNOLOGY PROCESS PROVIDED WITH COMPONENT MAIN BODY, MOUNTING BASE WHOSE OUTER WALL IS CONNECTED WITH CLAMPING PIECE THAT IS MOUNTED WITH CLAMPING SLOT, AND HOLE PART FORMED WITH CLAMPING SLOT | CN204851917U | VINEET BROWN KARL M PIPITONE J (US) | INFINERA CORP (US) | F16B-025/00 |
| 5279 | 3D printing | HIGH-TOUGHNESS PHOTOSENSITIVE RESIN USED FOR THREE-DIMENSIONAL PRINTING OF ELASTIC MODELS USING E.G. DIGITAL LIGHT PROCESSING RAPID PROTOTYPING PRINTER, CONTAINS MALLEABLE AND RIGID PHOTOSENSITIVE RESINS, PHOTOINITIATOR AND ADDITIVE | CN104765251A | ROSE P (US)\ LOPATA E (US)\ FELTS J (US) | HOFMANN RALF (US)\ BROWN KARL M (US)\ PIPITONE JOHN (US)\ MEHTA VINEET (US) | C08G-018/30\ C08G-018/48\ C08G-018/61\ C08G-018/67\ C08G-018/69\ C08G-018/75\ C08G-018/76\ G03F-007/004\ G03F-007/027 |
| 5280 | 3D printing | METHOD FOR MANUFACTURING FALSE TOOTH BRACKET BASED ON THREE DIMENSIONAL PRINTING LASER STEREO FORMING TECHNOLOGY, INVOLVES ANALYZING LOCALIZED CORROSION ELECTROCHEMICAL BEHAVIOR ON IMPEDANCE SPECTRUM TEST SURFACE OF DENTURE SUPPORT | CN104999078A | SANDHU VISHNU K AGARWAL GARO D (US) | WATKINS JOHNSON COMPANY INC US | A61C-013/02\ B22F-003/105\ B33Y-010/00 |
| 5281 | 3D printing | COMPUTER IMPLEMENTED METHOD FOR AUTOMATICALLY CONFIGURING FAILOVER, INVOLVES CONFIGURING FAILOVER OF HIGH-AVAILABILITY CLUSTER FUNCTIONALITY WITH HIGH-AVAILABILITY CONFIGURATION SCRIPT ACCORDING TO SERVICE LEVEL AGREEMENT | US9223606B1 | UEDA MASASHI (JP)\ TAKAGI TOMOKO (JP)\ ITOU NORIKAZU (JP) | MOSAID TECHNOLOGIES INC (CA) | G06F-009/455 |
| 5282 | 3D printing | SYSTEM FOR MANAGING BANDWIDTH BETWEEN ZONES WITH ONE TENANT IN NETWORK BASED ON PREDETERMINED SERVICE LEVEL AGREEMENT AND QUALITY OF SERVICE, HAS GLOBAL CONTROLLER FOR AUTOMATICALLY ADJUSTING BANDWIDTH SHAPING AND PROVISIONS | WO2015194933A1 | GRAETTINGER CEM BASCERI MARK V (US) | ISHIKAWAJIMA HARIMA HEAVY IND (JP)\ UEDA MASASHI (JP)\ TAKAGI TOMOKO (JP)\ ITOU NORIKAZU (JP) | G06Q-050/10 |
| 5283 | 3D printing | HEAD-UP DISPLAY (HUD) OF VEHICLE CARRIER DISPLAYING DEVICE, HAS REGULATING UNIT THAT REGULATES HEAD-UP DISPLAY PORTION ALONG REFLECTED DIRECTION SO AS TO CAPTURE IMAGE WITH PREDETERMINED DEPTH | CN105150935A | YAMAMOTO NAOKI (JP)\ SUZUKI NORIO (JP)\ UCHIYAMA HIROYUKI (JP)\ NISHITANI EISUKE (JP)\ KIMURA SHIN ICHIRO (JP)\ HOZAWA KAZUYUKI (JP) | MICRON TECHNOLOGY INC (US) | B60R-001/00\ G02B-027/01 |
| 5284 | 3D printing | THREE-DIMENSIONAL DIGITAL LIGHT PROCESSING PRINTER, HAS FRAME FORMED WITH PHOTOSENSITIVE RESIN GROOVE AND CONNECTED WITH PRINT PLATFORM, AND ELASTIC SUPPORTING PIECE FIXED WITH FASTENING PIECE AND TRIANGULAR-SHAPED STRUCTURE | CN204869689U | UEYAMA MUNETSUGU (JP)\ HAHAKURA SHUJI (JP)\ OHMATSU KAZUYA (JP)\ HASEGAWA KATSUYA (JP) | SUZUKI NORIO (JP)\ YAMAMOTO NAOKI (JP)\ UCHIYAMA HIROYUKI (JP)\ NISHITANI EISUKE (JP)\ KIMURA SHIN ICHIRO (JP)\ HOZAWA KAZUYUKI (JP) | B29C-067/00\ B33Y-030/00\ B33Y-040/00 |
| 5285 | 3D printing | COMPUTER-IMPLEMENTED METHOD FOR PREVENTING SERVICE LEVEL AGREEMENT (SLA)VIOLATION, INVOLVES MIGRATING VM TO TARGET PM IF PREDEFINED VM UTILIZATION THRESHOLD, RESPONSE TIME THRESHOLD AND TARGET PM UTILIZATION THRESHOLD ARE NOT VIOLATED | US2015372878A1 | FORBES LEONARD (US)\ AHN KIE (US) | SUMITOMO ELECTRIC INDUSTRIES (JP)\ INT SUPERCONDUCTIVITY TECH (JP)\ HAHAKURA SHUJI (JP)\ OHMATSU KAZUYA (JP)\ UEYAMA MUNETSUGU (JP)\ HASEGAWA KATSUYA (JP) | H04L-012/24\ H04L-012/26 |
| 5286 | 3D printing | METHOD FOR FABRICATING COMPOSITE PART FROM PREPREG SHEETS USING FUSED DEPOSITION MODELING, INVOLVES DELIVERING MATRIX AND FIBER MATERIAL TO LOCATION OF PRINTER, MELTING MATRIX MATERIAL, AND EMBEDDING FIBER MATERIAL WITHIN MATRIX MATERIAL | US2015367576A1 | SUZUKI NORIO (JP)\ TAKAMATSU AKIRA (JP)\ MARUYAMA HIROYUKI (JP)\ SATO HIDENORI (JP)\ SAIKAWA KENJI (JP) | MICRON TECHNOLOGY INC (US) | B29C-067/00\ B29C-070/30\ B29C-070/38 |
| 5287 | 3D printing | CODE PROCESSING WORK-PIECE MANUFACTURING METHOD, INVOLVES OBTAINING WORK-PIECE MODEL BY WORK-PIECE SCANNING DEVICE, AND PERFORMING THREE-DIMENSIONAL PRINT PROCESSING OPERATION TO MANUFACTURE CODE PROCESSING WORK-PIECE BY USING PRINT DEVICE | CN105138726A | NGO MINH VAN CHAN SIMON S BESS (US) | SAIKAWA TAKESHI (JP)\ SUZUKI NORIO (JP)\ ICHIZOE HIROYUKI (JP)\ TAKAMATSU AKIRA (JP)\ HOTTA KATSUHIKO (JP)\ SATO HIDENORI (JP)\ MARUYAMA HIROYUKI (JP) | G06F-017/50 |
| 5288 | 3D printing | NEAR-INFRARED SPECTRUM ANALYZER BASED ON DIGITAL LIGHT PROCESSING TECHNOLOGY, HAS CONDENSER LENS THAT INCLUDES COVER ON OUTSIDE OF LED CHIP OF TRANSPARENT CONE, SUCH THAT TRANSPARENT CONE IS WRAPPED IN SIDE OF SHADING LAYER | CN105092516A | TATSUYA HIUE (JP)\ FUMITOSHI ITO (JP)\ SHIRO KAMAHARA (JP) | ADVANCED MICRO DEVICES INC (US)\ NGO MINH-VAN (US)\ BESSER PAUL R (US)\ CHAN SIMON S (US)\ KING PAUL L (US)\ RYAN ERROL TODD (US)\ CHIU ROBERT J (US) | G01N-021/3577\ G01N-021/359 |
| 5289 | 3D printing | METHOD FOR EXECUTING IN VIRTUAL APPLIANCE IN FIRST NETWORK, INVOLVES SENDING RESOURCE AVAILABILITY INDEX TO MANAGEMENT APPLICATION EXECUTING IN SECOND NETWORK FOR APPROPRIATE MANAGEMENT AND ALLOCATION OF RESOURCES IN SECOND NETWORK | US2015365341A1 | YUICHI HOTTA KATSUHIKO SASAHAR (JP) | RENESAS TECH CORP (JP) | H04L-012/911\ H04L-029/08 |
| 5290 | 3D printing | STENT FOR MONITORING ENVIRONMENT INTO WHICH IT IS INSERTED IN BODY, HAS RESISTIVE MATERIAL THAT INCLUDES PLASTIC, METAL, CERAMIC, OR COMPOSITE, AND CONDUCTIVE MATERIAL INCLUDES PLASTIC, METAL, CERAMIC, OR COMPOSITE | WO2015183893A1 | NGO MINH VAN (US)\ QI XIANG (US)\ HAIHONG WANG (US) | RENESAS TECH CORP (JP) | A61B-005/05\ A61F-002/82 |
| 5291 | 3D printing | THREE-DIMENSIONAL NONVOLATILE MEMORY DEVICE I.E. NAND FLASH MEMORY, FOR USE IN SEMICONDUCTOR SYSTEM, HAS PAD, CONTACT PLUG AND BIT LINE IN STACKED CONFIGURATION OVER VERTICAL CHANNEL LAYER, AND COMMON SOURCE LINE | US2015340370A1\ CN105097817A\ KR2015134934A\ US9287286B2 | Empty Field US | GLOBALFOUNDRIES INC (KY) | G11C-016/06\ H01L-027/115\ H01L-021/8247\ H01L-029/792 |
| 5292 | 3D printing | INNER GLASS LAYER BONDING METHOD FOR DIGITAL LIGHT PROCESSING (DLP) SCREEN, INVOLVES DIVIDING INSIDE OF MARKED ADHESIVE INTO FOUR LONGER STRIP SLOTS FOR PRODUCING HOT-MELT ADHESIVE AFTER REMOVING EXCESS ADHESIVE RESIDUE | CN104745106A | PIERRE BLANCHARD (FR) | MAK CECILIA Y (US)\ LAW KAM S (US) | C09J-005/06 |
| 5293 | 3D printing | METHOD FOR PROVIDING DATA TO BATCH CONSUMER AND STREAMING CONSUMER, INVOLVES DELIVERING BATCH OF SET OF BATCHES IN FIRST DIRECTORY TO BATCH CONSUMER, AND DELIVERING DATA IN FIRST DIRECTORY AND BATCH OF SET OF BATCHES IN SECOND DIRECTORY | US2015358394A1 | XU JEFF J (TW)\ YEH CHIH CHIEH (TW)\ CHANG CHIH-HAO (TW)\ WANG CHIEN-HSUN (TW)\ CHANG CHIH-HSIANG (TW) | E2V SEMICONDUCTORS (FR) | G06F-017/30\ H04L-012/24\ H04L-029/06\ H04L-029/08 |
| 5294 | 3D printing | LIGHT SOURCE DEVICE OF PROJECTOR, HAS MAIN PORTION THAT IS COMPRISED WITH FLUORESCENT SUBSTANCE LAYER WHICH CARRIES OUT WAVELENGTH CONVERSION OF LIGHT TO WAVELENGTH RANGE AS WAVELENGTH RANGE OF LIGHT EMITTED FROM SECOND LIGHT SOURCE | JP2015219321A | XIAO MANCHAO (US)\ HOCHBERG ARTHUR KENNETH (US)\ CUTHILL KIRK SCOTT (US) | TAIWAN SEMICONDUCTOR MFG (TW)\ XU JEFF J (TW)\ CHANG CHIH-HAO (TW)\ WANG CHIEN-HSUN (TW)\ YEH CHIH CHIEH (TW)\ CHANG CHIH-HSIANG (TW) | F21S-002/00\ F21V-005/04\ F21V-009/08\ F21V-009/10\ F21V-009/16\ G03B-021/00\ G03B-021/14\ H04N-009/31 |
| 5295 | 3D printing | FILE SEMANTIC SYSTEM BASED REAL TIME STATE REDUNDANCY DATA WEIGHT REMOVING METHOD, INVOLVES CALCULATING CURRENT SYSTEM RESPONSE TIME ACCORDING TO REMOVING WEIGHT EXTENSION RATE, AND OBTAINING DYNAMIC REGULATING MECHANISM | CN105068757A | DAHL JEREMY E (US)\ CARLSON ROBERT M (US)\ LIU SHENGGAO (US) | HOCHBERG ARTHUR K (US)\ CUTHILL KIRK S (US)\ XIAO MANCHAO (US) | G06F-017/30\ G06F-003/06 |
| 5296 | 3D printing | DIGITAL LIGHT PROCESSING (DLP) LIGHT POLARIZATION TYPE THREE-DIMENSIONAL PROJECTION DEVICE HAS LIGHT WOTH SECOND POLARIZATION DIRECTION CONSTITUTING LEFT EYE IMAGE, AND MODULATED LIGHT IS PROJECTED ON SCREEN THROUGH PROJECTION LENS | CN105045019A | YOSHIDA YOSHIFUMI (JP)\ WAKE YOSHIKAZU (JP) | CHEVRON USA INC (US)\ DAHL JEREMY E (US)\ CARLSON ROBERT M (US)\ LIU SHENGGAO (US) | G02B-026/08\ G02B-027/28\ G03B-021/00\ G03B-021/20\ G03B-021/56 |
| 5297 | 3D printing | HIGH SPEED LIGHT CURING AGENT 3D PRINT DEVICE, HAS LIQUID OUTLET AND ENTRANCE ENDS OF PIPELINE CONNECTED WITH EACH OTHER, WHERE LIQUID ENTRANCE PIPELINE IS CONNECTED WITH CIRCULATION FLOW DRIVE ELEMENT OF PIPELINE | CN105014974A | WILMAN TSAI (US)\ CAYMAX MATHIEU (BE)\ CHEN PEIJUN JERRY (US)\ MAES JAN WILLEM (BE) | WAKE MIWA (JP)\ YOSHIDA YOSHIFUMI (JP) | B29C-067/00\ B33Y-010/00\ B33Y-030/00 |
| 5298 | 3D printing | DATA EXCAVATING BASED CLOUD SERVICE PERFORMANCE WARNING EVENT GENERATING METHOD, INVOLVES STORING VIRTUAL MACHINE COPY DECISION DATA IN SELF-ADAPTIVE LIBRARY DATA, AND PERFORMING FEEDBACK LEARNING PROCESS FOR UPDATING TRIGGERING DATA | CN105022823A | TSUYOSHI FUJIWARA (JP)\ KATSUHIKO ICHINOSE (JP)\ NAOHUMI OHASHI (JP) | IMEC INTER UNI MICRO ELECTR (BE)\ ASM INC (US)\ CHEN JERRY (BE)\ WILMAN TSAI (BE)\ CAYMAX MATTY (BE)\ MAES JAN-WILLEM (BE) | G06F-011/30\ G06F-017/30 |
| 5299 | 3D printing | DISPLAY SYSTEM FOR FOR PRESENTING AUGMENTED REALITY EXPERIENCES TO USERS, HAS A SPATIAL LIGHT MODULATOR THAT IS COUPLED TO IMAGE SOURCE FOR PROJECTING LIGHT, AND SPATIAL LIGHT MODULATOR IS PROVIDED WITH A DIGITAL LIGHT PROCESSING SYSTEM | US2015346495A1\ WO2015184412A1 | GRIVNA GORDON M (US) | RENESAS TECH CORP (JP) | G02B-027/01\ G06F-003/01\ G06T-019/00\ G02B-015/20\ G02B-027/22\ H04N-005/74 |
| 5300 | 3D printing | PLANAR INDUCTION HEATING BODY I.E. INDUCTION HEATING SEAMLESS TUBING, FOR USE AS IMAGE FIXING APPARATUSOF E.G. LASER PRINTER, HAS RESIN REGION, WHERE INTERSECTION OF BOUNDARY LINE OF METAL AND RESIN REGIONS AND STRAIGHT LINE IS PERFORMED | JP2015206883A | NAGANO SHOZO (JP)\ HARGARTER HINRICH (NL)\ LI JIANXING (US)\ BUEHLER JANE (US) | SEMICONDUCTOR COMPONENTS IND (US) | G03G-015/00\ G03G-015/20 |
| 5301 | 3D printing | SLA-TYPE THREE DIMENSIONAL PRINTER, HAS LIGHT SOURCE DEVICE FIXED WITH LIFTING PART AND CONTROLLING UNIT, AND SLA-TYPE THREE-DIMENSIONAL PRINTER MAIN BODY PROVIDED WITH MEASURING PART THAT IS LOCATED WITH BEARING BODY | CN204585858U | ALREUSEUGEULRENBI (US) | HONEYWELL INT INC (US) | B29C-067/00\ B33Y-050/02 |
| 5302 | 3D printing | SLA TYPE PRINTER, HAS PHOTOSENSITIVE RESIN CONNECTED WITH CURING AGENT THAT IS CONNECTED WITH LIGHT SOURCE DEVICE, AND LIGHT SOURCE DEVICE CONNECTED WITH LIFTING PART THAT IS CONNECTED WITH SLA TYPE PRINTER MAIN BODY | CN204585859U | SATO HIDENORI (JP)\ HAYASHI YOSHIYUKI (JP)\ ANDO TOSHIO (JP) | LUCENT TECHNOLOGIES INC (US) | B29C-067/00\ B33Y-030/00 |
| 5303 | 3D printing | LIGHT GUIDE LIGHT LASER SELF-ADAPTING HEADLAMP, HAS UNIFORM LIGHT SPOT CONNECTED TO DIGITAL LIGHT PROCESSING PROJECTION MODULE BY REFLECTION LENS, AND FINISH HEADLIGHT LIGHTING LIGHT CONNECTED TO PROJECT LIGHT SCREEN THROUGH PROJECTION LENS | CN204786017U | KAZUYUKI FUJII (JP)\ MINORU HANAZAKI (JP)\ GEN KAWAHARADA (JP)\ MASAKAZU TAKI (JP)\ MUTSUMI TSUDA (JP) | RENESAS TECH CORP (JP)\ HITACHI ULSI SYS CO LTD (JP) | F21S-008/10\ F21V-013/12\ F21V-005/04\ F21V-008/00 |
| 5304 | 3D printing | METHOD FOR CARGO HANDLING FOR PROVIDING ONLINE REAL-TIME ACCESS FOR STAKEHOLDERS, INVOLVES TRANSMITTING DATA FROM FIRST SMART DEVICE TO SECOND SMART DEVICE AND TO CUSTOMER SERVICE PORTAL FOR REAL-TIME ACCESS TO GOODS | US2015347960A1 | LI WEIMIN (US) | RENESAS TECH CORP (JP) | G06Q-010/08 |
| 5305 | 3D printing | CLOUD COMPUTING BASED SERVICE MANAGING SYSTEM, HAS REQUEST SCHEDULING AND RESOURCE MANAGEMENT MODULE FOR PROCESSING SCHEDULING STRATEGY FOR USER TERMINAL ACCORDING TO USER SERVICE REQUEST FOR RESOURCE DISPATCHING | CN105049516A | GATTIS PASCAL (FR) | MICRON TECHNOLOGY INC (US) | H04L-029/08 |
| 5306 | 3D printing | STEREOLITHOGRAPHY (SLA) THREE DIMENSIONAL 3D PRINTER HAS OPTICAL FIBER THAT IS SET ALONG TRANSFERRING FIBER ADAPTER, AND HINGE MECHANISM THAT IS OPENED TO REFLECT OUT | CN104890248A | WILLER JOSEF (DE)\ SCHLOESSER TILL (DE)\ HOFMANN FRANZ (DE)\ KRAUTSCHNEIDER WOLFGANG (DE) | ST MICROELECTRONICS SA (FR) | B29C-067/00\ B33Y-030/00 |
| 5307 | 3D printing | DEVICE USED FOR PROTECTING ENVIRONMENT FROM KITCHEN GARBAGE, COMPRISES BOX, BOX COVER, FILTER BOX, CLAPBOARD, HEATING PIPE, OIL HOLE, OIL PIPE, TELESCOPIC PIPE, HOGWASH WATER PIPE, WATER OUTLET, DRAIN PIPE, POLY CHAMBER, OIL CHAMBER AND SLA | CN104860421A | WALTER PETERS DAVID (US)\ HOUSTON MEIERE SCOTT (US) | INFINEON TECHNOLOGIES AG (DE)\ SCHLOESSER TILL (DE)\ HOFMANN FRANZ (DE)\ KRAUTSCHNEIDER WOLFGANG (DE)\ WILLER JOSEF (DE) | C02F-009/02 |
| 5308 | 3D printing | IMAGING DEVICE, HAS LINE SELECTING CIRCUIT FOR SELECTING SELECTION LINE, UNIT CELL CIRCUIT PROVIDED WITH IMAGE SENSOR, AND CONNECTION PART FOR SELECTING SIGNAL OUTPUT FROM VERTICAL SHIFT REGISTER CIRCUIT TO LINE SELECTING CIRCUIT | JP2015207911A | SALOT RAPHA L (FR)\ MARTINET SEBASTIEN (FR)\ BRUN JEAN (FR)\ POUPON GILLES (FR) | PRAXAIR TECHNOLOGY INC (US)\ THOMPSON DAVID M (US)\ PETERS DAVID WALTER (US)\ MEIERE SCOTT HOUSTON (US) | H01L-027/148\ H04N-005/372\ H04N-005/376 |
| 5309 | 3D printing | ILLUMINATION SYSTEM FOR SEQUENTIALLY PRODUCING COLOR LIGHTS FOR SINGLE-CHIP DIGITAL LIGHT PROCESSING PROJECTOR, HAS LIGHT GUIDING DEVICE CONNECTED TO THREE PATHS FOR GUIDING LIGHTS ALONG FIRST PATH OR GUIDING LIGHT TO CONVERSION ELEMENT | US2015338061A1 | YIEH ELLIE (US)\ NEMANI SRINIVAS (IN)\ XIA LI-QUN (CN) | COMMISSARIAT ENERGIE ATOMIQUE (FR)\ SALOT RAPHAEL (FR)\ MARTINET SEBASTIEN (FR)\ BRUN JEAN (FR)\ POUPON GILLES (FR) | F21V-007/04\ F21V-009/08\ F21V-009/14\ G03B-021/20 |
| 5310 | 3D printing | COMPUTER-IMPLEMENTED METHOD FOR ENABLING SELF-ADJUSTMENT IN VISUAL DISPLAY FOR PRESENTING E.G. IMAGES, INVOLVES CREATING DISPLAY CHARACTERISTICS SHIFT PROFILE FOR DISPLAY CHARACTERISTIC UPON WHICH SELF-ADJUSTMENT OF DISPLAY IS BASED | US2015339964A1 | YOSHIKO HARADA (JP)\ NAOTADA OGURA (JP) | APPLIED MATERIALS INC (US) | G09G-003/20\ G09G-003/36 |
| 5311 | 3D printing | PHOTOELECTRIC TECHNOLOGY BASED DIGITAL MICRO-MIRROR DEVICE BLUE LIGHT FLUORESCENT POWDER-TYPE LASER LAMP FOR VEHICLE, HAS FLUORESCENT MICRO-LENS ARRAY PLATE FIXED TO PROJECT LIGHT SCREEN BY PROJECTION LENS TO GENERATE VEHICLE LIGHT-TYPE | CN204740418U | XIANG QI (US) | YAMAHA CORP (JP) | G02B-027/09\ G03B-021/14\ G03B-021/20 |
| 5312 | 3D printing | IMAGE PROJECTOR I.E. DIGITAL LIGHT PROCESSING PROJECTOR, FOR GENERATING IMAGE BASED ON PICTURE DATA AT E.G. OFFICES, HAS FIRST CONTROLLER FOR ACTIVATING SECOND CONTROLLER WHEN LIGHT SOURCE IS LIGHTED AT INITIAL VOLTAGE | US2015334361A1\ JP2015215551A\ CN105093788A\ EP2957954A1 | YANG HAINING (US)\ SANDHU GURTEJ S (US) | GLOBALFOUNDRIES INC (KY) | G02B-026/08\ H04N-009/31\ G03B-021/00\ G03B-021/14\ H04N-005/74\ G06F-001/32 |
| 5313 | 3D printing | METHOD FOR END-TO-END NETWORK BANDWIDTH OPTIMIZATION, INVOLVES SELECTING PATH IN MULTIPLE PATHS DISTINCT AND ASSIGNING FLOW TO SELECTED PATH, IF PROJECTED UTILIZATION OF SHORTEST PATH IS GREATER THAN TARGET UTILIZATION THRESHOLD | US2015334002A1 | SATO HIDENORI (JP)\ ICHINOSE KATSUHIKO (JP)\ ISHII YUKINO (JP)\ JINBO TOMOKO (JP) | MICRON TECHNOLOGY INC (US) | H04L-012/729\ H04L-012/801 |
| 5314 | 3D printing | DISPLAY DEVICE FOR ELECTRONIC DEVICE, HAS CONTROLLER FOR PERFORMING FIRST OPERATION IN NON-DISPLAY INTERVAL BETWEEN FIRST DISPLAY INTERVAL AND SECOND DISPLAY INTERVAL, WHERE FIRST OPERATION IS DIFFERENT FROM SECOND OPERATION | US2015332647A1\ JP2015219245A\ CN105093716A\ KR2015130235A | GRILL ALFRED (US)\ JAHNES CHRISTOPHER VINCENT (US)\ PERRAUD LAURENT CLAUDE (FR)\ PATEL VISHNUBHAI VITTHALHAI (US) | RISA SCIENCE TECHNOLOGY CO LTD (JP) | G02F-001/1335\ G02F-001/1343\ G09G-003/20\ G09G-003/36\ G02F-001/133\ G09G-003/30 |
| 5315 | 3D printing | PATTERNING METHOD IN MANUFACTURING OF ELECTRO-OPTICAL APPARATUS E.G. FIELD EMISSION DISPLAY INVOLVES ETCHING EXPOSED FIRST FILM SO THAT ETCHING PRODUCT OF FIRST FILM IS ADHERED TO SIDE WALLS OF ETCHING HOLES | JP2015198156A | HSU SHENG TENG (US)\ ZHUANG WEI-WEI (US)\ CHARNESKI LAWRENCE J (US) | PERRAUD LAURENT CLAUDE (FR)\ GRILL ALFRED (US)\ JAHNES CHRISTOPHER VINCENT (US)\ PATEL VISHNUBHAI VITTHALHAI (US) | G02F-001/1368\ H01L-021/3065 |
| 5316 | 3D printing | ARRAY LIGHT SOURCE FOR ILLUMINATION OPTICAL SYSTEM, HAS SECOND SIDE SURFACE WHOSE SEVERAL REFLECTIVE AREA ADJACENT TO FIRST SIDE SURFACE REFLECTS LIGHT FROM SEMICONDUCTOR LASER ELEMENT OF SECOND ARRAY LIGHT SOURCE UNIT IN ONE DIRECTION | WO2015166596A1 | HAMADA YOSHITAKA (JP) | SHARP LAB OF AMERICA INC (US) | F21S-002/00\ H01S-005/022 |
| 5317 | 3D printing | METHOD FOR PROCESSING LIGHT-CURED ORTHOTIC COMPOSITE, INVOLVES TAKING DIGITAL MODEL OF DESIGN PHASE OF ORTHOTICS, PERFORMING THREE-DIMENSIONAL PRINTING PROCESS, FOLLOWED BY COATING WITH MOLD RELEASE AGENT AND PLACING IN OVEN | CN104814808A | ASANO ISAMU (JP)\ TAMARU TSUYOSHI (JP)\ AOKI HIDEO (JP)\ NAKAMURA YOSHITAKA (JP)\ FUKUDA NAOKI (JP)\ YAMADA SATORU (JP)\ GOTO HIDEKAZU (JP)\ KAWAKITA KEIZO (JP)\ TANAKA KATSUHIKO (JP)\ SAKUMA HIROSHI (JP)\ HIRASAWA MASAYOSHI (JP) | SHINETSU CHEMICAL CO (JP)\ HAMADA YOSHITAKA (JP) | A61F-002/02\ A61F-002/50 |
| 5318 | 3D printing | NEW GENOMIC DNA MULTIPLEX PCR PRIMER SET USEFUL FOR ANALYZING SWINE LEUKOCYTE ANTIGEN (SLA)-DQA, SLA-DQB1 AND SLA-DRB1 GENE, COMPRISES SPECIFIC BASE PAIR SEQUENCE | KR1548261B1 | VRTIS RAYMOND NICHOLAS (US)\ LUKAS AARON SCOTT (US)\ O'NEILL MARK LEONARD (CA)\ VINCENT JEAN LOUISE (CA)\ BITNER MARK DANIEL (US)\ PETERSON BRIAN KEITH (US)\ KARWACKI JR EUGENE JOSEPH US | HITACHI LTD (US) | C12N-015/11\ C12Q-001/68 |
| 5319 | 3D printing | DATA GENERATOR FOR DISPLAY DRIVER OF DISPLAY APPARATUS OF ELECTRONIC DEVICE, HAS BUFFER FOR PROVIDING PREVIOUS DISPLAY DATA TO OVER DRIVE DATA GENERATOR, STORING CURRENT DISPLAY DATA, AND OUTPUTTING CURRENT DISPLAY DATA AND OVER DRIVE DATA | US2015325217A1\ KR2015127333A | OOMORI KAZUTOSHI (JP)\ FUJIWARA TSUYOSHI (JP)\ ASAKA KATSUYUKI (JP)\ NARIYOSHI YASUHIRO (JP)\ HOSHINO YOSHINORI (JP) | AIR PROD & CHEM (US) | G09G-005/39\ G09G-005/06 |
| 5320 | 3D printing | SYSTEM FOR QUERY PLAN POST OPTIMIZATION ANALYSIS AND REOPTIMIZATION, HAS REOPTIMIZATION ENGINE THAT IS CONFIGURED TO APPLY CORRECTIVE ACTIONS TO REOPTIMIZE IDENTIFIED SUBOPTIMAL QUERY PLAN BEFORE EXECUTION | WO2015167466A1 | AHN KIE Y (US)\ FORBES LEONARD (US) | RENESAS TECH CORP (JP)\ HITACHI ULSI SYS CO LTD (JP)\ HITACHI TOBU SEMICONDUCTOR LTD (JP) | G06Q-010/06\ G06Q-010/10 |
| 5321 | 3D printing | MULTI-CLOUD FABRIC SYSTEM FOR USE IN DATA CENTER FOR HOUSING E.G. STORAGE SYSTEMS, HAS CONTROLLER FOR MONITORING SERVICE LEVEL AGREEMENT AND FOR MOVING RESOURCES ACROSS CLOUDS OF PRIVATE AND PUBLIC CLOUDS TO OPTIMIZE PERFORMANCE OF SYSTEM | US2015319050A1 | WELCH DAVID F (US)\ NAGARAJAN RADHAKRISHNAN L (US)\ CHIANG TING-KUANG (US)\ DOMINIC VINCENT G (US)\ KISH JR FRED A (US) | MICRON TECHNOLOGY INC (US) | H04L-012/24\ H04L-012/911\ H04L-029/08 |
| 5322 | 3D printing | METHOD FOR IMPROVING DATA COMMUNICATION IN NETWORK, INVOLVES PRIORITIZING ORIGINAL PACKETS WITH RESPECT TO QUEUING AND DE-QUEUING SUCH THAT SERVICE LEVEL (SL) PARAMETERS FOR ORIGINAL PACKETS ARE SATISFIED | US2015319098A1 | RICHARD A HOGLE US\ PATRICK JOSEPH HELLY US\ CE MA US\ LAURA JOY MILLER US | INFINERA CORP (US) | H04L-012/865 |
| 5323 | 3D printing | TRANSCEIVER FOR SELECTING CHANNEL STATE INFORMATION (CSI), HAS PROCESSING APPARATUS THAT IS PROVIDED USES ENHANCED SLOW LINK ADAPTATION (ESLA) ALGORITHM TO ADJUST CSI ADJUSTING FACTOR USING BLOCK ERROR RATE (BLER) TARGET | WO2015165514A1\ EP2959620A1\ CN105229956A | HAMAGUCHI MAKI (JP) | BOC GROUP INC (US) | H04L-001/00\ H04L-001/20 |
| 5324 | 3D printing | METHOD FOR MAXIMIZING SERVER UTILIZATION WITHIN DATACENTER FOR MANAGING COLLOCATION OF CRITICAL AND NON-CRITICAL WORKLOADS, INVOLVES ADJUSTING RESOURCE UTILIZATION AMONG CRITICAL AND NON-CRITICAL WORKLOADS OPERATING ON VIRTUAL MACHINES | US2015312167A1 | DAHL JEREMY E (US)\ CARLSON ROBERT M (US)\ LIU SHENGGAO (US) | KOBE STEEL LTD (JP) | G06F-009/455\ H04L-012/24\ H04L-012/911 |
| 5325 | 3D printing | SLICE DELAMINATION METHOD BASED ON STEREO LITHOGRAPHY (STL) MODE, INVOLVES GENERATING EACH SLICE COMBINED WITH ADJACENCY CORRESPONDING TO CONTOUR RING TO OBTAIN COMPLETE OUTLINE SECTION, AFTER EACH SLICE INTERSECTION | CN104899359A | SINGH BHANWAR (US)\ GHANDEHARI KOUROS (US)\ LAFONTAINE BRUNO (US) | CHEVRON USA INC (US)\ DAHL JEREMY E (US)\ CARLSON ROBERT M (US)\ LIU SHENGGAO (US) | G06F-017/50 |
| 5326 | 3D printing | APPARATUS FOR FACILITATING ACOUSTIC CONTROL USING HEAD-RELATED TRANSFER FUNCTION, HAS LOUDSPEAKER FOR EMITTING BINAURAL ACOUSTIC SIGNAL, WHERE CONTROL FILTER COEFFICIENT IS CALCULATED BASED ON HEAD-RELATED TRANSFER FUNCTION | US2015312695A1 | SUN SEY-PING (US)\ GARDNER MARK I (US)\ NGO MINH VAN (US) | ADVANCED MICRO DEVICES INC (US) | H04S-007/00 |
| 5327 | 3D printing | METHOD FOR TRANSMITTING VIDEOS PROVIDED BY CAMERAS FIXED AT MOBILE OBJECT TRAVELING ALONG PRE-DEFINED ROUTE, INVOLVES TRANSMITTING SCHEDULED VIDEO LAYERS WITH RESPECT TO ONE OF SERVICE LEVEL AGREEMENT (SLA) PARAMETERS | US2015304735A1\ US9173008B1 | NGUYEN TUE (US)\ SENZAKI YOSHIHIDE (US)\ CHARNESKI LAWRENCE JOEL (US)\ KOBAYASHI MASATO (US) | ADVANCED MICRO DEVICES INC (US) | H04N-021/236\ H04N-021/414\ H04N-021/4363\ H04N-021/647\ H04H-060/32 |
| 5328 | 3D printing | MOVING OBJECT HEADER CONTROLLING SYSTEM, HAS CONTROL MODULE FRO TRANSMITTING INFORMATION TO ONLINE DEVICE, MANAGING MODULE FOR OBTAINING REGISTER POST INFORMATION, AND DATA MODULE FOR TRANSMITTING CONFIGURATION INFORMATION TO MODULE | CN104954456A | TADASHI KOSHIISHI US\ MANY SHADIA US\ BETANCOURT GREGORY R US\ CHAO SANDAY US\ BHATTACHARYYA GAUTAM (US) | SHARP KK (JP)\ SHARP MICROELETRONICS TECHNOLO (US) | H04L-029/08 |
| 5329 | 3D printing | PROJECTION MICRO-STEREOLITHOGRAPHY SYSTEM FOR PRODUCING SUB-DIFFRACTION-LIMITED FEATURES, HAS FAR-FIELD SUPERLENS (FSL) THAT CONVERTS FAR-FIELD IMAGE PRODUCED BY SPATIAL LIGHT MODULATOR (SLM) AND IS REDUCED TO NEAR-FIELD IMAGE | US2015309473A1 | KAWAGUCHI TOSHIYUKI (JP)\ FUJIKI HIRONAO (JP)\ TANIGUCHI ATSUSHI (JP)\ GONDA TAKASHI (JP)\ TAHARA KAZUTOKI (JP) | LAM RESERCH CORP US | B29C-067/00\ G02B-001/00\ G02B-027/56\ G02B-005/00\ G03H-001/00\ G03H-001/02\ G03H-001/26 |
| 5330 | 3D printing | SYSTEM FOR AUTOMATICALLY AND DYNAMICALLY SCALING CLOUD INFRASTRUCTURE BASED ON PARAMETER ESTIMATES, HAS MODELING AND OPTIMIZATION COMPONENT FOR DETERMINING DIRECTIVES FOR SCALING APPLICATION DEPLOYMENT BASED ON ESTIMATED PARAMETERS | US2015312110A1\ US9300553B2 | TSUYOSHI NONAKA (JP)\ MASANORI IOKA (JP)\ SHINGO KAIMORI (JP)\ MASATO FUKAGAYA (JP) | SHINETSU POLYMER CO (JP)\ KAWAGUCHI TOSHIYUKI (JP)\ FUJIKI HIRONAO (JP)\ TANIGUCHI ATSUSHI (JP)\ GONDA TAKASHI (JP)\ TAHARA KAZUTOKI (JP) | H04L-012/24\ H04L-029/08\ G06F-009/44 |
| 5331 | 3D printing | METHOD FOR COMPENSATING IMAGE DISTORTION OF PROJECTIVE IMAGE DISPLAY DEVICE E.G. PROJECTOR, INVOLVES PRODUCING TURNAROUND TIME VALUE ABOUT PLACES USING LIGHT ACCEPTING POINT OF TIME OF LASER | KR2015116617A | MAA JER-SHEN (US)\ SHIEN TEN SUU US\ PAN WEI (US)\ EVANS DAVID RUSSELL (US) | SUMITOMO ELECTRIC WINTEC INC (JP)\ NONAKA TSUYOSHI (JP)\ KAIMORI SHINGO (JP)\ IOKA MASANORI (JP)\ FUKAGAYA MASATO (JP) | H04N-005/21\ H04N-005/74 |
| 5332 | 3D printing | METHOD FOR MANUFACTURING WINDING OF WINDING CARRIER E.G. STATOR, OF ELECTRIC MACHINE, INVOLVES RELEASING END SECTION BY MOVING HOLDING ELEMENT INTO UNLOCKING POSITION, AND BENDING REGION INTO BENDING DIRECTION AROUND BENDING ANGLE | DE102014208082A1\ WO2015165654A1 | YAMADA SATORU (JP)\ OYU KIYONORI (JP)\ KIMURA SHINICHIRO (JP) | SHARP LAB OF AMERICA INC (US) | H02K-015/085\ H02K-003/28\ H02K-015/00\ H02K-015/04 |
| 5333 | 3D printing | METHOD FOR PROVIDING ALERT ENRICHMENT IN CLOUD STORAGE SYSTEM, INVOLVES PERFORMING CROSS DOMAIN FOR MATCHING BETWEEN TWO DOMAINS, AND ENRICHING ALERT/EVENT WITH LOGICAL SERVICE TO PROVIDE CONTEXT WITHIN LOGICAL SERVICE | US9170951B1 | SOLAYAPPAN NARAYAN (US)\ PAZ DE ARAUJO CARLOS A (US)\ UCHIYAMA KIYOSHI (US) | HITACHI LTD (JP) | G06F-012/08\ G06F-009/54 |
| 5334 | 3D printing | METHOD FOR OPERATING MULTI-TENANCY STORAGE NODE, INVOLVES INSTALLING PARTITIONS OF GENERATED ADDITIONAL DATABASE AND SERVICE LEVEL AGREEMENT (SLA) ON THE OTHER STORAGE NODE | US2015302020A1 | SUZUKI MASAYUKI (JP) | SYMETRIX CORP (US)\ MATSUSHITA ELECTRIC IND CO LTD (US) | G06F-017/30\ H04L-012/24 |
| 5335 | 3D printing | METHOD FOR GENERATING CUSTOMIZED REPORT IN COMPUTING DEVICE IN INFORMATION TECHNOLOGY INFRASTRUCTURE SERVICE DELIVERY ENVIRONMENT, INVOLVES ANALYZING WHETHER SET OF ACTIVITIES IS COMPLIED WITH SERVICE LEVEL AGREEMENT BASED ON METRICS | IN201502817I1 | WEIMER RONALD A (US)\ DEBOER SCOTT J (US)\ GEALY DAN (US)\ AL-SHAREEF HUSAM N (US) | RENESAS TECH CORP (JP) | G06Q-010/00 |
| 5336 | 3D printing | COMPUTER PROGRAM PRODUCT FOR MANAGING ISOLATION REQUIREMENTS OF MULTI-NODE WORKLOAD APPLICATION, HAS SET OF INSTRUCTIONS FOR IDENTIFYING ISOLATION LEVEL REQUIRED TO MEET PERFORMANCE TARGETS, WHERE ISOLATION LEVEL INCLUDES ADJUSTMENT | US2015304173A1 | SENZAKI YOSHIHIDE (US) | MICRON TECHNOLOGY INC (US) | H04L-012/24\ H04L-012/26\ H04L-012/927\ H04L-029/08 |
| 5337 | 3D printing | METHOD FOR MANAGING OPERATION OF PROCESSOR FOR E.G. SERVICE PROVIDER COMPUTING SYSTEM, INVOLVES PROVIDING APPLICATION PERFORMANCE DATA TO CONTROLLER IN COMPUTING SYSTEM THAT IS DISTINCT FROM PROCESSOR CORES | US2015304177A1 | HSHIEH FWU-IUAN (US)\ OWYANG KING (US)\ HO YUEH-SE (US)\ DUN JOWEI (US)\ FUESSER HANS-JUERGEN (DE)\ ZACHAI REINHARD (DE)\ CHANG MIKE F (US) | SHARP KK (JP)\ SHARP MICROELETRONICS TECHNOLO (US) | H04L-012/24\ H04L-012/26 |
| 5338 | 3D printing | METHOD FOR MANAGING ISOLATION REQUIREMENT OF MULTI-NODE WORKLOAD APPLICATION, INVOLVES IDENTIFYING SECOND ISOLATION LEVEL WHICH INCLUDES ADJUSTMENT TO FIRST ISOLATION LEVEL FOR WORKLOAD NODE, THAT IS REQUIRED TO MEET PERFORMANCE TARGET | US2015304232A1 | GRILL ALFRED (US)\ PATEL VISHNUBHAI V (US)\ GATES STEPHEN M (US) | SILICONIX INC (US) | H04L-012/26\ H04L-012/851\ H04L-012/911 |
| 5339 | 3D printing | DYNAMIC LIQUID CIRCULATION COOLING SYSTEM OF DIGITAL LIGHT PROCESSING (DLP) BACK PROJECTION TV, HAS OPTICAL ENGINE MODULE WHOSE TOP PORTION IS PROVIDED WITH SUCTION AIR PIPE WHICH IS PROVIDED WITH FILTER COMPONENT | CN204634238U | RICHARDS CHRISTOPHER (CA)\ CAMERON ROB (CA)\ MIZELL JERRY (US) | IBM (US) | H04N-005/74\ H04N-009/31\ H05K-007/20 |
| 5340 | 3D printing | DISPLAY DEVICE I.E. LCD, FOR E.G. LAPTOP COMPUTER, HAS SENSING CIRCUIT COUPLED TO EACH OF LEDS AND ADAPTED TO DETECT SHADOW FROM OPERATOR FINGER AND RECEIVE ELECTRICAL SIGNAL FROM EACH LED WHEN LED IS OPERATED IN SENSE MODE | US2015301687A1 | THOMPSON DAVID M (US)\ PETERS DAVID WALTER (US) | PRAXAIR TECHNOLOGY INC (US)\ THOMPSON DAVID M (US)\ PETERS DAVID W (US) | G06F-003/038\ G06F-003/041\ G06F-003/042\ G09F-009/30\ G09F-009/33\ G09G-003/20\ G09G-003/30\ G09G-003/32\ H01L-027/32\ H01L-033/00\ H01L-051/50\ H05B-033/08 |
| 5341 | 3D printing | THREE-DIMENSIONAL PRINTING APPARATUS E.G. STEREO LITHOGRAPHY PRINTING APPARATUS, HAS MODELING PLATFORM WHICH DRIVES AND TILTS TANK AWAY FROM BASE TO RELEASE THREE-DIMENSIONAL OBJECT FROM BOTTOM OF TANK BY GRAVITY | US2015298396A1\ CN104999665A\ TW201540559A\ US9346218B2\ TW541142B1 | DELORENZO DAVID US\ MONTGOMERY STEPHEN US\ FITE ROBERT US | THOMPSON DAVID M (US)\ PETERS DAVID W (US) | B29C-067/00\ B29C-067/02\ B41M-003/06\ B29L-031/00\ B33Y-030/00 |
| 5342 | 3D printing | CUSTOMIZED DISPLAY FOR INTELLIGENT NON-SCREEN TV USED IN HOME, HAS LENS WHOSE FRONT-END IS PROVIDED WITH REFLECTION MIRRORS ARRANGED WITH DIGITAL LIGHT PROCESSING CONTROL MODULE | CN204613578U | OOTSUKA FUMIO (JP)\ ICHINOSE KATSUHITO US | INTEL CORP (US) | G03B-021/14\ H04N-005/74 |
| 5343 | 3D printing | DIGITAL LIGHT PROCESSING (DLP) BASED LIGHT CURING NOODLE FORMING DEVICE HAS RESIN GROOVE THAT IS FIXED ON MACHINE FRAME, AND DLP PROJECTION SYSTEM THAT IS PROVIDED BELOW RESIN GROOVE CONTAINING PHOTOSENSITIVE RESIN | CN204566705U | SUZUKI MASAYUKI (JP) | RENESAS TEHNOLOGY CORP (JP) | B29C-067/00\ B33Y-030/00 |
| 5344 | 3D printing | DIRECT LIGHT PROCESSING THREE DIMENSIONAL PRINTER INCLUDES MACHINE FRAME, CONTROL UNIT, FORMING GROOVE, SCREEN PROJECTION UNIT, PROJECTION SCREEN, SCANNING PROJECTION DEVICE, SCANNING PROJECTION LIGHT SOURCE AND FORMING LIGHT SOURCE | CN204505857U | Empty Field US | RENESAS TECH CORP (JP) | B22F-003/115\ B29C-067/00\ B33Y-030/00\ B33Y-040/00 |
| 5345 | 3D printing | DISPLAY DEVICE E.G. LCD DEVICE HAS SPECIFIC SUBSTRATE THAT INCLUDES SPECIFIC BASE SUBSTRATE AND SEALING UNIT WHICH IS PROVIDED ACCORDING TO EDGE OF SPECIFIC BASE SUBSTRATE AND ARRANGED ON PAD PORTION | KR2015111456A | KONECNI ANTHONY (US)\ HAVEMANN ROBERT H (US)\ DIXIT GIRISH A (US) | MACRONIX INT CO LTD (TW) | G02F-001/133\ G02F-001/1339\ G02F-001/1345\ G02F-001/136 |
| 5346 | 3D printing | SLA HETEROGENEOUS FACE MAP REDUCE COLLECTION BASED ENERGY CONSUMPTION MANAGING METHOD, INVOLVES STORING NODE RESPONSE RESULT IN COPY STORAGE STRATEGY, AND MINIMIZING ENERGY CONSUMPTION ACCORDING TO NODE RESPONSE RESULT | CN104852819A | KENJI KATO (JP)\ EIJI TAKAHASHI (JP) | TEXAS INSTRUMENTS INC (US) | G06F-017/30\ H04L-012/24\ H04L-029/08 |
| 5347 | 3D printing | METHOD OF LIVE MIGRATION OF CLOUD DATA THAT USES PRE-COPY LIVE MIGRATION TECHNIQUE TO ACHIEVE HIGH EFFICIENCY SERVICE MIGRATION | TW201525711A | MAROULIS PETER JAMES (US)\ KETKAR SUHAS NARAYAN (US)\ MCDERMOTT WAYNE THOMAS (US) | NISSIN ELECTRIC CO LTD (JP)\ KATO KENJI (JP)\ TAKAHASHI EIJI (JP) | G06F-015/16\ G06F-009/445 |
| 5348 | 3D printing | G-HOME NETWORK -ACCESS NETWORK CONCENTRATION EQUIPMENT, HAS SWITCH FOR CONNECTING PHYSICAL LAYER TO DOMAIN MASTERS AND END POINT, AND CONTROL UNIT FOR CONTROLLING SIGNAL-NOISE RATIO OFFSET FUNCTION AND CALCULATING ACTUAL VELOCITY OF SIGNAL | WO2015152570A1 | WON SEOK JUN (KR)\ PARK JUNG MIN (KR)\ KWON DAE JIN (KR)\ SONG MIN WOO (KR)\ KIM WEON HONG (KR)\ KIM JU YOUN (KR) | MAROULIS PETER J (US)\ KETKAR SUHAS N (US)\ MCDERMOTT WAYNE T (US) | H04L-012/24\ H04L-029/02\ H04L-029/12\ H04M-011/06 |
| 5349 | 3D printing | METHOD FOR USE IN ASSESSMENT OF SERVICE LEVEL AGREEMENT COMPLIANCE, INVOLVES EVALUATING COMPUTING SYSTEM BASED UPON FIRST PERFORMANCE AND COMPARING FIRST PERFORMANCE TO REQUIREMENT OF SERVICE LEVEL AGREEMENT | US2015288577A1 | SUSAN H DOWNEY (US)\ PETER R HARPER (US) | SAMSUNG ELECTRONICS CO LTD (KR) | H04L-012/24\ H04L-029/08 |
| 5350 | 3D printing | METHOD FOR ASSESSMENT OF CLOUD HOSTING SUITABILITY FOR MULTIPLE APPLICATIONS, INVOLVES RECORDING SECOND PERFORMANCE OF SYNTHETIC APPLICATION AND EVALUATING COMPUTING SYSTEM BASED UPON FIRST PERFORMANCE AND SECOND PERFORMANCE | US2015288746A1 | TSUJI NAOTO (JP)\ TAKAHASHI SATOSHI (JP)\ OZAKI NORITOSHI US | MOTOROLA INC (US) | H04L-012/24\ H04L-029/08 |
| 5351 | 3D printing | SYSTEM FOR CREATING UNIFIED CATALOG FOR DISTRIBUTING CATALOG INFORMATION FROM DISPARATE CATALOG SYSTEM, HAS PROCESSOR FOR DETERMINING ITEM IDENTIFIER FOR SELECTED ITEM AND SENDING ORDER FOR SELECTED ITEM TO SOURCE CATALOG | US2015287111A1 | TUENUYEN (US)\ RORENS J SHARNESKI (US) | ASM JAPAN KK US | G06Q-030/06 |
| 5352 | 3D printing | METHOD FOR PROVIDING SUPPORT FOR ELASTICITY IN NETWORK VIRTUALIZATION, INVOLVES PRODUCING VIRTUAL NETWORK TOPOLOGY BY OVERLAYING MULTI-DOMAIN NETWORK IN ASSOCIATING ELASTICITY PARAMETERS INFERRED FROM PHYSICAL PATHS WITH VIRTUAL LINKS | WO2015144257A1 | QINGYU LUO US\ HONGRONG TU US\ HAIQING CHEN US\ TIANYI BAO US\ WENZHI QIU US\ ZHENHUA YU (CN) | SHARP LAB OF AMERICA INC (US)\ SHARP KK (US) | H04L-012/717\ H04L-012/931 |
| 5353 | 3D printing | SHORT-FOCUS LENS FOR DIGITAL LIGHT PROCESSING PROJECTION DISPLAY, HAS CEMENTED LENS IN WHICH SPECIFIC DISTANCE RANGES BETWEEN CEMENTED LENS CENTER AND FOURTH OPTICAL LENS AND BETWEEN FOURTH AND FIFTH OPTICAL LENSES AND OPTICAL CENTER | CN204536641U | YAMAZAKI SHUNPEI (JP) | TAIWAN SEMICONDUCTOR MFG (TW)\ BAO TIEN-I (TW)\ YU CHEN-HUA (TW)\ CHIOU WEN-CHIH (TW)\ LO CHING-YU (TW)\ TU HUNG-JUNG (TW)\ CHEN HAI-CHING (TW) | G02B-013/18 |
| 5354 | 3D printing | METHOD FOR MATCHING MAINTENANCE OPERATIONS IN CLOUD COMPUTING ENVIRONMENT, INVOLVES OBTAINING SERVICE GRADE PROTOCOL SLA PARAMETERS INCLUDING MIDDLEWARE QUALITY OF SERVICE, AND DETERMINING WHETHER PARAMETERS EXCEED THRESHOLD VALUE | CN104796274A | ENICKS DARWIN G (US) | SEMICONDUCTOR ENERGY LAB (JP) | G06F-017/30\ H04L-012/24 |
| 5355 | 3D printing | SEMICONDUCTOR DEVICE HAS A CAPACITOR WITH TWO ELECTRODES, THREE SWITCHES THAT ARE ELECTRICALLY CONNECTED IN SERIES BETWEEN WIRINGS, AND GATE OF TRANSISTOR IS CONNECTED TO NODE, WHERE LIGHT EMITTING ELEMENT IS PROVIDED WITH PIXEL ELECTRODE | JP2015179259A\ US2015287749A1 | MOORE JOHN T (US)\ FISCHER MARK (US) | ATMEL CORP (US)\ ENICKS DARWIN G (US) | G09G-003/20\ G09G-003/30\ H01L-051/50\ H01L-027/12 |
| 5356 | 3D printing | METHOD FOR MONITORING CONNECTIVITY IN LONG TERM EVOLUTION NETWORK IN CELLULAR PHONE, INVOLVES MEASURING ROUND-TRIP TIME OF PACKET, AND PERFORMING FAULT ISOLATION AT BEARER PATH LAYER AFTER USER ENDPOINT IS WOKEN UP BY PROCESSOR | US2015280974A1 | AWANO YUJI (JP) | MOORE JOHN T (US)\ FISCHER MARK (US) | H04L-012/24\ H04L-012/26\ H04L-029/12\ H04W-024/06 |
| 5357 | 3D printing | METHOD FOR MANAGING NETWORK, INVOLVES OUTPUTTING DERIVED INFORMATION TO SUBSCRIBER AT SLOWER RATE THAN RATE AT WHICH KEY PERFORMANCE INDICATOR (KPI) INFORMATION IS RECEIVED BY SERVER DEVICE | US2015281004A1 | HWANG DU SEOP (KR)\ JUNG EUN AE (KR)\ YOO CHA YEONG (KR) | FUJITSU LTD (JP) | H04L-012/24 |
| 5358 | 3D printing | MULTI-CLOUD FABRIC SYSTEM HAS DISTRIBUTED ELASTIC SERVICE LEVEL AGREEMENT (SLA) ANALYZER THAT ANALYZES AND AGGREGATES PROCESSED LOGS AND STATISTICS FROM DISTRIBUTED ELASTIC LOG INDEXER AND DISTRIBUTED ELASTIC STATS PROCESSOR | US2015281006A1 | SUGIYAMA MASAO (JP)\ KANEKO YOSHIYUKI (JP)\ KONDO YOSHINORI (JP)\ HARASAWA MASAYOSHI (JP) | SAMSUNG ELECTRONICS CO LTD (US) | H04L-012/24\ H04L-029/08 |
| 5359 | 3D printing | METHOD FOR SHARING ONE OR MORE COMPUTATIONAL RESOURCES, INVOLVES ALLOCATING COMBINATION OF PORTION OF COMPUTATIONAL RESOURCES ASSOCIATED WITH EACH OF NEIGHBORING SECOND NODES BASED ON COMPARISON | US2015281117A1\ US9471369B2 | YAMAMOTO HIROHIKO (JP)\ KIKUCHI HIROAKI (JP)\ SAWADA TOSHIAKI (JP) | RENESAS TECH CORP (JP) | H04L-012/24\ H04L-012/911\ G06F-009/46 |
| 5360 | 3D printing | METHOD FOR DEFINING SUPPORTING STRUCTURE FOR THREE-DIMENSIONAL OBJECT TO BE PRODUCED THROUGH STEREO-LITHOGRAPHY PROCESS, INVOLVES DEFINING SPECIFIC PAIR OF SUPPORTING ELEMENTS FOR EACH VERTEX HAVING CONNECTED ACYCLIC GRAPH | WO2015145346A1\ TW201540484A\ CA2936171A1\ IT1423305B\ KR2016098427A\ SG11201605308A1 | SHIN JIN-HYUN (KR)\ YUN JAE-SUN (KR) | RENESAS TECH CORP (JP) | B29C-067/00\ G06F-017/50\ B29C-067/02\ B33Y-010/00\ B33Y-030/00\ B33Y-050/00\ G03F-009/00\ B29C-000/00\ B33Y-040/00 |
| 5361 | 3D printing | POWER CONVERTER DEVICE FOR DIRECT CURRENT MULTI-TERMINAL TRANSMISSION-AND-DISTRIBUTION SYSTEM, HAS MAXIMUM/MINIMUM POTENTIAL TERMINAL WHOSE UPPER/LOWER SIDE SWITCHING ELEMENT OPENING/CLOSING OPERATION IS CONTROLLED | WO2015145518A1 | HASHIMOTO KOJI (JP)\ TAKAHASHI KOJI (JP) | SAMSUNG ELECTRONICS CO LTD (KR) | H02M-007/12 |
| 5362 | 3D printing | METHOD FOR REMOVING AND RECOMBINING PORTION OF PROJECTED LIGHT STREAM OF PROGRAM E.G. MOVIE, HAS EMITTING FOCUSED PORTION THROUGH APERTURE HAVING SHAPE TO COMBINE FOCUSED PORTION WITH PROJECTED LIGHT STREAM ON DISPLAY SURFACE | US2015268547A1\ WO2015143338A1 | WATER LUR (CN)\ DAVID LEE (CN) | FUJITSU MICROELECTRONICS LTD (JP) | G03B-021/26\ H04N-005/74\ H04N-009/31 |
| 5363 | 3D printing | SEMICONDUCTOR MEMORY DEVICE FOR ELECTRONIC APPARATUS HAS CONTROL LOGIC UNIT THAT ISSUES COMMAND TO PERIPHERAL CIRCUIT UNIT TO DETERMINE WHETHER TO PERFORM DETRAP VOLTAGE APPLYING OPERATION BASED ON RESULT OF FIRST VERIFYING OPERATION | US2015270009A1\ KR2015109120A\ US9165671B2 | TAN CHUNG F (SG)\ LIU JINPING (SG)\ LEE HYEOK J (SG)\ INDAJANG BANGUN (SG)\ CHOR ENG F (SG)\ ONG SHIANG Y (SG) | UNITED MICROELECTRONICS CORP (TW) | G11C-016/04\ G11C-016/10\ G11C-016/34 |
| 5364 | 3D printing | STEREO LITHOGRAPHY APPARATUS-TYPE PRINTER, HAS CONTROL UNIT CONNECTED WITH LIFTING PART, AND STEREO LITHOGRAPHY APPARATUS-TYPE PRINTER MAIN BODY CONNECTED WITH LIQUID CRYSTAL PANEL THAT IS FIXED WITH HEAT RADIATING PART | CN104786504A | UENISHI TOSHIYA (JP)\ MEGURO SATOSHI (JP)\ KUBO MASAHARU (JP)\ KATO MASATAKA (JP)\ MIURA HIDEO (JP) | CHARTERED SEMICONDUCTOR MFG (SG) | B29C-067/00\ B33Y-030/00 |
| 5365 | 3D printing | DIGITAL LIGHT PROCESSING (DLP) LARGE SCREEN SPLICING DEVICE HAS PROJECTOR THAT IS INSTALLED IN RECTANGULAR BOX WHOSE UPPER PORTION IS EQUIPPED WITH INCLINED REFLECTION LENS, AND SCREEN THAT IS MOUNTED IN FRAME FRONT SURFACE OF BOX | CN204515335U | TSUGANE HIDEAKI (JP)\ YOSHIDA YASUKO (JP)\ ASHIHARA HIROSHI (JP)\ SAITO TATSUYUKI (JP)\ TANAKA UITSU (JP)\ SUZUKI HIDENORI (JP)\ OKUTANI KEN (JP) | RENESAS TECH CORP (JP) | G03B-021/28\ G03B-021/56 |
| 5366 | 3D printing | DLP TECHNOLOGY BASED SLA FORMING PANEL, HAS PANEL MAIN BODY AND LIFTING DEVICE CONNECTED WITH CONNECTING PIECE, WHERE LOWER SURFACE OF PANEL MAIN BODY IS FORMED WITH GROOVE OR ROUND HOLE AND PROVIDED WITH FRICTION LAYER | CN204451224U | SAIKAWA TAKESHI (JP)\ FURUKAWA RYOUICHI (JP)\ FUJIWARA TSUYOSHI (JP)\ KUNITOMO MASATO (JP) | RENESAS TECH CORP (JP)\ HITACHI ULSI SYS CO LTD (JP) | B29C-067/00\ B33Y-030/00 |
| 5367 | 3D printing | SELF-ADAPTIVE DISPLAY FOR SERVICE LEVEL AGREEMENT STATUS AND BUSINESS IMPACT ANALYSIS IN E.G. SMART PHONE, HAS GRAPH DISPLAY UNIT FOR SUMMARIZING GRAPH OF STATUS AND BUSINESS IMPACT INFORMATION OF RETURNED RESULTS | RD617036A | SHUE SHAU-LIN (TW)\ WANG MEI-YUN (TW)\ YU CHEN-HUA (TW) | RENESAS TECH CORP (US) | G06Q-000/00 |
| 5368 | 3D printing | MULTI-CLOUD FABRIC I.E. LAYER 4-7 FABRIC, FOR MANAGING SERVICE LEVEL AGREEMENT OF DATA CENTER USED TO HOUSE E.G. COMPUTER SYSTEMS, HAS AGENTS TO AUTOMATICALLY SCALE-UP OR SCALE-DOWN NETWORK SERVICE, NETWORK APPLICATION OR NETWORK SERVERS | US2015263906A1 | SUZUKI TATSUYA (JP)\ AOYAMA TOHRU (JP) | TAIWAN SEMICONDUCTOR MFG (US) | H04L-012/24 |
| 5369 | 3D printing | METHOD FOR LAUNCHING CONTROLLER UNIT IN MULTI-CLOUD SERVICE FABRIC IN E.G. DATA CENTER, INVOLVES LAUNCHING VALUE ADDED SERVICE UNIT, LAUNCHING PROFILER, AND SENDING TIER EVENT TO VALUE ADDED SERVICE UNIT WITH TIER INFORMATION | US2015264117A1 | TSUUE NUEN US\ LAWRENCE J CHARENISKIJ US\ SHIEN TEN SUU US | NEC ELECTRONICS CORP (JP) | H04L-012/24\ H04L-029/08 |
| 5370 | 3D printing | DEVICE USED FOR CULTURE AND/OR SUPPORT OF LIVING CELLS COMPRISES A MEMBRANE CONFIGURED TO SEPARATE THE CENTRAL CHANNEL TO FORM A MICROCHANNEL AND A MESOCHANNEL | WO2015138034A2\ WO2015138034A3\ CA2934662A1\ AU2014386209A1\ AU2014386209A2\ EP3083940A2\ US2016313306A1 | SUMI HIROFUMI (JP)\ YAMANE CHIGUSA (JP) | SHARP KK (JP)\ SHARP MICROELECT TECH INC (US) | C12M-003/02\ C12N-005/02\ C12M-001/12\ C12M-001/34\ C12M-001/42\ C12M-003/06\ G01N-033/50 |
| 5371 | 3D printing | METHOD FOR PREDICTING IMPACT OF SERVICE LEVEL AGREEMENT, INVOLVES CALIBRATING COST MODEL OF SERVICE LEVEL AGREEMENT, BY CALIBRATING WORKLOAD VOLUME FROM WORKLOAD DATA AND EFFORT TIME FROM EFFORT DATA TO MATCH BASELINE SERVICE PARAMETER | US2015262106A1 | LIU YONGXUN (JP)\ MATSUKAWA TAKASHI (JP)\ ENDO KAZUHIKO (JP)\ OUCHI SHINICHI (JP)\ SAKAMOTO KUNIHIRO (JP)\ MASAHARA MEISHOKU (JP) | SONY CORP (JP) | G06Q-010/06 |
| 5372 | 3D printing | SYSTEM FOR PROVIDING ITEM INFORMATION TO USER BY DISPLAYING INFORMATION ON VISUAL DISPLAY, HAS DISPLAY DEVICE FOR DISPLAYING INFORMATION ABOUT ITEMS MOVING ALONG PATH, WHERE SYSTEM CAUSES DISPLAY OF PORTION OF ITEM IMAGE DATA AND FEATURES | US2015262348A1 | PAISANT LAURENT (FR) | NAT INST OF ADVANCED IND SCIEN (JP)\ LIU YONGXUN (JP)\ MATSUKAWA TAKASHI (JP)\ ENDO KAZUHIKO (JP)\ OUCHI SHINICHI (JP)\ SAKAMOTO KUNIHIRO (JP)\ MASAHARA MEISHOKU (JP) | G06K-009/18\ G06T-007/00\ G06T-007/20\ H04N-007/18 |
| 5373 | 3D printing | THREE-DIMENSIONAL SLA-TYPE PRINTER, HAS LIGHT SOURCE DEVICE CONNECTED WITH PHOTOSENSITIVE RESIN LIFTING PART, WHERE LIGHT SOURCE DEVICE IS CONNECTED WITH MEASURING PART THAT IS CONNECTED WITH BEARING BODY | CN104772908A | KARCHER WOLFRAM (DE)\ ZEILER DIETER (DE)\ LEHR MATTHIAS (DE) | ATMEL NANTES SA (FR) | B29C-067/00\ B33Y-050/02 |
| 5374 | 3D printing | DEVICE FOR ADJUSTING RED, GREEN, BLUE AND WHITE BALANCE OF DIGITAL LIGHT PROJECTOR, HAS PERSONAL COMPUTER (PC) MACHINE CONNECTED WITH ILLUMINATION METER FOR SENDING MEASURED CHROMATICITY COORDINATE AND LUMINANCE TO PC MACHINE | CN104730823A | LEE GILL (US) | INFINEON TECHNOLOGIES AG (DE)\ KARCHER WOLFRAM (DE)\ LEHR MATTHIAS (DE)\ ZEILER DIETER (DE) | G03B-021/14\ G03B-021/20\ H04N-009/31 |
| 5375 | 3D printing | LIGHT SOURCE SYSTEM FOR IMAGING SYSTEM OF PROJECTION DEVICE E.G. DIGITAL LIGHT PROJECTOR, HAS LIGHT WAVE BAND SWITCHING MODE FIXED WITH WAVE BAND FOR TRANSMITTING RAY OF LIGHT TO LIGHT SOURCE MODULE THROUGH LIGHT FACE | CN104730826A | SAYAMA TAKASHI US\ NAGOYA TAKATOSHI (JP)\ TAKAMIZAWA SHOICHI (JP) | INFINEON TECHNOLOGIES CORP (US) | F21V-009/10\ F21Y-101/02\ G02B-027/10\ G03B-021/20 |
| 5376 | 3D printing | METHOD FOR ADJUSTING BANDWIDTH SCHEDULING STRATEGY FOR USE IN COMMUNICATION FIELD, INVOLVES ADJUSTING ORIGINAL BANDWIDTH SCHEDULING STRATEGY WHEN SERVICE QUALITY PARAMETER EXCEEDS REFERENCE VALUE OF SERVICE QUALITY PARAMETERS | CN104717158A | ROZENBLAT AVRAHAM (IL)\ HAIMSON SHAI (IL)\ DRORI ROTEM (IL)\ ROTLAIN MAOR (IL)\ HORVITZ DROR (IL) | SHINETSU HANDOTAI KK (JP)\ NAGOYA TAKATOSHI (JP)\ TAKAMIZAWA SHOICHI (JP)\ SAYAMA RYUJI (JP) | H04L-012/927 |
| 5377 | 3D printing | CONSTRUCTION OF HOLLOW TUBE MICRO MATRIX CERAMIC MATERIAL USED IN E.G. AVIATION BY DEPOSITING CERAMIC FILMS IN THREE-DIMENSIONAL MICRO-ARRAY MATRIX TEMPLATE, AND REMOVING HOLLOW TUBE MICRO-ARRAY CERAMIC MATERIALS FROM PREFORM STRUCTURE | CN104694904A | BERGENDAHL ALBERT S (US)\ CASTRUCCI PAUL C (US)\ FLEMING DANIEL J (US)\ XIAO T D (US) | ROZENBLAT AVRAHAM (IL)\ HAIMSON SHAI (IL)\ DRORI ROTEM (IL)\ ROTLAIN MAOR (IL)\ HORVITZ DROR (IL) | B29C-067/00\ C23C-014/06\ C23C-016/01\ C23C-016/30\ C23C-016/34\ C23C-018/12 |
| 5378 | 3D printing | DIGITAL LIGHT PROCESSING (DLP) THREE-DIMENSIONAL PRINTER HAS PROJECTION SCANNING DEVICE WHICH IS PROVIDED WITH DIGITAL MICRO-MIRROR WAFER THAT IS CONTROLLED BY LIGHT CONTROL UNIT PROVIDED FOR CONTROLLING SCANNING LIGHT SOURCE | CN104723560A | ITO HIROYASU (JP) | BERGENDAHL ALBERT S (US)\ CASTRUCCI PAUL C (US)\ FLEMING DANIEL J (US)\ XIAO T D (US) | B22F-003/115\ B29C-067/00\ B33Y-030/00\ B33Y-040/00 |
| 5379 | 3D printing | METHOD FOR MONITORING AND MANAGING OF RESOURCES FOR AN APPLICATION BY A SERVICE LEVEL AGREEMENT MANAGEMENT NODE, INVOLVES EVALUATING DEPENDENT METRIC INFLUENCE ON THE SERVICE LEVEL AGREEMENT METRIC | WO2015130199A1\ CN106030536A | OMI TOSHIHIKO (JP)\ MIMURO YOICHI (JP) | DENSO CORP (JP) | G06F-009/50 |
| 5380 | 3D printing | MULTI-LAYERED THREE DIMENSIONAL VARIABLE-RESISTANCE STORAGE DEVICE HAS GATING TUBE WHOSE GATE TERMINAL IS USED AS WORD LINE OF MAIN PORTION AND SOURCE TERMINAL IS USED AS SOURCE LINE OF MAIN PORTION | CN104716259A | Empty Field US | SEIKO INSTR INC (JP) | H01L-045/00 |
| 5381 | 3D printing | CLOUD COMPUTATION PLATFORM DATA BASE STANDARD TESTING SYSTEM, HAS IDENTITY VERIFICATION MECHANISM IN WEAK STATE, SO THAT INTRUDER OBTAINS USER ACCOUNT AND LOGIN OF CLIENT VIRTUAL MACHINE AT TIME OF APPLICATION PROGRAM INSPECTING | CN104683394A | OSANAI JUN (JP) | TEXAS INSTRUMENTS INC (US) | G06F-017/30\ H04L-029/06\ H04L-029/08 |
| 5382 | 3D printing | DLP MICRO-PROJECTOR MODULE GROUP SYSTEM, HAS LIGHT-EMITTING UNIT PROVIDED WITH COLLIMATING LENS GROUP, AND FREE CURVED SURFACE/PLANE FIXED WITH DLP LIGHT ADJUSTOR THAT IS CONNECTED WITH AND PROJECTION LENS DEVICE | CN204331264U\ WO2016115934A1 | ZHIGANG LIU (CN)\ XIN WANG (CN)\ ZEKUI LI (CN) | SEIKO INSTR INC (JP) | G03B-021/16\ G03B-021/20\ G03B-021/28 |
| 5383 | 3D printing | WAVEGUIDE APPARATUS FOR USE IN E.G. FIBER SCAN PROJECTORS, HAS DIFFRACTIVE OPTICAL ELEMENT FOR OPTICAL PATH TO PROVIDE SET OF OPTICAL PATHS BETWEEN EXTERIOR AND INTERIOR OF PLANAR WAVEGUIDE, WHERE PATHS ARE ORIENTED IN OPPOSITE DIRECTIONS | US2015247975A1 | KOU WANYON US\ MIIKA LEINIKKA US\ KILPELA OLLI V (FI)\ HUOTARI HANNU A (FI)\ TUOMINEN MARKO (FI) | ZHONGXIN INT IC MFG SHANGHAI (CN) | G02B-006/34 |
| 5384 | 3D printing | IMAGE PROJECTOR E.G. DIGITAL LIGHT PROCESSOR PROJECTOR HAS DETERMINATION UNIT THAT DETERMINES DISPLAY AREA BY GIVING PRIORITY TO REGION IN AMENDMENT IMAGE CORRESPONDING TO SMALL REGION OF DISTORTION IN CAPTURED IMAGE | JP2015158591A | BUDD RUSSELL A (US)\ PATEL CHIRAG S (US)\ BUCHWALTER LEENA PAIVIKKI (US) | KILPELA OLLI V (FI)\ KOH WONYONG (JP)\ HUOTARI HANNU A (FI)\ LEINIKKA MIIKA (FI)\ TUOMINEN MARKO (FI) | G03B-021/00\ G03B-021/14\ G09G-003/20\ G09G-005/00\ G09G-005/36\ G09G-005/377\ H04N-005/74 |
| 5385 | 3D printing | WATERPROOF COATED CIS WOOL FABRIC COMPRISES CIS WOOL FABRICS BASE LAYER, WHERE ONE SIDE OF THE CIS WOOL FABRIC IS ADHERED WITH WATERPROOF COATING, AND THE CIS WOOL FABRICS BASE LAYER IS BROKEN TWILL WEAVE FABRIC | CN104647817A\ CN104647817B | LEE KYOUNG-WOO (US)\ CHOI SEUNG-MAN (US) | IBM (US) | B32B-027/40\ B32B-007/12\ B32B-009/02\ D03D-015/00\ D06B-003/18 |
| 5386 | 3D printing | STEREO-LITHOGRAPHY MACHINE FOR PRODUCING THREE-DIMENSIONAL OBJECT, HAS LIGHT EMITTING UNIT AND OPTICAL UNIT, SUCH THAT RATIO BETWEEN MAXIMUM DIAMETER OF BEAM AND MINIMUM DIAMETER OF MINIMUM CROSS SECTION DOES NOT EXCEED SPECIFIC RANGE | WO2015128783A1\ TW201532790A\ CA2939498A1\ IN201627026687A\ IT1423557B | DAHL JEREMY E (US)\ CARLSON ROBERT M (US)\ LIU SHENGGAO (US) | SAMSUNG ELECTRONICS CO LTD (KR) | B29C-067/00\ G02B-026/08\ G02B-026/10\ G03F-007/207 |
| 5387 | 3D printing | PROJECTOR I.E. DIGITAL LIGHT PROCESSING PROJECTOR, FOR PROJECTING IMAGE ON SCREEN, HAS ESTABLISHMENT PART FOR ESTABLISHING WIRELESS CONNECTION WITH TERMINAL DEVICE BASED ON CONNECTION REQUEST AND CONNECTION INFORMATION FROM TERMINAL DEVICE | JP2015158596A | LENOBLE DAMIEN (FR) | CHEVRON USA INC (US)\ DAHL JEREMY E (US)\ CARLSON ROBERT M (US)\ LIU SHENGGAO (US) | G03B-021/14\ G09G-005/00\ H04N-005/74 |
| 5388 | 3D printing | ELECTRONIC DISPLAY SYSTEM E.G. REAR PROJECTION DISPLAY SYSTEM, FOR MOBILE DEVICE, HAS TRANSPARENT ADHESIVE LAYER RUNNING ALONG SEAM FOR COUPLING SHEETS TOGETHER AND COMPRISING MAJOR EDGE THAT OVERLAPS WITH LIGHT EMANATING IMAGING PORTION | WO2015127035A1\ CN106030504A | CHOI KYUNG-IN (KR)\ CHOI GIL-HEYUN (KR)\ KIM BYUNG-HEE (KR)\ KANG SANG-BUM (KR) | ST MICROELECTRONICS SA (FR) | G06F-003/147 |
| 5389 | 3D printing | COMPUTING PLATFORM FOR MONITORING REAL-TIME CONDITIONS OF E.G. AIRCRAFTS ON WORKSITE, HAS SENSING DEVICES INSTALLED ON OBJECTS ON WORKSITE, AND MOBILE DEVICE ARRANGED WITH MOBILE PROCESSOR TO PROVIDE MOBILE USER WITH MOBILE APPLICATION | US2015242769A1\ WO2015128743A2\ WO2015128743A3\ US9466038B2 | STAMPER ANTHONY K (US)\ WONG KWONG HON (US)\ BURRELL LLOYD G (US)\ GAMBINO JEFFREY P (US)\ LEE HYUN KOO (US)\ LEVY MARK D (US)\ LI BAOZHEN (US)\ LUCE STEPHEN E (US)\ MCDEVITT THOMAS L (US)\ YANKEE SALLY J (US)\ COONEY III EDWARD E (US)\ HEIDENREICH III JOHN E (US) | SAMSUNG ELECTRONICS CO LTD (KR) | G01D-021/00\ G06Q-010/06\ G06Q-000/00\ G06Q-010/00\ G08G-001/00\ G08G-001/16 |
| 5390 | 3D printing | COMPUTER-IMPLEMENTED METHOD FOR DYNAMICALLY MODIFYING QUALITY OF SERVICE LEVELS FOR APPLICATIONS IN COMPUTING ENVIRONMENT, INVOLVES ADJUSTING QUALITY OF SERVICE PARAMETERS IF CURRENT PERFORMANCE FAILS TO MEET SERVICE LEVEL REQUIREMENTS | US2015244589A1 | DING YI (US) | IBM (US) | H04L-012/24\ H04L-029/08 |
| 5391 | 3D printing | IMAGE PROJECTION SYSTEM E.G. CRYSTAL TYPE PROJECTOR SYSTEM, HAS PROJECTING UNIT FOR PROJECTING FIRST PROJECTION IMAGE BASED ON CONTENT DATA ON PROJECTED OBJECT AND INTERCONNECTING FIRST PROJECTION IMAGE TO SECOND PROJECTION IMAGE | US2015244998A1\ JP2015161830A | LEE TAI-PENG (US) | PROMOS TECHNOLOGIES INC (TW) | G03B-037/04\ H04N-009/31\ G03B-021/00\ G03B-021/14\ G03B-021/26\ H04N-005/74 |
| 5392 | 3D printing | DISPLAY SYSTEM FOR DIGITAL OPTICAL PROCESSING, HAS VIDEO CONNECTING INTERFACE THAT IS CONNECTED WITH VIDEO PROCESSOR, SUCH THAT DIGITAL LIGHT PROCESSING (DLP) CONTROL CIRCUIT AND OPTICAL ENGINE ARE CONNECTED WITH ELECTRICAL PROPERTY | CN204362227U\ HK1201020A2 | YOO JONG RYEOL (KR)\ CHOI SI YEONG US\ KIM CHEOL SEONG US\ LEE DEOK HYEONG US\ LEE BYEONG CHAN (KR) | PROMOS TECHNOLOGIES INC (TW) | G03B-021/00\ G03B-021/14\ H04N-009/31\ G09F-000/00 |
| 5393 | 3D printing | SYSTEM OF MANAGING ENERGY CONSUMPTION WITHIN COMPUTE ENVIRONMENT, HAS COOLING SYSTEM THAT IS CONTROLLED TO SELECTIVELY MODIFY TEMPERATURE OF NODE IN COMPUTE ENVIRONMENT BASED ON DATA | US2015234441A1\ US9411393B2 | DE SANTI GIORGIO (IT)\ ZANOTTI LUCA (IT) | SAMSUNG ELECTRONICS CO LTD (KR) | G05D-023/19\ G06F-001/32\ G06F-009/50\ G06F-001/20 |
| 5394 | 3D printing | COMPUTER IMPLEMENTED METHOD FOR STORING AND RETRIEVING E.G. ARRANGEMENT OF PARALLEL LINES, INVOLVES DEVELOPING ELECTRONIC FILE TO BE USED FOR PRINTING 3D OBJECT, AND EMBEDDING 3D SYMBOL MATRIX INTO 3D OBJECT BY UTILIZING ELECTRONIC FILE | US2015235069A1\ US9400910B2 | HONG CHANG-KI (KR)\ LEE JONG-WON (KR)\ KIM HO-YOUNG (KR) | ST MICROELECTRONICS SRL (IT) | G05B-019/18\ G06K-001/12\ G06K-019/06\ G06K-007/14\ B29C-067/00\ B33Y-010/00\ B33Y-050/00\ B33Y-099/00\ G05B-019/4099 |
| 5395 | 3D printing | SLA THEORY BASED MATERIAL EXPOSURE SYSTEM, HAS LIQUID STORAGE POOL WHOSE BOTTOM PART IS PROVIDED WITH SEALING PROPERTY MOVING BAFFLE PLATE, WHERE LIQUID STORAGE POOL IS CONNECTED WITH FLUID INFUSION PART THROUGH PIPELINE | CN104647758A | DE SANTI GIORGIO (IT)\ ZANOTTI LUCA (IT)\ CRISENZA GIUSEPPE (IT) | SAMSUNG ELECTRONICS CO LTD (US) | B29C-067/00\ B33Y-030/00 |
| 5396 | 3D printing | METHOD FOR DYNAMICALLY UPDATING POLICY ENFORCEMENT POINT AT SERVICE, INVOLVES PROPAGATING SERVICE LEVEL AGREEMENT TO ACTIVE SERVICE AND PROPAGATING POLICY TO CLIENT WITH SERVICE LEVEL AGREEMENT INSTALLED AT ACTIVE SERVICE | US2015229537A1\ US9444697B2 | ANNAPRAGADA RAO V (US)\ DUNTON SAMUEL VANCE (US)\ WELING MILIND GANESH (US)\ BOTHRA SUBHAS (US) | ST MICROELECTRONICS SRL (IT) | H04L-012/24\ G06F-015/173\ G06F-017/30 |
| 5397 | 3D printing | METHOD OF BROKERING COMPUTE RESOURCES, INVOLVES IDENTIFYING AND RESERVING NEW COMPUTE RESOURCES FROM COMPUTE RESOURCE ENVIRONMENTS AND MIGRATING WORKLOAD TO NEW COMPUTE RESOURCES IF THRESHOLD IS MET | US2015229586A1 | HSU SHENG TENG (US)\ NGUYEN TUE (US)\ CHARNESKI LAWRENCE J (US)\ SENZAKI YOSHIHIDE (US)\ KOBAYASHI MASATO (US) | VLSI TECHNOLOGY INC (US) | G06Q-020/14\ G06Q-030/02\ G06Q-030/04\ H04L-012/24\ H04L-012/911 |
| 5398 | 3D printing | CLOUD COMPUTING BASED SYSTEM FOR DETECTING INJECTION LOOPHOLE OF C SHARP OR ASP LANGUAGE, HAS LOAD BALANCE AND MAINTENANCE MANAGEMENT OPERATIONS PERFORMED BY UTILIZING SLA MONITORING AND CREDIT MANAGEMENT TECHNOLOGY | CN104636667A | WONG LAWRENCE D (US) | SHARP KK (JP)\ SHARP MICROELECT TECH INC (US) | G06F-021/57 |
| 5399 | 3D printing | NON-TRANSITORY COMPUTER-READABLE STORAGE MEDIUM FOR IMPLEMENTING AFFILIATE MANAGEMENT FUNCTIONALITY WITHIN CLOUD SERVICE BROKERAGE PLATFORM, INCLUDES INSTRUCTION FOR IMPLEMENTING BUDGETARY ACTION IN RESPONSE TO BUDGETARY IMPACT | US2015222723A1 | RAO SATYAVOLU S PAPA (US)\ GRUNOW STEPHAN (US)\ RUSSELL NOEL M (US) | INTEL CORP (US) | G06Q-030/06\ H04L-029/08 |
| 5400 | 3D printing | EYE-IMAGING SYSTEM FOR HEAD WORN DISPLAY, HAS PROCESSOR WHICH IS ADAPTED TO ANALYZE EYE-IMAGE LIGHT AND ASSESSES SHARPNESS OF EYE-IMAGE LIGHT AS INDICATION OF SHARPNESS OF CONTENT-IMAGE LIGHT | US2015205347A1 | IIJIMA SHINPEI (JP)\ KUROKI KEIJI (JP) | TEXAS INSTRUMENTS INC (US) | G06F-003/01\ G02B-027/01\ G06F-001/16\ G06F-003/00 |
| 5401 | 3D printing | SYSTEM FOR FACILITATING SYMMETRIC COHERENT REQUEST/RESPONSE POLICY ENFORCEMENT, HAS PROCESSOR FOR ENFORCING SERVICE LEVEL AGREEMENT POLICIES AND SERVICE LEVEL DEFINITION POLICIES ON RESPONSE MESSAGE USING IDENTIFIED PROCESSING RULES | US2015229538A1\ US9379998B2 | TSUGANE MASARU US\ MARUYAMA HIROYUKI (JP)\ OHASHI TADASHI (JP)\ FUJIWARA TAKESHI US | ELPIDA MEMORY INC (JP) | H04L-012/24\ H04L-012/917\ G06F-015/173\ H04L-029/06 |
| 5402 | 3D printing | METHOD FOR PERFORMING SYMMETRIC AND COHERENT ENFORCEMENT OF E.G. SERVICE LEVEL AGREEMENT POLICIES, INVOLVES ENFORCING SERVICE LEVEL AGREEMENT POLICIES ON RESPONSE MESSAGE USING IDENTIFIED POLICY ENFORCEMENT PROCESSING RULES | US2015229539A1 | JUNG JEONG HUI (KR)\ PARK IN SEONG (KR)\ YEO JAE HYEON (KR) | HITACHI LTD (JP)\ FUJIWARA TSUYOSHI (JP)\ OHASHI NAOHUMI (JP)\ MARUYAMA HIROYUKI (JP)\ TSUGANE KEN (JP) | H04L-012/24\ H04L-012/917 |
| 5403 | 3D printing | PHOSPHOR WHEEL FOR USE IN E.G. SINGLE-CHIP DIGITAL LIGHT PROCESSING PROJECTOR USED IN LIFE ENTERTAINMENT APPLICATION, HAS WAVELENGTH CONVERTING LAYER FORMED ON COATING REGION OF SURFACE FOR CONVERTING LIGHT WAVELENGTH OF LIGHT BEAM | US2015229892A1\ CN104834169A | LIU JIANYING (CN) | SAMSUNG ELECTRONICS CO LTD (KR) | F21V-014/08\ G02B-026/00\ H04N-009/31\ F21V-009/10\ G03B-021/14\ G03B-021/16\ G03B-021/20 |
| 5404 | 3D printing | COMPUTER SYSTEM FOR ALIGNING CONTENT DISPLAYED FROM PROJECTOR ONTO TOUCH SENSITIVE MAT, HAS COMPUTING DEVICE CAUSING PROJECTOR TO ADJUST SETTINGS FOR BORDER OF IMAGE REFLECTED ONTO MAT TO FIT WITHIN DETECTED BORDER OF MAT | WO2015116220A1\ TW201535206A\ CN105940359A | MARUYAMA HIROYUKI (JP)\ NAGOYA TAKATOSHI (JP)\ TAKAMIZAWA SHOICHI (JP)\ SAYAMA RYUJI (JP) | UNIV SHANGHAI (CN)\ LIU JIANYING (CN) | G06F-003/01\ G06F-003/042 |
| 5405 | 3D printing | LED DRIVER, HAS FLY-BACK ISOLATING CIRCUIT FOR GENERATING OUTPUT SIGNAL TO MCU CHIP THAT IS CONNECTED WITH POWER SUPPLY CIRCUIT, SECONDARY CIRCUIT WHOSE OUTPUT END IS CONNECTED WITH EXTERNAL LOAD, AND SHELL CONNECTED WITH DRIVING MODE | CN204362400U | YUE DUOFENG (US)\ CHEN PEIJUN J (US)\ MERCER DOUGLAS E (US)\ RUSSELL NOEL (US) | SHINETSU HANDOTAI KK (JP)\ NAGOYA TAKATOSHI (JP)\ TAKAMIZAWA SHOICHI (JP)\ SAYAMA RYUJI (JP)\ MARUYAMA HIROYUKI (JP) | H05B-037/02 |
| 5406 | 3D printing | SCRAPER VOLTAGE STABILIZING SYSTEM FOR STEREOLITHOGRAPHY LASER PRINTER, HAS DATA PROCESSING MODULE FOR RECEIVING MEASURED PRESSURE VALUE OF PRESSURE SENSOR, AND COMPARING MEASURED PRESSURE VALUE WITH PRESSURE REFERENCE PRESET VALUE | CN104626584A | HSU SHENG TENG (US)\ EVANS DAVID R (US)\ PAN WEI (US)\ BARROWCLIFF ROBERT (US) | TEXAS INSTRUMENTS INC (US) | B29C-067/00\ B33Y-030/00\ B33Y-050/02 |
| 5407 | 3D printing | METHOD FOR DYNAMICALLY ADJUSTING COMMUNICATION CHARACTERISTICS OF SATELLITE COMMUNICATION LINKS, INVOLVES CALCULATING TOTAL SIGNAL ATTENUATION USING NOWCASTING PREDICTION WHICH IS GENERATED USING RECEIVED WEATHER CONDITION | WO2015111050A1 | LEE TAI-PENG (MY)\ JANG CHUCK (US) | SHARP LAB OF AMERICA INC (US) | H01B-001/10 |
| 5408 | 3D printing | THREE DIMENSIONAL (3D) PRINTER FOR BUILDING COMPONENT LAYER BY LAYER, HAS KINEMATIC COUPLING BETWEEN BUILD PLATFORM AND MOVEABLE STAGE AND TWO FLEXURES FOR DIFFERENTIALLY CHANGING POSITION OF POINTS OF CONTACT | WO2015112998A1 | SANDHU GURTEJ (US)\ IRELAND PHILIP J (US)\ RHODES HOWARD E (US)\ SHARAN SUJIT (US)\ PRALL KIRK (US) | PROMOS TECHNOLOGIES INC (TW) | B29C-067/00 |
| 5409 | 3D printing | THREE DIMENSIONAL PRINTER E.G. CONTINUOUS CORE REINFORCED FILAMENT-EXTRUSION PRINTER, HAS RETURN DETECTION CIRCUIT RESPONDING TO INPUT ASSOCIATED WITH RETURN OF BUILD PLATFORM TO ACTIVATE PRINT RESUME CIRCUIT | US2015210007A1 | YAMADA KEIICHI (JP) | MICRON TECHNOLOGY INC (US) | B29C-067/00 |
| 5410 | 3D printing | PRESENTATION SYSTEM FOR PREDICTIVELY TRACKING EXPECTED PRINT JOBS, HAS CONTROL UNIT FOR ADDING RECEIVED PRINT JOB TO LIST OF ON-TIME RECEIVED PRINT JOBS IN RESPONSIVE TO DETERMINATION THAT RECEIVED JOB DOES NOT CORRESPONDED WITH PRINT JOB | US2015212775A1\ US9292245B2 | CHOI GIL-HEYUN (KR)\ KIM BYUNG-HEE (KR)\ LEE JONG-MYEONG (KR)\ LEE MYOUNG-BUM (KR) | RENESAS TECH CORP (JP) | G06F-003/12\ G06K-015/02 |
| 5411 | 3D printing | METHOD FOR CHANGING DISPLAY REFRESH RATE IN ACTIVE PERIOD OF DISPLAY DEVICE E.G. LCD, INVOLVES DELAYING THE CHANGING OF DISPLAY REFRESH RATE WHEN DETERMINING THAT DISPLAY IS NOT OPERATING IN ACTIVE PERIOD | US9087473B1 | HENLEY FRANCOIS J (US)\ CHEUNG NATHAN W (US) | SAMSUNG ELECTRONICS CO LTD (US) | G09G-005/00 |
| 5412 | 3D printing | OPTICAL WRITER FOR FORMING ELECTROSTATIC LATENT IMAGE ON IMAGE RETAINER, HAS RESTRAINING PLATES AND HOLDER COMPRISING LOWER LINEAR EXPANSION COEFFICIENT THAN LINEAR EXPANSION COEFFICIENT OF ROD LENS ARRAY | US9091957B1\ JP2015150802A\ US2015234307A1\ JP5999120B2 | FARRAR PAUL A (US) | SILICON GENESIS CORP (US) | B41J-002/385\ G03G-015/043\ B41J-002/447\ B41J-002/45\ G02B-027/18\ G02B-003/00\ H04N-001/036 |
| 5413 | 3D printing | COMPUTER-READABLE STORAGE MEDIUM STORING PROGRAM FOR IMPLEMENTING PRICING RULES MANAGEMENT FUNCTIONALITY, INCLUDES INSTRUCTIONS FOR APPLYING PRICING RULE TO CLOUD SERVICE PRICES WITH PRICING RULE CONFIGURATION INFORMATION AND CONTEXT | US2015206207A1 | THOMPSON DAVID M (US)\ PETERS DAVID WALTER (US) | MICRON TECHNOLOGY INC (US) | G06Q-030/02 |
| 5414 | 3D printing | PREDICTIVE SERVICE LEVEL AGREEMENT (SLA) ANOMALY DETECTION MECHANISM FOR MULTI-SUBSCRIBER INFORMATION TECHNOLOGY INFRASTRUCTURE, HAS SLA-AWARE ALERTS PRIORITIZATION MODULE THAT PRIORITIZES SLA ALERTS BASED ON SIGNIFICANCE OF ALERTS | US2015207696A1 | CHEN WEI-KUO (TW)\ CHEN CHING-YU (TW) | THOMPSON DAVID M (US)\ PETERS DAVID WALTER (US) | H04L-012/24 |
| 5415 | 3D printing | OPTICAL SWITCH FOR VEHICLE I.E. CAR, HAS TRANSLUCENT BODY FIXED WITH LOWER-SIDE OF PANEL FOR CHANGING PROGRESSIVE DIRECTION OF LIGHT TO OUTPUT LIGHT TO DLP ENGINE AND REFLECT LIGHT TO LOWER-SIDE OF PANEL | WO2015105231A1\ KR2015083244A\ KR1550576B1 | HAHN JENS (DE)\ FITZ CLEMENS (DE)\ BUERKE AXEL (DE)\ JAKUBOWSKI FRANK (DE)\ MONO TOBIAS (DE)\ REGUL JOERN (DE)\ SCHMIDBAUER SVEN (DE) | UNIV NAT CHIAO TUNG (TW) | H01H-013/83 |
| 5416 | 3D printing | LOW ENERGY CONSUMPTION CLOUD STORAGE VIDEO MONITORING SYSTEM, HAS MONITORING VIDEO BROWSING SERVER THAT RECEIVES HISTORY MONITORING VIDEO DATA, AND SLA INFORMATION MANAGEMENT SERVER CONNECTED WITH DATA ACCESS MANAGEMENT SERVER | CN104581073A | Empty Field US | QIMONDA AG (DE) | H04L-029/08\ H04N-007/18 |
| 5417 | 3D printing | DISPLAY SYSTEM FOR DISPLAYING IMAGE TO USER, HAS IMAGING ASSEMBLY, WHICH CREATES IMAGE FROM LIGHT, AND DIFFUSER ASSEMBLY WHICH REDUCES COHERENCE OF LIGHT AND HAS DIFFUSERS SEPARATED BY GAP | WO2015103633A1 | OFFENBERG DIRK (DE)\ SPERLICH HANS-PETER (DE)\ VOGT MIRKO (DE)\ CIGAL JEAN C (DE) | IBM (US) | G02B-027/18 |
| 5418 | 3D printing | MULTILEVEL DEVICE FOR USE IN NON-VOLATILE MEMORY DEVICE, HAS SECOND ELECTRICALLY CONDUCTIVE LAYER IN STACK DIFFERENT FROM FIRST ELECTRICALLY CONDUCTIVE LAYER, AND IS TOPMOST LAYER IN LATERALLY CENTRAL PORTION OF SECOND RECESSES | US2015194380A1\ WO2015105709A1\ US9230905B2 | LIN CHIH-CHENG (TW)\ TSENG CHIANG-HUNG (TW)\ PANG JIA-PANG (TW)\ WU TSE (TW) | QIMONDA AG (DE) | H01L-021/768\ H01L-023/50\ H01L-027/115\ H01L-027/24\ H01L-027/06 |
| 5419 | 3D printing | MOBILE TERMINAL E.G. MOBILE PHONE, HAS CONTROLLER THAT OVERWRITES FIRST PORTION OF PREVIOUSLY RECORDED PORTION OF VIDEO WITH ANOTHER PORTION OF VIDEO CURRENTLY BEING RECORDED | US2015194186A1\ KR2015082788A\ US9465468B2 | WATANABE KATSUYUKI (JP)\ TAN SHIRO (JP) | INNOLUX DISPLAY CORP (TW) | G06F-003/041\ G11B-027/034\ G11B-031/00\ G11B-033/10\ H04N-005/232\ H04N-005/77\ H04B-001/40\ G11B-027/00\ G11B-027/031\ G11B-027/34\ H04N-009/82 |
| 5420 | 3D printing | DISPLAY APPARATUS FOR E.G. LIQUID CRYSTAL PROJECTOR, FOR DISPLAYING DIRECTION OF INPUT IMAGE, HAS ROTATION CONTROLLER FOR CONTROLLING ROTATION UNIT DEPENDING ON IMAGE FORMAT, AND DISPLAY UNIT FOR DISPLAYING OUTPUT IMAGE FROM ROTATION UNIT | US2015193913A1\ JP2015129787A\ US9471960B2 | KIM DO-HYUNG (KR)\ LEE HYEON-DEOK (KR)\ LEE JU-BUM (KR) | FUJI PHOTO FILM CO LTD (JP) | G06T-011/60\ G06T-003/60\ G09G-005/22\ G09G-005/377\ G09G-005/38\ G09G-003/20\ G09G-005/00\ G09G-005/36\ G09G-005/391\ H04N-005/66 |
| 5421 | 3D printing | METHOD FOR ENABLING SERVICE LEVEL AGREEMENT (SLA) NEGOTIATION IN DATA CENTER, INVOLVES APPLYING REQUIRED QUALITY OF SERVICE PARAMETERS IN SLA NEGOTIATION FOR EGRESS TRAFFIC FLOW | EP2892183A1 | GUPTA SUBHASH (US)\ CHOOL SIMON US\ ZHOU MEI SHENG (SG) | SAMSUNG ELECTRONICS CO LTD (KR) | H04L-012/24\ H04L-012/46 |
| 5422 | 3D printing | PROJECTION TYPE DISPLAY APPARATUS E.G. LIQUID-CRYSTAL PROJECTOR HAS ROLL THAT IS PROVIDED IN ONE DUCT, AND OTHER DUCT THAT GUIDES COOLING AIR TOWARD UPPER PORTION OF VALVE ACCORDING TO DEAD WEIGHT OF WEIGHT | JP2015125208A | MARUYAMA SHINYA (JP) | CHARTERED SEMICONDUCTOR MFG (US) | G03B-021/00\ G03B-021/14\ G03B-021/16\ H04N-005/74 |
| 5423 | 3D printing | APPARATUS FOR ALIGNMENT OF DISPLAY E.G. ACTIVE MATRIX DISPLAY BASED ON EYE TRACKING, HAS ALIGNMENT MODULE THAT MOVES GRAPHICAL OBJECT TO REDUCE VIEWING ANGLE BETWEEN GRAPHICAL OBJECT AND ASPECT OF USER | US2015185832A1 | RAMANATHAN SASANGAN (US)\ LEE SANG-HYOEB (US) | NEC ELECTRONICS CORP (JP) | G06F-003/01\ G06F-003/0484\ G06K-009/00 |
| 5424 | 3D printing | DIGITAL LIGHT PROCESSING-TYPE THREE-DIMENSIONAL PRINTER, HAS RESIN RESERVOIR FORMED IN SIDE OF RESERVOIR MAIN BODY THAT IS PROVIDED WITH LIGHT CURING RESIN, AND PROJECTOR TRANSPORT UNIT COMBINED WITH LOWER PART OF PROJECTOR CASING PART | KR1533374B1 | GREGG JOHN N (US)\ NOAH CRAIG M (US)\ JACKSON ROBERT M (US) | NOVELLUS SYSTEMS INC (US) | B29C-067/00 |
| 5425 | 3D printing | METHOD FOR FACILITATING PREDICTIVE LEARNING MACHINE-BASED APPROACH TO DETECT TRAFFIC OUTSIDE OF SERVICE LEVEL AGREEMENTS, INVOLVES DETECTING WHETHER SERVICE LEVEL AGREEMENTS REQUIREMENT IS SATISFIED BASED ON PERFORMANCE METRIC | US2015195149A1\ WO2015103523A1\ US9338065B2 | LEE SANG WOO (US)\ CHOI GIL-HEYUN (KR)\ HAN SUNG-HO (KR)\ KIM RAK-HWAN (KR)\ CHOI KYOUNG-IN US | ADVANCED TECH MATERIALS (US) | H04L-012/24\ H04L-012/851\ G06Q-010/04\ G06N-007/00\ G06N-099/00\ H04L-012/26 |
| 5426 | 3D printing | STEREO LITHOGRAPHY SYSTEM FOR MANUFACTURING THREE-DIMENSIONAL COMPONENT, HAS CARRIAGE MOVING IN X-AXIS INDEPENDENT OF LINEAR DRIVES, WHERE ONE OF LINEAR DRIVES IS ARRANGED TO BE MOVABLE INDEPENDENT OF ANOTHER LINEAR DRIVE ALONG Y-AXIS | DE102013021961A1 | FUTASE TAKUYA (JP)\ MAKABE KAZUYA (JP)\ YAMAZUMI SAIGOU (JP) | SAMSUNG ELECTRONICS CO LTD (KR) | B29C-067/00 |
| 5427 | 3D printing | DISPLAY DEVICE E.G. ELECTROLUMINESCENCE (EL) PANEL TYPE DISPLAY DEVICE FOR USE IN ELECTRONIC APPLIANCE, HAS LIGHT EMITTING ELEMENT THAT IS PROVIDED TO EMIT WHITE LIGHT, AND COLOR FILTER THAT IS PROVIDED OVER LIGHT EMITTING ELEMENT | US2015187812A1 | ROZBICKI ROBERT (US)\ PRADHAN ANSHU A (US) | RENESAS TECH CORP (JP)\ FUTASE TAKUYA (JP)\ MAKABE KAZUYA (JP)\ YAMAZUMI SAIGOU (JP) | H01L-027/12 |
| 5428 | 3D printing | ASYNCHRONOUS ACCESS CONTROLLER APPARATUS FOR MANAGING WIRELESS LOCAL AREA NETWORKS (WLAN), HAS PASSWORD INJECTOR CONTROLLER THAT INJECTS NEW NETWORK ACCESS PASSWORD INTO NODES OF WLAN RESPONSIVE TO AFFIRMATIVE DETERMINATION | US9071972B1 | ROZBICKI ROBERT (US)\ KAILASAM SRIDHAR (US)\ YU CHENTAO (US)\ HAYDEN DOUGLAS (US) | NOVELLUS SYSTEMS INC (US)\ PRADHAN ANSHU A (US)\ ROZBICKI ROBERT (US) | H04K-001/00\ H04W-012/04\ H04W-012/08 |
| 5429 | 3D printing | SERVICE TEMPLATE GENERATOR FOR MANAGING SERVICE TEMPLATE, HAS SERVICE GENERATION MODULE THAT GENERATES SPECIFIC SERVICE TEMPLATES BASED ON GENERIC SERVICE TEMPLATE, AND SERVICE TEMPLATE CATALOG STORES SERVICE TEMPLATES | US2015180736A1\ WO2015094299A1\ US9385926B2\ CN105765568A\ DE112013007696T5 | HOPPER PETER J (US)\ FRENCH WILLIAM (US)\ GABRYS ANN (US) | NOVELLUS SYSTEMS INC (US) | H04L-012/24\ G06F-017/30\ G06F-015/173 |
| 5430 | 3D printing | ROUTER FOR CORE NETWORK FOR RECEIVING MULTI-PRIORITY TRAFFIC STREAM OF PACKETS FROM MULTIMEDIA TRANSMISSION NETWORK, HAS LOAN BUCKET FOR ASSOCIATING NON-CONFORMING PACKET WITH HIGHER DROP PRECEDENCE WHEN BANDWIDTH IS NOT BORROWED FOR PACKET | US2015188825A1 | JAIN MANOJ K (US)\ KODURI SREENIVASAN (US) | NAT SEMICONDUCTOR CORP (US)\ HOPPER PETER J (US)\ FRENCH WILLIAM (US)\ GABRYS ANN (US) | H04L-012/801\ H04L-012/819 |
| 5431 | 3D printing | NETWORK ELEMENT OF PROVIDER NETWORK FOR E.G. IN DATA CENTER, HAS SERVICE ASSURANCE AGENT (SAA) MODULE TO GENERATE SAA NOTIFICATION HAVING ADDRESSES OF LOCAL VIRTUAL MACHINE ASSOCIATED WITH NETWORK DEVICE AND REMOTE VIRTUAL MACHINE | US2015188780A1\ US9178775B2 | ROZBICKI ROBERT (US)\ VAN SCHRAVENDIJK BART (US)\ WU WEN (US)\ MOUNTSIER THOMAS (US) | TEXAS INSTRUMENTS INC (US)\ JAIN MANOJ K (US)\ KODURI SREENIVASAN (US) | H04L-012/24\ H04L-012/26\ H04L-012/707\ G01R-031/08 |
| 5432 | 3D printing | MANUAL STEREO-LITHOGRAPHY SYSTEM HAS CONTROL MODULE THAT IS PROVIDED FOR RECEIVING COMMAND FROM CONTROL DEVICE, FOR ADJUSTING HEIGHT OF LIFTING PLATFORM | CN104494147A | REKHI SANJAY (US)\ CHERUKUPALLI NAGENDRA (US)\ KESWICK PAUL D (US) | NOVELLUS SYSTEMS INC (US)\ ROZBICKI ROBERT (US)\ VAN SCHRAVENDIJK BART (US)\ MOUNTSIER THOMAS (US)\ WU WEN (US) | A21D-015/08\ B22F-003/115\ B29C-067/00\ B33Y-010/00\ B33Y-030/00 |
| 5433 | 3D printing | METHOD OF PREPARING PRINTING AND/OR EMBOSSING CYLINDER OR PRINTING AND/OR EMBOSSING SLEEVE FOR PRODUCING PRINTING OR EMBOSSING PATTERN ON E.G. PAPER, INVOLVES PRODUCING CYLINDER JACKET OUTER SURFACE BY A GENERATIVE MANUFACTURING METHOD | EP2886366A1\ WO2015091545A1 | SPREY HESSEL (BE)\ NAKANO AKINORI (JP) | CYPRESS SEMICONDUCTOR CORP (US)\ REKHI SANJAY (US)\ CHERUKUPALLI NAGENDRA (US)\ KESWICK PAUL D (US) | B22F-003/105\ B29C-067/00\ B41C-001/00\ B44B-005/02\ B44C-003/02\ G03F-007/00 |
| 5434 | 3D printing | METHOD FOR PREPARATION OF PRINTING OR EMBOSSING PLATE USED FOR PRODUCING PRINTING OR EMBOSSING PATTERN ON MATERIAL OR WEB OF MATERIAL SUCH AS PAPER, INVOLVES PRODUCING THE PLATE OUTER SURFACE BY A GENERATIVE MANUFACTURING METHOD | EP2886367A1\ WO2015091549A1 | BU HAOWEN (US)\ ROUSE RICHARD P (US)\ EKBOTE SHASHANK S (US) | ASM INT (NL) | B22F-003/105\ B29C-067/00\ B41C-001/00\ B44B-005/02\ B44C-003/02\ G03F-007/00 |
| 5435 | 3D printing | LINEAR DIGITAL LIGHT PROCESSING (DLP) MICRO-PROJECTOR HAS LIGHT OPTICAL SYSTEM THAT IS INCLUDED WITH BEAM SHAPING COMPONENT AND LIGHT BEAM GUIDING COMPONENT, AND SECONDARY FACE THAT IS PLATED WITH REFLECTION FILM | CN204270006U\ HK1199603A2 | ROZBICKI ROBERT (US)\ KAILASAM SRIDHAR (US)\ YU CHENTAO (US)\ HAYDEN DOUGLAS (US) | TEXAS INSTRUMENTS INC (US) | G03B-021/00\ G03B-021/20\ G03B-000/00 |
| 5436 | 3D printing | KIT USEFUL FOR DETECTING AUTOIMMUNE LIVER-RELATED AUTOANTIBODY PROFILE FOR DIAGNOSING AUTOIMMUNE HEPATITIS AND PRIMARY BILIARY CIRRHOSIS, COMPRISES FILM STRIP, ENZYME SOLUTION, AND SUBSTRATE AND ENZYME INCUBATION BUFFER SOLUTION | CN104459159A | BROWN KARL M (US)\ PIPITONE JOHN (US)\ MEHTA VINEET (US) | NOVELLUS SYSTEMS INC (US) | G01N-033/558\ G01N-033/576\ G01N-033/68 |
| 5437 | 3D printing | ILLUMINATION SYSTEM FOR PROJECTION DEVICE E.G. DIGITAL LIGHT PROCESSING (DLP) PROJECTOR, HAS WAVEBAND CONVERTING MODULE FROM WHICH LIGHT BEAM OF SPECIFIC WAVEBAND IS TRANSMITTED TO LIGHT CONSOLIDATING MODULE | US2015177599A1\ TW201525605A\ TW499858B1\ US9229301B2 | KARABACAK TANSEL (US)\ LU TOH-MING (US)\ BARTHEL JOHN ROBERT (US) | APPLIED MATERIALS INC (US) | F21K-099/00\ G03B-021/14\ G03B-021/20\ F21Y-101/02\ G03B-021/00 |
| 5438 | 3D printing | INTERCHANGEABLE CARTRIDGE FOR STEREO-LITHOGRAPHY MACHINE, HAS CONTAINER DELIMITED BY WALL AND PROVIDED WITH ACCESS OPENING, AND RESERVOIR COMPRISING TIGHT CONNECTION UNIT FOR CONNECTION TO EXTERNAL AIR FEEDING DEVICE | WO2015092717A1\ TW201524475A\ IT1421481B\ CA2927256A1\ KR2016072192A\ CN105793016A\ IN201627012894A\ SG11201603717A1\ US2016288421A1\ EP3083206A1 | XIANG QI (US) | RENSSELAER POLYTECH INST (US) | B29C-067/00\ A61C-013/14\ A61C-013/15\ A61C-013/16\ B33Y-030/00\ B33Y-050/02\ B33Y-040/00 |
| 5439 | 3D printing | COOLING DEVICE FOR USE IN E.G. PROJECTOR, HAS COOLING FAN SUPPORTED ON HOLDER THROUGH CUSHION CLAMPED IN BETWEEN PROTRUSION AND HOLDER | JP2015119021A | JUNKER KURT H (US)\ ENGBRECHT EDWARD R (US)\ EKERDT JOHN G (US)\ SUN YANG-MING (US) | GLOBALFOUNDRIES INC (KY) | G03B-021/00\ G03B-021/16\ H04N-005/74\ H05K-007/20 |
| 5440 | 3D printing | SEMICONDUCTOR MEMORY DEVICE E.G. NON-VOLATILE MEMORY DEVICES, HAS CHANNEL LAYER FORMED OF SILICON-GERMANIUM LAYER IN WHICH GERMANIUM CONCENTRATION OF PORTION IN CONTACT WITH TUNNEL INSULATING LAYER IS GREATER THAN THAT OF CENTER PORTION | US2015171106A1\ KR2015070819A\ CN104716142A | PAPA RAO SATYAVOLU S (US)\ GRUNOW STEPHAN (US)\ RUSSELL NOEL M (US) | SEMICONDUCTOR RES CORP (US) | H01L-027/115\ H01L-029/161\ H01L-021/8247\ H01L-029/36 |
| 5441 | 3D printing | NEW PRIMER SET USEFUL IN PCR AMPLIFICATION, OR REVERSE TRANSCRIPTION-PCR FOR AMPLIFICATION OF EXONS 1-4 OF SWINE LEUKOCYTE ANTIGEN 2 GENE, COMPRISING SPECIFIC BASE PAIR SEQUENCES | KR2015039595A | SCHLAF RUDIGER (US) | TEXAS INSTRUMENTS INC (US) | C12N-015/11\ C12Q-001/68 |
| 5442 | 3D printing | PRIMER SET USED IN GENOTYPING METHOD OF SWINE LEUKOCYTE ANTIGEN-1, COMPRISES GENOMIC PCR PRIMER, REVERSE TRANSCRIPTASE-PCR PRIMER AND SEQUENCING PRIMER | KR2015039157A | YU JENGYI (US) | UNIV SOUTH FLORIDA (US) | C12N-015/11\ C12Q-001/68 |
| 5443 | 3D printing | PRIMER SET USEFUL IN SWINE LEUKOCYTE ANTIGEN (SLA-3) GENOTYPING METHOD, COMPRISES GENOMIC PCR PRIMER HAVING SPECIFIC BASE PAIR SEQUENCE CAPABLE OF AMPLIFYING EXONS 2 AND 3 OF SLA-3 GENE | KR2015039158A | SOLAYAPPAN NARAYAN (US)\ PAZ DE ARAUJO CARLOS A (US)\ UCHIYAMA KIYOSHI (US) | CYPRESS SEMICONDUCTOR CORP (US) | C12Q-001/68 |
| 5444 | 3D printing | METHOD FOR FACILITATING QUALITY-OF-SERVICE CONFIGURATION IN E.G. WIRELESS OR POWERLINE COMMUNICATIONS NETWORKS, INVOLVES APPLYING QUALITY-OF-SERVICE CONFIGURATION PARAMETERS ACCORDING TO PARAMETERS FROM DISTRIBUTED INTELLIGENCE AGENT | US2015180800A1 | NA YUN-CHUNG (TW) | SYMETRIX CORP AND MATSUSHITA E (US) | H04L-012/721\ H04L-012/725\ H04L-012/931 |
| 5445 | 3D printing | PROJECTION APPARATUS OF PROJECTION-TYPE VIDEO DISPLAY APPARATUS, HAS ASPECT-RATIO CONVERSION OPTICAL ELEMENT THAT IS PROVIDED TO CONVERT ASPECT-RATIO OF IMAGE EMITTED FROM SECOND OPTICAL SYSTEM | JP2015114609A | WEI ZHANG (CN)\ QINGQING SUN US\ PENGFEI WANG US\ LIN CHEN US\ WEN YANG US | NAT UNIV TSINGHUA (TW) | G02B-013/18\ G02B-017/08\ G03B-021/00\ G03B-021/14\ H04N-005/74 |
| 5446 | 3D printing | MOBILE TERMINAL E.G. MOBILE PHONE HAS CONTROLLER THAT IS CONFIGURED TO CONTROL DISPLAY TO DISPLAY PRESCRIBED ONE OF STORED ONE IMAGE FRAGMENT ON PRESCRIBED REGION OF PREVIEW SCREEN | US2015172560A1\ KR2015068644A | TIAN WEIDONG (US)\ CHUANG MING-YEH (US)\ AGGARWAL RAJNI J (US) | UNIV FUDAN (CN)\ SUN QINGQING (CN)\ ZHANG WEI (CN)\ CHEN LIN (CN)\ YANG WEN (CN)\ WANG PENGFEI (CN) | G06K-009/00\ H04N-005/232\ H04N-005/247\ H04N-005/265\ G06F-003/048\ H04B-001/40 |
| 5447 | 3D printing | CULTURE MEDIUM USED FOR CULTURING NAVICULA SAPROPHILA FOR SYNTHESIZING HIGHLY UNSATURATED FATTY ACIDS, COMPRISES RICE STRAW, AMMONIUM NITRATE, SODIUM DIHYDROGEN PHOSPHATE, PROLINE, PYRUVATE, POTASSIUM CITRATE, GLYCINE BETAINE, AND MANNITOL | CN104388313A | Empty Field US | TEXAS INSTRUMENTS INC (US)\ TIAN WEIDONG (US)\ CHUANG MING-YEH (US)\ AGGARWAL RAJNI J (US) | C12N-001/12\ C12R-001/89 |
| 5448 | 3D printing | THREE-DIMENSIONAL PRINTING METHOD FOR FORMING THREE-DIMENSIONAL OBJECT ON BASE, INVOLVES PRINTING OBJECT HAVING OVERHANGING PORTION RELATIVE TO BASE, SUCH THAT SUPPORTING UNIT IS FILLED BETWEEN OVERHANGING PORTION AND BASE | US2015137423A1\ JP2015098165A\ TW201520076A\ CN104647753A\ JP5893092B2\ US9434109B2 | GANITZER PAUL (AT)\ RIEGER WALTER (AT)\ POELZL MARTIN (AT)\ HAEBERLEN OLIVER (AT) | LAVOIE ADRIEN R (US) | B29C-067/00\ B41M-003/06\ G06T-019/20\ B41J-027/00\ B33Y-010/00\ B33Y-050/00\ B29C-033/30\ B29C-033/58\ B29C-033/60\ B29C-041/02\ B29C-041/38 |
| 5449 | 3D printing | JOINT COMBINATION STRUCTURE FOR HIGH DEFINITION REAR PROJECTION OF DIGITAL LIGHT PROCESSING (DLP), HAS IMAGE SPLICING CONTROLLER AND TEMPERATURE CONTROL SYSTEM WHICH ARE ARRANGED IN FRAME BOX SET IN REAR SIDE OF PROJECTION SCREEN | CN204256356U | MAES JAN WILLEM (BE)\ WITTE HILDE D (BE)\ POMAREDE CHRISTOPHE (US) | INFINEON TECHNOLOGIES AG (DE) | G03B-021/54 |
| 5450 | 3D printing | PROJECTOR FOR DATA PROJECTOR APPARATUS OF DIGITAL LIGHT PROCESSING (DLP) SYSTEM, HAS SECOND SIGNAL GENERATION UNIT THAT GENERATES ELECTRICAL SIGNAL INPUT INTO LIGHT EMITTING ELEMENT BY SIGNAL WIDTH BASED ON SPACE OF ELECTRICAL SIGNAL | JP2015109659A | ROSA MICHEL A (AU)\ PEETERS ERIC (US) | MAES JAN W (BE)\ WITTE HILDE D (BE)\ POMAREDE CHRISTOPHE (US) | G03B-021/00\ G03B-021/14\ G09G-003/20\ G09G-003/34\ G09G-005/00\ H04N-009/31 |
| 5451 | 3D printing | WIDE-ANGLE PROJECTION OPTICAL SYSTEM FOR PROJECTION DISPLAY APPARATUS, HAS MANGIN MIRROR, WHICH HAS REFRACTING SURFACE AND REFLECTING SURFACE FOR REFRACTING LIGHT TWO TIMES AND REFLECTING LIGHT ONE TIME, WHICH PRODUCES ENLARGED REAL IMAGE | EP2881776A1\ JP2015108797A\ US2015160441A1\ TW201523026A\ TW497114B1\ US2016274344A1 | LEE CHUNG J (US)\ KUMAR ATUL (US)\ CHEN CHIEH (US)\ PIKOVSKY YURI (US) | PALO ALTO RES CT INC (US) | G02B-013/16\ G02B-013/22\ G02B-017/08\ G03B-021/28\ G02B-013/04\ G02B-027/18\ G02B-013/18\ G02B-027/00 |
| 5452 | 3D printing | MICRO ANGLE FORWARD/BACKWARD ROTATION OPTICAL SWITCH FOR VEHICLE, COMPRISES ROTATING UNIT, WHERE HOUSING IS PROVIDED WITH OPENING IN WHICH ONE SIDE IS OPENED, AND PREDETERMINED ACCOMMODATION SPACE IS PREPARED INSIDE OF HOUSING | KR1523296B1 | SANDHU GURTEJ S (US) | LEE CHUNG J (US)\ KUMAR ATUL (US)\ CHEN CHIEH (US)\ PIKOVSKY YURI (US) | B60Q-003/02\ B60R-016/02 |
| 5453 | 3D printing | SEMICONDUCTOR DEVICE, HAS FIRST TRANSISTORS SELF-ALIGNED TO ONE OF SECOND TRANSISTORS, WHERE SECOND TRANSISTORS ARE HORIZONTALLY ORIENTED TRANSISTORS, AND SECOND LAYER COMPRISING RESISTIVE-RAM CELLS | US2015123072A1 | SHUE SHAU-LIN (TW)\ WANG MEI-YUN (TW)\ CHANG CHIH-WEI (TW)\ LIN CHENG-TUNG (TW)\ WU CHII-MING (TW) | SANDHU GURTEJ S (US) | H01L-027/108\ H01L-027/24 |
| 5454 | 3D printing | POLYMERIZING OLEFIN COMPRISES SELECTING FIRST OLEFIN MONOMER AND ALPHA-OLEFIN COMONOMER, SELECTING CATALYST SYSTEMS COMPRISING PROCATALYST COMPRISING FIRST METAL, COCATALYST COMPRISING ALUMINUM, AND SELF-LIMITING AGENT AND POLYMERIZING | WO2015073221A1\ CN105705528A\ EP3068808A1 | NAM EUN SOO (KR)\ KIM HO YOUNG (KR)\ OH MYOUNG SOOK (KR)\ JUNG DONG YUN (KR)\ HONG SEON E (KR)\ CHO KYOUNG IK (KR) | WANG MEI-YUN (TW)\ CHANG CHIH-WEI (TW)\ WU CHII-MING (TW)\ LIN CHENG-TUNG (TW)\ SHUE SHAU-LIN (TW) | C08F-010/00\ C08F-004/649 |
| 5455 | 3D printing | METHOD FOR IDENTIFYING NETWORK TOPOLOGIES IN COMMUNICATION NETWORK, INVOLVES DETERMINING VALID COMBINATIONS OF NETWORK NODES, AND GENERATING PERFORMANCE MEASUREMENT COMMANDS FOR VALID COMBINATIONS OF NETWORK NODES USING PROCESSOR | US2015163108A1 | SAITO TSUYOSHI (JP) | KOREA ELECTRONICS TELECOMM (KR) | H04L-012/26 |
| 5456 | 3D printing | SYSTEM FOR PROVISIONING DYNAMIC SYSTEM LEVEL AGREEMENT IN LOCAL COMPUTING DEVICES WITHIN COMPUTING ENVIRONMENT, HAS SERVICE LEVEL AGREEMENT MODULE FOR AUTOMATICALLY DETERMINING SERVICE LEVEL AGREEMENT PROVISION FOR CUSTOMER | US2015156081A1 | YOU LU (US)\ LOPATIN SERGEY D (US)\ BESSER PAUL R (US)\ WANG PIN-CHIN CONNIE (US)\ MYERS ALLINE F (US)\ ROMERO JEREMIAS D (US)\ TRAN MINH Q (US) | TOSHIBA CORP (JP) | H04L-012/24 |
| 5457 | 3D printing | NON-TRANSITORY COMPUTER-READABLE STORAGE MEDIUM STORING INSTRUCTIONS FOR IMPLEMENTING POLICY MANAGEMENT FUNCTIONALITY, INCLUDES INSTRUCTIONS FOR APPLYING POLICY TO CLOUD SERVICE PLATFORM TASK | US2015156065A1 | YU JICK M (US) | ADVANCED MICRO DEVICES INC (US) | H04L-012/24\ H04L-012/26 |
| 5458 | 3D printing | METHOD FOR DYNAMIC SYSTEM LEVEL AGREEMENT PROVISIONING, INVOLVES EVALUATING UTILITY FUNCTIONS DEFINING SERVICE LEVEL VARIABLES, AND AUTOMATICALLY DETERMINING SERVICE LEVEL AGREEMENT PROVISION FOR CUSTOMER BASED ON UTILITY FUNCTIONS | US2015156080A1 | GRILL ALFRED (US)\ JAHNES CHRISTOPHER VINCENT (US)\ PATEL VISHNUBHAI VITTHALBHAI (US)\ PERRAUD LAURENT CLAUDE (FR) | INTEL CORP (US) | H04L-012/24\ H04L-029/08 |
| 5459 | 3D printing | METHOD FOR FACILITATING E.G. CONFIGURATION OF PATH FOR TRANSMISSION OF DATA OVER NETWORK, INVOLVES COMPARING REQUIREMENT ASSOCIATED WITH TRANSMITTING DATA AND COMPOSITE RESULT AND CONFIGURING NETWORK ELEMENT TO TRANSMIT DATA | US2015156082A1\ US9191282B2 | Empty Field US | IBM (US) | H04L-012/24\ G06F-015/16\ H04L-012/863 |
| 5460 | 3D printing | OPERATION ASSISTANCE SYSTEM HAS OPERATION SPECIFICATION UNIT THAT SPECIFIES OPERATION OF USER WITH RESPECT TO OPERATION TARGET IMAGE AND DISPLAYS POSITION OF OPERATION TARGET IMAGE IN IMAGE DISPLAY APPARATUS | JP2015103233A | CHOU JING-PEI (US)\ KAO CHIEN-TEH (US)\ LAI CHIUKIN (US)\ MOSELY RODERICK C (US)\ CHANG MEI (US) | TAIWAN SEMICONDUCTOR MFG (US) | G06F-003/01\ G06F-003/0346\ G06F-003/038\ G06F-003/0481 |
| 5461 | 3D printing | LIGHT MODULE FOR PROJECTION DEVICE, HAS SECOND OPTICAL PATH PROCEEDING FROM SECOND EXCITATION RADIATION SOURCE THAT COMPRISES SECOND DICHROIC MIRROR, SECOND FOCUSING DEVICE, IRRADIATION REGION, FIRST FOCUSING DEVICE AND DICHROIC MIRROR | DE102013224768A1\ US2015153636A1\ CN104698729A | Empty Field US | APPLIED MATERIALS INC (US) | G02B-005/08\ G03B-021/14\ G02B-001/10\ G03B-021/20\ G03B-021/28\ H01S-003/091\ G02B-027/00 |
| 5462 | 3D printing | RAPID PROTOTYPING AUTOMATIC PAVING MECHANISM USEFUL FOR SINTERING POWDER, COMPRISES A MOLDING CAVITY BODY, A MOLDING WORKBENCH, A POWER DEVICE, A MOVABLE BLADE, A MOVABLE PIPELINE AND A POWER PUMP | CN104439242A | LIU WEN-CHUN (TW)\ LAI CHIEN-HUNG (TW) | GOLDSTEIN AVERY N (US) | B22F-003/105\ B29C-067/00 |
| 5463 | 3D printing | COMPUTER PROGRAM PRODUCT FOR OPTIMIZING SERVICE POOLS SUPPORTING RESOURCE SHARING AND ENFORCING SERVICE LEVEL AGREEMENT (SLA), INCLUDES INSTRUCTION FOR ALLOCATING RESOURCE OCCUPIED BY GLOBAL STANDBY SERVICE INSTANCE FROM RESOURCE POOL | US2015149632A1 | CHAO TSAN-HUI (TW)\ HUNG TIAN-JIUE (TW)\ WU YA-WEN (TW)\ LI SHR-PING (TW)\ TZENG SHOU-YI (TW) | WALSIN ADVANCED ELECTRONICS (TW) | H04L-012/911 |
| 5464 | 3D printing | METHOD FOR MAKING THREE-DIMENSIONAL OBJECT FROM SOLIDIFIABLE MATERIAL, INVOLVES GENERATING SETS OF SOLIDIFICATION ENERGY SOURCE EVENT DATA BASED ON ACTIVE VOXELS, WHERE EACH SET OF SOURCE EVENT DATA CORRESPONDS TO SCAN LINE AND OBJECT LAYER | US2015145177A1\ WO2015080898A2\ WO2015080898A3\ EP3074879A2 | OMORI KAZUTOSHI US\ KITA KENTARO US\ MURATA SHUHEI (JP) | MACRONIX INT CO LTD (TW) | B29C-067/00\ G06F-017/00 |
| 5465 | 3D printing | LIGHT SOURCE SYSTEM FOR DISPLAY APPARATUS OF E.G. LCD PROJECTION DEVICE, HAS TOTAL REFLECTION SURFACES FORMED CORRESPONDING TO LIGHT INCIDENT ZONES, AND PROJECTED AREAS OF TOTAL SURFACES ON OUTPUT SURFACE ARE NOT OVERLAPPED WITH EACH OTHER | US2015146173A1\ TW201520601A | KONDO DAIYU US\ NIHEI MIZUHISA US\ SATO SHINTARO (JP) | RENESAS ELECTRONICS CORP (JP) | F21V-007/04\ G02B-027/22\ G03B-021/20\ G03B-021/60\ G02B-027/18 |
| 5466 | 3D printing | ERASE METHOD FOR NON-VOLATILE MEMORY DEVICE, INVOLVES ERASING MEMORY CELLS WHILE SEQUENTIALLY LOWERING POTENTIALS OF WORD LINES, WHEN POTENTIALS OF WORD LINES CONNECTED TO MEMORY CELLS ARE INCREASED | US2015146487A1\ KR2015059499A\ US9064584B2 | TSUBOTA TOSHIKI US\ NAGAHATA HIROYUKI US\ SUENAGA TOMOKO US | FUJITSU LTD (JP) | G11C-016/14\ G11C-011/56\ G11C-016/04\ G11C-016/16\ G11C-005/02\ G11C-005/06 |
| 5467 | 3D printing | METHOD FOR TREATING SUBSTRATE USING OPTICAL PROJECTION, INVOLVES VARYING AMPLITUDE OF LIGHT PROJECTED ON SURFACE OF SUBSTRATE BY LOCATION BASED ON SUBSTRATE SIGNATURE | US2015147827A1 | FURUKAWA RYOICHI (JP)\ SAIKAWA KENJI (JP)\ FUJIWARA TAKESHI US\ KUNITOMO MASATO (JP) | KYUSHU INST OF TECHNOLOGY US\ SANYU KOGYO KK US\ SUENAGA TOMOKO US | H01L-021/027\ H01L-021/66 |
| 5468 | 3D printing | MOBILE TERMINAL, HAS CONTROLLER ADAPTED TO RECEIVE MULTI-TAPPING INPUT ON OBJECT AND CONTROL WIRELESS COMMUNICATION UNIT TO TRANSMIT PRESCRIBED REQUEST MESSAGE AMONG STORED REQUEST MESSAGES TO COUNTERPART TERMINAL IN RESPONSE TO INPUT | US2015148083A1\ EP2879025A2\ KR2015062035A\ KR2015070837A\ EP2879025A3\ CN104683562A | SUZUKI NORIO (JP)\ TANIGUCHI YASUHIRO (JP)\ YADORI SHOJI (JP) | RENESAS TECH CORP (JP) | H04M-001/725\ H04W-004/12\ G06F-003/041\ G06F-003/0484\ H04M-001/2745\ H04B-001/40\ G06F-003/048 |
| 5469 | 3D printing | COMPUTER-IMPLEMENTED METHOD FOR MODIFYING CLOUD APPLICATION SERVICE LEVELS BASED UPON ANALYSIS OF DOCUMENTS FOR ORGANIZATIONAL IT SYSTEMS, INVOLVES PRIORITIZING DISPATCHING TECHNIQUES BASED ON SCORES OF CLOUD SERVICES | US2015142786A1\ US9300547B2 | TANABE YOSHIKAZU (JP)\ YAMAMOTO NAOKI (JP)\ ASANO ISAMU (JP)\ SAITO MASAYOSHI (JP)\ YOSHIDA MAKOTO (JP)\ KASHU NOBUYOSHI US | RENESAS TECH CORP (JP) | G06F-017/30\ H04L-012/24\ H04L-029/08 |
| 5470 | 3D printing | COMPUTER PROGRAM PRODUCT FOR SELECTING HOST FOR APPLICATION PATTERN COMPONENT, HAS COMPUTER READABLE STORAGE MEDIUM COMPRISING PROGRAM CODE TO SELECT HOST IN RESPONSE TO HOST BEING SUFFICIENT FOR SERVICE LEVEL AGREEMENT REQUIREMENT | US2015142950A1\ CN104660669A | TANABE YOSHIKAZU (JP)\ KASHU NOBUYOSHI US\ SAKAI SATORU (JP) | HITACHI LTD (JP) | H04L-012/24\ H04L-029/08 |
| 5471 | 3D printing | METHOD FOR MANAGING VIRTUAL MACHINES E.G. UNDER-PERFORMING VIRTUAL MACHINE, IN DISTRIBUTED COMPUTING ENVIRONMENT, INVOLVES RE-SUBSCRIBING NEW-SET OF VIRTUAL MACHINES FROM POOL OF VIRTUAL MACHINES OF DISTRIBUTED COMPUTING ENVIRONMENT | US2015143363A1\ US9262190B2 | NAITO KATSUYUKI US | RENESAS TECH CORP (JP) | G06F-009/455\ G06F-009/50 |
| 5472 | 3D printing | METHOD FOR PARENT-CHILD SCHEDULING AND MANAGEMENT OF PARENT-CHILD AND RECURSIVE TREE COMPUTATIONAL WORKLOAD, INVOLVES RECEIVING RESUME REQUEST FROM PARENT TASK, AND RETURNING RESOURCE YIELDED BY PARENT TASK TO PARENT TASK BY SCHEDULER | US2015143381A1\ US9417918B2 | ITO FUMITOSHI (JP)\ KANBARA SHIRO US\ HINOUE TATSUYA (JP) | TOSHIBA CORP (JP) | G06F-009/50\ G06F-009/48 |
| 5473 | 3D printing | NON-VOLATILE SEMICONDUCTOR DEVICE I.E. THREE-DIMENSIONALLY LAMINATED NOT-AND FLASH MEMORY, HAS CONTROL CIRCUIT FOR APPLYING FIRST VOLTAGE TO ONE OF GATE LINES DURING ERASE OPERATION, WHERE FIRST VOLTAGE IS HIGHER THAN SECOND VOLTAGE | US2015138883A1 | YAMAMOTO NAOKI (JP)\ SUZUKI NORIO (JP)\ UCHIYAMA HIROYUKI (JP)\ NISHITANI EISUKE (JP)\ KIMURA SHIN ICHIRO (JP)\ HOZAWA KAZUYUKI (JP) | RENESAS TECH CORP (JP) | G11C-016/04\ G11C-016/14\ G11C-016/34 |
| 5474 | 3D printing | METHOD FOR OFFERING CLOUD-BASED HARDWARE ENCRYPTION MODULE (HSM) SERVICES, INVOLVES ROUTING CRYPTOGRAPHY REQUESTS BETWEEN CLOUD-BASED APPLICATION AND VHSM BASED ON HANDLE FOR SOURCE VIRTUAL HSM AND HANDLE FOR CLOUD-BASED APPLICATION | US2015134953A1\ WO2015069460A1 | MORITA SHINYA US\ OZAKI TAKASHI US | SUZUKI NORIO (JP)\ YAMAMOTO NAOKI (JP)\ UCHIYAMA HIROYUKI (JP)\ NISHITANI EISUKE (JP)\ KIMURA SHIN ICHIRO (JP)\ HOZAWA KAZUYUKI (JP) | H04L-029/06\ H04L-009/08\ G06F-021/57\ G06F-021/60 |
| 5475 | 3D printing | COMPUTER-IMPLEMENTED METHOD FOR OPERATING MULTI-TENANCY STORAGE NODE USING STATIONARY DEVICE E.G. DESKTOP COMPUTER, INVOLVES INSTALLING PARTITIONS OF ADDITIONAL DATABASE AND ADDITIONAL SERVICE LEVEL AGREEMENT (SLA) ON STORAGE NODE | US2015127611A1\ US9135259B2 | KAMINAGA DODAI US\ SUWAUCHI NAOKATSU US\ ENDO FUMIAKI (JP) | HITACHI INT ELECTRIC INC US | G06F-017/30 |
| 5476 | 3D printing | STEREO VIEWER AND/OR STEREO VIEWFINDER USED FOR ELECTRONIC DEVICE, HAS PAIR OF LENS WHICH IS ATTACHED TO INNER WALL FACE OF HOOD THROUGH HINGE PORTION, AND MAGNIFIER EXPANDED IN PARALLEL IS PROVIDED WITH RESPECT TO ELECTRONIC DISPLAY | JP2015089111A\ JP5749842B2 | NOZAKI SHINJI US\ UCHIDA KAZUO US\ MORIZAKI HIROSHI US\ KAWASAKI TAKU US\ IBUKIYAMA MASAHIRO US | RENESAS TECH CORP (JP) | H04N-013/02\ G02B-027/22\ G03B-035/18 |
| 5477 | 3D printing | METHOD FOR OPTIMIZING COMPUTATION OF METRIC IN INFORMATION TECHNOLOGY ENABLED FRAMEWORK, INVOLVES EXECUTING QUERY ON MATERIALIZED VIEW TO COMPUTE METRIC, WHERE EXECUTION OF QUERY FACILITATES OPTIMIZATION OF COMPUTATION OF METRIC | EP2871586A1\ US2015134593A1\ IN201303541I3 | HANASHIMA TAKEO US\ MIYA HIRONOBU US\ NAKAO FUSAYUKI US\ MITSUTA TADAO US | DENKI KAGAKU KOGYO KK US | G06F-017/30\ G06Q-010/06\ H04L-012/26\ G06Q-010/00 |
| 5478 | 3D printing | METHOD FOR OPERATING E.G. GAS DISCHARGE LAMP FOR PROJECTION APPARATUS, INVOLVES DEVIATING REPETITIONS IN PREDETERMINED TIME INTERVAL OF COMMUTATIONS BY DIRECT CURRENT (DC) PHASE AND PROVIDING TIME INTERVAL AS MAXIMUM OF PRESET VALUE | DE102013223138A1\ US2015131063A1\ CN104640333A | JIROKU HIROAKI US | HITACHI INT ELECTRIC INC US | H05B-041/292\ H05B-041/36\ G03B-021/20\ H05B-041/16\ H05B-041/28 |
| 5479 | 3D printing | PROCESSING METHOD USED BY RESOURCE MANAGER IN COMMUNICATIONS NETWORK CONNECTED TO AT LEAST TWO HOSTS FOR ENABLING OF RESOURCE COMPONENT ALLOCATION DETERMINES METRIC OF RESOURCE COMPONENT FOR PERFORMANCE MONITORING OF APPLICATION AT HOSTS | WO2015060753A1\ CN105830392A\ EP3061209A1\ US2016261519A1 | JIROKU HIROAKI US | SEIKO EPSON CORP (JP) | H04L-012/26\ H04L-012/24\ H04L-012/911 |
| 5480 | 3D printing | THREE DIMENSIONAL STACKED NONVOLATILE SEMICONDUCTOR MEMORY E.G. BIT COST SCALABLE (BICS)-NOT-AND (NAND) FLASH MEMORY HAS CONTACT PLUG WHOSE LOWER END IS CONNECTED TO SOURCE DIFFUSION LAYER AND UPPER END IS CONNECTED TO SOURCE LINE | US2015115350A1\ US9437610B2 | MIZUNO KANEKAZU US\ INAGAKI TOMOYOSHI US | SEIKO EPSON CORP (JP) | H01L-023/535\ H01L-027/115\ H01L-029/423\ H01L-029/792 |
| 5481 | 3D printing | DEVICE FOR MAKING THREE-DIMENSIONAL PRODUCT, HAS CONTAINER WHICH IS PROVIDED WITH ADJUSTING PLATFORM ALONG HORIZONTAL PLANE, AND POINT LIGHT SOURCE WHICH IS CONNECTED WITH MACHINE ARM AND SUSPENDED PLATFORM | CN204196263U | MUKAI MIKIO US | HITACHI INT ELECTRIC INC US | B29C-067/00 |
| 5482 | 3D printing | LCD DEVICE I.E. IN-PLANE SWITCHING ACTIVE-MATRIX-TYPE LCD DEVICE, FOR ELECTRONIC DEVICES, HAS PIXEL ELECTRODE PARTS CONTAINING STANDARD ELECTRODE, WHERE PHASE OF PICTURE ELEMENT VOLTAGE AND REFERENCE VOLTAGE ARE INVERTED FOR FRAMES | JP2015087562A | URANO YUICHI US | SONY CORP (JP) | G02F-001/133\ G09G-003/20\ G09G-003/36 |
| 5483 | 3D printing | DIGITAL LIGHT PROCESSING TYPE PROJECTION SYSTEM INSTALLED IN SHOW WINDOW OF STORE, HAS SWITCHING CONTROL UNIT TO CONTROL SWITCHING OF EMISSION DIRECTION OF DIRECTION CHANGE UNIT BASED ON ACQUIRED ENVIRONMENT INFORMATION | JP2015084073A | VAUTIER MANON US | FUJI ELECTRIC CO LTD (JP) | G03B-021/00\ G03B-021/28\ H04N-005/74 |
| 5484 | 3D printing | THREE-DIMENSIONAL PRINTING METHOD FOR PREPARATION OF BASE METAL E.G. COPPER, INVOLVES STARTING PRINTING PROCESS BY THREE-DIMENSIONAL PRINTER, FOR PRINTING WORK PIECE OUTLINE, FOR FORMING BASE METAL BLANK DRIED IN HEATING FURNACE | CN104399986A | ZEQIANG YAO (CN)\ YAN YU (CN)\ BO YU (CN) | AIR LIQUIDE (FR) | B22F-003/22 |
| 5485 | 3D printing | HANDHELD DIGITAL LIGHT PROCESSING MULTIMEDIA PROJECTION SYSTEM, HAS FORMAT CONVERSION UNIT CONNECTED WITH VIDEO INPUT INTERFACE TO CONVERT VIDEO DATA INTO SIGNAL, AND INPUT MODULE CONNECTED WITH CENTER PROCESSOR | CN104394342A | ZHIXUAN PAN (CN)\ JUYING CHEN (CN) | SHANGHAI HUAHONG NEC ELECT CO (CN) | G08C-023/04\ H04N-005/74\ H04N-009/31\ H04W-088/02 |
| 5486 | 3D printing | LIGHT SOURCE DEVICE FOR E.G. LIQUID-CRYSTAL PROJECTOR, HAS CONTROL CIRCUIT WHICH DETERMINES LIGHT QUANTITY TARGET SIGNAL, SO THAT RATIO WITH DIFFERENT OBJECT OF WAVELENGTH BAND IS SET TO PREDETERMINED VALUE | WO2015056522A1\ JP2015099768A\ JP5811248B2\ US2016255313A1 | WEI ZHANG (CN)\ PEIXIN QIAN (CN)\ RONGHUA LIU (CN)\ HUIWANG LIN (CN)\ ZHIHONG LIU (CN)\ CHANGCHUN CHEN (CN) | SHANGHAI HUAHONG NEC ELECT CO (CN) | G03B-021/14\ G09G-003/20\ G09G-003/34\ H05B-037/02\ G03B-021/00\ H01L-033/00\ H01S-005/0683\ H01S-005/042\ H01S-005/0687\ H04N-009/31 |
| 5487 | 3D printing | METHOD FOR SOFTWARE DEFINED NETWORK (SDN) AWARE DATA REPLICATION, INVOLVES ESTABLISHING DATA REPLICATION AWARENESS OF SDN CONTROLLER AND SENDING REPLICATION DATA BETWEEN EACH OF SOURCE FILERS AND DESTINATION FILERS BY LOGICAL NETWORKS | WO2015057960A1\ US2015112933A1\ US9330156B2\ CN105659563A\ EP3058704A1 | QIAN PEIXIN LIANG (CN) | UNIV TSINGHUA (CN) | H04L-012/24\ H04L-029/08\ G06F-017/30\ G06F-017/00 |
| 5488 | 3D printing | STEREO LITHOGRAPHY MULTIMATERIAL SYSTEM FOR PRODUCING MACRO AND MICRO THREE-DIMENSIONAL MODELS, HAS MAGNET POSITIONS OPTICAL LIGHT, WHERE MASKS ARE GENERATED AND EXCESS MATERIAL IS REMOVED | PT107096A1 | YANG DEREN XI (CN) | UNIV TSINGHUA (CN) | B29C-067/00\ G06T-017/00 |
| 5489 | 3D printing | NEW ORGANIC FUNCTIONAL STRUCTURAL UNIT-CONTAINING COMPOUND FOR PREPARING MIXTURE FOR COMPOSITION USED AS PAINT OR PRINTING INK FOR MANUFACTURING ELECTRONIC DEVICE | CN104277064A | MIAO BINGYOU (CN) | UNIV ZHEJIANG (CN) | C07F-005/05\ C09K-011/06\ H01L-051/00\ H01L-051/54 |
| 5490 | 3D printing | METHOD FOR OPTIMIZING ALLOCATION OF CONFIGURATION ELEMENTS IN SERVICE ENGAGEMENT, INVOLVES OPTIMIZING MODEL TO OBTAIN OPTIMAL ALLOCATION OF CONFIGURATION ELEMENTS FOR SUBSET BY ALLOCATING ANOTHER SUBSET OF CONFIGURATION ELEMENTS | US2015106143A1 | JIA CHENG US\ RENCHENG WANG US\ LINHONG JI US\ WEIFENG LIU US\ CHUNCAI WANG US\ JIA LIN US\ YUCHUN SUN US\ DAOXIN HAO US | SHANGHAI IC R & D CT CO LTD (CN) | G06Q-010/06\ G06Q-030/02\ H04L-012/24\ H04L-012/911 |
| 5491 | 3D printing | METHOD FOR SELECTING OPTIMAL POLICY TO BE IMPLEMENTED IN PRODUCTION SUPPORT ENGAGEMENT, INVOLVES APPLYING CONSTRAINTS AND OBJECTIVES ON SET OF RELEVANT POLICIES SIMULATED USING OPTIMIZATION MODEL | US2015106165A1\ EP2863345A1 | QUAN WANG (CN)\ XINXIN LI US | UNIV TSINGHUA (CN) | G06Q-010/06 |
| 5492 | 3D printing | MOBILE WIRELESS SYNCHRONOUS TRANSMISSION PROJECTOR HAS POWER SUPPLY MANAGEMENT MODULE WHICH IS CONNECTED WITH WIRELESS FIDELITY MODULE, SCREEN, DECODER, DRIVE PLATE AND DIGITAL LIGHT PROCESSING MODULE GROUP | CN204190856U | ZHIQIANG XIAO (CN)\ XIANGDONG GAO (CN)\ JIANWEI WU US\ GENSHEN HONG US | SHANGHAI INST MICROSYS & INF (CN) | G06F-003/14\ H04N-005/74 |
| 5493 | 3D printing | STEREOLITHOGRAPHY DEVICE FOR MANUFACTURING THREE-DIMENSIONAL SHAPED ELEMENT, HAS STRUCTURAL UNIT WHICH IS INSERTED TOGETHER INTO FRAME ASSEMBLY, AND IS RELEASABLY SECURED BY FASTENING DEVICE AND REMOVED BY FRAME ASSEMBLY | DE102014215213A1\ WO2016016443A1 | TIE LI US\ WENRONG WANG US\ YUXIU ZHOU US\ YUELIN WANG US | CHINA ELECTRONICS TECHNOLOGY GROUP CORP NO 58 RES INST US | B29C-067/00 |
| 5494 | 3D printing | HOT MELTING PAD ULTRASONIC WELDING-TYPE POLYMER PROTECTING PLATE FOR USE CONSTRUCTION OF TUNNEL, HAS PLATE BODY PRESSED ON SHELLS, WHERE INNER PART OF EACH SHELL IS ARRANGED ON DISTRIBUTE AREA AND FLAT AREA THAT IS LOCATED ON MELTING PART | CN204175334U | HUALUN CHEN (CN)\ YU CHEN (CN)\ TAO XIONG (CN)\ XIAO LUO (CN)\ XIONGBIN CHEN (CN) | SHANGHAI INST MICROSYS & INF (CN) | E21F-016/02 |
| 5495 | 3D printing | FUNGICIDAL COMPOSITION, E.G. USED FOR TREATING DISEASES CAUSED BY PHYTOPATHOGENIC FUNGI AND BACTERIA ON USEFUL PLANTS OR ON THEIR PROPAGATION MATERIAL, INCLUDES CARBOXAMIDE COMPOUND, DITHIINO-CONTAINING COMPOUND AND FENPYRAZAMINE | WO2015049168A1\ CN105611836A\ EP3051945A1\ IN201617009205A\ US2016235064A1\ JP2016531883W | DEJIN GAO US\ YONGBIAN KUANG US\ YUE PAN US\ POREN TANG US\ YU TANG US\ ZHE YU US\ LIJIE ZHANG US\ RU HUANG (CN) | SHANGHAI HUAHONG NEC ELECT CO (CN) | A01N-043/26\ A01N-043/34\ A01N-043/42\ A01N-043/50\ A01N-043/54\ A01N-043/78\ A01N-043/90\ A01P-003/00\ A01N-025/02\ A01N-025/12\ A01N-025/14\ A01N-037/10\ A01N-037/18\ A01N-043/16\ A01N-043/56\ A01N-043/60\ A01N-043/80\ A01N-047/12\ A01N-037/36\ A01N-037/38\ A01N-037/44\ A01N-043/10\ A01N-043/836\ A01N-047/16\ A01N-047/24 |
| 5496 | 3D printing | SLA PLANTING BODY BONE ASSOCIATIVE DEVICE STERILIZING METHOD, INVOLVES FIXING PRESSURE STEAM STERILIZER WITH INNER CONTAINER TO PERFORM STERILIZING PROCESS, AND CONNECTING PRESSURE STEAM STERILIZER WITH SEAL CONTAINER DURING STERILIZATION | CN104353092A | HONGJUN LIU US\ YIZHU FU US\ GUOXING SHENG US\ DIANLI WANG US | UNIV BEIJING (CN) | A61L-002/07 |
| 5497 | 3D printing | OPTICAL ENGINE MODULE FOR USE IN DIGITAL LIGHT PROCESSING PROJECTION APPARATUS, HAS PHOSPHOR WHEEL AND HEAT-DISSIPATING PART PARTIALLY OVERLAPPED WITH EACH OTHER ALONG AIRFLOW EXITING DIRECTION | US2015092164A1\ CN104516178A\ CN104516178B\ US9442351B2 | WEI ZHANG (CN)\ SHIJIN DING US\ KEJIA QIAN US | NO 55 INST OF CHINA ELECTRONIC US | F21V-029/00\ F21V-009/08\ G03B-021/16\ H05K-007/20\ G03B-021/20\ G03B-033/08 |
| 5498 | 3D printing | METHOD FOR GENERATING THREE-DIMENSIONAL TOOLPATH INSTRUCTIONS FOR E.G. CONTINUOUS CORE REINFORCED FILAMENT-EXTRUSION PRINTER, INVOLVES GENERATING ANISOTROPIC FILL TOOL PATH FOR CONTROLLING PRINTER TO DEPOSIT ANISOTROPIC FILL MATERIAL | WO2015042422A1 | HELIN JI US | UNIV FUDAN (CN) | B29C-067/00 |
| 5499 | 3D printing | ADAPTIVE COLOR PROJECTION DEVICE FOR USE IN HOME, HAS PROJECTION UNIT MOUNTED WITH SHOOTING DEVICE THAT IS CONNECTED WITH LIGHT ANALYSIS MODULE, CONTROL MODULE, DIGITAL LIGHT PROCESSING DEVICE AND DIGITAL IMAGING MODULE | CN104361848A | HONGDONG LI (CN)\ XIANYI LU (CN)\ DONG LU (CN)\ SHAOHENG CHENG (CN)\ GUANGTIAN ZOU (CN) | HELIN JI US | G03B-021/00\ G09G-003/00 |
| 5500 | 3D printing | METHOD FOR PROCESSING UNBIND STEREO LITHOGRAPHY MODEL WITH BOUNDARY, INVOLVES PROCESSING TWO-DIMENSIONAL POLYGON TO REDUCE EMPTY TRAVEL, AND OUTPUTTING UNIVERSAL FILE BY THREE-DIMENSIONAL PRINTER | CN104331555A | WEI ZHANG (CN)\ ZHIQIANG XIAO (CN)\ BO ZHANG (CN)\ XIAORONG LUO (CN)\ HAO DENG (CN)\ HUANMEI GAO (CN)\ ZHENGCAI CHEN (CN)\ YUANGANG WANG (CN)\ TIANFEI LEI (CN)\ ZHAOJI LI (CN) | UNIV JILIN (CN) | G06F-017/50 |
| 5501 | 3D printing | INTERNALLY-FED HIGH-SPEED ROTARY PRINTING SYSTEM FOR PRINTING FLUID ONTO SURFACE OF WEB SUBSTRATE, HAS FLUID CHANNEL FOR PROVIDING FLUID COMMUNICATION OF FLUID FROM POSITION TO WEB SUBSTRATE CONTACTING SURFACE LOCATED AT DISTANCE | US2015090138A1\ WO2015048061A1\ US9085130B2\ CA2925744A1\ EP3049250A1\ JP2016532588W | WEIXIA OUYANG (CN)\ XINGLONG GUO (CN)\ ZONGSHENG LAI (CN)\ YONGHUA ZHANG (CN)\ CHAO WANG (CN)\ LEI LIU (CN) | UNIV ELECTRONIC SCIENCE & TECH (CN) | B41F-009/00\ B41F-009/06\ B41F-031/22\ B41J-002/315\ B41F-013/11\ B41F-031/26\ B41F-033/10 |
| 5502 | 3D printing | MICRO-REFLECTOR ARRAY ADJUSTABLE DIGITAL LIGHT PROCESSING POWER DISTRIBUTOR, HAS MICRO-REFLECTOR ARRAY FOR DEFLECTING LIGHT TO INPUT PORT THROUGH INCIDENT LIGHT SPOT, WHERE ARRAY IS UTILIZED FOR ADJUSTING LIGHT POWER TO OUTPUT PORT | CN104345390A | TAIJIANG CHEN (CN)\ YU LIU (CN) | UNIV EAST CHINA NORMAL (CN) | G02B-026/08\ G02B-006/28 |
| 5503 | 3D printing | SLA BASED VIRTUAL RESOURCE PRE-ALLOCATION METHOD INVOLVES PRE-ALLOCATING VIRTUAL RESOURCE THROUGH VIRTUAL CONTROL MACHINES, TO ESTABLISH MAPPING BETWEEN TENANT AND VIRTUAL MACHINES, AND ACHIEVE DYNAMIC ALLOCATION OF RESOURCES TO TENANT | CN104331332A | LINHONG YANG (CN)\ LIANG CHEN (CN) | SEMICONDUCTOR MFG INT SHANGHAI (CN) | G06F-009/455\ G06F-009/50 |
| 5504 | 3D printing | THREE-DIMENSIONAL PRODUCT MANUFACTURING METHOD, INVOLVES INJECTING UV RESIN INTO CONTAINER, LOCATING ADJUSTING PRINT PLATFORM WITH CONTAINER AND ARRANGED ON HORIZONTAL PLANE, AND CONNECTING INJECTION DEVICE WITH CONTAINER | CN104325643A | HONG LIU (CN)\ YI SHI (CN) | SEMICONDUCTOR MFG INT SHANGHAI (CN) | B29C-067/00 |
| 5505 | 3D printing | METHOD FOR ALLOCATING E.G. PROCESSING RESOURCE BETWEEN WEB SERVICES IN COMPOSITE SERVICE, INVOLVES ALLOCATING SET OF RESOURCES FROM PUBLISHER WEB SERVICE TO SUBSCRIBER WEB SERVICE ACCORDING TO RESOURCE ALLOCATION AND IDENTIFIER | US2015081914A1 | YINTANG YANG (CN)\ ZHENYU WU (CN)\ JIAYOU WANG (CN)\ JUNXING FU (CN)\ YUEJIN LI (CN) | USTC UNIV SCIENCE TECH CN (CN) | H04L-012/851\ H04L-012/911 |
| 5506 | 3D printing | INDOOR UNIT FOR AIR CONDITIONER, HAS PRINTED CIRCUIT BOARD ENCLOSED WITH SURROUNDING WALL BOARD OF ELECTRICAL-EQUIPMENT BOX MAIN BODY, AND SHUTTER BOARD FOR CLOSING SLIT ON ELECTRICAL-EQUIPMENT BOX COVER | JP2015059735A | YINTANG YANG (CN)\ ZHENYU WU (CN)\ JIAYOU WANG (CN)\ YUEJIN LI (CN) | UNIV XIDIAN (CN) | F24F-013/20 |
| 5507 | 3D printing | METHOD FOR CAPTURING IMAGE BY E.G. VISIBLE LIGHT CAMERA FOR INPUT AND PROJECTING CONTENT, INVOLVES CAPTURING IMAGE BY IMAGE CAPTURE DEVICE WHEN CAPTURE FRAME IS PROJECTED, AND PERFORMING COMPUTER VISION FUNCTION BASED ON CAPTURED IMAGE | US2015085155A1\ WO2015048067A1\ CN105637529A\ EP3049996A1\ IN201627005469A | ZHIQIANG XIAO (CN)\ JING XU (CN)\ XIANGDONG GAO (CN)\ LI JUN (CN) | UNIV XIDIAN (CN) | G06T-019/00\ H04N-005/232\ H04N-005/74\ G06F-003/14\ G06K-009/00\ G06K-009/20\ G06K-009/78\ H04N-009/00 |
| 5508 | 3D printing | COLOR WHEEL MODULE FOR USE IN DIGITAL LIGHT PROCESSING PROJECTION APPARATUS TO FILTER ILLUMINATION BEAM EMITTED BY LIGHT SOURCE, HAS COLOR WHEEL FOR DRIVING AIRFLOW THAT FLOWS THROUGH COLOR WHEEL AND IS DISCHARGED FROM AIRFLOW OUTLET | US2015085260A1\ CN104516079A\ US9405177B2 | ZHENMAO LIU (CN)\ SHENG TAO (CN)\ FANGKUI SUN (CN)\ FENG DING (CN)\ KAIGUO LI (CN)\ HUI ZHAO (CN)\ YAN JIN (CN) | WUXI ZHONGWEI MICROCHIPS CO LT (CN) | G02B-026/00\ G03B-021/16\ G02B-007/00\ G03B-021/00\ G03B-021/14\ H04N-009/31\ H05K-007/20 |
| 5509 | 3D printing | SYSTEM FOR E.G. DISCOVERING SERVICE LEVEL AGREEMENT INFORMATION BETWEEN SERVICE PROVIDER DOMAINS, HAS DATABASE FOR STORING AGREEMENT INFORMATION, AND SERVICE LEVEL AGREEMENT MANAGER FOR MONITORING SERVICES ESTABLISHED THROUGH DOMAIN | US2015089057A1 | WENZHONG SHEN (CN)\ XINYI CHEN (CN)\ HONG CHEN (CN) | HEILONGJIANG BADA UNIVERSAL SE (CN) | H04L-012/24 |
| 5510 | 3D printing | METHOD FOR MANAGING MULTI-LEVEL SERVICE LEVEL AGREEMENTS (SLA) IN CLOUD-BASED NETWORKS, INVOLVES ADJUSTING RESOURCES USED WITHIN CLOUD NETWORK FROM FIRST TO SECOND COMMITMENT LEVEL IN RESPONSE TO DETECTING TRIGGERING EVENT | US2015081917A1\ US9438484B2 | CHEN JIA (CN)\ WANG RENCHENG (CN)\ JI LINHONG (CN)\ LIU WEIFENG (CN)\ WANG CHUNCAI (CN)\ LIN JIA (CN)\ SUN YUCHUN (CN)\ HAO DAOXIN (CN) | UNIV SHANGHAI JIAOTONG (CN) | G06F-009/455\ H04L-012/24\ H04L-012/911\ G06F-015/173\ G06F-009/50\ G06Q-010/06 |
| 5511 | 3D printing | METHOD FOR MONITORING WORKER PERFORMING VIDEO SURVEILLANCE TASK BEING PRESENTED ON MOBILE PHONE, INVOLVES DETERMINING INATTENTION INSTANCE OF WORKER, AND COMMUNICATING INATTENTION INSTANCE OF WORKER TO CROWDSOURCING PLATFORM | US2015086072A1 | NANBA KENJI (JP)\ TAGO MASAMOTO (JP) | UNIV TSINGHUA (CN)\ CHEN JIA (CN)\ WANG RENCHENG (CN)\ JI LINHONG (CN)\ LIU WEIFENG (CN)\ WANG CHUNCAI (CN)\ LIN JIA (CN)\ SUN YUCHUN (CN)\ HAO DAOXIN (CN) | G06K-009/00\ G06T-007/20\ H04N-007/18 |
| 5512 | 3D printing | NON-TRANSITORY COMPUTER-READABLE MEDIA FOR SCHEDULING JOB TO RECOMPUTE METRICS BASED ON ELASTIC SERVICE LEVEL AGREEMENT, HAS SET OF INSTRUCTIONS FOR ADJUSTING SERVICE LEVEL AGREEMENT IN RESPONSE TO COMPUTATIONAL CAPACITY AVAILABILITY | US8990383B1 | SHANKER SUNIL (US)\ LANG CHI-I (US) | NEC CORP (JP)\ NANBA KENJI (JP)\ TAGO MASAMOTO (JP) | G06F-015/173 |
| 5513 | 3D printing | METHOD FOR DETERMINING PRE-DEFINABLE WAVE FORM OF LAMP CURRENT FOR OPERATING DISCHARGE LAMP OF PROJECTION ARRANGEMENT E.G. DIGITAL LIGHT PROCESSING PROJECTOR, INVOLVES PROVIDING FIRST WAVE FORM AS PRE-DEFINABLE WAVE FORM AT BALLAST | US2015077721A1\ EP2852261A1\ CN104470166A\ US9436072B2 | DALTON JEREMIE JAMES (US)\ GOPINATH SANJAY (US)\ BLACKBURN JASON M (US)\ DREWERY JOHN STEPHEN (US) | NOVELLUS SYSTEMS INC (US) | G03B-021/20\ H04N-009/31\ H05B-041/38\ G03B-021/00\ H05B-041/28\ G02B-026/00\ H05B-037/02\ H05B-041/288\ H05B-041/392 |
| 5514 | 3D printing | APPARATUS FOR ENHANCING CLOUD SERVICE THROUGH USE OF CLOUD SERVICES BROKERAGE (CSB) PLATFORM, HAS CSB PLATFORM THAT CONFIGURES PROCURED CLOUD RESOURCES TO EMBODY ONE OR MORE VIRTUAL MACHINES | US2015073960A1 | NGO MINH VAN (US)\ ZHAO LARRY (US)\ BESSER PAUL RAYMOND (US) | NOVELLUS SYSTEMS INC (US) | G06Q-040/00\ H04L-012/26 |
| 5515 | 3D printing | PREPARATION OF MULTIFUNCTIONAL POROUS COMPOSITE IMPLANT STRUCTURE USEFUL IN SANDBLASTED AND ACID-ETCHED SLA OSTEOGENIC ACTIVITY AND DENTAL IMPLANT, INVOLVES ETCHING PROCESSED IMPLANT INCLUDING PROJECTIONS USING OXALIC ACID SOLUTION | CN104233318A | PLAT MARINA V (US)\ GHANDEHARI KOUROS (US) | ADVANCED MICRO DEVICES INC (US) | A61L-027/06\ A61L-027/56\ C22F-001/18\ C23F-001/26\ C23F-001/38\ C23F-017/00 |
| 5516 | 3D printing | METHOD FOR PROVISIONING COMMUNICATION SERVICE USING PROXY SERVER IN CLOUD, INVOLVES DETECTING ATTACHMENT ASSOCIATED WITH MESSAGE AND ROUTING MESSAGE TO RECEIVER IN ACCORDANCE TO ZERO-RATED SERVICE LEVEL AGREEMENT (SLA) | US2015067066A1 | PRICE DAVID T (US)\ KALPATHY-CRAMER JAYASHREE (US) | ADVANCED MICRO DEVICES INC (US) | H04L-012/58 |
| 5517 | 3D printing | SYSTEM FOR ADVERTISEMENT OF SERVICE LEVEL AGREEMENT (SLA) ATTRIBUTES OF SERVICE AND TEST CAPABILITY OF ENDPOINT DEVICE, HAS FIRST ENDPOINT DEVICE TO TRANSMIT ENHANCED PROTOCOL DATA UNIT TO SECOND ENDPOINT DEVICE | US2015067117A1 | TAKAHASHI TAKAHIKO (JP)\ ITOH FUMIKAZU (JP)\ SHIMASE AKIRA (JP)\ HONGO MIKIO (JP)\ HARAICHI SATOSHI (JP)\ YAMAGUCHI HIROSHI (JP) | LSI LOGIC CORP (US) | H04L-012/24 |
| 5518 | 3D printing | METHOD FOR IMPROVING EFFICIENCY AND EFFECTIVENESS IN MULTIPLE INTERFACE NETWORK USED IN E.G. HOMES, INVOLVES TRANSMITTING CONTROL MESSAGE TO RECEIVER DEVICE, AND TRANSMITTING DATA TO RECEIVER DEVICE OVER LOW-POWER CHANNEL BY SENDER DEVICE | US2015071295A1 | KAILASAM SRIDHAR K (US)\ POWELL RONALD A (US)\ SETTLES E DERRYCK (US) | HITACHI LTD (JP) | H04J-003/06\ H04L-012/707\ H04L-012/725 |
| 5519 | 3D printing | METHOD FOR RAPID MOLDING OF STEREOLITHOGRAPHY (SLA) THREE-DIMENSIONAL PRINTER, HAS IRON PLATE THAT IS ARRANGED ON BOTTOM OF CABINET AND CONNECTED WITH POWER MACHINE, AND BRACKET THAT IS ARRANGED WITH VERTICAL SLIDING RAIL | CN204076846U | DANEK MICHAL (US)\ D COUTO GERARD C (US)\ TKACH GEORGE (US)\ WOITGE MICHAEL (DE) | NOVELLUS SYSTEMS INC (US) | B29C-067/00 |
| 5520 | 3D printing | FAST MOLDING STEREOLITHOGRAPHY (SLA) THREE DIMENSIONAL PRINTER HAS RESIN GROOVE WHOSE PORTION IS SET WITH HANGING PLATFORM, BRACKET THAT IS SET WITH VERTICAL SLIDING RAIL, AND HANGING PLATFORM THAT IS SLID UP AND DOWN ALONG SLIDE RAIL | CN104228068A | SETIADI DADI (US)\ LIU HONGYUE (US) | NOVELLUS SYSTEMS INC (US) | B29C-067/00 |
| 5521 | 3D printing | ELECTRONIC DEVICE E.G. MOBILE PHONE FOR CONTROLLING MICRODISPLAY, HAS DISPLAY CONTROLLER WITH CONTROL LOGIC TO DISPLAY ANIMATION ON MICRODISPLAY BY COMMENCING DISPLAY OF ANIMATION WHEN SIGNALS REPRESENTING START COMMAND ARE DETECTED | WO2015027316A1\ US2015062130A1 | ZENG XIANGLIN (US)\ CHANG-CHIEN PATTY (US) | SEAGATE TECHNOLOGY LLC (US) | G09G-005/36\ G09G-005/395\ H04W-088/02\ G06T-013/00 |
| 5522 | 3D printing | METHOD FOR HANDLING CONNECTIONS OF NETWORK E.G. LOCAL AREA NETWORK, INVOLVES PROCESSING PACKET ACCORDING TO SERVICE-LEVEL AGREEMENT TO OBTAIN PROCESSED PACKET, AND TRANSMITTING PROCESSED PACKET TO NETWORK BY NETWORK PROTOCOL STACK | US2015067182A1 | LAVOIE ADRIEN R (US)\ PLOMBON JOHN J (US)\ DOMINGUEZ JUAN E (US)\ HAN JOSEPH H (US)\ SIMKA HARSONO S (US) | NORTHROP GRUMMAN SPACE & MSN (US) | H04L-012/24\ H04L-029/08 |
| 5523 | 3D printing | MOBILE TERMINAL E.G. MOBILE PHONE, FOR USE IN AUDIO RECORDING APPLICATIONS, HAS CONTROLLER DISPLAYING VISUAL INDICATOR ON SYNTHESIZED IMAGE, WHERE INDICATOR IS DISPLAYED AT REGION OF SYNTHESIZED IMAGE THAT CORRESPONDS TO ANOTHER IMAGE | US2015062288A1\ KR2015027655A | ANTONELLI GEORGE A (US)\ SRIRAM MANDAYAM A (US) | HAN JOSEPH H (US)\ SIMKA HARSONO S (US)\ LAVOIE ADRIEN R (US)\ PLOMBON JOHN J (US)\ DOMINGUEZ JUAN E (US) | H04N-005/232\ H04N-005/265\ G06F-003/14\ H04B-001/40 |
| 5524 | 3D printing | METHOD FOR PROACTIVELY ESTABLISHING ASSOCIATIONS OF NETSURFING END-SYSTEM TO SHARED INTEGRATED ACCESS DEVICE OR NETWORK APPLICATION SERVICE, INVOLVES ESTABLISHING ASSOCIATION OF ACCESS DEVICE AND SERVICE WITH NETSURFING END-SYSTEM | US2015063309A1 | VANHAELEMEERSCH SERGE (BE)\ MEYNEN HERMAN (BE)\ DEMBOWSKI PHILIP DAVID (US) | ANTONELLI GEORGE A (US)\ SRIRAM MANDAYAM A (US) | H04W-036/00 |
| 5525 | 3D printing | METHOD FOR HANDLING CONNECTION REQUESTS IN COMPUTING SYSTEM, INVOLVES RECEIVING INCOMING DATA FROM NETWORK, PROCESSING PROTOCOL STACK ON OPERATING SYSTEM, AND PROVIDING PROCESSED INCOMING DATA TO APPLICATION | US2015063372A1 | CHEN YI-NAN (TW)\ WU WEN-CHIEH (TW)\ WU CHUN-YI (TW) | INTERUNIVERSTAIR MICROELEKTRON (US) | H04L-012/24 |
| 5526 | 3D printing | DEVICE FOR GENERATING AND STORING ENFORCEMENT POLICY INFORMATION ASSOCIATED WITH CUSTOMER OF CLOUD COMPUTING SERVICE, HAS TRAFFIC THAT IS MANAGED BASED ON COMPARING AGGREGATED PERFORMANCE METRIC AND MAXIMUM PERFORMANCE METRIC | US2015058474A1 | LO YUNG-TSUN (TW)\ TSAI RAYMOND (TW)\ HO WEN-YU (TW) | Empty Field US | H04L-012/851 |
| 5527 | 3D printing | POLYMERIZING ETHYLENE-BASED POLYMERS COMPRISES POLYMERIZING THE ETHYLENE AND OPTIONALLY ALPHA-OLEFIN COMONOMERS IN THE PRESENCE OF THE CATALYST SYSTEMS COMPRISING SELF-LIMITING AGENTS VIA A SLURRY OR GAS-PHASE POLYMERIZATION PROCESS | WO2015026731A1\ EP3036263A1\ CN105980422A | CHANG HUI-LIN (TW)\ KUO CHENG-TZU (TW)\ LIN CHAO-HSUN (TW)\ HSU CHIH-MING (TW) | Empty Field US | C08F-010/02\ C08F-002/40\ C08F-002/42\ C08F-210/16\ C08F-004/649\ C08F-004/65 |
| 5528 | 3D printing | 3D IC BASED SYSTEM, HAS LAYER COMPRISING THROUGH LAYER VIA WITH SPECIFIC RANGE DIAMETER, AND ONE OF SET OF TRANSISTORS FOR FORMING TWO STABLE STATE MEMORY CELLS COMPRISING BACK-BIAS REGION | US2015054090A1 | WU WEN-CHIEH (TW)\ WU CHUN-YI (TW) | Empty Field US | H01L-027/115\ H01L-029/78 |
| 5529 | 3D printing | SLA-08 LACTOBACILLUS PARTICLE ENCAPSULATION STRUCTURE FOR PROMOTING BREAST GROWTH TO ACHIEVE BREAST ENHANCEMENT | TW487080U | CHUANG PING (TW)\ CHEN LI-SEN (TW)\ HSU CHIEN-YU (TW) | NANYA TECHNOLOGY CORP (TW) | A61K-009/20 |
| 5530 | 3D printing | MOTOR E.G. DIGITAL LIGHT PROCESSING PROJECTOR DRIVE MOTOR, HAS MAGNETIC POSITION DETECTING MECHANISM THAT DETECTS ROTARY POSITION OF ROTOR HOLDER BY DETECTING MULTIPLE ALTERNATING MAGNETIC POLE SWITCHING UNITS BETWEEN MAGNETIC POLE | CN104242592A | WU CHING-YUAN (TW) | SPEED TECH CORP (TW) | H02K-029/08 |
| 5531 | 3D printing | COMPUTER-IMPLEMENTED METHOD FOR GENERATING SERVICE LEVEL AGREEMENT (SLA) REPORTS USING REUSABLE DATA MODULES IN COMPUTER SYSTEM, INVOLVES GENERATING SERVICE LEVEL AGREEMENT REPORT IN ACCORDANCE WITH REQUIREMENTS USING SPECIFIC RESULT | AU2015200213A1 | WU WEN-FA (TW)\ OU GENG-LIANG (TW)\ JOU CHANG-BIN (TW) | SILICON BASED TECH CORP (TW) | G06F-017/30\ G06Q-010/00 |
| 5532 | 3D printing | COLOR WHEEL UNIT FOR PROJECTOR, HAS CONTROL UNIT WHICH CONTROLS LIGHT EMISSION OF LIGHT EMITTING COMPONENT BASED ON CHANGE OF LIGHT RECEPTION INTENSITY CORRESPONDING TO BRIGHTNESS OF RECEIVED REFLECTED LIGHT | JP2015036776A | YU SANG SOO (KR)\ SHIN DONG IL (KR)\ PAEK SUNG KWAN (KR) | NAT SCIENCE COUNCIL (TW) | G03B-021/00\ G03B-021/14\ H04N-009/31 |
| 5533 | 3D printing | DISPLAY APPARATUS E.G. DIGITAL LIGHT PROCESSING (DLP) PROJECTOR, HAS CONTROLLER WHICH IS CONFIGURED TO TRANSMIT EXTENDED DISPLAY IDENTIFICATION DATA UNITS (EDIDS) CORRESPONDING TO ONE OF CONNECTORS TO EXTERNAL VIDEO SIGNAL SOURCE | US2015036054A1 | KIM JEONG JU (KR) | SAMSUNG TECHWIN CO LTD (KR) | G09G-005/00 |
| 5534 | 3D printing | METHOD FOR DYNAMICALLY MAPPING TRAFFIC TO DIFFERENT ROUTING TECHNOLOGIES, INVOLVES CREATING SECOND ROUTING TOPOLOGY, AND SECOND ROUTING TOPOLOGY MAP RECEIVER DEVICE THAT RECEIVE TRANSMISSION FROM SENDER DEVICE USING SECOND CHANNEL TYPE | US2015043384A1\ WO2015020718A1\ US9172613B2\ EP3031182A1\ JP2016527757W | Empty Field US | SAMSUNG ELECTRONICS CO LTD (KR) | H04L-012/24\ H04L-012/751\ H04W-040/12\ H04W-040/24\ H04L-012/26\ H04W-040/30\ H04W-088/06\ H04W-040/00 |
| 5535 | 3D printing | FORMING METHOD OF OBJECT, BY POSITIONING FOIL DRUMS, WELDING FOIL, CUTTING WELDED FOIL, RETRACTING FOIL DRUMS, CONSOLIDATING PORTION OF WELDED FOIL TO OBJECT, LOWERING BUILD PLATFORM, AND REPEATING PROCESSES UNTIL OBJECT IS COMPLETE | WO2015020954A1 | Empty Field US | SUMITOMO ELECTRIC INDUSTRIES (JP)\ INT SUPERCONDUCTIVITY TECH (JP) | B23K-020/10 |
| 5536 | 3D printing | METHOD FOR EXECUTING ENTERPRISE PROCESS ASSOCIATED WITH ONE OF E.G. TRAFFIC POLICE MONITORING IN ONE OF OFFLINE MODE AND ONLINE MODE THROUGH E.G. LAPTOP, INVOLVES SYNCHRONIZING LOCAL DATABASE STORING REPORT WITH CENTRALIZED DATABASE | US2015046393A1\ EP2835774A1\ SG201307215A1\ SG201307215B | SAYAMA HIROKAZU US | RENESAS ELECTRONICS CORP (JP) | G06F-017/30\ G06Q-010/10 |
| 5537 | 3D printing | METHOD FOR SERVICE LEVEL AGREEMENT ANALYSIS, INVOLVES RECEIVING SELECTION OF SERVICE LEVEL AGREEMENT TO BE ANALYZED, RECEIVING SELECTION OF GROUPING OPTION FOR SERVICE LEVEL AGREEMENT CLAUSE AND RECEIVING SELECTION OF SUB-GROUPING OPTION | WO2015012868A1\ CN105247506A\ US2016132798A1\ EP3025248A1 | HANAOKA KENICHI US\ OTSUKA MASASHI US | RENESAS ELECTRONICS CORP (JP) | G06F-017/00\ G06F-003/14\ G06Q-010/06\ G06Q-050/18 |
| 5538 | 3D printing | DIGITAL LIGHT PROCESSING (DLP) PROJECTOR HAS MAGNETIC SENSING COMPONENT TO SENSE HIGH MAGNETIC FIELD INTENSITY OF MAGNETIZING PORTION TO POSITION OF FILTER UNIT ACCORDING TO OUTPUT SIGNAL OF FILTER UNIT | CN104199248A | HAO CHAN US\ JOSHI POORAN CHANDRA US\ VOUTSAS APOSTOLOS T US | RENESAS TECH CORP (JP) | G03B-021/00\ G03B-021/14 |
| 5539 | 3D printing | METHOD FOR DETERMINING MECHANICAL PROPERTIES OF BRITTLE MATERIALS AT TENSION | RU2540460C1\ RU2013131669A | KONO MASAYUKI US\ NISHIDA TATSUO US\ NAKANISHI TOSHIO US | SHARP KK (JP) | G01N-001/28\ G01N-003/08 |
| 5540 | 3D printing | METHOD FOR PERFORMING ROUTE OPTIMIZATION OF SERVICES PROVIDED BY MULTIPLE SERVICE PROVIDERS FOR COMBINED LINKS, INVOLVES DISTRIBUTING DATA AMONG LINKS IN MANNER THAT MINIMIZES COST OF SERVICES PROVIDED BY COMPUTER NETWORK SERVICE PROVIDER | US2015046374A1 | HUANG KUN-YUNG (TW)\ LUH SONG-PING (TW) | TOKYO ELECTRON LTD (JP) | H04L-012/14 |
| 5541 | 3D printing | METHOD FOR MANAGING QUALITY OF SERVICE OF TENANT'S NETWORK IN VIRTUALIZED COMPUTING ENVIRONMENT, INVOLVES SETTING COMMUNICATION TRANSMISSION RATE TO RATE IN RESPONSE TO DETERMINE THAT AGGREGATED TRANSMISSION RATE IS ABOVE THRESHOLD | US2015040121A1 | OUCHI YOSHIFUMI US\ YAMAZUMI SAIGO US\ ARAI NOBORU US\ HIRATA KEIJI US | FUPO ELECTRONICS CORP (CN) | G06F-009/455 |
| 5542 | 3D printing | DIGITAL LIGHT PROCESSING LIGHT CURING DEMOULDING SINKING WORKING TABLE FOR THREE DIMENSIONAL PRINTER, COMPRISES WORKING TABLE THAT IS SET WITH UPPER PORTION FOR FIXING TRANSPARENT RESIN GROOVE OF MOUNTING BOTTOM FRAME | CN203994734U | MIYAMOTO YUUKI US\ HONDA TOMOO US | RENESAS TECH CORP (JP) | B29C-067/00 |
| 5543 | 3D printing | SYSTEM FOR ACHIEVING BUSINESS CONTINUITY AND APPLICATION CONTINUITY, HAS CONTINUITY GRID THAT IS PAIRED WITH CONTINUITY PEER GRIDS LOCATED IN SAME OR DIFFERENT CLOUD INFRASTRUCTURES TO SATISFY CONTINUITY REQUIREMENTS OF CONTINUITY GRID | US2015032817A1\ IN201303359I4 | TSUBOTA TOSHIKI US\ NAGAHATA HIROYUKI US\ SUENAGA TOMOKO US | RENESAS TECH CORP (JP) | H04L-029/08\ G06F-000/00 |
| 5544 | 3D printing | THREE-DIMENSIONAL STRUCTURE NONVOLATILE SEMICONDUCTOR MEMORY DEVICE, HAS SELECT TRANSISTOR INCLUDING GATE ELECTRODE EXTENDING IN STACKING DIRECTION AND GATE INSULATOR FILM PROVIDED BETWEEN GATE ELECTRODE AND LINE PORTIONS | US2015035037A1\ JP2015028966A | KAWAGUCHI KATSUO US | KYUSHU INST OF TECHNOLOGY US\ SANYU KOGYO KK US\ SUENAGA TOMOKO US | H01L-027/115\ H01L-021/336\ H01L-021/8247\ H01L-029/788\ H01L-029/792 |
| 5545 | 3D printing | METHOD FOR MEASURING SERVICE-SPECIFIC PACKET DATA UNIT LOSS FOR PSEUDOWIRES USED FOR SERVICE PROVIDER NETWORKS, INVOLVES SENDING PSEUDOWIRE PACKET LOSS DATA WITH DIFFERENCE VALUE FOR PSEUDOWIRE FROM LOCAL ROUTER TO MANAGEMENT ENTITY | US8948032B1 | SAKAGAMI MAMORU US | IBIDEN CO LTD US | H04L-012/26 |
| 5546 | 3D printing | ILLUMINATION OPTICAL SYSTEM FOR ELECTRONIC APPARATUS SUCH AS PROJECTOR, HAS REFLECTIVE AREA, FLUORESCENT SUBSTANCE REGION AND LIGHT TUNNEL WHICH ARE ARRANGED, SO THAT LIGHTS OF BOTH WAVELENGTH ZONES ARE TRANSMITTED THROUGH LIGHT TUNNEL | JP2015011302A | KAI NAOKI US | FUJITSU LTD (JP) | G03B-021/00\ G03B-021/14\ H04N-009/31 |
| 5547 | 3D printing | COMPUTER IMPLEMENTED METHOD FOR CONTROLLING HEAT DISSIPATION WITHIN DATA CENTER, INVOLVES PERFORMING SCHEDULE MODIFICATION FOR CANDIDATE PROCESSING JOBS BASED ON SERVICE LEVEL AGREEMENT (SLA) ANALYSIS | US2015026695A1\ US9219657B2 | TANABE YOSHIKAZU (JP)\ KASHU NOBUYOSHI US\ SAKAI SATORU (JP) | TOSHIBA CORP (JP) | G06F-009/50\ H04L-012/24\ G06F-001/32\ G06F-009/48\ G06F-009/52 |
| 5548 | 3D printing | PROJECTOR I.E. DIGITAL LIGHT PROCESSING, HAS CONTROL PART FOR CONTROLLING BRIGHTNESS OF LIGHT SOURCE DURING EMISSION PERIOD OF LIGHT AND PREDETERMINED WAVELENGTH RANGE OF LIGHT SOURCE WHEN BRIGHTNESS IS SMALLER THAN THRESHOLD VALUE | JP2015022273A | SUZUKI NORIO (JP)\ TAKAMATSU AKIRA (JP)\ SHIMODA MAKI (JP)\ IKEDA SHUJI (JP)\ ICHIZOE HIROYUKI (JP)\ OGISHIMA ATSUSHI (JP)\ OKAMOTO KEIJI (JP)\ HORIBE SHINICHI (JP)\ KOJIMA MASAYUKI (JP)\ WATANABE KOZO (JP)\ YOSHIDA YASUKO (JP)\ ISHITSUKA NORIO (JP) | RENESAS TECH CORP (JP) | G03B-021/00\ G03B-021/14\ H04N-009/31 |
| 5549 | 3D printing | SYSTEM FOR DISPLAYING ADVERTISEMENT AROUND PERIPHERY OF DISPLAY E.G. ORGANIC LED DISPLAY, HAS SHEET MATERIAL ON WHICH ADVERTISEMENT IS PRINTED AND VISIBLE THROUGH FRONT HOUSING WHEN FRONT HOUSING IS MOUNTED TO REAR HOUSING | US2015027016A1 | MASUDA AIKA US\ YUASA KAZUHIRO US | HITACHI LTD (JP)\ HITACHI ULSI SYS CO LTD (JP)\ SUZUKI NORIO (JP)\ ICHIZOE HIROYUKI (JP)\ KOJIMA MASAYUKI (JP)\ OKAMOTO KEIJI (JP)\ HORIBE SHINICHI (JP)\ WATANABE KOZO (JP)\ YOSHIDA YASUKO (JP)\ IKEDA SHUJI (JP)\ TAKAMATSU AKIRA (JP)\ ISHITSUKA NORIO (JP)\ OGISHIMA ATSUSHI (JP)\ SHIMODA MAKI (JP) | A47G-001/06\ G09F-023/00\ G09F-007/04 |
| 5550 | 3D printing | THREE-DIMENSIONAL PRINTER FOR ADDITIVE MANUFACTURING OF ELEMENT, HAS CONTROLLER TO EXECUTE INSTRUCTION THAT CAUSE FILAMENT DRIVE TO HOLD UNATTACHED TERMINAL END OF COMPOSITE FILAMENT IN COLD FEED ZONE BETWEEN DRIVE AND IRONING TIP | WO2015009938A1\ EP3022046A1\ CN105579220A\ US9370896B2\ JP2016531020W | HOSHIKAWA MAKI US\ HIRAI KENTARO US\ TANAKA EIJI US | HITACHI INT ELECTRIC INC US | B29C-067/00\ B29C-031/04\ B29C-065/40\ B29C-070/16\ B29C-070/40\ B29C-070/42\ B29C-070/52\ B29K-025/00\ B29K-063/00\ B29K-071/00\ B29K-077/00\ B29K-079/00\ B33Y-010/00\ B33Y-030/00\ B33Y-050/02 |
| 5551 | 3D printing | LIGHT SOURCE DEVICE FOR PROJECTOR, HAS DRIVE CIRCUIT THAT IS FEEDBACK-CONTROLLED SO THAT DIFFERENCE BETWEEN DEFINED TARGET AND COMPREHENSIVE HUE INSTRUCTION VALUES BECOMES SMALL WITH RESPECT TO R,G AND B COLOR OUTPUT LUMINOUS FLUXES | WO2015008829A1\ JP2015022137A\ JP5880496B2\ US2016131968A1 | SUMIKAWA MASAHITO US | MITSUI CHEMICALS INC US | G03B-021/00\ G03B-021/14\ G09G-003/20\ G09G-003/34\ G09G-003/36\ F21S-002/00\ H04N-009/31\ G03B-021/20\ H01S-005/0683 |
| 5552 | 3D printing | SOLAR LIGHT ASSEMBLY FOR COORDINATING SOLAR POWERED LIGHTING WITH GRID POWERED LIGHTING, HAS GENERALLY PARABOLIC REFLECTOR THAT IS SECURED TO MOUNT AND LIGHT CONVEYING UNIT IS CONNECTED BETWEEN FOCUS OF REFLECTOR AND LIGHT SENSING UNIT | US2015023049A1\ US9091404B2 | TAKEMURA YASUHIKO (JP)\ YAMAZAKI SHUNPEI (JP)\ UOJI HIDEKI US\ MASE AKIRA (JP) | SHARP KK (JP) | F21S-009/03\ F21V-007/06\ F21V-008/00\ F21V-023/04\ H05B-037/00 |
| 5553 | 3D printing | METHOD FOR MANAGING ALLOCATION OF FREQUENCY BANDS TO TRANSMIT HIGH FREQUENCIES IN COMMUNICATION NETWORK, INVOLVES ALLOCATING FREQUENCIES TO USERS, SO THAT CONFORMITY OF SPECTRUM RESULTING FROM SIMULTANEOUS USE OF FREQUENCIES IS MANAGED | EP2830342A1\ FR3009152A1\ US2015029960A1\ CA2857750A1\ SG10201404381A1\ FR3009152B1 | TANABE YOSHIKAZU (JP)\ KASHU NOBUYOSHI US\ SAKAI SATORU (JP) | SEMICONDUCTOR ENERGY LAB (JP) | H04L-005/00\ H04W-016/10\ H04W-016/14\ H04W-072/04\ H04W-028/16\ H04W-028/26 |
| 5554 | 3D printing | SOLAR ENERGY CONCENTRATOR FOR SPACE POWER WITH CORDS, FIBERS, OR WIRES STRENGTHENING STRETCHED LENS, HAS STRENGTHENING ELEMENT THAT IS MECHANICALLY ATTACHED TO REFRACTIVE OPTICAL ELEMENT TO RESIST PORTION OF TENSION FORCE | US2015022909A1 | OGASAWARA MAKOTO (JP)\ KANBARA SHIRO US | RENESAS TECH CORP (JP) | G02B-019/00\ G02B-007/02 |
| 5555 | 3D printing | METHOD FOR AUTOMATICALLY FOCUSING DIGITAL LIGHT PROCESSING PICO PROJECTOR IN PROJECTION SYSTEM FOR E.G. DIGITAL VIDEO CAMERA, INVOLVES COMPUTING DEPTH MAP FROM CAPTURED IMAGE, AND ADJUSTING FOCUS OF PROJECTOR BASED ON COMPUTED DEPTH MAP | US2015022697A1 | GOTO TOSHIHIRO US | RENESAS TECH CORP (JP) | G02B-026/08\ H04N-005/222\ H04N-005/232\ H04N-005/262\ H04N-005/74\ H04N-009/31 |
| 5556 | 3D printing | METHOD FOR PROBING NETWORK, INVOLVES INSTRUCTING MORE PARTICULAR NODES TO DESTINATION NODES ACCORDING TO PROBING PROFILES BASED ON INFORMATION THAT IS DESIRED FROM NODES OF COMPUTER NETWORK | US2015023186A1\ WO2015009667A1 | SAKASHITA MASAKI US | RENESAS TECH CORP (JP) | H04L-012/26\ H04L-012/24\ H04L-012/717\ H04L-012/721\ H04W-024/06 |
| 5557 | 3D printing | NON-TRANSITORY COMPUTER-READABLE STORAGE MEDIUM FOR E.G. CREATING DATA CENTERS TO SUPPLY SERVICES, HAS SET OF INSTRUCTIONS FOR ASSESSING CAPACITY PARAMETER OF CLOUD SERVICE OFFERINGS TO CHARACTERIZE ABILITY TO FULFILL RESOURCE REQUIREMENTS | US2015026349A1 | NAGAMURA HIROYUKI US | RENESAS TECH CORP (JP) | H04L-012/24\ H04L-029/08 |
| 5558 | 3D printing | OPTICAL TRANSMISSION SYSTEM E.G. FINANCIAL TRANSACTION SYSTEM FOR TRANSMITTING LARGE CAPACITY OF INFORMATION, DETERMINES ADJUSTMENT AMOUNT FOR OPTICAL TRANSMISSION DEVICE SO THAT CALCULATED TRANSMISSION QUALITY SATISFY THRESHOLD VALUE | US2015016818A1\ JP2015019337A\ US9419741B2 | HIROI MASAYUKI (JP) | RENESAS TECH CORP (JP)\ RENESAS NTHN JP SEMICONDUCTOR US | H04B-010/079\ H04J-014/02\ H04B-010/294\ H04L-012/26\ H04L-012/70 |
| 5559 | 3D printing | GENOMIC PCR BASED PRIMER, HAS PRIMER BODY FOR AMPLIFYING AND SEQUENCING PIG LEUKOCYTE ANTIGEN SLA-DQA ALLELE | KR2014134980A\ KR1522836B1 | ABE DAISUKE US | NEC CORP (US) | C12N-015/11\ C12Q-001/68 |
| 5560 | 3D printing | IMAGE PROJECTION APPARATUS I.E. REFLECTIVE LIQUID CRYSTAL PROJECTOR, FOR USE IN IMAGE DISPLAY SYSTEM TO PROJECT IMAGE ON SCREEN, HAS CORRECTION UNIT FOR CORRECTING BRIGHTNESS OF IMAGE PROJECTED ON PROJECTION SURFACE | US2015015470A1\ JP2015018051A | SHEPARD JOSEPH (US)\ DUTARTRE DIDIER (FR)\ BREIL NICOLAS (FR) | SEIKO EPSON CORP (JP) | G09G-003/20\ G09G-003/34\ G03B-021/00\ G03B-021/14\ H04N-005/74 |
| 5561 | 3D printing | ROBOTICALLY CONTROLLED E.G. CRT PROJECTOR/ELECTRONIC DISPLAY FOR USE IN STADIUM FOR E.G. REPLAYING SPORTING EVENT, HAS ELECTRONIC VIDEO PROJECTORS MOUNTED WITH MOVEABLE SUPPORT, WHERE PROJECTORS ARE MOVABLE IN THREE-DIMENSIONS | US2015015781A1 | EVERSMANN BJOERN-OLIVER (DE)\ HACKENSCHMIED PETER (DE)\ KUHN HARALD (DE)\ STRASSBURG MATTHIAS (DE) | IBM (US)\ ST MICROELECTRONICS SA (FR) | H04N-009/31 |
| 5562 | 3D printing | METHOD FOR GENERATING NON-UNIFORM STRIPE SEGMENT, INVOLVES OBTAINING SEGMENTED RELATION BETWEEN CERTAIN POINTS OF HORIZONTAL PIXEL COORDINATE OF STRIPE IMAGE TO PERFORM PHASE CONVERSION OF GREY SCALE OF PIXEL | CN104154879A | HUANG KUN-YUNG (TW)\ LUH SONG-PING (TW) | SIEMENS AG (DE) | G01B-011/25 |
| 5563 | 3D printing | DIGITAL LIGHT PROCESSING (DLP) PICO PROJECTOR HAS LIGHTING OPTICAL SYSTEM THAT IS PROVIDED WITH BEAM-SHAPING UNIT, WEDGE-SHAPED OPTICAL COMPONENT, AND LIGHT BEAM GUIDE UNIT | CN104155835A\ HK1199501A0\ CN104155835B\ WO2016023281A1\ US2016295181A1 | TONGQING LIU US\ HONG QIN US | FUPO ELECTRONICS CORP (TW) | G03B-021/14\ G03B-021/20\ G03B-000/00\ G03B-021/00\ G02B-001/11\ G02B-017/00\ G02B-017/08\ G02B-027/09\ G02B-027/14\ G02B-027/30\ H04N-009/31 |
| 5564 | 3D printing | PHOTOSENSITIVE RESIN MATERIAL USED FOR DIGITAL LIGHT PROCESSING 3D PRINTER INCLUDES PREPOLYMER, EPOXY RESIN, DILUENT, PHOTOSENSITIZER, DEFOAMER, CATIONIC PHOTOINITIATOR, POLYMERIZATION INHIBITOR, CATALYST, TOUGHENER AND CROSSLINKING AGENT | CN104151526A | GANG ZHENG US | WUXI SENCOCH SEMICONDUCTOR CO LTD US | C08G-059/17\ C08L-063/10\ C09D-163/10\ C09D-007/12 |
| 5565 | 3D printing | METHOD FOR WELDING METAL PORTION FOR MOLDING METAL POWDER-BASED SUPPORT STRUCTURE, USED IN INDUSTRIAL APPLICATION, INVOLVES PERFORMING SLICING OF STEREO-LITHOGRAPHY FORMAT TO OBTAIN LAYERED SURFACING MOLDING PATH TRAJECTORY DATA | CN104148776A | GENGHUI GAO (CN) | KAIFENG POWER SUPPLY CO HENAN US | B23K-037/00\ B23K-009/04\ B23K-009/133\ B23K-009/16 |
| 5566 | 3D printing | MULTI-TENANT PROPERTY BASED ISOLATION FRAME VIRTUALIZATION METHOD, INVOLVES DETERMINING APPLICATION ACCESS CONTROL BY USING BOUNDARY OF TENANT, DETERMINING DYNAMIC ALLOCATION OF VIRTUAL RESOURCE, AND OBTAINING TOTAL RESOURCE UTILIZATION | CN104142864A | YU WEIBIN LIAO (CN) | FUJIAN FUSHUN SEMICONDUCTOR MA (CN) | G06F-009/455\ G06F-009/50 |
| 5567 | 3D printing | METHOD FOR MAKING E.G. RUNNER FOR CASTING METAL SYSTEM, INVOLVES CREATING CASTING CONDUIT USING ADDITIVE PROCESS, WHERE SHAPE FOR INTERNAL STRUCTURE OF CONDUIT BODY IS PROVIDED BY COMPUTER AIDED DESIGN FILE, WHICH DIRECTS ADDITIVE PROCESS | GB2515773A | YU WEIBIN LIAO (CN) | INTEGRATED SERVICE TECHNOLOGY (CN) | B22C-009/04\ B22C-009/08 |
| 5568 | 3D printing | DEVICE FOR RECOGNIZING PATCHES IN STRUCTURED LIGHT PATTERN IN THREE-DIMENSIONAL CAMERA SYSTEM, HAS DETERMINATION MODULE FOR DETERMINING LOCATION FOR PATCH RELATIVE TO STRUCTURED LIGHT PATTERN BASED ON INFORMATION PROVIDED BY PATCH LABEL | US2015002628A1\ WO2014209746A1\ US9025863B2 | HU ZHENGJUN (CN) | INTEGRATED SERVICE TECHNOLOGY (CN) | G06T-007/00\ H04N-013/02\ H04N-005/262\ G06K-009/00 |
| 5569 | 3D printing | DISPLAY AUXILIARY DEVICE FOR USE IN PROJECTOR OF DIGITAL LIGHT PROCESSING (DLP), HAS SWITCHING CONTROL UNIT THAT PERFORMS SHUTTER SWITCHING OPERATION OF SHUTTER FOR LEFT-EYE AND RIGHT-EYE IMAGES TO BE SWITCHED AND DISPLAYED | EP2813888A1\ JP2015002410A\ CN104301710A | HU ZHENGJUN (CN) | SH INTEGRATED CIRCUIT RES & DE (CN) | G03B-035/16\ H04N-013/04\ G02B-027/22\ G03B-021/00\ G03B-021/14\ G03B-037/00\ G09F-009/00\ H04N-013/00 |
| 5570 | 3D printing | SLA MOLDING MACHINE MATERIAL CYLINDER CAPACITY CHANGING DEVICE, HAS WORKING TABLE WHOSE INNER PART IS ARRANGED WITH CYLINDER BODY, MOTOR FIXED ON UPPER PART OF BRACKET, AND TRAVEL SWITCH WHOSE LOWER PART IS FIXED WITH INDUCTION DEVICE | CN203919482U | LU HUOTIE (CN)\ LI DAWEI (CN)\ WANG GUANGZH (CN) | SHANGHAI IC RES & DEV CT CO LT (CN) | B29C-035/08 |
| 5571 | 3D printing | FACING CLOUD COMPUTING TENANT ADJUSTING METHOD FOR VIRTUAL MACHINE SET, INVOLVES ARRANGING VIRTUAL MACHINE ON VIRTUAL MACHINE PLATFORM WITH VIRTUAL MACHINE ARRANGEMENT SOFTWARE CODE, AND DETERMINING PRESET MONITORING PERIOD OF GROUP CLOUD | CN104135535A | YANG HAIGANG US\ YOU TIANYOU US\ JIANG YURONG US\ SONG GUILIN US\ WANG TIANXING US\ CHANG FANGGAO US | LIANHUA ELECTRONICS CO LTD (CN) | H04L-029/08 |
| 5572 | 3D printing | INTEGRATED STRUCTURE OF SPLICING AND BLACK CRYSTAL DIGITAL LIGHT PROCESSING (DLP) SCREENS, CONNECTS BOX CASE USING ALLOY NEEDLE AND CRYSTAL SCREEN THROUGH BOLT FIXING HOLE POSITION FIXED ON INNER SCREEN FRAME FIXED ON UPPER BOX CASE | CN203930318U | ZHONG HUICAI (CN)\ ZHU KAIGUI US\ CHEN FANGFANG US | UNIV HENAN NORMAL US | G03B-021/602 |
| 5573 | 3D printing | NETWORK SYSTEM FOR IMPROVING NETWORK COMMUNICATION PERFORMANCE, HAS CLIENT SITE NETWORK COMPONENT AND NETWORK SERVER COMPONENT BETWEEN WHICH DATA TRAFFIC IS CARRIED OVER BONDED/AGGREGATED CONNECTION | US2015003465A1\ US9264350B2\ WO2016041048A1\ TW201616834A | FAN MINGGUO US | UNIV BEIHANG US | H04L-012/723\ H04L-012/911\ H04L-012/24\ H04L-012/28\ H04L-012/70\ H04L-012/709\ H04L-029/06\ H04L-012/717 |
| 5574 | 3D printing | PRIMER SET FOR ANALYZING PIG LEUKOCYTE ANTIGEN SPECIFIC GENOTYPE IN PIG SARCOMA INDUSTRY, HAS PRIMER SET PART FOR ANALYZING SPECIFIC GENOTYPE THROUGH DIRECT SEQUENCING OF GENOMIC DNA TO AMPLIFY PIG LEUKOCYTE ANTIGEN SPECIFIC GENE | KR2014134976A\ KR1508342B1 | GUO HONG US\ HAN YUANYUAN US\ YIN FAZHANG US\ ZHANG XIMIN US\ FAN YEMING US | 41ST INST CHINA ELECTRONICS TECHNOLOGY GROUP CORP US | C12N-015/11\ C12Q-001/68 |
| 5575 | 3D printing | GENOTYPE SWINE LEUKOCYTE ANTIGEN-2 ANALYZING METHOD, INVOLVES AMPLIFYING EXXON OF SWINE LEUKOCYTE ANTIGEN-2, ANALYZING PCR PRODUCT WITH BASE SEQUENCE, AND AMPLIFYING GENOMIC DNA BASED ON ANALYZED PCR PRODUCT | KR2014134977A\ KR1511741B1 | MA XIAOLONG US\ YIN HUAXIANG (KR) | BEIJING NONFERROUS METAL US | C12N-015/11\ C12Q-001/68 |
| 5576 | 3D printing | NEW PCR-BASED GENOMIC PRIMER HAVING SEQUENCE, FOR AMPLIFYING OR ANALYZING PIG LEUKOCYTE ANTIGEN SLA-3 EXON 2-3 GENOTYPE | KR2014134979A\ KR1512825B1 | HEMING ZHANG (CN)\ RONGXI XUAN (CN)\ HUIYONG HU (CN)\ JIANJUN SONG (CN)\ BIN WANG US\ YI LV US\ HAIDONG WANG US\ YUE HAO (CN) | INST OF MICROELECTRONICS CAS (CN) | C12N-015/11\ C12Q-001/68 |
| 5577 | 3D printing | CLOUD SERVICE BASED CSP SERVICE QUALITY EVALUATING METHOD, INVOLVES OBTAINING SCORE OF CSP DIFFERENT SERVICE ITEMS CORRESPONDING TO USERS, DETERMINING CSP SERVICE QUALITY VALUE, AND MARKING DIFFERENT ITEMS OF USERS | CN104104545A | HEMING ZHANG (CN)\ BIN SHU (CN)\ RONGXI XUAN (CN)\ HUIYONG HU (CN)\ JIANJUN SONG (CN)\ BIN WANG US\ CHUNYU ZHOU US\ YUE HAO (CN) | UNIV XIDIAN (CN) | H04L-012/24\ H04L-029/08 |
| 5578 | 3D printing | DIGITAL LIGHT PROCESSING PROJECTOR, HAS PHOTO-ELECTRIC CONVERSION DEVICE CONNECTED WITH LIGHT END OF OPTICAL SYSTEM, AND PHOTO-ELECTRIC CONVERSION PART FOR CONVERTING LIGHT ENERGY INTO ELECTRIC ENERGY | CN104102078A\ CN104102078B | WEI ZHANG (CN)\ DONGPING WU US\ QINGQING SUN US\ PENG ZHOU US | UNIV XIDIAN (CN) | G03B-021/14\ G03B-021/20 |
| 5579 | 3D printing | METHOD FOR CACHING DATA IN SYSTEM OF BASE STATIONS, INVOLVES RECEIVING DATA FROM CONTENT PROVIDER AND TRANSMITTING RECEIVED DATA TO ONE OF BASE STATIONS FOR CACHING BASED ON PARAMETERS | US2015003234A1\ WO2014209584A1 | SHAOFENG DING US\ XINPING QU US\ FEI CHEN US\ YULONG JIANG US\ GUOPING RU US | UNIV FUDAN (CN) | H04W-028/02\ H04L-029/08\ H04W-004/00 |
| 5580 | 3D printing | TOOL FOR PRE-FORMING ELEMENT FOR FORMING SUBSEQUENT INTERNAL HIGH PRESSURE FOR MANUFACTURE OF IHU COMPONENT, INVOLVES PIPE ELEMENT TRANSFORMED SUCH THAT EVERY PART OF ELEMENT IS MATCHED WITH PRE-DETERMINED CAVITY OF BASE TUBE PART | DE102013212758A1\ WO2014206981A1\ CN105163879A | LE LI US\ SHUGEN PENG US | UNIV FUDAN (CN) | B21D-026/033\ B21D-026/047 |
| 5581 | 3D printing | METHOD FOR IMPROVING MULTICAST RATE CONTROL IN WIRELESS COMMUNICATION SYSTEMS, INVOLVES DETERMINING NEW TRANSMISSION RATE FOR SUBSEQUENT MULTICAST TRANSMISSIONS TO MULTICAST GROUP OF DEVICES USING MULTICAST RECEIVE QUALITY FEEDBACK | US2014376376A1 | JIAQING LI (CN) | SHANGHAI GRACE SEMICONDUCTOR US | H04L-012/18\ H04L-012/825 |
| 5582 | 3D printing | OPTICAL WRITE-IN APPARATUS OF IMAGE FORMING APPARATUS, HAS HEAT SINK WHICH IS AFFIXED, SUCH THAT CENTER PORTION OF AREA OF CONTACT SURFACE OF SUBSTRATE AND HEAT SINK IS LARGER THAN EDGE PORTIONS IN Y DIRECTION OF LIGHT EMISSION PORTION | JP2014237285A | HUALUN CHEN (CN)\ YU CHEN (CN)\ TAO XIONG (CN)\ XIAO LUO (CN)\ XIONGBIN CHEN (CN) | SH INTEGRATED CIRCUIT RES & DE (CN) | B41J-002/44\ B41J-002/45\ B41J-002/455\ H04N-001/036 |
| 5583 | 3D printing | SLA THEORY BASED ON THREE-DIMENSIONAL PRINTER LIQUID LEVEL CONTROL SYSTEM, HAS PERISTALTIC PUMP CONNECTED WITH CONVEYING HOSE, AND LIQUID SURFACE TRANSMITTING SIGNAL TO CREEP PUMP THAT IS FORMED WITH MAINTAIN RESIN GROOVE | CN203864020U | JIANXIN ZHANG (CN) | SHANGHAI HUAHONG NEC ELECT CO (CN) | B29C-067/00 |
| 5584 | 3D printing | DISPLAY DEVICE E.G. LCD DEVICE, HAS WIRING SUBSTRATE PLACED IN CONNECTION PORTION AND CONNECTED TO TERMINALS, WHERE ADHESIVE MATERIAL IS FORMED BETWEEN SUBSTRATES AND OUTSIDE OF ADHESIVE MATERIAL CONTACTS WITH ANOTHER ADHESIVE MATERIAL | US2014375941A1\ US9104049B2 | QIUDE LI (CN)\ YUANLI CAI (CN) | CHINTOP MICROELECTRONICS SHANG (CN) | G02F-001/1333\ G02F-001/1339 |
| 5585 | 3D printing | PROJECTION IMAGE CORRECTION SYSTEM FOR LIFTING-TYPE STEREO LITHOGRAPHY APPARATUS FOR CORRECTING IMAGE DATA TO BE OUTPUT TO PROJECTOR, HAS CORRECTION SECTION FOR TRANSFORMING IMAGE DATA TO BE OUTPUT TO PROJECTOR TO CORRECT IMAGE DATA | US2014379114A1\ JP2015007866A | LITIAN LIU (CN)\ ZEWEN LIU (CN)\ GUANGWEI HU (CN)\ ZHIJIAN LI (CN) | HEJIAN TECH SUZHOU CO LTD (CN) | B29C-067/00\ G03B-021/00\ G03B-021/14\ G06T-003/00\ G09G-005/00\ G09G-005/36\ H04N-005/74 |
| 5586 | 3D printing | PROGRAM FOR PREPARING FUNCTIONAL DESCRIPTION, INCLUDE INSTRUCTIONS FOR PERFORMING FUNCTIONAL DESCRIPTION FILE CREATION STEP CLASSIFIED BY IDENTIFIER TO PRODUCE FUNCTIONAL DESCRIPTION FILE CLASSIFIED BY IDENTIFIER | JP2014228954A | MEISTER PIERRE-ANDRE (CH)\ ZANETTA ANDRE (CH)\ FLEURY EMMANUEL (CH)\ BLONDEAU FABIEN (CH) | UNIV TSINGHUA (CN) | B41J-029/38\ G06F-003/12 |
| 5587 | 3D printing | REAR SCREEN TYPE DIGITAL LIGHT PROCESSING (DLP) PROJECTION DISPLAY DEVICE FOR USE IN E.G. OFFICE AND SCHOOL, HAS AUDIO DEVICE AND IMAGE OUTPUT DEVICE THAT ARE SYMMETRICALLY ARRANGED ON BOTH SIDES OF CONTROL UNIT | CN203851220U | AIME DELPHINE (FR)\ FROMENT BENOIT (BE) | ETA SA MFT HORLOGERE SUISSE (CH) | H04N-005/74 |
| 5588 | 3D printing | DISPLAY SYSTEM FOR E.G. IMAGE TRANSMISSION SYSTEM APPLICATION, HAS OPTICALLY REFLECTIVE SWITCHING DEVICE INCLUDING OPTICALLY REFLECTIVE LAYER THAT INCLUDES ELECTRO-CHROMIC MATERIAL, SUBSTRATE, ASSOCIATED EXCITATION UNIT AND CONTROLLER | US2014368898A1 | AIZAWA KEIICHI (JP)\ HOSAKA SHINYA (JP) | ST MICROELECTRONICS SA (FR) | G02F-001/15\ G02F-001/157\ G02F-001/163 |
| 5589 | 3D printing | TOOTH BRACKET FOR ORTHODONTIC TREATMENT, HAS LIGATION DEVICE WRAPPED AROUND TWO FIRST TABS OPPOSITE TO BRACKET GROOVE AND NOT AROUND TWO SECOND TABS OPPOSITE TO BRACKET GROOVE FOR PASSIVE LIGATION | US2014370455A1 | AJTKHOZHIN SABIR ABENOVICH (RU) | RENESAS ELECTRONICS CORP (JP)\ AIZAWA KEIICHI (JP)\ HOSAKA SHINYA (JP) | A61C-007/30 |
| 5590 | 3D printing | COMPUTER-IMPLEMENTED METHOD FOR MANAGING COMPUTER RESOURCES IN MULTI-CLOUD SYSTEMS, INVOLVES INITIATING CREDIT CHECK PROCESS, AND DETERMINING WHETHER VIRTUAL MACHINES ARE MOVED FROM FIRST COMPUTER SERVER TO SECOND COMPUTER SERVER | US8914511B1 | ANDERSON BRENT A (US)\ NOWAK EDWARD J (US)\ BRYANT ANDRES (US) | Empty Field US | G06F-015/173\ G06Q-010/00\ G06Q-010/06 |
| 5591 | 3D printing | IMAGE PROJECTOR E.G. DIGITAL LIGHT PROCESSING-TYPE LCOS-TYPE PROJECTOR, FOR PROJECTING IMAGE ON TRANSMISSIVE SCREEN, HAS REMOTE CONTROL ATTACHED TO MAIN BODY SUCH THAT ATTACHMENT OR DETACHMENT OF REMOTE CONTROL IS POSSIBLE | JP2014235344A | ANDERSON BRENT A (US)\ NOWAK EDWARD J (US)\ JOSEPH ALVIN J (US) | IBM (US) | G03B-021/00\ G03B-021/14\ G03B-021/62\ H04N-005/74 |
| 5592 | 3D printing | METHOD FOR IMAGING DNA, INVOLVES ALIGNING TRAPPING LINE TO COINCIDE WITH SCANNING LINE AND HAVING SCANNING EXCITATION FOCUS, AND RECORDING FLUORESCENCE RESPONSE AS FUNCTION OF DISTINCT SCANNING POSITIONS OF EXCITATION FOCUS ALONG LINE | WO2014196854A1\ NL2011087C | ANDERSON BRENT A NOWAK EDWARD (US) | IBM (US)\ ANDERSON BRENT A (US)\ NOWAK EDWARD J (US)\ JOSEPH ALVIN J (US) | B01L-003/00\ C12Q-001/68\ G01N-021/64\ G02B-021/00\ G02B-021/16\ G02B-021/32 |
| 5593 | 3D printing | ANALYZING HUMAN MALE SAMPLE, INVOLVES DETERMINING LEVELS OF X DIFFERENT BIOMARKERS E.G. REQUIRED FOR CELL DIFFERENTIATION (RCD)1 HOMOLOG, ACTIVATING TRANSCRIPTION FACTOR 4 AND DISTAL-LESS HOMEOBOX 3 IN SAMPLE | WO2014195730A2\ WO2014195730A3 | ANDERSON BRENT A NOWAK EDWARD (US) | IBM (US)\ ANDERSON BRENT A (US)\ NOWAK EDWARD J (US) | G01N-033/564\ G01N-033/68 |
| 5594 | 3D printing | NON-TRANSITORY COMPUTER-READABLE STORAGE MEDIUM FOR CONFIGURING CLOUD RESOURCES FOR CLOUD COMPUTING ARCHITECTURES, INCLUDES INSTRUCTIONS FOR INSTANTIATING ADDITIONAL VIRTUAL MACHINES WITHIN RESOURCE GROUP RESPONSE TO RECEIVING REQUEST | US2014365662A1 | AOYAMA ATSUYUKI US | IBM (US) | G06F-009/455\ H04L-012/24\ H04L-029/08 |
| 5595 | 3D printing | METHOD FOR GENERATING STRUCTURED LIGHTING PATTERN, INVOLVES DETECTING IMAGE DURING IMAGE INTEGRATION PERIOD OF CAMERA AND ITERATION OF STRUCTURED LIGHTING PATTERN IS GENERATED, WHICH INCORPORATES GRAY LEVEL VARIATIONS WITHIN TIME PERIOD | DE102014209471A1\ JP2014238400A\ US2014362203A1\ US9350921B2 | ASANO TETSUO US | FUJITSU LTD (JP) | G01B-011/00\ G01B-011/02\ G01B-011/25\ H04N-005/225\ H04N-005/235\ G01N-021/00\ H04N-005/265 |
| 5596 | 3D printing | METHOD FOR OPTIMIZING VIRTUAL MACHINE INSTANCES IN A NETWORKED COMPUTING ENVIRONMENT, INVOLVES OPTIMIZING SET OF VIRTUAL MACHINE INSTANCES BY SPLITTING SINGLE INSTANCE, WHICH HAS MULTIPLE UNIQUE PROJECTS, INTO MULTIPLE INSTANCES | US2014298337A1 | ASANO TETSUO US\ ISHIHARA HIDETOSHI US | SANYO ELECTRIC CO (JP) | G06F-009/455 |
| 5597 | 3D printing | COMPUTING DEVICE FOR OPERATING TOUCH DISPLAY E.G. LCD, TO IMPLEMENT TOUCH INPUT INTERPRETATION, HAS TOUCH INTERPRETER FOR INTERPRETING TOUCH INPUT AS NON-TAP GESTURE IF DISTANCE BETWEEN POINTS OF CONTACT IS MORE THAN THRESHOLD DISTANCE | US2014354595A1 | ASANO TETSUO US\ ISHIHARA HIDETOSHI US | SANYO ELECTRIC CO (JP) | G06F-003/044\ G06F-003/0485\ G06F-003/0488 |
| 5598 | 3D printing | SYSTEM FOR CONTROLLING MULTI-TENANT REQUESTS TO SHARED RESOURCES IN MULTI-TENANT ENVIRONMENT, HAS ADMISSION CONTROLLER FOR PROCESSING REQUESTS BY PROCESSING NODES FROM TENANT QUEUES BASED ON REFERENCE VALUE FOR TENANT RECEIVED BY CONTROLLER | US2014359113A1 | ASAYAMA HIDEKAZU US\ ADACHI HISASHI US\ TORIGOE KAZUNAO US\ MOTOYAMA TAMIO (JP)\ MATSUMOTO KOJI (JP) | SANYO ELECTRIC CO (JP) | H04L-012/24\ H04L-012/911 |
| 5599 | 3D printing | METHOD FOR MANAGING AVAILABILITY OF VIRTUAL MACHINES (VMS) IN CLOUD COMPUTING SERVICES, INVOLVES IDENTIFYING N VMS FROM AMONG FAILED VMS, SUSPENDING M VMS AND RESTARTING N VMS ON REMAINING FUNCTIONING HOSTS | US2014344805A1\ US9183034B2 | ASSEFA SOLOMON (US)\ CHEN KUAN-NENG (US)\ KOESTER STEVEN J (US)\ VLASOV YURII A (US) | SUMCO CORP (JP)\ ASAYAMA EIICHI (JP)\ ADACHI NAOSHI (JP)\ MOTOYAMA TAMIO (JP)\ MATSUMOTO KOJI (JP)\ TORIGOE KAZUHISA (JP) | G06F-009/455 |
| 5600 | 3D printing | SEMICONDUCTOR MEMORY DEVICE E.G. NONVOLATILE MEMORY DEVICE FOR E.G. MOBILE PHONE, HAS CHANNEL LAYER PROVIDED WITH THREE-DIMENSIONAL U-SHAPED STRUCTURE, AND PERIPHERAL CIRCUIT IS CONFIGURED TO PERFORM ERASE OPERATION ON PIPE CELL | US2014340966A1\ KR2014135402A\ US9147478B2 | ASSEFA SOLOMON (US)\ CHEN KUAN-NENG (US)\ KOESTER STEVEN J (US)\ VLASOV YURII A (US) | IBM (US)\ CHEN KUAN-NENG (US)\ ASSEFA SOLOMON (US)\ KOESTER STEVEN J (US)\ VLASOV YURI A (US) | G11C-016/04\ G11C-016/14\ G11C-016/06\ G11C-005/02\ H01L-027/115\ G11C-016/16\ G11C-016/34 |
| 5601 | 3D printing | METHOD FOR ESTABLISHING AND ENFORCING HOLISTIC QUALITY-OF-SERVICE POLICY WITHIN IN NETWORK NODE OF NETWORK, INVOLVES OUTPUTTING MARKED PACKETS BY NETWORK NODE TO ANOTHER NETWORK NODE OF MULTIPLE NETWORK NODES | US2014341027A1 | BACH KONRAD (DE) | IBM (US)\ CHEN KUAN-NENG (US)\ ASSEFA SOLOMON (US)\ KOESTER STEVEN J (US)\ VLASOV YURI A (US) | H04L-012/813 |
| 5602 | 3D printing | THREE-DIMENSIONAL DISPLAY APPARATUS HAS CONTROL UNIT THAT MOVES PARALLAX BARRIER, IN WHICH BARRIER AND SLIT FORMS PERIODICALLY IN PREDETERMINED ALIGN DIRECTION AND MAKES SWITCH LIQUID CRYSTAL PANEL TO DISPLAY PARALLAX BARRIER | WO2014181567A1\ CN105164573A\ US2016195731A1\ JP5969699B2 | BACH KONRAD (DE) | X FAB SEMICONDUCTOR FOUNDRIES (DE) | G02B-027/22\ G02F-001/13\ G02F-001/133\ G02F-001/1347\ G03B-035/24\ H04N-013/04\ G02F-001/29\ G02F-001/1333\ G09F-009/00 |
| 5603 | 3D printing | SYSTEM FOR PROVIDING SERVICE LEVEL AGREEMENT PROFILING IN COMMON LOGICAL PROCESS MODEL I.E. ENTERPRISE MODEL, HAS COMPUTER INCLUDING SERVICE LEVEL AGREEMENT PROFILE MODULE TO APPLY APPLICABLE PROFILE TO PARTICULAR PROCESS INSTANCE | US2014358624A1 | BAIRO MASAAKI US\ ABE TAKAHISA US | X FAB SEMICONDUCTOR FOUNDRIES (DE) | G06F-017/30\ G06F-021/62\ G06Q-010/06\ H04L-012/24\ H04L-029/06\ H04L-029/08 |
| 5604 | 3D printing | THREE-DIMENSIONAL NONVOLATILE NAND MEMORY FOR OPERATION OF RE-PROGRAMMABLE NONVOLATILE MEMORY SYSTEMS, HAS PARAMETER SELECTING CIRCUIT THAT SELECTS OPERATING PARAMETERS FOR MEMORY CELLS OF DIFFERENT PHYSICAL LEVELS OF PHYSICAL LEVELS | US8902661B1 | BAIRO MASAAKI US\ OISHI TETSUYA US\ YASUSHIGE HIROAKI US | SONY CORP (JP) | G11C-016/04\ G11C-016/10\ G11C-016/26\ G11C-029/00\ H01L-027/115 |
| 5605 | 3D printing | DIGITAL LIGHT PROCESSING (DLP) MICRO-PROJECTOR HAS FLY-EYE LENS OR ROD WHICH IS ARRANGED ALONG LIGHT PATH, AND DIGITAL MICROMIRROR DEVICE (DMD) CHIP WHICH IS USED FOR REFLECTING THE LIGHT INCIDENT ON LENS DEVICE TOWARDS THE FIELD LENS | CN203811977U | BALDWIN GREGORY CHARLES (US) | SONY CORP (JP) | G03B-021/00\ G03B-021/20 |
| 5606 | 3D printing | METHOD FOR SERVICE LEVEL ASSURANCE (SLA)-AWARE SYSTEM FOR RECOMMENDING BEST MATCH FOR CLOUD RESOURCE PROVISIONING, INVOLVES EVALUATING ARCHIVED DATA THAT PREDICTS TIME NEEDED FOR DEVICES TO FULFILL PROVISIONING REQUEST | US2014351441A1 | BAO RONGSHENG (CN) | TEXAS INSTRUMENTS INC (US)\ BALDWIN GREGORY CHARLES (US) | H04L-012/24 |
| 5607 | 3D printing | OPHTHALMOSCOPE FOR EXAMINING REAR EYE PORTION OF PATIENT, HAS ILLUMINATION DEVICE CREATING ILLUMINATION BEAM, AND MODULE HOUSING SUPPORTED AROUND SPACE AXIS, WHERE ILLUMINATION BEAM IS DIRECTED AT PATIENT'S EYE THROUGH VIEWING DIRECTION | DE102013007075A1 | BAUER FLORIAN (DE)\ GEORGAKOS GEORG (DE)\ PACHA CHRISTIAN (DE)\ BERTHOLD JORG (DE) | SHANGHAI BEILING CO LTD (CN) | A61B-003/12\ A61B-003/13\ A61B-003/135\ G02B-021/22\ G02B-021/24 |
| 5608 | 3D printing | APPARATUS FOR FACILITATING BOTTOM-UP FABRICATION OF SOLID OBJECTS, HAS ROTATION MECHANISM FOR VARYING RELATIVE ROTATIONAL POSITION OF VAT RELATIVE TO BUILD PLATFORM, WHERE ROTATION IS ABOUT AXIS, WHICH IS NORMAL TO WORKING SURFACE OF VAT | GB2514139A\ US2014339741A1\ WO2014186463A1\ EP2996852A1 | BEACH ROBERT (US)\ BRIDGER PAUL (US) | INFINEON TECHNOLOGIES AG (DE) | B29C-067/00\ B29C-067/24\ B29C-035/02\ B29C-035/08\ C08F-002/01 |
| 5609 | 3D printing | INTELLIGENT PROJECTOR HAS CENTRAL PROCESSOR THAT IS SERIALLY CONNECTED WITH BLUETOOTH MODULE USED FOR MOBILE TERMINAL, AND PROJECTOR UNIT WHICH IS PROVIDED WITH VIDEO DECODING MODULE THAT IS PROVIDED WITH AUDIO DECODER | CN203801020U | BEINTNER JOCHEN C (US) | INT RECTIFIER CORP (US) | H04N-005/74\ H04N-009/31 |
| 5610 | 3D printing | INTELLIGENT PROJECTING CAMERA, HAS DIGITAL LIGHT PROCESSING MACHINE AND ELECTRIC SOURCE MODULE FIXED IN INNER SURFACE OF PROJECTOR MAIN BODY, AND PLATE CARD CONNECTED WITH MULTI-INTERFACE MODULE AND FIXED WITH MULTI-INPUT/OUTPUT DEVICE | CN203801032U | BIN LI US\ MINGJIAN ZHAO US\ LINING LIU US\ ZHAOHUI WU US | IBM (US) | G03B-021/00\ G03B-021/14\ H04N-009/31 |
| 5611 | 3D printing | PROJECTOR ASSEMBLY E.G. LIQUID CRYSTAL ON SILICON PROJECTOR ASSEMBLY, HAS SCREEN THAT IS CHANGEABLE FROM OPAQUE TO TRANSPARENT UNDER PRESET CONDITIONS AND CONTROL DEVICE TO COORDINATES POWER, IF PROJECTING DEVICE DOES NOT PROJECT IMAGE | WO2014183037A1\ US2016048016A1\ EP2994796A1\ JP2016526181W | BO ZHANG (CN)\ BO LUO US\ MING QIAO (CN)\ LINGLI JIANG (CN)\ SHUANGLIANG DUAN (CN)\ DAPING FU US | UNIV SOUTH CHINA TECH (CN) | G02B-027/01\ G03B-021/56\ G03B-021/60\ G02F-001/13\ G02F-001/17\ G03B-021/14\ G03B-021/62\ B60K-035/00\ B60R-011/02\ G03B-021/00\ G09F-009/00\ H04N-005/74 |
| 5612 | 3D printing | METHOD FOR CONTROLLING ADAPTIVE LINE RATE OF ACCESS NETWORK BASED ON G.HN TECHNOLOGY, INVOLVES PERFORMING SPECIFIC FREQUENCY NOTCH FILTER PROCESSING ABOUT ACTUAL PERFORMANCE OF SUBSCRIBER TERMINAL BASED ON COMPARISON RESULT | KR1458380B1 | BO ZHANG (CN)\ MING QIAO (CN)\ LINGLI JIANG (CN)\ SHUANGLIANG DUAN (CN)\ BO CHEN (CN)\ LEI ZHAO (CN) | UNIV ELECTRONIC SCIENCE & TECH (CN) | H04L-029/02 |
| 5613 | 3D printing | METHOD FOR TRAINING CROWDWORKER E.G. HOME-BASED EMPLOYEE ON SKILL SETS IMPLEMENTED ON COMPUTING DEVICE, INVOLVES ASSIGNING TRAINING TASK SUCH THAT CROWDWORKER GETS TRAINED ON SKILL SETS WITHOUT VIOLATING SERVICE LEVEL AGREEMENT | US2014335485A1 | BOUCHIER DANIEL (FR)\ YAM VY (FR)\ CAMMILLERI VINCENZO DAVIDE (FR)\ FOSSARD FREDERIC (FR)\ RENARD CHARLES (FR) | UNIV ELECTRONIC SCIENCE & TECH (CN)\ SHENZHEN LAND HOPE MICRO ELECTRONICS CO LTD US | G09B-019/00 |
| 5614 | 3D printing | LIGHT FILTER SYSTEM FOR PROJECTOR E.G. DIGITAL LIGHT PROCESSING (DLP) PROJECTOR, HAS COLOR LIGHT PRODUCING UNIT WHICH IS PROVIDED FOR FORMING RED, GREEN AND BLUE COLORS | CN103995419A\ CN103995419B | BOUTROS KARIM S (US)\ KARAM NASSER H (US)\ KRUT DIMITRI (US)\ HADDAD MORAN (US) | UNIV PARIS SUD (FR)\ CENTRE NAT RECH SCIENT (FR)\ BOUCHIER DANIEL (FR)\ YAM VY (FR)\ CAMMILLERI VINCENZO DAVIDE (FR)\ FOSSARD FREDERIC (FR)\ RENARD CHARLES (FR) | G03B-021/14 |
| 5615 | 3D printing | MONITORING SYSTEM FOR MONITORING OPERATION OF DISTRIBUTED COMPUTING COMPONENTS, HAS EVENT PROCESSING ENGINE THAT EXECUTES QUERY ON INPUT EVENT AND PRODUCES OUTPUT EVENT INDICATING RELATIONSHIP BETWEEN SERVICE-LEVEL AGREEMENT VIOLATIONS | EP2801909A1\ US2014337510A1 | BRADDOCK WALTER DAVID IV (US)\ JOHNSON MARK (US) | BOEING CO (US) | G06F-011/34\ H04L-012/24 |
| 5616 | 3D printing | METHOD FOR OPERATING AUDIO SYSTEM IN VEHICLE, INVOLVES SPEAKERS THAT EMITS DIRECT AUDIO SIGNALS WHICH ARE SUM AND DIFFERENCE OF SIGNAL OF CENTER AUDIO CHANNEL AND SIDE SIGNAL OF RIGHT AND LEFT AUDIO CHANNELS, RESPECTIVELY | DE102013007689A1\ DE102013007689B4 | BRIET MICHEL (FR)\ VERDANT ARNAUD (FR) | OSEMI INC (US)\ BRADDOCK WALTER DAVID IV (US)\ JOHNSON MARK (US) | H04R-005/02\ H04S-001/00 |
| 5617 | 3D printing | THREE-DIMENSIONAL IMAGE DISPLAY APPARATUS HAS SHUTTER SWITCH SIGNAL WHICH ARE GIVEN AS OUTPUT TO SHUTTER SPECTACLES UNIT SO THAT SHUTTER FOR LEFT EYE AND SHUTTER FOR RIGHT EYES IS MADE INTO IMPERVIOUSNESS IN PREDETERMINED PERIOD | JP2014207508A | BROMBERGER CHRISTOPH DIPL-PHYS (DE) | ATMEL NANTES SA SA (FR) | G02B-027/22\ H04N-013/04 |
| 5618 | 3D printing | METHOD FOR ARRANGING PERFLUORINATED CARBON CHAIN ON SURFACE OF E.G. MICROELECTROMECHANICAL SYSTEM DEVICE, TO PROTECT DEVICE, INVOLVES ARRANGING COATING ON SURFACE OF COMPONENT, WHERE COATING COMPRISES AMPHIPHILIC COMPOUND | US2014329392A1\ US9150410B2 | BROMBERGER CHRISTOPH DIPL-PHYS (DE) | ATMEL GERMANY GMBH (DE) | B81C-001/00\ H01L-021/02\ B81B-003/00\ B81B-007/00\ H01L-021/31\ H01L-021/469\ H04W-004/02 |
| 5619 | 3D printing | GLASS COMPOSITION USED FOR E.G. PASTE COMPOSITION, CONTAINS SPECIFIED AMOUNT OF BORON TRIOXIDE, SODIUM OXIDE, ALUMINUM OXIDE, LITHIUM OXIDE, TITANIUM DIOXIDE, ZINC OXIDE, BARIUM OXIDE, TANTALUM PENTOXIDE AND NIOBIUM TRIOXIDE | WO2014172106A1\ US2014314969A1\ CN105143127A\ EP2986575A1\ JP2016515996W | BROWN DAVID E (US)\ LUNING SCOTT D (US) | ATMEL GERMANY GMBH (DE) | C03C-027/00\ C03C-003/14\ C03C-008/24\ C03C-003/21\ C03C-008/16 |
| 5620 | 3D printing | NON-TRANSITORY COMPUTER-READABLE STORAGE MEDIUM FOR ANALYZING EXPENDITURES OF CLOUD SERVICES, HAS SET OF INSTRUCTIONS FOR DETERMINING COST PARAMETER INDICATING RELATIONSHIP BETWEEN AMOUNT SPENT ON SERVICES AND ALLOCATED SERVICE PORTION | US2014324647A1 | BU HAOWEN (US)\ KHAMANKAR RAJESH B (US)\ GRIDER DOUGLAS TAD (US) | ADVANCED MICRO DEVICES INC (US) | G06Q-040/00 |
| 5621 | 3D printing | COMPUTER HARDWARE SYSTEM FOR GENERATING RESOURCE ASSOCIATED SERVICE LEVEL AGREEMENT BETWEEN ENTERPRISE AND CUSTOMER, HAS PROCESSOR PROVIDING USER WITH SERVICE LEVEL AGREEMENT BREACH VALUE AND RETRIEVING SELECTED BREACH VALUE FROM USER | US2014325071A1\ US9444696B2 | BU HAOWEN (US)\ YU SHAOFENG (US)\ PINTO ANGELO (US)\ VARGHESE AJITH (US) | TEXAS INSTRUMENTS INC (US)\ BU HAOWEN (US)\ KHAMANKAR RAJESH B (US)\ GRIDER DOUGLAS TAD (US) | H04L-012/24\ G06F-015/173\ H04L-012/26\ H04L-029/06\ H04L-029/08 |
| 5622 | 3D printing | SCREEN APPARATUS FOR USE IN HEAD-UP DISPLAY, HAS DRIVE UNIT WHICH DRIVES SCREEN AROUND THIRD SHAFT DIFFERING FROM DIRECTIONS OF FIRST SHAFT AND THE SECOND SHAFT THAT ARE ALONG TWO DIFFERENT DIRECTIONS | WO2014167672A1 | CAMPANELLA PINEDA HUMBERTO (ES)\ ESTEVE TINTO JAUME (ES)\ CABRUJA CASAS ENRIC (ES)\ MONTSERRAT MARTI JOSE (ES)\ TERES TERES LLUIS (ES)\ CARMONA FLORES MANUEL (DE) | TEXAS INSTRUMENTS INC (US) | G03B-021/14\ G03B-021/60 |
| 5623 | 3D printing | FLAT-PANEL COMPUTER INTEGRATED-TYPE FILM PROJECTOR HAS MAIN PLATE SET WITH SWITCHING CONTROL CIRCUIT, AND CONNECTED WITH DIGITAL LIGHT PROCESSING (DLP) PROJECTION MODULE CONSISTING OF PROJECTION LENS CONNECTED WITH RADIATING MODULE | CN203773220U | CAMPIDELLI YVES (FR)\ KERMARREC OLIVIER US | CONSEJO SUPERIOR INVESTIGACION (ES)\ EPSON EUROP ELECTRONICS GMBH (DE)\ CAMPANELLA PINEDA HUMBERTO (ES)\ ESTEVE TINTO JAUME (ES)\ CABRUJA CASAS ENRIC (ES)\ MONTSERRAT MARTI JOSE (ES)\ TERES TERES LLUIS (ES)\ CARMONA FLORES MANUEL (DE) | G03B-021/16\ G03B-029/00\ H05K-007/20 |
| 5624 | 3D printing | SPLICING SCREEN FOR DIGITAL LIGHT PROCESSING (DLP) PROJECTION, HAS SQUARE FRAME WHICH IS CONNECTED TO MAIN DISPLAY SCREEN AND BACK PROJECTION DISPLAY MODULE THROUGH ELASTIC ELEMENT FOR COMMUNICATING DISPLAY MODULE AND DISPLAY SCREEN | CN203773880U | CAO WANG US\ XU BAIXIN US\ ZHAO HAI US\ WANG XIAO US | STMICROELECTRONICS CROLLES SAS (FR) | G03B-021/56\ G09F-009/30 |
| 5625 | 3D printing | MODIFIED POLYETHER KETONE RESIN COMPOSITION FOR THREE-DIMENSIONAL PRINTING COMPRISES POLYETHER KETONE RESIN, CHAIN EXTENDER, END-CAPPING AGENT, CROSSLINKING AGENT, LOW-MOLECULAR-WEIGHT POLYMER, TOUGHENING AGENT, AND REINFORCING AGENT | CN103980690A\ CN103980690B | CHA WOOK JIN (KR) | WUXI CHINA RESOURCES SEMICO CO US | B29C-047/00\ B29C-067/00\ C08K-013/02\ C08K-003/04\ C08K-003/26\ C08K-003/30\ C08K-003/36\ C08K-005/101\ C08K-005/17\ C08K-005/29\ C08L-033/08\ C08L-067/02\ C08L-071/10 |
| 5626 | 3D printing | THREE-DIMENSIONAL VIDEO PROJECTOR HAS PROJECTION UNIT LOCATED INSIDE MIRROR WITH ARC-SHAPED REFLECTIVE SURFACE | JP2014202861A | CHAO GUO US\ NING AN US | DONGBU ELECTRONICS CO LTD (KR) | G02B-027/22\ G03B-035/18\ H04N-013/04 |
| 5627 | 3D printing | PROJECTOR FOR PROJECTING IMAGE ON PLANAR OBJECT E.G. SCREEN, OF DIGITAL LIGHT PROCESSING SYSTEM, HAS PROJECTION IMAGE DATA CORRECTION UNIT THAT CORRECTS PROJECTION IMAGE DATA SO THAT PROJECTION IMAGE IS PROJECTED ONLY ON PLANE RANGE | JP2014192808A | CHARLES HARRY K JR (US)\ BANDA CHARLES V (US)\ FRANCOMACARO ARTHUR S (US)\ KEENEY ALLEN C (US)\ LEHTONEN SEPPO J (US) | SHANGHAI LUNYI PHOTOELECTRIC TECHNOLOGY CO LTD US | G03B-021/00\ G03B-021/14\ G09G-005/00\ G09G-005/36\ H04N-005/74 |
| 5628 | 3D printing | COMPUTER-IMPLEMENTED METHOD FOR PREDICTING DIAGNOSIS OF HYPERTENSION PROBLEM OF OBJECT-ORIENTED PROGRAMMING LANGUAGE DATABASE CONNECTIVITY, IN CLOUD SERVICES, INVOLVES GENERATING OUTPUT BASED IN PART ON SET OF ANOMALIES | US2014310714A1 | CHATTERJEE AMITAVA (US)\ DROBNY VLADIMIR F (US)\ STEINMANN PHILLIPP (US)\ WISE RICK (US) | US NATIONAL SECURITY AGENCY (US)\ UNIV JOHNS HOPKINS (US) | G06F-009/48 |
| 5629 | 3D printing | COMPUTER-IMPLEMENTED METHOD FOR APPLYING THREAD OR STACK SEGMENT INTENSITY ANALYTICS, INVOLVES INCLUDING PARTICULAR FEATURE VECTOR AND IDENTIFYING STATE TRANSITION WITH ONE OF ENACTMENT OF DIRECTIVE AND ANOMALY | US2014310235A1\ WO2014168981A1\ WO2014169056A1\ CN105144112A\ CN105190564A\ EP2984567A1\ EP2984568A1\ JP2016517984W\ JP2016518660W | CHEN GUANFENG US\ ZHANG XIAOYU US\ BU HUIQIN US | TEXAS INSTRUMENTS INC (US) | G06F-017/30\ G06F-011/00\ G06F-011/07\ G06F-011/30\ G06F-011/34 |
| 5630 | 3D printing | PIEZOELECTRIC SURGICAL GUIDE I.E. DENTAL GUIDE, FOR TREATING JAWBONE OF HUMAN PATIENT BY DENTAL SURGEON, HAS HEAD GUIDE DEFINING PATH ACROSS SURFACE FROM POINT ON SURFACE TO DIFFERENT POINT WHEN GUIDE IS FITTED TO PART SURFACE OF HUMAN BODY | GB2513148A\ EP2792329A2\ EP2792329A3 | CHEN JUN (CN)\ HUI GUAN (CN) | WUXI CHINA RESOURCES SEMICO CO US | A61B-017/15\ A61C-001/08\ A61C-013/00\ A61C-013/20\ A61C-013/34\ A61C-008/00\ A61C-003/03\ A61C-007/00 |
| 5631 | 3D printing | PROJECTION DEVICE E.G. PICO PROJECTOR, FOR BEING USED ON E.G. WHITEBOARDS, HAS IMAGE SENSOR SENSING IMAGE PATTERN, AND STORAGE UNIT STORING INFORMATION OF PREVIOUS IMAGE PATTERN, CALIBRATION DRIVING PROCEDURE, AND CONVERSION TABLE | US2014307102A1\ CN104104891A | CHEN JUN (CN)\ KUI HU US\ DABAO ZHANG US | SHANGHAI MATRIX ENGINEERING IN (CN) | H04N-009/31\ H04N-005/74 |
| 5632 | 3D printing | OPERATION METHOD OF FIRST END DEVICE OF SERVICE PROVIDER NETWORK INVOLVES ANALYZING RECEIVED REPLY FRAME CORRESPONDING TO GENERATED MEASUREMENT FRAME, AND HIERARCHICALLY ACQUIRING SERVICE LEVEL AGREEMENT (SLA) METRICS FOR SECOND END DEVICE | US2014293799A1\ KR2014118100A | CHEN JUN (CN)\ KUI HU US\ DABAO ZHANG US | XINJIANG AKSU ELECTRIC POWER CO LTD US | H04W-024/08\ H04L-012/26 |
| 5633 | 3D printing | PROJECTOR E.G. DIGITAL LIGHT PROCESSING (DLP) PROJECTOR HAS DISTORTION CORRECTION UNIT TO PERFORM DISTORTION CORRECTION OF IMAGE SIGNAL OUTPUT FROM IMAGE OUTPUTTING APPARATUS BASED ON OUTPUT PIXEL COUNT SO AS TO PROJECT IMAGE SIGNAL | JP2014187530A | CHEN KANGMIN YAO (CN) | XINJIANG AKSU ELECTRIC POWER CO LTD US | G03B-021/14\ G09G-003/20\ G09G-003/34\ G09G-003/36\ H04N-005/74 |
| 5634 | 3D printing | METHOD FOR PREDICTING APPLICATION USAGE FOR MOBILE CLIENT E.G. SMARTPHONE, INVOLVES RETAINING PREFETCHED ADVERTISEMENTS IN ADVERTISEMENT CACHE OF MOBILE CLIENT AND DISPLAYING ON DISPLAY SCREEN DURING EXECUTION OF APPLICATIONS | US2014279026A1\ WO2014150630A2\ WO2014150630A3\ CN105074743A\ EP2973269A2 | CHEN SHANRONG US | SHANGHAI BEILING CO LTD (CN) | G06Q-030/02\ G06Q-000/00\ G06Q-010/10\ G06Q-050/30\ H04W-088/02 |
| 5635 | 3D printing | METHOD FOR DEPLOYING E.G. LINUX-APACHE-MYSQL-PHP SERVER PLATFORM IN CLOUD-COMPUTING ENVIRONMENT, INVOLVES RECEIVING DEPLOYMENT POLICY FOR EXECUTION OF DEPLOYMENT OF WORKLOAD IN ACCORDANCE WITH RESOURCE AGNOSTICALLY OF CLOUD PROVIDER | US2014280961A1 | CHEN SHIGUO US\ DENG LING US\ LIAO XINHUA US | UNIV JISHOU US | H04L-012/911 |
| 5636 | 3D printing | METHOD FOR FACILITATING WEIGHT-BASED COLLOCATION MANAGEMENT IN DATA CENTER, INVOLVES ACCESSING PERFORMANCE SPECIFICATION FOR APPLICATION, AND DETERMINING RESOURCE WEIGHT FOR RESOURCE FOR NEXT TIME INTERVAL BY PROCESSOR | US2014282503A1\ US9178763B2 | CHENG CHUN-FAI (TW)\ CHEN BWO-NING (TW)\ SU CHIN-TE (TW)\ HO HUANG-SHENG (TW) | CHEN SHIGUO US\ DENG LING US\ LIAO XINHUA US | G06F-009/455\ H04L-012/26\ G06F-011/30\ H04L-012/24 |
| 5637 | 3D printing | NON-TRANSITORY COMPUTER-READABLE STORAGE MEDIUM STORING INSTRUCTION FOR MANAGING VIRTUAL DATA CENTER (VDC) RESOURCE, INCLUDES INSTRUCTION FOR DISPLAYING ARCHITECTURAL LAYOUT BY CREATING VISUAL DEPICTION OF VDC RESOURCES AND APPLICATION | US2014282525A1\ US9454294B2 | CHENG KANGGUO (US)\ KHAKIFIROOZ ALI (US)\ KULKARNI PRANITA (US)\ SHAHIDI GHAVAM G (US) | TAIWAN SEMICONDUCTOR MFG (TW)\ CHENG CHUN-FAI (HK)\ CHEN BWO-NING (TW)\ SU CHIN-TE (TW)\ HO HUANG-SHENG (TW) | G06F-009/455\ G06F-003/0484\ G06F-009/445\ G06Q-030/06\ G06Q-040/00\ H04L-012/24\ H04L-012/26\ H04L-012/911\ H04L-029/08 |
| 5638 | 3D printing | METHOD FOR EFFICIENT MITIGATION OF DENIAL OF SERVICE ATTACKS IN SOFTWARE DEFINED NETWORK TO MAINTAIN SECURITY SERVICE LEVEL AGREEMENT GUARANTEED TO VIRTUAL MACHINE, INVOLVES EXECUTING MITIGATION ACTION TO MITIGATE DENIAL OF SERVICE ATTACK | US2014283051A1\ US9450981B2 | CHENG KANGGUO (US)\ KHAKIFIROOZ ALI (US)\ SHAHIDI GHAVAM G (US)\ KERBER PRANITA (US) | IBM (US)\ CHENG KANGGUO (US)\ KHAKIFIROOZ ALI (US)\ KULKARNI PRANITA (US)\ SHAHIDI GHAVAM G (US) | H04L-029/06\ H04L-012/24 |
| 5639 | 3D printing | COMMUNICATION NETWORK I.E. ETHERNET-BASED METRO AREA NETWORK, FOR PROVIDING E.G. ETHERNET LINE SERVICE, HAS CORE MULTIPLEXORS INCLUDING UPLINK PORT AND LINE PORTS, WHERE LINE PORTS INCLUDE ONE-TO-ONE LINE PORT | US2014270767A1 | CHIANG CHEN-DER (TW)\ TANG XIANG-FENG (TW)\ WENG BING-GUO (TW)\ SHI ZHI-CHANG (TW)\ GAO YAO-TANG (TW) | IBM (US)\ CHENG KANGGUO (US)\ KHAKIFIROOZ ALI (US)\ KULKARNI PRANITA (US)\ SHAHIDI GHAVAM G (US) | H04J-014/02\ H04L-012/42\ H04L-012/721\ H04L-012/937\ H04Q-011/00 |
| 5640 | 3D printing | METHOD FOR DYNAMICALLY ENABLING SELECTIVE ROUTING CAPABILITY IN SHARED-MEDIA COMMUNICATION NETWORK, INVOLVES NOTIFYING ONE OR MORE NEIGHBORING NODES IN SHARED-MEDIA COMMUNICATION NETWORK OF ENTERED SELECTIVE FORWARDING MODE | US2014269402A1\ WO2014149601A1\ CN105052091A\ EP2974172A1\ US9426020B2\ IN201502555P3 | CHIOU JING-HUNG (TW)\ YEN KAI-HSIANG (TW)\ WANG CHIN-HORNG (TW)\ CHEN JEN-YI (TW)\ FANG PEI-YI (TW) | CHUNG SHAN INST OF SCIENCE (TW) | H04L-012/26\ H04L-012/24\ H04L-012/703\ H04L-012/707\ H04L-012/721\ H04L-012/803\ H04W-028/02\ H04W-040/10\ H04W-052/02\ H04L-012/725\ H04L-012/753\ H04W-040/02 |
| 5641 | 3D printing | LIGHT SWITCH ARRAY BASED DIGITAL LIGHT PROCESSING DEVICE, HAS MICRO-REFLECTING MIRROR PROVIDED WITH WORKING STATE OF DEFLECTION DIRECTION AND CONNECTED TO INPUT OUTPUT UNIT THAT IS PROVIDED WITH TRIPLE-FIBER COLLIMATOR | CN103969749A | CHO DONG IL (KR) | IND TECH RES INST (TW) | G02B-006/35 |
| 5642 | 3D printing | THREE-DIMENSIONAL INFORMATION CARRIER, COMPRISES TWO-DIMENSIONAL CODE OR COLOR CODE FORMED ALONG XY-AXIS PLANE, WHERE TWO-DIMENSIONAL CODE, COLOR CODE OR PLANE ELEMENT COMBINATION CODE ARE SCANNED VIA COMPUTED TOMOGRAPHY SCANNING PROCESS | CN103971138A | CHO SEONG-HO (KR) | SEOUL NAT UNIV IND FOUNDATION (KR) | G06K-015/00 |
| 5643 | 3D printing | SERVICE RESOURCE FACTOR MEASURING METHOD, INVOLVES OBTAINING RESOURCE SERVICE INFORMATION, DETERMINING SERVICE RESOURCE FACTOR ACCORDING TO RATE OF RESOURCE SERVICE INFORMATION, AND CALCULATING BASIC SERVICE RESOURCE FACTOR EVALUATION VALUE | CN103973458A | CHO WON SEOK (KR)\ KWAK KUN HO (KR)\ JANG JAE HOON (KR)\ JUNG SOON MOON (KR)\ LIM HOON (KR)\ KIM SUNG JIN (KR)\ HWANG BYUNG JUN (KR)\ KIM JONG HYUK (KR)\ HAN MYANG SIK (KR) | SAMSUNG ELECTRONICS CO LTD (KR)\ CHO SEONG-HO (KR) | H04L-012/14\ H04L-029/08 |
| 5644 | 3D printing | SERVICE LEVEL AGREEMENT PROCESSING METHOD, INVOLVES PERFORMING SERVICE LEVEL AGREEMENT CONTROLLING PROCESS, RECEIVING RULE INFORMATION BY SERVICE END, AND PERFORMING SERVICE RULE UPDATING PROCESS WHEN SUBSCRIPTION INFORMATION IS OBTAINED | CN103973467A | CHOI GIL HEYUN (KR)\ LEE JONG MYEONG (KR)\ KANG PIL KYU (KR)\ BAE DAE LOK (KR) | SAMSUNG ELECTRONICS CO LTD (KR) | H04L-012/24 |
| 5645 | 3D printing | THREE-DIMENSIONAL PRINTER LIFTING MECHANISM HAS GUIDE RAIL SLIDE BLOCK THAT IS LENGTHWAYS CONNECTED TO Z-AXIS SUBSTRATE, AND Z-AXIS ELECTRIC MACHINE THAT IS ROTATED BY APIR OF SCREW | CN203752530U | CHOI GYU HWAN (KR) | SAMSUNG ELECTRONICS CO LTD (US) | B29C-067/00 |
| 5646 | 3D printing | SLA THEORY-BASED 3D PRINTER, HAS ELECTRIC CONTROL DEVICE INSTALLED ON RESIN CONTROL DEVICE, RESIN MOLDING PROCESSING ROOM INSTALLED ON LIGHT WAY CONTROL DEVICE, AND DISPLAY CONTROL SCREEN ROTATABLY FIXED WITH MAIN BODY DEVICE | CN203752531U | CHUNZU YANG US | SAMSUNG ELECTRO MECH (KR) | B29C-067/00 |
| 5647 | 3D printing | PORTABLE DIGITAL LIGHT PROCESSING (DLP) PROJECTOR USED IN OFFICE AND SCHOOL, HAS MACHINE DRIVE PLATE AND PLATE MACHINE, WHICH ARE LOCATED ON SAME PLANE, AND POWER SOURCE MODULE AND SOUND BOX WHICH ARE LOCATED ON SAME PLANE | CN203732866U | CLAMPITT DARWIN A (US)\ LYONSMITH SHAWN D (US)\ TSUI REGAN S (US) | CHUNZU YANG US | G03B-021/00\ G03B-021/14 |
| 5648 | 3D printing | FOCUSING STRUCTURE FOR DIGITAL LIGHT PROCESSING (DLP) PROJECTOR, HAS LIGHT MACHINE WHOSE FOCUSING DEFLECTOR POLE IS PASSED THROUGH THROUGH-HOLE OF FOCUSING SHIFTING SHEET AND FOCUSING DIAL TONGUE SHIFTING SHEET FROM OPENING OF COVER | CN203734754U | CLEMENS BRUCE M (US)\ GROVES JAMES R (US)\ HAYES GARRETT J (US)\ LI BINGRUI JOEL (US)\ SALLEO ALBERTO (US) | MICRON TECHNOLOGY INC (US) | H04N-005/74 |
| 5649 | 3D printing | DIGITAL LIGHT PROCESSING (DLP) BUILT-IN SOUND BOX HAS MICRO-SPEAKER WHICH IS SET IN SHELL, AND WHOSE INNER FRAME SEAL IS ADHERED TO INNER SIDE OF HOLE IN WHICH SIDE PANEL IS ARRANGED CORRESPONDING TO PNEUMA HOLE OF REAR SHELL PANEL | CN203734796U | COLOMA BERNARD US\ DELAUNAY PATRICK US | CLEMENS BRUCE M (US)\ GROVES JAMES R (US)\ HAYES GARRETT J (US)\ LI BINGRUI JOEL (US)\ SALLEO ALBERTO (US) | H04R-001/02 |
| 5650 | 3D printing | MICRO-PROJECTION FUNCTION INTEGRATED CLOUD COMPUTING MINIATURE INTELLIGENT TERMINAL HAS DIGITAL LIGHT PROCESSING PLATE WHICH IS SET IN LOWER PORTION OF LED CONTROL MODULE WHOSE LOWER PORTION IS SET WITH PROJECTION OPTICAL MODULE | CN203673388U | COOKE PAUL (US)\ LABYUK VICTOR (US)\ YE SHERRY QIANWEN (US)\ HOFFMAN RICHARD W JR (US) | ATMEL NANTES SA SA (FR) | G03B-029/00\ G06F-001/16 |
| 5651 | 3D printing | PROJECTION TYPE DISPLAY APPARATUS E.G. LCD PROJECTOR HAS DRIVE UNIT CHANGING OPTICAL PATH OF PROJECTED LIGHT SO THAT IMAGE DISPLAY POSITION IS NOT CHANGED WHILE MOVING PROJECTION LENS | JP2014182322A | COOPER GREGORY D (US)\ WEHRENBERG BRIAN L (US) | EMCORE CORP (US)\ COOKE PAUL (US)\ LABYUK VICTOR (US)\ YE SHERRY QIANWEN (US)\ HOFFMAN RICHARD W (US) | G03B-021/00\ G03B-021/14\ H04N-005/74 |
| 5652 | 3D printing | SERVICE GROUP CLIENT SLA LEVEL AIMING METHOD, INVOLVES DETERMINING PRIORITY OF SBC DIALOGUE GROUP NAME BY PRIVATE NETWORK CUSTOMER, AND DETERMINING GRAY LIST ACCORDING TO PRESET GROUP OF SERVICE LEVEL PROTOCOL | CN103916364A | CREMER SEBASTIEN (FR) | PIXELLIGENT TECHNOLOGIES LLC (US) | H04L-012/46\ H04L-029/06 |
| 5653 | 3D printing | INTELLIGENT PROJECTOR-ENABLED MOBILE PHONE, HAS DRIVING PLATE CONNECTED WITH DIGITAL LIGHT PROCESSING MACHINE, MOBILE PLATE AND DRIVING PLATE CARD CONNECTED TO CONTROL DRIVING PLATE, AND MANAGEMENT MODULE FIXED ON PHONE MAIN BODY | CN203708298U | CRIPE JERRY R (US)\ PHAM LE T (US)\ TOTH ANDREW G (US) | ST MICROELECTRONICS CROLLES 2 (FR) | H04M-001/02\ H04M-001/21 |
| 5654 | 3D printing | ROD-SHAPED OPTICAL SCANNING DEVICE FOR PRODUCING THREE DIMENSIONAL IMAGES OF BODY, HAS SEVERAL LENSES AND LIGHTING DEVICES ARRANGED ON SURFACE OF ROD-SHAPED HOLDER SO THAT LENSES AND LIGHTING DEVICES OVERLAP EACH OTHER DURING SCANNING | DE202013004557U1 | CZAGAS JOSEPH (US)\ WOODBURY DUSTIN (US)\ BEASOM JAMES (US) | RAYTHEON CO (US) | G02B-027/22\ G03B-037/04 |
| 5655 | 3D printing | DISPLAY APPARATUS FOR USE IN PROJECTOR DISPLAY, HAS ARRAY OF LIGHT MODULATORS THAT IS CONFIGURED TO MODULATE LIGHT EMITTED FROM LIGHT GUIDE PLATE TO FORM IMAGE ON APPARATUS MAIN PORTION | WO2014143713A1\ IN201502986P3\ EP2971948A1\ CN105308388A\ KR2016019886A\ US2016223736A1\ HK1215467A0 | DAI QING-LIANG (TW)\ LIU MAO-CHENG (TW) | INTERSIL INC (US) | F21V-008/00\ G02B-027/09\ G02B-027/48\ G02F-001/1335\ G03B-021/20\ H04N-009/31\ G09G-003/06\ F21V-000/00\ G02B-000/00\ G02F-000/00\ G03B-000/00\ H04N-000/00 |
| 5656 | 3D printing | METHOD FOR REDUCING CUSTOMER NOISE IN FACILITIES MANAGEMENT COMPUTING ENVIRONMENT, INVOLVES PLACING SERVICE REQUEST RECORD (SRR) IN THIRD ELECTRONIC QUEUE WHEN CUSTOMER SURVEY IS NOT COMPLETED WITHIN SURVEY TIME PERIOD | WO2014145690A1\ US2014278643A1\ CA2907331A1 | DAKE CHEN US\ BIN HAN US\ JIALIANG LUO US | UNIV NAT CHUNGHSING (TW) | G06F-017/30\ G06Q-010/06\ G06Q-010/08 |
| 5657 | 3D printing | DIGITAL LIGHT PROCESSING PROJECTOR APPARATUS FOR PROJECTING MONOCHROMATIC DISPLAY, COMPRISES LIGHT SOURCES, LIGHT SINK FOR CAPTURING MONOCHROMATIC LIGHT BEAMS, PROJECTION LENS, DIGITAL MICROMIRROR DEVICE CHIP, AND CONTROL UNIT | WO2014141272A2\ WO2014141272A3\ CN105209972A\ EP2972584A2\ US2016033756A1\ JP2016513818W | DEGROOTE STEFAN (BE)\ CHENG KAI (BE) | LIANYUNGANG JIERUI ELECTRONICS CO LTD US | G03B-021/00\ B33Y-030/00\ G02B-026/08\ G03F-007/20\ G03B-021/14 |
| 5658 | 3D printing | MULTI-COLOR ILLUMINATION APPARATUS FOR ILLUMINATING PHOSPHOR COLOR WHEEL IN DIGITAL LIGHT PROCESSING PROJECTION SYSTEM, HAS LIGHT SOURCES THAT ARE ARRANGED TO ILLUMINATE WHEEL AT DIFFERENT LOCATIONS TO GENERATE TIME-SEQUENCED BEAMS | EP2778741A1\ JP2014174555A\ US2014268066A1\ CN104049446A\ US9146452B2 | DEYUAN XIAO US\ JIANHUA ZHOU US\ JING CHEN US\ JIEXIN LUO US\ QINGQING WU US\ XI WANG US | IMEC (BE)\ CHENG KAI (BE)\ DEGROOTE STEFAN (BE) | G02B-026/00\ G03B-021/00\ G03B-021/14\ H04N-009/31\ F21V-009/10\ G03B-021/20 |
| 5659 | 3D printing | INFORMATION HANDLING SYSTEM E.G. EMBEDDED COMPUTER SYSTEM FOR MANAGING MAINTENANCE OPERATION, HAS REMOTE VIRTUALIZATION MANAGER THAT RECORDS INFORMATION ASSOCIATED WITH MODES OF HYPERVISOR AND PLACE HYPERVISOR IN MAINTENANCE MODE | US2014245296A1\ US9384021B2 | DORIS BRUCE B (US)\ BASKER VEERARAGHAVAN S (US)\ CHENG KANGGUO (US)\ FALTERMEIER JOHNATHAN E (US)\ KHAKIFIROOZ ALI (US) | SHANGHAI INST OF MICROELECTRONICS AND INFORMATION TECHNOLOGY CHINESE ACADEMY (CN) | G06F-009/455\ G06F-001/00\ G06F-013/28\ G06F-015/16 |
| 5660 | 3D printing | SYSTEM FOR FABRICATING THREE-DIMENSIONAL OBJECT COMPRISES PART BED SURFACE\ MATERIAL DEPOSITING UNIT CONFIGURED TO DEPOSIT THE MATERIALS ONE LAYER\ LASER-BASED THERMAL SOURCE\ DIGITAL LIGHT PROCESSING PROJECTOR\ AND CONTROLLER | US2014255666A1 | DORIS BRUCE B (US)\ CHENG KANGGUO (US)\ KULKARNI PRANITA (US)\ ADAM THOMAS N (US)\ HARAN BALASUBRAMANIAN S (US)\ REZNICEK ALEXANDER (US) | IBM (US)\ IBM UK (GB)\ CHENG KANGGUO (US)\ KHAKIFIROOZ ALI (US)\ DORIS BRUCE (US)\ BASKER VEERARAGHAVAN (US)\ FALTERMEIER JOHNATHAN (US) | B29C-067/00 |
| 5661 | 3D printing | METHOD FOR DYNAMICALLY CONTROLLING MULTI-TIER PROCESSING SYSTEM, INVOLVES COMPUTING CONTROL MODEL OF TIER BASED ON RECEIVED QUEUE LENGTHS AND ADJUSTING PARAMETERS OF CONTROL MODEL BASED ON RECEIVED CONTROL OBJECTIVE | US2014244980A1 | DORIS BRUCE B (US)\ ZHANG YING (US)\ CHENG KANG-GUO (CN) | IBM (US)\ CHENG KANGGUO (US)\ DORIS BRUCE B (US)\ KULKARNI PRANITA (US)\ ADAM THOMAS N (US)\ HARAN BALASUBRAMANIAN S (US)\ REZNICEK ALEXANDER (US) | G06F-009/30 |
| 5662 | 3D printing | METHOD FOR ENERGY EFFICIENT ALLOCATION OF STORAGE RESOURCES IN NETWORKED COMPUTING ENVIRONMENT, INVOLVES SELECTING STORAGE DEVICE FROM SET OF STORAGE DEVICES FOR HANDLING STORAGE WORKLOAD ACCORDING TO DEPLOYED ALLOCATION PLAN | US2014129717A1\ US9432300B2 | DOYLE BRIAN S (US)\ BAI GANG (US)\ LIANG CHUNLIN (US)\ FRASER DAVID B (US)\ CHENG PENG (US) | IBM (US)\ IBM UK (GB)\ CHENG KANGGUO (US)\ ZHANG YING (US)\ DORIS BRUCE (US) | H04L-012/911\ G06F-001/00\ G06F-001/32\ G06F-003/06 |
| 5663 | 3D printing | METHOD FOR PROVISIONING OF STORAGE FOR VIRTUALIZED APPLICATIONS BY MEETING SERVICE LEVEL AGREEMENT, INVOLVES QUANTIFYING PRIMARY RESOURCE USED BY SECONDARY APPLICATION WHEN SECONDARY APPLICATION IS RUNNING | US2014130055A1 | DU JUN US\ HAILING TU US\ YI WANG US\ FENG WEI US\ SHOUJING YUE US | INTEL CORP (US) | G06F-009/50 |
| 5664 | 3D printing | CLOUD COMPUTING RESOURCE DISTRIBUTING METHOD, INVOLVES CARRYING OUT AUTHENTICATION PROCESS, JUDGING WHETHER HOST MACHINE SATISFIES SLA REQUIREMENT, AND STOPPING RESOURCE DISTRIBUTION WHEN SLA REQUIREMENT IS NOT SATISFY | CN103905529A | DUNNE BRENDAN (FR) | BEIJING NONFERROUS METAL US | G06F-009/48\ H04L-012/911\ H04L-029/08 |
| 5665 | 3D printing | METHOD FOR GENERATING VOICE SERVICE SERVICE-LEVEL AGREEMENT THRESHOLD VALUE IN 2G/3G NETWORK, INVOLVES GENERATING SHORT TIME SERVICE LEVEL AGREEMENT THRESHOLD VALUE OR LONG TIME SERVICE LEVEL AGREEMENT THRESHOLD VALUE | CN103906145A | DUNNE BRENDAN (FR)\ CHANNON KEVIN (GB)\ CHRISTISON ERIC (GB)\ NICOL ROBERT (GB) | ST MICROELECTRONICS ROUSSET (FR) | H04W-028/24 |
| 5666 | 3D printing | LIGHT SOURCE DEVICE OF PROJECTOR E.G. DIGITAL LIGHT PROCESSING (DLP) PROJECTOR, HAS CONTROL CIRCUIT WHICH COMPARES LIGHT QUANTITY MEASUREMENT DATA WITH DATA RECEIVED AT NORMAL TIME, AND DETECTS ABNORMALITY | JP2014157184A | DURIEZ BLANDINE (GB)\ GRIDELET EVELYNE (GB)\ MERTENS HANS (GB)\ VANHOUCK TONY (GB)\ DONKERS JOHANNES JOSEPHUS THEODORUS (GB) | ST MICROELECTRONICS ROUSSET (FR)\ ST MICROELECTRONICS R&D LTD (GB) | F21S-002/00\ F21V-008/00\ G03B-021/00\ G03B-021/14\ G09G-003/20\ G09G-003/34\ G09G-003/36 |
| 5667 | 3D printing | COMPUTING SYSTEM FOR CONTENT ADAPTIVE POWER MANAGEMENT OF E.G. DIGITAL LIGHT PROCESSING PROJECTOR SYSTEM, HAS IMAGE PROCESSING BLOCK ADJUSTING PIXEL VALUES OF IMAGE DATA AND SENDING PIXEL VALUES AS PIXEL DATA ON EXTERNAL INTERFACE TO IMAGER | US2014247288A1\ WO2014137732A1\ KR2015103260A\ CN104956666A\ JP2016507998W | ECKSTEIN GERALD (DE)\ FREY ALEXANDER (DE)\ KUEHNE INGO (DE)\ MARINKOVIC DJORDJE (DE) | NXP BV (NL)\ DONKERS JOHANNES JOSEPHUS THEODORUS MARINUS (NL)\ DURIEZ BLANDINE (BE)\ MERTENS HANS (BE)\ VANHOUCKE TONY (BE)\ GRIDELET EVELYNE MARIE JOSEPHE FABIENNE (BE) | G09G-005/10\ G03B-021/00\ G09G-005/00\ H04N-005/74\ G09G-003/34\ H04N-005/58\ H04N-009/31\ G09G-003/20\ G09G-003/36 |
| 5668 | 3D printing | COMPUTER-IMPLEMENTED METHOD FOR PROTECTING AGAINST DATA LOSS IN NETWORKED COMPUTING ENVIRONMENT, INVOLVES REQUESTING INSURANCE POLICY FOR CLOUD CUSTOMER BASED ON INSURANCE COST ASSOCIATED WITH PARTICULAR PROVIDER IN RISK TABLE | US2014244311A1 | EICHHOLZ JOERG (DE) | SIEMENS AG (DE) | G06Q-040/08 |
| 5669 | 3D printing | FIBER SCANNING THREE-DIMENSIONAL (3D) PROJECTOR FOR MODULATING LIGHT, HAS SCANNER THAT INCLUDES ACTUATOR TO DRIVE FIBER BUNDLE AND SYNCHRONIZATION CONTROLLER SYNCHRONIZES DRIVING OF ACTUATOR AND DRIVING OF MODULATOR | US2014232993A1\ KR2014102992A\ US9445063B2 | EL FARHANE REBHA (FR) | FRAUNHOFER GES FORSCHUNG (DE) | H04N-009/31\ G03B-021/00\ G03B-021/14\ G02B-026/10\ G03B-021/20 |
| 5670 | 3D printing | METHOD FOR PROVISIONING LOGICAL STORAGE VOLUME FOR APPLICATION RUNNING IN COMPUTER SYSTEM, INVOLVES STORING UNIQUE IDENTIFIER FOR LOGICAL STORAGE VOLUME RECEIVED BY SYSTEM AND ASSOCIATING UNIQUE IDENTIFIER WITH APPLICATION RUNNING IN SYSTEM | US2014245016A1\ US8949570B2 | EN WILLIAM GEORGE (US)\ MASZARA WITOLD P (US)\ PELELLA MARIO M (US) | KONINKL PHILIPS ELECTRONICS NV (NL)\ EL FARHANE REBHA (FR) | G06F-003/06\ H04L-029/06\ G06F-012/02 |
| 5671 | 3D printing | THREE-DIMENSIONAL NONVOLATILE SEMICONDUCTOR MEMORY DEVICE I.E. BIT COST SCALABLE MEMORY, HAS TRANSISTOR CONNECTED BETWEEN END OF TRANSISTOR AND SOURCE LINE, AND CONTROLLER FOR CONTROLLING TRANSISTORS AFTER PERFORMING PROGRAM OPERATION | US2014233320A1\ JP2014164773A\ TW201434048A | ESSAIAN STEPAN (US) | ADVANCED MICRO DEVICES INC (US) | G11C-016/26\ G11C-016/02\ G11C-016/04\ G11C-016/06\ G11C-016/24\ G11C-008/14 |
| 5672 | 3D printing | CLOUD PLATFORM-BASED INFRASTRUCTURE-AS-A-SERVICE RESOURCE OPTIMIZING AND DEPLOYMENT METHOD, INVOLVES DETERMINING ANALYSIS APPLICATION INFORMATION BY MODE IDENTIFICATION MODULE THAT IS PROVIDED WITH CLASSIFICATION MODULE | CN103873569A | FAN YUAN-HENG (CN) | NAT SEMICONDUCTOR CORP (US) | G06F-009/50\ G06N-003/02\ H04L-029/08 |
| 5673 | 3D printing | METHOD FOR CONTROLLING DISPLAY I.E. LCD, IN E.G. DISPLAY APPARATUS OF ELECTRONIC DEVICES, INVOLVES INCREASING MODIFIED GRAY SCALE VALUES AND GRAY SCALE VALUES CORRESPONDING TO COLORS OTHER THAN SPECIFIC COLOR FOR WHITE-TENDING SUB FRAME | US2014225934A1\ TW201432666A\ TW485696B1\ US9129564B2 | FANG ZHENXIAN US\ LIU YING (US) | SEMICONDUCTOR MFG INT SHANGHAI (CN) | G09G-003/36\ G09G-005/30\ G09G-003/34\ G09G-005/10 |
| 5674 | 3D printing | METHOD FOR LINKING POLICY ASSERTION TO PROCESSING RULE CONTEXTS FOR SERVICE LEVEL AGREEMENT POLICY ENFORCEMENT WITHIN RUNTIME ENVIRONMENT, INVOLVES ENFORCING POLICY ENFORCEMENT RULES FROM POLICY DOMAINS DURING RUNTIME | US2014229595A1 | FASONG BAI US\ YUHONG GAO US\ LIANSHENG GE US\ FUQUAN HUANG US\ YUAN NI US\ LIANG YUAN US | UNIV HEILONGJIANG US | H04L-012/24 |
| 5675 | 3D printing | SYSTEM FOR VISUALIZING RUNTIME RESOURCE POLICY ATTACHMENTS AND APPLIED POLICY DETAILS REGARDING SERVICE LEVEL AGREEMENT, HAS PROCESSOR FOR DISPLAYING EFFECTIVE APPLIED POLICY CONFIGURATION ASSOCIATED WITH SELECTED POLICY RESOURCE | US2014229843A1\ US9430116B2 | FENG CHEN US\ BIAO WANG US\ XIANHENG XIA US\ LIANLIANG TAI US\ QINGWEI LIU US\ HONGFENG XIA US\ GUANGREN LI US | HUAINAN MINING GROUP CO LTD US\ NAT ENGINEERING RES CT FOR COAL GAS CONTROL US\ NANJING ZIHUAI APPLIED MINING ELECTRONIC HI TECH CO LTD US | G06F-003/048\ G06F-003/0482\ G06F-003/0484\ G06Q-010/06\ G06Q-030/00 |
| 5676 | 3D printing | METHOD FOR FACILITATING E.G. MONITORING OF SERVICE POLICY ENFORCEMENT OF CORE PROCESSING MODULE IN E.G. COMPUTING DEVICE, INVOLVES CORRELATING PER-TRANSACTION SERVICE POLICY ENFORCEMENT INFORMATION WITH POLICY IDENTIFICATION INFORMATION | US2014230006A1\ US9363289B2 | FENG RAN US\ SHENGKAI XUE US\ YUCHEN TANG US\ XIAO CAI US | HEFEI LIJIE SEMICONDUCTOR TECHNOLOGY CO LTD US\ LONTIUM SEMICONDUCTOR CORP US | H04L-029/06\ G06F-015/16 |
| 5677 | 3D printing | METHOD FOR PROVIDING MIXED CENTRALIZED/DISTRIBUTED ALGORITHM FOR RISK MITIGATION IN E.G. IEEE-61334 NETWORKS, INVOLVES INSTRUCTING PORTION OF TRAFFIC TO REROUTE OVER ALTERNATE ACCEPTABLE NODE WHEN PORTION OF TRAFFIC IS ROUTED OVER NODE | US2014219103A1\ WO2014123924A1\ EP2954649A1 | FENGYI HUANG US\ TAO LI US\ XUSHENG TANG US\ NAN JIANG US | UNIV SHANGHAI (CN) | H04L-012/803\ H04L-012/721\ H04L-012/729 |
| 5678 | 3D printing | PROJECTOR E.G. LIQUID CRYSTAL PROJECTOR FOR PROJECTING STILL IMAGE AND MOVING IMAGE, HAS SHRINKING UNIT WHICH SHRINKS PROJECTION IMAGE BASED ON ACQUIRED DISTANCE WHEN POSITIONS ARE NOT SIMULTANEOUSLY FOCUSING | JP2014142466A | FORBES LEONARD (US) | UNIV SOUTHEAST US\ S TEK SHANGHAI HIGH FREQUENCY COMM TECHNOLOGY CO LTD US | G03B-021/00\ G03B-021/14\ G09G-003/20\ G09G-003/36\ G09G-005/00\ H04N-005/74 |
| 5679 | 3D printing | PROJECTION APPARATUS E.G. DIGITAL LIGHT PROCESSING PROJECTOR APPARATUS, HAS MEASUREMENT UNIT MEASURES TO FORM OPTICAL IMAGE ACCORDING TO IMAGE SIGNAL INPUT DURING PERIOD, IN WHICH DIFFERENT TYPES OF ELEMENTS SIMULTANEOUSLY EMIT LIGHT | US8794765B2 | FORBES LEONARD (US) | MICRON TECHNOLOGY INC (US) | G03B-021/00 |
| 5680 | 3D printing | METHOD FOR GENERATING HOLISTIC QUALITY OF SERVICE POLICY IN E.G. NETWORK, INVOLVES OUTPUTTING MARKED PACKETS BY ONE NETWORK NODE TO OTHER NETWORK NODE, WHERE MARKED PACKETS PERMIT OTHER NETWORK NODE TO PROCESS MARKED PACKETS | US8797867B1 | FORRAI DAVID (US)\ ENDRES DARREL (US)\ JONES ROBERT (US)\ GARTER MICHAEL JAMES (US) | MICRON TECHNOLOGY INC (US) | H04L-012/26 |
| 5681 | 3D printing | NEW HYDROXY ALIPHATIC SUBSTITUTED PHENYL AMINOALKYL ETHER DERIVATIVE USEFUL FOR TREATING NERVOUS SYSTEM DISEASES, AND DEVELOPMENTAL, BEHAVIORAL AND MENTAL DISORDERS WITH COGNITIVE DEFICITS, LEARNING DEFICITS OR MEMORY DEFICITS | EP2745876A1\ WO2014096377A1\ AU2013366502A1\ CA2895592A1\ EP2945702A1\ US2015344408A1\ JP2016504992W | FOSSUM ERIC R (US)\ NIXON ROBERT (US) | FORRAI DAVID (US)\ ENDRES DARREL (US)\ JONES ROBERT (US)\ GARTER MICHAEL JAMES (US)\ L 3 COMM CINCINNATI ELECTRONICS CORP (US) | A61P-025/04\ A61P-025/16\ A61P-025/18\ A61P-025/24\ C07C-217/54\ C07D-209/30\ C07D-211/22\ C07D-265/30\ C07D-333/20\ C07D-339/04\ C07H-015/203\ A61K-031/138\ A61K-031/165\ A61K-031/381\ A61K-031/385\ A61K-031/4015\ A61K-031/4045\ A61K-031/451\ A61K-031/5375\ A61K-031/7032\ A61P-025/00\ C07C-217/44\ C07C-217/48\ C07D-207/27\ C07D-209/08\ A61K-031/192\ A61K-031/375\ A61K-031/7004\ A61K-045/06\ C07C-217/04\ C07C-059/68\ C07D-207/267\ C07D-307/62\ C07H-015/22\ A61K-031/404\ A61K-031/445\ A61K-045/00\ A61P-021/00\ A61P-025/08\ A61P-025/14\ A61P-025/20\ A61P-025/28\ A61P-029/00\ A61P-009/00\ C07C-217/18\ C07C-217/28 |
| 5682 | 3D printing | THREE-DIMENSIONAL IMAGE DISPLAY APPARATUS HAS CONTROL UNIT DRIVING SHUTTER DEVICE AT FREQUENCY HIGHER THAN LIGHT EMISSION FREQUENCY OF LED DISPLAY UNIT | JP2014137511A | FOSSUM ERIC R (US)\ NIXON ROBERT (US) | CALIFORNIA INST OF TECHN (US) | G02B-026/02\ G02B-027/22\ G02F-001/13\ G02F-001/17\ G03B-035/18\ H04N-013/04 |
| 5683 | 3D printing | DEVICE FOR ADJUSTING RECORDING POSITION OF LINE HEAD E.G. PRINT HEAD OF IMAGE FORMING APPARATUS, INVOLVES CONTROLLING RECORDING TIMING OF EACH HEAD MODULE BASED ON CORRECTED AMOUNT OF HEAD MODULE STORED IN STORAGE UNIT | WO2014112313A1\ JP2014138983A\ US2015306870A1\ EP2946928A1\ US9227394B2\ JP5968797B2 | FRESART EDOUARD DE (US)\ STEEL JOHN W (US)\ THEODORE DAVID (IN) | CALIFORNIA INST OF TECHN (US) | B41J-002/01\ B41J-002/045\ B41J-002/055 |
| 5684 | 3D printing | POLARIZED-LIGHT LAMINATED ELEMENT FOR SEMI-TRANSPARENT PROJECTOR SCREEN E.G. OPAQUE SCREEN, HAS DIFFUSION-POLARIZATION LAYER AND ABSORPTION-POLARIZATION LAYER WHICH ARE LAMINATED TOGETHER, SUCH THAT TRANSMISSION AXES ARE PARALLEL | WO2014112412A1\ JP2014197163A\ TW201435471A\ KR2015106442A\ CN104937456A\ US2015362728A1 | FREY DOUGLAS R (US) | MOTOROLA INC (US) | B32B-007/02\ G02B-005/30\ G03B-021/62\ G02F-001/13\ G03B-021/00\ H04N-005/74\ G02B-027/28\ G03B-021/604\ G03B-021/60\ G02B-027/01 |
| 5685 | 3D printing | ILLUMINATION SYSTEM OF PROJECTION DEVICE E.G. DIGITAL LIGHT PROCESSING (DLP) PROJECTION DISPLAY, TRANSFORMS FIRST WAVE BAND LIGHT INTO SECOND WAVE BAND LIGHT BY FIRST WAVE BAND TRANSFORMING AREA, WHICH IS REFLECTED BY MIRROR | US2014204349A1 | FROEHLICH THOMAS (CH) | SILICON LAB INC (US) | F21V-013/08\ F21V-017/02\ G03B-021/16\ G03B-021/28 |
| 5686 | 3D printing | SERVICE LEVEL AGREEMENT (SLA) GUARANTEE APPARATUS FOR USE IN SOFTWARE AS A SERVICE (SAAS) ENVIRONMENT, ADDS SMODEL TO PROCESS SLA IN FOUR KINDS OF DATA STRUCTURE AND EXPANDS WEB SERVICE DESCRIPTION LANGUAGE (WSDL) | KR2014086357A | FU SHI US | AUSTRIAMICROSYSTEMS AG (AT) | G06F-017/00\ G06F-017/20 |
| 5687 | 3D printing | COMPUTER-BASED METHOD FOR SWITCHING WEB SERVICE MESSAGE, INVOLVES ROUTING SERVICE CALL MESSAGE TO SERVICE PROVIDER BASED ON POSITIVE VERIFICATION THAT SERVICE CONSUMER REQUESTS SERVICE AND ATTRIBUTES OF SERVICE CALL MESSAGE | US8781104B1\ US2014198910A1 | FUJII TAKASHI US\ MORIMOTO SHIGEO US\ YADOGUCHI YASUHIRO US | SENODIA TECHNOLOGIES SHANGHAI CO LTD US | H04M-003/42\ H04M-003/523\ H04M-003/51 |
| 5688 | 3D printing | METHOD FOR PROVIDING PRIVILEGED NETWORK ROUTING SERVICES, INVOLVES DETERMINING WHETHER TO ROUTE TRAFFIC AT NETWORK BACKBONE USING RESERVED RESOURCES OVER PRIVILEGED NETWORK PATH, AND INITIATING TRAFFIC OVER PRIVILEGED NETWORK PATH | US2014192644A1 | FUJIOKA HIROSHI US\ OSHIMA MASAHARU US | RENESAS ELECTRONICS CORP (JP) | H04L-012/725 |
| 5689 | 3D printing | METHOD FOR PROVIDING CLOUD SERVICES TO CUSTOMERS IN COMPUTING CLOUD, INVOLVES PROVIDING CLOUD SERVICES BASED ON NEGOTIATED SERVICE LEVEL AGREEMENTS (SLAS) TO CUSTOMERS | US2014200947A1 | FUJIWARA HIROYUKI (JP)\ ABIKO ICHIMATSU (JP)\ SAKUTA MASAAKI (JP)\ OGIWARA MITSUHIKO US | JAPAN SCIENCE & TECH CORP US | G06Q-030/06 |
| 5690 | 3D printing | INFORMATION PROCESSING SYSTEM FOR MANAGING VIRTUAL MACHINES (VMS), HAS CIRCUITRY THAT GENERATES VALUE INDICATING WHETHER PERFORMANCE OF SYSTEM SATISFIES AGREEMENT AND DETERMINES VM TO BE STOPPED AMONG MULTIPLE VMS BASED ON VALUES | US2014196035A1\ JP2014134904A | FUJIWARA HIROYUKI (JP)\ ABIKO ICHIMATSU (JP)\ SAKUTA MASAAKI (JP)\ OGIWARA MITSUHIKO US | OKI DATA KK (JP)\ OKI DIGITAL IMAGING KK US | G06F-009/455\ G06F-009/46\ G06F-009/50 |
| 5691 | 3D printing | DISPLAY APPARATUS E.G. LIQUID CRYSTAL PROJECTOR DISPLAYS IMAGE CORRESPONDING TO VIDEO SIGNAL ON SAME REGION AS REGION DISPLAYED BY FIRST DISPLAYING CONDITION ACCORDING TO HAVING SWITCHED FROM FIRST TO SECOND DISPLAYING CONDITION | JP2014130182A | FUJIWARA HIROYUKI (JP)\ ABIKO ICHIMATSU (JP)\ SAKUTA MASAAKI (JP)\ OGIWARA MITSUHIKO US | OKI DATA KK (JP)\ OKI DIGITAL IMAGING KK US | G02F-001/133\ G09G-003/20\ G09G-003/36\ G09G-005/00\ G09G-005/36\ G09G-005/38\ H04N-005/74 |
| 5692 | 3D printing | METHOD FOR PERFORMING REMOTE POINT OF VIEW PROCESSING OF SCENE PRESENTED ON LCD DEVICE IN AUTOMOBILE, INVOLVES PROCESSING IMAGE FOR DISPLAY ON DISPLAY DEVICE RESPONSIVE TO DETECTED POSITION OF VIEWER'S HEAD RELATIVE TO DISPLAY DEVICE | EP2753085A1\ US2014191965A1\ HK1199783A0 | FUJIWARA HIROYUKI (JP)\ OGIWARA MITSUHIKO US | OKI DATA KK (JP)\ OKI DIGITAL IMAGING KK US | G06F-003/01\ G06F-003/0481\ H04N-013/04\ G06F-000/00\ H04N-000/00 |
| 5693 | 3D printing | TELECOMMUNICATION SYSTEM WITH SERVICE ADMISSION CONTROL FUNCTION, HAS SERVICE ACCESS CONTROL (SAC) ELEMENT TO DETERMINE SUFFICIENT CAPACITY OF COMPONENT TO HANDLE DATA FLOWS WITH DATA PATH OPERATING BASED ON SERVICE PERFORMANCE DATA | US8774003B1 | FUQIANG CAO US | OKI DATA KK (JP)\ OKI DIGITAL IMAGING KK US | H04L-012/26 |
| 5694 | 3D printing | METHOD FOR DISPLAYING VIDEO IN THREE DIMENSIONAL DISPLAY APPARATUS IN E.G. ELECTRONIC DEVICE, INVOLVES ADJUSTING DISTANCE ALONG HORIZONTAL DIRECTION OF POSITION FOR DISPLAYING VIDEO FOR RIGHT-LEFT EYES BASED ON POSITION OF USER'S FINGER TIP | JP2014127836A | FURUKAWA YUTAKA US\ YOKOYAMA TAKAMASA US | WUXI QUALCHIP TECHNOLOGIES INC US | G06F-003/041\ G06F-003/0488\ G09G-005/00\ G09G-005/36\ H04N-013/04 |
| 5695 | 3D printing | ELECTRO-WETTING CELL FOR THREE-DIMENSIONAL IMAGE DISPLAYER FOR SHOWING E.G. ANIMATION, INSERTS HORIZONTAL VOLTAGE LEVEL INTO DRIVING SECTION WHEN DRIVING SECTION CHANGES FROM ONE THRESHOLD VOLTAGE LEVEL TO OTHER THRESHOLD VOLTAGE LEVEL | US2014185126A1\ CN103901608A\ TW201426001A | GALY PHILIPPE (FR)\ ENTRINGER CHRISTOPHE (FR)\ JIMENEZ JEAN (FR) | PANASONIC CORP (JP) | G02B-026/00\ G02B-026/08\ G02B-027/22\ G09G-003/34 |
| 5696 | 3D printing | TRUE THREE-DIMENSIONAL IMAGE DISPLAY SYSTEM, HAS LIGHT BEAM IMAGE REFLECTED ON IMAGING REGION OF IMAGING DEVICE, LIGHT SOURCE UNIT LED OR PNEUMA LAMP, AND MICRO-LENS ARRANGED ON DIGITAL LIGHT PROCESSING DEVICE | CN103777454A | GAN GE US\ HU YANGWEI US | ST MICROELECTRONICS SA (FR)\ GALY PHILIPPE (FR)\ ENTRINGER CHRISTOPHE (FR)\ JIMENEZ JEAN (FR) | G02B-027/22\ G03B-035/18\ H04N-013/04 |
| 5697 | 3D printing | PROJECTOR, PARTICULARLY REFLECTION TYPE PROJECTOR FOR LIQUID-CRYSTAL DISPLAY SYSTEM, COMPRISES OPENING PORTION THAT INTAKES AIR PROVIDED IN SURFACE OF HOUSING, WHERE ANOTHER OPENING PORTION EXHAUSTS GAS PROVIDED IN SURFACE OF THE HOUSING | JP2014115360A | GASPER MARTIN J (US)\ MCMANUS MICHAEL J (US) | SHANGHAI KUNENG MICROELECTRONICS CO LTD US | G02F-001/13\ G02F-001/1333\ G02F-001/13357\ G03B-021/00\ G03B-021/14\ G03B-021/16 |
| 5698 | 3D printing | METHOD FOR PLACING TENANTS IN A CLOUD BASED DATABASE SERVER, INVOLVES ESTIMATING COST OF PLACING SET OF TENANTS ON SERVER USING SIMULATOR, AND SELECTING OPTIMAL SERVER FOR EACH TENANT BASED WITH COST-BASED SCHEDULER USING SIMULATOR | US2014188532A1 | GASPER MARTIN J (US)\ MCMANUS MICHAEL J (US) | LSI CORP (US)\ GASPER MARTIN J (US)\ MCMANUS MICHAEL J (US) | G06Q-010/06 |
| 5699 | 3D printing | METHOD FOR PROVIDING SMART OR INTELLIGENT DATA CENTER POSITIONING IN CLOUD COMPUTING, INVOLVES RANKING CANDIDATE DATA CENTERS IN LIST IN ACCORDANCE WITH ATTRIBUTES, AND SELECTING HIGHEST RANKED DATA CENTER IN LIST TO SERVE SERVICE REQUEST | US2014189092A1 | GIDON PIERRE (FR) | LSI CORP (US)\ GASPER MARTIN J (US)\ MCMANUS MICHAEL J (US) | H04L-012/24 |
| 5700 | 3D printing | CIRCUIT ARRANGEMENT FOR OPERATING PARALLEL STRANDS OF SEMICONDUCTOR LIGHT SOURCE, HAS CONTROL DEVICE CONFIGURED TO PROVIDE CLOCK SIGNALS, SUCH THAT SIGNALS ARE PHASE SHIFTED WITH OTHER TO CONTROL OUTPUTS AROUND PREDETERMINED DEGREES | DE102012224346A1\ US2014177664A1\ CN103889107A\ US9101009B2 | GLENN JACK L (US)\ CLEAR TROY D (US)\ GOSE MARK W (US)\ OSBORN DOUBLAS B (US)\ CAMPANILE NICHOLAS T (US) | COMMISSARIAT L éNERGIE ATOMIQUE ET AUX éNERGIES ALTERNATIVES (FR) | G03B-021/14\ H05B-037/02\ H05B-033/08 |
| 5701 | 3D printing | SYSTEM AND METHOD ENABLING BUNDLING OF MULTIPLE PRODUCT SERVICE LEVEL AGREEMENTS INTO A COMPREHENSIVE SUPPORT SERVICE LEVEL AGREEMENT | IN201100870I3 | GLENN JACK L (US)\ GOSE MARK W (US) | GLENN JACK L (US)\ CLEAR TROY D (US)\ GOSE MARK W (US)\ OSBORN DOUBLAS B (US)\ CAMPANILE NICHOLAS T (US) | G06F-015/173\ G06F-015/16 |
| 5702 | 3D printing | SYSTEM FOR PREDICTIVELY TRACKING EXPECTED PRINT JOBS, HAS CONTROL SYSTEM THAT DETERMINES WHETHER EXPECTED PRINT JOBS ARE RECEIVED AT PRESENTATION SYSTEM, AND GENERATES ALERT WHEN EXPECTED JOB IS NOT RECEIVED AT PRESENTATION SYSTEM | US2014176989A1\ US9036187B2 | GOLDBACH MATTHIAS (DE)\ DREESKORNFELD LARS (DE)\ LANDGRAF ERHARD (DE) | GLENN JACK L (US)\ GOSE MARK W (US) | G06F-003/12 |
| 5703 | 3D printing | METHOD FOR PROCESSING INPUT-OUTPUT (I/O) COMMANDS IN STORAGE AREA NETWORK (SAN) BASED STORAGE SYSTEM, INVOLVES RETRIEVING SECOND IDENTIFIER FROM I/O AT SYSTEM, AND TRANSLATING SECOND IDENTIFIER TO A LOGICAL STORAGE VOLUME IDENTIFIER | US2014181398A1\ US8914610B2 | GOSE MARK W (US)\ GRAWCOCK JOHN A (US) | QIMONDA AG (DE) | G06F-003/06\ G06F-012/02\ G06F-009/00\ H04L-029/06\ H04L-029/08\ H04L-029/12 |
| 5704 | 3D printing | SYSTEM FOR ESTABLISHING AND SUSTAINING AUTOMATIC LINK ESTABLISHMENT FOR HIGH FREQUENCY BROADBAND COMMUNICATION, HAS MASTER POST SELECTING SUB-BAND REPRESENTING REQUIRED CAPABILITY FOR TRIGGERING COMMUNICATION WITH CALLED POST | EP2744263A1\ FR2999844A1\ US2014169199A1\ FR2999844B1\ SG201309302A1\ ZA201309461A\ IL229970A1\ SG201309302B\ IL229970A\ US9474091B2 | GOURAUD PASCAL (FR)\ LE-GRATIET BERTRAND (FR) | GOSE MARK W (US)\ GRAWCOCK JOHN A (US) | H04W-028/18\ H04W-072/08\ H04W-076/02\ H04W-084/18\ H04L-005/00\ H04W-028/16\ H04W-024/08\ H04W-004/00\ H04W-028/24\ H04W-024/00 |
| 5705 | 3D printing | STAMP MANUFACTURING METHOD, INVOLVES CONVERTING INFORMATION IMAGE INTO TARGET BINARY IMAGE, CONVERTING BINARY IMAGE INTO BITMAP MATRIX BASED ON BITMAP MATRIX BODY, AND OBTAINING UNIT PIXEL BY BITMAP MATRIX | CN103770483A | GOUX LUDOVIC (BE)\ GERVAIS MONIQUE (FR) | ST MICROELECTRONICS CROLLES 2 (FR)\ GOURAUD PASCAL (FR)\ LE-GRATIET BERTRAND (FR) | B41K-001/02\ G06F-003/12 |
| 5706 | 3D printing | COMPUTER-IMPLEMENTED METHOD FOR PRIORITIZING DATA PACKETS ASSOCIATED WITH PROVIDER'S APPLICATIONS RUNNING IN E.G. COMPUTING ENVIRONMENT, INVOLVES DETERMINING WHETHER MODIFICATION TO PACKETS PRIORITY IS SAFE TO IMPLEMENT BASED ON EFFECT | US2014173098A1\ US9191283B2 | GU LIHUI (CN) | UNIV FRANCOIS RABELAIS UFR SCI (FR) | H04L-012/24\ G06F-015/173\ H04L-012/851\ H04L-012/859 |
| 5707 | 3D printing | METHOD FOR PROVIDING QUALITY OF SERVICE TREATMENTS FOR E.G. VOICE TYPE DATA FLOWS TO E.G. ROUTERS VIA LABEL-SWITCH PATH CHANNEL, INVOLVES TRANSMITTING PROCESSED DATA FLOWS TOWARD DESTINATION DEVICE BY NETWORK DEVICE AND VIA NETWORK CHANNEL | US2014169788A1\ US9191114B2 | GU XIAOFENG US\ LIANG HAILIAN US\ DONG SHURONG US\ DING SHENG US | WUXI CSMC TECH SEMICONDUCTOR US | H04B-010/27\ H04B-010/00\ H04L-012/00 |
| 5708 | 3D printing | AUTOMATIC FAILURE HANDLING CACHE SYSTEM FOR TRANSFERRING TRAFFIC OF END USER TO CACHE SERVER, HAS FIRST PBR ROUTER THAT IS ACCESSED TO CHANGE TRAFFIC FORWARDING DESTINATION OF FIRST PBR ROUTER INTO SECOND CACHE SERVE | WO2014087850A1\ JP2014112768A\ US2015347246A1 | GU XIAOFENG US\ LIANG HAILIAN US\ DONG SHURONG US\ DING SHENG US | UNIV JIANGNAN US | G06F-013/00\ H04L-012/707\ H04L-012/803\ G06F-011/20\ G06F-003/06 |
| 5709 | 3D printing | METHOD FOR DISTRIBUTING ROUTING INFORMATION, INVOLVES PLACING ROUTES IN UPDATE MESSAGES BASED ON SPECIFIC ORDER OF PRIORITY, SUCH THAT HIGHER PRIORITY ROUTES ARE PLACED AHEAD OF LOWER PRIORITY ROUTES WITHIN UPDATE MESSAGES | US8737406B1 | GUOHUA DING (CN)\ YE ZHOU (CN)\ JIE HE (CN)\ BIN HU (CN) | UNIV JIANGNAN US | H04L-012/28\ H04L-012/56\ H04L-029/06 |
| 5710 | 3D printing | MICROLENS ARRAY OF LIGHT INTENSITY DISTRIBUTION UNIFORMIZING ELEMENT USED IN PROJECTION APPARATUS, HAS MICROLENSES WHOSE LIGHT ENTRANCE SURFACE IS MADE OF RESIN AND LIGHT EXIT SURFACE IS MADE OF GLASS | US2014168615A1\ CN103885103A\ JP2014119702A\ CN103885103B | GUPTA SANDHYA (US)\ KOSIER STEVE L (US)\ BECKMAN JOHN C (US) | WUXI SI POWER MICRO ELECTRONIC (CN) | G02B-003/00\ G03B-021/14\ G02B-027/09\ G03B-021/20\ G02B-001/00\ G02B-001/04\ G02B-019/00 |
| 5711 | 3D printing | DISPLAY SYSTEM, HAS MULTIMEDIA DEVICE CONNECTED WITH DIGITAL LIGHT PROCESSING PROJECTOR, OBSERVATION UNIT ARRANGED ALONG PROJECTION DIRECTION OF DIGITAL LIGHT PROCESSING PROJECTOR, AND CONTROLLER CONNECTED WITH OBSERVATION UNIT | HK1185507A2 | HAEBERLEN OLIVER (AT) | POLARFAB LLC (US) | G02B-000/00 |
| 5712 | 3D printing | METHOD FOR DETECTING SCANNING ANGLE RANGE OF BEAM OF LASER PROJECTOR, INVOLVES STARTING UP PROJECTOR TO ENTER PROJECTION MODE, DETECTING ACTUAL TIME INTERVAL BETWEEN SENSING SIGNALS, AND CONTROLLING PROJECTOR TO STOP EMITTING BEAM | US2014168619A1\ CN103868675A\ US8905553B2\ CN103868675B | HAIOU LI US\ WEI HUANG US\ ZHAOJUN LIU US\ WANJUN CHEN US\ ZONGGUANG YU US | INFINEON TECHNOLOGIES AUSTRIA (AT) | G03B-021/20\ G01M-011/02\ G02B-026/10\ G03B-021/00\ G03B-021/14\ H04N-009/31 |
| 5713 | 3D printing | REAL-TIME SPECIFIC DIMENSION OPTICAL COHERENCE TOMOGRAPHY IMAGING SYSTEM USED IN E.G. CLINICAL APPLICATION, CALCULATES STRUCTURE INFORMATION AND DOPPLER INFORMATION TO PROVIDE DISPLAY OF COMBINED STRUCTURE AND FLOW OF OBJECT | US2014160487A1\ US9025159B2 | HAITING YAO US | WUXI JINGKAI TECHNOLOGY CO LTD US | G01B-009/02\ A61B-003/10\ A61B-005/00\ A61B-005/026\ G01B-009/021 |
| 5714 | 3D printing | LARGE SCALE CLUSTER MANAGEMENT METHOD, INVOLVES DETERMINING MULTIPLE SERVICE LEVELS OF MANAGEMENT OBJECT, PROVIDING RESOURCE UNIT WITH LARGE-SCALE CLUSTER, AND DETERMINING MANAGEMENT OBJECT TARGET PROPERTY | CN103763130A\ WO2015101089A1 | HAITING YAO US | DAILY SILVER MICROELECTRONICS CO LTD US | G06F-009/50\ H04L-012/24\ H04L-029/08 |
| 5715 | 3D printing | HIGH-VOLTAGE DISCHARGE LAMP E.G. METAL HALIDE LAMP USED IN IMAGE DISPLAY APPARATUS E.G. LIQUID-CRYSTAL PROJECTOR, HAS NOTCH THAT IS PROVIDED IN AREA TO WHICH STICKING AGENT IS ADHERED IN DIELECTRIC MATERIAL | JP2014110081A\ JP5789761B2 | HALLOCK ROBERT B (US)\ HEAD PAUL M (US) | DAILY SILVER IMP MICROELECTRONICS CO LTD US | F21S-002/00\ H01J-061/36\ H01J-061/54\ H01J-061/86 |
| 5716 | 3D printing | MONITOR AND CONTROL EQUIPMENT OF SUPERVISION AND CONTROL SYSTEM, DETERMINES SWITCHING DESTINATION COMMUNICATION CHANNEL BASED ON ESTIMATION INFLUENCE DEGREE REGARDING SWITCHING DESTINATION COMMUNICATION CHANNEL CANDIDATE | JP2014107624A | HAN SANG M (US)\ LI QIMING (US) | RAYTHEON CO (US)\ HALLOCK ROBERT B (US)\ HEAD PAUL M (US) | H04L-012/707\ H04M-011/00\ H04M-011/04 |
| 5717 | 3D printing | REGENERATIVE THERMAL OXIDIZER E.G. PULL-THROUGH REGENERATIVE THERMAL OXIDIZER HAS CONTROLLER THAT ACTUATES BALANCING DAMPER TO PROVIDE PREDETERMINED VOLUME OF AIR TO PURGE DUCT TO CLEAN HEAT-EXCHANGE MEDIA | US8740613B1 | HAN XIAO (CN)\ RU HUANG (CN) | STC UNM (US) | F27D-017/00 |
| 5718 | 3D printing | IMAGE DISPLAY APPARATUS E.G. LCD PROJECTOR, HAS MIRRORS WITH SURFACES WHERE SURFACE NORMAL LINE IN OPTICAL EFFECTIVE RANGE IS NOT PARALLEL TO X, Y, AND Z-AXES, AND FLAT SURFACES PERPENDICULAR TO X, Y, AND Z-AXES | JP2014098758A | HAO PINGHAI (US)\ HOU FAN-CHI (US)\ KHAN IMRAN (US) | UNIV BEIJING (CN) | G03B-021/00\ G03B-021/14\ H04N-005/74 |
| 5719 | 3D printing | PROJECTION-TYPE VIDEO DISPLAY APPARATUS FOR LCD SYSTEMS OR DIGITAL LIGHT PROCESSING SYSTEMS, HAS DETACHABLE POSITION ADJUSTMENT APPARATUS WHICH ADJUSTS POSITION OF PROJECTION UNIT VIA INSTALLATION BOARD WITH PILLAR BOLTS AND NUTS | JP2014095794A | HARDEE KIM C (US) | TEXAS INSTRUMENTS INC (US) | G03B-021/14\ H04N-005/74 |
| 5720 | 3D printing | LCD DEVICE E.G. ACTIVE MATRIX LCD DEVICE USED IN PORTABLE ELECTRONIC DEVICE E.G. CELLULAR PHONE, HAS SECOND ELECTRODE THAT IS CONNECTED TO WIRING TO WHICH POTENTIAL WHICH CHANGES BETWEEN FIRST AND SECOND POTENTIALS IS APPLIED | US2014152932A1\ KR2014070388A\ JP2014130345A\ CN103852943A | HASSIBI ARJANG (US)\ JANG BYUNGCHUL (US)\ MANICKAM ARUN (US) | SONY CORP (JP)\ UNITED MEMORIES INC (US) | G02F-001/133\ G02F-001/1343\ G02F-001/1368\ G09F-009/30\ G09G-003/20\ G09G-003/36\ H01L-029/786 |
| 5721 | 3D printing | TOUCH PANEL-EQUIPPED DISPLAY DEVICE E.G. IN-PLANE SWITCHING (IPS) SYSTEM LCD DEVICE FOR SMART PHONE, HAS FIRST OTHER-SIDE SUB-DETECTION LINES THAT ARE ARRANGED BETWEEN CORRESPONDING FIRST AND SECOND MAIN DETECTION LINE | US2014132557A1\ JP2014096089A\ TW201423540A\ US9235304B2\ TW506521B1 | HE JIQIANG (CN)\ HUANG WEIDONG (CN)\ XIE XIONGCAI (CN) | UNIV TEXAS (US)\ HASSIBI ARJANG (US)\ JANG BYUNGCHUL (US)\ MANICKAM ARUN (US) | G06F-003/044\ G06F-003/041 |
| 5722 | 3D printing | MANAGING METHOD FOR SERVER MANAGING SERVICE LEVEL AGREEMENT (SLA) WITH RESPECT TO DEVICES ON NETWORK, INVOLVES EXECUTING PRESCRIBED EVENT TO CONTROL DEVICES ACCORDING TO MONITORING RESULT AND SETTING OF SERVICE MANAGEMENT AUTOMATIC OPTION | EP2736192A1\ KR2014067822A\ US2014149584A1\ CN103840966A | HEIN JERRELL P (US)\ SOOCH NAVDEEP S (US) | TRULY SEMICONDUCTORS LTD (CN) | H04L-012/24\ H04L-012/26 |
| 5723 | 3D printing | DISTRIBUTED STORAGE SUBSYSTEM FOR ACHIEVING OPTIMAL RESOURCE ALLOCATION IN DISTRIBUTED COMPUTING ENVIRONMENT, HAS VIRTUAL DATA SPLIT LAYER (VDSL) THAT SPLITS INPUT FILE INTO NUMBER OF CHUNKS OF DATA, WITHOUT REWRITING INPUT FILE | US2014136779A1\ US9442954B2 | HEMING ZHANG (CN)\ BIN SHU (CN)\ RONGXI XUAN (CN)\ HUIYONG HU (CN)\ JIANJUN SONG (CN)\ BIN WANG US\ CHUNYU ZHOU US\ YUE HAO (CN) | SILICON LAB INC (US) | G06F-003/06\ G06F-012/00\ G06F-013/00\ G06F-017/30\ G06F-009/50\ H04L-029/08 |
| 5724 | 3D printing | METHOD FOR MANUFACTURING POLYMER SHELL DENTAL/ORTHODONTIC APPLIANCES FOR E.G. TEETH REPOSITIONING APPLICATIONS, INVOLVES SUBJECTING SHEET OF THERMOPLASTIC POLYMER FILM TO VACUUM MOLDING OR PRESSURE MOLDING UTILIZING POSITIVE TOOTH MODEL | GB2507986A\ WO2014076188A1 | HEMING ZHANG (CN)\ BIN SHU (CN)\ RONGXI XUAN (CN)\ HUIYONG HU (CN)\ JIANJUN SONG (CN)\ BIN WANG US\ HAIDONG WANG US\ YUE HAO (CN) | UNIV XIDIAN (CN) | A61C-007/08\ A61C-013/34 |
| 5725 | 3D printing | CLOUD ENVIRONMENT AUDIT LOG AND/OR SECURITY EVENT CONFIGURATION METHOD, INVOLVES CONFIGURING COMPUTING CLOUD SERVICE PROVIDER IN SERVICE LEVEL AGREEMENT TO COMPLETE CLOUD ENVIRONMENT AUDIT LOG AND/OR SECURITY EVENT CONFIGURATION OPERATION | CN103428177A | HEMING ZHANG (CN)\ BIN SHU (CN)\ RONGXI XUAN (CN)\ HUIYONG HU (CN)\ JIANJUN SONG (CN)\ BIN WANG US\ YI LV US\ YUE HAO (CN) | UNIV XIDIAN (CN) | H04L-012/24\ H04L-029/06\ H04L-029/08 |
| 5726 | 3D printing | REAL THREE-DIMENSIONAL IMAGE DISPLAY SYSTEM, HAS TWO-DIMENSIONAL ACOUSTO-OPTIC MODULATOR FOR MODULATING LIGHTING BEAM THAT IS REFLECTED TO IMAGING REGIONS OF IMAGING DEVICE, AND BEAM ADJUSTING ELEMENT FOR ADJUSTING LIGHTING BEAM | CN103713462A | HEMING ZHANG (CN)\ BIN SHU (CN)\ RONGXI XUAN (CN)\ HUIYONG HU (CN)\ JIANJUN SONG (CN)\ HAIDONG WANG US\ YUE HAO (CN) | UNIV XIDIAN (CN) | G02B-027/22\ G03B-035/18\ H04N-013/04 |
| 5727 | 3D printing | THREE-DIMENSIONAL IMAGE DISPLAY SYSTEM HAS DIGITAL LIGHT PROCESSING DEVICE WHICH RECEIVES LIGHT BEAM OF REAR DEFLECTION, AND ACOUSTO-OPTIC MODULATOR THAT GENERATES LIGHTING BEAM IN IMAGING REGION BY MODULATING LIGHT BEAM IMAGE | CN103713463A\ CN103713463B | HEMING ZHANG (CN)\ BIN SHU (CN)\ RONGXI XUAN (CN)\ HUIYONG HU (CN)\ JIANJUN SONG (CN)\ YI LV US\ CHUNYU ZHOU US\ YUE HAO (CN) | UNIV XIDIAN (CN) | G02B-027/22\ G03B-035/18\ H04N-013/04 |
| 5728 | 3D printing | PERFORMING THREE-DIMENSIONAL PRINTING PROCESS TO PRODUCE THREE-DIMENSIONAL PERIODIC STRUCTURE METAMATERIAL, COMPRISES ESTABLISHING METAL AND RESIN MATERIAL STRUCTURES, AND DISCRETIZING STRUCTURES INTO STEREO LITHOGRAPHY FORMAT FILE | CN103692654A\ CN103692654B | HEMING ZHANG (CN)\ BIN SHU (CN)\ RONGXI XUAN (CN)\ HUIYONG HU (CN)\ JIANJUN SONG (CN)\ YI LV US\ CHUNYU ZHOU US\ YUE HAO (CN) | UNIV XIDIAN (CN) | B22F-005/00\ B29C-067/00 |
| 5729 | 3D printing | SYSTEM FOR MEASURING COMPLIANCE WITH SERVICE LEVEL AGREEMENT (SLA), HAS SERVER WITH MONITORING DEVICE TO DETERMINE NONCOMPLIANCE OF NETWORK WITH SLA, WHEN FRAME LOSS IS DETERMINED AND COMMITTED INFORMATION RATE (CIR) IS NOT EXCEEDED | US2014126413A1\ US9049147B2 | HEMING ZHANG (CN)\ BIN SHU (CN)\ RONGXI XUAN (CN)\ HUIYONG HU (CN)\ JIANJUN SONG (CN)\ YI LV US\ CHUNYU ZHOU US\ YUE HAO (CN) | UNIV XIDIAN (CN) | H04L-012/24\ H04L-012/26\ G01R-005/12\ H04L-012/54\ H04L-012/801\ H04L-012/813\ H04L-012/815\ H04L-012/851 |
| 5730 | 3D printing | DISPLAY APPARATUS E.G. ACTIVE-MATRIX-TYPE ORGANIC ELECTROLUMINESCENCE (EL) DISPLAY APPARATUS HAS AUXILIARY CAPACITY THAT IS PROVIDED WITH FIRST ELECTRODE CONNECTED TO DRIVE TRANSISTOR SOURCE ELECTRODE AND SECOND ELECTRODE | JP2014081422A | HEMING ZHANG (CN)\ BIN SHU (CN)\ RONGXI XUAN (CN)\ HUIYONG HU (CN)\ JIANJUN SONG (CN)\ YI LV US\ HAIDONG WANG US\ YUE HAO (CN) | UNIV XIDIAN (CN) | G09F-009/30\ G09G-003/20\ G09G-003/30\ H01L-027/32\ H01L-051/50 |
| 5731 | 3D printing | METHOD FOR ALIGNING IMAGE OF PRINTED SUBSTRATE USING MOBILE DEVICE E.G. SMARTPHONE, INVOLVES INSTRUCTING USER OF MOBILE DEVICE BY PROCESSING DEVICE TO MOVE MOBILE DEVICE TO ALIGN MOBILE DEVICE AND PRINTED SUBSTRATE | US2014118560A1 | HEMING ZHANG (CN)\ BIN SHU (CN)\ RONGXI XUAN (CN)\ HUIYONG HU (CN)\ JIANJUN SONG (CN)\ YI LV US\ HAIDONG WANG US\ YUE HAO (CN) | UNIV XIDIAN (CN) | H04N-005/225 |
| 5732 | 3D printing | DEVICE FOR LAYER-WISE GENERATIVE MANUFACTURING OF THREE DIMENSIONAL COMPONENTS BY PHOTOPOLYMERIZATION, HAS EXPOSURE UNITS FORMING EXPOSURE STATIONS ALONG DIRECTION, WHERE RADIATION IS DIRECTED INTO MATERIAL IN STATIONS THROUGH DISK | DE102012021284A1\ DE102012021284B4 | HEMING ZHANG (CN)\ BIN SHU (CN)\ RONGXI XUAN (CN)\ HUIYONG HU (CN)\ JIANJUN SONG (CN)\ YI LV US\ HAIDONG WANG US\ YUE HAO (CN) | UNIV XIDIAN (CN) | B29C-067/00\ G03F-007/20 |
| 5733 | 3D printing | METHOD FOR PROCESSING THREE-DIMENSIONAL IMAGE FOR ELIMINATION OF DEPTH ARTIFACTS IN HUMAN-MACHINE INTERFACE APPLICATIONS, INVOLVES APPLYING SUPER RESOLUTION TECHNIQUE UTILIZING IMAGE TO RECONSTRUCT DEPTH INFORMATION OF DEFECTIVE PIXELS | WO2014065887A1\ CA2844705A1\ RU2012145349A\ US2014240467A1\ TW201421419A\ CN104025567A\ KR2015079638A\ JP2016502704W | HEMING ZHANG (CN)\ BIN SHU (CN)\ RONGXI XUAN (CN)\ HUIYONG HU (CN)\ JIANJUN SONG (CN)\ YI LV US\ YUCHEN LI US\ YUE HAO (CN) | UNIV XIDIAN (CN) | H04N-005/367\ G06T-003/40\ G06T-005/00\ G06T-017/00\ H04N-013/02\ H04N-005/232\ G06T-001/00\ H04N-013/00 |
| 5734 | 3D printing | METHOD FOR RENDERING OF PORTAL PAGE DISPLAYED ON CLIENT SYSTEM, INVOLVES PREPARING INTERMEDIATE RESULT OF PORTAL PAGE, BASED ON SERVER-SIDE AGGREGATION AND RENDERING, SUCH THAT CLIENT-SIDE PROCESSING COMPLETES INTERRUPTED AGGREGATION | US2014122999A1\ GB2507483A | HEMING ZHANG (CN)\ BIN SHU (CN)\ RONGXI XUAN (CN)\ HUIYONG HU (CN)\ JIANJUN SONG (CN)\ YI LV US\ YUCHEN LI US\ YUE HAO (CN) | UNIV XIDIAN (CN) | G06F-017/22\ G06F-017/30 |
| 5735 | 3D printing | DATA STORAGE SYSTEM QUALITY OF SERVICE (QOS) SERVER THAT OPTIMIZES REVENUE REALIZED UNDER SEVERAL SERVICE LEVEL AGREEMENTS SPECIFYING AVERAGE MAXIMUM RESPONSE TIME WITH STORAGE SERVICES CUSTOMER | US2014122685A1\ US9172618B2 | HEMING ZHANG (CN)\ BIN SHU (CN)\ RONGXI XUAN (CN)\ HUIYONG HU (CN)\ JIANJUN SONG (CN)\ YI LV US\ YUCHEN LI US\ YUE HAO (CN) | UNIV XIDIAN (CN) | H04L-012/24\ G06F-015/173\ G06Q-040/02\ H04L-012/801 |
| 5736 | 3D printing | MANUFACTURE OF TERMINAL-MODIFIED (HYDROGENATED) POLYBUTADIENE FOR POLYBUTADIENE COMPOSITION, INVOLVES REACTING (METH)ACRYLATE, DIISOCYANATE, AND HYDROXY-TERMINATED (HYDROGENATED) POLYBUTADIENE IN PRESENCE OF ORGANOMETALLIC COMPOUND | WO2014061251A1\ TW201428015A\ KR2015048802A\ EP2910578A1\ US2015240009A1\ CN104781288A\ TW504622B1\ JP5898781B2\ JP2014541940X | HEMING ZHANG (CN)\ BIN SHU (CN)\ RONGXI XUAN (CN)\ HUIYONG HU (CN)\ JIANJUN SONG (CN)\ YI LV US\ YUCHEN LI US\ YUE HAO (CN) | UNIV XIDIAN (CN) | C08F-299/00\ C08F-008/30\ C08C-019/38\ C08F-036/06\ C08L-015/00\ C08L-047/00\ C08F-136/06 |
| 5737 | 3D printing | FEEDBACK-TYPE DYNAMIC QUALITY CONTROL SERVICE PROVISIONING SYSTEM AND METHOD CAPABLE OF ENSURING THE SERVICE QUALITY BY DYNAMICALLY ADJUSTING THE PROVISIONING PARAMETER | TW201406099A | HEMING ZHANG (CN)\ BIN SHU (CN)\ RONGXI XUAN (CN)\ HUIYONG HU (CN)\ JIANJUN SONG (CN)\ YI LV US\ YUCHEN LI US\ YUE HAO (CN) | UNIV XIDIAN (CN) | H04L-012/24\ H04L-012/801 |
| 5738 | 3D printing | METHOD FOR FACILITATING DATA SHARING FOR CLOUD APPLICATIONS, INVOLVES DETERMINING COSTING FUNCTION THAT CONSIDERS RESOURCE COST OF CREATING AND MAINTAINING SHARING POTENTIAL PENALTIES TO BE PAID IF SERVICE LEVEL AGREEMENT IS BREACHED | US2014122374A1 | HEMING ZHANG (CN)\ BIN SHU (CN)\ RONGXI XUAN (CN)\ HUIYONG HU (CN)\ JIANJUN SONG (CN)\ YUCHEN LI US\ CHUNYU ZHOU US\ YUE HAO (CN) | UNIV XIDIAN (CN) | G06Q-030/02 |
| 5739 | 3D printing | METHOD FOR MEASURING END-TO-END SERVICE LEVEL AGREEMENT IN NETWORK, INVOLVES SENDING TEST CONDITION FOR SERVICE LEVEL AGREEMENT MEASUREMENT TO SERVER, SENDING TEST PACKET TO SERVER, AND OBTAINING PARAMETER VALUES FOR METRICS | US2014119221A1\ KR2014053654A\ US9426042B2 | HEMING ZHANG (CN)\ BIN SHU (CN)\ RONGXI XUAN (CN)\ HUIYONG HU (CN)\ JIANJUN SONG (CN)\ YUCHEN LI US\ HAIDONG WANG US\ YUE HAO (CN) | UNIV XIDIAN (CN) | H04L-012/24\ H04L-012/26 |
| 5740 | 3D printing | COMPUTERIZED METHOD FOR CREATING THREE-DIMENSIONAL SURFACE TEXTURE ON PORTION OF E.G. OUTSOLE OF SHOE, INVOLVES USING AUGMENTED DATA FILE TO INSTRUCT RAPID MANUFACTURING DEVICE TO MANUFACTURE PORTION OF SHOE WITH SURFACE TEXTURE | US2014114457A1\ WO2014066174A1\ EP2908681A1\ US9122819B2\ CN104754972A | HEMING ZHANG (CN)\ RONGXI XUAN (CN)\ HUIYONG HU (CN)\ JIANJUN SONG (CN)\ BIN WANG US\ HAIDONG WANG US\ CHUNYU ZHOU US\ YUE HAO (CN) | UNIV XIDIAN (CN) | G06F-017/50\ A43B-001/14\ G06F-017/00\ G06F-019/00 |
| 5741 | 3D printing | SYSTEM FOR PROVIDING ADVERTISING AROUND PERIPHERY OF DISPLAY DEVICE E.G. TV, HAS ADVERTISING PANEL THAT IS RETAINED BETWEEN FRONT AND REAR HOUSINGS, WHERE WIDTH OF FRONT HOUSING IS GREATER THAN WIDTH OF REAR HOUSING | US2014101980A1 | HEMING ZHANG (CN)\ RONGXI XUAN (CN)\ HUIYONG HU (CN)\ JIANJUN SONG (CN)\ BIN WANG US\ HAIDONG WANG US\ CHUNYU ZHOU US\ YUE HAO (CN) | UNIV XIDIAN (CN) | G09F-007/18 |
| 5742 | 3D printing | FRONT MAINTENANCE TYPE DIGITAL LIGHT PROCESSING (DLP) DISPLAY DEVICE HAS LOCATING BEARING THAT IS TIGHTLY MATCHED WITH SYNCHRONOUS BELT, SYNCHRONOUS WHEEL AND SYNCHRONOUS SHAFT AND BALL BEARING | CN103605257A | HEMING ZHANG (CN)\ RONGXI XUAN (CN)\ HUIYONG HU (CN)\ JIANJUN SONG (CN)\ BIN WANG US\ YI LV US\ CHUNYU ZHOU US\ YUE HAO (CN) | UNIV XIDIAN (CN) | G03B-021/58 |
| 5743 | 3D printing | COLOR WHEEL DEVICE FOR DIGITAL LIGHT PROCESSING (DLP) PROJECTION SYSTEM, HAS EXTERNAL RING THAT IS ORIENTED ALONG ANNULAR INNER RING AND IS PROVIDED WITH MULTI-SECTION FILTER, AND TRANSPARENT SHEET WHICH IS PROVIDED IN INNER RING | CN203453998U | HEMING ZHANG (CN)\ RONGXI XUAN (CN)\ HUIYONG HU (CN)\ JIANJUN SONG (CN)\ BIN WANG US\ YI LV US\ HAIDONG WANG US\ YUE HAO (CN) | UNIV XIDIAN (CN) | F21V-014/08\ G03B-021/14 |
| 5744 | 3D printing | VIDEO CONTROLLER OF VIDEO PROJECTOR USED FOR SYNCHRONIZING PRESENTATION OF IMAGES PROJECTED ONTO DISPLAY, PRESENTS LIGHT AND OF CONTROL SIGNALS THAT ARE BEING TIMED IN SYNCHRONY WITH FIXED TIME SEGMENTS | US2014098203A1 | HEMING ZHANG (CN)\ RONGXI XUAN (CN)\ HUIYONG HU (CN)\ JIANJUN SONG (CN)\ BIN WANG US\ YUCHEN LI US\ HAIDONG WANG US\ YUE HAO (CN) | UNIV XIDIAN (CN) | H04N-013/04 |
| 5745 | 3D printing | DISPLAY APPARATUS FOR USE IN PROJECTOR APPARATUS, HAS SUPPRESSION UNIT THAT SUPPRESSES SUPERIMPOSITION OF SYNCHRONIZING SIGNAL BY SUPERIMPOSITION UNIT | JP2014062979A | HEMING ZHANG (CN)\ RONGXI XUAN (CN)\ HUIYONG HU (CN)\ JIANJUN SONG (CN)\ YUCHEN LI US\ HAIDONG WANG US\ CHUNYU ZHOU US\ YUE HAO (CN) | UNIV XIDIAN (CN) | G02F-001/13\ G03B-021/00\ G03B-021/14\ G09G-003/20\ G09G-003/34\ G09G-003/36\ G09G-005/00\ G09G-005/18\ G09G-005/36\ H04N-013/04 |
| 5746 | 3D printing | METHOD FOR AUTOMATICALLY HANDLING CARGO, INVOLVES TRANSMITTING DATA FROM WAREHOUSE MANAGEMENT SYSTEM TO CUSTOMER SERVICE PORTAL FOR REAL-TIME ACCESS TO CUSTOMER SERVICE PORTAL FOR REAL-TIME DATA WHILE PROCESSING CARGO THROUGH LOGISTIC CHAIN | US2014101069A1 | HEMING ZHANG (CN)\ XIANYING DAI (CN)\ BIN SHU (CN)\ RONGXI XUAN (CN)\ HUIYONG HU (CN)\ JIANJUN SONG (CN)\ BIN WANG US\ YUE HAO (CN) | UNIV XIDIAN (CN) | G06Q-010/08 |
| 5747 | 3D printing | COMPUTER-IMPLEMENTED METHOD FOR CONFIGURING REQUEST-CENTRIC SYSTEM E.G. FILE SYSTEM, TO DETERMINE EXTERNAL OR INTERNAL WORKLOADS OF SYSTEM, INVOLVES DETERMINING OPTIMAL CONFIGURATION BASED ON MAPPING FUNCTION TO ALLOCATE RESOURCES | US2014095674A1 | HEMING ZHANG (CN)\ XIANYING DAI (CN)\ BIN SHU (CN)\ RONGXI XUAN (CN)\ HUIYONG HU (CN)\ JIANJUN SONG (CN)\ BIN WANG US\ YUE HAO (CN) | UNIV XIDIAN (CN) | G06F-015/177 |
| 5748 | 3D printing | TOUCH SENSING SYSTEM FOR E.G. DIGITAL MICROMIRROR DEVICE PROJECTOR, CONTROLS LIGHT SOURCE, SUCH THAT LIGHT SOURCE IS SELECTIVELY ILLUMINATED DURING TOUCH DETECTING INTERVALS IN SYNCHRONISM WITH MODULATED PROJECTED LIGHT OF TOUCH SHEET | WO2014049331A1\ GB2506849A\ US2015248189A1 | HEMING ZHANG (CN)\ XIANYING DAI (CN)\ BIN SHU (CN)\ RONGXI XUAN (CN)\ HUIYONG HU (CN)\ JIANJUN SONG (CN)\ CHUNYU ZHOU US\ YUE HAO (CN) | UNIV XIDIAN (CN) | G06F-001/16\ G06F-003/03\ G06F-003/0354\ G06F-003/042 |
| 5749 | 3D printing | COHERENT LIGHT SOURCE DEVICE FOR PROJECTOR E.G. LIQUID-CRYSTAL PROJECTOR, CHANGES CROSS SECTIONAL SHAPE OF REFLECTIVE SIDE SURFACE PROVIDED PERPENDICULAR TO AXIS TAKEN IN PROPAGATION DIRECTION OF LIGHT FROM INCIDENT END TO EMISSION END | WO2014050736A1\ JP2014071979A | HEMING ZHANG (CN)\ XIANYING DAI (CN)\ BIN SHU (CN)\ RONGXI XUAN (CN)\ HUIYONG HU (CN)\ JIANJUN SONG (CN)\ CHUNYU ZHOU US\ YUE HAO (CN) | UNIV XIDIAN (CN) | F21S-002/00\ F21Y-101/02\ G02B-019/00\ G02B-027/48\ G03B-021/14 |
| 5750 | 3D printing | LIGHT SOURCE DEVICE OF PROJECTOR, HAS HEAT SINK PROVIDED WITH NAIL THAT IS CAUGHT IN PRINTED WIRING BOARD SUCH THAT FORCE IS APPLIED TO DIRECTION WHICH STRETCHES LEAD WIRE OF LIGHT SOURCE ELEMENT, WHEN HEAT SINK IS REMOVED FROM HOLDER | JP2014059515A | HEMING ZHANG (CN)\ XIANYING DAI (CN)\ BIN SHU (CN)\ RONGXI XUAN (CN)\ HUIYONG HU (CN)\ JIANJUN SONG (CN)\ CHUNYU ZHOU US\ YUE HAO (CN) | UNIV XIDIAN (CN) | F21S-002/00\ F21V-025/00\ F21V-029/00\ G03B-021/00\ G03B-021/14\ G03B-021/16 |
| 5751 | 3D printing | DRIVE DEVICE FOR DISCHARGE LAMP OF LIGHT SOURCE DEVICE, HAS CONTROL UNIT THAT CONTROLS SUPPLY SECTION IN LOW FREQUENCY DRIVE TO BE IN ZERO ELECTRIC CURRENT STATE PROVIDED BETWEEN POSITIVE POLARITY STATE AND NEGATIVE POLARITY STATE | JP2014060094A | HEMING ZHANG (CN)\ XIANYING DAI (CN)\ BIN SHU (CN)\ RONGXI XUAN (CN)\ HUIYONG HU (CN)\ JIANJUN SONG (CN)\ YUCHEN LI US\ YUE HAO (CN) | UNIV XIDIAN (CN) | H05B-041/24 |
| 5752 | 3D printing | METHOD FOR MANAGING INPUT/OUTPUT (I/O) REQUEST PROCESSING, INVOLVES SERVICING I/O REQUEST AT ORCHESTRATION LAYER, AND PROVIDING POLICY WITH SERVICE LEVEL AGREEMENT AND INPUT WITH PROCESSING COST INFORMATION FOR PROCESSING RESOURCES | US8682955B1 | HEMING ZHANG (CN)\ XIANYING DAI (CN)\ BIN SHU (CN)\ RONGXI XUAN (CN)\ HUIYONG HU (CN)\ JIANJUN SONG (CN)\ YUCHEN LI US\ YUE HAO (CN) | UNIV XIDIAN (CN) | G06F-015/16\ G06F-003/00 |
| 5753 | 3D printing | SERVICE LEVEL AGREEMENT CONSTRAINTS BASED CLOUD SERVICE EVALUATION METHOD, INVOLVES PERFORMING NORMALIZATION TO OBTAIN FUZZY EVALUATION MATRIX, DETERMINING COMPONENT VALUE OF FUZZY EVALUATION MATRIX, AND DETERMINING BACKGROUND DATA | CN103561085A\ CN103561085B | HEMING ZHANG (CN)\ XIANYING DAI (CN)\ BIN SHU (CN)\ RONGXI XUAN (CN)\ HUIYONG HU (CN)\ JIANJUN SONG (CN)\ YUCHEN LI US\ YUE HAO (CN) | UNIV XIDIAN (CN) | H04L-029/06\ H04L-029/08 |
| 5754 | 3D printing | SERVICE LEVEL AGREEMENT CONSTRAINTS BASED VIRTUAL MACHINE REQUIREMENT PREDICTING METHOD, INVOLVES SUBMITTING CLOUD RESOURCE SERVICE LEVEL AGREEMENT TEMPLATE, AND OBTAINING FIRST-LAYER PREDICTION CONDITION | CN103559089A | HIROYUKI FUJIWARA (JP)\ MITSUHIKO OGIHARA (JP)\ ICHIMATSU ABIKO (JP)\ MASAAKI SAKUTA (JP) | UNIV XIDIAN (CN) | G06F-009/455\ G06F-009/50\ H04L-012/913 |
| 5755 | 3D printing | METHOD FOR CONTROLLING THREE-DIMENSIONAL MULTI-LAYER SPEAKER ARRANGEMENT, INVOLVES EXTRACTING TWO-DIMENSIONAL SOURCE POSITION FROM SOURCE POSITION AND CALCULATING LAYER SPECIFIC SPEAKER COEFFICIENTS USING TWO-DIMENSIONAL CALCULATOR | WO2014044332A1\ EP2898706A1\ CN104798383A\ US2015271622A1\ EP2898706B1 | HIROYUKI FUJIWARA (JP)\ MITSUHIKO OGIHARA (JP)\ ICHIMATSU ABIKO (JP)\ MASAAKI SAKUTA (JP) | OKI DATA KK (JP)\ OKI DIGITAL IMAGING KK US | H04S-007/00\ H04R-005/02\ H04R-027/00 |
| 5756 | 3D printing | ILLUMINATION OPTICAL DEVICE OF PROJECTOR, HAS CONTROL UNIT THAT CAUSES EACH LIGHT SOURCE UNIT TO LIGHT ACCORDING TO LIGHTING PATTERN INFORMATION SHOWING LIGHTING PATTERN SELECTED BY SELECTING SIGNAL RECEIVED BY CONTROL UNIT | WO2014041636A1\ US2015219984A1 | HISHIDA SHUNICHI US\ HANEDA HAJIME US\ OHASHI NAOKI US\ SAKAGUCHI ISAO US\ SAITO NORIKO US | OKI DATA KK (JP)\ OKI DIGITAL IMAGING KK US | G03B-021/14\ G02B-026/00\ G02B-027/14\ G03B-021/00\ G03B-021/20 |
| 5757 | 3D printing | MONITORING DEVICE FOR DIGITAL LIGHT PROCESSOR PROJECTION SYSTEM, HAS VOLTAGE AND CURRENT DETECTION UNIT TO PROVIDE VOLTAGE ABOUT EXTERNAL POWER SUPPLY SUPPLIED TO DIGITAL LIGHT PROCESSOR PROJECTION SYSTEM AND CONTROL UNIT EXTRACTS CURRENT | KR1372937B1 | HONGCHEN WANG US\ WENLI CHEN US\ XIANFENG GAN US | NAT INST FOR MATERIALS SCIENCE (JP) | H04N-017/00\ H04N-005/74 |
| 5758 | 3D printing | DISPLAY APPARATUS E.G. DIRECT-VIEW-TYPE DISPLAY APPARATUS, FOR USE IN DIGITAL LIGHT PROCESSING PROJECTOR, HAS DISPLAY UNIT TO DISPLAY IMAGE ON WHICH SYNCHRONIZATION SIGNAL IS SUPERPOSED BY SYNCHRONIZATION SIGNAL SUPERPOSITION UNIT | US2014078271A1\ JP2014077985A\ US9485498B2 | HONGNING CHEN US\ YONG CHEN US\ SHAOMING FANG US\ PENGFEI LIU US\ XINQIANG WANG US\ LIRONG ZHANG US\ YAMIN ZHAO US | IRAY TECHNOLOGY CO LTD US | H04N-013/04\ G02B-027/22\ G03B-021/00\ G03B-021/14\ G09F-009/30\ G09F-009/40\ G09G-005/00\ G09G-005/18\ G09G-005/36\ H04N-009/31 |
| 5759 | 3D printing | IMAGE DISPLAY APPARATUS FOR PROJECTOR THAT IS UTILIZED FOR DIGITAL LIGHT PROCESSING SYSTEM, HAS DISPLAY CONTROL PART FOR ADJUSTING CONTROL SIGNAL GENERATING PERIOD ACCORDING TO JUDGMENT RESULT OBTAINED BY JUDGMENT PART | JP2014056115A | HORSTMANN MANFRED (DE)\ WEI ANDY (DE)\ KAMMLER THORSTEN (DE)\ HOENTSCHEL JAN (DE) | FOUNDER GROUP (CN)\ SHENZHEN FOUNDER MICROELECTRON US | G03B-021/00\ G03B-021/14\ G09G-005/00\ G09G-005/08\ G09G-005/36\ H04N-005/74 |
| 5760 | 3D printing | LIGHT SOURCE PROCESSING DEVICE FOR USE IN HIGH-FRAME-FREQUENCY ONE GRAY SCALE DISPLAY IMAGING DEVICE, HAS DIGITAL LIGHT PROCESSING MODULE FOR RECEIVING OUTPUT SIGNAL OF BRIGHTNESS ADJUSTING DEVICE TO PRODUCE COLOR LIGHT | CN103543585A | HOUSTON THEODORE W (US) | HORSTMANN MANFRED (DE)\ KAMMLER THORSTEN (DE)\ WEI ANDY (DE)\ HOENTSCHEL JAN (DE) | G02B-007/00\ G03B-021/14\ G03B-021/20\ H04N-009/31 |
| 5761 | 3D printing | LIGHT PROCESSING DEVICE FOR USE IN HIGH-FRAME-FREQUENCY COLOR DISPLAY IMAGING DEVICE, HAS DIGITAL LIGHT PROCESSING MODULE LOCATED ON OUTPUT LIGHT PATH OF LIGHT SPLITTING MODULE THAT RECEIVES MULTI-COLOR LIGHT BEAMS | CN103543587A\ CN103543587B | HOWARD GREGORY E (US)\ BABCOCK JEFFREY A (US)\ PINTO ANGELO (US)\ SCHIEKOFER MANFRED (DE)\ BALSTER SCOTT G (DE)\ HAUSLER ALFRED (DE) | TEXAS INSTRUMENTS INC (US)\ HOUSTON THEODORE W (US) | G02B-027/10\ G02B-007/00\ G03B-021/14\ G03B-021/20\ H04N-009/31 |
| 5762 | 3D printing | METHOD FOR CONTROLLING MAXIMUM THROUGHPUT FOR FACILITATING COMMUNICATION WITHIN METRO ETHERNET NETWORK, INVOLVES COMMUNICATING MESSAGE INDICATING MAXIMUM THROUGHPUT, AMOUNT OF LOSS AND FRAMES PER SECOND | US2014082193A1\ US9137133B2 | HU BIN (US)\ LI WEI (CN)\ ZHAI YAHONG US\ LI PING US\ GU KE US | TEXAS INSTRUMENTS INC (US)\ BABCOCK JEFFREY A (US)\ PINTO ANGELO (US)\ SCHIEKOFER MANFRED (DE)\ BALSTER SCOTT G (DE)\ HOWARD GREGORY E (US)\ HAUSLER ALFRED (DE) | H04L-012/26\ H04L-012/24 |
| 5763 | 3D printing | METHOD FOR AUTOMATICALLY DETECTING SERVICE LEVEL AGREEMENT (SLA) SETTINGS IN ETHERNET VIRTUAL CIRCUIT, INVOLVES DETERMINING EFFECTIVE COMMITTED INFORMATION RATE AND EXCESS INFORMATION RATE VALUES ENFORCED FROM COLLECTED TRAFFIC SAMPLES | EP2706701A1\ US2014071822A1\ CN103684901A\ US8917596B2\ US2015071066A1\ US9407515B2\ US2016315831A1 | HU CHIA-HSIN (TW)\ CHANG SUN-JAY (TW)\ HORNG JAW-JUINN (TW)\ CHEN CHUNG-HUI (TW) | UNIV ELECTRONIC SCIENCE & TECH (CN) | H04L-012/24\ H04L-012/26\ H04L-012/70\ H04L-012/28\ G01R-031/08\ H04L-012/815\ H04L-012/801\ H04L-012/819\ H04L-012/851 |
| 5764 | 3D printing | LIGHT PROCESSING DEVICE OF HIGH-FRAME-FREQUENCY GRAY SCALE DISPLAY IMAGING DEVICE, HAS DIGITAL LIGHT PROCESSING MODULE THAT IS LOCATED ON OUTPUT LIGHT PATH OF BRIGHTNESS ADJUSTING DEVICE FOR OUTPUTTING COLOR LIGHT TO OTHER COLOR LIGHT | CN103543586A\ CN103543586B | HUAJIE CHEN (US)\ DURESETI CHIDAMBARRAO (US) | TAIWAN SEMICONDUCTOR MFG (TW)\ HU CHIA-HSIN (TW)\ CHANG SUN-JAY (TW)\ HORNG JAW-JUINN (TW)\ CHEN CHUNG-HUI (TW) | G02B-007/00\ G03B-021/14\ G03B-021/20\ H04N-009/31 |
| 5765 | 3D printing | METHOD FOR OPERATING INVERTER FOR CONTROLLING ELECTRICAL MACHINE OF MOTOR VEHICLE DRIVE STRAND, INVOLVES DETERMINING SIZE OF ELECTRIC CURRENT OF PHASE LINES BASED ON INPUT CURRENT OF INVERTER, AND DETECTING SWITCH-ON TIME OF SWITCHES | DE102012215811A1\ US2014062356A1 | HUAN WANG US\ JIAN XU US\ YIN LUO US\ ZHIQIANG GUAN US\ RONG WANG US\ XIANWEN ZENG US\ ZHIGONG WANG US | IBM (US)\ CHEN HUAJIE (US)\ CHIDAMBARRAO DURESETI (US)\ GLUSCHENKOV OLEG G (US)\ STEEGEN AN L (US)\ YANG HAINING S (US) | H02M-007/537\ H02M-007/44\ H02P-006/00 |
| 5766 | 3D printing | NONVOLATILE SEMICONDUCTOR MEMORY I.E. BIT COST SCALABLE-NAND FLASH MEMORY, HAS DRIVER PLACED ON P-TYPE SEMICONDUCTOR SUBSTRATE, SELECTED AND NON-SELECTED CELL UNITS CONNECTED TO BIT LINE, AND CONDUCTIVE LAYERS STACKED ON SUBSTRATE | US2014071758A1\ US8837218B2 | HUANG LI-PING (TW)\ FUNG KA-HING (TW)\ CHENG CHUN-FAI (HK) | NANJING SINO CHIP MICROELECTRONICS CO LTD US | G11C-016/04\ G11C-016/08\ G11C-005/02\ G11C-005/06\ H01L-027/115 |
| 5767 | 3D printing | PROJECTOR OF DIGITAL LIGHT PROCESSOR (DLP) SYSTEM, HAS DISPLAY CONTROL UNIT THAT CONTROLS DISPLAY GRADATION OF OPTICAL IMAGE IN SPOKE PERIOD BASED ON INFORMATION SHOWING LIGHT QUANTITY FOR COLOR LIGHT DURING SPOKE PERIOD | JP2014048543A | HUANG RU (CN)\ FAN JIEWEN (CN)\ LI MING (CN)\ LI JIA (CN)\ XU XIAOYAN (CN) | TAIWAN SEMICONDUCTOR MFG (TW)\ HUANG LI-PING (TW)\ CHENG CHUN-FAI (HK)\ FUNG KA-HING (TW) | G02B-026/02\ G03B-021/00\ G03B-021/14\ G09G-003/20\ G09G-003/34\ H04N-005/74 |
| 5768 | 3D printing | ANISOTROPIC LIGHT-DIFFUSION LAMINATED COMPONENT USED FOR PROJECTOR SCREEN, CONSISTS OF ANISOTROPIC LIGHT DIFFUSION LAYER FORMED BY CONTINUOUS PHASE AND ELONGATE DISPERSED PHASE CONTAINING RESINS, AND ISOTROPIC LIGHT DIFFUSION LAYER | JP2014038171A | HWANG BYUNG-JUN (KR)\ LIM HOON (KR)\ KWAK KUN-HO (KR)\ JANG JAE-HOON (KR)\ JUNG SOON-MOON (KR)\ CHO WON-SEOK (KR)\ KIM SUNG-JIN (KR)\ KIM JONG-HYUK (KR) | UNIV BEIJING (CN) | C08L-023/10\ C08L-069/00\ G02B-005/02 |
| 5769 | 3D printing | DIGITAL LIGHT PROCESSING (DLP) PROJECTOR HAS CONVEX LENS AND RIGHT ANGLE PRISM WHICH ARE ARRANGED CORRESPONDING TO ANGLE OF LIGHT MODULATOR AND PARALLEL TO EACH OTHER, WHOSE OPTICAL AXIS IS VERTICAL TO PROJECTION LENS SYSTEM | CN103529629A\ CN103529629B | HWANG BYUNG-JUN (KR)\ LIM HOON (KR)\ KWAK KUN-HO (KR)\ JANG JAE-HOON (KR)\ JUNG SOON-MOON (KR)\ CHO WON-SEOK (KR)\ KIM SUNG-JIN (KR)\ KIM JONG-HYUK (KR) | SAMSUNG ELECTRONICS CO LTD (KR) | G02B-027/09\ G02B-027/10\ G02B-003/02\ G03B-021/00\ G03B-021/20 |
| 5770 | 3D printing | SERVICE LEVEL DEFINED HOLIDAY RESPONSE TIMING METHOD, INVOLVES RECORDING EVENT CREATING DIFFERENCE TIME AND ACCUMULATED SUSPENSION TIME IN DATABASE TABLE, AND DESIGNING WORKING TIME FORMAT | CN103530759A | IKURA YOSHIHIRO US\ KUMAGAI NAOKI US\ HARADA YUICHI US\ JINBO SHINICHI US\ YOSHIDA KAZUHIKO US | SAMSUNG ELECTRONICS CO LTD (KR) | G06Q-010/10 |
| 5771 | 3D printing | METHOD FOR PROVIDING CLOUD SERVICES TO CUSTOMERS IN COMPUTING CLOUD, INVOLVES ALLOWING CUSTOMER TO NEGOTIATE TERMS OF SERVICE LEVEL AGREEMENT (SLA) BASED ON TRADEOFF REGARDING HIGH-LEVEL PARAMETERS AT SERVICE LAYER OF CLOUD | US2014074647A1 | IKURA YOSHIHIRO US\ KUMAGAI NAOKI US\ HARADA YUICHI US\ JINBO SHINICHI US\ YOSHIDA KAZUHIKO US | FUJI ELEC DEVICE TECH CO LTD (JP) | G06Q-030/06 |
| 5772 | 3D printing | COMPUTER-IMPLEMENTED METHOD FOR MONITORING CLOUD SERVICES TO DETERMINE STATE OF CLOUD COMPUTING RESOURCES AVAILABLE TO CONSUMER, INVOLVES ADJUSTING SERVICE LEVEL AGREEMENT RULE WHEN TO INCREASE NUMBER OF COMPUTE INSTANCES IN ZONES | US2014075005A1 | INAKANAKA HIROSHI US | FUJI ELECTRIC SYSTEMS CO LTD US | H04L-012/24 |
| 5773 | 3D printing | PROJECTION DEVICE E.G. DIGITAL LIGHT PROCESSING (DLP) PROJECTOR HAS SHADING ELEMENT WHICH IS CONFIGURED BETWEEN DIGITAL MICRO-MIRROR DEVICE (DMD) AND CONDENSING ELEMENT TO BLOCK OFF-STATE LIGHT | US2014071405A1\ CN103676426A\ US9033521B2 | ISHIDA MAKOTO (JP)\ SAWADA KAZUAKI (JP)\ AKAI DAISUKE (JP)\ YOKAWA MIKAKO (JP)\ HIRABAYASHI KEISUKE (JP) | SONY CORP (JP) | H04N-005/74\ H04N-009/31\ G03B-021/00\ G03B-021/14\ G03B-021/20 |
| 5774 | 3D printing | SHEET-SHAPED BOARD E.G. FLEXIBLE PRINTED CIRCUIT BOARD OF OPTICAL PICK-UP APPARATUS USED IN OPTICAL DISK RECORDING DEVICE, IS SUBJECTED TO BENDING PROCESS, SO THAT HOLLOW SECTIONS CONTAINING LANDS ARE FORMED IN SURFACE OF MAIN PORTION | JP2014044774A | ITO HIROYASU (JP) | NAT UNIV CORP TOYOHASHI UNIV T (JP) | G11B-007/22 |
| 5775 | 3D printing | METHOD FOR THREE-DIMENSIONAL SHAPE MEASUREMENT E.G. BEATING RABBIT HEART MEASUREMENT, INVOLVES PERFORMING FRINGE ANALYSIS TO RECONSTRUCT THREE-DIMENSIONAL SHAPE OF OBJECT AND FRINGE PATTERNS DISTORTED BY SURFACE GEOMETRY OF OBJECT | US2014064603A1\ US8929644B2 | IWAI YOSHITAKA US | DENSO CORP (JP) | G01B-011/25\ G06K-009/00 |
| 5776 | 3D printing | METHOD FOR CREATING SY SESSION AND RECOVERING FROM INCONSISTENT SESSION STATE BY POLICY AND CHARGING RULES NODE (PCRN) IN SUBSCRIBER NETWORK, INVOLVES RECEIVING SPENDING LIMIT ANSWER (SLA) WITH STATUS REPORTS FOR REQUIRED COUNTERS | US2014064151A1\ US8995305B2 | IWAI YOSHITAKA US\ YASUKAWA HISATADA (JP) | MATSUSHITA ELECTRIC IND CO LTD (JP) | H04W-004/24\ H04L-012/14\ H04L-012/16\ H04M-015/00 |
| 5777 | 3D printing | AUTOMATIC CLEANING TABLE FOR THREE-DIMENSIONAL PRINTER, COMPRISES AN INTERNAL COVER, WHICH IS EQUIPPED WITH X-AXIAL MOVING ROD AND Y-AXIAL MOVING ROD, WHERE THE X-AXIAL MOVING ROD IS MOVABLY CONNECTED WITH A VERTICAL DRIVE MOTOR | CN204844876U | IWASHITA SETSUYA (JP)\ HIGUCHI TAKAMITSU (JP)\ MIYAZAWA HIROMU (JP) |  | A09-D03\ A11-A\ A11-B16\ A12-W12F\ X21-A01F\ X21-A07\ X25-A08M2\ X25-H |
| 5778 | 3D printing | A GELATIN PREPARATION BASED ON 3D PRINTING INVOLVES SPREADING POWDER IN MOLDING CHAMBER, SPRAYING CURING AGENT, POLYMERIZING AND HEATING | CN105001652A | JACHEN C BEINTNER US | MATSUSHITA ELECTRIC IND CO LTD (JP) | A03-C01\ A04-D05A\ A08-A06\ A08-C\ A08-D\ A08-D02\ A08-E01\ A09-D01\ A11-B16\ A11-C02D\ X25-A08A\ X25-A08M2 |
| 5779 | 3D printing | PRODUCING THERMISTOR CERAMIC INK USED FOR INK-JET PRINTING, BY INTRODUCING METAL-OXIDE AND -SALT INTO CONVENTION OXIDE SOLID PHASE BALL MILL, CHEMICAL LIQUID PHASE REVERSE MICROEMULSION OR CHEMICAL LIQUID PHASE CO-PRECIPITATION METHOD | CN105153808A | JAIN AMITABH (US) | SEIKO EPSON CORP (US) | A12-E01\ G02-A04A\ G05-F03\ L03-G09X\ S06-G04 |
| 5780 | 3D printing | NANO ACTIVATED CARBON COMPOSITE BASE PAPER CONTAINS ALPINE RUSH PULP, BLEACHED CHEMICAL SOFTWOOD PULP, BASALT FIBER, DUCK FEATHER, CHESTNUT SHELL, MELON VINE, MAGNOLIA FLOWER, SILICONE OIL, ALUMINUM NITRIDE, AND NANO ACTIVATED CARBON | CN105155337A | JAIN AMITABH (US)\ PAL HIMADRI SEKHAR (US)\ CHOI YOUN SUNG (US) | IBM (US) | A04-B06\ A06-A00E\ A12-W06B\ A12-W06C\ F05-A06C\ F05-A06D\ F05-A06E\ D09-A |
| 5781 | 3D printing | COMPOSITE THIN FILM FOR FLUORESCENT UMBRELLA, COMPRISES POLYURETHANE FILM, FLUORESCENT COATING, AND POLYPROPYLENE THIN FILM FILLED WITH TITANIA-COATED HOLLOW GLASS MICROBEADS | CN105058928A | JAMES DAVIS ZACHARY (JP) | TEXAS INSTRUMENTS INC (US)\ JAIN AMITABH (US) | A04-G03E\ A05-G01E1\ A08-R\ A08-R04\ A11-B05\ A11-C04A\ A12-W\ A12-W07D\ G02-A02H\ G05-F |
| 5782 | 3D printing | MODULAR IRON-CARBON MICROELECTROLYSIS FILLER FOR TREATING E.G. ORGANIC WASTEWATER DISCHARGED FROM COKING PLANT, COMPRISES CAST IRON OR PIG IRON, NORMAL POWDERED COKE, COPPER, AND BINDER COMPRISING YELLOW SAND AND PORTLAND CEMENT | CN104961202A | JAMIN FEN F (US)\ CHEN HUAJIE (US)\ FREEMAN GREGORY G (US)\ AHLGREN DAVID C (US)\ SUBBANNA SESHADRI (US)\ JAGANNATHAN BASANTH (US)\ ANGELL DAVID (US)\ SCHONENBERG KATHRYN T (US)\ STEIN KENNETH J (US) | TEXAS INSTRUMENTS INC (US) | C11-C05\ D04-A01M\ D04-B06\ J04-X |
| 5783 | 3D printing | POLYCHROMATIC KETTLE CONSISTS OF KETTLE LID AND MAINFRAME HAVING OUTER SURFACE PRINTED WITH TEMPERATURE-SENSITIVE MATERIAL COMPRISING TEMPERATURE-SENSITIVE POWDER AND INK CONTAINING RESIN, BINDER AND LIQUID CRYSTAL CAPSULE | CN104983305A | JAVORKA PETER (DE)\ KRONHOLZ STEPHAN-DETLEF (DE)\ WIATR MACIEJ (DE) | SEIKO EPSON CORP (JP)\ DAVIS ZACHARY JAMES (ES) | A12-W07D\ G02-A04A |
| 5784 | 3D printing | METALLIZATION AND SINTERING OF CERAMIC COMPONENT INVOLVES CARRYING OUT HIGH-TEMPERATURE METALLIZATION OF CERAMIC SURFACE BY USING ACTIVATED MOLYBDENUM-MANGANESE METHOD, COATING NICKEL PASTE, CURING NICKEL PASTE, AND SINTERING | CN104987102A | JI YUN-HYUCK (KR)\ KIM BEOM-YONG (KR)\ LEE SEUNG-MI (KR) | IBM (US) | L02-A02\ L02-A04\ L02-A08\ L02-A11\ L02-J01A\ L02-J01C |
| 5785 | 3D printing | HEALTHCARE FOOD USEFUL FOR E.G. ENHANCING BONE DENSITY AND IMMUNITY, STIMULATING OSTEOBLAST PROLIFERATION AND SELF COLLAGEN SYNTHESIS AND REGULATING HORMONAL LEVELS IN THE BODY COMPRISES CALCIUM E.G. CALCIUM LACTATE AND MILK CALCIUM | CN104939054A | JIA PENG US\ DAMING HUANG US\ MINGFU LI US | GLOBALFOUNDRIES INC (KY) | A03-A00A\ A12-V01\ A12-W09\ D03-B\ D03-F06\ D03-H01T2B\ B04-A08G2\ B04-A10F\ B04-C01\ B04-C02E2\ B04-N02\ B05-A01B\ B05-C07\ B06-D01\ B07-D02\ B07-D03\ B10-A07A\ B10-A07C\ B10-A07D\ B10-B02\ B10-C02\ B10-C04D\ B10-C04E6\ B14-D01\ B14-G01\ B14-N01\ B14-S11\ B14-S13\ B14-S18 |
| 5786 | 3D printing | WEAR-RESISTANT PRINTING INK INCLUDES PROPYLENE GLYCOL METHYL ETHER ACETATE, NANO-ZINC OXIDE, PTFE POWDER, ROSIN-MODIFIED PHENOLIC RESIN, OXO-2-ETHYLHEXANOATE ALUMINUM HOMOPOLYMER, MINERAL OIL, SOYBEAN OIL AND T-BUTYL HYDROPEROXIDE | CN104877449A | JIANG YANFENG (CN)\ LI JINHONG (CN)\ ZHANG CHANGNIAN (CN) | SK HYNIX INC (KR) | A02-A01\ A03-A00A\ A04-E08\ A04-F04\ A04-F06E\ A06-D01\ A10-B01\ A10-E01\ A12-W07D\ G02-A04A |
| 5787 | 3D printing | PRODUCTION OF PACKAGING PAPER INVOLVES PUNCHING DIAMOND-SHAPED HOLES INTO POLYPROPYLENE FILM AFTER PRINTING PATTERN ON POLYPROPYLENE FILM, BONDING PUNCHED POLYPROPYLENE FILM WITH FOOD GRADE WHITE WOOD PULP PAPER AND CURING | CN104878658A | JIANG YU US\ GUO GUILIANG US\ YAN YUEPENG US | UNIV FUDAN (CN) | A04-G03E1\ A11-A05A\ A11-A05C\ A11-B09A2\ A11-C01C\ A11-C02\ A11-C04A\ A12-P01A\ F05-A06 |
| 5788 | 3D printing | RAPID WELDING DEVICE USED FOR SEALING METALLIC PRINTER, COMPRISES FORMING UNIT, WHICH IS MOUNTED WITH MOVEMENT UNIT, NOZZLE, WELDING MACHINE, WIRE, WELDING METAL FORMING HEAD AND PLATFORMS | CN105127415A | JIANGTAO XU US\ JING GAO US\ ZAIFENG SHI US\ YINGCONG YE US | ZHUOLI SCIENCE AND TECHNOLOGY (CN) | A04-E08B\ A12-H\ M22-H03G\ X25-A08B |
| 5789 | 3D printing | FLEXIBLE METAL POWDER ADHESIVE CLOTH FOR SILICON NITRIDE CERAMIC SURFACE COMPRISES BASE MATERIAL IS SILVER POWDER PARTICLES WHICH INCLUDES COPPER, TITANIUM,NICKEL, CERIUM, SCANDIUM | CN105057919A | JIANHONG YANG US\ SHUIYING JIA US | INST OF MICROELECTRONICS CAS (CN) | A12-W12G\ G03-B01\ G03-B02D3\ L02-A03\ L02-H02B2\ L02-J01C |
| 5790 | 3D printing | THREE-DIMENSIONAL HOLLOW TITANIUM DIOXIDE ASSEMBLY USED FOR MANUFACTURING LITHIUM BATTERY CATHODE MATERIAL, HAS THREE-DIMENSIONAL HOLLOW TITANIUM DIOXIDE HAVING HOLLOW HIGH-ENERGY CRYSTAL FACE | CN105133013A | JIANJUN LI US\ WEI LI (CN)\ PING LI (CN) | UNIV TIANJIN (CN) | A04-E10B\ A10-E05B\ A12-E06A\ A12-W11\ L03-A02B\ L03-A02D\ L03-E01B3\ L03-E01B5B\ L03-E08B\ L03-H05\ X16-B01F1\ X16-E01C1\ X16-E01G |
| 5791 | 3D printing | SCRATCH-RESISTANT PRINTING INK COMPRISES PRINTING INK BINDER, FILLER, LUBRICANT, PLASTICIZER, LIGHT AGENT, DISPERSING AGENT, PIGMENT AND SOLVENT | CN105086615A | JIANTAO BIAN US\ CHAO CHEN US\ XIANG CHENG US\ JING LU US\ HUANGPING YAN US | UNIV LANGZHOU US | A04-F04\ A04-G08A\ A06-A00E4\ A10-E\ A10-E07B\ A10-E23\ A12-W07D\ G02-A04A |
| 5792 | 3D printing | HIGH PERFORMANCE DURABLE PRINTING INK COMPRISES HIGH DURABILITY PRINTING INK BINDER, FILLER, REINFORCING AGENT, LUBRICANT, DISPERSANT, PIGMENT AND SOLVENT | CN105086620A | JIANTAO BIAN US\ ZENGFENG DI US\ MIAO ZHANG US | UNIV ELECTRONIC SCIENCE & TECH (CN) | A03-A04A\ A12-W07D\ G02-A04A |
| 5793 | 3D printing | FAST-DRYING PRINTING INK COMPRISES BINDER, FILLER, PLASTICIZER, SURFACTANT, ANTIFOAMING AGENT, PIGMENT AND SOLVENT | CN105086611A | JIANTAO BIAN US\ ZENGFENG DI US\ MIAO ZHANG US | UNIV XIAMEN (CN) | A05-G01E\ A10-E08A\ A10-E08B\ A12-W07D\ A12-W12C\ G02-A04A |
| 5794 | 3D printing | DURABLE ANTICORROSIVE PRINTING INK COMPRISES POLYMER ROSIN PENTAERYTHRITOL ESTER, ALUMINUM HYDROXIDE POWDER, CHLOROPHENOL, 2,6-DI-T-BUTYL-4-METHYLPHENOL, HIGH-DENSITY POLYETHYLENE WAX, PHTHALOCYANINE BLUE, AND ISOPROPANOL | CN105086599A | JIAYIN LI US | SHANGHAI INST MICROSYS & INF (CN)\ BIAN JIANTAO (CN)\ DI ZENGFENG (CN)\ ZHANG MIAO (CN) | A04-G02B\ A08-A06\ A08-M03\ A08-R01\ A08-S02\ A10-E07C\ A12-W07D\ G02-A04A\ G02-A04B |
| 5795 | 3D printing | ECONOMICAL PRINTING INK COMPRISES CHLORINATED POLYPROPYLENE RESIN, ALUMINUM HYDROXIDE POWDER, DIPROPYLENE GLYCOL DIBENZOATE, LAUROYLPROPYL DIMETHYLAMINE, POLYDIMETHYLSILOXANE, LINSEED OIL, BENZIDINE YELLOW, AND TOLUENE | CN105086619A | JIE LI (CN)\ YANGBO YI (CN)\ SHEN XU (CN)\ HAISONG LI (CN)\ WEIFENG SUN (CN)\ XIAOJUAN XIA (CN)\ LONGXING SHI (CN) | SHANGHAI INST MICROSYS & INF (CN) | A06-A00B\ A08-M03\ A08-P04\ A08-R\ A08-S02\ A10-E04A\ A12-W07D\ G02-A04A |
| 5796 | 3D printing | LIGHT-RESISTANT HIGH-PERFORMANCE PRINTING INK COMPRISES NITRILE RUBBER, CORK WOOD POWDER, 2,4-DIHYDROXY-BENZOPHENONE, ACETALDOXIME, COCAMIDOPROPYL-DIMETHYL-T-AMINE, CORN STARCH, CARBON BLACK, AND CYCLOHEXANONE | CN105086588A | JIEMING BOERSTLER DAVID W HAIL (US) | JIAYIN LI US | A03-A\ A04-B04\ A08-A03\ A08-A06\ A08-M03B\ A08-M10\ A08-R07\ A08-S02\ A12-W07D\ G02-A04A\ G02-A04B |
| 5797 | 3D printing | THREE-DIMENSIONAL LASER PRINTING DEVICE COMPRISES POWDER INJECTION MODULE GROUP, WORKING CAVITY, LASER GENERATOR, LASER SHOWER NOZZLE, ROLLER COMPONENT, POWDER COLLECTING BUCKET, POWDER PLATFORM, PISTON AND COMPUTER CONTROL SYSTEM | CN105081322A | JIN DA TANG (CN) | UNIV DONGNAN (CN) | M22-H03B\ S06-K07A\ V08-A09\ X25-A08B |
| 5798 | 3D printing | HIGH-PERFORMANCE FRAGRANCE FOR PRINTING INK, COMPRISES BINDER, FILLER, FRAGRANCE, LUBRICANT, THICKENING AGENT, ANTI-FOAMING AGENT, PIGMENT AND SOLVENT, WHERE BINDER IS ACRYLIC RESIN, AND FILLER IS DIATOMACEOUS EARTH POWDER | CN105062212A | JING ZHU US\ TIANXIANG HAN US\ QINSONG QIAN US\ SHENGLI LU US\ WEIFENG SUN (CN)\ LONGXING SHI (CN) | IBM (US) | A04-E08\ A04-F01A1\ A12-W07D\ G02-A02C\ G02-A04A\ G02-A04B |
| 5799 | 3D printing | GRAVURE PEARLESCENT INK USEFUL IN PAINT AND DYE, COMPRISES PEARL PULP, PEARL POWDER, ADDITIVE, BINDER AND SOLVENT | CN105111819A | JINJIN YU US\ YONGPING CHEN US\ HONGHUI YUAN US\ SHIJUN CHEN US\ RUOHAN DENG US | ZHONGXIN INT IC MFG SHANGHAI (CN) | A05-E01D\ A08-S02\ A12-W07D\ G02-A04A\ G02-A04B |
| 5800 | 3D printing | THREE-DIMENSIONAL PRINT POWDER COMPRISES STAINLESS STEEL POWDER, DISPERSANT FOR PREPARING STAINLESS STEEL POWDER, AND FERRIC HYDROXIDE POWDER | CN105057664A | JOHAUSSON T (DE)\ NORSTREM H (DE)\ ALGOTSSON P (DE) | UNIV SOUTHEAST US | A10-E05B\ A11-B16\ A12-W12F\ M22-H03B |
| 5801 | 3D printing | MANUFACTURING FACE DOUBLE LAYER WALL HOLLOW TURBINE BLADE COMPRISES E.G. USING THREE DIMENSION MODELING SOFTWARE, DESIGNING CONTAINING THE DOUBLE LAYER WALL HOLLOW TURBINE BLADE AND NEGATIVE RESIN AND PREPARING COMPRISING IMPINGEMENT HOLES | CN104907492A | JOLY JEAN-PIERRE (FR)\ FAYNOT OLIVIER (FR)\ CLAVELIER LAURENT (FR) | SHANGHAI TECH PHYSICS INST US | A02-A\ A02-A01\ A04-B\ A04-D04\ A04-F04A\ A04-G03C\ A08-S05\ A09-D03\ A10-B03\ A10-E05B\ A10-E09A\ A11-B16\ A11-C02B\ A12-A02\ A12-H\ A12-W12G\ A12-W16\ M22-B02\ M22-B03\ T01-J07B\ T01-J10C4\ X25-A01 |
| 5802 | 3D printing | MANUFACTURE OF COVERLAY COMBINED MAGNETIC SHEET AND FLEXIBLE PRINTED CIRCUIT BOARD INVOLVES COATING THERMOSETTING ADHESIVE TO ORIENTATION OF MAGNETIC SHEET POLYIMIDE FILM SURFACE | KR2015137905A | JONAK-AUER INGRID DR (AT)\ VESCOLI VERENA DR (AT)\ TEVA JORDI DR (AT) | INFINEON TECHNOLOGIES AG (DE) | A05-J01\ A08-R05\ A11-B03\ A11-B05D\ A11-B09A2\ A11-B13\ A11-C01C\ A12-E07A\ L03-H04E6A\ V04-R05D\ V04-U15 |
| 5803 | 3D printing | RESIN RUBBER COMPOSITE BONDED GRINDING WHEEL USED FOR POLISHING CERAMIC MATERIAL, COMPRISES WHEEL BASAL BODY AND ABRASIVE LAYER I.E. COMPOSITE BINDER E.G. POLYVINYL BUTYRAL RESIN AND CURING AGENT AND ABRASIVE MATERIAL E.G. DIAMOND POWDER | CN105058249A | JOODAKI MOJTABA (DE) | COMMISSARIAT ENERGIE ATOMIQUE (FR) | A03-B\ A05-A01E3\ A07-A01\ A08-C02\ A08-C03\ A08-C04\ A08-D\ A08-D03\ A08-D05\ A10-E02\ A11-B01\ A11-C01C\ A12-A03\ G03-A\ G03-B02D2 |
| 5804 | 3D printing | 3-DIMENSIONAL PRINTING METHOD OF NAIL BY SCANNING NAIL TO OBTAIN PRECISE MODELING DATA, DESIGNING NAIL PATTERN, TRANSFERRING PATTERN TO PRINTER, ADDING PRINTING POWDER MATERIAL TO CYLINDER OF PRINTER, AND PRINTING NAILS | CN105058797A | JUMPERTZ REINER (DE)\ SCHIMPF KLAUS (DE) | AUSTRIAMICROSYSTEMS AG (AT) | A09-D03\ A11-B03\ A12-V04C\ A12-W12F\ A12-W12G\ T04-M05\ X25-A08A\ X25-A08M2 |
| 5805 | 3D printing | ZIRCONIA FIBER BOARD COMPRISES SPECIFIED AMOUNT OF ZIRCONIA FIBER, ZIRCONIA HOLLOW SPHERE, ZIRCONIA COMPOSITE POWDER, STABILIZER, ORGANIC SPHERES, ORGANIC MICRO-POWDER AND BINDING AGENT | CN104788108A | JUN SI BEOM (KR) | JOODAKI MOJTABA (DE) | A10-E05B\ A12-W12G\ L02-B08\ L02-G01D |
| 5806 | 3D printing | THREE-DIMENSIONAL PRINTER HAS SHELL, POWDER SENDING POWDER DEVICE, WORKING CYLINDER DEVICE, PRINTING DEVICE, EXTERNAL POWDER STORAGE BUCKET, SCREW PUSH SENDING POWDER DEVICE, POWDER DELIVERING PIPE, ELECTRICAL MACHINE, AND SWITCHING BOARD | CN204773600U | KADO TETSUO US | TEXAS INSTRUMENTS DEUTSCHLAND (DE) | A11-B16\ A12-H02\ A12-W12F\ X25-A08B\ X25-A08M2 |
| 5807 | 3D printing | THREE-DIMENSIONAL PRINTING ELECTRON BEAM DEVICE COMPRISES COLD WATER CRUCIBLE, WATER CIRCULATION PASSAGE LAYING ON CRUCIBLE HOLDER, THE OUTER LAYER FOR SEPARATE HEAT MATERIAL, FEEDING DEVICE, FEEDING SHOWER NOZZLE, AND ELECTRONIC GUN | CN204773606U | KAIZHOU TAN US\ JING ZHANG US\ ZHENGFAN ZHANG US | SAMSUNG ELECTRONICS CO LTD (KR) | A09-D03\ A11-A\ A11-B16\ A11-C04A\ A11-C07\ A12-W12F\ X25-A08B\ X25-A08M2\ X25-C |
| 5808 | 3D printing | HIGH TEMPERATURE FLAME RETARDANT POLY VINYL CHLORIDE COMPLEX FLOORING COMPRISES E.G. MUTUALLY OVERLAPPED POLY VINYL CHLORIDE WEARABLE LAYER, A PRINTED PATTERN LAYER, SUBSTRATE LAYER AND POLY VINYL CHLORIDE FOAMING LAYER | CN104947915A | KANG KU CHANG (KR) | NAT INST OF ADV IND & TECHNOL US | A04-E02E1\ A05-C01B1\ A08-A01A\ A08-F\ A08-F05\ A08-M03\ A08-P01\ A08-R01\ A09-D03\ A11-A03\ A11-A04\ A11-B07\ A11-C01C\ A11-C02C\ A12-R03\ A12-R06\ A12-S04B\ A12-S08B\ A12-S08D |
| 5809 | 3D printing | PREPARING MAGNESITE BASED FLUORINE REMOVING ADSORBENT MATERIAL, COMPRISES E.G. WET BALL-MILLING NATURAL MAGNESITE POWDER AND BIOMASS POWDER, ADDING BINDER SOLUTION, STIRRING, IMMERSING POLYURETHANE FOAM SPONGE IN SLURRY AND CALCINING | CN104874368A | KANG TAE KYOUNG US\ SUE YOON MIN US | NO 24 RES INST CETC US | A10-E05B\ A12-S02\ A12-W11D\ A12-W11J\ D04-A01F3\ D04-A01P1\ D04-B07E\ D04-D |
| 5810 | 3D printing | METHOD FOR PREPARING DIE-CASTING ALUMINUM ALLOY EXTERIOR SURFACE ON MOBILE PHONE SHELL, INVOLVES UTILIZING SCREEN-PRINTING PROCESS WITH HIGH BRIGHTNESS ICON AND DIE-CASTING ALUMINUM ALLOY SUBSTRATE | CN105109260A | KANG TAEG-HYUN (KR)\ YUN SUNG-SON (KR) | KANG KU CHANG (KR) | W01-C01D3C |
| 5811 | 3D printing | COMPOSITION FOR TREATING LEUKEMIA, COMPRISES RAW MATERIALS, BINDERS AND LUBRICANTS, WHERE THE RAW MATERIALS COMPRISES IMATINIB AND DASATINIB DRUG | CN105055367A | KAVALIEROS JACK (US)\ CAPPELLANI ANNALISA (US)\ DATTA SUMAN (US)\ BRASK JUSTIN K (US)\ DOCZY MARK L (US)\ CHAU ROBERT S (US)\ METZ MATTHEW V (US)\ BARNS CHRIS E (US) | CURITEL COMM INC US | A03-A05\ A04-D05A\ A10-E02\ A12-V01\ B04-C03A\ B07-D04C\ B07-D10\ B07-D12\ B07-F01\ B10-C04E5\ B12-M11C\ B14-H01A |
| 5812 | 3D printing | CHARGING TYPE MAGNETIC MATERIAL 3D COLD PRINTING DEVICE HAS CONTROL CENTER, STEPPING MOTOR, LIQUID RESERVOIR CONNECTED TO CURING AGENT DRIVING DEVICE VIA CONVEYING PIPE, AND CHARGING BARREL CONNECTED TO MATERIAL SLURRY DRIVING DEVICE | CN105014074A | KAVALIEROS JACK (US)\ METZ MATTHEW (US)\ CHAU ROBERT (US)\ DOCZY MARK (US)\ BRASK JUSTIN (CA)\ DOYLE BRIAN (IE)\ DATTA SUMAN (IN)\ WANG EVERETT (US)\ MATAGNE PHILIPPE (BE)\ SHIFREN LUCIAN (ZA)\ JIN BEEN-YIH (US)\ STETTLER MARK (US)\ GILES MARTIN (GB) | KANG TAEG-HYUN (KR)\ YUN SUNG-SON (KR) | M22-H\ X25-A08B |
| 5813 | 3D printing | LOW CARBON GAS FUEL MANUFACTURING DEVICE COMPRISES NATURAL GAS STORAGE TANK, NATURAL GAS PROCESSING UNIT, HYDROGEN FLAME ARRESTOR, HYDROGEN STORAGE TANK, HYDROGEN PRETREATMENT UNIT, MIXER, CONTROL DEVICE, MIXED GAS TANK AND BYPASS VALVE | CN105038885A | KAZUYA AIZAWA US | KAVALIEROS JACK (US)\ CAPPELLANI ANNALISA (US)\ BRASK JUSTIN K (US)\ DOCZY MARK L (US)\ METZ MATTHEW V (US)\ DATTA SUMAN (US)\ BARNS CHRIS E (US)\ CHAU ROBERT S (US) | H06-A\ H06-P |
| 5814 | 3D printing | MAGNETIC HEAT TRANSFER PRINTING BELT, COMPRISES BASE FILM, HEAT-RESISTANT BACK COAT LAYER, RELEASE LAYER AND MAGNETIC INK LAYER, WHERE BASE FILM IS ARRANGED WITH RELEASE LAYER ON ONE SIDE AND OTHER SIDE WITH MAGNETIC INK LAYER | CN105034621A | KIM C HARDY US | KAVALIEROS JACK (US)\ BRASK JUSTIN K (US)\ DOCZY MARK L (US)\ METZ MATTHEW V (US)\ DATTA SUMAN (US)\ CHAU ROBERT S (US)\ DOYLE BRIAN S (US)\ WANG EVERETT X (US)\ JIN BEEN Y (US)\ GILES MARTIN D (US)\ MATAGNE PHILIPPE (US)\ SHIFREN LUCIAN (US)\ STETTLER MARK (US) | A04-G02E3\ A05-E01D3\ A10-E01\ A12-L05C1\ A12-W07D\ S06-D04\ S06-E03C\ S06-E04B1\ S06-E05\ S06-F03\ S06-K03\ S06-K05\ S06-K07A |
| 5815 | 3D printing | USE OF DOT MATRIX PRINTER YOKE BASE MATERIAL FOR PREPARING YOKE BASE PRODUCTS BY UNIFORMLY MIXING CARBONYL IRON POWDER WITH ALLOY POWDER, MIXING POWDER WITH BINDER, KNEADING, INJECTION MOLDING TO FORM BLANK BODY, AND DEGREASING | CN104962821A | KIM CHEON SU US\ YOO HYEON GYU US\ PARK PIL JAE (KR)\ PARK MUN YANG (KR) | SANKEN ELECTRIC CO LTD (JP) | A04-G02E\ A04-G03E\ A08-M03\ A08-S02\ A10-E05B\ A11-A03\ A11-B12A\ A12-W07F\ L03-B02A2\ L03-B02J\ L03-H05\ L04-A01A\ M22-H03B\ M27-A04\ V02-A02A\ V02-A09\ V02-C\ V02-H |
| 5816 | 3D printing | FILAMENT BONDED HONEYCOMB STRUCTURE HIGH ELASTIC FLAME RETARDANT NONWOVEN FABRIC COMPRISES SEQUENTIALLY STACKED PLURALITY OF COMPOSITE FIBER NETWORK SPRINKLED WITH MIXED POWDER OF HOT MELT POWDER AND FLAME RETARDANT POWDER | CN104818584A | KIM DONG SU (KR)\ KIM WEON CHAN (KR)\ KIM IN SU (KR)\ SEO SONG HA (KR)\ JUNG YEONG TAE (KR) | SONY CORP (JP)\ UNITED MEMORIES INC (US) | A05-E01B\ A11-C05B\ A11-C05C\ A12-S05B\ A12-S05E\ A12-S05G\ A12-S05R\ F01-E01\ F02-C01\ F02-C02B\ F03-C03 |
| 5817 | 3D printing | POWDER MATERIAL USED FOR POWDER LAMINATION-MODELING TO PRODUCE THREE-DIMENSIONAL MOLDED ARTICLE, COMPRISES PARTICLES WHICH FORM SECONDARY PARTICLES WITH WHICH PRIMARY PARTICLE THREE-DIMENSIONALLY BONDED WITH GAPS | WO2015194678A1 | KIM KI JOONG (KR)\ KIM YOUN SUK (KR)\ WON JUN GOO (KR) | KOREA ELECTRONICS TELECOMM (KR) | A11-A04\ A11-B09A2\ A11-B14\ A11-B16\ A11-C01C\ A12-S09A\ A12-W12F\ A12-W12G |
| 5818 | 3D printing | PREPARATION OF ZIRCONIUM OXIDE CERAMIC MOBILE PHONE BACK COVER BY STIRRING YTTRIUM OXIDE AND NANOZIRCONIA POWDERS IN WATER, ADDING DISPERSANT, ALUMINUM OXIDE, TITANIUM DIOXIDE AND SILICON DIOXIDE, DISSOLVING IN ORGANIC SOLVENT AND POLISHING | CN104961461A | KIM KI-CHUL (KR)\ LEE HO (KR)\ LEE JUNG-DEOG (KR) | KEC KK (KR) | A10-E05B\ A12-E12\ A12-W12G\ L02-A02\ L02-A03\ L02-A04\ L02-A08\ L02-A11\ L02-A12\ L02-G01A\ L02-G01B\ L02-G01C1\ L02-G01D\ L02-G01E\ L02-G08\ L02-G12B\ L02-G12E\ T01-J07B\ W01-C01D3C |
| 5819 | 3D printing | PREPARATION OF CERAMIC/RESIN COMPOSITE MATERIAL FOR MULTILAYERED DEVICE, INVOLVES MIXING CERAMIC POWDER AND DEIONIZED WATER, SCREEN PRINTING CERAMIC GREEN TAPE, PREFABRICATING RESIN MONOMER POWDER TO POROUS MATERIAL AND CURING MATERIAL | CN104945002A | KIM WAN SOO (KR) | SAMSUNG ELECTRO MECH (KR) | A10-E05B\ A12-W12G\ L02-A02\ L02-A03\ L02-A11\ L02-D14S\ L02-G01X\ L02-G12X |
| 5820 | 3D printing | OXIDE-ENHANCED-OXIDE-BASED COMPOSITE MATERIAL COATED WITH A HIGH-TEMPERATURE-RESISTANT LOW INFRARED EMISSIVITY COATING COMPRISES A COMPOSITE MATERIAL BASE AND A COATING, WHERE THE COMPOSITE MATERIAL BASE IS COATED WITH THE COATING | CN104858434A | KIM WEON CHAN (KR)\ KIM IN SU (KR)\ SONG IN HYUK (KR) | SAMSUNG ELECTRONICS CO LTD (KR)\ LEE HO (KR)\ KIM KI-CHUL (KR)\ LEE JUNG-DEOG (KR) | A03-A03\ A10-E05B\ A11-B05\ A12-B08\ M22-H02\ M22-H03B\ M22-H03F |
| 5821 | 3D printing | CLEANING MATERIAL FOR FUSED DEPOSITION MODELING 3-DIMENSIONAL PRINTER NOZZLE, COMPRISES POLYOLEFIN SUBSTRATE, ANTIOXIDANT, DISPERSING AGENT, INORGANIC POWDER AND CLEANING AGENT | CN104945732A | KIM YONG DON (KR)\ CHANG HOON (KR)\ PAK SEO IN (KR) | HYNIX SEMICONDUCTOR INC (KR) | A04-G01E\ A08-R01\ A08-S01\ A09-D02\ A11-B07D\ A11-B16\ A11-C\ A12-W12B |
| 5822 | 3D printing | POWDER APPARATUS FOR PRINTING MACHINE, HAS SECOND NOZZLES THAT ARE ARRANGED IN BLOCK INTERVALS WHICH ARE LARGER THAN DISTANCES BETWEEN SPECIFIC NOZZLES | DE102015209514A1 | KIM YONG-DON (KR)\ LEE EUNG-KYU (KR)\ BAE SUNG-RYOUL (KR)\ KIM SOO-BANG (KR)\ JANG DONG-EUN (KR) | KEC KK (KR) | S06-C03A\ S06-K07A\ S06-K99C |
| 5823 | 3D printing | POLYIMIDE POWDER USED FOR E.G. HEAT-RESISTANT INSULATING TAPE, HAS PRESET SPECIFIC SURFACE AREA, DOES NOT SHOW CRYSTALLINE SCATTERING PEAKS, AND HAS PRESET SOLUBILITY WITH RESPECT TO SOLVENT E.G. AMIDE SYSTEM | JP5833783B1 | KIM YOUNG PIL (KR)\ KIM JIN BUM (KR)\ JUNG IN SUN (KR)\ KIM KI HONG (KR)\ KYOUNG YONG KOO (KR) | SAMSUNG ELECTRONICS CO LTD (KR)\ KIM YONG-DON (KR)\ CHANG HOON (KR)\ PAK SEO-IN (KR) | A05-F01A\ A08-S02\ A10-D03\ F03-D\ G02-A05A\ G03-B04\ L03-B05K1\ L03-E05B2\ L03-G09\ L03-H04E6A\ S03-E06C\ U11-F02A1A\ V04-A08\ V04-M05\ V04-M06\ V04-R01A3\ V04-R01A4\ V04-R01A5A\ V04-S02\ V04-S10\ V04-S15\ V04-S30\ V04-X01B\ V04-X01F\ X12-C01D2\ X12-C03\ X15-A01A\ X15-A02A\ X15-A02X\ X16-B01F1\ X16-E\ X16-F02\ X16-F03A1\ X16-K02\ X16-L01 |
| 5824 | 3D printing | THREE-DIMENSIONAL (3D) PRINTING EQUIPMENT FOR MANUFACTURING COMPOSITE NOZZLE, HAS SLURRY STORAGE SYSTEM CONNECTED WITH QUANTITATIVE MATERIAL FEEDING SYSTEM, AND HEAT INITIATING SYSTEM FOR HEATING METAL MATERIAL SLURRY AND CURING AGENT | CN204818064U | KIM YOUNG-PIL (KR)\ KIM JIN-BUM (KR)\ LEE JUN-HO (KR)\ WON JUNG-YUN (KR)\ JUNG IN-SUN (KR) | SAMSUNG ELECTRONICS CO LTD (KR)\ KIM YONG-DON (KR)\ LEE EUNG-KYU (KR)\ BAE SUNG-RYOUL (KR)\ KIM SOO-BANG (KR)\ JANG DONG-EUN (KR) | M22-H03F\ X25-A08B |
| 5825 | 3D printing | MAGNETIC INSULATION CUSHION COMPRISES UPPER AND LOWER CUSHIONS, THE TWO CUSHIONS ARE BONDED WITH A ADHESIVE, AT LEAST ONE CUSHION IS MAGNETIC CUSHION WHICH IS MADE UP OF MAGNETIC POWDER AND FOAMED POLYURETHANE POWDER | CN105062038A | KINOSHITA MASAYA (JP)\ INABA SEIJIRO (JP)\ GOMI KEIZO (JP) | SAMSUNG ELECTRONICS CO LTD (KR) | A05-A01E2\ A08-M09A\ A09-A03\ A11-B08\ A11-B09A2\ A12-E08\ A12-S02\ G03-B01\ G03-B02 |
| 5826 | 3D printing | HIGH-TEMPERATURE COOKING BAG HAS CASTING POLYPROPYLENE FILM LAYER, POLYIMIDE FILM LAYER, POLYTETRAFLUOROETHYLENE FILM, SILICON COATING AND PRINTED LAYER OBTAINED BY FORMING POLYURETHANE COATING ON SILICON INK PRINTING PATTERN | CN105015119A | KO CHIH-HSIN (TW) | SAMSUNG ELECTRONICS CO LTD (KR) | A04-E08\ A04-G03E1\ A05-G01E1\ A05-J01B\ A12-D03\ A12-W07D\ G03-B02D3\ G03-B02E2\ G03-B02E4 |
| 5827 | 3D printing | THREE-DIMENSIONAL (3D) PRINT MATERIAL SLURRY SPRAYING-SHAPING-CURING METHOD INVOLVES PERFORMING CURING PROCESS ACCORDING TO MASS RATIO OF LIQUID CURING AGENT AND CURING AGENT POWDER, TO FORM CURING LAYER WITH PREDETERMINED THICKNESS | CN104959613A | KOELE WERNER (AT) | SONY CORP (JP) | A11-B16\ A11-C02D\ M22-H01\ X25-A08A |
| 5828 | 3D printing | IMPROVING PRECISION OF LASER SPOT DIRECT FORMING INVOLVES ESTABLISHING 3D MOLD OF PART, CONTROLLING COAXIAL POWDER FEEDING NOZZLE, LASER SPOT PROCESSING, SCANNING, FEEDING POWDER AND SINGLE-CHANNEL MEASURING CLADDING CROSS-SECTIONAL WIDTH | CN104923784A | KOIKE HIDEHARU (JP) | TAIWAN SEMICONDUCTOR MFG (TW) | M22-H03B\ T01-J10C4\ X25-A08A |
| 5829 | 3D printing | 3D PRINTING FUNCTIONALLY GRADED MATERIAL STRUCTURE INVOLVES 3D MODELING PART, GETTING PROFILE INFORMATION OF EACH MATERIAL COMPONENT, PRINT PATH PLANNING, DUSTING, PRINTING EACH PRINT LAYER, FORMING PISTON AND LOWERING PRINT LAYER HEIGHT | CN104923787A | KRONHOLZ STEPHAN (DE)\ WASYLUK JOANNA (DE)\ REIMER BERTHOLD (DE)\ METZGER SVEN (DE)\ NOWLING GREGORY (US)\ FOSTER JOHN (US)\ BESSER PAUL (US) | KOELE WERNER (AT) | M22-H03B\ T01-J08A\ X25-A08A |
| 5830 | 3D printing | MANUFACTURING ELECTRICAL CONTACTS INVOLVES MILLING SILVER METAL INGREDIENTS ACCORDING TO COMPOSITION AND RATIO REQUIREMENTS TO FORM COMPOSITE POWDERS | CN104907561A | KRUTSICK THOMAS J (US)\ BASTEK JOHN JOSEPH (US)\ PLUMMER ROBERT D (US) | WINBOND ELECTRONICS CORP (TW) | M22-H01\ M22-H02\ M22-H03B\ M22-H03F\ A12-W12F\ X25-A08A\ X25-A09 |
| 5831 | 3D printing | PREPARATION OF MICROWAVE DIELECTRIC CERAMIC/RESIN BICONTINUOUS COMPOSITE MATERIAL USED FOR PRINTED CIRCUIT BOARD SUBSTRATE, INVOLVES PREPARING POROUS CERAMIC COMPONENT USING CERAMIC POWDER, HEATING, POURING RESIN, MELTING AND CURING | CN104693688A | KUMAGAI NAOKI US\ YOSHIDA KAZUHIKO US\ ICHIMURA TAKESHI US\ FUJIHIRA TATSUHIKO US | GLOBALFOUNDRIES INC (KY)\ KRONHOLZ STEPHAN (DE)\ WASYLUK JOANNA (DE)\ REIMER BERTHOLD (DE)\ METZGER SVEN (DE)\ NOWLING GREGORY (US)\ FOSTER JOHN (US)\ BESSER PAUL (US) | A10-E05B\ A11-C02\ A12-E07A\ A12-W12G\ L02-A02\ L02-A03\ L02-A04\ L02-A05\ L02-D14S\ L03-H04E1\ L03-H04E5\ V04-Q30N\ V04-R07\ V04-T03B\ V04-T03J\ V04-T03P |
| 5832 | 3D printing | SILVER PASTE USED FOR FRONT SURFACE OF SOLAR BATTERY, COMPRISES SPECIFIED AMOUNT OF SILVER POWDER, GLASS POWDER CONTAINING SILICON DIOXIDE AND ZINC OXIDE, ORGANIC BINDER CONTAINING ORGANIC CARRIER AND ORGANIC SOLVENT, AND ADDITIVE | CN104681122A | KURANOUCHI ATSUSHI US | AGERE SYSTEMS INC (US) | A03-A03\ A03-A04A\ A04-F04\ A12-E11B\ A12-W16\ L03-A02\ L03-E05B\ L03-H05\ L04-A03D\ L04-C10E\ L04-C12A\ X15-A01A\ X15-A02F |
| 5833 | 3D printing | RAPID MACHINE HAND MANUFACTURE QUARTZ CRYSTAL RESONATOR, HAS SUBSTRATE FRAME FIXED WITH FOUNDATION BED BOTTOM PLATE, PIN FIXED IN COVER SHELL, AND CRYSTAL SUBSTRATE FRAME FORMED WITH CRYSTAL INSERTION SLOT AND MATCHED WITH CRYSTAL | CN204886894U | KWAK SANG HUN (KR)\ LIM GWANG HYEON (KR) | FUJI ELEC DEVICE TECH CO LTD (JP) | V06-V01B\ V06-V01E\ V06-V03A\ X25-A08 |
| 5834 | 3D printing | FINE SAND ROADBED MICROORGANISM GROUTING UNITE GEOGRID REINFORCEMENT STRUCTURE, HAS PVC PIPE CONNECTED WITH IRON SOIL PROCESSING GRID GRATE THROUGH WIRE, AND MICROORGANISM CEMENTATION SAND BODY FORMED AROUND PVC PIPE | CN204875409U | KWOK TSZ-MEI (TW)\ CHENG CHUN-FAI (HK)\ CHANG AN-SHEN (TW)\ LIN HUI-MIN (TW)\ LO HSIEN-CHING (TW) | SONY CORP (JP) | A04-E02E1\ A12-H02\ A12-R09 |
| 5835 | 3D printing | THREE-DIMENSIONAL PRINT DEVICE, HAS LIGHT BEAM CONNECTED WITH LIGHT SOURCE AND ADHESIVE BONDING PORTION AND FORMED WITH POWDER LAYER, AND MAIN BODY MATCHED WITH POWDER LAYER THAT IS FORMED WITH BONDING PORTION | CN204844872U | KWON GYU HYEONG US | DONGBU ELECTRONICS CO LTD (KR) | A11-A\ A11-B16\ A11-C01D\ A12-A05\ X25-A08B\ X25-A08M2 |
| 5836 | 3D printing | METHOD FOR MANUFACTURING COMPARTMENTALIZED COLOR PALETTE-TYPE MAKEUP PRODUCT USING THREE DIMENSIONAL PRINTING PROCESS, INVOLVES DEPOSITING MAKEUP POWDER LAYERS ON POWDER BED WITHIN PRINT CHAMBER TO CREATE THREE-DIMENSIONAL OBJECT | US2015366327A1 | LAERMER FRANZ (DE)\ BENZEL HUBERT (DE)\ ILLING MATTHIAS (DE)\ ARMBRUSTER SIMON (DE)\ SCHELLING CHRISTOPH (DE)\ BRASAS JOERG (DE)\ GRUNDMANN ARMIN (DE) | TAIWAN SEMICONDUCTOR MFG (TW)\ KWOK TSZ-MEI (TW)\ CHENG CHUN-FAI (HK)\ CHANG AN-SHEN (TW)\ LIN HUI-MIN (TW)\ LO HSIEN-CHING (TW) | A12-V04C\ S05-G02B\ T01-J05B4P\ T01-N01D\ T01-N01E\ T01-N03 |
| 5837 | 3D printing | COMPOSITION USED FOR FORMING ELECTROLESS PLATING BASE LAYER, COMPRISES METAL POWDER OR METAL OXIDE POWDER, BINDER RESIN, COMPOUND HAVING TRAPPING PROPERTY WITH RESPECT TO METAL AND METAL ION, IN SPECIFIED MASS RATIO | JP2015229788A | LAM YEE LOY (SG)\ CHAN YUEN CHUEN (SG) | SAMSUNG ELECTRONICS CO LTD (KR) | A12-E07A\ L03-H04E3\ M13-B\ M13-H03 |
| 5838 | 3D printing | TIN POWDER USED IN ELECTROCONDUCTIVE COMPOSITION, COMPRISES AGGREGATE OF PARTICLES OF TIN OR TIN-BASED ALLOY, HAS PRESET PRIMARY PARTICLE DIAMETER AND CONTAINS NITROGEN IN PARTICLE SURFACE AND VICINITY | JP2015229789A | LE LI US\ SHUGEN PENG US | BOSCH GMBH ROBERT (DE) | M22-H01\ M22-H02\ X24-A\ X25-A08 |
| 5839 | 3D printing | DIRECT INCIDENCE TYPE LIGHT AMPLIFICATION THREE-DIMENSIONAL SCANNING PROBE ARM, HAS PROCESSING SYSTEM THAT DETERMINES INCIDENCE POSITION CHANGE VALUE OF LASER BEAM AND THREE-DIMENSIONAL DISPLACEMENT CHANGE VALUE OF BALL | CN105136038A | LE LUO US\ GAOWEI XU US\ XIAO CHEN US\ JIWEI JIAO US\ BINWEI MI US | DENSELIGHT SEMICONDUCTORS PTE (SG)\ LAM YEE LOY (SG)\ CHAN YUEN CHUEN (SG)\ FINNIE PETER JOHN (GB) | S02-A03\ S02-A10B |
| 5840 | 3D printing | MICROCRYSTALLINE GRAPHENE-BASED ELECTRICALLY-CONDUCTIVE INK USEFUL AS ANODE MATERIAL IN LITHIUM BATTERIES AND FOR PRINTING DEVICE, COMPRISES MICROCRYSTALLINE GRAPHITE POWDER, BONDING ADDITIVES AND DISPERSANT | CN105062219A | LE TIEC YANNICK (FR)\ GRENOUILLET LAURENT (FR)\ POSSEME NICOLAS (FR)\ VINET MAUD (FR) | SHANGHAI GRACE SEMICONDUCTOR US | A12-E06A\ A12-W07D\ G02-A04A\ G02-A05B\ L03-A02B\ L03-E01B3\ L03-E08B\ X16-B01F1\ X16-E01 |
| 5841 | 3D printing | NEW SUBSTITUTED CARBAMATE DERIVATIVES ARE PHOSPHODIESTERASE-4 INHIBITORS USEFUL FOR PREVENTING OR TREATING RESPIRATORY TRACT DISEASES E.G. ASTHMA OR CHRONIC OBSTRUCTIVE PULMONARY DISEASE | WO2015185650A1 | LEE CHUNG-SUN (KR)\ JANG HYUNG-SUN (KR)\ KANG UN-BYOUNG (KR)\ KWON WOON-SEONG (KR)\ KWON YONG-CHAI (KR)\ LEE DONG-HO (KR) | SHANGHAI INST MICROSYS & INF (CN) | B07-D04B\ B14-D07A1\ B14-K01 |
| 5842 | 3D printing | CEREMONIAL ACTIVITY BONE MATERIAL INCENSE PAPER PREPARING METHOD, INVOLVES MAKING SUBSTRATE WITH INCENSE AND JOSS STICK PRODUCT PAPER, PREPARING INCENSE BINDING POWDER, AND REGULATING FLAVOR SPICE INTO SIDE SURFACE OF SLURRY COATING PAPER | CN104939653A | LEE HYO-SAN (KR)\ KO HYUNG-HO (KR)\ HONG CHANG-KI (KR)\ CHOI SANG-JUN (KR) | COMMISSARIAT ENERGIE ATOMIQUE (FR) | M22-H01\ M22-H03G |
| 5843 | 3D printing | NOZZLE APPARATUS FOR PREPARATION OF THREE-DIMENSIONAL (3D) PRINTING METAL POWDER, HAS AIR GUIDE JOINT THAT IS ARRANGED BETWEEN GUIDE PIPE AND AIR CAVITY WHOSE WALL IS PROVIDED WITH GUIDE HOLE CONNECTED WITH AIR GUIDE JOINT | CN204747507U | LEE JONG WOOK (KR)\ KIM YOUNG PIL (KR)\ KIM JIN BUM (KR)\ CHOI SI YOUNG (KR)\ LEE BYEONG CHAN (KR) | SAMSUNG ELECTRONICS CO LTD (KR) | L01-F04\ L01-G02\ X25-A05\ X25-A08B |
| 5844 | 3D printing | GLASS THREE-DIMENSIONAL PRINT DEVICE HAS LASER, GLASS POWDER SPRAYING DEVICE, AND PHOSPHATE LOW-MELTING POINT GLASS MATERIAL | CN104944750A | LEE KIM FUNG (US) | SAMSUNG ELECTRONICS CO LTD (KR) | M22-A01\ M22-A04\ M22-B01\ M22-D\ T01-J07B\ T01-J15H |
| 5845 | 3D printing | SAND CASTING METHOD FOR SPECIALLY-SHAPED TITANIUM OR TITANIUM ALLOY CAST INVOLVES PREPARING INLAY, MACHINE PROCESSING BLANK INLAY, CASTING EXTENSION, CASTING BASE SURFACE, INLAY BLANK PROCESSING, THINNING THE WALL, FUSING AND MILLING BLANK | CN104923727A | LEE KONG-SOO (KR)\ KIM KYOUNG-SEOK (KR)\ PARK SANG-JIN (KR)\ LEE CHANG-HOON (KR)\ JEONG JI-HYUN (KR)\ PARK JAE-HYUN (KR)\ OH JAE-HEE (KR) | SAMSUNG ELECTRONICS CO LTD (KR)\ CHOI SI-YOUNG (KR)\ KIM JIN-BUM (KR)\ KIM YOUNG-PIL (KR)\ LEE BYEONG-CHAN (KR)\ LEE JONG-WOOK (KR) | A09-D03\ A11-B16\ A12-D01\ A12-D04\ A12-V03D\ X25-A08A\ X25-A08M2\ X25-A08U2 |
| 5846 | 3D printing | METHOD FOR MANUFACTURING BODY-SUPPORTING ELEMENT IN FORM OF E.G. MATTRESS, INVOLVES PREPARING SUPPORTING ELEMENT ON BASIS OF PRINT DATA WHICH FORM SPECIFIC THREE-DIMENSIONAL SUPPORTING STRUCTURE OF PERSON BY THREE-DIMENSIONAL PRINTER | DE102015100816B3 | LEE KYOUNG HO (KR) | SILICON LAB INC (US) | M22-C01\ M22-D\ X25-A01\ X25-A08A\ X25-A08U3\ X25-H |
| 5847 | 3D printing | MULTI-CAVITY STRUCTURE VEHICLE EXHAUST GAS TREATMENT PROCESSING SYSTEM COMPONENT PRECISION CASTING RAPID PROTOTYPING COMPRISES MOLDING WATER SOLUBLE WAX TYPE CORE IN MOLD, PRESSING, COMBINING CASTING MOLD AND WAX PATTERN, AND CASTING | CN104646629A | LEMMERHIRT DAVID F (US)\ RICH COLLIN A (US) | SAMSUNG ELECTRONICS CO LTD (KR)\ LEE KONG-SOO (KR)\ KIM KYOUNG-SEOK (KR)\ PARK SANG-JIN (KR)\ LEE CHANG-HOON (KR)\ JEONG JI-HYUN (KR)\ PARK JAE-HYUN (KR)\ OH JAE-HEE (KR) | A11-B16\ X25-A08 |
| 5848 | 3D printing | 3D PRINT TECHNOLOGY BASED SMALL ELECTRONIC INSTRUMENT SHELL MANUFACTURING METHOD, INVOLVES DESIGNING ELECTRIC EQUIPMENT SHELL SHAPE ACCORDING TO ELECTRIC EQUIPMENT REQUIREMENTS, AND PERFORMING UTILIZED 3D PRINTER SHELL MANUFACTURING PROCESS | CN105034362A | LEOBANDUNG EFFENDI (US)\ LEE BYOUNG HUN (US)\ DIRAHOUI BACHIR (US)\ SU TAI-CHI (US) | SAMSUNG ELECTRO MECH (KR)\ LEE KYOUNG HO (KR) | D03-H01T2B\ D03-H01T2C\ D03-N |
| 5849 | 3D printing | HEALTH FOOD USED FOR ENHANCING BONE IMMUNITY, COMPRISES COLOSTRUM, COLOSTRUM BASIC PROTEIN, CALCIUM, VITAMIN D AND AUXILIARY MATERIAL | CN104939098A | LESLIE TERRENCE C (US) | SONETICS ULTRASOUND INC (US)\ LEMMERHIRT DAVID F (US)\ RICH COLLIN A (US) | A08-M01\ A08-S01\ A08-S02\ A08-S03\ A11-A03\ A12-W07D\ A12-W07F2\ G02-A04A |
| 5850 | 3D printing | ENVIRONMENTALLY-FRIENDLY WATER-BASED PLASTIC INK COMPRISES WATER-BASED INK BINDER, DISPERSING AGENT, DEFOAMING AGENT, PIGMENT POWDER, SURFACTANT, PH STABILIZER, ADHESION PROMOTER AND SOLVENT | CN104845439A | LI CUNDE (CN) | IBM (US) | A03-A03\ A10-E05B\ A12-B01A\ L01-H09A\ L01-H09C\ L01-H09D\ M13-C01\ M13-H02 |
| 5851 | 3D printing | HIGH TEMPERATURE RESISTANCE, HIGH BONDING STRENGTH AND LOW INFRARED EMISSIVITY COMPOSITE COATING COMPRISES MULTI-LAYER OVERLAPPED STRUCTURE INCLUDING NICKEL CHROMIUM ALUMINUM YTTRIUM METAL BONDING LAYER | CN104818482A | LI HAITING US\ HUANG HE US\ LIU XUANJIE US | MICRON TECHNOLOGY INC (US) | L02-A02\ L02-A03\ L02-A04\ L02-G01A\ L02-G01B1\ L02-G01D\ L02-G01E\ L02-G04\ L02-G10B\ L02-G12E1\ X25-A08 |
| 5852 | 3D printing | MANUFACTURE OF JADE PRODUCT E.G. CORUNDUM FOR E.G. JADE BRACELETS, INVOLVES MIXING POWDER MATERIAL FORMED OF MATRIX POWDER MATERIAL, PHASE SINTERED POWDER MATERIAL, COLORANT POWDER AND CRYSTAL GROWTH ADDITIVE, WITH BINDER, AND PRINTING | CN104802278A | LI JUNJUN (US)\ GAUTHIER JR ROBERT J (US)\ CLARK JR WILLIAM F (US) | HUATIAN MICRO ELECTRONICS CO L (CN) | F03-D |
| 5853 | 3D printing | FLEXIBLE CEMENTITIOUS COMPOSITE FOR CONSTRUCTION, HAS LIQUID BARRIER LAYER THAT IS PROVIDED ON SPECIFIC FACE OF NON-WOVEN LAYER SUCH THAT LIQUID BARRIER LAYER COMPRISES COEFFICIENT OF WATER PERMEABILITY LESS THAN SPECIFIC VALUE | WO2015187826A2 | LI JUNJUN (US)\ MISHRA RAHUL (US)\ GAUTHIER JR ROBERT J (US)\ CAMPI JR JOHN B (US) | SEMICONDUCTOR MFG INT SHANGHAI (CN) | V08-A02 |
| 5854 | 3D printing | CERAMIC POWDER SINTERING LASER FORMING MACHINE, HAS LIFTING DRIVE MECHANISM AND CYLINDER SCREW THREAD CONNECTION WITH EACH OTHER, AND LASER SINTERING DEVICE AND CYLINDER SCREW THREAD CONNECTION WITH EACH OTHER | CN204772942U | LI TIESHENG (US) | IBM (US)\ GAUTHIER JR ROBERT J (US)\ LI JUNJUN (US)\ CLARK JR WILLIAM F (US) | A09-D03\ A11-B01\ A11-B16\ A12-D05\ S06-G10\ S06-K02\ S06-K03\ S06-K07A\ S06-K99C\ T01-J07B\ T01-J12C\ T01-S03\ X25-A08\ X25-T04D |
| 5855 | 3D printing | THREE-DIMENSIONAL (3D) SHAPING APPARATUS E.G. 3D PRINTER FOR MODELING AND MANUFACTURING THREE-DIMENSIONAL OBJECT HAS CONTROL UNIT THAT CONTROLS DISCHARGE TIMING OF BINDER LIQUID BASED ON POSITION ON PLANE OF POWDER MATERIAL | JP2015217533A | LI XIAOCHAO US\ LIN YU US\ LIN CHUN US\ GUO DONGHUI US | IBM (US)\ LI JUNJUN (US)\ MISHRA RAHUL (US)\ CAMPI JOHN B (US)\ GAUTHIER ROBERT J (US) | A02-C\ A05-D02E1\ A08-A01B\ A08-A03\ A08-D\ A08-D01\ A08-E02\ A08-E04\ A08-F01\ A08-M\ A08-R06A\ A11-B05\ A11-C02B\ A11-C02C\ A12-R01A\ L02-D05\ L02-D07B\ L02-D14F\ L02-D14M |
| 5856 | 3D printing | CHIP FOR RESIN-BASED REINFORCED NATURAL STONE I.E. GRANITE, FOR INTERIOR BUILDING MATERIALS FOR BEAUTIFUL SURFACE PATTERNS WITH STEREOSCOPIC TEXTURE, HAS RESIN-BASED REINFORCED NATURAL STONE CHIP ELEMENT WHOSE SURFACE IS COATED WITH PIGMENT | US2015344377A1 | LIANG BO US | MONOLITHIC POWER SYSTEMS INC (US)\ LI TIESHENG (US) | F03-F16\ F03-F17\ F03-F31\ F03-F32 |
| 5857 | 3D printing | ENVIRONMENTALLY-FRIENDLY ROTARY SCREEN PRINTING PAINT COMPRISES PLANT PIGMENT, NATURAL THICKENER, WATER-SOLUBLE BINDER, WETTING AGENT, PEARL POWDER, NANO-TITANIA AND WATER | CN105040475A | LIANG CHA CHER US\ CHAN LAP (US) | UNIV XIAMEN (CN) | A11-B16\ S06-G\ S06-K01A |
| 5858 | 3D printing | THREE-DIMENSIONAL OBJECT MODELING APPARATUS USED FOR E.G. MOLTEN MATERIAL DEPOSITION METHOD, HAS FORMATION PORTION THAT IS FORMED IN MAIN PORTION, SO THAT DISCHARGE VOLUME OF INK PER UNIT VOLUME IS REDUCED | WO2015178443A1 | LIN CHIN-HSIANG (TW)\ LIN HSIEN-HSIN (TW)\ PAI YI-FANG (TW)\ YANG CHI-MING (TW)\ YEH MING-HSI (TW)\ CHANG CHIEN YING-HSUEH (TW) | ADVANCED SEMICONDUCTOR MFG CORP LTD US | A12-E14\ A12-W16\ T04-F02A2\ T04-K01C\ T04-K03B\ U12-A02A3\ U12-A02A4A\ U12-A02A8\ X15-A01A\ X15-A02A\ X15-A02D1 |
| 5859 | 3D printing | CONDUCTIVE COMPOSITION USED TO PRODUCE METAL FINE PATTERN FOR E.G. CRYSTALLINE SOLAR CELL ELECTRODE, THIN-FILM SOLAR CELL ELECTRODE AND DYE-SENSITIZED SOLAR CELL ELECTRODE, COMPRISES COPPER POWDER, METAL PRECURSOR, BINDER RESIN AND SOLVENT | WO2015178696A1 | LIN HE (CN) | CHARTERED SEMICONDUCTOR MFG (US)\ UNIV SINGAPORE (US) | A11-B16\ S06-G\ S06-K02C\ S06-K07 |
| 5860 | 3D printing | METHOD FOR PERFORMING THREE-DIMENSIONAL MODELING, INVOLVES FORMING LEVEL DIFFERENCE OF UNEVEN SHAPE IN STACKING SURFACE OF MODELING MATERIAL LAYER BASED ON MODELING DATA OF PREDETERMINED LAYER | JP2015212042A | LIN JIANFA US | TAIWAN SEMICONDUCTOR MFG (TW)\ LIN HSIEN-HSIN (TW)\ PAI YI-FANG (TW)\ YANG CHI-MING (TW)\ LIN CHIN-HSIANG (TW)\ YEH MING-HSI (TW)\ CHANG CHIEN YING-HSUEH (TW) | A05-G\ A05-G02\ A05-G03\ A05-G04\ A12-E11\ G02-A02C\ G02-A02D\ G02-A02H\ G02-A04A\ G03-B02D1\ L03-G02\ L03-G05\ U11-A07 |
| 5861 | 3D printing | ACTIVE ENERGY RAY-CURABLE RESIN COMPOSITION FOR E.G. COATING AGENT, COMPRISES SPECIFIED AMOUNT OF URETHANE (METH)ACRYLATE-TYPE COMPOUNDS DIFFERING IN ETHYLENICALLY UNSATURATED GROUP CONCENTRATION | JP2015199921A | LIN MEI-HSUAN (TW)\ LIN CHIH-HSUN (TW)\ CHAO CHIH-KANG (TW)\ WANG LING-SUNG (TW) | LIN HE (CN) | L02-A02\ L02-A04\ L02-G01A\ L02-G01B\ L02-G01X\ L02-G11\ L02-G12E\ L02-G12X\ L02-H02B2\ L02-J02C\ L04-C25\ L04-E03A |
| 5862 | 3D printing | CERAMIC SUBSTRATE USED IN LED COMPRISES E.G. ALUMINUM TITANIUM NITRIDE, ALUMINUM NITRIDE, ALUMINA POWDER, MCM-41 MOLECULAR SIEVE, SILICON CARBIDE, GLASS SINTERING AUXILIARY AGENT, IRON NANO-PARTICLE, RARE EARTH OXIDE AND ORGANIC SOLVENT | CN104961468A | LIN WEN (US)\ PEARCE CHARLES W (US) | QUZHOU OIL HYDRAULIC INDUSTRY CO LTD US | A05-A01E3\ A05-A04\ A07-A03B\ A08-D01\ A08-M10\ A08-P07\ A08-R06A\ A08-S02\ A11-A02\ A11-A04\ A11-C01C\ A11-C02\ A12-A04A\ A12-A05C\ G03-B01\ G03-B02E2 |
| 5863 | 3D printing | THIN STONE FIBERGLASS COMPOSITE DECORATIVE PANEL COMPRISES EPOXY ADHESIVE BONDED WITH STONE SURFACE LAYER AND INTERMEDIATE FIBERGLASS CLOTH LAYER | CN104947900A | LIN WENT T (US) | TAIWAN SEMICONDUCTOR MFG (TW)\ LIN MEI-HSUAN (TW)\ LIN CHIH-HSUN (TW)\ CHAO CHIH-KANG (TW)\ WANG LING-SUNG (TW) | A02-A02\ A04-H00H\ A08-C03\ A08-E02\ A08-S02\ A10-B04\ A11-B05C\ A12-B08\ A12-W07D\ G02-A03D\ G02-A04A\ G02-A04B |
| 5864 | 3D printing | AQUEOUS ALUMINUM POWDER PIGMENT USED FOR PREPARING PAINT OR PRINTING INK, COMPRISES COVERING OF SILICON DIOXIDE AND INTERNAL LAYER OF ALUMINUM POWDER, WHERE EXTERNAL SURFACE OF CLADDING LAYER HAS CONNECTING POINT | CN104817856A | LIN WENT T (US) | AGERE SYSTEMS INC (US) | A10-E05B\ A12-W12G\ L02-B08\ L02-C08\ L02-E03 |
| 5865 | 3D printing | ALUMINUM-SILICON LIGHTWEIGHT REFRACTORIES COMPRISE RAW MATERIAL INCLUDING CERAMIC POWDER AS BASIC FILLER, SOLVENT INCLUDING WATER, THICKENER, DISPERSING AGENT AND SILICA SOL, CALCIUM ALUMINATE CEMENT AND CERAMIC FIBERS | CN104788114A | LIN ZHILANG ZHANG (CN) | LIN WEN T (US) | A11-B16\ A11-C04A |
| 5866 | 3D printing | THREE-DIMENSIONAL PRINT FORMING METHOD USING METALLIC MATERIAL OR CERAMIC MATERIAL USING METAL OR CERAMIC POWDER AND BINDER AS RAW MATERIAL FOR PRINTING TO FORM A HIGHER-STRENGTH THREE-DIMENSIONAL PRODUCT | TW201532791A | LINPEI ZHU US\ KANGMIN CHEN US | LIN WEN T (US) | L03-H03A\ M22-H03B\ M22-H03F\ U11-C09G\ X25-A08 |
| 5867 | 3D printing | MANUFACTURING APPARATUS FOR THREE-DIMENSIONAL ADDITIVE MANUFACTURING OF MATERIALS, HAS COMPUTER UNIT THAT IS COUPLED TO ULTRASHORT PULSE LASER TO ADJUST PULSE REPETITION RATE AND AVERAGE POWER OF ULTRASHORT PULSE LASER | US2015343664A1 | LI-PING HUANG US\ CHIH-HSIANG HUANG US\ KA-HING FUNG US\ CHUNG-CHENG WU US\ HAITING WANG US | SHANGHAI INST MICROSYS & INF (CN) | A12-R01A\ G05-F\ L02-A02\ L02-A03\ L02-A04\ L02-D14A |
| 5868 | 3D printing | METHOD OF MANUFACTURING THREE-DIMENSIONAL MODEL USING LAYER STRUCTURE TECHNIQUE, INVOLVES INHIBITING OR STOPPING APPLICATION OF REACTION LIQUID BY PRESSURE HEAD, AND NOT APPLYING BINDER MATERIAL | DE102014007584A1 | LIU JIAZHOU (CN)\ GUO DAWEI (CN)\ WANG YANFENG (CN) | SHANGHAI BELLING CO LTD (CN) | D08-A02\ D08-A03 |
| 5869 | 3D printing | FABRICATION OF DENTAL RESTORATION MATERIAL USED IN DENTAL APPLICATIONS E.G. VENEERS, INVOLVES PROVIDING POWDER OF DENTAL MATERIAL, SELECTIVELY DEPOSITING BINDER INTO DENTAL MATERIAL AND SINTERING THREE-DIMENSIONAL UNFINISHED MODEL | WO2015168463A1 | LIU LIN-SHIH (US)\ CHAN MARK T (US)\ XU YANZHONG (US)\ WATT JEFFREY T (US)\ RAHIM IRFAN (US) | TAIWAN SEMICONDUCTOR MFG (TW)\ HUANG LI-PING (TW)\ HUANG CHIH-HSIANG (TW)\ FUNG KA HING (TW)\ WU CHUNG-CHENG (TW)\ WANG HAITING (TW) | A12-R01A\ L02-C02\ L02-D13A |
| 5870 | 3D printing | THREE-DIMENSIONAL PRINTING CEMENT-BASED MATERIAL COMPRISES POWDERED CEMENTITIOUS MATERIALS COMPRISING CEMENT, ACTIVE MATERIAL, WATER REDUCING AGENT, EARLY STRENGTH AGENT, RETARDER, EXTENDER, AND BINDER | CN104891891A | LIU RUOPENG US\ LUAN LIN US\ SUN HAOWEN US | LIU JIAZHOU (CN)\ GUO DAWEI (CN)\ WANG YANFENG (CN)\ BEKEN CORP (CN) | A05-G01C\ A11-B16\ A12-D01\ A12-R01 |
| 5871 | 3D printing | BUILT ENVIRONMENT PRODUCT E.G. BENCHTOP, FOR USE IN E.G. METAL FOUNDRY INDUSTRY, HAS CONCRETE BASED BINDER FOR BINDING POWDERED MINERAL WASTE, WHERE PORTION OF PARTICLES OF MINERAL WASTE MEDIA IS LESS THAN SPECIFIED RANGE IN DIAMETER | WO2015176141A1 | LIU YANXIANG (US)\ YANG XIAODONG (US)\ LIU JINPING (US) | LIU LIN-SHIH (US)\ CHAN MARK T (US)\ XU YANZHONG (US)\ RAHIM IRFAN (US)\ WATT JEFFREY T (US) | A04-E08\ A04-F04B\ A05-G01E\ A12-H11\ A12-L05C1\ A12-S02\ S06-E04A\ S06-E04C\ S06-K99C |
| 5872 | 3D printing | POWDER SENDING ROLLER SPONGE I.E. POLYURETHANE SPONGE FOR LASER PRINTER, HAS PROCESSING AGENT SELECTED AS MIXTURE OF AQUEOUS BINDER AND POWDER, WHERE POWDER IS SELECTED FROM ONE OF SILICON DIOXIDE OR FLUORINE CONTAINING POLYMER | WO2015172325A1 | LIXIA ZHAO (CN)\ ZHAOGENG YUAN (CN)\ BINGKE CHEN (CN) | KUANG CHI INST ADVANCED TECH US\ SHENZHEN KUANG CHI INNOVATE TECHNOLOGY CO LTD US | A03-A04A\ A11-B05\ A12-E06A\ A12-W07D1\ L03-E01B3\ L03-E01B5B\ L03-E08B\ L03-H05\ S06-G\ S06-K03G\ X16-E01C\ X16-E01G\ X16-E01J |
| 5873 | 3D printing | INKJET-PRINTABLE FORMULATION USEFUL FOR CATHODE MATERIAL IN FLEXIBLE BATTERY COMPRISES LITHIUM-PHOSPHATE COMPOUND CONTAINING NANOPARTICLES AND A BINDER COMPRISING CARBOXYMETHYLCELLULOSE | US2015325856A1 | LIXIN WANG (CN) | GLOBALFOUNDRIES INC (KY)\ LIU YANXIANG (US)\ YANG XIAODONG (US)\ LIU JINPING (US) | A09-D03\ A11-B16\ A12-H01\ S06-E06B1\ S06-E06B2\ S06-G10\ S06-K99C\ X25-A08\ X25-B01C3C |
| 5874 | 3D printing | 3D PRINTER MOTOR EXTRUSION HEAD, HAS SHOWER NOZZLE BASE ARRANGED WITH SHOWER NOZZLE BASE COVER PLATE, COVER SHELL PROVIDED WITH WIRE MATERIAL CONVEYOR BELT, AND STEERING SWINGING ROD CONNECTED WITH LEVELLING SENSOR THROUGH CONVEYOR BELT | CN204712472U | LLOYD NEIL (GB)\ READER ALEC (GB) | HEBEI PUXING ELECTRONICS TECHNOLOGY STOCK CO LTD US | A11-B16 |
| 5875 | 3D printing | POWDER MATERIAL SUPPLY APPARATUS OF THREE-DIMENSIONAL MODELING APPARATUS, HAS SUCTION DEVICE PROVIDED WITH SUCTION NOZZLE THAT ATTRACTS EXCESSIVE POWDER MATERIAL CUT WITH RUBBING BY PLANARIZATION ELEMENT | JP2015196254A | LOH WAH KIT (US)\ DENG XIAOWEI (US) | INST OF MICROELECTRONICS CAS (CN) | A05-A01E2\ A08-D01\ A08-E01\ A08-M09A\ A08-S02\ A11-C01A\ A11-C04A\ A12-E07A\ L03-H03\ L03-H04E3\ T04-K01C\ T04-K03B\ V04-A06\ V04-M01\ V04-R02A\ V04-S02\ V04-S10\ V04-S30\ W02-G05 |
| 5876 | 3D printing | METHOD FOR MANUFACTURING COPPER PLATING THIN FILM ADDER CIRCUIT FOR USE IN E.G. RFID LABEL AND ANTENNA, INVOLVES COATING ONE-WAY ELECTRIC CONDUCTION ON COPPER PLATING LAYER, COATING ANTI-OXIDATION LAYER, AND FINISHING PRODUCT LIBRARY | CN104968157A | LOH WAH KIT (US)\ SESHADRI ANAND (US) | INNOS LTD (GB)\ LLOYD NEIL (GB)\ READER ALEC (GB) | A11-B09C\ A11-C01D\ A12-E14\ X25-B01H |
| 5877 | 3D printing | THERMOPLASTIC CARBON FIBER PREPREG PREPARATION SYSTEM HAS INFRARED HEATER MOUNTED ON ELECTRODE OUTSIDE, STEEPING DEVICE PROVIDED WITH HOPPER AND HIGH-PRESSURE NOZZLE ARRANGED BELOW HOPPER AND VACUUM SUCTION DEVICE ARRANGED BELOW NOZZLE | CN104924487A | LU HANG US | TEXAS INSTRUMENTS INC (US) | A03-A05\ A04-F01A\ A08-M03\ A08-R\ A08-R06B\ A12-W07D\ G02-A02C\ G02-A04A\ G02-A04B |
| 5878 | 3D printing | MICROCRYSTALLINE WAX PRINTING INK COMPRISES MICROCRYSTALLINE WAX EMULSION, ANTI-FRICTION ADDITIVE, BLACK CARBON, ACRYLIC RESIN SOLUTION, PHTHALOCYANINE PIGMENTS, BINDER, CELLULOSE, TALC POWDER AND CALCIUM CARBONATE | CN104817875A | LYU HONG KUN (KR)\ JEON WAN BAE (KR) | TEXAS INSTRUMENTS INC (US)\ LOH WAH KIT (US)\ SESHADRI ANAND (US) | A02-A\ A02-A01\ A04-B\ A04-B09\ A04-D04A\ A08-P\ A08-S01\ A08-S02\ A10-B04\ A10-E05B\ A12-A02\ A12-W12G\ M22-B01\ M22-B03\ M22-D\ T01-J07B\ X25-A01 |
| 5879 | 3D printing | FAST PRECISION CASTING USED FOR COMPLEX PARTS INVOLVES PREPARING RESIN MOLD AND CERAMIC SLURRY, GEL-CASTING, DRYING, ROASTING AND PRECISION CASTING | CN104741530A | MA DONGSHENG (US)\ ZHANG CHUANG (US) | SHANGHAI JINGKE SCIENT INSTR CO LTD US | A05-C03\ A06-A00E2\ A08-D01\ A08-D03\ A11-B05\ A12-E01\ A12-T03D\ L03-H03\ L03-H04E7\ V04-R02\ V04-R04A5\ V04-S02\ V04-S15\ V04-T03A\ V04-T03P\ W01-C01B3E\ W01-C01D3C\ W01-C01G8\ X24-A01A |
| 5880 | 3D printing | ELECTRICALLY AND THERMALLY CONDUCTIVE COMPOSITE MATERIAL USED E.G. FOR 4G COMMUNICATION BASE STATIONS, COMPRISES SILVER POWDER, COPPER METAL, SILICONE RESINS, PHENOL, FORMALDEHYDE, PHENOLIC RESIN, NL CURING AGENT AND METHYL IMIDAZOLE | CN104725768A | MA GORDON (US)\ CHEN QIANG (SE) | DAEGU GYEONGBUK INST SCIENCE (KR) | F05-A04 |
| 5881 | 3D printing | PAPER-MAKING DUST COLLECTING DEVICE, HAS FAN WHOSE END IS CONNECTED WITH DUST PIPE AND ANOTHER END IS CONNECTED WITH HOSE, LOWER TUBE FIXED WITH NOZZLE, AND LOCATING BLOCK MOUNTED WITH HAIR BRUSH DEVICE THROUGH REGULATING BOLT | CN204367053U | MA JIANBIN US\ KUANG ZHANGQU US\ TANG MIAN US\ CHEN JIE US\ LIU ZHIBI US | UNIV LOUISIANA STATE (US) | A11-C01C\ A12-L05\ A12-L05C1\ S06-E04A\ S06-E04C |
| 5882 | 3D printing | MAGNETIC BONDING DEVICE HAS INJECTION PLASTIC ALUMINUM IRON BORON WHOSE OUTSIDE IS COVERED WITH IRON SHEET HAVING LOWER END WHICH IS EQUIPPED WITH HANDLE | CN204694992U | MAA JER-SHEN (US)\ HSU SHENG TENG (US)\ LEE JONG-JAN (US)\ TWEET DOUGLAS J (US) | INFINEON TECHNOLOGIES AG (DE) | A11-B16\ X25-A08 |
| 5883 | 3D printing | FOLDED PORTABLE 3D PRINTER, HAS MAIN SHELL FIXED WITH X-AXIS MOVEMENT MECHANISM AND FORMED WITH CONTAINING CAVITY, AND STRAIGHT LINE SLIDING RAIL LOCATING PLATE FIXED WITH STRAIGHT LINE SLIDING RAIL THAT IS MATCHED WITH CLAMPING SLOT | CN204658973U | MAA JER-SHEN (US)\ SHIEN TEN SUU US\ LEE JONG-JAN (US)\ TWEET DOUGLAS J (US) | BEIJING SUPERPIX MICROELECTRONIC TECHNOLOGY CO LTD US | S06-C03A\ S06-K07A |
| 5884 | 3D printing | FILLING LIQUID AND COSMETIC POWDER PRINTING MACHINE, HAS COSMETIC SUPPLY GROUP PROVIDED WITH STORAGE UNIT AND TRANSPORT UNIT, WHERE STORAGE UNIT IS CONNECTED WITH NOZZLE UNIT, AND CONTROL UNIT CONTROLLING OPERATION OF TRANSPORT UNIT | CN204702494U | MACK MICHAEL (US)\ PETERSON MARK (US)\ GLOECKNER STEFFEN (US)\ NARASIMHA ADITHYARAM (US)\ KOUMANS ROGER (US)\ DE DOBBELAERE PETER (US) | SHARP LAB OF AMERICA INC (US) | A09-D01\ A11-B16\ S06-G03\ S06-K07A\ S06-K99C\ T01-J08A |
| 5885 | 3D printing | THREE-DIMENSIONAL LAMINATION-MODELING APPARATUS E.G. INKJET PRINTER HAS PRINT HEAD THAT IS CONTROLLED SO THAT MOLDED ARTICLE IS FORMED IN VICINITY OF STAND-BY POSITION OF PRINT HEAD | JP2015193184A | MAEDA AKIYUKI US | SHARP LAB OF AMERICA INC (US) | A09-D02\ A11-B07\ A11-B16\ S06-E06B1\ S06-E06B2\ S06-G10\ S06-K03F\ S06-K07A\ S06-K99C\ X25-A08\ X25-B01C3C |
| 5886 | 3D printing | 3D PRINTER MOTOR EXTRUSION HEAD, HAS CONTROL CABLE CONNECTED WITH SHOWER NOZZLE CONTROLLER, AND STEERING ENGINE FIXED WITH FIXING FRAME AND STEERING SWINGING ROD THAT IS CONNECTED WITH LEVELING SENSOR | CN104890244A | MANTL SIEGFRIED (DE)\ LENSSEN DANIEL (DE)\ BAY HELGE (DE) | MACK MICHAEL (US)\ PETERSON MARK (US)\ GLOECKNER STEFFEN (US)\ NARASIMHA ADITHYARAM (US)\ KOUMANS ROGER (US)\ DE DOBBELAERE PETER (US)\ LUXTERA INC (US) | L03-A01A3\ L03-H02\ L03-H04E4\ M22-H02\ A12-E01\ A12-E07A\ V04-R01A4\ V04-R02B\ V04-R02G |
| 5887 | 3D printing | ELECTRICALLY CONDUCTIVE PASTE FOR FORMATION OF ELECTRICALLY CONDUCTIVE CIRCUIT OF PRINTED WIRING BOARD COMPRISES ELECTROCONDUCTIVE METAL POWDER, ELECTROCONDUCTIVE ORGANIC COMPOUND, AND BINDER | JP2015187946A | MANZHA N M (RU) | SHARP KK (JP) | L03-G05\ L03-H03A\ L03-J02\ M22-G03K\ M26-A\ M26-C01\ W01-C01D3C\ W01-C01G6C\ W01-C01G8\ W03-A09A1\ W03-A12A\ W03-A18C1 |
| 5888 | 3D printing | THREE-DIMENSIONAL METAL GLASS COMPONENT FOR E.G. CELL PHONE, HAS METALLIC GLASS SHELL FOR SURROUNDING ALLOY CORE, WHERE ALLOY CORE PROVIDES COMPRESSIVE FORCE ON METALLIC GLASS SHELL AT INTERFACE BETWEEN ALLOY CORE AND METALLIC GLASS SHELL | US2015314566A1 | MANZHA NIKOLAJ MIKHAJLOVICH (RU)\ MANZHA LJUBOV PAVLOVNA (RU)\ SHURCHKOV IGOR OLEGOVICH (RU)\ SULIMIN ALEKSANDR DMITRIEVICH (RU)\ JACHMENEV VLADIMIR VASIL EVICH (RU)\ KOVALENKO GALINA PETROVNA (RU) | FORSCHUNGSZENTRUM JUELICH GMBH (DE)\ MANTL SIEGFRIED (DE)\ LENSSEN DANIEL (DE)\ BAY HELGE (DE) | X25-A09\ X25-F01\ X25-P02 |
| 5889 | 3D printing | SEPARATION APPARATUS USED IN FOODSTUFFS, HAS FIRST CONVEYING UNIT AND SECOND CONVEYING UNIT FOR CONVEYING CONVEYANCE MEDIUM AND POWDER | JP2015193425A | MARSH JOHN HAIG (GB)\ MCDOUGALL STEWART DUNCAN (GB)\ HAMILTON CRAIG JAMES (GB)\ KOWALSKI OLEK PETER (GB) | Empty Field US | A12-L05\ A12-L05C2\ G06-C04\ G06-G05A\ S06-E04A1\ S06-E04B\ S06-E04C\ S06-E04D\ S06-K99B |
| 5890 | 3D printing | MAGENTA TONER FOR ELECTROSTATIC IMAGE DEVELOPMENT USED IN IMAGE FORMING APPARATUS E.G. ELECTROPHOTOGRAPHIC COPYING MACHINE, HAS PIGMENT DISPERSION LIQUID THAT IS FORMED WITH A PH VALUE OF PREDETERMINED VALUE | JP2015194744A | MARTY MICHEL (FR)\ AVENIER GREGORY (FR) | Empty Field US | A08-S02\ A12-W12F\ M22-H\ M22-H02 |
| 5891 | 3D printing | MANUFACTURE OF THREE-DIMENSIONAL METAL PARTS INVOLVES MIXING METAL POWDER AND BINDER, ADDING ORGANIC SOLVENT TO OBTAIN SLURRY, SPRAYING SLURRY ONTO SUBSTRATE, HEATING AND MELTING SLURRY TO FORM MATERIAL SLURRY LAYERS ON SUBSTRATE | CN104785786A | MARUYAMA HIROYUKI (JP)\ NAGOYA TAKATOSHI (JP)\ TAKAMIZAWA SHOICHI (JP)\ SAYAMA RYUJI (JP) | UNIV GLASGOW (GB)\ MARSH JOHN HAIG (GB)\ MCDOUGALL STEWART DUNCAN (GB)\ HAMILTON CRAIG JAMES (GB)\ KOWALSKI OLEK PETER (GB) | A11-B16\ A11-C04A |
| 5892 | 3D printing | MULTI-MATERIAL THREE DIMENSIONAL PRINTER, HAS POWDER REMOVER MOVABLE OVER PORTIONS OF BUILD PLATE TO REMOVE UNBOUND FIRST POWDER IN FIRST POWDER LAYER AND POWDER DISPENSER MOVABLE OVER PORTIONS OF BUILD PLATE TO PLACE SECOND POWDER LAYER | US2015314530A1 | MARYA J-S (JP)\ TWITT D J (JP)\ ANAU Y (JP) | ST MICROELECTRONICS SA (FR) | S06-G\ S06-K03\ S06-K06\ S06-K99C |
| 5893 | 3D printing | SMART PRINTER HAS DRUM WHICH COMPRISES HOLLOW HOUSING PROVIDED WITH POWDER FILLING DEVICE THAT COMPRISES STORAGE BOX THAT IS MUTUALLY CONNECTED WITH TUBE THAT IS FORMED WITH NOZZLE WHOSE OUTSIDE IS FIXED WITH HEATING COVER | CN204687555U | MASUI KENJI US | SHINETSU HANDOTAI KK (JP)\ NAGOYA TAKATOSHI (JP)\ TAKAMIZAWA SHOICHI (JP)\ SAYAMA RYUJI (JP)\ MARUYAMA HIROYUKI (JP) | A08-S02\ A12-L05C2\ G06-G05A\ G06-G08E\ S06-E04A1\ S06-E04D\ S06-K03\ S06-K06C |
| 5894 | 3D printing | MANUFACTURING METHOD OF TONER USED FOR COPIER, INVOLVES MIXING THE INORGANIC FINE PARTICLE AND HYDROPHOBIC DISPERSION MEDIUM SO AS TO SATISFY PREDETERMINED EXPRESSIONS WITH RESPECT TO RESIN SOLUTION, AND FORMING A DROPLET IS FORMED | JP2015191200A | MATSUOKA YUTAKA US\ OKUBO YUKIO US\ AMANO YOSHIAKI US\ TAKAGI AKIO US | SHARP LAB OF AMERICA INC (US) | A12-W11\ G01-B03\ L03-H04E\ L03-J01\ L03-J02\ L04-F05\ M22-H01\ M22-H02\ M22-H03B\ V04-X01B\ X25-H |
| 5895 | 3D printing | MANUFACTURE OF METAL POWDER E.G. COPPER POWDER USED IN SINTERING PASTE, INVOLVES PRECIPITATING METAL POWDER BY CONTACTING BASIC METAL SALT SOLUTION WITH REDUCING AGENT AND RECOVERING PRECIPITATED METAL POWDER FROM SOLVENT | WO2015162405A1 | MATSUYAMA HIROSHI US\ SUGAWARA AKI US\ KURAGANO TAKAAKI US | SHARP KK (JP) | A11-B16\ A11-C04B2\ A11-C04D\ A12-E01\ A12-L02B2\ L03-H04E5\ L04-A01A\ V04-R01A1\ V04-R01A4\ V04-R02B |
| 5896 | 3D printing | METHOD FOR FORMING WORKPIECE OF E.G. ELECTRONIC DEVICE, INVOLVES REMOVING LAYER OF MATERIAL ON TOP OF GRAPHENE SURFACE IN PREDETERMINED PATTERN, AND RELEASING GRAPHENE FROM CATALYTIC FILM SUCH THAT GRAPHENE IS IN FORM OF PATTERNED LAYER | WO2015164552A1 | MCKEE RODNEY A (US)\ WALKER FREDERICK J (US) | ANRITSU CORP US | A10-E01\ A11-A03\ A11-B05\ A12-W07D1\ G02-A04A\ G02-A04B\ G05-F03\ L01-G04F2\ L01-G04G1\ L01-G04G2\ L01-G09\ S06-G04\ S06-K01A |
| 5897 | 3D printing | COMPOSITION USED FOR DIGITALLY PRINTING ON CERAMIC ARTICLE OR GLASS ARTICLE SUBSTRATE AND AS INK JET INK, COMPRISES PARTICULATE SOLID, A NON-POLAR MEDIUM AND A DISPERSING AGENT COMPRISING SUBSTITUTED HYDROCARBYL COMPOUNDS | WO2015157071A1 | MCMONAGLE JASON (GB)\ ARNOLD RONALD (GB) | MITSUMI ELECTRIC CO (JP) | A11-B01\ A11-B16 |
| 5898 | 3D printing | THREE-DIMENSIONAL MOLDED ARTICLE, HAS THREE-DIMENSIONAL OBJECT PROVIDED WITH OUTER-LAYER IN WHICH BINDER IS COATED, AND OUTER-LAYER PROVIDED WITH POWDER MATERIAL IN WHICH BINDER IS NOT COATED | JP2015189035A | MEIKEI IEONG (US)\ VICTOR CHAN (US)\ GUARINI KATHRYN W (US) | MARTIN MARIETTA ENERGY SYSTEMS (US) | A09-D03\ A11-A\ A11-B01\ A11-B03\ A11-B16\ A12-E07A\ A12-P06B\ A12-W07D\ L03-H04E\ L03-J02\ V04-S15\ X25-A08 |
| 5899 | 3D printing | MULTI-TYPE MATERIAL POWDER THREE DIMENSIONAL PRINTER HAS SHOWER NOZZLE THAT COMPRISES MOVABLE BRACKET AND INK BOXES, AND SHOWER NOZZLE SUPPORT INSTALLED WITH DRIVE PLATE, AND MAIN CONTROL CIRCUIT BOARD FOR CONTROLLING PRECISE SPRINKLE | CN204658980U | MEIXNER LEONHARD (DE)\ FRIEDBERGER ALOIS DR RER NAT (DE)\ MARTIN ULRICH (DE) | FILTRONIC COMPOUND SEMICONDUCTORS LTD (GB)\ RFMD (GB) | A09-D03\ A11-B01\ A11-B03\ A11-B16\ X25-A08 |
| 5900 | 3D printing | THREE-DIMENSIONAL MOLD FORMING DEVICE, HAS POWDER DISPERSING DEVICE CONNECTED WITH POWDER CHAMBER, POWDER TRANSPORTING DEVICE CONNECTED WITH MOLDING POWDER PUSHING SCRAPE DEVICE, AND MOLDING POWDER LAYER FORMED WITH CAVITY | CN204640818U | MELLIN JONI (FI) | IBM (US) | S06-E04D\ S06-G04\ S06-G06\ S06-K06 |
| 5901 | 3D printing | UV-PRINTER, HAS INK BOX CONNECTED WITH CONTAINING CAVITY THAT IS PROVIDED WITH STORING INK, AND PUMP BODY PROVIDED WITH TRANSFUSION PIPE THAT IS CONNECTED WITH INFUSION CHAMBER | CN204605185U | MENUT OLIVIER (FR) | FRAUNHOFER GES FORSCHUNG (DE)\ EADS DEUTSCHLAND GMBH (DE) | L02-A02\ L02-A03\ L02-A08 |
| 5902 | 3D printing | THREE-DIMENSIONAL MICRO-JET PRINTING GRAPHITE POWDER MOLDING DEVICE HAS MOVEMENT PLATFORM SET WITH RACK, AND MOVEMENT MECHANISM CONTROL UNIT SET IN BOTTOM PORTION OF FRAME, AND AIR SUPPLY UNIT ARRANGED ALONG PERIPHERY OF FRAME | CN204607867U | MIKAWA TAKASHI (JP)\ YOSHIMURA TETSUZO (JP)\ IBARAGI OSAMU (JP) | MICRO ANALOG SYST OY (FI) | A11-C05A\ A12-S05G\ F02-C02\ F03-C01\ F03-C03\ F05-A07\ S06-G\ S06-K99C |
| 5903 | 3D printing | APPARATUS FOR MANUFACTURING SHEET FOR WASTE-INK ABSORBER OF INKJET PRINTER, COMPRISES MIXING UNIT WHICH MIXES FIBERS AND POWDER CONTAINING RESIN AND FLAME RETARDANT, AND BINDING UNIT WHICH BINDS FIBERS AND POWDER | JP2015182412A | MIKAWA TAKASHI (JP)\ YOSHIMURA TETSUZO (JP)\ IBARAGI OSAMU (JP) | ST MICROELECTRONICS SA (FR)\ MENUT OLIVIER (FR) | A05-A01E2\ A10-E05B\ A12-E07C\ L03-G10A\ L03-G10M\ L04-C13B\ L04-C16\ L04-C17\ L04-C21\ L04-E03\ L04-E05D\ M22-H02\ M22-H03B\ M22-H03F\ U11-E02A2\ U11-E02A3\ U12-A01A1E\ U12-A02A1\ U12-B03F1B |
| 5904 | 3D printing | SINTERING POWDER FOR E.G. SINTERED JOINT USED FOR LED, MICROELECTROMECHANICAL SYSTEM, ORGANIC LED, OR PHOTOVOLTAIC CELL COMPRISES METAL PARTICLES HAVING AN AVERAGE LONGEST DIMENSION WITHIN A GIVEN RANGE | WO2015155542A1 | MIN LI US | FUJITSU LTD (JP)\ NTT ADVANCED TECH KK US | L03-G02\ M13-H01\ M13-L\ M22-H03B\ T01-J08A\ V05-F04\ V05-F05A3\ V05-F05A7A\ V05-F05E5\ V05-F08D1\ V07-F01A1\ V07-K01A\ V08-A03A\ X25-A08\ X25-A09 |
| 5905 | 3D printing | LASER CLADDING DEPOSITION- ELECTRON BEAM ADDITIVE MANUFACTURING INTEGRATED PRINT DEVICE COMPRISES BOTTOM BEAM, MOBILE CROSS BEAM, WORKBENCH, MULTI-SHAFT ROBOT, LASER SHOWER NOZZLE, LASER, OPTICAL FIBER, FEEDER, POWER SUPPLY, AND CONTROLLER | CN204570038U | MINGXIAN LI US\ JIAOLIN HUANG US | FUJITSU LTD (JP)\ NTT ADVANCED TECH KK US | V02-G01B\ V02-G02A3\ V02-H\ X12-C01B\ X12-C01D\ X12-C03 |
| 5906 | 3D printing | METHOD OF MANUFACTURING ELECTRIC CURRENT MUTUAL INDUCTOR, INVOLVES ASSEMBLING AND CASTING CURRENT MUTUAL INDUCTOR ACCORDING TO CASTING PROCESS FOR MUTUAL INDUCTOR COIL AND OTHER ACCESSORIES | CN104835640A | MINGXIN ZHOU (CN) | SEMICONDUCTOR MFG INT SHANGHAI (CN) | A11-B02B\ F01-D06A\ F02-C01\ F03-D |
| 5907 | 3D printing | GASKET MATERIAL FOR HOME TEXTILE PRODUCT COMPRISES LAMINATED UPPER SPUNBONDED NONWOVEN FABRIC, MIDDLE BAMBOO FIBER MESH LAYER AND LOWER SPUNBONDED NONWOVEN FABRIC FORMING THREE-DIMENSIONAL STRUCTURE FILLED WITH NEGATIVE OXYGEN IONS POWDER | CN204547244U | MIRABEDINI MOHAMMAD R (US)\ SUVKHANOV AGAJAN (US) | HUAYING OPTOELECTRONICS CO LTD US | A05-E01D\ A11-C01C\ A12-H02\ A12-L05\ A12-L05C1\ S06-E06 |
| 5908 | 3D printing | METAL BASE FIXING FILM, HAS COVER FIXED ON METAL BASE LAYER, AND SURFACE GLUE LAYER CONNECTED WITH SPECIAL FLUORINE POLYESTER FILM LAYER, WHERE METAL BASE LAYER IS FORMED WITH STAINLESS STEEL PIPE | CN204613596U | MIRABEDINI MOHAMMAD R (US)\ SUVKHANOV AGAJAN (US) | TIANSHUI HUATIAN MICROELECTRON (CN) | A11-B14\ A12-E01\ A12-E02\ X12-D01F1\ X12-D07\ X25-A08 |
| 5909 | 3D printing | METHOD FOR MANUFACTURING SILVER METHOXY ELECTRIC CONTACT OF INTEGRATED MODULE, INVOLVES PROVIDING INTEGRATED MODULE WITH THREE-DIMENSIONAL MODEL, AND OBTAINING 3D PRINT OF INTEGRATED MODULE BY PERFORMING SELECTIVE LASER SINTERING PROCESS | CN104867620A | MITA KEIJI (JP) | LSI LOGIC CORP (US) | A09-D03\ A11-C01D\ A12-A05\ X25-A08 |
| 5910 | 3D printing | POWDER BED THREE DIMENSION PRINTER HAS ADHESIVE NOZZLE THAT IS SET WITH FLAT BELT GROUP, AND CONTROL UNIT THAT DRIVES ALONG FLAT PLANE ORTHOGONAL TO X, Y-AXIS MOVEMENT, FOR POWDER CYLINDER THAT IS ARRANGED ABOVE FORMING CYLINDER | CN204566707U | MITA KEIJI (JP) | LSI CORP (US) | A12-L05C2\ G02-A03\ G02-A07A\ G06-G05A\ S06-E04A1\ S06-K01A\ S06-K99C |
| 5911 | 3D printing | COLORING POWDER USED FOR E.G. POWDER COATING MATERIAL, COMPRISES BINDER RESIN AND COLORED RESIN PARTICLES CONTAINING COLORING AGENT, AND HAS PRESET CUTTING STRESS DIFFERENCE BETWEEN SHEAR STRESS MEASURED UNDER SPECIFIED CONDITIONS | JP2015183092A | MIYAGI HIROSHI (JP) | SANYO ELECTRIC CO (JP)\ SANYO SEMICONDUCTOR CO LTD (JP)\ SANYO SEMICONDUCTOR MFG CO LTD (JP) | A11-B09A2\ A11-C04B2\ A12-E08\ L03-B02B4\ V02-E01 |
| 5912 | 3D printing | FLEXIBLE MAGNETIC ATTRACTION SHEET HAS MAGNETIC LAYER WHICH IS PROVIDED ON BOTH SURFACES OF CONTACT BONDING LAYER SUCH THAT THE YOUNG'S MODULUS AND GURLEY RIGIDITY OF THE SHEET ARE SET TO SPECIFIC VALUES | WO2015151930A1 | MONIER MAHER (US)\ PIERRE BORDES JEAN (US)\ CHRISTOPHER LAMB (US)\ PATEL SANJAY J (US) | SANYO ELECTRIC CO (JP)\ SANYO HANDOTAI SEIZO KK US\ SANYO SEMICONDUCTOR CO LTD (JP) | J01-G06 |
| 5913 | 3D printing | GLUE PRINT ENGINE POWDER REMOVING MACHINE, HAS WIND CHANNEL WHOSE LOWER PART IS FIXED WITH METAL NET, AND SPRAY WATER POOL FORMED WITH WATER SHOWER NOZZLE AND FIXED WITH OVERFLOW PIPE THAT IS CONNECTED WITH SEDIMENT POOL | CN204563840U | MURATA TOMOHIRO US | TOYOTA JIDOSHOKKI KK (JP)\ NIIGATA SEIMITSU CO LTD (JP)\ MIYAGI HIROSHI (JP) | A04-D05A\ A05-E01D2\ A08-S02\ A11-A03\ A11-A04\ A12-E01\ L03-A01A3\ M22-H01\ U11-C01J8\ X12-D01F2\ X12-D02A1 |
| 5914 | 3D printing | PREPARING A PASTE COMPOSITION, USEFUL FOR FLEXIBLE SUBSTRATE HAVING CONDUCTIVE FILM BY PREPARING A PLATE SHAPED SILVER POWDER BY REDUCTION OF SILVER IN SILVER COMPOUND SOLUTION, AND MIXING E.G. FIRST PLATE-SHAPED SILVER POWDER WITH SOLVENT | KR2015108490A | NADD BRUNO C (FR) | NVIDIA CORP (US) | T01-J08A |
| 5915 | 3D printing | COAL MINE GAS MULTI-LAYER PROTECTING COMPREHENSIVE CONTROL DEVICE, HAS MICROPROCESSOR CONNECTED WITH AUTOMATIC WATER ANTI-EXPLOSION DOOR, AND MICROPROCESSOR CONNECTED WITH AUTOMATIC SPRINKLE POWDER EXPLOSION SUPPRESSION DEVICE | CN204552806U | NAKAJIMA KENSUKE US\ SUEMATSU KENJI US\ TSUTSUMI TSUNEJI US\ NISHINO TAMOTSU US\ YOSHIDA YUKIHISA US | PANASONIC CORP (JP) | M22-H\ X25-A08 |
| 5916 | 3D printing | LASER-BASED MELT PRINTING NOZZLE DEVICE COMPRISES PRINTING NOZZLE POLYHEDRAL STRUCTURE, OUTER PRINTING NOZZLE, INFRARED FIBER LASER, AND CONNECTED TO INNER PRINTING NOZZLE | CN204504221U | NAKAMURA HIROYUKI US | INTERNAT RECITIFIER CORP (US) | A05-G01E1\ A08-M10\ A12-R07\ F05-A06B\ G02-A05F |
| 5917 | 3D printing | WALLPAPER IS OBTAINED BY FORMING ADHESIVE LAYER, FORMING WOOD POWDER LAYER, MIXING E.G. NEUTRALIZING AGENT, FORMING NEUTRALIZING AGENT-ADSORBED TOP COAT LAYER, FORMING COLORED LAYER AND FORMING PRINTED LAYER IN ORDER ON SUPPORT | KR1548878B1 | NARIZUKA SHIGEYA US\ MARUYAMA TAKAHIRO US\ MORIWAKE TATSUYA US | MITSUBISHI ELECTRIC CORP (JP) | A09-D03\ A11-B01\ A11-B03\ A11-B16\ X25-A08 |
| 5918 | 3D printing | THREE-DIMENSIONAL MODEL FORMING DEVICE, HAS POWDER DISPERSING DEVICE PROVIDED WITH ENERGY PROJECTION DEVICE AND SCRAPE, LIFTING DEVICE CONNECTED WITH MOLDING POWDER CHAMBER, AND LIQUID INJECTION DEVICE CONNECTED WITH LIQUID SHOWER NOZZLE | CN104859152A | NAWATE MASAKATSU (JP)\ IMAHASHI MANABU (JP) | CANON KK (JP) |  |
| 5919 | 3D printing | PRESSURIZING DEVICE BUILDING COMPONENT 3D PRINT SHOWER NOZZLE, HAS RECEIVING TUBE PROVIDED WITH DRY POWDER ENTERING OPENING PART, STIRRING DEVICE FIXED MOTOR, AND FIBER CONNECTOR PROVIDED WITH PRESSURIZING DEVICE THAT IS FIXED TO FIBER RING | CN204566322U | NEIL Y KIM (US)\ PIETER VORENKAMP (US) | SUMITOMO ELECTRIC INDUSTRIES (JP)\ NARIZUKA SHIGEYA US\ MARUYAMA TAKAHIRO US | S06-E08\ S06-K01A\ S06-K07C1\ S06-K07C6 |
| 5920 | 3D printing | TEMPERATURE FLUORESCENT POWDER COLOR PRINT DEVICE, HAS FLUORESCENT POWDER INK BOX PROVIDED WITH COOL WHITE FLUORESCENT POWDER BOX AND WARM WHITE FLUORESCENT POWDER BOX, AND CIE CHROMATICITY CONTROLLER FOR CONTROLLING PRINT OPERATION | CN204566935U | NOGUCHI TAKASHI (KR) | NAWATE MASAKATSU (JP)\ IMAHASHI MANABU (JP) | A11-B05\ A11-B16\ A11-C02B\ A11-C02C\ S06-G10\ S06-K99C |
| 5921 | 3D printing | MODELING UNIT OF THREE-DIMENSIONAL LAMINATION-MODELING APPARATUS USED IN INK JET PRINTER HAS MOVING MECHANISM WHICH RECIPROCATES RE-COATER UNIT AND LINE HEAD UNIT INTEGRALLY WITH RESPECT TO POWDER LAYER | JP2015178247A | NOVOSIADLYI STEPAN PETROVYCH (UA)\ MELNYK LIUBOMYR VASYLIOVYCH (UA)\ VARVARUK VASYL MYKOLAIOVYCH (UA)\ KINDRAT TARAS PETROVYCH (UA) | BROADCOM CORPORATIO (US) | J01-G03\ J01-K04 |
| 5922 | 3D printing | PRINTING INK PARTICLE SCREENING DEVICE, HAS SIEVE SCREEN FORMED WITH POWDER CAVITY, AND CAVITY BLOCK DISCHARGE PORT FIXED ON UPPER PART OF VIBRATING SCREEN, WHERE OUTER SIDE OF DISCHARGING PORT IS INSTALLED WITH SEALING COVER | CN204544705U | OH MYOUNG-HWAN (KR)\ KO YOUNG-GUN (KR) | UNIV RYUKYUS US |  |
| 5923 | 3D printing | HIGH PRECISION STAMPING DEVICE, HAS CONTROL DEVICE PROVIDED ON FIXED FOUNDATION BED THAT IS CONNECTED WITH PHOTOELECTRIC DETECTING MACHINE, AND STAMPING ALUMINUM FOIL PROVIDED WITH BUCKLE THAT IS CONNECTED WITH PRESSING SHELL BODY | CN204547297U | OKAMURA SHINICHI US | VASYL STEFANYK PRYKARPATTIA NAT UNIVERSITY (UA) | A09-D03\ A11-B03\ A11-B16\ A12-L05\ T01-F06\ T01-F07\ T01-S03\ X25-A08 |
| 5924 | 3D printing | APPARATUS E.G. POWDER BASED THREE-DIMENSIONAL PRINTING APPARATUS, FOR GENERATING THREE-DIMENSIONAL OBJECT, HAS CONTROLLER FOR CAUSING DISTRIBUTOR TO DISTRIBUTE ADDITIONAL AMOUNT OF POWDER IN SUBSEQUENT LAYER BASED ON LAYER VOLUME CHANGE | WO2015152875A1 | OKASHIN YAMATO US | SAMSUNG ELECTRONICS CO LTD (US) | G06-G08\ S06-E04A\ S06-K99C |
| 5925 | 3D printing | METHOD FOR OBTAINING TONER USED IN PRINTER, INVOLVES MIXING DYE, MAGNETIC POWDER AND BINDERS AND PROCESSING UNIFORM MIXTURE BY SHOCK WAVES GENERATED BY SUPERSONIC WITH HOT AIR | RU2013133352A | OKAWA SHIGEAKI (JP)\ OKODA TOSHIYUKI (JP) | KONICA MINOLTA MED & GRAPHIC US | A11-A\ A11-A02C\ A11-C03A\ A12-D04\ A12-H07\ X25-A08\ X27-F01 |
| 5926 | 3D printing | ANTI-ULTRA-CRITICAL THREE-DIMENSION BASED CARBON DIOXIDE GAS SOLVENT PRINTING DEVICE, HAS INJECTION UNIT PROVIDED WITH AIR PUMP, PNEUMATIC NEEDLE VALVE, COOLING FAN AND HEATER, AND LIQUID BOOSTER PUMP FIXED WITH REFRIGERATOR | CN104816478A | OKUBO HIROAKI US\ FURUMIYA MASAYUKI (JP)\ NAKASHIBA YASUTAKA (JP) | SONY CORP (JP) | S02-A01 |
| 5927 | 3D printing | THREE DIMENSIONAL LOCATION DETECTION DEVICE OF MULTI-CAVITY CASTING STRUCTURE, HAS ONE DETECTION ROD FIXED IN TEST ELEMENT AND OTHER DETECTION ROD INSERTED IN SLOT OF BULGE PORTION OF TEST ELEMENT, BY CLAMPING DEVICE FIXED IN HOUSING | CN204535595U | OKUBO HIROAKI US\ YAMAMOTO RYOTA (JP)\ FURUMIYA MASAYUKI (JP)\ NAKASHIBA YASUTAKA (JP)\ KIKUCHI HIROMASA US | SANYO ELECTRIC CO (JP) | A08-E02\ F05-A06B\ G01-A10\ G01-B01\ G02-A03D\ G02-A04B\ G02-A07A\ D08-B\ D08-B03C\ D08-B09A1A\ D08-B09B2\ E12-A\ E12-B05\ E31-P02C\ E35-C04\ E35-F\ E35-H\ E35-K01\ E35-K04\ E35-L\ E35-U01\ X24-D03\ X24-D04 |
| 5928 | 3D printing | EFFECT PIGMENT USEFUL IN, E.G., DYES, BUTTON PASTES AND AUTOMOTIVE COATINGS, COMPRISES ALUMINUM OXIDE PLATELETS ON SURFACE OF TWO HIGH REFRACTIVE INDEX COATINGS, COMPRISING LAYERS OF TITANIUM DIOXIDE AND ILMENITE | WO2015139825A1 | OMI TOSHIHIKO (JP)\ WATANABE HITOMI (JP)\ ISHII KAZUTOSHI (JP)\ SAITOH NAOTO (JP) | RENESAS ELECTRONICS CORP (JP)\ FURUMIYA MASAYUKI (JP)\ OHKUBO HIROAKI (JP)\ NAKASHIBA YASUTAKA (JP) | V01-A\ X12-A |
| 5929 | 3D printing | INTEGRATED POWER CONTROL CEMENT RESISTOR ALUMINUM BRACKET, HAS ALUMINUM BRACKET BODY WHOSE END IS FORMED WITH GROOVE THAT IS MATCHED WITH RESISTANCE WIRE, AND CEMENT RESISTOR LEAD CONNECTED WITH PRINTED CIRCUIT PLATE | CN204496994U | ONO YOSHICHIKA US\ SOLANKI RAJENDRA (US)\ CONLEY JR JOHN F (US) | NEC ELECTRONICS CORP (JP) | A09-D03\ A11-B07\ A11-B14\ A11-B16\ A11-C04A\ A12-W12F\ X25-A08 |
| 5930 | 3D printing | INTEGRATED NUMBER CONTROL THREE-DIMENSIONAL PRINTING DEVICE INCLUDES WORKTABLE, PORTAL FRAME, HANGING DRIVING MECHANISM, LASER SINTERING METAL POWDER SHOWER NOZZLE COMPONENT, ROTATING TOWER AND CUTTING HEAD MAIN SHAFT ELECTRIC MACHINE | CN204470601U | OOTSUKA FUMIO (JP)\ ICHINOSE KATSUHITO US | SEIKO INSTR INC (JP) | A03-C02\ A04-B10\ A04-C04\ A04-G03C\ A08-M01D\ A08-R06A\ A08-S02\ A11-B15B\ A11-C05A1\ A11-C06\ A12-H04\ A12-S04A3\ A12-S05L\ F03-C02A\ D04-A01\ D04-D |
| 5931 | 3D printing | MELTBLOWN POLYPROPYLENE BLENDED MODIFIED VERMICULITE HIGHLY HYDROPHOBIC COMPOUND FILTER MATERIAL COMPRISES VERMICULITE, EXPANDED POLYSTYRENE-DIVINYL BENZENE MICROSPHERES, CARBONIZATION OIL PLANT HUSK POWDER, POLYPROPYLENE, AND MICROSILICA | CN104759157A | OSTERMAY INA (DE)\ KRONHOLZ STEPHAN (DE)\ OTT ANDREAS (DE) | SHARP LAB OF AMERICA INC (US) | E05-L02B\ E05-L02C\ E05-L03\ E06-D13\ E10-J01A\ E10-J02D1\ E11-F03\ E11-F07A\ E11-F07D\ E11-F08\ E11-F10C\ E11-F11\ E11-G03\ E11-Q01A\ E11-S\ E12-A10\ J01-E03C2\ J04-X\ N05-B\ N07-D03\ N07-D11A |
| 5932 | 3D printing | MICROPOROUS TRANSITION METAL ORGANIC FRAMEWORK MATERIAL COMPRISING TRANSITION METAL ION AND POLYCARBOXYLIC ACID LIGAND, USEFUL FOR ADSORPTION AND STORAGE OF METHANE AND ACETYLENE | CN104549164A | PAI CHIEN-SHING (US)\ LIU CHUN-TING (US) | RENESAS TEHNOLOGY CORP (JP) | A12-A03 |
| 5933 | 3D printing | SCREEN PRINTING-TYPE CASTING DIAMOND DRY GRINDING PLATE, HAS GRINDING WORKING LAYER INCLUDED WITH LIQUID BINDING AGENT, DIAMOND ABRASIVE, ENDOTHERMIC AGENT, FOAMING AGENT AND AUXILIARY MATERIAL AND FORMED ON SURFACE OF TEXTILE LAYER | CN104816257A | PAN ERIC TING-SHAN (US) | GLOBALFOUNDRIES INC (KY) | A09-D03\ A11-B16\ A11-C04\ A12-A03 |
| 5934 | 3D printing | APPARATUS FOR FABRICATING POLISHING LAYER OF POLISHING PAD USED FOR FINISHING OPERATIONS SUCH AS BUFFING, HAS UV RADIATION SOURCE THAT CURES PAD MATERIAL PRECURSOR TO SOLIDIFY PAD MATERIAL PRECURSOR | US2015273770A1 | PAN GUANGRAN (CN)\ SHI JINCHENG US | LUCENT TECHNOLOGIES INC (US) | L03-A01A3\ L03-A02\ L03-H04E3\ M22-H01\ V04-Q30N\ V04-R01A5A\ V04-R02B\ V04-R04A2\ V04-R04C |
| 5935 | 3D printing | ELECTROCONDUCTIVE PASTE FOR LAMINATED CERAMIC ELECTRONIC COMPONENT AND PRINTED WIRING BOARD, COMPRISES SILVER POWDER, GLASS FRIT, ORGANIC BINDER, AND POWDER CONTAINING ELEMENT CHOSEN FROM VANADIUM, CHROMIUM, IRON, MANGANESE AND COBALT | WO2015141816A1 | PARK DEUK HEE (KR)\ KWON KYOUNG SOO (KR)\ JEONG HA WOONG (KR)\ GO CHAE DONG (KR) | PAN ERIC TING-SHAN (US)\ ATHENAEUM LLC (US) | S06-E04A\ S06-E04C\ S06-K07C1\ S06-K07C5\ S06-K99F |
| 5936 | 3D printing | NOZZLE RECEIVER ARRANGED IN POWDER CONTAINER USED IN IMAGE FORMING APPARATUS, HAS SUPPORTER HAVING END SURFACE PORTION WHOSE PROJECTION AREA IS SMALLER THAN PROJECTION AREA OF SEALING PORTION OF CONTAINER SHUTTER | WO2015141861A1 | PARK DONG-GUN (KR)\ AHN YOUNG-JOON (KR)\ LEE CHOONG-HO (KR)\ KANG HEE-SOO (KR) | SHENZHEN FOUNDER MICROELECTRONICS CO LTD US\ UNIV PEKING FOUNDER GROUP CO (CN) | A11-B16 |
| 5937 | 3D printing | MANUFACTURE OF THREE-DIMENSIONAL STRUCTURE INVOLVES FORMING LAYERS USING THREE-DIMENSIONAL FORMATION COMPOSITION CONTAINING SOLVENT, DISCHARGING BINDING SOLUTION TO RESULTANT LAYERS AND PREVENTING PERMEATION OF SOLVENT FROM LAYERS | US2015258707A1 | PARK DONG-GUN (KR)\ LEE CHANG-SUB (KR)\ KIM SUNG-MIN (KR)\ CHOE JEONG-DONG (KR)\ LEE SHIN-AE (KR)\ KIM SEONG-HO (KR) | SAMSUNG ELECTRO MECH (KR) | W04-M01C1A\ X27-A03 |
| 5938 | 3D printing | THREE-DIMENSIONAL SPACE CAMERA ADJUSTING DEVICE FOR OPTICAL REMOTE SENSOR FLOOR FIELD, HAS SWITCHING BASE FIXED WITH FOUNDATION BED THAT IS FIXED WITH SHELL STRUCTURE, AND TRANSMISSION PLATE WHOSE END PART IS CONNECTED WITH LOCKING BOLT | CN104777581A | PARK DONG-GUN (KR)\ RI SEIEI US\ KIM SUNG-MIN (KR)\ RO KYOKAN US | SAMSUNG ELECTRONICS CO LTD (KR) | A04-G02E\ A12-W07F\ L02-A02\ L02-A03\ L02-A04\ L02-G01D\ L02-G12B |
| 5939 | 3D printing | PREPARATION OF 3-DIMENSIONAL PRINTER ZIRCONIUM DIOXIDE POWDER MOLDING MATERIAL BY ADDING PETROLEUM ETHER AND POLYETHYLENE IN REACTOR, HEATING WITH STIRRING AND ADDING PREPROCESSED ZIRCONIUM DIOXIDE POWDER AND AZOBISISOBUTYRONITRILE | CN104725039A | PARK GIL HO (KR) | SAMSUNG ELECTRONICS CO LTD (KR) | A03-A04A\ A04-F06E\ A05-F01E2\ A12-E01\ X12-D01F1\ X12-D07 |
| 5940 | 3D printing | PRINTING PASTE USED IN CURVED SURFACE OF MOVABLE COMPONENT, COMPRISES SUPERFINE PRECIOUS METAL POWDER COMPRISING MIXTURE OF SILVER POWDER AND PALLADIUM POWDER, ORGANIC BINDER, LEAD-FREE GLASS FRIT AND FUNCTIONAL ADDITIVES | CN104733075A | PARK HEE-SOOK (KR) | SAMSUNG ELECTRONICS CO LTD (KR) | A09-D01\ A11-B16\ A11-C02D\ X25-A08 |
| 5941 | 3D printing | METHOD FOR PRODUCING THREE-DIMENSIONAL OBJECT, INVOLVES PERFORMING DISCHARGE TREATMENT ON POWDERED RESIN MATERIAL AT TIMING WHILE FORMING UNIT LAYER AND TIMING AFTER FORMING UNIT LAYER AND BEFORE STARTING FORMING ANOTHER UNIT LAYER | EP2923824A1 | PARK KYUNG BAE (KR)\ KIM KYU SIK (KR)\ JIN YONG WAN (KR)\ CHOI WOONG (KR)\ LEE KWANG HEE (KR)\ KIM DO HWAN (KR) | DONGBU ELECTRONICS CO LTD (KR) | A12-R07\ A12-W07F |
| 5942 | 3D printing | SHEET MANUFACTURING APPARATUS FOR MANUFACTURING SHEET E.G. PAPER, HAS MIXING UNIT WHICH MIXES FIRST FIBER AND POWDER CONSISTING OF NATURAL FIBER AND RESIN INTEGRALLY | JP2015168255A | PARK KYUNG-BAE (KR)\ LIM HYUCK (KR)\ XIANYU WENXU (KR)\ CHO HANS S (KR)\ NOGUCHI TAKASHI (KR)\ PARK YOUNG-SOO (KR)\ ZHANG XIAOXIN (KR)\ YIN HUAXIANG (KR)\ KIM SUK-PIL (KR) | SAMSUNG ELECTRONICS CO LTD (KR) | A11-B09A2\ A11-B16\ A12-W12F |
| 5943 | 3D printing | MODELING TANK OF THREE-DIMENSIONAL MODELING APPARATUS E.G. THREE-DIMENSIONAL (3D) PRINTER, HAS OUTER WALLS THAT ARE ATTACHED TO FREE END PORTION OF SUPPORT BY ROTATING SHAFT PROVIDED IN EDGE PORTION OF SURFACE OPPOSITE TO SUPPORT | WO2015141776A1 | PARK SANG-JINE (KR)\ BAEK JAE-JIK (KR)\ CHO BYUNG-KWON (KR)\ YOON BO-UN (KR)\ HAN JEONG-NAM (KR) | SAMSUNG ELECTRONICS CO LTD (KR) | S06-E04A\ S06-E04C\ S06-K01A\ S06-K99F |
| 5944 | 3D printing | POWDER CONTAINER FOR USE IN IMAGE FORMING APPARATUS, HAS SCOOPING SURFACE INCLINED TOWARD UPSTREAM SIDE IN ROTATION DIRECTION OF POWDER STORAGE AND TANGENT TO EDGE OF INNER END PORTION IN CROSS-SECTION PERPENDICULAR TO ROTATION AXIS | WO2015141859A1 | PENDHARKAR SAMEER (US)\ DENISON MARIE (US) | SAMSUNG ELECTRONICS CO LTD (KR) | A11-B16\ X25-A08 |
| 5945 | 3D printing | HEIGHT ADJUSTING-TYPE THREE DIMENSION PRINTER, HAS FIRST LIFTING MODULE FIXED ON FOUNDATION BED THAT IS CONNECTED WITH WORKBENCH, WHERE FIRST LIFTING MODULE IS CONNECTED WITH SECOND LIFTING MODULE | CN104786499A | PENDHARKAR SAMEER (US)\ DENISON MARIE (US)\ HOWER PHILIP L (US) | YOON BO-UN (KR)\ BAEK JAE-JIK (KR)\ CHO BYUNG-KWON (KR)\ PARK SANG-JINE (KR)\ HAN JEONG-NAM (KR) | F03-D\ F05-A06\ F05-A06B |
| 5946 | 3D printing | WAVE-ABSORBING WALLPAPER HAS PAPER SUBSTRATE WHOSE WALL PORTION IS PROVIDED WITH COMPOSITE ANTI-MOISTURE LAYER, SOUND ABSORBING LAYER, WAVE-ABSORBING SUBSTRATE LAYER AND PRINT LAYER FROM BOTTOM TO TOP PORTIONS | CN204475049U | PENG LI (CN)\ YAQING CHI US\ BIN LIANG US\ YANKANG DU US\ JIANJUN CHEN US\ YIBAI HE US\ BIWEI LIU US\ ZHEN LIU US\ ZHENTAO LI US | TEXAS INSTRUMENTS INC (US)\ DENISON MARIE (US)\ PENDHARKAR SAMEER (US) | S06-E04D\ S06-G\ S06-K03\ S06-K06D |
| 5947 | 3D printing | SHOWER NOZZLE AUTOMATIC CLEANING DEVICE HAS BLOWING HOLES AND AIR PUMP WHICH COMMUNICATING WITH AIR INTAKE PORT AND AIR OUTLET PORT, IN WHICH BLOWING HOLE HAVING LONG AND NARROW PORES FOR BLOWING AIR FROM NARROW PORES OF BLOWING HOLE | CN204472113U | PENG LI (CN)\ YAQING CHI US\ BIN LIANG US\ YANKANG DU US\ JIANJUN CHEN US\ YIBAI HE US\ JUNRUI QIN US\ BIWEI LIU US\ ZONGLIN LIU US\ CHUNMEI HU US | TEXAS INSTRUMENTS INC (US)\ DENISON MARIE (US)\ PENDHARKAR SAMEER (US)\ HOWER PHILIP L (US) | A10-E05B\ A12-E07\ A12-W12G\ L02-A02\ L02-A03\ L02-A08\ L02-G01E\ L02-G10A\ L02-G10B\ L02-K\ L03-G05\ L03-G09B\ L03-G09V |
| 5948 | 3D printing | FERROELECTRIC SWITCHING FLUORESCENT CERAMIC MATERIAL USED FOR, E.G. PHOTOELECTRIC SENSOR COMPRISES BISMUTH SODIUM YTTERBIUM PRASEODYMIUM TITANATE CONTAINING COMPOUND | CN104710173A | PENG LI (CN)\ YAQING CHI US\ YONGJIE SUN US\ BIN LIANG US\ YANKANG DU US\ JIANJUN CHEN US\ YIBAI HE US\ JUNRUI QIN US\ BIWEI LIU US\ ZHEN LIU US | NAT UNIV DEFENSE TECHNOLOGY US | L03-G10\ L03-H04A\ M22-H01\ S06-K07A\ S06-K07B\ X25-B02A\ X25-C\ X25-H |
| 5949 | 3D printing | DEVICE USEFUL IN THE PREPARATION OF ULTRAFINE SPHERICAL METALLIC POWDER BY UNIFORM DROPLET EJECTION PROCESS, COMPRISES SHELL, CRUCIBLE, POWDER COLLECTING AREA, PIEZOELECTRIC CERAMIC, TRANSMISSION ROD, AIR INLET PIPE AND A MACHINE PUMP | CN104550988A | PENG LI (CN)\ YAQING CHI US\ YONGJIE SUN US\ BIN LIANG US\ YANKANG DU US\ XIANGYUAN LIU US\ JIANJUN CHEN US\ YIBAI HE US\ JUNRUI QIN US | NAT UNIV DEFENSE TECHNOLOGY US | T01-J08A2 |
| 5950 | 3D printing | FLAME DETECTING DEVICE HAS ELECTRIC SOURCE MODULE AND PHOTOELECTRIC CONVERSION MODULE THAT ARE PROVIDED INDEPENDENT OF PRINT CIRCUIT PLATE MODULE CONNECTED INTO PRINT CIRCUIT PLATE GROUP BY INSERTING COMBINATION | CN204460273U | PENG LI (CN)\ YAQING CHI US\ YONGJIE SUN US\ BIN LIANG US\ YANKANG DU US\ XIANGYUAN LIU US\ JIANJUN CHEN US\ YIBAI HE US\ JUNRUI QIN US | NAT UNIV DEFENSE TECHNOLOGY US | A09-D02\ A11-A03A\ A11-B07\ A11-B09D |
| 5951 | 3D printing | THREE-DIMENSIONAL STRUCTURE MANUFACTURING APPARATUS FOR MANUFACTURING E.G. THREE-DIMENSIONAL OBJECT, BY LAMINATING LAYERS, HAS CURING UNIT FOR BINDING THREE-DIMENSIONAL FORMATION POWDERS BY CURING DISCHARGED BINDING SOLUTION | US2015258723A1 | PENUNURI DAVID (US)\ EISENBEISER KURT W (US)\ FINDER JEFFREY M (US)\ VOIGHT STEVEN (US)\ SMITH STEVEN M (US)\ TALIN ALBERT ALEC (US) | NAT UNIV DEFENSE TECHNOLOGY US | U11-E01A\ U11-E02A2\ V07-F01A1\ V07-F02A\ V07-G03\ V07-G10C\ V07-G10D\ U12-A01B3\ V08-A01A\ V08-A04A\ W02-C04A1 |
| 5952 | 3D printing | LIGHT EMITTER PACKAGING STRUCTURE, HAS OPTICAL FIBER ABUTTING JOINT PIECE WHOSE INNER SIDE IS FIXED WITH LIGHT ISOLATOR CORRESPONDING TO SPHERICAL LENS SEAT, WHERE LIGHT ISOLATOR IS FORMED WITH LIGHT CONNECTING EXTERNAL OPTICAL FIBER | CN204479806U | PING-CHUAN HE ZHONG-XIANG JOSE (US) | NAT UNIV DEFENSE TECHNOLOGY US | L01-E05\ L01-E06\ L01-F04\ L01-G01A\ L01-H09\ A09-D03\ A11-B01\ A11-B16\ A12-W12F\ X25-A08 |
| 5953 | 3D printing | THREE-DIMENSIONAL MOLD FORMING DEVICE HAS POWDER SCRAPING PUSHING DEVICE THAT IS PROVIDED WITH DRIVING DEVICE CONNECTED WITH ROLLER, AND INJECTION DEVICE THAT IS ARRANGED WITH FIRST SHOWER NOZZLE AND SECOND SHOWER NOZZLE | CN204471892U | PINTO ANGELO (US)\ JOHNSON FRANK S (US) | MOTOROLA INC (US) | A09-D03\ A11-B16\ A11-C01C\ A12-W12F |
| 5954 | 3D printing | SELECTIVE REGIONS SOLID POWDER ADHESIVE THREE-DIMENSIONAL PRINTER COMPRISES MAIN MACHINE BODY, WORKING TABLE, WORKING FRAME, TRANSPARENT BAFFLE PLATE, ADHESIVE CHAMBER, POWDER MACHINE, SHOWER NOZZLE AND ADHESIVE CONDUIT | CN204431733U | QIAN PEIXIN LIANG (CN) | IBM (US)\ HE ZHONG-XIANG (US)\ RAMACHANDRAN VIDHYA (US)\ JOSEPH J ALVIN (US)\ ORNER A BRADLEY (US)\ ST ONGE A STEPHEN (US)\ WANG PING-CHUAN (US) | T04-L02\ U11-C13 |
| 5955 | 3D printing | 3D PRINTING SOLAR ENERGY BATTERY ELECTRODE, HAS 3D PRINTER CONNECTED WITH ELECTRIC CONDUCTION INK LINE, AND SUBSTRATE CONNECTED WITH ELECTRODE MAIN BODY THROUGH NOZZLE, WHERE ELECTRODE MAIN BODY IS FIXED WITH GRATE LINE | CN104752530A | QIGING OUYANG US\ ALFRED GRILL US\ CHU JACK O (US)\ DEHLINGER GABRIEL K (AT)\ KOESTER STEVEN J (US)\ SCHAUB JEREMY D (GB) | TEXAS INSTRUMENTS INC (US) | A09-D03\ A11-B16\ A12-W12F\ T01-J10B1\ T01-J15X |
| 5956 | 3D printing | METHOD FOR MANUFACTURING THREE-DIMENSIONAL STRUCTURE BASED ON MODEL OF THREE-DIMENSIONAL OBJECT GENERATED WITH THREE-DIMENSIONAL CAD SOFTWARE, INVOLVES PREPARING COMPOSITION CONTAINING NON-BOUND FORMATION POWDERS AND SOLVENT | US2015258705A1 | QINGLIN SONG US\ HONGPENG SUI US | UNIV TSINGHUA (CN) | S05-D03\ V06-V01N |
| 5957 | 3D printing | BLUETOOTH SWITCH CONTROL-BASED THREE DIMENSIONAL SCANNING ULTRASONIC IMAGING MECHANISM HAS ULTRASONIC WAVE PROBE WHOSE ONE SIDE IS PROVIDED WITH DEPTH SENSOR ALONG VERTICAL DIRECTION AND HEAD IS EXTENDED OUT OF MAIN PORTION | CN204445935U | QIUDE LI (CN)\ YUANLI CAI (CN) | IBM (US)\ GRILL ALFRED (US)\ KOESTER STEVEN J (US)\ CHU JACK O (US)\ DEHLINGER GABRIEL K (AT)\ OUYANG QIGING (US)\ SCHAUB JEREMY D (US) | A11-B05\ A11-C01C\ A12-E07A\ L03-H04E6A\ V02-H\ V04-R01A4\ V04-R02G\ V04-R05D\ V04-U15\ X12-C01D1 |
| 5958 | 3D printing | METHOD FOR MANUFACTURING FLEXIBLE PRINTED CIRCUIT BOARD COVERLAY DECOUPLING-TYPE MAGNETIC SHEET, INVOLVES PRESSURIZING SOFTMAGNETIC COMPOSITE MATERIAL AT CERTAIN TEMPERATURE RANGE, WHERE ALUMINUM THIN PLATE COATS WITH THERMOSETTING ADHESIVE | KR1549988B1 | QUANHONG CHE US\ ZHINONG LI US\ CHENXIAO YANG US | GOERTEK INC US | A07-A01\ A07-A02\ A07-A03\ A07-A04\ A08-E01\ A08-F03\ A08-M\ A08-M01\ A08-M02\ A08-M06\ A08-S01\ A08-S02\ A08-S03\ A11-A03\ A11-B05D\ A12-W07D\ F05-A06B\ G02-A04A\ G02-A04B\ G02-A05C |
| 5959 | 3D printing | MANUFACTURE OF PRINTING INK USED FOR E.G. TEXTILES, INVOLVES STIR-MIXING BASE BINDER WITH NICKEL-PHOSPHORUS-TYPE ALLOY POWDER, MIXING ANTIMICROBIAL BINDER WITH NICKEL-PHOSPHORUS MIXED/ALLOY POWDER, AND MIXING INK CONTAINING PIGMENT | JP2015160952A | RAMACHANDRAN VIDHYA (IN)\ ST ONGE A STEPHEN (US)\ HE ZHONG XI ANG (US)\ JOSEPH J ALVIN (IN)\ ORNER A BRADLEY (US)\ WANG PING CHUAN (US) | HEJIAN TECH SUZHOU CO LTD (CN) | A04-D05A\ A11-B15C\ A12-E10\ A12-S05E\ A12-S05L\ A12-W11K\ A12-W14\ F03-C08\ E12-A04\ E31-G\ U14-E05B |
| 5960 | 3D printing | PREPARATION OF BISMUTH SELENIDE-MICROSHEET USED IN E.G. THERMOELECTRIC MATERIAL, INVOLVES PREPARING NANOFIBER BY DISSOLVING BISMUTH NITRATE IN DIMETHYLFORMAMIDE, ADDING POLYVINYLPYRROLIDONE AND SPINNING, AND MIXING SELENIUM POWDER | CN104528663A | RAMDANI JAMAL (US)\ DROOPAD RAVINDRANATH (US)\ HILT LYNDEE L (US)\ EISENBEISER KURT WILLIAMSON (US) | SHENZHEN ZK SOFTWARE BIOMETRIC IDENTIFICATION TECHNOLOGY CO LTD US | A11-B16\ X25-A06\ X25-A08 |
| 5961 | 3D printing | THREE-DIMENSIONAL PRINTING DEVICE HAS LASER SINTERING ELEMENT ABOVE MOVING CARRIER FOR APPLYING LASER BEAM TO NON-SINTERED POWDER SPREAD ON CARRIER AND PRESSED SO AS TO SINTER PORTION OF LAYER | TW501934U | RAO BHASKER B (US)\ KALB JEFFREY CLIFFORD (US) | IBM (US) | A02-A06\ A05-J07\ A10-D\ F05-A06\ F05-B01\ G02-A04A\ G02-A05\ G02-A05E\ G02-A05K\ G03-B01\ G03-B02\ D04-A01\ D04-D\ H01-D11\ H07-A\ H07-L\ M22-H03F |
| 5962 | 3D printing | PRODUCING POLYAMINE USEFUL AS BUILDING BLOCK FOR E.G. MATERIAL FOR PRODUCING SYNTHETIC RUBBER AND RUBBER CHEMICALS AND FLAME RETARDANTS, COMPRISES PERFORMING POLYCONDENSATION OF DIAMINES IN LIQUID PHASE IN PRESENCE OF HYDROGEN AND CATALYST | WO2015128021A1 | REN JUNYAN US\ FU HAIPENG US\ MA SHUNLI US\ JIANG JIANBING US\ CAI DEYUN US\ LI NING US | PHISCARL SEMICONDUCTOR INC (US) | A11-A\ A11-C04A\ A12-H02 |
| 5963 | 3D printing | ULTRASONIC NOZZLE MICRO-JET PRINTING DEVICE, HAS AIR ROOM BODY CONNECTED WITH SHAFT THROUGH LOCKING BLOCK, LAVAL SPRAY PIPE CONNECTED WITH AIR ROOM BODY, AND ANNULAR MESH CONNECTED WITH LAVAL SPRAY PIPE | CN204414604U | RHEE HWA-SUNG (KR)\ LEE HO (KR)\ KIM MYUNG-SUN (KR)\ YI JI-HYE (KR) | MICRO DEVICES CORP CALIFORNIA (US) | A08-E01\ A08-S02\ A11-B05D\ A11-B06A\ A12-B02A\ A12-S04A1\ A12-S04D\ F02-G03\ F04-B01A |
| 5964 | 3D printing | VELVET SENSING GRINDING FLOWER LEATHER HAS BASE CLOTH LAYER, AND FIXED BONDED WET FABRIC LAYER INCLUDING VELVETY VENEER LAYER AND WEAR FLOWER LAYER | CN104611943A | RIAZIAT MAJID (US) | UNIV FUDAN (CN) | B11-C05\ B12-M11B |
| 5965 | 3D printing | METHOD FOR PREPARING DRUG USING THREE-DIMENSIONAL PRINTING TECHNIQUE, INVOLVES FORMING MICRO-NOZZLE IN WHICH DOSAGE IS FACILITATED ACCORDING TO PATIENT USING MICRO-NOZZLE WITH MONOLAYER | KR2015078774A | RILEY DEBORAH J (US)\ JAIN AMITABH (US) | SAMSUNG ELECTRONICS CO LTD (KR)\ KIM MYUNG-SUN (KR)\ RHEE HWA-SUNG (KR)\ LEE HO (KR)\ YI JI-HYE (KR) | A08-R06A\ D08-B\ D08-B01A\ D08-B01C\ D08-B03A\ D08-B09A2A\ E12-A08\ E31-P03\ L03-H04E6A\ L03-H04E8\ U11-A01A\ U11-A07\ V04-R01A4\ V04-R05A\ V04-X01B\ V04-X01F |
| 5966 | 3D printing | SILICA PARTICLES USED IN E.G. COSMETICS, COMPRISE PARTICLES HAVING POROUS OR NONPOROUS OUTER SHELL SILICA LAYER AND PRESET AVERAGE PARTICLE DIAMETER | JP2015155373A | RING ZOLTAN (US)\ SHEPPARD SCOTT THOMAS (US)\ HAGLEITNER HELMUT (US) | RIAZIAT MAJID (US)\ OEPIC SEMICONDUCTORS INC (US) | A09-D03\ A11-B03\ A11-B14\ A11-B16\ A11-C02B\ S06-E04B1\ S06-K07A |
| 5967 | 3D printing | 3D PRINT SYSTEM, HAS HIGH PRESSURE GENERATOR CONNECTED WITH SHOWER NOZZLE AND CURING MATERIAL ALLOWED TO SPRAY WITH CHARGE UNIT, AND SERVO MECHANISM CONNECTED WITH WORKBENCH FOR CONTROLLING THREE DIMENSION MOVEMENT | CN104690960A | RING ZOLTAN (US)\ SHEPPARD SCOTT THOMAS (US)\ HAGLEITNER HELMUT (US) | TEXAS INSTRUMENTS INC (US)\ RILEY DEBORAH J (US)\ JAIN AMITABH (US) | A11-B01\ A11-B09D\ A11-B16 |
| 5968 | 3D printing | COMPOSITION FOR THREE-DIMENSIONAL MODELING FOR MANUFACTURING THREE-DIMENSIONAL MOLDED ARTICLE, COMPRISES FIRST PARTICLE AND SECOND PARTICLE DIFFERENT FROM FIRST PARTICLE, WHERE MODELING IS FORMED BY FORMING LAYER, MOLDING AND LAMINATING | JP2015155168A | ROBERT GAUTHIER US\ JUNJUN LI US\ SOUVICK MITRA US\ STEPHEN PUTNAM CHRISTOPHER US\ MAHMOUD MOUSA US | SHEPPARD SCOTT (US)\ RING ZOLTAN (US)\ HAGLEITNER HELMUT (US) | A11-B01\ A11-B09D\ A11-B16 |
| 5969 | 3D printing | PARTICLE FOR MANUFACTURING THREE-DIMENSIONAL MOLDED ARTICLE, BY FORMING POWDER LAYER, MOLDING AND LAMINATING FOR CONTRACTION | JP2015155169A | ROBERT METZ MATTHEW DATTA SUMA (US) | SHEPPARD SCOTT (US)\ RING ZOLTAN (US)\ HAGLEITNER HELMUT (US) | U24-G01B\ U24-G05A\ X24-A01A\ U24-G04D\ V04-A01\ V04-M05\ V04-Q02A |
| 5970 | 3D printing | POWER TUBE CONNECTION STRUCTURE WITH POWER AMPLIFIER, HAS POWER PIPE WHOSE ONE END IS EXTENDED INTO MOUNTING GROOVE CLOSE TO POWER AMPLIFIER INPUT END AND SIDE WALL AND WHOSE UPPER WALL IS PROVIDED WITH SOLDERING FLUX ESCAPING CHANNEL | CN204408283U | ROBERT METZ MATTHEW DATTA SUMA (US) | IBM (US)\ IBM UK (GB)\ LI JUNJUN (US)\ MITRA SOUVICK (US)\ GAUTHIER JR ROBERT (US)\ PUTNAM CHRISTOPHER STEPHEN (US)\ MOUSA MAHMOUD (US) | S03-E14A2 |
| 5971 | 3D printing | SUPERFINE DRY POWDER FIRE EXTINGUISHING AGENT PERFORMANCE MEASURING DEVICE, HAS SHOWER NOZZLE ARRANGED ON VERTICAL RACK BODY, AND THREE-DIMENSIONAL UPPER SHELF UNIFORMLY SCATTERED WITH FUEL ARTICLE | CN104678060A | ROMAS GREGORY G (US)\ OGLESBY DARREL C JR (US)\ JASPER SCOTT F (US)\ NAJFUS PHILIP (US)\ GOVINDARAJU VENKATESH (US)\ YEH CHUN-LIANG (US)\ LISENBY JAMES (US) | KAVALIEROS JACK (US)\ BRASK JUSTIN K (US)\ DOCZY MARK L (US)\ METZ MATTHEW V (US)\ DATTA SUMAN (US)\ CHAU ROBERT S (US) | L03-A02B\ L03-E01B4\ L03-E01C\ L03-E04B\ L03-E08\ L03-H05\ X16-C01A\ X16-E06A1\ X16-E06A5A |
| 5972 | 3D printing | GRADIENT FUNCTIONAL ANODE ELECTRODE FOR USE IN CARBON-BASED SOLID OXIDE FUEL CELL, COMPRISES SEQUENTIALLY STACKED REFORMING CATALYST LAYER, TRANSITION LAYER AND ELECTROCHEMICAL REACTION LAYER ON ANODE STRUCTURE | CN104638277A | RONGJUN CHE US\ XIA LIN US\ YAFANG LUO US | KAVALIEROS JACK (US)\ BRASK JUSTIN K (US)\ DOCZY MARK L (US)\ METZ MATTHEW V (US)\ DATTA SUMAN (US)\ CHAU ROBERT S (US) | M22-H03B |
| 5973 | 3D printing | METHOD FOR MANUFACTURING THREE-DIMENSIONAL OBJECT, INVOLVES PERFORMING OVERALL DENSIFICATION PROCESSING ON ENCLOSURE AND MACHINING POWDER INSIDE ENCLOSURE, SO AS TO IMPLEMENT METALLURGICAL BONDING BETWEEN MACHINING POWDER AND ENCLOSURE | EP2910324A2 | RONGSHAN WEI US\ SHOUCHANG CHEN US\ JINFENG CHEN US\ MINGHUA HE US | TEXAS INSTRUMENTS INC (US) | U12-A01A\ X26-D02\ X26-H |
| 5974 | 3D printing | LARGE ANGLE LED LIGHT-EMITTING DEVICE, HAS ELECTRIC CONDUCTION CONNECTING PART CONNECTED WITH LIGHT SHELL THAT IS CONNECTED WITH THREE DIMENSIONAL SOLID LIGHT SOURCE BODY, WHICH IS CONNECTED WITH GEOMETRY CENTER REGION | CN204387748U | RONGSHAN WEI US\ SHOUCHANG CHEN US\ JINFENG CHEN US\ MINGHUA HE US | CHENGDU COPOO TECHNOLOGY CO US | M22-H02\ M22-H03E\ M22-H03G\ A11-C04A\ A12-H08\ A12-W12F\ A12-W12G |
| 5975 | 3D printing | METHOD FOR FORMING E.G. HOT GAS PATH COMPONENT, INVOLVES TREATING UNTREATED COMPONENT TO YIELD TREATED COMPONENT INCLUDING TREATED CREEP STRENGTH, WHERE TREATED CREEP STRENGTH IS GREATER THAN UNTREATED CREEP STRENGTH | US2015231796A1 | RONGSHAN WEI US\ SHOUCHANG CHEN US\ JINFENG CHEN US\ MINGHUA HE US | UNIV FUZHOU US | E06-F01\ E10-A07B\ E10-E04H\ E10-E04J\ L03-J02\ S06-G01\ S06-G03\ S06-G10\ S06-K03\ S06-K04 |
| 5976 | 3D printing | THREE-DIMENSIONAL PROTOTYPING COMPOSITION USEFUL FOR FORMING MOLDED CONSTRUCTION LAYER OF THREE-DIMENSIONAL PHYSICAL MODEL, COMPRISES A CONSTRUCTION POWDER MIXTURE OF POWDERY MOLDING MATERIAL AND POWDERY BINDING AGENT, AND INKJET LIQUID | US2015232648A1 | RONGSHAN WEI US\ SHOUCHANG CHEN US\ JINFENG CHEN US\ MINGHUA HE US | UNIV FUZHOU US | T04-F02A2\ T04-H03F\ T04-H04 |
| 5977 | 3D printing | TOUCH WINDOW FOR USE IN DISPLAY DEVICE, HAS SENSING ELECTRODE THAT IS ARRANGED ON SUBSTRATE, WHERE SENSING ELECTRODE IS PROVIDED WITH BINDER AND CONDUCTIVE POWDER IS DISPERSED IN BINDER, AND WIRING CONNECTS SENSING ELECTRODES | KR2015087944A | RONGSHENG BAO (CN) | UNIV FUZHOU US | A09-D03\ A11-B01\ A11-B16\ A12-W12F\ L01-E05\ L01-E06\ L01-F04\ L01-G01A\ L01-H09\ X25-A08 |
| 5978 | 3D printing | THREE-DIMENSIONAL MOLD AND FORMING DEVICE HAS FORMING POWDER DISPERSING DEVICE, SPRAYING DEVICE WHICH IS PROVIDED IN CAVITY, CONTROL DEVICE AND DRIVING DEVICE | CN104626592A | ROSS KEVIN (US) | UNIV FUZHOU US | A05-C01B\ A07-A04B\ A08-C07\ A08-C09A\ A08-D\ A08-D04A\ A08-R03\ A08-S02\ A10-E02\ A12-E10\ A12-W14\ L03-A02G\ L03-H04A\ S06-K03G\ X25-B01B\ X25-B01C |
| 5979 | 3D printing | HEAT GENERATION PASTE COMPOSITION FOR E.G. PLANE HEATER DEVICE, COMPRISES CONDUCTIVE POWDER PROVIDED WITH NANOTUBE PARTICLE, AND MIXING BINDER MIXED WITH ACETAL RESIN OR PHENOLIC RESIN, AND DISPERSING AGENT MIXED WITH ORGANIC SOLVENT | WO2015122641A1 | ROYBAL RICHARD G (US)\ ARSHAD SHARIQ (US)\ TANG SHAOPING (US)\ SALZMAN JAMES FRED (US) | SHANGHAI BEILING CO LTD (CN) | A09-D03\ A11-B14\ A11-B16\ A11-C04A\ A12-C04\ S06-G10\ S06-K07A4\ T01-J10C4\ X25-A06\ X25-A08 |
| 5980 | 3D printing | THREE DIMENSIONAL PRINT INSOLE MANUFACTURING METHOD, INVOLVES SCANNING FOOT DATA, AND OBTAINING FOOT DATA SIZE FROM ACQUIRED FOOT DATA AND PRINTING INSOLE PLANE FUSED WITH THERMOPLASTIC POWDER MATERIAL DEPOSITION FOR ACQUIRED FOOT DATA | CN104647757A | ROYTER YAKOV I (US)\ RAJAVEL RAJESH D (US)\ IONOV STANISLAV I (US) | PHILIPS ELECTRONICS NA (US) | A11-B05B1\ A12-V01\ A12-W15\ B04-C02A\ B04-C03\ B05-A01B\ B05-B02C\ B07-A02B\ B10-B02A\ B10-C04D\ B10-E04C\ B10-G02\ B12-M10A\ B12-M10A6\ B12-M11D |
| 5981 | 3D printing | DICLOFENAC SODIUM DUAL RELEASE PELLET COMPOSITION USEFUL FOR MAINTAINING PLASMA CONCENTRATION, COMPRISES SUSTAINED-RELEASE PELLET AND ENTERIC-COATED PELLET E.G. DICLOFENAC SODIUM, FILLER, ENTERIC MATERIAL AND SUSTAINED-RELEASE MATERIAL | CN104510724A | RU HUANG (CN)\ LE YE US\ YIXIAO WANG US\ HUAILIN LIAO US | TEXAS INSTRUMENTS INC (US)\ ROYBAL RICHARD G (US)\ ARSHAD SHARIQ (US)\ TANG SHAOPING (US)\ SALZMAN JAMES FRED (US) |  |
| 5982 | 3D printing | DRY POWDER INJECTION COVER SYSTEM, HAS 3D WIDE CLUSTER NOZZLE CONNECTED WITH EQUIPMENT ROOM BY LEVEL PIPELINE, AND DETECTOR CONNECTED WITH EQUIPMENT ROOM THROUGH CABLE, WHERE LEVEL PIPELINE IS PROVIDED WITH DISTRIBUTOR | CN204337574U | RUI GUAN US | HRL LAB LLC (US)\ ROYTER YAKOV (US)\ RAJAVEL RAJESH D (US)\ IONOVA IRINA (US)\ IONOVA SOPHI (US) | W04-M01G7C\ W04-M01L |
| 5983 | 3D printing | REAL-TIME THREE DIMENSIONAL ROAD INFORMATION COLLECTING DEVICE HAS HOUSING WHOSE INTERNAL PORTION IS SET WITH ELECTRICAL MACHINE, AND COVER PORTION THAT IS PIVOTED TO SIDE EDGE PORTION OF UPPER HOUSING | CN204377012U | RUPP THOMAS (AT)\ MAUDER ANTON (DE)\ GSCHWANDTNER ALEXANDER (DE)\ FOERG RAIMUND (DE) | UNIV BEIJING (CN) | T04-F02A2\ T04-H03F\ T04-H04 |
| 5984 | 3D printing | TOUCH WINDOW FOR DISPLAY DEVICE, IS ARRANGED ON SUBSTRATE, WHERE WIRING IS PROVIDED FOR ELECTRICALLY CONNECTING SENSING ELECTRODE, AND SENSING ELECTRODE SENSES POSITION | KR2015086140A | RUSSELL JAMES K (US) | SHANGHAI FUDAN MICROELECTRONIC GROUP CO LTD US | A08-M09A\ A09-A03\ A12-A05\ G03-B04\ L03-A02D\ L03-G05\ L03-H03\ L03-H04E |
| 5985 | 3D printing | CONDUCTIVE ADHESIVE USED FOR ADHESIVE FILM, COMPRISES METAL POWDER, CONDUCTIVE POLYMER AND MACROMOLECULAR BINDER | WO2015115781A1 | RYO KANDA (JP)\ SHIGEAKI OKAWA (JP)\ KAZUHIRO YOSHITAKA (JP) | INFINEON TECHNOLOGIES AUSTRIA (AT) | A04-F06E6\ A04-G06\ A05-H03\ A12-E13\ J04-C03\ S03-E04D1 |
| 5986 | 3D printing | SURFACE ENHANCED RAMAN SCATTERING SUBSTRATE, COMPRISES METAL NANO-UNIT, WHERE METAL NANO-UNIT IS FIXED BY ADHESIVE BONDING OF METAL POWDER PARTICLE, AND ADHESIVE IS INORGANIC ADHESIVE OR ORGANIC ADHESIVE | CN104568894A | RYO KANDA (JP)\ SHIGEAKI OKAWA (JP)\ KAZUHIRO YOSHITAKE (JP) | MICROCHIP TECH INC (US)\ RUSSELL JAMES K (US) | U11-C09A\ U12-A02A1\ U12-A02A4B\ X15-A01A\ X15-A02A |
| 5987 | 3D printing | METHOD FOR PREPARING SOLAR ENERGY BATTERY SUBSTRATE, INVOLVES SPRAYING ADHESIVE BY THREE DIMENSIONAL PRINTING DEVICE THROUGH NOZZLE ON SILVER POWDER PARTICLES, SO THAT SILVER POWDER PARTICLES ARE ADSORBED AND FIXED | CN104576816A | SAI TAIKI US\ KIN SOSHU US\ LEE NAE-IN (KR)\ BAE GEUM-JONG (KR)\ RHEE HWA-SUNG (KR) | SANYO ELECTRIC CO (JP) | M22-H03B\ V08-A02 |
| 5988 | 3D printing | METAL POWDER CONTINUOUS FEEDING SYSTEM FOR THREE-DIMENSIONAL PRINTER, HAS STORAGE BOX THAT IS USED FOR STORING METAL POWDER IN NOZZLE, TRANSPARENT HOUSING THAT IS SET INSIDE LASER, NOZZLE THAT IS FORMED ON BOTTOM PLATE BELOW LASER | CN104625057A | SAI TAIKI US\ SO GENSHO US\ BOKU SEIU US\ LEE GIL-GWANG (KR) | SANYO ELECTRIC CO (JP) | A09-D03\ A11-A05\ A11-B16\ A12-W12F |
| 5989 | 3D printing | INTEGRATED NUMBER CONTROL THREE-DIMENSIONAL PRINTING DEVICE HAS ROTARY TOWER WHOSE FRONT END IS SET WITH CUTTING HEAD SPINDLE MOTOR WHOSE OUTPUT SHAFT IS CONNECTED WITH MACHINING TOOL | CN104625061A | SALCEDO JAVIER A (US)\ LIOU JUIN J (US)\ BERNIER JOSEPH C (US)\ WHITSEY DONALD K (US) | SAMSUNG ELECTRONICS CO LTD (KR) | M22-H |
| 5990 | 3D printing | METAL POWDER FEEDING MOVEMENT DEVICE OF THREE-DIMENSIONAL PRINTER, HAS MOVING BLOCKS THAT ARE FIXEDLY MOUNTED WITH HORIZONTAL UPPER GUIDE RAIL MOUNTED WITH HORIZONTAL ROTATING MOTOR AND DRIVING CHAIN FIXEDLY MOUNTED ABOVE SHOWER NOZZLE | CN104625063A | SANDHU GURTEJ (US)\ RUEGER NEAL R (US) | SAMSUNG ELECTRONICS CO LTD (US) | A11-C02B\ G02-A02H\ G02-A04A\ G02-A05\ G03-B02E4 |
| 5991 | 3D printing | NEW URETHANE BOND-CONTAINING COMPOUND USED IN ACTIVE ENERGY RAY-HARDENING-TYPE COMPOSITION FOR E.G. COATING AGENT | JP2015143350A | SANO KOICHIRO US | UNIV CENTRAL FLORIDA (US) | A03-A03\ A05-J08\ A07-A01\ A08-M01\ A08-R05\ A08-S02\ A08-S03\ A11-B05B1\ A11-C02B\ A11-C02C\ A12-P06B\ A12-W07D |
| 5992 | 3D printing | MANUFACTURE OF PACKING BOX INVOLVES SPRAYING COLOR PAINT CONTAINING GOLD OR SILVER, TRANSPARENT INK, PEARL POWDER AND BINDER ON PAPER SUBSTRATE, FORMING PATTERN PRINTED LAYER, COATING INK LIQUID OF NITROCELLULOSE RESIN, AND ETCHING | KR2015078624A | SANO KOICHIRO US | MICRON TECHNOLOGY INC (US) | A08-C07\ A08-C09\ A08-D\ A08-D04\ A12-L02E\ G02-A02B1\ G02-A04A\ G02-A07A\ G04-B02\ G06-D\ G06-D05A\ G06-D06A\ G06-D06B\ G06-E\ G06-F03C\ G06-F03D\ G06-G17\ G06-G18\ L03-G02\ L03-H04E2\ L03-H04E3\ L03-H04E8\ U11-A06B\ V05-A01A3\ V07-F01A1\ V07-F02A\ V07-F02C\ V07-G15 |
| 5993 | 3D printing | REACTIVE MONOMER FOR RESIN COMPOSITION USED FOR ACTIVE ENERGY RAY POLYMERIZABLE RESIN COMPOSITION, IS OBTAINED BY REACTING UNSATURATED DOUBLE BOND-CONTAINING COMPOUND HAVING CARBOXYL GROUP, AND CYCLIC IMINE COMPOUND | JP2015129234A | SATO KEIMEI US | MATSUSHITA ELECTRIC IND CO LTD (JP) | A03-A02\ A12-E01\ A12-W12G\ L02-A02\ L02-A03\ L02-A04\ L02-A11\ L02-G02\ L02-J01C |
| 5994 | 3D printing | CERAMIC DISCHARGE CIRCUIT PIPE SEALING PROCESS BY SCREEN PRINTING SOLDER PASTE ON METALLIZED CERAMIC PIPE END, DRYING, HEATING IN HYDROGEN FURNACE OR VACUUM FURNACE, COATING SOLDER PASTE MELT, COOLING, WASHING CARBON BLACK, AND WELDING | CN104496513A | SAYAMA TAKASHI US\ NAGOYA TAKATOSHI (JP)\ TAKAMIZAWA SHOICHI (JP) | MATSUSHITA ELECTRIC IND CO LTD (JP) |  |
| 5995 | 3D printing | STEREOSCOPIC LIGHTING DISPLAY DEVICE, HAS SHELL PROVIDED WITH TRANSMISSION MECHANISM AND INNER PART PROVIDED WITH TRANSMISSION SHAFT, BRACKET GROUP FIXED ON FOUNDATION BED, AND LIGHT-EMITTING COMPONENT FIXED WITH ARC CONTROL UNIT | CN204348290U | SCHLIG EUGENE STEWART (US)\ SANFORD JAMES LAWRENCE (US) | MATSUSHITA ELECTRIC IND CO LTD (JP) | A04-F01A1\ A11-B05\ A12-R01\ G02-A02C\ G02-A04A\ G02-A04B |
| 5996 | 3D printing | FORMATION OF BUILDING PANEL E.G. LAMINATE FLOORS, INVOLVES PRINTING DIGITAL IMAGE ON SUBSTRATE USING INK CONTAINING SPECIFIC COLORANT, BINDER COMPRISING ACRYLIC RESIN DISPERSION AND VISCOSITY INCREASING SUBSTANCE | US2015210055A1 | SCHOELLKOPF JEAN PIERRE US\ CLERC SYLVAIN US\ JACQUET FRANCOIS (FR) | SHINETSU HANDOTAI KK (JP)\ NAGOYA TAKATOSHI (JP)\ TAKAMIZAWA SHOICHI (JP)\ SAYAMA RYUJI (JP) | A08-R\ A10-E01\ A12-E07A\ G03-B02E\ G03-B04\ L03-A03A\ L03-A03B\ L03-H04E3\ L03-H04E6A\ L03-H04E8\ L03-J02\ V04-R01A5A\ V04-R07\ V04-X01B |
| 5997 | 3D printing | INSULATING RESIN COMPOSITION FOR THERMAL RADIATION MATERIALS, COMPRISES BORON NITRIDE POWDER WITH AMINO GROUP INTRODUCED BY PLASMA-PROCESSING, AND RESIN REACTIVE WITH AMINO GROUP AND HAVING FUNCTIONAL GROUP WHICH FORMS CHEMICAL BOND | JP2015137335A | SCHULZE HANS-JOACHIM (DE)\ HILLE FRANK (DE)\ RUPP THOMAS (AT) | TPO DISPLAYS CORP (TW) | A09-D03\ A11-B03\ A11-B16\ A11-C01C\ A12-W12F |
| 5998 | 3D printing | SELECTIVE AREA BONDING BASED POWDER THREE-DIMENSIONAL (3D) PRINTER HAS SHOWER NOZZLE DRIVING BELT DEVICE THAT IS SET TO PERFORM RECIPROCATING MOVEMENT ON WORKING FRAME, AND LIGHT BAFFLE PLATE THAT IS SET FOR PREVENTING OUTLET OF POWDER | CN104608379A | SCOTT DAVID B (US)\ LIN HENG-CHIH (US) | ST MICROELECTRONICS SA (FR) | A05-E06A\ A05-E06B\ A05-H\ A05-J01B\ A06-A00E\ A07-A03\ A07-A03A\ A08-A03\ A08-M10\ A11-B01\ V04-R02B\ V04-R05A\ V04-S02\ V04-S10\ V04-S30\ V04-X01A\ V04-X01F\ W06-B01 |
| 5999 | 3D printing | THERMOPLASTIC COMPOSITION USED IN ARTICLE E.G. CALL BUTTON, COMPRISES POLY(ETHERIMIDE), POLYCARBONATE COMPONENT, COMPATIBILIZER POLYCARBONATE COMPONENT COMPRISING E.G. POLY(CARBONATE-ARYLATE ESTER), UV LIGHT STABILIZER AND TITANIUM DIOXIDE | WO2015106204A1 | SEKI AKINORI (JP)\ TANI YUKARI (JP)\ SHIBATA NORIYOSHI (JP) | INFINEON TECHNOLOGIES AG (DE) | A12-E10\ A12-T04C\ A12-W05\ L03-G10C\ L03-H04E\ L03-H05\ L04-C12B\ L04-C13A\ L04-C16\ L04-C17\ L04-C25\ V04-Q05\ V04-Q30B\ V04-R05E\ V04-T03P\ V04-X01B |
| 6000 | 3D printing | RESIN IMPREGNATED BORON NITRIDE SINTERED COMPACT USED AS HEAT RADIATING COMPONENT, HAS BORON NITRIDE SINTERED COMPACT BONDED THREE DIMENSIONALLY WITH RESIN, AND HAS PRESET ORIENTATION DEGREE OF BORON NITRIDE | JP2015124122A | SELIGSON DANIEL (US) | TEXAS INSTRUMENTS INC (US) | X26-H01 |
| 6001 | 3D printing | THREE-DIMENSIONAL LIGHTING AND WHITE LIGHT EMITTING LED LAMP STRIP FOR USE IN LED INDUSTRY, HAS TRANSPARENT BASE MATERIAL PROVIDED WITH LED CHIPS IN DIE BOND MANNER EXTERNALLY WRAPPED WITH YELLOW PHOSPHORUS FLUORESCENT POWDER LAYER | WO2015109888A1 | SERGIO MORINI (US) | TOYOTA MOTOR CORP US\ JAPAN FINE CERAMICS CT (JP) | A05-G01E\ A05-J05\ A06-A00E\ A10-E01\ A12-A02\ M22-B03\ M22-C01\ M22-D\ M22-G03N |
| 6002 | 3D printing | COLD CASTING RUBBER COMPRISES MANUFACTURING AND CASTING A MODEL HAVING SHAPE EXACTLY THE SAME AS MASTER MODEL, CURING TO FORM A RUBBER MOLD, CLAMPING THE RUBBER MOLD TIGHTLY, FREEZING IMMERSING IN TRIETHYLSILYL SLURRY, AND BAKING | CN104550700A | SEVILLANO BERASATEGUI JUAN FRANCISCO (ES)\ ASENSIO CERMENO ALEJANDRO (ES)\ GARCIA-ALONSO MONTOYA ANDRES (ES)\ PUENTE URRUZMENDI INIGO (ES)\ REBOLLO PIMENTEL IVAN (ES)\ PARDO SANCHEZ DANIEL (ES) | INTEL CORP (US) | M22-B03\ M22-D\ M22-G03E |
| 6003 | 3D printing | PREPARING GRAPHITE- AND CERAMIC-BASED CORE TITANIUM ALLOY CASTING, COMPRISES E.G. PREPARING CERAMIC CORE AND GRAPHITE SHELL, AND DEGASSING CERAMIC CORE-GRAPHITE SHELL BY PLACING IN VACUUM DEGASSING FURNACE, HEATING, COOLING, AND SMELTING | CN104550755A | SHANRONG CHEN US | INT RECTIFIER CORP (US) | A04-F04B\ A08-R01\ A12-W07D\ G02-A04A |
| 6004 | 3D printing | ANTI-COUNTERFEIT PRINTING INK COMPRISES DRY WHITE CREAM, FINE POWDER, INFRARED (IR) TRANSPARENT TONER, AND BINDER | CN104559464A | SHAW DONALD (CA)\ AL-KHALILI DHAMIN (CA)\ ROZON COME (CA) | FARSENS S L (ES)\ GARCIA-ALONSO MONTOYA ANDRES (ES)\ SEVILLANO BERASATEGUI JUAN FRANCISCO (ES)\ ASENSIO CERMENO ALEJANDRO (ES)\ PUENTE URRUZMENDI INIGO (ES)\ REBOLLO PIMENTEL IVAN (ES)\ PARDO SANCHEZ DANIEL (ES) | A08-E01\ A08-S01\ A11-B16 |
| 6005 | 3D printing | POWDER FOR MANUFACTURING THREE-DIMENSIONAL MOLDED ARTICLE WITH INK CONTAINING BINDER, COMPRISES POROUS PARTICLES | JP2015093481A | SHEALY JEFFREY B (US)\ POULTON MATTHEW (US)\ VETURY RAMAKRISHNA (US) | UNIV JISHOU US | A09-D01\ A11-B05\ A11-B16\ A11-C02B\ A11-C02C |
| 6006 | 3D printing | APPARATUS FOR GENERATING THREE-DIMENSIONAL OBJECT THROUGH SOLIDIFICATION OF POWDERED BUILD MATERIAL, HAS AGENT DISTRIBUTOR MOVABLE IN FIRST AXIS DIFFERENT TO SECOND AXIS TO DELIVER AGENT ONTO PORTIONS OF SUCCESSIVE LAYERS OF BUILD MATERIAL | WO2015106832A1 | SHEN YONG US\ XIA HONGFENG US\ CHEN FENG (SG) | QUEEN IN RIGHT OF CANADA AS RE (CA) | A03-C02\ A12-E07A\ A12-W12F\ L03-H04E6\ V04-A01\ V04-M05\ V04-R04A\ V04-R04C\ V04-T01C\ X24-A01A |
| 6007 | 3D printing | SOLDER COMPOSITION FOR MOUNTING ELECTRONIC COMPONENT ON PRINTED WIRING BOARD, CONTAINS DIESTER COMPOUND HAVING SULFUR BOND(S) AND ESTER BONDS AND/OR COMPOUND HAVING HINDERED PHENOL STRUCTURE WITH AMIDE BONDS, AS ANTIOXIDANT | JP2015123472A | SHENG YUAN US | RF MICRO DEVICES INC (US) | A09-D03\ S06-E05\ S06-G\ S06-K07A |
| 6008 | 3D printing | APPARATUS FOR MODELING THREE-DIMENSIONAL MOLDED ARTICLE, HAS PARTICLE LAYER FORMATION UNIT THAT IS PROVIDED FOR FORMING PARTICLE LAYER ON MODELING SURFACE BY HEATING PARTICLE IMAGE FORMED ON IMAGE CARRIER | JP2015131439A | SHENG YUAN US\ KEYONG HU US | LONTIUM SEMICONDUCTOR TECHNOLOGY HEFEI CO LTD US | A08-E01\ A08-M02\ A08-R01\ A09-A\ A11-B02\ A11-B07\ G05-F\ D09-A |
| 6009 | 3D printing | THREE-DIMENSIONAL PRINTING MATERIAL WITH ANTIBACTERIAL PROPERTY COMPRISES HIGH MOLECULAR WEIGHT POLYMER, PIGMENT POWDER, INORGANIC FILLER, ANTIBACTERIAL AGENT AND BINDER | CN104530668A | SHENGDONG ZHANG (CN)\ DINGYU LI (CN)\ WENXIN CHEN (CN)\ RUQI HAN (CN) | HANGZHOU XINSAI MICRO ELECTRONICS CO LTD US | A12-W12G\ A12-W14\ G05-F\ L02-A02\ L02-A03\ L02-A14\ L02-G01A\ L02-G01B\ L02-G01E\ L02-G01F\ L02-G11\ L02-G12C\ L02-G12E\ L02-H02B2\ L02-J02C\ X25-A08 |
| 6010 | 3D printing | COPPER-DOPED INORGANIC NANO COMPOSITE MATERIAL FOR RAPID PROTOTYPING THREE-DIMENSIONAL PRINTER COMPRISES CERAMIC PRECURSOR POWDER, NANOPOWDER ENHANCED MATERIAL, SURFACTANT, ORGANIC SOLVENT, INORGANIC BINDER, AND WARM CURING AGENT | CN104529474A | SHEPARD JOSEPH (US)\ DUTARTRE DIDIER (FR)\ BREIL NICOLAS (FR) | HANGZHOU XINSAI MICRO ELECTRONICS CO LTD US | A12-W12G\ A12-W14\ G05-F\ L02-A02\ L02-A03\ L02-A14\ L02-G01A\ L02-G01B\ L02-G01E\ L02-G01F\ L02-G11\ L02-G12C\ L02-G12E\ L02-H02B2\ L02-J02C |
| 6011 | 3D printing | MAGNESIUM-DOPED INORGANIC NANO COMPOSITE MATERIAL FOR THREE-DIMENSIONAL PRINTER COMPRISES CERAMIC PRECURSOR POWDER, NANOPOWDER ENHANCED MATERIAL, SURFACTANT, ORGANIC SOLVENT, INORGANIC ADHESIVE, AND WARM CURING AGENT | CN104529476A | SHIFU TANG US | UNIV BEIJING (CN) | A09-D01\ A09-D02\ A11-A\ A11-A03\ A11-A04\ A11-B07\ A11-B07C\ A11-B16\ A11-C04A\ A12-S09A |
| 6012 | 3D printing | THREE-DIMENSIONAL PRINTING ALLOYING AND BATCHING EXTRUDING DEVICE HAS MOLTEN METAL EXTRUDING SHOWER NOZZLE WHICH IS PROVIDED WITH SHAFT JOINT, MOTOR, SHOWER NOZZLE MAIN PORTION, AND SCREW ROD | CN104552944A | SHIFU TANG US | IBM (US)\ ST MICROELECTRONICS SA (FR) | L01-J04\ L01-L04\ L01-L05\ L01-L06\ L03-G05D\ L03-G05E\ L03-G09V\ L03-G10\ L03-H04E\ L04-C18\ U22-A04A2\ V04-Q30D\ V04-S15\ V05-A01A3\ V05-A01D1\ V05-D01C3\ V05-D05B7\ V05-D07A5\ V05-L07E7 |
| 6013 | 3D printing | GLASS PASTE USED FOR E.G. PLASMA DISPLAY PANEL, COMPRISES GLASS POWDER HAVING SPECIFIED AVERAGE PARTICLE DIAMETER AND VOLATILE ORGANIC SOLVENT, DOES NOT CONTAIN BINDER RESIN, AND HAS PRESET VISCOSITY MEASURED BY ROTATIONAL VISCOMETER | JP2015120622A | SHIGEAKI OHKAWA (JP)\ HIGICHIRO OKINO (JP) | SHIFU TANG US | A04-F06E\ A05-H03\ A12-E08B\ A12-W12G\ L02-A02\ L02-A03\ L03-B02C\ L03-B02J\ V02-A01B9\ V02-A09\ V02-G\ X12-C01D1 |
| 6014 | 3D printing | PREPARATION OF CERAMIC PRINTING PASTE USED FOR PRODUCTION OF CHIP INDUCTOR, INVOLVES ADDING CERAMIC POWDER MICROPARTICLES TO NITRATE, UNIFORMLY MIXING TO OBTAIN MIXED PARTICLES, ADDING BINDER, DISPERSANT AND PLASTICIZER, AND STIRRING | CN104529471A | SHIMA MASASHI (JP) | SHIFU TANG US | A12-W12G\ A12-W14\ L02-A02\ L02-A03\ L02-A14\ L02-G01B1\ L02-G01F\ L02-G12C\ L02-G12E1\ L02-H02B2\ L02-J02C\ S06-G10\ S06-K99C |
| 6015 | 3D printing | ZIRCONIUM-DOPED INORGANIC NANOCOMPOSITE MATERIAL USEFUL FOR THREE-DIMENSIONAL PRINTING, CONTAINS CERAMIC PRECURSOR POWDER, NANOPOWDER-REINFORCED MATERIAL, ZIRCONIUM POWDER, SURFACTANT, INORGANIC BINDER, AND LOW TEMPERATURE CURING AGENT | CN104529475A | SHIMA MASASHI (JP)\ SHIMAMUNE YOSUKE (JP)\ HATADA AKIYOSHI (JP)\ KATAKAMI AKIRA (JP)\ TAMURA NAOYOSHI (JP) | SENYO ELECTRIC CO LTD (JP) | A04-D05A\ A05-H03\ A12-W12C\ A12-W12G\ L02-A02\ L02-A03\ L02-A14\ L02-G01B1\ L02-G01F\ L02-G12C\ L02-G12E1\ L02-H02B2\ L02-J02C\ S06-G10\ S06-K99C |
| 6016 | 3D printing | TIN-DOPED INORGANIC NANOCOMPOSITE MATERIAL USEFUL FOR THREE-DIMENSIONAL PRINTING, CONTAINS CERAMIC PRECURSOR POWDER, NANOPOWDER-REINFORCED MATERIAL, TIN POWDER, SURFACTANT, INORGANIC BINDER AND LOW TEMPERATURE CURING AGENT | CN104529477A | SHIMA MASASHI (JP)\ SHIMAMUNE YOSUKE (JP)\ HATADA AKIYOSHI (JP)\ KATAKAMI AKIRA (JP)\ TAMURA NAOYOSHI (JP)\ OHTA HIROYUKI (JP) | FUJITSU SEMICONDUCTOR LTD (JP)\ SHIMA MASASHI (JP) | A11-C04A |
| 6017 | 3D printing | METHOD FOR THREE-DIMENSIONAL PRINTING ARTICLE, INVOLVES PRINTING BINDER FLUID WITH PARTICULATE MATTER CONTAINING DIFFERENT CHARACTERISTICS AND MEAN PARTICLE SIZE DIAMETER ON LAYER BY JET DEPOSITION | WO2015100086A1 | SHIMABUKURO RANDY L (US)\ RUSSELL STEPHEN D (US)\ OFFORD BRUCE W (US) | FUJITSU LTD (JP) | A09-D01\ A11-C04A |
| 6018 | 3D printing | METHOD FOR THREE-DIMENSIONALLY PRINTING ARTICLE FROM BUILD MATERIAL POWDER, INVOLVES SELECTIVELY PRINTING BINDER FLUID ON LAYER BY JET DEPOSITION, WHERE BINDER FLUID COMPRISES PARTICULATE MATTER THAT INCLUDES MEAN PARTICLE SIZE DIAMETER | WO2015100085A2 | SHIMADA HIROYUKI (JP) | FUJITSU LTD (JP) | A11-C04A\ A12-R01A\ A12-W12F |
| 6019 | 3D printing | THREE-DIMENSIONAL PRINTING APPARATUS HAS THIRD SOURCE SUPPLYING UNIT THAT SUPPLIES SYNTHETIC RESIN TO EXTRUSION HEAD AS PRINT RAW MATERIAL | KR1526827B1 | SHIMADA MASAO US | US NAVY (US) | A10-E02\ A12-W07D\ G02-A04A\ G02-A05F\ L02-G01B\ L02-G07C\ L02-K\ L03-E05B1\ L03-G05\ L03-J02\ T04-K01A\ T04-K01B\ T04-K03B\ U11-A08A\ U11-C08A4\ X15-A01A\ X15-A02D1\ X15-A05 |
| 6020 | 3D printing | SELF-REPAIRING SILICA BASED DIELECTRIC INK IS USED FOR E.G. FLEXIBLE PRINTING E.G. SCREEN PRINTING ON SUBSTRATE FOR ELECTRONIC COMPONENT, COMPRISES SPECIFIED AMOUNT OF SILICA COLLOIDAL SUSPENSION, SILICA DISPERSANT AND POLYMERIC BINDER | WO2015087350A1 | SHIN HARADA (JP)\ TAKEYOSHI MASUDA (JP) | SEIKO EPSON CORP (JP) | A05-A01E2\ A11-B05\ A12-E14\ L03-H04E4\ L03-H05\ L03-J02\ L04-C10E\ L04-C11C\ L04-C13B\ L04-C16\ L04-C21\ U11-A08B\ U11-C05E\ V04-R02G\ X15-A01A\ X15-A02A |
| 6021 | 3D printing | CONDUCTIVE PASTE COMPOSITION USED IN E.G. MANUFACTURE OF ELECTRODE, COMPRISES COPPER POWDER, SILVER POWDER, CARBON POWDER, GLASS POWDER AND BINDER, IN PRESET WEIGHT RATIO | US2015171238A1 | SHUGEN PENG US\ JIANHUA ZHOU US\ MINGHUI GAO US | NEC ELECTRONICS CORP (JP) | A05-G01B\ A05-G01E1\ A06-A00B\ A08-M\ A08-M01D\ A08-M06\ A08-M07\ A08-M10\ A08-S01\ A11-A03\ A12-B02A\ F03-C05\ F03-E01\ D07-B |
| 6022 | 3D printing | MATTE LEATHER PROCESSING AGENT COMPRISES E.G. AQUEOUS POLYURETHANE RESIN, DEIONIZED WATER, EMULSIFIED SILICONE OIL, ORGANOSILICONE FLATTING AGENT, NONIONIC AQUEOUS POLYURETHANE THICKENING AGENT, KAOLIN AND SILANE COUPLE AGENT | CN104499291A | SHYUE LAI LI US\ CHENG LIN JING US | SUMITOMO ELECTRIC INDUSTRIES (JP)\ HARADA SHIN (JP)\ MASUDA TAKEYOSHI (JP) | M22-G03F\ M22-H01\ M22-H03A |
| 6023 | 3D printing | DEVICE FOR CRUCIBLE-FREE MELTING OF MATERIAL AND FOR ATOMIZING MOLTEN MATERIAL TO PRODUCE E.G. METAL POWDER, HAS MATERIAL BAR AND INDUCTION COIL ARRANGED IN SUCH A WAY THAT MELT FLOW IS INDUCTIVELY HEATED BEFORE MELT FLOW ENTERS NOZZLE | DE102013022096A1 | SIEGEL PETER H (US) | SHANGHAI GRACE SEMICONDUCTOR US | A04-E10B\ A08-S02\ G02-A03\ G02-A07A\ G03-B02D2\ G04-B04\ G06-G05A\ J01-G\ S06-E01C1\ S06-E04A1\ S06-E04B1\ S06-E04D\ S06-E06D\ S06-K03G\ S06-K03H\ S06-K99B\ U11-C06A1A\ X16-B01F1\ X16-F02\ X16-F03B\ X25-A08 |
| 6024 | 3D printing | MANUFACTURE OF POLYVINYLIDENE-FLUORIDE RESIN PARTICLES USED FOR E.G. THICKENER, INVOLVES DEPOSITING RESIN PARTICLES BY ADDING SOLUTION CONTAINING ORGANIC SOLVENT, WATER AND POLYVINYLIDENE-FLUORIDE RESIN TO POOR SOLVENT | WO2015083489A1 | SILVERBROOK KIA (AU) | TAIWAN SEMICONDUCTOR MFG (TW)\ LAI LI-SHYUE (TW)\ LIN JING-CHENG (TW) | F01-E09\ F02-C02\ F04-C05A |
| 6025 | 3D printing | DUAL LAYER CARPET FOR USE IN CARPET SYSTEM IN CAR, HAS NEEDLE PUNCHED SURFACE LAYER AND ADJACENT NEEDLE PUNCHED CUSHIONING LAYER MADE OF STAPLE FIBERS, SUCH THAT SURFACE AND CUSHIONING LAYERS ARE MADE OF SPECIFIC HOLLOW STAPLE FIBERS | EP2873768A1 | SIN KIM YU (KR)\ SEOK LEE CHANG (KR)\ DU LEE KWANG (KR)\ SUN KIM HAK (KR)\ SOO YANG CHANG (KR) | NASA (US) | A08-C09\ A08-D05\ A12-W12G\ L02-A02\ L02-G01A\ L02-G01B\ L02-G11\ L02-G12E\ L02-H02A\ L02-H02B2\ L02-J02C |
| 6026 | 3D printing | CHROMIUM-DOPED INORGANIC NANOCOMPOSITE USED IN THREE-DIMENSIONAL PRINTING, COMPRISES CERAMIC PRECURSOR POWDER, REINFORCING NANO-POWDER HAVING PRESET PARTICLE SIZE, SURFACTANT, ORGANIC SOLVENT, INORGANIC BINDER AND CURING AGENT | CN104446394A | SINGH NARSINGH BAHADUR (US)\ WAGNER BRIAN (US)\ AUMER MIKE (US)\ THOMSON DARREN (US)\ KHALER DAVID (US)\ BERGHMANS ANDRE (US)\ KNUTESON DAVID (US) | ZAMTEC LTD (IE)\ SILVERBROOK KIA (AU) | A10-E05B\ A12-W12G\ L02-A02\ L02-A03\ L02-A04\ L02-A07\ L02-A08\ L02-A12\ L02-G02\ L02-J02A |
| 6027 | 3D printing | PRODUCTION OF MICROCRYSTALLINE GLASS-CERAMIC COMPOSITE BOARD BY FORMING BOTTOM GLAZE LAYER AND PRINTING LAYER ON BRICK SURFACE, SEQUENTIALLY SPRAYING LAYER OF HYDROPHOBIC AGENT, FRIT PARTICLE, AND FRIT PARTICLE BINDER, AND FIRING | CN104513070A | SORADA HARUYUKI (JP)\ ASAI AKIRA (JP)\ TAKAGI TAKESHI (JP)\ INOUE AKIRA (JP)\ KAWASHIMA YOSHIO (JP) | SAMSUNG ELECTRO MECH (KR) | A11-C01C\ A12-L04B\ S03-E13D\ S06-K03\ T01-J10C4 |
| 6028 | 3D printing | METHOD FOR PREPARATION OF COLUMNAR SECTION PROCESSING ROCK BODY SAMPLE, INVOLVES BONDING ADHESIVE GLUE WATER TO POWDER-SHAPED RAW MATERIAL COMPRISING COLUMNAR SECTION PROCESSING CYLINDER POWDER MATERIAL JOINTS AND POWDER MATERIAL | CN104515696A | SORADA HARUYUKI (JP)\ ASAI AKIRA (JP)\ TAKAGI TAKESHI (JP)\ INOUE AKIRA (JP)\ KAWASHIMA YOSHIO (JP) | NORTHROP GRUMMAN SYSTEMS CORP (US)\ SINGH NARSINGH BAHADUR (US)\ WAGNER BRIAN (US)\ AUMER MIKE (US)\ THOMSON DARREN (US)\ KHALER DAVID (US)\ BERGHMANS ANDRE (US)\ KNUTESON DAVID (US) | A05-E01D\ A12-L05C2\ G06-C04\ G06-G05A\ S06-E04A1\ S06-K01A |
| 6029 | 3D printing | TONER FOR DEVELOPING ELECTROSTATIC IMAGE, COMPRISES CRYSTALLINE POLYESTER WITH TERMINAL CRYSTAL NUCLEATING AGENT SITE, NONCRYSTALLINE POLYESTER, COLORING AGENT, AND HEXAGONAL BORON NITRIDE WITH PRESET DIAMETER AND CRYSTALLINITY DEGREE | JP2015118341A | SRIDHAR SEETHARAMAN (US)\ HAO PINGHAI (US)\ TODD JAMES ROBERT (US) | PANASONIC CORP (JP) | A08-C01\ A08-D01\ A12-W12C\ A12-W12G\ L02-A02\ L02-G01A\ L02-G01B\ L02-G11\ L02-G12E\ L02-H02A\ L02-H02B2\ L02-J02C |
| 6030 | 3D printing | CALCIUM-DOPED INORGANIC NANOCOMPOSITE FOR THREE-DIMENSIONAL PRINTING, COMPRISES PRESET AMOUNT OF CERAMIC PRECURSOR POWDER, NANO-REINFORCING MATERIAL, SURFACTANT, ORGANIC SOLVENT, INORGANIC BINDER AND LOW-TEMPERATURE CURING AGENT | CN104446392A | SRIKANTAM VAMSI K (US)\ BAER RICHARD L (GB) | PANASONIC CORP (JP) | S02-A03\ S02-A10C |
| 6031 | 3D printing | DENTAL THREE-DIMENSIONAL SCANNER, HAS BEARING DEVICE CONNECTED WITH INTERNAL SCAN ENGINE SHELL AND EQUIPPED WITH BOTTOM SURFACE OF SCANNING MACHINE SHELL, AND TRANSLATION MODULE AND ROTATING MODULE PLACED AT TRANSLATION STAGE | CN204286377U | STACEY WILLIAM F (US) | TEXAS INSTRUMENTS INC (US)\ HAO PINGHAI (US)\ SRIDHAR SEETHARAMAN (US)\ TODD JAMES ROBERT (US) | A11-B16 |
| 6032 | 3D printing | METHOD FOR PRODUCING THREE-DIMENSIONAL MODELS, INVOLVES PERFORMING LAYER CONSTRUCTION TECHNIQUE, AND REPEATING SIMULTANEOUSLY PARTICULATE BUILDING MATERIAL APPLYING STEP AND BINDER LIQUID APPLYING STEP UNTIL DESIRED OBJECT IS CREATED | DE102013021891A1 | STRUBEL MARTIN (CH) | BAER RICHARD L (US)\ SRIKANTAM VAMSI K (US) | A10-E05B\ A12-E07C\ A12-E14\ L03-A02\ X12-D01F1\ X12-D01F2\ X12-D07 |
| 6033 | 3D printing | ELECTROCONDUCTIVE COPPER PASTE FOR PREPARING SEMICONDUCTOR CERAMIC ELECTRODE, COMPRISES COPPER POWDER, GLASS POWDER, ORGANIC BINDER, REDUCING AGENT, NANO-SILICA AND SURFACTANT | CN104505137A | SU CHIEN-CHANG (TW)\ LIN HSIEN-HSIN (TW)\ SUNG HSUEH-CHANG (TW)\ PAI YI-FANG (TW)\ CHEN KUAN-YU (TW)\ KWOK TSZ-MEI (TW) | RAYTHEON CO (US) | G02-A05B\ L03-E05B2\ L04-C10F\ L04-C10J\ L04-C11C\ L04-E01\ U11-A08B2\ U11-C05C9\ U11-C05F6\ U11-C18B9\ U12-A02A1\ U12-A02A3\ U12-A02A4A\ X15-A01A\ X15-A02A\ X15-A02X |
| 6034 | 3D printing | CONDUCTIVE PASTE FOR FORMING CONDUCTIVE FILM, COMPRISES CONDUCTIVE POWDER, ADDITIVE CONTAINING TELLURIUM OR SELENIUM, ADDITIVE CONTAINING COMPOUND COMPRISING ELEMENT E.G. VANADIUM OR TUNGSTEN, GLASS FRIT, ORGANIC BINDER AND SOLVENT | US2015159025A1 | SUH DONGWOO (KR)\ KIM GYUNGOCK (KR) | STRUBEL MARTIN (CH) | J04-A\ M22-H01\ M22-H03G |
| 6035 | 3D printing | ATOMIZATION DEVICE FOR PREPARING SPHERICAL TITANIUM AND TITANIUM ALLOY POWDER COMPRISES CONTINUOUS FEEDER, METAL STRAIGHTENING MECHANISM, ATOMIZATION NOZZLE, HIGH FREQUENCY SMELTER, ATOMIZING ROOM, POWDER TANK, GUIDE DEVICE, AND VACUUM ROOM | CN104475744A | SUKEKAWA MITSUNARI (JP) | TAIWAN SEMINCONDUCTOR MFG COMPANY LTD (TW) | A05-A01E3\ A10-E05B\ A12-W12F\ M28-D\ T01-J15X\ X25-A08\ X25-C07\ X25-R02 |
| 6036 | 3D printing | THREE-DIMENSIONAL PRINT MANUFACTURE OF POROUS NIOBIUM PIECE INVOLVES SELECTING RAW MATERIAL, ADDING HIGH MOLECULAR ADHESIVE POWDER OR NIOBIUM POWDER SURFACE COATING ADHESIVE FILM, AND SELECTIVE LASER SINTERING MOLDING | CN104476653A | SUKHAREV VALERIY (US)\ MIRABEDINI MOHAMMAD R (US) | KOREA ELECTRONICS TELECOMM (KR)\ SUH DONGWOO (KR)\ KIM GYUNGOCK (KR) |  |
| 6037 | 3D printing | LARGE DISTANCE COVERAGE LONG TUBE NETWORK FIRE EXTINGUISHING DRY POWDER INJECTION SYSTEM, HAS DISTRIBUTION PIPE CONNECTED WITH DISTRIBUTOR, DETECTOR CONNECTED WITH EQUIPMENT ROOM THROUGH CABLE, AND TRANSFORMER BODY FORMED WITH NOZZLE | CN104511113A | SULIGOJ TOMISLAV (HR)\ BILJANOVIC PETAR (HR)\ WANG KANG L (US) | ELPIDA MEMORY INC (JP) | S06-G01\ S06-G03\ S06-G05\ S06-K02\ S06-K03\ S06-K07A\ S06-K99C |
| 6038 | 3D printing | PRINTING APPARATUS I.E. INK-JET PRINTER, HAS CONTROLLER FOR CAUSING DETECTION CONDITION MEMORY TO STORE CONDITION VALUE AS NEW-CONDITION VALUE WHEN LIQUID FAILURE FROM PRINTHEAD IS DETERMINED TO BE PRESENT | US9056512B1 | SUMIDA HITOSHI US | LSI LOGIC CORP (US) | V04-D03\ V04-S02\ V04-X01B\ V04-X01F\ V04-B01\ V04-M05\ V04-P05\ V04-Q02A4\ V04-Q02A5\ V04-R04B |
| 6039 | 3D printing | CONNECTOR SOCKET, HAS TWO BAFFLE PLATES FIXED WITH TWO SIDES OF INSULATION MAIN BODY, CLAPBOARD LOCATED BETWEEN TWO CONTACT TERMINALS, AND TONGUE PLATE FIXED TO FOUNDATION BED, WHERE TONGUE PLATE AND SHIELDING SHELL ARE FORMED WITH SLOT | CN204271328U | SUMMERFELT SCOTT R (US)\ RICKES JUERGEN T (US)\ MCADAMS HUGH P (US)\ GRACE JAMES W (US)\ LANHAM RALPH H R (US) | UNIV CALIFORNIA (US)\ SULIGOJ TOMISLAV (HR)\ BILJANOVIC PETAR (HR)\ WANG KANG L (US) | V04-D03\ V04-M01\ V04-P04\ V04-P06\ V04-S02\ V04-B01\ V04-M05\ V04-P05\ V04-Q02A4\ V04-Q02A5 |
| 6040 | 3D printing | CONNECTOR PLUG, HAS TWO BAFFLE PLATES CONNECTED WITH TONGUE PLATE AND FIXED WITH SHIELDING SHELL, WHERE OUTER SIDE OF SHIELDING SHELL IS FIXED WITH TONGUE PLATE AND BAFFLE PLATES ARE FORMED WITH LOCATION HOLE | CN204271341U | SUN MAOYOU US | FUJI ELEC DEVICE TECH CO LTD (JP) | S06-E06A\ S06-G\ S06-K03\ S06-K07A |
| 6041 | 3D printing | APPARATUS FOR TRANSFERRING, FOCUSING AND PURGING POWDER FOR DIRECT PRINTING AT LOW TEMPERATURE, HAS PRESSURE AND PURGING UNITS THAT ARE CLOSED AND OPERATED AND OPENED AND INTERRUPTED SIMULTANEOUSLY | US9056483B1 | SUN WEIFENG US\ ZHU JING US\ HAN TIANXIANG US\ QIAN QINSONG US\ LU SHENGLI US\ SHI LONGXING US | TEXAS INSTRUMENTS INC (US)\ AGILENT TECHNOLOGIES INC (US) | A12-E07A\ A12-R08\ V04-B01\ V04-D01\ V04-D02\ V04-D03\ V04-M05\ V04-M06\ V04-R04D |
| 6042 | 3D printing | PRINTED CIRCUIT BOARD CONNECTOR, HAS SHELL PROVIDED WITH SCREW HOLE, FOUNDATION BED PROVIDED WITH CONTACT HOLE, LINER PLATE PROVIDED WITH MULTIPLE PIN HOLES, WHERE BED IS FIXED ON HORIZONTAL SHELL, AND RUBBER PAD FIXED ON BED | CN104505627A | SUNG YOON JE (KR) | SUN MAOYOU US | M22-B03\ T01-J07B\ X25-A01 |
| 6043 | 3D printing | RAPID CASTING LIGHT-CURED THREE-DIMENSIONAL PRINTING TECHNOLOGY INVOLVES OPTIMIZING THREE-DIMENSIONAL MODEL, SCALING, CONVERTING TO STEREOLITHOGRAPHY FILE FORMAT, IMPORTING PROCESSING SOFTWARE, MODELING, LIGHT CURING AND SPRINKLING SAND | CN104493094A | SUYING YAO US\ JIANGTAO XU US\ JING GAO US\ ZAIFENG SHI US\ YUANQING LI US\ LONGFEI WANG US\ YIQIANG LI US | UNIV SOUTHEAST US | A03-A04A1\ A03-A05\ A04-F06E5\ A05-H03\ A08-S02\ A12-V01\ A12-W15\ B04-C02A1\ B04-C02A2\ B04-C03B\ B04-C03C\ B05-A01B\ B05-B02C\ B10-A09A\ B10-A17\ B12-M10A\ B12-M11R |
| 6044 | 3D printing | GUANFACINE HYDROCHLORIDE SUSTAINED-RELEASE PELLET COMPRISES COATING LAYER AND MEDICINE CONTAINING PELLETS, COATING LAYER COMPRISES EUDRAGIT AND TALCUM POWDER, AND MEDICINE CONTAINING PELLET COMPRISES GUANFACINE HYDROCHLORIDE | CN104473906A | SUZUKI NORIO (JP)\ SHIRASU TATSUMI (JP)\ YAMADA EIICHI (JP)\ KAWAKOE HIROUTO (JP)\ KIYODA SHOGO (JP) | LG ELECTRONICS INC (KR) | A12-W12G\ L02-A02\ L02-A03\ L02-A14\ L02-G01A\ L02-G01B\ L02-G01E\ L02-G01F\ L02-G12C\ L02-G12E\ L02-H02A\ L02-H02B2\ L02-J02C\ S06-G10 |
| 6045 | 3D printing | POTASSIUM-DOPED INORGANIC COMPOSITE NANOMATERIAL USED FOR 3D PRINTING COMPRISES CERAMIC PRECURSOR POWDER, NANOPOWDER REINFORCING MATERIAL, SURFACTANT, INORGANIC BINDER AND LOW TEMPERATURE CURING AGENT | CN104478414A | SUZUKI TAKAHITO (JP)\ FUJIWARA HIROYUKI (JP)\ ABIKO ICHIMATSU (JP)\ SAKUTA MASAAKI (JP)\ OGIWARA MITSUHIKO US\ CHIHARA SUSUMU (JP) | UNIV TIANJIN (CN) | A12-W07D1\ G02-A04A\ M22-H01\ S06-G\ S06-K07A |
| 6046 | 3D printing | INKJET RECORDING METHOD USED TO FORM RECORDING MATERIAL HAVING PATTERN (PRINTING PART) COMPRISES DISCHARGING COMPOSITION INCLUDING METAL POWDER, ORGANIC SOLVENT AND BINDER RESIN, IN WHICH METAL OR METAL ALLOY COMPRISES METAL POWDER SURFACE | US2015145935A1 | SUZUKI TAKAHITO (JP)\ FUJIWARA HIROYUKI (JP)\ OGIWARA MITSUHIKO US\ IGARI YUUKI US\ MUTO MASATAKA (JP) | SUZUKI NORIO (JP)\ KAWAGOE HIROTO (JP)\ SHIRASU TATSUMI (JP)\ KIYOTA SHOGO (JP)\ YAMADA EIICHI (JP)\ SUGINO YUJI (JP)\ KITANO MANABU (JP)\ SAKURAI YOSHIHIKO (JP)\ NAGANUMA TAKASHI (JP)\ ARAKAWA HISASHI (JP) | L03-E01B5B\ L03-E08B\ T04-L02\ X16-B01F1\ X16-E01G\ X16-E01J\ X16-E02\ X25-A08 |
| 6047 | 3D printing | THREE-DIMENSIONAL PRINTER SYSTEM FOR MANUFACTURING ELECTRODE OF SECONDARY BATTERY, HAS LASER CURING PART ABOVE CURRENT COLLECTOR DISTRIBUTION DEPARTMENT, AND GAS SUPPLY PART TO SUPPLY INERT GAS TO CURRENT COLLECTOR DISTRIBUTION DEPARTMENT | KR2015051648A | SUZUKI TATSUYA (JP)\ AOYAMA TOHRU (JP) | OKI DATA KK (JP)\ OKI DIGITAL IMAGING KK US | B05-B01B\ B06-H\ B07-D10\ B07-D12\ B07-D13\ B07-H\ B14-C01\ B14-J01A3\ B14-J01A4\ B14-J01B3\ B14-L01\ B14-L06\ B14-M01\ B14-N03\ B14-N16\ B14-N17\ B14-S18\ D09-A01 |
| 6048 | 3D printing | COMPOSITION USED E.G. FOR TREATING, AMELIORATING, PREVENTING OR LESSENING SYMPTOMS OF DEMENTIA, PARKINSON'S DISEASE OR ALLODYNIA, INCLUDES DIAZINE COMPOUND, AMINO-DIAZINE COMPOUND, AND 6-(4-METHOXYPHENYL)-N4-OCTYLPYRIMIDINE-2,4-DIAMINE | WO2015069752A1 | SYED AHMED RASHID (US)\ WEST BURNELL G (US) | SUZUKI TAKAHITO (JP)\ FUJIWARA HIROYUKI (JP)\ OGIHARA MITSUHIKO (JP)\ MUTO MASATAKA (JP)\ IGARI TOMOKI (JP) | A11-B14\ A11-C04A |
| 6049 | 3D printing | METHOD FOR PRODUCING E.G. PRINTING COMPONENT BY LAYER DESIGN TECHNOLOGY USING MATERIAL SYSTEM, INVOLVES USING PARTICULATE MATERIAL FOR CONSTITUTING MATERIAL LAYERS THAT ARE STACKED OR SOLIDIFIED BY ENTRY OF AQUEOUS LIQUID | DE102013020491A1 | TADA KENTOKU US | NEC ELECTRONICS CORP (JP) | A08-S02\ A12-E01\ A12-W16\ L03-E05B\ L03-H04B\ L03-H04E\ L03-H05\ T04-K03B\ U12-A01A\ X15-A01A\ X15-A02 |
| 6050 | 3D printing | GRAPHENE ELECTROCONDUTION SLURRY FOR USE IN E.G. SOLAR CELL, COMPRISES GRAPHENE WITH DISPERSANTS ADSORBED ON SURFACE, CONDUCTIVE FILLER, SOLVENT, ORGANIC BINDING AGENT, AUXILIARY FILLER AND FUNCTIONAL ADDITIVES | CN104464883A | TAI HSIN-CHIH (US)\ MAO DULI (US)\ VENEZIA VINCENT (US)\ KU KEH-CHIANG (US)\ CHEN GANG (US) | CREDENCE SYSTEMS CORP (US) | A11-B16\ A11-C02B |
| 6051 | 3D printing | THREE DIMENSIONAL MODELING APPARATUS HAS INTEGRAL HEAD STRUCTURE THAT IS PROVIDED WITH HARDENING ACCELERATION UNIT WHICH ACCELERATES HARDENING OF BINDER DISCHARGED BY POWDER MATERIAL | JP2015101011A | TAI STEPHEN (CN) | PANASONIC CORP (JP) | A05-E01D\ A12-L05\ A12-L05C2\ G06-G05A\ G06-G18\ S06-E04A1\ S06-K99B |
| 6052 | 3D printing | GRINDING TONER FOR DEVELOPING ELECTROSTATIC LATENT IMAGE, EXPOSES RATIO OF DISTANCE BETWEEN CONVEX PORTIONS OF MAGNETIC POWDER WHICH EXISTS IN MAGNETIC TONER PARTICLE SURFACE VICINITY FROM MAGNETIC TONER PARTICLE SURFACE | JP2015102762A | TAJIMA SHIGERU US | TAI HSIN-CHIH (US)\ MAO DULI (US)\ KU KEH-CHIANG (US)\ VENEZIA VINCENT (US)\ CHEN GANG (US) | E05-U06\ E12-C02\ E35-U01\ J04-A\ J04-B\ J04-C02A\ J04-C02B\ J04-E01\ N07-L03A |
| 6053 | 3D printing | SUPRAMOLECULAR HYBRID HYDROGEL USED FOR PREPARING GRAPHENE AEROGEL COMPRISES FERROCENE-AMINO ACID GEL FACTOR AND GRAPHENE OXIDE CO-ASSEMBLED BY NON-COVALENT BONDS, AND HAS THREE-DIMENSIONAL NETWORK-SHAPED STRUCTURE | CN104474980A | TAKASHI OHSAWA (JP) | MONTAGE TECHNOLOGY SHANGHAI CO (CN) | A04-F01A1\ A08-C01\ A08-M01D\ A12-B05\ G02-A02C\ G02-A02D\ G02-A03\ G02-A04A\ G03-B02D1\ L01-G04G1 |
| 6054 | 3D printing | ACTIVE-ENERGY RAY CURABLE RESIN COMPOSITION USED IN COATING AGENT COMPOSITION, CONTAINS UNSATURATED COMPOUND, PHOSPHORIC ACID-CONTAINING ETHYLENICALLY UNSATURATED COMPOUND, SILANE COUPLING AGENT AND FLUORINE-TYPE COMPOUND | JP2015083658A | TAKEUCHI NOBORU US\ KAWASHIMA RYOSUKE (JP) | SONY CORP (JP) | A04-G02C\ A08-R\ A11-A02C\ A11-A03A\ A11-A04\ A12-S09A |
| 6055 | 3D printing | UNIFORM MIXING OF WOOD POWDER AND INORGANIC STRENGTHENING PARTICLES INVOLVES MIXING PLASTICIZED WOOD POWDER WITH INORGANIC STRENGTHENING PARTICLES IN TWO-DIMENSIONAL MOTION MIXER AND MIXING OBTAINED MIXED POWDER WITH WOOD POWDER | CN104403338A | TALBOT ALEXANDRE US\ LENOBLE DAMIEN (FR)\ DUTARTRE DIDIER (FR) | TOSHIBA KK (JP) | A11-B16\ A12-H |
| 6056 | 3D printing | BUILDING CONTAINER FOR POWDER-BASED THREE-DIMENSIONAL PRINTER, HAS SIDE WALLS DECORATED WITH PLATFORM TO REDUCE SLIDING FRICTION BETWEEN DEVELOPED LAYERS AND SIDE WALLS, WHERE PLATFORM AND WALLS ARE PROVIDED WITH EQUAL VELOCITY | DE102013018031A1 | TAMURA MANABU US | SHARP KK (JP) | L02-A02\ L02-A03\ L02-A04\ L02-A08\ L02-A14\ L02-G07A\ L02-G07E\ L02-G10\ M22-H02\ M22-H03C |
| 6057 | 3D printing | MANUFACTURE OF BULK FULLY-DENSE NANOSTRUCTURED MATERIAL USED FOR E.G. CATHODE, INVOLVES PLACING POWDER COMPRISING NANOPARTICLES INSIDE ENCLOSURE, CLEANING NANOPARTICLES, PREPARING COMPACT POWDER, AND SINTERING AT PRESET CONDITIONS | US2015147590A1 | TAN CHUNG F (SG)\ LIU JINPING (SG)\ LEE HYEOK J (SG)\ INDAJANG BANGUN (SG)\ CHOR ENG F (SG)\ ONG SHIANG Y (SG) | ST MICROELECTRONICS SA (FR)\ IMEC INTER UNI MICRO ELECTR (BE)\ ST MICROELECTRONICS CROLLES 2 (FR) | A11-B05\ A11-C01D\ A12-W12F |
| 6058 | 3D printing | GENERATIVE MANUFACTURING OF COMPONENT, COMPRISES LAYER-WISE DEPOSITION OF POWDER MATERIAL ON SUBSTRATE OR PART OF PREVIOUSLY PREPARED COMPONENT AND LOCATION WISE BONDING OF POWDER MATERIAL TO COMPONENT, AND PRODUCING DOCUMENTATION BODY | EP2875933A1 | TANAHASHI KATSUTO US\ KANEDA HIROSHI US | ALPS ELECTRIC CO LTD (JP) | A04-F01A1\ A07-A05\ A08-S02\ A10-B04\ A11-B05D\ A11-B09A2\ A12-E01A |
| 6059 | 3D printing | PRODUCING POLYMER BINDER COMPOSITION, USEFUL FOR PRODUCING MAGNETIC SHEET FOR ELECTROMAGNETIC SHIELDING, COMPRISES E.G. PRODUCING COPOLYMER, MIXING THERMOPLASTIC RESIN WITH SOLVENT TO PRODUCE THERMOPLASTIC RESIN, AND MIXING WITH COPOLYMER | KR2015051953A | TANIZAWA YUKIHIKO (JP) | CHARTERED SEMICONDUCTOR MFG (SG) | T01-J07D1\ T05-G01\ T05-G02A\ V04-Q01\ V04-Q02A5\ V04-Q30B\ V04-T03J\ V04-U03\ W05-B03\ W05-B10C |
| 6060 | 3D printing | DRIVING SAFETY PRECAUTION DEVICE WITH RADIATING AND SHIELDING FUNCTION, FOR VEHICLE, HAS UPPER SHIELDING COVER WHOSE CONVEX PORTION CONTACTS PRINTED CIRCUIT BOARD WHOSE LOWER SURFACE CONTACTS BUMP OF LOWER SHIELDING COVER | CN204206718U | TAO HUN-JAN (TW)\ LIM PENG-SOON (MY)\ HOU YONG-TIAN (SG)\ JIN YING (SG) | FUJITSU LTD (JP) | A12-E01A\ A12-E07A\ L03-G06\ L03-H04E\ L03-H04E9\ V04-Q30F\ V04-R01A3\ V04-R03E\ V04-R05A\ V04-U |
| 6061 | 3D printing | MULTILAYER PRINTED CIRCUIT BOARD WITH TWO-LAYER PROTECTIVE COATING | RU2551838C1 | TAYLOR GEOFF W (US) | DENSO CORP (JP) | A09-D01\ A09-D03\ A11-B16\ A11-C04A\ A12-W07D |
| 6062 | 3D printing | METHOD OF MANUFACTURING THREE-DIMENSIONAL SHAPED OBJECT, INVOLVES DISCHARGING MULTIPLE DIFFERENT KINDS OF INKS ONTO DISCHARGE UNIT REGION WHICH IS NARROWER ON SURFACE OF LAYER WHERE INK IS DISCHARGED | US2015140295A1 | TAYLOR GEOFF W (US) | TAO HUN-JAN (TW)\ LIM PENG-SOON (MY)\ HOU YONG-TIAN (SG)\ YING JIN (SG) | A03-A04A\ A04-G02E\ A05-F05\ A10-E05B\ A12-W12G\ L02-A02\ L02-A03\ L02-G01B\ L02-G01C2\ L02-G01D\ L02-G04\ L02-G12B\ L02-G12D2\ L02-G12E\ L02-H02A\ L02-J01\ L02-J02C |
| 6063 | 3D printing | COLOR SELECTOR USED FOR ZIRCONIUM OXIDE CERAMIC NOZZLE INCLUDES SILICON CARBIDE, ZIRCONIUM OXIDE, STEEL FIBER, MAGNESITE, ACETYLENE, CHALK, CELLULOSE ETHER, PROPANETRIOL, ORGANIC MOLYBDENUM, ARAMID FIBER, CERIUM OXIDE AND CETYL SULFONATE | CN104446484A | TAYLOR GEOFF W (US) | UNIV CONNECTICUT CT FOR SCIENCE & TECHNOLOGY COM (US) | A12-W11\ A12-W14\ L02-A02\ L02-A14\ L02-G01A\ L02-G01B\ L02-G11\ L02-G12E\ L02-H02B2\ L02-J02C\ S06-G10\ S06-K99C |
| 6064 | 3D printing | COBALT-DOPED INORGANIC NANOCOMPOSITE MATERIAL USED IN 3D PRINTER, COMPRISES SPECIFIED AMOUNT OF CERAMIC PRECURSOR POWDER, REINFORCING MATERIAL NANOPOWDER, SURFACTANT, ORGANIC SOLVENT, INORGANIC BINDER AND LOW-TEMPERATURE CURING AGENT | CN104446517A | TIGELAAR HOWARD L (US)\ BARNA GABRIEL G (US)\ FAYNOT OLIVIER ALAIN (FR) | UNIV CONNECTICUT (US)\ OPEL INC (US)\ TAYLOR GEOFF W (US) | S03-C02X\ T01-C03A\ T01-C07C4A\ T01-F04\ T01-F06\ V04-A05\ V04-G15\ V04-M02\ V04-Q02B1\ V04-S30 |
| 6065 | 3D printing | MAGNETOTELLURIC DEVICE, HAS PRINTED PLATE MODULE COMPONENT PROVIDED WITH ARM MODULE, FPGA MODULE, MOTHER BOARD AND ELECTRIC SOURCE MODULE, AND MOUNTING FRAME FIXED WITH FUNCTION MODULE AND PROVIDED WITH FIXED SUPPORT | CN204228974U | TO SOHO US\ KO KENTOKU US\ O HEIKOKU US\ SHI SHISHO US\ KO YODO US\ RA SHUNKETSU US\ YO SANTOKU US | UNIV CONNECTICUT (US)\ OPEL INC (US) | A12-H04\ D04-A01F1\ D04-A01F2 |
| 6066 | 3D printing | DEVELOPING MACHINE WATER WASHING FILTERING SYSTEM, HAS FILTER LAYER PROVIDED WITH FILTER NETWORK, TANK BODY FORMED WITH DOUBLE INLET CHANNEL, AND PLASTIC TANK SHELL PROVIDED WITH BOTTOM WATER COVER | CN204219907U | TOKORO KAZUHIKO (JP)\ KIKUCHI KATSUYA (JP)\ AOYANAGI MASAHIRO US\ NAKAGAWA HIROSHI (JP)\ OKADA YOSHIKUNI US\ ITO HIDEO US\ MIKAWA TAKASHI (JP) | TEXAS INSTRUMENTS INC (US) | A08-R06A\ A11-B16 |
| 6067 | 3D printing | POWDER FOR THREE-DIMENSIONAL MODELING, HAS POWDER PARTICLE THAT IS POROUS AND HYDROPHOBIZATION TREATMENT IS GIVEN AND HYDROPHOBIZATION TREATMENT TRANSDUCES HYDROCARBON GROUP | JP2015093480A | TOYAMA HIROSHI (JP)\ NAKAMURA YUKIO (JP) | MINISTRY OF NAT DEFENSE CHUNG US | L01-G03\ L01-G05B\ L01-H09A\ L01-H09D |
| 6068 | 3D printing | PREPARATION OF ELECTROCONDUCTIVE GRID STRUCTURE USED FOR ELECTROMAGNETIC SHIELDING GLASS, INVOLVES FORMING GRID PATTERN USING PASTE CONTAINING GLASS POWDER AND BORON POWDER, ADHERING PATTERN ON GLASS SURFACE AND TEMPERING | CN104446001A | TOYAMA HIROSHI (JP)\ NAKAMURA YUKIO (JP) | NAT INST OF ADV IND & TECHNOL US | A12-W12G\ A12-W14\ L02-A02\ L02-A03\ L02-A14\ L02-G01A\ L02-G01B\ L02-G01E\ L02-G01F\ L02-G11\ L02-G12C\ L02-G12E\ L02-H02B2\ L02-J02C\ S06-G10 |
| 6069 | 3D printing | DOPED BARIUM INORGANIC COMPOSITE NANOMATERIAL USED FOR 3D PRINTING COMPRISES CERAMIC PRECURSOR POWDER, REINFORCING MATERIAL NANOPOWDER, SURFACTANT, ORGANIC SOLVENT, INORGANIC BINDER AND LOW TEMPERATURE CURING AGENT | CN104446516A | TOYAMA HIROSHI (JP)\ NAKAMURA YUKIO (JP) | OKI DATA KK (JP)\ OKI DIGITAL IMAGING KK US | L02-A02\ L02-A03\ L02-G01A\ L02-G01B\ L02-G11\ L02-G12E |
| 6070 | 3D printing | THREE-DIMENSIONAL CERAMIC BOARD COMPRISES ALUMINA FIBER, NON-ALKALI GLASS FIBER, WETTING AGENT, DISPERSANT, THIXOTROPIC AGENT, BINDER, ULTRAFINE SILICA POWDER, ETHANOL AND SILANE COUPLING AGENT, PREPARED BY PRE-MOLDING, DIPPING AND PRESSING | CN104446579A | TOYAMA HIROSHI (JP)\ NAKAMURA YUKIO (JP) | OKI DATA KK (JP)\ OKI DIGITAL IMAGING KK US | X25-A01\ X25-A03 |
| 6071 | 3D printing | ELECTRIC DRILL FOR CONNECTING A PROTECTIVE SHELL, COMPRISES ALLOY MATERIAL THAT IS EQUIPPED WITHIN DIE CASTING MACHINE, AND IS EQUIPPED WITH UPPER FRAME AND LOWER FRAME, WHERE SHAFT MACHINE THAT IS EQUIPPED WITH PRINT DEVICE | CN204209652U | TOYAMA HIROSHI (JP)\ NAKAMURA YUKIO (JP) | OKI DATA KK (JP)\ OKI DIGITAL IMAGING KK US | A10-E05B\ A12-W12F\ M22-H01\ M22-H03B\ M22-H03F\ M22-H03G\ M23-A01\ M23-A03\ M23-A04\ X24-A02A |
| 6072 | 3D printing | METHOD FOR FORMING E.G. SOLDERING TIP THAT IS USED IN SOLDERING/DE-SOLDERING WORKSTATION, INVOLVES SUBJECTING GREEN STATE ARTICLE DE-BINDING PROCESS, AND SINTERING DE-BOUND ARTICLE TO FUSE WITH POWDER METAL TO FINAL NET SHAPE | US2015125334A1 | TOYAMA HIROSHI (JP)\ NAKAMURA YUKIO (JP) | OKI DATA KK (JP)\ OKI DIGITAL IMAGING CORP US | A03-A04\ A10-E05B\ A12-W12G\ L02-A02\ L02-A03\ L02-A04 |
| 6073 | 3D printing | METHOD FOR CLADDING SILICON CARBIDE CERAMIC SURFACE INVOLVES MILLING MOLYBDENUM, NICKEL, COBALT AND SILICON, DRYING, SIEVING, ADDING ORGANIC BINDER, STIRRING, COATING RESULTANT PASTE ON SILICON CARBIDE CERAMIC, HEATING AND COOLING | CN104402533A | TOYAMA HIROSHI (JP)\ NAKAMURA YUKIO (JP) | OKI DATA KK (JP)\ OKI DIGITAL IMAGING CORP US | A08-E01\ A08-S01\ A08-S02\ A08-S03\ A11-C04A\ A12-S05U\ F03-C\ F03-E01\ F03-F27\ X25-T04B |
| 6074 | 3D printing | TEXTILE PRINTING FUNCTIONAL FABRIC, COMPROMISES E.G. FORMING RELEASING LAYER ON TRANSFER PAPER SURFACE, FORMING LIGHT PATTERN BY PRINTING LIGHT PAINT INK ON RELEASING LAYER, DRYING LIGHT PATTERN AND PRINTING PIGMENT IMAGE ON RELEASING LAYER | KR1515322B1 | TSAI CHUN HSIUNG (TW)\ LIU SU-HAO (TW)\ WANG TSAN-CHUN (TW) | OKI DATA KK (JP)\ OKI DIGITAL IMAGING CORP US | A10-E05B\ A12-E07B\ A12-E14\ L03-A02\ L03-B03G\ L03-H05\ V01-B01A3\ V01-B01D5\ V01-B01G1 |
| 6075 | 3D printing | HIGH-POROSITY AND HIGH-CONDUCTIVITY POROUS ELECTRODE FOR PSEUDO ELECTRIC SUPER CAPACITOR, HAS PRESSURE GRADING QUADRATIC ROLLER FORMED WITH MULTI-CARBON FILM, WHERE PORE-FORMING AGENT IS MIXED WITH CARBON MATERIAL AND BINDER | CN104425134A | TSUBAKI KOTARO US | OKI DATA KK (JP)\ OKI DIGITAL IMAGING KK US | A04-E02E1\ A08-E01\ A08-R08A\ A11-A01B\ A11-B09A1\ A11-C01A\ A11-C04\ A11-C05C\ A12-R03\ A12-S05N\ A12-S08C\ A12-S08D |
| 6076 | 3D printing | FORMATION OF DECORATIVE WEAR RESISTANT LAYER FOR FLOOR PANEL, INVOLVES PROVIDING SUBSTRATE AND TRANSPARENT LAYER OF THERMOPLASTIC MATERIAL, AND CONTINUOUS PRINT LAYER OF PARTICLES, PRINTING DIGITAL IMAGE OF COLOR PIGMENTS AND BONDING | WO2015060778A1 | TSUNG-LIN LEE US\ CHIEH YEH CHIH US | TAIWAN SEMICONDUCTOR MFG (TW)\ TSAI CHUN HSIUNG (TW)\ LIU SU-HAO (TW)\ WANG TSAN-CHUN (TW) | A08-S02\ A11-B05\ A11-B16\ G03-B02\ L02-A02\ L02-A03\ L02-A04 |
| 6077 | 3D printing | MANUFACTURING COMPONENT, E.G. THREE-DIMENSIONAL MOLDING, COMPRISES APPLYING PARTICLE LAYER ON BUILDING PLATFORM, APPLYING BINDER, SUBJECTING TO HEAT TREATMENT, AND LOWERING BUILDING PLATFORM TO LAYER THICKNESS OR RAISING POWDER COATER | DE102013018182A1 | TSUYOSHI TAKAHASHI (JP)\ KATSUYA OKABE (JP)\ AKIRA HATSUGAI (JP) | UNIV TOYO US | A04-G02E2\ A04-G03E1\ A04-G06\ A05-E04E\ A11-B09A1\ A11-C01A1\ A12-P02\ A12-S05G\ F03-D\ D09-B |
| 6078 | 3D printing | MANUFACTURE OF TRIPLE-LAYER LAMINATED NONWOVEN FABRIC FOR BAGS, INVOLVES HEAT-BONDING FILAMENT NONWOVEN FABRIC OF CORE-SHEATH TYPE LONG FIBER, WITH ULTRAFINE FIBER NONWOVEN FABRIC, AND HEAT-BONDING ANOTHER FILAMENT NONWOVEN FABRIC | JP2015080899A | TSUYOSHI TAKAHASHI (JP)\ TOSHIYUKI OKODA (JP) | TAIWAN SEMICONDUCTOR MFG (TW)\ LEE TSUNG-LIN (TW)\ YEH CHIH CHIEH (TW) | L03-J02\ L04-C21\ L04-E03\ U12-A01A1E |
| 6079 | 3D printing | OLED DEVICE STRUCTURE, HAS GLASS SUBSTRATE ARRANGED ON BOTTOM LAYER, WHERE UPPER PART OF SUBSTRATE IS FIXED WITH ANODE AND CATHODE LAYERS, ORGANIC LIGHT-EMITTING LAYER, ELECTRONIC INJECTION CACHE LAYER AND PACKAGING THIN FILM LAYER | CN104409651A | TSZ-MEI KWOK US\ HSIEN-HSIN LIN US\ HSUEH-CHANG SUNG US\ KUAN-YU CHEN US | SANYO ELECTRIC CO (JP) | A09-D01\ A10-B01\ A10-D\ A11-B12A\ A11-B16\ A12-A02\ A12-H05\ A12-L02A\ A12-V02A |
| 6080 | 3D printing | MOLD FOR MANUFACTURING SPECTACLE LENS FROM PLASTIC, HAS MOLD BODY PROVIDED WITH INTEGRATED BLOCK PIECE AND MOLD SHELL, WHICH IS MANUFACTURED BY PROTOTYPES AND 3D-DRUCK BY INJECTION-MOLDING, AND FUNCTION LAYER PROVIDED BETWEEN SHELL AND SLUG | DE102013222232A1 | TU KAO-WAY (TW)\ CHIEN FENG-TSO (TW)\ GONG ZHONG-YUAN (TW)\ SU SHIH-TZUNG (TW) | SANYO ELECTRIC CO (JP) | A04-F01A\ A08-C01\ A11-B05\ A12-W07D1\ M13-C01\ M13-H\ S06-G\ S06-K02\ S06-K03 |
| 6081 | 3D printing | MANUFACTURE OF HAIRLINE-FORMED STEEL PLATE INVOLVES INJECTING COMPOSITION CONTAINING METALLIC POWDER AND TRANSPARENT INK INTO INKJET PRINTING DEVICE, AND FORMING HAIRLINE COATING BY SPRAYING COMPOSITION ON SURFACE OF STEEL PLATE | KR1510556B1 | UEDA TETSUZO (JP)\ TANAKA TSUYOSHI (JP) | TAIWAN SEMICONDUCTOR MFG (TW)\ KWOK TSZ-MEI (TW)\ LIN HSIEN-HSIN (TW)\ SUNG HSUEH-CHANG (TW)\ CHEN KUAN-YU (TW) | A05-A01C\ A07-A05\ A08-S02\ A11-A03\ G02-A02G\ G02-A04A\ G03-B02E2\ G06-G05A\ L03-G02\ L03-G05\ L03-H04A\ L03-H04E2\ L03-H04E6A\ L03-H04E8\ U11-A09\ V04-Q30A\ V04-Q30B\ V04-Q30D\ V04-Q30S\ V04-R01A4\ V04-R04A2F\ V04-R04A5\ V04-S01A\ V04-S02A\ V04-S15\ V04-S30 |
| 6082 | 3D printing | EPOXY RESIN COMPOSITION FOR, E.G. ADHESIVE AGENTS, COMPRISES EPOXY RESIN, AND CORE-SHELL POLYMER, WITH CORE AND SHELL HAVING DIFFERING GLASS TRANSITION TEMPERATURE, AND SHELL COMPRISING MONOMER HAVING EPOXY GROUP | WO2015053289A1 | UEMICHI YUSUKE US\ AIZAWA TAKUYA US\ NAKAO SATORU US | CHINO EXCEL TECHNOLOGY CORP (TW) | A03-A00A\ A05-F05\ A11-B05\ A11-C\ A12-S05U\ A12-W07D\ F02-F01A\ F03-A02\ F03-C01\ F03-C03\ F03-C04\ F03-C05\ F03-C06\ F03-C07\ F03-F06\ F03-F14\ F03-F17\ F03-F18\ F03-F31\ S06-C03A\ S06-C05\ S06-K01A\ S06-K03\ S06-K07C1\ S06-K07C6 |
| 6083 | 3D printing | PRINTING MULTI-COMPONENT INTRINSIC FLAME RETARDANT FIBER FABRIC COMPRISES E.G. SELECTING BATCH MULTI-COMPONENT INTRINSIC FLAME RETARDANT FIBER FABRIC BLANK, DISTRIBUTING, SEWING, PRE-PROCESSING, DEHYDRATING, TWISTING, DRYING, AND SINGEING | CN104313924A | UKRAINTSEV VLADIMIR ALEXEEVICH (US)\ HALL CRAIG LAWRENCE (US) | UEDA TETSUZO (JP)\ TANAKA TSUYOSHI (JP) | A04-E10\ A05-E01D1\ A12-L05C2\ G06-F07\ G06-G05A\ G06-G08C\ G06-G08E\ S06-E04A1\ S06-E04D\ S06-E05\ S06-K03\ S06-K04B\ S06-K06C2\ S06-K99B |
| 6084 | 3D printing | MANUFACTURE OF POSITIVELY CHARGEABLE TONER FOR FORMING FUSED IMAGES, INVOLVES MELT-KNEADING POLYESTER RESIN BINDER, CHARGE CONTROL AGENT, AND FINE FLUORORESIN PARTICLES, AND RECYCLED POWDER, PULVERIZING, AND REMOVING RECYCLED POWDER | EP2863262A1 | VICTOR MOROZ US | FUJIKURA LTD US | L03-H03\ L03-H04E\ V04-B01\ V04-D03\ V04-D06B\ V04-M05\ V04-M06\ W01-C01A\ W01-C01D3C |
| 6085 | 3D printing | CONNECTION MODULE FOR WATERPROOF-TYPE MOBILE COMMUNICATION TERMINAL, HAS SHELL SECTION FIXED IN OPENING PART THAT IS FIXED IN LOWER SIDE OF MAIN PART, AND TERMINAL SUPPORTING PART ACCOMMODATING TERMINAL AMONG MULTIPLE TERMINALS | KR2015040737A | VILLA FLAVIO (IT) | TEXAS INSTRUMENTS INC (US) | M22-H03A |
| 6086 | 3D printing | THREE-DIMENSIONAL PRINTING ISOTROPIC BONDED PERMANENT MAGNET MANUFACTURING METHOD, INVOLVES PERFORMING HEATING AND CURING PROCESS TO OBTAIN THREE-DIMENSIONAL PRINTING ISOTROPY BONDED PERMANENT MAGNET PRODUCT | CN104384510A | VINET MAUD (FR)\ CLAVELIER LAURENT (FR)\ AUGENDRE EMMANUEL (FR)\ BATUDE PERRINE (FR) | SYNOPSYS INC (US)\ MOROZ VICTOR (US) | A08-M\ A08-M09A\ A08-S02\ A12-E07\ E10-C02A\ E10-C02B\ E10-C02F\ E10-C04D4\ E10-C04D5\ E12-B05\ L03-A01A2\ L03-A01A3\ L03-G09\ L03-H02\ L03-H03\ L03-H05\ V04-Q02A5\ V04-Q02A9\ V04-Q30F\ V04-R02P\ V04-S02\ V04-S15\ V04-U01\ V04-U03\ V04-X01B |
| 6087 | 3D printing | ELECTRICALLY CONDUCTIVE PASTE FOR USE DURING SUPERHEATED STEAM PROCESS AFTER PRINTING OR COATING E.G. CONDUCTIVE THIN FILM, COMPRISES ELECTROCONDUCTIVE METAL POWDER, RESIN BINDER, SOLVENT, AND ADDITIVE | JP2015076232A | WANG BIN US\ YAO SUYING (CN)\ XU JIANGTAO (CN)\ GAO JING (CN)\ SHI ZAIFENG (CN)\ GAO CEN (CN)\ LI JIAN (US) | ST MICROELECTRONICS SRL (IT) | A08-M09A\ A09-A03\ A11-B05B1\ A12-B01\ G06-G05\ G06-G08E\ S06-E04A1\ S06-E04C\ S06-K06C\ S06-K99C |
| 6088 | 3D printing | RECOATING OF SPENT LASER PRINTER TONER CARTRIDGE DOCTOR BAR E.G. TONER REGULATING COMPONENT, INVOLVES CLEANING DOCTOR BAR SURFACE WITH ORGANIC SOLVENT, AND COATING SURFACE WITH COATING CONTAINING CONDUCTIVE POWDER AND BINDER POWDER | US2015104224A1 | WANG HAITING (SG)\ FUNG KA-HING (TW)\ TSAI HAN-TING (TW) | COMMISSARIAT ENERGIE ATOMIQUE (FR)\ AUGENDRE EMMANUEL (FR)\ VINET MAUD (FR)\ CLAVELIER LAURENT (FR)\ BATUDE PERRINE (FR) | S02-A02\ S02-A10E\ V04-Q02A1\ V04-Q30G\ V04-T02\ W01-C01B3E\ W01-C01D3C\ X26-C01B\ X26-H01 |
| 6089 | 3D printing | SYSTEM FOR DETECTING MOBILE PHONE CASE STEEL PIECE FLATNESS, HAS PRINTED CIRCUIT BOARD (PCB) WHICH IS CONNECTED WITH LED LAMP, WHERE MIDDLE PORTION OF PCB IS INSTALLED WITH TRAVEL SWITCH OPPOSITE TO UPPER END OF THIMBLE | CN204202555U | WANG HONGCHEN (CN)\ CHEN WENLI (CN)\ GAN XIANFENG (CN) | UNIV TIANJIN (CN) | V04-N\ V04-Q02A1\ V04-Q02A4\ V04-Q30L\ V04-T02\ W01-D02\ X16-B01\ X22-B\ X27-A03\ X27-X01 |
| 6090 | 3D printing | SOLAR CHARGING INFANT STROLLER INFLATABLE LUMINOUS WHEEL HAS PRINTED CIRCUIT BOARD HAVING LIGHTING AND MICRO-VIBRATION SENSE SYSTEMS THAT ARE CONNECTED WITH STORAGE BATTERY, ELECTRIC SOURCE POWER SUPPLY UNIT AND LED LUMINOUS PORTION | CN204196577U | WANG HUAN US\ XU JIAN US\ LUO YIN US\ GUAN ZHIQIANG US\ WANG RONG US\ ZENG XIANWEN US\ WANG ZHIGONG US | TAIWAN SEMICONDUCTOR MFG (TW)\ WANG HAITING (TW)\ FUNG KA-HING (TW)\ TSAI HAN-TING (TW) | T01-F04\ V04-A01\ V04-B01\ V04-D03\ V04-D06B\ V04-M05\ V04-M30L\ V04-P05\ V04-Q30H\ V04-Q30K\ V04-R01A4\ V04-S01A\ W01-C05B1E\ W04-X02C\ X24-D03 |
| 6091 | 3D printing | CONNECTION MODULE FOR MOBILE COMMUNICATION TERMINAL, HAS SHELL COVER WHICH SURROUNDS MAIN HOUSING AND PRINTED CIRCUIT BOARD (PCB) IS MOUNTED IN LOWER-SIDE OF SHELL COVER | KR2015040207A | WANG PENGJUN US\ MEI FENGNA US | YANTAI RUICHUANG MICRO NANO TECHNOLOGY CO LTD US | A04-B01E\ A04-B03\ A04-C04\ A04-C04B\ A04-F01A1\ A04-F06E4\ A04-G01E\ A05-E01D\ A10-B05\ A10-E04A\ A12-L05C2\ G06-G05A\ S06-E04A1\ S06-K03\ T05-L07 |
| 6092 | 3D printing | FORGERY PREVENTION-TYPE POLYMERIZED TONER, HAS MAIN BODY COMPRISING BINDER RESIN, PIGMENT, PIGMENT STABILIZER, CHARGE CONTROL AGENT, WAX AND FLUORESCENT PAINT WITH SPECIFIC AMOUNT, WHERE FLUORESCENT PAINT IS COATED ON BINDER RESIN | KR2015034384A | WANG QUAN (CN)\ FAN CHUNHUI US | NANJING SINO CHIP MIRCOELECTRONICS CO LTD US | A03-C03\ A04-B03\ A07-A05\ A08-M03\ A08-R\ A11-B03\ A11-C01D\ A12-R\ A12-R09\ G03-B02A\ G03-B02B\ G03-B02D3\ G03-B04 |
| 6093 | 3D printing | QUICK PAVING BELT FOR CARPET COMPRISES GRAVEL SURFACE LAYER BONDED WITH ADHESIVE LAYER IN WHICH ADHESIVE LAYER CONTAINS STYRENE-BUTADIENE-STYRENE MODIFIED ASPHALT, RUBBER POWDER, AND ENVIRONMENT-FRIENDLY RUBBER OIL | CN104343073A | WANG QUAN (CN)\ FAN CHUNHUI US | UNIV NINGBO US | A02-A09\ A04-F01\ A04-H00H\ A08-C09\ A10-B06\ A10-E07B\ A11-B05\ A11-C02B\ A11-C02C\ A12-L02B2\ A12-L03D\ G02-A02D\ G02-A04A\ G02-A04B\ G03-B02E\ G05-A01\ G05-F\ G06-D01\ G06-D03\ G06-D05\ G06-D06A\ G06-D06B\ G06-E02\ G06-F03B\ G06-F03D\ G06-G17\ G06-G18\ G06-H07\ E05-G01\ E05-G02\ E06-A01\ E06-B01\ E06-D01\ E10-A09B1\ E10-A10A\ E10-A10D\ E10-A11B1\ E10-A15\ E10-A18A\ E10-G02G1\ E11-F06\ E11-P\ L03-B05K1\ L03-G02\ L03-G05B7\ L03-G05B9\ L03-G09P\ L03-H02\ L03-J02\ S06-C01\ S06-E03A2\ S06-E04B1\ S06-K01A\ S06-K03F\ S06-K07A4\ U11-A09\ U11-C18D\ V04-X01B\ V07-F02B\ V07-L02 |
| 6094 | 3D printing | NEW OXIME ESTER COMPOUND USED IN PHOTOPOLYMERIZABLE COMPOSITION FOR COATING SUBSTRATE FOR FORMING RELIEF IMAGES AND PREPARING PIGMENTED AND NON-PIGMENTED PAINTS AND VARNISH, POWDER COATING, PRINTING PLATE, ADHESIVE AND GEL COAT | WO2015036910A1 | WANG TAO (TW)\ LU SHEY-SHI (TW) | SH INTEGRATED CIRCUIT RES & DE (CN) | A05-G02\ A08-M\ A08-M09A\ A08-S02\ A09-A03\ A12-E07\ L03-A01A2\ L03-A01A3\ L03-H02\ L03-H03\ V04-Q02A5\ V04-Q02A9\ V04-Q30F\ V04-R02B\ V04-R02F\ V04-R02P\ V04-U01\ V04-U04 |
| 6095 | 3D printing | ELECTROCONDUCTIVE PASTE USED IN SUPERHEATED STEAM PROCESS IN FORMATION OF CONDUCTIVE THIN FILM, CONTAINS RESIN BINDER CONTAINING ELASTIC BINDER, AND HAS ELECTROCONDUCTIVITY BY MEANS OF SUPERHEATED STEAM PROCESS AFTER PRINTING | WO2015053160A1 | WANG WEIGUO HUANG (CN) | SH INTEGRATED CIRCUIT RES & DE (CN) | L03-B02A1\ L03-B02A5\ L03-B02X\ M22-H03B\ M22-H03C\ V02-A01A1\ V02-E01\ V02-H04\ X12-C01D1 |
| 6096 | 3D printing | LASER MOLDING HOT ISOSTATIC PRESSING SINTERING RARE EARTH PERMANENT MAGNET PROCESSING METHOD, INVOLVES PERFORMING SCREEN SINTERING PROCESS BY USING SINTERING LAYER, AND ATTACHING RHENIUM-IRON-BORON EARTH MAGNET WITH PRESSING MEDIUM | CN104377027A | WANG XIAOJUAN US\ YU LI US | UNIV NAT TAIWAN (TW) | A05-G01E\ A12-W07D\ G02-A04A |
| 6097 | 3D printing | SMOOTH LONG PRINTING INK USEFUL AS HIGH-GRADE INK, COMPRISES E.G. PIGMENT, CUTTLEFISH BONE POWDER, EGG WHITE, CALCINED EGG SHELL POWDER, CASHEW NUT SHELL LIQUID, POLYURETHANE RESIN, ISOPROPYL ALCOHOL, PROPYL ACETATE AND AUXILIARY AGENT | CN104356747A | WANG YANMING US\ MAO ZILI US\ MIAO ZHENLIN US\ NIU FENGJUAN US | SHANGHAI BEILING CO LTD (CN) | A03-A05\ A06-A00C\ A10-E05B\ A12-W12G\ L02-A02\ L02-A03\ L02-A04\ L02-A07\ L02-A08\ L02-A12\ L02-G02\ L02-J02A |
| 6098 | 3D printing | PRODUCTION OF MICROCRYSTALLINE GLASS CERAMIC COMPOSITE PLATE INVOLVES PROCESSING POWDER, PRESSING, FORMING GLAZE LAYER AND PRINTING LAYER, SINTERING, MIXING FRIT PARTICLES AND BINDER, COATING MIXTURE ON SINTERED COMPONENT AND SINTERING | CN104311159A | WANG YING (CN)\ YANG XIAOLIANG US\ CAO FEI US\ LIU YUNTAO US\ SHAO LEI US | ZHENGZHOU DANDIAN TECH SOFTWARE CO LTD US | L02-A02\ L02-A03\ L02-A04\ L02-B05\ L02-D13\ L02-G01A\ L02-G01B1\ L02-G11\ L02-G12E1 |
| 6099 | 3D printing | ZERO-VALENT IRON-COATED POROUS CERAMIC PRODUCT USED IN E.G. WASTEWATER TREATMENT APPLICATION, IS PREPARED BY SINTERING MIXTURE OF ALPHA-ALUMINUM OXIDE POWDER, CLAY, BENTONITE AND SURFACE-ACTIVATED BAMBOO CHARCOAL, AND MOLDING | CN104291797A | WANG YING (CN)\ YANG XIAOLIANG US\ CAO FEI US\ LIU YUNTAO US\ SHAO LEI US | HUNAN HUALEI OPTOELECTRONIC CORP US | A10-E05B\ A12-W12G\ L02-A02\ L02-A03\ L02-A04\ L02-A05\ L02-A07\ L02-A08\ L02-A12\ L02-G02\ L02-J02A |
| 6100 | 3D printing | PRODUCTION OF MICROCRYSTALLINE GLASS-CERAMIC COMPOSITE PLATE INVOLVES PRESSING POWDER, FORMING E.G. ENAMEL LAYER, SINTERING, APPLYING SUSPENSION OBTAINED USING FRIT PARTICLES AND BINDER, COOLING AND POLISHING SEMI-FINISHED PRODUCT | CN104291874A | WANG YONGHUAI US\ WANG JIANGUO US\ CHEN HANNING US | UNIV HARBIN ENG US | A09-D03\ S06-G |
| 6101 | 3D printing | THREE-DIMENSIONAL MODELING APPARATUS USED IN MOLTEN MATERIAL DEPOSITION SYSTEM, HAS MODELING MATERIAL THAT IS SUPPLIED ON MODELING STAGE FROM FORMATION PORTIONS, SO THAT THREE-DIMENSIONAL MOLDED ARTICLE IS MODELED | WO2015049834A1 | WANGCHENG SHI (CN)\ JIAMING XU US | UNIV HARBIN ENG US | A05-E06B\ A12-E07\ L03-A01A3\ L03-A02D\ L03-H02\ L03-H04E4\ L03-H05\ V04-R02B |
| 6102 | 3D printing | ELECTRICALLY CONDUCTIVE PASTE FOR ELECTRONIC MATERIAL HAS ELECTROCONDUCTIVE METAL POWDER, BAKING BINDER, RESIN BINDER, SOLVENT AND ADDITIVE, AND HAS PREDETERMINED ELECTROCONDUCTIVITY BY PERFORMING SUPERHEATED STEAM PROCESS | JP2015065123A | WANN CLEMENT HSINGJEN (US)\ KO CHIH-HSIN (TW) | BAOJI ZHONGLI GENERAL ELECTRIC APPLIANCE CO LTD US | A04-B01E\ A08-M09A\ A08-R05\ A09-A03\ A10-E24\ A12-E01\ L03-A01A3\ L03-A02E\ V04-R02P\ V04-X01B1\ X12-D01F1\ X12-D02A1 |
| 6103 | 3D printing | ELECTRICALLY CONDUCTIVE PASTE FOR ELECTROCONDUCTIVE FILM OR ELECTROCONDUCTIVE PATTERN CONTAINS ELECTROCONDUCTIVE FILLER AND RESIN, AND ELECTROCONDUCTIVE FILLER CONTAINS METAL POWDER OF PREDETERMINED AVERAGE PARTICLE SIZE | JP2015065139A | WANN CLEMENT HSINGJEN (US)\ KO CHIH-HSIN (TW) | SEMICONDUCTOR MFG INT SHANGHAI (CN)\ SEMICONDUCTOR MFG INT BEIJING (CN) | A05-G01E2\ A08-M01D\ A08-S02\ A12-E06A\ L03-E04B\ L03-E05B2\ L04-C10E\ L04-C11C\ L04-C14A\ L04-C16\ U11-A08B2\ X15-A01A\ X15-A02A\ X16-C01A |
| 6104 | 3D printing | ELECTROCONDUCTIVE PASTE COMPOSITION FOR FORMING SINTERED COMPONENTS, COMPRISES POLYURETHANE RESIN OBTAINED BY REACTING POLYOXYALKYLENE GLYCOL WITH DIISOCYANATE, SOLVENT, ELECTROCONDUCTIVE POWDER, COUPLING AGENT, AND BLOCK ISOCYANATE | JP2015063590A | WANN CLEMENT HSINGJEN (US)\ KO CHIH-HSIN (TW) | WANN CLEMENT HSINGJEN (US)\ KO CHIH-HSIN (TW) | L01-F03F\ L01-G02\ L01-H09E\ L01-L05\ S06-K03\ V07-F01A1\ V07-F01A3A\ X25-B02\ X25-C |
| 6105 | 3D printing | 3D PRINT-DOPED OPTICAL FIBER PREFABRICATED STICK MANUFACTURING SYSTEM, HAS PREFABRICATED ROD FIXED BETWEEN HEATING ROD AND HIGH FREQUENCY ELECTROMAGNETIC HEATING FURNACE, WHERE HEATING ROD AND PREFABRICATED ROD ARE FIXED WITH SECOND MOTOR | CN104355531A | WANN CLEMENT HSINGJEN (US)\ KO CHIH-HSIN (TW)\ WU CHENG-HSIEN (TW) | TAIWAN SEMICONDUCTOR MFG (TW) | A04-F01A\ A04-F04\ A12-W12F\ M23-A01\ M23-A02\ M23-A04\ V04-A01\ V04-R04A5\ V04-S15\ X24-A01A |
| 6106 | 3D printing | SOLDERING FLUX FOR USE IN SOLDER PASTE COMPOSITION I.E. UTILIZED FOR ELECTRONIC PRODUCTS, COMPRISES BASE RESIN, ACTIVATOR CONTAINING ACRYLIC RESIN, AND SOLVENT CONTAINING CYCLOHEXANE COMPOUND HAVING CARBOXYL GROUP IN CYCLOHEXYL SKELETON | US2015090366A1 | WANN CLEMENT HSINGJEN (US)\ KO CHIH-HSIN (TW)\ WU CHENG-HSIEN (TW) | WANN CLEMENT HSINGJEN (US)\ KO CHIH-HSIN (TW) | A03-A02\ A03-A03\ A08-S02\ A12-E07\ L02-G07\ L02-K\ L03-A01A3\ L03-H05\ V01-B03A5\ V01-B03B\ V01-B03C3A\ V01-B03D5\ V04-Q30B\ V04-R02P |
| 6107 | 3D printing | ELECTROCONDUCTIVE PASTE FOR CERAMIC ELECTRONIC COMPONENT, CONTAINS PRESET AMOUNT OF ORGANIC SOLVENT IN VEHICLE CONTAINING NICKEL POWDER HAVING PRESET AVERAGE PRIMARY GRAIN SIZE, AND CELLULOSE ACETATE BUTYRATE AS BINDER RESIN COMPONENT | WO2015040916A1 | WANN CLEMENT HSINGJEN (US)\ KO CHIH-HSIN (TW)\ WU CHENG-HSIEN (TW) | TAIWAN SEMICONDUCTOR MFG (TW)\ WANN CLEMENT HSINGJEN (US)\ KO CHIH-HSIN (TW)\ WU CHENG-HSIEN (TW) | A08-R03\ A10-E05B\ A11-B16\ T01-J10 |
| 6108 | 3D printing | METHOD FOR MAKING THREE DIMENSIONAL ARTICLE, INVOLVES DEPOSITING ALTERNATING LAYERS OF BINDER AND FILLER TO FORM ARTICLE, HEAT TREATING ARTICLE IN NON-OXIDIZING ENVIRONMENT, AND INFUSING ARTICLE WITH IMPREGNATING PITCH | WO2015038260A2 | WANWEN SHI US\ ZHIMING CHEN US\ ZHOUPING ZHANG US\ SHIGEN JIANG US\ TAN DU US | TAIWAN SEMICONDUCTOR MFG (TW)\ WANN CLEMENT HSINGJEN (US)\ KO CHIH-HSIN (TW)\ WU CHENG-HSIEN (TW) | A10-E05B\ A12-W12G\ L02-A02\ L02-A03\ L02-A04\ L02-A05\ L02-A07\ L02-A08\ L02-A12\ L02-D14S\ L02-G02\ L02-J02A |
| 6109 | 3D printing | PRODUCTION OF MICROCRYSTALLINE GLASS-CERAMIC COMPOSITE PLATE INVOLVES FORMING BRICK BY PRESSING POWDER MATERIAL, SINTERING, OBTAINING CLINKER PARTICLES, SPRAYING BINDER CONTAINING CELLULOSE THICKENER, SINTERING, COOLING, AND POLISHING | CN104291871A | WEE TEO LEE US\ FU CHONG YUNG US\ CHAN ALAIN (SG)\ ELGIN QUEK KIOK BOONE (SG) | TAIWAN SEMICONDUCTOR MFG (TW)\ WANN CLEMENT HSINGJEN (US)\ KO CHIH-HSIN (TW)\ WU CHENG-HSIEN (TW) | L03-E01B\ L03-E01D1\ L03-E01D5\ X16-B01F1\ X16-F01C\ X16-F03A3\ X16-X |
| 6110 | 3D printing | ALUMINUM DIE-CASTING LITHIUM BATTERY PROTECTIVE COVER HAS RING THAT IS CONNECTED ACROSS ANODE LUG AND CATHODE LUG, AND ALUMINUM SHELL COVER PLATE THAT IS PROVIDED WITH LUG IN INNER GROOVE OF RING | CN204179168U | WEI CHUANYONG US | SUZHOU HUAXIN MICROELECTRONICS CO LTD US | X26-D01A\ X26-D01B\ X26-F\ X26-H |
| 6111 | 3D printing | SHELL ARRANGED SHAPING MATERIAL LED LIGHT SOURCE, HAS LUMINOUS LIGHT SOURCE STRUCTURE PROVIDED WITH SINGLE-FACE LIGHT-EMITTING LIGHT SOURCE, DOUBLE SURFACE LIGHT-EMITTING SOURCE AND THREE-DIMENSIONAL MODELING LIGHT EMITTING SOURCE | CN204164792U | WEI RONGSHAN US\ CHEN SHOUCHANG US\ CHEN JINFENG US\ HE MINGHUA US | CHARTERED SEMICONDUCTOR MFG (SG) | G05-F |
| 6112 | 3D printing | LABEL FOR ADHERING TO PLASTIC BOTTLE, COMPRISES ORIENTED POLY PROPYLENE FILM LAYER, BONDING LAYER FORMED IN ONE SIDE OF ORIENTED POLY PROPYLENE FILM LAYER, AND PRINTING LAYER FORMED IN OTHER SIDE OF ORIENTED POLY PROPYLENE FILM LAYER | KR2015031811A | WEI RONGSHAN US\ CHEN SHOUCHANG US\ CHEN JINFENG US\ HE MINGHUA US | WEI CHUANYONG US | A04-E02E1\ A11-C03A\ A12-E07A\ L03-H04E\ V04-R01A6 |
| 6113 | 3D printing | HIGH DENSITY PRINTED CIRCUIT BOARD PRODUCTION METHOD USING WASTE POWDER GRANULES, INVOLVES ADDING PROPER AMOUNT OF AUXILIARY AGENT COMPOSING MIXED MATERIAL, AND COOLING AND CUTTING HIGH DENSITY PLATE USING CONTINUOUS EXTRUSION DIE HEAD | CN104327461A | WEI ZHANG (CN)\ PEIXIN QIAN (CN)\ RONGHUA LIU (CN)\ HUIWANG LIN (CN)\ ZHIHONG LIU (CN)\ CHANGCHUN CHEN (CN) | UNIV FUZHOU US | A10-E09B\ A12-E11B\ A12-W16\ X12-D01F1\ X12-D07\ X15-A01A\ X15-A02A\ X15-A02F |
| 6114 | 3D printing | METHOD FOR PREPARING NANOSILVER CONDUCTIVE PASTE UTILIZED IN NANOCRYSTALLINE SILICON SOLAR CELL, INVOLVES PREPARING NANOSILVER POWDER AND ORGANIC CARRIER, AND MIXING NANOSILVER POWDER WITH ORGANIC CARRIER, SURFACTANT AND GLASS POWDER | CN104282357A | WEI ZHOU (CN)\ ZHIHONG LIU (CN)\ LIREN YAN (CN) | UNIV FUZHOU US | A03-A03\ A08-S02\ A11-B05D\ A12-E01\ L03-A01A3\ S06-G |
| 6115 | 3D printing | ELECTRICALLY CONDUCTIVE PASTE FOR CERAMIC ELECTRONIC COMPONENT FORMATION COMPRISES NICKEL POWDER, BINDER RESIN COMPONENT, AND ORGANIC SOLVENT IN WHICH BINDER RESIN COMPONENT INCLUDES CELLULOSE ACETATE BUTYRATE AND SYNTHETIC MACROMOLECULE | JP2015060652A | WEIDONG NIE (CN)\ WEIMIN ZHU (CN)\ FAYOU YI (CN)\ DONGQIN CHEN (CN) | UNIV TSINGHUA (CN) | S06-G10\ S06-K03 |
| 6116 | 3D printing | CERAMIC CASTING TYPE 3D PRINTING METHOD, INVOLVES CONNECTING BINDER WITH INNER PART OF 3D PRINT HEAD, AND PERFORMING CASTING PROCESS BY TO ACHIEVE CERAMIC CASTING TYPE PRODUCT TO OBTAIN HIGH INTENSITY AND HARDNESS | CN104339437A | WEIGUO WU (CN) | UNIV TSINGHUA (CN) | W01-C01B3E\ W01-C01D3C |
| 6117 | 3D printing | DISPLAY DEVICE FOR MOBILE PHONE, HAS BONDING UNIT COMBINED WITH ACCOMMODATING CHAMBER THAT ACCOMMODATES SUBSTRATE, AND DECO LAYER FOR COVERING WIRING PART AND SUBSTRATE, WHERE BONDING UNIT IS OVERLAPPED IN PART OF DECO LAYER | KR2015030011A | WEIGUO WU (CN) | WUXI JINGYUAN MICROELECTRONICS (CN) | B06-H\ B07-H\ B10-A08\ B10-A10\ B10-A11B\ B10-A12C\ B10-A13D\ B10-A18\ B10-B01\ B14-C03\ B14-D07\ B14-E08\ B14-E10\ B14-G02\ B14-H01\ B14-J01\ B14-N03\ B14-N04\ B14-N17 |
| 6118 | 3D printing | NEW DIAMINE DERIVATIVE USEFUL IN PHARMACEUTICAL COMPOSITION FOR TREATMENT OF DISEASES INCLUDING ACUTE OR CHRONIC INFLAMMATION, ALLERGIC REACTIONS, ALLERGIC CONTACT DERMATITIS, ALLERGIC RHINITIS AND CROHN'S DISEASE | US2015080382A1 | WELCH DAVID F (US)\ NAGARAJAN RADHAKRISHNAN L (US)\ CHIANG TING-KUANG (US)\ DOMINIC VINCENT G (US)\ KISH JR FRED A (US) | GUANGZHOU NANKE INTEGRATION EL (CN) | A12-G\ F03-C\ F03-E01\ F03-F05\ F03-F10\ F03-F17\ F03-F32 |
| 6119 | 3D printing | NANO-FAR-INFRARED ANION PIGMENT PASTE USED FOR PRINTING TEXTILE, COMPRISES NANO FAR-INFRARED ANIONIC POWDER, COLORANT, SELF-CROSSLINKING BINDER, THICKENER AND WATER | CN104264503A | WELCH DAVID F (US)\ NAGARAJAN RADHAKRISHNAN L (US)\ CHIANG TING-KUANG (US)\ DOMINIC VINCENT G (US)\ KISH JR FRED A (US) | GUANGZHOU NANKER INTEGRATED EL (CN) | E11-Q03J\ E31-A03\ J04-C02\ J04-C04A\ S01-H03B\ S03-E02\ V04-Q08\ V04-Q30Q\ V04-S01A\ X27-A03 |
| 6120 | 3D printing | MULTI-REDUNDANCY HYDROGEN SENSOR, HAS SEAL JOINT AND PROBE CONNECTED WITH FOUNDATION BED, SHELL WHOSE TOP PART IS CONNECTED WITH ELECTRIC CONNECTOR, AND AIR INLET AND AIR OUTLET FORMED WITH GUIDE HOLE THAT IS MATCHED WITH DETECTING HOLE | CN204154671U | WEN OU US | INFINERA CORP (US) |  |
| 6121 | 3D printing | POLISHING CERAMIC TILE BRICK BODY TEXTURE PRINTING DEVICE, HAS PRINT OPERATION MODULE CONNECTED WITH BELT CONVEYING DEVICE, AND PRINT OPERATION MODULE CONNECTED WITH NOZZLE MOUNTING PLATE, WHERE BELT CONVEYING DEVICE IS PIPELINE | CN104325548A | WEN OU US | INFINERA CORP (US) | A05-G01E1\ A08-D05\ A10-D\ A10-E07B\ A10-E24\ G02-A02C\ G02-A02D\ G02-A03\ G02-A04A\ G03-B02D1\ G03-B04 |
| 6122 | 3D printing | ACTIVE ENERGY RAY-CURABLE RESIN COMPOSITION USED IN AGENT FOR COATING METALLIC SUBSTRATES, COMPRISES URETHANE (METH)ACRYLATE-TYPE COMPOUNDS, ACRYLIC RESIN, AND PHOSPHORIC ACID GROUP-CONTAINING ETHYLENICALLY UNSATURATED COMPOUND | JP2015052121A | WILLIAMS RICHARD K (US)\ DISNEY DONALD R (US)\ CHAN WAI T (CN) | JIANGSU INTERNET THINGS RES AND DEV CT US | A12-B\ A12-V01\ A12-W15\ B04-C03\ B12-M10\ B12-M11D |
| 6123 | 3D printing | PHARMACEUTICAL COMPOSITION CONTAINING FLOATING SUSTAINED-RELEASE PELLETS COMPRISES MEDICINE-CONTAINING PELLET CORE COATED WITH SUSTAINED-RELEASE COATING LAYER AND BLEACHING LAYER | CN104288107A | WILLIAMS RICHARD K (US)\ DISNEY DONALD RAY (US)\ CHAN WAI TIEN (CN) | JIANGSU INTERNET THINGS RES AND DEV CT US | A04-F04\ A05-G01E\ A05-H01B\ A10-E05B\ A12-R04\ A12-W12G\ L02-A03\ L02-A04\ L02-A07\ L02-A08\ L02-A12 |
| 6124 | 3D printing | SINTERING MICRO-CRYSTALLINE GLASS COMPOSITE PLATE MANUFACTURING METHOD, INVOLVES PERFORMING PRESSING-TYPE MOLDING PROCESS, AND PERFORMING POLISHING-TYPE GRINDING PROCESS BY UTILIZING MICRO-CRYSTALLINE GLASS COMPOSITE PLATE | CN104291877A | WILLIAMS RICHARD K (US)\ RYU HYUNGSIK (US)\ CHEN JUN-WEI (US)\ DISNEY DONALD R (US)\ CHAN WAI T (CN) | ADVANCED ANALOGIC TECH INC (US)\ ADVANCED ANALOGIC TECH INC (CN) | D04-A01\ D04-B03\ D04-B06B\ D04-B07D\ E10-C02A\ E10-C02D1\ E10-C02F\ E11-Q02B\ E24\ E31-F01B\ E31-P02D\ E34-D01\ F05-A02C |
| 6125 | 3D printing | SPONGE IRON-BASED WATER PURIFYING AGENT USED FOR E.G. PAPERMAKING WASTEWATER, COMPRISES ACTIVE COMPONENT CONTAINING SPONGE IRON, ZINC POWDER AND SILVER POWDER, BINDER AND PORE-FORMING ADDITIVE CONTAINING TARTARIC ACID AND CITRIC ACID | CN104261549A | WILLIAMS RICHARD K (US)\ RYU HYUNGSIK (US)\ CHEN JUN-WEI (US)\ DISNEY DONALD R (US)\ CHAN WAI T (CN) | DISNEY DONALD RAY (US)\ CHAN WAI TIEN (HK)\ WILLIAMS RICHARD KENT (US) | A03-A05A\ A03-C01\ A11-B05\ A12-S05Q\ A12-W07D\ F03-C05\ F03-F02\ F03-F03\ F03-F09\ F03-F11\ F03-F19\ F03-F31\ F03-F32\ S06-G10\ S06-H02A\ X25-H03 |
| 6126 | 3D printing | THERMAL FOAMED OR THERMAL BUBBLE PRINTER REACTIVE DYE INK DIGITAL PRINTING COMPRISES SOAKING TEXTILE IN POST-PROCESSING LIQUID HAVING E.G. SODIUM BICARBONATE, DRYING, ROLLING, PRINTING, EVAPORATING, FIXING COLOR, DRYING, SHAPING AND ROLLING | CN104213443A | WILLIAMS RICHARD K (US)\ RYU HYUNGSIK (US)\ CHEN JUN-WEI (US)\ DISNEY DONALD R (US)\ CHAN WAI T (CN) | ANVANCED ANALOGIC TECHNOLOGIES INC (US) | M22-H03B\ A11-C01C\ A12-L05\ A12-W12F\ S06-E04B1\ S06-E04D\ S06-G10\ S06-K03\ S06-K07A\ S06-K99C\ X25-A01\ X25-A08 |
| 6127 | 3D printing | METAL LASER SINTERING 3D PRINTER COMPRISES BASE, LIFTING WORKING TABLE, BRACKET, POWDER SPREADING DEVICE, EXTRUSION NOZZLE DEVICE, LASER PRINTING DEVICE, SHELL, COMPUTER CONTROL SYSTEM, | CN204018721U | WILLIAMS RICHARD K (US)\ RYU HYUNGSIK (US)\ CHEN JUN-WEI (US)\ DISNEY DONALD R (US)\ CHAN WAI T (CN) | ADVANCED ANALOGIC TECH INC (US)\ WILLIAMS RICHARD K (US)\ DISNEY DONALD R (US) | A05-H03\ A05-H04\ A10-E05B\ A12-W11\ E10-A09B4\ E10-G02E\ E11-F06\ E12-A10\ E31-P01\ E31-P02D\ J04-E04\ J04-E11\ N05-E02\ N06-E01\ N06-F\ N07-D07 |
| 6128 | 3D printing | SPHERICAL MESOPOROUS MONTMORILLONITE COMPOSITE CARRIER FOR PREPARING ETHYL ACETATE, CONTAINS SPHERICAL MESOPOROUS COMPOSITE MATERIAL AND MESOPOROUS MATERIAL WITH THREE-DIMENSIONAL CUBIC PORE STRUCTURE, AND HAS SPECIFIED PORE VOLUME | CN104248991A | WOO HYUK (KR) | ADVANCED ANALOGIC TECH INC (US)\ WILLIAMS RICHARD K (US)\ DISNEY DONALD R (US) | A11-B09D\ A11-C01D |
| 6129 | 3D printing | METHOD FOR MODELING THREE-DIMENSIONAL STRUCTURE, INVOLVES LAMINATING POWDER LAYER IN MODELING TANK, VIBRATING POWDER IN MODELING TANK BEFORE DRIPPING LIQUID TO POSITION OF POWDER LAYER, WHERE POWDER LAYER IS PROVIDED WITH POWDER | JP2015044304A | WU FANG (CA)\ PIMENOV KIRILL (CA)\ TOLSTIKHIN VALERY (CA)\ LOGVIN YURI (CA) | ADVANCED ANALOGIC TECH INC (US)\ WILLIAMS RICHARD K (US)\ DISNEY DONALD R (US) | M22-C01\ M22-D\ M22-G03D1\ M22-G03D2\ M22-G03H\ M22-G03N |
| 6130 | 3D printing | CASTING METHOD OF CAST ALLOY COMPONENT HAVING CONVOLUTED INTERNAL PASSAGEWAYS USED IN AEROSPACE AND POWER INDUSTRIES INVOLVES REMOVING CAST CERAMIC CORE FROM HARDENED ALLOY MATERIAL TO REVEAL CAST ALLOY COMPONENT HAVING INTERIOR PASSAGEWAY | US2015075746A1 | WU HANMING (CN)\ CHEN JOHN (CN)\ ZHU BEI (CN)\ GAO DA WEI (CN)\ BONFANTI PAOLO (CN) | DONGBUANAM SEMICONDUCTOR INC (KR) | A11-B16\ A11-C02\ A12-R01 |
| 6131 | 3D printing | THREE-DIMENSIONAL PRINTING METHOD FOR MAKING ARTICLES FROM POWDER BUILD MATERIAL E.G. CERAMIC, INVOLVES SPREADING LAYER OF BUILD MATERIAL POWDER PARTICLES, AND SELECTIVELY PRINTING FLUID ONTO LAYER OF BUILD MATERIAL POWDER PARTICLES | US2015069649A1 | WU HANMING (CN)\ CHEN JOHN (CN)\ ZHU BEI (CN)\ GAO DA WEI (CN)\ BONFANTI PAOLO (CN) | ONECHIP PHOTONICS INC (CA)\ LOGVIN YURI (CA)\ WU FANG (CA)\ PIMENOV KIRILL (CA)\ TOLSTIKHIN VALERY (CA) | A05-E04C\ A05-E04E\ A11-A05\ A11-B\ A11-B05\ A11-B12A\ A11-C01C\ A11-C04A\ A12-W07D\ A12-W07F\ W01-C01D3C |
| 6132 | 3D printing | METHOD FOR PREPARING PHOTOSENSITIVE PLASTIC MOBILE PHONE CASE, INVOLVES PREPARING PROTECTION INK LAYER WITH SPECIFIC THICKNESS, AND BONDING PREPARED PROTECTION INK LAYER INTO FINISHED PRODUCT AFTER INJECTION MOLDING OF PET SHEET | CN104260273A | WU HANMING ZHANG (CN) | SEMICONDUCTOR MFG INT SHANGHAI (CN)\ SEMICONDUCTOR MFG INT BEIJING (CN)\ WU HANMING (CN)\ CHEN JOHN (CN)\ GAO DA WEI (CN)\ ZHU BEI (CN)\ BONFANTI PAOLO (CN) | A03-A05\ A04-F04A\ A06-A00B\ A08-M03\ A08-M10\ A08-R01\ A08-S02\ A11-A03\ A11-B05D\ A12-B04\ A12-P06C\ G02-A02A\ G02-A02D2\ G02-A03D\ B04-C02A1\ B04-C03\ B11-C06D\ S06-K07A |
| 6133 | 3D printing | PUSH-THROUGH-PACK PRIMER USED IN LASER-PRINTING OF PHARMACEUTICAL BLISTER PACKAGE, COMPRISES PLANT-DERIVED CELLULOSE-BASED RESIN AS MATRIX RESIN, BINDER RESIN, ISOCYANATE CURING AGENT, LUBRICANT, INORGANIC POWDER AND LEVELING AGENT | CN104263092A | WU JIAN (SG)\ GU XIAOFENG US\ LIANG HAILIAN US\ DONG SHURONG US\ HUANG LONG US | SEMICONDUCTOR MFG INT SHANGHAI (CN) | A11-A05\ A11-B01\ A11-B09A2\ A11-C02D\ A12-E08\ L03-B02\ V02-E\ V02-H04\ X12-C01D1 |
| 6134 | 3D printing | MAGNETIC FIELD ORIENTATED THREE-DIMENSIONAL PRINTED ANISOTROPIC BONDED MAGNET IS SEMI-CONTINUOUS OR CONTINUOUS ORIENTATION-CHANGE ANISOTROPIC BONDED MAGNET, AND PREPARED BY FORMING SHEET LAYER BY MOLDING ORIENTATED MAGNET POWDER | CN104269265A | WU XINYAN US | ZHONGXIN INT IC MFG SHANGHAI (CN) | A04-F06E4\ A05-F01E\ A11-C01C\ A12-E11A\ A12-W11G\ A12-W14\ L03-H03A\ U11-A01D\ U11-A09\ U11-A14\ W04-D10\ W04-M01B1\ W04-M01D3 |
| 6135 | 3D printing | CARBON NANOCOMPOSITE MATERIAL, USEFUL FOR E.G. THREE DIMENSIONAL PRINTING MATERIAL, HEAT EXCHANGER, NOTEBOOK COMPUTER, HIGH-POWER LED LIGHT, AND DIGITAL CAMERA, COMPRISES CARBON-BASED MICRO-NANO POWDER FILLER, AND POLYMER BONDING AGENT | CN104130753A | WU YANGBO US\ FAN XIAOHUI US\ NI HAIYAN US\ HU JIANPING US | UNIV JIANGNAN US | S06-E04A\ S06-E04C\ S06-K02\ S06-K03A\ S06-K06C2\ S06-K99C |
| 6136 | 3D printing | LASER PRINTER CARBON POWDER BOX CABIN STRUCTURE, HAS CABIN SHELL BODY CONNECTED WITH SEAL COVER, TRANSFER BAG CONNECTED WITH BAG BODY, AND POWDER CABIN CONNECTED WITH BAG NOZZLE AND SEAL RING ALONG AXIAL DIRECTION | CN204044511U | XIA AN US\ RU HUANG (CN)\ FEI TAN US\ WEIKANG WU US\ LIANGXI HUANG US | WU XINYAN US | S06-K03\ S06-K99C |
| 6137 | 3D printing | LASER PRINTER CARBON POWDER BOX CHAMBER, HAS CHAMBER UNIT PROVIDED WITH SHELL THAT IS FORMED WITH POWDER OUTLET, TRANSFER BAG FILLED WITH CARBON POWDER, AND BAG BODY CONNECTED WITH BAG NOZZLE AND FILLED WITH CARBON POWDER | CN204044513U | XIA AN US\ RU HUANG (CN)\ QIANQIAN HUANG US\ FEI TAN US\ DONG YANG US\ XING ZHANG (CN) | UNIV NINGBO US | A11-A02C\ A11-A05A\ A11-B07\ A11-B17\ A11-C04A\ A12-H02\ A12-T01A |
| 6138 | 3D printing | INNER SICULA MOLDING TIRE EXTRUDING METHOD, INVOLVES COATING EXTERNAL SURFACE OF TIRE TUBE WITH SPRAY POWDER, PERFORMING SICULA CUTTING PROCESS, AND FIXING CUTTING SURFACE OF TIRE TUBE WITH PIPE JOINT | CN104275816A | XIA JUN US\ WU HAO US | UNIV BEIJING (CN) |  |
| 6139 | 3D printing | GLAZE POWDER HEAT INSULATING CORROSION-PROOF AND WATERPROOF ANTI-IMPACT ENAMEL CONTAINER, HAS MACHINE ARRANGED WITH HIGH TEMPERATURE RESISTANT CERAMIC CLOTH, AND DOOR LOCK PART CONNECTED WITH GLASS FIBER REINFORCED TYPE ENAMEL CONTAINER | CN104276364A | XIANG QI (US) | UNIV BEIJING (CN)\ HUANG RU (CN)\ HUANG QIANQIAN (CN)\ TAN FEI (CN)\ AN XIA (CN)\ YANG DONG (CN)\ ZHANG XING (CN) | F04-B01B |
| 6140 | 3D printing | THREE-DIMENSIONAL ARTIFICIAL LEATHER WITH COLORFUL EFFECT COMPRISES A MUTUALLY BONDED SURFACE LAYER AND BASE LAYER, WHERE THE SURFACE LAYER IS ADHERED WITH A PEARL POWDER LAYER, AND THE PEARL POWDER LAYER IS ADHERED WITH A TRANSPARENT FILM | CN104294644A | XIANZHOU LIU US | XIAMEN TIANMA MICRO ELECTRONICS CO LTD US | A03-A00A\ A03-A04A\ A04-F06E\ A06-A00E\ A10-E05B\ A12-W07D\ G02-A04A |
| 6141 | 3D printing | NON-PENETRATING SOLID COLOR PRINTING INK INCLUDES EDIBLE ALCOHOL, EDIBLE SALT, POTASSIUM SORBATE, CHAMOMILE ESSENTIAL OIL, SOYBEAN OIL, CASTOR OIL, BEAN DREGS, CASTOR SHELL, CHESTNUT SHELL, XANTHAN GUM, MALT SYRUP, AND CAPSANTHIN | CN104277578A | XIANZHOU LIU US\ YAZHEN WU US | GLOBALFOUNDRIES INC (KY) |  |
| 6142 | 3D printing | REPAIRING METHOD OF FLIP-CHIP BONDING DEVICE, INVOLVES SUBTRACTING PREVIOUSLY MEASURED THICKNESS OF ELEMENT AND WELDING POINT HEIGHT WITH VERNIER CALIPER MULTI-TIMES IN DIFFERENT PIN MAIN PORTION TOP SURFACE TO WELDING POINT SIZE | CN104259609A | XIAOYAN XU US\ RU HUANG (CN)\ SHOUBIN XUE US\ XING ZHANG (CN) | SHANGHAI GRACE SEMICONDUCTOR US | A02-A01\ A03-A04\ A03-A05A\ A04-F01A1\ A04-G07\ A05-G01E\ A08-E01\ A08-F01\ A08-M02\ A08-P08\ A08-S05\ A10-E04A\ A10-E09\ A12-C00C\ F01-F01\ F01-F02\ F01-G00G\ F01-H01\ F01-H03D\ F03-C02B\ F03-F03\ F03-F04\ F03-F09\ F03-F18\ F03-F33\ X25-T04B\ X25-T04D |
| 6143 | 3D printing | BAMBOO FIBER FABRIC PREPARED BY SPRAYING ANTIBACTERIAL SOLUTION ON SURFACE OF AN ANTIBACTERIAL BAMBOO FIBER, PERFORMING MICROWAVE TREATMENT OF THE SPRAYED BAMBOO FIBER, AND DYEING DISPERSE DYE ON SPRAYED ANTIBACTERIAL BAMBOO FIBER | CN104264444A | XIN PAN US\ QINGLIN SONG US | SHANGHAI GRACE SEMICONDUCTOR US | A11-B05\ A11-C02C\ A12-E07A\ L03-G06\ L03-H04E\ V04-Q02A5\ V04-R05D\ V04-S02\ V04-S10\ V04-U03\ V04-U15 |
| 6144 | 3D printing | MANUFACTURING COVER LAYER OF FLEXIBLE PRINTED CIRCUIT BOARD INVOLVES PREPARING MIXTURE OF METAL POWDER AND POLYMERIC BINDER FOR FIXING ELECTROMAGNETIC SHIELD, AND USING FLEXIBLE BASE LAYER FOR SUPPORTING POLYMERIC BINDER COVER LAYER | KR2015015208A | XINYU LIU (CN)\ HUANMING LIU US\ JINGTAO ZHOU US\ CHENGYUE YANG US\ HONGGANG LIU US\ HUAJUN SHEN US\ DEXIN WU US | UNIV BEIJING (CN) | A11-C02\ A12-W10A\ G02-A04A\ H01-C03\ L02-A04\ T01-C05A |
| 6145 | 3D printing | CERAMIC INK FOR ADDITIVE FORMATION OF PROPPANTS COMPRISES CERAMIC MATERIAL AND BINDING AGENT | WO2015023612A2 | XINYU LIU (CN)\ WEIJUN LUO US\ XIAOJUAN CHEN US\ LEI PANG US\ NING WANG US\ ZHONGZI CHEN US | GOERTEK INC US | A06-A00B\ A08-R\ L03-E05B\ L03-H04E1\ U11-A07\ U11-D01B\ U11-E02A1\ U12-A01A4\ U12-A02A4B\ U12-A02A4E\ X15-A01A\ X15-A02A |
| 6146 | 3D printing | SILICONE RESIN BASE MATERIAL FOR PRODUCTS E.G. LIGHT-EMITTING DIODE SUBSTRATE, IS OBTAINED BY MOLDING COMPOSITION CONTAINING POLYSILOXANE WHICH IS THREE-DIMENSIONALLY CROSSLINKED TO FORM SILICONE RESIN, BY ADDITION OR CONDENSATION | JP2015028188A | XU DANIEL (CN) | INST OF MICROELECTRONICS CAS (CN) | A09-D03\ A11-A02B\ A11-B01\ A11-C01D\ S06-E01\ S06-E04B1\ S06-G10\ S06-K07A\ S06-K99C |
| 6147 | 3D printing | LIGHT CURING 3D PRINTER, HAS SUPPORT CONNECTED WITH ELASTIC BLADE THAT IS FORMED WITH STRIP-SHAPED NOZZLE, AND COMPUTER CONTROL SYSTEM CONNECTED WITH LIQUID PHOTOSENSITIVE RESIN FEEDING SYSTEM AND ADJUSTABLE UV LIGHT OUTPUT SYSTEM | CN204095145U | XU HUA (CN) | INST OF MICROELECTRONICS CAS (CN) | A12-E08\ A12-V\ A12-V03C2\ A12-W14\ B11-C08A\ B11-C12\ B12-K04\ S03-E09X |
| 6148 | 3D printing | MULTIFUNCTIONAL SUPERPARAMAGNETIC NANOSYSTEM AS CONTRAST AGENT FOR MRI FORMED BY A CORE-SHELL STRUCTURE IN WHICH CORES ARE FORMED BY IRON OXIDE OR MIXED SPINELS NANOPARTICLES BEING COATED BY A HYBRID SILICA SHELL WITH INTERCONNECTED PORES | WO2015026252A1 | XU HUILONG US\ ZHANG ZHIYONG US\ PENG LIANMAO US\ WANG SHENG US | SEMICONDUCTOR MFG INT SHANGHAI (CN)\ XU DANIEL (CN) |  |
| 6149 | 3D printing | LASER PRINTER CARBON POWDER BOX STRUCTURE, HAS ELECTRIC CONDUCTION EDGE CABIN FIXEDLY CONNECTED WITH SEAL COVER THAT IS FORMED WITH FEEDING HOLE, AND TRANSFER BAG FIXEDLY CONNECTED WITH ELECTRIC CONDUCTION COVER AND BAG NOZZLE | CN204065664U | XU WENJING US\ YAO SUYING (CN)\ NIE KAIMING (CN)\ XU JIANGTAO (CN)\ GAO JING (CN)\ SHI ZAIFENG (CN)\ GAO CEN (CN) | UNIV TSINGHUA (CN)\ XU HUA (CN) | S06-E04A\ S06-E04C\ S06-E06\ S06-K02\ S06-K03A\ S06-K06C2\ S06-K99C |
| 6150 | 3D printing | IMPROVED LASER PRINTER CARBON POWDER BOX HAS OUTLET SEAL COVER WHOSE HOUSING SIDE WALL IS CONNECTED WITH CHAMBER, AND TRANSFER BAG WHICH IS SLEEVED IN INNER PORTION SET WITH POWDER AND BAG NOZZLE | CN204065665U | XU YANZHONG (US)\ WATT JEFFREY T (US) | UNIV BEIJING (CN) | A05-J\ A10-D\ A10-E05C\ A11-B05\ A12-E11\ A12-W07F\ A12-W16\ S06-G03\ S06-G06\ S06-K03\ S06-K06\ S06-K99C\ X15-A01A\ X15-A02 |
| 6151 | 3D printing | INKJET PRINT HEAD FOR USE IN INDUSTRIAL INKJET PRINTER USED IN E.G. DISPLAY APPARATUS, HAS PROTECTING LAYER ARRANGED ON INNER SURFACE OF MAIN BODY, INORGANIC LAYER ARRANGED ON PROTECTING LAYER, AND ORGANIC LAYER ARRANGED ON INORGANIC LAYER | US8960857B1 | XU YONG US\ WANG BO US\ WANG XI (CN) | UNIV TIANJIN (CN) | A11-A\ A11-B07C |
| 6152 | 3D printing | PLASTIC SILK EXTRUDER, HAS FEEDING SCREW ROD, ADD MATERIAL DEVICE, HEATING DEVICE, CHAIN WHEEL GROUP AND MOTOR THAT ARE CONNECTED WITH EACH OTHER, WHERE HEATING DEVICE IS CONNECTED WITH HALF REAR SURFACE OF MACHINE CYLINDER | CN204054572U | YAMADAYA MASAYUKI (JP) | ALTERA CORP (US) |  |
| 6153 | 3D printing | THREE-DIMENSIONAL PRINT PRINCIPLE BASED LED FLUORESCENT POWDER COATING SYSTEM, HAS MOVEMENT CONTROL MODULE FORMING THREE-DIMENSIONAL PRINCIPLE BASED FLUORESCENT POWDER SPRAY COATING LAYER ON LED CHIP THROUGH COATING CONTROL MODULE | CN204052092U | YAMAGISHI KAZUO US | AISINO CORP INC US | S06-G04\ S06-G06\ S06-K03\ S06-K07A\ S06-K99C |
| 6154 | 3D printing | INK STORAGE FUNCTIONAL PORTABLE CLOTHES SPRINKLE INK PRINTER, HAS INK BOX SHELL ARRANGED WITH INK OUTLET, AND BRACKET ARRANGED WITH CASTERS, TELESCOPIC POLE AND SUPPORTING ROD AND CONNECTED WITH INNER PART OF PEDESTAL | CN204054948U | YAN WANG US\ FENGYI HUANG US\ XUSHENG TANG US\ NAN JIANG US | FUJI ELECTRIC SYSTEMS CO LTD US |  |
| 6155 | 3D printing | MICRO-DECOMPRESSION PRESSURE PIPELINE STRUCTURE, HAS MAIN BODY FORMED WITH STRIP-SHAPED SOLID STRUCTURE BODY THAT IS FORMED AS TRIANGULAR OR HORN SHAPED THREE-DIMENSIONAL STRUCTURE BODY | CN204061860U | YANAGIDA YOHEI US | KANSAI NIPPON ELECTRIC US | A08-M09A\ A08-S02\ A09-A04\ A11-A03\ A11-A04\ A11-B05D\ A12-R07\ A12-S09A\ A12-W07F2\ G02-A05B |
| 6156 | 3D printing | SOFT MAGNETIC COATING FOR USE IN MAGNETIC COMPOSITE MATERIAL, COMPRISES FLAKY SOFT MAGNETIC POWDER, ADHESIVES, DILUENTS AND ADJUVANTS | WO2014201898A1 | YANCHEN WANG US | UNIV SOUTHEAST US\ S TEK SHANGHAI HIGH FREQUENCY COMM TECHNOLOGY CO LTD US | M22-H03B\ M22-H03F\ M22-H03G |
| 6157 | 3D printing | THREE-DIMENSIONAL LASER PUMP IMPELLER PRINTING APPARATUS HAS CLOSE CAVITY WHICH IS PROVIDED WITH GAS INLET AND AIR OUTLET, AND IS MOUNTED WITH INNER OXYGEN CONCENTRATION SENSOR | CN104226996A | YANCHEN WANG US | RENESAS TECH CORP (JP) | L03-G10C\ L03-H02\ M22-H03B\ S06-K03G\ S06-K07A\ T01-J07B\ T04-D07D3 |
| 6158 | 3D printing | METHOD OF PRINTING THREE-DIMENSIONAL METAL INVOLVES NESTING PRINT HEAD BETWEEN TWO PIECES OF HIGH MELTING POINT METAL BLOCK AND HIGH MELTING POINT SPHERICAL METAL BALLS, AND CONNECTING METAL BLOCKS INTO POWER INPUTS | CN104226997A | YANCHEN WANG US | YANCHEN WANG US | L02-A08\ L02-D09\ T01-J15X |
| 6159 | 3D printing | METHOD FOR PRINTING THREE-DIMENSIONAL CRAMIC MATRIX COMPOSITE NOZZLE SKIRT NIOBIUM ALLOY, INVOLVES REMOVING CONNECTOR UNDER HIGH TEMPERATURE UNTIL CONNECTOR IS PILED INTO THREE-DIMENSIONAL SOLID COMPONENTS | CN104211424A | YANG CHENG (CN)\ ZEXIN LIAO US\ ZHE ZHAO US\ FENG ZHOU US | YANCHEN WANG US | A06-A00B\ A08-S01\ A10-D\ L03-H04E9\ V04-Q02A4\ V04-R01A5\ V04-R03L\ V04-R05A\ V04-T03A\ V04-X01E |
| 6160 | 3D printing | PREPARATION OF ORGANIC-INORGANIC COMPLEX SOL USED FOR COMPOSITE FOR E.G. LED AND HEAT SINK, INVOLVES REACTING ALKYLSILANE AND ALUMINA SOL, ADDING SURFACTANT AND ORGANIC BINDER, MIXING, DRYING AT ROOM TEMPERATURE AND HEAT TREATING | KR2014139685A | YANG CHENG (CN)\ ZEXIN LIAO US\ ZHE ZHAO US\ FENG ZHOU US | YANCHEN WANG US | A04-G03E\ A10-E05B\ A12-E15\ A12-W07F\ A12-W12G\ L02-A02\ L02-A03\ L02-A04\ L02-G01E\ L02-G12A\ L03-G09A2\ L03-G10A1\ L03-G10C\ S06-E06A\ V06-V01E |
| 6161 | 3D printing | PREPARING PIEZOELECTRIC ELEMENT USED IN PRINT HEAD, INVOLVES HEATING PIEZOELECTRIC CERAMIC POWDER AND BINDER COMPRISING PARAFFIN WAX, POLYPROPYLENE AND OLEIC ACID, WHERE HEATED MIXTURE IS MIXED WITH BINDER TO OBTAIN ANOTHER MIXTURE | CN104230332A | YANG CHUN-CHU (TW) | UNIV FUDAN (CN)\ SHANGHAI SHENGRUIWO TECHNOLOGY DEV CO LTD US | A03-A04A\ A06-A00E1\ A10-E05B\ A12-W12G\ L02-A02\ L02-A03\ L02-A04\ L02-A05\ L02-A07\ L02-A12\ L02-G02 |
| 6162 | 3D printing | PRODUCTION OF MICROCRYSTALLINE GLASS CERAMIC COMPOSITE PLATE BY PROCESSING POWDER PRESSING FORMING BRICK, FORMING GLAZE AND FORMING PRINTING LAYER, SINTERING, CERAMIC BLANK AND SPRAYING LAYER OF BINDER | CN104230394A | YANG HAIGANG US\ ZHU WENRUI US | UNIV FUDAN (CN) | A10-E05B\ A11-B05\ A12-E10\ M22-H03B\ M22-H03F\ V04-X01B |
| 6163 | 3D printing | PREPARATION OF FLEXIBLE THERMOELECTRIC THIN FILM INCLUDES SYNTHESIZING THERMOELECTRIC MATERIAL INGOT, GRINDING, SIEVING, MIXING BINDER, DISPERSANT AND ORGANIC SOLVENT INTO SLURRY, MIXING WITH MATERIAL POWDER, AND DEPOSITING ON SUBSTRATE | CN104209524A | YANG HAINING (US)\ CHEN XIANGDONG (US)\ KO YOUNG G (US) | YANG CHUN-CHU (TW) | A10-E09B\ A12-A02\ G05-F\ M22-A01\ M22-A03\ M22-D |
| 6164 | 3D printing | REFRACTORY MATERIAL FOR THREE DIMENSIONAL POWDER PRINTER, HAS POWDER COMPONENT FOR FORMING MOLTEN METAL CASTING SHELL IN PRINTER, WHERE MATERIAL HAS HYDRAULIC BONDS WHEN LIQUID COMPONENT AND POWDER COMPONENT ARE COMBINED | US2015041098A1 | YANG HSIAO-LAN (TW) | CHINESE ACAD INST ELECTRONICS US | A11-B01\ X25-A08 |
| 6165 | 3D printing | METHOD FOR RECOVERY AND REGENERATION OF METAL POWDERS IN ELECTRON BEAM MELTING (EBM) APPLICATIONS, INVOLVES SUPPLYING METAL POWDERS HAVING PREDETERMINED LOW OXYGEN CONTENT AT ACCESS POINT ALONG RECOVERY AND REGENERATION PATH | EP2832528A1 | YANG SHAOJUN US | IBM (US)\ SAMSUNG ELECTRONICS CO LTD (KR) | A08-C01\ A08-D01\ A11-B05\ A11-B16\ A11-C04E\ A11-C06\ A12-V02\ D09-C01E\ B04-C02\ B04-C03\ B04-F01\ B04-H19\ B04-L05C\ B04-N02\ B11-C04\ S06-G10\ S06-K99C |
| 6166 | 3D printing | METHOD FOR FABRICATING E.G. BIOLOGICAL TISSUE OR TISSUE ENGINEERING SCAFFOLD DURING E.G. EMBEDDED FUSION MODELING FOR PRINTING SOLIDS, INVOLVES REMOVING SUPPORT MATERIAL TO RELEASE FABRICATED STRUCTURE FROM SUPPORT MATERIAL | WO2015017421A2 | YANGYUAN WANG US\ RU HUANG (CN)\ QIANQIAN HUANG US\ YINGXIN QIU US\ ZHAN ZHAN (CN) | TAIWAN SEMICONDUCTOR MFG (TW)\ YANG HSIAO-LAN (TW) | S06-D02A\ S06-E01D\ S06-E03A1\ S06-E03A2\ S06-G10\ S06-K07A\ T01-J08A |
| 6167 | 3D printing | 3D PRINTING PRINCIPLE BASED LED FLUORESCENT POWDER COATING SYSTEM, HAS LED CHIP WHOSE PROJECTION LIGHT POWDER LAYER IS COATED WITH PIEZOELECTRIC FLUORESCENT POWDER THROUGH COATING CONTROL MODULE AND MOTION CONTROL MODULE | CN104226568A | YANGYUAN WANG US\ RU HUANG (CN)\ QIANQIAN HUANG US\ ZHAN ZHAN (CN) | BEIJING ZHUOYUE MICROTECHNOLOGY CO LTD US | A12-R01A\ L02-C05\ L02-D14M\ L02-G08 |
| 6168 | 3D printing | PRINTING COMPOSITION USED IN THREE DIMENSIONAL PRINTING, COMPRISES GYPSUM POWDER, OXIDE POWDER, BINDER MATERIAL, REINFORCING AGENTS, SOLID LUBRICANTS, PRESERVATIVES, AND AUXILIARY AGENT | CN104230289A | YANGYUAN WANG US\ RU HUANG (CN)\ QIANQIAN HUANG US\ ZHAN ZHAN (CN) | UNIV BEIJING (CN)\ HUANG RU (CN)\ HUANG QIANQIAN (CN)\ ZHAN ZHAN (CN)\ WANG YANGYUAN (CN)\ QIU YINGXIN (CN) | M22-G03D1\ M22-G03D2\ V04-S01A\ W02-C03B1B\ W02-G03\ W03-A16A |
| 6169 | 3D printing | 4-OUTPUT I-TYPE SATELLITE RECEIVER STRUCTURE COMPRISES MACHINE SHELL MAIN BODY COMPRISING CIRCUIT PLATE MOUNTING GROOVE MOUNTED WITH PRINTED CIRCUIT BOARD PLATES ELECTRICALLY CONNECTED WITH COPPER BAR, AND F HEAD MOUNTING GROOVE WITH SLOTS | CN204013502U | YANGYUAN WANG US\ XIN HUANG US\ RU HUANG (CN)\ QIANQIAN HUANG US\ ZHAN ZHAN (CN) | UNIV BEIJING (CN)\ HUANG RU (CN)\ HUANG QIANQIAN (CN)\ ZHAN ZHAN (CN)\ WANG YANGYUAN (CN) | D03-P\ S06-D02A\ S06-E03A1\ S06-E03A3\ S06-K03\ S06-K07A1\ T01-J12\ V08-A04B\ V08-A09 |
| 6170 | 3D printing | MARKING A FOOD PRODUCT WITH A GRAPHICAL MARK COMPRISES PROVIDING A LASER LIGHT EMITTER, AND POSITIONING A WORKFRAME AT A USER-DEFINED DISTANCE FROM THE LASER LIGHT EMITTER | US2015030731A1 | YAN-MAN SO KOON-CHONG HSHIEH F (US) | UNIV BEIJING (CN) | A12-E13\ A12-W07D\ G02-A02E\ G02-A02F\ G02-A02G\ G02-A04A\ G02-A04B\ T01-J10B2A\ T04-D07F2 |
| 6171 | 3D printing | LUMINOUS PRINTING INK FOR FORMING INK LAYER OF FINGERPRINT RECOGNITION DEVICE, COMPRISES VARNISH, LUMINOUS POWDER CONTAINING RARE-EARTH ELEMENT-DOPED METAL ALUMINATE, BINDER CHOSEN FROM E.G. ALKYD RESIN AND EPOXY RESIN, AND ADDITIVE | CN104194485A | YAO SUYING (CN)\ NIE KAIMING (CN)\ XU JIANGTAO (CN)\ GAO JING (CN)\ SHI ZAIFENG (CN) | UNIV BEIJING (CN)\ HUANG RU (CN)\ HUANG XIN (CN)\ HUANG QIANQIAN (CN)\ ZHAN ZHAN (CN)\ WANG YANGYUAN (CN) | A11-B16 |
| 6172 | 3D printing | 3D-PRINTER POWDERED INSTANT INORGANIC BINDER MANUFACTURING METHOD, INVOLVES PERFORMING DRYING OPERATION FOR REMOVING UN-BONDED POWDER, AND PERFORMING HIGH-TEMPERATURE SINTERING OPERATION TO OBTAIN HIGH STRENGTH INORGANIC BINDER | CN104149348A | YAO SUYING (CN)\ NIE KAIMING (CN)\ XU JIANGTAO (CN)\ GAO JING (CN)\ SHI ZAIFENG (CN)\ GAO CEN (CN) | GEN SEMICONDUCTOR INC (US) | M22-H01\ M22-H03C\ M22-H03F\ A12-W12F |
| 6173 | 3D printing | THREE-DIMENSIONAL OBJECT MANUFACTURING DEVICE, HAS SUB-POWDER LAYER LAMINATED IN UPPER PART OF ANOTHER SUB-POWDER LAYER, AND WORKBENCH FORMED WITH HOLLOW SPACE THROUGH HIGH-PRESSURE WATER JETTING PROCESS | KR1473165B1 | YASUDA HIROSHI (US)\ STAUFER BERTHOLD (DE) | UNIV TIANJIN (CN) | M22-H03C\ M22-H03F\ A12-W12F |
| 6174 | 3D printing | THREE-DIMENSIONAL OBJECT MANUFACTURING DEVICE HAS POWDER FEEDER THAT SUPPLIES DIFFERENT KINDS OF POWDER TO INJECTION NOZZLE USED FOR SPRAYING POWDER AT SUBSONIC SPEED AND LAMINATING LAYER OF POWDER IN WORKBENCH | KR1473164B1 | YEGANG LV US\ SANNIAN SONG US\ FENG RAO US\ ZHITANG SONG US\ BO LIU US\ LIANGCAI WU US\ CHENG PENG (US) | YAO SUYING (CN)\ NIE KAIMING (CN)\ XU JIANGTAO (CN)\ GAO JING (CN)\ SHI ZAIFENG (CN)\ GAO CEN (CN)\ UNIV TIANJIN (CN) | A08-M01C\ A08-P01\ A11-B16\ S06-E04B1\ S06-G10 |
| 6175 | 3D printing | METHOD OF 3D PRINTING ON REFRACTORY ARTICLES INVOLVES MIXING THE POWDER IN 3D MODEL CYCLE COMPLETION | RU2535704C1 | YEO YEE-CHIA (US)\ YANG FU-LIANG (TW)\ LIN CHUN-CHIEH (TW)\ HU CHEN M (TW) | TEXAS INTRUMENTS INC (US)\ YASUDA HIROSHI (US)\ STAUFER BERTHOLD (DE) |  |
| 6176 | 3D printing | MAGNESIUM ALLOY DIE CASTING THIN COATING SPRAYING SHIELDING TOOL HAS COVER PLATE PROFILE WHOSE LOWER END FACE IS SET WITH MAGNESIUM ALLOY DIE-CASTING AND UPPER END FACE IS MOUNTED WITH HANDLE | CN204018096U | YI DAN US | SHANGHAI INST MICROSYS & INF (CN) | A08-S02\ A10-E05B\ A11-B05\ A12-E06A\ L03-E01B\ L03-E04A1\ X16-C01A |
| 6177 | 3D printing | SINTERED ZIRCONIA PLATE USED IN FLAT-TYPE ANODE-SUPPORTED SOLID OXIDE-FUEL CELL, HAS MAIN COMPONENT AND COVER COMPRISING YTTRIA-STABILIZED ZIRCONIA POROUS FILM PROVIDED ON UPPER AND LOWER SURFACES OF MAIN COMPONENT | CN104183860A | YI DING (CN)\ QIANG LIU (CN)\ XIN WANG (CN)\ YONGPING CHEN US\ HONGHUI YUAN US\ SHIJUN CHEN US\ XING XU US | TAIWAN SEMICONDUCTOR MFG (US) | B06-D13\ B06-D16\ B06-D17\ B14-C01\ B14-L06 |
| 6178 | 3D printing | NEW DIAZEPINE DERIVATIVE USED IN AGENT FOR PREVENTING AND/OR TREATING NOCICEPTIVE PAIN, INFLAMMATORY PAIN AND NEUROPATHIC PAIN | WO2015005467A1 | YI SHAN US | SHANGHAI GRACE SEMICONDUCTOR US | A12-E06A\ A12-E06B\ E31-H05\ E31-N04B\ L03-E01B\ L03-E01C3\ L03-H05\ X16-B01 |
| 6179 | 3D printing | PREPARATION OF INORGANIC SOLID ELECTROLYTE MEMBRANE/ELECTRODE COMPOSITE MATERIAL INVOLVES MIXING INORGANIC SOLID ELECTROLYTE POWDER MATERIAL AND BINDER AND ULTRASONICALLY-DISPERSING IN SOLVENT UNDER INERT ATMOSPHERE | CN104143656A | YI SHAN US\ CHENGQIONG TANG US | SHANGHAI TECH PHYSICS INST US | M22-H01\ M22-H03B\ M23-D05\ M23-H\ X24-D03 |
| 6180 | 3D printing | FEEDING SPACE LASER PROCESSING AND THREE-DIMENSIONAL FORMING METHOD, INVOLVES USING MACHINERY ARM TO DRIVE INNER POWDER FEEDING HEAD ACCORDING TO PRESET TRACK EDGE AREA AND FILLING AREA | CN104190927A | YOKOGAWA SEIICHI US\ INOUE TAKAHIRO (JP) | SHANGHAI GRACE SEMICONDUCTOR US | M22-H03A\ M22-H03B\ M22-H03G\ T01-J15X\ T01-N01D2 |
| 6181 | 3D printing | LASER MOLDING RHENIUM NOZZLE PREPARATION METHOD, INVOLVES PAVING ROLLER WITH MIXED POWDER OF SPECIFIC AMOUNT IN WORKING TABLE IN UNIFORM THICKNESS, AND PRE-HEATING WORKING TABLE TO SPECIFIC TEMPERATURE | CN104190933A | YOKOGAWA SEIICHI US\ INOUE TAKAHIRO (JP)\ KAWASHIMA RYOSUKE (JP) | SHANGHAI GRACE SEMICONDUCTOR US | M22-G03J\ M22-H03A\ M22-H03B\ M22-H03G\ T01-J07B\ T01-J15X\ T01-N01D2\ X25-A01\ X25-A02 |
| 6182 | 3D printing | LASER RAPID MOLDING NIOBIUM TUNGSTEN ALLOY PREPARATION METHOD, INVOLVES UNIFORMLY MIXING TUNGSTEN AND NIOBIUM POWDER UPON CERTAIN PROPORTION INTO POWDER JAR, PRE-HEATING WORKING TABLE, AND REDUCING WORKING PLATFORM INTO CERTAIN HEIGHT | CN104190934A | YONG HUANG US | SHARP KK (JP) | S03-E08\ V04-Q30Q\ V04-R03C4\ V06-V01N\ V06-V04G |
| 6183 | 3D printing | HIGH FREQUENCY ULTRASONIC SENSOR, HAS PRINTED CIRCUIT BOARD PLATE PROVIDED IN FIXED CONNECTION WITH ANODE PIN AND CATHODE PIN, AND METAL SHELL WHOSE INNER CAVITY IS PROVIDED WITH SOUND ABSORBING LAYER | CN104197970A | YONGLIANG LI US\ QIUXIA XU US | SHARP KK (JP) |  |
| 6184 | 3D printing | POWDER DEVICE FOR POWDERING OF SUBSTRATES IN PRINTING PRESS, COMPRISES FRONT-SIDE NOZZLE AND REAR-SIDE NOZZLE OF COMMON METERING UNIT AND COMMON MANIFOLD SUPPLIED WITH POWDER, WHERE POWDER IS DISCHARGED TO MANIFOLD | DE102014009427A1 | YONGLIN ZHANG US\ XIONGJIE WU US | YONG HUANG US | M22-H |
| 6185 | 3D printing | METAL THREE-DIMENSIONAL PRINTER INTEGRATED NOZZLE STRUCTURE INCLUDES CLADDING SPRAY NOZZLE, AND REINFORCING NOZZLE | CN203992399U | YOU YOUNG KUG (KR) | INST OF MICROELECTRONICS CAS (CN) | A08-C09\ A11-B05\ A11-C02B\ A12-L03D\ G02-A04A\ G02-A04B\ G03-B02E\ G05-A01\ G05-F\ G06-C16\ G06-D01\ G06-D03\ G06-D05\ G06-D06A\ G06-D06B\ G06-E02\ G06-F03B\ G06-F03D\ G06-G17\ G06-G18\ G06-H07\ D08-A\ D08-A05\ E06-D13\ E11-F06\ E12-A05\ E25\ E31-N04D\ L03-G02\ L03-G05B7\ L03-G09P\ L03-H02\ L03-J02\ U13-A01\ V04-X01B\ V07-F02B\ V07-L02 |
| 6186 | 3D printing | NEW 9H-CARBAZOLE COMPOUNDS, USEFUL FOR THE PHOTOPOLYMERIZATION OF THE COMPOSITION, FOR PRODUCING PAINTS, POWDER COATINGS, PRINTING INKS, PRINTING PLATES, ADHESIVES, MAGNETIC RECORDING MATERIALS, MICROMECHANICAL PARTS, AND WAVEGUIDES | WO2015004565A1 | YOULI ZHANG US | YONGLIN ZHANG US | A02-A\ A02-A00A\ A02-A09\ A04-B04\ A04-F01\ A04-G01B\ A07-A02\ A08-C07\ A08-M\ A08-M01D\ A08-R08B\ A10-E13\ A12-E07A\ A12-E14\ G03-B02D1\ L03-H04E6\ L03-H05\ M26-B05\ U11-C01J8\ V04-A01\ V04-A11\ V04-M05\ V04-Q02B\ V04-R01A4\ V04-R04A\ V04-R04C\ V04-T03J\ V04-T03P\ X24-A01A |
| 6187 | 3D printing | ANISOTROPIC ELECTROCONDUCTIVE PASTE FOR CONNECTING ELECTRODES OF PRINTED WIRING BOARD, CONTAINS THERMOPLASTIC RESIN, (METH)ACRYLATE REACTIVE DILUENT, RADICAL POLYMERIZATION INITIATOR, ACTIVATOR, SOLDER POWDER AND SILANE COUPLING AGENT | JP2015005435A | YOULI ZHANG US | YOU YOUNG KUG (KR) | A03-A04A\ A05-E01D\ A10-E05B\ A12-V02\ A12-W12G\ L02-A02\ L02-A03\ L02-A04\ L02-G03A1 |
| 6188 | 3D printing | MANUFACTURE OF POROUS CERAMIC SUPPORT FOR BONE SCAFFOLD, INVOLVES MIXING BINDER CONTAINING WATER AND METHYL CELLULOSE, AND CERAMIC POWDER, PRODUCING CERAMIC PASTE, EXTRUDING IN PRESENCE OF NONSOLVENT, MOLDING, DRYING, AND HEAT-TREATING | KR1481988B1 | YU BIN (US) | YOULI ZHANG US | A04-F05\ A04-G08A\ A05-F03\ A07-A03C\ A08-A03\ A08-A06\ A08-M\ A08-M06\ A08-M09C\ A08-M10\ A08-P08\ A08-S01\ A08-S02\ A08-S05\ A10-E01\ A10-E03\ A11-A03\ A11-A04\ A12-S09A |
| 6189 | 3D printing | PREPARATION OF NYLON POWDER USED FOR 3-DIMENSIONAL PRINTING, INVOLVES TAKING MODIFIED NYLON, MIXING MODIFIED NYLON WITH NYLON BINDER, AND ADDING ADDITIVES, ORGANIC SOLVENT, SURFACTANT, LIGHT ABSORBER, COMPATIBILIZER AND VISCOSITY REDUCER | CN104194326A | YU SHAOFENG (US)\ KIRKPATRICK BRIAN K (US)\ MEHRAD FREIDOON (US) | YOULI ZHANG US | V04-A01\ V04-M05\ V04-R04B\ V04-R04C\ V04-R07\ V04-X01A\ V06-V01E\ V06-V03A5 |
| 6190 | 3D printing | ASSEMBLING STRUCTURE FOR METAL SURFACE-MOUNT ELECTRONIC ELEMENT PACKAGING AND STICKING DEVICE, HAS LEAD THAT IS INSERTED INTO, AND CONNECTED TO WELDING DISCS, AND WHOSE LENGTH IS NOT GREATER THAN THICKNESS OF PCB CIRCUIT SUBSTRATE | CN203984371U | YU SHAOFENG (US)\ PINTO ANGELO (US)\ JOHNSON FRANK S (US)\ MCKEE BENJAMIN P (US) | ADVANCED MICRO DEVICES INC (US) | E31-N03B |
| 6191 | 3D printing | PREPARATION OF GRAPHITE MATERIAL USED FOR CAST STEEL TRAIN WHEEL CASTING MOLD, BY CRUSHING CALCINED PETROLEUM COKE, SIEVING, MIXING CARBONACEOUS PARTICLES, POWDER AND COAL TAR PITCH BINDER, KNEADING, MOLDING, BAKING, AND GRAPHITIZING | CN104150467A | YU TING US\ PENG BENXIAN US\ YU FENGQI US | TEXAS INSTRUMENTS INC (US) | S06-K03\ S06-K99C\ V04-A04C\ V04-M30L\ V04-T02\ V04-U03\ V04-U04 |
| 6192 | 3D printing | LASER PRINTER HAS PROTECTION ELEMENT THAT IS PROVIDED ALONG UPPER LIMIT IN GROOVE DIRECTION AND SPRING THAT IS PROVIDED WITH ONE END AND PROTECTIVE ELEMENT PROVIDED FOR ABUTTING OTHER END OF SPRING POWDER BOX MOUNTING POSITION ABUTMENT | CN203982081U | YU WANG (CN)\ WENLIAN WANG US | TEXAS INSTRUMENTS INC (US)\ YU SHAOFENG (US)\ PINTO ANGELO (US)\ JOHNSON FRANK S (US)\ MCKEE BENJAMIN P (US) | A09-D03\ A11-C04A\ A12-V02B |
| 6193 | 3D printing | DENTURE WAX TYPE THREE-DIMENSIONAL PRINTING FORMING TEST MACHINE, HAS TEMPERATURE CONTROL DEVICE MOUNTED AT BOTTOM PLATE OF SIDE WALL OF MACHINE FRAME, AND SPRAY HEAD WHOSE LOWER END PART IS PROVIDED WITH NOZZLE FOR INJECTING WAX POWDER | CN104175553A | YUAN GAOBIN US\ LI YUANQING US\ YAO SUYING (CN)\ NIE KAIMING (CN)\ XU JIANGTAO (CN)\ GAO JING (CN)\ SHI ZAIFENG (CN) | SHENZHEN INST OF ADV TECH CAS (CN) | L03-G02\ M22-H03B\ M22-H03G\ M23-D05\ M23-G\ M23-H\ V07-F01A1\ V07-G10D\ X24-D03\ X25-A03 |
| 6194 | 3D printing | SYNCHRONOUS POWDER FEEDING SPACE LASER PROCESSING AND THREE-DIMENSIONAL FORMING APPARATUS INCLUDES LIGHT POWDER FEED NOZZLE, LASER GENERATOR, ROBOTICS, CONTROL MODULES, TRANSMISSION FIBER, AIRBORNE POWDER FEEDER, AND AIR SUPPLY | CN203992397U | YUELIN ZHU US | UNIV NORTH CHINA US | B06-H\ B10-A15\ B10-A16\ B10-B01B\ B10-B04B\ B10-C02\ B10-C04A\ B10-E04A\ B10-G03\ B10-H02\ B10-J02\ B14-A02B1\ B14-A03B\ B14-H01\ C06-H\ C10-A15\ C10-A16\ C10-B01B\ C10-B04B\ C10-C02\ C10-E04A\ C10-G03\ C10-H02\ C10-J02\ C14-A02B1\ C14-A03B\ C14-H01\ T01-J06A\ T01-J13A |
| 6195 | 3D printing | PREPARING DIOXOLO COMPOUNDS OF BENZO-FUSED CYCLIC OLEFINS, USEFUL E.G. AS ANTIHIV AGENT, COMPRISES MIXING BENZO-FUSED COMPOUND WITH POWDERED OXONE AND SODIUM BICARBONATE IN SOLVENT, EVAPORATING ACETONE, AND PURIFICATION | WO2014207766A1 | YUICHI NIIMURA US\ TAKESHI NOBE US\ HIDEO NISIKAWA US\ FUMIHITO KATO US | UNIV TIANJIN (CN) | A04-E02E1\ A11-C01C\ A12-R03\ A05-G01E\ G05-F |
| 6196 | 3D printing | PVC WOOD-PLASTIC FLOOR, HAS PVC MAIN BODY LAYER COVERED WITH BOTTOM LAYER OF WOOD PLASTIC LAYER, AND ADJACENT TWO FLOOR BLOCKS COMBINED WITH SHORT HEAD STRAIGHT FALLING-TYPE LOCK CATCH STRUCTURE OR TYPE LOCKING STRUCTURE | CN203961205U | YUICHI WATANABE US\ AKIRA YAMANE US\ YASUO OISHIBASHI US | YUELIN ZHU US | S02-A03\ S02-A10C |
| 6197 | 3D printing | PORTABLE SELF-ADAPTION THREE-DIMENSIONAL RECONSTRUCTION FOCUSING TRANSMISSION STRUCTURE, HAS LIGHT SOURCE SLIDE BASE WHOSE SIDE PART IS FIXED WITH MAGNETIC GRID RULER READING HEAD, AND FIXED BASE FIXED WITH MAGNETIC GRID RULER | CN203964880U | YULIN ZHOU US | PANASONIC ELEC WORKS CO LTD (JP)\ NIIMURA YUICHI (JP)\ NOBE TAKESHI (JP)\ NISIKAWA HIDEO (JP)\ KATO FUMIHITO (JP) | T01-J10C4 |
| 6198 | 3D printing | PORTABLE SELF-ADAPTIVE THREE-DIMENSIONAL IMAGE RECONSTRUCTING-TYPE LIGHT SOURCE STRUCTURE, HAS LAMP TUBE REGULATING MECHANISM FIXED ON UPPER PART OF HOUSING, AND LAMP TUBE SUPPORT BASE CONNECTED WITH LAMP TUBE REGULATING MECHANISM | CN203966200U | YUMING ZHANG US\ QINGWEN SONG US\ YIMEN ZHANG US\ XIAOYAN TANG US\ RENXU JIA US\ YUEHU WANG US | WATANABE YUICHI (JP)\ YAMANE AKIRA (JP)\ OISHIBASHI YASUO (JP)\ ON SEMICONDUCTOR TRADING LTD (BM) | M13-C01\ M13-H03\ M13-L\ M13-M01\ M22-H02\ M22-H03B\ M22-H03F |
| 6199 | 3D printing | NET-SHAPED BONDING LAYER THERMAL BARRIER COATING STRUCTURE, HAS COATING STRUCTURE ELEMENT ARRANGED ON ALLOY SUBSTRATE, AND THERMAL BARRIER COATING LAYER FORMED BY USING AIR PLASMA SPRAYING PROCESS ACCORDING TO SPRAYING PARAMETER ADJUSTMENT | CN104164643A | YUNG FU CHONG US\ RANDALL CHER LIANG CHA US\ ALEX SEE US | YULIN ZHOU US | L02-A02\ L02-A04\ L02-G01B1\ L02-J02C |
| 6200 | 3D printing | METHOD FOR PREPARING CERAMIC HANDICRAFT ARTICLE, INVOLVES PRINTING PHOTOGRAPHIC IMAGE ON SURFACE OF CERAMIC PRODUCT, COATING LIGHT-COLORED ALCOHOL BASED RAW MATERIAL ON SURFACE OF CERAMIC PRODUCT, AND BAKING TO OBTAIN CERAMIC ARTICLE | CN104150968A | YUSONG HUANG US | UNIV XIDIAN (CN) | A12-H04\ A12-W11F\ D09-B01\ J01-D\ J01-E01B\ J01-E02D\ J01-E02E\ J01-F\ J01-G03B\ J01-H02B\ L03-H03A\ L03-J03\ S06-E07 |
| 6201 | 3D printing | FLAME-RETARDANT DEODORIZING FILTER TO BE INTEGRATED IN ELECTRONIC DEVICE, COMPRISES ADSORBATE CONTAINING METAL OXIDE PARTICLES CONSISTING OF METAL OXIDE AND MINERAL BINDER, AND CATALYST PARTICLES CONTAINING ACTIVATED CARBON PARTICLES | JP2015002946A | YUSONG HUANG US | CHARTERED SEMICONDUCTOR MFG (US) | L02-A02\ L02-A03\ L02-A04\ L02-A11\ L02-C05\ L02-J01C\ S06-G10\ S06-K99C |
| 6202 | 3D printing | WATER BASE INORGANIC BINDER BASED 3D PRINTING METHOD, INVOLVES STORING 3D PRINTER DRYING POWDER IN DRYING BOX, AND OBTAINING INORGANIC CERAMIC ADHESIVE COMPONENT WITH HIGH TEMPERATURE SINTERING PART | CN104150915A | YUWEN CHEN US\ XIAOLU HUANG US\ BINGYONG YAN US | YUSONG HUANG US |  |
| 6203 | 3D printing | 3D-REINFORCED CONCRETE STRUCTURE, HAS 3D-PRINTING MOLDING PART CONNECTED WITH CAST CONCRETE SHELL AND CONCAVE-CONVEX LINE, WHERE CONCAVO-CONVEX LINE IS FORMED AS ARC-SHAPED STRUCTURE | CN203947570U | YUWEN CHEN US\ XIAOLU HUANG US\ BINGYONG YAN US\ CIYUN QIU US | YUSONG HUANG US |  |
| 6204 | 3D printing | DISPOSABLE CUP MARK DEVICE, HAS INTERACTION MECHANISM FOR DRIVING ANNULAR FLEXIBLE COMPONENT, TELESCOPIC COMPONENT FIXED WITH RING THAT IS FORMED WITH ANNULAR CLAMPING GROOVE, AND ELASTIC MECHANISM FIXED WITH BASE ALONG VERTICAL DIRECTION | CN203934967U | YVHUA CHENG US\ MENGJUN JIA US\ WENZHENG WU US | SHANGHAI HUALI MICROELECT CORP (CN) | A10-E05B\ A12-W12G\ L02-A02\ L02-A03\ L02-A04\ L02-G01E\ L02-G12A |
| 6205 | 3D printing | RAPID LITHIUM TITANATE TRITIUM BREEDER PELLET PREPARATION METHOD INVOLVES MIXING LITHIUM TITANATE AND BINDER, PRINTING IN PRINTING DEVICE THROUGH STEREOLITHOGRAPHY, AND PERFORMING COLD ISOSTATIC PRESS DENSIFICATION AND DEBINDING TREATMENT | CN104140259A | ZEHONG LI (CN)\ BO ZHANG (CN)\ ZHAOJI LI (CN)\ XIAOSONG WANG (CN)\ YIMING WANG (CN)\ KUN YI (CN) | SHANGHAI HUALI MICROELECT CORP (CN) | A03-C02\ A06-A00E2\ A10-E09B\ A12-E01\ X12-D01F1\ X12-D01F2\ X12-D07 |
| 6206 | 3D printing | OYSTER SHELL/FLUORAPATITE COMPOSITE CONDUCTIVE SILVER PASTE FOR DEVICE, CONTAINS SILVER POWDER, KAOLIN, OYSTER SHELL, FLUORAPATITE, METHYLENEDIOXY NAPHTHALENE SULFONATE, HYDROGENATED CASTOR OIL, ALUMINUM POTASSIUM SULFATE AND ADDITIVE | CN104143373A | ZEHONG LI (CN)\ BO ZHANG (CN)\ ZHAOJI LI (CN)\ XIAOSONG WANG (CN)\ YIMING WANG (CN)\ ZHUO WANG (CN)\ JIAN YANG (CN) | SHANGHAI RES INST OF MICROELECTRONICS SHRIME PEKING UNIVERSITY US | A08-M\ A08-M01\ A08-M09C\ A09-A02\ A12-L03 |
| 6207 | 3D printing | EMBEDDED LAMP HOLDER, COMPRISES AN EMBEDDED BOTTOM SHELL CONTAINING A TRANSPARENT AGENT POWDER, BONDING AGENT, CERAMIC POWDER, COPPER POWDER, AND PLASTIC, PROVIDED WITH AN UPPER CONNECTING HOLE; AND AN EMBEDDED BLOCK | CN104141936A | ZEHONG LI (CN)\ BO ZHANG (CN)\ ZHAOJI LI (CN)\ YU WANG (CN)\ LIANGYAN REN (CN)\ HAIZHOU WU (CN) | ELECTRONIC SCIENCE TECHNOLOGY (CN) | A11-B16 |
| 6208 | 3D printing | THREE-DIMENSIONAL MODELING APPARATUS PROVIDES UPPER SURFACE OF TABLE WITH BINDER LIQUID, AND DISCHARGES BINDER LIQUID INTO POWDER MATERIAL, WHILE FOLLOWING MOVEMENT OF POWDER MATERIAL SUPPLY APPARATUSES AND MOVING | WO2014208741A1 | ZHANG BO US\ FANG JIAN US\ JIA YAOYAO US\ LI YUAN US\ YUAN TONGWEI US\ HUANG SHUAI US | UNIV ELECTRONIC SCIENCE & TECH (CN) |  |
| 6209 | 3D printing | METHOD FOR COATING PAPER-FEEDING ROLLER FOR CONVEYING SHEET-LIKE RECORDING MEDIA, SUCH AS PAPER IN PRINTER, INVOLVES SPRAYING WATER-BASED COATING MATERIAL ON OUTER PERIPHERAL SURFACE OF PAPER-FEEDING ROLLER | JP2015000358A | ZHANG BO US\ LI ZEHONG US\ JIANG GUANJUN US\ YU SHIJIANG US\ XIE JIAXIONG US\ ZHANG SHUAI US\ HU TAO US\ ZHANG CHAO US\ REN MIN US\ XIAO XUAN US\ LI TING (CN) | UNIV GUILIN ELECTRONIC TECH (CN) | S06-E06\ S06-G10\ S06-K03 |
| 6210 | 3D printing | 3D PRINTING BASED REINFORCED CONCRETE STRUCTURE FORMING METHOD, INVOLVES FORMING PRE-FABRICATED CONCRETE SHELL WITH CONCAVE LINE, AND FIXING STRUCTURE BEAM ON COLUMN STRUCTURE THAT IS FORMED WITH FLOOR SLAB CONSTRUCTION COLUMN STRUCTURE | CN104120788A | ZHANG GUANLUN LIU (CN) | UNIV ELECTRONIC SCIENCE & TECH (CN) | A08-C01\ A08-D01\ A08-S01\ A11-A03\ A11-B02D\ A11-B15B\ A12-L03A\ A12-S05L\ A12-S05U\ F01-D\ F01-D03\ F01-D04\ F03-C02A\ F03-C07\ L03-G02\ V07-F01A1\ V07-F01A3A |
| 6211 | 3D printing | COMPOSITE PHOSPHORESCENT POWDER USED IN PHOSPHORESCENT MASTERBATCH USED FOR MANUFACTURING OPTICAL FIBER, COMPRISES PHOSPHORESCENT MATERIAL CONTAINING RARE-EARTH ELEMENTS, AND DIAMMONIA-BONDED THREE-DIMENSIONAL STRUCTURED ORGANIC COMPOUND | CN104119886A | ZHANG HEMING US\ SONG JIANJUN US\ HU HUIYONG US\ XUAN RONGXI US\ HAO YUE US\ LI YUCHEN US\ WANG BIN US\ WANG HAIDONG US | UNIV ELECTRONIC SCIENCE & TECH (CN) | S03-E06B3A\ T01-J06A |
| 6212 | 3D printing | METHOD FOR MANUFACTURING DIAPHRAGM USED FOR PRODUCING FOR E.G. COMPUTER TOMOGRAPHY, INVOLVES PRODUCING DIAPHRAGM HAVING SEVERAL DIAPHRAGM APERTURES BY SCREEN PRINTING PROCESS | WO2014202424A1 | ZHANG HEMING US\ SONG JIANJUN US\ SHU BIN US\ HU HUIYONG US\ XUAN RONGXI US\ DAI XIANYING US\ HAO YUE US\ WANG HAIDONG US | TAIWAN SEMICONDUCTOR MFG (CN) | L03-H03A\ L03-H04E3\ L03-H04E4\ A12-E01\ A12-E07A\ V04-A03\ V04-M05\ V04-M30E\ V04-M30G\ V04-Q30C\ V04-Q30G\ V04-R01A5A\ V04-R05A\ W01-C01D3C\ W01-C01G8\ W01-C01P2 |
| 6213 | 3D printing | ROLL TO ROLL TYPE MANUFACTURING METHOD OF PRINTED CIRCUIT BOARD FOR E.G. SMART PHONE, INVOLVES REMOVING MICRO-BUBBLE GENERATED IN CONDUCTIVE PASTE AND LAMINATING CONDUCTIVE LAYER INTO SIDE OF INSULATING LAYER | KR1470957B1 | ZHANG HEMING US\ SONG JIANJUN US\ SHU BIN US\ HU HUIYONG US\ XUAN RONGXI US\ HAO YUE US\ LV YI US\ LI YUCHEN US | UNIV XIDIAN (CN) | A01-D13\ E10-J02C2\ E11-G02\ E31-K05C\ E31-P03\ E34-C02\ H04-B02\ H04-F02B\ H04-F05\ H04-F06\ J04-E04\ N03-C\ N03-C02\ N03-D\ N06-A\ N06-E01\ N07-F02 |
| 6214 | 3D printing | CRACKING CATALYST USED FOR PRODUCING PROPYLENE FROM TETRACARBON OLEFINS, COMPRISES SILICA-ALUMINA COMPOUND, ZSM-5 ZEOLITE, GROUP-VB OR GROUP-VIB TRANSITION METAL OXIDE AND BINDER | CN104107713A | ZHANG HEMING US\ SONG JIANJUN US\ SHU BIN US\ HU HUIYONG US\ XUAN RONGXI US\ HAO YUE US\ LV YI US\ WANG BIN US | UNIV XIDIAN (CN) | M22-G03J\ S06-G10\ S06-K02\ S06-K03A\ S06-K07A |
| 6215 | 3D printing | HIGH-EFFICIENCY PRINTING HEAD FOR 3D PRINTING DEVICE IN METAL CASTING SAND-TYPE POWDER PRODUCTION FIELD, HAS PAVING DEVICE AND PRINTING HEAD BODY THAT ARE ARRANGED ON PLATFORM, WHERE HEAD BODY IS PROVIDED WITH INDEPENDENT CONTROL NOZZLES | CN203887992U | ZHANG HEMING US\ ZHOU CHUNYU US\ SONG JIANJUN US\ SHU BIN US\ HU HUIYONG US\ XUAN RONGXI US\ DAI XIANYING US\ HAO YUE US | UNIV XIDIAN (CN) | M13-H\ M22-H03B\ M22-H03G\ S06-K99C |
| 6216 | 3D printing | LASER PRINTER 3D DECOMPRESSION SHAFT POWDER FEEDING NOZZLE, HAS END COVER WHOSE CENTER PART IS FIXED WITH POWDER FEEDING PIPE, AND PRESSURE RELIEF CHANNEL FIXED ON UPPER PART OF BAFFLE PLATE, WHERE BAFFLE PLATE IS FIXED WITH FEEDING PIPE | CN203890442U | ZHANG HEMING US\ ZHOU CHUNYU US\ SONG JIANJUN US\ SHU BIN US\ HU HUIYONG US\ XUAN RONGXI US\ HAO YUE US\ LV YI US | UNIV XIDIAN (CN) | M22-H01\ M22-H02\ M22-H03B\ M22-H03C\ M22-H03G |
| 6217 | 3D printing | PIM PROCESS BASED CABINET SHELF HANDLE MANUFACTURING METHOD, INVOLVES CARRYING OUT FINE PROCESSING AND SINTERING PROCESSES ON HANDLE AND CUSHION BLOCK, AND COMPARING CUSHION BLOCK AND HANDLE WITH FASTENING PIECE TO DETERMINE QUALITY OF SIZE | CN104107915A | ZHANG HEMING US\ ZHOU CHUNYU US\ SONG JIANJUN US\ SHU BIN US\ HU HUIYONG US\ XUAN RONGXI US\ HAO YUE US\ LV YI US | UNIV XIDIAN (CN) | J01-D01\ J01-E02B\ J01-E03C\ M22-H01\ M22-H02\ M22-H03B\ T01-J07B\ X25-A01 |
| 6218 | 3D printing | FORMING HIGH ABSORPTION POWDER MATERIAL BASED ON 3-DIMENSIONAL PRINTING TECHNOLOGY COMPRISES MIXING MAIN MATERIAL INCLUDING E.G. TOURMALINE AND AUXILIARY MATERIALS INCLUDING E.G. QUARTZ, SPRAYING ADHESIVE, UV CURING, MOLDING AND SINTERING | CN104096535A | ZHANG JIANPING (US) | UNIV XIDIAN (CN) | M22-H03B\ M22-H03G\ S06-G10\ S06-K07A |
| 6219 | 3D printing | LARGE TILTING ANGLE 3D PRINTING DEVICE USED FOR SUPPLYING POWDER COMPRISES MAIN DELIVERING PIPE, ELECTRIC CONTROL, DELIVERING PIPE, POWDER FEEDING NOZZLE, FOCUSING LENS AND INCLINED SPRAYING TUBE | CN203875342U | ZHANG JIANQIANG US\ XU KEN US | UNIV XIDIAN (CN) | L03-E04A1\ L03-E04H3\ X16-C01A\ X16-E06A |
| 6220 | 3D printing | ANODE PROTECTION LAYER FOR USE IN SOLID OXIDE FUEL CELL, COMPRISES PRECURSOR MATERIAL COMPRISING IN SITU REDUCTION SINTERED SPINEL COMPRISING GADOLINIUM-CERIUM-OXIDE COMPONENT, AND BARIUM-ZIRCONIUM-CERIUM-YTTRIUM-YTTERBIUM-OXIDE COMPONENT | CN104103838A | ZHANG LEI US\ XU CHENGKAI US\ TANG DEMING US | MICRON TECHNOLOGY INC (US) | A05-E04C\ A05-G01B\ A08-D01\ A08-P01\ A08-R01\ A08-S02\ A08-S08\ A09-D03\ A11-C04A\ A11-C05A\ A12-G03\ A12-S05F\ A12-S05Q\ A12-S05U |
| 6221 | 3D printing | METHOD FOR MANUFACTURING WIDE-DIGITAL PRINTED TERYLENE FABRIC, INVOLVES WEAVING FABRIC, PADDING WATERPROOFING AGENT, PROCESSING POLYURETHANE LAYER, PREPARING GLUE, AND PREHEATING AND PRINTING FLOWER DIRECTLY FOLLOWED BY FIXING AND COOLING | CN104088424A | ZHANG SHENGDONG CHEN (CN) | GUANGZHOU RISING MICRO ELECTRONICS CO LTD US | S06-G05\ S06-K03\ S06-K06D |
| 6222 | 3D printing | NOZZLE CLEANING DEVICE, HAS BLOWING DEVICE CONNECTED WITH NOZZLE MOUNTING PLATE AND FORMED WITH AIR INLET HOLE AND PNEUMATIC PUMP HOLE, WHERE UPPER SIDE OF BLOWING PNEUMATIC HOLE IS FORMED WITH LONG AND NARROW HOLE I.E. AIR BLOWING HOLE | CN104085196A | ZHANG SHENGDONG CHEN (CN) | SHANGHAI LEXVU OPTO MICROELECTRONICS TECHNOLOGY CO LTD US | A04-G07\ A04-G10\ A08-A01A1\ A08-C01\ A08-M\ A08-M01\ A08-M03B\ A08-P01\ A08-R03\ A08-S05\ A11-A04\ A11-B07\ A12-W07F\ G03-B02D2\ G03-B02D3 |
| 6223 | 3D printing | ENVIRONMENT-FRIENDLY HOT MELT GLUE USED FOR PRINTER MATERIAL ASSEMBLY, INCLUDES ETHYLENE-VINYL ACETATE COPOLYMER, POLY-4-METHYL-1-PENTANE DILUTED, POWDER NITRILE, THIODIPROPIONIC ACID BIS STEARYL ESTER, VALERIAN OIL AND EPOXY OCTYL STEARATE | CN104087205A | ZHANG XIAOLIN US\ SHEN JING US | UNIV BEIJING (CN) | A08-F01\ A09-D01\ A11-B03\ A11-B04\ A11-B04B\ A11-B12A\ A12-E01\ A12-H\ A12-H07\ A12-W12F\ F05-A06B\ T01-J07B\ X25-A03F\ X25-A06\ X25-L01A |
| 6224 | 3D printing | ADJUSTABLE ELECTROMAGNETIC VALVE CONTROLLING METHOD, INVOLVES PERFORMING CASTING MOLDING PROCESS, AND CONNECTING HIGH DENSITY MACHINE WITH AM BOTTOM PLATE, POINT PLATE, WIRE PLATE, PROTECTING POINT PLATE, CARRYING OUT PLATE AND STOP PLATE | CN104057245A | ZHANG XINWEI US\ FAN CHENGJIAN US\ XIAO JIANNONG US\ SU WEI US\ XIA CHANGFENG (US) | UNIV BEIJING (CN) |  |
| 6225 | 3D printing | PREPARATION METHOD FOR THREE-DIMENSIONAL ABRASIVE CARBIDE TOOL INVOLVES ADDING MIXING AGENT TO MIXTURE OF MATERIALS BASED ON SPECIFIC PROPORTION AND MIXING UNIFORMLY, POURING MIXTURE IN A MOLD, AND PRESSING INTO BLANK | CN104057404A | ZHANGMING ZHU US\ JUNYONG HU US | UNIV BEIHANG US | A03-A04A\ A10-E05B\ A12-E01\ A12-W12F\ A12-W12G\ L02-A03\ L02-A11\ L02-J01C |
| 6226 | 3D printing | MICROWAVE DEVICE ENERGY TRANSMITTING WINDOW CERAMIC CHIP METALLIZATION COATING COMPRISES METAL POWDER CONSISTING OF E.G. MOLYBDENUM AND MANGANESE(II) OXIDE AND BONDING AGENT CONSISTING OF TERPINEOL AND ETHYL CELLULOSE | CN104058794A | ZHAO CHAOSHENG US\ WANG XUELIANG US\ HE BING US\ JIANG YUNKUAN US\ ZHAO KUN US\ YU TIANYE US | WUXI CSMC TECH SEMICONDUCTOR US\ CSMC TECHNOLOGIES CORP US | A08-S02\ A10-E05\ A11-B05D\ A12-W12G\ G02-A04A |
| 6227 | 3D printing | SOLVENT-BASED INK USEFUL FOR MANUFACTURING THREE-DIMENSIONAL PRINTED METAL/CERAMIC COMPOSITE PRODUCT, COMPRISING PRINTING THE MOLDED PRODUCT, PLASTICIZING, DE-BONDING AND SINTERING | CN104059427A | ZHAO DEYI US\ WANG PENG US\ LI HAISONG US\ WANG ZHONGFANG US\ LU HONGLI US\ YUE HONGJU US\ WU LONGSHENG US | CHENGDU CHIP RAIL MICROELECTRONICS CO LTD US | A11-A05C\ A11-C04A\ A12-E08B\ U24-D01B\ U24-F02\ V02-G01A\ V02-G02B1\ V02-H01B\ V02-H01C3\ X12-C01D2\ X12-J |
| 6228 | 3D printing | MANUFACTURE OF LAMINATED COIL COMPONENT USED FOR E.G. VOLTAGE BOOSTER CIRCUIT, INVOLVES MIXING MAGNETIC POWDER, GLASS POWDER AND BINDER, MOLDING, PRINTING CONDUCTIVE PATTERN ON OBTAINED SHEET, AND LAMINATING WITH ANOTHER GREEN SHEET | JP2014236112A | ZHAO MINGJIAN US\ LI BIN US\ WU CHAOHUI US\ LI XIANBO US | HUBEI LIGHTNING PROT CT US | S06-E03A\ S06-G10\ X25-A08 |
| 6229 | 3D printing | POST-PROCESSING OBJECT METHOD FOR ADDITIVE MANUFACTURING PROCESSES USING MICROWAVE RADIATION, INVOLVES PROVIDING AND EXPOSING OBJECT IN ABSORPTIVE STATE TO UNIFORM FIELD OF MICROWAVE RADIATION | WO2014197086A1 | ZHAO YUE US\ LOU WENZHONG US\ SONG RONGCHANG US\ DING XURAN US | NO 771 INST OF NO 9 RES INST CHINA AEROSPACE SCIENCE & TECHNOLOGY CORP US | V04-Q02A1\ X27-H03 |
| 6230 | 3D printing | PET LUMINESCENT TAG HAS POINT CONTACT SWITCH THAT IS LOCATED BETWEEN BUTTON BATTERY AND PRINTED CIRCUIT BOARD PLATE, AND SILICON GLUE BUTTON KEY THAT IS PROVIDED ON CELL SURFACE CONNECTED TO SILICON GLUE KEY OF BOX MAIN UNIT | CN203839002U | ZHAOYUN ZHANG US\ BO PENG US\ ZHIGUI SHI US\ WEI SU US\ YANG GAO (CN) | UNIV SOUTH CHINA TECH (CN) | A10-E02\ A10-E05B\ A12-E15\ A12-W12G\ L03-G10\ V06-V01B1\ V06-V01E |
| 6231 | 3D printing | NIOBIC ACID-POTASSIUM-SODIUM BASED MULTI-LAYER PIEZOELECTRIC CERAMIC COMPONENT | CN104051606A | ZHE LIU US | BEIJING INST TECHNOLOGY US |  |
| 6232 | 3D printing | GLASS CURTAIN WALL THREE-DIMENSIONAL ADJUSTABLE BRACKET, HAS REGULATING PIECE CONNECTED WITH SCREW POLE THAT IS CONNECTED WITH TOP PART OF SUPPORT KEEL THROUGH SCREW THREAD, AND REGULATING MECHANISM CONNECTED WITH BOTTOM PART BASE | CN203834762U | ZHENG HONG US\ LI ZHAO US\ LI ZHEN US | INST ELECTRONIC ENG CHINA ACADEMY ENG PHYSICS US | F05-A02B\ F05-A06B |
| 6233 | 3D printing | METHOD FOR MAKING SYNTHETIC PAPER, INVOLVES LAMINATING FILMS WITH ROLL PAPER PRINTOUTS TO GENERATE PAPER WITH SPECIFIC THICKNESS BY SMOOTHING ADDITIVES CONTAINING SUBSTANCES, BINDERS, COLORING AGENTS, ANTISTATIC PLASTIC CEMENT AND OIL | ID501111A | ZHENG JUN-FEI (US)\ LIANG CHUNLIN (US) | ZHE LIU US | D01-B02\ D03-E\ D03-H01G\ D03-H01H\ D03-H02B2B\ D03-K09\ X25-A08 |
| 6234 | 3D printing | PREPARING EDIBLE PRODUCT, PREFERABLY FOOD PRODUCT INCLUDING BAKERY PRODUCT, AND CONFECTIONARY PRODUCT, INVOLVES PROVIDING EDIBLE POWDER COMPOSITION, AND SUBJECTING COMPOSITION TO SELECTIVE LASER SINTERING | WO2014193226A1 | ZHENG REN US\ HUALIN WU US\ SHAOJIAN HU US\ XI LI US\ YANLING SHI US | UNIV BEIHANG US | A12-E06\ L03-E04A1\ L03-E04B\ L03-E04H2\ X16-C01A1 |
| 6235 | 3D printing | SEGMENTED-IN-SERIES FLAT TUBULAR SOLID OXIDE FUEL CELL COMPACT STRUCTURE, HAS FLAT PIPE-TYPE CERAMIC SUPPORT UNIT FOR EXTRUDING KNEADING UNIT THAT IS PROVIDED WITH BASE, WHERE BASE IS MADE OF CERAMIC RAW MATERIAL AND PORE-FORMING AGENT | KR2014134917A | ZHENGHAO GAN US | INTEL CORP (US) | J01-K\ G06-G05A\ S06-E04A1\ S06-E04D\ S06-K99C |
| 6236 | 3D printing | PNEUMATIC CLASSIFIER FOR USE DURING MANUFACTURE OF FINE POWDERY TONER FOR E.G. COPY MACHINE, MAKES GAS WHICH FLOWS OUT OF CLASSIFICATION AREA INTO FINE POWDER DISCHARGE FLOW PATH TO FLOW BELOW IN VICINITY AT MATERIAL SUPPLY NOZZLE TIP | JP2014223618A | ZHENGQI JIN (CN)\ JINGANG WU (CN)\ JINGHUA NI US\ HONG ZHU US | UNIV EAST CHINA NORMAL (CN)\ SHANGHAI INTEGRATED CIRCUIT RES & DEV CT US | M22-H02\ M22-H03B\ M22-H03F\ A12-H05\ A12-W12F\ T01-J15X\ X25-A08 |
| 6237 | 3D printing | MANUFACTURING DEVICE OF THREE-DIMENSIONAL OBJECT, HAS CONTROL UNIT THAT COMPARES SHAPE OF POWDER LAMINATED LAYER AND THREE DIMENSIONAL CAD DATA OR OBJECT STORED IN EQUAL ELEVATION DATA AND CONTROLS PREDETERMINED SPEED OF POWDER FEEDER | KR1456830B1 | ZHENGSHENG HAN US\ JIAJUN LUO US\ JINSHUN BI (CN)\ CHAOHE HAI (CN) | SEMINCONDUCTOR MFG SHANGHAI INTERNAT CORP US | U11-D02B\ U12-A01A |
| 6238 | 3D printing | LARGE POWER LED LIGHTING DEVICE HAS MULTIPLE LED CHIPS THAT ARE CONNECTED IN SERIES THROUGH LEAD, AND IS FIXED TO MOUNTING GROOVE OF STRIP-SHAPED BRACKET THROUGH BONDING GLUE AND COATED WITH LED FLUORESCENT POWDER | CN203826377U | ZHENGSHENG HAN US\ JIAJUN LUO US\ JINSHUN BI (CN)\ CHAOHE HAI (CN) | SEMICONDUCTOR MFG INT SHANGHAI (CN)\ SEMICONDUCTOR MFG INT BEIJING (CN) | M22-H03B\ S06-G10 |
| 6239 | 3D printing | 3D PRINTING METHOD OF LARGE TILTING ANGLE PART COMPRISES DIVIDING PARTS ALONG BOUNDARIES, FORMING, SPRAYING, CONVEYING, PARTITIONING AND PRINTING | CN104014791A | ZHI JIN (CN)\ NING WANG US\ XIANTAI WANG US\ YONGBO SU US\ JIANNAN GUO US | INST OF MICROELECTRONICS CAS (CN) | A02-A00A\ A04-B09\ A04-F04A\ A10-B01\ A10-E01\ A12-W11D\ D03-K03\ D03-K04\ E06-A01\ E10-A15A\ E11-Q01A\ J04-B01\ J04-C01\ S03-E04D |
| 6240 | 3D printing | FLUORESCENT TOCOPHEROL IMPRINTED POLYMER PREPARED BY ADDING FLUORESCENT QUANTUM DOT NANOMATERIALS TO TOCOPHEROL, USING TEMPLATE MOLECULES TO FORM HYDROGEN BONDS WITH FUNCTIONAL MONOMER, AND CARRYING OUT THERMAL POLYMERIZATION REACTION | CN104017126A | ZHIFENG LUO US\ FENG WAN US\ XIAOPING ZHONG US | INST OF MICROELECTRONICS CAS (CN) | A12-E06A\ L03-E04\ X16-C01A\ X16-E01C1\ X16-E01G\ X16-E06A1 |
| 6241 | 3D printing | METHOD FOR MANUFACTURING NICKEL/STABILIZED ZIRCONIA CORE-SHELL COMPLEX FOR SOLID OXIDE FUEL CELL USED IN GAS TURBINE, INVOLVES MANUFACTURING CORE-SHELL POWDER INTO PELLETS, AND SINTERING CORE-SHELL POWDER AT HIGH TEMPERATURES | US2014342269A1 | ZHIQIANG XIAO (CN)\ JI ZHANG (CN)\ MINGFENG LIU (CN) | INST OF MICROELECTRONICS CAS (CN) | A05-A01E2\ A05-E01D2\ A05-F01B2\ A07-A03A\ A07-A03B\ A08-D\ A08-M03\ A08-M09A\ A08-M09A1\ A08-R01\ A09-A03\ A12-E14\ L03-A01A3\ L03-A01A5\ L03-A02B\ L03-A02E\ L03-H05\ L04-C10E\ L04-C10F\ L04-C11C\ U11-A08B\ U11-A14\ U11-C05E\ V05-A\ V05-L01\ X25-A08 |
| 6242 | 3D printing | CONDUCTIVE PASTE COMPOSITION FOR USE DURING MANUFACTURING ELECTRODE, COMPRISES ADDITIVE MADE OF ORGANIC COMPOUND, AND PLATE-SHAPED CONDUCTIVE METAL MADE OF INORGANIC MATERIAL THAT COMPRISES BINDER RESIN AND POLYESTER RESIN | KR1455797B1 | ZHIQIANG XIAO (CN)\ MING QIAO (CN)\ HONGLI JIANG (CN) | SHENZHEN TONGXINGDA TECHNOLOGY CO LTD US | A10-E05B\ A12-E11C\ L03-G05\ L03-H05\ L04-E03\ W03-A01B5\ W03-A01B8\ W03-A08J\ W03-G04\ W03-G08 |
| 6243 | 3D printing | METHOD FOR FABRICATING LIGHT EXTRACTION SUBSTRATE ARRANGED ON ONE SURFACE OF ORGANIC LIGHT-EMITTING DEVICE (OLED), INVOLVES FIRING PRINTED FRIT PASTE TO FORM LIGHT EXTRACTION LAYER IN WHICH SEVERAL PORES ARE FORMED AS BOTTOM SURFACE | US2014335637A1 | ZHIWEI WANG (CN)\ DEMING TANG US | WUXI ZHONGWEI MICROCHIPS CO LTD US | A12-L05C2\ G06-G05A\ S06-E04A1\ S06-E04C\ S06-E04D\ S06-K06C\ S06-K07A4D\ S06-K99C\ S06-K99D |
| 6244 | 3D printing | TONER USED FOR ELECTROPHOTOGRAPHIC CARTRIDGE FOR E.G. COPIER AND PRINTER, IS MAGNETIC TONER CONTAINING BINDER RESIN, WAX AND/OR MAGNETIC POWDER, AND HAS INORGANIC FINE PARTICLES HAVING SPECIFIED AVERAGE PRIMARY PARTICLE SIZE ON SURFACE | JP2014209186A | ZHIWEI WANG (CN)\ RONG YANG (CN)\ WANG CHEN (CN) | WUXI I CORE ELECTRONICS CO LTD (CN) | M22-H01\ M22-H03B\ M22-H03C\ M22-H03F\ T01-J15X |
| 6245 | 3D printing | PRODUCING THREE-DIMENSIONAL OBJECT, COMPRISES E.G. SUPPLYING POWDER TO SPRAY NOZZLE, HEATING POWDER BY HEATING UNIT, CONTROLLING POSITION OF SPRAY NOZZLE, STACKING INTO POWDER LAYER, AND SINTERING POWDER LAYER BY POWDER SINTERING DEVICE | KR1459284B1 | ZHIXIN KE US\ XINGREN WAN US | SHANGHAI LEXVU OPTO MICROELECTRONICS TECHNOLOGY CO LTD US | A11-B05D\ A12-F\ L02-A03\ L02-C05\ L02-D07A\ L02-K02\ S06-E04B1\ S06-E07\ S06-G10 |
| 6246 | 3D printing | FAST MOLDING POWDER MATERIAL FOR THREE-DIMENSIONAL PRINTING FOR MEDICAL MODEL COMPRISES MOLDING POWDER A E.G. ALPHA-CALCIUM SULFATE HEMIHYDRATE GYPSUM POWDER AND MOLDING POWDER B, WATER-SOLUBLE BINDER POWDER, ENHANCER, AND ADDITIVES | CN103992088A | ZHONG HUICAI (CN)\ ZHU KAIGUI US\ CHEN FANGFANG US | NANO BEIJING PHOTONICS INC (CN) | M22-H03B\ M22-H03G\ S06-K99C |
| 6247 | 3D printing | LASER 3-D PRINTER SIDE DECOMPRESSION SHAFT POWDER NOZZLE COMPRISES POWDER BARREL CAVITY, NOZZLE, FEEDING PIPE, RELIEF TUBE, PRESSURE BAFFLE AND END COVER | CN103990800A | ZHOU JUNMING CHEN (CN) | TAIWAN SEMICONDUCTOR MFG (TW) | A03-A04A1\ A12-V02B\ D08-A03\ L02-G03A\ L02-K02 |
| 6248 | 3D printing | THREE-DIMENSIONAL PORCELAIN TOOTH COMPRISES BASE LAYER, BONDING LAYER, DENTIN LAYER AND ENAMEL LAYER CONTAINING GLAZE COMPONENT AND SHINY-GLAZE COMPONENT | CN103989587A | ZHOU PENG (US)\ SUN QINGQING (CN)\ ZHANG WEI (CN)\ YANG WEN (CN)\ WANG PENGFEI (CN)\ ZHENG SHAN US | UNIV BEIHANG US | A09-D03\ A11-A\ A11-B01\ A11-C04A\ A12-H07\ A12-W12F |
| 6249 | 3D printing | FUSED DEPOSITION TYPE THREE-DIMENSIONAL PRINTER HAS DISTRIBUTION HOPPER CONNECTED TO FEEDER THROUGH PIPELINE SET WITH FIRST STOP VALVE CONTROLLED BY CONTROLLER, AND PROPORTIONING BIN PROVIDED TO CONTAIN ADDITIVE RAW MATERIAL | CN203779868U | ZHOU PING US\ LI YITIAN US\ WANG ZI US | CHINESE ACAD PHYSICS INST (CN) | A04-E10\ A08-S01\ A08-S02\ A11-C02C\ A12-L05C2\ S06-E04A\ S06-E06A\ S06-K99C |
| 6250 | 3D printing | METHOD FOR FORMING FLUOROPLASTIC TOPCOAT IN PRINTER FUSER ELEMENT USED IN ELECTROPHOTOGRAPHIC PRINTING DEVICE, INVOLVES HEATING COATED SUBSTRATE TO CURE COATING DISPERSION TO FORM FINAL COATING FILM ON SUBSTRATE | US2014322447A1 | ZHOU ZEKUN US\ SHI YUE US\ MING XIN US\ ZHANG BO US\ ZHANG QIYING US\ XU TIANHUI US\ GOU CHAO US\ CUI JIANAN US | UNIV FUDAN (CN) | A08-E02\ A12-B01\ F05-A06B\ G02-A03\ G02-A03D\ D08-B\ D08-B09A1A\ E10-E04H\ E12-A08\ E12-A14\ E25\ E31-P03\ E34-C01\ E35-U02\ S06-E06D\ S06-K01A\ S06-K99B\ X24-D03\ X24-D04 |
| 6251 | 3D printing | ALUMINA FLAKES HAVING A SPECIFIED PARTICLE THICKNESS, USED IN E.G. AUTOMOTIVE FINISHING, INDUSTRIAL COATINGS, POWDER COATINGS, PRINTING INKS, SECURITY PRINTING INKS, PLASTICS, CERAMIC MATERIALS, COSMETICS, GLASSES, PAPER, AND PAPER COATINGS | US2014322536A1 | ZHOUPING ZHANG US\ SHIGEN JIANG US\ TAN DU US\ MENG JIANG US\ SHAN ZHANG US | SHANGHAI HUAHONG NEC ELECT CO (CN) | A11-B01\ A11-B14\ A12-W12F |
| 6252 | 3D printing | METHOD OF MANUFACTURING THREE-DIMENSIONAL-SHAPED MOLDED ARTICLE USED AS METAL MOLD, PERFORMING SUCTION REMOVAL OF POWDER AROUND SOLIDIFICATION LAYER AND/OR MOLDED ARTICLE BY NOZZLE WHOSE TIP LEVEL IS LOWER THAN THAT OF CUTTING DEVICE | JP5612735B1 | ZHU XIAOHONG (FR)\ DEFAY EMMANUEL (FR)\ FRIBOURG-BLANC ERIC (FR) | UNIV ELECTRONIC SCIENCE & TECH (CN) | G02-A04A\ G02-A04B\ G05-F03\ S06-G\ S06-K01A |
| 6253 | 3D printing | INK COMPOSITION USED FOR PRINTING CERAMIC TILE, COMPRISES BLUE CERAMIC PIGMENT DISPERSED IN DISPERSION, IN WHICH BLUE CERAMIC PIGMENT CONTAINS MAGNESIUM-COBALT-ALUMINUM OXIDE POWDER AND/OR MAGNESIUM-NICKEL-ALUMINUM OXIDE POWDER | WO2014175519A1 | ZHU YUELIN US | SUZHOU HUAXIN MICRO ELECTRONICS CO LTD US | L03-E05A\ L03-H02\ L03-H05\ L04-A08\ U14-E05B\ U14-E05C |
| 6254 | 3D printing | FABRICATION METHOD FOR PRODUCING THICK FILM THERMOELECTRIC DEVICES WITH PRECURSOR MICROSTRUCTURE OF RELATIVELY LARGE FREE-VOLUME, INVOLVES SINTERING LAYER DURING SIMULTANEOUSLY VOLATILIZING AND REMOVING VEHICLE IN PASTE | US2014315345A1 | ZHUANG YIQI LI (CN) | COMMISSARIAT ENERGIE ATOMIQUE (FR) | A08-A03\ A08-A06\ A08-M01D\ A08-R01\ A11-A03\ A11-A04\ A11-B07\ A12-W07F\ A12-W14\ S06-E01A1\ S06-E01A2\ S06-E01A3\ S06-E02\ S06-E07\ S06-G10\ S06-K07A |
| 6255 | 3D printing | 3D PRINTING MICRO-NANO POWDER/POLYMER COMPOSITE MATERIAL CONTAINS MICRO-NANO POWDER AS MAIN BODY RAW MATERIAL, POLYMER RESIN AS BINDER, AND RAW MATERIAL WHICH CONSISTS OF MICRO-NANO POWDER, POLYMER RESIN, AND RADIATION SENSITIZER | JP2014212166A | CHIDAMBARRAO DURESETI (US)\ CHEN HUAJIE (US)\ PANDA SIDDHARTHA (US)\ OH SANG-HYUN (US)\ UTOMO HENRY K (US)\ RAUSCH WERNER A (US) | TEXAS INSTRUMENTS INC (US)\ MONTGOMERY CLINT L (US)\ KIRKPATRICK BRIAN K (US)\ XIONG WEIZE (US)\ PRINS STEVEN L (US) | A03-A04A\ A10-E05B\ A11-B05D\ A12-E14\ L04-C17A\ M22-H02\ M22-H03B\ X24-A |
| 6256 | 3D printing | PREPARATION OF NICKEL CONDUCTOR ELECTRODE USED FOR SOLDERING TIN, INVOLVES MIXING NICKEL, BORON, ALUMINUM AND ZINC POWDER, AGGLOMERATING, MIXING WITH GLASS POWDER, ETHYL CELLULOSE AND TERPINEOL BINDER AND PRINTING ON ALUMINA SUBSTRATE | KR2014124550A | Empty Field US | IBM (US) | L03-A01A2\ L03-A01A3\ L03-H02\ L03-H03\ L03-H04E3\ L03-H05\ M22-H01\ A11-C04A\ A12-E01\ A12-E01A\ A12-E02\ A12-E07A\ A12-E10\ A12-E14\ G02-A03\ G02-A06\ V04-R01A5A\ V04-R02B\ V04-U01\ V04-U04 |
| 6257 | 3D printing | ELECTROCONDUCTIVE PASTE USED FOR SUPERHEATED STEAM PROCESS FORMATION OF E.G. CONDUCTIVE THIN FILM, COMPRISES ELECTROCONDUCTIVE METAL POWDER, RESIN BINDER HAVING PRESET DECOMPOSITION START TEMPERATURE, AND SOLVENT | US2014319734A1 | VAWTER G ALLEN (US) | Empty Field US | A08-R03\ A11-C02B\ A12-S08C\ A12-S08D3\ A12-S08E\ A12-T03\ A12-T04D\ L03-G05\ S06-E04B1\ S06-E07\ S06-K07A\ U14-H01E\ W06-B01\ W06-B15D\ X15-A01A\ X15-A02\ X15-A05 |
| 6258 | 3D printing | METHOD FOR FORMING HIGH-STRENGTH GRAPHENE-REINFORCED POLYMER MATRIX COMPOSITE FOR E.G. AIRCRAFT PART FOR COMMERCIAL APPLICATION, INVOLVES APPLYING SUCCESSION OF SHEAR STRAIN EVENTS TO MOLTEN POLYMER PHASE | CN203799728U | RAHIM IRFAN (US)\ MUTHUKUMAR SRIRAM (US)\ VEST WILLIAM BRADLEY (US)\ WONG MYRON WAI (US) | SANDIA CORP (US)\ VAWTER G ALLEN (US) | A12-W07D\ A12-W07F\ G02-A04A\ G02-A04B\ G02-A05B\ L03-A01A\ S06-C02 |
| 6259 | 3D printing | CORE CELL COMPRISES A CONDUCTIVE INK FOR PRINTING PATTERN TO CONDUCT ELECTRICAL SIGNALS, AND A CORE CELL FOR PRINTED AS SEVERAL DOTS TO GROW NANOWIRES BY CONDUCTIVE INK AND SEVERAL CELLS COMPRISING THE DOTS | CN104003700A | WESSENDORF KURT O (US) | ALTERA CORP (US) | A05-E02C\ A05-H06\ A08-D01\ A08-S02\ A12-E14\ L03-A02D\ S06-C03\ V05-A\ X12-D01F1 |
| 6260 | 3D printing | CONDUCTIVE PASTE COMPOSITION USEFUL TO PREPARE ELECTRODE, AND FOR GRAVURE OFFSET PRINTING COMPRISES A BINDER RESIN COMPRISING POLYESTER RESIN AND PHENOXY RESIN, CONDUCTIVE POWDER, CURING AGENTS AND A SOLVENT | CN104004984A | KIM JOYCE (US) | SANDIA CORP (US) | A09-D01\ A11-B16 |
| 6261 | 3D printing | THREE-DIMENSIONAL PRINTER FOR CERAMIC ARTICLE PRECURSOR PREPARATION SYSTEM HAS COMPRESSOR THAT APPLIES COMPRESSIVE FORCE TO SUCCESSIVE ALTERNATING LAYERS OF CERAMIC PRECURSOR POWDER AND LIQUID BINDER SOLUTION BASED ON POSITION OF TAMPER | CN104005019A | PARK DONGWON (US)\ JOO YOUNGJOONG (US) | KIM JOYCE (US) | A12-E11C\ A12-E14\ U14-H01E\ U14-J05A\ X26-J |
| 6262 | 3D printing | THREE-DIMENSIONAL-TYPE INORGANIC EL ILLUMINANT I.E. LID-SHAPED LIGHT EMITTING COMPONENT, HAS BACK ELECTRODE LAYER, SUBSTANCE LAYER AND ELECTRODE LAYER FORMED ON SURFACE OF BASE MATERIAL, WHICH CONSISTS OF NON-CONDUCTIVE MATERIAL | CN104005020A | Empty Field US | UNIV ARIZONA (US) | S05-B |
| 6263 | 3D printing | POSTURE CORRECTION GIRDLE FOR ADOLESCENT PATIENT TO PROVIDE IDIOPATHIC SCOLIOSIS CORRECTION TO SPINE DURING RAPID PERIODS OF GROWTH IN HEALTHY CHILDREN, HAS POCKETS PLACED ON SHELL AT PREDETERMINED POSITION, AND PADDING INSERTED INTO POCKET | CN104005022A | Empty Field US | ANRITSU CORP US | M22-H03B\ M22-H03F\ X25-A01\ X25-A08\ X25-A09 |
| 6264 | 3D printing | MAKING COMBUSTION TURBINE COMPONENT INVOLVES ASSEMBLING METALLIC COMBUSTION TURBINE SUBCOMPONENT GREENBODIES AND BINDER TO FORM METALLIC GREENBODY ASSEMBLY; POSITIONING AND ACTIVATING THE BINDER AND SINTERING THE METALLIC GREENBODY ASSEMBLY | CN104005023A | Empty Field US | MINISTRY OF NAT DEFENSE CHUNG SHAN INST OF SCIENCE & TECHNOLOGY US |  |
| 6265 | 3D printing | MANUFACTURING METHOD OF STACKED HEAT EXCHANGER, INVOLVES DRAWING IN MELTED SOLDER MATERIAL OF ANNULAR PROTRUSION ACCORDING TO CAPILLARY PHENOMENA BETWEEN PERIPHERAL WALL THAT IS ADJOINED WITH PLATE, AND HARDENING MELTED SOLDER MATERIAL | CN104005024A | Empty Field US | MINISTRY OF NAT DEFENSE CHUNG SHAN INST OF SCIENCE & TECHNOLOGY US | A09-D03\ A11-B01\ A11-B16\ A11-C02B\ A11-C02D\ A12-W12F |
| 6266 | 3D printing | METHOD FOR MANUFACTURING THREE-DIMENSIONAL-SHAPED MOLDED ARTICLE USED AS E.G. METAL MOLD, INVOLVES CARRYING OUT SUCTION REMOVAL OF POWDER AROUND SOLIDIFICATION LAYER AND/OR ARTICLE BY SUCTION NOZZLE PRIOR TO SURFACE CUTTING PROCESS | CN203789961U | Empty Field US | MINISTRY OF NAT DEFENSE CHUNG SHAN INST OF SCIENCE & TECHNOLOGY US | A09-D03\ A11-B16\ L02-A02\ L02-A05\ M22-H |
| 6267 | 3D printing | THREE DIMENSIONAL (3D) PRINTING SYSTEM FOR PRINTING DESIRED 3D OBJECT INCLUDES INSERTION SYSTEM THAT INSERTS PORTION OF INHIBITOR MATERIAL BETWEEN PORTIONS OF DEPOSITED FUSIBLE POWDER AFTER HAVING BEEN DEPOSITED BY DEPOSITION SYSTEM | CN203791624U | Empty Field US | MINISTRY OF NAT DEFENSE CHUNG SHAN INST OF SCIENCE & TECHNOLOGY US | A05-E01D1\ A12-L05C2\ G06-G05A\ S06-E04A1\ S06-K99C |
| 6268 | 3D printing | MAGNETIC ONE-COMPONENT DEVELOPING TONER FOR FORMING IMAGE ONTO PAPER BY PRINTER, HAS TONER PARTICLES CONTAINING BINDER RESIN AND MAGNETIC POWDER, WHERE MASS OF TONER IS MEASURED AFTER STANDING IN DESICCATOR TOGETHER WITH DESICCANT | CN203791626U | Empty Field US | MINISTRY OF NAT DEFENSE CHUNG SHAN INST OF SCIENCE & TECHNOLOGY US | A03-C01\ A04-G07\ A05-E04E\ A12-E\ A12-W07D\ G02-A04A\ G02-A04B\ G02-A05B\ T04-K01C\ T04-K03B\ W02-B07A3A\ W02-B08L\ W02-B08P8E\ W02-G05A\ W06-A04B5\ W06-A04G7 |
| 6269 | 3D printing | CARBON-BASED ELECTRIC CONDUCTION INK FOR RADIO FREQUENCY IDENTIFICATION ANTENNA COMPRISES SUPER-CONDUCTIVE CARBON BLACK, ELECTRIC CONDUCTION GRAPHITE, CARRIER, BINDING AGENT, CARRIER SOLVENT, AND AUXILIARY AGENT | CN203791836U | ABE HIROMI (JP)\ IWAMA KATSUNARI US\ TSUKAMOTO HIROSHI (JP)\ YAJIMA AKIRA (JP) | RENESAS ELECTRONICS CORP (JP) | A12-H04\ D04-A01F1\ D04-A01F2 |
| 6270 | 3D printing | DEVELOPING MACHINE DEVELOPER FILTRATION SYSTEM, HAS A PLASTIC SHELL SET WITH A FILTER CORE COVER AND FILLED WITH A FOLDABLE COTTON FILTER CORE, WHILE THE INNER SIDE OF PLASTIC SHELL IS FORMED WITH AN INSULATION LAYER | CN203794990U | AHN KIE Y (US)\ FORBES LEONARD (US) | RENESAS TECH CORP (JP) |  |
| 6271 | 3D printing | INOCULATING DEVICE HAS TOWER WHOSE LOWER PORTION SIDE WALL IS EQUIPPED WITH MOUNTING PLATE UNIFORMLY DISTRIBUTED ALONG CIRCUMFERENTIAL DIRECTION AND PROVIDED WITH PNEUMATIC NOZZLE | CN203794991U | AHN KIE Y (US)\ FORBES LEONARD (US) | MICRON TECHNOLOGY INC (US) | V04-A01\ V04-M05\ V04-R04A1\ V04-R04A5A\ V04-R06D3 |
| 6272 | 3D printing | AUTOMATIC WIDTH REGULATING WAVE-SOLDERING PRODUCTION LINE OF PRINTED CIRCUIT BOARD (PCB) PLATE DURING COATING PROCESS, HAS BELT THAT IS ARRANGED WITH RECIPROCATING DRIVING MECHANISM, AND BELT FIXING SEAT THAT IS FIXED ON BELT WHEEL | CN203794992U | AKAISHI SUSUMU US\ YAMADA TETSUHIRO US\ UEMATSU SATORU (JP) | MICRON TECHNOLOGY INC (US) | U11-D01B\ U11-D03A9\ U11-D03B3\ U12-A01B\ U12-A01B3\ U12-A01C\ V07-F01A\ V07-G10C |
| 6273 | 3D printing | PREPARING SYNCHRONOUS BONDED ADHESIVE INVOLVES FORMING THREE-DIMENSIONAL MODEL, PLACING SUBSTRATE PARTICLES, ADHESIVE IN FEED CONTAINER, WITHOUT PRE-HEATING, COMPRESSING GAS, CONVEYING SPRAY GUN, CONTROLLING SHAPE OF MODEL, BONDING | CN203794993U | AKAIWA MASAYASU US | SEIKO EPSON CORP (JP) | A11-B05\ A12-V02\ D09-C01\ L02-A01\ L02-J02B\ L02-K02 |
| 6274 | 3D printing | PHOTOSENSITIVE SOL BONDING AGENT FOR USE IN THREE-DIMENSIONAL PRINTING METHOD, COMPRISES SOL BINDER, EXTERNAL ADHESIVE BOX, SPRAY PRINTING HEAD, MICRO-PUMP, PRINTING HEAD BRACKET, SPREADING ROLLER MOTION, PAVING ROLLER AND PRINTING HEAD | CH707950A2 | AKAMATSU KAZUO US\ EGUCHI KOJI US | RENESAS TECH CORP (JP) | A11-B01\ A11-B16\ A11-C02B\ A11-C02D\ A12-H05\ D09-C01D\ D09-C01E\ D09-C01F\ D09-C04B\ D09-D\ B04-C03\ B11-C05\ B11-C11\ L03-G09P\ L03-G10\ T01-J07B\ W01-A07H2A\ W04-H05\ W04-Y03C\ X24-D03 |
| 6275 | 3D printing | PREPARATION OF POWDER MATERIAL IN THREE-DIMENSIONAL PRINTER MOLDING APPLICATIONS, INVOLVES PRETREATING POWDER MATERIAL, MIXING WITH WATER, POLYOXYETHYLENE LAURYL ETHER AND POLYVINYL ALCOHOL, GRINDING, DRYING, AND RAPID MOLDING | KR2014114498A | AKASAKA YASUSHI US | DENSO CORP (JP) | A12-E02\ A12-E03\ X12-D03B1\ X12-D03C1\ X12-D03D\ X12-D03H |
| 6276 | 3D printing | OPTICAL WAVEGUIDE DEVICE HAS WIRING PATTERN THAT IS CONNECTED TO SIDE TERMINAL OF OPTICAL ELEMENT AND IS FORMED WITH RESPECT TO MAIN SURFACE OF OPTICAL ELEMENT, AND SIDE FACE OF ELEMENT, AND OUTERMOST SURFACE AT TOP OF WIRING BOARD | CN103924238A | ALFORD TERRY L (US)\ MISRA EKTA (US) | SEMICONDUCTOR LEADING EDGE TEC (JP) | A04-F08\ A10-E05B\ A12-R01\ A12-R06\ A12-R07\ A12-W12G\ L02-A03\ L02-A04\ L02-A07\ L02-A11\ L02-A12\ L02-F05\ L02-G01A\ L02-G01B1\ L02-G02\ L02-G08\ L02-G10B\ L02-G11\ L02-G12E1\ L02-J02C\ L02-K01\ X24-D03 |
| 6277 | 3D printing | MANUFACTURING COATING LAYER FOR SURFACE TREATMENT OF IMPLANT, INVOLVES DIPPING BIOACTIVE MATERIAL IN MEMBRANOUS POLYMERS CARRIER LAYER | JP2014215332A | ALLMAN DERRYL D J (US)\ SUN SEY-SHING (US)\ PRITCHARD DAVID (DE)\ PRICE DAVID T (US)\ BHATT HEMANSHU D (US)\ MAY CHARLES E (US)\ KWAK BYUNG-SUNG (US)\ BURKE PETER AUSTIN (US) | UNIV ARIZONA (US) | M13-C01\ M13-L\ M29-C\ M29-D |
| 6278 | 3D printing | MANUFACTURING METHOD FOR MOLDING AND CASTING CUSTOM SOFTENING POLYMERS OR PREPOLYMERS INTO COMPLEX SHAPED DEVICES E.G. EARPLUG, INVOLVES INJECTING POLYMER OR PREPOLYMER INTO CASING AND CURING PREPOLYMER IN SHORT PERIOD OF TIME | WO2014180812A1 | ALPERT CHARLES JAY (US)\ REN HAOXING (US)\ VILLARUBIA PAUL GERARD (US) | SUN SEY-SHING (US)\ ALLMAN DERRYL D J (US)\ BHATT HEMANSHU D (US)\ MAY CHARLES E (US)\ BURKE PETER AUSTIN (US)\ KWAK BYUNG-SUNG (US)\ PRICE DAVID T (US)\ PRITCHARD DAVID (DE) | M13-H01\ M26-B08\ M26-B08A\ M26-B08C\ M26-B09 |
| 6279 | 3D printing | MULTI-STRANDED CONDUCTOR CORE FIREPROOF CABLE USED IN HIGH RISE BUILDING FOR CONVEYING ELECTRIC POWER, HAS CABLE CONDUCTOR THAT IS INCLUDED WITH GROUP OF MULTIPLE LEADS THAT ARE TWISTED WITH EACH OTHER AND COVERED WITH WINDING LAYER | CN103992099A | ANDERSON BRENT A (US)\ NOWAK EDWARD J (US)\ BRYANT ANDRES (US) | VILLARRUBIA PAUL G (US)\ ALPERT CHARLES J (US)\ REN HAOXING (US) | M13-H01\ M26-B08\ M26-B08A\ M26-B08B\ M26-B08C\ M26-B08M\ M26-B08N\ M26-B08S\ M26-B12\ M26-B12C\ M26-B12M\ M26-B12N\ M26-B12S\ M26-B12T\ M27-A04\ M27-A04C\ M27-A04M\ M27-A04N\ M27-A04S\ M27-A04T |
| 6280 | 3D printing | PREPARATION OF ENVIRONMENT-FRIENDLY CERAMIC VITRIFIED TILE INVOLVES MILLING DRIED RED SHALE WASTE SLAG, WASTE GLASS, AND DOLOMITE, ADDING ALUMINA AND BINDER, MIXING, MOLDING, DRYING, SINTERING, POLISHING, CLEANING, AND SURFACE DECORATING | CN103993201A | AOCHI HIDEAKI (JP) | IBM (US)\ ANDERSON BRENT A (US)\ NOWAK EDWARD J (US)\ BRYANT ANDRES (US) | M13-H01 |
| 6281 | 3D printing | IMPROVEMENT OF CORROSION-RESISTANT PROPERTIES OF HEATING SURFACE COATING OF BOILER INVOLVES SPRAYING TO CLEAN, PERFORMING PRE-SHOT PEENING, PERFORMING SHOT PEENING, DROPPING SURFACE TEMPERATURE, AND HEATING | CN103993309A | ARENA PAOLO (IT)\ BRANCIFORTE MARCO (IT)\ OCCHIPINTI LUIGI (IT)\ BERNARDO GIOVANNI DI (IT) | TOSHIBA KK (JP) | M13-H01 |
| 6282 | 3D printing | PREPARING ALUMINUM ALLOY COMPOSITE COATING FOR USE IN ALUMINUM ALLOY ENGINES, INVOLVES TAKING ALUMINUM ALLOY PLATE WITH LASER CLADDING TO PERFORM SURFACE SHOT BLASTING PROCESS | CN103993311A | ARIMA YUTAKA (JP) | ST MICROELECTRONICS SRL (IT) | M13-H01\ M26-A02\ M26-A02N\ M26-B08 |
| 6283 | 3D printing | MANUFACTURING SCREW DRILL RADIAL BEARING INVOLVES APPLYING CLADDING WEAR-RESISTANT MATERIAL AFTER HEAT TREATMENT AND MECHANICAL PROCESSING OF INNER CYLINDRICAL SLEEVE BY LASER, WHERE POWDER MATERIAL IS IRON-BASED COMPOSITE POWDER | CN203782233U | ARNAL VINCENT (FR)\ TORRES JOAQUIN (FR)\ GOSSET LAURENT (FR)\ CHHUN SONARITH (NL) | KITAKYUSHU FOUNDATION US | M22-H03B\ T01-J07B\ X25-A01\ X25-B02A2\ X25-B04 |
| 6284 | 3D printing | MOLDING IRON-BASED POWDER LASER CLADDING PIPELINE INVOLVES PLANNING PIPELINE ACCORDING TO POSITION AND RELATIVE SIZE IN ANTICLOCKWISE DIRECTION | CN203785958U | ASANO TETSUO US | NXP BV (NL)\ ST MICROELECTRONICS CROLLES 2 (FR)\ GOSSET LAURENT (FR)\ TORRES JOAQUIN (FR)\ ARNAL VINCENT (FR)\ CHHUN SONARITH (NL) | M22-H03A\ M22-H03B\ S03-E06B1 |
| 6285 | 3D printing | PREPARING TITANIUM-ALUMINUM-NIOBIUM ALLOY COATING ON TITANIUM METAL SURFACE, INVOLVES MIXING TITANIUM POWDER, ALUMINUM POWDER, NIOBIUM POWDER, AND YTTRIUM OXIDE POWDER UNIFORMITY IN BALL MILL | CN103993308A | ASAYAMA EIICHI (JP)\ ONO TOSHIAKI (JP)\ TANAKA TADAMI (JP)\ UMENO SHIGERU (JP)\ NISHIKAWA HIDESHI (JP) | SANYO ELECTRIC CO (JP) | M23-D05\ X24-D03 |
| 6286 | 3D printing | PREPARING HIGH QUALITY NICKEL BASE ALLOY COATING ON TITANIUM METAL SURFACE, INVOLVES MIXING NICKEL BASE SELF-FLUXING ALLOY POWDER LANTHANUM OXIDE IN BALL MILL UNIFORMLY | CN103993310A | ASO HIROYUKI US\ NAKABAYASHI MASAAKI US | SUMITOMO MITSUBISHI SILICON (JP) | M13-H01 |
| 6287 | 3D printing | SPECIMEN BAG USED DURING LAPAROSCOPIC SURGERY, HAS SAMPLE BAG STRUCTURE THAT IS PROVIDED WITH PRESSING BELT, AND THAT IS PROVIDED WITH ARC-SHAPED PRESSING STRIP TO WHICH SKID-PROOF BELT IS SET | CN203782492U | AVANZINO STEVEN C (US)\ NGO MINH VAN (US)\ WANG PIN-CHIN CONNIE (US) | FUJITSU LTD (JP) | M13-H01\ M13-L |
| 6288 | 3D printing | TEMPERATURE CONTROL DEVICE FOR POWDER PRE-HEATING AND CONVEYING DEVICE OF THREE-DIMENSIONAL PRINTING, HAS MICROPROCESSOR WHICH CALCULATES HEATING TIME AND ELECTRIC CURRENT OF HEATING SYSTEM FOR HEATING POWDER JAR | CN103996734A | AVANZINO STEVEN C (US)\ NGO MINH VAN (US)\ WOO CHRISTY MEI-CHU (US)\ SANCHEZ JR JOHN E (US) | ADVANCED MICRO DEVICES INC (US) | M13-H01\ M22-H03B |
| 6289 | 3D printing | SINGLE LIGHT SOURCE AND DOUBLE SCANNING ELECTRON MICROSCOPE SPECTRAL SELECTIVE LASER MELTING MOLDING DEVICE INCLUDES FORMING CAVITY, OPTICAL SPLITTER, LASER EXPANDING MIRRORS, WORKING PLATFORM, INERT GAS CURTAIN AND HEATING SYSTEM | CN103990799A | BAILEY JAMES R (US)\ CRUTCHFIELD DAVID A (US) | ADVANCED MICRO DEVICES INC (US) | M13-H01\ M13-L |
| 6290 | 3D printing | VARIABLE ANGLE OF WORKTABLE USED FOR LASER PROCESSING COMPRISES BRACKET, WORKING TABLE MAIN BODY, BASE, AND SLIDING GUIDE RAIL | CN203778963U | BANDYOPADHYAY ABHIJIT (US)\ PETTI CHRISTOPHER J (US)\ KUMAR TANMAY (US) | LEXMARK INT INC (US) | M22-H03B\ S04-A04B\ X25-A08 |
| 6291 | 3D printing | NON-HORIZONTAL PLANE LASER CLADDING DEVICE COMPRISES BOTTOM BASE, BRACKET AND SUPPORT PLATES | CN203782232U | BAO XINGKUN US | SANDISK 3D LLC (US)\ BANDYOPADHYAY ABHIJIT (US)\ PETTI CHRISTOPHER J (US)\ KUMAR TANMAY (US) | L03-H04A\ L04-C07\ L04-D\ L04-E03\ U11-C07D2\ U11-C18B4\ U12-A01A2 |
| 6292 | 3D printing | POWDER FEEDER-BASED NOZZLE DEVICE USED FOR LASER CLADDING PROCESS, HAS POWDER BOX WHOSE LOWER PORTION IS PROVIDED WITH PALLET TO WHICH THROUGH HOLE IS PROVIDED, AND BOX STRUCTURE THAT IS SET WITH LASER CLADDING MECHANISM | US2014333977A1 | BEASOM JAMES DOUGLAS (US) | GUANGZHOU GUIZHIYUAN MICROELECTRONICS CO LTD US | M13-H01\ M26-B08\ M26-B08B\ M26-B08C\ M26-B08M\ M26-B08S |
| 6293 | 3D printing | THREE-DIMENSIONAL LASER CLADDING FORMING WORKING TABLE COMPRISES CLADDING PLATFORM, SUPPORT PLATFORM, LEVER MECHANISM AND REGULATING END | WO2014179679A1 | BESSER PAUL R (US)\ LOPATIN SERGEY (US)\ ERB DARRELL M (US) | INTERSIL INC (US) | V07-F02B\ V07-K01\ V07-K05\ V07-M |
| 6294 | 3D printing | PULSED LASER CLADDING-TYPE SCRAPER WITH ROTATING POWDER FEEDER FOR USE IN E.G. INDUSTRIAL APPLICATION, HAS DELIVERING PIPE THAT IS MOUNTED ON WORKING TABLE, AND SCRAPING PLATE THAT IS PROVIDED WITH POWDER GROOVE | EP2799179A1 | BING CAI US | ADVANCED MICRO DEVICES INC (US) |  |
| 6295 | 3D printing | MANUFACTURING E.G. CROWN, OF A WATCH, COMPRISES FORMING A COMPONENT BY SELECTIVE LASER SINTERING, POLISHING THE COMPONENT, IMMERSING THE COMPONENT IN A BATH COMPRISING POLISHING PARTICLES, AND MOVING THE COMPONENT WITH RESPECT TO PARTICLES | WO2014175447A1 | BING CAI US | UNIV XIANGFAN US | A10-E05B\ A12-E10\ A12-W12G\ L02-A02\ L02-A03\ L02-A04\ L02-A11\ L02-A12\ L02-E07\ L02-F05\ L02-G08\ L02-J02C |
| 6296 | 3D printing | METHOD FOR MANUFACTURING LIGHT EXTRACTION LAYER FOR LIGHT EMITTING APPARATUS, INVOLVES FORMING CONCAVO-CONVEX ON SURFACE OF LIGHT EXTRACTION LAYER, WHERE LIGHT EXTRACTION LAYER IS COATED ON SUBSTRATE OF LIGHT EMITTING APPARATUS | WO2014178706A1 | BINGYAN REN US\ LI REN US | UNIV XIANGFAN US | M13-H01\ M22-H01\ M22-H02\ M22-H03F\ M26-A01\ M26-A01B\ M26-A01C\ M26-A01J\ M26-A01N\ M26-A01S\ M26-A04\ M26-B08\ M26-B08B\ M26-B08C\ M26-B08J\ M26-B08N\ M26-B08S |
| 6297 | 3D printing | LASER CLADDING METHOD FOR PROCESSING STEEL SURFACE WITH NICKEL BASE ALLOY, INVOLVES POLISHING STEEL SURFACE TO REMOVE UNWANTED RUST AND IMPURITIES | WO2014176827A1 | BIZEN MITSUHIRO US | BINGYAN REN US\ LI REN US | M13-H01\ M22-H02\ M27-A04\ M27-A04B\ M27-A04C\ M27-A04M\ M27-A04N\ M27-A04S\ M27-B04\ M27-B04B\ M27-B04C\ M27-B04M\ M27-B04N\ M27-B04S\ M27-C03\ V08-A09 |
| 6298 | 3D printing | APPARATUS FOR READING IMAGE, WHICH IS DISPLAYED ON TWO-DIMENSIONAL ARRAY SHAPE DISPLAY-TYPE SPATIAL LIGHT MODULATOR FOR ELECTRONIC HOLOGRAPHY DISPLAY APPARATUS, HAS HALF-WAVE PLATE FOR INJECTING LIGHT INTO DEFLECTION BEAM SPLITTER | US2014327023A1 | BO LIU US\ SHUNLI CHEN US\ CHUNHAI LIU US\ YUAN WANG US\ JIJUN YANG US | SANYO ELECTRIC CO (JP)\ TOKYO SANYO ELECTRIC CO US | M13-H01\ M22-H02\ M22-H03A\ M26-A02\ M26-A02S\ M26-A02T\ M26-A04\ M26-B\ M26-B00S\ M26-B00T |
| 6299 | 3D printing | BEVERAGE BOTTLE FOR USE BY CHILDREN FOR CONTAINING BEVERAGE, HAS SIDEWALL COVERED WITH SHRINK-WRAP, WHERE SHRINK-WRAP IS DEPICTED WITH ILLUSTRATION COMPRISING SIMULATE RELIEF, AND RELIEF AND EMBOSSMENT ARE IN CORRESPONDENCE WITH EACH OTHER | JP5610031B1 | BONSHIHARA MANABU US\ KAMIBAYASHI KAZUTOSHI US\ HASHIMOTO SEIJI US | UNIV SICHUAN US | L04-C18A\ V08-A01A\ V08-A04A |
| 6300 | 3D printing | MANUFACTURE OF ENVIRONMENTALLY-FRIENDLY HONEYCOMB CERAMIC HEAT ACCUMULATOR INVOLVES MIXING RED SHALE WASTE, WASTE SLAG, WASTE GLASS, LIMESTONE, TALC, COKE POWDER AND POLYVINYL ACETATE, PRESS-MOLDING, HEATING, SINTERING AND COOLING | JP2014199413A | BOSELLI GIANLUCA (US)\ DUVVURY CHARVAKA (US) | ZYCUBE KK US | S03-F02\ X24-D03 |
| 6301 | 3D printing | NICKEL-BASED ALLOY POWDER FOR PREPARATION OF NICKEL-BASED AMORPHOUS COMPOSITE COATING E.G. NIOBIUM CARBIDE-REINFORCED AMORPHOUS COMPOSITE COATING COMPRISES IRON, BORON, SILICON, NIOBIUM, CARBON, AND NICKEL | CN103915561A | BRODSKY JONATHAN (US)\ STEINHOFF ROBERT (US)\ VROTSOS THOMAS A (US) | TEXAS INSTRUMENTS INC (US)\ BOSELLI GIANLUCA (US)\ DUVVURY CHARVAKA (US) | L04-A01A\ M22-H02\ M26-B08\ M26-B08B\ M26-B08C\ M26-B08M\ M26-B08N\ M26-B08R\ M26-B08S\ M26-B08V\ M26-B12\ M26-B12B\ M26-B12C\ M26-B12M\ M26-B12N\ M26-B12R\ M26-B12S\ M26-B12T\ M26-B12V\ V08-A04A\ V08-A04B\ V08-A04C2\ V08-A09 |
| 6302 | 3D printing | LASER REMANUFACTURE OF ROLLER INVOLVES MECHANICALLY CLEANING ROLLER AT ROOM TEMPERATURE, REMOVING OIL AND RUST, CLEANING WITH ALCOHOL AND ACETONE, PREHEATING ROLLER SUBSTRATE, AND ADJUSTING LASER CLADDING ALLOY POWDER FEED RATE | CA2841700A1 | BRUCE ALLEN BOECK US\ JEFF THOMAS WETZEL US\ TERRY GRANT SPARKS US | TEXAS INSTRUMENTS INC (US) | M22-H02\ M22-H03A\ M26-A02\ M26-A02N\ M26-A04\ M26-B13\ M26-B13N\ X25-A02A\ X25-A09 |
| 6303 | 3D printing | PREPARATION OF TITANIUM-SILICON ALLOY COATING COMPRISES MIXING SILICON POWDER AND TITANIUM POWDER, MILLING, DRYING, COMPACTING, LASER CLADDING AND CLEANING | JP2014199296A | BULLER JAMES F (US)\ LINN JACK H (US)\ LOWRY ROBERT K (US)\ ROUSE GEORGE V (US) | MOTOROLA INC (US) |  |
| 6304 | 3D printing | SMALL DIAMETER PIPE WALL LASER CLADDING EQUIPMENT INCLUDES MACHINE BODY, FIXED PIPE WORKPIECE SPINDLE, SEMICONDUCTOR LASER SYSTEM, INNER WALL CLADDING HEAD REFLECTING MIRROR, INVERTED CONE-SHAPED OUTLET, POWDER FEEDING ARM, SCREW AND MOTOR | US2014316786A1 | BUSSE BERND (DE)\ BERGMAN LARS-ERIK (SE) | INTERSIL CORP (US) | T01-J08A\ X14-E |
| 6305 | 3D printing | LASER CLADDING SAMPLE CUTTING TEST CLAMP, HAS CUTTING BUCKET PROVIDED WITH CUTTING EDGES, RIGHT ADJUSTING BLOCK LOCATED ON CUTTING BOX BODY, TRANSMISSION BOLT CONNECTED WITH TRANSMISSION NUT, AND PIN FIXED ON CLAMP BOX BODY | JP2014195861A | BUYNOSKI MATTHEW S (US)\ WANG PIN-CHIN CONNIE (US)\ MARATHE AMIT P (US)\ PANGRLE SUZETTE K (US) | AKZO NOBEL NV (NL)\ EKA CHEMICALS AB (SE)\ BUSSE BERND (DE)\ BERGMAN LARS ERIK (SE) | A09-D03\ A11-B01\ A11-B03\ A12-L03\ A12-W12F |
| 6306 | 3D printing | LASER CLADDING METHOD FOR ROLLER SHAFT COMPONENT INVOLVES CLEANING ROLLER SHAFT COMPONENT WITH ACETONE AND ALCOHOL, FEEDING LASER CLADDING ALLOY POWDER INTO ROLLER SHAFT COMPONENT THROUGH FEEDING DEVICE, AND PERFORMING LASER CLADDING | CN203764976U | CABRAL JR CYRIL (US)\ KALDOR STEFFEN K (US)\ KIM HYUNGJUN (US)\ ROSSNAGEL STEPHEN M (US) | ADVANCED MICRO DEVICES INC (US) | M23-D05\ M23-G\ T01-J07A\ T01-J07B\ X24-D03\ X26-C03 |
| 6307 | 3D printing | FORMATION OF NIOBIUM-BASED MULTI-ELEMENT POWDER FUSED ALLOY COATING INVOLVES PREPARING MULTI-ELEMENT MIXED POWDER SHEET BY MIXING NIOBIUM POWDER, TIN POWDER AND TITANIUM POWDER, PRESSING, AND PLACING SHEET IN TITANIUM METAL SURFACE | CN103981518A | CARROLL MICHAEL SCOTT (US)\ SANA PEYMAN (US)\ PATEL MINESH AMRAT (US)\ MA YI (US) | IBM (US) | M13-H01\ M13-L\ T01-F06\ T01-J07B\ X26-C03 |
| 6308 | 3D printing | SIDE PILE PLATE STRUCTURE FOR USE IN STEEP ROCK SLOPE BANK, HAS ANCHORED ROCK BODY WHOSE LOWER PART IS FIXED WITH REINFORCED CONCRETE PLATE INNER VERTICAL LAYERED FORMED WITH GEOTEXTILE GRID | CN103982653A | CAUBET PIERRE (FR)\ GREGOIRE MAGALI (FR) | AGERE SYSTEMS INC (US) | A12-E11A\ U12-A01A1E\ U12-A01A2\ V07-F02C\ V07-K01\ V07-M |
| 6309 | 3D printing | FLUORESCENT LAYER FOR NUCLEAR BATTERY THAT IS UTILIZED IN E.G. MICRO-CONTROLLER, HAS FLUORESCENT POWDER LAYER ADHERED ON GLASS SUBSTRATE | CN203774327U | CHAN ALBERT (US) | ST MICROELECTRONICS SA (FR) | A11-B14\ X25-A08 |
| 6310 | 3D printing | SELECTIVE LASER MELTING QUICK MOLDING DEVICE COMPRISES CONTROL SYSTEM FOR CONTROLLING STATE OF LASER AND LASER MODULE IN EACH WORK STATION, LASER, LIGHT GUIDE, WORK STATION, SCANNING SYSTEM, VIBRATION MIRROR SYSTEM AND AUXILIARY DEVICE | CN103976883A | CHAN VICTOR W C (US)\ KANIKE NARASIMHULU (US)\ SHANG HUILING (US)\ VIDYA VARADARAJAN (US)\ YUAN JUN (US)\ BOOTH JR ROGER ALLEN (US) | FUJITSU LTD (JP) | M22-H03B\ M23-D05\ X24-D03 |
| 6311 | 3D printing | COMPLEX PIECE COAXIAL POWDER FEEDING LASER CLADDING DEVICE COMPRISES CONDUCTIVE FIBER, LASER LIGHT PATH CAVITY, TWO-DIMENSIONAL LASER SENSOR, LASER COAXIAL POWDER FEEDING HEAD AND END COMPUTER CONTROL ANALYSIS SYSTEM | CN103980409A | CHANDA KAUSHIK (US)\ RAMANATH G (US)\ MURARKA SHYAM P (US)\ KRISHNAMOORTHY AHILA (IN) | IBM (US)\ CHAN VICTOR W C (US)\ KANIKE NARASIMHULU (US)\ SHANG HUILING (US)\ VIDYA VARADARAJAN (US)\ YUAN JUN (US)\ BOOTH JR ROGER ALLEN (US) | U11-C09G\ U12-A01B\ U14-H01E\ V07-F02B\ V07-G10D\ V07-K01\ V08-A01B\ V08-A04A |
| 6312 | 3D printing | COMPLEX STRUCTURE PRESET POWDER LASER CLADDING PROCESSING DEVICE INCLUDES CONDUCTIVE FIBER, LIGHT PATH CAVITY, LASER CLADDING HEAD, SPOT LASER SENSOR, PROGRAMMABLE LOGIC CONTROLLER CONTROL SYSTEM, CONNECTING PIECE AND PHOTOSENSITIVE ELEMENT | CN103980552A | CHANG AUGUSTINE WEI-CHUN (US) | RENSSELAER POLYTECH INST (US) | A11-B01\ A12-V02\ S05-B\ S05-Y03\ T01-J06A\ V08-A09\ X25-A08 |
| 6313 | 3D printing | DISPLAY DEVICE FOR MOBILE DEVICE, HAS SPATIAL LIGHT MODULATOR UNIT FOR GENERATING INTERFERENCE PATTERN AND DISPLAYING HOLOGRAM BASED ON INTERFERENCE PATTERN, WHERE BACKLIGHT UNIT IS MANUFACTURED AS ORGANIC LED INCLUDING QUANTUM DOTS | CN103980690A | CHANG CHYH-YIH (TW)\ JIANG HSIN-CHIN (TW)\ KER MING-DOU (TW) | SCHOTTKY LSI INC (US) | T01-H01B6A\ T01-J10C4\ T01-J10D\ V07-F02B\ V07-F02C\ V07-K01 |
| 6314 | 3D printing | METHOD FOR OPERATING ADDITIVE MANUFACTURING APPARATUS E.G. SELECTIVE LASER SINTERING (SLS) DEVICE, INVOLVES DIRECTING THIRD ENERGY BEAM ALONG OFFSET CONTOUR VECTOR HAVING UNPROCESSED POWDER REGION IN BUILD PLANE | CN103981519A | CHANG KUO-TUNG (US)\ SUN YU (US) | IND TECH RES INST (US) | L03-H04A\ L04-E03A\ A12-E07C\ U12-A01A |
| 6315 | 3D printing | PREPARATION OF COARSE-GRAINED FINAL STRUCTURE FOR HOT-GAS-CONVEYING WORKPIECE, INVOLVES APPLYING LAYER OF POWDER MIXTURE ON STRUCTURE, SOLIDIFYING, APPLYING ANOTHER LAYER OF ANOTHER POWDER MIXTURE ON OBTAINED STRUCTURE AND SOLIDIFYING | CN103982578A | CHANG MEI (US)\ TSENG JENNIFER (US)\ CHERN CHYI (US)\ CHEN LING (US)\ SMITH DAVID C (US)\ LITTAU KARL A (US)\ LIAO MARVIN (SG) | SPANSION LLC (US) | A08-R01\ A12-E06A\ L03-E01B5B\ L03-E03\ L03-E08\ L03-H03A\ L03-H05\ T01-J07D1\ T01-M06A1\ W01-C01D3C\ W01-C01E5B\ X16-B01F1\ X16-E01G\ X21-A01F\ X21-B01A |
| 6316 | 3D printing | SEMICONDUCTOR LASER APPARATUS HAS SPATIAL LIGHT MODULATOR WHICH IS PROVIDED WITH TRANSPARENT COMMON ELECTRODE, TRANSPARENT PIXEL ELECTRODES, AND LIQUID CRYSTAL LAYER ARRANGED BETWEEN COMMON ELECTRODE AND PIXEL ELECTRODES | CN103983203A | CHEN DAVID JIA (US)\ COUGHLIN JR TERRY C (US) | APPLIED MATERIALS INC (US) | A11-B05\ A11-C02B\ A11-C02C\ A12-L03\ A12-L03B\ E05-C\ E12-A04\ L03-G02A\ L03-G05A\ L03-G05B7B\ U11-A03A\ U11-C18D\ U14-K01A1G\ U14-K01A1J\ V07-F02B\ V07-K10A |
| 6317 | 3D printing | METHOD FOR MANUFACTURING CRANIOPLASTY PLATE FIT TO E.G. MERELY UNDESIRED SHAPED UNIT OF BONE STRUCTURE OR SKULL OF HUMAN PATIENT, INVOLVES FABRICATING CUSTOMIZED IMPLANT FROM BIOCOMPATIBLE PLATE THROUGH ADDITIVE MANUFACTURING TECHNIQUE | US2014293386A1 | CHEN JIA (CN)\ WANG RENCHENG (CN)\ JI LINHONG (CN)\ LIU WEIFENG (CN)\ WANG CHUNCAI (CN)\ LIN JIA (CN)\ SUN YUCHUN (CN)\ HAO DAOXIN (CN) | IBM (US) | A02-A01\ A04-D04A\ A04-F06E\ A08-C07\ A08-D03\ A10-E05B\ A11-C02\ A12-E15\ A12-W12G\ L03-G09A2\ L03-G10\ V06-V01E\ X24-D03 |
| 6318 | 3D printing | SYSTEM FOR GENERATING AND DISPLAYING HIGH RESOLUTION HOLOGRAMS ON E.G. LCD DEVICES, HAS HOLOGRAM MANAGEMENT COMPONENT INTERPOLATING DOWNSAMPLED HOLOGRAM TO FACILITATE GENERATION OF HOLOGRAM WITH RESOLUTION FOR DISPLAYING HOLOGRAPHIC IMAGE | WO2014172496A1 | CHEN JUN (CN)\ WEI YAN US\ QIXUE ZHANG US\ HONGHAI NIU US\ CHUANJUN BAI US\ LIANGGEN XIONG US\ KAI WANG (CN) | UNIV TSINGHUA (CN)\ CHEN JIA (CN)\ WANG RENCHENG (CN)\ JI LINHONG (CN)\ LIU WEIFENG (CN)\ WANG CHUNCAI (CN)\ LIN JIA (CN)\ SUN YUCHUN (CN)\ HAO DAOXIN (CN) | U23-P01\ U23-Q\ V07-F02A\ V07-F02C\ V07-K01\ V07-M\ V08-A01A\ V08-A03 |
| 6319 | 3D printing | FABRICATION METHOD OF LIGHT-EMITTING DEVICE USED IN LIGHTING SYSTEM INVOLVES DISPOSING LAYERED PHOSPHOR COMPOSITE INCLUDING YELLOW-EMITTING PHOSPHOR LAYER OVER SECOND PHOSPHOR LAYER FORMED BY MANGANESE-DOPED POTASSIUM FLUOROSILICATE (PFS) | WO2014169078A2 | CHEN LING (US)\ MARCADAL CHRISTOPHE (US) | NARI RELAYS ELECTRIC CO LTD US\ NANJING NARI RELAYS ENG TECH US | V07-F02A\ V07-G15\ V07-K05 |
| 6320 | 3D printing | POSITIVE ELECTRODE FOR LITHIUM ION SECONDARY BATTERY USED IN E.G. MOBILE TELEPHONE, HAS COATING LAYER MANUFACTURED ON COATED SURFACE OF ACTIVE MATERIAL LAYER, SUCH THAT MATERIAL OF COATING LAYER IS SPRAYED ON SURFACE OF MATERIAL LAYER | CN103938209A | CHEN TIAN-AN (US)\ O'BRIEN KEVIN P (US) | APPLIED MATERIALS INC (US) | T01-E01B\ T01-J03\ T01-J18\ T01-J30A\ T01-L02\ W04-V |
| 6321 | 3D printing | MANUFACTURE OF OPTICAL COMPENSATION FILM USED FOR POLARIZING PLATE, INVOLVES PROVIDING OPTICALLY-ANISOTROPIC LAYER COMPOSITION CONTAINING LIQUID CRYSTAL COMPOUND IN LAYERED FORM ON ORIENTED FILM, AND IRRADIATING ULTRAVIOLET RAY | US2014286813A1 | CHEN YI-NAN (TW)\ WU WEN-CHIEH (TW)\ WU CHUN-YI (TW) | INTEL CORP (US) |  |
| 6322 | 3D printing | PREPARATION OF PIEZOELECTRIC CERAMIC-POLYMER COMPOSITE MATERIAL INVOLVES MIXING CERAMIC POWDER, MONOMER, DISPERSING AGENT, CROSSLINKING AGENT AND WATER, VACUUM-DEFOAMING OBTAINED SLURRY, ADDING INITIATOR AND CATALYST, AND REACTING | US2014287165A1 | CHEN ZI-PING (TW)\ LIN KUN-HSIEN (TW)\ JIANG RYAN HSIN-CHIN (TW) | Empty Field US | L03-G02\ M13-H01\ M13-L\ M22-H03B\ V07-F01A1\ V07-K01A\ X25-A08\ X25-A09 |
| 6323 | 3D printing | LASER APPARATUS HAS SPATIAL LIGHT MODULATOR THAT IS ARRANGED TO ACT AS INTRA-CAVITY DIGITAL HOLOGRAPHIC MIRROR AND TO DISPLAY SELECTIVELY GRAY-SCALE IMAGE OF HOLOGRAM TO PHASE-MODULATE LASER LIGHT IN CAVITY | US2014299091A1 | CHENG KANGGUO (US)\ KHAKIFIROOZ ALI (US)\ KULKARNI PRANITA (US)\ CAI JIN (US) | AMAZING MICROELECTRONIC CORP (TW)\ CHEN ZI-PING (TW)\ LIN KUN-HSIEN (TW)\ JIANG RYAN HSIN-CHIN (TW) | M13-H01\ M26-B08\ M26-B08B\ M26-B08C\ M26-B08M\ M26-B08S\ M26-B08T |
| 6324 | 3D printing | OPTICAL OPERATING DEVICE I.E. OPTICAL SWITCH APPARATUS, FOR OPTICAL COMMUNICATION SYSTEM, HAS ANAMORPHIC OPTICAL SYSTEM EXPANDING BEAM DIAMETER OF LIGHT FROM SIDE OF OPTICAL INPUT-OUTPUT PORT, WHERE BEAM WAIST RADIUS IS SET TO SMALLER VALUE | US2014299742A1 | CHENG SHILIN US | IBM (US)\ CHENG KANGGUO (US)\ KHAKIFIROOZ ALI (US)\ KULKARNI PRANITA (US)\ CAI JIN (US) | M13-H01\ M13-H04 |
| 6325 | 3D printing | METHOD FOR CONSTRUCTING AND TRAINING STATISTICAL LANGUAGE MODEL (SLM), INVOLVES RANKING CANDIDATE ARTICULATIONS AND TRAINING SLM TO RECOGNIZE MACHINE TEXT-TO-SPEECH ARTICULATIONS, SUCH THAT SUBSET OF CHALLENGE ITEMS IS USED AS SEED SET | US2014301736A1 | CHENG YI-KAN (TW)\ LU LEE-CHUNG (TW)\ TIEN LI-CHUN (TW)\ HOU YUNG-CHIN (TW)\ GUO TA-PEN (US)\ TAI CHUN-HUI (TW)\ HOU YUAN-TE (TW) | LIANYUNGANG DONGHAI COUNTY HONGWEI QUARTZ PRODUCT CO LTD US | L04-C01\ L04-C02D\ L04-C04\ L04-F03\ U11-C01J1\ U11-C01J2\ U11-C02A2\ U11-D01C3 |
| 6326 | 3D printing | AUTOMATIC FLAT BOARD PUNCHING MACHINE HAS REGISTER ADJUSTMENT UNIT THAT IS CONVEYED BY PUNCHING UNIT, FOR ADJUSTING POSITION OF SHEET PROVIDED BETWEEN PUNCHING UNIT AND SHEET FEEDER | US8842359B1 | CHERN CHAN-HONG (US) | TAIWAN SEMICONDUCTOR MFG (TW)\ CHENG YI-KAN (TW)\ LU LEE-CHUNG (TW)\ GUO TA-PEN (US)\ TIEN LI-CHUN (TW)\ HOU YUNG-CHIN (TW)\ TAI CHUN-HUI (TW)\ HOU YUAN-TE (TW) | A04-F06E5\ A08-M\ A08-R\ A08-S05\ A09-D03\ A11-B16\ A12-V02B\ D08-A03 |
| 6327 | 3D printing | INTEGRATED PLATFORM FOR MANUFACTURING LASER FORMING EQUIPMENT, HAS SUB-CONTROLLER THAT CONTROLS REPAIRING SUB-PLATFORM LASER CLADDING DEPOSITION FORMING CLADDING SPRAY HEAD | US2014299405A1 | CHIDAMBARRAO DURESETI (US)\ JAMMY RAJARAO (US)\ DOKUMACI OMER H (US)\ DORIS BRUCE BENNETT (US)\ GLUSCHENKOV OLEG (US)\ MANDELMAN JACK ALLEN (US) | TAIWAN SEMICONDUCTOR MFG (TW)\ TAIWAN SEMICONDUCTOR MFG COPMANY (TW) | A02-A03\ A02-D01\ A04-F06E\ A08-A07\ A08-S05\ A10-B03\ A11-A04\ A11-B16\ A12-W07F\ X25-A08 |
| 6328 | 3D printing | LASER CLADDING METHOD OF PUMP MECHANICAL SEAL SLEEVE INVOLVES SURFACE-TREATING ABRASION PORTION OF SLEEVE BY LASER USING NICKEL-BASED COMPOSITE POWDER CONTAINING CARBON, CHROMIUM, SILICON AND IRON OXIDE | US2014313469A1 | CHIGIRA TAKASHI US\ ICHIDA MAKOTO (JP) | IBM (US) | A02-A03\ A05-E02B\ A08-D\ A08-M10\ A08-R\ A08-S02\ A10-E01\ A10-E05B\ A10-E08A\ A11-B01\ A11-C02D\ A12-W07\ G05-F\ S06-D02A\ S06-E03A1\ S06-E03A3\ S06-G10\ S06-K03\ S06-K07A\ S06-K99D\ T01-J05B4P\ T01-J15X |
| 6329 | 3D printing | CORROSION RESISTANT AND WEARABLE HYDRAULIC CYLINDER PISTON HAS HYDRAULIC CYLINDER COLUMN PORTION COATED WITH METAL-CERAMIC LAYER COMPRISING CHROMIUM, SILICON, IRON, CARBON, NICKEL AND MOLYBDENUM, ON SURFACE OF COLUMN | US2014294652A1 | CHO CHI O (KR)\ NAM SEOK HYUN (KR)\ YOON SANG HYUCK (KR)\ KIM HYUN JIN (KR)\ KIM HYUK HWAN (KR) | TOSHIBA CORP (JP)\ TOSHIBA DIGITAL MEDIA ENG US | A05-J10\ A08-A04\ A08-D01\ A08-M03\ A08-M09B\ A11-A03\ A11-B07\ A11-B16\ T01-J07B\ V08-A09\ X25-A08 |
| 6330 | 3D printing | HIGH-VOLTAGE ELECTRONIC STATIC DISCHARGE (ESD) PROTECTION CIRCUIT FOR TRANSMISSION LINE PULSE (TLP), HAS N-TYPE METAL OXIDE SEMICONDUCTOR TUBE WHICH IS FORMED WITH TWO N DIFFUSION AREAS, P DIFFUSION AREA AND POLYCRYSTALLINE | US2014297619A1 | CHO HAK JU (KR) | SAMSUNG ELECTRONICS CO LTD (KR) | L02-A03\ L02-K02\ M13-H01\ M13-H03 |
| 6331 | 3D printing | COMPOSITE MATERIAL USED FOR ARTIFICIAL TOOTH, COMPRISES POLY(METHYL METHACRYLATE), HYDROXYAPATITE NANOPARTICLES, MICROCRYSTALLINE WAX, PARAFFIN WAX AND EMULSIFYING AGENT CONTAINING SODIUM DODECYL SULFATE AND SORBITAN MONOSTEARATE | CN103966599A | CHO HAK JU (KR)\ AHN HYEONG GEUN (KR) | SAMSUNG ELECTRONICS CO LTD (US) | L02-A02B\ L02-A04\ L02-F05C\ L02-J02C |
| 6332 | 3D printing | POLYMETHACRYLATE POWDER, USEFUL FOR THREE-DIMENSIONAL PRINTING, COMPRISES METHACRYLIC ACID ESTER, OLEFIN UNSATURATED MONOMER, EMULSIFYING AGENT, OIL SOLUBLE-OXIDIZING AGENT, REDUCING AGENT, ION CHELATING AGENT, AND PH BUFFERING AGENT | EP2789597A1 | CHOI KISIK (US)\ KIM HOON (US) | SAMSUNG ELECTRONICS CO LTD (US) | S02-A03\ S02-A10B\ T01-F06\ T01-J08A2\ T01-J10D\ U13-A01\ U13-C04C\ U13-D02A |
| 6333 | 3D printing | COMPOSITION USEFUL FOR 3D PRINTING MATERIAL AND THREE-DIMENSIONAL PRODUCT COMPRISES CHITOSAN POLYMER, LOW DEGREE GRAFT COPOLYMER, AZIDE REAGENT, ALKYNYL AGENT, AND HALIDE REAGENT | WO2014164807A1 | CHOI SAM-JONG (KR)\ KIM YONG-KWON (KR)\ CHO KYOO-CHUL (KR)\ KIM KYUNG-SOO (KR)\ JUNG JAE-RYONG (KR)\ KANG TAE-SOO (KR)\ KIM SANG-SIG (KR) | GLOBALFOUNDRIES INC (KY)\ CHOI KISIK (US)\ KIM HOON (US) | V07-F02C\ V07-G15\ V07-K01\ V07-M\ V08-A09\ W04-Q01B1 |
| 6334 | 3D printing | MODIFIED POLYETHER KETONE RESIN COMPOSITION FOR THREE-DIMENSIONAL PRINTING COMPRISES POLYETHER KETONE RESIN, CHAIN EXTENDER, END-CAPPING AGENT, CROSSLINKING AGENT, LOW-MOLECULAR-WEIGHT POLYMER, TOUGHENING AGENT, AND REINFORCING AGENT | US2014259668A1 | CHOI SUNG-JE (KR)\ LIM HAN-JIN (KR) | SAMSUNG ELECTRONICS CO LTD (KR) | M22-H03B\ M22-H03F\ V08-A09\ X25-A08\ X25-A09 |
| 6335 | 3D printing | TITANIUM ALLOY SURFACE LASER CLADDING PROCESS FOR BIO-CERAMIC INVOLVES COATING BASE MATERIAL SURFACE WITH SODIUM OR MAGNESIUM OXIDE, SILICON HYDROXYAPATITE DOPED WITH ZINC OR ZIRCONIUM OXIDE AND RARE EARTH OXIDE | CN103962549A | CHON CHOUNG-KU (KR)\ CHOI DONG-HYUN (KR) | SAMSUNG ELECTRONICS CO LTD (US) | A10-E07C\ A10-E08A\ A12-V01\ B04-C03C\ B06-A03\ B10-A09A\ B12-M09\ B14-H01 |
| 6336 | 3D printing | PREPARATION OF AIRPLANE CARBON/CARBON COMPOSITE BRAKE MATERIAL FRICTION PAIR INVOLVES NEEDLING CARBON FELT IN PERFORMING HEAT TREATMENT, PERFORMING PRIMARY CHEMICAL VAPOR DEPOSITION, ROUGHING, AND PERFORMING INTERMEDIATE HEAT TREATMENT | CN103962550A | CHONG YON UK (KR) | SAMSUNG ELECTRONICS CO LTD (KR) | M27-A04\ M27-B04 |
| 6337 | 3D printing | LASER CLADDING MOLTEN POOL DEFOCUSING AMOUNT MEASURING DEVICE, HAS COMS IMAGE SENSOR CONVERTING OPTICAL IMAGE INTO DIGITAL IMAGE, AND COMMUNICATION MODULE RECEIVING MOLTEN POOL DEFOCUSING AMOUNT INFORMATION | CN103962556A | CHOU JING-PEI (US)\ KAO CHIEN-TEH (US)\ LAI CHIUKIN (US)\ MOSELY RODERICK C (US)\ CHANG MEI (US) | KOREA RES INST OF STANDARDS (KR) | M22-H01\ M22-H02\ M22-H03B\ M22-H03C\ M22-H03G |
| 6338 | 3D printing | HOLOGRAPHIC IMAGE DISPLAY DEVICE USED IN GAMING FIELD, HAS OPTICAL SWITCHING UNIT THAT BLOCKS LASER LIGHT EMITTED TOWARDS SPATIAL LIGHT MODULATOR (SLM) IN BLOCKING MODE, AND TRANSMITS LASER LIGHT EMITTED TO SLM IN TRANSMISSION MODE | CN103966566A | CHU CHEN-LIANG (TW)\ LIAO CHUN-TING (TW)\ HUANG TSUNG-YI (TW)\ CHEN FEI-YUH (TW) | APPLIED MATERIALS INC (US) | M13-H01 |
| 6339 | 3D printing | POWDER BED DEPOSITION APPARATUS E.G. DIRECT LASER SINTERING (DLS), HAS NONMETALLIC BARRIER LAYER AND ANCHOR THAT DEFINES REMOVABLE BUILD ASSEMBLY WITH POWDER BED WORKING SURFACE | CN103971138A | CHU DAVID C K (US) | TAIWAN SEMICONDUCTOR MFG (TW)\ CHU CHEN-LIANG (TW)\ LIAO CHUN-TING (TW)\ HUANG TSUNG-YI (TW)\ CHEN FEI-YUH (TW) | X24-D08C |
| 6340 | 3D printing | TREATING CANCER IN HUMAN SUBJECT INVOLVES ADMINISTERING NAPHTHOFURAN COMPOUND OR NAPHTHOFURAN SALT, SOLVATE, HYDRATE, OR NAPHTHOFURAN PRODRUG TO SUBJECT IN NEED OR SUBJECT DIAGNOSED OF CANCER | CN103972071A | CHUNG HENRY (US) | UNIV GEORGIA RES FOUND (US)\ CHU DAVID C K (US) | U23-H\ V07-K01\ V07-K02 |
| 6341 | 3D printing | Q235D CHANNEL LASER CLADDING METHOD INVOLVES COMBINING FIBER LASER DEVICE WITH KUKA ROBOT FOR MANUAL PROGRAMMING OR AUTOMATIC PROGRAMMING, AND COMBINING POWDER FEEDER WITH THE FIBER LASER DEVICE | CN103972216A | CHUNG HUA (US) | ALLIED SIGNAL INC (US) | W01-C01D3C\ W01-C01G8\ W01-C01P2\ W02-C04A1 |
| 6342 | 3D printing | METHOD FOR MANUFACTURING HIGH MELTING POINT METAL BASED OBJECT E.G. COLLIMATOR, INVOLVES PROVIDING HEATING PRESSURE TREATMENT TO GREEN OBJECT, AND CONTROLLING TEMPERATURE APPLIED TO GREEN OBJECT TO RE-SINTERING POINT OF GREEN OBJECT | CN103960800A | CHYAN OLIVER (US)\ PONNUSWAMY THOMAS (US) | APPLIED MATERIALS INC (US) | V07-K01 |
| 6343 | 3D printing | COMPOSITION FOR LASER CLADDING FOR FORMING COATING SURFACES ON SUBSTRATE, COMPRISES CLADDING POWDER PARTICLES, AND PRESET AMOUNT OF FLUX PARTICLES HAVING SPECIFIED AVERAGE PARTICLE SIZE ADHERED TO SURFACE OF CLADDING POWDER PARTICLES | CN103966597A | CIYUN QIU US | UNIV NORTH TEXAS (US) |  |
| 6344 | 3D printing | METHOD FOR MAKING MONOBLOC PISTON BODY FOR DIESEL FUELED COMPRESSION IGNITION INTERNAL COMBUSTION ENGINE IN VEHICLE, INVOLVES MELTING METALLIC POWDER THROUGH PROCESS TO BUILD REMAINDER OF PISTON BODY ONTO CAST OR FORGED PORTION OF BODY | CN203752535U | COLOMBO LUIGI (US)\ WAINERDI JAMES COOPER (US)\ TELLKAMP JOHN PAUL (US)\ DOERING ROBERT REID (US) | SEMICONDUCTOR MFG INT SHANGHAI (CN)\ SEMICONDUCTOR MFG INT BEIJING (CN)\ CHIU TZU YIN (CN) | V07-F02A\ V07-F02B\ V07-G15\ V07-K01\ V07-K05 |
| 6345 | 3D printing | PATTERN IRRADIATION APPARATUS I.E. MICROSCOPE APPARATUS, HAS ZERO-ORDER LIGHT CUT FILTER TO BLOCK ZERO-ORDER LIGHT, AND CONTROL DEVICE TO MAKE CORRESPONDENCE BETWEEN FOCUSING POSITION OF ZERO-ORDER LIGHT AND POSITION OF LIGHT CUT FILTER | WO2014169148A1 | CONN ROBERT O (US) | TEXAS INSTRUMENTS INC (US)\ WAINERDI JAMES COOPER (US)\ COLOMBO LUIGI (US)\ TELLKAMP JOHN PAUL (US)\ DOERING ROBERT REID (US) | M22-H03B\ M22-H03F\ X25-A01\ X25-A08\ X25-A09 |
| 6346 | 3D printing | MULTI-LEVEL OPTICAL SIGNAL GENERATOR FOR OPTICAL TRANSMITTER USED IN SMARTPHONE, COMBINES N NUMBER OF 2-LEVEL OPTICAL SIGNALS DIRECTLY MODULATED BY RESPECTIVE DIRECT MODULATION LASERS TO GENERATE 2N-LEVEL OPTICAL SIGNAL | CN103949638A | COOPER BRIAN E (US)\ LOVE SHAUN T (US)\ WEED STEVEN F (US) | XILINX INC (US) | T01-H03A\ T01-J05B3\ T01-J05B4B\ T01-J05B4M\ T01-J17 |
| 6347 | 3D printing | IMAGING SYSTEM, HAS MULTIPLE DEFLECTABLE RIBBONS SUPPORTED BY SUPPORT STRUCTURES ABOVE SURFACE OF DIE, WHERE SUPPORT STRUCTURES DIVIDE EACH DEFLECTABLE RIBBON INTO REGIONS ALONG LONG AXIS TO FORM MULTIPLE ONE-DIMENSIONAL DIFFRACTOR ARRAYS | CN103949639A | CORCHERO RICHARD (FR) | LEXMARK INT INC (US) | M13-H01\ M22-H01\ M27-A01\ M27-A01A\ M27-A01C\ M27-A01M\ M27-A01N\ M27-A01S\ M27-A01T\ M27-A01V\ M27-A04\ M27-A04A\ M27-A04C\ M27-A04M\ M27-A04N\ M27-A04S\ M27-A04T\ M27-A04V\ M27-A04Z\ M27-C01\ M27-C02 |
| 6348 | 3D printing | FIREARM SUPPRESSOR DEVICE FOR LARGE CALIBER RIFLE, HAS HOLES THAT ARE FORMED TO DISPERSE AND IMPEDE FORWARD MOVEMENT OF GAS FLOW THROUGH DEVICE SUFFICIENT TO REDUCE FIRING DECIBEL LEVEL RO SPECIFIC RANGE | CN103952695A | CORONEL PHILIPPE (FR)\ BERNARD EMILIE (FR)\ GUILLAUMOT BERNARD (FR)\ VIZIOZ CHRISTIAN (FR) | REHCA (FR) | L02-A11\ L02-J01C |
| 6349 | 3D printing | OPTICAL SWITCH E.G. FEW-MODE FIBER SWITCH, FOR USE IN INTEGRATED CIRCUIT CHIP, HAS WAVELENGTH DEMULTIPLEXER SEPARATING SPECTRAL COMPONENTS OF INCIDENT BEAM IN ANGLE, AND LENSES IMAGING INPUT ARRAY MODES OF FIBER TO SPATIAL LIGHT MODULATOR | CN103952697A | CUNNINGHAM JAMES A (US) | COMMISSARIAT ENERGIE ATOMIQUE (FR)\ ST MICROELECTRONICS CROLLES 2 (FR)\ ST MICROELECTRONICS GRENOBLE (FR) | M22-H03B\ V08-A09\ X25-A08 |
| 6350 | 3D printing | MAKING COMBUSTION TURBINE COMPONENT INVOLVES ASSEMBLING METALLIC COMBUSTION TURBINE SUBCOMPONENT GREENBODIES AND BINDER TO FORM METALLIC GREENBODY ASSEMBLY; POSITIONING AND ACTIVATING THE BINDER AND SINTERING THE METALLIC GREENBODY ASSEMBLY | CN103956333A | DE PAUW PIET (BE) | CUNNINGHAM JAMES A (US) | X24-B |
| 6351 | 3D printing | PERSISTENT STORED MODEL COMPILER OPTIMIZER FOR PROCESSING BULK DATA MANIPULATION LANGUAGE IN RELATIONAL DATABASE MANAGEMENT SYSTEM, HAS QUERY EXECUTOR FOR BATCH-PROCESSING DATA MANIPULATION LANGUAGES FORWARDED FROM VIRTUAL MACHINE | CN203741418U | DEBOER SCOTT J (US)\ AL-SHAREEF HUSAM N (US) | MELEXIS NV (BE)\ DE PAUW PIET (BE) | M22-H01\ M22-H02\ M27-A01\ M27-A01A\ M27-A01C\ M27-A01M\ M27-A01N\ M27-A01S\ M27-A01V\ M27-A01Z\ M27-A04\ M27-A04A\ M27-A04C\ M27-A04M\ M27-A04N\ M27-A04S\ M27-A04V\ M27-A04Z\ M27-C01\ M27-C02 |
| 6352 | 3D printing | ALLOY POWDER FOR LASER CLADDING, COMPRISES CARBON, SILICON, CHROMIUM, NICKEL, MOLYBDENUM, NIOBIUM, ALUMINUM, ZIRCONIUM, TITANIUM, VANADIUM AND IRON | CN203741419U | DEYUAN XIAO US\ MINGHUA JI US | MICRON TECHNOLOGY INC (US) | M22-H01\ M27-A01\ M27-A01A\ M27-A01C\ M27-A01M\ M27-A01N\ M27-A01S\ M27-A01T\ M27-A01Z\ M27-A04\ M27-A04A\ M27-A04C\ M27-A04M\ M27-A04N\ M27-A04S\ M27-A04T\ M27-A04Z\ M27-C01\ M27-C02 |
| 6353 | 3D printing | CONFIGURATION FOR JOINING CERAMIC LAYER TO METALLIC LAYER FOR USE IN HOT GAS ENVIRONMENT, HAS CERAMIC LAYER CONNECTED WITH INTERLOCKING ELEMENT OF INTERFACE LAYER AND BRAZING LAYER BY WHICH INTERFACE LAYER IS JOINED TO METALLIC LAYER | CN103949636A | DICKMANN RORY (DE) | SEMICONDUCTOR MFG INT SHANGHAI (CN) | M22-H03B |
| 6354 | 3D printing | SOLID FREEFORM OBJECT MANUFACTURING SYSTEM FOR SELECTIVE LASER MELTING POWDER BED ADDITIVE MANUFACTURING PROCESS COMPRISES MANUFACTURING CHAMBER CONTAINING POWDER BASED ADDITIVE MANUFACTURING DEVICE AND FIRST ENVIRONMENTAL CONTROL CHAMBER | CN103949637A | DIEHL LOTHAR (DE)\ RIEGEL JOHANN (DE) | INFINEON TECHNOLOGIES AG (DE) | L03-H04D\ M13-F02\ M13-G01\ M13-G03\ V06-V01N\ V06-V04C |
| 6355 | 3D printing | METHOD FOR REPAIRING BLADE TIP OF TURBINE BLADE USED IN GAS TURBINE ENGINE, INVOLVES BUILDING SQUEALER TIP USING WELDING PROCESS THAT FORMS MECHANICAL LOCKING BEAD OVER TIP PLATE | CN103951971A | DIJON JEAN (FR)\ PANTIGNY PHILIPPE (FR) | BOSCH GMBH ROBERT (DE)\ RIEGEL JOHANN (DE)\ DIEHL LOTHAR (DE) | S06-K01A\ S06-K07B\ S06-K99C\ S06-K99D\ T01-J06A\ T01-J07B |
| 6356 | 3D printing | ALLOY POWDER USEFUL FOR LASER CLADDING COMPRISES DISSIMILAR METAL POWDER AND HIGH VISCOSITY LIQUID FLUX | CN103952595A | DILLON MICHAEL N (US)\ OELTJEN BRET A (US) | COMMISSARIAT ENERGIE ATOMIQUE (FR)\ DIJON JEAN (FR)\ PANTIGNY PHILIPPE (FR) | L04-C11C\ L04-C12A\ L04-C12J\ L04-C16A\ L04-C26\ L04-E01B1\ L04-X06\ U11-C03A\ U11-C03J2A\ U11-C05B6\ U11-C05C\ U11-C18A3\ U12-D02A |
| 6357 | 3D printing | ALLOY POWDER USED FOR LASER CLADDING MATERIAL, COMPRISES HIGH-VISCOSITY LIQUID FLUX AND METAL POWDER CONTAINING CARBON, SILICON, CHROMIUM, NICKEL, MOLYBDENUM, NIOBIUM, ALUMINUM, ZIRCONIUM, TITANIUM AND IRON | CN103952694A | DIXIT GIRISH (US)\ PADHI DEENESH (US) | LSI LOGIC CORP (US) | L03-H02\ L03-H05\ L03-J01\ L04-A01A\ L04-C09B\ L04-C10F\ L04-C10L1\ L04-C16A\ L04-C18\ L04-C25\ L04-D02\ L04-D05\ L04-E\ L04-X05\ U11-C01A3\ U11-C01J4A\ U11-C03A\ U11-C03J2A\ U11-C03J8C\ U11-C06A1B\ U11-D02B\ U11-D03B2 |
| 6358 | 3D printing | SELECTIVE LASER MELTING OF TITANIUM POWDER INVOLVES DETERMINING OPTIMAL PROCESSING PARAMETERS, PREPARING THREE-DIMENSIONAL MODEL OF DESIRED STRUCTURE, PRE-PROCESSING, GENERATING PRINT FILE AND PRINTING USING TITANIUM POWDER AS RAW MATERIAL | CN103952699A | DOAN TRUNG T (US)\ CATHEY DAVID A (US) | APPLIED MATERIALS INC (US) | F02-B01\ F02-B03B\ F03-C01\ F03-C05\ F04-C03\ T01-J07B\ X25-T04B2\ X27-A03 |
| 6359 | 3D printing | MANUFACTURING ALLOY DIFFUSION BARRIER LAYER INVOLVES PLACING SINGLE-CRYSTAL SILICON SUBSTRATE SUCCESSIVELY IN ACETONE AND ETHANOL FOR ULTRASONIC CLEANING FOLLOWED BY SUFFICIENT WASHING OUT DRYING STANDBY | CN103938206A | DONO CHIAKI (JP) | MICRON TECHNOLOGY INC (US) | M13-C01\ M13-H01\ M13-L |
| 6360 | 3D printing | THREE-DIMENSIONAL INFORMATION CARRIER, COMPRISES TWO-DIMENSIONAL CODE OR COLOR CODE FORMED ALONG XY-AXIS PLANE, WHERE TWO-DIMENSIONAL CODE, COLOR CODE OR PLANE ELEMENT COMBINATION CODE ARE SCANNED VIA COMPUTED TOMOGRAPHY SCANNING PROCESS | CN103938207A | DORIS BRUCE B (US)\ CHENG KANGGUO (US)\ ZHANG YING (US) | ELPIDA MEMORY INC (JP) | A11-A05C\ A11-B14\ X25-A08 |
| 6361 | 3D printing | METHOD FOR MANUFACTURING NITROGEN-CONTAINING GATE OXIDE LAYER USED IN P-CHANNEL MOSFET SEMICONDUCTOR DEVICE, INVOLVES FORMING SEMICONDUCTOR SUBSTRATE BY THERMAL OXIDATION FOLLOWED BY INJECTING PLASMA OF NITROGEN INTO GATE OXIDE LAYER | CN103939327A | DREWERY JOHN S (US) | IBM (US)\ IBM UK (GB)\ CHENG KANGGUO (US)\ ZHANG YING (US)\ DORIS BRUCE (US) | S05-D05\ T01-J06A |
| 6362 | 3D printing | MANGANESE METASILICATE/COPPER GERMANIDE DIFFUSION BARRIER LAYER MANUFACTURING METHOD FOR SEMICONDUCTOR DEVICE, INVOLVES PERFORMING VACUUM FURNACE ANNEALING PROCESS, AND COOLING VACUUM FURNACE TO OBTAIN DIFFUSION BARRIER LAYER | CN103934566A | DRUMHELLER PAUL D (US) | NOVELLUS SYSTEMS INC (US) | L03-G02\ L03-H03A\ M22-H03A\ M22-H03B\ T01-J07B\ V07-F01A1\ V07-F02A\ V07-K05\ V07-N01\ X27-T |
| 6363 | 3D printing | CHILDREN AIR FABRIC LAYER MANUFACTURING METHOD FOR INFANT PUNCHING ADHESIVE WEARS, INVOLVES SELECTING ORGANIC LONG STAPLED COTTON, SILK OR WOOL CASHMERE AS OUTER LAYER RAW MATERIAL, AND SELECTING RAW MATERIAL FOR INNER AIR LAYER | CN103934569A | EDELSTEIN DANIEL C (US)\ NOGAMI TAKESHI (US)\ SHOBHA HOSADURGA K (US) | GORE ENTERPRISE HOLDINGS INC (US)\ DRUMHELLER PAUL D (US) | M22-H03B\ M26-A01\ M26-A01N\ M26-A01S\ M26-B13\ M26-B13N\ M26-B13S |
| 6364 | 3D printing | CAST IRON SHEAVE ELEVATOR REPAIR METHOD INVOLVES GRINDING TO-BE-WELDED PORTION, CLEANING TO-BE-WELDED PORTION USING ACETONE, SPRAYING IRON ALLOY POWDER AND NICKEL-BASED ALLOY POWDER TO TO-BE-WELDED PORTION AND RADIATING LASER RAYS | CN103934898A | ELIAN KLAUS (DE)\ HECKMANN NICOLE (DE)\ HOHLE CHRISTOPH (DE) | IBM (US)\ NOGAMI TAKESHI (US)\ EDELSTEIN DANIEL C (US)\ SHOBHA HOSADURGA K (US) | L02-G01A\ L02-G01C\ L02-G01D\ L02-G10B\ L02-G11\ L02-G12D\ M13-H01 |
| 6365 | 3D printing | FIBER IMPLANTED DEVICE FOR SELECTIVE LASER SINTERING, HAS VERTICAL MOVING MECHANISM THAT IS FIXED TO CONNECTING END OF MACHINE FRAME AND MOVABLE END OF CONNECTING BRACKET | CN103938208A | EMEL JANOV VIKTOR ANDREEVICH (BY)\ LEONOV NIKOLAJ IVANOVICH (BY)\ KOTOV VLADIMIR SEMENOVICH (BY)\ LEMESHEVSKAJA ALLA MIKHAJLOVNA (BY)\ DUDAR NATAL JA LEONIDOVNA (BY)\ SHVEDOV SERGEJ VASIL EVICH (BY) | INFINEON TECHNOLOGIES AG (DE) | M13-H01 |
| 6366 | 3D printing | METHOD FOR MEASURING REFRACTIVE CONDITION OF EYE, INVOLVES COMPUTING REFRACTIVE CONDITION BASED ON DIFFERENCES SUCH THAT LIGHT SENSOR AND EYE TOGETHER HAS CAMERA THAT IS NOT FOCUSED ON FIRST SPATIAL LIGHT MODULATOR (SLM) AND ON RETINA | CN103943550A | ENGELHARDT MANFRED (DE) | PROIZV RESPUB UNITARNOE PRED Z (BY) | L04-C05A\ L04-C06B\ L04-C07G\ L04-C09B\ L04-C10\ L04-C12\ L04-C12A\ L04-C13A\ L04-C13B\ L04-C27\ U11-C05B4\ U11-C05C\ U11-C07D3 |
| 6367 | 3D printing | SELECTIVE LASER MELTING MOLDING DEVICE INCLUDES MOLDING CAVITY, FIRST SCANNING MIRROR ARRAY, SECOND SCANNING MIRROR ARRAY, BEAM SPLITTER, FIRST LASER BEAM EXPANDER, SECOND LASER BEAM EXPANDER, LASER LIGHT SOURCE AND OXYGEN CONTENT SENSOR | CN103944065A | FARRAR PAUL A (US) | SIEMENS AG (DE)\ ENGELHARDT MANFRED (DE) | M13-H01 |
| 6368 | 3D printing | PREPARATION OF NIOBIUM-SILICON SUPERALLOY USING SELECTIVE LASER MELTING INVOLVES FORMING NIOBIUM-SILICON PARTS, DIVIDING INTO UNIFORM SLICES, PLACING IN SELECTIVE LASER MELTING DEVICE, FIXING SUBSTRATE, AND SELECTIVELY SCANNING LASER BEAM | DE102013206542A1 | FELK YACINE (FR)\ CHAABOUNI HAMED (FR)\ FARCY ALEXIS (FR) | MICRON TECHNOLOGY INC (US) | M13-H01 |
| 6369 | 3D printing | PREPARING AMORPHOUS CERAMIC COATING COMPOSITION INVOLVES PROCESSING LASER CLADDING OF CERAMIC MATERIAL TO OBTAIN AMORPHOUS FORM OF THE CERAMIC MATERIAL, WHERE CERAMIC MATERIAL COMPRISES ALUMINUM OXIDE, ZIRCONIA AND RARE EARTH METAL OXIDE | DE102013006299A1 | FENG HUANG US\ JINLONG LI US\ HONGBO LI US\ QUNJI XUE US | ST MICROELECTRONICS SA (FR)\ ST MICROELECTRONICS CROLLES 2 (FR) | A09-D03\ A11-B01\ A11-B14\ A12-R09\ A12-W12F\ X25-A01\ X25-A08 |
| 6370 | 3D printing | TUNGSTEN COPPER COMPOSITE MATERIAL COMPRISES OXYGEN-FREE COPPER BASE BODY AND TUNGSTEN COPPER SURFACE LAYER, AND TUNGSTEN COPPER COMPOSITE MATERIAL IS PREPARED BY MIXING TUNGSTEN POWDER AND COPPER POWDER UNIFORMLY | WO2014162926A1 | FENG YUXIANG US\ HUANG XIANGJUN US\ CHENG DEKAI US\ PAN ZHIJIAN US\ HUA QING US\ CHEN CHAO US | NINGBO INST MAT TECH & ENG CAS US | M22-H03B\ W06-B |
| 6371 | 3D printing | TSV BASED METAL LINE LAYER MANUFACTURING METHOD, INVOLVES PERFORMING PLANARIZATION PROCESS ON CT OXIDE LAYER, COATING METAL ELECTRIC CONDUCTION MATERIAL ON DIFFUSION BARRIER LAYER, AND FLATTENING SURFACE OF METAL LINE LAYER | WO2014165310A2 | FROMENT BENOIT (BE)\ CACHO FLORIAN (FR) | GUANGDONG MIDEA ELECTRIC APLNC US | A05-F01B1\ A08-A06\ A08-M06\ A08-R03A\ A08-S01\ A08-S02\ A12-S08C\ A12-S09\ X25-A08 |
| 6372 | 3D printing | LASER CLADDING COAXIAL PROTECTIVE GAS CONVEYING DEVICE COMPRISES SHARP-NOSE OUTER TUBE, SHARP INNER TUBE, CURRENT DIVIDER, AIR INLET, TAIL PIPE, MULTI-AIR OUTLETS AND LASER | WO2014158600A1 | FUJISHIMA NAOTO (JP)\ SALAMA C ANDRE T (CA) | ST MICROELECTRONICS CROLLES 2 (FR) | M13-H01\ M22-G03L\ M22-H\ M26-B08\ M26-B08A\ M26-B08B\ M26-B08C\ M26-B08H\ M26-B08M\ M26-B08T\ M26-B08X |
| 6373 | 3D printing | LASER CLADDING SYNCHRONOUS FEEDING DEVICE COMPRISES CLAMP, HORIZONTAL GUIDE RAIL, BOLT, L-SHAPED BRACKET, LEAD SCREW NUT, GUIDE SCREW, BEARING, POWDER GUN, FIXED FRAME, SLIDE GROOVE AND SCREW ROD | JP2014192070A | FUJISHIMA NAOTO (JP)\ SALAMA C ANDRE T (CA) | FUJI ELECTRIC CO LTD (JP) | M13-H01 |
| 6374 | 3D printing | LASER RAPID PROTOTYPING DEVICE COMPRISES CONTROL UNIT, GALVANOMETER SYSTEM, LASER, POWDER SUPPLY UNIT, MOLDING UNIT, AND PAVING ROLLER | WO2014148286A1 | FUKAURA YASUHIRO US | FUJI ELEC DEVICE TECH CO LTD (JP) | M13-H\ M13-L\ M22-H03B |
| 6375 | 3D printing | PROCESSING OF TITANIUM-NICKEL MEMORY ALLOY BASED ON SELECTIVE LASER MELTING TECHNOLOGY FOR E.G. MEDICAL EQUIPMENT INVOLVES MIXING NICKEL POWDER AND TITANIUM POWDER, LOW SPEED SCANNING USING LASER, AND PRE-HEATING | CN103927788A | FUKUDA KOJI (JP)\ YAMASHITA HIROKI (JP) | TOSHIBA CORP (JP) | L02-G01A\ L02-J01\ M13-H01\ M13-H04A |
| 6376 | 3D printing | CARBON FIBER-REINFORCED RESIN POWDER MATERIAL USEFUL FOR SELECTIVE LASER SINTERING, COMPRISES RESIN POWDER, CARBON FIBER, FLOW AUXILIARY AGENT, ANTIOXIDANT AND DISPERSANT | CN103928347A | FUKUNAGA TAKESHI (JP) | HITACHI LTD (JP)\ FUKUDA KOJI (JP)\ YAMASHITA HIROKI (JP) | L02-A02\ L02-A03\ L02-G01A\ L02-J01E\ M13-H01\ M13-H04A |
| 6377 | 3D printing | LASER CLADDING POWDER FOR REPAIRING DIRECTIONALLY SOLIDIFIED NICKEL-BASED SUPERALLOY BLADES COMPRISES CHROMIUM, COBALT, CARBON, MOLYBDENUM, TUNGSTEN, ALUMINUM, TANTALUM, BORON, RHENIUM, HAFNIUM, AND NICKEL | CN103920877A | FURUTA HIROSHI (JP)\ KOBAYASHI TAKAAKI (JP)\ AZUHATA HIROFUMI (JP)\ MORITA TOMOYA (JP)\ OKAMURA RYUICHI (JP)\ TAKAHASHI TOSHIFUMI (JP) | RENESAS ELECTRONICS CORP (JP) | M26-B\ M26-B00C\ M26-B00J\ M26-B00N\ M26-B00S |
| 6378 | 3D printing | LASER REPAIRING PROCESS FOR GLASS LINING MATERIAL, INVOLVES PROCESSING REPAIRING MATERIAL INTO POWDER SHAPE THROUGH LASER DEVICE BY UTILIZING LASER CLADDING TECHNOLOGY | CN203715724U | FURUTA HIROSHI (JP)\ YANAGISAWA MASAYUKI (JP)\ KITAJIMA HIROYASU (JP)\ IZUMI KATSUYA (JP) | RENESAS ELECTRONICS CORP (JP)\ FURUTA HIROSHI (JP)\ KOBAYASHI TAKAAKI (JP)\ AZUHATA HIROFUMI (JP)\ MORITA TOMOYA (JP)\ OKAMURA RYUICHI (JP)\ TAKAHASHI TOSHIFUMI (JP) | M23-D02A\ M23-D05\ M23-E01\ X24-C\ X24-D08C |
| 6379 | 3D printing | LASER MELTING VACUUM PROTECTIVE DEVICE COMPRISES SELECTIVE LASER MELTING MOLDING WORKING MECHANISM, CENTRAL CONTROL SYSTEM, POSITION MACHINE, SCREEN SYSTEM, LASER LENS AND OXYGEN SENSOR AIR INLET ELECTROMAGNETIC VALVE | CN203712095U | FURUYA SHIGEKI (JP)\ WATANABE HISAKI (JP)\ MOTOTANI ATSUSHI (JP) | RENESAS ELECTRONICS CORP (JP) | M23-D05\ M23-G\ T01-J10C4\ T01-J15X\ X24-D03\ X25-A08 |
| 6380 | 3D printing | METAL CERAMIC LASER CLADDING POWDER FOR MANUFACTURING THERMOELECTRIC COUPLE PROTECTING SLEEVE COMPRISES ALUMINA POWDER, TITANIUM POWDER, MOLYBDENUM POWDER, AND BORON POWDER | CN103909378A | FUSE KENICHI US | MATSUSHITA ELECTRIC IND CO LTD (US) |  |
| 6381 | 3D printing | LASER CLADDING OF AUSTENITIC STAINLESS STEEL SURFACE INVOLVES PERFORMING SURFACE OIL REMOVING TREATMENT AND TEXTURING OF AUSTENITIC STAINLESS STEEL BASE MATERIAL, AND MIXING ALUMINA POWDER, TITANIUM POWDER, AND MOLYBDENUM POWDER | JP2014192465A | FUTASE TAKUYA (JP)\ MAKABE KAZUYA (JP)\ YAMAZUMI SAIGOU (JP) | FUSE KENICHI US | M13-H01 |
| 6382 | 3D printing | MANUFACTURE OF WEAR RESISTANT CONCRETE PUMP SPECTACLE PLATE INVOLVES MIXING RAW MATERIAL, COVERING ON SURFACE OF SPECTACLE PLATE SUBSTRATE, FUSING BY LASER CLADDING, FORMING METAL-CERAMIC LAYER, AND FIXING ON SPECTACLE PLATE SUBSTRATE | DE102014104196A1 | GANAPATHIRAMAN RAMANATH (US)\ KANE RAVINDRA S (US)\ PETHURAJA GOPAL GANESAN (US) | RENESAS TECH CORP (JP)\ FUTASE TAKUYA (JP)\ MAKABE KAZUYA (JP)\ YAMAZUMI SAIGOU (JP) | L04-A01A\ L04-C10L2\ L04-C12A\ L04-C12B\ L04-C13B\ L04-C27\ L04-D03\ U11-C05B5\ U11-C05B9A\ U11-C05C6\ U11-C06A1A |
| 6383 | 3D printing | METHOD OF INCREASING HEAD WEAR RESISTANCE OF STIR FRICTION WELDING OF HIGH STRENGTH ALUMINUM ALLOY INVOLVES UNIFORMLY DEPOSITING STIR HEAD SHAFT SHOULDER AND STIR NEEDLE SURFACE WITH STRIPE-SHAPED LASER CLADDING WEAR-RESISTANT COATING | JP2014188871A | GAO CHAO US\ HUANG SUJUAN US | RENSSELAER POLYTECH INST (US) | V08-A04A\ V08-A06 |
| 6384 | 3D printing | LAYERED SLICING METHOD BASED ON SELECTIVE LASER SINTERING INVOLVES DETERMINING MAXIMUM AND MINIMUM THICKNESS VALUE OF LAYERED DIRECTION, SLICING FIRST LAYER IN LAMINATING DIRECTION, CALCULATING LOWER LAYER MINIMUM THICKNESS, AND SORTING | EP2784045A1 | GAUL STEPHEN JOSEPH (US)\ CHURCH MICHAEL D (US)\ VINSON JAMES EDWIN (US) | YANGZHOU JIANGXIN ELECTRONICS CO LTD US | M22-H03B |
| 6385 | 3D printing | MUDDY WATER SHIELD CLAY LAYER MANUFACTURING METHOD, INVOLVES DILUTING SELF-WORD CREATION SLURRY, ADDING WATER AND BENTONITE TO BENTONITE MIXING TANK, AND CONNECTING AIR CHAMBER WITH WASHING WATER PUMP | JP2014183284A | GILTON TERRY L (US) | INTERSIL INC (US) | M22-H01\ M22-H03F |
| 6386 | 3D printing | PERFORMING LASER CLADDING PROCESS USED FOR MANUFACTURING Q235D GRADE STEEL PLATE, INVOLVES USING FIBER LASER DEVICE THROUGH MANUAL PROGRAMMING TO CUT Q235D STEEL SUBSTRATE MATERIAL | WO2014149122A2 | GJUNTSEL GIZELA (DE)\ KHAKMANN KERSTIN (DE)\ BUNKE DITMAR (DE)\ BEMERMANN JORG (DE)\ SHTANDKE MIRKO (DE) | MICRON TECHNOLOGY INC (US) | S03-E04D\ V07-F02B\ V07-K01\ V07-K05 |
| 6387 | 3D printing | TOP METAL INTERCONNECTION LAYER MANUFACTURING METHOD, INVOLVES FORMING TOP METAL INTERCONNECT LAYER WITH UPPER MULTI-METAL INTERCONNECTION LAYER, AND COVERING PASSIVATION LAYER ON TOP METAL INTERCONNECTION LAYER | WO2014152984A1 | GONG DAWEI US\ MA QINGJIE US | SCHOELLER TECHNOCELL GMBH & CO KG (DE) | A11-B14\ T01-E01B\ T01-J05B4P |
| 6388 | 3D printing | SEMI-CONDUCTOR LASER CHANGING SLOW AXIS FIELD DISTRIBUTING METHOD, INVOLVES MEASURING WIDTH AND PITCH OF SEMI-CONDUCTOR LASER ARRAY DEVICE, PERFORMING PHOTO-ETCHING PROCESS, AND ADJUSTING BAR SLOW AXIS DIRECTION ACCORDING TO SPECIFIC FIELD | WO2014146188A1 | GRAUR IOANA (US)\ LIEBMANN LARS W (US) | CNAC CHONGQING MICROELECTRONICS CO LTD US |  |
| 6389 | 3D printing | PROCESSING EXCESS REUSABLE MATERIAL POWDER, WHICH IS ACCUMULATED AND COLLECTED DURING PRODUCTION OF THREE-DIMENSIONAL OBJECTS FROM POWDER MATERIAL HAVING DESIRED PARTICLE SIZE DISTRIBUTION | CN103911611A | GRAVEZ PIERRE (FR)\ THILL MICHEL (FR) | IBM (US) | A08-S02\ A10-E21B\ A11-B05D\ A12-E06A\ A12-E06B\ J04-E04D\ J04-E11\ L03-E04A\ L03-E04B1\ L03-E04B2\ L03-E04H2\ L03-E08B\ X16-C01\ X16-E06A5A\ X16-E06A5C |
| 6390 | 3D printing | PRODUCING MOLDED PART, COMPRISES PRESSURIZING PANEL, WHICH IS DEFORMED BY APPLIED PRESSURE AND APPLYING PARTIALLY THICKENED PORTIONS ON MOLDED PANEL, IN WHICH POWDER LAYER IS APPLIED IN PREDETERMINED WIDTH AND THICKNESS ON PANEL PORTIONS | CN103911612A | GUPTA SUBHASH (US)\ ZHOU MEI SHENG (SG)\ CHERN CHYI S (SG) | GEMALTO SA (FR) | U11-F02A1\ U12-A01A4 |
| 6391 | 3D printing | OPTICAL MODULE OF OBSERVATION APPARATUS, HAS POLARIZATION COMPONENTS THAT ARE MODULATED AND REFLECTED BY REFLECTIVE SPATIAL LIGHT MODULATORS RESPECTIVELY AFTER GOING THROUGH RESPECTIVE POLARIZING ELEMENTS | CN103909263A | HAMADA MOTOTSUGU (JP) | CHARTERED SEMICONDUCTOR MFG (SG) | T01-J10C4 |
| 6392 | 3D printing | LASER SINTERING SYSTEM FOR FABRICATING THREE DIMENSIONAL OBJECTS FROM DIGITAL DATA, HAS RETURN POWDER DEVICE SELECTIVELY MOVEABLE TO ENABLE PORTION OF POWDER TO BE TRANSFERRED FROM SIDE OF POWDER DISTRIBUTION DEVICE TO ANOTHER SIDE | CN103915571A | HANAOKA KAZUYA (JP)\ FUJII TERUYUKI (JP) | TOSHIBA KK (JP) | L04-A01A\ L04-C12C\ L04-C16\ U11-C05F1\ U11-C18A3\ U12-D02E\ U12-E02 |
| 6393 | 3D printing | GAS TURBINE ENGINE E.G. HIGH-BYPASS GEARED AIRCRAFT ENGINE, HAS COMPRESSOR SECTION INCLUDING COMPRESSOR CASE, AND FLANGE SEPARATED FROM COMPONENT BY AIR SEAL, WHERE AIR SEAL IS SNAP FIT TO FLANGE THAT INCLUDES TWO DISTINCT MATERIAL LAYERS | CN203695959U | HAPP THOMAS (DE)\ ANDRES DIETER (DE)\ MAJEWSKI PETRA (DE)\ RUF BERNHARD (DE) | SEMICONDUCTOR ENERGY LAB (JP)\ HANAOKA KAZUYA (JP)\ FUJII TERUYUKI (JP) | M22-H03B |
| 6394 | 3D printing | MANUFACTURE OF CATALYST INK FOR FORMING CATALYST LAYER, INVOLVES MIXING CATALYST DISPERSION LIQUID OBTAINED BY MIXING ELECTRODE CATALYST, WATER AND ALCOHOL, AND GELATINIZATION IONOMER SOLUTION OBTAINED BY MIXING IONOMER AND SOLVENT | RU2529827C1 | HASEGAWA TSUYOSHI (JP) | QIMONDA AG (DE) | M13-H01\ M13-L |
| 6395 | 3D printing | METHOD FOR MANUFACTURING OPTICAL SEMICONDUCTOR DEVICE E.G. LED DEVICE, INVOLVES PROVIDING TIME DURATION IN WHICH SEALING LAYER IS MANUFACTURED UNTIL OPTICAL-SEMICONDUCTOR ELEMENT IS SEALED BY SEALING LAYER IS SET TO 24 HOURS | JP2014183261A | HASEI HIRONOBU US | TATSUTA SYSTEM ELECTRONICS KK US | X24-D03 |
| 6396 | 3D printing | MUNICIPAL VERTICAL PLAN BASED DEM LAYER MANUFACTURING METHOD, INVOLVES BUILDING CHARACTERISTIC POINT, OBTAINING TERRAIN FEATURES CORRESPONDING TO SELECTIVE SAMPLING PROCESS, AND OBTAINING ALTITUDE POINT OF LOCAL AREA | WO2014150490A1 | HASHIMOTO KOJI (JP)\ TAKAHASHI KOJI (JP) | SEIKO EPSON CORP (JP) | M13-H01\ M13-L\ M23-D05\ M23-G\ X24-D03 |
| 6397 | 3D printing | METHOD FOR IMPROVING SURFACE OXIDATION LAYER FINFET STRUCTURE, INVOLVES FORMING FIN STRUCTURE WITH ISOLATION STRUCTURE THAT IS PROVIDED WITH SEMICONDUCTOR SUBSTRATE IN REACTION CHAMBER, AND FORMING FIN STRUCTURE SURFACE WITH OXIDE LAYER | WO2014151877A1 | HASHIMOTO TAKASHI (JP)\ MORIMOTO TAKASHI (JP) | FUJITSU LTD (JP) | L03-H04E\ L03-J\ M13-B\ M13-H02\ V04-R01A4\ V04-R02B\ V04-R07\ V04-V02\ V04-X01B\ V04-X01F\ X24-D03A\ X24-D03B\ X25-A02 |
| 6398 | 3D printing | DESIGNING OF SELECTIVE LASER MELTING METAL PARTS SUPPORT STRUCTURE INVOLVES DESIGNING SUPPORT STRUCTURE COMPRISING SUPPORT GRID THIN-WALLED STRUCTURE, AND SUPPORT GRID WALL PROVIDED ON EDGE OF SAWTOOTH CONNECTING STRUCTURE | EP2781691A1 | HAYAKAWA DAISUKE US | PANASONIC CORP (JP)\ MORIMOTO TAKASHI (JP)\ HASHIMOTO TAKASHI (JP) | L02-A02\ A12-P\ A12-W03\ A12-W12F\ T01-S03 |
| 6399 | 3D printing | LASER CLADDING CONTINUOUS NEGATIVE PRESSURE TYPE POWDER FEEDER COMPRISES STORAGE UNIT, PRE-HEATING UNIT, POWDER FEEDING UNIT, BOX BODY, MOTOR CONTROL UNIT, AND PNEUMATIC CONTROL UNIT, WHERE FEEDING UNIT COMPRISES END COVER AND STOREHOUSE | KR2014111095A | HAYAKAWA HISASHI US\ NISHITANI YUJI US\ ASAMI HIROSHI US | SUNX LTD US | A05-F03\ A08-M\ A11-B16\ X25-A08 |
| 6400 | 3D printing | LASER CLADDING ANTI-SPLASHING DEVICE INCLUDES AIR KNIFE CAVITY PROVIDED WITH CIRCULAR OPENING, AIR OUTLET, RADIATION-PROOF FRAME, CERAMIC NOZZLE, RADIATION-PROOF CERAMIC PLATE, AIR INLET, RECTANGULAR SIDEWALL AND AIR INLET JOINT | DE102013205244A1 | HAYASHIDA YOKO (JP) | SONY CORP (JP) | D09-C01\ L02-A04\ L02-G01X\ L02-G12X\ M22-H03B |
| 6401 | 3D printing | MANUFACTURE OF POSITIVE DISPLACEMENT BALL LARGE MODULUS GEAR FIBER LASER INVOLVES PLACING CLEAN SURFACE FLEXIBLE LASER PROCESSING SYSTEM, FEEDING POWDER MIXTURE, MULTI-CHANNEL AND MULTI-LAP CLADDING, AND TOOTH-SHAPED PROCESSING | JP2014179206A | HE KAI US\ SHEN YUAN US | ELPIDA MEMORY INC (JP) |  |
| 6402 | 3D printing | MANUFACTURE OF ELECTRIC CIRCUIT WIRING BOARD E.G. PRINTED WIRING BOARD, INVOLVES FORMING FILM CONTAINING METAL OXIDE PARTICLES, ON SUBSTRATE, AND TRANSFERRING METAL OXIDE PARTICLES OF INSULATING LAYER ON ELECTRIC CIRCUIT WIRING | WO2014145813A1 | HEIDLER RALF (US) | XI AN CHUANGXIN SCIENCE & TECHNOLOGY CO LTD US | M22-H01\ M22-H03B\ M22-H03F\ X25-A02 |
| 6403 | 3D printing | METHOD OF PRODUCING THREE-DIMENSIONAL COMPONENT BY LAYERED APPLICATION OF BUILDING MATERIAL, INVOLVES PRODUCING BOTH COMPONENTS AND A COMPONENT ASSOCIATED WITH IDENTIFICATION ELEMENT HOLDING INFORMATION OF COMPONENT, IN COMMON ENVELOPE | WO2014144319A1 | HILLIGER ANDREAS (JP) | SCHLUMOG OVERSEA S A (PA) | U23-G\ U23-H |
| 6404 | 3D printing | COMPOSITE MATERIAL POWDER USED FOR SELECTIVE LASER SINTERING METHOD COMPRISES SPHERICAL AGGREGATES WHICH HAVE NO MELTING POINT AND HAVE AVERAGE PARTICLE DIAMETER AND SPECIFIC GRAVITY WITHIN SPECIFIED RANGES AND POLYAMIDE | WO2014144630A1 | HILLIGER ANDREAS (JP)\ WELLHAUSEN UWE (JP) | INFINEON TECHNOLOGIES AG (DE) |  |
| 6405 | 3D printing | MANUFACTURE OF THREE-DIMENSIONAL ARTICLE INVOLVES USING HIGH-ENERGY SOURCE(S) AND PULVERULENT SUBSTRATE COMPRISING MAIN SUBSTRATE(S) AND ENERGY TRANSFERRING VECTOR(S) BY SELECTIVE LASER SINTERING/MELTING PROCESS | US2014251481A1 | HIRASAWA SHIGEKI US\ HORII YOICHI US\ SUKETA HIROKO US\ AKASHI TAKUYA US\ HOSHINO TAKASHI US\ MARUYAMA YUKINOBU US | INFINEON TECHNOLOGIES AG (DE)\ HILLIGER ANDREAS (DE)\ WELLHAUSEN UWE (DE) | M13-H |
| 6406 | 3D printing | HEAT RADIATOR COMPRISES THERMAL INTERFACE MATERIAL THAT IS ARRANGED ON THERMAL RADIATION BODY, AND THIN FILM PIECE THAT MAKES MAIN SURFACE PARALLEL TO AXIS OF GRAPHITE CRYSTAL AND SHAFT, WHERE COATING CONSISTS OF CARBON ALLOTROPE | KR2014087432A | HIRASHIMA HIROYUKI US\ KATSUYA MASASHI US | HITACHI LTD (JP) | M13-H01\ M26-B12\ M26-B12C\ M26-B12T |
| 6407 | 3D printing | MANUFACTURING METHOD FOR TURBINE ENGINE COMPONENT INVOLVES FORMING TURBINE ENGINE COMPONENT FROM POWDER USING DIRECT METAL LASER SINTERING PROCESS | AT13536U1 | HIRASHIMA HIROYUKI US\ KATSUYA MASASHI US | SHARP KK (JP) | L02-A04\ A04-E08\ A09-D03\ A11-B14\ A11-C\ A12-W12F\ X25-A08 |
| 6408 | 3D printing | OPTICAL ILLUMINATION SYSTEM HAS COHERENCE LIGHT SOURCE WHICH IS OPTICALLY COUPLED WITH PHASE MODULATING SPATIAL LIGHT MODULATOR (SLM), AND ILLUMINATES PORTION OF IMAGE PLANE BY DIRECTING OPTICAL SIGNAL TO PHASE MODULATING SLM | WO2014144482A1 | HIROSHI FURUTA (JP) | SHARP KK (JP) | U12-A01A1E\ U12-A01A2\ U12-A01A7 |
| 6409 | 3D printing | FITNESS APPARATUS FOR PHYSICAL FITNESS TESTING OR TRAINING, HAS VERTICAL FREE-STANDING POLE WHICH COMPRISES BASE FOR ABUTTING SUPPORT ON TOP SURFACE OF MAT WITH RADIAL DISTANCE GRADUATED MARKINGS, AND FIRST TARGET LOCATED PROXIMAL TO BASE | WO2014144255A2 | HIROSHIKI HAYASHI (JP)\ SHINICHI NAGAMORI (JP) | NEC ELECTRONICS CORP (JP) | M22-H03B\ S06-D03\ S06-E03B\ S06-G10\ S06-K07A\ S06-K99D\ T01-J07B\ X25-A01\ X25-A08 |
| 6410 | 3D printing | PAVING DEVICE USED FOR PERFORMING SELECTIVE LASER MELTING TECHNIQUE ON METALLIC BASE MATERIAL, COMPRISES HOPPER-TYPE BUNKER USED FOR HOLDING POWDER MATERIAL FOR LAYERING BASE MATERIAL AND OUTLET FUNNEL FOR REGULATING PRESSURE INSIDE BUNKER | WO2014141259A1 | HISAMOTO MASARU US\ SAITO SHINICHI US | USHIO ELECTRIC INC (JP) | T01-C08\ T01-J04A\ W06-A02E |
| 6411 | 3D printing | PROCESSING LASER REPAIRING OF CHROMIUM MOLYBDENUM VANADIUM STEEL GRADE, INVOLVES CLEANING COLD PRESSING MOULD SURFACE, AND THEN PERFORMING LASER CLADDING OF IRON-316 ALLOY POWDER AND TUNGSTEN CARBIDE COBALT ALLOY POWDER | WO2014144053A2 | HO HERBERT L (US)\ SHANK STEVEN M (US) | HITACHI LTD (JP) | E12-A04\ E31-N04B\ L03-G07\ L04-C16\ L04-C25\ L04-E03\ G03-B02E\ U11-D02B\ U12-A01A\ V04-T03A\ V04-X01B\ V04-X01F |
| 6412 | 3D printing | ROTARY POWDER SPREADING DEVICE USED IN SELECTIVE LASER SINTERING COMPRISES DRIVING DEVICE SUPPORTING SEAT, ROTARY DRIVING DEVICE, ROLLER, AND CYLINDER | JP2014175285A | HO JUNG-CHING J (US)\ SOWARDS JANE W (US)\ WU SHUXIAN (US) | IBM (US) | V08-A05 |
| 6413 | 3D printing | ACTIVE MATRIX ORGANIC LED DISPLAY PANEL FOR ACTIVE MATRIX ORGANIC LED DISPLAY DEVICE, HAS HOLE TRANSMISSION LAYER PROVIDED WITH FILM LAYER, AND CATHODE PROTECTION LAYER PROVIDED ON FILM LAYER | WO2014144230A1 | HOLLENBECK R GARY (US) | XILINX INC (US) | S05-D05\ U23-H\ V07-F02C\ V07-K01\ V07-K02\ V07-M |
| 6414 | 3D printing | SELECTIVE LASER SINTERING MOLDING 3D PRINTING DEVICE HAS LASER, SCANNING SYSTEM HAVING FOCUSING MIRROR, Y-AXIS GALVANOMETER AND X AXIS GALVANOMETER, WORKING CONTAINER WITH PRECISE ALTIMETER, NUMBER CONTROL MOVEMENT CONTROLLER, AND COMPUTER | WO2014144824A1 | HONG SAM-HYO (SE)\ HUME JEFFREY (US)\ HOYER HENRIK (US) | UPM PHARMACEUTICALS INC (US)\ HOLLENBECK R GARY (US) |  |
| 6415 | 3D printing | ACOUSTIC SOUNDER OF PULSED SOUND SOURCES DISTANCE FROM AN ACOUSTIC LOCATOR, ITS CORRECTED SOUND-RANGING ANGLE AND TOPOGRAPHIC COORDINATES OF THIS SS | EP2778779A1 | HONG SEONG IL (KR)\ KIM HWA YOUNG (KR) | INFINEON TECHNOLOGIES AG (DE)\ HONG SAM-HYO (SE)\ HOYER HENRIK (US)\ HUME JEFFREY (US) | S06-K02\ S06-K03A\ S06-K07A\ V08-A06 |
| 6416 | 3D printing | THERMAL INTERFACE MATERIAL USED FOR HEAT-DISSIPATION APPARATUS, HEAT SINK AND SEMICONDUCTOR LIGHT-EMITTING DEVICE E.G. LED ELEMENT, HAS GRAPHITE SHEET WITH FLARE SHAPE STRUCTURE WHICH IS PEELED IN LAYERED FORM | US2014255483A1 | HONGDA CHEN (CN)\ BEIJU HUANG (CN)\ XU ZHANG (CN) | LG DISPLAY CO LTD (KR) | M22-H03B\ M22-H03G\ X25-A08 |
| 6417 | 3D printing | METHOD FOR FORMING DIFFUSION COOLING HOLE IN SUBSTRATE, INVOLVES FORMING RAISED FEATURE ON SUBSTRATE SURFACE WITHIN ONE OF METERING AND DIFFUSING SECTIONS BY DEPOSITING MATERIAL ON SURFACE AND SELECTIVELY HEATING MATERIAL | DE102013003760A1 | HOPPER PETER J (US)\ VASHCHENKO VLADISLAV (US)\ STRACHAN ANDY (US)\ LINDORFER PHILIPP (US) | INST SEMICONDUCTORS CAS (CN) | L03-E01A\ L03-E01B5B\ L03-E01C2\ L03-E03\ L03-E08B\ L03-H05\ X16-B01F1\ X16-E01J\ X16-F02 |
| 6418 | 3D printing | PROVIDING METHOD OF IMAGE TO EYE OF PATIENT, INVOLVES ILLUMINATING SPATIAL LIGHT MODULATOR (SLM) WITH OPTICAL BEAM SUCH THAT PHASE MODULATED LIGHT FROM SLM IS RECEIVED BY EYE | DE102013003937A1 | HORIE SATOSHI US\ YONEZAWA TAKAHIRO US\ KIYOMURA HIROYUKI US\ TOKUNAGA TETSUYA US\ SASAOKA TATSUO US | NAT SEMICONDUCTOR CORP (US) | B01-B01\ B01-B02\ B01-D02\ B10-C02\ B10-E02\ B14-H01D1\ B14-H01F3\ B14-H01F4\ B14-L06\ B14-S08\ B15-C00 |
| 6419 | 3D printing | METHOD FOR RECONDITIONING HOT GAS PATH PART OF INDUSTRIAL GAS TURBINE TO FLEXIBLY ADAPT OPERATION REGIME OF GAS TURBINE, INVOLVES COATING HOT GAS PATH PART WITH METALLIC OVERLAY WITH DESIRED THERMO-MECHANICAL FATIGUE IN AREA | JP2014152743A | HORST HARTMANN (DE)\ OLAF ZEIKA (DE)\ ANDREA LUX (DE)\ STEFFEN WILLMANN (DE) | MATSUSHITA ELECTRIC IND CO LTD (JP) | A11-B14\ A12-W12F\ T01-J07B |
| 6420 | 3D printing | LASER SINTERING LOGIC SYSTEM, HAS SENSOR FIXED ON SUBSTRATE, LASER DRIVER COMPONENT RECEIVING PULSE SIGNAL FROM RECOGNITION PART, WHERE RECOGNITION PART IS CONNECTED WITH CONTROLLER PART, AND SENSOR DRIVER SENSING PULSE SIGNAL | KR1392335B1 | HOSHIKA HIROSHI US\ FUE KOICHIRO US\ SAKAI KEISUKE US\ YOSHIDA TAKAHARU US | NOVALED AG (DE)\ HARTMANN HORST (DE)\ ZEIKA OLAF (DE)\ LUX ANDREA (DE)\ WILLMANN STEFFEN (DE) | A12-W11\ M22-H03B\ T01-L02\ T01-N01D2 |
| 6421 | 3D printing | PREPARING ADDITIVE USEFUL IN THE MANUFACTURE OF COMPONENT E.G. OIL FILTER DEVICE OF MOTOR VEHICLE, WHERE THE METHOD COMPRISES PRODUCING THE COMPONENT BY LASER SINTERING PROCESS, CLEANING THE COMPONENT AND POST-PROCESSING THE COMPONENT | JP2014163428A | HOWARD GREGORY E (US)\ BABCOCK JEFFREY A (US)\ PINTO ANGELO (US)\ SCHIEKOFER MANFRED (DE)\ BALSTER SCOTT G (DE)\ HAUSLER ALFRED (DE) | MATSUSHITA ELECTRIC IND CO LTD (JP) | A12-H02C\ V08-A09\ X25-A08 |
| 6422 | 3D printing | NON-AQUEOUS ELECTROLYTE SECONDARY BATTERY E.G., LITHIUM ION SECONDARY BATTERY, INCLUDES SILICA BETWEEN POSITIVE ELECTRODE ACTIVE MATERIAL LAYER AND SEPARATOR OR IN POSITIVE ELECTRODE ACTIVE MATERIAL LAYER | WO2014135136A1 | HOZUMI MASATOSHI (JP) | TEXAS INSTRUMENTS INC (US)\ BABCOCK JEFFREY A (US)\ PINTO ANGELO (US)\ SCHIEKOFER MANFRED (DE)\ BALSTER SCOTT G (DE)\ HOWARD GREGORY E (US)\ HAUSLER ALFRED (DE) | S03-E04A5\ U14-K01A1A |
| 6423 | 3D printing | FORMING COMPOSITION OF ABIRATERONE ACETATE NANOPARTICLES TO TREAT CASTRATION RESISTANT PROSTATE CANCER INVOLVES DRY MILLING COMPOSITION OF ABIRATERONE ACETATE/MILLABLE GRINDING COMPOUND/FACILITATING AGENT/ANTIOXIDANT OR SEQUESTERING AGENT | DE202012013036U1 | HSIEN-WEI CHEN US\ JIAN-HONG LIN US\ TZU-LI LEE US | NEC ELECTRONICS CORP (JP) | M13-D02\ M22-H03B\ M22-H03E\ M22-H03F\ M23-D05\ V08-A09\ X24-D03\ M22-H01 |
| 6424 | 3D printing | LASER SINTERING SYSTEM FOR FABRICATING THREE DIMENSIONAL OBJECT BASED UPON DESIGN DATA AND SINTERABLE POWDER, HAS CHUTE THAT EXTENDS FROM HOPPER TOWARD SURFACE SINTERABLE POWDER IS DEPOSITED UPON AND IS FORMED BETWEEN DEVICE AND BED | CN103898502A | HSU YU-HAO (TW) | TAIWAN SEMICONDUCTOR MFG (TW)\ LEE TZU-LI (TW)\ CHEN HSIEN-WEI (TW)\ LIN JIAN-HONG (TW) | M23-D05\ X24-D03 |
| 6425 | 3D printing | CONSTRUCTION METHOD OR THREE-DIMENSIONAL STRUCTURE WITHIN LASER SINTERING APPARATUS INVOLVES UPDATING COMPUTER FILE WITH DATA PERTAINING TO BUILD CYCLE OVER COMPUTER NETWORK | CN103898503A | HSU YU-HAO (TW) | UNITED MICROELECTRONICS CORP (TW) | A09-D03\ A11-B14\ S03-B01\ U13-A01 |
| 6426 | 3D printing | ADDITIVE LAYER MANUFACTURING METHOD E.G. SELECTIVE LASER MELTING METHOD, FOR PRODUCING THREE-DIMENSIONAL OBJECT E.G. HYDRAULIC DISTRIBUTION SYSTEM, INVOLVES IRRADIATING SET OF LAYERS SUCH THAT POWDER MATERIAL IS LOCALLY MELTED OR SINTERED | CN203685834U | HSU YU-HAO (TW) | UNITED MICROELECTRONICS CORP (TW) | S05-G02B\ X27-A03 |
| 6427 | 3D printing | UV EXPOSING APPARATUS HAS CONTROL UNIT THAT CONTROLS BRING/TAKE UNIT, STAGE UNIT, OPTICAL SOURCE SECTION AND MEASURING UNIT, SO THAT OUTPUT ENERGY AND LIGHT EMISSION REGION OF OPTICAL SOURCE SECTION IS ESTABLISHED | CN203685897U | HU BINGHUA (US)\ PENDHARKAR SAMEER P (US) | UNITED MICROELECTRONICS CORP (TW) | A12-L03\ A12-L04\ V07-F02A\ V07-F02B\ V07-F02C\ V07-K01\ V07-M |
| 6428 | 3D printing | MANUFACTURING A MOLDED ARTICLE FROM METALLIC PENETRATING COMPOSITE MATERIAL, COMPRISES MANUFACTURING FRAME-SHAPED BODY WITH OPEN-CELL SKELETON STRUCTURE, AND MELTING POWDER AT PREDETERMINED LOCATIONS | CN103897386A | HU CHAO-KUN (US)\ GRECO STEPHEN (US)\ MCLAUGHLIN PAUL (US) | TEXAS INSTRUMENTS INC (US)\ HU BINGHUA (US)\ PENDHARKAR SAMEER P (US) | A12-D00D\ A12-E06A\ A12-E06B\ A12-E08B\ X16-C01\ X16-E06A5E\ X21-A01J\ X21-B01A |
| 6429 | 3D printing | MANUFACTURING APPARATUS FOR CONSTRUCTING THREE-DIMENSIONAL STRUCTURES BY FUSING AND ANNEALING POWERED MATERIAL, HAS ACTUATOR THAT MANEUVERS MIRROR TO SCAN ENERGY BEAMS ACROSS LENS OUTPUTTING ENERGY BEAMS TOWARD AND NORMAL TO PLATFORM | CN103895232A | HUA GUANG PING (SG)\ SONG JUN (SG)\ LO KENG FOO (SG)\ CAI JUN (US) | MCLAUGHLIN PAUL S (US)\ HU CHAO-KUN (US)\ GRECO STEPHEN E (US) | A05-J10\ A08-M09A1\ A11-B14\ A11-B16\ A12-E01\ X12-D01F1 |
| 6430 | 3D printing | METHOD FOR DETECTING TEMPERATURE AT LASER SINTERING SITE WITHIN FIELD OF VIEW OF IMAGE SENSOR, INVOLVES CORRELATING LIGHT INTENSITY OF PIXEL WITHIN FIRST DIGITAL IMAGE WITH FIRST TEMPERATURE AT FIRST TIME BASED ON FIRST SHUTTER SPEED | CN103901730A | HUANG CHENG (US)\ LIU YOWJUANG BILL (US) | CHARTERED SEMICONDUCTOR MFG (SG) | T01-J07B\ W04-X01K1\ X25-A08 |
| 6431 | 3D printing | SEXUAL BED (SLB) FOR ENHANCING PLEASURE OF LOVE MAKING AND INTERCOURSE, HAS SEXUAL ELECTRICALLY ELEVATION DEVICE (SED) FOR ELEVATING AND POSITIONING BODY OF USER IN DIFFERENT HEIGHTS AND DIFFERENT ANGLES | CN103903981A | HUANG CHIH-FENG (TW)\ CHIANG CHIU-CHIH (TW)\ WU YOU-KUO (TW)\ LIN LONG SHIH (TW) | ALTERA CORP (US) | W04-N05C |
| 6432 | 3D printing | MULTI-CHANNEL SIMULTANEOUS PHOTOMANIPULATION DEVICE FOR OPTICAL MICROSCOPES, HAS OPTICAL SYSTEM FOR COMBINING REFLECTED INPUT LIGHT BEAMS FROM SPATIAL LIGHT MODULATORS DEVICES, AND MICROSCOPE FOR DELIVERING COMBINED HOLOGRAM TO SAMPLE | CN103903986A | HUANG CHIH-FENG (TW)\ CHIANG CHIU-CHIH (TW)\ WU YOU-KUO (TW)\ LIN LONG-SHIH (TW) | SYSTEM GENERAL CORP (TW) | A10-E07\ A10-E21A\ A12-V01\ D09-C01D\ B04-C02A2\ B04-C02A3\ B05-B02C\ B06-D02\ B07-A02\ B10-A07B\ B10-A09B\ B10-C04E6\ B14-A04A\ B14-C03\ B14-D01E\ B14-D02A1\ B14-D02A2\ B14-E09\ B14-F01\ B14-F02C\ B14-F06\ B14-G02D\ B14-H01C\ B14-J01\ B14-J05\ B14-J07\ B14-K01\ B14-N01\ B14-N03\ B14-N06\ B14-N10\ B14-N11\ B14-N12\ B14-N13\ B14-N16\ B14-P02\ B14-S02\ B14-S04\ B14-S13\ B14-S20 |
| 6433 | 3D printing | GAS DIFFUSION LAYER FOR FUEL CELL, HAS THROUGH-HOLES FORMED IN STATE OF PENETRATING BOTTOM FACE OF GAS FLOW PATH AND BOTTOM SURFACE OF POROUS BLOCK | CN103904055A | HUANG CHUN-HSIEN (TW)\ CHEN MING-JUI (TW)\ WU TE-HUNG (TW)\ YANG YU-SHIANG (TW) | SYSTEM GENERAL CORP (TW) | M22-H03B\ S02-J01\ S02-J02\ S02-J04\ T01-J07B1\ V08-A09\ W06-B05\ W06-B08\ X24-D03 |
| 6434 | 3D printing | COMPOSITE POWDER, USEFUL FOR LASER SINTERING AND FOR FORMING THREE-DIMENSIONAL ARTICLE, COMPRISES A POLYMERIC MATRIX COMPRISING A POLYMERIC MATERIAL HAVING SPECIFIED MELT VISCOSITY, AND CARBON NANOPARTICLES DISPERSED IN POLYMERIC MATRIX | CN103904535A | HUANG CHUN-HSIEN (TW)\ CHEN MING-JUI (TW)\ WU TE-HUNG (TW)\ YANG YU-SHIANG (TW) | UNITED MICROELECTRONICS CORP (TW)\ HUANG CHUN-HSIEN (TW)\ CHEN MING-JUI (TW)\ WU TE-HUNG (TW)\ YANG YU-SHIANG (TW) | M22-H03B |
| 6435 | 3D printing | METHOD FOR MANUFACTURING E.G. CUSTOM GOLF CLUB FOR PLAYING GOLF BY GOLFER, INVOLVES MANUFACTURING CUSTOM GOLF CLUB HEAD BASED ON COMPUTER MODEL USING ADDITIVE LAYER MANUFACTURING PROCESSES AND USING POWDER MATERIAL AND HIGH ENERGY BEAM | CN103895224A | HUANG FUREN US\ HUANG SAIQIN US\ LIN JISHEN US\ LIN ZHIXIONG US\ CHEN LUNXING US\ YANG ZHONGWU US | UNITED MICROELECTRONICS CORP (TW) | X22-A03\ X22-A20C |
| 6436 | 3D printing | SYSTEM USEFUL FOR PRODUCING IMAGE HAVING HIGH DYNAMIC RANGE, HAS LIGHT SOURCE, SPATIAL LIGHT MODULATOR, AND SET OF SEQUENTIALLY-ARRANGED OPTICAL ELEMENTS | JP2014165148A | HUANG FUREN US\ HUANG SAIQIN US\ LIN JISHEN US\ LIN ZHIXIONG US\ CHEN LUNXING US\ YANG ZHONGWU US | FUJIAN ANGSTREM SEMICONDUCTOR CO LTD US | E05-E02D\ E12-A04\ E31-P03\ E34-B01\ E34-C02\ E35-C02\ E35-K02\ E35-L\ F03-C02A\ G02-A01\ G02-A05B\ G02-A06\ L02-A04\ X15-A01A\ X15-A02\ X15-A05 |
| 6437 | 3D printing | COMPOSITION FOR TREATING E.G. CYSTIC FIBROSIS COMPRISES SOLID DISPERSION OF AMORPHOUS N-(2,4-BIS(1,1-DIMETHYLETHYL)-5-HYDROXYPHENYL)-1,4-DIHYDRO-4-OXOQUINOLINE-3-CARBOXAMIDE, AND E.G. MANNITOL | ES2476142A2 | HUANG JUDY H (US) | FUJIAN ANGSTREM SEMICONDUCTOR CO LTD US | S03-B01 |
| 6438 | 3D printing | ASSESSING QUALITY OF COMPONENT MADE A GENERATIVE LASER SINTERING- AND/OR LASER MELTING PROCESS E.G. AIRCRAFT ENGINE COMPONENT, COMPRISES E.G. DETERMINING DIFFERENCE BETWEEN FIRST AND SECOND DATA SET, AND EVALUATING QUALITY OF COMPONENT | US2014240532A1 | HUANG YU-FONG (TW)\ HAN TZUNG-TING (TW)\ LU WEN-PIN (TW) | APPLIED MATERIALS INC (US) | A09-D03\ A11-B03\ A11-B14\ A12-L03\ V07-F02A |
| 6439 | 3D printing | EVALUATING STRUCTURAL QUALITY OF THREE-DIMENSIONAL COMPONENTS, WHICH ARE PRODUCED BY LASER SINTERING OR LASER MELTING METHOD, COMPRISES E.G. PRODUCING THE COMPONENT BY SUCCESSIVE SOLIDIFICATION OF SINGLE LAYERS MADE OF BUILDING MATERIAL | US2014242400A1 | HUANG YU-FONG (TW)\ HAN TZUNG-TING (TW)\ LU WEN-PIN (TW) | MACRONIX INT CO LTD (TW)\ HUANG YU-FONG (TW)\ HAN TZUNG-TING (TW)\ LU WEN-PIN (TW) | J01-F |
| 6440 | 3D printing | INTAKE DEVICE OF E.G. DIESEL ENGINE MOUNTED IN VEHICLE, HAS CONTROL UNIT THAT SUCKS GASES IN TUBE OF INTERNAL COMBUSTION ENGINE FROM SUCTION PORT AT LAYERED FORM, AND CONNECTION CHANNEL THAT CONNECTS CHANNELS UPSTREAM OF SUCTION PORT | EP2772331A1 | HUI SU KUO US\ NAN CHEN YI US\ WEN LIU HSIEN US | MACRONIX INT CO LTD (TW) | M13-H01\ M13-L |
| 6441 | 3D printing | MANUFACTURING SUPER-HYDROPHOBIC COATING LAYER USEFUL E.G. FOR AUTOMOTIVE GLASS COMPRISES PRODUCING CERAMIC PRECURSOR SOLUTION, FORMING CERAMIC COATING LAYER ON A SUBSTRATE, FORMING INTER-PARTICLE NECKING BY HEAT-TREATING AND FLUORINATING | WO2014131810A1 | HUMBERT BRUNO (FR) | NANYA TECHNOLOGY CORP (TW)\ SU KUO HUI (TW)\ CHEN YI NAN (TW)\ LIU HSIEN WEN (TW) | A03-A05\ A11-B05\ A12-B04C\ M13-H01\ M13-H03\ M26-B\ V08-A09 |
| 6442 | 3D printing | LAMINATION ELEMENT FOR BEARING PAD OF BEARING DEVICE, HAS HEAT INSULATION HOLDER WHICH IS INSERTED IN HOLE, AND HEAT DETECTION UNIT IS PROVIDED TO DETECT TEMPERATURE OF SURFACE IN INNER SIDE OF HEAT INSULATION HOLDER | JP2014156905A | HWANG SUN HWA (KR) | PEUGEOT CITROEN AUTOMOBILES SA (FR) |  |
| 6443 | 3D printing | LASER BEAM-DEFLECTING DEVICE FOR APPARATUS E.G. LASER SINTERING DEVICE, FOR CONSTRUCTING THREE-DIMENSIONAL OBJECTS, HAS HOUSING FOR ACCOMMODATING DEFLECTING MIRRORS, WHERE HOUSING AND/OR HOUSING PARTS ARE FORMED BY POWDER MATERIAL | CN103872579A | ICHINOSE KATSUHIKO (JP) | EDMI CO LTD (KR) |  |
| 6444 | 3D printing | FILTER UNIT FOR CONNECTING TO LASER SINTERING OR LASER MELTING PLANT, HAS INLET AND OUTLET PROVIDED AT FILTER MODULE FOR WETTING MEDIUM SO AS TO FLOOD OR MOISTEN FILTER BEFORE OPENING OF MODULE AND PARTLY LOADED WITH SUCKED OFF RESIDUES | WO2014125280A2 | ICHINOSE KATSUHIKO (JP)\ OTSUKA FUMIO (JP)\ WAKAHARA YOSHIFUMI US | RENESAS TECH CORP (JP) | A05-F03\ A08-E01\ A08-M06\ A08-M10\ X25-A08 |
| 6445 | 3D printing | COATING TURBINE BLADE SHROUD BY LASER CLADDING PROCESS INVOLVES CLEANING TURBINE BLADE SHROUD USING AN ACETONE OR ETHANOL, CLAMPING CLEANED TURBINE BLADE ON JIG, AND THEN COATING CLAMPED TURBINE BLADE SHROUD DESIRED MATERIAL | US2014246809A1 | IDA NOBUO US | RENESAS TECH CORP (JP) | A11-B14\ A11-C\ X25-A08\ X25-U01 |
| 6446 | 3D printing | LASER CLADDING USED FOR REPAIRING SHAFT, INVOLVES COATING SURFACE OF METAL USING METHYL CELLULOSE BINDER AND PASTE, AND THEN DRYING METAL TO OBTAIN PRODUCT | US2014231650A1 | IGUCHI DAISUKE US | NEC MICROSYSTEMS LTD US |  |
| 6447 | 3D printing | ENGINE CRANKSHAFT, HAS SHAFT MAIN BODY WHOSE TWO ENDS ARE CONNECTED WITH FLANGE, AND NITRIDING LAYER FORMED WITH SHAFT NECK, WHERE OUTER SURFACE OF SHAFT MAIN BODY ARRANGED WITH LASER CLADDING UNIT | WO2014125133A1 | IINUMA TOSHIHIKO (JP) | FUJI XEROX CO LTD (JP) | L04-C16A\ L04-C22\ L04-D05\ U11-C03J2 |
| 6448 | 3D printing | ENGINE CRANKSHAFT FLANGE, HAS CRANKSHAFT FLANGE MAIN BODY WHOSE END PART IS CONNECTED WITH SHAFT NECK, AND SCREW THREAD HOLE FORMED WITH CYLINDRICAL LUG BOSS, WHERE OUTER CIRCLE OF FLANGE MAIN BODY IS FORMED WITH LASER CLADDING COATING | WO2014125258A2 | IIZUKA KOJI US | TOSHIBA KK (JP) | L04-C11C1\ L04-C12A\ L04-C12B\ L04-C16A\ L04-C22\ U11-C01J4A\ U11-C03A\ U11-C03J2A\ U11-C03J8C\ U11-C05B5\ U11-C05B7 |
| 6449 | 3D printing | REINFORCING MATERIAL USEFUL IN SELECTIVE LASER SINTERING TECHNOLOGY, COMPRISES NYLON 12 (POLYAMIDE 12), AN INTENSITY MODIFIED MATERIAL, A NUCLEATING AGENT, A FLUIDITY MODIFIER, WHITENING OR DYEING AGENT AND A MODIFIED MATERIAL | WO2014125281A2 | IKEDA SHUJI (JP)\ ISHIBASHI KOKCHIRO US\ OSADA KENICHI (JP)\ MINAMI MASATAKA (JP) | RENESAS TECH CORP (JP) | L03-H02\ L04-C07\ L04-C10F\ L04-C12A\ L04-C12B\ L04-C13A\ U11-C01J3A\ U11-C05F1A\ U11-C07D4 |
| 6450 | 3D printing | SELECTIVE LASER SINTERING MATERIAL CLEANING MACHINE, HAS WORKING TABLE FIXED WITH UPPER PROTECTIVE WINDOW AND LOWER PROTECTIVE WINDOW, AND SCREENING DEVICE AND COLLECTING BOX INSTALLED ON LOWER END OF WORKING CHAMBER | US2014229594A1 | IKEDA TAKESHI (JP)\ NAKANISHI TSUTOMU (JP)\ OKAMOTO AKIRA (JP) | RENESAS TECH CORP (JP) | A12-L03A\ L03-F02\ L03-G02B\ L04-A01A\ V08-A04C2\ V08-A09 |
| 6451 | 3D printing | EXPOSURE DEVICE FOR COATING SUBSTRATE, HAS CONTROL UNIT FOR CONTROLLING SPACE LIGHT MODULATOR THAT IS MOUNTED ON MOVING MECHANISM, WHERE SPACE LIGHT MODULATOR, MOVING MECHANISM AND WORK PIECE TABLE ARE CONTROLLED BY CONTROL UNIT | CN103882424A | IKUMA MAKOTO US\ MIYAJI HIROYUKI US | TIF KK (JP) | A08-B01\ A11-B14\ A12-H05\ T01-J15X |
| 6452 | 3D printing | DEEP WELL ANNEALING METHOD, INVOLVES FORMING CHAMBER WITH HEATING SILICON SUBSTRATE LAYER, AND PRODUCING OXYGEN AND INERT GAS FROM REACTION GAS UNIT, WHERE INERT GAS IS PURE NITROGEN, AND OXYGEN FLOW RATE IS IN SPECIFIC RANGE | CN103886166A | IMAHASHI MANABU (JP) | MATSUSHITA ELECTRIC IND CO LTD (JP) | A10-E21B\ A11-C04E\ A12-E06A\ A12-M01\ A12-M02\ A12-W11K\ J04-E04\ J04-E11\ L03-E04A2\ L03-E04H3\ X16-C01C\ X16-C17\ X16-E06A1\ X16-E06A5A |
| 6453 | 3D printing | GRID MEDIUM LAYER MANUFACTURING METHOD, INVOLVES OBTAINING SEMICONDUCTOR SUBSTRATE BY USING HEAT OXIDATION AND HEAT ANNEALING PROCESS, AND FORMING SILICON OXYNITRIDE LAYER ON GATE MEDIUM LAYER | CN103881371A | ISHIBASHI KOICHIRO (JP) | PANASONIC CORP (JP)\ IMAHASHI MANABU (JP) | M22-H03B\ X24-D03 |
| 6454 | 3D printing | CONTACT HOLE ETCHING BARRIER LAYER STRUCTURE, HAS ACTIVE AREA LOCATED AT GATE STRUCTURE, WHERE ACTIVE AREA IS PROVIDED WITH METAL SILICIDE LAYER, AND CONTACT HOLE ETCHING BARRIER LAYER PROVIDED WITH FIRST AND SECOND NITRIDE STRESS FILM | CN103882324A | ISHII HIROSHI US | UNIV ELECTRO COMMUNICATIONS (JP)\ ISHIBASHI KOICHIRO (JP) | T01-J11A1\ T01-N01B3\ W04-N05 |
| 6455 | 3D printing | HIGH POWER FIBER LASER CLADDING LIGHT FILTERING DEVICE, HAS STRIPPING AREA FORMED WITH STRIPPING PROTECTIVE LAYER, OUTER LAYER AND INNER LAYER, AND EXPOSED INNER CLADDING PART COATED WITH FILTER MATERIAL | CN103883587A | ISHIZAKA TADAHIRO (US)\ HOSHINO SATOHIKO (US)\ HAMAMOTO KUZUHIRO (US)\ MIZUNO SHIGERU (US)\ MIZUSAWA YASUSHI (JP) | MITSUBISHI HEAVY IND LTD US | M22-H03F |
| 6456 | 3D printing | CERAMIC SLURRY INTEGRATED POROUS RESIN MOLD MANUFACTURING METHOD, INVOLVES PERFORMING GRANULE OR POWDER SINTERING PROCESS, AND PERFORMING PORE-FORMING AGENT REMOVING PROCESS IN WATER FLOW CHANNEL TO OBTAIN POROUS RESIN MOLD STRUCTURE | CN103876882A | IWATA HIROSHI (JP)\ OGURA TAKAYUKI (JP)\ SHIBATA AKIHIDE (JP) | TOKYO ELECTRON LTD (JP)\ ISHIZAKA TADAHIRO (US)\ HOSHINO SATOHIKO (US)\ HAMAMOTO KUZUHIRO (US)\ MIZUNO SHIGERU (US)\ MIZUSAWA YASUSHI (JP) | M23-D02A2\ M23-D02A4\ M23-D02B2\ X24-C09 |
| 6457 | 3D printing | MANUFACTURING CATHODE CATALYST LAYER USEFUL FOR MANUFACTURING POLYMER ELECTROLYTE FUEL CELL, INVOLVES FORMING PRECURSOR LAYER CONTAINING CATALYST SUPPORT PARTICLE AND IONOMER, AND IRRADIATING ULTRASONIC WAVE TO PRECURSOR LAYER | DE102013003303A1 | IWATA HIROSHI (JP)\ OGURA TAKAYUKI (JP)\ SHIBATA AKIHIDE (JP) | SHARP KK (JP) | X25-A08\ X25-A09 |
| 6458 | 3D printing | MANUFACTURING FOAM BY LASER CLADDING OR RECHARGE, MIXING PARTICLES OF FOAM PRECURSOR MATERIAL WITH CARRIER GAS TO FORM TWO-PHASE FLOW INCLUDING DUST OR GAS | KR1428469B1 | IWATA HIROSHI (JP)\ OGURA TAKAYUKI (JP)\ SHIBATA AKIHIDE (JP) | SHARP KK (JP) |  |
| 6459 | 3D printing | METHOD FOR LIGHT FIELD PHOTOGRAPHY OR IMAGING, INVOLVES PERFORMING CALCULATIONS BY COMPUTER TO COMPUTE SPECIFIC LIGHT FIELD FUNCTION OF LIGHT INCIDENT ON IMAGING SENSOR | CN103866318A | IWATA HIROSHI (JP)\ OGURA TAKAYUKI (JP)\ SHIBATA AKIHIDE (JP) | SHARP KK (JP) | U12-A01B1J\ U12-A01B2\ V08-A04A |
| 6460 | 3D printing | MANUFACTURE OF HYBRID COMPONENT BY ESTABLISHING CONTROLLED GRAIN ORIENTATION IN PRIMARY AND SECONDARY DIRECTION OF COMPONENT PART, WHERE CONTROLLED SECONDARY GRAIN ORIENTATION IS REALIZED BY APPLYING SPECIFIC SCANNING PATTERN OF ENERGY BEAM | CN103866319A | IWATA HIROSHI (JP)\ OGURA TAKAYUKI (JP)\ SHIBATA AKIHIDE (JP) | SHARP KK (JP) | M22-H03B\ X25-A08 |
| 6461 | 3D printing | STOPPER MADE OF BASE MATERIAL, USEFUL FOR CLOSING HOLE IN COMPONENT, COMPRISES STOPPER UPPER- AND LOWER SIDE, LATERAL CIRCUMFERENTIAL SURFACE, AND SEVERAL HALF CONES WITH HALF CONE BASE SURFACE, HALF CONE LATERAL SURFACE AND HALF CONE TIP | CN103866320A | IWATA HIROSHI (JP)\ OGURA TAKAYUKI (JP)\ SHIBATA AKIHIDE (JP) | SHARP KK (JP) | A09-D03\ A11-B03\ A11-B14\ V08-A09 |
| 6462 | 3D printing | GRIPPING APPARATUS E.G. HYDRAULIC GRIPPER, FOR GRIPPING THREE-DIMENSIONAL OBJECTS IN E.G. LABORATORY ENVIRONMENT, HAS GRIPPING UNIT PLACED UPON ACTUATION OF ACTUATORS, AND ACTUATING SOURCE PROVIDING ACTUATING MEDIUM FOR ACTUATING ACTUATOR | CN103860291A | IWATA HIROSHI (JP)\ OGURA TAKAYUKI (JP)\ SHIBATA AKIHIDE (JP)\ ADACHI KOUICHIROU (JP) | SHARP KK (JP) | W07-A01C\ W07-B\ W07-E06 |
| 6463 | 3D printing | COVERING STRUCTURE USED FOR STEAM TURBINE, HAS WATERPROOF SHEET THAT IS PROVIDED IN OUTERMOST LAYER OF OUTER SURFACE OF HEAT RETENTION BODY, AND PERFORMS WATER PROOFING ON BOTH SURFACES OF OUTER SURFACE AND INNER SURFACE | CN103871869A | IWATA HIROSHI (JP)\ OGURA TAKAYUKI (JP)\ SHIBATA AKIHIDE (JP)\ ADACHI KOUICHIROU (JP) | SHARP KK (JP) | H06-A04\ H06-P\ J04-C02\ J04-C02A\ J04-X03\ T01-J07B |
| 6464 | 3D printing | SEMICONDUCTOR LASER ARRAY DEVICE CHIP LIGHT FIELD DISTRIBUTION SLOW AXIS DIRECTION CHANGING METHOD, INVOLVES PERFORMING SEMICONDUCTOR LASER ARRAY DEVICE CHIP LIGHT FIELD DISTRIBUTION SLOW AXIS DIRECTION CHANGING PROCESS | CN103862042A | IWATA HIROSHI (JP)\ SHIBATA AKIHIDE (JP)\ ADACHI KOUICHIROU (JP) | SHARP KK (JP) | M22-H03B |
| 6465 | 3D printing | SELECTIVE LASER SOLIDIFICATION APPARATUS E.G. SELECTIVE LASER SINTERING HAS LASER SCANNING UNIT THAT SCANS LASER BEAM OVER POWDER LAYER TO SELECTIVELY SOLIDIFY PORTION OF POWDER LAYER TO FORM REQUIRED PATTERN | CN103862044A | IWATA HIROSHI (JP)\ SHIBATA AKIHIDE (JP)\ KAKIMOTO SEIZO (JP) | SHARP KK (JP)\ IWATA HIROSHI (JP)\ SHIBATA AKIHIDE (JP)\ ADACHI KOUICHIROU (JP) | M22-H03B |
| 6466 | 3D printing | APPARATUS, USEFUL FOR ADDITIVELY MANUFACTURING CELLULAR STRUCTURES, COMPRISES A TRANSLATION SYSTEM ASSOCIATED WITH ONE OF BUILD HEADS AND SUBSTRATE SUCH THAT A SPATIAL RELATIONSHIP BETWEEN THE BUILD HEADS AND THE SUBSTRATE IS CONTROLLED | CN103862046A | JAIN AMITABH (US) | SHARP KK (JP)\ IWATA HIROSHI (JP)\ SHIBATA AKIHIDE (JP)\ KAKIMOTO SEIZO (JP) | T01-J16C3\ T01-S03\ W01-A06A3 |
| 6467 | 3D printing | ELECTRO-OPTIC (EO) SENSOR FOR GUIDING MUNITIONS, E.G., SELF-PROPELLED MISSILES, HAS CELLS HAVING PIXEL UNIT WHICH CONVERTS INCIDENT OPTICAL RADIATION TO ELECTRICAL SIGNAL WHILE SUB-REGIONS ARE MAPPED TO DIFFERENT DETECTOR CELL | CN203649666U | JANG SYUN-MING (TW)\ LEE SHEN-NAN (TW)\ SONG JIN-YIING (TW) | TEXAS INSTRUMENTS INC (US)\ JAIN AMITABH (US) | V08-A04A\ V08-A04B\ V08-A04C\ V08-A09 |
| 6468 | 3D printing | PLANT FOR IMPROVING THE BIODEGRADABILITY OF SOURCES OF ORGANIC MATTER COMPRISES AN ENTRY FOR MEASURING AND CONTROLLING FLOW, CONDUCTIVITY, PH, TEMPERATURE, REDOX, AND CAVITATION FEATURE, A HEATING UNIT, AND A CAVITATION-DISPERSION REACTOR | CN203649913U | JANG SYUN-MING (TW)\ SHIEH JYU-HORNG (TW)\ CHOU CHUN-LI (TW) | JANG SYUN-MING (TW)\ LEE SHEN-NAN (TW)\ SONG JIN-YIING (TW) | T01-J15H |
| 6469 | 3D printing | SELECTIVE LASER SOLIDIFICATION APPARATUS E.G. FOR BUILDING OBJECTS HAS LASER SCANNING UNIT THAT SCANS LASER BEAM OVER POWDER LAYER, AND PROCESSING UNIT THAT SELECTS SCANNING SEQUENCE OF LASER BEAM BASED ON GAS FLOW DIRECTION | CN103871749A | JANG WOO JIN (US)\ CHO SUNG DONG (US)\ MOON BUM KI (US) | TAIWAN SEMICONDUCTOR MFG (TW) | A05-F03\ A08-R\ A09-D01\ A11-A03\ A11-B16\ T01-J07B |
| 6470 | 3D printing | SELECTIVE LASER SOLIDIFICATION APPARATUS FOR SELECTIVELY SOLIDIFYING POWDER LAYER ON POWDER BED HAS LASER SCANNING UNIT THAT SCANS LASER BEAM OVER POWDER LAYER TO SELECTIVELY SOLIDIFY AT LEAST PART OF POWDER LAYER TO FORM REQUIRED PATTERN | CN103866461A | JENSEN BRADLEY (US)\ CHU CHARLES Y (US) | SAMSUNG ELECTRONICS CO LTD (KR)\ INFINEON TECHNOLOGIES CORP (US) | M13-D03B\ M13-H01\ V08-A09 |
| 6471 | 3D printing | METHOD FOR APPLYING POLICY ATTACHMENT SERVICE LEVEL MANAGEMENT SEMANTICS WITHIN PEERED POLICY ENFORCEMENT DEPLOYMENT, INVOLVES SHARING RUNTIME POLICY ENFORCEMENT METRIC WITH OTHER PEERED POLICY ENFORCEMENT POINT PLATFORM | CN103864333A | JIA CHENG US\ RENCHENG WANG US\ LINHONG JI US\ WEIFENG LIU US\ CHUNCAI WANG US\ JIA LIN US\ YUCHUN SUN US\ DAOXIN HAO US | JENSEN BRADLEY (US)\ CHU CHARLES Y (US) |  |
| 6472 | 3D printing | PREPARING MILL GUIDE ROLLER INVOLVES SELECTING MATRIX GUIDE AND GUARD ROLLER, WHERE GUARD ROLLER IS MADE OF STEEL CARBON STEEL OR LOW ALLOY STEEL MATERIALS | CN203660167U | JIANG QIUZHI HUANG (CN) | UNIV TSINGHUA (CN) | A04-G02E3\ A12-V03D\ F02-C01\ F04-E\ X25-T09B |
| 6473 | 3D printing | HIGH-EFFICIENT GEOMETRICAL FEATURE DECOMPOSITION MIX MANUFACTURING AND REMOVING PART STRUCTURE PROCESSING METHOD, INVOLVES PRODUCING LASER SINTERING COAXIAL POWDER, AND PERFORMING INTERFERENCE CHECK SIMULATION ANALYSIS OPERATION | CN103845762A | JIE LUO US\ YUNCHUAN HUANG US\ LI MA US\ JI GAO US\ LEI AI US\ KEQIANG XIANG US\ WENCHANG LI (CN) | SYSTEM GENERAL CORP (CN) | L02-A02\ L02-A03\ L02-A05\ M22-D\ M22-G03H\ A05-F01E\ A11-B04B\ A11-C04A\ A12-A02\ A12-H05\ A12-W12F |
| 6474 | 3D printing | STONE-POLYMER COMPOSITE POWDER USED FOR LASER SINTERING THREE-DIMENSIONAL MANUFACTURE, COMPRISES NYLON 12 POWDER AND LIMESTONE POWDER, IN SPECIFIED RATIO | WO2014115729A1 | JIN YAN (CN) | CHENGDU CHENGDIAN SILICON SEA TECHNOLOGY CO LTD US | A11-B09A2\ A12-E11B\ A12-W16\ L03-E05B2\ L03-H05\ L04-A01A\ L04-A03A\ L04-A03D\ L04-C10E\ L04-C10F\ L04-C10K\ L04-C11C\ L04-C22\ U11-C01J2\ U11-C01J3B\ U11-C01J4A\ U11-C05C\ U11-C05E\ U11-C05F3B\ U11-C05F3D\ U11-C18B9\ U12-A02A2A\ U12-A02A3\ X15-A01A\ X15-A02A\ X15-A02B\ X15-A02C |
| 6475 | 3D printing | CORROSION-RESISTANT METAL SURFACE COATING COMPRISES MANGANESE POWDER, CHROMIUM POWDER, ALUMINUM POWDER, BORON IRON POWDER, NANO ALUMINUM OXIDE POWDER, FERROSILICON POWDER AND IRON AND UNAVOIDABLE IMPURITIES | CN103855603A | JING LUO US\ ZHONGJIE XUE US\ XINJIE ZHOU US\ YONGQIANG HU US\ YI ZHOU US | SHANGHAI HUAHONG NEC ELECT CO (CN) | M13-H\ M13-L\ M23-D05\ M23-G\ M23-H\ X24-D03 |
| 6476 | 3D printing | INNER HOLE SEALING UNIT OF HYDRAULIC SUPPORT OIL PROCESSING CYLINDER HAS INNER OPENING WHICH IS SET WITH GUIDE HOLE ON GUIDE SEAL SET WITH MOVABLE COLUMN POLE, AND INNER HOLE IS CLOSE TO OPENING POSITION | CN203635913U | JING LUO US\ ZHONGJIE XUE US\ XINJIE ZHOU US\ YONGQIANG HU US\ YI ZHOU US | 58TH RES INST CETC US | A10-E09B\ A12-W12F\ M13-H01\ M13-L\ M22-H02\ M26-A\ M26-A00B\ M26-A00C\ M26-A00N\ M26-A00S\ M26-B06\ M26-B08\ M26-B08B\ M26-B08C\ M26-B08S |
| 6477 | 3D printing | SELF-HEATING TYPE AROMATIC HOT PASTE E.G. NONWOVEN FABRICS LAYER, MANUFACTURED BY MANUFACTURING NONWOVEN FABRIC LAYER CONTAINING POLYETHYLENE FILM, HEATING RAW MATERIALS E.G. WORMWOOD POWDER, AND ADDING MIXTURE IN ZIPLOCK COVER | CN203635914U | JOBETTO HIROYASU (JP) | CHINA ELECTRONICS TECHNOLOGY GROUP CORP NO 58 RES INST US | L04-E03B\ L04-X\ M26-A\ M26-A00N\ M26-B08\ V08-A04A |
| 6478 | 3D printing | PRODUCING MOLDINGS TO MANUFACTURE HOLLOW GLASS SPHERES, COMPRISES PRODUCING WATER-SOLUBLE MOLD, SEALING SURFACE OF MOLD WITH MATERIAL, CREATING CAST OF MOLD PART, AND DISSOLVING CASTING MOLD WITH AQUEOUS SOLUTION AFTER CAST IS SOLIDIFIED | CN203635915U | JOUSSEAUME VINCENT (FR) | CASIO COMPUTER CO LTD (JP)\ JOBETTO HIROYASU (JP) | S05-X\ T01-J06A\ T01-J10C4 |
| 6479 | 3D printing | CIGS THIN-FILM SOLAR CELL MANUFACTURING METHOD, INVOLVES FORMING BACK SURFACE ELECTRODE LAYER IN UPPER PART OF ANOTHER BACK SURFACE ELECTRODE LAYER, MANUFACTURING SUBSTRATE, AND LAMINATING LATTER BACK SURFACE ELECTRODE LAYER ON SUBSTRATE | CN203635916U | JUNG JAE CHANG (KR)\ KIM SAM YOUNG (KR)\ LIM CHANG MOON (KR)\ MOON SEUNG CHAN (KR)\ BOK CHEOL-KYU US | COMMISSARIAT ENERGIE ATOMIQUE (FR)\ JOUSSEAUME VINCENT (FR) | A05-J01B\ A11-B05\ A11-C02C\ A11-C04D\ A11-C04E\ A12-E07C\ L03-H04D\ L04-C06B1\ L04-C06B2\ L04-C07D\ L04-C07G\ L04-C12E\ L04-C18\ U11-C04E1\ U11-C05B9A\ U11-C07A1 |
| 6480 | 3D printing | SELECTIVE LASER MELTING DEEP HOLE REPAIRING DEVICE INCLUDES BASE, X-AXIS GUIDE RAIL GROUP, X-AXIS MOTOR, BRACKET PLATFORM, TABLE, SILK, Z-AXIS SUPPORT BASE, Z-AXIS ELECTRIC ACTUATOR, CONNECTING PLATE, Y-AXIS BASE, AND Y-AXIS MOTOR | CN103849872A | JUNHAO CHU (CN)\ JINCHUN JIANG (CN)\ FUWEN SHI (CN) | HYNIX SEMICONDUCTOR INC (KR) | M22-H03B\ S05-C |
| 6481 | 3D printing | PREPARATION OF ZIRCONIUM ALLOY SURFACE NICKEL BASE COATING INVOLVES GRINDING ZIRCONIUM ALLOY SUBSTRATE SURFACE, REMOVING OIL, CLEANING, FULLY MIXING NICKEL BASE ALLOY POWDER AND ORGANIC BONDING AGENT, AND PERFORMING LASER CLADDING PROCESS | JP2014148593A | KADOTA JUNJI US\ FURUTA HIROSHI (JP)\ MIZUGUCHI ICHIRO (JP) | SHANGHAI SOLAR BATTERY RES AND (CN) | M22-H03B\ M22-H03C\ X25-A08 |
| 6482 | 3D printing | IMPROVEMENT OF NICKEL-BASED TUNGSTEN CARBIDE COATING INVOLVES ADDING RARE EARTH LANTHANUM OXIDE POWDER TO NICKEL BASED TUNGSTEN CARBIDE COATING POWDER, MIXING, GRINDING, CLEANING, AND PERFORMING SEMICONDUCTOR LASER CLADDING PROCESS | CN103833350A | KADOTA JUNJI US\ FURUTA HIROSHI (JP)\ MIZUGUCHI ICHIRO (JP) | RENESAS ELECTRONICS CORP (JP) | M22-H03B\ M22-H03G\ X25-B02A |
| 6483 | 3D printing | VENTRICULAR SEPTAL DEFECT PATCH MANUFACTURING METHOD, INVOLVES MANUFACTURING THREE-DIMENSIONAL SOLID MODEL, UTILIZING THREE-DIMENSIONAL SOLID MODEL TO CUT OUT OPERATION PATCH, AND PERFORMING POWDER SINTERING MOLDING PROCESS | WO2014117079A1 | KADOTA JUNJI US\ UCHIDA SHOZO US\ FURUTA HIROSHI (JP)\ MATSUSHIGE MUNEAKI (JP) | NEC ELECTRONICS CORP (JP) | M13-H01\ V08-A09 |
| 6484 | 3D printing | NON-PHOTOSENSITIVE POLYIMIDE PASSIVATION LAYER MANUFACTURING METHOD, INVOLVES REMOVING DEVELOPED PHOTORESIST, AND PERFORMING NON-PHOTOSENSITIVE POLYIMIDE PHOTO-RESIST CURING PROCESS TO FORM NON-PHOTOSENSITIVE PASSIVATION LAYER | KR2014065921A | KADOTA JUNJI US\ UCHIDA SHOZO US\ FURUTA HIROSHI (JP)\ MATSUSHIGE MUNEAKI (JP) | RENESAS ELECTRONICS CORP (JP)\ FURUTA HIROSHI (JP)\ UCHIDA SHOUZOU (JP)\ MATSUSHIGE MUNEAKI (JP)\ MONDEN JUNJI (JP) | V08-A04A\ V08-A09 |
| 6485 | 3D printing | METHOD FOR FORMING ULTRAFINE LASER DIRECT DIRECTIONAL GROWTH OF COLUMNAR CRYSTALS, INVOLVES OBSERVING FINAL MICROSTRUCTURE UNDER OPTICAL MICROSCOPE, AND RECORDING GROWTH STATUS COLUMN DIRECTIONAL GRAIN STRUCTURE | CN203625469U | KAI-MING CHING US | NEC ELECTRONICS CORP (JP) | L03-H05\ X16-E01C\ X16-E01G |
| 6486 | 3D printing | NEGATIVE PRESSURE TANK FOR SELECTIVE LASER SINTERING USED IN THREE-DIMENSIONAL PRINTING FIELD, HAS SUPPORTING PLATE WHOSE INNER WALL IS ADHERED WITH POWDER SIEVE HAVING PORES WHOSE DIAMETER IS LESS THAN DIAMETER OF POWDER | JP2014137511A | KAIZHOU TAN US\ JING ZHANG US\ ZHENGFAN ZHANG US | TAIWAN SEMICONDUCTOR MFG (TW)\ CHING KAI-MING (TW) | F02-A03 |
| 6487 | 3D printing | ELECTROMAGNETIC MODULATION MELTING TRANSMISSION DEVICE COMPRISES INDUCTION HEATING COIL, THREE-PHASE LINEAR INDUCTION HEATING COIL, AND NOZZLE | DE102013001374A1 | KAJITANI KAZUHIKO US\ OTSUKA KANJI (JP) | NO 24 RES INST CETC US | A11-B06A\ A12-R\ A12-R01A\ L02-A02C\ L02-D01\ L02-D14M |
| 6488 | 3D printing | STEAM TURBINE BLADE INLET EDGE LASER CLADDING GROOVE STRUCTURE COMPRISES GROOVE, TURBINE BLADE, BLADE ROOT EXTENDING SHROUD, ARC INNER SURFACE, AIR INLET, SMOOTH CIRCULAR ARC AND CONNECTING SIDE | DE102013201629A1 | KAMOSHIMA TAKAO US\ TAKADA YOSHIFUMI US\ FUJII YASUHISA US | ELPIDA MEMORY INC (JP) | V04-D03\ V04-M06\ V04-S02\ V04-X01A |
| 6489 | 3D printing | CONTINUOUS PROCESSING SHAFT LASER DEPOSITED MULTIFUNCTIONAL WORKING STATION, HAS MOVING DEVICE PROVIDED WITH X-AXIS GUIDE RAIL THAT IS INSTALLED ON GUIDE WAY, AND TWO TABLE SYSTEMS FIXED WITH TWO SIDES OF X-AXIS GUIDE RAIL | JP2014135357A | KANAMOTO TOSHIKI (JP)\ INABA HISATO (JP) | RENESAS TECH CORP (JP) | D09-C01E\ E10-E02F1\ E12-A10\ E35-C02\ X25-A08 |
| 6490 | 3D printing | DYE-SENSITIZED SOLAR ENERGY BATTERY LIGHT ANODE THIN FILM LIGHT SCATTERING LAYER MANUFACTURING METHOD, INVOLVES SPRAYING ETHANOL SOLUTION ON DYE-SENSITIZED SOLAR ENERGY BATTERY LIGHT ANODE THIN FILM BY USING TITANIUM SOURCE SPRAYER | US2014192146A1 | KANDA YASUO US | RENESAS ELECTRONICS CORP (JP) | X12-G04A1\ X22-X01B\ X22-X20A |
| 6491 | 3D printing | BI-DIRECTIONAL COMBED PURE COTTON OVER-TWISTED DOUBLE-LAYER COTTON SURFACE MATERIAL LAYER, COMPRISES WARP AND WEFT THAT ARE INTER-WEAVED WITH EACH OTHER, WHERE DENSITY AND WIDTH OF WEFT ARE IN SPECIFIC VALUE | CN203622602U | KANEKO YOSHIO (JP) | SONY CORP (JP) | U11-F01C3\ U11-F01C5\ U12-A01B1J\ V08-A04A\ V08-A06 |
| 6492 | 3D printing | MORTAR ADDITIVE USED FOR E.G. CONSTRUCTING BRICK, COMPRISES HIGHLY CONCENTRATED CURING LIQUID, CAUSTIC SODA, LATEX, SODIUM LAURYL SULFATE NEEDLE SURFACTANT, AND PURIFIED WATER | CN203625467U | KANG MI-HYUN (KR)\ LEE MEUNG-RYUL (KR)\ KIM YONG-HOAN (KR) | TOSHIBA KK (JP) | M22-H03B\ M22-H03G\ V06-M06E |
| 6493 | 3D printing | ANTI-ABLATION TYPE COMPOSITE PLATE FOR MISSILE, HAS HIGH TEMPERATURE RESISTANT ADHESIVE LAYER THAT IS PROVIDED TO CONNECT AN INSULATION ELEMENT AT INNER HOLE WHICH IS PROVIDED IN STAINLESS STEEL OR COPPER ALLOY SUBSTRATE | CN203625468U | KANG SEONG HO (KR)\ KOO BON CHEOL (KR) | SAMSUNG ELECTRONICS CO LTD (KR)\ KANG MI-HYUN (KR)\ LEE MEUNG-RYUL (KR)\ KIM YONG-HOAN (KR) | M22-H03B\ V06-M06E\ V06-M10 |
| 6494 | 3D printing | METHOD FOR MANUFACTURING THREE-DIMENSIONAL INTER-CONNECTED POROUS TISSUE SCAFFOLD BY UTILIZING SELECTIVE LASER SINTERING TECHNOLOGY, INVOLVES SUPERPOSING SINTERING LAYER TO OBTAIN POROUS BETA-TRICHLOROPHENOL BRACKET | CN103835935A | KARP JAMES (US)\ LI RICHARD C (US) | LG ELECTRONICS INC (KR) | M22-H03B\ M22-H03G\ X25-A01 |
| 6495 | 3D printing | METHOD FOR MANUFACTURING CYLINDRICAL CLADDING, INVOLVES EMBOSSING SHEET-LIKE BASE MATERIAL TO FORM UNEVEN SHAPE WHICH IS CURVED AND MOLDED IN CYLINDER SHAPE | CN103839989A | KATAOKA JUNNOSUKE US | XILINX INC (US)\ KARP JAMES (US)\ LI RICHARD C (US) | M22-H01\ M22-H03B |
| 6496 | 3D printing | SEMICONDUCTOR LASER STACK CHANGE SLOW AXIS DIRECTION LIGHT FIELD DISTRIBUTION CHANGING METHOD, INVOLVES FORMING SLOW AXIS DIRECTION DISTRIBUTE OPTICAL FIELD OF MULTI-SEMICONDUCTOR LASER ARRAY DEVICE ACCORDING TO CERTAIN SEQUENCE | CN103840032A | KATSUNAO KANARI US | CANON KK (JP) | M13-E01\ M13-E04\ M13-H01\ M13-L\ V08-A09 |
| 6497 | 3D printing | SELECTIVE LASER MELTING SPREADING DEVICE COMPRISES MAIN BODY, GUIDE RAIL, SLIDING BLOCK, SERVO DRIVE MOTOR, AND SCRAPING DEVICE | US2014203460A1 | KATSUYA ARAI (JP)\ TOSHIHIRO KOGAMI (JP)\ HIROAKI YABU (JP) | FUJITSU LTD JP US | A05-H03\ A12-D05B\ G02-A04A\ G02-A04B |
| 6498 | 3D printing | SELECTIVE LASER MELTING BIDIRECTIONAL SPREADING DEVICE COMPRISES MAIN BODY, SLIDE BLOCK, ELECTRICAL MACHINE GUIDE RAIL, POWDER SPREADING DEVICE, DESULFURIZING DEVICE, SERVO MOTOR, TRANSMISSION SHAFT, AND FLEXIBLE SCRAPING POWDER BRUSH | WO2014109675A1 | KAWAMURA HIROKI US | PANASONIC CORP (JP)\ ARAI KATSUYA (JP)\ KOGAMI TOSHIHIRO (JP)\ YABU HIROAKI (JP) | A10-E05B\ A10-E09B1\ A12-W12G\ L02-A02\ L02-A03\ L02-A04\ L02-B01\ L02-G01E\ L02-G10B\ L02-G12A\ L02-J02C |
| 6499 | 3D printing | SELECTIVE DOUBLE-LASER MELTING DEVICE COMPRISES OPTICAL SYSTEM, CENTRAL CONTROL MACHINE, SEAL FORMING ROOM, LIGHT VALVE STEERING UNIT, BEAM EXPANDING LENS UNIT, AND VIBRATION MIRROR UNIT | DE102013000511A1 | KAWANO HIDEO (JP)\ TAMEGAI HARUKO (JP)\ YASHIMA TOORU (JP) | SHARP KK (JP) | T01-S03\ W04-C |
| 6500 | 3D printing | LASER CLADDING DEVICE COMPRISES BASE, SUBSTRATE GROOVE, STAINLESS STEEL FOUNDATION, GUIDE POST, AND HOLLOW STAINLESS STEEL PLATE | CN103817328A | KAWASE TOMOO (JP)\ KUROKAWA HIDEKAZU US | RENESAS ELECTRONICS CORP (JP)\ KAWANO HIDEO (JP)\ TAMEGAI HARUKO (JP)\ YASHIMA TOORU (JP) | A12-V02\ D09-C01E |
| 6501 | 3D printing | PREPARATION OF COATING VIA NANOSECOND PULSED LASER CLADDING BY DEPOSITING METAL ON WORKPIECE SURFACE USING METAL ORGANIC CHEMICAL VAPOR DEPOSITION, SCANNING WORKPIECE SURFACE USING NANOSECOND LASER, COATING MOLTEN METAL AND FORMING COATING | CN103820780A | KEHAN ZHU (CN)\ SHURONG DONG (CN)\ YAN HAN (CN) | DENSO CORP (JP) | J04-X\ M13-H01 |
| 6502 | 3D printing | INK COMPOSITION FOR WRITING INSTRUMENTS E.G. PEN, COMPRISES LEUCO DYE, COLOR DEVELOPER, LIQUID MEDIUM, AND MICROCAPSULE CONTAINING POLAR COMPOUND AND COMPOUND HAVING HYDROXYL GROUP, CARBONYL GROUP, AMINO GROUP OR POLYOXYALKYLENE GROUP | CN103820849A | KENJO SEISHI US | UNIV ZHEJIANG (CN) | U12-A01A5B\ U12-A01A7\ U14-K01A3\ V07-K01B\ W03-A08E7C\ W03-A12A |
| 6503 | 3D printing | PREPARATION OF ALUMINUM-CALCIUM TITANATE CERAMIC INVOLVES MILLING CALCIUM CARBONATE, ALUMINUM CARBONATE AND TITANIUM DIOXIDE, ADDING BINDER AND PLASTICIZER, PRESSING AND CARRYING OUT DIRECT CARBON DIOXIDE LASER SINTERING PROCESS | CN103825964A | KER MING-DOU (TW)\ HSIAO YUAN-WEN (TW)\ WANG CHANG-TZU (TW) | KYOCERA KINSEKI CORP US | A12-E10\ A12-H05\ A12-H08\ A12-S08B\ A12-S08D1\ A12-S08D2\ M22-H03A\ M22-H03B |
| 6504 | 3D printing | NON-TRANSITORY COMPUTER-ACCESSIBLE MEDIUM FOR EXTENDED DEPTH OF FIELD IMAGING TO GENERATE IMAGES OF PORTIONS OF SAMPLE HAS INSTRUCTIONS EXECUTED BY COMPUTER HARDWARE ARRANGEMENT TO RECEIVE INFORMATION RELATED TO ELECTROMAGNETIC RADIATION | JP2014125873A | KER MING-DOU (TW)\ LIN GEENG-LIH (TW)\ JOU YEH-NING (TW) | UNITED MICROELECTRONICS CORP (TW)\ UNIV NAT CHIAO TUNG (TW) | M22-H03B |
| 6505 | 3D printing | GREEN BODY COMPOSITION USEFUL IN HARD TISSUE REGENERATION, COMPRISES CERAMIC POWDER E.G. MAGNESIUM PHOSPHATE, MAGNESIUM OXIDE OR MAGNESIUM SULFATE, AND A THICKENER E.G. HYDROXYPROPYL METHYLCELLULOSE, GELATIN, COLLAGEN OR CHITOSAN SOLUTION | CN103801658A | KERTESZ PHILIPPE US\ NAVARD GILLES US\ CABAN CHASTAS DANIEL US | VANGUARD INT SEMICONDUCT CORP (TW) | L03-H04A\ U11-C04C4\ U11-C04E1\ U11-C04E1A |
| 6506 | 3D printing | LASER CLADDING FORMING DEVICE SUITABLE FOR USE IN SMALL LABORATORIES COMPRISES LASER HEAD, LASER SENSOR, WORKING PLATFORM HAVING BOTTOM BASE, HYDRAULIC DRIVE SYSTEM, AND HYDRAULIC CYLINDER CONNECTED WITH ENERGY ACCUMULATOR | CN103805989A | KIKUCHI SHUICHI US\ FUJINO TSUTOMU US | THALES SA (FR) | V07-F02C\ V07-K01\ V07-M\ W03-A08B |
| 6507 | 3D printing | THREE-DIMENSIONAL IMAGE DISPLAY APPARATUS HAS CONTROL UNIT DRIVING SHUTTER DEVICE AT FREQUENCY HIGHER THAN LIGHT EMISSION FREQUENCY OF LED DISPLAY UNIT | CN103805990A | KIM DO-HYUNG (KR)\ KIM SUNG-BONG (KR)\ HONG JUNG-IN (KR) | SANYO ELECTRIC CO (JP) |  |
| 6508 | 3D printing | DEVICE FOR PRODUCING THREE-DIMENSIONAL OBJECT BY COMPACTING POWDERED LAYERS OF COMPOSITIONAL MATERIAL, COMPRISES CARRIER ARRANGED IN CONSTRUCTION CHAMBER, APPLICATION DEVICE FOR APPLYING COMPOSITIONAL MATERIAL LAYERS AND IRRADIATING DEVICE | CN103805992A | KIM SEONG-DONG (US)\ JEONG YONG-KUK (KR)\ YU DONG-HEE (US)\ YANG JONG-HO (KR) | SAMSUNG ELECTRONICS CO LTD (KR) | M13-H01\ V08-A09 |
| 6509 | 3D printing | GENERATIVE AND LAYER-WISE PRODUCTION OF COMPONENT BY E.G. LASER, COMPRISES LAYER-BY-LAYER MELTING OF METAL POWDER LOCATED IN SPACE OF COMPONENT BY LASER, WHERE ENERGY REQUIRED FOR MELTING IS REGULATED DEPENDING ON POSITION OF COMPONENT | JP2014129487A | KIM TAE S (US)\ LU ZHIJIAN (US) | IBM (US)\ SAMSUNG ELECTRONICS CO LTD (KR) | M13-H01\ X26-D01A |
| 6510 | 3D printing | ILLUMINATION OPTICAL SYSTEM FOR USE IN POLARIZED-LIGHT UNIT TO ILLUMINATE RETICLE SURFACE BY ILLUMINATION LIGHT FROM LIGHT SOURCE, HAS BIREFRINGENT PART WHOSE OPTICAL AXIS IS PARALLEL TO OPTICAL AXIS OF MAIN BODY | WO2014104902A1 | KIM YOUNG HO (KR)\ CHUNG HAN (KR)\ PARK JAE HONG (KR)\ SHIN MYOUNG SUB (KR) | TEXAS INSTRUMENTS INC (US) |  |
| 6511 | 3D printing | APPARATUS FOR DISPLAYING HOLOGRAM IMAGE HAS LIGHT SOURCE PROVIDER THAT IS PROVIDED TO CONTROL POSITION OF LIGHT SOURCE OF DISPLAY HOLOGRAM IMAGE BASED ON POSITION OF PUPIL OF USER | CN103761017A | KITAMURA AKIO (JP) | I3SYSTEM CORP (KR) | L04-A01A\ L04-B04A\ L04-C02\ L04-C10B\ L04-C11C1\ L04-C12A\ L04-C12B\ L04-C12J\ L04-E01\ L04-E01D\ U11-C01J2\ U11-C05B9A\ U11-C05F1A\ U11-C18A2\ U12-D01A1\ U12-E02 |
| 6512 | 3D printing | THREE-DIMENSIONAL (3D) CERAMIC PRINTER HAS SPREADING HOPPER WHOSE LEFT AND RIGHT SIDES ARE SYMMETRICALLY INSTALLED WITH SCRAPING PLATE, WHILE FIXING REAR SIDE OF SPREADING HOPPER ON SCREW ROD THROUGH NUT BLOCK | CN103794491A | KITCH VASSILI (US) | FUJI ELECTRIC CO LTD (US) | L03-E05B\ L04-A01A\ L04-C01\ L04-C05A\ L04-C07D\ L04-C09\ L04-C12B\ U11-C01B\ U11-C01J4A\ U11-C05B2\ U11-C05B5\ U11-C07A1\ U11-C07C1\ U11-C18B9\ U12-A02A1\ U12-A02A2X\ U12-A02A3\ U12-A02A4D\ X15-A01C\ X15-A02A |
| 6513 | 3D printing | HOLLOW LIGHT INNER LASER COAXIAL POWDER FEEDING DEVICE COMPRISES MAIN BODY HAVING INTERNAL MIX NOZZLE CAVITY AND AIR FLOW NOZZLE CAVITY CONNECTED TO EACH OTHER, AND EXTERNAL RING | CN103794593A | KLEIN RITA J (US) | NAT SEMICONDUCTOR CORP (US) | K05-B05A\ K05-B08\ X14-A02\ X14-B05\ X14-C04 |
| 6514 | 3D printing | PARALLEL ANNULAR LASER CLADDING COMPRISES SHELL WITH INCIDENT OPENING AND EMITTING PORT, BRACKET, AND CONICAL SURFACE MIRROR, IN WHICH INNER WALL OF CONICAL SURFACE AND LOWER CONICAL SURFACE REFLECTION MIRROR FORM ANNULAR PARALLEL LIGHT | CN103794717A | KLEIN RITA J (US) | MICRON TECHNOLOGY INC (US) | M22-H03B\ X25-A08 |
| 6515 | 3D printing | DIAPHRAGM PUMP MOVABLE COLUMN STRUCTURE, HAS CARD RING FIXED WITH UPPER PART OF OUTER COVER, WHERE OUTER SIDE LAYER OF OUTER COVER IS FORMED WITH INTER LAYER AND OUTER SIDE LAYER IS PLATING LAYER | CN103795674A | KOBAYASHI NAOHIRO (JP) | MICRON TECHNOLOGY INC (US)\ KLEIN RITA J (US) | A09-D03\ A11-B14\ A12-W12F |
| 6516 | 3D printing | LOW VOLTAGE IGBT BUFFERING LAYER, HAS P-PLUS-TYPE SUBSTRATE, N-PULSE BUFFERING LAYER AND N-DRIFT AREA, AND P-TYPE BASE REGION LOCATED IN N-DRIFT AREA, WHERE N-PLUS BUFFERING LAYER IS COATED WITH P-PLUS-TYPE SUBSTRATE | CN103796149A | KOBAYASHI NAOHIRO (JP) | RENESAS ELECTRONICS CORP (JP)\ KOBAYASHI NAOHIRO (JP) | M22-H03B\ V06-M06B\ V06-M06E\ X25-A08 |
| 6517 | 3D printing | PREPARATION OF DOUBLE LAYER ANTI-REFLECTION COATING USED FOR MONOCRYSTALLINE SILICON SOLAR CELLS COMPRISES TAKING SILICON SHEET, ETCHING, DIFFUSING, CLEANING AND PERFORMING PLASMA ENHANCED CHEMICAL VAPOR DEPOSITION | CN203593782U | KOBAYASHI TAKASHI (JP)\ KURATA HIDEAKI (JP)\ KOBAYASHI NAOKI (JP)\ KUME HITOSHI (JP)\ KIMURA KATSUTAKA (JP)\ SAEKI SHUNICHI (JP) | NEC ELECTRONICS CORP (JP) | M13-H01\ M13-L\ M23-D05\ X24-D03 |
| 6518 | 3D printing | REMOTELY OPERATED LASER SINTERING SYSTEM FOR REMOTELY PREPARING HIGH DENSITY FUEL PELLET, HAS LASER STRUCTURED TO DIRECT BEAM TO SINTER PATTERN IN LAYER OF FEED COMPOSITION, WHERE CONTROL UNITS FOR LASER ARE POSITIONED OUTSIDE OF ENCLOSURE | CN203596344U | KOBAYASHI TAKASHI (JP)\ KURATA HIDEAKI (JP)\ KUME HITOSHI (JP)\ KIMURA KATSUTAKA (JP)\ SAEKI SHUNICHI (JP)\ GOTO YASUSHI (JP)\ KURE TOKUO (JP) | RENESAS TECH CORP (JP) | L03-H05\ L04-B01\ L04-B02B\ L04-B03\ L04-B04\ L04-C01B\ L04-C02C\ L04-C09\ L04-C16\ L04-D01\ U11-C01B\ U11-C01J1\ U11-C01J4A\ U11-C02J1C |
| 6519 | 3D printing | METAL ARTICLES PRODUCTION FROM POWDERED MATERIAL BY SELECTIVE LASER SINTERING OF POWDER MATERIAL LAYERS | JP2014127598A | KOBAYASHI TSUGUO (JP) | RENESAS TECH CORP (US)\ HITACHI DEVICE ENG (US) | T01-N01A2L\ T01-N01D3A\ W01-A06A3\ W01-A06E1G |
| 6520 | 3D printing | DEVICE E.G. LASER SINTERING DEVICE FOR MANUFACTURING THREE-DIMENSIONAL OBJECT, HAS MULTIPLE CONVEYING ELEMENTS THAT ARE PROVIDED FOR TRANSPORTING OF DIFFERENT BUILDING MATERIALS TO ONE METERING CHAMBER THROUGH SEPARATE FEED LINES | WO2014103770A1 | KODAMA NORIYUKI US\ YOSHIZAWA YUTAKA US | TOSHIBA KK (JP) |  |
| 6521 | 3D printing | DEVICE FOR INCREASING MASS OF SELECTIVE LASER SINTERING METAL POWDER COMPONENT, HAS TOP POLE WHOSE LOWER END IS CONNECTED WITH LINEAR SERVO MOTOR FOR DRIVING FIXED BOTTOM PLATE | WO2014103033A1 | KOERNER HEINRICH (DE) | NEC ELECTRONICS CORP (JP) | A04-C03\ A06-A00E\ A07-A04F\ A08-M03B\ A11-B16\ A11-C02D\ A12-W12G\ M22-A04\ M22-B01\ M22-B03\ M22-D\ X25-A07\ X25-A08 |
| 6522 | 3D printing | PREPARATION OF COATING FOR ROLLER SURFACE INVOLVES PERFORMING DEGREASING AND RUST REMOVAL TREATMENT ON ROLLER PROCESSING SURFACE, ADJUSTING LIGHT BEAM SPOT SIZE, AND AUTOMATICALLY FEEDING ALLOY POWDER TO LASER MOLTEN POOL | WO2014103054A1 | KOIZUMI NORIOMI US | KOERNER HEINRICH (DE) | M13-H01\ M22-G03A\ M26-A02\ M26-A02C\ M26-B03\ V08-A01D\ V08-A09 |
| 6523 | 3D printing | PRODUCTION OF 12-INCH MONOCRYSTALLINE SILICON WAFER INVOLVES INTRODUCING HIGH-PURITY HYDROGEN CHLORIDE GAS INTO REACTION CAVITY, PURGING RESIDUAL HYDROCHLORIC ACID AND REACTION PRODUCT, PROVIDING SILICON WAFER ON BASE LAYER AND HEATING | JP3191474U | KOJI TAKAHASHI (JP)\ KOSHI HASHIMOTO (JP) | SEIKO EPSON CORP (JP) | M13-H01\ V08-A09 |
| 6524 | 3D printing | CLOUD PAAS PLATFORM BASED SLS TASK SCHEDULING DEVICE, HAS HARDWARE DEVICE WHOSE DOCUMENT IS STORED ON LEGAL STORAGE AND PROCESSING MODULE, AND SCHEDULING MODULE WHOSE EXTRACTION INFORMATION IS SENT TO SYSTEM MONITORING MODULE | KR2014062957A | KOJIMA SHUICHIRO (JP)\ SEIKE MAMORU (JP)\ ICHIHARA TAKASHI (JP) | FUJITSU SEMICONDUCTOR LTD (JP)\ HASHIMOTO HIROSHI (JP)\ TAKAHASHI KOJI (JP) | M13-H01\ M26-B\ M26-B00J\ M26-B00N |
| 6525 | 3D printing | WATERPROOF FLOOR STRUCTURE USED FOR ROOFTOP PARKING LOT OF BUILDING, HAS ANTISKID LAYER WHICH IS PROVIDED WITH COLORED LAYER FORMED IN LAYERED FORM BY COLOR DIFFERENT FROM ANOTHER AMONG LAMINATED STRUCTURES | CN103628055A | KOJIMA TOSHIO US | PANASONIC CORP (JP) | A04-G02E2\ A04-G11\ A05-F01B2\ A05-F01E3\ A07-A04E\ A08-M09B\ A08-M10\ A09-A05A\ A10-E23\ A12-P01B |
| 6526 | 3D printing | MANUFACTURE OF CERAMIC SHELL INVOLVES PERFORMING SELECTIVE LASER SINTERING RAPID PROTOTYPING OF ACRYLONITRILE BUTADIENE STYRENE POWDER AND SILICON RUBBER TO PRODUCE SILICONE RUBBER LAYER CONVEX MOLD AND UNIFORMLY COATING RELEASE AGENT | CN103526102A | KOLDIAEV VIKTOR (US) | RENESAS TECH CORP (JP) | M13-H01\ M23-D05\ M24-D02A\ X24-D03 |
| 6527 | 3D printing | LASER CLAD OF COPPER ALLOY CRYSTALLIZER SURFACE BY SELECTING COPPER ALLOY CRYSTALLIZER, SURFACE PREPROCESSING, APPLYING COATING LAYER ON SURFACE, AND LASER CLADDING | WO2014105113A1 | KOMATSU SHIGEYUKI (JP) | KOLDIAEV VIKTOR (US) | T04-F02A2\ T04-F02C\ T04-H04 |
| 6528 | 3D printing | LASER CLAD OF COPPER ALLOY SURFACE BY PULVERIZING ALUMINUM AND NICKEL TO OBTAIN METAL NICKEL BASE ALLOY, AND PULVERIZING CARBON, CHROMIUM, SILICON, TUNGSTEN, IRON AND NICKEL | WO2014094088A1 | KOMATSUBARA KOKI US | MATSUSHITA ELECTRIC IND CO LTD (JP) | L04-A01A\ L04-B04A\ L04-C02B\ L04-C12A\ L04-E\ U11-C01J4A\ U11-C02J1C\ U11-C05B4 |
| 6529 | 3D printing | ELECTRIC SPARK DEPOSITION AND LASER CLAD OF REINFORCED METAL WATER TURBINE ROTARY WHEEL BLADE SURFACE BY PREPARING IRON-NICKEL ALLOY POWDER, LASER CLADDING WITH CARBON DIOXIDE UNDER ARGON ATMOSPHERE, AND SURFACE POLISHING WITH SANDPAPER | US2014178241A1 | KOMIYA MANABU US\ OSAWA KOUICHI US\ TATSUKAWA NAOHISA US\ TOMITA YASUHIRO US\ FUKUSHIMA TETSUYUKI US\ SHISHIDO KATSUHIKO (JP) | OKI SEMICONDUCTOR CO LTD (JP)\ OKI SEMICONDUCTOR MIYAGI CO LT US | U11-C05C\ U11-C05F1\ U11-C18A3\ U11-G01\ U12-D02A\ U12-E02 |
| 6530 | 3D printing | POLYETHYLENE-TYPE STRUCTURE USEFUL IN E.G. DETERGENT AND AGROCHEMICAL COMPRISES RESIN COMPOSITION COMPRISING POLYETHYLENE, ACID-MODIFIED POLYETHYLENE, AND METAXYLYLENE GROUP CONTAINING POLYAMIDE | US2014176540A1 | KOMURO HIDEYUKI (JP)\ NOZOE KOJI (JP) | MATSUSHITA ELECTRIC IND CO LTD (JP)\ KOMIYA MANABU US\ OSAWA KOUICHI US\ SHISHIDO KATSUHIKO (JP)\ TATSUKAWA NAOHISA US\ TOMITA YASUHIRO US\ FUKUSHIMA TETSUYUKI US | L04-A01A\ L04-C12A\ L04-C12B\ L04-C12K\ L04-C13B |
| 6531 | 3D printing | DURABILITY REGENERATING AND INCREASING METHOD OF MILL ROLL, INVOLVES COVERING LATERAL WORKING SURFACES OF MILL ROLL VIA LASER CLADDING WITH LAYER OF METALLIC MATERIAL | US2014175343A1 | KONDO ATSUO US | FUJITSU SEMICONDUCTOR LTD (JP)\ KOMURO HIDEYUKI (JP)\ NOZOE KOJI (JP) | W01-A09D\ W02-K07C |
| 6532 | 3D printing | SINGLE LAYER MULTI-POINT CAPACITOR TYPE TOUCH SCREEN, HAS PANEL ADHERED ON SCREEN MODULE THROUGH TRANSPARENT OPTICAL GLUE, AND TOUCH CONTROL UNIT INDEPENDENTLY CONNECTED WITH PROCESSOR THROUGH DOWN-LEAD WIRE | CA2837408A1 | KONDO MASAKI (JP) | NGK INSULATORS LTD US | V06-V03A |
| 6533 | 3D printing | LOW-DIELECTRIC CONSTANT LAYER MANUFACTURING METHOD, INVOLVES FORMING MEDIUM LAYER ON SILICON DEPOSITION LAYER, AND FORMING LOW-DIELECTRIC CONSTANT LAYER AT UPPER END OF ANOTHER LOW-DIELECTRIC CONSTANT LAYER BY OMCTS AND ARGON PNEUMA | DE102012223556A1 | KONNO TOSHIAKI US\ HAGA NOBUAKI US | TOSHIBA KK (JP) | M13-G02A\ M13-L\ V05-F04B5C\ V05-F04K\ V05-F05C\ V05-F05E\ V05-F08D1A |
| 6534 | 3D printing | POWER MOS TRANSISTOR ARRAY, HAS CELL STRUCTURE PROVIDED WITH ACTIVE DEVICE AND METAL CONNECTING LAYER, AND METAL CONNECTING LAYER FORMED GROOVE-SHAPED HOLE AND METAL THROUGH HOLE, WHERE METAL THROUGH HOLE IS FIXED BETWEEN METAL LAYERS | CN103769586A | KOPLEY THOMAS EDWARD (US)\ VOOK DIETRICH W (US)\ DUNGAN THOMAS (US) | ALPS ELECTRIC CO LTD (JP) | U11-C05C\ U11-C05G2C\ V04-Q05\ V04-R01A3\ V04-U03\ V04-U15 |
| 6535 | 3D printing | MAGNETIC TUNNEL JUNCTION DEVICE DIELECTRIC LAYER MANUFACTURING METHOD, INVOLVES FIXING DIELECTRIC LAYER WITH SUBSTRATE, AND SELECTING TRACE METAL FROM ONE OF ALUMINUM, TANTALUM, CHROMIUM, COPPER, GOLD AND PLATINUM | CN103771837A | KORDIC SRDJAN (NL)\ MUTSAERS CORNELIS ADRIANUS (NL)\ KLEE MAREIKE KATHARINE (NL)\ GROEN WILHELM ALBERT (NL) | AVAGO TECHNOLOGIES GENERAL IP (SG) | T04-F02A2\ T04-F02C\ U11-C05C\ V06-M06F\ V06-U04B\ V06-V01C\ V06-V01E\ V06-V04G\ V06-V04Q |
| 6536 | 3D printing | MULTIPLE-INPUT AND MULTIPLE-OUTPUT-OFDM SYSTEM PAPR REDUCING METHOD, INVOLVES FORMING L-PACKETS OF ANTENNA BY UTILIZING MULTIPLE-INPUT AND MULTIPLE-OUTPUT-OFDM SYSTEM, AND RECEIVING MULTIPLE BASEBAND SIGNAL OF OFDM SYSTEM | CN103774025A | KOSCHINSKY FRANK (DE)\ MEYER MORITZ ANDREAS (DE)\ LANGER ECKHARD (DE) | PHILIPS CORP (US) | X16-B01\ X16-F06\ X16-K02\ X21-A01D\ X21-A01F\ X21-B01A |
| 6537 | 3D printing | WEDGE-SHAPED ACOUSTIC MATCHING LAYER MANUFACTURING METHOD, INVOLVES CREATING WEDGE-SHAPED STRUCTURE TEMPLATE, FILLING LIQUID MATERIAL IN MIDDLE PART OF MICRO-NEEDLE ARRAY, AND GRINDING SHEET STRUCTURE TO PREPARE ACOUSTIC MATCHING LAYER | CN103774138A | KOSHIZUKA KUNIHIRO US\ KITAMURA SHIGEHIRO US\ TAKIMOTO MASATAKA US\ KAWAMURA TOMONORI US | MEYER MORITZ ANDREAS (DE)\ LANGER ECKHARD (DE)\ KOSCHINSKY FRANK (DE) |  |
| 6538 | 3D printing | RADIO FREQUENCY MAGNETIC CONTROL SPUTTERING DEVICE COMPRISES SPUTTERING TARGET BASE BODY, COOLING DISK, SHIELDING COVER AND SUPPORTING MEMBER | CN103774139A | KOSUGE YOSHIE US | KONICA MINOLTA HOLDINGS INC US | A04-G02B\ A05-F01B2\ A07-A04E\ A10-E01 |
| 6539 | 3D printing | CERAMIC SUBSTRATE HAS COPPER LAYER ARRANGED ON SUBSTRATE, WHILE COVERING PLATE WITH THROUGH-HOLE IS FORMED COPPER LAYER | CN203578769U | KOZLOV ANTON VIKTOROVICH (RU)\ TIKHONOV ROBERT DMITRIEVICH (RU) | RENESAS ELECTRONICS CORP (JP) | X27-C05 |
| 6540 | 3D printing | SMALL-SIZED ACTUATOR E.G. ELECTROSTATIC TYPE SENSOR, FOR TOUCH SCREEN OF PORTABLE TERMINAL OF CELL BOARD COMPOSITE-BODY, HAS PIEZOELECTRIC MATERIAL LAYER INTERPOSED BETWEEN FIRST ELECTRODE LAYER AND SECOND ELECTRODE LAYER | CN203580143U | KUDO KOJI (JP) | G UCHREZHDENIE NP KOMPLEKS T T (RU)\ G OBRAZOVATEL NOE UCHREZHDENIE (RU) | U11-D01B1\ U12-A01A2\ U12-A01A4 |
| 6541 | 3D printing | BATTERY MODULE MOUNTED IN E.G. ELECTRIC VEHICLE HAS METAL PLATE WHICH IS ARRANGED FACING SURFACE OF SECONDARY BATTERY AND IS PROVIDED WITH HEAT CONDUCTIVITY DIFFERING FROM THAT OF OTHER METAL PLATE | WO2014098635A2 | KUJIRAI YUTAKA US\ OKUYAMA KOSUKE US\ OYU SHIZUNORI US\ NAGAI AKIRA US\ KUMOCHI TAKAHIRO US\ ICHINOSE TERUYUKI (JP)\ HATA KAZUHIRO (JP)\ UCHIYAMA HIROYUKI (JP) | FUJITSU LTD (US) | M13-H01\ M13-H03 |
| 6542 | 3D printing | BAMBOO POWDER CONTAINING MOLDED OBJECT USED FOR INTERIOR MATERIAL FOR VEHICLE, HAS SCALE-LIKE BAMBOO POWDER WHICH COMPRISES HOLE DERIVED FROM VASCULAR BUNDLE | WO2014100320A1 | KULAH HALUK (TR)\ KOYDEMIR HATICE CEYLAN (TR)\ OZGEN CANAN (TR) | KUJIRAI HIROSHI (JP)\ OKUYAMA KOUSUKE (JP)\ HATA KAZUHIRO (JP)\ OYU KIYONORI (JP)\ NAGAI RYO (JP)\ KUMAUCHI TAKAHIRO (JP)\ ICHISE TERUHISA (JP)\ UCHIYAMA HIROYUKI (JP) | A08-S02\ A12-W12F\ M13-H01\ M13-L\ M22-H02\ M22-H03F\ M26-A04\ M26-B08 |
| 6543 | 3D printing | POLYETHYLENE-TYPE STRUCTURE USED FOR HOLLOW MOLDED PRODUCT E.G. TANK, COMPRISES RESIN COMPOSITION CONTAINING SPECIFIED AMOUNT OF POLYETHYLENE, ACID-MODIFIED POLYETHYLENE HAVING ACID VALUE, AND METAXYLYLENE GROUP-CONTAINING POLYAMIDE | US2014166978A1 | KUMAGAI NAOKI US\ SAITO JUN US | KULAH HALUK (TR) | V08-A09 |
| 6544 | 3D printing | STRUCTURE OF BUCKET FOR E.G. GRILL, HAS PRIMARY NET ELEMENT WHICH IS ARRANGED FOR MAKING SMOKING ELEMENT AND CLIP TO CORRESPOND, AND SECONDARY NET ELEMENT, GUIDE ELEMENT AND PRIMARY NET ELEMENT THAT ARE STRADDLED BY MULTI-LAYERS | WO2014074379A1 | KUND MICHAEL (DE) | FUJI ELECTRIC CO LTD (JP) | L03-E04\ M22-H02\ M22-H03B\ M22-H03E\ M22-H03G\ X16-C17A |
| 6545 | 3D printing | METHOD FOR MANUFACTURING FLUORESCENCE LAYER UTILIZED IN LED PACKAGE ASSEMBLY, INVOLVES COATING RELEASE AGENT IN MULTIPLE ACCOMMODATING GROOVES FORMED IN TRAY, AND INJECTING FLUORESCENT MATERIAL IN ACCOMMODATING GROOVES | US2014168034A1 | KUNO ISAMU (JP)\ KATAGIRI TOMOHARU (JP) | QIMONDA AG (DE) | A08-R05\ M22-H02\ M22-H03B |
| 6546 | 3D printing | SURFACE LASER CLADDING OF ALLOY COMPOSITE COATING INVOLVES POLISHING ALUMINUM OR ALUMINUM ALLOY, WASHING, CLEANING, DRYING, COATING WITH RARE EARTH CERIUM OXIDE AND NICKEL-60 ALLOY POWDER WITH ADHESIVE, AND CARRYING OUT LASER CLADDING | US2014168783A1 | KUO CHEN-CHENG (TW)\ CHEN CHEN-SHIEN (TW)\ CHING KAI-MING (TW)\ CHEN CHIH-HUA (TW) | KAWASAKI MICROELECTRONICS KK US | T01-J10C4\ T01-J11A1\ T01-S03 |
| 6547 | 3D printing | PREPARATION METHOD FOR MICRO-NANO SILICIDE PARTICLE REINFORCED HIGH ABRASION LASER CLADDING COBALT-BASED ALLOY POWDER INVOLVES SIEVING, CRUSHING AND DRYING POWDER TO OBTAIN PREDEFINED PARTICLE SIZE WITHOUT COMPONENT SEGREGATION | CN103741137A | KURODA HIDEHIKO (JP) | TAIWAN SEMICONDUCTOR MFG (TW) | A05-E\ A08-D01\ A08-S02\ A12-L02B2\ A12-L03B\ G06-D06B\ G06-F03B\ G06-F03D\ L03-G05B9\ L03-G09P\ U11-A06 |
| 6548 | 3D printing | COMPONENT FOR GAS TURBINE ENGINE, HAS VASCULAR ENGINEERED LATTICE STRUCTURE FORMED INSIDE BODY PORTION AND INCLUDING ONE OF HOLLOW AND SOLID VASCULAR STRUCTURES FOR COMMUNICATING FLUID | US2014158667A1 | KURODA KENICHI (JP)\ MORIUCHI HISAHIRO US\ SHIRAI MASAKI US\ SAKAGUCHI JIRO US\ MATSUO AKINORI US\ YOSHIDA SEIJI US\ TAKEDA TOSHIFUMI (JP) | RENESAS ELECTRONICS CORP (JP) | M26-A01\ M26-A01B\ M26-A01C\ M26-A01N\ M26-B08\ M26-B08B\ M26-B08C\ M26-B08N\ D08-A\ D09-C01 |
| 6549 | 3D printing | OBTAINING MICROCHANNEL PLATES FOR CHEMICAL MICROREACTORS, INVOLVES ADDING METALLIC MATERIAL TO DEVICE CONTAINING YTTERBIUM, WHERE LASER DIRECT METAL LASER SINTERING IS PERFORMED FOR PROCESSING METALLIC MATERIAL | WO2014087961A1 | KURODA TAKAO (JP)\ ISHIDA HIDEKI (JP) | HITACHI LTD (JP)\ HITACHI ULSI SYS CO LTD (JP) | L03-E05B\ L03-H05\ L04-C10E\ L04-C11C\ L04-C13B\ L04-C16\ U11-C05C5\ U11-C18B9\ U12-A02A1\ U12-A02A3\ U14-H01E\ X15-A01A\ X15-A02A |
| 6550 | 3D printing | PULVERANT MATERIAL SUPPLY SYSTEM FOR ADDITIVE MANUFACTURING SYSTEM TO SUPPLY E.G. CERAMIC TO WORK STAGE, HAS INNER SHELL COMPRISING OPENINGS FOR PASSAGE OF FLUID AND FORMED FROM ALLOY, WHERE PASSAGE IS PROVIDED BETWEEN SHELLS | WO2014080765A1 | KUSAKI WATARU US\ KOITABASHI RYUJI US | PANASONIC CORP (JP) | M22-H03B\ A11-C01C\ A11-C07\ A12-W12F |
| 6551 | 3D printing | METHOD FOR RECONSTRUCTING LAYERED FORM OF THREE-DIMENSIONAL SCENE FROM LIGHT FIELD, INVOLVES ESTIMATING ATOM COEFFICIENTS FOR LAYER LIGHT FIELDS FOR LAYERS, AND LAYERS OF THREE-DIMENSIONAL SCENE FROM LAYER LIGHT FIELDS AND MASKS | KR2014060204A | KUSUHO JUNJI US | SHINETSU CHEMICAL CO (JP) | L02-A03\ L02-A04\ L02-A05\ L02-G01A\ L02-G01C1\ L02-G01D\ L02-G03A\ L02-G10B\ L02-G12B |
| 6552 | 3D printing | PHOTOSENSITIVE RESIN COMPOSITION, USEFUL FOR MANUFACTURING A LIGHT BLOCKING LAYER, COMPRISES A BINDER RESIN INCLUDING A CARDO-BASED RESIN, A PHOTOPOLYMERIZABLE MONOMER, A PHOTOPOLYMERIZATION INITIATOR, A COLORANT, AND A SOLVENT | US2014131876A1 | KWON CHUL-SOON (KR) | FUJITSU SEMICONDUCTOR LTD (JP) | M13-H01\ M26-A02\ M26-A02C\ M26-A02J\ M26-A02M\ M26-A02N\ M26-A02T\ M26-B12\ M26-B12C\ M26-B12J\ M26-B12M\ M26-B12N\ M26-B12T\ X27-L |
| 6553 | 3D printing | NOBLE-METAL DENTAL ALLOY FOR POWDER USED FOR PRODUCTION OF METALLIC COMPONENT E.G. DENTAL COMPONENT, AND FOR DENTAL RESTORATION, CONTAINS COBALT, NOBLE METALS CHOSEN FROM RUTHENIUM, PALLADIUM, IRIDIUM AND PLATINUM, CHROMIUM, AND BORON | US2014133020A1 | KWON OH-JUNG (US)\ KWON O SUNG (US)\ SUH BONG-SEOK (KR)\ YU DONG-HEE (KR)\ KIM SEONG-DONG (US)\ JEONG YONG-KUK (KR) | SAMSUNG ELECTRONICS CO LTD (KR) | M13-H01 |
| 6554 | 3D printing | METHOD FOR MANUFACTURING HETERO JUNCTION TYPE SOLAR CELL, INVOLVES FORMING METAL INTERCONNECTS ON SURFACE OF SOLAR CELL BY LOCAL APPLICATION OF CONDUCTIVE PASTE AND BY SINTERING WITH LASER RADIATION | WO2014097661A1 | LAI KEN K (TW)\ BYUN JEONG SOO (KR)\ WU FREDERICK C (CN)\ SRINIVAS RAMANUJAPURAM A (IN)\ GELATOS AVGERINOS (US) | INFINEON TECHNOLOGIES CORP (US) | M13-H01\ M22-H02 |
| 6555 | 3D printing | PRODUCTION OF THREE-DIMENSIONAL PRINTED PRODUCT INVOLVES MIXING METAL POWDER AND THERMOPLASTIC ORGANIC BINDER, LASER-SINTERING OR ELECTRIC BEAM-SINTERING TO THREE-DIMENSIONAL PRINTED METAL PRODUCT AND REMOVING GREEN COMPONENT | US2014170571A1 | LE TIEC YANNICK (FR)\ POSSEME NICOLAS (FR)\ VINET MAUD (FR)\ GRENOUILLET LAURENT (US) | SRINIVAS RAMANUJAPURAN A (US)\ LAI KEN K (US)\ BYUN JEONG SOO (US)\ WU FREDERICK C (US)\ GELATOS AVGERINOS (US)\ CHANG MEI (US)\ KORI MORIS (US)\ SINHA ASHOK K (US)\ CHUNG HUA (US)\ FANG HONGBIN (US)\ MAK ALFRED W (US)\ YANG MICHAEL X (US)\ XI MING (US) | M22-H03B\ X25-A08 |
| 6556 | 3D printing | ALUMINUM OXIDE/ZIRCONIUM OXIDE POWDER MATERIAL MOLDING FOR SELECTIVE LASER MELTING COMPRISES ALUMINUM OXIDE POWDER AND ZIRCONIUM OXIDE POWDER WITH ADDED YTTRIUM OXIDE STABILIZING AGENT | DE102012223239A1 | LEE GILL YONG (DE) | COMMISSARIAT ENERGIE ATOMIQUE (FR)\ VINET MAUD (FR)\ GRENOUILLET LAURENT (FR)\ POSSEME NICOLAS (FR)\ TIEC YANNICK LE (FR) | A11-B14\ A12-W12F |
| 6557 | 3D printing | COATING MATERIAL CONTAINS TUNGSTEN CARBIDE, NICKEL, CHROMIUM, MANGANESE, MOLYBDENUM, TITANIUM, AND BALANCE IRON | WO2014092961A1 | LEE JIAN-HSING (TW)\ WU YI-HSUN (TW) | LEE GILL YONG (DE) | M23-F\ X24-D03A\ X24-D08G |
| 6558 | 3D printing | METHOD OF FORMING SELF-LUBRICATING CLADDING ON TITANIUM ALLOY BASE MATERIAL, BY MIXING NICRBSI ALLOY POWDER AND NICKEL/MOLYBDENUM SULFIDE POWDER ACCORDING TO CERTAIN RATIO, AND ADDING VOLATILE ORGANIC SOLVENT TO OBTAIN CLADDING MATERIAL | WO2014086417A1 | LEE JIAN-HSING (TW)\ YU TA-LEE (TW)\ CHEN SHUI-HUNG (TW) | TAIWAN SEMICONDUCTOR MFG (US) | A05-J10\ A07-A03\ A09-C\ A11-A04\ A11-B16\ A12-S09A\ X25-A08 |
| 6559 | 3D printing | SURFACE STRENGTHENING METHOD OF TITANIUM ALLOY SURFACE BASE MATERIAL, BY MIXING NICRBSI ALLOY POWDER AND TITANIUM CARBIDE ACCORDING TO CERTAIN VOLUME PROPORTION, AND ADDING VOLATILE ORGANIC SOLVENT TO OBTAIN CLADDING MATERIAL | WO2014072699A1 | LEE KONG-SOO (KR) | TAIWAN SEMICONDUCTOR MFG (US) | L03-G05\ L03-H04A\ L04-E03A\ U12-A01A1A\ U12-A01A7 |
| 6560 | 3D printing | FOCUSING DEVICE OF SELECTIVE LASER SINTERING THREE-DIMENSIONAL DEVICE, HAS CARRYING DEVICE THAT IS PROVIDED WITH CARRIER PLATE PROVIDED WITH EMBEDDING PORT THAT IS MATCHED WITH OBJECTIVE LENS EMBEDDED IN CARRIER PLATE | EP2730353A1 | LEE KYU-MANN (KR) | SAMSUNG ELECTRONICS CO LTD (KR) | M23-D01A2\ M23-D01A4\ M23-D01B1\ M23-D01B2\ X24-B01\ X24-B03 |
| 6561 | 3D printing | PRINT TABLE FOR SELECTED LASER SINTERING (SLS) THREE-DIMENSIONAL PRINTER, HAS WORKING TABLE AND POWDER SUPPLY TABLE WHOSE LOWER ENDS ARE EMBEDDED IN SWING LEVER WHOSE LOWER END IS CONNECTED WITH BRACKET | US2014159612A1 | LEE PETER WUNG (US)\ HSU FU-CHANG (US) | SAMSUNG ELECTRONICS CO LTD (US) | T01-J10C\ V07-G04\ V07-K01 |
| 6562 | 3D printing | CLADDING METHOD FOR METALLIC COAT ON METAL ELEMENT USED IN REINSTATING WORN-OUT OR DAMAGED PARTS OF MACHINERY, INVOLVES CLADDING METAL ELEMENT VIA LASER CLADDING WITH AT LEAST TWO LAYERS OF METALLIC MATERIAL HAVING DIFFERENT THICKNESS | EP2738577A1 | LEE SEUNG-MOK (JP) | LEE PETER WUNG (US)\ HSU FU-CHANG (US)\ APLUS FLASH TECHNOLOGY INC US | T01-J10B2A |
| 6563 | 3D printing | PREPARING FEEDSTOCK FOR SELECTIVE LASER SINTERING COMPRISING POLYARYLETHERKETONE PARTICLES, INVOLVES DETERMINING PARTICLE SIZE DISTRIBUTION OF POLYARYLETHERKETONE PARTICLES IN FEEDSTOCK BASED ON NUMBER OF PARTICLES | JP2014102092A | LEE SEUNG-MOK (JP) | LEE SEUNG-MOK (JP) | M13-H01\ M13-L |
| 6564 | 3D printing | SEMICONDUCTOR LIGHT EMITTING DEVICE USED FOR E.G. DISPLAY APPARATUS, COMPRISES N-TYPE SEMICONDUCTOR LAYER, P-TYPE SEMICONDUCTOR LAYER AND LIGHT EMITTING PORTION INCLUDING PRIMARY LAYER CONTAINING ALUMINUM-GALLIUM NITRIDE | CN103595344A | LEE SUNG HO (KR) | LEE SEUNG-MOK (JP) | X24-D02 |
| 6565 | 3D printing | MANUFACTURING METHOD FOR MAKING ROTARY ARTICLE SUCH AS IMPELLER INVOLVES ROTATING ROTARY SUBSTRATE WHILE DEPOSITING LAYER BY LAYER FILLER METAL ON WELDING PATH OF ROTARY SUBSTRATE TO FORM ROTARY ARTICLE | CN103750923A | LEE TAE-JUNG (KR)\ JANG DONG-RYUL US\ LEE SOO-CHEOL (KR)\ KIM HYEON-CHEOL (KR) | LEE SUNG HO (KR) | V06-V01B\ V06-V04A1\ V06-V04M\ V06-V04R\ W01-C01A5\ W01-C01D3C\ W01-C01G8\ W01-C01P2 |
| 6566 | 3D printing | APPARATUS FOR PROVIDING NEAR-EYE PARALLAX BARRIER DISPLAYS, HAS ARRAY THAT PRODUCES LIGHT FIELD BY ALTERING LIGHT EMITTED BY DISPLAY TO SIMULATE OBJECT IN FOCUS TO OBSERVER WHILE ARRAY AND DISPLAY ARE LOCATED WITHIN NEAR-EYE RANGE | CN103751852A | LEE TUNG-HSING (TW)\ HSU TSE-HSIANG (TW)\ KO CHING-CHUNG (TW) | SAMSUNG ELECTRONICS CO LTD (KR) | L03-E05B\ L03-G05F\ L03-G09G\ L04-C22\ M11-A02\ M11-B05A\ U14-J02D2\ X15-A01A\ X15-A02F\ X25-R04 |
| 6567 | 3D printing | APPARATUS FOR PROVIDING NEAR-EYE DISPLAY TO PROJECT IMAGES DIRECTLY INTO VIEWER EYE, HAS MICRO- LENS ARRAY THAT IS OPERABLE TO PRODUCE LIGHT FIELD BY ALTERING LIGHT EMITTED BY DISPLAY TO SIMULATE OBJECT THAT IS IN FOCUS TO OBSERVER | CN103752818A | LEE WOOK-HYOUNG (KR) | MEDIATEK INC (TW)\ LEE TUNG-HSING (TW)\ HSU TSE-HSIANG (TW)\ KO CHING-CHUNG (TW) | V07-F02C\ V07-K01\ V07-M\ W03-A08B\ W03-A08E3\ W03-A12A |
| 6568 | 3D printing | LASER SURFACE CLADDING PROCESS OF DRILL BIT TOOTH SURFACE INVOLVES DEGREASING TOOTH SURFACE, PROCESSING USING DYE PENETRANT, COATING WITH NICKEL-BASED ALLOY POWDER, CLADDING, COATING WITH ALLOY POWDER, AND FURTHER CLADDING | CN103752823A | LEI BIQING US\ WEI SHOUGUO US | SAMSUNG ELECTRONICS CO LTD (KR) | U11-C05B9A\ U11-C05D3\ U11-C06A2\ U11-D01A |
| 6569 | 3D printing | CLOSED LOOP CONTROL METHOD FOR LAYER MANUFACTURING APPARATUS USED FOR LAYER-BY-LAYER MANUFACTURING OF THREE-DIMENSIONAL METALLIC WORK PIECE, INVOLVES TRANSLATING ELECTRON BEAM THROUGH SECOND PREDETERMINED RASTER PATTERN FRAME | CN103753021A | LEVIN SHARON (IL)\ NAOT IRA (IL)\ SHAPIRA SHYE (IL)\ STRAIN ROBERT J (US)\ NETZER YOSSI (IL) | YANGZHOU JIANGXIN ELECTRONIC CO LTD US | V07-F01A\ V07-K01 |
| 6570 | 3D printing | ELECTRO-ACOUSTIC TRANSDUCER FOR USE IN ELECTRONIC DEVICE, HAS VIBRATION PORTION THAT STACKS ON LAYERED FORM AND IS ARRANGED WITH PREDETERMINED SPACE ALONG VIBRATION DIRECTION SO THAT AREA EXPANDS OR SHRINKS SEQUENTIALLY | CN103757630A | LEVIN SHARON (IL)\ SHAPIRA SHYE (IL)\ LEE ZACHARY K (US) | TOWER SEMICONDUCTOR LTD (IL) | A08-R05\ L03-J\ M22-H01\ M22-H02\ M22-H03B\ X22-X10\ X22-X20A\ X25-A02D |
| 6571 | 3D printing | SUBSTRATE FOR FLEXIBLE DEVICE AND E.G. ORGANIC ELECTROLUMINESCENT ILLUMINATION, HAS GLASS LAYER BY WHICH BISMUTH TYPE GLASS HAVING ELECTRIC INSULATION ON SURFACE OF NICKEL-PLATING LAYER IS FORMED IN LAYERED FORM | CN103759535A | LI JUNJUN (US)\ GAUTHIER JR ROBERT J (US) | TOWER SEMICONDUCTOR LTD (IL)\ LEVIN SHARON (IL)\ LEE ZACHARY K (US)\ SHAPIRA SHYE (IL) | M11-G\ X24-D03 |
| 6572 | 3D printing | DIGITAL HOLOGRAM IMAGE DISPLAY DEVICE E.G. LCD DEVICE FOR DIFFRACTING THE LASER LIGHT, HAS FOCAL DISTANCE CHANGING UNIT TO CHANGE THE FOCAL DISTANCE OF LIGHT FROM SPATIAL LIGHT MODULATOR (SLM) DEPENDING ON DISTANCE BETWEEN SLM AND USER | CN203569189U | LI JUNJUN (US)\ GAUTHIER JR ROBERT J (US) | IBM (US) | M26-A01\ M26-A01C\ M26-A01M\ M26-A01N\ M26-A01T\ M26-B08\ M26-B08C\ M26-B08M\ M26-B08N\ M26-B08T\ D08-A02 |
| 6573 | 3D printing | SEMICONDUCTOR DEVICE, USEFUL IN SEMICONDUCTOR PACKAGE, COMPRISES DIE, CONDUCTIVE VIA FORMED IN DIE, PASSIVATION LAYER ARRANGED ON PORTION OF BACK SURFACE OF DIE, AND PROTECTION CAP ARRANGED ON PROTRUDED END OF CONDUCTIVE VIA | CN203569190U | LI PING US\ WANG JIANJUN US\ CHU JIANFEI US | IBM (US) | A03-A00A\ A12-W09\ A12-W11L\ D03-D03A\ D03-H02B2\ D05-A02C\ D05-A04D\ D05-B02\ D05-C\ D05-E\ D06-G |
| 6574 | 3D printing | TIME MULTIPLEXED AUTOSTEREOSCOPIC DISPLAY DEVICE FOR IMAGE PRESENTATION, HAS FOCUSING ELEMENT WITH POSITIVE OPTICAL POWER IN FIRST MERIDIANS EXTENDING ACROSS LIGHT EXTRACTION FEATURES, AND NO OPTICAL POWER IN SECOND MERIDIANS | DE102012222098A1 | LI WEI-MIN (FI) | NANJING ZHONGXU ELECTRONICS SCIENCE AND TECHNOLOGY CO LTD US | H01-B01D\ H01-B03C\ H01-B03D\ H01-E01\ A12-H\ A12-T02\ A12-W10A\ X15-C01B2 |
| 6575 | 3D printing | METHOD FOR MANUFACTURING METAL POWDER, INVOLVES STAMPING WORKPIECE MATERIAL WITH METAL LAYER OF THICKNESS CORRESPONDING TO AXIAL LENGTH OF METALLIC COLUMN-LIKE PARTICLE OF PUNCH AND DYE SO AS TO PRODUCE METALLIC COLUMN-LIKE PARTICLE | IN201402202I4 | LI YAN-NAN (TW)\ CHIANG HSUEH-LI (TW) | ASM INT (NL) | A09-D03\ A12-W12F\ T01-J07B\ T01-S03\ X25-A08 |
| 6576 | 3D printing | ELECTRODE ASSEMBLY FOR USE IN ELECTRIC-FIELD-BASED COMBUSTION CONTROL SYSTEM FOR CONTROLLING E.G. COMBUSTION CHARACTERISTICS OF FLAME, HAS VOLTAGE SOURCE COUPLED TO ELECTRODE, WHERE ELECTRODE AND VOLTAGE SOURCE APPLY ELECTRIC FIELD | JP2014097312A | LI ZHAOHUA US\ WANG LEKANG US\ HU QIAO US\ LIN DAOMING US\ ZHAO CHUNBO US\ CHEN XUKUN US | NOVATEK MICROELECTRONICS CORP (TW) | A09-D03\ A11-C04E\ M22-H01\ M22-H03B\ X25-A08\ X25-B04 |
| 6577 | 3D printing | NOBLE METAL-DENTAL ALLOY MADE OF E.G. COBALT, RUTHENIUM, RHODIUM, PALLADIUM, OSMIUM, IRIDIUM OR PLATINUM, CHROMIUM, MOLYBDENUM AND TUNGSTEN, USEFUL FOR PRODUCING METALLIC COMPONENT, PREFERABLY A DENTAL COMPONENT AS DENTAL RESTORATION | CN103681967A | LI ZHAOHUA US\ WANG LEKANG US\ HU QIAO US\ LIN DAOMING US\ ZHAO CHUNBO US\ CHEN XUKUN US | SHENZHEN MINGWEI ELECTRONIC CO US | U12-A01A |
| 6578 | 3D printing | SIMULTANEOUSLY LIQUEFYING/SACCHARIFYING STARCH COMPOSITION TO MAKE GLUCOSE COMPOSITION INVOLVES CONTACTING COMPOSITION WITH ASPERGILLUS FUMIGATUS GLUCOAMYLASE EXPRESSED IN TRICHODERMA REESEI AND ALPHA-AMYLASE; AND LIQUEFYING/SACCHARIFYING | JP2014087186A | LIANG BO US | SHENZHEN MINGWEI ELECTRONIC CO US | S01-H09\ S03-C02B\ S03-C06\ T01-J13 |
| 6579 | 3D printing | BUOY SYSTEM E.G. CATENARY ANCHOR LEG MOORING BUOY SYSTEM, FOR LOADING COMPRESSED NATURAL GAS FROM GAS WELL TO E.G. SHIP, HAS OFFSHORE WAVE ENERGY CONVERTER PROVIDED SUCH THAT SYSTEM GATHERS AND STORES WAVE ENERGY | CN103741134A | LIAO HONG US\ LUO BO US | ADVANCED SEMICONDUCTOR MFG CORP LTD US | K07-A02A\ K07-B01A\ X14-D |
| 6580 | 3D printing | METHOD OF FORMING METALLIC COMPONENT BY ADDITIVE LAYER MANUFACTURING (ALM), BY RELIEVING WORKPIECE OF STRESS IF MEASURED STRESSES TENDING TO DISTORT WORKPIECE ARE ABOVE THRESHOLD | CN103742517A | LIM GUNE HWAN (KR)\ YANG SEUNG SEOK (KR) | SICHUAN CHANGHONG ELECTRIC CO US | X15-A01C2\ X15-A01C3\ X15-A02 |
| 6581 | 3D printing | METHOD OF MANUFACTURING THREE-DIMENSIONAL OBJECT INVOLVES CONTROLLING TEMPERATURE OF GAS ATMOSPHERE INSIDE CHAMBER AND/OR OF LAYER BEING IRRADIATED, SUCH THAT IRRADIATING TWO DIFFERENT TEMPERATURES OF GAS ATMOSPHERE AND/OR OF LAYER | CN103745996A | LIN CHIANG-HUNG (TW) | MICO TN LTD (KR) | S05-F |
| 6582 | 3D printing | LIGHT-EMITTING DEVICE E.G. RECESSED LIGHT, HAS SOLID-STATE LIGHT-EMITTING STRUCTURES THAT ARE COUPLED IN PARALLEL FOR ALLOWING CURRENT REDISTRIBUTION IN RESPONSE TO FLUCTUATION DUE TO CHARACTERISTICS OF BALANCING RESISTOR ELEMENT | CN103746006A | LIN GENGLI ZHOU (CN) | NANYA TECHNOLOGY CORP (TW) | A12-V02\ D09-C01E |
| 6583 | 3D printing | MULTI-MODE-OBJECT DETECTOR FOR DETECTING OBJECTS, HAS TRANSMISSION- AND RECEIVING COIL AS SENSOR, OPTIONALLY CONNECTABLE ELECTRIC CIRCUIT COMPONENTS, TRANSMIT SIGNAL-GENERATOR, ELECTRONIC EVALUATION UNIT AND ELECTRONIC CONTROL UNIT | CN103737273A | LIN TING-YI (TW)\ CHIN TSUNG-SHUNE (TW)\ CHENG HUAI-YU (TW)\ FANG JAU-SHIUNG (TW)\ CHIU CHIN-FU (TW) | VANGUARD INT SEMICONDUCT CORP (CN) | M22-H03F |
| 6584 | 3D printing | STORAGE CONTAINER OF WASTE MATERIAL, HAS COVER STRUCTURE PORTION WHOSE UPPER SURFACE, BOTTOM FACE, AND SIDE SURFACE ARE COMPRISED FROM THREE LAYERS OF RADIATION-SHIELDING LAYER, SEALING LAYER, AND RADIOACTIVE-SUBSTANCE ADSORPTION LAYER | CN103741136A | LIN XIANGWU (CN) | CHIN TSUNG-SHUNE (TW) | M22-H03B\ T01-G02A2B\ T01-J07B\ X25-A08 |
| 6585 | 3D printing | RESONANT BACK-REFLECTED FILM SOLAR BATTERY STRUCTURE, HAS LIGHT FOCUSING PART COMPOSED OF TRIPLE PRISM AND LENS, AND SOLAR RESONANCE BACK-REFLECTED STRUCTURE AS SOLAR BATTERY WORKING MEDIUM OF SEMICONDUCTOR THIN FILM | CN103741138A | LIN XI-WEI (US)\ MOROZ VICTOR (US) | LIN XIANGWU (CN) | M23-D05\ V08-A09\ X24-D03 |
| 6586 | 3D printing | ARTIFICIAL TEMPORAL SELECTIVE LASER MELTING LOWER JAW JOINT, HAS FEMUR NECK CONVEX PART WHOSE UPPER END IS CONNECTED WITH TITANIUM PLATE THAT IS FIXED AT LOWER END OF CONDYLAR NECK PART, WHERE CONDYLAR NECK PART IS FORMED WITH NAIL HOLE | CN103722171A | LIN XI-WEI (US)\ PRAMANIK DIPANKAR (US)\ MOROZ VICTOR (US) | SYNOPSYS INC (US) | M13-H01\ M13-L\ X25-A02D |
| 6587 | 3D printing | THREE-DIMENSIONAL ARTIFICIAL RANDOM POROUS STRUCTURE ENGINEERING BRACKET TISSUE PREPARATION METHOD, INVOLVES ESTABLISHING ARTIFICIAL RANDOM POROUS STRUCTURE OF THREE-DIMENSIONAL INTERNAL AND EXTERNAL MODEL, AND OPERATING BRACKET | WO2014082870A1 | LIN XI-WEI (US)\ PRAMANIK DIPANKAR (US)\ MOROZ VICTOR (US) | SYNOPSYS INC (US)\ LIN XI-WEI (US)\ PRAMANIK DIPANKAR (US)\ MOROZ VICTOR (US) | A11-B09A1\ A12-E08B\ A12-E10\ A12-S08B |
| 6588 | 3D printing | IRON-BASED COMPOSITE POWDER USED FOR LASER CLADDING, COMPRISES CHROMIUM CARBIDE, CHROMIUM AND IRON COMPOUNDS AND NICKEL-MOLYBDENUM-SILICON-BORON-CONTAINING ELEMENT | US2014154088A1 | LIN XI-WEI (US)\ PRAMANIK DIPANKAR (US)\ MOROZ VICTOR (US) | SYNOPSYS INC (US) | M13-H01 |
| 6589 | 3D printing | SELECTIVE LASER SINTERING INVOLVES UTILIZING CROSS SECTIONAL PROFILE OF LASER SCAN PATH, WHERE THE LASER SCAN PATH COMPRISES THREE PARALLEL GROUPS OF LINEAR SCAN PATH AND TRIANGULAR GRID WITH INTEGRATED SCAN PATH | CN103658647A | LIU AI-SEN (TW) | SYNOPSYS INC (US) | M13-H01 |
| 6590 | 3D printing | LASER WELDING INVOLVES WELDING OF COPPER AND BRASS, WHERE WELDING IS PROCESSED AT COPPER WELDING JOINT FIXED AT THE JUNCTION, WHERE JUNCTION OF BRASS IS WELDED THROUGH LASER CLADDING METHOD FOR COATING IT WITH MIDDLE MATERIAL | CN203546146U | LIU BO US\ ZHANG YANPO US\ CHEN SHUNLI US | TAIWAN SEMICONDUCTOR MFG (TW) |  |
| 6591 | 3D printing | LASER AXIS CORRECTION OF SHAFT BY DETECTING AXIS, DETERMINING DEGREE OF BENDING, MACHINED GROOVE CLADDING, LASER CLADDING, ALIGNING, SYNCHRONIZING, CLADDING BY HAND, COOLING, DETECTING AXIS, CHECKING, MACHINING AND POLISHING | CN103726048A | LIU HUANG (SG)\ INDAJANG BANGUN (SG)\ LU WEI (SG) | UNIV SICHUAN US | S03-E06C\ S05-D02A\ S05-G02B2B |
| 6592 | 3D printing | METHOD FOR MANUFACTURING ONE-FREQUENCY VACUUM MELTING FURNACE COIL IN HEAT PRESERVATION LAYER, INVOLVES USING ANNULAR CAVITY COMPOSED OF GLASS FIBER REINFORCED PLASTIC MATERIAL | CN203546588U | LIU LIANJUN (CN) | GLOBALFOUNDRIES SINGAPORE PTE US | T05-H05E\ T05-H08A\ T05-H08C1 |
| 6593 | 3D printing | LASER CLADDING SYNCHRONOUS POWDER FEEDER MECHANICAL AUTOMATIC DEVICE HAS STORAGE CONTAINER HAVING SPIRAL CONVEYING MECHANISM WHICH IS CONNECTED WITH MIXER, WHERE TOP PART OF CONTAINER IS PROVIDED WITH CAM INDEXING FEEDING MECHANISM | CN103721547A | LIU YOWJUANG BILL (US)\ GREGOIRE FRANCOIS (US) | MYERSON ELECTRONICS TIANJIN CO LTD US | L03-H03\ L03-H05\ L04-C02\ L04-C04\ L04-C05A\ L04-C07\ L04-C09B\ L04-C10B\ L04-C10E\ L04-C10L1\ L04-C11C\ L04-C12A\ L04-C12B\ L04-C16A\ L04-C26\ U11-C01B\ U11-C02J1C\ U11-C05B2\ U11-C05B3\ U11-C05B5\ U11-C05C3\ U11-C05C5\ U11-C05E\ U11-C05F3B\ U11-C06A1A\ U11-C07C1\ U11-C07C2 |
| 6594 | 3D printing | LASER CLADDING SYNCHRONOUS POWDER FEEDER AUTOMATIC DEVICE COMPRISES POWDER STORAGE CONTAINER WHERE TOP PART IS PROVIDED WITH ARC INDEXING CAM FEEDING MECHANISM, AND MIXER WHICH IS CONNECTED TO SPIRAL FEEDING MECHANISM | CN103726047A | LIU YOW-JUANG BILL (US)\ JAYAPALAN JAYAKANNAN (US)\ GREGOIRE FRANCOIS (US)\ MCELHENY PETER JOHN (US) | ALTERA CORP (US) | V06-M03\ V06-M08A\ X11-H01\ X11-J02A |
| 6595 | 3D printing | DEFLECTOR FOR BALL SCREW, HAS DEFLECTOR MAIN PORTION WHICH IS PROVIDED WITH RETURN CHANNEL WHOSE LONGITUDINAL AXIS IS PROVIDED IN DIFFERENT DIRECTION, AND IS MADE UP OF FUSIBLE, SINTERABLE AND CURABLE POWDER | CN203546145U | LIVENGOOD RICHARD H (US)\ SLAWECKI DARREN (US) | ALTERA CORP (US) | M13-H01 |
| 6596 | 3D printing | THE CHASSIS AND TROLLEY FOR PEDIATRIC X-RAY IMAGING MACHINE WITH ANTICOLLAPSE PROTECTION | US2014145917A1 | LIXIN WANG (CN) | LIVENGOOD RICHARD H (US)\ SLAWECKI DARREN (US) |  |
| 6597 | 3D printing | GAME MACHINE E.G. PACHINKO MACHINE HAS CONTROL BOARDS WHICH ARE ARRANGED AT LAYERED FORM, AND BOARDS IN WHICH GAP IS FORMED, AND CLEARANCE GAP PREVENTION PORTION THAT IS FIXED WITH RESPECT TO ANOTHER ATTACHMENT PORTION | US2014146313A1 | LOISELET EMMANUEL (GB) | INST OF MICROELECTRONICS CAS (CN) | U11-C05B4 |
| 6598 | 3D printing | METHOD FOR PREPARING LOCAL BACK SURFACE PASSIVATION BATTERY COMPRISES POLISHING AND ETCHING P-TYPE POLYCRYSTALLINE SILICON CHIP, DIFFUSING, CLEANING, DRY OXIDIZING, ANNEALING, PLASMA-ENHANCED CHEMICAL VAPOR DEPOSITING, AND SINTERING | CN103709737A | LONG WUQIANG FENG (CN) | THALES HOLDINGS UK PLC (GB) | U11-C05B9A\ X15-A01A\ X15-A02A\ X15-A02C |
| 6599 | 3D printing | STATOR OF MOTOR SUCH AS BRUSHLESS MOTOR, HAS CONNECTION CONDUCTORS THAT ARE PROVIDED WITH IN-PHASE WINDINGS AND ARE PROVIDED NOT TO MUTUALLY OVERLAP WITH AXIAL DIRECTION OF CORE SO AS TO BE CONNECTED TO INSERTION CONDUCTORS | CN203535257U | LONG WUQIANG WEI (CN) | UNIV DALIAN TECH (CN) | M13-H01\ M13-L |
| 6600 | 3D printing | JAR BODY SURFACE ANTI-CORROSIVE WEARABLE LAYER MANUFACTURING DEVICE HAS UPPER UPRIGHT POST WHICH IS PROVIDED WITH Z-AXIS GUIDE RAIL, AND SLIDING PLATE WHICH CAN RELATIVELY SLIDE UP ON Z-AXIS GUIDE RAIL | JP2014097613A | LOOK KEVIN T (US)\ HSUEH SHIH-CHENG (US) | UNIV DALIAN SCIENCE & ENG (CN) | M13-H01 |
| 6601 | 3D printing | ENGINE CRANK SHAFT, HAS SHAFT MAIN BODY WHOSE OUTER SURFACE IS FORMED WITH LASER CLADDING COATING, FLANGE ARRANGED IN CLOSED POSITION TO END SURFACE OF MAIN BODY, AND SPACER FORMED WITH NITRIDE LAYER, WHERE COATING IS SET WITH INTERVAL | JP2014098055A | LOOK KEVIN T (US)\ HSUEH SHIH-CHENG (US) | XILINX INC (US) | M13-H01\ M13-L |
| 6602 | 3D printing | TRANSVERSE POWER DEVICE PART INSULATION BURIED LAYER, HAS ACTIVE LAYER PROVIDED WITH GRID REGION, AND SUPPORT SUBSTRATE PROVIDED WITH FIRST AND SECOND DOPED BURIED LAYERS, WHERE SECOND DOPED BURIED LAYER IS LOCATED BELOW SOURCE REGION | US8736932B1 | LOPATIN SERGEY (US)\ SHANMUGASUNDRAM ARULKUMAR (US)\ LUBOMIRSKY DMITRY (US)\ PANCHAM IAN A (US) | XILINX INC (US) | M22-H03B\ T01-G02A2B\ X25-A08 |
| 6603 | 3D printing | CRYSTAL SILICON SOLAR BATTERY PASSIVATION LAYER, HAS PASSIVATION LAYER PART MADE OF CERIUM OXIDE, WHERE PASSIVATION LAYER PART IS PROVIDED WITH P-TYPE BACKLIGHT SURFACE | RU139624U1 | LOPATIN SERGEY D (US)\ BESSER PAUL R (US)\ BUYNOSKI MATTHEW S (US)\ WANG PIN-CHIN CONNIE (US) | APPLIED MATERIALS INC (US) |  |
| 6604 | 3D printing | MANUFACTURE OF WEAR-RESISTANT LASER CLADDING TUNGSTEN CARBIDE CUTTER COATING INVOLVES DISSOLVING CUTTER, PRE-FORGING, FORGING, SLOW COOLING, ANNEALING, ROUGH MACHINING, AND PERFORMING HIGH TEMPERATURE VACUUM QUENCHING | WO2014075185A1 | LOVE DAVID G (US)\ MORESCO LARRY L (US) | ADVANCED MICRO DEVICES INC (US) | M22-H03B\ X24-D02\ X24-D03\ X25-A08 |
| 6605 | 3D printing | PREPARATION OF WAVE-SHAPED STEEL WEB PLATE COMPRISED USING LASER CLADDING FOR ALUMINUM-SILICON-ALUMINUM OXIDE ALLOY COATING TO POWDER COMPACT UPPER WAVE-SHAPED STEEL WEB PLATE SUBSTRATE | US2014141930A1 | LU JIONG-PING (US)\ ZHAO JIN (US) | FUJITSU LTD (JP) | L03-G02\ M22-H03B\ V07-F01A1\ V07-F02A |
| 6606 | 3D printing | STATIC MAGNETIC FIELD-LASER COMPOSITE COAXIAL CLADDING METHOD COMPRISES SURFACE POLISHING OF BASAL BODY, CLEANING, DRYING, FIXING, CLADDING END FACE OF MAGNETIC FIELD COIL TO BASAL BODY SURFACE, MELTING, AND CARRYING OUT LASER CLADDING | US2014140835A1 | LU LEE-CHUNG (TW)\ KUO CHIEN-CHIH (TW)\ LI JIAN-YI (TW)\ YANG SHENG-JIER (TW) | TEXAS INSTRUMENTS INC (US) | M13-H01\ M23-D05\ M23-H\ X24-D03A |
| 6607 | 3D printing | LASER SCANNING A CELLULAR SELECTIVE LASER SINTERING BY CALCULATING A LASER SCAN PATH IN CROSS-SECTIONAL PROFILE, SCANNING THREE PARALLEL DISCONTINUOUS LINE SCAN PATH OF HONEYCOMB MONOLITH, AND SCANNING METAL POWDER PARTS | CN103668179A | LU LEE-CHUNG (TW)\ KUO CHIEN-CHIH (TW)\ LI JIAN-YI (TW)\ YANG SHENG-JIER (TW) | TAIWAN SEMICONDUCTOR MFG (TW)\ LU LEE-CHUNG (TW)\ YANG SHENG-JIER (TW)\ KUO CHIEN-CHIH (TW)\ LI JIAN-YI (TW) | M13-H01\ M26-B08\ M26-B08A\ M26-B08B\ M26-B08C\ M26-B08N |
| 6608 | 3D printing | METAL SLEEVE FOR USE AS CARRIER OF ABRASIVE LAYER OF SAWING BEAD, HAS AXIAL BORE THAT IS OBTAINED BY METAL INJECTION MOLDING AND PROVIDED WITH AXIAL MIDDLE PORTION AND CHAMFERED OPENINGS AT BOTH END | CN103668184A | LU NING (US) | TAIWAN SEMICONDUCTOR MFG (TW)\ LU LEE-CHUNG (TW)\ YANG SHENG-JIER (TW)\ KUO CHIEN-CHIH (TW)\ LI JIAN-YI (TW) | A12-R09 |
| 6609 | 3D printing | METHOD FOR MANUFACTURING THREE-DIMENSIONAL METALLIC COMPONENT E.G. BLADE USED IN COMPRESSOR, INVOLVES REALIZING CONTROLLED SECONDARY GRAIN ORIENTATION BY APPLYING SPECIFIC SCANNING PATTERN OF ENERGY BEAM | CN103658520A | LUE HANG-TING (TW)\ LIEN HAO-MING (TW) | IBM (US)\ LU NING (US) | J01-E02\ J01-F |
| 6610 | 3D printing | LASER SELECTIVE MELTING (SLM) DEVICE HAS SCAN VIBRATION MIRROR UNIT, LIGHT STEERING UNIT, VARIABLE BEAM EXPANDING LENS UNIT AND SCAN VIBRATION MIRROR UNIT THAT ARE ARRANGED ON UPPER PORTION OF MOVABLE PANEL | CN103658646A | LUNDQUIST THEODORE R (US)\ WILSHER KENNETH R (US) | MACRONIX INTERNATL CO LTD US | M13-H01\ M24-D02A |
| 6611 | 3D printing | LASER CLADDING METAL POWDER FEEDING NOZZLE HAS FASTENING PLATE WHICH IS PROVIDED ON CARD BLOCK OF LOWER SHELL, WHILE SPRAY HEAD IS FIXED ON UPPER SHELL | WO2014070272A2 | MAA JER-SHEN (US)\ SHIEN TEN SUU US\ ZHANG FENGYAN (CN) | CREDENCE SYSTEMS CORP (US) | M13-H01 |
| 6612 | 3D printing | NICKEL BASED ALLOY POWDER USED FOR CONTINUOUS WAVE FIBER LASER CLADDING, COMPRISES SILICON CARBIDE, ANTIMONY, ALUMINUM OXIDE, CHROME, BORON, IRIDIUM, AND NICKEL | JP2014082122A | MABUCHI KEIJI (JP) | SHARP LAB OF AMERICA INC (US) | U12-A01A1E\ U12-A01A5B\ U12-A01A7 |
| 6613 | 3D printing | FES STEEL BRIDGE DECK PAVING STRUCTURE, HAS FRP PAVING LAYER FORMED WITH THREE-DIMENSIONAL LAYING GLASS FIBER CLOTH, WHERE FRP PAVING LAYER IS FORMED WITH ROUGH SURFACE OF BASE LAYER | GB2507468A | MAEDA AKIYUKI US | SONY CORP (JP)\ MABUCHI KEIJI (JP) | A06-A00E\ A12-L04\ S03-A02C\ S03-E04C\ S03-E13B\ S03-E13F |
| 6614 | 3D printing | WATER DIPPING WASHING-TYPE GAS IMPURITIES FILTER, HAS CYLINDER BODY EQUIPPED WITH INLET AND OUTLET, CYLINDER COVER MATCHED WITH FIXED SCREW, AND CYLINDRICAL GAS FILTER CHAMBER EQUIPPED WITH ANNULAR GAS FILTERING CHAMBER | WO2014057268A1 | MAEDA YUKIHIRO (JP)\ OOTANI YUJI (JP)\ NAKANO TETSUO (JP)\ NAGASAKA TAKASHI (JP) | SHARP KK (JP) | A08-A03\ A08-A06\ A11-A03\ A12-W11\ L03-F02A2\ V08-A09\ X25-A08 |
| 6615 | 3D printing | DIP-COATING METHOD INVOLVES POLISHING METAL SUBSTRATE MECHANICALLY, DEGREASING USING MIXTURE OF ACETONE, SATURATED ETHANOL SOLUTION OF SHELLAC AND ALCOHOL POWDER IN SPECIFIC MASS RATIO, NATURALLY-DRYING, AND FORMING COATING | EP2732889A2 | MAEMURA KIMIHIRO US | DENSO CORP (JP) | V07-F01A1\ V08-A04C2\ V08-A09 |
| 6616 | 3D printing | ANNULAR CLADDING DEVICE COMPRISES CONDUCTIVE FIBER, LIGHT WAY CONNECTING CAVITY, AND SLIDING MODULE CONNECTED WITH WORKPIECE SLIDING MODULE | EP2732890A2 | MAENO MUNEAKI (JP)\ SEI TOSHIKAZU (JP) | SEIKO EPSON CORP (JP) | A12-W07F1\ G05-F01 |
| 6617 | 3D printing | ORGANIC LED DISPLAY DEVICE, HAS ORGANIC LED THAT IS CONNECTED TO FIFTH NODE, WHERE LIGHT EMISSION OF ORGANIC LED IS CONTROLLED ACCORDING TO DIFFERENCE VOLTAGE BETWEEN HIGH-LEVEL SOURCE VOLTAGE AND DATA VOLTAGE | GB2507986A | MAMADA YOSHIZO US\ UMETSU KAZUYUKI US\ MIMORI MASA US\ WATANABE MASASHI US | TOSHIBA CORP (JP)\ TOSHIBA MICRO ELECTRONICS US | A08-M01C\ A08-R01\ A11-B01\ A11-B05\ A12-E01\ L03-A03A\ L03-H02\ U11-A07\ U11-C05B4 |
| 6618 | 3D printing | CUVETTE FOR USE WITH LIGHT SCATTERING DETECTOR, HAS TROUGH REGION WITHIN WHICH LIQUID IS INJECTED, WHICH IS NOT IN LIQUID CONTACT WITH SAMPLE MEASUREMENT CHAMBER | CN103656752A | MANDELMAN JACK A (US)\ JAMMY RAJARAO (US) | RENESAS TECH CORP (JP) | V07-F02C\ V07-K01\ V07-M\ V08-A09\ W04-P01E5 |
| 6619 | 3D printing | POLYMER COMPOSITE POWDER USED FOR SELECTIVE LASER SINTERING COMPRISES POLYMER POWDER, LASER ABSORBENT, ADDITIVES AND ANTIOXIDANT | CN103646908A | MANDELMAN JACK A (US)\ YANG CHIH-CHAO (US)\ HSU LOUIS LU-CHEN (US)\ TONTI WILLIAM R (US) | IBM (US) | M22-H03B\ M22-H03C |
| 6620 | 3D printing | HIGH-POWER OPTICAL FIBER DOUBLE-CLAD FILTER MODULE, HAS FIBER LASER CLADDING MODULE UNIT CONNECTED WITH FILTER THAT IS MATCHED WITH HIGH REFRACTIVE INDEX ADHESIVE AND CONNECTED WITH HEAT CONDUCTING ELEMENT | CN103621954A | MARATHE AMIT P (US) | YANG CHIH-CHAO (US)\ HSU LOUIS LU-CHEN (US)\ TONTI WILLIAM R (US)\ MANDELMAN JACK A (US) | A12-V\ A12-V01\ A12-V02\ D08-A03\ D09-C01C\ D09-C01D\ D09-D\ B01-D02\ B02-C01\ B02-G\ B02-M\ B02-P\ B02-R\ B02-T\ B02-V01\ B04-C02E\ B04-C03\ B05-A03A4\ B07-D04A\ B07-D04C\ B07-D09\ B10-A08\ B12-M11G\ B14-D07\ B14-L06\ B14-N17B\ C01-D02\ C02-C01\ C02-G\ C02-M\ C02-P\ C02-R\ C02-T\ C02-V01\ C04-C02E\ C04-C03\ C05-A03A4\ C07-D04A\ C07-D04C\ C07-D09\ C10-A08\ C12-M11G\ C14-D07\ C14-L06\ C14-N17B\ S05-X |
| 6621 | 3D printing | METHOD FOR MANUFACTURING HOLLOW PARTICLE CONTAINING LAYER THAT IS UTILIZED FOR SUBLIMATION TRANSFER RECORDING SYSTEM, INVOLVES PERFORMING SOLVENT ELIMINATION PROCESS TO REMOVE SOLVENT FROM COATING LAYER AFTER SURFACING PARTICLE | CN103567352A | MARK LOBODA (US) | ADVANCED MICRO DEVICES INC (US) | X15-B01A |
| 6622 | 3D printing | COMPOSITION USED FOR FILM AND SUBSTRATE FOR ELECTRONIC COMPONENT E.G. SEMICONDUCTOR ELEMENT AND LIGHT-EMITTING ELEMENT FOR ELECTRONIC CIRCUIT, COMPRISES RESIN MATERIAL, FILLER COMPRISING INORGANIC MATERIAL, AND ALUMINATE COUPLING AGENT | KR2014015837A | MARTINA DAMAYANTI US\ THIRUMANY SRITHARAN US\ MANG NG CHEE US | DOW CORNING (US)\ LOBODA MARK (US) | M23-A01\ M23-A03\ M23-D05\ X24-A09\ X24-D03 |
| 6623 | 3D printing | SYSTEM FOR PATH COMPENSATION OF MULTIPLE INCOHERENT OPTICAL BEAMS FOR HIGH BEAM QUALITY MULTIPLE INCOHERENT BEAM LASER SOURCE, HAS RELAY OPTICS RECEIVING DIFFRACTION CORRECTED FULL APERTURE COMPENSATED COMBINED BEAM FOR FAR FIELD EMISSION | CN103537794A | MARUO AKIHIKO EBINA YUTAKA (JP) | GLOBALFOUNDRIES SG PTE LTD (SG)\ UNIV NANYANG TECH (SG) | M13-H01\ M13-L\ M22-H02\ M27-A04\ M27-A04B\ M27-A04C\ M27-A04M\ M27-A04S\ M27-A04T\ M27-B04\ M27-B04B\ M27-B04C\ M27-B04M\ M27-B04S\ M27-B04T\ M27-C01\ M27-C02\ T01-J07D1 |
| 6624 | 3D printing | DEVICE FOR PRODUCING ARTICLE VIA LASER SINTERING, HAS WORKING CHAMBER AND/OR ADDITIONAL WORKING CHAMBER WHICH ARE PROVIDED FOR PRESSING AND CLEANING SCOOTER AND ARE PROVIDED WITH POSSIBILITY FOR VERTICAL DISPLACEMENT | CN103715303A | MARUYAMA HIROYUKI (JP)\ NAGOYA TAKATOSHI (JP)\ TAKAMIZAWA SHOICHI (JP)\ SAYAMA RYUJI (JP) | SEIKO EPSON CORP (JP)\ HALO LSI DESIGN & DEVICE TECH (US) | M13-H01\ M13-L\ M27-A\ M27-A00B\ M27-A00C\ M27-A00M\ M27-A00S\ M27-A00T\ M27-C01\ M27-C02 |
| 6625 | 3D printing | ANTIBIOTIC-ELUTING ARTICLE, USED AS ORTHOPAEDIC SKELETAL COMPONENT AND WOUND TREATMENT DEVICE FOR IMPLANTATION INTO MAMMALS, COMPRISES POLYMERIC MATERIAL WITH ANTIBIOTIC COMPOSITION, WHERE ARTICLE IS PRODUCED BY ADDITIVE PREPARING PROCESS | CN203509463U | MASUDA KAZUHIRO US | SHINETSU HANDOTAI KK (JP)\ NAGOYA TAKATOSHI (JP)\ TAKAMIZAWA SHOICHI (JP)\ SAYAMA RYUJI (JP)\ MARUYAMA HIROYUKI (JP) | M22-D\ T01-C08\ T01-J07B\ T01-J15X\ X25-A01 |
| 6626 | 3D printing | REMANUFACTURED PLANET CARRIER USED IN GEAR BOX OF WIND TURBINE, HA SPIN BORES AND SHAFT BORE WHOSE SURFACE LAYER IS PROVIDED WITH SPECIFIC VALUE OF HARDNESS AND SPECIFIC VALUE OF STRENGTH | CN203521157U | MASUO AKAMATSU (JP) | SEIKO EPSON CORP (JP) | M22-H03B\ V06-M06E\ V06-M10 |
| 6627 | 3D printing | SLURRY PUMP COMPONENT USEFUL AS AN IMPELLER OR A VOLUTE OF A SLURRY PUMP COMPRISES A BASE MEMBER AND A CLADDING SURFACE INCLUDING A BRAZING ALLOY INNER LAYER, A DUCTILE INTERMEDIATE LAYER AND A WEAR RESISTANT OUTER LAYER | CN103691784A | MATSUI KATSUNORI US | HITACHI LTD (JP) | X24-D03 |
| 6628 | 3D printing | REPAIRING AUTOMOBILE LASER MOLD, COMPRISES E.G. SUBJECTING MOLD TO SURFACE TREATMENT, DETECTING, AND ANALYZING THE MOLD, PERFORMING LOW TEMPERATURE HEATING, PROGRAMMING WEAR PARTS OF MOLD CAVITY, PERFORMING LASER CLADDING, AND HEATING | CN103691947A | MATSUMOTO KOJI (JP) | RICOH KK (JP) | L03-E01B5B\ L03-E01D5\ L03-E03\ L03-E08B\ L03-H05\ X16-B01F1\ X16-E02\ X16-F03\ X21-A01D\ X21-A01F\ X21-A05A\ X21-B01A |
| 6629 | 3D printing | LASER REPAIRING OF AUTOMOTIVE MOLD COMPRISES HEATING MOLD AT LOW-TEMPERATURE, REMOVING SURFACE MOISTURE, OIL AND GAS, PROGRAMMING MOLD CAVITY WEAR PARTS, LASER CLADDING PLANE AND LEVEL PARTS, AND CONDUCTING MOLD VIBRATION AGING TREATMENT | CN103696957A | MATSUMOTO YOSHINOBU US\ HARUYAMA SHINICHIRO US | SHARP KK (JP) | T01-J04B1\ T01-J04B2\ T01-J10B2\ V07-K01 |
| 6630 | 3D printing | SPLITTING METAL CASTING COMPRISES COMPLEXITY ANALYSIS OF CASTING MOLD BASED ON COMPUTER ASSISTED DESIGN TO OBTAIN CASTING TYPE MODULE, SPLITTING INTO SERIES OF SUB-MODELS SUITABLE FOR LASER SINTERING BY SPLIT PRINCIPLE AND DETECTING SIZE | CN103695899A | MATSUYAMA HIROSHI US\ SUGAWARA AKI US\ KURAGANO TAKAAKI US | NAKAGAWA KENKYUSHO KK US | J04-B\ J04-C01\ S03-E08 |
| 6631 | 3D printing | SELECTIVE LASER MELTING (SLM) BASED BIDIRECTIONAL POWDER DUSTING DEVICE HAS SUPPLY GEAR WHOSE TWO ENDS ARE PROVIDED WITH SERVO MOTOR TRANSMISSION SHAFTS, AND DE-SULFURIZE DEVICE WHOSE ENDS ARE PROVIDED WITH SCRAPING POWDER BRUSH | CN103695900A | MCCLURE BRENT A (US) | MITSUMI ELECTRIC CO (JP) | A09-D03\ A12-W12F |
| 6632 | 3D printing | METHOD FOR LASER CLADDING E.G. TURBINE VANES OF GAS TURBINE WITH FILLER LAYER BY LASER BEAM WELDING, INVOLVES MOVING SUBSTRATE AND LASER BEAM RELATIVE TO EACH OTHER, WHILE MAINTAINING UNIFORM OPTICAL ENERGY TRANSFER | CN103695901A | MCGAHAY VINCENT J (US)\ WANG YUN-YU (US)\ CHANDA KAUSHIK (US)\ FILIPPI RONALD G (US)\ WANG PING-CHUAN (US)\ DEMAREST JAMES J (US)\ IGGULDEN ROY C (US)\ KIEWRA EDWARD W (US) | MICRON TECHNOLOGY INC (US)\ MCCLURE BRENT A (US) | A11-B14\ D09-C01 |
| 6633 | 3D printing | LITHIUM ION SECONDARY BATTERY FOR USE IN VEHICLES E.G. PLUG-IN HYBRID VEHICLE, HAS POSITIVE ELECTRODE METALLIC SUBSTRATE COVERED WITH COATING LAYER, WHERE MELTING POINT OF COPPER IS HIGHER THAN THAT OF ALUMINUM METAL | CN103695939A | MCMONAGLE JASON (GB)\ ARNOLD RONALD (GB) | IBM (US) | A11-B08A\ A11-C04B2\ A12-V03C1\ S05-E\ V08-A09 |
| 6634 | 3D printing | METHOD FOR OPERATING LIQUID CRYSTAL ON SILICON SPATIAL LIGHT MODULATOR, INVOLVES PERFORMING OPTICAL CORRELATION BETWEEN INPUT IMAGE AND REFERENCE IMAGE, AND PERFORMING ANOTHER CORRELATION BETWEEN INPUT IMAGE AND ANOTHER REFERENCE IMAGE | CN103668176A | MENUT OLIVIER (FR)\ JAOUEN HERVE (FR) | FILTRONIC COMPOUND SEMICONDUCTORS LTD (GB)\ RFMD (GB) | B12-M16\ D09-C01D |
| 6635 | 3D printing | ACTUATION METHOD FOR PROBES IN PROBE SYSTEM INVOLVES CONTROLLING ACTUATION BEAMLETS SO THAT DIFFERENT AMOUNTS OF PHOTOTHERMAL ENERGY ARE DELIVERED TO AT LEAST TWO OF PROBES DURING ILLUMINATION SEQUENCE | CN103668188A | MERCHANT SAILESH MANSINH (US)\ SAXENA VIVEK (IN)\ ABDELGADIR MAHJOUB ALI (SD)\ LAYADI NACE (DZ)\ YIH PAI H (TW) | ST MICROELECTRONICS SA (FR) | U11-C08A3 |
| 6636 | 3D printing | MACHINE FOR MAKING THREE-DIMENSIONAL OBJECTS FROM POWDERED MATERIALS, SPACES APPLICATOR UNIT ACTING ON IT FOR BRINGING IT INTO RAISED POSITION AND SPACES IT AWAY FROM LOWERED POSITION BASED ON LINE GOING AWAY FROM WORKING SURFACE | CN103681288A | MICHAEL JACOB (DE) | LUCENT TECHNOLOGIES INC (US) | D03-A04\ D03-H02D\ E34-D02 |
| 6637 | 3D printing | MACHINE FOR MAKING THREE-DIMENSIONAL OBJECTS FROM POWDERED MATERIALS HAS FASTENING ELEMENT THAT IS INTERPOSED BETWEEN ANNULAR BASE BODY AND MOUNTING END OF MAIN BODY TO FIX ANNULAR BASE BODY TO MAIN BODY | CN103659024A | MINAMI SHINICHI (JP)\ KAMIGAKI YOSHIAKI (JP)\ KATO MASATAKA (JP)\ KATAYAMA KOZO (JP) | QIMONDA AG (DE) | A05-C01B1\ A08-D03\ A08-M01D\ A08-M03\ A11-A03\ A11-B05\ A12-A02\ A12-W12F\ M22-A01\ M22-A03\ M22-B01\ M22-B02\ X25-A08 |
| 6638 | 3D printing | METHOD FOR MANUFACTURING POLYMER SHELL DENTAL/ORTHODONTIC APPLIANCES FOR E.G. TEETH REPOSITIONING APPLICATIONS, INVOLVES SUBJECTING SHEET OF THERMOPLASTIC POLYMER FILM TO VACUUM MOLDING OR PRESSURE MOLDING UTILIZING POSITIVE TOOTH MODEL | JP2014084744A | MINAMI SHINICHI (JP)\ KAMIGAKI YOSHIAKI (JP)\ KATO MASATAKA (JP)\ KATAYAMA KOZO (JP) | RENESAS TECH CORP (JP) | A04-G\ A10-B01\ A12-E01\ E05-A\ E05-B01\ E05-B02\ E05-C02\ E05-D\ E05-E01C\ E05-E02C\ E05-E02D\ E05-F\ E05-L\ E05-M\ E05-N\ E05-V\ E10-J02A1\ E25-B03\ E31-Q02\ E34-C02\ E34-E01\ E35\ L03-E05B\ L03-H05\ L04-C06C\ U11-A01F\ U12-A01A1E\ U12-A02A2D\ U12-A02A3\ U12-B03C\ X15-A01A\ X15-A02A\ X15-A02B\ X15-A02C\ X15-A02F |
| 6639 | 3D printing | METHOD FOR MANUFACTURING ARTIFICIAL BONE BY UTILIZING GRAPHITE ALKENE TOUGHNESS BIOLOGICAL ACTIVITY CERAMIC MATERIAL, INVOLVES MIXING CERAMIC POWDER AND GRAPHITE ALKENE POWDER, AND FORMING GRAPHITE/CERAMIC ARTIFICIAL BONE POROUS STRUCTURE | EP2728114A1 | MINAMI SHINICHI (JP)\ KAMIGAKI YOSHIAKI (JP)\ KATO MASATAKA (JP)\ KATAYAMA KOZO (JP) | RENESAS TECH CORP (JP) | M23-D05\ M23-H\ V08-A04C\ V08-A09\ X24-D03 |
| 6640 | 3D printing | METHOD FOR ISOLATION OF DEVICE I.E.COMPLEMENTARY METAL-OXIDE-SEMICONDUCTOR DEVICE, INVOLVES UTILIZING HARP PROCESS BY DEPOSITING SILICON DIOXIDE LAYER IN SHALLOW TRENCH, PERFORMING ANNEALING PROCESS FOLLOWED BY PASSING NITROGEN AND HELIUM | KR1384347B1 | MING LI (CN)\ ZHANG FAN (SG) | RENESAS TECH CORP (JP) | L03-E05B\ L04-A01A\ L04-C16\ L04-C17\ L04-D05\ L04-D09\ X15-A01A\ X15-A02A |
| 6641 | 3D printing | PRODUCTION OF BRASSICA NAPOBRASSICA INVOLVES CLEANING FRESH B. NAPOBRASSICA, ADDING SALT, MARINATING TO OBTAIN BLANK, MARINATING WITH MISO SAUCE, SOY SAUCE MASH, AND WATER, SQUEEZING JUICE, CONCENTRATING, ADDING WATER, AND DESALINATING | CN103526198A | MING ZHOU US\ YUN SHEN US | LI MING (CN)\ INST MATERIALS RESEARCH & ENG (SG)\ ZHANG FAN (SG) | M22-H02\ M22-H03B\ M22-H03F\ X24-D03 |
| 6642 | 3D printing | PRODUCTION OF SELECTIVE LASER SINTERING RAPID PROTOTYPE COATED SAND INVOLVES FORMING COMPOSITION BY SIEVING CRUDE SAND, CRUSHING BONDING AGENT, MIXING CURING AGENT, LUBRICANT AND ADDITIVE WITH WATER, AND CARRYING OUT COATING PROCESS | CN103668036A | MINGHUA JI US | NANTONG MINGXING MICROELECTRONICS CO LTD US | L03-B03\ A04-E08\ A12-E01\ A12-E07B\ V01-B03D3 |
| 6643 | 3D printing | NEW NORBORNENE-BASED STYRENE DERIVATIVE USED FOR MANUFACTURE OF HOLE TRANSPORT POLYMER FOR ELECTRIC COMPONENT E.G. SOLAR CELL AND ORGANIC LIGHT-EMITTING DIODE | CN103668175A | MINOURA JUNJI US | SEMICONDUCTOR MFG INT SHANGHAI (CN) | A12-H\ A12-H05\ A12-W12F |
| 6644 | 3D printing | IMPLEMENTING TWO-DIMENSIONAL SEQUENTIAL LATERAL SOLIDIFICATION LASER CRYSTALLIZATION BY ONE-DIMENSIONAL PRECISE TRANSLATION OF A SAMPLE, COMPRISES E.G. USING MULTI-POINT LASER MASK, AND ADJUSTING TRANSLATIONAL PULSE DISTANCE TO FORM CRYSTAL | CN103668177A | MITA KEIJI (JP) | OKI SEMICONDUCTOR CO LTD (JP) | M22-H03B\ M22-H03G\ V06-M06B\ V06-M06E |
| 6645 | 3D printing | SOLAR BATTERY FILL DIFFUSION BONDING METHOD, INVOLVES PRE-PROCESSING SILICON SHEET, AND EXTENDING SILICON SHEET OUT OF OUTLET BOAT ACCORDING TO LOW TEMPERATURE AND HIGH TEMPERATURE DIFFUSION CONDITIONS | CN103668186A | MITA KEIJI (JP) | SANYO ELECTRIC CO (JP)\ SANYO SEMICONDUCTOR CO LTD (JP)\ SANYO SEMICONDUCTOR MFG CO LTD (JP) |  |
| 6646 | 3D printing | OPTIONAL-SHAPE COOLING WATERWAY INJECTION MOLD MANUFACTURING DEVICE COMPRISES BEAM FOCUSING SYSTEM, NEAR-WAVELENGTH COAXIAL VISUAL LOCATING SYSTEM, POWDER PAVING SYSTEM, GAS PROTECTIVE SYSTEM, AND LIGHT BEAM FOCUSING SYSTEM | CN103671350A | MITA KEIJI (JP) | SANYO ELECTRIC CO (JP)\ SANYO HANDOTAI SEIZO KK US\ SANYO SEMICONDUCTOR CO LTD (JP) | M13-H01\ M22-H01\ M27-A04\ M27-A04B\ M27-A04C\ M27-A04M\ M27-A04N\ M27-A04S\ M27-C02 |
| 6647 | 3D printing | LARGE BLOCKING CAPACITOR, HAS SHELL WHOSE INNER PART IS SLEEVED WITH TFE TUBE, WHERE SURFACE OF TFE TUBE IS PROVIDED WITH GROOVE AND SURFACE OF GROOVE IS COATED WITH ZINC COATING THAT IS COATED WITH COPPER COATING | CN103668180A | MITANI SHINICHIRO (JP)\ YANAGIDA YOHEI US\ ICHINOSE KATSUHIKO (JP)\ SAITO TOMOHIRO (JP) | SANYO ELECTRIC CO (JP)\ SANYO HANDOTAI SEIZO KK US\ SANYO SEMICONDUCTOR CO LTD (JP) | M13-H01\ M27-A04\ M27-B04\ M27-D01 |
| 6648 | 3D printing | CORRUGATED SANDWICH LAYER MANUFACTURING METHOD, INVOLVES LOCATING UPPER MOLD IN HEATING FURNACE, AND FORMING SUPER PLASTIC LAYER BY NON-UNIFORM-THICKNESS WALL PLATE TO OBTAIN CORRUGATED CLAMP | CN103668181A | MITSUO BABA US | RENESAS TECH CORP (JP) | M13-H01\ U11-C01X\ U11-C03D |
| 6649 | 3D printing | SELECTIVE LASER MELTING PAVING DEVICE COMPRISES MAIN BODY, GUIDING RAIL LINEAR MOTOR FOR DRIVING SPREADING BODY OF SPREADING DEVICE, SERVO DRIVING MOTOR, POWDER SUPPLY ROLLER, AND SCRAPING DEVICE, WHERE MAIN BODY HAS CYLINDRICAL SECTION | CN103668182A | MIYAGAWA SEIJI (JP) | RENESAS ELECTRONICS CORP (JP)\ BABA MITSUO (JP) | M13-H01\ M13-L\ M14-H\ M24-D02A\ M24-D07\ M24-E |
| 6650 | 3D printing | WEARABLE PLUNGER PISTON PUMP FOR USE IN HYDRAULIC MACHINE ENGINEERING MACHINERY, HAS CAM SHAFT WITH CAM SURFACE IN CIRCUMFERENTIAL DIRECTION WHICH IS COATED WITH CERAMIC WEAR-RESISTING COATING | CN103668185A | MIYAMOTO MARI US\ SHIMADA AKIRA (JP)\ TOKUDA YUICHI (JP) | NEC ELECTRONICS CORP (JP) | M13-H01\ M13-L |
| 6651 | 3D printing | LASER CLADDING METHOD INVOLVES MIXING CARBON, SILICON, BORON, CHROMIUM, NICKEL AND MANGANESE, AND THEN HEATING OBTAINED MATERIAL, WHERE OBTAINED MOLTEN LIQUID IS ATOMIZED BY PASSING MOLTEN LIQUID AT HIGH PRESSURE IN PRESENCE OF GAS | CN103676392A | MIYASHITA KATSURA (JP) | NIPPON STEEL CHEMICAL CO (JP) | M13-H01\ X24-D03 |
| 6652 | 3D printing | PROCESSING ROD INVOLVES COATING ROD, WHICH IS MADE UP OF STAINLESS STEEL, BY APPLYING LASER CLADDING TECHNIQUE, WHICH INVOLVES ADDING COATING POWDER TO SURFACE OF ROD TO OBTAIN DESIRED PRODUCT | CN103656737A | MIYAZAKI TOYOHIDE (JP)\ KOYAMA KENJI (JP) | TOSHIBA KK (US) | L04-B04\ L04-C06\ L04-C09\ L04-C11C\ L04-C12A\ L04-C12C1\ L04-C16A\ L04-D05\ U11-C06A1B\ U11-C08A6 |
| 6653 | 3D printing | MULTI-CHANNEL LASER CLADDING PROCESS FOR REMOVING OVERLAPPING HOLES INVOLVES CLADDING OF SINGLE-CHANNEL LASER TO DETERMINE ASPECT RATIO OF INITIAL VALUE AND FIXING BASIC PARAMETERS OF LASER CLADDING IN VARIOUS PARAMETERS | CN103659201A | MIYAZAWA MAKOTO US\ IZUMI KENJI US | MIYAZAKI TOYOHIDE (JP)\ KOYAMA KENJI (JP)\ CANON KK (JP) | V08-A09 |
| 6654 | 3D printing | LARGE SHEARING CUTTER LASER REPAIR EQUIPMENT REMANUFACTURING METHOD INVOLVES COATING INJURY AREA ABSORBING LAYER ON SURFACE OF CUTTER, AND DETERMINING TOOL DAMAGE ZONE ON SURFACE OF NON-DESTRUCTIVE TESTING TOOL | CN103664175A | MIZOBE TAKENORI US\ OTSUKA MASAYA US | ELPIDA MEMORY INC (JP)\ NEC MICROSYSTEMS LTD US | X11-A01B\ X11-A01C\ X11-C01\ X11-J01A\ X11-U01C |
| 6655 | 3D printing | LASER REPAIR METHOD OF WHEEL GEAR, INVOLVES TREATING SURFACE OF GEAR, TO ANALYZE GEAR FAILURE, AND OPTIMIZING PROCESS PARAMETERS AND LASER CLADDING LAYER ACCORDING TO RESULT OF GEAR FAILURE ANALYSIS | CN103664178A | MOORE PAUL M (US) | RICOH KK (JP) |  |
| 6656 | 3D printing | LASER CLADDING ETCHING METHOD FOR STEAM TURBINE BLADE, INVOLVES PERFORMING SURFACE ROUGHENING TREATMENT FOR BLADE GROOVE AND PRE-HEATING BLADE CLAMP USING PRE-HEATING AREA THROUGH PARAXIAL POWDER HEAD | CN103664185A | MORIMOTO HIROYUKI US | MICREL INC (US) | A12-H03\ A12-H11 |
| 6657 | 3D printing | HIGH RELIABILITY LOW TEMPERATURE GATE OXIDE LAYER MANUFACTURING METHOD, INVOLVES PROVIDING SILICON-ON-INSULATOR WAFER WITH CLEAN SURFACE, AND PLACING SILICON-ON-INSULATOR WAFER IN OXIDATION FURNACE TUBE | CN103664186A | MORISHITA YASUYUKI (JP) | ANALOGIST KK US | M13-H01\ M27-A\ M27-A00B\ M27-A00C\ M27-A00N\ M27-A00S\ M27-B\ M27-B00B\ M27-B00C\ M27-B00N\ M27-B00S |
| 6658 | 3D printing | GROOVE STRUCTURE USED FOR LASER CLADDING OF TURBINE BLADE INLET SIDE, HAS LASER CLADDING GROOVE WHOSE BACK-ARC SIDE CROSS-SECTION IS CONNECTED TO INTAKE SIDE EDGES OF CIRCULAR ARC SHAPED CURVED SURFACE TO FORM SMOOTH TRANSITION | CN103664187A | MORISHITA YASUYUKI (JP) | NEC ELECTRONICS CORP (JP) | M13-C01\ M13-H01\ M13-L\ M23-D05\ M23-G\ M26-B08\ M26-B08B\ M26-B08C\ M26-B08M\ M26-B08N\ M26-B08S\ M26-B08T\ X24-D03 |
| 6659 | 3D printing | DUST ADHESION PREVENTION APPARATUS FOR STATOR BLADE OF FURNACE TOP PRESSURE RECOVERY TURBINE HAS SLIT THAT EXTENDS IN CIRCUMFERENTIAL DIRECTION AT OUTER PERIPHERAL WALL UPSTREAM OF STATOR BLADE | CN103664188A | MORISHITA YASUYUKI (JP) | NEC ELECTRONICS CORP (JP) | M13-H01\ M13-L\ M23-D05\ M23-H\ X24-D03 |
| 6660 | 3D printing | PLATFORM COOLING DEVICE FOR COOLING BLADE ROOT PLATFORM OF BLADE OF GAS TURBINE, HAS FIRST PORTION POSITIONED AT BLADE ROOT PLATFORM, AND CHANNEL DIRECTING COOLING FLUID FROM INLET TO IMPINGEMENT HOLES FOR RELEASING FLUID AT FIRST PORTION | CN103667798A | MORIYAMA YOSHINARI US | NIPPON ELECTRIC CO (JP) | M13-H01\ M13-H04\ M13-L\ M26-B\ M26-B00A\ M26-B00N\ M26-B00Z\ M26-C01\ X24-D03 |
| 6661 | 3D printing | METHOD FOR MANUFACTURING BUFFER MATERIAL OF GUIDE ROLLER, INVOLVES INJECTING LIQUID URETHANE THERMOSETTING RESIN RAW MATERIAL ON EXTERNAL LAYER IN EXTERIOR OF INNER LAYER SEPARATED FROM EXTERNAL LAYER MANUFACTURE MOLDING | EP2727709A1 | MORIYAMA YOSHINARI US | MATSUSHITA ELECTRIC IND CO LTD (JP) | M13-H01\ X25-A02 |
| 6662 | 3D printing | PREPARATION METHOD FOR NIOBIUM CARBIDE (NBC) PARTICLES CONTAINING IRON-BASED RARE EARTH ELEMENT USED FOR LASER CLADDING COATING INVOLVES LASER CLADDING ALLOY POWDER BASED ON CHEMICAL COMPONENT AND CERIUM OXIDE | CN203484663U | MOU-SHIUNG LIN (TW)\ JIN-YUAN LEE (TW) | MATSUSHITA ELECTRIC IND CO LTD (JP) | M13-H01\ M13-L |
| 6663 | 3D printing | LASER REPAIRING OF VALVE SEAT, INVOLVES SURFACE-TREATMENT OF VALVE SEAT, FAILURE ANALYSIS, OPTIMIZATION OF PROCESS PARAMETERS ACCORDING TO FAILURE ANALYSIS, AND LASER CLADDING BY ALLOY-POWDER FEEDING METHOD USING CARBON DIOXIDE LASER | CN103639403A | MURAMOTO MAI (JP)\ MINAMOTO TAKATOSHI (JP) | MEGICA CORP (TW)\ LEE JIN-YUAN (TW)\ LIN MOU-SHIUNG (TW)\ LEI MING-TA (TW)\ HUANG CHING-CHENG (TW) | M13-H01\ M13-L\ M24-D02\ M24-D06\ M24-D07\ M24-E\ T01-J07D1 |
| 6664 | 3D printing | LASER REPAIRING PROCESS OF THIN-WALLED SLEEVE INVOLVES SURFACE-TREATING THIN-WALLED SLEEVE, FIXING INNER HOLE OF THIN-WALLED SLEEVE, OPTIMIZING PROCESS PARAMETERS, LASER CLADDING, ANALYZING ALLOY POWDER, AND DISMANTLING TOOL ASSEMBLY | CN103640353A | MURANAKA SEIJI (JP) | TOSHIBA KK (JP)\ MURAMOTO MAI (JP)\ MINAMOTO TAKATOSHI (JP) | M13-H01\ M13-L\ M22-H02\ M27-A04\ M27-A04B\ M27-A04C\ M27-A04M\ M27-A04S\ M27-A04T\ M27-B04\ M27-B04B\ M27-B04C\ M27-B04M\ M27-B04S\ M27-B04T\ M27-C01\ M27-C02 |
| 6665 | 3D printing | COATING METHOD OF AMORPHOUS ALLOY STEEL SURFACE INVOLVES MIXING ZIRCONIUM POWDER, ALUMINUM POWDER, NICKEL POWDER AND COPPER POWDER, COATING ON SURFACE OF CARBON STEEL PLATE, AND ANALYZING CORROSION RESISTANCE CHARACTERISTICS | CN103646916A | MUROOKA FUMIO US | RENESAS ELECTRONICS CORP (JP) | M13-H01\ M13-L\ M27-A04\ M27-A04B\ M27-A04C\ M27-A04M\ M27-A04S\ M27-A04T\ M27-B04\ M27-B04B\ M27-B04C\ M27-B04M\ M27-B04S\ M27-B04T\ M27-C01\ M27-C02\ T01-J07D1 |
| 6666 | 3D printing | TITANIUM ALLOY LASER CLADDING SURFACE HARDENING METHOD INVOLVES WIPING TITANIUM ALLOY SURFACE, FORMING PASTE BY SODIUM SILICATE SOLUTION, UNIFORMLY COATING OBTAINED PASTE ON RESULTANT TITANIUM ALLOY SURFACE, DRYING, AND LASER CLADDING | JP2014082003A | NADD BRUNO C (FR) | SHARP KK (JP) | M13-H01\ M13-L\ M22-H02\ M27-A04\ M27-A04B\ M27-A04C\ M27-A04M\ M27-A04S\ M27-A04T\ M27-B04\ M27-B04B\ M27-B04C\ M27-B04M\ M27-B04S\ M27-B04T\ M27-C01\ M27-C02\ T01-J07D1 |
| 6667 | 3D printing | HYDRAULIC OPENING AND CLOSING MACHINE OIL JAR PISTON ROD HAS LASER CLADDING ALLOY POWDER WHICH FORMS PARALLEL ANNULAR ELECTROMAGNETIC TRAVEL OF GROOVE | GB2507467A | NAGAMITSU MASATOMO US\ SAEKI TAKANORI (JP) | INTERNAT RECITIFIER CORP (US) | U14-K02A2 |
| 6668 | 3D printing | PROCESSING AN AUTOMOBILE MOLD, INVOLVES TREATING SURFACE OF MOLD BY SURFACE TREATMENT PROCESS, HEATING MOLD AT LOW TEMPERATURE TO REMOVE MOISTURE, GAS AND OIL IN THE MOLD AND PERFORMING LASER CLADDING ON WEAR PARTS OF THE MOLD | TW472317U | NAGASE SHUICHI (JP) | RENESAS ELECTRONICS CORP (JP) | L02-A02\ L02-A04\ L02-A08\ L02-G01X\ L02-G03A1\ L02-G12X\ D09-C01D |
| 6669 | 3D printing | LASER REPAIRING PROCESS OF AUTOMOBILE MOLD INVOLVES TREATING SURFACE OF MOLD FOR DETECTING AND ANALYSING, LOW-TEMPERATURE HEATING MOLD IN WHICH MOISTURE AND BASE OIL ARE REMOVED FROM SURFACE, AND LASER CLADDING RESULTANT PRODUCT | WO2014064228A1 | NAGATA KOICHIRO (JP)\ NIWAYAMA MASANORI US | ELPIDA MEMORY INC (JP) |  |
| 6670 | 3D printing | LASER REPAIR PROCESS FOR AUTOMOBILE E.G. CAR, INVOLVES PERFORMING MOLD DEFORMATION DETECTION AND PERMEATION FLAW DETECTION IN WHICH DEFORMATION ON MOLD SURFACE FOR MACHINERY PROCESS IS DETECTED | FR2997289A1 | NAKAJIMA KAZUHIRO (JP)\ KANBA KOJI US | MITSUBISHI ELECTRIC CORP (JP) | L02-A02\ L02-A03\ L02-A04\ L02-H02A\ L02-H02B2\ L02-J02C |
| 6671 | 3D printing | PROCESSING AN AUTOMOBILE MOLD, INVOLVES TREATING SURFACE OF MOLD BY SURFACE TREATMENT PROCESS, HEATING MOLD AT LOW TEMPERATURE TO REMOVE MOISTURE, GAS AND OIL IN THE MOLD AND PERFORMING LASER CLADDING ON WEAR PARTS OF THE MOLD | JP2014077859A | NAKAJIMA KAZUHIRO (JP)\ KANBA KOUJI (JP) | NEC ELECTRONICS CORP (JP) | L02-A02\ L02-A03\ L02-A04\ L02-H02A\ L02-H02B2\ L02-J02C |
| 6672 | 3D printing | PATTERN FILM LAYER MANUFACTURING METHOD, INVOLVES CONNECTING SUBSTRATE WITH FIRST AND SECOND SURFACES, DEPOSITING CHARGED PARTICLES ON FIRST SURFACE, AND FORMING PATTERN FILM LAYER BY CHARGED PARTICLES | JP2014078588A | NAKAMURA HIROYUKI US | NEC ELECTRONICS CORP (JP) | L02-A02\ L02-A03\ L02-A04\ L02-H02B1\ L02-H02B2\ L02-J02C |
| 6673 | 3D printing | PRODUCTION OF THREE DIMENSIONAL HYDROXYAPATITE/TRI-CALCIUM PHOSPHATE/TETRACALCIUM PHOSPHATE MULTIPHASE POROUS CERAMIC ARTIFICIAL BONE INVOLVES SELECTIVELY LASER SINTERING HYDROXYAPATITE POWDER, AND REMOVING NON-SINTERED POWDER | US2014103581A1 | NAKAMURA HIROYUKI US | CANON KK (JP) | L02-A02\ L02-A03\ L02-A04\ L02-H02B1\ L02-H02B2\ L02-J02C |
| 6674 | 3D printing | LASER CLADDING WATER-PROOF CORROSION TURBINE BLADE PROCESSING METHOD, INVOLVES OBTAINING RAW MATERIAL BY USING BLADE BODY, WHERE REAR END OF BLADE BODY IS PROVIDED WITH MOLDING PART AND FORMED WITH LASER CLADDING GROOVE | CN103526201A | NAKAMURA HIROYUKI US | CANON KK (JP) | L02-A02\ L02-A03\ L02-A04\ L02-H02B1\ L02-H02B2\ L02-J02C |
| 6675 | 3D printing | MANUFACTURE OF ALUMINUM OXY NITRIDE-TANTALUM CARBIDE-CONTAINING COMPOSITE SHEET INVOLVES MIXING ALUMINUM OXY NITRIDE AND TANTALUM CARBIDE CERAMIC POWDER, SINTERING AT PRESET TEMPERATURE UNDER ARGON ATMOSPHERE, AND SHAPING | KR2014047762A | NAKAMURA KAZUYOSHI US\ TAKIMOTO MICHIHITO US\ ISHIMURA DAICHI US\ KOBAYASHI HIROYUKI US | CANON KK (JP) | L02-A02\ L02-A03\ L02-A04\ L02-H02B1\ L02-H02B2\ L02-J02C |
| 6676 | 3D printing | MANUFACTURE OF ALUMINUM OXY NITRIDE-VANADIUM CARBIDE-CONTAINING COMPOSITE SHEET INVOLVES MIXING ALUMINUM OXY NITRIDE AND VANADIUM CARBIDE CERAMIC POWDER, SINTERING AT PRESET TEMPERATURE UNDER ARGON ATMOSPHERE, AND SHAPING | US2014118508A1 | NAKAMURA SATOSHI US | PANASONIC CORP (JP) | M13-H01\ M26-B08\ M26-B08A\ M26-B08B\ M26-B08C\ M26-B08O\ M26-B08T\ M26-B08V |
| 6677 | 3D printing | MANUFACTURE OF ALUMINUM OXY NITRIDE-TANTALUM DIBORIDE-CONTAINING COMPOSITE SHEET INVOLVES MIXING ALUMINUM OXY NITRIDE AND TANTALUM DIBORIDE CERAMIC POWDER, SINTERING AT PRESET TEMPERATURE UNDER ARGON ATMOSPHERE, AND SHAPING | JP2014078446A | NAKAMURA YOSHITAKA (JP)\ SAITO MASAYOSHI (JP)\ KOBAYASHI NOBUYOSHI (JP)\ FUKUDA TAKUYA (JP)\ FUKADA SHINICHI (JP)\ KAWAMOTO YOSHIFUMI (JP) | SEIKO EPSON CORP (JP) | A12-E06\ A12-E12\ A12-E15\ A12-L02A\ L03-G10\ L03-H03A\ T01-L02\ V06-V01B\ V06-V04L\ V06-V04M\ X25-A08 |
| 6678 | 3D printing | MANUFACTURE OF ALUMINUM OXY NITRIDE-VANADIUM DIBORIDE-CONTAINING COMPOSITE SHEET INVOLVES MIXING ALUMINUM OXY NITRIDE AND VANADIUM DIBORIDE CERAMIC POWDER, SINTERING AT PRESET TEMPERATURE UNDER ARGON ATMOSPHERE, AND SHAPING | US2014114457A1 | NAKAMURA YUKINARI US | HITACHI LTD (JP)\ FUKUDA TAKUYA (JP)\ KOBAYASHI NOBUYOSHI (JP)\ NAKAMURA YOSHITAKA (JP)\ SAITO MASAYOSHI (JP)\ FUKADA SHINICHI (JP)\ KAWAMOTO YOSHIFUMI (JP) | L02-A03\ L02-A04\ L02-K01\ A11-B14\ A12-W12F\ X25-A08 |
| 6679 | 3D printing | MANUFACTURE OF ALUMINUM OXY NITRIDE-TUNGSTEN DIBORIDE-CONTAINING COMPOSITE SHEET INVOLVES MIXING ALUMINUM OXY NITRIDE AND TUNGSTEN DIBORIDE CERAMIC POWDER, SINTERING AT PRESET TEMPERATURE UNDER ARGON ATMOSPHERE, AND SHAPING | WO2014061588A1 | NAKANO TADASHI US\ ONO HIDEAKI US | TOPPAN PRINTING CO LTD (JP) | M13-H01\ M22-H\ M22-H03F1\ M26-B08\ M26-B08A\ M26-B08B\ M26-B08C\ M26-B08S\ V08-A04C2\ V08-A09 |
| 6680 | 3D printing | MANUFACTURE OF ALUMINUM OXY NITRIDE-ZIRCONIUM DIBORIDE-CONTAINING COMPOSITE SHEET INVOLVES MIXING ALUMINUM OXY NITRIDE AND ZIRCONIUM DIBORIDE CERAMIC POWDER, SINTERING AT PRESET TEMPERATURE UNDER ARGON ATMOSPHERE, AND SHAPING | US2014109440A1 | NAKANO TAKASHI US | KAWASAKI STEEL CO US | G05-F |
| 6681 | 3D printing | METAL CERAMIC ALLOY POWDER USED FOR CONTINUOUS WAVE LASER CLADDING COMPRISES ALUMINUM OXIDE, TITANIUM DIOXIDE, CERIUM OXIDE, VANADIUM, CHROMIUM, BORON, AND NICKEL | US2014111128A1 | NAKATANI NAOTO US\ HERAI MASAYASU US | NEC CORP (US) | L03-H05\ L04-C07G\ L04-C12B\ L04-C12D\ L04-C13A\ L04-C26\ L04-D04\ U11-C01J2 |
| 6682 | 3D printing | LAYERWISE MANUFACTURING METHOD OF TANGIBLE PRODUCT, INVOLVES ADJUSTING DISTANCE BETWEEN MATERIAL PROVIDING DEVICE AND BUILDING PLATFORM BY MOVING PLATFORM RELATIVE TO CONVEYOR, PAST THE DEVICE REPEATEDLY TO FORM FIRST LAYER PRODUCT | JP2014075862A | NARASIMHAN GEETHAKRISHNAN (US)\ BANACHOWICZ BARTOSZ (US)\ KAPRE RAVINDRA (US) | NIPPON AVIONICS CO LTD US\ MISHIMA DAIJI US | U11-C05B4\ U11-D01C3\ X12-F01A\ X12-F09 |
| 6683 | 3D printing | FORMING WORKBENCH OF SELECTIVE LASER SINTERING DEVICE, HAS ALUMINUM CHUNK WHOSE UPPER SURFACE IS SET WITH UPPER GROOVE IN WHICH ORGANIC GLASS PLATE IS SET, AND ORGANIC GLASS COVER FRAME THAT IS SET ON ALUMINUM CHUNK | JP2014073984A | NARAYAN CHANDRASEKHAR (US)\ KUMAR KAUSHIK ARUN (US)\ DALTON TIMOTHY JOSEPH (US)\ PURUSHOTHAMAN SAMPATH (US)\ CHIRAS STEFANIE RUTH (US)\ LANE MICHAEL WAYNE (US)\ CHEN SHYNG-TSONG (US)\ MALONE KELLY (US)\ COLBURN MATTHEW EARL (US)\ HEDRICK JEFFREY CURTIS (US)\ HUANG ELBERT EMIN (US)\ NITTA SATYANARAYANA VENKATA (US)\ ROSENBURG ROBERT (US)\ TYBERG CHRISTY SENSENICH (US)\ YU ROY RONGQING (US) | NARASIMHAN GEETHAKRISHNAN (US)\ BANACHOWICZ BARTOSZ (US)\ KAPRE RAVINDRA (US) | T01-J04B1\ T01-J04B2\ T01-J10B2\ T04-D03A\ T04-D04\ V07-F02A\ V07-K01 |
| 6684 | 3D printing | CONTINUOUS WAVE FIBER LASER CLADDING COBALT-BASED ALLOY POWDER USED FOR WORK MOLD SURFACE OF BASE MATERIAL COMPRISES SILICON NITRIDE, ANTIMONY, BORON, SILICON, ALUMINUM OXIDE, CARBON, COBALT AND IMPURITY OF DIFFERENT WEIGHT PERCENTAGES | JP2014073985A | NARUTA YASUHISA (JP) | IBM (US) | U11-C09G\ U11-F02A4 |
| 6685 | 3D printing | MEDICAL FILM ANTISTATIC LAYER, HAS BASE MATERIAL PROVIDED WITH HALF-TRANSPARENT PET PLATE, WHERE WEIGHT PERCENT OF ADHESIVE, EXTINCTION POWDER, POLY-ANILINE ANTISTATIC AGENT AND DISPERSING AGENTS ARE IN SPECIFIC RANGE | US2014103474A1 | NAWATE MASAKATSU (JP) | NEC ELECTRONICS CORP (JP) | V07-F01A\ V07-K01\ W03-A08E\ W03-A09\ W03-A12A |
| 6686 | 3D printing | HDP PSG DI-ELECTRIC LAYER MANUFACTURING METHOD, INVOLVES GENERATING HYDROGEN PASSIVATION TREATMENT IN PRE-CARRYING UNIT, AND FORMING HIGH DENSITY PLASMA ON PHOSPHORUS SILICON GLASS LAYER | CN103571315A | NAWATE MASAKATSU (JP)\ IMAHASHI MANABU (JP) | MATSUSHITA ELECTRIC IND CO LTD (JP) | A05-F01E3\ A06-A00E3\ A12-V02\ D09-C01D\ S05-F\ T01-J06A\ X25-A08 |
| 6687 | 3D printing | ELECTRO-STATIC DISCHARGE (ESD) PROTECTION DEVICE FOR SEMICONDUCTOR DEVICE AND ELECTRONIC CIRCUIT, HAS PROTECTIVE FILM, WHICH AND COVERS END SURFACES IN WHICH FIRST AND SECOND DISCHARGE-ELECTRODE LAYERS PROTRUDED | CN103627856A | NEGORO TAKAAKI (JP) | NAWATE MASAKATSU (JP)\ IMAHASHI MANABU (JP) | A11-C01C\ A12-L05A\ A12-P\ A12-W03\ A12-W06\ A12-W07\ G03-B04\ G05-F |
| 6688 | 3D printing | METHOD FOR PERFORMING OPTICAL CORRELATION BETWEEN INPUT IMAGE AND REFERENCE IMAGE, INVOLVES APPLYING OUTPUT BEAM TO LENS, SUCH THAT CORRELATION PATTERN IS COMPRISED OF LENSING EFFECT TO MOVE FAR-FIELD AWAY FROM FOCUS OF ZERO-ORDER | KR1381114B1 | NEGORO TAKAAKI (JP)\ SHIMIZU EI US | RICOH CO LTD (JP)\ NEGORO TAKAAKI (JP) | U11-C05C |
| 6689 | 3D printing | TRANSPORT MECHANISM OF LASER SINTERING PACKAGING APPARATUS | RU137490U1 | NGO MINH VAN (US)\ ZHAO LARRY (US)\ BESSER PAUL RAYMOND (US) | RICOH KK (JP) | A09-D03\ A11-B16\ A11-C02B\ A11-C02D\ T01-J15X |
| 6690 | 3D printing | ILLUMINATION DEVICE FOR SPATIAL LIGHT MODULATOR DEVICE OF IMAGE DISPLAY DEVICE, HAS ANGULAR SPECTRUM OF PLANE WAVES THAT IS DECOUPLED FROM LIGHT DECOUPLER AND HAVING PREDEFINED VALUE WHICH DEPENDS ON USE REQUIREMENT | CN103572282A | NGUYEN TUE (US) | ADVANCED MICRO DEVICES INC (US) | M13-H01\ M13-L\ X24-D03\ X25-A03B2\ X25-A03C2 |
| 6691 | 3D printing | METHOD FOR PRODUCING TEMPORARY HIP PROSTHESIS OF PATIENT, INVOLVES PRODUCING PRE-MOLD FROM VIRTUAL MODEL BY SELECTIVE LASER SINTERING PROCESS, PRODUCING MOLD FROM PRE-MOLD, AND CASTING MEDICAL CEMENT IN MOLD TO OBTAIN TEMPORARY PROSTHESIS | IN201203926I4 | NI HENRI A III (US)\ MICHAEGEHE WENSENT J (US)\ THOMAN KURT A (US) | TEGAL CORP (US) | L04-A08\ L04-A99\ L04-C10D\ L04-C10L2\ L04-C16\ U11-A13\ U11-C01J2 |
| 6692 | 3D printing | LABEL RECORD E.G. VOUCHER USED FOR DELIVERY OF GOODS, HAS RESIN COATING LAYER THAT IS PROVIDED WITH MULTILAYER STRUCTURE TO WHICH COATING UNIT LAYERS OF SINGLE LAYERED FORM ARE STACKED UP IN THICKNESS DIRECTION | CN103551492A | NICHOLAS J HEATON (US) | IBM (US) | V07-F02C\ V07-K01\ V07-M |
| 6693 | 3D printing | METHOD FOR MANUFACTURING WIRING LAYER OF SEMICONDUCTOR DEVICE, INVOLVES REMOVING COPPER FILM ON UPPERMOST SURFACE OF INSULATING FILM FOR EMBEDDING COPPER FILM IN PRIMARY GROOVE AND FORMING DUMMY PATTERN WITH COPPER FILM IN SECONDARY GROOVE | WO2014041409A2 | NIIMIYABARA SHOZO US\ BABA TOMOYUKI US\ FURUYA AKIHIKO US\ O ZORIN US\ YASUDA KEIICHIRO US\ IKEDA HIDEO US\ HAGIWARA MUNEAKI US | SCHLUMBERGER TECHNOLOGY CORP (US) | L03-B03G2\ L03-E01A\ L03-E01B4C\ L03-E01B5B\ L03-E03\ L03-E08B\ L03-H05\ L03-J\ V01-B01A4\ V01-B01A5\ V01-B01D\ V01-B01G1\ V01-B01G3\ V01-B01G5\ V01-B04B5\ X16-B01A3\ X16-B01F1\ X16-E01G\ X16-L02\ X22-F01 |
| 6694 | 3D printing | CREATING THREE-DIMENSIONAL MODEL INVOLVES MOVING MASKLESS OPTICAL IMAGING SYSTEM WITH MICRON POSITIONING ACCURACY PROXIMATE PHOTOSENSITIVE MEDIUM, FOLLOWED BY EXPOSING TO ENERGY SOURCE, LOWERING PLATE AND APPLYING NEW LAYER OF MEDIUM | CN103628056A | NISHIKAWA HIDETOSHI (JP)\ SONODA MASAHIKO (JP) | JAPAN SCIENCE & TECH CORP US\ KUMABO METAL KK US\ OGATA KOGYO KK US\ NODAICHI DENSHI KK US\ TOPPAN PRINTING CO LTD (JP) | T01-E01B\ T01-J15X\ X25-A08\ X25-A09 |
| 6695 | 3D printing | LASER CLADDING METHOD FOR MACHINE INVOLVES ROOTING MACHINE'S CUTTING EDGE AFTER LASER CLADDING, TAKING SURFACE GRINDING MACHINE, AND CUTTING EDGE GROUP | CN103625219A | NISHIMAKI HIDEKATSU US\ OBAYASHI MASAYUKI US | ROHM CO LTD (US) | L03-H05\ L04-C10D\ L04-C12\ L04-C16\ L04-C17C\ L04-C22\ L04-C25\ U11-C05B4\ U11-C05C\ U11-D02B\ X21-A01F |
| 6696 | 3D printing | COPPER, INDIUM, GALLIUM, SELENIUM LIGHT ABSORPTION LAYER MANUFACTURING METHOD, INVOLVES COATING COPPER, INDIUM, GALLIUM, SELENIUM THIN FILM WITH SELENIUM PRECURSOR SOLUTION DURING SPIN COATING PROCESS OR SPRAY PROCESS | CN203476713U | NISHIMURA HIDETOSHI (JP)\ HAYASHI KOHTARO (JP) | RENESAS TECH CORP (JP) |  |
| 6697 | 3D printing | DIGITAL HOLOGRAM DISPLAY DEVICE FOR DISPLAYING THREE-DIMENSIONAL IMAGE, CHANGES PATH OF MODULATION LIGHT OUTPUTTED FROM ONE PIXEL, SO THAT PATH OF MODULATION LIGHT IS CORRESPONDED TO PATH OF OTHER LIGHT FOR COMBINING MODULATION LIGHTS | CN103624250A | NISHIMURA HISAHARU US\ OGURA HIROYOSHI US | PANASONIC CORP (JP)\ NISHIMURA HIDETOSHI (JP)\ HAYASHI KOHTARO (JP) | U24-F02\ V06-N03A\ V06-N03B\ X13-A03\ X13-H01B\ X13-H01F\ X13-H01G |
| 6698 | 3D printing | ELECTRICAL STORAGE APPARATUS E.G. LITHIUM ION SECONDARY BATTERY, FOR USE IN VEHICLE, HAS POSITIVE ELECTRODE INCLUDING POSITIVE ELECTRODE ACTIVE MATERIAL LAYER THAT IS FORMED ON POSITIVE ELECTRODE METAL FOIL, AND ELECTRODE ASSEMBLY LAMINATED | CN103611879A | NISHINOHARA KAZUMI (JP) | PANASONIC CORP (JP) | V06-M08A\ V06-M11B\ X11-J01B\ X11-J02A\ X11-J08B |
| 6699 | 3D printing | COMPUTERIZED METHOD FOR CREATING THREE-DIMENSIONAL SURFACE TEXTURE ON PORTION OF E.G. OUTSOLE OF SHOE, INVOLVES USING AUGMENTED DATA FILE TO INSTRUCT RAPID MANUFACTURING DEVICE TO MANUFACTURE PORTION OF SHOE WITH SURFACE TEXTURE | CN103600066A | NISHIYAMA SATOSHI US | TOSHIBA KK (JP) | D08-B01 |
| 6700 | 3D printing | POWER MODULE SUBSTRATE WITH HEAT SINK USED FOR ELECTRIC VEHICLE, HAS METAL LAYER OF COPPER OR ITS ALLOY, HEAT SINK FORMED FROM ALUMINUM OR ITS ALLOY, AND INTERMETALLIC COMPOUND LAYER, WHICH ARE DISTRIBUTED WITH OXIDE AT INTERFACE | CN103600067A | NOIRAY JEROME (FR)\ GRANNEMAN ERNST H A (NL) | CITIZEN WATCH CO LTD (US) | D08-B01 |
| 6701 | 3D printing | SHOE HAS LASER-SINTERED MIDSOLE PORTION WHICH IS REMOVABLY INSERTED INTO MIDSOLE HOUSING OF CUP OUTSOLE, SUCH THAT MIDSOLE PORTION IS VIEWABLE THROUGH PARTIALLY TRANSPARENT SIDE WALL OF CUP OUTSOLE | CN103600213A | NOMURA TOSHIO US | ASM INT NV US | U11-C05F6\ U11-C18B4 |
| 6702 | 3D printing | METHOD FOR ACTUATING E.G. CLOCKED FLYBACK CONVERTER OF THREE-PHASE MOTOR, INVOLVES GENERATING ACTUATION SIGNAL FROM OUTPUT VOLTAGE FOR SEMICONDUCTOR SWITCH OF CONVERTER CIRCUIT, AND LIMITING VOLTAGE WHEN VOLTAGE EXCEEDS SWITCHING THRESHOLD | CN103600214A | NONAKA JUNPEI US | FUJITSU LTD (JP) | A04-G05\ A05-D02E\ A05-J02\ A07-A04C\ A07-A04F\ A08-R\ A08-R03\ A08-R06A\ A08-S02\ A11-A03\ A11-A04\ A11-B07\ A12-B01G\ A12-B01H\ A12-B01V\ G02-A02D\ G02-A02E\ G02-A03A\ G02-A03D |
| 6703 | 3D printing | INNER ROTOR-TYPE MOTOR, HAS COILS, EACH WITH ELECTRIC WIRE AT FIRST LAYER THAT IS WOUND AROUND TOOTH ALIGNED WITH YOKE SIDE, WITH CLEARANCE GAP SEPARATING WIRE FROM CENTERLINE OF TEETH | CN103601476A | NOPPER MARKUS (DE)\ NOTHELFER UDO (DE)\ PREUSSE AXEL (DE) | NEC ELECTRONICS CORP (JP) | M24-D02A\ M24-D07\ M24-E\ T01-J07B\ V08-A04B\ V08-A04C\ V08-A09 |
| 6704 | 3D printing | SHEET-LIKE COSMETICS FOR APPLYING E.G. FOUNDATION AND LIP COLOR, HAVE FLOCKING LAYER FORMED BY CARRYING OUT ELECTROSTATIC FLOCKING OF SHORT FIBERS HAVING SPECIFIED SINGLE YARN FINENESS, ON SURFACE OF BASE MATERIAL HAVING FOAM SHEET | CN203450349U | NORIHITO NAKAMOTO (JP) | NOPPER MARKUS (DE)\ NOTHELFER UDO (DE)\ PREUSSE AXEL (DE) | A11-B05\ A12-B04C\ A12-B04D\ L03-H04E3\ L03-H04E4\ L03-H04E9\ L03-J\ S06-C02A\ S06-G\ S06-K03\ V04-Q02A5\ V04-Q30F\ V04-R01A4\ V04-R01A5A\ V04-R02F\ V04-R03\ V04-U15\ V04-X01D\ V04-X01F |
| 6705 | 3D printing | SHEET-LIKE COSMETICS FOR APPLYING E.G. FOUNDATION, HAVE FLOCKING LAYER FORMED BY CARRYING OUT ELECTROSTATIC FLOCKING OF SHORT FIBERS ON BASE MATERIAL HAVING FOAM SHEET WHERE PORTION(S) OF FLOCKING LAYER COMPRISES POWDER COSMETICS | CN103602857A | NORIYUKI MIURA (JP) | NEC ELECTRONICS CORP (JP) | M22-H03B |
| 6706 | 3D printing | TUNNELING MAGNETO-RESISTANCE SENSOR I.E. CURRENT-IN-PLANE TUNNELING MAGNETO-RESISTANCE SENSOR, HAS ELECTRODE ARRAY CONNECTED TO MAGNETO-RESISTANCE COMPONENT, AND MAGNETIC TUNNELING JUNCTION UNIT CONNECTED TO ELECTRODES OF ELECTRODE ARRAY | CN103602876A | NORIYUKI MIURA (JP) | OKI ELECTRIC IND CO LTD (JP) | M13-H01\ M13-L\ M26-B08\ M26-B08A\ M26-B08B\ M26-B08C\ M26-B08S\ M26-B08T |
| 6707 | 3D printing | HIGH-STRENGTH FINISH PAINT CONTAINS UNSATURATED POLYESTER RESIN, TRIGLYCIDYL ISOCYANURATE, POLYBENZIMIDAZOLE, TIN OXIDE, ANGELICA OIL, TITANIUM DIOXIDE, GLYCOL, NANO BENTONITE AND COMPOSITE FILLER | CN103602948A | NOSAKA NORIHITO (JP)\ WAKABAYASHI AKIRA (JP)\ OGIKUBO SHINYA (JP)\ HARA MINAKO (JP) | OKI SEMICONDUCTOR CO LTD (JP) | A10-E07C\ A12-V04C\ D08-B01C\ D08-B09A3\ B04-A08G1\ B04-A09\ B04-C03C\ B07-A02B\ B10-C02\ B10-C04D\ B10-E04B\ B10-E04C\ B12-M06\ B12-M09\ B12-M12B\ B14-N17\ B14-R01 |
| 6708 | 3D printing | LASER STRENGTHENING DEVICE INCLUDES COMPUTER CONTROL PROCESSING SYSTEM, CLADDING UNIT, LASER IMPACT STRENGTHENING UNIT, CENTRAL CONTROL PROCESSOR, CARBON DIOXIDE LASER CONTROLLER, LIGHT GUIDE TUBE, AND FIVE-SHAFT LINKAGE WORKING TABLE | CN103602977A | NOVININ F (NL)\ BUTTAJE B (NL) | FUJIFILM CORP (JP)\ NOSAKA NORIHITO (JP)\ WAKABAYASHI AKIRA (JP)\ OGIKUBO SHINYA (JP)\ HARA MINAKO (JP) | M22-A01\ M22-H02\ M22-H03B\ M26-A02\ M26-B10 |
| 6709 | 3D printing | MANUFACTURING PLATE, USEFUL FOR SHIELD CAN, COMPRISES PREPARING METAL PLATE, MANUFACTURING COMPLETE PLATE AND SHIELDING LAYER, SCREEN PRINTING ON METAL PLATE SURFACE, AND FORMING MULTIPLE PILOT PIN HOLES ON E.G. BOTH SIDE SURFACES OF PLATE | CN103602978A | NOWAK EDWARD J (US)\ LOGAN LYNDON R (US)\ ROBISON ROBERT R (US)\ WINSLOW JONATHAN K (US) | PHILIPS CORP (US) | M22-G03F\ V08-A09\ X25-A08 |
| 6710 | 3D printing | DEVICE FOR LASER SINTERING OF POWDER MATERIAL BILLET, HAS PLATFORM THAT IS PROVIDED WITH BASE LAYER FOR PLACING POWDER MATERIAL, AND VIBRO-UNIT IS ARRANGED INSIDE PLATFORM UNDER BASE LAYER AND IS RIGIDLY CONNECTED WITH LATTER | CN103602979A | NOZOE MITSUYOSHI US | IBM (US)\ NOWAK EDWARD J (US)\ LOGAN LYNDON R (US)\ ROBISON ROBERT R (US)\ WINSLOW II JONATHAN K (US) | M13-H01\ M26-B\ M26-B00C\ M26-B00N\ M26-B00T |
| 6711 | 3D printing | LASER CLADDING USED FOR COATING SURFACE OF PLUG, INCLUDES TRANSITIONAL ALLOY LAYER, AND HIGH STRENGTH ALLOY LAYER, WHERE TRANSITIONAL ALLOY LAYER COMPRISES CARBON, CHROMIUM, BORON, SILICON AND NICKEL | CN103602980A | OGATA KATSUYA (JP)\ ROKUGAWA HIROYUKI (JP) | MATSUSHITA ELECTRIC IND CO LTD (JP) | A11-B17\ A11-C01C\ A12-T01 |
| 6712 | 3D printing | DERMATOLOGICAL OR COSMETIC COMPOSITION USEFUL FOR E.G. CLEANSING SKIN, ANTISEB COMPRISING GLYCOLIC ACID, SODIUM LACTATE AND ALOE VERA AND SEBOKLEN COMPRISING COCO-GLUCOSIDE AND LAURYL GLUCOSIDE | CN103602858A | OH HYEOK-SANG (KR)\ JANG WOO-JIN (KR)\ MOON BUM-KI (US)\ CHOI JI-HONG (US)\ OH MINSEOK (US)\ CHENG TIEN-JEN (US) | FUJITSU SEMICONDUCTOR LTD (JP)\ OGATA KATSUYA (JP)\ ROKUGAWA HIROYUKI (JP) |  |
| 6713 | 3D printing | COATED SAND MATERIAL USEFUL FOR LASER SINTERING OF MAGNESIUM ALLOY, COMPRISES RAW SAND, ORGANIC BINDER, COUPLING AGENT, CURING AGENT AND A FLAME RETARDANT | CN103606803A | OH JOON-HAK US\ LEE JONG-SEO US\ KANG HA-YUN (KR) | SAMSUNG ELECTRONICS CO LTD (KR)\ OH HYEOK-SANG (KR)\ JANG WOO-JIN (KR)\ MOON BUM-KI (US)\ CHOI JI-HONG (US)\ OH MINSEOK (US)\ CHENG TIEN-JEN (US) | M13-H01\ M22-H |
| 6714 | 3D printing | METHOD FOR MANUFACTURING STEEL CASTS, PREFERABLY MANGANESE STEEL USED TO OBTAIN WEAR ELEMENTS, INVOLVES MAKING REINFORCEMENT INSERT AND PREPARING MOLD FOR CAST TO BE MANUFACTURED, IN WHICH REINFORCEMENT INSERT IS POSITIONED | CN103606805A | OH JUN HWAN (KR)\ CHUNG JU HYUCK (KR)\ KIM IL GOO (KR)\ KIM HYOUNG SIK (KR) | SAMSUNG DISPLAY CO LTD (KR) | M22-A04\ M22-C01\ M22-D\ M22-G03N\ A09-D03\ A11-B05\ A11-B14\ A11-B16\ A11-C02B\ A11-C02D\ A12-A02\ T01-J07B\ X25-A01\ X25-A08 |
| 6715 | 3D printing | TITANIUM ALLOY 15 SURFACE LASER CLADDING MATERIAL COMPRISES NICKEL-60 POWDER, TITANIUM DIBORIDE POWDER, AND COBALT-TUNGSTEN CARBIDE MIXTURE POWDER | CN203462127U | OHGURO TATSUYA (JP) | SAMSUNG ELECTRONICS CO LTD (KR) | M13-H01\ M22-H\ M22-H03F1\ M26-B08\ M26-B08A\ M26-B08M |
| 6716 | 3D printing | RADIAL TIRE CROWN BELT LAYER MANUFACTURING METHOD, INVOLVES PERFORMING STRIP BELT DIPPING OPERATION AND BAND SUB-ADHESIVE COATING OPERATION, WHERE ADHESIVE IS SELECTED FROM GROUP CONSISTING OF ADHESIVE GLUE, RUBBER AND AUXILIARY AGENT | CN203462128U | OHMI TADAHIRO (JP)\ TANAKA HIROAKI (JP) | TOSHIBA KK (JP) | M13-H01\ M22-H\ M22-H03F1\ M26-B08\ M26-B08A\ M26-B08C\ M26-B08M\ M26-B08S |
| 6717 | 3D printing | LASER STRENGTHENING COMPRESSOR SET INTEGRATED SELF-TEMPERATURE CONTROL SYSTEM, HAS HOT EXCHANGER CONNECTED WITH EXPANSION VALVE, AND LASER SINTERING DIAMOND HARDENED LAYER CONNECTED WITH INNER UPPER WALL SURFACE OF SEMI CLOSED COMPRESSOR | CN103615995A | OHMI TADAHIRO (JP)\ TANAKA HIROAKI (JP) | UNIV TOHOKU (JP) | L04-C26\ L04-E03B\ M22-H01\ X25-A03B2\ X25-Q02 |
| 6718 | 3D printing | NICKEL-BASED CERMET ALLOY POWDER USED IN CONTINUOUS FIBER LASER CLADDING PROCESS, COMPRISES SPECIFIED AMOUNT OF ALUMINUM OXIDE, LANTHANUM OXIDE, TITANIUM DIOXIDE, CALCIUM FLUORIDE, CERIUM OXIDE AND NICKEL | CN103614720A | OISHI HIDEO (JP)\ SEIKE TADAYOSHI (JP)\ HIROFUJI MASANORI (JP) | OHMI TADAHIRO (JP)\ UNIV TOHOKU (JP)\ TANAKA HIROAKI (JP) | X24-B |
| 6719 | 3D printing | MANUFACTURING SELECTIVE LASER SINTERING-BASED CASTING RESIN MOLD INVOLVES MANUFACTURING THREE-DIMENSIONAL MOLD WITH SELECTIVE LASER SINTERING DEVICE THAT IS BASED ON COMPUTER DESIGN | CN103614721A | OIZUMI MUNENORI (JP)\ AOKI KATSUHIRO (JP)\ FUKUDA YUKIO (JP) | MATSUSHITA ELECTRIC IND CO LTD (US) | L02-A02\ L02-A04\ L02-A11\ L02-G01A\ L02-G07D\ L02-G11\ L02-J01C\ L03-H04A |
| 6720 | 3D printing | COBALT ALLOY POWDER USED FOR LASER CLADDING COMPRISES SILICON CARBIDE, ANTIMONY, ALUMINUM OXIDE, LANTHANUM OXIDE, CALCIUM FLUORIDE, MANGANESE AND COBALT | CN103614723A | OKADA HIROSHI (JP) | TEXAS INSTRUMENTS INC (US) | A12-R\ A12-R06\ A12-T02\ A12-T04 |
| 6721 | 3D printing | COBALT ALLOY POWDER USED FOR CONTINUOUS LASER CLADDING COMPRISES SILICON CARBIDE, CALCIUM FLUORIDE, MOLYBDENUM, CHROMIUM, SILICON NITRIDE, SILICON, CARBON, ANTIMONY AND COBALT | CN103614731A | OKAMOTO NANA (JP)\ NISHIMURA HIDETOSHI (JP)\ TAMARU MASAKI (JP) | DENSO CORP (US) | M13-H01\ M22-H\ M22-H03F1\ M26-B08\ M26-B08C\ M26-B08M\ M26-B08N |
| 6722 | 3D printing | PREPARATION OF HOT BAR SHEARING MACHINE BLADES CUTTING EDGE BY SELECTING HOT KNIFE CUTTING BOARD MATERIAL, INK BLACKENING, DRYING, PREPARING TITANIUM CARBIDE-BASED CERAMIC METAL ALLOY POWDER, THERMAL ANNEALING, COOLING, AND FINISHING | CN103614779A | OKOROANYANWU UZODINMA (US) | PANASONIC CORP (JP)\ OKAMOTO NANA (JP)\ NISHIMURA HIDETOSHI (JP)\ TAMARU MASAKI (JP) | M13-H01\ M22-H\ M22-H03F1\ M26-B\ M26-B00C\ M26-B00N\ M26-B00S\ V08-A09 |
| 6723 | 3D printing | HYDRAULIC BRACKET UPRIGHT POST THREADED HOLE REPRODUCING HEAD MANUFACTURING METHOD, INVOLVES FORMING ARC WELDED STAINLESS STEEL WITH STAINLESS STEEL LAYER, AND DRILLING INNER HOLE IN REAR PROCESSING STAINLESS STEEL LAYER | CN103616077A | OKOROANYANWU UZODINMA (US)\ ACHETA ALDEN (US) | ADVANCED MICRO DEVICES INC (US) | G02-A01\ M13-F\ M13-H01 |
| 6724 | 3D printing | LED SUBSTRATE MATERIAL USED IN SEMICONDUCTOR LIGHTING INDUSTRY, HAS ALUMINA CERAMIC POWDER, SINTERING AUXILIARY AGENT, ORGANIC SOLVENT, BONDING AGENT, PLASTICIZER AND DISPERSANT IN SPECIFIED AMOUNTS | CN103616744A | OKUBO HIROAKI US\ FURUMIYA MASAYUKI (JP)\ NAKASHIBA YASUTAKA (JP) | ADVANCED MICRO DEVICES INC (US) | M13-H01\ V08-A09 |
| 6725 | 3D printing | MOBILE-TYPE SHUNT SHIP, HAS BLOWING PIPELINE FIXED ON FLOATING BODY, WHERE OUTPUT PORT OF PIPELINE IS FIXED ON DIVIDING INSULATING CABIN, AND FLOW OBSERVING PLATFORM ARRANGED ON SIDE OF FLOW COMPARTMENT | US2014106087A1 | OKUBO HIROAKI US\ NAKASHIBA YASUTAKA (JP) | RENESAS ELECTRONICS CORP (JP)\ FURUMIYA MASAYUKI (JP)\ OHKUBO HIROAKI (JP)\ NAKASHIBA YASUTAKA (JP) | M13-H01 |
| 6726 | 3D printing | ALLOY POWDER FOR CONTINUOUS WAVE LASER CLADDING HAS CHEMICAL COMPOSITION WHICH INCLUDES TITANIUM CARBIDE, LANTHANUM OXIDE, ALUMINUM OXIDE, CHROME, IRIDIUM, CARBON, MANGANESE AND NICKEL | US2014098409A1 | OKUDA MASAKI (JP) | NEC ELECTRONICS CORP (JP) | M13-H01\ M13-L\ M26-B08\ M26-B08B\ M26-B08C\ M26-B08S\ V08-A09 |
| 6727 | 3D printing | METAL-CERAMIC ALLOY POWDER USED FOR CONTINUOUS WAVE LASER CLADDING USED IN E.G. AVIATION HAS CHEMICAL COMPOSITION WHICH TITANIUM DIOXIDE, SILICON NITRIDE, CALCIUM FLUORIDE, ALUMINUM OXIDE, SILICON, CHROMIUM, IRIDIUM, CARBON AND COBALT | US2014099476A1 | OMI TOSHIHIKO (JP)\ WATANABE HITOMI (JP)\ ISHII KAZUTOSHI (JP)\ SAITOH NAOTO (JP) | FUJITSU MICROELECTRONICS LTD (JP) | M13-H01\ M13-L\ M22-H02\ M24-D01A1\ M24-D03\ M24-D07\ M24-E\ M27-A04\ M27-B04 |
| 6728 | 3D printing | NICKEL CERMET ALLOY POWDER FOR CONTINUOUS-WAVE FIBER LASER CLADDING CONTAINS ALUMINUM OXIDE, TITANIUM DIOXIDE, CALCIUM FLUORIDE, CERIUM OXIDE, SILICON NITRIDE, AND NICKEL BASE | GB2506745A | ONUMA HIDETO US\ HANAOKA KAZUYA (JP)\ FUJII TERUYUKI (JP) | SEIKO INSTR INC (JP) | M13-H01\ M26-B08\ M26-B12 |
| 6729 | 3D printing | COATING METHOD FOR PULSE CURRENT LASER CLADDING THINNING METAL SOLIDIFICATION TISSUE INVOLVES STOPPING LASER CLADDING OF PULSE CURRENT TO TERMINATE DELIVERY OF PULSED CURRENT TO CONDUCTIVE SUBSTRATE AT SAME TIME | CN103553632A | OOISHI MITSUMASA US | SEMICONDUCTOR ENERGY LAB (JP)\ HANAOKA KAZUYA (JP)\ OHNUMA HIDETO (JP)\ FUJII TERUYUKI (JP) | V07-F01A1\ V08-A04C2\ V08-A05 |
| 6730 | 3D printing | SYNCHRONIZED LASER CLADDING POWDER FEEDER MECHANICAL AUTOMATIC CONVERSION DEVICE HAS WORM DISK WITH DISK CONNECTED TO POWDER OUTLET TO ROTATE POWDER TUBE | CN103592324A | OOKA HIDEYUKI US | NEC ELECTRONICS CORP (JP) | L03-F02B\ L03-G02B\ L04-A01A\ A05-E01D\ A11-C04D\ A12-E11\ A12-L03C\ A12-L03D\ V07-F02B\ V07-K10C\ V08-A04C2\ V08-A05 |
| 6731 | 3D printing | CONTINUOUS WAVE LASER CLADDING FOR METAL CERAMIC ALLOY POWDER, HAS COMPONENTS WITH PREDETERMINED WEIGHT PERCENTAGES WHICH INCLUDE OF TITANIUM CARBIDE, LANTHANUM OXIDE, ALUMINUM OXIDE, SILICON NITRIDE, SILICON, BORON, CARBON AND NICKEL | JP2014063681A | ORTEGA MEL A (US) | NEC CORP (US) | M13-H01 |
| 6732 | 3D printing | LASER REPAIRING METHOD FOR FINISHING ROLLER ABRASION OF REAR, INVOLVES HARDNESS PROCESS CHECKING FOR FINISHING ROLLER PROCESSING PRECISION OF REAR OF QUALIFIED PRODUCT COATING ANTIRUST OIL AND PACKAGING | WO2014049842A1 | OSADA KENICHI (JP)\ YANAGISAWA KAZUMASA (JP)\ YAMAOKA MASANAO (JP) | INTEL CORP (US) | M13-H01\ M23-D05\ M23-H\ X24-D03 |
| 6733 | 3D printing | ALLOY POWDER, USEFUL FOR CONTINUOUS FIBER LASER CLADDING PROCESS OF METAL PARTS, COMPRISES ALUMINUM OXIDE, SILICON CARBIDE, TITANIUM DIOXIDE, CALCIUM FLUORIDE, CERIUM OXIDE, AND COBALT | DE102012217912A1 | OSAMU MURASAWA (JP) | RENESAS TECH CORP (JP) | S02-A05B\ S02-A10B\ T01-J04A |
| 6734 | 3D printing | HIGH-POWER LASER CLADDING OPTICAL FIBER STRIPPING DEVICE, HAS RADIATING DEVICE COATED WITH HIGH REFRACTIVE INDEX OPTICAL GEL, CONNECTED WITH BASE AND UPPER CONVEX PART AND LOCATED WITH CROSS SECTION PART OF COLUMN UNIT | CN103568326A | OSAWA TAKASHI US | SONY COMP ENTERTAINMENT INC (JP) | M13-H01\ M22-H\ M26-B08\ M26-B08C\ M26-B08J\ M26-B08M |
| 6735 | 3D printing | DUAL-CLAD FIBER LASER CLADDING OPTICAL FILTERING DEVICE, HAS UPPER GUIDE HOT MODULE FIXED ON LOWER GUIDE HOT MODULE, AND DUAL-CLAD LAYER WHOSE OUTER SURFACE IS COATED WITH HIGH REFRACTIVE INDEX POLYESTER MATERIAL | CN103576389A | OSAWA TAKASHI US | TOSHIBA CORP (JP) | M13-H01\ M22-H\ M26-B08\ M26-B08A\ M26-B08B |
| 6736 | 3D printing | BALL VALVE BALL BODY LASER CLADDING COATING PROCESSING AND LOCATING DEVICE, COMPRISES BALL VALVE BALL BODY, TWO LINKAGE END COVER, RECTANGULAR SWINGING FRAME, BOTTOM FRAME, ELECTRIC MACHINE, SUPPORTING PLATE AND TRANSMISSION SHAFT | CN103572281A | OTREMBA RALF (DE) | TOSHIBA CORP (JP) | M13-H01\ M13-H03\ M26-B08\ M26-B12\ M26-B12C\ M26-B12T |
| 6737 | 3D printing | BALL VALVE LASER CLADDING SPHERE LOCATING MECHANISM COMPRISES FIXING FRAME, ROTATING FRAME PROVIDED WITH ROTATING SHAFT, TWO DRIVING PARTS, BALL FIXING DEVICE, DRIVING PIECE SET ON FIXING FRAME, AND POWER OUTPUT SHAFT | CN103572283A | OTSUKA FUMIO (JP)\ YAMAMOTO TOMOSHI (JP)\ SAKAI SATORU (JP) | INFINEON TECHNOLOGIES AG (DE)\ OTREMBA RALF (DE) | M13-H01\ M13-L\ M14-H\ M24-D02A\ M24-D03\ M24-D07\ M24-E\ M27-A04\ M27-B04\ X25-A08 |
| 6738 | 3D printing | NON-DESTRUCTIVE ULTRA-SONIC SURFACE WAVE THIN CLADDING LAYER THICKNESS EVALUATING METHOD, INVOLVES CALCULATING AVERAGE VALUE AND LASER CLADDING LAYER THICKNESS VALUE, AND DETERMINING ULTRASONIC SURFACE WAVE PROPAGATION RANGE | CN103594354A | OWAKI YOSHIAKI (JP)\ KUWAHARA YASUHIRO (JP) | RENESAS TECH CORP (JP) | L03-H05\ L04-A01B\ L04-C01\ L04-C02C\ L04-C07B\ L04-C16\ U11-C01J1\ U11-C02J1C\ U11-C07A1\ U11-C09X\ U11-F02A1A |
| 6739 | 3D printing | ALLOY POWDER, USEFUL FOR CONTINUOUS-WAVE FIBER LASER CLADDING PROCESS, COMPRISES SILICON CARBIDE, CALCIUM FLUORIDE, SILICON NITRIDE, CHROMIUM NITRIDE, INDIUM NITRIDE, CARBON, MANGANESE, IRON, AND COBALT | CN103594623A | PALSULE CHINTAMANI (US)\ HADDAD HOMAYOON (US)\ NISHI YOSHIO (US) | PANASONIC CORP (JP)\ OWAKI YOSHIAKI (JP)\ KUWAHARA YASUHIRO (JP) | S02-C06D1\ S03-A02\ T01-J10A\ T01-J10B2\ W04-M01B\ W04-M01C5\ W04-M01D |
| 6740 | 3D printing | ALLOY POWDER, USEFUL FOR CONTINUOUS-WAVE FIBER LASER CLADDING PROCESS, COMPRISES SILICON CARBIDE, ANTIMONY, BORON, CALCIUM FLUORIDE, AND COBALT | CN203440446U | PARK BYUNG-JUN (KR) | PALSULE CHINTAMANI (US)\ HADDAD HOMAYOON (US)\ NISHI YOSHIO (US) | V08-A09 |
| 6741 | 3D printing | PREPARING REINFORCED TITANIUM CARBIDE ALLOY BY CLEANING SURFACE OF TITANIUM SAMPLE BODY, MIXING CLADDING MATERIAL POWDER, COBALT-BASED ALLOY AND CERAMIC HARD PHASE IN SAMPLE BODY, AND UNIFORMLY COATING ADHESIVE PASTE ON SURFACE OF BODY | CN103592233A | PARK BYUNG-JUN (KR) | SAMSUNG ELECTRONICS CO LTD (US) |  |
| 6742 | 3D printing | REPAIRING A COMPOSITE ROLLER BY FORMING A WORKING LAYER OF COMPOSITE ROLLER, COAXIAL POWDER-FEEDING LASER-CLADDING PROCESSING ON THE WORKING LAYER WITH A FEEDING POWDER AND THEN ROTATING THE LAYER, AND LASER RAPID PROTOTYPING ON THE LAYER | CN103567392A | PARK IL MUN (KR)\ KIM HEAN SIK (KR) | SAMSUNG ELECTRONICS CO LTD (KR) | V07-K01 |
| 6743 | 3D printing | IMPROVING N-TYPE DOPING CONCENTRATION UNIFORMITY OF SILICON CARBIDE EPITAXIAL SHEET, BY PLACING CARBIDE SUBSTRATE HAVING SURFACE MORPHOLOGY OF SILICON CARBIDE ON GRAPHITE COATING, AND HEATING SUBSTRATE TO SPECIFIC TEMPERATURE AND PRESSURE | JP2014063047A | PASSEMARD GERARD (FR)\ MAITREJEAN SYLVAIN (FR)\ IVANOVA-HRISTOVA VALENTINA (FR) | I & H CO LTD (KR) | M22-H02\ M22-H03B\ M22-H03F\ X25-A08 |
| 6744 | 3D printing | COLUMN VECTOR POLARIZED LIGHT POLARIZATION STATE MEASURING SYSTEM, HAS MAIN BODY PROVIDED WITH LIQUID CRYSTAL SPATIAL LIGHT MODULATOR, CCD CAMERA RECEIVING LIGHT INTENSITY, AND COMPUTER FOR REDUCING FAN ANGLE AND GRAY LEVEL MEASUREMENT | US2014095701A1 | PATRICK FRANKE (DE)\ WILHELM KUHNZ (DE) | PASSEMARD GERARD (FR)\ MAITREJEAN SYLVAIN (FR)\ IVANOVA-HRISTOVA VALENTINA (FR) | V07-G15\ V07-K01\ W01-A03E1\ W02-C04B4B\ W02-K04 |
| 6745 | 3D printing | HIGH POWER LASER CLADDING LIGHT SEGMENT PARTIAL PEELING METHOD, INVOLVES FORMING STRIPPING COATING LAYER AND COVERING LAYER ON SECTION, FILLING HOLE WALL OF RADIATING DEVICE WITH GRAPHITE, AND FIXING RADIATING DEVICE WITH THERMISTOR | DE102012109262A1 | PAZ DE ARAUJO CARLOS A (US)\ CUCHIARO JOSEPH D (US)\ FURUYA AKIRA (US)\ MIYASAKA YOICHI (US) | SCHERING AG (DE) | L02-A02\ L02-A03\ L02-A04\ L02-C05\ L02-H02B2 |
| 6746 | 3D printing | LASER CLADDING SYSTEM FOR USE IN GAS TURBINES OF E.G. JET ENGINES, HAS LASER CLADDING COMPONENT FOR CLADDING FIRST AREA OF ARTICLE, AND THERMAL TRACING COMPONENT HEATING SECOND AREA OF ARTICLE, WHERE SECOND AREA IS PROXIMATE TO FIRST AREA | EP2712722A1 | PENDHARKAR SAMEER (US)\ PAN SHANJEN (US)\ TODD JAMES R (US) | SYMETRIX CORP (US)\ NEC CORP (JP) | M11-A03\ M11-A07\ M11-B05\ M11-B07\ M26-B03\ M26-B03T\ S03-E06D\ S03-E13D\ X25-Q01\ X25-R04 |
| 6747 | 3D printing | LCD-BASED SPATIAL LIGHT MODULATOR, HAS DIELECTRIC LAYER PROVIDED BETWEEN THIN METAL FILM AND LAYER AT SIDE SURFACE OF ANOTHER LAYER SUCH THAT THIN METAL FILM INCLUDES ANISOTROPIC PERMITTIVITY DUE TO ELECTRIC FIELD | CN103451649A | PENG DONG-QING (TW)\ YE JIA-BIN (TW)\ XU GUO-ZHONG (TW)\ QIU JUN-HONG (TW)\ ZHANG RUO-PING (TW) | TEXAS INSTRUMENTS INC (US) | L03-A02B\ L03-E01B3\ L03-E01B5B\ L03-E01C2\ L03-E03\ L03-E08\ L03-H05\ A11-C01A1\ A11-C01C\ A12-E06A\ X16-B01F\ X16-E01C\ X16-F01\ X16-F05 |
| 6748 | 3D printing | METHOD FOR MAKING GAS TURBINE AIRFOIL E.G. SUPERALLOY BLADES, WITH CERAMIC THERMAL BARRIER COATINGS, INVOLVES APPLYING LASER ENERGY TO POWDER LAYER, AND REPEATING POWDER LAYER DELIVERING PROCESS FOR SUCCESSIVE SECTION PLANES | US2014083250A1 | PENNOCK JOHN LAURENCE (GB)\ STEELE COLIN FINDLAY (GB)\ STOJANOVIC GORAN (GB) | UNIV NAT CHENG KUNG (TW) | U14-F02B\ V02-E\ X12-C05A |
| 6749 | 3D printing | SWITCH DEVICE FOR OPTICAL SWITCH E.G., WAVELENGTH SELECTIVE SWITCHES, HAS SWITCH PLANE AND DISPERSION PLANE, AND OPTICS THAT PROVIDES IMAGING FUNCTION IN DISPERSION PLANE, AND FOURIER TRANSFORM IN SWITCH PLANE | EP2712698A1 | PERNYESZI JOSEPH (US) | WOLFSON MICROELECTRONICS PLC (GB) | T01-J10B2 |
| 6750 | 3D printing | PREPARATION OF COMPACT SILICON NITRIDE CERAMIC MATERIAL INVOLVES BALL MILLING AMINO SILICON NITRIDE CERAMIC POWDER, SINTERING AUXILIARY AGENT AND DEIONIZED WATER, OBTAINING CERAMIC SLURRY, MOLDING, DRYING AND SINTERING | US2014091314A1 | PFENNINGSTORF OLAF (GB)\ SCHNITT WOLFGANG (GB) | PERNYESZI JOSEPH (US)\ GEN MICROELECTRONICS CORP (US) | A05-E02B\ A05-E02C\ A09-D03\ A11-B14\ A12-V02\ D09-C01B\ D09-C01F\ S05-X |
| 6751 | 3D printing | MANUFACTURING METHOD FOR DETECT COINAGE PLATING LAYER OF COPPER-TIN ALLOY SPECTRUM STANDARD SAMPLE, INVOLVES COVERING UP STEEL CORE BY DOUBLE-METAL PLATING OF COPPER-TIN ALLOY PLATING LAYER CONSISTING OF TIN AND COPPER | DE102013010229A1 | PHAN TONY T (US)\ EL-HAMDI MOHAMED (US)\ HENDRIX LUTHER (US)\ MOORE BRADLEY T (US) | PFENNIGSTORF OLAF (DE)\ SCHNITT WOLFGANG (DE) | A12-L00L\ L03-G05B5\ L03-G05B8\ U14-K01A1J |
| 6752 | 3D printing | ELECTRODE ACTIVE MATERIAL FOR NON-AQUEOUS ELECTROLYTE SECONDARY BATTERY, HAS CARBON COATING LAYER CONNECTED WITH PART OF SURFACE OF LITHIUM IRON PHOSPHATE OLIVINE-TYPE STRUCTURE, WHERE COATING LAYER IS IN LINEAR SHAPE OR SCALE SHAPE | US2014083065A1 | PIO FEDERICO (IT) | SAMSUNG ELECTRONICS CO LTD (KR)\ SAMSUNG AUSTIN SEMICONDUCTOR (US) | M13-H01\ M13-M02\ M26-B08\ M26-B08A\ M26-B08B\ M26-B08C\ M26-B08S\ M26-B08T |
| 6753 | 3D printing | SUPERCONDUCTING COIL USED IN REFRIGERATOR, HAS METAL PLATE THAT IS PROVIDED WITH CONVEX PORTION EXTENDED ALONG SUPERCONDUCTING TWISTED WIRE, BETWEEN ADJACENT TWISTED WIRE THAT MUTUALLY ADJOIN METAL PLATE WITHIN SAME LAYER | CN103560076A | POLESE FRANK J (US)\ TESCH ROBERT J (US)\ NOOTENS STEPHEN P (US)\ BRADBURY WILLIAM L (US)\ DINESCU SORIN (US) | MICRON TECHNOLOGY INC (US)\ PIO FEDERICO (IT) | M13-H01\ M13-M02\ M26-B08\ M26-B08A\ M26-B08B\ M26-B08C\ M26-B08S\ M26-B08T\ X25-Q02 |
| 6754 | 3D printing | METHOD FOR GENERATING COMPONENT E.G. BURNER, INVOLVES GENERATING BASE AND TOP PORTIONS OF COMPONENT IN MUTUALLY DIFFERENT METHODS, POSITIONING THE PORTIONS RELATIVE TO EACH OTHER USING IMAGE PROCESSING SYSTEM, AND JOINING THE PORTIONS | CN203419984U | POSSLEY BRIAN D (US) | HARRIS JONATHAN H (US)\ TESCH ROBERT J (US)\ POLESE FRANK J (US)\ NOOTENS STEPHEN P (US)\ DINESCU SORIN (US)\ BRADBURY WILLIAM L (US) | L04-C01\ L04-C12A\ L04-C12B\ L04-C16\ U11-C01J4A\ U11-C03J8C\ U11-C05B5 |
| 6755 | 3D printing | METHOD FOR PREPARING BLOOD VESSEL STENT, INVOLVES SELECTING LASER SINTERING 3D TECHNOLOGY, PROCESSING RAW MATERIAL LAYER, FORMING BLOOD VESSEL BRACKET BY SINTERING FORMING PROCESS, AND SCANNING RAW MATERIAL LAYER BY LASER SCANNING PROCESS | CN104385605A | PRADEN ANNE-MARIE (FR) | INTEL CORP (US) | L04-C11C1\ L04-C12A\ L04-C12E\ L04-C12H\ L04-E01A\ U11-C05B4\ U11-C18A3 |
| 6756 | 3D printing | ALIGNMENT LAYER FOR SUBSTRATE OF DISPLAY DEVICE, HAS FRICTION ALIGNMENT LAYER AND OPTICAL ALIGNMENT LAYER FORMED ALONG DIRECTION FAR AWAY FROM SURFACE OF UPPER BASE PLATE, WHERE LAYERS ARE ORIENTED AT ANGLE | CN105055037A | PRADEN ANNE-MARIE (FR) | GEMPLUS (FR) | M13-H01 |
| 6757 | 3D printing | ANNEALING PIERCING PLUG AFTER LASER CLADDING, INVOLVES PERFORMING LASER CLADDING IN PLUG UPPER SURFACE IN TRANSITIONAL ALLOY LAYER, WHERE LASER CLADDING UPPER TRANSITIONAL ALLOY LAYER HIGH STRENGTH ALLOY LAYER | CN105063491A | PRAMANIK DIPANKAR (US)\ MOROZ VICTOR (US) | GEMPLUS (FR) | S03-E04A |
| 6758 | 3D printing | HARDENING LASER PERFORATION HEAD INVOLVES CLADDING TRANSITIONAL ALLOY LAYER OF UPPER SURFACE, WHERE UPPER TRANSITIONAL ALLOY LAYER INCLUDES HIGH STRENGTH ALLOY LAYER, AND TRANSITIONAL ALLOY LAYER WITH NICKEL BASE ALLOY | CN105154871A | PRAMANIK DIPANKAR (US)\ MOROZ VICTOR (US) | SYNOPSYS INC (US)\ PRAMANIK DIPANKAR (US)\ MOROZ VICTOR (US) | M22-D\ X25-A01\ X25-A08 |
| 6759 | 3D printing | DIELECTRIC LAYER MANUFACTURING METHOD, INVOLVES DEPOSITING SILICON NITRIDE LAYER IN FIRST SILICON DIOXIDE LAYER, AND ARRANGING END OF SILICON NITRIDE LAYER ON SECOND DIOXIDE LAYER, AND DEPOSITING THIRD DIOXIDE LAYER ON SECOND DIOXIDE LAYER | CN105055182A | PRAMONO EDDY (US) | SYNOPSYS INC (US) | S02-B08E\ T01-J07D3A\ T01-J10B3A\ T01-S03\ X22-E |
| 6760 | 3D printing | INSULATING COATING LAYER FOR ORGANIC FET, HAS MAIN COATING LAYER ADHERED WITH TWO INSULATING LAYERS, WHERE UPPER SURFACE OF ONE OF INSULATING LAYERS IS ADHERED WITH OTHER INSULATING LAYER BY UTILIZING SPIN COATING SOLUTION | CN105063613A | QIAO QIONGHUA XIAO (CN) | CADENCE DESIGN SYSTEMS INC (US) | T01-J03\ T01-N01D3A\ T01-N02B2B\ T01-S03\ W01-A06A3\ W01-A06B3 |
| 6761 | 3D printing | LASER CLADDING DEVICE COMPRISES MACHINE FRAME, SAW BLADE ROTATING DEVICE, FEEDING DEVICE, LASER, WORKING HEAD, CORNER MOTOR, BLADE SHAFT, BLADE PRESSURE PLATE, AND MACHINE FRAME | CN105112909A | QIU ZHIMING (CN) | SHANGHAI XIANJIN SEMICONDUCTOR (CN) | M22-H03A\ M22-H03B |
| 6762 | 3D printing | ATOM ABSORPTION INSTRUMENT FLAME BURNING HEAD MANUFACTURING METHOD, INVOLVES SECURELY WELDING LOCAL AREA OF PLATFORMS WITH EACH OTHER TO FORM THIN LAYER OF COMBUSTION HEAD FOR OBTAINING ATOM ABSORPTION FLAME BURNING HEAD | CN105039973A | RAMANATHAN SASANGAN (US)\ LEE SANG-HYOEB (US) | QIU ZHIMING (CN) | A11-C\ A12-H\ A12-W12F |
| 6763 | 3D printing | METHOD FOR MANUFACTURING DIESEL ENGINE JAR COVER, INVOLVES PERFORMING JAR COVER SAND TYPE OF LASER CALCINING FAST RAPID PROTOTYPING PROCESS | CN104910609A | RAO SATYAVOLU S PAPA (US)\ GRUNOW STEPHAN (US)\ RUSSELL NOEL M (US) | NOVELLUS SYSTEMS INC (US) | M13-H01\ M23-D05\ M23-H\ V08-A09\ X24-D03 |
| 6764 | 3D printing | MAP IMAGE DISPLAY SYSTEM FOR DISPLAYING MAP IMAGE FOR PROVIDING DRIVING GUIDANCE OF VEHICLE, HAS IMAGE DISPLAY UNIT TO DISPLAY MAP IMAGE OF AREA ON DISPLAY, WHERE DISPLAY-TARGET-OBJECT REDUCTION UNIT DECREASES TARGET OBJECT OF MAP IMAGE | CN104923797A | RAY SUDIPTA K (US)\ COHEN MITCHELL S (US)\ HERRON LESTER WYNN (US)\ INTERRANTE MARIO J (US)\ LOMBARDI THOMAS E (US)\ SHINDE SUBHASH L (US) | TEXAS INSTRUMENTS INC (US) | M25-F\ M25-J\ X25-C |
| 6765 | 3D printing | METHOD FOR SYNCHRONIZING SERVICE LEVEL MONITORING STATUSES OF SET OF APPLIANCES IN CLUSTER, INVOLVES UPDATING TOKEN WITH LOCAL STATISTICS FOR APPLIANCE, AND PASSING TOKEN TO NEXT APPLIANCE THAT IS MOST LIKELY TO REACH THRESHOLD | CN104876587A | RHODES HOWARD E (US) | INTERNAT BUSSINESS MACHINES CO (US) | X24-D03 |
| 6766 | 3D printing | DEVICE FOR STABILIZING POWDER BED FOR MANUFACTURING POWDER-BASED ADDITIVE COMPONENT USED IN PROTOTYPING APPLICATIONS, HAS CONNECTOR THAT IS CONNECTED TO SUCTION PUMP TO PRODUCE LOW PRESSURE SO THAT POWDER BED IS PRESSED AGAINST FILTER | CN105132912A | RHODES HOWARD E (US) | MICRON TECHNOLOGY INC (US) | L03-H03\ L04-A01A\ L04-A02B1\ L04-C11C1\ L04-D\ L04-E01\ L04-E08\ U11-C01B\ U11-C01J1\ U11-C01J3A\ U11-C01J4A\ U11-C01J6A\ U11-C05B2\ U11-C05B5\ U11-C05C3\ W01-C01D3C |
| 6767 | 3D printing | COMPOSITE PORTION FOR USE IN GAS TURBINE BURNER, COMPRISES CHANNEL THAT BECOMES NARROWER ALONG TAPERED PORTION THAT HAS PRIMARY SECTION AND SECONDARY SECTION WITHIN INTERFACE, IN WHICH PRIMARY PORTION AND SECONDARY PORTION ARE EQUIPPED | CN105081313A | RHODES HOWARD E (US) | RHODES HOWARD E (US) | M22-H03B\ M22-H03G\ T01-J07B\ X25-B02A |
| 6768 | 3D printing | BALL VALVE SPHERICAL SURFACE LASER CLADDING LOCATING MECHANISM, HAS DRIVE PIECE FIXED ON UPPER ROTATING FRAME IN TRANSMISSION MANNER, AND ROTATING FRAME FIXED WITH DRIVING UNIT POWER OUTPUT SHAFT | CN104876611A | RIECHERT HENNING (DE)\ WEBER WALTER MICHAEL (DE) | MICRON TECHNOLOGY INC (US) | A11-C05A\ A11-C05C\ A12-H04\ F03-A02\ F04-E05\ J01-F02\ J01-H02 |
| 6769 | 3D printing | SLURRY ON DEMAND CRUCIBLE APPARATUS FOR USE IN SYSTEM FOR PROCESSING SLM AND/OR SSM, HAS SUSCEPTOR STRUCTURE WHOSE PORTS ARE IN FLUIDIC COMMUNICATION WITH PASSAGEWAY TO ALLOW PASSAGE OF GAS/LIQUID BETWEEN PORTS TO EXTRACT CRUCIBLE HEAT | CN105127426A | RIKINO KUNITO US\ SASAKI YASUHIKO (JP)\ YANO KAZUO (JP)\ KATO NAOKI (JP) | QIMONDA AG (DE)\ RIECHERT HENNING (DE)\ WEBER WALTER MICHAEL (DE) | L04-C01\ L04-C03\ L04-C04\ L04-C07\ L04-C07C\ L04-C09B\ L04-C10B\ L04-C10L2\ L04-C12A\ L04-C12B\ L04-C16B\ L04-C18B\ U11-C01J2\ U11-C05B5 |
| 6770 | 3D printing | POWDER FEEDING DEVICE, USEFUL IN LASER CLADDING WELDING DEVICE, COMPRISES POWDER RESERVOIRS, FIRST POWDER CONVEYING DEVICE TO TRANSPORT POWDER TO SECOND RESERVOIR, AND SECOND POWDER CONVEYING DEVICE TO TRANSPORT POWDER TO TRANSFER LOCATION | CN204867410U | RO HYUN TAE (KR)\ SEOK KYUNG SOO (KR) | RENESAS TECH CORP (JP)\ HITACHI DEVICE ENG (JP) | M13-H01\ M13-L\ X25-A03A |
| 6771 | 3D printing | SEMICONDUCTOR APPARATUS USED IN E.G. POWER SUPPLY APPARATUS, INCLUDES SUBSTRATE, BUFFER LAYER FORMED OF ALUMINUM GALLIUM NITRIDE, STRAINED LAYER SUPPERLATTICE BUFFER LAYER FORMED OF LATTICE LAYERS, AND ELECTRON TRANSIT AND SUPPLY LAYERS | CN105108144A | ROBERT GAUTHIER US\ JUNJUN LI US\ SOUVICK MITRA US\ STEPHEN PUTNAM CHRISTOPHER US\ MAHMOUD MOUSA US | RO HYUN TAE (KR) | A11-A\ A11-A03\ A11-B01\ A11-B14\ A12-L02A\ A12-W12F\ A12-W14\ X25-A01 |
| 6772 | 3D printing | MAKING ELECTRICAL INDUCTOR COMPRISES FORMING ELECTRICAL INDUCTOR FROM ELECTRICALLY CONDUCTIVE MATERIAL BY LAYER-BY-LAYER PRODUCTION PROCESS INCLUDING SELECTIVE LASER MELTING, SPRAY FORMING AND/OR LASER WIRE SPRAYING | CN105112907A | ROBERTS CEREDIG (US)\ MOULI CHNDRA V US | IBM (US)\ IBM UK (GB)\ LI JUNJUN (US)\ MITRA SOUVICK (US)\ GAUTHIER JR ROBERT (US)\ PUTNAM CHRISTOPHER STEPHEN (US)\ MOUSA MAHMOUD (US) | D08-A03\ S05-E |
| 6773 | 3D printing | CONDUCTIVE FILTER MEDIUM FOR OPERATING ENVIRONMENTS NEEDING STATIC ELECTRICITY DISSIPATION TO REDUCE IGNITION SOURCES, HAS SUBSTRATE LAYER, CONDUCTIVE SCRIM AND TOP LAYER MANUFACTURED INTO FILTER MEDIUM BY MECHANICAL WEB FORMATION | CN105112908A | ROIZIN YAKOV (IL)\ PIKHAY EVGENY (IL)\ NAVEH ISHAI (US) | MOULI CHANDRA V (US)\ ROBERTS CEREDIG (US) | M13-H01\ M24-A05B\ M24-D01A\ M24-D02A\ M24-D02B\ M24-D04\ M24-D06\ M27-A04\ M27-A04C\ M27-A04M\ M27-A04N\ M27-A04S\ M27-A04T\ M27-A04V\ M27-C03\ X25-A02C\ X25-Q02 |
| 6774 | 3D printing | POLYCRYSTALLINE SILICON LAYER MANUFACTURING METHOD, INVOLVES FORMING AMORPHOUS SILICON LAYER ON BASE PLATE, DIVIDING AMORPHOUS SILICON LAYER INTO MULTIPLE AREAS, AND FORMING MULTIPLE CRYSTAL LAYERS BY AMORPHOUS SILICON LAYER | CN105039676A | ROIZIN YAKOV (IL)\ PIKHAY EVGENY (IL)\ NAVEH ISHAI (US) | TOWER SEMICONDUCTOR LTD (IL)\ PIKHAY EVGENY (IL)\ GUTMAN MICHA (IL)\ ROIZIN YAKOV (IL) | M13-H01 |
| 6775 | 3D printing | CORROSION-PROOF HYDRAULIC MANUFACTURING DEVICE COMPRISES MACHINE TOOL, HYDRAULIC-PRESSURE ROD BLANK SURFACE TURNING UNIT AND HYDRAULIC ROD SEMI-FINISHED PRODUCTS TAKING MACHINE LATHE CLAMP FOR MACHINING SEMI-FINISHED PRODUCTS | CN204851917U | ROIZIN YAKOV (IL)\ PIKHAY EVGENY (IL)\ NAVEH ISHAI (US) | TOWER SEMICONDUCTOR LTD (IL)\ PIKHAY EVGENY (IL)\ GUTMAN MICHA (IL)\ ROIZIN YAKOV (IL) | D09-D\ S06-B06B\ T01-N01E\ T04-M03\ T04-M05\ X25-A08A\ X25-A08U2 |
| 6776 | 3D printing | NANO-LEVEL COMPONENTS LASER SINTERING MOLDING DEVICE, HAS BUFFER CAVITY PROVIDED WITH CAVITY BODY IN BOTTOM WALL BY ARC TRANSITION FROM UPPER END TO LOWER END, AND CON-FOCAL LASER GROUP LOCATED IN AIR DYNAMICS LENS AND WORKTABLE | CN105112708A | ROSENBERG ROBERT (US)\ CHIRAS STEFANIE RUTH (US)\ LANE MICHAEL WAYNE (US)\ MALHOTRA SANDRA GUY (US)\ MC FEELY FENTON REED (US)\ SAMBUCETTI CARLOS JUAN (US)\ VEREECKEN PHILIPPE MARK (US) | TOWER SEMICONDUCTOR LTD (IL)\ PIKHAY EVGENY (IL)\ GUTMAN MICHA (IL)\ ROIZIN YAKOV (IL) | M13-H01 |
| 6777 | 3D printing | MANUFACTURING METHOD OF INDIVIDUAL DENTAL ABUTMENT FOR PATIENT WITH DENTITION DEFECT, INVOLVES TAKING TABLE MODEL SATISFYING PROPERTY OF DENTAL ABUTMENT MATERIAL FOR THREE-DIMENSIONAL SLICE DELAMINATION PROCESS | CN105132913A | ROSSNAGEL BRULEY JOHN CARRUTHE (US) | IBM (US) | M13-H01\ M22-H01\ M22-H02\ M27-A01\ M27-A01B\ M27-A01C\ M27-A01J\ M27-A01M\ M27-A01N\ M27-A01S\ M27-A04\ M27-A04B\ M27-A04C\ M27-A04M\ M27-A04N\ M27-A04S\ M27-C02 |
| 6778 | 3D printing | MANUFACTURE OF RADIAL FORGING MACHINE INVOLVES FORGING MATRIX MATERIAL, ANNEALING, AIR-COOLING, OIL-QUENCHING, TEMPERING, GRINDING MATRIX MATERIAL, CARRYING OUT LASER CLADDING PROCESS, ADDING TO VACUUM OVEN, HEAT PRESERVING AND COOLING | CN105112906A | ROY FRANCOIS (FR)\ TOURNIER ARNAUD (FR) | IBM (US) | M13-H01\ M13-L\ T01-J08F\ V08-A02 |
| 6779 | 3D printing | PREPARATION OF TITANIUM ALUMINUM BASE ALLOY GRADIENT COMBUSTION RETARDANT MATERIAL BY PREPARING FLAME RETARDANT MATERIAL, USING LASER CLADDING METHOD TO PREPARE TITANIUM ALLOY SUBSTRATE, AND TREATING FLAME RETARDANT LAYER IN ARGON GAS | CN105088226A | ROY FRANCOIS (FR)\ TOURNIER ARNAUD (FR)\ MARCELLIER YANN (FR) | ST MICROELECTRONICS SA (FR) | A05-G01B\ A08-A03\ A08-A06\ A08-R01\ A11-B16\ A11-C02B\ X25-A08C2\ X25-A09 |
| 6780 | 3D printing | THREE-DIMENSIONAL PRINTING METHOD FOR MANUFACTURING NURSING FILM, INVOLVES SCANNING NURSING FILM PORTION, PROCESSING DATA, PREPARING MEMBRANE MATERIALS AND MIXING FILM MATERIAL THOROUGHLY INTO 3D PRINTER | CN105060734A | ROYTER YAKOV I (US)\ RAJAVEL RAJESH D (US)\ IONOV STANISLAV I (US) | ST MICROELECTRONICS SA (FR) | M22-G03M\ M22-H01\ M22-H02\ M26-A01\ M26-A01N\ M26-B08\ X25-A08 |
| 6781 | 3D printing | PREPARATION OF TITANIUM ALLOY WEAR-RESISTANT COATING INVOLVES UNIFORMLY MIXING CERAMIC POWDER AND NICKEL-BASED SELF-FLUXING ALLOY POWDER, VACUUM-DRYING OBTAINED CLADDING POWDER, SUBJECTING TO SCANNING TRACK, AND SURFACE CLADDING | CN105039972A | ROZBICKI ROBERT (US)\ KAILASAM SRIDHAR (US)\ YU CHENTAO (US)\ HAYDEN DOUGLAS (US) | HRL LAB LLC (US)\ ROYTER YAKOV (US)\ RAJAVEL RAJESH D (US)\ IONOVA IRINA (US)\ IONOVA SOPHI (US) | A10-E05B\ A12-W12G\ L02-A02\ L02-A03\ L02-A04\ L02-A12\ L02-G03 |
| 6782 | 3D printing | LASER CLADDING COATING COMPRISES IRON-BASED ALLOY POWDER, CHROMIUM CARBIDE ALLOY POWDER, AND CERIUM (IV) OXIDE POWDER | CN105112910A | ROZBICKI ROBERT (US)\ KAILASAM SRIDHAR (US)\ YU CHENTAO (US)\ HAYDEN DOUGLAS (US) | NOVELLUS SYSTEMS INC (US) | M13-H |
| 6783 | 3D printing | HARD SEALING LAYER AND FORMING METHOD USING SIDE AXIS POWDERING PROCESS FOR PUMP BODY INVOLVES LASER IRRADIATION, POWDER SPRAYING AND LASER MELTING | CN105088225A | ROZBICKI ROBERT (US)\ KAILASAM SRIDHAR (US)\ YU CHENTAO (US)\ HAYDEN DOUGLAS (US) | NOVELLUS SYSTEMS INC (US) | A03-A00A\ A03-C02\ A11-B05\ A12-W12F\ M13-H01\ M22-H02\ M27-A04\ M27-A04B\ M27-A04C\ M27-A04N\ M27-A04S |
| 6784 | 3D printing | POLYURETHANE COMPOSITE MATERIAL USED FOR THREE-DIMENSIONAL PRINTING IN E.G. LASER SINTERING, IS PREPARED BY MIXING POLYURETHANE, INORGANIC FILLER, LIGHT STABILIZER AND ANTIOXIDANT, AND HAS AVERAGE PARTICLE DIAMETER OF PRESET VALUE | CN104934610A | RU HUANG (CN)\ YU CAO US\ XIAORUI CUI US\ JINZE YIN US\ ZIJUN WEI US\ HUABO ZHAO US\ QINQIN WEI (CN)\ YUNYI FU (CN)\ XING ZHANG (CN) | NOVELLUS SYSTEMS INC (US) | L02-A02\ L02-A03\ L02-A04\ L02-G07A\ L02-K\ L03-B02J\ L03-G06\ L03-G07\ X25-B\ X25-H03 |
| 6785 | 3D printing | PREPARATION OF NICKEL-BASED ALLOY POWDER INVOLVES SMELTING RAW MATERIAL ALLOY, MELTING, SPRAYING RESULTANT MOLTEN ALLOY, ATOMIZING, SOLIDIFYING, OBTAINING ALLOY POWDER, SIEVING AND PERFORMING SELECTIVE LASER MELTING PROCESS | CN204224703U | RUIPENG YANG US\ JIAXIANG NIE US\ XIANGTAO KONG US\ JIONGPING LU US | UNIV BEIJING (CN) | M22-H01\ M22-H03B\ U23-Q\ X25-A08A\ X25-A08C3 |
| 6786 | 3D printing | PREPARATION OF ANTI-PURPLE HALO TRANSPARENT CERAMIC PANEL INVOLVES MOLDING MIXED SLURRY PREPARED USING CERAMIC POWDER, SINTERING AID, BINDER, DISPERSING AGENT AND RARE-EARTH OXIDE, DEGREASING OBTAINED CERAMIC WHITEWARE, AND SINTERING | CN204842978U | RYO KANDA (JP)\ SHIGEAKI OKAWA (JP)\ KAZUHIRO YOSHITAKA (JP) | SEMICONDUCTOR MFG INT SHANGHAI (CN) | M22-H03A\ M22-H03B\ X25-A01\ X25-A08B |
| 6787 | 3D printing | EXTERNAL MOBILE SCANNING SYSTEM COMPRISES OUTSIDE COVER COMPONENT ON PLATFORM, WHICH IS LOCATED IN MOLDING CHAMBER AND SYNCHRONOUS MULTI-STATION LASER SCANNING GALVANOMETER THAT CONTAINS DUSTPROOF COVER, PROTECTION LENS AND LIGHT PATH | CN204825050U | RYO KANDA (JP)\ SHIGEAKI OKAWA (JP)\ KAZUHIRO YOSHITAKE (JP) | SANYO ELECTRIC CO (JP) | M22-H03B\ X25-A08A |
| 6788 | 3D printing | PREPARATION OF DOUBLE-COATED ORGANIC IRON-BASED ALLOY POWDER LASER CLADDING LAYER BY DISSOLVING ROSIN TO ANHYDROUS ETHANOL, WRAPPING IRON-BASED ALLOY POWDER, HEATING INDUSTRIAL STARCH IN PURIFIED WATER, STIRRING AND BALL-MILLING | CN204825051U | RYO SUI US\ RIN MEISHO US\ RIN ISHO US | SANYO ELECTRIC CO (JP) | M13-H01\ W06-C01 |
| 6789 | 3D printing | PREPARATION OF GRAPHENE-REINFORCED CERAMIC USED FOR E.G., SENSORS, INVOLVES DISPERSING MIXTURE OF CERAMIC POWDER, SINTERING AID, ETHANOL AND TITANATE-MODIFIED GRAPHENE PREPARED USING TITANATE, WATER AND GRAPHENE POWDER AND MILLING | CN204825052U | SADAKI FUTAGI (JP)\ SEIGO NAKAO (JP)\ DAICHI IMAMURA (JP) | LIAW CHORNG-WEI (TW)\ LIN MING-JANG (TW)\ LIN WEI-JYE (TW) | M13-H01\ M13-H04A\ M13-L\ M22-H02 |
| 6790 | 3D printing | SINTERING THREE-DIMENSIONAL STRUCTURE INVOLVES STRUCTURING POWDER MATERIAL, WHICH COMPRISES CAVITY FORMED IN AMORPHOUS STRUCTURE, WHERE POWDER MATERIAL IS SPREAD LAYER BY LAYER BY USING SELECTIVE LASER SINTERING | CN204779670U | SAITO TAKASHI US | PANASONIC CORP (JP)\ PANASONIC CORP (US) | M13-H01\ M13-L\ M24-D06\ T01-J08A\ V08-A02 |
| 6791 | 3D printing | DESKTOP SELECTIVE LASER MELTING MOLDING DEVICE COMPRISES A FORMING PLATFORM THAT IS FIXEDLY SET IN A CLOSED FORMING CHAMBER CAVITY, AND EXTRACTING DEVICE THAT IS PROVIDED WITH BASE AND GETTING POWDER CYLINDER, WHICH IS SET WITH FIXING PART | CN204779671U | SAITO TATSUYUKI (JP)\ NOGUCHI JUNJI (JP)\ OHASHI TADASHI (JP) | FUJI ELECTRIC CO LTD (JP)\ SAITO MASARU (JP) |  |
| 6792 | 3D printing | FORMING METHOD FOR NON-METAL PORTION FREE OF LASER CLADDING INVOLVES FORMING FORMING UNIT USING BRANCH STRUCTURE FROM SUBSTRATE OF TO-BE-FORMING UNIT, AND OBTAINING FORMING UNIT | CN105060941A | SAKAJIRI KYOHEI (US)\ COBB BAILEY (US) | RENESAS TECH CORP (JP) | M22-H01\ M22-H02\ M22-H03B\ M22-H03G\ M26-A01\ M26-A01A\ M26-B09\ M29-C\ T01-J07B3\ T01-N01D2\ X25-A02\ X25-A08A\ X25-A08C1\ X25-Q02 |
| 6793 | 3D printing | PREPARATION OF IN-SITU SYNTHESIZED TITANIUM BORIDE/TITANIUM CARBIDE ENHANCED TITANIUM NITRIDE/TITANIUM NITRIDE DUAL PHASE METAL COMPOUND BASED COATING BY GRINDING NICKEL-BASED ALLOY POWDER AND CARBIDE CERAMIC POWDER, AND LASER CLADDING | CN104911585A | SAKAMOTO HIDEO (JP) | MENTOR GRAPHICS CORP (US) | M13-H01\ M22-H02\ M22-H03G |
| 6794 | 3D printing | LASER CLADDING TUNGSTEN CARBIDE CERAMIC PARTICLE ENHANCED METAL BASE COATING COMPRISES ALLOY POWDER INCLUDING TUNGSTEN POWDER AND GRAPHITE | CN104911581A | SAKAMOTO TOSHITSUGU (JP) | RENESAS ELECTRONICS CORP (JP)\ SAKAMOTO HIDEO (JP) | M13-H01 |
| 6795 | 3D printing | LASER STRENGTHENING OF COMPOSITE REINFORCED METAL MATERIAL BY PROCESSING METAL MATERIAL, COATING METAL MATERIAL WITH METAL PLATING LAYER, FIXING PLATED METAL MATERIAL ON STAGE, AND UNIFORMLY LAYING METAL POWDER TO COATED METAL SURFACE | CN105132911A | SAKAMOTO TOSHITSUGU (JP)\ KAWAURA HISAO (JP) | NEC CORP (JP)\ SAKAMOTO TOSHITSUGU (JP) | M13-H01\ M22-H02\ M27-A04 |
| 6796 | 3D printing | BOLT INSTALLATION STRUCTURE, HAS SLS TECHNOLOGY PROCESS PROVIDED WITH COMPONENT MAIN BODY, MOUNTING BASE WHOSE OUTER WALL IS CONNECTED WITH CLAMPING PIECE THAT IS MOUNTED WITH CLAMPING SLOT, AND HOLE PART FORMED WITH CLAMPING SLOT | CN204779813U | SAKAMOTO TOSHITSUGU (JP)\ KAWAURA HISAO (JP)\ SUNAMURA HIROSHI (JP)\ INOUE NAOYA (JP) | NEC CORP (JP)\ SAKAMOTO TOSHITSUGU (JP)\ KAWAURA HISAO (JP) | L01-G04 |
| 6797 | 3D printing | RAPIDLY PRODUCING LASER REMELTING SCANNING CARBIDE DISPERSION REINFORCED ALUMINUM ALLOY COMPRISES E.G. CONVERTING THREE-DIMENSION COMPONENT INTO STEREOLITHOGRAPHY FORMAT, FORMING ON SUBSTRATE, QUENCHING AND CERAMIC BEAD BLAST TREATMENT | CN105033251A | SAKANO YOSHIO US | NEC CORP (JP)\ SAKAMOTO TOSHITSUGU (JP)\ KAWAURA HISAO (JP)\ SUNAMURA HIROSHI (JP)\ INOUE NAOYA (JP) | M13-H01\ M26-B08 |
| 6798 | 3D printing | LASER CLADDING ALLOY POWDER FOR HOT-DIE REPAIR-REMANUFACTURE, CONTAINS ELECTROLYTIC MANGANESE, FERROSILICON, FERROTITANIUM, METAL CHROMIUM, FERROCHROME, GRAPHITE, FERROVANADIUM, FERROMOLYBDENUM, TUNGSTEN POWDER, AND WASTE STEEL | CN104985183A | SAKURAI NAOKI US | RENESAS TECH CORP (JP) | M13-H01 |
| 6799 | 3D printing | PIPE INNER WALL LASER CLADDING DEVICE COMPRISES DRIVING DEVICE, CONNECTING PLATE INSTALLED ON OUTPUT SHAFT OF DRIVING DEVICE, INNER CHUCK, OUTER CHUCK, PARALLEL FRAMES, BALL LEAD SCREW, GUIDE SHAFT AND SUPPORTING DEVICE | CN104846368A | SANG WOO KYU (KR) | HITACHI LTD (JP) | L03-G05\ L03-X\ L04-C18A\ L04-D09\ T01-J08A |
| 6800 | 3D printing | REMANUFACTURE OF TITANIUM SPONGE REACTION TANK OUTER WALL COMPRISES TAKING TITANIUM SPONGE REACTION TANK, SCREENING FOR CRACKS, PEELING SURFACE, REMOVING CRACKS, PRE-HEATING, INJECTING CLADDING POWDER, AND LASER CLADDING | CN104805141A | SANO KOICHIRO US | LG DISPLAY CO LTD (KR) | L03-A02B\ L03-A02G\ L03-E01B3\ L03-E01B5B\ L03-E08B\ L03-H05\ X16-B01F1\ X16-E01C1\ X16-E01G\ X16-E01J |
| 6801 | 3D printing | ANTI-UV RADIATION, ANTI-ELECTROSTATIC COMPOSITE FILM LAYER MANUFACTURING METHOD BY COATING ANTI-ULTRAVIOLET COMPOSITE FILM LAYER ON GLASS SURFACE, COATING WITH ALUMINUM TITANIUM OXIDE, SILICON OXIDE, AND TITANIUM OXIDE FILM LAYERS | CN104831274A | SASAKI UICHIRO US\ SAKURAI YASUHIRO US | MATSUSHITA ELECTRIC IND CO LTD (JP) | M13-H01\ M13-L\ S02-A10C\ X25-A03C1 |
| 6802 | 3D printing | WEARABLE STEEL PIPE PLUG COMPRISES MAIN BODY THAT IS SET WITH NICKEL BASE ALLOY LAYER, WHERE TOP SURFACE OF WEARABLE STEEL PIPE PLUG IS SHAPED , AND IS PERFORMED WITH SURFACE LASER CLADDING PROCESS | CN105132824A | SATO HIDEFUMI US\ NAKANISHI TAKASHI US | COSMO ASSOCIE KK US | M22-H03B\ X25-A08 |
| 6803 | 3D printing | LASER CLADDING METHOD COMPRISES LASER CLADDING OLD COLUMN SURFACE OF HYDRAULIC PRESSURE BRACKET FOR LASER CLADDING WITH OUTPUT POWER OF LASER AND SCANNING LINEAR VELOCITY | CN105132909A | SATO JUNPEI US | TOSHIBA CORP (JP) | L03-G02\ M13-H01\ V07-F01A1\ V07-F02A\ V07-G10D\ W01-A07H4 |
| 6804 | 3D printing | MECHANICAL IMPACT DEVICE USED FOR REDUCING LASER CLADDING CRACK INCLUDES IMPACT HAMMER, SPRING, FIXED SLEEVE, CAM GROOVE, NUMERICAL CONTROL DEVICE, POWDER FEEDING LASER CLADDING HIGH POWER SEMICONDUCTOR DEVICE AND MECHANICAL IMPACT ASSEMBLY | CN105132915A | SATO KIYOSHI US\ KIKUIRI KATSUYA US\ KOBAYASHI KIYOSHI US\ UTSU YOSHITAKA US | TOSHIBA KK (JP)\ SATO JUMPEI (JP) | M13-C01\ M13-H |
| 6805 | 3D printing | PREPARATION OF SELF-SUPPORTING FLEXIBLE COMPOSITE ELECTRODE MATERIAL USED FOR LITHIUM ION BATTERY, INVOLVES USING TRANSITION METAL COMPLEX NANOFIBER, SOLVENT, AQUEOUS CARBON NANOTUBE DISPERSION AND GRAPHENE OXIDE SOLUTION | CN105018926A | SATO SHINYA (JP)\ SAIKI TAKAYUKI (JP)\ TAKAMIYA HIROYUKI (JP) | ALPS ELECTRIC CO LTD (JP) | M13-H01 |
| 6806 | 3D printing | THREE-DIMENSIONAL MEASUREMENT DEVICE USED FOR LASER CLADDING COMPRISES MOVABLE PLATFORM WHICH IS LOCATED ON MEASURING MECHANISM HAVING TWO SIDES RESPECTIVELY PROVIDED WITH LASER CLADDING MACHINE AND LASER MILLING MACHINE | CN105014070A | SATO TAKAO US\ CHIBA TOSHIHARU US\ INABA HISATO (JP) | SEIKO EPSON CORP (JP) | M24-D02A\ M24-D07\ M24-E |
| 6807 | 3D printing | LASER SINTERING HIGH PRECISION THREE DIMENSIONAL PRINTER COMPRISES AN UPPER AND BOTTOM PLATE CONNECTED WITH A LEAD SCREW BOLT, THROUGH LOWER BEARING BASE WHERE A PISTON PLATE AND OUTER SIDE CABIN IS MATCHED AND SET WITH O-TYPE SEALING RING | CN105154872A | SATOSHI SAKAI (JP)\ DAICHI MATSUMOTO (JP)\ KATSUYUKI ASAKA (JP)\ MASATOSHI HASEGAWA (JP)\ KAZUTAKA MORI (JP) | RENESAS TECH CORP (JP) | M24-D02A |
| 6808 | 3D printing | INNER HOLE FOR LASER CLADDING, COMPRISES PROTECTING LENS SET IN LASER BEAM ON LIGHT OUTLET PORT, AND LASER BEAM OUTLET WHICH IS SET IN LASER HEAD MAIN BODY WITH FRONT END AT SIDE OF LASER HEAD MAIN BODY FRONT END | CN105118787A | SCHAEPKENS MARC FLANAGAN KEVIN (US) | RENESAS TECH CORP (US) | A04-F06E\ A12-W12G\ D09-C\ L02-A03\ L02-B05\ L02-G01A\ L02-G01B1\ L02-G01C1\ L02-G12E1\ L02-J02C |
| 6809 | 3D printing | CORROSION RESISTANT DEVICE, COMPRISES A CORROSION RESISTANCE POWDER SPRAYING ROOM IN WHICH A BURNING ROOM IS CONNECTED TO THE LASER GRINDING ROOM, A LASER SINTERING IS CONNECTED TO A SURFACE GRINDER | CN105132916A | SCHEER HEINER (DE)\ GUENSCHEL HARALD (DE)\ DIEHL LOTHAR (DE)\ SCHEFFEL MARCUS (DE)\ SEILER THOMAS (DE)\ DOTTERWEICH THOMAS (DE) | SCHAEPKENS MARC (US)\ FLANAGAN KEVIN WARNER (US) | M13-H01\ M13-L |
| 6810 | 3D printing | FEEDING DEVICE, COMPRISES A MOVING PIECE THAT IS CONNECTED WITH A BUFFER COMPONENT WHICH IS PROVIDED WITH A SENDING POWDER PIPE AND A ROTATING HEAD, A ROTATING HEAD IS SET WITH AN END IN THE FEEDING PIPE | CN105039869A | SCHUELEIN MARK E (US) | BOSCH GMBH ROBERT (DE) | M13-H01\ M22-H02 |
| 6811 | 3D printing | DEVICE FOR STAMPING VEHICLE SURFACE, COMPRISES LEFT WORKING CAVITY AND RIGHT WORKING CAVITY THAT ARE PROVIDED WITH SPRAY GUN AND ROTATING PLATE CONNECTED WITH ROTATING MOTOR, WHERE THE LEFT WORKING CAVITY IS SET WITH THE FIRST CONTROL BASE | CN104760296A | SCHULZE HANS-JOACHIM (DE)\ HILLE FRANK (DE)\ RUPP THOMAS (AT) | INTEL CORP (US) | M13-H\ M13-L\ M22-H03A\ M22-H03B\ X25-A01 |
| 6812 | 3D printing | DEVICE FOR STAMPING VEHICLE SURFACE, COMPRISES WORKING CHAMBER PROVIDED WITH FIRST AND SECOND MOUNTING TRACK, WHERE THE UPPER PART OF FIRST MOUNTING PATHWAY IS CONNECTED WITH FIRST ROTATING ELECTRICAL MACHINE LOCATED IN WORKING CHAMBER | RU2568600C1 | SCHWARZ UWE (DE)\ SCHMIDT KONSTANZE (DE)\ HOELZER GISBERT (DE)\ WIEL APPO VAN DER (BE) | INFINEON TECHNOLOGIES AG (DE) | M13-H01\ M13-L |
| 6813 | 3D printing | PREPARATION OF CERAMIC COMPOSITE COATING INVOLVES TAKING COATING COMPONENTS INCLUDING NANO ALUMINA, MAGNESIUM OXIDE, SILICON CARBIDE CERAMIC, NANO SILVER NITRATE, TITANIUM CARBIDE, YTTRIUM OXIDE, ZIRCONIUM SILICATE, AND SODIUM SILICATE | CN105154869A | SCOTT T BECKER US | X FAB SEMICONDUCTOR FOUNDRIES (DE) | M22-H03B\ X25-A08B |
| 6814 | 3D printing | PREPARATION OF COMPOSITE LINER COVER INVOLVES SPREADING METAL OR ALLOY POWDER ON OUTER SIDE OF PRETREATED LINER BY LASER CLADDING OR CARRYING OUT MATERIAL CLADDING THROUGH LASER AFTER SPREADING METAL OR ALLOY POWDER | CN104959598A | SEIGO NAKAO (JP)\ DAICHI IMAMURA (JP) | TELA INNOVATIONS INC (US)\ BECKER SCOTT T (US)\ MALI JIM (US)\ LAMBERT CAROLE (US) | M22-H02\ D09-C01D\ S03-E06B3A\ S05-D02A1\ T01-J06A\ T01-J10B2\ T01-J15X\ T01-N01D2 |
| 6815 | 3D printing | COPPER-CONTAINING HIGH-ENTROPY ALLOY COATING WITH LIQUID PHASE SEPARATION TISSUE FOR IRON-BASED MATERIAL, COMPRISES HIGH-ENTROPY ALLOY COMPOSITION CONTAINING METAL/NONMETAL ELEMENTS, WHERE METAL ELEMENT(S) IS COPPER, AND YTTRIUM OXIDE | CN104961881A | SEKIGUCHI KANETAKA US | PANASONIC IP CORP AMERICA (US) | M13-H01\ M13-L |
| 6816 | 3D printing | CONTINUOUS-TYPE SELECTIVE LASER MELTING (SLM) DEVICE, HAS FORMING SHAPED PLATFORM THAT IS DRIVEN ALONG HORIZONTAL MOTION PLATFORM DRIVING MECHANISM, SUCH THAT PLATFORM IS CONNECTED TO DRIVE MECHANISM AND CONTROL SYSTEM | CN104878382A | SEKIGUCHI KANETAKA US | CITIZEN FINETECH MIYOTA CO LTD US | A04-F01A\ A12-W11L\ D05-A01A2\ D05-A01B3\ D05-C19\ E10-C04J2P\ E11-M |
| 6817 | 3D printing | LASER CLADDING MACHINE FOR SENDING POWDER PIPELINE THREE-WAY PIPE DEVICE COMPRISES THREE-WAY PIPE DEVICE MAIN BODY WITH THREE-WAY PIPE OF AIR INLET, DRILLED DOWN AIR OUTLET, THREE-WAY PIPE HOLE, AND CONTROL ONE-WAY VALVE STRUCTURE | CN104975288A | SEOL HYUN SU (KR) | CITIZEN MIYOTA CO LTD US | M13-H01\ M13-L\ M22-H01 |
| 6818 | 3D printing | SELECTIVE LASER MELTING AND FORMING DEVICE FOR DOUBLE PISTON USED IN ADDITIVE MANUFACTURING INCLUDES MAIN MACHINE BODY, AUXILIARY MACHINE BODY, CYLINDER BODY, SCRAPER GUIDE AND LIGHT PATH SYSTEM | CN104962778A | SHAOFENG DING US\ XINPING QU US\ FEI CHEN US\ YULONG JIANG US\ GUOPING RU US | SAMSUNG ELECTRONICS CO LTD (KR) | M13-H01\ M22-H01\ M27-A01\ M27-A01B\ M27-A01C\ M27-A01N\ M27-A01S\ M27-A04\ M27-A04B\ M27-A04C\ M27-A04N\ M27-A04S\ M27-C02 |
| 6819 | 3D printing | PREPARING BONE IMPLANTS FOR TREATING THE BONE DEFECT PATIENT, INVOLVES USING COMPUTERIZED TOMOGRAPHY SCANS IN PATIENTS TO OBTAIN CROSS-SECTIONAL IMAGES OF BONES AND SAVING AS DIGITAL IMAGING AND COMMUNICATIONS IN MEDICINE | CN104910616A | SHAOFENG DING US\ XINPING QU US\ QI XIE US | UNIV FUDAN (CN) | M13-H01\ M26-B08\ M26-B08C\ M26-B08M\ M26-B08S |
| 6820 | 3D printing | LASER CLADDING BASED CORROSION RESISTANT ALUMINUM ALLOY TWO-DIMENSION CODE MARKING METHOD INVOLVES CARRYING OUT MARKING PROCESS ON ALUMINUM ALLOY TEST PORTION TO MARK DM CODE | CN104962906A | SHAU JENG-JYE (US) | UNIV FUDAN (CN) | M13-H01 |
| 6821 | 3D printing | SECONDARY ENZYMATIC HYDROLYSIS PREPARATION OF LOW ENERGY SLS STRUCTURE LIPID USING LIPASE NOVOZYM 435 FIRST-STAGE SOYBEAN OIL COMPRISES REACTING LIPASE NOVOZYM 435 AND SOYBEAN OIL/ANHYDROUS ETHANOL (SUBSTRATE), AND GAS PHASE CHROMATOGRAPHY | CN104923788A | SHIBAYAMA AKINORI (JP) | Empty Field US | M13-H01\ M13-L\ M26-B08\ V08-A01C\ V08-A04C\ X24-B01\ X24-B06\ X24-C09 |
| 6822 | 3D printing | LASER HOT-MELT CLADDING METHOD OF OIL PIPE INVOLVES CLEANING OIL AND RUST ON OIL PIPE SURFACE, PERFORMING FLAW DETECTION ON CLEANED PIPE SURFACE, AND LASER-CLADDING PIPE WITH CUBIC BORON NITRIDE-ALUMINUM OXIDE NANO POWDER | CN104923789A | SHIBAYAMA AKINORI (JP) | SHIBAYAMA AKINORI (JP) | M22-H03B\ X25-A08A\ X25-A08C3 |
| 6823 | 3D printing | MARTENSITIC IRON BASED ALLOY POWDER FOR LASER CLADDING LAYER WITH HIGH HARDNESS AND ANTI-CRACKING ABILITY, COMPRISES SPECIFIED AMOUNT OF CHROMIUM, NICKEL, BORON, SILICON, CARBON AND IRON | CN104947103A | SHIGEAKI OHKAWA (JP)\ HIGICHIRO OKINO (JP) | MATSUSHITA ELECTRIC IND CO LTD (JP) | M13-H01\ M26-A\ M26-A00C\ M26-A00N\ M26-A00V\ M26-B08\ M26-B08C\ M26-B08V |
| 6824 | 3D printing | CRANKSHAFT REPAIRING LASER CLADDING MATERIAL USED FOR INTERNAL COMBUSTION ENGINE, COMPRISES ALLOY POWDER COMPRISING CARBON, SILICON, MANGANESE, SULFUR, PHOSPHORUS, NICKEL, CERIUM, NODULIZER AND GRAPHITE AGENT, AND COBALT | CN105127423A | SHIMA MASASHI (JP)\ SHIMAMUNE YOSUKE (JP)\ HATADA AKIYOSHI (JP)\ KATAKAMI AKIRA (JP)\ TAMURA NAOYOSHI (JP) | SENYO ELECTRIC CO LTD (JP) | L03-J02\ L04-C07B\ L04-C11C\ L04-C16\ L04-C17\ U11-C07A1\ U11-C07A4\ X24-D03\ X25-A02\ X25-A03B2 |
| 6825 | 3D printing | BALL VALVE MAIN SURFACE LOCATING DEVICE FOR USE DURING LASER CLADDING PROCESS, HAS SUPPORTING PLATFORM WHICH IS FIXEDLY PROVIDED WITH SPECIFIC ELECTRIC MACHINE CONNECTED WITH ROTOR DRIVE GEAR OF ROTATING SECTION AT UPPER PORTION | CN105128040A | SHIMA MASASHI (JP)\ SHIMAMUNE YOSUKE (JP)\ HATADA AKIYOSHI (JP)\ KATAKAMI AKIRA (JP)\ TAMURA NAOYOSHI (JP)\ OHTA HIROYUKI (JP) | FUJITSU LTD (JP) | M13-H01\ M22-H01\ M27-A01\ M27-A01C\ M27-A01J\ M27-A01N\ M27-A01S\ M27-A01V\ M27-A04\ M27-A04C\ M27-A04N\ M27-A04S\ M27-A04V |
| 6826 | 3D printing | REPAIRING SHIP TURBINE ROTOR INVOLVES CARRYING OUT HEAT RESISTANT MICRO-ARC WELDING TECHNOLOGY, REPAIRING ROTOR DISK DAMAGE DEFECTS OF TURBINE ROTOR, TRIMMING WORK-PIECE SURFACE TO OBTAIN SMOOTH SURFACE AND REMOVING OIL FROM SURFACE | CN105132910A | SHIMAMOTO SATOSHI US | FUJITSU LTD (JP) | M13-H01\ M22-H01\ M22-H03G\ M27-A01\ M27-A01C\ M27-A01J\ M27-A01M\ M27-A01N\ M27-A01S\ M27-A04\ M27-A04C\ M27-A04M\ M27-A04N\ M27-A04S\ M27-C01\ M27-C02\ M27-D01 |
| 6827 | 3D printing | SELECTIVE LASER SINTERING METHOD OF 3-DIMENSIONAL PRINT COMPRISES SELECTIVE BURNING OF MATERIAL IN POWDER LAYER USING LASER, DETERMINING BURNING AREA OF REDUNDANT ADHESIVE, AND REMOVING REDUNDANT ADHESIVE USING ANOTHER LASER | CN105136085A | SHIMIZU MASAHIRO (JP)\ TANAKA YOSHINORI (JP)\ ARIMA HIDEAKI (JP) | RENESAS TECH CORP (JP) | A05-F03\ A08-M01C\ A08-M01D\ A08-M09C\ A08-R\ A08-S02\ A09-A01A\ A11-A02C\ A11-A03\ A11-A04\ A11-B01\ X25-A08C2\ X25-A08C3\ X25-A08M2 |
| 6828 | 3D printing | PREPARING NICKEL-BASED ALLOY GRADIENT MATERIAL FOR PREPARING TITANIUM ALLOY, BY PREPARING NICKEL-BASED ALLOY AND INTERMEDIATE LAYER POWDER MATERIAL AND FORMING VANADIUM-CHROMIUM ALLOY LAYER ON TITANIUM ALLOY SUBSTRATE BY LASER CLADDING | CN105081567A | SHIMIZU TORU US | RENESAS TECH CORP (JP) |  |
| 6829 | 3D printing | PROCESSING ELECTRONIC PRODUCT BY LASER SINTERING OF COPPER INVOLVES FRONTAL-ETCHING COPPER PLATE, SOLID-CRYSTALLIZING, WIRE BONDING AND PRESS-MOLDING PLATE, COPPER-SINTERING MOLDED PRODUCT, AND CUTTING | CN105088005A | SHIN JAW-JUNG (TW)\ LIN BURN JENG (TW)\ LIU PEI-YI (TW)\ LIN SHY-JAY (TW)\ WANG WEN-CHUAN (TW) | SEIKO INSTR INC (US) | M13-H01\ M13-L\ T01-E01B |
| 6830 | 3D printing | TRAIN AXLE LASER CLADDING REPAIR ALLOY POWDER COMPRISES CARBON, CHROMIUM, NICKEL, BORON, SILICON, MANGANESE, VANADIUM, RARE EARTH LANTHANUM AND CERIUM, AND IRON | CN105063615A | SHIN JIN-HYUN (KR)\ YUN JAE-SUN (KR) | TAIWAN SEMICONDUCTOR MFG (TW)\ LIU PEI-YI (TW)\ LIN SHY-JAY (TW)\ WANG WEN-CHUAN (TW)\ SHIN JAW-JUNG (TW)\ LIN BURN JENG (TW) | M22-H03B\ T01-J07B\ X25-A08A |
| 6831 | 3D printing | ALLOY POWDER USEFUL IN LASER CLADDING FOR REMANUFACTURING FV520B STAINLESS STEEL COMPONENT AND REPAIRING SURFACE DAMAGE, CONTAINS CARBON, CHROMIUM, NICKEL, MOLYBDENUM, MANGANESE, NIOBIUM, COPPER, SILICON, BORON AND IRON | CN105057666A | SHINDO MASAO US\ KANEKO SHINICHI US\ NODA MASAAKI US | SAMSUNG ELECTRONICS CO LTD (KR) | A05-G01A\ A05-G01B\ A10-D\ A11-B01\ A11-B16\ X25-A08C3\ X25-A08C4\ X25-A09\ X25-H |
| 6832 | 3D printing | PREPARATION OF THERMAL-CONDUCTIVE FUNCTIONAL MATERIAL INVOLVES MIXING COUPLING AGENT-ALCOHOL SOLUTION WITH THERMAL-CONDUCTIVE FILLER POWDER, ADDING NYLON, EXTRUDING, PERFORMING SELECTIVE LASER SINTERING PROCESS AND HEAT TREATING | CN105002491A | SHINGO EGUCHI US | MATSUSHITA ELECTRIC IND CO LTD (JP) | M13-H01\ M22-H\ M22-H02\ M26-B08\ M26-B08C |
| 6833 | 3D printing | GAS TURBINE ENGINE COOLED BLADE | CN105002492A | SHINICHI NAGANO (JP) | SEMICONDUCTOR ENERGY LAB (JP)\ EGUCHI SHINGO (JP) | L03-B02A4\ L03-J01\ L03-X\ L04-A01A\ L04-C18\ L04-D09\ M27-A04\ M27-A04C\ M27-A04M\ M27-A04N\ M27-A04S\ M27-C02\ V02-A01A9\ V02-A02A9\ X25-A02E\ X25-A08A\ X25-Q01 |
| 6834 | 3D printing | LASER CLADDING SCANNING ROUTE OBTAINING METHOD INVOLVES OBTAINING NODE OF CHARACTERISTIC POINT INFORMATION OF ROUTE CONTROLLER, AND CONTROLLING LASER CLADDING WORK HEAD BY PATH CONTROLLER ACCORDING TO APPROXIMATION PROCESS | CN105002493A | SHINOHARA HIROFUMI (JP)\ NISHIDA YUKIO (JP)\ HORITA KATSUYUKI (JP)\ YAMASHITA TOMOHIRO (JP)\ ODA HIDEKAZU (JP) | ALPS ELECTRIC CO LTD (JP) | M13-H01\ M26-A\ M26-A00C\ M26-A00T\ M26-A00V\ M26-B06\ M26-B06C\ M26-B06V |
| 6835 | 3D printing | LASER SINTERING RAPID MANUFACTURING METHOD BASED ON POWER CHARACTERISTIC OF DECOMPOSING FILL BY ESTABLISHING THREE-DIMENSION MODEL, EXTRACTING SHELL THICKNESS AREA, COMPARING, PERFORMING DYNAMIC ANALYSIS, AND SELECTING FILLING ELEMENT | CN204703963U | SHINOHARA TSUNEO US | SHINOHARA HIROFUMI (JP)\ NISHIDA YUKIO (JP)\ HORITA KATSUYUKI (JP)\ YAMASHITA TOMOHIRO (JP)\ ODA HIDEKAZU (JP) | A05-F03\ A08-A06\ A08-M01D\ A08-M10\ A08-P01\ A08-S01\ A08-S02\ A09-D03\ A10-E18\ A11-A03\ X25-A08C3 |
| 6836 | 3D printing | THREE-DIMENSIONAL PRINTING DYNAMIC BOND-CONTAINING POLYURETHANE MATERIAL USED FOR MANUFACTURING E.G. SELECTIVE LASER SINTERING TECHNOLOGY INCLUDES DIISOCYANATE, POLYESTERPOLYOL/POLYETHER POLYOL AND DIISOCYANATE TRIMER CROSSLINKING AGENT | CN204712462U | SHINOHARA TSUNEO US | SHARP KK (JP) | M13-H01 |
| 6837 | 3D printing | LASER CLADDING ALLOY POWDER COMPRISES TITANIUM CARBIDE, FREE CARBON, AND NICKEL MATERIAL | CN105118772A | SHINOHARA TSUNEO US | SHARP KK (JP) | M22-H01\ M22-H03A\ M22-H03B\ X25-A01\ X25-A08B |
| 6838 | 3D printing | MANUFACTURE OF STRAIGHTENING ROLLER BY STRAIGHTENING ROLLER FOR DISASSEMBLY, ROLLER AND ROLLER NECK REAR SIDE TURNING, PREPARING METAL CERAMIC ALLOY POWDER, LASER CLADDING, DRAWING AND STRAIGHTENING ROLLER AND PRECISION STRAIGHTENING | CN105118900A | SHINPO HIROYUKI US\ NISHIMURA HIDETOSHI (JP) | SHARP KK (JP) | M22-G03J\ M22-H03A\ M22-H03B\ M22-H03E\ T01-J07B\ T06-A07A\ T06-D09\ X25-A01\ X25-A08B |
| 6839 | 3D printing | TITANIUM ALLOY COMPRISES VANADIUM AT HIGH CONTENT, CHROMIUM, AND TITANIUM AND IMPURITIES | CN204869693U | SHIRAISHI TAKANORI US | SHIMBO HIROYUKI (JP)\ NISHIMURA HIDETOSHI (JP) | M13-H01\ M22-H02\ M26-A\ M26-A00C\ M26-B08 |
| 6840 | 3D printing | LOW TEMPERATURE NYLON POWDER MATERIAL USED FOR SELECTIVE LASER SINTERING INCLUDES DODECANEDIOIC ACID, DEUTERATED TRIFLUOROACETIC ACID, ANTIOXIDANT, SOLVENT, NUCLEATING AGENT, PLASTICIZER AND SILANE COUPLING AGENT | JP2015229265A | SHOJI IZURU US | KYOCERA MITA CORP US | M22-H03B\ X25-A08B |
| 6841 | 3D printing | LASER SPOT REPAIR SYSTEM COMPRISES PROCESSING PLATFORM THAT IS SET WITH POWER MACHINE AND CARRIER ROLLER, WHERE POWER MACHINE SET WITH CHUCK, AND OTHER END OF POWER MACHINE IS SET WITH SUPPORTING ROLLER | US2015360288A1 | SHUAI CHENG US | DENSO CORP (JP) | M13-C01\ M13-H01\ M23-D05\ X24-D03\ X25-A02\ X25-A03B2 |
| 6842 | 3D printing | SINGLE CYLINDER SELECTIVE LASER MELTING DESKTOP FORMING APPARATUS INCLUDES MOLDING PLATFORMS, MOBILE PLATFORMS, LASERS, OPTICAL TRANSMISSION DEVICE, FORMING SUBSTRATE, POWDER CONTAINING DEVICE, DUSTING DEVICE, AND EXTRACTING DEVICE | CN204842975U | SHUE SHAU-LIN (TW)\ HUANG CHENG-LIN (TW)\ HSIEH CHING-HUA (TW) | SILERGY SEMICONDUCTOR TECHNOLOGY HANGZHOU (CN)\ SILERGY SEMICONDUCTOR TECHNOLOGY HANGZHOU LTD (CN) | M13-H01 |
| 6843 | 3D printing | SELECTIVE LASER MELTING COUPLING SHOCK EQUIPMENT INCLUDES SELECTIVE LASER MELTING SHOP INCLUDING MOLDING SYSTEM AND COMPUTER CONTROL SYSTEM, AND ULTRASONIC SHOCK WAVE TRANSMITTER | DE102014210986A1 | SHUE SHAU-LIN (TW)\ WU CHII-MING (TW)\ PENG CHAO-HSIEN (TW) | TAIWAN SEMICONDUCTOR MFG (TW) | M13-H01\ M13-L\ S02-A10B |
| 6844 | 3D printing | PETROLEUM DRILL LASER CLADDING WEAR-RESISTANT, CORROSION-PROOF COATING MATERIAL COMPRISES COBALT-BASED POWDER, ALUMINUM OXIDE AND LANTHANUM OXIDE | CN105061989A | SIM SANG PIL (KR)\ LEE CHANG HYUN (KR)\ LEE SEUNG KEUN (KR) | TAIWAN SEMICONDUCTOR MFG (US) | M23-D05\ M23-G\ M23-H\ X24-D03 |
| 6845 | 3D printing | SELECTIVE LASER MELTING THIRD DIMENSION PRINT DEVICE HAS UPPER FIRST AND SECOND WORKBENCH THAT IS EQUIPPED WITH WORKBENCH WITH EACH OF INDEPENDENT GALVANOMETER SYSTEM | CN104999180A | SIMON ANDREW H (US)\ BONILLA GRISELDA (US)\ CHANDA KAUSHIK (US)\ FILIPPI RONALD G (US)\ GRUNOW STEPHAN (US)\ WANG PING-CHUAN (US)\ HU CHAO-KUN (US)\ LUSTIG NAFTALI E (US) | SAMSUNG ELECTRONICS CO LTD (KR) | M13-H01\ M22-H03G\ M26-A\ M26-A00A\ M26-A00C\ M26-B03\ M26-B03A |
| 6846 | 3D printing | METHOD OF MANUFACTURING TITANIUM ALLOY LASER CLADDING HIGH-SPEED FOOD CUTTING TIP, INVOLVES FORMING BLADE EDGE LAYER BY LASER CLADDING HIGH-HARDNESS TOOL STEEL ON NICKEL ALLOY LAYER | CN104959600A | SING VIRAHAMPAL US\ HIROSE YUTAKA US | IBM (US)\ SIMON ANDREW H (US)\ BONILLA GRISELDA (US)\ CHANDA KAUSHIK (US)\ FILIPPI RONALD G (US)\ GRUNOW STEPHAN (US)\ WANG PING-CHUAN (US)\ HU CHAO-KUN (US)\ LUSTIG NAFTALI E (US) | M13-H01\ M13-L\ T01-E01B |
| 6847 | 3D printing | STATIC MAGNETIC FIELD-ASSISTED LASER CLADDING METHOD INVOLVES ADJUSTING DIRECT CURRENT POWER SOURCE TO OBTAIN CONSTANT MAGNETIC FIELD, AFTER LASER BEAM IS IRRADIATED ON CLADDING SUBSTRATE | CN104962909A | SINGH RANA P (US)\ GRUDOWSKI PAUL A (US)\ JALLEPALLI SRINIVAS (US)\ JEON YONGJOO (US)\ YEAP GEOFFREY C (US)\ BURNETT JAMES D (US) | PANASONIC CORP (JP) | M13-H\ M13-L\ M22-H03A\ M22-H03B\ X25-A01\ X25-A02 |
| 6848 | 3D printing | METHOD FOR MEASURING LASER BEAM AND LASER CLADDING DEVIATION OF POWDER CONE, INVOLVES METALLOGRAPHIC PREPARING OF CROSS-SECTION OF SAMPLE, AND MEASURING OFFSET DELTA OF CLADDING LAYER OF TWO SAMPLES IN TWO ORTHOGONAL DIRECTIONS | CN104943323A | SMAYLING MICHAEL C (US)\ BECKER SCOTT T (US) | Empty Field US | M13-H01\ M13-L |
| 6849 | 3D printing | SMOKE DUST TREATMENT DEVICE FOR SELECTIVE LASER MELTING, HAS CONTROL PIPE JOINT UNIT THAT IS SET IN CONTROL SYSTEM CORRESPONDING TO WORK STATION AND CONTROLLING GAS CIRCULATION SYSTEM WORK | CN104923781A | SOGO SEIJI (JP) | TELA INNOVATIONS INC (US)\ SMAYLING MICHAEL C (US)\ BECKER SCOTT T (US) | M13-H01\ M13-L |
| 6850 | 3D printing | NON-SPARKING ALUMINUM-BRONZE POWDER USED IN LASER CLADDING OF AXIAL FAN IMPELLER TUBE, COMPRISES SPECIFIED AMOUNT OF CARBON, SILICON, MANGANESE, SULFUR, CHROMIUM, NICKEL, COPPER, ALUMINUM AND IRON | CN104907561A | SOLAYAPPAN NARAYAN (US)\ JOSHI VIKRAM (US)\ PAZ DE ARAUJO CARLOS A (US)\ MCMILLAN LARRY D (US) | MATSUSHITA ELECTRONICS CORP (JP) | M13-H01\ M13-L |
| 6851 | 3D printing | LASER CLADDING SCANNING PATH ACQUISITION METHOD FOR REPAIRING FREE CURVED SURFACE, INVOLVES OBTAINING SINGLE DATA ARRAY INPUT IN ROUTE CONTROLLER ACCORDING TO APPROXIMATION PROCESS | CN105140191A | SOLAYAPPAN NARAYAN (US)\ JOSHI VIKRAM (US)\ PAZ DE ARAUJO CARLOS A (US)\ MCMILLAN LARRY D (US) | SYMETRIX CORP (US)\ SOLAYAPPAN NARAYAN (US)\ CELINSKA JOLANTA (US)\ JOSHI VIKRAM (US)\ PAZ DE ARAUJO CARLOS A (US)\ MCMILLAN LARRY D (US) |  |
| 6852 | 3D printing | NON-WELD METAL LATTICE STRUCTURE FORMING METHOD BASED ON SELECTIVE LASER MELTING, INVOLVES SEPARATING SUBSTRATE AND REMOVING SUBSTRATE WITHOUT LASER MELTING OF METAL POWDER MOLDING OF METAL LATTICE | CN105140359A | SONG JUN (SG)\ LO KENG-FOO (SG)\ HUA GUANG-PING (SG) | SYMETRIX CORP (US)\ SOLAYAPPAN NARAYAN (US)\ CELINSKA JOLANTA (US)\ JOSHI VIKRAM (US)\ PAZ DE ARAUJO CARLOS A (US)\ MCMILLAN LARRY D (US) | A09-D03\ A11-B01\ A11-B16\ A12-W12F\ X25-A01\ X25-A08B\ X25-A08M2 |
| 6853 | 3D printing | LASER CLADDING OF CHROMIUM-MOLYBDENUM-VANADIUM COLD WORK DIE STEEL INVOLVES CLEANING SURFACE OF STEEL USING SANDPAPER, CLEANING WITH ALCOHOL TO REMOVE OIL, DRYING NICKEL-CHROMIUM ALLOY, COAXIAL POWDER FEEDING AND LASER CLADDING | US2015370322A1 | SONG MING-HSIANG (TW)\ LEE JAM-WEM (TW) | CHARTERED SEMICONDUCTOR MFG (US) | L04-C02B\ L04-C06A\ L04-C07G\ L04-D04A\ L04-E\ U11-C04A1H\ U11-C04D |
| 6854 | 3D printing | METHOD FOR PREPARING CERAMIC PARTICLES REINFORCED METAL MATRIX COMPOSITE COATING, INVOLVES PROVIDING ALLOY OR METAL CERAMIC COMPOSITE POWDER INTO MIDDLE OF WELD POOL BY COAXIAL POWDER FEEDING NOZZLE, AND CARRYING OUT LASER CLADDING | US2015367576A1 | STAMPER ANTHONY K (US)\ CHINTHAKINDI ANIL K (US)\ COOLBAUGH DOUGLAS D (US)\ COTTE JOHN M (US)\ ESHUN EBENEZER E (US)\ HE ZHONG-XIANG (US)\ WHITE ERIC J (US) | TAIWAN SEMICONDUCTOR MFG (TW)\ SONG MING-HSIANG (TW)\ LEE JAM-WEM (TW) | E31-H04\ L03-H05\ L04-C02\ L04-C11\ L04-C16A\ L04-E03A\ U11-C01J3A\ U11-C01J6\ U11-C02J1A\ U11-C02J2\ U11-D02B\ U12-A01A1A\ U12-E01B2 |
| 6855 | 3D printing | MULTI-WAY LAP JOINT LASER CLADDING PROCESS BASED DIFFERENT WIDTH DAMAGE GROOVE REPAIRING METHOD INVOLVES CONTROLLING LASER LIGHT SPOT SIZE BY UNCHANGING OTHER PARAMETERS SUCH THAT WIDTH OF DAMAGE GROOVE IS CHANGED | CN204852780U | STAMPER ANTHONY K (US)\ GAMBINO JEFFREY P (US)\ MCDEVITT THOMAS L (US)\ HILL WILLIAM (US)\ WINSLOW ARTHUR C (US)\ ZWONIK ROBERT (US)\ MCAVEY KENNETH E JR (US) | IBM (US)\ CHINTHAKINDI ANIL K (US)\ COOLBAUGH DOUGLAS D (US)\ COTTE JOHN M (US)\ ESHUN EBENEZER E (US)\ HE ZHONG-XIANG (US)\ STAMPER ANTHONY K (US)\ WHITE ERIC J (US) | A11-B01\ A11-B16\ A11-C07\ X25-A08B\ X25-A08M2 |
| 6856 | 3D printing | INDUSTRIAL DUAL-REAR PRESSURE STEAM TURBINE FINAL-STAGE BLADE, HAS MOVABLE BLADE FIXED WITH BLADE ROOT THAT IS FIXED WITH FORMING STRUCTURE, AND TIP SHROUD RING FIXED WITH VARIABLE SECTION TORSION PART AND FOUR-FORK-TYPE BLADE ROOT | EP2957935A1 | STAUF GREGORY T (US)\ HENDRIX BRYAN C (US)\ ROEDER JEFFREY F (US)\ CHEN ING-SHIN (US) | STAMPER ANTHONY K (US)\ MCAVEY KENNETH E JR (US)\ GAMBINO JEFFREY P (US)\ HILL WILLIAM (US)\ MCDEVITT THOMAS L (US)\ WINSLOW ARTHUR C (US)\ ZWONIK ROBERT (US) | A11-C01A\ A12-T04D |
| 6857 | 3D printing | THREE-DIMENSIONAL PRINTER COMPRISES Z-AXIS GUIDE RAIL, FIRST Y-SHAFT GUIDE RAIL, FIRST DRIVING DEVICE INSTALLED ON Z-AXIS GUIDE RAIL, X-AXIS GUIDE RAIL PROVIDED ALONG THIRD DIRECTION OF FIRST Y-AXIS GUIDE RAIL, AND SECOND DRIVE DEVICE | JP2015230745A | STEEGEN AN L (US)\ WANG YUN-YU (US)\ KU VICTOR (US)\ NARAYANAN VIJAY (US)\ JAMISON PAUL C (US)\ LI YING (US)\ WONG KWONG H (US)\ CABRAL JR CYRIL (US) | ADVANCED TECH MATERIALS (US) | D09-C01\ M22-H03B\ M22-H03C\ T01-S03\ X25-A08A |
| 6858 | 3D printing | SEMICONDUCTOR DEVICE ION INJECTION BARRIER LAYER MANUFACTURING METHOD, INVOLVES FORMING ANTI-REFLECTION LAYER ON SEMICONDUCTOR SUBSTRATE, AND PERFORMING ETCHING PROCESS TO REMOVE RESIDUAL THICKENED PART | US2015362622A1 | STUHLFELNER FRIEDBERT (DE) | IBM (US)\ STEEGEN AN L (US)\ WANG YUN YU (US)\ KU VICTOR (US)\ WONG KWONG HON (US)\ LI YING (US)\ NARAYANAN VIJAY (US)\ CABRAL CYRIL JR (US)\ JAMISON PAUL (US) | A09-D03\ A11-A\ A11-B01\ A11-B14\ A11-B16\ A12-L02A\ A12-L03\ A12-L05\ A12-W12F\ X25-A01\ X25-A08C3\ X25-A08M2 |
| 6859 | 3D printing | GROWING GALLIUM NITRIDE BASED LED EPITAXIAL WAFERS, COMPRISES PLACING CARRYING OUT HIGH TEMPERATURE PURIFICATION OF SUBSTRATE, GROWING E.G. GALLIUM NITRIDE BUFFER LAYER, NON-DOPED GALLIUM NITRIDE LAYER AND N-TYPE GALLIUM NITRIDE LAYER | CN105141565A | SU CHIEN-CHANG (TW)\ TSAI CHUN HSIUNG (TW)\ LIU SU-HAO (TW)\ CHAN CHIEN-TAI (TW)\ WONG KING-YUEN (TW) | CONTINENTAL AUTOMOTIVE GMBH (DE) | U12-B03F1A\ U12-B03F1B |
| 6860 | 3D printing | SEALED SELECTIVE LASER MELTING DEVICE, HAS POWDER CYLINDER WHOSE LOWER PART IS FIXED WITH POWDER BED, TRANSITION FRAME CONNECTED WITH STEEL BELT, AND AUXILIARY CHAMBER AND ISOLATION DEVICE THAT ARE CONNECTED WITH EACH OTHER | CN105142342A | SUKEKAWA MITSUNARI (JP) | TAIWAN SEMICONDUCTOR MFG (TW)\ SU CHIEN-CHANG (TW)\ TSAI CHUN HSIUNG (TW)\ LIU SU-HAO (TW)\ CHAN CHIEN-TAI (TW)\ WONG KING-YUEN (TW) | A05-J10\ A08-R\ A11-A03\ A11-A04\ X25-A08A\ X25-A08C3 |
| 6861 | 3D printing | JOINT STRUCTURE OF RESIN COMPONENT AND METAL COMPONENT FOR MANUFACTURE OF MOTOR VEHICLE COMPONENTS, HAS SILANE COUPLING AGENT FORMED IN LAYERED FORM OVER WHOLE AREA FACING THERMOPLASTIC ELASTOMER AMONG SURFACES OF RESIN COMPONENT | CN204818061U | SUMIKAWA TAKASHI (JP)\ YAMASHITA KYOJI (JP)\ MOTOJIMA DAI (JP) | ELPIDA MEMORY INC (JP) | T01-J08A\ X24-D03\ X25-A08 |
| 6862 | 3D printing | ADDITIVE MANUFACTURING METHOD OF MULTILAYERED PORTION E.G. PROSTHETICS, PANEL INVOLVES FORMING MULTILAYERED PORTION USING ADDITIVE MANUFACTURING BASED ON OPTIMIZED POWDER MATERIAL AND OPTIMIZED LAYERS CONSTITUTING MULTILAYERED PORTION | CN105093701A | SUMMERFELT SCOTT R (US)\ AGGARWAL SANJEEV (US)\ CHI CHIU (US)\ SAKODA TOMOJUKI (JP)\ MOISE IV THEODORE S (US) | MATSUSHITA ELECTRIC IND CO LTD (JP) | E11-Q03J\ E31-D02\ J04-C04A\ J04-F\ M22-H03B\ M22-H03F\ S03-E02 |
| 6863 | 3D printing | SELECTIVE LASER SINTERING THREE DIMENSIONAL PRINTER PASSES LASER GENERATED BY LASER GENERATOR SEQUENTIALLY INTO FIRST REFLECTION MIRROR, SECOND REFLECTION MIRROR, THIRD REFLECTING MIRROR AND FOCUSING LENS | CN204770665U | SUMMERFELT SCOTT R (US)\ BONIFIELD THOMAS D (US) | TEXAS INSTRUMENTS INC (US)\ AGILENT TECHNOLOGIES INC (US) | A05-A01E4\ A11-B05B1\ A11-C04E\ A12-B01L\ M13-B\ M13-C01\ M13-H01 |
| 6864 | 3D printing | MICROMECHANICAL LAYER ARRANGEMENT, HAS INDEPENDENT STRUCTURED MECHANICAL ACTIVE FUNCTION LAYERS VERTICALLY ARRANGED AND FUNCTIONALLY COUPLED WITH EACH OTHER, WHERE FUNCTION LAYERS COMPRISE SPRING ELEMENT AND REFLECTIVE COATING | DE102014211174A1 | SUMMERFELT SCOTT R (US)\ CELII FRANCIS G (US)\ SAKODA TOMOYUKI (JP)\ CHI CHIU (US) | TEXAS INSTRUMENTS INC (US) | A08-R03A\ A11-B09C\ A12-S08C\ A12-S08E\ A12-S08F\ A12-S09 |
| 6865 | 3D printing | PREPARING POLYETHER ETHER KETONE/NANO-HYDROXYAPATITE COMPOSITE MATERIAL USING SELECTIVE LASER SINTERING, COMPRISES E.G. ADDING POLYETHER ETHER KETONE INTO REACTION PRECURSOR AQUEOUS SOLUTION 1, DISPERSING UNIFORMLY AND STIRRING | WO2015183893A1 | SUMMERFELT SCOTT R (US)\ COLOMBO LUIGI (US)\ AGGARWAL SANJEEV (US)\ MARTIN J SCOTT (US)\ MOISE IV THEODORE S (US) | TEXAS INSTRUMENTS INC (US)\ AGILENT TECHNOLOGIES INC (US) | M22-H03B\ T01-J07B3\ T01-J08A\ X25-A08A |
| 6866 | 3D printing | SYSTEM FOR PRINTING THREE-DIMENSIONAL NANOSECOND-PICOSECOND-FEMTOSECOND LASER COMPOSITE CERAMIC MICRO-STRUCTURE, MONITORS CERAMIC LAYER SIZE, SHAPE AND CRYSTAL PHASE STRUCTURE OF CHEMISTRY COMPONENT THAT CARRIES OUT WORKTABLE MOVEMENT | CN204799942U | SUMMERFELT SCOTT R (US)\ MOISE TED S (US)\ BASIM GUL B (US) | TEXAS INSTRUMENTS INC (US) | M22-H01\ M22-H02\ M22-H03B\ M22-H03F\ A12-W12F\ X25-A08A\ X25-A09 |
| 6867 | 3D printing | PREPARATION OF FLAT-PLATE OXYGEN SENSOR BY FEMTOSECOND LASER COMPOSITE TECHNOLOGY BY LOADING CURRENT LAYER RAW MATERIAL POWDER, PREHEATING UNDER VACUUM ENVIRONMENT, DETERMINING ORIGINAL LASER, SINTERING, MELTING, TEST VERIFYING AND CHECKING | CN204779812U | SUMMERFELT SCOTT R (US)\ SAKODA TOMOYUKI (JP)\ CHI CHIU (US) | TEXAS INSTRUMENTS INC (US)\ SUMMERFELT SCOTT R (US)\ MOISE TED S (US)\ BASIM GUL B (US) | A11-C01C\ A11-C04D\ A12-E04\ A12-E07C\ A12-H08\ L03-H05\ L04-C07\ L04-C13A\ L04-C17D1\ L04-C20A\ U11-C07A4\ U11-C07C2 |
| 6868 | 3D printing | PREPARING ANTI-FRICTION AND WEAR RESISTANT COATING FOR METAL SUBSTRATE SURFACE, INVOLVES MIXING WEAR RESISTANT POWDER AND METAL POWDER MASS TO OBTAIN CLADDING POWDER, WHERE WEAR RESISTANT POWDER IS TUNGSTEN CARBIDE POWDER | CN204779814U | SUN QINGQING (CN)\ ZHANG WEI (CN)\ LU HONGLIANG US\ ZHANG YUAN US\ ZHU SHANGBIN US\ GENG YANG US | TEXAS INSTRUMENTS INC (US)\ AGILENT TECHNOLOGIES INC (US) | L03-H05\ L04-C02B\ L04-C10D\ L04-E03\ U11-C01J2\ U11-C05C |
| 6869 | 3D printing | METHOD FOR PREPARING CARBON FIBER REINFORCED POLYMER COMPOSITE MATERIAL, INVOLVES LAYING PRE-FORMED CARBON FIBER KNITTING LAYER ON WORKING TABLE, AND DISPERSING POLYMER POWDER, FOLLOWED BY POWDER COMBINING, COOLING AND REPEATING PROCESS | CN204752849U | SUN SEY-SHING (US)\ BURKE PETER A (US)\ PRITCHARD DAVID (DE)\ PRICE DAVID T (US)\ BHATT HEMANSHU D (US)\ MAY CHARLES E (US)\ KWAK BYUNG-SUNG (US)\ ALLMAN DERRYL D (US) | UNIV FUDAN (CN) | T01-C04\ T01-C07C2\ T01-J10B3A |
| 6870 | 3D printing | SELF-PROPAGATING REACTION-BASED 3D PRINTING METHOD INVOLVES BUILDING DIMENSIONAL MEASUREMENT OF COMPONENTS AND MATERIAL ANALYSIS, INPUTTING MEASUREMENT DATA USING 3D MODELING SOFTWARE AND DIVIDING 3D MODEL INTO TWO-DIMENSIONAL CROSS SECTION | CN105117508A | SUNG WOO YONG (KR)\ LEE A RAM (KR)\ CHA TAE WOON (KR)\ KIM TAE GYUN (KR)\ LEE HYOUNG SUB (KR)\ CHAE SEUNG YEON (KR)\ CHOI SANG GUN (KR) | LSI LOGIC CORP (US) | A09-D03\ A11-B09C\ A11-B16\ X25-A08A\ X25-A08C1\ X25-A08C2\ X25-A08C3\ X25-A08M2 |
| 6871 | 3D printing | MANUFACTURING ELECTRICAL CONTACTS INVOLVES MILLING SILVER METAL INGREDIENTS ACCORDING TO COMPOSITION AND RATIO REQUIREMENTS TO FORM COMPOSITE POWDERS | CN104959599A | SUNGLYONG KIM US\ JONGJIB KIM US | SAMSUNG DISPLAY CO LTD (KR) |  |
| 6872 | 3D printing | REDISTRIBUTED LEAD LAYER MANUFACTURING METHOD, INVOLVES FORMING ADHESIVE LAYER ON CARRIER SURFACE, DEPOSITING METAL MATERIAL ON INNER PART OF LEADING-OUT THROUGH HOLE, AND FORMING LEAD LAYER ON SURFACE OF PLASTIC SEAL LAYER | CN104942287A | SUZUKI NOBUYUKI (JP) | FAIRCHILD SEMICONDUCTOR (US)\ KIM SUNGLYONG (US)\ KIM JONGJIB (US) | U14-H01E1\ V07-K01 |
| 6873 | 3D printing | COPPER INDIUM GALLIUM SELENIDE THIN FILM SOLAR ENERGY BATTERY BUFFER LAYER MANUFACTURING METHOD, INVOLVES FORMING CIGS THIN FILM SOLAR ENERGY BATTERY WITH BUFFER LAYER, AND DOPING METAL ION ON INDIUM ABSORB LAYER | CN104914527A | SYUN-MING JANG US\ HUI-LIN CHANG US\ YUNG-CHENG LU US | SUZUKI NOBUYUKI (JP) | L03-B03G2\ L03-E01B\ L03-E08\ L03-H05\ L03-J\ X16-B01A1\ X16-B01A3\ X16-B01F1\ X16-F03A3\ X21-A01D\ X21-A01F\ X21-B01A\ X24-D03 |
| 6874 | 3D printing | METHOD OF OPERATING VIDEO DEVICE, INVOLVES DISPLAYING ONE OR MORE OF MASKED IMAGE PIXELS ON MULTIPLE DISPLAY DEVICES BASED ON HEAD POSITION CHANGE | CN204471997U | TACHIBANA SUGURU (JP)\ KATO TATSUO (JP)\ MORISHITA TOMONARI (JP) | TAIWAN SEMICONDUCTOR MFG (TW) | S03-C09\ T01-J05A1 |
| 6875 | 3D printing | METHOD FOR FABRICATING COMPOSITE PART FROM PREPREG SHEETS USING FUSED DEPOSITION MODELING, INVOLVES DELIVERING MATRIX AND FIBER MATERIAL TO LOCATION OF PRINTER, MELTING MATRIX MATERIAL, AND EMBEDDING FIBER MATERIAL WITHIN MATRIX MATERIAL | CN105105838A | TADASHI CHIBA (JP) | FUJITSU LTD (JP) | W01-A09D\ W02-K07C |
| 6876 | 3D printing | ANTI-CORROSIVE OIL PIPE END ANTI-CORROSION SEALING STRUCTURE, HAS STEEL OIL PIPE BODY FORMED WITH CORROSION RESISTANT METAL ANTI-CORROSIVE LAYER, WHERE END OF STEEL OIL PIPE BODY IS FIXED WITH NON-METAL ANTICORROSIVE COATING PART | CN105108444A | TAGUCHI KAZUO (JP) | OKI ELECTRIC IND CO LTD (JP) | V04-Q02A\ V04-Q30J\ V04-Q30M\ V04-R01A3 |
| 6877 | 3D printing | NANOPHOTONIC DEVICE OF NANOPHOTONIC MATRIX ARRAY E.G. NANOPHOTONIC SPATIAL LIGHT MODULATOR (SLM), HAS NANOSCALE ELEMENT THAT IS SWITCHABLE BETWEEN TWO DIFFERENT STATES AND TWO STATES OF ELEMENT DIFFER BY REFRACTIVE INDEX VALUE | CN204832007U | TAGUCHI TAKAAKI US\ YANO KAZUYA US | SEIKO EPSON CORP (JP) | M22-H03B\ M22-H03C |
| 6878 | 3D printing | ELECTRICAL STORAGE APPARATUS MOUNTED IN VEHICLE E.G. ELECTRIC VEHICLE, HAS ELECTRICALLY-CONDUCTIVE ELEMENT AND CURRENT COLLECTION PORTION GROUP THAT ARE WELDED BY SEVERAL STEP PORTIONS | US2015352122A1 | TAGUCHI YOSHIHIDE (JP) | SEIKO NPC CORP US | U14-K01A1A\ U14-K01A1J |
| 6879 | 3D printing | PERFORMING METHOD FOR FULL-WAVEFIELD INVERSION REQUIRING VISCOACOUSTIC OR VISCOELASTIC WAVE PROPAGATION INVOLVES UPDATING INITIAL GEOPHYSICAL MODEL AND OBTAINING UPDATED GEOPHYSICAL MODEL USING, WITH A COMPUTER, GRADIENT OF COST FUNCTION | CN204805296U | TAKA OHAYASHI (JP)\ HIDEAKI KURATA (JP)\ NAOKI OHAYASHI (JP) | TOSHIBA KK (JP) | L03-G02\ M22-H01\ M22-H03B\ M22-H03E\ M22-H03F\ V07-F01A1\ V07-F01A3A\ V07-K05\ V08-A04C2\ X25-A08B |
| 6880 | 3D printing | METHOD FOR REDUCING ORTHOGONAL FREQUENCY DIVISION MULTIPLEXING SIGNAL PEAK-TO-AVERAGE POWER RATIO SELECTED MAPPING BLOCK, INVOLVES OBTAINING ORIGINAL BIT INFORMATION ACCORDING TO BASE BAND MODULATION PROCESS | WO2015185310A1 | TAKA OHAYASHI (JP)\ HIDEAKI KURATA (JP)\ NAOKI OHAYASHI (JP) | RENESAS TECH CORP (JP)\ HITACHI DEVICE ENG (JP) | M23-D05\ X24-D03 |
| 6881 | 3D printing | CAMERA HEAD MODULE SOFT PROTECTING LAYER MANUFACTURING METHOD, INVOLVES COVERING INK LAYER REGION WITH COVER FILM BY PRESS MOLDING ACCORDING TO SOFT BOARD CAMERA MODULE SIZE REQUIREMENT, AND COVERING PROTECTIVE LAYER WITH CAMERA MODULE | CN204807035U | TAKAHASHI HIROYUKI (JP)\ KITOU RYOUTAKA (JP) | RENESAS TECH CORP (JP)\ HITACHI DEVICE ENG (JP) | A11-B16\ A12-V02\ A12-V03B\ D09-C01F\ S05-G02X\ V08-A09 |
| 6882 | 3D printing | FLOATING COUNTERWEIGHT RESILIENT SEALING DEVICE USED FOR SELECTIVE LASER POWDER MATERIAL SINTERING APPARATUS, HAS SEALING PLATE THAT IS FIXEDLY CONNECTED ON UPPER AND LOWER ELASTIC PRESSING PLATES | EP2952341A1 | TAKAHASHI HIROYUKI (JP)\ YAMANO SEIYA (JP) | RENESAS ELECTRONICS CORP (JP)\ TAKAHASHI HIROYUKI (JP)\ KITOU RYOUTAKA (JP) | M22-H03B\ X25-A08 |
| 6883 | 3D printing | METHOD FOR MANUFACTURING LIQUID CRYSTAL ORIENTATION LAYER OF DISPLAY DEVICE, INVOLVES PERFORMING CURING PROCESS ACCORDING TO ELECTRIC/MAGNETIC FIELD SITE AND LIQUID CRYSTAL ORIENTATION LAYER MANUFACTURING PROCESS | CN204740459U | TAKANO MINORU US | RENESAS ELECTRONICS CORP (JP)\ TAKAHASHI HIROYUKI (JP)\ YAMANO SEIYA (JP) | M13-H01 |
| 6884 | 3D printing | ADDITIVE MANUFACTURING EQUIPMENT FOR SELECTIVE LASER MELTING METAL POWDER, HAS WORKING CYLINDER FORMED WITH PISTON INSTALLED WITH UPPER MOLD BASE PLATE, AND HIGH SPEED GRINDING HEAD MOUNTED ON XY WORKTABLE BY Y-AXIS SLIDE BLOCK | CN204792825U | TAKASE SATORU (JP) | MATSUSHITA ELECTRIC IND CO LTD (JP) | L03-G02\ M13-H01\ M13-L\ V07-F01A1\ X14-C06\ X25-A03A |
| 6885 | 3D printing | WHEEL FOR MOTOR CAR, HAS AMPLIFICATION PORTION WHICH IS MECHANICALLY-COUPLED OR CONNECTED WITH WHEEL TO INCREASE RIGIDITY OF WHEEL, AND PREPARED BY SELECTIVE LASER MELTING AND/OR LASER CLADDING | CN105058549A | TAKASHI OHSAWA (JP) | TOSHIBA KK (JP) | M13-H01 |
| 6886 | 3D printing | STENT FOR MONITORING ENVIRONMENT INTO WHICH IT IS INSERTED IN BODY, HAS RESISTIVE MATERIAL THAT INCLUDES PLASTIC, METAL, CERAMIC, OR COMPOSITE, AND CONDUCTIVE MATERIAL INCLUDES PLASTIC, METAL, CERAMIC, OR COMPOSITE | EP2949768A1 | TAKASHIMA DAIZABURO US | TOSHIBA KK (JP) | T01-G02A2B\ T01-J15X |
| 6887 | 3D printing | LASER SINTERING THREE-DIMENSIONAL PRINTER HAS MOTOR MOUNTING FRAME THAT IS FIXEDLY CONNECTED WITH OPTICAL GRATING RULER THAT IS MOUNTED ON VERTICAL BOTTOM PLATE LOWER END, AND CABIN CASING THAT IS SET IN PISTON PLATE | CN105014976A | TAKASU HIROAKI (JP) | TOSHIBA KK (JP) | M22-H03B\ X25-A02 |
| 6888 | 3D printing | BALL VALVE OF LASER CLADDING AND CLAMPING FIXTURE, HAS LOCKING SCREW THAT IS CONNECTED WITH SPRING GASKET, PRESSURE RING THAT IS CONNECTED WITH DISC SPRING, AND END COVER HOLE THAT IS CONNECTED WITH SCREW THREAD HOLE | CN104894555A | TAKEUCHI KAZUTAKA (JP) | TAKASU HIROAKI (JP) | M22-H03B\ S06-K99C\ X25-A08B\ X25-A08U1 |
| 6889 | 3D printing | LASER CLADDING REPAIRING SYSTEM USED FOR NUCLEAR POWER STATION, HAS FEEDER THAT IS CONNECTED WITH LATHE CHUCK PORTION THROUGH SHAFT JOINT CONNECTED WITH POWER DRIVE DEVICE, AND LASER CLADDING HEAD THAT IS CONNECTED TO OPTICAL FIBER | CN104894557A | TAKIMOTO TAKAHIRO (JP) | FUJITSU SEMICONDUCTOR LTD (JP) | A04-G05\ A05-A01E\ A06-A00E4\ A07-A04A\ A07-A04F\ A08-A06\ A08-F05\ A08-M\ A08-R01\ A08-R05\ A08-R06B\ A11-A03\ A11-B07\ A12-L03A\ A12-S08B\ A12-S08C\ A12-S08D\ V07-F01B4 |
| 6890 | 3D printing | PIPELINE INNER WALL LASER CLADDING DEVICE HAS GUIDE SHAFT THAT IS FIXED ON SUPPORTING DEVICE, AND PARALLEL FRAME THAT IS MOUNTED ON BALL LEAD SCREW AND GUIDE SHAFT OF LASER, AND POWDER SENDING DEVICE THAT IS INSTALLED AT PARALLEL FRAME | CN104890240A | TAMURA MANABU US | SHARP KK (JP) | A05-A01E\ A05-A01E2\ A06-A00E2\ A11-B09A1\ A11-C04A\ A12-E01\ A12-H00H\ A12-S08B\ A12-S08D1\ A12-S08D2\ A12-S08F\ A12-W07F1\ G05-F\ L03-J\ L04-A01A |
| 6891 | 3D printing | SELECTIVE LASER MELTING TECHNOLOGY BASED SCAN PATH GENERATION METHOD, INVOLVES ESTABLISHING OBJECT BUILDING DIGITAL MODEL, OBTAINING SCREEN DATA, AND DETERMINING STARTING POINT OF OBJECT BUILDING DIGITAL MODEL | CN104911562A | TAMURA MASAHIKO US\ YUKI TETSUO US | ALPS ELECTRIC CO LTD (JP) | S05-B01\ S05-D02A1 |
| 6892 | 3D printing | LASER METHOD FOR FORMING CURVED SURFACE ON THIN WALL METAL PORTION, INVOLVES OBTAINING RADIUS OF CURVATURE OF COMPLEX SURFACES OF THIN-WALLED METAL PORTION TO DETERMINE ANGLE BETWEEN LASER HEAD AND COMPLEX SURFACES OF METAL PORTION | CN104911583A | TAN QIANNING US\ QIAO AIGUO US\ LIU BAOSHENG US | HITACHI LTD (JP) | X24-D03 |
| 6893 | 3D printing | THREE DIMENSIONAL JEWELRY PRINTER USED FOR JEWELRY MANUFACTURING, COMPRISES FIVE PART OF MICROCOMPUTER, HELP STAMP PAD, PRINT HEAD, RAW MATERIAL TANK AND LASER SINTERING DEVICE, WHERE MICRO-COMPUTER CARRY OUT PRINT SCHEME | CN104878380A | TANABE AKIRA US | SHENZHEN CHIPSEA TECHNOLOGIES CO LTD US | S03-F08\ X24-D03 |
| 6894 | 3D printing | METHOD FOR MANUFACTURING PROTECTIVE LAYER THAT IS UTILIZED IN OPTICAL CABLE, INVOLVES WEIGHING RAW MATERIALS COMPRISING ORGANIC SILICON RESIN, POLYISOBUTENE, INORGANIC FILLER AND ANTIOXIDANTS FOLLOWED BY KNEADING AND EXTRUDING | CN104862694A | TANABE YOSHIKAZU (JP)\ YAMAMOTO NAOKI (JP)\ MITANI SHINICHIRO (JP)\ HANAOKA HIROKO US | NEC CORP (US) | B05-B02A3\ B06-F02\ B10-A09A\ B12-M11B\ B12-M11C\ B12-M12N\ B12-M12Z\ B14-C01\ C05-B02A3\ C06-F02\ C10-A09A\ C12-M11B\ C12-M11C\ C12-M12N\ C12-M12Z\ C14-C01 |
| 6895 | 3D printing | ENVIRONMENT-FRIENDLY WEAR-RESISTING COMPOSITE PLATE FOR E.G. ELECTRONIC DEVICE, HAS SUBSTRATE LAYER, ENHANCEMENT LAYER, WEARABLE LAYER AND TRANSFER PRINTING SURFACE LAYER WHICH ARE ARRANGED SEQUENTIALLY FROM UPPER SIDE TO LOWER SIDE | CN104862695A | TANAKA KATSUHIKO (JP) | RENESAS TECH CORP (JP) |  |
| 6896 | 3D printing | INDIVIDUAL JAW BONE SURGERY GUIDE PLATE MANUFACTURING METHOD, INVOLVES PERFORMING PATIENT JAW MOLD SCANNING PROCESS BY USING CT SCANNING PROCESS, AND SELECTING INDIVIDUAL JAW BONE SURGERY GUIDE PLATE BY USING SELECTIVE LASER MELTING DEVICE | CN105023836A | TANG SANH DANG (US)\ BRAUN CHRIS (US)\ GOOD FARRELL M (US) | RENESAS ELECTRONICS CORP (JP) | X24-D03\ X24-D09 |
| 6897 | 3D printing | METHOD FOR REPAIRING AND STRENGTHENING LASER CUTTING SURFACE OF HIGH TEMPERATURE SERVICE CUTTING DEVICE, INVOLVES COATING POLISHING-TOOL SURFACE WITH ABSORB LAYER AND RESTRAINT LAYER, AND PERFORMING SURFACE LASER IMPACT PROCESSING OPERATION | CN105092379A | TANG SANH DANG (US)\ BRAUN CHRIS (US)\ GOOD FARRELL M (US) | MICRON TECHNOLOGY INC (US) | S02-A03A\ S02-J04A5 |
| 6898 | 3D printing | LASER CLADDING LAYER INTENSITY MEASURING DEVICE, HAS REGULATING SCREW FIXED WITH LOWER BOX, CUTTING EDGE FIXED WITH LOCATING SUPPORT BARREL, AND CLAMP BOX MATCHED WITH GAP AND GAP OF SUPPORT BARREL | WO2015185502A1 | TANIMOTO YOSHIHIDE US | MICRON TECHNOLOGY INC (US) | X25-A08\ X25-A09 |
| 6899 | 3D printing | TABLET OR CAPSULE USED FOR TREATING PAIN, E.G. OSTEOARTHRITIS PAIN, IN ANIMAL INCLUDING MAN, CONTAINS GIVEN AMOUNT OF MELOXICAM | CN105044966A | TATSUMI KYOKA (JP) | FUNAI ELECTRIC CO US\ LEXMARK INT INC (US) | T06-A04B1\ V08-A03C |
| 6900 | 3D printing | WEARABLE SHAFT WITHOUT SHAFT TILE, HAS SHAFT BODY PROVIDED POWDER METALLURGY LAYER THAT IS PROVIDED WITH BASAL BODY AND WORKING PART, WHERE BASAL BODY AND WORKING PART ARE PROVIDED WITH SHAFT | CN204772942U | TELLKAMP JOHN P (US) | NEC ELECTRONICS CORP (JP) | L04-C10\ L04-C11C\ L04-E05D\ U11-C01J2 |
| 6901 | 3D printing | ASSEMBLY USEFUL FOR LASER CLADDING AND LASER BEAM BUILDUP WELDING, COMPRISES A WORKTOP MOUNTED ON A SPRING AND ADAPTED TO BE EXCITED TO OSCILLATE FOR SUPPORTING A WORKPIECE TO BE PROCESSED DURING THE LASER CLADDING | CN204749284U | TEST HOWARD R (US)\ AMADOR GONZALO (US)\ SUBIDO WILLMAR E (US) | TEXAS INSTRUMENTS INC (US) | T01-J15X\ X25-A08A |
| 6902 | 3D printing | EXPANSION LIGHT SOURCE INTERFEROMETER HAS BEAM EXPANDER THAT IS PROVIDED WITH FIRST LENS AND SECOND LENS, TO-BE-DETECTED MIRROR THAT IS SET WITH BEAM SPLITTER, AND PLANE REFLECTOR THAT IS SET WITH LENS | CN105068277A | THAKUR RANDHIR P S (US)\ SCHUEGRAF KLAUS FLORIAN (US) | TEXAS INSTRUMENTS INC (US) | M22-H02\ M22-H03B\ M22-H03G\ M26-B08\ X25-A08\ X25-H |
| 6903 | 3D printing | GAS TURBINE ENGINE COMPONENT E.G. BLADE OUTER AIR SEAL, FOR USE IN AIRCRAFT, HAS RETENTION INTERFACE FORMED ON SURFACE OF SUBSTRATE AND BY ADDITIVE MANUFACTURING PROCESS, AND THERMAL BARRIER LAYER FORMED ON RETENTION INTERFACE | DE102014007408A1 | THEAN VOON-YEW (US)\ NGUYEN BICH-YEN (US) | MICRON TECHNOLOGY INC (US) | A11-A02\ A11-B01\ A11-B16\ A11-C07\ A12-S09\ X25-A08 |
| 6904 | 3D printing | AUTOMATIC MULTI-WAVE LENGTH LASER WAVELENGTH SWITCHING CONTROL SYSTEM, HAS PHOTOELECTRIC SIGNAL CONVERTER FIXED WITH CONTROL CHIP THAT PROVIDES PHOTOELECTRIC SIGNAL VALUE, AND CONTROL ELECTRIC MACHINE CONNECTED WITH LASER OPTICAL DEVICE | JP2015210922A | TIANHE LI US\ JIANGUO CHEN US | THEAN VOON-YEW (US)\ NGUYEN BICH-YEN (US) | M22-H |
| 6905 | 3D printing | LIGHT TRANSPARENT FILM GENERATION CHIP, HAS PLANE GLASS FORMED WITH FIRST TRANSPARENT ELECTRIC CONDUCTION OXIDE LAYER, AND PHOTOELECTRIC CONVERSION LAYER FORMED WITH SECOND TRANSPARENT ELECTRIC CONDUCTION OXIDE LAYER | CN104966789A | TIKHONOV ROBERT DMITRIEVICH (RU) | SHENZHEN FOUNDER MICROELECTRONICS CO LTD US\ UNIV PEKING FOUNDER GROUP CO (CN) | M13-H01\ M22-D\ X25-A01\ X25-A02 |
| 6906 | 3D printing | 3D PRINT LASER PIEZOELECTRIC CERAMIC PREPARATION METHOD, INVOLVES COATING SINTERING CURING AGENT AFTER CERAMIC SINTERING PROCESS, AND CARRYING OUT POLARIZATION PROCESS AFTER CERAMIC SINTERING PROCESS | WO2015175682A1 | TIKHONOV ROBERT DMITRIEVICH (RU)\ AMELICHEV VLADIMIR VIKTOROVICH (RU)\ BUDANOV VLADIMIR MIKHAJLOVICH (RU)\ GUSEV DMITRIJ VALENTINOVICH (RU)\ SOKOLOV MIKHAIL EHDUARDOVICH (RU)\ SUKHANOV VLADIMIR SERGEEVICH (RU) | TIKHONOV ROBERT DMITRIEVICH (RU) | L03-G02\ L03-H03A\ A09-D03\ A12-L03C\ A12-W12F\ A12-W14\ T01-J07B\ T01-J08A1\ V07-F01A1\ V07-F02A\ V07-K05\ V08-A04C2\ X25-A08M2 |
| 6907 | 3D printing | NICKEL-BASE SUPERALLOY POWDER USED IN ADDITIVE MANUFACTURING OF THREE-DIMENSIONAL ARTICLES, COMPRISES CHEMICAL COMPOSITION HAVING SPECIFIED SIZE DISTRIBUTION AND SPHERICAL MORPHOLOGY AND WEIGHT RATIOS OF ALLOYING ELEMENTS E.G. CARBON | CN104953011A | TODA YUKITOSHI US\ TORII SABURO US | G UCHREZHDENIE NP KOMPLEKS T T (RU) | M13-E07 |
| 6908 | 3D printing | SEALED SELECTIVE LASER MELTING DEVICE, HAS FORMING CHAMBER FIXED TO UPPER PART OF POWDER CYLINDER, AND SCRAPER COMPONENT FIXED WITH TRANSITION FRAME BY SLIDE BLOCK CONNECTION, WHERE FRAME IS FIXED WITH STEEL BELT | CN104906768A | TOLPYGO SERGEY K (US)\ AMPARO DENIS (US)\ HUNT RICHARD (US)\ VIVALDA JOHN (US)\ YOHANNES DANIEL (US) | HITACHI LTD (JP) | M13-H01\ M13-L\ X25-D02A\ X25-F01 |
| 6909 | 3D printing | PREPARATION OF IRON-BASED NICKEL BORON CARBIDE LASER CLADDING MATERIAL INVOLVES TAKING STEEL SUBSTRATE, POLISHING WITH GRIT SANDPAPER, REMOVING SURFACE OIL AND RUST, DRYING NICKEL BORON CARBIDE POWDER AND IRON ALLOY AND LASER CLADDING | CN104905894A | TOLPYGO SERGEY K (US)\ AMPARO DENIS (US)\ HUNT RICHARD (US)\ VIVALDA JOHN (US)\ YOHANNES DANIEL (US) | TOLPYGO SERGEY K (US)\ AMPARO DENIS (US)\ HUNT RICHARD (US)\ VIVALDA JOHN (US)\ YOHANNES DANIEL (US)\ HYPRES INC (US) | M13-H01\ M13-L\ M14-H\ M24-D06\ M24-D07\ M24-E\ M26-B08\ M26-B08B\ M26-B08C\ M26-B08J\ M26-B08S\ M26-B08V\ M27-A04\ M27-A04B\ M27-A04C\ M27-A04M\ M27-A04S\ M27-A04T\ M27-A04V\ M27-C02\ M27-C03\ M27-C04 |
| 6910 | 3D printing | METAL MOLD COMPOSITE MOLDING METHOD INVOLVES PROVIDING MULTI-AXIS LASER FOR LASER CLADDING, PERFORMING LASER CLADDING PROCESS, AND OBTAINING COMPONENT USED FOR FORMING METAL MOLD | CN104807547A | TOMOAKI IKEGAMI US\ HIDETOSHI NISHIMURA US\ KAZUYUKI NAKANISHI US | TOLPYGO SERGEY K (US)\ AMPARO DENIS (US)\ HUNT RICHARD (US)\ VIVALDA JOHN (US)\ YOHANNES DANIEL (US)\ HYPRES INC (US) | M13-H01\ M24-D02A\ M27-A04\ M27-A04C\ M27-A04N |
| 6911 | 3D printing | SYSTEM FOR MANUFACTURING NANO POWDER SELECTIVE LASER MELTING ADDITIVE FOR USE IN AVIATION SPACEFLIGHT AREA, HAS COMPUTER WHICH IS CONNECTED WITH SINGLE MODE OPTICAL FIBER LASER, VIA LASER CONTROL LINE | CN104857557A | TOMOAKI IKEGAMI US\ HIDETOSHI NISHIMURA US\ KAZUYUKI NAKANISHI US | PANASONIC CORP (JP)\ IKEGAMI TOMOAKI (JP)\ NISHIMURA HIDETOSHI (JP)\ NAKANISHI KAZUYUKI (JP) | A03-A04A\ A11-B05\ A12-W12F\ G02-A01\ M13-H01\ M13-H03\ M26-A\ M26-A00A\ M26-A00C\ M26-A00N\ M26-B09\ M26-B09C\ M26-B09N |
| 6912 | 3D printing | METHOD FOR PURIFYING ATOMIC LAYER DEPOSITION (ALD) MACHINE CHAMBER, INVOLVES INTRODUCING HYDROGEN AND OXYGEN INTO CAVITY PORTION OF MACHINE AT SAME TIME WHILE PERFORMING DECOMPRESSION AND TEMPERATURE RISING OF CAVITY PORTION OF MACHINE | CN204586445U | TONOMURA OSAMU (JP)\ MIKI HIROSHI (JP)\ SEKIGUCHI TOMOKO (JP)\ TAKEDA KENICHI (JP) | PANASONIC CORP (JP)\ IKEGAMI TOMOAKI (JP)\ NISHIMURA HIDETOSHI (JP)\ NAKANISHI KAZUYUKI (JP) | U11-C01J1\ U11-C06A1A |
| 6913 | 3D printing | METHOD OF MANUFACTURING SCRAPER CONVEYOR USED IN COAL MINING FIELD, INVOLVES PERFORMING REMANUFACTURING FOR REPAIR AND/OR REPLACEMENT ACCORDING TO AMOUNT OF WEAR FAILURE, TO REPAIR LASER CLADDING | CN104799932A | TORII KENSUKE US | TONOMURA OSAMU (JP)\ MIKI HIROSHI (JP)\ SEKIGUCHI TOMOKO (JP)\ TAKEDA KENICHI (JP) | S03-F02C |
| 6914 | 3D printing | TRAMCAR WHEEL LASER MANUFACTURING AND STRENGTHENING METHOD BY FLAW DETECTING DAMAGED WHEEL, REMOVING DAMAGED LAYER, USING LASER CLADDING METHOD FOR SIZE RECOVERY AND PERFORMING FOLLOW-UP SURFACE STRENGTHENING TREATMENT | CN204773649U | TORII KENSUKE US | SEIKO EPSON CORP (JP) | A09-D03\ A11-B16\ T01-J15X\ T01-L02\ T01-S03\ X25-A08 |
| 6915 | 3D printing | PREPARING REINFORCED TAPPET COLUMN INVOLVES CLEANING FACE LIFTER AND THEN TREATING WITH PHOSPHATE BY HEATING TO IMPROVE STRENGTH | CN204790085U | TORRES JOAQUIM (FR)\ GOSSET LAURENT (FR) | SEIKO EPSON CORP (JP) | U14-K01A1C\ U14-K01A1J |
| 6916 | 3D printing | COMPOSITE COATING COMPRISES COMPOSITE POWDER FORMED BY LASER CLADDING, WHERE COMPOSITE POWDER COMPRISES NICKEL-CHROMIUM ALLOY, CHROMIUM CARBIDE, SILICON POWDER AND ALUMINUM POWDER | CN105067485A | TORRES JOAQUIN (FR)\ GOSSET LAURENT-GEORGES (FR) | KONINKL PHILIPS ELECTRONICS NV (NL)\ ST MICROELECTRONICS CROLLES 2 (FR) | V08-A02 |
| 6917 | 3D printing | POWER DEVICE BACK BUFFER LAYER MANUFACTURING METHOD, INVOLVES FORMING N-LAYER BY USING SINGLE CRYSTAL SILICON CHIP WHILE PERFORMING POLISHING AND CLEANING PROCESS, AND REMOVING EDGE OF SINGLE CRYSTAL SILICON CHIP BY PERFORMING CORROSION | CN105055035A | TORRES JOAQUIN (FR)\ GOSSET LAURENT-GEORGES (FR) | ST MICROELECTRONICS CROLLES 2 (FR) | A11-B03\ A11-B16\ X25-A08 |
| 6918 | 3D printing | LASER CLADDING LAYER AND SUBSTRATE COMBINED STRENGTH EVALUATION METHOD, INVOLVES EVALUATING COMBINED INTENSITY OF LASER CLADDING LAYER AND SUBSTRATE BY COMPARING MAXIMUM STRESS VALUES OF TWO GROUPS | DE102014210119A1 | TORRES JOAQUIN (FR)\ JOURDAN NICOLAS (FR) | ST MICROELECTRONICS CROLLES 2 (FR) | A11-B14\ A11-C04A\ A12-E\ A12-E12\ A12-L03\ A12-V03C2\ S05-G02G3\ W05-A10\ X27-A02D |
| 6919 | 3D printing | LAYERED MANUFACTURING METHOD FOR LAYER-BY-LAYER MANUFACTURING OF THREE-DIMENSIONAL PRODUCT, INVOLVES PROVIDING FILLER WITH FILLER MATERIAL DIFFERING FROM SUPPORT MATERIAL, SUCH THAT SUPPORT STRUCTURE IS FILLED WITH DISCRETE FILLER | DE102015209702A1 | TORRES JOAQUIN (FR)\ JOURDAN NICOLAS (FR)\ GOSSET LAURANT GEORGES (FR) | ST MICROELECTRONICS CROLLES 2 (FR)\ TORRES JOAQUIN (FR)\ JOURDAN NICOLAS (FR) | L02-A02\ L02-A04\ L02-A05\ L02-A08\ A09-D03\ A11-A05\ A11-B03\ A11-B14\ A11-B16\ A12-W12F\ T01-J07B |
| 6920 | 3D printing | METHOD FOR MANUFACTURING PHOTO-RESIST LAYER OF BASE PLATE, INVOLVES CONNECTING COLOR QUANTUM ROD WITH PART OF LONG SHAFT, AND PERFORMING SUBSTRATE MIXED MATERIAL COATING PROCESS ACCORDING TO VACUUM DRYING PROCESS | WO2015181772A1 | TOSHIHIKO TAKIMOTO TAKAHIRO NA (JP) | TORRES JOAQUIN (FR)\ JOURDAN NICOLAS (FR)\ GOSSET LAURANT GEORGES (FR) | L03-B03G2\ L03-E01A\ L03-E01B\ L03-E01C1\ L03-E01D1\ L03-E03\ L03-E08\ L03-H05\ X16-B01F1\ X16-F01C\ X16-F02\ X21-A01D\ X21-A01F\ X21-B01A |
| 6921 | 3D printing | CERAMIC POWDER SINTERING LASER FORMING MACHINE, HAS LIFTING DRIVE MECHANISM AND CYLINDER SCREW THREAD CONNECTION WITH EACH OTHER, AND LASER SINTERING DEVICE AND CYLINDER SCREW THREAD CONNECTION WITH EACH OTHER | CN105047608A | TOSHIKATSU KITAJIMA HIROYASU F (JP) | SHARP KK (JP) | U12-A01A1E |
| 6922 | 3D printing | SLS POWDER 3D PRINTER MAIN CYLINDER PISTON STRUCTURE, HAS FORMING CYLINDER DRIVING PISTON FIXED WITH BALL LEAD SCREW, AND BASE PLATE FORMED WITH LIMITING GROOVE, WHERE FORMING CYLINDER DRIVING PISTON IS FIXED WITH GROUP NEEDLE | CN105034394A | TOUBOU TETSUROU (JP)\ OKAMOTO NANA (JP)\ YANO JUNICHI (JP) | NEC ELECTRONICS CORP (JP) | A04-H00H\ A11-B16\ G06-G05\ X25-A08 |
| 6923 | 3D printing | INTELLIGENT GLASSES APPLYING SYSTEM, HAS INTELLIGENT GLASSES PROVIDED WITH GLASSES FRAME, PRESET JUDGMENT DATA STORAGE SUB MODULE FOR STORING PRESET JUDGMENT DATA, AND PROMPTING MODULE FOR RECEIVING EXCITATION STATE INTERVENTION SIGNAL | RU2569279C2 | TOYAMA HIROSHI (JP)\ NAKAMURA YUKIO (JP) | TOUBOU TETSUROU (JP)\ OKAMOTO NANA (JP)\ YANO JUNICHI (JP) | U11-C01J2\ U11-C05C\ U12-A01A2 |
| 6924 | 3D printing | DEVICE USEFUL E.G. FOR GENERATIVE PRODUCTION OF THREE DIMENSIONAL OBJECTS, COMPRISES HOUSING HAVING A PROCESS CHAMBER IN WHICH BUILDING SPACE OR INTERCHANGEABLE CONTAINER IS ARRANGED, AND MEMORY CHIP READABLE BY ELECTRONIC READING DEVICE | JP2015206061A | TRAN KHANH (US)\ ELLUL JOSEPH P (US)\ SRIVASTAVA ANURANJAN (US)\ IKEUCHI KIYOKO (US)\ BARRY SCOTT WILSON (US) | OKI DATA KK (JP)\ OKI DIGITAL IMAGING CORP US | A11-C01C\ A12-A04B\ A12-F01B |
| 6925 | 3D printing | ELECTRICAL STORAGE APPARATUS E.G. LITHIUM ION SECONDARY BATTERY, FOR E.G. ELECTRIC VEHICLE, HAS RESIN-MADE THICKNESS ADJUSTMENT PART POSITIONED BETWEEN LONG WALL PARTS OF CASE, AND PROVIDED WITH ADDITIVE TO RAISE THERMAL CONDUCTIVITY | CN104916662A | TSAI HAO-YI (TW)\ SUN SHI-CHUNG (TW) | MAXIM INTEGRATED PRODUCTS (US) | D09-C01D\ S05-F |
| 6926 | 3D printing | ELECTRIC CHARGE CONNECTING LAYER OF LAMINATED LAYER ORGANIC LED (OLED) DEVICE, HAS PAIR OF MATERIAL LAYERS PROVIDED WITH HEAVE PORTION AND RECESS, WHICH ARE EXTENDED WITH RESPECT TO EACH OTHER | CN104985182A | TSUCHIDA HIROMI (JP)\ MAEJIMA AKIHIRO (JP)\ KANEDA JINSAKU (JP)\ MAEDA EISAKU (JP) | PROMOS TECHNOLOGIES INC (US) | M23-D05\ M23-G\ S03-A03\ S03-B01\ T01-J07A\ T01-J13\ X24-D03 |
| 6927 | 3D printing | SOLUBLE POLYMERIC MATERIAL USED IN E.G. ADDITIVE MANUFACTURING SYSTEM, COMPRISES THERMOPLASTIC COPOLYMER CONTAINING CARBOXYLIC ACID GROUPS IN WHICH PORTION OF CARBOXYLIC ACID GROUPS ARE NEUTRALIZED TO FORM CARBOXYLIC ACID SALT GROUPS | CN104943051A | TSUKIOKA HIROO US\ ISHIKAWA YASUHISA US | TSUCHIDA HIROMI (JP)\ MAEJIMA AKIHIRO (JP)\ KANEDA JINSAKU (JP)\ MAEDA EISAKU (JP) | D09-C01D\ E34-C02\ T01-J06A\ T01-J15X\ X25-A08\ X25-Q02 |
| 6928 | 3D printing | BASE PLATE METAL LAYER MANUFACTURING METHOD FOR LED LAMP, INVOLVES FORMING METAL LAYER WITH BASE PLATE AND METAL CIRCUIT UNIT, AND FORMING FIRST DIVIDING REGION WITH METAL CIRCUIT UNIT AND SECOND DIVIDING REGION | CN104911582A | TSUNODA HIDEKI US\ ICHIKI SHUZO US | MITSUMI ELECTRIC CO (JP) |  |
| 6929 | 3D printing | TABLE TENNIS APPLIANCE INTEGRATED MULTIFUNCTION PLATE LAYER, HAS RACKET MAIN BODY PROVIDED WITH HANDLE, NEGATIVE ION ADHESIVE LAYER FIXED WITH CORE PLATE LAYER, AND COMPOSITE BOARD TEMPERATURE AND PRESSURE IS SPECIFIC IN VALUES | CN104874793A | TSUTOMU FURUKI (JP) | RENESAS TECH CORP (JP) | D09-C01D\ T01-J06A\ T01-J07B |
| 6930 | 3D printing | METAL POWDER LASER SINTERING BASED CRANIAL BONE REPAIRING PROSTHESIS TITANIUM ALLOY MATERIAL PREPARING METHOD, INVOLVES PERFORMING TITANIUM ALLOY MATERIAL SELECTING PROCESS, AND PERFORMING CRANIAL BONE WIRE BINDING PROCESS | CN104846364A | TSUYOSHI TAKAHASHI (JP)\ TOSHIYUKI OKODA (JP) | NEC ELECTRONICS CORP (JP) | A09-D03\ A11-B01\ A11-B14\ A11-B16\ A12-L05\ A12-R07\ V08-A02\ X25-A08 |
| 6931 | 3D printing | LASER CLADDING MOLTEN POOL TEMPERATURE DETECTION DEVICE COMPRISES VISIBLE LIGHT EMITTER, VISIBLE LIGHT, FIRST DETECTION SPOT, LASER CLADDING MOLTEN POOL, LIGHT SENSOR, REFLECTED LIGHT AND PROCESSOR | CN104746071A | TUMAKHA SERHII (US)\ ANG BOON Y (US)\ GHIA AMIT (US)\ DE JONG JAN L (US) | SANYO ELECTRIC CO (JP) | V07-F01A1\ V07-G10C\ V07-K01C2\ V08-A02\ V08-A04C2 |
| 6932 | 3D printing | PERSONALIZED BETA-TITANIUM-15MOLYBDENUM-ALLOY-ALUMINUM OXIDE CERAMIC TIBIAL PLATEAU SCAFFOLDS, COMPRISE POROUS BETA-TITANIUM-15MOLYBDENUM-ALLOY TRABECULAR BONE AND ALUMINUM OXIDE CERAMIC ARTICULAR SURFACE | CN104998551A | TUOMINEN MARKO (FI)\ HAUKKA SUVI (FI)\ SKARP JARMO (FI) | XILINX INC (US)\ TUMAKHA SERHII (US)\ ANG BOON Y (US)\ GHIA AMIT (US)\ DE JONG JAN L (US) | S03-E13D\ S03-F04 |
| 6933 | 3D printing | HEAT PRESERVATION CURTAIN, HAS HEAT SEPARATE LAYER FORMED AS FLOCCULENT FILLER LAYER, AND HEAT PRESERVATION BAG SECTION ARRANGED ALONG LONGER BOTTOM EDGE OF REFLECTING LAYER, WHERE TRAPEZOID SHORT EDGE IS FORMED WITH BOTTOM SEPARATE LAYER | CN105047537A | TWEET DOUGLAS J (US)\ EVANS DAVID RUSSELL (US)\ MA YANJUN (US) | ASM INTERNATIONAL NV (US) | S05-E |
| 6934 | 3D printing | POROUS STRUCTURE BONE CEMENT SCREW DESIGNING METHOD, INVOLVES REMOVING VACUUM CASTING MACHINE FROM BONE CEMENT WHEN CURING PROCESS IS PERFORMED, DISSOLVING SOLVENT IN BOLT MOLD, AND ARRANGING POROUS BONE CEMENT BOLT | CN204744450U | UEMATSU SATORU (JP) | SHARP LAB OF AMERICA INC (US) | M23-D05\ X24-D03 |
| 6935 | 3D printing | LASER SINTERING 3D PRINTER POWER SUPPLY SYSTEM, HAS PUMP CONNECTED WITH OUTER SIDE WALL OF DRIVING WHEEL, AND SCREW SHAFT FORMED WITH FIRST PUMP CAVITY, WHERE LOWER END OF FIRST PUMP CAVITY IS MATCHED WITH SECOND PUMP CAVITY | US2015341723A1 | UEMURA TOSHIFUMI (JP) | SEIKO EPSON CORP (JP) | M22-D\ M22-G02A\ M22-G02B\ M22-G03C\ M22-G03D1\ M22-G03K2\ X25-A01 |
| 6936 | 3D printing | OPTICAL FIBER COMBINER, HAS OUTPUT OPTICAL FIBER PROVIDED WITH FIBER CORE THAT IS COATED WITH CLADDING LAYER, AND INPUT OPTICAL FIBER PROVIDED WITH ANOTHER FIBER CORE, WHERE CLADDING LAYER IS COATED WITH COATING LAYER | GB2526328A | UENISHI TOSHIYA (JP)\ MEGURO SATOSHI (JP)\ KUBO MASAHARU (JP)\ KATO MASATAKA (JP)\ MIURA HIDEO (JP) | RENESAS ELECTRONICS CORP (JP)\ UEMURA TOSHIFUMI (JP) | X25-A08 |
| 6937 | 3D printing | CUTTER PROCESSING PART DIFFUSION LAYER BASED LASER CLADDING ADHERED PREPARING METHOD, INVOLVES OBTAINING CUTTING TOOL THAT IS FORMED WITH GROOVE, AND MATCHING DIFFUSION LAYER WITH SURFACE OXIDATION LAYER | JP2015193896A | UENO JOJI US\ SAKAINO YASUTAKA (JP)\ UMEZAWA YOSHIAKI (JP) | RENESAS TECH CORP (JP) | L03-G05B9\ L04-C07E\ L04-C11C3\ L04-C12\ U11-C05B4\ U11-C05C\ U14-K01A1B\ U14-K01A1J |
| 6938 | 3D printing | SELECTIVE LASER MELTING TECHNOLOGY BASED POSITIVE PURE TITANIUM ORTHODONTIC BRACKET GROOVE MANUFACTURING METHOD, INVOLVES OBTAINING OUTLINE INFORMATION OF SLICE LAYER, AND FORMING THIN WALL WITH TITANIUM POSITIVE ORTHODONTIC BRACKET GROOVE | CN104934843A | UENO KOICHIRO US | OKI ELECTRIC IND CO LTD (JP) | A09-D03\ A11-A02B\ A11-B03\ A11-B14\ A11-B16\ T01-J08A\ T06-A06A9\ X25-A08\ X25-B04 |
| 6939 | 3D printing | OPTICAL SYSTEM USEFUL FOR WELDING, PREFERABLY LASER CLADDING, COMPRISES A STATIC OUTER HOUSING HAVING TWO LENSES OR LENS PACKAGE MOVE ALONG HORIZONTAL DIRECTION AND/OR BY ROTATION AND AN OSCILLATING SOLIDIFICATION FRONT TO PRODUCE SUBSTRATE | US2015327986A1 | UENO TAKESHI US | ASAHI KASEI DENSHI KK (JP) | M22-H03B |
| 6940 | 3D printing | USE OF DIRECT METAL LASER SINTERING FOR PRODUCING CASTING DIE, PREFERABLY INGOT MOLD FOR PREVENTING AIR INCLUSIONS IN THE GRAVITATIONAL DIE CASTING TO PRODUCE PISTON FOR INTERNAL COMBUSTION ENGINE | CN104992987A | UFERT KLAUS (DE) | RICOH KK (JP) | M13-D03A\ M13-D04\ M24-D02A\ M27-A04 |
| 6941 | 3D printing | LASER OPERATING MACHINE FOR ADDITIVE MANUFACTURING OF METAL ALLOY OBJECTS IN AUTOMOTIVE AND AEROSPACE INDUSTRIES, HAS TOOL-CARRIER FRAME ASSOCIATED WITH SYSTEM OF MOVEMENT AXES AND MOVABLE WITH RESPECT TO OPTICAL LASER ASSEMBLY | CN204644469U | UGAJI MASAYA (JP)\ MINO TATSUJI US\ NAGAO NOBUAKI US\ TAKAHASHI KEIICHI US | QIMONDA AG (DE) | U12-A01A1E\ U12-A01A7 |
| 6942 | 3D printing | METHOD FOR MANUFACTURING ARRAY BASE PLATE, INVOLVES COVERING GRID ELECTRODE INSULATION GAP LAYER WITH ACTIVE REGION LAYER, AND CONNECTING SOURCE POLE WITH DRAIN ELECTRODE THAT IS CONNECTED WITH GRID ELECTRODE | CN204603301U | USAMI SHIRO (JP) | MATSUSHITA ELECTRIC IND CO LTD (JP) | M22-H02\ M22-H03B\ X25-A08\ X25-H |
| 6943 | 3D printing | LARGE SIZE SELECTIVE LASER SINTERING DIVIDING AREA PRE-HEATING METHOD, INVOLVES CALCULATING TEMPERATURE VALUE OF POWDER BED BODY, AND INCREASING POWDER BED BODY TEMPERATURE SITE HOMOGENEITY AFTER CONTROLLING HEATING LAYER | EP2946912A1 | USAMI SHIRO (JP) | USAMI SHIRO (JP) | A09-D01\ A11-A\ A11-B06A\ A11-C02D\ A12-S04A1\ A12-T04B |
| 6944 | 3D printing | METHOD OF LASER SINTERING AND DEVICE FOR ITS IMPLEMENTATION HAS ONE HEATING LASER BEAM, AND THE HEATING LASER BEAM IS DEFOCUSED OR FOCUSED MORE WIDELY | JP2015211107A | USAMI SHIRO (JP) | PANASONIC CORP (JP)\ USAMI SHIRO (JP) | M13-H01\ M13-L\ X25-A03F |
| 6945 | 3D printing | CASE-HARDENING-STEEL MATERIAL HAS GRANULAR CARBONIZED MATERIAL THAT IS PROVIDED IN SURFACE SIDE OF FERROUS MATERIAL IN FIRST REGION, AND SECOND REGION IN WHICH CARBONIZED MATERIAL OF LAYERED FORM OR PLATE SHAPE IS PROVIDED IN INSIDE | CN104439704A | USHIYAMA KOUJI US\ MIYASHITA KOJI US\ TOMOHIRO YASUHIKO US | MATSUSHITA ELECTRIC IND CO LTD (JP) | M22-H02\ M22-H03B\ M22-H03F\ M26-B08\ X25-A08\ X25-A09 |
| 6946 | 3D printing | ORGANIC LED (OLED) DISPLAY PANEL HAS GLASS CAPSULE THAT IS CONTACTED WITH BACK PLATE BY DIGGING REGION, AND METAL BLOCK AND INORGANIC PROTECTIVE LAYER ARE COVERED WITH REFLECTION SURFACE FOR REFLECTING INCIDENCE LASER TO DIGGING AREA | CN105014307A | USHIYAMA KOUJI US\ MIYASHITA KOJI US\ TOMOHIRO YASUHIKO US | SEIKO EPSON CORP (JP) | M13-H01\ M22-H\ M27-A01\ M27-A01B\ M27-A01C\ M27-A01J\ M27-A01M\ M27-A01N\ M27-A04\ M27-A04B\ M27-A04C\ M27-A04M\ M27-A04N\ M27-C02 |
| 6947 | 3D printing | PRECIPITATION STRENGTHENING OF GH4169 ALLOY LASER MELT COMPRISES ADDING PURE TITANIUM AND/OR PURE ALUMINUM NANO POWDER INTO GH4169 POWDER, THREE DIMENSIONAL GRAVITATIONAL MIXING, SELECTIVE LASER MELTING, FORMING AND HEAT TREATMENT | CN204735712U | USUJIMA AKIHIRO US\ SATO SHIGEO US | SEIKO EPSON CORP (JP) | M13-G03\ M13-H01\ M13-M01 |
| 6948 | 3D printing | VEHICLE HEAT SEPARATE FOAMING LAYER MANUFACTURING METHOD, INVOLVES STIRRING RAW MATERIAL WITH MOLD LIQUID IN FOAMING CHAMBER, AND PREPARING SPONGE FOAM WHILE PERFORMING CURING PROCESS | CN204735713U | VASKELIS ALGIRDAS (LT)\ JAGMINIENE ALDONA (LT)\ STANKEVICIENE INA (LT)\ NORKUS EUGENIJUS (LT) | FUJITSU LTD (JP) | F04-F01\ J01-C03\ X24-D08A |
| 6949 | 3D printing | CORRUGATED ROLLER HOT LASER CLADDING METHOD BY PRE-PROCESSING SURFACE OF CORRUGATED ROLLER, TAKING CORRUGATED ROLLER INSTALL CLAMP ON MACHINE TOOL, ADJUSTING LIGHT SPOT WIDTH OF CONTROL CABINET AND SUBJECTING CORRUGATED ROLLER TO LASER | US2015332673A1 | VENKATRAMAN CHANDRA (US)\ BRODBECK CYNDI L (US)\ KIRK MATTHEW P (US) | VASKELIS ALGIRDAS (LT)\ JAGMINIENE ALDONA (LT)\ STANKEVICIENE INA (LT)\ NORKUS EUGENIJUS (LT) | L03-H05\ L04-A02A1A\ L04-C07E\ L04-C07G\ U11-C01J3A |
| 6950 | 3D printing | DIRECT MANUFACTURE OF THREE-DIMENSIONAL PRINTING ENGINE CYLINDER HEAD VALVE SEAT INVOLVES REINFORCING ENGINE CYLINDER HEAD INLET AND VENT SEALING CONES IN SITU METAL MATRIX WITH LAMINATE CLADDING COATING | CN204690104U | VISOKAY MARK (US)\ MOISE THEODORE S (US)\ XING GUOQIANG (US)\ GAYNOR JUSTIN F (US)\ SUMMERFELT SCOTT R (US)\ GILBERT STEPHEN R (US)\ CELII FRANCIS (US)\ COLOMBO LUIGI (US) | BEKAERT SA NV (BE)\ VENKATRAMAN CHANDRA (US)\ BRODBECK CYNDI L (US)\ KIRK MATTHEW P (US) |  |
| 6951 | 3D printing | IRON-BASED POWDER USED FOR LASER CLADDING, CARBON, CHROMIUM, NICKEL, MOLYBDENUM, NIOBIUM, BORON AND BALANCED AMOUNT OF IRON | JP2015199199A | VOLDMAN STEVEN H (US) | TEXAS INSTRUMENTS INC (US) | W04-W05A |
| 6952 | 3D printing | METAL-BASED LUBRICATING COMPOSITE COATING COMPRISES BASAL BODY METAL SURFACE COATED WITH THERMAL BARRIER TRANSITION LAYER, LUBRICATING MIDDLE LAYER AND LUBRICATING WORKING LAYER | JP2015199979A | WADA MASATAKE (JP)\ IMAKITA NAOKI (JP) | VOLDMAN STEVEN H (US) | A11-B09A1\ A11-B16\ A12-W14 |
| 6953 | 3D printing | METHOD FOR MANUFACTURING INTERNAL-PRESSED TUBULAR FILM THAT IS UTILIZED IN FOOD INDUSTRY, INVOLVES OVERLAPPING NON-COATING PART AT EDGE OF COATING FILM LAYER TO OBTAIN TUBULAR FILM, AND CUTTING FILM ACCORDING TO NEEDED LENGTH | CN104972677A | WADA TOSHIO (JP)\ HATA YOJI (JP) | RENESAS ELECTRONICS CORP (JP)\ WADA MASATAKE (JP)\ IMAKITA NAOKI (JP) | A08-R05\ A12-T01\ M11-A01\ M11-A03\ M11-A04\ M11-A06\ M11-B04\ M24-D01A1\ M24-D02\ M24-D03\ M24-F03\ M24-F06\ M27-A04\ M27-A04C\ M27-A04M\ M27-A04S\ M27-B04\ M27-B04C\ M27-B04M\ M27-B04S\ M27-C03\ M27-C04 |
| 6954 | 3D printing | NON-CONTINUOUS EPITAXIAL LAYER MANUFACTURING METHOD, INVOLVES TRANSFERRING IMAGE TO UPPER PART OF SUBSTRATE, FORMING SUBSTRATE SURFACE WITH CONCAVE GROOVE OF SUBSTRATE, AND SEPARATING CONCAVE GROOVE FROM DISCONTINUOUS EPITAXIAL LAYER | US2015322557A1 | WAGNER JR LAWRENCE F (US)\ WOLF RANDY L (US) | NIPPON STEEL SEMICONDUCTOR CO US | L03-F02\ L03-G02\ V07-F01A\ V07-F02B\ V08-A01A2\ V08-A02B\ V08-A03D\ V08-A04C2 |
| 6955 | 3D printing | DENTAL IMPLANT SYSTEM, HAS MOUNTING SLIDE BLOCK FORMED WITH POROUS TANTALUM RING, HEAD PART WHOSE OUTER SIDE IS FORMED WITH SPRING THAT IS FORMED WITH GROOVE, AND TAIL PART FORMED WITH BULLET HEAD SHAPED | CN204686015U | WALD PHILLIP G (US)\ LANE RICHARD (US) | WAGNER JR LAWRENCE F (US)\ WOLF RANDY L (US) | A06-A00E3\ A11-B05\ A12-V02\ D09-C01\ S05-Y03 |
| 6956 | 3D printing | METHOD FOR PROVIDING MULTITASK LEARNING FOR BROADBAND SOURCE-LOCATION MAPPING OF ACOUSTIC SOURCES, INVOLVES MODELING DISCRETE-TIME FOURIER TRANSFORM VECTORS FOR MEASUREMENT BLOCK OF TIME SERIES DATA, WHERE VECTORS COMPRISE REPLICAS | WO2015163020A1 | WALD PHILLIP G (US)\ LANE RICHARD (US) | MICRON TECHNOLOGY INC (US) | U11-C01J2 |
| 6957 | 3D printing | METHOD FOR FORMING COMPOSITE COMPONENT, SUCH AS AIRCRAFT STRUCTURAL COMPONENT BY ADDITIVE LAYER MANUFACTURING, INVOLVES PROVIDING ELONGATE TAPE OF CARBON NANOTUBES AND FORMING ALIGNED TAPE INTO PORTIONS OF REQUIRED SIZE | CN204644468U | WANG CHIH-HAO (TW)\ WANG TA-WEI (TW) | MICRON TECHNOLOGY INC (US) | M13-H01\ M13-L |
| 6958 | 3D printing | MANUFACTURE OF ULTRA-FINE BRASS-PLATED STEEL WIRE USED FOR STEEL CORD, INVOLVES WIRE DRAWING HOT-ROLLED WIRE CONTAINING CARBON, SILICON, MANGANESE, CHROMIUM AND IRON, FORMING METAL PLATING, DIFFUSION-HEAT PROCESSING, AND WIRE DRAWING | CN204644473U | WANG HUIMIN (CN) | WANG CHIH-HAO (TW)\ WANG TA-WEI (TW) | M22-H01\ M22-H03B\ M22-H03C\ X25-A01\ X25-A08 |
| 6959 | 3D printing | FULL OPTICAL FIBER-BASED MID-INFRARED ENERGY PASSIVELY Q-SWITCHED FIBER LASER USED IN E.G. MILITARY FIELD, HAS SINGLE CONTINUOUS LASER CLADDING WHOSE INPUT PORTION IS OBTAINED, FOR OUTPUTTING PULSED LASER | CN104801703A | WANG MINGPU LI (CN) | GUANGQI SCIENCE & TECHNOLOGY C (CN) | A08-R03\ A11-B09A1\ A12-S08C\ A12-S08D3\ A12-T04 |
| 6960 | 3D printing | METHOD FOR CREATING TEXTURED SURFACE ON BREAST IMPLANT INVOLVES USING COMPUTER-CONTROLLED DEPOSIT MECHANISM TO DEPOSIT TEXTURING MATERIAL ONTO TACKY LAYER AND TEXTURING MATERIAL IS REMOVED FROM TACKY LAYER TO OBTAIN TEXTURED SURFACE | CN204701159U | WANG NAN XU (CN) | UNIV ZHONGNAN (CN) | U11-A13\ U11-C05C |
| 6961 | 3D printing | SOLAR ENERGY BATTERY FLUORINATED GRAPHENE HIGH FLAME-RETARDANT LAYER, HAS FILM LAYER ARRANGED ON CONTACT LAYER, AND REAR ELECTRODE CONNECTED WITH FLAME-RETARDANT LAYER THAT IS CONNECTED WITH TRANSPARENT ELECTRIC CONDUCTION THIN FILM LAYER | EP2942510A1 | WARREN ROBERT W (US)\ ABDALI USAMA K (US)\ SHEN HONG (CN)\ RAMANATHAN RAVI (IN)\ LUO QIULIANG (CN) | HUAHONG NEC ELECTRONICS CO LTD (CN) | M23-D05\ M23-G\ X24-D03 |
| 6962 | 3D printing | LASER CLADDING VERTICAL ANTI-SPLASHING DEVICE HAS LIGHT BLOCKING PLATE WHICH IS CONNECTED TO RING, FIXING ROD HAVING INSERT CONNECTED TO CYLINDER BLOCK, SPRING HOOK HAVING ROUND HOLE, AND BAFFLE HAVING ONE END CONNECTED TO CYLINDER BLOCK | US2015323817A1 | WATANABE RIE US\ KITAMURA YUICHI US | SKYWORKS SOLUTIONS INC (US)\ SHEN HONG (US)\ RAMANATHAN RAVI (US)\ LUO QIULIANG (US)\ WARREN ROBERT W (US)\ ABDALI USAMA K (US) |  |
| 6963 | 3D printing | PRINT DEVICE WITH INCREASED SELECTIVE LASER SINTERING 3D COMPONENT STRENGTH HAS POWDER HEATING UNIT, CARBON DIOXIDE LASER EMITTER, SCANNING GALVANOMETER, FORMING CYLINDER, POWDER CYLINDER, OPTICAL QUARTZ GLASS PLATE, HEATER AND THERMOCOUPLE | US2015323900A1 | WAYNE FREDERICK MARLIN US\ PAUL CLARK DAVID US | MATSUSHITA ELECTRIC IND CO LTD (JP) | L03-G02\ M22-H01\ M22-H03B\ M22-H03E\ M22-H03F\ T01-J07B\ V07-F01A1\ V07-K05\ V08-A04C2\ X25-A02D\ X25-A08 |
| 6964 | 3D printing | METHOD FOR FORMING STRUCTURAL AIRCRAFT COMPONENT BY ADDITIVE LAYER MANUFACTURING IN HIGH STRENGTH APPLICATIONS, INVOLVES TRANSFERRING PORTIONS AS REQUIRED TO COMPONENT BUILD LOCATION TO FORM COMPONENT LAYER BY LAYER | CN104977304A | WEI XUE (CN)\ SHAOQUN SHEN (CN)\ WENXIANG WANG (CN)\ MINHANG BAO (CN)\ QUAN WANG (CN)\ BING WANG (CN) | ADVANCED RISC MACH LTD (GB) | M22-H03B\ M22-H03G\ S03-B01\ X25-A08 |
| 6965 | 3D printing | METHOD FOR MANUFACTURING PHOTOELECTRIC CONVERSION APPARATUS, INVOLVES PREPARING ELECTRODE LAYER, WHERE THE PROCESS IS SCATTERED ON A MAIN SURFACE ON AN OPPOSITE SIDE TO THE ELECTRODE LAYER OF THE REGION | CN104952723A | WEI ZHANG (CN)\ PENGFEI WANG US\ SHIJIN DING US\ LONGLONG HU US\ CHUNXIAO YANG US | UNIV WENZHOU (CN) | T01-C08A\ T01-J03\ T01-J11A1\ T01-J12C\ T01-J16C3\ T01-J21\ T05-D02\ W04-V01\ W04-V04A6 |
| 6966 | 3D printing | CASTING METHOD OF TITANIUM ALUMINIDE MATERIAL INCLUDES TAKING TITANIUM ALUMINIDE NIOBIUM MATERIAL, MILLING, SCRUBBING WITH ACETONE, WELDING, LASER CLADDING, INSPECTING CRACKS AND DEFECTS, HARDENING, PERFORMING HEAT TREATMENT, AND GRINDING | WO2015165447A1 | WEI ZHANG (CN)\ QINGQING SUN US\ PENGFEI WANG US\ LIN CHEN US\ WEN YANG US | UNIV FUDAN (CN) | M13-H01 |
| 6967 | 3D printing | MOISTURE-PROOF ANTI-ABRASION RESISTANCE-TYPE WELDING STRIP PACKAGE BOX MANUFACTURING METHOD, INVOLVES FIXING DOUBLE UPPER HANDLE WITH ROUGH PACKAGE BOX, AND FIXING TINPLATE WITH UPPER END COVER AND OPENING END OF LOWER COVER BODY | US2015315710A1 | WEI ZHANG (CN)\ QINGQING SUN US\ PENGFEI WANG US\ SHIJIN DING US\ HONGLIANG LU US\ LIHENG XIE US | UNIV FUDAN (CN)\ SUN QINGQING (CN)\ ZHANG WEI (CN)\ CHEN LIN (CN)\ YANG WEN (CN)\ WANG PENGFEI (CN) | A11-A01\ A11-A01B\ A11-B09A2\ A11-B12A\ A11-C01C\ A12-A04A\ A12-D00D\ A12-E12 |
| 6968 | 3D printing | MOLTEN METAL POWDER MATERIAL MANUFACTURING FINISHING EQUIPMENT HAS LASER ENGRAVING MACHINE COMPOSITE THAT IS FORMED BY OPTICAL FIBER LASER GALVANOMETER SCANNING MECHANISM PROVIDED WITH WORKING CAVITY IN HORIZONTAL DIRECTION | WO2015167335A1 | WEI ZHANG (CN)\ XINPING QU US\ YULONG JIANG US\ GUOPING RU US\ QI XIE US\ HAO YU US\ BINGZONG LI US | UNIV FUDAN (CN) | M24-A02\ M24-A05 |
| 6969 | 3D printing | MOLTEN POOL TEMPERATURE DETECTING DEVICE HAS LENS THAT IS SCREWED WITH PROTECTIVE TUBE, AND PROTECTING CYLINDER THAT IS PROVIDED WITH NEUTRAL ATTENUATION SHEET AND PROTECTIVE ELEMENT, AND LASER HEAD THAT IS FIXED WITH CONNECTING BLOCK | CN204687962U | WEIDONG NIE (CN)\ WEIMIN ZHU (CN)\ FAYOU YI (CN)\ DONGQIN CHEN (CN) | UNIV FUDAN (CN) | A05-A01C\ A08-R03A\ A09-D01\ A11-B09A1\ A11-B16\ A11-C02D\ A12-S08C\ A12-T04\ F01-D09A\ F05-A06B\ T01-J07B\ T01-J07D1\ T01-J15X\ X25-A03C3 |
| 6970 | 3D printing | METHOD OF REVISING LANGUAGE MODEL SCORES BASED ON SEMANTIC CLASS HYPOTHESES SUCH AS YEAR CLASS, INVOLVES REVISING INITIAL SCORES FOR CANDIDATE WORD SEQUENCES BASED ON HYPOTHESES FOR SEMANTIC CLASS OF TOKEN IN EACH OF WORD SEQUENCES | CN204694872U | WEIDONG NIE (CN)\ XIAOJUN DENG (CN)\ HUIQIN BO (CN)\ WEI ZHANG (CN) | WUXI JINGYUAN MICROELECTRONICS (CN) | M26-B08\ M29-C |
| 6971 | 3D printing | ANNULAR NOZZLE OF LASER CLADDING HEAD, HAS OUTSIDE COLD WATER TANK WHICH IS PROVIDED WITH TWO C-SHAPED CIRCULAR ARC GROOVES, AND RESPECTIVE CONNECTED WITH INNER COOL WATER CHANNEL AND OUTSIDE COLD WATER CHANNEL | CN204695273U | WEILL MYLENE (FR)\ FORT PHILIPPE (FR)\ LEONETTI JEAN-PAUL (FR) | WUXI YOUDA ELECTRONICS CO LTD (CN) | M22-H03B |
| 6972 | 3D printing | IN-MOLD MOLDING OBJECT E.G. DOMESTIC ELECTRICAL APPLIANCE HAS TRANSFER LAYER THAT IS FORMED ON SURFACE, AND TWO LIQUID CURABLE RESIN LAYERS AND THERMOPLASTIC RESIN LAYER THAT ARE FORMED IN TRANSFER LAYER | DE102014208134A1 | WEIMER RONALD A (US) | UNIV MONTPELLIER 2 (FR)\ CENTRAL NAT DE LA RECH SCIENT (FR) | E10-D01C\ E11-D02\ J04-E04B\ N02-A01\ N02-F02\ N06-C08\ N07-B02 |
| 6973 | 3D printing | CHARGING-MATERIAL INSERTION METHOD OF BELL-LESS BLAST FURNACE INVOLVES INSERTING LAYERED FORM C-BATCH OF COKE, AND O-BATCH OF ORE BY TURNS, FORMING FINE GRANULES BY SIEVING, AND INSERTING FINE GRANULE ORE TO FURNACE WALL VICINITY | CN204658567U | WEIMER RONALD A (US)\ POWELL DON C (US)\ MOORE JOHN T (US)\ MCKEE JEFF A (US) | MICRON TECHNOLOGY INC (US) | M13-H01\ M13-L |
| 6974 | 3D printing | 3D PRINT TECHNOLOGY BASED VEHICLE PARTS CARBON FIBER COMPOSITE MATERIAL MOLDING METHOD, INVOLVES COATING EPOXY RESIN ON VEHICLE PARTS MAIN BODY, AND PERFORMING SAND PAPER GRINDING PROCESS BY POLISHING MACHINE | CN204639130U | WEIZHEN BA (CN)\ CHAOYANG CHEN (CN) | WEIMER RONALD A (US)\ POWELL DON C (US)\ MOORE JOHN T (US)\ MCKEE JEFF A (US) | M13-H01 |
| 6975 | 3D printing | POST-BUILT UP HEAT TREATMENT METHOD FOR ADDITIVELY MANUFACTURED COMPONENT MADE FROM GAMMA PRIME STRENGTHENED SUPERALLOY INVOLVES APPLYING TIME/TEMPERATURE STEPS TO COMPONENT DEPENDING ON PURPOSE OF HEAT TREATMENT | CN204639135U | WEN DIANZHONG (CN) | XINJIANG INST OF PHYSICS & CHE (CN) | A04-B02\ A04-B03\ A04-B06\ A04-B08\ A04-F06E\ A04-G06\ A05-E07\ A12-W12F\ M22-H01\ M22-H03B\ X25-A08 |
| 6976 | 3D printing | LASER SINTERING DEVICE USED IN SINTERING SYSTEM, HAS BURNING PLATFORM THAT IS PROVIDED WITH BURNING CHAMBER, AND BURNING CABIN GAS AIR INLET AND AIR OUTLET THAT ARE CONNECTED WITH ISOLATION COVER | CN204639137U | WEST JEFFREY A (US) | WEN DIANZHONG (CN) | A09-D03\ A11-A\ A11-B01\ A11-B03\ A11-B14\ A11-B16\ A11-C03A\ A12-H02\ X25-A08 |
| 6977 | 3D printing | SOLID CATALYST USED IN MANUFACTURE OF ALDEHYDE E.G. ACETALDEHYDE, HAS LAYERED FORM STRUCTURE HAVING SURFACE COATED WITH CATALYST POWDER LAYER OBTAINED BY ADHERING CATALYST POWDER TO OUTER SURFACE OF SUPPORT | CN104816093A | WEST JEFFREY A (US)\ TIWARI RAJESH (US) | TEXAS INSTRUMENTS INC (US)\ WEST JEFFREY A (US) | X25-A08\ X25-A09 |
| 6978 | 3D printing | LASER CLADDING BLOWING DEVICE HAS U TYPE CARD AND CONNECTING PLATE WHICH IS PROVIDED WITH VENT HOLE, AIR CURTAIN HAVING CURTAIN FRONT AIR CAVITY AND BACK AIR CURTAIN CAVITY, AND AIR OUTLET HOLE WHICH IS MUTUALLY CONNECTED TO AIR CAVITY | CN104805450A | WEST JEFFREY A (US)\ VINCENT PATRICIA DIANE (US)\ TUERCK ROBERT A (US) | TEXAS INSTRUMENTS INC (US)\ WEST JEFFREY A (US)\ TIWARI RAJESH (US) | U12-B03A\ U14-K01\ V07-F02B\ V07-F02C\ V07-K01\ V07-M |
| 6979 | 3D printing | AIR NOZZLE OR FEEDING HEAD LASER CLADDING AND FIXING DEVICE HAS FIXED FRAME, CONNECTING ROD HAVING FIRST AND SECOND CONNECTING ROD, FIXING END HEAD, SECOND POLE, AND CLAMPING RING WHICH IS SLEEVED ON LOCATION ROD CONNECTED WITH ONE OF RING | CN104775114A | WILLER JOSEF (DE)\ SELL BERNHARD (DE)\ CAPPELLANI ANNALISA (IT) | WEST JEFFREY A (US)\ VINCENT PATRICIA DIANE (US)\ TUERCK ROBERT A (US) | V07-F02A\ V07-K01\ V07-M |
| 6980 | 3D printing | FLEXIBLE METAL POWDER USED FOR THREE-DIMENSIONAL PRINTING TO OBTAIN FLEXIBLE METAL PRODUCT E.G. FLANGE COMPRISES METAL POWDER, RUBBER, METAL OXIDE, SOFTENING AGENT, INTERFACIAL MODIFIER, ANTIOXIDANT AND ACCELERATING AGENT | CN104788102A | WILLIAM WILLE (US)\ DANIEL EDELSTEIN (US)\ WILLIAM COTE (US)\ PETER BIOLSI (US)\ JOHN FRITCHE (US)\ ALLAN UPHAM (US) | SIEMENS AG (DE) | S03-E04F1\ S03-E04F2\ T01-J10B2 |
| 6981 | 3D printing | AUTOMATIC SLS MOLDING MACHINE POWDER RECYCLING DEVICE, HAS CYLINDERS WHOSE OUTER SIDES ARE FIXED TO UPPER END OF MICRO-VIBRATION MOTOR AND CONTROL BOARD, AND SENSORS WHOSE INPUT SIGNAL CONTROLS INLET OF ELECTROMAGNETIC THREE-WAY PIPE | CN104775119A | WILLIAMS RICHARD K (US)\ DISNEY DONALD RAY (US)\ CHAN WAI TIEN (CN) | IBM (US)\ IBM UK (GB)\ WILLE WILLIAM (US)\ EDELSTEIN DANIEL (US)\ COTE WILLIAM (US)\ BIOLSI PETER (US)\ FRITCHE JOHN (US)\ UPHAM ALLAN (US) | L04-A01A\ L04-C01D\ L04-C06B1\ L04-C06B2\ L04-C07A\ L04-C07B\ L04-C11C1\ L04-C12A\ U11-C01A3\ U11-C01J4A\ U11-C04A1B\ U11-C04A1C\ U11-C04E1\ U11-C05C2\ U11-C07A1 |
| 6982 | 3D printing | METHOD FOR OPERATING ADDITIVE MANUFACTURING SYSTEM, INVOLVES CONNECTING FIRST CONDUIT WITH SECOND FLUID CONDUIT BY FLUID COUPLING, WHERE FIRST CONDUIT INCLUDES CONFIGURATION THAT IS DIFFERENT THAN CONFIGURATION OF SECOND CONDUIT | CN104746068A | WILSHAW PETER RICHARD (GB)\ MALLIK KANAD (GB)\ FALSTER ROBERT JAMES (GB) | DISNEY DONALD RAY (US)\ CHAN WAI TIEN (HK)\ WILLIAMS RICHARD KENT (US) |  |
| 6983 | 3D printing | COMPLEX SPATIAL LIGHT MODULATOR FOR DISPLAYING DIGITAL HOLOGRAM, HAS POLARIZER PROVIDED ON PHASE TYPE MODULATOR, WHERE AMPLITUDE TYPE MODULATOR AND PHASE TYPE MODULATOR ARE COMMONLY AND ELECTRICALLY CONNECTED TO TFT LAYER | CN104970645A | WON SEOKJUN (KR)\ KANG HOKYU (KR) | ISIS INNOVATION (GB)\ WILSHAW PETER RICHARD (GB)\ MALLIK KANAD (GB)\ FALSTER ROBERT JAMES (GB) | M13-C01\ M13-H01\ M13-L |
| 6984 | 3D printing | METHOD FOR ENHANCING RESOLUTION AND WIDE VIEWING ANGLE OF DIGITAL HOLOGRAPHIC SYSTEM INVOLVES RECORDING TARGET IMAGE ON RECORDING MEDIUM USING CONDENSED BEAM OBTAINED FROM PARALLEL BEAM THAT LOADS MULTIPLE HOGELS PASSING THROUGH LENS | CN104979629A | WON SEOKJUN (KR)\ KANG HOKYU (KR) | SAMSUNG ELECTRONICS CO LTD (KR) | A11-B16\ A11-C02 |
| 6985 | 3D printing | SPACE LIGHT MODULATOR SUB-PIXEL STRUCTURE DEFECT DETECTING DEVICE, HAS SERVO SYSTEM CONNECTED WITH SPACE LIGHT MODULATOR SCREEN AND ADJUSTING RECOVERY SYSTEM, WHERE UPPER PART OF SCREEN IS CONNECTED WITH DISPLAY UNIT AND PROCESSING SYSTEM | WO2015166261A1 | WONG LAWRENCE D (US) | SAMSUNG ELECTRONICS CO LTD (KR) | A12-T04C\ A12-T04E\ X22-B02D\ X22-B03E |
| 6986 | 3D printing | GATE SIDE WALL LAYER MANUFACTURING METHOD, INVOLVES DEPOSITING MEDIUM LAYER WITH GRID ELECTRODE, REMOVING TOP MEDIUM LAYER BY DRY ETCHING, REMOVING PHOTO RESIST FROM SUBSTRATE SURFACE, AND DRY ETCHING TIME IS IN SPECIFIC RANGE | US2015316655A1 | WOO YOUNG-JIN (KR)\ PARK KONG-SOON (KR)\ KIM YOUNG-SIK (KR) | INTEL CORP (US)\ WONG LAWRENCE D (US) | A11-C01C\ A12-L02A\ A12-L03\ A12-V02A |
| 6987 | 3D printing | BEARING ELEMENT FOR E.G. ROLLING BEARING, PROVIDED WITH SUPPORT ELEMENT OVER WHICH FUNCTIONAL LAYER MANUFACTURED FROM HIGHER ALLOY MATERIAL IS ARRANGED AND CONNECTED WITH SUPPORT ELEMENT | CN104924615A | WU CHI-HSI (TW) | SILICON WORKS CO LTD (KR)\ WOO YOUNG-JIN (KR)\ PARK KONG-SOON (KR)\ KIM YOUNG-SIK (KR) | T04-F02A2\ T04-F02C\ T04-H04 |
| 6988 | 3D printing | METAL CLADDING APPARATUS FOR CLADDING WORKPIECE, ALLOWS CLADDING ADDITIONAL STRIPS ON WORKPIECE THROUGH REPEATED SWEEPING MOTIONS AND CONTROL SYSTEM CONTROLS AND COORDINATES MOTION OF CLADDING HEAD MOTION SYSTEM AND CLADDING HEAD | CN204696236U | WU CHUNG CHENG (TW)\ KESHAVARZI ALI (US)\ GUO TA PEN (TW)\ CHANG HELEN SHU HUI (TW)\ TSENG HSIANG JEN (TW)\ LIN SHYUE SHYH (TW)\ LU LEE CHUNG (TW)\ TIEN LI CHUN (TW)\ YANG JUNG CHAN (TW)\ CHEN SHU MIN (TW)\ CAO MIN (US)\ HOU YUNG CHIN (TW) | WU CHI-HSI (TW) |  |
| 6989 | 3D printing | METHOD FOR MAKING TANGIBLE PRODUCTS BY LAYERWISE MANUFACTURING, INVOLVES LEVELING LAYER OF CONSTRUCTION MATERIAL BY REMOVING SURPLUS OF CONSTRUCTION MATERIAL IN ORDER TO OBTAIN UNIFORM THICKNESS | DE102014208130A1 | WU CHUNG-CHENG (TW)\ LU LEE-CHUNG (TW)\ KESHAVARZI ALI (US)\ CAO MIN (US)\ GUO TA-PEN (TW)\ CHANG HELEN SHU-HUI (TW)\ TSENG HSIANG-JEN (TW)\ LIN SHYUE-SHYH (TW)\ TIEN LI-CHUN (TW)\ YANG JUNG-CHAN (TW)\ CHEN SHU-MIN (TW)\ HOU YUNG-CHIN (TW) | TAIWAN SEMICONDUCTOR MFG (TW) | M13-H01\ M13-L |
| 6990 | 3D printing | VEHICLE SAFETY DANGER INDICATOR HAS WARNING MAP OR DIRECTIONS LAYERS THAT ARE PRODUCED FIXED LICENSING PORTION WITH REFLECTIVE MATERIAL, AND SMALL MAGNETIC BLOCK WITH SOFT CLOTH WRAPPING THAT IS FIXED ON RECTANGULAR PLATE PORTION | DE102014208140A1 | WU CHUNG-CHENG (TW)\ LU LEE-CHUNG (TW)\ KESHAVARZI ALI (US)\ CAO MIN (US)\ GUO TA-PEN (TW)\ CHANG HELEN SHU-HUI (TW)\ TSENG HSIANG-JEN (TW)\ LIN SHYUE-SHYH (TW)\ TIEN LI-CHUN (TW)\ YANG JUNG-CHAN (TW)\ CHEN SHU-MIN (TW)\ HOU YUNG-CHIN (TW) | TAIWAN SEMICONDUCTOR MFG (TW)\ CAO MIN (TW)\ WANG SHYH-WEI (TW)\ WU CHUNG-CHENG (TW)\ KESHAVARZI ALI (US)\ GUO TA-PEN (US)\ TZENG JIANN-TYNG (TW)\ CHEN YEN-MING (TW)\ LIN SHYUE-SHYH (TW)\ YANG SHENG-JIER (TW)\ TSENG HSIANG-JEN (TW)\ SCOTT DAVID B (US)\ HING FUNG KA (TW) | M22-G03J\ M22-H03A\ M22-H03B\ M22-H03F\ X25-A01\ X25-A03B2\ X25-A03F\ X25-A08 |
| 6991 | 3D printing | DOPAMINE RESIN SURFACE ADHESIVE LAYER MANUFACTURING FOG-PROOF LENS, HAS SUBSTRATE FORMED WITH HARD COAT LAYER, WHICH IS MATCHED WITH REFLECTION FILM LAYER AND WATER-PROOF FILM LAYER, AND ADHESIVE LAYER MATCHED WITH ANTI-FOG FILM LAYER | CN204676955U | WU SCHYI-YI (US) | TAIWAN SEMICONDUCTOR MFG (TW)\ CAO MIN (TW)\ WANG SHYH-WEI (TW)\ WU CHUNG-CHENG (TW)\ KESHAVARZI ALI (US)\ GUO TA-PEN (US)\ TZENG JIANN-TYNG (TW)\ CHEN YEN-MING (TW)\ LIN SHYUE-SHYH (TW)\ YANG SHENG-JIER (TW)\ TSENG HSIANG-JEN (TW)\ SCOTT DAVID B (US)\ HING FUNG KA (TW) | M22-H02\ M22-H03B\ M22-H03C\ X25-A08 |
| 6992 | 3D printing | TOUCH SCREEN STRUCTURE, HAS SUBSTRATE CONNECTED WITH X-ELECTRODE THAT IS FORMED WITH SIO2 LAYER, WHERE Y-ELECTRODE IS CONNECTED WITH X-ELECTRODE AND Y-ELECTRODE IS FORMED WITH TRADITIONAL INSULATION FRAME BRIDGE LAYER | CN104898383A | WU WEN-FA (TW)\ OU GENG-LIANG (TW)\ JOU CHANG-BIN (TW) | MICREL INC (US) | M22-H03A\ M22-H03B\ M22-H03G |
| 6993 | 3D printing | DRILL HAS A MAIN BODY, WHICH HAS A CENTER AXIS AND A DRILL TIP HAVING AN END FACE, AND A SHANK, WHERE THE DRILL IS PRODUCED BY A LASER SINTERING PROCESS | CN104842065A | WU YUN (US)\ ZHU BEI (US)\ WU ZHIYUAN (US)\ HART MICHAEL J (US) | NAT SCIENCE COUNCIL (TW) | M23-D05\ X24-D03 |
| 6994 | 3D printing | LASER CLADDING DIRECT MOLDING ROTARY MOLD-CUTTING MOLD ROLLER HAS CLADDING LAYER, TRANSITION STRAIGHT FORM STRUCTURE AND BASAL PORTION WHICH ARE FORMED ORDERLY, AND TRANSITION LAYER WHICH IS CONNECTED WITH HOLLOW STRUCTURE | CN104758982A | WU ZHENCHENG LU (CN) | XILINX INC (US)\ WU YUN (US)\ ZHU BEI (US)\ WU ZHIYUAN (US)\ HART MICHAEL J (US) | M13-H01\ M13-L\ M14-A01\ M14-H\ M22-H02\ M26-B06\ M26-B06A\ M26-B06C\ M29-C\ X25-A03C2\ X25-A03C3 |
| 6995 | 3D printing | DEVICE FOR SINGLE CYLINDER TYPE SELECTIVE LASER MELTING AND MILLING COMPOSITE PROCESS, INCLUDES SCANNING VIBRATION MIRROR, OPTICAL LENS, LASER, X-AXIS GUIDE RAIL, Y-AXIS GUIDE RAIL, Y-AXIS GUIDE RAIL, FORMING CHAMBER AND MILLING CUTTER | CN104896455A | XIA AN US\ RU HUANG (CN)\ SHOUBIN XUE US\ SIHAO WANG US\ XING ZHANG (CN) | TAIWAN SEMICONDUCTOR MFG (TW) | M13-H01\ M22-H\ M27-A04\ M27-A04B\ M27-A04C\ M27-A04N\ M27-A04S\ M27-C03 |
| 6996 | 3D printing | CLEANING EQUIPMENT FOR SELECTIVE LASER SINTERING DEVICE, INCLUDES VIBRATION SIEVING DEVICE, HIGH PRESSURE GAS INJECTION DEVICE, POWDER SEPARATING DEVICE, DUST REMOVING FILTER, UPPER AND LOWER CONNECTING SOFT PIPE AND CLEANING WORKBENCH | CN104947934A | XIANG QI (US)\ PATON ERIC N (US)\ WANG HAIHONG (US) | UNIV BEIJING (CN) | A05-A01E\ A10-E05B\ A12-W12G\ L02-A01\ L02-A02\ L02-A03\ L02-A04\ L02-A13\ L02-A14\ L02-H02B2 |
| 6997 | 3D printing | SELECTIVE LASER MELTING DEVICE INCLUDES MACHINE FRAME, LIFTING SUPPORTING CYLINDER, MAIN PUSHING CYLINDER, FEEDSTOCK AIR CYLINDER, LIFTING AIR CYLINDER SUPPORT, FORMING SHELL, BOTTOM PLATE, FORMING WAY HOLE, FORMING TUBE AND FEEDING TUBE | CN104948144A | XIAO DE YUAN (CN)\ CHEN GUO QING (CN) | ADVANCED MICRO DEVICES INC (US) | M13-H01\ M13-L\ M27-A\ M27-A01\ M27-D04 |
| 6998 | 3D printing | LONG HOLE LASER CLADDING DEVICE COMPRISES VALVE BODY THAT PASSES INTO PROTECTIVE GAS AFTER STARTING LASER CLADDING, ALLOY SEALING SURFACE, AND HOLE LOCATED AT BOTTOM OF VALVE BODY | CN204665504U | XIAOYING LU US\ YUDE LI US\ LIFANG HU US\ QINGSEN MENG US\ CUIRONG LIU US | SEMICONDUCTOR MFG INT SHANGHAI (CN)\ XIAO DE YUAN (CN)\ CHEN GUO QING (CN) | M13-H01\ M26-B03\ X12-D01A |
| 6999 | 3D printing | PROTECTIVE COATING COMPRISES MICRO ALUMINUM POWDER, MICRO TITANIUM POWDER AND MICRO COPPER POWDER | CN204570039U | XIN CHEN (CN)\ YANG GAO (CN) | UNIV TAIYUAN TECHNOLOGY US |  |
| 7000 | 3D printing | LASER CLADDING MATERIAL COMPRISES BOTTOM LAYER AND BASE LAYER, WHERE BOTTOM LAYER COMPRISES CARBON, CHROMIUM, NICKEL, SILICON, BORON AND IRON | CN104831271A | XINCHAO WANG (CN)\ DONGMING FENG US\ WENYUAN WANG US\ XINMIN YE US\ ZHEN ZHU US | NANJING GAOHUA TECHNOLOGY CO L (CN) | W02-B07A3A\ W02-B08L |
| 7001 | 3D printing | PREPARATION OF SILICON NITRIDE NANOPOWDER BY STIRRING ETHANOL AND GAMMA-GLYCIDOXYPROPYL TRIMETHOXYSILANE IN REACTOR, ADDING SILICON NITRIDE CERAMIC NANOPOWDER, AND HEATING AND REFLUXING | CN104775118A | XIUHUA CHEN US\ WEIQIANG ZHANG US\ JINZHONG XIANG US\ ZAIYU ZHANG US\ LIHONG WANG US | JIANGYIN XINSHUN MICROELECTRONIC CO LTD US | A04-E\ A04-E10\ A12-T03A\ K04-F01\ W07-J07 |
| 7002 | 3D printing | IRON-BASED AMORPHOUS ALLOY COATING LASER CLADDING PREPARATION METHOD COMPRISES PRESETTING IRON BASE AMORPHOUS ALLOY POWDER ON TRANSITION LAYER SURFACE, USING LASER IRRADIATION MELTING ALLOY POWDER, COATING AND CHECKING | CN104711569A | XI-WEI LIN US\ VICTOR MOROZ US\ MUNKANG CHOI US | UNIV YUNNAN US | T04-K03B\ W06-A03A5A\ W06-A03A5C |
| 7003 | 3D printing | COPPER BASE COATING USED FOR IRON-BASED ALLOY SURFACE LASER CLADDING COMPRISES PURE COPPER POWDER, AND NANO-OXIDE MIXTURE COMPRISING CERIUM OXIDE, SAMARIUM OXIDE, ALUMINUM OXIDE AND/OR YTTRIUM OXIDE | CN104694918A | XU JEFF J (TW)\ YEH CHIH CHIEH (TW)\ CHANG CHIH-HAO (TW)\ WANG CHIEN-HSUN (TW)\ CHANG CHIH-HSIANG (TW) | SYNOPSYS INC (US)\ LIN XI-WEI (US)\ MOROZ VICTOR (US)\ CHOI MUNKANG (US) | A11-B16\ X25-A08 |
| 7004 | 3D printing | CUP BODY BOTTOM PRINT LAYER MANUFACTURING METHOD, INVOLVES CONNECTING MULTI-CUP BOTTOM WITH FIRST PROTECTIVE FILM COVER AND CUP BOTTOM PRINT LAYER, WHERE CUP BOTTOM PRINT LAYER IS ARRANGED AT INNER SURFACE OF CUP BODY | CN104694921A | XU JUNHAO US\ LIN CHENG-TUNG (TW)\ SHA MOKEI US\ WANG HSIANG-YI (TW)\ LEE WEN-CHIN (TW) | TAIWAN SEMICONDUCTOR MFG (TW)\ XU JEFF J (TW)\ CHANG CHIH-HAO (TW)\ WANG CHIEN-HSUN (TW)\ YEH CHIH CHIEH (TW)\ CHANG CHIH-HSIANG (TW) | W02-B07A3\ W02-B08L |
| 7005 | 3D printing | SELECTIVE LASER MELTING MANUFACTURING METHOD, INVOLVES MELTING THIN FILM PART INTO LIQUID STATE, CLEANING SURFACE BEADS OF BALL, PASSING LASER LIGHT TO THIN FILM TO REALIZE PATTERN, AND DETERMINING POSITION OF LASER LIGHT IN CURVED SURFACE | CN104694922A | XU XIANGMING (CN) | TAIWAN SEMICONDUCTOR MFG (TW)\ XU JEFF J (TW)\ LIN CHENG-TUNG (TW)\ WANG HSIANG-YI (TW)\ LEE WEN-CHIN (TW)\ HSIEH BETTY (TW) |  |
| 7006 | 3D printing | COMPOSITE REACTIVE MATERIAL USEFUL IN MUNITION, BOMBS AND GUN AMMUNITION, COMPRISES METAL LATTICE STRUCTURE HAVING INTERSTITIAL SPACES AND A POWDER COMPRISING METAL POWDER OR AT HALOGEN-CONTAINING POLYMER POWDER IN INTERSTITIAL SPACES | CN104930300A | XU XIANGMING LI (CN) | HUAHONG NEC ELECTRONICS CO LTD (CN) |  |
| 7007 | 3D printing | GLOBAL POSITION DETERMINING METHOD FOR GLOBAL POSITIONING SYSTEM (GPS) AND DOPPLER AUGMENTATION (GDAUG) RECEIVER (GDER), INVOLVES DETERMINING AGGREGATE RANGE BETWEEN GPS SATELLITE AND GDAUG SATELLITE (GSAT), AND DOPPLER SHIFTS OF SIGNAL | US2015309473A1 | YACH RANDY L (US) | HUAHONG NEC ELECTRONICS CO LTD (CN) |  |
| 7008 | 3D printing | SLS POWDER 3D PRINTER MAIN CYLINDER PISTON DEVICE, HAS ROLLER PIN MOUNTED IN STRAIGHT CAGE THAT IS ARRANGED BETWEEN MOLD BASE PLATE AND FORMING CYLINDER PISTON, AND BALL LEAD SCREW MOUNTED ON FORMING CYLINDER PISTON AND GUIDE COLUMN | WO2015163765A1 | YADORI SHOJI (JP)\ AJIKA NATSUO US | MICROCHIP TECH INC (US)\ YACH RANDY L (US) | T01-J04 |
| 7009 | 3D printing | SELECTIVE LASER MELTING (SLM) ANTENNA OBTAINED BY MANUFACTURING METHOD OF ANTENNA, HAS LASER TRANSPARENT MATERIAL THAT IS FORMED WITH THROUGH HOLE AFTER REMOVING MATERIAL, AND FEEDER TERMINAL THAT IS PROVIDED WITH THROUGH HOLE | US2015306667A1 | YAEGASHI TOSHITAKE (JP)\ OZAWA YOSHIO (JP) | GENUSION KK US | U11-C06A2\ X24-D03B |
| 7010 | 3D printing | TOOL FOR CUTTING PROCESSING OF WORKPIECE, HAS BASE IN WHICH CUTTING EDGE IS PROVIDED, IS MANUFACTURED BY LASER SINTERING PROCESS AND IS PROVIDED WITH CAVITY | US2015306858A1 | YAEGASHI TOSHITAKE (JP)\ OZAWA YOSHIO (JP) | YAEGASHI TOSHITAKE (JP)\ OZAWA YOSHIO (JP) | D09-C01D\ D09-C01E |
| 7011 | 3D printing | INTERFACE FOR CONNECTING ROTARY TOOL WITH TOOL HOLDER, HAS DAMPING CHAMBER WHICH IS ARRANGED IN CONTACT AREA BETWEEN OUTER SIDE OF ONE TOOL PORTION, AND INNER SIDE OF RECEIVING PORTION OF OTHER TOOL PORTION | DE102014005915A1 | YAMADA HIROSHI US | TOSHIBA CORP (JP) |  |
| 7012 | 3D printing | FRONT AND REAR MOVING THREE-DIMENSIONAL PARKING PAVILION, HAS BIG SUPPORTING FRAME WHOSE OUTSIDE PART IS CONNECTED WITH PORTAL FRAME, AND TRIANGULAR BRACKET CARRIER VEHICLE FRAME CONNECTED WITH REAR CAR | CN204641012U | YAMADA HIROSHI US | NIPPON TELEGRAPH & TELEPHONE (JP) |  |
| 7013 | 3D printing | METHOD FOR MANAGING DOUBLE ALIGNMENT PRECISION CONTROL LAYER, INVOLVES DETECTING CORRECTING MARK, CALCULATING DEVIATION VALUE BETWEEN FIRST AND SECOND MANUFACTURING LAYERS TO CENTER OF THIRD MANUFACTURING LAYER BY DETECTED INTERVAL | CN104886821A | YAMADA SACHIYO (JP) | NIPPON TELEGRAPH & TELEPHONE (JP) |  |
| 7014 | 3D printing | PRESSURE SENSOR BASED ASSISTANT FOCUSING DEVICE OF LASER PROCESSING HEAD FOR AEROSPACE, COMPRESSES COKE NEEDLE PRESSURE TO LINEAR SPRING BY CUSHION BLOCK FOR PRESSURE TRANSMISSION TO PRESSURE SENSOR BY DATA COLLECTING SYSTEM | WO2015160910A1 | YAMADA TOMOYUKI US\ AWAYA TOMOHARU US | SAMSUNG ELECTRONICS CO LTD (KR) |  |
| 7015 | 3D printing | ACETABULAR BONE SCAFFOLD, COMPRISES POROUS BETA-TITANIUM-MOLYBDENUM ALLOY ARTIFICIAL BONE TRABECULA, COBALT-CHROMIUM-MOLYBDENUM ALLOY SUPPORTING LAYER AND ALUMINA CERAMIC ARTICULAR SURFACE | CN204604457U | YAMAMOTO HIROHIKO (JP)\ KIKUCHI HIROAKI (JP)\ SAWADA TOSHIAKI (JP) | FUJITSU LTD (JP) | L03-G05A\ M13-H01\ M13-L\ U14-K01A1J |
| 7016 | 3D printing | QUICK HIGH-TEMPERATURE STEAM GENERATOR, HAS WATER INLET MATCHED WITH WATER CAVITY THAT IS FIXED WITH FIRST HEATING PIPE, AND STEAM PIPE WHOSE UPPER END IS CONNECTED WITH STEAM OUTLET | JP2015183246A | YAMAMOTO HIROSHI (JP) | RENESAS TECH CORP (JP) | M13-H01\ T01-J07B\ X25-A09 |
| 7017 | 3D printing | FLOOR LAYER MANUFACTURING METHOD, INVOLVES DETERMINING DECORATE LAYER DESIGN REQUIREMENT, PERFORMING REQUIREMENT CONCRETE CASTING PROCESS ACCORDING TO DECORATE LAYER DESIGN REQUIREMENT, AND PERFORMING FLOOR LAYER REMOLDING PROCESS | CN104875395A | YAMAMOTO NAOKI (JP)\ TADAKI YOSHITAKA (JP)\ KOGAYU HIROSHIGE (JP) | NEC ELECTRONICS CORP (JP) | M13-H01 |
| 7018 | 3D printing | MINE ULTRASONIC WAVE SUBMARINE NATURAL GAS AQUO-COMPLEX SURFACE LAYER MANUFACTURING METHOD, INVOLVES CONNECTING AQUO-COMPLEX NATURAL GAS DECOMPOSING DEVICE WITH END USER FOR SEPARATING WATER AND NATURAL GAS | CN204589304U | YAMAOKA TORU US\ MORINAGA MINORU US | HITACHI LTD (US) | M13-H |
| 7019 | 3D printing | COMPRESSOR FOR AIR CONDITIONER, HAS MAIN BODY FIXED WITH LIQUID STORAGE TANK, AND SOUND INSULATION ELEMENT PROVIDED WITH SILENCER SPONGE COTTON LAYER THAT IS FORMED WITH FASTENING STRIP | CN204589305U | YAMASHITA KYOJI (JP)\ ARAI KATSUYA (JP)\ OTANI KATSUHIRO (JP)\ IKOMA DAISAKU (JP) | MATSUSHITA ELECTRIC IND CO LTD (JP) | M13-H01\ X25-B02A |
| 7020 | 3D printing | LCD INTEGRATED MACHINE TOOL FOR FORMING PLATFORM FOR NUCLEAR POWER MAIN PIPELINE, HAS MACHINE TOOL DISC THAT IS FIXEDLY INSTALLED ON BED HEAD BOX, AND TAIL SEAT SLEEVE PORTION THAT IS FIXEDLY MOUNTED ON TAIL SEAT | CN204584274U | YAMASHITA YUHEI US\ NOBORI KAZUHIRO US\ KATO YASUSHI US\ DOI SATORU US\ ARAI YASUHIRO US | MATSUSHITA ELECTRIC IND CO LTD (JP) | L04-B01C\ L04-C18A\ L04-C25\ L04-E01C\ M13-H01\ M13-L |
| 7021 | 3D printing | LASER SHAFT MELT COATING PROCESS INVOLVES ACTIVATING LASER FEEDER, CONNECTING SIMULTANEOUS STARTING MACHINE TOOL WITH DRIVING SHAFT SERIES COMPONENT, AND USING LASER CLADDING LAYER FOR FORMING ON SURFACE OF SHAFT SERIES COMPONENT | WO2015151831A1 | YAMAZAKI HIROAKI US | MATSUSHITA ELECTRIC IND CO LTD (JP) | M13-H01\ V08-A01A1 |
| 7022 | 3D printing | LASER CLADDING POWDER PRESETTING METHOD BY CLADDING SURFACE OF PRE-PROCESSING WORK PIECE, CLEANING, USING ELECTRIC SPRAY GUN AND CLADDING WORKPIECE SURFACE, CLADDING POWDER COATING WORK-PIECE SURFACE, AND LASER CLADDING POWDER | CN204570038U | YAMAZAKI SHUNPEI (JP)\ EGUCHI SHINGO (JP)\ OIKAWA YOSHIAKI (JP) | NEC COMPOUND SEMICONDUCTOR US |  |
| 7023 | 3D printing | METAL SURFACE TREATMENT DEVICE LASER CLADDING COMPRISES VACUUM DEVICE, INPUT AND OUTPUT MOUTHS, HEAT MELTING DEVICE AND TRANSMISSION DEVICE | CN204570041U | YAMAZAKI TOMOYUKI (JP) | SEMICONDUCTOR ENERGY LAB (JP)\ EGUCHI SHINGO (JP)\ OIKAWA YOSHIAKI (JP)\ YAMAZAKI SHUNPEI (JP) | A12-L03\ V07-F02A\ V07-F02B\ V07-K01 |
| 7024 | 3D printing | METAL-CERAMIC COMPOSITE COATING METHOD INVOLVES USING HIGH FREQUENCY INDUCTION HEATING COIL IN WORKPIECE SURFACE TO GENERATE SKIN EFFECT, REACHING RED-HOT STATE AND POSITIONING LASER BEAM TO INDUCTION HEATING ZONE WHILE RAPID LASER CLADDING | CN104790033A | YAMAZUMI SAIGO US\ FUJIWARA TETSUO US\ AJIRO YUUJI US | JUJI ELECTRIC DEVICE (JP) | A09-D03\ A11-A05\ A11-B16\ A11-C06\ X25-A08 |
| 7025 | 3D printing | METHOD OF CONTROLLING LASER CLADDING ALLOY SINGLE CRYSTAL GROWTH PROCESS BY DRIVING COOLING DEVICE DURING LASER CLADDING TO COOL NICKEL BASE SINGLE-CRYSTAL TURBINE BLADE TIP IN AXIAL DIRECTION TO ENHANCE TEMPERATURE GRADIENT | CN104760088A | YANG CHIN-SHENG (TW) | TRECENTI TECHNOLOGIES INC US | M22-H03B\ M22-H03F\ M23-D05\ M23-H\ X24-D03A |
| 7026 | 3D printing | RING HOLE TYPE LASER COAXIAL POWDER FEEDING NOZZLE COMPRISES CONICAL NOZZLE MAIN BODY WITH LARGE DIAMETER END PROVIDED WITH LASER HEAD CONNECTED WITH CONNECTING SLEEVE, AND TAPERED UPPER NOZZLE MAIN BODY ARRANGED WITH FEEDING CHANNELS | CN104822223A | YANG CHIN-SHENG (TW) | UNITED MICROELECTRONICS CORP (TW)\ YANG CHIN-SHENG (TW) | A11-B09A2\ A11-C04A\ A12-H12\ A12-W12F |
| 7027 | 3D printing | ANTI-CORROSIVE OIL PIPE END ANTI-CORROSION SEALING STRUCTURE, HAS PIPE SECTION WHOSE SIDE END IS FIXED WITH ANTI-CORROSIVE LAYER, AND OIL PIPE BODY WHOSE INNER HOLE SURFACE IS UNIFORMLY COATED WITH NON-METAL ANTI-CORROSIVE COATING | CN204515735U | YANG YINTANG US\ WU XIAOPENG US\ YU XINHAI US\ CHAI CHANGCHUN US\ GAO HAIXIA US\ DONG GANG US | UNITED MICROELECTRONICS CORP (TW) | L02-A02\ L02-A04\ L02-A05\ M22-H03B |
| 7028 | 3D printing | PROJECTION MICRO-STEREOLITHOGRAPHY SYSTEM FOR PRODUCING SUB-DIFFRACTION-LIMITED FEATURES, HAS FAR-FIELD SUPERLENS (FSL) THAT CONVERTS FAR-FIELD IMAGE PRODUCED BY SPATIAL LIGHT MODULATOR (SLM) AND IS REDUCED TO NEAR-FIELD IMAGE | RU155048U1 | YANGYUAN WANG US\ RU HUANG (CN)\ QIANQIAN HUANG US\ ZHAN ZHAN (CN) | UNIV XIDIAN (CN) | A11-B09A2\ A11-C01C\ A12-H02\ A12-H07 |
| 7029 | 3D printing | PRODUCTION LINE FOR LAYERWISE MANUFACTURING OF TANGIBLE PRODUCTS, HAS CONVEYOR THAT CONVEYS BUILDING PLATFORMS TOWARD AND AWAY FROM DEPOSITION HEAD SUCH THAT TRANSPORTERS ARE MOVABLE AT VARIABLE SPEED ALONG TRAJECTORY OF CONVEYOR | WO2015160844A1 | YANNAN LI (CN)\ XUELI JIANG (CN) | UNIV BEIJING (CN) |  |
| 7030 | 3D printing | MANUFACTURING METHOD OF METAL PRODUCTS USING DIRECT METAL LASER SINTERING PROCESS, INVOLVES CONSTRUCTING PRODUCT, IN WHICH DOWNWARD FACING SURFACES OF PRODUCT IS MADE WITHOUT NEED TO CONSTRUCT TEMPORARY SUPPORT STRUCTURE | US2015294918A1 | YASUTAKA NAKASHIBA (JP)\ SUSUMU KUROSAWA (JP)\ YUKI FUJIMOTO (JP) | NOVATEK MICROELECTRONICS CORP (CN) | V04-T03 |
| 7031 | 3D printing | INFUSION BOLT FOR USE IN JOINING COMPOSITE MATERIALS USED IN TURBOMACHINEY, HAS HEAD COMPRISING AXIALLY ACCESSIBLE OPENING, AND SET OF AXIALLY EXTENDING APERTURES FLUIDLY CONNECTED WITH INNER CAVITY AND AXIALLY ACCESSIBLE OPENING IN HEAD | CN104882557A | YASUYUKI MINO SHINJI ISHII HIR (JP) | NEC ELECTRONICS CORP (US) | M13-H01\ M13-L |
| 7032 | 3D printing | DEVICE FOR GENERATIVE PRODUCTION OF THREE-DIMENSIONAL OBJECTS POWDER-LIKE BUILDING MATERIAL, HAS LASER BEAM FROM LASER SYSTEM PENETRATED THROUGH POWDER LAYER OF MELT POOL IS PARTIALLY REFLECTED AT REFLECTED RADIATION PORTIONS | CN104867620A | YASUYUKI MINO SHINJI ISHII HIR (JP) | PANASONIC CORP (JP) | M24-A02\ M24-A05 |
| 7033 | 3D printing | MOTOR INSULATION LAYER FORMING AIR DISTRIBUTION SYSTEM, HAS FIRST SWITCHING VALVE CONNECTED WITH RUBBER AIR BAG, SECOND SWITCHING VALVE CONNECTED WITH EMPTYING PIPE, AND THIRD SWITCHING VALVE CONNECTED WITH AIR COMPRESSOR | CN104867952A | YASUYUKI MORISHITA (JP) | PANASONIC CORP (JP) | A05-F01C\ A08-R03A\ A08-S02\ A11-A04\ A11-B14\ A11-B16\ A11-C02D\ A12-S08C\ X25-A08 |
| 7034 | 3D printing | ANTI-FOG HAZE PROTECTING MIST HEALTH CARE RESPIRATOR, HAS OUTER LAYER, JOINING MIDDLE LAYER AND INNER LAYER THAT ARE ARRANGED TOGETHER, WHERE INNER LAYER IS COVERED ON ANTI-FOG MIST HEALTH CARE CORE AND MADE OF BAMBOO FIBER CLOTH MATERIAL | CN104785779A | YEAP GEOFFREY C-F (US)\ SRINIVAS JALLEPALLI (US)\ YONGJOO JEON (US)\ DAVID BURNETT JAMES (US)\ SINGH RANA P (US)\ GRUDOWSKI PAUL A (US) | NEC ELECTRONICS CORP (JP) | M13-H01\ M13-L\ S03-B01A\ T06-B13B1\ T06-D10 |
| 7035 | 3D printing | COOLING SYSTEM FOR COOLING HEAT SOURCE, HAS MONOLITHIC STRUCTURE WITH MULTIPLE EMBEDDED COOLING CHANNELS ARE ARRANGED IN MULTI-PLANAR ARRANGEMENT USING SELECTIVE LASER MELTING (SLM) PROCESS TO CARRY FLUID THROUGHOUT STRUCTURE | CN204524771U | YEH CHIH CHIEH (TW) | MOTOROLA INC (US) | M13-H01\ M13-L |
| 7036 | 3D printing | ROTARY MOLD CUTTING DEVICE HAS TRANSITION LAYER THAT IS SET WITH BLADE BASAL PORTION, AND RING THAT IS LOCATED ON SIDE FACE OF ROLLER MAIN PORTION AND WHOSE OUTSIDE DIAMETER IS SET AT MAXIMUM DISTANCE FROM BLADE OF LARGE CENTER ROLLER | CN204529982U | YERIC GREG MUNSON (US)\ FREDERICK MARLIN WAYNE (US) | YEH CHIH CHIEH (TW) | M22-H03B\ M22-H03F\ V05-F04D1\ V05-F05C\ V05-F05E3\ X25-A08 |
| 7037 | 3D printing | METHOD OF INSERTION OF CHARGING-MATERIAL IN BELL-LESS BLAST FURNACE, INVOLVES EXTENDING CORE BATCH TO CENTER FROM FURNACE WALL IN HORIZONTAL TYPE TERRACE WITH RESPECT TO FURNACE-APERTURE RADIUS | CN204509128U | YIBIN LI US\ XIAODONG HE US\ HONGZHEN LU US\ YUFANG SUN US | FREDERICK MARLIN WAYNE (US)\ YERIC GREGORY MUNSON (US) | A11-B16\ A12-W12F\ X25-A08 |
| 7038 | 3D printing | SELECTIVE LASER SINTERING POLYMER/CARBON FIBER COMPOSITE POWDER MATERIAL PREPARATION METHOD, INVOLVES PERFORMING HEAT TREATMENT OF LIQUID PHASE OXIDATION MODIFIED CARBON FIBER, AND COOLING CARBON FIBER TO ROOM TEMPERATURE | CN104759756A | YINGXIANG LIN US | HARBIN INST OF TECHNOLOGY US | L03-G02\ M13-H01\ M13-L\ M22-H03B\ T01-J08A\ V05-F04\ V05-F05A3\ V05-F05A7A\ V05-F05E5\ V05-F08D1\ V07-F01A1\ V07-K01A\ V08-A03A\ X25-A08\ X25-A09 |
| 7039 | 3D printing | LASER CLADDING CHECKING, REGULATING AND CONTROLLING DEVICE HAS ANALOG/DIGITAL (A/D) CONVERTER WHICH IS CONNECTED WITH DISPLAY CONTROLLER, AND INPUT VALVE AND OUTPUT VALVE WHICH ARE CONNECTED WITH LIQUID NITROGEN DEWAR FLASK DIVIDER | JP2015179593A | YIQUAN LI (HK) | YINGXIANG LIN US | M13-H01\ M22-H03B |
| 7040 | 3D printing | LASER CLADDING COAXIAL THREE WAY POWER SUPPLY DEVICE INCLUDES COPPER MIRROR CONNECTING CYLINDER, ADJUSTABLE GUIDE LIGHT CYLINDER, COPPER CONNECTING CYLINDER, COPPER CONE, POWDER DEVICE, LENS BASE, WALL AND THREADED HOLE | CN204544543U | YI-WEN WU US\ MING-DA CHENG US\ WEN-HSIUNG LU US\ CHIH-WEI LIN US\ CHING-WEN CHEN US\ CHIA-TUNG CHANG US\ MING-CHE HO US\ CHUNG-SHI LIU (BE) | YIQUAN LI (HK) | U11-C01J2 |
[truncated: 5,895,801 more chars]
